# Comparative genomic analysis of translation initiation mechanisms for genes lacking the Shine–Dalgarno sequence in prokaryotes

**DOI:** 10.1093/nar/gkx124

**Published:** 2017-02-21

**Authors:** So Nakagawa, Yoshihito Niimura, Takashi Gojobori

**Affiliations:** 1 Department of Molecular Life Science, Tokai University School of Medicine, Isehara 259-1193, Japan; 2 Micro/Nano Technology Center, Tokai University, Hiratsuka 259-1292, Japan; 3 Department of Applied Biological Chemistry, Graduate School of Agricultural and Life Sciences, The University of Tokyo, Tokyo 113-8657, Japan; 4 King Abdullah University of Science and Technology, Computational Bioscience Research Center, Thuwal 23955-6900, Kingdom of Saudi Arabia

## Abstract

In prokaryotes, translation initiation is believed to occur through an interaction between the 3΄ tail of a 16S rRNA and a corresponding Shine–Dalgarno (SD) sequence in the 5΄ untranslated region (UTR) of an mRNA. However, some genes lack SD sequences (non-SD genes), and the fraction of non-SD genes in a genome varies depending on the prokaryotic species. To elucidate non-SD translation initiation mechanisms in prokaryotes from an evolutionary perspective, we statistically examined the nucleotide frequencies around the initiation codons in non-SD genes from 260 prokaryotes (235 bacteria and 25 archaea). We identified distinct nucleotide frequency biases upstream of the initiation codon in bacteria and archaea, likely because of the presence of leaderless mRNAs lacking a 5΄ UTR. Moreover, we observed overall similarities in the nucleotide patterns between upstream and downstream regions of the initiation codon in all examined phyla. Symmetric nucleotide frequency biases might facilitate translation initiation by preventing the formation of secondary structures around the initiation codon. These features are more prominent in species’ genomes that harbor large fractions of non-SD sequences, suggesting that a reduced stability around the initiation codon is important for efficient translation initiation in prokaryotes.

## INTRODUCTION

In prokaryotes (bacteria and archaea), translation initiation is generally thought to occur through a base-pairing interaction between the 3΄ tail of the 16S rRNA of the 30S (small) ribosomal subunit and the complementary sequence in the 5΄ untranslated region (UTR) of an mRNA (1–3). Subsequently, the 50S (large) ribosomal subunit docks to the 30S subunit to form a 70S ribosome, where an initiator tRNA is used for protein synthesis (2,3). The ribosome binding site in the mRNA is called the Shine–Dalgarno (SD) sequence (GGAGG) that is located approximately 10 nucleotides (nt) upstream of the initiation codon (1–3). Although this interaction process is thought to be a major initiator of prokaryotic translation (3–5), the fraction of genes with an SD sequence in a genome widely varies among species, ranging from 9% to 97% (6,7). In addition, previous work suggests that the SD sequence does not necessarily contribute to efficient translation initiation in species harboring a small fraction of genes with an SD sequence (6). Therefore, prokaryotes may employ additional translation initiation mechanisms.

Notably, at least two other prokaryotic translation initiation mechanisms have been identified. One mechanism depends on a leaderless mRNA that lacks a 5΄ upstream sequence and directly binds to a 70S ribosome to initiate translation (8–10). Leaderless mRNAs have been found in various species of prokaryotes (11–15). For example, approximately two-thirds or one-quarter of the transcripts examined were leaderless for *Halobacterium salinarum* or *Mycobacterium smegmatis* (belonging to Euryarchaeota or Mycoplasma), respectively (13,14). Further, Wurtzel *et al.* analyzed a transcriptome of a crenarchaeon species *Sulfolobus solfataricus* P2, and found 69% of protein-coding transcripts were leaderless (15). Another prokaryotic translation initiation mechanism is mediated by ribosomal protein S1 (RPS1), the largest component of the 30S ribosomal subunit (16). In *Escherichia coli*, RPS1 interacts with an mRNA at ∼11 nucleotides upstream of the SD sequence (17). Bound RPS1 then promotes translation initiation by unfolding the mRNA structure, particularly in mRNAs with a highly structured 5΄ UTR and no or a weak SD sequence (6,16–19). RPS1 genes have been identified only in bacterial genomes, suggesting that only bacteria use this mechanism (6,20).

Although these two translation initiation mechanisms may be essential in various bacteria and archaea, their usage is not well understood at a genomic level, especially in nonmodel organisms. Therefore, to better understand prokaryotic translation initiation mechanisms other than SD sequence interactions from an evolutionary perspective, in this study, we conducted extensive comparisons among genes without 5΄ UTR SD sequences using genome sequences from 260 prokaryotes belonging to 14 bacterial phyla and three archaeal phyla (Supplementary data). Nucleotide hybridization energy between the 5΄ UTR in an mRNA and 3΄ tail of 16S rRNA was used to define whether a gene does or does not contain an SD sequence. Nucleotide frequency biases at each position around the initiation codon were evaluated using *G*-statistics, a measure representing the deviation from the expected nucleotide frequency among the entire genomic sequence (6,21–23). From these analyses, we compared several features observed around the initiation codons that are related with translation initiation mechanisms in prokaryotes.

## MATERIALS AND METHODS

### Genomic data and 16S rRNA genes

For this study, genome sequences and gene annotations of 260 species (235 and 25 species of bacteria and archaea, respectively) were obtained from the Gene Trek in Prokaryote Space (GTPS) database at the DNA Data Bank of Japan, DDBJ (24). For each species, we selected protein-coding genes that initiated from the AUG, GUG, UUG, AUA, AUU or AUC codon (25) and ended with a stop codon, on the basis of the annotation. For the 16S rRNA genes, we utilized 13-nt sequences from the 3΄ tail of 16S rRNA, which we obtained in a previously study (6). The 3΄ tails of 16S rRNAs were determined manually, which would improve accuracy of determination of non-SD genes.

### Determination of non-SD genes

To identify genes that do not contain an SD sequence, we calculated the Gibbs free energy (Δ*G*) of the base pairing between 5΄ UTR of an mRNA and the complementary sequence at the 3΄ end of the 16S rRNA (anti-SD sequence) for each species. For this purpose, we used a computational program, free_scan, based on individual nearest-neighbor hydrogen bonding methods (26). This program computes Δ*G* from two input sequences, using a sliding window from the beginning to the end of the two sequences, without gaps (26). The minimum Δ*G* was assumed to be representative of the interaction between the two sequences. For a given mRNA, the region from –20 (i.e. 20 bp before the initiation codon) to –5 was examined: in this study, this region was designated the SD region. We then defined the threshold value as −0.8924 (kcal/mol), calculated as the mean energy value of the three-base interactions between SD and anti-SD sequences (GGA and CCU; GAG and CUC; AGG and UCC). If the Δ*G* between the 3΄ end of a 16S rRNA and the SD region of an mRNA was greater than −0.8924 (kcal/mol), the gene was assumed not to contain SD sequence. In this study, we denoted such genes as ‘non-SD genes’. To reduce false positives in the non-SD genes, we used a looser threshold energy value is looser than that used in our previous reports (6,26).

### Evaluation of nucleotide frequency biases using *G*-statistic

To examine nucleotide frequency biases around the initiation codons, we applied the *G*-statistic to a fraction of nucleotides at each position (6,21–23). All protein-coding genes from each species were aligned at the initiation codons without any gaps. At each position, the observed frequencies of nucleotides (A, U, G and C) were compared with the expected frequencies using the likelihood-ratio statistic, which is used to test goodness of fit (27). The expected frequencies were calculated for each species in four separate categories—upstream of the initiation codon, and the first, second and third positions in a codon in CDSs—because the nucleotide frequencies differed among these categories. The *G*-value at position *i* was calculated using the formula:

\begin{equation*}{G^{(i)}} = 2\sum\limits_{\rm{n}} {O_{\rm{n}}^{(i)}ln\left( {{\hbox{${O_{\rm{n}}^{(i)}}$} \!{\left/ {\vphantom {{O_{\rm{n}}^{(i)}} {E_{\rm{n}}^{(i)}}}}\right.} \!\lower0.7ex\hbox{${E_{\rm{n}}^{(i)}}$}}} \right)} ,\end{equation*}

where *O*_n_^(^*^i^*^)^ is the observed number of nucleotide n (A, U, G and C) at position *i*, and *E*_n_^(^*^i^*^)^ is the expected number of nucleotide n in the category to which position *i* belongs (100 nt upstream of the initiation codon, or the first, second or third position in a codon in CDSs). The *G*-value distribution was approximated by the χ^2^-distribution with three degrees of freedom. When *O*_n_^(^*^i^*^)^ was larger and smaller than *E*_n_, the values of *G*_n_^(^*^i^*^)^ [ = 2*O*_n_^(^*^i^*^)^ln(*O*_n_^(^*^i^*^)^/*E*_n_^(^*^i^*^)^)] became positive and negative, respectively. For this reason, we regarded each term in this formula as a measure of the bias for each nucleotide at a given position. Because the *G*_n_^(^*^i^*^)^ was proportional to the number of genes (*N*) when the fractions of the observed and expected numbers of nucleotides were identical, we defined a *g*_n_ value as *G*_n_^(^*^i^*^)^ divided by the number of genes *N*$(G^{(i)} /N = \sum\limits_{\rm{n}} g_{\rm{n}}^{(i)} )$. As this *g*_n_ value was not affected by the number of genes, we utilized *g*_n_ value for the comparisons of nucleotide frequency biases among species in this study.

### Cluster analysis of the patterns in nucleotide frequency biases

Using the Pearson's correlation coefficient, we quantified similarities in patterns of nucleotide frequency bias upstream of the initiation codons as described in our previous report (23). The correlation coefficient between species X and Y, *r*_XY_, was calculated using the *g*_n_ values from positions −40 to −1 in the 5΄ UTRs as follows:

\begin{equation*}{r_{{\rm{XY}}}} = \frac{{\sum\limits_i {\sum\limits_{\rm{n}} {\left( {g_{{\rm{Xn}}}^{(i)} - \overline {{g_{\rm{X}}}} } \right)\left( {g_{{\rm{Yn}}}^{(i)} - \overline {{g_{\rm{Y}}}} } \right)} } }}{{\sqrt {\sum\limits_i {\sum\limits_{\rm{n}} {{{\left( {g_{{\rm{Xn}}}^{(i)} - \overline {{g_{\rm{X}}}} } \right)}^2}} } } \sqrt {\sum\limits_i {\sum\limits_{\rm{n}} {\left( {g_{{\rm{Yn}}}^{(i)} - \overline {{g_{\rm{Y}}}} } \right)} } } }}\end{equation*}

where *g*_Xn_^(^*^i^*^)^ and *g*_Yn_^(^*^i^*^)^ represent the *g*_n_ values of nucleotide n (A, U, G or C) at position *i* (from –40 to –1) in species X and Y, respectively, and $\overline {{g_{\rm{X}}}}$ and $\overline {{g_{\rm{Y}}}}$ represent the average of *g*_n_ values among all positions and nucleotides in species X and Y, respectively. We calculated *r* values for all combinations among the 260 examined species (Supplementary data) and then defined the similarity score, *D*, as follows:

\begin{equation*}D = 1-r.\end{equation*}

Using the *D* scores, a cluster analysis was conducted using the group average method implemented in R (http://www.r-project.org/).

### Principal component analysis

We performed a principal component analysis (PCA) of *g*_n_ values from positions –40 to –1 (160 variables) in all species examined. The PCA analysis was conducted using the amap package in R.

### Evaluation of secondary structure

We calculated the minimum Δ*G* of a secondary structure in a given mRNA using the hybrid-ss-min program (version 3.5 with default parameters: NA (nucleic acid) = RNA, t (temperature) = 37, [Na^+^] = 1, [Mg^2+^] = 0, maxloop = 30, prefilter = 22) (28,29).

### Randomized sequence

We generated randomized sequences from position –20 to +20, assuming that each nucleotide at every single position appears independently for each species. Therefore, nucleotide fractions at each position were conserved between observed and randomized sequences for each species. The number of generated randomized sequences was a hundred-fold the number of genes in a given species.

## RESULTS

### Evaluation of nucleotide frequency biases for SD and non-SD genes

We applied the *G*-statistic separately to *E. coli* genes with or without SD sequences. As described in the Materials and Methods, we identified genes with and without SD sequences depending on the interaction energy between the SD region in the 5΄ UTR of an mRNA sequence and the 3΄ tail of a 16S rRNA. Here, we denoted the two groups of genes as ‘SD genes’ and ‘non-SD genes’ for each species. Nucleotide frequency biases around position –9 were prominent in SD genes, but those signals were hardly observed in non-SD genes (Supplementary Figure S1A). As noted, the biases observed around position –9 were due to SD sequences, and therefore these results strongly suggested that our method could effectively identify the SD sequence. We conducted the *G*-statistic for non-SD genes of 260 prokaryote species used in this study (see Materials and Methods and Supplementary data). Supplementary Figure S1 shows results of *G*-statistic of representative 6 species including *E. coli* as an example. In addition, *G*-statistics of all 260 species examined was provided as Supplementary Data.

### Difference in the nucleotide biases of non-SD genes between bacteria and archaea

**Figure 1.**
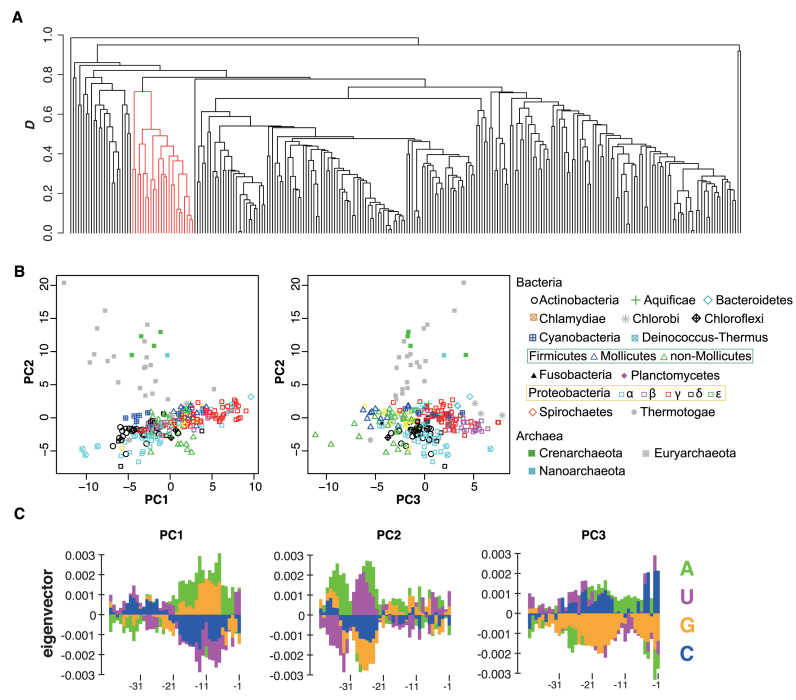
Cluster and principal component analyses of the upstream regions of non-Shine-Dalgarno (SD) genes for each species. (**A**) Cluster dendrogram showing similarities in the patterns of *g*_n_ values upstream of the initiation codon from –40 to –1 among 260 species according to the score *D*. Branches in red represent a cluster containing all archaeal species. (**B**) Scatter plots of principal components (PCs) 1 and 2, and 2 and 3. The symbols indicate each taxonomic group of species. Proteobacteria were subdivided into five classes (α, β, γ, δ, and ε), because of the large number of species included in this phylum (138 species). Moreover, Firmicutes were subdivided into two classes, Mollicutes and others, because the fractions of SD genes in Mollicutes including *Mycoplasma* spp., are significantly lower than those in other Firmicutes (6). (**C**) The eigenvectors of PC1, 2, and 3 from left to right. The color scheme for each nucleotide is shown in the bottom right. See Supplementary Figure S1 for details of *G*-statistics analysis.

Then, we compared the nucleotide frequency biases observed in the 5΄ UTRs of non-SD genes. As shown in Supplementary Figure S1, the nucleotide frequency patterns in non-SD genes varied among species. For a detailed comparison, the region from positions −40 to −1, where a unique pattern of nucleotide appearance were utilized. We defined a similarity score *D* based on the Pearson's correlation coefficient (see Materials and Methods) and conducted a cluster analysis using this score. The resulting dendrogram (Figure 1A) indicates that all archaea examined form a single cluster, suggesting that these species exhibited similar patterns of nucleotide frequency biases for non-SD genes.

We also conducted a principal component analysis (PCA) using *g*_n_ values from positions –40 to –1 in the 5΄ UTRs. Plots of the first three principal components (PCs) reveal differences in the patterns of nucleotide frequency biases between bacteria and archaea (Figure 1B for each clade and Supplementary Figure S2A for bacterial and archaeal clades). Moreover, species belonging to the same phylum tended to form clusters in the plot, indicating the similarity of nucleotide frequency biases among closely related species. We also compared variations of PC scores among various clades, and found that some clades showing highly variable SD content (such as Mollicutes and α Proteobacteria) previously reported (6) did not vary compared to those of other clades (Supplementary Figure S2B). The mean value and the standard deviation of each PC for each phylum are summarized in Table 1.

**Table 1.** Eigenvalues of three major principal components with standard deviations (sd) of Figure 1B

Phylum	# of species	PC 1 (sd)	PC 2 (sd)	PC 3 (sd)
Actinobacteria	22	-4.05 (2.02)	-2.44 (1.05)	-0.86 (1.09)
Aquificae	1	2.45 (–)	2.11 (–)	0.41 (–)
Bacteroidetes	4	6.64 (2.62)	1.94 (0.82)	3.22 (1.10)
Chlamydiae	7	1.56 (0.77)	0.01 (0.71)	-2.06 (0.73)
Chlorobi	3	1.15 (0.74)	1.07 (0.79)	6.26 (1.20)
Chloroflexi	2	0.39 (0.27)	-2.42 (0.58)	-2.34 (1.41)
Cyanobacteria	9	-1.05 (3.47)	0.43 (0.50)	0.96 (1.59)
Deinococcus-Thermus	3	-9.80 (0.79)	-4.89 (0.54)	3.71 (2.09)
Firmicutes	37	0.66 (1.83)	-0.96 (2.02)	-4.10 (2.23)
*Firmicutes excluding Mollicutes*	*25*	*-0.07 (1.67)*	*-1.67 (1.99)*	*-4.15 (2.36)*
*Mollicutes*	*12*	*2.18 (1.03)*	*0.52 (1.05)*	*-3.98 (1.94)*
Fusobacteria	1	-1.81 (–)	-0.80 (–)	-4.35 (–)
Planctomycetes	1	-2.08 (–)	-1.11 (–)	2.32 (–)
Proteobacteria	138	1.19 (4.00)	-0.93 (1.82)	1.33 (2.44)
*α proteobacteria*	*43*	*-0.56 (3.77)*	*-2.06 (2.03)*	*-0.52 (1.68)*
*β proteobacteria*	*25*	*-1.05 (2.01)*	*-1.16 (0.71)*	*4.28 (1.22)*
*γ proteobacteria*	*53*	*4.30 (3.06)*	*0.25 (1.20)*	*1.81 (2.07)*
*δ proteobacteria*	*11*	*-1.94 (3.06)*	*-1.92 (2.06)*	*0.38 (1.20)*
*ε proteobacteria*	*6*	*1.31 (1.87)*	*-0.41 (0.75)*	*-0.13 (1.67)*
Spirochaetes	6	0.97 (3.10)	-0.57 (2.08)	-3.25 (1.66)
Thermotogae	1	-4.94 (–)	-3.39 (–)	-5.58 (–)
Crenarchaeota	4	-2.78 (1.33)	11.4 (1.35)	-0.15 (2.52)
Euryarchaeota	20	-4.53 (3.90)	8.67 (4.49)	-1.00 (1.82)
Nanoarchaeota	1	-0.34 (–)	9.44 (–)	1.973 (–)

Figure 1C indicates the eigenvector of each PC. The proportions of variances explained by PC1, 2 and 3 were 0.036, 0.032 and 0.027, respectively. PC1 was similar to the SD sequence and likely represents genes with a weak SD-like signal. Bacteria and archaea clusters were separated mainly by a difference in the value of PC2 (Figure 1B), which corresponded to the U/A signal near position –25, A signal near position –30, and G/C signal near position –35. These nucleotide frequency biases were observed only in archaeal species (Figure 1C). PC3 was characterized by the C signals immediately upstream of the initiation codon, C and A signals near position –15, and U signals near position –25. These signals were observed in species belonging to Chlorobi, Deinococcus-Thermus, Bacteroidetes, Cyanobacteria and some species of Proteobacteria (Figure 1C), as indicated by positive mean PC3 scores (Table 1).

### Symmetrical nucleotide biases around the initiation codons in non-SD genes

**Figure 2.**
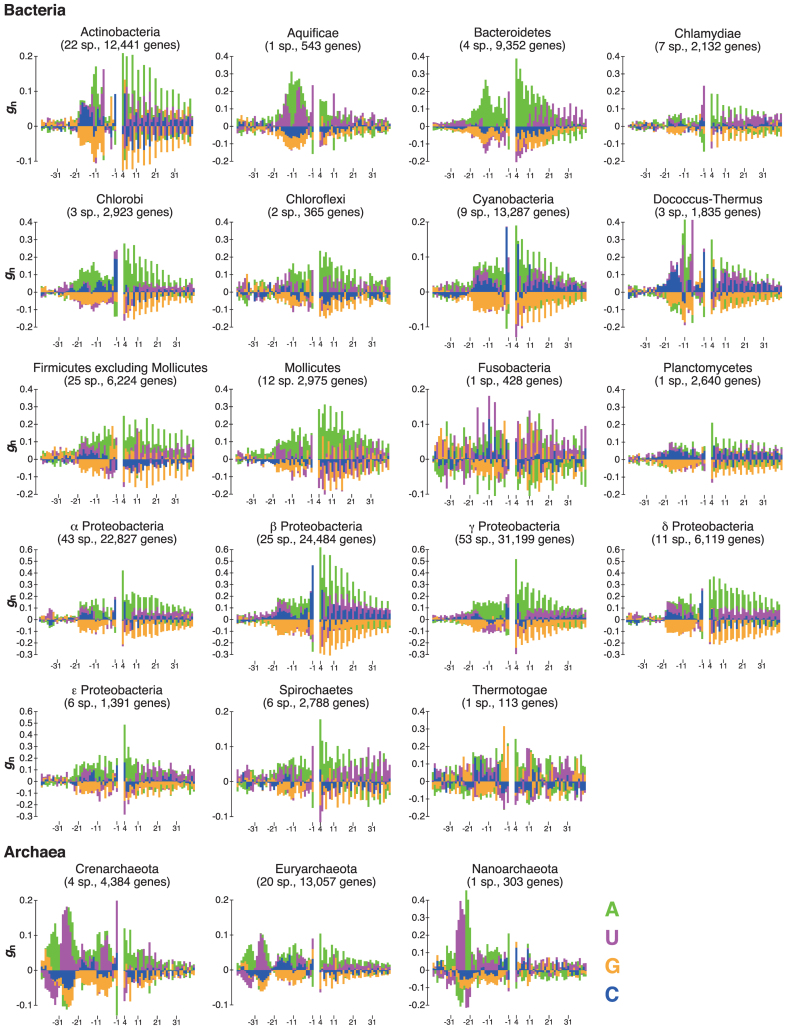
Nucleotide frequency biases around the initiation codons of non-SD genes for each taxonomic group of prokaryotes. The *g*_n_ value for each nucleotide at each position was calculated from the average fractions of *O*_n_/*N* and those of *E*_n_/*N* among all species belonging to a given bacterial or archaeal taxonomic group. Note that the *g*_n_ values at the initiation codon are not shown. The color scheme for each nucleotide is shown in the bottom right. The number of species used is shown for each group (sp., species).

As shown above, nucleotide frequency biases in the upstream regions of initiation codons were similar among closely related species (Figure 1B). Figure 2 presents the mean *g*_n_ values at each nucleotide position for both upstream and downstream regions of the initiation codon among the species belonging to each phylum. Note that in a coding region, nucleotide frequencies vary widely among the first, second, and third codon positions, and biases in the *g*_n_ values were corrected (see Materials and Methods). Figure 2 suggests that the nucleotide frequency biases observed upstream and downstream of the initiation codon for non-SD genes are similar. For example, in Aquificae and Bacteroidetes, U/A and A, respectively, were overrepresented in the 5΄ UTR near position –10; simultaneously, similar U/A and A patterns were also observed at many positions in the coding regions for each phylum. Note that this symmetrical pattern could not be identified without eliminating SD genes, as it was hidden by a strong signal from the SD sequence in the analysis of all protein-coding genes (Supplementary Figure S3).

The presence of symmetrical nucleotide frequency biases would hider formation of the mRNA secondary structure around the initiation codon. In fact, many genes in bacterial and archaeal species have been found to exhibit relatively relaxed structures around the initiation codon (30). Moreover, mRNA folding around the initiation codon is known to strongly influence the amount of protein produced (29,31–34). In particular, Scharff *et al.* experimentally verified in *E. coli* that the local absence of an RNA secondary structure facilitates the initiation of Shine–Dalgarno-independent translation (34). Therefore, we hypothesized that the symmetrical nucleotide frequency biases around the initiation codon observed in non-SD genes would affect efficient translation initiation by reducing mRNA stability in various species of prokaryotes.

### Secondary structure around the initiation codon

To test this hypothesis, we generated randomized sequences in which nucleotide fractions at each position were identical to those of non-SD genes for each species (see Materials and Methods). Because *g*_n_ values shown in Figure 2 displayed the mean values of nucleotide frequencies at each site of all non-SD genes, we could not guarantee that the nucleotide appearance is symmetrical for each sequence around the initiation codon. If this is the case, its average mRNA folding energy around the initiation codons tends to be weak for observed compared with that of randomized sequences.

We computed the mRNA folding energy (Δ*G*) values around the initiation codons (from positions –20 to +20) of actual and randomized sequences and compared the distributions. For example, as shown in Supplementary Figure S4, the distribution of Δ*G* values obtained from actual and randomized sequences of *Bacteroides fragilis* belonging to the phylum Bacteroidetes was compared using the Wilcoxon rank-sum test. We accordingly determined that for 70 out of 260 species, the Δ*G* values of non-SD genes were significantly weaker than those of randomized sequences (*P* < 0.01 with the Bonferroni correction). This tendency was prominent in the following phyla: Bacteroidetes (4/4), Chlorobi (3/3), Deinococcus-Thermus (3/3), Cyanobacteria (7/9) and β proteobacteria (20/25). However, two species in β proteobacteria exhibited the opposite tendency (*P* < 0.01 with the Bonferroni correction). These results are summarized in Table 2 and the Supplementary data for each phylum and each species, respectively. The results suggest a tendency toward a symmetrical nucleotide appearance around the initiation codon of each non-SD gene to reduce stability near the initiation codon. As a result, the nucleotide frequency biases observed upstream and downstream of the initiation codon are similar, as shown in Figure 2.

**Table 2.** Statistic analyses for the sequences around the initiation codon

		Secondary structure randomization		Secondary structure SD versus non-SD		Interaction between 16S rRNA and mRNA	
Phylum	# of species	*P* < 0.05	*P* < 0.01	*P* < 0.05	*P* < 0.01	*P* < 0.05	*P* < 0.01
Actinobacteria	22	0	0	0 (4)	0 (4)	7	6
Aquificae	1	0	0	1	1	0	0
Bacteroidetes	4	4	4	4	4	0 (1)	0
Chlamydiae	7	3	1	7	7	0	0
Chlorobi	3	3	3	3	3	0	0
Chloroflexi	2	0	0	0	0	0	0
Cyanobacteria	9	8	7	9	9	0 (2)	0 (1)
Deinococcus-Thermus	3	3	3	3	3	0	0
Firmicutes	37	1	1	1 (8)	1 (6)	19	15
*Firmicutes excluding Mollicutes*	*25*	*0*	*0*	*0 (8)*	*0 (6)*	*19*	*15*
*Mollicutes*	*12*	*1*	*1*	*1*	*1*	*0*	*0*
Fusobacteria	1	0 (1)	0	0 (1)	0 (1)	1	1
Planctomycetes	1	0	0	1	1	0	0
Proteobacteria	138	58 (3)	50 (2)	72 (8)	70 (6)	24 (1)	21 (1)
*α proteobacteria*	*43*	*7*	*4*	*9 (7)*	*8 (5)*	*11*	*10*
*β proteobacteria*	*25*	*21 (2)*	*20 (2)*	*22 (1)*	*22 (1)*	*1 (1)*	*1 (1)*
*γ proteobacteria*	*53*	*29 (1)*	*25*	*39*	*38*	*6*	*4*
*δ proteobacteria*	*11*	*1*	*1*	*2*	*2*	*3*	*3*
*ε proteobacteria*	*6*	*0*	*0*	*0*	*0*	*3*	*3*
Spirochaetes	6	0	0	1	1	1	0
Thermotogae	1	0	0	0	0	1	1
Crenarchaeota	4	1	0	4	4	0	0
Euryarchaeota	20	1	1	15	14	6	5
Nanoarchaeota	1	0	0	0	0	0	0

We applied a Bonferroni correction to each *p*-value. The number in a parenthesis indicates the number of species showing an opposite trend statistically.

We further analyzed the possibility that to ensure efficient translation initiation, secondary structures around the initiation codon are weaker in non-SD genes than in SD genes. We compared the folding energies (Δ*G*) of mRNA secondary structures in SD genes with those in non-SD genes for each species, and found that 118 species exhibited relatively relaxed structures around the initiation codons of non-SD genes, compared with SD genes (Wilcoxon rank sum tests, *P* < 0.01 with the Bonferroni correction). These results also support the hypothesis that a weak secondary structure around the initiation codon facilitates translation initiation in non-SD genes. However, 23 species belonging to Fusobacteria, Proteobacteria, Firmicutes (non-Mollicutes) or Actinobacteria exhibited the opposite tendency; in other words, SD genes contained a weaker structure around the initiation codon, compared with non-SD genes.

### Interaction between the 3΄ end of 16S rRNA and the initiation codon

A recent genome-wide ribosomal profiling analysis of *E. coli* and *B. subtilis* found that SD-like sequences (GGU, GGG, GGA, GUG, AGG, GAG) within coding sequences induce pervasive translation pausing, and that such nucleotide patterns are disfavored in the coding regions (35,36). Indeed, it was recently reported that highly expressed genes tend to contain fewer SD-like sequences in coding regions, which was observed in various species of prokaryotes (37,38). Interestingly, Starmer *et al.* previously reported that mRNAs encoded by 2420 of 58 550 genes from 18 species exhibited strong interactions between a single-stranded 16S rRNA tail and a sequence around the mRNA initiation codon (26). These results could indicate binding-induced ribosomal complex pausing at the initiation codon, which might play a role in efficient and accurate translation initiation. In particular, such interactions might be beneficial for mRNAs lacking the SD sequence. Therefore, we hypothesized that non-SD genes might harbor a sequence around the initiation codon that could interact with the 3΄ tail of a 16S rRNA.

**Figure 3.**
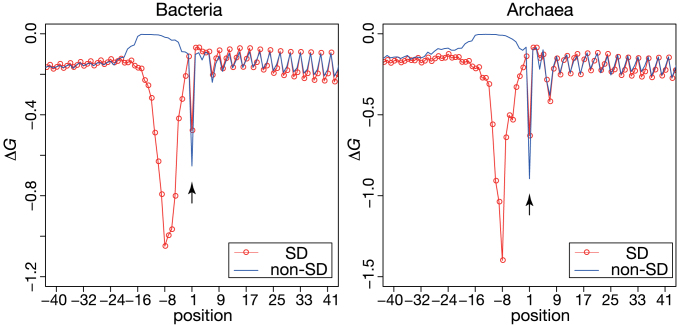
Average Gibbs energy changes of SD and non-SD genes in bacteria and archaea. The Gibbs energy changes, Δ*G*, of the interactions between the 3΄ tail of 16S rRNA and sequences around the initiation codons of mRNAs were calculated for both SD (red line with circle) and non-SD genes (blue line) in bacteria (left) and archaea (right). The decrease indicated by an arrow represents the position that includes the initiation codon. The upstream decreases near position 8 indicate the presence of SD sequences.

To test this hypothesis, we calculated the binding energy Δ*G* between 16S rRNA tails and mRNA sequences from position –100 to +100 from each species, using the procedure previously applied for the detection of SD sequences (see Materials and Methods). The average interaction energies of both SD and non-SD gene groups at each position (from –40 to +40) from bacteria and archaea are shown in Figure 3. At position +1, in agreement with Starmer *et al.* (26), peaks were observed for all species examined (Figure 3 and Supplementary Figure S5 for each phylum). Moreover, the average energy at position +1 was significantly stronger for non-SD genes than for SD genes in both bacteria and archaea. This result was statistically supported by Wilcoxon-paired signed-rank tests of the average changes in the Δ*G* of SD and non-SD genes for each species of bacteria and archaea (at position +1, *P* < 0.01 with the Bonferroni correction). This tendency was particularly evident in some species belonging to Actinobacteria, Firmicutes (non-Mollicutes), Fusobacteria, Proteobacteria and Thermotogae among bacteria, and five archaeal species of Euryarchaeota (Wilcoxon signed-rank test comparing SD and non-SD genes for each species, *P* < 0.01 with the Bonferroni correction), although each one species each from Cyanobacteria and β proteobacteria exhibited a different trend (Table 2; Supplementary Figure S5). The average Δ*G* interaction energies of both SD and non-SD genes for each species are summarized in the Supplementary data. These results support the idea that an interaction between 16S rRNA and mRNAs may be functional to identify the initiation codon position, although it might be caused by misannotation of the initiation codons. Further investigations are needed to verify that the interaction promotes efficient translation initiation of non-SD genes in some prokaryotic phyla.

## DISCUSSION

In this study, we demonstrated differences in the nucleotide frequencies upstream of the initiation codons of non-SD genes between bacteria and archaea. Previous studies statistically analyzed the nucleotide composition biases around the initiation codon in various prokaryote species (39). However, Hasan *et al.* did not observe nucleotide patterns before the initiation codon that could be distinguish between bacteria and archaea, despite the use of similar statistical methods (39). This discrepancy between our and their studies can be explained by nucleotide frequency biases resulting from large fractions of SD genes in multiple bacterial and archaeal species.

One major factor that differentiates the nucleotide frequency biases in bacteria and archaea comprises three position-dependent biases found in archaea: U/A around position –25, A around position –30, and G/C around position –35 (Figure 1B and C). These archaeal nucleotide frequency biases might correspond to the transcriptional initiation signals of leaderless mRNAs. In general, leaderless mRNAs contain literally no nucleotides upstream of the initiation codon, and therefore transcription initiation signals can be observed immediately before the initiation codon. Indeed, the nucleotide frequency biases obtained in this study appear to correspond to the two common archaeal transcriptional initiation signals: i) Box A for U/A around position –25, which is located about 26 nt upstream of the transcription initiation site (40,41) and ii) transcription factor B recognition element for A around position –30 and G/C around position –35, which are found immediately upstream of Box A (12,42). Therefore, the signals observed for non-SD genes in all examined archaeal species might be attributable to the transcriptional signals of leaderless mRNAs.

For bacteria, the Pribnow box, located at 10 nt upstream from the transcription initiation site, is known as the common transcriptional signal (43,44). This element has a consensus sequence of TATAAT in *E. coli* (45). Therefore, AT-rich biases observed at positions of approximately –20 to –10 in some bacterial phyla, indicated by PC 3 (Figure 1B and C), might correspond to the Pribnow box, thus suggesting that mRNAs possessing these signals might be leaderless. Indeed, these nucleotide frequency biases are prominent in Bacteroidetes, Cyanobacteria and Deinococcus-Thermus, which were reported to harbor a high fraction of leaderless mRNAs, in particular for single and proximal operon genes (46–48). However, Actinobacteria species that were also predicted to harbor high fractions of leaderless mRNAs do not exhibit this pattern of nucleotide frequency bias (Figure 1C). Therefore, the AT-rich biases observed at approximately –20 to –10 cannot be simply attributed to the transcriptional initiation signals of leaderless mRNAs.

The abovementioned nucleotide frequency biases might also correspond to RPS1 binding sites. RPS1 genes are found only in bacteria, and RPS1 is known to facilitate translation initiation by interacting with U-rich sequences located a few base pairs upstream of the SD sequence (6,16–19). However, RPS1 might not be functional with respect to translation initiation in some phyla of bacteria, such as Cyanobacteria, Fusobacteria, and some classes of Firmicutes (6,20). Given these features of RPS1, some of the areas of U-rich bias observed around position –10 in phyla such as Actinobacteria, Bacteroidetes, or Deinococcus-Thermus might be RPS1 binding sequences. However, the contributions of RPS1, as well as the Pribnow box, to the biases observed in our analysis remain unclear. In addition, it is known that archaeal translation initiation factors are more similar to eukaryotic homologs rather than bacterial ones, although the initiation mechanisms are totally different between archaea and eukaryotes (reviewed in 49). The differences of translation initiation factors between bacteria and archaea might be related the distinct patterns observed in this study.

Regarding efficient translation initiation, the relaxed structure observed around the initiation codon might work primarily to characterize nucleotide frequency biases in coding regions, together with symmetrical biases in upstream regions in a broad range of species of bacteria and archaea. As analyzed in this study, in many species of bacteria and archaea, non-SD genes tend to exhibit weaker secondary structures around the initiation codon compared with SD genes (Table 2). Our comparison between SD and non-SD genes suggested that such a relaxed structure could functionally promote effective translation initiation in a broad range of prokaryotic phyla. Moreover, interactions between 16S rRNA and mRNAs are prominent among non-SD genes in most phyla, although bacterial species belonging to Bacteroidetes, Chlorobi, and Cyanobacteria that harbor a small fraction of SD genes do not show this pattern. The relationship between these interactions and translation initiation remains unknown and further investigations are required.

**Figure 4.**
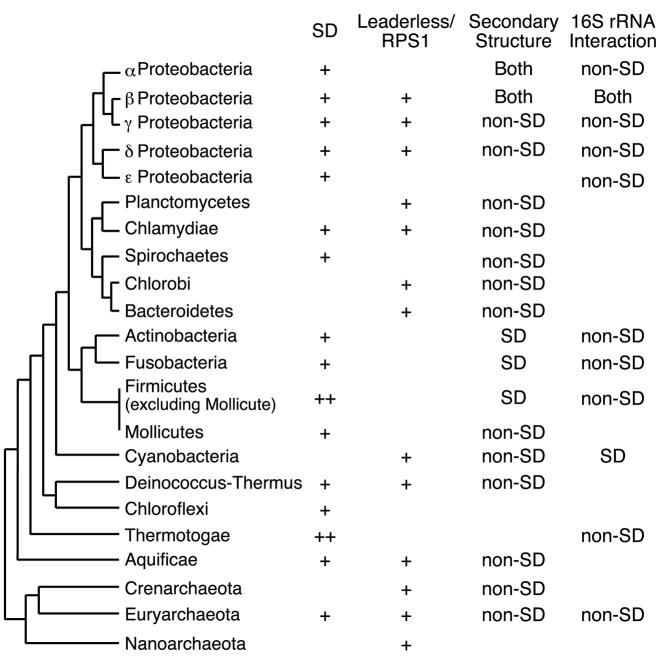
Cladogram representing translation initiation mechanism usage in prokaryotes. This phylogenetic classification follows that of Olsen *et al.* (50). For the SD column, the average fraction of SD genes in a species for each phyla (*R*_SD_) was determined by Nakagawa *et al.* (6) and are presented as follows: ++, *R*_SD_ > 0.8; +, *R*_SD_ > 0.5. For the leaderless/RPS1 column, + indicates a positive mean value of the principal component (PC) 3 (shown in Table 1) in a taxonomic group of bacteria, or if positive PC 2 for a given phylum of archaea. Note that the RPS1 signal is functional only in bacteria. For the secondary structure column, ‘non-SD’ is indicated if a species exhibited a statistically weaker structure around the initiation codon for non-SD genes (*P* < 0.01 with the Bonferroni correction, Table 2); ‘SD’ indicates a species exhibiting the opposite trend. For 16S rRNA interaction column, ‘non-SD’ indicates a species that exhibited a statistically stronger interaction between the tails of 16S rRNA and 5΄ UTRs of mRNAs from non-SD genes (*P* < 0.01 with the Bonferroni correction, Table 2); ‘SD’ indicates which species of Cyanobacteria exhibited the opposite trend.

Regarding the phylogeny of prokaryotes (50), we summarized the results obtained in this study in Figure 4 with SD gene usage in each phylum (6). Interestingly, the species harboring small fractions of SD genes that belong to Planctomycetes, Chlorobi, Bacteroidetes, Cyanobacteria, Crenarchaeota and Nanoarchaeota phyla tended to exhibit the nucleotide frequency bias due to leaderless mRNA and/or RPS1. These results suggest that the translation of non-SD genes in species of these phyla was actively initiated through mechanisms involving leaderless mRNA and/or RPS1 interactions. Moreover, the folding energies of non-SD genes were weaker than those of SD genes, particularly in the species having small fractions of SD genes. Those features were observed independently in multiple species belonging to different phyla of bacteria and archaea. An assumption that various prokaryotic translation initiation mechanisms work in a complementary manner might explain these findings. In addition, there may be as yet unknown translation initiation mechanisms, such as interactions between mRNA sequences around the initiation codon and the 3΄ tail of 16S rRNA. However, there are several limitations of this study that the misannotation of initiation codons in a genome may influence the results of comparisons. Indeed, species having high proportion of SD genes show lower mRNA folding stability in SD genes instead of non-SD genes, which might be explained by misannotation of the initiation codon of non-SD genes. The number of species and non-SD genes may not be enough to compare comprehensively among prokaryotes. Further studies are required for these points; however the results obtained in this study suggest that prokaryotes have implemented various translation initiation mechanisms that have been dynamically diversified through evolution.

## Supplementary Material

Supplementary Data
