# Supplementary material for: Comparative genomic analysis of translation initiation mechanisms for genes lacking the Shine–Dalgarno sequence in prokaryotes
Source: Nucleic Acids Res. 2017 Feb 21;45(7):3922–31. doi: 10.1093/nar/gkx124 (PMC5397173; doi:10.1093/nar/gkx124)

Comparative genomic analysis of translation initiation mechanisms for genes lacking the Shine–Dalgarno sequence in prokaryotes

So Nakagawa, Yoshihito Niimura and Takashi Gojobori

Supplementary Figures and Dataset

Figure S1. Nucleotide frequency biases upstream of the initiation codon in six species.

Each diagram shows g_n values from positions −40 to −1 for (A) *E. coli* in γ Proteobacteria, (B) *Bordetella bronchiseptica* in β proteobacteria, (C) *Chlorobium chlorochromatii* in Chlorobi, (D) *Thermus thermophilus* in Deinococcus-Thermus, (E) *Picrophilus torridus* in Euryarchaeota, and (F) *Sulfolobus acidocaldarius* in Crenarchaeota. We conducted *G*-tests of each position upstream of the initiation codon in all genes (left), SD genes (center), and non-SD genes (right). Numbers in parentheses represent the numbers of genes (*N*) used in each analysis. O_n/N shows frequency of nucleotides (n = A, U, G, and C) divided by the numbers of genes (*N*) appearing at each position in a 50nt region of the 5′ UTR. A, U, G, and C are shown in green, magenta, yellow, and blue, respectively. *G*/*N* shows *G*-values divided by *N* indicating the deviation from the expected values. Values of g_n indicated by colored bars above and below the horizontal line show positive and negative g_n values, respectively, and were drawn without overlapping. The color scheme is the same as that used in the diagrams in the top row.

Figure S2. Comparison of PC scores computed from the upstream regions of non-Shine-Dalgarno (SD) genes for each species

(A) Scatter plots of principal components (PCs) 1 and 2, and 2 and 3. A black circle or a red triangle indicates bacterial or archaeal species, respectively. Please see the Figure 1B as well. (B) Box plot of PC scores. A clade containing more than 8 species was shown. The number of species was given in a parenthesis for each clade. The middle line indicates the median and the upper and lower edges of the boxes represent the first and third quartiles, respectively. The ends of the vertical

lines indicate the minimum and maximum data values, unless outliers are present, in which the lines extend to a maximum of 1.5 times the interquartile range.

Figure S3. Nucleotide frequency biases around the initiation codon for each taxonomic group of prokaryotes.

Please see the Figure 2 for the details.

Figure S4. Distribution of mRNA folding energy around the initiation codon, obtained from observational and randomized non-SD gene sequences in *Bacteroides fragilis*.

The Gibbs energy changes, ΔG, in the mRNA tertiary structures around the initiation codons were calculated for both observational (orange) and randomized (green) sequences. The mean value is shown for each group.

Figure S5. Average Gibbs energy changes in SD and non-SD genes for each taxonomic group of prokaryotes.

Please see Figure 3 for the details.

Supplementary Dataset

G-statistic results of non-SD genes (G-stat_non-SD.pdf) and all protein-coding genes (G-stat_all.pdf) for 260 prokaryotes examined in this study.

Supplementary Figure S1

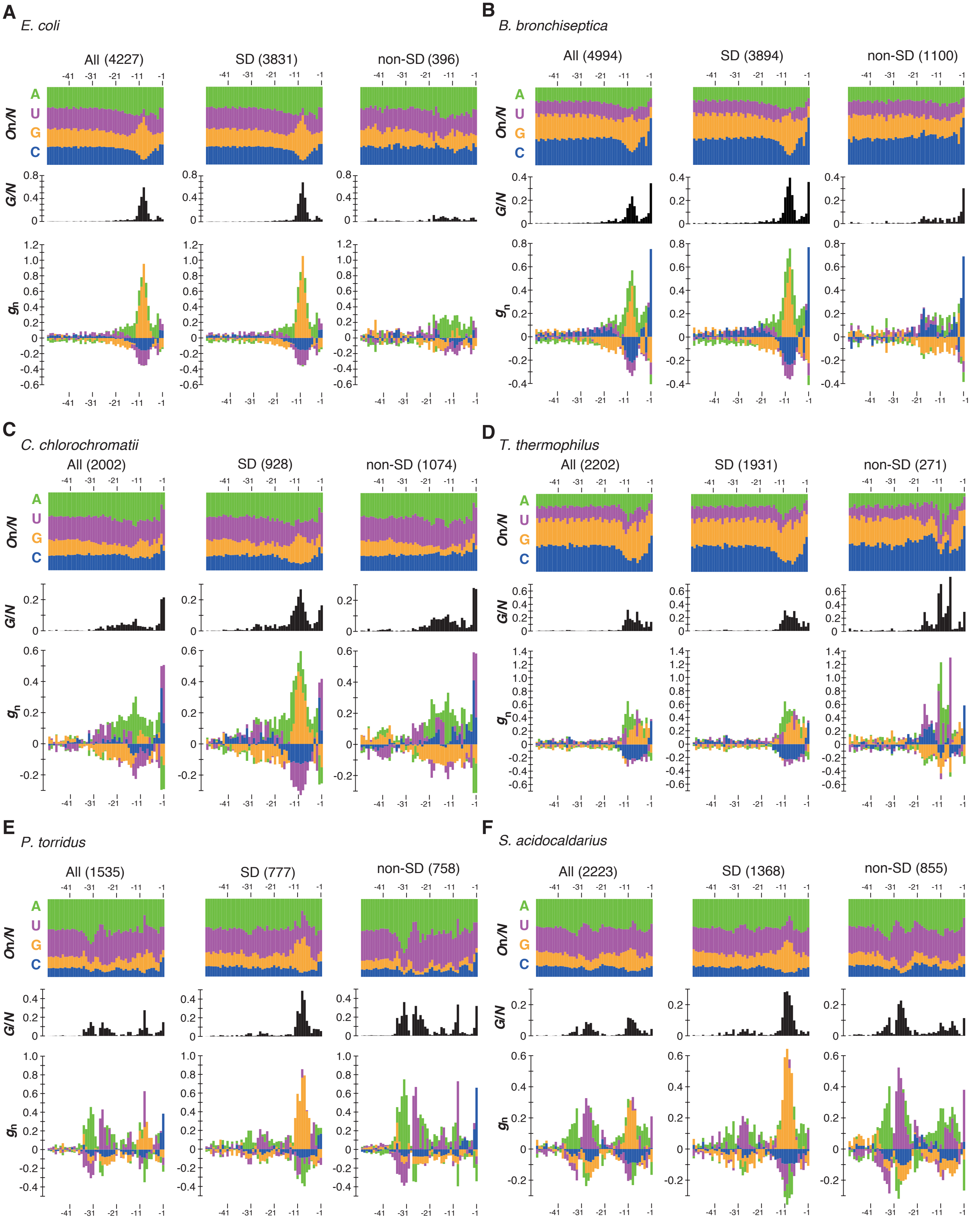

Supplementary Figure S2

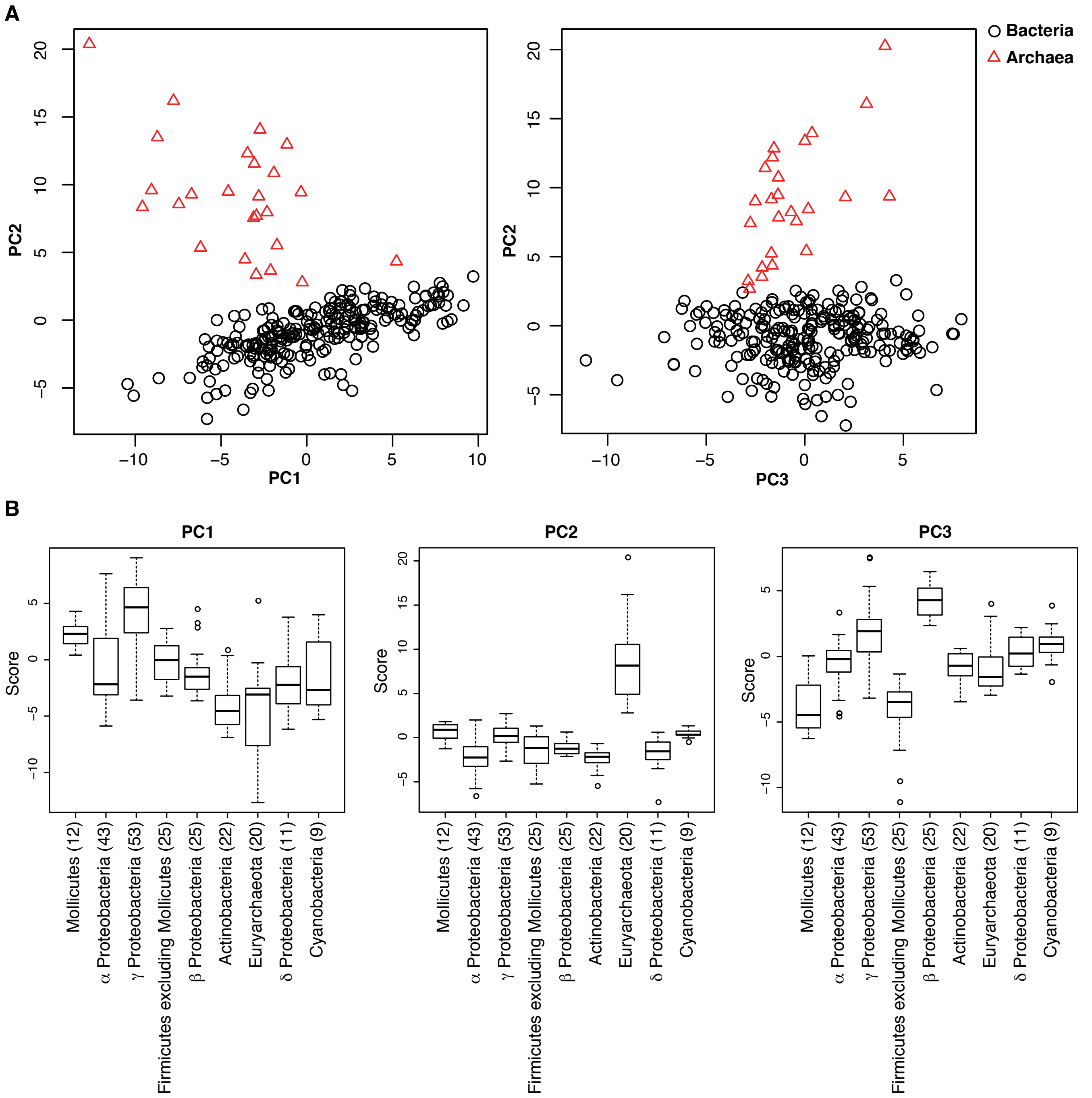

Supplementary Figure S3

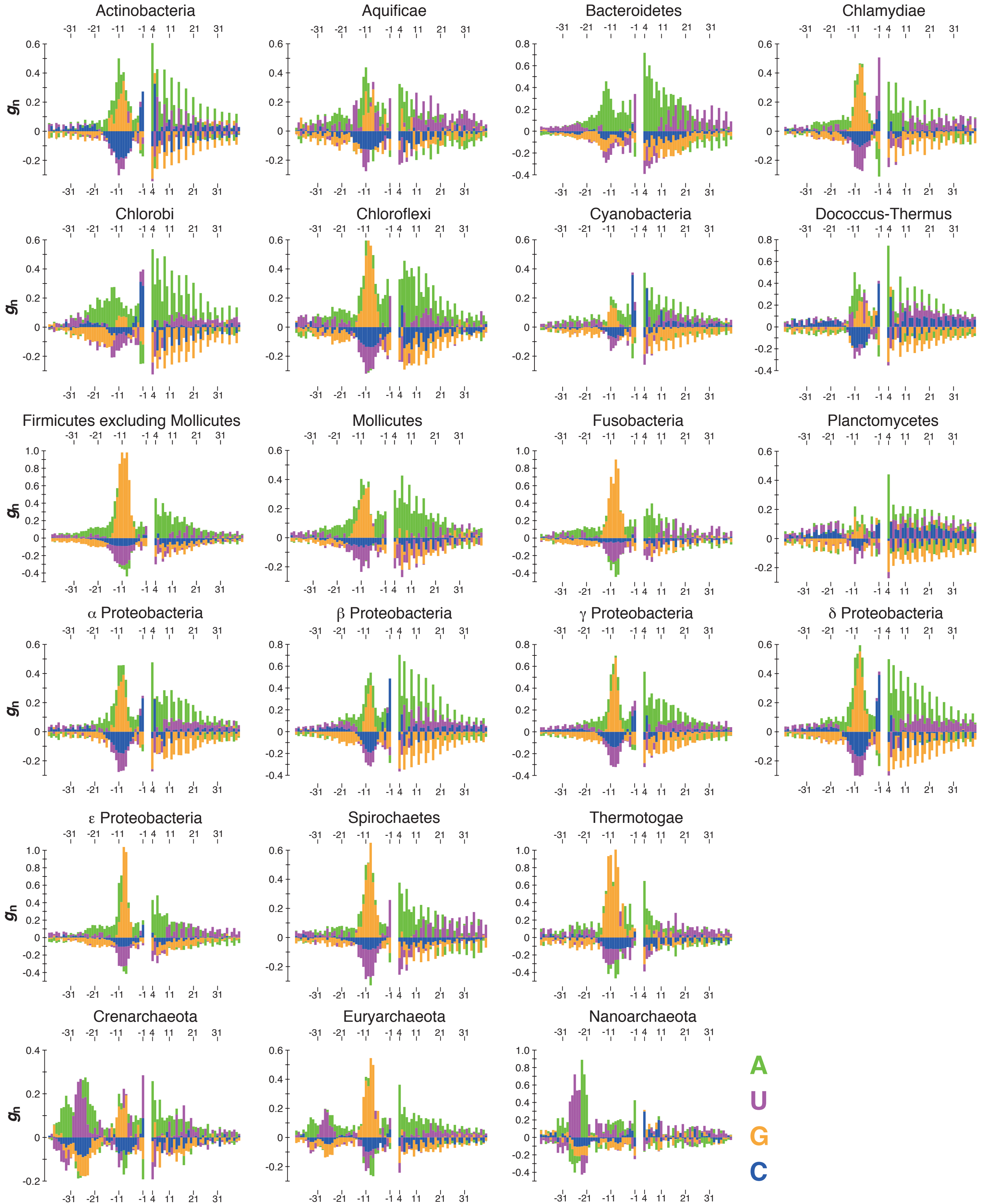

Supplementary Figure S4

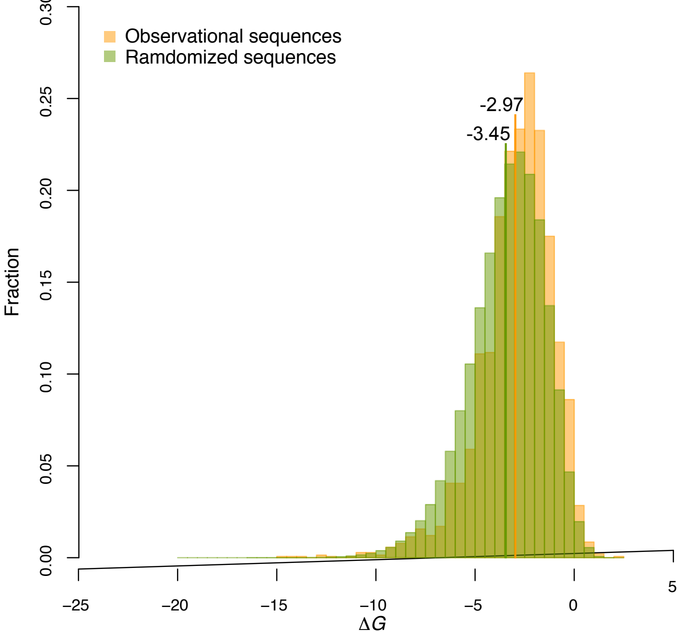

Supplementary Figure S5

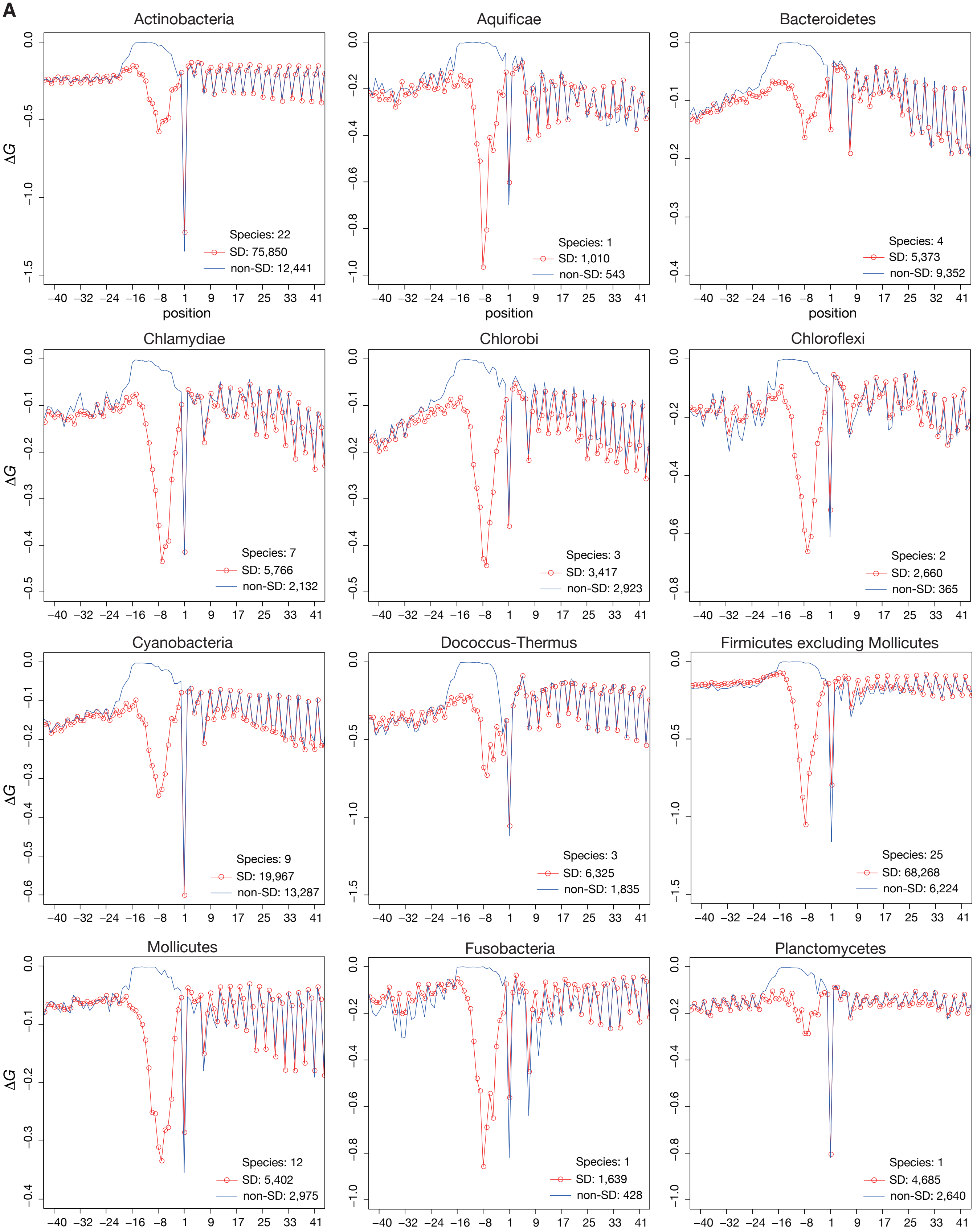

Supplementary Figure S5 (continued)

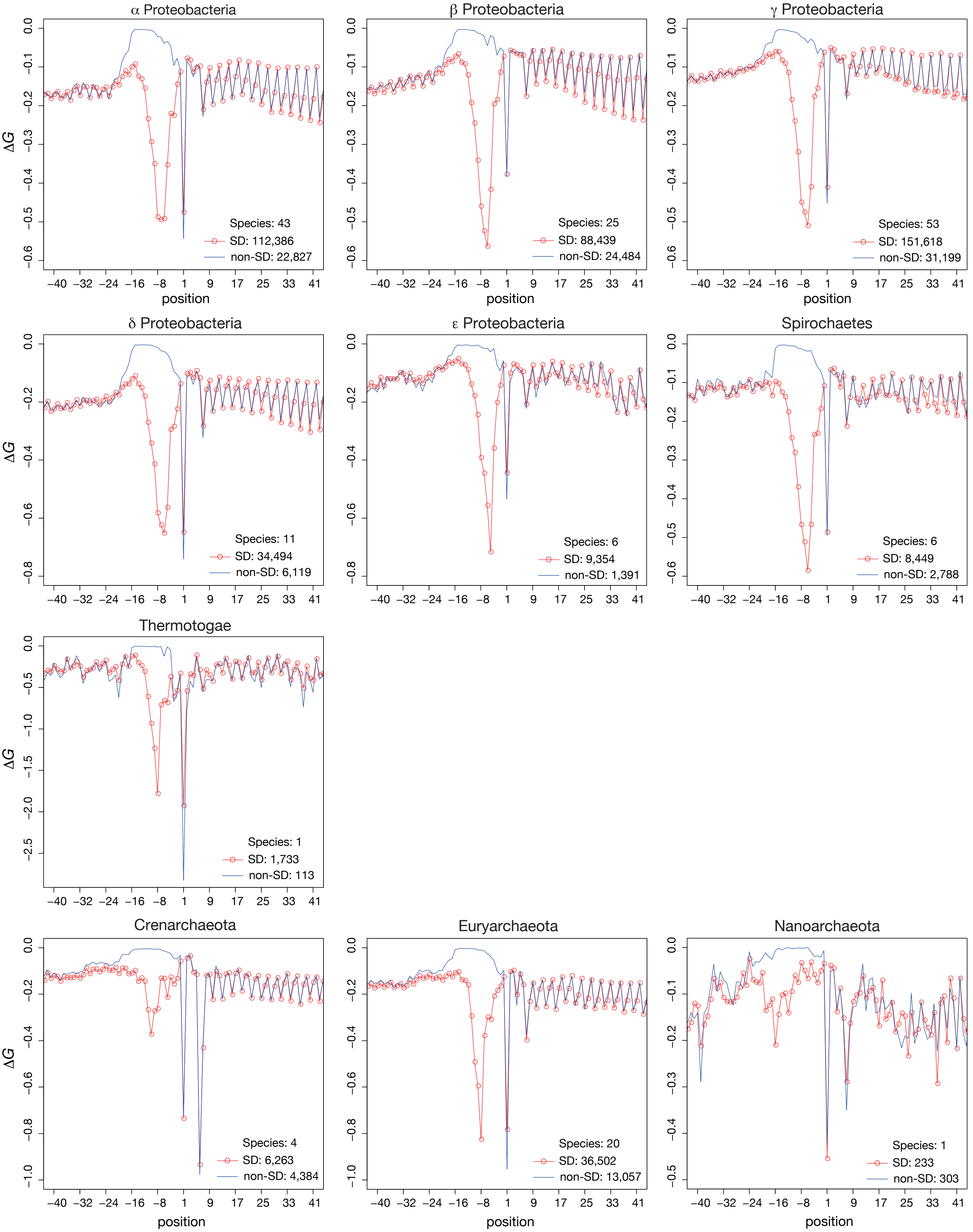

Supplement: Supplementary Data [file gkx124_Supp.zip › nar-02696-h-2016-File006.pdf]
