# Supplementary material for: Comparative genomic analysis of translation initiation mechanisms for genes lacking the Shine–Dalgarno sequence in prokaryotes
Source: Nucleic Acids Res. 2017 Feb 21;45(7):3922–31. doi: 10.1093/nar/gkx124 (PMC5397173; doi:10.1093/nar/gkx124)
Supplement: Supplementary Data [file nar-02696-h-2016-File008.pdf › nar-02696-h-2016-File008.pdf]

Suuplementary Data - *G*-statistic for all protein-coding genes

Aaeo_VF5
5UTR:

	50	49	48	47	46	45	44	43	42	41
a_O	496	475	471	498	524	501	483	537	499	508
	(31.9)	(30.6)	(30.3)	(32.1)	(33.7)	(32.3)	(31.1)	(34.6)	(32.1)	(32.7)
a_E	510.5	510.5	510.5	510.5	510.5	510.5	510.5	510.5	510.5	510.5
	(32.9)	(32.9)	(32.9)	(32.9)	(32.9)	(32.9)	(32.9)	(32.9)	(32.9)	(32.9)
	-28.6	-68.5	-75.9	-24.7	27.3	-18.8	-53.5	54.3	-22.8	-5.0
	-1.842	-4.410	-4.886	-1.591	1.760	-1.213	-3.446	3.498	-1.465	-0.322
t_O	408	454	458	425	459	470	429	438	490	438
	(26.3)	(29.2)	(29.5)	(27.4)	(29.6)	(30.3)	(27.6)	(28.2)	(31.6)	(28.2)
t_E	434.1	434.1	434.1	434.1	434.1	434.1	434.1	434.1	434.1	434.1
	(28.0)	(28.0)	(28.0)	(28.0)	(28.0)	(28.0)	(28.0)	(28.0)	(28.0)	(28.0)
	-50.6	40.7	49.1	-18.0	51.2	74.7	-10.1	7.8	118.7	7.8
	-3.258	2.621	3.161	-1.160	3.297	4.809	-0.653	0.505	7.644	0.505
g_O	349	330	309	336	307	317	351	314	293	341
	(22.5)	(21.2)	(19.9)	(21.6)	(19.8)	(20.4)	(22.6)	(20.2)	(18.9)	(22.0)
g_E	335.2	335.2	335.2	335.2	335.2	335.2	335.2	335.2	335.2	335.2
	(21.6)	(21.6)	(21.6)	(21.6)	(21.6)	(21.6)	(21.6)	(21.6)	(21.6)	(21.6)
	28.2	-10.3	-50.3	1.6	-53.9	-35.4	32.4	-41.0	-78.8	11.7
	1.816	-0.662	-3.236	0.106	-3.472	-2.277	2.085	-2.640	-5.075	0.756
c_O	300	294	315	294	263	265	290	264	271	266
	(19.3)	(18.9)	(20.3)	(18.9)	(16.9)	(17.1)	(18.7)	(17.0)	(17.5)	(17.1)
c_E	273.2	273.2	273.2	273.2	273.2	273.2	273.2	273.2	273.2	273.2
	(17.6)	(17.6)	(17.6)	(17.6)	(17.6)	(17.6)	(17.6)	(17.6)	(17.6)	(17.6)
	56.1	43.1	89.7	43.1	-20.0	-16.2	34.6	-18.1	-4.4	-14.2
	3.614	2.777	5.774	2.777	-1.290	-1.041	2.227	-1.166	-0.283	-0.916
N	1553	1553	1553	1553	1553	1553	1553	1553	1553	1553
X2	5.2	5.0	12.8	2.1	4.5	4.4	3.3	3.1	12.8	0.3
Gvalue	5.1	5.1	12.6	2.0	4.6	4.3	3.3	3.1	12.7	0.3
Gv/N	0.330	0.325	0.813	0.132	0.295	0.278	0.214	0.198	0.820	0.022
Prob	0.16285	0.16804	0.00553	0.56280	0.20526	0.22871	0.34536	0.38129	0.00525	0.95239

	40	39	38	37	36	35	34	33	32	31
a_O	524	495	469	532	515	495	539	510	549	538
	(33.7)	(31.9)	(30.2)	(34.3)	(33.2)	(31.9)	(34.7)	(32.8)	(35.4)	(34.6)
a_E	510.5	510.5	510.5	510.5	510.5	510.5	510.5	510.5	510.5	510.5
	(32.9)	(32.9)	(32.9)	(32.9)	(32.9)	(32.9)	(32.9)	(32.9)	(32.9)	(32.9)
	27.3	-30.5	-79.5	43.9	9.0	-30.5	58.5	-1.0	79.8	56.4
	1.760	-1.967	-5.122	2.825	0.581	-1.967	3.770	-0.066	5.139	3.634
t_O	434	473	408	449	469	428	465	474	434	490
	(27.9)	(30.5)	(26.3)	(28.9)	(30.2)	(27.6)	(29.9)	(30.5)	(27.9)	(31.6)
t_E	434.1	434.1	434.1	434.1	434.1	434.1	434.1	434.1	434.1	434.1
	(28.0)	(28.0)	(28.0)	(28.0)	(28.0)	(28.0)	(28.0)	(28.0)	(28.0)	(28.0)
	-0.2	81.2	-50.6	30.3	72.5	-12.1	63.9	83.4	-0.2	118.7
	-0.013	5.228	-3.258	1.951	4.671	-0.780	4.118	5.368	-0.013	7.644
g_O	318	306	390	315	290	364	299	303	302	268
	(20.5)	(19.7)	(25.1)	(20.3)	(18.7)	(23.4)	(19.3)	(19.5)	(19.4)	(17.3)
g_E	335.2	335.2	335.2	335.2	335.2	335.2	335.2	335.2	335.2	335.2
	(21.6)	(21.6)	(21.6)	(21.6)	(21.6)	(21.6)	(21.6)	(21.6)	(21.6)	(21.6)
	-33.5	-55.7	118.2	-39.1	-84.0	60.0	-68.3	-61.2	-63.0	-119.9
	-2.155	-3.589	7.608	-2.519	-5.407	3.867	-4.398	-3.939	-4.054	-7.720
c_O	277	279	286	257	279	266	250	266	268	257
	(17.8)	(18.0)	(18.4)	(16.5)	(18.0)	(17.1)	(16.1)	(17.1)	(17.3)	(16.5)
c_E	273.2	273.2	273.2	273.2	273.2	273.2	273.2	273.2	273.2	273.2
	(17.6)	(17.6)	(17.6)	(17.6)	(17.6)	(17.6)	(17.6)	(17.6)	(17.6)	(17.6)
	7.6	11.7	26.2	-31.4	11.7	-14.2	-44.4	-14.2	-10.3	-31.4
	0.491	0.753	1.685	-2.024	0.753	-0.916	-2.858	-0.916	-0.665	-2.024
N	1553	1553	1553	1553	1553	1553	1553	1553	1553	1553
X2	1.3	6.6	14.5	3.6	9.1	3.2	9.7	6.9	6.3	23.1
Gvalue	1.3	6.6	14.2	3.6	9.3	3.2	9.8	6.9	6.3	23.8
Gv/N	0.084	0.425	0.913	0.233	0.597	0.204	0.631	0.447	0.408	1.533
Prob	0.72872	0.08574	0.00268	0.30561	0.02581	0.36701	0.02040	0.07364	0.09660	0.00003

	30	29	28	27	26	25	24	23	22	21
a_O	500	527	521	545	561	557	566	596	579	585
	(32.2)	(33.9)	(33.5)	(35.1)	(36.1)	(35.9)	(36.4)	(38.4)	(37.3)	(37.7)
a_E	510.5	510.5	510.5	510.5	510.5	510.5	510.5	510.5	510.5	510.5
	(32.9)	(32.9)	(32.9)	(32.9)	(32.9)	(32.9)	(32.9)	(32.9)	(32.9)	(32.9)
	-20.8	33.5	21.2	71.3	105.8	97.1	116.8	184.6	145.8	159.4
	-1.339	2.158	1.365	4.588	6.814	6.252	7.521	11.884	9.387	10.261
t_O	468	457	508	418	409	465	472	414	483	441
	(30.1)	(29.4)	(32.7)	(26.9)	(26.3)	(29.9)	(30.4)	(26.7)	(31.1)	(28.4)
t_E	434.1	434.1	434.1	434.1	434.1	434.1	434.1	434.1	434.1	434.1
	(28.0)	(28.0)	(28.0)	(28.0)	(28.0)	(28.0)	(28.0)	(28.0)	(28.0)	(28.0)
	70.4	47.0	159.7	-31.6	-48.7	63.9	79.0	-39.3	103.1	13.9
	4.532	3.026	10.285	-2.034	-3.137	4.118	5.088	-2.528	6.640	0.896
g_O	288	324	262	305	315	291	247	302	259	259
	(18.5)	(20.9)	(16.9)	(19.6)	(20.3)	(18.7)	(15.9)	(19.4)	(16.7)	(16.7)
g_E	335.2	335.2	335.2	335.2	335.2	335.2	335.2	335.2	335.2	335.2
	(21.6)	(21.6)	(21.6)	(21.6)	(21.6)	(21.6)	(21.6)	(21.6)	(21.6)	(21.6)
	-87.4	-22.0	-129.1	-57.6	-39.1	-82.3	-150.8	-63.0	-133.6	-133.6
	-5.627	-1.416	-8.311	-3.706	-2.519	-5.297	-9.711	-4.054	-8.600	-8.600
c_O	297	245	262	285	268	240	268	241	232	268
	(19.1)	(15.8)	(16.9)	(18.4)	(17.3)	(15.5)	(17.3)	(15.5)	(14.9)	(17.3)
c_E	273.2	273.2	273.2	273.2	273.2	273.2	273.2	273.2	273.2	273.2
	(17.6)	(17.6)	(17.6)	(17.6)	(17.6)	(17.6)	(17.6)	(17.6)	(17.6)	(17.6)
	49.6	-53.4	-22.0	24.1	-10.3	-62.2	-10.3	-60.5	-75.9	-10.3
	3.193	-3.439	-1.414	1.551	-0.665	-4.006	-0.665	-3.893	-4.885	-0.665
N	1553	1553	1553	1553	1553	1553	1553	1553	1553	1553
X2	11.6	5.0	29.2	6.2	7.8	16.3	32.6	22.3	38.2	28.4
Gvalue	11.8	5.1	29.9	6.2	7.7	16.6	34.7	21.9	39.5	29.4
Gv/N	0.759	0.329	1.925	0.398	0.493	1.067	2.234	1.409	2.541	1.892
Prob	0.00814	0.16391	0.00000	0.10279	0.05366	0.00087	0.00000	0.00007	0.00000	0.00000

	20	19	18	17	16	15	14	13	12	11
a_O	577	551	505	543	508	511	604	648	688	626
	(37.2)	(35.5)	(32.5)	(35.0)	(32.7)	(32.9)	(38.9)	(41.7)	(44.3)	(40.3)
a_E	510.5	510.5	510.5	510.5	510.5	510.5	510.5	510.5	510.5	510.5
	(32.9)	(32.9)	(32.9)	(32.9)	(32.9)	(32.9)	(32.9)	(32.9)	(32.9)	(32.9)
	141.3	84.1	-11.0	67.0	-5.0	1.0	203.1	309.1	410.6	255.3
	9.098	5.416	-0.706	4.315	-0.322	0.063	13.081	19.902	26.437	16.441
t_O	418	473	496	437	557	577	451	456	322	290
	(26.9)	(30.5)	(31.9)	(28.1)	(35.9)	(37.2)	(29.0)	(29.4)	(20.7)	(18.7)

t_E	434.1	434.1	434.1	434.1	434.1	434.1	434.1	434.1	434.1	434.1
	(28.0)	(28.0)	(28.0)	(28.0)	(28.0)	(28.0)	(28.0)	(28.0)	(28.0)	(28.0)
	-31.6	81.2	132.2	5.8	277.7	328.4	34.4	44.9	-192.4	-234.0
	-2.034	5.228	8.515	0.375	17.882	21.146	2.218	2.890	-12.387	-15.066
g_O	304	271	287	333	289	264	307	312	412	509
	(19.6)	(17.5)	(18.5)	(21.4)	(18.6)	(17.0)	(19.8)	(20.1)	(26.5)	(32.8)
g_E	335.2	335.2	335.2	335.2	335.2	335.2	335.2	335.2	335.2	335.2
	(21.6)	(21.6)	(21.6)	(21.6)	(21.6)	(21.6)	(21.6)	(21.6)	(21.6)	(21.6)
	-59.4	-115.2	-89.1	-4.3	-85.7	-126.0	-53.9	-44.7	170.0	425.3
	-3.823	-7.418	-5.736	-0.280	-5.517	-8.116	-3.472	-2.880	10.949	27.386
c_O	254	258	265	240	199	201	191	137	131	128
	(16.4)	(16.6)	(17.1)	(15.5)	(12.8)	(12.9)	(12.3)	(8.8)	(8.4)	(8.2)
c_E	273.2	273.2	273.2	273.2	273.2	273.2	273.2	273.2	273.2	273.2
	(17.6)	(17.6)	(17.6)	(17.6)	(17.6)	(17.6)	(17.6)	(17.6)	(17.6)	(17.6)
	-37.0	-29.6	-16.2	-62.2	-126.1	-123.4	-136.7	-189.1	-192.6	-194.1
	-2.385	-1.903	-1.041	-4.006	-8.122	-7.945	-8.805	-12.178	-12.401	-12.499
N	1553	1553	1553	1553	1553	1553	1553	1553	1553	1553
X2	13.5	19.8	16.1	6.1	61.3	81.2	44.9	107.6	182.3	241.3
Gvalue	13.3	20.5	16.0	6.3	60.9	79.9	46.9	120.1	195.6	252.6
Gv/N	0.856	1.322	1.032	0.404	3.920	5.147	3.022	7.734	12.598	16.263
Prob	0.00405	0.00013	0.00112	0.09919	0.00000	0.00000	0.00000	0.00000	0.00000	0.00000

	10	9	8	7	6	5	4	3	2	1
a_O	652	537	442	547	557	559	615	540	536	474
	(42.0)	(34.6)	(28.5)	(35.2)	(35.9)	(36.0)	(39.6)	(34.8)	(34.5)	(30.5)
a_E	510.5	510.5	510.5	510.5	510.5	510.5	510.5	510.5	510.5	510.5
	(32.9)	(32.9)	(32.9)	(32.9)	(32.9)	(32.9)	(32.9)	(32.9)	(32.9)	(32.9)
	319.0	54.3	-127.4	75.5	97.1	101.4	229.0	60.7	52.2	-70.3
	20.541	3.498	-8.202	4.863	6.252	6.532	14.748	3.905	3.363	-4.530
t_O	359	455	472	524	531	431	470	349	448	586
	(23.1)	(29.3)	(30.4)	(33.7)	(34.2)	(27.8)	(30.3)	(22.5)	(28.8)	(37.7)
t_E	434.1	434.1	434.1	434.1	434.1	434.1	434.1	434.1	434.1	434.1
	(28.0)	(28.0)	(28.0)	(28.0)	(28.0)	(28.0)	(28.0)	(28.0)	(28.0)	(28.0)
	-136.4	42.8	79.0	197.3	214.0	-6.2	74.7	-152.3	28.2	351.7
	-8.782	2.755	5.088	12.701	13.778	-0.398	4.809	-9.807	1.818	22.643
g_O	422	449	516	315	310	360	256	416	339	266
	(27.2)	(28.9)	(33.2)	(20.3)	(20.0)	(23.2)	(16.5)	(26.8)	(21.8)	(17.1)
g_E	335.2	335.2	335.2	335.2	335.2	335.2	335.2	335.2	335.2	335.2
	(21.6)	(21.6)	(21.6)	(21.6)	(21.6)	(21.6)	(21.6)	(21.6)	(21.6)	(21.6)
	194.4	262.5	445.2	-39.1	-48.4	51.4	-138.0	179.7	7.7	-123.0
	12.518	16.905	28.670	-2.519	-3.118	3.312	-8.885	11.573	0.495	-7.919
c_O	120	112	123	167	155	203	212	248	230	227
	(7.7)	(7.2)	(7.9)	(10.8)	(10.0)	(13.1)	(13.7)	(16.0)	(14.8)	(14.6)
c_E	273.2	273.2	273.2	273.2	273.2	273.2	273.2	273.2	273.2	273.2
	(17.6)	(17.6)	(17.6)	(17.6)	(17.6)	(17.6)	(17.6)	(17.6)	(17.6)	(17.6)
	-197.5	-199.8	-196.3	-164.4	-175.7	-120.6	-107.5	-48.0	-79.2	-84.1
	-12.715	-12.862	-12.641	-10.587	-11.314	-7.765	-6.925	-3.092	-5.099	-5.417
N	1553	1553	1553	1553	1553	1553	1553	1553	1553	1553
X2	160.6	136.2	192.6	63.7	78.9	24.5	56.8	40.2	8.6	77.9
Gvalue	179.6	159.9	200.6	69.2	86.9	26.1	58.2	40.1	9.0	74.2
Gv/N	11.562	10.297	12.914	4.459	5.598	1.681	3.748	2.579	0.577	4.778
Prob	0.00000	0.00000	0.00000	0.00000	0.00000	0.00001	0.00000	0.00000	0.02981	0.00000

CDS_INITIAL:

	1	2	3	4	5	6	7	8	9	10
a_O	1270	0	0	718	642	573	689	605	561	545
	(81.8)	(0.0)	(0.0)	(46.2)	(41.3)	(36.9)	(44.4)	(39.0)	(36.1)	(35.1)
a_E	505.6	539.7	472.4	505.6	539.7	472.4	505.6	539.7	472.4	505.6
	(32.6)	(34.7)	(30.4)	(32.6)	(34.7)	(30.4)	(32.6)	(34.7)	(30.4)	(32.6)
	2339.4	0.0	0.0	503.6	223.0	221.2	426.5	138.3	192.9	81.8
	150.636	0.000	0.000	32.429	14.356	14.245	27.461	8.904	12.418	5.266
t_O	116	1553	1	160	343	357	237	519	293	313
	(7.5)	(100.0)	(0.1)	(10.3)	(22.1)	(23.0)	(15.3)	(33.4)	(18.9)	(20.2)
t_E	261.5	510.9	334.4	261.5	510.9	334.4	261.5	510.9	334.4	261.5
	(16.8)	(32.9)	(21.5)	(16.8)	(32.9)	(21.5)	(16.8)	(32.9)	(21.5)	(16.8)
	-188.6	3453.1	-11.6	-157.3	-273.4	46.6	-46.7	16.3	-77.5	112.4
	-12.145	222.347	-0.749	-10.126	-17.602	3.003	-3.008	1.049	-4.990	7.240
g_O	167	0	1552	484	334	387	434	235	433	427
	(10.8)	(0.0)	(99.9)	(31.2)	(21.5)	(24.9)	(27.9)	(15.1)	(27.9)	(27.5)
g_E	536.0	232.4	365.0	536.0	232.4	365.0	536.0	232.4	365.0	536.0
	(34.5)	(15.0)	(23.5)	(34.5)	(15.0)	(23.5)	(34.5)	(15.0)	(23.5)	(34.5)
	-389.5	0.0	4492.7	-98.8	242.3	45.3	-183.2	5.2	147.9	-194.1
	-25.079	0.000	289.293	-6.359	15.602	2.917	-11.797	0.338	9.526	-12.501
c_O	0	0	0	191	234	236	193	194	266	268
	(0.0)	(0.0)	(0.0)	(12.3)	(15.1)	(15.2)	(12.4)	(12.5)	(17.1)	(17.3)
c_E	249.9	270.0	381.2	249.9	270.0	381.2	249.9	270.0	381.2	249.9
	(16.1)	(17.4)	(24.5)	(16.1)	(17.4)	(24.5)	(16.1)	(17.4)	(24.5)	(16.1)
	0.0	0.0	0.0	-102.6	-67.0	-226.3	-99.7	-128.3	-191.4	37.6
	0.000	0.000	0.000	-6.608	-4.315	-14.570	-6.418	-8.261	-12.323	2.418
N	1553	1553	1553	1553	1553	1553	1553	1553	1553	1553
X2	1740.5	3167.6	5046.2	147.6	123.8	79.6	101.2	29.5	69.2	36.7
Gvalue	1761.3	3453.1	4481.1	145.0	124.9	86.9	96.9	31.5	71.9	37.6
Gv/N	113.411	222.347	288.545	9.336	8.041	5.595	6.238	2.030	4.631	2.423
Prob	1.00000	1.00000	1.00000	0.00000	0.00000	0.00000	0.00000	0.00000	0.00000	0.00000

	11	12	13	14	15	16	17	18	19	20
a_O	489	487	541	501	497	547	452	486	540	455
	(31.5)	(31.4)	(34.8)	(32.3)	(32.0)	(35.2)	(29.1)	(31.3)	(34.8)	(29.3)
a_E	539.7	472.4	505.6	539.7	472.4	505.6	539.7	472.4	505.6	539.7
	(34.7)	(30.4)	(32.6)	(34.7)	(30.4)	(32.6)	(34.7)	(30.4)	(32.6)	(34.7)
	-96.4	29.6	73.2	-74.5	50.4	86.1	-160.3	27.6	71.1	-155.3
	-6.209	1.908	4.714	-4.797	3.248	5.543	-10.319	1.776	4.577	-10.000
t_O	688	393	316	652	367	301	677	391	300	643
	(44.3)	(25.3)	(20.3)	(42.0)	(23.6)	(19.4)	(43.6)	(25.2)	(19.3)	(41.4)
t_E	510.9	334.4	261.5	510.9	334.4	261.5	510.9	334.4	261.5	510.9
	(32.9)	(21.5)	(16.8)	(32.9)	(21.5)	(16.8)	(32.9)	(21.5)	(16.8)	(32.9)
	409.5	126.9	119.5	318.0	68.2	84.6	381.1	122.2	82.3	295.7
	26.367	8.168	7.697	20.474	4.393	5.447	24.540	7.870	5.300	19.041
g_O	194	369	449	191	360	434	206	327	459	222
	(12.5)	(23.8)	(28.9)	(12.3)	(23.2)	(27.9)	(13.3)	(21.1)	(29.6)	(14.3)
g_E	232.4	365.0	536.0	232.4	365.0	536.0	232.4	365.0	536.0	232.4
	(15.0)	(23.5)	(34.5)	(15.0)	(23.5)	(34.5)	(15.0)	(23.5)	(34.5)	(15.0)
	-70.1	8.0	-159.0	-74.9	-9.9	-183.2	-49.7	-71.9	-142.3	-20.3
	-4.511	0.518	-10.240	-4.825	-0.640	-11.797	-3.198	-4.630	-9.166	-1.308

c_O	182	304	247	209	329	271	218	349	254	233
	(11.7)	(19.6)	(15.9)	(13.5)	(21.2)	(17.5)	(14.0)	(22.5)	(16.4)	(15.0)
c_E	270.0	381.2	249.9	270.0	381.2	249.9	270.0	381.2	249.9	270.0
	(17.4)	(24.5)	(16.1)	(17.4)	(24.5)	(16.1)	(17.4)	(24.5)	(16.1)	(17.4)
	−143.6	−137.5	−5.7	−107.1	−96.8	44.0	−93.3	−61.5	8.3	−68.7
	−9.247	−8.856	−0.367	−6.895	−6.236	2.834	−6.009	−3.962	0.537	−4.425
N	1553	1553	1553	1553	1553	1553	1553	1553	1553	1553
X2	101.2	26.4	28.0	62.9	11.7	30.5	81.3	16.6	19.1	53.0
Gvalue	99.4	27.0	28.0	61.5	11.9	31.5	77.9	16.4	19.4	51.4
Gv/N	6.400	1.738	1.805	3.957	0.766	2.027	5.014	1.053	1.248	3.308
Prob	0.00000	0.00001	0.00000	0.00000	0.00777	0.00000	0.00000	0.00096	0.00023	0.00000

	21	22	23	24	25	26	27	28	29	30
a_O	512	524	473	474	502	459	491	492	467	497
	(33.0)	(33.7)	(30.5)	(30.5)	(32.3)	(29.6)	(31.6)	(31.7)	(30.1)	(32.0)
a_E	472.4	505.6	539.7	472.4	505.6	539.7	472.4	505.6	539.7	472.4
	(30.4)	(32.6)	(34.7)	(30.4)	(32.6)	(34.7)	(30.4)	(32.6)	(34.7)	(30.4)
	82.4	37.4	−124.7	3.2	−7.2	−148.6	37.9	−26.8	−135.1	50.4
	5.307	2.411	−8.032	0.206	−0.463	−9.570	2.441	−1.728	−8.698	3.248
t_O	379	307	588	356	305	558	362	288	587	396
	(24.4)	(19.8)	(37.9)	(22.9)	(19.6)	(35.9)	(23.3)	(18.5)	(37.8)	(25.5)
t_E	334.4	261.5	510.9	334.4	261.5	510.9	334.4	261.5	510.9	334.4
	(21.5)	(16.8)	(32.9)	(21.5)	(16.8)	(32.9)	(21.5)	(16.8)	(32.9)	(21.5)
	94.8	98.4	165.3	44.5	93.8	98.4	57.4	55.5	163.0	133.8
	6.107	6.336	10.641	2.866	6.038	6.335	3.693	3.574	10.494	8.618
g_O	336	453	245	337	485	273	314	486	259	313
	(21.6)	(29.2)	(15.8)	(21.7)	(31.2)	(17.6)	(20.2)	(31.3)	(16.7)	(20.2)
g_E	365.0	536.0	232.4	365.0	536.0	232.4	365.0	536.0	232.4	365.0
	(23.5)	(34.5)	(15.0)	(23.5)	(34.5)	(15.0)	(23.5)	(34.5)	(15.0)	(23.5)
	−55.6	−152.4	25.9	−53.8	−97.0	87.9	−94.5	−95.2	56.2	−96.2
	−3.582	−9.813	1.667	−3.464	−6.243	5.662	−6.086	−6.127	3.616	−6.195
c_O	326	269	247	386	261	263	386	287	240	347
	(21.0)	(17.3)	(15.9)	(24.9)	(16.8)	(16.9)	(24.9)	(18.5)	(15.5)	(22.3)
c_E	381.2	249.9	270.0	381.2	249.9	270.0	381.2	249.9	270.0	381.2
	(24.5)	(16.1)	(17.4)	(24.5)	(16.1)	(17.4)	(24.5)	(16.1)	(17.4)	(24.5)
	−101.9	39.7	−44.0	9.7	22.8	−13.9	9.7	79.5	−56.6	−65.2
	−6.563	2.556	−2.835	0.627	1.465	−0.893	0.627	5.121	−3.643	−4.197
N	1553	1553	1553	1553	1553	1553	1553	1553	1553	1553
X2	19.5	22.9	22.5	3.6	12.6	23.7	10.2	13.2	27.5	23.1
Gvalue	19.7	23.1	22.4	3.6	12.4	23.8	10.5	13.0	27.5	22.9
Gv/N	1.268	1.490	1.441	0.234	0.797	1.534	0.675	0.840	1.769	1.475
Prob	0.00020	0.00004	0.00005	0.30377	0.00620	0.00003	0.01490	0.00456	0.00000	0.00004

	31	32	33	34	35	36	37	38	39	40
a_O	456	454	457	484	449	498	502	489	488	477
	(29.4)	(29.2)	(29.4)	(31.2)	(28.9)	(32.1)	(32.3)	(31.5)	(31.4)	(30.7)
a_E	505.6	539.7	472.4	505.6	539.7	472.4	505.6	539.7	472.4	505.6
	(32.6)	(34.7)	(30.4)	(32.6)	(34.7)	(30.4)	(32.6)	(34.7)	(30.4)	(32.6)
	−94.2	−157.0	−30.3	−42.3	−165.2	52.6	−7.2	−96.4	31.7	−55.6
	−6.064	−10.106	−1.951	−2.722	−10.635	3.384	−0.463	−6.209	2.041	−3.578
t_O	336	607	392	316	579	360	302	543	346	282
	(21.6)	(39.1)	(25.2)	(20.3)	(37.3)	(23.2)	(19.4)	(35.0)	(22.3)	(18.2)
t_E	261.5	510.9	334.4	261.5	510.9	334.4	261.5	510.9	334.4	261.5
	(16.8)	(32.9)	(21.5)	(16.8)	(32.9)	(21.5)	(16.8)	(32.9)	(21.5)	(16.8)
	168.3	209.2	124.5	119.5	144.9	53.1	86.9	66.1	23.5	42.5
	10.840	13.471	8.019	7.697	9.328	3.416	5.594	4.259	1.516	2.735
g_O	481	236	318	518	240	331	507	257	340	530
	(31.0)	(15.2)	(20.5)	(33.4)	(15.5)	(21.3)	(32.6)	(16.5)	(21.9)	(34.1)
g_E	536.0	232.4	365.0	536.0	232.4	365.0	536.0	232.4	365.0	536.0
	(34.5)	(15.0)	(23.5)	(34.5)	(15.0)	(23.5)	(34.5)	(15.0)	(23.5)	(34.5)
	−104.1	7.3	−87.7	−35.4	15.5	−64.7	−56.4	51.7	−48.2	−11.9
	−6.705	0.468	−5.645	−2.277	0.996	−4.168	−3.630	3.331	−3.107	−0.767
c_O	280	256	386	235	285	364	242	264	379	264
	(18.0)	(16.5)	(24.9)	(15.1)	(18.4)	(23.4)	(15.6)	(17.0)	(24.4)	(17.0)
c_E	249.9	270.0	381.2	249.9	270.0	381.2	249.9	270.0	381.2	249.9
	(16.1)	(17.4)	(24.5)	(16.1)	(17.4)	(24.5)	(16.1)	(17.4)	(24.5)	(16.1)
	63.8	−27.3	9.7	−28.8	30.8	−33.5	−15.5	−11.9	−4.3	29.1
	4.106	−1.759	0.627	−1.856	1.981	−2.160	−0.997	−0.767	−0.278	1.871
N	1553	1553	1553	1553	1553	1553	1553	1553	1553	1553
X2	35.3	32.5	16.5	13.7	25.4	7.3	8.1	9.5	2.6	4.1
Gvalue	33.8	32.2	16.3	13.1	25.9	7.3	7.8	9.5	2.7	4.1
Gv/N	2.177	2.074	1.049	0.842	1.669	0.472	0.504	0.614	0.172	0.262
Prob	0.00000	0.00000	0.00099	0.00448	0.00001	0.06223	0.04964	0.02290	0.44528	0.25487

	41	42	43	44	45	46	47	48	49	50
a_O	464	465	489	467	476	471	498	472	506	507
	(29.9)	(29.9)	(31.5)	(30.1)	(30.7)	(30.3)	(32.1)	(30.4)	(32.6)	(32.6)
a_E	539.7	472.4	505.6	539.7	472.4	505.6	539.7	472.4	505.6	539.7
	(34.7)	(30.4)	(32.6)	(34.7)	(30.4)	(32.6)	(34.7)	(30.4)	(32.6)	(34.7)
	−140.2	−14.7	−32.7	−135.1	7.2	−66.8	−80.0	−0.8	0.8	−63.3
	−9.027	−0.946	−2.103	−8.698	0.465	−4.301	−5.153	−0.052	0.051	−4.077
t_O	534	375	334	563	373	297	534	368	293	508
	(34.4)	(24.1)	(21.5)	(36.3)	(24.0)	(19.1)	(34.4)	(23.7)	(18.9)	(32.7)
t_E	510.9	334.4	261.5	510.9	334.4	261.5	510.9	334.4	261.5	510.9
	(32.9)	(21.5)	(16.8)	(32.9)	(21.5)	(16.8)	(32.9)	(21.5)	(16.8)	(32.9)
	47.2	85.9	163.4	109.3	81.4	75.5	47.2	70.4	66.6	−5.8
	3.039	5.530	10.519	7.038	5.243	4.863	3.039	4.534	4.286	−0.375
g_O	273	328	475	244	322	524	224	347	498	236
	(17.6)	(21.1)	(30.6)	(15.7)	(20.7)	(33.7)	(14.4)	(22.3)	(32.1)	(15.2)
g_E	232.4	365.0	536.0	232.4	365.0	536.0	232.4	365.0	536.0	232.4
	(15.0)	(23.5)	(34.5)	(15.0)	(23.5)	(34.5)	(15.0)	(23.5)	(34.5)	(15.0)
	87.9	−70.1	−114.8	23.8	−80.7	−23.7	−16.5	−35.1	−73.2	7.3
	5.662	−4.515	−7.389	1.532	−5.198	−1.526	−1.061	−2.260	−4.714	0.468
c_O	282	385	255	279	382	261	297	366	256	302
	(18.2)	(24.8)	(16.4)	(18.0)	(24.6)	(16.8)	(19.1)	(23.6)	(16.5)	(19.4)
c_E	270.0	381.2	249.9	270.0	381.2	249.9	270.0	381.2	249.9	270.0
	(17.4)	(24.5)	(16.1)	(17.4)	(24.5)	(16.1)	(17.4)	(24.5)	(16.1)	(17.4)
	24.5	7.7	10.4	18.2	1.7	22.8	56.6	−29.7	12.4	67.6
	1.576	0.496	0.668	1.175	0.108	1.465	3.642	−1.914	0.800	4.352
N	1553	1553	1553	1553	1553	1553	1553	1553	1553	1553
X2	19.3	8.8	27.7	16.0	9.5	7.9	7.3	4.9	6.6	5.8
Gvalue	19.4	8.8	26.3	16.3	9.6	7.8	7.2	4.8	6.5	5.7
Gv/N	1.250	0.565	1.694	1.047	0.618	0.501	0.467	0.308	0.422	0.369
Prob	0.00022	0.03245	0.00001	0.00100	0.02236	0.05063	0.06445	0.18884	0.08779	0.12521

	51	52	53	54	55	56	57	58	59	60
a_O	457	464	487	457	478	505	481	448	501	497
	(29.4)	(29.9)	(31.4)	(29.4)	(30.8)	(32.5)	(31.0)	(28.8)	(32.3)	(32.0)
a_E	472.4	505.6	539.7	472.4	505.6	539.7	472.4	505.6	539.7	472.4
	(30.4)	(32.6)	(34.7)	(30.4)	(32.6)	(34.7)	(30.4)	(32.6)	(34.7)	(30.4)
	-30.3	-79.7	-100.0	-30.3	-53.7	-67.1	17.3	-108.4	-74.5	50.4
	-1.951	-5.131	-6.440	-1.951	-3.456	-4.318	1.117	-6.979	-4.797	3.248
t_O	347	317	527	351	280	576	360	306	530	327
	(22.3)	(20.4)	(33.9)	(22.6)	(18.0)	(37.1)	(23.2)	(19.7)	(34.1)	(21.1)
t_E	334.4	261.5	510.9	334.4	261.5	510.9	334.4	261.5	510.9	334.4
	(21.5)	(16.8)	(32.9)	(21.5)	(16.8)	(32.9)	(21.5)	(16.8)	(32.9)	(21.5)
	25.6	121.9	32.7	34.0	38.2	138.1	53.1	96.1	38.9	-14.7
	1.649	7.851	2.104	2.186	2.459	8.894	3.416	6.186	2.503	-0.946
g_O	380	524	253	353	536	216	348	520	252	349
	(24.5)	(33.7)	(16.3)	(22.7)	(34.5)	(13.9)	(22.4)	(33.5)	(16.2)	(22.5)
g_E	365.0	536.0	232.4	365.0	536.0	232.4	365.0	536.0	232.4	365.0
	(23.5)	(34.5)	(15.0)	(23.5)	(34.5)	(15.0)	(23.5)	(34.5)	(15.0)	(23.5)
	30.6	-23.7	43.0	-23.6	0.0	-31.6	-33.2	-31.5	40.8	-31.3
	1.971	-1.526	2.768	-1.520	0.002	-2.035	-2.138	-2.028	2.629	-2.015
c_O	369	248	286	392	259	256	364	279	270	380
	(23.8)	(16.0)	(18.4)	(25.2)	(16.7)	(16.5)	(23.4)	(18.0)	(17.4)	(24.5)
c_E	381.2	249.9	270.0	381.2	249.9	270.0	381.2	249.9	270.0	381.2
	(24.5)	(16.1)	(17.4)	(24.5)	(16.1)	(17.4)	(24.5)	(16.1)	(17.4)	(24.5)
	-23.9	-3.7	32.9	22.0	18.6	-27.3	-33.5	61.5	-0.1	-2.3
	-1.541	-0.239	2.117	1.415	1.198	-1.759	-2.160	3.963	-0.003	-0.150
N	1553	1553	1553	1553	1553	1553	1553	1553	1553	1553
X2	2.0	15.5	8.4	2.0	3.1	12.4	3.7	18.0	5.1	2.2
Gvalue	2.0	14.8	8.5	2.0	3.1	12.2	3.7	17.7	5.2	2.1
Gv/N	0.127	0.954	0.549	0.130	0.202	0.783	0.235	1.143	0.332	0.138
Prob	0.57755	0.00198	0.03641	0.56826	0.37079	0.00685	0.30156	0.00050	0.16094	0.54377

	61	62	63	64	65	66	67	68	69	70
a_O	476	511	446	476	510	473	507	531	470	478
	(30.7)	(32.9)	(28.7)	(30.7)	(32.8)	(30.5)	(32.6)	(34.2)	(30.3)	(30.8)
a_E	505.6	539.7	472.4	505.6	539.7	472.4	505.6	539.7	472.4	505.6
	(32.6)	(34.7)	(30.4)	(32.6)	(34.7)	(30.4)	(32.6)	(34.7)	(30.4)	(32.6)
	-57.4	-55.8	-51.3	-57.4	-57.7	1.2	2.8	-17.2	-4.8	-53.7
	-3.699	-3.592	-3.304	-3.699	-3.713	0.077	0.180	-1.107	-0.309	-3.456
t_O	295	508	383	282	530	340	286	535	345	276
	(19.0)	(32.7)	(24.7)	(18.2)	(34.1)	(21.9)	(18.4)	(34.4)	(22.2)	(17.8)
t_E	261.5	510.9	334.4	261.5	510.9	334.4	261.5	510.9	334.4	261.5
	(16.8)	(32.9)	(21.5)	(16.8)	(32.9)	(21.5)	(16.8)	(32.9)	(21.5)	(16.8)
	71.0	-5.8	103.9	42.5	38.9	11.2	51.1	49.3	21.5	29.7
	4.573	-0.375	6.689	2.735	2.503	0.724	3.292	3.174	1.383	1.912
g_O	516	234	346	512	222	345	508	236	343	525
	(33.2)	(15.1)	(22.3)	(33.0)	(14.3)	(22.2)	(32.7)	(15.2)	(22.1)	(33.8)
g_E	536.0	232.4	365.0	536.0	232.4	365.0	536.0	232.4	365.0	536.0
	(34.5)	(15.0)	(23.5)	(34.5)	(15.0)	(23.5)	(34.5)	(15.0)	(23.5)	(34.5)
	-39.2	3.2	-37.0	-46.9	-20.3	-38.9	-54.5	7.3	-42.6	-21.7
	-2.525	0.208	-2.382	-3.019	-1.308	-2.504	-3.508	0.468	-2.746	-1.400
c_O	266	300	378	283	291	395	252	251	395	274
	(17.1)	(19.3)	(24.3)	(18.2)	(18.7)	(25.4)	(16.2)	(16.2)	(25.4)	(17.6)
c_E	249.9	270.0	381.2	249.9	270.0	381.2	249.9	270.0	381.2	249.9
	(16.1)	(17.4)	(24.5)	(16.1)	(17.4)	(24.5)	(16.1)	(17.4)	(24.5)	(16.1)
	33.3	63.2	-6.3	70.5	43.5	28.2	4.3	-36.7	28.2	50.5
	2.144	4.067	-0.406	4.538	2.803	1.814	0.276	-2.362	1.814	3.254
N	1553	1553	1553	1553	1553	1553	1553	1553	1553	1553
X2	7.8	4.9	9.5	8.8	4.4	1.7	3.8	2.7	2.2	4.9
Gvalue	7.7	4.8	9.3	8.6	4.4	1.7	3.7	2.7	2.2	4.8
Gv/N	0.493	0.308	0.597	0.556	0.285	0.110	0.240	0.173	0.141	0.310
Prob	0.05379	0.18795	0.02587	0.03464	0.21901	0.63563	0.29235	0.44249	0.53336	0.18652

	71	72	73	74	75	76	77	78	79	80
a_O	516	507	513	549	487	483	568	474	508	526
	(33.2)	(32.6)	(33.0)	(35.4)	(31.4)	(31.1)	(36.6)	(30.5)	(32.7)	(33.9)
a_E	539.7	472.4	505.6	539.7	472.4	505.6	539.7	472.4	505.6	539.7
	(34.7)	(30.4)	(32.6)	(34.7)	(30.4)	(32.6)	(34.7)	(30.4)	(32.6)	(34.7)
	-46.3	71.7	14.9	18.8	29.6	-44.2	58.1	3.2	4.8	-27.0
	-2.980	4.614	0.959	1.212	1.908	-2.845	3.743	0.206	0.309	-1.737
t_O	555	336	279	526	354	284	497	343	281	537
	(35.7)	(21.6)	(18.0)	(33.9)	(22.8)	(18.3)	(32.0)	(22.1)	(18.1)	(34.6)
t_E	510.9	334.4	261.5	510.9	334.4	261.5	510.9	334.4	261.5	510.9
	(32.9)	(21.5)	(16.8)	(32.9)	(21.5)	(16.8)	(32.9)	(21.5)	(16.8)	(32.9)
	91.9	3.2	36.1	30.6	40.3	46.8	-27.5	17.4	40.3	53.5
	5.915	0.203	2.322	1.971	2.593	3.013	-1.768	1.118	2.597	3.443
g_O	236	321	510	233	333	513	216	335	514	213
	(15.2)	(20.7)	(32.8)	(15.0)	(21.4)	(33.0)	(13.9)	(21.6)	(33.1)	(13.7)
g_E	232.4	365.0	536.0	232.4	365.0	536.0	232.4	365.0	536.0	232.4
	(15.0)	(23.5)	(34.5)	(15.0)	(23.5)	(34.5)	(15.0)	(23.5)	(34.5)	(15.0)
	7.3	-82.5	-50.7	1.2	-61.1	-45.0	-31.6	-57.5	-43.1	-37.1
	0.468	-5.310	-3.264	0.079	-3.935	-2.896	-2.035	-3.700	-2.773	-2.390
c_O	246	389	251	245	379	273	272	401	250	277
	(15.8)	(25.0)	(16.2)	(15.8)	(24.4)	(17.6)	(17.5)	(25.8)	(16.1)	(17.8)
c_E	270.0	381.2	249.9	270.0	381.2	249.9	270.0	381.2	249.9	270.0
	(17.4)	(24.5)	(16.1)	(17.4)	(24.5)	(16.1)	(17.4)	(24.5)	(16.1)	(17.4)
	-45.8	15.8	2.3	-47.7	-4.3	48.3	4.0	40.7	0.3	14.1
	-2.952	1.019	0.146	-3.069	-0.278	3.113	0.255	2.620	0.017	0.910
N	1553	1553	1553	1553	1553	1553	1553	1553	1553	1553
X2	7.0	8.0	2.5	2.9	4.4	6.1	3.0	3.7	2.4	3.5
Gvalue	7.0	8.2	2.5	3.0	4.5	6.0	3.0	3.8	2.3	3.5
Gv/N	0.452	0.526	0.163	0.193	0.288	0.385	0.196	0.243	0.151	0.226
Prob	0.07152	0.04258	0.46926	0.39197	0.21464	0.11257	0.38496	0.28688	0.50516	0.32049

	81	82	83	84	85	86	87	88	89	90
a_O	496	500	536	484	517	546	493	492	546	516
	(31.9)	(32.2)	(34.5)	(31.2)	(33.3)	(35.2)	(31.7)	(31.7)	(35.2)	(33.2)
a_E	472.4	505.6	539.7	472.4	505.6	539.7	472.4	505.6	539.7	472.4
	(30.4)	(32.6)	(34.7)	(30.4)	(32.6)	(34.7)	(30.4)	(32.6)	(34.7)	(30.4)
	48.3	-11.1	-7.3	23.5	23.0	12.7	42.1	-26.8	12.7	91.1
	3.113	-0.718	-0.470	1.511	1.484	0.821	2.709	-1.728	0.821	5.866
t_O	367	268	517	332	267	519	373	258	513	318
	(23.6)	(17.3)	(33.3)	(21.4)	(17.2)	(33.4)	(24.0)	(16.6)	(33.0)	(20.5)
t_E	334.4	261.5	510.9	334.4	261.5	510.9	334.4	261.5	510.9	334.4
	(21.5)	(16.8)	(32.9)	(21.5)	(16.8)	(32.9)	(21.5)	(16.8)	(32.9)	(21.5)
	68.2	13.1	12.2	-4.8	11.0	16.3	81.4	-7.0	4.2	-32.0

	4.393	0.842	0.788	−0.312	0.710	1.049	5.243	−0.453	0.269	−2.063
g_O	323	497	228	352	530	240	325	554	233	318
	(20.8)	(32.0)	(14.7)	(22.7)	(34.1)	(15.5)	(20.9)	(35.7)	(15.0)	(20.5)
g_E	365.0	536.0	232.4	365.0	536.0	232.4	365.0	536.0	232.4	365.0
	(23.5)	(34.5)	(15.0)	(23.5)	(34.5)	(15.0)	(23.5)	(34.5)	(15.0)	(23.5)
	−79.0	−75.1	−8.7	−25.5	−11.9	15.5	−75.4	36.6	1.2	−87.7
	−5.085	−4.834	−0.560	−1.644	−0.767	0.996	−4.858	2.358	0.079	−5.645
c_O	367	288	272	385	239	248	362	249	261	401
	(23.6)	(18.5)	(17.5)	(24.8)	(15.4)	(16.0)	(23.3)	(16.0)	(16.8)	(25.8)
c_E	381.2	249.9	270.0	381.2	249.9	270.0	381.2	249.9	270.0	381.2
	(24.5)	(16.1)	(17.4)	(24.5)	(16.1)	(17.4)	(24.5)	(16.1)	(17.4)	(24.5)
	−27.8	81.8	4.0	7.7	−21.3	−42.2	−37.3	−1.7	−17.7	40.7
	−1.790	5.268	0.255	0.496	−1.368	−2.718	−2.405	−0.111	−1.143	2.620
N	1553	1553	1553	1553	1553	1553	1553	1553	1553	1553
X2	9.7	8.9	0.2	0.8	0.9	2.2	10.7	1.0	0.4	11.9
Gvalue	9.8	8.7	0.2	0.8	0.9	2.3	10.7	1.0	0.4	12.1
Gv/N	0.631	0.558	0.013	0.052	0.059	0.148	0.689	0.066	0.025	0.777
Prob	0.02038	0.03398	0.97839	0.84777	0.82193	0.51340	0.01343	0.79654	0.94245	0.00715

	91	92	93	94	95	96	97	98	99	100
a_O	489	509	495	508	530	484	522	589	472	496
	(31.5)	(32.8)	(31.9)	(32.7)	(34.1)	(31.2)	(33.6)	(37.9)	(30.4)	(31.9)
a_E	505.6	539.7	472.4	505.6	539.7	472.4	505.6	539.7	472.4	505.6
	(32.6)	(34.7)	(30.4)	(32.6)	(34.7)	(30.4)	(32.6)	(34.7)	(30.4)	(32.6)
	−32.7	−59.6	46.3	4.8	−19.2	23.5	33.3	103.0	−0.8	−19.0
	−2.103	−3.835	2.978	0.309	−1.234	1.511	2.145	6.635	−0.052	−1.225
t_O	265	509	344	259	503	324	272	476	331	289
	(17.1)	(32.8)	(22.2)	(16.7)	(32.4)	(20.9)	(17.5)	(30.7)	(21.3)	(18.6)
t_E	261.5	510.9	334.4	261.5	510.9	334.4	261.5	510.9	334.4	261.5
	(16.8)	(32.9)	(21.5)	(16.8)	(32.9)	(21.5)	(16.8)	(32.9)	(21.5)	(16.8)
	7.0	−3.8	19.4	−5.1	−15.7	−20.5	21.3	−67.4	−6.8	57.7
	0.448	−0.246	1.250	−0.326	−1.012	−1.322	1.373	−4.339	−0.439	3.715
g_O	530	244	327	525	244	319	509	214	360	541
	(34.1)	(15.7)	(21.1)	(33.8)	(15.7)	(20.5)	(32.8)	(13.8)	(23.2)	(34.8)
g_E	536.0	232.4	365.0	536.0	232.4	365.0	536.0	232.4	365.0	536.0
	(34.5)	(15.0)	(23.5)	(34.5)	(15.0)	(23.5)	(34.5)	(15.0)	(23.5)	(34.5)
	−11.9	23.8	−71.9	−21.7	23.8	−85.9	−52.6	−35.3	−9.9	10.1
	−0.767	1.532	−4.630	−1.400	1.532	−5.534	−3.386	−2.272	−0.640	0.649
c_O	269	291	387	261	276	426	250	274	390	227
	(17.3)	(18.7)	(24.9)	(16.8)	(17.8)	(27.4)	(16.1)	(17.6)	(25.1)	(14.6)
c_E	249.9	270.0	381.2	249.9	270.0	381.2	249.9	270.0	381.2	249.9
	(16.1)	(17.4)	(24.5)	(16.1)	(17.4)	(24.5)	(16.1)	(17.4)	(24.5)	(16.1)
	39.7	43.5	11.8	22.8	12.1	94.7	0.3	8.0	17.9	−43.6
	2.556	2.803	0.757	1.465	0.778	6.101	0.017	0.515	1.151	−2.806
N	1553	1553	1553	1553	1553	1553	1553	1553	1553	1553
X2	2.1	4.0	5.4	0.8	1.0	11.7	2.3	8.4	0.3	5.2
Gvalue	2.1	3.9	5.5	0.8	1.0	11.7	2.3	8.4	0.3	5.2
Gv/N	0.135	0.254	0.356	0.048	0.064	0.756	0.149	0.539	0.020	0.333
Prob	0.55326	0.26772	0.13713	0.86095	0.80146	0.00830	0.50857	0.03887	0.95858	0.15933

	101	102	103	104	105	106	107	108	109	110
a_O	548	499	525	555	480	498	557	508	492	497
	(35.3)	(32.1)	(33.8)	(35.7)	(30.9)	(32.1)	(35.9)	(32.7)	(31.7)	(32.0)
a_E	539.7	472.4	505.6	539.7	472.4	505.6	539.7	472.4	505.6	539.7
	(34.7)	(30.4)	(32.6)	(34.7)	(30.4)	(32.6)	(34.7)	(30.4)	(32.6)	(34.7)
	16.8	54.7	39.5	31.1	15.3	−15.1	35.2	73.8	−26.8	−81.9
	1.082	3.520	2.545	2.003	0.986	−0.972	2.268	4.752	−1.728	−5.271
t_O	510	333	247	523	326	266	479	344	270	510
	(32.8)	(21.4)	(15.9)	(33.7)	(21.0)	(17.1)	(30.8)	(22.2)	(17.4)	(32.8)
t_E	510.9	334.4	261.5	510.9	334.4	261.5	510.9	334.4	261.5	510.9
	(32.9)	(21.5)	(16.8)	(32.9)	(21.5)	(16.8)	(32.9)	(21.5)	(16.8)	(32.9)
	−1.8	−2.8	−28.3	24.5	−16.6	9.0	−61.8	19.4	17.2	−1.8
	−0.118	−0.184	−1.820	1.574	−1.072	0.579	−3.979	1.250	1.107	−0.118
g_O	233	307	520	201	350	546	209	323	544	238
	(15.0)	(19.8)	(33.5)	(12.9)	(22.5)	(35.2)	(13.5)	(20.8)	(35.0)	(15.3)
g_E	232.4	365.0	536.0	232.4	365.0	536.0	232.4	365.0	536.0	232.4
	(15.0)	(23.5)	(34.5)	(15.0)	(23.5)	(34.5)	(15.0)	(23.5)	(34.5)	(15.0)
	1.2	−106.3	−31.5	−58.3	−29.4	20.2	−44.3	−79.0	16.1	11.4
	0.079	−6.842	−2.028	−3.756	−1.892	1.302	−2.855	−5.085	1.040	0.731
c_O	262	414	261	274	397	243	308	378	247	308
	(16.9)	(26.7)	(16.8)	(17.6)	(25.6)	(15.6)	(19.8)	(24.3)	(15.9)	(19.8)
c_E	270.0	381.2	249.9	270.0	381.2	249.9	270.0	381.2	249.9	270.0
	(17.4)	(24.5)	(16.1)	(17.4)	(24.5)	(16.1)	(17.4)	(24.5)	(16.1)	(17.4)
	−15.8	68.4	22.8	8.0	32.3	−13.5	81.1	−6.3	−5.7	81.1
	−1.018	4.406	1.465	0.515	2.081	−0.872	5.219	−0.406	−0.367	5.219
N	1553	1553	1553	1553	1553	1553	1553	1553	1553	1553
X2	0.4	13.5	2.5	5.0	1.6	0.6	10.2	7.8	0.8	8.9
Gvalue	0.4	14.0	2.5	5.2	1.6	0.6	10.1	7.9	0.8	8.7
Gv/N	0.024	0.900	0.163	0.336	0.104	0.037	0.652	0.512	0.051	0.561
Prob	0.94591	0.00294	0.47003	0.15647	0.65719	0.90398	0.01748	0.04717	0.85132	0.03344

	111	112	113	114	115	116	117	118	119	120
a_O	472	511	556	473	503	531	478	483	537	475
	(30.4)	(32.9)	(35.8)	(30.5)	(32.4)	(34.2)	(30.8)	(31.1)	(34.6)	(30.6)
a_E	472.4	505.6	539.7	472.4	505.6	539.7	472.4	505.6	539.7	472.4
	(30.4)	(32.6)	(34.7)	(30.4)	(32.6)	(34.7)	(30.4)	(32.6)	(34.7)	(30.4)
	−0.8	10.8	33.2	1.2	−5.2	−17.2	11.3	−44.2	−5.3	5.2
	−0.052	0.698	2.135	0.077	−0.335	−1.107	0.725	−2.845	−0.342	0.335
t_O	321	213	482	330	271	511	339	285	492	317
	(20.7)	(13.7)	(31.0)	(21.2)	(17.5)	(32.9)	(21.8)	(18.4)	(31.7)	(20.4)
t_E	334.4	261.5	510.9	334.4	261.5	510.9	334.4	261.5	510.9	334.4
	(21.5)	(16.8)	(32.9)	(21.5)	(16.8)	(32.9)	(21.5)	(16.8)	(32.9)	(21.5)
	−26.3	−87.5	−56.2	−8.8	19.3	0.2	9.2	49.0	−37.1	−33.9
	−1.694	−5.632	−3.617	−0.566	1.240	0.011	0.593	3.152	−2.390	−2.185
g_O	352	554	237	344	525	249	349	535	220	341
	(22.7)	(35.7)	(15.3)	(22.2)	(33.8)	(16.0)	(22.5)	(34.4)	(14.2)	(22.0)
g_E	365.0	536.0	232.4	365.0	536.0	232.4	365.0	536.0	232.4	365.0
	(23.5)	(34.5)	(15.0)	(23.5)	(34.5)	(15.0)	(23.5)	(34.5)	(15.0)	(23.5)
	−25.5	36.6	9.3	−40.8	−21.7	34.4	−31.3	−2.0	−24.1	−46.4
	−1.644	2.358	0.599	−2.625	−1.400	2.214	−2.015	−0.127	−1.552	−2.987
c_O	408	275	278	406	254	262	387	250	304	420
	(26.3)	(17.7)	(17.9)	(26.1)	(16.4)	(16.9)	(24.9)	(16.1)	(19.6)	(27.0)
c_E	381.2	249.9	270.0	381.2	249.9	270.0	381.2	249.9	270.0	381.2
	(24.5)	(16.1)	(17.4)	(24.5)	(16.1)	(17.4)	(24.5)	(16.1)	(17.4)	(24.5)
	55.5	52.7	16.2	51.3	8.3	−15.8	11.8	0.3	72.1	81.5
	3.575	3.395	1.042	3.300	0.537	−1.018	0.757	0.017	4.640	5.248

N	1553	1553	1553	1553	1553	1553	1553	1553	1553	1553
X2	2.9	12.2	2.5	2.9	0.6	1.6	0.9	3.1	5.6	6.5
Gvalue	2.9	12.7	2.5	2.9	0.6	1.5	0.9	3.1	5.5	6.4
Gv/N	0.184	0.820	0.160	0.185	0.042	0.099	0.060	0.198	0.354	0.411
Prob	0.41340	0.00526	0.47858	0.41115	0.88580	0.67293	0.81842	0.38072	0.13859	0.09446

	121	122	123	124	125	126	127	128	129	130
a_O	514	526	487	482	554	484	509	548	457	484
	(33.1)	(33.9)	(31.4)	(31.0)	(35.7)	(31.2)	(32.8)	(35.3)	(29.4)	(31.2)
a_E	505.6	539.7	472.4	505.6	539.7	472.4	505.6	539.7	472.4	505.6
	(32.6)	(34.7)	(30.4)	(32.6)	(34.7)	(30.4)	(32.6)	(34.7)	(30.4)	(32.6)
	16.9	-27.0	29.6	-46.1	29.0	23.5	6.8	16.8	-30.3	-42.3
	1.090	-1.737	1.908	-2.968	1.870	1.511	0.439	1.082	-1.951	-2.722
t_O	251	473	363	278	486	381	261	537	347	267
	(16.2)	(30.5)	(23.4)	(17.9)	(31.3)	(24.5)	(16.8)	(34.6)	(22.3)	(17.2)
t_E	261.5	510.9	334.4	261.5	510.9	334.4	261.5	510.9	334.4	261.5
	(16.8)	(32.9)	(21.5)	(16.8)	(32.9)	(21.5)	(16.8)	(32.9)	(21.5)	(16.8)
	-20.7	-72.9	59.5	33.9	-48.6	99.3	-1.1	53.5	25.6	11.0
	-1.330	-4.697	3.832	2.185	-3.129	6.397	-0.070	3.443	1.649	0.710
g_O	519	233	351	518	216	289	524	188	341	545
	(33.4)	(15.0)	(22.6)	(33.4)	(13.9)	(18.6)	(33.7)	(12.1)	(22.0)	(35.1)
g_E	536.0	232.4	365.0	536.0	232.4	365.0	536.0	232.4	365.0	536.0
	(34.5)	(15.0)	(23.5)	(34.5)	(15.0)	(23.5)	(34.5)	(15.0)	(23.5)	(34.5)
	-33.4	1.2	-27.5	-35.4	-31.6	-134.9	-23.7	-79.7	-46.4	18.2
	-2.152	0.079	-1.768	-2.277	-2.035	-8.690	-1.526	-5.132	-2.987	1.171
c_O	269	321	352	275	297	399	259	280	408	257
	(17.3)	(20.7)	(22.7)	(17.7)	(19.1)	(25.7)	(16.7)	(18.0)	(26.3)	(16.5)
c_E	249.9	270.0	381.2	249.9	270.0	381.2	249.9	270.0	381.2	249.9
	(16.1)	(17.4)	(24.5)	(16.1)	(17.4)	(24.5)	(16.1)	(17.4)	(24.5)	(16.1)
	39.7	111.0	-56.0	52.7	56.6	36.5	18.6	20.3	55.5	14.5
	2.556	7.148	-3.608	3.395	3.642	2.350	1.198	1.308	3.575	0.932
N	1553	1553	1553	1553	1553	1553	1553	1553	1553	1553
X2	2.6	12.8	5.7	5.3	5.4	23.4	0.6	10.3	4.4	1.4
Gvalue	2.5	12.3	5.7	5.2	5.4	24.4	0.6	10.9	4.4	1.4
Gv/N	0.164	0.792	0.364	0.334	0.348	1.569	0.040	0.701	0.286	0.090
Prob	0.46760	0.00641	0.12963	0.15826	0.14437	0.00002	0.89095	0.01240	0.21814	0.70498

	131	132	133	134	135	136	137	138	139	140
a_O	509	473	493	541	476	515	518	445	501	555
	(32.8)	(30.5)	(31.7)	(34.8)	(30.7)	(33.2)	(33.4)	(28.7)	(32.3)	(35.7)
a_E	539.7	472.4	505.6	539.7	472.4	505.6	539.7	472.4	505.6	539.7
	(34.7)	(30.4)	(32.6)	(34.7)	(30.4)	(32.6)	(34.7)	(30.4)	(32.6)	(34.7)
	-59.6	1.2	-24.9	2.7	7.2	19.0	-42.4	-53.2	-9.2	31.1
	-3.835	0.077	-1.603	0.172	0.465	1.221	-2.733	-3.425	-0.590	2.003
t_O	511	326	263	518	334	247	491	363	265	460
	(32.9)	(21.0)	(16.9)	(33.4)	(21.5)	(15.9)	(31.6)	(23.4)	(17.1)	(29.6)
t_E	510.9	334.4	261.5	510.9	334.4	261.5	510.9	334.4	261.5	510.9
	(32.9)	(21.5)	(16.8)	(32.9)	(21.5)	(16.8)	(32.9)	(21.5)	(16.8)	(32.9)
	0.2	-16.6	2.9	14.3	-0.9	-28.3	-39.0	59.5	7.0	-96.6
	0.011	-1.072	0.188	0.919	-0.055	-1.820	-2.514	3.832	0.448	-6.219
g_O	244	336	532	231	361	540	255	359	535	254
	(15.7)	(21.6)	(34.3)	(14.9)	(23.2)	(34.8)	(16.4)	(23.1)	(34.4)	(16.4)
g_E	232.4	365.0	536.0	232.4	365.0	536.0	232.4	365.0	536.0	232.4
	(15.0)	(23.5)	(34.5)	(15.0)	(23.5)	(34.5)	(15.0)	(23.5)	(34.5)	(15.0)
	23.8	-55.6	-7.9	-2.8	-8.0	8.1	47.3	-11.9	-2.0	45.2
	1.532	-3.582	-0.511	-0.179	-0.512	0.519	3.049	-0.766	-0.127	2.908
c_O	289	418	265	263	382	251	289	386	252	284
	(18.6)	(26.9)	(17.1)	(16.9)	(24.6)	(16.2)	(18.6)	(24.9)	(16.2)	(18.3)
c_E	270.0	381.2	249.9	270.0	381.2	249.9	270.0	381.2	249.9	270.0
	(17.4)	(24.5)	(16.1)	(17.4)	(24.5)	(16.1)	(17.4)	(24.5)	(16.1)	(17.4)
	39.3	77.1	31.2	-13.9	1.7	2.3	39.3	9.7	4.3	28.7
	2.527	4.966	2.007	-0.893	0.108	0.146	2.527	0.627	0.276	1.845
N	1553	1553	1553	1553	1553	1553	1553	1553	1553	1553
X2	3.7	6.1	1.3	0.3	0.1	1.0	5.2	4.2	0.1	8.2
Gvalue	3.7	6.0	1.3	0.3	0.1	1.0	5.1	4.2	0.1	8.3
Gv/N	0.235	0.388	0.081	0.019	0.005	0.066	0.329	0.268	0.007	0.537
Prob	0.30182	0.11001	0.74020	0.96118	0.99480	0.79334	0.16442	0.24508	0.99091	0.03941

	141	142	143	144	145	146	147	148	149	150
a_O	472	477	552	476	450	514	447	495	556	486
	(30.4)	(30.7)	(35.5)	(30.7)	(29.0)	(33.1)	(28.8)	(31.9)	(35.8)	(31.3)
a_E	472.4	505.6	539.7	472.4	505.3	539.3	472.1	505.3	539.3	472.1
	(30.4)	(32.6)	(34.7)	(30.4)	(32.6)	(34.7)	(30.4)	(32.6)	(34.7)	(30.4)
	-0.8	-55.6	24.9	7.2	-104.3	-49.4	-48.8	-20.4	33.9	28.2
	-0.052	-3.578	1.606	0.465	-6.719	-3.185	-3.147	-1.311	2.183	1.817
t_O	353	272	503	346	264	515	339	259	501	324
	(22.7)	(17.5)	(32.4)	(22.3)	(17.0)	(33.2)	(21.8)	(16.7)	(32.3)	(20.9)
t_E	334.4	261.5	510.9	334.4	261.4	510.6	334.2	261.4	510.6	334.2
	(21.5)	(16.8)	(32.9)	(21.5)	(16.8)	(32.9)	(21.5)	(16.8)	(32.9)	(21.5)
	38.2	21.3	-15.7	23.5	5.3	8.9	9.6	-4.7	-19.0	-20.1
	2.457	1.373	-1.012	1.516	0.340	0.571	0.621	-0.305	-1.224	-1.296
g_O	338	543	221	341	566	235	367	544	233	340
	(21.8)	(35.0)	(14.2)	(22.0)	(36.5)	(15.1)	(23.6)	(35.1)	(15.0)	(21.9)
g_E	365.0	536.0	232.4	365.0	535.6	232.2	364.8	535.6	232.2	364.8
	(23.5)	(34.5)	(15.0)	(23.5)	(34.5)	(15.0)	(23.5)	(34.5)	(15.0)	(23.5)
	-52.0	14.1	-22.2	-46.4	62.4	5.5	4.5	16.8	1.5	-47.8
	-3.345	0.909	-1.430	-2.987	4.021	0.358	0.289	1.086	0.098	-3.081
c_O	390	261	277	390	272	288	399	254	262	402
	(25.1)	(16.8)	(17.8)	(25.1)	(17.5)	(18.6)	(25.7)	(16.4)	(16.9)	(25.9)
c_E	381.2	249.9	270.0	381.2	249.7	269.9	380.9	249.7	269.9	380.9
	(24.5)	(16.1)	(17.4)	(24.5)	(16.1)	(17.4)	(24.5)	(16.1)	(17.4)	(24.5)
	17.9	22.8	14.1	17.9	46.5	37.5	37.0	8.7	-15.5	43.3
	1.151	1.465	0.910	1.151	2.998	2.416	2.384	0.558	-0.997	2.790
N	1553	1553	1553	1553	1552	1552	1552	1552	1552	1552
X2	3.2	2.6	1.1	2.2	9.8	2.5	2.3	0.4	0.9	3.6
Gvalue	3.3	2.6	1.1	2.2	9.9	2.5	2.3	0.4	0.9	3.6
Gv/N	0.210	0.170	0.074	0.144	0.640	0.159	0.147	0.028	0.060	0.231
Prob	0.35255	0.45002	0.76517	0.52404	0.01916	0.48034	0.51525	0.93281	0.81936	0.30964

Abor_SK2
5UTR:

	50	49	48	47	46	45	44	43	42	41
a_O	636	607	637	606	609	538	669	684	617	642
	(23.1)	(22.0)	(23.1)	(22.0)	(22.1)	(19.5)	(24.3)	(24.8)	(22.4)	(23.3)
a_E	675.9	675.9	675.9	675.9	675.9	675.9	675.9	675.9	675.9	675.9
	(24.5)	(24.5)	(24.5)	(24.5)	(24.5)	(24.5)	(24.5)	(24.5)	(24.5)	(24.5)
	-77.3	-130.5	-75.5	-132.3	-126.9	-245.5	-13.7	16.4	-112.5	-66.0
	-2.807	-4.736	-2.739	-4.801	-4.606	-8.910	-0.496	0.594	-4.082	-2.396
t_O	646	712	650	658	686	719	650	663	673	627
	(23.4)	(25.8)	(23.6)	(23.9)	(24.9)	(26.1)	(23.6)	(24.1)	(24.4)	(22.8)
t_E	650.6	650.6	650.6	650.6	650.6	650.6	650.6	650.6	650.6	650.6
	(23.6)	(23.6)	(23.6)	(23.6)	(23.6)	(23.6)	(23.6)	(23.6)	(23.6)	(23.6)
	-9.1	128.5	-1.1	15.0	72.8	143.8	-1.1	25.1	45.6	-46.3
	-0.330	4.665	-0.041	0.543	2.642	5.221	-0.041	0.912	1.657	-1.679
g_O	721	711	722	773	738	719	711	711	717	755
	(26.2)	(25.8)	(26.2)	(28.1)	(26.8)	(26.1)	(25.8)	(25.8)	(26.0)	(27.4)
g_E	725.5	725.5	725.5	725.5	725.5	725.5	725.5	725.5	725.5	725.5
	(26.3)	(26.3)	(26.3)	(26.3)	(26.3)	(26.3)	(26.3)	(26.3)	(26.3)	(26.3)
	-9.0	-28.7	-7.0	98.0	25.2	-13.0	-28.7	-28.7	-16.9	60.2
	-0.326	-1.043	-0.254	3.558	0.914	-0.470	-1.043	-1.043	-0.614	2.184
c_O	752	725	746	718	722	779	725	697	748	731
	(27.3)	(26.3)	(27.1)	(26.1)	(26.2)	(28.3)	(26.3)	(25.3)	(27.2)	(26.5)
c_E	703.1	703.1	703.1	703.1	703.1	703.1	703.1	703.1	703.1	703.1
	(25.5)	(25.5)	(25.5)	(25.5)	(25.5)	(25.5)	(25.5)	(25.5)	(25.5)	(25.5)
	101.2	44.6	88.5	30.2	38.4	159.8	44.6	-12.1	92.7	57.0
	3.674	1.617	3.211	1.096	1.393	5.800	1.617	-0.438	3.365	2.068
N	2755	2755	2755	2755	2755	2755	2755	2755	2755	2755
X2	5.8	13.8	4.9	10.7	9.3	43.6	1.0	0.7	8.9	4.9
Gvalue	5.8	13.9	4.9	10.9	9.5	45.2	1.0	0.7	9.0	4.9
Gv/N	0.210	0.503	0.177	0.397	0.343	1.641	0.038	0.025	0.325	0.177
Prob	0.12228	0.00309	0.18173	0.01212	0.02374	0.00000	0.79149	0.87829	0.02980	0.18166

	40	39	38	37	36	35	34	33	32	31
a_O	686	597	640	654	599	648	670	639	598	666
	(24.9)	(21.7)	(23.2)	(23.7)	(21.7)	(23.5)	(24.3)	(23.2)	(21.7)	(24.2)
a_E	675.9	675.9	675.9	675.9	675.9	675.9	675.9	675.9	675.9	675.9
	(24.5)	(24.5)	(24.5)	(24.5)	(24.5)	(24.5)	(24.5)	(24.5)	(24.5)	(24.5)
	20.4	-148.2	-69.8	-43.0	-144.6	-54.6	-11.7	-71.7	-146.4	-19.6
	0.741	-5.378	-2.534	-1.562	-5.250	-1.981	-0.424	-2.602	-5.314	-0.711
t_O	674	721	648	657	701	663	676	700	654	665
	(24.5)	(26.2)	(23.5)	(23.8)	(25.4)	(24.1)	(24.5)	(25.4)	(23.7)	(24.1)
t_E	650.6	650.6	650.6	650.6	650.6	650.6	650.6	650.6	650.6	650.6
	(23.6)	(23.6)	(23.6)	(23.6)	(23.6)	(23.6)	(23.6)	(23.6)	(23.6)	(23.6)
	47.7	148.2	-5.1	12.9	104.7	25.1	51.9	102.5	6.9	29.2
	1.732	5.381	-0.185	0.470	3.800	0.912	1.882	3.722	0.250	1.060
g_O	678	710	700	686	707	692	651	718	739	685
	(24.6)	(25.8)	(25.4)	(24.9)	(25.7)	(25.1)	(23.6)	(26.1)	(26.8)	(24.9)
g_E	725.5	725.5	725.5	725.5	725.5	725.5	725.5	725.5	725.5	725.5
	(26.3)	(26.3)	(26.3)	(26.3)	(26.3)	(26.3)	(26.3)	(26.3)	(26.3)	(26.3)
	-91.8	-30.7	-50.1	-76.8	-36.5	-65.4	-141.1	-14.9	27.2	-78.7
	-3.334	-1.114	-1.819	-2.789	-1.326	-2.376	-5.121	-0.542	0.988	-2.857
c_O	717	727	767	758	748	752	758	698	764	739
	(26.0)	(26.4)	(27.8)	(27.5)	(27.2)	(27.3)	(27.5)	(25.3)	(27.7)	(26.8)
c_E	703.1	703.1	703.1	703.1	703.1	703.1	703.1	703.1	703.1	703.1
	(25.5)	(25.5)	(25.5)	(25.5)	(25.5)	(25.5)	(25.5)	(25.5)	(25.5)	(25.5)
	28.2	48.7	133.5	114.1	92.7	101.2	114.1	-10.1	127.0	73.7
	1.022	1.767	4.847	4.140	3.365	3.674	4.140	-0.366	4.610	2.675
N	2755	2755	2755	2755	2755	2755	2755	2755	2755	2755
X2	4.4	18.0	8.6	7.2	16.0	6.3	13.0	5.9	14.5	4.6
Gvalue	4.4	18.1	8.5	7.2	16.2	6.3	13.1	5.8	14.7	4.6
Gv/N	0.161	0.657	0.309	0.260	0.588	0.229	0.477	0.212	0.535	0.166
Prob	0.21755	0.00042	0.03669	0.06708	0.00103	0.09771	0.00433	0.12023	0.00205	0.20570

	30	29	28	27	26	25	24	23	22	21
a_O	645	654	701	666	691	688	638	734	773	714
	(23.4)	(23.7)	(25.4)	(24.2)	(25.1)	(25.0)	(23.2)	(26.6)	(28.1)	(25.9)
a_E	675.9	675.9	675.9	675.9	675.9	675.9	675.9	675.9	675.9	675.9
	(24.5)	(24.5)	(24.5)	(24.5)	(24.5)	(24.5)	(24.5)	(24.5)	(24.5)	(24.5)
	-60.3	-43.0	51.2	-19.6	30.6	24.5	-73.6	121.1	207.6	78.4
	-2.189	-1.562	1.858	-0.711	1.111	0.888	-2.671	4.396	7.535	2.845
t_O	702	652	690	684	626	706	699	638	688	680
	(25.5)	(23.7)	(25.0)	(24.8)	(22.7)	(25.6)	(25.4)	(23.2)	(25.0)	(24.7)
t_E	650.6	650.6	650.6	650.6	650.6	650.6	650.6	650.6	650.6	650.6
	(23.6)	(23.6)	(23.6)	(23.6)	(23.6)	(23.6)	(23.6)	(23.6)	(23.6)	(23.6)
	106.8	2.9	81.2	68.6	-48.2	115.5	100.4	-24.9	77.0	60.2
	3.878	0.105	2.948	2.489	-1.749	4.191	3.644	-0.903	2.795	2.185
g_O	658	716	621	605	695	632	652	644	577	610
	(23.9)	(26.0)	(22.5)	(22.0)	(25.2)	(22.9)	(23.7)	(23.4)	(20.9)	(22.1)
g_E	725.5	725.5	725.5	725.5	725.5	725.5	725.5	725.5	725.5	725.5
	(26.3)	(26.3)	(26.3)	(26.3)	(26.3)	(26.3)	(26.3)	(26.3)	(26.3)	(26.3)
	-128.5	-18.9	-193.2	-219.8	-59.7	-174.4	-139.3	-153.5	-264.3	-211.6
	-4.665	-0.686	-7.012	-7.978	-2.168	-6.331	-5.057	-5.572	-9.594	-7.679
c_O	750	733	743	800	743	729	766	739	717	751
	(27.2)	(26.6)	(27.0)	(29.0)	(27.0)	(26.5)	(27.8)	(26.8)	(26.0)	(27.3)
c_E	703.1	703.1	703.1	703.1	703.1	703.1	703.1	703.1	703.1	703.1
	(25.5)	(25.5)	(25.5)	(25.5)	(25.5)	(25.5)	(25.5)	(25.5)	(25.5)	(25.5)
	96.9	61.1	82.1	206.7	82.1	52.8	131.4	73.7	28.2	99.1
	3.519	2.219	2.980	7.502	2.980	1.917	4.768	2.675	1.022	3.596
N	2755	2755	2755	2755	2755	2755	2755	2755	2755	2755
X2	14.9	2.1	20.6	35.2	4.8	18.0	18.8	16.2	46.8	25.1
Gvalue	14.9	2.1	21.3	35.9	4.8	18.4	18.9	16.4	48.4	26.1
Gv/N	0.543	0.076	0.774	1.301	0.174	0.667	0.685	0.597	1.758	0.946
Prob	0.00186	0.55169	0.00009	0.00000	0.18674	0.00037	0.00029	0.00092	0.00000	0.00001

	20	19	18	17	16	15	14	13	12	11
a_O	760	819	769	810	822	742	865	895	874	895
	(27.6)	(29.7)	(27.9)	(29.4)	(29.8)	(26.9)	(31.4)	(32.5)	(31.7)	(32.5)
a_E	675.9	675.9	675.9	675.9	675.9	675.9	675.9	675.9	675.9	675.9
	(24.5)	(24.5)	(24.5)	(24.5)	(24.5)	(24.5)	(24.5)	(24.5)	(24.5)	(24.5)
	178.3	314.6	198.5	293.3	321.8	138.5	426.8	502.7	449.4	502.7
	6.473	11.420	7.207	10.645	11.680	5.028	15.493	18.246	16.311	18.246
t_O	620	684	689	622	712	660	517	600	509	328
	(22.5)	(24.8)	(25.0)	(22.6)	(25.8)	(24.0)	(18.8)	(21.8)	(18.5)	(11.9)

```
t_E       650.6   650.6   650.6   650.6   650.6   650.6   650.6   650.6   650.6   650.6
        ( 23.6) ( 23.6) ( 23.6) ( 23.6) ( 23.6) ( 23.6) ( 23.6) ( 23.6) ( 23.6) ( 23.6)
          -59.7    68.6    79.1   -55.8   128.5    19.0  -237.6   -97.1  -249.8  -449.2
         -2.166   2.489   2.871  -2.027   4.665   0.690  -8.624  -3.524  -9.067 -16.306
g_O         662     533     603     651     608     688     768     687     867    1080
        ( 24.0) ( 19.3) ( 21.9) ( 23.6) ( 22.1) ( 25.0) ( 27.9) ( 24.9) ( 31.5) ( 39.2)
g_E       725.5   725.5   725.5   725.5   725.5   725.5   725.5   725.5   725.5   725.5
        ( 26.3) ( 26.3) ( 26.3) ( 26.3) ( 26.3) ( 26.3) ( 26.3) ( 26.3) ( 26.3) ( 26.3)
         -121.3  -328.7  -223.1  -141.1  -214.9   -73.0    87.4   -74.9   308.9   859.3
         -4.403 -11.931  -8.097  -5.121  -7.799  -2.651   3.173  -2.720  11.214  31.192
c_O         713     719     694     672     613     665     605     573     505     452
        ( 25.9) ( 26.1) ( 25.2) ( 24.4) ( 22.3) ( 24.1) ( 22.0) ( 20.8) ( 18.3) ( 16.4)
c_E       703.1   703.1   703.1   703.1   703.1   703.1   703.1   703.1   703.1   703.1
        ( 25.5) ( 25.5) ( 25.5) ( 25.5) ( 25.5) ( 25.5) ( 25.5) ( 25.5) ( 25.5) ( 25.5)
           20.0    32.2   -18.0   -60.7  -168.1   -74.0  -181.8  -234.4  -334.2  -399.4
          0.727   1.170  -0.653  -2.204  -6.100  -2.687  -6.597  -8.509 -12.130 -14.496

N          2755    2755    2755    2755    2755    2755    2755    2755    2755    2755
X2         17.6    83.5    35.9    36.9    68.0    10.6    96.5   101.1   172.3   493.8
Gvalue     17.4    86.7    36.6    35.6    67.4    10.5    94.9    96.2   174.3   513.4
Gv/N      0.631   3.148   1.328   1.292   2.446   0.380   3.445   3.493   6.327  18.636
Prob    0.00059 0.00000 0.00000 0.00000 0.00000 0.01491 0.00000 0.00000 0.00000 0.00000
        -------------------------------------------------------------------------------
             10       9       8       7       6       5       4       3       2       1
        -------------------------------------------------------------------------------
a_O         836     725     877     877     816     835     821     848     431     637
        ( 30.3) ( 26.3) ( 31.8) ( 31.8) ( 29.6) ( 30.3) ( 29.8) ( 30.8) ( 15.6) ( 23.1)
a_E       675.9   675.9   675.9   675.9   675.9   675.9   675.9   675.9   675.9   675.9
        ( 24.5) ( 24.5) ( 24.5) ( 24.5) ( 24.5) ( 24.5) ( 24.5) ( 24.5) ( 24.5) ( 24.5)
          355.5   101.7   456.9   456.9   307.5   353.1   319.4   384.8  -387.8   -75.5
         12.904   3.693  16.585  16.585  11.161  12.816  11.594  13.967 -14.076  -2.739
t_O         336     331     341     499     640     595     607     541     752     615
        ( 12.2) ( 12.0) ( 12.4) ( 18.1) ( 23.2) ( 21.6) ( 22.0) ( 19.6) ( 27.3) ( 22.3)
t_E       650.6   650.6   650.6   650.6   650.6   650.6   650.6   650.6   650.6   650.6
        ( 23.6) ( 23.6) ( 23.6) ( 23.6) ( 23.6) ( 23.6) ( 23.6) ( 23.6) ( 23.6) ( 23.6)
         -444.0  -447.3  -440.5  -264.7   -20.9  -106.2   -84.1  -199.5   217.9   -69.1
        -16.116 -16.237 -15.991  -9.608  -0.760  -3.856  -3.054  -7.243   7.910  -2.510
g_O        1224    1379    1201     887     776     665     560     720     514     371
        ( 44.4) ( 50.1) ( 43.6) ( 32.2) ( 28.2) ( 24.1) ( 20.3) ( 26.1) ( 18.7) ( 13.5)
g_E       725.5   725.5   725.5   725.5   725.5   725.5   725.5   725.5   725.5   725.5
        ( 26.3) ( 26.3) ( 26.3) ( 26.3) ( 26.3) ( 26.3) ( 26.3) ( 26.3) ( 26.3) ( 26.3)
         1280.3  1771.3  1210.7   356.5   104.4  -115.8  -290.0   -11.0  -354.3  -497.6
         46.472  64.294  43.945  12.941   3.790  -4.204 -10.527  -0.398 -12.860 -18.063
c_O         359     320     336     492     523     660     767     646    1058    1132
        ( 13.0) ( 11.6) ( 12.2) ( 17.9) ( 19.0) ( 24.0) ( 27.8) ( 23.4) ( 38.4) ( 41.1)
c_E       703.1   703.1   703.1   703.1   703.1   703.1   703.1   703.1   703.1   703.1
        ( 25.5) ( 25.5) ( 25.5) ( 25.5) ( 25.5) ( 25.5) ( 25.5) ( 25.5) ( 25.5) ( 25.5)
         -482.6  -503.8  -496.2  -351.3  -309.5   -83.4   133.5  -109.4   864.8  1078.3
        -17.517 -18.285 -18.009 -12.750 -11.233  -3.028   4.847  -3.969  31.390  39.141

N          2755    2755    2755    2755    2755    2755    2755    2755    2755    2755
X2        700.9   957.9   710.4   194.5    78.9    49.9    77.7    67.0   345.4   439.1
Gvalue    709.2   922.0   730.9   197.5    81.5    47.6    78.8    64.9   340.6   436.1
Gv/N     25.744  33.465  26.531   7.169   2.958   1.728   2.860   2.356  12.364  15.829
Prob    0.00000 0.00000 0.00000 0.00000 0.00000 0.00000 0.00000 0.00000 0.00000 0.00000
        -------------------------------------------------------------------------------

CDS_INITIAL:
        -------------------------------------------------------------------------------
              1       2       3       4       5       6       7       8       9      10
        -------------------------------------------------------------------------------
a_O        2261       0       0    1211     806     664     953     963     672     846
        ( 82.1) (  0.0) (  0.0) ( 44.0) ( 29.3) ( 24.1) ( 34.6) ( 35.0) ( 24.4) ( 30.7)
a_E       628.5   784.9   418.9   628.5   784.9   418.9   628.5   784.9   418.9   628.5
        ( 22.8) ( 28.5) ( 15.2) ( 22.8) ( 28.5) ( 15.2) ( 22.8) ( 28.5) ( 15.2) ( 22.8)
         5788.9     0.0     0.0  1588.4    42.7   611.8   793.3   393.8   635.2   502.7
        210.123   0.000   0.000  57.653   1.551  22.205  28.795  14.294  23.057  18.248
t_O         183    2755       0     337     500     786     400     740     743     489
        (  6.6) (100.0) (  0.0) ( 12.2) ( 18.1) ( 28.5) ( 14.5) ( 26.9) ( 27.0) ( 17.7)
t_E       413.4   814.0   630.8   413.4   814.0   630.8   413.4   814.0   630.8   413.4
        ( 15.0) ( 29.5) ( 22.9) ( 15.0) ( 29.5) ( 22.9) ( 15.0) ( 29.5) ( 22.9) ( 15.0)
         -298.3  6718.1     0.0  -137.7  -487.3   345.9   -26.4  -141.0   243.4   164.2
        -10.827 243.851   0.000  -5.000 -17.688  12.555  -0.958  -5.118   8.834   5.961
g_O         311       0    2755     642     561     575     808     443     653     684
        ( 11.3) (  0.0) (100.0) ( 23.3) ( 20.4) ( 20.9) ( 29.3) ( 16.1) ( 23.7) ( 24.8)
g_E      1018.0   525.1   840.3  1018.0   525.1   840.3  1018.0   525.1   840.3  1018.0
        ( 37.0) ( 19.1) ( 30.5) ( 37.0) ( 19.1) ( 30.5) ( 37.0) ( 19.1) ( 30.5) ( 37.0)
         -737.6     0.0  6542.8  -591.9    74.2  -436.3  -373.3  -150.6  -329.3  -544.0
        -26.772   0.000 237.489 -21.486   2.694 -15.835 -13.552  -5.467 -11.953 -19.745
c_O           0       0       0     565     888     730     594     609     687     736
        (  0.0) (  0.0) (  0.0) ( 20.5) ( 32.2) ( 26.5) ( 21.6) ( 22.1) ( 24.9) ( 26.7)
c_E       695.1   631.0   865.1   695.1   631.0   865.1   695.1   631.0   865.1   695.1
        ( 25.2) ( 22.9) ( 31.4) ( 25.2) ( 22.9) ( 31.4) ( 25.2) ( 22.9) ( 31.4) ( 25.2)
            0.0     0.0     0.0  -234.1   606.7  -247.9  -186.6   -43.3  -316.7    84.3
          0.000   0.000   0.000  -8.497  22.023  -8.997  -6.775  -1.571 -11.495   3.059

N          2755    2755    2755    2755    2755    2755    2755    2755    2755    2755
X2       5554.3  6569.8  6277.8   717.1   228.8   286.5   225.9    60.7   251.3   201.1
Gvalue   4753.1  6718.1  6542.8   624.6   236.4   273.5   206.9    58.9   232.6   207.3
Gv/N    172.525 243.851 237.489  22.671   8.579   9.928   7.511   2.139   8.443   7.523
Prob    1.00000 1.00000 1.00000 0.00000 0.00000 0.00000 0.00000 0.00000 0.00000 0.00000
        -------------------------------------------------------------------------------
             11      12      13      14      15      16      17      18      19      20
        -------------------------------------------------------------------------------
a_O         786     653     833     753     606     789     710     572     784     767
        ( 28.5) ( 23.7) ( 30.2) ( 27.3) ( 22.0) ( 28.6) ( 25.8) ( 20.8) ( 28.5) ( 27.8)
a_E       784.9   418.9   628.5   784.9   418.9   628.5   784.9   418.9   628.5   784.9
        ( 28.5) ( 15.2) ( 22.8) ( 28.5) ( 15.2) ( 22.8) ( 28.5) ( 15.2) ( 22.8) ( 28.5)
            2.2   579.8   469.2   -62.5   447.5   358.8  -142.5   356.4   346.5   -35.4
          0.078  21.045  17.031  -2.270  16.245  13.023  -5.171  12.935  12.579  -1.286
t_O         834     712     513     933     705     495     919     710     450     911
        ( 30.3) ( 25.8) ( 18.6) ( 33.9) ( 25.6) ( 18.0) ( 33.4) ( 25.8) ( 16.3) ( 33.1)
t_E       814.0   630.8   413.4   814.0   630.8   413.4   814.0   630.8   413.4   814.0
        ( 29.5) ( 22.9) ( 15.0) ( 29.5) ( 22.9) ( 15.0) ( 29.5) ( 22.9) ( 15.0) ( 29.5)
           40.6   172.5   221.4   254.7   156.9   178.3   223.1   168.1    76.3   205.2
          1.472   6.263   8.038   9.244   5.695   6.472   8.097   6.100   2.770   7.448
g_O         472     663     683     440     654     702     438     668     762     413
        ( 17.1) ( 24.1) ( 24.8) ( 16.0) ( 23.7) ( 25.5) ( 15.9) ( 24.2) ( 27.7) ( 15.0)
g_E       525.1   840.3  1018.0   525.1   840.3  1018.0   525.1   840.3  1018.0   525.1
        ( 19.1) ( 30.5) ( 37.0) ( 19.1) ( 30.5) ( 37.0) ( 19.1) ( 30.5) ( 37.0) ( 19.1)
         -100.6  -314.2  -545.2  -155.6  -327.8  -521.8  -158.9  -306.5  -441.4  -198.3
         -3.652 -11.405 -19.788  -5.647 -11.899 -18.940  -5.766 -11.126 -16.023  -7.199
```

c_O	663	727	726	629	790	769	688	805	759	664
	(24.1)	(26.4)	(26.4)	(22.8)	(28.7)	(27.9)	(25.0)	(29.2)	(27.5)	(24.1)
c_E	631.0	865.1	695.1	631.0	865.1	695.1	631.0	865.1	695.1	631.0
	(22.9)	(31.4)	(25.2)	(22.9)	(31.4)	(25.2)	(22.9)	(31.4)	(25.2)	(22.9)
	65.5	-252.8	63.3	-4.0	-143.4	155.5	118.9	-115.9	133.6	67.6
	2.379	-9.177	2.296	-0.147	-5.206	5.644	4.317	-4.206	4.850	2.455
N	2755	2755	2755	2755	2755	2755	2755	2755	2755	2755
X2	7.5	200.7	202.1	32.5	140.1	163.0	40.3	105.4	111.9	37.6
Gvalue	7.6	185.3	208.7	32.5	133.2	170.8	40.7	102.0	115.1	39.1
Gv/N	0.277	6.726	7.577	1.181	4.835	6.199	1.478	3.703	4.176	1.418
Prob	0.05403	0.00000	0.00000	0.00000	0.00000	0.00000	0.00000	0.00000	0.00000	0.00000

	21	22	23	24	25	26	27	28	29	30
a_O	581	749	751	521	678	679	510	616	673	457
	(21.1)	(27.2)	(27.3)	(18.9)	(24.6)	(24.6)	(18.5)	(22.4)	(24.4)	(16.6)
a_E	418.9	628.5	784.9	418.9	628.5	784.9	418.9	628.5	784.9	418.9
	(15.2)	(22.8)	(28.5)	(15.2)	(22.8)	(28.5)	(15.2)	(22.8)	(28.5)	(15.2)
	380.1	262.7	-66.4	227.3	102.7	-196.9	200.7	-24.8	-207.1	79.6
	13.797	9.534	-2.409	8.250	3.728	-7.146	7.286	-0.901	-7.516	2.888
t_O	719	472	904	696	456	903	688	448	932	675
	(26.1)	(17.1)	(32.8)	(25.3)	(16.6)	(32.8)	(25.0)	(16.3)	(33.8)	(24.5)
t_E	630.8	413.4	814.0	630.8	413.4	814.0	630.8	413.4	814.0	630.8
	(22.9)	(15.0)	(29.5)	(22.9)	(15.0)	(29.5)	(22.9)	(15.0)	(29.5)	(22.9)
	188.3	125.1	189.7	137.0	89.4	187.5	119.5	72.0	252.4	91.5
	6.835	4.541	6.885	4.973	3.246	6.805	4.339	2.613	9.162	3.322
g_O	660	795	446	711	831	512	741	927	509	747
	(24.0)	(28.9)	(16.2)	(25.8)	(30.2)	(18.6)	(26.9)	(33.6)	(18.5)	(27.1)
g_E	840.3	1018.0	525.1	840.3	1018.0	525.1	840.3	1018.0	525.1	840.3
	(30.5)	(37.0)	(19.1)	(30.5)	(37.0)	(19.1)	(30.5)	(37.0)	(19.1)	(30.5)
	-318.8	-393.1	-145.6	-237.6	-337.3	-25.8	-186.3	-173.6	-31.7	-175.8
	-11.570	-14.270	-5.285	-8.623	-12.244	-0.938	-6.763	-6.302	-1.150	-6.381
c_O	795	739	654	827	790	661	816	764	641	876
	(28.9)	(26.8)	(23.7)	(30.0)	(28.7)	(24.0)	(29.6)	(27.7)	(23.3)	(31.8)
c_E	865.1	695.1	631.0	865.1	695.1	631.0	865.1	695.1	631.0	865.1
	(31.4)	(25.2)	(22.9)	(31.4)	(25.2)	(22.9)	(31.4)	(25.2)	(22.9)	(31.4)
	-134.3	90.6	46.8	-74.4	202.3	61.3	-95.3	144.5	20.1	22.0
	-4.875	3.289	1.698	-2.702	7.344	2.227	-3.460	5.246	0.730	0.798
N	2755	2755	2755	2755	2755	2755	2755	2755	2755	2755
X2	119.4	83.0	24.2	53.2	55.6	25.8	39.5	18.1	33.7	17.1
Gvalue	115.3	85.3	24.5	52.3	57.1	26.1	38.6	18.1	33.8	17.3
Gv/N	4.187	3.095	0.889	1.898	2.074	0.948	1.402	0.656	1.226	0.628
Prob	0.00000	0.00000	0.00002	0.00000	0.00000	0.00001	0.00000	0.00042	0.00000	0.00061

	31	32	33	34	35	36	37	38	39	40
a_O	674	638	468	656	613	396	633	637	422	638
	(24.5)	(23.2)	(17.0)	(23.8)	(22.3)	(14.4)	(23.0)	(23.1)	(15.3)	(23.2)
a_E	628.5	784.9	418.9	628.5	784.9	418.9	628.5	784.9	418.9	628.5
	(22.8)	(28.5)	(15.2)	(22.8)	(28.5)	(15.2)	(22.8)	(28.5)	(15.2)	(22.8)
	94.1	-264.4	103.7	56.1	-303.1	-44.5	9.0	-266.0	6.2	19.1
	3.417	-9.599	3.766	2.036	-11.001	-1.616	0.325	-9.656	0.226	0.692
t_O	417	954	679	441	905	682	432	906	654	433
	(15.1)	(34.6)	(24.6)	(16.0)	(32.8)	(24.8)	(15.7)	(32.9)	(23.7)	(15.7)
t_E	413.4	814.0	630.8	413.4	814.0	630.8	413.4	814.0	630.8	413.4
	(15.0)	(29.5)	(22.9)	(15.0)	(29.5)	(22.9)	(15.0)	(29.5)	(22.9)	(15.0)
	7.2	302.9	100.1	57.0	191.9	106.5	38.0	194.1	47.3	40.1
	0.262	10.994	3.633	2.068	6.965	3.867	1.379	7.046	1.718	1.455
g_O	890	491	729	911	530	800	930	541	778	984
	(32.3)	(17.8)	(26.5)	(33.1)	(19.2)	(29.0)	(33.8)	(19.6)	(28.2)	(35.7)
g_E	1018.0	525.1	840.3	1018.0	525.1	840.3	1018.0	525.1	840.3	1018.0
	(37.0)	(19.1)	(30.5)	(37.0)	(19.1)	(30.5)	(37.0)	(19.1)	(30.5)	(37.0)
	-239.2	-65.9	-207.1	-202.3	9.9	-78.6	-168.2	32.3	-119.8	-66.8
	-8.682	-2.392	-7.518	-7.344	0.358	-2.852	-6.104	1.173	-4.349	-2.426
c_O	774	672	879	747	707	877	760	671	901	700
	(28.1)	(24.4)	(31.9)	(27.1)	(25.7)	(31.8)	(27.6)	(24.4)	(32.7)	(25.4)
c_E	695.1	631.0	865.1	695.1	631.0	865.1	695.1	631.0	865.1	695.1
	(25.2)	(22.9)	(31.4)	(25.2)	(22.9)	(31.4)	(25.2)	(22.9)	(31.4)	(25.2)
	166.5	84.6	28.1	107.7	160.7	24.0	135.8	82.4	73.3	9.9
	6.045	3.069	1.019	3.909	5.835	0.872	4.929	2.992	2.661	0.361
N	2755	2755	2755	2755	2755	2755	2755	2755	2755	2755
X2	28.4	56.5	24.4	18.2	57.0	7.5	14.5	41.3	7.0	2.2
Gvalue	28.7	57.1	24.8	18.4	59.4	7.5	14.6	42.8	7.1	2.2
Gv/N	1.042	2.072	0.900	0.669	2.157	0.271	0.529	1.554	0.257	0.081
Prob	0.00000	0.00000	0.00002	0.00036	0.00000	0.05871	0.00221	0.00000	0.06956	0.52434

	41	42	43	44	45	46	47	48	49	50
a_O	636	439	612	644	459	589	614	432	591	641
	(23.1)	(15.9)	(22.2)	(23.4)	(16.7)	(21.4)	(22.3)	(15.7)	(21.5)	(23.3)
a_E	784.9	418.9	628.5	784.9	418.9	628.5	784.9	418.9	628.5	784.9
	(28.5)	(15.2)	(22.8)	(28.5)	(15.2)	(22.8)	(28.5)	(15.2)	(22.8)	(28.5)
	-267.6	41.2	-32.6	-254.9	83.9	-76.5	-301.6	26.6	-72.8	-259.7
	-9.714	1.494	-1.185	-9.251	3.046	-2.778	-10.947	0.966	-2.642	-9.426
t_O	863	649	465	895	655	453	899	605	450	875
	(31.3)	(23.6)	(16.9)	(32.5)	(23.8)	(16.4)	(32.6)	(22.0)	(16.3)	(31.8)
t_E	814.0	630.8	413.4	814.0	630.8	413.4	814.0	630.8	413.4	814.0
	(29.5)	(22.9)	(15.0)	(29.5)	(22.9)	(15.0)	(29.5)	(22.9)	(15.0)	(29.5)
	101.0	37.0	109.4	169.9	49.4	82.9	178.7	-50.4	76.3	126.5
	3.665	1.343	3.970	6.166	1.793	3.007	6.485	-1.831	2.770	4.593
g_O	543	781	970	533	809	989	538	813	967	553
	(19.7)	(28.3)	(35.2)	(19.3)	(29.4)	(35.9)	(19.5)	(29.5)	(35.1)	(20.1)
g_E	525.1	840.3	1018.0	525.1	840.3	1018.0	525.1	840.3	1018.0	525.1
	(19.1)	(30.5)	(37.0)	(19.1)	(30.5)	(37.0)	(19.1)	(30.5)	(37.0)	(19.1)
	36.4	-114.3	-93.7	15.9	-61.4	-57.2	26.1	-53.7	-99.4	57.3
	1.322	-4.148	-3.401	0.579	-2.228	-2.075	0.949	-1.947	-3.608	2.079
c_O	713	886	708	683	832	724	704	905	747	686
	(25.9)	(32.2)	(25.7)	(24.8)	(30.2)	(26.3)	(25.6)	(32.8)	(27.1)	(24.9)
c_E	631.0	865.1	695.1	631.0	865.1	695.1	631.0	865.1	695.1	631.0
	(22.9)	(31.4)	(25.2)	(22.9)	(31.4)	(25.2)	(22.9)	(31.4)	(25.2)	(22.9)
	174.2	42.4	26.1	108.1	-64.9	59.1	154.1	81.7	107.7	114.6
	6.322	1.537	0.949	3.924	-2.355	2.145	5.593	2.964	3.909	4.160
N	2755	2755	2755	2755	2755	2755	2755	2755	2755	2755
X2	42.5	6.2	9.4	37.8	7.2	8.3	54.9	4.2	11.9	37.2
Gvalue	43.9	6.3	9.2	39.1	7.1	8.2	57.3	4.2	11.8	38.7
Gv/N	1.595	0.227	0.333	1.418	0.258	0.299	2.080	0.152	0.429	1.406
Prob	0.00000	0.09973	0.02719	0.00000	0.06872	0.04130	0.00000	0.24248	0.00802	0.00000

	51	52	53	54	55	56	57	58	59	60
a_O	401	588	654	419	603	645	402	583	687	422
	(14.6)	(21.3)	(23.7)	(15.2)	(21.9)	(23.4)	(14.6)	(21.2)	(24.9)	(15.3)
a_E	418.9	628.5	784.9	418.9	628.5	784.9	418.9	628.5	784.9	418.9
	(15.2)	(22.8)	(28.5)	(15.2)	(22.8)	(28.5)	(15.2)	(22.8)	(28.5)	(15.2)
	-35.0	-78.4	-238.7	0.2	-50.0	-253.3	-33.1	-87.7	-183.1	6.2
	-1.271	-2.846	-8.664	0.007	-1.816	-9.193	-1.202	-3.183	-6.646	0.226
t_O	666	430	867	655	459	864	627	406	866	688
	(24.2)	(15.6)	(31.5)	(23.8)	(16.7)	(31.4)	(22.8)	(14.7)	(31.4)	(25.0)
t_E	630.8	413.4	814.0	630.8	413.4	814.0	630.8	413.4	814.0	630.8
	(22.9)	(15.0)	(29.5)	(22.9)	(15.0)	(29.5)	(22.9)	(15.0)	(29.5)	(22.9)
	72.4	33.8	109.5	49.4	96.0	103.1	-7.5	-14.7	107.3	119.5
	2.629	1.228	3.973	1.793	3.486	3.742	-0.272	-0.533	3.896	4.339
g_O	787	1020	533	811	1039	550	825	1063	542	815
	(28.6)	(37.0)	(19.3)	(29.4)	(37.7)	(20.0)	(29.9)	(38.6)	(19.7)	(29.6)
g_E	840.3	1018.0	525.1	840.3	1018.0	525.1	840.3	1018.0	525.1	840.3
	(30.5)	(37.0)	(19.1)	(30.5)	(37.0)	(19.1)	(30.5)	(37.0)	(19.1)	(30.5)
	-103.1	4.0	15.9	-57.5	42.4	51.0	-30.3	92.0	34.4	-49.8
	-3.742	0.146	0.579	-2.088	1.540	1.851	-1.099	3.338	1.247	-1.807
c_O	901	717	701	870	654	696	901	703	660	830
	(32.7)	(26.0)	(25.4)	(31.6)	(23.7)	(25.3)	(32.7)	(25.5)	(24.0)	(30.1)
c_E	865.1	695.1	631.0	865.1	695.1	631.0	865.1	695.1	631.0	865.1
	(31.4)	(25.2)	(22.9)	(31.4)	(25.2)	(22.9)	(31.4)	(25.2)	(22.9)	(31.4)
	73.3	44.6	147.4	9.9	-79.6	136.4	73.3	16.0	59.3	-68.7
	2.661	1.618	5.352	0.359	-2.890	4.952	2.661	0.580	2.151	-2.494
N	2755	2755	2755	2755	2755	2755	2755	2755	2755	2755
X2	7.6	4.0	33.2	2.0	8.9	35.9	2.5	5.5	17.4	7.4
Gvalue	7.6	4.0	34.2	2.0	8.8	37.2	2.5	5.6	17.9	7.3
Gv/N	0.277	0.146	1.240	0.072	0.320	1.351	0.089	0.202	0.648	0.264
Prob	0.05425	0.25934	0.00000	0.57655	0.03195	0.00000	0.48176	0.13467	0.00047	0.06356

	61	62	63	64	65	66	67	68	69	70
a_O	594	636	408	635	675	401	614	724	410	568
	(21.6)	(23.1)	(14.8)	(23.0)	(24.5)	(14.6)	(22.3)	(26.3)	(14.9)	(20.6)
a_E	628.5	784.9	418.9	628.5	784.9	418.9	628.5	784.9	418.9	628.5
	(22.8)	(28.5)	(15.2)	(22.8)	(28.5)	(15.2)	(22.8)	(28.5)	(15.2)	(22.8)
	-67.1	-267.6	-21.5	13.0	-203.7	-35.0	-28.7	-117.0	-17.6	-115.1
	-2.437	-9.714	-0.781	0.471	-7.393	-1.271	-1.043	-4.246	-0.639	-4.176
t_O	467	881	668	420	848	665	439	837	679	438
	(17.0)	(32.0)	(24.2)	(15.2)	(30.8)	(24.1)	(15.9)	(30.4)	(24.6)	(15.9)
t_E	413.4	814.0	630.8	413.4	814.0	630.8	413.4	814.0	630.8	413.4
	(15.0)	(29.5)	(22.9)	(15.0)	(29.5)	(22.9)	(15.0)	(29.5)	(22.9)	(15.0)
	113.8	139.4	76.6	13.3	69.5	70.3	52.7	46.7	100.1	50.6
	4.132	5.061	2.782	0.482	2.522	2.552	1.914	1.696	3.633	1.837
g_O	1000	524	812	978	525	850	1008	504	852	999
	(36.3)	(19.0)	(29.5)	(35.5)	(19.1)	(30.9)	(36.6)	(18.3)	(30.9)	(36.3)
g_E	1018.0	525.1	840.3	1018.0	525.1	840.3	1018.0	525.1	840.3	1018.0
	(37.0)	(19.1)	(30.5)	(37.0)	(19.1)	(30.5)	(37.0)	(19.1)	(30.5)	(37.0)
	-35.7	-2.2	-55.6	-78.4	-0.2	19.6	-19.9	-41.3	23.6	-37.6
	-1.295	-0.079	-2.018	-2.846	-0.006	0.710	-0.722	-1.500	0.857	-1.366
c_O	694	714	867	722	707	839	694	690	814	750
	(25.2)	(25.9)	(31.5)	(26.2)	(25.7)	(30.5)	(25.2)	(25.0)	(29.5)	(27.2)
c_E	695.1	631.0	865.1	695.1	631.0	865.1	695.1	631.0	865.1	695.1
	(25.2)	(22.9)	(31.4)	(25.2)	(22.9)	(31.4)	(25.2)	(22.9)	(31.4)	(25.2)
	-2.1	176.4	3.9	54.9	160.7	-51.4	-2.1	123.3	-99.1	114.1
	-0.076	6.403	0.140	1.994	5.835	-1.864	-0.076	4.475	-3.596	4.143
N	2755	2755	2755	2755	2755	2755	2755	2755	2755	2755
X2	9.2	44.7	3.4	2.8	26.0	3.5	2.0	11.7	7.1	12.0
Gvalue	8.9	46.1	3.4	2.8	26.4	3.5	2.0	11.7	7.0	12.1
Gv/N	0.324	1.672	0.124	0.101	0.957	0.127	0.072	0.425	0.255	0.437
Prob	0.03038	0.00000	0.33267	0.42436	0.00001	0.31969	0.57425	0.00846	0.07097	0.00720

	71	72	73	74	75	76	77	78	79	80
a_O	677	413	581	725	425	585	723	395	597	713
	(24.6)	(15.0)	(21.1)	(26.3)	(15.4)	(21.2)	(26.2)	(14.3)	(21.7)	(25.9)
a_E	784.9	418.9	628.5	784.9	418.9	628.5	784.9	418.9	628.5	784.9
	(28.5)	(15.2)	(22.8)	(28.5)	(15.2)	(22.8)	(28.5)	(15.2)	(22.8)	(28.5)
	-200.3	-11.7	-91.4	-115.1	12.3	-84.0	-118.8	-46.4	-61.5	-137.0
	-7.269	-0.425	-3.317	-4.180	0.446	-3.049	-4.313	-1.684	-2.231	-4.974
t_O	877	631	444	816	665	410	822	647	399	817
	(31.8)	(22.9)	(16.1)	(29.6)	(24.1)	(14.9)	(29.8)	(23.5)	(14.5)	(29.7)
t_E	814.0	630.8	413.4	814.0	630.8	413.4	814.0	630.8	413.4	814.0
	(29.5)	(22.9)	(15.0)	(29.5)	(22.9)	(15.0)	(29.5)	(22.9)	(15.0)	(29.5)
	130.8	0.5	63.4	4.1	70.3	-6.8	16.1	32.9	-28.3	6.1
	4.749	0.018	2.301	0.148	2.552	-0.247	0.586	1.194	-1.028	0.221
g_O	542	856	1025	548	828	1053	503	858	1037	575
	(19.7)	(31.1)	(37.2)	(19.9)	(30.1)	(38.2)	(18.3)	(31.1)	(37.6)	(20.9)
g_E	525.1	840.3	1018.0	525.1	840.3	1018.0	525.1	840.3	1018.0	525.1
	(19.1)	(30.5)	(37.0)	(19.1)	(30.5)	(37.0)	(19.1)	(30.5)	(37.0)	(19.1)
	34.4	31.7	14.1	46.8	-24.4	71.2	-43.2	35.8	38.4	104.4
	1.247	1.152	0.510	1.699	-0.884	2.584	-1.569	1.300	1.392	3.790
c_O	659	855	705	666	837	707	707	855	722	650
	(23.9)	(31.0)	(25.6)	(24.2)	(30.4)	(25.7)	(25.7)	(31.0)	(26.2)	(23.6)
c_E	631.0	865.1	695.1	631.0	865.1	695.1	631.0	865.1	695.1	631.0
	(22.9)	(31.4)	(25.2)	(22.9)	(31.4)	(25.2)	(22.9)	(31.4)	(25.2)	(22.9)
	57.2	-20.0	20.0	71.9	-55.2	24.1	160.7	-20.0	54.9	38.5
	2.075	-0.727	0.727	2.608	-2.005	0.875	5.835	-0.727	1.994	1.398
N	2755	2755	2755	2755	2755	2755	2755	2755	2755	2755
X2	21.5	0.5	6.0	7.5	3.0	4.5	15.0	2.3	3.5	11.9
Gvalue	22.1	0.5	6.1	7.6	3.0	4.5	14.8	2.3	3.5	12.0
Gv/N	0.802	0.018	0.221	0.275	0.109	0.164	0.539	0.083	0.127	0.435
Prob	0.00006	0.92025	0.10735	0.05530	0.38914	0.21133	0.00196	0.51368	0.32044	0.00746

	81	82	83	84	85	86	87	88	89	90
a_O	397	592	715	421	579	757	421	607	776	415
	(14.4)	(21.5)	(26.0)	(15.3)	(21.0)	(27.5)	(15.3)	(22.0)	(28.2)	(15.1)
a_E	418.9	628.5	784.9	418.9	628.5	784.9	418.9	628.5	784.9	418.9
	(15.2)	(22.8)	(28.5)	(15.2)	(22.8)	(28.5)	(15.2)	(22.8)	(28.5)	(15.2)
	-42.6	-70.9	-133.4	4.2	-95.1	-54.8	4.2	-42.3	-17.7	-7.8
	-1.547	-2.574	-4.843	0.153	-3.451	-1.990	0.153	-1.537	-0.644	-0.282
t_O	617	416	818	659	462	858	626	442	831	641
	(22.4)	(15.1)	(29.7)	(23.9)	(16.8)	(31.1)	(22.7)	(16.0)	(30.2)	(23.3)
t_E	630.8	413.4	814.0	630.8	413.4	814.0	630.8	413.4	814.0	630.8
	(22.9)	(15.0)	(29.5)	(22.9)	(15.0)	(29.5)	(22.9)	(15.0)	(29.5)	(22.9)
	-27.2	5.2	8.1	57.7	102.7	90.4	-9.5	59.1	34.4	20.7

	-0.988	0.188	0.294	2.096	3.727	3.282	-0.344	2.146	1.249	0.750
g_O	904	1026	508	841	1029	506	825	978	521	860
	(32.8)	(37.2)	(18.4)	(30.5)	(37.4)	(18.4)	(29.9)	(35.5)	(18.9)	(31.2)
g_E	840.3	1018.0	525.1	840.3	1018.0	525.1	840.3	1018.0	525.1	840.3
	(30.5)	(37.0)	(19.1)	(30.5)	(37.0)	(19.1)	(30.5)	(37.0)	(19.1)	(30.5)
	132.2	16.1	-33.6	1.5	22.1	-37.5	-30.3	-78.4	-8.1	39.9
	4.797	0.583	-1.220	0.053	0.803	-1.360	-1.099	-2.846	-0.296	1.449
c_O	837	721	714	834	685	634	883	728	627	839
	(30.4)	(26.2)	(25.9)	(30.3)	(24.9)	(23.0)	(32.1)	(26.4)	(22.8)	(30.5)
c_E	865.1	695.1	631.0	865.1	695.1	631.0	865.1	695.1	631.0	865.1
	(31.4)	(25.2)	(22.9)	(31.4)	(25.2)	(22.9)	(31.4)	(25.2)	(22.9)	(31.4)
	-55.2	52.9	176.4	-61.0	-20.0	6.0	36.2	67.4	-8.0	-51.4
	-2.005	1.919	6.403	-2.215	-0.724	0.216	1.315	2.448	-0.291	-1.864
N	2755	2755	2755	2755	2755	2755	2755	2755	2755	2755
X2	7.2	3.2	17.7	2.4	9.9	4.1	0.7	5.8	0.5	1.5
Gvalue	7.1	3.2	17.5	2.4	9.8	4.1	0.7	5.8	0.5	1.5
Gv/N	0.258	0.116	0.634	0.087	0.355	0.147	0.025	0.211	0.019	0.053
Prob	0.06867	0.36143	0.00057	0.49600	0.02058	0.25485	0.87445	0.12123	0.91592	0.69262

	91	92	93	94	95	96	97	98	99	100
a_O	591	761	432	591	717	411	612	739	410	594
	(21.5)	(27.6)	(15.7)	(21.5)	(26.0)	(14.9)	(22.2)	(26.8)	(14.9)	(21.6)
a_E	628.5	784.9	418.9	628.5	784.9	418.9	628.5	784.9	418.9	628.5
	(22.8)	(28.5)	(15.2)	(22.8)	(28.5)	(15.2)	(22.8)	(28.5)	(15.2)	(22.8)
	-72.8	-47.1	26.6	-72.8	-129.8	-15.6	-32.6	-89.1	-17.6	-67.1
	-2.642	-1.710	0.966	-2.642	-4.711	-0.568	-1.185	-3.234	-0.639	-2.437
t_O	438	790	679	421	877	667	403	825	644	419
	(15.9)	(28.7)	(24.6)	(15.3)	(31.8)	(24.2)	(14.6)	(29.9)	(23.4)	(15.2)
t_E	413.4	814.0	630.8	413.4	814.0	630.8	413.4	814.0	630.8	413.4
	(15.0)	(29.5)	(22.9)	(15.0)	(29.5)	(22.9)	(15.0)	(29.5)	(22.9)	(15.0)
	50.6	-47.2	100.1	15.3	130.8	74.5	-20.6	22.2	26.8	11.3
	1.837	-1.714	3.633	0.556	4.749	2.705	-0.746	0.806	0.972	0.408
g_O	1021	540	813	1057	511	845	1004	555	835	1038
	(37.1)	(19.6)	(29.5)	(38.4)	(18.5)	(30.7)	(36.4)	(20.1)	(30.3)	(37.7)
g_E	1018.0	525.1	840.3	1018.0	525.1	840.3	1018.0	525.1	840.3	1018.0
	(37.0)	(19.1)	(30.5)	(37.0)	(19.1)	(30.5)	(37.0)	(19.1)	(30.5)	(37.0)
	6.0	30.2	-53.7	79.5	-27.8	9.5	-27.8	61.5	-10.5	40.4
	0.218	1.098	-1.947	2.885	-1.009	0.344	-1.009	2.232	-0.382	1.466
c_O	705	664	831	686	650	832	736	636	866	704
	(25.6)	(24.1)	(30.2)	(24.9)	(23.6)	(30.2)	(26.7)	(23.1)	(31.4)	(25.6)
c_E	695.1	631.0	865.1	695.1	631.0	865.1	695.1	631.0	865.1	695.1
	(25.2)	(22.9)	(31.4)	(25.2)	(22.9)	(31.4)	(25.2)	(22.9)	(31.4)	(25.2)
	20.0	67.6	-66.8	-18.0	38.5	-64.9	84.3	10.0	1.9	18.0
	0.727	2.455	-2.424	-0.653	1.398	-2.355	3.059	0.362	0.067	0.654
N	2755	2755	2755	2755	2755	2755	2755	2755	2755	2755
X2	3.9	3.6	6.3	4.0	11.7	3.5	3.3	4.6	0.5	2.5
Gvalue	3.9	3.6	6.3	4.0	11.8	3.5	3.3	4.6	0.5	2.5
Gv/N	0.141	0.129	0.227	0.146	0.427	0.127	0.118	0.167	0.018	0.091
Prob	0.27551	0.31273	0.09961	0.25925	0.00825	0.32060	0.35275	0.20343	0.91874	0.47271

	101	102	103	104	105	106	107	108	109	110
a_O	762	395	599	736	403	602	777	412	617	731
	(27.7)	(14.3)	(21.8)	(26.7)	(14.6)	(21.9)	(28.2)	(15.0)	(22.4)	(26.6)
a_E	784.9	418.9	628.3	784.6	418.7	628.3	784.6	418.7	628.1	784.4
	(28.5)	(15.2)	(22.8)	(28.5)	(15.2)	(22.8)	(28.5)	(15.2)	(22.8)	(28.5)
	-45.2	-46.4	-57.2	-94.2	-30.9	-51.5	-15.2	-13.4	-22.0	-103.0
	-1.639	-1.684	-2.078	-3.420	-1.122	-1.870	-0.552	-0.486	-0.798	-3.741
t_O	809	677	431	847	654	454	806	684	450	847
	(29.4)	(24.6)	(15.6)	(30.8)	(23.7)	(16.5)	(29.3)	(24.8)	(16.3)	(30.8)
t_E	814.0	630.8	413.3	813.7	630.5	413.3	813.7	630.5	413.1	813.4
	(29.5)	(22.9)	(15.0)	(29.5)	(22.9)	(15.0)	(29.5)	(22.9)	(15.0)	(29.5)
	-9.9	95.8	36.2	68.0	47.8	85.4	-15.3	111.4	77.0	68.6
	-0.359	3.477	1.315	2.469	1.736	3.100	-0.554	4.044	2.796	2.493
g_O	561	849	1043	519	820	1011	528	843	985	509
	(20.4)	(30.8)	(37.9)	(18.8)	(29.8)	(36.7)	(19.2)	(30.6)	(35.8)	(18.5)
g_E	525.1	840.3	1017.6	524.9	840.0	1017.6	524.9	840.0	1017.3	524.7
	(19.1)	(30.5)	(37.0)	(19.1)	(30.5)	(37.0)	(19.1)	(30.5)	(37.0)	(19.1)
	74.2	17.5	51.4	-11.7	-39.5	-13.2	6.2	6.1	-63.5	-30.9
	2.694	0.637	1.865	-0.426	-1.433	-0.480	0.226	0.221	-2.306	-1.124
c_O	623	834	681	652	877	687	643	815	701	666
	(22.6)	(30.3)	(24.7)	(23.7)	(31.8)	(24.9)	(23.3)	(29.6)	(25.5)	(24.2)
c_E	631.0	865.1	694.8	630.8	864.8	694.8	630.8	864.8	694.5	630.6
	(22.9)	(31.4)	(25.2)	(22.9)	(31.4)	(25.2)	(22.9)	(31.4)	(25.2)	(22.9)
	-16.0	-61.0	-27.3	43.1	24.7	-15.5	24.6	-96.6	13.0	72.8
	-0.579	-2.215	-0.992	1.565	0.895	-0.563	0.895	-3.508	0.471	2.645
N	2755	2755	2754	2754	2754	2754	2754	2754	2753	2753
X2	3.3	6.0	3.0	5.2	2.1	5.2	0.4	7.5	4.6	7.5
Gvalue	3.2	5.9	3.0	5.2	2.1	5.1	0.4	7.5	4.5	7.5
Gv/N	0.117	0.215	0.111	0.189	0.077	0.187	0.015	0.271	0.163	0.273
Prob	0.36022	0.11553	0.38501	0.15790	0.54913	0.16192	0.94025	0.05880	0.21314	0.05717

	111	112	113	114	115	116	117	118	119	120
a_O	397	579	783	383	610	809	414	617	780	425
	(14.4)	(21.0)	(28.4)	(13.9)	(22.2)	(29.4)	(15.0)	(22.4)	(28.3)	(15.4)
a_E	418.6	628.1	784.4	418.6	628.1	784.4	418.6	627.9	784.1	418.4
	(15.2)	(22.8)	(28.5)	(15.2)	(22.8)	(28.5)	(15.2)	(22.8)	(28.5)	(15.2)
	-42.1	-94.2	-2.7	-68.1	-35.6	50.1	-9.1	-21.5	-8.1	13.2
	-1.528	-3.423	-0.098	-2.473	-1.295	1.818	-0.332	-0.782	-0.295	0.480
t_O	668	477	815	669	434	809	664	391	790	634
	(24.3)	(17.3)	(29.6)	(24.3)	(15.8)	(29.4)	(24.1)	(14.2)	(28.7)	(23.0)
t_E	630.3	413.1	813.4	630.3	413.1	813.4	630.3	413.0	813.1	630.1
	(22.9)	(15.0)	(29.5)	(22.9)	(15.0)	(29.5)	(22.9)	(15.0)	(29.5)	(22.9)
	77.6	137.2	3.3	79.7	42.8	-8.7	69.2	-42.7	-45.5	7.9
	2.819	4.983	0.118	2.896	1.555	-0.317	2.513	-1.553	-1.653	0.287
g_O	818	973	512	843	1062	503	877	1017	518	824
	(29.7)	(35.3)	(18.6)	(30.6)	(38.6)	(18.3)	(31.9)	(37.0)	(18.8)	(29.9)
g_E	839.7	1017.3	524.7	839.7	1017.3	524.7	839.7	1016.9	524.5	839.4
	(30.5)	(37.0)	(19.1)	(30.5)	(37.0)	(19.1)	(30.5)	(37.0)	(19.1)	(30.5)
	-42.8	-86.6	-25.1	6.7	91.4	-42.5	76.3	0.2	-12.9	-30.4
	-1.553	-3.144	-0.912	0.243	3.321	-1.544	2.772	0.008	-0.470	-1.106
c_O	870	724	643	858	647	632	798	727	664	869
	(31.6)	(26.3)	(23.4)	(31.2)	(23.5)	(23.0)	(29.0)	(26.4)	(24.1)	(31.6)
c_E	864.4	694.5	630.6	864.4	694.5	630.6	864.4	694.3	630.3	864.1
	(31.4)	(25.2)	(22.9)	(31.4)	(25.2)	(22.9)	(31.4)	(25.2)	(22.9)	(31.4)
	11.1	60.1	25.1	-12.8	-91.8	2.9	-127.6	66.9	69.1	9.8
	0.405	2.185	0.912	-0.467	-3.333	0.104	-4.637	2.432	2.510	0.355

N	2753	2753	2753	2753	2753	2753	2753	2752	2752	2752
X2	4.0	16.9	0.6	5.5	6.8	1.7	8.6	2.9	2.6	0.4
Gvalue	3.9	16.5	0.6	5.5	6.8	1.7	8.7	2.9	2.5	0.4
Gv/N	0.143	0.601	0.020	0.200	0.248	0.062	0.316	0.105	0.092	0.016
Prob	0.26752	0.00088	0.90570	0.13823	0.07732	0.63607	0.03354	0.40808	0.46987	0.93252

	121	122	123	124	125	126	127	128	129	130
a_O	578	793	427	606	843	436	609	762	402	603
	(21.0)	(28.8)	(15.5)	(22.0)	(30.6)	(15.8)	(22.2)	(27.7)	(14.6)	(21.9)
a_E	627.6	783.8	418.3	627.6	783.8	418.3	627.2	783.2	418.0	627.2
	(22.8)	(28.5)	(15.2)	(22.8)	(28.5)	(15.2)	(22.8)	(28.5)	(15.2)	(22.8)
	−95.2	18.5	17.6	−42.5	122.8	36.2	−35.8	−41.8	−31.4	−47.4
	−3.461	0.674	0.640	−1.545	4.464	1.314	−1.303	−1.522	−1.140	−1.724
t_O	410	735	626	413	781	663	395	782	654	402
	(14.9)	(26.7)	(22.8)	(15.0)	(28.4)	(24.1)	(14.4)	(28.4)	(23.8)	(14.6)
t_E	412.8	812.8	629.8	412.8	812.8	629.8	412.5	812.2	629.4	412.5
	(15.0)	(29.5)	(22.9)	(15.0)	(29.5)	(22.9)	(15.0)	(29.5)	(22.9)	(15.0)
	−5.6	−147.9	−7.7	0.4	−62.3	68.0	−34.3	−59.2	50.2	−20.8
	−0.204	−5.375	−0.278	0.014	−2.265	2.473	−1.247	−2.155	1.826	−0.755
g_O	1088	541	840	1032	507	827	1025	524	835	1052
	(39.5)	(19.7)	(30.5)	(37.5)	(18.4)	(30.1)	(37.3)	(19.1)	(30.4)	(38.3)
g_E	1016.5	524.3	839.1	1016.5	524.3	839.1	1015.8	523.9	838.4	1015.8
	(37.0)	(19.1)	(30.5)	(37.0)	(19.1)	(30.5)	(37.0)	(19.1)	(30.5)	(37.0)
	147.9	33.9	1.9	31.2	−34.1	−23.9	18.5	0.1	−6.9	73.7
	5.375	1.231	0.069	1.134	−1.238	−0.870	0.674	0.004	−0.250	2.682
c_O	675	682	858	700	620	825	720	681	858	692
	(24.5)	(24.8)	(31.2)	(25.4)	(22.5)	(30.0)	(26.2)	(24.8)	(31.2)	(25.2)
c_E	694.0	630.1	863.8	694.0	630.1	863.8	693.5	629.7	863.2	693.5
	(25.2)	(22.9)	(31.4)	(25.2)	(22.9)	(31.4)	(25.2)	(22.9)	(31.4)	(25.2)
	−37.6	107.9	−11.6	12.0	−20.1	−75.9	53.9	106.8	−10.3	−3.1
	−1.365	3.924	−0.422	0.435	−0.729	−2.758	1.962	3.884	−0.376	−0.112
N	2751	2751	2751	2751	2751	2751	2749	2749	2749	2749
X2	9.5	12.4	0.2	1.0	6.5	4.4	2.4	5.9	1.6	2.5
Gvalue	9.5	12.5	0.2	1.0	6.4	4.4	2.4	5.8	1.6	2.5
Gv/N	0.345	0.454	0.009	0.038	0.231	0.160	0.086	0.211	0.059	0.091
Prob	0.02343	0.00589	0.97020	0.79165	0.09506	0.22132	0.49992	0.12206	0.65592	0.47642

	131	132	133	134	135	136	137	138	139	140
a_O	808	401	632	781	374	626	784	427	567	810
	(29.4)	(14.6)	(23.0)	(28.4)	(13.6)	(22.8)	(28.5)	(15.5)	(20.6)	(29.5)
a_E	783.2	418.0	627.2	783.2	418.0	626.7	782.6	417.7	626.5	782.4
	(28.5)	(15.2)	(22.8)	(28.5)	(15.2)	(22.8)	(28.5)	(15.2)	(22.8)	(28.5)
	50.4	−33.3	9.7	−4.4	−83.2	−1.4	2.7	18.8	−113.1	56.3
	1.832	−1.210	0.353	−0.161	−3.025	−0.052	0.099	0.686	−4.120	2.048
t_O	765	626	419	780	700	429	784	666	421	792
	(27.8)	(22.8)	(15.2)	(28.4)	(25.5)	(15.6)	(28.5)	(24.2)	(15.3)	(28.8)
t_E	812.2	629.4	412.5	812.2	629.4	412.2	811.6	628.9	412.1	811.3
	(29.5)	(22.9)	(15.0)	(29.5)	(22.9)	(15.0)	(29.5)	(22.9)	(15.0)	(29.5)
	−91.6	−6.7	13.1	−63.1	148.9	34.3	−54.3	76.3	18.1	−38.1
	−3.332	−0.245	0.476	−2.295	5.416	1.247	−1.975	2.777	0.658	−1.389
g_O	501	863	999	559	829	1006	523	828	1031	510
	(18.2)	(31.4)	(36.3)	(20.3)	(30.2)	(36.6)	(19.0)	(30.1)	(37.5)	(18.6)
g_E	523.9	838.4	1015.8	523.9	838.4	1015.0	523.6	837.8	1014.7	523.4
	(19.1)	(30.5)	(37.0)	(19.1)	(30.5)	(37.0)	(19.1)	(30.5)	(37.0)	(19.1)
	−44.9	49.8	−33.3	72.4	−18.8	−18.0	−1.1	−19.6	32.9	−26.4
	−1.632	1.812	−1.211	2.634	−0.683	−0.655	−0.041	−0.712	1.199	−0.961
c_O	675	859	699	629	846	686	656	826	727	634
	(24.6)	(31.2)	(25.4)	(22.9)	(30.8)	(25.0)	(23.9)	(30.1)	(26.5)	(23.1)
c_E	629.7	863.2	693.5	629.7	863.2	693.0	629.2	862.6	692.8	629.0
	(22.9)	(31.4)	(25.2)	(22.9)	(31.4)	(25.2)	(22.9)	(31.4)	(25.2)	(22.9)
	93.9	−8.4	11.0	−1.3	−34.0	−14.0	54.7	−71.6	70.1	10.1
	3.415	−0.304	0.399	−0.047	−1.238	−0.509	1.993	−2.605	2.553	0.368
N	2749	2749	2749	2749	2749	2747	2747	2747	2746	2746
X2	7.8	1.4	0.5	3.6	13.0	0.8	2.1	4.1	7.8	1.8
Gvalue	7.8	1.5	0.5	3.6	12.9	0.8	2.1	4.0	8.0	1.8
Gv/N	0.283	0.053	0.017	0.131	0.469	0.030	0.076	0.147	0.290	0.066
Prob	0.05071	0.69372	0.92753	0.30862	0.00487	0.84280	0.55624	0.25734	0.04699	0.61206

	141	142	143	144	145	146	147	148	149	150
a_O	429	591	757	398	584	753	416	612	768	413
	(15.6)	(21.5)	(27.6)	(14.5)	(21.3)	(27.5)	(15.2)	(22.3)	(28.0)	(15.1)
a_E	417.5	625.8	781.5	417.1	625.8	781.5	417.1	625.8	781.5	417.1
	(15.2)	(22.8)	(28.5)	(15.2)	(22.8)	(28.5)	(15.2)	(22.8)	(28.5)	(15.2)
	23.3	−67.6	−48.2	−37.3	−80.7	−56.0	−2.1	−27.3	−26.8	−8.1
	0.847	−2.466	−1.758	−1.358	−2.944	−2.040	−0.078	−0.995	−0.976	−0.295
t_O	614	434	844	637	428	845	645	357	801	634
	(22.4)	(15.8)	(30.8)	(23.2)	(15.6)	(30.8)	(23.5)	(13.0)	(29.2)	(23.1)
t_E	628.7	411.6	810.4	628.0	411.6	810.4	628.0	411.6	810.4	628.0
	(22.9)	(15.0)	(29.5)	(22.9)	(15.0)	(29.5)	(22.9)	(15.0)	(29.5)	(22.9)
	−29.0	46.0	68.5	18.1	33.4	70.6	34.4	−101.6	−18.7	12.0
	−1.058	1.676	2.498	0.660	1.218	2.574	1.256	−3.705	−0.683	0.439
g_O	880	987	501	870	996	531	857	1049	536	845
	(32.0)	(36.0)	(18.3)	(31.7)	(36.3)	(19.4)	(31.2)	(38.2)	(19.5)	(30.8)
g_E	837.5	1013.6	522.8	836.6	1013.6	522.8	836.6	1013.6	522.8	836.6
	(30.5)	(37.0)	(19.1)	(30.5)	(37.0)	(19.1)	(30.5)	(37.0)	(19.1)	(30.5)
	87.1	−52.4	−42.7	68.1	−34.8	16.5	41.3	72.1	26.7	16.9
	3.170	−1.911	−1.556	2.482	−1.269	0.603	1.504	2.628	0.975	0.615
c_O	823	731	641	838	735	614	825	725	638	851
	(30.0)	(26.6)	(23.4)	(30.6)	(26.8)	(22.4)	(30.1)	(26.4)	(23.3)	(31.0)
c_E	862.2	692.0	628.3	861.3	692.0	628.3	861.3	692.0	628.3	861.3
	(31.4)	(25.2)	(22.9)	(31.4)	(25.2)	(22.9)	(31.4)	(25.2)	(22.9)	(31.4)
	−76.7	80.1	25.7	−46.0	88.6	−28.2	−71.1	67.5	19.6	−20.5
	−2.792	2.921	0.937	−1.676	3.229	−1.029	−2.591	2.461	0.714	−0.747
N	2746	2743	2743	2743	2743	2743	2743	2743	2743	2743
X2	4.6	6.0	3.3	3.0	6.4	3.0	2.5	10.4	0.8	0.3
Gvalue	4.6	6.0	3.3	3.0	6.4	3.0	2.5	10.7	0.8	0.3
Gv/N	0.167	0.220	0.121	0.108	0.234	0.108	0.091	0.389	0.030	0.011
Prob	0.20432	0.11034	0.34394	0.39701	0.09278	0.39747	0.47465	0.01366	0.84353	0.95918

Acin_ADP1
5UTR:

	50	49	48	47	46	45	44	43	42	41
a_O	1025	1060	1028	1069	1086	1018	1032	1031	1033	1073
	(32.1)	(33.2)	(32.2)	(33.5)	(34.0)	(31.9)	(32.3)	(32.3)	(32.4)	(33.6)
a_E	1071.0	1071.0	1071.0	1071.0	1071.0	1071.0	1071.0	1071.0	1071.0	1071.0
	(33.5)	(33.5)	(33.5)	(33.5)	(33.5)	(33.5)	(33.5)	(33.5)	(33.5)	(33.5)
	-90.0	-21.9	-84.3	-4.0	30.2	-103.4	-76.6	-78.5	-74.7	4.0
	-2.820	-0.687	-2.640	-0.126	0.945	-3.237	-2.399	-2.459	-2.339	0.124
t_O	1073	1067	1026	1050	1049	1088	990	1065	1076	1038
	(33.6)	(33.4)	(32.1)	(32.9)	(32.9)	(34.1)	(31.0)	(33.4)	(33.7)	(32.5)
t_E	1029.7	1029.7	1029.7	1029.7	1029.7	1029.7	1029.7	1029.7	1029.7	1029.7
	(32.2)	(32.2)	(32.2)	(32.2)	(32.2)	(32.2)	(32.2)	(32.2)	(32.2)	(32.2)
	88.5	76.0	-7.3	41.1	39.0	119.9	-77.8	71.9	94.7	16.7
	2.770	2.380	-0.229	1.286	1.222	3.755	-2.436	2.251	2.966	0.524
g_O	544	527	585	555	541	547	604	547	524	544
	(17.0)	(16.5)	(18.3)	(17.4)	(16.9)	(17.1)	(18.9)	(17.1)	(16.4)	(17.0)
g_E	571.0	571.0	571.0	571.0	571.0	571.0	571.0	571.0	571.0	571.0
	(17.9)	(17.9)	(17.9)	(17.9)	(17.9)	(17.9)	(17.9)	(17.9)	(17.9)	(17.9)
	-52.7	-84.6	28.3	-31.6	-58.4	-47.0	67.8	-47.0	-90.1	-52.7
	-1.652	-2.648	0.886	-0.989	-1.830	-1.472	2.124	-1.472	-2.820	-1.652
c_O	551	539	554	519	517	540	567	550	560	538
	(17.3)	(16.9)	(17.4)	(16.3)	(16.2)	(16.9)	(17.8)	(17.2)	(17.5)	(16.8)
c_E	521.3	521.3	521.3	521.3	521.3	521.3	521.3	521.3	521.3	521.3
	(16.3)	(16.3)	(16.3)	(16.3)	(16.3)	(16.3)	(16.3)	(16.3)	(16.3)	(16.3)
	61.1	36.0	67.4	-4.6	-8.5	38.1	95.3	59.0	80.2	34.0
	1.913	1.128	2.112	-0.143	-0.268	1.193	2.985	1.847	2.513	1.063
N	3193	3193	3193	3193	3193	3193	3193	3193	3193	3193
X2	6.8	5.5	4.1	0.9	2.2	7.6	8.9	5.3	10.2	1.9
Gvalue	6.8	5.5	4.1	0.9	2.2	7.6	8.8	5.3	10.2	1.9
Gv/N	0.212	0.173	0.129	0.027	0.069	0.238	0.274	0.166	0.320	0.060
Prob	0.07975	0.13684	0.24930	0.83353	0.52974	0.05501	0.03277	0.15170	0.01687	0.59340

	40	39	38	37	36	35	34	33	32	31
a_O	1049	1049	1011	1054	980	1105	1129	1045	1066	1107
	(32.9)	(32.9)	(31.7)	(33.0)	(30.7)	(34.6)	(35.4)	(32.7)	(33.4)	(34.7)
a_E	1071.0	1071.0	1071.0	1071.0	1071.0	1071.0	1071.0	1071.0	1071.0	1071.0
	(33.5)	(33.5)	(33.5)	(33.5)	(33.5)	(33.5)	(33.5)	(33.5)	(33.5)	(33.5)
	-43.6	-43.6	-116.6	-33.8	-174.1	69.0	119.0	-51.4	-10.0	73.2
	-1.365	-1.365	-3.652	-1.058	-5.452	2.162	3.728	-1.610	-0.314	2.291
t_O	1087	1074	1015	1110	1135	1007	1062	1098	998	1043
	(34.0)	(33.6)	(31.8)	(34.8)	(35.5)	(31.5)	(33.3)	(34.4)	(31.3)	(32.7)
t_E	1029.7	1029.7	1029.7	1029.7	1029.7	1029.7	1029.7	1029.7	1029.7	1029.7
	(32.2)	(32.2)	(32.2)	(32.2)	(32.2)	(32.2)	(32.2)	(32.2)	(32.2)	(32.2)
	117.8	90.5	-29.1	166.8	221.1	-44.8	65.7	141.1	-62.4	26.8
	3.689	2.836	-0.912	5.223	6.924	-1.404	2.057	4.419	-1.953	0.840
g_O	490	538	607	516	540	541	464	524	548	487
	(15.3)	(16.8)	(19.0)	(16.2)	(16.9)	(16.9)	(14.5)	(16.4)	(17.2)	(15.3)
g_E	571.0	571.0	571.0	571.0	571.0	571.0	571.0	571.0	571.0	571.0
	(17.9)	(17.9)	(17.9)	(17.9)	(17.9)	(17.9)	(17.9)	(17.9)	(17.9)	(17.9)
	-150.0	-64.1	74.2	-104.6	-60.3	-58.4	-192.6	-90.1	-45.1	-155.0
	-4.696	-2.007	2.323	-3.275	-1.889	-1.830	-6.032	-2.820	-1.412	-4.855
c_O	567	532	560	513	538	540	538	526	581	556
	(17.8)	(16.7)	(17.5)	(16.1)	(16.8)	(16.9)	(16.8)	(16.5)	(18.2)	(17.4)
c_E	521.3	521.3	521.3	521.3	521.3	521.3	521.3	521.3	521.3	521.3
	(16.3)	(16.3)	(16.3)	(16.3)	(16.3)	(16.3)	(16.3)	(16.3)	(16.3)	(16.3)
	95.3	21.6	80.2	-16.4	34.0	38.1	34.0	9.5	126.0	71.7
	2.985	0.678	2.513	-0.515	1.063	1.193	1.063	0.296	3.947	2.245
N	3193	3193	3193	3193	3193	3193	3193	3193	3193	3193
X2	19.1	4.5	8.7	12.0	20.7	3.8	24.7	9.1	8.8	16.1
Gvalue	19.6	4.5	8.7	12.0	20.6	3.8	26.1	9.1	8.5	16.6
Gv/N	0.613	0.141	0.271	0.376	0.646	0.120	0.816	0.285	0.267	0.521
Prob	0.00021	0.21198	0.03409	0.00740	0.00013	0.27927	0.00001	0.02799	0.03606	0.00084

	30	29	28	27	26	25	24	23	22	21
a_O	1015	1092	1127	1072	1136	1133	1054	1094	1157	1084
	(31.8)	(34.2)	(35.3)	(33.6)	(35.6)	(35.5)	(33.0)	(34.3)	(36.2)	(33.9)
a_E	1071.0	1071.0	1071.0	1071.0	1071.0	1071.0	1071.0	1071.0	1071.0	1071.0
	(33.5)	(33.5)	(33.5)	(33.5)	(33.5)	(33.5)	(33.5)	(33.5)	(33.5)	(33.5)
	-109.1	42.4	114.8	2.0	133.8	127.5	-33.8	46.4	178.7	26.1
	-3.416	1.327	3.597	0.061	4.191	3.992	-1.058	1.455	5.596	0.818
t_O	1130	1020	1035	1046	980	1068	1116	1013	1099	1126
	(35.4)	(31.9)	(32.4)	(32.8)	(30.7)	(33.4)	(35.0)	(31.7)	(34.4)	(35.3)
t_E	1029.7	1029.7	1029.7	1029.7	1029.7	1029.7	1029.7	1029.7	1029.7	1029.7
	(32.2)	(32.2)	(32.2)	(32.2)	(32.2)	(32.2)	(32.2)	(32.2)	(32.2)	(32.2)
	210.1	-19.2	10.7	32.9	-96.9	78.1	179.7	-33.1	143.2	201.4
	6.581	-0.603	0.335	1.031	-3.035	2.445	5.628	-1.036	4.486	6.308
g_O	494	533	457	509	540	439	486	508	415	480
	(15.5)	(16.7)	(14.3)	(15.9)	(16.9)	(13.7)	(15.2)	(15.9)	(13.0)	(15.0)
g_E	571.0	571.0	571.0	571.0	571.0	571.0	571.0	571.0	571.0	571.0
	(17.9)	(17.9)	(17.9)	(17.9)	(17.9)	(17.9)	(17.9)	(17.9)	(17.9)	(17.9)
	-143.2	-73.5	-203.6	-117.0	-60.3	-230.8	-156.7	-118.8	-264.9	-166.7
	-4.483	-2.300	-6.376	-3.666	-1.889	-7.230	-4.908	-3.721	-8.296	-5.221
c_O	554	548	574	566	537	553	537	578	522	503
	(17.4)	(17.2)	(18.0)	(17.7)	(16.8)	(17.3)	(16.8)	(18.1)	(16.3)	(15.8)
c_E	521.3	521.3	521.3	521.3	521.3	521.3	521.3	521.3	521.3	521.3
	(16.3)	(16.3)	(16.3)	(16.3)	(16.3)	(16.3)	(16.3)	(16.3)	(16.3)	(16.3)
	67.4	54.8	110.6	93.1	31.9	65.3	31.9	119.4	1.4	-35.9
	2.112	1.715	3.463	2.917	0.999	2.045	0.999	3.739	0.045	-1.125
N	3193	3193	3193	3193	3193	3193	3193	3193	3193	3193
X2	25.1	4.4	31.1	10.8	8.5	37.5	20.6	13.9	54.2	24.3
Gvalue	25.4	4.4	32.5	11.0	8.5	40.0	21.1	13.9	58.4	24.9
Gv/N	0.794	0.139	1.018	0.344	0.266	1.253	0.662	0.437	1.830	0.780
Prob	0.00001	0.21818	0.00000	0.01182	0.03697	0.00000	0.00010	0.00298	0.00000	0.00002

	20	19	18	17	16	15	14	13	12	11
a_O	1154	1163	1130	1198	1224	1151	1184	1202	1091	1201
	(36.1)	(36.4)	(35.4)	(37.5)	(38.3)	(36.0)	(37.1)	(37.6)	(34.2)	(37.6)
a_E	1071.0	1071.0	1071.0	1071.0	1071.0	1071.0	1071.0	1071.0	1071.0	1071.0
	(33.5)	(33.5)	(33.5)	(33.5)	(33.5)	(33.5)	(33.5)	(33.5)	(33.5)	(33.5)
	172.2	191.6	121.1	268.5	326.8	165.8	237.5	277.4	40.3	275.1
	5.394	6.002	3.794	8.408	10.236	5.192	7.438	8.687	1.263	8.617
t_O	1008	1136	1099	1019	1109	1093	954	949	877	607
	(31.6)	(35.6)	(34.4)	(31.9)	(34.7)	(34.2)	(29.9)	(29.7)	(27.5)	(19.0)

t_E	1029.7	1029.7	1029.7	1029.7	1029.7	1029.7	1029.7	1029.7	1029.7	1029.7
	(32.2)	(32.2)	(32.2)	(32.2)	(32.2)	(32.2)	(32.2)	(32.2)	(32.2)	(32.2)
	-42.9	223.3	143.2	-21.2	164.6	130.5	-145.6	-154.8	-281.5	-641.6
	-1.343	6.993	4.486	-0.665	5.156	4.086	-4.561	-4.850	-8.816	-20.093
g_O	475	407	488	470	427	530	616	651	905	1076
	(14.9)	(12.7)	(15.3)	(14.7)	(13.4)	(16.6)	(19.3)	(20.4)	(28.3)	(33.7)
g_E	571.0	571.0	571.0	571.0	571.0	571.0	571.0	571.0	571.0	571.0
	(17.9)	(17.9)	(17.9)	(17.9)	(17.9)	(17.9)	(17.9)	(17.9)	(17.9)	(17.9)
	-174.9	-275.6	-153.3	-183.0	-248.2	-79.0	93.4	170.7	833.5	1363.5
	-5.478	-8.632	-4.802	-5.732	-7.773	-2.475	2.926	5.345	26.105	42.702
c_O	556	487	476	506	433	419	439	391	320	309
	(17.4)	(15.3)	(14.9)	(15.8)	(13.6)	(13.1)	(13.7)	(12.2)	(10.0)	(9.7)
c_E	521.3	521.3	521.3	521.3	521.3	521.3	521.3	521.3	521.3	521.3
	(16.3)	(16.3)	(16.3)	(16.3)	(16.3)	(16.3)	(16.3)	(16.3)	(16.3)	(16.3)
	71.7	-66.3	-86.5	-30.1	-160.7	-183.0	-150.8	-224.9	-312.3	-323.2
	2.245	-2.076	-2.710	-0.944	-5.033	-5.733	-4.724	-7.044	-9.781	-10.122
N	3193	3193	3193	3193	3193	3193	3193	3193	3193	3193
X2	25.3	68.2	23.9	33.5	79.2	32.9	34.0	66.1	296.1	722.3
Gvalue	26.1	73.0	24.5	34.1	82.6	34.2	34.4	68.3	280.1	673.9
Gv/N	0.818	2.287	0.768	1.068	2.585	1.071	1.078	2.139	8.771	21.104
Prob	0.00001	0.00000	0.00000	0.00000	0.00000	0.00000	0.00000	0.00000	0.00000	0.00000

	10	9	8	7	6	5	4	3	2	1
a_O	1116	1059	1212	1229	1245	1199	1182	1214	1026	930
	(35.0)	(33.2)	(38.0)	(38.5)	(39.0)	(37.6)	(37.0)	(38.0)	(32.1)	(29.1)
a_E	1071.0	1071.0	1071.0	1071.0	1071.0	1071.0	1071.0	1071.0	1071.0	1071.0
	(33.5)	(33.5)	(33.5)	(33.5)	(33.5)	(33.5)	(33.5)	(33.5)	(33.5)	(33.5)
	91.8	-23.9	299.8	338.2	374.8	270.7	233.1	304.3	-88.1	-262.6
	2.876	-0.749	9.388	10.592	11.738	8.477	7.300	9.529	-2.760	-8.224
t_O	621	679	675	909	995	988	1023	877	1048	1094
	(19.4)	(21.3)	(21.1)	(28.5)	(31.2)	(30.9)	(32.0)	(27.5)	(32.8)	(34.3)
t_E	1029.7	1029.7	1029.7	1029.7	1029.7	1029.7	1029.7	1029.7	1029.7	1029.7
	(32.2)	(32.2)	(32.2)	(32.2)	(32.2)	(32.2)	(32.2)	(32.2)	(32.2)	(32.2)
	-628.0	-565.4	-570.1	-226.6	-68.2	-81.6	-13.3	-281.5	37.0	132.6
	-19.669	-17.709	-17.854	-7.097	-2.135	-2.557	-0.416	-8.816	1.158	4.153
g_O	1188	1206	990	672	596	500	412	619	485	334
	(37.2)	(37.8)	(31.0)	(21.0)	(18.7)	(15.7)	(12.9)	(19.4)	(15.2)	(10.5)
g_E	571.0	571.0	571.0	571.0	571.0	571.0	571.0	571.0	571.0	571.0
	(17.9)	(17.9)	(17.9)	(17.9)	(17.9)	(17.9)	(17.9)	(17.9)	(17.9)	(17.9)
	1740.7	1803.3	1089.6	218.8	51.0	-132.8	-269.0	99.9	-158.4	-358.2
	54.515	56.477	34.123	6.854	1.598	-4.160	-8.423	3.128	-4.960	-11.219
c_O	268	249	316	383	357	506	576	483	634	835
	(8.4)	(7.8)	(9.9)	(12.0)	(11.2)	(15.8)	(18.0)	(15.1)	(19.9)	(26.2)
c_E	521.3	521.3	521.3	521.3	521.3	521.3	521.3	521.3	521.3	521.3
	(16.3)	(16.3)	(16.3)	(16.3)	(16.3)	(16.3)	(16.3)	(16.3)	(16.3)	(16.3)
	-356.6	-367.9	-316.4	-236.1	-270.3	-30.1	115.0	-73.7	248.2	786.8
	-11.169	-11.524	-9.908	-7.395	-8.465	-0.944	3.601	-2.308	7.773	24.641
N	3193	3193	3193	3193	3193	3193	3193	3193	3193	3193
X2	953.8	967.9	529.0	92.0	82.3	26.3	61.6	48.6	39.5	309.8
Gvalue	847.8	846.0	502.9	94.3	87.4	26.1	65.8	48.9	38.7	298.5
Gv/N	26.553	26.496	15.749	2.953	2.737	0.818	2.061	1.533	1.212	9.350
Prob	0.00000	0.00000	0.00000	0.00000	0.00000	0.00001	0.00000	0.00000	0.00000	0.00000

CDS_INITIAL:

	1	2	3	4	5	6	7	8	9	10
a_O	2531	0	0	1527	1089	1044	1319	1267	1087	1120
	(79.3)	(0.0)	(0.0)	(47.8)	(34.1)	(32.7)	(41.3)	(39.7)	(34.0)	(35.1)
a_E	872.6	1017.8	899.4	872.6	1017.8	899.4	872.6	1017.8	899.4	872.6
	(27.3)	(31.9)	(28.2)	(27.3)	(31.9)	(28.2)	(27.3)	(31.9)	(28.2)	(27.3)
	5390.5	0.0	0.0	1709.0	147.2	311.4	1089.9	554.9	411.9	559.1
	168.822	0.000	0.000	53.522	4.610	9.751	34.134	17.378	12.901	17.511
t_O	375	3193	1	556	745	1214	581	962	1152	767
	(11.7)	(100.0)	(0.0)	(17.4)	(23.3)	(38.0)	(18.2)	(30.1)	(36.1)	(24.0)
t_E	607.6	987.6	1211.1	607.6	987.6	1211.1	607.6	987.6	1211.1	607.6
	(19.0)	(30.9)	(37.9)	(19.0)	(30.9)	(37.9)	(19.0)	(30.9)	(37.9)	(19.0)
	-361.9	7493.8	-14.2	-98.7	-420.0	5.8	-52.0	-50.5	-115.2	357.4
	-11.336	234.694	-0.445	-3.091	-13.153	0.183	-1.629	-1.581	-3.609	11.192
g_O	287	0	3192	564	510	487	682	348	544	598
	(9.0)	(0.0)	(100.0)	(17.7)	(16.0)	(15.3)	(21.4)	(10.9)	(17.0)	(18.7)
g_E	1033.8	497.6	595.1	1033.8	497.6	595.1	1033.8	497.6	595.1	1033.8
	(32.4)	(15.6)	(18.6)	(32.4)	(15.6)	(18.6)	(32.4)	(15.6)	(18.6)	(32.4)
	-735.6	0.0	10723.1	-683.5	25.2	-195.2	-567.4	-248.8	-97.7	-654.7
	-23.038	0.000	335.831	-21.408	0.788	-6.115	-17.771	-7.793	-3.059	-20.506
c_O	0	0	0	546	849	448	611	616	410	708
	(0.0)	(0.0)	(0.0)	(17.1)	(26.6)	(14.0)	(19.1)	(19.3)	(12.8)	(22.2)
c_E	679.0	690.0	487.5	679.0	690.0	487.5	679.0	690.0	487.5	679.0
	(21.3)	(21.6)	(15.3)	(21.3)	(21.6)	(15.3)	(21.3)	(21.6)	(15.3)	(21.3)
	0.0	0.0	0.0	-238.0	352.0	-75.6	-128.9	-139.8	-141.9	59.3
	0.000	0.000	0.000	-7.454	11.025	-2.368	-4.036	-4.379	-4.444	1.857
N	3193	3193	3193	3193	3193	3193	3193	3193	3193	3193
X2	4459.4	7130.6	13928.4	734.7	101.5	46.1	356.1	114.6	58.7	296.9
Gvalue	4292.9	7493.8	10708.9	688.7	104.4	46.3	341.6	115.8	57.1	321.1
Gv/N	134.448	234.694	335.386	21.570	3.271	1.451	10.697	3.625	1.789	10.055
Prob	1.00000	1.00000	1.00000	0.00000	0.00000	0.00000	0.00000	0.00000	0.00000	0.00000

	11	12	13	14	15	16	17	18	19	20
a_O	1027	1093	1121	1072	1042	1062	1020	996	1070	1040
	(32.2)	(34.2)	(35.1)	(33.6)	(32.6)	(33.3)	(31.9)	(31.2)	(33.5)	(32.6)
a_E	1017.8	899.4	872.6	1017.8	899.4	872.6	1017.8	899.4	872.6	1017.8
	(31.9)	(28.2)	(27.3)	(31.9)	(28.2)	(27.3)	(31.9)	(28.2)	(27.3)	(31.9)
	18.4	426.2	561.6	111.2	306.8	417.2	4.3	203.3	436.4	44.8
	0.577	13.349	17.589	3.482	9.608	13.067	0.136	6.367	13.668	1.404
t_O	1119	1194	715	1134	1195	715	1183	1251	702	1084
	(35.0)	(37.4)	(22.4)	(35.5)	(37.4)	(22.4)	(37.0)	(39.2)	(22.0)	(33.9)
t_E	987.6	1211.1	607.6	987.6	1211.1	607.6	987.6	1211.1	607.6	987.6
	(30.9)	(37.9)	(19.0)	(30.9)	(37.9)	(19.0)	(30.9)	(37.9)	(19.0)	(30.9)
	279.6	-33.9	232.8	313.6	-32.0	232.8	427.2	81.1	202.8	202.0
	8.758	-1.062	7.290	9.821	-1.001	7.290	13.380	2.541	6.350	6.326
g_O	327	472	673	339	497	680	358	450	756	408
	(10.2)	(14.8)	(21.1)	(10.6)	(15.6)	(21.3)	(11.2)	(14.1)	(23.7)	(12.8)
g_E	497.6	595.1	1033.8	497.6	595.1	1033.8	497.6	595.1	1033.8	497.6
	(15.6)	(18.6)	(32.4)	(15.6)	(18.6)	(32.4)	(15.6)	(18.6)	(32.4)	(15.6)
	-274.5	-218.8	-577.8	-260.2	-179.1	-569.8	-235.7	-251.5	-473.2	-161.9
	-8.598	-6.851	-18.097	-8.148	-5.608	-17.844	-7.382	-7.877	-14.821	-5.072

c_O	720	434	684	648	459	736	632	496	665	661
	(22.5)	(13.6)	(21.4)	(20.3)	(14.4)	(23.1)	(19.8)	(15.5)	(20.8)	(20.7)
c_E	690.0	487.5	679.0	690.0	487.5	679.0	690.0	487.5	679.0	690.0
	(21.6)	(15.3)	(21.3)	(21.6)	(15.3)	(21.3)	(21.6)	(15.3)	(21.3)	(21.6)
	61.2	-100.8	10.1	-81.5	-55.2	118.7	-111.0	17.2	-27.6	-56.8
	1.917	-3.157	0.317	-2.551	-1.729	3.719	-3.478	0.540	-0.865	-1.780
N	3193	3193	3193	3193	3193	3193	3193	3193	3193	3193
X2	77.3	73.3	215.7	77.7	40.7	186.0	82.7	47.2	134.3	27.2
Gvalue	84.7	72.7	226.7	83.1	40.6	199.0	84.8	50.1	138.3	28.0
Gv/N	2.654	2.278	7.099	2.603	1.270	6.231	2.656	1.570	4.332	0.878
Prob	0.00000	0.00000	0.00000	0.00000	0.00000	0.00000	0.00000	0.00000	0.00000	0.00000

	21	22	23	24	25	26	27	28	29	30
a_O	1042	1003	1000	975	896	969	1012	893	940	953
	(32.6)	(31.4)	(31.3)	(30.5)	(28.1)	(30.3)	(31.7)	(28.0)	(29.4)	(29.8)
a_E	899.4	872.6	1017.8	899.4	872.6	1017.8	899.4	872.6	1017.8	899.4
	(28.2)	(27.3)	(31.9)	(28.2)	(27.3)	(31.9)	(28.2)	(27.3)	(31.9)	(28.2)
	306.8	279.4	-35.4	157.4	47.4	-95.3	238.8	41.3	-149.6	110.4
	9.608	8.750	-1.107	4.931	1.485	-2.984	7.479	1.293	-4.684	3.457
t_O	1222	701	1090	1162	698	1101	1163	674	1068	1134
	(38.3)	(22.0)	(34.1)	(36.4)	(21.9)	(34.5)	(36.4)	(21.1)	(33.4)	(35.5)
t_E	1211.1	607.6	987.6	1211.1	607.6	987.6	1211.1	607.6	987.6	1211.1
	(37.9)	(19.0)	(30.9)	(37.9)	(19.0)	(30.9)	(37.9)	(19.0)	(30.9)	(37.9)
	21.9	200.5	215.1	-96.2	193.6	239.4	-94.2	139.8	167.2	-149.2
	0.687	6.279	6.738	-3.011	6.064	7.498	-2.951	4.379	5.238	-4.671
g_O	436	804	389	537	867	433	528	921	483	580
	(13.7)	(25.2)	(12.2)	(16.8)	(27.2)	(13.6)	(16.5)	(28.8)	(15.1)	(18.2)
g_E	595.1	1033.8	497.6	595.1	1033.8	497.6	595.1	1033.8	497.6	595.1
	(18.6)	(32.4)	(15.6)	(18.6)	(32.4)	(15.6)	(18.6)	(32.4)	(15.6)	(18.6)
	-271.3	-404.3	-191.5	-110.3	-305.2	-120.4	-126.3	-212.9	-28.7	-29.8
	-8.496	-12.662	-5.998	-3.455	-9.558	-3.770	-3.956	-6.667	-0.899	-0.933
c_O	493	685	714	519	732	690	490	705	702	526
	(15.4)	(21.5)	(22.4)	(16.3)	(22.9)	(21.6)	(15.3)	(22.1)	(22.0)	(16.5)
c_E	487.5	679.0	690.0	487.5	679.0	690.0	487.5	679.0	690.0	487.5
	(15.3)	(21.3)	(21.6)	(15.3)	(21.3)	(21.6)	(15.3)	(21.3)	(21.6)	(15.3)
	11.2	12.1	48.8	65.1	110.1	-0.1	5.1	53.1	24.1	80.1
	0.350	0.380	1.527	2.039	3.449	-0.002	0.160	1.662	0.756	2.508
N	3193	3193	3193	3193	3193	3193	3193	3193	3193	3193
X2	65.3	85.0	35.5	16.1	45.1	23.8	23.6	21.0	13.1	11.5
Gvalue	68.6	87.7	37.0	16.1	46.0	23.7	23.4	21.3	13.1	11.5
Gv/N	2.148	2.746	1.160	0.503	1.440	0.742	0.732	0.666	0.411	0.360
Prob	0.00000	0.00000	0.00000	0.00110	0.00000	0.00003	0.00003	0.00009	0.00437	0.00928

	31	32	33	34	35	36	37	38	39	40
a_O	921	907	957	916	894	941	909	872	920	916
	(28.8)	(28.4)	(30.0)	(28.7)	(28.0)	(29.5)	(28.5)	(27.3)	(28.8)	(28.7)
a_E	872.6	1017.8	899.4	872.6	1017.8	899.4	872.6	1017.8	899.4	872.6
	(27.3)	(31.9)	(28.2)	(27.3)	(31.9)	(28.2)	(27.3)	(31.9)	(28.2)	(27.3)
	99.4	-209.1	118.9	88.9	-231.9	85.2	74.3	-269.7	41.7	88.9
	3.114	-6.550	3.723	2.785	-7.264	2.667	2.327	-8.446	1.307	2.785
t_O	694	1147	1190	683	1077	1179	685	1077	1229	659
	(21.7)	(35.9)	(37.3)	(21.4)	(33.7)	(36.9)	(21.5)	(33.7)	(38.5)	(20.6)
t_E	607.6	987.6	1211.1	607.6	987.6	1211.1	607.6	987.6	1211.1	607.6
	(19.0)	(30.9)	(37.9)	(19.0)	(30.9)	(37.9)	(19.0)	(30.9)	(37.9)	(19.0)
	184.5	343.3	-41.8	159.8	186.7	-63.3	164.3	186.7	36.1	107.0
	5.780	10.752	-1.309	5.004	5.848	-1.983	5.145	5.848	1.131	3.352
g_O	903	505	539	908	492	529	941	479	572	969
	(28.3)	(15.8)	(16.9)	(28.4)	(15.4)	(16.6)	(29.5)	(15.0)	(17.9)	(30.3)
g_E	1033.8	497.6	595.1	1033.8	497.6	595.1	1033.8	497.6	595.1	1033.8
	(32.4)	(15.6)	(18.6)	(32.4)	(15.6)	(18.6)	(32.4)	(15.6)	(18.6)	(32.4)
	-244.4	15.0	-106.7	-235.7	-11.1	-124.6	-177.1	-36.4	-45.3	-125.5
	-7.654	0.469	-3.343	-7.382	-0.347	-3.901	-5.546	-1.141	-1.418	-3.931
c_O	675	634	507	686	730	544	658	765	472	649
	(21.1)	(19.9)	(15.9)	(21.5)	(22.9)	(17.0)	(20.6)	(24.0)	(14.8)	(20.3)
c_E	679.0	690.0	487.5	679.0	690.0	487.5	679.0	690.0	487.5	679.0
	(21.3)	(21.6)	(15.3)	(21.3)	(21.6)	(15.3)	(21.3)	(21.6)	(15.3)	(21.3)
	-7.9	-107.4	39.9	14.2	82.2	119.4	-41.3	157.8	-30.4	-58.6
	-0.247	-3.363	1.249	0.443	2.575	3.740	-1.292	4.942	-0.952	-1.835
N	3193	3193	3193	3193	3193	3193	3193	3193	3193	3193
X2	31.6	42.5	10.1	26.9	25.5	16.7	20.4	37.8	2.1	11.9
Gvalue	31.7	41.8	10.2	27.2	25.9	16.7	20.2	38.4	2.1	11.9
Gv/N	0.993	1.308	0.320	0.851	0.812	0.523	0.633	1.203	0.067	0.371
Prob	0.00000	0.00000	0.01677	0.00001	0.00001	0.00081	0.00015	0.00000	0.54455	0.00791

	41	42	43	44	45	46	47	48	49	50
a_O	901	909	883	925	926	871	903	918	903	883
	(28.2)	(28.5)	(27.7)	(29.0)	(29.0)	(27.3)	(28.3)	(28.8)	(28.3)	(27.7)
a_E	1017.8	899.4	872.6	1017.8	899.4	872.6	1017.8	899.4	872.6	1017.8
	(31.9)	(28.2)	(27.3)	(31.9)	(28.2)	(27.3)	(31.9)	(28.2)	(27.3)	(31.9)
	-219.7	19.4	20.9	-176.9	54.0	-3.2	-216.2	37.6	61.8	-251.0
	-6.881	0.606	0.655	-5.541	1.692	-0.100	-6.771	1.179	1.937	-7.860
t_O	1073	1243	719	1053	1217	646	1060	1230	670	1070
	(33.6)	(38.9)	(22.5)	(33.0)	(38.1)	(20.2)	(33.2)	(38.5)	(21.0)	(33.5)
t_E	987.6	1211.1	607.6	987.6	1211.1	607.6	987.6	1211.1	607.6	987.6
	(30.9)	(37.9)	(19.0)	(30.9)	(37.9)	(19.0)	(30.9)	(37.9)	(19.0)	(30.9)
	178.1	64.7	242.1	135.1	11.9	79.2	150.1	38.1	131.0	171.6
	5.576	2.025	7.582	4.231	0.371	2.480	4.699	1.194	4.103	5.373
g_O	521	510	976	465	554	1001	504	564	986	516
	(16.3)	(16.0)	(30.6)	(14.6)	(17.4)	(31.3)	(15.8)	(17.7)	(30.9)	(16.2)
g_E	497.6	595.1	1033.8	497.6	595.1	1033.8	497.6	595.1	1033.8	497.6
	(15.6)	(18.6)	(32.4)	(15.6)	(18.6)	(32.4)	(15.6)	(18.6)	(32.4)	(15.6)
	48.0	-157.4	-112.4	-63.0	-79.3	-64.6	12.9	-60.5	-93.4	37.5
	1.502	-4.929	-3.520	-1.972	-2.483	-2.024	0.406	-1.896	-2.926	1.176
c_O	698	531	615	750	496	675	726	481	634	724
	(21.9)	(16.6)	(19.3)	(23.5)	(15.5)	(21.1)	(22.7)	(15.1)	(19.9)	(22.7)
c_E	690.0	487.5	679.0	690.0	487.5	679.0	690.0	487.5	679.0	690.0
	(21.6)	(15.3)	(21.3)	(21.6)	(15.3)	(21.3)	(21.6)	(15.3)	(21.3)	(21.6)
	16.0	90.9	-121.7	125.0	17.2	-7.9	73.8	-12.8	-86.9	69.6
	0.502	2.846	-3.811	3.915	0.540	-0.247	2.311	-0.401	-2.721	2.179
N	3193	3193	3193	3193	3193	3193	3193	3193	3193	3193
X2	22.0	17.0	29.8	20.1	3.8	3.5	20.2	2.4	12.7	27.1
Gvalue	22.3	17.5	28.9	20.2	3.9	3.5	20.6	2.4	12.5	27.7
Gv/N	0.699	0.548	0.906	0.633	0.121	0.108	0.645	0.076	0.393	0.868
Prob	0.00006	0.00055	0.00000	0.00015	0.27648	0.32628	0.00013	0.49050	0.00574	0.00000

	51	52	53	54	55	56	57	58	59	60
a_O	1001	845	922	966	869	850	887	820	891	954
	(31.3)	(26.5)	(28.9)	(30.3)	(27.2)	(26.6)	(27.8)	(25.7)	(27.9)	(29.9)
a_E	899.4	872.6	1017.8	899.4	872.6	1017.8	899.4	872.6	1017.8	899.4
	(28.2)	(27.3)	(31.9)	(28.2)	(27.3)	(31.9)	(28.2)	(27.3)	(31.9)	(28.2)
	214.3	-54.3	-182.3	138.1	-7.2	-306.3	-24.6	-102.0	-237.2	112.5
	6.713	-1.701	-5.711	4.324	-0.225	-9.594	-0.769	-3.193	-7.427	3.524
t_O	1176	660	1046	1172	724	1081	1260	667	1064	1176
	(36.8)	(20.7)	(32.8)	(36.7)	(22.7)	(33.9)	(39.5)	(20.9)	(33.3)	(36.8)
t_E	1211.1	607.6	987.6	1211.1	607.6	987.6	1211.1	607.6	987.6	1211.1
	(37.9)	(19.0)	(30.9)	(37.9)	(19.0)	(30.9)	(37.9)	(19.0)	(30.9)	(37.9)
	-69.1	109.2	120.3	-76.9	253.8	195.4	99.8	124.4	158.6	-69.1
	-2.165	3.420	3.766	-2.408	7.949	6.121	3.125	3.897	4.968	-2.165
g_O	537	1026	528	554	1011	516	590	1052	493	571
	(16.8)	(32.1)	(16.5)	(17.4)	(31.7)	(16.2)	(18.5)	(32.9)	(15.4)	(17.9)
g_E	595.1	1033.8	497.6	595.1	1033.8	497.6	595.1	1033.8	497.6	595.1
	(18.6)	(32.4)	(15.6)	(18.6)	(32.4)	(15.6)	(18.6)	(32.4)	(15.6)	(18.6)
	-110.3	-15.6	62.7	-79.3	-45.2	37.5	-10.1	36.6	-9.1	-47.2
	-3.455	-0.489	1.963	-2.483	-1.415	1.176	-0.318	1.147	-0.285	-1.478
c_O	479	662	697	501	589	746	456	654	745	492
	(15.0)	(20.7)	(21.8)	(15.7)	(18.4)	(23.4)	(14.3)	(20.5)	(23.3)	(15.4)
c_E	487.5	679.0	690.0	487.5	679.0	690.0	487.5	679.0	690.0	487.5
	(15.3)	(21.3)	(21.6)	(15.3)	(21.3)	(21.6)	(15.3)	(21.3)	(21.6)	(15.3)
	-16.8	-33.5	14.0	27.5	-167.4	116.4	-60.8	-49.0	114.2	9.1
	-0.525	-1.049	0.439	0.860	-5.244	3.644	-1.905	-1.534	3.577	0.286
N	3193	3193	3193	3193	3193	3193	3193	3193	3193	3193
X2	18.3	5.9	14.4	9.4	34.7	41.7	4.2	10.2	26.1	5.4
Gvalue	18.1	5.8	14.6	9.4	34.0	43.0	4.2	10.1	26.6	5.3
Gv/N	0.567	0.181	0.457	0.294	1.065	1.347	0.133	0.317	0.833	0.166
Prob	0.00042	0.12370	0.00219	0.02473	0.00000	0.00000	0.23681	0.01766	0.00001	0.15040

	61	62	63	64	65	66	67	68	69	70
a_O	851	911	871	841	950	930	814	956	888	867
	(26.7)	(28.5)	(27.3)	(26.3)	(29.8)	(29.1)	(25.5)	(29.9)	(27.8)	(27.2)
a_E	872.6	1017.8	899.4	872.6	1017.8	899.4	872.6	1017.8	899.4	872.6
	(27.3)	(31.9)	(28.2)	(27.3)	(31.9)	(28.2)	(27.3)	(31.9)	(28.2)	(27.3)
	-42.7	-202.0	-55.8	-62.0	-131.0	62.3	-113.2	-119.8	-22.6	-11.2
	-1.336	-6.328	-1.749	-1.943	-4.104	1.951	-3.544	-3.753	-0.708	-0.350
t_O	680	1046	1183	669	1021	1183	640	987	1253	626
	(21.3)	(32.8)	(37.0)	(21.0)	(32.0)	(37.0)	(20.0)	(30.9)	(39.2)	(19.6)
t_E	607.6	987.6	1211.1	607.6	987.6	1211.1	607.6	987.6	1211.1	607.6
	(19.0)	(30.9)	(37.9)	(19.0)	(30.9)	(37.9)	(19.0)	(30.9)	(37.9)	(19.0)
	153.1	120.3	-55.5	128.8	68.0	-55.5	66.5	-1.1	85.3	37.4
	4.795	3.766	-1.739	4.034	2.129	-1.739	2.083	-0.036	2.670	1.170
g_O	994	515	623	1028	454	591	1032	539	573	1004
	(31.1)	(16.1)	(19.5)	(32.2)	(14.2)	(18.5)	(32.3)	(16.9)	(17.9)	(31.4)
g_E	1033.8	497.6	595.1	1033.8	497.6	595.1	1033.8	497.6	595.1	1033.8
	(32.4)	(15.6)	(18.6)	(32.4)	(15.6)	(18.6)	(32.4)	(15.6)	(18.6)	(32.4)
	-78.1	35.5	57.1	-11.6	-83.2	-8.2	-3.7	86.2	-43.4	-58.8
	-2.447	1.111	1.788	-0.365	-2.606	-0.256	-0.115	2.700	-1.358	-1.842
c_O	668	721	516	655	768	489	707	711	479	696
	(20.9)	(22.6)	(16.2)	(20.5)	(24.1)	(15.3)	(22.1)	(22.3)	(15.0)	(21.8)
c_E	679.0	690.0	487.5	679.0	690.0	487.5	679.0	690.0	487.5	679.0
	(21.3)	(21.6)	(15.3)	(21.3)	(21.6)	(15.3)	(21.3)	(21.6)	(15.3)	(21.3)
	-21.7	63.3	58.7	-47.1	164.4	3.1	57.2	42.6	-16.8	34.5
	-0.681	1.983	1.840	-1.474	5.150	0.097	1.792	1.333	-0.525	1.081
N	3193	3193	3193	3193	3193	3193	3193	3193	3193	3193
X2	10.9	16.7	4.5	8.2	18.3	1.7	6.8	7.8	2.6	1.9
Gvalue	10.6	17.0	4.5	8.1	18.2	1.7	6.9	7.8	2.6	1.9
Gv/N	0.331	0.532	0.141	0.252	0.569	0.054	0.215	0.245	0.080	0.059
Prob	0.01426	0.00071	0.21322	0.04493	0.00041	0.63227	0.07616	0.04979	0.46512	0.59735

	71	72	73	74	75	76	77	78	79	80
a_O	932	863	887	953	910	882	957	935	824	947
	(29.2)	(27.0)	(27.8)	(29.9)	(28.5)	(27.6)	(30.0)	(29.3)	(25.8)	(29.7)
a_E	1017.8	899.4	872.3	1017.5	899.1	872.3	1017.5	899.1	872.3	1017.5
	(31.9)	(28.2)	(27.3)	(31.9)	(28.2)	(27.3)	(31.9)	(28.2)	(27.3)	(31.9)
	-164.2	-71.2	29.6	-124.8	22.0	19.5	-117.4	73.2	-93.9	-136.0
	-5.143	-2.231	0.927	-3.911	0.688	0.609	-3.677	2.294	-2.942	-4.261
t_O	1037	1239	607	1005	1194	660	992	1212	626	1055
	(32.5)	(38.8)	(19.0)	(31.5)	(37.4)	(20.7)	(31.1)	(38.0)	(19.6)	(33.1)
t_E	987.6	1211.1	607.4	987.3	1210.7	607.4	987.3	1210.7	607.4	987.3
	(30.9)	(37.9)	(19.0)	(30.9)	(37.9)	(19.0)	(30.9)	(37.9)	(19.0)	(30.9)
	101.3	56.5	-0.8	35.8	-33.2	109.6	9.5	2.6	37.7	140.0
	3.173	1.769	-0.026	1.122	-1.039	3.434	0.298	0.081	1.182	4.387
g_O	488	630	995	479	588	964	495	557	1030	481
	(15.3)	(19.7)	(31.2)	(15.0)	(18.4)	(30.2)	(15.5)	(17.4)	(32.3)	(15.1)
g_E	497.6	595.1	1033.5	497.4	594.9	1033.5	497.4	594.9	1033.5	497.4
	(15.6)	(18.6)	(32.4)	(15.6)	(18.6)	(32.4)	(15.6)	(18.6)	(32.4)	(15.6)
	-18.9	71.8	-75.6	-36.1	-13.7	-134.2	-4.8	-73.4	-7.0	-32.3
	-0.593	2.249	-2.368	-1.132	-0.430	-4.206	-0.151	-2.298	-0.220	-1.011
c_O	736	461	703	755	500	686	748	488	712	709
	(23.1)	(14.4)	(22.0)	(23.7)	(15.7)	(21.5)	(23.4)	(15.3)	(22.3)	(22.2)
c_E	690.0	487.5	678.7	689.8	487.3	678.7	689.8	487.3	678.7	689.8
	(21.6)	(15.3)	(21.3)	(21.6)	(15.3)	(21.3)	(21.6)	(15.3)	(21.3)	(21.6)
	94.9	-51.4	49.4	136.3	25.7	14.6	121.1	1.4	68.1	38.9
	2.973	-1.611	1.546	4.271	0.806	0.457	3.795	0.044	2.134	1.218
N	3193	3193	3192	3192	3192	3192	3192	3192	3192	3192
X2	13.0	5.6	2.5	11.2	0.8	9.4	8.5	3.9	4.9	10.6
Gvalue	13.1	5.6	2.6	11.2	0.8	9.4	8.5	3.9	4.9	10.6
Gv/N	0.409	0.175	0.080	0.350	0.024	0.294	0.266	0.122	0.154	0.333
Prob	0.00450	0.13278	0.46529	0.01089	0.85610	0.02447	0.03704	0.27395	0.17878	0.01394

	81	82	83	84	85	86	87	88	89	90
a_O	965	885	984	935	875	1042	935	815	994	906
	(30.2)	(27.7)	(30.8)	(29.3)	(27.4)	(32.6)	(29.3)	(25.5)	(31.1)	(28.4)
a_E	899.1	872.3	1017.5	899.1	872.3	1017.5	899.1	872.3	1017.5	899.1
	(28.2)	(27.3)	(31.9)	(28.2)	(27.3)	(31.9)	(28.2)	(27.3)	(31.9)	(28.2)
	136.5	25.5	-65.9	73.2	5.4	49.6	73.2	-110.8	-46.5	13.9
	4.278	0.800	-2.065	2.294	0.168	1.553	2.294	-3.471	-1.456	0.435
t_O	1188	625	993	1215	566	991	1254	629	993	1233
	(37.2)	(19.6)	(31.1)	(38.1)	(17.7)	(31.0)	(39.3)	(19.7)	(31.1)	(38.6)
t_E	1210.7	607.4	987.3	1210.7	607.4	987.3	1210.7	607.4	987.3	1210.7
	(37.9)	(19.0)	(30.9)	(37.9)	(19.0)	(30.9)	(37.9)	(19.0)	(30.9)	(37.9)
	-45.0	35.7	11.5	8.6	-79.9	7.5	88.1	43.9	11.5	45.0

	-1.409	1.118	0.361	0.270	-2.504	0.235	2.761	1.377	0.361	1.410
g_O	565	982	494	567	1033	462	531	1045	503	552
	(17.7)	(30.8)	(15.5)	(17.8)	(32.4)	(14.5)	(16.6)	(32.7)	(15.8)	(17.3)
g_E	594.9	1033.5	497.4	594.9	1033.5	497.4	594.9	1033.5	497.4	594.9
	(18.6)	(32.4)	(15.6)	(18.6)	(32.4)	(15.6)	(18.6)	(32.4)	(15.6)	(18.6)
	-58.3	-100.4	-6.8	-54.5	-1.0	-68.2	-120.7	23.1	11.2	-82.6
	-1.826	-3.146	-0.213	-1.707	-0.032	-2.138	-3.781	0.723	0.352	-2.589
c_O	474	700	721	475	718	697	472	703	702	501
	(14.8)	(21.9)	(22.6)	(14.9)	(22.5)	(21.8)	(14.8)	(22.0)	(22.0)	(15.7)
c_E	487.3	678.7	689.8	487.3	678.7	689.8	487.3	678.7	689.8	487.3
	(15.3)	(21.3)	(21.6)	(15.3)	(21.3)	(21.6)	(15.3)	(21.3)	(21.6)	(15.3)
	-26.2	43.2	63.8	-24.3	80.7	14.4	-30.1	49.4	24.6	27.8
	-0.822	1.352	1.997	-0.761	2.529	0.452	-0.943	1.546	0.770	0.870

N	3192	3192	3192	3192	3192	3192	3192	3192	3192	3192
X2	7.1	3.9	2.6	3.1	5.1	3.2	10.3	5.5	0.9	3.9
Gvalue	7.0	4.0	2.6	3.1	5.1	3.3	10.6	5.6	0.9	4.0
Gv/N	0.221	0.124	0.080	0.096	0.161	0.102	0.331	0.175	0.027	0.126
Prob	0.07056	0.26602	0.46424	0.38033	0.16284	0.35379	0.01438	0.13308	0.83569	0.25985

	91	92	93	94	95	96	97	98	99	100
a_O	868	1001	951	845	1023	958	892	983	856	871
	(27.2)	(31.4)	(29.8)	(26.5)	(32.1)	(30.0)	(28.0)	(30.8)	(26.8)	(27.3)
a_E	872.3	1017.5	899.1	872.1	1017.2	898.8	872.1	1017.2	898.8	872.1
	(27.3)	(31.9)	(28.2)	(27.3)	(31.9)	(28.2)	(27.3)	(31.9)	(28.2)	(27.3)
	-8.6	-32.8	106.8	-53.3	11.6	122.2	40.3	-67.2	-83.5	-2.1
	-0.270	-1.026	3.345	-1.669	0.365	3.830	1.264	-2.107	-2.618	-0.066
t_O	640	1011	1211	618	984	1204	614	1017	1266	620
	(20.1)	(31.7)	(37.9)	(19.4)	(30.8)	(37.7)	(19.2)	(31.9)	(39.7)	(19.4)
t_E	607.4	987.3	1210.7	607.2	986.9	1210.3	607.2	986.9	1210.3	607.2
	(19.0)	(30.9)	(37.9)	(19.0)	(30.9)	(37.9)	(19.0)	(30.9)	(37.9)	(19.0)
	66.9	48.1	0.6	21.8	-5.9	-12.6	13.6	61.0	113.9	25.8
	2.096	1.505	0.019	0.682	-0.185	-0.395	0.427	1.912	3.569	0.809
g_O	1027	480	573	1028	478	545	985	446	595	1066
	(32.2)	(15.0)	(18.0)	(32.2)	(15.0)	(17.1)	(30.9)	(14.0)	(18.6)	(33.4)
g_E	1033.5	497.4	594.9	1033.2	497.3	594.7	1033.2	497.3	594.7	1033.2
	(32.4)	(15.6)	(18.6)	(32.4)	(15.6)	(18.6)	(32.4)	(15.6)	(18.6)	(32.4)
	-13.0	-34.2	-43.0	-10.4	-37.8	-95.2	-94.1	-97.0	0.6	66.6
	-0.407	-1.072	-1.347	-0.325	-1.183	-2.982	-2.949	-3.041	0.017	2.089
c_O	657	700	457	700	706	484	700	745	474	634
	(20.6)	(21.9)	(14.3)	(21.9)	(22.1)	(15.2)	(21.9)	(23.3)	(14.9)	(19.9)
c_E	678.7	689.8	487.3	678.5	689.6	487.1	678.5	689.6	487.1	678.5
	(21.3)	(21.6)	(15.3)	(21.3)	(21.6)	(15.3)	(21.3)	(21.6)	(15.3)	(21.3)
	-42.8	20.5	-58.7	43.6	33.2	-6.3	43.6	115.1	-25.9	-86.1
	-1.341	0.643	-1.838	1.366	1.040	-0.197	1.366	3.608	-0.813	-2.698
N	3192	3192	3192	3191	3191	3191	3191	3191	3191	3191
X2	2.5	1.6	5.7	1.7	1.2	8.1	3.5	11.8	5.0	4.2
Gvalue	2.5	1.6	5.7	1.7	1.2	8.1	3.5	11.9	5.0	4.3
Gv/N	0.078	0.050	0.178	0.054	0.037	0.255	0.109	0.372	0.155	0.134
Prob	0.47798	0.65891	0.12813	0.62885	0.75680	0.04307	0.32284	0.00781	0.17530	0.23196

	101	102	103	104	105	106	107	108	109	110
a_O	1010	898	843	1045	919	827	1016	922	841	984
	(31.7)	(28.1)	(26.4)	(32.8)	(28.8)	(25.9)	(31.8)	(28.9)	(26.4)	(30.8)
a_E	1017.2	898.8	871.8	1016.9	898.5	871.8	1016.9	898.5	871.8	1016.9
	(31.9)	(28.2)	(27.3)	(31.9)	(28.2)	(27.3)	(31.9)	(28.2)	(27.3)	(31.9)
	-14.3	-1.6	-56.6	57.0	41.4	-87.2	-1.8	47.6	-60.5	-64.7
	-0.449	-0.051	-1.774	1.787	1.298	-2.734	-0.055	1.491	-1.895	-2.028
t_O	988	1254	591	997	1232	612	910	1202	623	986
	(31.0)	(39.3)	(18.5)	(31.3)	(38.6)	(19.2)	(28.5)	(37.7)	(19.5)	(30.9)
t_E	986.9	1210.3	607.0	986.6	1209.9	607.0	986.6	1209.9	607.0	986.6
	(30.9)	(37.9)	(19.0)	(30.9)	(37.9)	(19.0)	(30.9)	(37.9)	(19.0)	(30.9)
	2.1	88.9	-31.6	20.8	44.5	10.0	-147.2	-15.8	32.4	-1.3
	0.066	2.786	-0.992	0.653	1.395	0.313	-4.613	-0.497	1.014	-0.040
g_O	488	554	1054	450	564	1075	506	597	1051	514
	(15.3)	(17.4)	(33.0)	(14.1)	(17.7)	(33.7)	(15.9)	(18.7)	(32.9)	(16.1)
g_E	497.3	594.7	1032.9	497.1	594.5	1032.9	497.1	594.5	1032.9	497.1
	(15.6)	(18.6)	(32.4)	(15.6)	(18.6)	(32.4)	(15.6)	(18.6)	(32.4)	(15.6)
	-18.3	-78.6	42.7	-89.6	-59.5	86.0	18.0	4.9	36.6	34.4
	-0.575	-2.463	1.338	-2.808	-1.864	2.695	0.563	0.155	1.147	1.077
c_O	705	485	702	698	475	676	758	469	675	706
	(22.1)	(15.2)	(22.0)	(21.9)	(14.9)	(21.2)	(23.8)	(14.7)	(21.2)	(22.1)
c_E	689.6	487.1	678.3	689.4	487.0	678.3	689.4	487.0	678.3	689.4
	(21.6)	(15.3)	(21.3)	(21.6)	(15.3)	(21.3)	(21.6)	(15.3)	(21.3)	(21.6)
	31.1	-4.3	48.2	17.3	-23.7	-4.6	143.8	-35.3	-6.6	33.6
	0.976	-0.134	1.510	0.543	-0.743	-0.145	4.509	-1.107	-0.208	1.054
N	3191	3191	3190	3190	3190	3190	3190	3190	3190	3190
X2	0.6	4.4	2.6	5.5	2.7	4.1	12.9	1.3	1.8	2.0
Gvalue	0.6	4.4	2.6	5.6	2.8	4.1	12.9	1.3	1.8	2.0
Gv/N	0.018	0.139	0.083	0.175	0.086	0.128	0.404	0.042	0.058	0.064
Prob	0.90400	0.21936	0.45153	0.13296	0.43072	0.25246	0.00489	0.71880	0.60433	0.56406

	111	112	113	114	115	116	117	118	119	120
a_O	945	877	1030	914	819	1028	940	855	1088	906
	(29.6)	(27.5)	(32.3)	(28.7)	(25.7)	(32.2)	(29.5)	(26.8)	(34.1)	(28.4)
a_E	898.5	871.8	1016.9	898.5	871.8	1016.9	898.5	871.2	1016.2	898.0
	(28.2)	(27.3)	(31.9)	(28.2)	(27.3)	(31.9)	(28.2)	(27.3)	(31.9)	(28.2)
	95.3	10.5	26.4	31.2	-102.3	22.4	84.8	-32.2	148.5	16.1
	2.988	0.328	0.828	0.979	-3.207	0.701	2.659	-1.009	4.657	0.507
t_O	1191	648	961	1246	593	942	1206	597	932	1249
	(37.3)	(20.3)	(30.1)	(39.1)	(18.6)	(29.5)	(37.8)	(18.7)	(29.2)	(39.2)
t_E	1209.9	607.0	986.6	1209.9	607.0	986.6	1209.9	606.6	986.0	1209.2
	(37.9)	(19.0)	(30.9)	(37.9)	(19.0)	(30.9)	(37.9)	(19.0)	(30.9)	(37.9)
	-37.6	84.6	-50.6	73.2	-27.7	-87.2	-7.9	-19.1	-105.0	80.9
	-1.178	2.654	-1.586	2.294	-0.869	-2.734	-0.247	-0.600	-3.294	2.538
g_O	561	998	476	545	1098	507	557	1052	494	561
	(17.6)	(31.3)	(14.9)	(17.1)	(34.4)	(15.9)	(17.5)	(33.0)	(15.5)	(17.6)
g_E	594.5	1032.9	497.1	594.5	1032.9	497.1	594.5	1032.2	496.8	594.2
	(18.6)	(32.4)	(15.6)	(18.6)	(32.4)	(15.6)	(18.6)	(32.4)	(15.6)	(18.6)
	-65.1	-68.5	-41.3	-94.8	134.3	20.0	-72.7	39.9	-5.6	-64.4
	-2.042	-2.149	-1.294	-2.973	4.210	0.627	-2.277	1.253	-0.174	-2.021
c_O	493	667	723	485	680	713	487	684	674	472
	(15.5)	(20.9)	(22.7)	(15.2)	(21.3)	(22.4)	(15.3)	(21.5)	(21.1)	(14.8)
c_E	487.0	678.3	689.4	487.0	678.3	689.4	487.0	677.9	689.0	486.7
	(15.3)	(21.3)	(21.6)	(15.3)	(21.3)	(21.6)	(15.3)	(21.3)	(21.6)	(15.3)
	12.1	-22.5	68.8	-4.0	3.4	48.0	0.0	12.3	-29.6	-28.9
	0.379	-0.704	2.158	-0.125	0.105	1.506	0.000	0.385	-0.928	-0.907

N	3190	3190	3190	3190	3190	3190	3190	3188	3188	3188
X2	4.7	4.2	3.4	5.5	7.6	3.1	4.3	0.9	8.4	3.7
Gvalue	4.7	4.1	3.4	5.6	7.6	3.2	4.3	0.9	8.3	3.7
Gv/N	0.146	0.129	0.105	0.175	0.239	0.099	0.135	0.028	0.261	0.116
Prob	0.19814	0.24910	0.33906	0.13370	0.05465	0.36647	0.22892	0.82785	0.04002	0.29540

	121	122	123	124	125	126	127	128	129	130
a_O	828	1052	960	839	1050	907	822	1048	902	843
	(26.0)	(33.0)	(30.1)	(26.4)	(33.0)	(28.5)	(25.8)	(32.9)	(28.3)	(26.5)
a_E	871.0	1015.9	897.7	869.9	1014.6	896.6	869.6	1014.3	896.3	868.5
	(27.3)	(31.9)	(28.2)	(27.3)	(31.9)	(28.2)	(27.3)	(31.9)	(28.2)	(27.3)
	−83.8	73.4	128.9	−60.6	71.9	21.0	−92.5	68.5	11.5	−50.2
	−2.628	2.304	4.044	−1.905	2.260	0.660	−2.908	2.151	0.361	−1.581
t_O	580	940	1231	605	955	1204	596	979	1250	631
	(18.2)	(29.5)	(38.6)	(19.0)	(30.0)	(37.8)	(18.7)	(30.8)	(39.3)	(19.9)
t_E	606.5	985.7	1208.8	605.7	984.5	1207.3	605.5	984.2	1206.9	604.7
	(19.0)	(30.9)	(37.9)	(19.0)	(30.9)	(37.9)	(19.0)	(30.9)	(37.9)	(19.0)
	−51.7	−89.3	44.8	−1.4	−58.1	−6.6	−18.9	−10.3	87.7	53.6
	−1.624	−2.801	1.405	−0.044	−1.824	−0.206	−0.593	−0.324	2.756	1.688
g_O	1038	495	541	1067	483	613	1037	460	568	1043
	(32.6)	(15.5)	(17.0)	(33.5)	(15.2)	(19.3)	(32.6)	(14.5)	(17.9)	(32.8)
g_E	1031.9	496.6	594.0	1030.6	496.0	593.2	1030.3	495.9	593.0	1029.0
	(32.4)	(15.6)	(18.6)	(32.4)	(15.6)	(18.6)	(32.4)	(15.6)	(18.6)	(32.4)
	12.2	−3.3	−101.1	74.1	−25.7	40.2	13.5	−69.0	−49.0	28.2
	0.384	−0.102	−3.172	2.327	−0.807	1.263	0.424	−2.170	−1.540	0.888
c_O	741	700	455	672	695	459	727	695	462	661
	(23.3)	(22.0)	(14.3)	(21.1)	(21.8)	(14.4)	(22.8)	(21.8)	(14.5)	(20.8)
c_E	677.7	688.7	486.5	676.8	687.9	485.9	676.6	687.7	485.8	675.8
	(21.3)	(21.6)	(15.3)	(21.3)	(21.6)	(15.3)	(21.3)	(21.6)	(15.3)	(21.3)
	132.4	22.7	−61.0	−9.6	14.3	−52.3	104.4	14.8	−46.4	−29.2
	4.153	0.713	−1.913	−0.303	0.450	−1.644	3.282	0.464	−1.457	−0.919
N	3187	3187	3187	3183	3183	3183	3182	3182	3182	3178
X2	9.2	3.6	11.5	2.4	2.5	2.3	6.5	3.8	3.8	2.4
Gvalue	9.1	3.6	11.6	2.4	2.5	2.3	6.5	3.9	3.8	2.4
Gv/N	0.286	0.113	0.364	0.076	0.079	0.072	0.205	0.122	0.120	0.075
Prob	0.02797	0.30697	0.00890	0.49096	0.47042	0.51216	0.08932	0.27606	0.28243	0.49455

	131	132	133	134	135	136	137	138	139	140
a_O	1008	884	815	1042	885	824	1036	909	830	1005
	(31.7)	(27.8)	(25.7)	(32.8)	(27.9)	(26.0)	(32.7)	(28.7)	(26.2)	(31.7)
a_E	1013.1	895.1	867.4	1011.8	894.0	866.3	1010.5	892.9	866.0	1010.2
	(31.9)	(28.2)	(27.3)	(31.9)	(28.2)	(27.3)	(31.9)	(28.2)	(27.3)	(31.9)
	−10.1	−22.2	−101.6	61.3	−17.9	−82.5	51.6	32.5	−70.6	−10.3
	−0.317	−0.697	−3.200	1.933	−0.565	−2.603	1.629	1.025	−2.227	−0.326
t_O	966	1219	624	968	1237	615	935	1241	617	974
	(30.4)	(38.4)	(19.7)	(30.5)	(39.0)	(19.4)	(29.5)	(39.1)	(19.5)	(30.7)
t_E	982.9	1205.4	604.0	981.7	1203.9	603.2	980.5	1202.4	603.0	980.1
	(30.9)	(37.9)	(19.0)	(30.9)	(37.9)	(19.0)	(30.9)	(37.9)	(19.0)	(30.9)
	−33.6	27.4	40.7	−27.2	67.1	23.8	−88.8	78.5	28.3	−12.3
	−1.056	0.861	1.282	−0.857	2.116	0.750	−2.800	2.477	0.892	−0.387
g_O	465	593	1046	520	562	1052	474	547	1068	489
	(14.6)	(18.7)	(33.0)	(16.4)	(17.7)	(33.2)	(15.0)	(17.3)	(33.7)	(15.4)
g_E	495.2	592.3	1027.7	494.6	591.6	1026.4	494.0	590.8	1026.1	493.8
	(15.6)	(18.6)	(32.4)	(15.6)	(18.6)	(32.4)	(15.6)	(18.6)	(32.4)	(15.6)
	−58.6	1.4	36.9	52.1	−57.6	51.8	−39.1	−84.3	85.6	−9.6
	−1.843	0.044	1.164	1.641	−1.815	1.636	−1.235	−2.659	2.700	−0.303
c_O	739	482	689	644	490	679	725	473	654	701
	(23.3)	(15.2)	(21.7)	(20.3)	(15.4)	(21.4)	(22.9)	(14.9)	(20.6)	(22.1)
c_E	686.8	485.2	674.9	685.9	484.6	674.1	685.1	483.9	673.9	684.8
	(21.6)	(15.3)	(21.3)	(21.6)	(15.3)	(21.3)	(21.6)	(15.3)	(21.3)	(21.6)
	108.3	−6.3	28.5	−81.2	11.0	9.9	82.2	−21.6	−39.1	32.7
	3.407	−0.198	0.896	−2.560	0.345	0.312	2.592	−0.682	−1.235	1.031
N	3178	3178	3174	3174	3174	3170	3170	3170	3169	3169
X2	6.1	0.3	4.4	5.0	2.5	3.0	5.9	5.0	4.1	0.5
Gvalue	6.1	0.3	4.5	5.0	2.6	3.0	5.9	5.1	4.1	0.5
Gv/N	0.191	0.010	0.142	0.157	0.081	0.095	0.186	0.161	0.130	0.015
Prob	0.10806	0.95741	0.21191	0.17293	0.46503	0.39174	0.11748	0.16472	0.24842	0.92096

	141	142	143	144	145	146	147	148	149	150
a_O	892	824	1033	898	815	1044	940	830	1015	876
	(28.1)	(26.0)	(32.6)	(28.4)	(25.8)	(33.0)	(29.8)	(26.3)	(32.2)	(27.7)
a_E	892.6	864.9	1008.9	891.5	863.3	1007.0	889.8	862.8	1006.4	889.2
	(28.2)	(27.3)	(31.9)	(28.2)	(27.3)	(31.9)	(28.2)	(27.3)	(31.9)	(28.2)
	−1.2	−79.9	48.8	13.1	−93.9	75.4	103.2	−64.3	17.4	−26.3
	−0.038	−2.525	1.541	0.413	−2.971	2.385	3.267	−2.036	0.550	−0.832
t_O	1223	661	960	1188	603	971	1191	625	993	1261
	(38.6)	(20.9)	(30.3)	(37.5)	(19.1)	(30.7)	(37.7)	(19.8)	(31.5)	(39.9)
t_E	1202.0	602.3	978.9	1200.5	601.1	977.1	1198.2	600.7	976.4	1197.4
	(37.9)	(19.0)	(30.9)	(37.9)	(19.0)	(30.9)	(37.9)	(19.0)	(30.9)	(37.9)
	42.4	123.0	−37.4	−24.8	3.7	−12.1	−14.3	49.5	33.4	130.5
	1.338	3.886	−1.183	−0.783	0.119	−0.382	−0.454	1.567	1.058	4.132
g_O	589	1003	488	621	1054	498	561	1040	495	575
	(18.6)	(31.7)	(15.4)	(19.6)	(33.4)	(15.8)	(17.8)	(32.9)	(15.7)	(18.2)
g_E	590.6	1024.8	493.2	589.9	1022.8	492.3	588.8	1022.2	492.0	588.4
	(18.6)	(32.4)	(15.6)	(18.6)	(32.4)	(15.6)	(18.6)	(32.4)	(15.6)	(18.6)
	−3.2	−43.1	−10.4	63.9	63.3	11.5	−54.2	35.9	6.1	−26.5
	−0.102	−1.361	−0.327	2.018	2.003	0.365	−1.715	1.138	0.193	−0.838
c_O	465	677	684	458	687	646	467	662	654	445
	(14.7)	(21.4)	(21.6)	(14.5)	(21.7)	(20.4)	(14.8)	(21.0)	(20.7)	(14.1)
c_E	483.8	673.0	684.0	483.2	671.7	682.7	482.3	671.3	682.3	482.0
	(15.3)	(21.3)	(21.6)	(15.3)	(21.3)	(21.6)	(15.3)	(21.3)	(21.6)	(15.3)
	−36.8	8.0	0.0	−49.0	30.9	−71.4	−30.0	−18.5	−55.3	−71.0
	−1.162	0.253	0.001	−1.549	0.978	−2.259	−0.951	−0.585	−1.752	−2.249
N	3169	3165	3165	3165	3159	3159	3159	3157	3157	3157
X2	1.1	8.2	1.0	3.1	4.0	3.4	4.7	2.7	1.5	6.7
Gvalue	1.1	8.0	1.0	3.1	4.0	3.5	4.6	2.7	1.6	6.7
Gv/N	0.035	0.253	0.031	0.099	0.128	0.109	0.147	0.084	0.049	0.213
Prob	0.77478	0.04584	0.80283	0.37253	0.25658	0.32660	0.19995	0.44632	0.66875	0.08101

Adeh_2CPC
5UTR:

	50	49	48	47	46	45	44	43	42	41
a_O	537	575	467	513	578	455	539	561	465	508
	(12.4)	(13.2)	(10.7)	(11.8)	(13.3)	(10.5)	(12.4)	(12.9)	(10.7)	(11.7)
a_E	550.5	550.5	550.5	550.5	550.5	550.5	550.5	550.5	550.5	550.5
	(12.7)	(12.7)	(12.7)	(12.7)	(12.7)	(12.7)	(12.7)	(12.7)	(12.7)	(12.7)
	-26.6	50.1	-153.6	-72.3	56.4	-173.3	-22.7	21.2	-156.9	-81.6
	-0.613	1.153	-3.535	-1.665	1.298	-3.990	-0.523	0.489	-3.612	-1.878
t_O	466	598	458	524	622	484	462	614	527	528
	(10.7)	(13.8)	(10.5)	(12.1)	(14.3)	(11.1)	(10.6)	(14.1)	(12.1)	(12.2)
t_E	525.6	525.6	525.6	525.6	525.6	525.6	525.6	525.6	525.6	525.6
	(12.1)	(12.1)	(12.1)	(12.1)	(12.1)	(12.1)	(12.1)	(12.1)	(12.1)	(12.1)
	-112.2	154.4	-126.1	-3.2	209.5	-79.8	-119.2	190.9	2.8	4.8
	-2.581	3.553	-2.902	-0.073	4.822	-1.837	-2.742	4.394	0.065	0.111
g_O	1648	1492	1673	1600	1512	1683	1682	1623	1644	1723
	(37.9)	(34.3)	(38.5)	(36.8)	(34.8)	(38.7)	(38.7)	(37.4)	(37.8)	(39.7)
g_E	1647.6	1647.6	1647.6	1647.6	1647.6	1647.6	1647.6	1647.6	1647.6	1647.6
	(37.9)	(37.9)	(37.9)	(37.9)	(37.9)	(37.9)	(37.9)	(37.9)	(37.9)	(37.9)
	0.8	-296.0	51.2	-93.8	-259.7	71.6	69.6	-48.8	-7.2	154.2
	0.019	-6.812	1.179	-2.158	-5.977	1.648	1.601	-1.123	-0.165	3.550
c_O	1694	1680	1747	1708	1633	1723	1662	1547	1709	1586
	(39.0)	(38.7)	(40.2)	(39.3)	(37.6)	(39.7)	(38.3)	(35.6)	(39.3)	(36.5)
c_E	1621.3	1621.3	1621.3	1621.3	1621.3	1621.3	1621.3	1621.3	1621.3	1621.3
	(37.3)	(37.3)	(37.3)	(37.3)	(37.3)	(37.3)	(37.3)	(37.3)	(37.3)	(37.3)
	148.5	119.4	260.8	177.9	23.4	209.5	82.3	-145.2	180.0	-69.9
	3.418	2.748	6.002	4.093	0.538	4.823	1.894	-3.343	4.142	-1.609
N	4345	4345	4345	4345	4345	4345	4345	4345	4345	4345
X2	10.3	27.9	31.5	8.6	30.3	27.0	9.7	18.8	18.0	7.5
Gvalue	10.6	27.9	32.3	8.6	29.6	28.0	10.0	18.1	18.7	7.6
Gv/N	0.243	0.642	0.744	0.197	0.681	0.644	0.230	0.417	0.430	0.174
Prob	0.01431	0.00000	0.00000	0.03584	0.00000	0.00000	0.01865	0.00041	0.00032	0.05618

	40	39	38	37	36	35	34	33	32	31
a_O	526	436	489	550	444	530	535	434	538	566
	(12.1)	(10.0)	(11.3)	(12.7)	(10.2)	(12.2)	(12.3)	(10.0)	(12.4)	(13.0)
a_E	550.5	550.5	550.5	550.5	550.5	550.5	550.5	550.5	550.5	550.5
	(12.7)	(12.7)	(12.7)	(12.7)	(12.7)	(12.7)	(12.7)	(12.7)	(12.7)	(12.7)
	-47.9	-203.3	-115.8	-1.0	-190.9	-40.2	-30.5	-206.4	-24.7	31.5
	-1.101	-4.679	-2.666	-0.022	-4.393	-0.925	-0.702	-4.749	-0.568	0.724
t_O	616	542	605	666	586	645	635	466	482	575
	(14.2)	(12.5)	(13.9)	(15.3)	(13.5)	(14.8)	(14.6)	(10.7)	(11.1)	(13.2)
t_E	525.6	525.6	525.6	525.6	525.6	525.6	525.6	525.6	525.6	525.6
	(12.1)	(12.1)	(12.1)	(12.1)	(12.1)	(12.1)	(12.1)	(12.1)	(12.1)	(12.1)
	195.5	33.3	170.3	315.4	127.5	264.1	240.2	-112.2	-83.5	103.3
	4.501	0.767	3.918	7.258	2.935	6.078	5.527	-2.581	-1.921	2.378
g_O	1540	1692	1676	1524	1680	1632	1583	1683	1598	1540
	(35.4)	(38.9)	(38.6)	(35.1)	(38.7)	(37.6)	(36.4)	(38.7)	(36.8)	(35.4)
g_E	1647.6	1647.6	1647.6	1647.6	1647.6	1647.6	1647.6	1647.6	1647.6	1647.6
	(37.9)	(37.9)	(37.9)	(37.9)	(37.9)	(37.9)	(37.9)	(37.9)	(37.9)	(37.9)
	-208.0	90.0	57.3	-237.6	65.5	-31.0	-126.6	71.6	-97.7	-208.0
	-4.787	2.072	1.319	-5.470	1.507	-0.714	-2.914	1.648	-2.247	-4.787
c_O	1663	1675	1575	1605	1635	1538	1592	1762	1727	1664
	(38.3)	(38.6)	(36.2)	(36.9)	(37.6)	(35.4)	(36.6)	(40.6)	(39.7)	(38.3)
c_E	1621.3	1621.3	1621.3	1621.3	1621.3	1621.3	1621.3	1621.3	1621.3	1621.3
	(37.3)	(37.3)	(37.3)	(37.3)	(37.3)	(37.3)	(37.3)	(37.3)	(37.3)	(37.3)
	84.4	109.1	-91.4	-32.5	27.4	-162.3	-58.2	293.2	218.0	86.4
	1.942	2.510	-2.103	-0.749	0.631	-3.736	-1.339	6.747	5.018	1.989
N	4345	4345	4345	4345	4345	4345	4345	4345	4345	4345
X2	24.7	27.3	20.7	46.9	28.3	32.3	26.3	44.4	12.3	13.2
Gvalue	24.1	29.1	20.4	44.2	29.5	30.6	24.9	46.2	12.3	13.2
Gv/N	0.554	0.670	0.469	1.018	0.679	0.703	0.573	1.064	0.282	0.305
Prob	0.00002	0.00000	0.00014	0.00000	0.00000	0.00000	0.00002	0.00000	0.00657	0.00416

	30	29	28	27	26	25	24	23	22	21
a_O	450	470	499	426	471	512	420	541	562	430
	(10.4)	(10.8)	(11.5)	(9.8)	(10.8)	(11.8)	(9.7)	(12.5)	(12.9)	(9.9)
a_E	550.5	550.5	550.5	550.5	550.5	550.5	550.5	550.5	550.5	550.5
	(12.7)	(12.7)	(12.7)	(12.7)	(12.7)	(12.7)	(12.7)	(12.7)	(12.7)	(12.7)
	-181.4	-148.6	-98.0	-218.4	-146.9	-74.2	-227.3	-18.8	23.3	-212.4
	-4.175	-3.419	-2.255	-5.027	-3.381	-1.708	-5.230	-0.433	0.536	-4.889
t_O	451	508	570	461	456	559	478	452	594	499
	(10.4)	(11.7)	(13.1)	(10.6)	(10.5)	(12.9)	(11.0)	(10.4)	(13.7)	(11.5)
t_E	525.6	525.6	525.6	525.6	525.6	525.6	525.6	525.6	525.6	525.6
	(12.1)	(12.1)	(12.1)	(12.1)	(12.1)	(12.1)	(12.1)	(12.1)	(12.1)	(12.1)
	-138.1	-34.6	92.5	-120.9	-129.5	68.9	-90.7	-136.4	145.4	-51.8
	-3.177	-0.796	2.128	-2.782	-2.981	1.586	-2.088	-3.138	3.345	-1.192
g_O	1695	1676	1556	1723	1661	1520	1721	1572	1482	1618
	(39.0)	(38.6)	(35.8)	(39.7)	(38.2)	(35.0)	(39.6)	(36.2)	(34.1)	(37.2)
g_E	1647.6	1647.6	1647.6	1647.6	1647.6	1647.6	1647.6	1647.6	1647.6	1647.6
	(37.9)	(37.9)	(37.9)	(37.9)	(37.9)	(37.9)	(37.9)	(37.9)	(37.9)	(37.9)
	96.2	57.3	-178.0	154.2	26.9	-245.0	150.1	-147.6	-313.9	-58.6
	2.214	1.319	-4.096	3.550	0.620	-5.639	3.454	-3.398	-7.225	-1.349
c_O	1749	1691	1720	1735	1757	1754	1726	1780	1707	1798
	(40.3)	(38.9)	(39.6)	(39.9)	(40.4)	(40.4)	(39.7)	(41.0)	(39.3)	(41.4)
c_E	1621.3	1621.3	1621.3	1621.3	1621.3	1621.3	1621.3	1621.3	1621.3	1621.3
	(37.3)	(37.3)	(37.3)	(37.3)	(37.3)	(37.3)	(37.3)	(37.3)	(37.3)	(37.3)
	265.1	142.3	203.2	235.1	282.3	275.9	215.9	332.3	175.7	371.9
	6.101	3.274	4.676	5.411	6.498	6.349	4.969	7.649	4.045	8.559
N	4345	4345	4345	4345	4345	4345	4345	4345	4345	4345
X2	40.3	15.8	19.7	47.5	32.1	25.5	45.3	29.5	30.3	47.5
Gvalue	41.8	16.4	19.7	50.0	32.9	25.5	48.0	29.5	30.5	49.0
Gv/N	0.963	0.378	0.453	1.151	0.757	0.588	1.105	0.680	0.701	1.128
Prob	0.00000	0.00093	0.00020	0.00000	0.00000	0.00001	0.00000	0.00000	0.00000	0.00000

	20	19	18	17	16	15	14	13	12	11
a_O	549	530	485	585	583	598	810	906	874	1145
	(12.6)	(12.2)	(11.2)	(13.5)	(13.4)	(13.8)	(18.6)	(20.9)	(20.1)	(26.4)
a_E	550.5	550.5	550.5	550.5	550.5	550.5	550.5	550.5	550.5	550.5
	(12.7)	(12.7)	(12.7)	(12.7)	(12.7)	(12.7)	(12.7)	(12.7)	(12.7)	(12.7)
	-3.0	-40.2	-122.8	71.2	66.9	99.0	625.7	902.8	808.1	1677.1
	-0.068	-0.925	-2.827	1.638	1.540	2.279	14.401	20.779	18.598	38.599
t_O	543	604	474	532	630	574	521	566	543	364
	(12.5)	(13.9)	(10.9)	(12.2)	(14.5)	(13.2)	(12.0)	(13.0)	(12.5)	(8.4)

t_E	525.6	525.6	525.6	525.6	525.6	525.6	525.6	525.6	525.6	525.6
	(12.1)	(12.1)	(12.1)	(12.1)	(12.1)	(12.1)	(12.1)	(12.1)	(12.1)	(12.1)
	35.4	168.0	-97.9	12.9	228.3	101.1	-9.1	83.9	35.4	-267.4
	0.815	3.866	-2.254	0.297	5.255	2.328	-0.210	1.930	0.815	-6.155
g_O	1521	1457	1596	1676	1586	1695	1707	1569	1784	1901
	(35.0)	(33.5)	(36.7)	(38.6)	(36.5)	(39.0)	(39.3)	(36.1)	(41.1)	(43.8)
g_E	1647.6	1647.6	1647.6	1647.6	1647.6	1647.6	1647.6	1647.6	1647.6	1647.6
	(37.9)	(37.9)	(37.9)	(37.9)	(37.9)	(37.9)	(37.9)	(37.9)	(37.9)	(37.9)
	-243.2	-358.2	-101.5	57.3	-120.8	96.2	121.0	-153.4	283.8	544.0
	-5.597	-8.244	-2.337	1.319	-2.781	2.214	2.784	-3.529	6.532	12.519
c_O	1732	1754	1790	1552	1546	1478	1307	1304	1144	935
	(39.9)	(40.4)	(41.2)	(35.7)	(35.6)	(34.0)	(30.1)	(30.0)	(26.3)	(21.5)
c_E	1621.3	1621.3	1621.3	1621.3	1621.3	1621.3	1621.3	1621.3	1621.3	1621.3
	(37.3)	(37.3)	(37.3)	(37.3)	(37.3)	(37.3)	(37.3)	(37.3)	(37.3)	(37.3)
	228.7	275.9	354.3	-135.7	-147.1	-273.6	-563.4	-568.1	-797.9	-1029.4
	5.263	6.349	8.153	-3.123	-3.386	-6.298	-12.966	-13.074	-18.363	-23.691
N	4345	4345	4345	4345	4345	4345	4345	4345	4345	4345
X2	17.9	45.4	32.0	5.7	28.5	22.6	185.5	298.6	342.5	1021.3
Gvalue	17.9	45.4	32.0	5.7	27.3	22.7	174.2	265.2	329.4	924.3
Gv/N	0.413	1.046	0.735	0.131	0.627	0.523	4.008	6.105	7.582	21.272
Prob	0.00045	0.00000	0.00000	0.12739	0.00001	0.00005	0.00000	0.00000	0.00000	0.00000

	10	9	8	7	6	5	4	3	2	1
a_O	939	783	936	876	577	729	636	606	564	256
	(21.6)	(18.0)	(21.5)	(20.2)	(13.3)	(16.8)	(14.6)	(13.9)	(13.0)	(5.9)
a_E	550.5	550.5	550.5	550.5	550.5	550.5	550.5	550.5	550.5	550.5
	(12.7)	(12.7)	(12.7)	(12.7)	(12.7)	(12.7)	(12.7)	(12.7)	(12.7)	(12.7)
	1002.9	551.8	993.7	813.9	54.3	409.5	183.7	116.5	27.4	-392.0
	23.082	12.699	22.870	18.733	1.250	9.425	4.228	2.680	0.630	-9.022
t_O	414	355	436	622	443	481	478	355	406	125
	(9.5)	(8.2)	(10.0)	(14.3)	(10.2)	(11.1)	(11.0)	(8.2)	(9.3)	(2.9)
t_E	525.6	525.6	525.6	525.6	525.6	525.6	525.6	525.6	525.6	525.6
	(12.1)	(12.1)	(12.1)	(12.1)	(12.1)	(12.1)	(12.1)	(12.1)	(12.1)	(12.1)
	-197.6	-278.6	-163.0	209.5	-151.5	-85.3	-90.7	-278.6	-209.6	-359.1
	-4.548	-6.412	-3.750	4.822	-3.486	-1.963	-2.088	-6.412	-4.825	-8.264
g_O	2120	2351	2026	1743	1891	1514	1391	1716	1241	1260
	(48.8)	(54.1)	(46.6)	(40.1)	(43.5)	(34.8)	(32.0)	(39.5)	(28.6)	(29.0)
g_E	1647.6	1647.6	1647.6	1647.6	1647.6	1647.6	1647.6	1647.6	1647.6	1647.6
	(37.9)	(37.9)	(37.9)	(37.9)	(37.9)	(37.9)	(37.9)	(37.9)	(37.9)	(37.9)
	1068.9	1671.7	837.8	196.3	521.2	-256.0	-470.9	139.6	-703.4	-675.9
	24.602	38.475	19.281	4.517	11.994	-5.892	-10.839	3.214	-16.188	-15.555
c_O	872	856	947	1104	1434	1621	1840	1668	2134	2704
	(20.1)	(19.7)	(21.8)	(25.4)	(33.0)	(37.3)	(42.3)	(38.4)	(49.1)	(62.2)
c_E	1621.3	1621.3	1621.3	1621.3	1621.3	1621.3	1621.3	1621.3	1621.3	1621.3
	(37.3)	(37.3)	(37.3)	(37.3)	(37.3)	(37.3)	(37.3)	(37.3)	(37.3)	(37.3)
	-1081.7	-1093.5	-1018.4	-848.6	-352.2	-0.7	465.5	94.6	1172.6	2766.0
	-24.895	-25.168	-23.439	-19.530	-8.105	-0.016	10.714	2.178	26.987	63.660
N	4345	4345	4345	4345	4345	4345	4345	4345	4345	4345
X2	779.7	815.2	652.7	380.8	71.9	72.5	87.0	65.2	290.0	1277.0
Gvalue	792.6	851.3	650.1	371.1	71.8	67.5	87.5	72.1	286.9	1339.1
Gv/N	18.241	19.594	14.962	8.542	1.653	1.554	2.015	1.660	6.604	30.820
Prob	0.00000	0.00000	0.00000	0.00000	0.00000	0.00000	0.00000	0.00000	0.00000	0.00000

CDS_INITIAL:

	1	2	3	4	5	6	7	8	9	10
a_O	3008	0	0	1521	853	345	922	999	347	842
	(69.2)	(0.0)	(0.0)	(35.0)	(19.6)	(7.9)	(21.2)	(23.0)	(8.0)	(19.4)
a_E	613.0	930.3	48.8	613.0	930.3	48.8	613.0	930.3	48.8	613.0
	(14.1)	(21.4)	(1.1)	(14.1)	(21.4)	(1.1)	(14.1)	(21.4)	(1.1)	(14.1)
	9569.9	0.0	0.0	2764.7	-148.1	1349.7	752.8	142.3	1361.5	534.7
	220.251	0.000	0.000	63.629	-3.407	31.063	17.326	3.275	31.335	12.305
t_O	144	4345	0	303	543	254	366	831	311	457
	(3.3)	(100.0)	(0.0)	(7.0)	(12.5)	(5.8)	(8.4)	(19.1)	(7.2)	(10.5)
t_E	440.1	1162.6	71.1	440.1	1162.6	71.1	440.1	1162.6	71.1	440.1
	(10.1)	(26.8)	(1.6)	(10.1)	(26.8)	(1.6)	(10.1)	(26.8)	(1.6)	(10.1)
	-321.8	11456.8	0.0	-226.2	-826.8	646.8	-135.0	-558.0	917.9	34.4
	-7.405	263.678	0.000	-5.207	-19.028	14.886	-3.107	-12.843	21.125	0.792
g_O	1193	0	4345	1245	1119	1381	1547	1085	1549	1408
	(27.5)	(0.0)	(100.0)	(28.7)	(25.8)	(31.8)	(35.6)	(25.0)	(35.7)	(32.4)
g_E	1957.4	957.1	2013.7	1957.4	957.1	2013.7	1957.4	957.1	2013.7	1957.4
	(45.0)	(22.0)	(46.3)	(45.0)	(22.0)	(46.3)	(45.0)	(22.0)	(46.3)	(45.0)
	-1181.4	0.0	6683.0	-1126.7	349.6	-1041.7	-728.0	272.1	-812.8	-927.7
	-27.190	0.000	153.810	-25.931	8.047	-23.976	-16.755	6.262	-18.707	-21.352
c_O	0	0	0	1276	1830	2365	1510	1430	2138	1638
	(0.0)	(0.0)	(0.0)	(29.4)	(42.1)	(54.4)	(34.8)	(32.9)	(49.2)	(37.7)
c_E	1334.5	1294.9	2211.4	1334.5	1294.9	2211.4	1334.5	1294.9	2211.4	1334.5
	(30.7)	(29.8)	(50.9)	(30.7)	(29.8)	(50.9)	(30.7)	(29.8)	(50.9)	(30.7)
	0.0	0.0	0.0	-114.4	1265.8	317.6	373.1	283.7	-144.3	671.3
	0.000	0.000	0.000	-2.634	29.132	7.310	8.586	6.530	-3.322	15.449
N	4345	4345	4345	4345	4345	4345	4345	4345	4345	4345
X2	11190.7	11894.0	5030.3	1649.8	585.1	2478.3	277.4	130.8	2741.9	309.5
Gvalue	8066.7	11456.8	6683.0	1297.3	640.6	1272.3	262.9	140.0	1322.2	312.6
Gv/N	185.656	263.678	153.810	29.858	14.744	29.283	6.050	3.223	30.431	7.194
Prob	1.00000	1.00000	1.00000	0.00000	0.00000	0.00000	0.00000	0.00000	0.00000	0.00000

	11	12	13	14	15	16	17	18	19	20
a_O	801	249	868	852	268	813	882	278	869	797
	(18.4)	(5.7)	(20.0)	(19.6)	(6.2)	(18.7)	(20.3)	(6.4)	(20.0)	(18.3)
a_E	930.3	48.8	613.0	930.3	48.8	613.0	930.3	48.8	613.0	930.3
	(21.4)	(1.1)	(14.1)	(21.4)	(1.1)	(14.1)	(21.4)	(1.1)	(14.1)	(21.4)
	-239.8	811.7	604.0	-149.9	913.1	459.3	-94.1	967.5	606.7	-246.6
	-5.519	18.682	13.900	-3.449	21.014	10.570	-2.166	22.267	13.962	-5.675
t_O	1057	277	402	1199	260	444	1193	214	430	1277
	(24.3)	(6.4)	(9.3)	(27.6)	(6.0)	(10.2)	(27.5)	(4.9)	(9.9)	(29.4)
t_E	1162.6	71.1	440.1	1162.6	71.1	440.1	1162.6	71.1	440.1	1162.6
	(26.8)	(1.6)	(10.1)	(26.8)	(1.6)	(10.1)	(26.8)	(1.6)	(10.1)	(26.8)
	-201.3	753.4	-72.8	74.0	674.2	7.8	61.6	471.6	-20.0	239.8
	-4.632	17.339	-1.676	1.703	15.517	0.179	1.419	10.854	-0.460	5.518
g_O	942	1501	1515	902	1510	1557	898	1575	1597	871
	(21.7)	(34.5)	(34.9)	(20.8)	(34.8)	(35.8)	(20.7)	(36.2)	(36.8)	(20.0)
g_E	957.1	2013.7	1957.4	957.1	2013.7	1957.4	957.1	2013.7	1957.4	957.1
	(22.0)	(46.3)	(45.0)	(22.0)	(46.3)	(45.0)	(22.0)	(46.3)	(45.0)	(22.0)
	-30.1	-882.1	-776.3	-107.1	-869.4	-712.7	-114.6	-774.0	-650.0	-164.3
	-0.692	-20.302	-17.867	-2.464	-20.008	-16.402	-2.637	-17.814	-14.959	-3.781

c_O	1545	2318	1560	1392	2307	1531	1372	2278	1449	1400
	(35.6)	(53.3)	(35.9)	(32.0)	(53.1)	(35.2)	(31.6)	(52.4)	(33.3)	(32.2)
c_E	1294.9	2211.4	1334.5	1294.9	2211.4	1334.5	1294.9	2211.4	1334.5	1294.9
	(29.8)	(50.9)	(30.7)	(29.8)	(50.9)	(30.7)	(29.8)	(50.9)	(30.7)	(29.8)
	545.6	218.2	487.1	201.2	195.3	420.6	158.6	135.2	238.5	218.4
	12.556	5.023	11.210	4.631	4.494	9.679	3.650	3.111	5.489	5.027
N	4345	4345	4345	4345	4345	4345	4345	4345	4345	4345
X2	76.1	1553.5	247.5	18.2	1616.9	176.2	11.5	1461.6	183.4	46.6
Gvalue	74.5	901.2	241.9	18.3	913.2	174.9	11.6	800.3	175.2	47.3
Gv/N	1.714	20.742	5.567	0.420	21.017	4.026	0.266	18.418	4.032	1.089
Prob	0.00000	0.00000	0.00000	0.00039	0.00000	0.00000	0.00899	0.00000	0.00000	0.00000

	21	22	23	24	25	26	27	28	29	30
a_O	185	739	752	154	681	789	138	571	718	135
	(4.3)	(17.0)	(17.3)	(3.5)	(15.7)	(18.2)	(3.2)	(13.1)	(16.5)	(3.1)
a_E	48.8	613.0	930.3	48.8	613.0	930.3	48.8	613.0	930.3	48.8
	(1.1)	(14.1)	(21.4)	(1.1)	(14.1)	(21.4)	(1.1)	(14.1)	(21.4)	(1.1)
	493.2	276.4	-320.1	354.0	143.4	-260.0	287.0	-81.0	-372.0	274.8
	11.350	6.361	-7.366	8.148	3.300	-5.984	6.605	-1.863	-8.562	6.325
t_O	195	451	1264	170	426	1247	168	429	1242	181
	(4.5)	(10.4)	(29.1)	(3.9)	(9.8)	(28.7)	(3.9)	(9.9)	(28.6)	(4.2)
t_E	71.1	440.1	1162.6	71.1	440.1	1162.6	71.1	440.1	1162.6	71.1
	(1.6)	(10.1)	(26.8)	(1.6)	(10.1)	(26.8)	(1.6)	(10.1)	(26.8)	(1.6)
	393.5	22.0	211.5	296.4	-27.8	174.8	288.9	-22.0	164.2	338.2
	9.056	0.507	4.867	6.821	-0.639	4.024	6.649	-0.505	3.778	7.785
g_O	1650	1675	889	1681	1798	889	1719	1900	940	1756
	(38.0)	(38.6)	(20.5)	(38.7)	(41.4)	(20.5)	(39.6)	(43.7)	(21.6)	(40.4)
g_E	2013.7	1957.4	957.1	2013.7	1957.4	957.1	2013.7	1957.4	957.1	2013.7
	(46.3)	(45.0)	(22.0)	(46.3)	(45.0)	(22.0)	(46.3)	(45.0)	(22.0)	(46.3)
	-657.4	-522.0	-131.3	-607.1	-305.5	-131.3	-544.0	-113.1	-34.0	-480.9
	-15.129	-12.013	-3.023	-13.973	-7.030	-3.023	-12.520	-2.603	-0.782	-11.068
c_O	2315	1480	1440	2340	1440	1420	2320	1445	1445	2273
	(53.3)	(34.1)	(33.1)	(53.9)	(33.1)	(32.7)	(53.4)	(33.3)	(33.3)	(52.3)
c_E	2211.4	1334.5	1294.9	2211.4	1334.5	1294.9	2211.4	1334.5	1294.9	2211.4
	(50.9)	(30.7)	(29.8)	(50.9)	(30.7)	(29.8)	(50.9)	(30.7)	(29.8)	(50.9)
	212.0	306.3	305.8	264.5	219.1	261.8	222.4	229.9	316.9	124.9
	4.878	7.049	7.038	6.088	5.042	6.026	5.119	5.290	7.293	2.874
N	4345	4345	4345	4345	4345	4345	4345	4345	4345	4345
X2	666.7	82.8	64.1	426.9	29.3	44.5	343.6	14.0	71.6	356.9
Gvalue	441.2	82.7	65.9	307.8	29.2	45.3	254.3	13.8	75.0	257.0
Gv/N	10.155	1.904	1.516	7.084	0.673	1.043	5.853	0.318	1.726	5.915
Prob	0.00000	0.00000	0.00000	0.00000	0.00000	0.00000	0.00000	0.00315	0.00000	0.00000

	31	32	33	34	35	36	37	38	39	40
a_O	546	709	134	588	730	107	606	735	118	610
	(12.6)	(16.3)	(3.1)	(13.5)	(16.8)	(2.5)	(13.9)	(16.9)	(2.7)	(14.0)
a_E	613.0	930.3	48.8	613.0	930.3	48.8	613.0	930.3	48.8	613.0
	(14.1)	(21.4)	(1.1)	(14.1)	(21.4)	(1.1)	(14.1)	(21.4)	(1.1)	(14.1)
	-126.3	-385.3	270.8	-48.9	-354.0	168.1	-13.8	-346.4	208.4	-5.9
	-2.907	-8.867	6.232	-1.125	-8.148	3.868	-0.318	-7.973	4.797	-0.136
t_O	429	1243	162	392	1239	155	430	1279	131	402
	(9.9)	(28.6)	(3.7)	(9.0)	(28.5)	(3.6)	(9.9)	(29.4)	(3.0)	(9.3)
t_E	440.1	1162.6	71.1	440.1	1162.6	71.1	440.1	1162.6	71.1	440.1
	(10.1)	(26.8)	(1.6)	(10.1)	(26.8)	(1.6)	(10.1)	(26.8)	(1.6)	(10.1)
	-22.0	166.3	266.8	-90.8	157.8	241.6	-20.0	244.1	160.1	-72.8
	-0.505	3.827	6.141	-2.089	3.631	5.560	-0.460	5.619	3.685	-1.676
g_O	1889	945	1719	1895	956	1795	1906	883	1734	1926
	(43.5)	(21.7)	(39.6)	(43.6)	(22.0)	(41.3)	(43.9)	(20.3)	(39.9)	(44.3)
g_E	1957.4	957.1	2013.7	1957.4	957.1	2013.7	1957.4	957.1	2013.7	1957.4
	(45.0)	(22.0)	(46.3)	(45.0)	(22.0)	(46.3)	(45.0)	(22.0)	(46.3)	(45.0)
	-134.4	-24.1	-544.0	-122.8	-2.3	-412.7	-101.4	-142.4	-518.6	-62.3
	-3.093	-0.556	-12.520	-2.826	-0.053	-9.499	-2.335	-3.277	-11.936	-1.434
c_O	1481	1448	2330	1470	1420	2288	1403	1448	2362	1407
	(34.1)	(33.3)	(53.6)	(33.8)	(32.7)	(52.7)	(32.3)	(33.3)	(54.4)	(32.4)
c_E	1334.5	1294.9	2211.4	1334.5	1294.9	2211.4	1334.5	1294.9	2211.4	1334.5
	(30.7)	(29.8)	(50.9)	(30.7)	(29.8)	(50.9)	(30.7)	(29.8)	(50.9)	(30.7)
	308.5	323.5	243.4	284.3	261.8	155.8	140.4	323.5	311.2	148.8
	7.100	7.446	5.603	6.542	6.026	3.586	3.232	7.446	7.163	3.425
N	4345	4345	4345	4345	4345	4345	4345	4345	4345	4345
X2	26.1	76.5	314.5	22.0	60.2	194.9	5.2	76.5	197.7	7.8
Gvalue	25.8	80.4	237.0	21.8	63.2	152.7	5.1	78.8	161.1	7.8
Gv/N	0.594	1.851	5.455	0.502	1.456	3.515	0.118	1.814	3.708	0.179
Prob	0.00001	0.00000	0.00000	0.00007	0.00000	0.00000	0.16246	0.00000	0.00000	0.05057

	41	42	43	44	45	46	47	48	49	50
a_O	757	117	579	742	84	572	718	113	556	753
	(17.4)	(2.7)	(13.3)	(17.1)	(1.9)	(13.2)	(16.5)	(2.6)	(12.8)	(17.3)
a_E	930.3	48.8	613.0	930.3	48.8	613.0	930.3	48.8	613.0	930.3
	(21.4)	(1.1)	(14.1)	(21.4)	(1.1)	(14.1)	(21.4)	(1.1)	(14.1)	(21.4)
	-312.2	204.7	-66.0	-335.7	91.3	-79.1	-372.0	189.8	-108.4	-318.5
	-7.184	4.711	-1.519	-7.725	2.101	-1.821	-8.562	4.369	-2.496	-7.330
t_O	1195	130	437	1188	142	429	1222	139	455	1131
	(27.5)	(3.0)	(10.1)	(27.3)	(3.3)	(9.9)	(28.1)	(3.2)	(10.5)	(26.0)
t_E	1162.6	71.1	440.1	1162.6	71.1	440.1	1162.6	71.1	440.1	1162.6
	(26.8)	(1.6)	(10.1)	(26.8)	(1.6)	(10.1)	(26.8)	(1.6)	(10.1)	(26.8)
	65.8	156.9	-6.2	51.4	196.4	-22.0	121.8	186.4	30.3	-62.3
	1.513	3.611	-0.143	1.183	4.521	-0.505	2.804	4.289	0.696	-1.433
g_O	929	1776	1962	911	1798	1970	943	1735	1993	954
	(21.4)	(40.9)	(45.2)	(21.0)	(41.4)	(45.3)	(21.7)	(39.9)	(45.9)	(22.0)
g_E	957.1	2013.7	1957.4	957.1	2013.7	1957.4	957.1	2013.7	1957.4	957.1
	(22.0)	(46.3)	(45.0)	(22.0)	(46.3)	(45.0)	(22.0)	(46.3)	(45.0)	(22.0)
	-55.5	-446.2	9.2	-90.0	-407.4	25.3	-28.1	-516.9	71.8	-6.3
	-1.276	-10.269	0.212	-2.072	-9.377	0.582	-0.646	-11.897	1.653	-0.145
c_O	1464	2322	1367	1504	2321	1374	1462	2358	1341	1507
	(33.7)	(53.4)	(31.5)	(34.6)	(53.4)	(31.6)	(33.6)	(54.3)	(30.9)	(34.7)
c_E	1294.9	2211.4	1334.5	1294.9	2211.4	1334.5	1294.9	2211.4	1334.5	1294.9
	(29.8)	(50.9)	(30.7)	(29.8)	(50.9)	(30.7)	(29.8)	(50.9)	(30.7)	(29.8)
	359.3	226.6	65.7	450.2	224.5	80.1	354.8	302.7	13.0	457.1
	8.269	5.216	1.513	10.361	5.168	1.844	8.166	6.967	0.299	10.520
N	4345	4345	4345	4345	4345	4345	4345	4345	4345	4345
X2	56.1	177.7	2.7	74.7	124.6	4.3	73.3	197.6	6.5	69.4
Gvalue	57.4	142.0	2.7	75.9	104.8	4.3	76.5	162.0	6.6	70.0
Gv/N	1.321	3.269	0.063	1.746	2.413	0.099	1.761	3.727	0.153	1.612
Prob	0.00000	0.00000	0.43459	0.00000	0.00000	0.22894	0.00000	0.00000	0.08446	0.00000

	51	52	53	54	55	56	57	58	59	60
a_O	97	550	722	96	554	750	78	581	784	78
	(2.2)	(12.7)	(16.6)	(2.2)	(12.8)	(17.3)	(1.8)	(13.4)	(18.0)	(1.8)
a_E	48.8	613.0	930.3	48.8	613.0	930.3	48.8	613.0	930.3	48.8
	(1.1)	(14.1)	(21.4)	(1.1)	(14.1)	(21.4)	(1.1)	(14.1)	(21.4)	(1.1)
	133.3	-119.2	-366.1	130.0	-112.0	-323.2	73.2	-62.2	-268.3	73.2
	3.068	-2.743	-8.425	2.991	-2.579	-7.439	1.685	-1.432	-6.176	1.685
t_O	124	449	1200	124	455	1182	128	440	1153	108
	(2.9)	(10.3)	(27.6)	(2.9)	(10.5)	(27.2)	(2.9)	(10.1)	(26.5)	(2.5)
t_E	71.1	440.1	1162.6	71.1	440.1	1162.6	71.1	440.1	1162.6	71.1
	(1.6)	(10.1)	(26.8)	(1.6)	(10.1)	(26.8)	(1.6)	(10.1)	(26.8)	(1.6)
	137.9	17.9	76.0	137.9	30.3	39.2	150.5	-0.2	-19.1	90.3
	3.174	0.413	1.750	3.174	0.696	0.902	3.464	-0.006	-0.439	2.078
g_O	1842	1951	945	1786	1991	954	1824	1966	985	1901
	(42.4)	(44.9)	(21.7)	(41.1)	(45.8)	(22.0)	(42.0)	(45.2)	(22.7)	(43.8)
g_E	2013.7	1957.4	957.1	2013.7	1957.4	957.1	2013.7	1957.4	957.1	2013.7
	(46.3)	(45.0)	(22.0)	(46.3)	(45.0)	(22.0)	(46.3)	(45.0)	(22.0)	(46.3)
	-328.3	-12.8	-24.1	-428.6	67.8	-6.3	-360.9	17.2	56.5	-219.0
	-7.557	-0.294	-0.556	-9.865	1.560	-0.145	-8.307	0.396	1.300	-5.040
c_O	2282	1395	1478	2339	1345	1459	2315	1358	1423	2258
	(52.5)	(32.1)	(34.0)	(53.8)	(31.0)	(33.6)	(53.3)	(31.3)	(32.8)	(52.0)
c_E	2211.4	1334.5	1294.9	2211.4	1334.5	1294.9	2211.4	1334.5	1294.9	2211.4
	(50.9)	(30.7)	(29.8)	(50.9)	(30.7)	(29.8)	(50.9)	(30.7)	(29.8)	(50.9)
	143.4	123.7	390.8	262.4	21.0	348.1	212.0	47.4	268.4	94.2
	3.301	2.846	8.995	6.039	0.484	8.011	4.878	1.090	6.177	2.167
N	4345	4345	4345	4345	4345	4345	4345	4345	4345	4345
X2	103.9	9.4	73.9	118.2	6.8	56.1	85.7	2.1	36.6	43.9
Gvalue	86.3	9.6	76.7	101.7	7.0	57.8	74.7	2.1	37.5	38.7
Gv/N	1.987	0.221	1.765	2.340	0.161	1.329	1.720	0.049	0.863	0.890
Prob	0.00000	0.02231	0.00000	0.00000	0.07147	0.00000	0.00000	0.54314	0.00000	0.00000

	61	62	63	64	65	66	67	68	69	70
a_O	575	839	75	543	801	92	557	848	76	545
	(13.2)	(19.3)	(1.7)	(12.5)	(18.4)	(2.1)	(12.8)	(19.5)	(1.7)	(12.5)
a_E	613.0	930.3	48.8	613.0	930.3	48.8	613.0	930.3	48.8	613.0
	(14.1)	(21.4)	(1.1)	(14.1)	(21.4)	(1.1)	(14.1)	(21.4)	(1.1)	(14.1)
	-73.5	-173.4	64.5	-131.6	-239.8	116.7	-106.6	-157.2	67.4	-128.1
	-1.692	-3.991	1.484	-3.029	-5.519	2.686	-2.454	-3.617	1.551	-2.948
t_O	431	1152	109	426	1118	118	404	1182	123	424
	(9.9)	(26.5)	(2.5)	(9.8)	(25.7)	(2.7)	(9.3)	(27.2)	(2.8)	(9.8)
t_E	440.1	1162.6	71.1	440.1	1162.6	71.1	440.1	1162.6	71.1	440.1
	(10.1)	(26.8)	(1.6)	(10.1)	(26.8)	(1.6)	(10.1)	(26.8)	(1.6)	(10.1)
	-18.1	-21.0	93.1	-27.8	-87.4	119.6	-69.2	39.2	134.8	-31.6
	-0.415	-0.484	2.144	-0.639	-2.012	2.751	-1.592	0.902	3.103	-0.728
g_O	1962	944	1864	1987	998	1911	1999	961	1896	2004
	(45.2)	(21.7)	(42.9)	(45.7)	(23.0)	(44.0)	(46.0)	(22.1)	(43.6)	(46.1)
g_E	1957.4	957.1	2013.7	1957.4	957.1	2013.7	1957.4	957.1	2013.7	1957.4
	(45.0)	(22.0)	(46.3)	(45.0)	(22.0)	(46.3)	(45.0)	(22.0)	(46.3)	(45.0)
	9.2	-26.1	-288.0	59.6	83.4	-200.1	84.1	7.7	-228.4	94.3
	0.212	-0.601	-6.628	1.372	1.920	-4.605	1.935	0.178	-5.256	2.170
c_O	1377	1410	2297	1389	1428	2224	1385	1354	2250	1372
	(31.7)	(32.5)	(52.9)	(32.0)	(32.9)	(51.2)	(31.9)	(31.2)	(51.8)	(31.6)
c_E	1334.5	1294.9	2211.4	1334.5	1294.9	2211.4	1334.5	1294.9	2211.4	1334.5
	(30.7)	(29.8)	(50.9)	(30.7)	(29.8)	(50.9)	(30.7)	(29.8)	(50.9)	(30.7)
	86.3	240.0	174.5	111.2	279.3	25.3	102.8	120.8	77.9	76.0
	1.986	5.525	4.015	2.558	6.429	0.581	2.367	2.779	1.792	1.749
N	4345	4345	4345	4345	4345	4345	4345	4345	4345	4345
X2	3.9	19.5	48.7	11.1	35.1	74.5	10.9	10.3	60.6	10.3
Gvalue	3.9	19.5	44.1	11.4	35.6	61.4	11.1	10.5	51.7	10.6
Gv/N	0.091	0.448	1.015	0.262	0.818	1.414	0.255	0.242	1.189	0.243
Prob	0.26813	0.00022	0.00000	0.00974	0.00000	0.00000	0.01129	0.01474	0.00000	0.01430

	71	72	73	74	75	76	77	78	79	80
a_O	807	68	578	822	87	566	856	80	569	897
	(18.6)	(1.6)	(13.3)	(18.9)	(2.0)	(13.0)	(19.7)	(1.8)	(13.1)	(20.6)
a_E	930.3	48.8	613.0	930.3	48.8	613.0	930.3	48.8	613.0	930.3
	(21.4)	(1.1)	(14.1)	(21.4)	(1.1)	(14.1)	(21.4)	(1.1)	(14.1)	(21.4)
	-229.5	45.2	-67.9	-203.5	100.6	-90.2	-142.6	79.1	-84.7	-65.5
	-5.283	1.039	-1.562	-4.684	2.316	-2.076	-3.281	1.821	-1.949	-1.507
t_O	1171	130	394	1164	107	423	1138	99	418	1138
	(27.0)	(3.0)	(9.1)	(26.8)	(2.5)	(9.7)	(26.2)	(2.3)	(9.6)	(26.2)
t_E	1162.6	71.1	440.1	1162.6	71.1	440.1	1162.6	71.1	440.1	1162.6
	(26.8)	(1.6)	(10.1)	(26.8)	(1.6)	(10.1)	(26.8)	(1.6)	(10.1)	(26.8)
	16.9	156.9	-87.2	2.9	87.5	-33.6	-48.6	65.5	-43.1	-48.6
	0.389	3.611	-2.008	0.066	2.013	-0.773	-1.119	1.508	-0.992	-1.119
g_O	998	1900	1982	960	1926	1979	964	1907	2009	947
	(23.0)	(43.7)	(45.6)	(22.1)	(44.3)	(45.5)	(22.2)	(43.9)	(46.2)	(21.8)
g_E	957.1	2013.7	1957.4	957.1	2013.7	1957.4	957.1	2013.7	1957.4	957.1
	(22.0)	(46.3)	(45.0)	(22.0)	(46.3)	(45.0)	(22.0)	(46.3)	(45.0)	(22.0)
	83.4	-220.9	49.5	5.7	-171.5	43.4	13.7	-207.7	104.5	-20.2
	1.920	-5.083	1.139	0.131	-3.948	0.999	0.316	-4.779	2.406	-0.465
c_O	1369	2247	1391	1399	2225	1377	1387	2259	1349	1363
	(31.5)	(51.7)	(32.0)	(32.2)	(51.2)	(31.7)	(31.9)	(52.0)	(31.0)	(31.4)
c_E	1294.9	2211.4	1334.5	1294.9	2211.4	1334.5	1294.9	2211.4	1334.5	1294.9
	(29.8)	(50.9)	(30.7)	(29.8)	(50.9)	(30.7)	(29.8)	(50.9)	(30.7)	(29.8)
	152.3	71.8	115.3	216.3	27.3	86.3	190.5	96.2	29.1	139.6
	3.504	1.651	2.654	4.977	0.628	1.986	4.384	2.214	0.670	3.214
N	4345	4345	4345	4345	4345	4345	4345	4345	4345	4345
X2	22.4	63.3	9.5	21.0	52.0	5.9	13.1	37.6	5.8	5.4
Gvalue	23.1	52.9	9.7	21.3	43.8	5.9	13.1	33.2	5.9	5.4
Gv/N	0.531	1.218	0.223	0.490	1.009	0.137	0.301	0.765	0.135	0.123
Prob	0.00004	0.00000	0.02122	0.00009	0.00000	0.11446	0.00449	0.00000	0.11835	0.14754

	81	82	83	84	85	86	87	88	89	90
a_O	85	515	867	75	563	868	60	559	888	80
	(2.0)	(11.9)	(20.0)	(1.7)	(13.0)	(20.0)	(1.4)	(12.9)	(20.4)	(1.8)
a_E	48.8	613.0	930.3	48.8	613.0	930.3	48.8	613.0	930.3	48.8
	(1.1)	(14.1)	(21.4)	(1.1)	(14.1)	(21.4)	(1.1)	(14.1)	(21.4)	(1.1)
	94.4	-179.3	-122.3	64.5	-95.7	-120.4	24.8	-103.0	-82.7	79.1
	2.172	-4.128	-2.814	1.484	-2.203	-2.771	0.571	-2.371	-1.904	1.821
t_O	114	455	1116	112	444	1161	112	422	1109	85
	(2.6)	(10.5)	(25.7)	(2.6)	(10.2)	(26.7)	(2.6)	(9.7)	(25.5)	(2.0)
t_E	71.1	440.1	1162.6	71.1	440.1	1162.6	71.1	440.1	1162.6	71.1
	(1.6)	(10.1)	(26.8)	(1.6)	(10.1)	(26.8)	(1.6)	(10.1)	(26.8)	(1.6)
	107.6	30.3	-91.3	101.8	7.8	-3.1	101.8	-35.5	-104.6	30.4

	2.477	0.696	-2.100	2.343	0.179	-0.072	2.343	-0.817	-2.408	0.699
g_O	1955	1949	979	1888	1956	956	1930	1965	978	1958
	(45.0)	(44.9)	(22.5)	(43.5)	(45.0)	(22.0)	(44.4)	(45.2)	(22.5)	(45.1)
g_E	2013.7	1957.4	957.1	2013.7	1957.4	957.1	2013.7	1957.4	957.1	2013.7
	(46.3)	(45.0)	(22.0)	(46.3)	(45.0)	(22.0)	(46.3)	(45.0)	(22.0)	(46.3)
	-115.7	-16.8	44.2	-243.4	-2.8	-2.3	-163.9	15.2	42.2	-109.9
	-2.662	-0.386	1.017	-5.602	-0.065	-0.053	-3.772	0.350	0.970	-2.528
c_O	2191	1426	1383	2270	1382	1360	2243	1399	1370	2222
	(50.4)	(32.8)	(31.8)	(52.2)	(31.8)	(31.3)	(51.6)	(32.2)	(31.5)	(51.1)
c_E	2211.4	1334.5	1294.9	2211.4	1334.5	1294.9	2211.4	1334.5	1294.9	2211.4
	(50.9)	(30.7)	(29.8)	(50.9)	(30.7)	(29.8)	(50.9)	(30.7)	(29.8)	(50.9)
	-40.6	189.1	182.0	118.7	96.6	133.3	63.6	132.0	154.4	21.2
	-0.935	4.352	4.188	2.732	2.224	3.069	1.465	3.038	3.553	0.489
N	4345	4345	4345	4345	4345	4345	4345	4345	4345	4345
X2	54.7	22.5	12.7	47.0	5.8	7.4	30.0	8.6	9.2	24.3
Gvalue	45.7	23.2	12.7	41.6	5.9	7.5	26.4	8.7	9.2	20.9
Gv/N	1.052	0.535	0.291	0.958	0.136	0.172	0.607	0.201	0.211	0.480
Prob	0.00000	0.00004	0.00544	0.00000	0.11706	0.05778	0.00001	0.03285	0.02695	0.00011

	91	92	93	94	95	96	97	98	99	100
a_O	577	831	60	567	862	67	562	901	50	590
	(13.3)	(19.1)	(1.4)	(13.0)	(19.8)	(1.5)	(12.9)	(20.7)	(1.2)	(13.6)
a_E	613.0	930.3	48.8	613.0	930.3	48.8	613.0	930.3	48.8	613.0
	(14.1)	(21.4)	(1.1)	(14.1)	(21.4)	(1.1)	(14.1)	(21.4)	(1.1)	(14.1)
	-69.8	-187.7	24.8	-88.4	-131.5	42.5	-97.5	-57.7	2.5	-45.0
	-1.605	-4.319	0.571	-2.034	-3.027	0.978	-2.245	-1.329	0.056	-1.036
t_O	462	1188	85	450	1146	89	454	1103	90	436
	(10.6)	(27.3)	(2.0)	(10.4)	(26.4)	(2.0)	(10.4)	(25.4)	(2.1)	(10.0)
t_E	440.1	1162.6	71.1	440.1	1162.6	71.1	440.1	1162.6	71.1	440.1
	(10.1)	(26.8)	(1.6)	(10.1)	(26.8)	(1.6)	(10.1)	(26.8)	(1.6)	(10.1)
	44.8	51.4	30.4	20.0	-32.9	40.0	28.2	-116.0	42.4	-8.2
	1.032	1.183	0.699	0.460	-0.757	0.920	0.649	-2.671	0.976	-0.189
g_O	1944	976	1923	1969	1004	1950	1988	1005	1912	2043
	(44.7)	(22.5)	(44.3)	(45.3)	(23.1)	(44.9)	(45.8)	(23.1)	(44.0)	(47.0)
g_E	1957.4	957.1	2013.7	1957.4	957.1	2013.7	1957.4	957.1	2013.7	1957.4
	(45.0)	(22.0)	(46.3)	(45.0)	(22.0)	(46.3)	(45.0)	(22.0)	(46.3)	(45.0)
	-26.7	38.1	-177.3	23.3	96.0	-125.4	61.7	98.1	-198.2	174.9
	-0.615	0.876	-4.080	0.535	2.208	-2.885	1.419	2.257	-4.561	4.025
c_O	1362	1350	2277	1359	1333	2239	1341	1336	2293	1276
	(31.3)	(31.1)	(52.4)	(31.3)	(30.7)	(51.5)	(30.9)	(30.7)	(52.8)	(29.4)
c_E	1334.5	1294.9	2211.4	1334.5	1294.9	2211.4	1334.5	1294.9	2211.4	1334.5
	(30.7)	(29.8)	(50.9)	(30.7)	(29.8)	(50.9)	(30.7)	(29.8)	(50.9)	(30.7)
	55.5	112.4	133.1	49.4	77.2	55.5	13.0	83.4	166.2	-114.4
	1.278	2.587	3.064	1.137	1.777	1.278	0.299	1.919	3.824	-2.634
N	4345	4345	4345	4345	4345	4345	4345	4345	4345	4345
X2	3.9	13.9	11.3	4.2	8.7	13.7	5.2	7.7	13.2	7.2
Gvalue	3.9	14.2	11.0	4.3	8.7	12.6	5.3	7.7	12.9	7.2
Gv/N	0.089	0.328	0.254	0.098	0.201	0.291	0.122	0.177	0.296	0.166
Prob	0.27527	0.00260	0.01157	0.23382	0.03283	0.00551	0.15123	0.05306	0.00495	0.06570

	101	102	103	104	105	106	107	108	109	110
a_O	910	56	615	884	61	558	881	60	591	901
	(20.9)	(1.3)	(14.2)	(20.3)	(1.4)	(12.8)	(20.3)	(1.4)	(13.6)	(20.7)
a_E	930.3	48.8	613.0	930.3	48.8	613.0	930.3	48.8	613.0	930.3
	(21.4)	(1.1)	(14.1)	(21.4)	(1.1)	(14.1)	(21.4)	(1.1)	(14.1)	(21.4)
	-40.2	15.4	4.1	-90.3	27.3	-104.8	-96.0	24.8	-43.1	-57.7
	-0.926	0.355	0.094	-2.079	0.627	-2.412	-2.210	0.571	-0.992	-1.329
t_O	1116	99	438	1085	89	432	1164	94	449	1141
	(25.7)	(2.3)	(10.1)	(25.0)	(2.0)	(9.9)	(26.8)	(2.2)	(10.3)	(26.3)
t_E	1162.6	71.1	440.1	1162.6	71.1	440.1	1162.6	71.1	440.1	1162.6
	(26.8)	(1.6)	(10.1)	(26.8)	(1.6)	(10.1)	(26.8)	(1.6)	(10.1)	(26.8)
	-91.3	65.5	-4.2	-149.9	40.0	-16.1	2.9	52.5	17.9	-42.7
	-2.100	1.508	-0.097	-3.449	0.920	-0.370	0.066	1.208	0.413	-0.984
g_O	959	1927	1946	1037	1945	2041	975	1895	1984	970
	(22.1)	(44.3)	(44.8)	(23.9)	(44.8)	(47.0)	(22.4)	(43.6)	(45.7)	(22.3)
g_E	957.1	2013.7	1957.4	957.1	2013.7	1957.4	957.1	2013.7	1957.4	957.1
	(22.0)	(46.3)	(45.0)	(22.0)	(46.3)	(45.0)	(22.0)	(46.3)	(45.0)	(22.0)
	3.7	-169.6	-22.7	166.2	-135.0	170.7	36.0	-230.3	53.5	25.9
	0.085	-3.904	-0.523	3.825	-3.108	3.929	0.829	-5.300	1.232	0.595
c_O	1360	2263	1346	1339	2250	1314	1325	2296	1321	1333
	(31.3)	(52.1)	(31.0)	(30.8)	(51.8)	(30.2)	(30.5)	(52.8)	(30.4)	(30.7)
c_E	1294.9	2211.4	1334.5	1294.9	2211.4	1334.5	1294.9	2211.4	1334.5	1294.9
	(29.8)	(50.9)	(30.7)	(29.8)	(50.9)	(30.7)	(29.8)	(50.9)	(30.7)	(29.8)
	133.3	104.4	23.1	89.6	77.9	-40.7	60.8	172.4	-26.9	77.2
	3.069	2.402	0.531	2.062	1.792	-0.937	1.399	3.967	-0.619	1.777
N	4345	4345	4345	4345	4345	4345	4345	4345	4345	4345
X2	5.6	16.9	0.2	15.6	10.6	9.0	3.6	20.2	1.5	2.6
Gvalue	5.6	15.7	0.2	15.6	10.0	9.1	3.7	19.4	1.5	2.6
Gv/N	0.128	0.362	0.004	0.359	0.231	0.209	0.085	0.447	0.034	0.060
Prob	0.13525	0.00128	0.98042	0.00136	0.01824	0.02839	0.29714	0.00022	0.68903	0.45454

	111	112	113	114	115	116	117	118	119	120
a_O	53	534	907	65	587	894	68	625	983	70
	(1.2)	(12.3)	(20.9)	(1.5)	(13.5)	(20.6)	(1.6)	(14.4)	(22.6)	(1.6)
a_E	48.8	613.0	930.3	48.8	613.0	930.3	48.8	612.8	930.1	48.8
	(1.1)	(14.1)	(21.4)	(1.1)	(14.1)	(21.4)	(1.1)	(14.1)	(21.4)	(1.1)
	8.8	-147.3	-46.1	37.3	-50.8	-71.2	45.2	24.6	108.7	50.6
	0.202	-3.389	-1.060	0.858	-1.169	-1.639	1.039	0.567	2.503	1.164
t_O	88	468	1092	94	444	1141	82	422	1126	101
	(2.0)	(10.8)	(25.1)	(2.2)	(10.2)	(26.3)	(1.9)	(9.7)	(25.9)	(2.3)
t_E	71.1	440.1	1162.6	71.1	440.1	1162.6	71.1	440.0	1162.3	71.1
	(1.6)	(10.1)	(26.8)	(1.6)	(10.1)	(26.8)	(1.6)	(10.1)	(26.8)	(1.6)
	37.5	57.5	-136.8	52.5	7.8	-42.7	23.4	-35.3	-71.5	71.0
	0.864	1.323	-3.148	1.208	0.179	-0.984	0.538	-0.812	-1.645	1.633
g_O	1934	2015	1005	1906	1951	1039	1930	1978	912	2018
	(44.5)	(46.4)	(23.1)	(43.9)	(44.9)	(23.9)	(44.4)	(45.5)	(21.0)	(46.5)
g_E	2013.7	1957.4	957.1	2013.7	1957.4	957.1	2013.7	1957.0	956.9	2013.2
	(46.3)	(45.0)	(22.0)	(46.3)	(45.0)	(22.0)	(46.3)	(45.0)	(22.0)	(46.3)
	-156.2	116.9	98.1	-209.5	-12.8	170.5	-163.9	42.3	-87.7	9.5
	-3.595	2.690	2.257	-4.823	-0.294	3.924	-3.772	0.974	-2.019	0.219
c_O	2270	1328	1341	2280	1363	1271	2265	1319	1323	2155
	(52.2)	(30.6)	(30.9)	(52.5)	(31.4)	(29.3)	(52.1)	(30.4)	(30.5)	(49.6)
c_E	2211.4	1334.5	1294.9	2211.4	1334.5	1294.9	2211.4	1334.2	1294.6	2210.9
	(50.9)	(30.7)	(29.8)	(50.9)	(30.7)	(29.8)	(50.9)	(30.7)	(29.8)	(50.9)
	118.7	-13.0	93.7	139.3	57.6	-47.4	108.5	-30.3	57.3	-110.4
	2.732	-0.299	2.157	3.206	1.325	-1.092	2.497	-0.696	1.320	-2.541

N	4345	4345	4345	4345	4345	4345	4345	4344	4344	4344
X2	9.1	13.7	8.9	20.6	1.8	9.3	14.0	1.4	6.9	23.2
Gvalue	8.8	14.1	8.9	19.5	1.8	9.1	13.1	1.4	6.9	20.7
Gv/N	0.203	0.324	0.206	0.450	0.041	0.209	0.302	0.032	0.158	0.476
Prob	0.03177	0.00280	0.03016	0.00021	0.62081	0.02807	0.00435	0.70816	0.07660	0.00012

	121	122	123	124	125	126	127	128	129	130
a_O	583	914	62	560	971	49	633	960	69	633
	(13.4)	(21.0)	(1.4)	(12.9)	(22.4)	(1.1)	(14.6)	(22.1)	(1.6)	(14.6)
a_E	612.8	930.1	48.8	612.8	930.1	48.8	612.8	930.1	48.8	612.8
	(14.1)	(21.4)	(1.1)	(14.1)	(21.4)	(1.1)	(14.1)	(21.4)	(1.1)	(14.1)
	-58.1	-32.0	29.7	-100.9	83.5	0.4	41.0	60.7	47.9	41.0
	-1.339	-0.736	0.685	-2.323	1.923	0.010	0.945	1.398	1.102	0.945
t_O	419	1151	105	448	1132	91	429	1113	82	427
	(9.6)	(26.5)	(2.4)	(10.3)	(26.1)	(2.1)	(9.9)	(25.6)	(1.9)	(9.8)
t_E	440.0	1162.3	71.1	440.0	1162.3	71.1	440.0	1162.3	71.1	440.0
	(10.1)	(26.8)	(1.6)	(10.1)	(26.8)	(1.6)	(10.1)	(26.8)	(1.6)	(10.1)
	-41.0	-22.5	81.9	16.1	-59.8	44.9	-21.8	-96.5	23.4	-25.7
	-0.944	-0.518	1.886	0.371	-1.377	1.035	-0.501	-2.221	0.539	-0.590
g_O	1937	923	1951	2018	947	2008	1949	956	1942	1959
	(44.6)	(21.2)	(44.9)	(46.5)	(21.8)	(46.2)	(44.9)	(22.0)	(44.7)	(45.1)
g_E	1957.0	956.9	2013.2	1957.0	956.9	2013.2	1957.0	956.9	2013.2	1957.0
	(45.0)	(22.0)	(46.3)	(45.0)	(22.0)	(46.3)	(45.0)	(22.0)	(46.3)	(45.0)
	-39.7	-66.6	-122.5	124.0	-19.8	-10.5	-15.9	-1.9	-139.9	4.1
	-0.914	-1.534	-2.821	2.854	-0.455	-0.241	-0.366	-0.043	-3.221	0.094
c_O	1405	1356	2226	1318	1294	2196	1333	1315	2251	1325
	(32.3)	(31.2)	(51.2)	(30.3)	(29.8)	(50.6)	(30.7)	(30.3)	(51.8)	(30.5)
c_E	1334.2	1294.6	2210.9	1334.2	1294.6	2210.9	1334.2	1294.6	2210.9	1334.2
	(30.7)	(29.8)	(50.9)	(30.7)	(29.8)	(50.9)	(30.7)	(29.8)	(50.9)	(30.7)
	145.3	125.6	30.3	-32.2	-1.3	-29.7	-2.4	41.0	80.9	-18.4
	3.344	2.891	0.698	-0.742	-0.030	-0.684	-0.056	0.944	1.863	-0.423
N	4344	4344	4344	4344	4344	4344	4344	4344	4344	4344
X2	6.4	4.5	21.8	6.8	2.7	5.7	1.0	3.4	13.3	1.1
Gvalue	6.4	4.5	19.4	6.9	2.7	5.2	1.0	3.4	12.3	1.1
Gv/N	0.147	0.103	0.447	0.159	0.062	0.121	0.022	0.078	0.283	0.026
Prob	0.09412	0.21485	0.00022	0.07469	0.44503	0.15531	0.80856	0.33520	0.00646	0.77393

	131	132	133	134	135	136	137	138	139	140
a_O	922	59	588	872	42	569	945	52	602	936
	(21.2)	(1.4)	(13.5)	(20.1)	(1.0)	(13.1)	(21.8)	(1.2)	(13.9)	(21.5)
a_E	930.1	48.8	612.8	930.1	48.8	612.8	930.1	48.8	612.8	930.1
	(21.4)	(1.1)	(14.1)	(21.4)	(1.1)	(14.1)	(21.4)	(1.1)	(14.1)	(21.4)
	-16.2	22.5	-48.6	-112.5	-12.6	-84.4	30.0	6.7	-21.4	11.8
	-0.372	0.517	-1.119	-2.590	-0.289	-1.943	0.691	0.153	-0.493	0.272
t_O	1117	80	438	1136	91	425	1088	83	455	1134
	(25.7)	(1.8)	(10.1)	(26.2)	(2.1)	(9.8)	(25.0)	(1.9)	(10.5)	(26.1)
t_E	1162.3	71.1	440.0	1162.3	71.1	440.0	1162.3	71.1	440.0	1162.3
	(26.8)	(1.6)	(10.1)	(26.8)	(1.6)	(10.1)	(26.8)	(1.6)	(10.1)	(26.8)
	-88.8	18.9	-4.0	-52.0	44.9	-29.5	-143.8	25.7	30.5	-55.9
	-2.045	0.435	-0.093	-1.197	1.035	-0.680	-3.309	0.592	0.701	-1.287
g_O	952	1985	1996	993	1983	1999	976	1915	1908	946
	(21.9)	(45.7)	(45.9)	(22.9)	(45.6)	(46.0)	(22.5)	(44.1)	(43.9)	(21.8)
g_E	956.9	2013.2	1957.0	956.9	2013.2	1957.0	956.9	2013.2	1957.0	956.9
	(22.0)	(46.3)	(45.0)	(22.0)	(46.3)	(45.0)	(22.0)	(46.3)	(45.0)	(22.0)
	-9.8	-56.1	78.9	73.5	-60.0	85.0	38.5	-191.6	-96.7	-21.7
	-0.226	-1.291	1.815	1.692	-1.382	1.956	0.887	-4.411	-2.225	-0.500
c_O	1353	2220	1322	1343	2228	1351	1335	2294	1379	1328
	(31.1)	(51.1)	(30.4)	(30.9)	(51.3)	(31.1)	(30.7)	(52.8)	(31.7)	(30.6)
c_E	1294.6	2210.9	1334.2	1294.6	2210.9	1334.2	1294.6	2210.9	1334.2	1294.6
	(29.8)	(50.9)	(30.7)	(29.8)	(50.9)	(30.7)	(29.8)	(50.9)	(30.7)	(29.8)
	119.3	18.2	-24.3	98.5	34.3	33.8	82.0	169.3	91.1	67.6
	2.746	0.420	-0.560	2.267	0.790	0.778	1.887	3.897	2.096	1.555
N	4344	4344	4344	4344	4344	4344	4344	4344	4344	4344
X2	4.5	3.7	1.9	7.4	7.1	4.8	6.6	10.1	3.4	1.7
Gvalue	4.5	3.5	1.9	7.4	6.7	4.8	6.7	10.1	3.4	1.7
Gv/N	0.103	0.081	0.044	0.171	0.154	0.111	0.155	0.232	0.079	0.039
Prob	0.21432	0.31869	0.59052	0.05922	0.08218	0.18426	0.08159	0.01805	0.33157	0.63485

	141	142	143	144	145	146	147	148	149	150
a_O	42	565	898	50	616	927	33	572	922	53
	(1.0)	(13.0)	(20.7)	(1.2)	(14.2)	(21.3)	(0.8)	(13.2)	(21.2)	(1.2)
a_E	48.8	612.8	930.1	48.8	612.8	930.1	48.8	612.8	930.1	48.8
	(1.1)	(14.1)	(21.4)	(1.1)	(14.1)	(21.4)	(1.1)	(14.1)	(21.4)	(1.1)
	-12.6	-91.8	-63.1	2.5	6.4	-6.2	-25.8	-78.8	-16.2	8.8
	-0.289	-2.113	-1.453	0.057	0.147	-0.143	-0.594	-1.815	-0.372	0.203
t_O	78	457	1185	78	447	1149	85	450	1119	70
	(1.8)	(10.5)	(27.3)	(1.8)	(10.3)	(26.5)	(2.0)	(10.4)	(25.8)	(1.6)
t_E	71.1	440.0	1162.3	71.1	440.0	1162.3	71.1	440.0	1162.3	71.1
	(1.6)	(10.1)	(26.8)	(1.6)	(10.1)	(26.8)	(1.6)	(10.1)	(26.8)	(1.6)
	14.5	34.6	45.8	14.5	14.1	-26.5	30.4	20.2	-85.0	-2.2
	0.333	0.797	1.055	0.333	0.324	-0.609	0.700	0.465	-1.956	-0.050
g_O	1975	1978	949	1963	1985	918	1959	1964	958	2033
	(45.5)	(45.5)	(21.8)	(45.2)	(45.7)	(21.1)	(45.1)	(45.2)	(22.1)	(46.8)
g_E	2013.2	1957.0	956.9	2013.2	1957.0	956.9	2013.2	1957.0	956.9	2013.2
	(46.3)	(45.0)	(22.0)	(46.3)	(45.0)	(22.0)	(46.3)	(45.0)	(22.0)	(46.3)
	-75.7	42.3	-15.8	-99.2	56.5	-76.3	-107.0	14.1	2.1	39.7
	-1.744	0.974	-0.364	-2.284	1.300	-1.755	-2.463	0.325	0.049	0.914
c_O	2249	1344	1312	2253	1296	1350	2267	1358	1345	2188
	(51.8)	(30.9)	(30.2)	(51.9)	(29.8)	(31.1)	(52.2)	(31.3)	(31.0)	(50.4)
c_E	2210.9	1334.2	1294.6	2210.9	1334.2	1294.6	2210.9	1334.2	1294.6	2210.9
	(50.9)	(30.7)	(29.8)	(50.9)	(30.7)	(29.8)	(50.9)	(30.7)	(29.8)	(50.9)
	76.9	19.6	34.9	85.0	-75.3	113.0	113.6	48.0	102.6	-45.6
	1.769	0.452	0.804	1.957	-1.734	2.602	2.615	1.105	2.363	-1.049
N	4344	4344	4344	4344	4344	4344	4344	4344	4344	4344
X2	3.0	4.7	1.9	2.8	1.6	4.1	10.7	3.4	3.6	0.8
Gvalue	3.0	4.8	1.9	2.7	1.6	4.1	11.2	3.5	3.6	0.8
Gv/N	0.070	0.110	0.043	0.063	0.038	0.094	0.258	0.079	0.084	0.018
Prob	0.38789	0.18903	0.60199	0.43294	0.65219	0.25059	0.01063	0.32708	0.30309	0.84866

Aehr_HLHE1
5UTR:

	50	49	48	47	46	45	44	43	42	41
a_O	507	527	468	482	564	508	538	563	468	518
	(17.7)	(18.4)	(16.3)	(16.8)	(19.7)	(17.7)	(18.8)	(19.7)	(16.3)	(18.1)
a_E	542.6	542.6	542.6	542.6	542.6	542.6	542.6	542.6	542.6	542.6
	(18.9)	(18.9)	(18.9)	(18.9)	(18.9)	(18.9)	(18.9)	(18.9)	(18.9)	(18.9)
	-68.9	-30.8	-138.5	-114.2	43.5	-67.0	-9.2	41.5	-138.5	-48.1
	-2.404	-1.076	-4.834	-3.987	1.520	-2.339	-0.323	1.448	-4.834	-1.680
t_O	434	521	517	461	499	489	441	536	444	464
	(15.1)	(18.2)	(18.0)	(16.1)	(17.4)	(17.1)	(15.4)	(18.7)	(15.5)	(16.2)
t_E	474.2	474.2	474.2	474.2	474.2	474.2	474.2	474.2	474.2	474.2
	(16.6)	(16.6)	(16.6)	(16.6)	(16.6)	(16.6)	(16.6)	(16.6)	(16.6)	(16.6)
	-76.9	98.0	89.3	-26.1	50.8	30.0	-64.1	131.3	-58.5	-20.2
	-2.686	3.421	3.116	-0.911	1.774	1.047	-2.236	4.581	-2.042	-0.706
g_O	963	884	894	992	921	917	957	859	951	922
	(33.6)	(30.9)	(31.2)	(34.6)	(32.1)	(32.0)	(33.4)	(30.0)	(33.2)	(32.2)
g_E	938.1	938.1	938.1	938.1	938.1	938.1	938.1	938.1	938.1	938.1
	(32.7)	(32.7)	(32.7)	(32.7)	(32.7)	(32.7)	(32.7)	(32.7)	(32.7)	(32.7)
	50.5	-105.0	-86.0	110.9	-33.8	-41.7	38.2	-151.3	26.0	-31.9
	1.763	-3.664	-3.003	3.871	-1.181	-1.454	1.335	-5.280	0.909	-1.112
c_O	961	933	986	930	881	951	929	907	1002	961
	(33.5)	(32.6)	(34.4)	(32.5)	(30.8)	(33.2)	(32.4)	(31.7)	(35.0)	(33.5)
c_E	910.1	910.1	910.1	910.1	910.1	910.1	910.1	910.1	910.1	910.1
	(31.8)	(31.8)	(31.8)	(31.8)	(31.8)	(31.8)	(31.8)	(31.8)	(31.8)	(31.8)
	104.7	46.5	158.0	40.3	-57.2	83.7	38.3	-6.1	192.9	104.7
	3.654	1.621	5.516	1.407	-1.996	2.921	1.336	-0.213	6.732	3.654
N	2865	2865	2865	2865	2865	2865	2865	2865	2865	2865
X2	9.3	8.8	22.5	10.7	3.4	5.0	3.1	15.5	21.7	4.5
Gvalue	9.4	8.7	22.8	10.9	3.4	5.0	3.2	15.3	21.9	4.4
Gv/N	0.327	0.303	0.795	0.380	0.117	0.175	0.112	0.536	0.765	0.155
Prob	0.02483	0.03394	0.00004	0.01232	0.34043	0.17130	0.36244	0.00155	0.00007	0.21817

	40	39	38	37	36	35	34	33	32	31
a_O	568	481	513	580	462	546	576	475	517	571
	(19.8)	(16.8)	(17.9)	(20.2)	(16.1)	(19.1)	(20.1)	(16.6)	(18.0)	(19.9)
a_E	542.6	542.6	542.6	542.6	542.6	542.6	542.6	542.6	542.6	542.6
	(18.9)	(18.9)	(18.9)	(18.9)	(18.9)	(18.9)	(18.9)	(18.9)	(18.9)	(18.9)
	51.9	-116.0	-57.6	77.2	-148.7	6.7	68.7	-126.5	-50.0	58.2
	1.811	-4.049	-2.012	2.696	-5.189	0.235	2.399	-4.414	-1.747	2.031
t_O	513	543	474	517	489	462	524	517	444	524
	(17.9)	(19.0)	(16.5)	(18.0)	(17.1)	(16.1)	(18.3)	(18.0)	(15.5)	(18.3)
t_E	474.2	474.2	474.2	474.2	474.2	474.2	474.2	474.2	474.2	474.2
	(16.6)	(16.6)	(16.6)	(16.6)	(16.6)	(16.6)	(16.6)	(16.6)	(16.6)	(16.6)
	80.6	147.1	-0.5	89.3	30.0	-24.1	104.6	89.3	-58.5	104.6
	2.814	5.133	-0.016	3.116	1.047	-0.843	3.651	3.116	-2.042	3.651
g_O	838	937	905	868	924	906	847	884	929	859
	(29.2)	(32.7)	(31.6)	(30.3)	(32.3)	(31.6)	(29.6)	(30.9)	(32.4)	(30.0)
g_E	938.1	938.1	938.1	938.1	938.1	938.1	938.1	938.1	938.1	938.1
	(32.7)	(32.7)	(32.7)	(32.7)	(32.7)	(32.7)	(32.7)	(32.7)	(32.7)	(32.7)
	-189.1	-2.1	-65.0	-134.8	-27.9	-63.0	-173.0	-105.0	-18.1	-151.3
	-6.599	-0.075	-2.267	-4.704	-0.975	-2.200	-6.038	-3.664	-0.630	-5.280
c_O	946	904	973	900	990	951	918	989	975	911
	(33.0)	(31.6)	(34.0)	(31.4)	(34.6)	(33.2)	(32.0)	(34.5)	(34.0)	(31.8)
c_E	910.1	910.1	910.1	910.1	910.1	910.1	910.1	910.1	910.1	910.1
	(31.8)	(31.8)	(31.8)	(31.8)	(31.8)	(31.8)	(31.8)	(31.8)	(31.8)	(31.8)
	73.3	-12.1	130.1	-20.0	166.7	83.7	15.9	164.5	134.4	1.9
	2.558	-0.422	4.542	-0.698	5.819	2.921	0.557	5.743	4.691	0.066
N	2865	2865	2865	2865	2865	2865	2865	2865	2865	2865
X2	16.4	17.0	7.1	11.8	19.7	3.3	16.2	22.3	7.9	13.4
Gvalue	16.7	16.8	7.1	11.7	20.1	3.3	16.3	22.4	7.8	13.4
Gv/N	0.584	0.588	0.247	0.410	0.702	0.114	0.568	0.781	0.273	0.467
Prob	0.00080	0.00076	0.06923	0.00832	0.00016	0.35286	0.00100	0.00005	0.04997	0.00390

	30	29	28	27	26	25	24	23	22	21
a_O	501	523	600	534	545	630	538	563	665	591
	(17.5)	(18.3)	(20.9)	(18.6)	(19.0)	(22.0)	(18.8)	(19.7)	(23.2)	(20.6)
a_E	542.6	542.6	542.6	542.6	542.6	542.6	542.6	542.6	542.6	542.6
	(18.9)	(18.9)	(18.9)	(18.9)	(18.9)	(18.9)	(18.9)	(18.9)	(18.9)	(18.9)
	-80.0	-38.6	120.6	-17.1	4.7	188.1	-9.2	41.5	270.4	100.9
	-2.792	-1.346	4.209	-0.598	0.165	6.565	-0.323	1.448	9.440	3.522
t_O	513	435	539	444	429	499	505	416	492	512
	(17.9)	(15.2)	(18.8)	(15.5)	(15.0)	(17.4)	(17.6)	(14.5)	(17.2)	(17.9)
t_E	474.2	474.2	474.2	474.2	474.2	474.2	474.2	474.2	474.2	474.2
	(16.6)	(16.6)	(16.6)	(16.6)	(16.6)	(16.6)	(16.6)	(16.6)	(16.6)	(16.6)
	80.6	-75.1	138.0	-58.5	-86.0	50.8	63.5	-109.0	36.2	78.5
	2.814	-2.622	4.817	-2.042	-3.002	1.774	2.216	-3.804	1.263	2.739
g_O	881	886	803	877	906	767	829	871	767	769
	(30.8)	(30.9)	(28.0)	(30.6)	(31.6)	(26.8)	(28.9)	(30.4)	(26.8)	(26.8)
g_E	938.1	938.1	938.1	938.1	938.1	938.1	938.1	938.1	938.1	938.1
	(32.7)	(32.7)	(32.7)	(32.7)	(32.7)	(32.7)	(32.7)	(32.7)	(32.7)	(32.7)
	-110.6	-101.2	-249.7	-118.1	-63.0	-308.9	-204.9	-129.2	-308.9	-305.7
	-3.860	-3.532	-8.715	-4.121	-2.200	-10.780	-7.153	-4.511	-10.780	-10.668
c_O	970	1021	923	1010	985	969	993	1015	941	993
	(33.9)	(35.6)	(32.2)	(35.3)	(34.4)	(33.8)	(34.7)	(35.4)	(32.8)	(34.7)
c_E	910.1	910.1	910.1	910.1	910.1	910.1	910.1	910.1	910.1	910.1
	(31.8)	(31.8)	(31.8)	(31.8)	(31.8)	(31.8)	(31.8)	(31.8)	(31.8)	(31.8)
	123.7	234.9	26.1	210.5	155.9	121.6	173.2	221.5	62.9	173.2
	4.319	8.198	0.910	7.346	5.441	4.245	6.046	7.733	2.196	6.046
N	2865	2865	2865	2865	2865	2865	2865	2865	2865	2865
X2	13.8	20.4	34.5	17.0	11.6	50.4	22.3	24.8	60.5	45.3
Gvalue	13.8	20.0	35.0	16.8	11.6	51.7	22.5	24.8	60.7	46.9
Gv/N	0.481	0.698	1.221	0.585	0.404	1.803	0.787	0.865	2.119	1.639
Prob	0.00323	0.00017	0.00000	0.00079	0.00895	0.00000	0.00005	0.00002	0.00000	0.00000

	20	19	18	17	16	15	14	13	12	11
a_O	663	714	622	633	732	637	680	784	697	744
	(23.1)	(24.9)	(21.7)	(22.1)	(25.5)	(22.2)	(23.7)	(27.4)	(24.3)	(26.0)
a_E	542.6	542.6	542.6	542.6	542.6	542.6	542.6	542.6	542.6	542.6
	(18.9)	(18.9)	(18.9)	(18.9)	(18.9)	(18.9)	(18.9)	(18.9)	(18.9)	(18.9)
	265.6	391.9	169.8	195.0	438.2	204.3	306.9	577.0	349.0	469.6
	9.272	13.679	5.927	6.806	15.296	7.129	10.711	20.138	12.181	16.391
t_O	437	518	488	421	471	426	368	372	283	230
	(15.3)	(18.1)	(17.0)	(14.7)	(16.4)	(14.9)	(12.8)	(13.0)	(9.9)	(8.0)

t_E	474.2	474.2	474.2	474.2	474.2	474.2	474.2	474.2	474.2	474.2
	(16.6)	(16.6)	(16.6)	(16.6)	(16.6)	(16.6)	(16.6)	(16.6)	(16.6)	(16.6)
	-71.5	91.5	27.9	-100.2	-6.4	-91.4	-186.7	-180.6	-292.2	-332.9
	-2.494	3.192	0.975	-3.499	-0.225	-3.190	-6.515	-6.305	-10.199	-11.618
g_O	823	723	862	930	838	959	1051	1075	1316	1433
	(28.7)	(25.2)	(30.1)	(32.5)	(29.2)	(33.5)	(36.7)	(37.5)	(45.9)	(50.0)
g_E	938.1	938.1	938.1	938.1	938.1	938.1	938.1	938.1	938.1	938.1
	(32.7)	(32.7)	(32.7)	(32.7)	(32.7)	(32.7)	(32.7)	(32.7)	(32.7)	(32.7)
	-215.4	-376.6	-145.8	-16.1	-189.1	42.3	238.9	292.9	891.0	1214.3
	-7.519	-13.143	-5.089	-0.561	-6.599	1.477	8.340	10.225	31.100	42.385
c_O	942	910	893	881	824	843	766	634	569	458
	(32.9)	(31.8)	(31.2)	(30.8)	(28.8)	(29.4)	(26.7)	(22.1)	(19.9)	(16.0)
c_E	910.1	910.1	910.1	910.1	910.1	910.1	910.1	910.1	910.1	910.1
	(31.8)	(31.8)	(31.8)	(31.8)	(31.8)	(31.8)	(31.8)	(31.8)	(31.8)	(31.8)
	65.0	-0.1	-33.8	-57.2	-163.7	-129.1	-264.0	-458.3	-534.4	-629.0
	2.268	-0.004	-1.180	-1.996	-5.714	-4.504	-9.215	-15.998	-18.654	-21.953
N	2865	2865	2865	2865	2865	2865	2865	2865	2865	2865
X2	44.9	107.5	18.5	22.0	84.9	26.7	95.0	233.1	401.1	686.2
Gvalue	43.8	106.7	18.1	21.5	79.0	26.1	95.2	230.9	413.3	722.1
Gv/N	1.527	3.723	0.633	0.750	2.758	0.913	3.321	8.060	14.427	25.204
Prob	0.00000	0.00000	0.00041	0.00008	0.00000	0.00001	0.00000	0.00000	0.00000	0.00000

	10	9	8	7	6	5	4	3	2	1
a_O	778	686	724	753	600	669	654	652	361	392
	(27.2)	(23.9)	(25.3)	(26.3)	(20.9)	(23.4)	(22.8)	(22.8)	(12.6)	(13.7)
a_E	542.6	542.6	542.6	542.6	542.6	542.6	542.6	542.6	542.6	542.6
	(18.9)	(18.9)	(18.9)	(18.9)	(18.9)	(18.9)	(18.9)	(18.9)	(18.9)	(18.9)
	560.6	321.6	417.5	493.4	120.6	280.1	244.2	239.4	-294.3	-254.9
	19.567	11.227	14.573	17.221	4.209	9.776	8.522	8.356	-10.271	-8.899
t_O	235	281	330	518	455	454	403	345	560	378
	(8.2)	(9.8)	(11.5)	(18.1)	(15.9)	(15.8)	(14.1)	(12.0)	(19.5)	(13.2)
t_E	474.2	474.2	474.2	474.2	474.2	474.2	474.2	474.2	474.2	474.2
	(16.6)	(16.6)	(16.6)	(16.6)	(16.6)	(16.6)	(16.6)	(16.6)	(16.6)	(16.6)
	-330.0	-294.1	-239.3	91.5	-37.7	-39.6	-131.2	-219.5	186.2	-171.5
	-11.518	-10.266	-8.353	3.192	-1.315	-1.382	-4.579	-7.662	6.499	-5.985
g_O	1387	1499	1314	992	1088	911	861	1031	588	599
	(48.4)	(52.3)	(45.9)	(34.6)	(38.0)	(31.8)	(30.1)	(36.0)	(20.5)	(20.9)
g_E	938.1	938.1	938.1	938.1	938.1	938.1	938.1	938.1	938.1	938.1
	(32.7)	(32.7)	(32.7)	(32.7)	(32.7)	(32.7)	(32.7)	(32.7)	(32.7)	(32.7)
	1084.8	1405.2	885.7	110.9	322.6	-53.4	-147.6	194.8	-549.3	-537.4
	37.865	49.049	30.913	3.871	11.261	-1.862	-5.153	6.798	-19.173	-18.757
c_O	465	399	497	602	722	831	947	837	1356	1496
	(16.2)	(13.9)	(17.3)	(21.0)	(25.2)	(29.0)	(33.1)	(29.2)	(47.3)	(52.2)
c_E	910.1	910.1	910.1	910.1	910.1	910.1	910.1	910.1	910.1	910.1
	(31.8)	(31.8)	(31.8)	(31.8)	(31.8)	(31.8)	(31.8)	(31.8)	(31.8)	(31.8)
	-624.5	-658.0	-601.3	-497.6	-334.3	-151.0	75.4	-140.1	1081.5	1487.1
	-21.797	-22.966	-20.987	-17.367	-11.667	-5.272	2.630	-4.890	37.749	51.907
N	2865	2865	2865	2865	2865	2865	2865	2865	2865	2865
X2	655.3	739.0	442.6	193.0	69.7	37.9	41.4	72.3	425.5	561.2
Gvalue	691.0	774.8	462.6	198.2	71.3	36.1	40.7	74.6	424.1	523.4
Gv/N	24.118	27.043	16.146	6.918	2.488	1.261	1.421	2.603	14.804	18.267
Prob	0.00000	0.00000	0.00000	0.00000	0.00000	0.00000	0.00000	0.00000	0.00000	0.00000

CDS_INITIAL:

	1	2	3	4	5	6	7	8	9	10
a_O	2464	0	0	1223	747	470	786	986	577	801
	(86.0)	(0.0)	(0.0)	(42.7)	(26.1)	(16.4)	(27.4)	(34.4)	(20.1)	(28.0)
a_E	503.0	737.6	136.1	503.0	737.6	136.1	503.0	737.6	136.1	503.0
	(17.6)	(25.7)	(4.8)	(17.6)	(25.7)	(4.8)	(17.6)	(25.7)	(4.8)	(17.6)
	7830.0	0.0	0.0	2173.0	19.0	1164.8	701.6	572.5	1666.7	745.2
	273.297	0.000	0.000	75.847	0.663	40.658	24.487	19.982	58.176	26.012
t_O	97	2865	0	285	374	532	287	584	450	394
	(3.4)	(100.0)	(0.0)	(9.9)	(13.1)	(18.6)	(10.0)	(20.4)	(15.7)	(13.8)
t_E	327.0	806.7	246.0	327.0	806.7	246.0	327.0	806.7	246.0	327.0
	(11.4)	(28.2)	(8.6)	(11.4)	(28.2)	(8.6)	(11.4)	(28.2)	(8.6)	(11.4)
	-235.8	7262.0	0.0	-78.3	-575.0	820.5	-74.9	-377.3	543.4	146.9
	-8.229	253.474	0.000	-2.734	-20.069	28.639	-2.614	-13.170	18.966	5.127
g_O	304	0	2865	782	712	697	1004	597	922	807
	(10.6)	(0.0)	(100.0)	(27.3)	(24.9)	(24.3)	(35.0)	(20.8)	(32.2)	(28.2)
g_E	1159.9	628.7	1164.4	1159.9	628.7	1164.4	1159.9	628.7	1164.4	1159.9
	(40.5)	(21.9)	(40.6)	(40.5)	(21.9)	(40.6)	(40.5)	(21.9)	(40.6)	(40.5)
	-814.1	0.0	5159.2	-616.5	177.1	-715.3	-289.8	-61.8	-430.4	-585.4
	-28.416	0.000	180.076	-21.519	6.183	-24.968	-10.114	-2.157	-15.022	-20.434
c_O	0	0	0	575	1032	1166	788	698	916	863
	(0.0)	(0.0)	(0.0)	(20.1)	(36.0)	(40.7)	(27.5)	(24.4)	(32.0)	(30.1)
c_E	875.1	692.0	1318.5	875.1	692.0	1318.5	875.1	692.0	1318.5	875.1
	(30.5)	(24.2)	(46.0)	(30.5)	(24.2)	(46.0)	(30.5)	(24.2)	(46.0)	(30.5)
	0.0	0.0	0.0	-483.0	824.8	-286.6	-165.3	12.0	-667.2	-24.1
	0.000	0.000	0.000	-16.858	28.790	-10.002	-5.768	0.419	-23.289	-0.840
N	2865	2865	2865	2865	2865	2865	2865	2865	2865	2865
X2	9312.7	7310.1	4184.4	1261.8	410.3	1356.5	193.7	146.8	1770.3	297.7
Gvalue	6780.1	7262.0	5159.2	995.2	446.0	983.4	171.7	145.4	1112.5	282.6
Gv/N	236.652	253.474	180.076	34.735	15.567	34.326	5.992	5.074	38.831	9.865
Prob	1.00000	1.00000	1.00000	0.00000	0.00000	0.00000	0.00000	0.00000	0.00000	0.00000

	11	12	13	14	15	16	17	18	19	20
a_O	814	498	802	779	478	698	767	449	725	765
	(28.4)	(17.4)	(28.0)	(27.2)	(16.7)	(24.4)	(26.8)	(15.7)	(25.3)	(26.7)
a_E	737.6	136.1	503.0	737.6	136.1	503.0	737.6	136.1	503.0	737.6
	(25.7)	(4.8)	(17.6)	(25.7)	(4.8)	(17.6)	(25.7)	(4.8)	(17.6)	(25.7)
	160.5	1291.9	748.2	85.2	1200.8	457.3	60.0	1071.8	530.0	55.9
	5.604	45.092	26.114	2.973	41.913	15.960	2.096	37.408	18.498	1.951
t_O	663	439	338	800	399	360	839	373	367	830
	(23.1)	(15.3)	(11.8)	(27.9)	(13.9)	(12.6)	(29.3)	(13.0)	(12.8)	(29.0)
t_E	806.7	246.0	327.0	806.7	246.0	327.0	806.7	246.0	327.0	806.7
	(28.2)	(8.6)	(11.4)	(28.2)	(8.6)	(11.4)	(28.2)	(8.6)	(11.4)	(28.2)
	-260.1	508.4	22.4	-13.3	385.8	69.2	65.9	310.4	84.7	47.3
	-9.080	17.744	0.781	-0.466	13.466	2.417	2.299	10.834	2.957	1.650
g_O	601	820	858	522	878	831	526	846	881	531
	(21.0)	(28.6)	(29.9)	(18.2)	(30.6)	(29.0)	(18.4)	(29.5)	(30.8)	(18.5)
g_E	628.7	1164.4	1159.9	628.7	1164.4	1159.9	628.7	1164.4	1159.9	628.7
	(21.9)	(40.6)	(40.5)	(21.9)	(40.6)	(40.5)	(21.9)	(40.6)	(40.5)	(21.9)
	-54.2	-575.1	-517.3	-194.2	-495.7	-554.1	-187.7	-540.5	-484.5	-179.4
	-1.891	-20.072	-18.055	-6.778	-17.303	-19.342	-6.550	-18.865	-16.912	-6.261

c_O	787	1108	867	764	1110	976	733	1197	892	739
	(27.5)	(38.7)	(30.3)	(26.7)	(38.7)	(34.1)	(25.6)	(41.8)	(31.1)	(25.8)
c_E	692.0	1318.5	875.1	692.0	1318.5	875.1	692.0	1318.5	875.1	692.0
	(24.2)	(46.0)	(30.5)	(24.2)	(46.0)	(30.5)	(24.2)	(46.0)	(30.5)	(24.2)
	202.4	−385.4	−16.2	151.2	−382.1	213.0	84.3	−231.4	34.1	97.1
	7.065	−13.451	−0.564	5.277	−13.336	7.434	2.943	−8.076	1.190	3.388
N	2865	2865	2865	2865	2865	2865	2865	2865	2865	2865
X2	47.8	1248.9	256.7	28.0	1057.2	183.8	21.7	882.9	170.2	20.1
Gvalue	48.6	839.8	237.1	28.8	708.8	185.3	22.6	610.3	164.3	20.8
Gv/N	1.698	29.313	8.276	1.006	24.741	6.469	0.789	21.302	5.733	0.727
Prob	0.00000	0.00000	0.00000	0.00000	0.00000	0.00000	0.00005	0.00000	0.00000	0.00011

	21	22	23	24	25	26	27	28	29	30
a_O	387	698	721	336	633	651	293	545	637	265
	(13.5)	(24.4)	(25.2)	(11.7)	(22.1)	(22.7)	(10.2)	(19.0)	(22.2)	(9.2)
a_E	136.1	503.0	737.6	136.1	503.0	737.6	136.1	503.0	737.6	136.1
	(4.8)	(17.6)	(25.7)	(4.8)	(17.6)	(25.7)	(4.8)	(17.6)	(25.7)	(4.8)
	808.7	457.3	−32.7	607.2	290.9	−162.5	449.2	87.3	−186.7	353.1
	28.228	15.960	−1.143	21.194	10.155	−5.673	15.681	3.048	−6.518	12.324
t_O	389	320	883	332	348	870	323	343	879	298
	(13.6)	(11.2)	(30.8)	(11.6)	(12.1)	(30.4)	(11.3)	(12.0)	(30.7)	(10.4)
t_E	246.0	327.0	806.7	246.0	327.0	806.7	246.0	327.0	806.7	246.0
	(8.6)	(11.4)	(28.2)	(8.6)	(11.4)	(28.2)	(8.6)	(11.4)	(28.2)	(8.6)
	356.4	−13.8	159.6	199.0	43.3	131.4	175.8	32.8	150.9	114.2
	12.439	−0.483	5.571	6.944	1.513	4.588	6.137	1.145	5.267	3.986
g_O	827	946	542	962	948	587	1003	1040	626	1048
	(28.9)	(33.0)	(18.9)	(33.6)	(33.1)	(20.5)	(35.0)	(36.3)	(21.8)	(36.6)
g_E	1164.4	1159.9	628.7	1164.4	1159.9	628.7	1164.4	1159.9	628.7	1164.4
	(40.6)	(40.5)	(21.9)	(40.6)	(40.5)	(21.9)	(40.6)	(40.5)	(21.9)	(40.6)
	−565.9	−385.6	−160.9	−367.4	−382.4	−80.6	−299.3	−226.9	−5.4	−220.7
	−19.752	−13.459	−5.615	−12.822	−13.348	−2.813	−10.446	−7.919	−0.189	−7.704
c_O	1262	901	719	1235	936	757	1246	937	723	1254
	(44.0)	(31.4)	(25.1)	(43.1)	(32.7)	(26.4)	(43.5)	(32.7)	(25.2)	(43.8)
c_E	1318.5	875.1	692.0	1318.5	875.1	692.0	1318.5	875.1	692.0	1318.5
	(46.0)	(30.5)	(24.2)	(46.0)	(30.5)	(24.2)	(46.0)	(30.5)	(24.2)	(46.0)
	−110.5	52.5	55.0	−161.5	125.9	135.9	−140.9	128.0	63.3	−125.7
	−3.856	1.834	1.919	−5.638	4.395	4.742	−4.916	4.469	2.210	−4.388
N	2865	2865	2865	2865	2865	2865	2865	2865	2865	2865
X2	645.6	115.9	20.6	364.0	77.9	24.0	231.2	21.0	21.6	147.8
Gvalue	488.8	110.4	21.0	277.3	77.8	24.2	184.9	21.3	22.1	120.8
Gv/N	17.060	3.852	0.732	9.679	2.714	0.844	6.454	0.743	0.770	4.218
Prob	0.00000	0.00000	0.00011	0.00000	0.00000	0.00002	0.00000	0.00009	0.00006	0.00000

	31	32	33	34	35	36	37	38	39	40
a_O	503	624	227	513	620	206	526	664	177	489
	(17.6)	(21.8)	(7.9)	(17.9)	(21.6)	(7.2)	(18.4)	(23.2)	(6.2)	(17.1)
a_E	503.0	737.6	136.1	503.0	737.6	136.1	503.0	737.6	136.1	503.0
	(17.6)	(25.7)	(4.8)	(17.6)	(25.7)	(4.8)	(17.6)	(25.7)	(4.8)	(17.6)
	−0.1	−208.7	232.2	20.1	−215.3	170.7	47.0	−139.5	93.0	−27.7
	−0.003	−7.283	8.104	0.702	−7.515	5.958	1.639	−4.870	3.245	−0.966
t_O	340	847	324	324	850	316	339	892	305	356
	(11.9)	(29.6)	(11.3)	(11.3)	(29.7)	(11.0)	(11.8)	(31.1)	(10.6)	(12.4)
t_E	327.0	806.7	246.0	327.0	806.7	246.0	327.0	806.7	246.0	327.0
	(11.4)	(28.2)	(8.6)	(11.4)	(28.2)	(8.6)	(11.4)	(28.2)	(8.6)	(11.4)
	26.5	82.6	178.4	−6.0	88.9	158.2	24.5	179.3	131.0	60.5
	0.926	2.882	6.225	−0.208	3.102	5.520	0.854	6.259	4.574	2.112
g_O	1068	637	1023	1056	621	1031	1053	640	1092	1090
	(37.3)	(22.2)	(35.7)	(36.9)	(21.7)	(36.0)	(36.8)	(22.3)	(38.1)	(38.0)
g_E	1159.9	628.7	1164.4	1159.9	628.7	1164.4	1159.9	628.7	1164.4	1159.9
	(40.5)	(21.9)	(40.6)	(40.5)	(21.9)	(40.6)	(40.5)	(21.9)	(40.6)	(40.5)
	−176.2	16.7	−264.9	−198.1	−15.3	−250.9	−203.6	22.8	−140.2	−135.4
	−6.151	0.582	−9.245	−6.915	−0.535	−8.756	−7.105	0.795	−4.892	−4.727
c_O	954	757	1291	972	774	1312	947	669	1291	930
	(33.3)	(26.4)	(45.1)	(33.9)	(27.0)	(45.8)	(33.1)	(23.4)	(45.1)	(32.5)
c_E	875.1	692.0	1318.5	875.1	692.0	1318.5	875.1	692.0	1318.5	875.1
	(30.5)	(24.2)	(46.0)	(30.5)	(24.2)	(46.0)	(30.5)	(24.2)	(46.0)	(30.5)
	164.7	135.9	−54.3	204.1	173.3	−12.9	149.5	−45.3	−54.3	113.1
	5.748	4.742	−1.897	7.125	6.049	−0.450	5.219	−1.580	−1.897	3.949
N	2865	2865	2865	2865	2865	2865	2865	2865	2865	2865
X2	14.9	25.7	103.1	20.3	30.9	71.1	17.2	17.3	31.5	10.6
Gvalue	14.9	26.5	91.3	20.2	31.5	65.1	17.4	17.3	29.5	10.6
Gv/N	0.520	0.924	3.188	0.704	1.101	2.273	0.607	0.603	1.029	0.369
Prob	0.00191	0.00001	0.00000	0.00016	0.00000	0.00000	0.00059	0.00062	0.00000	0.01434

	41	42	43	44	45	46	47	48	49	50
a_O	581	183	458	653	175	467	626	162	494	594
	(20.3)	(6.4)	(16.0)	(22.8)	(6.1)	(16.3)	(21.8)	(5.7)	(17.2)	(20.7)
a_E	737.6	136.1	503.0	737.6	136.1	503.0	737.6	136.1	503.0	737.6
	(25.7)	(4.8)	(17.6)	(25.7)	(4.8)	(17.6)	(25.7)	(4.8)	(17.6)	(25.7)
	−277.2	108.3	−85.9	−159.0	87.9	−69.4	−205.3	56.4	−17.9	−257.2
	−9.677	3.781	−2.999	−5.551	3.069	−2.424	−7.166	1.968	−0.625	−8.976
t_O	887	261	354	856	256	329	823	286	343	869
	(31.0)	(9.1)	(12.4)	(29.9)	(8.9)	(11.5)	(28.7)	(10.0)	(12.0)	(30.3)
t_E	806.7	246.0	327.0	806.7	246.0	327.0	806.7	246.0	327.0	806.7
	(28.2)	(8.6)	(11.4)	(28.2)	(8.6)	(11.4)	(28.2)	(8.6)	(11.4)	(28.2)
	168.3	30.8	56.2	101.6	20.3	4.0	32.9	86.1	32.8	129.3
	5.876	1.075	1.961	3.545	0.709	0.141	1.149	3.005	1.145	4.513
g_O	640	1070	1127	644	1093	1152	633	1116	1130	641
	(22.3)	(37.3)	(39.3)	(22.5)	(38.2)	(40.2)	(22.1)	(39.0)	(39.4)	(22.4)
g_E	628.7	1164.4	1159.9	628.7	1164.4	1159.9	628.7	1164.4	1159.9	628.7
	(21.9)	(40.6)	(40.5)	(21.9)	(40.6)	(40.5)	(21.9)	(40.6)	(40.5)	(21.9)
	22.8	−180.9	−64.8	30.9	−138.3	−15.7	8.6	−94.7	−58.9	24.8
	0.795	−6.314	−2.261	1.080	−4.827	−0.547	0.300	−3.306	−2.057	0.866
c_O	757	1351	926	712	1341	917	783	1301	898	761
	(26.4)	(47.2)	(32.3)	(24.9)	(46.8)	(32.0)	(27.3)	(45.4)	(31.3)	(26.6)
c_E	692.0	1318.5	875.1	692.0	1318.5	875.1	692.0	1318.5	875.1	692.0
	(24.2)	(46.0)	(30.5)	(24.2)	(46.0)	(30.5)	(24.2)	(46.0)	(30.5)	(24.2)
	135.9	65.9	104.7	40.5	45.5	85.7	193.4	−34.7	46.4	144.6
	4.742	2.300	3.654	1.414	1.587	2.993	6.751	−1.211	1.618	5.047
N	2865	2865	2865	2865	2865	2865	2865	2865	2865	2865
X2	47.5	25.5	10.2	13.7	16.3	4.7	29.2	13.7	2.3	39.9
Gvalue	49.7	24.1	10.2	14.0	15.4	4.7	29.6	13.1	2.3	41.5
Gv/N	1.736	0.842	0.355	0.488	0.538	0.164	1.034	0.456	0.080	1.450
Prob	0.00000	0.00002	0.01715	0.00293	0.00149	0.19635	0.00000	0.00449	0.51187	0.00000

	51	52	53	54	55	56	57	58	59	60
a_O	195	488	613	168	504	618	167	516	637	145
	(6.8)	(17.0)	(21.4)	(5.9)	(17.6)	(21.6)	(5.8)	(18.0)	(22.2)	(5.1)
a_E	136.1	503.0	737.6	136.1	503.0	737.6	136.1	503.0	737.6	136.1
	(4.8)	(17.6)	(25.7)	(4.8)	(17.6)	(25.7)	(4.8)	(17.6)	(25.7)	(4.8)
	140.2	−29.6	−226.8	70.7	1.9	−218.6	68.3	26.3	−186.7	18.3
	4.893	−1.034	−7.916	2.468	0.067	−7.630	2.384	0.916	−6.518	0.640
t_O	290	348	917	265	320	858	265	354	854	291
	(10.1)	(12.1)	(32.0)	(9.2)	(11.2)	(29.9)	(9.2)	(12.4)	(29.8)	(10.2)
t_E	246.0	327.0	806.7	246.0	327.0	806.7	246.0	327.0	806.7	246.0
	(8.6)	(11.4)	(28.2)	(8.6)	(11.4)	(28.2)	(8.6)	(11.4)	(28.2)	(8.6)
	95.3	43.3	235.0	39.3	−13.8	105.8	39.3	56.2	97.3	97.7
	3.328	1.513	8.204	1.373	−0.483	3.693	1.373	1.961	3.397	3.409
g_O	1126	1101	578	1204	1167	679	1144	1166	623	1158
	(39.3)	(38.4)	(20.2)	(42.0)	(40.7)	(23.7)	(39.9)	(40.7)	(21.7)	(40.4)
g_E	1164.4	1159.9	628.7	1164.4	1159.9	628.7	1164.4	1159.9	628.7	1164.4
	(40.6)	(40.5)	(21.9)	(40.6)	(40.5)	(21.9)	(40.6)	(40.5)	(21.9)	(40.6)
	−75.5	−114.7	−97.2	80.6	14.3	104.5	−40.4	12.3	−11.4	−12.7
	−2.635	−4.003	−3.394	2.812	0.500	3.647	−1.410	0.430	−0.397	−0.444
c_O	1254	928	757	1228	874	710	1289	829	751	1271
	(43.8)	(32.4)	(26.4)	(42.9)	(30.5)	(24.8)	(45.0)	(28.9)	(26.2)	(44.4)
c_E	1318.5	875.1	692.0	1318.5	875.1	692.0	1318.5	875.1	692.0	1318.5
	(46.0)	(30.5)	(24.2)	(46.0)	(30.5)	(24.2)	(46.0)	(30.5)	(24.2)	(46.0)
	−125.7	108.9	135.9	−174.6	−2.2	36.4	−58.3	−89.8	122.8	−93.2
	−4.388	3.801	4.742	−6.093	−0.078	1.271	−2.033	−3.133	4.287	−3.253
N	2865	2865	2865	2865	2865	2865	2865	2865	2865	2865
X2	37.7	8.0	46.3	16.5	0.2	27.1	9.5	5.0	21.6	10.5
Gvalue	34.3	7.9	46.9	16.1	0.2	28.1	9.0	5.0	22.0	10.1
Gv/N	1.199	0.277	1.637	0.560	0.007	0.981	0.313	0.175	0.769	0.352
Prob	0.00000	0.04709	0.00000	0.00111	0.97797	0.00000	0.02962	0.17111	0.00006	0.01784

	61	62	63	64	65	66	67	68	69	70
a_O	481	601	149	478	627	161	462	611	159	461
	(16.8)	(21.0)	(5.2)	(16.7)	(21.9)	(5.6)	(16.1)	(21.3)	(5.5)	(16.1)
a_E	503.0	737.6	136.1	503.0	737.6	136.1	503.0	737.6	136.1	503.0
	(17.6)	(25.7)	(4.8)	(17.6)	(25.7)	(4.8)	(17.6)	(25.7)	(4.8)	(17.6)
	−43.1	−246.1	26.9	−48.8	−203.6	54.1	−78.6	−230.0	49.4	−80.5
	−1.504	−8.590	0.940	−1.704	−7.108	1.887	−2.745	−8.029	1.724	−2.809
t_O	290	875	274	334	844	269	343	870	269	334
	(10.1)	(30.5)	(9.6)	(11.7)	(29.5)	(9.4)	(12.0)	(30.4)	(9.4)	(11.7)
t_E	327.0	806.7	246.0	327.0	806.7	246.0	327.0	806.7	246.0	327.0
	(11.4)	(28.2)	(8.6)	(11.4)	(28.2)	(8.6)	(11.4)	(28.2)	(8.6)	(11.4)
	−69.6	142.2	59.0	14.2	76.3	48.0	32.8	131.4	48.0	14.2
	−2.430	4.964	2.059	0.495	2.663	1.675	1.145	4.588	1.675	0.495
g_O	1204	629	1132	1168	619	1170	1197	665	1189	1171
	(42.0)	(22.0)	(39.5)	(40.8)	(21.6)	(40.8)	(41.8)	(23.2)	(41.5)	(40.9)
g_E	1159.9	628.7	1164.4	1159.9	628.7	1164.4	1159.9	628.7	1164.4	1159.9
	(40.5)	(21.9)	(40.6)	(40.5)	(21.9)	(40.6)	(40.5)	(21.9)	(40.6)	(40.5)
	89.9	0.6	−63.9	16.3	−19.3	11.3	75.5	74.6	49.8	22.4
	3.139	0.020	−2.229	0.570	−0.673	0.393	2.634	2.605	1.737	0.782
c_O	890	760	1310	885	775	1265	863	719	1248	899
	(31.1)	(26.5)	(45.7)	(30.9)	(27.1)	(44.2)	(30.1)	(25.1)	(43.6)	(31.4)
c_E	875.1	692.0	1318.5	875.1	692.0	1318.5	875.1	692.0	1318.5	875.1
	(30.5)	(24.2)	(46.0)	(30.5)	(24.2)	(46.0)	(30.5)	(24.2)	(46.0)	(30.5)
	30.0	142.4	−16.9	19.9	175.5	−104.7	−24.1	55.0	−137.1	48.4
	1.048	4.971	−0.588	0.694	6.126	−3.655	−0.840	1.919	−4.785	1.690
N	2865	2865	2865	2865	2865	2865	2865	2865	2865	2865
X2	7.1	37.7	5.4	1.6	28.4	8.9	5.5	29.8	10.3	4.4
Gvalue	7.2	39.1	5.2	1.6	28.9	8.6	5.6	31.0	10.1	4.5
Gv/N	0.253	1.365	0.182	0.055	1.008	0.300	0.194	1.082	0.352	0.158
Prob	0.06455	0.00000	0.15714	0.66277	0.00000	0.03517	0.13510	0.00000	0.01793	0.21073

	71	72	73	74	75	76	77	78	79	80
a_O	642	128	462	658	134	467	638	143	472	653
	(22.4)	(4.5)	(16.1)	(23.0)	(4.7)	(16.3)	(22.3)	(5.0)	(16.5)	(22.8)
a_E	737.6	136.1	503.0	737.6	136.1	503.0	737.6	136.1	503.0	737.6
	(25.7)	(4.8)	(17.6)	(25.7)	(4.8)	(17.6)	(25.7)	(4.8)	(17.6)	(25.7)
	−178.2	−15.7	−78.6	−150.2	−4.2	−69.4	−185.0	14.1	−60.1	−159.0
	−6.219	−0.550	−2.745	−5.243	−0.147	−2.424	−6.458	0.492	−2.099	−5.551
t_O	868	283	317	849	264	327	839	257	331	839
	(30.3)	(9.9)	(11.1)	(29.6)	(9.2)	(11.4)	(29.3)	(9.0)	(11.6)	(29.3)
t_E	806.7	246.0	327.0	806.7	246.0	327.0	806.7	246.0	327.0	806.7
	(28.2)	(8.6)	(11.4)	(28.2)	(8.6)	(11.4)	(28.2)	(8.6)	(11.4)	(28.2)
	127.1	79.2	−19.7	86.8	37.2	0.0	65.9	22.4	8.1	65.9
	4.438	2.765	−0.687	3.029	1.298	0.001	2.299	0.782	0.282	2.299
g_O	634	1167	1190	630	1221	1163	645	1190	1155	615
	(22.1)	(40.7)	(41.5)	(22.0)	(42.6)	(40.6)	(22.5)	(41.5)	(40.3)	(21.5)
g_E	628.7	1164.4	1159.9	628.7	1164.4	1159.9	628.7	1164.4	1159.9	628.7
	(21.9)	(40.6)	(40.5)	(21.9)	(40.6)	(40.5)	(21.9)	(40.6)	(40.5)	(21.9)
	10.6	5.2	61.1	2.6	115.9	6.3	33.0	51.8	−9.7	−27.1
	0.370	0.183	2.131	0.090	4.047	0.220	1.151	1.808	−0.338	−0.947
c_O	721	1287	896	728	1246	908	743	1275	907	758
	(25.2)	(44.9)	(31.3)	(25.4)	(43.5)	(31.7)	(25.9)	(44.5)	(31.7)	(26.5)
c_E	692.0	1318.5	875.1	692.0	1318.5	875.1	692.0	1318.5	875.1	692.0
	(24.2)	(46.0)	(30.5)	(24.2)	(46.0)	(30.5)	(24.2)	(46.0)	(30.5)	(24.2)
	59.1	−62.2	42.3	73.8	−140.9	67.0	105.6	−85.5	64.9	138.0
	2.064	−2.170	1.475	2.575	−4.916	2.338	3.686	−2.983	2.266	4.818
N	2865	2865	2865	2865	2865	2865	2865	2865	2865	2865
X2	18.3	6.8	4.9	12.7	8.1	3.8	18.9	2.8	3.1	17.6
Gvalue	18.7	6.5	5.0	12.9	8.1	3.9	19.4	2.8	3.2	17.8
Gv/N	0.654	0.229	0.175	0.451	0.282	0.135	0.679	0.099	0.111	0.620
Prob	0.00031	0.08772	0.17014	0.00479	0.04439	0.27526	0.00022	0.41843	0.36568	0.00049

	81	82	83	84	85	86	87	88	89	90
a_O	149	487	674	153	469	670	136	468	715	145
	(5.2)	(17.0)	(23.5)	(5.3)	(16.4)	(23.4)	(4.7)	(16.3)	(25.0)	(5.1)
a_E	136.1	503.0	737.6	136.1	503.0	737.6	136.1	503.0	737.6	136.1
	(4.8)	(17.6)	(25.7)	(4.8)	(17.6)	(25.7)	(4.8)	(17.6)	(25.7)	(4.8)
	26.9	−31.6	−121.5	35.8	−65.7	−128.7	−0.2	−67.6	−44.4	18.3
	0.940	−1.102	−4.240	1.249	−2.294	−4.493	−0.008	−2.359	−1.550	0.640
t_O	255	326	804	279	350	825	271	305	782	251
	(8.9)	(11.4)	(28.1)	(9.7)	(12.2)	(28.8)	(9.5)	(10.6)	(27.3)	(8.8)
t_E	246.0	327.0	806.7	246.0	327.0	806.7	246.0	327.0	806.7	246.0
	(8.6)	(11.4)	(28.2)	(8.6)	(11.4)	(28.2)	(8.6)	(11.4)	(28.2)	(8.6)
	18.2	−2.0	−5.4	70.1	47.6	37.0	52.4	−42.5	−48.6	10.0

```
          0.637  -0.069  -0.188   2.448   1.662   1.292   1.828  -1.482  -1.698   0.350
g_O        1200    1162     648    1161    1167     660    1229    1201     647    1185
        ( 41.9) ( 40.6) ( 22.6) ( 40.5) ( 40.7) ( 23.0) ( 42.9) ( 41.9) ( 22.6) ( 41.4)
g_E      1164.4  1159.9   628.7  1164.4  1159.9   628.7  1164.4  1159.9   628.7  1164.4
        ( 40.6) ( 40.5) ( 21.9) ( 40.6) ( 40.5) ( 21.9) ( 40.6) ( 40.5) ( 21.9) ( 40.6)
           72.3     4.3    39.2    -6.8    14.3    64.1   132.8    83.7    37.1    41.6
          2.524   0.150   1.367  -0.236   0.500   2.237   4.634   2.922   1.295   1.452
c_O        1261     890     739    1272     879     710    1229     891     721    1284
        ( 44.0) ( 31.1) ( 25.8) ( 44.4) ( 30.7) ( 24.8) ( 42.9) ( 31.1) ( 25.2) ( 44.8)
c_E      1318.5   875.1   692.0  1318.5   875.1   692.0  1318.5   875.1   692.0  1318.5
        ( 46.0) ( 30.5) ( 24.2) ( 46.0) ( 30.5) ( 24.2) ( 46.0) ( 30.5) ( 24.2) ( 46.0)
         -112.4    30.0    97.1   -91.3     7.8    36.4  -172.7    32.1    59.1   -68.0
         -3.922   1.048   3.388  -3.185   0.272   1.271  -6.028   1.119   2.064  -2.374

N          2865    2865    2865    2865    2865    2865    2865    2865    2865    2865
X2          5.1     0.8     9.3     8.2     4.0     8.6    12.2     5.7     3.2     1.9
Gvalue      5.1     0.8     9.4     7.9     4.0     8.8    12.2     5.7     3.2     1.9
Gv/N      0.179   0.027   0.327   0.276   0.140   0.307   0.425   0.200   0.111   0.068
Prob    0.16294 0.85526 0.02493 0.04795 0.26138 0.03218 0.00678 0.12487 0.36408 0.58542
        -------------------------------------------------------------------------------
             91      92      93      94      95      96      97      98      99     100
        -------------------------------------------------------------------------------
a_O         469     740     154     462     665     144     462     679     131     503
        ( 16.4) ( 25.8) (  5.4) ( 16.1) ( 23.2) (  5.0) ( 16.1) ( 23.7) (  4.6) ( 17.6)
a_E       503.0   737.6   136.1   502.9   737.3   136.1   502.9   737.3   136.1   502.9
        ( 17.6) ( 25.7) (  4.8) ( 17.6) ( 25.7) (  4.8) ( 17.6) ( 25.7) (  4.8) ( 17.6)
          -65.7     4.9    38.0   -78.3  -137.3    16.3   -78.3  -111.9   -10.0     0.3
         -2.294   0.171   1.327  -2.734  -4.793   0.569  -2.734  -3.906  -0.348   0.009
t_O         319     801     273     320     860     245     331     826     253     326
        ( 11.1) ( 28.0) (  9.5) ( 11.2) ( 30.0) (  8.6) ( 11.6) ( 28.8) (  8.8) ( 11.4)
t_E       327.0   806.7   246.0   326.9   806.4   246.0   326.9   806.4   246.0   326.9
        ( 11.4) ( 28.2) (  8.6) ( 11.4) ( 28.2) (  8.6) ( 11.4) ( 28.2) (  8.6) ( 11.4)
          -15.8   -11.4    56.8   -13.6   110.6    -1.9     8.3    39.6    14.3    -1.7
         -0.551  -0.397   1.981  -0.475   3.863  -0.067   0.290   1.384   0.499  -0.061
g_O        1205     593    1160    1192     622    1199    1185     652    1213    1189
        ( 42.1) ( 20.7) ( 40.5) ( 41.6) ( 21.7) ( 41.9) ( 41.4) ( 22.8) ( 42.4) ( 41.5)
g_E      1159.9   628.7  1164.4  1159.5   628.5  1164.0  1159.5   628.5  1164.0  1159.5
        ( 40.5) ( 21.9) ( 40.6) ( 40.5) ( 21.9) ( 40.6) ( 40.5) ( 21.9) ( 40.6) ( 40.5)
           92.0   -69.4    -8.7    66.0   -12.9    71.1    51.7    47.9   100.1    59.8
          3.212  -2.421  -0.305   2.305  -0.451   2.482   1.804   1.672   3.495   2.089
c_O         872     731    1278     890     717    1276     886     707    1267     846
        ( 30.4) ( 25.5) ( 44.6) ( 31.1) ( 25.0) ( 44.6) ( 30.9) ( 24.7) ( 44.2) ( 29.5)
c_E       875.1   692.0  1318.5   874.8   691.8  1318.0   874.8   691.8  1318.0   874.8
        ( 30.5) ( 24.2) ( 46.0) ( 30.5) ( 24.2) ( 46.0) ( 30.5) ( 24.2) ( 46.0) ( 30.5)
           -6.2    80.1   -79.7    30.6    51.3   -82.6    22.5    30.8  -100.0   -56.7
         -0.217   2.796  -2.780   1.070   1.792  -2.886   0.787   1.074  -3.491  -1.978

N          2865    2865    2865    2864    2864    2864    2864    2864    2864    2864
X2          4.3     4.3     6.6     4.6    11.6     2.9     4.1     6.3     4.4     1.7
Gvalue      4.3     4.3     6.4     4.7    11.8     2.9     4.2     6.4     4.4     1.7
Gv/N      0.150   0.149   0.222   0.165   0.412   0.100   0.146   0.224   0.155   0.060
Prob    0.23086 0.23360 0.09475 0.19285 0.00813 0.41486 0.24391 0.09334 0.21890 0.63508
        -------------------------------------------------------------------------------
            101     102     103     104     105     106     107     108     109     110
        -------------------------------------------------------------------------------
a_O         694     140     475     698     138     478     706     112     458     723
        ( 24.2) (  4.9) ( 16.6) ( 24.4) (  4.8) ( 16.7) ( 24.7) (  3.9) ( 16.0) ( 25.2)
a_E       737.3   136.1   502.9   737.3   136.1   502.9   737.3   136.1   502.9   737.3
        ( 25.7) (  4.8) ( 17.6) ( 25.7) (  4.8) ( 17.6) ( 25.7) (  4.8) ( 17.6) ( 25.7)
          -84.0     8.0   -54.2   -76.5     3.9   -48.5   -61.3   -43.6   -85.6   -28.3
         -2.933   0.278  -1.891  -2.670   0.136  -1.693  -2.139  -1.523  -2.989  -0.989
t_O         827     244     354     832     276     324     790     275     311     810
        ( 28.9) (  8.5) ( 12.4) ( 29.1) (  9.6) ( 11.3) ( 27.6) (  9.6) ( 10.9) ( 28.3)
t_E       806.4   246.0   326.9   806.4   246.0   326.9   806.4   246.0   326.9   806.4
        ( 28.2) (  8.6) ( 11.4) ( 28.2) (  8.6) ( 11.4) ( 28.2) (  8.6) ( 11.4) ( 28.2)
           41.7    -3.9    56.4    52.0    63.6    -5.7   -32.5    61.4   -31.0     7.2
          1.455  -0.136   1.971   1.814   2.221  -0.200  -1.135   2.144  -1.081   0.251
g_O         644    1201    1132     629    1216    1228     633    1198    1230     625
        ( 22.5) ( 41.9) ( 39.5) ( 22.0) ( 42.5) ( 42.9) ( 22.1) ( 41.8) ( 42.9) ( 21.8)
g_E       628.5  1164.0  1159.5   628.5  1164.0  1159.5   628.5  1164.0  1159.5   628.5
        ( 21.9) ( 40.6) ( 40.5) ( 21.9) ( 40.6) ( 40.5) ( 21.9) ( 40.6) ( 40.5) ( 21.9)
           31.4    75.2   -54.2     1.0   106.3   141.1     9.0    69.0   145.3    -7.0
          1.096   2.626  -1.894   0.035   3.713   4.926   0.316   2.410   5.073  -0.243
c_O         699    1279     903     705    1234     834     735    1279     865     706
        ( 24.4) ( 44.7) ( 31.5) ( 24.6) ( 43.1) ( 29.1) ( 25.7) ( 44.7) ( 30.2) ( 24.7)
c_E       691.8  1318.0   874.8   691.8  1318.0   874.8   691.8  1318.0   874.8   691.8
        ( 24.2) ( 46.0) ( 30.5) ( 24.2) ( 46.0) ( 30.5) ( 24.2) ( 46.0) ( 30.5) ( 24.2)
           14.5   -76.8    57.3    26.7  -162.5   -79.7    89.1   -76.8   -19.5    28.7
          0.506  -2.683   2.000   0.932  -5.675  -2.782   3.110  -2.683  -0.681   1.003

N          2864    2864    2864    2864    2864    2864    2864    2864    2864    2864
X2          3.5     2.5     5.4     3.2    11.4     7.2     4.4     9.8     9.2     0.6
Gvalue      3.6     2.5     5.3     3.2    11.3     7.2     4.4    10.0     9.2     0.6
Gv/N      0.125   0.086   0.186   0.111   0.395   0.251   0.152   0.349   0.322   0.021
Prob    0.31187 0.48281 0.14992 0.36364 0.01012 0.06624 0.22496 0.01867 0.02639 0.89538
        -------------------------------------------------------------------------------
            111     112     113     114     115     116     117     118     119     120
        -------------------------------------------------------------------------------
a_O         135     491     740     134     471     744     143     486     745     139
        (  4.7) ( 17.1) ( 25.8) (  4.7) ( 16.5) ( 26.0) (  5.0) ( 17.0) ( 26.0) (  4.9)
a_E       136.1   502.9   737.3   136.1   502.7   737.0   136.0   502.7   737.0   136.0
        (  4.8) ( 17.6) ( 25.7) (  4.8) ( 17.6) ( 25.7) (  4.8) ( 17.6) ( 25.7) (  4.8)
           -2.1   -23.4     5.4    -4.1   -61.3    14.0    14.3   -32.8    16.0     6.0
         -0.075  -0.819   0.189  -0.144  -2.142   0.488   0.500  -1.146   0.559   0.210
t_O         282     357     789     280     324     797     270     326     809     244
        (  9.8) ( 12.5) ( 27.5) (  9.8) ( 11.3) ( 27.8) (  9.4) ( 11.4) ( 28.3) (  8.5)
t_E       246.0   326.9   806.4   246.0   326.8   806.1   245.9   326.8   806.1   245.9
        (  8.6) ( 11.4) ( 28.2) (  8.6) ( 11.4) ( 28.2) (  8.6) ( 11.4) ( 28.2) (  8.6)
           77.1    62.9   -34.5    72.6    -5.5   -18.2    50.6    -1.5     5.7    -3.7
          2.693   2.198  -1.203   2.535  -0.192  -0.635   1.766  -0.053   0.200  -0.130
g_O        1148    1155     636    1179    1195     641    1154    1200     609    1165
        ( 40.1) ( 40.3) ( 22.2) ( 41.2) ( 41.7) ( 22.4) ( 40.3) ( 41.9) ( 21.3) ( 40.7)
g_E      1164.0  1159.5   628.5  1164.0  1159.0   628.3  1163.6  1159.0   628.3  1163.6
        ( 40.6) ( 40.5) ( 21.9) ( 40.6) ( 40.5) ( 21.9) ( 40.6) ( 40.5) ( 21.9) ( 40.6)
          -31.7    -8.9    15.1    30.2    73.0    25.7   -19.1    83.3   -38.0     2.9
         -1.108  -0.310   0.527   1.056   2.550   0.898  -0.666   2.911  -1.326   0.100
c_O        1299     861     699    1271     873     681    1296     851     700    1315
        ( 45.4) ( 30.1) ( 24.4) ( 44.4) ( 30.5) ( 23.8) ( 45.3) ( 29.7) ( 24.4) ( 45.9)
c_E      1318.0   874.8   691.8  1318.0   874.5   691.5  1317.5   874.5   691.5  1317.5
        ( 46.0) ( 30.5) ( 24.2) ( 46.0) ( 30.5) ( 24.2) ( 46.0) ( 30.5) ( 24.2) ( 46.0)
          -37.7   -27.4    14.5   -92.3    -3.0   -20.9   -42.7   -46.4    17.0    -5.1
         -1.317  -0.957   0.506  -3.223  -0.105  -0.731  -1.492  -1.620   0.594  -0.177
```

N	2864	2864	2864	2864	2863	2863	2863	2863	2863	2863
X2	5.8	3.3	0.6	6.6	3.1	0.6	3.2	2.6	0.8	0.1
Gvalue	5.5	3.2	0.6	6.4	3.2	0.6	3.1	2.6	0.8	0.1
Gv/N	0.194	0.112	0.019	0.225	0.111	0.021	0.108	0.092	0.028	0.003
Prob	0.13593	0.35995	0.90708	0.09247	0.36603	0.89940	0.37960	0.45231	0.85036	0.99352

	121	122	123	124	125	126	127	128	129	130
a_O	474	750	135	457	739	156	489	748	127	494
	(16.6)	(26.2)	(4.7)	(16.0)	(25.8)	(5.5)	(17.1)	(26.1)	(4.4)	(17.3)
a_E	502.7	737.0	136.0	502.5	736.8	136.0	502.5	736.8	136.0	502.5
	(17.6)	(25.7)	(4.8)	(17.6)	(25.7)	(4.8)	(17.6)	(25.7)	(4.8)	(17.6)
	-55.7	26.1	-2.0	-86.8	4.4	42.9	-26.7	22.6	-17.3	-16.9
	-1.946	0.913	-0.071	-3.032	0.155	1.498	-0.931	0.790	-0.606	-0.590
t_O	307	793	256	340	776	249	321	783	257	319
	(10.7)	(27.7)	(8.9)	(11.9)	(27.1)	(8.7)	(11.2)	(27.4)	(9.0)	(11.1)
t_E	326.8	806.1	245.9	326.6	805.9	245.8	326.6	805.9	245.8	326.6
	(11.4)	(28.2)	(8.6)	(11.4)	(28.2)	(8.6)	(11.4)	(28.2)	(8.6)	(11.4)
	-38.3	-26.1	20.7	27.2	-58.6	6.5	-11.2	-45.1	22.9	-15.1
	-1.338	-0.910	0.722	0.952	-2.047	0.226	-0.391	-1.574	0.801	-0.528
g_O	1201	618	1201	1225	633	1186	1203	618	1169	1246
	(41.9)	(21.6)	(41.9)	(42.8)	(22.1)	(41.4)	(42.0)	(21.6)	(40.8)	(43.5)
g_E	1159.0	628.3	1163.6	1158.6	628.1	1163.2	1158.6	628.1	1163.2	1158.6
	(40.5)	(21.9)	(40.6)	(40.5)	(21.9)	(40.6)	(40.5)	(21.9)	(40.6)	(40.5)
	85.4	-20.4	76.1	136.4	9.9	46.1	90.4	-20.0	11.7	181.1
	2.983	-0.712	2.656	4.767	0.347	1.611	3.158	-0.697	0.409	6.329
c_O	881	702	1271	840	714	1271	849	713	1309	803
	(30.8)	(24.5)	(44.4)	(29.4)	(24.9)	(44.4)	(29.7)	(24.9)	(45.7)	(28.1)
c_E	874.5	691.5	1317.5	874.2	691.3	1317.1	874.2	691.3	1317.1	874.2
	(30.5)	(24.2)	(46.0)	(30.5)	(24.2)	(46.0)	(30.5)	(24.2)	(46.0)	(30.5)
	13.0	21.1	-91.4	-67.0	46.1	-90.5	-49.7	44.1	-16.1	-136.4
	0.456	0.736	-3.193	-2.342	1.612	-3.163	-1.735	1.540	-0.563	-4.767
N	2863	2863	2863	2862	2862	2862	2862	2862	2862	2862
X2	4.4	0.8	3.3	9.8	1.9	5.1	2.9	1.7	1.2	12.7
Gvalue	4.4	0.8	3.3	9.9	1.9	4.9	2.9	1.7	1.2	12.7
Gv/N	0.155	0.027	0.114	0.345	0.066	0.172	0.100	0.058	0.042	0.444
Prob	0.21788	0.85710	0.35123	0.01965	0.59286	0.17686	0.41127	0.64583	0.75556	0.00529

	131	132	133	134	135	136	137	138	139	140
a_O	693	136	479	806	150	458	745	136	489	722
	(24.2)	(4.8)	(16.7)	(28.2)	(5.2)	(16.0)	(26.0)	(4.8)	(17.1)	(25.2)
a_E	736.8	136.0	502.5	736.8	136.0	502.3	736.5	135.9	502.3	736.5
	(25.7)	(4.8)	(17.6)	(25.7)	(4.8)	(17.6)	(25.7)	(4.8)	(17.6)	(25.7)
	-84.9	0.0	-45.9	144.7	29.4	-84.6	17.0	0.1	-26.3	-28.8
	-2.967	0.002	-1.604	5.057	1.029	-2.958	0.596	0.005	-0.920	-1.005
t_O	787	276	304	751	255	358	813	245	332	813
	(27.5)	(9.6)	(10.6)	(26.2)	(8.9)	(12.5)	(28.4)	(8.6)	(11.6)	(28.4)
t_E	805.9	245.8	326.6	805.9	245.8	326.5	805.6	245.7	326.5	805.6
	(28.2)	(8.6)	(11.4)	(28.2)	(8.6)	(11.4)	(28.2)	(8.6)	(11.4)	(28.2)
	-37.3	64.0	-43.7	-105.9	18.8	65.9	14.9	-1.4	11.0	14.9
	-1.302	2.236	-1.526	-3.700	0.656	2.302	0.522	-0.049	0.385	0.522
g_O	630	1136	1185	617	1182	1186	595	1180	1185	612
	(22.0)	(39.7)	(41.4)	(21.6)	(41.3)	(41.5)	(20.8)	(41.2)	(41.4)	(21.4)
g_E	628.1	1163.2	1158.6	628.1	1163.2	1158.2	627.8	1162.8	1158.2	627.8
	(21.9)	(40.6)	(40.5)	(21.9)	(40.6)	(40.5)	(21.9)	(40.6)	(40.5)	(21.9)
	3.9	-53.7	53.3	-21.9	38.0	56.2	-63.9	34.7	54.1	-31.3
	0.136	-1.876	1.863	-0.766	1.327	1.964	-2.234	1.214	1.892	-1.093
c_O	752	1314	894	688	1275	859	708	1300	855	714
	(26.3)	(45.9)	(31.2)	(24.0)	(44.5)	(30.0)	(24.7)	(45.4)	(29.9)	(25.0)
c_E	691.3	1317.1	874.2	691.3	1317.1	873.9	691.1	1316.6	873.9	691.1
	(24.2)	(46.0)	(30.5)	(24.2)	(46.0)	(30.5)	(24.2)	(46.0)	(30.5)	(24.2)
	126.6	-6.1	40.1	-6.6	-82.8	-29.5	34.3	-33.0	-37.4	46.6
	4.423	-0.215	1.399	-0.230	-2.893	-1.032	1.199	-1.154	-1.306	1.630
N	2862	2862	2862	2862	2862	2861	2861	2861	2861	2861
X2	8.4	4.4	3.7	10.4	3.4	7.9	2.3	0.5	1.5	1.5
Gvalue	8.3	4.2	3.8	10.3	3.4	7.9	2.3	0.5	1.5	1.5
Gv/N	0.290	0.147	0.132	0.361	0.119	0.276	0.081	0.016	0.051	0.053
Prob	0.04046	0.23888	0.28787	0.01588	0.33365	0.04837	0.50768	0.92606	0.68843	0.67934

	141	142	143	144	145	146	147	148	149	150
a_O	127	491	772	124	461	722	134	479	743	110
	(4.4)	(17.2)	(27.0)	(4.3)	(16.1)	(25.2)	(4.7)	(16.7)	(26.0)	(3.8)
a_E	135.9	502.3	736.5	135.9	502.3	736.5	135.9	502.2	736.3	135.9
	(4.8)	(17.6)	(25.7)	(4.8)	(17.6)	(25.7)	(4.8)	(17.6)	(25.7)	(4.8)
	-17.3	-22.4	72.6	-22.8	-79.2	-28.8	-3.8	-45.2	13.5	-46.5
	-0.603	-0.784	2.539	-0.796	-2.767	-1.005	-0.134	-1.582	0.473	-1.625
t_O	247	345	791	251	332	809	249	340	856	244
	(8.6)	(12.1)	(27.6)	(8.8)	(11.6)	(28.3)	(8.7)	(11.9)	(29.9)	(8.5)
t_E	245.7	326.5	805.6	245.7	326.5	805.6	245.7	326.4	805.3	245.6
	(8.6)	(11.4)	(28.2)	(8.6)	(11.4)	(28.2)	(8.6)	(11.4)	(28.2)	(8.6)
	2.6	38.0	-28.9	10.7	11.0	6.9	6.6	27.7	104.5	-3.2
	0.091	1.327	-1.010	0.375	0.385	0.240	0.232	0.969	3.655	-0.112
g_O	1176	1141	597	1220	1199	640	1154	1152	589	1179
	(41.1)	(39.9)	(20.9)	(42.6)	(41.9)	(22.4)	(40.3)	(40.3)	(20.6)	(41.2)
g_E	1162.8	1158.2	627.8	1162.8	1158.2	627.8	1162.8	1157.8	627.6	1162.4
	(40.6)	(40.5)	(21.9)	(40.6)	(40.5)	(21.9)	(40.6)	(40.5)	(21.9)	(40.6)
	26.6	-34.2	-60.1	117.3	82.9	24.6	-17.4	-11.6	-74.8	33.5
	0.931	-1.196	-2.102	4.099	2.899	0.858	-0.610	-0.407	-2.616	1.173
c_O	1311	884	701	1266	869	690	1324	889	672	1327
	(45.8)	(30.9)	(24.5)	(44.3)	(30.4)	(24.1)	(46.3)	(31.1)	(23.5)	(46.4)
c_E	1316.6	873.9	691.1	1316.6	873.9	691.1	1316.6	873.6	690.8	1316.2
	(46.0)	(30.5)	(24.2)	(46.0)	(30.5)	(24.2)	(46.0)	(30.5)	(24.2)	(46.0)
	-11.2	20.3	20.0	-99.3	-9.8	-2.1	14.8	31.1	-37.1	21.8
	-0.392	0.711	0.700	-3.469	-0.341	-0.074	0.518	1.087	-1.298	0.761
N	2861	2861	2861	2861	2861	2861	2861	2860	2860	2860
X2	0.8	1.7	3.6	5.9	5.0	0.5	0.2	1.9	6.1	5.3
Gvalue	0.8	1.7	3.6	5.9	5.0	0.5	0.2	1.9	6.1	5.6
Gv/N	0.027	0.058	0.127	0.208	0.176	0.019	0.006	0.068	0.214	0.196
Prob	0.85398	0.64647	0.30439	0.11471	0.16922	0.91030	0.98088	0.58448	0.10531	0.13199

Afful_DSM4304
5UTR:

	50	49	48	47	46	45	44	43	42	41
a_O	712	730	711	797	772	750	784	798	714	715
	(29.6)	(30.3)	(29.5)	(33.1)	(32.1)	(31.2)	(32.6)	(33.2)	(29.7)	(29.7)
a_E	724.2	724.2	724.2	724.2	724.2	724.2	724.2	724.2	724.2	724.2
	(30.1)	(30.1)	(30.1)	(30.1)	(30.1)	(30.1)	(30.1)	(30.1)	(30.1)	(30.1)
	-24.2	11.6	-26.2	152.7	98.7	52.5	124.4	154.9	-20.3	-18.3
	-1.006	0.483	-1.088	6.342	4.099	2.181	5.168	6.434	-0.842	-0.760
t_O	652	704	692	561	664	652	593	662	670	626
	(27.1)	(29.2)	(28.7)	(23.3)	(27.6)	(27.1)	(24.6)	(27.5)	(27.8)	(26.0)
t_E	656.0	656.0	656.0	656.0	656.0	656.0	656.0	656.0	656.0	656.0
	(27.3)	(27.3)	(27.3)	(27.3)	(27.3)	(27.3)	(27.3)	(27.3)	(27.3)	(27.3)
	-8.1	99.3	73.9	-175.6	16.0	-8.1	-119.8	12.0	28.2	-58.7
	-0.335	4.127	3.068	-7.295	0.665	-0.335	-4.978	0.497	1.172	-2.438
g_O	591	509	551	618	555	539	613	492	571	657
	(24.6)	(21.1)	(22.9)	(25.7)	(23.1)	(22.4)	(25.5)	(20.4)	(23.7)	(27.3)
g_E	584.0	584.0	584.0	584.0	584.0	584.0	584.0	584.0	584.0	584.0
	(24.3)	(24.3)	(24.3)	(24.3)	(24.3)	(24.3)	(24.3)	(24.3)	(24.3)	(24.3)
	14.0	-140.0	-64.2	69.9	-56.6	-86.5	59.4	-168.7	-25.8	154.7
	0.583	-5.816	-2.665	2.903	-2.351	-3.593	2.466	-7.010	-1.071	6.427
c_O	452	464	453	431	416	466	417	455	452	409
	(18.8)	(19.3)	(18.8)	(17.9)	(17.3)	(19.4)	(17.3)	(18.9)	(18.8)	(17.0)
c_E	442.7	442.7	442.7	442.7	442.7	442.7	442.7	442.7	442.7	442.7
	(18.4)	(18.4)	(18.4)	(18.4)	(18.4)	(18.4)	(18.4)	(18.4)	(18.4)	(18.4)
	18.8	43.6	20.8	-23.1	-51.8	47.8	-49.9	24.9	18.8	-64.8
	0.779	1.810	0.864	-0.961	-2.152	1.984	-2.074	1.034	0.779	-2.692
N	2407	2407	2407	2407	2407	2407	2407	2407	2407	2407
X2	0.5	14.2	4.3	23.4	6.3	5.6	13.9	22.4	0.9	13.2
Gvalue	0.5	14.6	4.3	23.8	6.3	5.7	14.0	23.0	0.9	12.9
Gv/N	0.021	0.605	0.179	0.990	0.262	0.237	0.582	0.955	0.038	0.536
Prob	0.91713	0.00224	0.22895	0.00003	0.09805	0.12712	0.00290	0.00004	0.81923	0.00483

	40	39	38	37	36	35	34	33	32	31
a_O	804	747	761	706	724	888	962	1003	1065	946
	(33.4)	(31.0)	(31.6)	(29.3)	(30.1)	(36.9)	(40.0)	(41.7)	(44.2)	(39.3)
a_E	724.2	724.2	724.2	724.2	724.2	724.2	724.2	724.2	724.2	724.2
	(30.1)	(30.1)	(30.1)	(30.1)	(30.1)	(30.1)	(30.1)	(30.1)	(30.1)	(30.1)
	168.1	46.3	75.4	-36.0	-0.4	362.1	546.3	653.3	821.4	505.5
	6.982	1.923	3.133	-1.494	-0.017	15.044	22.696	27.141	34.127	21.000
t_O	680	695	598	648	561	466	540	568	435	525
	(28.3)	(28.9)	(24.8)	(26.9)	(23.3)	(19.4)	(22.4)	(23.6)	(18.1)	(21.8)
t_E	656.0	656.0	656.0	656.0	656.0	656.0	656.0	656.0	656.0	656.0
	(27.3)	(27.3)	(27.3)	(27.3)	(27.3)	(27.3)	(27.3)	(27.3)	(27.3)	(27.3)
	48.8	80.2	-110.8	-16.0	-175.6	-318.8	-210.2	-163.7	-357.5	-234.0
	2.027	3.332	-4.603	-0.664	-7.295	-13.244	-8.734	-6.801	-14.851	-9.720
g_O	505	527	628	609	618	654	532	468	573	553
	(21.0)	(21.9)	(26.1)	(25.3)	(25.7)	(27.2)	(22.1)	(19.4)	(23.8)	(23.0)
g_E	584.0	584.0	584.0	584.0	584.0	584.0	584.0	584.0	584.0	584.0
	(24.3)	(24.3)	(24.3)	(24.3)	(24.3)	(24.3)	(24.3)	(24.3)	(24.3)	(24.3)
	-146.8	-108.3	91.2	51.0	69.9	148.0	-99.3	-207.3	-21.9	-60.4
	-6.101	-4.499	3.788	2.119	2.903	6.149	-4.125	-8.613	-0.908	-2.509
c_O	418	438	420	444	504	399	373	368	334	383
	(17.4)	(18.2)	(17.4)	(18.4)	(20.9)	(16.6)	(15.5)	(15.3)	(13.9)	(15.9)
c_E	442.7	442.7	442.7	442.7	442.7	442.7	442.7	442.7	442.7	442.7
	(18.4)	(18.4)	(18.4)	(18.4)	(18.4)	(18.4)	(18.4)	(18.4)	(18.4)	(18.4)
	-48.0	-9.4	-44.3	2.6	130.7	-83.0	-127.8	-136.1	-188.2	-111.0
	-1.996	-0.390	-1.839	0.107	5.429	-3.447	-5.311	-5.652	-7.821	-4.611
N	2407	2407	2407	2407	2407	2407	2407	2407	2407	2407
X2	21.7	8.7	11.5	1.6	24.2	104.8	114.2	154.8	261.7	103.8
Gvalue	22.0	8.8	11.5	1.6	24.5	108.4	108.9	146.2	253.9	100.1
Gv/N	0.913	0.365	0.480	0.067	1.020	4.502	4.526	6.075	10.548	4.160
Prob	0.00007	0.03223	0.00910	0.65552	0.00002	0.00000	0.00000	0.00000	0.00000	0.00000

	30	29	28	27	26	25	24	23	22	21
a_O	715	686	682	632	867	885	846	891	850	731
	(29.7)	(28.5)	(28.3)	(26.3)	(36.0)	(36.8)	(35.1)	(37.0)	(35.3)	(30.4)
a_E	724.2	724.2	724.2	724.2	724.2	724.2	724.2	724.2	724.2	724.2
	(30.1)	(30.1)	(30.1)	(30.1)	(30.1)	(30.1)	(30.1)	(30.1)	(30.1)	(30.1)
	-18.3	-74.4	-81.9	-172.1	312.0	354.9	263.0	369.3	272.3	13.6
	-0.760	-3.090	-3.403	-7.152	12.964	14.744	10.927	15.345	11.311	0.567
t_O	699	730	975	988	817	890	798	695	694	711
	(29.0)	(30.3)	(40.5)	(41.0)	(33.9)	(37.0)	(33.2)	(28.9)	(28.8)	(29.5)
t_E	656.0	656.0	656.0	656.0	656.0	656.0	656.0	656.0	656.0	656.0
	(27.3)	(27.3)	(27.3)	(27.3)	(27.3)	(27.3)	(27.3)	(27.3)	(27.3)	(27.3)
	88.7	156.0	772.6	809.1	358.5	542.9	312.6	80.2	78.1	114.4
	3.684	6.479	32.099	33.614	14.895	22.555	12.989	3.332	3.244	4.753
g_O	566	568	444	453	410	369	438	486	468	473
	(23.5)	(23.6)	(18.4)	(18.8)	(17.0)	(15.3)	(18.2)	(20.2)	(19.4)	(19.7)
g_E	584.0	584.0	584.0	584.0	584.0	584.0	584.0	584.0	584.0	584.0
	(24.3)	(24.3)	(24.3)	(24.3)	(24.3)	(24.3)	(24.3)	(24.3)	(24.3)	(24.3)
	-35.5	-31.6	-243.4	-230.2	-290.1	-338.9	-252.1	-178.6	-207.3	-199.5
	-1.475	-1.313	-10.113	-9.563	-12.053	-14.078	-10.472	-7.420	-8.613	-8.287
c_O	427	423	306	334	313	263	325	335	395	492
	(17.7)	(17.6)	(12.7)	(13.9)	(13.0)	(10.9)	(13.5)	(13.9)	(16.4)	(20.4)
c_E	442.7	442.7	442.7	442.7	442.7	442.7	442.7	442.7	442.7	442.7
	(18.4)	(18.4)	(18.4)	(18.4)	(18.4)	(18.4)	(18.4)	(18.4)	(18.4)	(18.4)
	-30.9	-38.5	-226.0	-188.2	-217.1	-273.9	-200.9	-186.8	-90.1	103.9
	-1.283	-1.602	-9.391	-7.821	-9.018	-11.381	-8.347	-7.761	-3.743	4.315
N	2407	2407	2407	2407	2407	2407	2407	2407	2407	2407
X2	4.0	11.7	233.3	235.8	157.5	271.3	119.0	83.4	52.2	31.3
Gvalue	4.0	11.4	221.2	218.5	163.4	285.0	122.7	84.1	52.9	32.4
Gv/N	0.166	0.475	9.192	9.079	6.789	11.841	5.096	3.495	2.199	1.347
Prob	0.26161	0.00962	0.00000	0.00000	0.00000	0.00000	0.00000	0.00000	0.00000	0.00000

	20	19	18	17	16	15	14	13	12	11
a_O	712	697	652	752	773	689	746	709	723	764
	(29.6)	(29.0)	(27.1)	(31.2)	(32.1)	(28.6)	(31.0)	(29.5)	(30.0)	(31.7)
a_E	724.2	724.2	724.2	724.2	724.2	724.2	724.2	724.2	724.2	724.2
	(30.1)	(30.1)	(30.1)	(30.1)	(30.1)	(30.1)	(30.1)	(30.1)	(30.1)	(30.1)
	-24.2	-53.4	-137.0	56.6	100.8	-68.7	44.2	-30.1	-2.4	81.7
	-1.006	-2.218	-5.690	2.353	4.188	-2.853	1.838	-1.250	-0.100	3.395
t_O	640	749	737	647	697	725	574	666	582	462
	(26.6)	(31.1)	(30.6)	(26.9)	(29.0)	(30.1)	(23.8)	(27.7)	(24.2)	(19.2)

```
t_E        656.0   656.0   656.0   656.0   656.0   656.0   656.0   656.0   656.0   656.0
         ( 27.3) ( 27.3) ( 27.3) ( 27.3) ( 27.3) ( 27.3) ( 27.3) ( 27.3) ( 27.3) ( 27.3)
           -31.7   198.5   171.5   -18.0    84.4   144.9  -153.4    20.1  -139.4  -324.0
          -1.316   8.247   7.126  -0.746   3.508   6.021  -6.372   0.834  -5.791 -13.461
g_O          586     483     525     547     497     552     657     593     648     825
         ( 24.3) ( 20.1) ( 21.8) ( 22.7) ( 20.6) ( 22.9) ( 27.3) ( 24.6) ( 26.9) ( 34.3)
g_E        584.0   584.0   584.0   584.0   584.0   584.0   584.0   584.0   584.0   584.0
         ( 24.3) ( 24.3) ( 24.3) ( 24.3) ( 24.3) ( 24.3) ( 24.3) ( 24.3) ( 24.3) ( 24.3)
             3.9  -183.5  -111.9   -71.7  -160.4   -62.3   154.7    18.1   134.7   570.0
           0.164  -7.623  -4.648  -2.977  -6.664  -2.587   6.427   0.751   5.596  23.679
c_O          469     478     493     461     440     441     430     439     454     356
         ( 19.5) ( 19.9) ( 20.5) ( 19.2) ( 18.3) ( 18.3) ( 17.9) ( 18.2) ( 18.9) ( 14.8)
c_E        442.7   442.7   442.7   442.7   442.7   442.7   442.7   442.7   442.7   442.7
         ( 18.4) ( 18.4) ( 18.4) ( 18.4) ( 18.4) ( 18.4) ( 18.4) ( 18.4) ( 18.4) ( 18.4)
            54.1    73.3   106.1    37.3    -5.4    -3.4   -25.1    -7.4    22.8  -155.2
           2.247   3.045   4.407   1.550  -0.225  -0.143  -1.042  -0.308   0.949  -6.449

N           2407    2407    2407    2407    2407    2407    2407    2407    2407    2407
X2           2.2    34.5    28.9     4.3    18.8    10.7    20.4     0.6    15.7   176.0
Gvalue       2.1    34.9    28.7     4.3    19.4    10.5    20.5     0.6    15.7   172.5
Gv/N       0.089   1.452   1.194   0.180   0.806   0.438   0.851   0.027   0.654   7.165
Prob     0.54405 0.00000 0.00000 0.22874 0.00023 0.01447 0.00013 0.88708 0.00128 0.00000
         -------------------------------------------------------------------------------
              10       9       8       7       6       5       4       3       2       1
         -------------------------------------------------------------------------------
a_O          953     697     524     668     606     797     692     821     698     612
         ( 39.6) ( 29.0) ( 21.8) ( 27.8) ( 25.2) ( 33.1) ( 28.7) ( 34.1) ( 29.0) ( 25.4)
a_E        724.2   724.2   724.2   724.2   724.2   724.2   724.2   724.2   724.2   724.2
         ( 30.1) ( 30.1) ( 30.1) ( 30.1) ( 30.1) ( 30.1) ( 30.1) ( 30.1) ( 30.1) ( 30.1)
           523.3   -53.4  -339.1  -107.9  -216.0   152.7   -63.0   206.0   -51.5  -206.1
          21.739  -2.218 -14.089  -4.484  -8.973   6.342  -2.616   8.557  -2.138  -8.561
t_O          541     586     590     943     700     576     792     618     610     656
         ( 22.5) ( 24.3) ( 24.5) ( 39.2) ( 29.1) ( 23.9) ( 32.9) ( 25.7) ( 25.3) ( 27.3)
t_E        656.0   656.0   656.0   656.0   656.0   656.0   656.0   656.0   656.0   656.0
         ( 27.3) ( 27.3) ( 27.3) ( 27.3) ( 27.3) ( 27.3) ( 27.3) ( 27.3) ( 27.3) ( 27.3)
          -208.6  -132.3  -125.2   684.3    90.8  -149.9   298.3   -73.8   -88.8    -0.1
          -8.667  -5.497  -5.201  28.431   3.772  -6.227  12.394  -3.067  -3.688  -0.003
g_O          657     834     946     519     816     705     573     681     663     548
         ( 27.3) ( 34.6) ( 39.3) ( 21.6) ( 33.9) ( 29.3) ( 23.8) ( 28.3) ( 27.5) ( 22.8)
g_E        584.0   584.0   584.0   584.0   584.0   584.0   584.0   584.0   584.0   584.0
         ( 24.3) ( 24.3) ( 24.3) ( 24.3) ( 24.3) ( 24.3) ( 24.3) ( 24.3) ( 24.3) ( 24.3)
           154.7   594.3   912.5  -122.5   545.8   265.4   -21.9   209.2   168.2   -69.8
           6.427  24.690  37.910  -5.091  22.677  11.027  -0.908   8.692   6.987  -2.899
c_O          256     290     347     277     285     329     350     287     436     591
         ( 10.6) ( 12.0) ( 14.4) ( 11.5) ( 11.8) ( 13.7) ( 14.5) ( 11.9) ( 18.1) ( 24.6)
c_E        442.7   442.7   442.7   442.7   442.7   442.7   442.7   442.7   442.7   442.7
         ( 18.4) ( 18.4) ( 18.4) ( 18.4) ( 18.4) ( 18.4) ( 18.4) ( 18.4) ( 18.4) ( 18.4)
          -280.5  -245.4  -169.1  -259.8  -251.1  -195.3  -164.5  -248.8   -13.3   341.5
         -11.652 -10.194  -7.024 -10.793 -10.430  -8.116  -6.834 -10.337  -0.554  14.186

N           2407    2407    2407    2407    2407    2407    2407    2407    2407    2407
X2         180.3   168.2   307.0   199.2   170.6    71.3    49.2    86.0    15.0    69.3
Gvalue     188.9   163.2   279.1   194.1   169.6    72.9    49.0    92.6    14.6    65.5
Gv/N       7.848   6.780  11.596   8.063   7.046   3.027   2.036   3.845   0.607   2.722
Prob     0.00000 0.00000 0.00000 0.00000 0.00000 0.00000 0.00000 0.00000 0.00219 0.00000
         -------------------------------------------------------------------------------

CDS_INITIAL:
         -------------------------------------------------------------------------------
               1       2       3       4       5       6       7       8       9      10
         -------------------------------------------------------------------------------
a_O         1828       0       0    1047     913     652     814     803     629     850
         ( 75.9) (  0.0) (  0.0) ( 43.5) ( 37.9) ( 27.1) ( 33.8) ( 33.4) ( 26.1) ( 35.3)
a_E        759.0   741.9   472.6   759.0   741.9   472.6   759.0   741.9   472.6   759.0
         ( 31.5) ( 30.8) ( 19.6) ( 31.5) ( 30.8) ( 19.6) ( 31.5) ( 30.8) ( 19.6) ( 31.5)
          3213.7     0.0     0.0   673.7   378.9   419.6   114.0   127.0   359.6   192.6
         133.516   0.000   0.000  27.990  15.740  17.432   4.735   5.278  14.940   8.001
t_O           56    2407       0     222     651     507     386     821     558     389
         (  2.3) (100.0) (  0.0) (  9.2) ( 27.0) ( 21.1) ( 16.0) ( 34.1) ( 23.2) ( 16.2)
t_E        367.8   783.5   524.9   367.8   783.5   524.9   367.8   783.5   524.9   367.8
         ( 15.3) ( 32.6) ( 21.8) ( 15.3) ( 32.6) ( 21.8) ( 15.3) ( 32.6) ( 21.8) ( 15.3)
          -210.8  5403.0     0.0  -224.1  -241.2   -35.2    37.3    76.7    68.2    43.7
          -8.758 224.469   0.000  -9.311 -10.022  -1.463   1.552   3.188   2.834   1.814
g_O          523       0    2407     762     468     811     856     417     696     807
         ( 21.7) (  0.0) (100.0) ( 31.7) ( 19.4) ( 33.7) ( 35.6) ( 17.3) ( 28.9) ( 33.5)
g_E        904.9   422.8   743.3   904.9   422.8   743.3   904.9   422.8   743.3   904.9
         ( 37.6) ( 17.6) ( 30.9) ( 37.6) ( 17.6) ( 30.9) ( 37.6) ( 17.6) ( 30.9) ( 37.6)
          -573.4     0.0  5656.7  -261.9    95.0   141.4   -95.0   -11.6   -91.5  -184.7
         -23.822   0.000 235.012 -10.879   3.947   5.876  -3.948  -0.481  -3.801  -7.674
c_O            0       0       0     376     375     437     351     366     524     361
         (  0.0) (  0.0) (  0.0) ( 15.6) ( 15.6) ( 18.2) ( 14.6) ( 15.2) ( 21.8) ( 15.0)
c_E        375.4   458.7   666.2   375.4   458.7   666.2   375.4   458.7   666.2   375.4
         ( 15.6) ( 19.1) ( 27.7) ( 15.6) ( 19.1) ( 27.7) ( 15.6) ( 19.1) ( 27.7) ( 15.6)
             0.0     0.0     0.0     1.2  -151.1  -368.5   -47.2  -165.3  -251.6   -28.3
           0.000   0.000   0.000   0.049  -6.279 -15.310  -1.961  -6.867 -10.453  -1.174

N           2407    2407    2407    2407    2407    2407    2407    2407    2407    2407
X2        2306.7  4987.4  5387.7   189.6    82.0   153.7     9.1    25.6    87.2    23.3
Gvalue    2429.5  5403.0  5656.7   188.9    81.5   157.3     9.1    26.9    84.7    23.3
Gv/N     100.936 224.469 235.012   7.848   3.385   6.535   0.378   1.117   3.520   0.966
Prob     1.00000 1.00000 1.00000 0.00000 0.00000 0.00000 0.02803 0.00001 0.00000 0.00004
         -------------------------------------------------------------------------------
              11      12      13      14      15      16      17      18      19      20
         -------------------------------------------------------------------------------
a_O          692     593     818     730     536     818     681     526     746     696
         ( 28.7) ( 24.6) ( 34.0) ( 30.3) ( 22.3) ( 34.0) ( 28.3) ( 21.9) ( 31.0) ( 28.9)
a_E        741.9   472.6   759.0   741.9   472.6   759.0   741.9   472.6   759.0   741.9
         ( 30.8) ( 19.6) ( 31.5) ( 30.8) ( 19.6) ( 31.5) ( 30.8) ( 19.6) ( 31.5) ( 30.8)
           -96.4   269.1   122.6   -23.7   134.9   122.6  -116.7   112.6   -25.7   -89.0
          -4.006  11.181   5.092  -0.983   5.605   5.092  -4.849   4.678  -1.068  -3.696
t_O          967     555     393     925     635     391     960     629     415     916
         ( 40.2) ( 23.1) ( 16.3) ( 38.4) ( 26.4) ( 16.2) ( 39.9) ( 26.1) ( 17.2) ( 38.1)
t_E        783.5   524.9   367.8   783.5   524.9   367.8   783.5   524.9   367.8   783.5
         ( 32.6) ( 21.8) ( 15.3) ( 32.6) ( 21.8) ( 15.3) ( 32.6) ( 21.8) ( 15.3) ( 32.6)
           406.9    61.9    52.1   307.1   241.8    47.9   390.0   227.6   100.3   286.2
          16.906   2.570   2.167  12.759  10.045   1.990  16.204   9.454   4.166  11.891
g_O          369     748     839     383     718     812     356     664     838     393
         ( 15.3) ( 31.1) ( 34.9) ( 15.9) ( 29.8) ( 33.7) ( 14.8) ( 27.6) ( 34.8) ( 16.3)
g_E        422.8   743.3   904.9   422.8   743.3   904.9   422.8   743.3   904.9   422.8
         ( 17.6) ( 30.9) ( 37.6) ( 17.6) ( 30.9) ( 37.6) ( 17.6) ( 30.9) ( 37.6) ( 17.6)
          -100.5     9.5  -126.8   -75.8   -49.7  -175.8  -122.5  -149.8  -128.6   -57.5
          -4.175   0.393  -5.268  -3.149  -2.065  -7.305  -5.089  -6.223  -5.344  -2.389
```

c_O	379	511	357	369	518	386	410	588	408	402
	(15.7)	(21.2)	(14.8)	(15.3)	(21.5)	(16.0)	(17.0)	(24.4)	(17.0)	(16.7)
c_E	458.7	666.2	375.4	458.7	666.2	375.4	458.7	666.2	375.4	458.7
	(19.1)	(27.7)	(15.6)	(19.1)	(27.7)	(15.6)	(19.1)	(27.7)	(15.6)	(19.1)
	-144.7	-271.0	-35.9	-160.6	-260.7	21.5	-92.1	-146.8	67.9	-106.1
	-6.012	-11.261	-1.492	-6.673	-10.829	0.892	-3.825	-6.100	2.822	-4.409
N	2407	2407	2407	2407	2407	2407	2407	2407	2407	2407
X2	67.0	68.6	12.0	47.0	65.4	15.9	60.5	44.3	14.1	34.4
Gvalue	65.3	69.4	12.0	47.0	66.4	16.1	58.7	43.5	13.9	33.6
Gv/N	2.713	2.884	0.499	1.954	2.757	0.668	2.441	1.809	0.576	1.397
Prob	0.00000	0.00000	0.00738	0.00000	0.00000	0.00109	0.00000	0.00000	0.00310	0.00000

	21	22	23	24	25	26	27	28	29	30
a_O	543	740	672	537	805	721	552	713	723	520
	(22.6)	(30.7)	(27.9)	(22.3)	(33.4)	(30.0)	(22.9)	(29.6)	(30.0)	(21.6)
a_E	472.6	759.0	741.9	472.6	759.0	741.9	472.6	759.0	741.9	472.6
	(19.6)	(31.5)	(30.8)	(19.6)	(31.5)	(30.8)	(19.6)	(31.5)	(30.8)	(19.6)
	150.8	-37.4	-133.1	137.2	94.8	-41.3	171.4	-89.1	-37.4	99.4
	6.264	-1.556	-5.528	5.699	3.939	-1.714	7.122	-3.701	-1.553	4.129
t_O	626	407	873	562	413	844	536	436	804	588
	(26.0)	(16.9)	(36.3)	(23.3)	(17.2)	(35.1)	(22.3)	(18.1)	(33.4)	(24.4)
t_E	524.9	367.8	783.5	524.9	367.8	783.5	524.9	367.8	783.5	524.9
	(21.8)	(15.3)	(32.6)	(21.8)	(15.3)	(32.6)	(21.8)	(15.3)	(32.6)	(21.8)
	220.5	82.5	188.8	76.7	95.8	125.5	22.4	148.4	41.5	133.5
	9.161	3.427	7.845	3.188	3.980	5.215	0.931	6.165	1.724	5.545
g_O	638	868	441	693	808	434	706	857	440	697
	(26.5)	(36.1)	(18.3)	(28.8)	(33.6)	(18.0)	(29.3)	(35.6)	(18.3)	(29.0)
g_E	743.3	904.9	422.8	743.3	904.9	422.8	743.3	904.9	422.8	743.3
	(30.9)	(37.6)	(17.6)	(30.9)	(37.6)	(17.6)	(30.9)	(37.6)	(17.6)	(30.9)
	-194.9	-72.2	37.1	-97.1	-182.9	22.6	-72.7	-93.1	35.0	-89.6
	-8.097	-2.999	1.542	-4.033	-7.601	0.940	-3.019	-3.869	1.455	-3.723
c_O	600	392	421	615	381	408	613	401	440	602
	(24.9)	(16.3)	(17.5)	(25.6)	(15.8)	(17.0)	(25.5)	(16.7)	(18.3)	(25.0)
c_E	666.2	375.4	458.7	666.2	375.4	458.7	666.2	375.4	458.7	666.2
	(27.7)	(15.6)	(19.1)	(27.7)	(15.6)	(19.1)	(27.7)	(15.6)	(19.1)	(27.7)
	-125.6	33.9	-72.3	-98.3	11.3	-95.6	-102.0	52.9	-36.7	-122.0
	-5.217	1.408	-3.002	-4.086	0.467	-3.972	-4.238	2.197	-1.523	-5.068
N	2407	2407	2407	2407	2407	2407	2407	2407	2407	2407
X2	51.4	6.9	20.7	18.7	18.8	11.2	19.7	19.7	2.5	21.4
Gvalue	50.8	6.8	20.6	18.5	18.9	11.3	19.1	19.1	2.5	21.2
Gv/N	2.111	0.281	0.857	0.768	0.786	0.468	0.795	0.792	0.103	0.882
Prob	0.00000	0.07980	0.00013	0.00035	0.00028	0.01035	0.00026	0.00027	0.47878	0.00009

	31	32	33	34	35	36	37	38	39	40
a_O	753	693	532	715	679	569	772	750	539	806
	(31.3)	(28.8)	(22.1)	(29.7)	(28.2)	(23.6)	(32.1)	(31.2)	(22.4)	(33.5)
a_E	759.0	741.9	472.6	759.0	741.9	472.6	759.0	741.9	472.6	759.0
	(31.5)	(30.8)	(19.6)	(31.5)	(30.8)	(19.6)	(31.5)	(30.8)	(19.6)	(31.5)
	-11.9	-94.6	125.9	-85.3	-120.4	211.2	26.3	16.2	141.7	96.9
	-0.493	-3.929	5.232	-3.545	-5.001	8.775	1.093	0.674	5.887	4.027
t_O	412	858	568	413	829	554	400	808	532	364
	(17.1)	(35.6)	(23.6)	(17.2)	(34.4)	(23.0)	(16.6)	(33.6)	(22.1)	(15.1)
t_E	367.8	783.5	524.9	367.8	783.5	524.9	367.8	783.5	524.9	367.8
	(15.3)	(32.6)	(21.8)	(15.3)	(32.6)	(21.8)	(15.3)	(32.6)	(21.8)	(15.3)
	93.6	155.8	89.6	95.8	93.6	59.8	67.2	49.7	14.3	-7.5
	3.888	6.474	3.723	3.980	3.887	2.482	2.792	2.066	0.593	-0.312
g_O	847	437	703	901	449	711	837	433	733	833
	(35.2)	(18.2)	(29.2)	(37.4)	(18.7)	(29.5)	(34.8)	(18.0)	(30.5)	(34.6)
g_E	904.9	422.8	743.3	904.9	422.8	743.3	904.9	422.8	743.3	904.9
	(37.6)	(17.6)	(30.9)	(37.6)	(17.6)	(30.9)	(37.6)	(17.6)	(30.9)	(37.6)
	-111.9	28.8	-78.3	-7.7	53.9	-63.1	-130.5	20.6	-20.4	-137.8
	-4.650	1.197	-3.255	-0.319	2.240	-2.623	-5.421	0.855	-0.848	-5.727
c_O	395	419	604	378	450	573	398	416	603	404
	(16.4)	(17.4)	(25.1)	(15.7)	(18.7)	(23.8)	(16.5)	(17.3)	(25.1)	(16.8)
c_E	375.4	458.7	666.2	375.4	458.7	666.2	375.4	458.7	666.2	375.4
	(15.6)	(19.1)	(27.7)	(15.6)	(19.1)	(27.7)	(15.6)	(19.1)	(27.7)	(15.6)
	40.2	-75.9	-118.4	5.2	-17.3	-172.7	46.5	-81.3	-120.2	59.3
	1.669	-3.153	-4.918	0.215	-0.718	-7.175	1.932	-3.379	-4.993	2.463
N	2407	2407	2407	2407	2407	2407	2407	2407	2407	2407
X2	10.1	14.2	19.0	8.1	9.8	35.7	9.5	5.1	15.6	10.8
Gvalue	9.9	14.2	18.8	8.0	9.8	35.1	9.5	5.2	15.3	10.9
Gv/N	0.413	0.589	0.782	0.331	0.409	1.460	0.396	0.216	0.638	0.452
Prob	0.01900	0.00267	0.00030	0.04646	0.01997	0.00000	0.02312	0.15801	0.00154	0.01238

	41	42	43	44	45	46	47	48	49	50
a_O	721	525	755	690	548	720	678	514	747	696
	(30.0)	(21.8)	(31.4)	(28.7)	(22.8)	(29.9)	(28.2)	(21.4)	(31.0)	(28.9)
a_E	741.9	472.6	759.0	741.9	472.6	759.0	741.9	472.6	759.0	741.9
	(30.8)	(19.6)	(31.5)	(30.8)	(19.6)	(31.5)	(30.8)	(19.6)	(31.5)	(30.8)
	-41.3	110.4	-7.9	-100.1	162.2	-75.9	-122.2	86.3	-23.7	-89.0
	-1.714	4.586	-0.328	-4.160	6.739	-3.153	-5.076	3.585	-0.986	-3.696
t_O	784	571	402	804	572	382	855	578	373	780
	(32.6)	(23.7)	(16.7)	(33.4)	(23.8)	(15.9)	(35.5)	(24.0)	(15.5)	(32.4)
t_E	783.5	524.9	367.8	783.5	524.9	367.8	783.5	524.9	367.8	783.5
	(32.6)	(21.8)	(15.3)	(32.6)	(21.8)	(15.3)	(32.6)	(21.8)	(15.3)	(32.6)
	1.0	96.1	71.5	41.5	98.3	29.0	149.3	111.4	10.5	-7.0
	0.040	3.993	2.972	1.724	4.083	1.205	6.203	4.627	0.437	-0.291
g_O	441	730	836	442	693	867	410	752	873	436
	(18.3)	(30.3)	(34.7)	(18.4)	(28.8)	(36.0)	(17.0)	(31.2)	(36.3)	(18.1)
g_E	422.8	743.3	904.9	422.8	743.3	904.9	422.8	743.3	904.9	422.8
	(17.6)	(30.9)	(37.6)	(17.6)	(30.9)	(37.6)	(17.6)	(30.9)	(37.6)	(17.6)
	37.1	-26.3	-132.3	39.2	-97.1	-74.1	-25.3	17.5	-62.6	26.7
	1.542	-1.094	-5.498	1.628	-4.033	-3.078	-1.050	0.729	-2.599	1.111
c_O	461	581	414	471	594	438	464	563	414	495
	(19.2)	(24.1)	(17.2)	(19.6)	(24.7)	(18.2)	(19.3)	(23.4)	(17.2)	(20.6)
c_E	458.7	666.2	375.4	458.7	666.2	375.4	458.7	666.2	375.4	458.7
	(19.1)	(27.7)	(15.6)	(19.1)	(27.7)	(15.6)	(19.1)	(27.7)	(15.6)	(19.1)
	4.6	-159.0	81.0	24.9	-136.3	135.1	10.6	-189.5	81.0	75.4
	0.190	-6.605	3.365	1.034	-5.661	5.611	0.441	-7.873	3.365	3.131
N	2407	2407	2407	2407	2407	2407	2407	2407	2407	2407
X2	1.4	21.0	12.4	5.4	27.5	14.6	12.5	25.1	5.3	6.1
Gvalue	1.4	21.2	12.3	5.4	27.1	14.1	12.5	25.7	5.2	6.1
Gv/N	0.057	0.879	0.512	0.226	1.127	0.585	0.518	1.068	0.217	0.254
Prob	0.71084	0.00010	0.00635	0.14255	0.00001	0.00280	0.00596	0.00001	0.15541	0.10583

```
            51      52      53      54      55      56      57      58      59      60
       -------------------------------------------------------------------------------
a_O        520     789     703     508     809     712     502     756     724     529
       ( 21.6) ( 32.8) ( 29.2) ( 21.1) ( 33.6) ( 29.6) ( 20.9) ( 31.4) ( 30.1) ( 22.0)
a_E      472.6   759.0   741.9   472.6   759.0   741.9   472.6   759.0   741.9   472.6
       ( 19.6) ( 31.5) ( 30.8) ( 19.6) ( 31.5) ( 30.8) ( 19.6) ( 31.5) ( 30.8) ( 19.6)
          99.4    61.3   -75.8    73.4   103.3   -58.6    60.6    -5.9   -35.4   119.3
         4.129   2.545  -3.148   3.048   4.292  -2.436   2.516  -0.246  -1.472   4.954
t_O        525     364     786     557     395     817     581     345     785     569
       ( 21.8) ( 15.1) ( 32.7) ( 23.1) ( 16.4) ( 33.9) ( 24.1) ( 14.3) ( 32.6) ( 23.6)
t_E      524.9   367.8   783.5   524.9   367.8   783.5   524.9   367.8   783.5   524.9
       ( 21.8) ( 15.3) ( 32.6) ( 21.8) ( 15.3) ( 32.6) ( 21.8) ( 15.3) ( 32.6) ( 21.8)
           0.2    -7.5     5.0    66.1    56.4    68.4   118.0   -44.1     3.0    91.8
         0.007  -0.312   0.207   2.746   2.344   2.841   4.901  -1.832   0.123   3.813
g_O        721     878     425     733     821     424     707     948     429     696
       ( 30.0) ( 36.5) ( 17.7) ( 30.5) ( 34.1) ( 17.6) ( 29.4) ( 39.4) ( 17.8) ( 28.9)
g_E      743.3   904.9   422.8   743.3   904.9   422.8   743.3   904.9   422.8   743.3
       ( 30.9) ( 37.6) ( 17.6) ( 30.9) ( 37.6) ( 17.6) ( 30.9) ( 37.6) ( 17.6) ( 30.9)
         -43.9   -52.9     4.3   -20.4  -159.7     2.3   -70.8    88.3    12.4   -91.5
        -1.823  -2.198   0.181  -0.848  -6.634   0.097  -2.940   3.669   0.516  -3.801
c_O        641     376     493     609     382     454     617     358     469     613
       ( 26.6) ( 15.6) ( 20.5) ( 25.3) ( 15.9) ( 18.9) ( 25.6) ( 14.9) ( 19.5) ( 25.5)
c_E      666.2   375.4   458.7   666.2   375.4   458.7   666.2   375.4   458.7   666.2
       ( 27.7) ( 15.6) ( 19.1) ( 27.7) ( 15.6) ( 19.1) ( 27.7) ( 15.6) ( 19.1) ( 27.7)
         -49.4     1.2    71.1  -109.3    13.3    -9.4   -94.7   -34.0    20.8  -102.0
        -2.053   0.049   2.952  -4.542   0.552  -0.390  -3.933  -1.413   0.863  -4.238

N         2407    2407    2407    2407    2407    2407    2407    2407    2407    2407
X2         6.4     2.0     4.6     9.7    13.2     2.7    13.2     4.3     0.8    17.7
Gvalue     6.2     2.0     4.6     9.7    13.3     2.7    13.1     4.3     0.8    17.5
Gv/N     0.259   0.084   0.191   0.403   0.554   0.112   0.544   0.179   0.031   0.728
Prob   0.10051 0.56866 0.20369 0.02122 0.00397 0.44242 0.00441 0.23105 0.85953 0.00055
       -------------------------------------------------------------------------------
            61      62      63      64      65      66      67      68      69      70
       -------------------------------------------------------------------------------
a_O        724     709     546     765     703     546     738     751     484     736
       ( 30.1) ( 29.5) ( 22.7) ( 31.8) ( 29.2) ( 22.7) ( 30.7) ( 31.2) ( 20.1) ( 30.6)
a_E      759.0   741.9   472.6   759.0   741.9   472.6   759.0   741.9   472.6   759.0
       ( 31.5) ( 30.8) ( 19.6) ( 31.5) ( 30.8) ( 19.6) ( 31.5) ( 30.8) ( 19.6) ( 31.5)
         -68.3   -64.4   157.6    12.1   -75.8   157.6   -41.3    18.2    23.0   -45.2
        -2.837  -2.675   6.549   0.504  -3.148   6.549  -1.717   0.758   0.958  -1.879
t_O        415     774     534     368     819     516     395     770     562     376
       ( 17.2) ( 32.2) ( 22.2) ( 15.3) ( 34.0) ( 21.4) ( 16.4) ( 32.0) ( 23.3) ( 15.6)
t_E      367.8   783.5   524.9   367.8   783.5   524.9   367.8   783.5   524.9   367.8
       ( 15.3) ( 32.6) ( 21.8) ( 15.3) ( 32.6) ( 21.8) ( 15.3) ( 32.6) ( 21.8) ( 15.3)
         100.3   -18.9    18.3     0.5    72.6   -17.7    56.4   -26.8    76.7    16.6
         4.166  -0.786   0.761   0.019   3.014  -0.734   2.344  -1.113   3.188   0.691
g_O        900     437     694     878     417     726     888     428     728     931
       ( 37.4) ( 18.2) ( 28.8) ( 36.5) ( 17.3) ( 30.2) ( 36.9) ( 17.8) ( 30.2) ( 38.7)
g_E      904.9   422.8   743.3   904.9   422.8   743.3   904.9   422.8   743.3   904.9
       ( 37.6) ( 17.6) ( 30.9) ( 37.6) ( 17.6) ( 30.9) ( 37.6) ( 17.6) ( 30.9) ( 37.6)
          -9.7    28.8   -95.2   -52.9   -11.6   -34.2   -33.4    10.4   -30.2    53.0
        -0.402   1.197  -3.956  -2.198  -0.481  -1.419  -1.387   0.432  -1.257   2.204
c_O        368     487     633     396     468     619     386     458     633     364
       ( 15.3) ( 20.2) ( 26.3) ( 16.5) ( 19.4) ( 25.7) ( 16.0) ( 19.0) ( 26.3) ( 15.1)
c_E      375.4   458.7   666.2   375.4   458.7   666.2   375.4   458.7   666.2   375.4
       ( 15.6) ( 19.1) ( 27.7) ( 15.6) ( 19.1) ( 27.7) ( 15.6) ( 19.1) ( 27.7) ( 15.6)
         -14.7    58.3   -64.7    42.3    18.7   -91.0    21.5    -1.4   -64.7   -22.5
        -0.610   2.421  -2.688   1.756   0.779  -3.779   0.892  -0.060  -2.688  -0.934

N         2407    2407    2407    2407    2407    2407    2407    2407    2407    2407
X2         7.8     3.8    16.5     2.0     3.9    15.3     3.2     0.4     4.9     2.0
Gvalue     7.6     3.8    16.0     2.0     3.9    14.8     3.2     0.4     4.8     2.0
Gv/N     0.317   0.157   0.666   0.081   0.163   0.616   0.131   0.017   0.201   0.082
Prob   0.05431 0.28649 0.00112 0.58043 0.26912 0.00197 0.36707 0.93839 0.18462 0.57581
       -------------------------------------------------------------------------------
            71      72      73      74      75      76      77      78      79      80
       -------------------------------------------------------------------------------
a_O        739     514     771     756     496     747     755     493     778     740
       ( 30.7) ( 21.4) ( 32.0) ( 31.4) ( 20.6) ( 31.0) ( 31.4) ( 20.5) ( 32.3) ( 30.8)
a_E      741.9   472.6   759.0   741.9   472.6   759.0   741.9   472.6   758.6   741.6
       ( 30.8) ( 19.6) ( 31.5) ( 30.8) ( 19.6) ( 31.5) ( 30.8) ( 19.6) ( 31.5) ( 30.8)
          -5.8    86.3    24.3    28.4    47.9   -23.7    26.4    41.6    39.2    -3.2
        -0.243   3.585   1.008   1.180   1.991  -0.986   1.095   1.730   1.629  -0.135
t_O        809     516     361     769     544     397     778     558     386     800
       ( 33.6) ( 21.4) ( 15.0) ( 31.9) ( 22.6) ( 16.5) ( 32.3) ( 23.2) ( 16.0) ( 33.3)
t_E      783.5   524.9   367.8   783.5   524.9   367.8   783.5   524.9   367.6   783.2
       ( 32.6) ( 21.8) ( 15.3) ( 32.6) ( 21.8) ( 15.3) ( 32.6) ( 21.8) ( 15.3) ( 32.6)
          51.8   -17.7   -13.4   -28.8    38.9    60.7   -11.0    68.2    37.7    34.0
         2.152  -0.734  -0.557  -1.195   1.614   2.523  -0.457   2.834   1.566   1.412
g_O        436     749     905     429     752     900     440     752     854     437
       ( 18.1) ( 31.1) ( 37.6) ( 17.8) ( 31.2) ( 37.4) ( 18.3) ( 31.2) ( 35.5) ( 18.2)
g_E      422.8   743.3   904.9   422.8   743.3   904.9   422.8   743.3   904.5   422.7
       ( 17.6) ( 30.9) ( 37.6) ( 17.6) ( 30.9) ( 37.6) ( 17.6) ( 30.9) ( 37.6) ( 17.6)
          26.7    11.5     0.3    12.4    17.5    -9.7    35.0    17.5   -98.1    29.2
         1.111   0.477   0.012   0.516   0.729  -0.402   1.455   0.729  -4.077   1.212
c_O        423     628     370     453     615     363     434     604     388     429
       ( 17.6) ( 26.1) ( 15.4) ( 18.8) ( 25.6) ( 15.1) ( 18.0) ( 25.1) ( 16.1) ( 17.8)
c_E      458.7   666.2   375.4   458.7   666.2   375.4   458.7   666.2   375.3   458.5
       ( 19.1) ( 27.7) ( 15.6) ( 19.1) ( 27.7) ( 15.6) ( 19.1) ( 27.7) ( 15.6) ( 19.1)
         -68.6   -74.1   -10.8   -11.4   -98.3   -24.4   -48.1  -118.4    25.9   -57.1
        -2.850  -3.081  -0.447  -0.473  -4.086  -1.014  -1.998  -4.918   1.077  -2.374

N         2407    2407    2407    2407    2407    2407    2407    2407    2406    2406
X2         4.0     6.0     0.4     0.7     5.9     2.9     2.3     8.9     4.7     2.8
Gvalue     4.1     6.0     0.4     0.7     6.0     2.9     2.3     9.0     4.7     2.8
Gv/N     0.170   0.247   0.016   0.029   0.248   0.120   0.096   0.374   0.195   0.116
Prob   0.25143 0.11398 0.94149 0.87392 0.11334 0.40819 0.51011 0.02920 0.19577 0.42558
       -------------------------------------------------------------------------------
            81      82      83      84      85      86      87      88      89      90
       -------------------------------------------------------------------------------
a_O        513     729     753     498     772     753     505     742     748     533
       ( 21.3) ( 30.3) ( 31.3) ( 20.7) ( 32.1) ( 31.3) ( 21.0) ( 30.9) ( 31.1) ( 22.2)
a_E      472.4   758.3   741.3   472.2   758.0   741.0   472.0   757.4   740.4   471.6
       ( 19.6) ( 31.5) ( 30.8) ( 19.6) ( 31.5) ( 30.8) ( 19.6) ( 31.5) ( 30.8) ( 19.6)
          84.6   -57.5    23.6    52.9    28.2    24.2    68.2   -30.5    15.3   130.4
         3.515  -2.391   0.979   2.201   1.174   1.006   2.837  -1.268   0.637   5.429
t_O        540     388     808     563     385     775     558     353     800     566
       ( 22.4) ( 16.1) ( 33.6) ( 23.4) ( 16.0) ( 32.2) ( 23.2) ( 14.7) ( 33.3) ( 23.6)
t_E      524.7   367.5   782.9   524.5   367.3   782.5   524.3   367.0   781.9   523.8
       ( 21.8) ( 15.3) ( 32.6) ( 21.8) ( 15.3) ( 32.6) ( 21.8) ( 15.3) ( 32.6) ( 21.8)
          31.0    42.2    51.1    79.8    36.2   -15.0    69.6   -27.5    36.6    87.7
```

	1.290	1.754	2.123	3.318	1.506	-0.624	2.895	-1.144	1.525	3.649
g_O	701	911	414	720	899	435	705	914	415	683
	(29.1)	(37.9)	(17.2)	(29.9)	(37.4)	(18.1)	(29.3)	(38.1)	(17.3)	(28.4)
g_E	743.0	904.1	422.5	742.7	903.7	422.3	742.4	903.0	422.0	741.7
	(30.9)	(37.6)	(17.6)	(30.9)	(37.6)	(17.6)	(30.9)	(37.6)	(17.6)	(30.9)
	-81.5	13.9	-16.8	-44.6	-9.4	25.8	-72.8	22.2	-13.8	-112.7
	-3.389	0.576	-0.698	-1.856	-0.392	1.072	-3.028	0.924	-0.574	-4.692
c_O	652	377	430	624	348	441	636	393	439	620
	(27.1)	(15.7)	(17.9)	(25.9)	(14.5)	(18.3)	(26.5)	(16.4)	(18.3)	(25.8)
c_E	665.9	375.1	458.3	665.6	374.9	458.2	665.4	374.6	457.8	664.8
	(27.7)	(15.6)	(19.1)	(27.7)	(15.6)	(19.1)	(27.7)	(15.6)	(19.1)	(27.7)
	-27.5	3.8	-54.9	-80.6	-51.9	-33.7	-57.4	37.6	-36.8	-86.5
	-1.144	0.158	-2.282	-3.352	-2.159	-1.400	-2.388	1.566	-1.530	-3.602
N	2406	2405	2405	2405	2404	2404	2404	2402	2402	2402
X2	6.6	2.3	2.9	7.5	3.1	1.3	7.7	1.9	1.4	19.1
Gvalue	6.5	2.3	2.9	7.5	3.1	1.3	7.6	1.9	1.4	18.8
Gv/N	0.272	0.097	0.122	0.312	0.129	0.054	0.316	0.078	0.058	0.784
Prob	0.08799	0.50527	0.40051	0.05745	0.37582	0.73055	0.05491	0.59849	0.70791	0.00030

	91	92	93	94	95	96	97	98	99	100
a_O	724	717	510	753	776	485	744	766	479	736
	(30.1)	(29.9)	(21.2)	(31.3)	(32.3)	(20.2)	(31.0)	(31.9)	(20.0)	(30.7)
a_E	757.4	740.4	471.6	757.4	740.4	471.6	757.1	740.1	471.4	757.1
	(31.5)	(30.8)	(19.6)	(31.5)	(30.8)	(19.6)	(31.5)	(30.8)	(19.6)	(31.5)
	-65.3	-46.0	79.8	-8.7	72.9	27.1	-25.9	52.7	15.3	-41.5
	-2.718	-1.916	3.321	-0.364	3.035	1.129	-1.079	2.196	0.635	-1.730
t_O	346	786	542	385	737	559	391	775	573	385
	(14.4)	(32.7)	(22.6)	(16.0)	(30.7)	(23.3)	(16.3)	(32.3)	(23.9)	(16.0)
t_E	367.0	781.9	523.8	367.0	781.9	523.8	366.9	781.6	523.6	366.9
	(15.3)	(32.6)	(21.8)	(15.3)	(32.6)	(21.8)	(15.3)	(32.6)	(21.8)	(15.3)
	-40.8	8.2	37.0	36.9	-87.1	72.7	49.8	-13.1	103.3	37.2
	-1.698	0.343	1.539	1.534	-3.628	3.025	2.076	-0.544	4.303	1.548
g_O	962	444	677	908	437	719	901	409	698	914
	(40.0)	(18.5)	(28.2)	(37.8)	(18.2)	(29.9)	(37.5)	(17.0)	(29.1)	(38.1)
g_E	903.0	422.0	741.7	903.0	422.0	741.7	902.6	421.8	741.4	902.6
	(37.6)	(17.6)	(30.9)	(37.6)	(17.6)	(30.9)	(37.6)	(17.6)	(30.9)	(37.6)
	121.8	45.2	-123.7	10.1	30.6	-44.8	-3.2	-25.2	-84.3	23.0
	5.072	1.883	-5.148	0.420	1.275	-1.864	-0.133	-1.048	-3.509	0.956
c_O	370	455	673	356	452	639	365	451	651	366
	(15.4)	(18.9)	(28.0)	(14.8)	(18.8)	(26.6)	(15.2)	(18.8)	(27.1)	(15.2)
c_E	374.6	457.8	664.8	374.6	457.8	664.8	374.5	457.6	664.5	374.5
	(15.6)	(19.1)	(27.7)	(15.6)	(19.1)	(27.7)	(15.6)	(19.1)	(27.7)	(15.6)
	-9.2	-5.5	16.5	-36.3	-11.5	-50.6	-18.7	-13.1	-26.8	-16.8
	-0.384	-0.230	0.686	-1.512	-0.477	-2.107	-0.780	-0.544	-1.116	-0.698
N	2402	2402	2402	2402	2402	2402	2401	2401	2401	2401
X2	6.6	1.9	9.5	1.9	4.9	4.4	2.1	1.4	7.6	1.8
Gvalue	6.6	1.9	9.6	1.9	4.9	4.4	2.0	1.4	7.5	1.8
Gv/N	0.273	0.080	0.399	0.078	0.205	0.183	0.084	0.060	0.313	0.075
Prob	0.08753	0.58969	0.02243	0.60108	0.17798	0.22087	0.56679	0.69650	0.05724	0.61236

	101	102	103	104	105	106	107	108	109	110
a_O	750	482	725	721	476	724	802	517	743	795
	(31.2)	(20.1)	(30.2)	(30.0)	(19.8)	(30.2)	(33.4)	(21.5)	(31.0)	(33.2)
a_E	740.1	471.4	756.8	739.8	471.2	756.8	739.8	471.2	756.1	739.2
	(30.8)	(19.6)	(31.5)	(30.8)	(19.6)	(31.5)	(30.8)	(19.6)	(31.5)	(30.8)
	20.0	21.4	-62.2	-37.1	9.6	-64.1	129.5	95.8	-26.0	115.8
	0.832	0.890	-2.590	-1.544	0.399	-2.670	5.398	3.993	-1.085	4.829
t_O	756	593	364	800	586	408	737	518	407	743
	(31.5)	(24.7)	(15.2)	(33.3)	(24.4)	(17.0)	(30.7)	(21.6)	(17.0)	(31.0)
t_E	781.6	523.6	366.7	781.2	523.4	366.7	781.2	523.4	366.4	780.6
	(32.6)	(21.8)	(15.3)	(32.6)	(21.8)	(15.3)	(32.6)	(21.8)	(15.3)	(32.6)
	-50.3	147.6	-5.4	38.0	132.4	87.1	-85.9	-10.7	85.5	-73.3
	-2.094	6.147	-0.224	1.582	5.518	3.628	-3.580	-0.447	3.568	-3.058
g_O	404	684	938	394	693	902	404	672	874	412
	(16.8)	(28.5)	(39.1)	(16.4)	(28.9)	(37.6)	(16.8)	(28.0)	(36.4)	(17.2)
g_E	421.8	741.4	902.2	421.6	741.1	902.2	421.6	741.1	901.5	421.3
	(17.6)	(30.9)	(37.6)	(17.6)	(30.9)	(37.6)	(17.6)	(30.9)	(37.6)	(17.6)
	-34.8	-110.3	73.0	-53.4	-93.0	-0.4	-34.5	-131.6	-54.1	-18.3
	-1.449	-4.593	3.040	-2.223	-3.877	-0.018	-1.436	-5.483	-2.256	-0.763
c_O	491	642	373	485	645	366	457	693	374	448
	(20.4)	(26.7)	(15.5)	(20.2)	(26.9)	(15.2)	(19.0)	(28.9)	(15.6)	(18.7)
c_E	457.6	664.5	374.3	457.4	664.3	374.3	457.4	664.3	374.0	457.0
	(19.1)	(27.7)	(15.6)	(19.1)	(27.7)	(15.6)	(19.1)	(27.7)	(15.6)	(19.1)
	69.2	-44.3	-2.6	56.9	-37.9	-16.5	-0.8	58.7	-0.0	-17.8
	2.883	-1.845	-0.110	2.369	-1.581	-0.686	-0.032	2.447	-0.001	-0.744
N	2401	2401	2400	2400	2400	2400	2400	2400	2398	2398
X2	4.2	14.6	2.8	4.4	11.2	6.3	8.5	12.2	5.6	6.4
Gvalue	4.1	14.4	2.8	4.4	11.0	6.1	8.4	12.2	5.4	6.3
Gv/N	0.172	0.599	0.116	0.184	0.459	0.255	0.350	0.510	0.226	0.264
Prob	0.24865	0.00242	0.42736	0.22049	0.01165	0.10635	0.03856	0.00659	0.14370	0.09621

	111	112	113	114	115	116	117	118	119	120
a_O	461	750	725	465	742	757	475	744	747	470
	(19.2)	(31.3)	(30.3)	(19.4)	(31.0)	(31.6)	(19.8)	(31.1)	(31.2)	(19.6)
a_E	470.8	755.2	738.2	470.3	754.9	737.9	470.1	754.5	737.6	469.9
	(19.6)	(31.5)	(30.8)	(19.6)	(31.5)	(30.8)	(19.6)	(31.5)	(30.8)	(19.6)
	-19.5	-10.3	-26.2	-10.5	-25.5	38.6	9.9	-20.9	18.9	0.3
	-0.812	-0.431	-1.095	-0.436	-1.065	1.614	0.415	-0.875	0.789	0.011
t_O	525	337	808	598	385	761	539	385	747	535
	(21.9)	(14.1)	(33.7)	(25.0)	(16.1)	(31.8)	(22.5)	(16.1)	(31.2)	(22.4)
t_E	523.0	365.9	779.6	522.3	365.8	779.3	522.1	365.6	779.0	521.9
	(21.8)	(15.3)	(32.6)	(21.8)	(15.3)	(32.6)	(21.8)	(15.3)	(32.6)	(21.8)
	4.1	-55.5	57.8	161.9	39.4	-36.1	34.4	39.7	-62.6	26.6
	0.171	-2.318	2.414	6.759	1.647	-1.509	1.436	1.661	-2.615	1.112
g_O	720	881	374	655	889	442	718	885	414	721
	(30.0)	(36.8)	(15.6)	(27.3)	(37.1)	(18.5)	(30.0)	(37.0)	(17.3)	(30.1)
g_E	740.5	900.3	420.7	739.6	900.0	420.5	739.3	899.6	420.4	739.0
	(30.9)	(37.6)	(17.6)	(30.9)	(37.6)	(17.6)	(30.9)	(37.6)	(17.6)	(30.9)
	-40.4	-38.3	-88.1	-159.1	-21.8	44.0	-41.9	-28.9	-12.6	-35.5
	-1.686	-1.598	-3.677	-6.643	-0.910	1.837	-1.751	-1.209	-0.529	-1.483
c_O	692	427	488	677	378	434	662	379	485	667
	(28.9)	(17.8)	(20.4)	(28.3)	(15.8)	(18.1)	(27.7)	(15.8)	(20.3)	(27.9)
c_E	663.7	373.5	456.4	662.9	373.4	456.2	662.6	373.2	456.1	662.3
	(27.7)	(15.6)	(19.1)	(27.7)	(15.6)	(19.1)	(27.7)	(15.6)	(19.1)	(27.7)
	57.8	114.2	65.3	28.6	9.3	-43.4	-1.2	11.6	59.7	9.4
	2.410	4.769	2.725	1.192	0.388	-1.812	-0.050	0.486	2.494	0.393

N	2398	2395	2395	2395	2394	2394	2394	2393	2393	2393
X2	2.0	10.4	8.6	21.0	1.4	3.1	1.2	1.5	3.4	0.8
Gvalue	2.0	10.1	8.8	20.9	1.4	3.1	1.2	1.5	3.3	0.8
Gv/N	0.082	0.422	0.367	0.873	0.059	0.129	0.051	0.062	0.140	0.033
Prob	0.57695	0.01762	0.03226	0.00011	0.70474	0.37645	0.75000	0.68622	0.34144	0.84921

	121	122	123	124	125	126	127	128	129	130
a_O	763	724	463	754	744	513	754	760	493	746
	(31.9)	(30.3)	(19.3)	(31.6)	(31.1)	(21.5)	(31.6)	(31.8)	(20.6)	(31.2)
a_E	754.5	737.6	469.9	753.3	736.4	469.1	753.0	736.1	468.9	753.0
	(31.5)	(30.8)	(19.6)	(31.5)	(30.8)	(19.6)	(31.5)	(30.8)	(19.6)	(31.5)
	17.0	-27.0	-13.6	1.4	15.3	91.8	2.1	48.6	49.5	-13.9
	0.710	-1.127	-0.569	0.060	0.641	3.844	0.086	2.036	2.071	-0.581
t_O	390	787	542	427	726	516	414	775	568	356
	(16.3)	(32.9)	(22.6)	(17.9)	(30.4)	(21.6)	(17.3)	(32.5)	(23.8)	(14.9)
t_E	365.6	779.0	521.9	365.0	777.7	521.0	364.9	777.3	520.8	364.9
	(15.3)	(32.6)	(21.8)	(15.3)	(32.6)	(21.8)	(15.3)	(32.6)	(21.8)	(15.3)
	50.3	16.2	41.0	133.9	-99.8	-9.9	104.6	-4.7	98.6	-17.5
	2.103	0.676	1.715	5.606	-4.178	-0.416	4.380	-0.195	4.130	-0.734
g_O	865	423	730	848	444	712	831	399	687	901
	(36.1)	(17.7)	(30.5)	(35.5)	(18.6)	(29.8)	(34.8)	(16.7)	(28.8)	(37.7)
g_E	899.6	420.4	739.0	898.1	419.7	737.7	897.7	419.5	737.4	897.7
	(37.6)	(17.6)	(30.9)	(37.6)	(17.6)	(30.9)	(37.6)	(17.6)	(30.9)	(37.6)
	-67.8	5.3	-17.8	-97.3	50.0	-50.5	-128.3	-40.0	-97.3	6.6
	-2.835	0.220	-0.744	-4.074	2.095	-2.115	-5.374	-1.674	-4.075	0.276
c_O	375	459	658	360	475	648	389	454	640	385
	(15.7)	(19.2)	(27.5)	(15.1)	(19.9)	(27.1)	(16.3)	(19.0)	(26.8)	(16.1)
c_E	373.2	456.1	662.3	372.6	455.3	661.2	372.5	455.1	660.9	372.5
	(15.6)	(19.1)	(27.7)	(15.6)	(19.1)	(27.7)	(15.6)	(19.1)	(27.7)	(15.6)
	3.5	5.9	-8.6	-24.8	40.3	-26.2	33.8	-2.2	-41.2	25.5
	0.148	0.247	-0.360	-1.037	1.685	-1.095	1.416	-0.092	-1.725	1.068

N	2393	2393	2393	2389	2389	2389	2388	2388	2388	2388
X2	3.1	0.4	1.0	13.7	5.8	5.3	12.3	1.8	9.6	0.7
Gvalue	3.0	0.4	1.0	13.3	5.8	5.2	12.1	1.8	9.6	0.7
Gv/N	0.127	0.016	0.042	0.555	0.243	0.218	0.509	0.075	0.401	0.030
Prob	0.38564	0.94617	0.79999	0.00412	0.12105	0.15697	0.00689	0.61559	0.02253	0.87034

	131	132	133	134	135	136	137	138	139	140
a_O	765	484	733	758	530	761	736	486	749	768
	(32.0)	(20.3)	(30.7)	(31.7)	(22.2)	(31.9)	(30.8)	(20.4)	(31.4)	(32.2)
a_E	736.1	468.9	753.0	736.1	468.9	753.0	736.1	468.9	752.0	735.1
	(30.8)	(19.6)	(31.5)	(30.8)	(19.6)	(31.5)	(30.8)	(19.6)	(31.5)	(30.8)
	59.0	30.7	-39.4	44.5	129.9	16.1	-0.1	34.9	-6.0	67.1
	2.470	1.286	-1.650	1.863	5.439	0.676	-0.006	1.460	-0.253	2.815
t_O	753	530	370	741	521	395	775	537	382	761
	(31.5)	(22.2)	(15.5)	(31.0)	(21.8)	(16.5)	(32.5)	(22.5)	(16.0)	(31.9)
t_E	777.3	520.8	364.9	777.3	520.8	364.9	777.3	520.8	364.4	776.4
	(32.6)	(21.8)	(15.3)	(32.6)	(21.8)	(15.3)	(32.6)	(21.8)	(15.3)	(32.6)
	-47.9	18.6	10.3	-70.9	0.5	62.7	-4.7	33.0	36.0	-30.4
	-2.005	0.780	0.433	-2.971	0.019	2.625	-0.195	1.380	1.510	-1.275
g_O	404	725	936	420	702	882	440	735	894	397
	(16.9)	(30.4)	(39.2)	(17.6)	(29.4)	(36.9)	(18.4)	(30.8)	(37.5)	(16.6)
g_E	419.5	737.4	897.7	419.5	737.4	897.7	419.5	737.4	896.6	419.0
	(17.6)	(30.9)	(37.6)	(17.6)	(30.9)	(37.6)	(17.6)	(30.9)	(37.6)	(17.6)
	-30.4	-24.6	78.2	1.0	-69.1	-31.1	42.0	-4.8	-5.2	-42.8
	-1.273	-1.031	3.274	0.042	-2.894	-1.304	1.759	-0.202	-0.216	-1.793
c_O	466	649	349	469	635	350	437	630	360	459
	(19.5)	(27.2)	(14.6)	(19.6)	(26.6)	(14.7)	(18.3)	(26.4)	(15.1)	(19.2)
c_E	455.1	660.9	372.5	455.1	660.9	372.5	455.1	660.9	372.0	454.5
	(19.1)	(27.7)	(15.6)	(19.1)	(27.7)	(15.6)	(19.1)	(27.7)	(15.6)	(19.1)
	22.1	-23.6	-45.4	28.2	-50.8	-43.5	-35.5	-60.4	-23.6	9.0
	0.924	-0.990	-1.901	1.182	-2.129	-1.823	-1.485	-2.529	-0.989	0.377

N	2388	2388	2388	2388	2388	2388	2388	2388	2385	2385
X2	2.7	1.1	3.7	2.8	10.7	4.2	1.7	2.6	1.3	3.0
Gvalue	2.7	1.1	3.7	2.8	10.4	4.2	1.7	2.6	1.2	3.0
Gv/N	0.114	0.045	0.156	0.117	0.436	0.174	0.072	0.109	0.052	0.124
Prob	0.43514	0.78388	0.29268	0.42472	0.01544	0.24411	0.63180	0.45806	0.74209	0.39651

	141	142	143	144	145	146	147	148	149	150
a_O	485	718	728	500	737	742	465	726	734	464
	(20.3)	(30.2)	(30.6)	(21.0)	(31.0)	(31.2)	(19.6)	(30.6)	(30.9)	(19.5)
a_E	468.3	750.8	733.9	467.5	749.5	732.7	466.7	748.6	731.8	466.1
	(19.6)	(31.5)	(30.8)	(19.6)	(31.5)	(30.8)	(19.6)	(31.5)	(30.8)	(19.6)
	34.0	-64.1	-11.8	67.2	-24.8	18.8	-3.4	-44.4	4.5	-4.3
	1.426	-2.691	-0.495	2.822	-1.043	0.789	-0.145	-1.871	0.189	-0.179
t_O	588	371	770	529	388	781	567	368	764	577
	(24.7)	(15.6)	(32.3)	(22.2)	(16.3)	(32.9)	(23.9)	(15.5)	(32.2)	(24.3)
t_E	520.1	363.8	775.1	519.2	363.2	773.7	518.4	362.7	772.8	517.7
	(21.8)	(15.3)	(32.6)	(21.8)	(15.3)	(32.6)	(21.8)	(15.3)	(32.6)	(21.8)
	144.3	14.5	-10.1	19.7	51.3	14.6	101.7	10.6	-17.4	125.1
	6.049	0.611	-0.423	0.827	2.157	0.613	4.278	0.447	-0.735	5.270
g_O	687	918	430	718	887	455	713	892	417	672
	(28.8)	(38.6)	(18.1)	(30.2)	(37.3)	(19.1)	(30.0)	(37.6)	(17.6)	(28.3)
g_E	736.5	895.1	418.3	735.3	893.6	417.6	734.0	892.4	417.0	733.1
	(30.9)	(37.6)	(17.6)	(30.9)	(37.6)	(17.6)	(30.9)	(37.6)	(17.6)	(30.9)
	-95.6	46.4	23.8	-34.1	-13.1	78.1	-41.4	-0.9	-0.1	-116.9
	-4.007	1.950	0.999	-1.432	-0.551	3.287	-1.743	-0.038	-0.003	-4.926
c_O	625	374	453	634	365	399	632	388	459	661
	(26.2)	(15.7)	(19.0)	(26.6)	(15.4)	(16.8)	(26.6)	(16.3)	(19.3)	(27.8)
c_E	660.1	371.4	453.8	659.0	370.7	453.0	657.9	370.3	452.4	657.1
	(27.7)	(15.6)	(19.1)	(27.7)	(15.6)	(19.1)	(27.7)	(15.6)	(19.1)	(27.7)
	-68.3	5.3	-1.5	-49.0	-11.4	-101.3	-50.7	36.3	13.2	7.9
	-2.864	0.223	-0.064	-2.059	-0.479	-4.262	-2.135	1.529	0.557	0.333

N	2385	2381	2381	2381	2377	2377	2377	2374	2374	2374
X2	14.6	2.2	0.4	3.8	2.0	10.0	6.2	1.6	0.2	11.9
Gvalue	14.4	2.2	0.4	3.8	2.0	10.2	6.1	1.6	0.2	11.8
Gv/N	0.603	0.092	0.017	0.158	0.084	0.427	0.255	0.067	0.008	0.498
Prob	0.00242	0.53325	0.93851	0.28878	0.57134	0.01728	0.10818	0.65964	0.97732	0.00805

Amar_MARIES
5UTR:

	50	49	48	47	46	45	44	43	42	41
a_O	212	242	257	232	213	234	232	213	230	241
	(22.3)	(25.5)	(27.1)	(24.4)	(22.4)	(24.7)	(24.4)	(22.4)	(24.2)	(25.4)
a_E	231.3	231.3	231.3	231.3	231.3	231.3	231.3	231.3	231.3	231.3
	(24.4)	(24.4)	(24.4)	(24.4)	(24.4)	(24.4)	(24.4)	(24.4)	(24.4)	(24.4)
	-36.9	21.9	54.2	1.4	-35.1	5.5	1.4	-35.1	-2.6	19.8
	-3.891	2.309	5.709	0.150	-3.698	0.574	0.150	-3.698	-0.271	2.089
t_O	286	258	259	253	279	257	252	290	255	271
	(30.1)	(27.2)	(27.3)	(26.7)	(29.4)	(27.1)	(26.6)	(30.6)	(26.9)	(28.6)
t_E	270.9	270.9	270.9	270.9	270.9	270.9	270.9	270.9	270.9	270.9
	(28.5)	(28.5)	(28.5)	(28.5)	(28.5)	(28.5)	(28.5)	(28.5)	(28.5)	(28.5)
	31.0	-25.2	-23.3	-34.6	16.4	-27.1	-36.5	39.5	-30.9	0.1
	3.263	-2.659	-2.458	-3.651	1.726	-2.859	-3.847	4.157	-3.257	0.015
g_O	257	229	246	253	260	254	239	236	267	238
	(27.1)	(24.1)	(25.9)	(26.7)	(27.4)	(26.8)	(25.2)	(24.9)	(28.1)	(25.1)
g_E	253.8	253.8	253.8	253.8	253.8	253.8	253.8	253.8	253.8	253.8
	(26.7)	(26.7)	(26.7)	(26.7)	(26.7)	(26.7)	(26.7)	(26.7)	(26.7)	(26.7)
	6.5	-47.0	-15.3	-1.5	12.7	0.5	-28.6	-34.2	27.2	-30.5
	0.689	-4.953	-1.608	-0.158	1.333	0.053	-3.016	-3.607	2.864	-3.214
c_O	194	220	187	211	197	204	226	210	197	199
	(20.4)	(23.2)	(19.7)	(22.2)	(20.8)	(21.5)	(23.8)	(22.1)	(20.8)	(21.0)
c_E	193.0	193.0	193.0	193.0	193.0	193.0	193.0	193.0	193.0	193.0
	(20.3)	(20.3)	(20.3)	(20.3)	(20.3)	(20.3)	(20.3)	(20.3)	(20.3)	(20.3)
	1.9	57.5	-11.9	37.6	8.0	22.6	71.3	35.4	8.0	12.1
	0.205	6.064	-1.251	3.958	0.845	2.376	7.511	3.729	0.845	1.277
N	949	949	949	949	949	949	949	949	949	949
X2	2.5	7.3	3.8	2.9	1.9	1.4	7.8	5.5	1.7	1.6
Gvalue	2.5	7.2	3.7	2.8	2.0	1.4	7.6	5.5	1.7	1.6
Gv/N	0.266	0.760	0.392	0.299	0.206	0.145	0.797	0.582	0.182	0.167
Prob	0.47080	0.06532	0.29330	0.41674	0.58120	0.71196	0.05587	0.13747	0.63161	0.66318

	40	39	38	37	36	35	34	33	32	31
a_O	235	224	223	237	212	266	259	216	258	243
	(24.8)	(23.6)	(23.5)	(25.0)	(22.3)	(28.0)	(27.3)	(22.8)	(27.2)	(25.6)
a_E	231.3	231.3	231.3	231.3	231.3	231.3	231.3	231.3	231.3	231.3
	(24.4)	(24.4)	(24.4)	(24.4)	(24.4)	(24.4)	(24.4)	(24.4)	(24.4)	(24.4)
	7.5	-14.3	-16.3	11.6	-36.9	74.4	58.6	-29.5	56.4	24.0
	0.788	-1.512	-1.715	1.218	-3.891	7.838	6.176	-3.113	5.942	2.529
t_O	261	290	267	271	311	243	254	293	277	246
	(27.5)	(30.6)	(28.1)	(28.6)	(32.8)	(25.6)	(26.8)	(30.9)	(29.2)	(25.9)
t_E	270.9	270.9	270.9	270.9	270.9	270.9	270.9	270.9	270.9	270.9
	(28.5)	(28.5)	(28.5)	(28.5)	(28.5)	(28.5)	(28.5)	(28.5)	(28.5)	(28.5)
	-19.5	39.5	-7.8	0.1	85.8	-52.9	-32.8	45.9	12.3	-47.5
	-2.054	4.157	-0.822	0.015	9.040	-5.572	-3.454	4.836	1.293	-5.004
g_O	253	232	261	256	250	239	240	251	237	243
	(26.7)	(24.4)	(27.5)	(27.0)	(26.3)	(25.2)	(25.3)	(26.4)	(25.0)	(25.6)
g_E	253.8	253.8	253.8	253.8	253.8	253.8	253.8	253.8	253.8	253.8
	(26.7)	(26.7)	(26.7)	(26.7)	(26.7)	(26.7)	(26.7)	(26.7)	(26.7)	(26.7)
	-1.5	-41.6	14.7	4.5	-7.4	-28.6	-26.7	-5.5	-32.4	-21.0
	-0.158	-4.381	1.550	0.476	-0.784	-3.016	-2.818	-0.576	-3.411	-2.217
c_O	200	203	198	185	176	201	196	189	177	217
	(21.1)	(21.4)	(20.9)	(19.5)	(18.5)	(21.2)	(20.7)	(19.9)	(18.7)	(22.9)
c_E	193.0	193.0	193.0	193.0	193.0	193.0	193.0	193.0	193.0	193.0
	(20.3)	(20.3)	(20.3)	(20.3)	(20.3)	(20.3)	(20.3)	(20.3)	(20.3)	(20.3)
	14.2	20.4	10.1	-15.7	-32.5	16.3	6.0	-8.0	-30.7	50.8
	1.495	2.155	1.061	-1.657	-3.426	1.714	0.631	-0.840	-3.234	5.353
N	949	949	949	949	949	949	949	949	949	949
X2	0.7	4.0	0.7	0.5	9.1	9.3	5.2	2.9	5.7	6.3
Gvalue	0.7	4.0	0.7	0.5	8.9	9.1	5.1	2.9	5.6	6.3
Gv/N	0.072	0.418	0.073	0.053	0.939	0.964	0.535	0.306	0.591	0.661
Prob	0.87823	0.26468	0.87553	0.91922	0.03046	0.02737	0.16605	0.40740	0.13234	0.09904

	30	29	28	27	26	25	24	23	22	21
a_O	228	241	221	204	227	223	223	215	231	217
	(24.0)	(25.4)	(23.3)	(21.5)	(23.9)	(23.5)	(23.5)	(22.7)	(24.3)	(22.9)
a_E	231.3	231.3	231.3	231.3	231.3	231.3	231.3	231.3	231.3	231.3
	(24.4)	(24.4)	(24.4)	(24.4)	(24.4)	(24.4)	(24.4)	(24.4)	(24.4)	(24.4)
	-6.5	19.8	-20.1	-51.2	-8.5	-16.3	-16.3	-31.4	-0.6	-27.7
	-0.688	2.089	-2.120	-5.398	-0.896	-1.715	-1.715	-3.309	-0.061	-2.917
t_O	275	252	277	284	258	284	297	254	304	330
	(29.0)	(26.6)	(29.2)	(29.9)	(27.2)	(29.9)	(31.3)	(26.8)	(32.0)	(34.8)
t_E	270.9	270.9	270.9	270.9	270.9	270.9	270.9	270.9	270.9	270.9
	(28.5)	(28.5)	(28.5)	(28.5)	(28.5)	(28.5)	(28.5)	(28.5)	(28.5)	(28.5)
	8.2	-36.5	12.3	26.8	-25.2	26.8	54.6	-32.8	70.0	130.2
	0.864	-3.847	1.293	2.820	-2.659	2.820	5.750	-3.454	7.378	13.717
g_O	253	267	248	265	275	256	218	296	245	232
	(26.7)	(28.1)	(26.1)	(27.9)	(29.0)	(27.0)	(23.0)	(31.2)	(25.8)	(24.4)
g_E	253.8	253.8	253.8	253.8	253.8	253.8	253.8	253.8	253.8	253.8
	(26.7)	(26.7)	(26.7)	(26.7)	(26.7)	(26.7)	(26.7)	(26.7)	(26.7)	(26.7)
	-1.5	27.2	-11.4	23.0	44.2	4.5	-66.2	91.2	-17.2	-41.6
	-0.158	2.864	-1.198	2.423	4.661	0.476	-6.977	9.607	-1.812	-4.381
c_O	193	189	203	196	189	186	211	184	169	170
	(20.3)	(19.9)	(21.4)	(20.7)	(19.9)	(19.6)	(22.2)	(19.4)	(17.8)	(17.9)
c_E	193.0	193.0	193.0	193.0	193.0	193.0	193.0	193.0	193.0	193.0
	(20.3)	(20.3)	(20.3)	(20.3)	(20.3)	(20.3)	(20.3)	(20.3)	(20.3)	(20.3)
	-0.1	-8.0	20.4	6.0	-8.0	-13.8	37.6	-17.6	-44.9	-43.2
	-0.006	-0.840	2.155	0.631	-0.840	-1.454	3.958	-1.858	-4.735	-4.552
N	949	949	949	949	949	949	949	949	949	949
X2	0.1	2.5	1.2	4.4	2.6	1.2	9.5	9.7	7.3	18.4
Gvalue	0.1	2.5	1.2	4.5	2.5	1.2	9.6	9.4	7.3	17.7
Gv/N	0.012	0.266	0.130	0.475	0.266	0.126	1.017	0.986	0.770	1.867
Prob	0.99060	0.47142	0.74407	0.21120	0.47101	0.75290	0.02182	0.02488	0.06261	0.00050

	20	19	18	17	16	15	14	13	12	11
a_O	229	237	189	239	232	210	238	239	221	245
	(24.1)	(25.0)	(19.9)	(25.2)	(24.4)	(22.1)	(25.1)	(25.2)	(23.3)	(25.8)
a_E	231.3	231.3	231.3	231.3	231.3	231.3	231.3	231.3	231.3	231.3
	(24.4)	(24.4)	(24.4)	(24.4)	(24.4)	(24.4)	(24.4)	(24.4)	(24.4)	(24.4)
	-4.6	11.6	-76.3	15.7	1.4	-40.6	13.6	15.7	-20.1	28.2
	-0.480	1.218	-8.043	1.652	0.150	-4.274	1.434	1.652	-2.120	2.973
t_O	284	312	337	297	309	345	301	306	310	213
	(29.9)	(32.9)	(35.5)	(31.3)	(32.6)	(36.4)	(31.7)	(32.2)	(32.7)	(22.4)

```
t_E       270.9   270.9   270.9   270.9   270.9   270.9   270.9   270.9   270.9   270.9
        ( 28.5) ( 28.5) ( 28.5) ( 28.5) ( 28.5) ( 28.5) ( 28.5) ( 28.5) ( 28.5) ( 28.5)
           26.8    88.1   147.1    54.6    81.3   166.8    63.4    74.5    83.5  -102.5
          2.820   9.281  15.499   5.750   8.562  17.572   6.677   7.850   8.801 -10.799
g_O         265     226     244     259     236     230     261     233     268     329
        ( 27.9) ( 23.8) ( 25.7) ( 27.3) ( 24.9) ( 24.2) ( 27.5) ( 24.6) ( 28.2) ( 34.7)
g_E       253.8   253.8   253.8   253.8   253.8   253.8   253.8   253.8   253.8   253.8
        ( 26.7) ( 26.7) ( 26.7) ( 26.7) ( 26.7) ( 26.7) ( 26.7) ( 26.7) ( 26.7) ( 26.7)
           23.0   -52.3   -19.1    10.6   -34.2   -45.2    14.7   -39.8    29.3   170.9
          2.423  -5.516  -2.015   1.118  -3.607  -4.763   1.550  -4.189   3.086  18.007
c_O         171     174     179     154     172     164     149     171     150     162
        ( 18.0) ( 18.3) ( 18.9) ( 16.2) ( 18.1) ( 17.3) ( 15.7) ( 18.0) ( 15.8) ( 17.1)
c_E       193.0   193.0   193.0   193.0   193.0   193.0   193.0   193.0   193.0   193.0
        ( 20.3) ( 20.3) ( 20.3) ( 20.3) ( 20.3) ( 20.3) ( 20.3) ( 20.3) ( 20.3) ( 20.3)
          -41.4   -36.1   -27.0   -69.6   -39.7   -53.5   -77.2   -41.4   -75.7   -56.8
         -4.367  -3.806  -2.847  -7.331  -4.181  -5.633  -8.130  -4.367  -7.973  -5.983

N           949     949     949     949     949     949     949     949     949     949
X2          3.7    11.3    25.2    10.8     8.9    28.8    13.8     9.0    16.5    40.5
Gvalue      3.8    11.2    24.6    11.3     8.8    27.5    14.5     9.0    17.0    39.8
Gv/N      0.395   1.177   2.594   1.188   0.924   2.902   1.531   0.945   1.794   4.198
Prob    0.28968 0.01086 0.00002 0.01031 0.03254 0.00000 0.00227 0.02969 0.00070 0.00000
        -------------------------------------------------------------------------------
             10       9       8       7       6       5       4       3       2       1
        -------------------------------------------------------------------------------
a_O         220     212     215     245     179     220     243     214     218     214
        ( 23.2) ( 22.3) ( 22.7) ( 25.8) ( 18.9) ( 23.2) ( 25.6) ( 22.6) ( 23.0) ( 22.6)
a_E       231.3   231.3   231.3   231.3   231.3   231.3   231.3   231.3   231.3   231.3
        ( 24.4) ( 24.4) ( 24.4) ( 24.4) ( 24.4) ( 24.4) ( 24.4) ( 24.4) ( 24.4) ( 24.4)
          -22.0   -36.9   -31.4    28.2   -91.8   -22.0    24.0   -33.3   -25.8   -33.3
         -2.320  -3.891  -3.309   2.973  -9.668  -2.320   2.529  -3.504  -2.719  -3.504
t_O         249     250     220     275     288     252     329     288     244     363
        ( 26.2) ( 26.3) ( 23.2) ( 29.0) ( 30.3) ( 26.6) ( 34.7) ( 30.3) ( 25.7) ( 38.3)
t_E       270.9   270.9   270.9   270.9   270.9   270.9   270.9   270.9   270.9   270.9
        ( 28.5) ( 28.5) ( 28.5) ( 28.5) ( 28.5) ( 28.5) ( 28.5) ( 28.5) ( 28.5) ( 28.5)
          -42.0   -40.2   -91.6     8.2    35.2   -36.5   127.8    35.2   -51.1   212.4
         -4.429  -4.236  -9.655   0.864   3.708  -3.847  13.465   3.708  -5.384  22.380
g_O         327     352     383     300     312     291     226     303     279     185
        ( 34.5) ( 37.1) ( 40.4) ( 31.6) ( 32.9) ( 30.7) ( 23.8) ( 31.9) ( 29.4) ( 19.5)
g_E       253.8   253.8   253.8   253.8   253.8   253.8   253.8   253.8   253.8   253.8
        ( 26.7) ( 26.7) ( 26.7) ( 26.7) ( 26.7) ( 26.7) ( 26.7) ( 26.7) ( 26.7) ( 26.7)
          165.9   230.4   315.4   100.5   129.0    79.7   -52.3   107.5    52.9  -116.9
         17.477  24.279  33.230  10.586  13.588   8.400  -5.516  11.327   5.578 -12.320
c_O         153     135     131     129     170     186     151     144     208     187
        ( 16.1) ( 14.2) ( 13.8) ( 13.6) ( 17.9) ( 19.6) ( 15.9) ( 15.2) ( 21.9) ( 19.7)
c_E       193.0   193.0   193.0   193.0   193.0   193.0   193.0   193.0   193.0   193.0
        ( 20.3) ( 20.3) ( 20.3) ( 20.3) ( 20.3) ( 20.3) ( 20.3) ( 20.3) ( 20.3) ( 20.3)
          -71.1   -96.5  -101.6  -104.0   -43.2   -13.8   -74.2   -84.4    31.1   -11.9
         -7.494 -10.173 -10.702 -10.957  -4.552  -1.454  -7.815  -8.893   3.274  -1.251

N           949     949     949     949     949     949     949     949     949     949
X2         31.8    58.7    96.5    30.5    29.0     7.6    25.2    24.4     7.1    51.4
Gvalue     30.7    56.7    90.8    32.9    29.2     7.4    25.3    25.0     7.1    50.3
Gv/N      3.234   5.979   9.564   3.466   3.077   0.779   2.663   2.639   0.750   5.305
Prob    0.00000 0.00000 0.00000 0.00000 0.00000 0.06038 0.00001 0.00002 0.06835 0.00000
        -------------------------------------------------------------------------------

CDS_INITIAL:
        -------------------------------------------------------------------------------
              1       2       3       4       5       6       7       8       9      10
        -------------------------------------------------------------------------------
a_O         493       0       0     270     235     189     273     235     219     283
        ( 51.9) (  0.0) (  0.0) ( 28.5) ( 24.8) ( 19.9) ( 28.8) ( 24.8) ( 23.1) ( 29.8)
a_E       262.0   264.0   201.9   262.0   264.0   201.9   262.0   264.0   201.9   262.0
        ( 27.6) ( 27.8) ( 21.3) ( 27.6) ( 27.8) ( 21.3) ( 27.6) ( 27.8) ( 21.3) ( 27.6)
          623.4     0.0     0.0    16.3   -54.8   -25.0    22.5   -54.8    35.6    43.7
         65.686   0.000   0.000   1.714  -5.769  -2.635   2.369  -5.769   3.746   4.601
t_O         186     949       0     235     253     299     185     283     301     184
        ( 19.6) (100.0) (  0.0) ( 24.8) ( 26.7) ( 31.5) ( 19.5) ( 29.8) ( 31.7) ( 19.4)
t_E       164.2   288.5   246.7   164.2   288.5   246.7   164.2   288.5   246.7   164.2
        ( 17.3) ( 30.4) ( 26.0) ( 17.3) ( 30.4) ( 26.0) ( 17.3) ( 30.4) ( 26.0) ( 17.3)
           46.3  2260.1     0.0   168.5   -66.4   114.9    44.1   -10.9   119.7    41.9
          4.883 238.151   0.000  17.751  -6.999  12.112   4.647  -1.146  12.616   4.412
g_O         270       0     949     245     243     276     316     242     244     311
        ( 28.5) (  0.0) (100.0) ( 25.8) ( 25.6) ( 29.1) ( 33.3) ( 25.5) ( 25.7) ( 32.8)
g_E       348.4   183.9   269.8   348.4   183.9   269.8   348.4   183.9   269.8   348.4
        ( 36.7) ( 19.4) ( 28.4) ( 36.7) ( 19.4) ( 28.4) ( 36.7) ( 19.4) ( 28.4) ( 36.7)
         -137.6     0.0  2386.9  -172.5   135.4    12.4   -61.6   132.9   -49.1   -70.6
        -14.501   0.000 251.512 -18.175  14.271   1.312  -6.495  14.002  -5.177  -7.437
c_O           0       0       0     199     218     185     175     189     185     171
        (  0.0) (  0.0) (  0.0) ( 21.0) ( 23.0) ( 19.5) ( 18.4) ( 19.9) ( 19.5) ( 18.0)
c_E       174.4   212.6   230.5   174.4   212.6   230.5   174.4   212.6   230.5   174.4
        ( 18.4) ( 22.4) ( 24.3) ( 18.4) ( 22.4) ( 24.3) ( 18.4) ( 22.4) ( 24.3) ( 18.4)
            0.0     0.0     0.0    52.5    11.0   -81.4     1.1   -44.4   -81.4    -6.8
          0.000   0.000   0.000   5.527   1.157  -8.576   0.121  -4.683  -8.576  -0.715

N           949     949     949     949     949     949     949     949     949     949
X2        398.6  2172.8  2388.5    64.9    26.7    21.0     6.1    24.3    24.9     8.1
Gvalue    532.1  2260.1  2386.9    64.7    25.2    21.0     6.1    22.8    24.8     8.2
Gv/N     56.068 238.151 251.512   6.817   2.660   2.213   0.642   2.405   2.609   0.860
Prob    0.00000 1.00000 1.00000 0.00000 0.00001 0.00011 0.10730 0.00004 0.00002 0.04275
        -------------------------------------------------------------------------------
             11      12      13      14      15      16      17      18      19      20
        -------------------------------------------------------------------------------
a_O         221     234     252     220     220     305     228     228     258     239
        ( 23.3) ( 24.7) ( 26.6) ( 23.2) ( 23.2) ( 32.1) ( 24.0) ( 24.0) ( 27.2) ( 25.2)
a_E       264.0   201.9   262.0   264.0   201.9   262.0   264.0   201.9   262.0   264.0
        ( 27.8) ( 21.3) ( 27.6) ( 27.8) ( 21.3) ( 27.6) ( 27.8) ( 21.3) ( 27.6) ( 27.8)
          -78.6    69.0   -19.6   -80.3    37.7    92.7   -66.9    55.4    -7.9   -47.6
         -8.287   7.270  -2.064  -8.459   3.975   9.771  -7.051   5.836  -0.834  -5.018
t_O         342     304     212     332     298     198     333     256     228     343
        ( 36.0) ( 32.0) ( 22.3) ( 35.0) ( 31.4) ( 20.9) ( 35.1) ( 27.0) ( 24.0) ( 36.1)
t_E       288.5   246.7   164.2   288.5   246.7   164.2   288.5   246.7   164.2   288.5
        ( 30.4) ( 26.0) ( 17.3) ( 30.4) ( 26.0) ( 17.3) ( 30.4) ( 26.0) ( 17.3) ( 30.4)
          116.4   127.0   108.3    93.3   112.6    74.1    95.6    18.9   149.6   118.7
         12.264  13.377  11.412   9.829  11.862   7.807  10.070   1.994  15.769  12.511
g_O         205     257     301     204     243     293     225     283     296     186
        ( 21.6) ( 27.1) ( 31.7) ( 21.5) ( 25.6) ( 30.9) ( 23.7) ( 29.8) ( 31.2) ( 19.6)
g_E       183.9   269.8   348.4   183.9   269.8   348.4   183.9   269.8   348.4   183.9
        ( 19.4) ( 28.4) ( 36.7) ( 19.4) ( 28.4) ( 36.7) ( 19.4) ( 28.4) ( 36.7) ( 19.4)
           44.5   -25.1   -88.0    42.3   -50.9  -101.4    90.8    26.9   -96.4     4.2
          4.693  -2.642  -9.271   4.460  -5.366 -10.688   9.565   2.839 -10.162   0.445
```

c_O	181	154	184	193	188	153	163	182	167	181
	(19.1)	(16.2)	(19.4)	(20.3)	(19.8)	(16.1)	(17.2)	(19.2)	(17.6)	(19.1)
c_E	212.6	230.5	174.4	212.6	230.5	174.4	212.6	230.5	174.4	212.6
	(22.4)	(24.3)	(18.4)	(22.4)	(24.3)	(18.4)	(22.4)	(24.3)	(18.4)	(22.4)
	-58.2	-124.2	19.7	-37.3	-76.7	-40.1	-86.6	-86.0	-14.5	-58.2
	-6.134	-13.091	2.072	-3.930	-8.078	-4.226	-9.122	-9.064	-1.532	-6.134
N	949	949	949	949	949	949	949	949	949	949
X2	24.1	44.4	21.3	17.9	22.8	25.4	32.5	14.6	33.0	17.4
Gvalue	24.1	46.6	20.4	18.0	22.7	25.3	32.9	15.2	30.8	17.1
Gv/N	2.536	4.915	2.148	1.900	2.392	2.664	3.462	1.604	3.241	1.805
Prob	0.00002	0.00000	0.00014	0.00043	0.00005	0.00001	0.00000	0.00164	0.00000	0.00067

	21	22	23	24	25	26	27	28	29	30
a_O	206	278	219	198	260	228	193	290	213	194
	(21.7)	(29.3)	(23.1)	(20.9)	(27.4)	(24.0)	(20.3)	(30.6)	(22.4)	(20.4)
a_E	201.9	262.0	264.0	201.9	262.0	264.0	201.9	262.0	264.0	201.9
	(21.3)	(27.6)	(27.8)	(21.3)	(27.6)	(27.8)	(21.3)	(27.6)	(27.8)	(21.3)
	8.2	33.0	-81.9	-7.8	-4.0	-66.9	-17.5	58.9	-91.5	-15.5
	0.867	3.475	-8.631	-0.819	-0.417	-7.051	-1.839	6.208	-9.642	-1.637
t_O	282	224	306	281	175	300	274	180	307	280
	(29.7)	(23.6)	(32.2)	(29.6)	(18.4)	(31.6)	(28.9)	(19.0)	(32.3)	(29.5)
t_E	246.7	164.2	288.5	246.7	164.2	288.5	246.7	164.2	288.5	246.7
	(26.0)	(17.3)	(30.4)	(26.0)	(17.3)	(30.4)	(26.0)	(17.3)	(30.4)	(26.0)
	75.4	139.1	36.1	73.1	22.3	23.5	57.5	33.0	38.2	70.9
	7.945	14.657	3.800	7.706	2.346	2.474	6.058	3.482	4.024	7.469
g_O	262	297	219	258	352	226	268	302	196	258
	(27.6)	(31.3)	(23.1)	(27.2)	(37.1)	(23.8)	(28.2)	(31.8)	(20.7)	(27.2)
g_E	269.8	348.4	183.9	269.8	348.4	183.9	269.8	348.4	183.9	269.8
	(28.4)	(36.7)	(19.4)	(28.4)	(36.7)	(19.4)	(28.4)	(36.7)	(19.4)	(28.4)
	-15.5	-94.8	76.5	-23.2	7.3	93.2	-3.7	-86.3	25.0	-23.2
	-1.629	-9.985	8.062	-2.441	0.769	9.818	-0.388	-9.091	2.632	-2.441
c_O	199	150	205	212	162	195	214	177	233	217
	(21.0)	(15.8)	(21.6)	(22.3)	(17.1)	(20.5)	(22.6)	(18.7)	(24.6)	(22.9)
c_E	230.5	174.4	212.6	230.5	174.4	212.6	230.5	174.4	212.6	230.5
	(24.3)	(18.4)	(22.4)	(24.3)	(18.4)	(22.4)	(24.3)	(18.4)	(22.4)	(24.3)
	-58.5	-45.3	-14.9	-35.5	-23.9	-33.7	-31.8	5.2	42.7	-26.2
	-6.166	-4.770	-1.568	-3.741	-2.524	-3.547	-3.353	0.546	4.504	-2.763
N	949	949	949	949	949	949	949	949	949	949
X2	9.7	33.7	15.7	6.8	1.6	16.5	4.6	10.7	13.8	6.1
Gvalue	9.7	32.1	15.8	6.7	1.7	16.1	4.5	10.9	14.4	6.0
Gv/N	1.017	3.377	1.663	0.705	0.174	1.695	0.478	1.145	1.519	0.627
Prob	0.02174	0.00000	0.00126	0.08239	0.64730	0.00109	0.20889	0.01246	0.00239	0.11390

	31	32	33	34	35	36	37	38	39	40
a_O	234	207	208	276	221	176	250	207	175	247
	(24.7)	(21.8)	(21.9)	(29.1)	(23.3)	(18.5)	(26.3)	(21.8)	(18.4)	(26.0)
a_E	262.0	264.0	201.9	262.0	264.0	201.9	262.0	264.0	201.9	262.0
	(27.6)	(27.8)	(21.3)	(27.6)	(27.8)	(21.3)	(27.6)	(27.8)	(21.3)	(27.6)
	-52.9	-100.8	12.3	28.8	-78.6	-48.4	-23.4	-100.8	-50.1	-29.1
	-5.572	-10.617	1.299	3.030	-8.287	-5.097	-2.468	-10.617	-5.278	-3.067
t_O	176	317	260	198	310	276	204	321	280	214
	(18.5)	(33.4)	(27.4)	(20.9)	(32.7)	(29.1)	(21.5)	(33.8)	(29.5)	(22.6)
t_E	164.2	288.5	246.7	164.2	288.5	246.7	164.2	288.5	246.7	164.2
	(17.3)	(30.4)	(26.0)	(17.3)	(30.4)	(26.0)	(17.3)	(30.4)	(26.0)	(17.3)
	24.4	59.8	27.3	74.1	44.6	61.9	88.5	68.6	70.9	113.3
	2.571	6.296	2.874	7.807	4.698	6.525	9.327	7.224	7.469	11.943
g_O	352	220	268	329	218	280	337	205	279	306
	(37.1)	(23.2)	(28.2)	(34.7)	(23.0)	(29.5)	(35.5)	(21.6)	(29.4)	(32.2)
g_E	348.4	183.9	269.8	348.4	183.9	269.8	348.4	183.9	269.8	348.4
	(36.7)	(19.4)	(28.4)	(36.7)	(19.4)	(28.4)	(36.7)	(19.4)	(28.4)	(36.7)
	7.3	78.9	-3.7	-37.6	74.2	20.7	-22.4	44.5	18.6	-79.4
	0.769	8.310	-0.388	-3.966	7.815	2.180	-2.357	4.693	1.962	-8.363
c_O	187	205	213	146	200	217	158	216	215	182
	(19.7)	(21.6)	(22.4)	(15.4)	(21.1)	(22.9)	(16.6)	(22.8)	(22.7)	(19.2)
c_E	174.4	212.6	230.5	174.4	212.6	230.5	174.4	212.6	230.5	174.4
	(18.4)	(22.4)	(24.3)	(18.4)	(22.4)	(24.3)	(18.4)	(22.4)	(24.3)	(18.4)
	26.0	-14.9	-33.7	-51.9	-24.4	-26.2	-31.3	6.9	-30.0	15.5
	2.743	-1.568	-3.548	-5.474	-2.571	-2.763	-3.294	0.727	-3.158	1.630
N	949	949	949	949	949	949	949	949	949	949
X2	4.8	22.5	2.2	13.4	15.7	8.0	12.1	18.5	9.4	21.4
Gvalue	4.9	23.0	2.3	13.3	15.7	8.0	11.5	19.2	9.4	20.3
Gv/N	0.511	2.422	0.238	1.397	1.656	0.844	1.209	2.027	0.995	2.143
Prob	0.18298	0.00004	0.51968	0.00411	0.00129	0.04573	0.00942	0.00024	0.02399	0.00014

	41	42	43	44	45	46	47	48	49	50
a_O	203	161	255	233	193	205	217	184	245	192
	(21.4)	(17.0)	(26.9)	(24.6)	(20.3)	(21.6)	(22.9)	(19.4)	(25.8)	(20.2)
a_E	264.0	201.9	262.0	264.0	201.9	262.0	264.0	201.9	262.0	264.0
	(27.8)	(21.3)	(27.6)	(27.8)	(21.3)	(27.6)	(27.8)	(21.3)	(27.6)	(27.8)
	-106.7	-72.9	-13.8	-58.3	-17.5	-100.6	-85.1	-34.2	-32.9	-122.3
	-11.246	-7.685	-1.453	-6.140	-1.839	-10.597	-8.972	-3.605	-3.462	-12.891
t_O	320	301	166	302	278	227	311	267	169	341
	(33.7)	(31.7)	(17.5)	(31.8)	(29.3)	(23.9)	(32.8)	(28.1)	(17.8)	(35.9)
t_E	288.5	246.7	164.2	288.5	246.7	164.2	288.5	246.7	164.2	288.5
	(30.4)	(26.0)	(17.3)	(30.4)	(26.0)	(17.3)	(30.4)	(26.0)	(17.3)	(30.4)
	66.3	119.7	3.6	27.6	66.4	147.0	46.7	42.2	9.7	114.0
	6.991	12.616	0.378	2.913	6.995	15.490	4.925	4.447	1.023	12.018
g_O	237	256	369	185	276	339	220	305	348	223
	(25.0)	(27.0)	(38.9)	(19.5)	(29.1)	(35.7)	(23.2)	(32.1)	(36.7)	(23.5)
g_E	183.9	269.8	348.4	183.9	269.8	348.4	183.9	269.8	348.4	183.9
	(19.4)	(28.4)	(36.7)	(19.4)	(28.4)	(36.7)	(19.4)	(28.4)	(36.7)	(19.4)
	120.2	-27.0	42.5	2.2	12.4	-18.5	78.9	74.7	-0.7	86.0
	12.670	-2.842	4.474	0.233	1.312	-1.948	8.310	7.872	-0.078	9.060
c_O	189	231	159	229	202	178	201	193	187	193
	(19.9)	(24.3)	(16.8)	(24.1)	(21.3)	(18.8)	(21.2)	(20.3)	(19.7)	(20.3)
c_E	212.6	230.5	174.4	212.6	230.5	174.4	212.6	230.5	174.4	212.6
	(22.4)	(24.3)	(18.4)	(22.4)	(24.3)	(18.4)	(22.4)	(24.3)	(18.4)	(22.4)
	-44.4	1.0	-29.4	34.1	-53.4	7.2	-22.5	-68.6	26.0	-37.3
	-4.683	0.102	-3.103	3.591	-5.622	0.760	-2.372	-7.225	2.743	-3.930
N	949	949	949	949	949	949	949	949	949	949
X2	35.5	21.0	2.8	5.6	8.0	36.7	17.9	13.9	2.1	39.3
Gvalue	35.4	20.8	2.8	5.7	8.0	35.2	17.9	14.1	2.1	40.4
Gv/N	3.733	2.192	0.296	0.597	0.846	3.705	1.891	1.488	0.227	4.257
Prob	0.00000	0.00012	0.42144	0.12900	0.04534	0.00000	0.00045	0.00274	0.54189	0.00000

	51	52	53	54	55	56	57	58	59	60
a_O	191	235	220	173	242	234	202	236	209	199
	(20.1)	(24.8)	(23.2)	(18.2)	(25.5)	(24.7)	(21.3)	(24.9)	(22.0)	(21.0)
a_E	201.9	262.0	264.0	201.9	262.0	264.0	201.9	262.0	264.0	201.9
	(21.3)	(27.6)	(27.8)	(21.3)	(27.6)	(27.8)	(21.3)	(27.6)	(27.8)	(21.3)
	-21.2	-51.1	-80.3	-53.5	-38.4	-56.5	0.1	-49.3	-97.7	-5.8
	-2.239	-5.384	-8.459	-5.637	-4.048	-5.955	0.016	-5.196	-10.296	-0.612
t_O	278	167	318	259	194	329	264	187	317	271
	(29.3)	(17.6)	(33.5)	(27.3)	(20.4)	(34.7)	(27.8)	(19.7)	(33.4)	(28.6)
t_E	246.7	164.2	288.5	246.7	164.2	288.5	246.7	164.2	288.5	246.7
	(26.0)	(17.3)	(30.4)	(26.0)	(17.3)	(30.4)	(26.0)	(17.3)	(30.4)	(26.0)
	66.4	5.6	61.9	25.2	64.7	86.5	35.8	48.6	59.8	50.9
	6.995	0.592	6.527	2.653	6.815	9.111	3.768	5.121	6.296	5.363
g_O	274	369	195	281	339	195	257	359	207	273
	(28.9)	(38.9)	(20.5)	(29.6)	(35.7)	(20.5)	(27.1)	(37.8)	(21.8)	(28.8)
g_E	269.8	348.4	183.9	269.8	348.4	183.9	269.8	348.4	183.9	269.8
	(28.4)	(36.7)	(19.4)	(28.4)	(36.7)	(19.4)	(28.4)	(36.7)	(19.4)	(28.4)
	8.4	42.5	22.9	22.8	-18.5	22.9	-25.1	21.6	49.0	6.3
	0.882	4.474	2.409	2.399	-1.948	2.409	-2.642	2.274	5.162	0.669
c_O	206	178	216	236	174	191	226	167	216	206
	(21.7)	(18.8)	(22.8)	(24.9)	(18.3)	(20.1)	(23.8)	(17.6)	(22.8)	(21.7)
c_E	230.5	174.4	212.6	230.5	174.4	212.6	230.5	174.4	212.6	230.5
	(24.3)	(18.4)	(22.4)	(24.3)	(18.4)	(22.4)	(24.3)	(18.4)	(22.4)	(24.3)
	-46.3	7.2	6.9	11.1	-0.9	-40.9	-8.9	-14.5	6.9	-46.3
	-4.882	0.760	0.727	1.169	-0.090	-4.308	-0.943	-1.532	0.727	-4.882
N	949	949	949	949	949	949	949	949	949	949
X2	7.2	4.1	11.1	5.3	7.2	12.0	1.9	6.4	17.2	5.1
Gvalue	7.2	4.2	11.4	5.5	6.9	11.9	1.9	6.3	17.9	5.1
Gv/N	0.757	0.442	1.204	0.584	0.730	1.256	0.200	0.668	1.890	0.537
Prob	0.06635	0.24077	0.00965	0.13608	0.07438	0.00767	0.59459	0.09642	0.00045	0.16457

	61	62	63	64	65	66	67	68	69	70
a_O	243	205	166	235	204	181	253	221	172	230
	(25.6)	(21.6)	(17.5)	(24.8)	(21.5)	(19.1)	(26.7)	(23.3)	(18.1)	(24.2)
a_E	262.0	264.0	201.9	262.0	264.0	201.9	262.0	264.0	201.9	262.0
	(27.6)	(27.8)	(21.3)	(27.6)	(27.8)	(21.3)	(27.6)	(27.8)	(21.3)	(27.6)
	-36.6	-103.8	-65.0	-51.1	-105.2	-39.6	-17.7	-78.6	-55.2	-59.9
	-3.853	-10.933	-6.854	-5.384	-11.090	-4.173	-1.861	-8.287	-5.814	-6.312
t_O	185	293	273	178	326	256	174	319	285	186
	(19.5)	(30.9)	(28.8)	(18.8)	(34.4)	(27.0)	(18.3)	(33.6)	(30.0)	(19.6)
t_E	164.2	288.5	246.7	164.2	288.5	246.7	164.2	288.5	246.7	164.2
	(17.3)	(30.4)	(26.0)	(17.3)	(30.4)	(26.0)	(17.3)	(30.4)	(26.0)	(17.3)
	44.1	9.1	55.3	28.7	79.7	18.9	20.1	64.1	82.2	46.3
	4.647	0.958	5.825	3.024	8.399	1.994	2.123	6.759	8.665	4.883
g_O	352	235	301	356	209	287	331	180	266	367
	(37.1)	(24.8)	(31.7)	(37.5)	(22.0)	(30.2)	(34.9)	(19.0)	(28.0)	(38.7)
g_E	348.4	183.9	269.8	348.4	183.9	269.8	348.4	183.9	269.8	348.4
	(36.7)	(19.4)	(28.4)	(36.7)	(19.4)	(28.4)	(36.7)	(19.4)	(28.4)	(36.7)
	7.3	115.2	65.8	15.4	53.5	35.4	-33.9	-7.7	-7.6	38.2
	0.769	12.143	6.931	1.626	5.635	3.728	-3.568	-0.813	-0.805	4.030
c_O	169	216	209	180	210	225	191	229	226	166
	(17.8)	(22.8)	(22.0)	(19.0)	(22.1)	(23.7)	(20.1)	(24.1)	(23.8)	(17.5)
c_E	174.4	212.6	230.5	174.4	212.6	230.5	174.4	212.6	230.5	174.4
	(18.4)	(22.4)	(24.3)	(18.4)	(22.4)	(24.3)	(18.4)	(22.4)	(24.3)	(18.4)
	-10.7	6.9	-41.0	11.3	-5.1	-10.9	34.7	34.1	-8.9	-16.4
	-1.126	0.727	-4.316	1.193	-0.540	-1.149	3.653	3.591	-0.943	-1.733
N	949	949	949	949	949	949	949	949	949	949
X2	4.2	27.5	14.8	4.3	22.0	3.7	3.3	11.6	10.5	8.2
Gvalue	4.1	27.5	15.1	4.3	22.8	3.8	3.3	11.9	10.5	8.2
Gv/N	0.437	2.895	1.586	0.458	2.404	0.400	0.347	1.251	1.104	0.868
Prob	0.24615	0.00000	0.00177	0.22614	0.00004	0.28457	0.34883	0.00785	0.01495	0.04133

	71	72	73	74	75	76	77	78	79	80
a_O	255	175	236	220	158	216	212	150	213	226
	(26.9)	(18.4)	(24.9)	(23.2)	(16.6)	(22.8)	(22.3)	(15.8)	(22.4)	(23.8)
a_E	264.0	201.9	262.0	264.0	201.9	262.0	264.0	201.9	262.0	264.0
	(27.8)	(21.3)	(27.6)	(27.8)	(21.3)	(27.6)	(27.8)	(21.3)	(27.6)	(27.8)
	-17.8	-50.1	-49.3	-80.3	-77.5	-83.4	-93.1	-89.2	-88.2	-70.3
	-1.871	-5.278	-5.196	-8.459	-8.168	-8.787	-9.807	-9.397	-9.292	-7.408
t_O	276	261	187	285	247	189	324	259	209	304
	(29.1)	(27.5)	(19.7)	(30.0)	(26.0)	(19.9)	(34.1)	(27.3)	(22.0)	(32.0)
t_E	288.5	246.7	164.2	288.5	246.7	164.2	288.5	246.7	164.2	288.5
	(30.4)	(26.0)	(17.3)	(30.4)	(26.0)	(17.3)	(30.4)	(26.0)	(17.3)	(30.4)
	-24.4	29.4	48.6	-6.9	0.6	53.1	75.2	25.2	100.8	31.8
	-2.574	3.097	5.121	-0.731	0.061	5.599	7.927	2.653	10.622	3.355
g_O	183	278	356	222	288	360	182	317	359	195
	(19.3)	(29.3)	(37.5)	(23.4)	(30.3)	(37.9)	(19.2)	(33.4)	(37.8)	(20.5)
g_E	183.9	269.8	348.4	183.9	269.8	348.4	183.9	269.8	348.4	183.9
	(19.4)	(28.4)	(36.7)	(19.4)	(28.4)	(36.7)	(19.4)	(28.4)	(36.7)	(19.4)
	-1.8	16.6	15.4	83.6	37.5	23.6	-3.8	102.1	21.6	22.9
	-0.189	1.744	1.626	8.809	3.952	2.492	-0.398	10.760	2.274	2.409
c_O	235	235	170	222	256	184	231	223	168	224
	(24.8)	(24.8)	(17.9)	(23.4)	(27.0)	(19.4)	(24.3)	(23.5)	(17.7)	(23.6)
c_E	212.6	230.5	174.4	212.6	230.5	174.4	212.6	230.5	174.4	212.6
	(22.4)	(24.3)	(18.4)	(22.4)	(24.3)	(18.4)	(22.4)	(24.3)	(18.4)	(22.4)
	47.1	9.1	-8.7	19.3	53.7	19.7	38.4	-14.8	-12.6	23.4
	4.966	0.954	-0.921	2.029	5.657	2.072	4.046	-1.558	-1.329	2.471
N	949	949	949	949	949	949	949	949	949	949
X2	3.2	4.8	6.0	15.7	13.6	12.7	16.2	22.4	21.9	7.6
Gvalue	3.2	4.9	6.0	15.6	14.2	13.1	16.8	23.3	21.6	7.8
Gv/N	0.332	0.517	0.629	1.648	1.501	1.376	1.768	2.457	2.275	0.826
Prob	0.36881	0.17896	0.11299	0.00134	0.00259	0.00451	0.00079	0.00003	0.00008	0.04939

	81	82	83	84	85	86	87	88	89	90
a_O	169	250	225	191	216	229	195	241	222	176
	(17.8)	(26.3)	(23.7)	(20.1)	(22.8)	(24.1)	(20.5)	(25.4)	(23.4)	(18.5)
a_E	201.9	262.0	264.0	201.9	262.0	264.0	201.9	262.0	264.0	201.9
	(21.3)	(27.6)	(27.8)	(21.3)	(27.6)	(27.8)	(21.3)	(27.6)	(27.8)	(21.3)
	-60.2	-23.4	-72.0	-21.2	-83.4	-65.2	-13.6	-40.2	-77.0	-48.4
	-6.340	-2.468	-7.586	-2.239	-8.787	-6.870	-1.434	-4.241	-8.113	-5.097
t_O	286	191	300	269	174	296	264	161	289	263
	(30.1)	(20.1)	(31.6)	(28.3)	(18.3)	(31.2)	(27.8)	(17.0)	(30.5)	(27.7)
t_E	246.7	164.2	288.5	246.7	164.2	288.5	246.7	164.2	288.5	246.7
	(26.0)	(17.3)	(30.4)	(26.0)	(17.3)	(30.4)	(26.0)	(17.3)	(30.4)	(26.0)
	84.5	57.7	23.5	46.5	20.1	15.2	35.8	-6.4	1.0	33.6

```
          8.907   6.082   2.474   4.903   2.123   1.603   3.768  -0.671   0.108   3.543
g_O         275     359     193     256     381     184     271     375     209     293
        ( 29.0) ( 37.8) ( 20.3) ( 27.0) ( 40.1) ( 19.4) ( 28.6) ( 39.5) ( 22.0) ( 30.9)
g_E       269.8   348.4   183.9   269.8   348.4   183.9   269.8   348.4   183.9   269.8
        ( 28.4) ( 36.7) ( 19.4) ( 28.4) ( 36.7) ( 19.4) ( 28.4) ( 36.7) ( 19.4) ( 28.4)
           10.4    21.6    18.6   -27.0    68.2     0.2     2.3    55.2    53.5    48.2
          1.097   2.274   1.965  -2.842   7.189   0.021   0.244   5.822   5.635   5.083
c_O         219     149     231     233     178     240     219     172     229     217
        ( 23.1) ( 15.7) ( 24.3) ( 24.6) ( 18.8) ( 25.3) ( 23.1) ( 18.1) ( 24.1) ( 22.9)
c_E       230.5   174.4   212.6   230.5   174.4   212.6   230.5   174.4   212.6   230.5
        ( 24.3) ( 18.4) ( 22.4) ( 24.3) ( 18.4) ( 22.4) ( 24.3) ( 18.4) ( 22.4) ( 24.3)
          -22.4   -47.0    38.4     5.0     7.2    58.2   -22.4    -4.8    34.1   -26.2
         -2.365  -4.948   4.046   0.526   0.760   6.137  -2.365  -0.508   3.591  -2.763

N           949     949     949     949     949     949     949     949     949     949
X2         12.3     8.9     8.3     3.3    11.8     8.4     2.0     3.8    11.4     7.2
Gvalue     12.3     8.9     8.5     3.3    12.2     8.5     2.0     3.8    11.6     7.3
Gv/N      1.298   0.941   0.899   0.348   1.286   0.891   0.212   0.402   1.222   0.767
Prob    0.00637 0.03025 0.03629 0.34668 0.00672 0.03744 0.56942 0.28258 0.00890 0.06365
        --------------------------------------------------------------------------------
             91      92      93      94      95      96      97      98      99     100
        --------------------------------------------------------------------------------
a_O         259     262     175     229     235     152     256     210     160     224
        ( 27.3) ( 27.6) ( 18.4) ( 24.1) ( 24.8) ( 16.0) ( 27.0) ( 22.1) ( 16.9) ( 23.6)
a_E       262.0   264.0   201.9   262.0   264.0   201.9   262.0   264.0   201.9   262.0
        ( 27.6) ( 27.8) ( 21.3) ( 27.6) ( 27.8) ( 21.3) ( 27.6) ( 27.8) ( 21.3) ( 27.6)
           -5.9    -4.1   -50.1   -61.6   -54.8   -86.3   -11.8   -96.2   -74.5   -70.2
         -0.626  -0.427  -5.278  -6.495  -5.769  -9.098  -1.248 -10.134  -7.847  -7.395
t_O         191     294     276     165     315     284     177     303     276     176
        ( 20.1) ( 31.0) ( 29.1) ( 17.4) ( 33.2) ( 29.9) ( 18.7) ( 31.9) ( 29.1) ( 18.5)
t_E       164.2   288.5   246.7   164.2   288.5   246.7   164.2   288.5   246.7   164.2
        ( 17.3) ( 30.4) ( 26.0) ( 17.3) ( 30.4) ( 26.0) ( 17.3) ( 30.4) ( 26.0) ( 17.3)
           57.7    11.1    61.9     1.6    55.4    79.9    26.5    29.7    61.9    24.4
          6.082   1.173   6.525   0.166   5.836   8.424   2.797   3.134   6.525   2.571
g_O         347     190     309     379     221     269     358     201     287     363
        ( 36.6) ( 20.0) ( 32.6) ( 39.9) ( 23.3) ( 28.3) ( 37.7) ( 21.2) ( 30.2) ( 38.3)
g_E       348.4   183.9   269.8   348.4   183.9   269.8   348.4   183.9   269.8   348.4
        ( 36.7) ( 19.4) ( 28.4) ( 36.7) ( 19.4) ( 28.4) ( 36.7) ( 19.4) ( 28.4) ( 36.7)
           -2.7    12.4    83.7    63.9    81.2    -1.7    19.5    35.7    35.4    29.9
         -0.288   1.307   8.823   6.731   8.559  -0.178   2.057   3.766   3.728   3.147
c_O         152     203     189     176     178     244     158     235     226     186
        ( 16.0) ( 21.4) ( 19.9) ( 18.5) ( 18.8) ( 25.7) ( 16.6) ( 24.8) ( 23.8) ( 19.6)
c_E       174.4   212.6   230.5   174.4   212.6   230.5   174.4   212.6   230.5   174.4
        ( 18.4) ( 22.4) ( 24.3) ( 18.4) ( 22.4) ( 24.3) ( 18.4) ( 22.4) ( 24.3) ( 18.4)
          -41.8   -18.7   -75.1     3.2   -63.2    27.7   -31.3    47.1    -8.9    23.9
         -4.409  -1.972  -7.910   0.333  -6.659   2.923  -3.294   4.966  -0.943   2.518

N           949     949     949     949     949     949     949     949     949     949
X2          7.3     0.8    20.2     6.9    18.7    18.8     2.9    15.7    13.4     7.7
Gvalue      7.2     0.8    20.5     7.0    18.7    19.7     3.0    16.4    13.9     8.0
Gv/N      0.759   0.080   2.161   0.735   1.967   2.071   0.313   1.733   1.463   0.841
Prob    0.06564 0.85933 0.00013 0.07268 0.00032 0.00020 0.39617 0.00092 0.00307 0.04644
        --------------------------------------------------------------------------------
            101     102     103     104     105     106     107     108     109     110
        --------------------------------------------------------------------------------
a_O         253     180     256     264     162     264     229     171     240     225
        ( 26.7) ( 19.0) ( 27.0) ( 27.8) ( 17.1) ( 27.8) ( 24.1) ( 18.0) ( 25.3) ( 23.7)
a_E       264.0   201.9   262.0   264.0   201.9   262.0   264.0   201.9   262.0   264.0
        ( 27.8) ( 21.3) ( 27.6) ( 27.8) ( 21.3) ( 27.6) ( 27.8) ( 21.3) ( 27.6) ( 27.8)
          -21.6   -41.4   -11.8    -0.1   -71.4     4.0   -65.2   -56.9   -42.1   -72.0
         -2.276  -4.360  -1.248  -0.007  -7.521   0.426  -6.870  -5.991  -4.434  -7.586
t_O         293     266     170     303     265     191     317     253     181     320
        ( 30.9) ( 28.0) ( 17.9) ( 31.9) ( 27.9) ( 20.1) ( 33.4) ( 26.7) ( 19.1) ( 33.7)
t_E       288.5   246.7   164.2   288.5   246.7   164.2   288.5   246.7   164.2   288.5
        ( 30.4) ( 26.0) ( 17.3) ( 30.4) ( 26.0) ( 17.3) ( 30.4) ( 26.0) ( 17.3) ( 30.4)
            9.1    40.0    11.8    29.7    37.9    57.7    59.8    12.7    35.2    66.3
          0.958   4.220   1.241   3.134   3.993   6.082   6.296   1.342   3.713   6.991
g_O         211     260     342     184     283     335     197     300     359     191
        ( 22.2) ( 27.4) ( 36.0) ( 19.4) ( 29.8) ( 35.3) ( 20.8) ( 31.6) ( 37.8) ( 20.1)
g_E       183.9   269.8   348.4   183.9   269.8   348.4   183.9   269.8   348.4   183.9
        ( 19.4) ( 28.4) ( 36.7) ( 19.4) ( 28.4) ( 36.7) ( 19.4) ( 28.4) ( 36.7) ( 19.4)
           58.0   -19.3   -12.6     0.2    26.9   -26.2    27.1    63.6    21.6    14.5
          6.113  -2.037  -1.330   0.021   2.839  -2.763   2.857   6.698   2.274   1.525
c_O         192     243     181     198     239     159     206     225     169     213
        ( 20.2) ( 25.6) ( 19.1) ( 20.9) ( 25.2) ( 16.8) ( 21.7) ( 23.7) ( 17.8) ( 22.4)
c_E       212.6   230.5   174.4   212.6   230.5   174.4   212.6   230.5   174.4   212.6
        ( 22.4) ( 24.3) ( 18.4) ( 22.4) ( 24.3) ( 18.4) ( 22.4) ( 24.3) ( 18.4) ( 22.4)
          -39.1    25.6    13.4   -28.1    17.3   -29.4   -12.9   -10.9   -10.7     0.8
         -4.120   2.701   1.411  -2.964   1.820  -3.103  -1.365  -1.149  -1.126   0.089

N           949     949     949     949     949     949     949     949     949     949
X2          6.5     4.9     0.7     1.7    10.2     6.3     8.6     8.4     4.1     9.5
Gvalue      6.4     5.0     0.7     1.7    10.7     6.1     8.7     8.5     4.1     9.7
Gv/N      0.675   0.524   0.074   0.183   1.131   0.642   0.918   0.900   0.427   1.019
Prob    0.09336 0.17403 0.87293 0.62777 0.01324 0.10726 0.03332 0.03605 0.25596 0.02156
        --------------------------------------------------------------------------------
            111     112     113     114     115     116     117     118     119     120
        --------------------------------------------------------------------------------
a_O         179     270     254     181     261     237     196     222     249     181
        ( 18.9) ( 28.5) ( 26.8) ( 19.1) ( 27.5) ( 25.0) ( 20.7) ( 23.4) ( 26.2) ( 19.1)
a_E       201.9   262.0   264.0   201.9   262.0   264.0   201.9   262.0   264.0   201.9
        ( 21.3) ( 27.6) ( 27.8) ( 21.3) ( 27.6) ( 27.8) ( 21.3) ( 27.6) ( 27.8) ( 21.3)
          -43.1    16.3   -19.7   -39.6    -2.0   -51.2   -11.7   -73.5   -29.2   -39.6
         -4.546   1.714  -2.074  -4.173  -0.208  -5.395  -1.230  -7.749  -3.076  -4.173
t_O         273     190     296     259     175     301     266     163     284     262
        ( 28.8) ( 20.0) ( 31.2) ( 27.3) ( 18.4) ( 31.7) ( 28.0) ( 17.2) ( 29.9) ( 27.6)
t_E       246.7   164.2   288.5   246.7   164.2   288.5   246.7   164.2   288.5   246.7
        ( 26.0) ( 17.3) ( 30.4) ( 26.0) ( 17.3) ( 30.4) ( 26.0) ( 17.3) ( 30.4) ( 26.0)
           55.3    55.4    15.2    25.2    22.3    25.6    40.0    -2.4    -8.9    31.5
          5.825   5.840   1.603   2.653   2.346   2.693   4.220  -0.255  -0.939   3.320
g_O         248     328     203     249     335     193     248     385     211     260
        ( 26.1) ( 34.6) ( 21.4) ( 26.2) ( 35.3) ( 20.3) ( 26.1) ( 40.6) ( 22.2) ( 27.4)
g_E       269.8   348.4   183.9   269.8   348.4   183.9   269.8   348.4   183.9   269.8
        ( 28.4) ( 36.7) ( 19.4) ( 28.4) ( 36.7) ( 19.4) ( 28.4) ( 36.7) ( 19.4) ( 28.4)
          -41.9   -39.5    40.1   -40.0   -26.2    18.6   -41.9    77.0    58.0   -19.3
         -4.412  -4.165   4.228  -4.219  -2.763   1.965  -4.412   8.112   6.113  -2.037
c_O         249     161     196     260     178     218     239     179     205     246
        ( 26.2) ( 17.0) ( 20.7) ( 27.4) ( 18.8) ( 23.0) ( 25.2) ( 18.9) ( 21.6) ( 25.9)
c_E       230.5   174.4   212.6   230.5   174.4   212.6   230.5   174.4   212.6   230.5
        ( 24.3) ( 18.4) ( 22.4) ( 24.3) ( 18.4) ( 22.4) ( 24.3) ( 18.4) ( 22.4) ( 24.3)
           38.4   -25.8   -31.8    62.6     7.2    11.0    17.3     9.3   -14.9    32.0
          4.047  -2.718  -3.354   6.595   0.760   1.157   1.820   0.976  -1.568   3.370
```

N	949	949	949	949	949	949	949	949	949	949
X2	8.7	6.5	3.9	8.2	1.3	3.9	3.8	10.1	5.2	4.5
Gvalue	8.7	6.4	3.8	8.1	1.3	4.0	3.8	10.3	5.0	4.6
Gv/N	0.914	0.671	0.403	0.856	0.136	0.420	0.397	1.084	0.530	0.480
Prob	0.03393	0.09489	0.28083	0.04354	0.73161	0.26332	0.28713	0.01624	0.16982	0.20727

	121	122	123	124	125	126	127	128	129	130
a_O	262	241	168	239	248	178	249	236	199	229
	(27.6)	(25.4)	(17.7)	(25.2)	(26.2)	(18.8)	(26.3)	(24.9)	(21.0)	(24.2)
a_E	261.7	263.8	201.7	261.7	263.8	201.7	261.7	263.8	201.7	261.7
	(27.6)	(27.8)	(21.3)	(27.6)	(27.8)	(21.3)	(27.6)	(27.8)	(21.3)	(27.6)
	0.6	-43.5	-61.4	-43.4	-30.6	-44.5	-24.8	-52.5	-5.4	-61.2
	0.061	-4.588	-6.482	-4.577	-3.223	-4.696	-2.616	-5.536	-0.568	-6.451
t_O	147	288	279	186	259	262	165	313	280	179
	(15.5)	(30.4)	(29.4)	(19.6)	(27.3)	(27.6)	(17.4)	(33.0)	(29.5)	(18.9)
t_E	164.0	288.2	246.5	164.0	288.2	246.5	164.0	288.2	246.5	164.0
	(17.3)	(30.4)	(26.0)	(17.3)	(30.4)	(26.0)	(17.3)	(30.4)	(26.0)	(17.3)
	-32.2	-0.4	69.2	46.7	-55.3	32.1	1.9	51.7	71.5	31.2
	-3.402	-0.039	7.301	4.930	-5.834	3.381	0.203	5.455	7.539	3.296
g_O	352	195	272	353	213	269	348	188	259	333
	(37.1)	(20.6)	(28.7)	(37.2)	(22.5)	(28.4)	(36.7)	(19.8)	(27.3)	(35.1)
g_E	348.0	183.7	269.6	348.0	183.7	269.6	348.0	183.7	269.6	348.0
	(36.7)	(19.4)	(28.4)	(36.7)	(19.4)	(28.4)	(36.7)	(19.4)	(28.4)	(36.7)
	8.0	23.3	4.9	10.1	63.0	-1.1	-0.0	8.7	-20.7	-29.3
	0.848	2.454	0.517	1.062	6.649	-0.118	-0.000	0.916	-2.184	-3.096
c_O	187	224	229	170	228	239	186	211	210	207
	(19.7)	(23.6)	(24.2)	(17.9)	(24.1)	(25.2)	(19.6)	(22.3)	(22.2)	(21.8)
c_E	174.2	212.4	230.3	174.2	212.4	230.3	174.2	212.4	230.3	174.2
	(18.4)	(22.4)	(24.3)	(18.4)	(22.4)	(24.3)	(18.4)	(22.4)	(24.3)	(18.4)
	26.4	23.9	-2.5	-8.4	32.4	17.8	24.3	-2.7	-38.7	71.3
	2.787	2.523	-0.268	-0.884	3.420	1.875	2.562	-0.285	-4.083	7.523
N	948	948	948	948	948	948	948	948	948	948
X2	2.8	3.3	10.0	5.1	9.7	4.1	1.4	5.2	6.8	12.3
Gvalue	2.8	3.3	10.1	5.0	9.6	4.2	1.4	5.2	6.7	12.1
Gv/N	0.295	0.351	1.068	0.530	1.011	0.442	0.149	0.550	0.703	1.272
Prob	0.42468	0.34343	0.01751	0.17013	0.02242	0.24144	0.70338	0.15669	0.08327	0.00719

	131	132	133	134	135	136	137	138	139	140
a_O	233	182	256	250	182	259	238	189	250	259
	(24.6)	(19.2)	(27.0)	(26.4)	(19.2)	(27.3)	(25.1)	(20.0)	(26.4)	(27.3)
a_E	263.8	201.7	261.7	263.8	201.7	261.4	263.5	201.5	261.4	263.5
	(27.8)	(21.3)	(27.6)	(27.8)	(21.3)	(27.6)	(27.8)	(21.3)	(27.6)	(27.8)
	-57.8	-37.4	-11.3	-26.8	-37.4	-4.9	-48.4	-24.2	-22.4	-8.9
	-6.095	-3.949	-1.192	-2.825	-3.949	-0.512	-5.112	-2.556	-2.362	-0.938
t_O	306	261	180	301	293	187	301	260	182	318
	(32.3)	(27.5)	(19.0)	(31.8)	(30.9)	(19.7)	(31.8)	(27.5)	(19.2)	(33.6)
t_E	288.2	246.5	164.0	288.2	246.5	163.9	287.9	246.2	163.9	287.9
	(30.4)	(26.0)	(17.3)	(30.4)	(26.0)	(17.3)	(30.4)	(26.0)	(17.3)	(30.4)
	36.7	29.9	33.4	26.2	101.4	49.4	26.8	28.4	38.2	63.3
	3.872	3.158	3.526	2.763	10.694	5.215	2.833	2.996	4.034	6.683
g_O	205	282	337	189	264	340	195	240	351	160
	(21.6)	(29.7)	(35.5)	(19.9)	(27.8)	(35.9)	(20.6)	(25.3)	(37.1)	(16.9)
g_E	183.7	269.6	348.0	183.7	269.6	347.6	183.5	269.3	347.6	183.5
	(19.4)	(28.4)	(36.7)	(19.4)	(28.4)	(36.7)	(19.4)	(28.4)	(36.7)	(19.4)
	45.0	25.4	-21.7	10.7	-11.0	-15.1	23.7	-55.2	6.8	-43.9
	4.743	2.684	-2.284	1.133	-1.161	-1.595	2.501	-5.834	0.714	-4.633
c_O	204	223	175	208	209	161	213	258	164	210
	(21.5)	(23.5)	(18.5)	(21.9)	(22.0)	(17.0)	(22.5)	(27.2)	(17.3)	(22.2)
c_E	212.4	230.3	174.2	212.4	230.3	174.1	212.1	230.0	174.1	212.1
	(22.4)	(24.3)	(18.4)	(22.4)	(24.3)	(18.4)	(22.4)	(24.3)	(18.4)	(22.4)
	-16.4	-14.3	1.5	-8.6	-40.5	-25.1	1.7	59.2	-19.5	-4.2
	-1.727	-1.510	0.160	-0.909	-4.274	-2.652	0.184	6.252	-2.062	-0.447
N	948	948	948	948	948	947	947	947	947	947
X2	7.5	3.6	2.0	1.5	12.8	4.4	3.8	8.1	3.1	6.3
Gvalue	7.5	3.6	2.0	1.5	12.4	4.3	3.8	8.1	3.1	6.3
Gv/N	0.794	0.383	0.209	0.162	1.310	0.456	0.406	0.858	0.324	0.665
Prob	0.05692	0.30400	0.57553	0.67470	0.00607	0.22903	0.27877	0.04344	0.38105	0.09812

	141	142	143	144	145	146	147	148	149	150
a_O	179	227	251	170	240	230	193	259	256	177
	(18.9)	(24.0)	(26.5)	(18.0)	(25.3)	(24.3)	(20.4)	(27.3)	(27.0)	(18.7)
a_E	201.5	261.4	263.5	201.5	261.4	263.5	201.5	261.4	263.5	201.5
	(21.3)	(27.6)	(27.8)	(21.3)	(27.6)	(27.8)	(21.3)	(27.6)	(27.8)	(21.3)
	-42.4	-64.1	-24.4	-57.8	-41.1	-62.5	-16.6	-4.9	-14.7	-45.9
	-4.476	-6.771	-2.572	-6.103	-4.336	-6.601	-1.757	-0.512	-1.557	-4.846
t_O	257	184	313	261	172	309	267	188	286	230
	(27.1)	(19.4)	(33.1)	(27.6)	(18.2)	(32.6)	(28.2)	(19.9)	(30.2)	(24.3)
t_E	246.2	163.9	287.9	246.2	163.9	287.9	246.2	163.9	287.9	246.2
	(26.0)	(17.3)	(30.4)	(26.0)	(17.3)	(30.4)	(26.0)	(17.3)	(30.4)	(26.0)
	22.1	42.6	52.4	30.5	16.7	43.8	43.3	51.7	-3.7	-31.3
	2.332	4.503	5.530	3.219	1.759	4.620	4.575	5.455	-0.396	-3.305
g_O	285	362	184	264	376	182	252	344	183	313
	(30.1)	(38.2)	(19.4)	(27.9)	(39.7)	(19.2)	(26.6)	(36.3)	(19.3)	(33.1)
g_E	269.3	347.6	183.5	269.3	347.6	183.5	269.3	347.6	183.5	269.3
	(28.4)	(36.7)	(19.4)	(28.4)	(36.7)	(19.4)	(28.4)	(36.7)	(19.4)	(28.4)
	32.3	29.3	1.0	-10.4	59.0	-3.0	-33.4	-7.2	-1.0	94.2
	3.416	3.096	0.103	-1.103	6.228	-0.318	-3.529	-0.764	-0.108	9.946
c_O	226	174	199	252	159	226	235	156	222	227
	(23.9)	(18.4)	(21.0)	(26.6)	(16.8)	(23.9)	(24.8)	(16.5)	(23.4)	(24.0)
c_E	230.0	174.1	212.1	230.0	174.1	212.1	230.0	174.1	212.1	230.0
	(24.3)	(18.4)	(22.4)	(24.3)	(18.4)	(22.4)	(24.3)	(18.4)	(22.4)	(24.3)
	-8.0	-0.1	-25.4	46.0	-28.8	28.6	10.0	-34.2	20.2	-6.0
	-0.844	-0.013	-2.685	4.855	-3.039	3.023	1.061	-3.609	2.132	-0.636
N	947	947	947	947	947	947	947	947	947	947
X2	4.0	7.6	3.6	8.0	5.8	6.7	3.3	5.5	0.7	11.2
Gvalue	4.1	7.7	3.6	8.2	5.8	6.9	3.3	5.4	0.7	11.0
Gv/N	0.428	0.814	0.376	0.867	0.613	0.724	0.350	0.570	0.072	1.160
Prob	0.25602	0.05234	0.31275	0.04178	0.12170	0.07652	0.34567	0.14509	0.87782	0.01183

Apha_HZ
5UTR:

	50	49	48	47	46	45	44	43	42	41
a_O	353	349	358	362	378	374	353	371	343	366
	(27.9)	(27.6)	(28.3)	(28.6)	(29.9)	(29.6)	(27.9)	(29.4)	(27.1)	(29.0)
a_E	358.4	358.4	358.4	358.4	358.4	358.4	358.4	358.4	358.4	358.4
	(28.4)	(28.4)	(28.4)	(28.4)	(28.4)	(28.4)	(28.4)	(28.4)	(28.4)	(28.4)
	-10.8	-18.6	-0.9	7.1	40.2	31.8	-10.8	25.5	-30.2	15.3
	-0.855	-1.475	-0.071	0.565	3.177	2.514	-0.855	2.021	-2.391	1.208
t_O	418	421	422	374	400	403	419	399	414	393
	(33.1)	(33.3)	(33.4)	(29.6)	(31.6)	(31.9)	(33.1)	(31.6)	(32.8)	(31.1)
t_E	406.8	406.8	406.8	406.8	406.8	406.8	406.8	406.8	406.8	406.8
	(32.2)	(32.2)	(32.2)	(32.2)	(32.2)	(32.2)	(32.2)	(32.2)	(32.2)	(32.2)
	22.7	28.9	30.9	-62.9	-13.5	-7.6	24.7	-15.5	14.5	-27.2
	1.794	2.283	2.447	-4.977	-1.070	-0.601	1.956	-1.225	1.147	-2.149
g_O	269	296	286	295	281	303	281	267	312	287
	(21.3)	(23.4)	(22.6)	(23.3)	(22.2)	(24.0)	(22.2)	(21.1)	(24.7)	(22.7)
g_E	292.0	292.0	292.0	292.0	292.0	292.0	292.0	292.0	292.0	292.0
	(23.1)	(23.1)	(23.1)	(23.1)	(23.1)	(23.1)	(23.1)	(23.1)	(23.1)	(23.1)
	-44.2	8.0	-11.9	6.0	-21.6	22.4	-21.6	-47.8	41.3	-10.0
	-3.496	0.633	-0.944	0.473	-1.712	1.768	-1.712	-3.785	3.266	-0.789
c_O	224	198	198	233	205	184	211	227	195	218
	(17.7)	(15.7)	(15.7)	(18.4)	(16.2)	(14.6)	(16.7)	(18.0)	(15.4)	(17.2)
c_E	206.7	206.7	206.7	206.7	206.7	206.7	206.7	206.7	206.7	206.7
	(16.4)	(16.4)	(16.4)	(16.4)	(16.4)	(16.4)	(16.4)	(16.4)	(16.4)	(16.4)
	36.0	-17.0	-17.0	55.8	-3.4	-42.8	8.7	42.5	-22.7	23.2
	2.847	-1.348	-1.348	4.414	-0.269	-3.388	0.686	3.363	-1.799	1.835
N	1264	1264	1264	1264	1264	1264	1264	1264	1264	1264
X2	3.7	1.2	1.1	6.1	1.6	3.6	1.0	4.7	2.8	1.3
Gvalue	3.7	1.2	1.1	6.0	1.6	3.7	1.0	4.7	2.8	1.3
Gv/N	0.290	0.092	0.084	0.474	0.126	0.293	0.076	0.374	0.223	0.105
Prob	0.30046	0.76118	0.78729	0.11174	0.65973	0.29576	0.81220	0.19312	0.42129	0.72305

	40	39	38	37	36	35	34	33	32	31
a_O	358	354	378	383	326	344	367	380	374	357
	(28.3)	(28.0)	(29.9)	(30.3)	(25.8)	(27.2)	(29.0)	(30.1)	(29.6)	(28.2)
a_E	358.4	358.4	358.4	358.4	358.4	358.4	358.4	358.4	358.4	358.4
	(28.4)	(28.4)	(28.4)	(28.4)	(28.4)	(28.4)	(28.4)	(28.4)	(28.4)	(28.4)
	-0.9	-8.8	40.2	50.8	-61.9	-28.3	17.3	44.4	31.8	-2.9
	-0.071	-0.699	3.177	4.015	-4.894	-2.239	1.369	3.511	2.514	-0.229
t_O	415	420	385	394	424	411	402	408	401	432
	(32.8)	(33.2)	(30.5)	(31.2)	(33.5)	(32.5)	(31.8)	(32.3)	(31.7)	(34.2)
t_E	406.8	406.8	406.8	406.8	406.8	406.8	406.8	406.8	406.8	406.8
	(32.2)	(32.2)	(32.2)	(32.2)	(32.2)	(32.2)	(32.2)	(32.2)	(32.2)	(32.2)
	16.5	26.8	-42.4	-25.2	35.1	8.4	-9.6	2.4	-11.5	51.9
	1.308	2.119	-3.358	-1.996	2.775	0.665	-0.758	0.187	-0.914	4.106
g_O	288	264	294	279	302	294	266	275	271	275
	(22.8)	(20.9)	(23.3)	(22.1)	(23.9)	(23.3)	(21.0)	(21.8)	(21.4)	(21.8)
g_E	292.0	292.0	292.0	292.0	292.0	292.0	292.0	292.0	292.0	292.0
	(23.1)	(23.1)	(23.1)	(23.1)	(23.1)	(23.1)	(23.1)	(23.1)	(23.1)	(23.1)
	-8.0	-53.3	4.0	-25.5	20.3	4.0	-49.7	-33.0	-40.5	-33.0
	-0.633	-4.215	0.313	-2.015	1.605	0.313	-3.929	-2.614	-3.204	-2.614
c_O	203	226	207	208	212	215	229	201	218	200
	(16.1)	(17.9)	(16.4)	(16.5)	(16.8)	(17.0)	(18.1)	(15.9)	(17.2)	(15.8)
c_E	206.7	206.7	206.7	206.7	206.7	206.7	206.7	206.7	206.7	206.7
	(16.4)	(16.4)	(16.4)	(16.4)	(16.4)	(16.4)	(16.4)	(16.4)	(16.4)	(16.4)
	-7.3	40.3	0.6	2.6	10.7	16.9	46.9	-11.3	23.2	-13.2
	-0.581	3.191	0.046	0.205	0.848	1.338	3.711	-0.891	1.835	-1.044
N	1264	1264	1264	1264	1264	1264	1264	1264	1264	1264
X2	0.3	5.0	2.3	2.7	4.1	1.0	5.0	2.4	2.9	2.8
Gvalue	0.3	5.0	2.3	2.7	4.2	1.0	5.0	2.4	2.9	2.8
Gv/N	0.023	0.396	0.178	0.210	0.334	0.077	0.393	0.194	0.230	0.219
Prob	0.96249	0.17140	0.52161	0.44864	0.23898	0.80738	0.17374	0.48513	0.40626	0.42906

	30	29	28	27	26	25	24	23	22	21
a_O	351	359	345	360	374	377	324	356	340	328
	(27.8)	(28.4)	(27.3)	(28.5)	(29.6)	(29.8)	(25.6)	(28.2)	(26.9)	(25.9)
a_E	358.4	358.4	358.4	358.4	358.4	358.4	358.4	358.4	358.4	358.4
	(28.4)	(28.4)	(28.4)	(28.4)	(28.4)	(28.4)	(28.4)	(28.4)	(28.4)	(28.4)
	-14.7	1.1	-26.4	3.1	31.8	38.0	-65.5	-4.9	-35.9	-58.2
	-1.166	0.088	-2.087	0.246	2.514	3.010	-5.180	-0.386	-2.842	-4.607
t_O	425	370	419	409	358	385	450	422	439	435
	(33.6)	(29.3)	(33.1)	(32.4)	(28.3)	(30.5)	(35.6)	(33.4)	(34.7)	(34.4)
t_E	406.8	406.8	406.8	406.8	406.8	406.8	406.8	406.8	406.8	406.8
	(32.2)	(32.2)	(32.2)	(32.2)	(32.2)	(32.2)	(32.2)	(32.2)	(32.2)	(32.2)
	37.2	-70.2	24.7	4.4	-91.5	-42.4	90.8	30.9	66.8	58.3
	2.940	-5.554	1.956	0.346	-7.241	-3.358	7.183	2.447	5.289	4.610
g_O	287	290	286	297	320	290	284	277	292	298
	(22.7)	(22.9)	(22.6)	(23.5)	(25.3)	(22.9)	(22.5)	(21.9)	(23.1)	(23.6)
g_E	292.0	292.0	292.0	292.0	292.0	292.0	292.0	292.0	292.0	292.0
	(23.1)	(23.1)	(23.1)	(23.1)	(23.1)	(23.1)	(23.1)	(23.1)	(23.1)	(23.1)
	-10.0	-4.0	-11.9	10.0	58.5	-4.0	-15.8	-29.3	-0.1	12.1
	-0.789	-0.320	-0.944	0.793	4.632	-0.320	-1.253	-2.316	-0.004	0.955
c_O	201	245	214	198	212	212	206	209	193	203
	(15.9)	(19.4)	(16.9)	(15.7)	(16.8)	(16.8)	(16.3)	(16.5)	(15.3)	(16.1)
c_E	206.7	206.7	206.7	206.7	206.7	206.7	206.7	206.7	206.7	206.7
	(16.4)	(16.4)	(16.4)	(16.4)	(16.4)	(16.4)	(16.4)	(16.4)	(16.4)	(16.4)
	-11.3	83.3	14.8	-17.0	10.7	10.7	-1.4	4.6	-26.5	-7.3
	-0.891	6.588	1.174	-1.348	0.848	0.848	-0.112	0.365	-2.095	-0.581
N	1264	1264	1264	1264	1264	1264	1264	1264	1264	1264
X2	1.2	10.4	1.3	0.5	9.3	2.3	8.1	1.4	4.4	4.7
Gvalue	1.2	10.1	1.3	0.5	9.5	2.3	8.1	1.4	4.4	4.8
Gv/N	0.095	0.803	0.099	0.038	0.752	0.181	0.639	0.110	0.346	0.377
Prob	0.75232	0.01738	0.74063	0.92441	0.02328	0.51558	0.04445	0.70818	0.22349	0.19025

	20	19	18	17	16	15	14	13	12	11
a_O	354	366	333	369	367	345	396	372	328	361
	(28.0)	(29.0)	(26.3)	(29.2)	(29.0)	(27.3)	(31.3)	(29.4)	(25.9)	(28.6)
a_E	358.4	358.4	358.4	358.4	358.4	358.4	358.4	358.4	358.4	358.4
	(28.4)	(28.4)	(28.4)	(28.4)	(28.4)	(28.4)	(28.4)	(28.4)	(28.4)	(28.4)
	-8.8	15.3	-49.0	21.4	17.3	-26.4	78.9	27.6	-58.2	5.1
	-0.699	1.208	-3.880	1.694	1.369	-2.087	6.243	2.184	-4.607	0.405
t_O	437	434	440	447	479	441	383	396	435	350
	(34.6)	(34.3)	(34.8)	(35.4)	(37.9)	(34.9)	(30.3)	(31.3)	(34.4)	(27.7)

```
t_E       406.8   406.8   406.8   406.8   406.8   406.8   406.8   406.8   406.8   406.8
        ( 32.2) ( 32.2) ( 32.2) ( 32.2) ( 32.2) ( 32.2) ( 32.2) ( 32.2) ( 32.2) ( 32.2)
           62.6    56.1    69.0    84.2   156.5    71.2   -46.2   -21.3    58.3  -105.3
          4.949   4.442   5.459   6.662  12.380   5.630  -3.656  -1.689   4.610  -8.331
g_O         273     292     299     299     251     298     282     315     341     388
        ( 21.6) ( 23.1) ( 23.7) ( 23.7) ( 19.9) ( 23.6) ( 22.3) ( 24.9) ( 27.0) ( 30.7)
g_E       292.0   292.0   292.0   292.0   292.0   292.0   292.0   292.0   292.0   292.0
        ( 23.1) ( 23.1) ( 23.1) ( 23.1) ( 23.1) ( 23.1) ( 23.1) ( 23.1) ( 23.1) ( 23.1)
          -36.8    -0.1    14.1    14.1   -76.0    12.1   -19.7    47.7   105.7   220.5
         -2.910  -0.004   1.116   1.116  -6.013   0.955  -1.559   3.774   8.365  17.445
c_O         200     172     192     149     167     180     203     181     160     165
        ( 15.8) ( 13.6) ( 15.2) ( 11.8) ( 13.2) ( 14.2) ( 16.1) ( 14.3) ( 12.7) ( 13.1)
c_E       206.7   206.7   206.7   206.7   206.7   206.7   206.7   206.7   206.7   206.7
        ( 16.4) ( 16.4) ( 16.4) ( 16.4) ( 16.4) ( 16.4) ( 16.4) ( 16.4) ( 16.4) ( 16.4)
          -13.2   -63.2   -28.3   -97.6   -71.2   -49.8    -7.3   -48.1   -82.0   -74.4
         -1.044  -5.003  -2.242  -7.718  -5.637  -3.940  -0.581  -3.804  -6.484  -5.884

N          1264    1264    1264    1264    1264    1264    1264    1264    1264    1264
X2          3.8     7.8     5.7    20.6    26.4     6.9     5.7     5.8    23.3    47.9
Gvalue      3.7     8.1     5.7    22.2    26.5     7.0     5.6     5.9    23.8    46.0
Gv/N      0.295   0.642   0.453   1.755   2.100   0.557   0.446   0.467   1.884   3.636
Prob    0.29224 0.04360 0.12582 0.00006 0.00001 0.07072 0.13043 0.11675 0.00003 0.00000
        -------------------------------------------------------------------------------
             10       9       8       7       6       5       4       3       2       1
        -------------------------------------------------------------------------------
a_O         398     323     332     316     351     362     361     344     394     310
        ( 31.5) ( 25.6) ( 26.3) ( 25.0) ( 27.8) ( 28.6) ( 28.6) ( 27.2) ( 31.2) ( 24.5)
a_E       358.4   358.4   358.4   358.4   358.4   358.4   358.4   358.4   358.4   358.4
        ( 28.4) ( 28.4) ( 28.4) ( 28.4) ( 28.4) ( 28.4) ( 28.4) ( 28.4) ( 28.4) ( 28.4)
           83.3   -67.3   -50.9   -79.7   -14.7     7.1     5.1   -28.3    74.5   -90.0
          6.592  -5.322  -4.026  -6.302  -1.166   0.565   0.405  -2.239   5.896  -7.123
t_O         311     333     335     424     422     411     442     394     412     557
        ( 24.6) ( 26.3) ( 26.5) ( 33.5) ( 33.4) ( 32.5) ( 35.0) ( 31.2) ( 32.6) ( 44.1)
t_E       406.8   406.8   406.8   406.8   406.8   406.8   406.8   406.8   406.8   406.8
        ( 32.2) ( 32.2) ( 32.2) ( 32.2) ( 32.2) ( 32.2) ( 32.2) ( 32.2) ( 32.2) ( 32.2)
         -167.1  -133.3  -130.1    35.1    30.9     8.4    73.3   -25.2    10.4   350.0
        -13.216 -10.550 -10.296   2.775   2.447   0.665   5.801  -1.996   0.825  27.692
g_O         376     457     445     376     351     309     271     334     226     206
        ( 29.7) ( 36.2) ( 35.2) ( 29.7) ( 27.8) ( 24.4) ( 21.4) ( 26.4) ( 17.9) ( 16.3)
g_E       292.0   292.0   292.0   292.0   292.0   292.0   292.0   292.0   292.0   292.0
        ( 23.1) ( 23.1) ( 23.1) ( 23.1) ( 23.1) ( 23.1) ( 23.1) ( 23.1) ( 23.1) ( 23.1)
          190.1   409.3   374.9   190.1   129.1    34.9   -40.5    89.7  -115.9  -143.8
         15.036  32.383  29.659  15.036  10.216   2.762  -3.204   7.097  -9.166 -11.375
c_O         179     151     152     148     140     182     190     192     232     191
        ( 14.2) ( 11.9) ( 12.0) ( 11.7) ( 11.1) ( 14.4) ( 15.0) ( 15.2) ( 18.4) ( 15.1)
c_E       206.7   206.7   206.7   206.7   206.7   206.7   206.7   206.7   206.7   206.7
        ( 16.4) ( 16.4) ( 16.4) ( 16.4) ( 16.4) ( 16.4) ( 16.4) ( 16.4) ( 16.4) ( 16.4)
          -51.5   -94.8   -93.5   -98.9  -109.1   -46.3   -32.0   -28.3    53.6   -30.2
         -4.076  -7.503  -7.394  -7.824  -8.632  -3.666  -2.534  -2.242   4.237  -2.389

N          1264    1264    1264    1264    1264    1264    1264    1264    1264    1264
X2         54.8   125.1   109.2    46.6    34.2     4.0     5.9     8.1    21.6    88.5
Gvalue     54.8   113.9   100.4    46.6    36.2     4.1     5.9     7.8    22.7    86.0
Gv/N      4.336   9.009   7.943   3.686   2.864   0.326   0.468   0.620   1.793   6.806
Prob    0.00000 0.00000 0.00000 0.00000 0.00000 0.24833 0.11571 0.04958 0.00005 0.00000
        -------------------------------------------------------------------------------

CDS_INITIAL:
        -------------------------------------------------------------------------------
              1       2       3       4       5       6       7       8       9      10
        -------------------------------------------------------------------------------
a_O         793       0       2     354     393     337     415     410     333     415
        ( 62.7) (  0.0) (  0.2) ( 28.0) ( 31.1) ( 26.7) ( 32.8) ( 32.4) ( 26.3) ( 32.8)
a_E       375.1   373.4   327.7   375.1   373.4   327.7   375.1   373.4   327.7   375.1
        ( 29.7) ( 29.5) ( 25.9) ( 29.7) ( 29.5) ( 25.9) ( 29.7) ( 29.5) ( 25.9) ( 29.7)
         1187.4     0.0   -20.4   -41.0    40.3    18.9    83.9    76.7    10.7    83.9
         93.939   0.000  -1.614  -3.241   3.187   1.494   6.640   6.072   0.847   6.640
t_O         186    1264       1     276     378     461     291     369     487     278
        ( 14.7) (100.0) (  0.1) ( 21.8) ( 29.9) ( 36.5) ( 23.0) ( 29.2) ( 38.5) ( 22.0)
t_E       246.5   398.3   453.9   246.5   398.3   453.9   246.5   398.3   453.9   246.5
        ( 19.5) ( 31.5) ( 35.9) ( 19.5) ( 31.5) ( 35.9) ( 19.5) ( 31.5) ( 35.9) ( 19.5)
         -104.8  2919.2   -12.2    62.4   -39.6    14.2    96.6   -56.4    68.5    66.8
         -8.289 230.951  -0.968   4.935  -3.133   1.127   7.640  -4.466   5.418   5.288
g_O         285       0    1260     328     250     294     372     261     276     389
        ( 22.5) (  0.0) ( 99.7) ( 25.9) ( 19.8) ( 23.3) ( 29.4) ( 20.6) ( 21.8) ( 30.8)
g_E       438.9   227.4   296.8   438.9   227.4   296.8   438.9   227.4   296.8   438.9
        ( 34.7) ( 18.0) ( 23.5) ( 34.7) ( 18.0) ( 23.5) ( 34.7) ( 18.0) ( 23.5) ( 34.7)
         -246.1     0.0  3643.3  -191.0    47.5    -5.6  -123.0    72.0   -40.1   -93.8
        -19.467   0.000 288.235 -15.111   3.755  -0.444  -9.729   5.699  -3.176  -7.423
c_O           0       0       1     306     243     172     186     224     168     182
        (  0.0) (  0.0) (  0.1) ( 24.2) ( 19.2) ( 13.6) ( 14.7) ( 17.7) ( 13.3) ( 14.4)
c_E       203.5   264.9   185.6   203.5   264.9   185.6   203.5   264.9   185.6   203.5
        ( 16.1) ( 21.0) ( 14.7) ( 16.1) ( 21.0) ( 14.7) ( 16.1) ( 21.0) ( 14.7) ( 16.1)
            0.0     0.0   -10.4   249.5   -42.0   -26.1   -33.5   -75.2   -33.4   -40.7
          0.000   0.000  -0.826  19.741  -3.324  -2.065  -2.652  -5.950  -2.643  -3.221

N          1264    1264    1264    1264    1264    1264    1264    1264    1264    1264
X2        738.0  2747.0  4084.8    84.3     6.1     1.4    24.0    17.1     5.6    16.2
Gvalue    836.5  2919.2  3600.2    79.9     6.1     1.4    24.0    17.1     5.7    16.2
Gv/N     66.182 230.951 284.826   6.323   0.485   0.112   1.898   1.355   0.447   1.283
Prob    0.00000 1.00000 1.00000 0.00000 0.10572 0.70209 0.00003 0.00067 0.12989 0.00102
        -------------------------------------------------------------------------------
             11      12      13      14      15      16      17      18      19      20
        -------------------------------------------------------------------------------
a_O         360     364     376     359     335     412     369     365     379     352
        ( 28.5) ( 28.8) ( 29.7) ( 28.4) ( 26.5) ( 32.6) ( 29.2) ( 28.9) ( 30.0) ( 27.8)
a_E       373.4   327.7   375.1   373.4   327.7   375.1   373.4   327.7   375.1   373.4
        ( 29.5) ( 25.9) ( 29.7) ( 29.5) ( 25.9) ( 29.7) ( 29.5) ( 25.9) ( 29.7) ( 29.5)
          -26.2    76.5     1.8   -28.2    14.8    77.3    -8.7    78.7     7.9   -41.5
         -2.077   6.053   0.145  -2.229   1.170   6.119  -0.687   6.228   0.623  -3.282
t_O         429     458     261     431     462     277     448     490     293     465
        ( 33.9) ( 36.2) ( 20.6) ( 34.1) ( 36.6) ( 21.9) ( 35.4) ( 38.8) ( 23.2) ( 36.8)
t_E       398.3   453.9   246.5   398.3   453.9   246.5   398.3   453.9   246.5   398.3
        ( 31.5) ( 35.9) ( 19.5) ( 31.5) ( 35.9) ( 19.5) ( 31.5) ( 35.9) ( 19.5) ( 31.5)
           63.6     8.2    29.8    67.9    16.3    64.6   105.3    74.9   101.2   143.9
          5.035   0.647   2.359   5.376   1.288   5.111   8.330   5.928   8.010  11.386
g_O         248     268     423     217     286     378     232     257     392     232
        ( 19.6) ( 21.2) ( 33.5) ( 17.2) ( 22.6) ( 29.9) ( 18.4) ( 20.3) ( 31.0) ( 18.4)
g_E       227.4   296.8   438.9   227.4   296.8   438.9   227.4   296.8   438.9   227.4
        ( 18.0) ( 23.5) ( 34.7) ( 18.0) ( 23.5) ( 34.7) ( 18.0) ( 23.5) ( 34.7) ( 18.0)
           43.1   -54.7   -31.1   -20.2   -21.2  -112.9     9.4   -74.0   -88.5     9.4
          3.410  -4.331  -2.464  -1.601  -1.680  -8.929   0.742  -5.857  -7.004   0.742
```

c_O	227	174	204	257	181	197	215	152	200	215
	(18.0)	(13.8)	(16.1)	(20.3)	(14.3)	(15.6)	(17.0)	(12.0)	(15.8)	(17.0)
c_E	264.9	185.6	203.5	264.9	185.6	203.5	264.9	185.6	203.5	264.9
	(21.0)	(14.7)	(16.1)	(21.0)	(14.7)	(16.1)	(21.0)	(14.7)	(16.1)	(21.0)
	-70.2	-22.4	0.9	-15.6	-9.0	-12.9	-89.8	-60.7	-7.0	-89.8
	-5.552	-1.771	0.073	-1.238	-0.713	-1.018	-7.106	-4.798	-0.555	-7.106
N	1264	1264	1264	1264	1264	1264	1264	1264	1264	1264
X2	10.1	7.6	1.4	3.9	0.8	16.1	15.8	18.5	13.9	21.9
Gvalue	10.3	7.5	1.4	3.9	0.8	16.2	16.2	19.0	13.6	22.0
Gv/N	0.817	0.597	0.112	0.308	0.065	1.283	1.279	1.500	1.073	1.740
Prob	0.01602	0.05633	0.70108	0.27372	0.84546	0.00102	0.00105	0.00028	0.00357	0.00007

	21	22	23	24	25	26	27	28	29	30
a_O	333	375	361	347	369	315	333	364	322	332
	(26.3)	(29.7)	(28.6)	(27.5)	(29.2)	(24.9)	(26.3)	(28.8)	(25.5)	(26.3)
a_E	327.7	375.1	373.4	327.7	375.1	373.4	327.7	375.1	373.4	327.7
	(25.9)	(29.7)	(29.5)	(25.9)	(29.7)	(29.5)	(25.9)	(29.7)	(29.5)	(25.9)
	10.7	-0.2	-24.3	39.7	-12.1	-107.1	10.7	-21.8	-95.3	8.7
	0.847	-0.014	-1.924	3.144	-0.955	-8.472	0.847	-1.728	-7.541	0.687
t_O	459	295	424	443	289	448	429	259	463	448
	(36.3)	(23.3)	(33.5)	(35.0)	(22.9)	(35.4)	(33.9)	(20.5)	(36.6)	(35.4)
t_E	453.9	246.5	398.3	453.9	246.5	398.3	453.9	246.5	398.3	453.9
	(35.9)	(19.5)	(31.5)	(35.9)	(19.5)	(31.5)	(35.9)	(19.5)	(31.5)	(35.9)
	10.2	105.9	53.0	-21.6	91.9	105.3	-48.5	25.6	139.3	-11.8
	0.806	8.382	4.190	-1.709	7.272	8.330	-3.835	2.025	11.021	-0.932
g_O	275	398	228	272	410	248	302	428	223	294
	(21.8)	(31.5)	(18.0)	(21.5)	(32.4)	(19.6)	(23.9)	(33.9)	(17.6)	(23.3)
g_E	296.8	438.9	227.4	296.8	438.9	227.4	296.8	438.9	227.4	296.8
	(23.5)	(34.7)	(18.0)	(23.5)	(34.7)	(18.0)	(23.5)	(34.7)	(18.0)	(23.5)
	-42.0	-77.8	1.3	-47.5	-55.8	43.1	10.5	-21.5	-8.6	-5.6
	-3.322	-6.155	0.102	-3.758	-4.413	3.410	0.827	-1.697	-0.683	-0.444
c_O	197	196	251	202	196	253	200	213	256	190
	(15.6)	(15.5)	(19.9)	(16.0)	(15.5)	(20.0)	(15.8)	(16.9)	(20.3)	(15.0)
c_E	185.6	203.5	264.9	185.6	203.5	264.9	185.6	203.5	264.9	185.6
	(14.7)	(16.1)	(21.0)	(14.7)	(16.1)	(21.0)	(14.7)	(16.1)	(21.0)	(14.7)
	23.6	-14.8	-27.1	34.3	-14.8	-23.3	30.0	19.4	-17.6	9.0
	1.864	-1.171	-2.147	2.713	-1.171	-1.847	2.371	1.531	-1.391	0.711
N	1264	1264	1264	1264	1264	1264	1264	1264	1264	1264
X2	2.5	13.6	2.8	4.9	9.6	17.7	2.7	1.7	18.0	0.3
Gvalue	2.5	13.2	2.8	4.9	9.3	18.0	2.7	1.7	17.8	0.3
Gv/N	0.196	1.043	0.220	0.390	0.733	1.421	0.211	0.131	1.406	0.021
Prob	0.47936	0.00426	0.42632	0.17682	0.02604	0.00045	0.44602	0.64647	0.00049	0.96620

	31	32	33	34	35	36	37	38	39	40
a_O	348	316	325	357	324	326	379	312	329	383
	(27.5)	(25.0)	(25.7)	(28.2)	(25.6)	(25.8)	(30.0)	(24.7)	(26.0)	(30.3)
a_E	375.1	373.4	327.7	375.1	373.4	327.7	375.1	373.4	327.7	375.1
	(29.7)	(29.5)	(25.9)	(29.7)	(29.5)	(25.9)	(29.7)	(29.5)	(25.9)	(29.7)
	-52.2	-105.4	-5.4	-35.3	-91.9	-3.4	7.9	-112.0	2.6	16.0
	-4.127	-8.341	-0.424	-2.792	-7.270	-0.266	0.623	-8.864	0.208	1.265
t_O	293	433	459	281	423	461	262	447	491	275
	(23.2)	(34.3)	(36.3)	(22.2)	(33.5)	(36.5)	(20.7)	(35.4)	(38.8)	(21.8)
t_E	246.5	398.3	453.9	246.5	398.3	453.9	246.5	398.3	453.9	246.5
	(19.5)	(31.5)	(35.9)	(19.5)	(31.5)	(35.9)	(19.5)	(31.5)	(35.9)	(19.5)
	101.2	72.3	10.2	73.6	50.8	14.2	31.9	103.1	77.1	60.2
	8.010	5.718	0.806	5.822	4.022	1.127	2.526	8.153	6.098	4.759
g_O	436	253	281	420	244	300	435	241	276	387
	(34.5)	(20.0)	(22.2)	(33.2)	(19.3)	(23.7)	(34.4)	(19.1)	(21.8)	(30.6)
g_E	438.9	227.4	296.8	438.9	227.4	296.8	438.9	227.4	296.8	438.9
	(34.7)	(18.0)	(23.5)	(34.7)	(18.0)	(23.5)	(34.7)	(18.0)	(23.5)	(34.7)
	-5.7	54.1	-30.8	-36.9	34.5	6.4	-7.7	28.1	-40.1	-97.3
	-0.451	4.278	-2.435	-2.919	2.727	0.506	-0.608	2.222	-3.176	-7.701
c_O	187	262	199	206	273	177	188	264	168	219
	(14.8)	(20.7)	(15.7)	(16.3)	(21.6)	(14.0)	(14.9)	(20.9)	(13.3)	(17.3)
c_E	203.5	264.9	185.6	203.5	264.9	185.6	203.5	264.9	185.6	203.5
	(16.1)	(21.0)	(14.7)	(16.1)	(21.0)	(14.7)	(16.1)	(21.0)	(14.7)	(16.1)
	-31.7	-5.9	27.8	4.9	16.4	-16.7	-29.9	-1.9	-33.4	32.1
	-2.508	-0.463	2.201	0.391	1.294	-1.323	-2.363	-0.149	-2.643	2.537
N	1264	1264	1264	1264	1264	1264	1264	1264	1264	1264
X2	12.1	14.8	1.9	6.5	9.5	0.5	2.2	16.9	6.2	10.8
Gvalue	11.7	15.1	1.9	6.4	9.8	0.6	2.2	17.2	6.2	10.9
Gv/N	0.923	1.191	0.149	0.503	0.773	0.044	0.178	1.362	0.488	0.860
Prob	0.00861	0.00177	0.59612	0.09562	0.02068	0.90700	0.52289	0.00064	0.10382	0.01245

	41	42	43	44	45	46	47	48	49	50
a_O	334	308	386	335	329	321	321	300	335	315
	(26.4)	(24.4)	(30.5)	(26.5)	(26.0)	(25.4)	(25.4)	(23.7)	(26.5)	(24.9)
a_E	373.4	327.7	375.1	373.4	327.7	375.1	373.4	327.7	375.1	373.4
	(29.5)	(25.9)	(29.7)	(29.5)	(25.9)	(29.7)	(29.5)	(25.9)	(29.7)	(29.5)
	-74.4	-38.2	22.1	-72.6	2.6	-100.0	-97.0	-53.0	-75.7	-107.1
	-5.888	-3.020	1.752	-5.747	0.208	-7.909	-7.676	-4.190	-5.991	-8.472
t_O	434	437	254	398	480	293	399	468	306	436
	(34.3)	(34.6)	(20.1)	(31.5)	(38.0)	(23.2)	(31.6)	(37.0)	(24.2)	(34.5)
t_E	398.3	453.9	246.5	398.3	453.9	246.5	398.3	453.9	246.5	398.3
	(31.5)	(35.9)	(19.5)	(31.5)	(35.9)	(19.5)	(31.5)	(35.9)	(19.5)	(31.5)
	74.4	-33.2	15.2	-0.7	53.6	101.2	1.3	28.6	132.3	78.8
	5.889	-2.629	1.203	-0.052	4.241	8.010	0.106	2.260	10.467	6.234
g_O	257	300	452	255	293	445	248	302	428	244
	(20.3)	(23.7)	(35.8)	(20.2)	(23.2)	(35.2)	(19.6)	(23.9)	(33.9)	(19.3)
g_E	227.4	296.8	438.9	227.4	296.8	438.9	227.4	296.8	438.9	227.4
	(18.0)	(23.5)	(34.7)	(18.0)	(23.5)	(34.7)	(18.0)	(23.5)	(34.7)	(18.0)
	63.0	6.4	26.7	58.5	-7.6	12.4	43.1	10.5	-21.5	34.5
	4.983	0.506	2.110	4.629	-0.600	0.978	3.410	0.827	-1.697	2.727
c_O	239	219	172	276	162	205	296	194	195	269
	(18.9)	(17.3)	(13.6)	(21.8)	(12.8)	(16.2)	(23.4)	(15.3)	(15.4)	(21.3)
c_E	264.9	185.6	203.5	264.9	185.6	203.5	264.9	185.6	203.5	264.9
	(21.0)	(14.7)	(16.1)	(21.0)	(14.7)	(16.1)	(21.0)	(14.7)	(16.1)	(21.0)
	-49.3	72.6	-57.9	22.6	-44.0	2.9	65.6	17.3	-16.7	8.2
	-3.897	5.741	-4.582	1.785	-3.481	0.232	5.191	1.365	-1.323	0.647
N	1264	1264	1264	1264	1264	1264	1264	1264	1264	1264
X2	13.8	7.9	5.8	7.8	4.5	16.7	12.9	3.2	19.3	14.0
Gvalue	13.7	7.6	6.1	7.8	4.7	16.6	13.0	3.3	18.4	14.3
Gv/N	1.087	0.599	0.482	0.615	0.368	1.310	1.032	0.262	1.456	1.135
Prob	0.00328	0.05569	0.10727	0.05096	0.19922	0.00087	0.00455	0.34655	0.00036	0.00247

	51	52	53	54	55	56	57	58	59	60
a_O	319	357	346	351	328	330	301	340	324	292
	(25.2)	(28.2)	(27.4)	(27.8)	(25.9)	(26.1)	(23.8)	(26.9)	(25.6)	(23.1)
a_E	327.7	375.1	373.4	327.7	375.1	373.4	327.7	375.1	373.4	327.7
	(25.9)	(29.7)	(29.5)	(25.9)	(29.7)	(29.5)	(25.9)	(29.7)	(29.5)	(25.9)
	-17.1	-35.3	-52.7	48.2	-88.0	-81.5	-51.1	-66.8	-91.9	-67.3
	-1.356	-2.792	-4.167	3.817	-6.962	-6.447	-4.046	-5.283	-7.270	-5.328
t_O	477	231	452	442	235	437	458	291	451	484
	(37.7)	(18.3)	(35.8)	(35.0)	(18.6)	(34.6)	(36.2)	(23.0)	(35.7)	(38.3)
t_E	453.9	246.5	398.3	453.9	246.5	398.3	453.9	246.5	398.3	453.9
	(35.9)	(19.5)	(31.5)	(35.9)	(19.5)	(31.5)	(35.9)	(19.5)	(31.5)	(35.9)
	47.3	-30.0	114.3	-23.5	-22.5	81.0	8.2	96.6	112.0	62.1
	3.741	-2.375	9.040	-1.863	-1.778	6.406	0.647	7.640	8.862	4.912
g_O	281	468	225	303	465	246	291	452	231	299
	(22.2)	(37.0)	(17.8)	(24.0)	(36.8)	(19.5)	(23.0)	(35.8)	(18.3)	(23.7)
g_E	296.8	438.9	227.4	296.8	438.9	227.4	296.8	438.9	227.4	296.8
	(23.5)	(34.7)	(18.0)	(23.5)	(34.7)	(18.0)	(23.5)	(34.7)	(18.0)	(23.5)
	-30.8	60.2	-4.7	12.5	53.8	38.8	-11.5	26.7	7.3	4.4
	-2.435	4.760	-0.371	0.988	4.257	3.067	-0.911	2.110	0.581	0.347
c_O	187	208	241	168	236	251	214	181	258	189
	(14.8)	(16.5)	(19.1)	(13.3)	(18.7)	(19.9)	(16.9)	(14.3)	(20.4)	(15.0)
c_E	185.6	203.5	264.9	185.6	203.5	264.9	185.6	203.5	264.9	185.6
	(14.7)	(16.1)	(21.0)	(14.7)	(16.1)	(21.0)	(14.7)	(16.1)	(21.0)	(14.7)
	2.9	9.0	-45.7	-33.4	69.8	-27.1	61.0	-42.5	-13.7	6.9
	0.228	0.713	-3.612	-2.643	5.525	-2.147	4.828	-3.361	-1.084	0.549
N	1264	1264	1264	1264	1264	1264	1264	1264	1264	1264
X2	2.3	3.9	11.4	3.8	13.2	11.1	6.7	14.2	13.7	6.0
Gvalue	2.3	3.9	11.2	3.8	13.2	11.1	6.5	14.0	13.8	6.1
Gv/N	0.178	0.306	0.889	0.299	1.042	0.880	0.517	1.104	1.088	0.480
Prob	0.52124	0.27543	0.01050	0.28600	0.00429	0.01111	0.08820	0.00296	0.00326	0.10851

	61	62	63	64	65	66	67	68	69	70
a_O	350	316	303	352	299	352	345	378	329	369
	(27.7)	(25.0)	(24.0)	(27.8)	(23.7)	(27.8)	(27.3)	(29.9)	(26.0)	(29.2)
a_E	375.1	373.4	327.7	375.1	373.4	327.7	375.1	373.4	327.7	375.1
	(29.7)	(29.5)	(25.9)	(29.7)	(29.5)	(25.9)	(29.7)	(29.5)	(25.9)	(29.7)
	-48.5	-105.4	-47.5	-44.7	-132.8	50.4	-57.7	9.3	2.6	-12.1
	-3.834	-8.341	-3.755	-3.538	-10.508	3.986	-4.564	0.738	0.208	-0.955
t_O	267	436	463	264	438	423	277	409	470	245
	(21.1)	(34.5)	(36.6)	(20.9)	(34.7)	(33.5)	(21.9)	(32.4)	(37.2)	(19.4)
t_E	246.5	398.3	453.9	246.5	398.3	453.9	246.5	398.3	453.9	246.5
	(19.5)	(31.5)	(35.9)	(19.5)	(31.5)	(35.9)	(19.5)	(31.5)	(35.9)	(19.5)
	42.6	78.8	18.3	36.2	83.2	-59.7	64.6	21.6	32.7	-3.0
	3.373	6.234	1.449	2.863	6.579	-4.724	5.111	1.711	2.587	-0.238
g_O	460	236	336	433	254	315	445	212	297	449
	(36.4)	(18.7)	(26.6)	(34.3)	(20.1)	(24.9)	(35.2)	(16.8)	(23.5)	(35.5)
g_E	438.9	227.4	296.8	438.9	227.4	296.8	438.9	227.4	296.8	438.9
	(34.7)	(18.0)	(23.5)	(34.7)	(18.0)	(23.5)	(34.7)	(18.0)	(23.5)	(34.7)
	43.3	17.6	83.3	-11.6	56.3	37.5	12.4	-29.7	0.4	20.5
	3.424	1.393	6.592	-0.921	4.453	2.963	0.978	-2.346	0.029	1.622
c_O	187	276	162	215	273	174	197	265	168	201
	(14.8)	(21.8)	(12.8)	(17.0)	(21.6)	(13.8)	(15.6)	(21.0)	(13.3)	(15.9)
c_E	203.5	264.9	185.6	203.5	264.9	185.6	203.5	264.9	185.6	203.5
	(16.1)	(21.0)	(14.7)	(16.1)	(21.0)	(14.7)	(16.1)	(21.0)	(14.7)	(16.1)
	-31.7	22.6	-44.0	23.6	16.4	-22.4	-12.9	0.1	-33.4	-5.1
	-2.508	1.785	-3.481	1.863	1.294	-1.771	-1.018	0.009	-2.643	-0.400
N	1264	1264	1264	1264	1264	1264	1264	1264	1264	1264
X2	5.7	13.2	10.2	3.4	22.1	5.7	6.5	1.4	2.2	0.4
Gvalue	5.8	13.5	10.2	3.4	23.0	5.7	6.4	1.4	2.3	0.4
Gv/N	0.456	1.071	0.805	0.267	1.818	0.455	0.507	0.111	0.181	0.029
Prob	0.12397	0.00361	0.01714	0.33728	0.00004	0.12466	0.09357	0.70504	0.51546	0.94580

	71	72	73	74	75	76	77	78	79	80
a_O	316	316	347	322	300	391	357	330	350	349
	(25.0)	(25.0)	(27.5)	(25.5)	(23.7)	(30.9)	(28.2)	(26.1)	(27.7)	(27.6)
a_E	373.4	327.7	375.1	373.4	327.7	375.1	373.4	327.7	375.1	373.4
	(29.5)	(25.9)	(29.7)	(29.5)	(25.9)	(29.7)	(29.5)	(25.9)	(29.7)	(29.5)
	-105.4	-23.0	-54.0	-95.3	-53.0	32.5	-32.0	4.6	-48.5	-47.1
	-8.341	-1.816	-4.273	-7.541	-4.190	2.571	-2.532	0.367	-3.834	-3.727
t_O	422	455	273	428	480	264	415	466	276	406
	(33.4)	(36.0)	(21.6)	(33.9)	(38.0)	(20.9)	(32.8)	(36.9)	(21.8)	(32.1)
t_E	398.3	453.9	246.5	398.3	453.9	246.5	398.3	453.9	246.5	398.3
	(31.5)	(35.9)	(19.5)	(31.5)	(35.9)	(19.5)	(31.5)	(35.9)	(19.5)	(31.5)
	48.7	2.1	55.7	61.5	53.6	36.2	34.0	24.5	62.4	15.5
	3.854	0.169	4.409	4.865	4.241	2.863	2.692	1.935	4.935	1.225
g_O	260	286	437	262	284	416	243	286	448	237
	(20.6)	(22.6)	(34.6)	(20.7)	(22.5)	(32.9)	(19.2)	(22.6)	(35.4)	(18.8)
g_E	227.4	296.8	438.9	227.4	296.8	438.9	227.4	296.8	438.9	227.4
	(18.0)	(23.5)	(34.7)	(18.0)	(23.5)	(34.7)	(18.0)	(23.5)	(34.7)	(18.0)
	69.8	-21.2	-3.7	74.3	-25.1	-44.5	32.3	-21.2	18.5	19.7
	5.519	-1.680	-0.294	5.879	-1.984	-3.522	2.558	-1.680	1.461	1.558
c_O	266	207	207	252	200	193	249	182	190	272
	(21.0)	(16.4)	(16.4)	(19.9)	(15.8)	(15.3)	(19.7)	(14.4)	(15.0)	(21.5)
c_E	264.9	185.6	203.5	264.9	185.6	203.5	264.9	185.6	203.5	264.9
	(21.0)	(14.7)	(16.1)	(21.0)	(14.7)	(16.1)	(21.0)	(14.7)	(16.1)	(21.0)
	2.1	45.3	7.0	-25.2	30.0	-20.5	-30.9	-7.1	-26.2	14.3
	0.167	3.581	0.552	-1.997	2.371	-1.624	-2.445	-0.558	-2.070	1.131
N	1264	1264	1264	1264	1264	1264	1264	1264	1264	1264
X2	14.9	3.3	5.0	15.2	5.5	3.7	3.5	0.8	6.3	2.3
Gvalue	15.2	3.2	5.0	15.2	5.5	3.6	3.4	0.8	6.2	2.4
Gv/N	1.200	0.254	0.393	1.206	0.438	0.288	0.273	0.063	0.492	0.187
Prob	0.00168	0.36035	0.17374	0.00162	0.13659	0.30230	0.32758	0.84892	0.10130	0.50072

	81	82	83	84	85	86	87	88	89	90
a_O	299	357	345	297	347	327	325	349	339	319
	(23.7)	(28.2)	(27.3)	(23.5)	(27.5)	(25.9)	(25.7)	(27.6)	(26.8)	(25.2)
a_E	327.7	375.1	373.4	327.7	375.1	373.4	327.7	375.1	373.4	327.7
	(25.9)	(29.7)	(29.5)	(25.9)	(29.7)	(29.5)	(25.9)	(29.7)	(29.5)	(25.9)
	-54.8	-35.3	-54.5	-58.4	-54.0	-86.7	-5.4	-50.3	-65.5	-17.1
	-4.334	-2.792	-4.313	-4.621	-4.273	-6.861	-0.424	-3.981	-5.179	-1.356
t_O	483	259	383	460	263	445	429	302	403	452
	(38.2)	(20.5)	(30.3)	(36.4)	(20.8)	(35.2)	(33.9)	(23.9)	(31.9)	(35.8)
t_E	453.9	246.5	398.3	453.9	246.5	398.3	453.9	246.5	398.3	453.9
	(35.9)	(19.5)	(31.5)	(35.9)	(19.5)	(31.5)	(35.9)	(19.5)	(31.5)	(35.9)
	60.0	25.6	-30.1	12.2	34.1	98.6	-48.5	122.6	9.4	-3.9

```
            4.744   2.025  -2.378   0.967   2.694   7.801  -3.835   9.701   0.743  -0.305
g_O           296     447     269     309     427     262     304     415     260     287
          ( 23.4) ( 35.4) ( 21.3) ( 24.4) ( 33.8) ( 20.7) ( 24.1) ( 32.8) ( 20.6) ( 22.7)
g_E         296.8   438.9   227.4   296.8   438.9   227.4   296.8   438.9   227.4   296.8
          ( 23.5) ( 34.7) ( 18.0) ( 23.5) ( 34.7) ( 18.0) ( 23.5) ( 34.7) ( 18.0) ( 23.5)
             -1.6    16.4    90.5    24.9   -23.4    74.3    14.5   -46.4    69.8   -19.3
           -0.129   1.300   7.159   1.967  -1.851   5.879   1.150  -3.671   5.519  -1.527
c_O           186     201     267     198     227     230     206     198     262     206
          ( 14.7) ( 15.9) ( 21.1) ( 15.7) ( 18.0) ( 18.2) ( 16.3) ( 15.7) ( 20.7) ( 16.3)
c_E         185.6   203.5   264.9   185.6   203.5   264.9   185.6   203.5   264.9   185.6
          ( 14.7) ( 16.1) ( 21.0) ( 14.7) ( 16.1) ( 21.0) ( 14.7) ( 16.1) ( 21.0) ( 14.7)
              0.9    -5.1     4.1    25.7    49.5   -65.1    43.0   -10.9    -5.9    43.0
            0.069  -0.400   0.326   2.033   3.918  -5.147   3.406  -0.865  -0.463   3.406

N            1264    1264    1264    1264    1264    1264    1264    1264    1264    1264
X2            4.4     1.7    10.4     4.3     6.2    21.1     3.8    15.8     7.9     2.8
Gvalue        4.4     1.7    10.0     4.4     6.2    21.1     3.8    15.0     7.8     2.7
Gv/N        0.349   0.134   0.793   0.345   0.488   1.672   0.298   1.185   0.619   0.217
Prob      0.22005 0.63902 0.01835 0.22533 0.10382 0.00010 0.28829 0.00184 0.04967 0.43318
          -------------------------------------------------------------------------------
               91      92      93      94      95      96      97      98      99     100
          -------------------------------------------------------------------------------
a_O           376     389     326     396     358     324     344     356     315     315
          ( 29.7) ( 30.8) ( 25.8) ( 31.8) ( 28.7) ( 26.0) ( 27.9) ( 28.9) ( 25.5) ( 25.9)
a_E         375.1   373.4   327.7   370.0   368.3   323.3   365.9   364.2   319.7   360.5
          ( 29.7) ( 29.5) ( 25.9) ( 29.7) ( 29.5) ( 25.9) ( 29.7) ( 29.5) ( 25.9) ( 29.7)
              1.8    31.9    -3.4    53.7   -20.4     1.4   -42.4   -16.2    -9.2   -85.1
            0.145   2.525  -0.266   4.306  -1.636   0.115  -3.442  -1.316  -0.749  -7.002
t_O           291     398     474     269     403     442     259     398     443     268
          ( 23.0) ( 31.5) ( 37.5) ( 21.6) ( 32.3) ( 35.4) ( 21.0) ( 32.3) ( 35.9) ( 22.1)
t_E         246.5   398.3   453.9   243.2   393.0   447.8   240.5   388.6   442.8   237.0
          ( 19.5) ( 31.5) ( 35.9) ( 19.5) ( 31.5) ( 35.9) ( 19.5) ( 31.5) ( 35.9) ( 19.5)
             96.6    -0.7    41.0    54.3    20.3   -11.6    38.5    19.1     0.4    66.0
            7.640  -0.052   3.245   4.351   1.628  -0.928   3.119   1.549   0.033   5.431
g_O           429     230     265     400     265     278     433     225     295     419
          ( 33.9) ( 18.2) ( 21.0) ( 32.1) ( 21.3) ( 22.3) ( 35.1) ( 18.2) ( 23.9) ( 34.5)
g_E         438.9   227.4   296.8   433.0   224.3   292.8   428.1   221.8   289.5   421.8
          ( 34.7) ( 18.0) ( 23.5) ( 34.7) ( 18.0) ( 23.5) ( 34.7) ( 18.0) ( 23.5) ( 34.7)
            -19.5     5.3   -60.1   -63.3    88.4   -28.9     9.9     6.5    11.0    -5.7
           -1.543   0.420  -4.754  -5.080   7.087  -2.317   0.800   0.526   0.894  -0.467
c_O           168     247     199     182     221     203     197     254     180     213
          ( 13.3) ( 19.5) ( 15.7) ( 14.6) ( 17.7) ( 16.3) ( 16.0) ( 20.6) ( 14.6) ( 17.5)
c_E         203.5   264.9   185.6   200.8   261.4   183.1   198.5   258.4   181.0   195.7
          ( 16.1) ( 21.0) ( 14.7) ( 16.1) ( 21.0) ( 14.7) ( 16.1) ( 21.0) ( 14.7) ( 16.1)
            -64.5   -34.6    27.8   -35.8   -74.2    42.0    -3.1    -8.8    -2.0    36.2
           -5.101  -2.741   2.201  -2.870  -5.948   3.365  -0.250  -0.715  -0.164   2.979

N            1264    1264    1264    1247    1247    1247    1233    1233    1233    1215
X2           14.5     1.9     5.3     8.8    14.2     3.0     2.8     0.5     0.2    11.4
Gvalue       14.4     1.9     5.4     8.8    14.1     2.9     2.8     0.5     0.2    11.4
Gv/N        1.140   0.152   0.425   0.707   1.131   0.236   0.227   0.044   0.014   0.941
Prob      0.00239 0.58910 0.14642 0.03179 0.00276 0.40141 0.42391 0.91062 0.98135 0.00963
          -------------------------------------------------------------------------------
              101     102     103     104     105     106     107     108     109     110
          -------------------------------------------------------------------------------
a_O           357     321     310     347     311     326     367     313     318     335
          ( 29.4) ( 26.4) ( 25.8) ( 28.8) ( 25.9) ( 27.4) ( 30.8) ( 26.3) ( 27.1) ( 28.5)
a_E         358.9   315.0   357.0   355.3   311.9   353.4   351.8   308.8   348.4   346.8
          ( 29.5) ( 25.9) ( 29.7) ( 29.5) ( 25.9) ( 29.7) ( 29.5) ( 25.9) ( 29.7) ( 29.5)
             -3.8    12.1   -87.5   -16.5    -1.7   -52.7    31.0     8.5   -58.0   -23.2
           -0.311   1.000  -7.273  -1.371  -0.145  -4.422   2.606   0.716  -4.943  -1.973
t_O           415     439     258     384     450     257     362     418     247     341
          ( 34.2) ( 36.1) ( 21.4) ( 31.9) ( 37.4) ( 21.6) ( 30.4) ( 35.1) ( 21.0) ( 29.0)
t_E         382.9   436.3   234.6   379.1   432.0   232.3   375.3   427.7   229.0   370.0
          ( 31.5) ( 35.9) ( 19.5) ( 31.5) ( 35.9) ( 19.5) ( 31.5) ( 35.9) ( 19.5) ( 31.5)
             66.8     5.3    49.0     9.8    36.7    52.0   -26.2   -19.2    37.5   -55.6
            5.501   0.440   4.076   0.819   3.050   4.366  -2.198  -1.613   3.192  -4.737
g_O           235     291     442     197     285     409     238     254     416     240
          ( 19.3) ( 24.0) ( 36.7) ( 16.4) ( 23.7) ( 34.3) ( 20.0) ( 21.3) ( 35.4) ( 20.4)
g_E         218.5   285.3   417.7   216.4   282.5   413.5   214.2   279.7   407.6   211.2
          ( 18.0) ( 23.5) ( 34.7) ( 18.0) ( 23.5) ( 34.7) ( 18.0) ( 23.5) ( 34.7) ( 18.0)
             34.1    11.5    50.0   -37.0     5.0    -9.0    50.1   -48.9    16.9    61.4
            2.808   0.946   4.158  -3.074   0.419  -0.754   4.206  -4.107   1.443   5.233
c_O           208     164     193     275     157     199     224     206     193     258
          ( 17.1) ( 13.5) ( 16.0) ( 22.9) ( 13.1) ( 16.7) ( 18.8) ( 17.3) ( 16.4) ( 22.0)
c_E         254.7   178.4   193.7   252.2   176.6   191.8   249.6   174.8   189.0   246.1
          ( 21.0) ( 14.7) ( 16.1) ( 21.0) ( 14.7) ( 16.1) ( 21.0) ( 14.7) ( 16.1) ( 21.0)
            -84.2   -27.5    -1.4    47.7   -37.0    14.7   -48.6    67.6     8.0    24.4
           -6.932  -2.267  -0.119   3.964  -3.072   1.234  -4.077   5.672   0.680   2.079

N            1215    1215    1203    1203    1203    1191    1191    1191    1174    1174
X2           12.5     1.4     9.9     4.1     2.9     5.1     6.4     8.2     4.3     7.2
Gvalue       13.0     1.4    10.1     4.1     3.0     5.0     6.4     8.0     4.4     7.1
Gv/N        1.068   0.118   0.842   0.338   0.251   0.424   0.537   0.668   0.372   0.602
Prob      0.00470 0.69784 0.01754 0.25497 0.38788 0.16845 0.09365 0.04682 0.22430 0.06965
          -------------------------------------------------------------------------------
              111     112     113     114     115     116     117     118     119     120
          -------------------------------------------------------------------------------
a_O           285     327     350     283     319     300     290     318     316     304
          ( 24.3) ( 28.2) ( 30.2) ( 24.4) ( 27.9) ( 26.3) ( 25.4) ( 28.1) ( 27.9) ( 26.9)
a_E         304.4   343.6   342.1   300.2   338.9   337.3   296.1   335.6   334.1   293.2
          ( 25.9) ( 29.7) ( 29.5) ( 25.9) ( 29.7) ( 29.5) ( 25.9) ( 29.7) ( 29.5) ( 25.9)
            -37.5   -32.4    16.1   -33.4   -38.6   -70.4   -12.0   -34.3   -35.2    22.0
           -3.190  -2.802   1.388  -2.885  -3.378  -6.162  -1.050  -3.032  -3.109   1.943
t_O           392     248     348     438     255     370     439     232     367     380
          ( 33.4) ( 21.4) ( 30.1) ( 37.8) ( 22.3) ( 32.4) ( 38.4) ( 20.5) ( 32.4) ( 33.6)
t_E         421.6   225.8   364.9   415.9   222.7   359.9   410.1   220.6   356.4   406.2
          ( 35.9) ( 19.5) ( 31.5) ( 35.9) ( 19.5) ( 31.5) ( 35.9) ( 19.5) ( 31.5) ( 35.9)
            -57.1    46.4   -33.1    45.4    69.0    20.5    59.7    23.4    21.5   -50.6
           -4.863   4.010  -2.855   3.923   6.045   1.796   5.232   2.072   1.899  -4.475
g_O           296     376     211     275     390     200     265     402     214     264
          ( 25.2) ( 32.5) ( 18.2) ( 23.7) ( 34.2) ( 17.5) ( 23.2) ( 35.5) ( 18.9) ( 23.3)
g_E         275.7   402.1   208.3   271.9   396.5   205.4   268.2   392.7   203.4   265.6
          ( 23.5) ( 34.7) ( 18.0) ( 23.5) ( 34.7) ( 18.0) ( 23.5) ( 34.7) ( 18.0) ( 23.5)
             42.1   -50.4     5.5     6.2   -12.9   -10.7    -6.3    18.9    21.7    -3.2
            3.586  -4.352   0.471   0.534  -1.130  -0.935  -0.552   1.667   1.916  -0.280
c_O           201     207     249     162     178     272     148     179     234     183
          ( 17.1) ( 17.9) ( 21.5) ( 14.0) ( 15.6) ( 23.8) ( 13.0) ( 15.8) ( 20.7) ( 16.2)
c_E         172.3   186.5   242.7   170.0   183.9   239.4   167.7   182.1   237.1   166.0
          ( 14.7) ( 16.1) ( 21.0) ( 14.7) ( 16.1) ( 21.0) ( 14.7) ( 16.1) ( 21.0) ( 14.7)
             61.8    43.2    12.7   -15.6   -11.6    69.5   -36.9    -6.2    -6.1    35.6
            5.266   3.734   1.097  -1.349  -1.016   6.087  -3.232  -0.548  -0.539   3.148
```

```
N            1174    1158    1158    1158    1142    1142    1142    1131    1131    1131
X2            9.6     6.9     1.2     2.6     6.1     9.0     4.5     1.8     1.9     3.8
Gvalue        9.4     6.8     1.2     2.6     6.0     9.0     4.5     1.8     1.9     3.8
Gv/N        0.798   0.590   0.102   0.223   0.522   0.786   0.398   0.159   0.167   0.336
Prob      0.02477 0.07741 0.75865 0.46087 0.11374 0.02961 0.20792 0.61563 0.59618 0.28363
          -----------------------------------------------------------------------------
              121     122     123     124     125     126     127     128     129     130
          -----------------------------------------------------------------------------
a_O           308     321     276     309     344     241     299     298     275     300
          ( 27.4) ( 28.6) ( 24.6) ( 27.8) ( 31.0) ( 21.7) ( 27.3) ( 27.2) ( 25.1) ( 27.6)
a_E         333.2   331.7   291.1   329.4   327.9   287.8   325.5   324.0   284.4   322.6
          ( 29.7) ( 29.5) ( 25.9) ( 29.7) ( 29.5) ( 25.9) ( 29.7) ( 29.5) ( 25.9) ( 29.7)
            -48.5   -21.1   -29.5   -39.5    33.0   -85.5   -50.8   -49.9   -18.5   -43.5
           -4.321  -1.877  -2.624  -3.557   2.975  -7.701  -4.634  -4.551  -1.684  -4.003
t_O           266     355     419     232     306     437     247     369     386     239
          ( 23.7) ( 31.6) ( 37.3) ( 20.9) ( 27.6) ( 39.4) ( 22.5) ( 33.6) ( 35.2) ( 22.0)
t_E         219.0   353.9   403.3   216.5   349.8   398.6   213.9   345.7   394.0   212.0
          ( 19.5) ( 31.5) ( 35.9) ( 19.5) ( 31.5) ( 35.9) ( 19.5) ( 31.5) ( 35.9) ( 19.5)
            103.4     2.2    32.0    32.1   -81.9    80.3    71.0    48.1   -15.8    57.3
            9.208   0.197   2.851   2.895  -7.376   7.237   6.470   4.387  -1.436   5.273
g_O           385     213     258     389     218     247     370     210     269     360
          ( 34.3) ( 19.0) ( 23.0) ( 35.0) ( 19.6) ( 22.3) ( 33.7) ( 19.1) ( 24.5) ( 33.1)
g_E         389.9   202.0   263.7   385.4   199.7   260.7   380.9   197.3   257.6   377.4
          ( 34.7) ( 18.0) ( 23.5) ( 34.7) ( 18.0) ( 23.5) ( 34.7) ( 18.0) ( 23.5) ( 34.7)
             -9.8    22.6   -11.3     7.2    38.3   -26.6   -21.4    26.2    23.3   -34.0
           -0.868   2.012  -1.005   0.653   3.452  -2.395  -1.955   2.385   2.123  -3.128
c_O           164     234     170     180     242     185     181     220     167     188
          ( 14.6) ( 20.8) ( 15.1) ( 16.2) ( 21.8) ( 16.7) ( 16.5) ( 20.1) ( 15.2) ( 17.3)
c_E         180.8   235.4   164.9   178.7   232.7   163.0   176.6   229.9   161.0   175.0
          ( 16.1) ( 21.0) ( 14.7) ( 16.1) ( 21.0) ( 14.7) ( 16.1) ( 21.0) ( 14.7) ( 16.1)
            -32.1    -2.8    10.4     2.5    19.0    46.9     8.8   -19.4    12.1    26.9
           -2.854  -0.247   0.929   0.227   1.715   4.230   0.803  -1.773   1.105   2.471

N            1123    1123    1123    1110    1110    1110    1097    1097    1097    1087
X2           13.6     1.0     1.7     2.4     8.3    15.0     7.7     4.9     1.2     6.8
Gvalue       13.1     1.0     1.7     2.4     8.5    15.2     7.5     4.9     1.2     6.7
Gv/N        1.164   0.085   0.150   0.218   0.767   1.371   0.684   0.449   0.108   0.614
Prob      0.00448 0.81321 0.63974 0.49017 0.03650 0.00164 0.05738 0.17772 0.75537 0.08311
          -----------------------------------------------------------------------------
              131     132     133     134     135     136     137     138     139     140
          -----------------------------------------------------------------------------
a_O           302     241     298     318     292     318     310     261     296     305
          ( 27.8) ( 22.2) ( 27.7) ( 29.6) ( 27.2) ( 30.0) ( 29.3) ( 24.6) ( 28.2) ( 29.0)
a_E         321.1   281.8   318.7   317.2   278.4   314.3   312.8   274.5   311.9   310.4
          ( 29.5) ( 25.9) ( 29.7) ( 29.5) ( 25.9) ( 29.7) ( 29.5) ( 25.9) ( 29.7) ( 29.5)
            -37.0   -75.4   -40.0     1.5    27.8     7.5    -5.6   -26.4   -30.9   -10.8
           -3.405  -6.935  -3.728   0.141   2.587   0.712  -0.529  -2.493  -2.943  -1.028
t_O           308     408     243     342     372     244     328     388     219     349
          ( 28.3) ( 37.5) ( 22.6) ( 31.8) ( 34.6) ( 23.0) ( 31.0) ( 36.6) ( 20.8) ( 33.2)
t_E         342.6   390.4   209.5   338.5   385.7   206.5   333.7   380.3   205.0   331.2
          ( 31.5) ( 35.9) ( 19.5) ( 31.5) ( 35.9) ( 19.5) ( 31.5) ( 35.9) ( 19.5) ( 31.5)
            -65.5    36.1    72.2     7.1   -26.9    81.4   -11.4    15.5    29.0    36.5
           -6.025   3.317   6.722   0.664  -2.505   7.683  -1.072   1.467   2.759   3.475
g_O           232     274     369     225     238     344     196     248     355     187
          ( 21.3) ( 25.2) ( 34.4) ( 20.9) ( 22.2) ( 32.5) ( 18.5) ( 23.4) ( 33.8) ( 17.8)
g_E         195.5   255.3   372.9   193.2   252.2   367.7   190.5   248.7   364.9   189.0
          ( 18.0) ( 23.5) ( 34.7) ( 18.0) ( 23.5) ( 34.7) ( 18.0) ( 23.5) ( 34.7) ( 18.0)
             79.4    38.8    -7.7    68.6   -27.6   -45.8    11.2    -1.4   -19.5    -4.1
            7.302   3.573  -0.721   6.388  -2.569  -4.326   1.057  -0.128  -1.860  -0.387
c_O           245     164     164     189     172     153     225     162     181     210
          ( 22.5) ( 15.1) ( 15.3) ( 17.6) ( 16.0) ( 14.4) ( 21.2) ( 15.3) ( 17.2) ( 20.0)
c_E         227.8   159.6   172.9   225.1   157.7   170.5   222.0   155.5   169.2   220.3
          ( 21.0) ( 14.7) ( 16.1) ( 21.0) ( 14.7) ( 16.1) ( 21.0) ( 14.7) ( 16.1) ( 21.0)
             35.6     9.0   -17.4   -66.1    29.9   -33.2     6.1    13.3    24.3   -20.1
            3.272   0.825  -1.622  -6.155   2.786  -3.134   0.575   1.259   2.314  -1.913

N            1087    1087    1074    1074    1074    1059    1059    1059    1051    1051
X2           12.7     8.2     7.2    11.1     3.3    10.2     0.3     1.1     2.9     1.6
Gvalue       12.4     8.5     7.0    11.1     3.2     9.9     0.3     1.1     2.8     1.5
Gv/N        1.145   0.779   0.651   1.038   0.300   0.934   0.031   0.104   0.269   0.147
Prob      0.00600 0.03731 0.07212 0.01096 0.35849 0.01951 0.95555 0.77555 0.41830 0.67153
          -----------------------------------------------------------------------------
              141     142     143     144     145     146     147     148     149     150
          -----------------------------------------------------------------------------
a_O           266     293     287     282     298     283     289     271     309     247
          ( 25.3) ( 28.0) ( 27.5) ( 27.0) ( 28.8) ( 27.3) ( 27.9) ( 26.4) ( 30.1) ( 24.1)
a_E         272.5   310.1   308.7   270.9   307.1   305.7   268.3   304.8   303.4   266.2
          ( 25.9) ( 29.7) ( 29.5) ( 25.9) ( 29.7) ( 29.5) ( 25.9) ( 29.7) ( 29.5) ( 25.9)
            -12.8   -33.2   -41.8    22.6   -18.0   -43.7    42.9   -63.6    11.4   -37.1
           -1.216  -3.181  -4.000   2.165  -1.738  -4.224   4.146  -6.196   1.108  -3.609
t_O           401     228     353     364     211     339     382     230     292     384
          ( 38.2) ( 21.8) ( 33.8) ( 34.8) ( 20.4) ( 32.8) ( 36.9) ( 22.4) ( 28.4) ( 37.4)
t_E         377.4   203.8   329.3   375.3   201.9   326.2   371.7   200.3   323.6   368.8
          ( 35.9) ( 19.5) ( 31.5) ( 35.9) ( 19.5) ( 31.5) ( 35.9) ( 19.5) ( 31.5) ( 35.9)
             48.6    51.2    49.0   -22.2    18.7    26.2    20.9    63.6   -60.1    31.0
            4.621   4.896   4.692  -2.127   1.808   2.528   2.019   6.195  -5.851   3.016
g_O           239     344     192     234     362     181     203     357     187     255
          ( 22.7) ( 32.9) ( 18.4) ( 22.4) ( 35.0) ( 17.5) ( 19.6) ( 34.8) ( 18.2) ( 24.8)
g_E         246.8   362.8   188.0   245.4   359.4   186.2   243.0   356.6   184.7   241.2
          ( 23.5) ( 34.7) ( 18.0) ( 23.5) ( 34.7) ( 18.0) ( 23.5) ( 34.7) ( 18.0) ( 23.5)
            -15.4   -36.7     8.2   -22.2     5.3   -10.2   -73.1     0.8     4.6    28.4
           -1.461  -3.508   0.780  -2.129   0.513  -0.984  -7.062   0.083   0.445   2.770
c_O           145     180     213     165     164     232     161     169     239     141
          ( 13.8) ( 17.2) ( 20.4) ( 15.8) ( 15.8) ( 22.4) ( 15.6) ( 16.5) ( 23.3) ( 13.7)
c_E         154.3   168.3   219.0   153.4   166.7   216.9   151.9   165.4   215.3   150.8
          ( 14.7) ( 16.1) ( 21.0) ( 14.7) ( 16.1) ( 21.0) ( 14.7) ( 16.1) ( 21.0) ( 14.7)
            -18.0    24.2   -11.9    24.0    -5.3    31.1    18.6     7.3    50.0   -18.9
           -1.714   2.320  -1.140   2.300  -0.511   3.008   1.801   0.713   4.868  -1.839

N            1051    1045    1045    1045    1035    1035    1035    1027    1027    1027
X2            2.4     5.6     3.5     2.2     0.7     3.4     9.0     8.2     5.8     3.4
Gvalue        2.4     5.5     3.5     2.2     0.7     3.4     9.4     8.2     5.9     3.5
Gv/N        0.230   0.528   0.333   0.209   0.072   0.328   0.904   0.795   0.570   0.338
Prob      0.49060 0.13749 0.32373 0.53556 0.86253 0.33434 0.02487 0.04265 0.11877 0.32486
          -----------------------------------------------------------------------------
```

Atum_C58_CEREON
5UTR:

	50	49	48	47	46	45	44	43	42	41
a_O	1264	1245	1144	1159	1227	1177	1210	1226	1147	1155
	(23.8)	(23.5)	(21.6)	(21.9)	(23.2)	(22.2)	(22.8)	(23.1)	(21.6)	(21.8)
a_E	1236.3	1236.3	1236.3	1236.3	1236.3	1236.3	1236.3	1236.3	1236.3	1236.3
	(23.3)	(23.3)	(23.3)	(23.3)	(23.3)	(23.3)	(23.3)	(23.3)	(23.3)	(23.3)
	56.0	17.5	-177.5	-149.7	-18.5	-115.7	-52.0	-20.5	-172.0	-157.1
	1.057	0.329	-3.350	-2.824	-0.350	-2.183	-0.982	-0.387	-3.245	-2.965
t_O	1032	1257	1272	1130	1258	1206	1110	1267	1225	1058
	(19.5)	(23.7)	(24.0)	(21.3)	(23.7)	(22.8)	(20.9)	(23.9)	(23.1)	(20.0)
t_E	1171.0	1171.0	1171.0	1171.0	1171.0	1171.0	1171.0	1171.0	1171.0	1171.0
	(22.1)	(22.1)	(22.1)	(22.1)	(22.1)	(22.1)	(22.1)	(22.1)	(22.1)	(22.1)
	-260.9	178.1	210.4	-80.6	180.2	70.9	-118.9	199.6	110.4	-214.8
	-4.922	3.360	3.969	-1.521	3.400	1.339	-2.242	3.765	2.082	-4.053
g_O	1546	1324	1394	1568	1342	1387	1524	1316	1424	1587
	(29.2)	(25.0)	(26.3)	(29.6)	(25.3)	(26.2)	(28.8)	(24.8)	(26.9)	(29.9)
g_E	1451.6	1451.6	1451.6	1451.6	1451.6	1451.6	1451.6	1451.6	1451.6	1451.6
	(27.4)	(27.4)	(27.4)	(27.4)	(27.4)	(27.4)	(27.4)	(27.4)	(27.4)	(27.4)
	194.8	-243.7	-112.9	241.9	-210.7	-126.3	148.3	-258.1	-54.7	283.0
	3.675	-4.597	-2.130	4.564	-3.976	-2.383	2.799	-4.871	-1.032	5.340
c_O	1458	1474	1490	1443	1473	1530	1456	1491	1504	1500
	(27.5)	(27.8)	(28.1)	(27.2)	(27.8)	(28.9)	(27.5)	(28.1)	(28.4)	(28.3)
c_E	1441.0	1441.0	1441.0	1441.0	1441.0	1441.0	1441.0	1441.0	1441.0	1441.0
	(27.2)	(27.2)	(27.2)	(27.2)	(27.2)	(27.2)	(27.2)	(27.2)	(27.2)	(27.2)
	34.1	66.7	99.6	3.9	64.6	183.3	30.1	101.6	128.6	120.3
	0.644	1.258	1.879	0.074	1.219	3.458	0.567	1.918	2.427	2.270
N	5300	5300	5300	5300	5300	5300	5300	5300	5300	5300
X2	23.5	18.3	19.5	15.6	15.5	12.3	7.5	22.3	12.2	31.3
Gvalue	24.0	18.5	19.5	15.5	15.6	12.2	7.5	22.5	12.3	31.4
Gv/N	0.453	0.350	0.368	0.292	0.294	0.231	0.142	0.425	0.232	0.592
Prob	0.00002	0.00034	0.00022	0.00144	0.00139	0.00665	0.05734	0.00005	0.00642	0.00000

	40	39	38	37	36	35	34	33	32	31
a_O	1218	1114	1204	1282	1118	1184	1231	1155	1187	1257
	(23.0)	(21.0)	(22.7)	(24.2)	(21.1)	(22.3)	(23.2)	(21.8)	(22.4)	(23.7)
a_E	1236.3	1236.3	1236.3	1236.3	1236.3	1236.3	1236.3	1236.3	1236.3	1236.3
	(23.3)	(23.3)	(23.3)	(23.3)	(23.3)	(23.3)	(23.3)	(23.3)	(23.3)	(23.3)
	-36.3	-232.1	-63.8	93.1	-224.9	-102.4	-10.6	-157.1	-96.6	41.7
	-0.686	-4.379	-1.203	1.756	-4.244	-1.931	-0.200	-2.965	-1.823	0.787
t_O	1273	1206	1083	1268	1194	1115	1295	1216	1121	1289
	(24.0)	(22.8)	(20.4)	(23.9)	(22.5)	(21.0)	(24.4)	(22.9)	(21.2)	(24.3)
t_E	1171.0	1171.0	1171.0	1171.0	1171.0	1171.0	1171.0	1171.0	1171.0	1171.0
	(22.1)	(22.1)	(22.1)	(22.1)	(22.1)	(22.1)	(22.1)	(22.1)	(22.1)	(22.1)
	212.5	70.9	-169.3	201.7	46.4	-109.4	260.6	91.6	-97.9	247.4
	4.010	1.339	-3.194	3.806	0.875	-2.063	4.917	1.729	-1.848	4.668
g_O	1328	1430	1570	1295	1475	1481	1341	1440	1490	1279
	(25.1)	(27.0)	(29.6)	(24.4)	(27.8)	(27.9)	(25.3)	(27.2)	(28.1)	(24.1)
g_E	1451.6	1451.6	1451.6	1451.6	1451.6	1451.6	1451.6	1451.6	1451.6	1451.6
	(27.4)	(27.4)	(27.4)	(27.4)	(27.4)	(27.4)	(27.4)	(27.4)	(27.4)	(27.4)
	-236.4	-42.9	246.2	-295.7	47.2	59.4	-212.6	-23.1	77.8	-323.8
	-4.460	-0.809	4.645	-5.579	0.890	1.120	-4.011	-0.436	1.468	-6.110
c_O	1481	1550	1443	1455	1513	1520	1433	1489	1502	1475
	(27.9)	(29.2)	(27.2)	(27.5)	(28.5)	(28.7)	(27.0)	(28.1)	(28.3)	(27.8)
c_E	1441.0	1441.0	1441.0	1441.0	1441.0	1441.0	1441.0	1441.0	1441.0	1441.0
	(27.2)	(27.2)	(27.2)	(27.2)	(27.2)	(27.2)	(27.2)	(27.2)	(27.2)	(27.2)
	81.0	226.0	3.9	28.1	147.5	162.2	-16.0	97.5	124.5	68.7
	1.529	4.263	0.074	0.529	2.782	3.060	-0.303	1.840	2.348	1.296
N	5300	5300	5300	5300	5300	5300	5300	5300	5300	5300
X2	20.8	21.7	17.1	26.7	15.7	9.8	21.6	8.8	7.7	33.6
Gvalue	20.8	21.9	17.0	27.2	16.1	9.8	21.4	8.8	7.7	34.0
Gv/N	0.393	0.413	0.322	0.512	0.303	0.185	0.404	0.167	0.146	0.642
Prob	0.00011	0.00007	0.00069	0.00001	0.00110	0.02024	0.00009	0.03144	0.05235	0.00000

	30	29	28	27	26	25	24	23	22	21
a_O	1132	1258	1289	1119	1250	1352	1225	1327	1342	1206
	(21.4)	(23.7)	(24.3)	(21.1)	(23.6)	(25.5)	(23.1)	(25.0)	(25.3)	(22.8)
a_E	1236.3	1236.3	1236.3	1236.3	1236.3	1236.3	1236.3	1236.3	1236.3	1236.3
	(23.3)	(23.3)	(23.3)	(23.3)	(23.3)	(23.3)	(23.3)	(23.3)	(23.3)	(23.3)
	-199.5	43.8	107.6	-223.1	27.5	241.9	-22.5	187.9	220.2	-59.9
	-3.765	0.826	2.030	-4.210	0.520	4.564	-0.425	3.545	4.154	-1.129
t_O	1247	1081	1302	1218	1074	1230	1191	1060	1245	1245
	(23.5)	(20.4)	(24.6)	(23.0)	(20.3)	(23.2)	(22.5)	(20.0)	(23.5)	(23.5)
t_E	1171.0	1171.0	1171.0	1171.0	1171.0	1171.0	1171.0	1171.0	1171.0	1171.0
	(22.1)	(22.1)	(22.1)	(22.1)	(22.1)	(22.1)	(22.1)	(22.1)	(22.1)	(22.1)
	156.7	-173.0	276.0	95.8	-185.8	120.8	40.2	-211.2	152.5	152.5
	2.957	-3.264	5.208	1.807	-3.506	2.280	0.759	-3.985	2.877	2.877
g_O	1397	1522	1266	1422	1479	1268	1347	1414	1236	1382
	(26.4)	(28.7)	(23.9)	(26.8)	(27.9)	(23.9)	(25.4)	(26.7)	(23.3)	(26.1)
g_E	1451.6	1451.6	1451.6	1451.6	1451.6	1451.6	1451.6	1451.6	1451.6	1451.6
	(27.4)	(27.4)	(27.4)	(27.4)	(27.4)	(27.4)	(27.4)	(27.4)	(27.4)	(27.4)
	-107.1	144.1	-346.4	-58.6	55.3	-343.0	-201.5	-74.2	-397.5	-135.8
	-2.022	2.720	-6.536	-1.106	1.043	-6.471	-3.802	-1.401	-7.500	-2.563
c_O	1524	1439	1443	1541	1497	1450	1537	1499	1477	1467
	(28.8)	(27.2)	(27.2)	(29.1)	(28.2)	(27.4)	(29.0)	(28.3)	(27.9)	(27.7)
c_E	1441.0	1441.0	1441.0	1441.0	1441.0	1441.0	1441.0	1441.0	1441.0	1441.0
	(27.2)	(27.2)	(27.2)	(27.2)	(27.2)	(27.2)	(27.2)	(27.2)	(27.2)	(27.2)
	170.6	-4.1	3.9	206.7	114.1	18.0	198.2	118.2	72.8	52.4
	3.219	-0.077	0.074	3.900	2.152	0.339	3.739	2.231	1.374	0.988
N	5300	5300	5300	5300	5300	5300	5300	5300	5300	5300
X2	20.6	10.7	40.6	20.6	10.9	37.1	14.4	20.5	46.6	9.2
Gvalue	20.6	10.8	41.2	20.7	11.1	37.8	14.4	20.7	48.0	9.2
Gv/N	0.390	0.205	0.777	0.391	0.209	0.712	0.272	0.390	0.906	0.173
Prob	0.00012	0.01257	0.00000	0.00012	0.01128	0.00000	0.00238	0.00012	0.00000	0.02697

	20	19	18	17	16	15	14	13	12	11
a_O	1338	1414	1316	1441	1507	1398	1492	1630	1425	1485
	(25.2)	(26.7)	(24.8)	(27.2)	(28.4)	(26.4)	(28.2)	(30.8)	(26.9)	(28.0)
a_E	1236.3	1236.3	1236.3	1236.3	1236.3	1236.3	1236.3	1236.3	1236.3	1236.3
	(23.3)	(23.3)	(23.3)	(23.3)	(23.3)	(23.3)	(23.3)	(23.3)	(23.3)	(23.3)
	211.5	379.8	164.4	441.6	596.8	343.7	561.0	901.2	404.8	544.4
	3.991	7.166	3.102	8.331	11.260	6.484	10.584	17.005	7.638	10.271
t_O	1110	1272	1293	1051	1238	1288	888	1042	927	709
	(20.9)	(24.0)	(24.4)	(19.8)	(23.4)	(24.3)	(16.8)	(19.7)	(17.5)	(13.4)

t_E	1171.0	1171.0	1171.0	1171.0	1171.0	1171.0	1171.0	1171.0	1171.0	1171.0
	(22.1)	(22.1)	(22.1)	(22.1)	(22.1)	(22.1)	(22.1)	(22.1)	(22.1)	(22.1)
	-118.9	210.4	256.2	-227.3	137.7	245.2	-491.4	-243.3	-433.3	-711.5
	-2.242	3.969	4.834	-4.289	2.598	4.627	-9.271	-4.591	-8.175	-13.425
g_O	1483	1203	1286	1438	1199	1373	1570	1393	1838	2088
	(28.0)	(22.7)	(24.3)	(27.1)	(22.6)	(25.9)	(29.6)	(26.3)	(34.7)	(39.4)
g_E	1451.6	1451.6	1451.6	1451.6	1451.6	1451.6	1451.6	1451.6	1451.6	1451.6
	(27.4)	(27.4)	(27.4)	(27.4)	(27.4)	(27.4)	(27.4)	(27.4)	(27.4)	(27.4)
	63.5	-452.0	-311.6	-27.1	-458.5	-152.9	246.2	-114.8	867.6	1518.1
	1.197	-8.528	-5.879	-0.511	-8.650	-2.885	4.645	-2.166	16.369	28.644
c_O	1369	1411	1405	1370	1356	1241	1350	1235	1110	1018
	(25.8)	(26.6)	(26.5)	(25.8)	(25.6)	(23.4)	(25.5)	(23.3)	(20.9)	(19.2)
c_E	1441.0	1441.0	1441.0	1441.0	1441.0	1441.0	1441.0	1441.0	1441.0	1441.0
	(27.2)	(27.2)	(27.2)	(27.2)	(27.2)	(27.2)	(27.2)	(27.2)	(27.2)	(27.2)
	-140.4	-59.5	-71.2	-138.5	-165.0	-370.9	-176.2	-381.1	-579.4	-707.6
	-2.649	-1.122	-1.343	-2.614	-3.112	-6.999	-3.325	-7.191	-10.933	-13.350
N	5300	5300	5300	5300	5300	5300	5300	5300	5300	5300
X2	15.8	77.4	37.6	49.8	112.1	64.9	136.7	171.4	258.6	635.5
Gvalue	15.7	78.7	37.9	48.6	111.0	65.1	139.6	162.0	259.7	643.4
Gv/N	0.297	1.486	0.715	0.917	2.094	1.228	2.633	3.056	4.899	12.139
Prob	0.00129	0.00000	0.00000	0.00000	0.00000	0.00000	0.00000	0.00000	0.00000	0.00000

	10	9	8	7	6	5	4	3	2	1
a_O	1502	1338	1547	1529	1425	1546	1444	1409	1325	1301
	(28.3)	(25.2)	(29.2)	(28.8)	(26.9)	(29.2)	(27.2)	(26.6)	(25.0)	(24.5)
a_E	1236.3	1236.3	1236.3	1236.5	1236.5	1236.5	1236.5	1236.5	1236.5	1236.5
	(23.3)	(23.3)	(23.3)	(23.3)	(23.3)	(23.3)	(23.3)	(23.3)	(23.3)	(23.3)
	584.8	211.5	693.6	649.2	404.3	690.6	447.9	367.9	183.1	132.2
	11.034	3.991	13.088	12.247	7.627	13.028	8.450	6.941	3.454	2.494
t_O	851	913	764	1121	1204	951	1156	973	1054	917
	(16.1)	(17.2)	(14.4)	(21.1)	(22.7)	(17.9)	(21.8)	(18.4)	(19.9)	(17.3)
t_E	1171.0	1171.0	1171.0	1171.3	1171.3	1171.3	1171.3	1171.3	1171.3	1171.3
	(22.1)	(22.1)	(22.1)	(22.1)	(22.1)	(22.1)	(22.1)	(22.1)	(22.1)	(22.1)
	-543.3	-454.5	-652.6	-98.3	66.4	-396.2	-30.3	-360.9	-222.4	-448.8
	-10.252	-8.576	-12.313	-1.855	1.252	-7.475	-0.572	-6.808	-4.195	-8.467
g_O	1988	2071	1891	1482	1440	1504	1305	1568	1285	1140
	(37.5)	(39.1)	(35.7)	(28.0)	(27.2)	(28.4)	(24.6)	(29.6)	(24.2)	(21.5)
g_E	1451.6	1451.6	1451.6	1451.9	1451.9	1451.9	1451.9	1451.9	1451.9	1451.9
	(27.4)	(27.4)	(27.4)	(27.4)	(27.4)	(27.4)	(27.4)	(27.4)	(27.4)	(27.4)
	1250.3	1471.9	1000.1	60.9	-23.7	106.1	-278.4	241.3	-313.8	-551.4
	23.590	27.772	18.869	1.148	-0.447	2.001	-5.251	4.552	-5.920	-10.401
c_O	959	978	1098	1169	1232	1300	1396	1351	1637	1943
	(18.1)	(18.5)	(20.7)	(22.1)	(23.2)	(24.5)	(26.3)	(25.5)	(30.9)	(36.7)
c_E	1441.0	1441.0	1441.0	1441.3	1441.3	1441.3	1441.3	1441.3	1441.3	1441.3
	(27.2)	(27.2)	(27.2)	(27.2)	(27.2)	(27.2)	(27.2)	(27.2)	(27.2)	(27.2)
	-781.1	-758.2	-597.0	-489.6	-386.6	-268.3	-89.2	-174.8	416.8	1160.7
	-14.737	-14.305	-11.265	-9.236	-7.294	-5.061	-1.682	-3.298	7.863	21.895
N	5300	5300	5300	5301	5301	5301	5301	5301	5301	5301
X2	504.0	478.3	434.2	123.4	60.1	134.6	51.3	72.6	63.8	300.2
Gvalue	510.7	470.7	444.1	122.1	60.4	132.2	50.0	73.5	63.7	292.7
Gv/N	9.635	8.882	8.379	2.304	1.139	2.493	0.944	1.386	1.202	5.521
Prob	0.00000	0.00000	0.00000	0.00000	0.00000	0.00000	0.00000	0.00000	0.00000	0.00000

CDS_INITIAL:

	1	2	3	4	5	6	7	8	9	10
a_O	3360	0	0	2246	1314	1133	1639	1595	1081	1621
	(63.4)	(0.0)	(0.0)	(42.4)	(24.8)	(21.4)	(30.9)	(30.1)	(20.4)	(30.6)
a_E	1222.5	1361.6	548.3	1222.5	1361.6	548.3	1222.5	1361.6	548.3	1222.5
	(23.1)	(25.7)	(10.3)	(23.1)	(25.7)	(10.3)	(23.1)	(25.7)	(10.3)	(23.1)
	6794.4	0.0	0.0	2732.4	-93.5	1644.6	961.2	504.7	1467.5	914.8
	128.172	0.000	0.000	51.545	-1.764	31.024	18.132	9.521	27.684	17.257
t_O	1058	5301	0	643	1094	1104	807	1356	1211	862
	(20.0)	(100.0)	(0.0)	(12.1)	(20.6)	(20.8)	(15.2)	(25.6)	(22.8)	(16.3)
t_E	726.3	1571.4	955.2	726.3	1571.4	955.2	726.3	1571.4	955.2	726.3
	(13.7)	(29.6)	(18.0)	(13.7)	(29.6)	(18.0)	(13.7)	(29.6)	(18.0)	(13.7)
	795.9	12891.5	0.0	-156.7	-792.3	319.6	170.0	-399.8	574.6	295.2
	15.014	243.190	0.000	-2.956	-14.946	6.029	3.207	-7.541	10.840	5.569
g_O	883	0	5300	1244	1064	1494	1690	1057	1476	1525
	(16.7)	(0.0)	(100.0)	(23.5)	(20.1)	(28.2)	(31.9)	(19.9)	(27.8)	(28.8)
g_E	2037.1	993.9	1696.3	2037.1	993.9	1696.3	2037.1	993.9	1696.3	2037.1
	(38.4)	(18.8)	(32.0)	(38.4)	(18.8)	(32.0)	(38.4)	(18.8)	(32.0)	(38.4)
	-1476.3	0.0	12076.1	-1227.1	144.9	-379.5	-631.4	130.0	-410.7	-883.1
	-27.850	0.000	227.809	-23.148	2.734	-7.158	-11.911	2.453	-7.747	-16.659
c_O	0	0	1	1168	1829	1570	1165	1293	1533	1293
	(0.0)	(0.0)	(0.0)	(22.0)	(34.5)	(29.6)	(22.0)	(24.4)	(28.9)	(24.4)
c_E	1315.1	1374.1	2101.2	1315.1	1374.1	2101.2	1315.1	1374.1	2101.2	1315.1
	(24.8)	(25.9)	(39.6)	(24.8)	(25.9)	(39.6)	(24.8)	(25.9)	(39.6)	(24.8)
	0.0	0.0	-15.3	-277.1	1046.1	-915.0	-282.4	-157.3	-966.6	-43.8
	0.000	0.000	-0.289	-5.227	19.734	-17.262	-5.327	-2.967	-18.234	-0.827
N	5301	5301	5301	5301	5301	5301	5301	5301	5301	5301
X2	5858.0	12581.9	11258.6	1191.8	302.2	805.0	227.2	78.3	768.2	284.4
Gvalue	6114.0	12891.5	12060.8	1071.5	305.2	669.7	217.4	77.7	664.9	283.1
Gv/N	115.336	243.190	227.520	20.214	5.758	12.634	4.101	1.465	12.543	5.341
Prob	1.00000	1.00000	1.00000	0.00000	0.00000	0.00000	0.00000	0.00000	0.00000	0.00000

	11	12	13	14	15	16	17	18	19	20
a_O	1393	1111	1571	1309	1066	1556	1384	1016	1628	1363
	(26.3)	(21.0)	(29.6)	(24.7)	(20.1)	(29.4)	(26.1)	(19.2)	(30.7)	(25.7)
a_E	1361.6	548.3	1222.5	1361.6	548.3	1222.5	1361.6	548.3	1222.5	1361.6
	(25.7)	(10.3)	(23.1)	(25.7)	(10.3)	(23.1)	(25.7)	(10.3)	(23.1)	(25.7)
	63.5	1569.1	788.1	-103.1	1417.4	750.8	45.2	1253.3	932.8	2.8
	1.199	29.600	14.868	-1.945	26.738	14.163	0.852	23.642	17.596	0.053
t_O	1480	1241	858	1594	1232	906	1584	1168	887	1466
	(27.9)	(23.4)	(16.2)	(30.1)	(23.2)	(17.1)	(29.9)	(22.0)	(16.7)	(27.7)
t_E	1571.4	955.2	726.3	1571.4	955.2	726.3	1571.4	955.2	726.3	1571.4
	(29.6)	(18.0)	(13.7)	(29.6)	(18.0)	(13.7)	(29.6)	(18.0)	(13.7)	(29.6)
	-177.3	649.6	285.9	45.6	627.0	400.5	25.4	469.8	354.5	-203.5
	-3.345	12.254	5.393	0.860	11.827	7.555	0.478	8.862	6.688	-3.839
g_O	1002	1387	1546	949	1372	1493	943	1452	1472	1029
	(18.9)	(26.2)	(29.2)	(17.9)	(25.9)	(28.2)	(17.8)	(27.4)	(27.8)	(19.4)
g_E	993.9	1696.3	2037.1	993.9	1696.3	2037.1	993.9	1696.3	2037.1	993.9
	(18.8)	(32.0)	(38.4)	(18.8)	(32.0)	(38.4)	(18.8)	(32.0)	(38.4)	(18.8)
	16.2	-558.4	-853.0	-87.8	-582.2	-927.9	-99.2	-451.6	-956.5	71.3
	0.305	-10.534	-16.091	-1.657	-10.983	-17.504	-1.872	-8.519	-18.044	1.345

c_O	1426	1562	1326	1449	1631	1346	1390	1665	1314	1443
	(26.9)	(29.5)	(25.0)	(27.3)	(30.8)	(25.4)	(26.2)	(31.4)	(24.8)	(27.2)
c_E	1374.1	2101.2	1315.1	1374.1	2101.2	1315.1	1374.1	2101.2	1315.1	1374.1
	(25.9)	(39.6)	(24.8)	(25.9)	(39.6)	(24.8)	(25.9)	(39.6)	(24.8)	(25.9)
	105.8	−926.3	21.9	153.8	−826.2	62.5	32.0	−774.8	−2.2	141.2
	1.995	−17.475	0.413	2.902	−15.587	1.180	0.604	−14.615	−0.041	2.664
N	5301	5301	5301	5301	5301	5301	5301	5301	5301	5301
X2	8.1	857.7	241.7	8.5	736.2	281.5	3.3	572.0	326.8	11.8
Gvalue	8.1	733.9	243.0	8.5	635.9	285.9	3.3	496.7	328.6	11.9
Gv/N	0.154	13.845	4.583	0.160	11.995	5.394	0.062	9.370	6.198	0.224
Prob	0.04321	0.00000	0.00000	0.03744	0.00000	0.00000	0.34638	0.00000	0.00000	0.00791

	21	22	23	24	25	26	27	28	29	30
a_O	1000	1454	1333	910	1401	1289	912	1355	1242	886
	(18.9)	(27.4)	(25.1)	(17.2)	(26.4)	(24.3)	(17.2)	(25.6)	(23.4)	(16.7)
a_E	548.3	1222.5	1361.6	548.3	1222.5	1361.6	548.3	1222.5	1361.6	548.3
	(10.3)	(23.1)	(25.7)	(10.3)	(23.1)	(25.7)	(10.3)	(23.1)	(25.7)	(10.3)
	1201.8	504.4	−56.6	922.0	382.0	−141.2	928.0	278.9	−228.4	850.3
	22.671	9.515	−1.067	17.393	7.205	−2.664	17.507	5.262	−4.308	16.041
t_O	1176	850	1509	1163	810	1549	1130	818	1505	1082
	(22.2)	(16.0)	(28.5)	(21.9)	(15.3)	(29.2)	(21.3)	(15.4)	(28.4)	(20.4)
t_E	955.2	726.3	1571.4	955.2	726.3	1571.4	955.2	726.3	1571.4	955.2
	(18.0)	(13.7)	(29.6)	(18.0)	(13.7)	(29.6)	(18.0)	(13.7)	(29.6)	(18.0)
	489.0	267.3	−122.2	457.8	176.6	−44.4	379.7	194.4	−129.9	269.7
	9.225	5.042	−2.306	8.636	3.332	−0.838	7.163	3.668	−2.450	5.087
g_O	1395	1652	965	1471	1738	1061	1445	1793	1106	1518
	(26.3)	(31.2)	(18.2)	(27.7)	(32.8)	(20.0)	(27.3)	(33.8)	(20.9)	(28.6)
g_E	1696.3	2037.1	993.9	1696.3	2037.1	993.9	1696.3	2037.1	993.9	1696.3
	(32.0)	(38.4)	(18.8)	(32.0)	(38.4)	(18.8)	(32.0)	(38.4)	(18.8)	(32.0)
	−545.6	−692.3	−57.0	−419.3	−552.0	138.5	−463.4	−457.7	236.3	−337.2
	−10.292	−13.060	−1.076	−7.909	−10.413	2.613	−8.741	−8.635	4.457	−6.360
c_O	1730	1345	1494	1757	1352	1402	1814	1335	1448	1815
	(32.6)	(25.4)	(28.2)	(33.1)	(25.5)	(26.4)	(34.2)	(25.2)	(27.3)	(34.2)
c_E	2101.2	1315.1	1374.1	2101.2	1315.1	1374.1	2101.2	1315.1	1374.1	2101.2
	(39.6)	(24.8)	(25.9)	(39.6)	(24.8)	(25.9)	(39.6)	(24.8)	(25.9)	(39.6)
	−672.5	60.5	250.0	−628.6	74.8	56.4	−533.1	40.1	151.7	−531.4
	−12.686	1.141	4.716	−11.858	1.412	1.064	−10.057	0.757	2.862	−10.025
N	5301	5301	5301	5301	5301	5301	5301	5301	5301	5301
X2	542.2	138.4	14.4	370.1	80.7	9.3	349.7	55.5	29.9	282.5
Gvalue	472.7	139.8	14.1	331.9	81.4	9.2	311.2	55.8	29.8	251.4
Gv/N	8.918	2.638	0.267	6.262	1.536	0.174	5.871	1.052	0.561	4.742
Prob	0.00000	0.00000	0.00272	0.00000	0.00000	0.02616	0.00000	0.00000	0.00000	0.00000

	31	32	33	34	35	36	37	38	39	40
a_O	1352	1307	817	1363	1216	812	1334	1236	746	1253
	(25.5)	(24.7)	(15.4)	(25.7)	(22.9)	(15.3)	(25.2)	(23.3)	(14.1)	(23.6)
a_E	1222.5	1361.6	548.3	1222.5	1361.6	548.3	1222.5	1361.6	548.3	1222.5
	(23.1)	(25.7)	(10.3)	(23.1)	(25.7)	(10.3)	(23.1)	(25.7)	(10.3)	(23.1)
	272.3	−107.0	651.6	296.6	−275.0	637.7	233.0	−239.2	459.3	61.8
	5.137	−2.018	12.292	5.596	−5.188	12.029	4.394	−4.513	8.665	1.166
t_O	801	1521	1079	777	1520	1089	873	1611	1151	830
	(15.1)	(28.7)	(20.4)	(14.7)	(28.7)	(20.5)	(16.5)	(30.4)	(21.7)	(15.7)
t_E	726.3	1571.4	955.2	726.3	1571.4	955.2	726.3	1571.4	955.2	726.3
	(13.7)	(29.6)	(18.0)	(13.7)	(29.6)	(18.0)	(13.7)	(29.6)	(18.0)	(13.7)
	156.8	−99.1	262.9	104.8	−101.0	285.5	321.1	80.3	429.2	221.5
	2.957	−1.870	4.960	1.977	−1.906	5.385	6.058	1.514	8.096	4.178
g_O	1780	1045	1570	1815	1087	1491	1796	1044	1535	1867
	(33.6)	(19.7)	(29.6)	(34.2)	(20.5)	(28.1)	(33.9)	(19.7)	(29.0)	(35.2)
g_E	2037.1	993.9	1696.3	2037.1	993.9	1696.3	2037.1	993.9	1696.3	2037.1
	(38.4)	(18.8)	(32.0)	(38.4)	(18.8)	(32.0)	(38.4)	(18.8)	(32.0)	(38.4)
	−480.3	104.7	−243.0	−419.1	194.6	−384.7	−452.5	102.6	−306.8	−325.6
	−9.061	1.975	−4.583	−7.905	3.670	−7.257	−8.536	1.935	−5.787	−6.142
c_O	1368	1428	1835	1346	1478	1909	1298	1410	1869	1351
	(25.8)	(26.9)	(34.6)	(25.4)	(27.9)	(36.0)	(24.5)	(26.6)	(35.3)	(25.5)
c_E	1315.1	1374.1	2101.2	1315.1	1374.1	2101.2	1315.1	1374.1	2101.2	1315.1
	(24.8)	(25.9)	(39.6)	(24.8)	(25.9)	(39.6)	(24.8)	(25.9)	(39.6)	(24.8)
	107.9	109.9	−497.1	62.5	215.5	−366.2	−34.0	72.7	−437.7	72.8
	2.036	2.073	−9.377	1.180	4.065	−6.908	−0.641	1.372	−8.256	1.373
N	5301	5301	5301	5301	5301	5301	5301	5301	5301	5301
X2	56.0	8.5	190.8	44.6	33.8	188.0	68.6	16.0	152.4	30.7
Gvalue	56.7	8.5	174.5	44.9	34.0	172.3	67.6	16.4	144.1	30.5
Gv/N	1.070	0.161	3.292	0.847	0.641	3.250	1.276	0.309	2.719	0.575
Prob	0.00000	0.03646	0.00000	0.00000	0.00000	0.00000	0.00000	0.00096	0.00000	0.00000

	41	42	43	44	45	46	47	48	49	50
a_O	1194	760	1329	1269	802	1263	1195	803	1299	1235
	(22.5)	(14.3)	(25.1)	(23.9)	(15.1)	(23.8)	(22.5)	(15.1)	(24.5)	(23.3)
a_E	1361.6	548.3	1222.5	1361.6	548.3	1222.5	1361.6	548.3	1222.5	1361.6
	(25.7)	(10.3)	(23.1)	(25.7)	(10.3)	(23.1)	(25.7)	(10.3)	(23.1)	(25.7)
	−313.6	496.2	222.1	−178.7	609.9	82.4	−311.9	612.7	157.8	−241.0
	−5.917	9.361	4.190	−3.372	11.506	1.554	−5.884	11.558	2.976	−4.547
t_O	1572	1077	763	1512	1031	776	1584	1071	796	1525
	(29.7)	(20.3)	(14.4)	(28.5)	(19.4)	(14.6)	(29.9)	(20.2)	(15.0)	(28.8)
t_E	1571.4	955.2	726.3	1571.4	955.2	726.3	1571.4	955.2	726.3	1571.4
	(29.6)	(18.0)	(13.7)	(29.6)	(18.0)	(13.7)	(29.6)	(18.0)	(13.7)	(29.6)
	1.3	258.5	75.2	−116.5	157.4	102.7	25.4	245.0	145.8	−91.4
	0.024	4.876	1.418	−2.197	2.969	1.937	0.478	4.623	2.751	−1.723
g_O	1070	1554	1905	1028	1608	1917	1110	1540	1868	1079
	(20.2)	(29.3)	(35.9)	(19.4)	(30.3)	(36.2)	(20.9)	(29.1)	(35.2)	(20.4)
g_E	993.9	1696.3	2037.1	993.9	1696.3	2037.1	993.9	1696.3	2037.1	993.9
	(18.8)	(32.0)	(38.4)	(18.8)	(32.0)	(38.4)	(18.8)	(32.0)	(38.4)	(18.8)
	157.8	−272.3	−255.5	69.3	−171.9	−233.0	245.2	−297.7	−323.8	177.2
	2.976	−5.137	−4.819	1.306	−3.243	−4.395	4.625	−5.617	−6.108	3.342
c_O	1465	1910	1304	1492	1860	1345	1412	1887	1338	1462
	(27.6)	(36.0)	(24.6)	(28.1)	(35.1)	(25.4)	(26.6)	(35.6)	(25.2)	(27.6)
c_E	1374.1	2101.2	1315.1	1374.1	2101.2	1315.1	1374.1	2101.2	1315.1	1374.1
	(25.9)	(39.6)	(24.8)	(25.9)	(39.6)	(24.8)	(25.9)	(39.6)	(24.8)	(25.9)
	187.7	−364.4	−22.1	245.7	−453.5	60.5	76.9	−405.7	46.2	181.3
	3.541	−6.874	−0.417	4.634	−8.555	1.141	1.450	−7.653	0.872	3.421
N	5301	5301	5301	5301	5301	5301	5301	5301	5301	5301
X2	32.5	126.6	19.8	19.8	155.7	12.5	35.1	168.6	25.9	26.0
Gvalue	33.1	118.0	19.7	19.7	141.9	12.6	35.5	154.3	26.0	26.1
Gv/N	0.624	2.226	0.372	0.372	2.677	0.237	0.669	2.911	0.491	0.493
Prob	0.00000	0.00000	0.00020	0.00019	0.00000	0.00572	0.00000	0.00000	0.00001	0.00001

	51	52	53	54	55	56	57	58	59	60
a_O	753	1262	1209	723	1252	1138	738	1294	1182	720
	(14.2)	(23.8)	(22.8)	(13.6)	(23.6)	(21.5)	(13.9)	(24.4)	(22.3)	(13.6)
a_E	548.3	1222.5	1361.6	548.3	1222.5	1361.6	548.3	1222.5	1361.6	548.3
	(10.3)	(23.1)	(25.7)	(10.3)	(23.1)	(25.7)	(10.3)	(23.1)	(25.7)	(10.3)
	477.7	80.3	-287.4	399.9	59.8	-408.3	438.5	147.2	-334.4	392.3
	9.012	1.515	-5.422	7.544	1.128	-7.702	8.272	2.776	-6.308	7.400
t_O	1062	783	1542	1062	815	1622	1074	793	1599	1071
	(20.0)	(14.8)	(29.1)	(20.0)	(15.4)	(30.6)	(20.3)	(15.0)	(30.2)	(20.2)
t_E	955.2	726.3	1571.4	955.2	726.3	1571.4	955.2	726.3	1571.4	955.2
	(18.0)	(13.7)	(29.6)	(18.0)	(13.7)	(29.6)	(18.0)	(13.7)	(29.6)	(18.0)
	225.1	117.6	-58.2	225.1	187.7	102.9	251.7	139.3	55.7	245.0
	4.246	2.219	-1.098	4.246	3.542	1.941	4.749	2.627	1.052	4.623
g_O	1621	1935	1072	1651	1929	1021	1545	1942	1082	1620
	(30.6)	(36.5)	(20.2)	(31.1)	(36.4)	(19.3)	(29.1)	(36.6)	(20.4)	(30.6)
g_E	1696.3	2037.1	993.9	1696.3	2037.1	993.9	1696.3	2037.1	993.9	1696.3
	(32.0)	(38.4)	(18.8)	(32.0)	(38.4)	(18.8)	(32.0)	(38.4)	(18.8)	(32.0)
	-147.2	-199.0	162.1	-89.4	-210.4	54.8	-288.7	-185.7	183.7	-149.1
	-2.777	-3.754	3.057	-1.686	-3.969	1.034	-5.446	-3.503	3.465	-2.813
c_O	1865	1321	1478	1865	1305	1520	1944	1272	1438	1890
	(35.2)	(24.9)	(27.9)	(35.2)	(24.6)	(28.7)	(36.7)	(24.0)	(27.1)	(35.7)
c_E	2101.2	1315.1	1374.1	2101.2	1315.1	1374.1	2101.2	1315.1	1374.1	2101.2
	(39.6)	(24.8)	(25.9)	(39.6)	(24.8)	(25.9)	(39.6)	(24.8)	(25.9)	(39.6)
	-444.7	11.8	215.5	-444.7	-20.1	306.8	-302.3	-84.8	130.7	-400.3
	-8.389	0.223	4.065	-8.389	-0.379	5.787	-5.702	-1.599	2.466	-7.552
N	5301	5301	5301	5301	5301	5301	5301	5301	5301	5301
X2	118.2	10.8	31.6	95.3	17.4	54.6	105.6	16.2	34.9	92.4
Gvalue	110.9	10.8	32.0	90.9	17.0	56.2	99.3	16.0	35.8	87.8
Gv/N	2.091	0.204	0.603	1.714	0.321	1.061	1.873	0.302	0.675	1.657
Prob	0.00000	0.01281	0.00000	0.00000	0.00069	0.00000	0.00000	0.00114	0.00000	0.00000

	61	62	63	64	65	66	67	68	69	70
a_O	1198	1237	736	1245	1200	723	1219	1229	667	1199
	(22.6)	(23.3)	(13.9)	(23.5)	(22.6)	(13.6)	(23.0)	(23.2)	(12.6)	(22.6)
a_E	1222.5	1361.6	548.3	1222.5	1361.6	548.3	1222.5	1361.6	548.3	1222.5
	(23.1)	(25.7)	(10.3)	(23.1)	(25.7)	(10.3)	(23.1)	(25.7)	(10.3)	(23.1)
	-48.4	-237.4	433.3	45.5	-303.2	399.9	-6.9	-251.8	261.4	-46.5
	-0.914	-4.479	8.174	0.858	-5.720	7.544	-0.131	-4.751	4.931	-0.877
t_O	797	1569	1119	767	1642	1081	795	1564	1071	783
	(15.0)	(29.6)	(21.1)	(14.5)	(31.0)	(20.4)	(15.0)	(29.5)	(20.2)	(14.8)
t_E	726.3	1571.4	955.2	726.3	1571.4	955.2	726.3	1571.4	955.2	726.3
	(13.7)	(29.6)	(18.0)	(13.7)	(29.6)	(18.0)	(13.7)	(29.6)	(18.0)	(13.7)
	148.0	-4.7	354.1	83.6	144.4	267.4	143.6	-14.7	245.0	117.6
	2.792	-0.089	6.681	1.577	2.724	5.045	2.710	-0.277	4.623	2.219
g_O	1960	967	1649	1961	983	1600	1998	1020	1630	1949
	(37.0)	(18.2)	(31.1)	(37.0)	(18.5)	(30.2)	(37.7)	(19.2)	(30.7)	(36.8)
g_E	2037.1	993.9	1696.3	2037.1	993.9	1696.3	2037.1	993.9	1696.3	2037.1
	(38.4)	(18.8)	(32.0)	(38.4)	(18.8)	(32.0)	(38.4)	(18.8)	(32.0)	(38.4)
	-151.3	-53.2	-93.3	-149.3	-21.8	-187.0	-77.5	52.8	-130.0	-172.4
	-2.853	-1.003	-1.759	-2.817	-0.411	-3.528	-1.461	0.996	-2.452	-3.251
c_O	1346	1528	1797	1328	1476	1897	1289	1488	1933	1370
	(25.4)	(28.8)	(33.9)	(25.1)	(27.8)	(35.8)	(24.3)	(28.1)	(36.5)	(25.8)
c_E	1315.1	1374.1	2101.2	1315.1	1374.1	2101.2	1315.1	1374.1	2101.2	1315.1
	(24.8)	(25.9)	(39.6)	(24.8)	(25.9)	(39.6)	(24.8)	(25.9)	(39.6)	(24.8)
	62.5	324.4	-562.0	25.9	211.2	-387.8	-51.7	237.0	-322.5	112.1
	1.180	6.121	-10.602	0.489	3.984	-7.316	-0.975	4.471	-6.083	2.114
N	5301	5301	5301	5301	5301	5301	5301	5301	5301	5301
X2	11.0	29.4	137.7	5.7	30.0	97.5	7.8	23.1	55.8	11.0
Gvalue	10.8	29.1	132.2	5.7	30.6	92.5	7.6	23.3	54.0	10.9
Gv/N	0.204	0.550	2.494	0.107	0.577	1.745	0.143	0.439	1.018	0.205
Prob	0.01267	0.00000	0.00000	0.12969	0.00000	0.00000	0.05546	0.00004	0.00000	0.01232

	71	72	73	74	75	76	77	78	79	80
a_O	1222	654	1247	1249	665	1220	1229	643	1169	1271
	(23.1)	(12.3)	(23.5)	(23.6)	(12.5)	(23.0)	(23.2)	(12.1)	(22.1)	(24.0)
a_E	1361.6	548.3	1222.5	1361.6	548.3	1222.5	1361.6	548.3	1222.5	1361.6
	(25.7)	(10.3)	(23.1)	(25.7)	(10.3)	(23.1)	(25.7)	(10.3)	(23.1)	(25.7)
	-264.4	230.5	49.6	-215.6	256.6	-4.9	-251.8	204.8	-104.6	-175.0
	-4.987	4.349	0.935	-4.067	4.841	-0.093	-4.751	3.864	-1.972	-3.302
t_O	1570	1036	786	1620	1087	796	1599	1080	780	1604
	(29.6)	(19.5)	(14.8)	(30.6)	(20.5)	(15.0)	(30.2)	(20.4)	(14.7)	(30.3)
t_E	1571.4	955.2	726.3	1571.4	955.2	726.3	1571.4	955.2	726.3	1571.4
	(29.6)	(18.0)	(13.7)	(29.6)	(18.0)	(13.7)	(29.6)	(18.0)	(13.7)	(29.6)
	-2.7	168.2	124.1	98.8	280.9	145.8	55.7	265.2	111.2	65.9
	-0.052	3.173	2.341	1.863	5.300	2.751	1.052	5.002	2.098	1.244
g_O	1055	1673	1992	996	1659	1905	1038	1704	1984	967
	(19.9)	(31.6)	(37.6)	(18.8)	(31.3)	(35.9)	(19.6)	(32.1)	(37.4)	(18.2)
g_E	993.9	1696.3	2037.1	993.9	1696.3	2037.1	993.9	1696.3	2037.1	993.9
	(18.8)	(32.0)	(38.4)	(18.8)	(32.0)	(38.4)	(18.8)	(32.0)	(38.4)	(18.8)
	125.8	-46.3	-89.2	4.1	-73.8	-255.5	90.0	15.4	-104.8	-53.2
	2.373	-0.873	-1.683	0.077	-1.392	-4.819	1.698	0.291	-1.977	-1.003
c_O	1454	1938	1276	1436	1890	1380	1435	1874	1368	1459
	(27.4)	(36.6)	(24.1)	(27.1)	(35.7)	(26.0)	(27.1)	(35.4)	(25.8)	(27.5)
c_E	1374.1	2101.2	1315.1	1374.1	2101.2	1315.1	1374.1	2101.2	1315.1	1374.1
	(25.9)	(39.6)	(24.8)	(25.9)	(39.6)	(24.8)	(25.9)	(39.6)	(24.8)	(25.9)
	164.4	-313.3	-77.0	126.6	-400.3	133.0	124.5	-428.8	107.9	175.0
	3.101	-5.910	-1.453	2.388	-7.552	2.508	2.348	-8.089	2.036	3.301
N	5301	5301	5301	5301	5301	5301	5301	5301	5301	5301
X2	22.7	40.2	7.6	13.6	65.0	18.5	18.0	57.2	9.8	12.7
Gvalue	23.1	39.2	7.4	13.8	63.4	18.4	18.4	56.6	9.7	12.7
Gv/N	0.435	0.739	0.140	0.261	1.196	0.347	0.347	1.069	0.184	0.240
Prob	0.00004	0.00000	0.05907	0.00316	0.00000	0.00036	0.00036	0.00000	0.02090	0.00529

	81	82	83	84	85	86	87	88	89	90
a_O	687	1238	1297	649	1213	1253	683	1272	1201	667
	(13.0)	(23.4)	(24.5)	(12.2)	(22.9)	(23.6)	(12.9)	(24.0)	(22.7)	(12.6)
a_E	548.3	1222.5	1361.6	548.3	1222.5	1361.6	548.3	1222.5	1361.6	548.3
	(10.3)	(23.1)	(25.7)	(10.3)	(23.1)	(25.7)	(10.3)	(23.1)	(25.7)	(10.3)
	309.8	31.3	-126.1	218.8	-18.9	-208.3	300.0	101.1	-301.4	261.4
	5.844	0.590	-2.378	4.128	-0.356	-3.929	5.660	1.906	-5.687	4.931
t_O	1029	777	1587	991	763	1579	1020	781	1627	1041
	(19.4)	(14.7)	(29.9)	(18.7)	(14.4)	(29.8)	(19.2)	(14.7)	(30.7)	(19.6)
t_E	955.2	726.3	1571.4	955.2	726.3	1571.4	955.2	726.3	1571.4	955.2
	(18.0)	(13.7)	(29.6)	(18.0)	(13.7)	(29.6)	(18.0)	(13.7)	(29.6)	(18.0)
	153.1	104.8	31.4	72.9	75.2	15.3	133.8	113.3	113.2	179.0

	2.888	1.977	0.593	1.375	1.418	0.289	2.525	2.138	2.136	3.377
g_O	1622	1933	974	1680	2041	1009	1675	1951	1047	1644
	(30.6)	(36.5)	(18.4)	(31.7)	(38.5)	(19.0)	(31.6)	(36.8)	(19.8)	(31.0)
g_E	1696.3	2037.1	993.9	1696.3	2037.1	993.9	1696.3	2037.1	993.9	1696.3
	(32.0)	(38.4)	(18.8)	(32.0)	(38.4)	(18.8)	(32.0)	(38.4)	(18.8)	(32.0)
	−145.3	−202.8	−39.5	−32.4	7.8	30.3	−42.3	−168.5	108.9	−103.0
	−2.741	−3.826	−0.745	−0.612	0.147	0.572	−0.799	−3.179	2.054	−1.942
c_O	1963	1353	1443	1981	1284	1460	1923	1297	1426	1949
	(37.0)	(25.5)	(27.2)	(37.4)	(24.2)	(27.5)	(36.3)	(24.5)	(26.9)	(36.8)
c_E	2101.2	1315.1	1374.1	2101.2	1315.1	1374.1	2101.2	1315.1	1374.1	2101.2
	(39.6)	(24.8)	(25.9)	(39.6)	(24.8)	(25.9)	(39.6)	(24.8)	(25.9)	(39.6)
	−267.0	76.9	141.2	−233.3	−61.4	177.1	−340.8	−35.9	105.8	−293.0
	−5.037	1.451	2.664	−4.401	−1.159	3.341	−6.428	−0.678	1.995	−5.528
N	5301	5301	5301	5301	5301	5301	5301	5301	5301	5301
X2	53.1	10.1	7.1	26.9	2.7	14.3	52.8	10.0	25.7	46.0
Gvalue	50.6	10.1	7.1	25.9	2.6	14.4	50.8	9.9	26.4	44.4
Gv/N	0.954	0.191	0.133	0.489	0.050	0.272	0.958	0.187	0.498	0.838
Prob	0.00000	0.01735	0.06967	0.00001	0.45006	0.00238	0.00000	0.01910	0.00001	0.00000

	91	92	93	94	95	96	97	98	99	100
a_O	1221	1317	631	1273	1217	663	1277	1281	649	1257
	(23.0)	(24.8)	(11.9)	(24.0)	(23.0)	(12.5)	(24.1)	(24.2)	(12.3)	(23.8)
a_E	1222.5	1361.6	548.3	1221.3	1360.3	547.8	1220.2	1359.0	547.3	1220.2
	(23.1)	(25.7)	(10.3)	(23.1)	(25.7)	(10.3)	(23.1)	(25.7)	(10.3)	(23.1)
	−2.9	−87.7	177.2	105.5	−271.0	253.1	116.3	−151.5	221.3	74.8
	−0.055	−1.654	3.344	1.993	−5.116	4.779	2.198	−2.863	4.182	1.413
t_O	798	1574	1053	788	1586	975	764	1589	1008	812
	(15.1)	(29.7)	(19.9)	(14.9)	(29.9)	(18.4)	(14.4)	(30.0)	(19.1)	(15.3)
t_E	726.3	1571.4	955.2	725.6	1569.9	954.3	725.0	1568.4	953.4	725.0
	(13.7)	(29.6)	(18.0)	(13.7)	(29.6)	(18.0)	(13.7)	(29.6)	(18.0)	(13.7)
	150.2	5.3	205.2	129.9	32.4	41.8	80.1	41.5	112.2	184.1
	2.833	0.099	3.872	2.453	0.612	0.789	1.515	0.784	2.121	3.480
g_O	1977	932	1588	1993	1030	1622	1965	993	1613	1989
	(37.3)	(17.6)	(30.0)	(37.6)	(19.4)	(30.6)	(37.1)	(18.8)	(30.5)	(37.6)
g_E	2037.1	993.9	1696.3	2035.2	993.0	1694.7	2033.3	992.1	1693.1	2033.3
	(38.4)	(18.8)	(32.0)	(38.4)	(18.8)	(32.0)	(38.4)	(18.8)	(32.0)	(38.4)
	−118.4	−120.0	−209.5	−83.5	75.3	−142.2	−134.2	1.9	−156.3	−87.6
	−2.234	−2.263	−3.953	−1.577	1.423	−2.686	−2.537	0.035	−2.955	−1.655
c_O	1305	1478	2029	1242	1463	2036	1285	1428	2021	1233
	(24.6)	(27.9)	(38.3)	(23.5)	(27.6)	(38.4)	(24.3)	(27.0)	(38.2)	(23.3)
c_E	1315.1	1374.1	2101.2	1313.9	1372.8	2099.2	1312.6	1371.5	2097.2	1312.6
	(24.8)	(25.9)	(39.6)	(24.8)	(25.9)	(39.6)	(24.8)	(25.9)	(39.6)	(24.8)
	−20.1	215.5	−141.8	−139.7	186.2	−124.4	−54.6	115.3	−149.6	−154.3
	−0.379	4.065	−2.675	−2.638	3.516	−2.349	−1.033	2.179	−2.827	−2.916
N	5301	5301	5301	5296	5296	5296	5291	5291	5291	5291
X2	8.9	13.2	31.9	12.3	22.6	29.7	7.6	7.1	28.6	17.4
Gvalue	8.7	13.1	31.1	12.3	23.0	28.2	7.6	7.1	27.6	17.1
Gv/N	0.165	0.247	0.587	0.231	0.434	0.533	0.143	0.135	0.521	0.322
Prob	0.03325	0.00444	0.00000	0.00657	0.00004	0.00000	0.05563	0.06775	0.00000	0.00069

	101	102	103	104	105	106	107	108	109	110
a_O	1290	606	1207	1230	617	1199	1270	605	1210	1269
	(24.4)	(11.5)	(22.8)	(23.3)	(11.7)	(22.7)	(24.0)	(11.5)	(22.9)	(24.1)
a_E	1359.0	547.3	1219.2	1358.0	546.9	1217.9	1356.5	546.2	1216.2	1354.7
	(25.7)	(10.3)	(23.1)	(25.7)	(10.3)	(23.1)	(25.7)	(10.3)	(23.1)	(25.7)
	−134.5	123.5	−24.3	−243.5	148.9	−37.4	−167.3	123.6	−12.4	−165.8
	−2.542	2.335	−0.461	−4.606	2.816	−0.708	−3.167	2.341	−0.236	−3.143
t_O	1602	1042	790	1560	991	783	1585	1000	776	1595
	(30.3)	(19.7)	(14.9)	(29.5)	(18.7)	(14.8)	(30.0)	(18.9)	(14.7)	(30.2)
t_E	1568.4	953.4	724.4	1567.2	952.7	723.6	1565.4	951.6	722.6	1563.4
	(29.6)	(18.0)	(13.7)	(29.6)	(18.0)	(13.7)	(29.6)	(18.0)	(13.7)	(29.6)
	67.9	185.1	136.9	−14.4	78.1	123.6	39.4	99.2	110.6	63.9
	1.283	3.499	2.590	−0.272	1.477	2.340	0.745	1.878	2.097	1.212
g_O	1001	1629	1983	1045	1658	1996	1027	1644	1969	992
	(18.9)	(30.8)	(37.5)	(19.8)	(31.4)	(37.8)	(19.4)	(31.1)	(37.3)	(18.8)
g_E	992.1	1693.1	2031.7	991.3	1691.8	2029.4	990.2	1689.9	2026.7	988.9
	(18.8)	(32.0)	(38.4)	(18.8)	(32.0)	(38.4)	(18.8)	(32.0)	(38.4)	(18.8)
	17.9	−125.7	−96.3	110.2	−67.0	−66.3	75.0	−90.5	−113.8	6.2
	0.339	−2.377	−1.821	2.084	−1.266	−1.255	1.419	−1.714	−2.158	0.118
c_O	1398	2014	1307	1452	2021	1303	1399	2032	1319	1418
	(26.4)	(38.1)	(24.7)	(27.5)	(38.2)	(24.7)	(26.5)	(38.5)	(25.0)	(26.9)
c_E	1371.5	2097.2	1311.6	1370.5	2095.6	1310.1	1368.9	2093.2	1308.4	1367.1
	(25.9)	(39.6)	(24.8)	(25.9)	(39.6)	(24.8)	(25.9)	(39.6)	(24.8)	(25.9)
	53.5	−163.0	−9.2	167.8	−146.5	−14.2	60.8	−120.6	21.3	103.7
	1.011	−3.081	−0.174	3.174	−2.771	−0.269	1.152	−2.285	0.404	1.966
N	5291	5291	5287	5287	5287	5281	5281	5281	5274	5274
X2	4.8	20.3	7.2	19.9	13.9	5.8	7.8	11.8	5.7	8.0
Gvalue	4.9	19.9	7.1	20.1	13.5	5.6	7.9	11.6	5.6	8.1
Gv/N	0.092	0.376	0.134	0.380	0.256	0.107	0.149	0.219	0.107	0.153
Prob	0.18159	0.00018	0.06927	0.00016	0.00364	0.13079	0.04851	0.00893	0.13131	0.04495

	111	112	113	114	115	116	117	118	119	120
a_O	636	1213	1283	619	1221	1291	550	1259	1334	608
	(12.1)	(23.0)	(24.3)	(11.7)	(23.2)	(24.5)	(10.4)	(23.9)	(25.3)	(11.5)
a_E	545.5	1215.8	1354.1	545.3	1215.1	1353.4	545.0	1214.4	1352.6	544.7
	(10.3)	(23.1)	(25.7)	(10.3)	(23.1)	(25.7)	(10.3)	(23.1)	(25.7)	(10.3)
	195.2	−5.5	−138.5	156.9	11.9	−121.8	10.0	90.8	−36.9	133.7
	3.701	−0.105	−2.627	2.976	0.225	−2.312	0.190	1.725	−0.702	2.539
t_O	952	797	1539	1018	785	1580	1015	745	1522	976
	(18.1)	(15.1)	(29.2)	(19.3)	(14.9)	(30.0)	(19.3)	(14.1)	(28.9)	(18.5)
t_E	950.4	722.4	1562.8	950.0	721.9	1561.9	949.5	721.5	1561.0	948.9
	(18.0)	(13.7)	(29.6)	(18.0)	(13.7)	(29.6)	(18.0)	(13.7)	(29.6)	(18.0)
	3.3	156.7	−47.2	140.8	131.5	36.4	135.5	47.7	−77.0	54.9
	0.062	2.973	−0.895	2.670	2.495	0.692	2.572	0.905	−1.462	1.043
g_O	1589	2001	1063	1636	1999	1020	1619	2037	1026	1628
	(30.1)	(38.0)	(20.2)	(31.0)	(37.9)	(19.4)	(30.7)	(38.7)	(19.5)	(30.9)
g_E	1687.7	2026.0	988.5	1687.0	2024.8	987.9	1686.1	2023.7	987.4	1685.1
	(32.0)	(38.4)	(18.8)	(32.0)	(38.4)	(18.8)	(32.0)	(38.4)	(18.8)	(32.0)
	−191.4	−49.6	154.5	−100.5	−51.3	65.1	−131.4	26.8	78.7	−112.2
	−3.630	−0.941	2.930	−1.906	−0.973	1.236	−2.494	0.508	1.495	−2.131
c_O	2097	1261	1387	1999	1264	1378	2085	1225	1384	2054
	(39.8)	(23.9)	(26.3)	(37.9)	(24.0)	(26.2)	(39.6)	(23.3)	(26.3)	(39.0)
c_E	2090.5	1307.9	1366.6	2089.7	1307.2	1365.8	2088.5	1306.4	1365.0	2087.3
	(39.6)	(24.8)	(25.9)	(39.6)	(24.8)	(25.9)	(39.6)	(24.8)	(25.9)	(39.6)
	13.1	−92.1	41.2	−177.3	−84.9	24.5	−6.9	−157.6	38.2	−66.0
	0.249	−1.747	0.781	−3.364	−1.611	0.465	−0.132	−2.994	0.726	−1.254

N	5274	5272	5272	5272	5269	5269	5269	5266	5266	5266
X2	20.8	9.7	10.0	20.3	7.3	4.2	7.2	7.6	3.0	10.6
Gvalue	20.1	9.5	9.9	19.8	7.2	4.3	7.2	7.6	3.0	10.3
Gv/N	0.382	0.180	0.189	0.376	0.136	0.081	0.136	0.145	0.057	0.196
Prob	0.00016	0.02357	0.01900	0.00018	0.06718	0.23411	0.06643	0.05399	0.39275	0.01586

	121	122	123	124	125	126	127	128	129	130
a_O	1156	1280	589	1175	1359	619	1222	1334	606	1139
	(22.0)	(24.3)	(11.2)	(22.3)	(25.8)	(11.8)	(23.2)	(25.4)	(11.5)	(21.7)
a_E	1213.0	1351.1	544.1	1212.8	1350.8	544.0	1212.1	1350.0	543.7	1212.1
	(23.1)	(25.7)	(10.3)	(23.1)	(25.7)	(10.3)	(23.1)	(25.7)	(10.3)	(23.1)
	-111.3	-138.3	93.5	-74.4	16.4	160.0	19.9	-31.9	131.6	-141.7
	-2.116	-2.630	1.777	-1.414	0.313	3.042	0.379	-0.606	2.503	-2.696
t_O	730	1561	1012	794	1494	1000	736	1438	986	772
	(13.9)	(29.7)	(19.2)	(15.1)	(28.4)	(19.0)	(14.0)	(27.4)	(18.8)	(14.7)
t_E	720.7	1559.2	947.8	720.6	1558.9	947.7	720.2	1558.0	947.1	720.2
	(13.7)	(29.6)	(18.0)	(13.7)	(29.6)	(18.0)	(13.7)	(29.6)	(18.0)	(13.7)
	18.7	3.6	132.6	154.1	-127.1	107.5	32.0	-230.6	79.3	107.3
	0.355	0.068	2.520	2.930	-2.417	2.045	0.609	-4.387	1.510	2.042
g_O	2057	1001	1655	2009	1024	1671	1987	1049	1639	2052
	(39.1)	(19.0)	(31.5)	(38.2)	(19.5)	(31.8)	(37.8)	(20.0)	(31.2)	(39.0)
g_E	2021.4	986.3	1683.2	2021.0	986.1	1682.9	2019.8	985.5	1681.9	2019.8
	(38.4)	(18.8)	(32.0)	(38.4)	(18.8)	(32.0)	(38.4)	(18.8)	(32.0)	(38.4)
	71.9	29.7	-55.9	-23.9	77.3	-23.6	-65.1	131.0	-84.7	64.9
	1.367	0.565	-1.062	-0.454	1.470	-0.449	-1.239	2.492	-1.611	1.234
c_O	1317	1418	2004	1281	1382	1969	1311	1435	2025	1293
	(25.0)	(27.0)	(38.1)	(24.4)	(26.3)	(37.4)	(24.9)	(27.3)	(38.5)	(24.6)
c_E	1304.9	1363.5	2084.9	1304.7	1363.2	2084.5	1303.9	1362.4	2083.3	1303.9
	(24.8)	(25.9)	(39.6)	(24.8)	(25.9)	(39.6)	(24.8)	(25.9)	(39.6)	(24.8)
	24.3	111.2	-158.6	-46.9	37.9	-224.5	14.2	148.9	-115.0	-21.8
	0.461	2.115	-3.016	-0.892	0.720	-4.269	0.270	2.834	-2.188	-0.414
N	5260	5260	5260	5259	5259	5259	5256	5256	5256	5256
X2	3.5	6.1	11.7	9.2	4.5	19.7	1.0	17.4	11.5	8.7
Gvalue	3.6	6.2	11.5	8.9	4.5	19.3	1.0	17.5	11.2	8.7
Gv/N	0.068	0.117	0.219	0.170	0.085	0.368	0.019	0.333	0.213	0.166
Prob	0.31080	0.10323	0.00924	0.03021	0.21320	0.00023	0.80107	0.00056	0.01062	0.03289

	131	132	133	134	135	136	137	138	139	140
a_O	1327	613	1206	1309	614	1203	1343	604	1227	1274
	(25.2)	(11.7)	(23.0)	(24.9)	(11.7)	(22.9)	(25.6)	(11.5)	(23.4)	(24.3)
a_E	1350.0	543.7	1210.9	1348.7	543.1	1210.5	1348.2	542.9	1209.1	1346.7
	(25.7)	(10.3)	(23.1)	(25.7)	(10.3)	(23.1)	(25.7)	(10.3)	(23.1)	(25.7)
	-45.7	147.2	-9.8	-78.3	150.6	-14.9	-10.4	128.7	36.1	-141.4
	-0.869	2.800	-0.188	-1.491	2.868	-0.284	-0.199	2.453	0.688	-2.697
t_O	1563	991	748	1483	974	786	1530	1012	737	1544
	(29.7)	(18.9)	(14.2)	(28.2)	(18.5)	(15.0)	(29.1)	(19.3)	(14.1)	(29.4)
t_E	1558.0	947.1	719.5	1556.5	946.2	719.2	1556.0	945.9	718.4	1554.2
	(29.6)	(18.0)	(13.7)	(29.6)	(18.0)	(13.7)	(29.6)	(18.0)	(13.7)	(29.6)
	10.0	89.8	58.2	-143.6	56.4	139.6	-51.5	136.8	37.7	-20.3
	0.189	1.708	1.107	-2.734	1.074	2.660	-0.981	2.606	0.719	-0.387
g_O	973	1645	2011	1074	1647	1993	1020	1637	2007	1013
	(18.5)	(31.3)	(38.3)	(20.5)	(31.4)	(38.0)	(19.4)	(31.2)	(38.3)	(19.3)
g_E	985.5	1681.9	2017.9	984.6	1680.3	2017.1	984.2	1679.7	2014.8	983.1
	(18.8)	(32.0)	(38.4)	(18.8)	(32.0)	(38.4)	(18.8)	(32.0)	(38.4)	(18.8)
	-24.9	-73.0	-13.8	186.7	-65.9	-48.0	72.9	-84.2	-15.6	60.8
	-0.473	-1.389	-0.262	3.556	-1.256	-0.914	1.389	-1.605	-0.298	1.159
c_O	1393	2007	1286	1385	2016	1267	1356	1996	1272	1412
	(26.5)	(38.2)	(24.5)	(26.4)	(38.4)	(24.1)	(25.8)	(38.0)	(24.3)	(26.9)
c_E	1362.4	2083.3	1302.7	1361.1	2081.3	1302.2	1360.6	2080.5	1300.7	1359.1
	(25.9)	(39.6)	(24.8)	(25.9)	(39.6)	(24.8)	(25.9)	(39.6)	(24.8)	(25.9)
	61.8	-149.8	-33.2	48.2	-128.6	-69.4	-9.2	-165.6	-56.8	107.9
	1.176	-2.850	-0.632	0.917	-2.449	-1.323	-0.175	-3.155	-1.083	2.058
N	5256	5256	5251	5251	5251	5249	5249	5249	5243	5243
X2	1.3	14.5	1.4	13.2	12.8	7.5	1.8	16.0	1.4	7.0
Gvalue	1.3	14.1	1.4	13.0	12.4	7.3	1.8	15.7	1.4	7.0
Gv/N	0.024	0.269	0.026	0.248	0.236	0.139	0.033	0.299	0.027	0.134
Prob	0.74066	0.00272	0.71159	0.00460	0.00610	0.06250	0.62409	0.00129	0.70300	0.07186

	141	142	143	144	145	146	147	148	149	150
a_O	577	1158	1287	578	1144	1271	599	1177	1256	576
	(11.0)	(22.1)	(24.6)	(11.0)	(21.9)	(24.3)	(11.5)	(22.5)	(24.0)	(11.0)
a_E	542.3	1207.0	1344.4	541.4	1206.1	1343.4	541.0	1205.4	1342.6	540.7
	(10.3)	(23.1)	(25.7)	(10.3)	(23.1)	(25.7)	(10.3)	(23.1)	(25.7)	(10.3)
	71.5	-96.0	-112.3	75.6	-120.9	-140.7	122.1	-56.1	-167.5	72.9
	1.364	-1.834	-2.145	1.445	-2.312	-2.691	2.334	-1.074	-3.204	1.395
t_O	987	729	1566	989	786	1609	951	767	1530	933
	(18.8)	(13.9)	(29.9)	(18.9)	(15.0)	(30.8)	(18.2)	(14.7)	(29.3)	(17.8)
t_E	944.8	717.2	1551.5	943.2	716.6	1550.3	942.4	716.2	1549.4	941.9
	(18.0)	(13.7)	(29.6)	(18.0)	(13.7)	(29.6)	(18.0)	(13.7)	(29.6)	(18.0)
	86.3	23.9	29.1	93.9	145.3	119.5	17.2	105.1	-38.6	-17.7
	1.646	0.456	0.556	1.794	2.778	2.286	0.329	2.011	-0.739	-0.339
g_O	1709	2057	1013	1632	2017	934	1630	2004	1053	1651
	(32.6)	(39.3)	(19.4)	(31.2)	(38.6)	(17.9)	(31.2)	(38.3)	(20.1)	(31.6)
g_E	1677.7	2011.4	981.4	1674.9	2009.8	980.6	1673.6	2008.7	980.1	1672.6
	(32.0)	(38.4)	(18.8)	(32.0)	(38.4)	(18.8)	(32.0)	(38.4)	(18.8)	(32.0)
	63.1	92.3	64.2	-84.6	14.4	-91.0	-86.0	-9.3	151.1	-43.0
	1.203	1.764	1.227	-1.617	0.275	-1.740	-1.645	-0.179	2.892	-0.822
c_O	1970	1290	1368	2035	1283	1416	2050	1279	1388	2067
	(37.6)	(24.6)	(26.1)	(38.9)	(24.5)	(27.1)	(39.2)	(24.5)	(26.6)	(39.5)
c_E	2078.2	1298.5	1356.7	2074.6	1297.5	1355.7	2073.0	1296.7	1354.9	2071.8
	(39.6)	(24.8)	(25.9)	(39.6)	(24.8)	(25.9)	(39.6)	(24.8)	(25.9)	(39.6)
	-210.6	-16.9	22.6	-78.4	-28.8	123.3	-45.8	-35.2	67.0	-9.6
	-4.017	-0.323	0.433	-1.499	-0.551	2.357	-0.875	-0.674	1.282	-0.184
N	5243	5234	5234	5234	5230	5230	5230	5227	5227	5227
X2	10.3	3.3	3.7	6.6	10.1	11.0	7.7	4.5	12.1	2.7
Gvalue	10.3	3.3	3.7	6.5	10.0	11.1	7.5	4.5	12.1	2.6
Gv/N	0.197	0.063	0.071	0.124	0.190	0.211	0.143	0.085	0.231	0.050
Prob	0.01588	0.34824	0.29325	0.09036	0.01896	0.01140	0.05777	0.21671	0.00721	0.45106

Avar_ATCC29413
5UTR:

	50	49	48	47	46	45	44	43	42	41
a_O	1908	1832	1809	1842	1906	1868	1819	1900	1753	1859
	(33.7)	(32.4)	(32.0)	(32.6)	(33.7)	(33.0)	(32.2)	(33.6)	(31.0)	(32.9)
a_E	1881.4	1881.4	1881.4	1881.4	1881.4	1881.4	1881.4	1881.4	1881.4	1881.4
	(33.3)	(33.3)	(33.3)	(33.3)	(33.3)	(33.3)	(33.3)	(33.3)	(33.3)	(33.3)
	53.6	-97.5	-142.0	-78.0	49.5	-26.7	-122.7	37.4	-247.8	-44.5
	0.947	-1.723	-2.510	-1.378	0.875	-0.472	-2.169	0.661	-4.381	-0.787
t_O	1692	1705	1790	1789	1704	1757	1774	1755	1821	1764
	(29.9)	(30.1)	(31.6)	(31.6)	(30.1)	(31.1)	(31.4)	(31.0)	(32.2)	(31.2)
t_E	1729.5	1729.5	1729.5	1729.5	1729.5	1729.5	1729.5	1729.5	1729.5	1729.5
	(30.6)	(30.6)	(30.6)	(30.6)	(30.6)	(30.6)	(30.6)	(30.6)	(30.6)	(30.6)
	-74.2	-48.6	123.1	121.0	-50.6	55.4	90.1	51.4	187.8	69.7
	-1.311	-0.860	2.176	2.140	-0.895	0.980	1.594	0.908	3.319	1.232
g_O	1026	1002	994	1012	979	994	1023	1011	1051	1000
	(18.1)	(17.7)	(17.6)	(17.9)	(17.3)	(17.6)	(18.1)	(17.9)	(18.6)	(17.7)
g_E	1025.3	1025.3	1025.3	1025.3	1025.3	1025.3	1025.3	1025.3	1025.3	1025.3
	(18.1)	(18.1)	(18.1)	(18.1)	(18.1)	(18.1)	(18.1)	(18.1)	(18.1)	(18.1)
	1.4	-46.1	-61.7	-26.5	-90.5	-61.7	-4.6	-28.4	52.0	-50.0
	0.024	-0.815	-1.090	-0.468	-1.600	-1.090	-0.082	-0.503	0.919	-0.884
c_O	1031	1118	1064	1014	1068	1038	1041	991	1032	1034
	(18.2)	(19.8)	(18.8)	(17.9)	(18.9)	(18.3)	(18.4)	(17.5)	(18.2)	(18.3)
c_E	1020.8	1020.8	1020.8	1020.8	1020.8	1020.8	1020.8	1020.8	1020.8	1020.8
	(18.0)	(18.0)	(18.0)	(18.0)	(18.0)	(18.0)	(18.0)	(18.0)	(18.0)	(18.0)
	20.5	203.4	88.2	-13.5	96.6	34.7	40.8	-58.7	22.5	26.6
	0.363	3.596	1.560	-0.239	1.707	0.614	0.722	-1.038	0.399	0.470
N	5657	5657	5657	5657	5657	5657	5657	5657	5657	5657
X2	1.3	11.4	7.7	3.1	5.0	1.8	3.6	1.6	14.4	1.8
Gvalue	1.3	11.2	7.7	3.1	5.0	1.8	3.6	1.6	14.5	1.8
Gv/N	0.023	0.197	0.136	0.054	0.088	0.032	0.064	0.029	0.256	0.031
Prob	0.73025	0.01085	0.05297	0.38031	0.17378	0.61800	0.30409	0.65123	0.00231	0.62535

	40	39	38	37	36	35	34	33	32	31
a_O	1832	1882	1839	1923	1871	1899	1925	1831	1987	1956
	(32.4)	(33.3)	(32.5)	(34.0)	(33.1)	(33.6)	(34.0)	(32.4)	(35.1)	(34.6)
a_E	1881.4	1881.4	1881.4	1881.4	1881.4	1881.4	1881.4	1881.4	1881.4	1881.4
	(33.3)	(33.3)	(33.3)	(33.3)	(33.3)	(33.3)	(33.3)	(33.3)	(33.3)	(33.3)
	-97.5	1.2	-83.8	84.1	-20.7	35.4	88.2	-99.4	217.0	152.1
	-1.723	0.021	-1.482	1.487	-0.367	0.625	1.559	-1.758	3.836	2.689
t_O	1785	1785	1724	1757	1819	1766	1862	1819	1711	1725
	(31.6)	(31.6)	(30.5)	(31.1)	(32.2)	(31.2)	(32.9)	(32.2)	(30.2)	(30.5)
t_E	1729.5	1729.5	1729.5	1729.5	1729.5	1729.5	1729.5	1729.5	1729.5	1729.5
	(30.6)	(30.6)	(30.6)	(30.6)	(30.6)	(30.6)	(30.6)	(30.6)	(30.6)	(30.6)
	112.8	112.8	-11.0	55.4	183.6	73.8	274.9	183.6	-36.8	-9.0
	1.994	1.994	-0.194	0.980	3.245	1.304	4.860	3.245	-0.650	-0.159
g_O	998	999	1025	969	945	958	876	977	911	892
	(17.6)	(17.7)	(18.1)	(17.1)	(16.7)	(16.9)	(15.5)	(17.3)	(16.1)	(15.8)
g_E	1025.3	1025.3	1025.3	1025.3	1025.3	1025.3	1025.3	1025.3	1025.3	1025.3
	(18.1)	(18.1)	(18.1)	(18.1)	(18.1)	(18.1)	(18.1)	(18.1)	(18.1)	(18.1)
	-53.9	-52.0	-0.6	-109.5	-154.2	-130.1	-275.7	-94.3	-215.4	-248.5
	-0.953	-0.918	-0.011	-1.935	-2.725	-2.300	-4.874	-1.667	-3.807	-4.393
c_O	1042	991	1069	1008	1022	1034	994	1030	1048	1084
	(18.4)	(17.5)	(18.9)	(17.8)	(18.1)	(18.3)	(17.6)	(18.2)	(18.5)	(19.2)
c_E	1020.8	1020.8	1020.8	1020.8	1020.8	1020.8	1020.8	1020.8	1020.8	1020.8
	(18.0)	(18.0)	(18.0)	(18.0)	(18.0)	(18.0)	(18.0)	(18.0)	(18.0)	(18.0)
	42.9	-58.7	98.7	-25.4	2.4	26.6	-52.9	18.5	55.1	130.3
	0.758	-1.038	1.744	-0.449	0.043	0.470	-0.934	0.327	0.975	2.303
N	5657	5657	5657	5657	5657	5657	5657	5657	5657	5657
X2	4.2	3.3	3.3	4.6	11.0	5.5	33.6	8.3	19.6	24.2
Gvalue	4.2	3.3	3.2	4.7	11.1	5.6	34.5	8.3	20.0	24.9
Gv/N	0.075	0.059	0.057	0.082	0.196	0.099	0.610	0.147	0.353	0.440
Prob	0.23634	0.34461	0.35855	0.19842	0.01132	0.13168	0.00000	0.03996	0.00017	0.00002

	30	29	28	27	26	25	24	23	22	21
a_O	1907	1960	1939	1912	1945	2040	1949	1969	1924	1905
	(33.7)	(34.6)	(34.3)	(33.8)	(34.4)	(36.1)	(34.5)	(34.8)	(34.0)	(33.7)
a_E	1881.4	1881.4	1881.4	1881.4	1881.4	1881.4	1881.4	1881.4	1881.4	1881.4
	(33.3)	(33.3)	(33.3)	(33.3)	(33.3)	(33.3)	(33.3)	(33.3)	(33.3)	(33.3)
	51.5	160.4	116.9	61.7	129.3	330.2	137.6	179.2	86.2	47.5
	0.911	2.836	2.067	1.090	2.286	5.837	2.432	3.168	1.523	0.839
t_O	1816	1744	1869	1792	1680	1764	1809	1670	1845	1846
	(32.1)	(30.8)	(33.0)	(31.7)	(29.7)	(31.2)	(32.0)	(29.5)	(32.6)	(32.6)
t_E	1729.5	1729.5	1729.5	1729.5	1729.5	1729.5	1729.5	1729.5	1729.5	1729.5
	(30.6)	(30.6)	(30.6)	(30.6)	(30.6)	(30.6)	(30.6)	(30.6)	(30.6)	(30.6)
	177.3	29.1	290.0	127.2	-97.6	69.7	162.6	-116.9	238.6	240.7
	3.134	0.515	5.126	2.249	-1.725	1.232	2.875	-2.067	4.217	4.255
g_O	906	921	851	886	1003	838	809	893	823	860
	(16.0)	(16.3)	(15.0)	(15.7)	(17.7)	(14.8)	(14.3)	(15.8)	(14.5)	(15.2)
g_E	1025.3	1025.3	1025.3	1025.3	1025.3	1025.3	1025.3	1025.3	1025.3	1025.3
	(18.1)	(18.1)	(18.1)	(18.1)	(18.1)	(18.1)	(18.1)	(18.1)	(18.1)	(18.1)
	-224.2	-197.6	-317.2	-258.8	-44.1	-338.1	-383.4	-246.8	-361.8	-302.4
	-3.963	-3.494	-5.606	-4.575	-0.780	-5.977	-6.777	-4.362	-6.395	-5.346
c_O	1028	1032	998	1067	1029	1015	1090	1125	1065	1046
	(18.2)	(18.2)	(17.6)	(18.9)	(18.2)	(17.9)	(19.3)	(19.9)	(18.8)	(18.5)
c_E	1020.8	1020.8	1020.8	1020.8	1020.8	1020.8	1020.8	1020.8	1020.8	1020.8
	(18.0)	(18.0)	(18.0)	(18.0)	(18.0)	(18.0)	(18.0)	(18.0)	(18.0)	(18.0)
	14.5	22.5	-45.1	94.5	16.5	-11.5	143.0	218.7	90.3	51.0
	0.256	0.399	-0.797	1.670	0.292	-0.204	2.528	3.866	1.597	0.902
N	5657	5657	5657	5657	5657	5657	5657	5657	5657	5657
X2	18.6	14.1	43.2	23.8	4.1	48.3	56.4	33.8	50.5	35.4
Gvalue	19.1	14.5	44.7	24.6	4.1	50.2	59.8	34.2	53.2	36.8
Gv/N	0.338	0.256	0.790	0.436	0.073	0.888	1.058	0.605	0.941	0.651
Prob	0.00026	0.00232	0.00000	0.00002	0.24970	0.00000	0.00000	0.00000	0.00000	0.00000

	20	19	18	17	16	15	14	13	12	11
a_O	1910	1981	1974	2028	1952	1914	2028	1947	1844	2055
	(33.8)	(35.0)	(34.9)	(35.8)	(34.5)	(33.8)	(35.8)	(34.4)	(32.6)	(36.3)
a_E	1881.4	1881.4	1881.4	1881.4	1881.4	1881.4	1881.4	1881.4	1881.4	1881.4
	(33.3)	(33.3)	(33.3)	(33.3)	(33.3)	(33.3)	(33.3)	(33.3)	(33.3)	(33.3)
	57.6	204.4	189.7	304.3	143.8	65.8	304.3	133.5	-74.1	362.7
	1.019	3.613	3.353	5.380	2.542	1.162	5.380	2.359	-1.309	6.412
t_O	1789	1866	1705	1704	1875	1849	1585	1622	1466	1255
	(31.6)	(33.0)	(30.1)	(30.1)	(33.1)	(32.7)	(28.0)	(28.7)	(25.9)	(22.2)

```
t_E      1729.5  1729.5  1729.5  1729.5  1729.5  1729.5  1729.5  1729.5  1729.5  1729.5
        ( 30.6) ( 30.6) ( 30.6) ( 30.6) ( 30.6) ( 30.6) ( 30.6) ( 30.6) ( 30.6) ( 30.6)
          121.0   283.5   -48.6   -50.6   302.9   247.1  -276.6  -208.2  -484.6  -804.9
          2.140   5.012  -0.860  -0.895   5.355   4.368  -4.889  -3.680  -8.567 -14.229
g_O         912     879     926     886     820     994    1113    1179    1543    1571
        ( 16.1) ( 15.5) ( 16.4) ( 15.7) ( 14.5) ( 17.6) ( 19.7) ( 20.8) ( 27.3) ( 27.8)
g_E      1025.3  1025.3  1025.3  1025.3  1025.3  1025.3  1025.3  1025.3  1025.3  1025.3
        ( 18.1) ( 18.1) ( 18.1) ( 18.1) ( 18.1) ( 18.1) ( 18.1) ( 18.1) ( 18.1) ( 18.1)
         -213.6  -270.7  -188.7  -258.8  -366.5   -61.7   182.7   329.3  1261.3  1340.7
         -3.776  -4.785  -3.335  -4.575  -6.478  -1.090   3.229   5.822  22.297  23.700
c_O        1046     931    1052    1039    1010     900     931     909     804     776
        ( 18.5) ( 16.5) ( 18.6) ( 18.4) ( 17.9) ( 15.9) ( 16.5) ( 16.1) ( 14.2) ( 13.7)
c_E      1020.8  1020.8  1020.8  1020.8  1020.8  1020.8  1020.8  1020.8  1020.8  1020.8
        ( 18.0) ( 18.0) ( 18.0) ( 18.0) ( 18.0) ( 18.0) ( 18.0) ( 18.0) ( 18.0) ( 18.0)
           51.0  -171.4    63.4    36.8   -21.5  -226.7  -171.4  -210.9  -383.9  -425.5
          0.902  -3.030   1.120   0.650  -0.379  -4.007  -3.030  -3.727  -6.786  -7.522

N          5657    5657    5657    5657    5657    5657    5657    5657    5657    5657
X2         15.6    44.8    15.5    31.1    56.1    24.1    38.9    44.2   348.3   495.3
Gvalue     16.1    45.8    15.7    31.7    58.8    24.5    39.0    43.8   318.8   473.0
Gv/N      0.284   0.809   0.278   0.560   1.040   0.433   0.689   0.774   5.635   8.361
Prob    0.00109 0.00000 0.00129 0.00000 0.00000 0.00002 0.00000 0.00000 0.00000 0.00000
        -------------------------------------------------------------------------------
             10       9       8       7       6       5       4       3       2       1
        -------------------------------------------------------------------------------
a_O        1982    1922    2202    2206    2264    2117    2540    2232    1604    1542
        ( 35.0) ( 34.0) ( 38.9) ( 39.0) ( 40.0) ( 37.4) ( 44.9) ( 39.5) ( 28.4) ( 27.3)
a_E      1881.4  1881.4  1881.4  1881.4  1881.4  1881.4  1881.4  1881.4  1881.4  1881.4
        ( 33.3) ( 33.3) ( 33.3) ( 33.3) ( 33.3) ( 33.3) ( 33.3) ( 33.3) ( 33.3) ( 33.3)
          206.5    82.1   693.0   702.2   838.2   499.5  1524.7   762.8  -511.7  -613.5
          3.650   1.451  12.250  12.413  14.817   8.830  26.953  13.484  -9.046 -10.845
t_O        1315    1423    1504    1642    1649    1679    1444    1443    2098    2317
        ( 23.2) ( 25.2) ( 26.6) ( 29.0) ( 29.1) ( 29.7) ( 25.5) ( 25.5) ( 37.1) ( 41.0)
t_E      1729.5  1729.5  1729.5  1729.5  1729.5  1729.5  1729.5  1729.5  1729.5  1729.5
        ( 30.6) ( 30.6) ( 30.6) ( 30.6) ( 30.6) ( 30.6) ( 30.6) ( 30.6) ( 30.6) ( 30.6)
         -720.6  -555.1  -420.2  -170.5  -157.2   -99.5  -521.0  -522.7   810.5  1355.2
        -12.738  -9.813  -7.428  -3.014  -2.779  -1.759  -9.210  -9.239  14.327  23.956
g_O        1580    1541    1206    1012     986     994     822     945     569     635
        ( 27.9) ( 27.2) ( 21.3) ( 17.9) ( 17.4) ( 17.6) ( 14.5) ( 16.7) ( 10.1) ( 11.2)
g_E      1025.3  1025.3  1025.3  1025.3  1025.3  1025.3  1025.3  1025.3  1025.3  1025.3
        ( 18.1) ( 18.1) ( 18.1) ( 18.1) ( 18.1) ( 18.1) ( 18.1) ( 18.1) ( 18.1) ( 18.1)
         1366.5  1255.7   391.5   -26.5   -77.1   -61.7  -363.4  -154.2  -670.1  -608.5
         24.155  22.197   6.920  -0.468  -1.363  -1.090  -6.423  -2.725 -11.846 -10.757
c_O         780     771     745     797     758     867     851    1037    1386    1163
        ( 13.8) ( 13.6) ( 13.2) ( 14.1) ( 13.4) ( 15.3) ( 15.0) ( 18.3) ( 24.5) ( 20.6)
c_E      1020.8  1020.8  1020.8  1020.8  1020.8  1020.8  1020.8  1020.8  1020.8  1020.8
        ( 18.0) ( 18.0) ( 18.0) ( 18.0) ( 18.0) ( 18.0) ( 18.0) ( 18.0) ( 18.0) ( 18.0)
         -419.7  -432.7  -469.3  -394.5  -451.2  -283.1  -309.6    32.7   847.8   303.4
         -7.419  -7.650  -8.295  -6.973  -7.976  -5.005  -5.473   0.578  14.987   5.363

N          5657    5657    5657    5657    5657    5657    5657    5657    5657    5657
X2        461.6   375.7   190.4   109.7   150.7    55.1   346.2   119.3   453.2   429.2
Gvalue    432.6   349.9   195.0   110.8   152.7    55.2   330.7   118.7   476.4   436.5
Gv/N      7.648   6.185   3.446   1.959   2.699   0.976   5.847   2.097   8.422   7.717
Prob    0.00000 0.00000 0.00000 0.00000 0.00000 0.00000 0.00000 0.00000 0.00000 0.00000
        -------------------------------------------------------------------------------

CDS_INITIAL:
        -------------------------------------------------------------------------------
              1       2       3       4       5       6       7       8       9      10
        -------------------------------------------------------------------------------
a_O        5012       0       0    2600    1812    1963    2366    2180    2039    2030
        ( 88.6) (  0.0) (  0.0) ( 46.0) ( 32.0) ( 34.7) ( 41.8) ( 38.5) ( 36.0) ( 35.9)
a_E      1562.4  1741.6  1731.2  1562.4  1741.6  1731.2  1562.4  1741.6  1731.2  1562.4
        ( 27.6) ( 30.8) ( 30.6) ( 27.6) ( 30.8) ( 30.6) ( 27.6) ( 30.8) ( 30.6) ( 27.6)
        11684.3     0.0     0.0  2648.4   143.6   493.3  1963.8   978.9   667.3  1063.0
        206.545   0.000   0.000  46.816   2.539   8.720  34.714  17.305  11.796  18.791
t_O         185    5657       0     840    1241    2074     946    1584    1883    1165
        (  3.3) (100.0) (  0.0) ( 14.8) ( 21.9) ( 36.7) ( 16.7) ( 28.0) ( 33.3) ( 20.6)
t_E      1092.6  1708.2  1915.0  1092.6  1708.2  1915.0  1092.6  1708.2  1915.0  1092.6
        ( 19.3) ( 30.2) ( 33.9) ( 19.3) ( 30.2) ( 33.9) ( 19.3) ( 30.2) ( 33.9) ( 19.3)
         -657.1 13548.1     0.0  -441.6  -793.0   330.8  -272.5  -239.1   -63.5   149.6
        -11.616 239.492   0.000  -7.807 -14.019   5.847  -4.818  -4.227  -1.123   2.644
g_O         460       0    5657    1230     807     778    1216     679     749    1196
        (  8.1) (  0.0) (100.0) ( 21.7) ( 14.3) ( 13.8) ( 21.5) ( 12.0) ( 13.2) ( 21.1)
g_E      1828.0   942.6   960.6  1828.0   942.6   960.6  1828.0   942.6   960.6  1828.0
        ( 32.3) ( 16.7) ( 17.0) ( 32.3) ( 16.7) ( 17.0) ( 32.3) ( 16.7) ( 17.0) ( 32.3)
        -1269.4     0.0 20061.2  -974.6  -250.8  -328.0  -991.4  -445.5  -372.7 -1014.7
        -22.439   0.000 354.626 -17.229  -4.433  -5.798 -17.525  -7.876  -6.588 -17.938
c_O           0       0       0     987    1797     842    1129    1214     986    1266
        (  0.0) (  0.0) (  0.0) ( 17.4) ( 31.8) ( 14.9) ( 20.0) ( 21.5) ( 17.4) ( 22.4)
c_E      1174.1  1264.6  1050.2  1174.1  1264.6  1050.2  1174.1  1264.6  1050.2  1174.1
        ( 20.8) ( 22.4) ( 18.6) ( 20.8) ( 22.4) ( 18.6) ( 20.8) ( 22.4) ( 18.6) ( 20.8)
            0.0     0.0     0.0  -342.7  1262.9  -372.1   -88.4   -99.1  -124.4   190.8
          0.000   0.000   0.000  -6.057  22.324  -6.577  -1.563  -1.752  -2.198   3.373

N          5657    5657    5657    5657    5657    5657    5657    5657    5657    5657
X2      10568.3 13077.2 27658.5   972.9   374.3   120.2   639.6   195.1   105.8   370.4
Gvalue   9757.8 13548.1 20061.2   889.5   362.7   124.0   611.4   195.2   106.7   388.7
Gv/N    172.491 239.492 354.626  15.723   6.412   2.192  10.808   3.450   1.887   6.871
Prob    1.00000 1.00000 1.00000 0.00000 0.00000 0.00000 0.00000 0.00000 0.00000 0.00000
        -------------------------------------------------------------------------------
             11      12      13      14      15      16      17      18      19      20
        -------------------------------------------------------------------------------
a_O        1865    1967    1948    1838    1907    1863    1806    1880    1848    1779
        ( 33.0) ( 34.8) ( 34.4) ( 32.5) ( 33.7) ( 32.9) ( 31.9) ( 33.2) ( 32.7) ( 31.4)
a_E      1741.6  1731.2  1562.4  1741.6  1731.2  1562.4  1741.6  1731.2  1562.4  1741.6
        ( 30.8) ( 30.6) ( 27.6) ( 30.8) ( 30.6) ( 27.6) ( 30.8) ( 30.6) ( 27.6) ( 30.8)
          255.4   502.3   859.4   198.1   368.9   655.7   131.2   310.0   620.6    75.6
          4.514   8.880  15.193   3.501   6.520  11.591   2.319   5.480  10.970   1.337
t_O        1715    1877    1248    1904    1960    1226    1902    1897    1192    1828
        ( 30.3) ( 33.2) ( 22.1) ( 33.7) ( 34.6) ( 21.7) ( 33.6) ( 33.5) ( 21.1) ( 32.3)
t_E      1708.2  1915.0  1092.6  1708.2  1915.0  1092.6  1708.2  1915.0  1092.6  1708.2
        ( 30.2) ( 33.9) ( 19.3) ( 30.2) ( 33.9) ( 19.3) ( 30.2) ( 33.9) ( 19.3) ( 30.2)
           13.6   -75.3   332.0   413.3    91.0   282.5   408.8   -35.9   207.6   247.8
          0.241  -1.331   5.869   7.305   1.608   4.994   7.227  -0.635   3.671   4.381
g_O         643     749    1239     627     803    1295     738     816    1359     774
        ( 11.4) ( 13.2) ( 21.9) ( 11.1) ( 14.2) ( 22.9) ( 13.0) ( 14.4) ( 24.0) ( 13.7)
g_E       942.6   960.6  1828.0   942.6   960.6  1828.0   942.6   960.6  1828.0   942.6
        ( 16.7) ( 17.0) ( 32.3) ( 16.7) ( 17.0) ( 32.3) ( 16.7) ( 17.0) ( 32.3) ( 16.7)
         -492.0  -372.7  -963.7  -511.3  -287.7  -892.8  -361.2  -266.2  -805.8  -305.1
         -8.696  -6.588 -17.035  -9.039  -5.086 -15.781  -6.386  -4.705 -14.244  -5.394
```

c_O	1434	1064	1222	1288	987	1273	1211	1064	1258	1276
	(25.3)	(18.8)	(21.6)	(22.8)	(17.4)	(22.5)	(21.4)	(18.8)	(22.2)	(22.6)
c_E	1264.6	1050.2	1174.1	1264.6	1050.2	1174.1	1264.6	1050.2	1174.1	1264.6
	(22.4)	(18.6)	(20.8)	(22.4)	(18.6)	(20.8)	(22.4)	(18.6)	(20.8)	(22.4)
	360.6	27.8	97.7	47.3	-122.5	205.9	-104.8	27.8	173.7	23.0
	6.375	0.492	1.728	0.836	-2.165	3.640	-1.853	0.492	3.070	0.406
N	5657	5657	5657	5657	5657	5657	5657	5657	5657	5657
X2	126.7	79.6	309.0	133.9	48.6	237.9	71.1	34.9	187.6	39.5
Gvalue	137.7	82.1	325.5	147.3	49.6	251.4	73.9	35.7	196.1	41.3
Gv/N	2.433	1.452	5.754	2.604	0.877	4.444	1.306	0.632	3.467	0.729
Prob	0.00000	0.00000	0.00000	0.00000	0.00000	0.00000	-0.00000	0.00000	0.00000	0.00000

	21	22	23	24	25	26	27	28	29	30
a_O	1809	1813	1751	1891	1741	1681	1805	1705	1796	1861
	(32.0)	(32.0)	(31.0)	(33.4)	(30.8)	(29.7)	(31.9)	(30.1)	(31.7)	(32.9)
a_E	1731.2	1562.4	1741.6	1731.2	1562.4	1741.6	1731.2	1562.4	1741.6	1731.2
	(30.6)	(27.6)	(30.8)	(30.6)	(27.6)	(30.8)	(30.6)	(27.6)	(30.8)	(30.6)
	159.0	539.5	18.9	333.9	376.9	-119.1	150.7	297.9	110.5	269.1
	2.811	9.536	0.333	5.902	6.663	-2.105	2.664	5.266	1.953	4.756
t_O	1941	1141	1732	1850	1190	1873	1902	1163	1774	1875
	(34.3)	(20.2)	(30.6)	(32.7)	(21.0)	(33.1)	(33.6)	(20.6)	(31.4)	(33.1)
t_E	1915.0	1092.6	1708.2	1915.0	1092.6	1708.2	1915.0	1092.6	1708.2	1915.0
	(33.9)	(19.3)	(30.2)	(33.9)	(19.3)	(30.2)	(33.9)	(19.3)	(30.2)	(33.9)
	52.3	99.0	47.9	-127.8	203.3	345.0	-26.0	145.3	134.1	-79.2
	0.924	1.750	0.848	-2.260	3.594	6.099	-0.459	2.569	2.371	-1.401
g_O	808	1402	825	832	1474	800	865	1614	877	878
	(14.3)	(24.8)	(14.6)	(14.7)	(26.1)	(14.1)	(15.3)	(28.5)	(15.5)	(15.5)
g_E	960.6	1828.0	942.6	960.6	1828.0	942.6	960.6	1828.0	942.6	960.6
	(17.0)	(32.3)	(16.7)	(17.0)	(32.3)	(16.7)	(17.0)	(32.3)	(16.7)	(17.0)
	-279.5	-743.9	-220.0	-239.1	-634.5	-262.5	-181.3	-401.8	-126.6	-157.8
	-4.941	-13.150	-3.888	-4.227	-11.216	-4.641	-3.205	-7.103	-2.238	-2.790
c_O	1099	1301	1349	1084	1252	1303	1085	1175	1210	1043
	(19.4)	(23.0)	(23.8)	(19.2)	(22.1)	(23.0)	(19.2)	(20.8)	(21.4)	(18.4)
c_E	1050.2	1174.1	1264.6	1050.2	1174.1	1264.6	1050.2	1174.1	1264.6	1050.2
	(18.6)	(20.8)	(22.4)	(18.6)	(20.8)	(22.4)	(18.6)	(20.8)	(22.4)	(18.6)
	99.9	267.1	174.4	68.7	160.9	78.0	70.8	1.8	-106.7	-14.3
	1.765	4.721	3.083	1.215	2.844	1.379	1.251	0.032	-1.887	-0.253
N	5657	5657	5657	5657	5657	5657	5657	5657	5657	5657
X2	30.3	155.3	20.7	35.3	102.8	40.8	13.9	42.6	11.2	17.7
Gvalue	31.7	161.6	21.2	35.7	106.6	41.5	14.2	43.2	11.3	17.7
Gv/N	0.560	2.857	0.375	0.630	1.885	0.733	0.251	0.763	0.199	0.313
Prob	0.00000	0.00000	0.00009	0.00000	0.00000	0.00000	0.00268	0.00000	0.01042	0.00051

	31	32	33	34	35	36	37	38	39	40
a_O	1648	1681	1726	1688	1645	1747	1645	1640	1758	1567
	(29.1)	(29.7)	(30.5)	(29.8)	(29.1)	(30.9)	(29.1)	(29.0)	(31.1)	(27.7)
a_E	1562.4	1741.6	1731.2	1562.4	1741.6	1731.2	1562.4	1741.6	1731.2	1562.4
	(27.6)	(30.8)	(30.6)	(27.6)	(30.8)	(30.6)	(27.6)	(30.8)	(30.6)	(27.6)
	175.9	-119.1	-10.4	261.1	-187.7	31.7	169.5	-197.1	54.0	9.3
	3.109	-2.105	-0.184	4.615	-3.319	0.561	2.997	-3.485	0.954	0.164
t_O	1143	1790	2032	1091	1776	1956	1149	1776	1966	1123
	(20.2)	(31.6)	(35.9)	(19.3)	(31.4)	(34.6)	(20.3)	(31.4)	(34.8)	(19.9)
t_E	1092.6	1708.2	1915.0	1092.6	1708.2	1915.0	1092.6	1708.2	1915.0	1092.6
	(19.3)	(30.2)	(33.9)	(19.3)	(30.2)	(33.9)	(19.3)	(30.2)	(33.9)	(19.3)
	103.2	167.5	240.9	-3.1	138.3	82.8	115.7	138.3	103.3	61.7
	1.823	2.961	4.259	-0.055	2.444	1.463	2.046	2.444	1.825	1.091
g_O	1628	870	842	1651	911	906	1720	891	886	1729
	(28.8)	(15.4)	(14.9)	(29.2)	(16.1)	(16.0)	(30.4)	(15.8)	(15.7)	(30.6)
g_E	1828.0	942.6	960.6	1828.0	942.6	960.6	1828.0	942.6	960.6	1828.0
	(32.3)	(16.7)	(17.0)	(32.3)	(16.7)	(17.0)	(32.3)	(16.7)	(17.0)	(32.3)
	-377.2	-139.5	-221.8	-336.2	-62.2	-106.0	-209.4	-100.4	-143.2	-192.5
	-6.668	-2.467	-3.922	-5.943	-1.100	-1.873	-3.702	-1.775	-2.531	-3.402
c_O	1238	1316	1057	1227	1325	1048	1143	1350	1047	1238
	(21.9)	(23.3)	(18.7)	(21.7)	(23.4)	(18.5)	(20.2)	(23.9)	(18.5)	(21.9)
c_E	1174.1	1264.6	1050.2	1174.1	1264.6	1050.2	1174.1	1264.6	1050.2	1174.1
	(20.8)	(22.4)	(18.6)	(20.8)	(22.4)	(18.6)	(20.8)	(22.4)	(18.6)	(20.8)
	131.2	104.9	13.7	108.2	123.7	-4.4	-61.4	176.5	-6.4	131.2
	2.320	1.855	0.242	1.912	2.187	-0.077	-1.085	3.120	-0.112	2.320
N	5657	5657	5657	5657	5657	5657	5657	5657	5657	5657
X2	32.4	13.7	21.8	29.6	12.0	4.1	14.5	17.2	7.6	9.7
Gvalue	33.0	13.8	22.3	29.9	12.0	4.2	14.5	17.2	7.7	9.7
Gv/N	0.584	0.244	0.395	0.529	0.213	0.074	0.256	0.305	0.136	0.172
Prob	0.00000	0.00319	0.00006	0.00000	0.00727	0.24290	0.00230	0.00063	0.05240	0.02103

	41	42	43	44	45	46	47	48	49	50
a_O	1650	1747	1478	1639	1781	1549	1545	1733	1544	1513
	(29.2)	(30.9)	(26.1)	(29.0)	(31.5)	(27.4)	(27.3)	(30.6)	(27.3)	(26.7)
a_E	1741.6	1731.2	1562.4	1741.6	1731.2	1562.4	1741.6	1731.2	1562.4	1741.6
	(30.8)	(30.6)	(27.6)	(30.8)	(30.6)	(27.6)	(30.8)	(30.6)	(27.6)	(30.8)
	-178.3	31.7	-164.1	-199.0	101.0	-26.6	-370.1	3.6	-36.5	-425.8
	-3.152	0.561	-2.901	-3.518	1.785	-0.471	-6.543	0.063	-0.646	-7.527
t_O	1770	1841	1125	1732	1854	1102	1819	1891	1165	1908
	(31.3)	(32.5)	(19.9)	(30.6)	(32.8)	(19.5)	(32.2)	(33.4)	(20.6)	(33.7)
t_E	1708.2	1915.0	1092.6	1708.2	1915.0	1092.6	1708.2	1915.0	1092.6	1708.2
	(30.2)	(33.9)	(19.3)	(30.2)	(33.9)	(19.3)	(30.2)	(33.9)	(19.3)	(30.2)
	125.8	-145.2	65.8	47.9	-120.1	18.9	228.7	-47.8	149.6	422.1
	2.224	-2.566	1.163	0.848	-2.123	0.335	4.042	-0.845	2.644	7.462
g_O	904	995	1834	913	979	1779	973	983	1793	951
	(16.0)	(17.6)	(32.4)	(16.1)	(17.3)	(31.4)	(17.2)	(17.4)	(31.7)	(16.8)
g_E	942.6	960.6	1828.0	942.6	960.6	1828.0	942.6	960.6	1828.0	942.6
	(16.7)	(17.0)	(32.3)	(16.7)	(17.0)	(32.3)	(16.7)	(17.0)	(32.3)	(16.7)
	-75.7	70.1	12.1	-58.4	37.2	-96.6	61.7	45.4	-69.3	16.8
	-1.338	1.239	0.214	-1.032	0.658	-1.708	1.090	0.802	-1.224	0.297
c_O	1333	1074	1220	1373	1043	1227	1320	1050	1155	1285
	(23.6)	(19.0)	(21.6)	(24.3)	(18.4)	(21.7)	(23.3)	(18.6)	(20.4)	(22.7)
c_E	1264.6	1050.2	1174.1	1264.6	1050.2	1174.1	1264.6	1050.2	1174.1	1264.6
	(22.4)	(18.6)	(20.8)	(22.4)	(18.6)	(20.8)	(22.4)	(18.6)	(20.8)	(22.4)
	140.5	48.2	93.6	225.9	-14.3	108.2	113.3	-0.4	-37.9	41.2
	2.484	0.851	1.654	3.993	-0.253	1.912	2.002	-0.007	-0.670	0.728
N	5657	5657	5657	5657	5657	5657	5657	5657	5657	5657
X2	12.3	4.8	7.3	16.6	3.8	3.9	32.8	0.8	6.0	53.8
Gvalue	12.4	4.8	7.4	16.5	3.8	3.9	33.5	0.8	5.9	54.3
Gv/N	0.218	0.085	0.131	0.291	0.067	0.068	0.592	0.015	0.104	0.960
Prob	0.00625	0.18705	0.06057	0.00090	0.28553	0.27604	0.00000	0.84348	0.11653	0.00000

	51	52	53	54	55	56	57	58	59	60
a_O	1737	1504	1611	1694	1489	1605	1708	1530	1588	1700
	(30.7)	(26.6)	(28.5)	(29.9)	(26.3)	(28.4)	(30.2)	(27.0)	(28.1)	(30.1)
a_E	1731.2	1562.4	1741.6	1731.2	1562.4	1741.6	1731.2	1562.4	1741.6	1731.2
	(30.6)	(27.6)	(30.8)	(30.6)	(27.6)	(30.8)	(30.6)	(27.6)	(30.8)	(30.6)
	11.6	-114.5	-251.1	-73.6	-143.2	-262.2	-46.1	-64.1	-293.2	-61.9
	0.205	-2.025	-4.439	-1.301	-2.532	-4.635	-0.815	-1.133	-5.183	-1.093
t_O	1899	1148	1818	1906	1154	1793	1915	1218	1815	1906
	(33.6)	(20.3)	(32.1)	(33.7)	(20.4)	(31.7)	(33.9)	(21.5)	(32.1)	(33.7)
t_E	1915.0	1092.6	1708.2	1915.0	1092.6	1708.2	1915.0	1092.6	1708.2	1915.0
	(33.9)	(19.3)	(30.2)	(33.9)	(19.3)	(30.2)	(33.9)	(19.3)	(30.2)	(33.9)
	-31.9	113.6	226.5	-18.0	126.3	173.8	-0.1	264.7	220.2	-18.0
	-0.565	2.009	4.004	-0.319	2.232	3.072	-0.001	4.680	3.892	-0.319
g_O	923	1785	904	1024	1778	939	953	1769	922	1008
	(16.3)	(31.6)	(16.0)	(18.1)	(31.4)	(16.6)	(16.8)	(31.3)	(16.3)	(17.8)
g_E	960.6	1828.0	942.6	960.6	1828.0	942.6	960.6	1828.0	942.6	960.6
	(17.0)	(32.3)	(16.7)	(17.0)	(32.3)	(16.7)	(17.0)	(32.3)	(16.7)	(17.0)
	-73.6	-84.9	-75.7	131.0	-98.5	-7.3	-15.1	-116.0	-40.8	97.2
	-1.302	-1.501	-1.338	2.315	-1.742	-0.129	-0.266	-2.051	-0.722	1.718
c_O	1098	1220	1324	1033	1236	1320	1081	1140	1332	1043
	(19.4)	(21.6)	(23.4)	(18.3)	(21.8)	(23.3)	(19.1)	(20.2)	(23.5)	(18.4)
c_E	1050.2	1174.1	1264.6	1050.2	1174.1	1264.6	1050.2	1174.1	1264.6	1050.2
	(18.6)	(20.8)	(22.4)	(18.6)	(20.8)	(22.4)	(18.6)	(20.8)	(22.4)	(18.6)
	97.8	93.6	121.6	-34.1	127.0	113.3	62.5	-67.2	138.4	-14.3
	1.728	1.654	2.150	-0.603	2.245	2.002	1.105	-1.188	2.446	-0.253
N	5657	5657	5657	5657	5657	5657	5657	5657	5657	5657
X2	3.8	7.8	21.2	5.3	11.5	17.4	1.3	18.0	24.3	3.0
Gvalue	3.8	7.8	21.3	5.2	11.5	17.6	1.3	17.5	24.5	3.0
Gv/N	0.067	0.137	0.377	0.092	0.203	0.310	0.022	0.309	0.433	0.052
Prob	0.28539	0.05115	0.00009	0.15562	0.00940	0.00054	0.73681	0.00056	0.00002	0.39734

	61	62	63	64	65	66	67	68	69	70
a_O	1511	1616	1732	1576	1542	1743	1552	1598	1728	1472
	(26.7)	(28.6)	(30.6)	(27.9)	(27.3)	(30.8)	(27.4)	(28.2)	(30.5)	(26.0)
a_E	1562.4	1741.6	1731.2	1562.4	1741.6	1731.2	1562.4	1741.6	1731.2	1562.4
	(27.6)	(30.8)	(30.6)	(27.6)	(30.8)	(30.6)	(27.6)	(30.8)	(30.6)	(27.6)
	-101.0	-241.9	1.6	27.4	-375.4	23.7	-20.7	-275.0	-6.4	-175.4
	-1.786	-4.276	0.028	0.484	-6.636	0.418	-0.365	-4.861	-0.113	-3.101
t_O	1158	1840	1893	1098	1829	1883	1089	1758	1883	1101
	(20.5)	(32.5)	(33.5)	(19.4)	(32.3)	(33.3)	(19.3)	(31.1)	(33.3)	(19.5)
t_E	1092.6	1708.2	1915.0	1092.6	1708.2	1915.0	1092.6	1708.2	1915.0	1092.6
	(19.3)	(30.2)	(33.9)	(19.3)	(30.2)	(33.9)	(19.3)	(30.2)	(33.9)	(19.3)
	134.7	273.5	-43.8	10.9	250.0	-63.5	-7.1	101.1	-63.5	16.9
	2.381	4.835	-0.775	0.192	4.419	-1.123	-0.126	1.786	-1.123	0.299
g_O	1795	927	1001	1804	937	1056	1846	979	1088	1826
	(31.7)	(16.4)	(17.7)	(31.9)	(16.6)	(18.7)	(32.6)	(17.3)	(19.2)	(32.3)
g_E	1828.0	942.6	960.6	1828.0	942.6	960.6	1828.0	942.6	960.6	1828.0
	(32.3)	(16.7)	(17.0)	(32.3)	(16.7)	(17.0)	(32.3)	(16.7)	(17.0)	(32.3)
	-65.3	-31.0	82.6	-47.6	-11.3	200.1	36.3	74.1	271.1	-3.9
	-1.155	-0.549	1.459	-0.842	-0.199	3.536	0.641	1.310	4.792	-0.069
c_O	1193	1274	1031	1179	1349	975	1170	1322	958	1258
	(21.1)	(22.5)	(18.2)	(20.8)	(23.8)	(17.2)	(20.7)	(23.4)	(16.9)	(22.2)
c_E	1174.1	1264.6	1050.2	1174.1	1264.6	1050.2	1174.1	1264.6	1050.2	1174.1
	(20.8)	(22.4)	(18.6)	(20.8)	(22.4)	(18.6)	(20.8)	(22.4)	(18.6)	(20.8)
	38.1	18.9	-38.0	9.8	174.4	-144.9	-8.2	117.4	-176.0	173.7
	0.674	0.335	-0.672	0.174	3.083	-2.561	-0.144	2.076	-3.112	3.070
N	5657	5657	5657	5657	5657	5657	5657	5657	5657	5657
X2	6.5	19.6	2.3	0.5	37.1	15.5	0.3	17.3	25.5	11.3
Gvalue	6.5	19.5	2.3	0.5	37.7	15.3	0.3	17.6	25.1	11.3
Gv/N	0.114	0.345	0.040	0.009	0.666	0.271	0.005	0.311	0.444	0.199
Prob	0.09161	0.00021	0.51510	0.92295	0.00000	0.00156	0.96514	0.00054	0.00001	0.01042

	71	72	73	74	75	76	77	78	79	80
a_O	1616	1755	1514	1595	1724	1497	1662	1789	1584	1634
	(28.6)	(31.0)	(26.8)	(28.2)	(30.5)	(26.5)	(29.4)	(31.6)	(28.0)	(28.9)
a_E	1741.6	1731.2	1562.4	1741.6	1731.2	1562.4	1741.6	1731.2	1562.4	1741.6
	(30.8)	(30.6)	(27.6)	(30.8)	(30.6)	(27.6)	(30.8)	(30.6)	(27.6)	(30.8)
	-241.9	47.9	-95.2	-280.5	-14.4	-128.0	-155.5	117.5	43.6	-208.4
	-4.276	0.847	-1.683	-4.958	-0.254	-2.262	-2.749	2.077	0.770	-3.684
t_O	1756	1910	1204	1802	1913	1092	1739	1845	1140	1814
	(31.0)	(33.8)	(21.3)	(31.9)	(33.8)	(19.3)	(30.7)	(32.6)	(20.2)	(32.1)
t_E	1708.2	1915.0	1092.6	1708.2	1915.0	1092.6	1708.2	1915.0	1092.6	1708.2
	(30.2)	(33.9)	(19.3)	(30.2)	(33.9)	(19.3)	(30.2)	(33.9)	(19.3)	(30.2)
	96.9	-10.1	233.9	192.7	-4.1	-1.1	62.2	-137.5	96.9	218.0
	1.714	-0.178	4.134	3.406	-0.072	-0.020	1.099	-2.430	1.713	3.854
g_O	962	969	1803	948	997	1861	935	1038	1783	862
	(17.0)	(17.1)	(31.9)	(16.8)	(17.6)	(32.9)	(16.5)	(18.3)	(31.5)	(15.2)
g_E	942.6	960.6	1828.0	942.6	960.6	1828.0	942.6	960.6	1828.0	942.6
	(16.7)	(17.0)	(32.3)	(16.7)	(17.0)	(32.3)	(16.7)	(17.0)	(32.3)	(16.7)
	39.1	16.9	-49.6	10.7	74.2	66.7	-15.2	161.0	-88.8	-154.2
	0.691	0.300	-0.877	0.190	1.312	1.178	-0.269	2.845	-1.570	-2.726
c_O	1323	1023	1136	1312	1023	1207	1321	985	1150	1347
	(23.4)	(18.1)	(20.1)	(23.2)	(18.1)	(21.3)	(23.4)	(17.4)	(20.3)	(23.8)
c_E	1264.6	1050.2	1174.1	1264.6	1050.2	1174.1	1264.6	1050.2	1174.1	1264.6
	(22.4)	(18.6)	(20.8)	(22.4)	(18.6)	(20.8)	(22.4)	(18.6)	(20.8)	(22.4)
	119.5	-53.7	-74.9	96.6	-53.7	66.7	115.3	-126.2	-47.7	170.1
	2.113	-0.949	-1.325	1.708	-0.949	1.180	2.039	-2.232	-0.843	3.007
N	5657	5657	5657	5657	5657	5657	5657	5657	5657	5657
X2	13.5	1.1	14.4	19.3	2.1	4.3	6.8	14.8	4.0	25.5
Gvalue	13.7	1.1	14.1	19.5	2.1	4.3	6.8	14.7	3.9	25.6
Gv/N	0.242	0.020	0.249	0.346	0.037	0.076	0.120	0.260	0.070	0.452
Prob	0.00340	0.77165	0.00277	0.00021	0.55046	0.23259	0.07888	0.00208	0.26792	0.00001

	81	82	83	84	85	86	87	88	89	90
a_O	1714	1507	1632	1742	1492	1659	1708	1528	1713	1808
	(30.3)	(26.6)	(28.8)	(30.8)	(26.4)	(29.3)	(30.2)	(27.0)	(30.3)	(32.0)
a_E	1731.2	1562.4	1741.6	1731.2	1562.4	1741.6	1731.2	1562.1	1741.3	1730.9
	(30.6)	(27.6)	(30.8)	(30.6)	(27.6)	(30.8)	(30.6)	(27.6)	(30.8)	(30.6)
	-34.3	-108.8	-212.1	21.6	-137.5	-161.2	-46.1	-67.4	-56.1	157.6
	-0.605	-1.923	-3.750	0.383	-2.431	-2.850	-0.815	-1.192	-0.992	2.786
t_O	1946	1102	1785	1952	1130	1756	1904	1066	1737	1860
	(34.4)	(19.5)	(31.6)	(34.5)	(20.0)	(31.0)	(33.7)	(18.8)	(30.7)	(32.9)
t_E	1915.0	1092.6	1708.2	1915.0	1092.6	1708.2	1915.0	1092.4	1707.9	1914.7
	(33.9)	(19.3)	(30.2)	(33.9)	(19.3)	(30.2)	(33.9)	(19.3)	(30.2)	(33.9)
	62.4	18.9	157.0	74.6	76.1	96.9	-22.0	-52.1	58.7	-107.8

	1.103	0.335	2.776	1.319	1.346	1.714	−0.389	−0.921	1.038	−1.906
g_O	1041	1836	929	1008	1837	930	1061	1802	919	1007
	(18.4)	(32.5)	(16.4)	(17.8)	(32.5)	(16.4)	(18.8)	(31.9)	(16.2)	(17.8)
g_E	960.6	1828.0	942.6	960.6	1828.0	942.6	960.6	1827.6	942.5	960.4
	(17.0)	(32.3)	(16.7)	(17.0)	(32.3)	(16.7)	(17.0)	(32.3)	(16.7)	(17.0)
	167.4	16.1	−27.1	97.2	18.1	−25.1	211.0	−50.9	−46.4	95.4
	2.960	0.285	−0.479	1.718	0.320	−0.444	3.730	−0.900	−0.820	1.687
c_O	956	1212	1311	955	1198	1312	984	1260	1287	981
	(16.9)	(21.4)	(23.2)	(16.9)	(21.2)	(23.2)	(17.4)	(22.3)	(22.8)	(17.3)
c_E	1050.2	1174.1	1264.6	1050.2	1174.1	1264.6	1050.2	1173.9	1264.3	1050.0
	(18.6)	(20.8)	(22.4)	(18.6)	(20.8)	(22.4)	(18.6)	(20.8)	(22.4)	(18.6)
	−179.7	77.0	94.5	−181.5	48.3	96.6	−128.1	178.4	45.7	−133.4
	−3.176	1.362	1.671	−3.208	0.854	1.708	−2.265	3.154	0.808	−2.358
N	5657	5657	5657	5657	5657	5657	5657	5656	5656	5656
X2	15.9	3.3	12.3	11.8	5.0	7.2	15.0	8.1	1.9	11.8
Gvalue	15.9	3.3	12.3	12.0	5.0	7.2	14.8	7.9	1.9	11.8
Gv/N	0.282	0.059	0.218	0.212	0.089	0.128	0.261	0.140	0.034	0.209
Prob	0.00117	0.34579	0.00633	0.00745	0.17075	0.06480	0.00200	0.04767	0.58307	0.00801

	91	92	93	94	95	96	97	98	99	100
a_O	1478	1737	1774	1474	1717	1750	1491	1684	1703	1548
	(26.1)	(30.7)	(31.4)	(26.1)	(30.4)	(30.9)	(26.4)	(29.8)	(30.1)	(27.4)
a_E	1562.1	1741.3	1730.9	1562.1	1741.3	1730.9	1561.8	1741.0	1730.6	1561.8
	(27.6)	(30.8)	(30.6)	(27.6)	(30.8)	(30.6)	(27.6)	(30.8)	(30.6)	(27.6)
	−163.6	−8.6	87.3	−171.1	−48.2	38.4	−138.4	−112.1	−54.8	−27.5
	−2.892	−0.151	1.543	−3.026	−0.853	0.679	−2.447	−1.982	−0.968	−0.487
t_O	1145	1710	1919	1094	1664	1868	1117	1775	2008	1104
	(20.2)	(30.2)	(33.9)	(19.3)	(29.4)	(33.0)	(19.8)	(31.4)	(35.5)	(19.5)
t_E	1092.4	1707.9	1914.7	1092.4	1707.9	1914.7	1092.2	1707.6	1914.4	1092.2
	(19.3)	(30.2)	(33.9)	(19.3)	(30.2)	(33.9)	(19.3)	(30.2)	(33.9)	(19.3)
	107.7	4.2	8.6	3.2	−86.6	−92.3	50.2	137.5	191.8	23.8
	1.905	0.075	0.152	0.057	−1.532	−1.631	0.888	2.431	3.391	0.420
g_O	1882	903	1003	1862	1015	1047	1823	955	970	1854
	(33.3)	(16.0)	(17.7)	(32.9)	(17.9)	(18.5)	(32.2)	(16.9)	(17.2)	(32.8)
g_E	1827.6	942.5	960.4	1827.6	942.5	960.4	1827.3	942.3	960.2	1827.3
	(32.3)	(16.7)	(17.0)	(32.3)	(16.7)	(17.0)	(32.3)	(16.7)	(17.0)	(32.3)
	110.3	−77.3	87.1	69.4	150.5	180.8	−8.6	25.5	19.7	53.8
	1.951	−1.366	1.540	1.226	2.661	3.197	−0.152	0.452	0.348	0.951
c_O	1151	1306	960	1226	1260	991	1224	1241	974	1149
	(20.4)	(23.1)	(17.0)	(21.7)	(22.3)	(17.5)	(21.6)	(21.9)	(17.2)	(20.3)
c_E	1173.9	1264.3	1050.0	1173.9	1264.3	1050.0	1173.7	1264.1	1049.8	1173.7
	(20.8)	(22.4)	(18.6)	(20.8)	(22.4)	(18.6)	(20.8)	(22.4)	(18.6)	(20.8)
	−45.3	84.7	−172.1	106.5	−8.7	−114.6	102.8	−45.8	−146.0	−48.8
	−0.801	1.497	−3.042	1.883	−0.153	−2.027	1.817	−0.810	−2.582	−0.864
N	5656	5656	5656	5656	5656	5656	5655	5655	5655	5655
X2	9.1	3.0	10.7	7.9	7.1	12.5	5.9	5.1	10.6	1.2
Gvalue	9.2	3.0	10.9	8.0	6.9	12.3	6.0	5.1	10.7	1.2
Gv/N	0.162	0.054	0.192	0.141	0.123	0.218	0.105	0.090	0.189	0.021
Prob	0.02728	0.38424	0.01239	0.04624	0.07400	0.00636	0.11366	0.16406	0.01371	0.76252

	101	102	103	104	105	106	107	108	109	110
a_O	1672	1744	1511	1727	1737	1530	1651	1730	1498	1691
	(29.6)	(30.8)	(26.7)	(30.5)	(30.7)	(27.1)	(29.2)	(30.6)	(26.5)	(29.9)
a_E	1741.0	1730.6	1561.8	1741.0	1730.6	1561.5	1740.7	1730.3	1561.0	1740.1
	(30.8)	(30.6)	(27.6)	(30.8)	(30.6)	(27.6)	(30.8)	(30.6)	(27.6)	(30.8)
	−135.2	26.9	−100.0	−27.8	12.8	−62.4	−174.6	−0.6	−123.4	−96.7
	−2.391	0.476	−1.768	−0.492	0.227	−1.104	−3.089	−0.010	−2.183	−1.711
t_O	1753	1970	1098	1751	1976	1150	1799	1995	1101	1765
	(31.0)	(34.8)	(19.4)	(31.0)	(34.9)	(20.3)	(31.8)	(35.3)	(19.5)	(31.2)
t_E	1707.6	1914.4	1092.2	1707.6	1914.4	1092.0	1707.3	1914.0	1091.6	1706.7
	(30.2)	(33.9)	(19.3)	(30.2)	(33.9)	(19.3)	(30.2)	(33.9)	(19.3)	(30.2)
	92.0	112.9	11.7	87.9	125.2	119.0	188.3	165.3	18.9	118.6
	1.627	1.996	0.206	1.555	2.215	2.106	3.330	2.924	0.334	2.099
g_O	1016	997	1882	902	1013	1787	917	941	1864	954
	(18.0)	(17.6)	(33.3)	(16.0)	(17.9)	(31.6)	(16.2)	(16.6)	(33.0)	(16.9)
g_E	942.3	960.2	1827.3	942.3	960.2	1827.0	942.1	960.1	1826.3	941.8
	(16.7)	(17.0)	(32.3)	(16.7)	(17.0)	(32.3)	(16.7)	(17.0)	(32.3)	(16.7)
	153.0	74.9	111.0	−78.9	108.4	−79.1	−49.6	−37.7	76.1	24.5
	2.705	1.325	1.963	−1.395	1.917	−1.399	−0.878	−0.667	1.346	0.434
c_O	1214	944	1164	1275	929	1187	1287	988	1189	1242
	(21.5)	(16.7)	(20.6)	(22.5)	(16.4)	(21.0)	(22.8)	(17.5)	(21.0)	(22.0)
c_E	1264.1	1049.8	1173.7	1264.1	1049.8	1173.5	1263.9	1049.6	1173.1	1263.4
	(22.4)	(18.6)	(20.8)	(22.4)	(18.6)	(20.8)	(22.4)	(18.6)	(20.8)	(22.4)
	−98.2	−200.6	−19.3	21.9	−227.2	27.2	46.6	−119.6	32.1	−42.5
	−1.737	−3.547	−0.341	0.386	−4.017	0.481	0.825	−2.115	0.568	−0.753
N	5655	5655	5655	5655	5655	5654	5654	5654	5652	5652
X2	11.7	13.8	3.4	3.0	18.8	4.8	10.6	7.4	3.6	3.9
Gvalue	11.6	14.1	3.4	3.1	19.3	4.7	10.6	7.5	3.6	3.9
Gv/N	0.205	0.250	0.060	0.054	0.341	0.083	0.188	0.132	0.064	0.069
Prob	0.00889	0.00272	0.33354	0.38393	0.00024	0.19453	0.01385	0.05884	0.30254	0.27361

	111	112	113	114	115	116	117	118	119	120
a_O	1652	1493	1667	1710	1507	1552	1690	1601	1688	1745
	(29.2)	(26.4)	(29.5)	(30.3)	(26.7)	(27.5)	(29.9)	(28.4)	(29.9)	(30.9)
a_E	1729.7	1560.4	1739.4	1729.1	1559.6	1738.5	1728.2	1559.6	1738.5	1728.2
	(30.6)	(27.6)	(30.8)	(30.6)	(27.6)	(30.8)	(30.6)	(27.6)	(30.8)	(30.6)
	−151.8	−131.9	−141.8	−37.9	−103.4	−352.3	−75.5	83.9	−99.5	33.9
	−2.686	−2.335	−2.510	−0.671	−1.832	−6.238	−1.336	1.485	−1.763	0.600
t_O	1947	1097	1692	1919	1108	1833	1916	1018	1632	1954
	(34.4)	(19.4)	(29.9)	(34.0)	(19.6)	(32.5)	(33.9)	(18.0)	(28.9)	(34.6)
t_E	1913.3	1091.2	1706.1	1912.7	1090.6	1705.2	1911.7	1090.6	1705.2	1911.7
	(33.9)	(19.3)	(30.2)	(33.9)	(19.3)	(30.2)	(33.9)	(19.3)	(30.2)	(33.9)
	67.9	11.6	−28.0	12.7	35.0	265.0	8.7	−140.3	−143.2	85.6
	1.201	0.205	−0.496	0.224	0.620	4.693	0.154	−2.485	−2.535	1.516
g_O	1017	1852	1023	1036	1841	960	1020	1851	991	994
	(18.0)	(32.8)	(18.1)	(18.3)	(32.6)	(17.0)	(18.1)	(32.8)	(17.5)	(17.6)
g_E	959.7	1825.7	941.5	959.4	1824.7	941.0	958.9	1824.7	941.0	958.9
	(17.0)	(32.3)	(16.7)	(17.0)	(32.3)	(16.7)	(17.0)	(32.3)	(16.7)	(17.0)
	117.9	53.0	169.9	159.2	32.7	38.4	126.1	52.9	102.6	71.5
	2.086	0.938	3.007	2.818	0.579	0.680	2.233	0.937	1.818	1.267
c_O	1036	1208	1268	985	1191	1302	1021	1177	1336	954
	(18.3)	(21.4)	(22.4)	(17.4)	(21.1)	(23.1)	(18.1)	(20.8)	(23.7)	(16.9)
c_E	1049.3	1172.6	1263.0	1048.9	1172.0	1262.3	1048.3	1172.0	1262.3	1048.3
	(18.6)	(20.8)	(22.4)	(18.6)	(20.8)	(22.4)	(18.6)	(20.8)	(22.4)	(18.6)
	−26.3	71.8	10.0	−123.8	38.3	80.6	−53.9	10.0	151.6	−179.9
	−0.466	1.270	0.177	−2.191	0.678	1.427	−0.955	0.177	2.684	−3.186

```
N          5652    5650    5650    5650    5647    5647    5647    5647    5647    5647
X2          7.7     4.4    10.2    10.2     2.5    31.2     5.5     6.3    11.6    10.9
Gvalue      7.7     4.4    10.1    10.2     2.5    31.7     5.4     6.4    11.5    11.1
Gv/N      0.135   0.078   0.178   0.180   0.045   0.562   0.096   0.114   0.204   0.197
Prob    0.05377 0.21947 0.01807 0.01719 0.47163 0.00000 0.14508 0.09225 0.00931 0.01109
        -------------------------------------------------------------------------------
            121     122     123     124     125     126     127     128     129     130
        -------------------------------------------------------------------------------
a_O        1502    1716    1789    1519    1710    1826    1539    1675    1723    1540
        ( 26.6) ( 30.4) ( 31.7) ( 26.9) ( 30.3) ( 32.4) ( 27.3) ( 29.7) ( 30.5) ( 27.3)
a_E      1558.8  1737.6  1727.2  1558.8  1737.6  1727.2  1558.2  1737.0  1726.6  1558.0
        ( 27.6) ( 30.8) ( 30.6) ( 27.6) ( 30.8) ( 30.6) ( 27.6) ( 30.8) ( 30.6) ( 27.6)
         -111.5   -42.9   125.7   -78.5   -54.7   203.1   -38.2  -121.7    -7.2   -35.7
         -1.975  -0.760   2.227  -1.392  -0.970   3.598  -0.677  -2.157  -0.128  -0.633
t_O        1091    1687    1927    1088    1691    1949    1099    1689    1981    1120
        ( 19.3) ( 29.9) ( 34.1) ( 19.3) ( 30.0) ( 34.5) ( 19.5) ( 29.9) ( 35.1) ( 19.9)
t_E      1090.1  1704.3  1910.6  1090.1  1704.3  1910.6  1089.7  1703.7  1910.0  1089.5
        ( 19.3) ( 30.2) ( 33.9) ( 19.3) ( 30.2) ( 33.9) ( 19.3) ( 30.2) ( 33.9) ( 19.3)
            1.9   -34.4    32.9    -4.1   -26.4    77.5    18.7   -29.2   144.7    61.9
          0.033  -0.609   0.582  -0.073  -0.468   1.373   0.332  -0.517   2.564   1.097
g_O        1889     987    1001    1878     947     981    1857     921     980    1820
        ( 33.5) ( 17.5) ( 17.7) ( 33.3) ( 16.8) ( 17.4) ( 32.9) ( 16.3) ( 17.4) ( 32.3)
g_E      1823.8   940.5   958.4  1823.8   940.5   958.4  1823.1   940.1   958.0  1822.8
        ( 32.3) ( 16.7) ( 17.0) ( 32.3) ( 16.7) ( 17.0) ( 32.3) ( 16.7) ( 17.0) ( 32.3)
          132.8    95.3    87.2   110.1    13.1    45.8    68.4   -37.9    44.5    -5.6
          2.353   1.689   1.544   1.950   0.232   0.812   1.212  -0.672   0.788  -0.099
c_O        1162    1254     927    1159    1296     888    1147    1357     958    1161
        ( 20.6) ( 22.2) ( 16.4) ( 20.5) ( 23.0) ( 15.7) ( 20.3) ( 24.1) ( 17.0) ( 20.6)
c_E      1171.4  1261.7  1047.8  1171.4  1261.7  1047.8  1171.0  1261.2  1047.4  1170.8
        ( 20.8) ( 22.4) ( 18.6) ( 20.8) ( 22.4) ( 18.6) ( 20.8) ( 22.4) ( 18.6) ( 20.8)
          -18.7   -15.3  -227.1   -24.7    69.6  -293.8   -47.5   198.7  -170.9   -19.5
         -0.332  -0.271  -4.023  -0.437   1.233  -5.206  -0.841   3.521  -3.030  -0.345

N          5644    5644    5644    5644    5644    5644    5642    5642    5642    5641
X2          4.5     2.8    18.2     2.8     1.5    31.3     1.4    10.0    10.8     1.1
Gvalue      4.5     2.8    18.7     2.8     1.5    32.5     1.4     9.9    11.0     1.1
Gv/N      0.079   0.049   0.331   0.049   0.027   0.577   0.025   0.175   0.195   0.020
Prob    0.21438 0.43090 0.00032 0.43066 0.67875 0.00000 0.69659 0.01984 0.01186 0.76722
        -------------------------------------------------------------------------------
            131     132     133     134     135     136     137     138     139     140
        -------------------------------------------------------------------------------
a_O        1728    1701    1558    1683    1753    1500    1652    1746    1491    1707
        ( 30.6) ( 30.2) ( 27.6) ( 29.8) ( 31.1) ( 26.6) ( 29.3) ( 31.0) ( 26.5) ( 30.3)
a_E      1736.7  1726.3  1557.4  1736.1  1725.7  1556.8  1735.4  1725.1  1555.7  1734.2
        ( 30.8) ( 30.6) ( 27.6) ( 30.8) ( 30.6) ( 27.6) ( 30.8) ( 30.6) ( 27.6) ( 30.8)
          -17.3   -50.3     1.2  -104.5    55.0  -111.6  -162.8    42.1  -126.8   -54.0
         -0.307  -0.891   0.021  -1.853   0.976  -1.980  -2.888   0.746  -2.250  -0.958
t_O        1697    1908    1138    1623    1976    1113    1694    1920    1142    1746
        ( 30.1) ( 33.8) ( 20.2) ( 28.8) ( 35.0) ( 19.7) ( 30.1) ( 34.1) ( 20.3) ( 31.0)
t_E      1703.4  1909.6  1089.1  1702.8  1908.9  1088.7  1702.2  1908.3  1087.9  1700.9
        ( 30.2) ( 33.9) ( 19.3) ( 30.2) ( 33.9) ( 19.3) ( 30.2) ( 33.9) ( 19.3) ( 30.2)
          -12.7    -3.2   100.0  -155.7   136.4    49.1   -16.3    23.5   110.8    91.3
         -0.225  -0.058   1.773  -2.761   2.419   0.871  -0.289   0.417   1.967   1.621
g_O         964    1029    1792     989     948    1881     982    1007    1850     928
        ( 17.1) ( 18.2) ( 31.8) ( 17.5) ( 16.8) ( 33.4) ( 17.4) ( 17.9) ( 32.8) ( 16.5)
g_E       940.0   957.8  1822.1   939.6   957.5  1821.5   939.3   957.2  1820.2   938.6
        ( 16.7) ( 17.0) ( 32.3) ( 16.7) ( 17.0) ( 32.3) ( 16.7) ( 17.0) ( 32.3) ( 16.7)
           48.6   147.5   -59.8   101.3   -18.9   120.9    87.3   102.2    60.1   -21.2
          0.862   2.614  -1.060   1.796  -0.335   2.145   1.548   1.813   1.066  -0.376
c_O        1252    1003    1151    1344     962    1143    1309     964    1150    1252
        ( 22.2) ( 17.8) ( 20.4) ( 23.8) ( 17.1) ( 20.3) ( 23.2) ( 17.1) ( 20.4) ( 22.2)
c_E      1261.0  1047.2  1170.4  1260.5  1046.8  1169.9  1260.1  1046.5  1169.1  1259.2
        ( 22.4) ( 18.6) ( 20.8) ( 22.4) ( 18.6) ( 20.8) ( 22.4) ( 18.6) ( 20.8) ( 22.4)
          -17.9   -86.5   -38.4   172.3  -162.6   -53.3    99.7  -158.3   -37.9   -14.4
         -0.318  -1.534  -0.681   3.056  -2.884  -0.945   1.768  -2.808  -0.673  -0.255

N          5641    5641    5639    5639    5639    5637    5637    5637    5633    5633
X2          0.7     7.5     3.0    13.5     9.8     5.2     7.9     9.4     6.2     1.8
Gvalue      0.7     7.4     3.0    13.4     9.9     5.2     7.9     9.6     6.2     1.8
Gv/N      0.013   0.132   0.053   0.237   0.176   0.092   0.140   0.169   0.110   0.032
Prob    0.86378 0.05945 0.39352 0.00387 0.01924 0.15856 0.04810 0.02278 0.10340 0.62046
        -------------------------------------------------------------------------------
            141     142     143     144     145     146     147     148     149     150
        -------------------------------------------------------------------------------
a_O        1674    1516    1689    1655    1508    1609    1709    1495    1740    1696
        ( 29.7) ( 26.9) ( 30.0) ( 29.4) ( 26.8) ( 28.6) ( 30.3) ( 26.6) ( 30.9) ( 30.1)
a_E      1723.9  1555.5  1733.9  1723.6  1555.5  1733.9  1723.6  1554.4  1732.7  1722.3
        ( 30.6) ( 27.6) ( 30.8) ( 30.6) ( 27.6) ( 30.8) ( 30.6) ( 27.6) ( 30.8) ( 30.6)
          -98.3   -77.9   -88.6  -134.4   -93.5  -240.6   -29.0  -116.4    14.7   -52.3
         -1.745  -1.384  -1.574  -2.386  -1.660  -4.272  -0.515  -2.069   0.261  -0.929
t_O        1956    1124    1727    1966    1087    1689    1982    1126    1680    1968
        ( 34.7) ( 20.0) ( 30.7) ( 34.9) ( 19.3) ( 30.0) ( 35.2) ( 20.0) ( 29.9) ( 35.0)
t_E      1906.9  1087.7  1700.6  1906.6  1087.7  1700.6  1906.6  1087.0  1699.4  1905.2
        ( 33.9) ( 19.3) ( 30.2) ( 33.9) ( 19.3) ( 30.2) ( 33.9) ( 19.3) ( 30.2) ( 33.9)
           99.4    73.7    53.1   120.7    -1.5   -23.2   153.8    79.4   -38.6   127.6
          1.765   1.309   0.943   2.143  -0.026  -0.412   2.731   1.412  -0.687   2.267
g_O        1010    1871     943     990    1835     984     929    1834     967    1014
        ( 17.9) ( 33.2) ( 16.7) ( 17.6) ( 32.6) ( 17.5) ( 16.5) ( 32.6) ( 17.2) ( 18.0)
g_E       956.5  1819.9   938.5   956.3  1819.9   938.5   956.3  1818.6   937.8   955.6
        ( 17.0) ( 32.3) ( 16.7) ( 17.0) ( 32.3) ( 16.7) ( 17.0) ( 32.3) ( 16.7) ( 17.0)
          110.0   103.7     9.1    68.5    30.4    93.2   -53.8    30.9    59.3   120.2
          1.952   1.840   0.161   1.217   0.539   1.655  -0.956   0.550   1.053   2.136
c_O         993    1121    1273    1021    1202    1350    1012    1173    1241     950
        ( 17.6) ( 19.9) ( 22.6) ( 18.1) ( 21.3) ( 24.0) ( 18.0) ( 20.8) ( 22.1) ( 16.9)
c_E      1045.7  1168.9  1259.0  1045.5  1168.9  1259.0  1045.5  1168.1  1258.1  1044.8
        ( 18.6) ( 20.8) ( 22.4) ( 18.6) ( 20.8) ( 22.4) ( 18.6) ( 20.8) ( 22.4) ( 18.6)
         -102.8   -93.8    28.2   -48.5    67.1   188.5   -66.0     9.9   -33.9  -180.7
         -1.824  -1.666   0.501  -0.861   1.192   3.346  -1.172   0.175  -0.603  -3.211

N          5633    5632    5632    5632    5632    5632    5632    5628    5628    5628
X2          8.4     5.6     1.7     6.3     2.5    17.9     5.0     3.8     1.4    14.6
Gvalue      8.4     5.6     1.8     6.4     2.5    17.9     4.9     3.8     1.4    14.8
Gv/N      0.148   0.100   0.031   0.113   0.045   0.318   0.088   0.068   0.025   0.263
Prob    0.03924 0.13172 0.62446 0.09574 0.47213 0.00046 0.17592 0.28013 0.70880 0.00198
        -------------------------------------------------------------------------------
```

Ayel_PHYTOPLASMA
5UTR:

	50	49	48	47	46	45	44	43	42	41
a_O	305	313	315	303	309	295	317	321	308	318
	(44.0)	(45.2)	(45.5)	(43.7)	(44.6)	(42.6)	(45.7)	(46.3)	(44.4)	(45.9)
a_E	309.8	309.8	309.8	309.8	309.8	309.8	309.8	309.8	309.8	309.8
	(44.7)	(44.7)	(44.7)	(44.7)	(44.7)	(44.7)	(44.7)	(44.7)	(44.7)	(44.7)
	-9.6	6.4	10.4	-13.5	-1.7	-29.0	14.5	22.7	-3.7	16.5
	-1.386	0.917	1.502	-1.952	-0.242	-4.179	2.090	3.278	-0.529	2.386
t_O	220	215	223	224	237	236	215	214	234	231
	(31.7)	(31.0)	(32.2)	(32.3)	(34.2)	(34.1)	(31.0)	(30.9)	(33.8)	(33.3)
t_E	226.9	226.9	226.9	226.9	226.9	226.9	226.9	226.9	226.9	226.9
	(32.7)	(32.7)	(32.7)	(32.7)	(32.7)	(32.7)	(32.7)	(32.7)	(32.7)	(32.7)
	-13.6	-23.2	-7.8	-5.8	20.6	18.5	-23.2	-25.1	14.4	8.2
	-1.966	-3.348	-1.121	-0.837	2.973	2.672	-3.348	-3.620	2.075	1.188
g_O	61	65	53	65	50	71	54	61	56	61
	(8.8)	(9.4)	(7.6)	(9.4)	(7.2)	(10.2)	(7.8)	(8.8)	(8.1)	(8.8)
g_E	71.6	71.6	71.6	71.6	71.6	71.6	71.6	71.6	71.6	71.6
	(10.3)	(10.3)	(10.3)	(10.3)	(10.3)	(10.3)	(10.3)	(10.3)	(10.3)	(10.3)
	-19.6	-12.6	-31.9	-12.6	-35.9	-1.2	-30.5	-19.6	-27.5	-19.6
	-2.823	-1.817	-4.603	-1.817	-5.183	-0.175	-4.399	-2.823	-3.974	-2.823
c_O	107	100	102	101	97	91	107	97	95	83
	(15.4)	(14.4)	(14.7)	(14.6)	(14.0)	(13.1)	(15.4)	(14.0)	(13.7)	(12.0)
c_E	84.6	84.6	84.6	84.6	84.6	84.6	84.6	84.6	84.6	84.6
	(12.2)	(12.2)	(12.2)	(12.2)	(12.2)	(12.2)	(12.2)	(12.2)	(12.2)	(12.2)
	50.2	33.4	38.1	35.7	26.5	13.2	50.2	26.5	22.0	-3.2
	7.243	4.816	5.495	5.154	3.819	1.906	7.243	3.819	3.169	-0.466
N	693	693	693	693	693	693	693	693	693	693
X2	7.8	4.1	8.6	4.0	8.8	1.6	11.0	4.5	4.9	1.9
Gvalue	7.4	3.9	8.8	3.8	9.5	1.6	11.0	4.5	5.1	2.0
Gv/N	1.067	0.568	1.272	0.548	1.366	0.224	1.586	0.654	0.741	0.285
Prob	0.06027	0.26850	0.03182	0.28392	0.02367	0.66985	0.01178	0.20971	0.16235	0.57792

	40	39	38	37	36	35	34	33	32	31
a_O	296	301	316	324	303	305	329	304	289	307
	(42.7)	(43.4)	(45.6)	(46.8)	(43.7)	(44.0)	(47.5)	(43.9)	(41.7)	(44.3)
a_E	309.8	309.8	309.8	309.8	309.8	309.8	309.8	309.8	309.8	309.8
	(44.7)	(44.7)	(44.7)	(44.7)	(44.7)	(44.7)	(44.7)	(44.7)	(44.7)	(44.7)
	-27.1	-17.4	12.4	29.0	-13.5	-9.6	39.5	-11.6	-40.2	-5.7
	-3.904	-2.514	1.795	4.179	-1.952	-1.386	5.697	-1.669	-5.807	-0.816
t_O	246	235	221	238	221	237	238	245	258	262
	(35.5)	(33.9)	(31.9)	(34.3)	(31.9)	(34.2)	(34.3)	(35.4)	(37.2)	(37.8)
t_E	226.9	226.9	226.9	226.9	226.9	226.9	226.9	226.9	226.9	226.9
	(32.7)	(32.7)	(32.7)	(32.7)	(32.7)	(32.7)	(32.7)	(32.7)	(32.7)	(32.7)
	39.7	16.4	-11.7	22.7	-11.7	20.6	22.7	37.6	66.2	75.3
	5.732	2.373	-1.686	3.275	-1.686	2.973	3.275	5.420	9.558	10.869
g_O	59	60	69	50	67	71	43	72	61	42
	(8.5)	(8.7)	(10.0)	(7.2)	(9.7)	(10.2)	(6.2)	(10.4)	(8.8)	(6.1)
g_E	71.6	71.6	71.6	71.6	71.6	71.6	71.6	71.6	71.6	71.6
	(10.3)	(10.3)	(10.3)	(10.3)	(10.3)	(10.3)	(10.3)	(10.3)	(10.3)	(10.3)
	-22.9	-21.2	-5.1	-35.9	-8.9	-1.2	-43.9	0.8	-19.6	-44.8
	-3.298	-3.063	-0.739	-5.183	-1.287	-0.175	-6.329	0.113	-2.823	-6.467
c_O	92	97	87	81	102	80	83	72	85	82
	(13.3)	(14.0)	(12.6)	(11.7)	(14.7)	(11.5)	(12.0)	(10.4)	(12.3)	(11.8)
c_E	84.6	84.6	84.6	84.6	84.6	84.6	84.6	84.6	84.6	84.6
	(12.2)	(12.2)	(12.2)	(12.2)	(12.2)	(12.2)	(12.2)	(12.2)	(12.2)	(12.2)
	15.4	26.5	4.8	-7.1	38.1	-9.0	-3.2	-23.3	0.7	-5.2
	2.217	3.819	0.693	-1.025	5.495	-1.299	-0.466	-3.358	0.107	-0.747
N	693	693	693	693	693	693	693	693	693	693
X2	5.1	4.2	0.4	7.9	4.2	0.8	13.2	3.4	7.2	17.8
Gvalue	5.2	4.3	0.4	8.6	4.0	0.8	15.1	3.5	7.2	19.7
Gv/N	0.747	0.614	0.063	1.245	0.571	0.113	2.176	0.505	1.034	2.839
Prob	0.15932	0.23491	0.93196	0.03469	0.26638	0.85412	0.00175	0.32038	0.06677	0.00020

	30	29	28	27	26	25	24	23	22	21
a_O	297	343	326	340	376	343	342	346	354	369
	(42.9)	(49.5)	(47.0)	(49.1)	(54.3)	(49.5)	(49.4)	(49.9)	(51.1)	(53.2)
a_E	309.8	309.8	309.8	309.8	309.8	309.8	309.8	309.8	309.8	309.8
	(44.7)	(44.7)	(44.7)	(44.7)	(44.7)	(44.7)	(44.7)	(44.7)	(44.7)	(44.7)
	-25.1	69.7	33.1	63.2	145.5	69.7	67.5	76.4	94.3	129.0
	-3.628	10.065	4.783	9.115	21.001	10.065	9.747	11.022	13.612	18.609
t_O	255	220	233	217	187	222	219	207	209	209
	(36.8)	(31.7)	(33.6)	(31.3)	(27.0)	(32.0)	(31.6)	(29.9)	(30.2)	(30.2)
t_E	226.9	226.9	226.9	226.9	226.9	226.9	226.9	226.9	226.9	226.9
	(32.7)	(32.7)	(32.7)	(32.7)	(32.7)	(32.7)	(32.7)	(32.7)	(32.7)	(32.7)
	59.5	-13.6	12.3	-19.4	-72.4	-9.7	-15.6	-38.0	-34.4	-34.4
	8.586	-1.966	1.778	-2.799	-10.442	-1.404	-2.245	-5.489	-4.962	-4.962
g_O	73	51	41	56	62	49	58	55	43	38
	(10.5)	(7.4)	(5.9)	(8.1)	(8.9)	(7.1)	(8.4)	(7.9)	(6.2)	(5.5)
g_E	71.6	71.6	71.6	71.6	71.6	71.6	71.6	71.6	71.6	71.6
	(10.3)	(10.3)	(10.3)	(10.3)	(10.3)	(10.3)	(10.3)	(10.3)	(10.3)	(10.3)
	2.8	-34.6	-45.7	-27.5	-17.9	-37.2	-24.5	-29.0	-43.9	-48.2
	0.405	-4.996	-6.599	-3.974	-2.578	-5.365	-3.528	-4.189	-6.329	-6.949
c_O	68	79	93	80	68	79	74	85	87	77
	(9.8)	(11.4)	(13.4)	(11.5)	(9.8)	(11.4)	(10.7)	(12.3)	(12.6)	(11.1)
c_E	84.6	84.6	84.6	84.6	84.6	84.6	84.6	84.6	84.6	84.6
	(12.2)	(12.2)	(12.2)	(12.2)	(12.2)	(12.2)	(12.2)	(12.2)	(12.2)	(12.2)
	-29.8	-10.9	17.5	-9.0	-29.8	-10.9	-19.9	0.7	4.8	-14.6
	-4.294	-1.570	2.531	-1.299	-4.294	-1.570	-2.867	0.107	0.693	-2.100
N	693	693	693	693	693	693	693	693	693	693
X2	7.3	10.1	14.9	7.0	25.7	11.2	7.5	9.8	19.2	29.2
Gvalue	7.4	10.6	17.3	7.2	25.5	12.0	7.7	10.1	20.9	31.9
Gv/N	1.070	1.533	2.494	1.042	3.687	1.725	1.107	1.452	3.015	4.598
Prob	0.05987	0.01393	0.00062	0.06509	0.00001	0.00753	0.05332	0.01807	0.00011	0.00000

	20	19	18	17	16	15	14	13	12	11
a_O	348	374	336	357	386	378	385	371	351	334
	(50.2)	(54.0)	(48.5)	(51.5)	(55.7)	(54.5)	(55.6)	(53.5)	(50.6)	(48.2)
a_E	309.8	309.8	309.8	309.8	309.8	309.8	309.8	309.8	309.8	309.8
	(44.7)	(44.7)	(44.7)	(44.7)	(44.7)	(44.7)	(44.7)	(44.7)	(44.7)	(44.7)
	80.8	140.8	54.5	101.2	169.7	150.3	167.2	133.7	87.6	50.2
	11.665	20.314	7.860	14.597	24.484	21.691	24.132	19.288	12.635	7.238
t_O	195	207	204	187	170	155	125	122	120	110
	(28.1)	(29.9)	(29.4)	(27.0)	(24.5)	(22.4)	(18.0)	(17.6)	(17.3)	(15.9)

```
t_E       226.9   226.9   226.9   226.9   226.9   226.9   226.9   226.9   226.9   226.9
        ( 32.7) ( 32.7) ( 32.7) ( 32.7) ( 32.7) ( 32.7) ( 32.7) ( 32.7) ( 32.7) ( 32.7)
          -59.1   -38.0   -43.4   -72.4   -98.2  -118.2  -149.1  -151.4  -152.9  -159.3
         -8.531  -5.489  -6.269 -10.442 -14.169 -17.051 -21.511 -21.850 -22.064 -22.988
g_O          68      42      79      82      79     111     124     141     192     202
        (  9.8) (  6.1) ( 11.4) ( 11.8) ( 11.4) ( 16.0) ( 17.9) ( 20.3) ( 27.7) ( 29.1)
g_E        71.6    71.6    71.6    71.6    71.6    71.6    71.6    71.6    71.6    71.6
        ( 10.3) ( 10.3) ( 10.3) ( 10.3) ( 10.3) ( 10.3) ( 10.3) ( 10.3) ( 10.3) ( 10.3)
           -7.0   -44.8    15.5    22.2    15.5    97.3   136.2   191.1   378.7   419.0
         -1.015  -6.467   2.239   3.206   2.239  14.041  19.648  27.570  54.650  60.456
c_O          82      70      74      67      58      49      59      59      30      47
        ( 11.8) ( 10.1) ( 10.7) (  9.7) (  8.4) (  7.1) (  8.5) (  8.5) (  4.3) (  6.8)
c_E        84.6    84.6    84.6    84.6    84.6    84.6    84.6    84.6    84.6    84.6
        ( 12.2) ( 12.2) ( 12.2) ( 12.2) ( 12.2) ( 12.2) ( 12.2) ( 12.2) ( 12.2) ( 12.2)
           -5.2   -26.6   -19.9   -31.3   -43.8   -53.6   -42.6   -42.6   -62.2   -55.3
         -0.747  -3.834  -2.867  -4.517  -6.325  -7.728  -6.143  -6.143  -8.979  -7.978

N           693     693     693     693     693     693     693     693     693     693
X2          9.5    29.8     6.6    19.4    42.1    74.5   110.1   135.6   293.5   316.3
Gvalue      9.5    31.3     6.7    19.7    43.2    75.9   111.8   130.7   251.2   254.5
Gv/N      1.371   4.523   0.964   2.844   6.229  10.953  16.127  18.866  36.242  36.728
Prob    0.02331 0.00000 0.08287 0.00019 0.00000 0.00000 0.00000 0.00000 0.00000 0.00000
        -------------------------------------------------------------------------------
             10       9       8       7       6       5       4       3       2       1
        -------------------------------------------------------------------------------
a_O         286     314     277     271     297     334     332     349     346     349
        ( 41.3) ( 45.3) ( 40.0) ( 39.1) ( 42.9) ( 48.2) ( 47.9) ( 50.4) ( 49.9) ( 50.4)
a_E       309.8   309.8   309.8   309.8   309.8   309.8   309.8   309.8   309.8   309.8
        ( 44.7) ( 44.7) ( 44.7) ( 44.7) ( 44.7) ( 44.7) ( 44.7) ( 44.7) ( 44.7) ( 44.7)
          -45.8     8.4   -62.1   -72.6   -25.1    50.2    45.9    83.1    76.4    83.1
         -6.608   1.209  -8.957 -10.475  -3.628   7.238   6.619  11.987  11.022  11.987
t_O         160     172     202     263     246     231     273     230     241     246
        ( 23.1) ( 24.8) ( 29.1) ( 38.0) ( 35.5) ( 33.3) ( 39.4) ( 33.2) ( 34.8) ( 35.5)
t_E       226.9   226.9   226.9   226.9   226.9   226.9   226.9   226.9   226.9   226.9
        ( 32.7) ( 32.7) ( 32.7) ( 32.7) ( 32.7) ( 32.7) ( 32.7) ( 32.7) ( 32.7) ( 32.7)
         -111.8   -95.3   -47.0    77.6    39.7     8.2   100.9     6.2    29.0    39.7
        -16.135 -13.755  -6.782  11.200   5.732   1.188  14.566   0.895   4.187   5.732
g_O         209     167     159      98      86      61      34      55      44      38
        ( 30.2) ( 24.1) ( 22.9) ( 14.1) ( 12.4) (  8.8) (  4.9) (  7.9) (  6.3) (  5.5)
g_E        71.6    71.6    71.6    71.6    71.6    71.6    71.6    71.6    71.6    71.6
        ( 10.3) ( 10.3) ( 10.3) ( 10.3) ( 10.3) ( 10.3) ( 10.3) ( 10.3) ( 10.3) ( 10.3)
          447.7   282.8   253.7    61.5    31.5   -19.6   -50.7   -29.0   -42.9   -48.2
         64.606  40.811  36.603   8.873   4.545  -2.823  -7.309  -4.189  -6.185  -6.949
c_O          38      40      55      61      64      67      54      59      62      60
        (  5.5) (  5.8) (  7.9) (  8.8) (  9.2) (  9.7) (  7.8) (  8.5) (  8.9) (  8.7)
c_E        84.6    84.6    84.6    84.6    84.6    84.6    84.6    84.6    84.6    84.6
        ( 12.2) ( 12.2) ( 12.2) ( 12.2) ( 12.2) ( 12.2) ( 12.2) ( 12.2) ( 12.2) ( 12.2)
          -60.9   -60.0   -47.4   -39.9   -35.8   -31.3   -48.5   -42.6   -38.6   -41.3
         -8.781  -8.651  -6.841  -5.764  -5.161  -4.517  -7.002  -6.143  -5.568  -5.956

N           693     693     693     693     693     693     693     693     693     693
X2        310.9   164.0   123.2    26.9    10.1     7.2    41.8    16.6    21.8    29.5
Gvalue    229.3   135.9    97.2    26.6    10.3     7.5    47.6    17.7    24.0    33.4
Gv/N     33.081  19.613  14.024   3.834   1.488   1.086   6.874   2.551   3.457   4.814
Prob    0.00000 0.00000 0.00000 0.00001 0.01609 0.05695 0.00000 0.00051 0.00003 0.00000
        -------------------------------------------------------------------------------

CDS_INITIAL:
        -------------------------------------------------------------------------------
              1       2       3       4       5       6       7       8       9      10
        -------------------------------------------------------------------------------
a_O         514       0       0     341     314     364     398     332     321     349
        ( 74.2) (  0.0) (  0.0) ( 49.2) ( 45.3) ( 52.5) ( 57.4) ( 47.9) ( 46.3) ( 50.4)
a_E       265.8   277.6   275.1   265.8   277.6   275.1   265.8   277.6   275.1   265.8
        ( 38.4) ( 40.1) ( 39.7) ( 38.4) ( 40.1) ( 39.7) ( 38.4) ( 40.1) ( 39.7) ( 38.4)
          677.9     0.0     0.0   169.9    77.5   203.9   321.3   118.9    99.1   190.0
         97.819   0.000   0.000  24.513  11.181  29.417  46.365  17.163  14.296  27.424
t_O         129     693       0     164     220     234     141     203     254     186
        ( 18.6) (100.0) (  0.0) ( 23.7) ( 31.7) ( 33.8) ( 20.3) ( 29.3) ( 36.7) ( 26.8)
t_E       159.5   226.3   282.2   159.5   226.3   282.2   159.5   226.3   282.2   159.5
        ( 23.0) ( 32.7) ( 40.7) ( 23.0) ( 32.7) ( 40.7) ( 23.0) ( 32.7) ( 40.7) ( 23.0)
          -54.8  1551.0     0.0     9.0   -12.5   -87.6   -34.9   -44.2   -53.5    57.1
         -7.913 223.809   0.000   1.302  -1.801 -12.646  -5.029  -6.373  -7.714   8.234
g_O          50       0     693     104      47      52      83      59      39      84
        (  7.2) (  0.0) (100.0) ( 15.0) (  6.8) (  7.5) ( 12.0) (  8.5) (  5.6) ( 12.1)
g_E       164.0    70.4    46.6   164.0    70.4    46.6   164.0    70.4    46.6   164.0
        ( 23.7) ( 10.2) (  6.7) ( 23.7) ( 10.2) (  6.7) ( 23.7) ( 10.2) (  6.7) ( 23.7)
         -118.8     0.0  3740.2   -94.7   -38.0    11.3  -113.0   -20.8   -14.0  -112.4
        -17.137   0.000 539.715 -13.663  -5.480   1.633 -16.307  -3.007  -2.014 -16.213
c_O           0       0       0      84     112      43      71      99      79      74
        (  0.0) (  0.0) (  0.0) ( 12.1) ( 16.2) (  6.2) ( 10.2) ( 14.3) ( 11.4) ( 10.7)
c_E       103.7   118.7    89.1   103.7   118.7    89.1   103.7   118.7    89.1   103.7
        ( 15.0) ( 17.1) ( 12.9) ( 15.0) ( 17.1) ( 12.9) ( 15.0) ( 17.1) ( 12.9) ( 15.0)
            0.0     0.0     0.0   -35.4   -13.1   -62.6   -53.8   -36.0   -19.0   -49.9
          0.000   0.000   0.000  -5.102  -1.884  -9.038  -7.758  -5.191  -2.737  -7.202

N           693     693     693     693     693     693     693     693     693     693
X2        420.5  1428.9  9604.0    47.1    13.1    61.4   118.2    18.2    12.9    77.9
Gvalue    504.3  1551.0  3740.2    48.9    14.0    64.9   119.7    18.0    12.7    84.8
Gv/N     72.769 223.809 539.715   7.050   2.016   9.366  17.270   2.592   1.831  12.243
Prob    0.00000 1.00000 1.00000 0.00000 0.00294 0.00000 0.00000 0.00045 0.00535 0.00000
        -------------------------------------------------------------------------------
             11      12      13      14      15      16      17      18      19      20
        -------------------------------------------------------------------------------
a_O         301     314     339     328     320     328     339     321     313     308
        ( 43.4) ( 45.3) ( 48.9) ( 47.3) ( 46.2) ( 47.3) ( 48.9) ( 46.3) ( 45.2) ( 44.4)
a_E       277.6   275.1   265.8   277.6   275.1   265.8   277.6   275.1   265.8   277.6
        ( 40.1) ( 39.7) ( 38.4) ( 40.1) ( 39.7) ( 38.4) ( 40.1) ( 39.7) ( 38.4) ( 40.1)
           48.8    83.1   164.9   109.6    96.8   137.9   135.6    99.1   102.3    64.1
          7.045  11.986  23.794  15.809  13.963  19.899  19.566  14.296  14.760   9.252
t_O         266     244     148     217     252     172     229     279     144     240
        ( 38.4) ( 35.2) ( 21.4) ( 31.3) ( 36.4) ( 24.8) ( 33.0) ( 40.3) ( 20.8) ( 34.6)
t_E       226.3   282.2   159.5   226.3   282.2   159.5   226.3   282.2   159.5   226.3
        ( 32.7) ( 40.7) ( 23.0) ( 32.7) ( 40.7) ( 23.0) ( 32.7) ( 40.7) ( 23.0) ( 32.7)
           85.9   -71.0   -22.2   -18.3   -57.0    25.8     5.4    -6.3   -29.5    28.2
         12.399 -10.239  -3.209  -2.636  -8.229   3.730   0.776  -0.915  -4.261   4.063
g_O          42      37     132      46      52      98      44      28     117      68
        (  6.1) (  5.3) ( 19.0) (  6.6) (  7.5) ( 14.1) (  6.3) (  4.0) ( 16.9) (  9.8)
g_E        70.4    46.6   164.0    70.4    46.6   164.0    70.4    46.6   164.0    70.4
        ( 10.2) (  6.7) ( 23.7) ( 10.2) (  6.7) ( 23.7) ( 10.2) (  6.7) ( 23.7) ( 10.2)
          -43.4   -17.1   -57.2   -39.1    11.3  -100.9   -41.4   -28.6   -79.0    -4.7
         -6.260  -2.472  -8.259  -5.649   1.633 -14.555  -5.968  -4.123 -11.394  -0.680
```

```
c_O          84       98       74      102       69       95       81       65      119       77
       ( 12.1) ( 14.1) ( 10.7) ( 14.7) ( 10.0) ( 13.7) ( 11.7) (  9.4) ( 17.2) ( 11.1)
c_E       118.7     89.1    103.7    118.7     89.1    103.7    118.7     89.1    103.7    118.7
       ( 17.1) ( 12.9) ( 15.0) ( 17.1) ( 12.9) ( 15.0) ( 17.1) ( 12.9) ( 15.0) ( 17.1)
          -58.1     18.7    -49.9    -31.0    -35.2    -16.6    -61.9    -41.0     32.8    -66.7
         -8.387    2.701   -7.202   -4.469   -5.085   -2.397   -8.938   -5.911    4.734   -9.622

N           693      693      693      693      693      693      693      693      693      693
X2         30.5     13.6     35.7     20.4     15.7     42.8     35.5     21.7     25.6     18.9
Gvalue     33.2     13.7     35.5     21.2     15.8     46.3     37.7     23.2     26.6     20.9
Gv/N      4.796    1.975    5.123    3.055    2.282    6.677    5.436    3.347    3.839    3.013
Prob    0.00000  0.00336  0.00000  0.00010  0.00124  0.00000  0.00000  0.00004  0.00001  0.00011
        -------------------------------------------------------------------------------------
             21       22       23       24       25       26       27       28       29       30
        -------------------------------------------------------------------------------------
a_O         320      341      284      315      314      278      299      337      294      305
       ( 46.2) ( 49.2) ( 41.0) ( 45.5) ( 45.3) ( 40.1) ( 43.1) ( 48.6) ( 42.4) ( 44.0)
a_E       275.1    265.8    277.6    275.1    265.8    277.6    275.1    265.8    277.6    275.1
       ( 39.7) ( 38.4) ( 40.1) ( 39.7) ( 38.4) ( 40.1) ( 39.7) ( 38.4) ( 40.1) ( 39.7)
           96.8    169.9     13.0     85.3    104.6      0.9     49.8    159.9     33.9     62.9
         13.963   24.513    1.882   12.313   15.097    0.129    7.190   23.078    4.885    9.083
t_O         264      154      258      260      146      227      263      158      244      262
       ( 38.1) ( 22.2) ( 37.2) ( 37.5) ( 21.1) ( 32.8) ( 38.0) ( 22.8) ( 35.2) ( 37.8)
t_E       282.2    159.5    226.3    282.2    159.5    226.3    282.2    159.5    226.3    282.2
       ( 40.7) ( 23.0) ( 32.7) ( 40.7) ( 23.0) ( 32.7) ( 40.7) ( 23.0) ( 32.7) ( 40.7)
          -35.2    -10.9     67.6    -42.6    -25.9      1.3    -37.0     -3.1     36.7    -38.9
         -5.076   -1.573    9.752   -6.145   -3.739    0.194   -5.345   -0.445    5.294   -5.613
g_O          40      112       69       46      131       72       34      108       76       51
       (  5.8) ( 16.2) ( 10.0) (  6.6) ( 18.9) ( 10.4) (  4.9) ( 15.6) ( 11.0) (  7.4)
g_E        46.6    164.0     70.4     46.6    164.0     70.4     46.6    164.0     70.4     46.6
       (  6.7) ( 23.7) ( 10.2) (  6.7) ( 23.7) ( 10.2) (  6.7) ( 23.7) ( 10.2) (  6.7)
          -12.3    -85.4     -2.8     -1.3    -58.8      3.2    -21.5    -90.2     11.6      9.1
         -1.773  -12.319   -0.399   -0.183   -8.484    0.468   -3.102  -13.012    1.680    1.315
c_O          69       86       82       72      102      116       97       90       79       75
       ( 10.0) ( 12.4) ( 11.8) ( 10.4) ( 14.7) ( 16.7) ( 14.0) ( 13.0) ( 11.4) ( 10.8)
c_E        89.1    103.7    118.7     89.1    103.7    118.7     89.1    103.7    118.7     89.1
       ( 12.9) ( 15.0) ( 17.1) ( 12.9) ( 15.0) ( 17.1) ( 12.9) ( 15.0) ( 17.1) ( 12.9)
          -35.2    -32.2    -60.7    -30.6     -3.3     -5.4     16.5    -25.5    -64.4    -25.8
         -5.085   -4.640   -8.758   -4.422   -0.480   -0.777    2.386   -3.675   -9.287   -3.723

N           693      693      693      693      693      693      693      693      693      693
X2         14.0     40.9     16.0     10.8     16.5      0.1      7.5     40.0     16.1      7.3
Gvalue     14.1     41.4     17.2     10.8     16.6      0.1      7.8     41.2     17.8      7.4
Gv/N      2.029    5.981    2.478    1.563    2.393    0.015    1.129    5.946    2.572    1.063
Prob    0.00282  0.00000  0.00065  0.01267  0.00086  0.99160  0.04978  0.00000  0.00048  0.06112
        -------------------------------------------------------------------------------------
             31       32       33       34       35       36       37       38       39       40
        -------------------------------------------------------------------------------------
a_O         301      249      256      318      274      262      302      292      283      294
       ( 43.4) ( 35.9) ( 36.9) ( 45.9) ( 39.5) ( 37.8) ( 43.6) ( 42.1) ( 40.8) ( 42.4)
a_E       265.8    277.6    275.1    265.8    277.6    275.1    265.8    277.6    275.1    265.8
       ( 38.4) ( 40.1) ( 39.7) ( 38.4) ( 40.1) ( 39.7) ( 38.4) ( 40.1) ( 39.7) ( 38.4)
           74.8    -54.1    -36.8    114.0     -7.1    -25.6     77.1     29.6     16.0     59.3
         10.799   -7.801   -5.316   16.451   -1.019   -3.689   11.124    4.276    2.313    8.551
t_O         163      254      293      170      223      286      145      231      284      192
       ( 23.5) ( 36.7) ( 42.3) ( 24.5) ( 32.2) ( 41.3) ( 20.9) ( 33.3) ( 41.0) ( 27.7)
t_E       159.5    226.3    282.2    159.5    226.3    282.2    159.5    226.3    282.2    159.5
       ( 23.0) ( 32.7) ( 40.7) ( 23.0) ( 32.7) ( 40.7) ( 23.0) ( 32.7) ( 40.7) ( 23.0)
            7.0     58.6     22.0     21.6     -6.6      7.7    -27.7      9.4      3.6     71.1
          1.007    8.456    3.179    3.113   -0.953    1.108   -4.001    1.362    0.525   10.259
g_O         147       80       54      118       78       43      129       56       43      122
       ( 21.2) ( 11.5) (  7.8) ( 17.0) ( 11.3) (  6.2) ( 18.6) (  8.1) (  6.2) ( 17.6)
g_E       164.0     70.4     46.6    164.0     70.4     46.6    164.0     70.4     46.6    164.0
       ( 23.7) ( 10.2) (  6.7) ( 23.7) ( 10.2) (  6.7) ( 23.7) ( 10.2) (  6.7) ( 23.7)
          -32.1     20.5     15.8    -77.6     16.0     -7.0    -61.9    -25.6     -7.0    -72.1
         -4.632    2.953    2.284  -11.201    2.309   -1.008   -8.927   -3.698   -1.008  -10.407
c_O          82      110       90       87      118      102      117      114       83       85
       ( 11.8) ( 15.9) ( 13.0) ( 12.6) ( 17.0) ( 14.7) ( 16.9) ( 16.5) ( 12.0) ( 12.3)
c_E       103.7    118.7     89.1    103.7    118.7     89.1    103.7    118.7     89.1    103.7
       ( 15.0) ( 17.1) ( 12.9) ( 15.0) ( 17.1) ( 12.9) ( 15.0) ( 17.1) ( 12.9) ( 15.0)
          -38.5    -16.8      1.9    -30.5     -1.4     27.6     28.3     -9.3    -11.7    -33.8
         -5.551   -2.423    0.268   -4.404   -0.208    3.989    4.082   -1.336   -1.692   -4.873

N           693      693      693      693      693      693      693      693      693      693
X2         11.0      8.3      2.9     26.5      0.9      2.8     15.4      4.0      0.9     23.7
Gvalue     11.2      8.2      2.9     27.4      0.9      2.8     15.8      4.2      1.0     24.5
Gv/N      1.622    1.184    0.416    3.959    0.129    0.399    2.277    0.605    0.137    3.530
Prob    0.01048  0.04190  0.41033  0.00000  0.82724  0.42886  0.00126  0.24129  0.81280  0.00002
        -------------------------------------------------------------------------------------
             41       42       43       44       45       46       47       48       49       50
        -------------------------------------------------------------------------------------
a_O         283      281      286      245      281      306      258      264      255      265
       ( 40.8) ( 40.5) ( 41.3) ( 35.4) ( 40.5) ( 44.2) ( 37.2) ( 38.1) ( 36.8) ( 38.2)
a_E       277.6    275.1    265.8    277.6    275.1    265.8    277.6    275.1    265.8    277.6
       ( 40.1) ( 39.7) ( 38.4) ( 40.1) ( 39.7) ( 38.4) ( 40.1) ( 39.7) ( 38.4) ( 40.1)
           11.0     11.9     41.9    -61.1     11.9     86.2    -37.7    -21.7    -21.2    -24.5
          1.588    1.722    6.041   -8.821    1.722   12.433   -5.439   -3.137   -3.057   -3.539
t_O         233      286      196      242      264      168      243      281      215      249
       ( 33.6) ( 41.3) ( 28.3) ( 34.9) ( 38.1) ( 24.2) ( 35.1) ( 40.5) ( 31.0) ( 35.9)
t_E       226.3    282.2    159.5    226.3    282.2    159.5    226.3    282.2    159.5    226.3
       ( 32.7) ( 40.7) ( 23.0) ( 32.7) ( 40.7) ( 23.0) ( 32.7) ( 40.7) ( 23.0) ( 32.7)
           13.5      7.7     80.7     32.4    -35.2     17.3     34.5     -2.4    128.3     47.5
          1.954    1.108   11.639    4.676   -5.076    2.502    4.985   -0.342   18.508    6.860
g_O          90       42      116       73       52      131       72       53      116       73
       ( 13.0) (  6.1) ( 16.7) ( 10.5) (  7.5) ( 18.9) ( 10.4) (  7.6) ( 16.7) ( 10.5)
g_E        70.4     46.6    164.0     70.4     46.6    164.0     70.4     46.6    164.0     70.4
       ( 10.2) (  6.7) ( 23.7) ( 10.2) (  6.7) ( 23.7) ( 10.2) (  6.7) ( 23.7) ( 10.2)
           44.2     -8.8    -80.3      5.3     11.3    -58.8      3.2     13.6    -80.3      5.3
          6.381   -1.270  -11.584    0.765    1.633   -8.484    0.468    1.955  -11.584    0.765
c_O          87       84       95      133       96       88      120       95      107      106
       ( 12.6) ( 12.1) ( 13.7) ( 19.2) ( 13.9) ( 12.7) ( 17.3) ( 13.7) ( 15.4) ( 15.3)
c_E       118.7     89.1    103.7    118.7     89.1    103.7    118.7     89.1    103.7    118.7
       ( 17.1) ( 12.9) ( 15.0) ( 17.1) ( 12.9) ( 15.0) ( 17.1) ( 12.9) ( 15.0) ( 17.1)
          -54.1     -9.9    -16.6     30.2     14.4    -28.9      2.6     12.2      6.7    -24.0
         -7.806   -1.422   -2.397    4.359    2.074   -4.164    0.370    1.766    0.974   -3.468

N           693      693      693      693      693      693      693      693      693      693
X2         14.2      0.9     24.6      6.7      2.5     15.5      2.7      1.7     33.8      4.3
Gvalue     14.7      0.9     25.6      6.8      2.4     15.8      2.7      1.7     33.6      4.3
Gv/N      2.116    0.137    3.700    0.979    0.352    2.287    0.384    0.242    4.842    0.618
Prob    0.00213  0.81347  0.00001  0.07909  0.48574  0.00122  0.44709  0.64253  0.00000  0.23224
        -------------------------------------------------------------------------------------
```

	51	52	53	54	55	56	57	58	59	60
a_O	295	254	238	287	276	256	284	266	223	274
	(42.6)	(36.7)	(34.3)	(41.4)	(39.8)	(36.9)	(41.0)	(38.4)	(32.2)	(39.5)
a_E	275.1	265.8	277.6	275.1	265.8	277.6	275.1	265.8	277.6	275.1
	(39.7)	(38.4)	(40.1)	(39.7)	(38.4)	(40.1)	(39.7)	(38.4)	(40.1)	(39.7)
	41.2	-23.1	-73.2	24.3	20.8	-41.4	18.1	0.4	-97.6	-2.2
	5.947	-3.333	-10.560	3.508	2.995	-5.972	2.610	0.053	-14.084	-0.316
t_O	274	204	279	273	180	252	277	193	263	279
	(39.5)	(29.4)	(40.3)	(39.4)	(26.0)	(36.4)	(40.0)	(27.8)	(38.0)	(40.3)
t_E	282.2	159.5	226.3	282.2	159.5	226.3	282.2	159.5	226.3	282.2
	(40.7)	(23.0)	(32.7)	(40.7)	(23.0)	(32.7)	(40.7)	(23.0)	(32.7)	(40.7)
	-16.1	100.3	116.7	-18.1	43.4	54.2	-10.3	73.5	79.0	-6.3
	-2.328	14.469	16.847	-2.608	6.265	7.814	-1.483	10.602	11.398	-0.915
g_O	47	137	76	51	141	58	37	142	103	46
	(6.8)	(19.8)	(11.0)	(7.4)	(20.3)	(8.4)	(5.3)	(20.5)	(14.9)	(6.6)
g_E	46.6	164.0	70.4	46.6	164.0	70.4	46.6	164.0	70.4	46.6
	(6.7)	(23.7)	(10.2)	(6.7)	(23.7)	(10.2)	(6.7)	(23.7)	(10.2)	(6.7)
	0.7	-49.2	11.6	9.1	-42.5	-22.5	-17.1	-40.8	78.4	-1.3
	0.104	-7.102	1.680	1.315	-6.138	-3.242	-2.472	-5.892	11.313	-0.183
c_O	77	98	100	82	96	127	95	92	104	94
	(11.1)	(14.1)	(14.4)	(11.8)	(13.9)	(18.3)	(13.7)	(13.3)	(15.0)	(13.6)
c_E	89.1	103.7	118.7	89.1	103.7	118.7	89.1	103.7	118.7	89.1
	(12.9)	(15.0)	(17.1)	(12.9)	(15.0)	(17.1)	(12.9)	(15.0)	(17.1)	(12.9)
	-22.4	-11.0	-34.3	-13.6	-14.8	17.1	12.2	-22.0	-27.5	10.1
	-3.237	-1.593	-4.953	-1.958	-2.132	2.470	1.766	-3.173	-3.974	1.460
N	693	693	693	693	693	693	693	693	693	693
X2	3.3	17.7	21.3	1.8	6.8	7.3	2.8	11.3	33.6	0.3
Gvalue	3.4	16.9	20.9	1.8	6.9	7.4	2.9	11.0	32.2	0.3
Gv/N	0.486	2.441	3.014	0.257	0.990	1.070	0.420	1.590	4.653	0.046
Prob	0.33866	0.00073	0.00011	0.61863	0.07648	0.05981	0.40530	0.01163	0.00000	0.95684

	61	62	63	64	65	66	67	68	69	70
a_O	286	252	283	267	242	303	246	247	272	270
	(41.3)	(36.4)	(40.8)	(38.5)	(34.9)	(43.7)	(35.5)	(35.6)	(39.2)	(39.0)
a_E	265.8	277.6	275.1	265.8	277.6	275.1	265.8	277.6	275.1	265.8
	(38.4)	(40.1)	(39.7)	(38.4)	(40.1)	(39.7)	(38.4)	(40.1)	(39.7)	(38.4)
	41.9	-48.7	16.0	2.4	-66.3	58.5	-38.1	-57.6	-6.2	8.4
	6.041	-7.024	2.313	0.343	-9.573	8.448	-5.500	-8.313	-0.889	1.217
t_O	180	249	267	199	273	270	208	279	286	183
	(26.0)	(35.9)	(38.5)	(28.7)	(39.4)	(39.0)	(30.0)	(40.3)	(41.3)	(26.4)
t_E	159.5	226.3	282.2	159.5	226.3	282.2	159.5	226.3	282.2	159.5
	(23.0)	(32.7)	(40.7)	(23.0)	(32.7)	(40.7)	(23.0)	(32.7)	(40.7)	(23.0)
	43.4	47.5	-29.5	87.9	102.4	-23.8	110.3	116.7	7.7	50.2
	6.265	6.860	-4.263	12.690	14.772	-3.440	15.919	16.847	1.108	7.243
g_O	140	68	47	133	58	48	159	75	51	151
	(20.2)	(9.8)	(6.8)	(19.2)	(8.4)	(6.9)	(22.9)	(10.8)	(7.4)	(21.8)
g_E	164.0	70.4	46.6	164.0	70.4	46.6	164.0	70.4	46.6	164.0
	(23.7)	(10.2)	(6.7)	(23.7)	(10.2)	(6.7)	(23.7)	(10.2)	(6.7)	(23.7)
	-44.2	-4.7	0.7	-55.7	-22.5	2.8	-9.8	9.5	9.1	-24.9
	-6.382	-0.680	0.104	-8.032	-3.242	0.398	-1.409	1.371	1.315	-3.588
c_O	87	124	96	94	120	72	80	92	84	89
	(12.6)	(17.9)	(13.9)	(13.6)	(17.3)	(10.4)	(11.5)	(13.3)	(12.1)	(12.8)
c_E	103.7	118.7	89.1	103.7	118.7	89.1	103.7	118.7	89.1	103.7
	(15.0)	(17.1)	(12.9)	(15.0)	(17.1)	(12.9)	(15.0)	(17.1)	(12.9)	(15.0)
	-30.5	10.8	14.4	-18.4	2.6	-30.6	-41.5	-46.9	-9.9	-27.2
	-4.404	1.556	2.074	-2.658	0.370	-4.422	-5.986	-6.771	-1.422	-3.921
N	693	693	693	693	693	693	693	693	693	693
X2	10.3	4.9	1.6	16.5	16.4	6.7	21.7	21.9	0.8	6.6
Gvalue	10.5	4.9	1.6	16.2	16.1	6.8	21.0	21.7	0.8	6.6
Gv/N	1.520	0.713	0.229	2.342	2.327	0.984	3.024	3.134	0.112	0.951
Prob	0.01452	0.17620	0.66277	0.00102	0.00107	0.07795	0.00011	0.00007	0.85507	0.08615

	71	72	73	74	75	76	77	78	79	80
a_O	245	267	255	264	240	267	257	270	292	270
	(35.4)	(38.5)	(36.8)	(38.1)	(34.6)	(38.5)	(37.1)	(39.0)	(42.1)	(39.0)
a_E	277.6	275.1	265.8	277.6	275.1	265.8	277.6	275.1	265.8	277.6
	(40.1)	(39.7)	(38.4)	(40.1)	(39.7)	(38.4)	(40.1)	(39.7)	(38.4)	(40.1)
	-61.1	-16.0	-21.2	-26.4	-65.5	2.4	-39.5	-10.1	54.9	-14.9
	-8.821	-2.302	-3.057	-3.814	-9.454	0.343	-5.706	-1.457	7.918	-2.150
t_O	286	309	183	251	316	175	272	312	158	236
	(41.3)	(44.6)	(26.4)	(36.2)	(45.6)	(25.3)	(39.2)	(45.0)	(22.8)	(34.1)
t_E	226.3	282.2	159.5	226.3	282.2	159.5	226.3	282.2	159.5	226.3
	(32.7)	(40.7)	(23.0)	(32.7)	(40.7)	(23.0)	(32.7)	(40.7)	(23.0)	(32.7)
	133.9	56.1	50.2	51.9	71.5	32.4	100.0	62.7	-3.1	19.8
	19.315	8.095	7.243	7.495	10.321	4.668	14.429	9.043	-0.445	2.850
g_O	59	38	171	65	47	131	58	33	133	80
	(8.5)	(5.5)	(24.7)	(9.4)	(6.8)	(18.9)	(8.4)	(4.8)	(19.2)	(11.5)
g_E	70.4	46.6	164.0	70.4	46.6	164.0	70.4	46.6	164.0	70.4
	(10.2)	(6.7)	(23.7)	(10.2)	(6.7)	(23.7)	(10.2)	(6.7)	(23.7)	(10.2)
	-20.8	-15.6	14.4	-10.4	0.7	-58.8	-22.5	-22.8	-55.7	20.5
	-3.007	-2.247	2.075	-1.496	0.104	-8.484	-3.242	-3.295	-8.032	2.953
c_O	103	79	84	113	90	120	106	78	110	107
	(14.9)	(11.4)	(12.1)	(16.3)	(13.0)	(17.3)	(15.3)	(11.3)	(15.9)	(15.4)
c_E	118.7	89.1	103.7	118.7	89.1	103.7	118.7	89.1	103.7	118.7
	(17.1)	(12.9)	(15.0)	(17.1)	(12.9)	(15.0)	(17.1)	(12.9)	(15.0)	(17.1)
	-29.3	-19.0	-35.4	-11.2	1.9	35.1	-24.0	-20.7	13.0	-22.2
	-4.223	-2.737	-5.102	-1.611	0.268	5.063	-3.468	-2.989	1.879	-3.211
N	693	693	693	693	693	693	693	693	693	693
X2	23.5	5.5	7.9	4.0	8.5	10.7	14.3	8.6	8.8	3.1
Gvalue	22.6	5.6	8.0	4.0	8.6	11.0	14.0	9.0	9.1	3.1
Gv/N	3.264	0.809	1.159	0.574	1.240	1.590	2.013	1.302	1.319	0.443
Prob	0.00005	0.13244	0.04539	0.26422	0.03523	0.01162	0.00297	0.02897	0.02744	0.38140

	81	82	83	84	85	86	87	88	89	90
a_O	242	244	252	256	271	275	275	264	257	294
	(34.9)	(35.2)	(36.4)	(36.9)	(39.1)	(39.7)	(39.7)	(38.1)	(37.1)	(42.4)
a_E	275.1	265.8	277.6	275.1	265.8	277.6	275.1	265.8	277.6	275.1
	(39.7)	(38.4)	(40.1)	(39.7)	(38.4)	(40.1)	(39.7)	(38.4)	(40.1)	(39.7)
	-62.0	-41.8	-48.7	-36.8	10.5	-5.1	-0.2	-3.6	-39.5	39.1
	-8.953	-6.030	-7.024	-5.316	1.511	-0.733	-0.028	-0.522	-5.706	5.638
t_O	297	205	264	318	186	237	291	191	264	288
	(42.9)	(29.6)	(38.1)	(45.9)	(26.8)	(34.2)	(42.0)	(27.6)	(38.1)	(41.6)
t_E	282.2	159.5	226.3	282.2	159.5	226.3	282.2	159.5	226.3	282.2
	(40.7)	(23.0)	(32.7)	(40.7)	(23.0)	(32.7)	(40.7)	(23.0)	(32.7)	(40.7)
	30.4	102.8	81.3	76.0	57.1	21.8	17.9	68.7	81.3	11.7

```
          4.385  14.830  11.731  10.965   8.234   3.151   2.583   9.918  11.731   1.695
g_O          50     141      64      38     140      64      43     138      60      33
        (  7.2) ( 20.3) (  9.2) (  5.5) ( 20.2) (  9.2) (  6.2) ( 19.9) (  8.7) (  4.8)
g_E        46.6   164.0    70.4    46.6   164.0    70.4    46.6   164.0    70.4    46.6
        (  6.7) ( 23.7) ( 10.2) (  6.7) ( 23.7) ( 10.2) (  6.7) ( 23.7) ( 10.2) (  6.7)
            7.0   -42.5   -12.2   -15.6   -44.2   -12.2    -7.0   -47.6   -19.2   -22.8
          1.004  -6.138  -1.759  -2.247  -6.382  -1.759  -1.008  -6.864  -2.767  -3.295
c_O         104     103     113      81      96     117      84     100     112      78
        ( 15.0) ( 14.9) ( 16.3) ( 11.7) ( 13.9) ( 16.9) ( 12.1) ( 14.4) ( 16.2) ( 11.3)
c_E        89.1   103.7   118.7    89.1   103.7   118.7    89.1   103.7   118.7    89.1
        ( 12.9) ( 15.0) ( 17.1) ( 12.9) ( 15.0) ( 17.1) ( 12.9) ( 15.0) ( 17.1) ( 12.9)
           32.2    -1.4   -11.2   -15.4   -14.8    -3.4    -9.9    -7.2   -13.1   -20.7
          4.650  -0.195  -1.611  -2.221  -2.132  -0.494  -1.422  -1.042  -1.884  -2.989

N           693     693     693     693     693     693     693     693     693     693
X2          7.5    18.0     9.5     8.2     8.6     1.1     0.8    10.5     9.7     6.8
Gvalue      7.5    17.1     9.3     8.2     8.5     1.1     0.9    10.3     9.5     7.3
Gv/N      1.086   2.466   1.336   1.181   1.231   0.165   0.124   1.489   1.373   1.050
Prob    0.05693 0.00068 0.02606 0.04231 0.03625 0.76652 0.83533 0.01605 0.02317 0.06366
        -------------------------------------------------------------------------------
             91      92      93      94      95      96      97      98      99     100
        -------------------------------------------------------------------------------
a_O         246     260     265     292     271     258     264     273     300     269
        ( 35.5) ( 37.5) ( 38.2) ( 42.1) ( 39.1) ( 37.2) ( 38.1) ( 39.4) ( 43.3) ( 38.8)
a_E       265.8   277.6   275.1   265.8   277.6   275.1   265.8   277.6   275.1   265.8
        ( 38.4) ( 40.1) ( 39.7) ( 38.4) ( 40.1) ( 39.7) ( 38.4) ( 40.1) ( 39.7) ( 38.4)
          -38.1   -34.0   -19.8    54.9   -12.9   -33.1    -3.6    -9.0    52.0     6.4
         -5.500  -4.902  -2.860   7.918  -1.869  -4.778  -0.522  -1.303   7.503   0.925
t_O         180     244     272     152     228     299     178     248     282     202
        ( 26.0) ( 35.2) ( 39.2) ( 21.9) ( 32.9) ( 43.1) ( 25.7) ( 35.8) ( 40.7) ( 29.1)
t_E       159.5   226.3   282.2   159.5   226.3   282.2   159.5   226.3   282.2   159.5
        ( 23.0) ( 32.7) ( 40.7) ( 23.0) ( 32.7) ( 40.7) ( 23.0) ( 32.7) ( 40.7) ( 23.0)
           43.4    36.7   -20.0   -14.7     3.4    34.6    39.0    45.4    -0.4    95.3
          6.265   5.294  -2.886  -2.126   0.484   4.994   5.622   6.545  -0.054  13.753
g_O         165      71      66     153      66      42     132      55      42     133
        ( 23.8) ( 10.2) (  9.5) ( 22.1) (  9.5) (  6.1) ( 19.0) (  7.9) (  6.1) ( 19.2)
g_E       164.0    70.4    46.6   164.0    70.4    46.6   164.0    70.4    46.6   164.0
        ( 23.7) ( 10.2) (  6.7) ( 23.7) ( 10.2) (  6.7) ( 23.7) ( 10.2) (  6.7) ( 23.7)
            2.1     1.2    45.8   -21.2    -8.5    -8.8   -57.2   -27.1    -8.8   -55.7
          0.302   0.175   6.613  -3.054  -1.228  -1.270  -8.259  -3.918  -1.270  -8.032
c_O         102     118      90      96     128      94     119     117      69      89
        ( 14.7) ( 17.0) ( 13.0) ( 13.9) ( 18.5) ( 13.6) ( 17.2) ( 16.9) ( 10.0) ( 12.8)
c_E       103.7   118.7    89.1   103.7   118.7    89.1   103.7   118.7    89.1   103.7
        ( 15.0) ( 17.1) ( 12.9) ( 15.0) ( 17.1) ( 12.9) ( 15.0) ( 17.1) ( 12.9) ( 15.0)
           -3.3    -1.4     1.9   -14.8    19.3    10.1    32.8    -3.4   -35.2   -27.2
         -0.480  -0.208   0.268  -2.132   2.779   1.460   4.734  -0.494  -5.085  -3.921

N           693     693     693     693     693     693     693     693     693     693
X2          4.1     2.5     8.8     4.2     1.2     2.8    10.6     5.5     7.2    19.3
Gvalue      4.1     2.5     7.9     4.2     1.2     2.8    10.9     5.8     7.6    18.9
Gv/N      0.587   0.359   1.135   0.605   0.167   0.406   1.574   0.831   1.093   2.725
Prob    0.25451 0.47728 0.04884 0.24126 0.76373 0.42139 0.01224 0.12411 0.05565 0.00029
        -------------------------------------------------------------------------------
            101     102     103     104     105     106     107     108     109     110
        -------------------------------------------------------------------------------
a_O         267     291     261     255     265     279     256     274     302     293
        ( 38.5) ( 42.0) ( 37.7) ( 36.8) ( 38.2) ( 40.3) ( 36.9) ( 39.5) ( 43.6) ( 42.3)
a_E       277.6   275.1   265.8   277.6   275.1   265.8   277.6   275.1   265.8   277.6
        ( 40.1) ( 39.7) ( 38.4) ( 40.1) ( 39.7) ( 38.4) ( 40.1) ( 39.7) ( 38.4) ( 40.1)
          -20.7    32.7    -9.5   -43.2   -19.8    27.0   -41.4    -2.2    77.1    31.7
         -2.987   4.720  -1.377  -6.237  -2.860   3.898  -5.972  -0.316  11.124   4.580
t_O         259     280     160     223     305     161     271     307     138     209
        ( 37.4) ( 40.4) ( 23.1) ( 32.2) ( 44.0) ( 23.2) ( 39.1) ( 44.3) ( 19.9) ( 30.2)
t_E       226.3   282.2   159.5   226.3   282.2   159.5   226.3   282.2   159.5   226.3
        ( 32.7) ( 40.7) ( 23.0) ( 32.7) ( 40.7) ( 23.0) ( 32.7) ( 40.7) ( 23.0) ( 32.7)
           69.8    -4.4     0.9    -6.6    47.4     2.9    97.6    51.7   -40.0   -33.3
         10.079  -0.629   0.130  -0.953   6.843   0.421  14.088   7.467  -5.779  -4.804
g_O          54      45     170      69      38     152      56      37     154      68
        (  7.8) (  6.5) ( 24.5) ( 10.0) (  5.5) ( 21.9) (  8.1) (  5.3) ( 22.2) (  9.8)
g_E        70.4    46.6   164.0    70.4    46.6   164.0    70.4    46.6   164.0    70.4
        ( 10.2) (  6.7) ( 23.7) ( 10.2) (  6.7) ( 23.7) ( 10.2) (  6.7) ( 23.7) ( 10.2)
          -28.6    -3.2    12.3    -2.8   -15.6   -23.0   -25.6   -17.1   -19.3    -4.7
         -4.132  -0.465   1.776  -0.399  -2.247  -3.322  -3.698  -2.472  -2.785  -0.680
c_O         113      77     102     146      85     101     110      75      99     123
        ( 16.3) ( 11.1) ( 14.7) ( 21.1) ( 12.3) ( 14.6) ( 15.9) ( 10.8) ( 14.3) ( 17.7)
c_E       118.7    89.1   103.7   118.7    89.1   103.7   118.7    89.1   103.7   118.7
        ( 17.1) ( 12.9) ( 15.0) ( 17.1) ( 12.9) ( 15.0) ( 17.1) ( 12.9) ( 15.0) ( 17.1)
          -11.2   -22.4    -3.3    60.4    -8.0    -5.3   -16.8   -25.8    -9.1     8.7
         -1.611  -3.237  -0.480   8.714  -1.149  -0.763  -2.423  -3.723  -1.319   1.256

N           693     693     693     693     693     693     693     693     693     693
X2          9.2     2.6     0.3     8.2     4.0     1.6    14.1     6.4     8.7     2.4
Gvalue      9.3     2.7     0.3     7.8     4.1     1.6    13.8     6.6     8.6     2.4
Gv/N      1.349   0.389   0.049   1.125   0.587   0.234   1.996   0.956   1.241   0.352
Prob    0.02502 0.44131 0.95306 0.05041 0.25394 0.65476 0.00314 0.08497 0.03512 0.48607
        -------------------------------------------------------------------------------
            111     112     113     114     115     116     117     118     119     120
        -------------------------------------------------------------------------------
a_O         274     271     275     285     281     269     262     281     295     284
        ( 39.5) ( 39.1) ( 39.7) ( 41.1) ( 40.5) ( 38.8) ( 37.8) ( 40.6) ( 42.6) ( 41.0)
a_E       275.1   265.8   277.6   275.1   265.8   277.6   275.1   265.4   277.2   274.7
        ( 39.7) ( 38.4) ( 40.1) ( 39.7) ( 38.4) ( 40.1) ( 39.7) ( 38.4) ( 40.1) ( 39.7)
           -2.2    10.5    -5.1    20.2    31.2   -16.8   -25.6    32.0    36.8    18.9
         -0.316   1.511  -0.733   2.909   4.505  -2.430  -3.689   4.629   5.321   2.733
t_O         296     185     245     288     155     228     296     163     220     272
        ( 42.7) ( 26.7) ( 35.4) ( 41.6) ( 22.4) ( 32.9) ( 42.7) ( 23.6) ( 31.8) ( 39.3)
t_E       282.2   159.5   226.3   282.2   159.5   226.3   282.2   159.3   226.0   281.8
        ( 40.7) ( 23.0) ( 32.7) ( 40.7) ( 23.0) ( 32.7) ( 40.7) ( 23.0) ( 32.7) ( 40.7)
           28.3    54.8    38.8    11.7    -9.0     3.4    28.3     7.4   -11.8   -19.2
          4.082   7.902   5.605   1.695  -1.294   0.484   4.082   1.076  -1.711  -2.777
g_O          35     132      71      38     158      67      42     151      57      43
        (  5.1) ( 19.0) ( 10.2) (  5.5) ( 22.8) (  9.7) (  6.1) ( 21.8) (  8.2) (  6.2)
g_E        46.6   164.0    70.4    46.6   164.0    70.4    46.6   163.7    70.3    46.6
        (  6.7) ( 23.7) ( 10.2) (  6.7) ( 23.7) ( 10.2) (  6.7) ( 23.7) ( 10.2) (  6.7)
          -20.1   -57.2     1.2   -15.6   -11.7    -6.6    -8.8   -24.4   -23.9    -6.9
         -2.900  -8.259   0.175  -2.247  -1.688  -0.956  -1.270  -3.530  -3.454  -0.992
c_O          88     105     102      82      99     129      93      97     120      93
        ( 12.7) ( 15.2) ( 14.7) ( 11.8) ( 14.3) ( 18.6) ( 13.4) ( 14.0) ( 17.3) ( 13.4)
c_E        89.1   103.7   118.7    89.1   103.7   118.7    89.1   103.5   118.6    88.9
        ( 12.9) ( 15.0) ( 17.1) ( 12.9) ( 15.0) ( 17.1) ( 12.9) ( 15.0) ( 17.1) ( 12.9)
           -2.1     2.7   -31.0   -13.6    -9.1    21.4     8.0   -12.6     2.9     8.3
         -0.308   0.384  -4.469  -1.958  -1.319   3.091   1.157  -1.826   0.421   1.198
```

N	693	693	693	693	693	693	693	692	692	692
X2	3.6	10.4	3.9	2.6	1.4	1.3	1.9	2.4	3.8	1.1
Gvalue	3.9	10.7	4.0	2.8	1.4	1.3	1.9	2.4	4.0	1.1
Gv/N	0.558	1.538	0.578	0.398	0.204	0.189	0.281	0.349	0.577	0.162
Prob	0.27643	0.01374	0.26112	0.43043	0.70183	0.72674	0.58344	0.49069	0.26214	0.77270

	121	122	123	124	125	126	127	128	129	130
a_O	267	298	283	301	265	264	287	256	269	259
	(38.6)	(43.1)	(40.9)	(43.5)	(38.3)	(38.2)	(41.5)	(37.0)	(38.9)	(37.4)
a_E	265.4	277.2	274.7	265.4	277.2	274.7	265.4	277.2	274.7	265.4
	(38.4)	(40.1)	(39.7)	(38.4)	(40.1)	(39.7)	(38.4)	(40.1)	(39.7)	(38.4)
	3.1	43.2	16.8	75.7	−23.8	−21.0	44.8	−40.6	−11.3	−12.7
	0.455	6.247	2.435	10.940	−3.434	−3.032	6.480	−5.874	−1.630	−1.836
t_O	167	186	271	120	213	295	155	233	281	170
	(24.1)	(26.9)	(39.2)	(17.3)	(30.8)	(42.6)	(22.4)	(33.7)	(40.6)	(24.6)
t_E	159.3	226.0	281.8	159.3	226.0	281.8	159.3	226.0	281.8	159.3
	(23.0)	(32.7)	(40.7)	(23.0)	(32.7)	(40.7)	(23.0)	(32.7)	(40.7)	(23.0)
	15.7	−72.5	−21.1	−68.0	−25.2	27.0	−8.5	14.2	−1.6	22.1
	2.273	−10.472	−3.055	−9.830	−3.647	3.909	−1.231	2.054	−0.225	3.188
g_O	163	74	37	161	74	56	153	75	38	165
	(23.6)	(10.7)	(5.3)	(23.3)	(10.7)	(8.1)	(22.1)	(10.8)	(5.5)	(23.8)
g_E	163.7	70.3	46.6	163.7	70.3	46.6	163.7	70.3	46.6	163.7
	(23.7)	(10.2)	(6.7)	(23.7)	(10.2)	(6.7)	(23.7)	(10.2)	(6.7)	(23.7)
	−1.4	7.6	−17.0	−5.4	7.6	20.6	−20.7	9.7	−15.5	2.6
	−0.208	1.099	−2.461	−0.780	1.099	2.984	−2.995	1.404	−2.234	0.371
c_O	95	134	101	110	140	77	97	128	104	98
	(13.7)	(19.4)	(14.6)	(15.9)	(20.2)	(11.1)	(14.0)	(18.5)	(15.0)	(14.2)
c_E	103.5	118.6	88.9	103.5	118.6	88.9	103.5	118.6	88.9	103.5
	(15.0)	(17.1)	(12.9)	(15.0)	(17.1)	(12.9)	(15.0)	(17.1)	(12.9)	(15.0)
	−16.3	32.8	25.7	13.3	46.6	−22.2	−12.6	19.6	32.5	−10.8
	−2.360	4.744	3.710	1.928	6.729	−3.210	−1.826	2.837	4.700	−1.554
N	692	692	692	692	692	692	692	692	692	692
X2	1.1	10.9	4.3	14.9	5.4	4.5	3.0	2.9	4.2	1.2
Gvalue	1.1	11.2	4.3	15.6	5.2	4.5	3.0	2.9	4.2	1.2
Gv/N	0.159	1.617	0.628	2.258	0.746	0.651	0.428	0.421	0.610	0.169
Prob	0.77718	0.01073	0.22620	0.00135	0.16039	0.21177	0.39726	0.40517	0.23855	0.76063

	131	132	133	134	135	136	137	138	139	140
a_O	277	266	275	277	261	290	262	266	272	273
	(40.0)	(38.4)	(39.7)	(40.0)	(37.7)	(42.0)	(37.9)	(38.5)	(39.4)	(39.5)
a_E	277.2	274.7	265.4	277.2	274.7	265.0	276.8	274.3	265.0	276.8
	(40.1)	(39.7)	(38.4)	(40.1)	(39.7)	(38.4)	(40.1)	(39.7)	(38.4)	(40.1)
	−0.3	−17.1	19.5	−0.3	−26.7	52.2	−28.7	−16.4	14.1	−7.5
	−0.044	−2.474	2.815	−0.044	−3.859	7.552	−4.154	−2.367	2.038	−1.078
t_O	220	288	157	218	289	142	215	296	188	227
	(31.8)	(41.6)	(22.7)	(31.5)	(41.8)	(20.5)	(31.1)	(42.8)	(27.2)	(32.9)
t_E	226.0	281.8	159.3	226.0	281.8	159.1	225.7	281.4	159.1	225.7
	(32.7)	(40.7)	(23.0)	(32.7)	(40.7)	(23.0)	(32.7)	(40.7)	(23.0)	(32.7)
	−11.8	12.6	−4.6	−15.7	14.6	−32.3	−20.8	30.0	62.8	2.7
	−1.711	1.817	−0.665	−2.271	2.113	−4.670	−3.015	4.342	9.086	0.385
g_O	68	42	138	68	43	138	69	37	139	70
	(9.8)	(6.1)	(19.9)	(9.8)	(6.2)	(20.0)	(10.0)	(5.4)	(20.1)	(10.1)
g_E	70.3	46.6	163.7	70.3	46.6	163.5	70.2	46.5	163.5	70.2
	(10.2)	(6.7)	(23.7)	(10.2)	(6.7)	(23.7)	(10.2)	(6.7)	(23.7)	(10.2)
	−4.5	−8.7	−47.2	−4.5	−6.9	−46.8	−2.4	−16.9	−45.1	−0.4
	−0.652	−1.254	−6.817	−0.652	−0.992	−6.769	−0.342	−2.449	−6.527	−0.056
c_O	127	96	122	129	99	121	145	92	92	121
	(18.4)	(13.9)	(17.6)	(18.6)	(14.3)	(17.5)	(21.0)	(13.3)	(13.3)	(17.5)
c_E	118.6	88.9	103.5	118.6	88.9	103.4	118.4	88.8	103.4	118.4
	(17.1)	(12.9)	(15.0)	(17.1)	(12.9)	(15.0)	(17.1)	(12.9)	(15.0)	(17.1)
	17.5	14.7	40.1	21.8	21.2	38.1	58.8	6.5	−21.5	5.3
	2.527	2.117	5.789	3.149	3.064	5.512	8.512	0.937	−3.105	0.766
N	692	692	692	692	692	691	691	691	691	691
X2	0.8	1.4	7.7	1.3	2.3	11.2	7.3	3.1	10.4	0.1
Gvalue	0.8	1.4	7.8	1.3	2.3	11.2	6.9	3.2	10.3	0.1
Gv/N	0.119	0.206	1.122	0.182	0.326	1.625	1.001	0.464	1.492	0.017
Prob	0.84353	0.69984	0.05121	0.73928	0.52109	0.01056	0.07464	0.36113	0.01612	0.98973

	141	142	143	144	145	146	147	148	149	150
a_O	270	248	298	299	271	255	241	257	277	271
	(39.1)	(35.9)	(43.1)	(43.3)	(39.2)	(36.9)	(34.9)	(37.2)	(40.1)	(39.2)
a_E	274.3	265.0	276.8	274.3	265.0	276.8	274.3	265.0	276.8	274.3
	(39.7)	(38.4)	(40.1)	(39.7)	(38.4)	(40.1)	(39.7)	(38.4)	(40.1)	(39.7)
	−8.5	−33.0	44.1	51.6	12.0	−41.7	−62.4	−15.8	0.5	−6.6
	−1.236	−4.772	6.380	7.461	1.742	−6.041	−9.029	−2.294	0.072	−0.950
t_O	287	160	197	271	159	218	295	178	236	287
	(41.5)	(23.2)	(28.5)	(39.2)	(23.0)	(31.5)	(42.7)	(25.8)	(34.2)	(41.5)
t_E	281.4	159.1	225.7	281.4	159.1	225.7	281.4	159.1	225.7	281.4
	(40.7)	(23.0)	(32.7)	(40.7)	(23.0)	(32.7)	(40.7)	(23.0)	(32.7)	(40.7)
	11.4	1.8	−53.5	−20.4	−0.2	−15.1	27.9	40.0	21.1	11.4
	1.645	0.264	−7.748	−2.946	−0.026	−2.183	4.038	5.787	3.056	1.645
g_O	50	171	87	35	166	90	54	151	64	43
	(7.2)	(24.7)	(12.6)	(5.1)	(24.0)	(13.0)	(7.8)	(21.9)	(9.3)	(6.2)
g_E	46.5	163.5	70.2	46.5	163.5	70.2	46.5	163.5	70.2	46.5
	(6.7)	(23.7)	(10.2)	(6.7)	(23.7)	(10.2)	(6.7)	(23.7)	(10.2)	(6.7)
	7.2	15.4	37.4	−19.9	5.1	44.7	16.1	−24.0	−11.8	−6.7
	1.049	2.225	5.405	−2.879	0.734	6.475	2.335	−3.472	−1.711	−0.975
c_O	84	112	109	86	95	128	101	105	114	90
	(12.2)	(16.2)	(15.8)	(12.4)	(13.7)	(18.5)	(14.6)	(15.2)	(16.5)	(13.0)
c_E	88.8	103.4	118.4	88.8	103.4	118.4	88.8	103.4	118.4	88.8
	(12.9)	(15.0)	(17.1)	(12.9)	(15.0)	(17.1)	(12.9)	(15.0)	(17.1)	(12.9)
	−9.4	17.9	−18.0	−5.5	−16.1	20.0	26.0	3.3	−8.6	2.4
	−1.356	2.597	−2.605	−0.802	−2.324	2.894	3.757	0.473	−1.244	0.344
N	691	691	691	691	691	691	691	691	691	691
X2	0.7	2.2	10.0	5.5	0.9	8.3	7.6	3.5	1.2	0.4
Gvalue	0.7	2.2	9.9	5.8	0.9	7.9	7.6	3.4	1.2	0.4
Gv/N	0.102	0.313	1.433	0.832	0.126	1.144	1.102	0.494	0.173	0.063
Prob	0.87270	0.53865	0.01943	0.12430	0.83280	0.04794	0.05469	0.33200	0.75470	0.93218

Azoa_EBN1
5UTR:

	50	49	48	47	46	45	44	43	42	41
a_O	840	906	784	826	831	788	914	877	803	853
	(18.4)	(19.9)	(17.2)	(18.1)	(18.2)	(17.3)	(20.0)	(19.2)	(17.6)	(18.7)
a_E	913.9	913.9	913.9	913.9	913.9	913.9	913.9	913.9	913.9	913.9
	(20.0)	(20.0)	(20.0)	(20.0)	(20.0)	(20.0)	(20.0)	(20.0)	(20.0)	(20.0)
	-141.6	-15.7	-240.4	-167.0	-158.0	-233.6	0.2	-72.3	-207.7	-117.6
	-3.103	-0.344	-5.267	-3.660	-3.462	-5.118	0.005	-1.583	-4.552	-2.577
t_O	754	902	859	838	948	818	852	947	858	825
	(16.5)	(19.8)	(18.8)	(18.4)	(20.8)	(17.9)	(18.7)	(20.7)	(18.8)	(18.1)
t_E	835.2	835.2	835.2	835.2	835.2	835.2	835.2	835.2	835.2	835.2
	(18.3)	(18.3)	(18.3)	(18.3)	(18.3)	(18.3)	(18.3)	(18.3)	(18.3)	(18.3)
	-154.2	138.8	48.3	5.6	240.2	-34.0	33.9	237.9	46.2	-20.3
	-3.379	3.041	1.058	0.123	5.263	-0.746	0.744	5.213	1.013	-0.444
g_O	1529	1340	1447	1463	1346	1419	1430	1396	1378	1511
	(33.5)	(29.4)	(31.7)	(32.1)	(29.5)	(31.1)	(31.3)	(30.6)	(30.2)	(33.1)
g_E	1406.7	1406.7	1406.7	1406.7	1406.7	1406.7	1406.7	1406.7	1406.7	1406.7
	(30.8)	(30.8)	(30.8)	(30.8)	(30.8)	(30.8)	(30.8)	(30.8)	(30.8)	(30.8)
	255.0	-130.1	81.8	114.9	-118.7	24.8	47.1	-21.2	-56.7	216.2
	5.588	-2.851	1.793	2.518	-2.600	0.543	1.031	-0.465	-1.243	4.738
c_O	1441	1416	1474	1437	1439	1539	1368	1344	1525	1375
	(31.6)	(31.0)	(32.3)	(31.5)	(31.5)	(33.7)	(30.0)	(29.4)	(33.4)	(30.1)
c_E	1408.2	1408.2	1408.2	1408.2	1408.2	1408.2	1408.2	1408.2	1408.2	1408.2
	(30.9)	(30.9)	(30.9)	(30.9)	(30.9)	(30.9)	(30.9)	(30.9)	(30.9)	(30.9)
	66.3	15.5	134.5	58.1	62.2	273.3	-79.3	-125.5	242.9	-65.7
	1.452	0.341	2.947	1.273	1.362	5.988	-1.738	-2.750	5.323	-1.440
N	4564	4564	4564	4564	4564	4564	4564	4564	4564	4564
X2	25.3	8.6	23.4	11.3	26.0	29.9	1.9	19.5	24.3	12.7
Gvalue	25.4	8.5	24.2	11.6	25.7	30.4	1.9	18.9	24.7	12.6
Gv/N	0.557	0.187	0.531	0.253	0.562	0.667	0.041	0.414	0.540	0.277
Prob	0.00001	0.03629	0.00002	0.00907	0.00001	0.00000	0.59718	0.00029	0.00002	0.00553

	40	39	38	37	36	35	34	33	32	31
a_O	899	814	882	864	831	894	945	807	926	926
	(19.7)	(17.8)	(19.3)	(18.9)	(18.2)	(19.6)	(20.7)	(17.7)	(20.3)	(20.3)
a_E	913.9	913.9	913.9	913.9	913.9	913.9	913.9	913.9	913.9	913.9
	(20.0)	(20.0)	(20.0)	(20.0)	(20.0)	(20.0)	(20.0)	(20.0)	(20.0)	(20.0)
	-29.5	-188.4	-62.7	-97.0	-158.0	-39.3	63.3	-200.8	24.4	24.4
	-0.647	-4.129	-1.373	-2.125	-3.462	-0.862	1.386	-4.399	0.534	0.534
t_O	863	842	855	955	845	848	979	819	828	1007
	(18.9)	(18.4)	(18.7)	(20.9)	(18.5)	(18.6)	(21.5)	(17.9)	(18.1)	(22.1)
t_E	835.2	835.2	835.2	835.2	835.2	835.2	835.2	835.2	835.2	835.2
	(18.3)	(18.3)	(18.3)	(18.3)	(18.3)	(18.3)	(18.3)	(18.3)	(18.3)	(18.3)
	56.5	13.7	40.1	256.0	19.7	25.8	311.0	-32.1	-14.3	376.7
	1.238	0.299	0.878	5.609	0.432	0.565	6.815	-0.703	-0.314	8.255
g_O	1324	1426	1416	1346	1397	1369	1244	1450	1340	1201
	(29.0)	(31.2)	(31.0)	(29.5)	(30.6)	(30.0)	(27.3)	(31.8)	(29.4)	(26.3)
g_E	1406.7	1406.7	1406.7	1406.7	1406.7	1406.7	1406.7	1406.7	1406.7	1406.7
	(30.8)	(30.8)	(30.8)	(30.8)	(30.8)	(30.8)	(30.8)	(30.8)	(30.8)	(30.8)
	-160.4	38.9	18.7	-118.7	-19.3	-74.3	-305.7	88.0	-130.1	-379.7
	-3.514	0.853	0.411	-2.600	-0.422	-1.628	-6.699	1.928	-2.851	-8.319
c_O	1478	1482	1411	1399	1491	1453	1396	1488	1470	1430
	(32.4)	(32.5)	(30.9)	(30.7)	(32.7)	(31.8)	(30.6)	(32.6)	(32.2)	(31.3)
c_E	1408.2	1408.2	1408.2	1408.2	1408.2	1408.2	1408.2	1408.2	1408.2	1408.2
	(30.9)	(30.9)	(30.9)	(30.9)	(30.9)	(30.9)	(30.9)	(30.9)	(30.9)	(30.9)
	142.9	151.3	5.5	-18.4	170.3	90.9	-24.4	163.9	126.2	43.8
	3.131	3.315	0.121	-0.404	3.731	1.992	-0.534	3.592	2.764	0.960
N	4564	4564	4564	4564	4564	4564	4564	4564	4564	4564
X2	9.5	15.1	1.6	22.6	12.6	3.1	44.7	18.7	6.1	65.9
Gvalue	9.5	15.5	1.7	21.9	12.7	3.1	44.2	19.1	6.1	65.3
Gv/N	0.208	0.339	0.036	0.480	0.278	0.067	0.968	0.418	0.134	1.430
Prob	0.02319	0.00147	0.64610	0.00007	0.00531	0.38310	0.00000	0.00026	0.10680	0.00000

	30	29	28	27	26	25	24	23	22	21
a_O	799	871	899	820	963	950	837	954	974	959
	(17.5)	(19.1)	(19.7)	(18.0)	(21.1)	(20.8)	(18.3)	(20.9)	(21.3)	(21.0)
a_E	913.9	913.9	913.9	913.9	913.9	913.9	913.9	913.9	913.9	913.9
	(20.0)	(20.0)	(20.0)	(20.0)	(20.0)	(20.0)	(20.0)	(20.0)	(20.0)	(20.0)
	-214.7	-83.7	-29.5	-177.8	100.8	73.6	-147.1	82.0	124.1	92.4
	-4.704	-1.835	-0.647	-3.895	2.209	1.613	-3.224	1.796	2.719	2.025
t_O	878	877	999	857	872	970	887	835	995	898
	(19.2)	(19.2)	(21.9)	(18.8)	(19.1)	(21.3)	(19.4)	(18.3)	(21.8)	(19.7)
t_E	835.2	835.2	835.2	835.2	835.2	835.2	835.2	835.2	835.2	835.2
	(18.3)	(18.3)	(18.3)	(18.3)	(18.3)	(18.3)	(18.3)	(18.3)	(18.3)	(18.3)
	87.8	85.7	357.8	44.2	75.2	290.3	106.7	-0.4	348.4	130.2
	1.923	1.877	7.840	0.968	1.648	6.360	2.339	-0.009	7.633	2.853
g_O	1344	1349	1192	1379	1234	1196	1337	1267	1141	1189
	(29.4)	(29.6)	(26.1)	(30.2)	(27.0)	(26.2)	(29.3)	(27.8)	(25.0)	(26.1)
g_E	1406.7	1406.7	1406.7	1406.7	1406.7	1406.7	1406.7	1406.7	1406.7	1406.7
	(30.8)	(30.8)	(30.8)	(30.8)	(30.8)	(30.8)	(30.8)	(30.8)	(30.8)	(30.8)
	-122.5	-112.9	-394.8	-54.8	-323.2	-388.1	-135.8	-265.0	-477.7	-399.8
	-2.684	-2.474	-8.649	-1.200	-7.082	-8.503	-2.976	-5.806	-10.466	-8.759
c_O	1543	1467	1474	1508	1495	1448	1503	1508	1454	1518
	(33.8)	(32.1)	(32.3)	(33.0)	(32.8)	(31.7)	(32.9)	(33.0)	(31.9)	(33.3)
c_E	1408.2	1408.2	1408.2	1408.2	1408.2	1408.2	1408.2	1408.2	1408.2	1408.2
	(30.9)	(30.9)	(30.9)	(30.9)	(30.9)	(30.9)	(30.9)	(30.9)	(30.9)	(30.9)
	282.0	119.9	134.5	206.4	178.7	80.6	195.7	206.4	93.0	227.8
	6.179	2.627	2.947	4.522	3.916	1.766	4.289	4.522	2.037	4.992
N	4564	4564	4564	4564	4564	4564	4564	4564	4564	4564
X2	32.3	8.9	68.2	17.8	30.8	55.9	19.5	22.7	86.2	49.2
Gvalue	32.6	8.9	68.0	18.0	31.5	56.4	19.6	23.0	87.8	50.7
Gv/N	0.714	0.195	1.491	0.395	0.691	1.237	0.428	0.504	1.924	1.111
Prob	0.00000	0.03041	0.00000	0.00044	0.00000	0.00000	0.00021	0.00004	0.00000	0.00000

	20	19	18	17	16	15	14	13	12	11
a_O	963	1010	955	1052	1083	1047	1208	1243	1139	1310
	(21.1)	(22.1)	(20.9)	(23.0)	(23.7)	(22.9)	(26.5)	(27.2)	(25.0)	(28.7)
a_E	913.9	913.9	913.9	913.9	913.9	913.9	913.9	913.9	913.9	913.9
	(20.0)	(20.0)	(20.0)	(20.0)	(20.0)	(20.0)	(20.0)	(20.0)	(20.0)	(20.0)
	100.8	202.0	84.0	296.1	367.7	284.7	674.1	764.6	501.6	943.4
	2.209	4.426	1.841	6.488	8.057	6.239	14.770	16.753	10.990	20.670
t_O	914	978	925	858	913	783	676	768	562	458
	(20.0)	(21.4)	(20.3)	(18.8)	(20.0)	(17.2)	(14.8)	(16.8)	(12.3)	(10.0)

```
t_E       835.2   835.2   835.2   835.2   835.2   835.2   835.2   835.2   835.2   835.2
        ( 18.3) ( 18.3) ( 18.3) ( 18.3) ( 18.3) ( 18.3) ( 18.3) ( 18.3) ( 18.3) ( 18.3)
          164.8   308.7   188.9    46.2   162.6  -101.1  -285.9  -128.8  -445.3  -550.3
          3.611   6.765   4.139   1.013   3.563  -2.214  -6.265  -2.823  -9.757 -12.058
g_O        1201    1095    1231    1221    1208    1395    1359    1397    1855    1980
        ( 26.3) ( 24.0) ( 27.0) ( 26.8) ( 26.5) ( 30.6) ( 29.8) ( 30.6) ( 40.6) ( 43.4)
g_E      1406.7  1406.7  1406.7  1406.7  1406.7  1406.7  1406.7  1406.7  1406.7  1406.7
        ( 30.8) ( 30.8) ( 30.8) ( 30.8) ( 30.8) ( 30.8) ( 30.8) ( 30.8) ( 30.8) ( 30.8)
         -379.7  -548.5  -328.4  -345.7  -367.8   -23.2   -93.7   -19.3  1026.4  1353.8
         -8.319 -12.018  -7.196  -7.574  -8.060  -0.509  -2.053  -0.422  22.490  29.663
c_O        1486    1481    1453    1433    1360    1339    1321    1156    1008     816
        ( 32.6) ( 32.4) ( 31.8) ( 31.4) ( 29.8) ( 29.3) ( 28.9) ( 25.3) ( 22.1) ( 17.9)
c_E      1408.2  1408.2  1408.2  1408.2  1408.2  1408.2  1408.2  1408.2  1408.2  1408.2
        ( 30.9) ( 30.9) ( 30.9) ( 30.9) ( 30.9) ( 30.9) ( 30.9) ( 30.9) ( 30.9) ( 30.9)
          159.7   149.2    90.9    49.9   -94.8  -135.0  -169.0  -456.3  -674.1  -890.6
          3.499   3.269   1.992   1.094  -2.078  -2.959  -3.702  -9.999 -14.770 -19.513

N          4564    4564    4564    4564    4564    4564    4564    4564    4564    4564
X2         44.4   107.3    34.9    46.4    68.2    26.2   132.0   169.2   401.5   824.8
Gvalue     45.7   111.4    35.5    46.6    67.7    25.4   125.5   160.2   408.6   856.3
Gv/N      1.001   2.441   0.777   1.021   1.484   0.557   2.750   3.510   8.953  18.763
Prob    0.00000 0.00000 0.00000 0.00000 0.00000 0.00001 0.00000 0.00000 0.00000 0.00000
        -------------------------------------------------------------------------------
             10       9       8       7       6       5       4       3       2       1
        -------------------------------------------------------------------------------
a_O        1174    1164    1355    1241    1159    1142    1065    1209     942     857
        ( 25.7) ( 25.5) ( 29.7) ( 27.2) ( 25.4) ( 25.0) ( 23.3) ( 26.5) ( 20.6) ( 18.8)
a_E       913.9   913.9   913.9   913.9   913.9   913.9   913.9   913.9   913.9   913.9
        ( 20.0) ( 20.0) ( 20.0) ( 20.0) ( 20.0) ( 20.0) ( 20.0) ( 20.0) ( 20.0) ( 20.0)
          588.1   563.2  1067.3   759.4   550.8   508.9   325.9   676.6    57.1  -110.2
         12.885  12.339  23.386  16.639  12.068  11.151   7.141  14.826   1.251  -2.414
t_O         473     426     544     818     772     829     762     641     904     429
        ( 10.4) (  9.3) ( 11.9) ( 17.9) ( 16.9) ( 18.2) ( 16.7) ( 14.0) ( 19.8) (  9.4)
t_E       835.2   835.2   835.2   835.2   835.2   835.2   835.2   835.2   835.2   835.2
        ( 18.3) ( 18.3) ( 18.3) ( 18.3) ( 18.3) ( 18.3) ( 18.3) ( 18.3) ( 18.3) ( 18.3)
         -537.9  -573.6  -466.4   -34.0  -121.5   -12.4  -139.8  -339.3   143.1  -571.6
        -11.785 -12.568 -10.220  -0.746  -2.662  -0.271  -3.063  -7.434   3.136 -12.524
g_O        2208    2226    1840    1525    1400    1221    1253    1473     980    1287
        ( 48.4) ( 48.8) ( 40.3) ( 33.4) ( 30.7) ( 26.8) ( 27.5) ( 32.3) ( 21.5) ( 28.2)
g_E      1406.7  1406.7  1406.7  1406.7  1406.7  1406.7  1406.7  1406.7  1406.7  1406.7
        ( 30.8) ( 30.8) ( 30.8) ( 30.8) ( 30.8) ( 30.8) ( 30.8) ( 30.8) ( 30.8) ( 30.8)
         1991.0  2043.4   988.3   246.4   -13.3  -345.7  -289.9   135.8  -708.4  -228.8
         43.625  44.772  21.653   5.398  -0.291  -7.574  -6.352   2.975 -15.521  -5.014
c_O         709     748     825     980    1233    1372    1484    1241    1738    1991
        ( 15.5) ( 16.4) ( 18.1) ( 21.5) ( 27.0) ( 30.1) ( 32.5) ( 27.2) ( 38.1) ( 43.6)
c_E      1408.2  1408.2  1408.2  1408.2  1408.2  1408.2  1408.2  1408.2  1408.2  1408.2
        ( 30.9) ( 30.9) ( 30.9) ( 30.9) ( 30.9) ( 30.9) ( 30.9) ( 30.9) ( 30.9) ( 30.9)
         -973.1  -946.5  -882.3  -710.6  -327.7   -71.6   155.5  -313.8   731.3  1378.9
        -21.321 -20.739 -19.331 -15.570  -7.181  -1.568   3.407  -6.876  16.023  30.213

N          4564    4564    4564    4564    4564    4564    4564    4564    4564    4564
X2       1034.8  1055.7   689.5   257.6    92.4    82.4    52.3   163.4   213.2   452.4
Gvalue   1068.2  1086.5   706.8   261.1    88.3    79.4    51.8   159.3   223.1   468.3
Gv/N     23.404  23.805  15.487   5.721   1.934   1.739   1.134   3.491   4.889  10.261
Prob    0.00000 0.00000 0.00000 0.00000 0.00000 0.00000 0.00000 0.00000 0.00000 0.00000
        -------------------------------------------------------------------------------

CDS_INITIAL:
        -------------------------------------------------------------------------------
              1       2       3       4       5       6       7       8       9      10
        -------------------------------------------------------------------------------
a_O        3461       0       0    2035    1402     876    1298    1328     809    1247
        ( 75.8) (  0.0) (  0.0) ( 44.6) ( 30.7) ( 19.2) ( 28.4) ( 29.1) ( 17.7) ( 27.3)
a_E       901.6  1144.2   382.6   901.6  1144.2   382.6   901.6  1144.2   382.6   901.6
        ( 19.8) ( 25.1) (  8.4) ( 19.8) ( 25.1) (  8.4) ( 19.8) ( 25.1) (  8.4) ( 19.8)
         9311.0     0.0     0.0  3313.3   569.8  1451.2   946.0   395.7  1211.4   808.8
        204.010   0.000   0.000  72.596  12.484  31.796  20.727   8.669  26.543  17.722
t_O         368    4564       1     454     747     841     584    1131     789     681
        (  8.1) (100.0) (  0.0) (  9.9) ( 16.4) ( 18.4) ( 12.8) ( 24.8) ( 17.3) ( 14.9)
t_E       577.3  1296.2   463.1   577.3  1296.2   463.1   577.3  1296.2   463.1   577.3
        ( 12.6) ( 28.4) ( 10.1) ( 12.6) ( 28.4) ( 10.1) ( 12.6) ( 28.4) ( 10.1) ( 12.6)
         -331.4 11489.8   -12.3  -218.1  -823.4  1003.5    13.5  -308.4   840.8   225.1
         -7.260 251.749  -0.269  -4.779 -18.041  21.988   0.297  -6.758  18.422   4.932
g_O         735       0    4561     978     960    1133    1426     854    1317    1248
        ( 16.1) (  0.0) ( 99.9) ( 21.4) ( 21.0) ( 24.8) ( 31.2) ( 18.7) ( 28.9) ( 27.3)
g_E      1793.4   945.4  1716.9  1793.4   945.4  1716.9  1793.4   945.4  1716.9  1793.4
        ( 39.3) ( 20.7) ( 37.6) ( 39.3) ( 20.7) ( 37.6) ( 39.3) ( 20.7) ( 37.6) ( 39.3)
        -1311.3     0.0  8912.4 -1186.1    29.3  -941.9  -653.9  -173.7  -698.4  -905.0
        -28.731   0.000 195.275 -25.988   0.643 -20.637 -14.326  -3.807 -15.303 -19.830
c_O           0       0       2    1097    1455    1714    1256    1251    1649    1388
        (  0.0) (  0.0) (  0.0) ( 24.0) ( 31.9) ( 37.6) ( 27.5) ( 27.4) ( 36.1) ( 30.4)
c_E      1291.7  1178.1  2001.4  1291.7  1178.1  2001.4  1291.7  1178.1  2001.4  1291.7
        ( 28.3) ( 25.8) ( 43.9) ( 28.3) ( 25.8) ( 43.9) ( 28.3) ( 25.8) ( 43.9) ( 28.3)
            0.0     0.0   -27.6  -358.4   614.2  -531.3   -70.4   150.1  -638.7   199.6
          0.000   0.000  -0.605  -7.853  13.458 -11.642  -1.542   3.290 -13.994   4.374

N          4564    4564    4564    4564    4564    4564    4564    4564    4564    4564
X2       9257.6 11505.9  7552.4  1851.2   356.1  1184.3   250.6    63.9   859.6   324.0
Gvalue   7668.4 11489.8  8872.5  1550.7   389.9   981.5   235.3    63.6   715.1   328.5
Gv/N    168.019 251.749 194.401  33.976   8.543  21.505   5.156   1.394  15.668   7.198
Prob    1.00000 1.00000 1.00000 1.00000 0.00000 0.00000 0.00000 0.00000 0.00000 0.00000
        -------------------------------------------------------------------------------
             11      12      13      14      15      16      17      18      19      20
        -------------------------------------------------------------------------------
a_O        1198     793    1153    1212     793    1170    1158     672    1070    1091
        ( 26.2) ( 17.4) ( 25.3) ( 26.6) ( 17.4) ( 25.6) ( 25.4) ( 14.7) ( 23.4) ( 23.9)
a_E      1144.2   382.6   901.6  1144.2   382.6   901.6  1144.2   382.6   901.6  1144.2
        ( 25.1) (  8.4) ( 19.8) ( 25.1) (  8.4) ( 19.8) ( 25.1) (  8.4) ( 19.8) ( 25.1)
          110.1  1155.8   567.1   139.5  1155.8   609.8    27.8   756.9   366.4  -103.9
          2.412  25.324  12.427   3.058  25.324  13.360   0.608  16.584   8.029  -2.276
t_O        1257     816     637    1280     783     649    1361     808     657    1354
        ( 27.5) ( 17.9) ( 14.0) ( 28.0) ( 17.2) ( 14.2) ( 29.8) ( 17.7) ( 14.4) ( 29.7)
t_E      1296.2   463.1   577.3  1296.2   463.1   577.3  1296.2   463.1   577.3  1296.2
        ( 28.4) ( 10.1) ( 12.6) ( 28.4) ( 10.1) ( 12.6) ( 28.4) ( 10.1) ( 12.6) ( 28.4)
          -77.2   924.5   125.4   -32.2   822.4   152.0   132.7   899.5   170.0   118.1
         -1.692  20.256   2.749  -0.706  18.020   3.331   2.909  19.708   3.725   2.588
g_O         831    1299    1312     811    1237    1369     802    1267    1430     868
        ( 18.2) ( 28.5) ( 28.7) ( 17.8) ( 27.1) ( 30.0) ( 17.6) ( 27.8) ( 31.3) ( 19.0)
g_E       945.4  1716.9  1793.4   945.4  1716.9  1793.4   945.4  1716.9  1793.4   945.4
        ( 20.7) ( 37.6) ( 39.3) ( 20.7) ( 37.6) ( 39.3) ( 20.7) ( 37.6) ( 39.3) ( 20.7)
         -214.4  -724.7  -820.2  -248.8  -811.1  -739.4  -263.9  -770.0  -647.7  -148.4
         -4.699 -15.878 -17.972  -5.451 -17.771 -16.201  -5.783 -16.871 -14.191  -3.251
```

c_O	1278	1656	1462	1261	1751	1376	1243	1817	1407	1251
	(28.0)	(36.3)	(32.0)	(27.6)	(38.4)	(30.1)	(27.2)	(39.8)	(30.8)	(27.4)
c_E	1178.1	2001.4	1291.7	1178.1	2001.4	1291.7	1178.1	2001.4	1291.7	1178.1
	(25.8)	(43.9)	(28.3)	(25.8)	(43.9)	(28.3)	(25.8)	(43.9)	(28.3)	(25.8)
	208.0	-627.4	362.2	171.4	-468.0	174.0	133.2	-351.2	240.6	150.1
	4.556	-13.746	7.935	3.756	-10.254	3.813	2.919	-7.695	5.272	3.290
N	4564	4564	4564	4564	4564	4564	4564	4564	4564	4564
X2	26.0	870.3	228.0	29.2	826.5	194.8	28.7	610.6	126.4	15.9
Gvalue	26.4	728.2	234.5	29.9	699.2	196.4	29.8	535.2	129.4	16.0
Gv/N	0.578	15.956	5.139	0.656	15.319	4.303	0.653	11.726	2.835	0.350
Prob	0.00001	0.00000	0.00000	0.00000	0.00000	0.00000	0.00000	0.00000	0.00000	0.00114

	21	22	23	24	25	26	27	28	29	30
a_O	624	1033	1106	627	912	1003	555	961	954	494
	(13.7)	(22.6)	(24.2)	(13.7)	(20.0)	(22.0)	(12.2)	(21.1)	(20.9)	(10.8)
a_E	382.6	901.6	1144.2	382.6	901.6	1144.2	382.6	901.6	1144.2	382.6
	(8.4)	(19.8)	(25.1)	(8.4)	(19.8)	(25.1)	(8.4)	(19.8)	(25.1)	(8.4)
	610.4	281.1	-75.1	619.3	20.9	-264.2	412.8	122.6	-346.9	252.4
	13.373	6.158	-1.646	13.569	0.458	-5.789	9.045	2.687	-7.600	5.530
t_O	714	625	1393	708	663	1304	665	656	1397	681
	(15.6)	(13.7)	(30.5)	(15.5)	(14.5)	(28.6)	(14.6)	(14.4)	(30.6)	(14.9)
t_E	463.1	577.3	1296.2	463.1	577.3	1296.2	463.1	577.3	1296.2	463.1
	(10.1)	(12.6)	(28.4)	(10.1)	(12.6)	(28.4)	(10.1)	(12.6)	(28.4)	(10.1)
	618.2	99.3	200.6	601.1	183.6	15.6	481.2	167.8	209.2	525.2
	13.546	2.176	4.396	13.170	4.023	0.342	10.544	3.676	4.584	11.507
g_O	1327	1508	830	1435	1554	920	1436	1568	969	1455
	(29.1)	(33.0)	(18.2)	(31.4)	(34.0)	(20.2)	(31.5)	(34.4)	(21.2)	(31.9)
g_E	1716.9	1793.4	945.4	1716.9	1793.4	945.4	1716.9	1793.4	945.4	1716.9
	(37.6)	(39.3)	(20.7)	(37.6)	(39.3)	(20.7)	(37.6)	(39.3)	(20.7)	(37.6)
	-683.7	-522.8	-216.2	-514.8	-445.4	-50.2	-513.1	-421.3	47.7	-481.7
	-14.980	-11.456	-4.737	-11.279	-9.759	-1.100	-11.243	-9.230	1.045	-10.553
c_O	1899	1398	1235	1794	1435	1337	1908	1379	1244	1934
	(41.6)	(30.6)	(27.1)	(39.3)	(31.4)	(29.3)	(41.8)	(30.2)	(27.3)	(42.4)
c_E	2001.4	1291.7	1178.1	2001.4	1291.7	1178.1	2001.4	1291.7	1178.1	2001.4
	(43.9)	(28.3)	(25.8)	(43.9)	(28.3)	(25.8)	(43.9)	(28.3)	(25.8)	(43.9)
	-199.4	221.1	116.4	-392.4	302.0	338.2	-182.3	180.4	135.3	-132.4
	-4.369	4.845	2.551	-8.599	6.616	7.411	-3.994	3.953	2.965	-2.901
N	4564	4564	4564	4564	4564	4564	4564	4564	4564	4564
X2	382.0	77.3	25.3	353.3	60.7	39.6	216.0	48.9	43.7	177.2
Gvalue	345.5	78.7	25.8	313.2	61.1	39.4	198.6	49.5	45.4	163.5
Gv/N	7.571	1.724	0.564	6.862	1.339	0.864	4.352	1.084	0.994	3.583
Prob	0.00000	0.00000	0.00001	0.00000	0.00000	0.00000	0.00000	0.00000	0.00000	0.00000

	31	32	33	34	35	36	37	38	39	40
a_O	898	1027	523	892	986	457	864	982	446	877
	(19.7)	(22.5)	(11.5)	(19.5)	(21.6)	(10.0)	(18.9)	(21.5)	(9.8)	(19.2)
a_E	901.6	1144.2	382.6	901.6	1144.2	382.6	901.6	1144.2	382.6	901.6
	(19.8)	(25.1)	(8.4)	(19.8)	(25.1)	(8.4)	(19.8)	(25.1)	(8.4)	(19.8)
	-7.2	-222.0	326.9	-19.1	-293.4	162.3	-73.6	-300.2	136.7	-48.5
	-0.158	-4.863	7.162	-0.419	-6.429	3.557	-1.613	-6.578	2.995	-1.063
t_O	614	1344	593	576	1308	635	656	1358	613	656
	(13.5)	(29.4)	(13.0)	(12.6)	(28.7)	(13.9)	(14.4)	(29.8)	(13.4)	(14.4)
t_E	577.3	1296.2	463.1	577.3	1296.2	463.1	577.3	1296.2	463.1	577.3
	(12.6)	(28.4)	(10.1)	(12.6)	(28.4)	(10.1)	(12.6)	(28.4)	(10.1)	(12.6)
	75.8	97.3	293.2	-2.5	23.7	400.9	167.8	126.5	343.8	167.8
	1.660	2.132	6.425	-0.055	0.519	8.784	3.676	2.771	7.533	3.676
g_O	1705	952	1441	1751	1048	1519	1697	936	1530	1629
	(37.4)	(20.9)	(31.6)	(38.4)	(23.0)	(33.3)	(37.2)	(20.5)	(33.5)	(35.7)
g_E	1793.4	945.4	1716.9	1793.4	945.4	1716.9	1793.4	945.4	1716.9	1793.4
	(39.3)	(20.7)	(37.6)	(39.3)	(20.7)	(37.6)	(39.3)	(20.7)	(37.6)	(39.3)
	-172.5	13.2	-504.9	-83.9	215.9	-372.1	-187.6	-18.8	-352.7	-313.3
	-3.778	0.288	-11.062	-1.838	4.729	-8.152	-4.111	-0.412	-7.727	-6.865
c_O	1347	1241	2007	1345	1222	1953	1347	1288	1975	1402
	(29.5)	(27.2)	(44.0)	(29.5)	(26.8)	(42.8)	(29.5)	(28.2)	(43.3)	(30.7)
c_E	1291.7	1178.1	2001.4	1291.7	1178.1	2001.4	1291.7	1178.1	2001.4	1291.7
	(28.3)	(25.8)	(43.9)	(28.3)	(25.8)	(43.9)	(28.3)	(25.8)	(43.9)	(28.3)
	113.0	129.0	11.3	108.8	89.3	-95.5	113.0	229.7	-52.4	229.8
	2.475	2.827	0.248	2.384	1.957	-2.093	2.475	5.032	-1.147	5.035
N	4564	4564	4564	4564	4564	4564	4564	4564	4564	4564
X2	9.1	17.2	132.3	3.3	34.7	102.2	19.9	36.3	79.7	35.9
Gvalue	9.1	17.5	126.5	3.3	35.4	95.6	19.5	37.1	75.4	35.7
Gv/N	0.199	0.384	2.772	0.072	0.776	2.095	0.427	0.813	1.653	0.782
Prob	0.02831	0.00055	0.00000	0.34920	0.00000	0.00000	0.00022	0.00000	0.00000	0.00000

	41	42	43	44	45	46	47	48	49	50
a_O	959	408	865	977	456	880	984	501	839	895
	(21.0)	(8.9)	(19.0)	(21.4)	(10.0)	(19.3)	(21.6)	(11.0)	(18.4)	(19.6)
a_E	1144.2	382.6	901.6	1144.2	382.6	901.6	1144.2	382.6	901.6	1144.2
	(25.1)	(8.4)	(19.8)	(25.1)	(8.4)	(19.8)	(25.1)	(8.4)	(19.8)	(25.1)
	-338.7	52.4	-71.7	-306.7	160.0	-42.7	-296.8	270.1	-120.8	-439.7
	-7.420	1.148	-1.571	-6.763	3.505	-0.935	-6.504	5.917	-2.646	-9.634
t_O	1352	607	618	1356	568	594	1344	585	578	1361
	(29.6)	(13.3)	(13.5)	(29.7)	(12.4)	(13.0)	(29.4)	(12.8)	(12.7)	(29.8)
t_E	1296.2	463.1	577.3	1296.2	463.1	577.3	1296.2	463.1	577.3	1296.2
	(28.4)	(10.1)	(12.6)	(28.4)	(10.1)	(12.6)	(28.4)	(10.1)	(12.6)	(28.4)
	113.9	328.5	84.3	122.3	231.9	34.0	97.3	273.4	1.5	132.7
	2.496	7.197	1.847	2.679	5.082	0.744	2.132	5.990	0.032	2.909
g_O	972	1603	1702	980	1579	1741	956	1617	1756	1000
	(21.3)	(35.1)	(37.3)	(21.5)	(34.6)	(38.1)	(20.9)	(35.4)	(38.5)	(21.9)
g_E	945.4	1716.9	1793.4	945.4	1716.9	1793.4	945.4	1716.9	1793.4	945.4
	(20.7)	(37.6)	(39.3)	(20.7)	(37.6)	(39.3)	(20.7)	(37.6)	(39.3)	(20.7)
	53.9	-220.1	-178.1	70.4	-264.4	-103.3	21.2	-193.9	-74.1	112.2
	1.180	-4.822	-3.903	1.542	-5.794	-2.264	0.465	-4.248	-1.624	2.458
c_O	1281	1946	1379	1251	1961	1349	1280	1861	1391	1308
	(28.1)	(42.6)	(30.2)	(27.4)	(43.0)	(29.6)	(28.0)	(40.8)	(30.5)	(28.7)
c_E	1178.1	2001.4	1291.7	1178.1	2001.4	1291.7	1178.1	2001.4	1291.7	1178.1
	(25.8)	(43.9)	(28.3)	(25.8)	(43.9)	(28.3)	(25.8)	(43.9)	(28.3)	(25.8)
	214.5	-109.2	180.4	150.1	-79.9	117.1	212.3	-270.6	206.1	273.5
	4.699	-2.392	3.953	3.290	-1.750	2.566	4.651	-5.930	4.515	5.993
N	4564	4564	4564	4564	4564	4564	4564	4564	4564	4564
X2	42.1	55.5	14.9	33.0	49.7	5.1	33.1	84.4	12.8	75.0
Gvalue	43.6	51.6	14.8	34.1	47.6	5.1	34.0	78.9	12.7	78.8
Gv/N	0.955	1.131	0.325	0.747	1.043	0.111	0.744	1.729	0.278	1.727
Prob	0.00000	0.00000	0.00196	0.00000	0.00000	0.16763	0.00000	0.00000	0.00536	0.00000

	51	52	53	54	55	56	57	58	59	60
a_O	431	863	935	469	833	1001	469	856	956	464
	(9.4)	(18.9)	(20.5)	(10.3)	(18.3)	(21.9)	(10.3)	(18.8)	(20.9)	(10.2)
a_E	382.6	901.6	1144.2	382.6	901.6	1144.2	382.6	901.6	1144.2	382.6
	(8.4)	(19.8)	(25.1)	(8.4)	(19.8)	(25.1)	(8.4)	(19.8)	(25.1)	(8.4)
	102.6	-75.5	-377.6	190.9	-131.9	-267.7	190.9	-88.9	-343.6	178.9
	2.248	-1.655	-8.273	4.183	-2.889	-5.865	4.183	-1.947	-7.528	3.920
t_O	582	620	1334	537	627	1363	551	624	1335	573
	(12.8)	(13.6)	(29.2)	(11.8)	(13.7)	(29.9)	(12.1)	(13.7)	(29.3)	(12.6)
t_E	463.1	577.3	1296.2	463.1	577.3	1296.2	463.1	577.3	1296.2	463.1
	(10.1)	(12.6)	(28.4)	(10.1)	(12.6)	(28.4)	(10.1)	(12.6)	(28.4)	(10.1)
	266.0	88.6	76.7	159.0	103.6	136.9	191.5	97.2	78.7	244.0
	5.828	1.940	1.679	3.484	2.271	3.000	4.196	2.129	1.725	5.347
g_O	1622	1745	1016	1654	1749	935	1637	1764	966	1604
	(35.5)	(38.2)	(22.3)	(36.2)	(38.3)	(20.5)	(35.9)	(38.7)	(21.2)	(35.1)
g_E	1716.9	1793.4	945.4	1716.9	1793.4	945.4	1716.9	1793.4	945.4	1716.9
	(37.6)	(39.3)	(20.7)	(37.6)	(39.3)	(20.7)	(37.6)	(39.3)	(20.7)	(37.6)
	-184.5	-95.6	146.3	-123.5	-87.8	-20.8	-156.0	-58.4	41.6	-218.2
	-4.042	-2.094	3.204	-2.705	-1.923	-0.455	-3.419	-1.280	0.910	-4.781
c_O	1929	1336	1279	1904	1355	1265	1907	1320	1307	1923
	(42.3)	(29.3)	(28.0)	(41.7)	(29.7)	(27.7)	(41.8)	(28.9)	(28.6)	(42.1)
c_E	2001.4	1291.7	1178.1	2001.4	1291.7	1178.1	2001.4	1291.7	1178.1	2001.4
	(43.9)	(28.3)	(25.8)	(43.9)	(28.3)	(25.8)	(43.9)	(28.3)	(25.8)	(43.9)
	-142.1	90.1	210.1	-189.9	129.7	180.0	-184.2	57.2	271.3	-153.6
	-3.113	1.975	4.604	-4.161	2.841	3.943	-4.036	1.254	5.945	-3.365
N	4564	4564	4564	4564	4564	4564	4564	4564	4564	4564
X2	44.5	7.6	53.2	38.3	13.7	27.9	44.3	7.2	46.7	53.9
Gvalue	42.1	7.6	55.4	36.5	13.7	28.5	42.2	7.1	48.0	51.1
Gv/N	0.922	0.166	1.215	0.800	0.300	0.624	0.924	0.156	1.052	1.120
Prob	0.00000	0.05531	0.00000	0.00000	0.00336	0.00000	0.00000	0.06773	0.00000	0.00000

	61	62	63	64	65	66	67	68	69	70
a_O	814	973	458	810	1035	473	824	987	427	876
	(17.8)	(21.3)	(10.0)	(17.7)	(22.7)	(10.4)	(18.1)	(21.6)	(9.4)	(19.2)
a_E	901.6	1144.2	382.6	901.6	1144.2	382.6	901.6	1144.2	382.6	901.6
	(19.8)	(25.1)	(8.4)	(19.8)	(25.1)	(8.4)	(19.8)	(25.1)	(8.4)	(19.8)
	-166.4	-315.4	164.7	-173.6	-207.6	200.6	-148.3	-291.7	93.7	-50.5
	-3.646	-6.911	3.608	-3.803	-4.549	4.394	-3.250	-6.392	2.053	-1.106
t_O	588	1224	585	630	1251	570	597	1286	571	636
	(12.9)	(26.8)	(12.8)	(13.8)	(27.4)	(12.5)	(13.1)	(28.2)	(12.5)	(13.9)
t_E	577.3	1296.2	463.1	577.3	1296.2	463.1	577.3	1296.2	463.1	577.3
	(12.6)	(28.4)	(10.1)	(12.6)	(28.4)	(10.1)	(12.6)	(28.4)	(10.1)	(12.6)
	21.7	-140.3	273.4	110.1	-88.8	236.8	40.1	-20.4	239.2	123.3
	0.475	-3.075	5.990	2.413	-1.947	5.188	0.879	-0.446	5.240	2.701
g_O	1812	969	1634	1832	978	1670	1783	1030	1673	1755
	(39.7)	(21.2)	(35.8)	(40.1)	(21.4)	(36.6)	(39.1)	(22.6)	(36.7)	(38.5)
g_E	1793.4	945.4	1716.9	1793.4	945.4	1716.9	1793.4	945.4	1716.9	1793.4
	(39.3)	(20.7)	(37.6)	(39.3)	(20.7)	(37.6)	(39.3)	(20.7)	(37.6)	(39.3)
	37.3	47.7	-161.7	77.9	66.2	-92.5	-20.8	176.5	-86.7	-76.1
	0.817	1.045	-3.544	1.708	1.451	-2.027	-0.456	3.866	-1.899	-1.666
c_O	1350	1398	1887	1292	1300	1851	1360	1261	1893	1297
	(29.6)	(30.6)	(41.3)	(28.3)	(28.5)	(40.6)	(29.8)	(27.6)	(41.5)	(28.4)
c_E	1291.7	1178.1	2001.4	1291.7	1178.1	2001.4	1291.7	1178.1	2001.4	1291.7
	(28.3)	(25.8)	(43.9)	(28.3)	(25.8)	(43.9)	(28.3)	(25.8)	(43.9)	(28.3)
	119.2	478.4	-222.0	0.6	255.9	-289.1	140.2	171.4	-210.7	10.7
	2.612	10.482	-4.865	0.014	5.607	-6.335	3.071	3.756	-4.617	0.233
N	4564	4564	4564	4564	4564	4564	4564	4564	4564	4564
X2	11.5	71.3	57.5	15.0	25.7	58.6	11.0	35.1	37.3	7.5
Gvalue	11.8	70.4	54.3	15.1	25.7	55.7	11.2	35.8	35.4	7.4
Gv/N	0.258	1.542	1.189	0.332	0.562	1.220	0.245	0.784	0.777	0.161
Prob	0.00815	0.00000	0.00000	0.00170	0.00001	0.00000	0.01089	0.00000	0.00000	0.06101

	71	72	73	74	75	76	77	78	79	80
a_O	1045	406	863	1046	445	846	1041	442	890	1049
	(22.9)	(8.9)	(18.9)	(22.9)	(9.8)	(18.5)	(22.8)	(9.7)	(19.5)	(23.0)
a_E	1144.2	382.6	901.6	1144.2	382.6	901.6	1144.2	382.6	901.6	1144.2
	(25.1)	(8.4)	(19.8)	(25.1)	(8.4)	(19.8)	(25.1)	(8.4)	(19.8)	(25.1)
	-189.5	48.1	-75.5	-187.7	134.4	-107.7	-196.8	127.5	-23.1	-182.2
	-4.153	1.054	-1.655	-4.113	2.944	-2.360	-4.312	2.794	-0.505	-3.993
t_O	1223	606	625	1297	569	636	1298	592	598	1300
	(26.8)	(13.3)	(13.7)	(28.4)	(12.5)	(13.9)	(28.4)	(13.0)	(13.1)	(28.5)
t_E	1296.2	463.1	577.3	1296.2	463.1	577.3	1296.2	463.1	577.3	1296.2
	(28.4)	(10.1)	(12.6)	(28.4)	(10.1)	(12.6)	(28.4)	(10.1)	(12.6)	(28.4)
	-142.2	325.9	99.3	1.6	234.3	123.3	3.6	290.7	42.2	7.6
	-3.116	7.142	2.176	0.034	5.135	2.701	0.078	6.370	0.925	0.166
g_O	964	1682	1758	954	1631	1799	967	1672	1761	954
	(21.1)	(36.9)	(38.5)	(20.9)	(35.7)	(39.4)	(21.2)	(36.6)	(38.6)	(20.9)
g_E	945.4	1716.9	1793.4	945.4	1716.9	1793.4	945.4	1716.9	1793.4	945.4
	(20.7)	(37.6)	(39.3)	(20.7)	(37.6)	(39.3)	(20.7)	(37.6)	(39.3)	(20.7)
	37.5	-69.1	-70.2	17.2	-167.4	11.1	43.6	-88.6	-64.3	17.2
	0.821	-1.514	-1.538	0.377	-3.669	0.244	0.955	-1.942	-1.409	0.377
c_O	1332	1870	1318	1267	1919	1283	1258	1858	1315	1261
	(29.2)	(41.0)	(28.9)	(27.8)	(42.0)	(28.1)	(27.6)	(40.7)	(28.8)	(27.6)
c_E	1178.1	2001.4	1291.7	1178.1	2001.4	1291.7	1178.1	2001.4	1291.7	1178.1
	(25.8)	(43.9)	(28.3)	(25.8)	(43.9)	(28.3)	(25.8)	(43.9)	(28.3)	(25.8)
	327.0	-253.9	53.2	184.3	-161.3	-17.3	165.0	-276.2	47.0	171.4
	7.165	-5.563	1.165	4.037	-3.534	-0.379	3.616	-6.051	1.031	3.756
N	4564	4564	4564	4564	4564	4564	4564	4564	4564	4564
X2	33.2	54.8	6.8	15.2	42.1	9.5	15.2	56.5	1.9	13.8
Gvalue	32.7	51.1	6.8	15.3	40.0	9.4	15.4	53.4	1.9	13.9
Gv/N	0.717	1.119	0.148	0.335	0.877	0.205	0.337	1.170	0.042	0.305
Prob	0.00000	0.00000	0.08004	0.00158	0.00000	0.02485	0.00151	0.00000	0.59452	0.00300

	81	82	83	84	85	86	87	88	89	90
a_O	452	907	1071	422	819	1044	452	843	1002	421
	(9.9)	(19.9)	(23.5)	(9.2)	(17.9)	(22.9)	(9.9)	(18.5)	(22.0)	(9.2)
a_E	382.6	901.4	1143.9	382.6	901.4	1143.9	382.6	901.4	1143.9	382.6
	(8.4)	(19.8)	(25.1)	(8.4)	(19.8)	(25.1)	(8.4)	(19.8)	(25.1)	(8.4)
	150.6	11.2	-141.1	82.8	-157.0	-190.9	150.8	-112.9	-265.5	80.6
	3.300	0.246	-3.093	1.815	-3.442	-4.184	3.305	-2.475	-5.819	1.767
t_O	571	556	1282	565	609	1335	522	614	1265	559
	(12.5)	(12.2)	(28.1)	(12.4)	(13.3)	(29.3)	(11.4)	(13.5)	(27.7)	(12.3)
t_E	463.1	577.1	1295.9	463.0	577.1	1295.9	463.0	577.1	1295.9	463.0
	(10.1)	(12.6)	(28.4)	(10.1)	(12.6)	(28.4)	(10.1)	(12.6)	(28.4)	(10.1)
	239.2	-41.5	-27.7	225.0	65.5	79.3	125.2	76.0	-61.1	210.6

	5.240	-0.909	-0.608	4.930	1.434	1.738	2.744	1.666	-1.340	4.616
g_O	1640	1798	956	1674	1805	968	1695	1835	949	1648
	(35.9)	(39.4)	(21.0)	(36.7)	(39.6)	(21.2)	(37.1)	(40.2)	(20.8)	(36.1)
g_E	1716.9	1793.0	945.2	1716.5	1793.0	945.2	1716.5	1793.0	945.2	1716.5
	(37.6)	(39.3)	(20.7)	(37.6)	(39.3)	(20.7)	(37.6)	(39.3)	(20.7)	(37.6)
	-150.3	9.9	21.6	-84.0	24.0	46.1	-42.8	84.9	7.5	-134.3
	-3.293	0.217	0.474	-1.841	0.526	1.010	-0.938	1.860	0.165	-2.943
c_O	1901	1302	1254	1902	1330	1216	1894	1271	1347	1935
	(41.7)	(28.5)	(27.5)	(41.7)	(29.1)	(26.6)	(41.5)	(27.9)	(29.5)	(42.4)
c_E	2001.4	1291.4	1177.9	2000.9	1291.4	1177.9	2000.9	1291.4	1177.9	2000.9
	(43.9)	(28.3)	(25.8)	(43.9)	(28.3)	(25.8)	(43.9)	(28.3)	(25.8)	(43.9)
	-195.6	21.3	157.1	-192.9	78.3	77.5	-208.0	-40.5	361.4	-129.6
	-4.285	0.466	3.442	-4.227	1.717	1.698	-4.559	-0.887	7.921	-2.841
N	4564	4563	4563	4563	4563	4563	4563	4563	4563	4563
X2	46.2	0.9	9.8	32.5	10.5	11.7	26.1	7.4	42.6	28.7
Gvalue	43.9	0.9	9.8	30.9	10.7	11.9	25.2	7.5	42.3	27.4
Gv/N	0.961	0.020	0.216	0.678	0.235	0.261	0.553	0.164	0.928	0.600
Prob	0.00000	0.82088	0.01995	0.00000	0.01332	0.00763	0.00001	0.05826	0.00000	0.00000

	91	92	93	94	95	96	97	98	99	100
a_O	838	1104	440	868	986	414	866	1021	414	870
	(18.4)	(24.2)	(9.6)	(19.0)	(21.6)	(9.1)	(19.0)	(22.4)	(9.1)	(19.1)
a_E	901.4	1143.9	382.6	901.4	1143.9	382.6	901.2	1143.7	382.5	900.8
	(19.8)	(25.1)	(8.4)	(19.8)	(25.1)	(8.4)	(19.8)	(25.1)	(8.4)	(19.8)
	-122.2	-78.5	123.1	-65.6	-293.0	65.4	-69.0	-231.7	65.6	-60.6
	-2.679	-1.720	2.698	-1.437	-6.421	1.434	-1.513	-5.080	1.438	-1.328
t_O	557	1289	555	629	1338	544	611	1281	547	595
	(12.2)	(28.2)	(12.2)	(13.8)	(29.3)	(11.9)	(13.4)	(28.1)	(12.0)	(13.0)
t_E	577.1	1295.9	463.0	577.1	1295.9	463.0	577.0	1295.7	462.9	576.8
	(12.6)	(28.4)	(10.1)	(12.6)	(28.4)	(10.1)	(12.6)	(28.4)	(10.1)	(12.6)
	-39.6	-13.8	201.2	108.2	85.5	175.4	69.9	-29.1	182.6	37.1
	-0.867	-0.303	4.409	2.372	1.873	3.844	1.533	-0.639	4.003	0.813
g_O	1834	932	1683	1821	949	1640	1835	962	1689	1805
	(40.2)	(20.4)	(36.9)	(39.9)	(20.8)	(35.9)	(40.2)	(21.1)	(37.0)	(39.6)
g_E	1793.0	945.2	1716.5	1793.0	945.2	1716.5	1792.7	945.0	1716.2	1791.9
	(39.3)	(20.7)	(37.6)	(39.3)	(20.7)	(37.6)	(39.3)	(20.7)	(37.6)	(39.3)
	82.8	-26.3	-66.4	56.3	7.5	-149.6	85.7	34.2	-53.9	26.4
	1.815	-0.576	-1.455	1.235	0.165	-3.278	1.878	0.751	-1.181	0.578
c_O	1334	1238	1885	1245	1290	1965	1250	1298	1912	1290
	(29.2)	(27.1)	(41.3)	(27.3)	(28.3)	(43.1)	(27.4)	(28.5)	(41.9)	(28.3)
c_E	1291.4	1177.9	2000.9	1291.4	1177.9	2000.9	1291.1	1177.6	2000.5	1290.6
	(28.3)	(25.8)	(43.9)	(28.3)	(25.8)	(43.9)	(28.3)	(25.8)	(43.9)	(28.3)
	86.6	123.3	-225.0	-91.1	234.6	-71.2	-80.9	252.7	-173.0	-1.1
	1.898	2.701	-4.931	-1.997	5.141	-1.560	-1.774	5.538	-3.792	-0.024
N	4563	4563	4563	4563	4563	4563	4562	4562	4562	4560
X2	7.5	4.7	34.3	8.0	33.9	20.8	5.7	25.9	22.2	1.7
Gvalue	7.6	4.7	32.9	7.9	34.6	20.0	5.7	26.0	21.4	1.7
Gv/N	0.167	0.102	0.721	0.173	0.758	0.439	0.124	0.571	0.468	0.038
Prob	0.05509	0.19908	0.00000	0.04809	0.00000	0.00017	0.12859	0.00001	0.00009	0.62949

	101	102	103	104	105	106	107	108	109	110
a_O	1091	426	885	1083	451	846	1068	405	862	1110
	(23.9)	(9.3)	(19.4)	(23.8)	(9.9)	(18.6)	(23.4)	(8.9)	(18.9)	(24.4)
a_E	1143.2	382.3	900.8	1143.2	382.3	900.4	1142.7	382.1	900.0	1142.2
	(25.1)	(8.4)	(19.8)	(25.1)	(8.4)	(19.8)	(25.1)	(8.4)	(19.8)	(25.1)
	-102.0	92.2	-31.4	-117.2	149.1	-105.5	-144.4	47.1	-74.4	-63.5
	-2.236	2.022	-0.688	-2.569	3.269	-2.314	-3.168	1.033	-1.634	-1.393
t_O	1300	594	620	1264	554	608	1331	575	600	1250
	(28.5)	(13.0)	(13.6)	(27.7)	(12.1)	(13.3)	(29.2)	(12.6)	(13.2)	(27.4)
t_E	1295.1	462.7	576.8	1295.1	462.7	576.5	1294.5	462.5	576.3	1293.9
	(28.4)	(10.1)	(12.6)	(28.4)	(10.1)	(12.6)	(28.4)	(10.1)	(12.6)	(28.4)
	9.9	296.8	89.6	-61.4	199.5	64.7	74.0	250.4	48.5	-86.4
	0.216	6.508	1.966	-1.347	4.376	1.419	1.623	5.493	1.064	-1.896
g_O	948	1627	1775	964	1661	1858	969	1665	1804	975
	(20.8)	(35.7)	(38.9)	(21.1)	(36.4)	(40.8)	(21.3)	(36.5)	(39.6)	(21.4)
g_E	944.6	1715.4	1791.9	944.6	1715.4	1791.1	944.2	1714.6	1790.3	943.8
	(20.7)	(37.6)	(39.3)	(20.7)	(37.6)	(39.3)	(20.7)	(37.6)	(39.3)	(20.7)
	6.8	-172.2	-33.6	39.2	-107.1	136.3	50.2	-97.8	27.5	63.4
	0.149	-3.776	-0.736	0.859	-2.348	2.990	1.102	-2.147	0.604	1.393
c_O	1221	1913	1280	1249	1894	1246	1190	1913	1290	1221
	(26.8)	(42.0)	(28.1)	(27.4)	(41.5)	(27.3)	(26.1)	(42.0)	(28.3)	(26.8)
c_E	1177.1	1999.6	1290.6	1177.1	1999.6	1290.0	1176.6	1998.7	1289.4	1176.1
	(25.8)	(43.9)	(28.3)	(25.8)	(43.9)	(28.3)	(25.8)	(43.9)	(28.3)	(25.8)
	89.4	-169.4	-21.0	148.1	-205.5	-86.5	27.0	-167.7	1.2	91.5
	1.961	-3.715	-0.461	3.248	-4.507	-1.897	0.592	-3.680	0.025	2.009
N	4560	4560	4560	4560	4560	4558	4558	4558	4556	4556
X2	4.1	50.6	3.8	8.7	37.7	9.0	6.7	33.8	2.7	5.1
Gvalue	4.1	47.4	3.7	8.7	36.0	9.0	6.8	31.9	2.7	5.1
Gv/N	0.089	1.040	0.081	0.190	0.790	0.198	0.149	0.700	0.059	0.113
Prob	0.25424	0.00000	0.29706	0.03389	0.00000	0.02884	0.07824	0.00000	0.44022	0.16171

	111	112	113	114	115	116	117	118	119	120
a_O	429	841	1127	432	824	1096	440	892	1122	408
	(9.4)	(18.5)	(24.8)	(9.5)	(18.1)	(24.1)	(9.7)	(19.6)	(24.7)	(9.0)
a_E	382.0	899.4	1141.4	381.7	899.0	1140.9	381.5	898.1	1139.7	381.1
	(8.4)	(19.8)	(25.1)	(8.4)	(19.8)	(25.1)	(8.4)	(19.8)	(25.1)	(8.4)
	99.6	-113.0	-28.7	106.9	-143.6	-88.1	125.4	-12.1	-35.1	55.6
	2.187	-2.482	-0.630	2.348	-3.156	-1.935	2.756	-0.265	-0.772	1.223
t_O	575	641	1238	576	647	1273	550	601	1258	522
	(12.6)	(14.1)	(27.2)	(12.7)	(14.2)	(28.0)	(12.1)	(13.2)	(27.7)	(11.5)
t_E	462.3	575.9	1293.1	462.0	575.6	1292.5	461.8	575.0	1291.1	461.3
	(10.1)	(12.6)	(28.4)	(10.1)	(12.6)	(28.4)	(10.1)	(12.6)	(28.4)	(10.1)
	250.9	137.4	-107.8	254.1	151.3	-38.8	192.3	53.2	-65.4	129.1
	5.507	3.017	-2.368	5.581	3.324	-0.852	4.225	1.170	-1.438	2.840
g_O	1607	1845	953	1590	1821	991	1600	1762	937	1646
	(35.3)	(40.5)	(20.9)	(34.9)	(40.0)	(21.8)	(35.2)	(38.8)	(20.6)	(36.2)
g_E	1713.9	1789.1	943.2	1712.8	1788.3	942.8	1712.0	1786.4	941.7	1710.1
	(37.6)	(39.3)	(20.7)	(37.6)	(39.3)	(20.7)	(37.6)	(39.3)	(20.7)	(37.6)
	-207.0	113.5	19.8	-236.5	65.9	98.9	-216.5	-48.4	-9.4	-125.8
	-4.543	2.493	0.434	-5.195	1.449	2.174	-4.758	-1.065	-0.207	-2.768
c_O	1945	1226	1235	1955	1259	1191	1961	1291	1229	1970
	(42.7)	(26.9)	(27.1)	(42.9)	(27.7)	(26.2)	(43.1)	(28.4)	(27.0)	(43.3)
c_E	1997.8	1288.6	1175.3	1996.5	1288.0	1174.8	1995.7	1286.6	1173.5	1993.5
	(43.9)	(28.3)	(25.8)	(43.9)	(28.3)	(25.8)	(43.9)	(28.3)	(25.8)	(43.9)
	-104.3	-122.1	122.4	-82.2	-57.4	32.7	-68.7	8.8	113.6	-46.6
	-2.289	-2.681	2.688	-1.805	-1.260	0.718	-1.510	0.194	2.499	-1.026

N	4556	4553	4553	4553	4551	4551	4551	4546	4546	4546
X2	41.3	15.9	5.7	44.4	16.4	4.8	33.7	1.6	3.8	12.6
Gvalue	39.3	15.8	5.6	42.3	16.2	4.7	32.5	1.5	3.7	12.2
Gv/N	0.862	0.347	0.124	0.929	0.356	0.104	0.714	0.034	0.082	0.269
Prob	0.00000	0.00125	0.12995	0.00000	0.00103	0.19175	0.00000	0.67094	0.29082	0.00664

	121	122	123	124	125	126	127	128	129	130
a_O	828	1129	378	811	1167	426	865	1110	458	916
	(18.2)	(24.9)	(8.3)	(17.9)	(25.7)	(9.4)	(19.1)	(24.5)	(10.1)	(20.2)
a_E	897.5	1138.9	380.9	896.9	1138.2	380.6	895.9	1136.9	380.2	895.1
	(19.8)	(25.1)	(8.4)	(19.8)	(25.1)	(8.4)	(19.8)	(25.1)	(8.4)	(19.8)
	-133.4	-19.8	-5.7	-163.2	58.4	96.0	-60.7	-53.2	170.5	42.3
	-2.936	-0.435	-0.126	-3.595	1.286	2.114	-1.338	-1.173	3.760	0.934
t_O	613	1238	539	621	1192	539	587	1277	533	530
	(13.5)	(27.3)	(11.9)	(13.7)	(26.3)	(11.9)	(12.9)	(28.2)	(11.8)	(11.7)
t_E	574.6	1290.3	461.0	574.2	1289.4	460.7	573.6	1288.0	460.2	573.1
	(12.6)	(28.4)	(10.1)	(12.6)	(28.4)	(10.1)	(12.6)	(28.4)	(10.1)	(12.6)
	79.3	-102.4	168.6	97.3	-187.3	169.3	27.1	-21.9	156.6	-82.9
	1.745	-2.253	3.711	2.142	-4.125	3.729	0.598	-0.482	3.454	-1.829
g_O	1775	967	1658	1823	995	1626	1816	939	1609	1769
	(39.1)	(21.3)	(36.5)	(40.2)	(21.9)	(35.8)	(40.0)	(20.7)	(35.5)	(39.0)
g_E	1785.2	941.1	1709.0	1784.0	940.5	1707.9	1782.0	939.4	1706.0	1780.5
	(39.3)	(20.7)	(37.6)	(39.3)	(20.7)	(37.6)	(39.3)	(20.7)	(37.6)	(39.3)
	-20.3	52.5	-100.5	78.8	112.2	-159.8	68.5	-0.9	-188.4	-22.9
	-0.447	1.156	-2.212	1.736	2.470	-3.519	1.512	-0.019	-4.154	-0.505
c_O	1327	1209	1968	1285	1186	1949	1267	1209	1935	1316
	(29.2)	(26.6)	(43.3)	(28.3)	(26.1)	(42.9)	(27.9)	(26.7)	(42.7)	(29.0)
c_E	1285.7	1172.7	1992.1	1284.9	1171.9	1990.8	1283.5	1170.7	1988.6	1282.3
	(28.3)	(25.8)	(43.9)	(28.3)	(25.8)	(43.9)	(28.3)	(25.8)	(43.9)	(28.3)
	83.8	73.7	-48.0	0.2	28.3	-82.8	-32.7	77.9	-105.8	68.2
	1.845	1.622	-1.057	0.005	0.623	-1.823	-0.722	1.719	-2.333	1.505
N	4543	4543	4543	4540	4540	4540	4535	4535	4535	4531
X2	9.3	4.0	15.0	12.9	11.4	23.5	2.2	2.0	34.4	4.7
Gvalue	9.4	4.1	14.4	13.1	11.5	22.7	2.2	2.0	33.0	4.8
Gv/N	0.207	0.089	0.316	0.287	0.254	0.500	0.049	0.044	0.727	0.105
Prob	0.02444	0.25609	0.00244	0.00452	0.00912	0.00005	0.52357	0.57664	0.00000	0.19032

	131	132	133	134	135	136	137	138	139	140
a_O	1160	461	846	1168	423	852	1127	409	833	1137
	(25.6)	(10.2)	(18.7)	(25.8)	(9.3)	(18.8)	(24.9)	(9.0)	(18.4)	(25.2)
a_E	1135.9	379.9	894.9	1135.7	379.8	893.9	1134.4	379.4	892.7	1132.9
	(25.1)	(8.4)	(19.8)	(25.1)	(8.4)	(19.8)	(25.1)	(8.4)	(19.8)	(25.1)
	48.7	178.5	-95.1	65.6	91.2	-81.8	-14.8	61.5	-115.3	8.2
	1.074	3.939	-2.098	1.447	2.013	-1.808	-0.327	1.360	-2.553	0.181
t_O	1211	479	604	1235	534	605	1273	517	573	1215
	(26.7)	(10.6)	(13.3)	(27.3)	(11.8)	(13.4)	(28.1)	(11.4)	(12.7)	(26.9)
t_E	1286.8	459.8	573.0	1286.6	459.7	572.3	1285.1	459.1	571.6	1283.4
	(28.4)	(10.1)	(12.6)	(28.4)	(10.1)	(12.6)	(28.4)	(10.1)	(12.6)	(28.4)
	-147.1	39.3	63.7	-101.0	160.1	67.2	-24.2	122.7	2.9	-133.2
	-3.247	0.867	1.407	-2.230	3.535	1.484	-0.534	2.712	0.063	-2.947
g_O	967	1656	1797	960	1644	1756	977	1537	1868	944
	(21.3)	(36.5)	(39.7)	(21.2)	(36.3)	(38.8)	(21.6)	(34.0)	(41.3)	(20.9)
g_E	938.6	1704.5	1780.1	938.4	1704.1	1778.1	937.4	1702.2	1775.8	936.1
	(20.7)	(37.6)	(39.3)	(20.7)	(37.6)	(39.3)	(20.7)	(37.6)	(39.3)	(20.7)
	57.6	-95.6	34.0	43.7	-118.1	-44.0	80.9	-313.9	189.2	15.8
	1.272	-2.110	0.750	0.964	-2.607	-0.971	1.788	-6.937	4.187	0.350
c_O	1193	1935	1283	1167	1929	1312	1148	2062	1245	1223
	(26.3)	(42.7)	(28.3)	(25.8)	(42.6)	(29.0)	(25.4)	(45.6)	(27.6)	(27.1)
c_E	1169.6	1986.9	1282.1	1169.4	1986.4	1280.6	1168.1	1984.3	1279.0	1166.5
	(25.8)	(43.9)	(28.3)	(25.8)	(43.9)	(28.3)	(25.8)	(43.9)	(28.3)	(25.8)
	47.2	-102.4	1.9	-4.7	-113.2	63.5	-39.8	158.5	-67.0	115.6
	1.042	-2.260	0.041	-0.104	-2.499	1.403	-0.879	3.503	-1.482	2.559
N	4531	4531	4530	4530	4530	4525	4525	4525	4519	4519
X2	6.3	20.9	4.5	3.5	20.7	4.9	2.2	28.7	9.7	6.5
Gvalue	6.4	19.8	4.5	3.5	20.0	4.9	2.2	28.9	9.7	6.5
Gv/N	0.141	0.436	0.100	0.077	0.441	0.107	0.048	0.638	0.215	0.144
Prob	0.09442	0.00019	0.20932	0.32018	0.00017	0.18208	0.53920	0.00000	0.02120	0.09012

	141	142	143	144	145	146	147	148	149	150
a_O	387	855	1161	403	859	1090	392	825	1099	417
	(8.6)	(18.9)	(25.7)	(8.9)	(19.0)	(24.1)	(8.7)	(18.3)	(24.4)	(9.2)
a_E	378.9	892.3	1132.4	378.7	891.7	1131.7	378.4	890.9	1130.7	378.1
	(8.4)	(19.8)	(25.1)	(8.4)	(19.8)	(25.1)	(8.4)	(19.8)	(25.1)	(8.4)
	16.4	-73.1	57.9	50.1	-64.2	-81.8	27.6	-126.9	-62.4	81.7
	0.364	-1.617	1.282	1.110	-1.423	-1.812	0.611	-2.813	-1.384	1.810
t_O	521	614	1288	528	574	1254	520	604	1294	524
	(11.5)	(13.6)	(28.5)	(11.7)	(12.7)	(27.8)	(11.5)	(13.4)	(28.7)	(11.6)
t_E	458.5	571.3	1282.9	458.3	570.9	1282.0	458.0	570.4	1280.9	457.6
	(10.1)	(12.6)	(28.4)	(10.1)	(12.6)	(28.4)	(10.1)	(12.6)	(28.4)	(10.1)
	133.1	88.5	10.3	149.4	6.1	-55.4	132.0	69.1	26.4	141.9
	2.945	1.959	0.228	3.308	0.136	-1.228	2.923	1.531	0.585	3.147
g_O	1708	1780	930	1684	1768	976	1682	1862	968	1644
	(37.8)	(39.4)	(20.6)	(37.3)	(39.2)	(21.6)	(37.3)	(41.3)	(21.5)	(36.5)
g_E	1700.0	1775.0	935.7	1699.2	1773.8	935.1	1698.1	1772.2	934.3	1696.6
	(37.6)	(39.3)	(20.7)	(37.6)	(39.3)	(20.7)	(37.6)	(39.3)	(20.7)	(37.6)
	16.1	10.1	-11.4	-30.3	-11.6	83.6	-32.0	184.0	68.7	-103.5
	0.356	0.223	-0.252	-0.671	-0.256	1.852	-0.710	4.080	1.523	-2.296
c_O	1903	1268	1138	1902	1313	1194	1920	1219	1149	1925
	(42.1)	(28.1)	(25.2)	(42.1)	(29.1)	(26.5)	(42.5)	(27.0)	(25.5)	(42.7)
c_E	1981.6	1278.4	1166.0	1980.7	1277.5	1165.2	1979.4	1276.4	1164.2	1977.7
	(43.9)	(28.3)	(25.8)	(43.9)	(28.3)	(25.8)	(43.9)	(28.3)	(25.8)	(43.9)
	-154.1	-20.7	-55.3	-154.3	71.9	58.2	-117.1	-112.2	-30.2	-103.9
	-3.410	-0.458	-1.225	-3.416	1.593	1.290	-2.593	-2.487	-0.670	-2.305
N	4519	4517	4517	4517	4514	4514	4514	4510	4510	4510
X2	11.8	4.8	1.4	15.4	2.2	4.6	10.8	14.0	2.4	16.7
Gvalue	11.5	4.8	1.4	14.9	2.2	4.6	10.5	14.0	2.4	16.1
Gv/N	0.255	0.106	0.032	0.330	0.049	0.103	0.232	0.311	0.054	0.358
Prob	0.00923	0.18753	0.69407	0.00188	0.52663	0.20023	0.01500	0.00286	0.48766	0.00107

Babo_BIOVAR1_9941
5UTR:

	50	49	48	47	46	45	44	43	42	41
a_O	697	764	661	719	773	713	757	794	706	722
	(22.7)	(24.9)	(21.6)	(23.5)	(25.2)	(23.3)	(24.7)	(25.9)	(23.0)	(23.6)
a_E	759.3	759.3	759.3	759.3	759.3	759.3	759.3	759.3	759.3	759.3
	(24.8)	(24.8)	(24.8)	(24.8)	(24.8)	(24.8)	(24.8)	(24.8)	(24.8)	(24.8)
	-119.3	9.5	-183.3	-78.4	27.7	-89.7	-4.6	71.0	-102.7	-72.7
	-3.894	0.309	-5.981	-2.558	0.904	-2.927	-0.149	2.317	-3.353	-2.373
t_O	745	711	787	660	735	729	717	731	745	737
	(24.3)	(23.2)	(25.7)	(21.5)	(24.0)	(23.8)	(23.4)	(23.9)	(24.3)	(24.1)
t_E	715.6	715.6	715.6	715.6	715.6	715.6	715.6	715.6	715.6	715.6
	(23.4)	(23.4)	(23.4)	(23.4)	(23.4)	(23.4)	(23.4)	(23.4)	(23.4)	(23.4)
	60.1	-9.1	149.8	-106.7	39.4	27.1	2.9	31.2	60.1	43.5
	1.961	-0.297	4.889	-3.482	1.286	0.885	0.094	1.019	1.961	1.420
g_O	854	771	833	874	758	810	851	785	815	844
	(27.9)	(25.2)	(27.2)	(28.5)	(24.7)	(26.4)	(27.8)	(25.6)	(26.6)	(27.5)
g_E	825.4	825.4	825.4	825.4	825.4	825.4	825.4	825.4	825.4	825.4
	(26.9)	(26.9)	(26.9)	(26.9)	(26.9)	(26.9)	(26.9)	(26.9)	(26.9)	(26.9)
	58.1	-105.2	15.2	99.9	-129.2	-30.6	51.9	-78.8	-20.7	37.6
	1.897	-3.433	0.496	3.262	-4.217	-0.998	1.695	-2.573	-0.676	1.226
c_O	768	818	783	811	798	812	739	754	798	761
	(25.1)	(26.7)	(25.6)	(26.5)	(26.0)	(26.5)	(24.1)	(24.6)	(26.0)	(24.8)
c_E	763.7	763.7	763.7	763.7	763.7	763.7	763.7	763.7	763.7	763.7
	(24.9)	(24.9)	(24.9)	(24.9)	(24.9)	(24.9)	(24.9)	(24.9)	(24.9)	(24.9)
	8.6	112.3	39.0	97.4	70.1	99.5	-48.7	-19.3	70.1	-5.5
	0.279	3.665	1.274	3.179	2.286	3.248	-1.588	-0.631	2.286	-0.178
N	3064	3064	3064	3064	3064	3064	3064	3064	3064	3064
X2	7.3	7.5	20.4	12.2	7.8	6.4	1.6	4.0	6.6	2.9
Gvalue	7.5	7.5	20.8	12.3	7.9	6.4	1.6	4.0	6.7	2.9
Gv/N	0.243	0.245	0.678	0.401	0.259	0.209	0.052	0.132	0.218	0.095
Prob	0.05884	0.05763	0.00012	0.00649	0.04706	0.09330	0.65863	0.25795	0.08312	0.40386

	40	39	38	37	36	35	34	33	32	31
a_O	730	699	699	705	641	687	730	646	732	771
	(23.8)	(22.8)	(22.8)	(23.0)	(20.9)	(22.4)	(23.8)	(21.1)	(23.9)	(25.2)
a_E	759.3	759.3	759.3	759.3	759.3	759.3	759.3	759.3	759.3	759.3
	(24.8)	(24.8)	(24.8)	(24.8)	(24.8)	(24.8)	(24.8)	(24.8)	(24.8)	(24.8)
	-57.4	-115.6	-115.6	-104.6	-217.1	-137.4	-57.4	-208.7	-53.6	23.6
	-1.874	-3.774	-3.774	-3.413	-7.085	-4.486	-1.874	-6.813	-1.748	0.771
t_O	753	739	695	734	746	721	801	792	727	735
	(24.6)	(24.1)	(22.7)	(24.0)	(24.3)	(23.5)	(26.1)	(25.8)	(23.7)	(24.0)
t_E	715.6	715.6	715.6	715.6	715.6	715.6	715.6	715.6	715.6	715.6
	(23.4)	(23.4)	(23.4)	(23.4)	(23.4)	(23.4)	(23.4)	(23.4)	(23.4)	(23.4)
	76.8	47.6	-40.5	37.4	62.2	10.9	180.7	160.8	23.1	39.4
	2.507	1.555	-1.323	1.219	2.029	0.356	5.897	5.247	0.753	1.286
g_O	762	795	859	783	791	844	783	840	791	761
	(24.9)	(25.9)	(28.0)	(25.6)	(25.8)	(27.5)	(25.6)	(27.4)	(25.8)	(24.8)
g_E	825.4	825.4	825.4	825.4	825.4	825.4	825.4	825.4	825.4	825.4
	(26.9)	(26.9)	(26.9)	(26.9)	(26.9)	(26.9)	(26.9)	(26.9)	(26.9)	(26.9)
	-121.9	-59.7	68.5	-82.6	-67.4	37.6	-82.6	29.4	-67.4	-123.7
	-3.977	-1.949	2.235	-2.697	-2.200	1.226	-2.697	0.959	-2.200	-4.037
c_O	819	831	811	842	886	812	750	786	814	797
	(26.7)	(27.1)	(26.5)	(27.5)	(28.9)	(26.5)	(24.5)	(25.7)	(26.6)	(26.0)
c_E	763.7	763.7	763.7	763.7	763.7	763.7	763.7	763.7	763.7	763.7
	(24.9)	(24.9)	(24.9)	(24.9)	(24.9)	(24.9)	(24.9)	(24.9)	(24.9)	(24.9)
	114.4	140.3	97.4	164.3	263.1	99.5	-27.2	45.2	103.8	68.0
	3.735	4.579	3.179	5.362	8.588	3.248	-0.888	1.475	3.387	2.218
N	3064	3064	3064	3064	3064	3064	3064	3064	3064	3064
X2	12.0	12.6	9.7	14.6	40.7	10.4	13.8	26.0	5.9	7.2
Gvalue	12.0	12.6	9.7	14.4	40.8	10.6	13.4	26.6	5.9	7.3
Gv/N	0.391	0.410	0.318	0.471	1.332	0.344	0.438	0.868	0.192	0.238
Prob	0.00745	0.00566	0.02102	0.00238	0.00000	0.01440	0.00379	0.00001	0.11816	0.06300

	30	29	28	27	26	25	24	23	22	21
a_O	715	725	750	723	790	817	785	837	818	769
	(23.3)	(23.7)	(24.5)	(23.6)	(25.8)	(26.7)	(25.6)	(27.3)	(26.7)	(25.1)
a_E	759.3	759.3	759.3	759.3	759.3	759.3	759.3	759.3	759.3	759.3
	(24.8)	(24.8)	(24.8)	(24.8)	(24.8)	(24.8)	(24.8)	(24.8)	(24.8)	(24.8)
	-85.9	-67.0	-18.4	-70.8	62.7	119.7	52.3	163.1	121.9	19.6
	-2.804	-2.186	-0.602	-2.311	2.045	3.907	1.707	5.324	3.977	0.639
t_O	748	692	736	727	634	646	702	667	751	737
	(24.4)	(22.6)	(24.0)	(23.7)	(20.7)	(21.1)	(22.9)	(21.8)	(24.5)	(24.1)
t_E	715.6	715.6	715.6	715.6	715.6	715.6	715.6	715.6	715.6	715.6
	(23.4)	(23.4)	(23.4)	(23.4)	(23.4)	(23.4)	(23.4)	(23.4)	(23.4)	(23.4)
	66.3	-46.3	41.5	23.1	-153.4	-132.1	-26.9	-93.7	72.6	43.5
	2.165	-1.512	1.353	0.753	-5.008	-4.312	-0.877	-3.060	2.370	1.420
g_O	794	852	784	772	849	763	743	764	709	761
	(25.9)	(27.8)	(25.6)	(25.2)	(27.7)	(24.9)	(24.2)	(24.9)	(23.1)	(24.8)
g_E	825.4	825.4	825.4	825.4	825.4	825.4	825.4	825.4	825.4	825.4
	(26.9)	(26.9)	(26.9)	(26.9)	(26.9)	(26.9)	(26.9)	(26.9)	(26.9)	(26.9)
	-61.6	54.0	-80.7	-103.3	47.8	-120.0	-156.3	-118.2	-215.6	-123.7
	-2.012	1.762	-2.635	-3.372	1.560	-3.917	-5.102	-3.857	-7.037	-4.037
c_O	807	795	794	842	791	838	834	796	786	797
	(26.3)	(25.9)	(25.9)	(27.5)	(25.8)	(27.3)	(27.2)	(26.0)	(25.7)	(26.0)
c_E	763.7	763.7	763.7	763.7	763.7	763.7	763.7	763.7	763.7	763.7
	(24.9)	(24.9)	(24.9)	(24.9)	(24.9)	(24.9)	(24.9)	(24.9)	(24.9)	(24.9)
	88.9	63.8	61.7	164.3	55.5	155.5	146.8	65.9	45.2	68.0
	2.903	2.082	2.014	5.362	1.811	5.076	4.792	2.150	1.475	2.218
N	3064	3064	3064	3064	3064	3064	3064	3064	3064	3064
X2	7.7	4.5	4.0	13.4	12.2	23.1	15.8	17.2	23.4	7.2
Gvalue	7.7	4.5	4.0	13.2	12.5	23.1	15.9	17.1	24.1	7.4
Gv/N	0.251	0.146	0.130	0.432	0.409	0.754	0.519	0.558	0.785	0.240
Prob	0.05260	0.21541	0.26237	0.00415	0.00578	0.00004	0.00118	0.00067	0.00002	0.06143

	20	19	18	17	16	15	14	13	12	11
a_O	867	897	829	875	923	908	945	1010	1004	955
	(28.3)	(29.3)	(27.1)	(28.6)	(30.1)	(29.6)	(30.8)	(33.0)	(32.8)	(31.2)
a_E	759.3	759.3	759.3	759.3	759.3	759.3	759.3	759.3	759.3	759.3
	(24.8)	(24.8)	(24.8)	(24.8)	(24.8)	(24.8)	(24.8)	(24.8)	(24.8)	(24.8)
	230.0	299.0	145.7	248.2	360.4	324.8	413.6	576.4	561.0	438.0
	7.508	9.760	4.754	8.102	11.764	10.602	13.497	18.811	18.309	14.296
t_O	676	734	750	665	687	713	572	576	498	382
	(22.1)	(24.0)	(24.5)	(21.7)	(22.4)	(23.3)	(18.7)	(18.8)	(16.3)	(12.5)

t_E	715.6	715.6	715.6	715.6	715.6	715.6	715.6	715.6	715.6	715.6
	(23.4)	(23.4)	(23.4)	(23.4)	(23.4)	(23.4)	(23.4)	(23.4)	(23.4)	(23.4)
	-76.9	37.4	70.5	-97.5	-56.0	-5.1	-256.2	-249.9	-361.0	-479.5
	-2.510	1.219	2.301	-3.181	-1.827	-0.167	-8.361	-8.157	-11.782	-15.650
g_O	756	666	789	779	774	797	946	891	1111	1306
	(24.7)	(21.7)	(25.8)	(25.4)	(25.3)	(26.0)	(30.9)	(29.1)	(36.3)	(42.6)
g_E	825.4	825.4	825.4	825.4	825.4	825.4	825.4	825.4	825.4	825.4
	(26.9)	(26.9)	(26.9)	(26.9)	(26.9)	(26.9)	(26.9)	(26.9)	(26.9)	(26.9)
	-132.8	-285.9	-71.2	-90.2	-99.6	-55.9	258.0	136.2	660.2	1198.4
	-4.336	-9.330	-2.325	-2.944	-3.250	-1.823	8.419	4.446	21.546	39.113
c_O	765	767	696	745	680	646	601	587	451	421
	(25.0)	(25.0)	(22.7)	(24.3)	(22.2)	(21.1)	(19.6)	(19.2)	(14.7)	(13.7)
c_E	763.7	763.7	763.7	763.7	763.7	763.7	763.7	763.7	763.7	763.7
	(24.9)	(24.9)	(24.9)	(24.9)	(24.9)	(24.9)	(24.9)	(24.9)	(24.9)	(24.9)
	2.5	6.6	-129.3	-37.0	-157.9	-216.3	-288.0	-309.0	-475.1	-501.5
	0.083	0.214	-4.219	-1.207	-5.154	-7.059	-9.400	-10.084	-15.507	-16.367
N	3064	3064	3064	3064	3064	3064	3064	3064	3064	3064
X2	23.3	56.3	15.7	24.3	48.8	48.3	126.5	156.1	371.9	639.5
Gvalue	22.8	57.1	15.7	23.6	47.0	47.6	127.3	153.7	385.0	655.5
Gv/N	0.746	1.863	0.511	0.770	1.533	1.552	4.155	5.015	12.566	21.393
Prob	0.00004	0.00000	0.00132	0.00003	0.00000	0.00000	0.00000	0.00000	0.00000	0.00000

	10	9	8	7	6	5	4	3	2	1
a_O	853	879	1002	947	977	936	837	975	771	798
	(27.8)	(28.7)	(32.7)	(30.9)	(31.9)	(30.5)	(27.3)	(31.8)	(25.2)	(26.0)
a_E	759.3	759.3	759.3	759.3	759.3	759.3	759.3	759.3	759.3	759.3
	(24.8)	(24.8)	(24.8)	(24.8)	(24.8)	(24.8)	(24.8)	(24.8)	(24.8)	(24.8)
	198.6	257.4	555.9	418.4	492.6	391.7	163.1	487.6	23.6	79.4
	6.480	8.401	18.142	13.657	16.078	12.784	5.324	15.915	0.771	2.591
t_O	387	420	453	641	626	621	732	542	731	546
	(12.6)	(13.7)	(14.8)	(20.9)	(20.4)	(20.3)	(23.9)	(17.7)	(23.9)	(17.8)
t_E	715.6	715.6	715.6	715.6	715.6	715.6	715.6	715.6	715.6	715.6
	(23.4)	(23.4)	(23.4)	(23.4)	(23.4)	(23.4)	(23.4)	(23.4)	(23.4)	(23.4)
	-475.7	-447.6	-414.2	-141.1	-167.4	-176.0	33.3	-301.1	31.2	-295.3
	-15.526	-14.607	-13.518	-4.604	-5.464	-5.745	1.085	-9.828	1.019	-9.639
g_O	1441	1429	1184	987	860	820	729	838	644	619
	(47.0)	(46.6)	(38.6)	(32.2)	(28.1)	(26.8)	(23.8)	(27.3)	(21.0)	(20.2)
g_E	825.4	825.4	825.4	825.4	825.4	825.4	825.4	825.4	825.4	825.4
	(26.9)	(26.9)	(26.9)	(26.9)	(26.9)	(26.9)	(26.9)	(26.9)	(26.9)	(26.9)
	1605.8	1568.5	854.3	352.9	70.6	-10.8	-181.1	25.3	-319.7	-356.3
	52.409	51.193	27.880	11.517	2.303	-0.353	-5.912	0.827	-10.434	-11.628
c_O	383	336	425	489	601	687	766	709	918	1101
	(12.5)	(11.0)	(13.9)	(16.0)	(19.6)	(22.4)	(25.0)	(23.1)	(30.0)	(35.9)
c_E	763.7	763.7	763.7	763.7	763.7	763.7	763.7	763.7	763.7	763.7
	(24.9)	(24.9)	(24.9)	(24.9)	(24.9)	(24.9)	(24.9)	(24.9)	(24.9)	(24.9)
	-528.7	-551.8	-498.2	-436.0	-288.0	-145.5	4.5	-105.4	337.8	805.4
	-17.254	-18.009	-16.260	-14.231	-9.400	-4.748	0.148	-3.441	11.025	26.286
N	3064	3064	3064	3064	3064	3064	3064	3064	3064	3064
X2	811.3	821.9	479.9	184.6	109.8	61.4	19.6	107.5	71.6	242.7
Gvalue	800.0	826.6	497.7	194.2	107.8	59.4	19.8	106.4	72.9	233.2
Gv/N	26.109	26.978	16.244	6.339	3.517	1.938	0.647	3.472	2.380	7.610
Prob	0.00000	0.00000	0.00000	0.00000	0.00000	0.00000	0.00019	0.00000	0.00000	0.00000

CDS_INITIAL:

	1	2	3	4	5	6	7	8	9	10
a_O	2521	0	1	1333	882	726	973	1066	698	934
	(82.3)	(0.0)	(0.0)	(43.5)	(28.8)	(23.7)	(31.8)	(34.8)	(22.8)	(30.5)
a_E	707.5	808.1	350.2	707.5	808.1	350.2	707.5	808.1	350.2	707.5
	(23.1)	(26.4)	(11.4)	(23.1)	(26.4)	(11.4)	(23.1)	(26.4)	(11.4)	(23.1)
	6406.5	0.0	-11.7	1688.6	154.3	1058.6	620.0	590.5	962.8	518.7
	209.089	0.000	-0.382	55.113	5.037	34.548	20.235	19.271	31.424	16.930
t_O	260	3064	2	364	508	697	438	763	793	527
	(8.5)	(100.0)	(0.1)	(11.9)	(16.6)	(22.7)	(14.3)	(24.9)	(25.9)	(17.2)
t_E	411.7	905.5	642.4	411.7	905.5	642.4	411.7	905.5	642.4	411.7
	(13.4)	(29.6)	(21.0)	(13.4)	(29.6)	(21.0)	(13.4)	(29.6)	(21.0)	(13.4)
	-238.9	7470.1	-23.1	-89.6	-587.2	113.8	54.3	-261.3	334.1	260.3
	-7.799	243.801	-0.754	-2.923	-19.166	3.714	1.773	-8.527	10.905	8.497
g_O	283	0	3061	754	575	710	1006	509	769	836
	(9.2)	(0.0)	(99.9)	(24.6)	(18.8)	(23.2)	(32.8)	(16.6)	(25.1)	(27.3)
g_E	1180.0	570.4	963.6	1180.0	570.4	963.6	1180.0	570.4	963.6	1180.0
	(38.5)	(18.6)	(31.4)	(38.5)	(18.6)	(31.4)	(38.5)	(18.6)	(31.4)	(38.5)
	-808.1	0.0	7076.1	-675.4	9.3	-433.6	-321.0	-115.9	-346.9	-576.2
	-26.375	0.000	230.944	-22.042	0.304	-14.153	-10.475	-3.781	-11.322	-18.806
c_O	0	0	0	613	1099	931	647	726	804	767
	(0.0)	(0.0)	(0.0)	(20.0)	(35.9)	(30.4)	(21.1)	(23.7)	(26.2)	(25.0)
c_E	764.8	780.0	1107.9	764.8	780.0	1107.9	764.8	780.0	1107.9	764.8
	(25.0)	(25.5)	(36.2)	(25.0)	(25.5)	(36.2)	(25.0)	(25.5)	(36.2)	(25.0)
	0.0	0.0	0.0	-271.3	753.5	-323.9	-216.5	-104.3	-515.5	4.4
	0.000	0.000	0.000	-8.854	24.592	-10.570	-7.065	-3.402	-16.825	0.142
N	3064	3064	3064	3064	3064	3064	3064	3064	3064	3064
X2	6150.6	7304.0	6660.0	742.4	311.7	502.9	145.1	115.1	503.4	205.1
Gvalue	5359.4	7470.1	7041.3	652.4	329.9	414.9	136.9	109.1	434.5	207.2
Gv/N	174.915	243.801	229.808	21.293	10.767	13.540	4.468	3.561	14.182	6.763
Prob	1.00000	1.00000	1.00000	0.00000	0.00000	0.00000	0.00000	0.00000	0.00000	0.00000

	11	12	13	14	15	16	17	18	19	20
a_O	876	714	966	793	571	913	866	622	956	857
	(28.6)	(23.3)	(31.5)	(25.9)	(18.6)	(29.8)	(28.3)	(20.3)	(31.2)	(28.0)
a_E	808.1	350.2	707.5	808.1	350.2	707.5	808.1	350.2	707.5	808.1
	(26.4)	(11.4)	(23.1)	(26.4)	(11.4)	(23.1)	(26.4)	(11.4)	(23.1)	(26.4)
	141.3	1017.3	601.6	-30.0	558.3	465.5	119.8	714.6	575.5	100.7
	4.612	33.200	19.634	-0.978	18.221	15.194	3.910	23.322	18.781	3.285
t_O	902	800	516	984	838	515	965	776	465	936
	(29.4)	(26.1)	(16.8)	(32.1)	(27.3)	(16.8)	(31.5)	(25.3)	(15.2)	(30.5)
t_E	905.5	642.4	411.7	905.5	642.4	411.7	905.5	642.4	411.7	905.5
	(29.6)	(21.0)	(13.4)	(29.6)	(21.0)	(13.4)	(29.6)	(21.0)	(13.4)	(29.6)
	-7.0	351.1	233.1	163.7	445.6	230.7	122.9	293.3	113.3	62.0
	-0.227	11.460	7.609	5.341	14.543	7.529	4.010	9.574	3.698	2.025
g_O	485	716	853	468	717	891	464	685	817	507
	(15.8)	(23.4)	(27.8)	(15.3)	(23.4)	(29.1)	(15.1)	(22.4)	(26.7)	(16.5)
g_E	570.4	963.6	1180.0	570.4	963.6	1180.0	570.4	963.6	1180.0	570.4
	(18.6)	(31.4)	(38.5)	(18.6)	(31.4)	(38.5)	(18.6)	(31.4)	(38.5)	(18.6)
	-157.2	-425.3	-553.6	-185.1	-423.8	-500.6	-191.5	-467.5	-600.7	-119.4
	-5.132	-13.879	-18.068	-6.042	-13.833	-16.338	-6.250	-15.257	-19.605	-3.897

c_O	801	834	729	819	938	745	769	981	826	764
	(26.1)	(27.2)	(23.8)	(26.7)	(30.6)	(24.3)	(25.1)	(32.0)	(27.0)	(24.9)
c_E	780.0	1107.9	764.8	780.0	1107.9	764.8	780.0	1107.9	764.8	780.0
	(25.5)	(36.2)	(25.0)	(25.5)	(36.2)	(25.0)	(25.5)	(36.2)	(25.0)	(25.5)
	42.5	-473.6	-69.9	79.8	-312.2	-39.1	-21.9	-238.6	127.1	-31.8
	1.386	-15.458	-2.283	2.605	-10.191	-1.277	-0.716	-7.788	4.149	-1.036
N	3064	3064	3064	3064	3064	3064	3064	3064	3064	3064
X2	19.1	547.9	213.2	27.4	287.9	156.9	28.0	333.8	210.7	11.4
Gvalue	19.6	469.5	211.2	28.4	267.8	156.5	29.2	301.8	215.2	11.6
Gv/N	0.639	15.323	6.893	0.927	8.740	5.108	0.954	9.851	7.024	0.377
Prob	0.00021	0.00000	0.00000	0.00000	0.00000	0.00000	0.00000	0.00000	0.00000	0.00905

	21	22	23	24	25	26	27	28	29	30
a_O	567	804	806	526	768	757	480	757	745	495
	(18.5)	(26.2)	(26.3)	(17.2)	(25.1)	(24.7)	(15.7)	(24.7)	(24.3)	(16.2)
a_E	350.2	707.5	808.1	350.2	707.5	808.1	350.2	707.5	808.1	350.2
	(11.4)	(23.1)	(26.4)	(11.4)	(23.1)	(26.4)	(11.4)	(23.1)	(26.4)	(11.4)
	546.4	205.5	-4.2	427.9	126.0	-98.9	302.7	102.3	-121.2	342.6
	17.833	6.708	-0.138	13.967	4.111	-3.229	9.878	3.339	-3.955	11.181
t_O	809	528	936	764	440	908	757	457	930	729
	(26.4)	(17.2)	(30.5)	(24.9)	(14.4)	(29.6)	(24.7)	(14.9)	(30.4)	(23.8)
t_E	642.4	411.7	905.5	642.4	411.7	905.5	642.4	411.7	905.5	642.4
	(21.0)	(13.4)	(29.6)	(21.0)	(13.4)	(29.6)	(21.0)	(13.4)	(29.6)	(21.0)
	373.2	262.8	62.0	265.0	58.6	5.0	248.6	95.5	49.7	184.5
	12.180	8.578	2.025	8.648	1.912	0.164	8.114	3.117	1.622	6.021
g_O	713	972	530	750	1049	573	809	1075	561	834
	(23.3)	(31.7)	(17.3)	(24.5)	(34.2)	(18.7)	(26.4)	(35.1)	(18.3)	(27.2)
g_E	963.6	1180.0	570.4	963.6	1180.0	570.4	963.6	1180.0	570.4	963.6
	(31.4)	(38.5)	(18.6)	(31.4)	(38.5)	(18.6)	(31.4)	(38.5)	(18.6)	(31.4)
	-429.5	-376.9	-77.8	-375.9	-246.9	5.3	-282.9	-200.3	-18.6	-240.9
	-14.016	-12.302	-2.539	-12.267	-8.057	0.173	-9.233	-6.539	-0.606	-7.862
c_O	975	760	792	1024	807	826	1018	775	828	1006
	(31.8)	(24.8)	(25.8)	(33.4)	(26.3)	(27.0)	(33.2)	(25.3)	(27.0)	(32.8)
c_E	1107.9	764.8	780.0	1107.9	764.8	780.0	1107.9	764.8	780.0	1107.9
	(36.2)	(25.0)	(25.5)	(36.2)	(25.0)	(25.5)	(36.2)	(25.0)	(25.5)	(36.2)
	-249.1	-9.6	24.1	-161.2	86.6	94.6	-172.2	20.5	98.8	-194.1
	-8.131	-0.314	0.786	-5.262	2.828	3.086	-5.621	0.669	3.225	-6.334
N	3064	3064	3064	3064	3064	3064	3064	3064	3064	3064
X2	258.5	82.7	4.1	165.0	24.0	6.0	100.7	17.9	8.7	98.3
Gvalue	241.0	81.8	4.1	155.8	24.3	6.0	96.1	18.0	8.8	92.1
Gv/N	7.866	2.670	0.135	5.086	0.794	0.195	3.138	0.586	0.286	3.006
Prob	0.00000	0.00000	0.24776	0.00000	0.00002	0.11263	0.00000	0.00045	0.03258	0.00000

	31	32	33	34	35	36	37	38	39	40
a_O	760	735	449	748	704	442	682	677	468	675
	(24.8)	(24.0)	(14.7)	(24.4)	(23.0)	(14.4)	(22.3)	(22.1)	(15.3)	(22.0)
a_E	707.5	808.1	350.2	707.5	808.1	350.2	707.5	808.1	350.2	707.5
	(23.1)	(26.4)	(11.4)	(23.1)	(26.4)	(11.4)	(23.1)	(26.4)	(11.4)	(23.1)
	108.7	-139.4	223.2	83.2	-194.2	205.8	-50.1	-239.7	271.4	-63.5
	3.549	-4.550	7.283	2.716	-6.338	6.716	-1.636	-7.823	8.858	-2.074
t_O	450	968	755	461	928	694	472	941	691	440
	(14.7)	(31.6)	(24.6)	(15.0)	(30.3)	(22.7)	(15.4)	(30.7)	(22.6)	(14.4)
t_E	411.7	905.5	642.4	411.7	905.5	642.4	411.7	905.5	642.4	411.7
	(13.4)	(29.6)	(21.0)	(13.4)	(29.6)	(21.0)	(13.4)	(29.6)	(21.0)	(13.4)
	80.1	129.3	244.0	104.4	45.6	107.3	129.1	72.4	100.9	58.6
	2.616	4.218	7.963	3.406	1.488	3.503	4.214	2.363	3.292	1.912
g_O	1084	555	827	1123	607	865	1176	642	886	1172
	(35.4)	(18.1)	(27.0)	(36.7)	(19.8)	(28.2)	(38.4)	(21.0)	(28.9)	(38.3)
g_E	1180.0	570.4	963.6	1180.0	570.4	963.6	1180.0	570.4	963.6	1180.0
	(38.5)	(18.6)	(31.4)	(38.5)	(18.6)	(31.4)	(38.5)	(18.6)	(31.4)	(38.5)
	-183.9	-30.3	-252.8	-111.2	75.6	-186.7	-8.0	151.9	-148.7	-15.9
	-6.003	-0.989	-8.251	-3.628	2.467	-6.093	-0.260	4.959	-4.854	-0.519
c_O	770	806	1033	732	825	1063	734	804	1019	777
	(25.1)	(26.3)	(33.7)	(23.9)	(26.9)	(34.7)	(24.0)	(26.2)	(33.3)	(25.4)
c_E	764.8	780.0	1107.9	764.8	780.0	1107.9	764.8	780.0	1107.9	764.8
	(25.0)	(25.5)	(36.2)	(25.0)	(25.5)	(36.2)	(25.0)	(25.5)	(36.2)	(25.0)
	10.4	52.8	-144.6	-64.2	92.5	-87.9	-60.4	48.6	-170.4	24.6
	0.339	1.722	-4.718	-2.096	3.018	-2.869	-1.971	1.588	-5.562	0.801
N	3064	3064	3064	3064	3064	3064	3064	3064	3064	3064
X2	15.3	12.2	72.0	12.4	18.9	40.1	11.0	32.4	56.7	3.7
Gvalue	15.3	12.3	69.8	12.2	19.4	38.5	10.6	33.3	53.1	3.7
Gv/N	0.500	0.402	2.277	0.398	0.634	1.258	0.347	1.086	1.734	0.120
Prob	0.00156	0.00637	0.00000	0.00676	0.00022	0.00000	0.01379	0.00000	0.00000	0.29883

	41	42	43	44	45	46	47	48	49	50
a_O	695	452	677	672	438	706	724	451	684	688
	(22.7)	(14.8)	(22.1)	(21.9)	(14.3)	(23.0)	(23.6)	(14.7)	(22.3)	(22.5)
a_E	808.1	350.2	707.5	808.1	350.2	707.5	808.1	350.2	707.5	808.1
	(26.4)	(11.4)	(23.1)	(26.4)	(11.4)	(23.1)	(26.4)	(11.4)	(23.1)	(26.4)
	-209.6	230.7	-59.7	-247.9	196.0	-3.1	-159.2	228.2	-46.3	-221.4
	-6.841	7.528	-1.949	-8.091	6.396	-0.100	-5.195	7.447	-1.510	-7.227
t_O	931	676	473	910	725	410	909	742	451	906
	(30.4)	(22.1)	(15.4)	(29.7)	(23.7)	(13.4)	(29.7)	(24.2)	(14.7)	(29.6)
t_E	905.5	642.4	411.7	905.5	642.4	411.7	905.5	642.4	411.7	905.5
	(29.6)	(21.0)	(13.4)	(29.6)	(21.0)	(13.4)	(29.6)	(21.0)	(13.4)	(29.6)
	51.7	69.0	131.4	9.1	175.5	-3.3	7.0	214.0	82.3	1.0
	1.689	2.252	4.289	0.296	5.727	-0.108	0.230	6.984	2.687	0.034
g_O	595	865	1156	615	872	1174	638	829	1160	621
	(19.4)	(28.2)	(37.7)	(20.1)	(28.5)	(38.3)	(20.8)	(27.1)	(37.9)	(20.3)
g_E	570.4	963.6	1180.0	570.4	963.6	1180.0	570.4	963.6	1180.0	570.4
	(18.6)	(31.4)	(38.5)	(18.6)	(31.4)	(38.5)	(18.6)	(31.4)	(38.5)	(18.6)
	50.3	-186.7	-47.5	92.7	-174.1	-11.9	143.0	-249.4	-39.6	105.7
	1.643	-6.093	-1.550	3.025	-5.684	-0.390	4.668	-8.140	-1.293	3.448
c_O	843	1071	758	867	1029	774	793	1042	769	849
	(27.5)	(35.0)	(24.7)	(28.3)	(33.6)	(25.3)	(25.9)	(34.0)	(25.1)	(27.7)
c_E	780.0	1107.9	764.8	780.0	1107.9	764.8	780.0	1107.9	764.8	780.0
	(25.5)	(36.2)	(25.0)	(25.5)	(36.2)	(25.0)	(25.5)	(36.2)	(25.0)	(25.5)
	130.9	-72.5	-13.6	183.3	-152.0	18.5	26.1	-127.7	8.4	143.8
	4.271	-2.366	-0.443	5.981	-4.960	0.603	0.853	-4.169	0.273	4.694
N	3064	3064	3064	3064	3064	3064	3064	3064	3064	3064
X2	22.7	42.7	11.0	36.1	47.0	0.2	17.0	67.2	4.9	28.4
Gvalue	23.3	40.5	10.6	37.1	45.3	0.2	17.0	65.0	4.8	29.1
Gv/N	0.762	1.322	0.346	1.211	1.479	0.005	0.556	2.122	0.157	0.950
Prob	0.00003	0.00000	0.01406	0.00000	0.00000	0.98520	0.00070	0.00000	0.18702	0.00000

	51	52	53	54	55	56	57	58	59	60
a_O	452	686	672	407	694	724	396	680	719	418
	(14.8)	(22.4)	(21.9)	(13.3)	(22.7)	(23.6)	(12.9)	(22.2)	(23.5)	(13.6)
a_E	350.2	707.5	808.1	350.2	707.5	808.1	350.2	707.5	808.1	350.2
	(11.4)	(23.1)	(26.4)	(11.4)	(23.1)	(26.4)	(11.4)	(23.1)	(26.4)	(11.4)
	230.7	-42.4	-247.9	122.3	-26.8	-159.2	97.3	-54.0	-168.0	147.9
	7.528	-1.384	-8.091	3.993	-0.875	-5.195	3.177	-1.762	-5.484	4.828
t_O	685	438	936	631	430	909	688	485	951	672
	(22.4)	(14.3)	(30.5)	(20.6)	(14.0)	(29.7)	(22.5)	(15.8)	(31.0)	(21.9)
t_E	642.4	411.7	905.5	642.4	411.7	905.5	642.4	411.7	905.5	642.4
	(21.0)	(13.4)	(29.6)	(21.0)	(13.4)	(29.6)	(21.0)	(13.4)	(29.6)	(21.0)
	88.1	54.3	62.0	-22.5	37.5	7.0	94.5	159.0	93.3	60.6
	2.874	1.773	2.025	-0.735	1.223	0.230	3.083	5.190	3.044	1.979
g_O	887	1178	640	981	1180	582	925	1133	603	959
	(28.9)	(38.4)	(20.9)	(32.0)	(38.5)	(19.0)	(30.2)	(37.0)	(19.7)	(31.3)
g_E	963.6	1180.0	570.4	963.6	1180.0	570.4	963.6	1180.0	570.4	963.6
	(31.4)	(38.5)	(18.6)	(31.4)	(38.5)	(18.6)	(31.4)	(38.5)	(18.6)	(31.4)
	-146.9	-4.0	147.5	35.2	0.0	23.5	-75.6	-92.1	67.1	-9.1
	-4.794	-0.130	4.813	1.148	0.001	0.768	-2.467	-3.005	2.191	-0.298
c_O	1040	762	816	1045	760	849	1055	766	791	1015
	(33.9)	(24.9)	(26.6)	(34.1)	(24.8)	(27.7)	(34.4)	(25.0)	(25.8)	(33.1)
c_E	1107.9	764.8	780.0	1107.9	764.8	780.0	1107.9	764.8	780.0	1107.9
	(36.2)	(25.0)	(25.5)	(36.2)	(25.0)	(25.5)	(36.2)	(25.0)	(25.5)	(36.2)
	-131.5	-5.6	73.5	-122.1	-9.6	143.8	-103.2	2.4	22.1	-177.7
	-4.291	-0.184	2.400	-3.985	-0.314	4.694	-3.367	0.077	0.720	-5.800
N	3064	3064	3064	3064	3064	3064	3064	3064	3064	3064
X2	42.7	2.4	34.1	13.3	1.1	15.1	13.3	16.0	14.1	22.3
Gvalue	40.3	2.3	35.2	12.9	1.1	15.3	13.0	15.3	14.4	21.7
Gv/N	1.317	0.076	1.148	0.421	0.036	0.498	0.426	0.501	0.472	0.709
Prob	0.00000	0.50744	0.00000	0.00485	0.77796	0.00161	0.00455	0.00155	0.00235	0.00007

	61	62	63	64	65	66	67	68	69	70
a_O	643	711	396	690	696	412	669	729	418	682
	(21.0)	(23.2)	(12.9)	(22.5)	(22.7)	(13.4)	(21.8)	(23.8)	(13.6)	(22.3)
a_E	707.5	808.1	350.2	707.5	808.1	350.2	707.5	808.1	350.2	707.5
	(23.1)	(26.4)	(11.4)	(23.1)	(26.4)	(11.4)	(23.1)	(26.4)	(11.4)	(23.1)
	-123.0	-182.1	97.3	-34.6	-207.9	133.9	-74.9	-150.2	147.9	-50.1
	-4.014	-5.942	3.177	-1.130	-6.786	4.370	-2.446	-4.903	4.828	-1.636
t_O	455	968	705	454	924	677	430	883	675	453
	(14.8)	(31.6)	(23.0)	(14.8)	(30.2)	(22.1)	(14.0)	(28.8)	(22.0)	(14.8)
t_E	411.7	905.5	642.4	411.7	905.5	642.4	411.7	905.5	642.4	411.7
	(13.4)	(29.6)	(21.0)	(13.4)	(29.6)	(21.0)	(13.4)	(29.6)	(21.0)	(13.4)
	91.1	129.3	131.2	88.9	37.4	71.1	37.5	-44.4	66.9	86.7
	2.973	4.218	4.282	2.901	1.221	2.321	1.223	-1.449	2.184	2.830
g_O	1187	574	959	1138	602	1004	1157	554	942	1219
	(38.7)	(18.7)	(31.3)	(37.1)	(19.6)	(32.8)	(37.8)	(18.1)	(30.7)	(39.8)
g_E	1180.0	570.4	963.6	1180.0	570.4	963.6	1180.0	570.4	963.6	1180.0
	(38.5)	(18.6)	(31.4)	(38.5)	(18.6)	(31.4)	(38.5)	(18.6)	(31.4)	(38.5)
	14.1	7.3	-9.1	-82.5	65.0	82.5	-45.5	-32.2	-42.7	79.3
	0.459	0.239	-0.298	-2.691	2.122	2.694	-1.486	-1.052	-1.392	2.588
c_O	779	811	1004	782	842	971	808	898	1029	710
	(25.4)	(26.5)	(32.8)	(25.5)	(27.5)	(31.7)	(26.4)	(29.3)	(33.6)	(23.2)
c_E	764.8	780.0	1107.9	764.8	780.0	1107.9	764.8	780.0	1107.9	764.8
	(25.0)	(25.5)	(36.2)	(25.0)	(25.5)	(36.2)	(25.0)	(25.5)	(36.2)	(25.0)
	28.6	63.1	-197.7	34.7	128.7	-256.1	88.8	252.9	-152.0	-105.6
	0.934	2.060	-6.452	1.134	4.201	-8.358	2.897	8.254	-4.960	-3.447
N	3064	3064	3064	3064	3064	3064	3064	3064	3064	3064
X2	10.8	17.2	21.9	6.7	22.6	31.4	5.8	26.6	20.9	10.3
Gvalue	10.8	17.6	21.7	6.5	23.2	31.5	5.8	26.0	20.2	10.3
Gv/N	0.352	0.575	0.710	0.214	0.758	1.027	0.189	0.850	0.660	0.335
Prob	0.01293	0.00053	0.00007	0.08786	0.00004	0.00000	0.12245	0.00001	0.00015	0.01652

	71	72	73	74	75	76	77	78	79	80
a_O	683	429	639	757	410	676	731	421	683	798
	(22.3)	(14.0)	(20.9)	(24.7)	(13.4)	(22.1)	(23.9)	(13.7)	(22.3)	(26.0)
a_E	808.1	350.2	707.5	808.1	350.2	707.5	808.1	350.2	707.5	808.1
	(26.4)	(11.4)	(23.1)	(26.4)	(11.4)	(23.1)	(26.4)	(11.4)	(23.1)	(26.4)
	-229.8	174.1	-130.2	-98.9	129.3	-61.6	-146.6	155.0	-48.2	-20.1
	-7.499	5.683	-4.249	-3.229	4.219	-2.012	-4.786	5.060	-1.573	-0.656
t_O	955	688	442	867	713	443	957	646	447	913
	(31.2)	(22.5)	(14.4)	(28.3)	(23.3)	(14.5)	(31.2)	(21.1)	(14.6)	(29.8)
t_E	905.5	642.4	411.7	905.5	642.4	411.7	905.5	642.4	411.7	905.5
	(29.6)	(21.0)	(13.4)	(29.6)	(21.0)	(13.4)	(29.6)	(21.0)	(13.4)	(29.6)
	101.7	94.5	62.9	-75.3	148.8	65.0	105.9	7.3	73.6	15.1
	3.319	3.083	2.052	-2.458	4.856	2.122	3.457	0.238	2.403	0.493
g_O	588	917	1214	592	971	1164	574	938	1180	538
	(19.2)	(29.9)	(39.6)	(19.3)	(31.7)	(38.0)	(18.7)	(30.6)	(38.5)	(17.6)
g_E	570.4	963.6	1180.0	570.4	963.6	1180.0	570.4	963.6	1180.0	570.4
	(18.6)	(31.4)	(38.5)	(18.6)	(31.4)	(38.5)	(18.6)	(31.4)	(38.5)	(18.6)
	35.8	-90.9	69.0	44.1	14.9	-31.8	7.3	-50.5	0.0	-62.8
	1.169	-2.965	2.252	1.439	0.487	-1.036	0.239	-1.647	0.001	-2.051
c_O	838	1030	769	848	970	781	802	1059	754	815
	(27.3)	(33.6)	(25.1)	(27.7)	(31.7)	(25.5)	(26.2)	(34.6)	(24.6)	(26.6)
c_E	780.0	1107.9	764.8	780.0	1107.9	764.8	780.0	1107.9	764.8	780.0
	(25.5)	(36.2)	(25.0)	(25.5)	(36.2)	(25.0)	(25.5)	(36.2)	(25.0)	(25.5)
	120.1	-150.1	8.4	141.7	-257.8	32.7	44.5	-95.5	-21.5	71.5
	3.920	-4.900	0.273	4.624	-8.414	1.067	1.453	-3.118	-0.701	2.332
N	3064	3064	3064	3064	3064	3064	3064	3064	3064	3064
X2	26.9	28.7	9.9	11.6	35.2	4.4	10.9	17.2	4.0	3.6
Gvalue	27.9	27.6	10.0	11.5	35.1	4.3	11.1	16.3	4.0	3.6
Gv/N	0.909	0.901	0.328	0.376	1.147	0.141	0.363	0.533	0.129	0.118
Prob	0.00000	0.00000	0.01822	0.00918	0.00000	0.22969	0.01111	0.00097	0.26523	0.30749

	81	82	83	84	85	86	87	88	89	90
a_O	427	663	757	402	687	789	385	697	737	418
	(13.9)	(21.6)	(24.7)	(13.1)	(22.4)	(25.8)	(12.6)	(22.8)	(24.1)	(13.6)
a_E	350.2	707.5	808.1	350.2	707.5	808.1	350.2	707.3	807.9	350.1
	(11.4)	(23.1)	(26.4)	(11.4)	(23.1)	(26.4)	(11.4)	(23.1)	(26.4)	(11.4)
	169.3	-86.2	-98.9	110.9	-40.5	-37.8	72.9	-20.5	-135.3	148.2
	5.526	-2.813	-3.229	3.619	-1.321	-1.233	2.381	-0.668	-4.417	4.839
t_O	677	413	890	654	455	938	683	428	932	634
	(22.1)	(13.5)	(29.0)	(21.3)	(14.8)	(30.6)	(22.3)	(14.0)	(30.4)	(20.7)
t_E	642.4	411.7	905.5	642.4	411.7	905.5	642.4	411.5	905.2	642.1
	(21.0)	(13.4)	(29.6)	(21.0)	(13.4)	(29.6)	(21.0)	(13.4)	(29.6)	(21.0)
	71.1	2.7	-30.7	23.5	91.1	66.2	83.8	33.6	54.4	-16.2

	2.321	0.088	−1.002	0.767	2.973	2.160	2.735	1.097	1.776	−0.529
g_O	944	1219	604	992	1179	580	945	1234	580	937
	(30.8)	(39.8)	(19.7)	(32.4)	(38.5)	(18.9)	(30.8)	(40.3)	(18.9)	(30.6)
g_E	963.6	1180.0	570.4	963.6	1180.0	570.4	963.6	1179.6	570.2	963.3
	(31.4)	(38.5)	(18.6)	(31.4)	(38.5)	(18.6)	(31.4)	(38.5)	(18.6)	(31.4)
	−38.7	79.3	69.2	57.7	−2.0	19.5	−36.8	111.3	19.8	−51.8
	−1.264	2.588	2.260	1.883	−0.064	0.635	−1.200	3.633	0.647	−1.691
c_O	1016	769	813	1016	743	757	1051	704	814	1074
	(33.2)	(25.1)	(26.5)	(33.2)	(24.2)	(24.7)	(34.3)	(23.0)	(26.6)	(35.1)
c_E	1107.9	764.8	780.0	1107.9	764.8	780.0	1107.9	764.6	779.8	1107.5
	(36.2)	(25.0)	(25.5)	(36.2)	(25.0)	(25.5)	(36.2)	(25.0)	(25.5)	(36.2)
	−175.9	8.4	67.3	−175.9	−43.0	−45.4	−110.8	−116.2	69.9	−66.0
	−5.741	0.273	2.196	−5.741	−1.404	−1.482	−3.615	−3.794	2.282	−2.154
N	3064	3064	3064	3064	3064	3064	3064	3063	3063	3063
X2	26.7	4.1	6.9	16.3	5.8	2.5	9.3	8.1	8.7	15.0
Gvalue	25.8	4.2	6.9	16.2	5.6	2.5	9.2	8.2	8.8	14.2
Gv/N	0.842	0.136	0.225	0.528	0.184	0.080	0.300	0.268	0.289	0.465
Prob	0.00001	0.24399	0.07548	0.00104	0.13038	0.48265	0.02678	0.04199	0.03151	0.00259

	91	92	93	94	95	96	97	98	99	100
a_O	691	775	406	677	776	391	672	773	385	735
	(22.6)	(25.3)	(13.3)	(22.2)	(25.4)	(12.8)	(22.1)	(25.4)	(12.7)	(24.2)
a_E	707.1	807.6	350.0	704.3	804.4	348.6	702.7	802.6	347.8	701.1
	(23.1)	(26.4)	(11.4)	(23.1)	(26.4)	(11.4)	(23.1)	(26.4)	(11.4)	(23.1)
	−31.8	−63.8	120.6	−53.5	−55.8	89.8	−60.0	−58.1	78.2	69.5
	−1.038	−2.085	3.938	−1.755	−1.831	2.943	−1.972	−1.908	2.571	2.289
t_O	454	893	706	470	913	704	464	899	683	419
	(14.8)	(29.2)	(23.1)	(15.4)	(29.9)	(23.1)	(15.2)	(29.5)	(22.4)	(13.8)
t_E	411.4	904.9	641.9	409.8	901.3	639.4	408.8	899.3	638.0	407.9
	(13.4)	(29.6)	(21.0)	(13.4)	(29.6)	(21.0)	(13.4)	(29.6)	(21.0)	(13.4)
	89.5	−23.6	134.3	128.9	23.5	135.5	117.5	−0.6	93.2	22.5
	2.923	−0.772	4.386	4.226	0.769	4.441	3.860	−0.018	3.063	0.741
g_O	1167	592	932	1133	574	906	1120	575	934	1164
	(38.1)	(19.3)	(30.4)	(37.1)	(18.8)	(29.7)	(36.8)	(18.9)	(30.7)	(38.3)
g_E	1179.2	570.0	962.9	1174.6	567.7	959.2	1171.9	566.4	957.0	1169.2
	(38.5)	(18.6)	(31.4)	(38.5)	(18.6)	(31.4)	(38.5)	(18.6)	(31.4)	(38.5)
	−24.3	44.9	−60.9	−81.7	12.6	−103.3	−101.5	17.2	−45.4	−10.4
	−0.794	1.466	−1.988	−2.679	0.412	−3.388	−3.334	0.566	−1.491	−0.342
c_O	750	802	1018	770	787	1049	787	796	1041	718
	(24.5)	(26.2)	(33.2)	(25.2)	(25.8)	(34.4)	(25.9)	(26.2)	(34.2)	(23.6)
c_E	764.3	779.5	1107.1	761.3	776.5	1102.8	759.6	774.7	1100.3	757.8
	(25.0)	(25.5)	(36.2)	(25.0)	(25.5)	(36.2)	(25.0)	(25.5)	(36.2)	(25.0)
	−28.4	45.6	−170.9	17.4	21.2	−104.9	55.8	43.2	−115.3	−77.5
	−0.927	1.488	−5.582	0.572	0.695	−3.441	1.834	1.419	−3.789	−2.554
N	3062	3062	3062	3050	3050	3050	3043	3043	3043	3036
X2	5.2	3.0	23.5	11.5	1.4	17.2	12.1	1.8	10.9	4.1
Gvalue	5.0	3.0	23.1	11.1	1.4	16.9	11.8	1.8	10.8	4.1
Gv/N	0.164	0.097	0.754	0.364	0.045	0.556	0.388	0.060	0.354	0.134
Prob	0.16935	0.39605	0.00004	0.01116	0.71092	0.00073	0.00810	0.61211	0.01310	0.25396

	101	102	103	104	105	106	107	108	109	110
a_O	766	389	713	727	375	689	795	391	678	739
	(25.2)	(12.8)	(23.6)	(24.0)	(12.4)	(22.8)	(26.3)	(12.9)	(22.5)	(24.6)
a_E	800.7	347.0	698.5	797.8	345.7	697.4	796.5	345.2	695.1	793.9
	(26.4)	(11.4)	(23.1)	(26.4)	(11.4)	(23.1)	(26.4)	(11.4)	(23.1)	(26.4)
	−67.9	88.9	29.2	−135.2	60.9	−16.6	−3.0	97.5	−33.7	−105.9
	−2.238	2.928	0.967	−4.469	2.014	−0.551	−0.100	3.228	−1.120	−3.517
t_O	867	653	434	922	669	411	840	678	453	904
	(28.6)	(21.5)	(14.3)	(30.5)	(22.1)	(13.6)	(27.8)	(22.5)	(15.0)	(30.0)
t_E	897.2	636.5	406.4	894.0	634.2	405.7	892.5	633.1	404.4	889.5
	(29.6)	(21.0)	(13.4)	(29.6)	(21.0)	(13.4)	(29.6)	(21.0)	(13.4)	(29.6)
	−59.4	33.4	57.0	57.0	71.5	10.6	−101.8	92.8	102.8	29.2
	−1.956	1.102	1.884	1.883	2.364	0.350	−3.371	3.074	3.416	0.970
g_O	586	940	1100	603	944	1167	594	888	1111	553
	(19.3)	(31.0)	(36.4)	(19.9)	(31.2)	(38.6)	(19.7)	(29.4)	(36.9)	(18.4)
g_E	565.1	954.8	1165.0	563.1	951.3	1163.0	562.2	949.7	1159.2	560.3
	(18.6)	(31.4)	(38.5)	(18.6)	(31.4)	(38.5)	(18.6)	(31.4)	(38.5)	(18.6)
	42.5	−29.3	−126.2	82.6	−14.6	7.9	65.4	−119.4	−94.3	−14.5
	1.399	−0.965	−4.173	2.730	−0.481	0.263	2.167	−3.952	−3.134	−0.482
c_O	817	1054	778	773	1037	753	791	1063	768	814
	(26.9)	(34.7)	(25.7)	(25.6)	(34.3)	(24.9)	(26.2)	(35.2)	(25.5)	(27.0)
c_E	772.9	1097.7	755.1	770.1	1093.8	753.8	768.8	1092.0	751.3	766.3
	(25.5)	(36.2)	(25.0)	(25.5)	(36.2)	(25.0)	(25.5)	(36.2)	(25.0)	(25.5)
	90.6	−85.7	46.5	5.8	−110.5	−1.7	44.9	−57.1	33.7	98.3
	2.985	−2.823	1.538	0.191	−3.654	−0.055	1.488	−1.892	1.119	3.266
N	3036	3036	3025	3025	3025	3020	3020	3020	3010	3010
X2	5.8	7.5	6.5	10.0	7.4	0.2	5.5	14.0	8.6	7.1
Gvalue	5.8	7.3	6.5	10.1	7.3	0.2	5.6	13.8	8.4	7.1
Gv/N	0.191	0.241	0.215	0.335	0.243	0.006	0.184	0.457	0.280	0.237
Prob	0.12249	0.06264	0.08930	0.01751	0.06181	0.98028	0.13546	0.00317	0.03775	0.06806

	111	112	113	114	115	116	117	118	119	120
a_O	369	645	808	354	696	781	358	653	767	388
	(12.3)	(21.5)	(26.9)	(11.8)	(23.3)	(26.1)	(12.0)	(21.9)	(25.7)	(13.0)
a_E	344.0	693.4	792.0	343.2	691.1	789.4	342.1	690.0	788.1	341.5
	(11.4)	(23.1)	(26.4)	(11.4)	(23.1)	(26.4)	(11.4)	(23.1)	(26.4)	(11.4)
	51.7	−93.4	32.3	21.9	9.8	−16.7	32.6	−71.9	−41.6	99.0
	1.718	−3.111	1.074	0.728	0.326	−0.558	1.088	−2.408	−1.392	3.314
t_O	664	448	873	699	426	880	670	413	857	650
	(22.1)	(14.9)	(29.1)	(23.3)	(14.2)	(29.4)	(22.4)	(13.8)	(28.7)	(21.8)
t_E	631.0	403.5	887.5	629.6	402.1	884.5	627.5	401.4	883.0	626.4
	(21.0)	(13.4)	(29.6)	(21.0)	(13.4)	(29.6)	(21.0)	(13.4)	(29.6)	(21.0)
	67.6	93.8	−28.7	146.2	49.2	−9.0	87.9	23.4	−51.3	48.0
	2.246	3.124	−0.955	4.870	1.642	−0.300	2.936	0.784	−1.716	1.607
g_O	908	1177	579	872	1102	601	878	1183	577	928
	(30.2)	(39.2)	(19.3)	(29.0)	(36.8)	(20.1)	(29.3)	(39.6)	(19.3)	(31.1)
g_E	946.6	1156.5	559.0	944.4	1152.6	557.1	941.2	1150.7	556.2	939.7
	(31.4)	(38.5)	(18.6)	(31.4)	(38.5)	(18.6)	(31.4)	(38.5)	(18.6)	(31.4)
	−75.6	41.4	40.7	−139.1	−99.0	91.1	−122.1	65.5	42.4	−23.2
	−2.511	1.378	1.356	−4.631	−3.309	3.043	−4.081	2.191	1.417	−0.776
c_O	1069	733	743	1078	769	731	1087	739	787	1022
	(35.5)	(24.4)	(24.7)	(35.9)	(25.7)	(24.4)	(36.3)	(24.7)	(26.3)	(34.2)
c_E	1088.3	749.6	764.5	1085.8	747.1	762.0	1082.2	745.9	760.7	1080.4
	(36.2)	(25.0)	(25.5)	(36.2)	(25.0)	(25.5)	(36.2)	(25.0)	(25.5)	(36.2)
	−38.3	−32.8	−42.4	−15.6	44.4	−60.7	9.6	−13.6	53.5	−113.6
	−1.274	−1.093	−1.412	−0.518	1.485	−2.027	0.322	−0.456	1.791	−3.801

N	3010	3003	3003	3003	2993	2993	2993	2988	2988	2988
X2	5.5	9.0	1.9	13.6	4.3	4.8	7.9	3.3	3.0	10.5
Gvalue	5.4	8.9	1.9	13.5	4.3	4.8	7.9	3.3	3.0	10.3
Gv/N	0.179	0.298	0.062	0.449	0.144	0.159	0.265	0.111	0.101	0.344
Prob	0.14456	0.03006	0.59875	0.00371	0.22899	0.19069	0.04776	0.34645	0.39003	0.01623

	121	122	123	124	125	126	127	128	129	130
a_O	688	774	396	661	797	375	645	777	395	649
	(23.1)	(25.9)	(13.3)	(22.2)	(26.7)	(12.6)	(21.7)	(26.2)	(13.3)	(21.9)
a_E	689.1	787.0	341.1	688.1	786.0	340.6	686.1	783.6	339.6	685.1
	(23.1)	(26.4)	(11.4)	(23.1)	(26.4)	(11.4)	(23.1)	(26.4)	(11.4)	(23.1)
	−2.1	−25.8	118.3	−53.2	22.2	72.2	−79.6	−13.1	119.4	−70.3
	−0.071	−0.865	3.964	−1.785	0.746	2.421	−2.680	−0.442	4.020	−2.370
t_O	426	847	684	451	834	687	429	865	655	419
	(14.3)	(28.4)	(22.9)	(15.1)	(28.0)	(23.1)	(14.4)	(29.1)	(22.0)	(14.1)
t_E	400.9	881.8	625.6	400.4	880.7	624.7	399.2	878.0	622.9	398.6
	(13.4)	(29.6)	(21.0)	(13.4)	(29.6)	(21.0)	(13.4)	(29.6)	(21.0)	(13.4)
	51.7	−68.3	122.1	107.4	−90.8	130.5	61.8	−25.8	65.9	41.8
	1.733	−2.289	4.092	3.604	−3.047	4.380	2.082	−0.869	2.218	1.408
g_O	1120	563	918	1154	580	872	1187	540	904	1182
	(37.5)	(18.9)	(30.8)	(38.7)	(19.5)	(29.3)	(40.0)	(18.2)	(30.4)	(39.8)
g_E	1149.2	555.5	938.4	1147.6	554.7	937.2	1144.2	553.0	934.3	1142.6
	(38.5)	(18.6)	(31.4)	(38.5)	(18.6)	(31.4)	(38.5)	(18.6)	(31.4)	(38.5)
	−57.6	15.2	−40.4	12.8	51.7	−125.7	87.2	−25.8	−59.7	80.1
	−1.931	0.509	−1.353	0.428	1.735	−4.217	2.936	−0.868	−2.008	2.699
c_O	750	800	986	714	769	1046	710	789	1017	717
	(25.1)	(26.8)	(33.0)	(24.0)	(25.8)	(35.1)	(23.9)	(26.6)	(34.2)	(24.2)
c_E	744.9	759.7	1078.9	743.9	758.7	1077.5	741.6	756.4	1074.2	740.6
	(25.0)	(25.5)	(36.2)	(25.0)	(25.5)	(36.2)	(25.0)	(25.5)	(36.2)	(25.0)
	10.3	82.8	−177.6	−58.5	20.8	−62.1	−61.8	66.7	−111.4	−46.5
	0.346	2.773	−5.953	−1.963	0.699	−2.083	−2.082	2.244	−3.749	−1.566

N	2984	2984	2984	2980	2980	2980	2971	2971	2971	2967
X2	2.3	3.8	22.8	8.7	3.9	15.1	7.6	2.0	14.7	5.1
Gvalue	2.3	3.8	22.4	8.5	3.9	14.9	7.6	1.9	14.3	5.1
Gv/N	0.078	0.128	0.751	0.285	0.132	0.501	0.257	0.066	0.482	0.171
Prob	0.50820	0.28192	0.00005	0.03703	0.26727	0.00187	0.05420	0.58344	0.00250	0.16702

	131	132	133	134	135	136	137	138	139	140
a_O	770	404	657	768	393	649	744	346	632	741
	(26.0)	(13.6)	(22.2)	(26.0)	(13.3)	(22.0)	(25.2)	(11.7)	(21.5)	(25.2)
a_E	782.5	339.1	683.3	780.4	338.2	681.9	778.8	337.5	679.1	775.7
	(26.4)	(11.4)	(23.1)	(26.4)	(11.4)	(23.1)	(26.4)	(11.4)	(23.1)	(26.4)
	−24.9	141.5	−51.6	−24.7	118.0	−64.2	−68.1	17.2	−90.9	−67.8
	−0.838	4.768	−1.742	−0.833	3.989	−2.174	−2.306	0.582	−3.091	−2.305
t_O	887	634	442	856	632	430	818	654	428	881
	(29.9)	(21.4)	(14.9)	(28.9)	(21.4)	(14.6)	(27.7)	(22.1)	(14.6)	(30.0)
t_E	876.8	622.0	397.6	874.5	620.3	396.7	872.7	619.1	395.1	869.1
	(29.6)	(21.0)	(13.4)	(29.6)	(21.0)	(13.4)	(29.6)	(21.0)	(13.4)	(29.6)
	20.5	24.2	93.7	−36.5	23.5	69.2	−105.9	71.8	68.4	23.9
	0.690	0.815	3.166	−1.234	0.795	2.344	−3.585	2.430	2.326	0.812
g_O	556	922	1121	560	894	1167	594	904	1156	591
	(18.7)	(31.1)	(37.9)	(18.9)	(30.2)	(39.5)	(20.1)	(30.6)	(39.3)	(20.1)
g_E	552.3	933.1	1139.5	550.8	930.5	1137.2	549.7	928.7	1132.6	547.5
	(18.6)	(31.4)	(38.5)	(18.6)	(31.4)	(38.5)	(18.6)	(31.4)	(38.5)	(18.6)
	7.4	−22.0	−36.8	18.5	−71.6	60.3	92.1	−48.7	47.2	90.5
	0.250	−0.741	−1.243	0.626	−2.421	2.042	3.119	−1.648	1.606	3.076
c_O	754	1007	739	775	1040	707	797	1049	725	728
	(25.4)	(33.9)	(25.0)	(26.2)	(35.1)	(23.9)	(27.0)	(35.5)	(24.7)	(24.8)
c_E	755.3	1072.8	738.6	753.3	1069.9	737.1	751.8	1067.7	734.1	748.7
	(25.5)	(36.2)	(25.0)	(25.5)	(36.2)	(25.0)	(25.5)	(36.2)	(25.0)	(25.5)
	−2.7	−127.5	0.8	44.0	−59.0	−59.0	93.1	−37.1	−18.1	−40.9
	−0.091	−4.296	0.026	1.487	−1.993	−1.997	3.153	−1.258	−0.616	−1.390

N	2967	2967	2959	2959	2959	2953	2953	2953	2941	2941
X2	0.3	16.8	6.3	1.4	11.4	6.4	11.3	3.2	6.6	5.7
Gvalue	0.3	16.2	6.1	1.4	11.0	6.3	11.2	3.1	6.6	5.7
Gv/N	0.012	0.545	0.207	0.046	0.370	0.215	0.380	0.106	0.225	0.193
Prob	0.95100	0.00105	0.10580	0.71444	0.01200	0.09594	0.01055	0.37118	0.08567	0.12775

	141	142	143	144	145	146	147	148	149	150
a_O	334	649	751	347	669	743	338	686	767	373
	(11.4)	(22.1)	(25.6)	(11.8)	(22.8)	(25.4)	(11.5)	(23.5)	(26.2)	(12.8)
a_E	336.1	677.3	773.6	335.2	676.6	772.8	334.9	674.7	770.7	334.0
	(11.4)	(23.1)	(26.4)	(11.4)	(23.1)	(26.4)	(11.4)	(23.1)	(26.4)	(11.4)
	−4.3	−55.4	−44.5	23.9	−15.1	−58.4	6.3	22.7	−7.3	82.4
	−0.145	−1.888	−1.516	0.816	−0.515	−1.993	0.213	0.777	−0.250	2.822
t_O	638	425	840	628	428	869	619	391	845	641
	(21.7)	(14.5)	(28.6)	(21.4)	(14.6)	(29.7)	(21.1)	(13.4)	(28.9)	(21.9)
t_E	616.6	394.1	866.8	614.9	393.7	865.9	614.3	392.6	863.5	612.6
	(21.0)	(13.4)	(29.6)	(21.0)	(13.4)	(29.6)	(21.0)	(13.4)	(29.6)	(21.0)
	43.6	64.3	−52.7	26.5	71.6	6.2	9.5	−3.2	−36.6	58.1
	1.482	2.191	−1.797	0.903	2.444	0.213	0.324	−0.108	−1.254	1.989
g_O	953	1077	560	939	1109	573	873	1127	558	905
	(32.4)	(36.7)	(19.1)	(32.0)	(37.8)	(19.6)	(29.8)	(38.6)	(19.1)	(31.0)
g_E	924.9	1129.5	546.0	922.4	1128.4	545.4	921.4	1125.3	543.9	918.9
	(31.4)	(38.5)	(18.6)	(31.4)	(38.5)	(18.6)	(31.4)	(38.5)	(18.6)	(31.4)
	57.1	−102.6	28.4	33.6	−38.4	56.6	−94.3	3.4	28.5	−27.6
	1.940	−3.498	0.969	1.144	−1.311	1.930	−3.217	0.116	0.976	−0.945
c_O	1016	782	782	1019	724	745	1100	718	752	1003
	(34.5)	(26.7)	(26.7)	(34.7)	(24.7)	(25.4)	(37.5)	(24.6)	(25.7)	(34.3)
c_E	1063.4	732.1	746.7	1060.5	731.4	745.9	1059.4	729.4	743.9	1056.5
	(36.2)	(25.0)	(25.5)	(36.2)	(25.0)	(25.5)	(36.2)	(25.0)	(25.5)	(36.2)
	−92.6	103.1	72.3	−81.4	−14.7	−1.9	82.7	−22.6	16.3	−104.3
	−3.150	3.514	2.464	−2.774	−0.501	−0.063	2.823	−0.773	0.558	−3.569

N	2941	2933	2933	2933	2930	2930	2930	2922	2922	2922
X2	3.7	9.5	3.5	2.6	3.5	2.6	4.2	0.4	0.9	8.8
Gvalue	3.7	9.4	3.5	2.6	3.4	2.5	4.2	0.4	0.9	8.7
Gv/N	0.127	0.320	0.119	0.090	0.116	0.087	0.143	0.013	0.030	0.296
Prob	0.29091	0.02473	0.32055	0.45235	0.33293	0.46679	0.24151	0.94555	0.83341	0.03416

Bafz_PKO
5UTR:

	50	49	48	47	46	45	44	43	42	41
a_O	454	456	459	467	487	444	507	476	478	506
	(37.4)	(37.6)	(37.8)	(38.5)	(40.1)	(36.6)	(41.8)	(39.2)	(39.4)	(41.7)
a_E	483.6	483.6	483.6	483.6	483.6	483.6	483.6	483.6	483.6	483.6
	(39.8)	(39.8)	(39.8)	(39.8)	(39.8)	(39.8)	(39.8)	(39.8)	(39.8)	(39.8)
	-57.3	-53.5	-47.8	-32.5	6.9	-75.8	48.0	-15.0	-11.0	45.9
	-4.717	-4.407	-3.940	-2.680	0.570	-6.242	3.955	-1.234	-0.909	3.783
t_O	464	486	462	466	437	488	426	472	454	445
	(38.2)	(40.0)	(38.1)	(38.4)	(36.0)	(40.2)	(35.1)	(38.9)	(37.4)	(36.7)
t_E	439.8	439.8	439.8	439.8	439.8	439.8	439.8	439.8	439.8	439.8
	(36.2)	(36.2)	(36.2)	(36.2)	(36.2)	(36.2)	(36.2)	(36.2)	(36.2)	(36.2)
	49.6	97.0	45.4	53.8	-5.7	101.4	-27.2	66.6	28.8	10.4
	4.087	7.990	3.740	4.435	-0.467	8.353	-2.245	5.487	2.369	0.854
g_O	183	163	175	174	164	168	157	156	148	175
	(15.1)	(13.4)	(14.4)	(14.3)	(13.5)	(13.8)	(12.9)	(12.9)	(12.2)	(14.4)
g_E	179.2	179.2	179.2	179.2	179.2	179.2	179.2	179.2	179.2	179.2
	(14.8)	(14.8)	(14.8)	(14.8)	(14.8)	(14.8)	(14.8)	(14.8)	(14.8)	(14.8)
	7.6	-30.9	-8.4	-10.3	-29.1	-21.7	-41.6	-43.3	-56.7	-8.4
	0.628	-2.549	-0.689	-0.849	-2.399	-1.791	-3.425	-3.567	-4.668	-0.689
c_O	113	109	118	107	126	114	124	110	134	88
	(9.3)	(9.0)	(9.7)	(8.8)	(10.4)	(9.4)	(10.2)	(9.1)	(11.0)	(7.2)
c_E	111.4	111.4	111.4	111.4	111.4	111.4	111.4	111.4	111.4	111.4
	(9.2)	(9.2)	(9.2)	(9.2)	(9.2)	(9.2)	(9.2)	(9.2)	(9.2)	(9.2)
	3.3	-4.7	13.6	-8.6	31.1	5.3	26.6	-2.7	49.6	-41.5
	0.270	-0.387	1.124	-0.706	2.561	0.438	2.194	-0.225	4.083	-3.415
N	1214	1214	1214	1214	1214	1214	1214	1214	1214	1214
X2	3.2	7.9	2.9	2.4	3.3	9.3	5.8	5.5	10.6	6.1
Gvalue	3.2	7.8	2.9	2.4	3.2	9.2	5.8	5.6	10.6	6.5
Gv/N	0.268	0.647	0.235	0.200	0.265	0.758	0.479	0.460	0.875	0.534
Prob	0.35475	0.04922	0.41481	0.48869	0.35933	0.02678	0.12078	0.13387	0.01399	0.09058

	40	39	38	37	36	35	34	33	32	31
a_O	515	480	468	494	493	514	474	470	511	495
	(42.4)	(39.5)	(38.6)	(40.7)	(40.6)	(42.3)	(39.0)	(38.7)	(42.1)	(40.8)
a_E	483.6	483.6	483.6	483.6	483.6	483.6	483.6	483.6	483.6	483.6
	(39.8)	(39.8)	(39.8)	(39.8)	(39.8)	(39.8)	(39.8)	(39.8)	(39.8)	(39.8)
	64.9	-7.1	-30.6	21.1	19.1	62.8	-18.9	-26.7	56.4	23.2
	5.346	-0.583	-2.520	1.740	1.572	5.171	-1.558	-2.201	4.648	1.908
t_O	449	477	479	451	456	468	468	457	426	451
	(37.0)	(39.3)	(39.5)	(37.1)	(37.6)	(38.6)	(38.6)	(37.6)	(35.1)	(37.1)
t_E	439.8	439.8	439.8	439.8	439.8	439.8	439.8	439.8	439.8	439.8
	(36.2)	(36.2)	(36.2)	(36.2)	(36.2)	(36.2)	(36.2)	(36.2)	(36.2)	(36.2)
	18.5	77.4	81.7	22.6	32.9	58.1	58.1	35.0	-27.2	22.6
	1.524	6.373	6.730	1.861	2.710	4.784	4.784	2.881	-2.245	1.861
g_O	136	164	160	161	156	147	162	163	154	161
	(11.2)	(13.5)	(13.2)	(13.3)	(12.9)	(12.1)	(13.3)	(13.4)	(12.7)	(13.3)
g_E	179.2	179.2	179.2	179.2	179.2	179.2	179.2	179.2	179.2	179.2
	(14.8)	(14.8)	(14.8)	(14.8)	(14.8)	(14.8)	(14.8)	(14.8)	(14.8)	(14.8)
	-75.1	-29.1	-36.3	-34.5	-43.3	-58.3	-32.7	-30.9	-46.7	-34.5
	-6.184	-2.399	-2.992	-2.845	-3.567	-4.801	-2.697	-2.549	-3.849	-2.845
c_O	114	93	107	108	109	85	110	124	123	107
	(9.4)	(7.7)	(8.8)	(8.9)	(9.0)	(7.0)	(9.1)	(10.2)	(10.1)	(8.8)
c_E	111.4	111.4	111.4	111.4	111.4	111.4	111.4	111.4	111.4	111.4
	(9.2)	(9.2)	(9.2)	(9.2)	(9.2)	(9.2)	(9.2)	(9.2)	(9.2)	(9.2)
	5.3	-33.5	-8.6	-6.6	-4.7	-45.9	-2.7	26.6	24.4	-8.6
	0.438	-2.762	-0.706	-0.547	-0.387	-3.784	-0.225	2.194	2.012	-0.706
N	1214	1214	1214	1214	1214	1214	1214	1214	1214	1214
X2	12.7	7.5	6.2	2.5	3.8	15.8	3.7	4.0	6.8	2.6
Gvalue	13.6	7.6	6.2	2.5	4.0	16.6	3.7	3.9	6.9	2.6
Gv/N	1.123	0.628	0.511	0.208	0.327	1.370	0.303	0.325	0.566	0.218
Prob	0.00344	0.05442	0.10194	0.47019	0.26466	0.00084	0.29766	0.26798	0.07601	0.44984

	30	29	28	27	26	25	24	23	22	21
a_O	494	500	530	506	506	511	498	524	526	512
	(40.7)	(41.2)	(43.7)	(41.7)	(41.7)	(42.1)	(41.0)	(43.2)	(43.3)	(42.2)
a_E	483.6	483.6	483.6	483.6	483.6	483.6	483.6	483.6	483.6	483.6
	(39.8)	(39.8)	(39.8)	(39.8)	(39.8)	(39.8)	(39.8)	(39.8)	(39.8)	(39.8)
	21.1	33.4	97.2	45.9	45.9	56.4	29.3	84.2	88.5	58.5
	1.740	2.755	8.008	3.783	3.783	4.648	2.415	6.935	7.291	4.822
t_O	451	449	452	492	453	455	463	432	429	461
	(37.1)	(37.0)	(37.2)	(40.5)	(37.3)	(37.5)	(38.1)	(35.6)	(35.3)	(38.0)
t_E	439.8	439.8	439.8	439.8	439.8	439.8	439.8	439.8	439.8	439.8
	(36.2)	(36.2)	(36.2)	(36.2)	(36.2)	(36.2)	(36.2)	(36.2)	(36.2)	(36.2)
	22.6	18.5	24.6	110.3	26.7	30.8	47.5	-15.5	-21.4	43.3
	1.861	1.524	2.030	9.083	2.199	2.539	3.913	-1.281	-1.764	3.568
g_O	153	150	140	138	141	138	138	146	148	150
	(12.6)	(12.4)	(11.5)	(11.4)	(11.6)	(11.4)	(11.4)	(12.0)	(12.2)	(12.4)
g_E	179.2	179.2	179.2	179.2	179.2	179.2	179.2	179.2	179.2	179.2
	(14.8)	(14.8)	(14.8)	(14.8)	(14.8)	(14.8)	(14.8)	(14.8)	(14.8)	(14.8)
	-48.4	-53.4	-69.2	-72.2	-67.7	-72.2	-72.2	-59.9	-56.7	-53.4
	-3.988	-4.399	-5.697	-5.943	-5.573	-5.943	-5.943	-4.932	-4.668	-4.399
c_O	116	115	92	78	114	110	115	112	111	91
	(9.6)	(9.5)	(7.6)	(6.4)	(9.4)	(9.1)	(9.5)	(9.2)	(9.1)	(7.5)
c_E	111.4	111.4	111.4	111.4	111.4	111.4	111.4	111.4	111.4	111.4
	(9.2)	(9.2)	(9.2)	(9.2)	(9.2)	(9.2)	(9.2)	(9.2)	(9.2)	(9.2)
	9.4	7.4	-35.2	-55.6	5.3	-2.7	7.4	1.3	-0.7	-36.8
	0.778	0.607	-2.896	-4.577	0.438	-0.225	0.607	0.103	-0.061	-3.029
N	1214	1214	1214	1214	1214	1214	1214	1214	1214	1214
X2	4.5	5.6	16.8	26.7	9.7	11.6	11.3	9.7	9.4	11.2
Gvalue	4.7	5.9	17.5	28.5	10.3	12.4	12.1	10.0	9.7	11.7
Gv/N	0.390	0.487	1.444	2.345	0.847	1.019	0.993	0.825	0.797	0.961
Prob	0.19225	0.11613	0.00055	0.00000	0.01631	0.00621	0.00721	0.01841	0.02148	0.00860

	20	19	18	17	16	15	14	13	12	11
a_O	510	532	482	534	477	496	516	558	509	546
	(42.0)	(43.8)	(39.7)	(44.0)	(39.3)	(40.9)	(42.5)	(46.0)	(41.9)	(45.0)
a_E	483.6	483.6	483.6	483.6	483.6	483.6	483.6	483.6	483.6	483.6
	(39.8)	(39.8)	(39.8)	(39.8)	(39.8)	(39.8)	(39.8)	(39.8)	(39.8)	(39.8)
	54.3	101.6	-3.1	106.0	-13.0	25.2	67.0	159.8	52.2	132.6
	4.474	8.369	-0.255	8.730	-1.072	2.077	5.521	13.164	4.301	10.925
t_O	416	436	471	421	461	436	384	350	325	236
	(34.3)	(35.9)	(38.8)	(34.7)	(38.0)	(35.9)	(31.6)	(28.8)	(26.8)	(19.4)

```
t_E       439.8   439.8   439.8   439.8   439.8   439.8   439.8   439.8   439.8   439.8
        ( 36.2) ( 36.2) ( 36.2) ( 36.2) ( 36.2) ( 36.2) ( 36.2) ( 36.2) ( 36.2) ( 36.2)
          -46.4    -7.7    64.5   -36.9    43.3    -7.7  -104.3  -159.9  -196.7  -293.9
         -3.820  -0.631   5.310  -3.037   3.568  -0.631  -8.590 -13.175 -16.202 -24.206
g_O         184     157     180     171     185     200     233     241     328     381
        ( 15.2) ( 12.9) ( 14.8) ( 14.1) ( 15.2) ( 16.5) ( 19.2) ( 19.9) ( 27.0) ( 31.4)
g_E       179.2   179.2   179.2   179.2   179.2   179.2   179.2   179.2   179.2   179.2
        ( 14.8) ( 14.8) ( 14.8) ( 14.8) ( 14.8) ( 14.8) ( 14.8) ( 14.8) ( 14.8) ( 14.8)
            9.7   -41.6     1.5   -16.1    11.7    43.9   122.3   142.7   396.5   574.6
          0.796  -3.425   0.127  -1.324   0.966   3.613  10.071  11.757  32.657  47.335
c_O         104      89      81      88      91      82      81      65      52      51
        (  8.6) (  7.3) (  6.7) (  7.2) (  7.5) (  6.8) (  6.7) (  5.4) (  4.3) (  4.2)
c_E       111.4   111.4   111.4   111.4   111.4   111.4   111.4   111.4   111.4   111.4
        (  9.2) (  9.2) (  9.2) (  9.2) (  9.2) (  9.2) (  9.2) (  9.2) (  9.2) (  9.2)
          -14.2   -39.9   -51.6   -41.5   -36.8   -50.2   -51.6   -70.0   -79.2   -79.7
         -1.174  -3.288  -4.249  -3.415  -3.029  -4.136  -4.249  -5.767  -6.525  -6.562

N          1214    1214    1214    1214    1214    1214    1214    1214    1214    1214
X2          3.4    12.1    10.5    11.4     5.0    10.5    33.7    70.4   186.5   362.4
Gvalue      3.4    12.4    11.3    11.6     5.3    11.2    33.4    72.6   172.8   333.8
Gv/N      0.277   1.025   0.933   0.954   0.433   0.923   2.753   5.980  14.231  27.492
Prob    0.33901 0.00601 0.01009 0.00896 0.15424 0.01066 0.00000 0.00000 0.00000 0.00000
        -------------------------------------------------------------------------------
             10       9       8       7       6       5       4       3       2       1
        -------------------------------------------------------------------------------
a_O         467     432     479     484     460     490     525     500     468     430
        ( 38.5) ( 35.6) ( 39.5) ( 39.8) ( 37.9) ( 40.3) ( 43.2) ( 41.2) ( 38.5) ( 35.4)
a_E       483.6   483.6   483.6   484.0   484.0   484.0   484.0   484.0   484.0   484.0
        ( 39.8) ( 39.8) ( 39.8) ( 39.8) ( 39.8) ( 39.8) ( 39.8) ( 39.8) ( 39.8) ( 39.8)
          -32.5   -97.4    -9.1     0.1   -46.7    12.2    85.5    32.6   -31.4  -101.6
         -2.680  -8.023  -0.746   0.008  -3.843   1.002   7.036   2.685  -2.582  -8.366
t_O         253     232     276     371     387     461     472     482     521     586
        ( 20.8) ( 19.1) ( 22.7) ( 30.5) ( 31.9) ( 37.9) ( 38.8) ( 39.7) ( 42.9) ( 48.2)
t_E       439.8   439.8   439.8   440.2   440.2   440.2   440.2   440.2   440.2   440.2
        ( 36.2) ( 36.2) ( 36.2) ( 36.2) ( 36.2) ( 36.2) ( 36.2) ( 36.2) ( 36.2) ( 36.2)
         -279.8  -296.8  -257.2  -126.9   -99.7    42.6    65.8    87.4   175.6   335.3
        -23.051 -24.449 -21.190 -10.446  -8.206   3.502   5.418   7.196  14.451  27.595
g_O         444     492     392     313     284     185     132     145     136     107
        ( 36.6) ( 40.5) ( 32.3) ( 25.8) ( 23.4) ( 15.2) ( 10.9) ( 11.9) ( 11.2) (  8.8)
g_E       179.2   179.2   179.2   179.4   179.4   179.4   179.4   179.4   179.4   179.4
        ( 14.8) ( 14.8) ( 14.8) ( 14.8) ( 14.8) ( 14.8) ( 14.8) ( 14.8) ( 14.8) ( 14.8)
          805.6   993.7   613.6   348.5   261.0    11.4   -81.0   -61.7   -75.3  -110.6
         66.355  81.850  50.540  28.683  21.480   0.940  -6.664  -5.078  -6.197  -9.100
c_O          50      58      67      47      84      79      86      88      90      92
        (  4.1) (  4.8) (  5.5) (  3.9) (  6.9) (  6.5) (  7.1) (  7.2) (  7.4) (  7.6)
c_E       111.4   111.4   111.4   111.5   111.5   111.5   111.5   111.5   111.5   111.5
        (  9.2) (  9.2) (  9.2) (  9.2) (  9.2) (  9.2) (  9.2) (  9.2) (  9.2) (  9.2)
          -80.1   -75.7   -68.1   -81.2   -47.5   -54.4   -44.6   -41.6   -38.5   -35.3
         -6.597  -6.234  -5.609  -6.681  -3.912  -4.477  -3.672  -3.424  -3.169  -2.907

N          1214    1214    1214    1215    1215    1215    1215    1215    1215    1215
X2        504.9   675.1   331.3   147.7    75.4    10.7    24.1    16.0    30.0    86.9
Gvalue    413.1   523.8   279.2   140.5    67.1    11.8    25.7    16.8    30.4    87.8
Gv/N     34.028  43.143  22.994  11.565   5.519   0.967   2.118   1.379   2.503   7.222
Prob    0.00000 0.00000 0.00000 0.00000 0.00000 0.00828 0.00001 0.00079 0.00000 0.00000
        -------------------------------------------------------------------------------

CDS_INITIAL:
        -------------------------------------------------------------------------------
              1       2       3       4       5       6       7       8       9      10
        -------------------------------------------------------------------------------
a_O         906       0       0     640     623     562     698     602     578     591
        ( 74.6) (  0.0) (  0.0) ( 52.7) ( 51.3) ( 46.3) ( 57.4) ( 49.5) ( 47.6) ( 48.6)
a_E       485.0   463.8   450.4   485.0   463.8   450.4   485.0   463.8   450.4   485.0
        ( 39.9) ( 38.2) ( 37.1) ( 39.9) ( 38.2) ( 37.1) ( 39.9) ( 38.2) ( 37.1) ( 39.9)
         1132.2     0.0     0.0   354.9   367.7   248.7   508.1   314.0   288.2   233.6
         93.182   0.000   0.000  29.208  30.259  20.470  41.822  25.841  23.723  19.223
t_O         207    1215       0     179     303     469     223     368     435     274
        ( 17.0) (100.0) (  0.0) ( 14.7) ( 24.9) ( 38.6) ( 18.4) ( 30.3) ( 35.8) ( 22.6)
t_E       274.7   415.4   520.8   274.7   415.4   520.8   274.7   415.4   520.8   274.7
        ( 22.6) ( 34.2) ( 42.9) ( 22.6) ( 34.2) ( 42.9) ( 22.6) ( 34.2) ( 42.9) ( 22.6)
         -117.1  2607.7     0.0  -153.3  -191.3   -98.3   -92.9   -89.3  -156.7    -1.3
         -9.636 214.629   0.000 -12.615 -15.742  -8.093  -7.648  -7.346 -12.895  -0.108
g_O         102       0    1215     270     139      88     223     127     131     243
        (  8.4) (  0.0) (100.0) ( 22.2) ( 11.4) (  7.2) ( 18.4) ( 10.5) ( 10.8) ( 20.0)
g_E       322.7   143.9   134.9   322.7   143.9   134.9   322.7   143.9   134.9   322.7
        ( 26.6) ( 11.8) ( 11.1) ( 26.6) ( 11.8) ( 11.1) ( 26.6) ( 11.8) ( 11.1) ( 26.6)
         -235.0     0.0  5341.1   -96.3    -9.6   -75.2  -164.9   -31.7    -7.7  -137.9
        -19.339   0.000 439.595  -7.928  -0.793  -6.188 -13.568  -2.612  -0.632 -11.349
c_O           0       0       0     126     150      96      71     118      71     107
        (  0.0) (  0.0) (  0.0) ( 10.4) ( 12.3) (  7.9) (  5.8) (  9.7) (  5.8) (  8.8)
c_E       132.6   191.8   108.8   132.6   191.8   108.8   132.6   191.8   108.8   132.6
        ( 10.9) ( 15.8) (  9.0) ( 10.9) ( 15.8) (  9.0) ( 10.9) ( 15.8) (  9.0) ( 10.9)
            0.0     0.0     0.0   -12.8   -73.8   -24.1   -88.7  -114.7   -60.6   -45.9
          0.000   0.000   0.000  -1.057  -6.075  -1.981  -7.299  -9.440  -4.991  -3.776

N          1215    1215    1215    1215    1215    1215    1215    1215    1215    1215
X2        665.6  2338.3  9728.2    91.8    94.4    50.6   162.6    77.0    63.5    47.8
Gvalue    780.1  2607.7  5341.1    92.4    92.9    51.1   161.7    78.3    63.2    48.5
Gv/N     64.207 214.629 439.595   7.609   7.650   4.207  13.307   6.444   5.205   3.989
Prob    0.00000 1.00000 1.00000 0.00000 0.00000 0.00000 0.00000 0.00000 0.00000 0.00000
        -------------------------------------------------------------------------------
             11      12      13      14      15      16      17      18      19      20
        -------------------------------------------------------------------------------
a_O         511     536     568     463     528     529     442     485     536     398
        ( 42.1) ( 44.1) ( 46.7) ( 38.1) ( 43.5) ( 43.5) ( 36.4) ( 39.9) ( 44.1) ( 32.8)
a_E       463.8   450.4   485.0   463.8   450.4   485.0   463.8   450.4   485.0   463.8
        ( 38.2) ( 37.1) ( 39.9) ( 38.2) ( 37.1) ( 39.9) ( 38.2) ( 37.1) ( 39.9) ( 38.2)
           99.0   186.4   179.4    -1.6   167.8    91.8   -42.6    71.7   107.1  -121.8
          8.150  15.343  14.763  -0.134  13.807   7.555  -3.505   5.901   8.815 -10.026
t_O         434     471     316     513     489     334     492     520     314     531
        ( 35.7) ( 38.8) ( 26.0) ( 42.2) ( 40.2) ( 27.5) ( 40.5) ( 42.8) ( 25.8) ( 43.7)
t_E       415.4   520.8   274.7   415.4   520.8   274.7   415.4   520.8   274.7   415.4
        ( 34.2) ( 42.9) ( 22.6) ( 34.2) ( 42.9) ( 22.6) ( 34.2) ( 42.9) ( 22.6) ( 34.2)
           37.9   -94.7    88.6   216.4   -61.7   130.7   166.4    -1.7    84.1   260.6
          3.121  -7.798   7.294  17.811  -5.077  10.755  13.697  -0.138   6.919  21.450
g_O         129     114     227     105     102     238     113     128     246     116
        ( 10.6) (  9.4) ( 18.7) (  8.6) (  8.4) ( 19.6) (  9.3) ( 10.5) ( 20.2) (  9.5)
g_E       143.9   134.9   322.7   143.9   134.9   322.7   143.9   134.9   322.7   143.9
        ( 11.8) ( 11.1) ( 26.6) ( 11.8) ( 11.1) ( 26.6) ( 11.8) ( 11.1) ( 26.6) ( 11.8)
          -28.2   -38.4  -159.7   -66.2   -57.0  -145.0   -54.6   -13.4  -133.6   -50.0
         -2.321  -3.159 -13.147  -5.447  -4.694 -11.930  -4.496  -1.106 -10.992  -4.115
```

c_O	141	94	104	134	96	114	168	82	119	170
	(11.6)	(7.7)	(8.6)	(11.0)	(7.9)	(9.4)	(13.8)	(6.7)	(9.8)	(14.0)
c_E	191.8	108.8	132.6	191.8	108.8	132.6	191.8	108.8	132.6	191.8
	(15.8)	(9.0)	(10.9)	(15.8)	(9.0)	(10.9)	(15.8)	(9.0)	(10.9)	(15.8)
	-86.8	-27.5	-50.5	-96.2	-24.1	-34.4	-44.6	-46.4	-25.7	-41.1
	-7.146	-2.265	-4.157	-7.915	-1.981	-2.834	-3.670	-3.820	-2.117	-3.382
N	1215	1215	1215	1215	1215	1215	1215	1215	1215	1215
X2	20.6	26.3	55.0	50.9	24.8	41.7	24.7	9.6	30.6	49.4
Gvalue	21.9	25.8	57.7	52.4	25.0	43.1	24.6	10.2	31.9	47.7
Gv/N	1.804	2.121	4.753	4.315	2.056	3.546	2.026	0.838	2.625	3.927
Prob	0.00007	0.00001	0.00000	0.00000	0.00002	0.00000	0.00002	0.01711	0.00000	0.00000

	21	22	23	24	25	26	27	28	29	30
a_O	500	547	419	499	482	401	483	527	401	457
	(41.2)	(45.0)	(34.5)	(41.1)	(39.7)	(33.0)	(39.8)	(43.4)	(33.0)	(37.6)
a_E	450.4	485.0	463.8	450.4	485.0	463.8	450.4	485.0	463.8	450.4
	(37.1)	(39.9)	(38.2)	(37.1)	(39.9)	(38.2)	(37.1)	(39.9)	(38.2)	(37.1)
	104.4	131.5	-85.2	102.2	-6.1	-116.7	67.4	87.5	-116.7	13.2
	8.590	10.825	-7.008	8.409	-0.498	-9.606	5.548	7.198	-9.606	1.087
t_O	487	318	511	505	360	525	508	327	512	517
	(40.1)	(26.2)	(42.1)	(41.6)	(29.6)	(43.2)	(41.8)	(26.9)	(42.1)	(42.6)
t_E	520.8	274.7	415.4	520.8	274.7	415.4	520.8	274.7	415.4	520.8
	(42.9)	(22.6)	(34.2)	(42.9)	(22.6)	(34.2)	(42.9)	(22.6)	(34.2)	(42.9)
	-65.4	93.2	211.6	-31.2	194.8	245.7	-25.4	114.1	214.0	-7.6
	-5.385	7.670	17.413	-2.567	16.034	20.226	-2.087	9.390	17.612	-0.629
g_O	125	246	120	104	255	118	134	248	137	149
	(10.3)	(20.2)	(9.9)	(8.6)	(21.0)	(9.7)	(11.0)	(20.4)	(11.3)	(12.3)
g_E	134.9	322.7	143.9	134.9	322.7	143.9	134.9	322.7	143.9	134.9
	(11.1)	(26.6)	(11.8)	(11.1)	(26.6)	(11.8)	(11.1)	(26.6)	(11.8)	(11.1)
	-19.1	-133.6	-43.6	-54.1	-120.1	-46.8	-1.8	-130.6	-13.5	29.6
	-1.568	-10.992	-3.588	-4.453	-9.886	-3.854	-0.147	-10.751	-1.108	2.439
c_O	103	104	165	107	118	171	90	113	165	92
	(8.5)	(8.6)	(13.6)	(8.8)	(9.7)	(14.1)	(7.4)	(9.3)	(13.6)	(7.6)
c_E	108.8	132.6	191.8	108.8	132.6	191.8	108.8	132.6	191.8	108.8
	(9.0)	(10.9)	(15.8)	(9.0)	(10.9)	(15.8)	(9.0)	(10.9)	(15.8)	(9.0)
	-11.3	-50.5	-49.7	-3.6	-27.5	-39.3	-34.2	-36.1	-49.7	-30.9
	-0.932	-4.157	-4.094	-0.297	-2.264	-3.237	-2.813	-2.973	-4.094	-2.543
N	1215	1215	1215	1215	1215	1215	1215	1215	1215	1215
X2	8.7	39.2	34.0	12.8	42.4	44.3	5.9	33.8	35.0	4.2
Gvalue	8.6	40.7	33.1	13.3	41.1	42.9	6.1	34.8	34.1	4.3
Gv/N	0.705	3.346	2.724	1.092	3.386	3.529	0.501	2.863	2.805	0.353
Prob	0.03556	0.00000	0.00000	0.00410	0.00000	0.00000	0.10760	0.00000	0.00000	0.23146

	31	32	33	34	35	36	37	38	39	40
a_O	469	382	450	474	383	478	514	402	444	479
	(38.6)	(31.4)	(37.0)	(39.0)	(31.5)	(39.3)	(42.3)	(33.1)	(36.5)	(39.4)
a_E	485.0	463.8	450.4	485.0	463.8	450.4	485.0	463.8	450.4	485.0
	(39.9)	(38.2)	(37.1)	(39.9)	(38.2)	(37.1)	(39.9)	(38.2)	(37.1)	(39.9)
	-31.5	-148.3	-0.9	-21.8	-146.6	56.8	59.6	-115.0	-12.8	-12.0
	-2.596	-12.203	-0.073	-1.796	-12.070	4.672	4.907	-9.465	-1.053	-0.987
t_O	331	528	548	355	511	514	335	496	530	349
	(27.2)	(43.5)	(45.1)	(29.2)	(42.1)	(42.3)	(27.6)	(40.8)	(43.6)	(28.7)
t_E	274.7	415.4	520.8	274.7	415.4	520.8	274.7	415.4	520.8	274.7
	(22.6)	(34.2)	(42.9)	(22.6)	(34.2)	(42.9)	(22.6)	(34.2)	(42.9)	(22.6)
	123.5	253.2	55.7	182.2	211.6	-13.6	133.1	175.8	18.5	167.2
	10.167	20.837	4.586	14.994	17.413	-1.118	10.952	14.469	1.521	13.762
g_O	287	146	125	265	153	130	238	129	138	257
	(23.6)	(12.0)	(10.3)	(21.8)	(12.6)	(10.7)	(19.6)	(10.6)	(11.4)	(21.2)
g_E	322.7	143.9	134.9	322.7	143.9	134.9	322.7	143.9	134.9	322.7
	(26.6)	(11.8)	(11.1)	(26.6)	(11.8)	(11.1)	(26.6)	(11.8)	(11.1)	(26.6)
	-67.3	4.2	-19.1	-104.4	18.8	-9.6	-145.0	-28.2	6.3	-117.0
	-5.542	0.348	-1.568	-8.596	1.545	-0.792	-11.930	-2.321	0.516	-9.633
c_O	128	159	92	121	168	93	128	188	103	130
	(10.5)	(13.1)	(7.6)	(10.0)	(13.8)	(7.7)	(10.5)	(15.5)	(8.5)	(10.7)
c_E	132.6	191.8	108.8	132.6	191.8	108.8	132.6	191.8	108.8	132.6
	(10.9)	(15.8)	(9.0)	(10.9)	(15.8)	(9.0)	(10.9)	(15.8)	(9.0)	(10.9)
	-9.0	-59.7	-30.9	-22.1	-44.6	-29.2	-9.0	-7.6	-11.3	-5.1
	-0.741	-4.914	-2.543	-1.821	-3.670	-2.405	-0.741	-0.626	-0.932	-0.421
N	1215	1215	1215	1215	1215	1215	1215	1215	1215	1215
X2	16.2	50.6	4.7	35.1	39.6	4.3	37.4	25.5	0.6	33.6
Gvalue	15.6	49.4	4.9	33.8	39.1	4.3	38.7	25.0	0.6	33.0
Gv/N	1.288	4.068	0.402	2.781	3.218	0.357	3.188	2.058	0.053	2.720
Prob	0.00134	0.00000	0.18058	0.00000	0.00000	0.22675	0.00000	0.00002	0.88709	0.00000

	41	42	43	44	45	46	47	48	49	50
a_O	395	464	496	388	469	457	411	448	471	380
	(32.5)	(38.2)	(40.8)	(31.9)	(38.6)	(37.6)	(33.8)	(36.9)	(38.8)	(31.3)
a_E	463.8	450.4	485.0	463.8	450.4	485.0	463.8	450.4	485.0	463.8
	(38.2)	(37.1)	(39.9)	(38.2)	(37.1)	(39.9)	(38.2)	(37.1)	(39.9)	(38.2)
	-126.9	27.5	22.2	-138.5	37.9	-54.4	-99.4	-4.9	-27.7	-151.5
	-10.442	2.265	1.825	-11.399	3.117	-4.479	-8.179	-0.401	-2.277	-12.467
t_O	515	532	319	514	509	329	442	538	330	470
	(42.4)	(43.8)	(26.3)	(42.3)	(41.9)	(27.1)	(36.4)	(44.3)	(27.2)	(38.7)
t_E	415.4	520.8	274.7	415.4	520.8	274.7	415.4	520.8	274.7	415.4
	(34.2)	(42.9)	(22.6)	(34.2)	(42.9)	(22.6)	(34.2)	(42.9)	(22.6)	(34.2)
	221.3	22.6	95.5	218.8	-23.4	118.8	54.8	34.9	121.2	116.0
	18.210	1.857	7.859	18.010	-1.926	9.777	4.508	2.871	9.972	9.545
g_O	146	134	275	133	132	297	147	122	291	175
	(12.0)	(11.0)	(22.6)	(10.9)	(10.9)	(24.4)	(12.1)	(10.0)	(24.0)	(14.4)
g_E	143.9	134.9	322.7	143.9	134.9	322.7	143.9	134.9	322.7	143.9
	(11.8)	(11.1)	(26.6)	(11.8)	(11.1)	(26.6)	(11.8)	(11.1)	(26.6)	(11.8)
	4.2	-1.8	-88.0	-21.0	-5.7	-49.3	6.3	-24.5	-60.2	68.5
	0.348	-0.147	-7.244	-1.724	-0.472	-4.061	0.516	-2.018	-4.956	5.637
c_O	159	85	125	180	105	132	215	107	123	190
	(13.1)	(7.0)	(10.3)	(14.8)	(8.6)	(10.9)	(17.7)	(8.8)	(10.1)	(15.6)
c_E	191.8	108.8	132.6	191.8	108.8	132.6	191.8	108.8	132.6	191.8
	(15.8)	(9.0)	(10.9)	(15.8)	(9.0)	(10.9)	(15.8)	(9.0)	(10.9)	(15.8)
	-59.7	-42.0	-14.7	-22.9	-7.5	-1.2	49.0	-3.6	-18.5	-3.7
	-4.914	-3.457	-1.212	-1.888	-0.618	-0.096	4.034	-0.297	-1.519	-0.301
N	1215	1215	1215	1215	1215	1215	1215	1215	1215	1215
X2	39.7	5.9	14.9	37.3	1.2	14.4	10.6	1.8	15.4	29.0
Gvalue	38.9	6.3	14.9	36.4	1.2	13.9	10.7	1.9	14.8	29.3
Gv/N	3.202	0.518	1.228	3.000	0.101	1.141	0.879	0.155	1.219	2.413
Prob	0.00000	0.09833	0.00188	0.00000	0.74734	0.00309	0.01363	0.59812	0.00198	0.00000

	51	52	53	54	55	56	57	58	59	60
a_O	451	481	400	429	462	409	435	471	437	438
	(37.1)	(39.7)	(33.0)	(35.4)	(38.2)	(33.8)	(36.0)	(38.9)	(36.1)	(36.2)
a_E	450.4	484.2	463.0	449.7	483.0	461.9	448.6	483.0	461.9	448.6
	(37.1)	(39.9)	(38.2)	(37.1)	(39.9)	(38.2)	(37.1)	(39.9)	(38.2)	(37.1)
	1.1	−6.5	−117.1	−40.4	−41.2	−99.5	−26.8	−23.8	−48.4	−20.9
	0.092	−0.532	−9.653	−3.334	−3.401	−8.223	−2.212	−1.965	−4.003	−1.730
t_O	509	345	477	528	309	440	549	331	443	560
	(41.9)	(28.4)	(39.3)	(43.5)	(25.5)	(36.4)	(45.4)	(27.4)	(36.6)	(46.3)
t_E	520.8	274.2	414.8	520.0	273.5	413.7	518.7	273.5	413.7	518.7
	(42.9)	(22.6)	(34.2)	(42.9)	(22.6)	(34.2)	(42.9)	(22.6)	(34.2)	(42.9)
	−23.4	158.5	133.4	16.2	75.4	54.2	62.3	126.3	60.5	85.8
	−1.926	13.064	10.996	1.332	6.228	4.476	5.153	10.434	5.004	7.092
g_O	121	257	162	143	311	156	124	288	121	112
	(10.0)	(21.2)	(13.4)	(11.8)	(25.7)	(12.9)	(10.2)	(23.8)	(10.0)	(9.3)
g_E	134.9	322.2	143.7	134.7	321.4	143.3	134.3	321.4	143.3	134.3
	(11.1)	(26.6)	(11.8)	(11.1)	(26.6)	(11.8)	(11.1)	(26.6)	(11.8)	(11.1)
	−26.3	−116.2	38.9	17.2	−20.4	26.5	−19.9	−63.2	−40.9	−40.7
	−2.166	−9.579	3.209	1.414	−1.690	2.188	−1.642	−5.222	−3.384	−3.367
c_O	134	130	174	113	128	205	102	120	209	100
	(11.0)	(10.7)	(14.3)	(9.3)	(10.6)	(16.9)	(8.4)	(9.9)	(17.3)	(8.3)
c_E	108.8	132.4	191.5	108.6	132.0	191.1	108.4	132.0	191.1	108.4
	(9.0)	(10.9)	(15.8)	(9.0)	(10.9)	(15.8)	(9.0)	(10.9)	(15.8)	(9.0)
	55.8	−4.7	−33.4	8.9	−8.0	28.9	−12.4	−22.9	37.5	−16.1
	4.591	−0.387	−2.753	0.733	−0.657	2.388	−1.022	−1.896	3.102	−1.329
N	1215	1213	1213	1213	1210	1210	1210	1210	1210	1210
X2	7.5	31.5	21.9	1.8	6.0	9.9	3.4	16.9	8.6	7.9
Gvalue	7.2	31.1	21.8	1.8	5.8	10.0	3.4	16.3	8.7	8.1
Gv/N	0.591	2.566	1.798	0.146	0.480	0.829	0.277	1.351	0.719	0.666
Prob	0.06642	0.00000	0.00007	0.62196	0.12131	0.01836	0.34034	0.00097	0.03365	0.04474

	61	62	63	64	65	66	67	68	69	70
a_O	487	417	437	478	458	456	477	429	435	490
	(40.2)	(34.5)	(36.1)	(39.5)	(37.9)	(37.7)	(39.5)	(35.5)	(36.0)	(40.6)
a_E	483.0	461.9	448.6	483.0	461.9	448.6	482.6	461.5	448.2	482.2
	(39.9)	(38.2)	(37.1)	(39.9)	(38.2)	(37.1)	(39.9)	(38.2)	(37.1)	(39.9)
	8.0	−85.3	−22.9	−10.0	−7.8	14.9	−11.2	−62.7	−26.0	15.6
	0.657	−7.049	−1.891	−0.829	−0.643	1.235	−0.928	−5.186	−2.154	1.295
t_O	304	434	536	281	417	511	308	430	545	294
	(25.1)	(35.9)	(44.3)	(23.2)	(34.5)	(42.2)	(25.5)	(35.6)	(45.1)	(24.3)
t_E	273.5	413.7	518.7	273.5	413.7	518.7	273.3	413.4	518.3	273.1
	(22.6)	(34.2)	(42.9)	(22.6)	(34.2)	(42.9)	(22.6)	(34.2)	(42.9)	(22.6)
	64.2	41.5	35.2	15.1	6.5	−15.3	73.6	33.9	54.8	43.4
	5.308	3.430	2.908	1.252	0.541	−1.262	6.090	2.801	4.535	3.594
g_O	292	155	128	329	152	134	296	151	129	317
	(24.1)	(12.8)	(10.6)	(27.2)	(12.6)	(11.1)	(24.5)	(12.5)	(10.7)	(26.2)
g_E	321.4	143.3	134.3	321.4	143.3	134.3	321.1	143.2	134.2	320.9
	(26.6)	(11.8)	(11.1)	(26.6)	(11.8)	(11.1)	(26.6)	(11.8)	(11.1)	(26.6)
	−56.0	24.3	−12.4	15.4	17.9	−0.7	−48.2	16.0	−10.3	−7.7
	−4.629	2.010	−1.023	1.272	1.480	−0.057	−3.990	1.327	−0.849	−0.636
c_O	127	204	109	122	183	109	128	199	100	107
	(10.5)	(16.9)	(9.0)	(10.1)	(15.1)	(9.0)	(10.6)	(16.5)	(8.3)	(8.9)
c_E	132.0	191.1	108.4	132.0	191.1	108.4	131.9	190.9	108.3	131.8
	(10.9)	(15.8)	(9.0)	(10.9)	(15.8)	(9.0)	(10.9)	(15.8)	(9.0)	(10.9)
	−9.9	26.8	1.3	−19.3	−15.8	1.3	−7.7	16.6	−15.9	−44.6
	−0.817	2.211	0.104	−1.595	−1.302	0.104	−0.640	1.369	−1.316	−3.696
N	1210	1210	1210	1210	1210	1210	1209	1209	1209	1208
X2	6.3	7.2	1.2	1.2	0.9	0.2	6.6	3.7	2.6	6.4
Gvalue	6.3	7.3	1.2	1.2	0.9	0.2	6.4	3.8	2.6	6.7
Gv/N	0.519	0.602	0.098	0.101	0.076	0.020	0.532	0.311	0.216	0.557
Prob	0.09889	0.06353	0.75753	0.74890	0.82064	0.97072	0.09229	0.28811	0.45631	0.08091

	71	72	73	74	75	76	77	78	79	80
a_O	466	441	474	464	472	496	464	459	488	462
	(38.6)	(36.5)	(39.2)	(38.4)	(39.1)	(41.1)	(38.4)	(38.0)	(40.5)	(38.4)
a_E	461.1	447.8	482.2	461.1	447.8	481.8	460.8	447.5	480.6	459.6
	(38.2)	(37.1)	(39.9)	(38.2)	(37.1)	(39.9)	(38.2)	(37.1)	(39.9)	(38.2)
	9.8	−13.6	−16.3	5.7	49.6	28.7	6.5	23.3	14.8	4.8
	0.809	−1.125	−1.353	0.475	4.104	2.380	0.539	1.934	1.231	0.397
t_O	429	520	302	448	507	276	419	519	301	437
	(35.5)	(43.0)	(25.0)	(37.1)	(42.0)	(22.9)	(34.7)	(43.0)	(25.0)	(36.3)
t_E	413.1	517.8	273.1	413.1	517.8	272.8	412.7	517.4	272.2	411.7
	(34.2)	(42.9)	(22.6)	(34.2)	(42.9)	(22.6)	(34.2)	(42.9)	(22.6)	(34.2)
	32.5	4.3	60.8	72.8	−21.4	6.3	12.7	3.2	60.6	52.2
	2.690	0.359	5.034	6.024	−1.775	0.525	1.050	0.264	5.034	4.332
g_O	129	135	298	130	129	297	131	108	301	130
	(10.7)	(11.2)	(24.7)	(10.8)	(10.7)	(24.6)	(10.9)	(8.9)	(25.0)	(10.8)
g_E	143.1	134.1	320.9	143.1	134.1	320.6	143.0	134.0	319.8	142.6
	(11.8)	(11.1)	(26.6)	(11.8)	(11.1)	(26.6)	(11.8)	(11.1)	(26.6)	(11.8)
	−26.7	1.8	−44.1	−24.9	−10.0	−45.4	−22.9	−46.6	−36.5	−24.0
	−2.211	0.146	−3.647	−2.062	−0.832	−3.762	−1.895	−3.862	−3.029	−1.997
c_O	184	112	134	166	100	138	193	121	114	175
	(15.2)	(9.3)	(11.1)	(13.7)	(8.3)	(11.4)	(16.0)	(10.0)	(9.5)	(14.5)
c_E	190.7	108.2	131.8	190.7	108.2	131.7	190.6	108.1	131.4	190.1
	(15.8)	(9.0)	(10.9)	(15.8)	(9.0)	(10.9)	(15.8)	(9.0)	(10.9)	(15.8)
	−13.2	7.7	4.4	−46.1	−15.7	12.9	4.9	27.3	−32.4	−29.0
	−1.095	0.641	0.364	−3.817	−1.304	1.066	0.404	2.260	−2.688	−2.406
N	1208	1208	1208	1208	1208	1207	1207	1207	1204	1204
X2	2.3	0.3	4.9	7.4	2.3	2.5	1.1	6.9	6.6	3.9
Gvalue	2.3	0.3	4.8	7.5	2.3	2.5	1.2	7.2	6.6	3.9
Gv/N	0.193	0.021	0.398	0.619	0.194	0.209	0.098	0.596	0.548	0.325
Prob	0.50662	0.96870	0.18642	0.05801	0.50438	0.47081	0.75852	0.06606	0.08581	0.27047

	81	82	83	84	85	86	87	88	89	90
a_O	461	486	458	453	464	467	434	497	425	455
	(38.3)	(40.5)	(38.1)	(37.7)	(38.7)	(38.9)	(36.2)	(41.6)	(35.6)	(38.1)
a_E	446.4	479.4	458.5	445.3	478.6	457.7	444.5	477.1	456.2	443.0
	(37.1)	(39.9)	(38.2)	(37.1)	(39.9)	(38.2)	(37.1)	(39.9)	(38.2)	(37.1)
	29.7	13.2	−0.9	15.6	−28.8	18.8	−20.8	40.7	−60.2	24.3
	2.470	1.099	−0.078	1.301	−2.406	1.566	−1.733	3.407	−5.036	2.030
t_O	515	290	432	500	276	402	510	302	430	504
	(42.8)	(24.1)	(36.0)	(41.6)	(23.0)	(33.5)	(42.5)	(25.3)	(36.0)	(42.2)
t_E	516.1	271.5	410.7	514.8	271.0	410.0	514.0	270.1	408.6	512.3
	(42.9)	(22.6)	(34.2)	(42.9)	(22.6)	(34.2)	(42.9)	(22.6)	(34.2)	(42.9)
	−2.2	38.3	43.8	−29.2	10.0	−15.8	−7.9	67.3	43.9	−16.4

```
         -0.186   3.185   3.644  -2.435   0.835  -1.318  -0.661   5.636   3.672  -1.372
g_O         127     322     134     138     327     144     135     277     154     131
        ( 10.5) ( 26.8) ( 11.2) ( 11.5) ( 27.3) ( 12.0) ( 11.3) ( 23.2) ( 12.9) ( 11.0)
g_E       133.7   319.0   142.2   133.3   318.5   142.0   133.1   317.4   141.5   132.7
        ( 11.1) ( 26.6) ( 11.8) ( 11.1) ( 26.6) ( 11.8) ( 11.1) ( 26.6) ( 11.8) ( 11.1)
          -13.0     6.0   -16.0     9.5    17.3     4.0     3.8   -75.4    26.0    -3.3
         -1.081   0.501  -1.332   0.789   1.441   0.335   0.315  -6.313   2.176  -0.279
c_O         101     103     177     110     132     186     120     119     186     105
        (  8.4) (  8.6) ( 14.7) (  9.2) ( 11.0) ( 15.5) ( 10.0) ( 10.0) ( 15.6) (  8.8)
c_E       107.8   131.1   189.6   107.6   130.8   189.3   107.4   130.4   188.7   107.0
        (  9.0) ( 10.9) ( 15.8) (  9.0) ( 10.9) ( 15.8) (  9.0) ( 10.9) ( 15.8) (  9.0)
          -13.2   -49.6   -24.4     4.9     2.3    -6.6    26.7   -21.8    -5.3    -4.0
         -1.099  -4.132  -2.032   0.410   0.195  -0.548   2.223  -1.822  -0.446  -0.336

N          1204    1201    1201    1201    1199    1199    1199    1195    1195    1195
X2          1.2     7.4     2.4     0.8     0.8     0.4     1.8    10.7     4.4     0.5
Gvalue      1.3     7.8     2.4     0.8     0.8     0.4     1.7    10.8     4.4     0.5
Gv/N      0.105   0.653   0.203   0.065   0.065   0.036   0.145   0.908   0.367   0.043
Prob    0.73891 0.04946 0.48659 0.85399 0.85429 0.93399 0.62928 0.01258 0.22249 0.91567
        -------------------------------------------------------------------------------
             91      92      93      94      95      96      97      98      99     100
        -------------------------------------------------------------------------------
a_O         488     434     431     458     468     450     465     445     439     487
        ( 41.0) ( 36.5) ( 36.2) ( 38.6) ( 39.4) ( 37.9) ( 39.2) ( 37.6) ( 37.0) ( 41.1)
a_E       475.1   454.3   441.2   474.3   453.5   440.4   473.1   452.4   439.3   473.1
        ( 39.9) ( 38.2) ( 37.1) ( 39.9) ( 38.2) ( 37.1) ( 39.9) ( 38.2) ( 37.1) ( 39.9)
           26.2   -39.6   -20.1   -32.0    29.4    19.3   -16.0   -14.6    -0.6    28.3
          2.205  -3.330  -1.690  -2.690   2.479   1.628  -1.349  -1.232  -0.054   2.387
t_O         287     441     515     305     387     509     285     416     512     277
        ( 24.1) ( 37.1) ( 43.3) ( 25.7) ( 32.6) ( 42.8) ( 24.1) ( 35.1) ( 43.2) ( 23.4)
t_E       269.0   406.9   510.1   268.6   406.2   509.3   267.9   405.2   508.0   267.9
        ( 22.6) ( 34.2) ( 42.9) ( 22.6) ( 34.2) ( 42.9) ( 22.6) ( 34.2) ( 42.9) ( 22.6)
           37.2    71.0     9.8    77.6   -37.5    -0.5    35.3    21.9     8.1    18.6
          3.123   5.965   0.824   6.535  -3.157  -0.044   2.981   1.849   0.682   1.566
g_O         298     145     131     308     158     124     314     140     116     302
        ( 25.0) ( 12.2) ( 11.0) ( 25.9) ( 13.3) ( 10.4) ( 26.5) ( 11.8) (  9.8) ( 25.5)
g_E       316.1   140.9   132.1   315.6   140.7   131.9   314.8   140.3   131.6   314.8
        ( 26.6) ( 11.8) ( 11.1) ( 26.6) ( 11.8) ( 11.1) ( 26.6) ( 11.8) ( 11.1) ( 26.6)
          -35.1     8.2    -2.2   -14.9    36.6   -15.3    -1.5    -0.7   -29.2   -25.0
         -2.950   0.692  -0.188  -1.256   3.084  -1.289  -0.127  -0.058  -2.466  -2.108
c_O         117     170     113     117     175     105     121     184     118     119
        (  9.8) ( 14.3) (  9.5) (  9.8) ( 14.7) (  8.8) ( 10.2) ( 15.5) ( 10.0) ( 10.0)
c_E       129.9   187.9   106.6   129.6   187.6   106.4   129.3   187.1   106.1   129.3
        ( 10.9) ( 15.8) (  9.0) ( 10.9) ( 15.8) (  9.0) ( 10.9) ( 15.8) (  9.0) ( 10.9)
          -24.4   -34.0    13.2   -24.0   -24.3    -2.8   -16.1    -6.2    25.0   -19.8
         -2.050  -2.859   1.111  -2.020  -2.045  -0.234  -1.357  -0.519   2.111  -1.669

N          1190    1190    1190    1188    1188    1188    1185    1185    1185    1185
X2          3.9     5.6     0.7     6.9     4.3     0.7     1.8     0.5     3.2     2.1
Gvalue      3.9     5.6     0.7     6.8     4.3     0.7     1.8     0.5     3.2     2.1
Gv/N      0.328   0.469   0.056   0.569   0.361   0.060   0.148   0.039   0.273   0.176
Prob    0.27257 0.13416 0.87983 0.08007 0.23186 0.87133 0.62410 0.92781 0.35730 0.55536
        -------------------------------------------------------------------------------
            101     102     103     104     105     106     107     108     109     110
        -------------------------------------------------------------------------------
a_O         459     429     468     466     451     502     489     455     479     500
        ( 38.7) ( 36.2) ( 39.6) ( 39.4) ( 38.1) ( 42.5) ( 41.4) ( 38.5) ( 40.7) ( 42.4)
a_E       452.4   439.3   472.3   451.6   438.6   471.5   450.8   437.8   470.3   449.7
        ( 38.2) ( 37.1) ( 39.9) ( 38.2) ( 37.1) ( 39.9) ( 38.2) ( 37.1) ( 39.9) ( 38.2)
           13.4   -20.4    -8.5    29.3    25.2    63.0    79.5    35.0    17.6   106.1
          1.129  -1.721  -0.717   2.473   2.129   5.335   6.729   2.962   1.497   9.003
t_O         421     530     288     382     495     241     368     505     268     355
        ( 35.5) ( 44.7) ( 24.3) ( 32.3) ( 41.8) ( 20.4) ( 31.2) ( 42.8) ( 22.8) ( 30.1)
t_E       405.2   508.0   267.4   404.5   507.1   267.0   403.8   506.3   266.3   402.8
        ( 34.2) ( 42.9) ( 22.6) ( 34.2) ( 42.9) ( 22.6) ( 34.2) ( 42.9) ( 22.6) ( 34.2)
           32.2    45.0    42.7   -43.7   -23.9   -49.3   -68.4    -2.5     3.4   -89.7
          2.720   3.796   3.609  -3.697  -2.024  -4.177  -5.789  -0.214   0.291  -7.613
g_O         136     131     302     138     128     320     135     126     320     148
        ( 11.5) ( 11.1) ( 25.5) ( 11.7) ( 10.8) ( 27.1) ( 11.4) ( 10.7) ( 27.2) ( 12.6)
g_E       140.3   131.6   314.2   140.1   131.3   313.7   139.9   131.1   312.9   139.5
        ( 11.8) ( 11.1) ( 26.6) ( 11.8) ( 11.1) ( 26.6) ( 11.8) ( 11.1) ( 26.6) ( 11.8)
           -8.6    -1.1   -24.0    -4.2    -6.6    12.7    -9.6   -10.0    14.4    17.5
         -0.722  -0.096  -2.026  -0.354  -0.558   1.079  -0.811  -0.851   1.220   1.483
c_O         169      95     125     197     109     118     189      95     111     175
        ( 14.3) (  8.0) ( 10.6) ( 16.7) (  9.2) ( 10.0) ( 16.0) (  8.0) (  9.4) ( 14.9)
c_E       187.1   106.1   129.1   186.8   106.0   128.9   186.5   105.8   128.5   186.0
        ( 15.8) (  9.0) ( 10.9) ( 15.8) (  9.0) ( 10.9) ( 15.8) (  9.0) ( 10.9) ( 15.8)
          -34.4   -21.1    -8.1    21.0     6.2   -20.8     5.1   -20.4   -32.6   -21.3
         -2.903  -1.777  -0.681   1.773   0.522  -1.762   0.431  -1.728  -2.766  -1.811

N          1185    1185    1183    1183    1183    1181    1181    1181    1178    1178
X2          2.6     2.4     2.2     2.3     0.8     5.5     6.6     2.0     2.7    12.5
Gvalue      2.7     2.4     2.2     2.3     0.8     5.6     6.6     2.0     2.8    12.5
Gv/N      0.224   0.202   0.186   0.195   0.069   0.476   0.561   0.170   0.241   1.062
Prob    0.44827 0.49382 0.53281 0.51043 0.84632 0.13165 0.08485 0.57099 0.41620 0.00583
        -------------------------------------------------------------------------------
            111     112     113     114     115     116     117     118     119     120
        -------------------------------------------------------------------------------
a_O         442     439     457     441     457     416     445     480     477     454
        ( 37.5) ( 37.3) ( 38.9) ( 37.5) ( 39.0) ( 35.5) ( 37.9) ( 41.0) ( 40.8) ( 38.8)
a_E       436.7   469.5   448.9   436.0   468.3   447.8   434.9   467.1   446.6   433.8
        ( 37.1) ( 39.9) ( 38.2) ( 37.1) ( 39.9) ( 38.2) ( 37.1) ( 39.9) ( 38.2) ( 37.1)
           10.6   -58.9    16.3    10.1   -22.3   -61.2    20.5    26.2    62.7    41.4
          0.901  -5.010   1.385   0.858  -1.898  -5.222   1.747   2.240   5.363   3.539
t_O         525     263     389     511     257     387     469     246     389     489
        ( 44.6) ( 22.4) ( 33.1) ( 43.5) ( 21.9) ( 33.0) ( 40.0) ( 21.0) ( 33.2) ( 41.8)
t_E       505.0   265.8   402.1   504.1   265.2   401.1   502.8   264.5   400.1   501.5
        ( 42.9) ( 22.6) ( 34.2) ( 42.9) ( 22.6) ( 34.2) ( 42.9) ( 22.6) ( 34.2) ( 42.9)
           40.8    -5.7   -25.8    13.9   -16.1   -27.7   -65.3   -35.6   -21.8   -24.8
          3.466  -0.481  -2.193   1.178  -1.370  -2.359  -5.570  -3.047  -1.864  -2.118
g_O         109     333     135     128     328     162     135     294     140     126
        (  9.3) ( 28.3) ( 11.5) ( 10.9) ( 28.0) ( 13.8) ( 11.5) ( 25.1) ( 12.0) ( 10.8)
g_E       130.8   312.4   139.3   130.6   311.6   138.9   130.2   310.8   138.6   129.9
        ( 11.1) ( 26.6) ( 11.8) ( 11.1) ( 26.6) ( 11.8) ( 11.1) ( 26.6) ( 11.8) ( 11.1)
          -39.7    42.6    -8.4    -5.1    33.7    49.8     9.7   -32.6     2.9    -7.7
         -3.373   3.623  -0.717  -0.433   2.875   4.244   0.827  -2.788   0.246  -0.657
c_O         102     141     195      96     131     208     124     150     164     101
        (  8.7) ( 12.0) ( 16.6) (  8.2) ( 11.2) ( 17.7) ( 10.6) ( 12.8) ( 14.0) (  8.6)
c_E       105.5   128.3   185.7   105.3   128.0   185.2   105.1   127.7   184.7   104.8
        (  9.0) ( 10.9) ( 15.8) (  9.0) ( 10.9) ( 15.8) (  9.0) ( 10.9) ( 15.8) (  9.0)
           -6.9    26.6    19.1   -17.8     6.1    48.3    41.1    48.3   -39.1    -7.4
         -0.585   2.258   1.624  -1.514   0.517   4.116   3.505   4.132  -3.338  -0.636
```

N	1178	1176	1176	1176	1173	1173	1173	1170	1170	1170
X2	4.6	4.6	1.2	1.0	1.5	9.4	6.1	6.5	4.7	1.5
Gvalue	4.8	4.6	1.2	1.1	1.4	9.1	6.0	6.3	4.8	1.5
Gv/N	0.409	0.391	0.099	0.090	0.124	0.780	0.508	0.538	0.407	0.129
Prob	0.18580	0.20370	0.76033	0.78838	0.69410	0.02743	0.11346	0.09816	0.19007	0.68142

	121	122	123	124	125	126	127	128	129	130
a_O	498	460	448	478	463	441	442	448	424	435
	(42.8)	(39.5)	(38.5)	(41.2)	(39.9)	(38.0)	(38.1)	(38.6)	(36.6)	(37.6)
a_E	464.7	444.3	431.5	463.5	443.2	430.4	463.1	442.8	430.1	461.9
	(39.9)	(38.2)	(37.1)	(39.9)	(38.2)	(37.1)	(39.9)	(38.2)	(37.1)	(39.9)
	69.0	31.9	33.5	29.5	40.5	21.4	−41.2	10.4	−12.0	−52.2
	5.926	2.737	2.882	2.540	3.486	1.844	−3.550	0.899	−1.036	−4.509
t_O	244	401	494	262	354	475	273	406	513	270
	(21.0)	(34.5)	(42.4)	(22.6)	(30.5)	(40.9)	(23.5)	(35.0)	(44.2)	(23.3)
t_E	263.1	398.0	499.0	262.4	397.0	497.7	262.2	396.6	497.3	261.5
	(22.6)	(34.2)	(42.9)	(22.6)	(34.2)	(42.9)	(22.6)	(34.2)	(42.9)	(22.6)
	−36.8	6.0	−9.9	−0.9	−81.1	−44.3	22.0	18.9	32.0	17.2
	−3.164	0.516	−0.851	−0.077	−6.988	−3.818	1.896	1.632	2.756	1.485
g_O	310	140	117	310	143	114	305	120	136	314
	(26.6)	(12.0)	(10.1)	(26.7)	(12.3)	(9.8)	(26.3)	(10.3)	(11.7)	(27.1)
g_E	309.2	137.9	129.2	308.4	137.5	128.9	308.1	137.4	128.8	307.3
	(26.6)	(11.8)	(11.1)	(26.6)	(11.8)	(11.1)	(26.6)	(11.8)	(11.1)	(26.6)
	1.7	4.3	−23.3	3.3	11.2	−28.0	−6.2	−32.5	14.8	13.5
	0.142	0.371	−2.000	0.280	0.966	−2.413	−0.534	−2.799	1.277	1.168
c_O	112	163	105	111	201	131	140	186	87	138
	(9.6)	(14.0)	(9.0)	(9.6)	(17.3)	(11.3)	(12.1)	(16.0)	(7.5)	(11.9)
c_E	127.0	183.8	104.3	126.7	183.3	104.0	126.6	183.2	103.9	126.3
	(10.9)	(15.8)	(9.0)	(10.9)	(15.8)	(9.0)	(10.9)	(15.8)	(9.0)	(10.9)
	−28.2	−39.1	1.5	−29.4	37.0	60.5	28.2	5.7	−30.9	24.5
	−2.422	−3.362	0.129	−2.528	3.189	5.212	2.432	0.494	−2.662	2.122
N	1164	1164	1164	1161	1161	1161	1160	1160	1160	1157
X2	5.6	3.0	1.8	2.4	7.5	10.0	2.9	2.5	3.7	3.1
Gvalue	5.6	3.0	1.9	2.5	7.6	9.6	2.8	2.6	3.9	3.1
Gv/N	0.482	0.262	0.161	0.214	0.652	0.825	0.243	0.226	0.335	0.265
Prob	0.13206	0.38459	0.59932	0.47732	0.05568	0.02248	0.42065	0.45355	0.27426	0.38086

	131	132	133	134	135	136	137	138	139	140
a_O	465	446	443	449	434	435	418	400	499	434
	(40.2)	(38.5)	(38.4)	(38.9)	(37.6)	(37.8)	(36.3)	(34.7)	(43.4)	(37.8)
a_E	441.7	428.9	461.1	440.9	428.2	459.9	439.8	427.1	458.7	438.6
	(38.2)	(37.1)	(39.9)	(38.2)	(37.1)	(39.9)	(38.2)	(37.1)	(39.9)	(38.2)
	47.9	34.8	−35.4	16.3	11.7	−48.4	−42.4	−52.4	84.1	−9.2
	4.137	3.007	−3.069	1.414	1.011	−4.201	−3.683	−4.550	7.316	−0.800
t_O	391	490	247	386	508	272	412	502	258	383
	(33.8)	(42.4)	(21.4)	(33.4)	(44.0)	(23.6)	(35.8)	(43.6)	(22.5)	(33.3)
t_E	395.6	496.0	261.1	394.9	495.1	260.4	393.9	493.8	259.7	392.9
	(34.2)	(42.9)	(22.6)	(34.2)	(42.9)	(22.6)	(34.2)	(42.9)	(22.6)	(34.2)
	−9.2	−11.9	−27.4	−17.7	26.1	23.7	37.0	16.5	−3.5	−19.5
	−0.793	−1.027	−2.373	−1.529	2.260	2.055	3.212	1.430	−0.301	−1.698
g_O	111	139	304	133	123	312	134	127	281	138
	(9.6)	(12.0)	(26.3)	(11.5)	(10.6)	(27.1)	(11.6)	(11.0)	(24.5)	(12.0)
g_E	137.0	128.5	306.8	136.8	128.2	306.0	136.4	127.9	305.2	136.1
	(11.8)	(11.1)	(26.6)	(11.8)	(11.1)	(26.6)	(11.8)	(11.1)	(26.6)	(11.8)
	−46.8	21.9	−5.5	−7.5	−10.3	12.1	−4.8	−1.8	−46.4	3.9
	−4.042	1.895	−0.480	−0.648	−0.888	1.054	−0.419	−0.156	−4.039	0.336
c_O	190	82	161	187	90	133	188	123	111	194
	(16.4)	(7.1)	(13.9)	(16.2)	(7.8)	(11.5)	(16.3)	(10.7)	(9.7)	(16.9)
c_E	182.7	103.6	126.0	182.4	103.4	125.7	181.9	103.2	125.4	181.4
	(15.8)	(9.0)	(10.9)	(15.8)	(9.0)	(10.9)	(15.8)	(9.0)	(10.9)	(15.8)
	14.9	−38.4	78.8	9.4	−25.1	15.0	12.4	43.2	−27.0	26.0
	1.290	−3.318	6.825	0.812	−2.170	1.302	1.078	3.753	−2.354	2.264
N	1157	1157	1155	1155	1155	1152	1152	1152	1149	1149
X2	6.5	6.1	11.2	0.6	2.4	2.4	2.2	5.7	7.1	1.2
Gvalue	6.8	6.4	10.4	0.6	2.5	2.4	2.2	5.5	7.1	1.2
Gv/N	0.592	0.557	0.903	0.050	0.213	0.209	0.188	0.476	0.622	0.103
Prob	0.07699	0.09172	0.01526	0.90236	0.48327	0.49146	0.53974	0.13973	0.06751	0.75799

	141	142	143	144	145	146	147	148	149	150
a_O	423	456	437	432	464	450	422	458	466	412
	(36.8)	(39.7)	(38.1)	(37.6)	(40.6)	(39.3)	(36.9)	(40.1)	(40.8)	(36.0)
a_E	426.0	458.3	438.2	425.6	456.7	436.7	424.1	456.3	436.3	423.8
	(37.1)	(39.9)	(38.2)	(37.1)	(39.9)	(38.2)	(37.1)	(39.9)	(38.2)	(37.1)
	−5.9	−4.6	−2.5	12.9	14.7	27.0	−4.2	3.4	61.3	−23.2
	−0.516	−0.398	−0.215	1.122	1.288	2.358	−0.370	0.299	5.365	−2.027
t_O	497	276	388	476	270	388	483	262	360	484
	(43.3)	(24.0)	(33.8)	(41.5)	(23.6)	(33.9)	(42.2)	(22.9)	(31.5)	(42.3)
t_E	492.5	259.5	392.5	492.1	258.6	391.2	490.4	258.4	390.8	490.0
	(42.9)	(22.6)	(34.2)	(42.9)	(22.6)	(34.2)	(42.9)	(22.6)	(34.2)	(42.9)
	8.9	34.0	−9.0	−31.7	23.3	−6.3	−14.7	7.3	−59.2	−11.9
	0.779	2.962	−0.786	−2.761	2.035	−0.552	−1.284	0.638	−5.176	−1.039
g_O	110	311	141	127	270	142	124	302	138	137
	(9.6)	(27.1)	(12.3)	(11.1)	(23.6)	(12.4)	(10.8)	(26.4)	(12.1)	(12.0)
g_E	127.6	304.9	136.0	127.5	303.9	135.5	127.0	303.6	135.4	126.9
	(11.1)	(26.6)	(11.8)	(11.1)	(26.6)	(11.8)	(11.1)	(26.6)	(11.8)	(11.1)
	−32.6	12.3	10.3	−0.9	−63.8	13.3	−6.0	−3.2	5.3	21.0
	−2.837	1.069	0.893	−0.080	−5.577	1.165	−0.521	−0.279	0.464	1.835
c_O	119	105	182	113	140	164	115	121	179	110
	(10.4)	(9.1)	(15.9)	(9.8)	(12.2)	(14.3)	(10.1)	(10.6)	(15.7)	(9.6)
c_E	102.9	125.3	181.3	102.8	124.8	180.6	102.5	124.7	180.5	102.4
	(9.0)	(10.9)	(15.8)	(9.0)	(10.9)	(15.8)	(9.0)	(10.9)	(15.8)	(9.0)
	34.6	−37.1	1.5	21.3	32.1	−31.7	26.6	−7.3	−2.9	15.8
	3.009	−3.229	0.129	1.859	2.806	−2.769	2.321	−0.642	−0.256	1.383
N	1149	1148	1148	1148	1144	1144	1144	1143	1143	1143
X2	5.0	4.5	0.2	1.6	6.2	2.3	1.7	0.2	4.5	1.8
Gvalue	5.0	4.6	0.2	1.6	6.3	2.3	1.7	0.2	4.5	1.7
Gv/N	0.435	0.403	0.021	0.140	0.551	0.202	0.146	0.016	0.397	0.152
Prob	0.17216	0.20085	0.97029	0.65772	0.09769	0.50978	0.64348	0.98109	0.20922	0.62818

Bant_STERNE
5UTR:

	50	49	48	47	46	45	44	43	42	41
a_O	1864	1912	1932	1873	1932	1874	1919	1928	1887	1897
	(35.3)	(36.2)	(36.5)	(35.4)	(36.5)	(35.5)	(36.3)	(36.5)	(35.7)	(35.9)
a_E	1952.5	1952.5	1952.5	1952.5	1952.5	1952.5	1952.5	1952.5	1952.5	1952.5
	(36.9)	(36.9)	(36.9)	(36.9)	(36.9)	(36.9)	(36.9)	(36.9)	(36.9)	(36.9)
	-172.9	-80.1	-40.7	-155.7	-40.7	-153.7	-66.4	-48.6	-128.7	-109.3
	-3.270	-1.515	-0.770	-2.945	-0.770	-2.908	-1.255	-0.920	-2.435	-2.069
t_O	1738	1807	1854	1794	1794	1829	1811	1768	1790	1738
	(32.9)	(34.2)	(35.1)	(33.9)	(33.9)	(34.6)	(34.3)	(33.4)	(33.9)	(32.9)
t_E	1665.7	1665.7	1665.7	1665.7	1665.7	1665.7	1665.7	1665.7	1665.7	1665.7
	(31.5)	(31.5)	(31.5)	(31.5)	(31.5)	(31.5)	(31.5)	(31.5)	(31.5)	(31.5)
	147.7	294.2	397.1	266.2	266.2	342.1	302.9	210.7	257.6	147.7
	2.793	5.566	7.512	5.036	5.036	6.471	5.730	3.986	4.873	2.793
g_O	921	851	834	901	869	894	897	918	926	971
	(17.4)	(16.1)	(15.8)	(17.0)	(16.4)	(16.9)	(17.0)	(17.4)	(17.5)	(18.4)
g_E	1031.8	1031.8	1031.8	1031.8	1031.8	1031.8	1031.8	1031.8	1031.8	1031.8
	(19.5)	(19.5)	(19.5)	(19.5)	(19.5)	(19.5)	(19.5)	(19.5)	(19.5)	(19.5)
	-209.2	-327.8	-354.9	-244.2	-298.4	-256.2	-251.1	-214.5	-200.3	-117.9
	-3.957	-6.201	-6.714	-4.619	-5.644	-4.847	-4.750	-4.057	-3.789	-2.229
c_O	763	716	666	718	691	689	659	672	683	680
	(14.4)	(13.5)	(12.6)	(13.6)	(13.1)	(13.0)	(12.5)	(12.7)	(12.9)	(12.9)
c_E	636.1	636.1	636.1	636.1	636.1	636.1	636.1	636.1	636.1	636.1
	(12.0)	(12.0)	(12.0)	(12.0)	(12.0)	(12.0)	(12.0)	(12.0)	(12.0)	(12.0)
	277.7	169.5	61.3	174.0	114.5	110.2	46.7	73.9	97.3	90.8
	5.253	3.207	1.159	3.292	2.166	2.084	0.883	1.398	1.840	1.719
N	5286	5286	5286	5286	5286	5286	5286	5286	5286	5286
X2	44.4	54.5	60.8	40.2	40.5	42.0	31.7	21.2	25.8	11.3
Gvalue	43.3	55.8	62.7	40.4	41.6	42.3	32.1	21.5	25.9	11.3
Gv/N	0.819	1.057	1.186	0.764	0.787	0.799	0.608	0.406	0.490	0.214
Prob	0.00000	0.00000	0.00000	0.00000	0.00000	0.00000	0.00000	0.00008	0.00001	0.01021

	40	39	38	37	36	35	34	33	32	31
a_O	1967	2007	1978	2015	2008	2054	2084	2022	2050	1985
	(37.2)	(38.0)	(37.4)	(38.1)	(38.0)	(38.9)	(39.4)	(38.3)	(38.8)	(37.6)
a_E	1952.5	1952.5	1952.5	1952.5	1952.5	1952.5	1952.5	1952.5	1952.5	1952.5
	(36.9)	(36.9)	(36.9)	(36.9)	(36.9)	(36.9)	(36.9)	(36.9)	(36.9)	(36.9)
	29.2	110.6	51.4	127.0	112.6	208.3	271.7	141.5	199.9	65.6
	0.552	2.092	0.972	2.403	2.131	3.940	5.141	2.677	3.781	1.241
t_O	1759	1785	1756	1765	1771	1691	1759	1729	1637	1784
	(33.3)	(33.8)	(33.2)	(33.4)	(33.5)	(32.0)	(33.3)	(32.7)	(31.0)	(33.7)
t_E	1665.7	1665.7	1665.7	1665.7	1665.7	1665.7	1665.7	1665.7	1665.7	1665.7
	(31.5)	(31.5)	(31.5)	(31.5)	(31.5)	(31.5)	(31.5)	(31.5)	(31.5)	(31.5)
	191.7	246.9	185.4	204.4	217.1	50.9	191.7	128.9	-56.9	244.8
	3.626	4.671	3.507	3.866	4.107	0.964	3.626	2.439	-1.077	4.630
g_O	906	854	909	850	879	919	842	915	954	903
	(17.1)	(16.2)	(17.2)	(16.1)	(16.6)	(17.4)	(15.9)	(17.3)	(18.0)	(17.1)
g_E	1031.8	1031.8	1031.8	1031.8	1031.8	1031.8	1031.8	1031.8	1031.8	1031.8
	(19.5)	(19.5)	(19.5)	(19.5)	(19.5)	(19.5)	(19.5)	(19.5)	(19.5)	(19.5)
	-235.5	-322.9	-230.3	-329.4	-281.7	-212.7	-342.2	-219.8	-149.5	-240.7
	-4.455	-6.110	-4.356	-6.232	-5.329	-4.024	-6.475	-4.157	-2.828	-4.554
c_O	654	640	643	656	628	622	601	620	645	614
	(12.4)	(12.1)	(12.2)	(12.4)	(11.9)	(11.8)	(11.4)	(11.7)	(12.2)	(11.6)
c_E	636.1	636.1	636.1	636.1	636.1	636.1	636.1	636.1	636.1	636.1
	(12.0)	(12.0)	(12.0)	(12.0)	(12.0)	(12.0)	(12.0)	(12.0)	(12.0)	(12.0)
	36.4	7.9	14.0	40.5	-16.0	-27.8	-68.2	-31.7	18.0	-43.3
	0.688	0.150	0.264	0.766	-0.303	-0.526	-1.289	-0.600	0.341	-0.820
N	5286	5286	5286	5286	5286	5286	5286	5286	5286	5286
X2	21.2	40.7	19.9	40.6	31.0	18.3	50.9	18.5	11.4	25.8
Gvalue	21.7	42.4	20.4	42.5	32.0	18.7	53.0	19.0	11.4	26.3
Gv/N	0.411	0.803	0.387	0.804	0.605	0.353	1.003	0.359	0.216	0.498
Prob	0.00007	0.00000	0.00014	0.00000	0.00000	0.00032	0.00000	0.00028	0.00962	0.00001

	30	29	28	27	26	25	24	23	22	21
a_O	2036	2028	2073	2126	2112	2156	2072	2171	2148	2167
	(38.5)	(38.4)	(39.2)	(40.2)	(40.0)	(40.8)	(39.2)	(41.1)	(40.6)	(41.0)
a_E	1952.5	1952.5	1952.5	1952.5	1952.5	1952.5	1952.5	1952.5	1952.5	1952.5
	(36.9)	(36.9)	(36.9)	(36.9)	(36.9)	(36.9)	(36.9)	(36.9)	(36.9)	(36.9)
	170.6	153.9	248.4	362.0	331.8	427.6	246.2	460.7	410.0	451.8
	3.227	2.912	4.698	6.849	6.276	8.089	4.658	8.715	7.757	8.547
t_O	1760	1676	1731	1749	1684	1680	1809	1723	1761	1782
	(33.3)	(31.7)	(32.7)	(33.1)	(31.9)	(31.8)	(34.2)	(32.6)	(33.3)	(33.7)
t_E	1665.7	1665.7	1665.7	1665.7	1665.7	1665.7	1665.7	1665.7	1665.7	1665.7
	(31.5)	(31.5)	(31.5)	(31.5)	(31.5)	(31.5)	(31.5)	(31.5)	(31.5)	(31.5)
	193.8	20.6	133.1	170.7	36.8	28.7	298.5	116.5	195.9	240.5
	3.666	0.390	2.518	3.228	0.695	0.543	5.648	2.204	3.706	4.550
g_O	865	928	833	835	884	806	799	799	742	777
	(16.4)	(17.6)	(15.8)	(15.8)	(16.7)	(15.2)	(15.1)	(15.1)	(14.0)	(14.7)
g_E	1031.8	1031.8	1031.8	1031.8	1031.8	1031.8	1031.8	1031.8	1031.8	1031.8
	(19.5)	(19.5)	(19.5)	(19.5)	(19.5)	(19.5)	(19.5)	(19.5)	(19.5)	(19.5)
	-305.0	-196.7	-356.5	-353.3	-273.3	-398.0	-408.5	-408.5	-489.2	-440.7
	-5.769	-3.721	-6.744	-6.684	-5.169	-7.530	-7.729	-7.729	-9.255	-8.337
c_O	625	654	649	576	606	644	606	593	635	560
	(11.8)	(12.4)	(12.3)	(10.9)	(11.5)	(12.2)	(11.5)	(11.2)	(12.0)	(10.6)
c_E	636.1	636.1	636.1	636.1	636.1	636.1	636.1	636.1	636.1	636.1
	(12.0)	(12.0)	(12.0)	(12.0)	(12.0)	(12.0)	(12.0)	(12.0)	(12.0)	(12.0)
	-21.9	36.4	26.1	-114.3	-58.7	16.0	-58.7	-83.1	-2.1	-142.6
	-0.415	0.688	0.495	-2.162	-1.110	0.302	-1.110	-1.573	-0.040	-2.698
N	5286	5286	5286	5286	5286	5286	5286	5286	5286	5286
X2	36.1	13.9	48.5	62.8	35.8	70.8	73.6	81.8	106.4	103.7
Gvalue	37.5	14.3	51.1	65.1	36.6	74.2	77.6	85.5	114.6	109.0
Gv/N	0.709	0.270	0.967	1.232	0.692	1.403	1.467	1.617	2.168	2.062
Prob	0.00000	0.00258	0.00000	0.00000	0.00000	0.00000	0.00000	0.00000	0.00000	0.00000

	20	19	18	17	16	15	14	13	12	11
a_O	2216	2231	2207	2328	2434	2588	2459	2159	1612	1505
	(41.9)	(42.2)	(41.8)	(44.0)	(46.0)	(49.0)	(46.5)	(40.8)	(30.5)	(28.5)
a_E	1952.5	1952.5	1952.5	1952.5	1952.5	1952.5	1952.5	1952.5	1952.5	1952.5
	(36.9)	(36.9)	(36.9)	(36.9)	(36.9)	(36.9)	(36.9)	(36.9)	(36.9)	(36.9)
	561.1	595.0	540.9	819.1	1073.1	1458.5	1134.4	434.2	-617.8	-783.5
	10.615	11.257	10.232	15.495	20.301	27.593	21.460	8.214	-11.687	-14.822
t_O	1666	1653	1643	1428	1348	1159	867	707	609	510
	(31.5)	(31.3)	(31.1)	(27.0)	(25.5)	(21.9)	(16.4)	(13.4)	(11.5)	(9.6)

```
t_E      1665.7  1665.7  1665.7  1665.7  1665.7  1665.7  1665.7  1665.7  1665.7  1665.7
        ( 31.5) ( 31.5) ( 31.5) ( 31.5) ( 31.5) ( 31.5) ( 31.5) ( 31.5) ( 31.5) ( 31.5)
            0.6   -25.3   -45.1  -439.8  -570.6  -840.7 -1132.3 -1211.8 -1225.5 -1207.3
          0.011  -0.479  -0.854  -8.320 -10.794 -15.905 -21.420 -22.924 -23.185 -22.839
g_O         866     880     974    1081    1119    1245    1674    2203    2852    3085
        ( 16.4) ( 16.6) ( 18.4) ( 20.5) ( 21.2) ( 23.6) ( 31.7) ( 41.7) ( 54.0) ( 58.4)
g_E      1031.8  1031.8  1031.8  1031.8  1031.8  1031.8  1031.8  1031.8  1031.8  1031.8
        ( 19.5) ( 19.5) ( 19.5) ( 19.5) ( 19.5) ( 19.5) ( 19.5) ( 19.5) ( 19.5) ( 19.5)
         -303.3  -280.0  -112.2   100.8   181.7   467.8  1620.3  3342.2  5799.6  6758.0
         -5.738  -5.297  -2.123   1.907   3.437   8.850  30.653  63.228 109.717 127.847
c_O         538     522     462     449     385     294     286     217     213     186
        ( 10.2) (  9.9) (  8.7) (  8.5) (  7.3) (  5.6) (  5.4) (  4.1) (  4.0) (  3.5)
c_E       636.1   636.1   636.1   636.1   636.1   636.1   636.1   636.1   636.1   636.1
        ( 12.0) ( 12.0) ( 12.0) ( 12.0) ( 12.0) ( 12.0) ( 12.0) ( 12.0) ( 12.0) ( 12.0)
         -180.2  -206.3  -295.4  -312.7  -386.6  -453.8  -457.2  -466.7  -466.0  -457.4
         -3.408  -3.903  -5.589  -5.917  -7.313  -8.584  -8.649  -8.829  -8.817  -8.653

N          5286    5286    5286    5286    5286    5286    5286    5286    5286    5286
X2         77.3    82.6    84.4   163.5   285.8   589.0  1106.8  2179.4  4222.5  5309.0
Gvalue     78.2    83.4    88.1   167.4   297.6   631.9  1165.2  2097.9  3490.3  4309.8
Gv/N      1.480   1.577   1.667   3.166   5.631  11.953  22.044  39.688  66.028  81.532
Prob    0.00000 0.00000 0.00000 0.00000 0.00000 0.00000 0.00000 1.00000 1.00000 1.00000
        -------------------------------------------------------------------------------
             10       9       8       7       6       5       4       3       2       1
        -------------------------------------------------------------------------------
a_O        1620    1323    1543    1959    2153    2115    2018    2359    2311    1820
        ( 30.6) ( 25.0) ( 29.2) ( 37.1) ( 40.7) ( 40.0) ( 38.2) ( 44.6) ( 43.7) ( 34.4)
a_E      1952.5  1952.5  1952.5  1952.5  1952.5  1952.5  1952.5  1952.5  1952.5  1952.5
        ( 36.9) ( 36.9) ( 36.9) ( 36.9) ( 36.9) ( 36.9) ( 36.9) ( 36.9) ( 36.9) ( 36.9)
         -604.8 -1029.8  -726.3    13.1   421.0   338.2   133.2   892.4   779.2  -255.7
        -11.442 -19.482 -13.741   0.247   7.964   6.399   2.521  16.882  14.741  -4.838
t_O         662     874    1058    1386    1409    1633    1696    1263    1354    1931
        ( 12.5) ( 16.5) ( 20.0) ( 26.2) ( 26.7) ( 30.9) ( 32.1) ( 23.9) ( 25.6) ( 36.5)
t_E      1665.7  1665.7  1665.7  1665.7  1665.7  1665.7  1665.7  1665.7  1665.7  1665.7
        ( 31.5) ( 31.5) ( 31.5) ( 31.5) ( 31.5) ( 31.5) ( 31.5) ( 31.5) ( 31.5) ( 31.5)
        -1221.7 -1127.3  -960.4  -509.6  -471.7   -64.8    61.1  -699.1  -561.1   570.7
        -23.112 -21.327 -18.169  -9.640  -8.923  -1.226   1.156 -13.226 -10.614  10.797
g_O        2809    2847    2350    1548    1333    1016     943    1006     906     771
        ( 53.1) ( 53.9) ( 44.5) ( 29.3) ( 25.2) ( 19.2) ( 17.8) ( 19.0) ( 17.1) ( 14.6)
g_E      1031.8  1031.8  1031.8  1031.8  1031.8  1031.8  1031.8  1031.8  1031.8  1031.8
        ( 19.5) ( 19.5) ( 19.5) ( 19.5) ( 19.5) ( 19.5) ( 19.5) ( 19.5) ( 19.5) ( 19.5)
         5626.8  5779.5  3868.8  1256.1   683.0   -31.3  -169.6   -50.9  -235.5  -449.2
        106.448 109.335  73.190  23.762  12.920  -0.591  -3.209  -0.962  -4.455  -8.498
c_O         195     242     335     393     391     522     629     658     715     764
        (  3.7) (  4.6) (  6.3) (  7.4) (  7.4) (  9.9) ( 11.9) ( 12.4) ( 13.5) ( 14.5)
c_E       636.1   636.1   636.1   636.1   636.1   636.1   636.1   636.1   636.1   636.1
        ( 12.0) ( 12.0) ( 12.0) ( 12.0) ( 12.0) ( 12.0) ( 12.0) ( 12.0) ( 12.0) ( 12.0)
         -461.1  -467.7  -429.6  -378.4  -380.5  -206.3   -14.0    44.6   167.3   280.0
         -8.723  -8.848  -8.127  -7.159  -7.198  -3.903  -0.266   0.844   3.165   5.298

N          5286    5286    5286    5286    5286    5286    5286    5286    5286    5286
X2       4028.7  4017.1  2134.4   398.2   242.5    34.9    10.5   183.4   149.3   142.9
Gvalue   3339.2  3154.6  1752.5   381.1   251.8    35.9    10.7   187.0   149.9   145.8
Gv/N     63.171  59.678  33.154   7.210   4.763   0.678   0.202   3.538   2.836   2.758
Prob    1.00000 1.00000 1.00000 0.00000 0.00000 0.00000 0.01369 0.00000 0.00000 0.00000
        -------------------------------------------------------------------------------

CDS_INITIAL:
        -------------------------------------------------------------------------------
              1       2       3       4       5       6       7       8       9      10
        -------------------------------------------------------------------------------
a_O        4230       0       0    2796    2587    2271    2484    2373    2295    2170
        ( 80.0) (  0.0) (  0.0) ( 52.9) ( 48.9) ( 43.0) ( 47.0) ( 44.9) ( 43.4) ( 41.1)
a_E      1677.5  1782.1  2000.6  1677.5  1782.1  2000.6  1677.5  1782.1  2000.6  1677.5
        ( 31.7) ( 33.7) ( 37.8) ( 31.7) ( 33.7) ( 37.8) ( 31.7) ( 33.7) ( 37.8) ( 31.7)
         7824.6     0.0     0.0  2856.9  1928.4   575.8  1950.3  1359.1   630.1  1117.2
        148.026   0.000   0.000  54.046  36.481  10.893  36.895  25.711  11.920  21.135
t_O         521    5286       0     747    1084    1634     899    1442    1617    1181
        (  9.9) (100.0) (  0.0) ( 14.1) ( 20.5) ( 30.9) ( 17.0) ( 27.3) ( 30.6) ( 22.3)
t_E      1057.5  1715.0  1897.9  1057.5  1715.0  1897.9  1057.5  1715.0  1897.9  1057.5
        ( 20.0) ( 32.4) ( 35.9) ( 20.0) ( 32.4) ( 35.9) ( 20.0) ( 32.4) ( 35.9) ( 20.0)
         -737.7 11900.4     0.0  -519.3  -994.6  -489.3  -292.0  -500.0  -518.0   260.9
        -13.955 225.130   0.000  -9.825 -18.815  -9.257  -5.524  -9.459  -9.800   4.935
g_O         535       0    5286    1208     782     870    1157     729     887    1174
        ( 10.1) (  0.0) (100.0) ( 22.9) ( 14.8) ( 16.5) ( 21.9) ( 13.8) ( 16.8) ( 22.2)
g_E      1739.0   756.9   831.7  1739.0   756.9   831.7  1739.0   756.9   831.7  1739.0
        ( 32.9) ( 14.3) ( 15.7) ( 32.9) ( 14.3) ( 15.7) ( 32.9) ( 14.3) ( 15.7) ( 32.9)
        -1261.3     0.0 19551.7  -880.3    51.0    78.4  -942.9   -54.8   114.3  -922.5
        -23.862   0.000 369.878 -16.653   0.964   1.483 -17.838  -1.037   2.162 -17.453
c_O           0       0       0     535     833     511     746     742     487     761
        (  0.0) (  0.0) (  0.0) ( 10.1) ( 15.8) (  9.7) ( 14.1) ( 14.0) (  9.2) ( 14.4)
c_E       812.0  1032.0   555.8   812.0  1032.0   555.8   812.0  1032.0   555.8   812.0
        ( 15.4) ( 19.5) ( 10.5) ( 15.4) ( 19.5) ( 10.5) ( 15.4) ( 19.5) ( 10.5) ( 15.4)
            0.0     0.0     0.0  -446.4  -356.9   -85.9  -126.4  -489.5  -128.7   -98.7
          0.000   0.000   0.000  -8.445  -6.751  -1.625  -2.392  -9.261  -2.435  -1.867

N          5286    5286    5286    5286    5286    5286    5286    5286    5286    5286
X2       5801.7 11006.6 28311.4  1093.6   634.9    78.6   611.7   321.9    97.1   345.8
Gvalue   5825.7 11900.4 19551.7  1010.9   627.9    79.0   588.9   314.7    97.6   356.9
Gv/N    110.209 225.130 369.878  19.123  11.879   1.494  11.141   5.953   1.847   6.751
Prob    1.00000 1.00000 1.00000 0.00000 0.00000 0.00000 0.00000 0.00000 0.00000 0.00000
        -------------------------------------------------------------------------------
             11      12      13      14      15      16      17      18      19      20
        -------------------------------------------------------------------------------
a_O        1901    2319    2182    1952    2237    2017    1811    2193    1921    1765
        ( 36.0) ( 43.9) ( 41.3) ( 36.9) ( 42.3) ( 38.2) ( 34.3) ( 41.5) ( 36.3) ( 33.4)
a_E      1782.1  2000.6  1677.5  1782.1  2000.6  1677.5  1782.1  2000.6  1677.5  1782.1
        ( 33.7) ( 37.8) ( 31.7) ( 33.7) ( 37.8) ( 31.7) ( 33.7) ( 37.8) ( 31.7) ( 33.7)
          245.6   685.0  1147.5   355.5   499.7   743.5    58.3   402.7   520.8   -34.0
          4.646  12.958  21.708   6.726   9.453  14.065   1.102   7.618   9.852  -0.644
t_O        1955    1736    1136    1989    1775    1225    2009    1791    1152    2016
        ( 37.0) ( 32.8) ( 21.5) ( 37.6) ( 33.6) ( 23.2) ( 38.0) ( 33.9) ( 21.8) ( 38.1)
t_E      1715.0  1897.9  1057.5  1715.0  1897.9  1057.5  1715.0  1897.9  1057.5  1715.0
        ( 32.4) ( 35.9) ( 20.0) ( 32.4) ( 35.9) ( 20.0) ( 32.4) ( 35.9) ( 20.0) ( 32.4)
          512.1  -309.6   162.7   589.6  -237.7   360.2   635.8  -207.7   197.2   652.0
          9.688  -5.857   3.077  11.154  -4.497   6.814  12.027  -3.929   3.730  12.334
g_O         632     802    1182     592     829    1302     683     809    1418     693
        ( 12.0) ( 15.2) ( 22.4) ( 11.2) ( 15.7) ( 24.6) ( 12.9) ( 15.3) ( 26.8) ( 13.1)
g_E       756.9   831.7  1739.0   756.9   831.7  1739.0   756.9   831.7  1739.0   756.9
        ( 14.3) ( 15.7) ( 32.9) ( 14.3) ( 15.7) ( 32.9) ( 14.3) ( 15.7) ( 32.9) ( 14.3)
         -228.0   -58.3  -912.8  -291.0    -5.3  -753.7  -140.4   -44.7  -578.8  -122.3
         -4.313  -1.102 -17.268  -5.505  -0.101 -14.258  -2.656  -0.846 -10.949  -2.314
```

```
c_O         798     429     786     753     445     742     783     493     795     812
          ( 15.1) (  8.1) ( 14.9) ( 14.2) (  8.4) ( 14.0) ( 14.8) (  9.3) ( 15.0) ( 15.4)
c_E      1032.0   555.8   812.0  1032.0   555.8   812.0  1032.0   555.8   812.0  1032.0
          ( 19.5) ( 10.5) ( 15.4) ( 19.5) ( 10.5) ( 15.4) ( 19.5) ( 10.5) ( 15.4) ( 19.5)
         -410.4  -222.2   -51.1  -474.6  -197.9  -133.7  -432.4  -118.2   -33.6  -389.3
         -7.763  -4.203  -0.967  -8.979  -3.743  -2.530  -8.179  -2.237  -0.635  -7.365

N          5286    5286    5286    5286    5286    5286    5286    5286    5286    5286
X2        115.2    94.5   336.8   171.3    58.0   211.1   118.2    32.2   103.4   105.3
Gvalue    119.3    94.9   346.3   179.5    58.8   216.3   121.3    32.1   105.6   106.3
Gv/N      2.257   1.795   6.550   3.396   1.112   4.092   2.294   0.607   1.998   2.012
Prob    0.00000 0.00000 0.00000 0.00000 0.00000 0.00000 0.00000 0.00000 0.00000 0.00000
        -------------------------------------------------------------------------------
             21      22      23      24      25      26      27      28      29      30
        -------------------------------------------------------------------------------
a_O        2157    1831    1709    2189    1820    1760    2149    1765    1737    2074
          ( 40.8) ( 34.6) ( 32.3) ( 41.4) ( 34.4) ( 33.3) ( 40.7) ( 33.4) ( 32.9) ( 39.2)
a_E      2000.6  1677.5  1782.1  2000.6  1677.5  1782.1  2000.6  1677.5  1782.1  2000.6
          ( 37.8) ( 31.7) ( 33.7) ( 37.8) ( 31.7) ( 33.7) ( 37.8) ( 31.7) ( 33.7) ( 37.8)
          324.7   320.6  -143.2   394.0   296.8   -43.9   307.5   179.5   -89.0   149.4
          6.143   6.066  -2.708   7.453   5.614  -0.831   5.818   3.396  -1.684   2.827
t_O        1868    1188    1926    1747    1151    1893    1828    1095    1896    1856
          ( 35.3) ( 22.5) ( 36.4) ( 33.0) ( 21.8) ( 35.8) ( 34.6) ( 20.7) ( 35.9) ( 35.1)
t_E      1897.9  1057.5  1715.0  1897.9  1057.5  1715.0  1897.9  1057.5  1715.0  1897.9
          ( 35.9) ( 20.0) ( 32.4) ( 35.9) ( 20.0) ( 32.4) ( 35.9) ( 20.0) ( 32.4) ( 35.9)
          -59.4   276.5   447.0  -289.5   195.0   373.9  -137.2    76.3   380.5   -82.9
         -1.123   5.230   8.456  -5.477   3.689   7.073  -2.596   1.443   7.198  -1.569
g_O         749    1510     710     788    1580     760     772    1635     716     827
          ( 14.2) ( 28.6) ( 13.4) ( 14.9) ( 29.9) ( 14.4) ( 14.6) ( 30.9) ( 13.5) ( 15.6)
g_E       831.7  1739.0   756.9   831.7  1739.0   756.9   831.7  1739.0   756.9   831.7
          ( 15.7) ( 32.9) ( 14.3) ( 15.7) ( 32.9) ( 14.3) ( 15.7) ( 32.9) ( 14.3) ( 15.7)
         -156.8  -426.5   -90.9   -85.0  -303.0     6.1  -114.9  -201.7   -79.6    -9.3
         -2.967  -8.068  -1.720  -1.608  -5.733   0.116  -2.174  -3.816  -1.506  -0.176
c_O         512     757     941     562     735     873     537     791     937     529
          (  9.7) ( 14.3) ( 17.8) ( 10.6) ( 13.9) ( 16.5) ( 10.2) ( 15.0) ( 17.7) ( 10.0)
c_E       555.8   812.0  1032.0   555.8   812.0  1032.0   555.8   812.0  1032.0   555.8
          ( 10.5) ( 15.4) ( 19.5) ( 10.5) ( 15.4) ( 19.5) ( 10.5) ( 15.4) ( 19.5) ( 10.5)
          -84.1  -106.1  -173.7    12.5  -146.4  -292.1   -37.0   -41.4  -180.9   -52.3
         -1.590  -2.008  -3.286   0.236  -2.769  -5.526  -0.699  -0.783  -3.423  -0.989

N          5286    5286    5286    5286    5286    5286    5286    5286    5286    5286
X2         24.4    64.0    39.9    32.1    42.2    43.3    18.5    12.7    31.2     4.9
Gvalue     24.4    64.5    39.2    31.9    42.4    44.0    18.4    12.7    30.9     4.9
Gv/N      0.462   1.220   0.742   0.604   0.801   0.833   0.348   0.240   0.584   0.093
Prob    0.00002 0.00000 0.00000 0.00000 0.00000 0.00000 0.00037 0.00534 0.00000 0.17680
        -------------------------------------------------------------------------------
             31      32      33      34      35      36      37      38      39      40
        -------------------------------------------------------------------------------
a_O        1752    1668    2012    1733    1597    2024    1799    1576    2008    1713
          ( 33.1) ( 31.6) ( 38.1) ( 32.8) ( 30.2) ( 38.3) ( 34.0) ( 29.8) ( 38.0) ( 32.4)
a_E      1677.5  1782.1  2000.6  1677.5  1782.1  2000.6  1677.5  1782.1  2000.6  1677.5
          ( 31.7) ( 33.7) ( 37.8) ( 31.7) ( 33.7) ( 37.8) ( 31.7) ( 33.7) ( 37.8) ( 31.7)
          152.3  -220.7    22.8   112.8  -350.3    47.1   251.6  -387.4    14.8    71.7
          2.880  -4.176   0.432   2.134  -6.626   0.890   4.760  -7.328   0.280   1.357
t_O        1145    1907    1859    1178    1893    1824    1122    1990    1890    1170
          ( 21.7) ( 36.1) ( 35.2) ( 22.3) ( 35.8) ( 34.5) ( 21.2) ( 37.6) ( 35.8) ( 22.1)
t_E      1057.5  1715.0  1897.9  1057.5  1715.0  1897.9  1057.5  1715.0  1897.9  1057.5
          ( 20.0) ( 32.4) ( 35.9) ( 20.0) ( 32.4) ( 35.9) ( 20.0) ( 32.4) ( 35.9) ( 20.0)
          182.0   404.7   -77.0   254.2   373.9  -144.9   132.8   591.9   -15.8   236.5
          3.444   7.657  -1.458   4.809   7.073  -2.742   2.513  11.198  -0.299   4.475
g_O        1614     780     813    1620     765     867    1584     799     835    1601
          ( 30.5) ( 14.8) ( 15.4) ( 30.6) ( 14.5) ( 16.4) ( 30.0) ( 15.1) ( 15.8) ( 30.3)
g_E      1739.0   756.9   831.7  1739.0   756.9   831.7  1739.0   756.9   831.7  1739.0
          ( 32.9) ( 14.3) ( 15.7) ( 32.9) ( 14.3) ( 15.7) ( 32.9) ( 14.3) ( 15.7) ( 32.9)
         -240.8    46.8   -36.9  -229.7    16.2    72.2  -295.8    86.4     6.7  -264.8
         -4.556   0.886  -0.698  -4.346   0.307   1.365  -5.596   1.635   0.126  -5.009
c_O         775     931     602     755    1031     571     781     921     553     802
          ( 14.7) ( 17.6) ( 11.4) ( 14.3) ( 19.5) ( 10.8) ( 14.8) ( 17.4) ( 10.5) ( 15.2)
c_E       812.0  1032.0   555.8   812.0  1032.0   555.8   812.0  1032.0   555.8   812.0
          ( 15.4) ( 19.5) ( 10.5) ( 15.4) ( 19.5) ( 10.5) ( 15.4) ( 19.5) ( 10.5) ( 15.4)
          -72.2  -191.7    96.1  -109.8    -1.9    30.8   -60.7  -209.6    -5.6   -19.8
         -1.366  -3.627   1.819  -2.078  -0.037   0.583  -1.149  -3.964  -0.106  -0.375

N          5286    5286    5286    5286    5286    5286    5286    5286    5286    5286
X2         21.2    39.4     5.1    27.7    37.8     5.1    27.7    82.2     0.1    23.8
Gvalue     21.2    39.1     5.0    27.5    37.9     5.1    27.9    81.4     0.1    23.7
Gv/N      0.402   0.740   0.095   0.520   0.717   0.096   0.528   1.540   0.002   0.448
Prob    0.00009 0.00000 0.16972 0.00000 0.00000 0.16587 0.00000 0.00000 0.99325 0.00003
        -------------------------------------------------------------------------------
             41      42      43      44      45      46      47      48      49      50
        -------------------------------------------------------------------------------
a_O        1550    1988    1727    1580    2107    1639    1662    2016    1645    1496
          ( 29.3) ( 37.6) ( 32.7) ( 29.9) ( 39.9) ( 31.0) ( 31.4) ( 38.1) ( 31.1) ( 28.3)
a_E      1782.1  2000.6  1677.5  1782.1  2000.6  1677.5  1782.1  2000.6  1677.5  1782.1
          ( 33.7) ( 37.8) ( 31.7) ( 33.7) ( 37.8) ( 31.7) ( 33.7) ( 37.8) ( 31.7) ( 33.7)
         -432.6   -25.1   100.4  -380.4   218.3   -76.1  -231.9    30.9   -64.4  -523.6
         -8.183  -0.476   1.900  -7.196   4.131  -1.440  -4.387   0.585  -1.218  -9.905
t_O        1940    1955    1161    1952    1817    1190    1810    1874    1178    1963
          ( 36.7) ( 37.0) ( 22.0) ( 36.9) ( 34.4) ( 22.5) ( 34.2) ( 35.5) ( 22.3) ( 37.1)
t_E      1715.0  1897.9  1057.5  1715.0  1897.9  1057.5  1715.0  1897.9  1057.5  1715.0
          ( 32.4) ( 35.9) ( 20.0) ( 32.4) ( 35.9) ( 20.0) ( 32.4) ( 35.9) ( 20.0) ( 32.4)
          478.3   115.9   216.8   505.3  -158.3   280.9   195.2   -47.5   254.2   530.3
          9.049   2.192   4.101   9.560  -2.996   5.315   3.692  -0.899   4.809  10.031
g_O         834     847    1672     754     841    1628     836     823    1712     788
          ( 15.8) ( 16.0) ( 31.6) ( 14.3) ( 15.9) ( 30.8) ( 15.8) ( 15.6) ( 32.4) ( 14.9)
g_E       756.9   831.7  1739.0   756.9   831.7  1739.0   756.9   831.7  1739.0   756.9
          ( 14.3) ( 15.7) ( 32.9) ( 14.3) ( 15.7) ( 32.9) ( 14.3) ( 15.7) ( 32.9) ( 14.3)
          161.7    31.0  -131.4    -5.9    18.8  -214.8   166.1   -17.2   -53.6    63.4
          3.060   0.586  -2.486  -0.111   0.355  -4.064   3.143  -0.326  -1.015   1.199
c_O         962     496     726    1000     521     829     978     573     751    1039
          ( 18.2) (  9.4) ( 13.7) ( 18.9) (  9.9) ( 15.7) ( 18.5) ( 10.8) ( 14.2) ( 19.7)
c_E      1032.0   555.8   812.0  1032.0   555.8   812.0  1032.0   555.8   812.0  1032.0
          ( 19.5) ( 10.5) ( 15.4) ( 19.5) ( 10.5) ( 15.4) ( 19.5) ( 10.5) ( 15.4) ( 19.5)
         -135.1  -112.9  -162.5   -62.9   -67.4    34.4  -105.1    34.9  -117.2    14.1
         -2.556  -2.136  -3.074  -1.191  -1.275   0.651  -1.988   0.661  -2.218   0.267

N          5286    5286    5286    5286    5286    5286    5286    5286    5286    5286
X2         72.3     8.5    23.3    56.7    11.4    24.9    24.4     1.0    19.4    83.1
Gvalue     72.4     8.7    23.3    56.2    11.4    24.4    24.3     1.0    19.0    84.2
Gv/N      1.369   0.165   0.441   1.063   0.215   0.462   0.460   0.020   0.359   1.592
Prob    0.00000 0.03298 0.00003 0.00000 0.00979 0.00002 0.00002 0.79197 0.00028 0.00000
        -------------------------------------------------------------------------------
```

	51	52	53	54	55	56	57	58	59	60
a_O	1997	1715	1523	1970	1652	1607	2008	1755	1654	1963
	(37.8)	(32.4)	(28.8)	(37.3)	(31.3)	(30.4)	(38.0)	(33.2)	(31.3)	(37.1)
a_E	2000.6	1677.5	1782.1	2000.6	1677.5	1782.1	2000.6	1677.5	1782.1	2000.6
	(37.8)	(31.7)	(33.7)	(37.8)	(31.7)	(33.7)	(37.8)	(31.7)	(33.7)	(37.8)
	-7.2	75.8	-478.6	-60.8	-50.6	-332.4	14.8	158.5	-246.8	-74.5
	-0.136	1.435	-9.053	-1.149	-0.957	-6.288	0.280	2.999	-4.668	-1.410
t_O	1868	1242	1984	1887	1187	1907	1939	1146	1859	1939
	(35.3)	(23.5)	(37.5)	(35.7)	(22.5)	(36.1)	(36.7)	(21.7)	(35.2)	(36.7)
t_E	1897.9	1057.5	1715.0	1897.9	1057.5	1715.0	1897.9	1057.5	1715.0	1897.9
	(35.9)	(20.0)	(32.4)	(35.9)	(20.0)	(32.4)	(35.9)	(20.0)	(32.4)	(35.9)
	-59.4	399.4	578.2	-21.8	274.2	404.7	83.0	184.2	299.8	83.0
	-1.123	7.557	10.937	-0.412	5.188	7.657	1.571	3.484	5.671	1.571
g_O	850	1560	793	857	1631	763	802	1651	805	805
	(16.1)	(29.5)	(15.0)	(16.2)	(30.9)	(14.4)	(15.2)	(31.2)	(15.2)	(15.2)
g_E	831.7	1739.0	756.9	831.7	1739.0	756.9	831.7	1739.0	756.9	831.7
	(15.7)	(32.9)	(14.3)	(15.7)	(32.9)	(14.3)	(15.7)	(32.9)	(14.3)	(15.7)
	37.1	-339.0	73.8	51.4	-209.2	12.2	-58.3	-171.5	99.1	-52.5
	0.701	-6.412	1.397	0.973	-3.958	0.230	-1.102	-3.245	1.875	-0.993
c_O	571	769	986	572	816	1009	537	734	968	579
	(10.8)	(14.5)	(18.7)	(10.8)	(15.4)	(19.1)	(10.2)	(13.9)	(18.3)	(11.0)
c_E	555.8	812.0	1032.0	555.8	812.0	1032.0	555.8	812.0	1032.0	555.8
	(10.5)	(15.4)	(19.5)	(10.5)	(15.4)	(19.5)	(10.5)	(15.4)	(19.5)	(10.5)
	30.8	-83.6	-89.9	32.9	8.1	-45.4	-37.0	-148.2	-123.9	47.4
	0.583	-1.582	-1.700	0.622	0.153	-0.860	-0.699	-2.804	-2.344	0.896
N	5286	5286	5286	5286	5286	5286	5286	5286	5286	5286
X2	1.3	53.7	83.6	1.8	23.0	39.3	2.6	22.9	28.3	3.4
Gvalue	1.3	52.7	83.6	1.8	22.5	39.1	2.6	23.0	28.2	3.4
Gv/N	0.024	0.997	1.581	0.033	0.426	0.740	0.050	0.435	0.534	0.065
Prob	0.73054	0.00000	0.00000	0.62247	0.00005	0.00000	0.45329	0.00004	0.00000	0.33215

	61	62	63	64	65	66	67	68	69	70
a_O	1551	1593	1994	1602	1669	1887	1684	1757	2018	1674
	(29.3)	(30.1)	(37.7)	(30.3)	(31.6)	(35.7)	(31.9)	(33.2)	(38.2)	(31.7)
a_E	1677.5	1782.1	2000.6	1677.5	1782.1	2000.6	1677.5	1782.1	2000.6	1677.5
	(31.7)	(33.7)	(37.8)	(31.7)	(33.7)	(37.8)	(31.7)	(33.7)	(37.8)	(31.7)
	-243.2	-357.4	-13.2	-147.5	-218.9	-220.6	13.0	-49.8	34.9	-7.0
	-4.601	-6.761	-0.250	-2.791	-4.140	-4.174	0.246	-0.943	0.661	-0.132
t_O	1261	1840	1948	1234	1864	2003	1143	1755	1896	1117
	(23.9)	(34.8)	(36.9)	(23.3)	(35.3)	(37.9)	(21.6)	(33.2)	(35.9)	(21.1)
t_E	1057.5	1715.0	1897.9	1057.5	1715.0	1897.9	1057.5	1715.0	1897.9	1057.5
	(20.0)	(32.4)	(35.9)	(20.0)	(32.4)	(35.9)	(20.0)	(32.4)	(35.9)	(20.0)
	443.8	258.9	101.5	380.9	310.6	215.9	177.7	80.9	-3.8	122.3
	8.397	4.898	1.919	7.206	5.876	4.084	3.362	1.531	-0.073	2.313
g_O	1720	828	820	1685	781	849	1667	778	850	1697
	(32.5)	(15.7)	(15.5)	(31.9)	(14.8)	(16.1)	(31.5)	(14.7)	(16.1)	(32.1)
g_E	1739.0	756.9	831.7	1739.0	756.9	831.7	1739.0	756.9	831.7	1739.0
	(32.9)	(14.3)	(15.7)	(32.9)	(14.3)	(15.7)	(32.9)	(14.3)	(15.7)	(32.9)
	-37.8	148.6	-23.2	-106.4	48.9	35.0	-141.0	42.7	37.1	-83.0
	-0.716	2.811	-0.438	-2.012	0.925	0.663	-2.668	0.808	0.701	-1.571
c_O	754	1025	524	765	972	547	792	996	522	798
	(14.3)	(19.4)	(9.9)	(14.5)	(18.4)	(10.3)	(15.0)	(18.8)	(9.9)	(15.1)
c_E	812.0	1032.0	555.8	812.0	1032.0	555.8	812.0	1032.0	555.8	812.0
	(15.4)	(19.5)	(10.5)	(15.4)	(19.5)	(10.5)	(15.4)	(19.5)	(10.5)	(15.4)
	-111.7	-13.9	-61.7	-91.2	-116.4	-17.5	-39.4	-70.7	-65.5	-27.7
	-2.113	-0.263	-1.168	-1.725	-2.202	-0.330	-0.746	-1.337	-1.239	-0.524
N	5286	5286	5286	5286	5286	5286	5286	5286	5286	5286
X2	53.0	35.9	3.3	37.2	24.4	12.8	10.4	3.1	2.6	4.6
Gvalue	51.1	36.2	3.4	35.9	24.2	12.8	10.3	3.1	2.7	4.6
Gv/N	0.967	0.685	0.063	0.678	0.458	0.242	0.194	0.059	0.050	0.086
Prob	0.00000	0.00000	0.34058	0.00000	0.00002	0.00512	0.01635	0.37188	0.44836	0.20713

	71	72	73	74	75	76	77	78	79	80
a_O	1712	2005	1662	1770	2036	1740	1824	1996	1604	1817
	(32.4)	(37.9)	(31.4)	(33.5)	(38.5)	(32.9)	(34.5)	(37.8)	(30.3)	(34.4)
a_E	1782.1	2000.6	1677.5	1782.1	2000.6	1677.5	1782.1	2000.6	1677.5	1782.1
	(33.7)	(37.8)	(31.7)	(33.7)	(37.8)	(31.7)	(33.7)	(37.8)	(31.7)	(33.7)
	-137.4	8.8	-30.9	-24.1	71.4	127.3	84.8	-9.2	-143.7	70.5
	-2.599	0.166	-0.584	-0.456	1.351	2.408	1.604	-0.174	-2.719	1.333
t_O	1716	1894	1141	1797	1875	1103	1759	1889	1116	1706
	(32.5)	(35.8)	(21.6)	(34.0)	(35.5)	(20.9)	(33.3)	(35.7)	(21.1)	(32.3)
t_E	1715.0	1897.9	1057.5	1715.0	1897.9	1057.5	1715.0	1897.9	1057.5	1715.0
	(32.4)	(35.9)	(20.0)	(32.4)	(35.9)	(20.0)	(32.4)	(35.9)	(20.0)	(32.4)
	2.0	-7.8	173.4	167.9	-45.6	92.9	89.1	-17.8	120.2	-17.9
	0.038	-0.148	3.281	3.176	-0.862	1.758	1.686	-0.337	2.273	-0.339
g_O	788	835	1691	724	863	1699	694	828	1762	764
	(14.9)	(15.8)	(32.0)	(13.7)	(16.3)	(32.1)	(13.1)	(15.7)	(33.3)	(14.5)
g_E	756.9	831.7	1739.0	756.9	831.7	1739.0	756.9	831.7	1739.0	756.9
	(14.3)	(15.7)	(32.9)	(14.3)	(15.7)	(32.9)	(14.3)	(15.7)	(32.9)	(14.3)
	63.4	6.7	-94.7	-64.4	63.8	-79.1	-120.5	-7.3	46.3	14.2
	1.199	0.126	-1.792	-1.219	1.208	-1.497	-2.279	-0.138	0.875	0.269
c_O	1070	552	792	995	512	744	1009	573	804	999
	(20.2)	(10.4)	(15.0)	(18.8)	(9.7)	(14.1)	(19.1)	(10.8)	(15.2)	(18.9)
c_E	1032.0	555.8	812.0	1032.0	555.8	812.0	1032.0	555.8	812.0	1032.0
	(19.5)	(10.5)	(15.4)	(19.5)	(10.5)	(15.4)	(19.5)	(10.5)	(15.4)	(19.5)
	77.4	-7.6	-39.4	-72.6	-84.1	-130.1	-45.4	34.9	-15.9	-64.9
	1.465	-0.143	-0.746	-1.374	-1.590	-2.461	-0.860	0.661	-0.300	-1.227
N	5286	5286	5286	5286	5286	5286	5286	5286	5286	5286
X2	5.4	0.1	8.6	6.8	5.5	10.9	7.9	0.6	6.8	1.9
Gvalue	5.4	0.1	8.4	6.7	5.6	11.0	8.0	0.6	6.8	1.9
Gv/N	0.103	0.001	0.159	0.127	0.106	0.208	0.151	0.011	0.129	0.035
Prob	0.14247	0.99643	0.03839	0.08076	0.13205	0.01166	0.04609	0.89742	0.07765	0.60255

	81	82	83	84	85	86	87	88	89	90
a_O	2007	1668	1810	2034	1734	1781	2004	1629	1748	1996
	(38.0)	(31.6)	(34.2)	(38.5)	(32.8)	(33.7)	(37.9)	(30.8)	(33.1)	(37.8)
a_E	2000.6	1677.5	1782.1	2000.6	1677.5	1782.1	2000.6	1677.5	1782.1	2000.6
	(37.8)	(31.7)	(33.7)	(37.8)	(31.7)	(33.7)	(37.8)	(31.7)	(33.7)	(37.8)
	12.8	-18.9	56.2	67.3	114.9	-2.2	6.8	-95.6	-67.5	-9.2
	0.242	-0.358	1.064	1.274	2.173	-0.041	0.128	-1.808	-1.278	-0.174
t_O	1914	1123	1678	1886	1089	1804	1901	1041	1756	1925
	(36.2)	(21.2)	(31.7)	(35.7)	(20.6)	(34.1)	(36.0)	(19.7)	(33.2)	(36.4)
t_E	1897.9	1057.5	1715.0	1897.9	1057.5	1715.0	1897.9	1057.5	1715.0	1897.9
	(35.9)	(20.0)	(32.4)	(35.9)	(20.0)	(32.4)	(35.9)	(20.0)	(32.4)	(35.9)
	32.3	135.0	-73.2	-23.8	63.9	182.5	6.2	-32.8	83.0	54.5

	0.611	2.553	-1.385	-0.450	1.209	3.453	0.116	-0.620	1.570	1.032
g_O	798	1697	771	805	1631	720	838	1793	768	817
	(15.1)	(32.1)	(14.6)	(15.2)	(30.9)	(13.6)	(15.9)	(33.9)	(14.5)	(15.5)
g_E	831.7	1739.0	756.9	831.7	1739.0	756.9	831.7	1739.0	756.9	831.7
	(15.7)	(32.9)	(14.3)	(15.7)	(32.9)	(14.3)	(15.7)	(32.9)	(14.3)	(15.7)
	-65.9	-83.0	28.4	-52.5	-209.2	-72.0	12.7	109.6	22.3	-29.1
	-1.248	-1.571	0.537	-0.993	-3.958	-1.363	0.241	2.074	0.422	-0.550
c_O	567	798	1027	561	832	981	543	823	1014	548
	(10.7)	(15.1)	(19.4)	(10.6)	(15.7)	(18.6)	(10.3)	(15.6)	(19.2)	(10.4)
c_E	555.8	812.0	1032.0	555.8	812.0	1032.0	555.8	812.0	1032.0	555.8
	(10.5)	(15.4)	(19.5)	(10.5)	(15.4)	(19.5)	(10.5)	(15.4)	(19.5)	(10.5)
	22.6	-27.7	-9.9	10.4	40.6	-99.4	-25.3	22.2	-35.6	-15.5
	0.428	-0.524	-0.188	0.198	0.767	-1.880	-0.479	0.420	-0.674	-0.293
N	5286	5286	5286	5286	5286	5286	5286	5286	5286	5286
X2	1.7	5.4	1.5	1.5	10.0	8.9	0.4	3.5	2.1	0.8
Gvalue	1.8	5.3	1.5	1.5	10.2	8.9	0.4	3.5	2.1	0.8
Gv/N	0.033	0.100	0.029	0.029	0.192	0.169	0.007	0.066	0.040	0.014
Prob	0.62329	0.15149	0.67712	0.67263	0.01730	0.03018	0.94918	0.32306	0.55088	0.85778

	91	92	93	94	95	96	97	98	99	100
a_O	1658	1770	2098	1704	1813	2019	1649	1779	1999	1706
	(31.4)	(33.5)	(39.7)	(32.2)	(34.3)	(38.2)	(31.2)	(33.7)	(37.8)	(32.3)
a_E	1677.5	1782.1	2000.6	1677.5	1782.1	2000.6	1677.5	1782.1	2000.6	1677.5
	(31.7)	(33.7)	(37.8)	(31.7)	(33.7)	(37.8)	(31.7)	(33.7)	(37.8)	(31.7)
	-38.8	-24.1	199.4	53.4	62.3	36.9	-56.5	-6.2	-3.2	57.5
	-0.733	-0.456	3.773	1.011	1.179	0.699	-1.069	-0.117	-0.061	1.087
t_O	1056	1732	1817	1064	1629	1899	1057	1738	1897	1046
	(20.0)	(32.8)	(34.4)	(20.1)	(30.8)	(35.9)	(20.0)	(32.9)	(35.9)	(19.8)
t_E	1057.5	1715.0	1897.9	1057.5	1715.0	1897.9	1057.5	1715.0	1897.9	1057.5
	(20.0)	(32.4)	(35.9)	(20.0)	(32.4)	(35.9)	(20.0)	(32.4)	(35.9)	(20.0)
	-3.0	34.2	-158.3	13.0	-167.6	2.2	-1.0	46.3	-1.8	-22.9
	-0.057	0.647	-2.996	0.246	-3.171	0.041	-0.019	0.876	-0.035	-0.433
g_O	1767	772	805	1728	766	848	1771	783	851	1761
	(33.4)	(14.6)	(15.2)	(32.7)	(14.5)	(16.0)	(33.5)	(14.8)	(16.1)	(33.3)
g_E	1739.0	756.9	831.7	1739.0	756.9	831.7	1739.0	756.9	831.7	1739.0
	(32.9)	(14.3)	(15.7)	(32.9)	(14.3)	(15.7)	(32.9)	(14.3)	(15.7)	(32.9)
	56.4	30.4	-52.5	-22.0	18.2	33.0	64.5	53.0	39.1	44.2
	1.067	0.576	-0.993	-0.416	0.345	0.624	1.221	1.003	0.740	0.837
c_O	805	1012	566	790	1078	520	809	986	539	773
	(15.2)	(19.1)	(10.7)	(14.9)	(20.4)	(9.8)	(15.3)	(18.7)	(10.2)	(14.6)
c_E	812.0	1032.0	555.8	812.0	1032.0	555.8	812.0	1032.0	555.8	812.0
	(15.4)	(19.5)	(10.5)	(15.4)	(19.5)	(10.5)	(15.4)	(19.5)	(10.5)	(15.4)
	-13.9	-39.6	20.6	-43.3	94.1	-69.2	-5.9	-89.9	-33.1	-76.0
	-0.262	-0.748	0.389	-0.820	1.780	-1.310	-0.112	-1.700	-0.626	-1.438
N	5286	5286	5286	5286	5286	5286	5286	5286	5286	5286
X2	0.7	0.9	9.2	1.1	7.0	2.8	1.1	3.3	1.0	2.8
Gvalue	0.7	0.9	9.2	1.1	7.0	2.8	1.1	3.3	1.0	2.8
Gv/N	0.014	0.018	0.174	0.021	0.133	0.054	0.020	0.062	0.018	0.053
Prob	0.86441	0.81639	0.02655	0.77080	0.07032	0.41613	0.78134	0.35052	0.81072	0.42608

	101	102	103	104	105	106	107	108	109	110
a_O	1774	1956	1640	1823	1994	1665	1781	1952	1582	1812
	(33.6)	(37.0)	(31.0)	(34.5)	(37.7)	(31.5)	(33.7)	(36.9)	(29.9)	(34.3)
a_E	1782.1	2000.6	1677.5	1782.1	2000.6	1677.5	1782.1	2000.6	1677.2	1781.8
	(33.7)	(37.8)	(31.7)	(33.7)	(37.8)	(31.7)	(33.7)	(37.8)	(31.7)	(33.7)
	-16.2	-88.2	-74.2	82.7	-13.2	-24.9	-2.2	-96.0	-184.9	61.0
	-0.306	-1.669	-1.403	1.565	-0.250	-0.471	-0.041	-1.817	-3.498	1.154
t_O	1667	1933	1101	1660	1912	1093	1721	1928	1075	1696
	(31.5)	(36.6)	(20.8)	(31.4)	(36.2)	(20.7)	(32.6)	(36.5)	(20.3)	(32.1)
t_E	1715.0	1897.9	1057.5	1715.0	1897.9	1057.5	1715.0	1897.9	1057.3	1714.7
	(32.4)	(35.9)	(20.0)	(32.4)	(35.9)	(20.0)	(32.4)	(35.9)	(20.0)	(32.4)
	-94.6	70.8	88.7	-108.2	28.3	72.2	12.0	60.6	35.7	-37.1
	-1.790	1.339	1.679	-2.047	0.535	1.365	0.228	1.147	0.675	-0.703
g_O	784	812	1728	742	864	1716	761	836	1831	761
	(14.8)	(15.4)	(32.7)	(14.0)	(16.3)	(32.5)	(14.4)	(15.8)	(34.6)	(14.4)
g_E	756.9	831.7	1739.0	756.9	831.7	1739.0	756.9	831.7	1738.7	756.8
	(14.3)	(15.7)	(32.9)	(14.3)	(15.7)	(32.9)	(14.3)	(15.7)	(32.9)	(14.3)
	55.1	-38.9	-22.0	-29.6	65.9	-45.7	8.2	8.7	189.4	8.4
	1.042	-0.735	-0.416	-0.559	1.247	-0.865	0.154	0.164	3.584	0.160
c_O	1061	585	817	1061	516	812	1023	570	797	1016
	(20.1)	(11.1)	(15.5)	(20.1)	(9.8)	(15.4)	(19.4)	(10.8)	(15.1)	(19.2)
c_E	1032.0	555.8	812.0	1032.0	555.8	812.0	1032.0	555.8	811.8	1031.8
	(19.5)	(10.5)	(15.4)	(19.5)	(10.5)	(15.4)	(19.5)	(10.5)	(15.4)	(19.5)
	58.9	59.9	10.1	58.9	-76.7	0.1	-17.9	28.8	-29.4	-31.3
	1.113	1.133	0.191	1.113	-1.451	0.001	-0.338	0.544	-0.555	-0.593
N	5286	5286	5286	5286	5286	5286	5286	5286	5285	5285
X2	3.2	3.6	2.7	3.8	4.2	1.6	0.1	2.0	10.9	1.0
Gvalue	3.2	3.6	2.7	3.8	4.3	1.6	0.1	2.0	10.9	1.0
Gv/N	0.060	0.069	0.051	0.072	0.081	0.030	0.002	0.039	0.206	0.019
Prob	0.36789	0.30515	0.43847	0.28158	0.23201	0.66448	0.98910	0.56267	0.01234	0.80600

	111	112	113	114	115	116	117	118	119	120
a_O	1976	1676	1821	1962	1597	1829	2017	1656	1809	1970
	(37.4)	(31.7)	(34.5)	(37.1)	(30.2)	(34.6)	(38.2)	(31.3)	(34.2)	(37.3)
a_E	2000.2	1676.9	1781.4	1999.9	1676.5	1781.1	1999.5	1676.5	1781.1	1999.5
	(37.8)	(31.7)	(33.7)	(37.8)	(31.7)	(33.7)	(37.8)	(31.7)	(33.7)	(37.8)
	-48.2	-1.7	80.0	-75.0	-155.3	97.1	35.2	-40.8	56.3	-58.5
	-0.911	-0.033	1.515	-1.419	-2.939	1.838	0.666	-0.773	1.065	-1.108
t_O	1933	1077	1644	1934	1055	1644	1891	1116	1682	1940
	(36.6)	(20.4)	(31.1)	(36.6)	(20.0)	(31.1)	(35.8)	(21.1)	(31.8)	(36.7)
t_E	1897.6	1057.1	1714.3	1897.2	1056.9	1714.0	1896.8	1056.9	1714.0	1896.8
	(35.9)	(20.0)	(32.4)	(35.9)	(20.0)	(32.4)	(35.9)	(20.0)	(32.4)	(35.9)
	71.5	40.2	-137.8	74.3	-3.8	-137.1	-11.7	121.4	-63.4	87.3
	1.353	0.760	-2.607	1.406	-0.072	-2.596	-0.221	2.298	-1.201	1.652
g_O	813	1755	768	827	1790	764	818	1718	757	820
	(15.4)	(33.2)	(14.5)	(15.7)	(33.9)	(14.5)	(15.5)	(32.5)	(14.3)	(15.5)
g_E	831.5	1738.4	756.6	831.3	1738.0	756.5	831.2	1738.0	756.5	831.2
	(15.7)	(32.9)	(14.3)	(15.7)	(32.9)	(14.3)	(15.7)	(32.9)	(14.3)	(15.7)
	-36.6	33.4	22.9	-8.7	105.5	15.1	-26.2	-39.8	1.0	-22.2
	-0.693	0.633	0.433	-0.164	1.996	0.285	-0.495	-0.754	0.019	-0.421
c_O	563	776	1051	561	841	1046	557	793	1035	553
	(10.7)	(14.7)	(19.9)	(10.6)	(15.9)	(19.8)	(10.5)	(15.0)	(19.6)	(10.5)
c_E	555.7	811.7	1031.6	555.6	811.5	1031.4	555.5	811.5	1031.4	555.5
	(10.5)	(15.4)	(19.5)	(10.5)	(15.4)	(19.5)	(10.5)	(15.4)	(19.5)	(10.5)
	14.7	-69.7	39.2	10.9	60.0	29.4	3.0	-36.6	7.2	-5.0
	0.278	-1.320	0.742	0.206	1.137	0.557	0.057	-0.693	0.137	-0.094

N	5285	5284	5284	5284	5283	5283	5283	5283	5283	5283
X2	1.5	2.1	4.3	1.5	6.4	4.4	0.4	4.2	1.0	1.6
Gvalue	1.5	2.1	4.3	1.5	6.4	4.5	0.4	4.2	1.1	1.6
Gv/N	0.028	0.040	0.082	0.028	0.122	0.084	0.007	0.079	0.020	0.030
Prob	0.69084	0.54765	0.22773	0.68103	0.09221	0.21606	0.94316	0.24532	0.78910	0.66541

	121	122	123	124	125	126	127	128	129	130
a_O	1633	1757	1977	1594	1766	2000	1728	1809	2010	1609
	(30.9)	(33.3)	(37.4)	(30.2)	(33.4)	(37.9)	(32.7)	(34.2)	(38.1)	(30.5)
a_E	1676.5	1781.1	1999.5	1676.2	1780.7	1999.1	1676.2	1780.7	1999.1	1676.2
	(31.7)	(33.7)	(37.8)	(31.7)	(33.7)	(37.8)	(31.7)	(33.7)	(37.8)	(31.7)
	-86.0	-47.8	-44.7	-160.4	-29.4	1.8	105.1	57.0	21.9	-131.7
	-1.627	-0.906	-0.846	-3.036	-0.556	0.034	1.990	1.078	0.414	-2.494
t_O	1109	1679	1911	1118	1668	1911	1071	1645	1899	1097
	(21.0)	(31.8)	(36.2)	(21.2)	(31.6)	(36.2)	(20.3)	(31.1)	(36.0)	(20.8)
t_E	1056.9	1714.0	1896.8	1056.7	1713.7	1896.5	1056.7	1713.7	1896.5	1056.7
	(20.0)	(32.4)	(35.9)	(20.0)	(32.4)	(35.9)	(20.0)	(32.4)	(35.9)	(20.0)
	106.7	-69.3	28.4	126.1	-90.2	29.1	28.8	-134.6	5.0	82.1
	2.020	-1.312	0.538	2.387	-1.707	0.552	0.545	-2.548	0.095	1.554
g_O	1751	823	818	1742	767	865	1701	805	815	1803
	(33.1)	(15.6)	(15.5)	(33.0)	(14.5)	(16.4)	(32.2)	(15.2)	(15.4)	(34.1)
g_E	1738.0	756.5	831.2	1737.7	756.4	831.0	1737.7	756.4	831.0	1737.7
	(32.9)	(14.3)	(15.7)	(32.9)	(14.3)	(15.7)	(32.9)	(14.3)	(15.7)	(32.9)
	26.0	138.7	-26.2	8.6	21.4	69.3	-72.6	100.3	-31.8	133.0
	0.492	2.625	-0.495	0.163	0.406	1.312	-1.375	1.900	-0.601	2.518
c_O	790	1024	577	828	1081	506	782	1023	558	773
	(15.0)	(19.4)	(10.9)	(15.7)	(20.5)	(9.6)	(14.8)	(19.4)	(10.6)	(14.6)
c_E	811.5	1031.4	555.5	811.4	1031.2	555.4	811.4	1031.2	555.4	811.4
	(15.4)	(19.5)	(10.5)	(15.4)	(19.5)	(10.5)	(15.4)	(19.5)	(10.5)	(15.4)
	-42.4	-14.7	43.9	33.6	102.0	-94.2	-57.6	-16.3	5.3	-74.9
	-0.803	-0.279	0.830	0.637	1.931	-1.784	-1.091	-0.309	0.099	-1.417
N	5283	5283	5283	5282	5282	5282	5282	5282	5282	5282
X2	4.4	6.9	1.4	7.9	3.9	5.9	3.6	6.4	0.4	8.5
Gvalue	4.3	6.8	1.4	7.9	3.9	6.0	3.6	6.4	0.4	8.5
Gv/N	0.082	0.128	0.026	0.150	0.073	0.114	0.069	0.121	0.007	0.161
Prob	0.22714	0.07919	0.70737	0.04728	0.27598	0.11125	0.30416	0.09512	0.94303	0.03648

	131	132	133	134	135	136	137	138	139	140
a_O	1789	1972	1657	1862	1954	1706	1728	2012	1664	1806
	(33.9)	(37.3)	(31.4)	(35.3)	(37.0)	(32.3)	(32.7)	(38.1)	(31.5)	(34.2)
a_E	1780.7	1999.1	1676.2	1780.7	1999.1	1675.9	1780.4	1998.7	1675.3	1779.7
	(33.7)	(37.8)	(31.7)	(33.7)	(37.8)	(31.7)	(33.7)	(37.8)	(31.7)	(33.7)
	16.5	-53.8	-38.2	166.2	-89.2	60.7	-103.3	26.7	-22.5	52.9
	0.313	-1.019	-0.724	3.146	-1.688	1.150	-1.955	0.505	-0.426	1.002
t_O	1691	1923	1130	1685	1975	1077	1738	1906	1086	1713
	(32.0)	(36.4)	(21.4)	(31.9)	(37.4)	(20.4)	(32.9)	(36.1)	(20.6)	(32.4)
t_E	1713.7	1896.5	1056.7	1713.7	1896.5	1056.5	1713.4	1896.1	1056.1	1712.7
	(32.4)	(35.9)	(20.0)	(32.4)	(35.9)	(20.0)	(32.4)	(35.9)	(20.0)	(32.4)
	-45.1	53.4	151.6	-56.9	160.2	41.4	49.6	19.8	60.6	0.6
	-0.854	1.011	2.869	-1.077	3.034	0.784	0.939	0.375	1.148	0.010
g_O	777	816	1684	761	795	1745	793	831	1705	724
	(14.7)	(15.4)	(31.9)	(14.4)	(15.1)	(33.0)	(15.0)	(15.7)	(32.3)	(13.7)
g_E	756.4	831.0	1737.7	756.4	831.0	1737.4	756.2	830.9	1736.7	755.9
	(14.3)	(15.7)	(32.9)	(14.3)	(15.7)	(32.9)	(14.3)	(15.7)	(32.9)	(14.3)
	41.8	-29.8	-105.7	9.3	-70.5	15.3	75.3	0.2	-62.9	-62.5
	0.792	-0.564	-2.002	0.176	-1.334	0.289	1.426	0.005	-1.191	-1.184
c_O	1025	571	811	974	558	753	1022	532	824	1036
	(19.4)	(10.8)	(15.4)	(18.4)	(10.6)	(14.3)	(19.4)	(10.1)	(15.6)	(19.6)
c_E	1031.2	555.4	811.4	1031.2	555.4	811.2	1031.0	555.3	810.9	1030.6
	(19.5)	(10.5)	(15.4)	(19.5)	(10.5)	(15.4)	(19.5)	(10.5)	(15.4)	(19.5)
	-12.4	31.7	-0.7	-111.2	5.3	-112.1	-17.9	-45.6	26.4	10.8
	-0.234	0.600	-0.013	-2.104	0.099	-2.123	-0.339	-0.863	0.501	0.205
N	5282	5282	5282	5282	5282	5281	5281	5281	5279	5279
X2	0.9	1.4	7.0	7.4	5.8	5.1	3.8	1.1	1.7	1.8
Gvalue	0.9	1.4	6.9	7.4	5.8	5.2	3.8	1.1	1.7	1.8
Gv/N	0.018	0.027	0.130	0.140	0.110	0.099	0.071	0.021	0.032	0.034
Prob	0.81683	0.69461	0.07620	0.06028	0.12022	0.15476	0.28976	0.77011	0.63514	0.61884

	141	142	143	144	145	146	147	148	149	150
a_O	1961	1708	1768	1985	1616	1782	1990	1648	1792	1981
	(37.1)	(32.4)	(33.5)	(37.6)	(30.6)	(33.8)	(37.7)	(31.2)	(34.0)	(37.6)
a_E	1998.0	1675.0	1779.4	1997.6	1675.0	1779.4	1997.6	1674.0	1778.4	1996.4
	(37.8)	(31.7)	(33.7)	(37.8)	(31.7)	(33.7)	(37.8)	(31.7)	(33.7)	(37.8)
	-73.2	66.7	-22.7	-25.1	-115.8	5.2	-15.1	-51.6	27.3	-30.8
	-1.387	1.264	-0.431	-0.475	-2.194	0.099	-0.287	-0.978	0.518	-0.583
t_O	1928	1079	1797	1937	1126	1726	1871	1115	1801	1960
	(36.5)	(20.4)	(34.0)	(36.7)	(21.3)	(32.7)	(35.4)	(21.1)	(34.1)	(37.2)
t_E	1895.4	1055.9	1712.4	1895.1	1055.9	1712.4	1895.1	1055.3	1711.4	1894.0
	(35.9)	(20.0)	(32.4)	(35.9)	(20.0)	(32.4)	(35.9)	(20.0)	(32.4)	(35.9)
	65.7	46.7	173.3	84.8	144.7	27.3	-47.8	122.7	183.8	134.3
	1.245	0.885	3.284	1.607	2.742	0.517	-0.906	2.326	3.483	2.546
g_O	864	1640	717	812	1776	766	815	1680	729	771
	(16.4)	(31.1)	(13.6)	(15.4)	(33.6)	(14.5)	(15.4)	(31.8)	(13.8)	(14.6)
g_E	830.6	1736.4	755.8	830.4	1736.4	755.8	830.4	1735.4	755.4	829.9
	(15.7)	(32.9)	(14.3)	(15.7)	(32.9)	(14.3)	(15.7)	(32.9)	(14.3)	(15.7)
	68.2	-187.3	-75.6	-36.4	80.1	20.6	-30.5	-109.0	-51.8	-113.6
	1.292	-3.549	-1.431	-0.690	1.518	0.390	-0.578	-2.067	-0.982	-2.153
c_O	526	851	996	544	760	1004	602	832	953	563
	(10.0)	(16.1)	(18.9)	(10.3)	(14.4)	(19.0)	(11.4)	(15.8)	(18.1)	(10.7)
c_E	555.1	810.7	1030.4	555.0	810.7	1030.4	555.0	810.3	1029.8	554.6
	(10.5)	(15.4)	(19.5)	(10.5)	(15.4)	(19.5)	(10.5)	(15.4)	(19.5)	(10.5)
	-56.6	82.5	-67.7	-21.7	-98.2	-52.1	98.0	44.0	-147.8	16.8
	-1.072	1.563	-1.282	-0.411	-1.861	-0.988	1.856	0.835	-2.801	0.319
N	5279	5278	5278	5278	5278	5278	5278	5275	5275	5275
X2	4.1	8.5	7.4	1.6	10.8	0.9	4.6	6.1	11.4	6.7
Gvalue	4.1	8.6	7.4	1.6	10.8	0.9	4.5	6.1	11.5	6.8
Gv/N	0.078	0.162	0.140	0.031	0.205	0.018	0.085	0.115	0.218	0.129
Prob	0.24851	0.03560	0.06090	0.65259	0.01290	0.81775	0.21209	0.10750	0.00921	0.07827

Baph_BP
5UTR:

	50	49	48	47	46	45	44	43	42	41
a_O	210	208	224	214	193	215	216	187	212	203
	(41.4)	(41.0)	(44.2)	(42.2)	(38.1)	(42.4)	(42.6)	(36.9)	(41.8)	(40.0)
a_E	213.8	213.8	213.8	213.8	213.8	213.8	213.8	213.8	213.8	213.8
	(42.2)	(42.2)	(42.2)	(42.2)	(42.2)	(42.2)	(42.2)	(42.2)	(42.2)	(42.2)
	-7.6	-11.5	20.8	0.4	-39.5	2.4	4.4	-50.1	-3.6	-21.1
	-1.492	-2.263	4.112	0.073	-7.798	0.468	0.866	-9.885	-0.713	-4.157
t_O	200	199	197	213	211	197	207	218	202	202
	(39.4)	(39.3)	(38.9)	(42.0)	(41.6)	(38.9)	(40.8)	(43.0)	(39.8)	(39.8)
t_E	199.1	199.1	199.1	199.1	199.1	199.1	199.1	199.1	199.1	199.1
	(39.3)	(39.3)	(39.3)	(39.3)	(39.3)	(39.3)	(39.3)	(39.3)	(39.3)	(39.3)
	1.7	-0.3	-4.2	28.7	24.4	-4.2	16.0	39.5	5.8	5.8
	0.342	-0.054	-0.838	5.655	4.817	-0.838	3.163	7.783	1.138	1.138
g_O	50	50	42	44	51	50	44	54	51	51
	(9.9)	(9.9)	(8.3)	(8.7)	(10.1)	(9.9)	(8.7)	(10.7)	(10.1)	(10.1)
g_E	52.7	52.7	52.7	52.7	52.7	52.7	52.7	52.7	52.7	52.7
	(10.4)	(10.4)	(10.4)	(10.4)	(10.4)	(10.4)	(10.4)	(10.4)	(10.4)	(10.4)
	-5.3	-5.3	-19.1	-15.9	-3.3	-5.3	-15.9	2.6	-3.3	-3.3
	-1.037	-1.037	-3.760	-3.131	-0.659	-1.037	-3.131	0.520	-0.659	-0.659
c_O	47	50	44	36	52	45	40	48	42	51
	(9.3)	(9.9)	(8.7)	(7.1)	(10.3)	(8.9)	(7.9)	(9.5)	(8.3)	(10.1)
c_E	41.3	41.3	41.3	41.3	41.3	41.3	41.3	41.3	41.3	41.3
	(8.2)	(8.2)	(8.2)	(8.2)	(8.2)	(8.2)	(8.2)	(8.2)	(8.2)	(8.2)
	12.0	19.0	5.5	-10.0	23.8	7.6	-2.7	14.3	1.3	21.4
	2.375	3.747	1.079	-1.967	4.701	1.502	-0.523	2.824	0.259	4.220
N	507	507	507	507	507	507	507	507	507	507
X2	1.0	2.1	2.9	3.1	5.5	0.5	1.8	6.3	0.1	2.9
Gvalue	1.0	2.0	3.0	3.2	5.4	0.5	1.9	6.3	0.1	2.7
Gv/N	0.188	0.394	0.592	0.629	1.061	0.095	0.374	1.242	0.024	0.542
Prob	0.81282	0.57328	0.39115	0.36327	0.14609	0.92251	0.59438	0.09812	0.98907	0.43211

	40	39	38	37	36	35	34	33	32	31
a_O	189	233	220	225	223	226	217	224	206	206
	(37.3)	(46.0)	(43.4)	(44.4)	(44.0)	(44.6)	(42.8)	(44.2)	(40.6)	(40.6)
a_E	213.8	213.8	213.8	213.8	213.8	213.8	213.8	213.8	213.8	213.8
	(42.2)	(42.2)	(42.2)	(42.2)	(42.2)	(42.2)	(42.2)	(42.2)	(42.2)	(42.2)
	-46.6	40.0	12.5	22.9	18.8	25.0	6.4	20.8	-15.3	-15.3
	-9.198	7.898	2.474	4.525	3.700	4.941	1.265	4.112	-3.026	-3.026
t_O	228	194	208	199	210	204	212	207	210	213
	(45.0)	(38.3)	(41.0)	(39.3)	(41.4)	(40.2)	(41.8)	(40.8)	(41.4)	(42.0)
t_E	199.1	199.1	199.1	199.1	199.1	199.1	199.1	199.1	199.1	199.1
	(39.3)	(39.3)	(39.3)	(39.3)	(39.3)	(39.3)	(39.3)	(39.3)	(39.3)	(39.3)
	61.7	-10.1	18.1	-0.3	22.3	9.8	26.5	16.0	22.3	28.7
	12.174	-2.000	3.573	-0.054	4.400	1.942	5.235	3.163	4.400	5.655
g_O	50	42	39	41	34	47	36	37	55	49
	(9.9)	(8.3)	(7.7)	(8.1)	(6.7)	(9.3)	(7.1)	(7.3)	(10.8)	(9.7)
g_E	52.7	52.7	52.7	52.7	52.7	52.7	52.7	52.7	52.7	52.7
	(10.4)	(10.4)	(10.4)	(10.4)	(10.4)	(10.4)	(10.4)	(10.4)	(10.4)	(10.4)
	-5.3	-19.1	-23.5	-20.6	-29.8	-10.8	-27.4	-26.2	4.7	-7.1
	-1.037	-3.760	-4.631	-4.060	-5.878	-2.122	-5.412	-5.162	0.927	-1.407
c_O	40	38	40	42	40	30	42	39	36	39
	(7.9)	(7.5)	(7.9)	(8.3)	(7.9)	(5.9)	(8.3)	(7.7)	(7.1)	(7.7)
c_E	41.3	41.3	41.3	41.3	41.3	41.3	41.3	41.3	41.3	41.3
	(8.2)	(8.2)	(8.2)	(8.2)	(8.2)	(8.2)	(8.2)	(8.2)	(8.2)	(8.2)
	-2.7	-6.4	-2.7	1.3	-2.7	-19.3	1.3	-4.6	-10.0	-4.6
	-0.523	-1.266	-0.523	0.259	-0.523	-3.797	0.259	-0.900	-1.967	-0.900
N	507	507	507	507	507	507	507	507	507	507
X2	7.2	4.3	4.2	3.2	7.7	4.5	6.2	5.6	1.7	1.6
Gvalue	7.2	4.4	4.5	3.4	8.6	4.9	6.8	6.1	1.7	1.6
Gv/N	1.416	0.872	0.893	0.670	1.699	0.964	1.347	1.212	0.334	0.322
Prob	0.06641	0.21948	0.20983	0.33405	0.03489	0.18032	0.07747	0.10472	0.63818	0.65153

	30	29	28	27	26	25	24	23	22	21
a_O	218	229	194	219	210	219	207	226	209	218
	(43.0)	(45.2)	(38.3)	(43.2)	(41.4)	(43.2)	(40.8)	(44.6)	(41.2)	(43.0)
a_E	213.8	213.8	213.8	213.8	213.8	213.8	213.8	213.8	213.8	213.8
	(42.2)	(42.2)	(42.2)	(42.2)	(42.2)	(42.2)	(42.2)	(42.2)	(42.2)	(42.2)
	8.4	31.4	-37.7	10.5	-7.6	10.5	-13.4	25.0	-9.5	8.4
	1.667	6.198	-7.443	2.070	-1.492	2.070	-2.645	4.941	-1.878	1.667
t_O	216	197	223	210	207	214	232	189	225	224
	(42.6)	(38.9)	(44.0)	(41.4)	(40.8)	(42.2)	(45.8)	(37.3)	(44.4)	(44.2)
t_E	199.1	199.1	199.1	199.1	199.1	199.1	199.1	199.1	199.1	199.1
	(39.3)	(39.3)	(39.3)	(39.3)	(39.3)	(39.3)	(39.3)	(39.3)	(39.3)	(39.3)
	35.1	-4.2	50.5	22.3	16.0	30.8	70.9	-19.7	55.0	52.7
	6.926	-0.838	9.957	4.400	3.163	6.077	13.979	-3.895	10.838	10.397
g_O	40	41	43	33	41	32	33	46	34	37
	(7.9)	(8.1)	(8.5)	(6.5)	(8.1)	(6.3)	(6.5)	(9.1)	(6.7)	(7.3)
g_E	52.7	52.7	52.7	52.7	52.7	52.7	52.7	52.7	52.7	52.7
	(10.4)	(10.4)	(10.4)	(10.4)	(10.4)	(10.4)	(10.4)	(10.4)	(10.4)	(10.4)
	-22.1	-20.6	-17.5	-30.9	-20.6	-31.9	-30.9	-12.5	-29.8	-26.2
	-4.351	-4.060	-3.450	-6.093	-4.060	-6.297	-6.093	-2.467	-5.878	-5.162
c_O	33	40	47	45	49	42	35	46	39	28
	(6.5)	(7.9)	(9.3)	(8.9)	(9.7)	(8.3)	(6.9)	(9.1)	(7.7)	(5.5)
c_E	41.3	41.3	41.3	41.3	41.3	41.3	41.3	41.3	41.3	41.3
	(8.2)	(8.2)	(8.2)	(8.2)	(8.2)	(8.2)	(8.2)	(8.2)	(8.2)	(8.2)
	-14.9	-2.7	12.0	7.6	16.6	1.3	-11.7	9.8	-4.6	-21.8
	-2.936	-0.523	2.375	1.502	3.282	0.259	-2.302	1.934	-0.900	-4.306
N	507	507	507	507	507	507	507	507	507	507
X2	6.3	3.7	7.3	8.4	4.4	9.4	14.0	2.6	10.2	12.2
Gvalue	6.6	3.9	7.3	9.5	4.5	10.7	14.9	2.6	11.1	13.2
Gv/N	1.306	0.776	1.438	1.879	0.892	2.108	2.939	0.513	2.183	2.595
Prob	0.08491	0.26855	0.06312	0.02307	0.21010	0.01353	0.00190	0.45731	0.01138	0.00431

	20	19	18	17	16	15	14	13	12	11
a_O	217	209	223	241	225	209	225	210	214	197
	(42.8)	(41.2)	(44.0)	(47.5)	(44.4)	(41.2)	(44.4)	(41.4)	(42.2)	(38.9)
a_E	213.8	213.8	213.8	213.8	213.8	213.8	213.8	213.8	213.8	213.8
	(42.2)	(42.2)	(42.2)	(42.2)	(42.2)	(42.2)	(42.2)	(42.2)	(42.2)	(42.2)
	6.4	-9.5	18.8	57.7	22.9	-9.5	22.9	-7.6	0.4	-32.3
	1.265	-1.878	3.700	11.378	4.525	-1.878	4.525	-1.492	0.073	-6.365
t_O	204	225	206	183	182	202	167	179	152	159
	(40.2)	(44.4)	(40.6)	(36.1)	(35.9)	(39.8)	(32.9)	(35.3)	(30.0)	(31.4)

t_E	199.1	199.1	199.1	199.1	199.1	199.1	199.1	199.1	199.1	199.1
	(39.3)	(39.3)	(39.3)	(39.3)	(39.3)	(39.3)	(39.3)	(39.3)	(39.3)	(39.3)
	9.8	55.0	14.0	-30.9	-32.8	5.8	-58.8	-38.2	-82.1	-71.6
	1.942	10.838	2.754	-6.100	-6.460	1.138	-11.594	-7.527	-16.196	-14.118
g_O	47	41	40	54	56	61	86	91	115	123
	(9.3)	(8.1)	(7.9)	(10.7)	(11.0)	(12.0)	(17.0)	(17.9)	(22.7)	(24.3)
g_E	52.7	52.7	52.7	52.7	52.7	52.7	52.7	52.7	52.7	52.7
	(10.4)	(10.4)	(10.4)	(10.4)	(10.4)	(10.4)	(10.4)	(10.4)	(10.4)	(10.4)
	-10.8	-20.6	-22.1	2.6	6.8	17.8	84.2	99.4	179.5	208.5
	-2.122	-4.060	-4.351	0.520	1.342	3.520	16.615	19.609	35.400	41.126
c_O	39	32	38	29	44	35	29	27	26	28
	(7.7)	(6.3)	(7.5)	(5.7)	(8.7)	(6.9)	(5.7)	(5.3)	(5.1)	(5.5)
c_E	41.3	41.3	41.3	41.3	41.3	41.3	41.3	41.3	41.3	41.3
	(8.2)	(8.2)	(8.2)	(8.2)	(8.2)	(8.2)	(8.2)	(8.2)	(8.2)	(8.2)
	-4.6	-16.4	-6.4	-20.6	5.5	-11.7	-20.6	-23.0	-24.1	-21.8
	-0.900	-3.236	-1.266	-4.058	1.079	-2.302	-4.058	-4.540	-4.759	-4.306
N	507	507	507	507	507	507	507	507	507	507
X2	0.9	8.2	4.0	8.5	2.4	2.4	30.5	34.9	90.5	107.5
Gvalue	0.9	8.4	4.2	8.8	2.5	2.4	27.8	30.7	73.6	82.8
Gv/N	0.186	1.665	0.837	1.739	0.486	0.478	5.488	6.051	14.518	16.336
Prob	0.81555	0.03774	0.23645	0.03183	0.48203	0.48933	0.00000	0.00000	0.00000	0.00000

	10	9	8	7	6	5	4	3	2	1
a_O	231	200	223	256	252	271	255	281	215	144
	(45.6)	(39.4)	(44.0)	(50.5)	(49.7)	(53.5)	(50.3)	(55.4)	(42.4)	(28.4)
a_E	213.8	213.8	213.8	213.8	213.8	213.8	213.8	213.8	213.8	213.8
	(42.2)	(42.2)	(42.2)	(42.2)	(42.2)	(42.2)	(42.2)	(42.2)	(42.2)	(42.2)
	35.7	-26.7	18.8	92.2	82.8	128.5	89.8	153.6	2.4	-113.8
	7.044	-5.270	3.700	18.184	16.334	25.337	17.719	30.288	0.468	-22.455
t_O	120	129	128	140	171	174	188	157	208	284
	(23.7)	(25.4)	(25.2)	(27.6)	(33.7)	(34.3)	(37.1)	(31.0)	(41.0)	(56.0)
t_E	199.1	199.1	199.1	199.1	199.1	199.1	199.1	199.1	199.1	199.1
	(39.3)	(39.3)	(39.3)	(39.3)	(39.3)	(39.3)	(39.3)	(39.3)	(39.3)	(39.3)
	-121.6	-112.0	-113.1	-98.7	-52.1	-47.0	-21.6	-74.7	18.1	201.6
	-23.976	-22.094	-22.316	-19.459	-10.275	-9.262	-4.268	-14.724	3.573	39.770
g_O	129	153	128	78	48	27	28	34	37	26
	(25.4)	(30.2)	(25.2)	(15.4)	(9.5)	(5.3)	(5.5)	(6.7)	(7.3)	(5.1)
g_E	52.7	52.7	52.7	52.7	52.7	52.7	52.7	52.7	52.7	52.7
	(10.4)	(10.4)	(10.4)	(10.4)	(10.4)	(10.4)	(10.4)	(10.4)	(10.4)	(10.4)
	231.0	326.1	227.2	61.2	-9.0	-36.1	-35.4	-29.8	-26.2	-36.7
	45.555	64.329	44.809	12.065	-1.768	-7.123	-6.985	-5.878	-5.162	-7.246
c_O	27	25	28	33	36	35	36	35	47	53
	(5.3)	(4.9)	(5.5)	(6.5)	(7.1)	(6.9)	(7.1)	(6.9)	(9.3)	(10.5)
c_E	41.3	41.3	41.3	41.3	41.3	41.3	41.3	41.3	41.3	41.3
	(8.2)	(8.2)	(8.2)	(8.2)	(8.2)	(8.2)	(8.2)	(8.2)	(8.2)	(8.2)
	-23.0	-25.2	-21.8	-14.9	-10.0	-11.7	-10.0	-11.7	12.0	26.3
	-4.540	-4.962	-4.306	-2.936	-1.967	-2.302	-1.967	-2.302	2.375	5.190
N	507	507	507	507	507	507	507	507	507	507
X2	148.3	223.0	137.7	39.7	11.9	32.0	20.8	37.6	5.9	75.8
Gvalue	122.1	162.3	111.0	39.8	11.8	33.7	22.8	37.4	6.4	77.4
Gv/N	24.084	32.002	21.887	7.854	2.323	6.650	4.499	7.385	1.255	15.258
Prob	0.00000	0.00000	0.00000	0.00000	0.00818	0.00000	0.00004	0.00000	0.09533	0.00000

CDS_INITIAL:

	1	2	3	4	5	6	7	8	9	10
a_O	471	0	1	282	227	225	290	249	233	266
	(92.9)	(0.0)	(0.2)	(55.6)	(44.8)	(44.4)	(57.2)	(49.1)	(46.0)	(52.5)
a_E	201.2	177.2	213.0	201.2	177.2	213.0	201.2	177.2	213.0	201.2
	(39.7)	(35.0)	(42.0)	(39.7)	(35.0)	(42.0)	(39.7)	(35.0)	(42.0)	(39.7)
	801.3	0.0	-10.7	190.5	112.3	24.6	212.1	169.3	41.7	148.6
	158.047	0.000	-2.115	37.565	22.154	4.845	41.831	33.387	8.229	29.304
t_O	12	507	4	104	141	210	88	150	208	103
	(2.4)	(100.0)	(0.8)	(20.5)	(27.8)	(41.4)	(17.4)	(29.6)	(41.0)	(20.3)
t_E	125.5	174.2	217.6	125.5	174.2	217.6	125.5	174.2	217.6	125.5
	(24.8)	(34.4)	(42.9)	(24.8)	(34.4)	(42.9)	(24.8)	(34.4)	(42.9)	(24.8)
	-56.3	1083.1	-32.0	-39.1	-59.7	-14.9	-62.5	-44.9	-18.7	-40.7
	-11.112	213.636	-6.306	-7.714	-11.768	-2.939	-12.326	-8.858	-3.696	-8.032
g_O	24	0	502	73	39	35	66	47	38	74
	(4.7)	(0.0)	(99.0)	(14.4)	(7.7)	(6.9)	(13.0)	(9.3)	(7.5)	(14.6)
g_E	117.6	67.0	44.4	117.6	67.0	44.4	117.6	67.0	44.4	117.6
	(23.2)	(13.2)	(8.7)	(23.2)	(13.2)	(8.7)	(23.2)	(13.2)	(8.7)	(23.2)
	-76.3	0.0	2436.0	-69.6	-42.2	-16.6	-76.2	-33.3	-11.8	-68.5
	-15.044	0.000	480.477	-13.726	-8.323	-3.271	-15.035	-6.571	-2.319	-13.517
c_O	0	0	0	48	100	37	63	61	28	64
	(0.0)	(0.0)	(0.0)	(9.5)	(19.7)	(7.3)	(12.4)	(12.0)	(5.5)	(12.6)
c_E	62.7	88.5	32.0	62.7	88.5	32.0	62.7	88.5	32.0	62.7
	(12.4)	(17.5)	(6.3)	(12.4)	(17.5)	(6.3)	(12.4)	(17.5)	(6.3)	(12.4)
	0.0	0.0	0.0	-25.7	24.3	10.7	0.6	-45.5	-7.5	2.6
	0.000	0.000	0.000	-5.065	4.802	2.113	0.110	-8.965	-1.479	0.509
N	507	507	507	507	507	507	507	507	507	507
X2	601.7	968.4	5174.3	56.5	33.5	3.7	73.0	46.9	3.7	41.1
Gvalue	668.7	1083.1	2393.3	56.1	34.8	3.8	73.9	45.6	3.7	41.9
Gv/N	131.890	213.636	472.057	11.060	6.865	0.749	14.580	8.993	0.735	8.264
Prob	0.00000	0.00000	1.00000	0.00000	0.00000	0.28446	0.00000	0.00000	0.29273	0.00000

	11	12	13	14	15	16	17	18	19	20
a_O	211	209	246	198	208	231	169	236	218	190
	(41.6)	(41.2)	(48.5)	(39.1)	(41.0)	(45.6)	(33.3)	(46.5)	(43.0)	(37.5)
a_E	177.2	213.0	201.2	177.2	213.0	201.2	177.2	213.0	201.2	177.2
	(35.0)	(42.0)	(39.7)	(35.0)	(42.0)	(39.7)	(35.0)	(42.0)	(39.7)	(35.0)
	73.6	-8.0	98.9	43.8	-10.0	63.8	-16.1	48.3	35.0	26.4
	14.508	-1.581	19.516	8.648	-1.967	12.593	-3.177	9.526	6.903	5.207
t_O	183	240	136	195	237	133	210	190	140	197
	(36.1)	(47.3)	(26.8)	(38.5)	(46.7)	(26.2)	(41.4)	(37.5)	(27.6)	(38.9)
t_E	174.2	217.6	125.5	174.2	217.6	125.5	174.2	217.6	125.5	174.2
	(34.4)	(42.9)	(24.8)	(34.4)	(42.9)	(24.8)	(34.4)	(42.9)	(24.8)	(34.4)
	18.0	47.1	21.8	43.9	40.5	15.4	78.4	-51.5	30.6	48.4
	3.549	9.284	4.305	8.667	7.992	3.040	15.473	-10.160	6.032	9.549
g_O	49	30	71	46	27	83	59	47	93	48
	(9.7)	(5.9)	(14.0)	(9.1)	(5.3)	(16.4)	(11.6)	(9.3)	(18.3)	(9.5)
g_E	67.0	44.4	117.6	67.0	44.4	117.6	67.0	44.4	117.6	67.0
	(13.2)	(8.7)	(23.2)	(13.2)	(8.7)	(23.2)	(13.2)	(8.7)	(23.2)	(13.2)
	-30.6	-23.5	-71.6	-34.6	-26.8	-57.8	-15.0	5.4	-43.6	-32.0
	-6.045	-4.628	-14.128	-6.821	-5.288	-11.403	-2.956	1.073	-8.604	-6.312

```
c_O          64       28       54       68       35       60       69       34       56       72
        ( 12.6) (  5.5) ( 10.7) ( 13.4) (  6.9) ( 11.8) ( 13.6) (  6.7) ( 11.0) ( 14.2)
c_E        88.5     32.0     62.7     88.5     32.0     62.7     88.5     32.0     62.7     88.5
        ( 17.5) (  6.3) ( 12.4) ( 17.5) (  6.3) ( 12.4) ( 17.5) (  6.3) ( 12.4) ( 17.5)
          -41.5     -7.5    -16.2    -35.9      6.2     -5.3    -34.4      4.1    -12.7    -29.8
         -8.194   -1.479   -3.189   -7.080    1.232   -1.050   -6.787    0.808   -2.504   -5.873

N           507      507      507      507      507      507      507      507      507      507
X2         18.5      7.5     30.5     16.2      8.9     15.2     13.0      6.3      8.9     12.4
Gvalue     19.4      8.1     33.0     17.3     10.0     16.1     12.9      6.3      9.3     13.0
Gv/N      3.818    1.595    6.503    3.413    1.969    3.179    2.553    1.246    1.828    2.571
Prob    0.00023  0.04425  0.00000  0.00061  0.01872  0.00107  0.00476  0.09710  0.02596  0.00456
        -------------------------------------------------------------------------------------
             21       22       23       24       25       26       27       28       29       30
        -------------------------------------------------------------------------------------
a_O         221      237      187      228      209      163      213      220      178      228
        ( 43.6) ( 46.7) ( 36.9) ( 45.0) ( 41.2) ( 32.1) ( 42.0) ( 43.4) ( 35.1) ( 45.0)
a_E       213.0    201.2    177.2    213.0    201.2    177.2    213.0    201.2    177.2    213.0
        ( 42.0) ( 39.7) ( 35.0) ( 42.0) ( 39.7) ( 35.0) ( 42.0) ( 39.7) ( 35.0) ( 42.0)
           16.2     77.7     20.0     30.9     15.9    -27.3     -0.1     39.3      1.5     30.9
          3.195   15.317    3.951    6.101    3.142   -5.388   -0.018    7.759    0.297    6.101
t_O         217      111      184      221      144      182      213      118      175      207
        ( 42.8) ( 21.9) ( 36.3) ( 43.6) ( 28.4) ( 35.9) ( 42.0) ( 23.3) ( 34.5) ( 40.8)
t_E       217.6    125.5    174.2    217.6    125.5    174.2    217.6    125.5    174.2    217.6
        ( 42.9) ( 24.8) ( 34.4) ( 42.9) ( 24.8) ( 34.4) ( 42.9) ( 24.8) ( 34.4) ( 42.9)
           -1.2    -27.3     20.1      6.9     39.6     15.9     -9.1    -14.6      1.6    -20.6
         -0.230   -5.381    3.963    1.358    7.805    3.136   -1.789   -2.873    0.308   -4.072
g_O          39      104       50       32       87       69       50      107       70       38
        (  7.7) ( 20.5) (  9.9) (  6.3) ( 17.2) ( 13.6) (  9.9) ( 21.1) ( 13.8) (  7.5)
g_E        44.4    117.6     67.0     44.4    117.6     67.0     44.4    117.6     67.0     44.4
        (  8.7) ( 23.2) ( 13.2) (  8.7) ( 23.2) ( 13.2) (  8.7) ( 23.2) ( 13.2) (  8.7)
          -10.0    -25.5    -29.3    -20.9    -52.4      4.1     12.0    -20.2      6.2    -11.8
         -1.980   -5.035   -5.770   -4.122  -10.337    0.804    2.362   -3.980    1.213   -2.319
c_O          30       55       86       26       67       93       31       62       84       34
        (  5.9) ( 10.8) ( 17.0) (  5.1) ( 13.2) ( 18.3) (  6.1) ( 12.2) ( 16.6) (  6.7)
c_E        32.0     62.7     88.5     32.0     62.7     88.5     32.0     62.7     88.5     32.0
        (  6.3) ( 12.4) ( 17.5) (  6.3) ( 12.4) ( 17.5) (  6.3) ( 12.4) ( 17.5) (  6.3)
           -3.9    -14.5     -5.0    -10.8      8.8      9.1     -2.0     -1.4     -8.8      4.1
         -0.768   -2.850   -0.987   -2.134    1.744    1.803   -0.393   -0.283   -1.744    0.808

N           507      507      507      507      507      507      507      507      507      507
X2          1.1     10.6      5.5      5.7     11.3      1.8      0.8      3.2      0.4      2.6
Gvalue      1.1     10.4      5.9      6.1     11.9      1.8      0.8      3.2      0.4      2.6
Gv/N      0.217    2.051    1.157    1.204    2.354    0.355    0.161    0.623    0.074    0.518
Prob    0.77732  0.01546  0.11823  0.10671  0.00762  0.61487  0.84499  0.36812  0.94503  0.45257
        -------------------------------------------------------------------------------------
             31       32       33       34       35       36       37       38       39       40
        -------------------------------------------------------------------------------------
a_O         210      164      223      203      167      205      188      160      221      200
        ( 41.4) ( 32.3) ( 44.0) ( 40.0) ( 32.9) ( 40.4) ( 37.1) ( 31.6) ( 43.6) ( 39.4)
a_E       201.2    177.2    213.0    201.2    177.2    213.0    201.2    177.2    213.0    201.2
        ( 39.7) ( 35.0) ( 42.0) ( 39.7) ( 35.0) ( 42.0) ( 39.7) ( 35.0) ( 42.0) ( 39.7)
           18.0    -25.5     20.4      3.6    -19.9    -15.8    -25.5    -32.8     16.2     -2.4
          3.552   -5.026    4.017    0.719   -3.924   -3.114   -5.027   -6.462    3.195   -0.466
t_O         117      178      197      123      186      232      122      184      213      120
        ( 23.1) ( 35.1) ( 38.9) ( 24.3) ( 36.7) ( 45.8) ( 24.1) ( 36.3) ( 42.0) ( 23.7)
t_E       125.5    174.2    217.6    125.5    174.2    217.6    125.5    174.2    217.6    125.5
        ( 24.8) ( 34.4) ( 42.9) ( 24.8) ( 34.4) ( 42.9) ( 24.8) ( 34.4) ( 42.9) ( 24.8)
          -16.4      7.6    -39.2     -5.0     24.3     29.8     -6.9     20.1     -9.1    -10.8
         -3.242    1.506   -7.723   -0.981    4.800    5.872   -1.366    3.963   -1.789   -2.126
g_O         115       75       54      113       75       38      122       80       33      125
        ( 22.7) ( 14.8) ( 10.7) ( 22.3) ( 14.8) (  7.5) ( 24.1) ( 15.8) (  6.5) ( 24.7)
g_E       117.6     67.0     44.4    117.6     67.0     44.4    117.6     67.0     44.4    117.6
        ( 23.2) ( 13.2) (  8.7) ( 23.2) ( 13.2) (  8.7) ( 23.2) ( 13.2) (  8.7) ( 23.2)
           -5.1     16.9     21.2     -9.0     16.9    -11.8      9.0     28.4    -19.5     15.3
         -1.007    3.341    4.190   -1.771    3.341   -2.319    1.776    5.601   -3.850    3.017
c_O          65       90       33       68       79       32       75       83       40       62
        ( 12.8) ( 17.8) (  6.5) ( 13.4) ( 15.6) (  6.3) ( 14.8) ( 16.4) (  7.9) ( 12.2)
c_E        62.7     88.5     32.0     62.7     88.5     32.0     62.7     88.5     32.0     62.7
        ( 12.4) ( 17.5) (  6.3) ( 12.4) ( 17.5) (  6.3) ( 12.4) ( 17.5) (  6.3) ( 12.4)
            4.6      2.9      2.0     11.0    -18.0     -0.0     26.8    -10.7     17.8     -1.4
          0.915    0.581    0.395    2.167   -3.553   -0.005    5.289   -2.115    3.515   -0.283

N           507      507      507      507      507      507      507      507      507      507
X2          1.1      2.1      4.5      0.7      3.4      2.2      3.5      5.1      5.3      0.7
Gvalue      1.1      2.0      4.5      0.7      3.4      2.2      3.4      5.0      5.4      0.7
Gv/N      0.219    0.403    0.879    0.134    0.665    0.434    0.672    0.987    1.071    0.142
Prob    0.77454  0.56359  0.21603  0.87790  0.33782  0.53178  0.33297  0.17139  0.14291  0.86853
        -------------------------------------------------------------------------------------
             41       42       43       44       45       46       47       48       49       50
        -------------------------------------------------------------------------------------
a_O         158      226      193      164      213      191      150      196      190      170
        ( 31.2) ( 44.6) ( 38.1) ( 32.3) ( 42.0) ( 37.7) ( 29.6) ( 38.7) ( 37.5) ( 33.5)
a_E       177.2    213.0    201.2    177.2    213.0    201.2    177.2    213.0    201.2    177.2
        ( 35.0) ( 42.0) ( 39.7) ( 35.0) ( 42.0) ( 39.7) ( 35.0) ( 42.0) ( 39.7) ( 35.0)
          -36.3     26.7    -16.0    -25.5     -0.1    -19.8    -50.1    -32.7    -21.7    -14.2
         -7.165    5.262   -3.162   -5.026   -0.018   -3.914   -9.877   -6.448   -4.287   -2.800
t_O         187      222      135      187      209      120      172      219      139      172
        ( 36.9) ( 43.8) ( 26.6) ( 36.9) ( 41.2) ( 23.7) ( 33.9) ( 43.2) ( 27.4) ( 33.9)
t_E       174.2    217.6    125.5    174.2    217.6    125.5    174.2    217.6    125.5    174.2
        ( 34.4) ( 42.9) ( 24.8) ( 34.4) ( 42.9) ( 24.8) ( 34.4) ( 42.9) ( 24.8) ( 34.4)
           26.5      8.9     19.7     26.5    -16.8    -10.8     -4.4      2.8     28.4     -4.4
          5.221    1.760    3.880    5.221   -3.318   -2.126   -0.871    0.561    5.596   -0.871
g_O          73       31      120       81       44      130       85       49      108       67
        ( 14.4) (  6.1) ( 23.7) ( 16.0) (  8.7) ( 25.6) ( 16.8) (  9.7) ( 21.3) ( 13.2)
g_E        67.0     44.4    117.6     67.0     44.4    117.6     67.0     44.4    117.6     67.0
        ( 13.2) (  8.7) ( 23.2) ( 13.2) (  8.7) ( 23.2) ( 13.2) (  8.7) ( 23.2) ( 13.2)
           12.5    -22.2      4.9     30.8     -0.7     26.1     40.5      9.8    -18.4      0.0
          2.474   -4.382    0.964    6.068   -0.141    5.149    7.984    1.924   -3.621    0.004
c_O          89       28       59       75       41       66      100       43       70       98
        ( 17.6) (  5.5) ( 11.6) ( 14.8) (  8.1) ( 13.0) ( 19.7) (  8.5) ( 13.8) ( 19.3)
c_E        88.5     32.0     62.7     88.5     32.0     62.7     88.5     32.0     62.7     88.5
        ( 17.5) (  6.3) ( 12.4) ( 17.5) (  6.3) ( 12.4) ( 17.5) (  6.3) ( 12.4) ( 17.5)
            0.9     -7.5     -7.2    -24.9     20.3      6.7     24.3     25.4     15.4     19.9
          0.182   -1.479   -1.424   -4.910    4.002    1.326    4.802    5.005    3.032    3.925

N           507      507      507      507      507      507      507      507      507      507
X2          3.6      5.4      1.3      6.9      2.9      2.2     10.5      5.6      3.7      1.3
Gvalue      3.6      5.9      1.3      6.9      2.7      2.2     10.3      5.3      3.6      1.3
Gv/N      0.712    1.161    0.259    1.353    0.525    0.435    2.038    1.042    0.720    0.258
Prob    0.30660  0.11722  0.72628  0.07649  0.44681  0.53062  0.01595  0.15227  0.30184  0.72777
        -------------------------------------------------------------------------------------
```

	51	52	53	54	55	56	57	58	59	60
a_O	229	186	173	220	199	156	214	196	186	221
	(45.2)	(36.7)	(34.1)	(43.4)	(39.3)	(30.8)	(42.2)	(38.7)	(36.7)	(43.6)
a_E	213.0	201.2	177.2	213.0	201.2	177.2	213.0	201.2	177.2	213.0
	(42.0)	(39.7)	(35.0)	(42.0)	(39.7)	(35.0)	(42.0)	(39.7)	(35.0)	(42.0)
	33.1	−29.2	−8.4	14.1	−4.3	−39.8	1.9	−10.2	17.9	16.2
	6.523	−5.758	−1.656	2.787	−0.857	−7.858	0.377	−2.019	3.536	3.195
t_O	204	140	178	211	136	200	216	126	185	212
	(40.2)	(27.6)	(35.1)	(41.6)	(26.8)	(39.4)	(42.6)	(24.9)	(36.5)	(41.8)
t_E	217.6	125.5	174.2	217.6	125.5	174.2	217.6	125.5	174.2	217.6
	(42.9)	(24.8)	(34.4)	(42.9)	(24.8)	(34.4)	(42.9)	(24.8)	(34.4)	(42.9)
	−26.3	30.6	7.6	−13.0	21.8	55.2	−3.2	1.0	22.2	−11.0
	−5.187	6.032	1.506	−2.557	4.305	10.887	−0.622	0.192	4.381	−2.174
g_O	42	119	71	50	109	70	42	129	59	39
	(8.3)	(23.5)	(14.0)	(9.9)	(21.5)	(13.8)	(8.3)	(25.4)	(11.6)	(7.7)
g_E	44.4	117.6	67.0	44.4	117.6	67.0	44.4	117.6	67.0	44.4
	(8.7)	(23.2)	(13.2)	(8.7)	(23.2)	(13.2)	(8.7)	(23.2)	(13.2)	(8.7)
	−4.6	2.9	8.3	12.0	−16.5	6.2	−4.6	23.9	−15.0	−10.0
	−0.905	0.563	1.628	2.362	−3.258	1.213	−0.905	4.717	−2.956	−1.980
c_O	32	62	85	26	63	81	35	56	77	35
	(6.3)	(12.2)	(16.8)	(5.1)	(12.4)	(16.0)	(6.9)	(11.0)	(15.2)	(6.9)
c_E	32.0	62.7	88.5	32.0	62.7	88.5	32.0	62.7	88.5	32.0
	(6.3)	(12.4)	(17.5)	(6.3)	(12.4)	(17.5)	(6.3)	(12.4)	(17.5)	(6.3)
	−0.0	−1.4	−6.9	−10.8	0.6	−14.4	6.2	−12.7	−21.5	6.2
	−0.005	−0.283	−1.368	−2.134	0.110	−2.844	1.232	−2.504	−4.241	1.232
N	507	507	507	507	507	507	507	507	507	507
X2	2.2	2.8	0.6	2.3	1.5	7.1	0.4	2.0	3.6	1.4
Gvalue	2.2	2.8	0.6	2.3	1.5	7.1	0.4	2.0	3.6	1.4
Gv/N	0.426	0.555	0.111	0.458	0.300	1.398	0.082	0.386	0.719	0.273
Prob	0.53995	0.42142	0.90492	0.50826	0.67773	0.06913	0.93736	0.58094	0.30233	0.70949

	61	62	63	64	65	66	67	68	69	70
a_O	196	162	208	206	168	238	190	163	206	188
	(38.7)	(32.0)	(41.0)	(40.6)	(33.1)	(46.9)	(37.5)	(32.1)	(40.6)	(37.1)
a_E	201.2	177.2	213.0	201.2	177.2	213.0	201.2	177.2	213.0	201.2
	(39.7)	(35.0)	(42.0)	(39.7)	(35.0)	(42.0)	(39.7)	(35.0)	(42.0)	(39.7)
	−10.2	−29.1	−10.0	9.7	−18.0	52.7	−21.7	−27.3	−13.9	−25.5
	−2.019	−5.749	−1.967	1.922	−3.551	10.399	−4.287	−5.388	−2.733	−5.027
t_O	120	160	216	128	184	198	127	191	232	124
	(23.7)	(31.6)	(42.6)	(25.2)	(36.3)	(39.1)	(25.0)	(37.7)	(45.8)	(24.5)
t_E	125.5	174.2	217.6	125.5	174.2	217.6	125.5	174.2	217.6	125.5
	(24.8)	(34.4)	(42.9)	(24.8)	(34.4)	(42.9)	(24.8)	(34.4)	(42.9)	(24.8)
	−10.8	−27.3	−3.2	5.0	20.1	−37.3	3.0	35.1	29.8	−3.0
	−2.126	−5.375	−0.622	0.991	3.963	−7.367	0.590	6.927	5.872	−0.593
g_O	124	80	45	111	58	47	115	64	43	136
	(24.5)	(15.8)	(8.9)	(21.9)	(11.4)	(9.3)	(22.7)	(12.6)	(8.5)	(26.8)
g_E	117.6	67.0	44.4	117.6	67.0	44.4	117.6	67.0	44.4	117.6
	(23.2)	(13.2)	(8.7)	(23.2)	(13.2)	(8.7)	(23.2)	(13.2)	(8.7)	(23.2)
	13.2	28.4	1.3	−12.8	−16.7	5.4	−5.1	−5.8	−2.7	39.6
	2.600	5.601	0.255	−2.522	−3.297	1.073	−1.007	−1.153	−0.527	7.808
c_O	67	105	38	62	97	24	75	89	26	59
	(13.2)	(20.7)	(7.5)	(12.2)	(19.1)	(4.7)	(14.8)	(17.6)	(5.1)	(11.6)
c_E	62.7	88.5	32.0	62.7	88.5	32.0	62.7	88.5	32.0	62.7
	(12.4)	(17.5)	(6.3)	(12.4)	(17.5)	(6.3)	(12.4)	(17.5)	(6.3)	(12.4)
	8.8	35.8	13.0	−1.4	17.7	−13.8	26.8	0.9	−10.8	−7.2
	1.744	7.063	2.570	−0.283	3.492	−2.727	5.289	0.182	−2.134	−1.424
N	507	507	507	507	507	507	507	507	507	507
X2	1.0	8.1	1.3	0.5	3.0	6.8	3.1	2.9	2.4	4.0
Gvalue	1.0	7.8	1.2	0.5	3.1	7.0	3.0	2.9	2.4	3.9
Gv/N	0.199	1.540	0.236	0.108	0.607	1.378	0.586	0.568	0.477	0.764
Prob	0.79876	0.05014	0.75368	0.90847	0.37969	0.07239	0.39647	0.41025	0.48992	0.27551

	71	72	73	74	75	76	77	78	79	80
a_O	147	219	192	163	225	197	177	213	203	171
	(29.0)	(43.2)	(37.9)	(32.1)	(44.4)	(38.9)	(34.9)	(42.0)	(40.0)	(33.7)
a_E	177.2	213.0	201.2	177.2	213.0	201.2	177.2	213.0	201.2	177.2
	(35.0)	(42.0)	(39.7)	(35.0)	(42.0)	(39.7)	(35.0)	(42.0)	(39.7)	(35.0)
	−55.0	12.1	−17.9	−27.3	24.6	−8.3	−0.5	−0.1	3.6	−12.3
	−10.851	2.381	−3.539	−5.388	4.845	−1.634	−0.098	−0.018	0.719	−2.421
t_O	189	221	122	179	210	120	177	216	128	190
	(37.3)	(43.6)	(24.1)	(35.3)	(41.4)	(23.7)	(34.9)	(42.6)	(25.2)	(37.5)
t_E	174.2	217.6	125.5	174.2	217.6	125.5	174.2	217.6	125.5	174.2
	(34.4)	(42.9)	(24.8)	(34.4)	(42.9)	(24.8)	(34.4)	(42.9)	(24.8)	(34.4)
	30.8	6.9	−6.9	9.7	−14.9	−10.8	5.6	−3.2	5.0	32.9
	6.070	1.358	−1.366	1.910	−2.939	−2.126	1.105	−0.622	0.991	6.498
g_O	78	38	124	67	41	122	59	45	105	64
	(15.4)	(7.5)	(24.5)	(13.2)	(8.1)	(24.1)	(11.6)	(8.9)	(20.7)	(12.6)
g_E	67.0	44.4	117.6	67.0	44.4	117.6	67.0	44.4	117.6	67.0
	(13.2)	(8.7)	(23.2)	(13.2)	(8.7)	(23.2)	(13.2)	(8.7)	(23.2)	(13.2)
	23.7	−11.8	13.2	0.0	−6.5	9.0	−15.0	1.3	−23.8	−5.8
	4.682	−2.319	2.600	0.004	−1.273	1.776	−2.956	0.255	−4.687	−1.153
c_O	93	29	69	98	31	68	94	33	71	82
	(18.3)	(5.7)	(13.6)	(19.3)	(6.1)	(13.4)	(18.5)	(6.5)	(14.0)	(16.2)
c_E	88.5	32.0	62.7	88.5	32.0	62.7	88.5	32.0	62.7	88.5
	(17.5)	(6.3)	(12.4)	(17.5)	(6.3)	(12.4)	(17.5)	(6.3)	(12.4)	(17.5)
	9.1	−5.7	13.2	19.9	−2.0	11.0	11.3	2.0	17.6	−12.6
	1.803	−1.131	2.597	3.925	−0.393	2.167	2.219	0.395	3.472	−2.482
N	507	507	507	507	507	507	507	507	507	507
X2	8.4	1.4	1.5	2.3	1.2	0.9	1.3	0.1	2.5	2.3
Gvalue	8.6	1.5	1.5	2.3	1.2	0.9	1.4	0.1	2.5	2.2
Gv/N	1.704	0.290	0.291	0.450	0.240	0.183	0.270	0.010	0.495	0.442
Prob	0.03445	0.68939	0.68738	0.51560	0.74844	0.81823	0.71306	0.99699	0.47358	0.52385

	81	82	83	84	85	86	87	88	89	90
a_O	224	215	172	204	184	166	209	212	181	208
	(44.2)	(42.4)	(33.9)	(40.2)	(36.3)	(32.7)	(41.2)	(41.8)	(35.7)	(41.0)
a_E	213.0	201.2	177.2	213.0	201.2	177.2	213.0	201.2	177.2	213.0
	(42.0)	(39.7)	(35.0)	(42.0)	(39.7)	(35.0)	(42.0)	(39.7)	(35.0)	(42.0)
	22.5	28.6	−10.3	−17.7	−32.9	−21.8	−8.0	22.2	7.6	−10.0
	4.430	5.633	−2.039	−3.492	−6.481	−4.293	−1.581	4.379	1.496	−1.967
t_O	212	108	169	215	134	185	233	133	162	218
	(41.8)	(21.3)	(33.3)	(42.4)	(26.4)	(36.5)	(46.0)	(26.2)	(32.0)	(43.0)
t_E	217.6	125.5	174.2	217.6	125.5	174.2	217.6	125.5	174.2	217.6
	(42.9)	(24.8)	(34.4)	(42.9)	(24.8)	(34.4)	(42.9)	(24.8)	(34.4)	(42.9)
	−11.0	−32.5	−10.3	−5.1	17.5	22.2	31.9	15.4	−23.6	0.8

	-2.174	-6.403	-2.029	-1.013	3.458	4.381	6.292	3.040	-4.648	0.165
g_O	36	115	69	54	115	60	38	107	65	42
	(7.1)	(22.7)	(13.6)	(10.7)	(22.7)	(11.8)	(7.5)	(21.1)	(12.8)	(8.3)
g_E	44.4	117.6	67.0	44.4	117.6	67.0	44.4	117.6	67.0	44.4
	(8.7)	(23.2)	(13.2)	(8.7)	(23.2)	(13.2)	(8.7)	(23.2)	(13.2)	(8.7)
	-15.0	-5.1	4.1	21.2	-5.1	-13.2	-11.8	-20.2	-3.9	-4.6
	-2.965	-1.007	0.804	4.190	-1.007	-2.609	-2.319	-3.980	-0.774	-0.905
c_O	35	69	97	34	74	96	27	55	99	39
	(6.9)	(13.6)	(19.1)	(6.7)	(14.6)	(18.9)	(5.3)	(10.8)	(19.5)	(7.7)
c_E	32.0	62.7	88.5	32.0	62.7	88.5	32.0	62.7	88.5	32.0
	(6.3)	(12.4)	(17.5)	(6.3)	(12.4)	(17.5)	(6.3)	(12.4)	(17.5)	(6.3)
	6.2	13.2	17.7	4.1	24.5	15.5	-9.2	-14.5	22.1	15.4
	1.232	2.597	3.492	0.808	4.827	3.064	-1.814	-2.850	4.361	3.037
N	507	507	507	507	507	507	507	507	507	507
X2	2.6	4.1	1.2	2.6	4.1	2.7	2.9	2.9	2.2	1.8
Gvalue	2.7	4.2	1.2	2.5	4.0	2.8	2.9	3.0	2.2	1.7
Gv/N	0.523	0.820	0.229	0.493	0.798	0.543	0.578	0.588	0.435	0.330
Prob	0.44830	0.24478	0.76293	0.47526	0.25652	0.43165	0.40217	0.39420	0.53062	0.64278

	91	92	93	94	95	96	97	98	99	100
a_O	217	177	224	181	178	210	210	182	217	216
	(42.8)	(34.9)	(44.2)	(35.7)	(35.1)	(41.4)	(41.4)	(35.9)	(42.8)	(42.6)
a_E	201.2	177.2	213.0	201.2	177.2	213.0	201.2	177.2	213.0	201.2
	(39.7)	(35.0)	(42.0)	(39.7)	(35.0)	(42.0)	(39.7)	(35.0)	(42.0)	(39.7)
	32.8	-0.5	22.5	-38.3	1.5	-6.0	18.0	9.6	8.0	30.7
	6.478	-0.098	4.430	-7.549	0.297	-1.193	3.552	1.899	1.574	6.054
t_O	127	183	216	137	176	227	120	165	226	105
	(25.0)	(36.1)	(42.6)	(27.0)	(34.7)	(44.8)	(23.7)	(32.5)	(44.6)	(20.7)
t_E	125.5	174.2	217.6	125.5	174.2	217.6	125.5	174.2	217.6	125.5
	(24.8)	(34.4)	(42.9)	(24.8)	(34.4)	(42.9)	(24.8)	(34.4)	(42.9)	(24.8)
	3.0	18.0	-3.2	24.0	3.6	19.2	-10.8	-17.9	17.2	-37.5
	0.590	3.549	-0.622	4.733	0.705	3.794	-2.126	-3.540	3.384	-7.392
g_O	106	66	40	126	78	35	105	75	43	123
	(20.9)	(13.0)	(7.9)	(24.9)	(15.4)	(6.9)	(20.7)	(14.8)	(8.5)	(24.3)
g_E	117.6	67.0	44.4	117.6	67.0	44.4	117.6	67.0	44.4	117.6
	(23.2)	(13.2)	(8.7)	(23.2)	(13.2)	(8.7)	(23.2)	(13.2)	(8.7)	(23.2)
	-22.0	-2.0	-8.3	17.4	23.7	-16.6	-23.8	16.9	-2.7	11.1
	-4.335	-0.388	-1.632	3.438	4.682	-3.271	-4.687	3.341	-0.527	2.187
c_O	57	81	27	63	75	35	72	85	21	63
	(11.2)	(16.0)	(5.3)	(12.4)	(14.8)	(6.9)	(14.2)	(16.8)	(4.1)	(12.4)
c_E	62.7	88.5	32.0	62.7	88.5	32.0	62.7	88.5	32.0	62.7
	(12.4)	(17.5)	(6.3)	(12.4)	(17.5)	(6.3)	(12.4)	(17.5)	(6.3)	(12.4)
	-10.9	-14.4	-9.2	0.6	-24.9	6.2	19.9	-6.9	-17.7	0.6
	-2.151	-2.844	-1.814	0.110	-4.910	1.232	3.918	-1.368	-3.493	0.110
N	507	507	507	507	507	507	507	507	507	507
X2	2.9	1.1	1.8	3.7	3.9	2.7	3.3	1.7	4.2	4.7
Gvalue	2.9	1.1	1.8	3.7	3.9	2.8	3.3	1.7	4.8	4.9
Gv/N	0.581	0.219	0.362	0.731	0.774	0.561	0.657	0.333	0.938	0.959
Prob	0.39973	0.77440	0.60681	0.29485	0.26983	0.41602	0.34312	0.63956	0.19072	0.18201

	101	102	103	104	105	106	107	108	109	110
a_O	204	219	218	166	206	212	160	190	187	197
	(40.2)	(43.2)	(43.0)	(32.7)	(40.6)	(41.8)	(31.6)	(37.5)	(36.9)	(38.9)
a_E	177.2	213.0	201.2	177.2	213.0	201.2	177.2	213.0	201.2	177.2
	(35.0)	(42.0)	(39.7)	(35.0)	(42.0)	(39.7)	(35.0)	(42.0)	(39.7)	(35.0)
	57.4	12.1	35.0	-21.8	-13.9	22.2	-32.8	-43.5	-27.3	41.6
	11.312	2.381	6.903	-4.293	-2.733	4.379	-6.462	-8.581	-5.394	8.210
t_O	151	216	117	191	222	125	194	237	134	164
	(29.8)	(42.6)	(23.1)	(37.7)	(43.8)	(24.7)	(38.3)	(46.7)	(26.4)	(32.3)
t_E	174.2	217.6	125.5	174.2	217.6	125.5	174.2	217.6	125.5	174.2
	(34.4)	(42.9)	(24.8)	(34.4)	(42.9)	(24.8)	(34.4)	(42.9)	(24.8)	(34.4)
	-43.2	-3.2	-16.4	35.1	8.9	-1.0	41.7	40.5	17.5	-19.8
	-8.521	-0.622	-3.242	6.927	1.760	-0.202	8.229	7.992	3.458	-3.912
g_O	66	42	115	54	49	111	64	42	127	57
	(13.0)	(8.3)	(22.7)	(10.7)	(9.7)	(21.9)	(12.6)	(8.3)	(25.0)	(11.2)
g_E	67.0	44.4	117.6	67.0	44.4	117.6	67.0	44.4	117.6	67.0
	(13.2)	(8.7)	(23.2)	(13.2)	(8.7)	(23.2)	(13.2)	(8.7)	(23.2)	(13.2)
	-2.0	-4.6	-5.1	-23.3	9.8	-12.8	-5.8	-4.6	19.6	-18.4
	-0.388	-0.905	-1.007	-4.592	1.924	-2.522	-1.153	-0.905	3.861	-3.631
c_O	86	30	57	96	30	59	89	38	59	89
	(17.0)	(5.9)	(11.2)	(18.9)	(5.9)	(11.6)	(17.6)	(7.5)	(11.6)	(17.6)
c_E	88.5	32.0	62.7	88.5	32.0	62.7	88.5	32.0	62.7	88.5
	(17.5)	(6.3)	(12.4)	(17.5)	(6.3)	(12.4)	(17.5)	(6.3)	(12.4)	(17.5)
	-5.0	-3.9	-10.9	15.5	-3.9	-7.2	0.9	13.0	-7.2	0.9
	-0.987	-0.768	-2.151	3.064	-0.768	-1.424	0.182	2.570	-1.424	0.182
N	507	507	507	507	507	507	507	507	507	507
X2	7.2	0.4	2.6	5.5	0.9	1.2	4.1	5.5	2.5	4.3
Gvalue	7.2	0.4	2.6	5.6	0.9	1.2	4.0	5.5	2.5	4.3
Gv/N	1.416	0.085	0.504	1.106	0.182	0.232	0.797	1.076	0.502	0.850
Prob	0.06639	0.93329	0.46553	0.13236	0.81969	0.75925	0.25734	0.14135	0.46696	0.23008

	111	112	113	114	115	116	117	118	119	120
a_O	198	206	177	205	185	179	223	225	199	203
	(39.1)	(40.6)	(34.9)	(40.4)	(36.5)	(35.3)	(44.0)	(44.5)	(39.3)	(40.1)
a_E	213.0	201.2	177.2	213.0	201.2	177.2	213.0	200.8	176.9	212.6
	(42.0)	(39.7)	(35.0)	(42.0)	(39.7)	(35.0)	(42.0)	(39.7)	(35.0)	(42.0)
	-29.0	9.7	-0.5	-15.8	-31.0	3.5	20.4	51.2	46.9	-18.8
	-5.721	1.922	-0.098	-3.114	-6.121	0.694	4.017	10.125	9.260	-3.717
t_O	239	130	185	224	141	170	206	108	173	229
	(47.1)	(25.6)	(36.5)	(44.2)	(27.8)	(33.5)	(40.6)	(21.3)	(34.2)	(45.3)
t_E	217.6	125.5	174.2	217.6	125.5	174.2	217.6	125.3	173.9	217.2
	(42.9)	(24.8)	(34.4)	(42.9)	(24.8)	(34.4)	(42.9)	(24.8)	(34.4)	(42.9)
	44.9	9.1	22.2	13.0	32.8	-8.3	-22.5	-32.0	-1.8	24.3
	8.851	1.801	4.381	2.568	6.471	-1.645	-4.445	-6.331	-0.346	4.808
g_O	47	97	57	46	123	71	43	108	59	44
	(9.3)	(19.1)	(11.2)	(9.1)	(24.3)	(14.0)	(8.5)	(21.3)	(11.7)	(8.7)
g_E	44.4	117.6	67.0	44.4	117.6	67.0	44.4	117.3	66.9	44.3
	(8.7)	(23.2)	(13.2)	(8.7)	(23.2)	(13.2)	(8.7)	(23.2)	(13.2)	(8.7)
	5.4	-37.3	-18.4	3.3	11.1	8.3	-2.7	-17.9	-14.8	-0.5
	1.073	-7.362	-3.631	0.660	2.187	1.628	-0.527	-3.544	-2.916	-0.106
c_O	23	74	88	32	58	87	35	65	75	30
	(4.5)	(14.6)	(17.4)	(6.3)	(11.4)	(17.2)	(6.9)	(12.8)	(14.8)	(5.9)
c_E	32.0	62.7	88.5	32.0	62.7	88.5	32.0	62.6	88.4	31.9
	(6.3)	(12.4)	(17.5)	(6.3)	(12.4)	(17.5)	(6.3)	(12.4)	(17.5)	(6.3)
	-15.2	24.5	-1.1	-0.0	-9.1	-3.1	6.2	4.9	-24.6	-3.8
	-3.000	4.827	-0.212	-0.005	-1.791	-0.602	1.232	0.967	-4.861	-0.747

N	507	507	507	507	507	507	507	506	506	506
X2	5.9	5.9	2.2	0.6	3.8	0.4	1.4	6.1	5.7	1.2
Gvalue	6.1	6.0	2.2	0.6	3.8	0.4	1.4	6.2	5.8	1.2
Gv/N	1.203	1.188	0.439	0.110	0.747	0.075	0.276	1.218	1.137	0.237
Prob	0.10679	0.11054	0.52662	0.90658	0.28552	0.94381	0.70594	0.10397	0.12436	0.75290

	121	122	123	124	125	126	127	128	129	130
a_O	187	168	209	186	173	219	204	173	213	211
	(37.0)	(33.2)	(41.3)	(36.8)	(34.2)	(43.3)	(40.3)	(34.2)	(42.1)	(41.7)
a_E	200.8	176.9	212.6	200.8	176.9	212.6	200.8	176.9	212.6	200.8
	(39.7)	(35.0)	(42.0)	(39.7)	(35.0)	(42.0)	(39.7)	(35.0)	(42.0)	(39.7)
	-26.6	-17.3	-7.2	-28.5	-7.7	12.9	6.5	-7.7	0.7	20.9
	-5.258	-3.427	-1.421	-5.624	-1.524	2.557	1.280	-1.524	0.148	4.137
t_O	115	164	238	133	171	203	114	155	225	118
	(22.7)	(32.4)	(47.0)	(26.3)	(33.8)	(40.1)	(22.5)	(30.6)	(44.5)	(23.3)
t_E	125.3	173.9	217.2	125.3	173.9	217.2	125.3	173.9	217.2	125.3
	(24.8)	(34.4)	(42.9)	(24.8)	(34.4)	(42.9)	(24.8)	(34.4)	(42.9)	(24.8)
	-19.7	-19.2	43.6	15.9	-5.7	-27.4	-21.5	-35.6	16.0	-14.1
	-3.887	-3.791	8.623	3.149	-1.128	-5.408	-4.246	-7.041	3.157	-2.787
g_O	125	70	36	129	72	55	120	77	36	112
	(24.7)	(13.8)	(7.1)	(25.5)	(14.2)	(10.9)	(23.7)	(15.2)	(7.1)	(22.1)
g_E	117.3	66.9	44.3	117.3	66.9	44.3	117.3	66.9	44.3	117.3
	(23.2)	(13.2)	(8.7)	(23.2)	(13.2)	(8.7)	(23.2)	(13.2)	(8.7)	(23.2)
	15.8	6.4	-14.9	24.4	10.7	23.9	5.4	21.7	-14.9	-10.4
	3.121	1.270	-2.942	4.827	2.108	4.718	1.060	4.298	-2.942	-2.065
c_O	79	104	23	58	90	29	68	101	32	65
	(15.6)	(20.6)	(4.5)	(11.5)	(17.8)	(5.7)	(13.4)	(20.0)	(6.3)	(12.8)
c_E	62.6	88.4	31.9	62.6	88.4	31.9	62.6	88.4	31.9	62.6
	(12.4)	(17.5)	(6.3)	(12.4)	(17.5)	(6.3)	(12.4)	(17.5)	(6.3)	(12.4)
	36.8	33.9	-15.1	-8.8	3.3	-5.6	11.3	27.0	0.1	4.9
	7.267	6.697	-2.988	-1.749	0.652	-1.110	2.225	5.335	0.020	0.967
N	506	506	506	506	506	506	506	506	506	506
X2	6.6	3.9	6.1	3.1	0.6	4.0	1.6	5.5	1.8	1.3
Gvalue	6.3	3.8	6.4	3.1	0.6	3.8	1.6	5.4	1.9	1.3
Gv/N	1.243	0.749	1.272	0.603	0.109	0.756	0.318	1.069	0.382	0.253
Prob	0.09842	0.28513	0.09230	0.38373	0.90774	0.28084	0.65737	0.14431	0.58660	0.73408

	131	132	133	134	135	136	137	138	139	140
a_O	178	221	193	175	185	192	187	207	195	179
	(35.2)	(43.7)	(38.1)	(34.6)	(36.6)	(37.9)	(37.0)	(40.9)	(38.5)	(35.4)
a_E	176.9	212.6	200.8	176.9	212.6	200.8	176.9	212.6	200.8	176.9
	(35.0)	(42.0)	(39.7)	(35.0)	(42.0)	(39.7)	(35.0)	(42.0)	(39.7)	(35.0)
	2.2	17.1	-15.3	-3.8	-51.5	-17.2	20.8	-11.1	-11.4	4.2
	0.437	3.374	-3.018	-0.746	-10.177	-3.396	4.105	-2.194	-2.254	0.835
t_O	185	199	125	171	245	127	162	225	128	172
	(36.6)	(39.3)	(24.7)	(33.8)	(48.4)	(25.1)	(32.0)	(44.5)	(25.3)	(34.0)
t_E	173.9	217.2	125.3	173.9	217.2	125.3	173.9	217.2	125.3	173.9
	(34.4)	(42.9)	(24.8)	(34.4)	(42.9)	(24.8)	(34.4)	(42.9)	(24.8)	(34.4)
	22.9	-34.7	-0.5	-5.7	59.1	3.5	-22.9	16.0	5.5	-3.7
	4.534	-6.867	-0.105	-1.128	11.684	0.690	-4.531	3.157	1.092	-0.738
g_O	71	55	118	77	40	128	74	54	101	69
	(14.0)	(10.9)	(23.3)	(15.2)	(7.9)	(25.3)	(14.6)	(10.7)	(20.0)	(13.6)
g_E	66.9	44.3	117.3	66.9	44.3	117.3	66.9	44.3	117.3	66.9
	(13.2)	(8.7)	(23.2)	(13.2)	(8.7)	(23.2)	(13.2)	(8.7)	(23.2)	(13.2)
	8.5	23.9	1.3	21.7	-8.1	22.2	15.0	21.5	-30.3	4.4
	1.687	4.718	0.258	4.298	-1.604	4.396	2.968	4.240	-5.989	0.860
c_O	72	31	70	83	36	59	83	20	82	86
	(14.2)	(6.1)	(13.8)	(16.4)	(7.1)	(11.7)	(16.4)	(4.0)	(16.2)	(17.0)
c_E	88.4	31.9	62.6	88.4	31.9	62.6	88.4	31.9	62.6	88.4
	(17.5)	(6.3)	(12.4)	(17.5)	(6.3)	(12.4)	(17.5)	(6.3)	(12.4)	(17.5)
	-29.5	-1.9	15.6	-10.4	8.6	-7.0	-10.4	-18.7	44.3	-4.7
	-5.828	-0.370	3.092	-2.055	1.698	-1.380	-2.055	-3.703	8.751	-0.922
N	506	506	506	506	506	506	506	506	506	506
X2	4.0	4.5	1.2	1.9	8.1	1.6	2.5	7.0	8.5	0.2
Gvalue	4.2	4.3	1.2	1.9	8.1	1.6	2.5	7.6	8.1	0.2
Gv/N	0.828	0.855	0.228	0.369	1.601	0.309	0.487	1.500	1.599	0.035
Prob	0.24146	0.22810	0.76432	0.60052	0.04395	0.66757	0.48143	0.05531	0.04413	0.98122

	141	142	143	144	145	146	147	148	149	150
a_O	215	197	172	238	213	173	226	198	168	213
	(42.5)	(38.9)	(34.0)	(47.0)	(42.2)	(34.3)	(44.8)	(39.2)	(33.3)	(42.2)
a_E	212.6	200.8	176.9	212.6	200.4	176.5	212.2	200.4	176.5	212.2
	(42.0)	(39.7)	(35.0)	(42.0)	(39.7)	(35.0)	(42.0)	(39.7)	(35.0)	(42.0)
	4.8	-7.5	-9.7	53.7	26.0	-7.0	28.5	-4.8	-16.7	1.6
	0.943	-1.483	-1.909	10.605	5.148	-1.391	5.637	-0.941	-3.302	0.315
t_O	216	112	169	197	139	182	216	118	176	208
	(42.7)	(22.1)	(33.4)	(38.9)	(27.5)	(36.0)	(42.8)	(23.4)	(34.9)	(41.2)
t_E	217.2	125.3	173.9	217.2	125.0	173.5	216.7	125.0	173.5	216.7
	(42.9)	(24.8)	(34.4)	(42.9)	(24.8)	(34.4)	(42.9)	(24.8)	(34.4)	(42.9)
	-2.3	-25.1	-9.6	-38.4	29.5	17.3	-1.4	-13.6	5.0	-17.1
	-0.455	-4.955	-1.901	-7.584	5.836	3.433	-0.287	-2.700	0.983	-3.385
g_O	43	135	83	39	102	54	42	117	77	48
	(8.5)	(26.7)	(16.4)	(7.7)	(20.2)	(10.7)	(8.3)	(23.2)	(15.2)	(9.5)
g_E	44.3	117.3	66.9	44.3	117.1	66.7	44.2	117.1	66.7	44.2
	(8.7)	(23.2)	(13.2)	(8.7)	(23.2)	(13.2)	(8.7)	(23.2)	(13.2)	(8.7)
	-2.5	37.8	35.9	-9.9	-28.2	-22.9	-4.3	-0.2	22.1	8.0
	-0.495	7.477	7.095	-1.954	-5.583	-4.526	-0.843	-0.046	4.367	1.575
c_O	32	62	82	32	51	96	21	72	84	36
	(6.3)	(12.3)	(16.2)	(6.3)	(10.1)	(19.0)	(4.2)	(14.3)	(16.6)	(7.1)
c_E	31.9	62.6	88.4	31.9	62.5	88.2	31.9	62.5	88.2	31.9
	(6.3)	(12.4)	(17.5)	(6.3)	(12.4)	(17.5)	(6.3)	(12.4)	(17.5)	(6.3)
	0.1	-1.2	-12.3	0.1	-20.7	16.3	-17.5	20.4	-8.2	8.7
	0.020	-0.235	-2.423	0.020	-4.099	3.226	-3.474	4.047	-1.619	1.730
N	506	506	506	506	505	505	505	505	505	505
X2	0.1	4.1	4.6	5.5	6.4	3.6	4.7	1.9	2.2	1.2
Gvalue	0.1	4.1	4.4	5.5	6.6	3.7	5.2	1.8	2.2	1.2
Gv/N	0.014	0.804	0.862	1.087	1.302	0.742	1.034	0.359	0.429	0.235
Prob	0.99524	0.25440	0.22499	0.13868	0.08670	0.28993	0.15631	0.61187	0.53902	0.75565

Bavi_197N
5UTR:

	50	49	48	47	46	45	44	43	42	41
a_O	591	644	614	650	623	585	614	629	548	602
	(19.4)	(21.2)	(20.2)	(21.4)	(20.5)	(19.2)	(20.2)	(20.7)	(18.0)	(19.8)
a_E	639.9	639.9	639.9	639.9	639.9	639.9	639.9	639.9	639.9	639.9
	(21.0)	(21.0)	(21.0)	(21.0)	(21.0)	(21.0)	(21.0)	(21.0)	(21.0)	(21.0)
	-94.0	8.2	-50.7	20.4	-33.3	-104.9	-50.7	-21.6	-169.9	-73.5
	-3.090	0.271	-1.668	0.669	-1.097	-3.451	-1.668	-0.711	-5.588	-2.417
t_O	567	687	647	583	725	662	597	651	674	662
	(18.6)	(22.6)	(21.3)	(19.2)	(23.8)	(21.8)	(19.6)	(21.4)	(22.2)	(21.8)
t_E	656.7	656.7	656.7	656.7	656.7	656.7	656.7	656.7	656.7	656.7
	(21.6)	(21.6)	(21.6)	(21.6)	(21.6)	(21.6)	(21.6)	(21.6)	(21.6)	(21.6)
	-166.6	61.9	-19.3	-138.9	143.4	10.6	-113.9	-11.4	35.0	10.6
	-5.479	2.035	-0.636	-4.567	4.715	0.347	-3.745	-0.376	1.150	0.347
g_O	949	827	886	883	826	877	905	879	900	854
	(31.2)	(27.2)	(29.1)	(29.0)	(27.2)	(28.8)	(29.8)	(28.9)	(29.6)	(28.1)
g_E	852.0	852.0	852.0	852.0	852.0	852.0	852.0	852.0	852.0	852.0
	(28.0)	(28.0)	(28.0)	(28.0)	(28.0)	(28.0)	(28.0)	(28.0)	(28.0)	(28.0)
	204.7	-49.2	69.4	63.2	-51.1	50.8	109.3	54.9	98.7	4.1
	6.732	-1.618	2.282	2.077	-1.682	1.670	3.594	1.806	3.246	0.134
c_O	934	883	894	925	867	917	925	882	919	923
	(30.7)	(29.0)	(29.4)	(30.4)	(28.5)	(30.2)	(30.4)	(29.0)	(30.2)	(30.4)
c_E	892.4	892.4	892.4	892.4	892.4	892.4	892.4	892.4	892.4	892.4
	(29.3)	(29.3)	(29.3)	(29.3)	(29.3)	(29.3)	(29.3)	(29.3)	(29.3)	(29.3)
	85.1	-18.7	3.2	66.4	-50.1	49.9	66.4	-20.7	54.0	62.3
	2.799	-0.614	0.106	2.183	-1.646	1.641	2.183	-0.679	1.776	2.047
N	3041	3041	3041	3041	3041	3041	3041	3041	3041	3041
X2	29.0	2.3	2.6	10.8	9.1	6.2	11.0	1.2	17.2	3.3
Gvalue	29.3	2.2	2.6	11.1	8.8	6.3	11.1	1.2	17.8	3.4
Gv/N	0.962	0.074	0.084	0.364	0.291	0.207	0.364	0.040	0.584	0.111
Prob	0.00000	0.52454	0.46591	0.01140	0.03149	0.09816	0.01129	0.75130	0.00049	0.33728

	40	39	38	37	36	35	34	33	32	31
a_O	660	553	620	640	554	591	677	590	620	644
	(21.7)	(18.2)	(20.4)	(21.0)	(18.2)	(19.4)	(22.3)	(19.4)	(20.4)	(21.2)
a_E	639.9	639.9	639.9	639.9	639.9	639.9	639.9	639.9	639.9	639.9
	(21.0)	(21.0)	(21.0)	(21.0)	(21.0)	(21.0)	(21.0)	(21.0)	(21.0)	(21.0)
	40.8	-161.4	-39.2	0.2	-159.7	-94.0	76.3	-95.8	-39.2	8.2
	1.342	-5.308	-1.288	0.007	-5.252	-3.090	2.509	-3.150	-1.288	0.271
t_O	713	684	605	653	694	634	676	671	640	707
	(23.4)	(22.5)	(19.9)	(21.5)	(22.8)	(20.8)	(22.2)	(22.1)	(21.0)	(23.2)
t_E	656.7	656.7	656.7	656.7	656.7	656.7	656.7	656.7	656.7	656.7
	(21.6)	(21.6)	(21.6)	(21.6)	(21.6)	(21.6)	(21.6)	(21.6)	(21.6)	(21.6)
	117.2	55.6	-99.3	-7.5	76.6	-44.7	39.1	28.8	-33.0	104.3
	3.854	1.830	-3.265	-0.245	2.519	-1.469	1.285	0.948	-1.087	3.429
g_O	832	867	913	797	850	926	751	871	864	793
	(27.4)	(28.5)	(30.0)	(26.2)	(28.0)	(30.5)	(24.7)	(28.6)	(28.4)	(26.1)
g_E	852.0	852.0	852.0	852.0	852.0	852.0	852.0	852.0	852.0	852.0
	(28.0)	(28.0)	(28.0)	(28.0)	(28.0)	(28.0)	(28.0)	(28.0)	(28.0)	(28.0)
	-39.5	30.3	126.3	-106.3	-3.9	154.3	-189.5	38.5	24.2	-113.8
	-1.298	0.997	4.154	-3.496	-0.129	5.074	-6.231	1.265	0.797	-3.741
c_O	836	937	903	951	943	890	937	909	917	897
	(27.5)	(30.8)	(29.7)	(31.3)	(31.0)	(29.3)	(30.8)	(29.9)	(30.2)	(29.5)
c_E	892.4	892.4	892.4	892.4	892.4	892.4	892.4	892.4	892.4	892.4
	(29.3)	(29.3)	(29.3)	(29.3)	(29.3)	(29.3)	(29.3)	(29.3)	(29.3)	(29.3)
	-109.1	91.4	21.3	121.0	104.0	-4.8	91.4	33.5	49.9	9.2
	-3.589	3.006	0.702	3.979	3.421	-0.157	3.006	1.103	1.641	0.304
N	3041	3041	3041	3041	3041	3041	3041	3041	3041	3041
X2	9.5	15.4	9.2	7.4	16.5	11.0	16.9	4.9	1.9	8.0
Gvalue	9.4	15.9	9.2	7.4	17.0	10.9	17.3	5.0	1.9	8.0
Gv/N	0.310	0.524	0.303	0.244	0.558	0.358	0.570	0.166	0.062	0.262
Prob	0.02414	0.00116	0.02663	0.05979	0.00071	0.01232	0.00060	0.16939	0.59398	0.04640

	30	29	28	27	26	25	24	23	22	21
a_O	556	652	628	563	605	625	591	625	668	624
	(18.3)	(21.4)	(20.7)	(18.5)	(19.9)	(20.6)	(19.4)	(20.6)	(22.0)	(20.5)
a_E	639.9	639.9	639.9	639.9	639.9	639.9	639.9	639.9	639.9	639.9
	(21.0)	(21.0)	(21.0)	(21.0)	(21.0)	(21.0)	(21.0)	(21.0)	(21.0)	(21.0)
	-156.3	24.4	-23.6	-144.2	-67.9	-29.5	-94.0	-29.5	57.4	-31.4
	-5.139	0.803	-0.775	-4.741	-2.232	-0.968	-3.090	-0.968	1.888	-1.033
t_O	687	678	716	667	634	723	682	659	737	719
	(22.6)	(22.3)	(23.5)	(21.9)	(20.8)	(23.8)	(22.4)	(21.7)	(24.2)	(23.6)
t_E	656.7	656.7	656.7	656.7	656.7	656.7	656.7	656.7	656.7	656.7
	(21.6)	(21.6)	(21.6)	(21.6)	(21.6)	(21.6)	(21.6)	(21.6)	(21.6)	(21.6)
	61.9	43.2	123.7	20.7	-44.7	139.0	51.5	4.5	170.0	130.2
	2.035	1.421	4.068	0.680	-1.469	4.571	1.693	0.149	5.589	4.283
g_O	847	846	785	792	837	764	791	783	681	705
	(27.9)	(27.8)	(25.8)	(26.0)	(27.5)	(25.1)	(26.0)	(25.7)	(22.4)	(23.2)
g_E	852.0	852.0	852.0	852.0	852.0	852.0	852.0	852.0	852.0	852.0
	(28.0)	(28.0)	(28.0)	(28.0)	(28.0)	(28.0)	(28.0)	(28.0)	(28.0)	(28.0)
	-9.9	-11.9	-128.5	-115.6	-29.7	-166.5	-117.5	-132.2	-305.1	-267.0
	-0.326	-0.391	-4.227	-3.802	-0.976	-5.476	-3.863	-4.347	-10.032	-8.780
c_O	951	865	912	1019	965	929	977	974	955	993
	(31.3)	(28.4)	(30.0)	(33.5)	(31.7)	(30.5)	(32.1)	(32.0)	(31.4)	(32.7)
c_E	892.4	892.4	892.4	892.4	892.4	892.4	892.4	892.4	892.4	892.4
	(29.3)	(29.3)	(29.3)	(29.3)	(29.3)	(29.3)	(29.3)	(29.3)	(29.3)	(29.3)
	121.0	-53.9	39.6	270.4	151.0	74.7	177.0	170.5	129.5	212.2
	3.979	-1.773	1.304	8.891	4.965	2.456	5.820	5.606	4.259	6.977
N	3041	3041	3041	3041	3041	3041	3041	3041	3041	3041
X2	16.3	1.8	11.3	31.6	8.9	17.6	17.1	13.4	49.7	43.0
Gvalue	16.7	1.8	11.3	31.3	8.8	17.7	17.0	13.3	51.8	44.0
Gv/N	0.549	0.059	0.370	1.029	0.288	0.583	0.561	0.439	1.704	1.447
Prob	0.00082	0.61501	0.01044	0.00000	0.03274	0.00050	0.00069	0.00395	0.00000	0.00000

	20	19	18	17	16	15	14	13	12	11
a_O	645	649	634	719	745	674	746	817	829	861
	(21.2)	(21.3)	(20.8)	(23.6)	(24.5)	(22.2)	(24.5)	(26.9)	(27.3)	(28.3)
a_E	639.9	639.9	639.9	639.9	639.9	639.9	639.9	639.9	639.9	639.9
	(21.0)	(21.0)	(21.0)	(21.0)	(21.0)	(21.0)	(21.0)	(21.0)	(21.0)	(21.0)
	10.2	18.3	-11.7	167.6	226.6	70.0	228.9	399.2	429.3	511.1
	0.337	0.603	-0.386	5.511	7.451	2.301	7.527	13.128	14.116	16.806
t_O	748	825	800	754	825	793	660	698	562	434
	(24.6)	(27.1)	(26.3)	(24.8)	(27.1)	(26.1)	(21.7)	(23.0)	(18.5)	(14.3)

```
t_E        656.7   656.7   656.7   656.7   656.7   656.7   656.7   656.7   656.7   656.7
        ( 21.6) ( 21.6) ( 21.6) ( 21.6) ( 21.6) ( 21.6) ( 21.6) ( 21.6) ( 21.6) ( 21.6)
           194.7   376.4   315.7   208.3   376.4   299.0     6.5    85.1  -175.1  -359.6
           6.401  12.376  10.382   6.848  12.376   9.833   0.215   2.797  -5.758 -11.824
g_O          725     645     631     634     654     704     742     747     950    1147
        ( 23.8) ( 21.2) ( 20.7) ( 20.8) ( 21.5) ( 23.2) ( 24.4) ( 24.6) ( 31.2) ( 37.7)
g_E        852.0   852.0   852.0   852.0   852.0   852.0   852.0   852.0   852.0   852.0
        ( 28.0) ( 28.0) ( 28.0) ( 28.0) ( 28.0) ( 28.0) ( 28.0) ( 28.0) ( 28.0) ( 28.0)
          -234.0  -359.0  -378.9  -374.7  -345.9  -268.6  -205.1  -196.4   206.9   682.1
          -7.695 -11.806 -12.460 -12.322 -11.374  -8.833  -6.744  -6.460   6.805  22.431
c_O          923     922     976     934     817     870     893     779     700     599
        ( 30.4) ( 30.3) ( 32.1) ( 30.7) ( 26.9) ( 28.6) ( 29.4) ( 25.6) ( 23.0) ( 19.7)
c_E        892.4   892.4   892.4   892.4   892.4   892.4   892.4   892.4   892.4   892.4
        ( 29.3) ( 29.3) ( 29.3) ( 29.3) ( 29.3) ( 29.3) ( 29.3) ( 29.3) ( 29.3) ( 29.3)
            62.3    60.2   174.8    85.1  -144.2   -44.2     1.2  -211.7  -340.0  -477.6
           2.047   1.979   5.749   2.799  -4.743  -1.454   0.040  -6.962 -11.179 -15.704

N           3041    3041    3041    3041    3041    3041    3041    3041    3041    3041
X2          32.7    94.5    96.5    81.9   112.7    56.3    31.8    78.9   122.3   350.6
Gvalue      33.1    95.9    99.9    86.3   112.8    56.2    31.6    76.1   121.1   356.1
Gv/N       1.090   3.153   3.285   2.838   3.710   1.847   1.038   2.503   3.984  11.708
Prob     0.00000 0.00000 0.00000 0.00000 0.00000 0.00000 0.00000 0.00000 0.00000 0.00000
         -------------------------------------------------------------------------------
              10       9       8       7       6       5       4       3       2       1
         -------------------------------------------------------------------------------
a_O          827     813     873     906     847     699     788     898     543     429
        ( 27.2) ( 26.7) ( 28.7) ( 29.8) ( 27.9) ( 23.0) ( 25.9) ( 29.5) ( 17.9) ( 14.1)
a_E        639.9   639.9   639.9   639.9   639.9   639.9   639.9   639.9   639.9   639.9
        ( 21.0) ( 21.0) ( 21.0) ( 21.0) ( 21.0) ( 21.0) ( 21.0) ( 21.0) ( 21.0) ( 21.0)
           424.2   389.3   542.3   630.1   475.0   123.5   328.1   608.6  -178.3  -343.1
          13.951  12.802  17.835  20.720  15.619   4.061  10.789  20.013  -5.864 -11.282
t_O          458     399     484     627     620     726     607     530     876     504
        ( 15.1) ( 13.1) ( 15.9) ( 20.6) ( 20.4) ( 23.9) ( 20.0) ( 17.4) ( 28.8) ( 16.6)
t_E        656.7   656.7   656.7   656.7   656.7   656.7   656.7   656.7   656.7   656.7
        ( 21.6) ( 21.6) ( 21.6) ( 21.6) ( 21.6) ( 21.6) ( 21.6) ( 21.6) ( 21.6) ( 21.6)
          -330.1  -397.7  -295.4   -58.1   -71.4   145.6   -95.6  -227.3   504.7  -266.8
         -10.856 -13.077  -9.715  -1.911  -2.347   4.787  -3.144  -7.474  16.597  -8.774
g_O         1228    1327    1138     875     784     688     581     876     519     420
        ( 40.4) ( 43.6) ( 37.4) ( 28.8) ( 25.8) ( 22.6) ( 19.1) ( 28.8) ( 17.1) ( 13.8)
g_E        852.0   852.0   852.0   852.0   852.0   852.0   852.0   852.0   852.0   852.0
        ( 28.0) ( 28.0) ( 28.0) ( 28.0) ( 28.0) ( 28.0) ( 28.0) ( 28.0) ( 28.0) ( 28.0)
           897.9  1176.1   658.8    46.7  -130.4  -294.1  -444.8    48.7  -514.5  -594.1
          29.526  38.673  21.666   1.535  -4.287  -9.672 -14.627   1.602 -16.918 -19.537
c_O          528     502     546     633     790     928    1065     737    1103    1688
        ( 17.4) ( 16.5) ( 18.0) ( 20.8) ( 26.0) ( 30.5) ( 35.0) ( 24.2) ( 36.3) ( 55.5)
c_E        892.4   892.4   892.4   892.4   892.4   892.4   892.4   892.4   892.4   892.4
        ( 29.3) ( 29.3) ( 29.3) ( 29.3) ( 29.3) ( 29.3) ( 29.3) ( 29.3) ( 29.3) ( 29.3)
          -554.2  -577.6  -536.5  -434.8  -192.6    72.6   376.6  -282.0   467.4  2151.9
         -18.224 -18.994 -17.642 -14.297  -6.332   2.388  12.385  -9.273  15.371  70.761

N           3041    3041    3041    3041    3041    3041    3041    3041    3041    3041
X2         429.6   583.6   360.8   188.0    86.3    45.7   157.6   156.3   267.7  1033.4
Gvalue     437.8   590.1   369.3   183.9    80.7    47.6   164.3   148.0   279.3   947.8
Gv/N      14.396  19.404  12.143   6.046   2.653   1.564   5.403   4.868   9.185  31.168
Prob     0.00000 0.00000 0.00000 0.00000 0.00000 0.00000 0.00000 0.00000 0.00000 0.00000
         -------------------------------------------------------------------------------

CDS_INITIAL:
         -------------------------------------------------------------------------------
               1       2       3       4       5       6       7       8       9      10
         -------------------------------------------------------------------------------
a_O         2860       0       0    1410     888     585     980     968     653     841
        ( 94.0) (  0.0) (  0.0) ( 46.4) ( 29.2) ( 19.2) ( 32.2) ( 31.8) ( 21.5) ( 27.7)
a_E        620.4   761.3   276.2   620.4   761.3   276.2   620.4   761.3   276.2   620.4
        ( 20.4) ( 25.0) (  9.1) ( 20.4) ( 25.0) (  9.1) ( 20.4) ( 25.0) (  9.1) ( 20.4)
          8741.2     0.0     0.0  2315.1   273.4   878.2   896.0   465.0  1123.9   511.7
         287.445   0.000   0.000  76.129   8.991  28.879  29.465  15.292  36.959  16.825
t_O          181    3041       0     429     462     784     452     737     678     558
        (  6.0) (100.0) (  0.0) ( 14.1) ( 15.2) ( 25.8) ( 14.9) ( 24.2) ( 22.3) ( 18.3)
t_E        413.4   884.7   491.0   413.4   884.7   491.0   413.4   884.7   491.0   413.4
        ( 13.6) ( 29.1) ( 16.1) ( 13.6) ( 29.1) ( 16.1) ( 13.6) ( 29.1) ( 16.1) ( 13.6)
          -299.0  7509.4     0.0    31.8  -600.3   733.8    80.7  -269.2   437.6   334.7
          -9.832 246.938   0.000   1.044 -19.741  24.130   2.653  -8.854  14.390  11.007
g_O            0       0    3041     530     579     597     806     500     657     731
        (  0.0) (  0.0) (100.0) ( 17.4) ( 19.0) ( 19.6) ( 26.5) ( 16.4) ( 21.6) ( 24.0)
g_E       1175.5   606.3  1053.0  1175.5   606.3  1053.0  1175.5   606.3  1053.0  1175.5
        ( 38.7) ( 19.9) ( 34.6) ( 38.7) ( 19.9) ( 34.6) ( 38.7) ( 19.9) ( 34.6) ( 38.7)
             0.0     0.0  6450.0  -844.4   -53.3  -677.6  -608.3  -192.7  -619.9  -694.5
           0.000   0.000 212.102 -27.766  -1.754 -22.283 -20.003  -6.338 -20.384 -22.837
c_O            0       0       0     672    1112    1075     803     836    1053     911
        (  0.0) (  0.0) (  0.0) ( 22.1) ( 36.6) ( 35.4) ( 26.4) ( 27.5) ( 34.6) ( 30.0)
c_E        831.7   788.7  1220.8   831.7   788.7  1220.8   831.7   788.7  1220.8   831.7
        ( 27.3) ( 25.9) ( 40.1) ( 27.3) ( 25.9) ( 40.1) ( 27.3) ( 25.9) ( 40.1) ( 27.3)
             0.0     0.0     0.0  -286.5   764.0  -273.5   -56.4    97.4  -311.4   166.0
           0.000   0.000   0.000  -9.423  25.123  -8.992  -1.854   3.202 -10.240   5.457

N           3041    3041    3041    3041    3041    3041    3041    3041    3041    3041
X2       10222.3  7411.9  5740.9  1390.6   356.8   735.1   329.1   102.2   757.4   304.6
Gvalue    8442.2  7509.4  6450.0  1216.0   383.8   660.9   312.0   100.4   630.2   317.8
Gv/N     277.613 246.938 212.102  39.985  12.619  21.734  10.261   3.302  20.724  10.452
Prob     1.00000 1.00000 1.00000 0.00000 0.00000 0.00000 0.00000 0.00000 0.00000 0.00000
         -------------------------------------------------------------------------------
              11      12      13      14      15      16      17      18      19      20
         -------------------------------------------------------------------------------
a_O          720     512     822     769     522     767     785     476     770     691
        ( 23.7) ( 16.8) ( 27.0) ( 25.3) ( 17.2) ( 25.2) ( 25.8) ( 15.7) ( 25.3) ( 22.7)
a_E        761.3   276.2   620.4   761.3   276.2   620.4   761.3   276.2   620.4   761.3
        ( 25.0) (  9.1) ( 20.4) ( 25.0) (  9.1) ( 20.4) ( 25.0) (  9.1) ( 20.4) ( 25.0)
           -80.3   632.1   462.5    15.5   664.7   325.4    48.1   518.3   332.6  -133.9
          -2.641  20.787  15.210   0.509  21.857  10.699   1.583  17.043  10.938  -4.403
t_O          926     778     538    1014     747     561    1016     713     502     981
        ( 30.5) ( 25.6) ( 17.7) ( 33.3) ( 24.6) ( 18.4) ( 33.4) ( 23.4) ( 16.5) ( 32.3)
t_E        884.7   491.0   413.4   884.7   491.0   413.4   884.7   491.0   413.4   884.7
        ( 29.1) ( 16.1) ( 13.6) ( 29.1) ( 16.1) ( 13.6) ( 29.1) ( 16.1) ( 13.6) ( 29.1)
            84.5   716.2   283.4   276.6   626.9   342.5   281.2   532.0   194.9   202.7
           2.778  23.552   9.321   9.097  20.616  11.264   9.246  17.493   6.410   6.666
g_O          432     627     775     400     640     769     408     718     895     494
        ( 14.2) ( 20.6) ( 25.5) ( 13.2) ( 21.0) ( 25.3) ( 13.4) ( 23.6) ( 29.4) ( 16.2)
g_E        606.3  1053.0  1175.5   606.3  1053.0  1175.5   606.3  1053.0  1175.5   606.3
        ( 19.9) ( 34.6) ( 38.7) ( 19.9) ( 34.6) ( 38.7) ( 19.9) ( 34.6) ( 38.7) ( 19.9)
          -292.8  -650.2  -645.7  -332.7  -637.4  -652.6  -323.2  -549.9  -488.0  -202.4
          -9.630 -21.381 -21.233 -10.941 -20.960 -21.461 -10.628 -18.084 -16.047  -6.654
```

c_O	963	1124	906	858	1132	944	832	1134	874	875
	(31.7)	(37.0)	(29.8)	(28.2)	(37.2)	(31.0)	(27.4)	(37.3)	(28.7)	(28.8)
c_E	788.7	1220.8	831.7	788.7	1220.8	831.7	788.7	1220.8	831.7	788.7
	(25.9)	(40.1)	(27.3)	(25.9)	(40.1)	(27.3)	(25.9)	(40.1)	(27.3)	(25.9)
	384.5	-185.7	155.1	144.5	-171.0	239.2	88.9	-167.3	86.7	181.7
	12.645	-6.107	5.099	4.752	-5.623	7.864	2.924	-5.501	2.852	5.975
N	3041	3041	3041	3041	3041	3041	3041	3041	3041	3041
X2	92.8	549.2	246.1	95.3	520.8	243.1	87.5	357.8	124.1	47.2
Gvalue	95.9	512.5	255.4	103.9	483.2	254.4	95.0	333.0	126.3	48.2
Gv/N	3.153	16.852	8.397	3.417	15.891	8.365	3.125	10.951	4.155	1.583
Prob	0.00000	0.00000	0.00000	0.00000	0.00000	0.00000	0.00000	0.00000	0.00000	0.00000

	21	22	23	24	25	26	27	28	29	30
a_O	398	665	686	371	635	662	324	604	654	356
	(13.1)	(21.9)	(22.6)	(12.2)	(20.9)	(21.8)	(10.7)	(19.9)	(21.5)	(11.7)
a_E	276.2	620.4	761.3	276.2	620.4	761.3	276.2	620.4	761.3	276.2
	(9.1)	(20.4)	(25.0)	(9.1)	(20.4)	(25.0)	(9.1)	(20.4)	(25.0)	(9.1)
	290.9	92.3	-142.9	219.0	29.5	-185.0	103.5	-32.4	-198.7	180.8
	9.566	3.035	-4.699	7.203	0.970	-6.085	3.404	-1.065	-6.534	5.945
t_O	657	525	963	635	491	916	622	465	957	565
	(21.6)	(17.3)	(31.7)	(20.9)	(16.1)	(30.1)	(20.5)	(15.3)	(31.5)	(18.6)
t_E	491.0	413.4	884.7	491.0	413.4	884.7	491.0	413.4	884.7	491.0
	(16.1)	(13.6)	(29.1)	(16.1)	(13.6)	(29.1)	(16.1)	(13.6)	(29.1)	(16.1)
	382.7	250.9	163.3	326.6	168.9	63.7	294.2	109.4	150.3	158.6
	12.585	8.251	5.371	10.741	5.554	2.094	9.675	3.596	4.944	5.217
g_O	717	947	502	839	1025	586	871	1062	559	863
	(23.6)	(31.1)	(16.5)	(27.6)	(33.7)	(19.3)	(28.6)	(34.9)	(18.4)	(28.4)
g_E	1053.0	1175.5	606.3	1053.0	1175.5	606.3	1053.0	1175.5	606.3	1053.0
	(34.6)	(38.7)	(19.9)	(34.6)	(38.7)	(19.9)	(34.6)	(38.7)	(19.9)	(34.6)
	-551.2	-409.4	-189.5	-381.3	-280.8	-39.9	-330.6	-215.6	-90.8	-343.5
	-18.125	-13.462	-6.232	-12.538	-9.235	-1.312	-10.872	-7.091	-2.986	-11.296
c_O	1269	904	890	1196	890	877	1224	910	871	1257
	(41.7)	(29.7)	(29.3)	(39.3)	(29.3)	(28.8)	(40.2)	(29.9)	(28.6)	(41.3)
c_E	1220.8	831.7	788.7	1220.8	831.7	788.7	1220.8	831.7	788.7	1220.8
	(40.1)	(27.3)	(25.9)	(40.1)	(27.3)	(25.9)	(40.1)	(27.3)	(25.9)	(40.1)
	98.3	150.7	215.1	-49.1	120.6	186.1	6.4	163.8	172.9	73.5
	3.232	4.957	7.072	-1.614	3.966	6.120	0.211	5.386	5.685	2.416
N	3041	3041	3041	3041	3041	3041	3041	3041	3041	3041
X2	219.0	84.0	45.3	118.8	38.3	24.6	74.7	25.2	33.3	69.6
Gvalue	220.7	84.6	46.0	115.3	38.2	24.9	73.5	25.1	33.7	69.4
Gv/N	7.258	2.781	1.512	3.791	1.256	0.818	2.417	0.826	1.109	2.282
Prob	0.00000	0.00000	0.00000	0.00000	0.00000	0.00002	0.00000	0.00001	0.00000	0.00000

	31	32	33	34	35	36	37	38	39	40
a_O	583	634	309	584	580	295	585	631	324	571
	(19.2)	(20.8)	(10.2)	(19.2)	(19.1)	(9.7)	(19.2)	(20.7)	(10.7)	(18.8)
a_E	620.4	761.3	276.2	620.4	761.3	276.2	620.4	761.3	276.2	620.4
	(20.4)	(25.0)	(9.1)	(20.4)	(25.0)	(9.1)	(20.4)	(25.0)	(9.1)	(20.4)
	-72.5	-232.0	69.4	-70.7	-315.5	38.9	-68.8	-236.9	103.5	-94.8
	-2.385	-7.630	2.283	-2.323	-10.376	1.280	-2.262	-7.790	3.404	-3.117
t_O	467	933	587	444	921	503	435	951	537	476
	(15.4)	(30.7)	(19.3)	(14.6)	(30.3)	(16.5)	(14.3)	(31.3)	(17.7)	(15.7)
t_E	413.4	884.7	491.0	413.4	884.7	491.0	413.4	884.7	491.0	413.4
	(13.6)	(29.1)	(16.1)	(13.6)	(29.1)	(16.1)	(13.6)	(29.1)	(16.1)	(13.6)
	113.8	99.2	209.7	63.4	74.1	24.3	44.3	137.4	96.2	134.2
	3.744	3.262	6.894	2.085	2.435	0.799	1.456	4.520	3.163	4.413
g_O	1105	592	880	1126	636	1001	1130	582	956	1132
	(36.3)	(19.5)	(28.9)	(37.0)	(20.9)	(32.9)	(37.2)	(19.1)	(31.4)	(37.2)
g_E	1175.5	606.3	1053.0	1175.5	606.3	1053.0	1175.5	606.3	1053.0	1175.5
	(38.7)	(19.9)	(34.6)	(38.7)	(19.9)	(34.6)	(38.7)	(19.9)	(34.6)	(38.7)
	-136.7	-28.2	-315.9	-96.9	60.9	-101.5	-89.2	-47.6	-184.8	-85.3
	-4.494	-0.929	-10.389	-3.185	2.001	-3.336	-2.933	-1.565	-6.078	-2.806
c_O	886	882	1265	887	904	1242	891	877	1224	862
	(29.1)	(29.0)	(41.6)	(29.2)	(29.7)	(40.8)	(29.3)	(28.8)	(40.2)	(28.3)
c_E	831.7	788.7	1220.8	831.7	788.7	1220.8	831.7	788.7	1220.8	831.7
	(27.3)	(25.9)	(40.1)	(27.3)	(25.9)	(40.1)	(27.3)	(25.9)	(40.1)	(27.3)
	112.1	197.2	90.0	114.2	246.7	42.8	122.8	186.1	6.4	61.7
	3.686	6.485	2.959	3.756	8.112	1.406	4.037	6.120	0.211	2.029
N	3041	3041	3041	3041	3041	3041	3041	3041	3041	3041
X2	17.0	35.3	52.7	10.2	63.0	4.5	9.1	38.1	21.5	16.1
Gvalue	16.8	36.1	53.1	10.1	66.1	4.5	9.1	39.1	21.3	15.8
Gv/N	0.551	1.188	1.747	0.332	2.173	0.149	0.299	1.285	0.699	0.519
Prob	0.00079	0.00000	0.00000	0.01771	0.00000	0.20977	0.02817	0.00000	0.00009	0.00125

	41	42	43	44	45	46	47	48	49	50
a_O	636	293	595	611	314	582	671	333	576	636
	(20.9)	(9.6)	(19.6)	(20.1)	(10.3)	(19.1)	(22.1)	(11.0)	(18.9)	(20.9)
a_E	761.3	276.2	620.4	761.3	276.2	620.4	761.3	276.2	620.4	761.3
	(25.0)	(9.1)	(20.4)	(25.0)	(9.1)	(20.4)	(25.0)	(9.1)	(20.4)	(25.0)
	-228.7	34.7	-49.8	-268.8	80.6	-74.4	-169.4	124.6	-85.6	-228.7
	-7.522	1.140	-1.637	-8.838	2.652	-2.447	-5.572	4.099	-2.814	-7.522
t_O	964	527	416	956	506	403	915	536	457	938
	(31.7)	(17.3)	(13.7)	(31.4)	(16.6)	(13.3)	(30.1)	(17.6)	(15.0)	(30.8)
t_E	884.7	491.0	413.4	884.7	491.0	413.4	884.7	491.0	413.4	884.7
	(29.1)	(16.1)	(13.6)	(29.1)	(16.1)	(13.6)	(29.1)	(16.1)	(13.6)	(29.1)
	165.5	74.6	5.2	148.2	30.5	-20.6	61.6	94.0	91.6	109.7
	5.442	2.453	0.171	4.873	1.002	-0.676	2.026	3.091	3.013	3.609
g_O	592	979	1138	620	1006	1169	640	974	1190	623
	(19.5)	(32.2)	(37.4)	(20.4)	(33.1)	(38.4)	(21.0)	(32.0)	(39.1)	(20.5)
g_E	606.3	1053.0	1175.5	606.3	1053.0	1175.5	606.3	1053.0	1175.5	606.3
	(19.9)	(34.6)	(38.7)	(19.9)	(34.6)	(38.7)	(19.9)	(34.6)	(38.7)	(19.9)
	-28.2	-142.7	-73.8	27.7	-91.9	-12.9	69.3	-152.0	29.2	33.9
	-0.929	-4.694	-2.426	0.912	-3.023	-0.425	2.278	-4.998	0.960	1.114
c_O	849	1242	892	854	1215	887	815	1198	818	844
	(27.9)	(40.8)	(29.3)	(28.1)	(40.0)	(29.2)	(26.8)	(39.4)	(26.9)	(27.8)
c_E	788.7	1220.8	831.7	788.7	1220.8	831.7	788.7	1220.8	831.7	788.7
	(25.9)	(40.1)	(27.3)	(25.9)	(40.1)	(27.3)	(25.9)	(40.1)	(27.3)	(25.9)
	125.1	42.8	124.9	135.8	-11.6	114.2	53.5	-45.2	-27.1	114.4
	4.113	1.406	4.107	4.467	-0.381	3.756	1.758	-1.486	-0.893	3.761
N	3041	3041	3041	3041	3041	3041	3041	3041	3041	3041
X2	32.7	9.2	6.6	41.1	7.8	6.4	14.5	22.2	8.2	28.2
Gvalue	33.6	9.3	6.6	43.0	7.6	6.3	14.9	21.5	8.1	29.3
Gv/N	1.105	0.305	0.215	1.414	0.249	0.208	0.490	0.707	0.267	0.962
Prob	0.00000	0.02577	0.08763	0.00000	0.05563	0.09664	0.00191	0.00008	0.04386	0.00000

	51	52	53	54	55	56	57	58	59	60
a_O	306	570	612	313	562	654	306	574	641	283
	(10.1)	(18.7)	(20.1)	(10.3)	(18.5)	(21.5)	(10.1)	(18.9)	(21.1)	(9.3)
a_E	276.2	620.4	761.3	276.2	620.4	761.3	276.2	620.4	761.3	276.2
	(9.1)	(20.4)	(25.0)	(9.1)	(20.4)	(25.0)	(9.1)	(20.4)	(25.0)	(9.1)
	62.8	−96.6	−267.2	78.4	−111.2	−198.7	62.8	−89.3	−220.5	13.8
	2.065	−3.177	−8.786	2.577	−3.655	−6.534	2.065	−2.936	−7.251	0.455
t_O	527	478	964	514	443	910	528	441	938	512
	(17.3)	(15.7)	(31.7)	(16.9)	(14.6)	(29.9)	(17.4)	(14.5)	(30.8)	(16.8)
t_E	491.0	413.4	884.7	491.0	413.4	884.7	491.0	413.4	884.7	491.0
	(16.1)	(13.6)	(29.1)	(16.1)	(13.6)	(29.1)	(16.1)	(13.6)	(29.1)	(16.1)
	74.6	138.8	165.5	47.1	61.3	51.3	76.7	57.0	109.7	42.9
	2.453	4.564	5.442	1.548	2.014	1.687	2.523	1.874	3.609	1.410
g_O	986	1155	605	989	1208	616	1006	1189	602	1017
	(32.4)	(38.0)	(19.9)	(32.5)	(39.7)	(20.3)	(33.1)	(39.1)	(19.8)	(33.4)
g_E	1053.0	1175.5	606.3	1053.0	1175.5	606.3	1053.0	1175.5	606.3	1053.0
	(34.6)	(38.7)	(19.9)	(34.6)	(38.7)	(19.9)	(34.6)	(38.7)	(19.9)	(34.6)
	−129.7	−40.6	−2.6	−124.1	65.9	19.6	−91.9	27.2	−8.5	−70.8
	−4.265	−1.335	−0.085	−4.081	2.168	0.644	−3.023	0.894	−0.281	−2.329
c_O	1222	838	860	1225	828	861	1201	837	860	1229
	(40.2)	(27.6)	(28.3)	(40.3)	(27.2)	(28.3)	(39.5)	(27.5)	(28.3)	(40.4)
c_E	1220.8	831.7	788.7	1220.8	831.7	788.7	1220.8	831.7	788.7	1220.8
	(40.1)	(27.3)	(25.9)	(40.1)	(27.3)	(25.9)	(40.1)	(27.3)	(25.9)	(40.1)
	2.4	12.7	148.8	8.4	−7.4	151.0	−39.3	10.7	148.8	16.5
	0.079	0.417	4.895	0.277	−0.242	4.966	−1.292	0.350	4.895	0.541
N	3041	3041	3041	3041	3041	3041	3041	3041	3041	3041
X2	10.1	14.6	42.8	9.9	8.5	22.6	8.4	5.5	28.7	2.4
Gvalue	10.1	14.2	44.6	9.8	8.7	23.2	8.3	5.6	29.5	2.4
Gv/N	0.331	0.468	1.466	0.321	0.285	0.763	0.273	0.183	0.971	0.077
Prob	0.01812	0.00262	0.00000	0.02072	0.03419	0.00004	0.04038	0.13549	0.00000	0.50184

	61	62	63	64	65	66	67	68	69	70
a_O	563	659	285	517	639	283	553	722	300	577
	(18.5)	(21.7)	(9.4)	(17.0)	(21.0)	(9.3)	(18.2)	(23.7)	(9.9)	(19.0)
a_E	620.4	761.3	276.2	620.4	761.3	276.2	620.4	761.3	276.2	620.4
	(20.4)	(25.0)	(9.1)	(20.4)	(25.0)	(9.1)	(20.4)	(25.0)	(9.1)	(20.4)
	−109.3	−190.2	18.0	−188.5	−223.8	13.8	−127.2	−76.5	49.7	−83.7
	−3.596	−6.254	0.590	−6.200	−7.360	0.455	−4.184	−2.517	1.633	−2.753
t_O	407	907	508	428	906	494	422	895	509	421
	(13.4)	(29.8)	(16.7)	(14.1)	(29.8)	(16.2)	(13.9)	(29.4)	(16.7)	(13.8)
t_E	413.4	884.7	491.0	413.4	884.7	491.0	413.4	884.7	491.0	413.4
	(13.6)	(29.1)	(16.1)	(13.6)	(29.1)	(16.1)	(13.6)	(29.1)	(16.1)	(13.6)
	−12.7	45.2	34.6	29.7	43.1	6.0	17.4	20.7	36.7	15.3
	−0.418	1.485	1.137	0.976	1.417	0.198	0.571	0.681	1.205	0.504
g_O	1203	635	1065	1203	633	1076	1231	589	1037	1189
	(39.6)	(20.9)	(35.0)	(39.6)	(20.8)	(35.4)	(40.5)	(19.4)	(34.1)	(39.1)
g_E	1175.5	606.3	1053.0	1175.5	606.3	1053.0	1175.5	606.3	1053.0	1175.5
	(38.7)	(19.9)	(34.6)	(38.7)	(19.9)	(34.6)	(38.7)	(19.9)	(34.6)	(38.7)
	55.7	58.8	24.1	55.7	54.6	46.4	113.6	−34.1	−31.8	27.2
	1.831	1.932	0.791	1.831	1.795	1.527	3.736	−1.121	−1.047	0.894
c_O	868	840	1183	893	863	1188	835	835	1195	854
	(28.5)	(27.6)	(38.9)	(29.4)	(28.4)	(39.1)	(27.5)	(27.5)	(39.3)	(28.1)
c_E	831.7	788.7	1220.8	831.7	788.7	1220.8	831.7	788.7	1220.8	831.7
	(27.3)	(25.9)	(40.1)	(27.3)	(25.9)	(40.1)	(27.3)	(25.9)	(40.1)	(27.3)
	74.2	105.9	−74.4	127.0	155.4	−64.7	6.6	95.2	−51.1	45.2
	2.440	3.481	−2.447	4.177	5.109	−2.128	0.218	3.132	−1.679	1.487
N	3041	3041	3041	3041	3041	3041	3041	3041	3041	3041
X2	7.6	19.0	2.2	22.9	28.3	1.6	10.1	5.4	3.5	3.9
Gvalue	7.8	19.6	2.2	23.8	29.2	1.6	10.4	5.3	3.4	4.0
Gv/N	0.256	0.644	0.072	0.784	0.962	0.052	0.341	0.176	0.113	0.131
Prob	0.05061	0.00021	0.53589	0.00003	0.00000	0.66560	0.01561	0.14807	0.32765	0.26163

	71	72	73	74	75	76	77	78	79	80
a_O	663	271	570	711	297	549	694	307	588	702
	(21.8)	(8.9)	(18.7)	(23.4)	(9.8)	(18.1)	(22.8)	(10.1)	(19.3)	(23.1)
a_E	761.3	276.2	620.4	761.3	276.2	620.2	761.1	276.1	620.2	761.1
	(25.0)	(9.1)	(20.4)	(25.0)	(9.1)	(20.4)	(25.0)	(9.1)	(20.4)	(25.0)
	−183.3	−10.2	−96.6	−97.2	43.2	−133.9	−128.0	65.2	−62.7	−113.4
	−6.028	−0.336	−3.177	−3.196	1.421	−4.405	−4.211	2.145	−2.063	−3.730
t_O	884	541	418	894	488	412	886	523	444	900
	(29.1)	(17.8)	(13.7)	(29.4)	(16.0)	(13.6)	(29.1)	(17.2)	(14.6)	(29.6)
t_E	884.7	491.0	413.4	884.7	491.0	413.3	884.4	490.8	413.3	884.4
	(29.1)	(16.1)	(13.6)	(29.1)	(16.1)	(13.6)	(29.1)	(16.1)	(13.6)	(29.1)
	−1.4	104.9	9.2	18.7	−6.0	−2.5	3.2	66.4	63.7	31.4
	−0.046	3.451	0.304	0.615	−0.196	−0.084	0.105	2.184	2.095	1.035
g_O	631	1025	1230	576	1073	1166	623	999	1190	594
	(20.7)	(33.7)	(40.4)	(18.9)	(35.3)	(38.4)	(20.5)	(32.9)	(39.1)	(19.5)
g_E	606.3	1053.0	1175.5	606.3	1053.0	1175.1	606.1	1052.7	1175.1	606.1
	(19.9)	(34.6)	(38.7)	(19.9)	(34.6)	(38.7)	(19.9)	(34.6)	(38.7)	(19.9)
	50.4	−55.3	111.5	−59.0	40.3	−18.1	34.3	−104.6	30.0	−23.9
	1.658	−1.819	3.667	−1.941	1.325	−0.596	1.128	−3.441	0.987	−0.787
c_O	863	1204	823	860	1183	913	837	1211	818	844
	(28.4)	(39.6)	(27.1)	(28.3)	(38.9)	(30.0)	(27.5)	(39.8)	(26.9)	(27.8)
c_E	788.7	1220.8	831.7	788.7	1220.8	831.4	788.4	1220.4	831.4	788.4
	(25.9)	(40.1)	(27.3)	(25.9)	(40.1)	(27.3)	(25.9)	(40.1)	(27.3)	(25.9)
	155.4	−33.4	−17.3	148.8	−74.4	170.9	100.0	−18.7	−26.6	114.9
	5.109	−1.097	−0.568	4.895	−2.447	5.623	3.291	−0.616	−0.875	3.781
N	3041	3041	3041	3041	3041	3040	3040	3040	3040	3040
X2	20.7	6.2	6.8	11.4	3.1	16.3	9.4	8.4	4.4	9.0
Gvalue	21.1	6.0	6.8	11.3	3.1	16.3	9.5	8.3	4.3	9.0
Gv/N	0.693	0.198	0.225	0.371	0.102	0.537	0.312	0.272	0.143	0.298
Prob	0.00010	0.11110	0.07702	0.01024	0.37481	0.00097	0.02344	0.04086	0.22714	0.02868

	81	82	83	84	85	86	87	88	89	90
a_O	284	566	738	278	583	712	275	544	695	265
	(9.3)	(18.6)	(24.3)	(9.1)	(19.2)	(23.4)	(9.0)	(17.9)	(22.9)	(8.7)
a_E	276.1	620.2	761.1	276.1	620.0	760.8	276.0	620.0	760.8	276.0
	(9.1)	(20.4)	(25.0)	(9.1)	(20.4)	(25.0)	(9.1)	(20.4)	(25.0)	(9.1)
	16.1	−103.5	−45.4	3.9	−71.8	−94.4	−2.0	−142.3	−125.7	−21.5
	0.529	−3.406	−1.493	0.127	−2.361	−3.106	−0.065	−4.682	−4.137	−0.708
t_O	524	427	878	524	472	943	514	431	888	540
	(17.2)	(14.0)	(28.9)	(17.2)	(15.5)	(31.0)	(16.9)	(14.2)	(29.2)	(17.8)
t_E	490.8	413.3	884.4	490.8	413.1	884.1	490.7	413.1	884.1	490.7
	(16.1)	(13.6)	(29.1)	(16.1)	(13.6)	(29.1)	(16.1)	(13.6)	(29.1)	(16.1)
	68.5	27.9	−12.8	68.5	125.7	121.6	47.7	36.5	7.8	103.5

	2.254	0.918	−0.420	2.254	4.138	4.001	1.571	1.201	0.256	3.404
g_O	1032	1196	616	1023	1172	613	1023	1200	638	1042
	(33.9)	(39.3)	(20.3)	(33.7)	(38.6)	(20.2)	(33.7)	(39.5)	(21.0)	(34.3)
g_E	1052.7	1175.1	606.1	1052.7	1174.7	605.9	1052.3	1174.7	605.9	1052.3
	(34.6)	(38.7)	(19.9)	(34.6)	(38.7)	(19.9)	(34.6)	(38.7)	(19.9)	(34.6)
	−41.0	42.2	20.0	−58.5	−5.4	14.3	−57.9	51.1	65.9	−20.6
	−1.348	1.387	0.657	−1.926	−0.178	0.471	−1.904	1.682	2.168	−0.677
c_O	1200	851	808	1215	812	771	1227	864	818	1192
	(39.5)	(28.0)	(26.6)	(40.0)	(26.7)	(25.4)	(40.4)	(28.4)	(26.9)	(39.2)
c_E	1220.4	831.4	788.4	1220.4	831.1	788.2	1220.0	831.1	788.2	1220.0
	(40.1)	(27.3)	(25.9)	(40.1)	(27.3)	(25.9)	(40.1)	(27.3)	(25.9)	(40.1)
	−40.5	39.6	39.6	−10.8	−37.8	−34.0	14.0	67.0	60.7	−55.4
	−1.331	1.304	1.302	−0.355	−1.245	−1.119	0.462	2.205	1.999	−1.821
N	3040	3040	3040	3040	3039	3039	3039	3039	3039	3039
X2	3.2	6.0	1.4	3.1	11.0	7.5	2.0	11.9	8.5	6.1
Gvalue	3.2	6.2	1.4	3.1	10.7	7.5	2.0	12.3	8.7	6.0
Gv/N	0.104	0.203	0.046	0.101	0.353	0.247	0.065	0.405	0.285	0.197
Prob	0.36618	0.10400	0.70696	0.38024	0.01334	0.05764	0.58048	0.00640	0.03409	0.11196

	91	92	93	94	95	96	97	98	99	100
a_O	581	738	297	565	755	283	596	699	295	575
	(19.1)	(24.3)	(9.8)	(18.6)	(24.8)	(9.3)	(19.6)	(23.0)	(9.7)	(18.9)
a_E	620.0	760.8	276.0	620.0	760.8	276.0	620.0	760.8	276.0	620.0
	(20.4)	(25.0)	(9.1)	(20.4)	(25.0)	(9.1)	(20.4)	(25.0)	(9.1)	(20.4)
	−75.5	−44.9	43.6	−105.0	−11.6	14.2	−47.1	−118.4	39.3	−86.7
	−2.485	−1.478	1.435	−3.455	−0.380	0.468	−1.549	−3.897	1.294	−2.852
t_O	382	855	526	432	926	479	428	928	488	412
	(12.6)	(28.1)	(17.3)	(14.2)	(30.5)	(15.8)	(14.1)	(30.5)	(16.1)	(13.6)
t_E	413.1	884.1	490.7	413.1	884.1	490.7	413.1	884.1	490.7	413.1
	(13.6)	(29.1)	(16.1)	(13.6)	(29.1)	(16.1)	(13.6)	(29.1)	(16.1)	(13.6)
	−59.9	−57.3	73.1	38.6	85.7	−23.1	30.3	89.9	−5.3	−2.3
	−1.970	−1.885	2.407	1.269	2.820	−0.759	0.996	2.958	−0.175	−0.075
g_O	1239	615	1015	1195	570	1067	1155	586	1034	1222
	(40.8)	(20.2)	(33.4)	(39.3)	(18.8)	(35.1)	(38.0)	(19.3)	(34.0)	(40.2)
g_E	1174.7	605.9	1052.3	1174.7	605.9	1052.3	1174.7	605.9	1052.3	1174.7
	(38.7)	(19.9)	(34.6)	(38.7)	(19.9)	(34.6)	(38.7)	(19.9)	(34.6)	(38.7)
	132.0	18.4	−73.3	40.9	−69.6	29.5	−39.1	−39.1	−36.4	96.5
	4.345	0.604	−2.414	1.347	−2.291	0.971	−1.286	−1.287	−1.197	3.174
c_O	837	831	1201	847	788	1210	860	826	1222	830
	(27.5)	(27.3)	(39.5)	(27.9)	(25.9)	(39.8)	(28.3)	(27.2)	(40.2)	(27.3)
c_E	831.1	788.2	1220.0	831.1	788.2	1220.0	831.1	788.2	1220.0	831.1
	(27.3)	(25.9)	(40.1)	(27.3)	(25.9)	(40.1)	(27.3)	(25.9)	(40.1)	(27.3)
	11.8	87.9	−37.7	32.0	−0.4	−19.9	58.7	77.4	4.0	−2.3
	0.387	2.893	−1.241	1.054	−0.012	−0.655	1.932	2.547	0.132	−0.075
N	3039	3039	3039	3039	3039	3039	3039	3039	3039	3039
X2	8.4	4.1	5.8	6.4	4.2	0.7	2.8	9.7	1.6	5.2
Gvalue	8.4	4.1	5.7	6.5	4.2	0.7	2.8	9.8	1.6	5.2
Gv/N	0.277	0.134	0.187	0.215	0.137	0.024	0.092	0.321	0.053	0.172
Prob	0.03821	0.25288	0.12801	0.08852	0.24402	0.86322	0.42454	0.02081	0.65466	0.15553

	101	102	103	104	105	106	107	108	109	110
a_O	741	299	601	733	284	565	728	295	566	745
	(24.4)	(9.8)	(19.8)	(24.1)	(9.3)	(18.6)	(24.0)	(9.7)	(18.6)	(24.5)
a_E	760.8	276.0	619.8	760.5	275.9	619.6	760.3	275.8	619.4	760.0
	(25.0)	(9.1)	(20.4)	(25.0)	(9.1)	(20.4)	(25.0)	(9.1)	(20.4)	(25.0)
	−39.1	47.9	−37.0	−54.1	16.5	−104.2	−63.2	39.7	−102.1	−29.8
	−1.286	1.576	−1.219	−1.780	0.542	−3.432	−2.081	1.307	−3.361	−0.981
t_O	896	527	403	846	483	440	926	521	416	846
	(29.5)	(17.3)	(13.3)	(27.8)	(15.9)	(14.5)	(30.5)	(17.2)	(13.7)	(27.9)
t_E	884.1	490.7	413.0	883.8	490.5	412.9	883.5	490.4	412.7	883.2
	(29.1)	(16.1)	(13.6)	(29.1)	(16.1)	(13.6)	(29.1)	(16.1)	(13.6)	(29.1)
	23.9	75.3	−19.8	−74.0	−14.9	56.0	86.9	63.2	6.6	−72.9
	0.787	2.477	−0.650	−2.436	−0.491	1.844	2.862	2.080	0.216	−2.401
g_O	598	1001	1221	631	1056	1167	576	996	1207	615
	(19.7)	(32.9)	(40.2)	(20.8)	(34.8)	(38.4)	(19.0)	(32.8)	(39.8)	(20.3)
g_E	605.9	1052.3	1174.3	605.7	1052.0	1173.9	605.5	1051.7	1173.6	605.3
	(19.9)	(34.6)	(38.7)	(19.9)	(34.6)	(38.7)	(19.9)	(34.6)	(38.7)	(19.9)
	−15.7	−100.1	95.2	51.7	8.0	−13.8	−57.5	−108.3	67.8	19.6
	−0.516	−3.295	3.133	1.701	0.264	−0.456	−1.894	−3.566	2.234	0.645
c_O	804	1212	813	828	1215	865	807	1225	847	830
	(26.5)	(39.9)	(26.8)	(27.3)	(40.0)	(28.5)	(26.6)	(40.3)	(27.9)	(27.3)
c_E	788.2	1220.0	830.9	787.9	1219.6	830.6	787.7	1219.2	830.3	787.4
	(25.9)	(40.1)	(27.3)	(25.9)	(40.1)	(27.3)	(25.9)	(40.1)	(27.3)	(25.9)
	31.9	−15.9	−35.3	82.1	−9.2	70.2	39.1	11.6	33.7	87.4
	1.051	−0.525	−1.164	2.704	−0.302	2.312	1.288	0.383	1.110	2.880
N	3039	3039	3038	3038	3038	3037	3037	3037	3036	3036
X2	1.1	7.2	3.1	5.7	0.4	8.1	5.3	6.2	5.9	4.3
Gvalue	1.1	7.1	3.0	5.7	0.4	8.2	5.3	6.2	6.0	4.3
Gv/N	0.036	0.233	0.100	0.188	0.013	0.268	0.176	0.204	0.199	0.142
Prob	0.77780	0.06899	0.38571	0.12714	0.94349	0.04297	0.14907	0.10185	0.10946	0.22975

	111	112	113	114	115	116	117	118	119	120
a_O	295	584	788	281	590	745	279	619	827	289
	(9.7)	(19.2)	(26.0)	(9.3)	(19.4)	(24.6)	(9.2)	(20.4)	(27.3)	(9.5)
a_E	275.7	619.2	759.8	275.6	619.0	759.5	275.5	619.0	759.5	275.5
	(9.1)	(20.4)	(25.0)	(9.1)	(20.4)	(25.0)	(9.1)	(20.4)	(25.0)	(9.1)
	39.9	−68.3	57.4	10.9	−56.6	−28.8	7.0	0.0	140.7	27.6
	1.314	−2.252	1.892	0.358	−1.866	−0.950	0.230	0.001	4.638	0.909
t_O	515	393	806	500	436	878	486	378	871	488
	(17.0)	(12.9)	(26.6)	(16.5)	(14.4)	(28.9)	(16.0)	(12.5)	(28.7)	(16.1)
t_E	490.2	412.6	883.0	490.0	412.5	882.7	489.9	412.5	882.7	489.9
	(16.1)	(13.6)	(29.1)	(16.1)	(13.6)	(29.1)	(16.1)	(13.6)	(29.1)	(16.1)
	50.9	−38.2	−147.0	20.1	48.4	−9.3	−7.7	−66.0	−23.2	−3.7
	1.675	−1.260	−4.844	0.664	1.595	−0.307	−0.254	−2.174	−0.764	−0.123
g_O	1008	1189	630	1002	1191	621	1012	1223	574	1006
	(33.2)	(39.2)	(20.8)	(33.0)	(39.3)	(20.5)	(33.4)	(40.3)	(18.9)	(33.2)
g_E	1051.3	1173.2	605.1	1051.0	1172.8	604.9	1050.6	1172.8	604.9	1050.6
	(34.6)	(38.7)	(19.9)	(34.6)	(38.7)	(19.9)	(34.6)	(38.7)	(19.9)	(34.6)
	−84.8	31.9	50.8	−95.6	36.7	32.6	−75.8	102.6	−60.2	−87.3
	−2.793	1.050	1.675	−3.150	1.210	1.076	−2.498	3.380	−1.984	−2.878
c_O	1218	869	811	1252	817	790	1257	814	762	1251
	(40.1)	(28.6)	(26.7)	(41.3)	(26.9)	(26.0)	(41.4)	(26.8)	(25.1)	(41.2)
c_E	1218.8	830.0	787.2	1218.4	829.8	786.9	1218.0	829.8	786.9	1218.0
	(40.1)	(27.3)	(25.9)	(40.1)	(27.3)	(25.9)	(40.1)	(27.3)	(25.9)	(40.1)
	−1.6	79.7	48.4	68.1	−25.3	6.2	79.3	−31.2	−49.0	66.9
	−0.052	2.626	1.595	2.245	−0.836	0.205	2.612	−1.030	−1.615	2.205

N	3036	3035	3035	3035	3034	3034	3034	3034	3034	3034
X2	4.4	5.0	9.5	3.5	3.2	0.7	2.7	5.3	8.5	3.5
Gvalue	4.4	5.0	9.7	3.5	3.2	0.7	2.7	5.4	8.4	3.5
Gv/N	0.144	0.165	0.319	0.117	0.105	0.024	0.091	0.177	0.276	0.114
Prob	0.22478	0.17197	0.02157	0.31541	0.36505	0.86315	0.43227	0.14571	0.03884	0.32550

	121	122	123	124	125	126	127	128	129	130
a_O	605	769	274	575	767	281	554	766	268	600
	(19.9)	(25.4)	(9.0)	(19.0)	(25.3)	(9.3)	(18.3)	(25.3)	(8.9)	(19.8)
a_E	618.8	759.3	275.4	618.4	758.8	275.3	617.2	757.3	274.7	616.7
	(20.4)	(25.0)	(9.1)	(20.4)	(25.0)	(9.1)	(20.4)	(25.0)	(9.1)	(20.4)
	-27.3	19.5	-2.9	-83.6	16.5	11.6	-119.6	17.5	-13.3	-33.0
	-0.899	0.644	-0.095	-2.759	0.544	0.383	-3.954	0.579	-0.438	-1.093
t_O	413	814	553	463	821	480	416	864	504	414
	(13.6)	(26.8)	(18.2)	(15.3)	(27.1)	(15.8)	(13.8)	(28.6)	(16.7)	(13.7)
t_E	412.3	882.4	489.7	412.0	881.8	489.4	411.2	880.0	488.4	411.0
	(13.6)	(29.1)	(16.1)	(13.6)	(29.1)	(16.1)	(13.6)	(29.1)	(16.1)	(13.6)
	1.4	-131.3	134.4	108.0	-117.3	-18.6	9.6	-31.8	31.7	6.1
	0.045	-4.329	4.433	3.562	-3.870	-0.613	0.317	-1.051	1.047	0.202
g_O	1170	641	1025	1192	592	1069	1231	602	1052	1193
	(38.6)	(21.1)	(33.8)	(39.3)	(19.5)	(35.3)	(40.7)	(19.9)	(34.8)	(39.5)
g_E	1172.4	604.7	1050.3	1171.6	604.3	1049.6	1169.3	603.1	1047.5	1168.5
	(38.7)	(19.9)	(34.6)	(38.7)	(19.9)	(34.6)	(38.7)	(19.9)	(34.6)	(38.7)
	-4.8	74.7	-49.9	41.1	-24.3	39.2	126.6	-2.2	9.0	49.5
	-0.158	2.465	-1.646	1.356	-0.803	1.294	4.185	-0.073	0.298	1.636
c_O	845	809	1181	801	851	1201	824	793	1201	816
	(27.9)	(26.7)	(38.9)	(26.4)	(28.1)	(39.6)	(27.2)	(26.2)	(39.7)	(27.0)
c_E	829.5	786.6	1217.6	829.0	786.1	1216.8	827.3	784.6	1214.4	826.8
	(27.3)	(25.9)	(40.1)	(27.3)	(25.9)	(40.1)	(27.3)	(25.9)	(40.1)	(27.3)
	31.3	45.4	-72.1	-55.0	135.0	-31.4	-6.6	17.0	-26.6	-21.4
	1.032	1.496	-2.376	-1.813	4.454	-1.035	-0.219	0.561	-0.880	-0.708
N	3033	3033	3033	3031	3031	3031	3025	3025	3025	3023
X2	0.6	8.2	9.9	10.6	9.9	0.9	9.8	0.5	0.8	1.1
Gvalue	0.6	8.3	9.6	10.5	9.8	0.9	10.0	0.5	0.8	1.1
Gv/N	0.020	0.275	0.316	0.346	0.325	0.028	0.329	0.016	0.027	0.037
Prob	0.89568	0.03963	0.02249	0.01491	0.01993	0.83431	0.01888	0.92182	0.84353	0.76960

	131	132	133	134	135	136	137	138	139	140
a_O	782	305	570	747	279	609	772	281	586	715
	(25.9)	(10.1)	(18.9)	(24.7)	(9.2)	(20.2)	(25.6)	(9.3)	(19.4)	(23.7)
a_E	756.8	274.5	616.1	756.0	274.3	615.9	755.8	274.2	615.9	755.8
	(25.0)	(9.1)	(20.4)	(25.0)	(9.1)	(20.4)	(25.0)	(9.1)	(20.4)	(25.0)
	51.2	64.2	-88.7	-18.0	9.6	-13.8	32.8	13.8	-58.4	-79.3
	1.695	2.124	-2.938	-0.595	0.317	-0.456	1.085	0.458	-1.934	-2.628
t_O	824	523	408	827	486	405	811	523	449	901
	(27.3)	(17.3)	(13.5)	(27.4)	(16.1)	(13.4)	(26.9)	(17.3)	(14.9)	(29.8)
t_E	879.5	488.1	410.6	878.6	487.6	410.4	878.3	487.4	410.4	878.3
	(29.1)	(16.1)	(13.6)	(29.1)	(16.1)	(13.6)	(29.1)	(16.1)	(13.6)	(29.1)
	-107.4	72.3	-5.1	-100.1	-3.2	-10.8	-129.3	73.6	80.7	46.0
	-3.551	2.390	-0.169	-3.314	-0.106	-0.357	-4.283	2.439	2.672	1.523
g_O	626	1008	1191	650	962	1187	639	1030	1145	595
	(20.7)	(33.3)	(39.4)	(21.5)	(31.9)	(39.3)	(21.2)	(34.1)	(37.9)	(19.7)
g_E	602.7	1046.8	1167.4	602.1	1045.8	1167.0	601.9	1045.4	1167.0	601.9
	(19.9)	(34.6)	(38.7)	(19.9)	(34.6)	(38.7)	(19.9)	(34.6)	(38.7)	(19.9)
	47.5	-76.2	47.7	99.5	-160.6	40.4	76.4	-30.6	-43.5	-13.7
	1.571	-2.519	1.581	3.295	-5.319	1.337	2.532	-1.014	-1.442	-0.455
c_O	791	1187	851	796	1293	818	797	1185	839	808
	(26.2)	(39.3)	(28.2)	(26.4)	(42.8)	(27.1)	(26.4)	(39.3)	(27.8)	(26.8)
c_E	784.0	1213.6	825.9	783.3	1212.4	825.7	783.0	1212.0	825.7	783.0
	(25.9)	(40.1)	(27.3)	(25.9)	(40.1)	(27.3)	(25.9)	(40.1)	(27.3)	(25.9)
	14.0	-52.6	50.9	25.7	166.5	-15.3	28.2	-53.3	26.9	50.8
	0.463	-1.739	1.684	0.850	5.513	-0.506	0.936	-1.767	0.890	1.682
N	3023	3023	3020	3020	3020	3019	3019	3019	3019	3019
X2	5.3	7.9	4.7	7.2	12.2	0.6	8.0	3.6	5.7	3.7
Gvalue	5.4	7.7	4.8	7.1	12.2	0.6	8.1	3.5	5.6	3.7
Gv/N	0.177	0.256	0.159	0.236	0.405	0.019	0.269	0.117	0.186	0.122
Prob	0.14755	0.05158	0.18804	0.06821	0.00663	0.90484	0.04342	0.31628	0.13121	0.29655

	141	142	143	144	145	146	147	148	149	150
a_O	271	591	778	258	594	739	276	611	735	269
	(9.0)	(19.6)	(25.8)	(8.6)	(19.7)	(24.5)	(9.2)	(20.3)	(24.4)	(8.9)
a_E	274.2	615.5	755.3	274.0	615.3	755.0	273.9	615.3	755.0	273.9
	(9.1)	(20.4)	(25.0)	(9.1)	(20.4)	(25.0)	(9.1)	(20.4)	(25.0)	(9.1)
	-6.3	-48.1	46.1	-31.0	-41.9	-31.7	4.2	-8.6	-39.5	-9.7
	-0.209	-1.593	1.528	-1.028	-1.389	-1.052	0.140	-0.285	-1.311	-0.322
t_O	514	417	851	505	399	894	506	416	887	491
	(17.0)	(13.8)	(28.2)	(16.7)	(13.2)	(29.6)	(16.8)	(13.8)	(29.4)	(16.3)
t_E	487.4	410.1	877.7	487.1	410.0	877.4	487.0	410.0	877.4	487.0
	(16.1)	(13.6)	(29.1)	(16.1)	(13.6)	(29.1)	(16.1)	(13.6)	(29.1)	(16.1)
	54.5	13.8	-52.6	36.4	-21.7	33.5	38.8	12.1	19.2	8.1
	1.806	0.458	-1.744	1.207	-0.720	1.109	1.287	0.400	0.638	0.269
g_O	1042	1188	584	1041	1160	588	1077	1116	582	1055
	(34.5)	(39.4)	(19.4)	(34.5)	(38.5)	(19.5)	(35.7)	(37.0)	(19.3)	(35.0)
g_E	1045.4	1166.2	601.5	1044.7	1165.8	601.3	1044.4	1165.8	601.3	1044.4
	(34.6)	(38.7)	(19.9)	(34.6)	(38.7)	(19.9)	(34.6)	(38.7)	(19.9)	(34.6)
	-6.8	44.0	-34.5	-7.4	-11.6	-26.3	66.2	-97.5	-38.0	21.3
	-0.226	1.458	-1.143	-0.247	-0.385	-0.872	2.197	-3.232	-1.259	0.708
c_O	1192	821	804	1213	863	795	1157	873	812	1201
	(39.5)	(27.2)	(26.6)	(40.2)	(28.6)	(26.4)	(38.4)	(28.9)	(26.9)	(39.8)
c_E	1212.0	825.1	782.5	1211.2	824.9	782.2	1210.8	824.9	782.2	1210.8
	(40.1)	(27.3)	(25.9)	(40.1)	(27.3)	(25.9)	(40.1)	(27.3)	(25.9)	(40.1)
	-39.6	-8.2	43.6	3.7	78.0	25.8	-105.1	99.1	60.7	-19.5
	-1.312	-0.273	1.446	0.122	2.587	0.854	-3.485	3.284	2.012	-0.645
N	3019	3017	3017	3017	3016	3016	3016	3016	3016	3016
X2	1.8	1.5	2.6	1.6	2.8	1.2	4.2	5.1	2.4	0.3
Gvalue	1.8	1.5	2.6	1.6	2.8	1.2	4.2	5.0	2.4	0.3
Gv/N	0.060	0.051	0.086	0.054	0.093	0.038	0.139	0.167	0.079	0.010
Prob	0.61496	0.67552	0.45775	0.65586	0.42145	0.76300	0.24235	0.16924	0.49613	0.95852

Bbac_HD100
5UTR:

	50	49	48	47	46	45	44	43	42	41
a_O	947	947	898	960	989	939	986	1051	986	971
	(26.5)	(26.5)	(25.1)	(26.9)	(27.7)	(26.3)	(27.6)	(29.4)	(27.6)	(27.2)
a_E	991.0	991.0	991.0	991.0	991.0	991.0	991.0	991.0	991.0	991.0
	(27.7)	(27.7)	(27.7)	(27.7)	(27.7)	(27.7)	(27.7)	(27.7)	(27.7)	(27.7)
	-85.9	-85.9	-176.9	-60.9	-3.9	-101.1	-9.9	123.6	-9.9	-39.5
	-2.404	-2.404	-4.949	-1.705	-0.110	-2.829	-0.277	3.459	-0.277	-1.105
t_O	974	972	993	919	974	994	962	998	972	941
	(27.2)	(27.2)	(27.8)	(25.7)	(27.2)	(27.8)	(26.9)	(27.9)	(27.2)	(26.3)
t_E	944.5	944.5	944.5	944.5	944.5	944.5	944.5	944.5	944.5	944.5
	(26.4)	(26.4)	(26.4)	(26.4)	(26.4)	(26.4)	(26.4)	(26.4)	(26.4)	(26.4)
	59.9	55.8	99.4	-50.3	59.9	101.5	35.3	109.9	55.8	-7.0
	1.675	1.559	2.781	-1.408	1.675	2.839	0.987	3.075	1.559	-0.197
g_O	870	803	829	841	772	820	814	748	812	846
	(24.3)	(22.5)	(23.2)	(23.5)	(21.6)	(22.9)	(22.8)	(20.9)	(22.7)	(23.7)
g_E	844.2	844.2	844.2	844.2	844.2	844.2	844.2	844.2	844.2	844.2
	(23.6)	(23.6)	(23.6)	(23.6)	(23.6)	(23.6)	(23.6)	(23.6)	(23.6)	(23.6)
	52.3	-80.4	-30.2	-6.4	-138.1	-47.7	-59.3	-181.0	-63.2	3.6
	1.464	-2.249	-0.844	-0.180	-3.862	-1.335	-1.660	-5.064	-1.768	0.100
c_O	784	853	855	855	840	822	813	778	805	817
	(21.9)	(23.9)	(23.9)	(23.9)	(23.5)	(23.0)	(22.7)	(21.8)	(22.5)	(22.9)
c_E	795.3	795.3	795.3	795.3	795.3	795.3	795.3	795.3	795.3	795.3
	(22.2)	(22.2)	(22.2)	(22.2)	(22.2)	(22.2)	(22.2)	(22.2)	(22.2)	(22.2)
	-22.4	119.5	123.8	123.8	91.9	54.3	35.8	-34.2	19.5	44.0
	-0.628	3.342	3.462	3.462	2.570	1.519	1.001	-0.957	0.546	1.230
N	3575	3575	3575	3575	3575	3575	3575	3575	3575	3575
X2	3.8	8.9	16.0	6.2	9.6	6.9	1.8	18.0	2.2	1.0
Gvalue	3.8	8.9	16.1	6.1	9.7	6.9	1.8	18.3	2.2	1.0
Gv/N	0.107	0.249	0.450	0.169	0.273	0.193	0.051	0.513	0.061	0.028
Prob	0.28030	0.03057	0.00108	0.10878	0.02088	0.07484	0.60792	0.00038	0.53586	0.79921

	40	39	38	37	36	35	34	33	32	31
a_O	967	935	998	944	918	950	968	936	1071	1057
	(27.0)	(26.2)	(27.9)	(26.4)	(25.7)	(26.6)	(27.1)	(26.2)	(30.0)	(29.6)
a_E	991.0	991.0	991.0	991.0	991.0	991.0	991.0	991.0	991.0	991.0
	(27.7)	(27.7)	(27.7)	(27.7)	(27.7)	(27.7)	(27.7)	(27.7)	(27.7)	(27.7)
	-47.3	-108.7	14.1	-91.7	-140.4	-80.2	-45.4	-106.8	166.4	136.4
	-1.324	-3.041	0.395	-2.564	-3.928	-2.243	-1.269	-2.988	4.654	3.815
t_O	1031	935	930	1008	1026	1080	1060	992	919	1035
	(28.8)	(26.2)	(26.0)	(28.2)	(28.7)	(30.2)	(29.7)	(27.7)	(25.7)	(29.0)
t_E	944.5	944.5	944.5	944.5	944.5	944.5	944.5	944.5	944.5	944.5
	(26.4)	(26.4)	(26.4)	(26.4)	(26.4)	(26.4)	(26.4)	(26.4)	(26.4)	(26.4)
	180.6	-18.9	-28.8	131.1	169.8	289.5	244.5	97.3	-50.3	189.4
	5.053	-0.530	-0.806	3.668	4.750	8.099	6.840	2.722	-1.408	5.297
g_O	749	866	849	789	770	800	772	805	786	715
	(21.0)	(24.2)	(23.7)	(22.1)	(21.5)	(22.4)	(21.6)	(22.5)	(22.0)	(20.0)
g_E	844.2	844.2	844.2	844.2	844.2	844.2	844.2	844.2	844.2	844.2
	(23.6)	(23.6)	(23.6)	(23.6)	(23.6)	(23.6)	(23.6)	(23.6)	(23.6)	(23.6)
	-179.3	44.1	9.6	-106.7	-141.7	-86.1	-138.1	-76.6	-112.3	-237.6
	-5.015	1.234	0.268	-2.986	-3.964	-2.408	-3.862	-2.142	-3.142	-6.645
c_O	828	839	798	834	861	745	775	842	799	768
	(23.2)	(23.5)	(22.3)	(23.3)	(24.1)	(20.8)	(21.7)	(23.6)	(22.3)	(21.5)
c_E	795.3	795.3	795.3	795.3	795.3	795.3	795.3	795.3	795.3	795.3
	(22.2)	(22.2)	(22.2)	(22.2)	(22.2)	(22.2)	(22.2)	(22.2)	(22.2)	(22.2)
	66.7	89.8	5.4	79.3	136.7	-97.3	-40.1	96.1	7.4	-53.7
	1.866	2.511	0.151	2.217	3.823	-2.723	-1.121	2.688	0.207	-1.501
N	3575	3575	3575	3575	3575	3575	3575	3575	3575	3575
X2	20.6	6.2	0.3	12.0	24.4	26.6	21.3	10.0	11.2	33.8
Gvalue	20.8	6.2	0.3	12.0	24.4	25.9	21.0	10.0	11.1	34.5
Gv/N	0.581	0.174	0.009	0.335	0.681	0.724	0.587	0.280	0.311	0.966
Prob	0.00012	0.10080	0.95805	0.00745	0.00002	0.00001	0.00011	0.01861	0.01109	0.00000

	30	29	28	27	26	25	24	23	22	21
a_O	999	1047	1031	990	975	1031	970	1002	1077	1017
	(27.9)	(29.3)	(28.8)	(27.7)	(27.3)	(28.8)	(27.1)	(28.0)	(30.1)	(28.4)
a_E	991.0	991.0	991.0	991.0	991.0	991.0	991.0	991.0	991.0	991.0
	(27.7)	(27.7)	(27.7)	(27.7)	(27.7)	(27.7)	(27.7)	(27.7)	(27.7)	(27.7)
	16.1	115.2	81.7	-1.9	-31.7	81.7	-41.5	22.2	179.3	52.8
	0.452	3.222	2.285	-0.054	-0.886	2.285	-1.160	0.621	5.017	1.476
t_O	1018	954	1039	973	913	1024	939	960	983	979
	(28.5)	(26.7)	(29.1)	(27.2)	(25.5)	(28.6)	(26.3)	(26.9)	(27.5)	(27.4)
t_E	944.5	944.5	944.5	944.5	944.5	944.5	944.5	944.5	944.5	944.5
	(26.4)	(26.4)	(26.4)	(26.4)	(26.4)	(26.4)	(26.4)	(26.4)	(26.4)	(26.4)
	152.5	19.1	198.1	57.8	-62.0	165.5	-11.0	31.2	78.5	70.2
	4.267	0.533	5.542	1.617	-1.734	4.628	-0.308	0.873	2.196	1.964
g_O	781	791	728	795	878	729	831	816	715	751
	(21.8)	(22.1)	(20.4)	(22.2)	(24.6)	(20.4)	(23.2)	(22.8)	(20.0)	(21.0)
g_E	844.2	844.2	844.2	844.2	844.2	844.2	844.2	844.2	844.2	844.2
	(23.6)	(23.6)	(23.6)	(23.6)	(23.6)	(23.6)	(23.6)	(23.6)	(23.6)	(23.6)
	-121.6	-103.0	-215.7	-95.5	68.9	-213.9	-26.2	-55.5	-237.6	-175.7
	-3.401	-2.881	-6.032	-2.672	1.927	-5.985	-0.734	-1.552	-6.645	-4.916
c_O	777	783	777	817	809	791	835	797	800	828
	(21.7)	(21.9)	(21.7)	(22.9)	(22.6)	(22.1)	(23.4)	(22.3)	(22.4)	(23.2)
c_E	795.3	795.3	795.3	795.3	795.3	795.3	795.3	795.3	795.3	795.3
	(22.2)	(22.2)	(22.2)	(22.2)	(22.2)	(22.2)	(22.2)	(22.2)	(22.2)	(22.2)
	-36.2	-24.4	-36.2	44.0	27.6	-8.6	81.3	3.4	9.4	66.7
	-1.012	-0.683	-1.012	1.230	0.773	-0.240	2.276	0.095	0.264	1.866
N	3575	3575	3575	3575	3575	3575	3575	3575	3575	3575
X2	10.9	6.8	27.5	4.3	2.9	24.1	2.7	1.3	28.8	13.6
Gvalue	10.9	6.8	28.0	4.4	2.9	24.6	2.6	1.3	29.7	13.9
Gv/N	0.305	0.191	0.782	0.122	0.081	0.689	0.074	0.037	0.831	0.390
Prob	0.01217	0.07769	0.00000	0.22465	0.40876	0.00002	0.45113	0.72143	0.00000	0.00298

	20	19	18	17	16	15	14	13	12	11
a_O	1065	1082	1032	1052	1155	1079	1175	1223	1196	1453
	(29.8)	(30.3)	(28.9)	(29.4)	(32.3)	(30.2)	(32.9)	(34.2)	(33.5)	(40.6)
a_E	991.0	991.0	991.0	991.0	991.0	991.0	991.0	991.0	991.0	991.0
	(27.7)	(27.7)	(27.7)	(27.7)	(27.7)	(27.7)	(27.7)	(27.7)	(27.7)	(27.7)
	153.5	190.2	83.8	125.8	353.8	183.7	400.3	514.6	449.8	1112.2
	4.293	5.320	2.343	3.518	9.898	5.138	11.198	14.395	12.583	31.109
t_O	953	997	1019	978	948	987	803	861	817	541
	(26.7)	(27.9)	(28.5)	(27.4)	(26.5)	(27.6)	(22.5)	(24.1)	(22.9)	(15.1)

t_E	944.5	944.5	944.5	944.5	944.5	944.5	944.5	944.5	944.5	944.5
	(26.4)	(26.4)	(26.4)	(26.4)	(26.4)	(26.4)	(26.4)	(26.4)	(26.4)	(26.4)
	17.0	107.8	154.7	68.1	7.0	86.8	-260.7	-159.4	-237.0	-603.0
	0.477	3.016	4.327	1.906	0.195	2.429	-7.292	-4.459	-6.629	-16.866
g_O	767	689	706	753	774	798	889	842	1020	1130
	(21.5)	(19.3)	(19.7)	(21.1)	(21.7)	(22.3)	(24.9)	(23.6)	(28.5)	(31.6)
g_E	844.2	844.2	844.2	844.2	844.2	844.2	844.2	844.2	844.2	844.2
	(23.6)	(23.6)	(23.6)	(23.6)	(23.6)	(23.6)	(23.6)	(23.6)	(23.6)	(23.6)
	-147.2	-280.0	-252.5	-172.2	-134.4	-89.9	91.9	-4.4	385.9	658.9
	-4.116	-7.831	-7.062	-4.817	-3.760	-2.514	2.570	-0.124	10.793	18.431
c_O	790	807	818	792	698	711	708	649	542	451
	(22.1)	(22.6)	(22.9)	(22.2)	(19.5)	(19.9)	(19.8)	(18.2)	(15.2)	(12.6)
c_E	795.3	795.3	795.3	795.3	795.3	795.3	795.3	795.3	795.3	795.3
	(22.2)	(22.2)	(22.2)	(22.2)	(22.2)	(22.2)	(22.2)	(22.2)	(22.2)	(22.2)
	-10.6	23.6	46.0	-6.6	-182.2	-159.3	-164.6	-263.9	-415.7	-511.7
	-0.296	0.659	1.288	-0.184	-5.096	-4.457	-4.605	-7.381	-11.627	-14.312
N	3575	3575	3575	3575	3575	3575	3575	3575	3575	3575
X2	12.7	40.0	30.9	14.8	44.9	21.2	67.3	88.6	176.9	633.6
Gvalue	12.8	41.6	32.0	15.1	44.2	21.3	66.9	86.9	183.0	656.5
Gv/N	0.358	1.164	0.896	0.422	1.237	0.597	1.871	2.430	5.120	18.363
Prob	0.00509	0.00000	0.00000	0.00173	0.00000	0.00009	0.00000	0.00000	0.00000	0.00000

	10	9	8	7	6	5	4	3	2	1
a_O	1171	1046	1138	1093	905	1075	1150	1034	903	877
	(32.8)	(29.3)	(31.8)	(30.6)	(25.3)	(30.1)	(32.2)	(28.9)	(25.3)	(24.5)
a_E	991.0	991.0	991.0	991.0	991.0	991.0	991.0	991.0	991.0	991.0
	(27.7)	(27.7)	(27.7)	(27.7)	(27.7)	(27.7)	(27.7)	(27.7)	(27.7)	(27.7)
	391.0	113.1	314.9	214.2	-164.2	175.0	342.3	87.9	-167.9	-214.3
	10.936	3.163	8.808	5.993	-4.594	4.895	9.576	2.459	-4.696	-5.994
t_O	586	528	570	832	929	938	1003	954	1094	1214
	(16.4)	(14.8)	(15.9)	(23.3)	(26.0)	(26.2)	(28.1)	(26.7)	(30.6)	(34.0)
t_E	944.5	944.5	944.5	944.5	944.5	944.5	944.5	944.5	944.5	944.5
	(26.4)	(26.4)	(26.4)	(26.4)	(26.4)	(26.4)	(26.4)	(26.4)	(26.4)	(26.4)
	-559.5	-614.1	-575.7	-211.1	-30.8	-13.0	120.5	19.1	321.5	609.4
	-15.649	-17.179	-16.105	-5.904	-0.861	-0.363	3.371	0.533	8.992	17.047
g_O	1378	1639	1458	1168	1203	891	632	795	610	461
	(38.5)	(45.8)	(40.8)	(32.7)	(33.7)	(24.9)	(17.7)	(22.2)	(17.1)	(12.9)
g_E	844.2	844.2	844.2	844.2	844.2	844.2	844.2	844.2	844.2	844.2
	(23.6)	(23.6)	(23.6)	(23.6)	(23.6)	(23.6)	(23.6)	(23.6)	(23.6)	(23.6)
	1350.4	2174.7	1593.3	758.3	852.1	96.1	-366.0	-95.5	-396.4	-557.8
	37.773	60.831	44.569	21.213	23.835	2.688	-10.237	-2.672	-11.089	-15.603
c_O	440	362	409	482	538	671	790	792	968	1023
	(12.3)	(10.1)	(11.4)	(13.5)	(15.0)	(18.8)	(22.1)	(22.2)	(27.1)	(28.6)
c_E	795.3	795.3	795.3	795.3	795.3	795.3	795.3	795.3	795.3	795.3
	(22.2)	(22.2)	(22.2)	(22.2)	(22.2)	(22.2)	(22.2)	(22.2)	(22.2)	(22.2)
	-520.9	-569.8	-544.0	-482.7	-420.6	-228.1	-10.6	-6.6	380.4	515.1
	-14.571	-15.940	-15.216	-13.503	-11.764	-6.380	-0.296	-0.184	10.642	14.409
N	3575	3575	3575	3575	3575	3575	3575	3575	3575	3575
X2	665.0	1171.0	804.2	271.5	243.4	29.2	82.5	4.8	133.9	329.1
Gvalue	661.0	1103.8	788.5	278.8	236.5	30.1	86.3	4.9	137.6	352.5
Gv/N	18.489	30.876	22.056	7.798	6.616	0.841	2.415	0.136	3.849	9.859
Prob	0.00000	0.00000	0.00000	0.00000	0.00000	0.00000	0.00000	0.18091	0.00000	0.00000

CDS_INITIAL:

	1	2	3	4	5	6	7	8	9	10
a_O	2710	0	0	1880	1475	1263	1493	1316	1104	1270
	(75.8)	(0.0)	(0.0)	(52.6)	(41.3)	(35.3)	(41.8)	(36.8)	(30.9)	(35.5)
a_E	972.9	1108.6	595.9	972.9	1108.6	595.9	972.9	1108.6	595.9	972.9
	(27.2)	(31.0)	(16.7)	(27.2)	(31.0)	(16.7)	(27.2)	(31.0)	(16.7)	(27.2)
	5552.5	0.0	0.0	2476.9	842.4	1897.6	1278.8	451.4	1361.6	676.9
	155.313	0.000	0.000	69.285	23.564	53.078	35.772	12.626	38.086	18.935
t_O	414	3575	2	475	665	938	693	977	879	734
	(11.6)	(100.0)	(0.1)	(13.3)	(18.6)	(26.2)	(19.4)	(27.3)	(24.6)	(20.5)
t_E	617.6	1060.1	887.9	617.6	1060.1	887.9	617.6	1060.1	887.9	617.6
	(17.3)	(29.7)	(24.8)	(17.3)	(29.7)	(24.8)	(17.3)	(29.7)	(24.8)	(17.3)
	-331.2	8691.4	-24.4	-249.4	-620.3	103.0	159.6	-159.6	-17.7	253.4
	-9.265	243.116	-0.682	-6.977	-17.350	2.881	4.464	-4.463	-0.495	7.088
g_O	451	0	3573	755	630	718	728	504	846	734
	(12.6)	(0.0)	(99.9)	(21.1)	(17.6)	(20.1)	(20.4)	(14.1)	(23.7)	(20.5)
g_E	1224.8	582.8	1028.4	1224.8	582.8	1028.4	1224.8	582.8	1028.4	1224.8
	(34.3)	(16.3)	(28.8)	(34.3)	(16.3)	(28.8)	(34.3)	(16.3)	(28.8)	(34.3)
	-901.2	0.0	8900.0	-730.6	98.1	-515.9	-757.5	-146.5	-330.3	-751.7
	-25.208	0.000	248.950	-20.436	2.743	-14.430	-21.189	-4.097	-9.238	-21.026
c_O	0	0	0	465	805	656	661	778	746	837
	(0.0)	(0.0)	(0.0)	(13.0)	(22.5)	(18.3)	(18.5)	(21.8)	(20.9)	(23.4)
c_E	759.6	823.5	1062.9	759.6	823.5	1062.9	759.6	823.5	1062.9	759.6
	(21.2)	(23.0)	(29.7)	(21.2)	(23.0)	(29.7)	(21.2)	(23.0)	(29.7)	(21.2)
	0.0	0.0	0.0	-456.5	-36.5	-633.1	-183.9	-88.4	-528.2	162.3
	0.000	0.000	0.000	-12.768	-1.021	-17.710	-5.144	-2.471	-14.774	4.541
N	3575	3575	3575	3575	3575	3575	3575	3575	3575	3575
X2	4417.4	8480.8	8839.3	1173.2	272.6	999.1	501.6	58.5	560.2	317.2
Gvalue	4320.0	8691.4	8875.6	1040.4	283.7	851.5	497.0	57.0	485.4	341.0
Gv/N	120.841	243.116	248.268	29.103	7.937	23.819	13.903	1.595	13.579	9.537
Prob	1.00000	1.00000	1.00000	0.00000	0.00000	0.00000	0.00000	0.00000	0.00000	0.00000

	11	12	13	14	15	16	17	18	19	20
a_O	1025	942	1227	968	866	1065	940	821	1082	899
	(28.7)	(26.3)	(34.3)	(27.1)	(24.2)	(29.8)	(26.3)	(23.0)	(30.3)	(25.1)
a_E	1108.6	595.9	972.9	1108.6	595.9	972.9	1108.6	595.9	972.9	1108.6
	(31.0)	(16.7)	(27.2)	(31.0)	(16.7)	(27.2)	(31.0)	(16.7)	(27.2)	(31.0)
	-160.7	862.8	569.5	-262.6	647.5	192.7	-310.1	526.2	230.0	-376.8
	-4.496	24.135	15.929	-7.344	18.112	5.390	-8.675	14.720	6.434	-10.540
t_O	1325	1075	781	1409	1103	781	1391	1123	807	1420
	(37.1)	(30.1)	(21.8)	(39.4)	(30.9)	(21.8)	(38.9)	(31.4)	(22.6)	(39.7)
t_E	1060.1	887.9	617.6	1060.1	887.9	617.6	1060.1	887.9	617.6	1060.1
	(29.7)	(24.8)	(17.3)	(29.7)	(24.8)	(17.3)	(29.7)	(24.8)	(17.3)	(29.7)
	591.0	411.1	366.6	801.7	478.6	366.6	755.7	527.6	431.6	830.0
	16.532	11.500	10.254	22.425	13.387	10.254	21.138	14.758	12.074	23.218
g_O	480	793	831	459	808	908	513	823	890	457
	(13.4)	(22.2)	(23.2)	(12.8)	(22.6)	(25.4)	(14.3)	(23.0)	(24.9)	(12.8)
g_E	582.8	1028.4	1224.8	582.8	1028.4	1224.8	582.8	1028.4	1224.8	582.8
	(16.3)	(28.8)	(34.3)	(16.3)	(28.8)	(34.3)	(16.3)	(28.8)	(34.3)	(16.3)
	-186.3	-412.2	-644.7	-219.3	-389.7	-543.6	-130.9	-366.7	-568.4	-222.3
	-5.212	-11.530	-18.035	-6.133	-10.901	-15.204	-3.662	-10.256	-15.900	-6.218

c_O	745	765	736	739	798	821	731	808	796	799
	(20.8)	(21.4)	(20.6)	(20.7)	(22.3)	(23.0)	(20.4)	(22.6)	(22.3)	(22.3)
c_E	823.5	1062.9	759.6	823.5	1062.9	759.6	823.5	1062.9	759.6	823.5
	(23.0)	(29.7)	(21.2)	(23.0)	(29.7)	(21.2)	(23.0)	(29.7)	(21.2)	(23.0)
	-149.2	-503.2	-46.6	-159.9	-457.5	127.5	-174.1	-443.1	74.4	-48.2
	-4.173	-14.074	-1.302	-4.474	-12.796	3.567	-4.870	-12.393	2.082	-1.348
N	3575	3575	3575	3575	3575	3575	3575	3575	3575	3575
X2	98.1	377.8	237.0	167.6	287.8	138.8	147.7	249.4	163.6	189.7
Gvalue	94.8	358.6	244.8	160.0	278.9	143.2	140.5	244.1	167.7	182.8
Gv/N	2.651	10.031	6.846	4.474	7.802	4.007	3.930	6.829	4.690	5.113
Prob	0.00000	0.00000	0.00000	0.00000	0.00000	0.00000	0.00000	0.00000	0.00000	0.00000

	21	22	23	24	25	26	27	28	29	30
a_O	816	1030	881	826	1102	871	871	1085	867	758
	(22.8)	(28.8)	(24.6)	(23.1)	(30.8)	(24.4)	(24.4)	(30.3)	(24.3)	(21.2)
a_E	595.9	972.9	1108.6	595.9	972.9	1108.6	595.9	972.9	1108.6	595.9
	(16.7)	(27.2)	(31.0)	(16.7)	(27.2)	(31.0)	(16.7)	(27.2)	(31.0)	(16.7)
	513.1	117.5	-404.9	539.5	274.7	-420.2	661.3	236.7	-426.2	364.8
	14.351	3.287	-11.326	15.090	7.683	-11.753	18.497	6.620	-11.923	10.205
t_O	1089	750	1367	1037	699	1430	1049	760	1425	1047
	(30.5)	(21.0)	(38.2)	(29.0)	(19.6)	(40.0)	(29.3)	(21.3)	(39.9)	(29.3)
t_E	887.9	617.6	1060.1	887.9	617.6	1060.1	887.9	617.6	1060.1	887.9
	(24.8)	(17.3)	(29.7)	(24.8)	(17.3)	(29.7)	(24.8)	(17.3)	(29.7)	(24.8)
	444.7	291.3	695.1	322.0	173.0	856.0	349.8	315.3	843.0	345.2
	12.438	8.148	19.443	9.006	4.840	23.943	9.785	8.819	23.580	9.655
g_O	753	994	486	783	973	458	806	995	504	883
	(21.1)	(27.8)	(13.6)	(21.9)	(27.2)	(12.8)	(22.5)	(27.8)	(14.1)	(24.7)
g_E	1028.4	1224.8	582.8	1028.4	1224.8	582.8	1028.4	1224.8	582.8	1028.4
	(28.8)	(34.3)	(16.3)	(28.8)	(34.3)	(16.3)	(28.8)	(34.3)	(16.3)	(28.8)
	-469.3	-415.1	-176.6	-426.9	-447.9	-220.8	-392.7	-413.6	-146.5	-269.1
	-13.128	-11.612	-4.940	-11.940	-12.529	-6.175	-10.986	-11.568	-4.097	-7.528
c_O	917	801	841	929	801	816	849	735	779	887
	(25.7)	(22.4)	(23.5)	(26.0)	(22.4)	(22.8)	(23.7)	(20.6)	(21.8)	(24.8)
c_E	1062.9	759.6	823.5	1062.9	759.6	823.5	1062.9	759.6	823.5	1062.9
	(29.7)	(21.2)	(23.0)	(29.7)	(21.2)	(23.0)	(29.7)	(21.2)	(23.0)	(29.7)
	-270.8	84.9	35.5	-250.1	84.9	-14.8	-381.5	-48.5	-86.5	-320.9
	-7.573	2.375	0.992	-6.997	2.375	-0.415	-10.671	-1.356	-2.419	-8.976
N	3575	3575	3575	3575	3575	3575	3575	3575	3575	3575
X2	220.6	77.5	152.0	189.3	81.9	206.8	247.4	89.7	191.3	122.3
Gvalue	217.6	78.6	149.0	184.4	84.7	200.2	236.9	89.9	183.8	120.0
Gv/N	6.088	2.198	4.169	5.159	2.368	5.599	6.626	2.515	5.142	3.356
Prob	0.00000	0.00000	0.00000	0.00000	0.00000	0.00000	0.00000	0.00000	0.00000	0.00000

	31	32	33	34	35	36	37	38	39	40
a_O	1004	783	706	961	762	727	987	830	700	953
	(28.1)	(21.9)	(19.7)	(26.9)	(21.3)	(20.3)	(27.6)	(23.2)	(19.6)	(26.7)
a_E	972.9	1108.6	595.9	972.9	1108.6	595.9	972.9	1108.6	595.9	972.9
	(27.2)	(31.0)	(16.7)	(27.2)	(31.0)	(16.7)	(27.2)	(31.0)	(16.7)	(27.2)
	63.2	-544.5	239.4	-23.6	-571.4	289.2	28.4	-480.4	225.5	-39.4
	1.768	-15.231	6.698	-0.661	-15.982	8.089	0.795	-13.439	6.307	-1.101
t_O	759	1500	1103	788	1455	1041	758	1358	1058	801
	(21.2)	(42.0)	(30.9)	(22.0)	(40.7)	(29.1)	(21.2)	(38.0)	(29.6)	(22.4)
t_E	617.6	1060.1	887.9	617.6	1060.1	887.9	617.6	1060.1	887.9	617.6
	(17.3)	(29.7)	(24.8)	(17.3)	(29.7)	(24.8)	(17.3)	(29.7)	(24.8)	(17.3)
	312.9	1041.2	478.6	383.9	921.4	331.2	310.5	672.6	370.9	416.5
	8.752	29.126	13.387	10.739	25.772	9.265	8.684	18.813	10.375	11.650
g_O	1070	494	897	1094	541	926	1114	518	901	1076
	(29.9)	(13.8)	(25.1)	(30.6)	(15.1)	(25.9)	(31.2)	(14.5)	(25.2)	(30.1)
g_E	1224.8	582.8	1028.4	1224.8	582.8	1028.4	1224.8	582.8	1028.4	1224.8
	(34.3)	(16.3)	(28.8)	(34.3)	(16.3)	(28.8)	(34.3)	(16.3)	(28.8)	(34.3)
	-289.2	-163.4	-245.2	-247.2	-80.6	-194.2	-211.3	-122.2	-238.2	-278.8
	-8.090	-4.570	-6.858	-6.914	-2.254	-5.431	-5.911	-3.417	-6.664	-7.799
c_O	742	798	869	732	817	881	716	869	916	745
	(20.8)	(22.3)	(24.3)	(20.5)	(22.9)	(24.6)	(20.0)	(24.3)	(25.6)	(20.8)
c_E	759.6	823.5	1062.9	759.6	823.5	1062.9	759.6	823.5	1062.9	759.6
	(21.2)	(23.0)	(29.7)	(21.2)	(23.0)	(29.7)	(21.2)	(23.0)	(29.7)	(21.2)
	-34.9	-50.1	-350.0	-54.3	-12.9	-330.7	-84.7	93.6	-272.5	-29.0
	-0.976	-1.402	-9.791	-1.518	-0.360	-9.250	-2.370	2.617	-7.621	-0.812
N	3575	3575	3575	3575	3575	3575	3575	3575	3575	3575
X2	53.3	292.5	124.6	62.1	258.5	96.6	44.6	163.4	86.9	73.2
Gvalue	52.0	283.2	122.8	58.9	256.6	95.6	42.8	163.5	85.7	69.3
Gv/N	1.454	7.923	3.436	1.646	7.177	2.673	1.198	4.574	2.397	1.938
Prob	0.00000	0.00000	0.00000	0.00000	0.00000	0.00000	0.00000	0.00000	0.00000	0.00000

	41	42	43	44	45	46	47	48	49	50
a_O	815	749	959	842	701	899	839	652	925	866
	(22.8)	(21.0)	(26.8)	(23.6)	(19.6)	(25.1)	(23.5)	(18.2)	(25.9)	(24.2)
a_E	1108.6	595.9	972.9	1108.6	595.9	972.9	1108.6	595.9	972.9	1108.6
	(31.0)	(16.7)	(27.2)	(31.0)	(16.7)	(27.2)	(31.0)	(16.7)	(27.2)	(31.0)
	-501.5	342.6	-27.6	-463.2	227.8	-142.0	-467.6	117.4	-93.4	-427.7
	-14.028	9.583	-0.771	-12.957	6.372	-3.972	-13.079	3.283	-2.612	-11.965
t_O	1287	1027	737	1259	992	751	1233	992	755	1228
	(36.0)	(28.7)	(20.6)	(35.2)	(27.7)	(21.0)	(34.5)	(27.7)	(21.1)	(34.3)
t_E	1060.1	887.9	617.6	1060.1	887.9	617.6	1060.1	887.9	617.6	1060.1
	(29.7)	(24.8)	(17.3)	(29.7)	(24.8)	(17.3)	(29.7)	(24.8)	(17.3)	(29.7)
	499.2	299.0	260.5	432.9	220.0	293.7	372.5	220.0	303.3	361.0
	13.963	8.362	7.285	12.110	6.153	8.214	10.420	6.153	8.483	10.099
g_O	553	893	1088	522	954	1178	556	959	1136	553
	(15.5)	(25.0)	(30.4)	(14.6)	(26.7)	(33.0)	(15.6)	(26.8)	(31.8)	(15.5)
g_E	582.8	1028.4	1224.8	582.8	1028.4	1224.8	582.8	1028.4	1224.8	582.8
	(16.3)	(28.8)	(34.3)	(16.3)	(28.8)	(34.3)	(16.3)	(28.8)	(34.3)	(16.3)
	-58.1	-252.1	-257.8	-115.1	-143.2	-91.9	-52.4	-133.9	-171.1	-58.1
	-1.625	-7.050	-7.211	-3.219	-4.005	-2.569	-1.466	-3.746	-4.785	-1.625
c_O	920	906	791	952	928	747	947	972	759	928
	(25.7)	(25.3)	(22.1)	(26.6)	(26.0)	(20.9)	(26.5)	(27.2)	(21.2)	(26.0)
c_E	823.5	1062.9	759.6	823.5	1062.9	759.6	823.5	1062.9	759.6	823.5
	(23.0)	(29.7)	(21.2)	(23.0)	(29.7)	(21.2)	(23.0)	(29.7)	(21.2)	(23.0)
	204.0	-289.4	64.0	276.2	-251.9	-25.1	264.8	-173.8	-1.3	221.8
	5.706	-8.094	1.790	7.726	-7.045	-0.702	7.406	-4.860	-0.036	6.205
N	3575	3575	3575	3575	3575	3575	3575	3575	3575	3575
X2	139.2	102.1	39.8	127.8	53.2	36.4	113.5	29.9	39.4	94.5
Gvalue	143.6	100.1	39.1	130.8	52.7	34.7	117.3	29.7	37.5	97.0
Gv/N	4.016	2.801	1.093	3.659	1.474	0.971	3.282	0.830	1.049	2.714
Prob	0.00000	0.00000	0.00000	0.00000	0.00000	0.00000	0.00000	0.00000	0.00000	0.00000

	51	52	53	54	55	56	57	58	59	60
a_O	658	914	824	675	890	934	632	900	946	654
	(18.4)	(25.6)	(23.0)	(18.9)	(24.9)	(26.1)	(17.7)	(25.2)	(26.5)	(18.3)
a_E	595.9	972.9	1108.6	595.9	972.9	1108.6	595.9	972.9	1108.6	595.9
	(16.7)	(27.2)	(31.0)	(16.7)	(27.2)	(31.0)	(16.7)	(27.2)	(31.0)	(16.7)
	130.5	-114.1	-488.9	168.3	-158.5	-320.1	74.4	-140.2	-300.1	121.7
	3.651	-3.193	-13.676	4.708	-4.434	-8.955	2.081	-3.921	-8.394	3.405
t_O	937	716	1179	941	692	1122	946	712	1101	893
	(26.2)	(20.0)	(33.0)	(26.3)	(19.4)	(31.4)	(26.5)	(19.9)	(30.8)	(25.0)
t_E	887.9	617.6	1060.1	887.9	617.6	1060.1	887.9	617.6	1060.1	887.9
	(24.8)	(17.3)	(29.7)	(24.8)	(17.3)	(29.7)	(24.8)	(17.3)	(29.7)	(24.8)
	100.9	211.6	250.6	109.3	157.4	127.3	119.9	202.5	83.3	10.2
	2.822	5.920	7.010	3.058	4.402	3.561	3.355	5.664	2.330	0.287
g_O	970	1211	549	952	1195	574	991	1212	545	1002
	(27.1)	(33.9)	(15.4)	(26.6)	(33.4)	(16.1)	(27.7)	(33.9)	(15.2)	(28.0)
g_E	1028.4	1224.8	582.8	1028.4	1224.8	582.8	1028.4	1224.8	582.8	1028.4
	(28.8)	(34.3)	(16.3)	(28.8)	(34.3)	(16.3)	(28.8)	(34.3)	(16.3)	(28.8)
	-113.3	-27.5	-65.6	-146.9	-58.9	-17.5	-73.3	-25.5	-73.1	-52.0
	-3.170	-0.770	-1.836	-4.109	-1.649	-0.490	-2.051	-0.714	-2.046	-1.455
c_O	1010	734	1023	1007	798	945	1006	751	983	1026
	(28.3)	(20.5)	(28.6)	(28.2)	(22.3)	(26.4)	(28.1)	(21.0)	(27.5)	(28.7)
c_E	1062.9	759.6	823.5	1062.9	759.6	823.5	1062.9	759.6	823.5	1062.9
	(29.7)	(21.2)	(23.0)	(29.7)	(21.2)	(23.0)	(29.7)	(21.2)	(23.0)	(29.7)
	-103.1	-50.4	444.0	-108.8	78.6	260.2	-110.7	-17.2	348.2	-72.5
	-2.883	-1.410	12.418	-3.042	2.199	7.279	-3.095	-0.481	9.739	-2.027
N	3575	3575	3575	3575	3575	3575	3575	3575	3575	3575
X2	15.1	20.3	136.7	22.3	18.7	49.2	10.4	20.1	58.8	7.7
Gvalue	15.0	19.6	140.0	22.0	18.5	49.9	10.3	19.6	58.3	7.5
Gv/N	0.419	0.547	3.916	0.615	0.518	1.395	0.289	0.547	1.630	0.210
Prob	0.00183	0.00021	0.00000	0.00007	0.00034	0.00000	0.01587	0.00021	0.00000	0.05760

	61	62	63	64	65	66	67	68	69	70
a_O	889	936	673	933	989	654	892	1014	630	917
	(24.9)	(26.2)	(18.8)	(26.1)	(27.7)	(18.3)	(25.0)	(28.4)	(17.6)	(25.7)
a_E	972.9	1108.6	595.9	972.9	1108.6	595.9	972.9	1108.6	595.9	972.9
	(27.2)	(31.0)	(16.7)	(27.2)	(31.0)	(16.7)	(27.2)	(31.0)	(16.7)	(27.2)
	-160.3	-316.8	163.8	-78.1	-225.8	121.7	-154.9	-180.9	70.2	-108.5
	-4.485	-8.862	4.582	-2.185	-6.316	3.405	-4.332	-5.060	1.963	-3.035
t_O	667	1120	936	607	1104	859	714	1099	967	681
	(18.7)	(31.3)	(26.2)	(17.0)	(30.9)	(24.0)	(20.0)	(30.7)	(27.0)	(19.0)
t_E	617.6	1060.1	887.9	617.6	1060.1	887.9	617.6	1060.1	887.9	617.6
	(17.3)	(29.7)	(24.8)	(17.3)	(29.7)	(24.8)	(17.3)	(29.7)	(24.8)	(17.3)
	102.6	123.1	98.8	-21.1	89.5	-56.8	207.1	79.2	165.1	133.0
	2.870	3.443	2.763	-0.590	2.505	-1.590	5.792	2.214	4.617	3.721
g_O	1246	552	1029	1251	571	1051	1250	574	996	1222
	(34.9)	(15.4)	(28.8)	(35.0)	(16.0)	(29.4)	(35.0)	(16.1)	(27.9)	(34.2)
g_E	1224.8	582.8	1028.4	1224.8	582.8	1028.4	1224.8	582.8	1028.4	1224.8
	(34.3)	(16.3)	(28.8)	(34.3)	(16.3)	(28.8)	(34.3)	(16.3)	(28.8)	(34.3)
	42.7	-60.0	1.3	52.9	-23.4	45.8	50.8	-17.5	-63.7	-5.7
	1.194	-1.678	0.036	1.479	-0.655	1.281	1.422	-0.490	-1.781	-0.158
c_O	773	967	937	784	911	1011	719	888	982	755
	(21.6)	(27.0)	(26.2)	(21.9)	(25.5)	(28.3)	(20.1)	(24.8)	(27.5)	(21.1)
c_E	759.6	823.5	1062.9	759.6	823.5	1062.9	759.6	823.5	1062.9	759.6
	(21.2)	(23.0)	(29.7)	(21.2)	(23.0)	(29.7)	(21.2)	(23.0)	(29.7)	(21.2)
	26.9	310.8	-236.2	49.5	184.1	-101.2	-79.1	134.0	-155.4	-9.3
	0.753	8.693	-6.608	1.384	5.149	-2.830	-2.212	3.749	-4.348	-0.259
N	3575	3575	3575	3575	3575	3575	3575	3575	3575	3575
X2	11.8	56.9	27.5	3.2	24.3	9.6	24.5	14.7	16.2	9.7
Gvalue	11.9	57.0	27.7	3.2	24.4	9.5	24.0	14.8	16.1	9.6
Gv/N	0.333	1.596	0.774	0.089	0.683	0.266	0.670	0.414	0.451	0.269
Prob	0.00776	0.00000	0.00000	0.36625	0.00002	0.02319	0.00003	0.00201	0.00108	0.02230

	71	72	73	74	75	76	77	78	79	80
a_O	987	593	878	1025	658	968	1045	619	937	1053
	(27.6)	(16.6)	(24.6)	(28.7)	(18.4)	(27.1)	(29.2)	(17.3)	(26.2)	(29.5)
a_E	1108.6	595.9	972.9	1108.6	595.9	972.9	1108.6	595.9	972.9	1108.6
	(31.0)	(16.7)	(27.2)	(31.0)	(16.7)	(27.2)	(31.0)	(16.7)	(27.2)	(31.0)
	-229.3	-5.7	-180.2	-160.7	130.5	-9.7	-123.5	47.1	-70.4	-108.4
	-6.415	-0.161	-5.041	-4.496	3.651	-0.273	-3.454	1.318	-1.970	-3.031
t_O	1153	960	674	1048	920	656	1100	984	663	1087
	(32.3)	(26.9)	(18.9)	(29.3)	(25.7)	(18.3)	(30.8)	(27.5)	(18.5)	(30.4)
t_E	1060.1	887.9	617.6	1060.1	887.9	617.6	1060.1	887.9	617.6	1060.1
	(29.7)	(24.8)	(17.3)	(29.7)	(24.8)	(17.3)	(29.7)	(24.8)	(17.3)	(29.7)
	193.7	149.9	117.7	-24.1	65.4	79.1	81.2	202.3	94.0	54.4
	5.417	4.194	3.293	-0.674	1.828	2.212	2.272	5.658	2.629	1.522
g_O	553	1020	1267	575	978	1189	593	957	1227	585
	(15.5)	(28.5)	(35.4)	(16.1)	(27.4)	(33.3)	(16.6)	(26.8)	(34.3)	(16.4)
g_E	582.8	1028.4	1224.8	582.8	1028.4	1224.8	582.8	1028.4	1224.8	582.8
	(16.3)	(28.8)	(34.3)	(16.3)	(28.8)	(34.3)	(16.3)	(28.8)	(34.3)	(16.3)
	-58.1	-16.6	85.8	-15.5	-98.2	-70.6	20.5	-137.6	4.3	4.4
	-1.625	-0.465	2.399	-0.435	-2.747	-1.975	0.574	-3.850	0.121	0.122
c_O	882	1002	756	927	1019	762	837	1015	748	850
	(24.7)	(28.0)	(21.1)	(25.9)	(28.5)	(21.3)	(23.4)	(28.4)	(20.9)	(23.8)
c_E	823.5	1062.9	759.6	823.5	1062.9	759.6	823.5	1062.9	759.6	823.5
	(23.0)	(29.7)	(21.2)	(23.0)	(29.7)	(21.2)	(23.0)	(29.7)	(21.2)	(23.0)
	121.2	-118.2	-7.3	219.6	-85.9	4.7	27.3	-93.6	-23.1	53.9
	3.389	-3.306	-0.204	6.143	-2.403	0.132	0.764	-2.617	-0.647	1.509
N	3575	3575	3575	3575	3575	3575	3575	3575	3575	3575
X2	27.2	9.4	15.9	19.6	11.9	3.5	5.5	18.4	4.8	4.3
Gvalue	27.4	9.3	16.0	19.2	11.8	3.4	5.6	18.2	4.8	4.4
Gv/N	0.766	0.261	0.448	0.538	0.329	0.096	0.157	0.509	0.134	0.122
Prob	0.00000	0.02508	0.00112	0.00025	0.00828	0.33041	0.13279	0.00040	0.18881	0.22451

	81	82	83	84	85	86	87	88	89	90
a_O	637	963	1043	546	914	994	607	960	1066	597
	(17.8)	(26.9)	(29.2)	(15.3)	(25.6)	(27.8)	(17.0)	(26.9)	(29.8)	(16.7)
a_E	595.9	972.9	1108.6	595.9	972.9	1108.6	595.9	972.9	1108.6	595.9
	(16.7)	(27.2)	(31.0)	(16.7)	(27.2)	(31.0)	(16.7)	(27.2)	(31.0)	(16.7)
	85.0	-19.7	-127.2	-95.5	-114.1	-216.9	22.4	-25.6	-83.5	2.2
	2.378	-0.550	-3.559	-2.670	-3.193	-6.068	0.628	-0.716	-2.337	0.063
t_O	957	643	1110	986	654	1147	932	671	1044	987
	(26.8)	(18.0)	(31.0)	(27.6)	(18.3)	(32.1)	(26.1)	(18.8)	(29.2)	(27.6)
t_E	887.9	617.6	1060.1	887.9	617.6	1060.1	887.9	617.6	1060.1	887.9
	(24.8)	(17.3)	(29.7)	(24.8)	(17.3)	(29.7)	(24.8)	(17.3)	(29.7)	(24.8)
	143.5	51.8	102.1	206.7	74.8	180.7	90.4	111.2	-32.0	208.9

	4.013	1.448	2.855	5.781	2.093	5.054	2.528	3.111	-0.895	5.843
g_O	959	1220	557	1028	1229	546	976	1210	599	956
	(26.8)	(34.1)	(15.6)	(28.8)	(34.4)	(15.3)	(27.3)	(33.8)	(16.8)	(26.7)
g_E	1028.4	1224.8	582.8	1028.4	1224.8	582.8	1028.4	1224.8	582.8	1028.4
	(28.8)	(34.3)	(16.3)	(28.8)	(34.3)	(16.3)	(28.8)	(34.3)	(16.3)	(28.8)
	-133.9	-9.7	-50.5	-0.7	8.3	-71.3	-102.0	-29.5	32.8	-139.5
	-3.746	-0.270	-1.412	-0.020	0.233	-1.994	-2.853	-0.825	0.917	-3.902
c_O	1022	749	865	1015	778	888	1060	734	866	1035
	(28.6)	(21.0)	(24.2)	(28.4)	(21.8)	(24.8)	(29.7)	(20.5)	(24.2)	(29.0)
c_E	1062.9	759.6	823.5	1062.9	759.6	823.5	1062.9	759.6	823.5	1062.9
	(29.7)	(21.2)	(23.0)	(29.7)	(21.2)	(23.0)	(29.7)	(21.2)	(23.0)	(29.7)
	-80.2	-21.1	85.2	-93.6	37.1	134.0	-5.7	-50.4	87.3	-55.0
	-2.242	-0.592	2.382	-2.617	1.039	3.749	-0.161	-1.410	2.441	-1.539
N	3575	3575	3575	3575	3575	3575	3575	3575	3575	3575
X2	14.5	1.3	9.5	17.2	6.2	26.4	5.1	5.8	4.5	16.9
Gvalue	14.4	1.3	9.5	16.9	6.2	26.5	5.1	5.7	4.5	16.6
Gv/N	0.403	0.036	0.266	0.474	0.173	0.742	0.142	0.160	0.126	0.465
Prob	0.00241	0.72960	0.02339	0.00072	0.10258	0.00001	0.16591	0.12648	0.21134	0.00084

	91	92	93	94	95	96	97	98	99	100
a_O	926	1055	629	936	1095	635	955	1049	584	919
	(25.9)	(29.5)	(17.6)	(26.2)	(30.7)	(17.8)	(26.8)	(29.4)	(16.4)	(25.8)
a_E	972.9	1108.6	595.9	971.8	1107.4	595.2	969.6	1104.9	593.9	969.1
	(27.2)	(31.0)	(16.7)	(27.2)	(31.0)	(16.7)	(27.2)	(31.0)	(16.7)	(27.2)
	-91.5	-104.6	68.1	-70.3	-24.6	82.2	-29.0	-108.9	-19.6	-97.5
	-2.559	-2.925	1.904	-1.967	-0.688	2.301	-0.814	-3.056	-0.550	-2.738
t_O	651	1043	904	642	1062	964	631	1098	934	672
	(18.2)	(29.2)	(25.3)	(18.0)	(29.7)	(27.0)	(17.7)	(30.8)	(26.2)	(18.9)
t_E	617.6	1060.1	887.9	616.9	1058.9	886.9	615.6	1056.6	884.9	615.2
	(17.3)	(29.7)	(24.8)	(17.3)	(29.7)	(24.8)	(17.3)	(29.7)	(24.8)	(17.3)
	68.5	-34.0	32.5	51.1	6.1	160.7	31.3	84.5	100.9	118.7
	1.916	-0.950	0.909	1.432	0.172	4.501	0.878	2.371	2.831	3.332
g_O	1224	544	983	1181	542	938	1190	567	986	1181
	(34.2)	(15.2)	(27.5)	(33.1)	(15.2)	(26.3)	(33.4)	(15.9)	(27.7)	(33.2)
g_E	1224.8	582.8	1028.4	1223.5	582.2	1027.2	1220.7	580.9	1024.9	1220.0
	(34.3)	(16.3)	(28.8)	(34.3)	(16.3)	(28.8)	(34.3)	(16.3)	(28.8)	(34.3)
	-1.7	-75.0	-88.7	-83.4	-77.5	-170.4	-60.7	-27.4	-76.3	-76.8
	-0.047	-2.098	-2.480	-2.337	-2.171	-4.772	-1.703	-0.769	-2.142	-2.157
c_O	774	933	1059	812	872	1034	787	849	1059	789
	(21.7)	(26.1)	(29.6)	(22.7)	(24.4)	(29.0)	(22.1)	(23.8)	(29.7)	(22.2)
c_E	759.6	823.5	1062.9	758.8	822.5	1061.7	757.1	820.7	1059.3	756.7
	(21.2)	(23.0)	(29.7)	(21.2)	(23.0)	(29.7)	(21.2)	(23.0)	(29.7)	(21.2)
	29.0	233.1	-7.7	110.0	101.9	-54.6	61.0	57.6	-0.6	66.0
	0.810	6.519	-0.217	3.082	2.852	-1.530	1.711	1.616	-0.017	1.854
N	3575	3575	3575	3571	3571	3571	3563	3563	3563	3561
X2	4.3	20.0	4.1	7.5	5.9	17.8	2.6	5.8	4.4	10.5
Gvalue	4.3	19.5	4.1	7.5	5.9	17.8	2.6	5.8	4.3	10.3
Gv/N	0.121	0.546	0.116	0.209	0.165	0.499	0.072	0.162	0.122	0.291
Prob	0.22720	0.00021	0.24645	0.05816	0.11645	0.00048	0.46609	0.12293	0.22747	0.01585

	101	102	103	104	105	106	107	108	109	110
a_O	1041	655	919	1050	578	882	1049	635	922	1113
	(29.2)	(18.4)	(25.9)	(29.6)	(16.3)	(24.8)	(29.5)	(17.9)	(26.0)	(31.4)
a_E	1104.3	593.5	966.9	1101.8	592.2	966.1	1100.8	591.7	964.7	1099.3
	(31.0)	(16.7)	(27.2)	(31.0)	(16.7)	(27.2)	(31.0)	(16.7)	(27.2)	(31.0)
	-122.8	129.1	-93.4	-101.1	-28.1	-160.6	-101.2	89.7	-83.5	27.6
	-3.449	3.624	-2.628	-2.845	-0.790	-4.525	-2.851	2.526	-2.356	0.778
t_O	1056	937	635	1058	952	618	1042	926	644	1028
	(29.7)	(26.3)	(17.9)	(29.8)	(26.8)	(17.4)	(29.4)	(26.1)	(18.2)	(29.0)
t_E	1056.0	884.4	613.8	1053.6	882.4	613.3	1052.7	881.7	612.4	1051.2
	(29.7)	(24.8)	(17.3)	(29.7)	(24.8)	(17.3)	(29.7)	(24.8)	(17.3)	(29.7)
	0.1	108.2	43.1	8.8	144.5	9.4	-21.3	90.8	64.7	-45.9
	0.002	3.040	1.212	0.248	4.067	0.265	-0.600	2.559	1.825	-1.296
g_O	596	932	1218	598	955	1236	585	935	1246	562
	(16.7)	(26.2)	(34.3)	(16.8)	(26.9)	(34.8)	(16.5)	(26.3)	(35.1)	(15.9)
g_E	580.5	1024.3	1217.3	579.2	1022.0	1216.3	578.7	1021.2	1214.6	577.9
	(16.3)	(28.8)	(34.3)	(16.3)	(28.8)	(34.3)	(16.3)	(28.8)	(34.3)	(16.3)
	31.3	-176.1	1.4	38.1	-129.6	39.8	12.6	-164.8	63.7	-31.4
	0.880	-4.944	0.040	1.073	-3.646	1.121	0.354	-4.643	1.797	-0.886
c_O	868	1037	781	847	1068	814	874	1054	733	842
	(24.4)	(29.1)	(22.0)	(23.8)	(30.1)	(22.9)	(24.6)	(29.7)	(20.7)	(23.8)
c_E	820.2	1058.7	755.0	818.4	1056.3	754.3	817.7	1055.4	753.3	816.5
	(23.0)	(29.7)	(21.2)	(23.0)	(29.7)	(21.2)	(23.0)	(29.7)	(21.2)	(23.0)
	98.3	-43.0	52.9	58.2	23.5	123.9	116.4	-2.9	-40.0	51.7
	2.760	-1.207	1.490	1.638	0.660	3.491	3.279	-0.081	-1.128	1.458
N	3561	3561	3553	3553	3553	3550	3550	3550	3545	3545
X2	6.8	18.3	4.0	4.1	10.4	12.4	6.5	12.7	4.9	1.9
Gvalue	6.8	18.3	4.0	4.1	10.3	12.5	6.4	12.8	4.9	1.9
Gv/N	0.192	0.513	0.113	0.115	0.290	0.352	0.182	0.360	0.138	0.054
Prob	0.07744	0.00039	0.25906	0.25290	0.01608	0.00587	0.09172	0.00516	0.18106	0.58999

	111	112	113	114	115	116	117	118	119	120
a_O	626	957	1108	594	907	1123	608	944	1157	620
	(17.7)	(27.0)	(31.3)	(16.8)	(25.6)	(31.7)	(17.2)	(26.7)	(32.7)	(17.5)
a_E	590.9	964.4	1099.0	590.7	963.6	1098.1	590.2	962.3	1096.5	589.4
	(16.7)	(27.2)	(31.0)	(16.7)	(27.2)	(31.0)	(16.7)	(27.2)	(31.0)	(16.7)
	72.3	-14.8	18.1	6.6	-109.9	50.5	36.1	-36.2	124.3	62.8
	2.039	-0.419	0.511	0.186	-3.103	1.425	1.020	-1.024	3.515	1.776
t_O	900	597	1036	967	607	997	945	603	1029	915
	(25.4)	(16.8)	(29.2)	(27.3)	(17.1)	(28.2)	(26.7)	(17.1)	(29.1)	(25.9)
t_E	880.4	612.3	1050.9	880.2	611.8	1050.0	879.4	610.9	1048.6	878.2
	(24.8)	(17.3)	(29.7)	(24.8)	(17.3)	(29.7)	(24.8)	(17.3)	(29.7)	(24.8)
	39.6	-30.2	-29.6	181.9	-9.5	-103.4	135.9	-15.7	-38.8	75.1
	1.116	-0.851	-0.837	5.133	-0.268	-2.919	3.837	-0.443	-1.096	2.124
g_O	944	1187	548	969	1250	556	966	1208	523	980
	(26.6)	(33.5)	(15.5)	(27.3)	(35.3)	(15.7)	(27.3)	(34.2)	(14.8)	(27.7)
g_E	1019.7	1214.2	577.8	1019.4	1213.2	577.3	1018.6	1211.5	576.5	1017.1
	(28.8)	(34.3)	(16.3)	(28.8)	(34.3)	(16.3)	(28.8)	(34.3)	(16.3)	(28.8)
	-145.7	-53.8	-58.0	-98.3	74.7	-41.8	-102.4	-6.9	-101.8	-72.9
	-4.109	-1.518	-1.636	-2.775	2.110	-1.180	-2.891	-0.196	-2.879	-2.062
c_O	1075	803	852	1014	777	865	1022	781	827	1021
	(30.3)	(22.7)	(24.0)	(28.6)	(21.9)	(24.4)	(28.9)	(22.1)	(23.4)	(28.9)
c_E	1054.0	753.1	816.3	1053.7	752.4	815.6	1052.8	751.4	814.5	1051.3
	(29.7)	(21.2)	(23.0)	(29.7)	(21.2)	(23.0)	(29.7)	(21.2)	(23.0)	(29.7)
	42.5	103.1	72.9	-77.8	49.9	101.7	-60.6	60.4	25.2	-59.7
	1.199	2.910	2.057	-2.196	1.410	2.872	-1.712	1.709	0.714	-1.688

N	3545	3544	3544	3544	3541	3541	3541	3536	3536	3536
X2	8.6	4.4	3.4	12.6	5.3	7.0	9.0	1.6	8.9	5.4
Gvalue	8.7	4.3	3.4	12.4	5.3	7.0	9.0	1.6	9.0	5.3
Gv/N	0.244	0.121	0.096	0.349	0.151	0.198	0.253	0.046	0.253	0.151
Prob	0.03411	0.23111	0.33586	0.00624	0.14900	0.07147	0.02969	0.65587	0.02987	0.14864

	121	122	123	124	125	126	127	128	129	130
a_O	924	1103	657	876	1125	647	913	1097	587	932
	(26.1)	(31.2)	(18.6)	(24.8)	(31.9)	(18.3)	(25.9)	(31.1)	(16.6)	(26.4)
a_E	962.0	1096.2	589.2	960.6	1094.6	588.4	959.8	1093.7	587.9	959.0
	(27.2)	(31.0)	(16.7)	(27.2)	(31.0)	(16.7)	(27.2)	(31.0)	(16.7)	(27.2)
	-74.5	13.7	143.1	-161.6	61.6	122.9	-91.3	6.6	-1.8	-53.2
	-2.107	0.386	4.048	-4.578	1.744	3.482	-2.589	0.187	-0.050	-1.511
t_O	611	1003	881	620	988	862	625	1006	879	595
	(17.3)	(28.4)	(24.9)	(17.6)	(28.0)	(24.4)	(17.7)	(28.5)	(24.9)	(16.9)
t_E	610.7	1048.3	878.0	609.9	1046.8	876.7	609.3	1045.9	876.0	608.8
	(17.3)	(29.7)	(24.8)	(17.3)	(29.7)	(24.8)	(17.3)	(29.7)	(24.8)	(17.3)
	0.6	-88.5	6.1	20.5	-114.2	-29.2	31.7	-78.2	6.1	-27.3
	0.016	-2.505	0.173	0.580	-3.235	-0.827	0.899	-2.218	0.172	-0.775
g_O	1246	597	944	1282	578	925	1203	576	988	1229
	(35.2)	(16.9)	(26.7)	(36.3)	(16.4)	(26.2)	(34.1)	(16.3)	(28.0)	(34.9)
g_E	1211.1	576.3	1016.8	1209.4	575.5	1015.4	1208.4	575.0	1014.5	1207.4
	(34.3)	(16.3)	(28.8)	(34.3)	(16.3)	(28.8)	(34.3)	(16.3)	(28.8)	(34.3)
	70.7	42.1	-140.3	149.4	5.0	-172.5	-10.8	2.0	-52.4	43.7
	2.001	1.192	-3.970	4.233	0.143	-4.887	-0.305	0.057	-1.485	1.239
c_O	754	832	1053	752	839	1096	786	848	1073	768
	(21.3)	(23.5)	(29.8)	(21.3)	(23.8)	(31.0)	(22.3)	(24.0)	(30.4)	(21.8)
c_E	751.1	814.2	1051.0	750.1	813.1	1049.5	749.4	812.4	1048.6	748.8
	(21.2)	(23.0)	(29.7)	(21.2)	(23.0)	(29.7)	(21.2)	(23.0)	(29.7)	(21.2)
	5.7	35.9	4.0	3.8	52.6	95.0	74.9	72.7	49.3	38.9
	0.162	1.016	0.114	0.109	1.491	2.692	2.122	2.062	1.399	1.103

N	3535	3535	3535	3530	3530	3530	3527	3527	3527	3524
X2	2.5	3.1	13.0	12.0	5.0	16.2	4.5	3.1	1.3	2.0
Gvalue	2.5	3.1	12.9	12.1	5.0	16.2	4.5	3.1	1.3	2.0
Gv/N	0.071	0.089	0.364	0.344	0.142	0.460	0.128	0.088	0.036	0.056
Prob	0.47051	0.36991	0.00491	0.00695	0.16987	0.00102	0.21231	0.37786	0.73494	0.58141

	131	132	133	134	135	136	137	138	139	140
a_O	1146	621	942	1121	612	961	1064	593	941	1113
	(32.5)	(17.6)	(26.8)	(31.8)	(17.4)	(27.3)	(30.2)	(16.9)	(26.8)	(31.7)
a_E	1092.8	587.4	958.2	1091.9	586.9	957.6	1091.2	586.5	956.3	1089.7
	(31.0)	(16.7)	(27.2)	(31.0)	(16.7)	(27.2)	(31.0)	(16.7)	(27.2)	(31.0)
	109.0	69.1	-32.1	59.1	51.3	6.7	-53.8	13.0	-30.3	47.1
	3.093	1.962	-0.912	1.678	1.457	0.191	-1.528	0.369	-0.863	1.341
t_O	997	866	629	999	885	632	1039	876	591	1014
	(28.3)	(24.6)	(17.9)	(28.4)	(25.1)	(18.0)	(29.5)	(24.9)	(16.8)	(28.9)
t_E	1045.0	875.2	608.3	1044.1	874.5	608.0	1043.5	874.0	607.1	1042.0
	(29.7)	(24.8)	(17.3)	(29.7)	(24.8)	(17.3)	(29.7)	(24.8)	(17.3)	(29.7)
	-93.8	-18.3	42.1	-88.2	21.2	49.0	-9.0	4.0	-31.8	-55.3
	-2.661	-0.521	1.196	-2.506	0.601	1.393	-0.256	0.115	-0.904	-1.574
g_O	584	959	1160	542	970	1163	577	961	1230	574
	(16.6)	(27.2)	(32.9)	(15.4)	(27.5)	(33.0)	(16.4)	(27.3)	(35.0)	(16.3)
g_E	574.5	1013.7	1206.3	574.0	1012.8	1205.6	573.7	1012.2	1203.9	572.9
	(16.3)	(28.8)	(34.3)	(16.3)	(28.8)	(34.3)	(16.3)	(28.8)	(34.3)	(16.3)
	19.1	-106.4	-90.9	-62.2	-83.8	-83.8	6.6	-99.9	52.7	2.2
	0.543	-3.018	-2.581	-1.767	-2.380	-2.381	0.188	-2.837	1.499	0.064
c_O	797	1078	790	859	1054	763	839	1089	752	813
	(22.6)	(30.6)	(22.4)	(24.4)	(29.9)	(21.7)	(23.8)	(30.9)	(21.4)	(23.1)
c_E	811.7	1047.7	748.2	811.0	1046.8	747.7	810.6	1046.2	746.7	809.4
	(23.0)	(29.7)	(21.2)	(23.0)	(29.7)	(21.2)	(23.0)	(29.7)	(21.2)	(23.0)
	-29.1	61.4	85.9	98.8	14.4	30.8	57.9	87.3	10.7	7.2
	-0.827	1.743	2.441	2.805	0.409	0.876	1.645	2.480	0.303	0.205

N	3524	3524	3521	3521	3521	3519	3519	3519	3514	3514
X2	5.2	5.8	5.1	7.4	3.1	2.8	1.7	4.4	1.3	1.3
Gvalue	5.2	5.9	5.1	7.4	3.1	2.8	1.7	4.4	1.3	1.3
Gv/N	0.148	0.166	0.144	0.209	0.087	0.079	0.049	0.126	0.036	0.036
Prob	0.15678	0.11873	0.16672	0.06143	0.38066	0.42578	0.63459	0.21772	0.73535	0.73513

	141	142	143	144	145	146	147	148	149	150
a_O	607	920	1135	611	920	1112	565	919	1110	575
	(17.3)	(26.2)	(32.3)	(17.4)	(26.2)	(31.7)	(16.1)	(26.2)	(31.6)	(16.4)
a_E	585.7	955.7	1089.1	585.4	955.7	1089.1	585.4	954.9	1088.1	584.9
	(16.7)	(27.2)	(31.0)	(16.7)	(27.2)	(31.0)	(16.7)	(27.2)	(31.0)	(16.7)
	43.3	-70.1	93.8	52.4	-70.1	46.4	-40.0	-70.5	44.2	-19.6
	1.233	-1.997	2.671	1.491	-1.997	1.320	-1.140	-2.009	1.259	-0.558
t_O	872	618	1026	877	613	1045	944	626	1075	930
	(24.8)	(17.6)	(29.2)	(25.0)	(17.5)	(29.8)	(26.9)	(17.8)	(30.6)	(26.5)
t_E	872.7	606.7	1041.4	872.2	606.7	1041.4	872.2	606.2	1040.6	871.5
	(24.8)	(17.3)	(29.7)	(24.8)	(17.3)	(29.7)	(24.8)	(17.3)	(29.7)	(24.8)
	-1.5	22.7	-30.7	9.5	12.6	7.1	149.3	40.2	70.0	120.8
	-0.042	0.647	-0.873	0.272	0.358	0.203	4.250	1.145	1.996	3.444
g_O	952	1204	539	929	1199	553	969	1192	558	928
	(27.1)	(34.3)	(15.3)	(26.5)	(34.1)	(15.7)	(27.6)	(34.0)	(15.9)	(26.4)
g_E	1010.8	1203.3	572.6	1010.2	1203.3	572.6	1010.2	1202.2	572.1	1009.4
	(28.8)	(34.3)	(16.3)	(28.8)	(34.3)	(16.3)	(28.8)	(34.3)	(16.3)	(28.8)
	-114.1	1.5	-65.1	-155.7	-8.5	-38.4	-80.8	-20.4	-27.8	-156.0
	-3.248	0.043	-1.854	-4.435	-0.242	-1.094	-2.299	-0.580	-0.792	-4.446
c_O	1083	770	812	1095	780	802	1034	772	766	1076
	(30.8)	(21.9)	(23.1)	(31.2)	(22.2)	(22.8)	(29.4)	(22.0)	(21.8)	(30.7)
c_E	1044.7	746.3	808.9	1044.1	746.3	808.9	1044.1	745.6	808.3	1043.3
	(29.7)	(21.2)	(23.0)	(29.7)	(21.2)	(23.0)	(29.7)	(21.2)	(23.0)	(29.7)
	77.9	48.2	6.1	104.1	69.0	-13.8	-20.2	53.7	-82.3	66.5
	2.217	1.373	0.174	2.965	1.964	-0.394	-0.575	1.530	-2.344	1.895

N	3514	3512	3512	3512	3512	3512	3512	3509	3509	3509
X2	5.6	2.3	4.1	10.2	2.9	1.2	8.4	3.0	4.1	11.7
Gvalue	5.6	2.3	4.2	10.3	2.9	1.2	8.3	3.0	4.2	11.8
Gv/N	0.160	0.066	0.118	0.293	0.084	0.035	0.235	0.086	0.119	0.335
Prob	0.13056	0.51086	0.24481	0.01631	0.40166	0.74645	0.04074	0.38915	0.24453	0.00821

Bbro_RB50
5UTR:

	50	49	48	47	46	45	44	43	42	41
a_O	861	977	839	869	901	811	903	919	812	858
	(17.2)	(19.6)	(16.8)	(17.4)	(18.0)	(16.2)	(18.1)	(18.4)	(16.3)	(17.2)
a_E	946.0	946.0	946.0	946.0	946.0	946.0	946.0	946.0	946.0	946.0
	(18.9)	(18.9)	(18.9)	(18.9)	(18.9)	(18.9)	(18.9)	(18.9)	(18.9)	(18.9)
	−162.2	62.9	−201.5	−147.6	−87.9	−249.8	−84.1	−53.3	−248.1	−167.6
	−3.248	1.260	−4.035	−2.956	−1.760	−5.002	−1.684	−1.067	−4.968	−3.356
t_O	802	927	837	853	963	847	812	934	840	823
	(16.1)	(18.6)	(16.8)	(17.1)	(19.3)	(17.0)	(16.3)	(18.7)	(16.8)	(16.5)
t_E	877.1	877.1	877.1	877.1	877.1	877.1	877.1	877.1	877.1	877.1
	(17.6)	(17.6)	(17.6)	(17.6)	(17.6)	(17.6)	(17.6)	(17.6)	(17.6)	(17.6)
	−143.6	102.5	−78.4	−47.6	179.9	−59.2	−125.3	117.4	−72.6	−104.8
	−2.876	2.053	−1.569	−0.953	3.602	−1.185	−2.509	2.350	−1.455	−2.099
g_O	1650	1558	1597	1593	1567	1621	1616	1487	1588	1664
	(33.0)	(31.2)	(32.0)	(31.9)	(31.4)	(32.5)	(32.4)	(29.8)	(31.8)	(33.3)
g_E	1538.4	1538.4	1538.4	1538.4	1538.4	1538.4	1538.4	1538.4	1538.4	1538.4
	(30.8)	(30.8)	(30.8)	(30.8)	(30.8)	(30.8)	(30.8)	(30.8)	(30.8)	(30.8)
	231.2	39.5	119.5	111.2	57.8	169.6	159.1	−101.0	100.9	261.3
	4.629	0.792	2.393	2.227	1.158	3.397	3.187	−2.022	2.020	5.232
c_O	1681	1532	1721	1679	1563	1715	1663	1654	1754	1649
	(33.7)	(30.7)	(34.5)	(33.6)	(31.3)	(34.3)	(33.3)	(33.1)	(35.1)	(33.0)
c_E	1632.5	1632.5	1632.5	1632.5	1632.5	1632.5	1632.5	1632.5	1632.5	1632.5
	(32.7)	(32.7)	(32.7)	(32.7)	(32.7)	(32.7)	(32.7)	(32.7)	(32.7)	(32.7)
	98.5	−194.6	181.8	94.4	−136.0	169.1	61.6	43.3	251.9	33.2
	1.972	−3.898	3.639	1.889	−2.722	3.387	1.234	0.867	5.043	0.665
N	4994	4994	4994	4994	4994	4994	4994	4994	4994	4994
X2	23.6	10.3	21.0	10.2	14.0	28.9	11.3	6.5	31.2	22.0
Gvalue	23.9	10.4	21.4	10.4	13.9	29.8	11.4	6.4	32.0	22.0
Gv/N	0.478	0.207	0.428	0.207	0.277	0.596	0.228	0.128	0.640	0.441
Prob	0.00003	0.01580	0.00009	0.01580	0.00311	0.00000	0.00989	0.09335	0.00000	0.00006

	40	39	38	37	36	35	34	33	32	31
a_O	993	820	892	905	812	896	955	820	847	946
	(19.9)	(16.4)	(17.9)	(18.1)	(16.3)	(17.9)	(19.1)	(16.4)	(17.0)	(18.9)
a_E	946.0	946.0	946.0	946.0	946.0	946.0	946.0	946.0	946.0	946.0
	(18.9)	(18.9)	(18.9)	(18.9)	(18.9)	(18.9)	(18.9)	(18.9)	(18.9)	(18.9)
	96.2	−234.5	−104.9	−80.3	−248.1	−97.4	18.0	−234.5	−187.3	−0.1
	1.927	−4.695	−2.101	−1.607	−4.968	−1.950	0.361	−4.695	−3.751	−0.002
t_O	925	880	808	973	841	828	990	871	792	969
	(18.5)	(17.6)	(16.2)	(19.5)	(16.8)	(16.6)	(19.8)	(17.4)	(15.9)	(19.4)
t_E	877.1	877.1	877.1	877.1	877.1	877.1	877.1	877.1	877.1	877.1
	(17.6)	(17.6)	(17.6)	(17.6)	(17.6)	(17.6)	(17.6)	(17.6)	(17.6)	(17.6)
	98.3	5.8	−132.6	201.9	−70.7	−95.4	239.7	−12.2	−161.7	193.1
	1.969	0.116	−2.656	4.042	−1.416	−1.911	4.800	−0.244	−3.238	3.866
g_O	1486	1572	1604	1497	1571	1540	1422	1570	1606	1429
	(29.8)	(31.5)	(32.1)	(30.0)	(31.5)	(30.8)	(28.5)	(31.4)	(32.2)	(28.6)
g_E	1538.4	1538.4	1538.4	1538.4	1538.4	1538.4	1538.4	1538.4	1538.4	1538.4
	(30.8)	(30.8)	(30.8)	(30.8)	(30.8)	(30.8)	(30.8)	(30.8)	(30.8)	(30.8)
	−102.9	68.0	134.0	−81.6	66.0	3.3	−223.7	63.9	138.2	−210.8
	−2.061	1.362	2.684	−1.634	1.321	0.066	−4.479	1.280	2.768	−4.220
c_O	1590	1722	1690	1619	1770	1730	1627	1733	1749	1650
	(31.8)	(34.5)	(33.8)	(32.4)	(35.4)	(34.6)	(32.6)	(34.7)	(35.0)	(33.0)
c_E	1632.5	1632.5	1632.5	1632.5	1632.5	1632.5	1632.5	1632.5	1632.5	1632.5
	(32.7)	(32.7)	(32.7)	(32.7)	(32.7)	(32.7)	(32.7)	(32.7)	(32.7)	(32.7)
	−83.8	183.9	117.0	−26.8	286.3	200.8	−10.9	207.1	241.2	35.2
	−1.679	3.682	2.344	−0.538	5.733	4.020	−0.219	4.147	4.829	0.705
N	4994	4994	4994	4994	4994	4994	4994	4994	4994	4994
X2	7.8	22.4	13.4	13.5	32.8	11.2	23.4	23.7	29.9	17.6
Gvalue	7.8	23.2	13.5	13.2	33.4	11.2	23.1	24.3	30.3	17.5
Gv/N	0.156	0.464	0.270	0.263	0.669	0.225	0.462	0.488	0.608	0.350
Prob	0.05082	0.00004	0.00366	0.00431	0.00000	0.01063	0.00004	0.00002	0.00000	0.00057

	30	29	28	27	26	25	24	23	22	21
a_O	829	851	940	823	903	894	885	918	1041	934
	(16.6)	(17.0)	(18.8)	(16.5)	(18.1)	(17.9)	(17.7)	(18.4)	(20.8)	(18.7)
a_E	946.0	946.0	946.0	946.0	946.0	946.0	946.0	946.0	946.0	946.0
	(18.9)	(18.9)	(18.9)	(18.9)	(18.9)	(18.9)	(18.9)	(18.9)	(18.9)	(18.9)
	−219.0	−180.2	−12.0	−229.3	−84.1	−101.2	−118.1	−55.2	199.1	−23.9
	−4.385	−3.608	−0.241	−4.592	−1.684	−2.026	−2.364	−1.106	3.988	−0.479
t_O	860	885	1036	860	829	989	871	911	1003	900
	(17.2)	(17.7)	(20.7)	(17.2)	(16.6)	(19.8)	(17.4)	(18.2)	(20.1)	(18.0)
t_E	877.1	877.1	877.1	877.1	877.1	877.1	877.1	877.1	877.1	877.1
	(17.6)	(17.6)	(17.6)	(17.6)	(17.6)	(17.6)	(17.6)	(17.6)	(17.6)	(17.6)
	−33.9	15.8	344.9	−33.9	−93.6	237.5	−12.2	69.1	269.0	46.4
	−0.679	0.317	6.907	−0.679	−1.873	4.755	−0.244	1.383	5.387	0.928
g_O	1500	1523	1359	1524	1484	1395	1412	1378	1239	1319
	(30.0)	(30.5)	(27.2)	(30.5)	(29.7)	(27.9)	(28.3)	(27.6)	(24.8)	(26.4)
g_E	1538.4	1538.4	1538.4	1538.4	1538.4	1538.4	1538.4	1538.4	1538.4	1538.4
	(30.8)	(30.8)	(30.8)	(30.8)	(30.8)	(30.8)	(30.8)	(30.8)	(30.8)	(30.8)
	−75.8	−30.6	−336.9	−28.6	−106.8	−272.9	−242.0	−303.4	−536.3	−405.8
	−1.517	−0.612	−6.747	−0.572	−2.138	−5.465	−4.847	−6.075	−10.738	−8.127
c_O	1805	1735	1659	1787	1778	1716	1826	1787	1711	1841
	(36.1)	(34.7)	(33.2)	(35.8)	(35.6)	(34.4)	(36.6)	(35.8)	(34.3)	(36.9)
c_E	1632.5	1632.5	1632.5	1632.5	1632.5	1632.5	1632.5	1632.5	1632.5	1632.5
	(32.7)	(32.7)	(32.7)	(32.7)	(32.7)	(32.7)	(32.7)	(32.7)	(32.7)	(32.7)
	362.7	211.3	53.5	323.2	303.6	171.2	409.1	323.2	160.8	442.6
	7.262	4.232	1.071	6.472	6.080	3.429	8.192	6.472	3.219	8.863
N	4994	4994	4994	4994	4994	4994	4994	4994	4994	4994
X2	34.0	16.2	50.2	31.1	19.5	34.8	37.3	33.5	89.6	58.7
Gvalue	34.0	16.4	49.4	31.4	19.2	34.6	36.8	33.6	92.7	59.2
Gv/N	0.682	0.329	0.990	0.629	0.385	0.693	0.737	0.674	1.855	1.185
Prob	0.00000	0.00093	0.00000	0.00000	0.00025	0.00000	0.00000	0.00000	0.00000	0.00000

	20	19	18	17	16	15	14	13	12	11
a_O	1015	1053	988	1102	1130	1069	1222	1304	1289	1376
	(20.3)	(21.1)	(19.8)	(22.1)	(22.6)	(21.4)	(24.5)	(26.1)	(25.8)	(27.6)
a_E	946.0	946.0	946.0	946.0	946.0	946.0	946.0	946.0	946.0	946.0
	(18.9)	(18.9)	(18.9)	(18.9)	(18.9)	(18.9)	(18.9)	(18.9)	(18.9)	(18.9)
	142.8	225.6	85.8	336.3	401.6	261.3	625.6	836.9	797.5	1031.0
	2.860	4.517	1.717	6.735	8.041	5.231	12.526	16.759	15.969	20.646
t_O	955	1017	951	979	1072	975	828	934	644	532
	(19.1)	(20.4)	(19.0)	(19.6)	(21.5)	(19.5)	(16.6)	(18.7)	(12.9)	(10.7)

```
t_E        877.1    877.1    877.1    877.1    877.1    877.1    877.1    877.1    877.1    877.1
         ( 17.6)  ( 17.6)  ( 17.6)  ( 17.6)  ( 17.6)  ( 17.6)  ( 17.6)  ( 17.6)  ( 17.6)  ( 17.6)
           162.5    301.0    153.8    215.2    430.2    206.3    -95.4    117.4   -397.9   -532.0
           3.253    6.027    3.080    4.308    8.614    4.131   -1.911    2.350   -7.968  -10.653
g_O         1273     1154     1222     1208     1103     1303     1330     1321     1751     2000
         ( 25.5)  ( 23.1)  ( 24.5)  ( 24.2)  ( 22.1)  ( 26.1)  ( 26.6)  ( 26.5)  ( 35.1)  ( 40.0)
g_E       1538.4   1538.4   1538.4   1538.4   1538.4   1538.4   1538.4   1538.4   1538.4   1538.4
         ( 30.8)  ( 30.8)  ( 30.8)  ( 30.8)  ( 30.8)  ( 30.8)  ( 30.8)  ( 30.8)  ( 30.8)  ( 30.8)
          -482.1   -663.5   -562.7   -584.1   -733.9   -432.7   -387.1   -402.5    453.4   1049.7
          -9.653  -13.286  -11.267  -11.695  -14.696   -8.665   -7.752   -8.059    9.079   21.020
c_O         1751     1770     1833     1705     1689     1647     1614     1435     1310     1086
         ( 35.1)  ( 35.4)  ( 36.7)  ( 34.1)  ( 33.8)  ( 33.0)  ( 32.3)  ( 28.7)  ( 26.2)  ( 21.7)
c_E       1632.5   1632.5   1632.5   1632.5   1632.5   1632.5   1632.5   1632.5   1632.5   1632.5
         ( 32.7)  ( 32.7)  ( 32.7)  ( 32.7)  ( 32.7)  ( 32.7)  ( 32.7)  ( 32.7)  ( 32.7)  ( 32.7)
           245.4    286.3    424.7    148.2    115.0     29.2    -36.8   -370.0   -576.6   -885.3
           4.915    5.733    8.505    2.968    2.302    0.584   -0.736   -7.410  -11.546  -17.727

N           4994     4994     4994     4994     4994     4994     4994     4994     4994     4994
X2          66.3    142.0     97.8    111.7    204.2     63.0    111.7    193.7    279.4    652.7
Gvalue      68.7    149.4    101.6    115.6    212.8     64.0    106.2    181.8    276.4    663.5
Gv/N       1.375    2.991    2.035    2.315    4.261    1.281    2.127    3.640    5.534   13.285
Prob     0.00000  0.00000  0.00000  0.00000  0.00000  0.00000  0.00000  0.00000  0.00000  0.00000
         ------------------------------------------------------------------------------------------
              10        9        8        7        6        5        4        3        2        1
         ------------------------------------------------------------------------------------------
a_O         1319     1238     1483     1379     1213     1207     1234     1263      897      705
         ( 26.4)  ( 24.8)  ( 29.7)  ( 27.6)  ( 24.3)  ( 24.2)  ( 24.7)  ( 25.3)  ( 18.0)  ( 14.1)
a_E        946.0    946.0    946.0    946.0    946.0    946.0    946.0    946.0    946.0    946.0
         ( 18.9)  ( 18.9)  ( 18.9)  ( 18.9)  ( 18.9)  ( 18.9)  ( 18.9)  ( 18.9)  ( 18.9)  ( 18.9)
           876.7    666.0   1333.3   1039.3    603.0    588.1    655.8    729.9    -95.5   -414.7
          17.556   13.335   26.699   20.811   12.075   11.776   13.132   14.616   -1.912   -8.303
t_O          571      491      615      835      794      847      836      675      971      548
         ( 11.4)  (  9.8)  ( 12.3)  ( 16.7)  ( 15.9)  ( 17.0)  ( 16.7)  ( 13.5)  ( 19.4)  ( 11.0)
t_E        877.1    877.1    877.1    877.1    877.1    877.1    877.1    877.1    877.1    877.1
         ( 17.6)  ( 17.6)  ( 17.6)  ( 17.6)  ( 17.6)  ( 17.6)  ( 17.6)  ( 17.6)  ( 17.6)  ( 17.6)
          -490.2   -569.8   -436.7    -82.2   -158.1    -59.2    -80.3   -353.6    197.5   -515.5
          -9.816  -11.409   -8.744   -1.646   -3.166   -1.185   -1.608   -7.081    3.954  -10.323
g_O         2177     2415     1921     1633     1508     1138     1094     1814      944      709
         ( 43.6)  ( 48.4)  ( 38.5)  ( 32.7)  ( 30.2)  ( 22.8)  ( 21.9)  ( 36.3)  ( 18.9)  ( 14.2)
g_E       1538.4   1538.4   1538.4   1538.4   1538.4   1538.4   1538.4   1538.4   1538.4   1538.4
         ( 30.8)  ( 30.8)  ( 30.8)  ( 30.8)  ( 30.8)  ( 30.8)  ( 30.8)  ( 30.8)  ( 30.8)  ( 30.8)
          1511.8   2178.2    853.4    195.0    -60.1   -686.1   -745.8    598.0   -922.0  -1098.4
          30.273   43.617   17.089    3.904   -1.204  -13.738  -14.935   11.974  -18.462  -21.995
c_O          927      850      975     1147     1479     1802     1830     1242     2182     3032
         ( 18.6)  ( 17.0)  ( 19.5)  ( 23.0)  ( 29.6)  ( 36.1)  ( 36.6)  ( 24.9)  ( 43.7)  ( 60.7)
c_E       1632.5   1632.5   1632.5   1632.5   1632.5   1632.5   1632.5   1632.5   1632.5   1632.5
         ( 32.7)  ( 32.7)  ( 32.7)  ( 32.7)  ( 32.7)  ( 32.7)  ( 32.7)  ( 32.7)  ( 32.7)  ( 32.7)
         -1049.2  -1109.5  -1005.1   -809.7   -292.1    356.1    418.0   -679.1   1266.2   3754.4
         -21.009  -22.216  -20.125  -16.213   -5.848    7.130    8.371  -13.598   25.354   75.177

N           4994     4994     4994     4994     4994     4994     4994     4994     4994     4994
X2         823.9   1134.7    743.1    350.4     98.2    194.8    241.8    295.6    427.2   1831.8
Gvalue     849.2   1165.0    745.0    342.4     92.7    198.9    247.7    295.2    446.2   1725.8
Gv/N      17.004   23.328   14.918    6.857    1.857    3.982    4.961    5.911    8.934   34.557
Prob     0.00000  0.00000  0.00000  0.00000  0.00000  0.00000  0.00000  0.00000  0.00000  1.00000
         ------------------------------------------------------------------------------------------

CDS_INITIAL:
         ------------------------------------------------------------------------------------------
               1        2        3        4        5        6        7        8        9       10
         ------------------------------------------------------------------------------------------
a_O         4245        0        0     2261     1528      878     1529     1453      936     1368
         ( 85.0)  (  0.0)  (  0.0)  ( 45.3)  ( 30.6)  ( 17.6)  ( 30.6)  ( 29.1)  ( 18.7)  ( 27.4)
a_E        936.5   1196.6    243.5    936.5   1196.6    243.5    936.5   1196.6    243.5    936.5
         ( 18.8)  ( 24.0)  (  4.9)  ( 18.8)  ( 24.0)  (  4.9)  ( 18.8)  ( 24.0)  (  4.9)  ( 18.8)
         12831.0      0.0      0.0   3985.5    747.0   2252.2   1499.0    564.1   2520.8   1036.7
         256.928    0.000    0.000   79.807   14.958   45.099   30.015   11.296   50.476   20.759
t_O          264     4994        0      601      744      937      592     1131      816      747
         (  5.3)  (100.0)  (  0.0)  ( 12.0)  ( 14.9)  ( 18.8)  ( 11.9)  ( 22.6)  ( 16.3)  ( 15.0)
t_E        591.9   1418.5    316.2    591.9   1418.5    316.2    591.9   1418.5    316.2    591.9
         ( 11.9)  ( 28.4)  (  6.3)  ( 11.9)  ( 28.4)  (  6.3)  ( 11.9)  ( 28.4)  (  6.3)  ( 11.9)
          -426.3  12571.2      0.0     18.4   -960.2   2035.9      0.2   -512.3   1547.3    347.8
          -8.536  251.727    0.000    0.368  -19.228   40.766    0.005  -10.259   30.983    6.963
g_O          485        0     4994      977      926     1197     1492      863     1354     1273
         (  9.7)  (  0.0)  (100.0)  ( 19.6)  ( 18.5)  ( 24.0)  ( 29.9)  ( 17.3)  ( 27.1)  ( 25.5)
g_E       2022.1   1023.4   2035.7   2022.1   1023.4   2035.7   2022.1   1023.4   2035.7   2022.1
         ( 40.5)  ( 20.5)  ( 40.8)  ( 40.5)  ( 20.5)  ( 40.8)  ( 40.5)  ( 20.5)  ( 40.8)  ( 40.5)
         -1384.9      0.0   8963.4  -1421.4   -185.3  -1271.2   -907.3   -294.3  -1104.2  -1178.2
         -27.732    0.000  179.484  -28.462   -3.710  -25.455  -18.167   -5.893  -22.111  -23.593
c_O            0        0        0     1155     1796     1982     1381     1547     1888     1606
         (  0.0)  (  0.0)  (  0.0)  ( 23.1)  ( 36.0)  ( 39.7)  ( 27.7)  ( 31.0)  ( 37.8)  ( 32.2)
c_E       1443.4   1355.4   2398.7   1443.4   1355.4   2398.7   1443.4   1355.4   2398.7   1443.4
         ( 28.9)  ( 27.1)  ( 48.0)  ( 28.9)  ( 27.1)  ( 48.0)  ( 28.9)  ( 27.1)  ( 48.0)  ( 28.9)
             0.0      0.0      0.0   -515.0   1011.0   -756.4   -122.1    409.0   -904.0    342.8
           0.000    0.000    0.000  -10.312   20.243  -15.146   -2.446    8.190  -18.101    6.864

N           4994     4994     4994     4994     4994     4994     4994     4994     4994     4994
X2       14481.1  12587.9   7257.6   2471.0    565.0   3290.4    516.5    165.4   3096.8    535.3
Gvalue   11019.7  12571.2   8963.4   2067.6    612.5   2260.5    469.8    166.5   2059.9    549.0
Gv/N     220.660  251.727  179.484   41.401   12.264   45.265    9.407    3.334   41.248   10.993
Prob     1.00000  1.00000  1.00000  1.00000  0.00000  1.00000  0.00000  0.00000  1.00000  0.00000
         ------------------------------------------------------------------------------------------
              11       12       13       14       15       16       17       18       19       20
         ------------------------------------------------------------------------------------------
a_O         1119      787     1281     1254      750     1209     1192      703     1200     1137
         ( 22.4)  ( 15.8)  ( 25.7)  ( 25.1)  ( 15.0)  ( 24.2)  ( 23.9)  ( 14.1)  ( 24.0)  ( 22.8)
a_E       1196.6    243.5    936.5   1196.6    243.5    936.5   1196.6    243.5    936.5   1196.6
         ( 24.0)  (  4.9)  ( 18.8)  ( 24.0)  (  4.9)  ( 18.8)  ( 24.0)  (  4.9)  ( 18.8)  ( 24.0)
          -150.1   1846.6    802.4    117.4   1687.5    617.4     -9.3   1490.8    594.9   -116.2
          -3.006   36.976   16.068    2.352   33.791   12.364   -0.185   29.852   11.913   -2.328
t_O         1415      876      783     1490      893      815     1538      854      728     1540
         ( 28.3)  ( 17.5)  ( 15.7)  ( 29.8)  ( 17.9)  ( 16.3)  ( 30.8)  ( 17.1)  ( 14.6)  ( 30.8)
t_E       1418.5    316.2    591.9   1418.5    316.2    591.9   1418.5    316.2    591.9   1418.5
         ( 28.4)  (  6.3)  ( 11.9)  ( 28.4)  (  6.3)  ( 11.9)  ( 28.4)  (  6.3)  ( 11.9)  ( 28.4)
            -7.0   1785.4    438.2    146.5   1854.4    521.4    248.8   1697.1    301.4    253.1
          -0.140   35.751    8.775    2.934   37.132   10.441    4.982   33.983    6.035    5.068
g_O          793     1246     1401      752     1258     1391      780     1321     1488      803
         ( 15.9)  ( 24.9)  ( 28.1)  ( 15.1)  ( 25.2)  ( 27.9)  ( 15.6)  ( 26.5)  ( 29.8)  ( 16.1)
g_E       1023.4   2035.7   2022.1   1023.4   2035.7   2022.1   1023.4   2035.7   2022.1   1023.4
         ( 20.5)  ( 40.8)  ( 40.5)  ( 20.5)  ( 40.8)  ( 40.5)  ( 20.5)  ( 40.8)  ( 40.5)  ( 20.5)
          -404.6  -1223.3  -1028.3   -463.5  -1210.9  -1040.8   -423.7  -1142.5   -912.8   -389.6
          -8.101  -24.495  -20.590   -9.281  -24.248  -20.842   -8.485  -22.877  -18.278   -7.800
```

c_O	1667	2085	1529	1498	2093	1579	1484	2116	1578	1514
	(33.4)	(41.8)	(30.6)	(30.0)	(41.9)	(31.6)	(29.7)	(42.4)	(31.6)	(30.3)
c_E	1355.4	2398.7	1443.4	1355.4	2398.7	1443.4	1355.4	2398.7	1443.4	1355.4
	(27.1)	(48.0)	(28.9)	(27.1)	(48.0)	(28.9)	(27.1)	(48.0)	(28.9)	(27.1)
	689.8	-584.4	176.1	299.6	-570.6	283.5	269.0	-530.7	281.3	335.0
	13.813	-11.703	3.526	6.000	-11.426	5.676	5.386	-10.626	5.633	6.708
N	4994	4994	4994	4994	4994	4994	4994	4994	4994	4994
X2	128.6	2551.8	384.3	93.3	2442.1	373.1	80.2	2066.3	259.1	79.4
Gvalue	128.1	1824.3	388.5	100.1	1760.3	381.5	84.8	1514.8	264.8	82.3
Gv/N	2.566	36.529	7.779	2.005	35.249	7.639	1.698	30.332	5.302	1.648
Prob	0.00000	1.00000	0.00000	0.00000	1.00000	0.00000	0.00000	1.00000	0.00000	0.00000

	21	22	23	24	25	26	27	28	29	30
a_O	619	1020	1072	498	964	1062	447	895	970	417
	(12.4)	(20.4)	(21.5)	(10.0)	(19.3)	(21.3)	(9.0)	(17.9)	(19.4)	(8.4)
a_E	243.5	936.5	1196.6	243.5	936.5	1196.6	243.5	936.5	1196.6	243.5
	(4.9)	(18.8)	(24.0)	(4.9)	(18.8)	(24.0)	(4.9)	(18.8)	(24.0)	(4.9)
	1155.1	174.1	-235.8	712.7	55.7	-253.5	543.1	-81.2	-407.3	448.7
	23.130	3.487	-4.722	14.271	1.116	-5.077	10.875	-1.626	-8.157	8.985
t_O	708	793	1500	676	757	1462	611	663	1479	526
	(14.2)	(15.9)	(30.0)	(13.5)	(15.2)	(29.3)	(12.2)	(13.3)	(29.6)	(10.5)
t_E	316.2	591.9	1418.5	316.2	591.9	1418.5	316.2	591.9	1418.5	316.2
	(6.3)	(11.9)	(28.4)	(6.3)	(11.9)	(28.4)	(6.3)	(11.9)	(28.4)	(6.3)
	1141.5	463.9	167.6	1027.4	372.5	88.3	805.0	150.5	123.5	535.5
	22.857	9.290	3.356	20.572	7.460	1.768	16.120	3.013	2.474	10.722
g_O	1369	1689	930	1575	1709	950	1603	1894	980	1725
	(27.4)	(33.8)	(18.6)	(31.5)	(34.2)	(19.0)	(32.1)	(37.9)	(19.6)	(34.5)
g_E	2035.7	2022.1	1023.4	2035.7	2022.1	1023.4	2035.7	2022.1	1023.4	2035.7
	(40.8)	(40.5)	(20.5)	(40.8)	(40.5)	(20.5)	(40.8)	(40.5)	(20.5)	(40.8)
	-1086.3	-608.1	-178.1	-808.2	-575.1	-141.5	-766.0	-248.0	-85.0	-571.3
	-21.752	-12.177	-3.566	-16.183	-11.515	-2.833	-15.339	-4.966	-1.702	-11.440
c_O	2298	1492	1492	2245	1564	1520	2333	1542	1565	2326
	(46.0)	(29.9)	(29.9)	(45.0)	(31.3)	(30.4)	(46.7)	(30.9)	(31.3)	(46.6)
c_E	2398.7	1443.4	1355.4	2398.7	1443.4	1355.4	2398.7	1443.4	1355.4	2398.7
	(48.0)	(28.9)	(27.1)	(48.0)	(28.9)	(27.1)	(48.0)	(28.9)	(27.1)	(48.0)
	-197.1	98.7	286.5	-297.3	250.9	348.4	-129.5	203.7	450.0	-143.1
	-3.946	1.977	5.736	-5.953	5.025	6.976	-2.594	4.079	9.011	-2.866
N	4994	4994	4994	4994	4994	4994	4994	4994	4994	4994
X2	1287.3	132.3	40.0	789.6	105.4	41.7	538.8	25.2	79.8	312.5
Gvalue	1013.3	128.7	40.2	634.6	104.1	41.7	452.6	25.0	81.2	269.8
Gv/N	20.290	2.577	0.804	12.707	2.085	0.835	9.062	0.500	1.626	5.402
Prob	0.00000	0.00000	0.00000	0.00000	0.00000	0.00000	0.00000	0.00002	0.00000	0.00000

	31	32	33	34	35	36	37	38	39	40
a_O	894	962	405	864	907	358	879	936	362	830
	(17.9)	(19.3)	(8.1)	(17.3)	(18.2)	(7.2)	(17.6)	(18.7)	(7.2)	(16.6)
a_E	936.5	1196.6	243.5	936.5	1196.6	243.5	936.5	1196.6	243.5	936.5
	(18.8)	(24.0)	(4.9)	(18.8)	(24.0)	(4.9)	(18.8)	(24.0)	(4.9)	(18.8)
	-83.1	-419.9	412.2	-139.3	-502.7	276.0	-111.5	-459.9	287.1	-200.5
	-1.664	-8.409	8.253	-2.790	-10.066	5.527	-2.232	-9.208	5.750	-4.014
t_O	692	1481	542	609	1496	487	641	1477	497	649
	(13.9)	(29.7)	(10.9)	(12.2)	(30.0)	(9.8)	(12.8)	(29.6)	(10.0)	(13.0)
t_E	591.9	1418.5	316.2	591.9	1418.5	316.2	591.9	1418.5	316.2	591.9
	(11.9)	(28.4)	(6.3)	(11.9)	(28.4)	(6.3)	(11.9)	(28.4)	(6.3)	(11.9)
	216.3	127.7	584.2	34.7	159.2	420.7	102.2	119.4	449.6	119.6
	4.331	2.557	11.699	0.696	3.187	8.425	2.047	2.390	9.002	2.395
g_O	1919	1055	1712	1966	1034	1787	2035	1013	1814	2039
	(38.4)	(21.1)	(34.3)	(39.4)	(20.7)	(35.8)	(40.7)	(20.3)	(36.3)	(40.8)
g_E	2022.1	1023.4	2035.7	2022.1	1023.4	2035.7	2022.1	1023.4	2035.7	2022.1
	(40.5)	(20.5)	(40.8)	(40.5)	(20.5)	(40.8)	(40.5)	(20.5)	(40.8)	(40.5)
	-200.9	64.1	-592.9	-110.7	21.2	-465.6	25.8	-20.8	-418.3	33.9
	-4.024	1.283	-11.872	-2.217	0.425	-9.324	0.516	-0.416	-8.375	0.678
c_O	1489	1496	2335	1555	1557	2362	1439	1568	2321	1476
	(29.8)	(30.0)	(46.8)	(31.1)	(31.2)	(47.3)	(28.8)	(31.4)	(46.5)	(29.6)
c_E	1443.4	1355.4	2398.7	1443.4	1355.4	2398.7	1443.4	1355.4	2398.7	1443.4
	(28.9)	(27.1)	(48.0)	(28.9)	(27.1)	(48.0)	(28.9)	(27.1)	(48.0)	(28.9)
	92.6	295.2	-125.7	231.5	431.7	-72.8	-8.9	456.9	-152.8	65.9
	1.853	5.912	-2.516	4.636	8.645	-1.458	-0.177	9.148	-3.060	1.319
N	4994	4994	4994	4994	4994	4994	4994	4994	4994	4994
X2	25.6	64.3	321.6	16.3	104.4	177.1	7.7	92.6	187.8	18.5
Gvalue	24.8	67.1	277.8	16.2	109.4	158.3	7.7	95.6	165.6	18.8
Gv/N	0.496	1.344	5.564	0.325	2.191	3.170	0.154	1.915	3.317	0.377
Prob	0.00002	0.00000	0.00000	0.00101	0.00000	0.00000	0.05326	0.00000	0.00000	0.00030

	41	42	43	44	45	46	47	48	49	50
a_O	947	390	892	984	389	835	984	352	870	962
	(19.0)	(7.8)	(17.9)	(19.7)	(7.8)	(16.7)	(19.7)	(7.0)	(17.4)	(19.3)
a_E	1196.6	243.5	936.5	1196.6	243.5	936.5	1196.6	243.5	936.5	1196.6
	(24.0)	(4.9)	(18.8)	(24.0)	(4.9)	(18.8)	(24.0)	(4.9)	(18.8)	(24.0)
	-443.1	367.5	-86.9	-385.0	364.5	-191.7	-385.0	259.5	-128.2	-419.9
	-8.873	7.358	-1.741	-7.710	7.299	-3.838	-7.710	5.196	-2.568	-8.409
t_O	1489	505	618	1481	438	607	1498	475	642	1496
	(29.8)	(10.1)	(12.4)	(29.7)	(8.8)	(12.2)	(30.0)	(9.5)	(12.9)	(30.0)
t_E	1418.5	316.2	591.9	1418.5	316.2	591.9	1418.5	316.2	591.9	1418.5
	(28.4)	(6.3)	(11.9)	(28.4)	(6.3)	(11.9)	(28.4)	(6.3)	(11.9)	(28.4)
	144.4	472.9	53.4	127.7	285.5	30.6	163.4	386.7	104.4	159.2
	2.892	9.470	1.069	2.557	5.717	0.613	3.271	7.742	2.090	3.187
g_O	1021	1800	1948	1046	1815	2022	1004	1903	2068	1024
	(20.4)	(36.0)	(39.0)	(20.9)	(36.3)	(40.5)	(20.1)	(38.1)	(41.4)	(20.5)
g_E	1023.4	2035.7	2022.1	1023.4	2035.7	2022.1	1023.4	2035.7	2022.1	1023.4
	(20.5)	(40.8)	(40.5)	(20.5)	(40.8)	(40.5)	(20.5)	(40.8)	(40.5)	(20.5)
	-4.9	-442.9	-145.5	45.6	-416.5	-0.3	-38.5	-256.5	92.7	1.1
	-0.097	-8.869	-2.914	0.914	-8.340	-0.006	-0.771	-5.136	1.857	0.023
c_O	1537	2299	1536	1483	2352	1530	1508	2264	1414	1512
	(30.8)	(46.0)	(30.8)	(29.7)	(47.1)	(30.6)	(30.2)	(45.3)	(28.3)	(30.3)
c_E	1355.4	2398.7	1443.4	1355.4	2398.7	1443.4	1355.4	2398.7	1443.4	1355.4
	(27.1)	(48.0)	(28.9)	(27.1)	(48.0)	(28.9)	(27.1)	(48.0)	(28.9)	(27.1)
	386.4	-195.2	190.9	266.8	-92.4	178.2	321.7	-261.7	-58.3	330.6
	7.738	-3.908	3.823	5.342	-1.851	3.569	6.442	-5.239	-1.167	6.619
N	4994	4994	4994	4994	4994	4994	4994	4994	4994	4994
X2	79.9	232.4	11.9	53.0	158.7	16.6	59.8	144.4	10.6	68.3
Gvalue	82.9	202.3	11.9	55.1	141.1	16.9	61.6	128.0	10.6	70.9
Gv/N	1.660	4.051	0.237	1.103	2.825	0.338	1.233	2.563	0.212	1.420
Prob	0.00000	0.00000	0.00790	0.00000	0.00000	0.00074	0.00000	0.00000	0.01403	0.00000

	51	52	53	54	55	56	57	58	59	60
a_O	343	834	977	304	856	992	308	849	967	292
	(6.9)	(16.7)	(19.6)	(6.1)	(17.1)	(19.9)	(6.2)	(17.0)	(19.4)	(5.8)
a_E	243.5	936.5	1196.6	243.5	936.5	1196.6	243.5	936.5	1196.6	243.5
	(4.9)	(18.8)	(24.0)	(4.9)	(18.8)	(24.0)	(4.9)	(18.8)	(24.0)	(4.9)
	235.1	-193.4	-396.2	135.0	-153.9	-372.1	144.8	-166.6	-412.1	106.1
	4.707	-3.873	-7.934	2.703	-3.083	-7.451	2.899	-3.337	-8.251	2.125
t_O	465	637	1478	416	587	1432	420	620	1434	407
	(9.3)	(12.8)	(29.6)	(8.3)	(11.8)	(28.7)	(8.4)	(12.4)	(28.7)	(8.1)
t_E	316.2	591.9	1418.5	316.2	591.9	1418.5	316.2	591.9	1418.5	316.2
	(6.3)	(11.9)	(28.4)	(6.3)	(11.9)	(28.4)	(6.3)	(11.9)	(28.4)	(6.3)
	358.7	93.6	121.5	228.3	-9.7	27.1	238.5	57.6	31.2	205.5
	7.183	1.874	2.432	4.571	-0.195	0.543	4.776	1.153	0.624	4.116
g_O	1852	2093	1031	1883	2107	1054	1928	2096	1057	1951
	(37.1)	(41.9)	(20.6)	(37.7)	(42.2)	(21.1)	(38.6)	(42.0)	(21.2)	(39.1)
g_E	2035.7	2022.1	1023.4	2035.7	2022.1	1023.4	2035.7	2022.1	1023.4	2035.7
	(40.8)	(40.5)	(20.5)	(40.8)	(40.5)	(20.5)	(40.8)	(40.5)	(20.5)	(40.8)
	-350.2	144.2	15.2	-293.6	173.2	62.0	-209.5	150.4	68.2	-165.7
	-7.013	2.887	0.304	-5.878	3.469	1.242	-4.195	3.011	1.366	-3.319
c_O	2334	1430	1508	2391	1444	1516	2338	1429	1536	2344
	(46.7)	(28.6)	(30.2)	(47.9)	(28.9)	(30.4)	(46.8)	(28.6)	(30.8)	(46.9)
c_E	2398.7	1443.4	1355.4	2398.7	1443.4	1355.4	2398.7	1443.4	1355.4	2398.7
	(48.0)	(28.9)	(27.1)	(48.0)	(28.9)	(27.1)	(48.0)	(28.9)	(27.1)	(48.0)
	-127.6	-26.7	321.7	-15.3	1.1	339.5	-119.8	-28.7	384.2	-108.1
	-2.555	-0.536	6.442	-0.307	0.023	6.797	-2.399	-0.575	7.693	-2.165
N	4994	4994	4994	4994	4994	4994	4994	4994	4994	4994
X2	129.0	17.3	60.0	58.0	10.5	55.1	58.4	12.4	69.4	40.5
Gvalue	116.0	17.6	62.1	54.3	10.7	56.5	54.0	12.6	71.5	37.8
Gv/N	2.322	0.352	1.244	1.088	0.214	1.132	1.081	0.252	1.432	0.757
Prob	0.00000	0.00053	0.00000	0.00000	0.01355	0.00000	0.00000	0.00565	0.00000	0.00000

	61	62	63	64	65	66	67	68	69	70
a_O	801	980	306	809	1019	297	825	1020	297	904
	(16.0)	(19.6)	(6.1)	(16.2)	(20.4)	(5.9)	(16.5)	(20.4)	(5.9)	(18.1)
a_E	936.5	1196.6	243.5	936.5	1196.6	243.5	936.5	1196.6	243.5	936.5
	(18.8)	(24.0)	(4.9)	(18.8)	(24.0)	(4.9)	(18.8)	(24.0)	(4.9)	(18.8)
	-250.4	-391.4	139.9	-236.9	-327.5	118.0	-209.2	-325.8	118.0	-63.9
	-5.015	-7.838	2.801	-4.743	-6.558	2.363	-4.190	-6.524	2.363	-1.280
t_O	599	1436	400	628	1421	394	626	1442	381	583
	(12.0)	(28.8)	(8.0)	(12.6)	(28.5)	(7.9)	(12.5)	(28.9)	(7.6)	(11.7)
t_E	591.9	1418.5	316.2	591.9	1418.5	316.2	591.9	1418.5	316.2	591.9
	(11.9)	(28.4)	(6.3)	(11.9)	(28.4)	(6.3)	(11.9)	(28.4)	(6.3)	(11.9)
	14.3	35.2	188.1	74.4	5.0	173.4	70.2	47.4	142.1	-17.6
	0.287	0.705	3.767	1.490	0.100	3.472	1.405	0.949	2.846	-0.353
g_O	2114	1012	1991	2081	1014	1991	2081	1009	1965	2028
	(42.3)	(20.3)	(39.9)	(41.7)	(20.3)	(39.9)	(41.7)	(20.2)	(39.3)	(40.6)
g_E	2022.1	1023.4	2035.7	2022.1	1023.4	2035.7	2022.1	1023.4	2035.7	2022.1
	(40.5)	(20.5)	(40.8)	(40.5)	(20.5)	(40.8)	(40.5)	(20.5)	(40.8)	(40.5)
	187.8	-22.7	-88.3	119.4	-18.8	-88.3	119.4	-28.7	-138.8	11.7
	3.761	-0.455	-1.769	2.391	-0.376	-1.769	2.391	-0.574	-2.780	0.235
c_O	1480	1566	2297	1476	1540	2312	1462	1523	2351	1479
	(29.6)	(31.4)	(46.0)	(29.6)	(30.8)	(46.3)	(29.3)	(30.5)	(47.1)	(29.6)
c_E	1443.4	1355.4	2398.7	1443.4	1355.4	2398.7	1443.4	1355.4	2398.7	1443.4
	(28.9)	(27.1)	(48.0)	(28.9)	(27.1)	(48.0)	(28.9)	(27.1)	(48.0)	(28.9)
	74.0	452.3	-199.0	65.9	393.2	-170.2	37.4	355.1	-94.4	72.0
	1.483	9.057	-3.985	1.319	7.874	-3.408	0.748	7.110	-1.890	1.442
N	4994	4994	4994	4994	4994	4994	4994	4994	4994	4994
X2	24.8	72.3	43.6	22.0	51.6	35.0	17.2	47.4	28.5	2.2
Gvalue	25.8	73.3	40.7	22.8	51.9	32.9	17.7	48.0	26.9	2.2
Gv/N	0.516	1.468	0.814	0.457	1.040	0.659	0.355	0.960	0.538	0.043
Prob	0.00001	0.00000	0.00000	0.00004	0.00000	0.00000	0.00050	0.00000	0.00001	0.53909

	71	72	73	74	75	76	77	78	79	80
a_O	1089	306	892	1103	310	834	1103	270	901	1084
	(21.8)	(6.1)	(17.9)	(22.1)	(6.2)	(16.7)	(22.1)	(5.4)	(18.0)	(21.7)
a_E	1196.6	243.5	936.5	1196.6	243.5	936.5	1196.6	243.5	936.5	1196.6
	(24.0)	(4.9)	(18.8)	(24.0)	(4.9)	(18.8)	(24.0)	(4.9)	(18.8)	(24.0)
	-205.3	139.9	-86.9	-179.7	149.7	-193.4	-179.7	55.8	-69.7	-214.3
	-4.111	2.801	-1.741	-3.599	2.999	-3.873	-3.599	1.118	-1.396	-4.292
t_O	1389	407	591	1428	368	626	1420	387	605	1466
	(27.8)	(8.1)	(11.8)	(28.6)	(7.4)	(12.5)	(28.4)	(7.7)	(12.1)	(29.4)
t_E	1418.5	316.2	591.9	1418.5	316.2	591.9	1418.5	316.2	591.9	1418.5
	(28.4)	(6.3)	(11.9)	(28.4)	(6.3)	(11.9)	(28.4)	(6.3)	(11.9)	(28.4)
	-58.4	205.5	-1.8	19.1	111.7	70.2	3.0	156.4	26.5	96.6
	-1.169	4.116	-0.035	0.382	2.237	1.405	0.060	3.133	0.531	1.934
g_O	1047	1950	2006	968	1966	2024	1015	1959	2006	1015
	(21.0)	(39.0)	(40.2)	(19.4)	(39.4)	(40.5)	(20.3)	(39.2)	(40.2)	(20.3)
g_E	1023.4	2035.7	2022.1	1023.4	2035.7	2022.1	1023.4	2035.7	2022.1	1023.4
	(20.5)	(40.8)	(40.5)	(20.5)	(40.8)	(40.5)	(20.5)	(40.8)	(40.5)	(20.5)
	47.7	-167.7	-32.2	-107.8	-136.9	3.7	-16.8	-150.4	-32.2	-16.8
	0.955	-3.357	-0.644	-2.159	-2.741	0.074	-0.336	-3.011	-0.644	-0.336
c_O	1469	2331	1505	1495	2350	1510	1456	2378	1482	1429
	(29.4)	(46.7)	(30.1)	(29.9)	(47.1)	(30.2)	(29.2)	(47.6)	(29.7)	(28.6)
c_E	1355.4	2398.7	1443.4	1355.4	2398.7	1443.4	1355.4	2398.7	1443.4	1355.4
	(27.1)	(48.0)	(28.9)	(27.1)	(48.0)	(28.9)	(27.1)	(48.0)	(28.9)	(27.1)
	236.4	-133.4	125.7	293.0	-96.4	136.2	208.4	-41.2	78.1	151.1
	4.734	-2.672	2.517	5.868	-1.930	2.726	4.174	-0.825	1.565	3.025
N	4994	4994	4994	4994	4994	4994	4994	4994	4994	4994
X2	20.4	47.7	4.9	24.8	30.0	16.3	14.9	21.8	2.8	16.3
Gvalue	20.4	44.3	4.9	24.6	28.2	16.6	14.9	20.7	2.8	16.5
Gv/N	0.409	0.887	0.098	0.492	0.564	0.333	0.298	0.414	0.056	0.331
Prob	0.00014	0.00000	0.18138	0.00002	0.00000	0.00085	0.00192	0.00012	0.42260	0.00089

	81	82	83	84	85	86	87	88	89	90
a_O	324	842	1150	273	860	1157	300	884	1126	279
	(6.5)	(16.9)	(23.0)	(5.5)	(17.2)	(23.2)	(6.0)	(17.7)	(22.5)	(5.6)
a_E	243.5	936.5	1196.6	243.5	936.5	1196.6	243.5	936.5	1196.6	243.5
	(4.9)	(18.8)	(24.0)	(4.9)	(18.8)	(24.0)	(4.9)	(18.8)	(24.0)	(4.9)
	185.1	-179.2	-91.4	62.5	-146.7	-77.9	125.2	-102.1	-137.0	76.0
	3.707	-3.588	-1.831	1.251	-2.937	-1.561	2.508	-2.044	-2.744	1.521
t_O	371	648	1376	371	579	1417	367	591	1422	367
	(7.4)	(13.0)	(27.6)	(7.4)	(11.6)	(28.4)	(7.3)	(11.8)	(28.5)	(7.3)
t_E	316.2	591.9	1418.5	316.2	591.9	1418.5	316.2	591.9	1418.5	316.2
	(6.3)	(11.9)	(28.4)	(6.3)	(11.9)	(28.4)	(6.3)	(11.9)	(28.4)	(6.3)
	118.6	117.4	-83.7	118.6	-25.5	-3.0	109.4	-1.8	7.0	109.4

	2.376	2.351	-1.676	2.376	-0.510	-0.060	2.191	-0.035	0.140	2.191
g_O	1981	2058	1027	1982	2041	1047	2004	2031	998	2019
	(39.7)	(41.2)	(20.6)	(39.7)	(40.9)	(21.0)	(40.1)	(40.7)	(20.0)	(40.4)
g_E	2035.7	2022.1	1023.4	2035.7	2022.1	1023.4	2035.7	2022.1	1023.4	2035.7
	(40.8)	(40.5)	(20.5)	(40.8)	(40.5)	(20.5)	(40.8)	(40.5)	(20.5)	(40.8)
	-107.8	72.3	7.1	-105.9	37.9	47.7	-62.8	17.8	-50.2	-33.2
	-2.159	1.449	0.143	-2.120	0.759	0.955	-1.258	0.355	-1.006	-0.664
c_O	2318	1446	1441	2368	1514	1373	2323	1488	1448	2329
	(46.4)	(29.0)	(28.9)	(47.4)	(30.3)	(27.5)	(46.5)	(29.8)	(29.0)	(46.6)
c_E	2398.7	1443.4	1355.4	2398.7	1443.4	1355.4	2398.7	1443.4	1355.4	2398.7
	(48.0)	(28.9)	(27.1)	(48.0)	(28.9)	(27.1)	(48.0)	(28.9)	(27.1)	(48.0)
	-158.6	5.1	176.4	-61.0	144.5	35.4	-148.9	90.5	191.3	-137.3
	-3.176	0.103	3.533	-1.221	2.894	0.708	-2.983	1.812	3.831	-2.750
N	4994	4994	4994	4994	4994	4994	4994	4994	4994	4994
X2	40.3	15.5	8.5	14.9	10.2	2.1	24.2	4.4	11.1	15.5
Gvalue	37.3	15.7	8.4	14.3	10.3	2.1	22.9	4.4	11.1	14.9
Gv/N	0.747	0.314	0.169	0.286	0.206	0.042	0.458	0.088	0.222	0.298
Prob	0.00000	0.00132	0.03787	0.00257	0.01629	0.55301	0.00004	0.22080	0.01128	0.00191

	91	92	93	94	95	96	97	98	99	100
a_O	875	1141	254	891	1102	252	930	1096	252	912
	(17.5)	(22.8)	(5.1)	(17.8)	(22.1)	(5.0)	(18.6)	(22.0)	(5.0)	(18.3)
a_E	936.5	1196.6	243.5	936.5	1196.6	243.5	936.4	1196.4	243.4	936.4
	(18.8)	(24.0)	(4.9)	(18.8)	(24.0)	(4.9)	(18.8)	(24.0)	(4.9)	(18.8)
	-118.9	-108.6	21.5	-88.8	-181.6	17.3	-12.7	-192.1	17.4	-48.1
	-2.382	-2.175	0.430	-1.779	-3.636	0.347	-0.254	-3.848	0.349	-0.963
t_O	563	1405	371	561	1477	357	554	1470	365	587
	(11.3)	(28.1)	(7.4)	(11.2)	(29.6)	(7.1)	(11.1)	(29.4)	(7.3)	(11.8)
t_E	591.9	1418.5	316.2	591.9	1418.5	316.2	591.8	1418.2	316.1	591.8
	(11.9)	(28.4)	(6.3)	(11.9)	(28.4)	(6.3)	(11.9)	(28.4)	(6.3)	(11.9)
	-56.3	-26.9	118.6	-60.1	119.4	86.7	-73.1	105.4	105.0	-9.5
	-1.128	-0.538	2.376	-1.204	2.390	1.736	-1.463	2.112	2.102	-0.190
g_O	2053	1011	1965	2065	966	2017	2004	980	1971	2085
	(41.1)	(20.2)	(39.3)	(41.3)	(19.3)	(40.4)	(40.1)	(19.6)	(39.5)	(41.8)
g_E	2022.1	1023.4	2035.7	2022.1	1023.4	2035.7	2021.7	1023.2	2035.3	2021.7
	(40.5)	(20.5)	(40.8)	(40.5)	(20.5)	(40.8)	(40.5)	(20.5)	(40.8)	(40.5)
	62.2	-24.7	-138.8	86.6	-111.6	-37.1	-35.3	-84.6	-126.5	128.5
	1.245	-0.495	-2.780	1.734	-2.234	-0.744	-0.707	-1.694	-2.533	2.573
c_O	1503	1437	2404	1477	1449	2368	1505	1447	2405	1409
	(30.1)	(28.8)	(48.1)	(29.6)	(29.0)	(47.4)	(30.1)	(29.0)	(48.2)	(28.2)
c_E	1443.4	1355.4	2398.7	1443.4	1355.4	2398.7	1443.1	1355.2	2398.2	1443.1
	(28.9)	(27.1)	(48.0)	(28.9)	(27.1)	(48.0)	(28.9)	(27.1)	(48.0)	(28.9)
	121.6	168.0	10.7	67.9	193.5	-61.0	126.3	189.8	13.6	-67.5
	2.434	3.363	0.213	1.360	3.874	-1.221	2.530	3.801	0.273	-1.352
N	4994	4994	4994	4994	4994	4994	4993	4993	4993	4993
X2	8.4	7.8	12.4	5.5	19.6	6.1	5.3	18.4	9.9	3.5
Gvalue	8.5	7.7	11.9	5.6	19.7	5.9	5.3	18.5	9.6	3.5
Gv/N	0.169	0.155	0.239	0.111	0.394	0.118	0.106	0.370	0.192	0.069
Prob	0.03738	0.05212	0.00759	0.13467	0.00020	0.11570	0.15270	0.00035	0.02265	0.32718

	101	102	103	104	105	106	107	108	109	110
a_O	1148	255	859	1143	246	934	1125	270	857	1155
	(23.0)	(5.1)	(17.2)	(22.9)	(4.9)	(18.7)	(22.5)	(5.4)	(17.2)	(23.1)
a_E	1196.4	243.4	936.4	1196.4	243.4	936.2	1196.2	243.4	936.2	1196.2
	(24.0)	(4.9)	(18.8)	(24.0)	(4.9)	(18.8)	(24.0)	(4.9)	(18.8)	(24.0)
	-94.8	23.7	-148.1	-104.4	5.2	-4.3	-138.0	56.0	-151.4	-80.9
	-1.899	0.474	-2.967	-2.090	0.103	-0.087	-2.764	1.123	-3.034	-1.620
t_O	1469	368	557	1453	360	583	1490	365	589	1437
	(29.4)	(7.4)	(11.2)	(29.1)	(7.2)	(11.7)	(29.8)	(7.3)	(11.8)	(28.8)
t_E	1418.2	316.1	591.8	1418.2	316.1	591.6	1417.9	316.1	591.6	1417.9
	(28.4)	(6.3)	(11.9)	(28.4)	(6.3)	(11.9)	(28.4)	(6.3)	(11.9)	(28.4)
	103.4	111.9	-67.4	70.4	93.6	-17.2	147.7	105.1	-5.3	38.4
	2.070	2.240	-1.351	1.410	1.875	-0.344	2.959	2.106	-0.106	0.769
g_O	980	2024	2086	1016	2064	2016	948	2001	2050	1025
	(19.6)	(40.5)	(41.8)	(20.3)	(41.3)	(40.4)	(19.0)	(40.1)	(41.1)	(20.5)
g_E	1023.2	2035.3	2021.7	1023.2	2035.3	2021.3	1023.0	2034.8	2021.3	1023.0
	(20.5)	(40.8)	(40.5)	(20.5)	(40.8)	(40.5)	(20.5)	(40.8)	(40.5)	(20.5)
	-84.6	-22.4	130.5	-14.4	57.9	-10.7	-144.4	-67.1	57.7	4.0
	-1.694	-0.449	2.615	-0.289	1.160	-0.213	-2.893	-1.345	1.157	0.079
c_O	1396	2346	1491	1381	2323	1459	1429	2356	1496	1375
	(28.0)	(47.0)	(29.9)	(27.7)	(46.5)	(29.2)	(28.6)	(47.2)	(30.0)	(27.5)
c_E	1355.2	2398.2	1443.1	1355.2	2398.2	1442.9	1354.9	2397.7	1442.9	1354.9
	(27.1)	(48.0)	(28.9)	(27.1)	(48.0)	(28.9)	(27.1)	(48.0)	(28.9)	(27.1)
	82.9	-103.3	97.3	52.2	-148.0	32.5	152.2	-82.7	108.2	40.5
	1.660	-2.068	1.948	1.045	-2.964	0.650	3.049	-1.657	2.168	0.812
N	4993	4993	4993	4993	4993	4992	4992	4992	4992	4992
X2	6.8	10.3	12.1	3.8	8.9	0.3	17.5	11.8	9.1	2.0
Gvalue	6.9	9.8	12.2	3.8	8.6	0.3	17.5	11.3	9.2	2.0
Gv/N	0.137	0.197	0.245	0.076	0.173	0.007	0.351	0.227	0.185	0.040
Prob	0.07673	0.02004	0.00658	0.28314	0.03444	0.95510	0.00055	0.01010	0.02624	0.57473

	111	112	113	114	115	116	117	118	119	120
a_O	263	905	1159	257	887	1181	266	864	1213	288
	(5.3)	(18.1)	(23.2)	(5.1)	(17.8)	(23.7)	(5.3)	(17.3)	(24.3)	(5.8)
a_E	243.4	936.2	1196.2	243.4	936.0	1195.9	243.3	936.0	1195.9	243.3
	(4.9)	(18.8)	(24.0)	(4.9)	(18.8)	(24.0)	(4.9)	(18.8)	(24.0)	(4.9)
	40.8	-61.3	-73.1	28.0	-95.3	-29.6	47.4	-138.3	34.4	97.1
	0.817	-1.228	-1.465	0.560	-1.910	-0.594	0.949	-2.770	0.689	1.945
t_O	368	568	1412	365	602	1426	348	571	1418	352
	(7.4)	(11.4)	(28.3)	(7.3)	(12.1)	(28.6)	(7.0)	(11.4)	(28.4)	(7.1)
t_E	316.1	591.6	1417.9	316.1	591.5	1417.7	316.0	591.5	1417.7	316.0
	(6.3)	(11.9)	(28.4)	(6.3)	(11.9)	(28.4)	(6.3)	(11.9)	(28.4)	(6.3)
	112.0	-46.3	-11.8	105.1	21.1	16.7	67.2	-40.3	0.7	76.0
	2.244	-0.928	-0.237	2.106	0.424	0.336	1.346	-0.808	0.014	1.522
g_O	1980	2051	982	2017	2079	1021	1990	2052	1014	2018
	(39.7)	(41.1)	(19.7)	(40.4)	(41.7)	(20.5)	(39.9)	(41.1)	(20.3)	(40.4)
g_E	2034.8	2021.3	1023.0	2034.8	2020.9	1022.8	2034.4	2020.9	1022.8	2034.4
	(40.8)	(40.5)	(20.5)	(40.8)	(40.5)	(20.5)	(40.8)	(40.5)	(20.5)	(40.8)
	-108.2	59.8	-80.4	-35.5	117.8	-3.6	-87.9	62.6	-17.6	-32.7
	-2.167	1.197	-1.610	-0.712	2.360	-0.073	-1.761	1.255	-0.352	-0.656
c_O	2381	1468	1439	2353	1423	1363	2387	1504	1346	2333
	(47.7)	(29.4)	(28.8)	(47.1)	(28.5)	(27.3)	(47.8)	(30.1)	(27.0)	(46.7)
c_E	2397.7	1442.9	1354.9	2397.7	1442.6	1354.6	2397.2	1442.6	1354.6	2397.2
	(48.0)	(28.9)	(27.1)	(48.0)	(28.9)	(27.1)	(48.0)	(28.9)	(27.1)	(48.0)
	-33.3	50.7	173.3	-88.6	-38.9	16.8	-20.4	125.4	-17.2	-126.7
	-0.668	1.016	3.473	-1.775	-0.779	0.337	-0.409	2.513	-0.344	-2.539

N	4992	4992	4992	4992	4991	4991	4991	4991	4991	4991
X2	11.7	2.9	8.0	9.3	4.7	0.3	6.4	9.3	0.4	14.2
Gvalue	11.3	2.9	8.0	9.0	4.7	0.3	6.2	9.5	0.4	13.6
Gv/N	0.225	0.058	0.160	0.180	0.094	0.006	0.124	0.190	0.007	0.272
Prob	0.01043	0.41121	0.04651	0.02981	0.19406	0.96172	0.10190	0.02378	0.94554	0.00356

	121	122	123	124	125	126	127	128	129	130
a_O	847	1157	250	873	1194	270	848	1231	289	924
	(17.0)	(23.2)	(5.0)	(17.5)	(24.0)	(5.4)	(17.0)	(24.7)	(5.8)	(18.5)
a_E	935.6	1195.4	243.2	934.9	1194.5	243.0	934.5	1194.0	242.9	934.3
	(18.8)	(24.0)	(4.9)	(18.8)	(24.0)	(4.9)	(18.8)	(24.0)	(4.9)	(18.8)
	-168.5	-75.6	13.7	-119.5	-1.0	56.8	-164.7	75.1	100.3	-20.5
	-3.378	-1.516	0.275	-2.398	-0.019	1.139	-3.305	1.508	2.013	-0.411
t_O	578	1312	334	594	1320	342	606	1343	317	560
	(11.6)	(26.3)	(6.7)	(11.9)	(26.5)	(6.9)	(12.2)	(27.0)	(6.4)	(11.2)
t_E	591.3	1417.1	315.9	590.8	1415.9	315.6	590.6	1415.4	315.5	590.5
	(11.9)	(28.4)	(6.3)	(11.9)	(28.4)	(6.3)	(11.9)	(28.4)	(6.3)	(11.9)
	-26.3	-202.2	37.3	6.4	-185.2	54.9	31.3	-141.0	3.0	-59.3
	-0.527	-4.052	0.748	0.128	-3.716	1.102	0.627	-2.829	0.061	-1.191
g_O	2093	1106	2016	2097	1025	2023	2086	1061	1988	2072
	(42.0)	(22.2)	(40.4)	(42.1)	(20.6)	(40.6)	(41.9)	(21.3)	(39.9)	(41.6)
g_E	2020.1	1022.4	2033.6	2018.5	1021.6	2032.0	2017.7	1021.2	2031.2	2017.3
	(40.5)	(20.5)	(40.8)	(40.5)	(20.5)	(40.8)	(40.5)	(20.5)	(40.8)	(40.5)
	148.4	173.8	-35.1	160.0	6.8	-17.9	138.9	81.2	-85.4	110.9
	2.974	3.484	-0.703	3.210	0.137	-0.360	2.788	1.629	-1.714	2.226
c_O	1471	1414	2389	1421	1446	2350	1443	1348	2389	1426
	(29.5)	(28.3)	(47.9)	(28.5)	(29.0)	(47.1)	(29.0)	(27.1)	(47.9)	(28.6)
c_E	1442.0	1354.1	2396.3	1440.8	1353.0	2394.4	1440.3	1352.4	2393.4	1440.0
	(28.9)	(27.1)	(48.0)	(28.9)	(27.1)	(48.0)	(28.9)	(27.1)	(48.0)	(28.9)
	58.6	122.5	-14.5	-39.4	192.3	-87.9	5.5	-8.9	-8.8	-27.8
	1.175	2.455	-0.291	-0.790	3.857	-1.763	0.110	-0.178	-0.176	-0.558
N	4989	4989	4989	4985	4985	4985	4983	4983	4983	4982
X2	11.9	18.5	1.4	7.4	12.9	6.1	10.7	6.4	9.7	3.3
Gvalue	12.1	18.5	1.4	7.5	12.9	5.9	11.0	6.4	9.2	3.3
Gv/N	0.243	0.371	0.028	0.150	0.259	0.118	0.220	0.129	0.184	0.067
Prob	0.00689	0.00034	0.70937	0.05778	0.00482	0.11661	0.01198	0.09171	0.02717	0.34499

	131	132	133	134	135	136	137	138	139	140
a_O	1216	264	856	1196	284	864	1175	256	888	1117
	(24.4)	(5.3)	(17.2)	(24.0)	(5.7)	(17.4)	(23.6)	(5.1)	(17.8)	(22.5)
a_E	1193.8	242.9	933.5	1192.8	242.7	933.2	1192.3	242.6	933.0	1192.1
	(24.0)	(4.9)	(18.8)	(24.0)	(4.9)	(18.8)	(24.0)	(4.9)	(18.8)	(24.0)
	44.9	44.0	-148.5	6.4	89.3	-133.1	-34.4	27.5	-87.8	-145.3
	0.901	0.883	-2.982	0.129	1.793	-2.674	-0.691	0.553	-1.764	-2.921
t_O	1364	323	648	1373	306	625	1316	335	573	1399
	(27.4)	(6.5)	(13.0)	(27.6)	(6.1)	(12.6)	(26.4)	(6.7)	(11.5)	(28.1)
t_E	1415.1	315.4	590.0	1414.0	315.2	589.7	1413.4	315.0	589.6	1413.1
	(28.4)	(6.3)	(11.9)	(28.4)	(6.3)	(11.9)	(28.4)	(6.3)	(11.9)	(28.4)
	-100.3	15.3	121.6	-80.7	-18.1	72.6	-187.9	41.2	-32.8	-28.1
	-2.014	0.308	2.442	-1.622	-0.363	1.459	-3.776	0.827	-0.659	-0.564
g_O	1101	2024	2092	1018	1956	2056	1042	1999	2031	1042
	(22.1)	(40.6)	(42.0)	(20.4)	(39.3)	(41.3)	(20.9)	(40.2)	(40.8)	(20.9)
g_E	1021.0	2030.8	2015.7	1020.2	2029.1	2014.9	1019.7	2028.3	2014.5	1019.5
	(20.5)	(40.8)	(40.5)	(20.5)	(40.8)	(40.5)	(20.5)	(40.8)	(40.5)	(20.5)
	166.2	-13.5	155.5	-4.3	-143.6	83.1	45.0	-58.2	33.2	45.4
	3.335	-0.271	3.124	-0.086	-2.885	1.670	0.904	-1.170	0.668	0.913
c_O	1301	2371	1382	1391	2432	1431	1443	2386	1483	1417
	(26.1)	(47.6)	(27.8)	(27.9)	(48.9)	(28.8)	(29.0)	(48.0)	(29.8)	(28.5)
c_E	1352.2	2392.9	1438.8	1351.1	2391.0	1438.2	1350.5	2390.0	1437.9	1350.3
	(27.1)	(48.0)	(28.9)	(27.1)	(48.0)	(28.9)	(27.1)	(48.0)	(28.9)	(27.1)
	-100.4	-43.6	-111.4	81.0	82.7	-14.4	191.1	-8.1	91.5	136.7
	-2.015	-0.876	-2.237	1.627	1.661	-0.290	3.841	-0.162	1.839	2.748
N	4982	4982	4978	4978	4978	4976	4976	4976	4975	4975
X2	10.5	2.2	17.3	2.4	10.6	8.1	13.8	2.4	4.2	8.7
Gvalue	10.4	2.2	17.3	2.4	10.3	8.2	13.8	2.4	4.2	8.7
Gv/N	0.208	0.044	0.347	0.048	0.207	0.165	0.277	0.048	0.085	0.175
Prob	0.01577	0.53449	0.00062	0.49756	0.01621	0.04213	0.00320	0.49402	0.23965	0.03342

	141	142	143	144	145	146	147	148	149	150
a_O	238	890	1188	222	865	1150	221	868	1191	243
	(4.8)	(17.9)	(23.9)	(4.5)	(17.4)	(23.1)	(4.4)	(17.5)	(24.0)	(4.9)
a_E	242.6	932.8	1191.8	242.5	932.6	1191.6	242.5	932.4	1191.4	242.4
	(4.9)	(18.8)	(24.0)	(4.9)	(18.8)	(24.0)	(4.9)	(18.8)	(24.0)	(4.9)
	-9.0	-83.6	-7.7	-39.2	-130.2	-81.7	-41.0	-124.3	-0.7	1.2
	-0.181	-1.681	-0.154	-0.789	-2.618	-1.644	-0.824	-2.500	-0.015	0.024
t_O	333	628	1367	300	609	1399	315	629	1355	322
	(6.7)	(12.6)	(27.5)	(6.0)	(12.2)	(28.1)	(6.3)	(12.7)	(27.3)	(6.5)
t_E	315.0	589.5	1412.8	314.9	589.4	1412.5	314.8	589.3	1412.3	314.8
	(6.3)	(11.9)	(28.4)	(6.3)	(11.9)	(28.4)	(6.3)	(11.9)	(28.4)	(6.3)
	37.1	79.4	-90.1	-29.1	39.9	-26.9	0.3	82.1	-112.2	14.6
	0.745	1.597	-1.812	-0.585	0.802	-0.542	0.006	1.651	-2.256	0.294
g_O	2013	1969	1108	2035	2040	1055	2052	2005	1075	1945
	(40.5)	(39.6)	(22.3)	(40.9)	(41.0)	(21.2)	(41.3)	(40.3)	(21.6)	(39.1)
g_E	2027.9	2014.0	1019.3	2027.5	2013.6	1019.1	2027.1	2013.2	1018.9	2026.7
	(40.8)	(40.5)	(20.5)	(40.8)	(40.5)	(20.5)	(40.8)	(40.5)	(20.5)	(40.8)
	-29.7	-89.1	184.8	15.0	53.1	73.0	50.1	-16.4	115.2	-160.0
	-0.597	-1.791	3.716	0.302	1.067	1.468	1.008	-0.331	2.317	-3.219
c_O	2391	1487	1311	2417	1459	1369	2385	1470	1351	2462
	(48.1)	(29.9)	(26.4)	(48.6)	(29.3)	(27.5)	(48.0)	(29.6)	(27.2)	(49.5)
c_E	2389.6	1437.7	1350.0	2389.1	1437.4	1349.7	2388.6	1437.1	1349.5	2388.1
	(48.0)	(28.9)	(27.1)	(48.0)	(28.9)	(27.1)	(48.0)	(28.9)	(27.1)	(48.0)
	2.9	100.4	-76.9	56.2	43.6	38.8	-7.2	66.6	3.1	150.0
	0.058	2.018	-1.545	1.129	0.877	0.781	-0.144	1.339	0.062	3.018
N	4975	4974	4974	4974	4973	4973	4973	4972	4972	4972
X2	1.2	7.2	10.3	2.8	6.2	3.1	2.2	7.9	5.4	5.7
Gvalue	1.2	7.1	10.2	2.9	6.3	3.1	2.3	8.0	5.4	5.8
Gv/N	0.024	0.144	0.204	0.057	0.127	0.063	0.046	0.160	0.108	0.116
Prob	0.75078	0.06745	0.01733	0.41442	0.09634	0.37322	0.51868	0.04687	0.14562	0.12355

Bbur_B31
5UTR:

	50	49	48	47	46	45	44	43	42	41
a_O	663	635	654	671	629	591	664	606	628	634
	(40.6)	(38.9)	(40.0)	(41.1)	(38.5)	(36.2)	(40.6)	(37.1)	(38.4)	(38.8)
a_E	658.9	658.9	658.9	658.9	658.9	658.9	658.9	658.9	658.9	658.9
	(40.3)	(40.3)	(40.3)	(40.3)	(40.3)	(40.3)	(40.3)	(40.3)	(40.3)	(40.3)
	8.1	-47.0	-9.9	24.3	-58.5	-128.6	10.2	-101.5	-60.4	-48.9
	0.498	-2.877	-0.603	1.489	-3.581	-7.872	0.621	-6.213	-3.697	-2.995
t_O	573	629	621	588	618	650	569	626	635	612
	(35.1)	(38.5)	(38.0)	(36.0)	(37.8)	(39.8)	(34.8)	(38.3)	(38.9)	(37.5)
t_E	581.8	581.8	581.8	581.8	581.8	581.8	581.8	581.8	581.8	581.8
	(35.6)	(35.6)	(35.6)	(35.6)	(35.6)	(35.6)	(35.6)	(35.6)	(35.6)	(35.6)
	-17.4	98.2	81.1	12.5	74.7	144.2	-25.2	91.8	111.2	62.0
	-1.064	6.011	4.961	0.768	4.571	8.824	-1.545	5.616	6.806	3.796
g_O	227	227	199	220	217	213	253	182	183	224
	(13.9)	(13.9)	(12.2)	(13.5)	(13.3)	(13.0)	(15.5)	(11.1)	(11.2)	(13.7)
g_E	232.2	232.2	232.2	232.2	232.2	232.2	232.2	232.2	232.2	232.2
	(14.2)	(14.2)	(14.2)	(14.2)	(14.2)	(14.2)	(14.2)	(14.2)	(14.2)	(14.2)
	-10.3	-10.3	-61.4	-23.7	-29.4	-36.8	43.4	-88.7	-87.1	-16.1
	-0.629	-0.629	-3.758	-1.453	-1.798	-2.250	2.657	-5.426	-5.333	-0.986
c_O	171	143	160	155	170	180	148	220	188	164
	(10.5)	(8.8)	(9.8)	(9.5)	(10.4)	(11.0)	(9.1)	(13.5)	(11.5)	(10.0)
c_E	161.1	161.1	161.1	161.1	161.1	161.1	161.1	161.1	161.1	161.1
	(9.9)	(9.9)	(9.9)	(9.9)	(9.9)	(9.9)	(9.9)	(9.9)	(9.9)	(9.9)
	20.4	-34.1	-2.2	-12.0	18.3	39.9	-25.1	137.1	58.1	5.9
	1.249	-2.086	-0.134	-0.732	1.119	2.445	-1.536	8.391	3.554	0.359
N	1634	1634	1634	1634	1634	1634	1634	1634	1634	1634
X2	0.9	6.9	7.4	1.2	5.1	18.8	3.2	40.0	21.2	2.9
Gvalue	0.9	6.8	7.6	1.2	5.1	18.7	3.2	38.7	21.7	2.8
Gv/N	0.053	0.419	0.467	0.072	0.312	1.147	0.197	2.368	1.329	0.174
Prob	0.83228	0.07692	0.05445	0.75975	0.16502	0.00031	0.35813	0.00000	0.00007	0.41589

	40	39	38	37	36	35	34	33	32	31
a_O	657	635	632	679	673	681	676	668	717	704
	(40.2)	(38.9)	(38.7)	(41.6)	(41.2)	(41.7)	(41.4)	(40.9)	(43.9)	(43.1)
a_E	658.9	658.9	658.9	658.9	658.9	658.9	658.9	658.9	658.9	658.9
	(40.3)	(40.3)	(40.3)	(40.3)	(40.3)	(40.3)	(40.3)	(40.3)	(40.3)	(40.3)
	-3.9	-47.0	-52.8	40.7	28.4	44.8	34.5	18.2	121.1	93.1
	-0.238	-2.877	-3.230	2.492	1.739	2.744	2.114	1.116	7.410	5.699
t_O	621	624	613	556	597	602	604	589	531	600
	(38.0)	(38.2)	(37.5)	(34.0)	(36.5)	(36.8)	(37.0)	(36.0)	(32.5)	(36.7)
t_E	581.8	581.8	581.8	581.8	581.8	581.8	581.8	581.8	581.8	581.8
	(35.6)	(35.6)	(35.6)	(35.6)	(35.6)	(35.6)	(35.6)	(35.6)	(35.6)	(35.6)
	81.1	87.5	64.1	-50.4	30.9	41.2	45.3	14.6	-97.0	37.0
	4.961	5.353	3.924	-3.082	1.889	2.520	2.773	0.891	-5.934	2.267
g_O	182	235	250	189	203	212	197	217	220	192
	(11.1)	(14.4)	(15.3)	(11.6)	(12.4)	(13.0)	(12.1)	(13.3)	(13.5)	(11.8)
g_E	232.2	232.2	232.2	232.2	232.2	232.2	232.2	232.2	232.2	232.2
	(14.2)	(14.2)	(14.2)	(14.2)	(14.2)	(14.2)	(14.2)	(14.2)	(14.2)	(14.2)
	-88.7	5.6	36.9	-77.8	-54.6	-38.6	-64.8	-29.4	-23.7	-73.0
	-5.426	0.345	2.260	-4.762	-3.339	-2.361	-3.964	-1.798	-1.453	-4.467
c_O	174	140	139	210	161	139	157	160	166	138
	(10.6)	(8.6)	(8.5)	(12.9)	(9.9)	(8.5)	(9.6)	(9.8)	(10.2)	(8.4)
c_E	161.1	161.1	161.1	161.1	161.1	161.1	161.1	161.1	161.1	161.1
	(9.9)	(9.9)	(9.9)	(9.9)	(9.9)	(9.9)	(9.9)	(9.9)	(9.9)	(9.9)
	26.8	-39.3	-41.0	111.3	-0.2	-41.0	-8.1	-2.2	10.0	-42.7
	1.641	-2.405	-2.510	6.814	-0.012	-2.510	-0.495	-0.134	0.609	-2.614
N	1634	1634	1634	1634	1634	1634	1634	1634	1634	1634
X2	14.5	6.7	7.2	24.6	4.4	6.2	6.7	1.2	10.3	13.9
Gvalue	15.3	6.8	7.3	23.9	4.5	6.4	7.0	1.2	10.3	14.5
Gv/N	0.938	0.416	0.445	1.462	0.277	0.393	0.429	0.076	0.633	0.885
Prob	0.00155	0.07851	0.06360	0.00003	0.20967	0.09303	0.07158	0.74383	0.01592	0.00234

	30	29	28	27	26	25	24	23	22	21
a_O	689	687	702	721	642	681	703	781	684	667
	(42.2)	(42.0)	(43.0)	(44.1)	(39.3)	(41.7)	(43.0)	(47.8)	(41.9)	(40.8)
a_E	658.9	658.9	658.9	658.9	658.9	658.9	658.9	658.9	658.9	658.5
	(40.3)	(40.3)	(40.3)	(40.3)	(40.3)	(40.3)	(40.3)	(40.3)	(40.3)	(40.3)
	61.5	57.3	88.9	129.8	-33.4	44.8	91.0	265.4	51.1	17.0
	3.761	3.506	5.439	7.942	-2.047	2.744	5.569	16.245	3.124	1.043
t_O	593	612	558	602	610	618	595	494	615	639
	(36.3)	(37.5)	(34.1)	(36.8)	(37.3)	(37.8)	(36.4)	(30.2)	(37.6)	(39.1)
t_E	581.8	581.8	581.8	581.8	581.8	581.8	581.8	581.8	581.8	581.4
	(35.6)	(35.6)	(35.6)	(35.6)	(35.6)	(35.6)	(35.6)	(35.6)	(35.6)	(35.6)
	22.7	62.0	-46.5	41.2	57.8	74.7	26.8	-161.6	68.3	120.7
	1.389	3.796	-2.848	2.520	3.539	4.571	1.639	-9.888	4.182	7.392
g_O	197	197	225	172	212	178	179	205	172	199
	(12.1)	(12.1)	(13.8)	(10.5)	(13.0)	(10.9)	(11.0)	(12.5)	(10.5)	(12.2)
g_E	232.2	232.2	232.2	232.2	232.2	232.2	232.2	232.2	232.2	232.1
	(14.2)	(14.2)	(14.2)	(14.2)	(14.2)	(14.2)	(14.2)	(14.2)	(14.2)	(14.2)
	-64.8	-64.8	-14.2	-103.2	-38.6	-94.6	-93.2	-51.1	-103.2	-61.2
	-3.964	-3.964	-0.867	-6.318	-2.361	-5.791	-5.701	-3.126	-6.318	-3.745
c_O	155	138	149	139	170	157	157	154	163	128
	(9.5)	(8.4)	(9.1)	(8.5)	(10.4)	(9.6)	(9.6)	(9.4)	(10.0)	(7.8)
c_E	161.1	161.1	161.1	161.1	161.1	161.1	161.1	161.1	161.1	161.0
	(9.9)	(9.9)	(9.9)	(9.9)	(9.9)	(9.9)	(9.9)	(9.9)	(9.9)	(9.9)
	-12.0	-42.7	-23.3	-41.0	18.3	-8.1	-8.1	-13.9	3.8	-58.7
	-0.732	-2.614	-1.423	-2.510	1.119	-0.495	-0.495	-0.849	0.234	-3.596
N	1634	1634	1634	1634	1634	1634	1634	1634	1634	1633
X2	7.2	11.4	4.9	25.2	4.1	15.8	15.5	39.3	18.5	17.3
Gvalue	7.4	11.8	4.9	26.7	4.1	16.8	16.5	38.9	20.0	17.9
Gv/N	0.455	0.724	0.300	1.634	0.250	1.029	1.012	2.382	1.223	1.094
Prob	0.05945	0.00798	0.17963	0.00001	0.25273	0.00077	0.00088	0.00000	0.00017	0.00047

	20	19	18	17	16	15	14	13	12	11
a_O	671	712	664	710	755	686	745	769	703	721
	(41.1)	(43.6)	(40.6)	(43.5)	(46.2)	(42.0)	(45.6)	(47.1)	(43.0)	(44.1)
a_E	658.9	658.9	658.9	658.9	658.9	658.9	658.9	658.9	658.9	658.9
	(40.3)	(40.3)	(40.3)	(40.3)	(40.3)	(40.3)	(40.3)	(40.3)	(40.3)	(40.3)
	24.3	110.3	10.2	106.0	205.5	55.2	182.9	237.5	91.0	129.8
	1.489	6.749	0.621	6.485	12.575	3.379	11.193	14.538	5.569	7.942
t_O	617	593	597	547	558	576	467	453	478	309
	(37.8)	(36.3)	(36.5)	(33.5)	(34.1)	(35.3)	(28.6)	(27.7)	(29.3)	(18.9)

t_E	581.8	581.8	581.8	581.8	581.8	581.8	581.8	581.8	581.8	581.8
	(35.6)	(35.6)	(35.6)	(35.6)	(35.6)	(35.6)	(35.6)	(35.6)	(35.6)	(35.6)
	72.6	22.7	30.9	-67.4	-46.5	-11.5	-205.2	-226.7	-187.8	-391.0
	4.441	1.389	1.889	-4.125	-2.848	-0.702	-12.560	-13.871	-11.494	-23.930
g_O	213	172	239	232	190	246	301	301	371	507
	(13.0)	(10.5)	(14.6)	(14.2)	(11.6)	(15.1)	(18.4)	(18.4)	(22.7)	(31.0)
g_E	232.2	232.2	232.2	232.2	232.2	232.2	232.2	232.2	232.2	232.2
	(14.2)	(14.2)	(14.2)	(14.2)	(14.2)	(14.2)	(14.2)	(14.2)	(14.2)	(14.2)
	-36.8	-103.2	13.8	-0.4	-76.2	28.4	156.2	156.2	347.7	791.9
	-2.250	-6.318	0.845	-0.024	-4.664	1.739	9.561	9.561	21.280	48.461
c_O	133	157	134	145	131	126	121	111	82	97
	(8.1)	(9.6)	(8.2)	(8.9)	(8.0)	(7.7)	(7.4)	(6.8)	(5.0)	(5.9)
c_E	161.1	161.1	161.1	161.1	161.1	161.1	161.1	161.1	161.1	161.1
	(9.9)	(9.9)	(9.9)	(9.9)	(9.9)	(9.9)	(9.9)	(9.9)	(9.9)	(9.9)
	-51.0	-8.1	-49.4	-30.5	-54.2	-61.9	-69.3	-82.7	-110.7	-98.4
	-3.120	-0.495	-3.020	-1.868	-3.316	-3.790	-4.239	-5.060	-6.778	-6.023
N	1634	1634	1634	1634	1634	1634	1634	1634	1634	1634
X2	8.8	20.2	5.2	7.6	28.3	9.6	64.2	82.8	143.3	484.5
Gvalue	9.2	21.6	5.5	7.6	28.5	10.2	64.6	84.4	140.1	432.2
Gv/N	0.560	1.325	0.335	0.468	1.747	0.626	3.955	5.167	8.577	26.450
Prob	0.02727	0.00008	0.14053	0.05402	0.00000	0.01672	0.00000	0.00000	0.00000	0.00000

	10	9	8	7	6	5	4	3	2	1
a_O	664	553	706	692	650	743	767	711	686	630
	(40.7)	(33.9)	(43.2)	(42.4)	(39.8)	(45.5)	(46.9)	(43.5)	(42.0)	(38.6)
a_E	658.5	658.5	658.9	658.9	658.9	658.9	658.9	658.9	658.9	658.9
	(40.3)	(40.3)	(40.3)	(40.3)	(40.3)	(40.3)	(40.3)	(40.3)	(40.3)	(40.3)
	11.0	-193.2	97.4	67.7	-17.8	178.4	232.9	108.1	55.2	-56.6
	0.671	-11.830	5.961	4.146	-1.087	10.918	14.255	6.617	3.379	-3.464
t_O	398	372	351	508	548	506	569	611	640	756
	(24.4)	(22.8)	(21.5)	(31.1)	(33.5)	(31.0)	(34.8)	(37.4)	(39.2)	(46.3)
t_E	581.4	581.4	581.8	581.8	581.8	581.8	581.8	581.8	581.8	581.8
	(35.6)	(35.6)	(35.6)	(35.6)	(35.6)	(35.6)	(35.6)	(35.6)	(35.6)	(35.6)
	-301.7	-332.2	-354.7	-137.7	-65.5	-141.2	-25.2	59.9	122.1	396.1
	-18.474	-20.345	-21.708	-8.430	-4.010	-8.641	-1.545	3.667	7.474	24.242
g_O	471	613	489	335	319	241	158	175	188	133
	(28.8)	(37.5)	(29.9)	(20.5)	(19.5)	(14.7)	(9.7)	(10.7)	(11.5)	(8.1)
g_E	232.1	232.1	232.2	232.2	232.2	232.2	232.2	232.2	232.2	232.2
	(14.2)	(14.2)	(14.2)	(14.2)	(14.2)	(14.2)	(14.2)	(14.2)	(14.2)	(14.2)
	666.8	1190.9	728.4	245.6	202.6	17.9	-121.7	-99.0	-79.4	-148.2
	40.834	72.928	44.577	15.029	12.401	1.097	-7.445	-6.058	-4.859	-9.071
c_O	100	95	88	99	117	144	140	137	120	115
	(6.1)	(5.8)	(5.4)	(6.1)	(7.2)	(8.8)	(8.6)	(8.4)	(7.3)	(7.0)
c_E	161.0	161.0	161.1	161.1	161.1	161.1	161.1	161.1	161.1	161.1
	(9.9)	(9.9)	(9.9)	(9.9)	(9.9)	(9.9)	(9.9)	(9.9)	(9.9)	(9.9)
	-95.2	-100.2	-106.4	-96.4	-74.8	-32.3	-39.3	-44.4	-70.7	-77.5
	-5.832	-6.138	-6.513	-5.900	-4.580	-1.977	-2.405	-2.717	-4.326	-4.745
N	1633	1633	1634	1634	1634	1634	1634	1634	1634	1634
X2	327.0	744.7	412.1	80.5	46.6	22.7	44.5	23.3	25.8	109.0
Gvalue	280.9	565.3	364.7	79.2	44.5	22.8	46.7	24.7	27.3	113.8
Gv/N	17.199	34.615	22.317	4.845	2.723	1.397	2.860	1.510	1.668	6.962
Prob	0.00000	0.00000	0.00000	0.00000	0.00000	0.00004	0.00000	0.00002	0.00001	0.00000

CDS_INITIAL:

	1	2	3	4	5	6	7	8	9	10
a_O	1083	0	0	831	772	680	889	765	712	770
	(66.3)	(0.0)	(0.0)	(50.9)	(47.2)	(41.6)	(54.4)	(46.8)	(43.6)	(47.1)
a_E	654.9	633.6	605.6	654.9	633.6	605.6	654.9	633.2	605.6	654.9
	(40.1)	(38.8)	(37.1)	(40.1)	(38.8)	(37.1)	(40.1)	(38.8)	(37.1)	(40.1)
	1089.5	0.0	0.0	395.8	305.0	157.5	543.4	289.2	230.4	249.3
	66.678	0.000	0.000	24.223	18.665	9.638	33.255	17.711	14.099	15.259
t_O	388	1634	0	224	424	633	325	551	581	385
	(23.7)	(100.0)	(0.0)	(13.7)	(25.9)	(38.7)	(19.9)	(33.7)	(35.6)	(23.6)
t_E	363.0	548.4	683.7	363.0	548.4	683.7	363.0	548.1	683.7	363.0
	(22.2)	(33.6)	(41.8)	(22.2)	(33.6)	(41.8)	(22.2)	(33.6)	(41.8)	(22.2)
	51.7	3567.8	0.0	-216.2	-218.2	-97.6	-71.8	5.9	-189.1	45.4
	3.167	218.350	0.000	-13.234	-13.353	-5.970	-4.395	0.358	-11.576	2.777
g_O	163	0	1634	366	234	182	315	184	215	323
	(10.0)	(0.0)	(100.0)	(22.4)	(14.3)	(11.1)	(19.3)	(11.3)	(13.2)	(19.8)
g_E	432.8	194.6	186.6	432.8	194.6	186.6	432.8	194.5	186.6	432.8
	(26.5)	(11.9)	(11.4)	(26.5)	(11.9)	(11.4)	(26.5)	(11.9)	(11.4)	(26.5)
	-318.3	0.0	7090.7	-122.7	86.2	-9.1	-200.1	-20.5	60.9	-189.0
	-19.482	0.000	433.945	-7.510	5.275	-0.558	-12.249	-1.253	3.726	-11.568
c_O	0	0	0	213	204	139	105	133	126	156
	(0.0)	(0.0)	(0.0)	(13.0)	(12.5)	(8.5)	(6.4)	(8.1)	(7.7)	(9.5)
c_E	183.3	257.3	158.0	183.3	257.3	158.0	183.3	257.2	158.0	183.3
	(11.2)	(15.7)	(9.7)	(11.2)	(15.7)	(9.7)	(11.2)	(15.7)	(9.7)	(11.2)
	0.0	0.0	0.0	63.9	-94.7	-35.7	-117.0	-175.4	-57.1	-50.4
	0.000	0.000	0.000	3.910	-5.798	-2.183	-7.163	-10.740	-3.493	-3.083
N	1634	1634	1634	1634	1634	1634	1634	1633	1634	1634
X2	633.1	3234.5	12673.1	115.7	77.5	15.3	153.2	87.9	44.9	53.5
Gvalue	822.9	3567.8	7090.7	120.7	78.2	15.1	154.4	99.2	45.0	55.3
Gv/N	50.362	218.350	433.945	7.390	4.789	0.926	9.447	6.076	2.756	3.385
Prob	0.00000	1.00000	1.00000	0.00000	0.00000	0.00170	0.00000	0.00000	0.00000	0.00000

	11	12	13	14	15	16	17	18	19	20
a_O	624	673	789	681	724	739	625	668	801	597
	(38.2)	(41.2)	(48.3)	(41.7)	(44.3)	(45.2)	(38.2)	(40.9)	(49.0)	(36.5)
a_E	633.2	605.6	654.9	633.6	605.6	654.9	633.6	605.6	654.9	633.6
	(38.8)	(37.1)	(40.1)	(38.8)	(37.1)	(40.1)	(38.8)	(37.1)	(40.1)	(38.8)
	-18.3	141.9	294.0	98.2	258.5	178.6	-17.1	130.9	322.6	-71.1
	-1.123	8.687	17.990	6.010	15.818	10.928	-1.048	8.012	19.743	-4.351
t_O	613	646	417	619	597	426	630	614	395	664
	(37.5)	(39.5)	(25.5)	(37.9)	(36.5)	(26.1)	(38.6)	(37.6)	(24.2)	(40.6)
t_E	548.1	683.7	363.0	548.4	683.7	363.0	548.4	683.7	363.0	548.4
	(33.6)	(41.8)	(22.2)	(33.6)	(41.8)	(22.2)	(33.6)	(41.8)	(22.2)	(33.6)
	137.2	-73.3	115.7	149.9	-161.9	136.4	174.7	-132.1	66.8	254.0
	8.404	-4.486	7.083	9.173	-9.909	8.349	10.694	-8.081	4.089	15.543
g_O	209	169	280	160	192	313	174	195	310	170
	(12.8)	(10.3)	(17.1)	(9.8)	(11.8)	(19.2)	(10.6)	(11.9)	(19.0)	(10.4)
g_E	194.5	186.6	432.8	194.6	186.6	432.8	194.6	186.6	432.8	194.6
	(11.9)	(11.4)	(26.5)	(11.9)	(11.4)	(26.5)	(11.9)	(11.4)	(26.5)	(11.9)
	30.0	-33.5	-243.9	-62.7	10.9	-202.9	-39.0	17.1	-206.9	-46.0
	1.838	-2.051	-14.924	-3.838	0.668	-12.415	-2.387	1.049	-12.662	-2.816

c_O	187	146	148	174	121	156	205	157	128	203
	(11.5)	(8.9)	(9.1)	(10.6)	(7.4)	(9.5)	(12.5)	(9.6)	(7.8)	(12.4)
c_E	257.2	158.0	183.3	257.3	158.0	183.3	257.3	158.0	183.3	257.3
	(15.7)	(9.7)	(11.2)	(15.7)	(9.7)	(11.2)	(15.7)	(9.7)	(11.2)	(15.7)
	-119.2	-23.1	-63.4	-136.2	-64.6	-50.4	-93.2	-2.1	-92.0	-96.3
	-7.297	-1.415	-3.878	-8.333	-3.954	-3.083	-5.703	-0.126	-5.629	-5.891
N	1633	1634	1634	1634	1634	1634	1634	1634	1634	1634
X2	28.0	12.1	96.3	45.8	43.0	59.0	25.1	13.9	87.0	41.1
Gvalue	29.8	12.0	102.4	49.2	42.8	61.7	25.4	13.9	90.5	40.6
Gv/N	1.822	0.735	6.270	3.012	2.622	3.779	1.555	0.854	5.542	2.485
Prob	0.00000	0.00737	0.00000	0.00000	0.00000	0.00000	0.00001	0.00297	0.00000	0.00000

	21	22	23	24	25	26	27	28	29	30
a_O	620	730	604	664	651	557	658	692	539	636
	(37.9)	(44.7)	(37.0)	(40.6)	(39.8)	(34.1)	(40.3)	(42.4)	(33.0)	(38.9)
a_E	605.6	654.9	633.6	605.6	654.9	633.6	605.6	654.9	633.6	605.6
	(37.1)	(40.1)	(38.8)	(37.1)	(40.1)	(38.8)	(37.1)	(40.1)	(38.8)	(37.1)
	29.0	158.5	-57.8	122.2	-7.8	-143.6	109.1	76.3	-174.4	62.2
	1.778	9.700	-3.540	7.476	-0.476	-8.787	6.678	4.667	-10.671	3.807
t_O	667	426	687	702	479	687	674	431	702	663
	(40.8)	(26.1)	(42.0)	(43.0)	(29.3)	(42.0)	(41.2)	(26.4)	(43.0)	(40.6)
t_E	683.7	363.0	548.4	683.7	363.0	548.4	683.7	363.0	548.4	683.7
	(41.8)	(22.2)	(33.6)	(41.8)	(22.2)	(33.6)	(41.8)	(22.2)	(33.6)	(41.8)
	-33.0	136.4	309.6	37.1	265.7	309.6	-19.3	148.1	346.6	-40.8
	-2.020	8.349	18.945	2.269	16.263	18.945	-1.180	9.063	21.214	-2.496
g_O	205	325	136	167	351	202	162	336	165	188
	(12.5)	(19.9)	(8.3)	(10.2)	(21.5)	(12.4)	(9.9)	(20.6)	(10.1)	(11.5)
g_E	186.6	432.8	194.6	186.6	432.8	194.6	186.6	432.8	194.6	186.6
	(11.4)	(26.5)	(11.9)	(11.4)	(26.5)	(11.9)	(11.4)	(26.5)	(11.9)	(11.4)
	38.5	-186.2	-97.5	-37.1	-147.1	15.0	-45.8	-170.1	-54.5	2.8
	2.357	-11.394	-5.968	-2.270	-9.000	0.918	-2.805	-10.411	-3.336	0.170
c_O	142	153	207	101	153	188	140	175	228	147
	(8.7)	(9.4)	(12.7)	(6.2)	(9.4)	(11.5)	(8.6)	(10.7)	(14.0)	(9.0)
c_E	158.0	183.3	257.3	158.0	183.3	257.3	158.0	183.3	257.3	158.0
	(9.7)	(11.2)	(15.7)	(9.7)	(11.2)	(15.7)	(9.7)	(11.2)	(15.7)	(9.7)
	-30.4	-55.3	-90.1	-90.4	-55.3	-118.0	-33.9	-16.3	-55.2	-21.3
	-1.859	-3.387	-5.513	-5.534	-3.387	-7.223	-2.076	-0.997	-3.376	-1.302
N	1634	1634	1634	1634	1634	1634	1634	1634	1634	1634
X2	4.2	51.4	63.9	28.8	57.6	63.2	10.0	36.9	65.0	2.9
Gvalue	4.2	53.4	64.1	31.7	55.6	63.0	10.1	37.9	62.6	2.9
Gv/N	0.256	3.267	3.924	1.940	3.400	3.853	0.617	2.322	3.832	0.179
Prob	0.24190	0.00000	0.00000	0.00000	0.00000	0.00000	0.01790	0.00000	0.00000	0.40270

	31	32	33	34	35	36	37	38	39	40
a_O	652	550	630	661	533	670	686	548	596	720
	(39.9)	(33.7)	(38.6)	(40.5)	(32.6)	(41.0)	(42.0)	(33.6)	(36.5)	(44.1)
a_E	654.9	633.6	605.6	654.9	633.6	605.6	654.9	633.2	605.6	654.9
	(40.1)	(38.8)	(37.1)	(40.1)	(38.8)	(37.1)	(40.1)	(38.8)	(37.1)	(40.1)
	-5.8	-155.7	49.7	12.3	-184.3	135.3	63.7	-158.4	-19.1	136.5
	-0.354	-9.528	3.040	0.750	-11.282	8.281	3.896	-9.703	-1.171	8.352
t_O	429	694	678	449	711	657	423	650	666	398
	(26.3)	(42.5)	(41.5)	(27.5)	(43.5)	(40.2)	(25.9)	(39.8)	(40.8)	(24.4)
t_E	363.0	548.4	683.7	363.0	548.4	683.7	363.0	548.1	683.7	363.0
	(22.2)	(33.6)	(41.8)	(22.2)	(33.6)	(41.8)	(22.2)	(33.6)	(41.8)	(22.2)
	143.4	326.8	-11.4	191.0	369.2	-52.4	129.5	221.7	-34.9	73.3
	8.776	19.999	-0.695	11.690	22.595	-3.204	7.924	13.577	-2.139	4.488
g_O	373	183	213	371	154	167	345	188	211	346
	(22.8)	(11.2)	(13.0)	(22.7)	(9.4)	(10.2)	(21.1)	(11.5)	(12.9)	(21.2)
g_E	432.8	194.6	186.6	432.8	194.6	186.6	432.8	194.5	186.6	432.8
	(26.5)	(11.9)	(11.4)	(26.5)	(11.9)	(11.4)	(26.5)	(11.9)	(11.4)	(26.5)
	-110.9	-22.6	56.3	-114.3	-72.1	-37.1	-156.4	-12.8	51.8	-154.9
	-6.788	-1.381	3.447	-6.996	-4.414	-2.270	-9.574	-0.785	3.171	-9.479
c_O	180	207	113	153	236	140	180	247	161	170
	(11.0)	(12.7)	(6.9)	(9.4)	(14.4)	(8.6)	(11.0)	(15.1)	(9.9)	(10.4)
c_E	183.3	257.3	158.0	183.3	257.3	158.0	183.3	257.2	158.0	183.3
	(11.2)	(15.7)	(9.7)	(11.2)	(15.7)	(9.7)	(11.2)	(15.7)	(9.7)	(11.2)
	-6.6	-90.1	-75.8	-55.3	-40.8	-33.9	-6.6	-19.9	6.0	-25.7
	-0.404	-5.513	-4.639	-3.387	-2.498	-2.076	-0.404	-1.220	0.367	-1.571
N	1634	1634	1634	1634	1634	1634	1634	1633	1634	1634
X2	20.3	60.2	17.6	34.3	74.4	12.0	29.3	31.0	3.9	28.2
Gvalue	20.1	58.4	18.8	33.6	71.9	11.9	30.1	30.5	3.7	29.2
Gv/N	1.229	3.576	1.153	2.056	4.400	0.731	1.842	1.870	0.228	1.790
Prob	0.00016	0.00000	0.00029	0.00000	0.00000	0.00758	0.00000	0.00000	0.29240	0.00000

	41	42	43	44	45	46	47	48	49	50
a_O	561	611	693	525	640	615	595	617	662	578
	(34.3)	(37.4)	(42.4)	(32.1)	(39.2)	(37.6)	(36.4)	(37.8)	(40.5)	(35.4)
a_E	633.6	605.6	654.9	633.6	605.6	654.9	633.6	605.6	654.9	633.6
	(38.8)	(37.1)	(40.1)	(38.8)	(37.1)	(40.1)	(38.8)	(37.1)	(40.1)	(38.8)
	-136.6	10.8	78.4	-197.5	70.6	-77.3	-74.8	22.9	14.3	-106.2
	-8.359	0.658	4.796	-12.085	4.322	-4.732	-4.580	1.403	0.874	-6.500
t_O	658	676	412	674	658	454	575	693	375	571
	(40.3)	(41.4)	(25.2)	(41.2)	(40.3)	(27.8)	(35.2)	(42.4)	(22.9)	(34.9)
t_E	548.4	683.7	363.0	548.4	683.7	363.0	548.4	683.7	363.0	548.4
	(33.6)	(41.8)	(22.2)	(33.6)	(41.8)	(22.2)	(33.6)	(41.8)	(22.2)	(33.6)
	239.7	-15.3	104.4	277.9	-50.4	203.2	54.4	18.7	24.5	46.1
	14.672	-0.938	6.389	17.010	-3.086	12.435	3.331	1.145	1.497	2.820
g_O	221	205	345	177	199	413	203	175	423	234
	(13.5)	(12.5)	(21.1)	(10.8)	(12.2)	(25.3)	(12.4)	(10.7)	(25.9)	(14.3)
g_E	194.6	186.6	432.8	194.6	186.6	432.8	194.6	186.6	432.8	194.6
	(11.9)	(11.4)	(26.5)	(11.9)	(11.4)	(26.5)	(11.9)	(11.4)	(26.5)	(11.9)
	56.1	38.5	-156.4	-33.6	25.6	-38.7	17.1	-22.5	-19.4	86.2
	3.436	2.357	-9.574	-2.058	1.565	-2.367	1.045	-1.377	-1.185	5.275
c_O	194	142	184	258	137	152	261	149	174	251
	(11.9)	(8.7)	(11.3)	(15.8)	(8.4)	(9.3)	(16.0)	(9.1)	(10.6)	(15.4)
c_E	257.3	158.0	183.3	257.3	158.0	183.3	257.3	158.0	183.3	257.3
	(15.7)	(9.7)	(11.2)	(15.7)	(9.7)	(11.2)	(15.7)	(9.7)	(11.2)	(15.7)
	-109.6	-30.4	1.3	1.4	-39.1	-57.0	7.4	-17.5	-18.2	-12.5
	-6.707	-1.859	0.082	0.084	-2.395	-3.487	0.454	-1.073	-1.113	-0.764
N	1634	1634	1634	1634	1634	1634	1634	1634	1634	1634
X2	49.4	3.6	26.7	49.0	6.5	31.5	4.1	1.6	1.2	13.9
Gvalue	49.7	3.6	27.7	48.2	6.6	30.2	4.1	1.6	1.2	13.6
Gv/N	3.041	0.219	1.694	2.951	0.406	1.849	0.250	0.098	0.072	0.831
Prob	0.00000	0.31143	0.00000	0.00000	0.08475	0.00000	0.25311	0.65886	0.75830	0.00354

	51	52	53	54	55	56	57	58	59	60
a_O	630	607	506	573	630	581	632	651	582	579
	(38.6)	(37.1)	(31.0)	(35.1)	(38.6)	(35.6)	(38.7)	(39.8)	(35.6)	(35.4)
a_E	605.6	654.9	633.6	605.6	654.9	633.6	605.6	654.9	633.6	605.6
	(37.1)	(40.1)	(38.8)	(37.1)	(40.1)	(38.8)	(37.1)	(40.1)	(38.8)	(37.1)
	49.7	-92.2	-227.6	-63.5	-48.8	-100.8	53.8	-7.8	-98.9	-52.1
	3.040	-5.643	-13.930	-3.886	-2.989	-6.166	3.295	-0.476	-6.054	-3.188
t_O	641	453	654	701	433	588	644	435	570	692
	(39.2)	(27.7)	(40.0)	(42.9)	(26.5)	(36.0)	(39.4)	(26.6)	(34.9)	(42.4)
t_E	683.7	363.0	548.4	683.7	363.0	548.4	683.7	363.0	548.4	683.7
	(41.8)	(22.2)	(33.6)	(41.8)	(22.2)	(33.6)	(41.8)	(22.2)	(33.6)	(41.8)
	-82.7	200.7	230.3	35.0	152.8	82.0	-77.1	157.5	44.0	16.7
	-5.060	12.285	14.094	2.143	9.350	5.016	-4.716	9.638	2.693	1.021
g_O	201	342	224	196	374	202	202	378	196	196
	(12.3)	(20.9)	(13.7)	(12.0)	(22.9)	(12.4)	(12.4)	(23.1)	(12.0)	(12.0)
g_E	186.6	432.8	194.6	186.6	432.8	194.6	186.6	432.8	194.6	186.6
	(11.4)	(26.5)	(11.9)	(11.4)	(26.5)	(11.9)	(11.4)	(26.5)	(11.9)	(11.4)
	29.8	-161.1	62.9	19.2	-109.2	15.0	32.0	-102.3	2.7	19.2
	1.827	-9.856	3.852	1.177	-6.684	0.918	1.958	-6.263	0.167	1.177
c_O	162	232	250	164	197	263	156	170	286	167
	(9.9)	(14.2)	(15.3)	(10.0)	(12.1)	(16.1)	(9.5)	(10.4)	(17.5)	(10.2)
c_E	158.0	183.3	257.3	158.0	183.3	257.3	158.0	183.3	257.3	158.0
	(9.7)	(11.2)	(15.7)	(9.7)	(11.2)	(15.7)	(9.7)	(11.2)	(15.7)	(9.7)
	8.0	109.2	-14.4	12.2	28.3	11.5	-4.0	-25.7	60.4	18.4
	0.492	6.685	-0.883	0.744	1.734	0.703	-0.247	-1.571	3.699	1.128
N	1634	1634	1634	1634	1634	1634	1634	1634	1634	1634
X2	4.9	57.8	50.7	2.9	23.5	7.6	4.7	22.2	8.3	2.3
Gvalue	4.9	56.7	51.2	2.9	23.0	7.7	4.7	21.7	8.3	2.3
Gv/N	0.298	3.471	3.133	0.178	1.410	0.470	0.290	1.328	0.505	0.138
Prob	0.18147	0.00000	0.00000	0.40531	0.00004	0.05295	0.19159	0.00008	0.04098	0.52147

	61	62	63	64	65	66	67	68	69	70
a_O	682	553	607	644	606	577	662	643	592	663
	(41.7)	(33.8)	(37.1)	(39.4)	(37.1)	(35.3)	(40.5)	(39.4)	(36.2)	(40.6)
a_E	654.9	633.6	605.6	654.9	633.6	605.6	654.9	633.6	605.6	654.9
	(40.1)	(38.8)	(37.1)	(40.1)	(38.8)	(37.1)	(40.1)	(38.8)	(37.1)	(40.1)
	55.3	-150.5	2.7	-21.6	-54.0	-55.9	14.3	18.9	-27.0	16.3
	3.385	-9.212	0.166	-1.323	-3.306	-3.422	0.874	1.156	-1.651	0.997
t_O	392	593	678	420	565	733	392	557	712	369
	(24.0)	(36.3)	(41.5)	(25.7)	(34.6)	(44.9)	(24.0)	(34.1)	(43.6)	(22.6)
t_E	363.0	548.4	683.7	363.0	548.4	683.7	363.0	548.4	683.7	363.0
	(22.2)	(33.6)	(41.8)	(22.2)	(33.6)	(41.8)	(22.2)	(33.6)	(41.8)	(22.2)
	60.3	92.7	-11.4	122.6	33.7	102.1	60.3	17.3	57.7	12.2
	3.692	5.673	-0.695	7.502	2.060	6.246	3.692	1.059	3.534	0.744
g_O	399	224	172	410	187	175	412	186	178	400
	(24.4)	(13.7)	(10.5)	(25.1)	(11.4)	(10.7)	(25.2)	(11.4)	(10.9)	(24.5)
g_E	432.8	194.6	186.6	432.8	194.6	186.6	432.8	194.6	186.6	432.8
	(26.5)	(11.9)	(11.4)	(26.5)	(11.9)	(11.4)	(26.5)	(11.9)	(11.4)	(26.5)
	-64.9	62.9	-28.1	-44.4	-15.0	-22.5	-40.6	-16.9	-16.8	-63.0
	-3.971	3.852	-1.717	-2.715	-0.916	-1.377	-2.483	-1.034	-1.030	-3.858
c_O	161	264	177	160	276	149	168	248	152	202
	(9.9)	(16.2)	(10.8)	(9.8)	(16.9)	(9.1)	(10.3)	(15.2)	(9.3)	(12.4)
c_E	183.3	257.3	158.0	183.3	257.3	158.0	183.3	257.3	158.0	183.3
	(11.2)	(15.7)	(9.7)	(11.2)	(15.7)	(9.7)	(11.2)	(15.7)	(9.7)	(11.2)
	-41.8	13.5	40.1	-43.6	38.7	-17.5	-29.3	-18.3	-11.8	39.2
	-2.560	0.828	2.456	-2.666	2.368	-1.073	-1.796	-1.120	-0.724	2.397
N	1634	1634	1634	1634	1634	1634	1634	1634	1634	1634
X2	8.8	18.5	3.5	13.3	3.4	6.1	4.7	1.0	2.1	4.6
Gvalue	8.9	18.6	3.4	13.0	3.3	6.1	4.7	1.0	2.1	4.6
Gv/N	0.546	1.141	0.209	0.798	0.205	0.374	0.286	0.061	0.129	0.281
Prob	0.03041	0.00032	0.33162	0.00456	0.34114	0.10623	0.19725	0.80063	0.55147	0.20450

	71	72	73	74	75	76	77	78	79	80
a_O	627	602	621	618	619	666	634	633	645	642
	(38.4)	(36.8)	(38.0)	(37.8)	(37.9)	(40.8)	(38.8)	(38.8)	(39.5)	(39.3)
a_E	633.6	605.6	654.9	633.6	605.6	654.9	633.6	605.3	654.9	633.6
	(38.8)	(37.1)	(40.1)	(38.8)	(37.1)	(40.1)	(38.8)	(37.1)	(40.1)	(38.8)
	-13.2	-7.3	-66.0	-30.9	27.0	22.4	0.8	56.7	-19.7	16.9
	-0.807	-0.445	-4.040	-1.889	1.653	1.370	0.046	3.472	-1.203	1.032
t_O	565	716	400	593	678	366	548	691	403	577
	(34.6)	(43.8)	(24.5)	(36.3)	(41.5)	(22.4)	(33.5)	(42.3)	(24.7)	(35.3)
t_E	548.4	683.7	363.0	548.4	683.7	363.0	548.4	683.3	363.0	548.4
	(33.6)	(41.8)	(22.2)	(33.6)	(41.8)	(22.2)	(33.6)	(41.8)	(22.2)	(33.6)
	33.7	66.1	77.7	92.7	-11.4	6.1	-0.8	15.5	84.3	58.6
	2.060	4.045	4.756	5.673	-0.695	0.372	-0.051	0.950	5.160	3.588
g_O	166	162	385	175	181	415	179	183	408	170
	(10.2)	(9.9)	(23.6)	(10.7)	(11.1)	(25.4)	(11.0)	(11.2)	(25.0)	(10.4)
g_E	194.6	186.6	432.8	194.6	186.6	432.8	194.6	186.5	432.8	194.6
	(11.9)	(11.4)	(26.5)	(11.9)	(11.4)	(26.5)	(11.9)	(11.4)	(26.5)	(11.9)
	-52.8	-45.8	-90.1	-37.2	-11.1	-34.8	-30.0	-6.9	-48.1	-46.0
	-3.234	-2.805	-5.514	-2.278	-0.677	-2.133	-1.835	-0.425	-2.946	-2.816
c_O	276	154	228	248	156	187	273	126	178	245
	(16.9)	(9.4)	(14.0)	(15.2)	(9.5)	(11.4)	(16.7)	(7.7)	(10.9)	(15.0)
c_E	257.3	158.0	183.3	257.3	158.0	183.3	257.3	157.9	183.3	257.3
	(15.7)	(9.7)	(11.2)	(15.7)	(9.7)	(11.2)	(15.7)	(9.7)	(11.2)	(15.7)
	38.7	-8.0	99.4	-18.3	-4.0	7.4	32.3	-56.9	-10.5	-24.0
	2.368	-0.487	6.085	-1.120	-0.247	0.453	1.977	-3.486	-0.643	-1.471
N	1634	1634	1634	1634	1634	1634	1634	1633	1634	1634
X2	6.1	4.9	21.7	6.3	0.5	1.0	2.2	7.9	6.1	5.3
Gvalue	6.3	5.0	21.0	6.3	0.5	1.0	2.2	8.3	6.0	5.4
Gv/N	0.387	0.308	1.286	0.386	0.033	0.063	0.136	0.511	0.368	0.333
Prob	0.09670	0.16976	0.00010	0.09739	0.91066	0.79471	0.52625	0.03936	0.11081	0.14265

	81	82	83	84	85	86	87	88	89	90
a_O	584	635	601	650	656	640	590	636	603	618
	(35.7)	(38.9)	(36.8)	(39.8)	(40.2)	(39.2)	(36.1)	(38.9)	(36.9)	(37.8)
a_E	605.6	654.9	633.2	605.6	654.5	633.6	605.3	654.9	633.6	605.6
	(37.1)	(40.1)	(38.8)	(37.1)	(40.1)	(38.8)	(37.1)	(40.1)	(38.8)	(37.1)
	-42.5	-39.2	-62.8	91.9	3.0	12.8	-30.2	-37.3	-59.7	25.0
	-2.601	-2.398	-3.846	5.623	0.184	0.784	-1.847	-2.280	-3.656	1.528
t_O	699	386	610	653	382	565	716	421	563	671
	(42.8)	(23.6)	(37.4)	(40.0)	(23.4)	(34.6)	(43.8)	(25.8)	(34.5)	(41.1)
t_E	683.7	363.0	548.1	683.7	362.7	548.4	683.3	363.0	548.4	683.7
	(41.8)	(22.2)	(33.6)	(41.8)	(22.2)	(33.6)	(41.8)	(22.2)	(33.6)	(41.8)
	30.9	47.5	130.6	-60.0	39.5	33.7	67.0	124.9	29.5	-25.2

```
          1.893   2.906   7.996  -3.673   2.419   2.060   4.101   7.643   1.808  -1.541
g_O         192     422     182     173     448     203     176     373     203     185
        ( 11.8) ( 25.8) ( 11.1) ( 10.6) ( 27.4) ( 12.4) ( 10.8) ( 22.8) ( 12.4) ( 11.3)
g_E       186.6   432.8   194.5   186.6   432.5   194.6   186.5   432.8   194.6   186.6
        ( 11.4) ( 26.5) ( 11.9) ( 11.4) ( 26.5) ( 11.9) ( 11.4) ( 26.5) ( 11.9) ( 11.4)
           10.9   -21.3   -24.2   -26.2    31.5    17.1   -20.4  -110.9    17.1    -3.2
          0.668  -1.305  -1.483  -1.604   1.928   1.045  -1.249  -6.788   1.045  -0.197
c_O         159     191     240     158     147     226     151     204     265     160
        (  9.7) ( 11.7) ( 14.7) (  9.7) (  9.0) ( 13.8) (  9.2) ( 12.5) ( 16.2) (  9.8)
c_E       158.0   183.3   257.2   158.0   183.2   257.3   157.9   183.3   257.3   158.0
        (  9.7) ( 11.2) ( 15.7) (  9.7) ( 11.2) ( 15.7) (  9.7) ( 11.2) ( 15.7) (  9.7)
            1.9    15.6   -33.2    -0.1   -64.8   -58.7   -13.6    43.6    15.6     4.0
          0.119   0.958  -2.030  -0.004  -3.966  -3.590  -0.830   2.667   0.954   0.242

N          1634    1634    1633    1634    1633    1634    1633    1634    1634    1634
X2          1.3     2.7    10.6     5.6     8.7     4.7     2.8    20.4     2.5     0.5
Gvalue      1.3     2.6    10.4     5.6     9.2     4.9     2.8    20.3     2.5     0.5
Gv/N      0.078   0.161   0.638   0.342   0.566   0.299   0.174   1.242   0.151   0.032
Prob    0.73336 0.45226 0.01538 0.13331 0.02626 0.17995 0.41656 0.00015 0.48072 0.91305
        -------------------------------------------------------------------------------
             91      92      93      94      95      96      97      98      99     100
        -------------------------------------------------------------------------------
a_O         656     639     618     625     649     580     619     608     644     664
        ( 40.1) ( 39.1) ( 37.8) ( 38.6) ( 40.0) ( 35.8) ( 38.5) ( 37.9) ( 40.1) ( 41.6)
a_E       654.9   633.6   605.6   649.7   628.6   600.8   643.7   622.8   595.3   639.3
        ( 40.1) ( 38.8) ( 37.1) ( 40.1) ( 38.8) ( 37.1) ( 40.1) ( 38.8) ( 37.1) ( 40.1)
            2.2    10.8    25.0   -48.4    41.5   -40.9   -48.4   -29.2   101.4    50.4
          0.135   0.661   1.528  -2.988   2.559  -2.525  -3.014  -1.817   6.311   3.160
t_O         392     579     656     417     550     705     385     576     645     367
        ( 24.0) ( 35.4) ( 40.1) ( 25.7) ( 33.9) ( 43.5) ( 24.0) ( 35.9) ( 40.2) ( 23.0)
t_E       363.0   548.4   683.7   360.1   544.1   678.3   356.8   539.0   672.0   354.3
        ( 22.2) ( 33.6) ( 41.8) ( 22.2) ( 33.6) ( 41.8) ( 22.2) ( 33.6) ( 41.8) ( 22.2)
           60.3    62.8   -54.3   122.4    12.0    54.5    58.7    76.4   -52.9    25.8
          3.692   3.846  -3.322   7.550   0.738   3.363   3.654   4.760  -3.293   1.620
g_O         408     188     194     384     201     169     408     164     174     389
        ( 25.0) ( 11.5) ( 11.9) ( 23.7) ( 12.4) ( 10.4) ( 25.4) ( 10.2) ( 10.8) ( 24.4)
g_E       432.8   194.6   186.6   429.4   193.1   185.1   425.4   191.3   183.4   422.5
        ( 26.5) ( 11.9) ( 11.4) ( 26.5) ( 11.9) ( 11.4) ( 26.5) ( 11.9) ( 11.4) ( 26.5)
          -48.1   -13.0    15.1   -85.7    16.1   -30.8   -34.0   -50.5   -18.3   -64.2
         -2.946  -0.799   0.921  -5.289   0.996  -1.901  -2.119  -3.145  -1.142  -4.026
c_O         178     228     166     195     221     167     194     258     143     175
        ( 10.9) ( 14.0) ( 10.2) ( 12.0) ( 13.6) ( 10.3) ( 12.1) ( 16.1) (  8.9) ( 11.0)
c_E       183.3   257.3   158.0   181.9   255.3   156.8   180.2   252.9   155.3   179.0
        ( 11.2) ( 15.7) (  9.7) ( 11.2) ( 15.7) (  9.7) ( 11.2) ( 15.7) (  9.7) ( 11.2)
          -10.5   -55.2    16.3    27.2   -63.7    21.1    28.6    10.3   -23.6    -7.8
         -0.643  -3.376   0.999   1.676  -3.931   1.302   1.784   0.640  -1.472  -0.491

N          1634    1634    1634    1621    1621    1621    1606    1606    1606    1595
X2          3.9     5.3     2.1    15.7     5.7     3.8     5.0     6.9     6.5     4.1
Gvalue      3.9     5.4     2.1    15.4     5.9     3.9     4.9     7.0     6.5     4.2
Gv/N      0.237   0.332   0.127   0.950   0.362   0.239   0.305   0.438   0.404   0.264
Prob    0.27593 0.14324 0.55759 0.00150 0.11850 0.27563 0.17999 0.07087 0.09042 0.24005
        -------------------------------------------------------------------------------
            101     102     103     104     105     106     107     108     109     110
        -------------------------------------------------------------------------------
a_O         627     594     671     631     592     681     671     625     646     660
        ( 39.3) ( 37.2) ( 42.3) ( 39.8) ( 37.3) ( 43.3) ( 42.7) ( 39.8) ( 41.4) ( 42.3)
a_E       618.5   591.2   635.7   615.0   587.9   629.7   609.2   582.3   625.6   605.3
        ( 38.8) ( 37.1) ( 40.1) ( 38.8) ( 37.1) ( 40.1) ( 38.8) ( 37.1) ( 40.1) ( 38.8)
           17.1     5.6    72.6    32.4     8.3   106.8   129.7    88.5    41.4   114.2
          1.073   0.353   4.578   2.042   0.525   6.797   8.255   5.631   2.650   7.314
t_O         582     685     361     503     668     311     458     626     356     492
        ( 36.5) ( 42.9) ( 22.8) ( 31.7) ( 42.1) ( 19.8) ( 29.2) ( 39.8) ( 22.8) ( 31.5)
t_E       535.3   667.4   352.3   532.3   663.6   349.0   527.3   657.3   346.8   523.9
        ( 33.6) ( 41.8) ( 22.2) ( 33.6) ( 41.8) ( 22.2) ( 33.6) ( 41.8) ( 22.2) ( 33.6)
           97.3    35.7    17.6   -57.0     8.8   -71.7  -129.0   -61.2    18.7   -61.8
          6.100   2.237   1.110  -3.592   0.554  -4.561  -8.212  -3.894   1.200  -3.962
g_O         186     186     361     190     163     425     204     162     396     195
        ( 11.7) ( 11.7) ( 22.8) ( 12.0) ( 10.3) ( 27.1) ( 13.0) ( 10.3) ( 25.4) ( 12.5)
g_E       190.0   182.2   420.1   188.9   181.1   416.1   187.1   179.4   413.5   185.9
        ( 11.9) ( 11.4) ( 26.5) ( 11.9) ( 11.4) ( 26.5) ( 11.9) ( 11.4) ( 26.5) ( 11.9)
           -7.9     7.8  -109.4     2.2   -34.4    18.0    35.2   -33.1   -34.2    18.5
         -0.495   0.486  -6.900   0.136  -2.168   1.144   2.241  -2.107  -2.189   1.188
c_O         200     130     193     262     163     154     238     158     163     214
        ( 12.5) (  8.2) ( 12.2) ( 16.5) ( 10.3) (  9.8) ( 15.1) ( 10.1) ( 10.4) ( 13.7)
c_E       251.2   154.3   177.9   249.8   153.4   176.3   247.4   151.9   175.1   245.8
        ( 15.7) (  9.7) ( 11.2) ( 15.7) (  9.7) ( 11.2) ( 15.7) (  9.7) ( 11.2) ( 15.7)
          -91.1   -44.5    31.3    25.1    19.8   -41.6   -18.4    12.4   -23.4   -59.3
         -5.714  -2.789   1.976   1.581   1.249  -2.647  -1.173   0.787  -1.501  -3.801

N          1595    1595    1586    1586    1586    1571    1571    1571    1561    1561
X2         14.7     4.4    11.8     2.6     2.5    11.3    17.2     6.6     2.5    11.4
Gvalue     15.4     4.6    12.1     2.7     2.5    11.5    17.4     6.6     2.5    11.5
Gv/N      0.964   0.288   0.764   0.167   0.159   0.732   1.110   0.418   0.161   0.738
Prob    0.00152 0.20465 0.00702 0.44834 0.47035 0.00932 0.00058 0.08713 0.47353 0.00919
        -------------------------------------------------------------------------------
            111     112     113     114     115     116     117     118     119     120
        -------------------------------------------------------------------------------
a_O         585     562     610     586     619     553     568     628     618     602
        ( 37.5) ( 36.2) ( 39.3) ( 37.7) ( 40.1) ( 35.8) ( 36.8) ( 40.8) ( 40.2) ( 39.2)
a_E       578.2   622.4   602.2   575.6   618.8   598.7   572.3   616.4   596.0   569.7
        ( 37.1) ( 40.1) ( 38.8) ( 37.1) ( 40.1) ( 38.8) ( 37.1) ( 40.1) ( 38.8) ( 37.1)
           13.6  -114.8    15.7    20.9     0.3   -87.9    -8.5    23.4    44.8    66.4
          0.875  -7.392   1.009   1.348   0.022  -5.691  -0.553   1.519   2.914   4.321
t_O         691     391     520     623     336     518     636     327     508     600
        ( 44.3) ( 25.2) ( 33.5) ( 40.1) ( 21.8) ( 33.5) ( 41.2) ( 21.3) ( 33.1) ( 39.0)
t_E       652.7   345.0   521.2   649.8   343.0   518.2   646.0   341.6   515.9   643.1
        ( 41.8) ( 22.2) ( 33.6) ( 41.8) ( 22.2) ( 33.6) ( 41.8) ( 22.2) ( 33.6) ( 41.8)
           78.7    97.9    -2.5   -52.5   -13.8    -0.4   -19.9   -28.7   -15.6   -83.3
          5.046   6.306  -0.158  -3.381  -0.895  -0.027  -1.291  -1.863  -1.015  -5.418
g_O         135     399     191     173     405     203     171     407     186     170
        (  8.7) ( 25.7) ( 12.3) ( 11.1) ( 26.2) ( 13.1) ( 11.1) ( 26.5) ( 12.1) ( 11.1)
g_E       178.2   411.3   185.0   177.4   409.0   183.9   176.3   407.4   183.1   175.5
        ( 11.4) ( 26.5) ( 11.9) ( 11.4) ( 26.5) ( 11.9) ( 11.4) ( 26.5) ( 11.9) ( 11.4)
          -74.9   -24.3    12.2    -8.6    -7.9    40.1   -10.5    -0.7     5.9   -10.9
         -4.802  -1.565   0.786  -0.555  -0.510   2.596  -0.681  -0.048   0.382  -0.709
c_O         149     201     232     171     184     270     169     176     225     165
        (  9.6) ( 12.9) ( 14.9) ( 11.0) ( 11.9) ( 17.5) ( 10.9) ( 11.4) ( 14.6) ( 10.7)
c_E       150.9   174.2   244.6   150.2   173.2   243.1   149.3   172.6   242.0   148.7
        (  9.7) ( 11.2) ( 15.7) (  9.7) ( 11.2) ( 15.7) (  9.7) ( 11.2) ( 15.7) (  9.7)
           -3.7    57.4   -24.5    44.4    22.2    56.6    41.8     6.9   -32.9    34.4
         -0.239   3.697  -1.576   2.856   1.437   3.664   2.709   0.451  -2.138   2.240
```

N	1560	1553	1553	1553	1544	1544	1544	1538	1537	1537
X2	12.8	16.5	0.9	4.3	0.8	8.4	2.9	0.9	2.2	6.7
Gvalue	13.7	16.2	1.0	4.2	0.8	8.4	2.8	0.9	2.2	6.7
Gv/N	0.880	1.046	0.061	0.269	0.054	0.542	0.184	0.060	0.143	0.434
Prob	0.00330	0.00101	0.81264	0.24339	0.84065	0.03910	0.41739	0.82028	0.53246	0.08324

	121	122	123	124	125	126	127	128	129	130
a_O	613	612	579	617	634	569	589	654	554	592
	(40.2)	(40.1)	(37.9)	(40.5)	(41.7)	(37.4)	(38.9)	(43.1)	(36.5)	(39.1)
a_E	611.6	591.7	565.6	610.0	590.2	564.1	607.6	587.9	561.9	606.4
	(40.1)	(38.8)	(37.1)	(40.1)	(38.8)	(37.1)	(40.1)	(38.8)	(37.1)	(40.1)
	2.8	41.2	27.1	14.1	90.8	9.8	−36.6	139.4	−15.7	−28.5
	0.182	2.700	1.775	0.924	5.965	0.642	−2.417	9.198	−1.036	−1.881
t_O	349	485	644	348	478	605	353	494	665	328
	(22.9)	(31.8)	(42.2)	(22.9)	(31.4)	(39.8)	(23.3)	(32.6)	(43.9)	(21.7)
t_E	339.0	512.2	638.5	338.1	510.8	636.8	336.8	508.8	634.3	336.1
	(22.2)	(33.6)	(41.8)	(22.2)	(33.6)	(41.8)	(22.2)	(33.6)	(41.8)	(22.2)
	20.3	−52.9	11.0	20.1	−63.5	−62.1	33.3	−29.2	62.8	−16.0
	1.333	−3.465	0.722	1.321	−4.172	−4.078	2.194	−1.926	4.142	−1.057
g_O	401	195	156	426	181	165	370	166	173	415
	(26.3)	(12.8)	(10.2)	(28.0)	(11.9)	(10.8)	(24.4)	(10.9)	(11.4)	(27.4)
g_E	404.2	181.8	174.3	403.1	181.3	173.8	401.5	180.6	173.1	400.7
	(26.5)	(11.9)	(11.4)	(26.5)	(11.9)	(11.4)	(26.5)	(11.9)	(11.4)	(26.5)
	−6.4	27.4	−34.6	47.0	−0.6	−17.2	−60.5	−28.0	−0.3	29.0
	−0.416	1.795	−2.266	3.089	−0.039	−1.130	−3.993	−1.844	−0.019	1.917
c_O	163	234	147	131	229	183	204	202	124	178
	(10.7)	(15.3)	(9.6)	(8.6)	(15.0)	(12.0)	(13.5)	(13.3)	(8.2)	(11.8)
c_E	171.2	240.3	147.6	170.8	239.7	147.2	170.1	238.7	146.6	169.8
	(11.2)	(15.7)	(9.7)	(11.2)	(15.7)	(9.7)	(11.2)	(15.7)	(9.7)	(11.2)
	−16.0	−12.5	−1.2	−69.5	−20.9	79.7	74.2	−67.5	−41.6	16.9
	−1.051	−0.816	−0.077	−4.564	−1.372	5.235	4.892	−4.453	−2.741	1.116
N	1526	1526	1526	1522	1522	1522	1516	1516	1516	1513
X2	0.7	3.3	2.3	10.9	5.8	10.8	10.6	14.7	5.1	1.4
Gvalue	0.7	3.3	2.4	11.7	5.8	10.2	10.2	14.8	5.3	1.4
Gv/N	0.047	0.214	0.154	0.770	0.382	0.670	0.676	0.976	0.347	0.095
Prob	0.86791	0.35326	0.50257	0.00841	0.12103	0.01699	0.01662	0.00201	0.15401	0.69707

	131	132	133	134	135	136	137	138	139	140
a_O	614	579	593	613	552	590	566	515	581	590
	(40.6)	(38.3)	(39.3)	(40.7)	(36.6)	(39.2)	(37.6)	(34.2)	(38.7)	(39.3)
a_E	586.7	560.8	604.0	584.4	558.6	603.2	583.6	557.8	601.2	581.7
	(38.8)	(37.1)	(40.1)	(38.8)	(37.1)	(40.1)	(38.8)	(37.1)	(40.1)	(38.8)
	55.8	37.0	−21.8	58.6	−13.1	−26.1	−34.7	−82.3	−39.7	16.8
	3.691	2.445	−1.446	3.890	−0.867	−1.735	−2.303	−5.467	−2.647	1.120
t_O	490	589	339	481	673	330	515	641	377	472
	(32.4)	(38.9)	(22.5)	(31.9)	(44.7)	(21.9)	(34.2)	(42.6)	(25.1)	(31.5)
t_E	507.8	633.1	334.8	505.8	630.6	334.3	505.1	629.7	333.2	503.4
	(33.6)	(41.8)	(22.2)	(33.6)	(41.8)	(22.2)	(33.6)	(41.8)	(22.2)	(33.6)
	−35.0	−85.0	8.5	−48.3	87.7	−8.6	19.9	22.7	93.1	−60.9
	−2.312	−5.619	0.566	−3.208	5.817	−0.570	1.326	1.511	6.207	−4.059
g_O	157	188	366	165	165	399	165	160	358	140
	(10.4)	(12.4)	(24.3)	(10.9)	(10.9)	(26.5)	(11.0)	(10.6)	(23.9)	(9.3)
g_E	180.2	172.8	399.2	179.5	172.1	398.6	179.3	171.9	397.3	178.7
	(11.9)	(11.4)	(26.5)	(11.9)	(11.4)	(26.5)	(11.9)	(11.4)	(26.5)	(11.9)
	−43.3	31.7	−63.5	−27.8	−13.9	0.7	−27.4	−22.9	−74.6	−68.3
	−2.863	2.095	−4.212	−1.846	−0.924	0.050	−1.819	−1.523	−4.972	−4.554
c_O	252	157	209	248	117	186	259	189	184	298
	(16.7)	(10.4)	(13.9)	(16.5)	(7.8)	(12.4)	(17.2)	(12.6)	(12.3)	(19.9)
c_E	238.3	146.3	169.1	237.3	145.7	168.9	237.0	145.6	168.3	236.2
	(15.7)	(9.7)	(11.2)	(15.7)	(9.7)	(11.2)	(15.7)	(9.7)	(11.2)	(15.7)
	28.2	22.1	88.6	21.8	−51.4	36.0	46.0	98.7	32.8	138.5
	1.867	1.461	5.878	1.449	−3.412	2.390	3.055	6.560	2.188	9.232
N	1513	1513	1507	1507	1507	1505	1505	1505	1500	1500
X2	5.7	5.8	12.4	4.3	8.9	2.1	3.9	17.3	11.8	26.6
Gvalue	5.8	5.8	11.8	4.3	9.3	2.0	3.9	16.3	11.6	26.1
Gv/N	0.383	0.382	0.786	0.285	0.614	0.135	0.258	1.080	0.777	1.739
Prob	0.12227	0.12245	0.00793	0.23133	0.02609	0.56603	0.27505	0.00100	0.00868	0.00001

	141	142	143	144	145	146	147	148	149	150
a_O	565	620	604	609	574	575	586	633	597	553
	(37.7)	(41.4)	(40.4)	(40.7)	(38.5)	(38.6)	(39.3)	(42.6)	(40.2)	(37.2)
a_E	556.0	600.0	580.1	554.9	597.6	578.2	552.6	595.2	575.8	550.4
	(37.1)	(40.1)	(38.8)	(37.1)	(40.1)	(38.8)	(37.1)	(40.1)	(38.8)	(37.1)
	18.2	40.7	48.7	113.4	−46.2	−6.3	68.7	78.0	43.1	5.2
	1.213	2.717	3.259	7.574	−3.101	−0.424	4.607	5.252	2.901	0.349
t_O	603	365	439	599	379	489	593	307	481	618
	(40.2)	(24.4)	(29.3)	(40.0)	(25.4)	(32.8)	(39.8)	(20.7)	(32.4)	(41.6)
t_E	627.6	332.5	502.1	626.4	331.2	500.4	623.9	329.9	498.4	621.4
	(41.8)	(22.2)	(33.6)	(41.8)	(22.2)	(33.6)	(41.8)	(22.2)	(33.6)	(41.8)
	−48.3	68.0	−117.9	−53.5	102.2	−22.6	−60.2	−44.1	−34.2	−6.7
	−3.220	4.542	−7.882	−3.577	6.853	−1.515	−4.037	−2.971	−2.303	−0.451
g_O	159	357	201	145	357	174	168	396	199	148
	(10.6)	(23.8)	(13.4)	(9.7)	(23.9)	(11.7)	(11.3)	(26.7)	(13.4)	(10.0)
g_E	171.3	396.5	178.2	171.0	394.9	177.6	170.3	393.3	176.9	169.6
	(11.4)	(26.5)	(11.9)	(11.4)	(26.5)	(11.9)	(11.4)	(26.5)	(11.9)	(11.4)
	−23.7	−74.9	48.4	−47.8	−72.1	−7.1	−4.5	5.4	46.9	−40.3
	−1.581	−5.006	3.235	−3.192	−4.834	−0.479	−0.304	0.361	3.156	−2.715
c_O	173	155	252	144	181	253	144	149	208	166
	(11.5)	(10.4)	(16.8)	(9.6)	(12.1)	(17.0)	(9.7)	(10.0)	(14.0)	(11.2)
c_E	145.1	168.0	235.6	144.8	167.3	234.8	144.2	166.6	233.9	143.6
	(9.7)	(11.2)	(15.7)	(9.7)	(11.2)	(15.7)	(9.7)	(11.2)	(15.7)	(9.7)
	60.9	−24.9	33.9	−1.6	28.5	37.8	−0.4	−33.3	−48.7	48.1
	4.061	−1.663	2.269	−0.104	1.912	2.534	−0.027	−2.242	−3.282	3.237
N	1500	1497	1496	1497	1491	1491	1491	1485	1485	1485
X2	7.4	8.8	13.0	10.4	12.6	1.8	3.6	5.9	7.0	6.3
Gvalue	7.1	8.8	13.2	10.5	12.4	1.7	3.6	5.9	7.0	6.2
Gv/N	0.473	0.590	0.881	0.701	0.831	0.116	0.239	0.399	0.472	0.419
Prob	0.06897	0.03161	0.00428	0.01477	0.00616	0.63037	0.31322	0.11520	0.07164	0.10117

Bcen_AU1054
5UTR:

	50	49	48	47	46	45	44	43	42	41
a_O	1147	1165	1086	1133	1133	1056	1130	1182	1043	1152
	(17.7)	(18.0)	(16.8)	(17.5)	(17.5)	(16.3)	(17.4)	(18.2)	(16.1)	(17.8)
a_E	1229.8	1229.8	1229.8	1229.8	1229.8	1229.8	1229.8	1229.8	1229.8	1229.8
	(19.0)	(19.0)	(19.0)	(19.0)	(19.0)	(19.0)	(19.0)	(19.0)	(19.0)	(19.0)
	-159.9	-126.1	-270.1	-185.8	-185.8	-321.8	-191.3	-93.7	-343.7	-150.6
	-2.469	-1.948	-4.170	-2.868	-2.868	-4.968	-2.953	-1.447	-5.306	-2.325
t_O	1046	1189	1126	1054	1163	1100	1069	1253	1201	1097
	(16.1)	(18.4)	(17.4)	(16.3)	(18.0)	(17.0)	(16.5)	(19.3)	(18.5)	(16.9)
t_E	1157.7	1157.7	1157.7	1157.7	1157.7	1157.7	1157.7	1157.7	1157.7	1157.7
	(17.9)	(17.9)	(17.9)	(17.9)	(17.9)	(17.9)	(17.9)	(17.9)	(17.9)	(17.9)
	-212.2	63.5	-62.4	-197.7	10.7	-112.4	-170.3	198.3	88.3	-118.1
	-3.276	0.981	-0.964	-3.053	0.166	-1.735	-2.630	3.062	1.363	-1.823
g_O	2090	2023	2086	2116	2027	2104	2127	1929	2017	2119
	(32.3)	(31.2)	(32.2)	(32.7)	(31.3)	(32.5)	(32.8)	(29.8)	(31.1)	(32.7)
g_E	1983.0	1983.0	1983.0	1983.0	1983.0	1983.0	1983.0	1983.0	1983.0	1983.0
	(30.6)	(30.6)	(30.6)	(30.6)	(30.6)	(30.6)	(30.6)	(30.6)	(30.6)	(30.6)
	219.6	80.7	211.2	274.6	88.9	249.1	298.1	-106.6	68.5	281.0
	3.390	1.246	3.260	4.240	1.372	3.846	4.603	-1.646	1.057	4.339
c_O	2194	2100	2179	2174	2154	2217	2151	2113	2216	2109
	(33.9)	(32.4)	(33.6)	(33.6)	(33.3)	(34.2)	(33.2)	(32.6)	(34.2)	(32.6)
c_E	2106.5	2106.5	2106.5	2106.5	2106.5	2106.5	2106.5	2106.5	2106.5	2106.5
	(32.5)	(32.5)	(32.5)	(32.5)	(32.5)	(32.5)	(32.5)	(32.5)	(32.5)	(32.5)
	178.6	-13.0	147.5	137.2	96.1	226.7	90.0	13.0	224.6	5.0
	2.758	-0.200	2.277	2.118	1.483	3.500	1.389	0.201	3.468	0.078
N	6477	6477	6477	6477	6477	6477	6477	6477	6477	6477
X2	25.7	5.1	25.5	28.0	9.7	40.6	26.3	11.2	36.3	17.4
Gvalue	26.1	5.1	26.1	28.3	9.9	41.7	26.4	11.0	37.7	17.4
Gv/N	0.403	0.079	0.403	0.436	0.153	0.643	0.408	0.170	0.582	0.268
Prob	0.00001	0.16197	0.00001	0.00000	0.01958	0.00000	0.00001	0.01153	0.00000	0.00059

	40	39	38	37	36	35	34	33	32	31
a_O	1138	1132	1132	1233	1054	1211	1181	1077	1206	1210
	(17.6)	(17.5)	(17.5)	(19.0)	(16.3)	(18.7)	(18.2)	(16.6)	(18.6)	(18.7)
a_E	1229.8	1229.8	1229.8	1229.8	1229.8	1229.8	1229.8	1229.8	1229.8	1229.8
	(19.0)	(19.0)	(19.0)	(19.0)	(19.0)	(19.0)	(19.0)	(19.0)	(19.0)	(19.0)
	-176.6	-187.6	-187.6	6.4	-325.2	-37.3	-95.7	-285.8	-47.2	-39.3
	-2.726	-2.897	-2.897	0.099	-5.021	-0.576	-1.477	-4.412	-0.728	-0.607
t_O	1263	1171	1128	1233	1215	1137	1289	1232	1148	1307
	(19.5)	(18.1)	(17.4)	(19.0)	(18.8)	(17.6)	(19.9)	(19.0)	(17.7)	(20.2)
t_E	1157.7	1157.7	1157.7	1157.7	1157.7	1157.7	1157.7	1157.7	1157.7	1157.7
	(17.9)	(17.9)	(17.9)	(17.9)	(17.9)	(17.9)	(17.9)	(17.9)	(17.9)	(17.9)
	220.0	26.9	-58.5	155.5	117.5	-40.9	277.1	153.4	-19.2	317.2
	3.397	0.415	-0.904	2.401	1.814	-0.632	4.278	2.368	-0.297	4.897
g_O	1939	2003	2017	1884	2007	1961	1874	1985	1953	1709
	(29.9)	(30.9)	(31.1)	(29.1)	(31.0)	(30.3)	(28.9)	(30.6)	(30.2)	(26.4)
g_E	1983.0	1983.0	1983.0	1983.0	1983.0	1983.0	1983.0	1983.0	1983.0	1983.0
	(30.6)	(30.6)	(30.6)	(30.6)	(30.6)	(30.6)	(30.6)	(30.6)	(30.6)	(30.6)
	-87.1	40.1	68.5	-193.1	48.2	-43.9	-212.0	3.9	-59.6	-508.4
	-1.345	0.619	1.057	-2.981	0.744	-0.677	-3.273	0.060	-0.921	-7.849
c_O	2137	2171	2200	2127	2201	2168	2133	2183	2170	2251
	(33.0)	(33.5)	(34.0)	(32.8)	(34.0)	(33.5)	(32.9)	(33.7)	(33.5)	(34.8)
c_E	2106.5	2106.5	2106.5	2106.5	2106.5	2106.5	2106.5	2106.5	2106.5	2106.5
	(32.5)	(32.5)	(32.5)	(32.5)	(32.5)	(32.5)	(32.5)	(32.5)	(32.5)	(32.5)
	61.5	131.0	191.1	41.2	193.2	124.8	53.4	155.8	128.9	298.7
	0.949	2.022	2.951	0.636	2.983	1.927	0.824	2.405	1.990	4.612
N	6477	6477	6477	6477	6477	6477	6477	6477	6477	6477
X2	17.9	10.1	13.3	10.1	32.5	2.7	23.2	26.5	2.9	67.4
Gvalue	17.8	10.3	13.4	10.0	33.7	2.7	22.8	27.3	2.9	68.3
Gv/N	0.274	0.159	0.207	0.155	0.520	0.041	0.352	0.421	0.045	1.054
Prob	0.00049	0.01616	0.00380	0.01822	0.00000	0.44283	0.00004	0.00001	0.40759	0.00000

	30	29	28	27	26	25	24	23	22	21
a_O	1080	1169	1218	1082	1177	1238	1235	1309	1358	1239
	(16.7)	(18.0)	(18.8)	(16.7)	(18.2)	(19.1)	(19.1)	(20.2)	(21.0)	(19.1)
a_E	1229.8	1229.8	1229.8	1229.8	1229.8	1229.8	1229.8	1229.8	1229.8	1229.8
	(19.0)	(19.0)	(19.0)	(19.0)	(19.0)	(19.0)	(19.0)	(19.0)	(19.0)	(19.0)
	-280.6	-118.6	-23.5	-277.1	-103.3	16.4	10.4	163.4	269.3	18.4
	-4.332	-1.831	-0.363	-4.278	-1.595	0.254	0.161	2.522	4.158	0.285
t_O	1164	1155	1334	1263	1160	1298	1192	1184	1293	1301
	(18.0)	(17.8)	(20.6)	(19.5)	(17.9)	(20.0)	(18.4)	(18.3)	(20.0)	(20.1)
t_E	1157.7	1157.7	1157.7	1157.7	1157.7	1157.7	1157.7	1157.7	1157.7	1157.7
	(17.9)	(17.9)	(17.9)	(17.9)	(17.9)	(17.9)	(17.9)	(17.9)	(17.9)	(17.9)
	12.7	-5.3	378.3	220.0	4.7	297.1	69.7	53.3	285.9	303.8
	0.197	-0.082	5.841	3.397	0.073	4.586	1.076	0.823	4.415	4.690
g_O	1956	1874	1739	1831	1882	1659	1774	1694	1554	1588
	(30.2)	(28.9)	(26.8)	(28.3)	(29.1)	(25.6)	(27.4)	(26.2)	(24.0)	(24.5)
g_E	1983.0	1983.0	1983.0	1983.0	1983.0	1983.0	1983.0	1983.0	1983.0	1983.0
	(30.6)	(30.6)	(30.6)	(30.6)	(30.6)	(30.6)	(30.6)	(30.6)	(30.6)	(30.6)
	-53.7	-212.0	-456.8	-292.1	-196.9	-592.0	-395.2	-533.8	-757.7	-705.6
	-0.830	-3.273	-7.052	-4.510	-3.039	-9.140	-6.102	-8.241	-11.699	-10.894
c_O	2277	2279	2186	2301	2258	2282	2276	2290	2272	2349
	(35.2)	(35.2)	(33.8)	(35.5)	(34.9)	(35.2)	(35.1)	(35.4)	(35.1)	(36.3)
c_E	2106.5	2106.5	2106.5	2106.5	2106.5	2106.5	2106.5	2106.5	2106.5	2106.5
	(32.5)	(32.5)	(32.5)	(32.5)	(32.5)	(32.5)	(32.5)	(32.5)	(32.5)	(32.5)
	354.5	358.8	162.0	406.5	313.7	365.3	352.3	382.6	343.7	511.9
	5.473	5.539	2.501	6.275	4.843	5.639	5.439	5.906	5.306	7.904
N	6477	6477	6477	6477	6477	6477	6477	6477	6477	6477
X2	32.5	23.1	60.0	57.0	18.3	84.6	36.7	63.8	135.0	124.4
Gvalue	32.9	22.9	60.0	57.2	18.2	86.8	37.2	65.5	141.2	128.6
Gv/N	0.508	0.354	0.927	0.884	0.281	1.339	0.574	1.011	2.180	1.985
Prob	0.00000	0.00004	0.00000	0.00000	0.00040	0.00000	0.00000	0.00000	0.00000	0.00000

	20	19	18	17	16	15	14	13	12	11
a_O	1412	1400	1365	1483	1480	1508	1634	1669	1638	1767
	(21.8)	(21.6)	(21.1)	(22.9)	(22.9)	(23.3)	(25.2)	(25.8)	(25.3)	(27.3)
a_E	1229.8	1229.8	1229.8	1229.8	1229.8	1229.8	1229.8	1229.8	1229.8	1229.8
	(19.0)	(19.0)	(19.0)	(19.0)	(19.0)	(19.0)	(19.0)	(19.0)	(19.0)	(19.0)
	390.1	362.9	284.7	555.3	548.1	615.0	928.7	1019.3	939.0	1280.8
	6.023	5.603	4.396	8.573	8.463	9.496	14.338	15.737	14.497	19.775
t_O	1317	1435	1373	1318	1417	1273	1111	1126	847	688
	(20.3)	(22.2)	(21.2)	(20.3)	(21.9)	(19.7)	(17.2)	(17.4)	(13.1)	(10.6)

```
t_E      1157.7  1157.7  1157.7  1157.7  1157.7  1157.7  1157.7  1157.7  1157.7  1157.7
        ( 17.9) ( 17.9) ( 17.9) ( 17.9) ( 17.9) ( 17.9) ( 17.9) ( 17.9) ( 17.9) ( 17.9)
          339.7   616.4   468.5   342.0   572.9   241.8   -91.4   -62.4  -529.3  -716.0
          5.245   9.517   7.233   5.279   8.845   3.734  -1.411  -0.964  -8.172 -11.055
g_O        1557    1463    1514    1540    1440    1628    1776    1838    2240    2602
        ( 24.0) ( 22.6) ( 23.4) ( 23.8) ( 22.2) ( 25.1) ( 27.4) ( 28.4) ( 34.6) ( 40.2)
g_E      1983.0  1983.0  1983.0  1983.0  1983.0  1983.0  1983.0  1983.0  1983.0  1983.0
        ( 30.6) ( 30.6) ( 30.6) ( 30.6) ( 30.6) ( 30.6) ( 30.6) ( 30.6) ( 30.6) ( 30.6)
         -753.2  -889.9  -817.2  -778.8  -921.6  -642.4  -391.7  -279.2   545.8  1413.6
        -11.629 -13.740 -12.617 -12.024 -14.228  -9.917  -6.047  -4.311   8.427  21.825
c_O        2191    2179    2225    2136    2140    2068    1956    1844    1752    1420
        ( 33.8) ( 33.6) ( 34.4) ( 33.0) ( 33.0) ( 31.9) ( 30.2) ( 28.5) ( 27.0) ( 21.9)
c_E      2106.5  2106.5  2106.5  2106.5  2106.5  2106.5  2106.5  2106.5  2106.5  2106.5
        ( 32.5) ( 32.5) ( 32.5) ( 32.5) ( 32.5) ( 32.5) ( 32.5) ( 32.5) ( 32.5) ( 32.5)
          172.4   147.5   243.6    59.4    67.6   -76.3  -290.0  -490.8  -645.7 -1120.0
          2.661   2.277   3.760   0.918   1.043  -1.178  -4.477  -7.578  -9.969 -17.292

N          6477    6477    6477    6477    6477    6477    6477    6477    6477    6477
X2        143.9   228.9   172.5   173.7   258.2   138.7   167.1   201.0   311.8   842.1
Gvalue    149.0   236.9   179.6   177.9   267.0   138.2   155.6   186.8   309.8   858.4
Gv/N      2.300   3.657   2.773   2.746   4.122   2.134   2.403   2.885   4.784  13.253
Prob    0.00000 0.00000 0.00000 0.00000 0.00000 0.00000 0.00000 0.00000 0.00000 0.00000
        -------------------------------------------------------------------------------
             10       9       8       7       6       5       4       3       2       1
        -------------------------------------------------------------------------------
a_O        1583    1654    1874    1746    1771    1548    1439    1760    1146     828
        ( 24.4) ( 25.5) ( 28.9) ( 27.0) ( 27.3) ( 23.9) ( 22.2) ( 27.2) ( 17.7) ( 12.8)
a_E      1229.8  1229.8  1229.8  1229.8  1229.8  1229.8  1229.8  1229.8  1229.8  1229.8
        ( 19.0) ( 19.0) ( 19.0) ( 19.0) ( 19.0) ( 19.0) ( 19.0) ( 19.0) ( 19.0) ( 19.0)
          799.3   980.3  1578.7  1223.8  1291.7   712.4   452.1  1261.8  -161.8  -655.1
         12.341  15.135  24.374  18.895  19.943  10.999   6.980  19.481  -2.498 -10.115
t_O         718     637     772    1002    1021    1185    1136     958    1307     492
        ( 11.1) (  9.8) ( 11.9) ( 15.5) ( 15.8) ( 18.3) ( 17.5) ( 14.8) ( 20.2) (  7.6)
t_E      1157.7  1157.7  1157.7  1157.7  1157.7  1157.7  1157.7  1157.7  1157.7  1157.7
        ( 17.9) ( 17.9) ( 17.9) ( 17.9) ( 17.9) ( 17.9) ( 17.9) ( 17.9) ( 17.9) ( 17.9)
         -685.9  -761.1  -625.6  -289.4  -256.5    55.3   -42.9  -362.7   317.2  -842.0
        -10.590 -11.750  -9.658  -4.468  -3.960   0.854  -0.662  -5.600   4.897 -13.000
g_O        2987    3096    2539    2141    1774    1557    1430    1945    1251    1745
        ( 46.1) ( 47.8) ( 39.2) ( 33.1) ( 27.4) ( 24.0) ( 22.1) ( 30.0) ( 19.3) ( 26.9)
g_E      1983.0  1983.0  1983.0  1983.0  1983.0  1983.0  1983.0  1983.0  1983.0  1983.0
        ( 30.6) ( 30.6) ( 30.6) ( 30.6) ( 30.6) ( 30.6) ( 30.6) ( 30.6) ( 30.6) ( 30.6)
         2447.2  2758.4  1254.9   328.2  -395.2  -753.2  -935.1   -75.4 -1152.7  -446.3
         37.782  42.587  19.375   5.067  -6.102 -11.629 -14.437  -1.164 -17.796  -6.891
c_O        1189    1090    1292    1588    1911    2187    2472    1814    2773    3412
        ( 18.4) ( 16.8) ( 19.9) ( 24.5) ( 29.5) ( 33.8) ( 38.2) ( 28.0) ( 42.8) ( 52.7)
c_E      2106.5  2106.5  2106.5  2106.5  2106.5  2106.5  2106.5  2106.5  2106.5  2106.5
        ( 32.5) ( 32.5) ( 32.5) ( 32.5) ( 32.5) ( 32.5) ( 32.5) ( 32.5) ( 32.5) ( 32.5)
        -1360.0 -1436.3 -1263.1  -897.4  -372.2   164.1   791.1  -542.3  1524.6  3291.0
        -20.997 -22.175 -19.502 -13.855  -5.747   2.533  12.213  -8.373  23.539  50.811

N          6477    6477    6477    6477    6477    6477    6477    6477    6477    6477
X2       1176.3  1495.6   936.7   377.8   294.5   177.6   253.6   304.3   506.1  1351.7
Gvalue   1200.5  1541.3   945.0   365.3   267.7   178.6   265.2   281.4   527.4  1347.6
Gv/N     18.535  23.797  14.589   5.639   4.133   2.757   4.094   4.344   8.143  20.807
Prob    0.00000 1.00000 0.00000 0.00000 0.00000 0.00000 0.00000 0.00000 0.00000 0.00000
        -------------------------------------------------------------------------------

CDS_INITIAL:
        -------------------------------------------------------------------------------
              1       2       3       4       5       6       7       8       9      10
        -------------------------------------------------------------------------------
a_O        5742       0       0    2981    2075    1300    2000    1926    1124    1824
        ( 88.7) (  0.0) (  0.0) ( 46.0) ( 32.0) ( 20.1) ( 30.9) ( 29.7) ( 17.4) ( 28.2)
a_E      1255.7  1544.8   362.7  1255.7  1544.8   362.7  1255.7  1544.8   362.7  1255.7
        ( 19.4) ( 23.9) (  5.6) ( 19.4) ( 23.9) (  5.6) ( 19.4) ( 23.9) (  5.6) ( 19.4)
        17457.1     0.0     0.0  5154.6  1224.6  3319.0  1861.8   849.6  2542.6  1362.0
        269.524   0.000   0.000  79.583  18.906  51.242  28.745  13.117  39.256  21.028
t_O         219    6477       0     776     841    1158     852    1420    1125     959
        (  3.4) (100.0) (  0.0) ( 12.0) ( 13.0) ( 17.9) ( 13.2) ( 21.9) ( 17.4) ( 14.8)
t_E       817.4  1832.5   474.2   817.4  1832.5   474.2   817.4  1832.5   474.2   817.4
        ( 12.6) ( 28.3) (  7.3) ( 12.6) ( 28.3) (  7.3) ( 12.6) ( 28.3) (  7.3) ( 12.6)
         -576.9 16355.3     0.0   -80.7 -1310.0  2067.6    70.6  -724.3  1943.6   306.4
         -8.907 252.513   0.000  -1.246 -20.226  31.922   1.090 -11.183  30.008   4.730
g_O         516       0    6477    1199    1166    1684    1889    1059    1736    1644
        (  8.0) (  0.0) (100.0) ( 18.5) ( 18.0) ( 26.0) ( 29.2) ( 16.4) ( 26.8) ( 25.4)
g_E      2606.7  1282.2  2737.7  2606.7  1282.2  2737.7  2606.7  1282.2  2737.7  2606.7
        ( 40.2) ( 19.8) ( 42.3) ( 40.2) ( 19.8) ( 42.3) ( 40.2) ( 19.8) ( 42.3) ( 40.2)
        -1671.6     0.0 11155.1 -1862.3  -221.5 -1636.7 -1216.7  -405.0 -1581.6 -1515.6
        -25.808   0.000 172.226 -28.753  -3.420 -25.269 -18.785  -6.254 -24.419 -23.400
c_O           0       0       0    1521    2395    2335    1736    2072    2492    2050
        (  0.0) (  0.0) (  0.0) ( 23.5) ( 37.0) ( 36.1) ( 26.8) ( 32.0) ( 38.5) ( 31.7)
c_E      1797.2  1817.5  2902.3  1797.2  1817.5  2902.3  1797.2  1817.5  2902.3  1797.2
        ( 27.7) ( 28.1) ( 44.8) ( 27.7) ( 28.1) ( 44.8) ( 27.7) ( 28.1) ( 44.8) ( 27.7)
            0.0     0.0     0.0  -507.5  1321.6 -1015.7  -120.2   543.1  -759.7   539.7
          0.000   0.000   0.000  -7.836  20.405 -15.682  -1.856   8.384 -11.729   8.332

N          6477    6477    6477    6477    6477    6477    6477    6477    6477    6477
X2      19940.7 16415.8  8846.5  3175.3   912.5  3924.4   642.3   261.4  2915.4   672.9
Gvalue  15208.6 16355.3 11155.1  2704.0  1014.7  2734.1   595.5   263.3  2144.9   692.4
Gv/N    234.810 252.513 172.226  41.748  15.666  42.213   9.195   4.065  33.115  10.690
Prob    1.00000 1.00000 1.00000 1.00000 0.00000 1.00000 0.00000 0.00000 1.00000 0.00000
        -------------------------------------------------------------------------------
             11      12      13      14      15      16      17      18      19      20
        -------------------------------------------------------------------------------
a_O        1553     981    1737    1598     940    1583    1549     884    1507    1484
        ( 24.0) ( 15.1) ( 26.8) ( 24.7) ( 14.5) ( 24.4) ( 23.9) ( 13.6) ( 23.3) ( 22.9)
a_E      1544.8   362.7  1255.7  1544.8   362.7  1255.7  1544.8   362.7  1255.7  1544.8
        ( 23.9) (  5.6) ( 19.4) ( 23.9) (  5.6) ( 19.4) ( 23.9) (  5.6) ( 19.4) ( 23.9)
           16.5  1952.1  1127.2   108.2  1790.3   733.4     8.4  1575.0   549.9  -119.1
          0.254  30.140  17.403   1.671  27.641  11.322   0.130  24.317   8.489  -1.839
t_O        1763    1153     960    1907    1192    1052    1950    1100     964    1822
        ( 27.2) ( 17.8) ( 14.8) ( 29.4) ( 18.4) ( 16.2) ( 30.1) ( 17.0) ( 14.9) ( 28.1)
t_E      1832.5   474.2   817.4  1832.5   474.2   817.4  1832.5   474.2   817.4  1832.5
        ( 28.3) (  7.3) ( 12.6) ( 28.3) (  7.3) ( 12.6) ( 28.3) (  7.3) ( 12.6) ( 28.3)
         -136.4  2048.7   308.7   151.9  2197.3   530.8   242.3  1851.0   318.0   -21.0
         -2.105  31.630   4.766   2.346  33.924   8.196   3.741  28.578   4.910  -0.324
g_O        1024    1748    1793     962    1702    1894    1015    1786    2051    1123
        ( 15.8) ( 27.0) ( 27.7) ( 14.9) ( 26.3) ( 29.2) ( 15.7) ( 27.6) ( 31.7) ( 17.3)
g_E      1282.2  2737.7  2606.7  1282.2  2737.7  2606.7  1282.2  2737.7  2606.7  1282.2
        ( 19.8) ( 42.3) ( 40.2) ( 19.8) ( 42.3) ( 40.2) ( 19.8) ( 42.3) ( 40.2) ( 19.8)
         -460.5 -1568.5 -1341.9  -552.8 -1618.0 -1209.9  -474.4 -1525.8  -983.5  -297.7
         -7.110 -24.216 -20.718  -8.534 -24.981 -18.680  -7.324 -23.557 -15.185  -4.597
```

c_O	2137	2595	1987	2010	2643	1948	1963	2707	1955	2048
	(33.0)	(40.1)	(30.7)	(31.0)	(40.8)	(30.1)	(30.3)	(41.8)	(30.2)	(31.6)
c_E	1817.5	2902.3	1797.2	1817.5	2902.3	1797.2	1817.5	2902.3	1797.2	1817.5
	(28.1)	(44.8)	(27.7)	(28.1)	(44.8)	(27.7)	(28.1)	(44.8)	(27.7)	(28.1)
	692.1	-580.9	399.0	404.7	-494.8	314.0	302.3	-377.2	329.1	489.0
	10.686	-8.969	6.161	6.248	-7.639	4.848	4.668	-5.824	5.082	7.551
N	6477	6477	6477	6477	6477	6477	6477	6477	6477	6477
X2	110.8	2415.8	483.4	105.2	2420.2	360.2	74.9	1918.9	208.9	51.4
Gvalue	111.7	1851.4	493.1	112.1	1874.8	368.3	78.7	1523.0	213.5	51.2
Gv/N	1.725	28.585	7.613	1.731	28.946	5.686	1.216	23.515	3.296	0.790
Prob	0.00000	1.00000	0.00000	0.00000	1.00000	0.00000	0.00000	1.00000	0.00000	0.00000

	21	22	23	24	25	26	27	28	29	30
a_O	807	1300	1427	695	1228	1281	626	1223	1299	619
	(12.5)	(20.1)	(22.0)	(10.7)	(19.0)	(19.8)	(9.7)	(18.9)	(20.1)	(9.6)
a_E	362.7	1255.7	1544.8	362.7	1255.7	1544.8	362.7	1255.7	1544.8	362.7
	(5.6)	(19.4)	(23.9)	(5.6)	(19.4)	(23.9)	(5.6)	(19.4)	(23.9)	(5.6)
	1290.8	90.2	-226.3	903.9	-54.8	-479.7	683.3	-64.5	-450.2	661.7
	19.928	1.392	-3.495	13.956	-0.846	-7.406	10.549	-0.996	-6.951	10.217
t_O	970	936	1811	908	939	1827	794	875	1862	778
	(15.0)	(14.5)	(28.0)	(14.0)	(14.5)	(28.2)	(12.3)	(13.5)	(28.7)	(12.0)
t_E	474.2	817.4	1832.5	474.2	817.4	1832.5	474.2	817.4	1832.5	474.2
	(7.3)	(12.6)	(28.3)	(7.3)	(12.6)	(28.3)	(7.3)	(12.6)	(28.3)	(7.3)
	1388.2	253.6	-42.8	1179.6	260.4	-11.0	818.4	119.1	59.4	770.2
	21.433	3.915	-0.661	18.211	4.021	-0.170	12.636	1.839	0.918	11.892
g_O	1957	2190	1191	2073	2336	1267	2152	2394	1243	2200
	(30.2)	(33.8)	(18.4)	(32.0)	(36.1)	(19.6)	(33.2)	(37.0)	(19.2)	(34.0)
g_E	2737.7	2606.7	1282.2	2737.7	2606.7	1282.2	2737.7	2606.7	1282.2	2737.7
	(42.3)	(40.2)	(19.8)	(42.3)	(40.2)	(19.8)	(42.3)	(40.2)	(19.8)	(42.3)
	-1314.0	-763.0	-175.7	-1153.1	-512.3	-30.2	-1036.1	-407.6	-77.2	-962.1
	-20.287	-11.780	-2.713	-17.803	-7.910	-0.466	-15.997	-6.293	-1.191	-14.855
c_O	2743	2051	2048	2801	1974	2102	2905	1985	2073	2880
	(42.3)	(31.7)	(31.6)	(43.2)	(30.5)	(32.5)	(44.9)	(30.6)	(32.0)	(44.5)
c_E	2902.3	1797.2	1817.5	2902.3	1797.2	1817.5	2902.3	1797.2	1817.5	2902.3
	(44.8)	(27.7)	(28.1)	(44.8)	(27.7)	(28.1)	(44.8)	(27.7)	(28.1)	(44.8)
	-309.8	541.9	489.0	-199.1	370.5	611.4	5.3	394.6	545.3	-44.5
	-4.782	8.367	7.551	-3.074	5.721	9.439	0.082	6.093	8.419	-0.687
N	6477	6477	6477	6477	6477	6477	6477	6477	6477	6477
X2	1293.9	121.2	44.9	866.1	64.2	89.8	532.0	41.9	76.7	481.5
Gvalue	1055.3	122.7	44.2	731.3	63.9	90.4	470.9	41.7	77.4	425.3
Gv/N	16.292	1.895	0.682	11.290	0.986	1.396	7.271	0.643	1.195	6.567
Prob	0.00000	0.00000	0.00000	0.00000	0.00000	0.00000	0.00000	0.00000	0.00000	0.00000

	31	32	33	34	35	36	37	38	39	40
a_O	1166	1290	585	1175	1288	556	1139	1216	530	1127
	(18.0)	(19.9)	(9.0)	(18.1)	(19.9)	(8.6)	(17.6)	(18.8)	(8.2)	(17.4)
a_E	1255.7	1544.8	362.7	1255.7	1544.8	362.7	1255.7	1544.8	362.7	1255.7
	(19.4)	(23.9)	(5.6)	(19.4)	(23.9)	(5.6)	(19.4)	(23.9)	(5.6)	(19.4)
	-172.8	-465.0	559.3	-156.1	-468.3	475.0	-222.2	-582.0	402.0	-243.7
	-2.668	-7.180	8.635	-2.410	-7.230	7.334	-3.430	-8.986	6.207	-3.763
t_O	862	1797	696	864	1794	724	837	1878	683	813
	(13.3)	(27.7)	(10.7)	(13.3)	(27.7)	(11.2)	(12.9)	(29.0)	(10.5)	(12.6)
t_E	817.4	1832.5	474.2	817.4	1832.5	474.2	817.4	1832.5	474.2	817.4
	(12.6)	(28.3)	(7.3)	(12.6)	(28.3)	(7.3)	(12.6)	(28.3)	(7.3)	(12.6)
	91.6	-70.3	534.0	95.8	-76.2	612.6	39.6	92.1	498.3	-8.8
	1.414	-1.086	8.245	1.479	-1.177	9.458	0.612	1.422	7.693	-0.136
g_O	2540	1285	2176	2610	1320	2281	2633	1330	2357	2620
	(39.2)	(19.8)	(33.6)	(40.3)	(20.4)	(35.2)	(40.7)	(20.5)	(36.4)	(40.5)
g_E	2606.7	1282.2	2737.7	2606.7	1282.2	2737.7	2606.7	1282.2	2737.7	2606.7
	(40.2)	(19.8)	(42.3)	(40.2)	(19.8)	(42.3)	(40.2)	(19.8)	(42.3)	(40.2)
	-131.7	5.6	-999.4	6.6	76.7	-832.6	52.8	97.4	-705.9	26.6
	-2.034	0.087	-15.430	0.101	1.185	-12.855	0.815	1.504	-10.898	0.411
c_O	1909	2105	3020	1828	2075	2916	1868	2053	2907	1917
	(29.5)	(32.5)	(46.6)	(28.2)	(32.0)	(45.0)	(28.8)	(31.7)	(44.9)	(29.6)
c_E	1797.2	1817.5	2902.3	1797.2	1817.5	2902.3	1797.2	1817.5	2902.3	1797.2
	(27.7)	(28.1)	(44.8)	(27.7)	(28.1)	(44.8)	(27.7)	(28.1)	(44.8)	(27.7)
	230.5	618.2	240.0	62.2	549.8	27.4	144.4	500.3	9.3	247.5
	3.558	9.545	3.706	0.960	8.489	0.423	2.230	7.724	0.144	3.821
N	6477	6477	6477	6477	6477	6477	6477	6477	6477	6477
X2	17.5	88.2	360.0	8.4	81.1	310.8	14.4	103.4	222.0	21.3
Gvalue	17.5	88.5	334.0	8.4	82.1	282.4	14.7	107.7	203.8	21.6
Gv/N	0.270	1.366	5.156	0.130	1.267	4.360	0.227	1.663	3.147	0.333
Prob	0.00056	0.00000	0.00000	0.03781	0.00000	0.00000	0.00211	0.00000	0.00000	0.00008

	41	42	43	44	45	46	47	48	49	50
a_O	1275	542	1126	1283	548	1133	1274	558	1189	1215
	(19.7)	(8.4)	(17.4)	(19.8)	(8.5)	(17.5)	(19.7)	(8.6)	(18.4)	(18.8)
a_E	1544.8	362.7	1255.7	1544.8	362.7	1255.7	1544.8	362.7	1255.7	1544.8
	(23.9)	(5.6)	(19.4)	(23.9)	(5.6)	(19.4)	(23.9)	(5.6)	(19.4)	(23.9)
	-489.4	435.4	-245.5	-476.5	452.3	-233.0	-491.1	480.7	-129.8	-583.5
	-7.557	6.722	-3.790	-7.356	6.983	-3.597	-7.582	7.422	-2.004	-9.009
t_O	1857	688	831	1756	679	779	1839	633	827	1868
	(28.7)	(10.6)	(12.8)	(27.1)	(10.5)	(12.0)	(28.4)	(9.8)	(12.8)	(28.8)
t_E	1832.5	474.2	817.4	1832.5	474.2	817.4	1832.5	474.2	817.4	1832.5
	(28.3)	(7.3)	(12.6)	(28.3)	(7.3)	(12.6)	(28.3)	(7.3)	(12.6)	(28.3)
	49.3	512.0	27.4	-149.8	487.4	-75.0	13.0	365.6	19.3	71.6
	0.761	7.905	0.423	-2.313	7.525	-1.158	0.200	5.644	0.298	1.106
g_O	1251	2279	2716	1289	2405	2697	1284	2390	2582	1328
	(19.3)	(35.2)	(41.9)	(19.9)	(37.1)	(41.6)	(19.8)	(36.9)	(39.9)	(20.5)
g_E	1282.2	2737.7	2606.7	1282.2	2737.7	2606.7	1282.2	2737.7	2606.7	1282.2
	(19.8)	(42.3)	(40.2)	(19.8)	(42.3)	(40.2)	(19.8)	(42.3)	(40.2)	(19.8)
	-61.6	-835.9	223.1	13.7	-623.3	183.6	3.6	-649.3	-49.2	93.2
	-0.951	-12.906	3.444	0.211	-9.623	2.835	0.056	-10.024	-0.760	1.440
c_O	2094	2968	1804	2149	2845	1868	2080	2896	1879	2066
	(32.3)	(45.8)	(27.9)	(33.2)	(43.9)	(28.8)	(32.1)	(44.7)	(29.0)	(31.9)
c_E	1817.5	2902.3	1797.2	1817.5	2902.3	1797.2	1817.5	2902.3	1797.2	1817.5
	(28.1)	(44.8)	(27.7)	(28.1)	(44.8)	(27.7)	(28.1)	(44.8)	(27.7)	(28.1)
	593.1	132.8	13.7	720.1	-113.5	144.4	561.2	-12.6	167.3	529.5
	9.156	2.051	0.211	11.117	-1.753	2.230	8.664	-0.195	2.584	8.175
N	6477	6477	6477	6477	6477	6477	6477	6477	6477	6477
X2	90.3	263.3	18.2	108.1	224.6	19.7	85.4	202.5	7.6	106.7
Gvalue	91.3	244.3	18.7	107.5	202.9	20.1	86.7	184.4	7.6	110.9
Gv/N	1.410	3.772	0.288	1.659	3.133	0.310	1.339	2.847	0.118	1.711
Prob	0.00000	0.00000	0.00032	0.00000	0.00000	0.00016	0.00000	0.00000	0.05444	0.00000

	51	52	53	54	55	56	57	58	59	60
a_O	472	1186	1264	490	1179	1289	459	1148	1336	456
	(7.3)	(18.3)	(19.5)	(7.6)	(18.2)	(19.9)	(7.1)	(17.7)	(20.6)	(7.0)
a_E	362.7	1255.7	1544.8	362.7	1255.7	1544.8	362.7	1255.7	1544.8	362.7
	(5.6)	(19.4)	(23.9)	(5.6)	(19.4)	(23.9)	(5.6)	(19.4)	(23.9)	(5.6)
	248.6	-135.4	-507.1	294.8	-148.6	-466.7	216.1	-205.9	-388.0	208.8
	3.839	-2.091	-7.830	4.551	-2.294	-7.205	3.337	-3.179	-5.990	3.223
t_O	641	822	1807	631	858	1836	600	840	1790	596
	(9.9)	(12.7)	(27.9)	(9.7)	(13.2)	(28.3)	(9.3)	(13.0)	(27.6)	(9.2)
t_E	474.2	817.4	1832.5	474.2	817.4	1832.5	474.2	817.4	1832.5	474.2
	(7.3)	(12.6)	(28.3)	(7.3)	(12.6)	(28.3)	(7.3)	(12.6)	(28.3)	(7.3)
	386.3	9.2	-50.7	360.4	83.2	7.0	282.3	45.8	-84.0	272.4
	5.964	0.142	-0.783	5.565	1.284	0.108	4.358	0.707	-1.298	4.206
g_O	2384	2644	1335	2490	2636	1324	2354	2667	1286	2486
	(36.8)	(40.8)	(20.6)	(38.4)	(40.7)	(20.4)	(36.3)	(41.2)	(19.9)	(38.4)
g_E	2737.7	2606.7	1282.2	2737.7	2606.7	1282.2	2737.7	2606.7	1282.2	2737.7
	(42.3)	(40.2)	(19.8)	(42.3)	(40.2)	(19.8)	(42.3)	(40.2)	(19.8)	(42.3)
	-659.6	75.1	107.8	-472.3	58.9	85.0	-711.0	121.9	7.6	-479.6
	-10.184	1.159	1.664	-7.292	0.909	1.312	-10.977	1.883	0.118	-7.404
c_O	2980	1825	2071	2866	1804	2028	3064	1822	2065	2939
	(46.0)	(28.2)	(32.0)	(44.2)	(27.9)	(31.3)	(47.3)	(28.1)	(31.9)	(45.4)
c_E	2902.3	1797.2	1817.5	2902.3	1797.2	1817.5	2902.3	1797.2	1817.5	2902.3
	(44.8)	(27.7)	(28.1)	(44.8)	(27.7)	(28.1)	(44.8)	(27.7)	(28.1)	(44.8)
	157.4	56.1	540.8	-72.2	13.7	444.5	332.2	50.0	527.2	73.8
	2.430	0.866	8.349	-1.115	0.211	6.862	5.129	0.772	8.140	1.139
N	6477	6477	6477	6477	6477	6477	6477	6477	6477	6477
X2	139.4	4.9	88.9	119.4	7.1	68.1	121.7	11.6	62.9	78.9
Gvalue	132.7	4.9	90.8	110.7	7.1	69.7	119.6	11.9	62.9	75.4
Gv/N	2.049	0.076	1.401	1.709	0.110	1.077	1.847	0.183	0.970	1.164
Prob	0.00000	0.17725	0.00000	0.00000	0.06821	0.00000	0.00000	0.00790	0.00000	-0.00000

	61	62	63	64	65	66	67	68	69	70
a_O	1117	1312	465	1161	1373	460	1091	1374	462	1091
	(17.2)	(20.3)	(7.2)	(17.9)	(21.2)	(7.1)	(16.8)	(21.2)	(7.1)	(16.8)
a_E	1255.7	1544.8	362.7	1255.7	1544.8	362.7	1255.7	1544.8	362.7	1255.7
	(19.4)	(23.9)	(5.6)	(19.4)	(23.9)	(5.6)	(19.4)	(23.9)	(5.6)	(19.4)
	-261.5	-428.6	231.1	-182.1	-323.7	218.6	-306.8	-321.9	223.6	-306.8
	-4.037	-6.617	3.567	-2.811	-4.998	3.375	-4.736	-4.971	3.452	-4.736
t_O	820	1859	580	776	1747	608	843	1705	600	827
	(12.7)	(28.7)	(9.0)	(12.0)	(27.0)	(9.4)	(13.0)	(26.3)	(9.3)	(12.8)
t_E	817.4	1832.5	474.2	817.4	1832.5	474.2	817.4	1832.5	474.2	817.4
	(12.6)	(28.3)	(7.3)	(12.6)	(28.3)	(7.3)	(12.6)	(28.3)	(7.3)	(12.6)
	5.2	53.3	233.5	-80.7	-167.0	302.1	52.0	-246.0	282.3	19.3
	0.080	0.824	3.606	-1.246	-2.578	4.665	0.802	-3.797	4.358	0.298
g_O	2620	1298	2461	2759	1275	2520	2649	1352	2526	2711
	(40.5)	(20.0)	(38.0)	(42.6)	(19.7)	(38.9)	(40.9)	(20.9)	(39.0)	(41.9)
g_E	2606.7	1282.2	2737.7	2606.7	1282.2	2737.7	2606.7	1282.2	2737.7	2606.7
	(40.2)	(19.8)	(42.3)	(40.2)	(19.8)	(42.3)	(40.2)	(19.8)	(42.3)	(40.2)
	26.6	31.8	-524.5	313.3	-14.3	-417.7	85.2	143.4	-406.6	212.7
	0.411	0.491	-8.098	4.837	-0.221	-6.448	1.316	2.213	-6.278	3.283
c_O	1920	2008	2971	1781	2082	2889	1894	2046	2889	1848
	(29.6)	(31.0)	(45.9)	(27.5)	(32.1)	(44.6)	(29.2)	(31.6)	(44.6)	(28.5)
c_E	1797.2	1817.5	2902.3	1797.2	1817.5	2902.3	1797.2	1817.5	2902.3	1797.2
	(27.7)	(28.1)	(44.8)	(27.7)	(28.1)	(44.8)	(27.7)	(28.1)	(44.8)	(27.7)
	253.9	400.3	138.9	-32.2	565.7	-26.6	198.8	484.6	-26.6	103.1
	3.920	6.180	2.145	-0.497	8.734	-0.411	3.069	7.481	-0.411	1.592
N	6477	6477	6477	6477	6477	6477	6477	6477	6477	6477
X2	23.8	55.6	82.0	18.3	61.6	81.2	28.3	60.3	77.0	27.3
Gvalue	24.2	56.9	79.0	18.3	60.7	76.5	29.2	60.0	72.6	28.3
Gv/N	0.374	0.878	1.220	0.283	0.937	1.181	0.451	0.927	1.121	0.436
Prob	0.00002	0.00000	0.00000	0.00038	0.00000	-0.00000	0.00000	0.00000	0.00000	0.00000

	71	72	73	74	75	76	77	78	79	80
a_O	1397	453	1182	1345	465	1151	1353	421	1181	1436
	(21.6)	(7.0)	(18.2)	(20.8)	(7.2)	(17.8)	(20.9)	(6.5)	(18.2)	(22.2)
a_E	1544.8	362.7	1255.7	1544.8	362.7	1255.7	1544.8	362.7	1255.7	1544.8
	(23.9)	(5.6)	(19.4)	(23.9)	(5.6)	(19.4)	(23.9)	(5.6)	(19.4)	(23.9)
	-281.0	201.4	-143.0	-372.5	231.1	-200.4	-358.7	125.5	-144.9	-209.7
	-4.338	3.109	-2.207	-5.752	3.567	-3.094	-5.538	1.937	-2.236	-3.238
t_O	1763	608	823	1837	601	845	1826	587	858	1798
	(27.2)	(9.4)	(12.7)	(28.4)	(9.3)	(13.0)	(28.2)	(9.1)	(13.2)	(27.8)
t_E	1832.5	474.2	817.4	1832.5	474.2	817.4	1832.5	474.2	817.4	1832.5
	(28.3)	(7.3)	(12.6)	(28.3)	(7.3)	(12.6)	(28.3)	(7.3)	(12.6)	(28.3)
	-136.4	302.1	11.2	9.0	284.7	56.1	-13.0	250.4	83.2	-68.4
	-2.105	4.665	0.173	0.138	4.396	0.866	-0.201	3.866	1.284	-1.056
g_O	1331	2501	2643	1327	2528	2591	1283	2593	2588	1306
	(20.5)	(38.6)	(40.8)	(20.5)	(39.0)	(40.0)	(19.8)	(40.0)	(40.0)	(20.2)
g_E	1282.2	2737.7	2606.7	1282.2	2737.7	2606.7	1282.2	2737.7	2606.7	1282.2
	(19.8)	(42.3)	(40.2)	(19.8)	(42.3)	(40.2)	(19.8)	(42.3)	(40.2)	(19.8)
	99.5	-452.4	73.1	91.2	-403.0	-31.4	1.6	-281.7	-37.3	48.1
	1.536	-6.984	1.128	1.408	-6.221	-0.484	0.025	-4.349	-0.576	0.742
c_O	1986	2915	1829	1968	2883	1890	2015	2876	1850	1937
	(30.7)	(45.0)	(28.2)	(30.4)	(44.5)	(29.2)	(31.1)	(44.4)	(28.6)	(29.9)
c_E	1817.5	2902.3	1797.2	1817.5	2902.3	1797.2	1817.5	2902.3	1797.2	1817.5
	(28.1)	(44.8)	(27.7)	(28.1)	(44.8)	(27.7)	(28.1)	(44.8)	(27.7)	(28.1)
	352.1	25.4	64.2	313.1	-38.5	190.4	415.7	-52.4	107.2	246.7
	5.437	0.392	0.992	4.834	-0.595	2.939	6.418	-0.809	1.655	3.808
N	6477	6477	6477	6477	6477	6477	6477	6477	6477	6477
X2	34.3	80.7	5.4	39.9	78.9	14.5	45.3	44.1	8.1	16.6
Gvalue	34.3	76.6	5.5	40.7	74.3	14.7	45.6	41.8	8.2	16.6
Gv/N	0.529	1.182	0.085	0.629	1.147	0.227	0.704	0.646	0.126	0.257
Prob	0.00000	-0.00000	0.13787	0.00000	0.00000	0.00208	0.00000	0.00000	0.04226	0.00084

	81	82	83	84	85	86	87	88	89	90
a_O	446	1179	1423	421	1172	1471	446	1174	1467	426
	(6.9)	(18.2)	(22.0)	(6.5)	(18.1)	(22.7)	(6.9)	(18.1)	(22.6)	(6.6)
a_E	362.7	1255.7	1544.8	362.7	1255.7	1544.8	362.7	1255.7	1544.8	362.7
	(5.6)	(19.4)	(23.9)	(5.6)	(19.4)	(23.9)	(5.6)	(19.4)	(23.9)	(5.6)
	184.4	-148.6	-233.7	125.5	-161.7	-144.0	184.4	-158.0	-151.6	137.0
	2.847	-2.294	-3.608	1.937	-2.496	-2.223	2.847	-2.439	-2.340	2.116
t_O	586	830	1854	535	850	1851	574	806	1788	587
	(9.0)	(12.8)	(28.6)	(8.3)	(13.1)	(28.6)	(8.9)	(12.4)	(27.6)	(9.1)
t_E	474.2	817.4	1832.5	474.2	817.4	1832.5	474.2	817.4	1832.5	474.2
	(7.3)	(12.6)	(28.3)	(7.3)	(12.6)	(28.3)	(7.3)	(12.6)	(28.3)	(7.3)
	248.0	25.4	43.2	129.0	66.5	37.1	219.2	-22.7	-88.0	250.4

	3.829	0.392	0.667	1.992	1.026	0.573	3.384	-0.350	-1.358	3.866
g_O	2512	2641	1301	2518	2611	1268	2578	2598	1241	2473
	(38.8)	(40.8)	(20.1)	(38.9)	(40.3)	(19.6)	(39.8)	(40.1)	(19.2)	(38.2)
g_E	2737.7	2606.7	1282.2	2737.7	2606.7	1282.2	2737.7	2606.7	1282.2	2737.7
	(42.3)	(40.2)	(19.8)	(42.3)	(40.2)	(19.8)	(42.3)	(40.2)	(19.8)	(42.3)
	-432.3	69.0	37.9	-421.3	8.6	-28.2	-309.9	-17.4	-81.0	-503.0
	-6.674	1.065	0.585	-6.505	0.132	-0.436	-4.785	-0.269	-1.251	-7.766
c_O	2933	1827	1899	3003	1844	1887	2879	1899	1981	2991
	(45.3)	(28.2)	(29.3)	(46.4)	(28.5)	(29.1)	(44.4)	(29.3)	(30.6)	(46.2)
c_E	2902.3	1797.2	1817.5	2902.3	1797.2	1817.5	2902.3	1797.2	1817.5	2902.3
	(44.8)	(27.7)	(28.1)	(44.8)	(27.7)	(28.1)	(44.8)	(27.7)	(28.1)	(44.8)
	61.7	60.2	166.6	204.8	94.9	141.6	-46.5	209.3	341.3	180.0
	0.952	0.929	2.572	3.162	1.465	2.186	-0.718	3.232	5.269	2.779
N	6477	6477	6477	6477	6477	6477	6477	6477	6477	6477
X2	64.4	5.8	13.8	38.3	8.1	6.5	49.6	11.3	21.0	66.2
Gvalue	61.8	5.9	14.0	38.0	8.2	6.5	47.2	11.3	20.7	64.5
Gv/N	0.954	0.091	0.216	0.586	0.127	0.101	0.728	0.174	0.320	0.996
Prob	0.00000	0.11575	0.00292	0.00000	0.04195	0.08777	0.00000	0.01027	0.00012	0.00000

	91	92	93	94	95	96	97	98	99	100
a_O	1130	1471	442	1178	1460	421	1231	1402	383	1240
	(17.4)	(22.7)	(6.8)	(18.2)	(22.5)	(6.5)	(19.0)	(21.6)	(5.9)	(19.2)
a_E	1255.7	1544.8	362.7	1255.5	1544.5	362.7	1255.5	1544.5	362.7	1255.3
	(19.4)	(23.9)	(5.6)	(19.4)	(23.9)	(5.6)	(19.4)	(23.9)	(5.6)	(19.4)
	-238.4	-144.0	174.8	-150.1	-164.4	125.6	-48.5	-271.5	41.8	-30.4
	-3.680	-2.223	2.698	-2.318	-2.538	1.940	-0.749	-4.193	0.646	-0.470
t_O	755	1743	550	818	1865	545	812	1860	539	781
	(11.7)	(26.9)	(8.5)	(12.6)	(28.8)	(8.4)	(12.5)	(28.7)	(8.3)	(12.1)
t_E	817.4	1832.5	474.2	817.3	1832.2	474.2	817.3	1832.2	474.2	817.2
	(12.6)	(28.3)	(7.3)	(12.6)	(28.3)	(7.3)	(12.6)	(28.3)	(7.3)	(12.6)
	-119.9	-174.6	163.0	1.4	66.1	151.8	-10.5	55.9	138.2	-70.7
	-1.852	-2.696	2.517	0.022	1.021	2.343	-0.163	0.864	2.133	-1.092
g_O	2741	1298	2560	2674	1168	2548	2668	1302	2515	2681
	(42.3)	(20.0)	(39.5)	(41.3)	(18.0)	(39.3)	(41.2)	(20.1)	(38.8)	(41.4)
g_E	2606.7	1282.2	2737.7	2606.3	1282.0	2737.3	2606.3	1282.0	2737.3	2605.9
	(40.2)	(19.8)	(42.3)	(40.2)	(19.8)	(42.3)	(40.2)	(19.8)	(42.3)	(40.2)
	275.3	31.8	-343.7	137.1	-217.5	-365.2	124.8	40.3	-426.0	152.3
	4.251	0.491	-5.306	2.117	-3.359	-5.639	1.927	0.623	-6.579	2.352
c_O	1851	1965	2925	1806	1983	2962	1765	1912	3039	1773
	(28.6)	(30.3)	(45.2)	(27.9)	(30.6)	(45.7)	(27.3)	(29.5)	(46.9)	(27.4)
c_E	1797.2	1817.5	2902.3	1796.9	1817.2	2901.9	1796.9	1817.2	2901.9	1796.6
	(27.7)	(28.1)	(44.8)	(27.7)	(28.1)	(44.8)	(27.7)	(28.1)	(44.8)	(27.7)
	109.3	306.6	45.5	18.3	346.2	121.5	-63.2	194.4	280.6	-46.9
	1.687	4.734	0.703	0.282	5.346	1.876	-0.976	3.002	4.333	-0.725
N	6477	6477	6477	6476	6476	6476	6476	6476	6476	6475
X2	25.9	20.1	41.2	6.6	30.5	34.3	2.5	18.8	34.5	4.3
Gvalue	26.3	19.9	39.7	6.7	30.4	33.7	2.5	19.2	34.5	4.3
Gv/N	0.406	0.307	0.612	0.103	0.470	0.520	0.039	0.296	0.534	0.066
Prob	0.00001	0.00018	0.00000	0.08303	0.00000	0.00000	0.46936	0.00025	0.00000	0.23413

	101	102	103	104	105	106	107	108	109	110
a_O	1473	422	1256	1438	405	1234	1506	430	1173	1427
	(22.7)	(6.5)	(19.4)	(22.2)	(6.3)	(19.1)	(23.3)	(6.6)	(18.1)	(22.0)
a_E	1544.3	362.6	1255.3	1544.3	362.6	1255.3	1544.3	362.6	1255.3	1544.3
	(23.9)	(5.6)	(19.4)	(23.9)	(5.6)	(19.4)	(23.9)	(5.6)	(19.4)	(23.9)
	-139.3	128.1	1.4	-205.1	89.6	-42.2	-75.7	146.6	-159.1	-225.5
	-2.151	1.978	0.021	-3.168	1.384	-0.652	-1.168	2.265	-2.457	-3.482
t_O	1852	544	809	1745	529	849	1821	526	790	1831
	(28.6)	(8.4)	(12.5)	(26.9)	(8.2)	(13.1)	(28.1)	(8.1)	(12.2)	(28.3)
t_E	1832.0	474.1	817.2	1832.0	474.1	817.2	1832.0	474.1	817.2	1832.0
	(28.3)	(7.3)	(12.6)	(28.3)	(7.3)	(12.6)	(28.3)	(7.3)	(12.6)	(28.3)
	40.3	149.7	-16.2	-169.7	115.9	64.9	-21.8	109.3	-53.4	-1.9
	0.623	2.311	-0.251	-2.621	1.791	1.002	-0.337	1.688	-0.825	-0.029
g_O	1241	2534	2638	1339	2549	2678	1220	2534	2673	1305
	(19.2)	(39.1)	(40.7)	(20.7)	(39.4)	(41.4)	(18.8)	(39.1)	(41.3)	(20.2)
g_E	1281.8	2736.9	2605.9	1281.8	2736.9	2605.9	1281.8	2736.9	2605.9	1281.8
	(19.8)	(42.3)	(40.2)	(19.8)	(42.3)	(40.2)	(19.8)	(42.3)	(40.2)	(19.8)
	-80.3	-390.3	64.6	116.9	-362.6	146.1	-120.6	-390.3	135.9	46.8
	-1.240	-6.028	0.997	1.806	-5.599	2.257	-1.862	-6.028	2.098	0.723
c_O	1909	2975	1772	1953	2992	1714	1928	2985	1839	1912
	(29.5)	(45.9)	(27.4)	(30.2)	(46.2)	(26.5)	(29.8)	(46.1)	(28.4)	(29.5)
c_E	1816.9	2901.4	1796.6	1816.9	2901.4	1796.6	1816.9	2901.4	1796.6	1816.9
	(28.1)	(44.8)	(27.7)	(28.1)	(44.8)	(27.7)	(28.1)	(44.8)	(27.7)	(28.1)
	188.7	149.0	-48.9	282.0	183.9	-161.4	228.8	169.5	85.8	195.0
	2.914	2.301	-0.755	4.356	2.841	-2.492	3.533	2.618	1.325	3.011
N	6475	6475	6475	6475	6475	6475	6475	6475	6475	6475
X2	9.5	36.9	0.8	24.2	27.0	7.4	10.8	35.7	9.0	14.3
Gvalue	9.5	36.4	0.8	24.1	26.9	7.4	10.7	35.1	9.1	14.5
Gv/N	0.146	0.561	0.013	0.373	0.416	0.115	0.165	0.542	0.141	0.223
Prob	0.02374	0.00000	0.84607	0.00002	0.00001	0.05959	0.01344	0.00000	0.02754	0.00235

	111	112	113	114	115	116	117	118	119	120
a_O	397	1250	1444	379	1200	1540	420	1202	1624	425
	(6.1)	(19.3)	(22.3)	(5.9)	(18.5)	(23.8)	(6.5)	(18.6)	(25.1)	(6.6)
a_E	362.6	1255.1	1544.1	362.5	1255.1	1544.1	362.5	1254.7	1543.6	362.4
	(5.6)	(19.4)	(23.9)	(5.6)	(19.4)	(23.9)	(5.6)	(19.4)	(23.9)	(5.6)
	72.0	-10.2	-193.5	33.7	-107.8	-8.1	123.6	-103.2	164.9	135.4
	1.112	-0.158	-2.989	0.520	-1.665	-0.125	1.909	-1.595	2.548	2.092
t_O	511	786	1799	531	819	1752	511	842	1772	534
	(7.9)	(12.1)	(27.8)	(8.2)	(12.7)	(27.1)	(7.9)	(13.0)	(27.4)	(8.3)
t_E	474.1	817.0	1831.7	474.0	817.0	1831.7	474.0	816.8	1831.1	473.9
	(7.3)	(12.6)	(28.3)	(7.3)	(12.6)	(28.3)	(7.3)	(12.6)	(28.3)	(7.3)
	76.6	-60.9	-64.8	120.6	3.9	-155.8	76.8	51.2	-116.3	127.6
	1.183	-0.940	-1.000	1.862	0.061	-2.407	1.186	0.791	-1.797	1.971
g_O	2578	2663	1358	2566	2661	1298	2616	2586	1287	2605
	(39.8)	(41.1)	(21.0)	(39.6)	(41.1)	(20.0)	(40.4)	(40.0)	(19.9)	(40.3)
g_E	2736.9	2605.5	1281.6	2736.5	2605.5	1281.6	2736.5	2604.7	1281.2	2735.6
	(42.3)	(40.2)	(19.8)	(42.3)	(40.2)	(19.8)	(42.3)	(40.2)	(19.8)	(42.3)
	-308.3	116.2	157.3	-330.1	112.1	33.0	-235.5	-37.3	11.6	-254.9
	-4.762	1.795	2.429	-5.098	1.732	0.510	-3.638	-0.576	0.180	-3.938
c_O	2989	1775	1873	2998	1794	1884	2927	1842	1789	2908
	(46.2)	(27.4)	(28.9)	(46.3)	(27.7)	(29.1)	(45.2)	(28.5)	(27.6)	(44.9)
c_E	2901.4	1796.3	1816.7	2901.0	1796.3	1816.7	2901.0	1795.8	1816.1	2900.1
	(44.8)	(27.7)	(28.1)	(44.8)	(27.7)	(28.1)	(44.8)	(27.7)	(28.1)	(44.8)
	177.7	-42.4	114.4	197.2	-4.7	137.1	52.3	93.6	-53.8	15.8
	2.745	-0.655	1.767	3.047	-0.072	2.118	0.807	1.446	-0.831	0.245

N	6475	6474	6474	6474	6474	6474	6474	6472	6472	6472
X2	18.0	2.7	13.4	21.5	3.6	6.2	17.5	4.3	6.5	24.7
Gvalue	18.0	2.7	13.4	21.4	3.6	6.2	17.1	4.3	6.5	23.9
Gv/N	0.278	0.042	0.207	0.330	0.056	0.096	0.264	0.067	0.100	0.370
Prob	0.00044	0.43545	0.00382	0.00009	0.30343	0.10219	0.00068	0.22777	0.09048	0.00003

	121	122	123	124	125	126	127	128	129	130
a_O	1131	1545	416	1188	1606	398	1182	1560	424	1152
	(17.5)	(23.9)	(6.4)	(18.4)	(24.8)	(6.2)	(18.3)	(24.1)	(6.6)	(17.8)
a_E	1254.7	1543.6	362.4	1254.3	1543.1	362.3	1254.1	1542.9	362.3	1254.1
	(19.4)	(23.9)	(5.6)	(19.4)	(23.9)	(5.6)	(19.4)	(23.9)	(5.6)	(19.4)
	-234.8	2.8	114.7	-129.1	128.3	74.8	-140.1	34.4	133.5	-195.7
	-3.628	0.044	1.772	-1.995	1.983	1.156	-2.165	0.532	2.063	-3.026
t_O	758	1691	490	797	1764	518	808	1806	512	821
	(11.7)	(26.1)	(7.6)	(12.3)	(27.3)	(8.0)	(12.5)	(27.9)	(7.9)	(12.7)
t_E	816.8	1831.1	473.9	816.5	1830.5	473.7	816.4	1830.3	473.7	816.4
	(12.6)	(28.3)	(7.3)	(12.6)	(28.3)	(7.3)	(12.6)	(28.3)	(7.3)	(12.6)
	-113.2	-269.2	32.8	-38.6	-130.6	92.6	-16.7	-48.2	79.7	9.2
	-1.750	-4.160	0.507	-0.596	-2.019	1.431	-0.258	-0.745	1.232	0.142
g_O	2729	1362	2577	2673	1273	2603	2587	1236	2545	2717
	(42.2)	(21.0)	(39.8)	(41.3)	(19.7)	(40.2)	(40.0)	(19.1)	(39.3)	(42.0)
g_E	2604.7	1281.2	2735.6	2603.9	1280.8	2734.8	2603.5	1280.6	2734.3	2603.5
	(40.2)	(19.8)	(42.3)	(40.2)	(19.8)	(42.3)	(40.2)	(19.8)	(42.3)	(40.2)
	254.4	166.6	-307.8	140.0	-15.6	-257.1	-32.9	-87.6	-365.3	231.9
	3.931	2.574	-4.756	2.164	-0.240	-3.973	-0.509	-1.355	-5.646	3.584
c_O	1854	1874	2989	1812	1827	2951	1892	1867	2988	1779
	(28.6)	(29.0)	(46.2)	(28.0)	(28.2)	(45.6)	(29.2)	(28.9)	(46.2)	(27.5)
c_E	1795.8	1816.1	2900.1	1795.2	1815.5	2899.2	1794.9	1815.3	2898.7	1794.9
	(27.7)	(28.1)	(44.8)	(27.7)	(28.1)	(44.8)	(27.7)	(28.1)	(44.8)	(27.7)
	118.3	117.6	180.5	33.7	23.0	104.5	199.3	104.9	181.2	-31.8
	1.828	1.817	2.789	0.521	0.355	1.616	3.080	1.622	2.801	-0.491
N	6472	6472	6472	6470	6470	6470	6469	6469	6469	6469
X2	24.2	17.7	20.4	6.0	5.1	14.9	9.6	3.5	29.5	13.4
Gvalue	24.7	17.8	20.2	6.0	5.1	14.8	9.6	3.5	29.1	13.6
Gv/N	0.381	0.275	0.312	0.093	0.079	0.229	0.148	0.055	0.450	0.210
Prob	0.00002	0.00048	0.00016	0.11083	0.16483	0.00200	0.02251	0.31501	0.00000	0.00350

	131	132	133	134	135	136	137	138	139	140
a_O	1524	432	1165	1544	397	1229	1498	375	1243	1511
	(23.6)	(6.7)	(18.0)	(23.9)	(6.1)	(19.0)	(23.2)	(5.8)	(19.2)	(23.4)
a_E	1542.9	362.3	1253.9	1542.6	362.2	1253.4	1541.9	362.0	1253.2	1541.7
	(23.9)	(5.6)	(19.4)	(23.9)	(5.6)	(19.4)	(23.9)	(5.6)	(19.4)	(23.9)
	-37.5	152.1	-171.4	2.7	72.8	-48.3	-86.6	26.4	-20.3	-60.8
	-0.580	2.352	-2.650	0.042	1.126	-0.746	-1.339	0.408	-0.313	-0.940
t_O	1743	490	852	1760	530	789	1787	490	803	1770
	(26.9)	(7.6)	(13.2)	(27.2)	(8.2)	(12.2)	(27.6)	(7.6)	(12.4)	(27.4)
t_E	1830.3	473.7	816.3	1830.0	473.6	815.9	1829.1	473.4	815.8	1828.8
	(28.3)	(7.3)	(12.6)	(28.3)	(7.3)	(12.6)	(28.3)	(7.3)	(12.6)	(28.3)
	-170.3	33.3	73.0	-137.2	119.3	-52.9	-83.3	33.9	-25.3	-115.8
	-2.632	0.514	1.128	-2.122	1.845	-0.818	-1.288	0.524	-0.392	-1.791
g_O	1297	2637	2670	1371	2560	2667	1327	2655	2668	1308
	(20.0)	(40.8)	(41.3)	(21.2)	(39.6)	(41.3)	(20.5)	(41.1)	(41.3)	(20.2)
g_E	1280.6	2734.3	2603.1	1280.4	2733.9	2601.9	1279.8	2732.7	2601.5	1279.6
	(19.8)	(42.3)	(40.2)	(19.8)	(42.3)	(40.2)	(19.8)	(42.3)	(40.2)	(19.8)
	33.0	-191.2	135.5	187.5	-336.5	131.8	96.1	-153.1	134.7	57.4
	0.510	-2.955	2.095	2.898	-5.203	2.039	1.486	-2.368	2.084	0.888
c_O	1905	2910	1781	1793	2981	1780	1853	2945	1750	1875
	(29.4)	(45.0)	(27.5)	(27.7)	(46.1)	(27.5)	(28.7)	(45.6)	(27.1)	(29.0)
c_E	1815.3	2898.7	1794.7	1815.0	2898.3	1793.8	1814.1	2897.0	1793.6	1813.9
	(28.1)	(44.8)	(27.7)	(28.1)	(44.8)	(27.7)	(28.1)	(44.8)	(27.7)	(28.1)
	183.8	22.5	-27.2	-43.7	167.7	-27.6	78.5	96.9	-86.1	124.3
	2.842	0.349	-0.421	-0.676	2.593	-0.426	1.215	1.499	-1.331	1.923
N	6469	6469	6468	6468	6468	6465	6465	6465	6464	6464
X2	9.0	17.5	9.7	9.4	23.5	3.1	4.8	4.1	3.0	5.2
Gvalue	9.0	16.7	9.8	9.2	23.4	3.1	4.8	4.1	3.0	5.2
Gv/N	0.140	0.259	0.152	0.143	0.361	0.048	0.074	0.063	0.047	0.080
Prob	0.02885	0.00080	0.02021	0.02623	0.00003	0.37704	0.18814	0.25535	0.38605	0.15829

	141	142	143	144	145	146	147	148	149	150
a_O	412	1212	1572	370	1143	1432	375	1218	1506	387
	(6.4)	(18.8)	(24.3)	(5.7)	(17.7)	(22.2)	(5.8)	(18.9)	(23.3)	(6.0)
a_E	362.0	1252.8	1541.2	361.9	1252.4	1540.7	361.8	1251.4	1539.5	361.5
	(5.6)	(19.4)	(23.9)	(5.6)	(19.4)	(23.9)	(5.6)	(19.4)	(23.9)	(5.6)
	106.7	-80.2	62.2	16.4	-208.9	-209.6	27.0	-66.0	-66.3	52.8
	1.650	-1.242	0.963	0.255	-3.234	-3.245	0.417	-1.022	-1.028	0.818
t_O	526	757	1824	522	834	1863	511	812	1895	531
	(8.1)	(11.7)	(28.2)	(8.1)	(12.9)	(28.8)	(7.9)	(12.6)	(29.4)	(8.2)
t_E	473.3	815.5	1828.3	473.1	815.3	1827.7	473.0	814.6	1826.3	472.6
	(7.3)	(12.6)	(28.3)	(7.3)	(12.6)	(28.3)	(7.3)	(12.6)	(28.3)	(7.3)
	111.1	-112.7	-8.5	102.6	37.9	71.3	79.0	-5.3	140.0	123.7
	1.719	-1.745	-0.132	1.588	0.587	1.103	1.223	-0.082	2.168	1.916
g_O	2587	2694	1238	2667	2664	1292	2623	2623	1265	2578
	(40.0)	(41.7)	(19.2)	(41.3)	(41.2)	(20.0)	(40.6)	(40.6)	(19.6)	(39.9)
g_E	2732.2	2600.7	1279.2	2731.4	2599.9	1278.8	2730.5	2597.9	1277.8	2728.4
	(42.3)	(40.2)	(19.8)	(42.3)	(40.2)	(19.8)	(42.3)	(40.2)	(19.8)	(42.3)
	-282.6	189.9	-81.1	-127.2	129.8	26.5	-210.8	50.5	-25.5	-292.4
	-4.372	2.939	-1.255	-1.969	2.009	0.410	-3.263	0.782	-0.396	-4.530
c_O	2939	1799	1828	2903	1819	1873	2951	1802	1789	2959
	(45.5)	(27.8)	(28.3)	(44.9)	(28.2)	(29.0)	(45.7)	(27.9)	(27.7)	(45.8)
c_E	2896.5	1793.0	1813.3	2895.6	1792.5	1812.7	2894.7	1791.1	1811.3	2892.5
	(44.8)	(27.7)	(28.1)	(44.8)	(27.7)	(28.1)	(44.8)	(27.7)	(28.1)	(44.8)
	85.6	12.0	29.5	14.8	53.5	122.5	113.7	21.9	-44.4	134.6
	1.324	0.186	0.457	0.229	0.828	1.896	1.759	0.340	-0.688	2.085
N	6464	6462	6462	6462	6460	6460	6460	6455	6455	6455
X2	21.1	8.9	2.1	6.8	12.0	10.5	8.9	1.2	3.7	18.8
Gvalue	20.8	9.0	2.1	6.6	12.2	10.7	8.8	1.2	3.7	18.7
Gv/N	0.321	0.139	0.032	0.102	0.189	0.165	0.137	0.019	0.057	0.289
Prob	0.00012	0.02963	0.55544	0.08541	0.00662	0.01375	0.03159	0.74860	0.29639	0.00032

Bcer_ATCC14579
5UTR:

	50	49	48	47	46	45	44	43	42	41
a_O	1837	1845	1861	1888	1887	1931	1969	1907	1883	1913
	(35.0)	(35.1)	(35.4)	(35.9)	(35.9)	(36.7)	(37.5)	(36.3)	(35.8)	(36.4)
a_E	1943.9	1943.9	1943.9	1943.9	1943.9	1943.9	1943.9	1943.9	1943.9	1943.9
	(37.0)	(37.0)	(37.0)	(37.0)	(37.0)	(37.0)	(37.0)	(37.0)	(37.0)	(37.0)
	-207.8	-192.7	-162.3	-110.2	-112.2	-25.8	50.5	-73.1	-119.9	-61.3
	-3.955	-3.667	-3.088	-2.097	-2.134	-0.490	0.961	-1.392	-2.282	-1.167
t_O	1745	1799	1862	1763	1808	1777	1790	1818	1775	1769
	(33.2)	(34.2)	(35.4)	(33.5)	(34.4)	(33.8)	(34.1)	(34.6)	(33.8)	(33.7)
t_E	1657.2	1657.2	1657.2	1657.2	1657.2	1657.2	1657.2	1657.2	1657.2	1657.2
	(31.5)	(31.5)	(31.5)	(31.5)	(31.5)	(31.5)	(31.5)	(31.5)	(31.5)	(31.5)
	180.3	295.5	434.0	218.3	315.0	248.1	276.1	336.8	243.9	231.1
	3.430	5.623	8.259	4.154	5.995	4.722	5.253	6.409	4.641	4.397
g_O	927	923	866	876	873	840	836	889	899	914
	(17.6)	(17.6)	(16.5)	(16.7)	(16.6)	(16.0)	(15.9)	(16.9)	(17.1)	(17.4)
g_E	1023.7	1023.7	1023.7	1023.7	1023.7	1023.7	1023.7	1023.7	1023.7	1023.7
	(19.5)	(19.5)	(19.5)	(19.5)	(19.5)	(19.5)	(19.5)	(19.5)	(19.5)	(19.5)
	-183.9	-191.1	-289.7	-272.9	-278.0	-332.2	-338.6	-250.8	-233.5	-207.2
	-3.500	-3.637	-5.513	-5.194	-5.290	-6.322	-6.444	-4.773	-4.444	-3.942
c_O	746	688	666	728	687	707	660	641	698	659
	(14.2)	(13.1)	(12.7)	(13.9)	(13.1)	(13.5)	(12.6)	(12.2)	(13.3)	(12.5)
c_E	630.2	630.2	630.2	630.2	630.2	630.2	630.2	630.2	630.2	630.2
	(12.0)	(12.0)	(12.0)	(12.0)	(12.0)	(12.0)	(12.0)	(12.0)	(12.0)	(12.0)
	251.6	120.7	73.5	210.0	118.5	162.5	60.9	21.7	142.6	58.8
	4.788	2.296	1.399	3.995	2.255	3.093	1.159	0.413	2.713	1.119
N	5255	5255	5255	5255	5255	5255	5255	5255	5255	5255
X2	40.9	32.4	55.2	44.8	42.7	51.1	46.8	34.2	32.8	21.1
Gvalue	40.1	32.3	55.6	45.1	43.3	52.7	48.8	34.6	33.0	21.4
Gv/N	0.762	0.615	1.057	0.858	0.825	1.002	0.929	0.658	0.628	0.407
Prob	0.00000	0.00000	0.00000	0.00000	0.00000	0.00000	0.00000	0.00000	0.00000	0.00009

	40	39	38	37	36	35	34	33	32	31
a_O	1969	1959	1988	2010	2004	2006	2067	1991	2053	2034
	(37.5)	(37.3)	(37.8)	(38.2)	(38.1)	(38.2)	(39.3)	(37.9)	(39.1)	(38.7)
a_E	1943.9	1943.9	1943.9	1943.9	1943.9	1943.9	1943.9	1943.9	1943.9	1943.9
	(37.0)	(37.0)	(37.0)	(37.0)	(37.0)	(37.0)	(37.0)	(37.0)	(37.0)	(37.0)
	50.5	30.3	89.2	134.4	122.0	126.1	253.8	95.3	224.2	184.3
	0.961	0.576	1.697	2.557	2.322	2.400	4.830	1.813	4.266	3.507
t_O	1731	1811	1753	1796	1734	1715	1721	1778	1669	1726
	(32.9)	(34.5)	(33.4)	(34.2)	(33.0)	(32.6)	(32.7)	(33.8)	(31.8)	(32.8)
t_E	1657.2	1657.2	1657.2	1657.2	1657.2	1657.2	1657.2	1657.2	1657.2	1657.2
	(31.5)	(31.5)	(31.5)	(31.5)	(31.5)	(31.5)	(31.5)	(31.5)	(31.5)	(31.5)
	150.9	321.5	197.1	289.0	157.2	117.7	130.1	250.3	23.8	140.5
	2.872	6.119	3.751	5.500	2.991	2.239	2.476	4.763	0.452	2.674
g_O	905	853	904	843	885	894	857	864	929	871
	(17.2)	(16.2)	(17.2)	(16.0)	(16.8)	(17.0)	(16.3)	(16.4)	(17.7)	(16.6)
g_E	1023.7	1023.7	1023.7	1023.7	1023.7	1023.7	1023.7	1023.7	1023.7	1023.7
	(19.5)	(19.5)	(19.5)	(19.5)	(19.5)	(19.5)	(19.5)	(19.5)	(19.5)	(19.5)
	-223.0	-311.2	-224.8	-327.4	-257.7	-242.2	-304.6	-293.0	-180.3	-281.4
	-4.244	-5.921	-4.278	-6.230	-4.903	-4.609	-5.797	-5.577	-3.431	-5.354
c_O	650	632	610	606	632	640	610	622	604	624
	(12.4)	(12.0)	(11.6)	(11.5)	(12.0)	(12.2)	(11.6)	(11.8)	(11.5)	(11.9)
c_E	630.2	630.2	630.2	630.2	630.2	630.2	630.2	630.2	630.2	630.2
	(12.0)	(12.0)	(12.0)	(12.0)	(12.0)	(12.0)	(12.0)	(12.0)	(12.0)	(12.0)
	40.1	3.5	-39.8	-47.5	3.5	19.7	-39.8	-16.4	-51.4	-12.4
	0.764	0.067	-0.758	-0.905	0.067	0.374	-0.758	-0.312	-0.978	-0.236
N	5255	5255	5255	5255	5255	5255	5255	5255	5255	5255
X2	18.0	42.9	21.2	46.7	24.2	20.6	38.0	35.0	16.1	29.9
Gvalue	18.5	44.2	21.7	48.4	25.1	21.3	39.5	36.2	16.2	31.0
Gv/N	0.352	0.840	0.412	0.922	0.477	0.405	0.751	0.688	0.309	0.590
Prob	0.00035	0.00000	0.00008	0.00000	0.00002	0.00009	0.00000	0.00000	0.00101	0.00000

	30	29	28	27	26	25	24	23	22	21
a_O	2042	2047	2104	2070	2103	2104	2075	2176	2088	2164
	(38.9)	(39.0)	(40.0)	(39.4)	(40.0)	(40.0)	(39.5)	(41.4)	(39.7)	(41.2)
a_E	1943.9	1943.9	1943.9	1943.9	1943.9	1943.9	1943.9	1943.9	1943.9	1943.9
	(37.0)	(37.0)	(37.0)	(37.0)	(37.0)	(37.0)	(37.0)	(37.0)	(37.0)	(37.0)
	201.0	211.5	333.0	260.2	330.8	333.0	270.8	490.8	298.6	464.2
	3.825	4.025	6.337	4.951	6.296	6.337	5.153	9.340	5.682	8.833
t_O	1762	1619	1649	1741	1689	1674	1818	1646	1777	1699
	(33.5)	(30.8)	(31.4)	(33.1)	(32.1)	(31.9)	(34.6)	(31.3)	(33.8)	(32.3)
t_E	1657.2	1657.2	1657.2	1657.2	1657.2	1657.2	1657.2	1657.2	1657.2	1657.2
	(31.5)	(31.5)	(31.5)	(31.5)	(31.5)	(31.5)	(31.5)	(31.5)	(31.5)	(31.5)
	216.2	-75.4	-16.3	171.9	64.3	33.9	336.8	-22.2	248.1	84.7
	4.114	-1.435	-0.310	3.270	1.223	0.644	6.409	-0.423	4.722	1.612
g_O	843	932	860	838	850	833	787	834	786	836
	(16.0)	(17.7)	(16.4)	(15.9)	(16.2)	(15.9)	(15.0)	(15.9)	(15.0)	(15.9)
g_E	1023.7	1023.7	1023.7	1023.7	1023.7	1023.7	1023.7	1023.7	1023.7	1023.7
	(19.5)	(19.5)	(19.5)	(19.5)	(19.5)	(19.5)	(19.5)	(19.5)	(19.5)	(19.5)
	-327.4	-174.9	-299.7	-335.4	-316.1	-343.4	-413.9	-341.8	-415.3	-338.6
	-6.230	-3.328	-5.703	-6.383	-6.015	-6.535	-7.875	-6.505	-7.903	-6.444
c_O	608	657	642	606	613	644	575	599	604	556
	(11.6)	(12.5)	(12.2)	(11.5)	(11.7)	(12.3)	(10.9)	(11.4)	(11.5)	(10.6)
c_E	630.2	630.2	630.2	630.2	630.2	630.2	630.2	630.2	630.2	630.2
	(12.0)	(12.0)	(12.0)	(12.0)	(12.0)	(12.0)	(12.0)	(12.0)	(12.0)	(12.0)
	-43.7	54.6	23.7	-47.5	-34.0	27.8	-105.5	-60.9	-51.4	-139.4
	-0.831	1.040	0.452	-0.905	-0.647	0.529	-2.007	-1.159	-0.978	-2.652
N	5255	5255	5255	5255	5255	5255	5255	5255	5255	5255
X2	44.3	15.7	39.6	47.0	43.6	49.2	84.0	64.5	75.6	69.1
Gvalue	46.1	15.8	40.8	49.0	45.1	51.3	88.3	65.9	80.0	70.9
Gv/N	0.877	0.302	0.776	0.933	0.857	0.975	1.680	1.253	1.523	1.349
Prob	0.00000	0.00122	0.00000	0.00000	0.00000	0.00000	0.00000	0.00000	0.00000	0.00000

	20	19	18	17	16	15	14	13	12	11
a_O	2245	2240	2259	2336	2414	2567	2455	2181	1661	1553
	(42.7)	(42.6)	(43.0)	(44.5)	(45.9)	(48.8)	(46.7)	(41.5)	(31.6)	(29.6)
a_E	1943.9	1943.9	1943.9	1943.9	1943.9	1943.9	1943.9	1943.9	1943.9	1943.9
	(37.0)	(37.0)	(37.0)	(37.0)	(37.0)	(37.0)	(37.0)	(37.0)	(37.0)	(37.0)
	646.6	635.1	678.7	858.4	1045.6	1427.4	1146.1	502.0	-522.5	-697.4
	12.304	12.086	12.915	16.335	19.898	27.163	21.810	9.552	-9.943	-13.270
t_O	1601	1578	1596	1419	1296	1106	895	760	566	554
	(30.5)	(30.0)	(30.4)	(27.0)	(24.7)	(21.0)	(17.0)	(14.5)	(10.8)	(10.5)

t_E	1657.2	1657.2	1657.2	1657.2	1657.2	1657.2	1657.2	1657.2	1657.2	1657.2
	(31.5)	(31.5)	(31.5)	(31.5)	(31.5)	(31.5)	(31.5)	(31.5)	(31.5)	(31.5)
	-110.4	-154.5	-120.0	-440.3	-637.2	-894.4	-1102.7	-1184.9	-1216.1	-1214.0
	-2.101	-2.940	-2.284	-8.379	-12.125	-17.021	-20.984	-22.548	-23.141	-23.102
g_O	861	919	939	1088	1144	1282	1629	2113	2804	2966
	(16.4)	(17.5)	(17.9)	(20.7)	(21.8)	(24.4)	(31.0)	(40.2)	(53.4)	(56.4)
g_E	1023.7	1023.7	1023.7	1023.7	1023.7	1023.7	1023.7	1023.7	1023.7	1023.7
	(19.5)	(19.5)	(19.5)	(19.5)	(19.5)	(19.5)	(19.5)	(19.5)	(19.5)	(19.5)
	-298.0	-198.3	-162.2	132.6	254.3	576.9	1513.5	3062.6	5650.9	6310.5
	-5.671	-3.773	-3.086	2.523	4.838	10.979	28.802	58.280	107.533	120.086
c_O	548	518	461	412	401	300	276	201	224	182
	(10.4)	(9.9)	(8.8)	(7.8)	(7.6)	(5.7)	(5.3)	(3.8)	(4.3)	(3.5)
c_E	630.2	630.2	630.2	630.2	630.2	630.2	630.2	630.2	630.2	630.2
	(12.0)	(12.0)	(12.0)	(12.0)	(12.0)	(12.0)	(12.0)	(12.0)	(12.0)	(12.0)
	-153.2	-203.2	-288.3	-350.3	-362.6	-445.4	-455.8	-459.4	-463.4	-452.1
	-2.916	-3.867	-5.486	-6.665	-6.900	-8.476	-8.673	-8.742	-8.819	-8.604
N	5255	5255	5255	5255	5255	5255	5255	5255	5255	5255
X2	85.1	79.6	105.8	192.9	289.9	621.3	1041.9	1966.1	4117.7	4817.1
Gvalue	84.9	79.2	108.2	200.4	300.1	664.5	1101.1	1920.3	3448.9	3947.0
Gv/N	1.615	1.507	2.059	3.814	5.711	12.646	20.954	36.542	65.630	75.110
Prob	0.00000	0.00000	0.00000	0.00000	0.00000	0.00000	0.00000	1.00000	1.00000	1.00000

	10	9	8	7	6	5	4	3	2	1
a_O	1670	1357	1491	1850	2138	2101	1968	2346	2302	1851
	(31.8)	(25.8)	(28.4)	(35.2)	(40.7)	(40.0)	(37.5)	(44.6)	(43.8)	(35.2)
a_E	1943.9	1943.9	1943.9	1943.9	1943.9	1943.9	1943.9	1943.9	1943.9	1943.9
	(37.0)	(37.0)	(37.0)	(37.0)	(37.0)	(37.0)	(37.0)	(37.0)	(37.0)	(37.0)
	-507.3	-975.5	-791.0	-183.2	406.9	326.5	48.5	882.1	778.4	-181.3
	-9.653	-18.563	-15.052	-3.487	7.744	6.214	0.922	16.786	14.813	-3.451
t_O	657	828	1091	1322	1327	1600	1703	1264	1323	1942
	(12.5)	(15.8)	(20.8)	(25.2)	(25.3)	(30.4)	(32.4)	(24.1)	(25.2)	(37.0)
t_E	1657.2	1657.2	1657.2	1657.2	1657.2	1657.2	1657.2	1657.2	1657.2	1657.2
	(31.5)	(31.5)	(31.5)	(31.5)	(31.5)	(31.5)	(31.5)	(31.5)	(31.5)	(31.5)
	-1215.7	-1149.0	-912.1	-597.4	-589.7	-112.3	92.9	-684.6	-595.9	616.1
	-23.134	-21.865	-17.357	-11.369	-11.221	-2.137	1.769	-13.028	-11.339	11.723
g_O	2735	2840	2350	1700	1382	1046	941	1023	916	682
	(52.0)	(54.0)	(44.7)	(32.4)	(26.3)	(19.9)	(17.9)	(19.5)	(17.4)	(13.0)
g_E	1023.7	1023.7	1023.7	1023.7	1023.7	1023.7	1023.7	1023.7	1023.7	1023.7
	(19.5)	(19.5)	(19.5)	(19.5)	(19.5)	(19.5)	(19.5)	(19.5)	(19.5)	(19.5)
	5375.5	5795.9	3905.8	1724.6	829.6	45.1	-158.5	-1.4	-203.6	-554.0
	102.293	110.293	74.325	32.818	15.786	0.859	-3.016	-0.026	-3.875	-10.542
c_O	193	230	323	383	408	508	643	622	714	780
	(3.7)	(4.4)	(6.1)	(7.3)	(7.8)	(9.7)	(12.2)	(11.8)	(13.6)	(14.8)
c_E	630.2	630.2	630.2	630.2	630.2	630.2	630.2	630.2	630.2	630.2
	(12.0)	(12.0)	(12.0)	(12.0)	(12.0)	(12.0)	(12.0)	(12.0)	(12.0)	(12.0)
	-456.8	-463.7	-431.8	-381.5	-354.8	-219.1	25.8	-16.4	178.2	332.6
	-8.693	-8.824	-8.217	-7.260	-6.752	-4.169	0.491	-0.312	3.391	6.329
N	5255	5255	5255	5255	5255	5255	5255	5255	5255	5255
X2	3806.4	4069.0	2167.2	616.1	288.9	38.9	8.5	176.6	155.8	203.0
Gvalue	3195.8	3207.7	1770.8	562.4	292.0	40.3	8.7	179.7	157.1	213.3
Gv/N	60.814	61.040	33.698	10.702	5.556	0.766	0.165	3.420	2.989	4.060
Prob	1.00000	1.00000	1.00000	0.00000	0.00000	0.00000	0.03393	0.00000	0.00000	0.00000

CDS_INITIAL:

	1	2	3	4	5	6	7	8	9	10
a_O	3957	0	0	2775	2500	2218	2446	2338	2321	2211
	(75.3)	(0.0)	(0.0)	(52.8)	(47.6)	(42.2)	(46.5)	(44.5)	(44.2)	(42.1)
a_E	1671.3	1785.3	1993.6	1671.3	1785.3	1993.6	1671.3	1785.3	1993.6	1671.3
	(31.8)	(34.0)	(37.9)	(31.8)	(34.0)	(37.9)	(31.8)	(34.0)	(37.9)	(31.8)
	6820.8	0.0	0.0	2814.0	1683.5	473.2	1863.0	1261.2	705.9	1237.4
	129.796	0.000	0.000	53.549	32.037	9.004	35.452	24.000	13.432	23.546
t_O	670	5255	1	750	1187	1645	868	1463	1592	1105
	(12.7)	(100.0)	(0.0)	(14.3)	(22.6)	(31.3)	(16.5)	(27.8)	(30.3)	(21.0)
t_E	1049.2	1694.1	1898.2	1049.2	1694.1	1898.2	1049.2	1694.1	1898.2	1049.2
	(20.0)	(32.2)	(36.1)	(20.0)	(32.2)	(36.1)	(20.0)	(32.2)	(36.1)	(20.0)
	-601.0	11897.7	-15.1	-503.5	-844.5	-471.1	-329.1	-429.1	-560.2	114.6
	-11.436	226.407	-0.287	-9.582	-16.070	-8.965	-6.262	-8.166	-10.660	2.181
g_O	628	0	5254	1168	753	903	1196	684	901	1184
	(12.0)	(0.0)	(100.0)	(22.2)	(14.3)	(17.2)	(22.8)	(13.0)	(17.1)	(22.5)
g_E	1728.4	752.6	819.3	1728.4	752.6	819.3	1728.4	752.6	819.3	1728.4
	(32.9)	(14.3)	(15.6)	(32.9)	(14.3)	(15.6)	(32.9)	(14.3)	(15.6)	(32.9)
	-1271.6	0.0	19526.9	-915.5	0.9	175.7	-880.7	-130.7	171.3	-895.8
	-24.197	0.000	371.587	-17.421	0.017	3.343	-16.760	-2.486	3.259	-17.046
c_O	0	0	0	562	815	489	745	770	441	755
	(0.0)	(0.0)	(0.0)	(10.7)	(15.5)	(9.3)	(14.2)	(14.7)	(8.4)	(14.4)
c_E	806.1	1023.1	543.8	806.1	1023.1	543.8	806.1	1023.1	543.8	806.1
	(15.3)	(19.5)	(10.3)	(15.3)	(19.5)	(10.3)	(15.3)	(19.5)	(10.3)	(15.3)
	0.0	0.0	0.0	-405.5	-370.6	-104.0	-117.5	-437.6	-184.9	-98.9
	0.000	0.000	0.000	-7.716	-7.053	-1.979	-2.235	-8.328	-3.518	-1.882
N	5255	5255	5255	5255	5255	5255	5255	5255	5255	5255
X2	4769.5	11045.8	28437.6	1069.7	480.2	73.1	559.0	271.5	130.8	351.9
Gvalue	4948.2	11897.7	19511.8	989.6	469.4	73.8	535.7	263.8	132.0	357.3
Gv/N	94.162	226.407	371.300	18.831	8.932	1.404	10.195	5.020	2.513	6.798
Prob	1.00000	1.00000	1.00000	0.00000	0.00000	0.00000	0.00000	0.00000	0.00000	0.00000

	11	12	13	14	15	16	17	18	19	20
a_O	1943	2270	2125	1948	2246	1985	1818	2169	1966	1752
	(37.0)	(43.2)	(40.4)	(37.1)	(42.7)	(37.8)	(34.6)	(41.3)	(37.4)	(33.3)
a_E	1785.3	1993.6	1671.3	1785.3	1993.6	1671.3	1785.3	1993.6	1671.3	1785.3
	(34.0)	(37.9)	(31.8)	(34.0)	(37.9)	(31.8)	(34.0)	(37.9)	(31.8)	(34.0)
	329.0	589.5	1020.6	339.8	535.5	682.8	66.0	365.8	638.5	-66.0
	6.260	11.217	19.422	6.467	10.190	12.994	1.256	6.961	12.149	-1.255
t_O	1876	1731	1130	1945	1772	1192	1958	1813	1101	2026
	(35.7)	(32.9)	(21.5)	(37.0)	(33.7)	(22.7)	(37.3)	(34.5)	(21.0)	(38.6)
t_E	1694.1	1898.2	1049.2	1694.1	1898.2	1049.2	1694.1	1898.2	1049.2	1694.1
	(32.2)	(36.1)	(20.0)	(32.2)	(36.1)	(20.0)	(32.2)	(36.1)	(20.0)	(32.2)
	382.7	-319.3	167.7	537.3	-243.9	304.3	567.0	-166.6	106.2	725.0
	7.282	-6.076	3.192	10.224	-4.641	5.791	10.789	-3.171	2.021	13.796
g_O	622	812	1218	616	757	1305	693	797	1417	666
	(11.8)	(15.5)	(23.2)	(11.7)	(14.4)	(24.8)	(13.2)	(15.2)	(27.0)	(12.7)
g_E	752.6	819.3	1728.4	752.6	819.3	1728.4	752.6	819.3	1728.4	752.6
	(14.3)	(15.6)	(32.9)	(14.3)	(15.6)	(32.9)	(14.3)	(15.6)	(32.9)	(14.3)
	-237.0	-14.5	-852.5	-246.7	-119.7	-733.4	-114.3	-44.0	-563.0	-162.7
	-4.510	-0.277	-16.223	-4.694	-2.279	-13.956	-2.174	-0.837	-10.713	-3.097

c_O	814	442	782	746	480	773	786	476	771	811
	(15.5)	(8.4)	(14.9)	(14.2)	(9.1)	(14.7)	(15.0)	(9.1)	(14.7)	(15.4)
c_E	1023.1	543.8	806.1	1023.1	543.8	806.1	1023.1	543.8	806.1	1023.1
	(19.5)	(10.3)	(15.3)	(19.5)	(10.3)	(15.3)	(19.5)	(10.3)	(15.3)	(19.5)
	-372.2	-183.3	-47.5	-471.2	-119.9	-64.8	-414.4	-126.9	-68.7	-376.8
	-7.082	-3.488	-0.904	-8.967	-2.281	-1.234	-7.886	-2.414	-1.307	-7.170
N	5255	5255	5255	5255	5255	5255	5255	5255	5255	5255
X2	98.8	72.2	280.8	151.8	52.6	183.4	101.4	28.3	112.1	119.6
Gvalue	102.5	72.3	288.3	159.2	51.9	188.9	104.3	28.3	113.0	119.5
Gv/N	1.950	1.376	5.487	3.029	0.989	3.594	1.985	0.539	2.151	2.274
Prob	0.00000	0.00000	0.00000	0.00000	0.00000	0.00000	0.00000	0.00000	0.00000	0.00000

	21	22	23	24	25	26	27	28	29	30
a_O	2144	1828	1670	2146	1782	1715	2104	1751	1712	2093
	(40.8)	(34.8)	(31.8)	(40.8)	(33.9)	(32.6)	(40.0)	(33.3)	(32.6)	(39.8)
a_E	1993.6	1671.3	1785.3	1993.6	1671.3	1785.3	1993.6	1671.3	1785.3	1993.6
	(37.9)	(31.8)	(34.0)	(37.9)	(31.8)	(34.0)	(37.9)	(31.8)	(34.0)	(37.9)
	311.9	327.6	-223.0	316.2	228.5	-137.8	226.8	163.1	-143.5	203.7
	5.935	6.233	-4.243	6.017	4.348	-2.622	4.316	3.103	-2.731	3.876
t_O	1883	1173	1958	1816	1163	1912	1849	1119	1861	1827
	(35.8)	(22.3)	(37.3)	(34.6)	(22.1)	(36.4)	(35.2)	(21.3)	(35.4)	(34.8)
t_E	1898.2	1049.2	1694.1	1898.2	1049.2	1694.1	1898.2	1049.2	1694.1	1898.2
	(36.1)	(20.0)	(32.2)	(36.1)	(20.0)	(32.2)	(36.1)	(20.0)	(32.2)	(36.1)
	-30.4	261.7	567.0	-160.9	239.6	462.7	-97.2	144.2	349.8	-139.8
	-0.578	4.981	10.789	-3.061	4.559	8.805	-1.850	2.744	6.656	-2.660
g_O	724	1493	709	752	1555	730	777	1623	746	762
	(13.8)	(28.4)	(13.5)	(14.3)	(29.6)	(13.9)	(14.8)	(30.9)	(14.2)	(14.5)
g_E	819.3	1728.4	752.6	819.3	1728.4	752.6	819.3	1728.4	752.6	819.3
	(15.6)	(32.9)	(14.3)	(15.6)	(32.9)	(14.3)	(15.6)	(32.9)	(14.3)	(15.6)
	-179.1	-437.1	-84.5	-128.9	-328.8	-44.4	-82.4	-204.2	-13.1	-110.5
	-3.408	-8.319	-1.609	-2.453	-6.256	-0.845	-1.568	-3.886	-0.248	-2.103
c_O	504	761	918	541	755	898	525	762	936	573
	(9.6)	(14.5)	(17.5)	(10.3)	(14.4)	(17.1)	(10.0)	(14.5)	(17.8)	(10.9)
c_E	543.8	806.1	1023.1	543.8	806.1	1023.1	543.8	806.1	1023.1	543.8
	(10.3)	(15.3)	(19.5)	(10.3)	(15.3)	(19.5)	(10.3)	(15.3)	(19.5)	(10.3)
	-76.7	-87.7	-199.0	-5.7	-98.9	-234.2	-37.0	-85.8	-166.5	59.8
	-1.460	-1.668	-3.786	-0.108	-1.882	-4.456	-0.705	-1.632	-3.168	1.139
N	5255	5255	5255	5255	5255	5255	5255	5255	5255	5255
X2	25.5	63.9	61.9	20.8	40.3	46.8	10.2	17.3	26.9	13.2
Gvalue	25.7	64.5	60.5	20.7	40.4	46.3	10.2	17.3	26.7	13.2
Gv/N	0.490	1.228	1.151	0.394	0.769	0.882	0.194	0.329	0.507	0.252
Prob	0.00001	0.00000	0.00000	0.00012	0.00000	0.00000	0.01712	0.00061	0.00001	0.00418

	31	32	33	34	35	36	37	38	39	40
a_O	1739	1663	2050	1705	1581	2052	1701	1560	1993	1718
	(33.1)	(31.6)	(39.0)	(32.4)	(30.1)	(39.0)	(32.4)	(29.7)	(37.9)	(32.7)
a_E	1671.3	1785.3	1993.6	1671.3	1785.3	1993.6	1671.3	1785.3	1993.6	1671.3
	(31.8)	(34.0)	(37.9)	(31.8)	(34.0)	(37.9)	(31.8)	(34.0)	(37.9)	(31.8)
	138.0	-236.0	114.4	68.0	-384.3	118.5	59.8	-420.9	-1.2	94.6
	2.626	-4.491	2.177	1.294	-7.312	2.255	1.139	-8.009	-0.023	1.800
t_O	1116	1873	1893	1145	1896	1827	1127	1946	1944	1212
	(21.2)	(35.6)	(36.0)	(21.8)	(36.1)	(34.8)	(21.4)	(37.0)	(37.0)	(23.1)
t_E	1049.2	1694.1	1898.2	1049.2	1694.1	1898.2	1049.2	1694.1	1898.2	1049.2
	(20.0)	(32.2)	(36.1)	(20.0)	(32.2)	(36.1)	(20.0)	(32.2)	(36.1)	(20.0)
	137.8	376.1	-10.5	200.2	427.0	-139.8	161.3	539.6	92.6	349.7
	2.623	7.157	-0.199	3.809	8.125	-2.660	3.070	10.267	1.762	6.655
g_O	1635	730	754	1615	765	846	1604	818	807	1564
	(31.1)	(13.9)	(14.3)	(30.7)	(14.6)	(16.1)	(30.5)	(15.6)	(15.4)	(29.8)
g_E	1728.4	752.6	819.3	1728.4	752.6	819.3	1728.4	752.6	819.3	1728.4
	(32.9)	(14.3)	(15.6)	(32.9)	(14.3)	(15.6)	(32.9)	(14.3)	(15.6)	(32.9)
	-181.6	-44.4	-125.3	-219.2	25.1	54.3	-239.6	136.4	-24.4	-312.6
	-3.456	-0.845	-2.384	-4.170	0.478	1.032	-4.559	2.596	-0.465	-5.949
c_O	765	989	558	790	1013	530	823	931	511	761
	(14.6)	(18.8)	(10.6)	(15.0)	(19.3)	(10.1)	(15.7)	(17.7)	(9.7)	(14.5)
c_E	806.1	1023.1	543.8	806.1	1023.1	543.8	806.1	1023.1	543.8	806.1
	(15.3)	(19.5)	(10.3)	(15.3)	(19.5)	(10.3)	(15.3)	(19.5)	(10.3)	(15.3)
	-80.1	-67.0	28.7	-31.9	-20.0	-27.3	34.1	-175.6	-63.7	-87.7
	-1.524	-1.275	0.545	-0.607	-0.381	-0.520	0.649	-3.341	-1.212	-1.668
N	5255	5255	5255	5255	5255	5255	5255	5255	5255	5255
X2	14.1	29.1	7.2	17.2	47.7	5.6	15.6	79.9	3.3	44.7
Gvalue	14.1	28.7	7.3	17.1	47.8	5.6	15.7	79.5	3.3	44.1
Gv/N	0.269	0.546	0.139	0.325	0.909	0.107	0.298	1.513	0.063	0.839
Prob	0.00271	0.00000	0.06265	0.00067	0.00000	0.13169	0.00132	0.00000	0.34691	0.00000

	41	42	43	44	45	46	47	48	49	50
a_O	1536	1994	1732	1619	2025	1679	1626	1992	1653	1528
	(29.2)	(37.9)	(33.0)	(30.8)	(38.5)	(32.0)	(30.9)	(37.9)	(31.5)	(29.1)
a_E	1785.3	1993.6	1671.3	1785.3	1993.6	1671.3	1785.3	1993.6	1671.3	1785.3
	(34.0)	(37.9)	(31.8)	(34.0)	(37.9)	(31.8)	(34.0)	(37.9)	(31.8)	(34.0)
	-462.0	0.8	123.5	-316.6	63.3	15.4	-303.9	-3.2	-36.5	-475.6
	-8.792	0.015	2.350	-6.025	1.205	0.292	-5.784	-0.061	-0.694	-9.050
t_O	1914	1913	1148	1915	1845	1194	1840	1882	1144	1904
	(36.4)	(36.4)	(21.8)	(36.4)	(35.1)	(22.7)	(35.0)	(35.8)	(21.8)	(36.2)
t_E	1694.1	1898.2	1049.2	1694.1	1898.2	1049.2	1694.1	1898.2	1049.2	1694.1
	(32.2)	(36.1)	(20.0)	(32.2)	(36.1)	(20.0)	(32.2)	(36.1)	(20.0)	(32.2)
	467.2	29.6	206.7	469.5	-105.0	308.8	304.0	-32.4	198.0	444.8
	8.891	0.564	3.933	8.933	-1.998	5.876	5.786	-0.616	3.768	8.465
g_O	818	821	1629	789	841	1624	803	830	1707	796
	(15.6)	(15.6)	(31.0)	(15.0)	(16.0)	(30.9)	(15.3)	(15.8)	(32.5)	(15.1)
g_E	752.6	819.3	1728.4	752.6	819.3	1728.4	752.6	819.3	1728.4	752.6
	(14.3)	(15.6)	(32.9)	(14.3)	(15.6)	(32.9)	(14.3)	(15.6)	(32.9)	(14.3)
	136.4	3.4	-192.9	74.6	44.0	-202.3	104.2	21.5	-42.5	89.4
	2.596	0.065	-3.672	1.420	0.837	-3.850	1.983	0.410	-0.809	1.700
c_O	987	527	746	932	544	758	986	551	751	1027
	(18.8)	(10.0)	(14.2)	(17.7)	(10.4)	(14.4)	(18.8)	(10.5)	(14.3)	(19.5)
c_E	1023.1	543.8	806.1	1023.1	543.8	806.1	1023.1	543.8	806.1	1023.1
	(19.5)	(10.3)	(15.3)	(19.5)	(10.3)	(15.3)	(19.5)	(10.3)	(15.3)	(19.5)
	-70.8	-33.2	-115.6	-173.8	0.3	-93.3	-72.8	14.4	-106.4	7.9
	-1.348	-0.631	-2.200	-3.307	0.006	-1.775	-1.385	0.274	-2.024	0.150
N	5255	5255	5255	5255	5255	5255	5255	5255	5255	5255
X2	70.3	0.6	21.7	54.2	2.6	29.2	31.5	0.4	12.8	65.6
Gvalue	70.8	0.6	21.6	53.7	2.6	28.5	31.5	0.4	12.6	66.5
Gv/N	1.347	0.012	0.412	1.022	0.049	0.543	0.600	0.007	0.241	1.265
Prob	0.00000	0.88595	0.00008	0.00000	0.46286	0.00000	0.00000	0.94568	0.00546	0.00000

	51	52	53	54	55	56	57	58	59	60
a_O	2009	1658	1546	1987	1628	1629	1963	1700	1633	1924
	(38.2)	(31.6)	(29.4)	(37.8)	(31.0)	(31.0)	(37.4)	(32.4)	(31.1)	(36.6)
a_E	1993.6	1671.3	1785.3	1993.6	1671.3	1785.3	1993.6	1671.0	1785.0	1993.2
	(37.9)	(31.8)	(34.0)	(37.9)	(31.8)	(34.0)	(37.9)	(31.8)	(34.0)	(37.9)
	30.9	-26.6	-445.0	-13.2	-85.5	-298.5	-60.7	58.5	-290.6	-136.0
	0.588	-0.506	-8.468	-0.251	-1.628	-5.680	-1.156	1.113	-5.531	-2.589
t_O	1893	1208	1933	1858	1161	1858	1909	1189	1798	1953
	(36.0)	(23.0)	(36.8)	(35.4)	(22.1)	(35.4)	(36.3)	(22.6)	(34.2)	(37.2)
t_E	1898.2	1049.2	1694.1	1898.2	1049.2	1694.1	1898.2	1049.0	1693.8	1897.9
	(36.1)	(20.0)	(32.2)	(36.1)	(20.0)	(32.2)	(36.1)	(20.0)	(32.2)	(36.1)
	-10.5	340.6	510.0	-79.6	235.2	343.2	21.6	298.0	214.8	111.8
	-0.199	6.481	9.706	-1.515	4.476	6.531	0.410	5.672	4.087	2.128
g_O	806	1601	782	823	1635	749	829	1649	819	802
	(15.3)	(30.5)	(14.9)	(15.7)	(31.1)	(14.3)	(15.8)	(31.4)	(15.6)	(15.3)
g_E	819.3	1728.4	752.6	819.3	1728.4	752.6	819.3	1728.1	752.4	819.1
	(15.6)	(32.9)	(14.3)	(15.6)	(32.9)	(14.3)	(15.6)	(32.9)	(14.3)	(15.6)
	-26.4	-245.1	60.0	7.4	-181.6	-7.1	19.5	-154.4	138.9	-33.9
	-0.502	-4.665	1.142	0.141	-3.456	-0.135	0.371	-2.939	2.644	-0.646
c_O	547	788	994	587	831	1019	554	716	1004	575
	(10.4)	(15.0)	(18.9)	(11.2)	(15.8)	(19.4)	(10.5)	(13.6)	(19.1)	(10.9)
c_E	543.8	806.1	1023.1	543.8	806.1	1023.1	543.8	806.0	1022.9	543.7
	(10.3)	(15.3)	(19.5)	(10.3)	(15.3)	(19.5)	(10.3)	(15.3)	(19.5)	(10.3)
	6.3	-35.8	-57.3	89.6	50.5	-8.1	20.5	-169.5	-37.4	64.3
	0.120	-0.682	-1.090	1.706	0.962	-0.154	0.390	-3.226	-0.712	1.223
N	5255	5255	5255	5255	5255	5255	5255	5254	5254	5254
X2	0.4	33.9	67.7	4.3	18.9	29.6	0.8	32.9	25.6	6.2
Gvalue	0.4	33.1	67.8	4.2	18.6	29.5	0.8	32.5	25.7	6.1
Gv/N	0.007	0.629	1.290	0.081	0.353	0.561	0.016	0.619	0.489	0.117
Prob	0.94664	0.00000	0.00000	0.23723	0.00034	0.00000	0.84100	0.00000	0.00001	0.10492

	61	62	63	64	65	66	67	68	69	70
a_O	1615	1646	1980	1569	1691	1890	1660	1788	2047	1661
	(30.7)	(31.3)	(37.7)	(29.9)	(32.2)	(36.0)	(31.6)	(34.0)	(39.0)	(31.6)
a_E	1671.0	1785.0	1993.2	1671.0	1785.0	1993.2	1671.0	1785.0	1993.2	1671.0
	(31.8)	(34.0)	(37.9)	(31.8)	(34.0)	(37.9)	(31.8)	(34.0)	(37.9)	(31.8)
	-110.1	-266.8	-26.3	-197.7	-182.9	-201.0	-22.0	6.1	109.0	-20.0
	-2.096	-5.078	-0.501	-3.763	-3.481	-3.826	-0.418	0.116	2.075	-0.380
t_O	1187	1819	1934	1248	1854	1967	1099	1718	1863	1145
	(22.6)	(34.6)	(36.8)	(23.8)	(35.3)	(37.4)	(20.9)	(32.7)	(35.5)	(21.8)
t_E	1049.0	1693.8	1897.9	1049.0	1693.8	1897.9	1049.0	1693.8	1897.9	1049.0
	(20.0)	(32.2)	(36.1)	(20.0)	(32.2)	(36.1)	(20.0)	(32.2)	(36.1)	(20.0)
	293.5	259.5	72.9	433.7	335.2	140.7	102.4	48.8	-69.1	200.6
	5.586	4.939	1.388	8.254	6.379	2.678	1.949	0.929	-1.316	3.818
g_O	1692	826	824	1651	755	847	1713	757	812	1656
	(32.2)	(15.7)	(15.7)	(31.4)	(14.4)	(16.1)	(32.6)	(14.4)	(15.5)	(31.5)
g_E	1728.1	752.4	819.1	1728.1	752.4	819.1	1728.1	752.4	819.1	1728.1
	(32.9)	(14.3)	(15.6)	(32.9)	(14.3)	(15.6)	(32.9)	(14.3)	(15.6)	(32.9)
	-71.3	154.2	9.7	-150.6	5.2	56.6	-30.0	9.2	-14.2	-141.1
	-1.358	2.934	0.185	-2.867	0.099	1.078	-0.571	0.175	-0.271	-2.685
c_O	760	963	516	786	954	550	782	991	532	792
	(14.5)	(18.3)	(9.8)	(15.0)	(18.2)	(10.5)	(14.9)	(18.9)	(10.1)	(15.1)
c_E	806.0	1022.9	543.7	806.0	1022.9	543.7	806.0	1022.9	543.7	806.0
	(15.3)	(19.5)	(10.3)	(15.3)	(19.5)	(10.3)	(15.3)	(19.5)	(10.3)	(15.3)
	-89.2	-116.2	-54.1	-39.4	-133.0	12.6	-47.2	-62.7	-23.2	-27.7
	-1.699	-2.211	-1.029	-0.750	-2.531	0.239	-0.898	-1.194	-0.442	-0.527
N	5254	5254	5254	5254	5254	5254	5254	5254	5254	5254
X2	23.4	30.8	2.2	47.9	24.7	8.9	3.3	1.4	2.4	12.1
Gvalue	22.7	30.7	2.2	45.9	24.5	8.9	3.3	1.4	2.4	11.9
Gv/N	0.433	0.584	0.043	0.874	0.466	0.170	0.062	0.026	0.046	0.226
Prob	0.00005	0.00000	0.52413	0.00000	0.00002	0.03017	0.35135	0.70981	0.49330	0.00778

	71	72	73	74	75	76	77	78	79	80
a_O	1693	1959	1684	1818	2061	1685	1811	1984	1627	1796
	(32.2)	(37.3)	(32.1)	(34.6)	(39.2)	(32.1)	(34.5)	(37.8)	(31.0)	(34.2)
a_E	1785.0	1993.2	1671.0	1785.0	1993.2	1671.0	1785.0	1993.2	1671.0	1785.0
	(34.0)	(37.9)	(31.8)	(34.0)	(37.9)	(31.8)	(34.0)	(37.9)	(31.8)	(34.0)
	-179.1	-67.8	26.1	66.7	137.8	28.1	52.5	-18.4	-86.9	22.2
	-3.408	-1.291	0.496	1.270	2.624	0.534	0.999	-0.350	-1.654	0.422
t_O	1706	1899	1092	1729	1832	1113	1771	1909	1083	1687
	(32.5)	(36.1)	(20.8)	(32.9)	(34.9)	(21.2)	(33.7)	(36.3)	(20.6)	(32.1)
t_E	1693.8	1897.9	1049.0	1693.8	1897.9	1049.0	1693.8	1897.9	1049.0	1693.8
	(32.2)	(36.1)	(20.0)	(32.2)	(36.1)	(20.0)	(32.2)	(36.1)	(20.0)	(32.2)
	24.6	2.2	87.8	71.2	-129.5	131.9	157.9	22.3	69.2	-13.5
	0.467	0.042	1.671	1.355	-2.464	2.511	3.006	0.424	1.316	-0.257
g_O	781	837	1683	712	807	1697	719	822	1732	777
	(14.9)	(15.9)	(32.0)	(13.6)	(15.4)	(32.3)	(13.7)	(15.6)	(33.0)	(14.8)
g_E	752.4	819.1	1728.1	752.4	819.1	1728.1	752.4	819.1	1728.1	752.4
	(14.3)	(15.6)	(32.9)	(14.3)	(15.6)	(32.9)	(14.3)	(15.6)	(32.9)	(14.3)
	58.3	36.1	-88.9	-78.6	-24.1	-61.5	-65.3	5.7	7.9	50.0
	1.109	0.687	-1.692	-1.496	-0.459	-1.171	-1.243	0.109	0.150	0.951
c_O	1074	559	795	995	554	759	953	539	812	994
	(20.4)	(10.6)	(15.1)	(18.9)	(10.5)	(14.4)	(18.1)	(10.3)	(15.5)	(18.9)
c_E	1022.9	543.7	806.0	1022.9	543.7	806.0	1022.9	543.7	806.0	1022.9
	(19.5)	(10.3)	(15.3)	(19.5)	(10.3)	(15.3)	(19.5)	(10.3)	(15.3)	(19.5)
	104.8	30.9	-21.8	-55.0	20.7	-91.1	-134.9	-9.5	12.1	-56.9
	1.994	0.589	-0.414	-1.046	0.394	-1.734	-2.567	-0.180	0.231	-1.083
N	5254	5254	5254	5254	5254	5254	5254	5254	5254	5254
X2	8.5	1.4	3.2	4.3	5.0	7.3	10.2	0.2	2.3	1.7
Gvalue	8.5	1.4	3.2	4.3	5.0	7.3	10.2	0.2	2.3	1.7
Gv/N	0.162	0.027	0.060	0.082	0.095	0.139	0.195	0.003	0.044	0.033
Prob	0.03681	0.70515	0.36511	0.22961	0.17424	0.06290	0.01664	0.98391	0.50928	0.63404

	81	82	83	84	85	86	87	88	89	90
a_O	2011	1721	1856	2040	1666	1792	2013	1650	1785	1955
	(38.3)	(32.8)	(35.3)	(38.8)	(31.7)	(34.1)	(38.3)	(31.4)	(34.0)	(37.2)
a_E	1993.2	1671.0	1785.0	1993.2	1671.0	1785.0	1993.2	1671.0	1785.0	1993.2
	(37.9)	(31.8)	(34.0)	(37.9)	(31.8)	(34.0)	(37.9)	(31.8)	(34.0)	(37.9)
	35.7	101.4	144.9	94.7	-10.0	14.1	39.8	-41.8	0.1	-75.7
	0.680	1.931	2.758	1.802	-0.191	0.269	0.757	-0.795	0.002	-1.441
t_O	1897	1105	1666	1879	1103	1728	1897	1050	1697	1950
	(36.1)	(21.0)	(31.7)	(35.8)	(21.0)	(32.9)	(36.1)	(20.0)	(32.3)	(37.1)
t_E	1897.9	1049.0	1693.8	1897.9	1049.0	1693.8	1897.9	1049.0	1693.8	1897.9
	(36.1)	(20.0)	(32.2)	(36.1)	(20.0)	(32.2)	(36.1)	(20.0)	(32.2)	(36.1)
	-1.8	115.0	-55.1	-37.6	110.8	69.2	-1.8	2.1	6.5	105.6

	-0.034	2.189	-1.048	-0.715	2.109	1.316	-0.034	0.039	0.123	2.011
g_O	805	1630	731	805	1656	738	795	1761	773	783
	(15.3)	(31.0)	(13.9)	(15.3)	(31.5)	(14.0)	(15.1)	(33.5)	(14.7)	(14.9)
g_E	819.1	1728.1	752.4	819.1	1728.1	752.4	819.1	1728.1	752.4	819.1
	(15.6)	(32.9)	(14.3)	(15.6)	(32.9)	(14.3)	(15.6)	(32.9)	(14.3)	(15.6)
	-28.1	-190.4	-42.2	-28.1	-141.1	-28.5	-47.6	66.5	41.7	-70.7
	-0.534	-3.625	-0.803	-0.534	-2.685	-0.543	-0.906	1.266	0.794	-1.345
c_O	541	798	1001	530	829	996	549	793	999	566
	(10.3)	(15.2)	(19.1)	(10.1)	(15.8)	(19.0)	(10.4)	(15.1)	(19.0)	(10.8)
c_E	543.7	806.0	1022.9	543.7	806.0	1022.9	543.7	806.0	1022.9	543.7
	(10.3)	(15.3)	(19.5)	(10.3)	(15.3)	(19.5)	(10.3)	(15.3)	(19.5)	(10.3)
	-5.5	-15.8	-43.3	-27.1	46.7	-53.0	10.6	-25.7	-47.2	45.4
	-0.104	-0.301	-0.824	-0.517	0.890	-1.009	0.201	-0.489	-0.898	0.864
N	5254	5254	5254	5254	5254	5254	5254	5254	5254	5254
X2	0.4	10.1	4.4	1.9	6.5	1.7	1.0	1.1	1.1	4.7
Gvalue	0.4	10.2	4.3	1.9	6.5	1.7	1.0	1.1	1.1	4.7
Gv/N	0.008	0.194	0.083	0.036	0.123	0.032	0.018	0.021	0.021	0.089
Prob	0.93644	0.01716	0.22748	0.59888	0.09159	0.63581	0.80955	0.77700	0.77080	0.19735

	91	92	93	94	95	96	97	98	99	100
a_O	1666	1798	2062	1725	1813	1986	1645	1779	1989	1709
	(31.7)	(34.2)	(39.2)	(32.8)	(34.5)	(37.8)	(31.3)	(33.9)	(37.9)	(32.6)
a_E	1671.0	1785.0	1993.2	1671.0	1785.0	1993.2	1670.1	1783.9	1992.1	1669.4
	(31.8)	(34.0)	(37.9)	(31.8)	(34.0)	(37.9)	(31.8)	(34.0)	(37.9)	(31.8)
	-10.0	26.2	139.9	109.7	56.5	-14.4	-49.8	-9.9	-6.2	80.1
	-0.191	0.499	2.663	2.088	1.076	-0.274	-0.948	-0.188	-0.117	1.525
t_O	1050	1671	1849	1058	1661	1870	1025	1714	1900	1060
	(20.0)	(31.8)	(35.2)	(20.1)	(31.6)	(35.6)	(19.5)	(32.6)	(36.2)	(20.2)
t_E	1049.0	1693.8	1897.9	1049.0	1693.8	1897.9	1048.4	1692.8	1896.8	1048.0
	(20.0)	(32.2)	(36.1)	(20.0)	(32.2)	(36.1)	(20.0)	(32.2)	(36.1)	(20.0)
	2.1	-45.2	-96.5	18.1	-64.9	-55.4	-46.2	42.7	6.4	24.2
	0.039	-0.861	-1.837	0.345	-1.235	-1.054	-0.880	0.813	0.122	0.461
g_O	1720	779	826	1695	736	847	1793	746	815	1750
	(32.7)	(14.8)	(15.7)	(32.3)	(14.0)	(16.1)	(34.1)	(14.2)	(15.5)	(33.3)
g_E	1728.1	752.4	819.1	1728.1	752.4	819.1	1727.1	752.0	818.7	1726.4
	(32.9)	(14.3)	(15.6)	(32.9)	(14.3)	(15.6)	(32.9)	(14.3)	(15.6)	(32.9)
	-16.1	54.1	13.8	-65.5	-32.5	56.6	134.4	-11.9	-7.3	47.5
	-0.306	1.030	0.262	-1.246	-0.618	1.078	2.559	-0.227	-0.140	0.905
c_O	818	1006	517	776	1044	551	788	1012	547	730
	(15.6)	(19.1)	(9.8)	(14.8)	(19.9)	(10.5)	(15.0)	(19.3)	(10.4)	(13.9)
c_E	806.0	1022.9	543.7	806.0	1022.9	543.7	805.5	1022.3	543.4	805.2
	(15.3)	(19.5)	(10.3)	(15.3)	(19.5)	(10.3)	(15.3)	(19.5)	(10.3)	(15.3)
	24.3	-33.5	-52.2	-58.8	42.7	14.6	-34.6	-20.5	7.2	-143.1
	0.462	-0.637	-0.993	-1.119	0.813	0.278	-0.659	-0.390	0.136	-2.727
N	5254	5254	5254	5254	5254	5254	5251	5251	5251	5249
X2	0.2	1.6	5.0	3.6	1.9	1.5	3.8	0.4	0.1	8.4
Gvalue	0.2	1.6	5.0	3.6	1.9	1.5	3.8	0.4	0.1	8.6
Gv/N	0.004	0.031	0.095	0.068	0.036	0.028	0.072	0.008	0.001	0.165
Prob	0.97214	0.65686	0.17093	0.31222	0.59972	0.68904	0.28723	0.93406	0.99707	0.03448

	101	102	103	104	105	106	107	108	109	110
a_O	1758	1980	1640	1813	1975	1701	1808	1945	1587	1725
	(33.5)	(37.7)	(31.3)	(34.6)	(37.6)	(32.4)	(34.5)	(37.1)	(30.3)	(32.9)
a_E	1783.3	1991.3	1668.8	1782.6	1990.6	1668.8	1782.6	1990.6	1667.5	1781.2
	(34.0)	(37.9)	(31.8)	(34.0)	(37.9)	(31.8)	(34.0)	(37.9)	(31.8)	(34.0)
	-50.1	-22.6	-57.1	61.4	-31.0	65.0	51.2	-90.1	-157.1	-110.6
	-0.955	-0.430	-1.088	1.170	-0.591	1.239	0.976	-1.717	-2.996	-2.110
t_O	1686	1892	1129	1649	1924	1030	1703	1952	1086	1699
	(32.1)	(36.0)	(21.5)	(31.4)	(36.7)	(19.6)	(32.5)	(37.2)	(20.7)	(32.4)
t_E	1692.2	1896.1	1047.6	1691.5	1895.4	1047.6	1691.5	1895.4	1046.8	1690.2
	(32.2)	(36.1)	(20.0)	(32.2)	(36.1)	(20.0)	(32.2)	(36.1)	(20.0)	(32.2)
	-12.3	-8.2	169.0	-83.9	57.7	-34.8	23.1	115.0	79.9	17.6
	-0.234	-0.155	3.222	-1.600	1.100	-0.664	0.439	2.191	1.524	0.336
g_O	785	819	1721	740	808	1702	753	789	1775	759
	(15.0)	(15.6)	(32.8)	(14.1)	(15.4)	(32.4)	(14.4)	(15.0)	(33.9)	(14.5)
g_E	751.7	818.4	1725.8	751.4	818.1	1725.8	751.4	818.1	1724.4	750.8
	(14.3)	(15.6)	(32.9)	(14.3)	(15.6)	(32.9)	(14.3)	(15.6)	(32.9)	(14.3)
	68.1	1.3	-9.5	-22.6	-20.0	-47.2	3.2	-57.1	102.6	16.4
	1.297	0.024	-0.181	-0.432	-0.381	-0.899	0.061	-1.088	1.957	0.313
c_O	1020	558	757	1045	540	814	983	561	795	1060
	(19.4)	(10.6)	(14.4)	(19.9)	(10.3)	(15.5)	(18.7)	(10.7)	(15.2)	(20.2)
c_E	1021.9	543.2	804.9	1021.5	543.0	804.9	1021.5	543.0	804.3	1020.7
	(19.5)	(10.3)	(15.3)	(19.5)	(10.3)	(15.3)	(19.5)	(10.3)	(15.3)	(19.5)
	-3.8	29.9	-92.9	47.5	-6.0	18.3	-75.5	36.5	-18.4	80.0
	-0.072	0.570	-1.770	0.906	-0.115	0.349	-1.440	0.696	-0.352	1.526
N	5249	5249	5247	5247	5247	5247	5247	5247	5243	5243
X2	1.9	0.5	9.7	2.3	0.7	1.3	1.9	4.4	6.9	3.4
Gvalue	1.8	0.5	9.6	2.3	0.7	1.3	1.9	4.4	7.0	3.4
Gv/N	0.035	0.009	0.183	0.044	0.013	0.026	0.036	0.083	0.133	0.065
Prob	0.60636	0.92504	0.02237	0.51175	0.87471	0.71850	0.59076	0.22510	0.07251	0.33144

	111	112	113	114	115	116	117	118	119	120
a_O	1991	1657	1809	1950	1575	1798	2005	1625	1799	1899
	(38.0)	(31.6)	(34.5)	(37.2)	(30.1)	(34.3)	(38.3)	(31.1)	(34.4)	(36.3)
a_E	1989.0	1666.6	1780.2	1987.9	1665.3	1778.8	1986.4	1663.7	1777.1	1984.5
	(37.9)	(31.8)	(34.0)	(37.9)	(31.8)	(34.0)	(37.9)	(31.8)	(34.0)	(37.9)
	3.9	-19.1	58.1	-75.1	-175.6	38.5	37.4	-76.5	44.0	-167.2
	0.075	-0.364	1.108	-1.433	-3.354	0.736	0.714	-1.463	0.841	-3.197
t_O	1955	1063	1643	1890	1073	1605	1888	1107	1646	1940
	(37.3)	(20.3)	(31.4)	(36.1)	(20.5)	(30.7)	(36.1)	(21.2)	(31.5)	(37.1)
t_E	1893.9	1046.2	1689.3	1892.8	1045.4	1688.0	1891.4	1044.4	1686.4	1889.6
	(36.1)	(20.0)	(32.2)	(36.1)	(20.0)	(32.2)	(36.1)	(20.0)	(32.2)	(36.1)
	124.1	33.9	-91.2	-5.7	56.0	-161.8	-6.8	128.9	-79.7	102.2
	2.367	0.648	-1.741	-0.108	1.069	-3.090	-0.129	2.465	-1.524	1.953
g_O	785	1748	758	839	1756	789	781	1710	770	824
	(15.0)	(33.4)	(14.5)	(16.0)	(33.5)	(15.1)	(14.9)	(32.7)	(14.7)	(15.8)
g_E	817.4	1723.4	750.4	817.0	1722.1	749.8	816.3	1720.5	749.1	815.6
	(15.6)	(32.9)	(14.3)	(15.6)	(32.9)	(14.3)	(15.6)	(32.9)	(14.3)	(15.6)
	-63.6	49.4	15.3	44.7	68.4	80.3	-69.1	-20.9	42.3	17.0
	-1.212	0.944	0.291	0.852	1.306	1.534	-1.320	-0.400	0.809	0.324
c_O	512	772	1030	561	832	1044	562	789	1016	568
	(9.8)	(14.7)	(19.7)	(10.7)	(15.9)	(19.9)	(10.7)	(15.1)	(19.4)	(10.9)
c_E	542.6	803.8	1020.1	542.3	803.2	1019.4	541.9	802.4	1018.4	541.4
	(10.3)	(15.3)	(19.5)	(10.3)	(15.3)	(19.5)	(10.3)	(15.3)	(19.5)	(10.3)
	-59.5	-62.3	19.8	38.0	58.6	49.9	41.0	-26.6	-4.8	54.6
	-1.134	-1.190	0.378	0.726	1.120	0.952	0.782	-0.509	-0.091	1.043

N	5243	5240	5240	5240	5236	5236	5236	5231	5231	5231
X2	5.0	1.9	1.9	2.0	7.3	6.9	2.5	4.9	1.8	6.4
Gvalue	5.0	1.9	1.9	2.0	7.4	7.0	2.5	4.9	1.8	6.4
Gv/N	0.096	0.037	0.037	0.037	0.141	0.133	0.047	0.093	0.035	0.123
Prob	0.17066	0.58319	0.59061	0.58108	0.06035	0.07339	0.48069	0.18076	0.60980	0.09178

	121	122	123	124	125	126	127	128	129	130
a_O	1542	1773	1954	1598	1764	1943	1605	1840	1950	1605
	(29.5)	(33.9)	(37.4)	(30.6)	(33.7)	(37.2)	(30.7)	(35.2)	(37.3)	(30.8)
a_E	1662.8	1776.1	1983.4	1662.4	1775.8	1983.0	1661.2	1774.4	1981.5	1659.6
	(31.8)	(34.0)	(37.9)	(31.8)	(34.0)	(37.9)	(31.8)	(34.0)	(37.9)	(31.8)
	-232.5	-6.2	-58.3	-126.3	-23.5	-79.1	-110.4	133.6	-62.4	-107.3
	-4.448	-0.119	-1.115	-2.417	-0.449	-1.514	-2.114	2.557	-1.195	-2.057
t_O	1127	1601	1921	1116	1660	1894	1069	1580	1968	1108
	(21.6)	(30.6)	(36.7)	(21.4)	(31.8)	(36.2)	(20.5)	(30.3)	(37.7)	(21.2)
t_E	1043.8	1685.4	1888.5	1043.6	1685.1	1888.1	1042.8	1683.8	1886.7	1041.8
	(20.0)	(32.2)	(36.1)	(20.0)	(32.2)	(36.1)	(20.0)	(32.2)	(36.1)	(20.0)
	172.9	-164.5	65.6	149.8	-49.8	11.7	53.1	-201.0	166.1	136.6
	3.308	-3.146	1.254	2.865	-0.952	0.225	1.017	-3.849	3.180	2.617
g_O	1750	823	795	1729	742	868	1760	788	815	1723
	(33.5)	(15.7)	(15.2)	(33.1)	(14.2)	(16.6)	(33.7)	(15.1)	(15.6)	(33.0)
g_E	1719.5	748.7	815.1	1719.2	748.5	814.9	1717.9	748.0	814.3	1716.2
	(32.9)	(14.3)	(15.6)	(32.9)	(14.3)	(15.6)	(32.9)	(14.3)	(15.6)	(32.9)
	61.5	155.8	-39.7	19.7	-13.0	109.5	85.3	82.2	1.4	13.6
	1.177	2.980	-0.759	0.377	-0.249	2.095	1.633	1.573	0.026	0.261
c_O	809	1031	558	784	1061	522	789	1015	490	782
	(15.5)	(19.7)	(10.7)	(15.0)	(20.3)	(10.0)	(15.1)	(19.4)	(9.4)	(15.0)
c_E	802.0	1017.8	541.1	801.8	1017.6	541.0	801.2	1016.8	540.5	800.4
	(15.3)	(19.5)	(10.3)	(15.3)	(19.5)	(10.3)	(15.3)	(19.5)	(10.3)	(15.3)
	14.1	26.6	34.4	-35.2	88.6	-37.2	-24.2	-3.7	-96.2	-36.4
	0.270	0.508	0.658	-0.674	1.695	-0.712	-0.464	-0.070	-1.842	-0.698
N	5228	5228	5228	5227	5227	5227	5223	5223	5223	5218
X2	16.0	11.8	2.0	8.0	2.4	4.9	3.8	11.0	8.7	6.5
Gvalue	16.1	11.6	2.0	7.9	2.3	4.9	3.8	11.0	8.8	6.4
Gv/N	0.307	0.222	0.039	0.151	0.045	0.093	0.073	0.211	0.169	0.123
Prob	0.00110	0.00882	0.56876	0.04813	0.50600	0.18052	0.28539	0.01154	0.03153	0.09400

	131	132	133	134	135	136	137	138	139	140
a_O	1785	1901	1610	1839	1945	1673	1733	1996	1595	1765
	(34.2)	(36.4)	(30.9)	(35.3)	(37.3)	(32.2)	(33.3)	(38.4)	(30.7)	(34.0)
a_E	1772.7	1979.6	1656.4	1769.3	1975.8	1652.9	1765.6	1971.6	1651.6	1764.2
	(34.0)	(37.9)	(31.8)	(34.0)	(37.9)	(31.8)	(34.0)	(37.9)	(31.8)	(34.0)
	24.6	-154.0	-91.5	142.1	-61.1	40.5	-64.6	49.1	-111.3	1.5
	0.472	-2.951	-1.756	2.728	-1.172	0.778	-1.242	0.945	-2.143	0.030
t_O	1658	1972	1132	1620	1909	1102	1684	1895	1078	1696
	(31.8)	(37.8)	(21.7)	(31.1)	(36.7)	(21.2)	(32.4)	(36.5)	(20.8)	(32.7)
t_E	1682.2	1884.9	1039.8	1678.9	1881.3	1037.6	1675.4	1877.3	1036.8	1674.1
	(32.2)	(36.1)	(20.0)	(32.2)	(36.1)	(20.0)	(32.2)	(36.1)	(20.0)	(32.2)
	-48.0	178.2	192.4	-115.8	55.9	132.8	17.3	35.6	84.0	44.1
	-0.919	3.415	3.694	-2.223	1.073	2.554	0.332	0.684	1.618	0.849
g_O	768	813	1652	780	808	1667	781	808	1724	731
	(14.7)	(15.6)	(31.7)	(15.0)	(15.5)	(32.1)	(15.0)	(15.5)	(33.2)	(14.1)
g_E	747.3	813.5	1712.9	745.8	812.0	1709.3	744.2	810.3	1708.0	743.7
	(14.3)	(15.6)	(32.9)	(14.3)	(15.6)	(32.9)	(14.3)	(15.6)	(32.9)	(14.3)
	42.1	-1.1	-119.7	69.9	-7.9	-83.6	75.3	-4.5	32.2	-25.1
	0.806	-0.021	-2.298	1.342	-0.152	-1.608	1.449	-0.087	0.619	-0.484
c_O	1007	532	814	969	546	755	999	498	796	1001
	(19.3)	(10.2)	(15.6)	(18.6)	(10.5)	(14.5)	(19.2)	(9.6)	(15.3)	(19.3)
c_E	1015.9	540.0	798.9	1013.9	539.0	797.2	1011.8	537.8	796.6	1011.0
	(19.5)	(10.3)	(15.3)	(19.5)	(10.3)	(15.3)	(19.5)	(10.3)	(15.3)	(19.5)
	-17.6	-15.9	30.5	-87.8	14.1	-82.2	-25.4	-76.7	-1.2	-19.9
	-0.338	-0.305	0.585	-1.686	0.271	-1.581	-0.488	-1.475	-0.023	-0.383
N	5218	5218	5208	5208	5208	5197	5197	5197	5193	5193
X2	1.1	7.3	11.9	8.4	1.0	7.5	2.6	3.4	3.7	0.6
Gvalue	1.1	7.2	11.7	8.4	1.0	7.5	2.6	3.5	3.7	0.6
Gv/N	0.021	0.139	0.225	0.161	0.019	0.144	0.050	0.067	0.072	0.012
Prob	0.78148	0.06447	0.00834	0.03905	0.80157	0.05771	0.45801	0.32056	0.29202	0.89598

	141	142	143	144	145	146	147	148	149	150
a_O	1939	1717	1784	1937	1629	1776	1902	1634	1731	1925
	(37.3)	(33.1)	(34.4)	(37.3)	(31.4)	(34.3)	(36.7)	(31.6)	(33.5)	(37.2)
a_E	1970.1	1650.7	1763.2	1968.9	1648.1	1760.5	1965.9	1645.3	1757.4	1962.5
	(37.9)	(31.8)	(34.0)	(37.9)	(31.8)	(34.0)	(37.9)	(31.8)	(34.0)	(37.9)
	-61.7	135.3	41.8	-63.4	-38.0	31.2	-125.7	-22.4	-52.5	-74.3
	-1.187	2.607	0.806	-1.221	-0.734	0.601	-2.426	-0.434	-1.014	-1.435
t_O	1882	1054	1718	1923	1057	1694	1928	1091	1743	1915
	(36.2)	(20.3)	(33.1)	(37.1)	(20.4)	(32.7)	(37.2)	(21.1)	(33.7)	(37.0)
t_E	1875.9	1036.2	1673.1	1874.8	1034.6	1670.6	1871.9	1032.8	1667.7	1868.6
	(36.1)	(20.0)	(32.2)	(36.1)	(20.0)	(32.2)	(36.1)	(20.0)	(32.2)	(36.1)
	12.3	35.9	90.9	97.7	45.3	47.2	113.9	119.6	154.0	93.9
	0.237	0.692	1.752	1.882	0.874	0.911	2.198	2.313	2.978	1.815
g_O	861	1623	740	781	1736	762	798	1622	701	770
	(16.6)	(31.3)	(14.3)	(15.0)	(33.5)	(14.7)	(15.4)	(31.4)	(13.6)	(14.9)
g_E	809.6	1707.0	743.2	809.2	1704.4	742.1	807.9	1701.4	740.8	806.5
	(15.6)	(32.9)	(14.3)	(15.6)	(32.9)	(14.3)	(15.6)	(32.9)	(14.3)	(15.6)
	105.9	-163.8	-6.5	-55.3	63.8	40.3	-19.7	-155.1	-77.4	-71.4
	2.040	-3.156	-0.125	-1.066	1.232	0.778	-0.381	-2.997	-1.497	-1.379
c_O	511	796	948	549	760	950	554	826	998	563
	(9.8)	(15.3)	(18.3)	(10.6)	(14.7)	(18.3)	(10.7)	(16.0)	(19.3)	(10.9)
c_E	537.4	796.1	1010.4	537.1	794.9	1008.9	536.3	793.5	1007.1	535.4
	(10.3)	(15.3)	(19.5)	(10.3)	(15.3)	(19.5)	(10.3)	(15.3)	(19.5)	(10.3)
	-51.5	-0.3	-120.9	24.0	-68.3	-114.2	36.0	66.2	-18.1	56.7
	-0.993	-0.005	-2.329	0.463	-1.317	-2.204	0.694	1.281	-0.350	1.096
N	5193	5190	5190	5190	5182	5182	5182	5173	5173	5173
X2	5.1	7.1	5.3	3.0	2.8	4.4	4.5	8.4	6.0	4.9
Gvalue	5.0	7.1	5.4	3.0	2.8	4.5	4.5	8.4	6.0	4.9
Gv/N	0.097	0.138	0.104	0.058	0.055	0.087	0.086	0.162	0.116	0.096
Prob	0.16987	0.06761	0.14550	0.39098	0.41623	0.21275	0.21529	0.03888	0.11089	0.17595

Bcic_HC
5UTR:

	50	49	48	47	46	45	44	43	42	41
a_O	227	219	221	222	236	220	236	226	229	218
	(38.2)	(36.8)	(37.1)	(37.3)	(39.7)	(37.0)	(39.7)	(38.0)	(38.5)	(36.6)
a_E	228.4	228.4	228.4	228.4	228.4	228.4	228.4	228.4	228.4	228.4
	(38.4)	(38.4)	(38.4)	(38.4)	(38.4)	(38.4)	(38.4)	(38.4)	(38.4)	(38.4)
	−2.7	−18.3	−14.5	−12.5	15.6	−16.4	15.6	−4.7	1.3	−20.2
	−0.453	−3.078	−2.431	−2.105	2.614	−2.755	2.614	−0.786	0.218	−3.399
t_O	221	209	225	214	206	235	213	224	220	216
	(37.1)	(35.1)	(37.8)	(36.0)	(34.6)	(39.5)	(35.8)	(37.6)	(37.0)	(36.3)
t_E	210.4	210.4	210.4	210.4	210.4	210.4	210.4	210.4	210.4	210.4
	(35.4)	(35.4)	(35.4)	(35.4)	(35.4)	(35.4)	(35.4)	(35.4)	(35.4)	(35.4)
	21.7	−2.9	30.1	7.2	−8.8	51.9	5.2	28.0	19.6	11.3
	3.641	−0.479	5.063	1.210	−1.473	8.723	0.869	4.705	3.289	1.897
g_O	77	89	71	76	76	69	88	71	75	90
	(12.9)	(15.0)	(11.9)	(12.8)	(12.8)	(11.6)	(14.8)	(11.9)	(12.6)	(15.1)
g_E	82.8	82.8	82.8	82.8	82.8	82.8	82.8	82.8	82.8	82.8
	(13.9)	(13.9)	(13.9)	(13.9)	(13.9)	(13.9)	(13.9)	(13.9)	(13.9)	(13.9)
	−11.2	12.8	−21.9	−13.1	−13.1	−25.2	10.6	−21.9	−14.9	14.9
	−1.890	2.148	−3.679	−2.200	−2.200	−4.238	1.789	−3.679	−2.505	2.510
c_O	70	78	78	83	77	71	58	74	71	71
	(11.8)	(13.1)	(13.1)	(13.9)	(12.9)	(11.9)	(9.7)	(12.4)	(11.9)	(11.9)
c_E	73.4	73.4	73.4	73.4	73.4	73.4	73.4	73.4	73.4	73.4
	(12.3)	(12.3)	(12.3)	(12.3)	(12.3)	(12.3)	(12.3)	(12.3)	(12.3)	(12.3)
	−6.6	9.5	9.5	20.4	7.4	−4.7	−27.3	1.2	−4.7	−4.7
	−1.111	1.600	1.600	3.435	1.245	−0.788	−4.587	0.208	−0.788	−0.788
N	595	595	595	595	595	595	595	595	595	595
X2	1.1	1.1	3.2	2.1	1.1	5.6	3.8	2.6	1.3	1.3
Gvalue	1.1	1.1	3.3	2.0	1.1	5.6	4.1	2.7	1.3	1.3
Gv/N	0.186	0.190	0.553	0.341	0.185	0.941	0.685	0.448	0.214	0.219
Prob	0.77476	0.76971	0.34939	0.56682	0.77642	0.13268	0.25320	0.44642	0.73524	0.72826

	40	39	38	37	36	35	34	33	32	31
a_O	237	211	213	220	232	225	216	212	236	249
	(39.8)	(35.5)	(35.8)	(37.0)	(39.0)	(37.8)	(36.3)	(35.6)	(39.7)	(41.8)
a_E	228.4	228.4	228.4	228.4	228.4	228.4	228.4	228.4	228.4	228.4
	(38.4)	(38.4)	(38.4)	(38.4)	(38.4)	(38.4)	(38.4)	(38.4)	(38.4)	(38.4)
	17.6	−33.3	−29.6	−16.4	7.4	−6.7	−24.0	−31.5	15.6	43.1
	2.961	−5.605	−4.983	−2.755	1.236	−1.118	−4.037	−5.295	2.614	7.245
t_O	215	244	221	230	216	216	221	227	206	203
	(36.1)	(41.0)	(37.1)	(38.7)	(36.3)	(36.3)	(37.1)	(38.2)	(34.6)	(34.1)
t_E	210.4	210.4	210.4	210.4	210.4	210.4	210.4	210.4	210.4	210.4
	(35.4)	(35.4)	(35.4)	(35.4)	(35.4)	(35.4)	(35.4)	(35.4)	(35.4)	(35.4)
	9.2	72.2	21.7	40.9	11.3	11.3	21.7	34.4	−8.8	−14.6
	1.552	12.139	3.641	6.875	1.897	1.897	3.641	5.783	−1.473	−2.453
g_O	75	67	74	64	68	74	65	77	74	68
	(12.6)	(11.3)	(12.4)	(10.8)	(11.4)	(12.4)	(10.9)	(12.9)	(12.4)	(11.4)
g_E	82.8	82.8	82.8	82.8	82.8	82.8	82.8	82.8	82.8	82.8
	(13.9)	(13.9)	(13.9)	(13.9)	(13.9)	(13.9)	(13.9)	(13.9)	(13.9)	(13.9)
	−14.9	−28.4	−16.7	−33.0	−26.8	−16.7	−31.5	−11.2	−16.7	−26.8
	−2.505	−4.778	−2.805	−5.549	−4.510	−2.805	−5.297	−1.890	−2.805	−4.510
c_O	68	73	87	81	79	80	93	79	79	75
	(11.4)	(12.3)	(14.6)	(13.6)	(13.3)	(13.4)	(15.6)	(13.3)	(13.3)	(12.6)
c_E	73.4	73.4	73.4	73.4	73.4	73.4	73.4	73.4	73.4	73.4
	(12.3)	(12.3)	(12.3)	(12.3)	(12.3)	(12.3)	(12.3)	(12.3)	(12.3)	(12.3)
	−10.4	−0.8	29.6	16.0	11.7	13.8	44.1	11.7	11.7	3.3
	−1.742	−0.129	4.977	2.689	1.958	2.321	7.406	1.958	1.958	0.549
N	595	595	595	595	595	595	595	595	595	595
X2	1.6	9.7	5.0	7.2	3.3	1.7	10.3	3.3	1.7	4.8
Gvalue	1.6	9.7	4.9	7.5	3.5	1.8	10.2	3.3	1.7	4.9
Gv/N	0.268	1.628	0.830	1.259	0.581	0.295	1.711	0.557	0.293	0.831
Prob	0.66110	0.02142	0.17632	0.05783	0.32672	0.62533	0.01708	0.34601	0.62716	0.17585

	30	29	28	27	26	25	24	23	22	21
a_O	235	239	239	211	233	220	248	221	220	229
	(39.5)	(40.2)	(40.2)	(35.5)	(39.2)	(37.0)	(41.7)	(37.1)	(37.0)	(38.5)
a_E	228.4	228.4	228.4	228.4	228.4	228.4	228.4	228.4	228.4	228.4
	(38.4)	(38.4)	(38.4)	(38.4)	(38.4)	(38.4)	(38.4)	(38.4)	(38.4)	(38.4)
	13.5	21.8	21.8	−33.3	9.4	−16.4	40.9	−14.5	−16.4	1.3
	2.267	3.662	3.662	−5.605	1.578	−2.755	6.881	−2.431	−2.755	0.218
t_O	223	214	218	250	233	250	219	219	223	234
	(37.5)	(36.0)	(36.6)	(42.0)	(39.2)	(42.0)	(36.8)	(36.8)	(37.5)	(39.3)
t_E	210.4	210.4	210.4	210.4	210.4	210.4	210.4	210.4	210.4	210.4
	(35.4)	(35.4)	(35.4)	(35.4)	(35.4)	(35.4)	(35.4)	(35.4)	(35.4)	(35.4)
	25.9	7.2	15.4	86.2	47.5	86.2	17.5	17.5	25.9	49.7
	4.349	1.210	2.590	14.479	7.979	14.479	2.938	2.938	4.349	8.350
g_O	74	68	70	61	55	57	52	70	73	68
	(12.4)	(11.4)	(11.8)	(10.3)	(9.2)	(9.6)	(8.7)	(11.8)	(12.3)	(11.4)
g_E	82.8	82.8	82.8	82.8	82.8	82.8	82.8	82.8	82.8	82.8
	(13.9)	(13.9)	(13.9)	(13.9)	(13.9)	(13.9)	(13.9)	(13.9)	(13.9)	(13.9)
	−16.7	−26.8	−23.6	−37.3	−45.0	−42.6	−48.4	−23.6	−18.5	−26.8
	−2.805	−4.510	−3.961	−6.274	−7.571	−7.162	−8.138	−3.961	−3.101	−4.510
c_O	63	74	68	73	74	68	76	85	79	64
	(10.6)	(12.4)	(11.4)	(12.3)	(12.4)	(11.4)	(12.8)	(14.3)	(13.3)	(10.8)
c_E	73.4	73.4	73.4	73.4	73.4	73.4	73.4	73.4	73.4	73.4
	(12.3)	(12.3)	(12.3)	(12.3)	(12.3)	(12.3)	(12.3)	(12.3)	(12.3)	(12.3)
	−19.2	1.2	−10.4	−0.8	1.2	−10.4	5.3	25.0	11.7	−17.5
	−3.231	0.208	−1.742	−0.129	0.208	−1.742	0.895	4.199	1.958	−2.943
N	595	595	595	595	595	595	595	595	595	595
X2	3.4	3.2	3.2	14.5	11.9	16.2	13.6	4.4	2.7	6.5
Gvalue	3.4	3.4	3.3	14.7	13.1	16.8	15.3	4.4	2.7	6.6
Gv/N	0.580	0.569	0.548	2.472	2.195	2.821	2.576	0.745	0.451	1.115
Prob	0.32737	0.33593	0.35282	0.00208	0.00451	0.00078	0.00156	0.21840	0.44339	0.08452

	20	19	18	17	16	15	14	13	12	11
a_O	225	255	255	262	260	247	231	245	237	236
	(37.8)	(42.9)	(42.9)	(44.0)	(43.7)	(41.5)	(38.8)	(41.2)	(39.9)	(39.7)
a_E	228.4	228.4	228.4	228.4	228.4	228.4	228.4	228.4	228.0	228.4
	(38.4)	(38.4)	(38.4)	(38.4)	(38.4)	(38.4)	(38.4)	(38.4)	(38.4)	(38.4)
	−6.7	56.3	56.3	72.0	67.5	38.8	5.3	34.5	18.4	15.6
	−1.118	9.461	9.461	12.106	11.343	6.518	0.895	5.795	3.101	2.614
t_O	215	211	203	208	201	205	201	192	158	133
	(36.1)	(35.5)	(34.1)	(35.0)	(33.8)	(34.5)	(33.8)	(32.3)	(26.6)	(22.4)

t_E	210.4	210.4	210.4	210.4	210.4	210.4	210.4	210.4	210.1	210.4
	(35.4)	(35.4)	(35.4)	(35.4)	(35.4)	(35.4)	(35.4)	(35.4)	(35.4)	(35.4)
	9.2	1.1	-14.6	-4.8	-18.4	-10.7	-18.4	-35.2	-90.0	-122.0
	1.552	0.192	-2.453	-0.812	-3.098	-1.802	-3.098	-5.916	-15.155	-20.511
g_O	79	65	72	65	72	83	99	119	152	182
	(13.3)	(10.9)	(12.1)	(10.9)	(12.1)	(13.9)	(16.6)	(20.0)	(25.6)	(30.6)
g_E	82.8	82.8	82.8	82.8	82.8	82.8	82.8	82.8	82.7	82.8
	(13.9)	(13.9)	(13.9)	(13.9)	(13.9)	(13.9)	(13.9)	(13.9)	(13.9)	(13.9)
	-7.5	-31.5	-20.2	-31.5	-20.2	0.3	35.3	86.2	185.1	286.5
	-1.259	-5.297	-3.392	-5.297	-3.392	0.056	5.933	14.491	31.153	48.156
c_O	76	64	65	60	62	60	64	39	47	44
	(12.8)	(10.8)	(10.9)	(10.1)	(10.4)	(10.1)	(10.8)	(6.6)	(7.9)	(7.4)
c_E	73.4	73.4	73.4	73.4	73.4	73.4	73.4	73.4	73.3	73.4
	(12.3)	(12.3)	(12.3)	(12.3)	(12.3)	(12.3)	(12.3)	(12.3)	(12.3)	(12.3)
	5.3	-17.5	-15.8	-24.2	-20.9	-24.2	-17.5	-49.3	-41.7	-45.0
	0.895	-2.943	-2.651	-4.061	-3.513	-4.061	-2.943	-8.287	-7.024	-7.565
N	595	595	595	595	595	595	595	595	594	595
X2	0.4	8.2	5.7	11.3	8.0	4.1	4.8	34.7	80.8	159.2
Gvalue	0.4	8.4	5.7	11.5	8.0	4.2	4.7	36.2	71.7	135.0
Gv/N	0.071	1.412	0.965	1.935	1.340	0.711	0.787	6.084	12.075	22.693
Prob	0.93599	0.03841	0.12493	0.00925	0.04655	0.23774	0.19663	0.00000	0.00000	0.00000

	10	9	8	7	6	5	4	3	2	1
a_O	253	241	251	275	254	254	267	273	215	179
	(42.5)	(40.5)	(42.2)	(46.2)	(42.7)	(42.7)	(44.9)	(45.9)	(36.1)	(30.1)
a_E	228.4	228.4	228.4	228.4	228.4	228.4	228.4	228.4	228.4	228.4
	(38.4)	(38.4)	(38.4)	(38.4)	(38.4)	(38.4)	(38.4)	(38.4)	(38.4)	(38.4)
	51.9	26.0	47.5	102.2	54.1	54.1	83.5	97.5	-25.9	-87.2
	8.717	4.367	7.979	17.183	9.088	9.088	14.033	16.388	-4.354	-14.651
t_O	125	133	152	174	216	220	224	186	240	259
	(21.0)	(22.4)	(25.5)	(29.2)	(36.3)	(37.0)	(37.6)	(31.3)	(40.3)	(43.5)
t_E	210.4	210.4	210.4	210.4	210.4	210.4	210.4	210.4	210.4	210.4
	(35.4)	(35.4)	(35.4)	(35.4)	(35.4)	(35.4)	(35.4)	(35.4)	(35.4)	(35.4)
	-130.2	-122.0	-98.9	-66.2	11.3	19.6	28.0	-45.9	63.1	107.6
	-21.884	-20.511	-16.619	-11.118	1.897	3.289	4.705	-7.716	10.607	18.080
g_O	183	180	130	96	72	59	57	67	64	54
	(30.8)	(30.3)	(21.8)	(16.1)	(12.1)	(9.9)	(9.6)	(11.3)	(10.8)	(9.1)
g_E	82.8	82.8	82.8	82.8	82.8	82.8	82.8	82.8	82.8	82.8
	(13.9)	(13.9)	(13.9)	(13.9)	(13.9)	(13.9)	(13.9)	(13.9)	(13.9)	(13.9)
	290.1	279.4	117.2	28.3	-20.2	-40.0	-42.6	-28.4	-33.0	-46.2
	48.758	46.958	19.694	4.760	-3.392	-6.729	-7.162	-4.778	-5.549	-7.766
c_O	34	41	62	50	53	62	47	69	76	103
	(5.7)	(6.9)	(10.4)	(8.4)	(8.9)	(10.4)	(7.9)	(11.6)	(12.8)	(17.3)
c_E	73.4	73.4	73.4	73.4	73.4	73.4	73.4	73.4	73.4	73.4
	(12.3)	(12.3)	(12.3)	(12.3)	(12.3)	(12.3)	(12.3)	(12.3)	(12.3)	(12.3)
	-52.3	-47.7	-20.9	-38.4	-34.5	-20.9	-41.9	-8.5	5.3	69.8
	-8.792	-8.023	-3.513	-6.448	-5.797	-3.513	-7.039	-1.429	0.895	11.738
N	595	595	595	595	595	595	595	595	595	595
X2	179.6	157.5	47.1	25.4	10.1	11.9	25.0	14.9	9.3	43.9
Gvalue	159.4	135.6	44.9	26.0	10.7	12.7	27.0	14.7	9.5	44.0
Gv/N	26.798	22.792	7.541	4.376	1.795	2.135	4.538	2.466	1.599	7.400
Prob	0.00000	0.00000	0.00000	0.00001	0.01358	0.00533	0.00001	0.00212	0.02321	0.00000

CDS_INITIAL:

	1	2	3	4	5	6	7	8	9	10
a_O	518	0	1	279	191	230	296	267	244	274
	(87.1)	(0.0)	(0.2)	(46.9)	(32.1)	(38.7)	(49.7)	(44.9)	(41.0)	(46.1)
a_E	195.0	188.3	218.1	195.0	188.3	218.1	195.0	188.3	218.1	195.0
	(32.8)	(31.6)	(36.7)	(32.8)	(31.6)	(36.7)	(32.8)	(31.6)	(36.7)	(32.8)
	1012.2	0.0	-10.8	199.9	5.5	24.3	247.1	186.6	54.7	186.4
	170.122	0.000	-1.810	33.601	0.924	4.091	41.533	31.355	9.187	31.333
t_O	29	595	0	110	192	245	101	174	245	133
	(4.9)	(100.0)	(0.0)	(18.5)	(32.3)	(41.2)	(17.0)	(29.2)	(41.2)	(22.4)
t_E	115.0	195.5	259.2	115.0	195.5	259.2	115.0	195.5	259.2	115.0
	(19.3)	(32.8)	(43.6)	(19.3)	(32.8)	(43.6)	(19.3)	(32.8)	(43.6)	(19.3)
	-79.9	1324.7	0.0	-9.8	-6.9	-27.6	-26.2	-40.5	-27.6	38.6
	-13.430	222.645	0.000	-1.649	-1.151	-4.645	-4.412	-6.801	-4.645	6.495
g_O	48	0	594	110	70	51	95	75	58	78
	(8.1)	(0.0)	(99.8)	(18.5)	(11.8)	(8.6)	(16.0)	(12.6)	(9.7)	(13.1)
g_E	169.0	95.5	64.0	169.0	95.5	64.0	169.0	95.5	64.0	169.0
	(28.4)	(16.1)	(10.8)	(28.4)	(16.1)	(10.8)	(28.4)	(16.1)	(10.8)	(28.4)
	-120.8	0.0	2646.4	-94.5	-43.5	-23.2	-109.5	-36.3	-11.5	-120.6
	-20.311	0.000	444.768	-15.883	-7.317	-3.899	-18.398	-6.101	-1.927	-20.275
c_O	0	0	0	96	142	69	103	79	48	110
	(0.0)	(0.0)	(0.0)	(16.1)	(23.9)	(11.6)	(17.3)	(13.3)	(8.1)	(18.5)
c_E	116.0	115.7	53.6	116.0	115.7	53.6	116.0	115.7	53.6	116.0
	(19.5)	(19.5)	(9.0)	(19.5)	(19.5)	(9.0)	(19.5)	(19.5)	(9.0)	(19.5)
	0.0	0.0	0.0	-36.3	58.1	34.8	-24.4	-60.3	-10.6	-11.6
	0.000	0.000	0.000	-6.100	9.760	5.853	-4.108	-10.141	-1.784	-1.956
N	595	595	595	595	595	595	595	595	595	595
X2	802.1	1216.3	4915.8	60.5	12.9	8.5	87.9	51.4	5.0	84.2
Gvalue	811.5	1324.7	2635.6	59.3	13.2	8.3	87.0	49.5	4.9	92.8
Gv/N	136.381	222.645	442.958	9.970	2.216	1.399	14.615	8.313	0.830	15.597
Prob	0.00000	0.00000	1.00000	0.00000	0.00426	0.03976	0.00000	0.00000	0.17628	0.00000

	11	12	13	14	15	16	17	18	19	20
a_O	205	235	240	200	200	232	190	239	219	188
	(34.5)	(39.5)	(40.3)	(33.6)	(33.7)	(39.0)	(31.9)	(40.2)	(36.8)	(31.6)
a_E	188.3	218.1	195.0	188.3	217.8	195.0	188.3	218.1	195.0	188.3
	(31.6)	(36.7)	(32.8)	(31.6)	(36.7)	(32.8)	(31.6)	(36.7)	(32.8)	(31.6)
	34.9	35.0	99.7	24.2	-34.1	80.7	3.5	43.6	50.9	-0.5
	5.866	5.879	16.757	4.063	-5.735	13.555	0.584	7.335	8.550	-0.091
t_O	229	262	141	232	270	129	237	252	131	221
	(38.5)	(44.0)	(23.7)	(39.0)	(45.5)	(21.7)	(39.8)	(42.4)	(22.0)	(37.1)
t_E	195.5	259.2	115.0	195.5	258.8	115.0	195.5	259.2	115.0	195.5
	(32.8)	(43.6)	(19.3)	(32.8)	(43.6)	(19.3)	(32.8)	(43.6)	(19.3)	(32.8)
	72.5	5.6	57.4	79.5	22.9	29.6	91.4	-14.2	34.1	54.3
	12.192	0.940	9.654	13.367	3.858	4.976	15.353	-2.392	5.730	9.124
g_O	62	48	106	67	58	117	81	49	132	74
	(10.4)	(8.1)	(17.8)	(11.3)	(9.8)	(19.7)	(13.6)	(8.2)	(22.2)	(12.4)
g_E	95.5	64.0	169.0	95.5	63.9	169.0	95.5	64.0	169.0	95.5
	(16.1)	(10.8)	(28.4)	(16.1)	(10.8)	(28.4)	(16.1)	(10.8)	(28.4)	(16.1)
	-53.6	-27.7	-98.9	-47.5	-11.3	-86.1	-26.7	-26.2	-65.3	-37.8
	-9.010	-4.648	-16.625	-7.990	-1.898	-14.467	-4.493	-4.405	-10.970	-6.353

c_O	99	50	108	96	66	117	87	55	113	112
	(16.6)	(8.4)	(18.2)	(16.1)	(11.1)	(19.7)	(14.6)	(9.2)	(19.0)	(18.8)
c_E	115.7	53.6	116.0	115.7	53.5	116.0	115.7	53.6	116.0	115.7
	(19.5)	(9.0)	(19.5)	(19.5)	(9.0)	(19.5)	(19.5)	(9.0)	(19.5)	(19.5)
	-30.9	-7.0	-15.4	-35.9	27.7	2.1	-49.7	2.8	-5.9	-7.4
	-5.199	-1.172	-2.587	-6.034	4.657	0.346	-8.347	0.473	-0.988	-1.237
N	595	595	595	595	594	595	595	595	595	595
X2	21.4	5.6	40.3	19.5	5.4	24.7	18.2	5.8	13.4	8.3
Gvalue	22.9	5.9	42.8	20.3	5.2	26.2	18.4	6.0	13.8	8.6
Gv/N	3.849	0.999	7.199	3.405	0.883	4.409	3.097	1.010	2.323	1.444
Prob	0.00004	0.11435	0.00000	0.00015	0.15477	0.00001	0.00036	0.11103	0.00316	0.03526

	21	22	23	24	25	26	27	28	29	30
a_O	215	216	189	220	233	161	211	218	192	223
	(36.1)	(36.3)	(31.8)	(37.0)	(39.2)	(27.1)	(35.5)	(36.6)	(32.3)	(37.5)
a_E	218.1	195.0	188.3	218.1	195.0	188.3	218.1	195.0	188.3	218.1
	(36.7)	(32.8)	(31.6)	(36.7)	(32.8)	(31.6)	(36.7)	(32.8)	(31.6)	(36.7)
	-6.2	44.2	1.5	3.7	83.0	-50.4	-14.1	48.6	7.5	9.8
	-1.049	7.432	0.246	0.626	13.950	-8.468	-2.362	8.176	1.266	1.650
t_O	252	110	207	259	112	204	255	113	214	242
	(42.4)	(18.5)	(34.8)	(43.5)	(18.8)	(34.3)	(42.9)	(19.0)	(36.0)	(40.7)
t_E	259.2	115.0	195.5	259.2	115.0	195.5	259.2	115.0	195.5	259.2
	(43.6)	(19.3)	(32.8)	(43.6)	(19.3)	(32.8)	(43.6)	(19.3)	(32.8)	(43.6)
	-14.2	-9.8	23.8	-0.4	-6.0	17.5	-8.4	-4.0	38.8	-33.3
	-2.392	-1.649	3.993	-0.073	-1.000	2.934	-1.406	-0.671	6.520	-5.591
g_O	65	146	82	62	126	101	50	141	90	68
	(10.9)	(24.5)	(13.8)	(10.4)	(21.2)	(17.0)	(8.4)	(23.7)	(15.1)	(11.4)
g_E	64.0	169.0	95.5	64.0	169.0	95.5	64.0	169.0	95.5	64.0
	(10.8)	(28.4)	(16.1)	(10.8)	(28.4)	(16.1)	(10.8)	(28.4)	(16.1)	(10.8)
	2.0	-42.8	-25.1	-4.0	-74.0	11.2	-24.7	-51.1	-10.7	8.2
	0.330	-7.186	-4.211	-0.670	-12.441	1.889	-4.156	-8.592	-1.805	1.376
c_O	63	123	117	54	124	129	79	123	99	62
	(10.6)	(20.7)	(19.7)	(9.1)	(20.8)	(21.7)	(13.3)	(20.7)	(16.6)	(10.4)
c_E	53.6	116.0	115.7	53.6	116.0	115.7	53.6	116.0	115.7	53.6
	(9.0)	(19.5)	(19.5)	(9.0)	(19.5)	(19.5)	(9.0)	(19.5)	(19.5)	(9.0)
	20.3	14.5	2.5	0.8	16.6	28.0	61.3	14.5	-30.9	18.0
	3.417	2.431	0.426	0.131	2.788	4.703	10.295	2.431	-5.199	3.030
N	595	595	595	595	595	595	595	595	595	595
X2	1.9	6.0	2.6	0.1	19.0	6.2	15.4	7.8	4.6	2.8
Gvalue	1.8	6.1	2.7	0.1	19.6	6.3	14.1	8.0	4.7	2.8
Gv/N	0.306	1.028	0.454	0.014	3.297	1.058	2.371	1.344	0.782	0.465
Prob	0.61083	0.10612	0.44020	0.99374	0.00020	0.09811	0.00276	0.04612	0.19896	0.42884

	31	32	33	34	35	36	37	38	39	40
a_O	195	173	207	190	161	227	215	165	216	190
	(32.8)	(29.1)	(34.8)	(31.9)	(27.1)	(38.2)	(36.1)	(27.7)	(36.3)	(31.9)
a_E	195.0	188.3	218.1	195.0	188.3	218.1	195.0	188.3	218.1	195.0
	(32.8)	(31.6)	(36.7)	(32.8)	(31.6)	(36.7)	(32.8)	(31.6)	(36.7)	(32.8)
	0.0	-29.3	-21.7	-9.8	-50.4	18.1	42.0	-43.5	-4.3	-9.8
	0.005	-4.919	-3.649	-1.654	-8.468	3.036	7.062	-7.317	-0.717	-1.654
t_O	116	214	268	121	213	236	107	202	246	128
	(19.5)	(36.0)	(45.0)	(20.3)	(35.8)	(39.7)	(18.0)	(33.9)	(41.3)	(21.5)
t_E	115.0	195.5	259.2	115.0	195.5	259.2	115.0	195.5	259.2	115.0
	(19.3)	(32.8)	(43.6)	(19.3)	(32.8)	(43.6)	(19.3)	(32.8)	(43.6)	(19.3)
	2.0	38.8	17.9	12.3	36.6	-44.3	-15.5	13.3	-25.7	27.4
	0.332	6.520	3.002	2.063	6.154	-7.444	-2.598	2.236	-4.328	4.602
g_O	157	91	58	158	111	63	156	108	70	168
	(26.4)	(15.3)	(9.7)	(26.6)	(18.7)	(10.6)	(26.2)	(18.2)	(11.8)	(28.2)
g_E	169.0	95.5	64.0	169.0	95.5	64.0	169.0	95.5	64.0	169.0
	(28.4)	(16.1)	(10.8)	(28.4)	(16.1)	(10.8)	(28.4)	(16.1)	(10.8)	(28.4)
	-23.2	-8.8	-11.5	-21.3	33.3	-2.0	-25.0	26.5	12.5	-2.0
	-3.894	-1.487	-1.927	-3.582	5.598	-0.342	-4.204	4.452	2.099	-0.343
c_O	127	117	62	126	110	69	117	120	63	109
	(21.3)	(19.7)	(10.4)	(21.2)	(18.5)	(11.6)	(19.7)	(20.2)	(10.6)	(18.3)
c_E	116.0	115.7	53.6	116.0	115.7	53.6	116.0	115.7	53.6	116.0
	(19.5)	(19.5)	(9.0)	(19.5)	(19.5)	(9.0)	(19.5)	(19.5)	(9.0)	(19.5)
	23.1	2.5	18.0	20.9	-11.2	34.8	2.1	8.7	20.3	-13.5
	3.876	0.426	3.030	3.511	-1.881	5.853	0.346	1.458	3.417	-2.273
N	595	595	595	595	595	595	595	595	595	595
X2	1.9	3.2	2.7	2.0	8.3	6.9	3.6	4.9	2.9	2.0
Gvalue	1.9	3.2	2.7	2.0	8.4	6.6	3.6	4.9	2.8	2.0
Gv/N	0.319	0.540	0.455	0.338	1.404	1.103	0.605	0.829	0.471	0.332
Prob	0.59326	0.36009	0.43880	0.56991	0.03924	0.08724	0.30816	0.17667	0.42294	0.57725

	41	42	43	44	45	46	47	48	49	50
a_O	177	213	192	169	198	191	173	209	192	182
	(29.7)	(35.8)	(32.3)	(28.4)	(33.3)	(32.1)	(29.1)	(35.1)	(32.3)	(30.6)
a_E	188.3	218.1	195.0	188.3	218.1	195.0	188.3	218.1	195.0	188.3
	(31.6)	(36.7)	(32.8)	(31.6)	(36.7)	(32.8)	(31.6)	(36.7)	(32.8)	(31.6)
	-21.9	-10.2	-5.9	-36.5	-38.4	-7.9	-29.3	-17.9	-5.9	-12.3
	-3.672	-1.709	-0.996	-6.134	-6.449	-1.326	-4.919	-3.008	-0.996	-2.072
t_O	208	260	131	199	262	141	206	254	122	203
	(35.0)	(43.7)	(22.0)	(33.4)	(44.0)	(23.7)	(34.6)	(42.7)	(20.5)	(34.1)
t_E	195.5	259.2	115.0	195.5	259.2	115.0	195.5	259.2	115.0	195.5
	(32.8)	(43.6)	(19.3)	(32.8)	(43.6)	(19.3)	(32.8)	(43.6)	(19.3)	(32.8)
	25.9	1.6	34.1	7.2	5.6	57.4	21.6	-10.3	14.4	15.4
	4.349	0.263	5.730	1.202	0.940	9.654	3.638	-1.736	2.418	2.584
g_O	92	61	151	119	78	145	114	61	151	93
	(15.5)	(10.3)	(25.4)	(20.0)	(13.1)	(24.4)	(19.2)	(10.3)	(25.4)	(15.6)
g_E	95.5	64.0	169.0	95.5	64.0	169.0	95.5	64.0	169.0	95.5
	(16.1)	(10.8)	(28.4)	(16.1)	(10.8)	(28.4)	(16.1)	(10.8)	(28.4)	(16.1)
	-6.9	-5.9	-34.1	52.3	30.8	-44.5	40.3	-5.9	-34.1	-5.0
	-1.166	-0.993	-5.723	8.786	5.176	-7.472	6.772	-0.993	-5.723	-0.840
c_O	118	61	121	108	57	118	102	71	130	117
	(19.8)	(10.3)	(20.3)	(18.2)	(9.6)	(19.8)	(17.1)	(11.9)	(21.8)	(19.7)
c_E	115.7	53.6	116.0	115.7	53.6	116.0	115.7	53.6	116.0	115.7
	(19.5)	(9.0)	(19.5)	(19.5)	(9.0)	(19.5)	(19.5)	(9.0)	(19.5)	(19.5)
	4.6	15.8	10.3	-15.0	7.0	4.1	-25.8	39.9	29.7	2.5
	0.767	2.647	1.725	-2.513	1.174	0.686	-4.333	6.704	4.988	0.426
N	595	595	595	595	595	595	595	595	595	595
X2	1.7	1.3	4.4	8.3	5.2	9.4	7.0	6.3	4.1	0.6
Gvalue	1.7	1.2	4.4	8.0	5.0	9.2	6.9	5.8	4.1	0.6
Gv/N	0.278	0.209	0.736	1.341	0.842	1.543	1.158	0.967	0.687	0.098
Prob	0.64742	0.74237	0.22328	0.04644	0.17112	0.02701	0.07544	0.12427	0.25223	0.90096

	51	52	53	54	55	56	57	58	59	60
a_O	226	184	210	210	180	173	209	187	174	230
	(38.0)	(30.9)	(35.3)	(35.3)	(30.3)	(29.1)	(35.1)	(31.4)	(29.2)	(38.7)
a_E	218.1	195.0	188.3	218.1	195.0	188.3	218.1	195.0	188.3	218.1
	(36.7)	(32.8)	(31.6)	(36.7)	(32.8)	(31.6)	(36.7)	(32.8)	(31.6)	(36.7)
	16.0	-21.3	45.9	-16.0	-28.8	-29.3	-17.9	-15.6	-27.4	24.3
	2.687	-3.586	7.710	-2.686	-4.838	-4.919	-3.008	-2.628	-4.610	4.091
t_O	244	123	197	265	120	212	255	123	200	231
	(41.0)	(20.7)	(33.1)	(44.5)	(20.2)	(35.6)	(42.9)	(20.7)	(33.6)	(38.8)
t_E	259.2	115.0	195.5	259.2	115.0	195.5	259.2	115.0	195.5	259.2
	(43.6)	(19.3)	(32.8)	(43.6)	(19.3)	(32.8)	(43.6)	(19.3)	(32.8)	(43.6)
	-29.5	16.5	3.1	11.7	10.2	34.5	-8.4	16.5	9.2	-53.2
	-4.962	2.775	0.521	1.965	1.711	5.790	-1.406	2.775	1.545	-8.949
g_O	60	182	90	59	174	95	68	169	105	60
	(10.1)	(30.6)	(15.1)	(9.9)	(29.2)	(16.0)	(11.4)	(28.4)	(17.6)	(10.1)
g_E	64.0	169.0	95.5	64.0	169.0	95.5	64.0	169.0	95.5	64.0
	(10.8)	(28.4)	(16.1)	(10.8)	(28.4)	(16.1)	(10.8)	(28.4)	(16.1)	(10.8)
	-7.8	26.9	-10.7	-9.6	10.1	-1.1	8.2	-0.0	19.8	-7.8
	-1.310	4.525	-1.805	-1.621	1.697	-0.179	1.376	-0.008	3.335	-1.310
c_O	65	106	98	61	121	115	63	116	116	74
	(10.9)	(17.8)	(16.5)	(10.3)	(20.3)	(19.3)	(10.6)	(19.5)	(19.5)	(12.4)
c_E	53.6	116.0	115.7	53.6	116.0	115.7	53.6	116.0	115.7	53.6
	(9.0)	(19.5)	(19.5)	(9.0)	(19.5)	(19.5)	(9.0)	(19.5)	(19.5)	(9.0)
	25.0	-19.1	-32.6	15.8	10.3	-1.5	20.3	0.0	0.5	47.7
	4.209	-3.205	-5.481	2.647	1.725	-0.248	3.417	0.008	0.087	8.017
N	595	595	595	595	595	595	595	595	595	595
X2	3.8	3.0	5.6	1.8	1.7	2.6	2.3	0.9	2.1	11.7
Gvalue	3.7	3.0	5.6	1.8	1.8	2.6	2.3	0.9	2.1	11.0
Gv/N	0.624	0.509	0.946	0.305	0.295	0.444	0.379	0.147	0.357	1.850
Prob	0.29409	0.38727	0.13130	0.61151	0.62480	0.44991	0.52115	0.83183	0.54711	0.01170

	61	62	63	64	65	66	67	68	69	70
a_O	187	174	215	210	176	191	205	175	220	204
	(31.4)	(29.2)	(36.1)	(35.3)	(29.6)	(32.1)	(34.5)	(29.4)	(37.0)	(34.3)
a_E	195.0	188.3	218.1	195.0	188.3	218.1	195.0	188.3	218.1	195.0
	(32.8)	(31.6)	(36.7)	(32.8)	(31.6)	(36.7)	(32.8)	(31.6)	(36.7)	(32.8)
	-15.6	-27.4	-6.2	31.2	-23.7	-50.8	20.5	-25.6	3.7	18.4
	-2.628	-4.610	-1.049	5.237	-3.987	-8.531	3.451	-4.299	0.626	3.099
t_O	140	204	264	130	189	274	115	214	242	108
	(23.5)	(34.3)	(44.4)	(21.8)	(31.8)	(46.1)	(19.3)	(36.0)	(40.7)	(18.2)
t_E	115.0	195.5	259.2	115.0	195.5	259.2	115.0	195.5	259.2	115.0
	(19.3)	(32.8)	(43.6)	(19.3)	(32.8)	(43.6)	(19.3)	(32.8)	(43.6)	(19.3)
	55.0	17.5	9.7	31.8	-12.7	30.4	-0.0	38.8	-33.3	-13.6
	9.251	2.934	1.622	5.352	-2.134	5.108	-0.005	6.520	-5.591	-2.285
g_O	164	107	61	141	95	71	159	93	78	159
	(27.6)	(18.0)	(10.3)	(23.7)	(16.0)	(11.9)	(26.7)	(15.6)	(13.1)	(26.7)
g_E	169.0	95.5	64.0	169.0	95.5	64.0	169.0	95.5	64.0	169.0
	(28.4)	(16.1)	(10.8)	(28.4)	(16.1)	(10.8)	(28.4)	(16.1)	(10.8)	(28.4)
	-9.9	24.3	-5.9	-51.1	-1.1	14.7	-19.4	-5.0	30.8	-19.4
	-1.663	4.077	-0.993	-8.592	-0.179	2.467	-3.267	-0.840	5.176	-3.267
c_O	104	110	55	114	135	59	116	113	55	124
	(17.5)	(18.5)	(9.2)	(19.2)	(22.7)	(9.9)	(19.5)	(19.0)	(9.2)	(20.8)
c_E	116.0	115.7	53.6	116.0	115.7	53.6	116.0	115.7	53.6	116.0
	(19.5)	(19.5)	(9.0)	(19.5)	(19.5)	(9.0)	(19.5)	(19.5)	(9.0)	(19.5)
	-22.7	-11.2	2.8	-3.9	41.6	11.3	0.0	-5.4	2.8	16.6
	-3.810	-1.881	0.473	-0.659	6.985	1.899	0.008	-0.910	0.473	2.788
N	595	595	595	595	595	595	595	595	595	595
X2	7.1	3.1	0.3	7.8	4.2	5.5	1.1	2.8	4.2	2.0
Gvalue	6.8	3.1	0.3	8.0	4.1	5.6	1.1	2.8	4.1	2.0
Gv/N	1.149	0.520	0.053	1.338	0.685	0.943	0.187	0.470	0.684	0.336
Prob	0.07731	0.37768	0.95733	0.04683	0.25330	0.13207	0.77410	0.42390	0.25395	0.57311

	71	72	73	74	75	76	77	78	79	80
a_O	181	216	196	180	225	185	186	230	204	173
	(30.4)	(36.3)	(32.9)	(30.3)	(37.8)	(31.1)	(31.3)	(38.7)	(34.3)	(29.1)
a_E	188.3	218.1	195.0	188.3	218.1	195.0	188.3	218.1	195.0	188.3
	(31.6)	(36.7)	(32.8)	(31.6)	(36.7)	(32.8)	(31.6)	(36.7)	(32.8)	(31.6)
	-14.3	-4.3	2.0	-16.2	13.9	-19.4	-4.5	24.3	18.4	-29.3
	-2.396	-0.717	0.342	-2.718	2.340	-3.269	-0.758	4.091	3.099	-4.919
t_O	215	244	128	211	261	117	183	249	107	226
	(36.1)	(41.0)	(21.5)	(35.5)	(43.9)	(19.7)	(30.8)	(41.8)	(18.0)	(38.0)
t_E	195.5	259.2	115.0	195.5	259.2	115.0	195.5	259.2	115.0	195.5
	(32.8)	(43.6)	(19.3)	(32.8)	(43.6)	(19.3)	(32.8)	(43.6)	(19.3)	(32.8)
	41.0	-29.5	27.4	32.3	3.6	4.0	-24.1	-20.0	-15.5	65.6
	6.887	-4.962	4.602	5.427	0.601	0.673	-4.051	-3.366	-2.598	11.030
g_O	95	75	157	78	65	176	102	62	164	84
	(16.0)	(12.6)	(26.4)	(13.1)	(10.9)	(29.6)	(17.1)	(10.4)	(27.6)	(14.1)
g_E	95.5	64.0	169.0	95.5	64.0	169.0	95.5	64.0	169.0	95.5
	(16.1)	(10.8)	(28.4)	(16.1)	(10.8)	(28.4)	(16.1)	(10.8)	(28.4)	(16.1)
	-1.1	23.7	-23.2	-31.6	2.0	14.2	13.4	-4.0	-9.9	-21.6
	-0.179	3.988	-3.894	-5.316	0.330	2.393	2.245	-0.670	-1.663	-3.633
c_O	104	60	114	126	44	117	124	54	120	112
	(17.5)	(10.1)	(19.2)	(21.2)	(7.4)	(19.7)	(20.8)	(9.1)	(20.2)	(18.8)
c_E	115.7	53.6	116.0	115.7	53.6	116.0	115.7	53.6	116.0	115.7
	(19.5)	(9.0)	(19.5)	(19.5)	(9.0)	(19.5)	(19.5)	(9.0)	(19.5)	(19.5)
	-22.2	13.5	-3.9	21.4	-17.4	2.1	17.1	0.8	8.2	-7.4
	-3.739	2.271	-0.659	3.597	-2.922	0.346	2.873	0.131	1.376	-1.237
N	595	595	595	595	595	595	595	595	595	595
X2	3.4	3.6	2.4	5.7	2.0	0.8	1.8	1.1	1.3	7.5
Gvalue	3.4	3.4	2.3	5.9	2.1	0.8	1.8	1.1	1.3	7.4
Gv/N	0.574	0.579	0.391	0.990	0.349	0.142	0.310	0.186	0.214	1.242
Prob	0.33223	0.32771	0.50698	0.11697	0.55664	0.83809	0.60570	0.77497	0.73595	0.06045

	81	82	83	84	85	86	87	88	89	90
a_O	229	196	195	222	176	169	237	192	183	202
	(38.6)	(32.9)	(32.8)	(37.3)	(29.6)	(28.4)	(39.8)	(32.3)	(30.8)	(33.9)
a_E	217.8	195.0	188.3	218.1	195.0	188.3	218.1	195.0	188.3	218.1
	(36.7)	(32.8)	(31.6)	(36.7)	(32.8)	(31.6)	(36.7)	(32.8)	(31.6)	(36.7)
	23.0	2.0	13.7	7.8	-36.1	-36.5	39.3	-5.9	-10.4	-31.1
	3.874	0.342	2.302	1.307	-6.060	-6.134	6.604	-0.996	-1.746	-5.221
t_O	254	114	182	269	136	215	251	128	210	285
	(42.8)	(19.2)	(30.6)	(45.2)	(22.9)	(36.1)	(42.2)	(21.5)	(35.3)	(47.9)
t_E	258.8	115.0	195.5	259.2	115.0	195.5	259.2	115.0	195.5	259.2
	(43.6)	(19.3)	(32.8)	(43.6)	(19.3)	(32.8)	(43.6)	(19.3)	(32.8)	(43.6)
	-9.5	-2.0	-26.0	19.9	45.6	41.0	-16.2	27.4	30.1	54.0

	-1.595	-0.340	-4.364	3.349	7.661	6.887	-2.718	4.602	5.066	9.084
g_O	58	157	96	59	165	86	53	159	87	57
	(9.8)	(26.4)	(16.1)	(9.9)	(27.7)	(14.5)	(8.9)	(26.7)	(14.6)	(9.6)
g_E	63.9	169.0	95.5	64.0	169.0	95.5	64.0	169.0	95.5	64.0
	(10.8)	(28.4)	(16.1)	(10.8)	(28.4)	(16.1)	(10.8)	(28.4)	(16.1)	(10.8)
	-11.3	-23.2	0.9	-9.6	-7.9	-18.1	-20.0	-19.4	-16.3	-13.3
	-1.898	-3.894	0.157	-1.621	-1.336	-3.039	-3.367	-3.267	-2.736	-2.227
c_O	53	128	122	45	118	125	54	116	115	51
	(8.9)	(21.5)	(20.5)	(7.6)	(19.8)	(21.0)	(9.1)	(19.5)	(19.3)	(8.6)
c_E	53.5	116.0	115.7	53.6	116.0	115.7	53.6	116.0	115.7	53.6
	(9.0)	(19.5)	(19.5)	(9.0)	(19.5)	(19.5)	(9.0)	(19.5)	(19.5)	(9.0)
	-1.0	25.3	12.9	-15.8	4.1	19.2	0.8	0.0	-1.5	-5.1
	-0.175	4.244	2.160	-2.649	0.686	3.234	0.131	0.008	-0.248	-0.856
N	594	595	595	595	595	595	595	595	595	595
X2	1.2	2.1	1.5	2.2	5.8	5.6	3.8	2.1	2.0	4.7
Gvalue	1.2	2.1	1.5	2.3	5.7	5.6	3.9	2.1	2.0	4.6
Gv/N	0.207	0.352	0.256	0.387	0.951	0.948	0.650	0.347	0.335	0.780
Prob	0.74620	0.55271	0.67743	0.51221	0.12948	0.13039	0.27593	0.55881	0.57321	0.20022

	91	92	93	94	95	96	97	98	99	100
a_O	185	172	227	185	150	197	179	203	203	180
	(31.1)	(28.9)	(38.2)	(31.1)	(25.2)	(33.1)	(30.1)	(34.2)	(34.2)	(30.4)
a_E	195.0	188.3	218.1	195.0	188.3	218.1	194.7	188.0	217.8	194.3
	(32.8)	(31.6)	(36.7)	(32.8)	(31.6)	(36.7)	(32.8)	(31.6)	(36.7)	(32.8)
	-19.4	-31.1	18.1	-19.4	-68.2	-40.2	-30.0	31.3	-28.5	-27.6
	-3.269	-5.225	3.036	-3.269	-11.458	-6.751	-5.054	5.264	-4.803	-4.650
t_O	117	194	255	124	226	277	121	187	273	116
	(19.7)	(32.6)	(42.9)	(20.8)	(38.0)	(46.6)	(20.4)	(31.5)	(46.0)	(19.6)
t_E	115.0	195.5	259.2	115.0	195.5	259.2	114.8	195.1	258.8	114.6
	(19.3)	(32.8)	(43.6)	(19.3)	(32.8)	(43.6)	(19.3)	(32.8)	(43.6)	(19.3)
	4.0	-2.9	-8.4	18.7	65.6	36.8	12.7	-15.9	29.2	2.8
	0.673	-0.488	-1.406	3.135	11.030	6.178	2.135	-2.679	4.916	0.465
g_O	169	98	62	177	101	66	169	109	65	188
	(28.4)	(16.5)	(10.4)	(29.7)	(17.0)	(11.1)	(28.5)	(18.4)	(10.9)	(31.7)
g_E	169.0	95.5	64.0	169.0	95.5	64.0	168.7	95.4	63.9	168.5
	(28.4)	(16.1)	(10.8)	(28.4)	(16.1)	(10.8)	(28.4)	(16.1)	(10.8)	(28.4)
	-0.0	5.0	-4.0	16.3	11.2	4.0	0.5	29.1	2.2	41.3
	-0.008	0.840	-0.670	2.744	1.889	0.674	0.088	4.901	0.367	6.960
c_O	124	131	51	109	118	55	125	95	53	109
	(20.8)	(22.0)	(8.6)	(18.3)	(19.8)	(9.2)	(21.0)	(16.0)	(8.9)	(18.4)
c_E	116.0	115.7	53.6	116.0	115.7	53.6	115.8	115.5	53.5	115.6
	(19.5)	(19.5)	(9.0)	(19.5)	(19.5)	(9.0)	(19.5)	(19.5)	(9.0)	(19.5)
	16.6	32.4	-5.1	-13.5	4.6	2.8	19.2	-37.2	-1.0	-12.8
	2.788	5.453	-0.856	-2.273	0.767	0.473	3.224	-6.263	-0.175	-2.157
N	595	595	595	595	595	595	594	594	594	593
X2	1.1	3.5	0.6	2.0	12.9	3.4	2.3	7.1	1.8	3.7
Gvalue	1.1	3.5	0.6	2.0	13.3	3.4	2.3	7.3	1.8	3.7
Gv/N	0.184	0.580	0.104	0.337	2.229	0.573	0.393	1.224	0.306	0.618
Prob	0.77780	0.32715	0.89233	0.57167	0.00411	0.33290	0.50551	0.06381	0.61128	0.29969

	101	102	103	104	105	106	107	108	109	110
a_O	176	202	201	164	205	179	179	199	204	179
	(29.7)	(34.1)	(34.0)	(27.7)	(34.6)	(30.2)	(30.2)	(33.6)	(34.5)	(30.3)
a_E	187.6	217.4	194.0	187.3	217.0	194.0	187.3	217.0	194.0	187.0
	(31.6)	(36.7)	(32.8)	(31.6)	(36.7)	(32.8)	(31.6)	(36.7)	(32.8)	(31.6)
	-22.5	-29.7	14.2	-43.6	-23.4	-28.8	-16.3	-34.5	20.5	-15.7
	-3.800	-5.009	2.406	-7.366	-3.954	-4.867	-2.748	-5.835	3.463	-2.650
t_O	172	272	123	203	263	121	192	266	115	196
	(29.0)	(45.9)	(20.8)	(34.3)	(44.4)	(20.4)	(32.4)	(44.9)	(19.4)	(33.2)
t_E	194.8	258.3	114.4	194.5	257.9	114.4	194.5	257.9	114.4	194.1
	(32.8)	(43.6)	(19.3)	(32.8)	(43.6)	(19.3)	(32.8)	(43.6)	(19.3)	(32.8)
	-42.8	28.0	17.8	17.4	10.3	13.5	-4.9	16.4	1.1	3.7
	-7.221	4.725	2.999	2.944	1.736	2.280	-0.829	2.775	0.191	0.632
g_O	116	56	156	111	69	176	109	61	150	90
	(19.6)	(9.4)	(26.4)	(18.8)	(11.7)	(29.7)	(18.4)	(10.3)	(25.3)	(15.2)
g_E	95.2	63.8	168.2	95.1	63.7	168.2	95.1	63.7	168.2	94.9
	(16.1)	(10.8)	(28.4)	(16.1)	(10.8)	(28.4)	(16.1)	(10.8)	(28.4)	(16.1)
	45.8	-14.6	-23.4	34.4	11.0	16.0	29.8	-5.3	-34.3	-9.5
	7.726	-2.466	-3.959	5.816	1.862	2.706	5.042	-0.894	-5.795	-1.612
c_O	129	63	112	114	55	116	112	66	123	126
	(21.8)	(10.6)	(18.9)	(19.3)	(9.3)	(19.6)	(18.9)	(11.1)	(20.8)	(21.3)
c_E	115.4	53.4	115.4	115.2	53.3	115.4	115.2	53.3	115.4	115.0
	(19.5)	(9.0)	(19.5)	(19.5)	(9.0)	(19.5)	(19.5)	(9.0)	(19.5)	(19.5)
	28.9	20.8	-6.7	-2.3	3.4	1.2	-6.2	28.1	15.7	23.1
	4.865	3.500	-1.129	-0.389	0.569	0.206	-1.052	4.748	2.653	3.909
N	593	593	592	592	592	592	592	592	592	591
X2	9.5	4.5	1.9	6.0	1.3	1.9	2.5	4.9	3.0	1.7
Gvalue	9.3	4.4	1.9	5.9	1.3	1.9	2.4	4.7	3.0	1.6
Gv/N	1.570	0.750	0.317	1.005	0.213	0.325	0.413	0.794	0.513	0.279
Prob	0.02543	0.21718	0.59798	0.11408	0.73851	0.58856	0.48488	0.19494	0.38572	0.64827

	111	112	113	114	115	116	117	118	119	120
a_O	237	207	178	212	186	197	215	201	204	214
	(40.0)	(35.0)	(30.1)	(35.8)	(31.4)	(33.3)	(36.3)	(34.0)	(34.5)	(36.2)
a_E	217.0	194.0	187.3	217.0	194.0	187.3	217.0	193.7	187.0	216.7
	(36.7)	(32.8)	(31.6)	(36.7)	(32.8)	(31.6)	(36.7)	(32.8)	(31.6)	(36.7)
	41.7	26.8	-18.2	-10.0	-15.7	19.9	-4.1	14.9	35.5	-5.3
	7.042	4.535	-3.069	-1.684	-2.647	3.353	-0.688	2.525	6.005	-0.901
t_O	247	117	196	263	130	216	268	107	205	267
	(41.7)	(19.8)	(33.1)	(44.4)	(22.0)	(36.5)	(45.3)	(18.1)	(34.7)	(45.2)
t_E	257.9	114.4	194.5	257.9	114.4	194.5	257.9	114.2	194.1	257.5
	(43.6)	(19.3)	(32.8)	(43.6)	(19.3)	(32.8)	(43.6)	(19.3)	(32.8)	(43.6)
	-21.4	5.2	3.1	10.3	33.2	45.4	20.6	-14.0	22.3	19.4
	-3.607	0.876	0.519	1.736	5.601	7.662	3.474	-2.371	3.775	3.282
g_O	56	159	108	53	175	94	59	170	80	64
	(9.5)	(26.9)	(18.2)	(9.0)	(29.6)	(15.9)	(10.0)	(28.8)	(13.5)	(10.8)
g_E	63.7	168.2	95.1	63.7	168.2	95.1	63.7	167.9	94.9	63.6
	(10.8)	(28.4)	(16.1)	(10.8)	(28.4)	(16.1)	(10.8)	(28.4)	(16.1)	(10.8)
	-14.4	-17.8	27.6	-19.5	13.9	-2.1	-9.1	4.3	-27.3	0.8
	-2.438	-3.012	4.659	-3.294	2.353	-0.354	-1.529	0.720	-4.622	0.137
c_O	52	109	110	64	101	85	50	113	102	46
	(8.8)	(18.4)	(18.6)	(10.8)	(17.1)	(14.4)	(8.4)	(19.1)	(17.3)	(7.8)
c_E	53.3	115.4	115.2	53.3	115.4	115.2	53.3	115.2	115.0	53.3
	(9.0)	(19.5)	(19.5)	(9.0)	(19.5)	(19.5)	(9.0)	(19.5)	(19.5)	(9.0)
	-2.6	-12.4	-10.1	23.3	-26.9	-51.6	-6.5	-4.4	-24.4	-13.5
	-0.447	-2.098	-1.703	3.939	-4.545	-8.720	-1.093	-0.736	-4.129	-2.279

N	592	592	592	592	592	592	592	591	591	591
X2	3.3	1.8	2.5	4.1	4.5	10.8	1.0	0.8	6.0	1.4
Gvalue	3.3	1.8	2.4	4.1	4.5	11.5	1.0	0.8	6.1	1.4
Gv/N	0.550	0.301	0.407	0.697	0.762	1.942	0.165	0.137	1.030	0.240
Prob	0.35400	0.61944	0.49237	0.24806	0.21140	0.00932	0.80635	0.84672	0.10751	0.70099

	121	122	123	124	125	126	127	128	129	130
a_O	201	187	220	184	186	216	202	194	213	189
	(34.0)	(31.6)	(37.2)	(31.1)	(31.5)	(36.5)	(34.2)	(32.9)	(36.1)	(32.0)
a_E	193.7	187.0	216.7	193.7	187.0	216.7	193.3	186.7	216.3	193.3
	(32.8)	(31.6)	(36.7)	(32.8)	(31.6)	(36.7)	(32.8)	(31.6)	(36.7)	(32.8)
	14.9	-0.0	6.7	-18.9	-2.0	-1.4	17.7	14.9	-6.6	-8.6
	2.525	-0.001	1.133	-3.191	-0.339	-0.229	2.998	2.527	-1.114	-1.457
t_O	99	191	263	117	189	280	116	179	257	114
	(16.8)	(32.3)	(44.5)	(19.8)	(32.0)	(47.4)	(19.7)	(30.3)	(43.6)	(19.3)
t_E	114.2	194.1	257.5	114.2	194.1	257.5	114.0	193.8	257.0	114.0
	(19.3)	(32.8)	(43.6)	(19.3)	(32.8)	(43.6)	(19.3)	(32.8)	(43.6)	(19.3)
	-28.4	-6.2	11.2	5.6	-10.1	47.0	3.9	-28.5	-0.1	-0.1
	-4.798	-1.055	1.890	0.944	-1.717	7.947	0.667	-4.824	-0.013	-0.017
g_O	161	94	58	168	106	54	154	91	64	174
	(27.2)	(15.9)	(9.8)	(28.4)	(17.9)	(9.1)	(26.1)	(15.4)	(10.8)	(29.5)
g_E	167.9	94.9	63.6	167.9	94.9	63.6	167.6	94.7	63.5	167.6
	(28.4)	(16.1)	(10.8)	(28.4)	(16.1)	(10.8)	(28.4)	(16.1)	(10.8)	(28.4)
	-13.5	-1.8	-10.7	0.2	23.5	-17.7	-26.1	-7.3	1.0	13.0
	-2.282	-0.300	-1.808	0.038	3.971	-2.989	-4.419	-1.240	0.174	2.209
c_O	130	119	50	122	110	41	118	126	56	113
	(22.0)	(20.1)	(8.5)	(20.6)	(18.6)	(6.9)	(20.0)	(21.4)	(9.5)	(19.2)
c_E	115.2	115.0	53.3	115.2	115.0	53.3	115.0	114.8	53.2	115.0
	(19.5)	(19.5)	(9.0)	(19.5)	(19.5)	(9.0)	(19.5)	(19.5)	(9.0)	(19.5)
	31.4	8.2	-6.3	14.0	-9.7	-21.4	6.1	23.5	5.8	-4.0
	5.318	1.390	-1.066	2.369	-1.643	-3.628	1.029	3.988	0.988	-0.673

N	591	591	591	591	591	591	590	590	590	590
X2	4.5	0.2	0.9	1.0	1.7	6.2	1.6	2.7	0.2	0.4
Gvalue	4.5	0.2	0.9	1.0	1.6	6.5	1.6	2.7	0.2	0.4
Gv/N	0.764	0.034	0.149	0.161	0.273	1.101	0.276	0.451	0.035	0.064
Prob	0.21084	0.97764	0.83060	0.81294	0.65655	0.08935	0.65317	0.44725	0.97693	0.94543

	131	132	133	134	135	136	137	138	139	140
a_O	192	232	176	191	229	174	195	226	178	186
	(32.5)	(39.3)	(29.8)	(32.4)	(38.8)	(29.5)	(33.1)	(38.3)	(30.2)	(31.5)
a_E	186.7	216.3	193.3	186.7	216.3	193.3	186.7	216.3	193.3	186.7
	(31.6)	(36.7)	(32.8)	(31.6)	(36.7)	(32.8)	(31.6)	(36.7)	(32.8)	(31.6)
	10.8	32.5	-33.1	8.7	26.1	-36.7	17.0	19.8	-29.4	-1.4
	1.826	5.507	-5.608	1.479	4.425	-6.218	2.880	3.357	-4.990	-0.233
t_O	177	244	123	183	249	111	196	259	128	172
	(30.0)	(41.4)	(20.8)	(31.0)	(42.2)	(18.8)	(33.2)	(43.9)	(21.7)	(29.2)
t_E	193.8	257.0	114.0	193.8	257.0	114.0	193.8	257.0	114.0	193.8
	(32.8)	(43.6)	(19.3)	(32.8)	(43.6)	(19.3)	(32.8)	(43.6)	(19.3)	(32.8)
	-32.1	-25.4	18.6	-21.0	-15.8	-6.0	4.4	3.9	29.5	-41.1
	-5.445	-4.306	3.150	-3.561	-2.682	-1.020	0.745	0.667	5.007	-6.962
g_O	102	58	161	92	69	173	89	49	163	102
	(17.3)	(9.8)	(27.3)	(15.6)	(11.7)	(29.3)	(15.1)	(8.3)	(27.6)	(17.3)
g_E	94.7	63.5	167.6	94.7	63.5	167.6	94.7	63.5	167.6	94.7
	(16.1)	(10.8)	(28.4)	(16.1)	(10.8)	(28.4)	(16.1)	(10.8)	(28.4)	(16.1)
	15.1	-10.5	-12.9	-5.4	11.5	11.0	-11.1	-25.4	-9.1	15.1
	2.556	-1.778	-2.194	-0.912	1.947	1.859	-1.883	-4.303	-1.539	2.556
c_O	119	56	130	124	43	132	110	56	121	130
	(20.2)	(9.5)	(22.0)	(21.0)	(7.3)	(22.4)	(18.6)	(9.5)	(20.5)	(22.0)
c_E	114.8	53.2	115.0	114.8	53.2	115.0	114.8	53.2	115.0	114.8
	(19.5)	(9.0)	(19.5)	(19.5)	(9.0)	(19.5)	(19.5)	(9.0)	(19.5)	(19.5)
	8.6	5.8	31.9	19.2	-18.2	36.4	-9.3	5.8	12.3	32.4
	1.461	0.988	5.402	3.252	-3.092	6.168	-1.582	0.988	2.085	5.492

N	590	590	590	590	590	590	590	590	590	590
X2	2.3	2.4	4.5	1.5	3.4	4.7	0.9	3.9	3.4	5.0
Gvalue	2.4	2.4	4.4	1.5	3.5	4.7	0.9	4.2	3.3	5.0
Gv/N	0.398	0.411	0.751	0.257	0.598	0.789	0.160	0.709	0.564	0.854
Prob	0.50289	0.48956	0.21866	0.67842	0.31705	0.19887	0.81477	0.24241	0.34381	0.16911

	141	142	143	144	145	146	147	148	149	150
a_O	220	201	176	207	188	193	207	185	176	216
	(37.3)	(34.1)	(29.8)	(35.1)	(31.9)	(32.8)	(35.1)	(31.4)	(29.9)	(36.7)
a_E	216.3	193.3	186.7	216.3	193.0	186.4	215.9	193.0	186.4	215.9
	(36.7)	(32.8)	(31.6)	(36.7)	(32.8)	(31.6)	(36.7)	(32.8)	(31.6)	(36.7)
	7.4	15.6	-20.8	-18.2	-9.9	13.5	-17.5	-15.7	-20.2	0.1
	1.261	2.645	-3.517	-3.087	-1.682	2.290	-2.973	-2.665	-3.422	0.019
t_O	252	120	211	271	112	191	270	105	183	269
	(42.7)	(20.3)	(35.8)	(45.9)	(19.0)	(32.4)	(45.8)	(17.8)	(31.1)	(45.7)
t_E	257.0	114.0	193.8	257.0	113.9	193.5	256.6	113.9	193.5	256.6
	(43.6)	(19.3)	(32.8)	(43.6)	(19.3)	(32.8)	(43.6)	(19.3)	(32.8)	(43.6)
	-10.0	12.2	35.9	28.7	-3.7	-4.9	27.5	-17.0	-20.4	25.4
	-1.691	2.069	6.077	4.859	-0.625	-0.838	4.666	-2.887	-3.462	4.309
g_O	68	151	98	58	173	85	57	181	102	58
	(11.5)	(25.6)	(16.6)	(9.8)	(29.4)	(14.4)	(9.7)	(30.7)	(17.3)	(9.8)
g_E	63.5	167.6	94.7	63.5	167.3	94.6	63.4	167.3	94.6	63.4
	(10.8)	(28.4)	(16.1)	(10.8)	(28.4)	(16.1)	(10.8)	(28.4)	(16.1)	(10.8)
	9.3	-31.5	6.6	-10.5	11.6	-18.1	-12.1	28.5	15.4	-10.3
	1.583	-5.340	1.127	-1.778	1.962	-3.079	-2.054	4.831	2.619	-1.747
c_O	50	118	105	54	116	120	55	118	128	46
	(8.5)	(20.0)	(17.8)	(9.2)	(19.7)	(20.4)	(9.3)	(20.0)	(21.7)	(7.8)
c_E	53.2	115.0	114.8	53.2	114.8	114.6	53.1	114.8	114.6	53.1
	(9.0)	(19.5)	(19.5)	(9.0)	(19.5)	(19.5)	(9.0)	(19.5)	(19.5)	(9.0)
	-6.1	6.1	-18.7	1.7	2.4	11.1	3.9	6.5	28.4	-13.2
	-1.039	1.029	-3.166	0.287	0.407	1.886	0.667	1.099	4.816	-2.233

N	590	590	590	590	589	589	589	589	589	589
X2	0.7	2.3	3.1	1.6	0.4	1.5	1.8	2.2	3.3	2.0
Gvalue	0.7	2.4	3.1	1.7	0.4	1.5	1.8	2.2	3.3	2.0
Gv/N	0.113	0.404	0.521	0.280	0.062	0.258	0.305	0.377	0.552	0.347
Prob	0.88092	0.49669	0.38062	0.64732	0.94734	0.67741	0.61546	0.52751	0.35450	0.56277

Bcla_KSMK16
5UTR:

	50	49	48	47	46	45	44	43	42	41
a_O	1218	1244	1243	1267	1303	1302	1232	1268	1260	1276
	(29.8)	(30.4)	(30.4)	(31.0)	(31.9)	(31.9)	(30.1)	(31.0)	(30.8)	(31.2)
a_E	1314.3	1314.3	1314.3	1314.3	1314.3	1314.3	1314.3	1314.3	1314.3	1314.3
	(32.2)	(32.2)	(32.2)	(32.2)	(32.2)	(32.2)	(32.2)	(32.2)	(32.2)	(32.2)
	-185.4	-136.8	-138.7	-92.9	-22.5	-24.5	-159.3	-90.9	-106.3	-75.5
	-4.535	-3.346	-3.393	-2.272	-0.551	-0.599	-3.899	-2.225	-2.602	-1.847
t_O	1150	1201	1220	1162	1196	1249	1208	1190	1176	1158
	(28.1)	(29.4)	(29.9)	(28.4)	(29.3)	(30.6)	(29.6)	(29.1)	(28.8)	(28.3)
t_E	1099.0	1099.0	1099.0	1099.0	1099.0	1099.0	1099.0	1099.0	1099.0	1099.0
	(26.9)	(26.9)	(26.9)	(26.9)	(26.9)	(26.9)	(26.9)	(26.9)	(26.9)	(26.9)
	104.4	213.2	254.9	129.6	202.3	319.6	228.5	189.4	159.3	121.1
	2.553	5.217	6.236	3.170	4.951	7.820	5.591	4.633	3.898	2.964
g_O	967	868	848	941	855	835	894	859	909	918
	(23.7)	(21.2)	(20.7)	(23.0)	(20.9)	(20.4)	(21.9)	(21.0)	(22.2)	(22.5)
g_E	975.9	975.9	975.9	975.9	975.9	975.9	975.9	975.9	975.9	975.9
	(23.9)	(23.9)	(23.9)	(23.9)	(23.9)	(23.9)	(23.9)	(23.9)	(23.9)	(23.9)
	-17.8	-203.4	-238.3	-68.6	-226.2	-260.4	-156.8	-219.2	-129.1	-112.3
	-0.435	-4.978	-5.830	-1.678	-5.535	-6.372	-3.836	-5.364	-3.160	-2.749
c_O	752	774	776	717	733	701	753	770	742	735
	(18.4)	(18.9)	(19.0)	(17.5)	(17.9)	(17.2)	(18.4)	(18.8)	(18.2)	(18.0)
c_E	697.8	697.8	697.8	697.8	697.8	697.8	697.8	697.8	697.8	697.8
	(17.1)	(17.1)	(17.1)	(17.1)	(17.1)	(17.1)	(17.1)	(17.1)	(17.1)	(17.1)
	112.5	160.5	164.9	38.9	72.2	6.4	114.7	151.6	91.2	76.4
	2.753	3.926	4.034	0.953	1.766	0.157	2.806	3.710	2.231	1.869
N	4087	4087	4087	4087	4087	4087	4087	4087	4087	4087
X2	13.7	33.5	42.7	7.1	25.4	41.0	27.2	30.6	15.0	9.7
Gvalue	13.8	33.5	42.8	7.1	25.8	41.1	27.1	30.8	15.0	9.7
Gv/N	0.337	0.819	1.047	0.173	0.632	1.007	0.662	0.754	0.367	0.237
Prob	0.00326	0.00000	0.00000	0.07009	0.00001	0.00000	0.00001	0.00000	0.00182	0.02132

	40	39	38	37	36	35	34	33	32	31
a_O	1291	1332	1361	1380	1366	1347	1371	1354	1399	1429
	(31.6)	(32.6)	(33.3)	(33.8)	(33.4)	(33.0)	(33.5)	(33.1)	(34.2)	(35.0)
a_E	1314.3	1314.3	1314.3	1314.3	1314.3	1314.3	1314.3	1314.3	1314.3	1314.3
	(32.2)	(32.2)	(32.2)	(32.2)	(32.2)	(32.2)	(32.2)	(32.2)	(32.2)	(32.2)
	-46.2	35.6	95.0	134.6	105.4	66.2	115.8	80.6	174.7	239.1
	-1.130	0.872	2.325	3.294	2.579	1.620	2.834	1.972	4.276	5.851
t_O	1223	1187	1134	1170	1166	1121	1185	1171	1070	1113
	(29.9)	(29.0)	(27.7)	(28.6)	(28.5)	(27.4)	(29.0)	(28.7)	(26.2)	(27.2)
t_E	1099.0	1099.0	1099.0	1099.0	1099.0	1099.0	1099.0	1099.0	1099.0	1099.0
	(26.9)	(26.9)	(26.9)	(26.9)	(26.9)	(26.9)	(26.9)	(26.9)	(26.9)	(26.9)
	261.5	182.9	71.1	146.5	138.0	44.5	178.6	148.6	-57.2	28.2
	6.399	4.475	1.740	3.585	3.377	1.088	4.370	3.637	-1.400	0.690
g_O	865	856	900	851	852	875	850	858	900	884
	(21.2)	(20.9)	(22.0)	(20.8)	(20.8)	(21.4)	(20.8)	(21.0)	(22.0)	(21.6)
g_E	975.9	975.9	975.9	975.9	975.9	975.9	975.9	975.9	975.9	975.9
	(23.9)	(23.9)	(23.9)	(23.9)	(23.9)	(23.9)	(23.9)	(23.9)	(23.9)	(23.9)
	-208.7	-224.5	-145.8	-233.1	-231.4	-191.0	-234.8	-221.0	-145.8	-174.9
	-5.107	-5.492	-3.567	-5.704	-5.662	-4.674	-5.746	-5.407	-3.567	-4.279
c_O	708	712	692	686	703	744	681	704	718	661
	(17.3)	(17.4)	(16.9)	(16.8)	(17.2)	(18.2)	(16.7)	(17.2)	(17.6)	(16.2)
c_E	697.8	697.8	697.8	697.8	697.8	697.8	697.8	697.8	697.8	697.8
	(17.1)	(17.1)	(17.1)	(17.1)	(17.1)	(17.1)	(17.1)	(17.1)	(17.1)	(17.1)
	20.6	28.7	-11.5	-23.4	10.5	95.4	-33.2	12.5	41.0	-71.6
	0.503	0.702	-0.282	-0.572	0.256	2.335	-0.812	0.305	1.003	-1.752
N	4087	4087	4087	4087	4087	4087	4087	4087	4087	4087
X2	27.2	22.3	8.7	24.1	21.9	14.8	25.8	20.2	12.7	20.8
Gvalue	27.2	22.8	8.9	24.6	22.5	15.1	26.4	20.7	12.8	20.8
Gv/N	0.665	0.557	0.217	0.603	0.550	0.368	0.645	0.507	0.312	0.510
Prob	0.00001	0.00005	0.03125	0.00002	0.00005	0.00177	0.00001	0.00012	0.00518	0.00011

	30	29	28	27	26	25	24	23	22	21
a_O	1354	1426	1474	1383	1455	1522	1451	1470	1457	1495
	(33.1)	(34.9)	(36.1)	(33.8)	(35.6)	(37.2)	(35.5)	(36.0)	(35.6)	(36.6)
a_E	1314.3	1314.3	1314.3	1314.3	1314.3	1314.3	1314.3	1314.3	1314.3	1314.3
	(32.2)	(32.2)	(32.2)	(32.2)	(32.2)	(32.2)	(32.2)	(32.2)	(32.2)	(32.2)
	80.6	232.6	338.1	140.9	296.0	446.6	287.1	329.2	300.4	385.2
	1.972	5.692	8.272	3.448	7.241	10.928	7.026	8.054	7.349	9.424
t_O	1150	1082	1117	1168	1139	1175	1192	1168	1198	1175
	(28.1)	(26.5)	(27.3)	(28.6)	(27.9)	(28.7)	(29.2)	(28.6)	(29.3)	(28.7)
t_E	1099.0	1099.0	1099.0	1099.0	1099.0	1099.0	1099.0	1099.0	1099.0	1099.0
	(26.9)	(26.9)	(26.9)	(26.9)	(26.9)	(26.9)	(26.9)	(26.9)	(26.9)	(26.9)
	104.4	-33.7	36.3	142.3	81.5	157.2	193.7	142.3	206.7	157.2
	2.553	-0.825	0.889	3.481	1.993	3.845	4.739	3.481	5.057	3.845
g_O	902	900	800	830	773	708	764	795	712	760
	(22.1)	(22.0)	(19.6)	(20.3)	(18.9)	(17.3)	(18.7)	(19.5)	(17.4)	(18.6)
g_E	975.9	975.9	975.9	975.9	975.9	975.9	975.9	975.9	975.9	975.9
	(23.9)	(23.9)	(23.9)	(23.9)	(23.9)	(23.9)	(23.9)	(23.9)	(23.9)	(23.9)
	-142.1	-145.8	-318.0	-268.8	-360.4	-454.4	-374.1	-326.0	-449.0	-380.1
	-3.477	-3.567	-7.782	-6.578	-8.818	-11.119	-9.153	-7.977	-10.986	-9.300
c_O	681	679	696	706	720	682	680	654	720	657
	(16.7)	(16.6)	(17.0)	(17.3)	(17.6)	(16.7)	(16.6)	(16.0)	(17.6)	(16.1)
c_E	697.8	697.8	697.8	697.8	697.8	697.8	697.8	697.8	697.8	697.8
	(17.1)	(17.1)	(17.1)	(17.1)	(17.1)	(17.1)	(17.1)	(17.1)	(17.1)	(17.1)
	-33.2	-37.1	-3.6	16.5	45.1	-31.2	-35.1	-84.8	45.1	-79.1
	-0.812	-0.907	-0.087	0.404	1.104	-0.764	-0.859	-2.074	1.104	-1.937
N	4087	4087	4087	4087	4087	4087	4087	4087	4087	4087
X2	9.6	16.2	51.4	29.8	59.4	112.0	68.6	59.1	96.5	80.3
Gvalue	9.7	16.1	52.8	30.9	62.2	118.1	71.6	60.6	103.2	83.1
Gv/N	0.237	0.393	1.291	0.755	1.521	2.890	1.753	1.484	2.524	2.033
Prob	0.02157	0.00109	0.00000	0.00000	0.00000	0.00000	0.00000	0.00000	0.00000	0.00000

	20	19	18	17	16	15	14	13	12	11
a_O	1547	1474	1618	1791	1928	1982	1944	1764	1275	1142
	(37.9)	(36.1)	(39.6)	(43.8)	(47.2)	(48.5)	(47.6)	(43.2)	(31.2)	(27.9)
a_E	1314.3	1314.3	1314.3	1314.3	1314.3	1314.3	1314.3	1314.3	1314.3	1314.3
	(32.2)	(32.2)	(32.2)	(32.2)	(32.2)	(32.2)	(32.2)	(32.2)	(32.2)	(32.2)
	504.4	338.1	672.7	1108.5	1477.5	1628.4	1521.9	1038.2	-77.4	-321.0
	12.341	8.272	16.460	27.123	36.152	39.844	37.238	25.403	-1.894	-7.853
t_O	1090	1103	1058	803	781	698	501	378	295	253
	(26.7)	(27.0)	(25.9)	(19.6)	(19.1)	(17.1)	(12.3)	(9.2)	(7.2)	(6.2)

t_E	1099.0	1099.0	1099.0	1099.0	1099.0	1099.0	1099.0	1099.0	1099.0	1099.0
	(26.9)	(26.9)	(26.9)	(26.9)	(26.9)	(26.9)	(26.9)	(26.9)	(26.9)	(26.9)
	-17.9	8.0	-80.4	-504.0	-533.5	-633.7	-787.1	-806.8	-776.0	-743.2
	-0.438	0.197	-1.968	-12.331	-13.054	-15.505	-19.259	-19.742	-18.986	-18.184
g_O	809	868	854	1013	990	1082	1402	1778	2363	2543
	(19.8)	(21.2)	(20.9)	(24.8)	(24.2)	(26.5)	(34.3)	(43.5)	(57.8)	(62.2)
g_E	975.9	975.9	975.9	975.9	975.9	975.9	975.9	975.9	975.9	975.9
	(23.9)	(23.9)	(23.9)	(23.9)	(23.9)	(23.9)	(23.9)	(23.9)	(23.9)	(23.9)
	-303.5	-203.4	-227.9	75.6	28.4	223.3	1015.8	2133.1	4179.2	4871.0
	-7.426	-4.978	-5.577	1.849	0.694	5.464	24.855	52.193	102.257	119.182
c_O	641	642	557	480	388	325	240	167	154	149
	(15.7)	(15.7)	(13.6)	(11.7)	(9.5)	(8.0)	(5.9)	(4.1)	(3.8)	(3.6)
c_E	697.8	697.8	697.8	697.8	697.8	697.8	697.8	697.8	697.8	697.8
	(17.1)	(17.1)	(17.1)	(17.1)	(17.1)	(17.1)	(17.1)	(17.1)	(17.1)	(17.1)
	-108.8	-107.0	-251.0	-359.2	-455.4	-496.7	-512.3	-477.6	-465.4	-460.1
	-2.663	-2.618	-6.142	-8.788	-11.144	-12.152	-12.535	-11.686	-11.387	-11.258
N	4087	4087	4087	4087	4087	4087	4087	4087	4087	4087
X2	74.4	35.8	115.3	322.0	516.3	696.2	1113.4	1689.8	2984.6	3621.8
Gvalue	74.1	35.7	113.3	321.0	516.9	721.4	1238.3	1886.9	2860.5	3346.7
Gv/N	1.814	0.873	2.773	7.853	12.648	17.650	30.300	46.168	69.990	81.887
Prob	0.00000	0.00000	0.00000	0.00000	0.00000	0.00000	0.00000	1.00000	1.00000	1.00000

	10	9	8	7	6	5	4	3	2	1
a_O	1236	965	998	1244	1467	1450	1415	1467	1385	1369
	(30.2)	(23.6)	(24.4)	(30.4)	(35.9)	(35.5)	(34.6)	(35.9)	(33.9)	(33.5)
a_E	1314.3	1314.3	1314.3	1314.3	1314.3	1314.3	1314.3	1314.3	1314.3	1314.3
	(32.2)	(32.2)	(32.2)	(32.2)	(32.2)	(32.2)	(32.2)	(32.2)	(32.2)	(32.2)
	-151.8	-596.2	-549.5	-136.8	322.5	285.0	208.9	322.5	145.1	111.6
	-3.715	-14.589	-13.445	-3.346	7.891	6.972	5.112	7.891	3.551	2.732
t_O	399	522	740	887	882	1121	1089	847	875	949
	(9.8)	(12.8)	(18.1)	(21.7)	(21.6)	(27.4)	(26.6)	(20.7)	(21.4)	(23.2)
t_E	1099.0	1099.0	1099.0	1099.0	1099.0	1099.0	1099.0	1099.0	1099.0	1099.0
	(26.9)	(26.9)	(26.9)	(26.9)	(26.9)	(26.9)	(26.9)	(26.9)	(26.9)	(26.9)
	-808.5	-777.2	-585.3	-380.2	-388.0	44.5	-19.9	-441.2	-398.9	-278.5
	-19.783	-19.017	-14.322	-9.302	-9.494	1.088	-0.487	-10.795	-9.759	-6.815
g_O	2280	2363	2002	1536	1276	905	937	940	822	857
	(55.8)	(57.8)	(49.0)	(37.6)	(31.2)	(22.1)	(22.9)	(23.0)	(20.1)	(21.0)
g_E	975.9	975.9	975.9	975.9	975.9	975.9	975.9	975.9	975.9	975.9
	(23.9)	(23.9)	(23.9)	(23.9)	(23.9)	(23.9)	(23.9)	(23.9)	(23.9)	(23.9)
	3869.4	4179.2	2877.0	1393.3	684.2	-136.6	-76.3	-70.5	-282.2	-222.7
	94.676	102.257	70.393	34.092	16.741	-3.341	-1.866	-1.725	-6.904	-5.450
c_O	172	237	347	420	462	611	646	833	1005	912
	(4.2)	(5.8)	(8.5)	(10.3)	(11.3)	(14.9)	(15.8)	(20.4)	(24.6)	(22.3)
c_E	697.8	697.8	697.8	697.8	697.8	697.8	697.8	697.8	697.8	697.8
	(17.1)	(17.1)	(17.1)	(17.1)	(17.1)	(17.1)	(17.1)	(17.1)	(17.1)	(17.1)
	-481.7	-511.9	-484.8	-426.4	-381.0	-162.3	-99.6	295.1	733.3	488.3
	-11.787	-12.524	-11.863	-10.434	-9.323	-3.971	-2.438	7.220	17.942	11.948
N	4087	4087	4087	4087	4087	4087	4087	4087	4087	4087
X2	2589.3	2671.5	1448.6	476.7	232.5	30.4	13.2	103.0	209.0	103.0
Gvalue	2427.3	2293.9	1257.3	449.9	237.7	30.5	13.1	105.9	197.4	98.7
Gv/N	59.390	56.127	30.763	11.009	5.816	0.747	0.321	2.590	4.830	2.416
Prob	1.00000	1.00000	0.00000	0.00000	0.00000	0.00000	0.00436	0.00000	0.00000	0.00000

CDS_INITIAL:

	1	2	3	4	5	6	7	8	9	10
a_O	3063	0	0	1912	1848	1530	1750	1728	1646	1479
	(74.9)	(0.0)	(0.0)	(46.8)	(45.2)	(37.4)	(42.8)	(42.3)	(40.3)	(36.2)
a_E	1111.9	1287.5	1105.2	1111.9	1287.5	1105.2	1111.9	1287.5	1105.2	1111.9
	(27.2)	(31.5)	(27.0)	(27.2)	(31.5)	(27.0)	(27.2)	(31.5)	(27.0)	(27.2)
	6207.9	0.0	0.0	2073.1	1335.7	995.2	1587.5	1016.9	1311.3	844.0
	151.893	0.000	0.000	50.723	32.681	24.351	38.844	24.882	32.084	20.651
t_O	456	4087	0	491	729	1206	592	1035	1037	813
	(11.2)	(100.0)	(0.0)	(12.0)	(17.8)	(29.5)	(14.5)	(25.3)	(25.4)	(19.9)
t_E	735.7	1273.2	1177.6	735.7	1273.2	1177.6	735.7	1273.2	1177.6	735.7
	(18.0)	(31.2)	(28.8)	(18.0)	(31.2)	(28.8)	(18.0)	(31.2)	(28.8)	(18.0)
	-436.3	9533.1	0.0	-397.1	-813.0	57.4	-257.3	-428.8	-263.8	162.4
	-10.674	233.254	0.000	-9.717	-19.893	1.404	-6.296	-10.491	-6.454	3.974
g_O	568	0	4087	1200	635	679	1014	561	847	998
	(13.9)	(0.0)	(100.0)	(29.4)	(15.5)	(16.6)	(24.8)	(13.7)	(20.7)	(24.4)
g_E	1458.5	636.0	922.6	1458.5	636.0	922.6	1458.5	636.0	922.6	1458.5
	(35.7)	(15.6)	(22.6)	(35.7)	(15.6)	(22.6)	(35.7)	(15.6)	(22.6)	(35.7)
	-1071.3	0.0	12166.4	-468.2	-2.0	-416.3	-737.2	-140.8	-144.7	-757.3
	-26.212	0.000	297.685	-11.456	-0.048	-10.185	-18.038	-3.444	-3.541	-18.530
c_O	0	0	0	484	875	672	731	763	557	797
	(0.0)	(0.0)	(0.0)	(11.8)	(21.4)	(16.4)	(17.9)	(18.7)	(13.6)	(19.5)
c_E	780.9	890.3	881.6	780.9	890.3	881.6	780.9	890.3	881.6	780.9
	(19.1)	(21.8)	(21.6)	(19.1)	(21.8)	(21.6)	(19.1)	(21.8)	(21.6)	(19.1)
	0.0	0.0	0.0	-463.1	-30.3	-364.9	-96.6	-235.4	-511.5	32.5
	0.000	0.000	0.000	-11.331	-0.742	-8.928	-2.363	-5.761	-12.516	0.795
N	4087	4087	4087	4087	4087	4087	4087	4087	4087	4087
X2	4854.9	9032.3	14018.8	815.9	476.9	278.1	533.0	222.3	407.1	275.1
Gvalue	4700.3	9533.1	12166.4	744.6	490.4	271.5	496.4	211.9	391.2	281.6
Gv/N	115.006	233.254	297.685	18.220	11.999	6.643	12.147	5.186	9.572	6.890
Prob	1.00000	1.00000	1.00000	0.00000	0.00000	0.00000	0.00000	0.00000	0.00000	0.00000

	11	12	13	14	15	16	17	18	19	20
a_O	1373	1528	1503	1443	1472	1399	1330	1351	1276	1292
	(33.6)	(37.4)	(36.8)	(35.3)	(36.0)	(34.2)	(32.5)	(33.1)	(31.2)	(31.6)
a_E	1287.5	1105.2	1111.9	1287.5	1105.2	1111.9	1287.5	1105.2	1111.9	1287.5
	(31.5)	(27.0)	(27.2)	(31.5)	(27.0)	(27.2)	(31.5)	(27.0)	(27.2)	(31.5)
	176.5	989.9	906.1	329.0	843.7	642.8	86.4	542.6	351.4	9.0
	4.319	24.221	22.170	8.051	20.644	15.727	2.113	13.276	8.598	0.220
t_O	1368	1117	781	1399	1175	800	1447	1216	824	1447
	(33.5)	(27.3)	(19.1)	(34.2)	(28.7)	(19.6)	(35.4)	(29.8)	(20.2)	(35.4)
t_E	1273.2	1177.6	735.7	1273.2	1177.6	735.7	1273.2	1177.6	735.7	1273.2
	(31.2)	(28.8)	(18.0)	(31.2)	(28.8)	(18.0)	(31.2)	(28.8)	(18.0)	(31.2)
	196.5	-118.1	93.3	263.6	-5.3	134.0	370.3	78.0	186.8	370.3
	4.807	-2.890	2.283	6.450	-0.129	3.279	9.060	1.907	4.570	9.060
g_O	535	848	1053	504	825	1110	565	879	1225	599
	(13.1)	(20.7)	(25.8)	(12.3)	(20.2)	(27.2)	(13.8)	(21.5)	(30.0)	(14.7)
g_E	636.0	922.6	1458.5	636.0	922.6	1458.5	636.0	922.6	1458.5	636.0
	(15.6)	(22.6)	(35.7)	(15.6)	(22.6)	(35.7)	(15.6)	(22.6)	(35.7)	(15.6)
	-185.0	-142.9	-686.1	-234.5	-184.4	-606.2	-133.7	-85.0	-427.4	-71.8
	-4.527	-3.497	-16.787	-5.737	-4.512	-14.832	-3.272	-2.080	-10.459	-1.756

```
c_O          811     594     750     741     615     778     745     641     762     749
         ( 19.8) ( 14.5) ( 18.4) ( 18.1) ( 15.0) ( 19.0) ( 18.2) ( 15.7) ( 18.6) ( 18.3)
c_E        890.3   881.6   780.9   890.3   881.6   780.9   890.3   881.6   780.9   890.3
         ( 21.8) ( 21.6) ( 19.1) ( 21.8) ( 21.6) ( 19.1) ( 21.8) ( 21.6) ( 19.1) ( 21.8)
          -151.3  -469.1   -60.6  -272.0  -442.9    -5.8  -265.5  -408.6   -37.4  -258.9
          -3.702 -11.478  -1.483  -6.655 -10.838  -0.143  -6.495  -9.997  -0.915  -6.334

N           4087    4087    4087    4087    4087    4087    4087    4087    4087    4087
X2          35.8   264.7   254.4    83.6   212.7   163.1    56.8   123.6    72.7    48.3
Gvalue      36.7   259.8   252.7    86.2   211.1   164.8    57.4   127.0    73.3    48.6
Gv/N       0.898   6.357   6.184   2.109   5.165   4.032   1.406   3.106   1.794   1.190
Prob     0.00000 0.00000 0.00000 0.00000 0.00000 0.00000 0.00000 0.00000 0.00000 0.00000
         -------------------------------------------------------------------------------
              21      22      23      24      25      26      27      28      29      30
         -------------------------------------------------------------------------------
a_O         1312    1289    1274    1271    1301    1227    1260    1153    1256    1244
         ( 32.1) ( 31.5) ( 31.2) ( 31.1) ( 31.8) ( 30.0) ( 30.8) ( 28.2) ( 30.7) ( 30.4)
a_E       1105.2  1111.9  1287.5  1105.2  1111.9  1287.5  1105.2  1111.9  1287.5  1105.2
         ( 27.0) ( 27.2) ( 31.5) ( 27.0) ( 27.2) ( 31.5) ( 27.0) ( 27.2) ( 31.5) ( 27.0)
           450.1   381.1   -26.9   355.3   408.8  -118.1   330.3    83.8   -62.3   294.3
          11.012   9.325  -0.658   8.694  10.002  -2.891   8.082   2.050  -1.523   7.202
t_O         1187     784    1400    1159     755    1394    1171     799    1343    1173
         ( 29.0) ( 19.2) ( 34.3) ( 28.4) ( 18.5) ( 34.1) ( 28.7) ( 19.5) ( 32.9) ( 28.7)
t_E       1177.6   735.7  1273.2  1177.6   735.7  1273.2  1177.6   735.7  1273.2  1177.6
         ( 28.8) ( 18.0) ( 31.2) ( 28.8) ( 18.0) ( 31.2) ( 28.8) ( 18.0) ( 31.2) ( 28.8)
            18.8    99.7   265.8   -37.0    39.1   252.7   -13.2   131.9   143.3    -9.3
           0.460   2.439   6.504  -0.905   0.956   6.183  -0.324   3.226   3.507  -0.227
g_O          850    1223     615     887    1258     652     923    1351     667     835
         ( 20.8) ( 29.9) ( 15.0) ( 21.7) ( 30.8) ( 16.0) ( 22.6) ( 33.1) ( 16.3) ( 20.4)
g_E        922.6  1458.5   636.0   922.6  1458.5   636.0   922.6  1458.5   636.0   922.6
         ( 22.6) ( 35.7) ( 15.6) ( 22.6) ( 35.7) ( 15.6) ( 22.6) ( 35.7) ( 15.6) ( 22.6)
          -139.2  -430.7   -41.3   -69.7  -372.1    32.4     0.9  -206.9    63.5  -166.5
          -3.407 -10.539  -1.010  -1.706  -9.104   0.793   0.022  -5.062   1.554  -4.074
c_O          738     791     798     770     773     814     733     784     821     835
         ( 18.1) ( 19.4) ( 19.5) ( 18.8) ( 18.9) ( 19.9) ( 17.9) ( 19.2) ( 20.1) ( 20.4)
c_E        881.6   780.9   890.3   881.6   780.9   890.3   881.6   780.9   890.3   881.6
         ( 21.6) ( 19.1) ( 21.8) ( 21.6) ( 19.1) ( 21.8) ( 21.6) ( 19.1) ( 21.8) ( 21.6)
          -262.4    20.3  -174.7  -208.4   -15.8  -145.8  -270.6     6.2  -133.0   -90.7
          -6.421   0.496  -4.273  -5.100  -0.386  -3.568  -6.621   0.151  -3.255  -2.219

N           4087    4087    4087    4087    4087    4087    4087    4087    4087    4087
X2          67.9    69.5    23.0    40.7    60.3    21.2    46.8    14.9    11.5    28.2
Gvalue      67.2    70.3    23.0    40.2    60.0    21.1    47.4    14.9    11.6    27.9
Gv/N       1.644   1.721   0.562   0.983   1.468   0.517   1.159   0.365   0.283   0.682
Prob     0.00000 0.00000 0.00004 0.00000 0.00000 0.00010 0.00000 0.00187 0.00905 0.00000
         -------------------------------------------------------------------------------
              31      32      33      34      35      36      37      38      39      40
         -------------------------------------------------------------------------------
a_O         1159    1214    1201    1207    1197    1202    1140    1132    1147    1161
         ( 28.4) ( 29.7) ( 29.4) ( 29.5) ( 29.3) ( 29.4) ( 27.9) ( 27.7) ( 28.1) ( 28.4)
a_E       1111.9  1287.5  1105.2  1111.9  1287.5  1105.2  1111.9  1287.5  1105.2  1111.9
         ( 27.2) ( 31.5) ( 27.0) ( 27.2) ( 31.5) ( 27.0) ( 27.2) ( 31.5) ( 27.0) ( 27.2)
            96.3  -142.8   199.7   198.2  -174.5   201.8    57.0  -291.4    85.2   100.4
           2.355  -3.493   4.885   4.850  -4.270   4.938   1.394  -7.131   2.083   2.457
t_O          775    1331    1161     776    1368    1234     793    1431    1242     779
         ( 19.0) ( 32.6) ( 28.4) ( 19.0) ( 33.5) ( 30.2) ( 19.4) ( 35.0) ( 30.4) ( 19.1)
t_E        735.7  1273.2  1177.6   735.7  1273.2  1177.6   735.7  1273.2  1177.6   735.7
         ( 18.0) ( 31.2) ( 28.8) ( 18.0) ( 31.2) ( 28.8) ( 18.0) ( 31.2) ( 28.8) ( 18.0)
            80.6   118.2   -33.0    82.7   196.5   115.4   118.9   334.4   132.2    89.1
           1.973   2.891  -0.809   2.024   4.807   2.823   2.910   8.182   3.234   2.179
g_O         1400     688     910    1359     645     878    1408     665     887    1351
         ( 34.3) ( 16.8) ( 22.3) ( 33.3) ( 15.8) ( 21.5) ( 34.5) ( 16.3) ( 21.7) ( 33.1)
g_E       1458.5   636.0   922.6  1458.5   636.0   922.6  1458.5   636.0   922.6  1458.5
         ( 35.7) ( 15.6) ( 22.6) ( 35.7) ( 15.6) ( 22.6) ( 35.7) ( 15.6) ( 22.6) ( 35.7)
          -114.6   108.2   -24.9  -192.1    18.1   -86.9   -99.2    59.3   -69.7  -206.9
          -2.805   2.647  -0.610  -4.699   0.444  -2.127  -2.428   1.452  -1.706  -5.062
c_O          753     854     815     745     877     773     746     859     811     796
         ( 18.4) ( 20.9) ( 19.9) ( 18.2) ( 21.5) ( 18.9) ( 18.3) ( 21.0) ( 19.8) ( 19.5)
c_E        780.9   890.3   881.6   780.9   890.3   881.6   780.9   890.3   881.6   780.9
         ( 19.1) ( 21.8) ( 21.6) ( 19.1) ( 21.8) ( 21.6) ( 19.1) ( 21.8) ( 21.6) ( 19.1)
           -54.8   -71.1  -128.0   -70.2   -26.4  -203.2   -68.3   -61.5  -135.4    30.4
          -1.342  -1.739  -3.133  -1.717  -0.645  -4.973  -1.670  -1.504  -3.313   0.745

N           4087    4087    4087    4087    4087    4087    4087    4087    4087    4087
X2           7.4    12.6    13.7    18.8    13.7    26.7     8.5    40.8    12.1    12.9
Gvalue       7.4    12.5    13.6    18.7    13.7    27.0     8.4    40.8    12.2    13.1
Gv/N       0.182   0.306   0.334   0.458   0.336   0.662   0.206   0.998   0.299   0.319
Prob     0.05961 0.00584 0.00343 0.00031 0.00329 0.00001 0.03826 0.00000 0.00668 0.00452
         -------------------------------------------------------------------------------
              41      42      43      44      45      46      47      48      49      50
         -------------------------------------------------------------------------------
a_O         1151    1183    1122    1228    1176    1121    1168    1152    1124    1157
         ( 28.2) ( 28.9) ( 27.5) ( 30.0) ( 28.8) ( 27.4) ( 28.6) ( 28.2) ( 27.5) ( 28.3)
a_E       1287.5  1105.2  1111.9  1287.5  1105.2  1111.9  1287.5  1105.2  1111.9  1287.5
         ( 31.5) ( 27.0) ( 27.2) ( 31.5) ( 27.0) ( 27.2) ( 31.5) ( 27.0) ( 27.2) ( 31.5)
          -258.0   160.9    20.4  -116.2   146.0    18.4  -227.6    95.5    24.4  -247.3
          -6.313   3.938   0.499  -2.844   3.573   0.449  -5.568   2.338   0.597  -6.052
t_O         1435    1170     786    1385    1213     747    1359    1133     791    1417
         ( 35.1) ( 28.6) ( 19.2) ( 33.9) ( 29.7) ( 18.3) ( 33.3) ( 27.7) ( 19.4) ( 34.7)
t_E       1273.2  1177.6   735.7  1273.2  1177.6   735.7  1273.2  1177.6   735.7  1273.2
         ( 31.2) ( 28.8) ( 18.0) ( 31.2) ( 28.8) ( 18.0) ( 31.2) ( 28.8) ( 18.0) ( 31.2)
           343.3   -15.2   103.9   233.1    71.8    22.7   177.2   -87.6   114.6   303.2
           8.400  -0.373   2.543   5.704   1.756   0.556   4.337  -2.143   2.804   7.420
g_O          662     883    1355     640     937    1425     677     973    1411     661
         ( 16.2) ( 21.6) ( 33.2) ( 15.7) ( 22.9) ( 34.9) ( 16.6) ( 23.8) ( 34.5) ( 16.2)
g_E        636.0   922.6  1458.5   636.0   922.6  1458.5   636.0   922.6  1458.5   636.0
         ( 15.6) ( 22.6) ( 35.7) ( 15.6) ( 22.6) ( 35.7) ( 15.6) ( 22.6) ( 35.7) ( 15.6)
            53.1   -77.4  -199.5     8.0    29.1   -66.2    84.6   103.6   -93.4    51.0
           1.299  -1.893  -4.881   0.197   0.713  -1.620   2.070   2.535  -2.286   1.248
c_O          839     851     824     834     761     794     883     829     761     852
         ( 20.5) ( 20.8) ( 20.2) ( 20.4) ( 18.6) ( 19.4) ( 21.6) ( 20.3) ( 18.6) ( 20.8)
c_E        890.3   881.6   780.9   890.3   881.6   780.9   890.3   881.6   780.9   890.3
         ( 21.8) ( 21.6) ( 19.1) ( 21.8) ( 21.6) ( 19.1) ( 21.8) ( 21.6) ( 19.1) ( 21.8)
           -99.6   -60.1    88.5  -108.9  -223.9    26.4   -14.5  -102.0   -39.3   -74.9
          -2.436  -1.471   2.165  -2.665  -5.478   0.645  -0.355  -2.496  -0.962  -1.833

N           4087    4087    4087    4087    4087    4087    4087    4087    4087    4087
X2          39.1     8.3    13.3    16.2    22.3     1.2    19.6     9.6     6.3    32.1
Gvalue      38.8     8.2    13.3    16.0    23.0     1.2    19.8     9.6     6.3    32.0
Gv/N       0.950   0.201   0.326   0.391   0.563   0.030   0.483   0.234   0.153   0.783
Prob     0.00000 0.04211 0.00401 0.00114 0.00004 0.74333 0.00019 0.02249 0.09950 0.00000
         -------------------------------------------------------------------------------
```

	51	52	53	54	55	56	57	58	59	60
a_O	1138	1147	1207	1052	1102	1135	1174	1189	1214	1136
	(27.8)	(28.1)	(29.5)	(25.7)	(27.0)	(27.8)	(28.7)	(29.1)	(29.7)	(27.8)
a_E	1105.2	1111.9	1287.5	1105.2	1111.9	1287.5	1105.2	1111.9	1287.5	1105.2
	(27.0)	(27.2)	(31.5)	(27.0)	(27.2)	(31.5)	(27.0)	(27.2)	(31.5)	(27.0)
	66.6	71.4	-155.9	-103.8	-19.6	-286.2	141.8	159.5	-142.8	62.4
	1.628	1.747	-3.814	-2.540	-0.480	-7.003	3.469	3.903	-3.493	1.528
t_O	1227	779	1382	1208	789	1385	1139	781	1377	1210
	(30.0)	(19.1)	(33.8)	(29.6)	(19.3)	(33.9)	(27.9)	(19.1)	(33.7)	(29.6)
t_E	1177.6	735.7	1273.2	1177.6	735.7	1273.2	1177.6	735.7	1273.2	1177.6
	(28.8)	(18.0)	(31.2)	(28.8)	(18.0)	(31.2)	(28.8)	(18.0)	(31.2)	(28.8)
	100.8	89.1	226.6	61.5	110.3	233.1	-76.0	93.3	215.8	65.6
	2.465	2.179	5.545	1.505	2.700	5.704	-1.860	2.283	5.281	1.605
g_O	925	1402	667	979	1415	694	917	1352	686	906
	(22.6)	(34.3)	(16.3)	(24.0)	(34.6)	(17.0)	(22.4)	(33.1)	(16.8)	(22.2)
g_E	922.6	1458.5	636.0	922.6	1458.5	636.0	922.6	1458.5	636.0	922.6
	(22.6)	(35.7)	(15.6)	(22.6)	(35.7)	(15.6)	(22.6)	(35.7)	(15.6)	(22.6)
	4.9	-110.8	63.5	116.3	-85.7	121.2	-11.1	-205.0	103.9	-32.8
	0.120	-2.711	1.554	2.845	-2.097	2.965	-0.271	-5.017	2.541	-0.803
c_O	797	759	831	848	781	873	857	765	810	835
	(19.5)	(18.6)	(20.3)	(20.7)	(19.1)	(21.4)	(21.0)	(18.7)	(19.8)	(20.4)
c_E	881.6	780.9	890.3	881.6	780.9	890.3	881.6	780.9	890.3	881.6
	(21.6)	(19.1)	(21.8)	(21.6)	(19.1)	(21.8)	(21.6)	(19.1)	(21.8)	(21.6)
	-160.8	-43.2	-114.5	-65.9	0.2	-34.2	-48.5	-31.5	-153.1	-90.7
	-3.935	-1.058	-2.802	-1.613	0.004	-0.838	-1.187	-0.771	-3.746	-2.219
N	4087	4087	4087	4087	4087	4087	4087	4087	4087	4087
X2	11.2	6.5	19.8	8.1	5.2	33.5	6.3	16.2	23.8	4.5
Gvalue	11.4	6.4	19.7	8.1	5.2	33.8	6.2	16.3	23.8	4.5
Gv/N	0.279	0.157	0.482	0.197	0.126	0.828	0.152	0.398	0.583	0.111
Prob	0.00974	0.09221	0.00019	0.04474	0.15995	0.00000	0.10206	0.00100	0.00003	0.20889

	61	62	63	64	65	66	67	68	69	70
a_O	1105	1217	1127	1118	1264	1197	1175	1319	1123	1079
	(27.0)	(29.8)	(27.6)	(27.4)	(30.9)	(29.3)	(28.7)	(32.3)	(27.5)	(26.4)
a_E	1111.9	1287.5	1105.2	1111.9	1287.5	1105.2	1111.9	1287.5	1105.2	1111.9
	(27.2)	(31.5)	(27.0)	(27.2)	(31.5)	(27.0)	(27.2)	(31.5)	(27.0)	(27.2)
	-13.7	-137.1	44.0	12.3	-46.6	191.0	129.8	63.7	35.9	-64.7
	-0.334	-3.355	1.077	0.301	-1.140	4.674	3.176	1.559	0.878	-1.584
t_O	792	1372	1240	812	1314	1150	765	1249	1201	754
	(19.4)	(33.6)	(30.3)	(19.9)	(32.2)	(28.1)	(18.7)	(30.6)	(29.4)	(18.4)
t_E	735.7	1273.2	1177.6	735.7	1273.2	1177.6	735.7	1273.2	1177.6	735.7
	(18.0)	(31.2)	(28.8)	(18.0)	(31.2)	(28.8)	(18.0)	(31.2)	(28.8)	(18.0)
	116.8	205.1	128.0	160.2	82.9	-54.6	59.7	-48.0	47.2	37.0
	2.857	5.017	3.131	3.920	2.028	-1.337	1.461	-1.173	1.154	0.906
g_O	1442	668	897	1428	629	926	1393	652	898	1504
	(35.3)	(16.3)	(21.9)	(34.9)	(15.4)	(22.7)	(34.1)	(16.0)	(22.0)	(36.8)
g_E	1458.5	636.0	922.6	1458.5	636.0	922.6	1458.5	636.0	922.6	1458.5
	(35.7)	(15.6)	(22.6)	(35.7)	(15.6)	(22.6)	(35.7)	(15.6)	(22.6)	(35.7)
	-32.8	65.6	-50.4	-60.4	-13.9	6.9	-128.0	32.4	-48.4	92.4
	-0.803	1.605	-1.233	-1.477	-0.340	0.169	-3.132	0.793	-1.185	2.261
c_O	748	830	823	729	880	814	754	867	865	750
	(18.3)	(20.3)	(20.1)	(17.8)	(21.5)	(19.9)	(18.4)	(21.2)	(21.2)	(18.4)
c_E	780.9	890.3	881.6	780.9	890.3	881.6	780.9	890.3	881.6	780.9
	(19.1)	(21.8)	(21.6)	(19.1)	(21.8)	(21.6)	(19.1)	(21.8)	(21.6)	(19.1)
	-64.4	-116.4	-113.2	-100.3	-20.5	-129.9	-52.9	-46.0	-32.9	-60.6
	-1.577	-2.848	-2.770	-2.455	-0.500	-3.178	-1.295	-1.124	-0.805	-1.483
N	4087	4087	4087	4087	4087	4087	4087	4087	4087	4087
X2	5.9	17.2	8.3	12.0	1.9	13.5	8.6	2.2	1.7	4.1
Gvalue	5.8	17.2	8.4	11.9	1.9	13.4	8.6	2.2	1.7	4.1
Gv/N	0.143	0.420	0.205	0.290	0.047	0.328	0.210	0.055	0.042	0.100
Prob	0.11978	0.00065	0.03892	0.00789	0.58879	0.00383	0.03517	0.52393	0.63265	0.25326

	71	72	73	74	75	76	77	78	79	80
a_O	1292	1162	1099	1318	1218	1082	1317	1149	1094	1331
	(31.6)	(28.4)	(26.9)	(32.2)	(29.8)	(26.5)	(32.2)	(28.1)	(26.8)	(32.6)
a_E	1287.5	1105.2	1111.9	1287.5	1105.2	1111.9	1287.5	1105.2	1111.9	1287.5
	(31.5)	(27.0)	(27.2)	(31.5)	(27.0)	(27.2)	(31.5)	(27.0)	(27.2)	(31.5)
	9.0	116.5	-25.6	61.7	236.7	-58.9	59.6	89.3	-35.4	88.4
	0.220	2.850	-0.625	1.509	5.792	-1.441	1.459	2.185	-0.867	2.163
t_O	1238	1156	765	1316	1184	728	1310	1143	777	1263
	(30.3)	(28.3)	(18.7)	(32.2)	(29.0)	(17.8)	(32.1)	(28.0)	(19.0)	(30.9)
t_E	1273.2	1177.6	735.7	1273.2	1177.6	735.7	1273.2	1177.6	735.7	1273.2
	(31.2)	(28.8)	(18.0)	(31.2)	(28.8)	(18.0)	(31.2)	(28.8)	(18.0)	(31.2)
	-69.4	-42.9	59.7	87.0	12.8	-15.4	74.6	-68.3	84.8	-20.3
	-1.699	-1.049	1.461	2.129	0.312	-0.376	1.826	-1.670	2.076	-0.497
g_O	631	921	1479	607	901	1464	587	953	1465	653
	(15.4)	(22.5)	(36.2)	(14.9)	(22.0)	(35.8)	(14.4)	(23.3)	(35.8)	(16.0)
g_E	636.0	922.6	1458.5	636.0	922.6	1458.5	636.0	922.6	1458.5	636.0
	(15.6)	(22.6)	(35.7)	(15.6)	(22.6)	(35.7)	(15.6)	(22.6)	(35.7)	(15.6)
	-9.9	-3.1	41.3	-56.6	-42.6	11.0	-94.1	61.9	13.0	34.5
	-0.243	-0.076	1.010	-1.386	-1.042	0.270	-2.303	1.514	0.319	0.843
c_O	926	848	744	846	784	813	873	842	751	840
	(22.7)	(20.7)	(18.2)	(20.7)	(19.2)	(19.9)	(21.4)	(20.6)	(18.4)	(20.6)
c_E	890.3	881.6	780.9	890.3	881.6	780.9	890.3	881.6	780.9	890.3
	(21.8)	(21.6)	(19.1)	(21.8)	(21.6)	(19.1)	(21.8)	(21.6)	(19.1)	(21.8)
	72.8	-65.9	-72.1	-86.3	-184.0	65.5	-34.2	-77.4	-58.7	-97.7
	1.782	-1.613	-1.763	-2.112	-4.502	1.601	-0.838	-1.894	-1.436	-2.390
N	4087	4087	4087	4087	4087	4087	4087	4087	4087	4087
X2	2.5	4.6	3.3	5.7	22.9	2.2	5.8	5.5	3.8	4.8
Gvalue	2.5	4.6	3.4	5.7	22.9	2.2	5.9	5.5	3.8	4.9
Gv/N	0.060	0.112	0.082	0.140	0.561	0.054	0.145	0.136	0.092	0.119
Prob	0.48413	0.20612	0.33933	0.12602	0.00004	0.52983	0.11480	0.13611	0.28938	0.18076

	81	82	83	84	85	86	87	88	89	90
a_O	1202	1091	1312	1132	1113	1309	1111	1065	1277	1093
	(29.4)	(26.7)	(32.1)	(27.7)	(27.2)	(32.0)	(27.2)	(26.1)	(31.3)	(26.7)
a_E	1105.2	1111.6	1287.2	1104.9	1111.6	1287.2	1104.9	1111.6	1287.2	1104.9
	(27.0)	(27.2)	(31.5)	(27.0)	(27.2)	(31.5)	(27.0)	(27.2)	(31.5)	(27.0)
	201.8	-40.8	50.1	54.8	2.8	44.0	12.2	-91.2	-20.3	-23.7
	4.938	-0.998	1.225	1.341	0.069	1.076	0.298	-2.232	-0.497	-0.581
t_O	1184	724	1314	1168	711	1273	1196	747	1322	1242
	(29.0)	(17.7)	(32.2)	(28.6)	(17.4)	(31.2)	(29.3)	(18.3)	(32.4)	(30.4)
t_E	1177.6	735.5	1272.9	1177.4	735.5	1272.9	1177.4	735.5	1272.9	1177.4
	(28.8)	(18.0)	(31.2)	(28.8)	(18.0)	(31.2)	(28.8)	(18.0)	(31.2)	(28.8)
	12.8	-22.9	83.5	-18.6	-48.2	0.2	37.6	23.1	100.1	132.8

	0.312	−0.560	2.044	−0.456	−1.181	0.005	0.920	0.565	2.449	3.250
g_O	885	1451	641	889	1475	664	860	1491	653	923
	(21.7)	(35.5)	(15.7)	(21.8)	(36.1)	(16.3)	(21.0)	(36.5)	(16.0)	(22.6)
g_E	922.6	1458.1	635.8	922.3	1458.1	635.8	922.3	1458.1	635.8	922.3
	(22.6)	(35.7)	(15.6)	(22.6)	(35.7)	(15.6)	(22.6)	(35.7)	(15.6)	(22.6)
	−73.6	−14.3	10.4	−65.4	33.9	57.6	−120.3	66.4	34.8	1.4
	−1.800	−0.349	0.254	−1.601	0.830	1.409	−2.945	1.626	0.852	0.033
c_O	816	820	819	897	787	840	919	783	834	828
	(20.0)	(20.1)	(20.0)	(22.0)	(19.3)	(20.6)	(22.5)	(19.2)	(20.4)	(20.3)
c_E	881.6	780.7	890.1	881.4	780.7	890.1	881.4	780.7	890.1	881.4
	(21.6)	(19.1)	(21.8)	(21.6)	(19.1)	(21.8)	(21.6)	(19.1)	(21.8)	(21.6)
	−126.2	80.5	−136.3	31.5	12.6	−97.3	76.8	4.5	−108.5	−103.5
	−3.088	1.970	−3.336	0.771	0.308	−2.380	1.880	0.111	−2.656	−2.532
N	4087	4086	4086	4086	4086	4086	4086	4086	4086	4086
X2	14.9	2.6	7.5	2.2	1.1	4.4	6.1	2.9	6.0	6.9
Gvalue	14.8	2.5	7.7	2.2	1.1	4.5	6.2	2.9	6.0	6.9
Gv/N	0.363	0.062	0.188	0.054	0.026	0.109	0.152	0.071	0.147	0.169
Prob	0.00197	0.46753	0.05355	0.52687	0.78339	0.21521	0.10141	0.40743	0.11068	0.07457

	91	92	93	94	95	96	97	98	99	100
a_O	1094	1236	1143	1140	1320	1162	1114	1325	1107	1142
	(26.8)	(30.2)	(28.0)	(27.9)	(32.3)	(28.4)	(27.3)	(32.4)	(27.1)	(27.9)
a_E	1111.6	1287.2	1104.9	1111.6	1287.2	1104.9	1111.6	1287.2	1104.9	1111.6
	(27.2)	(31.5)	(27.0)	(27.2)	(31.5)	(27.0)	(27.2)	(31.5)	(27.0)	(27.2)
	−34.9	−100.3	77.4	57.6	66.4	117.0	4.8	76.7	4.1	61.7
	−0.854	−2.456	1.895	1.408	1.626	2.864	0.118	1.877	0.101	1.509
t_O	763	1309	1172	723	1277	1169	715	1228	1188	746
	(18.7)	(32.0)	(28.7)	(17.7)	(31.3)	(28.6)	(17.5)	(30.1)	(29.1)	(18.3)
t_E	735.5	1272.9	1177.4	735.5	1272.9	1177.4	735.5	1272.9	1177.4	735.5
	(18.0)	(31.2)	(28.8)	(18.0)	(31.2)	(28.8)	(18.0)	(31.2)	(28.8)	(18.0)
	55.9	73.2	−10.7	−24.9	8.2	−16.6	−40.5	−88.2	21.4	21.1
	1.369	1.792	−0.261	−0.608	0.201	−0.407	−0.991	−2.158	0.524	0.516
g_O	1463	650	937	1437	587	850	1493	687	916	1469
	(35.8)	(15.9)	(22.9)	(35.2)	(14.4)	(20.8)	(36.5)	(16.8)	(22.4)	(36.0)
g_E	1458.1	635.8	922.3	1458.1	635.8	922.3	1458.1	635.8	922.3	1458.1
	(35.7)	(15.6)	(22.6)	(35.7)	(15.6)	(22.6)	(35.7)	(15.6)	(22.6)	(35.7)
	9.7	28.6	29.6	−42.0	−93.8	−138.8	70.5	106.3	−12.6	21.8
	0.238	0.701	0.724	−1.027	−2.296	−3.398	1.726	2.603	−0.309	0.533
c_O	766	891	834	786	902	905	764	846	875	729
	(18.7)	(21.8)	(20.4)	(19.2)	(22.1)	(22.1)	(18.7)	(20.7)	(21.4)	(17.8)
c_E	780.7	890.1	881.4	780.7	890.1	881.4	780.7	890.1	881.4	780.7
	(19.1)	(21.8)	(21.6)	(19.1)	(21.8)	(21.6)	(19.1)	(21.8)	(21.6)	(19.1)
	−29.2	1.9	−92.2	10.6	24.0	47.8	−33.1	−85.9	−12.7	−100.0
	−0.714	0.046	−2.256	0.259	0.588	1.171	−0.810	−2.103	−0.312	−2.446
N	4086	4086	4086	4086	4086	4086	4086	4086	4086	4086
X2	1.6	3.4	4.1	1.3	4.8	9.3	1.8	9.0	0.2	4.5
Gvalue	1.6	3.4	4.1	1.3	4.9	9.4	1.8	8.9	0.2	4.6
Gv/N	0.039	0.083	0.102	0.031	0.119	0.230	0.043	0.219	0.005	0.112
Prob	0.66201	0.33489	0.24591	0.73418	0.18300	0.02431	0.62108	0.03021	0.97922	0.20702

	101	102	103	104	105	106	107	108	109	110
a_O	1239	1115	1110	1305	1137	1119	1307	1126	1064	1300
	(30.3)	(27.3)	(27.2)	(31.9)	(27.8)	(27.4)	(32.0)	(27.6)	(26.0)	(31.8)
a_E	1287.2	1104.9	1111.3	1286.9	1104.7	1111.3	1286.9	1104.7	1111.3	1286.9
	(31.5)	(27.0)	(27.2)	(31.5)	(27.0)	(27.2)	(31.5)	(27.0)	(27.2)	(31.5)
	−94.6	20.2	−2.6	36.5	65.6	15.4	40.5	43.1	−92.6	26.4
	−2.315	0.495	−0.064	0.893	1.606	0.378	0.992	1.055	−2.266	0.645
t_O	1271	1203	752	1253	1185	752	1234	1205	748	1230
	(31.1)	(29.4)	(18.4)	(30.7)	(29.0)	(18.4)	(30.2)	(29.5)	(18.3)	(30.1)
t_E	1272.9	1177.4	735.4	1272.6	1177.1	735.4	1272.6	1177.1	735.4	1272.6
	(31.2)	(28.8)	(18.0)	(31.2)	(28.8)	(18.0)	(31.2)	(28.8)	(18.0)	(31.2)
	−3.8	51.9	33.7	−38.9	15.9	33.7	−76.0	56.5	25.5	−83.7
	−0.093	1.269	0.824	−0.951	0.390	0.824	−1.860	1.384	0.624	−2.050
g_O	663	894	1442	651	891	1510	614	850	1485	651
	(16.2)	(21.9)	(35.3)	(15.9)	(21.8)	(37.0)	(15.0)	(20.8)	(36.4)	(15.9)
g_E	635.8	922.3	1457.8	635.7	922.1	1457.8	635.7	922.1	1457.8	635.7
	(15.6)	(22.6)	(35.7)	(15.6)	(22.6)	(35.7)	(15.6)	(22.6)	(35.7)	(15.6)
	55.5	−55.8	−31.4	31.0	−61.1	106.3	−42.6	−138.4	54.9	31.0
	1.358	−1.365	−0.769	0.759	−1.497	2.601	−1.043	−3.388	1.345	0.759
c_O	913	874	781	876	872	704	930	904	788	904
	(22.3)	(21.4)	(19.1)	(21.4)	(21.3)	(17.2)	(22.8)	(22.1)	(19.3)	(22.1)
c_E	890.1	881.4	780.5	889.8	881.2	780.5	889.8	881.2	780.5	889.8
	(21.8)	(21.6)	(19.1)	(21.8)	(21.6)	(19.1)	(21.8)	(21.6)	(19.1)	(21.8)
	46.5	−14.7	0.9	−27.5	−18.3	−145.3	82.1	46.2	15.0	28.5
	1.137	−0.360	0.022	−0.673	−0.447	−3.557	2.009	1.132	0.367	0.698
N	4086	4086	4085	4085	4085	4085	4085	4085	4085	4085
X2	3.6	1.6	0.5	1.1	2.1	9.8	4.0	7.3	2.8	2.2
Gvalue	3.6	1.6	0.5	1.1	2.1	10.0	4.0	7.4	2.8	2.2
Gv/N	0.087	0.039	0.013	0.028	0.053	0.246	0.099	0.182	0.069	0.053
Prob	0.31290	0.66230	0.90838	0.76749	0.54235	0.01824	0.25853	0.05901	0.41770	0.53897

	111	112	113	114	115	116	117	118	119	120
a_O	1104	1121	1328	1087	1044	1265	1172	1140	1300	1087
	(27.0)	(27.4)	(32.5)	(26.6)	(25.6)	(31.0)	(28.7)	(27.9)	(31.8)	(26.6)
a_E	1104.7	1111.3	1286.9	1104.7	1111.0	1286.6	1104.4	1111.0	1286.6	1104.4
	(27.0)	(27.2)	(31.5)	(27.0)	(27.2)	(31.5)	(27.0)	(27.2)	(31.5)	(27.0)
	−1.3	19.5	83.5	−35.0	−130.0	−42.8	139.3	58.7	27.0	−34.5
	−0.032	0.476	2.045	−0.858	−3.182	−1.048	3.410	1.437	0.661	−0.845
t_O	1202	755	1229	1242	757	1249	1180	739	1255	1175
	(29.4)	(18.5)	(30.1)	(30.4)	(18.5)	(30.6)	(28.9)	(18.1)	(30.7)	(28.8)
t_E	1177.1	735.4	1272.6	1177.1	735.2	1272.3	1176.8	735.2	1272.3	1176.8
	(28.8)	(18.0)	(31.2)	(28.8)	(18.0)	(31.2)	(28.8)	(18.0)	(31.2)	(28.8)
	50.4	39.8	−85.7	133.4	44.3	−46.1	6.5	7.7	−34.3	−3.5
	1.234	0.974	−2.097	3.265	1.084	−1.129	0.158	0.188	−0.840	−0.087
g_O	907	1430	684	892	1506	645	888	1478	670	936
	(22.2)	(35.0)	(16.7)	(21.8)	(36.9)	(15.8)	(21.7)	(36.2)	(16.4)	(22.9)
g_E	922.1	1457.8	635.7	922.1	1457.4	635.5	921.9	1457.4	635.5	921.9
	(22.6)	(35.7)	(15.6)	(22.6)	(35.7)	(15.6)	(22.6)	(35.7)	(15.6)	(22.6)
	−30.0	−55.0	100.2	−59.2	98.7	19.1	−66.5	41.4	70.8	28.5
	−0.733	−1.347	2.454	−1.449	2.418	0.468	−1.628	1.014	1.733	0.697
c_O	872	779	844	864	777	925	844	727	859	886
	(21.3)	(19.1)	(20.7)	(21.2)	(19.0)	(22.6)	(20.7)	(17.8)	(21.0)	(21.7)
c_E	881.2	780.5	889.8	881.2	780.4	889.6	881.0	780.4	889.6	881.0
	(21.6)	(19.1)	(21.8)	(21.6)	(19.1)	(21.8)	(21.6)	(19.1)	(21.8)	(21.6)
	−18.3	−3.1	−89.3	−34.0	−6.7	72.1	−72.3	−103.0	−60.2	10.1
	−0.447	−0.075	−2.186	−0.833	−0.164	1.766	−1.771	−2.521	−1.474	0.248

N	4085	4085	4085	4085	4084	4084	4084	4084	4084	4084
X2	0.9	1.1	8.8	5.2	6.3	2.3	6.9	4.7	3.3	0.5
Gvalue	0.9	1.1	8.8	5.1	6.4	2.3	6.9	4.8	3.3	0.5
Gv/N	0.021	0.028	0.215	0.126	0.156	0.057	0.169	0.117	0.080	0.013
Prob	0.83280	0.76732	0.03211	0.16235	0.09427	0.50859	0.07521	0.18778	0.35047	0.91398

	121	122	123	124	125	126	127	128	129	130
a_O	1115	1335	1136	1062	1343	1136	1061	1261	1119	1084
	(27.3)	(32.7)	(27.8)	(26.0)	(32.9)	(27.8)	(26.0)	(30.9)	(27.4)	(26.6)
a_E	1110.8	1286.3	1104.1	1110.5	1285.9	1103.9	1110.5	1285.9	1103.9	1110.2
	(27.2)	(31.5)	(27.0)	(27.2)	(31.5)	(27.0)	(27.2)	(31.5)	(27.0)	(27.2)
	8.5	99.3	64.7	−94.8	116.6	65.2	−96.8	−49.4	30.5	−51.8
	0.208	2.432	1.584	−2.323	2.857	1.598	−2.370	−1.210	0.747	−1.270
t_O	778	1238	1222	726	1168	1170	730	1189	1201	772
	(19.1)	(30.3)	(29.9)	(17.8)	(28.6)	(28.7)	(17.9)	(29.1)	(29.4)	(18.9)
t_E	735.0	1272.0	1176.5	734.8	1271.6	1176.2	734.8	1271.6	1176.2	734.6
	(18.0)	(31.2)	(28.8)	(18.0)	(31.2)	(28.8)	(18.0)	(31.2)	(28.8)	(18.0)
	88.5	−67.0	92.8	−17.5	−198.6	−12.4	−9.6	−159.8	50.1	76.6
	2.167	−1.641	2.272	−0.429	−4.866	−0.303	−0.235	−3.915	1.228	1.877
g_O	1422	618	863	1498	634	906	1509	654	924	1470
	(34.8)	(15.1)	(21.1)	(36.7)	(15.5)	(22.2)	(37.0)	(16.0)	(22.6)	(36.0)
g_E	1457.1	635.4	921.6	1456.7	635.2	921.4	1456.7	635.2	921.4	1456.4
	(35.7)	(15.6)	(22.6)	(35.7)	(15.6)	(22.6)	(35.7)	(15.6)	(22.6)	(35.7)
	−69.3	−34.3	−113.5	83.7	−2.4	−30.6	106.4	38.1	5.2	27.4
	−1.697	−0.839	−2.779	2.051	−0.059	−0.749	2.607	0.934	0.126	0.672
c_O	768	892	862	796	937	870	782	978	838	755
	(18.8)	(21.8)	(21.1)	(19.5)	(23.0)	(21.3)	(19.2)	(24.0)	(20.5)	(18.5)
c_E	780.2	889.4	880.7	780.0	889.2	880.5	780.0	889.2	880.5	779.8
	(19.1)	(21.8)	(21.6)	(19.1)	(21.8)	(21.6)	(19.1)	(21.8)	(21.6)	(19.1)
	−24.1	5.2	−37.1	32.4	98.1	−20.9	4.1	186.2	−83.0	−48.8
	−0.591	0.127	−0.908	0.793	2.404	−0.513	0.100	4.561	−2.032	−1.195
N	4083	4083	4083	4082	4082	4082	4082	4082	4082	4081
X2	3.6	3.2	6.8	3.7	13.6	1.4	4.1	15.3	2.8	3.4
Gvalue	3.5	3.2	6.9	3.7	13.7	1.3	4.1	15.1	2.8	3.4
Gv/N	0.086	0.079	0.168	0.092	0.336	0.033	0.101	0.370	0.069	0.084
Prob	0.31739	0.35812	0.07631	0.29072	0.00333	0.71825	0.24767	0.00172	0.42003	0.33178

	131	132	133	134	135	136	137	138	139	140
a_O	1346	1111	1096	1311	1140	1112	1297	1145	1072	1314
	(33.0)	(27.2)	(26.9)	(32.1)	(27.9)	(27.3)	(31.8)	(28.1)	(26.3)	(32.2)
a_E	1285.6	1103.6	1109.7	1285.0	1103.0	1109.4	1284.7	1102.8	1108.6	1283.7
	(31.5)	(27.0)	(27.2)	(31.5)	(27.0)	(27.2)	(31.5)	(27.0)	(27.2)	(31.5)
	123.5	14.9	−27.2	52.5	75.1	5.2	24.8	86.1	−72.0	61.2
	3.027	0.365	−0.667	1.288	1.842	0.127	0.607	2.110	−1.766	1.503
t_O	1259	1155	735	1322	1189	754	1289	1178	785	1280
	(30.9)	(28.3)	(18.0)	(32.4)	(29.1)	(18.5)	(31.6)	(28.9)	(19.3)	(31.4)
t_E	1271.3	1175.9	734.3	1270.7	1175.3	734.1	1270.4	1175.0	733.6	1269.5
	(31.2)	(28.8)	(18.0)	(31.2)	(28.8)	(18.0)	(31.2)	(28.8)	(18.0)	(31.2)
	−24.6	−41.4	1.4	104.6	27.5	40.3	37.5	5.9	106.4	21.1
	−0.602	−1.016	0.035	2.565	0.674	0.989	0.919	0.145	2.611	0.519
g_O	647	939	1437	606	900	1445	614	929	1426	617
	(15.9)	(23.0)	(35.2)	(14.9)	(22.1)	(35.4)	(15.1)	(22.8)	(35.0)	(15.1)
g_E	635.1	921.2	1455.6	634.7	920.7	1455.3	634.6	920.5	1454.2	634.1
	(15.6)	(22.6)	(35.7)	(15.6)	(22.6)	(35.7)	(15.6)	(22.6)	(35.7)	(15.6)
	24.1	35.9	−37.1	−56.2	−41.0	−20.5	−40.5	17.0	−55.9	−33.8
	0.591	0.881	−0.908	−1.377	−1.006	−0.503	−0.993	0.418	−1.372	−0.829
c_O	829	876	811	840	850	767	878	826	792	864
	(20.3)	(21.5)	(19.9)	(20.6)	(20.8)	(18.8)	(21.5)	(20.3)	(19.4)	(21.2)
c_E	889.0	880.3	779.4	888.5	879.9	779.2	888.3	879.7	778.6	887.7
	(21.8)	(21.6)	(19.1)	(21.8)	(21.6)	(19.1)	(21.8)	(21.6)	(19.1)	(21.8)
	−115.8	−8.6	64.5	−94.4	−58.7	−24.2	−20.5	−104.0	27.0	−46.7
	−2.838	−0.211	1.581	−2.314	−1.440	−0.594	−0.503	−2.550	0.662	−1.146
N	4081	4081	4079	4079	4079	4078	4078	4078	4075	4075
X2	7.2	0.8	1.7	6.5	2.9	0.8	1.2	5.0	5.6	1.9
Gvalue	7.3	0.8	1.7	6.6	2.9	0.8	1.2	5.0	5.5	1.9
Gv/N	0.178	0.019	0.041	0.162	0.071	0.020	0.029	0.123	0.136	0.047
Prob	0.06360	0.85261	0.64260	0.08623	0.41045	0.84804	0.75675	0.17001	0.13704	0.59376

	141	142	143	144	145	146	147	148	149	150
a_O	1119	1093	1347	1093	1151	1276	1108	1123	1291	1064
	(27.5)	(26.8)	(33.1)	(26.8)	(28.3)	(31.4)	(27.2)	(27.6)	(31.7)	(26.1)
a_E	1102.0	1108.0	1283.1	1101.4	1107.2	1282.2	1100.6	1107.0	1281.8	1100.3
	(27.0)	(27.2)	(31.5)	(27.0)	(27.2)	(31.5)	(27.0)	(27.2)	(31.5)	(27.0)
	34.3	−29.9	130.9	−16.8	89.2	−12.3	14.8	32.3	18.4	−71.5
	0.843	−0.734	3.214	−0.412	2.193	−0.302	0.364	0.794	0.451	−1.756
t_O	1171	735	1262	1197	742	1316	1228	756	1274	1229
	(28.7)	(18.0)	(31.0)	(29.4)	(18.2)	(32.3)	(30.2)	(18.6)	(31.3)	(30.2)
t_E	1174.2	733.2	1268.8	1173.6	732.7	1267.9	1172.7	732.5	1267.6	1172.5
	(28.8)	(18.0)	(31.2)	(28.8)	(18.0)	(31.2)	(28.8)	(18.0)	(31.2)	(28.8)
	−6.4	3.6	−13.7	47.3	18.8	98.0	113.1	47.8	12.8	115.8
	−0.156	0.089	−0.335	1.160	0.462	2.407	2.778	1.175	0.315	2.845
g_O	946	1457	574	909	1427	593	877	1445	622	906
	(23.2)	(35.8)	(14.1)	(22.3)	(35.1)	(14.6)	(21.5)	(35.5)	(15.3)	(22.3)
g_E	919.8	1453.5	633.8	919.4	1452.4	633.3	918.7	1452.1	633.2	918.5
	(22.6)	(35.7)	(15.6)	(22.6)	(35.7)	(15.6)	(22.6)	(35.7)	(15.6)	(22.6)
	53.1	7.0	−113.8	−20.7	−50.4	−78.1	−81.5	−14.1	−22.2	−24.8
	1.302	0.172	−2.794	−0.507	−1.239	−1.918	−2.003	−0.347	−0.545	−0.610
c_O	839	788	890	874	750	885	857	745	882	870
	(20.6)	(19.3)	(21.9)	(21.5)	(18.4)	(21.7)	(21.1)	(18.3)	(21.7)	(21.4)
c_E	879.0	778.2	887.2	878.6	777.7	886.6	877.9	777.5	886.4	877.7
	(21.6)	(19.1)	(21.8)	(21.6)	(19.1)	(21.8)	(21.6)	(19.1)	(21.8)	(21.6)
	−78.2	19.6	5.5	−9.1	−54.4	−3.2	−41.4	−63.6	−8.7	−15.4
	−1.919	0.482	0.136	−0.225	−1.335	−0.078	−1.017	−1.563	−0.214	−0.378
N	4075	4073	4073	4073	4070	4070	4070	4069	4069	4069
X2	2.8	0.3	8.9	0.7	3.3	4.4	5.0	2.4	0.3	4.2
Gvalue	2.9	0.3	9.0	0.7	3.3	4.5	5.0	2.4	0.3	4.1
Gv/N	0.070	0.008	0.221	0.016	0.080	0.110	0.124	0.059	0.008	0.102
Prob	0.41409	0.95238	0.02921	0.88033	0.35166	0.21589	0.16889	0.49551	0.95664	0.24710

Bflo
5UTR:

	50	49	48	47	46	45	44	43	42	41
a_O	240	228	235	223	250	214	235	231	243	243
	(41.2)	(39.1)	(40.3)	(38.3)	(42.9)	(36.7)	(40.3)	(39.6)	(41.7)	(41.7)
a_E	236.0	236.0	236.0	236.0	236.0	236.0	236.0	236.0	236.0	236.0
	(40.5)	(40.5)	(40.5)	(40.5)	(40.5)	(40.5)	(40.5)	(40.5)	(40.5)	(40.5)
	8.1	-15.7	-2.0	-25.2	28.9	-41.8	-2.0	-9.9	14.2	14.2
	1.391	-2.691	-0.335	-4.328	4.950	-7.178	-0.335	-1.690	2.444	2.444
t_O	244	249	237	237	233	264	221	232	239	233
	(41.9)	(42.7)	(40.7)	(40.7)	(40.0)	(45.3)	(37.9)	(39.8)	(41.0)	(40.0)
t_E	232.6	232.6	232.6	232.6	232.6	232.6	232.6	232.6	232.6	232.6
	(39.9)	(39.9)	(39.9)	(39.9)	(39.9)	(39.9)	(39.9)	(39.9)	(39.9)	(39.9)
	23.3	33.9	8.9	8.9	0.8	66.9	-22.6	-1.2	13.0	0.8
	4.005	5.820	1.524	1.524	0.137	11.468	-3.878	-0.206	2.225	0.137
g_O	63	60	63	73	61	63	74	68	57	63
	(10.8)	(10.3)	(10.8)	(12.5)	(10.5)	(10.8)	(12.7)	(11.7)	(9.8)	(10.8)
g_E	71.2	71.2	71.2	71.2	71.2	71.2	71.2	71.2	71.2	71.2
	(12.2)	(12.2)	(12.2)	(12.2)	(12.2)	(12.2)	(12.2)	(12.2)	(12.2)	(12.2)
	-15.5	-20.6	-15.5	3.6	-18.9	-15.5	5.6	-6.3	-25.4	-15.5
	-2.654	-3.531	-2.654	0.615	-3.244	-2.654	0.968	-1.083	-4.358	-2.654
c_O	36	46	48	50	39	42	53	52	44	44
	(6.2)	(7.9)	(8.2)	(8.6)	(6.7)	(7.2)	(9.1)	(8.9)	(7.5)	(7.5)
c_E	43.2	43.2	43.2	43.2	43.2	43.2	43.2	43.2	43.2	43.2
	(7.4)	(7.4)	(7.4)	(7.4)	(7.4)	(7.4)	(7.4)	(7.4)	(7.4)	(7.4)
	-13.1	5.8	10.1	14.6	-8.0	-2.3	21.7	19.3	1.6	1.6
	-2.249	0.995	1.739	2.511	-1.365	-0.403	3.722	3.311	0.280	0.280
N	583	583	583	583	583	583	583	583	583	583
X2	2.8	3.4	1.6	1.9	2.7	7.3	2.9	2.1	3.2	1.2
Gvalue	2.9	3.5	1.6	1.9	2.8	7.2	2.8	1.9	3.5	1.2
Gv/N	0.494	0.592	0.273	0.322	0.477	1.234	0.476	0.333	0.592	0.208
Prob	0.41094	0.32682	0.66088	0.59872	0.42630	0.06588	0.42760	0.58450	0.32730	0.75004

	40	39	38	37	36	35	34	33	32	31
a_O	245	227	257	251	211	234	215	222	241	223
	(42.0)	(38.9)	(44.1)	(43.1)	(36.2)	(40.1)	(36.9)	(38.1)	(41.3)	(38.3)
a_E	236.0	236.0	236.0	236.0	236.0	236.0	236.0	236.0	236.0	236.0
	(40.5)	(40.5)	(40.5)	(40.5)	(40.5)	(40.5)	(40.5)	(40.5)	(40.5)	(40.5)
	18.4	-17.6	43.9	31.0	-47.2	-3.9	-40.0	-27.1	10.1	-25.2
	3.153	-3.021	7.523	5.313	-8.099	-0.676	-6.867	-4.651	1.740	-4.328
t_O	229	255	223	220	269	244	260	256	241	252
	(39.3)	(43.7)	(38.3)	(37.7)	(46.1)	(41.9)	(44.6)	(43.9)	(41.3)	(43.2)
t_E	232.6	232.6	232.6	232.6	232.6	232.6	232.6	232.6	232.6	232.6
	(39.9)	(39.9)	(39.9)	(39.9)	(39.9)	(39.9)	(39.9)	(39.9)	(39.9)	(39.9)
	-7.1	46.9	-18.8	-24.5	78.2	23.3	57.9	49.1	17.1	40.4
	-1.225	8.043	-3.224	-4.203	13.417	4.005	9.933	8.418	2.933	6.925
g_O	74	61	57	69	61	66	59	57	60	71
	(12.7)	(10.5)	(9.8)	(11.8)	(10.5)	(11.3)	(10.1)	(9.8)	(10.3)	(12.2)
g_E	71.2	71.2	71.2	71.2	71.2	71.2	71.2	71.2	71.2	71.2
	(12.2)	(12.2)	(12.2)	(12.2)	(12.2)	(12.2)	(12.2)	(12.2)	(12.2)	(12.2)
	5.6	-18.9	-25.4	-4.4	-18.9	-10.1	-22.2	-25.4	-20.6	-0.5
	0.968	-3.244	-4.358	-0.753	-3.244	-1.727	-3.813	-4.358	-3.531	-0.079
c_O	35	40	46	43	42	39	49	48	41	37
	(6.0)	(6.9)	(7.9)	(7.4)	(7.2)	(6.7)	(8.4)	(8.2)	(7.0)	(6.3)
c_E	43.2	43.2	43.2	43.2	43.2	43.2	43.2	43.2	43.2	43.2
	(7.4)	(7.4)	(7.4)	(7.4)	(7.4)	(7.4)	(7.4)	(7.4)	(7.4)	(7.4)
	-14.7	-6.1	5.8	-0.4	-2.3	-8.0	12.4	10.1	-4.3	-11.4
	-2.525	-1.053	0.995	-0.065	-0.403	-1.365	2.122	1.739	-0.732	-1.963
N	583	583	583	583	583	583	583	583	583	583
X2	2.1	4.2	5.3	1.7	9.8	1.4	8.0	6.6	2.3	3.2
Gvalue	2.2	4.2	5.5	1.7	9.7	1.4	8.0	6.7	2.4	3.2
Gv/N	0.371	0.725	0.935	0.292	1.671	0.237	1.374	1.148	0.410	0.555
Prob	0.53896	0.23827	0.14144	0.63628	0.02090	0.71010	0.04578	0.08230	0.49542	0.35667

	30	29	28	27	26	25	24	23	22	21
a_O	222	264	250	238	245	236	235	266	239	229
	(38.1)	(45.3)	(42.9)	(40.8)	(42.0)	(40.5)	(40.3)	(45.6)	(41.0)	(39.3)
a_E	236.0	236.0	236.0	236.0	236.0	236.0	236.0	236.0	236.0	236.0
	(40.5)	(40.5)	(40.5)	(40.5)	(40.5)	(40.5)	(40.5)	(40.5)	(40.5)	(40.5)
	-27.1	59.2	28.9	4.1	18.4	0.0	-2.0	63.7	6.1	-13.8
	-4.651	10.162	4.950	0.696	3.153	0.007	-0.335	10.927	1.043	-2.359
t_O	266	245	228	236	232	246	236	211	248	248
	(45.6)	(42.0)	(39.1)	(40.5)	(39.8)	(42.2)	(40.5)	(36.2)	(42.5)	(42.5)
t_E	232.6	232.6	232.6	232.6	232.6	232.6	232.6	232.6	232.6	232.6
	(39.9)	(39.9)	(39.9)	(39.9)	(39.9)	(39.9)	(39.9)	(39.9)	(39.9)	(39.9)
	71.4	25.4	-9.1	6.8	-1.2	27.6	6.8	-41.1	31.8	31.8
	12.244	4.365	-1.562	1.175	-0.206	4.727	1.175	-7.055	5.454	5.454
g_O	56	43	62	67	68	61	65	63	53	62
	(9.6)	(7.4)	(10.6)	(11.5)	(11.7)	(10.5)	(11.1)	(10.8)	(9.1)	(10.6)
g_E	71.2	71.2	71.2	71.2	71.2	71.2	71.2	71.2	71.2	71.2
	(12.2)	(12.2)	(12.2)	(12.2)	(12.2)	(12.2)	(12.2)	(12.2)	(12.2)	(12.2)
	-26.9	-43.4	-17.2	-8.2	-6.3	-18.9	-11.9	-15.5	-31.3	-17.2
	-4.621	-7.445	-2.952	-1.407	-1.083	-3.244	-2.041	-2.654	-5.375	-2.952
c_O	39	31	43	42	38	40	47	43	43	44
	(6.7)	(5.3)	(7.4)	(7.2)	(6.5)	(6.9)	(8.1)	(7.4)	(7.4)	(7.5)
c_E	43.2	43.2	43.2	43.2	43.2	43.2	43.2	43.2	43.2	43.2
	(7.4)	(7.4)	(7.4)	(7.4)	(7.4)	(7.4)	(7.4)	(7.4)	(7.4)	(7.4)
	-8.0	-20.6	-0.4	-2.3	-9.7	-6.1	7.9	-0.4	-0.4	1.6
	-1.365	-3.527	-0.065	-0.403	-1.669	-1.053	1.363	-0.065	-0.065	0.280
N	583	583	583	583	583	583	583	583	583	583
X2	9.3	18.6	2.1	0.4	1.1	2.5	0.9	6.8	5.7	2.4
Gvalue	9.4	20.7	2.2	0.4	1.1	2.5	0.9	6.7	6.2	2.5
Gv/N	1.606	3.555	0.371	0.061	0.196	0.436	0.162	1.154	1.057	0.424
Prob	0.02482	0.00012	0.53974	0.94915	0.76718	0.46734	0.81536	0.08108	0.10403	0.48023

	20	19	18	17	16	15	14	13	12	11
a_O	271	256	229	246	243	270	259	249	248	255
	(46.5)	(43.9)	(39.3)	(42.2)	(41.7)	(46.3)	(44.4)	(42.7)	(42.5)	(43.7)
a_E	236.0	236.0	236.0	236.0	236.0	236.0	236.0	236.0	236.0	236.0
	(40.5)	(40.5)	(40.5)	(40.5)	(40.5)	(40.5)	(40.5)	(40.5)	(40.5)	(40.5)
	75.0	41.7	-13.8	20.5	14.2	72.7	48.2	26.7	24.6	39.5
	12.864	7.151	-2.359	3.509	2.444	12.474	8.270	4.588	4.227	6.781
t_O	228	240	236	239	221	205	198	188	180	161
	(39.1)	(41.2)	(40.5)	(41.0)	(37.9)	(35.2)	(34.0)	(32.2)	(30.9)	(27.6)

```
t_E       232.6   232.6   232.6   232.6   232.6   232.6   232.6   232.6   232.6   232.6
        ( 39.9) ( 39.9) ( 39.9) ( 39.9) ( 39.9) ( 39.9) ( 39.9) ( 39.9) ( 39.9) ( 39.9)
           -9.1    15.0     6.8    13.0   -22.6   -51.8   -63.8   -80.0   -92.3  -118.5
         -1.562   2.579   1.175   2.225  -3.878  -8.883 -10.939 -13.729 -15.830 -20.321
g_O          51      47      74      74      76      71      99     121     133     137
        (  8.7) (  8.1) ( 12.7) ( 12.7) ( 13.0) ( 12.2) ( 17.0) ( 20.8) ( 22.8) ( 23.5)
g_E        71.2    71.2    71.2    71.2    71.2    71.2    71.2    71.2    71.2    71.2
        ( 12.2) ( 12.2) ( 12.2) ( 12.2) ( 12.2) ( 12.2) ( 12.2) ( 12.2) ( 12.2) ( 12.2)
          -34.1   -39.1     5.6     5.6     9.9    -0.5    65.2   128.2   166.1   179.2
         -5.845  -6.704   0.968   0.968   1.690  -0.079  11.181  21.995  28.491  30.740
c_O          33      40      44      24      43      37      27      25      22      30
        (  5.7) (  6.9) (  7.5) (  4.1) (  7.4) (  6.3) (  4.6) (  4.3) (  3.8) (  5.1)
c_E        43.2    43.2    43.2    43.2    43.2    43.2    43.2    43.2    43.2    43.2
        (  7.4) (  7.4) (  7.4) (  7.4) (  7.4) (  7.4) (  7.4) (  7.4) (  7.4) (  7.4)
          -17.8    -6.1     1.6   -28.2    -0.4   -11.4   -25.4   -27.3   -29.7   -21.9
         -3.046  -1.053   0.280  -4.838  -0.065  -1.963  -4.351  -4.689  -5.091  -3.750

N           583     583     583     583     583     583     583     583     583     583
X2         13.4    10.4     0.4     9.2     1.1     9.1    24.3    51.7    76.5    88.3
Gvalue     14.1    11.5     0.4    10.9     1.1     9.0    24.3    47.6    68.8    78.4
Gv/N      2.410   1.973   0.065   1.866   0.190   1.549   4.160   8.164  11.796  13.450
Prob    0.00284 0.00929 0.94446 0.01241 0.77502 0.02888 0.00002 0.00000 0.00000 0.00000
        -------------------------------------------------------------------------------
             10       9       8       7       6       5       4       3       2       1
        -------------------------------------------------------------------------------
a_O         242     222     257     244     271     266     255     307     247     148
        ( 41.5) ( 38.1) ( 44.1) ( 41.9) ( 46.5) ( 45.6) ( 43.7) ( 52.7) ( 42.4) ( 25.4)
a_E       236.0   236.0   236.0   236.0   236.0   236.0   236.0   236.0   236.0   236.0
        ( 40.5) ( 40.5) ( 40.5) ( 40.5) ( 40.5) ( 40.5) ( 40.5) ( 40.5) ( 40.5) ( 40.5)
           12.2   -27.1    43.9    16.3    75.0    63.7    39.5   161.5    22.5  -138.1
          2.091  -4.651   7.523   2.798  12.864  10.927   6.781  27.709   3.867 -23.687
t_O         156     186     174     221     205     230     243     198     251     355
        ( 26.8) ( 31.9) ( 29.8) ( 37.9) ( 35.2) ( 39.5) ( 41.7) ( 34.0) ( 43.1) ( 60.9)
t_E       232.6   232.6   232.6   232.6   232.6   232.6   232.6   232.6   232.6   232.6
        ( 39.9) ( 39.9) ( 39.9) ( 39.9) ( 39.9) ( 39.9) ( 39.9) ( 39.9) ( 39.9) ( 39.9)
         -124.6   -83.2  -101.0   -22.6   -51.8    -5.2    21.3   -63.8    38.2   300.2
        -21.378 -14.266 -17.326  -3.878  -8.883  -0.887   3.646 -10.939   6.556  51.490
g_O         158     147     125      85      76      59      49      45      47      27
        ( 27.1) ( 25.2) ( 21.4) ( 14.6) ( 13.0) ( 10.1) (  8.4) (  7.7) (  8.1) (  4.6)
g_E        71.2    71.2    71.2    71.2    71.2    71.2    71.2    71.2    71.2    71.2
        ( 12.2) ( 12.2) ( 12.2) ( 12.2) ( 12.2) ( 12.2) ( 12.2) ( 12.2) ( 12.2) ( 12.2)
          251.8   213.0   140.6    30.0     9.9   -22.2   -36.7   -41.3   -39.1   -52.4
         43.182  36.537  24.117   5.154   1.690  -3.813  -6.288  -7.090  -6.704  -8.985
c_O          27      28      27      33      31      28      36      33      38      53
        (  4.6) (  4.8) (  4.6) (  5.7) (  5.3) (  4.8) (  6.2) (  5.7) (  6.5) (  9.1)
c_E        43.2    43.2    43.2    43.2    43.2    43.2    43.2    43.2    43.2    43.2
        (  7.4) (  7.4) (  7.4) (  7.4) (  7.4) (  7.4) (  7.4) (  7.4) (  7.4) (  7.4)
          -25.4   -24.3   -25.4   -17.8   -20.6   -24.3   -13.1   -17.8    -9.7    21.7
         -4.351  -4.163  -4.351  -3.046  -3.527  -4.163  -2.249  -3.046  -1.669   3.722

N           583     583     583     583     583     583     583     583     583     583
X2        137.1    96.1    63.3     5.9    12.2    11.3    10.1    38.6    10.8   126.9
Gvalue    113.9    78.5    58.1     6.0    12.5    12.0    11.0    38.7    12.0   131.4
Gv/N     19.544  13.457   9.962   1.026   2.144   2.065   1.890   6.634   2.050  22.539
Prob    0.00000 0.00000 0.00000 0.11246 0.00585 0.00726 0.01162 0.00000 0.00754 0.00000
        -------------------------------------------------------------------------------

CDS_INITIAL:
        -------------------------------------------------------------------------------
              1       2       3       4       5       6       7       8       9      10
        -------------------------------------------------------------------------------
a_O         524       0       0     311     244     220     292     252     239     280
        ( 89.9) (  0.0) (  0.0) ( 53.3) ( 41.9) ( 37.7) ( 50.1) ( 43.2) ( 41.0) ( 48.0)
a_E       210.9   197.0   227.5   210.9   197.0   227.5   210.9   197.0   227.5   210.9
        ( 36.2) ( 33.8) ( 39.0) ( 36.2) ( 33.8) ( 39.0) ( 36.2) ( 33.8) ( 39.0) ( 36.2)
          953.5     0.0     0.0   241.4   104.4   -14.7   189.9   124.1    23.6   158.6
        163.558   0.000   0.000  41.414  17.904  -2.518  32.569  21.280   4.057  27.200
t_O          20     583       0     127     204     274     140     193     248     151
        (  3.4) (100.0) (  0.0) ( 21.8) ( 35.0) ( 47.0) ( 24.0) ( 33.1) ( 42.5) ( 25.9)
t_E       145.4   199.2   262.4   145.4   199.2   262.4   145.4   199.2   262.4   145.4
        ( 24.9) ( 34.2) ( 45.0) ( 24.9) ( 34.2) ( 45.0) ( 24.9) ( 34.2) ( 45.0) ( 24.9)
          -79.4  1252.1     0.0   -34.4     9.7    23.7   -10.6   -12.2   -28.0    11.4
        -13.612 214.763   0.000  -5.903   1.662   4.062  -1.827  -2.098  -4.805   1.948
g_O          39       0     583      72      52      50      84      62      52      82
        (  6.7) (  0.0) (100.0) ( 12.3) (  8.9) (  8.6) ( 14.4) ( 10.6) (  8.9) ( 14.1)
g_E       146.1    82.9    62.2   146.1    82.9    62.2   146.1    82.9    62.2   146.1
        ( 25.1) ( 14.2) ( 10.7) ( 25.1) ( 14.2) ( 10.7) ( 25.1) ( 14.2) ( 10.7) ( 25.1)
         -103.0     0.0  2609.2  -101.9   -48.6   -21.8   -93.0   -36.1   -18.6   -94.7
        -17.670   0.000 447.540 -17.479  -8.329  -3.747 -15.950  -6.190  -3.197 -16.248
c_O           0       0       0      73      83      39      67      76      44      70
        (  0.0) (  0.0) (  0.0) ( 12.5) ( 14.2) (  6.7) ( 11.5) ( 13.0) (  7.5) ( 12.0)
c_E        80.5   103.8    30.9    80.5   103.8    30.9    80.5   103.8    30.9    80.5
        ( 13.8) ( 17.8) (  5.3) ( 13.8) ( 17.8) (  5.3) ( 13.8) ( 17.8) (  5.3) ( 13.8)
            0.0     0.0     0.0   -14.3   -37.2    18.1   -24.6   -47.4    31.0   -19.6
          0.000   0.000   0.000  -2.455  -6.375   3.107  -4.225  -8.135   5.326  -3.362

N           583     583     583     583     583     583     583     583     583     583
X2        731.8  1123.2  4880.7    88.1    27.0     5.3    60.0    28.3     8.6    52.3
Gvalue    771.2  1252.1  2609.2    90.8    28.3     5.3    61.6    28.3     8.0    55.6
Gv/N    132.276 214.763 447.540  15.577   4.861   0.904  10.568   4.857   1.380   9.538
Prob    0.00000 0.00000 1.00000 0.00000 0.00000 0.15299 0.00000 0.00000 0.04513 0.00000
        -------------------------------------------------------------------------------
             11      12      13      14      15      16      17      18      19      20
        -------------------------------------------------------------------------------
a_O         229     232     269     222     228     249     209     228     236     206
        ( 39.3) ( 39.8) ( 46.1) ( 38.1) ( 39.1) ( 42.7) ( 35.8) ( 39.1) ( 40.5) ( 35.3)
a_E       197.0   227.5   210.9   197.0   227.5   210.9   197.0   227.5   210.9   197.0
        ( 33.8) ( 39.0) ( 36.2) ( 33.8) ( 39.0) ( 36.2) ( 33.8) ( 39.0) ( 36.2) ( 33.8)
           68.9     9.2   130.8    53.0     1.1    82.6    24.7     1.1    53.0    18.4
         11.819   1.572  22.433   9.094   0.185  14.165   4.235   0.185   9.085   3.152
t_O         221     279     146     216     280     155     233     269     147     227
        ( 37.9) ( 47.9) ( 25.0) ( 37.0) ( 48.0) ( 26.6) ( 40.0) ( 46.1) ( 25.2) ( 38.9)
t_E       199.2   262.4   145.4   199.2   262.4   145.4   199.2   262.4   145.4   199.2
        ( 34.2) ( 45.0) ( 24.9) ( 34.2) ( 45.0) ( 24.9) ( 34.2) ( 45.0) ( 24.9) ( 34.2)
           45.9    34.2     1.1    35.0    36.3    19.8    73.0    13.3     3.2    59.3
          7.869   5.867   0.197   5.995   6.232   3.390  12.522   2.289   0.542  10.168
g_O          58      46      97      60      50     100      71      55     128      79
        (  9.9) (  7.9) ( 16.6) ( 10.3) (  8.6) ( 17.2) ( 12.2) (  9.4) ( 22.0) ( 13.6)
g_E        82.9    62.2   146.1    82.9    62.2   146.1    82.9    62.2   146.1    82.9
        ( 14.2) ( 10.7) ( 25.1) ( 14.2) ( 10.7) ( 25.1) ( 14.2) ( 10.7) ( 25.1) ( 14.2)
          -41.5   -27.8   -79.5   -38.9   -21.8   -75.8   -22.1   -13.5   -33.9    -7.7
         -7.118  -4.763 -13.630  -6.665  -3.747 -13.006  -3.787  -2.324  -5.808  -1.320
```

c_O	75	26	71	85	25	79	70	31	72	71
	(12.9)	(4.5)	(12.2)	(14.6)	(4.3)	(13.6)	(12.0)	(5.3)	(12.3)	(12.2)
c_E	103.8	30.9	80.5	103.8	30.9	80.5	103.8	30.9	80.5	103.8
	(17.8)	(5.3)	(13.8)	(17.8)	(5.3)	(13.8)	(17.8)	(5.3)	(13.8)	(17.8)
	-48.8	-9.0	-17.9	-34.0	-10.6	-3.0	-55.2	0.2	-16.1	-54.0
	-8.369	-1.545	-3.065	-5.835	-1.822	-0.517	-9.468	0.028	-2.762	-9.257
N	583	583	583	583	583	583	583	583	583	583
X2	23.1	6.1	33.6	14.3	4.7	22.1	19.2	1.0	6.1	14.9
Gvalue	24.5	6.6	34.6	15.1	4.9	23.5	20.4	1.0	6.2	16.0
Gv/N	4.202	1.131	5.935	2.589	0.847	4.032	3.502	0.178	1.056	2.743
Prob	0.00002	0.08612	0.00000	0.00174	0.17640	0.00003	0.00014	0.79277	0.10420	0.00114

	21	22	23	24	25	26	27	28	29	30
a_O	229	220	189	235	235	183	201	223	181	227
	(39.3)	(37.7)	(32.4)	(40.3)	(40.3)	(31.4)	(34.5)	(38.3)	(31.0)	(38.9)
a_E	227.5	210.9	197.0	227.5	210.9	197.0	227.5	210.9	197.0	227.5
	(39.0)	(36.2)	(33.8)	(39.0)	(36.2)	(33.8)	(39.0)	(36.2)	(33.8)	(39.0)
	3.1	18.5	-15.7	15.3	50.7	-27.0	-49.7	24.8	-30.7	-0.9
	0.529	3.170	-2.692	2.628	8.704	-4.632	-8.528	4.250	-5.264	-0.159
t_O	266	160	229	278	143	215	300	153	222	269
	(45.6)	(27.4)	(39.3)	(47.7)	(24.5)	(36.9)	(51.5)	(26.2)	(38.1)	(46.1)
t_E	262.4	145.4	199.2	262.4	145.4	199.2	262.4	145.4	199.2	262.4
	(45.0)	(24.9)	(34.2)	(45.0)	(24.9)	(34.2)	(45.0)	(24.9)	(34.2)	(45.0)
	7.2	30.6	63.8	32.1	-4.8	32.8	80.3	15.5	48.1	13.3
	1.240	5.242	10.947	5.504	-0.826	5.625	13.778	2.664	8.248	2.289
g_O	61	129	75	35	119	82	55	122	76	61
	(10.5)	(22.1)	(12.9)	(6.0)	(20.4)	(14.1)	(9.4)	(20.9)	(13.0)	(10.5)
g_E	62.2	146.1	82.9	62.2	146.1	82.9	62.2	146.1	82.9	62.2
	(10.7)	(25.1)	(14.2)	(10.7)	(25.1)	(14.2)	(10.7)	(25.1)	(14.2)	(10.7)
	-2.4	-32.1	-15.1	-40.3	-48.8	-1.9	-13.5	-44.0	-13.3	-2.4
	-0.410	-5.509	-2.590	-6.906	-8.376	-0.322	-2.324	-7.546	-2.280	-0.410
c_O	27	74	90	35	86	103	27	85	104	26
	(4.6)	(12.7)	(15.4)	(6.0)	(14.8)	(17.7)	(4.6)	(14.6)	(17.8)	(4.5)
c_E	30.9	80.5	103.8	30.9	80.5	103.8	30.9	80.5	103.8	30.9
	(5.3)	(13.8)	(17.8)	(5.3)	(13.8)	(17.8)	(5.3)	(13.8)	(17.8)	(5.3)
	-7.3	-12.5	-25.7	8.7	11.3	-1.7	-7.3	9.2	0.3	-9.0
	-1.255	-2.144	-4.413	1.489	1.942	-0.283	-1.255	1.579	0.058	-1.545
N	583	583	583	583	583	583	583	583	583	583
X2	0.6	4.4	7.4	13.6	8.2	2.3	9.8	5.3	4.5	1.0
Gvalue	0.6	4.4	7.3	15.8	8.4	2.3	9.7	5.5	4.4	1.0
Gv/N	0.103	0.759	1.251	2.715	1.444	0.387	1.670	0.947	0.763	0.174
Prob	0.89598	0.21912	0.06310	0.00123	0.03809	0.52062	0.02092	0.13727	0.21685	0.79730

	31	32	33	34	35	36	37	38	39	40
a_O	212	188	229	210	160	226	212	185	222	197
	(36.4)	(32.2)	(39.3)	(36.0)	(27.4)	(38.8)	(36.4)	(31.7)	(38.1)	(33.8)
a_E	210.9	197.0	227.5	210.9	197.0	227.5	210.9	197.0	227.5	210.9
	(36.2)	(33.8)	(39.0)	(36.2)	(33.8)	(39.0)	(36.2)	(33.8)	(39.0)	(36.2)
	2.1	-17.6	3.1	-1.9	-66.6	-2.9	2.1	-23.3	-10.8	-27.0
	0.361	-3.020	0.529	-0.325	-11.422	-0.500	0.361	-3.993	-1.851	-4.624
t_O	147	221	266	164	228	264	160	210	266	144
	(25.2)	(37.9)	(45.6)	(28.1)	(39.1)	(45.3)	(27.4)	(36.0)	(45.6)	(24.7)
t_E	145.4	199.2	262.4	145.4	199.2	262.4	145.4	199.2	262.4	145.4
	(24.9)	(34.2)	(45.0)	(24.9)	(34.2)	(45.0)	(24.9)	(34.2)	(45.0)	(24.9)
	3.2	45.9	7.2	39.4	61.5	3.2	30.6	22.1	7.2	-2.8
	0.542	7.869	1.240	6.762	10.557	0.547	5.242	3.799	1.240	-0.487
g_O	142	81	55	130	90	56	143	90	67	149
	(24.4)	(13.9)	(9.4)	(22.3)	(15.4)	(9.6)	(24.5)	(15.4)	(11.5)	(25.6)
g_E	146.1	82.9	62.2	146.1	82.9	62.2	146.1	82.9	62.2	146.1
	(25.1)	(14.2)	(10.7)	(25.1)	(14.2)	(10.7)	(25.1)	(14.2)	(10.7)	(25.1)
	-8.1	-3.8	-13.5	-30.4	14.7	-11.8	-6.1	14.7	9.9	5.9
	-1.387	-0.659	-2.324	-5.208	2.521	-2.020	-1.053	2.521	1.706	1.004
c_O	82	93	33	79	105	37	68	98	28	93
	(14.1)	(16.0)	(5.7)	(13.6)	(18.0)	(6.3)	(11.7)	(16.8)	(4.8)	(16.0)
c_E	80.5	103.8	30.9	80.5	103.8	30.9	80.5	103.8	30.9	80.5
	(13.8)	(17.8)	(5.3)	(13.8)	(17.8)	(5.3)	(13.8)	(17.8)	(5.3)	(13.8)
	3.0	-20.5	4.3	-3.0	2.4	13.3	-23.0	-11.3	-5.6	26.8
	0.512	-3.514	0.737	-0.517	0.404	2.279	-3.942	-1.943	-0.952	4.597
N	583	583	583	583	583	583	583	583	583	583
X2	0.2	4.0	1.0	4.2	11.7	1.8	3.5	2.2	0.8	2.9
Gvalue	0.2	3.9	1.1	4.2	12.0	1.8	3.5	2.2	0.8	2.9
Gv/N	0.028	0.675	0.183	0.713	2.059	0.306	0.608	0.384	0.142	0.490
Prob	0.98296	0.26845	0.78534	0.24531	0.00737	0.61823	0.31539	0.52426	0.84329	0.41436

	41	42	43	44	45	46	47	48	49	50
a_O	187	236	207	169	229	200	174	257	227	177
	(32.1)	(40.5)	(35.5)	(29.0)	(39.3)	(34.3)	(29.8)	(44.1)	(38.9)	(30.4)
a_E	197.0	227.5	210.9	197.0	227.5	210.9	197.0	227.5	210.9	197.0
	(33.8)	(39.0)	(36.2)	(33.8)	(39.0)	(36.2)	(33.8)	(39.0)	(36.2)	(33.8)
	-19.5	17.4	-7.8	-51.8	3.1	-21.3	-43.2	62.8	33.3	-37.9
	-3.346	2.983	-1.342	-8.892	0.529	-3.657	-7.415	10.764	5.710	-6.504
t_O	204	257	158	207	253	157	211	241	132	209
	(35.0)	(44.1)	(27.1)	(35.5)	(43.4)	(26.9)	(36.2)	(41.3)	(22.6)	(35.8)
t_E	199.2	262.4	145.4	199.2	262.4	145.4	199.2	262.4	145.4	199.2
	(34.2)	(45.0)	(24.9)	(34.2)	(45.0)	(24.9)	(34.2)	(45.0)	(24.9)	(34.2)
	9.7	-10.7	26.2	15.9	-18.5	24.0	24.3	-41.0	-25.6	20.0
	1.662	-1.837	4.494	2.723	-3.170	4.124	4.161	-7.037	-4.387	3.439
g_O	91	62	144	106	63	150	89	64	152	94
	(15.6)	(10.6)	(24.7)	(18.2)	(10.8)	(25.7)	(15.3)	(11.0)	(26.1)	(16.1)
g_E	82.9	62.2	146.1	82.9	62.2	146.1	82.9	62.2	146.1	82.9
	(14.2)	(10.7)	(25.1)	(14.2)	(10.7)	(25.1)	(14.2)	(10.7)	(25.1)	(14.2)
	16.9	-0.4	-4.2	52.0	1.6	7.9	12.5	3.6	12.0	23.5
	2.894	-0.071	-0.716	8.919	0.273	1.355	2.152	0.623	2.063	4.035
c_O	101	28	74	101	38	76	109	21	72	103
	(17.3)	(4.8)	(12.7)	(17.3)	(6.5)	(13.0)	(18.7)	(3.6)	(12.3)	(17.7)
c_E	103.8	30.9	80.5	103.8	30.9	80.5	103.8	30.9	80.5	103.8
	(17.8)	(5.3)	(13.8)	(17.8)	(5.3)	(13.8)	(17.8)	(5.3)	(13.8)	(17.8)
	-5.6	-5.6	-12.5	-5.6	15.7	-8.8	10.6	-16.2	-16.1	-1.7
	-0.957	-0.952	-2.144	-0.957	2.688	-1.506	1.817	-2.787	-2.762	-0.283
N	583	583	583	583	583	583	583	583	583	583
X2	1.5	0.7	1.7	10.8	2.0	1.8	4.1	8.8	3.6	4.0
Gvalue	1.5	0.7	1.7	10.5	1.9	1.8	4.2	9.1	3.6	4.0
Gv/N	0.252	0.122	0.292	1.793	0.321	0.315	0.715	1.564	0.625	0.686
Prob	0.68968	0.86987	0.63591	0.01510	0.59930	0.60679	0.24383	0.02777	0.30291	0.26172

	51	52	53	54	55	56	57	58	59	60
a_O	231	214	185	220	207	186	206	218	170	225
	(39.6)	(36.7)	(31.7)	(37.7)	(35.5)	(31.9)	(35.3)	(37.4)	(29.2)	(38.6)
a_E	227.5	210.9	197.0	227.5	210.9	197.0	227.5	210.9	197.0	227.5
	(39.0)	(36.2)	(33.8)	(39.0)	(36.2)	(33.8)	(39.0)	(36.2)	(33.8)	(39.0)
	7.1	6.1	-23.3	-14.7	-7.8	-21.4	-40.8	14.3	-50.1	-4.9
	1.223	1.054	-3.993	-2.518	-1.342	-3.671	-7.004	2.459	-8.600	-0.840
t_O	256	155	214	249	147	220	277	142	231	267
	(43.9)	(26.6)	(36.7)	(42.7)	(25.2)	(37.7)	(47.5)	(24.4)	(39.6)	(45.8)
t_E	262.4	145.4	199.2	262.4	145.4	199.2	262.4	145.4	199.2	262.4
	(45.0)	(24.9)	(34.2)	(45.0)	(24.9)	(34.2)	(45.0)	(24.9)	(34.2)	(45.0)
	-12.7	19.8	30.6	-26.1	3.2	43.7	30.0	-6.8	68.4	9.3
	-2.172	3.390	5.257	-4.481	0.542	7.491	5.142	-1.162	11.732	1.588
g_O	73	131	102	84	141	87	57	140	78	68
	(12.5)	(22.5)	(17.5)	(14.4)	(24.2)	(14.9)	(9.8)	(24.0)	(13.4)	(11.7)
g_E	62.2	146.1	82.9	62.2	146.1	82.9	62.2	146.1	82.9	62.2
	(10.7)	(25.1)	(14.2)	(10.7)	(25.1)	(14.2)	(10.7)	(25.1)	(14.2)	(10.7)
	23.4	-28.6	42.2	50.5	-10.0	8.3	-10.0	-11.9	-9.6	12.1
	4.006	-4.904	7.236	8.654	-1.720	1.425	-1.710	-2.049	-1.645	2.077
c_O	23	83	82	30	88	90	43	83	104	23
	(3.9)	(14.2)	(14.1)	(5.1)	(15.1)	(15.4)	(7.4)	(14.2)	(17.8)	(3.9)
c_E	30.9	80.5	103.8	30.9	80.5	103.8	30.9	80.5	103.8	30.9
	(5.3)	(13.8)	(17.8)	(5.3)	(13.8)	(17.8)	(5.3)	(13.8)	(17.8)	(5.3)
	-13.6	5.0	-38.7	-1.8	15.6	-25.7	28.4	5.0	0.3	-13.6
	-2.334	0.864	-6.640	-0.310	2.682	-4.413	4.866	0.864	0.058	-2.334
N	583	583	583	583	583	583	583	583	583	583
X2	4.1	2.3	10.8	8.6	1.0	4.8	8.0	0.6	9.1	2.7
Gvalue	4.2	2.4	10.8	7.8	0.9	4.8	7.5	0.6	9.0	2.9
Gv/N	0.723	0.404	1.860	1.346	0.162	0.832	1.293	0.111	1.545	0.490
Prob	0.23940	0.50223	0.01259	0.04934	0.81433	0.18314	0.05652	0.88523	0.02917	0.41414

	61	62	63	64	65	66	67	68	69	70
a_O	210	182	225	209	188	230	212	161	241	202
	(36.0)	(31.2)	(38.6)	(35.8)	(32.2)	(39.5)	(36.4)	(27.6)	(41.3)	(34.6)
a_E	210.9	197.0	227.5	210.9	197.0	227.5	210.9	197.0	227.5	210.9
	(36.2)	(33.8)	(39.0)	(36.2)	(33.8)	(39.0)	(36.2)	(33.8)	(39.0)	(36.2)
	-1.9	-28.9	-4.9	-3.9	-17.6	5.1	2.1	-65.0	27.9	-17.5
	-0.325	-4.949	-0.840	-0.666	-3.020	0.875	0.361	-11.149	4.780	-3.004
t_O	151	214	265	146	206	276	151	232	244	148
	(25.9)	(36.7)	(45.5)	(25.0)	(35.3)	(47.3)	(25.9)	(39.8)	(41.9)	(25.4)
t_E	145.4	199.2	262.4	145.4	199.2	262.4	145.4	199.2	262.4	145.4
	(24.9)	(34.2)	(45.0)	(24.9)	(34.2)	(45.0)	(24.9)	(34.2)	(45.0)	(24.9)
	11.4	30.6	5.2	1.1	13.8	27.9	11.4	70.7	-35.5	5.2
	1.948	5.257	0.893	0.197	2.368	4.781	1.948	12.126	-6.089	0.890
g_O	153	87	60	136	88	45	142	81	75	164
	(26.2)	(14.9)	(10.3)	(23.3)	(15.1)	(7.7)	(24.4)	(13.9)	(12.9)	(28.1)
g_E	146.1	82.9	62.2	146.1	82.9	62.2	146.1	82.9	62.2	146.1
	(25.1)	(14.2)	(10.7)	(25.1)	(14.2)	(10.7)	(25.1)	(14.2)	(10.7)	(25.1)
	14.1	8.3	-4.3	-19.5	10.4	-29.1	-8.1	-3.8	28.1	37.9
	2.421	1.425	-0.744	-3.343	1.786	-4.999	-1.387	-0.659	4.811	6.501
c_O	69	100	33	92	101	32	78	109	23	69
	(11.8)	(17.2)	(5.7)	(15.8)	(17.3)	(5.5)	(13.4)	(18.7)	(3.9)	(11.8)
c_E	80.5	103.8	30.9	80.5	103.8	30.9	80.5	103.8	30.9	80.5
	(13.8)	(17.8)	(5.3)	(13.8)	(17.8)	(5.3)	(13.8)	(17.8)	(5.3)	(13.8)
	-21.3	-7.5	4.3	24.5	-5.6	2.2	-5.0	10.6	-13.6	-21.3
	-3.655	-1.289	0.737	4.206	-0.957	0.377	-0.851	1.817	-2.334	-3.655
N	583	583	583	583	583	583	583	583	583	583
X2	2.2	2.6	0.3	2.4	1.0	5.5	0.4	12.3	6.8	4.3
Gvalue	2.3	2.6	0.3	2.3	1.0	6.0	0.4	12.4	6.8	4.3
Gv/N	0.389	0.443	0.046	0.394	0.176	1.034	0.071	2.135	1.168	0.733
Prob	0.51853	0.46032	0.96582	0.51260	0.79463	0.11018	0.93773	0.00600	0.07830	0.23361

	71	72	73	74	75	76	77	78	79	80
a_O	198	233	209	195	231	209	193	216	199	190
	(34.0)	(40.0)	(35.8)	(33.4)	(39.6)	(35.8)	(33.1)	(37.0)	(34.1)	(32.6)
a_E	197.0	227.5	210.9	197.0	227.5	210.9	197.0	227.5	210.9	197.0
	(33.8)	(39.0)	(36.2)	(33.8)	(39.0)	(36.2)	(33.8)	(39.0)	(36.2)	(33.8)
	2.0	11.2	-3.9	-4.0	7.1	-3.9	-7.9	-22.3	-23.2	-13.8
	0.339	1.922	-0.666	-0.687	1.223	-0.666	-1.363	-3.832	-3.981	-2.363
t_O	219	255	144	200	266	162	209	271	155	191
	(37.6)	(43.7)	(24.7)	(34.3)	(45.6)	(27.8)	(35.8)	(46.5)	(26.6)	(32.8)
t_E	199.2	262.4	145.4	199.2	262.4	145.4	199.2	262.4	145.4	199.2
	(34.2)	(45.0)	(24.9)	(34.2)	(45.0)	(24.9)	(34.2)	(45.0)	(24.9)	(34.2)
	41.5	-14.6	-2.8	1.6	7.2	35.0	20.0	17.5	19.8	-16.1
	7.114	-2.506	-0.487	0.271	1.240	5.998	3.439	2.994	3.390	-2.759
g_O	78	70	138	80	65	135	81	66	144	94
	(13.4)	(12.0)	(23.7)	(13.7)	(11.1)	(23.2)	(13.9)	(11.3)	(24.7)	(16.1)
g_E	82.9	62.2	146.1	82.9	62.2	146.1	82.9	62.2	146.1	82.9
	(14.2)	(10.7)	(25.1)	(14.2)	(10.7)	(25.1)	(14.2)	(10.7)	(25.1)	(14.2)
	-9.6	16.5	-15.7	-5.8	5.7	-21.3	-3.8	7.8	-4.2	23.5
	-1.645	2.834	-2.701	-0.992	0.979	-3.660	-0.659	1.340	-0.716	4.035
c_O	88	25	92	108	21	77	100	30	85	108
	(15.1)	(4.3)	(15.8)	(18.5)	(3.6)	(13.2)	(17.2)	(5.1)	(14.6)	(18.5)
c_E	103.8	30.9	80.5	103.8	30.9	80.5	103.8	30.9	80.5	103.8
	(17.8)	(5.3)	(13.8)	(17.8)	(5.3)	(13.8)	(17.8)	(5.3)	(13.8)	(17.8)
	-29.1	-10.6	24.5	8.5	-16.2	-6.9	-7.5	-1.8	9.2	8.5
	-4.994	-1.822	4.206	1.459	-2.787	-1.181	-1.289	-0.310	1.579	1.459
N	583	583	583	583	583	583	583	583	583	583
X2	4.7	2.5	2.1	0.3	3.4	2.9	0.7	1.1	1.6	2.2
Gvalue	4.8	2.5	2.1	0.3	3.8	2.9	0.7	1.1	1.6	2.2
Gv/N	0.816	0.428	0.352	0.051	0.655	0.491	0.128	0.192	0.271	0.373
Prob	0.19062	0.47606	0.56127	0.96100	0.28188	0.41357	0.86272	0.77227	0.66348	0.53717

	81	82	83	84	85	86	87	88	89	90
a_O	226	196	180	223	188	178	218	190	195	213
	(38.8)	(33.6)	(30.9)	(38.3)	(32.2)	(30.5)	(37.4)	(32.6)	(33.4)	(36.5)
a_E	227.5	210.9	197.0	227.5	210.9	197.0	227.5	210.9	197.0	227.5
	(39.0)	(36.2)	(33.8)	(39.0)	(36.2)	(33.8)	(39.0)	(36.2)	(33.8)	(39.0)
	-2.9	-28.8	-32.5	-8.8	-43.3	-36.1	-18.5	-39.7	-4.0	-28.0
	-0.500	-4.942	-5.577	-1.516	-7.428	-6.197	-3.178	-6.818	-0.687	-4.800
t_O	257	168	198	264	156	231	254	163	207	277
	(44.1)	(28.8)	(34.0)	(45.3)	(26.8)	(39.6)	(43.6)	(28.0)	(35.5)	(47.5)
t_E	262.4	145.4	199.2	262.4	145.4	199.2	262.4	145.4	199.2	262.4
	(45.0)	(24.9)	(34.2)	(45.0)	(24.9)	(34.2)	(45.0)	(24.9)	(34.2)	(45.0)
	-10.7	48.5	-2.4	3.2	21.9	68.4	-16.5	37.2	15.9	30.0

	-1.837	8.316	-0.415	0.547	3.756	11.732	-2.838	6.379	2.723	5.142
g_O	70	143	88	70	154	80	86	150	85	66
	(12.0)	(24.5)	(15.1)	(12.0)	(26.4)	(13.7)	(14.8)	(25.7)	(14.6)	(11.3)
g_E	62.2	146.1	82.9	62.2	146.1	82.9	62.2	146.1	82.9	62.2
	(10.7)	(25.1)	(14.2)	(10.7)	(25.1)	(14.2)	(10.7)	(25.1)	(14.2)	(10.7)
	16.5	-6.1	10.4	16.5	16.2	-5.8	55.7	7.9	4.2	7.8
	2.834	-1.053	1.786	2.834	2.781	-0.992	9.555	1.355	0.714	1.340
c_O	30	76	117	26	85	94	25	80	96	27
	(5.1)	(13.0)	(20.1)	(4.5)	(14.6)	(16.1)	(4.3)	(13.7)	(16.5)	(4.6)
c_E	30.9	80.5	103.8	30.9	80.5	103.8	30.9	80.5	103.8	30.9
	(5.3)	(13.8)	(17.8)	(5.3)	(13.8)	(17.8)	(5.3)	(13.8)	(17.8)	(5.3)
	-1.8	-8.8	27.9	-9.0	9.2	-18.7	-10.6	-1.0	-15.1	-7.3
	-0.310	-1.506	4.793	-1.545	1.579	-3.207	-1.822	-0.178	-2.582	-1.255
N	583	583	583	583	583	583	583	583	583	583
X2	1.1	4.9	3.5	1.9	3.9	7.9	10.9	4.3	1.0	2.5
Gvalue	1.1	4.7	3.4	1.9	4.0	7.8	10.0	4.3	1.0	2.5
Gv/N	0.186	0.814	0.588	0.320	0.687	1.336	1.716	0.738	0.168	0.426
Prob	0.78023	0.19139	0.33035	0.60132	0.26067	0.05065	0.01852	0.23063	0.80669	0.47857

	91	92	93	94	95	96	97	98	99	100
a_O	218	185	228	204	181	214	228	193	222	216
	(37.4)	(31.7)	(39.1)	(35.0)	(31.0)	(36.7)	(39.1)	(33.1)	(38.1)	(37.0)
a_E	210.9	197.0	227.5	210.9	197.0	227.5	210.9	197.0	227.5	210.9
	(36.2)	(33.8)	(39.0)	(36.2)	(33.8)	(39.0)	(36.2)	(33.8)	(39.0)	(36.2)
	14.3	-23.3	1.1	-13.7	-30.7	-26.1	35.4	-7.9	-10.8	10.2
	2.459	-3.993	0.185	-2.344	-5.264	-4.479	6.079	-1.363	-1.851	1.753
t_O	144	212	266	151	206	280	134	201	254	140
	(24.7)	(36.4)	(45.6)	(25.9)	(35.3)	(48.0)	(23.0)	(34.5)	(43.6)	(24.0)
t_E	145.4	199.2	262.4	145.4	199.2	262.4	145.4	199.2	262.4	145.4
	(24.9)	(34.2)	(45.0)	(24.9)	(34.2)	(45.0)	(24.9)	(34.2)	(45.0)	(24.9)
	-2.8	26.4	7.2	11.4	13.8	36.3	-21.9	3.6	-16.5	-10.6
	-0.487	4.524	1.240	1.948	2.368	6.232	-3.762	0.616	-2.838	-1.827
g_O	134	74	58	139	85	62	144	85	72	156
	(23.0)	(12.7)	(9.9)	(23.8)	(14.6)	(10.6)	(24.7)	(14.6)	(12.3)	(26.8)
g_E	146.1	82.9	62.2	146.1	82.9	62.2	146.1	82.9	62.2	146.1
	(25.1)	(14.2)	(10.7)	(25.1)	(14.2)	(10.7)	(25.1)	(14.2)	(10.7)	(25.1)
	-23.2	-16.9	-8.1	-13.9	4.2	-0.4	-4.2	4.2	21.1	20.5
	-3.975	-2.897	-1.394	-2.376	0.714	-0.071	-0.716	0.714	3.611	3.508
c_O	87	112	31	89	111	27	77	104	35	71
	(14.9)	(19.2)	(5.3)	(15.3)	(19.0)	(4.6)	(13.2)	(17.8)	(6.0)	(12.2)
c_E	80.5	103.8	30.9	80.5	103.8	30.9	80.5	103.8	30.9	80.5
	(13.8)	(17.8)	(5.3)	(13.8)	(17.8)	(5.3)	(13.8)	(17.8)	(5.3)	(13.8)
	13.5	17.0	0.2	17.8	14.8	-7.3	-6.9	0.3	8.7	-17.9
	2.310	2.910	0.028	3.057	2.543	-1.255	-1.181	0.058	1.489	-3.065
N	583	583	583	583	583	583	583	583	583	583
X2	1.8	3.2	0.3	1.7	2.1	2.5	2.5	0.1	2.5	2.1
Gvalue	1.8	3.2	0.3	1.7	2.1	2.5	2.5	0.1	2.4	2.2
Gv/N	0.306	0.545	0.059	0.284	0.360	0.426	0.420	0.026	0.410	0.369
Prob	0.61787	0.36477	0.95201	0.64661	0.55157	0.47776	0.48414	0.98535	0.49597	0.54113

	101	102	103	104	105	106	107	108	109	110
a_O	202	248	209	189	216	223	182	234	216	201
	(34.6)	(42.5)	(35.8)	(32.4)	(37.0)	(38.3)	(31.2)	(40.1)	(37.0)	(34.5)
a_E	197.0	227.5	210.9	197.0	227.5	210.9	197.0	227.5	210.9	197.0
	(33.8)	(39.0)	(36.2)	(33.8)	(39.0)	(36.2)	(33.8)	(39.0)	(36.2)	(33.8)
	10.1	42.9	-3.9	-15.7	-22.3	24.8	-28.9	13.3	10.2	8.1
	1.732	7.354	-0.666	-2.692	-3.832	4.250	-4.949	2.275	1.753	1.381
t_O	196	247	163	213	271	156	205	258	144	202
	(33.6)	(42.4)	(28.0)	(36.5)	(46.5)	(26.8)	(35.2)	(44.3)	(24.7)	(34.6)
t_E	199.2	262.4	145.4	199.2	262.4	145.4	199.2	262.4	145.4	199.2
	(34.2)	(45.0)	(24.9)	(34.2)	(45.0)	(24.9)	(34.2)	(45.0)	(24.9)	(34.2)
	-6.4	-29.9	37.2	28.5	17.5	21.9	11.7	-8.7	-2.8	5.6
	-1.093	-5.128	6.379	4.890	2.994	3.756	2.014	-1.500	-0.487	0.963
g_O	78	56	133	73	71	128	85	56	130	72
	(13.4)	(9.6)	(22.8)	(12.5)	(12.2)	(22.0)	(14.6)	(9.6)	(22.3)	(12.3)
g_E	82.9	62.2	146.1	82.9	62.2	146.1	82.9	62.2	146.1	82.9
	(14.2)	(10.7)	(25.1)	(14.2)	(10.7)	(25.1)	(14.2)	(10.7)	(25.1)	(14.2)
	-9.6	-11.8	-25.0	-18.6	18.8	-33.9	4.2	-11.8	-30.4	-20.4
	-1.645	-2.020	-4.287	-3.198	3.220	-5.808	0.714	-2.020	-5.208	-3.495
c_O	107	32	78	108	25	76	111	35	93	108
	(18.4)	(5.5)	(13.4)	(18.5)	(4.3)	(13.0)	(19.0)	(6.0)	(16.0)	(18.5)
c_E	103.8	30.9	80.5	103.8	30.9	80.5	103.8	30.9	80.5	103.8
	(17.8)	(5.3)	(13.8)	(17.8)	(5.3)	(13.8)	(17.8)	(5.3)	(13.8)	(17.8)
	6.4	2.2	-5.0	8.5	-10.6	-8.8	14.8	8.7	26.8	8.5
	1.104	0.377	-0.851	1.459	-1.822	-1.506	2.543	1.489	4.597	1.459
N	583	583	583	583	583	583	583	583	583	583
X2	0.6	3.4	3.4	2.6	3.2	4.0	1.9	1.4	3.8	1.7
Gvalue	0.6	3.4	3.4	2.7	3.3	4.0	1.9	1.4	3.8	1.8
Gv/N	0.098	0.584	0.575	0.458	0.560	0.691	0.322	0.243	0.655	0.308
Prob	0.90239	0.33360	0.34042	0.44508	0.35230	0.25859	0.59864	0.70124	0.28166	0.61573

	111	112	113	114	115	116	117	118	119	120
a_O	216	194	182	217	197	204	230	221	205	223
	(37.0)	(33.3)	(31.2)	(37.2)	(33.8)	(35.0)	(39.5)	(38.0)	(35.2)	(38.3)
a_E	227.5	210.9	197.0	227.5	210.9	197.0	227.5	210.6	196.7	227.1
	(39.0)	(36.2)	(33.8)	(39.0)	(36.2)	(33.8)	(39.0)	(36.2)	(33.8)	(39.0)
	-22.3	-32.5	-28.9	-20.4	-27.0	14.2	5.1	21.3	17.0	-8.1
	-3.832	-5.575	-4.949	-3.505	-4.624	2.439	0.875	3.665	2.920	-1.387
t_O	276	150	215	271	149	203	268	138	202	269
	(47.3)	(25.7)	(36.9)	(46.5)	(25.6)	(34.8)	(46.0)	(23.7)	(34.7)	(46.2)
t_E	262.4	145.4	199.2	262.4	145.4	199.2	262.4	145.2	198.9	262.0
	(45.0)	(24.9)	(34.2)	(45.0)	(24.9)	(34.2)	(45.0)	(24.9)	(34.2)	(45.0)
	27.9	9.3	32.8	17.5	7.2	7.6	11.3	-14.0	6.3	14.3
	4.781	1.593	5.625	2.994	1.241	1.311	1.938	-2.405	1.084	2.451
g_O	58	161	85	65	152	76	70	143	72	59
	(9.9)	(27.6)	(14.6)	(11.1)	(26.1)	(13.0)	(12.0)	(24.6)	(12.4)	(10.1)
g_E	62.2	146.1	82.9	62.2	146.1	82.9	62.2	145.9	82.8	62.1
	(10.7)	(25.1)	(14.2)	(10.7)	(25.1)	(14.2)	(10.7)	(25.1)	(14.2)	(10.7)
	-8.1	31.3	4.2	5.7	12.0	-13.3	16.5	-5.6	-20.1	-6.0
	-1.394	5.363	0.714	0.979	2.063	-2.280	2.834	-0.970	-3.459	-1.039
c_O	33	78	101	30	85	100	15	80	103	31
	(5.7)	(13.4)	(17.3)	(5.1)	(14.6)	(17.2)	(2.6)	(13.7)	(17.7)	(5.3)
c_E	30.9	80.5	103.8	30.9	80.5	103.8	30.9	80.4	103.7	30.9
	(5.3)	(13.8)	(17.8)	(5.3)	(13.8)	(17.8)	(5.3)	(13.8)	(17.8)	(5.3)
	4.3	-5.0	-5.6	-1.8	9.2	-7.5	-21.7	-0.8	-1.3	0.3
	0.737	-0.851	-0.957	-0.310	1.579	-1.289	-3.722	-0.131	-0.223	0.046

N	583	583	583	583	583	583	583	582	582	582
X2	1.7	3.1	2.5	0.9	1.5	1.0	9.3	0.9	1.8	0.4
Gvalue	1.7	3.1	2.5	0.9	1.5	1.1	11.2	0.9	1.9	0.4
Gv/N	0.293	0.530	0.433	0.157	0.259	0.181	1.925	0.159	0.322	0.072
Prob	0.63518	0.37768	0.47134	0.82108	0.67991	0.78728	0.01058	0.81931	0.59868	0.93627

	121	122	123	124	125	126	127	128	129	130
a_O	204	200	198	233	213	242	203	187	215	226
	(35.1)	(34.4)	(34.0)	(40.0)	(36.6)	(41.6)	(34.9)	(32.1)	(36.9)	(38.8)
a_E	210.6	196.7	227.1	210.6	196.7	227.1	210.6	196.7	227.1	210.6
	(36.2)	(33.8)	(39.0)	(36.2)	(33.8)	(39.0)	(36.2)	(33.8)	(39.0)	(36.2)
	-13.0	6.7	-54.3	47.1	34.0	30.8	-14.9	-18.9	-23.5	31.9
	-2.228	1.152	-9.322	8.098	5.836	5.295	-2.560	-3.242	-4.036	5.485
t_O	134	202	291	122	191	246	148	205	273	141
	(23.0)	(34.7)	(50.0)	(21.0)	(32.8)	(42.3)	(25.4)	(35.2)	(46.9)	(24.2)
t_E	145.2	198.9	262.0	145.2	198.9	262.0	145.2	198.9	262.0	145.2
	(24.9)	(34.2)	(45.0)	(24.9)	(34.2)	(45.0)	(24.9)	(34.2)	(45.0)	(24.9)
	-21.5	6.3	61.2	-42.4	-15.4	-30.9	5.7	12.4	22.5	-8.2
	-3.689	1.084	10.513	-7.292	-2.651	-5.314	0.979	2.138	3.873	-1.415
g_O	151	86	59	132	75	64	141	82	56	136
	(25.9)	(14.8)	(10.1)	(22.7)	(12.9)	(11.0)	(24.2)	(14.1)	(9.6)	(23.4)
g_E	145.9	82.8	62.1	145.9	82.8	62.1	145.9	82.8	62.1	145.9
	(25.1)	(14.2)	(10.7)	(25.1)	(14.2)	(10.7)	(25.1)	(14.2)	(10.7)	(25.1)
	10.5	6.5	-6.0	-26.3	-14.8	3.9	-9.5	-1.6	-11.6	-19.0
	1.800	1.120	-1.039	-4.527	-2.551	0.662	-1.639	-0.274	-1.990	-3.269
c_O	93	94	34	95	103	30	90	108	38	79
	(16.0)	(16.2)	(5.8)	(16.3)	(17.7)	(5.2)	(15.5)	(18.6)	(6.5)	(13.6)
c_E	80.4	103.7	30.9	80.4	103.7	30.9	80.4	103.7	30.9	80.4
	(13.8)	(17.8)	(5.3)	(13.8)	(17.8)	(5.3)	(13.8)	(17.8)	(5.3)	(13.8)
	27.1	-18.4	6.6	31.7	-1.3	-1.7	20.3	8.9	15.8	-2.7
	4.660	-3.157	1.130	5.455	-0.223	-0.293	3.495	1.525	2.715	-0.471

N	582	582	582	582	582	582	582	582	582	582
X2	3.2	1.1	7.4	10.1	2.4	2.0	1.6	0.9	3.4	1.9
Gvalue	3.2	1.2	7.5	10.1	2.4	2.0	1.6	0.9	3.3	1.9
Gv/N	0.542	0.198	1.282	1.734	0.412	0.350	0.275	0.148	0.561	0.331
Prob	0.36820	0.76387	0.05849	0.01782	0.49397	0.56513	0.65894	0.83539	0.35221	0.58755

	131	132	133	134	135	136	137	138	139	140
a_O	200	214	219	199	206	210	199	242	211	185
	(34.4)	(36.8)	(37.6)	(34.2)	(35.4)	(36.1)	(34.2)	(41.6)	(36.3)	(31.8)
a_E	196.7	227.1	210.6	196.7	227.1	210.6	196.7	227.1	210.6	196.7
	(33.8)	(39.0)	(36.2)	(33.8)	(39.0)	(36.2)	(33.8)	(39.0)	(36.2)	(33.8)
	6.7	-25.4	17.2	4.7	-40.1	-1.2	4.7	30.8	0.8	-22.6
	1.152	-4.360	2.948	0.804	-6.895	-0.202	0.804	5.295	0.142	-3.891
t_O	205	271	153	181	267	148	202	249	165	214
	(35.2)	(46.6)	(26.3)	(31.1)	(45.9)	(25.4)	(34.7)	(42.8)	(28.4)	(36.8)
t_E	198.9	262.0	145.2	198.9	262.0	145.2	198.9	262.0	145.2	198.9
	(34.2)	(45.0)	(24.9)	(34.2)	(45.0)	(24.9)	(34.2)	(45.0)	(24.9)	(34.2)
	12.4	18.4	16.1	-34.1	10.2	5.7	6.3	-25.3	42.2	31.4
	2.138	3.159	2.759	-5.857	1.748	0.979	1.084	-4.342	7.257	5.392
g_O	84	69	119	86	71	138	90	58	136	79
	(14.4)	(11.9)	(20.4)	(14.8)	(12.2)	(23.7)	(15.5)	(10.0)	(23.4)	(13.6)
g_E	82.8	62.1	145.9	82.8	62.1	145.9	82.8	62.1	145.9	82.8
	(14.2)	(10.7)	(25.1)	(14.2)	(10.7)	(25.1)	(14.2)	(10.7)	(25.1)	(14.2)
	2.4	14.5	-48.4	6.5	19.0	-15.3	15.0	-7.9	-19.0	-7.4
	0.415	2.498	-8.321	1.120	3.267	-2.624	2.578	-1.362	-3.269	-1.276
c_O	93	28	91	116	38	86	91	33	70	104
	(16.0)	(4.8)	(15.6)	(19.9)	(6.5)	(14.8)	(15.6)	(5.7)	(12.0)	(17.9)
c_E	103.7	30.9	80.4	103.7	30.9	80.4	103.7	30.9	80.4	103.7
	(17.8)	(5.3)	(13.8)	(17.8)	(5.3)	(13.8)	(17.8)	(5.3)	(13.8)	(17.8)
	-20.2	-5.5	22.6	26.1	15.8	11.6	-23.7	4.4	-19.4	0.7
	-3.465	-0.938	3.880	4.487	2.715	1.996	-4.071	0.758	-3.327	0.120

N	582	582	582	582	582	582	582	582	582	582
X2	1.4	2.1	7.1	3.2	5.0	0.9	2.2	2.0	4.7	2.0
Gvalue	1.4	2.1	7.4	3.2	4.9	0.9	2.3	2.0	4.7	2.0
Gv/N	0.240	0.359	1.266	0.554	0.837	0.150	0.394	0.349	0.803	0.345
Prob	0.70689	0.55393	0.06103	0.35870	0.18169	0.83255	0.51331	0.56541	0.19715	0.57065

	141	142	143	144	145	146	147	148	149	150
a_O	234	192	188	230	188	202	223	203	186	217
	(40.2)	(33.0)	(32.4)	(39.6)	(32.4)	(34.8)	(38.4)	(34.9)	(32.0)	(37.3)
a_E	227.1	210.2	196.3	226.7	210.2	196.3	226.7	210.2	196.3	226.7
	(39.0)	(36.2)	(33.8)	(39.0)	(36.2)	(33.8)	(39.0)	(36.2)	(33.8)	(39.0)
	14.1	-34.8	-16.3	6.7	-42.0	11.5	-7.3	-14.2	-20.1	-18.9
	2.416	-5.994	-2.808	1.150	-7.232	1.977	-1.257	-2.444	-3.463	-3.261
t_O	262	144	210	253	150	201	265	140	196	272
	(45.0)	(24.8)	(36.1)	(43.5)	(25.8)	(34.6)	(45.6)	(24.1)	(33.7)	(46.8)
t_E	262.0	144.9	198.5	261.5	144.9	198.5	261.5	144.9	198.5	261.5
	(45.0)	(24.9)	(34.2)	(45.0)	(24.9)	(34.2)	(45.0)	(24.9)	(34.2)	(45.0)
	0.1	-1.9	23.6	-16.7	10.3	5.0	7.0	-9.7	-5.0	21.4
	0.014	-0.319	4.060	-2.881	1.776	0.856	1.209	-1.667	-0.865	3.682
g_O	65	165	92	60	147	77	51	158	83	61
	(11.2)	(28.4)	(15.8)	(10.3)	(25.3)	(13.3)	(8.8)	(27.2)	(14.3)	(10.5)
g_E	62.1	145.6	82.7	62.0	145.6	82.7	62.0	145.6	82.7	62.0
	(10.7)	(25.1)	(14.2)	(10.7)	(25.1)	(14.2)	(10.7)	(25.1)	(14.2)	(10.7)
	5.9	41.3	19.7	-3.9	2.8	-10.9	-19.9	25.8	0.7	-2.0
	1.019	7.104	3.390	-0.675	0.484	-1.880	-3.427	4.445	0.117	-0.340
c_O	21	80	91	38	96	101	42	80	116	31
	(3.6)	(13.8)	(15.7)	(6.5)	(16.5)	(17.4)	(7.2)	(13.8)	(20.0)	(5.3)
c_E	30.9	80.2	103.5	30.8	80.2	103.5	30.8	80.2	103.5	30.8
	(5.3)	(13.8)	(17.8)	(5.3)	(13.8)	(17.8)	(5.3)	(13.8)	(17.8)	(5.3)
	-16.2	-0.5	-23.4	15.9	34.4	-4.9	26.0	-0.5	26.5	0.4
	-2.779	-0.084	-4.024	2.743	5.924	-0.841	4.478	-0.084	4.563	0.065

N	582	581	581	581	581	581	581	581	581	581
X2	3.5	4.2	3.6	2.1	5.6	0.6	6.1	1.5	2.1	0.9
Gvalue	3.9	4.1	3.6	2.0	5.5	0.6	5.8	1.4	2.0	0.9
Gv/N	0.670	0.708	0.619	0.336	0.953	0.112	1.003	0.249	0.352	0.147
Prob	0.27261	0.24968	0.30861	0.58228	0.13655	0.88521	0.12034	0.69419	0.56279	0.83699

Bfra_NCTC9343
5UTR:

	50	49	48	47	46	45	44	43	42	41
a_O	1331	1275	1363	1335	1344	1348	1353	1399	1396	1424
	(31.5)	(30.2)	(32.2)	(31.6)	(31.8)	(31.9)	(32.0)	(33.1)	(33.0)	(33.7)
a_E	1485.4	1485.4	1485.4	1485.4	1485.4	1485.4	1485.4	1485.4	1485.4	1485.4
	(35.1)	(35.1)	(35.1)	(35.1)	(35.1)	(35.1)	(35.1)	(35.1)	(35.1)	(35.1)
	-292.2	-389.5	-234.4	-285.0	-268.9	-261.7	-252.6	-167.7	-173.3	-120.2
	-6.912	-9.214	-5.546	-6.743	-6.361	-6.191	-5.976	-3.967	-4.100	-2.844
t_O	1305	1372	1249	1304	1294	1330	1287	1295	1300	1253
	(30.9)	(32.5)	(29.5)	(30.8)	(30.6)	(31.5)	(30.4)	(30.6)	(30.8)	(29.6)
t_E	1289.1	1289.1	1289.1	1289.1	1289.1	1289.1	1289.1	1289.1	1289.1	1289.1
	(30.5)	(30.5)	(30.5)	(30.5)	(30.5)	(30.5)	(30.5)	(30.5)	(30.5)	(30.5)
	32.0	171.0	-79.0	30.0	9.8	83.1	-4.2	11.8	21.9	-71.2
	0.757	4.046	-1.868	0.709	0.232	1.965	-0.100	0.279	0.518	-1.684
g_O	832	852	868	815	821	826	867	819	830	859
	(19.7)	(20.2)	(20.5)	(19.3)	(19.4)	(19.5)	(20.5)	(19.4)	(19.6)	(20.3)
g_E	758.8	758.8	758.8	758.8	758.8	758.8	758.8	758.8	758.8	758.8
	(18.0)	(18.0)	(18.0)	(18.0)	(18.0)	(18.0)	(18.0)	(18.0)	(18.0)	(18.0)
	153.2	197.3	233.3	116.4	129.3	140.1	231.1	125.0	148.8	213.0
	3.624	4.669	5.520	2.754	3.059	3.315	5.467	2.957	3.521	5.040
c_O	759	728	747	773	768	723	720	714	701	691
	(18.0)	(17.2)	(17.7)	(18.3)	(18.2)	(17.1)	(17.0)	(16.9)	(16.6)	(16.3)
c_E	693.7	693.7	693.7	693.7	693.7	693.7	693.7	693.7	693.7	693.7
	(16.4)	(16.4)	(16.4)	(16.4)	(16.4)	(16.4)	(16.4)	(16.4)	(16.4)	(16.4)
	136.6	70.3	110.7	167.4	156.4	59.9	53.7	41.3	14.7	-5.3
	3.232	1.664	2.618	3.960	3.699	1.417	1.269	0.976	0.349	-0.126
N	4227	4227	4227	4227	4227	4227	4227	4227	4227	4227
X2	29.5	48.3	31.1	28.6	26.5	21.2	28.2	10.4	12.2	16.8
Gvalue	29.6	49.2	30.6	28.7	26.6	21.4	27.9	10.4	12.1	16.3
Gv/N	0.701	1.164	0.725	0.680	0.629	0.506	0.660	0.246	0.287	0.385
Prob	0.00000	0.00000	0.00000	0.00000	0.00001	0.00009	0.00000	0.01551	0.00693	0.00099

	40	39	38	37	36	35	34	33	32	31
a_O	1387	1372	1401	1449	1392	1460	1451	1462	1473	1458
	(32.8)	(32.5)	(33.1)	(34.3)	(32.9)	(34.5)	(34.3)	(34.6)	(34.8)	(34.5)
a_E	1485.4	1485.4	1485.4	1485.4	1485.4	1485.4	1485.4	1485.4	1485.4	1485.4
	(35.1)	(35.1)	(35.1)	(35.1)	(35.1)	(35.1)	(35.1)	(35.1)	(35.1)	(35.1)
	-190.1	-217.9	-163.9	-71.9	-180.8	-50.4	-68.0	-46.4	-24.7	-54.3
	-4.498	-5.155	-3.878	-1.701	-4.277	-1.191	-1.608	-1.098	-0.584	-1.284
t_O	1327	1323	1364	1305	1361	1289	1432	1393	1358	1393
	(31.4)	(31.3)	(32.3)	(30.9)	(32.2)	(30.5)	(33.9)	(33.0)	(32.1)	(33.0)
t_E	1289.1	1289.1	1289.1	1289.1	1289.1	1289.1	1289.1	1289.1	1289.1	1289.1
	(30.5)	(30.5)	(30.5)	(30.5)	(30.5)	(30.5)	(30.5)	(30.5)	(30.5)	(30.5)
	76.9	68.7	154.1	32.0	147.7	-0.2	301.1	215.9	141.4	215.9
	1.819	1.625	3.645	0.757	3.495	-0.005	7.123	5.109	3.345	5.109
g_O	824	828	802	757	796	775	680	755	706	661
	(19.5)	(19.6)	(19.0)	(17.9)	(18.8)	(18.3)	(16.1)	(17.9)	(16.7)	(15.6)
g_E	758.8	758.8	758.8	758.8	758.8	758.8	758.8	758.8	758.8	758.8
	(18.0)	(18.0)	(18.0)	(18.0)	(18.0)	(18.0)	(18.0)	(18.0)	(18.0)	(18.0)
	135.8	144.5	88.8	-3.7	76.1	32.7	-149.2	-7.6	-101.9	-182.5
	3.212	3.418	2.100	-0.086	1.801	0.773	-3.529	-0.181	-2.410	-4.317
c_O	689	704	660	716	678	703	664	617	690	715
	(16.3)	(16.7)	(15.6)	(16.9)	(16.0)	(16.6)	(15.7)	(14.6)	(16.3)	(16.9)
c_E	693.7	693.7	693.7	693.7	693.7	693.7	693.7	693.7	693.7	693.7
	(16.4)	(16.4)	(16.4)	(16.4)	(16.4)	(16.4)	(16.4)	(16.4)	(16.4)	(16.4)
	-9.3	20.8	-65.7	45.4	-31.0	18.8	-58.0	-144.5	-7.3	43.3
	-0.220	0.492	-1.554	1.073	-0.733	0.444	-1.373	-3.419	-0.173	1.025
N	4227	4227	4227	4227	4227	4227	4227	4227	4227	4227
X2	13.3	16.0	13.2	1.8	12.1	0.9	26.1	17.2	7.5	22.1
Gvalue	13.2	16.0	13.2	1.8	12.1	0.9	25.9	17.4	7.5	22.5
Gv/N	0.313	0.380	0.313	0.043	0.286	0.021	0.612	0.410	0.178	0.532
Prob	0.00414	0.00111	0.00416	0.61274	0.00710	0.82448	0.00001	0.00060	0.05737	0.00005

	30	29	28	27	26	25	24	23	22	21
a_O	1447	1475	1427	1461	1500	1524	1508	1551	1591	1664
	(34.2)	(34.9)	(33.8)	(34.6)	(35.5)	(36.1)	(35.7)	(36.7)	(37.6)	(39.4)
a_E	1485.4	1485.4	1485.4	1485.4	1485.4	1485.4	1485.4	1485.4	1485.4	1485.4
	(35.1)	(35.1)	(35.1)	(35.1)	(35.1)	(35.1)	(35.1)	(35.1)	(35.1)	(35.1)
	-75.8	-20.7	-114.5	-48.4	29.4	78.2	45.5	134.1	218.5	377.9
	-1.793	-0.490	-2.708	-1.145	0.694	1.850	1.078	3.172	5.170	8.939
t_O	1456	1380	1464	1438	1375	1484	1469	1403	1392	1372
	(34.4)	(32.6)	(34.6)	(34.0)	(32.5)	(35.1)	(34.8)	(33.2)	(32.9)	(32.5)
t_E	1289.1	1289.1	1289.1	1289.1	1289.1	1289.1	1289.1	1289.1	1289.1	1289.1
	(30.5)	(30.5)	(30.5)	(30.5)	(30.5)	(30.5)	(30.5)	(30.5)	(30.5)	(30.5)
	354.5	188.0	372.5	314.4	177.4	417.9	383.8	237.6	213.8	171.0
	8.387	4.449	8.813	7.437	4.197	9.886	9.080	5.620	5.058	4.046
g_O	670	730	698	712	694	620	647	646	593	546
	(15.9)	(17.3)	(16.5)	(16.8)	(16.4)	(14.7)	(15.3)	(15.3)	(14.0)	(12.9)
g_E	758.8	758.8	758.8	758.8	758.8	758.8	758.8	758.8	758.8	758.8
	(18.0)	(18.0)	(18.0)	(18.0)	(18.0)	(18.0)	(18.0)	(18.0)	(18.0)	(18.0)
	-166.8	-56.5	-116.6	-90.7	-124.0	-250.5	-206.3	-208.0	-292.4	-359.4
	-3.947	-1.338	-2.760	-2.146	-2.932	-5.927	-4.881	-4.920	-6.919	-8.503
c_O	654	642	638	616	658	599	603	627	651	645
	(15.5)	(15.2)	(15.1)	(14.6)	(15.6)	(14.2)	(14.3)	(14.8)	(15.4)	(15.3)
c_E	693.7	693.7	693.7	693.7	693.7	693.7	693.7	693.7	693.7	693.7
	(16.4)	(16.4)	(16.4)	(16.4)	(16.4)	(16.4)	(16.4)	(16.4)	(16.4)	(16.4)
	-77.0	-99.4	-106.7	-146.3	-69.5	-175.8	-168.9	-126.7	-82.7	-93.8
	-1.822	-2.351	-2.525	-3.461	-1.643	-4.159	-3.997	-2.998	-1.955	-2.220
N	4227	4227	4227	4227	4227	4227	4227	4227	4227	4227
X2	35.3	11.4	35.4	29.2	13.2	68.8	53.8	36.1	54.6	89.9
Gvalue	34.9	11.4	34.7	29.0	13.3	69.7	54.1	36.9	57.2	95.6
Gv/N	0.825	0.270	0.820	0.685	0.315	1.650	1.280	0.874	1.354	2.262
Prob	0.00000	0.00978	0.00000	0.00000	0.00401	0.00000	0.00000	0.00000	0.00000	0.00000

	20	19	18	17	16	15	14	13	12	11
a_O	1771	1789	1722	1765	1754	1901	2301	2515	2378	2211
	(41.9)	(42.3)	(40.7)	(41.8)	(41.5)	(45.0)	(54.4)	(59.5)	(56.3)	(52.3)
a_E	1485.4	1485.4	1485.4	1485.4	1485.4	1485.4	1485.4	1485.4	1485.4	1485.4
	(35.1)	(35.1)	(35.1)	(35.1)	(35.1)	(35.1)	(35.1)	(35.1)	(35.1)	(35.1)
	622.9	665.4	509.0	608.8	583.1	937.9	2014.1	2648.8	2238.1	1758.9
	14.736	15.742	12.043	14.403	13.794	22.189	47.649	62.663	52.947	41.611
t_O	1209	1248	1284	1303	1436	1427	1117	965	937	965
	(28.6)	(29.5)	(30.4)	(30.8)	(34.0)	(33.8)	(26.4)	(22.8)	(22.2)	(22.8)

```
t_E      1289.1  1289.1  1289.1  1289.1  1289.1  1289.1  1289.1  1289.1  1289.1  1289.1
        ( 30.5) ( 30.5) ( 30.5) ( 30.5) ( 30.5) ( 30.5) ( 30.5) ( 30.5) ( 30.5) ( 30.5)
         -155.1   -80.9   -10.2    27.9   309.9   290.0  -320.1  -558.9  -597.8  -558.9
         -3.670  -1.914  -0.241   0.661   7.332   6.862  -7.574 -13.222 -14.144 -13.222
g_O         546     447     468     452     385     406     389     364     462     514
        ( 12.9) ( 10.6) ( 11.1) ( 10.7) (  9.1) (  9.6) (  9.2) (  8.6) ( 10.9) ( 12.2)
g_E       758.8   758.8   758.8   758.8   758.8   758.8   758.8   758.8   758.8   758.8
        ( 18.0) ( 18.0) ( 18.0) ( 18.0) ( 18.0) ( 18.0) ( 18.0) ( 18.0) ( 18.0) ( 18.0)
         -359.4  -473.1  -452.4  -468.4  -522.5  -507.8  -519.9  -534.8  -458.5  -400.5
         -8.503 -11.193 -10.702 -11.080 -12.360 -12.014 -12.298 -12.652 -10.847  -9.474
c_O         701     743     753     707     652     493     420     383     450     537
        ( 16.6) ( 17.6) ( 17.8) ( 16.7) ( 15.4) ( 11.7) (  9.9) (  9.1) ( 10.6) ( 12.7)
c_E       693.7   693.7   693.7   693.7   693.7   693.7   693.7   693.7   693.7   693.7
        ( 16.4) ( 16.4) ( 16.4) ( 16.4) ( 16.4) ( 16.4) ( 16.4) ( 16.4) ( 16.4) ( 16.4)
           14.7   102.1   123.6    26.9   -80.8  -336.7  -421.5  -455.0  -389.5  -274.9
          0.349   2.415   2.924   0.637  -1.911  -7.966  -9.971 -10.763  -9.214  -6.504

N          4227    4227    4227    4227    4227    4227    4227    4227    4227    4227
X2        119.7   195.0   154.2   177.1   252.0   353.1   759.0  1139.7   834.3   550.3
Gvalue    123.1   213.5   170.1   195.3   289.8   383.4   752.7  1100.1   792.3   524.6
Gv/N      2.912   5.051   4.023   4.621   6.855   9.071  17.806  26.025  18.743  12.411
Prob    0.00000 0.00000 0.00000 0.00000 0.00000 0.00000 0.00000 0.00000 0.00000 0.00000
        -------------------------------------------------------------------------------
             10       9       8       7       6       5       4       3       2       1
        -------------------------------------------------------------------------------
a_O        1934    1809    1850    1872    2078    1919    1895    2109    1557    1174
        ( 45.8) ( 42.8) ( 43.8) ( 44.3) ( 49.2) ( 45.4) ( 44.8) ( 49.9) ( 36.8) ( 27.8)
a_E      1485.4  1485.4  1485.4  1485.4  1485.4  1485.4  1485.4  1485.4  1485.4  1485.4
        ( 35.1) ( 35.1) ( 35.1) ( 35.1) ( 35.1) ( 35.1) ( 35.1) ( 35.1) ( 35.1) ( 35.1)
         1020.8   713.1   812.2   866.1  1395.3   983.0   923.0  1478.5   146.6  -552.4
         24.149  16.870  19.214  20.489  33.009  23.255  21.836  34.979   3.468 -13.068
t_O        1154    1187    1200    1289    1075    1057    1062     766    1352    2033
        ( 27.3) ( 28.1) ( 28.4) ( 30.5) ( 25.4) ( 25.0) ( 25.1) ( 18.1) ( 32.0) ( 48.1)
t_E      1289.1  1289.1  1289.1  1289.1  1289.1  1289.1  1289.1  1289.1  1289.1  1289.1
        ( 30.5) ( 30.5) ( 30.5) ( 30.5) ( 30.5) ( 30.5) ( 30.5) ( 30.5) ( 30.5) ( 30.5)
         -255.5  -195.9  -171.9    -0.2  -390.5  -419.7  -411.6  -797.4   128.8  1852.3
         -6.045  -4.635  -4.067  -0.005  -9.238  -9.928  -9.738 -18.865   3.047  43.821
g_O         543     644     645     562     596     692     665     821     571     394
        ( 12.8) ( 15.2) ( 15.3) ( 13.3) ( 14.1) ( 16.4) ( 15.7) ( 19.4) ( 13.5) (  9.3)
g_E       758.8   758.8   758.8   758.8   758.8   758.8   758.8   758.8   758.8   758.8
        ( 18.0) ( 18.0) ( 18.0) ( 18.0) ( 18.0) ( 18.0) ( 18.0) ( 18.0) ( 18.0) ( 18.0)
         -363.4  -211.3  -209.7  -337.5  -287.9  -127.6  -175.5   129.3  -324.8  -516.5
         -8.598  -5.000  -4.960  -7.985  -6.811  -3.018  -4.153   3.059  -7.683 -12.218
c_O         596     587     532     504     478     559     605     531     747     626
        ( 14.1) ( 13.9) ( 12.6) ( 11.9) ( 11.3) ( 13.2) ( 14.3) ( 12.6) ( 17.7) ( 14.8)
c_E       693.7   693.7   693.7   693.7   693.7   693.7   693.7   693.7   693.7   693.7
        ( 16.4) ( 16.4) ( 16.4) ( 16.4) ( 16.4) ( 16.4) ( 16.4) ( 16.4) ( 16.4) ( 16.4)
         -180.9  -196.0  -282.3  -322.0  -356.0  -241.3  -165.5  -283.8   110.7  -128.5
         -4.279  -4.637  -6.679  -7.617  -8.422  -5.709  -3.915  -6.714   2.618  -3.040

N          4227    4227    4227    4227    4227    4227    4227    4227    4227    4227
X2        224.8   112.4   150.4   203.5   374.0   200.4   175.9   517.3    57.1   676.6
Gvalue    220.9   109.8   148.3   206.4   360.9   194.4   170.4   526.6    61.3   654.9
Gv/N      5.227   2.598   3.508   4.883   8.537   4.600   4.030  12.458   1.450  15.494
Prob    0.00000 0.00000 0.00000 0.00000 0.00000 0.00000 0.00000 0.00000 0.00000 0.00000
        -------------------------------------------------------------------------------

CDS_INITIAL:
        -------------------------------------------------------------------------------
              1       2       3       4       5       6       7       8       9      10
        -------------------------------------------------------------------------------
a_O        3804       0       0    2631    2462    2128    2261    2036    1919    1962
        ( 90.0) (  0.0) (  0.0) ( 62.2) ( 58.2) ( 50.3) ( 53.5) ( 48.2) ( 45.4) ( 46.4)
a_E      1271.7  1409.8  1025.5  1271.7  1409.8  1025.5  1271.7  1409.8  1025.5  1271.7
        ( 30.1) ( 33.4) ( 24.3) ( 30.1) ( 33.4) ( 24.3) ( 30.1) ( 33.4) ( 24.3) ( 30.1)
         8336.2     0.0     0.0  3825.6  2745.2  3106.7  2602.2  1496.6  2404.8  1701.5
        197.212   0.000   0.000  90.503  64.945  73.496  61.562  35.406  56.891  40.254
t_O         271    4227       0     363     748    1077     598    1023     934     876
        (  6.4) (100.0) (  0.0) (  8.6) ( 17.7) ( 25.5) ( 14.1) ( 24.2) ( 22.1) ( 20.7)
t_E       816.2  1273.1  1276.2   816.2  1273.1  1276.2   816.2  1273.1  1276.2   816.2
        ( 19.3) ( 30.1) ( 30.2) ( 19.3) ( 30.1) ( 30.2) ( 19.3) ( 30.1) ( 30.2) ( 19.3)
         -597.6 10145.0     0.0  -588.2  -795.6  -365.6  -372.0  -447.5  -583.2   123.9
        -14.137 240.005   0.000 -13.916 -18.822  -8.649  -8.801 -10.587 -13.796   2.932
g_O         152       0    4227     900     475     587     731     493     802     772
        (  3.6) (  0.0) (100.0) ( 21.3) ( 11.2) ( 13.9) ( 17.3) ( 11.7) ( 19.0) ( 18.3)
g_E      1359.5   685.3   987.9  1359.5   685.3   987.9  1359.5   685.3   987.9  1359.5
        ( 32.2) ( 16.2) ( 23.4) ( 32.2) ( 16.2) ( 23.4) ( 32.2) ( 16.2) ( 23.4) ( 32.2)
         -666.1     0.0 12289.1  -742.4  -348.2  -611.2  -907.1  -324.7  -334.4  -873.7
        -15.757   0.000 290.729 -17.564  -8.237 -14.458 -21.459  -7.682  -7.912 -20.670
c_O           0       0       0     333     542     435     637     675     572     617
        (  0.0) (  0.0) (  0.0) (  7.9) ( 12.8) ( 10.3) ( 15.1) ( 16.0) ( 13.5) ( 14.6)
c_E       779.6   858.8   937.3   779.6   858.8   937.3   779.6   858.8   937.3   779.6
        ( 18.4) ( 20.3) ( 22.2) ( 18.4) ( 20.3) ( 22.2) ( 18.4) ( 20.3) ( 22.2) ( 18.4)
            0.0     0.0     0.0  -566.6  -498.9  -667.9  -257.4  -325.1  -565.0  -288.7
          0.000   0.000   0.000 -13.404 -11.803 -15.800  -6.090  -7.691 -13.366  -6.830

N          4227    4227    4227    4227    4227    4227    4227    4227    4227    4227
X2       7258.9  9807.5 13859.0  2115.8  1183.3  1648.1  1144.6   420.6  1047.5   666.9
Gvalue   7072.5 10145.0 12289.1  1928.3  1102.5  1462.1  1065.7   399.3   922.2   663.0
Gv/N    167.318 240.005 290.729  45.620  26.083  34.589  25.212   9.446  21.818  15.685
Prob    1.00000 1.00000 1.00000 1.00000 0.00000 0.00000 0.00000 0.00000 0.00000 0.00000
        -------------------------------------------------------------------------------
             11      12      13      14      15      16      17      18      19      20
        -------------------------------------------------------------------------------
a_O        1640    1799    1862    1537    1608    1628    1357    1614    1726    1296
        ( 38.8) ( 42.6) ( 44.1) ( 36.4) ( 38.0) ( 38.5) ( 32.1) ( 38.2) ( 40.8) ( 30.7)
a_E      1409.8  1025.5  1271.7  1409.8  1025.5  1271.7  1409.8  1025.5  1271.7  1409.8
        ( 33.4) ( 24.3) ( 30.1) ( 33.4) ( 24.3) ( 30.1) ( 33.4) ( 24.3) ( 30.1) ( 33.4)
          496.1  2022.1  1420.0   265.5  1446.4   804.3  -103.6  1463.9  1054.5  -218.2
         11.736  47.837  33.593   6.282  34.219  19.027  -2.451  34.631  24.946  -5.161
t_O        1585    1257     944    1702    1380    1157    1796    1316    1038    1791
        ( 37.5) ( 29.7) ( 22.3) ( 40.3) ( 32.6) ( 27.4) ( 42.5) ( 31.1) ( 24.6) ( 42.4)
t_E      1273.1  1276.2   816.2  1273.1  1276.2   816.2  1273.1  1276.2   816.2  1273.1
        ( 30.1) ( 30.2) ( 19.3) ( 30.1) ( 30.2) ( 19.3) ( 30.1) ( 30.2) ( 19.3) ( 30.1)
          694.6   -38.2   274.7   988.3   215.8   807.5  1236.0    80.8   499.1  1222.6
         16.433  -0.903   6.498  23.381   5.105  19.103  29.240   1.911  11.807  28.923
g_O         401     576     820     415     634     817     454     587     773     442
        (  9.5) ( 13.6) ( 19.4) (  9.8) ( 15.0) ( 19.3) ( 10.7) ( 13.9) ( 18.3) ( 10.5)
g_E       685.3   987.9  1359.5   685.3   987.9  1359.5   685.3   987.9  1359.5   685.3
        ( 16.2) ( 23.4) ( 32.2) ( 16.2) ( 23.4) ( 32.2) ( 16.2) ( 23.4) ( 32.2) ( 16.2)
         -429.8  -621.5  -829.1  -416.3  -562.4  -832.1  -373.8  -611.2  -872.8  -387.6
        -10.167 -14.703 -19.615  -9.848 -13.306 -19.685  -8.844 -14.458 -20.649  -9.171
```

c_O	601	595	601	573	605	625	620	710	690	698
	(14.2)	(14.1)	(14.2)	(13.6)	(14.3)	(14.8)	(14.7)	(16.8)	(16.3)	(16.5)
c_E	858.8	937.3	779.6	858.8	937.3	779.6	858.8	937.3	779.6	858.8
	(20.3)	(22.2)	(18.4)	(20.3)	(22.2)	(18.4)	(20.3)	(22.2)	(18.4)	(20.3)
	−429.0	−540.8	−312.8	−463.7	−529.7	−276.4	−404.0	−394.4	−168.6	−289.4
	−10.150	−12.794	−7.400	−10.971	−12.532	−6.538	−9.558	−9.331	−3.988	−6.847
N	4227	4227	4227	4227	4227	4227	4227	4227	4227	4227
X2	309.3	880.4	549.1	357.7	583.8	489.3	361.2	556.7	485.9	336.3
Gvalue	331.9	821.6	552.7	373.8	570.1	503.3	354.5	539.1	512.1	327.3
Gv/N	7.851	19.438	13.076	8.844	13.486	11.907	8.387	12.753	12.116	7.744
Prob	0.00000	0.00000	0.00000	0.00000	0.00000	0.00000	0.00000	0.00000	0.00000	0.00000

	21	22	23	24	25	26	27	28	29	30
a_O	1506	1497	1148	1337	1353	1106	1186	1258	1053	1183
	(35.6)	(35.4)	(27.2)	(31.6)	(32.0)	(26.2)	(28.1)	(29.8)	(24.9)	(28.0)
a_E	1025.5	1271.7	1409.8	1025.5	1271.7	1409.8	1025.5	1271.7	1409.8	1025.5
	(24.3)	(30.1)	(33.4)	(24.3)	(30.1)	(33.4)	(24.3)	(30.1)	(33.4)	(24.3)
	1157.3	488.4	−471.7	709.1	167.7	−536.9	344.8	−27.2	−614.6	337.9
	27.379	11.554	−11.159	16.777	3.968	−12.701	8.157	−0.644	−14.539	7.994
t_O	1272	1019	1771	1293	985	1647	1307	1038	1702	1313
	(30.1)	(24.1)	(41.9)	(30.6)	(23.3)	(39.0)	(30.9)	(24.6)	(40.3)	(31.1)
t_E	1276.2	816.2	1273.1	1276.2	816.2	1273.1	1276.2	816.2	1273.1	1276.2
	(30.2)	(19.3)	(30.1)	(30.2)	(19.3)	(30.1)	(30.2)	(19.3)	(30.1)	(30.2)
	−8.4	452.3	1169.1	33.8	370.4	848.2	62.3	499.1	988.3	74.6
	−0.200	10.701	27.659	0.799	8.762	20.066	1.474	11.807	23.381	1.765
g_O	683	969	563	770	1111	620	869	1143	697	860
	(16.2)	(22.9)	(13.3)	(18.2)	(26.3)	(14.7)	(20.6)	(27.0)	(16.5)	(20.3)
g_E	987.9	1359.5	685.3	987.9	1359.5	685.3	987.9	1359.5	685.3	987.9
	(23.4)	(32.2)	(16.2)	(23.4)	(32.2)	(16.2)	(23.4)	(32.2)	(16.2)	(23.4)
	−504.2	−656.2	−221.3	−383.8	−448.5	−124.1	−222.9	−396.5	23.6	−238.5
	−11.928	−15.524	−5.236	−9.079	−10.610	−2.937	−5.274	−9.380	0.559	−5.643
c_O	766	742	745	827	778	854	865	788	775	871
	(18.1)	(17.6)	(17.6)	(19.6)	(18.4)	(20.2)	(20.5)	(18.6)	(18.3)	(20.6)
c_E	937.3	779.6	858.8	937.3	779.6	858.8	937.3	779.6	858.8	937.3
	(22.2)	(18.4)	(20.3)	(22.2)	(18.4)	(20.3)	(22.2)	(18.4)	(20.3)	(22.2)
	−309.2	−73.4	−211.8	−207.1	−3.3	−9.6	−138.9	16.8	−159.1	−127.8
	−7.315	−1.738	−5.011	−4.899	−0.078	−0.226	−3.286	0.397	−3.765	−3.024
N	4227	4227	4227	4227	4227	4227	4227	4227	4227	4227
X2	350.5	204.3	280.2	155.9	85.5	181.5	45.7	95.0	243.2	46.5
Gvalue	335.5	211.0	264.3	152.0	86.3	177.6	45.3	92.2	238.3	46.2
Gv/N	7.936	4.993	6.254	3.597	2.042	4.202	1.071	2.180	5.637	1.093
Prob	0.00000	0.00000	0.00000	0.00000	0.00000	0.00000	0.00000	0.00000	0.00000	0.00000

	31	32	33	34	35	36	37	38	39	40
a_O	1265	1009	1111	1183	962	1072	1182	991	1079	1235
	(29.9)	(23.9)	(26.3)	(28.0)	(22.8)	(25.4)	(28.0)	(23.4)	(25.5)	(29.2)
a_E	1271.7	1409.8	1025.5	1271.7	1409.8	1025.5	1271.7	1409.8	1025.5	1271.7
	(30.1)	(33.4)	(24.3)	(30.1)	(33.4)	(24.3)	(30.1)	(33.4)	(24.3)	(30.1)
	−13.3	−675.0	177.8	−171.0	−735.3	95.0	−172.9	−698.6	109.6	−72.3
	−0.316	−15.969	4.207	−4.046	−17.396	2.247	−4.090	−16.528	2.594	−1.711
t_O	1048	1803	1240	951	1726	1289	971	1609	1268	948
	(24.8)	(42.7)	(29.3)	(22.5)	(40.8)	(30.5)	(23.0)	(38.1)	(30.0)	(22.4)
t_E	816.2	1273.1	1276.2	816.2	1273.1	1276.2	816.2	1273.1	1276.2	816.2
	(19.3)	(30.1)	(30.2)	(19.3)	(30.1)	(30.2)	(19.3)	(30.1)	(30.2)	(19.3)
	524.0	1254.8	−71.4	290.8	1050.6	25.7	337.3	753.5	−16.4	283.9
	12.397	29.686	−1.689	6.879	24.854	0.608	7.980	17.826	−0.388	6.715
g_O	1122	642	914	1213	689	966	1274	739	966	1288
	(26.5)	(15.2)	(21.6)	(28.7)	(16.3)	(22.9)	(30.1)	(17.5)	(22.9)	(30.5)
g_E	1359.5	685.3	987.9	1359.5	685.3	987.9	1359.5	685.3	987.9	1359.5
	(32.2)	(16.2)	(23.4)	(32.2)	(16.2)	(23.4)	(32.2)	(16.2)	(23.4)	(32.2)
	−430.8	−83.8	−142.2	−276.6	7.5	−43.3	−165.5	111.6	−43.3	−139.1
	−10.192	−1.982	−3.363	−6.543	0.177	−1.026	−3.915	2.639	−1.026	−3.292
c_O	792	773	962	880	850	900	800	888	914	756
	(18.7)	(18.3)	(22.8)	(20.8)	(20.1)	(21.3)	(18.9)	(21.0)	(21.6)	(17.9)
c_E	779.6	858.8	937.3	779.6	858.8	937.3	779.6	858.8	937.3	779.6
	(18.4)	(20.3)	(22.2)	(18.4)	(20.3)	(22.2)	(18.4)	(20.3)	(22.2)	(18.4)
	24.9	−162.7	50.0	213.1	−17.5	−73.1	41.2	59.4	−46.0	−46.6
	0.589	−3.850	1.184	5.041	−0.414	−1.730	0.975	1.405	−1.089	−1.102
N	4227	4227	4227	4227	4227	4227	4227	4227	4227	4227
X2	107.6	345.8	14.3	57.2	303.5	4.2	41.6	218.2	3.9	26.8
Gvalue	104.7	333.3	14.3	56.2	305.2	4.2	40.2	225.8	3.9	25.8
Gv/N	2.478	7.886	0.338	1.331	7.220	0.099	0.950	5.341	0.091	0.611
Prob	0.00000	0.00000	0.00254	0.00000	0.00000	0.24112	0.00000	0.00000	0.27627	0.00001

	41	42	43	44	45	46	47	48	49	50
a_O	986	1052	1188	1077	1112	1166	1046	1098	1202	1051
	(23.3)	(24.9)	(28.1)	(25.5)	(26.3)	(27.6)	(24.7)	(26.0)	(28.4)	(24.9)
a_E	1409.8	1025.5	1271.7	1409.8	1025.5	1271.7	1409.8	1025.5	1271.7	1409.8
	(33.4)	(24.3)	(30.1)	(33.4)	(24.3)	(30.1)	(33.4)	(24.3)	(30.1)	(33.4)
	−705.1	53.6	−161.7	−580.0	180.0	−202.3	−624.4	149.9	−135.5	−617.4
	−16.681	1.268	−3.826	−13.722	4.258	−4.787	−14.772	3.546	−3.205	−14.606
t_O	1677	1278	906	1566	1239	981	1517	1266	988	1556
	(39.7)	(30.2)	(21.4)	(37.0)	(29.3)	(23.2)	(35.9)	(30.0)	(23.4)	(36.8)
t_E	1273.1	1276.2	816.2	1273.1	1276.2	816.2	1273.1	1276.2	816.2	1273.1
	(30.1)	(30.2)	(19.3)	(30.1)	(30.2)	(19.3)	(30.1)	(30.2)	(19.3)	(30.1)
	924.2	3.6	189.2	648.5	−73.4	360.9	531.8	−20.4	377.5	624.4
	21.863	0.084	4.475	15.342	−1.735	8.537	12.580	−0.482	8.931	14.772
g_O	685	949	1298	708	984	1312	707	978	1272	736
	(16.2)	(22.5)	(30.7)	(16.7)	(23.3)	(31.0)	(16.7)	(23.1)	(30.1)	(17.4)
g_E	685.3	987.9	1359.5	685.3	987.9	1359.5	685.3	987.9	1359.5	685.3
	(16.2)	(23.4)	(32.2)	(16.2)	(23.4)	(32.2)	(16.2)	(23.4)	(32.2)	(16.2)
	−0.6	−76.3	−120.1	46.2	−7.8	−93.3	44.1	−19.7	−169.2	105.1
	−0.013	−1.805	−2.842	1.093	−0.185	−2.207	1.044	−0.467	−4.003	2.487
c_O	879	948	835	876	892	768	957	885	765	884
	(20.8)	(22.4)	(19.8)	(20.7)	(21.1)	(18.2)	(22.6)	(20.9)	(18.1)	(20.9)
c_E	858.8	937.3	779.6	858.8	937.3	779.6	858.8	937.3	779.6	858.8
	(20.3)	(22.2)	(18.4)	(20.3)	(22.2)	(18.4)	(20.3)	(22.2)	(18.4)	(20.3)
	40.9	21.5	114.5	34.7	−88.4	−23.1	207.2	−101.6	−29.0	51.1
	0.967	0.509	2.710	0.822	−2.091	−0.547	4.902	−2.405	−0.687	1.210
N	4227	4227	4227	4227	4227	4227	4227	4227	4227	4227
X2	256.0	2.3	22.1	147.0	10.6	43.9	152.5	8.2	45.9	158.7
Gvalue	259.4	2.4	21.8	149.4	10.4	42.1	158.7	8.2	43.8	163.3
Gv/N	6.136	0.056	0.517	3.535	0.247	0.997	3.754	0.193	1.036	3.863
Prob	0.00000	0.50221	0.00007	0.00000	0.01525	0.00000	0.00000	0.04284	0.00000	0.00000

	51	52	53	54	55	56	57	58	59	60
a_O	1055	1215	1094	1078	1130	1147	1086	1165	1218	1105
	(25.0)	(28.7)	(25.9)	(25.5)	(26.7)	(27.1)	(25.7)	(27.6)	(28.8)	(26.1)
a_E	1025.5	1271.7	1409.8	1025.5	1271.7	1409.8	1025.5	1271.7	1409.8	1025.5
	(24.3)	(30.1)	(33.4)	(24.3)	(30.1)	(33.4)	(24.3)	(30.1)	(33.4)	(24.3)
	59.7	−110.8	−554.9	107.5	−267.0	−473.3	124.4	−204.2	−356.2	164.9
	1.413	−2.621	−13.128	2.544	−6.316	−11.196	2.943	−4.830	−8.428	3.901
t_O	1280	970	1435	1303	977	1373	1304	965	1316	1273
	(30.3)	(22.9)	(33.9)	(30.8)	(23.1)	(32.5)	(30.8)	(22.8)	(31.1)	(30.1)
t_E	1276.2	816.2	1273.1	1276.2	816.2	1273.1	1276.2	816.2	1273.1	1276.2
	(30.2)	(19.3)	(30.1)	(30.2)	(19.3)	(30.1)	(30.2)	(19.3)	(30.1)	(30.2)
	7.6	335.0	343.5	54.1	351.4	207.4	56.2	323.3	87.2	−6.4
	0.179	7.924	8.127	1.280	8.314	4.907	1.328	7.647	2.063	−0.152
g_O	959	1318	744	896	1365	771	939	1311	725	958
	(22.7)	(31.2)	(17.6)	(21.2)	(32.3)	(18.2)	(22.2)	(31.0)	(17.2)	(22.7)
g_E	987.9	1359.5	685.3	987.9	1359.5	685.3	987.9	1359.5	685.3	987.9
	(23.4)	(32.2)	(16.2)	(23.4)	(32.2)	(16.2)	(23.4)	(32.2)	(16.2)	(23.4)
	−57.0	−81.7	122.3	−175.0	11.1	181.7	−95.4	−95.2	81.7	−58.9
	−1.348	−1.933	2.894	−4.140	0.262	4.300	−2.256	−2.253	1.933	−1.394
c_O	933	724	954	950	755	936	898	786	968	891
	(22.1)	(17.1)	(22.6)	(22.5)	(17.9)	(22.1)	(21.2)	(18.6)	(22.9)	(21.1)
c_E	937.3	779.6	858.8	937.3	779.6	858.8	937.3	779.6	858.8	937.3
	(22.2)	(18.4)	(20.3)	(22.2)	(18.4)	(20.3)	(22.2)	(18.4)	(20.3)	(22.2)
	−8.6	−107.2	200.6	25.6	−48.5	161.1	−76.9	12.8	231.7	−90.3
	−0.203	−2.537	4.745	0.605	−1.148	3.812	−1.820	0.302	5.482	−2.136
N	4227	4227	4227	4227	4227	4227	4227	4227	4227	4227
X2	1.7	36.8	106.9	12.0	48.3	74.5	8.2	37.9	43.7	9.4
Gvalue	1.7	35.2	111.6	12.2	47.0	77.0	8.2	36.6	44.4	9.3
Gv/N	0.041	0.834	2.639	0.289	1.112	1.823	0.195	0.867	1.050	0.219
Prob	0.63167	0.00000	0.00000	0.00674	0.00000	0.00000	0.04147	0.00000	0.00000	0.02612

	61	62	63	64	65	66	67	68	69	70
a_O	1231	1273	1055	1223	1334	1094	1294	1429	1118	1207
	(29.1)	(30.1)	(25.0)	(28.9)	(31.6)	(25.9)	(30.6)	(33.8)	(26.4)	(28.6)
a_E	1271.7	1409.8	1025.5	1271.7	1409.8	1025.5	1271.7	1409.8	1025.5	1271.7
	(30.1)	(33.4)	(24.3)	(30.1)	(33.4)	(24.3)	(30.1)	(33.4)	(24.3)	(30.1)
	−80.1	−259.9	59.7	−95.5	−147.5	141.4	45.0	38.6	193.0	−126.0
	−1.894	−6.148	1.413	−2.259	−3.489	3.345	1.065	0.914	4.566	−2.981
t_O	875	1303	1276	852	1317	1211	849	1295	1226	854
	(20.7)	(30.8)	(30.2)	(20.2)	(31.2)	(28.6)	(20.1)	(30.6)	(29.0)	(20.2)
t_E	816.2	1273.1	1276.2	816.2	1273.1	1276.2	816.2	1273.1	1276.2	816.2
	(19.3)	(30.1)	(30.2)	(19.3)	(30.1)	(30.2)	(19.3)	(30.1)	(30.2)	(19.3)
	121.8	60.5	−0.4	73.2	89.3	−127.1	66.9	44.1	−98.4	77.4
	2.881	1.431	−0.011	1.731	2.112	−3.006	1.584	1.044	−2.329	1.830
g_O	1345	712	985	1329	664	970	1324	624	972	1336
	(31.8)	(16.8)	(23.3)	(31.4)	(15.7)	(22.9)	(31.3)	(14.8)	(23.0)	(31.6)
g_E	1359.5	685.3	987.9	1359.5	685.3	987.9	1359.5	685.3	987.9	1359.5
	(32.2)	(16.2)	(23.4)	(32.2)	(16.2)	(23.4)	(32.2)	(16.2)	(23.4)	(32.2)
	−28.8	54.5	−5.8	−60.3	−41.9	−35.5	−70.0	−116.9	−31.6	−46.6
	−0.682	1.289	−0.138	−1.426	−0.991	−0.840	−1.657	−2.766	−0.747	−1.101
c_O	776	939	911	823	912	952	760	879	911	830
	(18.4)	(22.2)	(21.6)	(19.5)	(21.6)	(22.5)	(18.0)	(20.8)	(21.6)	(19.6)
c_E	779.6	858.8	937.3	779.6	858.8	937.3	779.6	858.8	937.3	779.6
	(18.4)	(20.3)	(22.2)	(18.4)	(20.3)	(22.2)	(18.4)	(20.3)	(22.2)	(18.4)
	−7.3	167.7	−51.9	89.1	109.6	29.6	−38.8	40.9	−51.9	103.9
	−0.172	3.967	−1.227	2.107	2.594	0.701	−0.918	0.967	−1.227	2.458
N	4227	4227	4227	4227	4227	4227	4227	4227	4227	4227
X2	5.7	22.5	1.6	6.5	9.5	8.5	3.1	6.6	11.3	8.7
Gvalue	5.6	22.7	1.6	6.5	9.5	8.4	3.1	6.8	11.1	8.7
Gv/N	0.133	0.537	0.038	0.154	0.226	0.199	0.074	0.160	0.263	0.205
Prob	0.13117	0.00005	0.66123	0.08993	0.02287	0.03811	0.37273	0.08010	0.01119	0.03408

	71	72	73	74	75	76	77	78	79	80
a_O	1384	1107	1248	1379	1136	1274	1368	1111	1273	1397
	(32.7)	(26.2)	(29.5)	(32.6)	(26.9)	(30.1)	(32.4)	(26.3)	(30.1)	(33.0)
a_E	1409.8	1025.5	1271.7	1409.8	1025.5	1271.7	1409.8	1025.5	1271.7	1409.8
	(33.4)	(24.3)	(30.1)	(33.4)	(24.3)	(30.1)	(33.4)	(24.3)	(30.1)	(33.4)
	−51.1	169.2	−46.9	−60.9	232.4	4.6	−82.4	177.8	2.6	−25.5
	−1.210	4.003	−1.110	−1.442	5.498	0.110	−1.949	4.207	0.062	−0.603
t_O	1329	1273	862	1265	1317	811	1311	1243	798	1273
	(31.4)	(30.1)	(20.4)	(29.9)	(31.2)	(19.2)	(31.0)	(29.4)	(18.9)	(30.1)
t_E	1273.1	1276.2	816.2	1273.1	1276.2	816.2	1273.1	1276.2	816.2	1273.1
	(30.1)	(30.2)	(19.3)	(30.1)	(30.2)	(19.3)	(30.1)	(30.2)	(19.3)	(30.1)
	114.2	−6.4	94.2	−16.2	82.8	−10.3	76.9	−65.6	−36.0	−0.2
	2.701	−0.152	2.228	−0.383	1.960	−0.244	1.819	−1.551	−0.851	−0.005
g_O	639	952	1382	671	950	1365	664	960	1384	648
	(15.1)	(22.5)	(32.7)	(15.9)	(22.5)	(32.3)	(15.7)	(22.7)	(32.7)	(15.3)
g_E	685.3	987.9	1359.5	685.3	987.9	1359.5	685.3	987.9	1359.5	685.3
	(16.2)	(23.4)	(32.2)	(16.2)	(23.4)	(32.2)	(16.2)	(23.4)	(32.2)	(16.2)
	−89.4	−70.5	45.4	−28.3	−74.4	11.1	−41.9	−55.0	49.5	−72.5
	−2.114	−1.668	1.074	−0.668	−1.759	0.262	−0.991	−1.302	1.170	−1.715
c_O	875	895	735	912	824	777	884	913	772	909
	(20.7)	(21.2)	(17.4)	(21.6)	(19.5)	(18.4)	(20.9)	(21.6)	(18.3)	(21.5)
c_E	858.8	937.3	779.6	858.8	937.3	779.6	858.8	937.3	779.6	858.8
	(20.3)	(22.2)	(18.4)	(20.3)	(22.2)	(18.4)	(20.3)	(22.2)	(18.4)	(20.3)
	32.7	−82.7	−86.7	109.6	−212.3	−5.3	51.1	−48.0	−15.2	103.3
	0.774	−1.956	−2.051	2.594	−5.023	−0.125	1.210	−1.135	−0.360	2.443
N	4227	4227	4227	4227	4227	4227	4227	4227	4227	4227
X2	6.4	9.7	5.9	4.3	28.4	0.1	3.8	9.4	0.9	5.1
Gvalue	6.4	9.6	5.9	4.3	28.5	0.1	3.8	9.2	0.9	5.1
Gv/N	0.151	0.226	0.141	0.101	0.675	0.002	0.089	0.218	0.022	0.120
Prob	0.09390	0.02256	0.11421	0.23481	0.00000	0.99532	0.28757	0.02632	0.81958	0.16738

	81	82	83	84	85	86	87	88	89	90
a_O	1065	1308	1368	1065	1271	1412	1081	1182	1404	1031
	(25.2)	(30.9)	(32.4)	(25.2)	(30.1)	(33.4)	(25.6)	(28.0)	(33.2)	(24.4)
a_E	1025.5	1271.7	1409.8	1025.5	1271.7	1409.8	1025.5	1271.7	1409.8	1025.5
	(24.3)	(30.1)	(33.4)	(24.3)	(30.1)	(33.4)	(24.3)	(30.1)	(33.4)	(24.3)
	80.4	73.7	−82.4	80.4	−1.4	4.4	113.8	−172.9	−11.6	10.9
	1.902	1.742	−1.949	1.902	−0.032	0.104	2.693	−4.090	−0.274	0.259
t_O	1279	808	1332	1292	865	1311	1274	829	1240	1304
	(30.3)	(19.1)	(31.5)	(30.6)	(20.5)	(31.0)	(30.1)	(19.6)	(29.3)	(30.8)
t_E	1276.2	816.2	1273.1	1276.2	816.2	1273.1	1276.2	816.2	1273.1	1276.2
	(30.2)	(19.3)	(30.1)	(30.2)	(19.3)	(30.1)	(30.2)	(19.3)	(30.1)	(30.2)
	5.6	−16.3	120.5	31.7	100.5	76.9	−4.4	25.8	−65.4	56.2

	0.132	−0.385	2.850	0.751	2.378	1.819	−0.105	0.611	−1.546	1.328
g_O	940	1338	668	976	1333	665	960	1427	685	946
	(22.2)	(31.7)	(15.8)	(23.1)	(31.5)	(15.7)	(22.7)	(33.8)	(16.2)	(22.4)
g_E	987.9	1359.5	685.3	987.9	1359.5	685.3	987.9	1359.5	685.3	987.9
	(23.4)	(32.2)	(16.2)	(23.4)	(32.2)	(16.2)	(23.4)	(32.2)	(16.2)	(23.4)
	−93.5	−42.6	−34.1	−23.7	−52.4	−39.9	−55.0	138.3	−0.6	−82.0
	−2.211	−1.008	−0.807	−0.561	−1.241	−0.945	−1.302	3.273	−0.013	−1.941
c_O	943	773	859	894	758	839	912	789	898	946
	(22.3)	(18.3)	(20.3)	(21.1)	(17.9)	(19.8)	(21.6)	(18.7)	(21.2)	(22.4)
c_E	937.3	779.6	858.8	937.3	779.6	858.8	937.3	779.6	858.8	937.3
	(22.2)	(18.4)	(20.3)	(22.2)	(18.4)	(20.3)	(22.2)	(18.4)	(20.3)	(22.2)
	11.4	−13.2	0.4	−84.6	−42.7	−39.1	−49.9	18.8	80.2	17.5
	0.270	−0.313	0.009	−2.001	−1.010	−0.926	−1.181	0.445	1.896	0.413
N	4227	4227	4227	4227	4227	4227	4227	4227	4227	4227
X2	3.9	1.5	4.4	3.9	4.0	2.2	4.5	10.0	2.7	2.5
Gvalue	3.9	1.5	4.4	3.9	4.0	2.2	4.4	10.1	2.7	2.5
Gv/N	0.092	0.036	0.103	0.092	0.094	0.052	0.105	0.239	0.063	0.059
Prob	0.27224	0.68050	0.22379	0.27588	0.26257	0.53474	0.21810	0.01781	0.44783	0.47278

	91	92	93	94	95	96	97	98	99	100
a_O	1224	1412	1053	1259	1325	1122	1287	1406	1030	1222
	(29.0)	(33.4)	(24.9)	(29.8)	(31.4)	(26.6)	(30.5)	(33.3)	(24.4)	(28.9)
a_E	1271.7	1409.8	1025.5	1271.1	1409.1	1025.1	1271.1	1409.1	1025.1	1271.1
	(30.1)	(33.4)	(24.3)	(30.1)	(33.4)	(24.3)	(30.1)	(33.4)	(24.3)	(30.1)
	−93.6	4.4	55.6	−24.1	−163.2	202.8	32.0	−6.3	9.9	−96.2
	−2.213	0.104	1.316	−0.569	−3.862	4.799	0.758	−0.149	0.234	−2.278
t_O	822	1202	1290	785	1362	1259	816	1269	1295	768
	(19.4)	(28.4)	(30.5)	(18.6)	(32.2)	(29.8)	(19.3)	(30.0)	(30.7)	(18.2)
t_E	816.2	1273.1	1276.2	815.8	1272.5	1275.6	815.8	1272.5	1275.6	815.8
	(19.3)	(30.1)	(30.2)	(19.3)	(30.1)	(30.2)	(19.3)	(30.1)	(30.2)	(19.3)
	11.7	−138.2	27.7	−60.4	185.1	−33.0	0.4	−7.0	39.1	−92.7
	0.276	−3.269	0.655	−1.430	4.382	−0.782	0.010	−0.166	0.924	−2.195
g_O	1431	704	956	1409	672	983	1331	646	1010	1450
	(33.9)	(16.7)	(22.6)	(33.3)	(15.9)	(23.3)	(31.5)	(15.3)	(23.9)	(34.3)
g_E	1359.5	685.3	987.9	1358.8	685.0	987.5	1358.8	685.0	987.5	1358.8
	(32.2)	(16.2)	(23.4)	(32.2)	(16.2)	(23.4)	(32.2)	(16.2)	(23.4)	(32.2)
	146.7	38.0	−62.8	102.1	−25.7	−8.9	−55.1	−75.6	45.6	188.3
	3.471	0.898	−1.486	2.418	−0.607	−0.210	−1.304	−1.790	1.079	4.457
c_O	750	909	928	772	866	861	791	904	890	785
	(17.7)	(21.5)	(22.0)	(18.3)	(20.5)	(20.4)	(18.7)	(21.4)	(21.1)	(18.6)
c_E	779.6	858.8	937.3	779.3	858.4	936.9	779.3	858.4	936.9	779.3
	(18.4)	(20.3)	(22.2)	(18.4)	(20.3)	(22.2)	(18.4)	(20.3)	(22.2)	(18.4)
	−58.2	103.3	−18.5	−14.5	15.3	−145.4	23.6	93.6	−91.3	11.5
	−1.376	2.443	−0.438	−0.343	0.362	−3.442	0.559	2.215	−2.162	0.272
N	4227	4227	4227	4225	4225	4225	4225	4225	4225	4225
X2	6.7	7.4	2.0	3.2	11.6	15.5	0.9	4.7	3.2	10.9
Gvalue	6.7	7.4	2.0	3.2	11.6	15.4	0.9	4.7	3.2	10.8
Gv/N	0.158	0.176	0.048	0.076	0.274	0.366	0.022	0.110	0.076	0.256
Prob	0.08238	0.05918	0.56995	0.36323	0.00892	0.00147	0.81377	0.19876	0.36007	0.01285

	101	102	103	104	105	106	107	108	109	110
a_O	1450	1074	1248	1369	1054	1256	1392	1036	1268	1355
	(34.3)	(25.4)	(29.5)	(32.4)	(25.0)	(29.8)	(33.0)	(24.5)	(30.0)	(32.1)
a_E	1409.1	1025.1	1270.8	1408.8	1024.8	1269.9	1407.8	1024.1	1269.6	1407.5
	(33.4)	(24.3)	(30.1)	(33.4)	(24.3)	(30.1)	(33.4)	(24.3)	(30.1)	(33.4)
	82.9	100.2	−45.2	−78.5	59.2	−27.6	−31.4	24.0	−3.2	−103.0
	1.962	2.371	−1.069	−1.858	1.401	−0.654	−0.745	0.567	−0.075	−2.440
t_O	1242	1319	860	1307	1268	813	1324	1275	754	1322
	(29.4)	(31.2)	(20.4)	(30.9)	(30.0)	(19.3)	(31.4)	(30.2)	(17.9)	(31.3)
t_E	1272.5	1275.6	815.6	1272.2	1275.3	815.0	1271.3	1274.4	814.8	1271.0
	(30.1)	(30.2)	(19.3)	(30.1)	(30.2)	(19.3)	(30.1)	(30.2)	(19.3)	(30.1)
	−60.3	88.2	91.2	70.5	−14.6	−4.0	107.5	1.2	−117.0	104.0
	−1.427	2.088	2.158	1.670	−0.345	−0.096	2.548	0.028	−2.773	2.465
g_O	658	967	1362	637	1010	1338	646	980	1406	618
	(15.6)	(22.9)	(32.2)	(15.1)	(23.9)	(31.7)	(15.3)	(23.2)	(33.3)	(14.6)
g_E	685.0	987.5	1358.5	684.8	987.2	1357.6	684.3	986.5	1357.2	684.1
	(16.2)	(23.4)	(32.2)	(16.2)	(23.4)	(32.2)	(16.2)	(23.4)	(32.2)	(16.2)
	−52.8	−40.5	7.0	−92.2	46.1	−38.8	−74.4	−13.0	99.3	−125.7
	−1.250	−0.958	0.165	−2.182	1.091	−0.920	−1.763	−0.308	2.352	−2.978
c_O	875	865	754	911	892	814	859	930	792	925
	(20.7)	(20.5)	(17.9)	(21.6)	(21.1)	(19.3)	(20.4)	(22.0)	(18.8)	(21.9)
c_E	858.4	936.9	779.1	858.2	936.6	778.5	857.6	936.0	778.4	857.4
	(20.3)	(22.2)	(18.4)	(20.3)	(22.2)	(18.4)	(20.3)	(22.2)	(18.4)	(20.3)
	33.5	−138.1	−49.4	108.8	−87.1	72.5	2.8	−11.9	27.5	140.4
	0.794	−3.268	−1.169	2.576	−2.063	1.718	0.067	−0.282	0.652	3.328
N	4225	4225	4224	4224	4224	4221	4221	4221	4220	4220
X2	3.3	9.7	3.6	8.7	3.5	2.1	4.5	0.2	6.5	15.7
Gvalue	3.3	9.8	3.6	8.7	3.5	2.0	4.5	0.2	6.6	15.8
Gv/N	0.078	0.233	0.085	0.205	0.084	0.048	0.107	0.005	0.157	0.375
Prob	0.34696	0.01994	0.30665	0.03388	0.31443	0.56593	0.21050	0.97437	0.08467	0.00124

	111	112	113	114	115	116	117	118	119	120
a_O	1062	1225	1360	1028	1236	1394	1069	1256	1426	1080
	(25.2)	(29.0)	(32.2)	(24.4)	(29.3)	(33.0)	(25.3)	(29.8)	(33.8)	(25.6)
a_E	1023.9	1269.3	1407.1	1023.6	1269.0	1406.8	1023.4	1268.1	1405.8	1022.6
	(24.3)	(30.1)	(33.4)	(24.3)	(30.1)	(33.4)	(24.3)	(30.1)	(33.4)	(24.3)
	77.7	−87.0	−92.7	8.8	−65.1	−25.5	93.3	−24.0	40.7	117.9
	1.841	−2.062	−2.197	0.209	−1.543	−0.604	2.211	−0.570	0.965	2.797
t_O	1294	790	1268	1289	770	1267	1276	794	1250	1280
	(30.7)	(18.7)	(30.1)	(30.6)	(18.3)	(30.0)	(30.3)	(18.8)	(29.7)	(30.4)
t_E	1274.1	814.6	1270.7	1273.8	814.4	1270.4	1273.5	813.9	1269.5	1272.6
	(30.2)	(19.3)	(30.1)	(30.2)	(19.3)	(30.1)	(30.2)	(19.3)	(30.1)	(30.2)
	40.1	−48.5	−5.4	30.6	−86.4	−6.8	5.0	−39.2	−38.7	14.8
	0.950	−1.150	−0.128	0.724	−2.049	−0.161	0.118	−0.931	−0.918	0.352
g_O	988	1439	657	978	1434	675	966	1428	668	960
	(23.4)	(34.1)	(15.6)	(23.2)	(34.0)	(16.0)	(22.9)	(33.9)	(15.8)	(22.8)
g_E	986.3	1356.9	684.0	986.1	1356.6	683.8	985.8	1355.6	683.3	985.1
	(23.4)	(32.2)	(16.2)	(23.4)	(32.2)	(16.2)	(23.4)	(32.2)	(16.2)	(23.4)
	3.4	169.0	−52.9	−16.0	159.2	−17.5	−39.2	148.5	−30.3	−49.6
	0.081	4.007	−1.253	−0.380	3.773	−0.415	−0.930	3.524	−0.719	−1.176
c_O	876	765	934	924	778	882	907	737	871	895
	(20.8)	(18.1)	(22.1)	(21.9)	(18.4)	(20.9)	(21.5)	(17.5)	(20.7)	(21.2)
c_E	935.8	778.2	857.2	935.5	778.0	857.0	935.3	777.4	856.4	934.6
	(22.2)	(18.4)	(20.3)	(22.2)	(18.4)	(20.3)	(22.2)	(18.4)	(20.3)	(22.2)
	−115.6	−26.1	160.3	−22.9	0.0	50.8	−55.8	−78.7	29.5	−77.6
	−2.740	−0.619	3.800	−0.543	0.001	1.204	−1.322	−1.868	0.700	−1.841

N	4220	4219	4219	4219	4218	4218	4218	4215	4215	4215
X2	5.6	7.5	9.5	0.4	7.7	1.0	3.3	6.6	1.2	5.6
Gvalue	5.6	7.4	9.4	0.4	7.7	1.0	3.3	6.5	1.2	5.6
Gv/N	0.133	0.176	0.222	0.010	0.182	0.023	0.078	0.155	0.028	0.132
Prob	0.13187	0.05994	0.02476	0.93859	0.05333	0.80991	0.35084	0.08803	0.75657	0.13545

	121	122	123	124	125	126	127	128	129	130
a_O	1222	1366	1027	1218	1365	1046	1217	1386	1021	1193
	(29.0)	(32.4)	(24.4)	(28.9)	(32.4)	(24.8)	(28.9)	(32.9)	(24.3)	(28.4)
a_E	1267.2	1404.8	1021.9	1266.6	1404.1	1021.4	1266.6	1404.1	1021.4	1266.0
	(30.1)	(33.4)	(24.3)	(30.1)	(33.4)	(24.3)	(30.1)	(33.4)	(24.3)	(30.1)
	-88.7	-76.5	10.2	-95.3	-77.2	49.7	-97.2	-36.0	-0.8	-141.7
	-2.106	-1.817	0.242	-2.263	-1.833	1.181	-2.308	-0.856	-0.020	-3.366
t_O	769	1281	1321	798	1223	1255	820	1297	1307	815
	(18.3)	(30.4)	(31.4)	(19.0)	(29.0)	(29.8)	(19.5)	(30.8)	(31.0)	(19.4)
t_E	813.3	1268.6	1271.7	812.9	1268.0	1271.1	812.9	1268.0	1271.1	812.5
	(19.3)	(30.1)	(30.2)	(19.3)	(30.1)	(30.2)	(19.3)	(30.1)	(30.2)	(19.3)
	-86.1	24.9	100.5	-29.5	-88.4	-32.0	14.3	58.7	72.8	5.0
	-2.045	0.592	2.386	-0.701	-2.099	-0.760	0.339	1.394	1.730	0.118
g_O	1427	679	977	1445	651	985	1350	661	957	1449
	(33.9)	(16.1)	(23.2)	(34.3)	(15.5)	(23.4)	(32.1)	(15.7)	(22.7)	(34.4)
g_E	1354.7	682.8	984.4	1354.0	682.5	983.9	1354.0	682.5	983.9	1353.4
	(32.2)	(16.2)	(23.4)	(32.2)	(16.2)	(23.4)	(32.2)	(16.2)	(23.4)	(32.2)
	148.5	-7.7	-14.8	187.9	-61.6	2.1	-8.0	-42.4	-53.1	197.9
	3.525	-0.182	-0.351	4.464	-1.462	0.050	-0.190	-1.006	-1.262	4.702
c_O	794	886	887	749	971	924	823	866	925	751
	(18.9)	(21.0)	(21.1)	(17.8)	(23.1)	(21.9)	(19.5)	(20.6)	(22.0)	(17.8)
c_E	776.9	855.8	934.0	776.5	855.3	933.5	776.5	855.3	933.5	776.1
	(18.4)	(20.3)	(22.2)	(18.4)	(20.3)	(22.2)	(18.4)	(20.3)	(22.2)	(18.4)
	34.6	61.6	-91.6	-54.0	246.3	-19.0	95.7	21.4	-17.0	-49.5
	0.822	1.461	-2.174	-1.284	5.850	-0.451	2.273	0.509	-0.404	-1.175
N	4212	4212	4212	4210	4210	4210	4210	4210	4210	4208
X2	8.3	2.3	4.4	9.2	19.8	0.9	4.8	1.7	1.8	11.8
Gvalue	8.3	2.3	4.4	9.1	19.2	0.9	4.8	1.7	1.8	11.7
Gv/N	0.196	0.054	0.104	0.217	0.455	0.021	0.113	0.041	0.043	0.279
Prob	0.04099	0.51612	0.22394	0.02762	0.00025	0.82782	0.18952	0.63431	0.60879	0.00839

	131	132	133	134	135	136	137	138	139	140
a_O	1400	1050	1242	1407	1017	1217	1370	1037	1226	1388
	(33.3)	(25.0)	(29.6)	(33.5)	(24.2)	(29.0)	(32.6)	(24.7)	(29.2)	(33.1)
a_E	1403.5	1020.9	1264.5	1401.8	1019.7	1262.7	1399.8	1018.3	1261.8	1398.8
	(33.4)	(24.3)	(30.1)	(33.4)	(24.3)	(30.1)	(33.4)	(24.3)	(30.1)	(33.4)
	-6.9	58.9	-44.5	10.4	-5.4	-89.7	-59.0	37.8	-70.5	-21.5
	-0.165	1.401	-1.059	0.248	-0.130	-2.136	-1.405	0.901	-1.681	-0.513
t_O	1256	1293	811	1217	1291	867	1269	1287	827	1269
	(29.8)	(30.7)	(19.3)	(29.0)	(30.7)	(20.7)	(30.2)	(30.7)	(19.7)	(30.3)
t_E	1267.4	1270.5	811.5	1265.9	1269.0	810.4	1264.1	1267.2	809.8	1263.2
	(30.1)	(30.2)	(19.3)	(30.1)	(30.2)	(19.3)	(30.1)	(30.2)	(19.3)	(30.1)
	-22.7	45.4	-1.1	-95.9	44.4	117.1	9.9	40.0	34.7	11.7
	-0.539	1.079	-0.026	-2.281	1.057	2.790	0.235	0.953	0.828	0.278
g_O	661	948	1386	694	987	1307	645	958	1394	623
	(15.7)	(22.5)	(33.0)	(16.5)	(23.5)	(31.1)	(15.4)	(22.8)	(33.2)	(14.9)
g_E	682.2	983.5	1351.8	681.4	982.3	1349.8	680.4	980.9	1348.9	679.9
	(16.2)	(23.4)	(32.2)	(16.2)	(23.4)	(32.2)	(16.2)	(23.4)	(32.2)	(16.2)
	-41.7	-69.7	69.3	25.5	9.4	-84.3	-69.0	-45.3	91.8	-108.9
	-0.992	-1.655	1.650	0.606	0.224	-2.008	-1.643	-1.079	2.188	-2.598
c_O	891	917	764	885	908	806	913	915	747	914
	(21.2)	(21.8)	(18.2)	(21.1)	(21.6)	(19.2)	(21.8)	(21.8)	(17.8)	(21.8)
c_E	854.9	933.1	775.2	853.9	932.0	774.1	852.7	930.7	773.6	852.1
	(20.3)	(22.2)	(18.4)	(20.3)	(22.2)	(18.4)	(20.3)	(22.2)	(18.4)	(20.3)
	73.6	-31.9	-22.3	63.3	-47.3	65.1	124.8	-31.0	-52.2	128.2
	1.750	-0.758	-0.530	1.505	-1.127	1.550	2.972	-0.740	-1.245	3.057
N	4208	4208	4203	4203	4203	4197	4197	4197	4194	4194
X2	2.3	2.8	1.4	3.3	1.0	8.3	6.8	1.5	3.8	9.4
Gvalue	2.3	2.8	1.4	3.3	1.0	8.2	6.7	1.5	3.8	9.4
Gv/N	0.054	0.066	0.034	0.078	0.025	0.196	0.160	0.035	0.091	0.224
Prob	0.51698	0.42500	0.69969	0.35024	0.79343	0.04193	0.08208	0.69262	0.28372	0.02433

	141	142	143	144	145	146	147	148	149	150
a_O	1070	1299	1398	1016	1185	1365	1007	1152	1358	1028
	(25.5)	(31.0)	(33.3)	(24.2)	(28.3)	(32.6)	(24.0)	(27.5)	(32.4)	(24.6)
a_E	1017.5	1261.2	1398.1	1017.1	1260.0	1396.8	1016.1	1259.7	1396.5	1015.8
	(24.3)	(30.1)	(33.4)	(24.3)	(30.1)	(33.4)	(24.3)	(30.1)	(33.4)	(24.3)
	107.6	76.8	-0.3	-2.1	-145.4	-62.9	-18.1	-205.8	-75.9	24.5
	2.565	1.832	-0.006	-0.050	-3.471	-1.501	-0.432	-4.916	-1.812	0.584
t_O	1266	809	1285	1339	807	1319	1299	870	1244	1323
	(30.2)	(19.3)	(30.7)	(31.9)	(19.3)	(31.5)	(31.0)	(20.8)	(29.7)	(31.6)
t_E	1266.3	809.4	1262.6	1265.7	808.7	1261.4	1264.4	808.5	1261.1	1264.1
	(30.2)	(19.3)	(30.1)	(30.2)	(19.3)	(30.1)	(30.2)	(19.3)	(30.1)	(30.2)
	-0.5	-0.8	45.3	150.9	-3.3	117.9	70.0	127.7	-33.9	120.4
	-0.012	-0.020	1.079	3.599	-0.079	2.814	1.672	3.049	-0.810	2.876
g_O	977	1352	689	913	1399	609	961	1388	659	926
	(23.3)	(32.3)	(16.4)	(21.8)	(33.4)	(14.5)	(22.9)	(33.2)	(15.7)	(22.1)
g_E	980.2	1348.2	679.6	979.7	1346.9	679.0	978.8	1346.6	678.8	978.6
	(23.4)	(32.2)	(16.2)	(23.4)	(32.2)	(16.2)	(23.4)	(32.2)	(16.2)	(23.4)
	-6.4	7.6	18.9	-128.8	106.1	-132.4	-35.3	84.0	-39.0	-102.3
	-0.153	0.180	0.451	-3.073	2.534	-3.162	-0.842	2.007	-0.931	-2.442
c_O	881	732	820	924	797	895	921	777	926	910
	(21.0)	(17.5)	(19.6)	(22.0)	(19.0)	(21.4)	(22.0)	(18.6)	(22.1)	(21.7)
c_E	930.0	773.2	851.7	929.5	772.5	850.9	928.7	772.3	850.7	928.4
	(22.2)	(18.4)	(20.3)	(22.2)	(18.4)	(20.3)	(22.2)	(18.4)	(20.3)	(22.2)
	-95.4	-80.1	-62.2	-11.1	49.9	90.5	-15.3	9.5	157.1	-36.5
	-2.274	-1.912	-1.483	-0.264	1.191	2.161	-0.364	0.227	3.753	-0.872
N	4194	4192	4192	4192	4188	4188	4188	4187	4187	4187
X2	5.3	3.3	1.7	8.8	7.3	12.9	1.4	15.2	8.5	6.1
Gvalue	5.3	3.4	1.7	8.9	7.3	13.0	1.4	15.3	8.4	6.1
Gv/N	0.126	0.080	0.041	0.211	0.175	0.311	0.034	0.366	0.200	0.145
Prob	0.15130	0.33806	0.63263	0.03124	0.06259	0.00455	0.70405	0.00155	0.03903	0.10740

Bgar_PBI
5UTR:

	50	49	48	47	46	45	44	43	42	41
a_O	365	364	342	360	388	365	385	351	375	382
	(39.2)	(39.1)	(36.7)	(38.6)	(41.6)	(39.2)	(41.3)	(37.7)	(40.2)	(41.0)
a_E	367.6	367.6	367.6	367.6	367.6	367.6	367.6	367.6	367.6	367.6
	(39.4)	(39.4)	(39.4)	(39.4)	(39.4)	(39.4)	(39.4)	(39.4)	(39.4)	(39.4)
	-5.2	-7.1	-49.4	-15.0	41.9	-5.2	35.6	-32.4	15.0	29.4
	-0.554	-0.767	-5.296	-1.612	4.499	-0.554	3.823	-3.479	1.606	3.152
t_O	347	371	377	347	330	351	345	376	365	348
	(37.2)	(39.8)	(40.5)	(37.2)	(35.4)	(37.7)	(37.0)	(40.3)	(39.2)	(37.3)
t_E	342.5	342.5	342.5	342.5	342.5	342.5	342.5	342.5	342.5	342.5
	(36.8)	(36.8)	(36.8)	(36.8)	(36.8)	(36.8)	(36.8)	(36.8)	(36.8)	(36.8)
	9.0	59.2	72.3	9.0	-24.6	17.1	4.9	70.1	46.4	11.0
	0.963	6.354	7.755	0.963	-2.641	1.838	0.530	7.520	4.974	1.181
g_O	126	118	121	144	120	122	126	121	99	128
	(13.5)	(12.7)	(13.0)	(15.5)	(12.9)	(13.1)	(13.5)	(13.0)	(10.6)	(13.7)
g_E	138.4	138.4	138.4	138.4	138.4	138.4	138.4	138.4	138.4	138.4
	(14.8)	(14.8)	(14.8)	(14.8)	(14.8)	(14.8)	(14.8)	(14.8)	(14.8)	(14.8)
	-23.6	-37.6	-32.4	11.5	-34.2	-30.7	-23.6	-32.4	-66.3	-19.9
	-2.530	-4.031	-3.481	1.235	-3.666	-3.294	-2.530	-3.481	-7.111	-2.138
c_O	94	79	92	81	94	94	76	84	93	74
	(10.1)	(8.5)	(9.9)	(8.7)	(10.1)	(10.1)	(8.2)	(9.0)	(10.0)	(7.9)
c_E	83.5	83.5	83.5	83.5	83.5	83.5	83.5	83.5	83.5	83.5
	(9.0)	(9.0)	(9.0)	(9.0)	(9.0)	(9.0)	(9.0)	(9.0)	(9.0)	(9.0)
	22.2	-8.8	17.8	-4.9	22.2	22.2	-14.3	1.0	20.0	-17.9
	2.387	-0.941	1.912	-0.530	2.387	2.387	-1.537	0.105	2.148	-1.920
N	932	932	932	932	932	932	932	932	932	932
X2	2.5	5.6	8.3	0.5	5.3	3.5	2.6	6.2	13.9	2.5
Gvalue	2.5	5.7	8.3	0.5	5.4	3.5	2.7	6.2	15.1	2.6
Gv/N	0.266	0.616	0.890	0.056	0.579	0.376	0.286	0.666	1.617	0.275
Prob	0.47884	0.12498	0.04037	0.91477	0.14504	0.31988	0.44627	0.10203	0.00176	0.46336

	40	39	38	37	36	35	34	33	32	31
a_O	381	384	351	387	382	384	369	374	391	372
	(40.9)	(41.2)	(37.7)	(41.5)	(41.0)	(41.2)	(39.6)	(40.1)	(42.0)	(39.9)
a_E	367.6	367.6	367.6	367.6	367.6	367.6	367.6	367.6	367.6	367.6
	(39.4)	(39.4)	(39.4)	(39.4)	(39.4)	(39.4)	(39.4)	(39.4)	(39.4)	(39.4)
	27.3	33.5	-32.4	39.8	29.4	33.5	2.8	12.9	48.3	8.9
	2.930	3.599	-3.479	4.273	3.152	3.599	0.303	1.387	5.180	0.952
t_O	346	346	375	351	344	363	364	363	318	345
	(37.1)	(37.1)	(40.2)	(37.7)	(36.9)	(38.9)	(39.1)	(38.9)	(34.1)	(37.0)
t_E	342.5	342.5	342.5	342.5	342.5	342.5	342.5	342.5	342.5	342.5
	(36.8)	(36.8)	(36.8)	(36.8)	(36.8)	(36.8)	(36.8)	(36.8)	(36.8)	(36.8)
	7.0	7.0	67.9	17.1	2.9	42.1	44.2	42.1	-47.3	4.9
	0.746	0.746	7.286	1.838	0.314	4.519	4.746	4.519	-5.073	0.530
g_O	121	120	131	107	116	125	117	120	129	140
	(13.0)	(12.9)	(14.1)	(11.5)	(12.4)	(13.4)	(12.6)	(12.9)	(13.8)	(15.0)
g_E	138.4	138.4	138.4	138.4	138.4	138.4	138.4	138.4	138.4	138.4
	(14.8)	(14.8)	(14.8)	(14.8)	(14.8)	(14.8)	(14.8)	(14.8)	(14.8)	(14.8)
	-32.4	-34.2	-14.3	-55.0	-40.9	-25.4	-39.2	-34.2	-18.1	3.3
	-3.481	-3.666	-1.537	-5.902	-4.388	-2.724	-4.210	-3.666	-1.939	0.354
c_O	84	82	75	87	90	60	82	75	94	75
	(9.0)	(8.8)	(8.0)	(9.3)	(9.7)	(6.4)	(8.8)	(8.0)	(10.1)	(8.0)
c_E	83.5	83.5	83.5	83.5	83.5	83.5	83.5	83.5	83.5	83.5
	(9.0)	(9.0)	(9.0)	(9.0)	(9.0)	(9.0)	(9.0)	(9.0)	(9.0)	(9.0)
	1.0	-3.0	-16.1	7.1	13.5	-39.7	-3.0	-16.1	22.2	-16.1
	0.105	-0.321	-1.730	0.764	1.445	-4.257	-0.321	-1.730	2.387	-1.730
N	932	932	932	932	932	932	932	932	932	932
X2	2.7	3.2	5.1	8.5	4.7	9.9	4.7	4.6	5.2	1.0
Gvalue	2.8	3.3	5.0	9.1	4.9	10.6	4.8	4.8	5.2	1.0
Gv/N	0.300	0.358	0.541	0.974	0.524	1.137	0.518	0.511	0.555	0.106
Prob	0.42408	0.34265	0.16887	0.02832	0.18070	0.01410	0.18454	0.19026	0.15939	0.80417

	30	29	28	27	26	25	24	23	22	21
a_O	377	377	391	376	398	396	367	412	387	380
	(40.5)	(40.5)	(42.0)	(40.3)	(42.7)	(42.5)	(39.4)	(44.2)	(41.5)	(40.8)
a_E	367.6	367.6	367.6	367.6	367.6	367.6	367.6	367.6	367.6	367.6
	(39.4)	(39.4)	(39.4)	(39.4)	(39.4)	(39.4)	(39.4)	(39.4)	(39.4)	(39.4)
	19.1	19.1	48.3	17.0	63.3	59.0	-1.2	94.0	39.8	25.2
	2.045	2.045	5.180	1.825	6.789	6.326	-0.127	10.084	4.273	2.708
t_O	361	356	333	391	351	348	361	328	340	372
	(38.7)	(38.2)	(35.7)	(42.0)	(37.7)	(37.3)	(38.7)	(35.2)	(36.5)	(39.9)
t_E	342.5	342.5	342.5	342.5	342.5	342.5	342.5	342.5	342.5	342.5
	(36.8)	(36.8)	(36.8)	(36.8)	(36.8)	(36.8)	(36.8)	(36.8)	(36.8)	(36.8)
	37.9	27.4	-18.8	103.5	17.1	11.0	37.9	-28.5	-5.1	61.4
	4.066	2.944	-2.018	11.102	1.838	1.181	4.066	-3.053	-0.543	6.586
g_O	109	126	120	100	110	115	109	108	123	109
	(11.7)	(13.5)	(12.9)	(10.7)	(11.8)	(12.3)	(11.7)	(11.6)	(13.2)	(11.7)
g_E	138.4	138.4	138.4	138.4	138.4	138.4	138.4	138.4	138.4	138.4
	(14.8)	(14.8)	(14.8)	(14.8)	(14.8)	(14.8)	(14.8)	(14.8)	(14.8)	(14.8)
	-52.0	-23.6	-34.2	-64.9	-50.5	-42.5	-52.0	-53.5	-28.9	-52.0
	-5.579	-2.530	-3.666	-6.968	-5.415	-4.564	-5.579	-5.741	-3.106	-5.579
c_O	85	73	88	65	73	73	95	84	82	71
	(9.1)	(7.8)	(9.4)	(7.0)	(7.8)	(7.8)	(10.2)	(9.0)	(8.8)	(7.6)
c_E	83.5	83.5	83.5	83.5	83.5	83.5	83.5	83.5	83.5	83.5
	(9.0)	(9.0)	(9.0)	(9.0)	(9.0)	(9.0)	(9.0)	(9.0)	(9.0)	(9.0)
	3.0	-19.6	9.2	-32.6	-19.6	-19.6	24.5	1.0	-3.0	-23.0
	0.323	-2.107	0.989	-3.495	-2.107	-2.107	2.628	0.105	-0.321	-2.473
N	932	932	932	932	932	932	932	932	932	932
X2	7.5	3.2	4.4	21.8	9.9	7.5	8.8	12.6	2.8	11.1
Gvalue	8.0	3.3	4.5	23.0	10.3	7.8	9.2	13.0	2.8	11.6
Gv/N	0.855	0.352	0.485	2.465	1.105	0.837	0.989	1.395	0.303	1.242
Prob	0.04669	0.35022	0.21074	0.00004	0.01622	0.05041	0.02655	0.00463	0.41928	0.00898

	20	19	18	17	16	15	14	13	12	11
a_O	367	410	399	396	377	376	409	422	381	421
	(39.4)	(44.0)	(42.8)	(42.5)	(40.5)	(40.3)	(43.9)	(45.3)	(40.9)	(45.2)
a_E	367.6	367.6	367.6	367.6	367.6	367.6	367.6	367.6	367.6	367.6
	(39.4)	(39.4)	(39.4)	(39.4)	(39.4)	(39.4)	(39.4)	(39.4)	(39.4)	(39.4)
	-1.2	89.5	65.4	59.0	19.1	17.0	87.3	116.5	27.3	114.2
	-0.127	9.607	7.020	6.326	2.045	1.825	9.369	12.500	2.930	12.256
t_O	339	329	344	315	337	342	295	279	252	182
	(36.4)	(35.3)	(36.9)	(33.8)	(36.2)	(36.7)	(31.7)	(29.9)	(27.0)	(19.5)

t_E	342.5	342.5	342.5	342.5	342.5	342.5	342.5	342.5	342.5	342.5
	(36.8)	(36.8)	(36.8)	(36.8)	(36.8)	(36.8)	(36.8)	(36.8)	(36.8)	(36.8)
	-7.0	-26.5	2.9	-52.8	-11.0	-1.1	-88.2	-114.5	-154.7	-230.2
	-0.756	-2.847	0.314	-5.666	-1.179	-0.116	-9.459	-12.284	-16.600	-24.698
g_O	151	133	119	144	149	155	174	185	256	296
	(16.2)	(14.3)	(12.8)	(15.5)	(16.0)	(16.6)	(18.7)	(19.8)	(27.5)	(31.8)
g_E	138.4	138.4	138.4	138.4	138.4	138.4	138.4	138.4	138.4	138.4
	(14.8)	(14.8)	(14.8)	(14.8)	(14.8)	(14.8)	(14.8)	(14.8)	(14.8)	(14.8)
	26.4	-10.5	-35.9	11.5	22.1	35.2	79.8	107.5	315.0	450.2
	2.833	-1.128	-3.849	1.235	2.369	3.777	8.558	11.533	33.803	48.306
c_O	75	60	70	77	69	59	54	46	43	33
	(8.0)	(6.4)	(7.5)	(8.3)	(7.4)	(6.3)	(5.8)	(4.9)	(4.6)	(3.5)
c_E	83.5	83.5	83.5	83.5	83.5	83.5	83.5	83.5	83.5	83.5
	(9.0)	(9.0)	(9.0)	(9.0)	(9.0)	(9.0)	(9.0)	(9.0)	(9.0)	(9.0)
	-16.1	-39.7	-24.7	-12.5	-26.3	-41.0	-47.1	-54.9	-57.1	-61.3
	-1.730	-4.257	-2.651	-1.341	-2.826	-4.399	-5.052	-5.886	-6.125	-6.575
N	932	932	932	932	932	932	932	932	932	932
X2	2.1	12.3	7.6	5.1	3.7	9.4	30.9	52.4	144.1	293.2
Gvalue	2.1	12.8	7.8	5.2	3.8	10.1	31.8	54.6	130.6	273.0
Gv/N	0.221	1.375	0.834	0.554	0.409	1.088	3.416	5.862	14.008	29.290
Prob	0.56066	0.00506	0.05088	0.16008	0.28292	0.01741	0.00000	0.00000	0.00000	0.00000

	10	9	8	7	6	5	4	3	2	1
a_O	373	316	394	363	341	368	370	376	348	333
	(40.0)	(33.9)	(42.3)	(38.9)	(36.6)	(39.5)	(39.7)	(40.3)	(37.3)	(35.7)
a_E	367.6	367.6	367.6	367.6	367.6	367.6	367.6	367.6	367.6	367.6
	(39.4)	(39.4)	(39.4)	(39.4)	(39.4)	(39.4)	(39.4)	(39.4)	(39.4)	(39.4)
	10.9	-95.6	54.7	-9.1	-51.2	0.8	4.8	17.0	-38.1	-65.8
	1.169	-10.255	5.866	-0.979	-5.494	0.088	0.519	1.825	-4.090	-7.062
t_O	185	175	196	272	309	362	373	400	431	465
	(19.8)	(18.8)	(21.0)	(29.2)	(33.2)	(38.8)	(40.0)	(42.9)	(46.2)	(49.9)
t_E	342.5	342.5	342.5	342.5	342.5	342.5	342.5	342.5	342.5	342.5
	(36.8)	(36.8)	(36.8)	(36.8)	(36.8)	(36.8)	(36.8)	(36.8)	(36.8)	(36.8)
	-227.9	-235.1	-218.8	-125.4	-63.7	40.0	63.6	124.1	198.0	284.3
	-24.456	-25.221	-23.481	-13.459	-6.833	4.292	6.819	13.311	21.247	30.499
g_O	338	409	292	256	220	137	114	100	90	75
	(36.3)	(43.9)	(31.3)	(27.5)	(23.6)	(14.7)	(12.2)	(10.7)	(9.7)	(8.0)
g_E	138.4	138.4	138.4	138.4	138.4	138.4	138.4	138.4	138.4	138.4
	(14.8)	(14.8)	(14.8)	(14.8)	(14.8)	(14.8)	(14.8)	(14.8)	(14.8)	(14.8)
	603.8	886.6	436.2	315.0	204.1	-2.7	-44.2	-64.9	-77.4	-91.9
	64.785	95.128	46.801	33.803	21.895	-0.290	-4.738	-6.968	-8.306	-9.856
c_O	36	32	50	41	62	65	75	56	63	59
	(3.9)	(3.4)	(5.4)	(4.4)	(6.7)	(7.0)	(8.0)	(6.0)	(6.8)	(6.3)
c_E	83.5	83.5	83.5	83.5	83.5	83.5	83.5	83.5	83.5	83.5
	(9.0)	(9.0)	(9.0)	(9.0)	(9.0)	(9.0)	(9.0)	(9.0)	(9.0)	(9.0)
	-60.6	-61.4	-51.3	-58.3	-36.9	-32.6	-16.1	-44.8	-35.5	-41.0
	-6.500	-6.587	-5.504	-6.259	-3.963	-3.495	-1.730	-4.802	-3.810	-4.399
N	932	932	932	932	932	932	932	932	932	932
X2	387.6	650.3	248.6	136.2	58.9	5.2	7.9	29.5	45.8	83.2
Gvalue	326.2	494.6	220.7	122.1	52.2	5.5	8.1	31.4	47.0	85.6
Gv/N	34.998	53.065	23.683	13.106	5.605	0.595	0.870	3.367	5.041	9.183
Prob	0.00000	0.00000	0.00000	0.00000	0.00000	0.13604	0.04377	0.00000	0.00000	0.00000

CDS_INITIAL:

	1	2	3	4	5	6	7	8	9	10
a_O	714	0	0	492	448	415	515	453	449	447
	(76.6)	(0.0)	(0.0)	(52.8)	(48.1)	(44.5)	(55.3)	(48.6)	(48.2)	(48.0)
a_E	370.0	349.8	342.7	370.0	349.8	342.7	370.0	349.8	342.7	370.0
	(39.7)	(37.5)	(36.8)	(39.7)	(37.5)	(36.8)	(39.7)	(37.5)	(36.8)	(39.7)
	938.7	0.0	0.0	280.4	221.7	158.8	340.5	234.2	242.6	169.0
	100.714	0.000	0.000	30.082	23.786	17.043	36.537	25.130	26.027	18.129
t_O	139	932	0	150	257	380	172	291	333	213
	(14.9)	(100.0)	(0.0)	(16.1)	(27.6)	(40.8)	(18.5)	(31.2)	(35.7)	(22.9)
t_E	210.7	321.8	403.5	210.7	321.8	403.5	210.7	321.8	403.5	210.7
	(22.6)	(34.5)	(43.3)	(22.6)	(34.5)	(43.3)	(22.6)	(34.5)	(43.3)	(22.6)
	-115.6	1982.2	0.0	-101.9	-115.6	-45.7	-69.7	-58.6	-127.9	4.7
	-12.402	212.677	0.000	-10.932	-12.402	-4.899	-7.484	-6.284	-13.727	0.505
g_O	79	0	932	193	104	79	191	100	100	195
	(8.5)	(0.0)	(100.0)	(20.7)	(11.2)	(8.5)	(20.5)	(10.7)	(10.7)	(20.9)
g_E	249.9	112.2	103.0	249.9	112.2	103.0	249.9	112.2	103.0	249.9
	(26.8)	(12.0)	(11.1)	(26.8)	(12.0)	(11.1)	(26.8)	(12.0)	(11.1)	(26.8)
	-181.9	0.0	4105.4	-99.7	-15.7	-41.9	-102.6	-23.0	-5.9	-96.7
	-19.521	0.000	440.490	-10.696	-1.686	-4.500	-11.012	-2.463	-0.638	-10.375
c_O	0	0	0	97	123	58	54	88	50	77
	(0.0)	(0.0)	(0.0)	(10.4)	(13.2)	(6.2)	(5.8)	(9.4)	(5.4)	(8.3)
c_E	101.4	148.2	82.7	101.4	148.2	82.7	101.4	148.2	82.7	101.4
	(10.9)	(15.9)	(8.9)	(10.9)	(15.9)	(8.9)	(10.9)	(15.9)	(8.9)	(10.9)
	0.0	0.0	0.0	-8.7	-45.9	-41.2	-68.1	-91.8	-50.4	-42.5
	0.000	0.000	0.000	-0.933	-4.924	-4.422	-7.307	-9.846	-5.405	-4.556
N	932	932	932	932	932	932	932	932	932	932
X2	562.4	1767.2	7499.9	70.8	45.5	29.6	100.0	59.2	58.3	34.0
Gvalue	641.1	1982.2	4105.4	70.1	44.5	30.0	100.0	60.9	58.3	34.5
Gv/N	68.791	212.677	440.490	7.521	4.774	3.223	10.735	6.537	6.257	3.703
Prob	0.00000	1.00000	1.00000	0.00000	0.00000	0.00000	0.00000	0.00000	0.00000	0.00000

	11	12	13	14	15	16	17	18	19	20
a_O	372	397	433	344	409	399	331	384	408	316
	(39.9)	(42.6)	(46.5)	(36.9)	(43.9)	(42.8)	(35.5)	(41.2)	(43.8)	(33.9)
a_E	349.8	342.7	370.0	349.8	342.7	370.0	349.8	342.7	370.0	349.8
	(37.5)	(36.8)	(39.7)	(37.5)	(36.8)	(39.7)	(37.5)	(36.8)	(39.7)	(37.5)
	45.8	116.7	136.1	-11.5	144.6	60.2	-36.6	87.4	79.7	-64.2
	4.911	12.527	14.605	-1.236	15.519	6.456	-3.925	9.373	8.555	-6.892
t_O	349	368	247	406	393	259	388	368	246	396
	(37.4)	(39.5)	(26.5)	(43.6)	(42.2)	(27.8)	(41.6)	(39.5)	(26.4)	(42.5)
t_E	321.8	403.5	210.7	321.8	403.5	210.7	321.8	403.5	210.7	321.8
	(34.5)	(43.3)	(22.6)	(34.5)	(43.3)	(22.6)	(34.5)	(43.3)	(22.6)	(34.5)
	56.6	-67.8	78.6	188.7	-20.8	107.0	145.2	-67.8	76.3	164.3
	6.076	-7.278	8.435	20.248	-2.229	11.481	15.575	-7.278	8.186	17.630
g_O	115	98	177	83	75	188	91	113	194	98
	(12.3)	(10.5)	(19.0)	(8.9)	(8.0)	(20.2)	(9.8)	(12.1)	(20.8)	(10.5)
g_E	112.2	103.0	249.9	112.2	103.0	249.9	112.2	103.0	249.9	112.2
	(12.0)	(11.1)	(26.8)	(12.0)	(11.1)	(26.8)	(12.0)	(11.1)	(26.8)	(12.0)
	5.7	-9.8	-122.1	-50.0	-47.6	-107.0	-38.1	20.9	-98.2	-26.5
	0.617	-1.050	-13.096	-5.363	-5.108	-11.477	-4.083	2.243	-10.536	-2.838

c_O	96	69	75	99	55	86	122	67	84	122
	(10.3)	(7.4)	(8.0)	(10.6)	(5.9)	(9.2)	(13.1)	(7.2)	(9.0)	(13.1)
c_E	148.2	82.7	101.4	148.2	82.7	101.4	148.2	82.7	101.4	148.2
	(15.9)	(8.9)	(10.9)	(15.9)	(8.9)	(10.9)	(15.9)	(8.9)	(10.9)	(15.9)
	-83.4	-25.1	-45.3	-79.9	-44.9	-28.4	-47.5	-28.3	-31.7	-47.5
	-8.949	-2.689	-4.862	-8.575	-4.820	-3.049	-5.098	-3.034	-3.402	-5.098
N	932	932	932	932	932	932	932	932	932	932
X2	22.2	14.3	45.1	46.1	30.0	31.0	23.3	12.1	25.3	26.8
Gvalue	24.7	14.1	47.4	47.3	31.3	31.8	23.0	12.2	26.1	26.1
Gv/N	2.654	1.510	5.082	5.075	3.361	3.411	2.469	1.304	2.803	2.802
Prob	0.00002	0.00281	0.00000	0.00000	0.00000	0.00000	0.00004	0.00688	0.00001	0.00001

	21	22	23	24	25	26	27	28	29	30
a_O	359	410	309	359	374	321	375	392	308	340
	(38.5)	(44.0)	(33.2)	(38.5)	(40.1)	(34.4)	(40.2)	(42.1)	(33.0)	(36.5)
a_E	342.7	370.0	349.8	342.7	370.0	349.8	342.7	370.0	349.8	342.7
	(36.8)	(39.7)	(37.5)	(36.8)	(39.7)	(37.5)	(36.8)	(39.7)	(37.5)	(36.8)
	33.3	84.1	-76.7	33.3	8.0	-55.2	67.5	45.2	-78.4	-5.4
	3.576	9.027	-8.225	3.576	0.858	-5.920	7.245	4.854	-8.412	-0.580
t_O	394	236	392	403	261	375	401	234	388	407
	(42.3)	(25.3)	(42.1)	(43.2)	(28.0)	(40.2)	(43.0)	(25.1)	(41.6)	(43.7)
t_E	403.5	210.7	321.8	403.5	210.7	321.8	403.5	210.7	321.8	403.5
	(43.3)	(22.6)	(34.5)	(43.3)	(22.6)	(34.5)	(43.3)	(22.6)	(34.5)	(43.3)
	-18.8	53.6	154.7	-1.1	111.8	114.7	-5.0	49.2	145.2	7.0
	-2.020	5.752	16.598	-0.113	12.001	12.311	-0.540	5.276	15.575	0.749
g_O	93	210	96	90	209	106	88	217	115	111
	(10.0)	(22.5)	(10.3)	(9.7)	(22.4)	(11.4)	(9.4)	(23.3)	(12.3)	(11.9)
g_E	103.0	249.9	112.2	103.0	249.9	112.2	103.0	249.9	112.2	103.0
	(11.1)	(26.8)	(12.0)	(11.1)	(26.8)	(12.0)	(11.1)	(26.8)	(12.0)	(11.1)
	-19.0	-73.0	-29.9	-24.3	-74.7	-12.0	-27.7	-61.2	5.7	16.6
	-2.041	-7.833	-3.205	-2.609	-8.010	-1.285	-2.975	-6.568	0.617	1.778
c_O	86	76	135	80	88	130	68	89	121	74
	(9.2)	(8.2)	(14.5)	(8.6)	(9.4)	(13.9)	(7.3)	(9.5)	(13.0)	(7.9)
c_E	82.7	101.4	148.2	82.7	101.4	148.2	82.7	101.4	148.2	82.7
	(8.9)	(10.9)	(15.9)	(8.9)	(10.9)	(15.9)	(8.9)	(10.9)	(15.9)	(8.9)
	6.6	-43.9	-25.2	-5.4	-25.0	-34.1	-26.7	-23.3	-49.1	-16.5
	0.713	-4.710	-2.708	-0.579	-2.686	-3.660	-2.863	-2.500	-5.270	-1.773
N	932	932	932	932	932	932	932	932	932	932
X2	2.1	20.1	23.6	2.5	20.5	13.7	7.9	9.7	23.7	1.6
Gvalue	2.1	20.8	22.9	2.6	20.2	13.5	8.1	9.9	23.4	1.6
Gv/N	0.228	2.235	2.460	0.276	2.163	1.445	0.866	1.062	2.509	0.173
Prob	0.54744	0.00011	0.00004	0.46225	0.00016	0.00372	0.04463	0.01947	0.00003	0.65648

	31	32	33	34	35	36	37	38	39	40
a_O	356	316	346	379	292	363	416	314	345	381
	(38.2)	(33.9)	(37.1)	(40.7)	(31.3)	(38.9)	(44.6)	(33.7)	(37.0)	(40.9)
a_E	370.0	349.8	342.7	370.0	349.8	342.7	370.0	349.8	342.7	370.0
	(39.7)	(37.5)	(36.8)	(39.7)	(37.5)	(36.8)	(39.7)	(37.5)	(36.8)	(39.7)
	-27.5	-64.2	6.6	18.2	-105.5	41.7	97.4	-67.8	4.6	22.3
	-2.951	-6.892	0.708	1.950	-11.318	4.479	10.456	-7.276	0.492	2.391
t_O	265	388	416	255	392	393	244	358	404	262
	(28.4)	(41.6)	(44.6)	(27.4)	(42.1)	(42.2)	(26.2)	(38.4)	(43.3)	(28.1)
t_E	210.7	321.8	403.5	210.7	321.8	403.5	210.7	321.8	403.5	210.7
	(22.6)	(34.5)	(43.3)	(22.6)	(34.5)	(43.3)	(22.6)	(34.5)	(43.3)	(22.6)
	121.6	145.2	25.3	97.4	154.7	-20.8	71.7	76.3	0.9	114.3
	13.050	15.575	2.718	10.452	16.598	-2.229	7.692	8.188	0.102	12.262
g_O	219	122	95	212	110	98	185	111	100	192
	(23.5)	(13.1)	(10.2)	(22.7)	(11.8)	(10.5)	(19.8)	(11.9)	(10.7)	(20.6)
g_E	249.9	112.2	103.0	249.9	112.2	103.0	249.9	112.2	103.0	249.9
	(26.8)	(12.0)	(11.1)	(26.8)	(12.0)	(11.1)	(26.8)	(12.0)	(11.1)	(26.8)
	-57.8	20.5	-15.4	-69.7	-4.3	-9.8	-111.2	-2.3	-5.9	-101.2
	-6.197	2.201	-1.651	-7.477	-0.459	-1.050	-11.933	-0.248	-0.638	-10.854
c_O	92	106	75	86	138	78	87	149	83	97
	(9.9)	(11.4)	(8.0)	(9.2)	(14.8)	(8.4)	(9.3)	(16.0)	(8.9)	(10.4)
c_E	101.4	148.2	82.7	101.4	148.2	82.7	101.4	148.2	82.7	101.4
	(10.9)	(15.9)	(8.9)	(10.9)	(15.9)	(8.9)	(10.9)	(15.9)	(8.9)	(10.9)
	-18.0	-71.1	-14.7	-28.4	-19.7	-9.2	-26.7	1.5	0.5	-8.7
	-1.930	-7.627	-1.581	-3.049	-2.117	-0.988	-2.868	0.166	0.055	-0.933
N	932	932	932	932	932	932	932	932	932	932
X2	19.2	29.8	1.8	17.6	25.6	2.0	29.9	7.8	0.1	26.4
Gvalue	18.4	30.4	1.8	17.5	25.2	2.0	31.2	7.7	0.1	26.7
Gv/N	1.971	3.257	0.193	1.877	2.704	0.212	3.347	0.830	0.011	2.865
Prob	0.00037	0.00000	0.61455	0.00056	0.00001	0.57655	0.00000	0.05172	0.99114	0.00001

	41	42	43	44	45	46	47	48	49	50
a_O	300	366	357	292	366	349	314	351	372	291
	(32.2)	(39.3)	(38.3)	(31.3)	(39.3)	(37.5)	(33.7)	(37.7)	(40.0)	(31.3)
a_E	349.8	342.7	370.0	349.8	342.7	369.6	349.4	342.3	369.2	349.1
	(37.5)	(36.8)	(39.7)	(37.5)	(36.8)	(39.7)	(37.5)	(36.8)	(39.7)	(37.5)
	-92.2	48.1	-25.6	-105.5	48.1	-40.1	-67.1	17.5	5.6	-105.9
	-9.888	5.163	-2.744	-11.318	5.163	-4.304	-7.212	1.882	0.599	-11.384
t_O	387	398	253	405	394	249	333	390	240	354
	(41.5)	(42.7)	(27.1)	(43.5)	(42.3)	(26.7)	(35.8)	(41.9)	(25.8)	(38.1)
t_E	321.8	403.5	210.7	321.8	403.5	210.4	321.5	403.1	210.2	321.1
	(34.5)	(43.3)	(22.6)	(34.5)	(43.3)	(22.6)	(34.5)	(43.3)	(22.6)	(34.5)
	142.8	-11.0	92.7	186.3	-18.8	83.8	23.5	-25.8	63.6	69.0
	15.320	-1.178	9.943	19.984	-2.020	9.001	2.523	-2.767	6.840	7.422
g_O	118	98	222	101	93	235	109	104	220	135
	(12.7)	(10.5)	(23.8)	(10.8)	(10.0)	(25.2)	(11.7)	(11.2)	(23.7)	(14.5)
g_E	112.2	103.0	249.9	112.2	103.0	249.6	112.0	102.9	249.3	111.9
	(12.0)	(11.1)	(26.8)	(12.0)	(11.1)	(26.8)	(12.0)	(11.1)	(26.8)	(12.0)
	12.0	-9.8	-52.5	-21.2	-19.0	-28.3	-6.0	2.2	-55.1	50.6
	1.285	-1.050	-5.634	-2.272	-2.041	-3.043	-0.644	0.236	-5.922	5.443
c_O	127	70	100	134	79	98	175	86	98	150
	(13.6)	(7.5)	(10.7)	(14.4)	(8.5)	(10.5)	(18.8)	(9.2)	(10.5)	(16.1)
c_E	148.2	82.7	101.4	148.2	82.7	101.3	148.1	82.7	101.2	147.9
	(15.9)	(8.9)	(10.9)	(15.9)	(8.9)	(10.9)	(15.9)	(8.9)	(10.9)	(15.9)
	-39.3	-23.4	-2.9	-27.0	-7.3	-6.6	58.5	6.8	-6.4	4.2
	-4.212	-2.512	-0.309	-2.902	-0.785	-0.706	6.282	0.733	-0.684	0.453
N	932	932	932	932	932	931	931	931	930	930
X2	23.6	3.9	12.1	33.5	3.0	9.2	9.0	0.8	7.8	17.8
Gvalue	23.3	3.9	11.7	32.6	3.0	8.8	8.8	0.8	7.8	18.0
Gv/N	2.505	0.423	1.256	3.493	0.317	0.948	0.949	0.085	0.834	1.934
Prob	0.00003	0.26765	0.00846	0.00000	0.39894	0.03169	0.03151	0.85134	0.05136	0.00044

	51	52	53	54	55	56	57	58	59	60
a_O	345	365	312	320	360	300	321	330	320	333
	(37.1)	(39.3)	(33.6)	(34.4)	(38.8)	(32.3)	(34.6)	(35.6)	(34.5)	(35.9)
a_E	342.0	368.8	348.7	341.6	368.8	348.7	341.6	368.4	348.3	341.2
	(36.8)	(39.7)	(37.5)	(36.8)	(39.7)	(37.5)	(36.8)	(39.7)	(37.5)	(36.8)
	6.1	-7.6	-69.4	-41.8	-17.4	-90.2	-40.0	-72.7	-54.2	-16.3
	0.652	-0.820	-7.466	-4.502	-1.878	-9.712	-4.301	-7.835	-5.845	-1.755
t_O	406	256	359	407	243	366	434	272	337	420
	(43.7)	(27.6)	(38.6)	(43.8)	(26.2)	(39.4)	(46.7)	(29.3)	(36.3)	(45.3)
t_E	402.7	210.0	320.8	402.2	210.0	320.8	402.2	209.8	320.4	401.8
	(43.3)	(22.6)	(34.5)	(43.3)	(22.6)	(34.5)	(43.3)	(22.6)	(34.5)	(43.3)
	6.7	101.5	80.8	9.6	71.0	96.6	66.0	141.4	34.0	37.2
	0.721	10.920	8.703	1.033	7.640	10.394	7.103	15.232	3.663	4.011
g_O	93	211	125	109	233	115	97	234	102	92
	(10.0)	(22.7)	(13.5)	(11.7)	(25.1)	(12.4)	(10.4)	(25.2)	(11.0)	(9.9)
g_E	102.8	249.1	111.8	102.7	249.1	111.8	102.7	248.8	111.7	102.6
	(11.1)	(26.8)	(12.0)	(11.1)	(26.8)	(12.0)	(11.1)	(26.8)	(12.0)	(11.1)
	-18.6	-70.0	27.9	13.0	-31.1	6.5	-11.0	-28.7	-18.5	-20.0
	-2.003	-7.534	3.003	1.401	-3.344	0.699	-1.189	-3.092	-1.993	-2.157
c_O	86	97	133	93	93	148	77	92	169	83
	(9.2)	(10.4)	(14.3)	(10.0)	(10.0)	(15.9)	(8.3)	(9.9)	(18.2)	(8.9)
c_E	82.6	101.1	147.8	82.5	101.1	147.8	82.5	101.0	147.6	82.4
	(8.9)	(10.9)	(15.9)	(8.9)	(10.9)	(15.9)	(8.9)	(10.9)	(15.9)	(8.9)
	7.0	-8.1	-28.0	22.3	-15.6	0.5	-10.6	-17.2	45.8	1.2
	0.754	-0.869	-3.011	2.404	-1.676	0.054	-1.139	-1.853	4.934	0.133
N	930	929	929	929	929	929	929	928	928	928
X2	1.1	16.1	11.4	3.2	7.1	13.3	4.4	24.2	7.1	2.1
Gvalue	1.2	15.8	11.4	3.1	6.9	13.3	4.4	22.8	7.0	2.1
Gv/N	0.125	1.697	1.228	0.336	0.741	1.434	0.475	2.452	0.758	0.231
Prob	0.76270	0.00127	0.00970	0.37300	0.07577	0.00398	0.22063	0.00005	0.07070	0.54235

	61	62	63	64	65	66	67	68	69	70
a_O	369	311	324	354	326	357	361	310	339	373
	(39.8)	(33.5)	(34.9)	(38.2)	(35.2)	(38.6)	(39.0)	(33.5)	(36.6)	(40.3)
a_E	368.4	348.3	341.2	367.6	347.6	340.5	367.2	347.2	340.1	367.2
	(39.7)	(37.5)	(36.8)	(39.7)	(37.5)	(36.8)	(39.7)	(37.5)	(36.8)	(39.7)
	1.1	-70.5	-33.6	-26.8	-41.7	33.8	-12.4	-70.2	-2.3	11.6
	0.122	-7.593	-3.621	-2.890	-4.508	3.647	-1.338	-7.592	-0.246	1.255
t_O	236	339	420	233	349	398	231	337	416	233
	(25.4)	(36.5)	(45.3)	(25.2)	(37.7)	(43.0)	(25.0)	(36.4)	(45.0)	(25.2)
t_E	209.8	320.4	401.8	209.3	319.7	400.9	209.1	319.4	400.5	209.1
	(22.6)	(34.5)	(43.3)	(22.6)	(34.5)	(43.3)	(22.6)	(34.5)	(43.3)	(22.6)
	55.6	38.2	37.2	50.0	61.1	-5.8	46.1	36.2	31.6	50.5
	5.996	4.117	4.011	5.397	6.602	-0.630	4.980	3.911	3.416	5.457
g_O	219	124	113	248	111	109	247	122	93	235
	(23.6)	(13.4)	(12.2)	(26.8)	(12.0)	(11.8)	(26.7)	(13.2)	(10.1)	(25.4)
g_E	248.8	111.7	102.6	248.3	111.4	102.4	248.0	111.3	102.2	248.0
	(26.8)	(12.0)	(11.1)	(26.8)	(12.0)	(11.1)	(26.8)	(12.0)	(11.1)	(26.8)
	-55.9	26.0	21.9	-0.5	-0.9	13.7	-2.0	22.4	-17.6	-25.3
	-6.021	2.797	2.358	-0.056	-0.095	1.481	-0.214	2.417	-1.905	-2.734
c_O	104	154	71	91	140	62	86	156	77	84
	(11.2)	(16.6)	(7.7)	(9.8)	(15.1)	(6.7)	(9.3)	(16.9)	(8.3)	(9.1)
c_E	101.0	147.6	82.4	100.8	147.3	82.2	100.7	147.1	82.1	100.7
	(10.9)	(15.9)	(8.9)	(10.9)	(15.9)	(8.9)	(10.9)	(15.9)	(8.9)	(10.9)
	6.1	13.1	-21.1	-18.6	-14.2	-35.0	-27.1	18.3	-9.9	-30.4
	0.653	1.411	-2.276	-2.009	-1.531	-3.778	-2.932	1.978	-1.072	-3.291
N	928	928	928	926	926	926	925	925	925	925
X2	6.9	6.7	4.3	4.1	4.4	6.2	4.6	6.5	1.8	6.3
Gvalue	7.0	6.8	4.4	4.1	4.3	6.7	4.6	6.6	1.8	6.4
Gv/N	0.750	0.732	0.472	0.441	0.468	0.720	0.496	0.714	0.193	0.687
Prob	0.07310	0.07894	0.22356	0.25259	0.22769	0.08340	0.20464	0.08560	0.61833	0.09565

	71	72	73	74	75	76	77	78	79	80
a_O	329	328	367	351	347	355	326	334	367	333
	(35.6)	(35.5)	(39.7)	(37.9)	(37.5)	(38.4)	(35.2)	(36.1)	(39.7)	(36.0)
a_E	347.2	340.1	367.2	347.2	340.1	367.2	347.2	340.1	367.2	347.2
	(37.5)	(36.8)	(39.7)	(37.5)	(36.8)	(39.7)	(37.5)	(36.8)	(39.7)	(37.5)
	-35.4	-23.8	-0.5	7.7	13.9	-24.1	-41.0	-12.2	-0.5	-27.8
	-3.826	-2.578	-0.052	0.831	1.498	-2.602	-4.437	-1.316	-0.052	-3.002
t_O	351	426	230	346	399	230	344	412	237	345
	(37.9)	(46.1)	(24.9)	(37.4)	(43.1)	(24.9)	(37.2)	(44.5)	(25.6)	(37.3)
t_E	319.4	400.5	209.1	319.4	400.5	209.1	319.4	400.5	209.1	319.4
	(34.5)	(43.3)	(22.6)	(34.5)	(43.3)	(22.6)	(34.5)	(43.3)	(22.6)	(34.5)
	66.3	52.6	43.9	55.4	-3.0	43.9	51.1	23.3	59.4	53.2
	7.163	5.686	4.742	5.987	-0.323	4.742	5.521	2.523	6.423	5.754
g_O	112	95	238	108	105	245	100	102	235	103
	(12.1)	(10.3)	(25.7)	(11.7)	(11.4)	(26.5)	(10.8)	(11.0)	(25.4)	(11.1)
g_E	111.3	102.2	248.0	111.3	102.2	248.0	111.3	102.2	248.0	111.3
	(12.0)	(11.1)	(26.8)	(12.0)	(11.1)	(26.8)	(12.0)	(11.1)	(26.8)	(12.0)
	1.4	-14.0	-19.6	-6.5	5.6	-5.9	-21.4	-0.5	-25.3	-16.0
	0.148	-1.509	-2.116	-0.707	0.604	-0.643	-2.318	-0.052	-2.734	-1.730
c_O	133	76	90	120	74	95	155	77	86	144
	(14.4)	(8.2)	(9.7)	(13.0)	(8.0)	(10.3)	(16.8)	(8.3)	(9.3)	(15.6)
c_E	147.1	82.1	100.7	147.1	82.1	100.7	147.1	82.1	100.7	147.1
	(15.9)	(8.9)	(10.9)	(15.9)	(8.9)	(10.9)	(15.9)	(8.9)	(10.9)	(15.9)
	-26.8	-11.8	-20.2	-48.9	-15.4	-11.0	16.2	-9.9	-27.1	-6.2
	-2.900	-1.273	-2.183	-5.286	-1.666	-1.194	1.750	-1.072	-2.932	-0.666
N	925	925	925	925	925	925	925	925	925	925
X2	5.4	3.0	3.6	7.4	1.0	2.9	4.8	0.8	6.6	3.3
Gvalue	5.4	3.0	3.6	7.6	1.0	2.8	4.8	0.8	6.5	3.3
Gv/N	0.584	0.327	0.390	0.826	0.113	0.303	0.516	0.083	0.705	0.356
Prob	0.14455	0.38811	0.30656	0.05416	0.78973	0.42299	0.18895	0.85767	0.08886	0.34872

	81	82	83	84	85	86	87	88	89	90
a_O	326	352	317	335	369	330	341	382	314	343
	(35.2)	(38.1)	(34.3)	(36.2)	(40.0)	(35.8)	(36.9)	(41.4)	(34.1)	(37.2)
a_E	340.1	367.2	347.2	340.1	366.4	346.4	339.4	366.1	346.1	339.0
	(36.8)	(39.7)	(37.5)	(36.8)	(39.7)	(37.5)	(36.8)	(39.7)	(37.5)	(36.8)
	-27.7	-29.8	-57.7	-10.2	5.1	-32.1	3.2	32.6	-61.0	8.0
	-2.993	-3.226	-6.233	-1.103	0.555	-3.474	0.346	3.534	-6.620	0.864
t_O	430	260	347	408	222	323	392	216	336	402
	(46.5)	(28.1)	(37.5)	(44.1)	(24.1)	(35.0)	(42.5)	(23.4)	(36.4)	(43.6)
t_E	400.5	209.1	319.4	400.5	208.6	318.7	399.6	208.4	318.4	399.2
	(43.3)	(22.6)	(34.5)	(43.3)	(22.6)	(34.5)	(43.3)	(22.6)	(34.5)	(43.3)
	61.1	113.3	57.5	15.1	27.6	8.7	-15.1	15.5	36.3	5.6

	6.609	12.253	6.221	1.638	2.989	0.938	-1.637	1.678	3.932	0.610
g_O	91	228	102	91	229	122	101	232	122	101
	(9.8)	(24.6)	(11.0)	(9.8)	(24.8)	(13.2)	(10.9)	(25.2)	(13.2)	(11.0)
g_E	102.2	248.0	111.3	102.2	247.5	111.1	102.0	247.2	111.0	101.9
	(11.1)	(26.8)	(12.0)	(11.1)	(26.8)	(12.0)	(11.1)	(26.8)	(12.0)	(11.1)
	-21.2	-38.3	-17.8	-21.2	-35.5	22.9	-2.0	-29.4	23.1	-1.8
	-2.292	-4.143	-1.928	-2.292	-3.846	2.479	-0.220	-3.191	2.511	-0.197
c_O	78	85	159	91	103	148	89	92	150	76
	(8.4)	(9.2)	(17.2)	(9.8)	(11.2)	(16.0)	(9.6)	(10.0)	(16.3)	(8.2)
c_E	82.1	100.7	147.1	82.1	100.5	146.8	81.9	100.4	146.6	81.9
	(8.9)	(10.9)	(15.9)	(8.9)	(10.9)	(15.9)	(8.9)	(10.9)	(15.9)	(8.9)
	-8.0	-28.8	24.7	18.7	5.1	2.4	14.7	-16.0	6.8	-11.3
	-0.868	-3.113	2.671	2.020	0.555	0.262	1.593	-1.736	0.738	-1.223
N	925	925	925	925	923	923	923	922	922	922
X2	4.2	17.1	6.8	2.4	2.3	1.9	0.8	2.6	5.1	0.5
Gvalue	4.2	16.4	6.8	2.4	2.3	1.9	0.8	2.6	5.2	0.5
Gv/N	0.455	1.771	0.731	0.263	0.253	0.206	0.082	0.285	0.560	0.055
Prob	0.23939	0.00095	0.07988	0.48823	0.50635	0.59349	0.86012	0.45255	0.16001	0.91823

	91	92	93	94	95	96	97	98	99	100
a_O	355	332	322	342	350	330	351	346	340	380
	(38.5)	(36.0)	(34.9)	(37.1)	(38.0)	(35.8)	(38.2)	(37.6)	(37.0)	(41.3)
a_E	366.1	346.1	339.0	365.7	345.7	338.7	365.3	345.3	338.3	364.9
	(39.7)	(37.5)	(36.8)	(39.7)	(37.5)	(36.8)	(39.7)	(37.5)	(36.8)	(39.7)
	-21.8	-27.5	-33.2	-45.7	8.7	-17.1	-27.9	1.4	3.4	30.9
	-2.361	-2.986	-3.601	-4.967	0.945	-1.858	-3.038	0.152	0.370	3.362
t_O	230	343	401	229	305	403	227	340	395	215
	(24.9)	(37.2)	(43.5)	(24.9)	(33.1)	(43.8)	(24.7)	(37.0)	(42.9)	(23.4)
t_E	208.4	318.4	399.2	208.2	318.0	398.8	207.9	317.7	398.3	207.7
	(22.6)	(34.5)	(43.3)	(22.6)	(34.5)	(43.3)	(22.6)	(34.5)	(43.3)	(22.6)
	45.4	51.2	3.6	43.7	-25.5	8.5	39.8	46.2	-6.6	14.8
	4.920	5.548	0.392	4.741	-2.766	0.925	4.326	5.023	-0.721	1.611
g_O	230	111	107	254	137	106	253	95	89	238
	(24.9)	(12.0)	(11.6)	(27.6)	(14.9)	(11.5)	(27.5)	(10.3)	(9.7)	(25.9)
g_E	247.2	111.0	101.9	246.9	110.8	101.8	246.7	110.7	101.7	246.4
	(26.8)	(12.0)	(11.1)	(26.8)	(12.0)	(11.1)	(26.8)	(12.0)	(11.1)	(26.8)
	-33.2	0.1	10.4	14.4	58.1	8.6	12.9	-29.1	-23.7	-16.5
	-3.596	0.009	1.131	1.559	6.305	0.931	1.398	-3.162	-2.579	-1.793
c_O	107	136	92	96	129	82	89	139	96	86
	(11.6)	(14.8)	(10.0)	(10.4)	(14.0)	(8.9)	(9.7)	(15.1)	(10.4)	(9.4)
c_E	100.4	146.6	81.9	100.3	146.5	81.8	100.1	146.3	81.7	100.0
	(10.9)	(15.9)	(8.9)	(10.9)	(15.9)	(8.9)	(10.9)	(15.9)	(8.9)	(10.9)
	13.7	-20.5	21.5	-8.3	-32.8	0.5	-21.0	-14.3	31.0	-26.0
	1.487	-2.222	2.332	-0.903	-3.559	0.051	-2.282	-1.551	3.372	-2.829
N	922	922	922	921	921	921	920	920	920	919
X2	4.2	3.3	2.4	4.0	8.8	0.4	3.7	4.2	4.1	3.1
Gvalue	4.2	3.2	2.3	4.0	8.5	0.4	3.7	4.3	4.1	3.2
Gv/N	0.451	0.350	0.254	0.431	0.924	0.048	0.403	0.462	0.442	0.351
Prob	0.24515	0.35745	0.50535	0.26525	0.03662	0.93179	0.29469	0.23532	0.25434	0.35776

	101	102	103	104	105	106	107	108	109	110
a_O	349	331	349	333	344	378	350	362	371	392
	(38.0)	(36.0)	(38.0)	(36.2)	(37.4)	(41.2)	(38.2)	(39.5)	(40.5)	(42.8)
a_E	344.9	337.9	364.9	344.9	337.9	364.1	344.2	337.2	363.7	343.8
	(37.5)	(36.8)	(39.7)	(37.5)	(36.8)	(39.7)	(37.5)	(36.8)	(39.7)	(37.5)
	8.2	-13.7	-31.0	-23.4	12.2	28.4	11.7	51.4	14.8	102.9
	0.892	-1.494	-3.375	-2.550	1.332	3.097	1.281	5.603	1.617	11.229
t_O	330	418	224	314	411	189	300	403	211	277
	(35.9)	(45.5)	(24.4)	(34.2)	(44.7)	(20.6)	(32.7)	(43.9)	(23.0)	(30.2)
t_E	317.3	397.9	207.7	317.3	397.9	207.3	316.6	397.0	207.0	316.3
	(34.5)	(43.3)	(22.6)	(34.5)	(43.3)	(22.6)	(34.5)	(43.3)	(22.6)	(34.5)
	25.9	41.2	33.8	-6.6	26.6	-34.9	-32.4	12.0	8.0	-73.5
	2.815	4.483	3.678	-0.718	2.898	-3.804	-3.529	1.311	0.872	-8.020
g_O	113	93	239	106	94	263	108	81	262	111
	(12.3)	(10.1)	(26.0)	(11.5)	(10.2)	(28.7)	(11.8)	(8.8)	(28.6)	(12.1)
g_E	110.6	101.6	246.4	110.6	101.6	245.8	110.4	101.4	245.6	110.2
	(12.0)	(11.1)	(26.8)	(12.0)	(11.1)	(26.8)	(12.0)	(11.1)	(26.8)	(12.0)
	4.9	-16.4	-14.5	-9.0	-14.6	35.5	-4.7	-36.3	33.9	1.5
	0.529	-1.786	-1.582	-0.979	-1.586	3.869	-0.508	-3.961	3.703	0.167
c_O	127	77	107	166	70	87	159	71	72	136
	(13.8)	(8.4)	(11.6)	(18.1)	(7.6)	(9.5)	(17.3)	(7.7)	(7.9)	(14.8)
c_E	146.2	81.6	100.0	146.2	81.6	99.8	145.8	81.4	99.7	145.7
	(15.9)	(8.9)	(10.9)	(15.9)	(8.9)	(10.9)	(15.9)	(8.9)	(10.9)	(15.9)
	-35.7	-8.9	14.4	42.3	-21.4	-23.9	27.5	-19.4	-46.9	-18.7
	-3.884	-0.970	1.568	4.598	-2.334	-2.608	2.996	-2.119	-5.118	-2.042
N	919	919	919	919	919	917	917	917	916	916
X2	3.1	2.1	2.7	3.3	2.8	5.0	2.2	7.3	9.0	12.3
Gvalue	3.2	2.2	2.6	3.2	2.8	5.1	2.2	7.7	9.8	12.2
Gv/N	0.351	0.234	0.287	0.351	0.310	0.554	0.239	0.835	1.073	1.334
Prob	0.35761	0.54166	0.45041	0.35826	0.41615	0.16620	0.53384	0.05368	0.02008	0.00667

	111	112	113	114	115	116	117	118	119	120
a_O	351	358	338	340	375	342	337	374	366	350
	(38.3)	(39.1)	(36.9)	(37.2)	(41.1)	(37.5)	(36.9)	(41.0)	(40.1)	(38.4)
a_E	336.8	363.3	343.4	336.5	362.5	342.7	335.7	362.1	342.3	335.4
	(36.8)	(39.7)	(37.5)	(36.8)	(39.7)	(37.5)	(36.8)	(39.7)	(37.5)	(36.8)
	28.9	-10.5	-10.8	7.1	25.5	-1.3	2.5	24.2	49.0	29.9
	3.158	-1.144	-1.176	0.777	2.790	-0.148	0.279	2.657	5.373	3.279
t_O	393	206	303	393	178	297	381	185	300	386
	(42.9)	(22.5)	(33.1)	(43.0)	(19.5)	(32.5)	(41.7)	(20.3)	(32.9)	(42.3)
t_E	396.6	206.8	315.9	396.2	206.4	315.2	395.3	206.1	314.9	394.9
	(43.3)	(22.6)	(34.5)	(43.3)	(22.6)	(34.5)	(43.3)	(22.6)	(34.5)	(43.3)
	-7.2	-1.6	-25.3	-6.3	-52.6	-35.4	-28.1	-40.0	-29.1	-17.5
	-0.782	-0.179	-2.769	-0.689	-5.766	-3.879	-3.075	-4.390	-3.189	-1.923
g_O	100	240	113	101	262	122	100	243	113	95
	(10.9)	(26.2)	(12.3)	(11.0)	(28.7)	(13.4)	(11.0)	(26.6)	(12.4)	(10.4)
g_E	101.2	245.3	110.1	101.1	244.8	109.9	100.9	244.5	109.8	100.8
	(11.1)	(26.8)	(12.0)	(11.1)	(26.8)	(12.0)	(11.1)	(26.8)	(12.0)	(11.1)
	-2.5	-10.5	5.8	-0.3	35.6	25.5	-1.8	-3.0	6.6	-11.3
	-0.271	-1.148	0.639	-0.030	3.903	2.798	-0.200	-0.329	0.722	-1.236
c_O	72	111	161	81	98	152	95	110	133	81
	(7.9)	(12.1)	(17.6)	(8.9)	(10.7)	(16.6)	(10.4)	(12.1)	(14.6)	(8.9)
c_E	81.3	99.6	145.5	81.2	99.4	145.2	81.1	99.3	145.0	81.0
	(8.9)	(10.9)	(15.9)	(8.9)	(10.9)	(15.9)	(8.9)	(10.9)	(15.9)	(8.9)
	-17.5	24.1	32.5	-0.5	-2.7	13.9	30.2	22.6	-23.1	0.1
	-1.914	2.630	3.557	-0.051	-0.300	1.523	3.304	2.475	-2.529	0.007

N	916	915	915	915	913	913	913	912	912	912
X2	1.7	1.5	2.3	0.1	5.6	2.7	2.9	3.7	3.4	1.2
Gvalue	1.7	1.5	2.3	0.1	5.7	2.7	2.8	3.8	3.4	1.2
Gv/N	0.191	0.159	0.250	0.007	0.627	0.294	0.308	0.413	0.378	0.128
Prob	0.62634	0.69292	0.51472	0.99585	0.12588	0.44317	0.42203	0.28815	0.32794	0.75984

	121	122	123	124	125	126	127	128	129	130
a_O	382	339	351	393	356	348	365	342	331	354
	(42.0)	(37.3)	(38.6)	(43.2)	(39.2)	(38.3)	(40.2)	(37.6)	(36.4)	(38.9)
a_E	361.3	341.5	334.6	360.9	341.2	334.3	360.9	341.2	334.3	360.9
	(39.7)	(37.5)	(36.8)	(39.7)	(37.5)	(36.8)	(39.7)	(37.5)	(36.8)	(39.7)
	42.6	−5.1	33.5	67.0	30.3	28.0	8.3	1.7	−6.5	−13.6
	4.681	−0.558	3.685	7.370	3.332	3.085	0.910	0.182	−0.713	−1.501
t_O	196	321	399	185	284	382	198	314	405	209
	(21.5)	(35.3)	(43.8)	(20.4)	(31.2)	(42.0)	(21.8)	(34.5)	(44.6)	(23.0)
t_E	205.7	314.2	394.0	205.5	313.9	393.6	205.5	313.9	393.6	205.5
	(22.6)	(34.5)	(43.3)	(22.6)	(34.5)	(43.3)	(22.6)	(34.5)	(43.3)	(22.6)
	−18.9	13.7	10.1	−38.8	−56.8	−22.8	−14.7	0.3	23.2	7.1
	−2.079	1.509	1.106	−4.270	−6.248	−2.508	−1.612	0.030	2.551	0.785
g_O	236	110	81	245	122	92	248	101	97	246
	(25.9)	(12.1)	(8.9)	(27.0)	(13.4)	(10.1)	(27.3)	(11.1)	(10.7)	(27.1)
g_E	244.0	109.5	100.6	243.7	109.4	100.5	243.7	109.4	100.5	243.7
	(26.8)	(12.0)	(11.1)	(26.8)	(12.0)	(11.1)	(26.8)	(12.0)	(11.1)	(26.8)
	−15.7	1.0	−35.1	2.6	26.6	−16.2	8.7	−16.1	−6.8	4.6
	−1.723	0.107	−3.855	0.286	2.928	−1.783	0.954	−1.774	−0.751	0.508
c_O	96	140	79	86	147	87	98	152	76	100
	(10.5)	(15.4)	(8.7)	(9.5)	(16.2)	(9.6)	(10.8)	(16.7)	(8.4)	(11.0)
c_E	99.1	144.7	80.8	98.9	144.6	80.7	98.9	144.6	80.7	98.9
	(10.9)	(15.9)	(8.9)	(10.9)	(15.9)	(8.9)	(10.9)	(15.9)	(8.9)	(10.9)
	−6.0	−9.3	−3.5	−24.1	4.9	13.1	−1.9	15.2	−9.1	2.1
	−0.661	−1.022	−0.389	−2.653	0.539	1.439	−0.207	1.676	−1.003	0.233
N	910	910	910	909	909	909	909	909	909	909
X2	2.0	0.3	4.7	6.6	5.0	2.1	0.4	1.0	0.8	0.2
Gvalue	2.0	0.3	5.0	6.7	5.0	2.1	0.4	1.0	0.8	0.2
Gv/N	0.218	0.036	0.547	0.733	0.551	0.233	0.045	0.114	0.084	0.025
Prob	0.57496	0.95562	0.17330	0.08338	0.17092	0.54870	0.93911	0.79188	0.85866	0.97334

	131	132	133	134	135	136	137	138	139	140
a_O	360	340	344	366	325	343	305	295	393	345
	(39.6)	(37.4)	(37.9)	(40.4)	(35.8)	(37.8)	(33.6)	(32.5)	(43.3)	(38.0)
a_E	341.2	334.3	360.1	340.4	333.5	360.1	340.4	333.5	360.1	340.4
	(37.5)	(36.8)	(39.7)	(37.5)	(36.8)	(39.7)	(37.5)	(36.8)	(39.7)	(37.5)
	38.7	11.6	−31.5	53.0	−16.8	−33.4	−67.0	−72.4	68.7	9.2
	4.254	1.274	−3.469	5.847	−1.855	−3.679	−7.390	−7.984	7.578	1.016
t_O	292	382	198	294	419	204	351	406	205	305
	(32.1)	(42.0)	(21.8)	(32.4)	(46.2)	(22.5)	(38.7)	(44.8)	(22.6)	(33.6)
t_E	313.9	393.6	205.0	313.2	392.7	205.0	313.2	392.7	205.0	313.2
	(34.5)	(43.3)	(22.6)	(34.5)	(43.3)	(22.6)	(34.5)	(43.3)	(22.6)	(34.5)
	−42.2	−22.8	−13.8	−37.1	54.3	−2.0	80.0	27.0	−0.0	−16.1
	−4.639	−2.508	−1.519	−4.096	5.989	−0.222	8.826	2.981	−0.002	−1.779
g_O	100	114	246	108	90	242	111	106	212	101
	(11.0)	(12.5)	(27.1)	(11.9)	(9.9)	(26.7)	(12.2)	(11.7)	(23.4)	(11.1)
g_E	109.4	100.5	243.2	109.2	100.3	243.2	109.2	100.3	243.2	109.2
	(12.0)	(11.1)	(26.8)	(12.0)	(11.1)	(26.8)	(12.0)	(11.1)	(26.8)	(12.0)
	−18.0	28.8	5.7	−2.3	−19.4	−2.3	3.7	11.8	−58.2	−15.7
	−1.975	3.168	0.629	−0.253	−2.141	−0.257	0.411	1.303	−6.412	−1.729
c_O	157	73	119	139	73	118	140	100	97	156
	(17.3)	(8.0)	(13.1)	(15.3)	(8.0)	(13.0)	(15.4)	(11.0)	(10.7)	(17.2)
c_E	144.6	80.7	98.7	144.3	80.5	98.7	144.3	80.5	98.7	144.3
	(15.9)	(8.9)	(10.9)	(15.9)	(8.9)	(10.9)	(15.9)	(8.9)	(10.9)	(15.9)
	25.9	−14.6	44.4	−10.3	−14.3	42.1	−8.4	43.3	−3.4	24.4
	2.849	−1.611	4.901	−1.137	−1.579	4.640	−0.923	4.777	−0.378	2.693
N	909	909	907	907	907	907	907	907	907	907
X2	4.4	3.0	5.2	3.3	3.7	4.6	8.4	9.9	7.0	1.8
Gvalue	4.5	2.9	4.9	3.3	3.8	4.4	8.4	9.8	7.1	1.8
Gv/N	0.490	0.324	0.541	0.362	0.414	0.482	0.923	1.077	0.786	0.202
Prob	0.21678	0.40015	0.17853	0.35049	0.28921	0.22380	0.03889	0.02061	0.06797	0.60777

	141	142	143	144	145	146	147	148	149	150
a_O	332	360	342	334	364	337	308	354	349	327
	(36.6)	(39.7)	(37.7)	(36.8)	(40.1)	(37.2)	(34.0)	(39.1)	(38.5)	(36.1)
a_E	333.5	360.1	340.4	333.5	360.1	340.4	333.5	359.7	340.0	333.2
	(36.8)	(39.7)	(37.5)	(36.8)	(39.7)	(37.5)	(36.8)	(39.7)	(37.5)	(36.8)
	−3.0	−0.2	3.2	1.0	7.9	−6.8	−49.0	−11.3	18.1	−12.2
	−0.335	−0.021	0.349	0.105	0.866	−0.751	−5.407	−1.248	2.002	−1.346
t_O	408	212	303	396	208	311	411	207	298	380
	(45.0)	(23.4)	(33.4)	(43.7)	(22.9)	(34.3)	(45.3)	(22.8)	(32.9)	(41.9)
t_E	392.7	205.0	313.2	392.7	205.0	313.2	392.7	204.8	312.8	392.3
	(43.3)	(22.6)	(34.5)	(43.3)	(22.6)	(34.5)	(43.3)	(22.6)	(34.5)	(43.3)
	31.2	14.2	−20.0	6.6	6.0	−4.3	37.4	4.5	−28.9	−24.2
	3.438	1.567	−2.206	0.730	0.664	−0.478	4.127	0.492	−3.194	−2.666
g_O	89	251	108	102	229	120	102	237	119	109
	(9.8)	(27.7)	(11.9)	(11.2)	(25.2)	(13.2)	(11.2)	(26.2)	(13.1)	(12.0)
g_E	100.3	243.2	109.2	100.3	243.2	109.2	100.3	242.9	109.0	100.1
	(11.1)	(26.8)	(12.0)	(11.1)	(26.8)	(12.0)	(11.1)	(26.8)	(12.0)	(11.1)
	−21.2	15.9	−2.3	3.5	−27.5	22.7	3.5	−11.7	20.8	18.5
	−2.337	1.755	−0.253	0.389	−3.031	2.507	0.389	−1.286	2.298	2.039
c_O	78	84	154	75	106	139	86	108	140	90
	(8.6)	(9.3)	(17.0)	(8.3)	(11.7)	(15.3)	(9.5)	(11.9)	(15.5)	(9.9)
c_E	80.5	98.7	144.3	80.5	98.7	144.3	80.5	98.6	144.1	80.4
	(8.9)	(10.9)	(15.9)	(8.9)	(10.9)	(15.9)	(8.9)	(10.9)	(15.9)	(8.9)
	−5.0	−27.1	20.1	−10.7	15.1	−10.3	11.3	19.6	−8.1	20.2
	−0.547	−2.992	2.221	−1.175	1.661	−1.137	1.248	2.166	−0.890	2.233
N	907	907	907	907	907	907	907	906	906	906
X2	1.9	2.7	1.0	0.4	1.4	1.3	3.2	1.1	2.0	2.4
Gvalue	2.0	2.8	1.0	0.4	1.5	1.3	3.2	1.1	1.9	2.4
Gv/N	0.219	0.309	0.110	0.049	0.160	0.142	0.357	0.124	0.215	0.260
Prob	0.57484	0.42342	0.80172	0.93051	0.69378	0.73219	0.35621	0.77119	0.58287	0.50137

Bhen_HOUSTON1
5UTR:

	50	49	48	47	46	45	44	43	42	41
a_O	520	475	469	492	496	466	506	471	500	474
	(35.4)	(32.3)	(31.9)	(33.4)	(33.7)	(31.7)	(34.4)	(32.0)	(34.0)	(32.2)
a_E	497.8	497.8	497.8	497.8	497.8	497.8	497.8	497.8	497.8	497.8
	(33.8)	(33.8)	(33.8)	(33.8)	(33.8)	(33.8)	(33.8)	(33.8)	(33.8)	(33.8)
	45.4	-44.5	-55.9	-11.5	-3.6	-61.5	16.6	-52.1	4.4	-46.4
	3.086	-3.027	-3.799	-0.783	-0.243	-4.181	1.125	-3.543	0.301	-3.156
t_O	462	470	499	453	451	519	453	477	487	471
	(31.4)	(32.0)	(33.9)	(30.8)	(30.7)	(35.3)	(30.8)	(32.4)	(33.1)	(32.0)
t_E	471.5	471.5	471.5	471.5	471.5	471.5	471.5	471.5	471.5	471.5
	(32.1)	(32.1)	(32.1)	(32.1)	(32.1)	(32.1)	(32.1)	(32.1)	(32.1)	(32.1)
	-18.8	-3.0	56.6	-36.3	-40.1	99.6	-36.3	11.0	31.5	-1.0
	-1.280	-0.205	3.844	-2.467	-2.727	6.772	-2.467	0.751	2.140	-0.069
g_O	285	297	261	296	263	284	256	292	250	298
	(19.4)	(20.2)	(17.7)	(20.1)	(17.9)	(19.3)	(17.4)	(19.9)	(17.0)	(20.3)
g_E	282.3	282.3	282.3	282.3	282.3	282.3	282.3	282.3	282.3	282.3
	(19.2)	(19.2)	(19.2)	(19.2)	(19.2)	(19.2)	(19.2)	(19.2)	(19.2)	(19.2)
	5.4	30.2	-41.0	28.1	-37.2	3.4	-50.1	19.7	-60.8	32.3
	0.369	2.050	-2.784	1.907	-2.532	0.232	-3.404	1.341	-4.130	2.193
c_O	204	229	242	230	261	202	256	231	234	228
	(13.9)	(15.6)	(16.5)	(15.6)	(17.7)	(13.7)	(17.4)	(15.7)	(15.9)	(15.5)
c_E	219.4	219.4	219.4	219.4	219.4	219.4	219.4	219.4	219.4	219.4
	(14.9)	(14.9)	(14.9)	(14.9)	(14.9)	(14.9)	(14.9)	(14.9)	(14.9)	(14.9)
	-29.7	19.6	47.5	21.7	90.6	-33.4	79.0	23.8	30.2	17.5
	-2.019	1.333	3.226	1.475	6.161	-2.269	5.370	1.618	2.050	1.192
N	1471	1471	1471	1471	1471	1471	1471	1471	1471	1471
X2	2.3	2.2	7.2	2.0	10.1	8.2	9.4	2.5	5.2	2.3
Gvalue	2.3	2.2	7.2	2.0	9.7	8.1	9.2	2.5	5.3	2.3
Gv/N	0.157	0.152	0.488	0.133	0.659	0.553	0.625	0.168	0.361	0.159
Prob	0.51203	0.52585	0.06662	0.58025	0.02135	0.04334	0.02683	0.48177	0.15051	0.50372

	40	39	38	37	36	35	34	33	32	31
a_O	490	479	474	476	471	475	490	497	500	501
	(33.3)	(32.6)	(32.2)	(32.4)	(32.0)	(32.3)	(33.3)	(33.8)	(34.0)	(34.1)
a_E	497.8	497.8	497.8	497.8	497.8	497.8	497.8	497.8	497.8	497.8
	(33.8)	(33.8)	(33.8)	(33.8)	(33.8)	(33.8)	(33.8)	(33.8)	(33.8)	(33.8)
	-15.5	-36.9	-46.4	-42.6	-52.1	-44.5	-15.5	-1.6	4.4	6.4
	-1.051	-2.506	-3.156	-2.897	-3.543	-3.027	-1.051	-0.107	0.301	0.438
t_O	474	510	471	477	546	475	503	469	492	473
	(32.2)	(34.7)	(32.0)	(32.4)	(37.1)	(32.3)	(34.2)	(31.9)	(33.4)	(32.2)
t_E	471.5	471.5	471.5	471.5	471.5	471.5	471.5	471.5	471.5	471.5
	(32.1)	(32.1)	(32.1)	(32.1)	(32.1)	(32.1)	(32.1)	(32.1)	(32.1)	(32.1)
	5.0	80.0	-1.0	11.0	160.2	7.0	65.0	-5.0	41.9	3.0
	0.339	5.441	-0.069	0.751	10.889	0.476	4.421	-0.340	2.846	0.203
g_O	274	249	286	258	237	293	236	272	251	275
	(18.6)	(16.9)	(19.4)	(17.5)	(16.1)	(19.9)	(16.0)	(18.5)	(17.1)	(18.7)
g_E	282.3	282.3	282.3	282.3	282.3	282.3	282.3	282.3	282.3	282.3
	(19.2)	(19.2)	(19.2)	(19.2)	(19.2)	(19.2)	(19.2)	(19.2)	(19.2)	(19.2)
	-16.4	-62.5	7.4	-46.4	-82.9	21.8	-84.6	-20.2	-59.0	-14.4
	-1.112	-4.249	0.506	-3.157	-5.636	1.482	-5.748	-1.375	-4.010	-0.980
c_O	233	233	240	260	217	228	242	233	228	222
	(15.8)	(15.8)	(16.3)	(17.7)	(14.8)	(15.5)	(16.5)	(15.8)	(15.5)	(15.1)
c_E	219.4	219.4	219.4	219.4	219.4	219.4	219.4	219.4	219.4	219.4
	(14.9)	(14.9)	(14.9)	(14.9)	(14.9)	(14.9)	(14.9)	(14.9)	(14.9)	(14.9)
	28.0	28.0	43.1	88.3	-4.8	17.5	47.5	28.0	17.5	5.2
	1.905	1.905	2.928	6.002	-0.325	1.192	3.226	1.905	1.192	0.356
N	1471	1471	1471	1471	1471	1471	1471	1471	1471	1471
X2	1.2	8.6	3.1	10.6	20.5	1.8	12.1	1.2	4.7	0.2
Gvalue	1.2	8.7	3.1	10.3	20.4	1.8	12.5	1.2	4.8	0.2
Gv/N	0.082	0.591	0.209	0.698	1.385	0.124	0.848	0.083	0.328	0.017
Prob	0.75102	0.03361	0.37935	0.01637	0.00014	0.61086	0.00591	0.74792	0.18494	0.96976

	30	29	28	27	26	25	24	23	22	21
a_O	474	515	481	487	512	504	532	495	552	549
	(32.2)	(35.0)	(32.7)	(33.1)	(34.8)	(34.3)	(36.2)	(33.7)	(37.5)	(37.3)
a_E	497.8	497.8	497.8	497.8	497.8	497.8	497.8	497.8	497.8	497.8
	(33.8)	(33.8)	(33.8)	(33.8)	(33.8)	(33.8)	(33.8)	(33.8)	(33.8)	(33.8)
	-46.4	35.0	-33.0	-21.3	28.8	12.5	70.7	-5.6	114.1	107.5
	-3.156	2.380	-2.244	-1.451	1.959	0.850	4.808	-0.378	7.758	7.309
t_O	519	485	526	509	485	474	462	480	482	480
	(35.3)	(33.0)	(35.8)	(34.6)	(33.0)	(32.2)	(31.4)	(32.6)	(32.8)	(32.6)
t_E	471.5	471.5	471.5	471.5	471.5	471.5	471.5	471.5	471.5	471.5
	(32.1)	(32.1)	(32.1)	(32.1)	(32.1)	(32.1)	(32.1)	(32.1)	(32.1)	(32.1)
	99.6	27.4	115.0	77.9	27.4	5.0	-18.8	17.1	21.2	17.1
	6.772	1.860	7.821	5.295	1.860	0.339	-1.280	1.165	1.442	1.165
g_O	259	264	239	252	254	251	251	240	219	228
	(17.6)	(17.9)	(16.2)	(17.1)	(17.3)	(17.1)	(17.1)	(16.3)	(14.9)	(15.5)
g_E	282.3	282.3	282.3	282.3	282.3	282.3	282.3	282.3	282.3	282.3
	(19.2)	(19.2)	(19.2)	(19.2)	(19.2)	(19.2)	(19.2)	(19.2)	(19.2)	(19.2)
	-44.6	-35.4	-79.6	-57.2	-53.7	-59.0	-59.0	-77.9	-111.2	-97.4
	-3.033	-2.406	-5.411	-3.890	-3.648	-4.010	-4.010	-5.297	-7.560	-6.622
c_O	219	207	225	223	220	242	226	256	218	214
	(14.9)	(14.1)	(15.3)	(15.2)	(15.0)	(16.5)	(15.4)	(17.4)	(14.8)	(14.5)
c_E	219.4	219.4	219.4	219.4	219.4	219.4	219.4	219.4	219.4	219.4
	(14.9)	(14.9)	(14.9)	(14.9)	(14.9)	(14.9)	(14.9)	(14.9)	(14.9)	(14.9)
	-0.8	-24.1	11.3	7.3	1.2	47.5	13.4	79.0	-2.8	-10.7
	-0.054	-1.637	0.771	0.493	0.082	3.226	0.911	5.370	-0.190	-0.725
N	1471	1471	1471	1471	1471	1471	1471	1471	1471	1471
X2	7.8	2.9	13.6	6.5	3.6	5.9	6.2	12.6	20.3	16.0
Gvalue	7.8	2.9	13.8	6.6	3.7	5.9	6.3	12.6	21.3	16.6
Gv/N	0.528	0.197	0.938	0.447	0.253	0.404	0.428	0.859	1.450	1.126
Prob	0.05113	0.40766	0.00320	0.08677	0.29301	0.11414	0.09811	0.00548	0.00009	0.00087

	20	19	18	17	16	15	14	13	12	11
a_O	558	552	563	572	569	584	600	621	632	566
	(37.9)	(37.5)	(38.3)	(38.9)	(38.7)	(39.7)	(40.8)	(42.2)	(43.0)	(38.5)
a_E	497.8	497.8	497.8	497.8	497.8	497.8	497.8	497.8	497.8	497.8
	(33.8)	(33.8)	(33.8)	(33.8)	(33.8)	(33.8)	(33.8)	(33.8)	(33.8)	(33.8)
	127.4	114.1	138.6	159.0	152.2	186.6	224.1	274.7	301.7	145.4
	8.663	7.758	9.423	10.807	10.344	12.682	15.235	18.672	20.512	9.882
t_O	456	494	466	429	496	442	365	327	287	240
	(31.0)	(33.6)	(31.7)	(29.2)	(33.7)	(30.0)	(24.8)	(22.2)	(19.5)	(16.3)

```
t_E        471.5    471.5    471.5    471.5    471.5    471.5    471.5    471.5    471.5    471.5
         ( 32.1)  ( 32.1)  ( 32.1)  ( 32.1)  ( 32.1)  ( 32.1)  ( 32.1)  ( 32.1)  ( 32.1)  ( 32.1)
           -30.5     46.0    -11.0    -81.1     50.2    -57.1   -186.9   -239.4   -285.0   -324.1
          -2.074    3.130   -0.745   -5.511    3.415   -3.884  -12.706  -16.271  -19.372  -22.036
g_O          246      240      232      249      232      271      325      381      472      570
         ( 16.7)  ( 16.3)  ( 15.8)  ( 16.9)  ( 15.8)  ( 18.4)  ( 22.1)  ( 25.9)  ( 32.1)  ( 38.7)
g_E        282.3    282.3    282.3    282.3    282.3    282.3    282.3    282.3    282.3    282.3
         ( 19.2)  ( 19.2)  ( 19.2)  ( 19.2)  ( 19.2)  ( 19.2)  ( 19.2)  ( 19.2)  ( 19.2)  ( 19.2)
           -67.7    -77.9    -91.1    -62.5    -91.1    -22.1     91.6    228.5    485.2    801.0
          -4.604   -5.297   -6.190   -4.249   -6.190   -1.505    6.224   15.532   32.986   54.455
c_O          211      185      210      221      174      174      181      142       80       95
         ( 14.3)  ( 12.6)  ( 14.3)  ( 15.0)  ( 11.8)  ( 11.8)  ( 12.3)  (  9.7)  (  5.4)  (  6.5)
c_E        219.4    219.4    219.4    219.4    219.4    219.4    219.4    219.4    219.4    219.4
         ( 14.9)  ( 14.9)  ( 14.9)  ( 14.9)  ( 14.9)  ( 14.9)  ( 14.9)  ( 14.9)  ( 14.9)  ( 14.9)
           -16.5    -63.1    -18.4      3.2    -80.7    -80.7    -69.6   -123.6   -161.4   -159.0
          -1.120   -4.290   -1.250    0.218   -5.485   -5.485   -4.735   -8.400  -10.973  -10.811

N           1471     1471     1471     1471     1471     1471     1471     1471     1471     1471
X2          12.8     18.7     18.0     18.8     29.8     26.6     58.2    136.6    324.4    486.8
Gvalue      12.7     19.1     18.2     18.6     30.7     26.6     59.1    140.2    340.6    463.2
Gv/N       0.865    1.301    1.238    1.265    2.084    1.808    4.017    9.533   23.152   31.491
Prob     0.00526  0.00026  0.00040  0.00033  0.00000  0.00001  0.00000  0.00000  0.00000  0.00000
         ------------------------------------------------------------------------------------------
              10        9        8        7        6        5        4        3        2        1
         ------------------------------------------------------------------------------------------
a_O          567      522      574      593      586      591      599      676      468      482
         ( 38.5)  ( 35.5)  ( 39.0)  ( 40.3)  ( 39.8)  ( 40.2)  ( 40.7)  ( 46.0)  ( 31.8)  ( 32.8)
a_E        497.8    497.8    497.8    497.8    497.8    497.8    497.8    497.8    497.8    497.8
         ( 33.8)  ( 33.8)  ( 33.8)  ( 33.8)  ( 33.8)  ( 33.8)  ( 33.8)  ( 33.8)  ( 33.8)  ( 33.8)
           147.6     49.6    163.5    207.6    191.2    202.9    221.7    413.7    -57.8    -31.1
          10.036    3.370   11.117   14.111   12.998   13.792   15.073   28.126   -3.927   -2.112
t_O          201      261      276      388      456      436      456      376      562      487
         ( 13.7)  ( 17.7)  ( 18.8)  ( 26.4)  ( 31.0)  ( 29.6)  ( 31.0)  ( 25.6)  ( 38.2)  ( 33.1)
t_E        471.5    471.5    471.5    471.5    471.5    471.5    471.5    471.5    471.5    471.5
         ( 32.1)  ( 32.1)  ( 32.1)  ( 32.1)  ( 32.1)  ( 32.1)  ( 32.1)  ( 32.1)  ( 32.1)  ( 32.1)
          -342.8   -308.7   -295.6   -151.3    -30.5    -68.3    -30.5   -170.2    197.3     31.5
         -23.301  -20.987  -20.096  -10.283   -2.074   -4.641   -2.074  -11.571   13.415    2.140
g_O          616      609      468      370      302      277      220      229      184      213
         ( 41.9)  ( 41.4)  ( 31.8)  ( 25.2)  ( 20.5)  ( 18.8)  ( 15.0)  ( 15.6)  ( 12.5)  ( 14.5)
g_E        282.3    282.3    282.3    282.3    282.3    282.3    282.3    282.3    282.3    282.3
         ( 19.2)  ( 19.2)  ( 19.2)  ( 19.2)  ( 19.2)  ( 19.2)  ( 19.2)  ( 19.2)  ( 19.2)  ( 19.2)
           961.3    936.5    473.1    200.2     40.7    -10.5   -109.7    -95.8   -157.5   -120.0
          65.350   63.661   32.165   13.609    2.770   -0.714   -7.458   -6.515  -10.708   -8.157
c_O           87       79      153      120      127      167      196      190      257      289
         (  5.9)  (  5.4)  ( 10.4)  (  8.2)  (  8.6)  ( 11.4)  ( 13.3)  ( 12.9)  ( 17.5)  ( 19.6)
c_E        219.4    219.4    219.4    219.4    219.4    219.4    219.4    219.4    219.4    219.4
         ( 14.9)  ( 14.9)  ( 14.9)  ( 14.9)  ( 14.9)  ( 14.9)  ( 14.9)  ( 14.9)  ( 14.9)  ( 14.9)
          -160.9   -161.4   -110.3   -144.8   -138.9    -91.1    -44.2    -54.7     81.3    159.3
         -10.941  -10.971   -7.498   -9.845   -9.440   -6.196   -3.005   -3.717    5.527   10.826

N           1471     1471     1471     1471     1471     1471     1471     1471     1471     1471
X2         639.2    563.1    235.0    105.3     56.4     32.7     37.3     97.1     59.8     40.1
Gvalue     605.2    515.9    230.8    111.7     62.6     33.0     37.3     93.0     63.4     39.7
Gv/N      41.143   35.073   15.687    7.592    4.254    2.240    2.536    6.323    4.307    2.697
Prob     0.00000  0.00000  0.00000  0.00000  0.00000  0.00000  0.00000  0.00000  0.00000  0.00000
         ------------------------------------------------------------------------------------------

CDS_INITIAL:
         ------------------------------------------------------------------------------------------
               1        2        3        4        5        6        7        8        9       10
         ------------------------------------------------------------------------------------------
a_O         1299        0        0      670      503      538      607      632      533      588
         ( 88.3)  (  0.0)  (  0.0)  ( 45.5)  ( 34.2)  ( 36.6)  ( 41.3)  ( 43.0)  ( 36.2)  ( 40.0)
a_E        418.3    452.3    430.7    418.3    452.3    430.7    418.3    452.3    430.7    418.3
         ( 28.4)  ( 30.7)  ( 29.3)  ( 28.4)  ( 30.7)  ( 29.3)  ( 28.4)  ( 30.7)  ( 29.3)  ( 28.4)
          2943.6      0.0      0.0    631.1    107.0    239.5    451.9    423.0    227.3    400.3
         200.111    0.000    0.000   42.902    7.273   16.278   30.719   28.755   15.450   27.215
t_O           81     1471        0      206      336      526      233      408      558      334
         (  5.5)  (100.0)  (  0.0)  ( 14.0)  ( 22.8)  ( 35.8)  ( 15.8)  ( 27.7)  ( 37.9)  ( 22.7)
t_E        274.5    451.9    615.5    274.5    451.9    615.5    274.5    451.9    615.5    274.5
         ( 18.7)  ( 30.7)  ( 41.8)  ( 18.7)  ( 30.7)  ( 41.8)  ( 18.7)  ( 30.7)  ( 41.8)  ( 18.7)
          -197.7   3472.6      0.0   -118.3   -199.1   -165.3    -76.5    -83.3   -109.4    130.9
         -13.443  236.067    0.000   -8.045  -13.534  -11.236   -5.198   -5.663   -7.439    8.901
g_O           91        0     1471      356      206      177      362      190      221      280
         (  6.2)  (  0.0)  (100.0)  ( 24.2)  ( 14.0)  ( 12.0)  ( 24.6)  ( 12.9)  ( 15.0)  ( 19.0)
g_E        482.5    239.6    235.1    482.5    239.6    235.1    482.5    239.6    235.1    482.5
         ( 32.8)  ( 16.3)  ( 16.0)  ( 32.8)  ( 16.3)  ( 16.0)  ( 32.8)  ( 16.3)  ( 16.0)  ( 32.8)
          -303.6      0.0   5395.3   -216.4    -62.3   -100.4   -208.0    -88.2    -27.2   -304.7
         -20.638    0.000  366.780  -14.713   -4.238   -6.826  -14.138   -5.997   -1.852  -20.714
c_O            0        0        0      239      426      230      269      241      159      269
         (  0.0)  (  0.0)  (  0.0)  ( 16.2)  ( 29.0)  ( 15.6)  ( 18.3)  ( 16.4)  ( 10.8)  ( 18.3)
c_E        295.6    327.2    189.8    295.6    327.2    189.8    295.6    327.2    189.8    295.6
         ( 20.1)  ( 22.2)  ( 12.9)  ( 20.1)  ( 22.2)  ( 12.9)  ( 20.1)  ( 22.2)  ( 12.9)  ( 20.1)
             0.0      0.0      0.0   -101.7    224.7     88.4    -50.8   -147.4    -56.3    -50.8
           0.000    0.000    0.000   -6.911   15.276    6.006   -3.454  -10.023   -3.828   -3.454

N           1471     1471     1471     1471     1471     1471     1471     1471     1471     1471
X2        2603.6   3317.8   7734.9    212.5     69.9     62.6    123.8    108.7     35.5    169.0
Gvalue    2442.3   3472.6   5395.3    194.7     70.3     62.1    116.6    104.0     34.3    175.8
Gv/N     166.030  236.067  366.780   13.233    4.777    4.223    7.928    7.071    2.331   11.948
Prob     1.00000  1.00000  1.00000  0.00000  0.00000  0.00000  0.00000  0.00000  0.00000  0.00000
         ------------------------------------------------------------------------------------------
              11       12       13       14       15       16       17       18       19       20
         ------------------------------------------------------------------------------------------
a_O          521      522      549      476      477      556      507      489      545      508
         ( 35.4)  ( 35.5)  ( 37.3)  ( 32.4)  ( 32.4)  ( 37.8)  ( 34.5)  ( 33.2)  ( 37.0)  ( 34.5)
a_E        452.3    430.7    418.3    452.3    430.7    418.3    452.3    430.7    418.3    452.3
         ( 30.7)  ( 29.3)  ( 28.4)  ( 30.7)  ( 29.3)  ( 28.4)  ( 30.7)  ( 29.3)  ( 28.4)  ( 30.7)
           147.4    200.8    298.4     48.7     97.5    316.3    115.9    124.2    288.3    118.1
          10.023   13.651   20.287    3.312    6.628   21.503    7.876    8.447   19.597    8.028
t_O          472      580      326      543      596      307      496      590      297      506
         ( 32.1)  ( 39.4)  ( 22.2)  ( 36.9)  ( 40.5)  ( 20.9)  ( 33.7)  ( 40.1)  ( 20.2)  ( 34.4)
t_E        451.9    615.5    274.5    451.9    615.5    274.5    451.9    615.5    274.5    451.9
         ( 30.7)  ( 41.8)  ( 18.7)  ( 30.7)  ( 41.8)  ( 18.7)  ( 30.7)  ( 41.8)  ( 18.7)  ( 30.7)
            41.2    -68.9    112.0    199.6    -38.3     68.6     92.5    -49.9     46.7    114.5
           2.799   -4.683    7.614   13.566   -2.607    4.663    6.286   -3.392    3.174    7.786
g_O          182      184      308      174      191      323      186      177      322      192
         ( 12.4)  ( 12.5)  ( 20.9)  ( 11.8)  ( 13.0)  ( 22.0)  ( 12.6)  ( 12.0)  ( 21.9)  ( 13.1)
g_E        239.6    235.1    482.5    239.6    235.1    482.5    239.6    235.1    482.5    239.6
         ( 16.3)  ( 16.0)  ( 32.8)  ( 16.3)  ( 16.0)  ( 32.8)  ( 16.3)  ( 16.0)  ( 32.8)  ( 16.3)
          -100.2    -90.1   -276.5   -111.4    -79.3   -259.2    -94.3   -100.4   -260.4    -85.1
          -6.809   -6.126  -18.794   -7.573   -5.389  -17.621   -6.409   -6.826  -17.702   -5.787
```

c_O	296	185	288	278	207	285	282	215	307	265
	(20.1)	(12.6)	(19.6)	(18.9)	(14.1)	(19.4)	(19.2)	(14.6)	(20.9)	(18.0)
c_E	327.2	189.8	295.6	327.2	189.8	295.6	327.2	189.8	295.6	327.2
	(22.2)	(12.9)	(20.1)	(22.2)	(12.9)	(20.1)	(22.2)	(12.9)	(20.1)	(22.2)
	-59.4	-9.5	-15.1	-90.7	35.9	-20.9	-83.9	53.6	23.1	-111.8
	-4.038	-0.645	-1.026	-6.164	2.441	-1.421	-5.705	3.643	1.573	-7.601
N	1471	1471	1471	1471	1471	1471	1471	1471	1471	1471
X2	28.2	32.6	113.7	45.0	15.4	102.2	29.2	26.6	94.0	34.7
Gvalue	29.1	32.3	118.9	46.2	15.8	104.8	30.1	27.5	97.7	35.7
Gv/N	1.976	2.198	8.081	3.140	1.073	7.125	2.049	1.872	6.642	2.426
Prob	0.00000	0.00000	0.00000	0.00000	0.00126	0.00000	0.00000	0.00000	0.00000	0.00000

	21	22	23	24	25	26	27	28	29	30
a_O	473	488	485	482	465	455	424	432	443	429
	(32.2)	(33.2)	(33.0)	(32.8)	(31.6)	(30.9)	(28.8)	(29.4)	(30.1)	(29.2)
a_E	430.7	418.3	452.3	430.7	418.3	452.3	430.7	418.3	452.3	430.7
	(29.3)	(28.4)	(30.7)	(29.3)	(28.4)	(30.7)	(29.3)	(28.4)	(30.7)	(29.3)
	88.7	150.3	67.8	108.6	98.3	5.5	-13.2	27.7	-18.3	-3.3
	6.031	10.218	4.609	7.381	6.684	0.374	-0.898	1.886	-1.246	-0.225
t_O	580	296	485	588	288	505	621	301	475	618
	(39.4)	(20.1)	(33.0)	(40.0)	(19.6)	(34.3)	(42.2)	(20.5)	(32.3)	(42.0)
t_E	615.5	274.5	451.9	615.5	274.5	451.9	615.5	274.5	451.9	615.5
	(41.8)	(18.7)	(30.7)	(41.8)	(18.7)	(30.7)	(41.8)	(18.7)	(30.7)	(41.8)
	-68.9	44.5	68.7	-53.7	27.6	112.3	11.1	55.4	47.5	5.0
	-4.683	3.028	4.668	-3.652	1.873	7.635	0.753	3.764	3.226	0.343
g_O	211	366	191	214	404	222	219	426	240	198
	(14.3)	(24.9)	(13.0)	(14.5)	(27.5)	(15.1)	(14.9)	(29.0)	(16.3)	(13.5)
g_E	235.1	482.5	239.6	235.1	482.5	239.6	235.1	482.5	239.6	235.1
	(16.0)	(32.8)	(16.3)	(16.0)	(32.8)	(16.3)	(16.0)	(32.8)	(16.3)	(16.0)
	-45.6	-202.2	-86.7	-40.2	-143.4	-34.0	-31.0	-106.0	0.7	-67.9
	-3.097	-13.748	-5.892	-2.730	-9.749	-2.309	-2.106	-7.209	0.048	-4.618
c_O	207	321	310	187	314	289	207	312	313	226
	(14.1)	(21.8)	(21.1)	(12.7)	(21.3)	(19.6)	(14.1)	(21.2)	(21.3)	(15.4)
c_E	189.8	295.6	327.2	189.8	295.6	327.2	189.8	295.6	327.2	189.8
	(12.9)	(20.1)	(22.2)	(12.9)	(20.1)	(22.2)	(12.9)	(20.1)	(22.2)	(12.9)
	35.9	52.8	-33.6	-5.6	37.8	-71.8	35.9	33.6	-27.9	78.9
	2.441	3.591	-2.281	-0.379	2.572	-4.883	2.441	2.284	-1.893	5.363
N	1471	1471	1471	1471	1471	1471	1471	1471	1471	1471
X2	10.2	43.6	15.6	9.3	19.8	12.0	2.8	10.5	2.0	12.8
Gvalue	10.2	45.4	16.2	9.1	20.3	12.0	2.8	10.7	2.0	12.7
Gv/N	0.692	3.089	1.104	0.620	1.380	0.817	0.189	0.726	0.135	0.863
Prob	0.01709	0.00000	0.00101	0.02771	0.00015	0.00731	0.42561	0.01358	0.57534	0.00535

	31	32	33	34	35	36	37	38	39	40
a_O	482	393	436	443	407	417	440	415	465	449
	(32.8)	(26.7)	(29.6)	(30.1)	(27.7)	(28.3)	(29.9)	(28.2)	(31.6)	(30.5)
a_E	418.3	452.3	430.7	418.3	452.3	430.7	418.3	452.3	430.7	418.3
	(28.4)	(30.7)	(29.3)	(28.4)	(30.7)	(29.3)	(28.4)	(30.7)	(29.3)	(28.4)
	136.5	-110.4	10.7	50.7	-85.8	-26.9	44.4	-71.4	71.3	63.5
	9.282	-7.504	0.731	3.449	-5.834	-1.827	3.019	-4.851	4.850	4.317
t_O	315	537	604	308	512	619	336	519	599	292
	(21.4)	(36.5)	(41.1)	(20.9)	(34.8)	(42.1)	(22.8)	(35.3)	(40.7)	(19.9)
t_E	274.5	451.9	615.5	274.5	451.9	615.5	274.5	451.9	615.5	274.5
	(18.7)	(30.7)	(41.8)	(18.7)	(30.7)	(41.8)	(18.7)	(30.7)	(41.8)	(18.7)
	86.6	185.4	-22.8	70.8	128.0	7.1	135.7	143.8	-32.5	36.0
	5.887	12.605	-1.547	4.815	8.699	0.479	9.227	9.776	-2.211	2.447
g_O	413	222	226	438	221	228	431	241	214	431
	(28.1)	(15.1)	(15.4)	(29.8)	(15.0)	(15.5)	(29.3)	(16.4)	(14.5)	(29.3)
g_E	482.5	239.6	235.1	482.5	239.6	235.1	482.5	239.6	235.1	482.5
	(32.8)	(16.3)	(16.0)	(32.8)	(16.3)	(16.0)	(32.8)	(16.3)	(16.0)	(32.8)
	-128.4	-34.0	-17.7	-84.7	-35.8	-13.9	-97.2	2.7	-40.2	-97.2
	-8.729	-2.309	-1.206	-5.757	-2.434	-0.944	-6.609	0.184	-2.730	-6.609
c_O	261	319	205	282	331	207	264	296	193	299
	(17.7)	(21.7)	(13.9)	(19.2)	(22.5)	(14.1)	(17.9)	(20.1)	(13.1)	(20.3)
c_E	295.6	327.2	189.8	295.6	327.2	189.8	295.6	327.2	189.8	295.6
	(20.1)	(22.2)	(12.9)	(20.1)	(22.2)	(12.9)	(20.1)	(22.2)	(12.9)	(20.1)
	-65.1	-16.3	31.6	-26.6	7.6	35.9	-59.8	-59.4	6.4	6.7
	-4.423	-1.106	2.146	-1.812	0.514	2.441	-4.064	-4.038	0.438	0.459
N	1471	1471	1471	1471	1471	1471	1471	1471	1471	1471
X2	29.7	25.3	1.8	10.3	14.0	2.2	23.8	16.0	5.1	8.9
Gvalue	29.7	24.8	1.8	10.2	13.9	2.2	23.1	15.8	5.1	9.0
Gv/N	2.016	1.685	0.124	0.694	0.945	0.148	1.573	1.072	0.347	0.613
Prob	0.00000	0.00002	0.61067	0.01683	0.00305	0.53508	0.00004	0.00127	0.16383	0.02912

	41	42	43	44	45	46	47	48	49	50
a_O	451	429	416	401	407	448	389	460	453	399
	(30.7)	(29.2)	(28.3)	(27.3)	(27.7)	(30.5)	(26.4)	(31.3)	(30.8)	(27.1)
a_E	452.3	430.7	418.3	452.3	430.7	418.3	452.3	430.7	418.3	452.3
	(30.7)	(29.3)	(28.4)	(30.7)	(29.3)	(28.4)	(30.7)	(29.3)	(28.4)	(30.7)
	-2.5	-3.3	-4.7	-96.5	-46.0	61.4	-117.2	60.6	72.1	-100.0
	-0.171	-0.225	-0.318	-6.558	-3.127	4.171	-7.969	4.122	4.901	-6.797
t_O	490	599	315	483	628	295	506	590	300	480
	(33.3)	(40.7)	(21.4)	(32.8)	(42.7)	(20.1)	(34.4)	(40.1)	(20.4)	(32.6)
t_E	451.9	615.5	274.5	451.9	615.5	274.5	451.9	615.5	274.5	451.9
	(30.7)	(41.8)	(18.7)	(30.7)	(41.8)	(18.7)	(30.7)	(41.8)	(18.7)	(30.7)
	79.4	-32.5	86.6	64.4	25.3	42.4	114.5	-49.9	53.2	58.0
	5.399	-2.211	5.887	4.377	1.719	2.882	7.786	-3.392	3.616	3.943
g_O	221	242	472	253	246	421	248	224	459	267
	(15.0)	(16.5)	(32.1)	(17.2)	(16.7)	(28.6)	(16.9)	(15.2)	(31.2)	(18.2)
g_E	239.6	235.1	482.5	239.6	235.1	482.5	239.6	235.1	482.5	239.6
	(16.3)	(16.0)	(32.8)	(16.3)	(16.0)	(32.8)	(16.3)	(16.0)	(32.8)	(16.3)
	-35.8	14.1	-20.7	27.4	22.4	-114.7	17.0	-21.6	-45.8	57.7
	-2.434	0.959	-1.407	1.865	1.523	-7.800	1.155	-1.467	-3.111	3.923
c_O	309	201	268	334	190	307	328	197	259	325
	(21.0)	(13.7)	(18.2)	(22.7)	(12.9)	(20.9)	(22.3)	(13.4)	(17.6)	(22.1)
c_E	327.2	189.8	295.6	327.2	189.8	295.6	327.2	189.8	295.6	327.2
	(22.2)	(12.9)	(20.1)	(22.2)	(12.9)	(20.1)	(22.2)	(12.9)	(20.1)	(22.2)
	-35.4	23.0	-52.6	13.7	0.4	23.1	1.5	14.7	-68.5	-4.5
	-2.409	1.566	-3.577	0.929	0.026	1.573	0.104	0.996	-4.660	-0.303
N	1471	1471	1471	1471	1471	1471	1471	1471	1471	1471
X2	5.7	1.3	8.8	8.8	2.1	11.9	15.6	3.8	10.9	11.2
Gvalue	5.7	1.3	8.6	9.0	2.1	12.2	15.8	3.8	11.0	11.3
Gv/N	0.385	0.089	0.585	0.613	0.141	0.826	1.076	0.260	0.747	0.767
Prob	0.12919	0.72843	0.03513	0.02916	0.55603	0.00687	0.00123	0.28132	0.01180	0.01033

	51	52	53	54	55	56	57	58	59	60
a_O	453	427	382	426	418	397	416	411	415	394
	(30.8)	(29.0)	(26.0)	(29.0)	(28.4)	(27.0)	(28.3)	(27.9)	(28.2)	(26.8)
a_E	430.7	418.3	452.3	430.7	418.3	452.3	430.7	418.3	452.3	430.7
	(29.3)	(28.4)	(30.7)	(29.3)	(28.4)	(30.7)	(29.3)	(28.4)	(30.7)	(29.3)
	45.8	17.5	-129.0	-9.3	-0.7	-103.5	-28.8	-14.6	-71.4	-70.1
	3.115	1.188	-8.769	-0.630	-0.047	-7.034	-1.959	-0.990	-4.851	-4.766
t_O	569	312	492	631	317	467	624	293	461	615
	(38.7)	(21.2)	(33.4)	(42.9)	(21.5)	(31.7)	(42.4)	(19.9)	(31.3)	(41.8)
t_E	615.5	274.5	451.9	615.5	274.5	451.9	615.5	274.5	451.9	615.5
	(41.8)	(18.7)	(30.7)	(41.8)	(18.7)	(30.7)	(41.8)	(18.7)	(30.7)	(41.8)
	-89.4	79.8	83.8	31.4	91.2	30.8	17.1	38.1	18.5	-1.0
	-6.075	5.425	5.694	2.136	6.197	2.093	1.166	2.591	1.256	-0.066
g_O	225	445	255	219	435	272	236	467	234	268
	(15.3)	(30.3)	(17.3)	(14.9)	(29.6)	(18.5)	(16.0)	(31.7)	(15.9)	(18.2)
g_E	235.1	482.5	239.6	235.1	482.5	239.6	235.1	482.5	239.6	235.1
	(16.0)	(32.8)	(16.3)	(16.0)	(32.8)	(16.3)	(16.0)	(32.8)	(16.3)	(16.0)
	-19.7	-71.9	31.7	-31.0	-90.1	68.9	1.9	-30.4	-11.2	70.3
	-1.337	-4.890	2.153	-2.106	-6.124	4.683	0.129	-2.068	-0.759	4.780
c_O	224	287	342	195	301	335	195	300	361	194
	(15.2)	(19.5)	(23.2)	(13.3)	(20.5)	(22.8)	(13.3)	(20.4)	(24.5)	(13.2)
c_E	189.8	295.6	327.2	189.8	295.6	327.2	189.8	295.6	327.2	189.8
	(12.9)	(20.1)	(22.2)	(12.9)	(20.1)	(22.2)	(12.9)	(20.1)	(22.2)	(12.9)
	74.2	-17.0	30.2	10.5	10.8	15.7	10.5	8.8	70.9	8.5
	5.045	-1.158	2.051	0.716	0.735	1.068	0.716	0.597	4.819	0.576
N	1471	1471	1471	1471	1471	1471	1471	1471	1471	1471
X2	11.3	8.4	16.1	1.7	11.3	11.8	0.8	1.9	6.9	7.8
Gvalue	11.0	8.3	16.6	1.7	11.2	11.9	0.8	1.9	6.8	7.7
Gv/N	0.748	0.565	1.129	0.116	0.760	0.810	0.052	0.130	0.465	0.525
Prob	0.01174	0.04000	0.00085	0.63667	0.01081	0.00769	0.85740	0.59177	0.07703	0.05211

	61	62	63	64	65	66	67	68	69	70
a_O	398	401	421	399	391	447	392	440	442	423
	(27.1)	(27.3)	(28.6)	(27.1)	(26.6)	(30.4)	(26.6)	(29.9)	(30.0)	(28.8)
a_E	418.3	452.3	430.7	418.3	452.3	430.7	418.3	452.3	430.7	418.3
	(28.4)	(30.7)	(29.3)	(28.4)	(30.7)	(29.3)	(28.4)	(30.7)	(29.3)	(28.4)
	-39.7	-96.5	-19.1	-37.8	-113.8	33.3	-51.0	-24.2	23.0	9.4
	-2.698	-6.558	-1.299	-2.569	-7.737	2.263	-3.467	-1.644	1.562	0.636
t_O	318	510	622	296	498	582	311	468	605	280
	(21.6)	(34.7)	(42.3)	(20.1)	(33.9)	(39.6)	(21.1)	(31.8)	(41.1)	(19.0)
t_E	274.5	451.9	615.5	274.5	451.9	615.5	274.5	451.9	615.5	274.5
	(18.7)	(30.7)	(41.8)	(18.7)	(30.7)	(41.8)	(18.7)	(30.7)	(41.8)	(18.7)
	93.4	123.5	13.1	44.5	96.9	-65.1	77.5	32.9	-20.8	11.0
	6.352	8.394	0.891	3.028	6.584	-4.426	5.271	2.234	-1.413	0.749
g_O	448	263	239	469	246	241	466	231	253	483
	(30.5)	(17.9)	(16.2)	(31.9)	(16.7)	(16.4)	(31.7)	(15.7)	(17.2)	(32.8)
g_E	482.5	239.6	235.1	482.5	239.6	235.1	482.5	239.6	235.1	482.5
	(32.8)	(16.3)	(16.0)	(32.8)	(16.3)	(16.0)	(32.8)	(16.3)	(16.0)	(32.8)
	-66.4	48.9	8.0	-26.5	12.9	12.0	-32.4	-17.0	37.2	1.1
	-4.514	3.325	0.542	-1.804	0.875	0.819	-2.199	-1.155	2.531	0.073
c_O	307	297	189	307	336	201	302	332	171	285
	(20.9)	(20.2)	(12.8)	(20.9)	(22.8)	(13.7)	(20.5)	(22.6)	(11.6)	(19.4)
c_E	295.6	327.2	189.8	295.6	327.2	189.8	295.6	327.2	189.8	295.6
	(20.1)	(22.2)	(12.9)	(20.1)	(22.2)	(12.9)	(20.1)	(22.2)	(12.9)	(20.1)
	23.1	-57.6	-1.6	23.1	17.8	23.0	12.8	9.6	-35.7	-20.9
	1.573	-3.915	-0.109	1.573	1.207	1.566	0.873	0.652	-2.426	-1.421
N	1471	1471	1471	1471	1471	1471	1471	1471	1471	1471
X2	10.8	18.4	0.4	3.4	13.4	3.3	7.2	1.3	3.7	0.5
Gvalue	10.5	18.3	0.4	3.4	13.7	3.3	7.0	1.3	3.7	0.5
Gv/N	0.714	1.245	0.024	0.228	0.929	0.222	0.478	0.088	0.254	0.037
Prob	0.01476	0.00038	0.94908	0.34036	0.00341	0.35257	0.07070	0.73145	0.29084	0.90830

	71	72	73	74	75	76	77	78	79	80
a_O	407	414	415	414	406	378	398	419	373	406
	(27.7)	(28.1)	(28.2)	(28.1)	(27.6)	(25.7)	(27.1)	(28.5)	(25.4)	(27.6)
a_E	452.3	430.7	418.3	452.3	430.7	418.3	452.3	430.7	418.3	452.3
	(30.7)	(29.3)	(28.4)	(30.7)	(29.3)	(28.4)	(30.7)	(29.3)	(28.4)	(30.7)
	-85.8	-32.7	-6.7	-73.2	-47.9	-76.7	-101.7	-23.0	-85.6	-87.6
	-5.834	-2.221	-0.453	-4.975	-3.255	-5.212	-6.915	-1.564	-5.819	-5.956
t_O	501	619	296	449	626	326	471	637	295	460
	(34.1)	(42.1)	(20.1)	(30.5)	(42.6)	(22.2)	(32.0)	(43.3)	(20.1)	(31.3)
t_E	451.9	615.5	274.5	451.9	615.5	274.5	451.9	615.5	274.5	451.9
	(30.7)	(41.8)	(18.7)	(30.7)	(41.8)	(18.7)	(30.7)	(41.8)	(18.7)	(30.7)
	103.5	7.1	44.5	-5.7	21.2	112.0	39.1	43.8	42.4	16.4
	7.033	0.479	3.028	-0.387	1.442	7.614	2.657	2.976	2.882	1.117
g_O	229	249	451	263	246	454	243	219	483	268
	(15.6)	(16.9)	(30.7)	(17.9)	(16.7)	(30.9)	(16.5)	(14.9)	(32.8)	(18.2)
g_E	239.6	235.1	482.5	239.6	235.1	482.5	239.6	235.1	482.5	239.6
	(16.3)	(16.0)	(32.8)	(16.3)	(16.0)	(32.8)	(16.3)	(16.0)	(32.8)	(16.3)
	-20.8	28.7	-60.8	48.9	22.4	-55.2	6.7	-31.0	1.1	59.9
	-1.415	1.952	-4.135	3.325	1.523	-3.753	0.459	-2.106	0.073	4.074
c_O	334	189	309	345	193	313	359	196	320	337
	(22.7)	(12.8)	(21.0)	(23.5)	(13.1)	(21.3)	(24.4)	(13.3)	(21.8)	(22.9)
c_E	327.2	189.8	295.6	327.2	189.8	295.6	327.2	189.8	295.6	327.2
	(22.2)	(12.9)	(20.1)	(22.2)	(12.9)	(20.1)	(22.2)	(12.9)	(20.1)	(22.2)
	13.7	-1.6	27.3	36.5	6.4	35.7	66.5	12.6	50.7	19.8
	0.929	-0.109	1.856	2.479	0.438	2.428	4.521	0.856	3.444	1.347
N	1471	1471	1471	1471	1471	1471	1471	1471	1471	1471
X2	10.5	1.5	4.4	6.5	2.2	16.2	10.4	2.4	8.4	8.5
Gvalue	10.5	1.5	4.4	6.5	2.2	15.8	10.6	2.4	8.5	8.6
Gv/N	0.712	0.101	0.296	0.442	0.148	1.076	0.722	0.162	0.581	0.582
Prob	0.01498	0.68495	0.22578	0.08962	0.53704	0.00123	0.01397	0.49664	0.03600	0.03568

	81	82	83	84	85	86	87	88	89	90
a_O	432	387	443	409	410	432	412	392	410	457
	(29.4)	(26.3)	(30.1)	(27.8)	(27.9)	(29.4)	(28.0)	(26.6)	(27.9)	(31.1)
a_E	430.7	418.3	452.3	430.7	418.3	452.3	430.7	418.3	452.3	430.7
	(29.3)	(28.4)	(30.7)	(29.3)	(28.4)	(30.7)	(29.3)	(28.4)	(30.7)	(29.3)
	2.7	-60.3	-18.3	-42.2	-16.5	-39.6	-36.5	-51.0	-80.4	54.3
	0.182	-4.098	-1.246	-2.870	-1.124	-2.691	-2.481	-3.467	-5.468	3.689
t_O	626	326	449	624	301	499	637	306	452	599
	(42.6)	(22.2)	(30.5)	(42.4)	(20.5)	(33.9)	(43.3)	(20.8)	(30.7)	(40.7)
t_E	615.5	274.5	451.9	615.5	274.5	451.9	615.5	274.5	451.9	615.5
	(41.8)	(18.7)	(30.7)	(41.8)	(18.7)	(30.7)	(41.8)	(18.7)	(30.7)	(41.8)
	21.2	112.0	-5.7	17.1	55.4	99.0	43.8	66.4	0.3	-32.5

	1.442	7.614	−0.387	1.166	3.764	6.733	2.976	4.512	0.020	−2.211
g_O	214	455	228	249	456	234	245	480	251	259
	(14.5)	(30.9)	(15.5)	(16.9)	(31.0)	(15.9)	(16.7)	(32.6)	(17.1)	(17.6)
g_E	235.1	482.5	239.6	235.1	482.5	239.6	235.1	482.5	239.6	235.1
	(16.0)	(32.8)	(16.3)	(16.0)	(32.8)	(16.3)	(16.0)	(32.8)	(16.3)	(16.0)
	−40.2	−53.3	−22.7	28.7	−51.4	−11.2	20.3	−4.9	23.2	50.3
	−2.730	−3.625	−1.545	1.952	−3.497	−0.759	1.381	−0.334	1.579	3.417
c_O	199	303	351	189	304	306	177	293	358	156
	(13.5)	(20.6)	(23.9)	(12.8)	(20.7)	(20.8)	(12.0)	(19.9)	(24.3)	(10.6)
c_E	189.8	295.6	327.2	189.8	295.6	327.2	189.8	295.6	327.2	189.8
	(12.9)	(20.1)	(22.2)	(12.9)	(20.1)	(22.2)	(12.9)	(20.1)	(22.2)	(12.9)
	18.8	14.9	49.2	−1.6	16.9	−41.1	−24.7	−5.3	64.3	−61.2
	1.280	1.012	3.345	−0.109	1.152	−2.792	−1.681	−0.358	4.373	−4.160
N	1471	1471	1471	1471	1471	1471	1471	1471	1471	1471
X2	2.5	13.7	2.5	2.0	4.4	7.3	2.8	5.3	7.4	10.5
Gvalue	2.6	13.3	2.5	2.0	4.4	7.2	2.9	5.2	7.4	10.8
Gv/N	0.174	0.903	0.168	0.139	0.296	0.491	0.195	0.354	0.504	0.734
Prob	0.46361	0.00408	0.48084	0.56398	0.22601	0.06518	0.41316	0.15741	0.05988	0.01286

	91	92	93	94	95	96	97	98	99	100
a_O	441	413	438	388	456	435	429	411	415	401
	(30.0)	(28.1)	(29.8)	(26.4)	(31.0)	(29.6)	(29.2)	(27.9)	(28.2)	(27.3)
a_E	418.3	452.3	430.7	418.3	452.3	430.7	418.3	452.3	430.7	418.3
	(28.4)	(30.7)	(29.3)	(28.4)	(30.7)	(29.3)	(28.4)	(30.7)	(29.3)	(28.4)
	46.5	−75.0	14.8	−58.4	7.5	8.7	21.6	−78.6	−30.7	−34.0
	3.162	−5.099	1.006	−3.973	0.511	0.593	1.467	−5.345	−2.090	−2.309
t_O	265	493	585	334	457	606	276	482	619	293
	(18.0)	(33.5)	(39.8)	(22.7)	(31.1)	(41.2)	(18.8)	(32.8)	(42.1)	(19.9)
t_E	274.5	451.9	615.5	274.5	451.9	615.5	274.5	451.9	615.5	274.5
	(18.7)	(30.7)	(41.8)	(18.7)	(30.7)	(41.8)	(18.7)	(30.7)	(41.8)	(18.7)
	−18.8	85.9	−59.4	130.9	10.3	−18.8	2.9	62.3	7.1	38.1
	−1.275	5.841	−4.040	8.901	0.704	−1.279	0.198	4.232	0.479	2.591
g_O	466	236	268	469	226	253	479	247	246	455
	(31.7)	(16.0)	(18.2)	(31.9)	(15.4)	(17.2)	(32.6)	(16.8)	(16.7)	(30.9)
g_E	482.5	239.6	235.1	482.5	239.6	235.1	482.5	239.6	235.1	482.5
	(32.8)	(16.3)	(16.0)	(32.8)	(16.3)	(16.0)	(32.8)	(16.3)	(16.0)	(32.8)
	−32.4	−7.2	70.3	−26.5	−26.5	37.2	−6.9	14.9	22.4	−53.3
	−2.199	−0.492	4.780	−1.804	−1.802	2.531	−0.469	1.015	1.523	−3.625
c_O	299	329	180	280	332	177	287	331	191	322
	(20.3)	(22.4)	(12.2)	(19.0)	(22.6)	(12.0)	(19.5)	(22.5)	(13.0)	(21.9)
c_E	295.6	327.2	189.8	295.6	327.2	189.8	295.6	327.2	189.8	295.6
	(20.1)	(22.2)	(12.9)	(20.1)	(22.2)	(12.9)	(20.1)	(22.2)	(12.9)	(20.1)
	6.7	3.5	−19.1	−30.4	9.6	−24.7	−17.0	7.6	2.4	55.0
	0.459	0.240	−1.298	−2.070	0.652	−1.681	−1.158	0.514	0.163	3.739
N	1471	1471	1471	1471	1471	1471	1471	1471	1471	1471
X2	2.2	7.2	6.8	16.3	0.9	2.4	0.6	6.0	1.1	5.9
Gvalue	2.1	7.2	6.6	15.5	1.0	2.4	0.6	6.1	1.1	5.8
Gv/N	0.146	0.490	0.448	1.055	0.065	0.164	0.038	0.416	0.075	0.396
Prob	0.54247	0.06539	0.08609	0.00143	0.81315	0.49145	0.90625	0.10603	0.77554	0.12076

	101	102	103	104	105	106	107	108	109	110
a_O	476	455	414	432	383	408	465	447	444	432
	(32.4)	(30.9)	(28.1)	(29.4)	(26.0)	(27.7)	(31.6)	(30.4)	(30.2)	(29.4)
a_E	452.3	430.7	418.3	452.3	430.7	418.3	452.3	430.7	418.3	452.3
	(30.7)	(29.3)	(28.4)	(30.7)	(29.3)	(28.4)	(30.7)	(29.3)	(28.4)	(30.7)
	48.7	50.0	−8.6	−39.6	−89.8	−20.4	25.8	33.3	52.8	−39.6
	3.312	3.401	−0.588	−2.691	−6.107	−1.389	1.757	2.263	3.593	−2.691
t_O	451	601	308	464	632	244	450	593	307	477
	(30.7)	(40.9)	(20.9)	(31.5)	(43.0)	(16.6)	(30.6)	(40.3)	(20.9)	(32.4)
t_E	451.9	615.5	274.5	451.9	615.5	274.5	451.9	615.5	274.5	451.9
	(30.7)	(41.8)	(18.7)	(30.7)	(41.8)	(18.7)	(30.7)	(41.8)	(18.7)	(30.7)
	−1.7	−28.6	70.8	24.6	33.5	−57.6	−3.7	−44.1	68.6	51.7
	−0.116	−1.946	4.815	1.673	2.275	−3.913	−0.252	−3.000	4.663	3.512
g_O	249	237	470	256	271	484	247	242	445	218
	(16.9)	(16.1)	(32.0)	(17.4)	(18.4)	(32.9)	(16.8)	(16.5)	(30.3)	(14.8)
g_E	239.6	235.1	482.5	239.6	235.1	482.5	239.6	235.1	482.5	239.6
	(16.3)	(16.0)	(32.8)	(16.3)	(16.0)	(32.8)	(16.3)	(16.0)	(32.8)	(16.3)
	19.1	3.9	−24.6	33.8	77.1	3.1	14.9	14.1	−71.9	−41.3
	1.296	0.266	−1.672	2.297	5.244	0.210	1.015	0.959	−4.890	−2.806
c_O	295	178	279	319	185	335	309	189	275	344
	(20.1)	(12.1)	(19.0)	(21.7)	(12.6)	(22.8)	(21.0)	(12.8)	(18.7)	(23.4)
c_E	327.2	189.8	295.6	327.2	189.8	295.6	327.2	189.8	295.6	327.2
	(22.2)	(12.9)	(20.1)	(22.2)	(12.9)	(20.1)	(22.2)	(12.9)	(20.1)	(22.2)
	−61.2	−22.9	−32.3	−16.3	−9.5	83.7	−35.4	−1.6	−39.8	34.4
	−4.160	−1.554	−2.198	−1.106	−0.645	5.692	−2.409	−0.109	−2.707	2.336
N	1471	1471	1471	1471	1471	1471	1471	1471	1471	1471
X2	4.8	2.5	5.4	2.6	11.3	8.9	1.6	1.7	9.8	5.1
Gvalue	4.9	2.5	5.2	2.5	11.3	8.8	1.6	1.7	9.7	5.2
Gv/N	0.331	0.167	0.357	0.173	0.767	0.599	0.110	0.112	0.659	0.351
Prob	0.18133	0.48253	0.15471	0.46723	0.01030	0.03184	0.65427	0.64794	0.02131	0.16067

	111	112	113	114	115	116	117	118	119	120
a_O	414	391	454	425	409	422	430	448	455	425
	(28.1)	(26.6)	(30.9)	(28.9)	(27.8)	(28.7)	(29.2)	(30.5)	(30.9)	(28.9)
a_E	430.7	418.3	452.3	430.7	418.3	452.3	430.7	418.3	452.3	430.7
	(29.3)	(28.4)	(30.7)	(29.3)	(28.4)	(30.7)	(29.3)	(28.4)	(30.7)	(29.3)
	−32.7	−52.9	3.5	−11.2	−18.5	−58.4	−1.3	61.4	5.5	−11.2
	−2.221	−3.594	0.238	−0.764	−1.257	−3.973	−0.090	4.171	0.374	−0.764
t_O	642	298	459	622	277	459	631	263	452	624
	(43.6)	(20.3)	(31.2)	(42.3)	(18.8)	(31.2)	(42.9)	(17.9)	(30.7)	(42.4)
t_E	615.5	274.5	451.9	615.5	274.5	451.9	615.5	274.5	451.9	615.5
	(41.8)	(18.7)	(30.7)	(41.8)	(18.7)	(30.7)	(41.8)	(18.7)	(30.7)	(41.8)
	54.2	48.9	14.4	13.1	4.9	14.4	31.4	−22.6	0.3	17.1
	3.682	3.321	0.979	0.891	0.335	0.979	2.136	−1.537	0.020	1.166
g_O	221	470	231	262	482	245	211	503	226	257
	(15.0)	(32.0)	(15.7)	(17.8)	(32.8)	(16.7)	(14.3)	(34.2)	(15.4)	(17.5)
g_E	235.1	482.5	239.6	235.1	482.5	239.6	235.1	482.5	239.6	235.1
	(16.0)	(32.8)	(16.3)	(16.0)	(32.8)	(16.3)	(16.0)	(32.8)	(16.3)	(16.0)
	−27.2	−24.6	−17.0	56.9	−0.9	10.8	−45.6	41.9	−26.5	45.9
	−1.852	−1.672	−1.155	3.867	−0.063	0.736	−3.097	2.851	−1.802	3.120
c_O	194	312	327	162	303	345	199	257	338	165
	(13.2)	(21.2)	(22.2)	(11.0)	(20.6)	(23.5)	(13.5)	(17.5)	(23.0)	(11.2)
c_E	189.8	295.6	327.2	189.8	295.6	327.2	189.8	295.6	327.2	189.8
	(12.9)	(20.1)	(22.2)	(12.9)	(20.1)	(22.2)	(12.9)	(20.1)	(22.2)	(12.9)
	8.5	33.6	−0.5	−51.3	14.9	36.5	18.8	−72.0	21.9	−46.2
	0.576	2.284	−0.033	−3.489	1.012	2.479	1.280	−4.895	1.487	−3.142

N	1471	1471	1471	1471	1471	1471	1471	1471	1471	1471
X2	2.7	5.0	0.4	7.3	0.4	3.2	3.3	8.5	1.1	5.5
Gvalue	2.7	5.0	0.4	7.4	0.4	3.2	3.4	8.7	1.2	5.6
Gv/N	0.185	0.339	0.030	0.504	0.028	0.221	0.230	0.591	0.079	0.379
Prob	0.43543	0.17255	0.93286	0.05992	0.93729	0.35483	0.33713	0.03362	0.76278	0.13428

	121	122	123	124	125	126	127	128	129	130
a_O	414	447	455	408	451	417	405	428	441	409
	(28.1)	(30.4)	(30.9)	(27.7)	(30.7)	(28.3)	(27.6)	(29.1)	(30.0)	(27.8)
a_E	418.3	452.3	430.7	418.3	452.3	430.7	417.8	451.6	430.1	417.8
	(28.4)	(30.7)	(29.3)	(28.4)	(30.7)	(29.3)	(28.4)	(30.7)	(29.3)	(28.4)
	-8.6	-10.5	50.0	-20.4	-2.5	-26.9	-25.2	-46.0	22.1	-17.4
	-0.588	-0.710	3.401	-1.389	-0.171	-1.827	-1.713	-3.133	1.506	-1.183
t_O	306	416	588	308	454	619	288	454	630	286
	(20.8)	(28.3)	(40.0)	(20.9)	(30.9)	(42.1)	(19.6)	(30.9)	(42.9)	(19.5)
t_E	274.5	451.9	615.5	274.5	451.9	615.5	274.2	451.2	614.6	274.2
	(18.7)	(30.7)	(41.8)	(18.7)	(30.7)	(41.8)	(18.7)	(30.7)	(41.8)	(18.7)
	66.4	-68.8	-53.7	70.8	4.3	7.1	28.3	5.5	31.1	24.2
	4.512	-4.676	-3.652	4.815	0.292	0.479	1.929	0.377	2.116	1.644
g_O	452	258	223	495	245	231	487	246	204	482
	(30.7)	(17.5)	(15.2)	(33.7)	(16.7)	(15.7)	(33.2)	(16.7)	(13.9)	(32.8)
g_E	482.5	239.6	235.1	482.5	239.6	235.1	481.8	239.3	234.7	481.8
	(32.8)	(16.3)	(16.0)	(32.8)	(16.3)	(16.0)	(32.8)	(16.3)	(16.0)	(32.8)
	-59.0	38.1	-23.5	25.4	10.8	-8.0	10.4	13.5	-57.3	0.4
	-4.008	2.588	-1.596	1.727	0.736	-0.546	0.711	0.922	-3.897	0.027
c_O	299	350	205	260	321	204	289	341	194	292
	(20.3)	(23.8)	(13.9)	(17.7)	(21.8)	(13.9)	(19.7)	(23.2)	(13.2)	(19.9)
c_E	295.6	327.2	189.8	295.6	327.2	189.8	295.2	326.8	189.5	295.2
	(20.1)	(22.2)	(12.9)	(20.1)	(22.2)	(12.9)	(20.1)	(22.2)	(12.9)	(20.1)
	6.7	47.1	31.6	-66.8	-12.4	29.4	-12.4	29.0	9.0	-6.4
	0.459	3.200	2.146	-4.542	-0.840	2.000	-0.841	1.975	0.613	-0.439
N	1471	1471	1471	1471	1471	1471	1469	1469	1469	1469
X2	5.6	5.9	4.4	9.0	0.3	1.6	1.3	2.1	4.8	0.7
Gvalue	5.5	5.9	4.4	9.0	0.3	1.6	1.3	2.1	5.0	0.7
Gv/N	0.375	0.401	0.300	0.611	0.017	0.106	0.086	0.141	0.338	0.049
Prob	0.13738	0.11643	0.22045	0.02952	0.96879	0.66755	0.73641	0.55804	0.17396	0.86743

	131	132	133	134	135	136	137	138	139	140
a_O	439	428	394	436	449	374	441	432	417	471
	(29.9)	(29.1)	(26.8)	(29.7)	(30.6)	(25.5)	(30.0)	(29.4)	(28.4)	(32.1)
a_E	451.6	430.1	417.8	451.6	430.1	417.5	451.3	429.8	417.5	451.3
	(30.7)	(29.3)	(28.4)	(30.7)	(29.3)	(28.4)	(30.7)	(29.3)	(28.4)	(30.7)
	-24.9	-4.1	-46.2	-30.7	38.7	-82.3	-20.4	4.4	-1.0	40.2
	-1.697	-0.282	-3.143	-2.092	2.633	-5.606	-1.392	0.303	-0.067	2.737
t_O	446	620	302	482	587	276	424	618	310	461
	(30.4)	(42.2)	(20.6)	(32.8)	(40.0)	(18.8)	(28.9)	(42.1)	(21.1)	(31.4)
t_E	451.2	614.6	274.2	451.2	614.6	274.0	450.9	614.2	274.0	450.9
	(30.7)	(41.8)	(18.7)	(30.7)	(41.8)	(18.7)	(30.7)	(41.8)	(18.7)	(30.7)
	-10.4	10.8	58.4	63.6	-54.0	4.0	-52.2	7.6	76.6	20.4
	-0.709	0.732	3.974	4.327	-3.678	0.275	-3.558	0.515	5.215	1.387
g_O	245	213	469	232	255	545	238	215	457	211
	(16.7)	(14.5)	(31.9)	(15.8)	(17.4)	(37.1)	(16.2)	(14.6)	(31.1)	(14.4)
g_E	239.3	234.7	481.8	239.3	234.7	481.5	239.2	234.6	481.5	239.2
	(16.3)	(16.0)	(32.8)	(16.3)	(16.0)	(32.8)	(16.3)	(16.0)	(32.8)	(16.3)
	11.5	-41.4	-25.3	-14.4	42.2	135.1	-2.3	-37.5	-47.7	-52.9
	0.782	-2.817	-1.720	-0.982	2.875	9.202	-0.158	-2.552	-3.248	-3.601
c_O	339	208	304	319	178	273	365	203	284	325
	(23.1)	(14.2)	(20.7)	(21.7)	(12.1)	(18.6)	(24.9)	(13.8)	(19.3)	(22.1)
c_E	326.8	189.5	295.2	326.8	189.5	295.0	326.6	189.4	295.0	326.6
	(22.2)	(12.9)	(20.1)	(22.2)	(12.9)	(20.1)	(22.2)	(12.9)	(20.1)	(22.2)
	24.9	38.6	17.8	-15.4	-22.4	-42.4	81.2	28.1	-21.7	-3.1
	1.692	2.631	1.210	-1.048	-1.523	-2.888	5.532	1.915	-1.476	-0.214
N	1469	1469	1469	1469	1469	1468	1468	1468	1468	1468
X2	1.0	3.9	4.8	3.0	4.5	14.6	6.4	2.6	6.4	4.4
Gvalue	1.0	3.9	4.7	3.0	4.5	14.4	6.2	2.7	6.2	4.5
Gv/N	0.068	0.264	0.321	0.205	0.307	0.983	0.425	0.182	0.424	0.309
Prob	0.80073	0.27539	0.19402	0.38969	0.21195	0.00237	0.10050	0.44602	0.10122	0.20966

	141	142	143	144	145	146	147	148	149	150
a_O	425	433	446	455	425	458	466	417	448	400
	(29.0)	(29.5)	(30.4)	(31.0)	(29.0)	(31.2)	(31.7)	(28.4)	(30.6)	(27.3)
a_E	429.8	417.5	451.3	429.8	417.5	451.3	429.8	416.9	450.7	429.2
	(29.3)	(28.4)	(30.7)	(29.3)	(28.4)	(30.7)	(29.3)	(28.4)	(30.7)	(29.3)
	-9.5	31.6	-10.6	51.9	15.1	13.4	75.4	0.1	-5.4	-56.4
	-0.648	2.151	-0.722	3.535	1.032	0.915	5.137	0.010	-0.370	-3.844
t_O	630	296	440	617	260	436	579	284	469	608
	(42.9)	(20.2)	(30.0)	(42.0)	(17.7)	(29.7)	(39.4)	(19.4)	(32.0)	(41.5)
t_E	614.2	274.0	450.9	614.2	274.0	450.9	614.2	273.6	450.3	613.4
	(41.8)	(18.7)	(30.7)	(41.8)	(18.7)	(30.7)	(41.8)	(18.7)	(30.7)	(41.8)
	31.9	45.7	-21.6	5.6	-27.2	-29.4	-68.4	21.2	38.1	-10.7
	2.176	3.116	-1.471	0.378	-1.856	-2.000	-4.659	1.443	2.601	-0.732
g_O	246	433	260	212	480	255	217	482	231	257
	(16.8)	(29.5)	(17.7)	(14.4)	(32.7)	(17.4)	(14.8)	(32.9)	(15.8)	(17.5)
g_E	234.6	481.5	239.2	234.6	481.5	239.2	234.6	480.8	238.8	234.3
	(16.0)	(32.8)	(16.3)	(16.0)	(32.8)	(16.3)	(16.0)	(32.8)	(16.3)	(16.0)
	23.4	-91.9	43.4	-42.9	-2.9	32.7	-33.8	2.4	-15.4	47.6
	1.594	-6.260	2.959	-2.922	-0.201	2.228	-2.302	0.161	-1.051	3.250
c_O	167	306	322	184	303	319	206	283	318	201
	(11.4)	(20.8)	(21.9)	(12.5)	(20.6)	(21.7)	(14.0)	(19.3)	(21.7)	(13.7)
c_E	189.4	295.0	326.6	189.4	295.0	326.6	189.4	294.6	326.1	189.2
	(12.9)	(20.1)	(22.2)	(12.9)	(20.1)	(22.2)	(12.9)	(20.1)	(22.2)	(12.9)
	-42.1	22.3	-9.1	-10.7	16.1	-15.0	34.6	-22.8	-16.0	24.4
	-2.866	1.520	-0.619	-0.728	1.099	-1.020	2.355	-1.556	-1.095	1.665
N	1468	1468	1468	1468	1468	1468	1468	1466	1466	1466
X2	3.7	7.6	2.2	3.8	1.1	1.8	7.8	0.9	1.3	5.0
Gvalue	3.8	7.7	2.2	3.9	1.1	1.8	7.8	0.9	1.2	4.9
Gv/N	0.257	0.528	0.147	0.263	0.073	0.123	0.531	0.059	0.085	0.337
Prob	0.28766	0.05154	0.53985	0.27634	0.78239	0.61459	0.05053	0.83550	0.74223	0.17571

Bjap_USDA110
5UTR:

	50	49	48	47	46	45	44	43	42	41
a_O	1625	1682	1528	1701	1651	1567	1747	1696	1505	1765
	(19.5)	(20.2)	(18.4)	(20.5)	(19.9)	(18.8)	(21.0)	(20.4)	(18.1)	(21.2)
a_E	1696.4	1696.4	1696.4	1696.4	1696.4	1696.4	1696.4	1696.4	1696.4	1696.4
	(20.4)	(20.4)	(20.4)	(20.4)	(20.4)	(20.4)	(20.4)	(20.4)	(20.4)	(20.4)
	-139.7	-28.6	-319.4	9.3	-89.5	-248.6	102.8	-0.7	-360.3	140.0
	-1.680	-0.344	-3.841	0.112	-1.076	-2.989	1.236	-0.009	-4.332	1.683
t_O	1542	1720	1690	1498	1684	1675	1452	1750	1671	1462
	(18.5)	(20.7)	(20.3)	(18.0)	(20.2)	(20.1)	(17.5)	(21.0)	(20.1)	(17.6)
t_E	1601.6	1601.6	1601.6	1601.6	1601.6	1601.6	1601.6	1601.6	1601.6	1601.6
	(19.3)	(19.3)	(19.3)	(19.3)	(19.3)	(19.3)	(19.3)	(19.3)	(19.3)	(19.3)
	-116.9	245.4	181.7	-200.3	169.1	150.2	-284.7	310.2	141.9	-266.6
	-1.405	2.951	2.184	-2.408	2.033	1.806	-3.423	3.730	1.706	-3.205
g_O	2667	2431	2496	2591	2392	2428	2563	2326	2553	2532
	(32.1)	(29.2)	(30.0)	(31.2)	(28.8)	(29.2)	(30.8)	(28.0)	(30.7)	(30.4)
g_E	2507.7	2507.7	2507.7	2507.7	2507.7	2507.7	2507.7	2507.7	2507.7	2507.7
	(30.2)	(30.2)	(30.2)	(30.2)	(30.2)	(30.2)	(30.2)	(30.2)	(30.2)	(30.2)
	328.6	-151.0	-23.3	169.4	-225.9	-156.8	111.9	-349.9	91.5	48.9
	3.950	-1.815	-0.280	2.037	-2.717	-1.885	1.345	-4.207	1.100	0.588
c_O	2483	2484	2603	2527	2590	2647	2555	2545	2588	2558
	(29.9)	(29.9)	(31.3)	(30.4)	(31.1)	(31.8)	(30.7)	(30.6)	(31.1)	(30.8)
c_E	2511.4	2511.4	2511.4	2511.4	2511.4	2511.4	2511.4	2511.4	2511.4	2511.4
	(30.2)	(30.2)	(30.2)	(30.2)	(30.2)	(30.2)	(30.2)	(30.2)	(30.2)	(30.2)
	-56.5	-54.5	186.5	31.3	159.6	278.4	88.0	67.7	155.5	94.1
	-0.679	-0.655	2.242	0.376	1.919	3.347	1.058	0.813	1.870	1.131
N	8317	8317	8317	8317	8317	8317	8317	8317	8317	8317
X2	15.7	11.5	25.0	9.6	13.3	23.1	17.5	27.4	27.8	16.0
Gvalue	15.5	11.3	25.4	9.7	13.3	23.2	17.9	27.3	28.5	16.4
Gv/N	0.187	0.136	0.306	0.117	0.159	0.279	0.215	0.328	0.343	0.197
Prob	0.00142	0.01001	0.00001	0.02136	0.00412	0.00004	0.00046	0.00001	0.00000	0.00095

	40	39	38	37	36	35	34	33	32	31
a_O	1736	1510	1603	1742	1566	1642	1675	1503	1781	1732
	(20.9)	(18.2)	(19.3)	(20.9)	(18.8)	(19.7)	(20.1)	(18.1)	(21.4)	(20.8)
a_E	1696.4	1696.4	1696.4	1696.4	1696.4	1696.4	1696.4	1696.4	1696.4	1696.4
	(20.4)	(20.4)	(20.4)	(20.4)	(20.4)	(20.4)	(20.4)	(20.4)	(20.4)	(20.4)
	80.2	-351.5	-181.5	92.5	-250.4	-107.0	-42.5	-363.8	173.4	72.0
	0.964	-4.226	-2.182	1.112	-3.011	-1.286	-0.511	-4.374	2.085	0.866
t_O	1732	1582	1480	1726	1689	1581	1797	1709	1475	1724
	(20.8)	(19.0)	(17.8)	(20.8)	(20.3)	(19.0)	(21.6)	(20.5)	(17.7)	(20.7)
t_E	1601.6	1601.6	1601.6	1601.6	1601.6	1601.6	1601.6	1601.6	1601.6	1601.6
	(19.3)	(19.3)	(19.3)	(19.3)	(19.3)	(19.3)	(19.3)	(19.3)	(19.3)	(19.3)
	271.2	-38.9	-233.6	258.3	179.6	-40.9	413.8	221.9	-242.8	254.0
	3.261	-0.467	-2.809	3.106	2.159	-0.491	4.976	2.668	-2.920	3.054
g_O	2328	2584	2609	2323	2454	2601	2386	2472	2411	2312
	(28.0)	(31.1)	(31.4)	(27.9)	(29.5)	(31.3)	(28.7)	(29.7)	(29.0)	(27.8)
g_E	2507.7	2507.7	2507.7	2507.7	2507.7	2507.7	2507.7	2507.7	2507.7	2507.7
	(30.2)	(30.2)	(30.2)	(30.2)	(30.2)	(30.2)	(30.2)	(30.2)	(30.2)	(30.2)
	-346.2	154.9	206.7	-355.4	-106.2	190.1	-237.4	-70.8	-189.6	-375.7
	-4.162	1.863	2.485	-4.273	-1.277	2.285	-2.854	-0.852	-2.279	-4.517
c_O	2521	2641	2625	2526	2608	2493	2459	2633	2650	2549
	(30.3)	(31.8)	(31.6)	(30.4)	(31.4)	(30.0)	(29.6)	(31.7)	(31.9)	(30.6)
c_E	2511.4	2511.4	2511.4	2511.4	2511.4	2511.4	2511.4	2511.4	2511.4	2511.4
	(30.2)	(30.2)	(30.2)	(30.2)	(30.2)	(30.2)	(30.2)	(30.2)	(30.2)	(30.2)
	19.2	265.8	232.3	29.3	196.9	-36.7	-103.7	249.0	284.7	75.8
	0.231	3.196	2.793	0.352	2.367	-0.441	-1.247	2.994	3.423	0.911
N	8317	8317	8317	8317	8317	8317	8317	8317	8317	8317
X2	24.5	29.7	23.6	24.6	19.7	5.6	31.1	35.6	25.6	25.9
Gvalue	24.5	30.4	23.8	24.7	19.8	5.6	30.3	36.3	25.7	26.1
Gv/N	0.295	0.365	0.286	0.297	0.238	0.067	0.364	0.436	0.309	0.314
Prob	0.00002	0.00000	0.00003	0.00002	0.00019	0.13323	0.00000	0.00000	0.00001	0.00001

	30	29	28	27	26	25	24	23	22	21
a_O	1558	1724	1791	1656	1850	1844	1642	1806	1761	1645
	(18.7)	(20.7)	(21.5)	(19.9)	(22.2)	(22.2)	(19.7)	(21.7)	(21.2)	(19.8)
a_E	1696.4	1696.4	1696.4	1696.4	1696.4	1696.4	1696.4	1696.4	1696.4	1696.4
	(20.4)	(20.4)	(20.4)	(20.4)	(20.4)	(20.4)	(20.4)	(20.4)	(20.4)	(20.4)
	-265.1	55.7	194.4	-79.8	320.8	307.8	-107.0	226.2	131.7	-101.2
	-3.188	0.670	2.338	-0.959	3.857	3.700	-1.286	2.720	1.583	-1.216
t_O	1717	1523	1714	1564	1495	1660	1584	1475	1729	1594
	(20.6)	(18.3)	(20.6)	(18.8)	(18.0)	(20.0)	(19.0)	(17.7)	(20.8)	(19.2)
t_E	1601.6	1601.6	1601.6	1601.6	1601.6	1601.6	1601.6	1601.6	1601.6	1601.6
	(19.3)	(19.3)	(19.3)	(19.3)	(19.3)	(19.3)	(19.3)	(19.3)	(19.3)	(19.3)
	239.0	-153.2	232.6	-74.2	-205.9	119.0	-34.9	-242.8	264.8	-15.1
	2.874	-1.842	2.797	-0.892	-2.475	1.431	-0.420	-2.920	3.183	-0.181
g_O	2332	2512	2324	2411	2445	2232	2394	2398	2204	2433
	(28.0)	(30.2)	(27.9)	(29.0)	(29.4)	(26.8)	(28.8)	(28.8)	(26.5)	(29.3)
g_E	2507.7	2507.7	2507.7	2507.7	2507.7	2507.7	2507.7	2507.7	2507.7	2507.7
	(30.2)	(30.2)	(30.2)	(30.2)	(30.2)	(30.2)	(30.2)	(30.2)	(30.2)	(30.2)
	-338.7	8.7	-353.6	-189.6	-123.8	-519.9	-222.1	-214.5	-569.0	-147.1
	-4.073	0.104	-4.251	-2.279	-1.488	-6.251	-2.671	-2.579	-6.841	-1.769
c_O	2710	2558	2488	2686	2527	2581	2697	2638	2623	2645
	(32.6)	(30.8)	(29.9)	(32.3)	(30.4)	(31.0)	(32.4)	(31.7)	(31.5)	(31.8)
c_E	2511.4	2511.4	2511.4	2511.4	2511.4	2511.4	2511.4	2511.4	2511.4	2511.4
	(30.2)	(30.2)	(30.2)	(30.2)	(30.2)	(30.2)	(30.2)	(30.2)	(30.2)	(30.2)
	412.5	94.1	-46.6	361.1	31.3	141.1	384.6	259.5	228.1	274.2
	4.960	1.131	-0.560	4.341	0.376	1.697	4.624	3.120	2.742	3.297
N	8317	8317	8317	8317	8317	8317	8317	8317	8317	8317
X2	47.6	5.2	26.8	17.7	22.7	47.2	20.8	28.3	54.3	10.9
Gvalue	47.6	5.2	26.9	17.5	22.4	48.0	20.6	28.4	55.6	10.8
Gv/N	0.573	0.063	0.324	0.210	0.270	0.577	0.247	0.341	0.668	0.130
Prob	0.00000	0.15554	0.00001	0.00056	0.00005	0.00000	0.00013	0.00000	0.00000	0.01263

	20	19	18	17	16	15	14	13	12	11
a_O	1986	1955	1738	2005	1975	1777	1989	2201	1909	2189
	(23.9)	(23.5)	(20.9)	(24.1)	(23.7)	(21.4)	(23.9)	(26.5)	(23.0)	(26.3)
a_E	1696.4	1696.4	1696.4	1696.4	1696.4	1696.4	1696.4	1696.4	1696.4	1696.4
	(20.4)	(20.4)	(20.4)	(20.4)	(20.4)	(20.4)	(20.4)	(20.4)	(20.4)	(20.4)
	626.1	554.8	84.3	670.3	600.7	165.0	633.1	1146.4	450.9	1116.2
	7.528	6.671	1.013	8.059	7.223	1.984	7.612	13.784	5.421	13.421
t_O	1503	1707	1658	1518	1755	1711	1347	1510	1463	1028
	(18.1)	(20.5)	(19.9)	(18.3)	(21.1)	(20.6)	(16.2)	(18.2)	(17.6)	(12.4)

t_E	1601.6	1601.6	1601.6	1601.6	1601.6	1601.6	1601.6	1601.6	1601.6	1601.6
	(19.3)	(19.3)	(19.3)	(19.3)	(19.3)	(19.3)	(19.3)	(19.3)	(19.3)	(19.3)
	-190.9	217.7	114.9	-162.7	321.1	226.2	-466.3	-177.8	-264.8	-911.6
	-2.296	2.617	1.381	-1.956	3.861	2.720	-5.607	-2.138	-3.183	-10.960
g_O	2319	2190	2330	2393	2180	2440	2644	2514	3083	3493
	(27.9)	(26.3)	(28.0)	(28.8)	(26.2)	(29.3)	(31.8)	(30.2)	(37.1)	(42.0)
g_E	2507.7	2507.7	2507.7	2507.7	2507.7	2507.7	2507.7	2507.7	2507.7	2507.7
	(30.2)	(30.2)	(30.2)	(30.2)	(30.2)	(30.2)	(30.2)	(30.2)	(30.2)	(30.2)
	-362.8	-593.3	-342.5	-224.0	-610.5	-133.5	279.9	12.7	1273.6	2315.2
	-4.362	-7.134	-4.118	-2.694	-7.341	-1.605	3.366	0.152	15.313	27.837
c_O	2509	2465	2591	2401	2407	2389	2337	2092	1862	1607
	(30.2)	(29.6)	(31.2)	(28.9)	(28.9)	(28.7)	(28.1)	(25.2)	(22.4)	(19.3)
c_E	2511.4	2511.4	2511.4	2511.4	2511.4	2511.4	2511.4	2511.4	2511.4	2511.4
	(30.2)	(30.2)	(30.2)	(30.2)	(30.2)	(30.2)	(30.2)	(30.2)	(30.2)	(30.2)
	-4.8	-91.9	161.7	-215.9	-204.4	-238.7	-336.4	-764.5	-1114.2	-1435.0
	-0.058	-1.105	1.944	-2.596	-2.458	-2.870	-4.045	-9.192	-13.396	-17.253

N	8317	8317	8317	8317	8317	8317	8317	8317	8317	8317
X2	69.7	87.5	18.1	70.6	107.6	19.1	110.5	225.4	338.6	1061.3
Gvalue	67.6	87.3	18.4	67.7	106.9	19.0	110.3	216.8	345.5	1084.9
Gv/N	0.813	1.049	0.221	0.814	1.286	0.228	1.326	2.606	4.154	13.044
Prob	0.00000	0.00000	0.00037	0.00000	0.00000	0.00027	0.00000	0.00000	0.00000	0.00000

	10	9	8	7	6	5	4	3	2	1
a_O	2067	2051	2373	2018	1906	1937	1867	1811	1709	1569
	(24.9)	(24.7)	(28.5)	(24.3)	(22.9)	(23.3)	(22.4)	(21.8)	(20.5)	(18.9)
a_E	1696.4	1696.4	1696.4	1696.4	1696.4	1696.4	1696.4	1696.4	1696.4	1696.4
	(20.4)	(20.4)	(20.4)	(20.4)	(20.4)	(20.4)	(20.4)	(20.4)	(20.4)	(20.4)
	816.9	778.7	1593.1	700.7	444.2	513.9	357.9	236.8	25.4	-244.9
	9.822	9.363	19.154	8.425	5.340	6.179	4.303	2.848	0.305	-2.945
t_O	1136	1187	1121	1620	1650	1481	1692	1380	1522	857
	(13.7)	(14.3)	(13.5)	(19.5)	(19.8)	(17.8)	(20.3)	(16.6)	(18.3)	(10.3)
t_E	1601.6	1601.6	1601.6	1601.6	1601.6	1601.6	1601.6	1601.6	1601.6	1601.6
	(19.3)	(19.3)	(19.3)	(19.3)	(19.3)	(19.3)	(19.3)	(19.3)	(19.3)	(19.3)
	-780.3	-711.1	-799.8	37.1	98.3	-231.8	185.9	-410.9	-155.1	-1071.8
	-9.383	-8.550	-9.617	0.446	1.182	-2.787	2.235	-4.941	-1.865	-12.886
g_O	3589	3539	3121	2660	2581	2515	2181	2856	1952	2196
	(43.2)	(42.6)	(37.5)	(32.0)	(31.0)	(30.2)	(26.2)	(34.3)	(23.5)	(26.4)
g_E	2507.7	2507.7	2507.7	2507.7	2507.7	2507.7	2507.7	2507.7	2507.7	2507.7
	(30.2)	(30.2)	(30.2)	(30.2)	(30.2)	(30.2)	(30.2)	(30.2)	(30.2)	(30.2)
	2573.4	2438.3	1365.7	313.7	148.8	14.7	-608.8	742.9	-978.0	-582.9
	30.942	29.317	16.421	3.772	1.789	0.176	-7.320	8.933	-11.759	-7.009
c_O	1525	1540	1702	2019	2180	2384	2577	2270	3134	3695
	(18.3)	(18.5)	(20.5)	(24.3)	(26.2)	(28.7)	(31.0)	(27.3)	(37.7)	(44.4)
c_E	2511.4	2511.4	2511.4	2511.4	2511.4	2511.4	2511.4	2511.4	2511.4	2511.4
	(30.2)	(30.2)	(30.2)	(30.2)	(30.2)	(30.2)	(30.2)	(30.2)	(30.2)	(30.2)
	-1521.5	-1506.3	-1324.3	-881.2	-617.0	-248.2	132.9	-458.8	1388.2	2853.6
	-18.294	-18.111	-15.923	-10.596	-7.419	-2.984	1.598	-5.517	16.691	34.310
N	8317	8317	8317	8317	8317	8317	8317	8317	8317	8317
X2	1070.0	981.3	824.9	167.0	73.2	49.7	66.5	110.0	281.5	952.3
Gvalue	1088.5	999.6	834.7	170.3	74.3	48.5	67.9	110.0	280.5	954.0
Gv/N	13.088	12.018	10.036	2.048	0.893	0.584	0.816	1.323	3.372	11.471
Prob	0.00000	0.00000	0.00000	0.00000	0.00000	0.00000	0.00000	0.00000	0.00000	0.00000

CDS_INITIAL:

	1	2	3	4	5	6	7	8	9	10
a_O	6204	0	0	3309	1838	1348	2297	2356	1391	2157
	(74.6)	(0.0)	(0.0)	(39.8)	(22.1)	(16.2)	(27.6)	(28.3)	(16.7)	(25.9)
a_E	1823.5	2043.2	552.8	1823.5	2043.2	552.8	1823.5	2043.2	552.8	1823.5
	(21.9)	(24.6)	(6.6)	(21.9)	(24.6)	(6.6)	(21.9)	(24.6)	(6.6)	(21.9)
	15193.0	0.0	0.0	3943.7	-389.1	2403.0	1060.6	671.2	2567.0	724.7
	182.674	0.000	0.000	47.417	-4.678	28.892	12.752	8.071	30.864	8.713
t_O	31	8317	1	951	1587	1360	1111	1885	1509	1250
	(0.4)	(100.0)	(0.0)	(11.4)	(19.1)	(16.4)	(13.4)	(22.7)	(18.1)	(15.0)
t_E	1084.0	2390.1	895.1	1084.0	2390.1	895.1	1084.0	2390.1	895.1	1084.0
	(13.0)	(28.7)	(10.8)	(13.0)	(28.7)	(10.8)	(13.0)	(28.7)	(10.8)	(13.0)
	-220.4	20741.7	-13.6	-248.9	-1299.8	1137.7	54.7	-895.1	1576.2	356.2
	-2.650	249.389	-0.163	-2.993	-15.628	13.680	0.658	-10.762	18.951	4.283
g_O	2082	0	8316	2009	1826	2538	2758	1731	2614	2478
	(25.0)	(0.0)	(100.0)	(24.2)	(22.0)	(30.5)	(33.2)	(20.8)	(31.4)	(29.8)
g_E	3240.8	1637.2	3070.7	3240.8	1637.2	3070.7	3240.8	1637.2	3070.7	3240.8
	(39.0)	(19.7)	(36.9)	(39.0)	(19.7)	(36.9)	(39.0)	(19.7)	(36.9)	(39.0)
	-1842.5	0.0	16570.1	-1921.3	398.7	-967.1	-889.8	193.0	-841.8	-1330.0
	-22.153	0.000	199.232	-23.101	4.793	-11.628	-10.698	2.320	-10.122	-15.991
c_O	0	0	0	2048	3066	3071	2151	2345	2803	2432
	(0.0)	(0.0)	(0.0)	(24.6)	(36.9)	(36.9)	(25.9)	(28.2)	(33.7)	(29.2)
c_E	2168.8	2246.5	3798.4	2168.8	2246.5	3798.4	2168.8	2246.5	3798.4	2168.8
	(26.1)	(27.0)	(45.7)	(26.1)	(27.0)	(45.7)	(26.1)	(27.0)	(45.7)	(26.1)
	0.0	0.0	0.0	-234.7	1907.1	-1305.6	-35.4	201.3	-1703.5	557.2
	0.000	0.000	0.000	-2.822	22.930	-15.698	-0.426	2.420	-20.483	6.699
N	8317	8317	8317	8317	8317	8317	8317	8317	8317	8317
X2	14129.4	20623.7	14204.2	1701.4	611.2	1616.8	195.7	164.3	2020.5	297.9
Gvalue	13130.1	20741.7	16556.5	1538.8	616.9	1268.0	190.1	170.3	1597.8	308.1
Gv/N	157.871	249.389	199.068	18.501	7.417	15.246	2.286	2.048	19.211	3.704
Prob	1.00000	1.00000	1.00000	1.00000	0.00000	0.00000	0.00000	0.00000	1.00000	0.00000

	11	12	13	14	15	16	17	18	19	20
a_O	1891	1262	2208	1937	1251	2237	1979	1294	2221	2000
	(22.7)	(15.2)	(26.5)	(23.3)	(15.0)	(26.9)	(23.8)	(15.6)	(26.7)	(24.0)
a_E	2043.2	552.8	1823.5	2043.2	552.8	1823.5	2043.2	552.8	1823.5	2043.2
	(24.6)	(6.6)	(21.9)	(24.6)	(6.6)	(21.9)	(24.6)	(6.6)	(21.9)	(24.6)
	-292.8	2083.3	845.0	-206.8	2043.2	914.5	-126.3	2200.9	876.0	-85.5
	-3.520	25.048	10.160	-2.486	24.567	10.995	-1.519	26.463	10.533	-1.028
t_O	2337	1592	1281	2365	1475	1269	2389	1474	1181	2359
	(28.1)	(19.1)	(15.4)	(28.4)	(17.7)	(15.3)	(28.7)	(17.7)	(14.2)	(28.4)
t_E	2390.1	895.1	1084.0	2390.1	895.1	1084.0	2390.1	895.1	1084.0	2390.1
	(28.7)	(10.8)	(13.0)	(28.7)	(10.8)	(13.0)	(28.7)	(10.8)	(13.0)	(28.7)
	-105.1	1833.3	427.8	-50.0	1473.4	400.0	-2.3	1470.4	202.5	-61.9
	-1.264	22.043	5.144	-0.602	17.716	4.809	-0.028	17.680	2.434	-0.744
g_O	1632	2515	2588	1572	2409	2524	1630	2312	2726	1612
	(19.6)	(30.2)	(31.1)	(18.9)	(29.0)	(30.3)	(19.6)	(27.8)	(32.8)	(19.4)
g_E	1637.2	3070.7	3240.8	1637.2	3070.7	3240.8	1637.2	3070.7	3240.8	1637.2
	(19.7)	(36.9)	(39.0)	(19.7)	(36.9)	(39.0)	(19.7)	(36.9)	(39.0)	(19.7)
	-10.3	-1004.1	-1164.2	-127.7	-1169.3	-1261.8	-14.3	-1312.3	-943.1	-49.9
	-0.124	-12.073	-13.998	-1.535	-14.059	-15.172	-0.172	-15.778	-11.339	-0.600

```
c_O       2457    2948    2240    2443    3182    2287    2319    3237    2189    2346
        ( 29.5) ( 35.4) ( 26.9) ( 29.4) ( 38.3) ( 27.5) ( 27.9) ( 38.9) ( 26.3) ( 28.2)
c_E     2246.5  3798.4  2168.8  2246.5  3798.4  2168.8  2246.5  3798.4  2168.8  2246.5
        ( 27.0) ( 45.7) ( 26.1) ( 27.0) ( 45.7) ( 26.1) ( 27.0) ( 45.7) ( 26.1) ( 27.0)
         440.1 -1494.3   144.8   409.7 -1126.8   242.8   147.3 -1035.3    40.6   203.3
         5.292 -17.967   1.741   4.926 -13.548   2.919   1.771 -12.448   0.489   2.445

N         8317    8317    8317    8317    8317    8317    8317    8317    8317    8317
X2        32.3  1743.2   250.7    25.6  1500.0   290.3     4.4  1638.4   177.3     6.1
Gvalue    32.0  1418.2   253.4    25.2  1220.5   295.4     4.4  1323.8   176.1     6.1
Gv/N     0.384  17.052   3.047   0.303  14.675   3.551   0.053  15.916   2.117   0.073
Prob   0.00000 0.00000 0.00000 0.00001 0.00000 0.00000 0.22276 0.00000 0.00000 0.10876
       -------------------------------------------------------------------------------
            21      22      23      24      25      26      27      28      29      30
       -------------------------------------------------------------------------------
a_O       1192    2027    1953    1064    1874    1775    1012    1847    1820    1011
        ( 14.3) ( 24.4) ( 23.5) ( 12.8) ( 22.5) ( 21.3) ( 12.2) ( 22.2) ( 21.9) ( 12.2)
a_E      552.8  1823.5  2043.2   552.8  1823.5  2043.2   552.8  1823.5  2043.2   552.8
        (  6.6) ( 21.9) ( 24.6) (  6.6) ( 21.9) ( 24.6) (  6.6) ( 21.9) ( 24.6) (  6.6)
        1831.7   429.0  -176.3  1393.3   102.5  -499.5  1223.8    47.4  -421.1  1220.5
        22.023   5.158  -2.120  16.752   1.232  -6.006  14.714   0.569  -5.063  14.675
t_O       1370    1167    2288    1365    1175    2252    1293    1115    2341    1347
        ( 16.5) ( 14.0) ( 27.5) ( 16.4) ( 14.1) ( 27.1) ( 15.5) ( 13.4) ( 28.1) ( 16.2)
t_E      895.1  1084.0  2390.1   895.1  1084.0  2390.1   895.1  1084.0  2390.1   895.1
        ( 10.8) ( 13.0) ( 28.7) ( 10.8) ( 13.0) ( 28.7) ( 10.8) ( 13.0) ( 28.7) ( 10.8)
        1166.2   172.2  -199.9  1151.9   189.5  -268.2   951.0    62.9   -97.3  1101.0
        14.022   2.071  -2.403  13.850   2.278  -3.224  11.435   0.756  -1.170  13.238
g_O       2460    2839    1647    2520    2942    1794    2609    3100    1764    2558
        ( 29.6) ( 34.1) ( 19.8) ( 30.3) ( 35.4) ( 21.6) ( 31.4) ( 37.3) ( 21.2) ( 30.8)
g_E     3070.7  3240.8  1637.2  3070.7  3240.8  1637.2  3070.7  3240.8  1637.2  3070.7
        ( 36.9) ( 39.0) ( 19.7) ( 36.9) ( 39.0) ( 19.7) ( 36.9) ( 39.0) ( 19.7) ( 36.9)
       -1091.0  -751.5    19.7  -996.1  -569.1   328.2  -850.2  -275.3   263.3  -934.6
       -13.117  -9.036   0.237 -11.977  -6.843   3.947 -10.223  -3.311   3.165 -11.237
c_O       3295    2284    2429    3368    2326    2496    3403    2255    2392    3401
        ( 39.6) ( 27.5) ( 29.2) ( 40.5) ( 28.0) ( 30.0) ( 40.9) ( 27.1) ( 28.8) ( 40.9)
c_E     3798.4  2168.8  2246.5  3798.4  2168.8  2246.5  3798.4  2168.8  2246.5  3798.4
        ( 45.7) ( 26.1) ( 27.0) ( 45.7) ( 26.1) ( 27.0) ( 45.7) ( 26.1) ( 27.0) ( 45.7)
        -936.8   236.5   379.4  -810.0   325.6   525.7  -748.0   175.8   300.2  -751.6
       -11.264   2.843   4.562  -9.739   3.915   6.321  -8.994   2.114   3.610  -9.037

N         8317    8317    8317    8317    8317    8317    8317    8317    8317    8317
X2      1179.1    85.0    23.2   866.8    48.0    85.9   668.8    10.7    44.6   735.0
Gvalue   970.1    86.1    23.0   739.1    48.4    86.3   576.5    10.8    45.1   635.3
Gv/N    11.663   1.036   0.276   8.886   0.582   1.038   6.932   0.130   0.543   7.639
Prob   0.00000 0.00000 0.00004 0.00000 0.00000 0.00000 0.00000 0.01303 0.00000 0.00000
       -------------------------------------------------------------------------------
            31      32      33      34      35      36      37      38      39      40
       -------------------------------------------------------------------------------
a_O       1919    1783     962    1798    1752     949    1776    1736     896    1770
        ( 23.1) ( 21.4) ( 11.6) ( 21.6) ( 21.1) ( 11.4) ( 21.4) ( 20.9) ( 10.8) ( 21.3)
a_E     1823.5  2043.2   552.8  1823.5  2043.2   552.8  1823.5  2043.2   552.8  1823.5
        ( 21.9) ( 24.6) (  6.6) ( 21.9) ( 24.6) (  6.6) ( 21.9) ( 24.6) (  6.6) ( 21.9)
         196.0  -485.7  1065.8   -50.6  -538.8  1025.6   -93.7  -565.7   865.3  -105.4
         2.356  -5.840  12.815  -0.608  -6.478  12.331  -1.126  -6.802  10.404  -1.267
t_O       1124    2374    1283    1139    2419    1223    1183    2291    1234    1139
        ( 13.5) ( 28.5) ( 15.4) ( 13.7) ( 29.1) ( 14.7) ( 14.2) ( 27.5) ( 14.8) ( 13.7)
t_E     1084.0  2390.1   895.1  1084.0  2390.1   895.1  1084.0  2390.1   895.1  1084.0
        ( 13.0) ( 28.7) ( 10.8) ( 13.0) ( 28.7) ( 10.8) ( 13.0) ( 28.7) ( 10.8) ( 13.0)
          81.5   -32.2   923.8   112.8    58.1   763.4   206.8  -194.1   792.4   112.8
         0.980  -0.387  11.107   1.356   0.698   9.179   2.487  -2.334   9.527   1.356
g_O       3023    1750    2764    3094    1747    2687    3080    1874    2781    3103
        ( 36.3) ( 21.0) ( 33.2) ( 37.2) ( 21.0) ( 32.3) ( 37.0) ( 22.5) ( 33.4) ( 37.3)
g_E     3240.8  1637.2  3070.7  3240.8  1637.2  3070.7  3240.8  1637.2  3070.7  3240.8
        ( 39.0) ( 19.7) ( 36.9) ( 39.0) ( 19.7) ( 36.9) ( 39.0) ( 19.7) ( 36.9) ( 39.0)
        -420.6   233.3  -581.7  -286.8   226.9  -717.3  -313.4   506.4  -551.2  -269.6
        -5.057   2.805  -6.994  -3.448   2.728  -8.625  -3.769   6.089  -6.627  -3.242
c_O       2251    2410    3308    2286    2399    3458    2278    2416    3406    2305
        ( 27.1) ( 29.0) ( 39.8) ( 27.5) ( 28.8) ( 41.6) ( 27.4) ( 29.0) ( 41.0) ( 27.7)
c_E     2168.8  2246.5  3798.4  2168.8  2246.5  3798.4  2168.8  2246.5  3798.4  2168.8
        ( 26.1) ( 27.0) ( 45.7) ( 26.1) ( 27.0) ( 45.7) ( 26.1) ( 27.0) ( 45.7) ( 26.1)
         167.5   338.6  -914.5   240.7   315.1  -649.3   223.9   351.5  -742.7   280.8
         2.014   4.071 -10.995   2.894   3.789  -7.806   2.692   4.226  -8.930   3.377

N         8317    8317    8317    8317    8317    8317    8317    8317    8317    8317
X2        24.2    52.9   564.8    16.1    59.6   482.4    23.8    97.4   409.2    18.8
Gvalue    24.4    54.0   493.4    16.1    61.3   422.4    23.6    98.1   363.8    18.7
Gv/N     0.294   0.649   5.932   0.193   0.737   5.079   0.283   1.179   4.375   0.224
Prob   0.00002 0.00000 0.00000 0.00109 0.00000 0.00000 0.00003 0.00000 0.00000 0.00032
       -------------------------------------------------------------------------------
            41      42      43      44      45      46      47      48      49      50
       -------------------------------------------------------------------------------
a_O       1772     913    1758    1690     872    1826    1667     888    1781    1674
        ( 21.3) ( 11.0) ( 21.1) ( 20.3) ( 10.5) ( 22.0) ( 20.0) ( 10.7) ( 21.4) ( 20.1)
a_E     2043.2   552.8  1823.5  2043.2   552.8  1823.5  2043.2   552.8  1823.5  2043.2
        ( 24.6) (  6.6) ( 21.9) ( 24.6) (  6.6) ( 21.9) ( 24.6) (  6.6) ( 21.9) ( 24.6)
        -504.7   916.1  -128.6  -641.5   794.8     5.1  -678.4   841.7   -83.9  -667.2
        -6.068  11.014  -1.546  -7.713   9.556   0.061  -8.157  10.120  -1.009  -8.023
t_O       2350    1235    1200    2366    1236    1093    2432    1086    1143    2433
        ( 28.3) ( 14.8) ( 14.4) ( 28.4) ( 14.9) ( 13.1) ( 29.2) ( 13.1) ( 13.7) ( 29.3)
t_E     2390.1   895.1  1084.0  2390.1   895.1  1084.0  2390.1   895.1  1084.0  2390.1
        ( 28.7) ( 10.8) ( 13.0) ( 28.7) ( 10.8) ( 13.0) ( 28.7) ( 10.8) ( 13.0) ( 28.7)
         -79.6   795.0   244.0   -48.1   797.7    18.1    84.4   419.9   121.2    86.5
        -0.957   9.559   2.934  -0.578   9.591   0.218   1.015   5.048   1.457   1.040
g_O       1719    2703    3166    1786    2788    3210    1805    2866    3200    1762
        ( 20.7) ( 32.5) ( 38.1) ( 21.5) ( 33.5) ( 38.6) ( 21.7) ( 34.5) ( 38.5) ( 21.2)
g_E     1637.2  3070.7  3240.8  1637.2  3070.7  3240.8  1637.2  3070.7  3240.8  1637.2
        ( 19.7) ( 36.9) ( 39.0) ( 19.7) ( 36.9) ( 39.0) ( 19.7) ( 36.9) ( 39.0) ( 19.7)
         167.7  -689.5  -147.8   310.8  -538.5   -61.3   352.3  -395.4   -81.0   259.0
         2.016  -8.290  -1.777   3.737  -6.475  -0.737   4.236  -4.755  -0.974   3.114
c_O       2476    3466    2193    2475    3421    2188    2413    3477    2193    2448
        ( 29.8) ( 41.7) ( 26.4) ( 29.8) ( 41.1) ( 26.3) ( 29.0) ( 41.8) ( 26.4) ( 29.4)
c_E     2246.5  3798.4  2168.8  2246.5  3798.4  2168.8  2246.5  3798.4  2168.8  2246.5
        ( 27.0) ( 45.7) ( 26.1) ( 27.0) ( 45.7) ( 26.1) ( 27.0) ( 45.7) ( 26.1) ( 27.0)
         481.7  -634.7    48.7   479.5  -715.9    38.6   345.0  -614.7    48.7   420.6
         5.792  -7.632   0.586   5.765  -8.608   0.464   4.149  -7.391   0.586   5.057

N         8317    8317    8317    8317    8317    8317    8317    8317    8317    8317
X2        64.2   436.8    16.8    98.1   377.6     0.5    99.5   284.7     5.0    95.1
Gvalue    65.1   386.9    16.4   100.8   338.0     0.5   103.4   251.4     4.9    98.7
Gv/N     0.783   4.651   0.197   1.212   4.064   0.007   1.243   3.022   0.059   1.187
Prob   0.00000 0.00000 0.00095 0.00000 0.00000 0.90969 0.00000 0.00000 0.17640 0.00000
       -------------------------------------------------------------------------------
```

	51	52	53	54	55	56	57	58	59	60
a_O	839	1796	1709	813	1761	1770	789	1688	1764	787
	(10.1)	(21.6)	(20.5)	(9.8)	(21.2)	(21.3)	(9.5)	(20.3)	(21.2)	(9.5)
a_E	552.8	1823.5	2043.2	552.8	1823.5	2043.2	552.8	1823.5	2043.2	552.8
	(6.6)	(21.9)	(24.6)	(6.6)	(21.9)	(24.6)	(6.6)	(21.9)	(24.6)	(6.6)
	700.0	-54.5	-610.5	627.1	-122.8	-508.1	561.3	-260.6	-518.4	555.9
	8.416	-0.656	-7.340	7.540	-1.476	-6.109	6.749	-3.134	-6.233	6.684
t_O	1128	1089	2394	1139	1137	2481	1118	1107	2341	1116
	(13.6)	(13.1)	(28.8)	(13.7)	(13.7)	(29.8)	(13.4)	(13.3)	(28.1)	(13.4)
t_E	895.1	1084.0	2390.1	895.1	1084.0	2390.1	895.1	1084.0	2390.1	895.1
	(10.8)	(13.0)	(28.7)	(10.8)	(13.0)	(28.7)	(10.8)	(13.0)	(28.7)	(10.8)
	521.7	10.1	7.7	548.9	108.6	185.1	497.2	46.5	-97.3	492.3
	6.273	0.121	0.093	6.600	1.306	2.226	5.978	0.559	-1.170	5.919
g_O	2875	3196	1717	2952	3205	1702	2970	3309	1731	3032
	(34.6)	(38.4)	(20.6)	(35.5)	(38.5)	(20.5)	(35.7)	(39.8)	(20.8)	(36.5)
g_E	3070.7	3240.8	1637.2	3070.7	3240.8	1637.2	3070.7	3240.8	1637.2	3070.7
	(36.9)	(39.0)	(19.7)	(36.9)	(39.0)	(19.7)	(36.9)	(39.0)	(19.7)	(36.9)
	-378.6	-88.9	163.5	-232.7	-71.2	132.2	-198.1	137.9	193.0	-76.9
	-4.553	-1.069	1.966	-2.798	-0.856	1.590	-2.381	1.658	2.320	-0.925
c_O	3475	2236	2497	3413	2214	2364	3440	2213	2481	3382
	(41.8)	(26.9)	(30.0)	(41.0)	(26.6)	(28.4)	(41.4)	(26.6)	(29.8)	(40.7)
c_E	3798.4	2168.8	2246.5	3798.4	2168.8	2246.5	3798.4	2168.8	2246.5	3798.4
	(45.7)	(26.1)	(27.0)	(45.7)	(26.1)	(27.0)	(45.7)	(26.1)	(27.0)	(45.7)
	-618.4	136.5	528.0	-730.2	91.4	241.0	-681.8	89.4	492.7	-785.3
	-7.435	1.641	6.348	-8.780	1.099	2.898	-8.197	1.074	5.924	-9.442
N	8317	8317	8317	8317	8317	8317	8317	8317	8317	8317
X2	248.7	3.1	86.5	232.6	6.1	48.7	193.5	12.9	69.0	199.8
Gvalue	224.7	3.1	88.7	213.0	6.0	50.3	178.6	13.1	70.0	186.0
Gv/N	2.701	0.038	1.066	2.561	0.073	0.604	2.148	0.158	0.841	2.236
Prob	0.00000	0.37298	0.00000	0.00000	0.10922	0.00000	0.00000	0.00436	0.00000	0.00000

	61	62	63	64	65	66	67	68	69	70
a_O	1702	1760	752	1711	1703	790	1704	1813	773	1686
	(20.5)	(21.2)	(9.0)	(20.6)	(20.5)	(9.5)	(20.5)	(21.8)	(9.3)	(20.3)
a_E	1823.5	2043.2	552.8	1823.5	2043.2	552.8	1823.5	2043.2	552.8	1823.5
	(21.9)	(24.6)	(6.6)	(21.9)	(24.6)	(6.6)	(21.9)	(24.6)	(6.6)	(21.9)
	-234.7	-525.2	462.7	-217.9	-620.3	564.0	-230.9	-433.4	518.2	-264.3
	-2.821	-6.315	5.564	-2.619	-7.458	6.781	-2.777	-5.211	6.231	-3.178
t_O	1098	2293	1138	1074	2426	1097	1067	2328	1102	1084
	(13.2)	(27.6)	(13.7)	(12.9)	(29.2)	(13.2)	(12.8)	(28.0)	(13.2)	(13.0)
t_E	1084.0	2390.1	895.1	1084.0	2390.1	895.1	1084.0	2390.1	895.1	1084.0
	(13.0)	(28.7)	(10.8)	(13.0)	(28.7)	(10.8)	(13.0)	(28.7)	(10.8)	(13.0)
	28.2	-190.3	546.4	-19.9	72.2	446.2	-33.7	-122.7	458.3	0.0
	0.339	-2.288	6.570	-0.239	0.869	5.365	-0.405	-1.475	5.510	0.000
g_O	3202	1755	3013	3285	1724	3032	3283	1758	2935	3338
	(38.5)	(21.1)	(36.2)	(39.5)	(20.7)	(36.5)	(39.5)	(21.1)	(35.3)	(40.1)
g_E	3240.8	1637.2	3070.7	3240.8	1637.2	3070.7	3240.8	1637.2	3070.7	3240.8
	(39.0)	(19.7)	(36.9)	(39.0)	(19.7)	(36.9)	(39.0)	(19.7)	(36.9)	(39.0)
	-77.1	244.0	-114.3	89.0	178.2	-76.9	85.0	250.4	-265.3	197.3
	-0.927	2.933	-1.374	1.071	2.143	-0.925	1.022	3.011	-3.190	2.373
c_O	2315	2509	3414	2247	2464	3398	2263	2418	3507	2209
	(27.8)	(30.2)	(41.0)	(27.0)	(29.6)	(40.9)	(27.2)	(29.1)	(42.2)	(26.6)
c_E	2168.8	2246.5	3798.4	2168.8	2246.5	3798.4	2168.8	2246.5	3798.4	2168.8
	(26.1)	(27.0)	(45.7)	(26.1)	(27.0)	(45.7)	(26.1)	(27.0)	(45.7)	(26.1)
	302.1	554.5	-728.4	159.2	455.4	-756.9	192.5	355.8	-559.8	81.2
	3.632	6.668	-8.758	1.915	5.476	-9.101	2.314	4.278	-6.730	0.976
N	8317	8317	8317	8317	8317	8317	8317	8317	8317	8317
X2	18.6	82.4	177.6	10.5	82.8	190.0	12.7	49.6	163.8	14.0
Gvalue	18.6	83.0	166.4	10.6	85.5	176.4	12.9	50.1	151.5	14.3
Gv/N	0.223	0.998	2.001	0.127	1.029	2.121	0.155	0.602	1.821	0.172
Prob	0.00034	0.00000	0.00000	0.01432	0.00000	0.00000	0.00496	0.00000	0.00000	0.00257

	71	72	73	74	75	76	77	78	79	80
a_O	1759	766	1744	1855	751	1676	1829	654	1713	1899
	(21.1)	(9.2)	(21.0)	(22.3)	(9.0)	(20.2)	(22.0)	(7.9)	(20.6)	(22.8)
a_E	2043.2	552.8	1823.5	2043.2	552.8	1823.5	2043.2	552.8	1823.5	2043.2
	(24.6)	(6.6)	(21.9)	(24.6)	(6.6)	(21.9)	(24.6)	(6.6)	(21.9)	(24.6)
	-526.9	499.6	-155.4	-358.5	460.1	-282.7	-405.1	219.8	-214.1	-278.0
	-6.335	6.007	-1.869	-4.310	5.532	-3.399	-4.871	2.643	-2.574	-3.342
t_O	2352	1075	1132	2413	1048	1143	2452	1043	1085	2387
	(28.3)	(12.9)	(13.6)	(29.0)	(12.6)	(13.7)	(29.5)	(12.5)	(13.0)	(28.7)
t_E	2390.1	895.1	1084.0	2390.1	895.1	1084.0	2390.1	895.1	1084.0	2390.1
	(28.7)	(10.8)	(13.0)	(28.7)	(10.8)	(13.0)	(28.7)	(10.8)	(13.0)	(28.7)
	-75.7	393.7	98.1	45.9	330.5	121.2	125.3	319.0	2.0	-6.3
	-0.910	4.734	1.180	0.552	3.974	1.457	1.506	3.835	0.024	-0.076
g_O	1751	2971	3177	1643	3058	3191	1683	3084	3323	1575
	(21.1)	(35.7)	(38.2)	(19.8)	(36.8)	(38.4)	(20.2)	(37.1)	(40.0)	(18.9)
g_E	1637.2	3070.7	3240.8	1637.2	3070.7	3240.8	1637.2	3070.7	3240.8	1637.2
	(19.7)	(36.9)	(39.0)	(19.7)	(36.9)	(39.0)	(19.7)	(36.9)	(39.0)	(19.7)
	235.4	-196.1	-126.3	11.7	-25.3	-98.8	93.0	26.7	166.5	-121.9
	2.831	-2.358	-1.518	0.141	-0.305	-1.188	1.118	0.321	2.002	-1.466
c_O	2455	3505	2264	2406	3460	2307	2353	3536	2196	2456
	(29.5)	(42.1)	(27.2)	(28.9)	(41.6)	(27.7)	(28.3)	(42.5)	(26.4)	(29.5)
c_E	2246.5	3798.4	2168.8	2246.5	3798.4	2168.8	2246.5	3798.4	2168.8	2246.5
	(27.0)	(45.7)	(26.1)	(27.0)	(45.7)	(26.1)	(27.0)	(45.7)	(26.1)	(27.0)
	435.8	-563.4	194.6	330.1	-645.6	285.1	218.0	-506.2	54.8	438.0
	5.240	-6.775	2.340	3.969	-7.763	3.428	2.621	-6.086	0.659	5.266
N	8317	8317	8317	8317	8317	8317	8317	8317	8317	8317
X2	67.4	144.2	11.0	28.9	127.3	24.7	30.4	61.1	9.1	32.1
Gvalue	68.6	133.8	11.0	29.2	119.7	24.8	31.1	59.3	9.2	31.8
Gv/N	0.825	1.608	0.132	0.351	1.439	0.298	0.374	0.713	0.111	0.382
Prob	0.00000	0.00000	0.01175	0.00000	0.00000	0.00002	0.00000	0.00000	0.02624	0.00000

	81	82	83	84	85	86	87	88	89	90
a_O	708	1715	1861	663	1734	1873	715	1757	1854	712
	(8.5)	(20.6)	(22.4)	(8.0)	(20.8)	(22.5)	(8.6)	(21.1)	(22.3)	(8.6)
a_E	552.8	1823.5	2043.2	552.8	1823.5	2043.2	552.8	1823.5	2043.2	552.8
	(6.6)	(21.9)	(24.6)	(6.6)	(21.9)	(24.6)	(6.6)	(21.9)	(24.6)	(6.6)
	350.3	-210.4	-347.6	240.9	-174.5	-325.8	367.8	-130.5	-360.3	360.3
	4.212	-2.529	-4.180	2.897	-2.098	-3.917	4.423	-1.569	-4.332	4.332
t_O	997	1094	2410	1092	1128	2387	1000	1064	2373	1035
	(12.0)	(13.2)	(29.0)	(13.1)	(13.6)	(28.7)	(12.0)	(12.8)	(28.5)	(12.4)
t_E	895.1	1084.0	2390.1	895.1	1084.0	2390.1	895.1	1084.0	2390.1	895.1
	(10.8)	(13.0)	(28.7)	(10.8)	(13.0)	(28.7)	(10.8)	(13.0)	(28.7)	(10.8)
	215.0	20.1	39.9	434.2	89.8	-6.3	221.6	-39.6	-34.2	300.6

	2.584	0.242	0.479	5.221	1.080	-0.076	2.665	-0.476	-0.411	3.614
g_O	3088	3269	1648	3050	3247	1720	3041	3321	1689	2986
	(37.1)	(39.3)	(19.8)	(36.7)	(39.0)	(20.7)	(36.6)	(39.9)	(20.3)	(35.9)
g_E	3070.7	3240.8	1637.2	3070.7	3240.8	1637.2	3070.7	3240.8	1637.2	3070.7
	(36.9)	(39.0)	(19.7)	(36.9)	(39.0)	(19.7)	(36.9)	(39.0)	(19.7)	(36.9)
	34.7	56.7	21.8	-41.3	12.5	169.8	-59.1	162.4	105.3	-167.0
	0.417	0.682	0.262	-0.496	0.150	2.042	-0.711	1.953	1.266	-2.008
c_O	3524	2239	2398	3512	2208	2337	3561	2175	2401	3584
	(42.4)	(26.9)	(28.8)	(42.2)	(26.5)	(28.1)	(42.8)	(26.2)	(28.9)	(43.1)
c_E	3798.4	2168.8	2246.5	3798.4	2168.8	2246.5	3798.4	2168.8	2246.5	3798.4
	(45.7)	(26.1)	(27.0)	(45.7)	(26.1)	(27.0)	(45.7)	(26.1)	(27.0)	(45.7)
	-528.4	142.7	313.0	-550.6	79.2	184.6	-459.6	12.5	319.4	-416.4
	-6.353	1.716	3.763	-6.620	0.952	2.220	-5.525	0.150	3.840	-5.006
N	8317	8317	8317	8317	8317	8317	8317	8317	8317	8317
X2	75.1	9.1	26.7	87.0	6.9	22.0	75.0	4.8	29.9	82.1
Gvalue	71.6	9.2	27.0	83.4	6.9	22.3	70.8	4.8	30.2	77.5
Gv/N	0.860	0.110	0.324	1.002	0.083	0.268	0.851	0.058	0.363	0.931
Prob	0.00000	0.02710	0.00001	0.00000	0.07370	0.00006	0.00000	0.18615	0.00000	0.00000

	91	92	93	94	95	96	97	98	99	100
a_O	1700	1963	672	1744	1898	630	1779	1811	638	1777
	(20.4)	(23.6)	(8.1)	(21.0)	(22.8)	(7.6)	(21.4)	(21.8)	(7.7)	(21.4)
a_E	1823.5	2043.2	552.8	1823.2	2042.9	552.8	1823.2	2042.9	552.8	1823.0
	(21.9)	(24.6)	(6.6)	(21.9)	(24.6)	(6.6)	(21.9)	(24.6)	(6.6)	(21.9)
	-238.4	-157.2	262.3	-155.0	-279.4	164.8	-87.4	-436.5	183.0	-90.9
	-2.866	-1.890	3.154	-1.864	-3.359	1.981	-1.051	-5.249	2.200	-1.093
t_O	1140	2391	1065	1116	2406	1030	1087	2428	1040	1068
	(13.7)	(28.7)	(12.8)	(13.4)	(28.9)	(12.4)	(13.1)	(29.2)	(12.5)	(12.8)
t_E	1084.0	2390.1	895.1	1083.9	2389.9	895.0	1083.9	2389.9	895.0	1083.7
	(13.0)	(28.7)	(10.8)	(13.0)	(28.7)	(10.8)	(13.0)	(28.7)	(10.8)	(13.0)
	114.9	1.7	370.1	65.2	32.4	289.4	6.3	76.9	312.3	-31.2
	1.381	0.020	4.450	0.784	0.389	3.480	0.076	0.925	3.755	-0.375
g_O	3249	1678	2971	3279	1668	3039	3248	1705	2948	3284
	(39.1)	(20.2)	(35.7)	(39.4)	(20.1)	(36.5)	(39.1)	(20.5)	(35.4)	(39.5)
g_E	3240.8	1637.2	3070.7	3240.4	1637.0	3070.3	3240.4	1637.0	3070.3	3240.0
	(39.0)	(19.7)	(36.9)	(39.0)	(19.7)	(36.9)	(39.0)	(19.7)	(36.9)	(39.0)
	16.5	82.7	-196.1	77.7	62.7	-62.3	15.2	138.9	-239.7	88.6
	0.198	0.994	-2.358	0.934	0.753	-0.750	0.183	1.670	-2.883	1.066
c_O	2228	2285	3609	2177	2344	3617	2202	2372	3690	2186
	(26.8)	(27.5)	(43.4)	(26.2)	(28.2)	(43.5)	(26.5)	(28.5)	(44.4)	(26.3)
c_E	2168.8	2246.5	3798.4	2168.5	2246.2	3797.9	2168.5	2246.2	3797.9	2168.2
	(26.1)	(27.0)	(45.7)	(26.1)	(27.0)	(45.7)	(26.1)	(27.0)	(45.7)	(26.1)
	120.1	77.7	-369.1	17.0	199.7	-353.0	67.5	258.5	-212.7	35.6
	1.444	0.934	-4.438	0.205	2.402	-4.245	0.812	3.108	-2.558	0.429
N	8317	8317	8317	8316	8316	8316	8316	8316	8316	8315
X2	12.9	4.8	70.6	4.9	15.2	40.1	1.6	36.8	44.6	2.1
Gvalue	13.0	4.9	67.3	4.9	15.4	38.8	1.6	37.7	42.9	2.1
Gv/N	0.157	0.058	0.809	0.059	0.185	0.467	0.020	0.453	0.515	0.026
Prob	0.00458	0.18263	0.00000	0.17690	0.00149	0.00000	0.65389	0.00000	0.00000	0.54361

	101	102	103	104	105	106	107	108	109	110
a_O	1981	642	1791	1915	632	1750	1954	662	1696	1900
	(23.8)	(7.7)	(21.5)	(23.0)	(7.6)	(21.1)	(23.5)	(8.0)	(20.4)	(22.9)
a_E	2042.7	552.7	1823.0	2042.7	552.7	1822.6	2042.2	552.6	1822.6	2042.2
	(24.6)	(6.6)	(21.9)	(24.6)	(6.6)	(21.9)	(24.6)	(6.6)	(21.9)	(24.6)
	-121.5	192.3	-63.5	-247.2	169.5	-142.3	-172.6	239.2	-244.2	-274.3
	-1.461	2.313	-0.764	-2.974	2.038	-1.711	-2.076	2.878	-2.937	-3.299
t_O	2366	954	1069	2390	1004	1114	2378	1062	1182	2396
	(28.5)	(11.5)	(12.9)	(28.7)	(12.1)	(13.4)	(28.6)	(12.8)	(14.2)	(28.8)
t_E	2389.6	894.9	1083.7	2389.6	894.9	1083.5	2389.0	894.7	1083.5	2389.0
	(28.7)	(10.8)	(13.0)	(28.7)	(10.8)	(13.0)	(28.7)	(10.8)	(13.0)	(28.7)
	-46.9	122.0	-29.2	0.9	231.0	61.9	-21.9	364.1	205.8	14.0
	-0.564	1.467	-0.352	0.010	2.778	0.745	-0.264	4.380	2.475	0.169
g_O	1701	3036	3275	1707	3016	3270	1682	3016	3252	1667
	(20.5)	(36.5)	(39.4)	(20.5)	(36.3)	(39.3)	(20.2)	(36.3)	(39.1)	(20.1)
g_E	1636.8	3070.0	3240.0	1636.8	3070.0	3239.2	1636.4	3069.2	3239.2	1636.4
	(19.7)	(36.9)	(39.0)	(19.7)	(36.9)	(39.0)	(19.7)	(36.9)	(39.0)	(19.7)
	131.0	-67.5	70.4	143.4	-107.0	61.9	92.5	-105.5	25.6	61.8
	1.575	-0.812	0.846	1.725	-1.286	0.744	1.113	-1.269	0.308	0.744
c_O	2267	3683	2180	2303	3663	2179	2299	3573	2183	2350
	(27.3)	(44.3)	(26.2)	(27.7)	(44.1)	(26.2)	(27.7)	(43.0)	(26.3)	(28.3)
c_E	2246.0	3797.4	2168.2	2246.0	3797.4	2167.7	2245.4	3796.5	2167.7	2245.4
	(27.0)	(45.7)	(26.1)	(27.0)	(45.7)	(26.1)	(27.0)	(45.7)	(26.1)	(27.0)
	42.3	-225.4	23.6	115.5	-264.1	22.6	108.4	-433.6	30.7	214.0
	0.508	-2.711	0.283	1.389	-3.176	0.272	1.304	-5.216	0.369	2.574
N	8315	8315	8315	8315	8315	8313	8313	8313	8313	8313
X2	4.8	22.2	1.2	12.4	30.4	4.1	6.4	67.0	17.9	15.4
Gvalue	4.8	21.4	1.2	12.6	29.4	4.1	6.4	64.2	17.9	15.5
Gv/N	0.058	0.257	0.015	0.151	0.354	0.050	0.078	0.773	0.215	0.187
Prob	0.18696	0.00009	0.75122	0.00568	0.00000	0.24743	0.09182	0.00000	0.00047	0.00142

	111	112	113	114	115	116	117	118	119	120
a_O	683	1683	1914	596	1735	1960	631	1711	1973	643
	(8.2)	(20.2)	(23.0)	(7.2)	(20.9)	(23.6)	(7.6)	(20.6)	(23.7)	(7.7)
a_E	552.6	1822.4	2042.0	552.5	1822.4	2042.0	552.5	1822.4	2042.0	552.5
	(6.6)	(21.9)	(24.6)	(6.6)	(21.9)	(24.6)	(6.6)	(21.9)	(24.6)	(6.6)
	289.5	-267.8	-247.7	90.3	-170.5	-160.6	167.6	-215.8	-135.6	195.1
	3.482	-3.222	-2.980	1.087	-2.051	-1.932	2.017	-2.596	-1.631	2.347
t_O	1003	1163	2378	979	1080	2408	1005	1147	2293	1003
	(12.1)	(14.0)	(28.6)	(11.8)	(13.0)	(29.0)	(12.1)	(13.8)	(27.6)	(12.1)
t_E	894.7	1083.3	2388.7	894.6	1083.3	2388.7	894.6	1083.3	2388.7	894.6
	(10.8)	(13.0)	(28.7)	(10.8)	(13.0)	(28.7)	(10.8)	(13.0)	(28.7)	(10.8)
	229.2	165.1	-21.4	176.6	-6.7	38.7	233.9	131.0	-187.5	229.5
	2.758	1.986	-0.257	2.124	-0.080	0.466	2.815	1.576	-2.256	2.761
g_O	2941	3329	1667	3079	3241	1660	3052	3247	1739	2994
	(35.4)	(40.1)	(20.1)	(37.0)	(39.0)	(20.0)	(36.7)	(39.1)	(20.9)	(36.0)
g_E	3069.2	3238.8	1636.2	3068.8	3238.8	1636.2	3068.8	3238.8	1636.2	3068.8
	(36.9)	(39.0)	(19.7)	(36.9)	(39.0)	(19.7)	(36.9)	(39.0)	(19.7)	(36.9)
	-251.0	182.8	62.2	20.3	4.3	48.0	-33.6	16.4	212.0	-147.9
	-3.019	2.200	0.749	0.245	0.052	0.577	-0.404	0.197	2.550	-1.779
c_O	3686	2137	2353	3658	2256	2284	3624	2207	2307	3672
	(44.3)	(25.7)	(28.3)	(44.0)	(27.1)	(27.5)	(43.6)	(26.6)	(27.8)	(44.2)
c_E	3796.5	2167.5	2245.1	3796.1	2167.5	2245.1	3796.1	2167.5	2245.1	3796.1
	(45.7)	(26.1)	(27.0)	(45.7)	(26.1)	(27.0)	(45.7)	(26.1)	(27.0)	(45.7)
	-217.8	-60.5	220.8	-271.1	180.6	78.4	-336.2	79.8	125.4	-244.0
	-2.620	-0.728	2.656	-3.261	2.173	0.943	-4.045	0.960	1.509	-2.936

N	8313	8312	8312	8312	8312	8312	8312	8312	8312	8312
X2	52.5	19.5	13.8	16.4	7.8	4.5	32.7	11.3	14.3	33.8
Gvalue	49.9	19.6	13.9	16.2	7.8	4.5	31.8	11.4	14.3	32.7
Gv/N	0.600	0.236	0.167	0.195	0.094	0.054	0.382	0.137	0.172	0.393
Prob	0.00000	0.00021	0.00302	0.00104	0.04949	0.21194	0.00000	0.00994	0.00257	0.00000

	121	122	123	124	125	126	127	128	129	130
a_O	1651	1936	662	1773	2077	668	1736	2003	634	1726
	(19.9)	(23.3)	(8.0)	(21.3)	(25.0)	(8.0)	(20.9)	(24.1)	(7.6)	(20.8)
a_E	1822.4	2042.0	552.5	1822.2	2041.7	552.4	1821.9	2041.5	552.4	1821.7
	(21.9)	(24.6)	(6.6)	(21.9)	(24.6)	(6.6)	(21.9)	(24.6)	(6.6)	(21.9)
	-326.1	-206.3	239.4	-97.0	71.2	253.8	-167.7	-76.2	174.8	-186.3
	-3.923	-2.482	2.880	-1.167	0.856	3.053	-2.019	-0.917	2.103	-2.242
t_O	1071	2264	977	1175	2297	990	1141	2306	978	1101
	(12.9)	(27.2)	(11.8)	(14.1)	(27.6)	(11.9)	(13.7)	(27.7)	(11.8)	(13.3)
t_E	1083.3	2388.7	894.6	1083.2	2388.4	894.5	1083.1	2388.1	894.4	1082.9
	(13.0)	(28.7)	(10.8)	(13.0)	(28.7)	(10.8)	(13.0)	(28.7)	(10.8)	(13.0)
	-24.5	-242.8	172.2	191.2	-179.3	200.9	118.9	-161.4	174.9	36.4
	-0.295	-2.921	2.072	2.300	-2.157	2.418	1.431	-1.942	2.104	0.438
g_O	3358	1765	3027	3224	1644	2988	3197	1689	2956	3274
	(40.4)	(21.2)	(36.4)	(38.8)	(19.8)	(36.0)	(38.5)	(20.3)	(35.6)	(39.4)
g_E	3238.8	1636.2	3068.8	3238.4	1636.0	3068.5	3238.0	1635.8	3068.1	3237.7
	(39.0)	(19.7)	(36.9)	(39.0)	(19.7)	(36.9)	(39.0)	(19.7)	(36.9)	(39.0)
	242.7	267.5	-83.1	-28.8	16.1	-158.8	-81.6	108.1	-220.1	73.1
	2.920	3.219	-1.000	-0.347	0.193	-1.911	-0.982	1.301	-2.648	0.880
c_O	2232	2347	3646	2139	2293	3665	2236	2312	3742	2208
	(26.9)	(28.2)	(43.9)	(25.7)	(27.6)	(44.1)	(26.9)	(27.8)	(45.0)	(26.6)
c_E	2167.5	2245.1	3796.1	2167.2	2244.9	3795.6	2166.9	2244.6	3795.2	2166.7
	(26.1)	(27.0)	(45.7)	(26.1)	(27.0)	(45.7)	(26.1)	(27.0)	(45.7)	(26.1)
	131.0	208.3	-294.1	-56.0	97.3	-256.7	140.3	136.8	-105.6	83.4
	1.576	2.505	-3.539	-0.674	1.170	-3.088	1.688	1.646	-1.270	1.004
N	8312	8312	8312	8311	8311	8311	8310	8310	8310	8309
X2	22.6	26.8	35.8	9.5	5.2	41.0	9.9	7.3	24.7	6.5
Gvalue	23.0	26.7	34.4	9.3	5.2	39.2	9.9	7.3	24.0	6.6
Gv/N	0.277	0.321	0.413	0.112	0.063	0.471	0.119	0.088	0.289	0.080
Prob	0.00004	0.00001	0.00000	0.02510	0.15673	0.00000	0.01977	0.06282	0.00003	0.08549

	131	132	133	134	135	136	137	138	139	140
a_O	2084	629	1817	1985	627	1751	1997	658	1743	2045
	(25.1)	(7.6)	(21.9)	(23.9)	(7.5)	(21.1)	(24.0)	(7.9)	(21.0)	(24.6)
a_E	2041.2	552.3	1821.7	2041.2	552.3	1821.5	2041.0	552.2	1821.5	2041.0
	(24.6)	(6.6)	(21.9)	(24.6)	(6.6)	(21.9)	(24.6)	(6.6)	(21.9)	(24.6)
	86.4	163.6	-9.4	-110.9	159.1	-138.2	-87.0	230.6	-153.6	8.0
	1.040	1.969	-0.113	-1.335	1.914	-1.664	-1.047	2.776	-1.848	0.097
t_O	2269	1009	1120	2323	980	1095	2336	936	1147	2296
	(27.3)	(12.1)	(13.5)	(28.0)	(11.8)	(13.2)	(28.1)	(11.3)	(13.8)	(27.6)
t_E	2387.8	894.3	1082.9	2387.8	894.3	1082.8	2387.6	894.1	1082.8	2387.6
	(28.7)	(10.8)	(13.0)	(28.7)	(10.8)	(13.0)	(28.7)	(10.8)	(13.0)	(28.7)
	-231.7	243.6	75.4	-127.9	179.5	24.5	-102.0	85.6	132.1	-179.6
	-2.788	2.932	0.907	-1.540	2.160	0.295	-1.228	1.031	1.590	-2.161
g_O	1633	3086	3152	1664	3072	3243	1654	2999	3248	1706
	(19.7)	(37.1)	(37.9)	(20.0)	(37.0)	(39.0)	(19.9)	(36.1)	(39.1)	(20.5)
g_E	1635.6	3067.7	3237.7	1635.6	3067.7	3237.3	1635.4	3067.4	3237.3	1635.4
	(19.7)	(36.9)	(39.0)	(19.7)	(36.9)	(39.0)	(19.7)	(36.9)	(39.0)	(19.7)
	-5.2	36.6	-169.0	57.3	8.5	11.5	37.4	-135.2	21.5	144.2
	-0.062	0.441	-2.034	0.690	0.103	0.138	0.451	-1.627	0.259	1.736
c_O	2323	3585	2220	2337	3630	2219	2321	3715	2170	2261
	(28.0)	(43.1)	(26.7)	(28.1)	(43.7)	(26.7)	(27.9)	(44.7)	(26.1)	(27.2)
c_E	2244.3	3794.7	2166.7	2244.3	3794.7	2166.4	2244.1	3794.2	2166.4	2244.1
	(27.0)	(45.7)	(26.1)	(27.0)	(45.7)	(26.1)	(27.0)	(45.7)	(26.1)	(27.0)
	160.0	-407.6	107.9	189.1	-322.1	106.4	156.5	-156.8	7.2	34.0
	1.926	-4.905	1.299	2.276	-3.877	1.281	1.883	-1.888	0.086	0.409
N	8309	8309	8309	8309	8309	8308	8308	8308	8308	8308
X2	9.6	37.1	4.9	7.6	25.5	4.2	4.9	25.4	7.2	6.7
Gvalue	9.6	36.2	4.9	7.6	24.9	4.2	4.9	24.2	7.2	6.7
Gv/N	0.116	0.436	0.058	0.092	0.300	0.050	0.059	0.291	0.087	0.081
Prob	0.02193	0.00000	0.18281	0.05491	0.00002	0.24299	0.17965	0.00002	0.06562	0.08216

	141	142	143	144	145	146	147	148	149	150
a_O	606	1698	1969	610	1770	2031	581	1701	2012	611
	(7.3)	(20.4)	(23.7)	(7.3)	(21.3)	(24.4)	(7.0)	(20.5)	(24.2)	(7.4)
a_E	552.2	1821.5	2041.0	552.2	1821.5	2041.0	552.2	1821.3	2040.7	552.2
	(6.6)	(21.9)	(24.6)	(6.6)	(21.9)	(24.6)	(6.6)	(21.9)	(24.6)	(6.6)
	112.6	-238.4	-141.4	121.4	-101.5	-19.9	59.0	-232.4	-57.1	123.7
	1.355	-2.870	-1.702	1.461	-1.222	-0.240	0.710	-2.798	-0.687	1.489
t_O	961	1122	2343	992	1123	2368	1020	1118	2347	1007
	(11.6)	(13.5)	(28.2)	(11.9)	(13.5)	(28.5)	(12.3)	(13.5)	(28.3)	(12.1)
t_E	894.1	1082.8	2387.6	894.1	1082.8	2387.6	894.1	1082.7	2387.3	894.0
	(10.8)	(13.0)	(28.7)	(10.8)	(13.0)	(28.7)	(10.8)	(13.0)	(28.7)	(10.8)
	138.6	79.8	-88.3	206.0	81.8	-39.0	268.6	71.8	-79.9	239.6
	1.668	0.960	-1.063	2.480	0.985	-0.469	3.234	0.864	-0.961	2.885
g_O	2997	3304	1710	3027	3260	1639	2901	3277	1721	3018
	(36.1)	(39.8)	(20.6)	(36.4)	(39.2)	(19.7)	(34.9)	(39.4)	(20.7)	(36.3)
g_E	3067.4	3237.3	1635.4	3067.4	3237.3	1635.4	3067.4	3236.9	1635.2	3067.0
	(36.9)	(39.0)	(19.7)	(36.9)	(39.0)	(19.7)	(36.9)	(39.0)	(19.7)	(36.9)
	-139.1	134.8	152.6	-80.2	45.6	7.2	-323.6	80.7	176.0	-97.2
	-1.675	1.623	1.836	-0.965	0.549	0.087	-3.894	0.972	2.119	-1.170
c_O	3744	2184	2286	3679	2155	2270	3806	2211	2227	3671
	(45.1)	(26.3)	(27.5)	(44.3)	(25.9)	(27.3)	(45.8)	(26.6)	(26.8)	(44.2)
c_E	3794.2	2166.4	2244.1	3794.2	2166.4	2244.1	3794.2	2166.2	2243.8	3793.8
	(45.7)	(26.1)	(27.0)	(45.7)	(26.1)	(27.0)	(45.7)	(26.1)	(27.0)	(45.7)
	-99.8	35.3	84.6	-226.9	-22.8	52.2	23.6	90.6	-33.5	-241.6
	-1.201	0.425	1.019	-2.732	-0.274	0.628	0.283	1.091	-0.403	-2.908
N	8308	8308	8308	8308	8308	8308	8308	8307	8307	8307
X2	12.5	11.3	7.6	20.8	3.2	0.5	28.3	10.5	5.7	25.3
Gvalue	12.2	11.5	7.5	20.2	3.2	0.5	27.6	10.7	5.6	24.6
Gv/N	0.147	0.138	0.091	0.244	0.038	0.006	0.333	0.129	0.068	0.296
Prob	0.00659	0.00940	0.05659	0.00015	0.36720	0.91534	0.00000	0.01358	0.13039	0.00002

Blon_NCC2705
5UTR:

	50	49	48	47	46	45	44	43	42	41
a_O	433	374	385	425	406	388	404	416	376	430
	(25.1)	(21.7)	(22.3)	(24.6)	(23.5)	(22.5)	(23.4)	(24.1)	(21.8)	(24.9)
a_E	432.5	432.5	432.5	432.5	432.5	432.5	432.5	432.5	432.5	432.5
	(25.0)	(25.0)	(25.0)	(25.0)	(25.0)	(25.0)	(25.0)	(25.0)	(25.0)	(25.0)
	1.0	-108.7	-89.6	-14.8	-51.3	-84.2	-55.1	-32.3	-105.3	-5.0
	0.059	-6.293	-5.186	-0.860	-2.972	-4.878	-3.188	-1.873	-6.095	-0.288
t_O	388	403	361	335	392	372	350	386	386	388
	(22.5)	(23.3)	(20.9)	(19.4)	(22.7)	(21.5)	(20.3)	(22.4)	(22.4)	(22.5)
t_E	358.4	358.4	358.4	358.4	358.4	358.4	358.4	358.4	358.4	358.4
	(20.8)	(20.8)	(20.8)	(20.8)	(20.8)	(20.8)	(20.8)	(20.8)	(20.8)	(20.8)
	61.6	94.6	5.2	-45.2	70.3	27.7	-16.6	57.3	57.3	61.6
	3.567	5.475	0.303	-2.618	4.069	1.606	-0.960	3.318	3.318	3.567
g_O	458	479	474	503	437	477	539	435	469	458
	(26.5)	(27.7)	(27.4)	(29.1)	(25.3)	(27.6)	(31.2)	(25.2)	(27.2)	(26.5)
g_E	478.1	478.1	478.1	478.1	478.1	478.1	478.1	478.1	478.1	478.1
	(27.7)	(27.7)	(27.7)	(27.7)	(27.7)	(27.7)	(27.7)	(27.7)	(27.7)	(27.7)
	-39.3	1.8	-8.1	51.1	-78.5	-2.2	129.3	-82.2	-18.0	-39.3
	-2.277	0.105	-0.472	2.959	-4.548	-0.126	7.485	-4.758	-1.043	-2.277
c_O	448	471	507	464	492	490	434	490	496	451
	(25.9)	(27.3)	(29.4)	(26.9)	(28.5)	(28.4)	(25.1)	(28.4)	(28.7)	(26.1)
c_E	458.0	458.0	458.0	458.0	458.0	458.0	458.0	458.0	458.0	458.0
	(26.5)	(26.5)	(26.5)	(26.5)	(26.5)	(26.5)	(26.5)	(26.5)	(26.5)	(26.5)
	-19.8	26.3	103.0	12.0	70.4	66.1	-46.8	66.1	79.0	-14.0
	-1.149	1.523	5.964	0.696	4.076	3.829	-2.709	3.829	4.575	-0.808
N	1727	1727	1727	1727	1727	1727	1727	1727	1727	1727
X2	3.5	13.8	10.5	3.0	10.8	7.3	11.1	8.9	12.8	3.4
Gvalue	3.5	14.0	10.5	3.0	10.8	7.4	10.9	8.9	13.0	3.4
Gv/N	0.200	0.810	0.610	0.176	0.626	0.431	0.628	0.515	0.755	0.195
Prob	0.32601	0.00291	0.01456	0.38478	0.01279	0.05922	0.01256	0.03067	0.00456	0.33928

	40	39	38	37	36	35	34	33	32	31
a_O	438	406	436	430	411	430	419	415	413	431
	(25.4)	(23.5)	(25.2)	(24.9)	(23.8)	(24.9)	(24.3)	(24.0)	(23.9)	(25.0)
a_E	432.5	432.5	432.5	432.5	432.5	432.5	432.5	432.5	432.5	432.5
	(25.0)	(25.0)	(25.0)	(25.0)	(25.0)	(25.0)	(25.0)	(25.0)	(25.0)	(25.0)
	11.1	-51.3	7.0	-5.0	-41.9	-5.0	-26.6	-34.3	-38.1	-3.0
	0.642	-2.972	0.408	-0.288	-2.426	-0.288	-1.538	-1.984	-2.205	-0.172
t_O	392	388	355	408	360	346	370	380	378	381
	(22.7)	(22.5)	(20.6)	(23.6)	(20.8)	(20.0)	(21.4)	(22.0)	(21.9)	(22.1)
t_E	358.4	358.4	358.4	358.4	358.4	358.4	358.4	358.4	358.4	358.4
	(20.8)	(20.8)	(20.8)	(20.8)	(20.8)	(20.8)	(20.8)	(20.8)	(20.8)	(20.8)
	70.3	61.6	-6.7	105.8	3.2	-24.3	23.6	44.5	40.3	46.6
	4.069	3.567	-0.391	6.126	0.187	-1.410	1.366	2.577	2.332	2.699
g_O	410	437	476	404	484	504	453	458	495	447
	(23.7)	(25.3)	(27.6)	(23.4)	(28.0)	(29.2)	(26.2)	(26.5)	(28.7)	(25.9)
g_E	478.1	478.1	478.1	478.1	478.1	478.1	478.1	478.1	478.1	478.1
	(27.7)	(27.7)	(27.7)	(27.7)	(27.7)	(27.7)	(27.7)	(27.7)	(27.7)	(27.7)
	-126.0	-78.5	-4.2	-136.1	11.9	53.2	-48.8	-39.3	34.4	-60.1
	-7.295	-4.548	-0.242	-7.878	0.689	3.080	-2.828	-2.277	1.993	-3.481
c_O	487	496	460	485	472	447	485	474	441	468
	(28.2)	(28.7)	(26.6)	(28.1)	(27.3)	(25.9)	(28.1)	(27.4)	(25.5)	(27.1)
c_E	458.0	458.0	458.0	458.0	458.0	458.0	458.0	458.0	458.0	458.0
	(26.5)	(26.5)	(26.5)	(26.5)	(26.5)	(26.5)	(26.5)	(26.5)	(26.5)	(26.5)
	59.7	79.0	3.9	55.5	28.4	-21.8	55.5	32.5	-33.4	20.2
	3.459	4.575	0.229	3.214	1.642	-1.262	3.214	1.881	-1.935	1.167
N	1727	1727	1727	1727	1727	1727	1727	1727	1727	1727
X2	14.8	10.7	0.1	20.0	1.6	2.1	3.7	3.4	3.2	3.7
Gvalue	15.1	10.7	0.1	20.3	1.6	2.1	3.7	3.4	3.2	3.7
Gv/N	0.875	0.622	0.005	1.174	0.092	0.121	0.214	0.197	0.184	0.213
Prob	0.00172	0.01322	0.99432	0.00015	0.66217	0.55293	0.29628	0.33378	0.36497	0.29755

	30	29	28	27	26	25	24	23	22	21
a_O	405	464	417	400	478	430	465	480	496	483
	(23.5)	(26.9)	(24.1)	(23.2)	(27.7)	(24.9)	(26.9)	(27.8)	(28.7)	(28.0)
a_E	432.5	432.5	432.5	432.5	432.5	432.5	432.5	432.5	432.5	432.5
	(25.0)	(25.0)	(25.0)	(25.0)	(25.0)	(25.0)	(25.0)	(25.0)	(25.0)	(25.0)
	-53.2	65.3	-30.4	-62.5	95.6	-5.0	67.4	100.1	135.9	106.7
	-3.080	3.779	-1.761	-3.618	5.538	-0.288	3.903	5.794	7.870	6.178
t_O	369	340	405	321	315	377	318	345	341	344
	(21.4)	(19.7)	(23.5)	(18.6)	(18.2)	(21.8)	(18.4)	(20.0)	(19.7)	(19.9)
t_E	358.4	358.4	358.4	358.4	358.4	358.4	358.4	358.4	358.4	358.4
	(20.8)	(20.8)	(20.8)	(20.8)	(20.8)	(20.8)	(20.8)	(20.8)	(20.8)	(20.8)
	21.5	-35.8	99.0	-70.7	-81.3	38.2	-76.0	-26.3	-33.9	-28.2
	1.247	-2.074	5.735	-4.096	-4.708	2.210	-4.403	-1.521	-1.964	-1.633
g_O	450	475	440	498	480	425	443	451	390	434
	(26.1)	(27.5)	(25.5)	(28.8)	(27.8)	(24.6)	(25.7)	(26.1)	(22.6)	(25.1)
g_E	478.1	478.1	478.1	478.1	478.1	478.1	478.1	478.1	478.1	478.1
	(27.7)	(27.7)	(27.7)	(27.7)	(27.7)	(27.7)	(27.7)	(27.7)	(27.7)	(27.7)
	-54.5	-6.2	-73.1	40.6	3.8	-100.1	-67.5	-52.6	-158.8	-84.0
	-3.156	-0.357	-4.231	2.353	0.222	-5.793	-3.911	-3.047	-9.198	-4.863
c_O	503	448	465	508	454	495	501	451	500	466
	(29.1)	(25.9)	(26.9)	(29.4)	(26.3)	(28.7)	(29.0)	(26.1)	(29.0)	(27.0)
c_E	458.0	458.0	458.0	458.0	458.0	458.0	458.0	458.0	458.0	458.0
	(26.5)	(26.5)	(26.5)	(26.5)	(26.5)	(26.5)	(26.5)	(26.5)	(26.5)	(26.5)
	94.2	-19.8	14.0	105.2	-8.0	76.8	89.9	-14.0	87.7	16.1
	5.456	-1.149	0.813	6.092	-0.465	4.450	5.203	-0.808	5.077	0.931
N	1727	1727	1727	1727	1727	1727	1727	1727	1727	1727
X2	8.1	3.5	9.8	12.6	10.1	9.9	13.6	7.4	30.2	10.7
Gvalue	8.1	3.4	9.6	12.6	10.2	10.0	13.7	7.2	30.8	10.6
Gv/N	0.467	0.199	0.556	0.731	0.588	0.579	0.792	0.418	1.785	0.614
Prob	0.04483	0.32818	0.02229	0.00551	0.01732	0.01858	0.00339	0.06527	0.00000	0.01408

	20	19	18	17	16	15	14	13	12	11
a_O	507	483	476	546	558	501	601	661	699	694
	(29.4)	(28.0)	(27.6)	(31.6)	(32.3)	(29.0)	(34.8)	(38.3)	(40.5)	(40.2)
a_E	432.5	432.5	432.5	432.5	432.5	432.5	432.5	432.5	432.5	432.5
	(25.0)	(25.0)	(25.0)	(25.0)	(25.0)	(25.0)	(25.0)	(25.0)	(25.0)	(25.0)
	161.2	106.7	91.3	254.5	284.4	147.3	395.5	560.8	671.2	656.4
	9.333	6.178	5.284	14.737	16.465	8.532	22.901	32.472	38.863	38.008
t_O	372	379	366	302	320	315	305	289	221	190
	(21.5)	(21.9)	(21.2)	(17.5)	(18.5)	(18.2)	(17.7)	(16.7)	(12.8)	(11.0)

```
t_E       358.4   358.4   358.4   358.4   358.4   358.4   358.4   358.4   358.4   358.4
        ( 20.8) ( 20.8) ( 20.8) ( 20.8) ( 20.8) ( 20.8) ( 20.8) ( 20.8) ( 20.8) ( 20.8)
           27.7    42.4    15.4  -103.4   -72.5   -81.3   -98.4  -124.4  -213.7  -241.1
          1.606   2.454   0.891  -5.987  -4.199  -4.708  -5.698  -7.202 -12.373 -13.963
g_O         414     452     469     473     455     562     524     485     572     642
        ( 24.0) ( 26.2) ( 27.2) ( 27.4) ( 26.3) ( 32.5) ( 30.3) ( 28.1) ( 33.1) ( 37.2)
g_E       478.1   478.1   478.1   478.1   478.1   478.1   478.1   478.1   478.1   478.1
        ( 27.7) ( 27.7) ( 27.7) ( 27.7) ( 27.7) ( 27.7) ( 27.7) ( 27.7) ( 27.7) ( 27.7)
         -119.2   -50.7   -18.0   -10.1   -45.0   181.8    96.1    13.9   205.2   378.5
         -6.901  -2.937  -1.043  -0.586  -2.608  10.524   5.564   0.806  11.880  21.917
c_O         434     413     416     406     394     349     297     292     235     201
        ( 25.1) ( 23.9) ( 24.1) ( 23.5) ( 22.8) ( 20.2) ( 17.2) ( 16.9) ( 13.6) ( 11.6)
c_E       458.0   458.0   458.0   458.0   458.0   458.0   458.0   458.0   458.0   458.0
        ( 26.5) ( 26.5) ( 26.5) ( 26.5) ( 26.5) ( 26.5) ( 26.5) ( 26.5) ( 26.5) ( 26.5)
          -46.8   -85.5   -80.1   -97.9  -118.7  -189.8  -257.3  -262.9  -313.7  -331.1
         -2.709  -4.950  -4.637  -5.669  -6.871 -10.988 -14.900 -15.223 -18.162 -19.172

N          1727    1727    1727    1727    1727    1727    1727    1727    1727    1727
X2         23.2    12.9     8.6    44.6    50.6    56.8   134.6   194.5   343.9   437.7
Gvalue     23.0    12.9     8.6    43.1    48.1    58.0   135.9   187.4   349.0   462.7
Gv/N      1.329   0.746   0.495   2.493   2.787   3.360   7.868  10.852  20.208  26.790
Prob    0.00004 0.00491 0.03588 0.00000 0.00000 0.00000 0.00000 0.00000 0.00000 0.00000
        -------------------------------------------------------------------------------
             10       9       8       7       6       5       4       3       2       1
        -------------------------------------------------------------------------------
a_O         631     549     527     564     522     510     544     575     338     339
        ( 36.5) ( 31.8) ( 30.5) ( 32.7) ( 30.2) ( 29.5) ( 31.5) ( 33.3) ( 19.6) ( 19.6)
a_E       432.5   432.5   432.5   432.5   432.5   432.5   432.5   432.5   432.5   432.5
        ( 25.0) ( 25.0) ( 25.0) ( 25.0) ( 25.0) ( 25.0) ( 25.0) ( 25.0) ( 25.0) ( 25.0)
          476.7   261.9   208.3   299.5   196.4   168.1   249.6   327.5  -166.6  -165.1
         27.604  15.166  12.062  17.341  11.371   9.736  14.451  18.965  -9.649  -9.562
t_O         179     208     229     241     296     338     341     269     477     434
        ( 10.4) ( 12.0) ( 13.3) ( 14.0) ( 17.1) ( 19.6) ( 19.7) ( 15.6) ( 27.6) ( 25.1)
t_E       358.4   358.4   358.4   358.4   358.4   358.4   358.4   358.4   358.4   358.4
        ( 20.8) ( 20.8) ( 20.8) ( 20.8) ( 20.8) ( 20.8) ( 20.8) ( 20.8) ( 20.8) ( 20.8)
         -248.5  -226.3  -205.1  -191.3  -113.2   -39.6   -33.9  -154.4   272.7   166.2
        -14.391 -13.106 -11.878 -11.075  -6.556  -2.293  -1.964  -8.938  15.793   9.621
g_O         692     764     758     646     598     495     389     496     331     310
        ( 40.1) ( 44.2) ( 43.9) ( 37.4) ( 34.6) ( 28.7) ( 22.5) ( 28.7) ( 19.2) ( 18.0)
g_E       478.1   478.1   478.1   478.1   478.1   478.1   478.1   478.1   478.1   478.1
        ( 27.7) ( 27.7) ( 27.7) ( 27.7) ( 27.7) ( 27.7) ( 27.7) ( 27.7) ( 27.7) ( 27.7)
          511.8   716.3   698.7   388.9   267.7    34.4  -160.4    36.5  -243.4  -268.6
         29.634  41.475  40.457  22.518  15.498   1.993  -9.290   2.112 -14.094 -15.553
c_O         225     206     213     276     311     384     453     387     581     644
        ( 13.0) ( 11.9) ( 12.3) ( 16.0) ( 18.0) ( 22.2) ( 26.2) ( 22.4) ( 33.6) ( 37.3)
c_E       458.0   458.0   458.0   458.0   458.0   458.0   458.0   458.0   458.0   458.0
        ( 26.5) ( 26.5) ( 26.5) ( 26.5) ( 26.5) ( 26.5) ( 26.5) ( 26.5) ( 26.5) ( 26.5)
         -319.9  -329.2  -326.2  -279.6  -240.8  -135.4   -10.0  -130.4   276.3   438.9
        -18.522 -19.063 -18.886 -16.190 -13.943  -7.840  -0.579  -7.552  16.001  25.414

N          1727    1727    1727    1727    1727    1727    1727    1727    1727    1727
X2        395.2   404.2   362.3   209.8   106.7    27.6    46.3    80.9   138.2   170.8
Gvalue    420.1   422.6   375.7   217.5   110.0    27.6    45.2    79.2   139.0   171.3
Gv/N     24.325  24.473  21.755  12.594   6.370   1.596   2.618   4.588   8.051   9.920
Prob    0.00000 0.00000 0.00000 0.00000 0.00000 0.00000 0.00000 0.00000 0.00000 0.00000
        -------------------------------------------------------------------------------

CDS_INITIAL:
        -------------------------------------------------------------------------------
              1       2       3       4       5       6       7       8       9      10
        -------------------------------------------------------------------------------
a_O        1282       0       0     668     322     368     558     644     378     587
        ( 74.2) (  0.0) (  0.0) ( 38.7) ( 18.6) ( 21.3) ( 32.3) ( 37.3) ( 21.9) ( 34.0)
a_E       405.7   482.4   157.5   405.7   482.4   157.5   405.7   482.4   157.5   405.7
        ( 23.5) ( 27.9) (  9.1) ( 23.5) ( 27.9) (  9.1) ( 23.5) ( 27.9) (  9.1) ( 23.5)
         2950.0     0.0     0.0   666.2  -260.4   624.6   355.7   372.0   661.9   433.6
        170.814   0.000   0.000  38.575 -15.076  36.169  20.595  21.543  38.326  25.110
t_O         162    1727       0     179     289     420     242     403     415     272
        (  9.4) (100.0) (  0.0) ( 10.4) ( 16.7) ( 24.3) ( 14.0) ( 23.3) ( 24.0) ( 15.7)
t_E       247.7   478.5   250.8   247.7   478.5   250.8   247.7   478.5   250.8   247.7
        ( 14.3) ( 27.7) ( 14.5) ( 14.3) ( 27.7) ( 14.5) ( 14.3) ( 27.7) ( 14.5) ( 14.3)
         -137.6  4433.0     0.0  -116.3  -291.5   433.2   -11.3  -138.4   418.1    50.9
         -7.968 256.690   0.000  -6.736 -16.877  25.086  -0.655  -8.016  24.212   2.945
g_O         283       0    1727     570     375     391     607     255     444     490
        ( 16.4) (  0.0) (100.0) ( 33.0) ( 21.7) ( 22.6) ( 35.1) ( 14.8) ( 25.7) ( 28.4)
g_E       675.6   304.6   549.3   675.6   304.6   549.3   675.6   304.6   549.3   675.6
        ( 39.1) ( 17.6) ( 31.8) ( 39.1) ( 17.6) ( 31.8) ( 39.1) ( 17.6) ( 31.8) ( 39.1)
         -492.5     0.0  3956.6  -193.7   155.9  -265.8  -130.0   -90.7  -189.0  -314.7
        -28.517   0.000 229.104 -11.218   9.027 -15.392  -7.525  -5.251 -10.942 -18.225
c_O           0       0       0     310     741     548     320     425     490     378
        (  0.0) (  0.0) (  0.0) ( 18.0) ( 42.9) ( 31.7) ( 18.5) ( 24.6) ( 28.4) ( 21.9)
c_E       398.0   461.4   769.5   398.0   461.4   769.5   398.0   461.4   769.5   398.0
        ( 23.0) ( 26.7) ( 44.6) ( 23.0) ( 26.7) ( 44.6) ( 23.0) ( 26.7) ( 44.6) ( 23.0)
            0.0     0.0     0.0  -154.9   702.0  -372.0  -139.6   -69.9  -442.3   -38.9
          0.000   0.000   0.000  -8.969  40.648 -21.540  -8.082  -4.047 -25.608  -2.255

N          1727    1727    1727    1727    1727    1727    1727    1727    1727    1727
X2       2548.4  4505.9  3702.8   224.6   314.1   504.9    79.5    77.0   538.0   135.4
Gvalue   2319.9  4433.0  3956.6   201.2   306.1   420.1    74.8    73.0   448.8   130.8
Gv/N    134.328 256.690 229.104  11.652  17.722  24.324   4.334   4.229  25.988   7.575
Prob    1.00000 1.00000 1.00000 0.00000 0.00000 0.00000 0.00000 0.00000 0.00000 0.00000
        -------------------------------------------------------------------------------
             11      12      13      14      15      16      17      18      19      20
        -------------------------------------------------------------------------------
a_O         481     349     604     541     327     537     535     324     502     531
        ( 27.9) ( 20.2) ( 35.0) ( 31.3) ( 18.9) ( 31.1) ( 31.0) ( 18.8) ( 29.1) ( 30.7)
a_E       482.4   157.5   405.7   482.4   157.5   405.7   482.4   157.5   405.7   482.4
        ( 27.9) (  9.1) ( 23.5) ( 27.9) (  9.1) ( 23.5) ( 27.9) (  9.1) ( 23.5) ( 27.9)
           -2.9   555.4   480.7   124.0   477.8   301.1   110.7   467.4   213.8   101.9
         -0.166  32.159  27.834   7.178  27.666  17.435   6.408  27.066  12.380   5.898
t_O         471     366     242     459     366     226     436     397     260     469
        ( 27.3) ( 21.2) ( 14.0) ( 26.6) ( 21.2) ( 13.1) ( 25.2) ( 23.0) ( 15.1) ( 27.2)
t_E       478.5   250.8   247.7   478.5   250.8   247.7   478.5   250.8   247.7   478.5
        ( 27.7) ( 14.5) ( 14.3) ( 27.7) ( 14.5) ( 14.3) ( 27.7) ( 14.5) ( 14.3) ( 27.7)
          -14.9   276.8   -11.3   -38.2   276.8   -41.5   -81.1   364.8    25.2   -18.8
         -0.864  16.028  -0.655  -2.213  16.028  -2.402  -4.698  21.123   1.456  -1.091
g_O         274     436     495     255     445     561     292     448     565     259
        ( 15.9) ( 25.2) ( 28.7) ( 14.8) ( 25.8) ( 32.5) ( 16.9) ( 25.9) ( 32.7) ( 15.0)
g_E       304.6   549.3   675.6   304.6   549.3   675.6   304.6   549.3   675.6   304.6
        ( 17.6) ( 31.8) ( 39.1) ( 17.6) ( 31.8) ( 39.1) ( 17.6) ( 31.8) ( 39.1) ( 17.6)
          -58.1  -201.4  -307.9   -90.7  -187.4  -208.5   -24.7  -182.6  -202.0   -84.0
         -3.362 -11.663 -17.829  -5.251 -10.850 -12.075  -1.431 -10.575 -11.696  -4.866
```

```
c_O          501      576      386      472      589      403      464      558      400      468
         ( 29.0) ( 33.4) ( 22.4) ( 27.3) ( 34.1) ( 23.3) ( 26.9) ( 32.3) ( 23.2) ( 27.1)
c_E        461.4    769.5    398.0    461.4    769.5    398.0    461.4    769.5    398.0    461.4
         ( 26.7) ( 44.6) ( 23.0) ( 26.7) ( 44.6) ( 23.0) ( 26.7) ( 44.6) ( 23.0) ( 26.7)
            82.4   -333.6    -23.6     21.4   -314.8     10.1      5.2   -358.6      4.1     13.2
           4.774  -19.317   -1.366    1.238  -18.230    0.585    0.299  -20.765    0.235    0.767

N           1727     1727     1727     1727     1727     1727     1727     1727     1727     1727
X2           6.6    357.8    145.7     16.2    297.5     63.9     10.0    338.1     41.6     12.0
Gvalue       6.6    297.2    137.9     16.4    252.4     61.2     10.0    291.0     41.0     12.2
Gv/N       0.383   17.208    7.984    0.952   14.613    3.543    0.577   16.850    2.375    0.707
Prob     0.08541  0.00000  0.00000  0.00092  0.00000  0.00000  0.01883  0.00000  0.00000  0.00667
         -------------------------------------------------------------------------------------------
              21       22       23       24       25       26       27       28       29       30
         -------------------------------------------------------------------------------------------
a_O          308      469      463      271      466      462      261      422      467      256
         ( 17.8) ( 27.2) ( 26.8) ( 15.7) ( 27.0) ( 26.8) ( 15.1) ( 24.4) ( 27.0) ( 14.8)
a_E        157.5    405.7    482.4    157.5    405.7    482.4    157.5    405.7    482.4    157.5
         (  9.1) ( 23.5) ( 27.9) (  9.1) ( 23.5) ( 27.9) (  9.1) ( 23.5) ( 27.9) (  9.1)
           413.2    136.0    -38.1    294.2    129.1    -40.0    263.7     33.2    -30.4    248.7
          23.923    7.873   -2.205   17.033    7.476   -2.316   15.268    1.923   -1.759   14.402
t_O          314      280      503      310      225      469      344      229      437      337
         ( 18.2) ( 16.2) ( 29.1) ( 18.0) ( 13.0) ( 27.2) ( 19.9) ( 13.3) ( 25.3) ( 19.5)
t_E        250.8    247.7    478.5    250.8    247.7    478.5    250.8    247.7    478.5    250.8
         ( 14.5) ( 14.3) ( 27.7) ( 14.5) ( 14.3) ( 27.7) ( 14.5) ( 14.3) ( 27.7) ( 14.5)
           141.2     68.6     50.2    131.5    -43.3    -18.8    217.5    -36.0    -79.3    199.2
           8.178    3.971    2.907    7.614   -2.507   -1.091   12.595   -2.084   -4.593   11.536
g_O          471      559      296      518      643      299      517      625      332      494
         ( 27.3) ( 32.4) ( 17.1) ( 30.0) ( 37.2) ( 17.3) ( 29.9) ( 36.2) ( 19.2) ( 28.6)
g_E        549.3    675.6    304.6    549.3    675.6    304.6    549.3    675.6    304.6    549.3
         ( 31.8) ( 39.1) ( 17.6) ( 31.8) ( 39.1) ( 17.6) ( 31.8) ( 39.1) ( 17.6) ( 31.8)
          -144.8   -211.8    -17.0    -60.8    -63.6    -11.1    -62.6    -97.3     57.1   -104.8
          -8.387  -12.263   -0.984   -3.518   -3.681   -0.645   -3.627   -5.633    3.309   -6.069
c_O          634      419      465      628      393      497      605      451      491      640
         ( 36.7) ( 24.3) ( 26.9) ( 36.4) ( 22.8) ( 28.8) ( 35.0) ( 26.1) ( 28.4) ( 37.1)
c_E        769.5    398.0    461.4    769.5    398.0    461.4    769.5    398.0    461.4    769.5
         ( 44.6) ( 23.0) ( 26.7) ( 44.6) ( 23.0) ( 26.7) ( 44.6) ( 23.0) ( 26.7) ( 44.6)
          -245.5     43.1      7.2   -255.2     -9.9     73.8   -291.0    112.8     61.0   -235.8
         -14.217    2.498    0.415  -14.774   -0.573    4.274  -16.848    6.532    3.532  -13.654

N           1727     1727     1727     1727     1727     1727     1727     1727     1727     1727
X2         194.8     35.3      2.3    123.6     12.7      3.9    139.7     12.9      8.5    118.6
Gvalue     164.0     35.9      2.3    109.7     12.4      3.8    127.6     12.8      8.5    107.3
Gv/N       9.497    2.079    0.133    6.354    0.715    0.223    7.388    0.738    0.490    6.215
Prob     0.00000  0.00000  0.51263  0.00000  0.00626  0.27840  0.00000  0.00520  0.03746  0.00000
         -------------------------------------------------------------------------------------------
              31       32       33       34       35       36       37       38       39       40
         -------------------------------------------------------------------------------------------
a_O          442      451      242      427      431      237      441      460      244      411
         ( 25.6) ( 26.1) ( 14.0) ( 24.7) ( 25.0) ( 13.7) ( 25.5) ( 26.6) ( 14.1) ( 23.8)
a_E        405.7    482.4    157.5    405.7    482.4    157.5    405.7    482.4    157.5    405.7
         ( 23.5) ( 27.9) (  9.1) ( 23.5) ( 27.9) (  9.1) ( 23.5) ( 27.9) (  9.1) ( 23.5)
            75.7    -60.8    207.9     43.7    -97.2    193.7     73.6    -43.8    213.6     10.6
           4.385   -3.519   12.038    2.529   -5.627   11.217    4.259   -2.537   12.370    0.616
t_O          246      459      312      223      452      287      213      459      294      225
         ( 14.2) ( 26.6) ( 18.1) ( 12.9) ( 26.2) ( 16.6) ( 12.3) ( 26.6) ( 17.0) ( 13.0)
t_E        247.7    478.5    250.8    247.7    478.5    250.8    247.7    478.5    250.8    247.7
         ( 14.3) ( 27.7) ( 14.5) ( 14.3) ( 27.7) ( 14.5) ( 14.3) ( 27.7) ( 14.5) ( 14.3)
            -3.4    -38.2    136.4    -46.9    -51.5     77.5    -64.3    -38.2     93.5    -43.3
          -0.199   -2.213    7.895   -2.715   -2.984    4.487   -3.725   -2.213    5.417   -2.507
g_O          615      350      526      674      339      505      659      319      510      697
         ( 35.6) ( 20.3) ( 30.5) ( 39.0) ( 19.6) ( 29.2) ( 38.2) ( 18.5) ( 29.5) ( 40.4)
g_E        675.6    304.6    549.3    675.6    304.6    549.3    675.6    304.6    549.3    675.6
         ( 39.1) ( 17.6) ( 31.8) ( 39.1) ( 17.6) ( 31.8) ( 39.1) ( 17.6) ( 31.8) ( 39.1)
          -115.6     97.2    -45.6     -3.2     72.5    -84.9    -32.8     29.4    -75.7     43.5
          -6.692    5.629   -2.639   -0.183    4.198   -4.916   -1.897    1.704   -4.383    2.519
c_O          424      467      647      403      505      698      414      489      679      394
         ( 24.6) ( 27.0) ( 37.5) ( 23.3) ( 29.2) ( 40.4) ( 24.0) ( 28.3) ( 39.3) ( 22.8)
c_E        398.0    461.4    769.5    398.0    461.4    769.5    398.0    461.4    769.5    398.0
         ( 23.0) ( 26.7) ( 44.6) ( 23.0) ( 26.7) ( 44.6) ( 23.0) ( 26.7) ( 44.6) ( 23.0)
            53.7     11.2   -224.3     10.1     91.1   -136.1     32.7     56.8   -169.8     -7.9
           3.110    0.649  -12.988    0.585    5.277   -7.879    1.892    3.287   -9.834   -0.458

N           1727     1727     1727     1727     1727     1727     1727     1727     1727     1727
X2          10.4      9.7     80.8      3.7     14.9     55.6      9.0      4.2     68.4      2.9
Gvalue      10.4      9.4     74.4      3.7     14.9     50.2      9.1      4.2     61.6      2.9
Gv/N       0.604    0.545    4.307    0.215    0.864    2.908    0.529    0.240    3.569    0.170
Prob     0.01522  0.02424  0.00000  0.29350  0.00189  0.00000  0.02745  0.24560  0.00000  0.40228
         -------------------------------------------------------------------------------------------
              41       42       43       44       45       46       47       48       49       50
         -------------------------------------------------------------------------------------------
a_O          448      225      414      493      205      408      475      202      425      412
         ( 25.9) ( 13.0) ( 24.0) ( 28.5) ( 11.9) ( 23.6) ( 27.5) ( 11.7) ( 24.6) ( 23.9)
a_E        482.4    157.5    405.7    482.4    157.5    405.7    482.4    157.5    405.5    482.2
         ( 27.9) (  9.1) ( 23.5) ( 27.9) (  9.1) ( 23.5) ( 27.9) (  9.1) ( 23.5) ( 27.9)
           -66.4    160.5     16.7     21.4    108.1      4.6    -14.8    100.5     40.0   -129.6
          -3.842    9.295    0.969    1.237    6.259    0.265   -0.854    5.822    2.316   -7.507
t_O          484      336      252      449      299      239      447      324      246      458
         ( 28.0) ( 19.5) ( 14.6) ( 26.0) ( 17.3) ( 13.8) ( 25.9) ( 18.8) ( 14.3) ( 26.5)
t_E        478.5    250.8    247.7    478.5    250.8    247.7    478.5    250.8    247.6    478.2
         ( 27.7) ( 14.5) ( 14.3) ( 27.7) ( 14.5) ( 14.3) ( 27.7) ( 14.5) ( 14.3) ( 27.7)
            11.0    196.6      8.6    -57.2    105.2    -17.1    -60.9    166.1     -3.2    -39.6
           0.639   11.386    0.499   -3.311    6.093   -0.992   -3.527    9.615   -0.183   -2.295
g_O          318      508      657      312      530      687      327      496      666      345
         ( 18.4) ( 29.4) ( 38.0) ( 18.1) ( 30.7) ( 39.8) ( 18.9) ( 28.7) ( 38.6) ( 20.0)
g_E        304.6    549.3    675.6    304.6    549.3    675.6    304.6    549.3    675.2    304.4
         ( 17.6) ( 31.8) ( 39.1) ( 17.6) ( 31.8) ( 39.1) ( 17.6) ( 31.8) ( 39.1) ( 17.6)
            27.3    -79.4    -36.7     14.9    -37.9     23.0     46.4   -101.2    -18.3     86.3
           1.583   -4.597   -2.122    0.865   -2.194    1.333    2.685   -5.862   -1.058    4.999
c_O          477      658      404      473      693      393      478      705      389      511
         ( 27.6) ( 38.1) ( 23.4) ( 27.4) ( 40.1) ( 22.8) ( 27.7) ( 40.8) ( 22.5) ( 29.6)
c_E        461.4    769.5    398.0    461.4    769.5    398.0    461.4    769.5    397.7    461.2
         ( 26.7) ( 44.6) ( 23.0) ( 26.7) ( 44.6) ( 23.0) ( 26.7) ( 44.6) ( 23.0) ( 26.7)
            31.7   -205.9     12.1     23.4   -145.1     -9.9     33.7   -123.4    -17.3    104.9
           1.834  -11.924    0.703    1.357   -8.399   -0.573    1.953   -7.143   -1.002    6.077

N           1727     1727     1727     1727     1727     1727     1727     1727     1726     1726
X2           3.6     77.2      0.8      2.5     31.9      0.6      4.4     44.5      1.3     21.9
Gvalue       3.7     71.8      0.8      2.6     30.4      0.6      4.4     42.0      1.3     22.0
Gv/N       0.213    4.160    0.049    0.148    1.758    0.033    0.257    2.432    0.073    1.274
Prob     0.29813  0.00000  0.83794  0.46557  0.00000  0.90142  0.21846  0.00000  0.73990  0.00007
         -------------------------------------------------------------------------------------------
```

	51	52	53	54	55	56	57	58	59	60
a_O	192	423	474	223	393	442	209	405	417	233
	(11.1)	(24.5)	(27.5)	(12.9)	(22.8)	(25.6)	(12.1)	(23.5)	(24.2)	(13.5)
a_E	157.4	405.5	482.2	157.4	405.5	482.2	157.4	405.5	482.2	157.4
	(9.1)	(23.5)	(27.9)	(9.1)	(23.5)	(27.9)	(9.1)	(23.5)	(27.9)	(9.1)
	76.3	35.8	-16.2	155.4	-24.6	-76.9	118.5	-1.0	-121.1	182.8
	4.420	2.073	-0.937	9.002	-1.424	-4.454	6.866	-0.055	-7.015	10.590
t_O	309	245	473	294	227	470	309	262	492	294
	(17.9)	(14.2)	(27.4)	(17.0)	(13.2)	(27.2)	(17.9)	(15.2)	(28.5)	(17.0)
t_E	250.6	247.6	478.2	250.6	247.6	478.2	250.6	247.6	478.2	250.6
	(14.5)	(14.3)	(27.7)	(14.5)	(14.3)	(27.7)	(14.5)	(14.3)	(27.7)	(14.5)
	129.4	-5.1	-10.4	93.9	-39.4	-16.3	129.4	29.7	27.9	93.9
	7.499	-0.297	-0.604	5.439	-2.283	-0.947	7.499	1.719	1.617	5.439
g_O	540	643	302	532	668	324	538	641	336	543
	(31.3)	(37.3)	(17.5)	(30.8)	(38.7)	(18.8)	(31.2)	(37.1)	(19.5)	(31.5)
g_E	549.0	675.2	304.4	549.0	675.2	304.4	549.0	675.2	304.4	549.0
	(31.8)	(39.1)	(17.6)	(31.8)	(39.1)	(17.6)	(31.8)	(39.1)	(17.6)	(31.8)
	-17.8	-62.8	-4.9	-33.4	-14.3	40.3	-21.7	-66.6	66.3	-11.9
	-1.031	-3.640	-0.282	-1.936	-0.829	2.337	-1.258	-3.860	3.840	-0.688
c_O	685	415	477	677	438	490	670	418	481	656
	(39.7)	(24.0)	(27.6)	(39.2)	(25.4)	(28.4)	(38.8)	(24.2)	(27.9)	(38.0)
c_E	769.0	397.7	461.2	769.0	397.7	461.2	769.0	397.7	461.2	769.0
	(44.6)	(23.0)	(26.7)	(44.6)	(23.0)	(26.7)	(44.6)	(23.0)	(26.7)	(44.6)
	-158.5	35.2	32.2	-172.6	84.4	59.4	-184.7	41.5	40.5	-208.5
	-9.183	2.042	1.867	-9.997	4.893	3.444	-10.701	2.405	2.348	-12.082
N	1726	1726	1726	1726	1726	1726	1726	1726	1726	1726
X2	30.5	3.1	0.8	46.4	6.2	6.5	43.5	3.6	13.3	60.5
Gvalue	29.4	3.1	0.8	43.3	6.2	6.6	41.5	3.6	13.6	56.2
Gv/N	1.705	0.178	0.044	2.508	0.357	0.381	2.406	0.209	0.790	3.259
Prob	0.00000	0.38082	0.86044	0.00000	0.10373	0.08651	0.00000	0.30801	0.00346	0.00000

	61	62	63	64	65	66	67	68	69	70
a_O	386	417	193	357	456	200	435	466	206	401
	(22.4)	(24.2)	(11.2)	(20.7)	(26.4)	(11.6)	(25.2)	(27.0)	(11.9)	(23.2)
a_E	405.5	482.2	157.4	405.5	482.2	157.4	405.5	482.2	157.4	405.5
	(23.5)	(27.9)	(9.1)	(23.5)	(27.9)	(9.1)	(23.5)	(27.9)	(9.1)	(23.5)
	-38.0	-121.1	78.7	-90.9	-50.9	95.8	61.1	-31.8	110.9	-8.9
	-2.202	-7.015	4.560	-5.267	-2.947	5.551	3.542	-1.840	6.423	-0.516
t_O	263	482	278	253	481	297	237	459	268	237
	(15.2)	(27.9)	(16.1)	(14.7)	(27.9)	(17.2)	(13.7)	(26.6)	(15.5)	(13.7)
t_E	247.6	478.2	250.6	247.6	478.2	250.6	247.6	478.2	250.6	247.6
	(14.3)	(27.7)	(14.5)	(14.3)	(27.7)	(14.5)	(14.3)	(27.7)	(14.5)	(14.3)
	31.8	7.5	57.7	11.0	5.5	100.9	-20.7	-37.7	36.0	-20.7
	1.841	0.437	3.341	0.635	0.321	5.844	-1.199	-2.184	2.083	-1.199
g_O	652	363	567	682	330	543	683	317	540	675
	(37.8)	(21.0)	(32.9)	(39.5)	(19.1)	(31.5)	(39.6)	(18.4)	(31.3)	(39.1)
g_E	675.2	304.4	549.0	675.2	304.4	549.0	675.2	304.4	549.0	675.2
	(39.1)	(17.6)	(31.8)	(39.1)	(17.6)	(31.8)	(39.1)	(17.6)	(31.8)	(39.1)
	-45.6	127.7	36.6	13.7	53.2	-11.9	15.7	25.6	-17.8	-0.4
	-2.641	7.399	2.123	0.793	3.082	-0.688	0.910	1.485	-1.031	-0.022
c_O	425	464	688	434	459	686	371	484	712	413
	(24.6)	(26.9)	(39.9)	(25.1)	(26.6)	(39.7)	(21.5)	(28.0)	(41.3)	(23.9)
c_E	397.7	461.2	769.0	397.7	461.2	769.0	397.7	461.2	769.0	397.7
	(23.0)	(26.7)	(44.6)	(23.0)	(26.7)	(44.6)	(23.0)	(26.7)	(44.6)	(23.0)
	56.3	5.7	-153.2	75.7	-4.3	-156.7	-51.7	46.8	-109.7	31.1
	3.264	0.330	-8.875	4.387	-0.250	-9.080	-2.993	2.711	-6.355	1.801
N	1726	1726	1726	1726	1726	1726	1726	1726	1726	1726
X2	4.6	20.1	20.2	9.3	3.6	29.1	4.5	3.0	20.6	1.1
Gvalue	4.5	19.9	19.8	9.4	3.6	28.1	4.5	3.0	19.3	1.1
Gv/N	0.262	1.152	1.149	0.547	0.206	1.627	0.260	0.171	1.120	0.063
Prob	0.21017	0.00018	0.00018	0.02402	0.31319	0.00000	0.21335	0.39846	0.00023	0.78047

	71	72	73	74	75	76	77	78	79	80
a_O	459	191	426	483	192	388	458	195	425	468
	(26.6)	(11.1)	(24.7)	(28.0)	(11.1)	(22.5)	(26.5)	(11.3)	(24.6)	(27.1)
a_E	482.2	157.4	405.5	482.2	157.4	405.5	482.2	157.4	405.5	482.2
	(27.9)	(9.1)	(23.5)	(27.9)	(9.1)	(23.5)	(27.9)	(9.1)	(23.5)	(27.9)
	-45.2	73.9	42.1	1.7	76.3	-34.2	-47.1	83.5	40.0	-27.9
	-2.618	4.282	2.437	0.098	4.420	-1.981	-2.728	4.840	2.316	-1.616
t_O	459	308	240	463	285	231	464	283	245	420
	(26.6)	(17.8)	(13.9)	(26.8)	(16.5)	(13.4)	(26.9)	(16.4)	(14.2)	(24.3)
t_E	478.2	250.6	247.6	478.2	250.6	247.6	478.2	250.6	247.6	478.2
	(27.7)	(14.5)	(14.3)	(27.7)	(14.5)	(14.3)	(27.7)	(14.5)	(14.3)	(27.7)
	-37.7	127.0	-14.9	-30.0	73.3	-32.0	-28.1	68.8	-5.1	-109.1
	-2.184	7.359	-0.865	-1.737	4.246	-1.855	-1.625	3.985	-0.297	-6.320
g_O	354	544	636	315	548	664	339	527	659	342
	(20.5)	(31.5)	(36.8)	(18.3)	(31.7)	(38.5)	(19.6)	(30.5)	(38.2)	(19.8)
g_E	304.4	549.0	675.2	304.4	549.0	675.2	304.4	549.0	675.2	304.4
	(17.6)	(31.8)	(39.1)	(17.6)	(31.8)	(39.1)	(17.6)	(31.8)	(39.1)	(17.6)
	106.8	-9.9	-76.1	21.5	-1.9	-22.2	72.9	-43.0	-32.0	79.6
	6.186	-0.573	-4.407	1.244	-0.112	-1.286	4.223	-2.494	-1.854	4.610
c_O	454	683	424	465	701	443	465	721	397	496
	(26.3)	(39.6)	(24.6)	(26.9)	(40.6)	(25.7)	(26.9)	(41.8)	(23.0)	(28.7)
c_E	461.2	769.0	397.7	461.2	769.0	397.7	461.2	769.0	397.7	461.2
	(26.7)	(44.6)	(23.0)	(26.7)	(44.6)	(23.0)	(26.7)	(44.6)	(23.0)	(26.7)
	-14.2	-162.0	54.2	7.7	-129.8	95.5	7.7	-93.0	-1.5	72.2
	-0.823	-9.387	3.140	0.447	-7.522	5.531	0.447	-5.386	-0.087	4.186
N	1726	1726	1726	1726	1726	1726	1726	1726	1726	1726
X2	10.1	30.0	5.3	0.9	18.3	7.2	5.6	17.0	1.4	14.8
Gvalue	9.7	29.0	5.3	0.9	17.8	7.1	5.5	16.3	1.3	14.8
Gv/N	0.561	1.680	0.305	0.051	1.032	0.408	0.317	0.945	0.078	0.860
Prob	0.02141	0.00000	0.15293	0.82878	0.00048	0.07030	0.14027	0.00098	0.71849	0.00196

	81	82	83	84	85	86	87	88	89	90
a_O	184	417	427	216	398	465	199	411	450	185
	(10.7)	(24.2)	(24.7)	(12.5)	(23.1)	(26.9)	(11.5)	(23.8)	(26.1)	(10.7)
a_E	157.4	405.5	482.2	157.4	405.5	482.2	157.4	405.5	482.2	157.4
	(9.1)	(23.5)	(27.9)	(9.1)	(23.5)	(27.9)	(9.1)	(23.5)	(27.9)	(9.1)
	57.5	23.4	-103.7	136.7	-14.8	-33.7	93.3	11.1	-62.1	59.8
	3.329	1.354	-6.011	7.921	-0.859	-1.952	5.407	0.644	-3.599	3.463
t_O	291	260	484	287	254	468	317	261	458	272
	(16.9)	(15.1)	(28.0)	(16.6)	(14.7)	(27.1)	(18.4)	(15.1)	(26.5)	(15.8)
t_E	250.6	247.6	478.2	250.6	247.6	478.2	250.6	247.6	478.2	250.6
	(14.5)	(14.3)	(27.7)	(14.5)	(14.3)	(27.7)	(14.5)	(14.3)	(27.7)	(14.5)
	87.0	25.5	11.6	77.8	13.0	-20.3	149.0	27.6	-39.6	44.5

	5.038	1.475	0.671	4.509	0.753	−1.174	8.632	1.596	−2.295	2.581
g_O	540	654	347	522	660	327	541	642	315	533
	(31.3)	(37.9)	(20.1)	(30.2)	(38.2)	(18.9)	(31.3)	(37.2)	(18.3)	(30.9)
g_E	549.0	675.2	304.4	549.0	675.2	304.4	549.0	675.2	304.4	549.0
	(31.8)	(39.1)	(17.6)	(31.8)	(39.1)	(17.6)	(31.8)	(39.1)	(17.6)	(31.8)
	−17.8	−41.7	90.8	−52.6	−30.0	46.7	−15.8	−64.7	21.5	−31.5
	−1.031	−2.417	5.261	−3.047	−1.740	2.708	−0.917	−3.750	1.244	−1.823
c_O	711	395	468	701	414	466	669	412	503	736
	(41.2)	(22.9)	(27.1)	(40.6)	(24.0)	(27.0)	(38.8)	(23.9)	(29.1)	(42.6)
c_E	769.0	397.7	461.2	769.0	397.7	461.2	769.0	397.7	461.2	769.0
	(44.6)	(23.0)	(26.7)	(44.6)	(23.0)	(26.7)	(44.6)	(23.0)	(26.7)	(44.6)
	−111.5	−5.5	13.8	−129.8	33.2	9.7	−186.4	29.0	87.4	−64.6
	−6.462	−0.317	0.798	−7.522	1.921	0.564	−10.801	1.681	5.062	−3.742
N	1726	1726	1726	1726	1726	1726	1726	1726	1726	1726
X2	15.5	1.6	12.4	34.4	1.3	2.6	41.7	2.9	7.2	8.5
Gvalue	15.1	1.6	12.4	32.1	1.3	2.5	40.1	3.0	7.1	8.3
Gv/N	0.874	0.094	0.720	1.861	0.076	0.146	2.322	0.171	0.412	0.479
Prob	0.00175	0.65296	0.00606	0.00000	0.72832	0.47157	0.00000	0.39890	0.06845	0.04092

	91	92	93	94	95	96	97	98	99	100
a_O	410	440	209	409	466	192	383	435	186	430
	(23.8)	(25.5)	(12.1)	(23.7)	(27.0)	(11.1)	(22.2)	(25.2)	(10.8)	(24.9)
a_E	405.5	482.2	157.4	405.5	482.2	157.4	405.5	482.2	157.4	405.5
	(23.5)	(27.9)	(9.1)	(23.5)	(27.9)	(9.1)	(23.5)	(27.9)	(9.1)	(23.5)
	9.1	−80.5	118.5	7.1	−31.8	76.3	−43.7	−89.5	62.1	50.5
	0.527	−4.665	6.866	0.410	−1.840	4.420	−2.531	−5.188	3.598	2.926
t_O	253	467	286	257	474	292	262	480	254	259
	(14.7)	(27.1)	(16.6)	(14.9)	(27.5)	(16.9)	(15.2)	(27.8)	(14.7)	(15.0)
t_E	247.6	478.2	250.6	247.6	478.2	250.6	247.6	478.2	250.6	247.6
	(14.3)	(27.7)	(14.5)	(14.3)	(27.7)	(14.5)	(14.3)	(27.7)	(14.5)	(14.3)
	11.0	−22.2	75.6	19.2	−8.4	89.3	29.7	3.5	6.8	23.4
	0.635	−1.287	4.377	1.112	−0.489	5.172	1.719	0.204	0.395	1.353
g_O	687	323	513	613	330	542	696	316	570	659
	(39.8)	(18.7)	(29.7)	(35.5)	(19.1)	(31.4)	(40.3)	(18.3)	(33.0)	(38.2)
g_E	675.2	304.4	549.0	675.2	304.4	549.0	675.2	304.4	549.0	675.2
	(39.1)	(17.6)	(31.8)	(39.1)	(17.6)	(31.8)	(39.1)	(17.6)	(31.8)	(39.1)
	23.8	38.2	−69.5	−118.5	53.2	−13.9	42.3	23.5	42.9	−32.0
	1.380	2.214	−4.028	−6.864	3.082	−0.802	2.448	1.364	2.483	−1.854
c_O	376	496	718	447	456	700	385	495	716	378
	(21.8)	(28.7)	(41.6)	(25.9)	(26.4)	(40.6)	(22.3)	(28.7)	(41.5)	(21.9)
c_E	397.7	461.2	769.0	397.7	461.2	769.0	397.7	461.2	769.0	397.7
	(23.0)	(26.7)	(44.6)	(23.0)	(26.7)	(44.6)	(23.0)	(26.7)	(44.6)	(23.0)
	−42.3	72.2	−98.6	104.4	−10.3	−131.6	−25.1	70.1	−102.3	−38.5
	−2.450	4.186	−5.711	6.047	−0.595	−7.627	−1.453	4.062	−5.926	−2.231
N	1726	1726	1726	1726	1726	1726	1726	1726	1726	1726
X2	1.6	7.7	27.7	12.2	2.8	20.7	3.1	7.5	9.7	3.4
Gvalue	1.6	7.7	26.0	12.2	2.7	20.1	3.1	7.6	9.5	3.4
Gv/N	0.092	0.449	1.505	0.704	0.158	1.162	0.182	0.442	0.550	0.195
Prob	0.66285	0.05167	0.00001	0.00686	0.43502	0.00016	0.37029	0.05420	0.02344	0.33920

	101	102	103	104	105	106	107	108	109	110
a_O	461	191	404	432	193	426	462	179	413	428
	(26.7)	(11.1)	(23.4)	(25.0)	(11.2)	(24.7)	(26.8)	(10.4)	(23.9)	(24.8)
a_E	482.2	157.4	405.5	482.2	157.4	405.2	481.9	157.3	405.2	481.9
	(27.9)	(9.1)	(23.5)	(27.9)	(9.1)	(23.5)	(27.9)	(9.1)	(23.5)	(27.9)
	−41.4	73.9	−3.0	−94.9	78.7	42.6	−38.9	46.2	15.7	−101.5
	−2.397	4.282	−0.171	−5.498	4.560	2.467	−2.256	2.680	0.908	−5.884
t_O	496	282	259	487	277	231	472	287	245	470
	(28.7)	(16.3)	(15.0)	(28.2)	(16.0)	(13.4)	(27.4)	(16.6)	(14.2)	(27.2)
t_E	478.2	250.6	247.6	478.2	250.6	247.4	478.0	250.5	247.4	478.0
	(27.7)	(14.5)	(14.3)	(27.7)	(14.5)	(14.3)	(27.7)	(14.5)	(14.3)	(27.7)
	36.2	66.5	23.4	17.7	55.5	−31.8	−11.9	78.2	−4.8	−15.8
	2.096	3.856	1.353	1.024	3.213	−1.841	−0.687	4.530	−0.281	−0.915
g_O	326	502	648	311	571	627	320	507	671	336
	(18.9)	(29.1)	(37.5)	(18.0)	(33.1)	(36.3)	(18.6)	(29.4)	(38.9)	(19.5)
g_E	304.4	549.0	675.2	304.4	549.0	674.8	304.3	548.7	674.8	304.3
	(17.6)	(31.8)	(39.1)	(17.6)	(31.8)	(39.1)	(17.6)	(31.8)	(39.1)	(17.6)
	44.6	−89.8	−53.3	13.3	44.9	−92.1	32.3	−80.1	−7.6	66.7
	2.584	−5.203	−3.087	0.768	2.603	−5.341	1.870	−4.641	−0.439	3.865
c_O	443	751	415	496	685	441	471	752	396	491
	(25.7)	(43.5)	(24.0)	(28.7)	(39.7)	(25.6)	(27.3)	(43.6)	(23.0)	(28.5)
c_E	461.2	769.0	397.7	461.2	769.0	397.5	460.9	768.6	397.5	460.9
	(26.7)	(44.6)	(23.0)	(26.7)	(44.6)	(23.0)	(26.7)	(44.6)	(23.0)	(26.7)
	−35.6	−35.6	35.2	72.2	−158.5	91.6	20.4	−32.8	−3.0	62.1
	−2.062	−2.063	2.042	4.186	−9.183	5.308	1.185	−1.900	−0.176	3.602
N	1726	1726	1726	1726	1726	1725	1725	1725	1725	1725
X2	3.8	15.5	2.4	8.2	20.9	10.3	1.9	11.8	0.2	11.4
Gvalue	3.8	15.0	2.4	8.3	20.6	10.2	1.9	11.6	0.2	11.5
Gv/N	0.221	0.872	0.137	0.480	1.193	0.593	0.112	0.670	0.012	0.668
Prob	0.28274	0.00177	0.49887	0.04063	0.00013	0.01676	0.58792	0.00908	0.97778	0.00922

	111	112	113	114	115	116	117	118	119	120
a_O	192	401	443	176	383	471	167	388	465	195
	(11.1)	(23.2)	(25.7)	(10.2)	(22.2)	(27.3)	(9.7)	(22.5)	(27.0)	(11.3)
a_E	157.3	405.2	481.9	157.3	405.0	481.6	157.2	405.0	481.6	157.2
	(9.1)	(23.5)	(27.9)	(9.1)	(23.5)	(27.9)	(9.1)	(23.5)	(27.9)	(9.1)
	76.5	−8.4	−74.5	39.5	−42.8	−21.0	20.2	−33.3	−32.6	84.0
	4.436	−0.489	−4.321	2.291	−2.483	−1.216	1.169	−1.931	−1.892	4.872
t_O	261	243	493	310	253	463	265	273	507	247
	(15.1)	(14.1)	(28.6)	(18.0)	(14.7)	(26.9)	(15.4)	(15.8)	(29.4)	(14.3)
t_E	250.5	247.4	478.0	250.5	247.3	477.7	250.3	247.3	477.7	250.3
	(14.5)	(14.3)	(27.7)	(14.5)	(14.3)	(27.7)	(14.5)	(14.3)	(27.7)	(14.5)
	21.5	−8.8	30.5	132.2	11.5	−28.9	30.2	54.0	60.4	−6.6
	1.246	−0.510	1.771	7.664	0.670	−1.677	1.752	3.132	3.503	−0.383
g_O	552	656	312	523	671	335	548	642	312	576
	(32.0)	(38.0)	(18.1)	(30.3)	(38.9)	(19.4)	(31.8)	(37.2)	(18.1)	(33.4)
g_E	548.7	674.8	304.3	548.7	674.4	304.1	548.3	674.4	304.1	548.3
	(31.8)	(39.1)	(17.6)	(31.8)	(39.1)	(17.6)	(31.8)	(39.1)	(17.6)	(31.8)
	6.7	−37.1	15.7	−50.1	−6.8	64.9	−0.7	−63.2	16.0	56.7
	0.389	−2.149	0.908	−2.903	−0.395	3.762	−0.039	−3.668	0.929	3.289
c_O	720	425	477	716	417	455	744	421	440	706
	(41.7)	(24.6)	(27.7)	(41.5)	(24.2)	(26.4)	(43.2)	(24.4)	(25.5)	(41.0)
c_E	768.6	397.5	460.9	768.6	397.3	460.6	768.1	397.3	460.6	768.1
	(44.6)	(23.0)	(26.7)	(44.6)	(23.0)	(26.7)	(44.6)	(23.0)	(26.7)	(44.6)
	−94.0	56.8	32.8	−101.5	40.4	−11.2	−47.5	48.8	−40.3	−119.1
	−5.449	3.294	1.900	−5.882	2.343	−0.649	−2.754	2.831	−2.338	−6.907

N	1725	1725	1725	1725	1724	1724	1724	1724	1724	1724
X2	11.2	2.5	4.4	21.2	2.3	3.9	2.2	6.4	3.5	15.5
Gvalue	10.7	2.5	4.4	20.2	2.3	3.8	2.2	6.3	3.5	15.0
Gv/N	0.622	0.146	0.258	1.170	0.135	0.220	0.128	0.364	0.202	0.871
Prob	0.01325	0.47330	0.21734	0.00016	0.50707	0.28383	0.53074	0.09877	0.32290	0.00181

	121	122	123	124	125	126	127	128	129	130
a_O	412	482	174	401	482	188	412	460	193	421
	(23.9)	(28.0)	(10.1)	(23.3)	(28.0)	(10.9)	(23.9)	(26.7)	(11.2)	(24.4)
a_E	405.0	481.6	157.2	405.0	481.6	157.2	405.0	481.6	157.2	405.0
	(23.5)	(27.9)	(9.1)	(23.5)	(27.9)	(9.1)	(23.5)	(27.9)	(9.1)	(23.5)
	14.1	0.8	35.3	−8.0	0.8	67.2	14.1	−42.2	79.1	32.6
	0.818	0.047	2.047	−0.463	0.047	3.899	0.818	−2.448	4.591	1.891
t_O	241	455	265	237	444	289	260	469	255	271
	(14.0)	(26.4)	(15.4)	(13.7)	(25.8)	(16.8)	(15.1)	(27.2)	(14.8)	(15.7)
t_E	247.3	477.7	250.3	247.3	477.7	250.3	247.3	477.7	250.3	247.3
	(14.3)	(27.7)	(14.5)	(14.3)	(27.7)	(14.5)	(14.3)	(27.7)	(14.5)	(14.3)
	−12.4	−44.3	30.2	−20.2	−64.9	83.0	26.1	−17.2	9.4	49.6
	−0.721	−2.568	1.752	−1.169	−3.767	4.817	1.511	−0.998	0.548	2.878
g_O	676	286	522	669	338	504	664	327	533	637
	(39.2)	(16.6)	(30.3)	(38.8)	(19.6)	(29.2)	(38.5)	(19.0)	(30.9)	(36.9)
g_E	674.4	304.1	548.3	674.4	304.1	548.3	674.4	304.1	548.3	674.4
	(39.1)	(17.6)	(31.8)	(39.1)	(17.6)	(31.8)	(39.1)	(17.6)	(31.8)	(39.1)
	3.2	−35.1	−51.4	−10.8	71.5	−85.0	−20.7	47.5	−30.2	−72.7
	0.185	−2.035	−2.980	−0.625	4.145	−4.929	−1.198	2.755	−1.754	−4.217
c_O	395	501	763	417	460	743	388	468	743	395
	(22.9)	(29.1)	(44.3)	(24.2)	(26.7)	(43.1)	(22.5)	(27.1)	(43.1)	(22.9)
c_E	397.3	460.6	768.1	397.3	460.6	768.1	397.3	460.6	768.1	397.3
	(23.0)	(26.7)	(44.6)	(23.0)	(26.7)	(44.6)	(23.0)	(26.7)	(44.6)	(23.0)
	−4.6	84.2	−10.2	40.4	−1.3	−49.4	−18.4	14.9	−49.4	−4.6
	−0.265	4.883	−0.592	2.343	−0.073	−2.866	−1.065	0.862	−2.866	−0.265
N	1724	1724	1724	1724	1724	1724	1724	1724	1724	1724
X2	0.3	5.7	4.0	1.5	6.2	16.4	1.2	3.0	9.5	5.0
Gvalue	0.3	5.6	3.9	1.5	6.1	15.9	1.1	2.9	8.9	5.0
Gv/N	0.017	0.327	0.226	0.086	0.353	0.921	0.066	0.171	0.518	0.287
Prob	0.96032	0.13088	0.27312	0.68686	0.10767	0.00120	0.76687	0.40038	0.03014	0.17517

	131	132	133	134	135	136	137	138	139	140
a_O	462	175	398	464	169	415	489	160	373	440
	(26.8)	(10.2)	(23.1)	(26.9)	(9.8)	(24.1)	(28.4)	(9.3)	(21.6)	(25.5)
a_E	481.6	157.2	405.0	481.6	157.2	404.8	481.3	157.1	404.8	481.3
	(27.9)	(9.1)	(23.5)	(27.9)	(9.1)	(23.5)	(27.9)	(9.1)	(23.5)	(27.9)
	−38.4	37.5	−13.9	−34.5	24.4	20.7	15.5	5.8	−61.0	−79.0
	−2.227	2.175	−0.806	−2.004	1.416	1.202	0.899	0.336	−3.540	−4.584
t_O	471	279	274	499	295	245	440	292	275	469
	(27.3)	(16.2)	(15.9)	(28.9)	(17.1)	(14.2)	(25.5)	(16.9)	(16.0)	(27.2)
t_E	477.7	250.3	247.3	477.7	250.3	247.2	477.4	250.2	247.2	477.4
	(27.7)	(14.5)	(14.3)	(27.7)	(14.5)	(14.3)	(27.7)	(14.5)	(14.3)	(27.7)
	−13.3	60.5	56.2	43.6	96.9	−4.3	−71.8	90.3	58.7	−16.7
	−0.770	3.510	3.260	2.527	5.620	−0.248	−4.168	5.239	3.408	−0.967
g_O	325	516	674	316	529	640	327	540	670	316
	(18.9)	(29.9)	(39.1)	(18.3)	(30.7)	(37.1)	(19.0)	(31.3)	(38.9)	(18.3)
g_E	304.1	548.3	674.4	304.1	548.3	674.0	303.9	548.0	674.0	303.9
	(17.6)	(31.8)	(39.1)	(17.6)	(31.8)	(39.1)	(17.6)	(31.8)	(39.1)	(17.6)
	43.2	−62.7	−0.8	24.3	−38.0	−66.3	47.9	−15.9	−8.0	24.6
	2.507	−3.638	−0.048	1.408	−2.203	−3.847	2.779	−0.924	−0.465	1.430
c_O	466	754	378	445	731	423	467	731	405	498
	(27.0)	(43.7)	(21.9)	(25.8)	(42.4)	(24.6)	(27.1)	(42.4)	(23.5)	(28.9)
c_E	460.6	768.1	397.3	460.6	768.1	397.1	460.4	767.7	397.1	460.4
	(26.7)	(44.6)	(23.0)	(26.7)	(44.6)	(23.0)	(26.7)	(44.6)	(23.0)	(26.7)
	10.8	−28.0	−37.6	−30.7	−72.4	53.5	13.4	−71.6	16.0	78.3
	0.627	−1.623	−2.182	−1.782	−4.201	3.108	0.776	−4.154	0.931	4.543
N	1724	1724	1724	1724	1724	1723	1723	1723	1723	1723
X2	2.4	7.5	3.9	2.6	11.3	3.7	4.9	8.9	5.8	7.3
Gvalue	2.4	7.3	3.9	2.6	10.9	3.7	4.9	8.6	5.8	7.3
Gv/N	0.138	0.424	0.224	0.150	0.633	0.214	0.287	0.498	0.335	0.422
Prob	0.49909	0.06259	0.27701	0.46017	0.01221	0.29805	0.17632	0.03539	0.12332	0.06367

	141	142	143	144	145	146	147	148	149	150
a_O	163	414	417	198	416	462	176	382	461	180
	(9.5)	(24.0)	(24.2)	(11.5)	(24.1)	(26.8)	(10.2)	(22.2)	(26.8)	(10.4)
a_E	157.1	404.8	481.3	157.1	404.8	481.3	157.1	404.8	481.3	157.1
	(9.1)	(23.5)	(27.9)	(9.1)	(23.5)	(27.9)	(9.1)	(23.5)	(27.9)	(9.1)
	12.0	18.7	−119.6	91.6	22.8	−37.9	39.9	−44.2	−39.8	48.9
	0.694	1.083	−6.943	5.314	1.321	−2.197	2.317	−2.568	−2.308	2.839
t_O	271	253	462	265	245	492	262	260	472	255
	(15.7)	(14.7)	(26.8)	(15.4)	(14.2)	(28.6)	(15.2)	(15.1)	(27.4)	(14.8)
t_E	250.2	247.2	477.4	250.2	247.2	477.4	250.2	247.2	477.4	250.2
	(14.5)	(14.3)	(27.7)	(14.5)	(14.3)	(27.7)	(14.5)	(14.3)	(27.7)	(14.5)
	43.3	11.8	−30.3	30.5	−4.3	29.6	24.2	26.4	−10.8	9.7
	2.515	0.687	−1.759	1.771	−0.248	1.719	1.404	1.530	−0.624	0.565
g_O	534	687	320	520	659	310	535	690	313	534
	(31.0)	(39.9)	(18.6)	(30.2)	(38.2)	(18.0)	(31.1)	(40.0)	(18.2)	(31.0)
g_E	548.0	674.0	303.9	548.0	674.0	303.9	548.0	674.0	303.9	548.0
	(31.8)	(39.1)	(17.6)	(31.8)	(39.1)	(17.6)	(31.8)	(39.1)	(17.6)	(31.8)
	−27.7	26.2	33.0	−54.6	−29.7	12.3	−25.7	32.3	18.4	−27.7
	−1.606	1.521	1.916	−3.167	−1.724	0.713	−1.493	1.877	1.070	−1.606
c_O	755	369	524	740	403	459	750	391	477	754
	(43.8)	(21.4)	(30.4)	(42.9)	(23.4)	(26.6)	(43.5)	(22.7)	(27.7)	(43.8)
c_E	767.7	397.1	460.4	767.7	397.1	460.4	767.7	397.1	460.4	767.7
	(44.6)	(23.0)	(26.7)	(44.6)	(23.0)	(26.7)	(44.6)	(23.0)	(26.7)	(44.6)
	−25.1	−54.1	135.7	−54.3	12.0	−2.7	−34.9	−12.0	33.9	−27.1
	−1.459	−3.139	7.876	−3.154	0.695	−0.157	−2.028	−0.698	1.966	−1.573
N	1723	1723	1723	1723	1723	1723	1723	1723	1723	1723
X2	2.5	2.6	18.7	13.9	0.8	1.3	3.5	2.4	1.8	4.0
Gvalue	2.5	2.6	18.8	13.1	0.8	1.4	3.5	2.4	1.8	3.9
Gv/N	0.144	0.152	1.089	0.763	0.044	0.078	0.200	0.141	0.104	0.225
Prob	0.47972	0.45303	0.00031	0.00434	0.86069	0.71671	0.32697	0.48775	0.61604	0.27503

Bmal_ATCC23344
5UTR:

	50	49	48	47	46	45	44	43	42	41
a_O	840	936	773	752	781	778	858	837	789	824
	(16.7)	(18.6)	(15.4)	(15.0)	(15.5)	(15.5)	(17.1)	(16.7)	(15.7)	(16.4)
a_E	910.1	910.1	910.1	910.1	910.1	910.1	910.1	910.1	910.1	910.1
	(18.1)	(18.1)	(18.1)	(18.1)	(18.1)	(18.1)	(18.1)	(18.1)	(18.1)	(18.1)
	-134.7	52.4	-252.5	-287.1	-239.0	-244.1	-101.3	-140.3	-225.4	-163.9
	-2.682	1.044	-5.026	-5.714	-4.758	-4.859	-2.015	-2.792	-4.487	-3.262
t_O	820	884	813	767	968	851	756	938	779	766
	(16.3)	(17.6)	(16.2)	(15.3)	(19.3)	(16.9)	(15.0)	(18.7)	(15.5)	(15.2)
t_E	846.1	846.1	846.1	846.1	846.1	846.1	846.1	846.1	846.1	846.1
	(16.8)	(16.8)	(16.8)	(16.8)	(16.8)	(16.8)	(16.8)	(16.8)	(16.8)	(16.8)
	-51.4	77.4	-64.9	-150.6	260.5	9.8	-170.3	193.4	-128.8	-152.4
	-1.024	1.541	-1.292	-2.998	5.186	0.195	-3.389	3.849	-2.563	-3.033
g_O	1633	1563	1770	1795	1692	1625	1695	1543	1778	1684
	(32.5)	(31.1)	(35.2)	(35.7)	(33.7)	(32.3)	(33.7)	(30.7)	(35.4)	(33.5)
g_E	1610.0	1610.0	1610.0	1610.0	1610.0	1610.0	1610.0	1610.0	1610.0	1610.0
	(32.0)	(32.0)	(32.0)	(32.0)	(32.0)	(32.0)	(32.0)	(32.0)	(32.0)	(32.0)
	46.4	-92.5	335.5	390.6	168.2	30.2	174.5	-131.1	353.0	151.4
	0.924	-1.842	6.678	7.774	3.348	0.602	3.473	-2.609	7.027	3.014
c_O	1731	1641	1668	1710	1583	1770	1715	1706	1678	1750
	(34.5)	(32.7)	(33.2)	(34.0)	(31.5)	(35.2)	(34.1)	(34.0)	(33.4)	(34.8)
c_E	1657.8	1657.8	1657.8	1657.8	1657.8	1657.8	1657.8	1657.8	1657.8	1657.8
	(33.0)	(33.0)	(33.0)	(33.0)	(33.0)	(33.0)	(33.0)	(33.0)	(33.0)	(33.0)
	149.6	-33.4	20.5	106.1	-146.1	231.9	116.4	97.8	40.7	189.5
	2.979	-0.664	0.408	2.112	-2.908	4.616	2.317	1.948	0.810	3.772
N	5024	5024	5024	5024	5024	5024	5024	5024	5024	5024
X2	9.8	4.0	37.9	57.8	43.4	27.0	19.1	20.0	39.2	24.3
Gvalue	9.9	4.0	38.6	59.0	43.6	27.8	19.4	19.9	39.6	24.7
Gv/N	0.197	0.079	0.768	1.174	0.867	0.553	0.386	0.396	0.788	0.491
Prob	0.01959	0.26670	0.00000	0.00000	0.00000	0.00000	0.00023	0.00018	0.00000	0.00002

	40	39	38	37	36	35	34	33	32	31
a_O	828	737	897	1000	748	794	821	832	1050	921
	(16.5)	(14.7)	(17.9)	(19.9)	(14.9)	(15.8)	(16.3)	(16.6)	(20.9)	(18.3)
a_E	910.1	910.1	910.1	910.1	910.1	910.1	910.1	910.1	910.1	910.1
	(18.1)	(18.1)	(18.1)	(18.1)	(18.1)	(18.1)	(18.1)	(18.1)	(18.1)	(18.1)
	-156.7	-311.0	-26.1	188.3	-293.5	-216.8	-169.3	-149.4	300.2	21.8
	-3.118	-6.191	-0.520	3.748	-5.842	-4.315	-3.369	-2.974	5.975	0.434
t_O	980	797	789	838	866	901	888	913	780	853
	(19.5)	(15.9)	(15.7)	(16.7)	(17.2)	(17.9)	(17.7)	(18.2)	(15.5)	(17.0)
t_E	846.1	846.1	846.1	846.1	846.1	846.1	846.1	846.1	846.1	846.1
	(16.8)	(16.8)	(16.8)	(16.8)	(16.8)	(16.8)	(16.8)	(16.8)	(16.8)	(16.8)
	287.9	-95.3	-110.3	-16.2	40.2	113.2	85.8	138.9	-126.9	13.8
	5.731	-1.898	-2.195	-0.322	0.801	2.254	1.708	2.765	-2.527	0.275
g_O	1480	1688	1641	1531	1748	1632	1604	1544	1537	1533
	(29.5)	(33.6)	(32.7)	(30.5)	(34.8)	(32.5)	(31.9)	(30.7)	(30.6)	(30.5)
g_E	1610.0	1610.0	1610.0	1610.0	1610.0	1610.0	1610.0	1610.0	1610.0	1610.0
	(32.0)	(32.0)	(32.0)	(32.0)	(32.0)	(32.0)	(32.0)	(32.0)	(32.0)	(32.0)
	-249.1	159.8	62.7	-154.0	287.6	44.4	-11.9	-129.2	-142.6	-150.2
	-4.959	3.181	1.248	-3.065	5.724	0.883	-0.237	-2.571	-2.838	-2.989
c_O	1736	1802	1697	1655	1662	1697	1711	1735	1657	1717
	(34.6)	(35.9)	(33.8)	(32.9)	(33.1)	(33.8)	(34.1)	(34.5)	(33.0)	(34.2)
c_E	1657.8	1657.8	1657.8	1657.8	1657.8	1657.8	1657.8	1657.8	1657.8	1657.8
	(33.0)	(33.0)	(33.0)	(33.0)	(33.0)	(33.0)	(33.0)	(33.0)	(33.0)	(33.0)
	160.1	300.7	79.4	-5.5	8.5	79.4	108.2	158.0	-1.5	120.6
	3.187	5.984	1.580	-0.110	0.169	1.580	2.153	3.145	-0.031	2.400
N	5024	5024	5024	5024	5024	5024	5024	5024	5024	5024
X2	42.8	52.1	5.6	12.8	41.2	19.6	12.5	18.3	30.0	6.0
Gvalue	42.2	54.1	5.7	12.6	42.8	20.2	12.8	18.3	29.1	6.0
Gv/N	0.840	1.077	0.113	0.251	0.851	0.402	0.254	0.365	0.580	0.120
Prob	0.00000	0.00000	0.12979	0.00556	0.00000	0.00015	0.00513	0.00037	0.00000	0.11088

	30	29	28	27	26	25	24	23	22	21
a_O	809	924	882	898	848	853	938	896	961	941
	(16.1)	(18.4)	(17.6)	(17.9)	(16.9)	(17.0)	(18.7)	(17.8)	(19.1)	(18.7)
a_E	910.1	910.1	910.1	910.1	910.1	910.1	910.1	910.1	910.1	910.1
	(18.1)	(18.1)	(18.1)	(18.1)	(18.1)	(18.1)	(18.1)	(18.1)	(18.1)	(18.1)
	-190.6	27.9	-55.4	-24.1	-120.0	-110.6	56.5	-28.1	104.5	62.7
	-3.794	0.556	-1.103	-0.480	-2.388	-2.202	1.125	-0.559	2.080	1.249
t_O	954	854	984	887	977	1022	990	867	953	1082
	(19.0)	(17.0)	(19.6)	(17.7)	(19.4)	(20.3)	(19.7)	(17.3)	(19.0)	(21.5)
t_E	846.1	846.1	846.1	846.1	846.1	846.1	846.1	846.1	846.1	846.1
	(16.8)	(16.8)	(16.8)	(16.8)	(16.8)	(16.8)	(16.8)	(16.8)	(16.8)	(16.8)
	229.0	15.8	297.1	83.7	281.0	386.0	310.9	42.3	226.7	532.1
	4.557	0.315	5.914	1.666	5.594	7.684	6.189	0.841	4.513	10.592
g_O	1631	1511	1397	1514	1549	1474	1482	1514	1348	1256
	(32.5)	(30.1)	(27.8)	(30.1)	(30.8)	(29.3)	(29.5)	(30.1)	(26.8)	(25.0)
g_E	1610.0	1610.0	1610.0	1610.0	1610.0	1610.0	1610.0	1610.0	1610.0	1610.0
	(32.0)	(32.0)	(32.0)	(32.0)	(32.0)	(32.0)	(32.0)	(32.0)	(32.0)	(32.0)
	42.4	-191.7	-396.4	-186.1	-119.6	-260.1	-245.5	-186.1	-478.8	-623.7
	0.843	-3.816	-7.891	-3.704	-2.380	-5.177	-4.886	-3.704	-9.530	-12.414
c_O	1630	1735	1761	1725	1650	1675	1614	1747	1762	1745
	(32.4)	(34.5)	(35.1)	(34.3)	(32.8)	(33.3)	(32.1)	(34.8)	(35.1)	(34.7)
c_E	1657.8	1657.8	1657.8	1657.8	1657.8	1657.8	1657.8	1657.8	1657.8	1657.8
	(33.0)	(33.0)	(33.0)	(33.0)	(33.0)	(33.0)	(33.0)	(33.0)	(33.0)	(33.0)
	-55.1	158.0	212.8	137.2	-15.5	34.6	-86.4	183.2	214.9	179.0
	-1.096	3.145	4.235	2.730	-0.309	0.689	-1.719	3.646	4.277	3.562
N	5024	5024	5024	5024	5024	5024	5024	5024	5024	5024
X2	25.7	10.0	57.9	10.6	26.8	51.8	36.6	11.3	65.5	149.2
Gvalue	25.6	10.0	58.0	10.6	26.0	49.9	35.6	11.3	67.3	150.2
Gv/N	0.510	0.200	1.155	0.212	0.517	0.994	0.710	0.225	1.340	2.989
Prob	0.00001	0.01825	0.00000	0.01388	0.00001	0.00000	0.00000	0.01027	0.00000	0.00000

	20	19	18	17	16	15	14	13	12	11
a_O	962	953	966	1119	1118	1040	1237	1160	1313	1407
	(19.1)	(19.0)	(19.2)	(22.3)	(22.3)	(20.7)	(24.6)	(23.1)	(26.1)	(28.0)
a_E	910.1	910.1	910.1	910.1	910.1	910.1	910.1	910.1	910.1	910.1
	(18.1)	(18.1)	(18.1)	(18.1)	(18.1)	(18.1)	(18.1)	(18.1)	(18.1)	(18.1)
	106.6	87.7	115.1	462.3	459.9	277.4	759.1	562.8	962.3	1225.8
	2.122	1.745	2.290	9.202	9.154	5.522	15.110	11.201	19.155	24.399
t_O	1077	1125	1075	992	965	926	902	843	706	598
	(21.4)	(22.4)	(21.4)	(19.7)	(19.2)	(18.4)	(18.0)	(16.8)	(14.1)	(11.9)

```
t_E        846.1   846.1   846.1   846.1   846.1   846.1   846.1   846.1   846.1   846.1
         ( 16.8) ( 16.8) ( 16.8) ( 16.8) ( 16.8) ( 16.8) ( 16.8) ( 16.8) ( 16.8) ( 16.8)
           519.7   641.0   514.7   315.6   253.7   167.1   115.4    -6.2  -255.6  -415.1
          10.344  12.758  10.246   6.281   5.050   3.326   2.296  -0.124  -5.088  -8.262
g_O         1283    1336    1293    1225    1346    1549    1371    1483    1713    1878
         ( 25.5) ( 26.6) ( 25.7) ( 24.4) ( 26.8) ( 30.8) ( 27.3) ( 29.5) ( 34.1) ( 37.4)
g_E       1610.0  1610.0  1610.0  1610.0  1610.0  1610.0  1610.0  1610.0  1610.0  1610.0
         ( 32.0) ( 32.0) ( 32.0) ( 32.0) ( 32.0) ( 32.0) ( 32.0) ( 32.0) ( 32.0) ( 32.0)
          -582.5  -498.4  -567.0  -669.5  -482.1  -119.6  -440.6  -243.6   212.5   578.4
         -11.594  -9.921 -11.285 -13.326  -9.595  -2.380  -8.769  -4.849   4.230  11.513
c_O         1702    1610    1690    1688    1595    1509    1514    1538    1292    1141
         ( 33.9) ( 32.0) ( 33.6) ( 33.6) ( 31.7) ( 30.0) ( 30.1) ( 30.6) ( 25.7) ( 22.7)
c_E       1657.8  1657.8  1657.8  1657.8  1657.8  1657.8  1657.8  1657.8  1657.8  1657.8
         ( 33.0) ( 33.0) ( 33.0) ( 33.0) ( 33.0) ( 33.0) ( 33.0) ( 33.0) ( 33.0) ( 33.0)
            89.6   -94.2    65.1    61.0  -123.1  -283.8  -274.7  -230.7  -644.1  -852.5
           1.784  -1.874   1.295   1.214  -2.451  -5.648  -5.468  -4.591 -12.821 -16.968

N           5024    5024    5024    5024    5024    5024    5024    5024    5024    5024
X2         133.5   141.9   128.4   165.7   109.8    41.7   169.0    87.3   288.8   549.7
Gvalue     133.4   136.1   127.9   169.4   108.5    41.1   159.2    82.2   275.1   536.6
Gv/N       2.656   2.709   2.546   3.372   2.159   0.819   3.169   1.637   5.475  10.681
Prob     0.00000 0.00000 0.00000 0.00000 0.00000 0.00000 0.00000 0.00000 0.00000 0.00000
         -------------------------------------------------------------------------------
              10       9       8       7       6       5       4       3       2       1
         -------------------------------------------------------------------------------
a_O         1220    1267    1395    1174    1259    1199    1055    1265     947     712
         ( 24.3) ( 25.2) ( 27.8) ( 23.4) ( 25.1) ( 23.9) ( 21.0) ( 25.2) ( 18.8) ( 14.2)
a_E        910.1   910.1   910.1   910.1   910.1   910.1   910.1   910.1   910.1   910.1
         ( 18.1) ( 18.1) ( 18.1) ( 18.1) ( 18.1) ( 18.1) ( 18.1) ( 18.1) ( 18.1) ( 18.1)
           714.9   838.2  1191.4   597.7   817.0   661.0   311.6   832.9    75.2  -349.6
          14.230  16.685  23.715  11.897  16.262  13.156   6.203  16.579   1.496  -6.959
t_O          571     528     649     815     764     897     794     679     937     415
         ( 11.4) ( 10.5) ( 12.9) ( 16.2) ( 15.2) ( 17.9) ( 15.8) ( 13.5) ( 18.7) (  8.3)
t_E        846.1   846.1   846.1   846.1   846.1   846.1   846.1   846.1   846.1   846.1
         ( 16.8) ( 16.8) ( 16.8) ( 16.8) ( 16.8) ( 16.8) ( 16.8) ( 16.8) ( 16.8) ( 16.8)
          -449.1  -498.0  -344.3   -61.1  -156.0   104.8  -101.0  -298.8   191.2  -591.3
          -8.939  -9.912  -6.852  -1.216  -3.105   2.085  -2.010  -5.948   3.806 -11.769
g_O         2188    2215    1907    1689    1628    1253    1321    1675    1051    1465
         ( 43.6) ( 44.1) ( 38.0) ( 33.6) ( 32.4) ( 24.9) ( 26.3) ( 33.3) ( 20.9) ( 29.2)
g_E       1610.0  1610.0  1610.0  1610.0  1610.0  1610.0  1610.0  1610.0  1610.0  1610.0
         ( 32.0) ( 32.0) ( 32.0) ( 32.0) ( 32.0) ( 32.0) ( 32.0) ( 32.0) ( 32.0) ( 32.0)
          1342.5  1413.4   645.8   161.9    36.3  -628.2  -522.6   132.7  -896.4  -276.5
          26.721  28.132  12.854   3.223   0.722 -12.503 -10.403   2.641 -17.843  -5.503
c_O         1045    1014    1073    1346    1373    1675    1854    1405    2089    2432
         ( 20.8) ( 20.2) ( 21.4) ( 26.8) ( 27.3) ( 33.3) ( 36.9) ( 28.0) ( 41.6) ( 48.4)
c_E       1657.8  1657.8  1657.8  1657.8  1657.8  1657.8  1657.8  1657.8  1657.8  1657.8
         ( 33.0) ( 33.0) ( 33.0) ( 33.0) ( 33.0) ( 33.0) ( 33.0) ( 33.0) ( 33.0) ( 33.0)
          -964.4  -996.9  -933.5  -560.8  -517.6    34.6   414.8  -464.9   966.0  1864.1
         -19.197 -19.843 -18.582 -11.163 -10.302   0.689   8.257  -9.253  19.228  37.104

N           5024    5024    5024    5024    5024    5024    5024    5024    5024    5024
X2         629.0   736.9   565.3   140.1   190.8   174.1   101.4   212.5   317.5   637.4
Gvalue     643.8   756.7   559.4   137.7   179.7   172.2   102.8   201.9   335.9   646.7
Gv/N      12.815  15.062  11.135   2.741   3.577   3.427   2.047   4.019   6.687  12.872
Prob     0.00000 0.00000 0.00000 0.00000 0.00000 0.00000 0.00000 0.00000 0.00000 0.00000
         -------------------------------------------------------------------------------

CDS_INITIAL:
         -------------------------------------------------------------------------------
               1       2       3       4       5       6       7       8       9      10
         -------------------------------------------------------------------------------
a_O         3930       0       0    2086    1470     804    1458    1479     791    1348
         ( 78.2) (  0.0) (  0.0) ( 41.5) ( 29.3) ( 16.0) ( 29.0) ( 29.4) ( 15.7) ( 26.8)
a_E        929.9  1187.2   243.8   929.9  1187.2   243.8   929.9  1187.2   243.8   929.9
         ( 18.5) ( 23.6) (  4.9) ( 18.5) ( 23.6) (  4.9) ( 18.5) ( 23.6) (  4.9) ( 18.5)
         11329.0     0.0     0.0  3370.8   628.2  1918.8  1311.5   650.1  1862.0  1001.1
         225.498   0.000   0.000  67.094  12.504  38.192  26.106  12.940  37.061  19.927
t_O          342    5024       0     645     774     847     626    1077     822     733
         (  6.8) (100.0) (  0.0) ( 12.8) ( 15.4) ( 16.9) ( 12.5) ( 21.4) ( 16.4) ( 14.6)
t_E        627.3  1368.5   349.6   627.3  1368.5   349.6   627.3  1368.5   349.6   627.3
         ( 12.5) ( 27.2) (  7.0) ( 12.5) ( 27.2) (  7.0) ( 12.5) ( 27.2) (  7.0) ( 12.5)
          -415.0 13067.8     0.0    35.8  -882.2  1499.0    -2.7  -515.9  1405.5   228.2
          -8.259 260.107   0.000   0.713 -17.559  29.836  -0.053 -10.269  27.975   4.543
g_O          752       0    5024     927    1066    1531    1615     879    1578    1376
         ( 15.0) (  0.0) (100.0) ( 18.5) ( 21.2) ( 30.5) ( 32.1) ( 17.5) ( 31.4) ( 27.4)
g_E       2040.4  1043.1  2163.2  2040.4  1043.1  2163.2  2040.4  1043.1  2163.2  2040.4
         ( 40.6) ( 20.8) ( 43.1) ( 40.6) ( 20.8) ( 43.1) ( 40.6) ( 20.8) ( 43.1) ( 40.6)
         -1501.3     0.0  8467.0 -1462.7    46.4 -1058.4  -755.2  -300.8  -995.4 -1084.2
         -29.882   0.000 168.532 -29.115   0.923 -21.066 -15.033  -5.988 -19.814 -21.581
c_O            0       0       0    1366    1714    1842    1325    1589    1833    1567
         (  0.0) (  0.0) (  0.0) ( 27.2) ( 34.1) ( 36.7) ( 26.4) ( 31.6) ( 36.5) ( 31.2)
c_E       1426.4  1425.3  2267.4  1426.4  1425.3  2267.4  1426.4  1425.3  2267.4  1426.4
         ( 28.4) ( 28.4) ( 45.1) ( 28.4) ( 28.4) ( 45.1) ( 28.4) ( 28.4) ( 45.1) ( 28.4)
             0.0     0.0     0.0  -118.2   632.3  -765.5  -195.4   345.5  -779.7   294.7
           0.000   0.000   0.000  -2.352  12.585 -15.237  -3.889   6.877 -15.520   5.865

N           5024    5024    5024    5024    5024    5024    5024    5024    5024    5024
X2       12049.3 13420.4  6644.4  2048.1   384.6  2259.5   395.9   178.4  2108.0   436.0
Gvalue    9412.8 13067.8  8467.0  1825.7   424.7  1593.9   358.3   178.9  1492.3   439.8
Gv/N     187.357 260.107 168.532  36.341   8.454  31.725   7.131   3.561  29.703   8.754
Prob     1.00000 1.00000 1.00000 1.00000 0.00000 1.00000 0.00000 0.00000 1.00000 0.00000
         -------------------------------------------------------------------------------
              11      12      13      14      15      16      17      18      19      20
         -------------------------------------------------------------------------------
a_O         1127     736    1162    1149     636    1218    1212     642    1152    1269
         ( 22.4) ( 14.6) ( 23.1) ( 22.9) ( 12.7) ( 24.2) ( 24.1) ( 12.8) ( 22.9) ( 25.3)
a_E       1187.2   243.8   929.9  1187.2   243.8   929.9  1187.2   243.8   929.9  1187.2
         ( 23.6) (  4.9) ( 18.5) ( 23.6) (  4.9) ( 18.5) ( 23.6) (  4.9) ( 18.5) ( 23.6)
          -117.3  1626.4   517.9   -75.1  1219.7   657.5    50.2  1243.2   493.5   169.2
          -2.334  32.373  10.309  -1.495  24.277  13.088   0.998  24.746   9.824   3.367
t_O         1332     878     805    1405     886     744    1402     786     709    1311
         ( 26.5) ( 17.5) ( 16.0) ( 28.0) ( 17.6) ( 14.8) ( 27.9) ( 15.6) ( 14.1) ( 26.1)
t_E       1368.5   349.6   627.3  1368.5   349.6   627.3  1368.5   349.6   627.3  1368.5
         ( 27.2) (  7.0) ( 12.5) ( 27.2) (  7.0) ( 12.5) ( 27.2) (  7.0) ( 12.5) ( 27.2)
           -71.9  1617.0   401.5    74.0  1647.8   253.8    67.9  1273.5   173.5  -112.5
          -1.432  32.185   7.991   1.474  32.798   5.052   1.351  25.349   3.454  -2.239
g_O          963    1384    1472     963    1496    1560     863    1599    1708     917
         ( 19.2) ( 27.5) ( 29.3) ( 19.2) ( 29.8) ( 31.1) ( 17.2) ( 31.8) ( 34.0) ( 18.3)
g_E       1043.1  2163.2  2040.4  1043.1  2163.2  2040.4  1043.1  2163.2  2040.4  1043.1
         ( 20.8) ( 43.1) ( 40.6) ( 20.8) ( 43.1) ( 40.6) ( 20.8) ( 43.1) ( 40.6) ( 20.8)
          -153.8 -1236.2  -961.3  -153.8 -1103.4  -837.6  -327.1  -966.4  -607.5  -236.2
          -3.061 -24.605 -19.135  -3.061 -21.962 -16.673  -6.510 -19.236 -12.092  -4.702
```

c_O	1602	2026	1585	1507	2006	1502	1547	1997	1455	1527
	(31.9)	(40.3)	(31.5)	(30.0)	(39.9)	(29.9)	(30.8)	(39.7)	(29.0)	(30.4)
c_E	1425.3	2267.4	1426.4	1425.3	2267.4	1426.4	1425.3	2267.4	1426.4	1425.3
	(28.4)	(45.1)	(28.4)	(28.4)	(45.1)	(28.4)	(28.4)	(45.1)	(28.4)	(28.4)
	374.5	-456.2	334.3	168.0	-491.5	155.2	253.5	-507.3	57.8	210.5
	7.453	-9.080	6.653	3.344	-9.783	3.089	5.046	-10.097	1.151	4.190
N	5024	5024	5024	5024	5024	5024	5024	5024	5024	5024
X2	32.1	2098.7	284.3	13.0	1689.8	228.1	42.8	1374.5	118.4	30.5
Gvalue	31.4	1551.0	292.3	13.1	1272.6	228.9	44.5	1043.1	117.4	30.9
Gv/N	0.626	30.872	5.819	0.261	25.330	4.556	0.885	20.763	2.337	0.616
Prob	0.00000	1.00000	0.00000	0.00440	0.00000	0.00000	0.00000	0.00000	0.00000	0.00000

	21	22	23	24	25	26	27	28	29	30
a_O	653	962	1098	485	997	1183	553	1052	1037	424
	(13.0)	(19.1)	(21.9)	(9.7)	(19.8)	(23.5)	(11.0)	(20.9)	(20.6)	(8.4)
a_E	243.8	929.9	1187.2	243.8	929.9	1187.2	243.8	929.9	1187.2	243.8
	(4.9)	(18.5)	(23.6)	(4.9)	(18.5)	(23.6)	(4.9)	(18.5)	(23.6)	(4.9)
	1286.7	65.4	-171.5	667.2	139.0	-8.3	905.8	259.6	-280.5	469.3
	25.612	1.301	-3.413	13.280	2.767	-0.166	18.030	5.168	-5.583	9.341
t_O	746	698	1340	629	666	1227	622	641	1426	565
	(14.8)	(13.9)	(26.7)	(12.5)	(13.3)	(24.4)	(12.4)	(12.8)	(28.4)	(11.2)
t_E	349.6	627.3	1368.5	349.6	627.3	1368.5	349.6	627.3	1368.5	349.6
	(7.0)	(12.5)	(27.2)	(7.0)	(12.5)	(27.2)	(7.0)	(12.5)	(27.2)	(7.0)
	1130.8	149.0	-56.3	738.8	79.7	-267.8	716.7	27.6	117.5	542.4
	22.508	2.966	-1.121	14.706	1.586	-5.330	14.265	0.550	2.338	10.796
g_O	1587	1737	964	1763	1782	1024	1725	1761	1044	1682
	(31.6)	(34.6)	(19.2)	(35.1)	(35.5)	(20.4)	(34.3)	(35.1)	(20.8)	(33.5)
g_E	2163.2	2040.4	1043.1	2163.2	2040.4	1043.1	2163.2	2040.4	1043.1	2163.2
	(43.1)	(40.6)	(20.8)	(43.1)	(40.6)	(20.8)	(43.1)	(40.6)	(20.8)	(43.1)
	-983.1	-559.3	-152.0	-721.2	-482.6	-37.8	-780.9	-518.7	1.9	-846.3
	-19.567	-11.133	-3.025	-14.356	-9.607	-0.752	-15.543	-10.325	0.038	-16.846
c_O	2038	1627	1622	2147	1579	1590	2124	1570	1517	2353
	(40.6)	(32.4)	(32.3)	(42.7)	(31.4)	(31.6)	(42.3)	(31.2)	(30.2)	(46.8)
c_E	2267.4	1426.4	1425.3	2267.4	1426.4	1425.3	2267.4	1426.4	1425.3	2267.4
	(45.1)	(28.4)	(28.4)	(45.1)	(28.4)	(28.4)	(45.1)	(28.4)	(28.4)	(45.1)
	-434.8	428.2	419.4	-234.4	321.0	347.7	-277.6	301.2	189.2	174.3
	-8.655	8.524	8.348	-4.665	6.390	6.922	-5.526	5.996	3.766	3.470
N	5024	5024	5024	5024	5024	5024	5024	5024	5024	5024
X2	1312.9	82.4	40.4	542.3	56.3	34.0	702.2	69.1	27.3	376.2
Gvalue	999.6	83.3	39.6	450.4	57.1	33.9	564.1	69.8	28.0	339.7
Gv/N	19.897	1.658	0.788	8.965	1.136	0.674	11.227	1.390	0.558	6.761
Prob	0.00000	0.00000	0.00000	0.00000	0.00000	0.00000	0.00000	0.00000	0.00000	0.00000

	31	32	33	34	35	36	37	38	39	40
a_O	879	977	469	821	1000	400	805	937	371	843
	(17.5)	(19.4)	(9.3)	(16.3)	(19.9)	(8.0)	(16.0)	(18.7)	(7.4)	(16.8)
a_E	929.9	1187.2	243.8	929.9	1187.2	243.8	929.9	1187.2	243.8	929.9
	(18.5)	(23.6)	(4.9)	(18.5)	(23.6)	(4.9)	(18.5)	(23.6)	(4.9)	(18.5)
	-98.9	-380.7	613.7	-204.5	-343.2	396.1	-232.2	-443.5	311.5	-165.4
	-1.969	-7.578	12.216	-4.070	-6.830	7.884	-4.621	-8.827	6.201	-3.291
t_O	746	1359	488	633	1300	500	750	1332	579	571
	(14.8)	(27.1)	(9.7)	(12.6)	(25.9)	(10.0)	(14.9)	(26.5)	(11.5)	(11.4)
t_E	627.3	1368.5	349.6	627.3	1368.5	349.6	627.3	1368.5	349.6	627.3
	(12.5)	(27.2)	(7.0)	(12.5)	(27.2)	(7.0)	(12.5)	(27.2)	(7.0)	(12.5)
	258.5	-18.9	325.5	11.4	-133.4	357.8	267.9	-71.9	584.2	-107.4
	5.145	-0.375	6.479	0.227	-2.656	7.122	5.333	-1.432	11.628	-2.138
g_O	2024	1029	1680	2102	1197	1894	1986	1201	1817	1964
	(40.3)	(20.5)	(33.4)	(41.8)	(23.8)	(37.7)	(39.5)	(23.9)	(36.2)	(39.1)
g_E	2040.4	1043.1	2163.2	2040.4	1043.1	2163.2	2040.4	1043.1	2163.2	2040.4
	(40.6)	(20.8)	(43.1)	(40.6)	(20.8)	(43.1)	(40.6)	(20.8)	(43.1)	(40.6)
	-32.7	-27.9	-849.3	125.0	329.6	-503.3	-107.4	338.7	-633.7	-150.0
	-0.651	-0.556	-16.905	2.488	6.560	-10.019	-2.137	6.741	-12.614	-2.985
c_O	1375	1659	2387	1468	1527	2230	1483	1554	2257	1646
	(27.4)	(33.0)	(47.5)	(29.2)	(30.4)	(44.4)	(29.5)	(30.9)	(44.9)	(32.8)
c_E	1426.4	1425.3	2267.4	1426.4	1425.3	2267.4	1426.4	1425.3	2267.4	1426.4
	(28.4)	(28.4)	(45.1)	(28.4)	(28.4)	(45.1)	(28.4)	(28.4)	(45.1)	(28.4)
	-100.9	503.8	245.3	84.4	210.5	-74.3	115.5	268.7	-20.8	471.4
	-2.008	10.028	4.883	1.681	4.190	-1.478	2.298	5.348	-0.415	9.384
N	5024	5024	5024	5024	5024	5024	5024	5024	5024	5024
X2	27.2	75.8	377.0	15.9	62.9	198.9	44.5	89.2	272.3	49.9
Gvalue	26.0	76.3	335.2	16.4	63.5	176.3	43.8	91.9	241.2	48.7
Gv/N	0.517	1.518	6.672	0.326	1.263	3.509	0.872	1.830	4.801	0.969
Prob	0.00001	0.00000	0.00000	0.00095	0.00000	0.00000	0.00000	0.00000	0.00000	0.00000

	41	42	43	44	45	46	47	48	49	50
a_O	888	384	864	1017	388	888	1031	415	790	941
	(17.7)	(7.6)	(17.2)	(20.2)	(7.7)	(17.7)	(20.5)	(8.3)	(15.7)	(18.7)
a_E	1187.2	243.8	929.9	1187.2	243.8	929.9	1187.2	243.8	929.7	1186.9
	(23.6)	(4.9)	(18.5)	(23.6)	(4.9)	(18.5)	(23.6)	(4.9)	(18.5)	(23.6)
	-515.7	348.9	-127.0	-314.7	360.6	-81.8	-290.8	441.5	-257.2	-437.0
	-10.264	6.945	-2.527	-6.264	7.177	-1.629	-5.789	8.788	-5.121	-8.700
t_O	1463	452	649	1280	465	713	1283	498	600	1323
	(29.1)	(9.0)	(12.9)	(25.5)	(9.3)	(14.2)	(25.5)	(9.9)	(11.9)	(26.3)
t_E	1368.5	349.6	627.3	1368.5	349.6	627.3	1368.5	349.6	627.2	1368.2
	(27.2)	(7.0)	(12.5)	(27.2)	(7.0)	(12.5)	(27.2)	(7.0)	(12.5)	(27.2)
	195.5	232.2	44.1	-171.1	265.2	182.5	-165.5	352.4	-53.2	-88.9
	3.890	4.622	0.878	-3.405	5.280	3.633	-3.294	7.014	-1.059	-1.769
g_O	1097	1883	2141	1061	1926	2059	1177	1835	2091	1174
	(21.8)	(37.5)	(42.6)	(21.1)	(38.3)	(41.0)	(23.4)	(36.5)	(41.6)	(23.4)
g_E	1043.1	2163.2	2040.4	1043.1	2163.2	2040.4	1043.1	2163.2	2040.0	1042.8
	(20.8)	(43.1)	(40.6)	(20.8)	(43.1)	(40.6)	(20.8)	(43.1)	(40.6)	(20.8)
	110.6	-522.4	206.0	36.2	-447.3	37.3	284.4	-603.8	103.2	278.1
	2.202	-10.397	4.101	0.720	-8.903	0.743	5.661	-12.018	2.055	5.537
c_O	1576	2305	1370	1666	2245	1364	1533	2276	1542	1585
	(31.4)	(45.9)	(27.3)	(33.2)	(44.7)	(27.1)	(30.5)	(45.3)	(30.7)	(31.6)
c_E	1425.3	2267.4	1426.4	1425.3	2267.4	1426.4	1425.3	2267.4	1426.1	1425.0
	(28.4)	(45.1)	(28.4)	(28.4)	(45.1)	(28.4)	(28.4)	(45.1)	(28.4)	(28.4)
	316.8	75.7	-110.5	519.9	-44.7	-122.0	223.3	17.2	241.0	337.3
	6.306	1.508	-2.199	10.349	-0.889	-2.428	4.446	0.342	4.798	6.715
N	5024	5024	5024	5024	5024	5024	5024	5024	5023	5023
X2	100.7	147.5	12.6	71.1	149.6	16.5	51.2	233.0	32.9	86.9
Gvalue	107.2	134.5	12.7	70.3	133.9	16.1	51.4	207.2	33.8	89.6
Gv/N	2.134	2.677	0.252	1.400	2.665	0.320	1.023	4.125	0.672	1.783
Prob	0.00000	0.00000	0.00544	0.00000	0.00000	0.00111	0.00000	0.00000	0.00000	0.00000

	51	52	53	54	55	56	57	58	59	60
a_O	361	795	1057	341	781	1001	393	859	913	317
	(7.2)	(15.8)	(21.0)	(6.8)	(15.5)	(19.9)	(7.8)	(17.1)	(18.2)	(6.3)
a_E	243.7	929.7	1186.9	243.7	929.7	1186.9	243.7	929.7	1186.9	243.7
	(4.9)	(18.5)	(23.6)	(4.9)	(18.5)	(23.6)	(4.9)	(18.5)	(23.6)	(4.9)
	283.6	-248.8	-245.1	229.0	-272.2	-341.1	375.5	-135.9	-479.1	166.6
	5.645	-4.954	-4.880	4.559	-5.419	-6.791	7.475	-2.705	-9.539	3.317
t_O	492	649	1377	554	640	1375	454	640	1322	509
	(9.8)	(12.9)	(27.4)	(11.0)	(12.7)	(27.4)	(9.0)	(12.7)	(26.3)	(10.1)
t_E	349.5	627.2	1368.2	349.5	627.2	1368.2	349.5	627.2	1368.2	349.5
	(7.0)	(12.5)	(27.2)	(7.0)	(12.5)	(27.2)	(7.0)	(12.5)	(27.2)	(7.0)
	336.4	44.3	17.7	510.3	25.9	13.6	237.4	25.9	-90.8	382.6
	6.697	0.883	0.352	10.159	0.515	0.272	4.727	0.515	-1.808	7.617
g_O	1885	2052	1050	2002	2165	1083	1787	2099	1231	2009
	(37.5)	(40.9)	(20.9)	(39.9)	(43.1)	(21.6)	(35.6)	(41.8)	(24.5)	(40.0)
g_E	2162.7	2040.0	1042.8	2162.7	2040.0	1042.8	2162.7	2040.0	1042.8	2162.7
	(43.1)	(40.6)	(20.8)	(43.1)	(40.6)	(20.8)	(43.1)	(40.6)	(20.8)	(43.1)
	-518.2	24.0	14.4	-309.2	257.5	81.8	-682.0	119.7	408.4	-296.3
	-10.316	0.478	0.286	-6.156	5.126	1.629	-13.578	2.382	8.130	-5.898
c_O	2285	1527	1539	2126	1437	1564	2389	1425	1557	2188
	(45.5)	(30.4)	(30.6)	(42.3)	(28.6)	(31.1)	(47.6)	(28.4)	(31.0)	(43.6)
c_E	2267.0	1426.1	1425.0	2267.0	1426.1	1425.0	2267.0	1426.1	1425.0	2267.0
	(45.1)	(28.4)	(28.4)	(45.1)	(28.4)	(28.4)	(45.1)	(28.4)	(28.4)	(45.1)
	36.2	208.8	236.9	-273.0	21.9	291.1	250.5	-2.2	275.8	-155.2
	0.720	4.157	4.715	-5.435	0.436	5.795	4.987	-0.044	5.491	-3.090
N	5023	5023	5023	5023	5023	5023	5023	5023	5023	5023
X2	150.3	27.5	23.4	179.1	31.8	44.3	194.4	7.3	111.0	108.4
Gvalue	138.0	28.3	23.8	157.0	33.0	45.5	181.3	7.5	114.3	97.7
Gv/N	2.747	0.564	0.473	3.127	0.657	0.905	3.610	0.149	2.275	1.946
Prob	0.00000	0.00000	0.00003	0.00000	0.00000	0.00000	0.00000	0.05846	0.00000	0.00000

	61	62	63	64	65	66	67	68	69	70
a_O	842	912	306	759	1001	339	840	992	330	909
	(16.8)	(18.2)	(6.1)	(15.1)	(19.9)	(6.7)	(16.7)	(19.7)	(6.6)	(18.1)
a_E	929.7	1186.9	243.7	929.7	1186.9	243.7	929.7	1186.9	243.7	929.7
	(18.5)	(23.6)	(4.9)	(18.5)	(23.6)	(4.9)	(18.5)	(23.6)	(4.9)	(18.5)
	-166.8	-480.6	139.2	-307.9	-341.1	223.7	-170.4	-356.0	200.0	-40.9
	-3.321	-9.568	2.771	-6.130	-6.791	4.453	-3.393	-7.087	3.981	-0.814
t_O	636	1485	536	649	1209	518	648	1326	437	628
	(12.7)	(29.6)	(10.7)	(12.9)	(24.1)	(10.3)	(12.9)	(26.4)	(8.7)	(12.5)
t_E	627.2	1368.2	349.5	627.2	1368.2	349.5	627.2	1368.2	349.5	627.2
	(12.5)	(27.2)	(7.0)	(12.5)	(27.2)	(7.0)	(12.5)	(27.2)	(7.0)	(12.5)
	17.7	243.3	458.3	44.3	-299.1	407.5	42.3	-83.1	195.2	1.6
	0.353	4.844	9.124	0.883	-5.955	8.113	0.842	-1.654	3.886	0.032
g_O	2035	1064	2037	2050	1033	2084	2038	1195	1976	2000
	(40.5)	(21.2)	(40.6)	(40.8)	(20.6)	(41.5)	(40.6)	(23.8)	(39.3)	(39.8)
g_E	2040.0	1042.8	2162.7	2040.0	1042.8	2162.7	2040.0	1042.8	2162.7	2040.0
	(40.6)	(20.8)	(43.1)	(40.6)	(20.8)	(43.1)	(40.6)	(20.8)	(43.1)	(40.6)
	-10.0	42.7	-244.0	20.0	-19.6	-154.6	-4.0	325.5	-356.8	-79.2
	-0.200	0.851	-4.858	0.398	-0.390	-3.077	-0.080	6.480	-7.104	-1.578
c_O	1510	1562	2144	1565	1780	2082	1497	1510	2280	1486
	(30.1)	(31.1)	(42.7)	(31.2)	(35.4)	(41.4)	(29.8)	(30.1)	(45.4)	(29.6)
c_E	1426.1	1425.0	2267.0	1426.1	1425.0	2267.0	1426.1	1425.0	2267.0	1426.1
	(28.4)	(28.4)	(45.1)	(28.4)	(28.4)	(45.1)	(28.4)	(28.4)	(45.1)	(28.4)
	172.7	286.7	-239.2	290.9	791.9	-354.4	145.3	174.9	26.1	122.3
	3.437	5.708	-4.762	5.792	15.765	-7.056	2.892	3.483	0.520	2.435
N	5023	5023	5023	5023	5023	5023	5023	5023	5023	5023
X2	13.3	87.3	129.3	45.7	136.2	136.4	12.9	60.6	68.6	3.8
Gvalue	13.5	92.2	114.3	47.4	132.0	122.2	13.1	61.4	64.4	3.7
Gv/N	0.269	1.835	2.276	0.943	2.629	2.432	0.261	1.223	1.282	0.074
Prob	0.00364	0.00000	0.00000	0.00000	0.00000	0.00000	0.00443	0.00000	0.00000	0.29131

	71	72	73	74	75	76	77	78	79	80
a_O	1077	327	841	1123	429	797	1003	330	801	1144
	(21.4)	(6.5)	(16.7)	(22.4)	(8.5)	(15.9)	(20.0)	(6.6)	(15.9)	(22.8)
a_E	1186.9	243.7	929.7	1186.9	243.7	929.7	1186.9	243.7	929.7	1186.9
	(23.6)	(4.9)	(18.5)	(23.6)	(4.9)	(18.5)	(23.6)	(4.9)	(18.5)	(23.6)
	-209.4	192.2	-168.6	-124.4	485.1	-245.5	-337.8	200.0	-238.7	-84.3
	-4.168	3.826	-3.357	-2.476	9.657	-4.887	-6.725	3.981	-4.752	-1.679
t_O	1269	414	588	1356	413	604	1482	410	693	1279
	(25.3)	(8.2)	(11.7)	(27.0)	(8.2)	(12.0)	(29.5)	(8.2)	(13.8)	(25.5)
t_E	1368.2	349.5	627.2	1368.2	349.5	627.2	1368.2	349.5	627.2	1368.2
	(27.2)	(7.0)	(12.5)	(27.2)	(7.0)	(12.5)	(27.2)	(7.0)	(12.5)	(27.2)
	-191.0	140.1	-75.9	-24.3	137.8	-45.5	236.8	130.8	138.3	-172.4
	-3.803	2.790	-1.511	-0.483	2.743	-0.907	4.715	2.604	2.753	-3.433
g_O	1166	2026	2106	1101	2005	1920	1087	2116	1980	1157
	(23.2)	(40.3)	(41.9)	(21.9)	(39.9)	(38.2)	(21.6)	(42.1)	(39.4)	(23.0)
g_E	1042.8	2162.7	2040.0	1042.8	2162.7	2040.0	1042.8	2162.7	2040.0	1042.8
	(20.8)	(43.1)	(40.6)	(20.8)	(43.1)	(40.6)	(20.8)	(43.1)	(40.6)	(20.8)
	260.3	-264.6	134.1	119.5	-303.7	-232.8	90.1	-92.4	-118.3	240.4
	5.182	-5.268	2.669	2.379	-6.045	-4.635	1.795	-1.840	-2.354	4.785
c_O	1511	2256	1488	1443	2176	1702	1451	2167	1549	1443
	(30.1)	(44.9)	(29.6)	(28.7)	(43.3)	(33.9)	(28.9)	(43.1)	(30.8)	(28.7)
c_E	1425.0	2267.0	1426.1	1425.0	2267.0	1426.1	1425.0	2267.0	1426.1	1425.0
	(28.4)	(45.1)	(28.4)	(28.4)	(45.1)	(28.4)	(28.4)	(45.1)	(28.4)	(28.4)
	177.1	-21.9	126.5	36.2	-178.3	602.0	52.4	-195.5	256.1	36.2
	3.525	-0.436	2.518	0.721	-3.549	11.986	1.044	-3.892	5.099	0.721
N	5023	5023	5023	5023	5023	5023	5023	5023	5023	5023
X2	37.1	49.0	15.7	7.0	167.5	80.2	40.3	46.4	37.1	20.1
Gvalue	37.0	45.8	16.0	7.0	140.9	78.2	41.6	42.8	37.5	19.8
Gv/N	0.736	0.911	0.319	0.140	2.805	1.557	0.829	0.853	0.746	0.394
Prob	0.00000	0.00000	0.00113	0.07102	0.00000	0.00000	0.00000	0.00000	0.00000	0.00019

	81	82	83	84	85	86	87	88	89	90
a_O	332	870	1114	321	874	1216	293	848	1097	303
	(6.6)	(17.3)	(22.2)	(6.4)	(17.4)	(24.2)	(5.8)	(16.9)	(21.8)	(6.0)
a_E	243.7	929.7	1186.9	243.7	929.7	1186.9	243.7	929.7	1186.9	243.7
	(4.9)	(18.5)	(23.6)	(4.9)	(18.5)	(23.6)	(4.9)	(18.5)	(23.6)	(4.9)
	205.2	-115.5	-141.3	176.7	-108.0	58.8	107.8	-156.0	-172.9	131.9
	4.085	-2.299	-2.813	3.519	-2.149	1.171	2.147	-3.105	-3.442	2.625
t_O	522	680	1228	387	599	1252	367	583	1296	502
	(10.4)	(13.5)	(24.4)	(7.7)	(11.9)	(24.9)	(7.3)	(11.6)	(25.8)	(10.0)
t_E	349.5	627.2	1368.2	349.5	627.2	1368.2	349.5	627.2	1368.2	349.5
	(7.0)	(12.5)	(27.2)	(7.0)	(12.5)	(27.2)	(7.0)	(12.5)	(27.2)	(7.0)
	418.7	109.9	-265.5	78.8	-55.1	-222.2	35.8	-85.2	-140.5	363.4

	8.335	2.188	-5.286	1.569	-1.097	-4.424	0.712	-1.696	-2.797	7.235
g_O	1867	2018	1242	2020	2084	1065	2054	2084	1040	2038
	(37.2)	(40.2)	(24.7)	(40.2)	(41.5)	(21.2)	(40.9)	(41.5)	(20.7)	(40.6)
g_E	2162.7	2040.0	1042.8	2162.7	2040.0	1042.8	2162.7	2040.0	1042.8	2162.7
	(43.1)	(40.6)	(20.8)	(43.1)	(40.6)	(20.8)	(43.1)	(40.6)	(20.8)	(43.1)
	-549.0	-43.8	434.1	-275.8	88.9	44.8	-211.9	88.9	-5.7	-242.1
	-10.930	-0.872	8.643	-5.491	1.770	0.891	-4.218	1.770	-0.113	-4.820
c_O	2302	1455	1439	2295	1466	1490	2309	1508	1590	2180
	(45.8)	(29.0)	(28.6)	(45.7)	(29.2)	(29.7)	(46.0)	(30.0)	(31.7)	(43.4)
c_E	2267.0	1426.1	1425.0	2267.0	1426.1	1425.0	2267.0	1426.1	1425.0	2267.0
	(45.1)	(28.4)	(28.4)	(45.1)	(28.4)	(28.4)	(45.1)	(28.4)	(28.4)	(45.1)
	70.6	58.4	28.1	56.4	80.9	132.9	84.8	168.4	348.4	-170.6
	1.405	1.163	0.560	1.122	1.611	2.646	1.688	3.353	6.936	-3.396
N	5023	5023	5023	5023	5023	5023	5023	5023	5023	5023
X2	158.0	9.1	57.0	38.3	6.7	14.0	17.1	15.9	29.7	91.4
Gvalue	145.4	9.1	55.4	36.1	6.7	14.3	16.5	16.1	29.3	82.6
Gv/N	2.895	0.180	1.103	0.719	0.134	0.284	0.329	0.321	0.583	1.644
Prob	0.00000	0.02848	0.00000	0.00000	0.08068	0.00258	0.00088	0.00106	0.00000	0.00000

	91	92	93	94	95	96	97	98	99	100
a_O	877	1188	310	840	1035	312	943	1112	289	818
	(17.5)	(23.7)	(6.2)	(16.7)	(20.6)	(6.2)	(18.8)	(22.2)	(5.8)	(16.3)
a_E	929.7	1186.9	243.7	928.6	1185.5	243.5	928.2	1185.1	243.4	927.6
	(18.5)	(23.6)	(4.9)	(18.5)	(23.6)	(4.9)	(18.5)	(23.6)	(4.9)	(18.5)
	-102.3	2.1	149.1	-168.4	-281.1	154.8	29.8	-141.5	99.4	-205.8
	-2.037	0.042	2.968	-3.357	-5.602	3.085	0.595	-2.822	1.981	-4.106
t_O	620	1188	423	735	1394	448	576	1296	464	602
	(12.3)	(23.7)	(8.4)	(14.7)	(27.8)	(8.9)	(11.5)	(25.8)	(9.3)	(12.0)
t_E	627.2	1368.2	349.5	626.5	1366.6	349.1	626.2	1366.0	349.0	625.8
	(12.5)	(27.2)	(7.0)	(12.5)	(27.2)	(7.0)	(12.5)	(27.2)	(7.0)	(12.5)
	-14.3	-335.5	161.4	234.9	55.4	223.4	-96.3	-136.4	264.3	-46.7
	-0.285	-6.680	3.213	4.682	1.105	4.453	-1.920	-2.719	5.271	-0.933
g_O	2130	1141	2167	1983	1042	1985	2105	1135	1951	2132
	(42.4)	(22.7)	(43.1)	(39.5)	(20.8)	(39.6)	(42.0)	(22.6)	(38.9)	(42.5)
g_E	2040.0	1042.8	2162.7	2037.6	1041.6	2160.1	2036.8	1041.2	2159.3	2035.6
	(40.6)	(20.8)	(43.1)	(40.6)	(20.8)	(43.1)	(40.6)	(20.8)	(43.1)	(40.6)
	183.9	205.3	8.6	-107.7	0.8	-335.7	138.7	195.8	-395.8	197.4
	3.661	4.087	0.170	-2.146	0.016	-6.691	2.766	3.905	-7.892	3.938
c_O	1396	1506	2123	1459	1546	2272	1391	1472	2311	1460
	(27.8)	(30.0)	(42.3)	(29.1)	(30.8)	(45.3)	(27.7)	(29.4)	(46.1)	(29.1)
c_E	1426.1	1425.0	2267.0	1424.4	1423.3	2264.3	1423.8	1422.7	2263.4	1423.0
	(28.4)	(28.4)	(45.1)	(28.4)	(28.4)	(45.1)	(28.4)	(28.4)	(45.1)	(28.4)
	-59.5	166.5	-278.6	70.1	255.7	15.5	-64.9	100.2	96.2	75.0
	-1.185	3.315	-5.547	1.396	5.096	0.308	-1.294	1.998	1.919	1.497
N	5023	5023	5023	5017	5017	5017	5015	5015	5015	5012
X2	7.7	37.6	42.6	29.6	30.2	61.5	7.3	18.2	67.6	19.4
Gvalue	7.7	38.3	40.4	28.8	30.8	58.0	7.4	18.1	64.2	19.9
Gv/N	0.153	0.763	0.804	0.575	0.614	1.156	0.147	0.362	1.279	0.397
Prob	0.05315	0.00000	0.00000	0.00000	0.00000	0.00000	0.06033	0.00041	0.00000	0.00018

	101	102	103	104	105	106	107	108	109	110
a_O	1237	268	862	1150	320	934	1178	256	891	1172
	(24.7)	(5.3)	(17.2)	(23.0)	(6.4)	(18.7)	(23.5)	(5.1)	(17.8)	(23.4)
a_E	1184.3	243.2	926.9	1183.4	243.0	926.0	1182.2	242.8	925.8	1182.0
	(23.6)	(4.9)	(18.5)	(23.6)	(4.9)	(18.5)	(23.6)	(4.9)	(18.5)	(23.6)
	107.6	52.0	-125.2	-65.8	176.1	16.1	-8.4	27.2	-68.3	-19.9
	2.147	1.038	-2.499	-1.315	3.517	0.322	-0.168	0.543	-1.365	-0.397
t_O	1325	381	610	1243	433	785	1310	497	676	1351
	(26.4)	(7.6)	(12.2)	(24.8)	(8.6)	(15.7)	(26.2)	(9.9)	(13.5)	(27.0)
t_E	1365.2	348.8	625.3	1364.1	348.5	624.7	1362.7	348.2	624.6	1362.5
	(27.2)	(7.0)	(12.5)	(27.2)	(7.0)	(12.5)	(27.2)	(7.0)	(12.5)	(27.2)
	-79.2	67.3	-30.3	-231.1	188.0	358.6	-103.4	353.8	107.0	-22.8
	-1.580	1.344	-0.605	-4.615	3.754	7.168	-2.067	7.072	2.138	-0.457
g_O	1017	2009	2190	1036	2104	1958	1053	2014	2035	1038
	(20.3)	(40.1)	(43.7)	(20.7)	(42.0)	(39.1)	(21.0)	(40.3)	(40.7)	(20.8)
g_E	1040.6	2158.0	2033.9	1039.7	2156.3	2031.9	1038.7	2154.1	2031.5	1038.5
	(20.8)	(43.1)	(40.6)	(20.8)	(43.1)	(40.6)	(20.8)	(43.1)	(40.6)	(20.8)
	-46.6	-287.4	323.8	-7.5	-103.3	-145.1	28.8	-270.9	7.0	-1.0
	-0.930	-5.735	6.466	-0.149	-2.062	-2.900	0.576	-5.415	0.140	-0.020
c_O	1433	2354	1346	1579	2151	1326	1462	2236	1400	1441
	(28.6)	(47.0)	(26.9)	(31.5)	(43.0)	(26.5)	(29.2)	(44.7)	(28.0)	(28.8)
c_E	1421.9	2262.0	1421.8	1420.8	2260.2	1420.4	1419.3	2258.0	1420.1	1419.1
	(28.4)	(45.1)	(28.4)	(28.4)	(45.1)	(28.4)	(28.4)	(45.1)	(28.4)	(28.4)
	22.3	187.6	-147.6	333.5	-213.1	-182.4	86.6	-43.7	-40.0	44.2
	0.445	3.744	-2.946	6.659	-4.255	-3.646	1.731	-0.874	-0.799	0.884
N	5012	5012	5008	5008	5008	5003	5003	5003	5002	5002
X2	4.1	19.5	20.9	29.3	51.4	50.2	3.5	73.7	5.8	0.5
Gvalue	4.1	19.6	20.8	29.1	47.8	47.2	3.5	66.4	5.7	0.5
Gv/N	0.082	0.390	0.416	0.580	0.955	0.944	0.071	1.326	0.115	0.010
Prob	0.24812	0.00021	0.00011	0.00000	0.00000	0.00000	0.31447	0.00000	0.12501	0.91465

	111	112	113	114	115	116	117	118	119	120
a_O	282	823	1121	312	960	1074	289	841	1128	356
	(5.6)	(16.5)	(22.4)	(6.2)	(19.2)	(21.5)	(5.8)	(16.9)	(22.6)	(7.1)
a_E	242.7	925.1	1181.0	242.5	924.7	1180.6	242.4	923.2	1178.7	242.0
	(4.9)	(18.5)	(23.6)	(4.9)	(18.5)	(23.6)	(4.9)	(18.5)	(23.6)	(4.9)
	84.6	-192.4	-117.0	157.2	72.0	-203.2	101.5	-156.9	-99.1	274.7
	1.691	-3.850	-2.340	3.144	1.440	-4.067	2.033	-3.145	-1.988	5.507
t_O	399	775	1348	381	633	1362	408	710	1222	369
	(8.0)	(15.5)	(27.0)	(7.6)	(12.7)	(27.3)	(8.2)	(14.2)	(24.5)	(7.4)
t_E	348.1	624.1	1361.4	347.8	623.8	1360.8	347.7	622.8	1358.7	347.1
	(7.0)	(12.5)	(27.2)	(7.0)	(12.5)	(27.2)	(7.0)	(12.5)	(27.2)	(7.0)
	108.9	335.7	-26.6	69.5	18.5	2.3	130.6	186.0	-259.1	45.1
	2.178	6.717	-0.533	1.390	0.370	0.047	2.614	3.729	-5.194	0.905
g_O	2080	2070	1065	1956	1938	1046	1942	2031	1154	2073
	(41.6)	(41.4)	(21.3)	(39.1)	(38.8)	(20.9)	(38.9)	(40.7)	(23.1)	(41.6)
g_E	2153.7	2029.9	1037.7	2152.0	2029.1	1037.2	2151.1	2025.8	1035.6	2147.7
	(43.1)	(40.6)	(20.8)	(43.1)	(40.6)	(20.8)	(43.1)	(40.6)	(20.8)	(43.1)
	-144.8	81.1	55.4	-373.5	-178.0	17.6	-397.2	10.4	249.9	-146.7
	-2.895	1.622	1.108	-7.473	-3.562	0.352	-7.950	0.209	5.010	-2.941
c_O	2241	1330	1464	2349	1465	1514	2357	1406	1484	2190
	(44.8)	(26.6)	(29.3)	(47.0)	(29.3)	(30.3)	(47.2)	(28.2)	(29.8)	(43.9)
c_E	2257.5	1419.0	1417.9	2255.7	1418.4	1417.4	2254.8	1416.2	1415.1	2251.2
	(45.1)	(28.4)	(28.4)	(45.1)	(28.4)	(28.4)	(45.1)	(28.4)	(28.4)	(45.1)
	-32.9	-172.3	93.6	190.4	94.7	199.7	209.0	-20.2	141.1	-120.7
	-0.658	-3.447	1.873	3.810	1.895	3.998	4.183	-0.406	2.829	-2.420

N	5002	4998	4998	4998	4996	4996	4996	4988	4988	4988
X2	16.4	54.1	5.4	44.8	7.1	16.3	44.4	19.6	32.8	59.3
Gvalue	15.8	52.1	5.4	43.5	7.1	16.4	43.9	19.3	32.8	52.4
Gv/N	0.316	1.042	0.109	0.871	0.143	0.329	0.879	0.387	0.658	1.051
Prob	0.00124	0.00000	0.14272	0.00000	0.06796	0.00092	0.00000	0.00024	0.00000	0.00000

	121	122	123	124	125	126	127	128	129	130
a_O	822	1102	290	865	1123	268	888	1217	276	827
	(16.5)	(22.1)	(5.8)	(17.4)	(22.6)	(5.4)	(17.9)	(24.5)	(5.5)	(16.7)
a_E	922.1	1177.3	241.8	921.2	1176.1	241.5	920.6	1175.4	241.4	919.1
	(18.5)	(23.6)	(4.9)	(18.5)	(23.6)	(4.9)	(18.5)	(23.6)	(4.9)	(18.5)
	-188.9	-145.6	105.5	-108.8	-103.7	55.8	-64.1	84.7	74.0	-174.7
	-3.792	-2.922	2.118	-2.187	-2.084	1.121	-1.288	1.703	1.488	-3.518
t_O	562	1193	372	619	1219	506	602	1404	394	737
	(11.3)	(23.9)	(7.5)	(12.4)	(24.5)	(10.2)	(12.1)	(28.2)	(7.9)	(14.8)
t_E	622.1	1357.0	346.7	621.5	1355.7	346.3	621.1	1354.8	346.1	620.1
	(12.5)	(27.2)	(7.0)	(12.5)	(27.2)	(7.0)	(12.5)	(27.2)	(7.0)	(12.5)
	-114.2	-307.4	52.4	-4.9	-259.1	383.7	-37.6	100.1	102.1	254.6
	-2.292	-6.170	1.052	-0.099	-5.205	7.709	-0.755	2.012	2.052	5.127
g_O	2096	1128	2063	2033	1096	2073	2002	991	2069	2092
	(42.1)	(22.6)	(41.4)	(40.8)	(22.0)	(41.7)	(40.2)	(19.9)	(41.6)	(42.1)
g_E	2023.4	1034.3	2145.1	2021.3	1033.3	2142.9	2020.1	1032.7	2141.6	2016.9
	(40.6)	(20.8)	(43.1)	(40.6)	(20.8)	(43.1)	(40.6)	(20.8)	(43.1)	(40.6)
	147.8	195.6	-161.0	23.4	129.1	-137.5	-36.1	-81.6	-142.8	153.0
	2.968	3.925	-3.231	0.470	2.595	-2.763	-0.725	-1.641	-2.870	3.081
c_O	1502	1559	2257	1460	1539	2130	1482	1362	2235	1310
	(30.1)	(31.3)	(45.3)	(29.3)	(30.9)	(42.8)	(29.8)	(27.4)	(44.9)	(26.4)
c_E	1414.5	1413.4	2248.5	1413.0	1412.0	2246.2	1412.2	1411.1	2244.9	1409.9
	(28.4)	(28.4)	(45.1)	(28.4)	(28.4)	(45.1)	(28.4)	(28.4)	(45.1)	(28.4)
	180.4	305.7	17.1	95.5	265.2	-226.3	143.0	-96.5	-19.7	-192.6
	3.621	6.137	0.343	1.918	5.328	-4.547	2.876	-1.940	-0.396	-3.878
N	4982	4982	4982	4977	4977	4977	4974	4974	4974	4966
X2	24.7	48.1	14.6	5.1	31.4	84.8	5.4	6.6	14.1	41.2
Gvalue	25.2	48.3	14.1	5.1	31.5	75.6	5.3	6.7	13.6	40.4
Gv/N	0.505	0.970	0.282	0.103	0.633	1.518	0.107	0.134	0.274	0.813
Prob	0.00001	0.00000	0.00282	0.16336	0.00000	0.00000	0.14973	0.08377	0.00348	0.00000

	131	132	133	134	135	136	137	138	139	140
a_O	1063	291	945	1226	308	839	1054	283	929	1101
	(21.4)	(5.9)	(19.1)	(24.7)	(6.2)	(16.9)	(21.3)	(5.7)	(18.8)	(22.3)
a_E	1173.5	241.0	917.8	1171.8	240.6	916.4	1169.9	240.3	915.2	1168.5
	(23.6)	(4.9)	(18.5)	(23.6)	(4.9)	(18.5)	(23.6)	(4.9)	(18.5)	(23.6)
	-210.2	109.8	55.1	110.8	152.0	-148.0	-220.0	92.7	27.7	-131.0
	-4.233	2.210	1.111	2.235	3.066	-2.989	-4.443	1.872	0.560	-2.650
t_O	1394	341	600	1204	412	674	1360	355	677	1320
	(28.1)	(6.9)	(12.1)	(24.3)	(8.3)	(13.6)	(27.5)	(7.2)	(13.7)	(26.7)
t_E	1352.7	345.6	619.2	1350.8	345.1	618.2	1348.6	344.5	617.5	1346.9
	(27.2)	(7.0)	(12.5)	(27.2)	(7.0)	(12.5)	(27.2)	(7.0)	(12.5)	(27.2)
	83.9	-9.1	-37.8	-277.0	146.0	116.5	22.9	21.3	124.6	-53.4
	1.690	-0.183	-0.763	-5.585	2.945	2.352	0.463	0.429	2.521	-1.079
g_O	1053	2076	2064	1142	2021	2060	1127	2110	2016	1070
	(21.2)	(41.8)	(41.6)	(23.0)	(40.8)	(41.6)	(22.8)	(42.6)	(40.8)	(21.6)
g_E	1031.0	2138.2	2014.0	1029.6	2135.2	2010.8	1027.9	2131.7	2008.3	1026.7
	(20.8)	(43.1)	(40.6)	(20.8)	(43.1)	(40.6)	(20.8)	(43.1)	(40.6)	(20.8)
	44.4	-122.5	101.2	236.7	-222.1	99.6	207.5	-43.2	15.3	88.5
	0.895	-2.468	2.040	4.774	-4.479	2.013	4.190	-0.873	0.310	1.790
c_O	1456	2258	1350	1387	2218	1378	1410	2203	1323	1454
	(29.3)	(45.5)	(27.2)	(28.0)	(44.7)	(27.8)	(28.5)	(44.5)	(26.8)	(29.4)
c_E	1408.8	2241.3	1407.9	1406.9	2238.1	1405.7	1404.6	2234.5	1403.9	1402.9
	(28.4)	(45.1)	(28.4)	(28.4)	(45.1)	(28.4)	(28.4)	(45.1)	(28.4)	(28.4)
	95.9	33.6	-113.4	-39.4	-40.0	-54.8	10.8	-62.5	-157.1	104.1
	1.931	0.677	-2.287	-0.795	-0.807	-1.106	0.219	-1.263	-3.178	2.104
N	4966	4966	4959	4959	4959	4951	4951	4951	4945	4945
X2	13.7	12.4	5.0	31.0	38.1	13.3	21.2	8.6	10.6	8.1
Gvalue	14.0	11.7	5.0	31.2	35.9	13.4	21.3	8.2	10.6	8.2
Gv/N	0.282	0.236	0.102	0.628	0.724	0.270	0.430	0.165	0.214	0.165
Prob	0.00288	0.00832	0.16857	0.00000	0.00000	0.00393	0.00009	0.04255	0.01437	0.04271

	141	142	143	144	145	146	147	148	149	150
a_O	275	830	1230	362	803	1088	332	851	1097	267
	(5.6)	(16.8)	(24.9)	(7.3)	(16.3)	(22.1)	(6.7)	(17.3)	(22.2)	(5.4)
a_E	240.0	914.0	1166.9	239.6	913.2	1165.9	239.4	912.7	1165.2	239.3
	(4.9)	(18.5)	(23.6)	(4.9)	(18.5)	(23.6)	(4.9)	(18.5)	(23.6)	(4.9)
	75.0	-159.9	129.6	298.7	-206.6	-150.5	217.0	-119.1	-132.3	58.5
	1.516	-3.239	2.625	6.049	-4.186	-3.050	4.399	-2.414	-2.684	1.187
t_O	438	632	1312	390	609	1415	318	666	1268	465
	(8.9)	(12.8)	(26.6)	(7.9)	(12.3)	(28.7)	(6.4)	(13.5)	(25.7)	(9.4)
t_E	344.1	616.6	1345.0	343.6	616.1	1344.0	343.3	615.7	1343.1	343.1
	(7.0)	(12.5)	(27.2)	(7.0)	(12.5)	(27.2)	(7.0)	(12.5)	(27.2)	(7.0)
	211.3	31.2	-65.3	98.7	-14.1	145.8	-48.8	104.6	-146.0	282.6
	4.274	0.632	-1.322	2.000	-0.286	2.955	-0.989	2.121	-2.961	5.732
g_O	2144	1967	1065	1911	2155	989	2038	1936	1106	1988
	(43.4)	(39.8)	(21.6)	(38.7)	(43.7)	(20.0)	(41.3)	(39.3)	(22.4)	(40.3)
g_E	2129.1	2005.5	1025.2	2126.1	2003.9	1024.4	2124.4	2002.7	1023.7	2123.1
	(43.1)	(40.6)	(20.8)	(43.1)	(40.6)	(20.8)	(43.1)	(40.6)	(20.8)	(43.1)
	29.8	-76.3	81.1	-407.7	313.4	-69.5	-169.2	-131.1	170.9	-261.4
	0.603	-1.544	1.643	-8.257	6.351	-1.409	-3.430	-2.658	3.467	-5.302
c_O	2088	1509	1331	2275	1367	1442	2246	1478	1460	2211
	(42.2)	(30.6)	(27.0)	(46.1)	(27.7)	(29.2)	(45.5)	(30.0)	(29.6)	(44.8)
c_E	2231.8	1402.0	1400.9	2228.6	1400.8	1399.8	2226.8	1400.0	1398.9	2225.5
	(45.1)	(28.4)	(28.4)	(45.1)	(28.4)	(28.4)	(45.1)	(28.4)	(28.4)	(45.1)
	-278.1	222.1	-136.3	93.7	-66.8	85.7	38.5	160.3	124.8	-28.8
	-5.624	4.497	-2.759	1.898	-1.354	1.738	0.781	3.251	2.531	-0.585
N	4945	4938	4938	4938	4934	4934	4934	4931	4931	4931
X2	40.1	17.0	9.3	91.5	25.6	11.5	41.3	14.8	17.5	55.2
Gvalue	38.0	17.1	9.2	83.4	25.9	11.5	37.6	14.8	17.4	50.9
Gv/N	0.769	0.345	0.187	1.690	0.525	0.233	0.761	0.300	0.353	1.032
Prob	0.00000	0.00069	0.02617	0.00000	0.00001	0.00924	0.00000	0.00202	0.00058	0.00000

Bmel_ABORTUS2308
5UTR:

	50	49	48	47	46	45	44	43	42	41
a_O	704	762	657	700	754	705	748	796	723	725
	(23.2)	(25.1)	(21.7)	(23.1)	(24.9)	(23.2)	(24.7)	(26.2)	(23.8)	(23.9)
a_E	750.7	750.7	750.7	750.7	750.7	750.7	750.7	750.7	750.7	750.7
	(24.7)	(24.7)	(24.7)	(24.7)	(24.7)	(24.7)	(24.7)	(24.7)	(24.7)	(24.7)
	-90.5	22.7	-175.2	-98.0	6.6	-88.6	-5.5	93.2	-54.4	-50.6
	-2.982	0.748	-5.776	-3.228	0.216	-2.921	-0.180	3.072	-1.794	-1.667
t_O	718	695	789	655	727	712	706	708	720	720
	(23.7)	(22.9)	(26.0)	(21.6)	(24.0)	(23.5)	(23.3)	(23.3)	(23.7)	(23.7)
t_E	705.1	705.1	705.1	705.1	705.1	705.1	705.1	705.1	705.1	705.1
	(23.2)	(23.2)	(23.2)	(23.2)	(23.2)	(23.2)	(23.2)	(23.2)	(23.2)	(23.2)
	26.0	-20.1	177.4	-96.6	44.4	13.8	1.8	5.8	30.1	30.1
	0.857	-0.662	5.846	-3.184	1.464	0.456	0.058	0.190	0.991	0.991
g_O	864	771	818	866	761	819	844	775	796	836
	(28.5)	(25.4)	(27.0)	(28.5)	(25.1)	(27.0)	(27.8)	(25.5)	(26.2)	(27.6)
g_E	821.1	821.1	821.1	821.1	821.1	821.1	821.1	821.1	821.1	821.1
	(27.1)	(27.1)	(27.1)	(27.1)	(27.1)	(27.1)	(27.1)	(27.1)	(27.1)	(27.1)
	88.1	-97.0	-6.1	92.3	-115.6	-4.1	46.5	-89.5	-49.3	30.2
	2.903	-3.197	-0.201	3.042	-3.811	-0.136	1.533	-2.949	-1.626	0.994
c_O	748	806	770	813	792	798	736	755	795	753
	(24.7)	(26.6)	(25.4)	(26.8)	(26.1)	(26.3)	(24.3)	(24.9)	(26.2)	(24.8)
c_E	757.1	757.1	757.1	757.1	757.1	757.1	757.1	757.1	757.1	757.1
	(25.0)	(25.0)	(25.0)	(25.0)	(25.0)	(25.0)	(25.0)	(25.0)	(25.0)	(25.0)
	-18.1	100.9	26.0	115.9	71.4	84.0	-41.6	-4.2	77.7	-8.2
	-0.596	3.326	0.858	3.818	2.354	2.768	-1.371	-0.138	2.561	-0.269
N	3034	3034	3034	3034	3034	3034	3034	3034	3034	3034
X2	5.5	6.5	21.9	13.6	6.7	5.1	1.2	5.3	4.0	1.5
Gvalue	5.5	6.5	22.1	13.6	6.8	5.1	1.2	5.3	4.0	1.5
Gv/N	0.182	0.215	0.727	0.448	0.223	0.168	0.041	0.176	0.131	0.049
Prob	0.13733	0.08871	0.00006	0.00350	0.07938	0.16548	0.74364	0.14932	0.26265	0.68313

	40	39	38	37	36	35	34	33	32	31
a_O	733	711	708	687	635	671	711	644	731	745
	(24.2)	(23.4)	(23.3)	(22.6)	(20.9)	(22.1)	(23.4)	(21.2)	(24.1)	(24.6)
a_E	750.7	750.7	750.7	750.7	750.7	750.7	750.7	750.7	750.7	750.7
	(24.7)	(24.7)	(24.7)	(24.7)	(24.7)	(24.7)	(24.7)	(24.7)	(24.7)	(24.7)
	-35.0	-77.3	-83.0	-121.9	-212.6	-150.7	-77.3	-197.5	-38.9	-11.4
	-1.155	-2.548	-2.735	-4.017	-7.008	-4.966	-2.548	-6.510	-1.283	-0.376
t_O	739	735	680	744	742	705	785	755	714	714
	(24.4)	(24.2)	(22.4)	(24.5)	(24.5)	(23.2)	(25.9)	(24.9)	(23.5)	(23.5)
t_E	705.1	705.1	705.1	705.1	705.1	705.1	705.1	705.1	705.1	705.1
	(23.2)	(23.2)	(23.2)	(23.2)	(23.2)	(23.2)	(23.2)	(23.2)	(23.2)	(23.2)
	69.4	61.0	-49.3	79.9	75.7	-0.2	168.5	103.2	17.9	17.9
	2.286	2.011	-1.626	2.632	2.494	-0.008	5.553	3.402	0.589	0.589
g_O	759	792	857	779	793	844	788	841	805	762
	(25.0)	(26.1)	(28.2)	(25.7)	(26.1)	(27.8)	(26.0)	(27.7)	(26.5)	(25.1)
g_E	821.1	821.1	821.1	821.1	821.1	821.1	821.1	821.1	821.1	821.1
	(27.1)	(27.1)	(27.1)	(27.1)	(27.1)	(27.1)	(27.1)	(27.1)	(27.1)	(27.1)
	-119.3	-57.1	73.4	-81.9	-55.1	46.5	-64.8	40.4	-31.8	-113.8
	-3.932	-1.881	2.420	-2.700	-1.818	1.533	-2.135	1.330	-1.048	-3.750
c_O	803	796	789	824	864	814	750	794	784	813
	(26.5)	(26.2)	(26.0)	(27.2)	(28.5)	(26.8)	(24.7)	(26.2)	(25.8)	(26.8)
c_E	757.1	757.1	757.1	757.1	757.1	757.1	757.1	757.1	757.1	757.1
	(25.0)	(25.0)	(25.0)	(25.0)	(25.0)	(25.0)	(25.0)	(25.0)	(25.0)	(25.0)
	94.5	79.8	65.1	139.6	228.3	118.0	-14.1	75.6	54.8	115.9
	3.116	2.630	2.147	4.600	7.523	3.889	-0.465	2.491	1.805	3.818
N	3034	3034	3034	3034	3034	3034	3034	3034	3034	3034
X2	9.5	6.4	6.2	15.6	35.8	13.4	12.5	21.0	1.9	8.5
Gvalue	9.6	6.4	6.3	15.6	36.1	13.6	12.3	21.6	1.9	8.5
Gv/N	0.315	0.211	0.206	0.515	1.191	0.448	0.405	0.714	0.062	0.281
Prob	0.02262	0.09383	0.09951	0.00136	0.00000	0.00351	0.00648	0.00008	0.59426	0.03608

	30	29	28	27	26	25	24	23	22	21
a_O	690	717	751	738	780	794	777	816	804	771
	(22.7)	(23.6)	(24.8)	(24.3)	(25.7)	(26.2)	(25.6)	(26.9)	(26.5)	(25.4)
a_E	750.7	750.7	750.7	750.7	750.7	750.7	750.7	750.7	750.7	750.7
	(24.7)	(24.7)	(24.7)	(24.7)	(24.7)	(24.7)	(24.7)	(24.7)	(24.7)	(24.7)
	-116.4	-65.9	0.5	-25.2	59.7	89.0	53.4	136.1	110.2	41.1
	-3.837	-2.173	0.018	-0.832	1.967	2.933	1.762	4.484	3.633	1.354
t_O	741	679	722	717	639	635	684	648	750	726
	(24.4)	(22.4)	(23.8)	(23.6)	(21.1)	(20.9)	(22.5)	(21.4)	(24.7)	(23.9)
t_E	705.1	705.1	705.1	705.1	705.1	705.1	705.1	705.1	705.1	705.1
	(23.2)	(23.2)	(23.2)	(23.2)	(23.2)	(23.2)	(23.2)	(23.2)	(23.2)	(23.2)
	73.6	-51.3	34.2	24.0	-125.8	-133.0	-41.6	-109.5	92.6	42.4
	2.424	-1.690	1.126	0.790	-4.148	-4.384	-1.371	-3.609	3.051	1.397
g_O	787	857	782	760	850	769	751	786	703	755
	(25.9)	(28.2)	(25.8)	(25.0)	(28.0)	(25.3)	(24.8)	(25.9)	(23.2)	(24.9)
g_E	821.1	821.1	821.1	821.1	821.1	821.1	821.1	821.1	821.1	821.1
	(27.1)	(27.1)	(27.1)	(27.1)	(27.1)	(27.1)	(27.1)	(27.1)	(27.1)	(27.1)
	-66.7	73.4	-76.2	-117.5	58.9	-100.7	-134.0	-68.6	-218.3	-126.7
	-2.198	2.420	-2.513	-3.872	1.941	-3.321	-4.415	-2.261	-7.194	-4.175
c_O	816	781	779	819	765	836	822	784	777	782
	(26.9)	(25.7)	(25.7)	(27.0)	(25.2)	(27.6)	(27.1)	(25.8)	(25.6)	(25.8)
c_E	757.1	757.1	757.1	757.1	757.1	757.1	757.1	757.1	757.1	757.1
	(25.0)	(25.0)	(25.0)	(25.0)	(25.0)	(25.0)	(25.0)	(25.0)	(25.0)	(25.0)
	122.3	48.6	44.4	128.7	15.9	165.8	135.2	54.8	40.3	50.6
	4.031	1.601	1.465	4.244	0.524	5.464	4.457	1.805	1.330	1.669
N	3034	3034	3034	3034	3034	3034	3034	3034	3034	3034
X2	12.7	4.8	2.9	10.0	8.4	21.0	13.1	12.8	24.1	7.3
Gvalue	12.7	4.8	2.9	10.0	8.6	21.0	13.1	12.7	24.9	7.4
Gv/N	0.420	0.159	0.096	0.330	0.284	0.692	0.432	0.420	0.820	0.245
Prob	0.00521	0.18574	0.40453	0.01853	0.03478	0.00011	0.00439	0.00524	0.00002	0.05941

	20	19	18	17	16	15	14	13	12	11
a_O	852	880	832	867	907	886	921	996	970	917
	(28.1)	(29.0)	(27.4)	(28.6)	(29.9)	(29.2)	(30.4)	(32.8)	(32.0)	(30.2)
a_E	750.7	750.7	750.7	750.7	750.7	750.7	750.7	750.7	750.7	750.7
	(24.7)	(24.7)	(24.7)	(24.7)	(24.7)	(24.7)	(24.7)	(24.7)	(24.7)	(24.7)
	215.6	279.6	171.0	249.7	343.0	293.6	376.5	563.1	497.1	366.9
	7.107	9.216	5.637	8.230	11.306	9.676	12.410	18.561	16.385	12.093
t_O	682	725	741	663	686	694	569	566	484	398
	(22.5)	(23.9)	(24.4)	(21.9)	(22.6)	(22.9)	(18.8)	(18.7)	(16.0)	(13.1)

```
t_E       705.1   705.1   705.1   705.1   705.1   705.1   705.1   705.1   705.1   705.1
        ( 23.2) ( 23.2) ( 23.2) ( 23.2) ( 23.2) ( 23.2) ( 23.2) ( 23.2) ( 23.2) ( 23.2)
          -45.5    40.3    73.6   -81.7   -37.7   -22.1  -244.1  -248.8  -364.2  -455.2
         -1.499   1.329   2.424  -2.692  -1.243  -0.727  -8.045  -8.200 -12.005 -15.005
g_O         731     657     794     759     758     792     946     870    1116    1287
        ( 24.1) ( 21.7) ( 26.2) ( 25.0) ( 25.0) ( 26.1) ( 31.2) ( 28.7) ( 36.8) ( 42.4)
g_E       821.1   821.1   821.1   821.1   821.1   821.1   821.1   821.1   821.1   821.1
        ( 27.1) ( 27.1) ( 27.1) ( 27.1) ( 27.1) ( 27.1) ( 27.1) ( 27.1) ( 27.1) ( 27.1)
         -169.9  -292.9   -53.2  -119.3  -121.1   -57.1   268.0   100.7   685.0  1156.9
         -5.599  -9.654  -1.754  -3.932  -3.993  -1.881   8.833   3.320  22.578  38.133
c_O         769     772     667     745     683     662     598     602     464     432
        ( 25.3) ( 25.4) ( 22.0) ( 24.6) ( 22.5) ( 21.8) ( 19.7) ( 19.8) ( 15.3) ( 14.2)
c_E       757.1   757.1   757.1   757.1   757.1   757.1   757.1   757.1   757.1   757.1
        ( 25.0) ( 25.0) ( 25.0) ( 25.0) ( 25.0) ( 25.0) ( 25.0) ( 25.0) ( 25.0) ( 25.0)
           24.0    30.1  -169.0   -24.0  -140.7  -177.7  -282.1  -276.0  -454.3  -484.8
          0.791   0.992  -5.570  -0.791  -4.637  -5.857  -9.299  -9.096 -14.975 -15.977

N          3034    3034    3034    3034    3034    3034    3034    3034    3034    3034
X2         24.5    55.9    22.2    25.4    45.1    37.5   117.3   142.3   352.8   574.6
Gvalue     24.3    57.1    22.4    24.7    43.5    36.7   118.3   139.1   363.6   583.9
Gv/N      0.801   1.883   0.737   0.815   1.433   1.210   3.900   4.585  11.983  19.244
Prob    0.00002 0.00000 0.00005 0.00002 0.00000 0.00000 0.00000 0.00000 0.00000 0.00000
        -------------------------------------------------------------------------------
             10       9       8       7       6       5       4       3       2       1
        -------------------------------------------------------------------------------
a_O         841     865     994     894     934     915     826     950     735     776
        ( 27.7) ( 28.5) ( 32.8) ( 29.5) ( 30.8) ( 30.2) ( 27.2) ( 31.3) ( 24.2) ( 25.6)
a_E       750.7   750.7   750.7   750.7   750.7   750.7   750.7   750.7   750.7   750.7
        ( 24.7) ( 24.7) ( 24.7) ( 24.7) ( 24.7) ( 24.7) ( 24.7) ( 24.7) ( 24.7) ( 24.7)
          191.0   245.1   558.0   312.3   408.0   362.1   157.8   447.3   -31.1    51.4
          6.295   8.079  18.392  10.293  13.449  11.935   5.203  14.743  -1.026   1.694
t_O         380     411     465     652     643     633     733     555     739     555
        ( 12.5) ( 13.5) ( 15.3) ( 21.5) ( 21.2) ( 20.9) ( 24.2) ( 18.3) ( 24.4) ( 18.3)
t_E       705.1   705.1   705.1   705.1   705.1   705.1   705.1   705.1   705.1   705.1
        ( 23.2) ( 23.2) ( 23.2) ( 23.2) ( 23.2) ( 23.2) ( 23.2) ( 23.2) ( 23.2) ( 23.2)
         -469.8  -443.7  -387.2  -102.1  -118.6  -136.6    56.8  -265.7    69.4  -265.7
        -15.485 -14.624 -12.762  -3.366  -3.909  -4.502   1.874  -8.759   2.286  -8.759
g_O        1422    1398    1154     969     857     798     720     844     654     608
        ( 46.9) ( 46.1) ( 38.0) ( 31.9) ( 28.2) ( 26.3) ( 23.7) ( 27.8) ( 21.6) ( 20.0)
g_E       821.1   821.1   821.1   821.1   821.1   821.1   821.1   821.1   821.1   821.1
        ( 27.1) ( 27.1) ( 27.1) ( 27.1) ( 27.1) ( 27.1) ( 27.1) ( 27.1) ( 27.1) ( 27.1)
         1562.0  1488.0   785.6   321.1    73.4   -45.5  -189.1    46.5  -297.6  -365.3
         51.483  49.045  25.894  10.582   2.420  -1.499  -6.234   1.533  -9.807 -12.041
c_O         391     360     421     519     600     688     755     685     906    1095
        ( 12.9) ( 11.9) ( 13.9) ( 17.1) ( 19.8) ( 22.7) ( 24.9) ( 22.6) ( 29.9) ( 36.1)
c_E       757.1   757.1   757.1   757.1   757.1   757.1   757.1   757.1   757.1   757.1
        ( 25.0) ( 25.0) ( 25.0) ( 25.0) ( 25.0) ( 25.0) ( 25.0) ( 25.0) ( 25.0) ( 25.0)
         -516.7  -535.2  -494.1  -391.9  -279.1  -131.7    -4.2  -137.1   325.4   808.2
        -17.031 -17.641 -16.286 -12.918  -9.198  -4.340  -0.138  -4.518  10.724  26.637

N          3034    3034    3034    3034    3034    3034    3034    3034    3034    3034
X2        777.6   753.8   444.8   132.9    84.4    50.3    21.1    92.4    65.2   238.9
Gvalue    766.4   754.2   462.3   139.3    83.8    48.4    21.4    91.0    66.0   228.5
Gv/N     25.261  24.859  15.238   4.591   2.762   1.595   0.705   2.999   2.177   7.531
Prob    0.00000 0.00000 0.00000 0.00000 0.00000 0.00000 0.00009 0.00000 0.00000 0.00000
        -------------------------------------------------------------------------------

CDS_INITIAL:
        -------------------------------------------------------------------------------
              1       2       3       4       5       6       7       8       9      10
        -------------------------------------------------------------------------------
a_O        2464       0       0    1305     850     708     950    1045     694     932
        ( 81.2) (  0.0) (  0.0) ( 43.0) ( 28.0) ( 23.3) ( 31.3) ( 34.4) ( 22.9) ( 30.7)
a_E       699.9   800.8   346.9   699.9   800.8   346.9   699.9   800.8   346.9   699.9
        ( 23.1) ( 26.4) ( 11.4) ( 23.1) ( 26.4) ( 11.4) ( 23.1) ( 26.4) ( 11.4) ( 23.1)
         6202.4     0.0     0.0  1626.1   101.4  1010.0   580.5   556.3   962.3   533.8
        204.429   0.000   0.000  53.595   3.343  33.289  19.133  18.337  31.717  17.595
t_O         264    3034       0     358     509     698     432     761     764     508
        (  8.7) (100.0) (  0.0) ( 11.8) ( 16.8) ( 23.0) ( 14.2) ( 25.1) ( 25.2) ( 16.7)
t_E       407.6   896.6   635.0   407.6   896.6   635.0   407.6   896.6   635.0   407.6
        ( 13.4) ( 29.6) ( 20.9) ( 13.4) ( 29.6) ( 20.9) ( 13.4) ( 29.6) ( 20.9) ( 13.4)
         -229.4  7397.2     0.0   -93.0  -576.3   132.0    50.1  -249.5   282.5   223.6
         -7.561 243.810   0.000  -3.065 -18.996   4.350   1.652  -8.225   9.311   7.370
g_O         306       0    3034     767     572     719     986     517     762     835
        ( 10.1) (  0.0) (100.0) ( 25.3) ( 18.9) ( 23.7) ( 32.5) ( 17.0) ( 25.1) ( 27.5)
g_E      1167.5   565.3   954.2  1167.5   565.3   954.2  1167.5   565.3   954.2  1167.5
        ( 38.5) ( 18.6) ( 31.5) ( 38.5) ( 18.6) ( 31.5) ( 38.5) ( 18.6) ( 31.5) ( 38.5)
         -819.5     0.0  7019.1  -644.5    13.4  -407.0  -333.3   -92.4  -342.8  -559.8
        -27.011   0.000 231.349 -21.244   0.443 -13.415 -10.984  -3.045 -11.299 -18.451
c_O           0       0       0     604    1103     909     666     711     814     759
        (  0.0) (  0.0) (  0.0) ( 19.9) ( 36.4) ( 30.0) ( 22.0) ( 23.4) ( 26.8) ( 25.0)
c_E       758.9   771.3  1097.8   758.9   771.3  1097.8   758.9   771.3  1097.8   758.9
        ( 25.0) ( 25.4) ( 36.2) ( 25.0) ( 25.4) ( 36.2) ( 25.0) ( 25.4) ( 36.2) ( 25.0)
            0.0     0.0     0.0  -275.8   789.0  -343.1  -174.0  -115.8  -486.9     0.2
          0.000   0.000   0.000  -9.090  26.007 -11.308  -5.733  -3.817 -16.049   0.006

N          3034    3034    3034    3034    3034    3034    3034    3034    3034    3034
X2       5891.6  7233.0  6612.8   698.2   313.3   472.4   130.4   103.8   485.4   196.4
Gvalue   5153.5  7397.2  7019.1   612.7   327.6   391.9   123.4    98.6   415.0   197.8
Gv/N    169.857 243.810 231.349  20.196  10.797  12.916   4.067   3.250  13.680   6.519
Prob    1.00000 1.00000 1.00000 0.00000 0.00000 0.00000 0.00000 0.00000 0.00000 0.00000
        -------------------------------------------------------------------------------
             11      12      13      14      15      16      17      18      19      20
        -------------------------------------------------------------------------------
a_O         868     688     935     795     554     897     878     605     920     845
        ( 28.6) ( 22.7) ( 30.8) ( 26.2) ( 18.3) ( 29.6) ( 28.9) ( 19.9) ( 30.3) ( 27.9)
a_E       800.8   346.9   699.9   800.8   346.9   699.9   800.8   346.9   699.9   800.8
        ( 26.4) ( 11.4) ( 23.1) ( 26.4) ( 11.4) ( 23.1) ( 26.4) ( 11.4) ( 23.1) ( 26.4)
          139.9   942.0   541.6   -11.5   518.5   445.1   161.7   672.8   503.1    90.8
          4.612  31.049  17.850  -0.379  17.091  14.671   5.329  22.176  16.582   2.994
t_O         887     776     526     981     812     514     950     757     476     907
        ( 29.2) ( 25.6) ( 17.3) ( 32.3) ( 26.8) ( 16.9) ( 31.3) ( 25.0) ( 15.7) ( 29.9)
t_E       896.6   635.0   407.6   896.6   635.0   407.6   896.6   635.0   407.6   896.6
        ( 29.6) ( 20.9) ( 13.4) ( 29.6) ( 20.9) ( 13.4) ( 29.6) ( 20.9) ( 13.4) ( 29.6)
          -19.1   311.1   268.1   176.5   399.2   238.3   110.0   266.0   147.6    21.0
         -0.628  10.254   8.838   5.819  13.158   7.855   3.624   8.766   4.864   0.691
g_O         477     735     847     479     727     877     447     684     830     513
        ( 15.7) ( 24.2) ( 27.9) ( 15.8) ( 24.0) ( 28.9) ( 14.7) ( 22.5) ( 27.4) ( 16.9)
g_E       565.3   954.2  1167.5   565.3   954.2  1167.5   565.3   954.2  1167.5   565.3
        ( 18.6) ( 31.5) ( 38.5) ( 18.6) ( 31.5) ( 38.5) ( 18.6) ( 31.5) ( 38.5) ( 18.6)
         -162.1  -383.7  -543.7  -158.7  -395.4  -501.9  -209.9  -455.5  -566.4   -99.6
         -5.342 -12.647 -17.920  -5.232 -13.034 -16.542  -6.920 -15.012 -18.670  -3.284
```

c_O	802	835	726	779	941	746	759	988	808	769
	(26.4)	(27.5)	(23.9)	(25.7)	(31.0)	(24.6)	(25.0)	(32.6)	(26.6)	(25.3)
c_E	771.3	1097.8	758.9	771.3	1097.8	758.9	771.3	1097.8	758.9	771.3
	(25.4)	(36.2)	(25.0)	(25.4)	(36.2)	(25.0)	(25.4)	(36.2)	(25.0)	(25.4)
	62.6	-457.0	-64.4	15.4	-290.0	-25.6	-24.4	-208.2	101.3	-4.6
	2.062	-15.061	-2.122	0.509	-9.560	-0.844	-0.806	-6.863	3.338	-0.153
N	3034	3034	3034	3034	3034	3034	3034	3034	3034	3034
X2	20.8	479.8	202.8	21.2	249.4	155.8	35.6	302.9	181.4	7.4
Gvalue	21.4	412.5	201.6	21.7	232.2	155.9	37.2	275.1	185.5	7.5
Gv/N	0.705	13.596	6.646	0.716	7.655	5.139	1.227	9.068	6.115	0.248
Prob	0.00009	0.00000	0.00000	0.00007	0.00000	0.00000	0.00000	0.00000	0.00000	0.05691

	21	22	23	24	25	26	27	28	29	30
a_O	558	807	795	526	756	748	466	762	735	480
	(18.4)	(26.6)	(26.2)	(17.3)	(24.9)	(24.7)	(15.4)	(25.1)	(24.2)	(15.8)
a_E	346.9	699.9	800.8	346.9	699.9	800.8	346.9	699.9	800.8	346.9
	(11.4)	(23.1)	(26.4)	(11.4)	(23.1)	(26.4)	(11.4)	(23.1)	(26.4)	(11.4)
	530.3	229.8	-11.5	437.8	116.6	-102.0	274.9	129.5	-126.0	311.6
	17.479	7.574	-0.379	14.429	3.842	-3.362	9.062	4.270	-4.153	10.271
t_O	783	498	931	749	443	910	745	463	907	719
	(25.8)	(16.4)	(30.7)	(24.7)	(14.6)	(30.0)	(24.6)	(15.3)	(29.9)	(23.7)
t_E	635.0	407.6	896.6	635.0	407.6	896.6	635.0	407.6	896.6	635.0
	(20.9)	(13.4)	(29.6)	(20.9)	(13.4)	(29.6)	(20.9)	(13.4)	(29.6)	(20.9)
	328.0	199.4	70.1	247.2	73.7	27.0	237.9	117.9	21.0	178.6
	10.810	6.572	2.312	8.149	2.429	0.891	7.843	3.886	0.691	5.885
g_O	712	975	511	748	1032	569	799	1050	571	828
	(23.5)	(32.1)	(16.8)	(24.7)	(34.0)	(18.8)	(26.3)	(34.6)	(18.8)	(27.3)
g_E	954.2	1167.5	565.3	954.2	1167.5	565.3	954.2	1167.5	565.3	954.2
	(31.5)	(38.5)	(18.6)	(31.5)	(38.5)	(18.6)	(31.5)	(38.5)	(18.6)	(31.5)
	-417.0	-351.4	-103.2	-364.3	-254.7	7.4	-283.7	-222.8	11.4	-235.0
	-13.743	-11.583	-3.403	-12.006	-8.395	0.243	-9.351	-7.344	0.376	-7.744
c_O	981	754	797	1011	803	807	1024	759	821	1007
	(32.3)	(24.9)	(26.3)	(33.3)	(26.5)	(26.6)	(33.8)	(25.0)	(27.1)	(33.2)
c_E	1097.8	758.9	771.3	1097.8	758.9	771.3	1097.8	758.9	771.3	1097.8
	(36.2)	(25.0)	(25.4)	(36.2)	(25.0)	(25.4)	(36.2)	(25.0)	(25.4)	(36.2)
	-220.7	-9.8	52.2	-166.5	90.7	73.0	-142.5	0.2	102.5	-173.9
	-7.274	-0.323	1.720	-5.489	2.989	2.405	-4.697	0.006	3.378	-5.730
N	3034	3034	3034	3034	3034	3034	3034	3034	3034	3034
X2	236.8	68.2	7.4	164.3	25.9	5.4	90.1	24.9	8.8	86.3
Gvalue	220.6	68.0	7.6	154.2	26.3	5.4	86.7	24.8	8.9	81.4
Gv/N	7.272	2.241	0.250	5.083	0.865	0.178	2.857	0.817	0.292	2.682
Prob	0.00000	0.00000	0.05536	0.00000	0.00001	0.14436	0.00000	0.00002	0.03111	0.00000

	31	32	33	34	35	36	37	38	39	40
a_O	750	725	454	754	688	420	682	672	463	690
	(24.7)	(23.9)	(15.0)	(24.9)	(22.7)	(13.8)	(22.5)	(22.1)	(15.3)	(22.7)
a_E	699.9	800.8	346.9	699.9	800.8	346.9	699.9	800.8	346.9	699.9
	(23.1)	(26.4)	(11.4)	(23.1)	(26.4)	(11.4)	(23.1)	(26.4)	(11.4)	(23.1)
	103.7	-144.1	244.2	112.3	-208.9	160.5	-35.3	-235.6	267.2	-19.7
	3.418	-4.751	8.048	3.700	-6.884	5.290	-1.165	-7.766	8.807	-0.648
t_O	447	948	740	458	927	696	456	940	674	437
	(14.7)	(31.2)	(24.4)	(15.1)	(30.6)	(22.9)	(15.0)	(31.0)	(22.2)	(14.4)
t_E	407.6	896.6	635.0	407.6	896.6	635.0	407.6	896.6	635.0	407.6
	(13.4)	(29.6)	(20.9)	(13.4)	(29.6)	(20.9)	(13.4)	(29.6)	(20.9)	(13.4)
	82.4	105.7	226.4	106.7	61.9	127.6	102.2	88.9	80.3	60.8
	2.715	3.485	7.462	3.516	2.039	4.205	3.369	2.930	2.645	2.003
g_O	1072	565	818	1098	620	867	1162	625	878	1143
	(35.3)	(18.6)	(27.0)	(36.2)	(20.4)	(28.6)	(38.3)	(20.6)	(28.9)	(37.7)
g_E	1167.5	565.3	954.2	1167.5	565.3	954.2	1167.5	565.3	954.2	1167.5
	(38.5)	(18.6)	(31.5)	(38.5)	(18.6)	(31.5)	(38.5)	(18.6)	(31.5)	(38.5)
	-183.0	-0.6	-252.0	-134.8	114.5	-166.2	-11.0	125.4	-146.2	-48.6
	-6.033	-0.021	-8.306	-4.444	3.773	-5.478	-0.364	4.135	-4.818	-1.600
c_O	765	796	1022	724	799	1051	734	797	1019	764
	(25.2)	(26.2)	(33.7)	(23.9)	(26.3)	(34.6)	(24.2)	(26.3)	(33.6)	(25.2)
c_E	758.9	771.3	1097.8	758.9	771.3	1097.8	758.9	771.3	1097.8	758.9
	(25.0)	(25.4)	(36.2)	(25.0)	(25.4)	(36.2)	(25.0)	(25.4)	(36.2)	(25.0)
	12.2	50.1	-146.2	-68.2	56.3	-91.6	-49.0	52.2	-151.8	10.2
	0.403	1.652	-4.820	-2.248	1.857	-3.018	-1.615	1.720	-5.003	0.337
N	3034	3034	3034	3034	3034	3034	3034	3034	3034	3034
X2	15.3	10.9	75.1	16.1	23.2	31.2	7.0	30.0	53.0	2.8
Gvalue	15.3	11.1	72.3	15.9	23.8	30.3	6.8	30.9	49.5	2.8
Gv/N	0.503	0.365	2.384	0.524	0.785	1.000	0.225	1.019	1.631	0.091
Prob	0.00160	0.01128	0.00000	0.00118	0.00003	0.00000	0.07732	0.00000	0.00000	0.43038

	41	42	43	44	45	46	47	48	49	50
a_O	709	435	687	648	424	675	710	453	672	705
	(23.4)	(14.3)	(22.6)	(21.4)	(14.0)	(22.2)	(23.4)	(14.9)	(22.1)	(23.2)
a_E	800.8	346.9	699.9	800.8	346.9	699.9	800.8	346.9	699.9	800.8
	(26.4)	(11.4)	(23.1)	(26.4)	(11.4)	(23.1)	(26.4)	(11.4)	(23.1)	(26.4)
	-172.6	196.8	-25.6	-274.3	170.1	-48.9	-170.8	241.6	-54.7	-179.6
	-5.689	6.485	-0.843	-9.043	5.606	-1.612	-5.631	7.965	-1.802	-5.920
t_O	911	677	478	915	700	419	915	742	452	882
	(30.0)	(22.3)	(15.8)	(30.2)	(23.1)	(13.8)	(30.2)	(24.5)	(14.9)	(29.1)
t_E	896.6	635.0	407.6	896.6	635.0	407.6	896.6	635.0	407.6	896.6
	(29.6)	(20.9)	(13.4)	(29.6)	(20.9)	(13.4)	(29.6)	(20.9)	(13.4)	(29.6)
	29.1	86.6	152.2	37.2	136.3	23.0	37.2	231.0	93.4	-28.9
	0.958	2.855	5.016	1.227	4.494	0.759	1.227	7.614	3.077	-0.953
g_O	587	870	1128	607	899	1156	633	803	1142	613
	(19.3)	(28.7)	(37.2)	(20.0)	(29.6)	(38.1)	(20.9)	(26.5)	(37.6)	(20.2)
g_E	565.3	954.2	1167.5	565.3	954.2	1167.5	565.3	954.2	1167.5	565.3
	(18.6)	(31.5)	(38.5)	(18.6)	(31.5)	(38.5)	(18.6)	(31.5)	(38.5)	(18.6)
	44.2	-160.8	-77.7	86.4	-107.2	-23.0	143.2	-277.1	-50.5	99.3
	1.456	-5.299	-2.562	2.846	-3.533	-0.757	4.718	-9.133	-1.665	3.272
c_O	827	1052	741	864	1011	784	776	1036	768	834
	(27.3)	(34.7)	(24.4)	(28.5)	(33.3)	(25.8)	(25.6)	(34.1)	(25.3)	(27.5)
c_E	771.3	1097.8	758.9	771.3	1097.8	758.9	771.3	1097.8	758.9	771.3
	(25.4)	(36.2)	(25.0)	(25.4)	(36.2)	(25.0)	(25.4)	(36.2)	(25.0)	(25.4)
	115.3	-89.6	-35.4	196.1	-166.5	51.0	9.4	-120.0	18.3	130.3
	3.800	-2.955	-1.167	6.462	-5.489	1.681	0.309	-3.957	0.603	4.295
N	3034	3034	3034	3034	3034	3034	3034	3034	3034	3034
X2	15.6	34.5	14.1	43.7	33.8	2.1	18.8	77.9	6.6	20.8
Gvalue	15.9	33.0	13.5	45.3	32.7	2.1	18.9	75.5	6.5	21.1
Gv/N	0.525	1.087	0.446	1.493	1.078	0.071	0.623	2.489	0.213	0.694
Prob	0.00118	0.00000	0.00364	0.00000	0.00000	0.54289	0.00029	0.00000	0.09134	0.00010

	51	52	53	54	55	56	57	58	59	60
a_O	464	695	670	407	686	732	402	657	710	408
	(15.3)	(22.9)	(22.1)	(13.4)	(22.6)	(24.1)	(13.2)	(21.7)	(23.4)	(13.4)
a_E	346.9	699.9	800.8	346.9	699.9	800.8	346.9	699.9	800.8	346.9
	(11.4)	(23.1)	(26.4)	(11.4)	(23.1)	(26.4)	(11.4)	(23.1)	(26.4)	(11.4)
	269.8	-9.8	-238.9	129.9	-27.5	-131.5	118.4	-83.1	-170.8	132.3
	8.892	-0.322	-7.875	4.283	-0.907	-4.333	3.903	-2.740	-5.631	4.359
t_O	676	441	934	622	439	903	691	463	907	669
	(22.3)	(14.5)	(30.8)	(20.5)	(14.5)	(29.8)	(22.8)	(15.3)	(29.9)	(22.1)
t_E	635.0	407.6	896.6	635.0	407.6	896.6	635.0	407.6	896.6	635.0
	(20.9)	(13.4)	(29.6)	(20.9)	(13.4)	(29.6)	(20.9)	(13.4)	(29.6)	(20.9)
	84.5	69.4	76.4	-25.8	65.1	12.9	116.7	117.9	21.0	69.7
	2.785	2.286	2.517	-0.851	2.144	0.425	3.847	3.886	0.691	2.297
g_O	874	1149	620	962	1173	573	917	1167	596	946
	(28.8)	(37.9)	(20.4)	(31.7)	(38.7)	(18.9)	(30.2)	(38.5)	(19.6)	(31.2)
g_E	954.2	1167.5	565.3	954.2	1167.5	565.3	954.2	1167.5	565.3	954.2
	(31.5)	(38.5)	(18.6)	(31.5)	(38.5)	(18.6)	(31.5)	(38.5)	(18.6)	(31.5)
	-153.5	-36.8	114.5	15.6	11.0	15.5	-73.0	-1.1	63.0	-16.4
	-5.059	-1.212	3.773	0.515	0.361	0.510	-2.405	-0.035	2.076	-0.539
c_O	1020	749	810	1043	736	826	1024	747	821	1011
	(33.6)	(24.7)	(26.7)	(34.4)	(24.3)	(27.2)	(33.8)	(24.6)	(27.1)	(33.3)
c_E	1097.8	758.9	771.3	1097.8	758.9	771.3	1097.8	758.9	771.3	1097.8
	(36.2)	(25.0)	(25.4)	(36.2)	(25.0)	(25.4)	(36.2)	(25.0)	(25.4)	(36.2)
	-149.9	-19.7	79.3	-106.8	-45.1	113.1	-142.5	-23.6	102.5	-166.5
	-4.942	-0.649	2.612	-3.520	-1.487	3.729	-4.697	-0.779	3.378	-5.489
N	3034	3034	3034	3034	3034	3034	3034	3034	3034	3034
X2	54.4	3.2	30.1	13.5	3.4	9.9	20.1	10.3	15.3	19.5
Gvalue	50.8	3.1	31.2	13.0	3.4	10.0	19.6	10.1	15.6	19.1
Gv/N	1.676	0.103	1.028	0.427	0.111	0.330	0.648	0.332	0.514	0.629
Prob	0.00000	0.37389	0.00000	0.00473	0.34021	0.01839	0.00020	0.01798	0.00137	0.00026

	61	62	63	64	65	66	67	68	69	70
a_O	645	713	406	674	685	425	679	705	405	683
	(21.3)	(23.5)	(13.4)	(22.2)	(22.6)	(14.0)	(22.4)	(23.2)	(13.3)	(22.5)
a_E	699.9	800.8	346.9	699.9	800.8	346.9	699.9	800.8	346.9	699.9
	(23.1)	(26.4)	(11.4)	(23.1)	(26.4)	(11.4)	(23.1)	(26.4)	(11.4)	(23.1)
	-105.4	-165.6	127.6	-50.8	-213.9	172.5	-41.2	-179.6	125.3	-33.4
	-3.473	-5.457	4.207	-1.676	-7.051	5.685	-1.357	-5.920	4.130	-1.101
t_O	443	959	683	460	902	658	426	895	679	432
	(14.6)	(31.6)	(22.5)	(15.2)	(29.7)	(21.7)	(14.0)	(29.5)	(22.4)	(14.2)
t_E	407.6	896.6	635.0	407.6	896.6	635.0	407.6	896.6	635.0	407.6
	(13.4)	(29.6)	(20.9)	(13.4)	(29.6)	(20.9)	(13.4)	(29.6)	(20.9)	(13.4)
	73.7	129.1	99.5	111.2	10.9	46.7	37.5	-3.2	90.9	50.1
	2.429	4.255	3.278	3.664	0.358	1.541	1.237	-0.104	2.996	1.652
g_O	1158	563	951	1135	586	970	1127	544	922	1221
	(38.2)	(18.6)	(31.3)	(37.4)	(19.3)	(32.0)	(37.1)	(17.9)	(30.4)	(40.2)
g_E	1167.5	565.3	954.2	1167.5	565.3	954.2	1167.5	565.3	954.2	1167.5
	(38.5)	(18.6)	(31.5)	(38.5)	(18.6)	(31.5)	(38.5)	(18.6)	(31.5)	(38.5)
	-19.0	-4.6	-6.4	-64.2	42.1	31.8	-79.6	-41.8	-63.3	109.3
	-0.626	-0.153	-0.212	-2.115	1.388	1.049	-2.625	-1.379	-2.088	3.604
c_O	788	799	994	765	861	981	802	890	1028	698
	(26.0)	(26.3)	(32.8)	(25.2)	(28.4)	(32.3)	(26.4)	(29.3)	(33.9)	(23.0)
c_E	758.9	771.3	1097.8	758.9	771.3	1097.8	758.9	771.3	1097.8	758.9
	(25.0)	(25.4)	(36.2)	(25.0)	(25.4)	(36.2)	(25.0)	(25.4)	(36.2)	(25.0)
	59.3	56.3	-197.4	12.2	189.4	-220.7	88.6	254.7	-135.1	-116.8
	1.954	1.857	-6.508	0.403	6.242	-7.274	2.920	8.396	-4.451	-3.850
N	3034	3034	3034	3034	3034	3034	3034	3034	3034	3034
X2	8.6	15.0	23.5	8.6	28.0	31.1	5.3	30.5	18.3	9.2
Gvalue	8.6	15.2	23.2	8.4	28.4	30.3	5.3	30.1	17.8	9.3
Gv/N	0.283	0.502	0.765	0.276	0.937	1.000	0.174	0.994	0.587	0.306
Prob	0.03536	0.00163	0.00004	0.03865	0.00000	0.00000	0.15305	0.00000	0.00048	0.02585

	71	72	73	74	75	76	77	78	79	80
a_O	696	425	641	732	391	650	718	414	679	787
	(22.9)	(14.0)	(21.1)	(24.1)	(12.9)	(21.4)	(23.7)	(13.6)	(22.4)	(25.9)
a_E	800.8	346.9	699.9	800.8	346.9	699.9	800.8	346.9	699.9	800.8
	(26.4)	(11.4)	(23.1)	(26.4)	(11.4)	(23.1)	(26.4)	(11.4)	(23.1)	(26.4)
	-195.2	172.5	-112.7	-131.5	93.5	-96.2	-156.7	146.3	-41.2	-27.3
	-6.434	5.685	-3.715	-4.333	3.081	-3.170	-5.164	4.822	-1.357	-0.900
t_O	938	693	447	864	710	418	913	649	443	912
	(30.9)	(22.8)	(14.7)	(28.5)	(23.4)	(13.8)	(30.1)	(21.4)	(14.6)	(30.1)
t_E	896.6	635.0	407.6	896.6	635.0	407.6	896.6	635.0	407.6	896.6
	(29.6)	(20.9)	(13.4)	(29.6)	(20.9)	(13.4)	(29.6)	(20.9)	(13.4)	(29.6)
	84.7	121.1	82.4	-64.0	158.4	21.0	33.1	28.2	73.7	31.1
	2.792	3.990	2.715	-2.108	5.222	0.691	1.092	0.930	2.429	1.025
g_O	573	919	1184	605	944	1189	596	937	1173	539
	(18.9)	(30.3)	(39.0)	(19.9)	(31.1)	(39.2)	(19.6)	(30.9)	(38.7)	(17.8)
g_E	565.3	954.2	1167.5	565.3	954.2	1167.5	565.3	954.2	1167.5	565.3
	(18.6)	(31.5)	(38.5)	(18.6)	(31.5)	(38.5)	(18.6)	(31.5)	(38.5)	(18.6)
	15.5	-69.1	33.2	82.1	-20.3	43.3	63.0	-34.1	11.0	-51.4
	0.510	-2.278	1.093	2.705	-0.670	1.428	2.076	-1.125	0.361	-1.694
c_O	827	997	762	833	989	777	807	1034	739	796
	(27.3)	(32.9)	(25.1)	(27.5)	(32.6)	(25.6)	(26.6)	(34.1)	(24.4)	(26.2)
c_E	771.3	1097.8	758.9	771.3	1097.8	758.9	771.3	1097.8	758.9	771.3
	(25.4)	(36.2)	(25.0)	(25.4)	(36.2)	(25.0)	(25.4)	(36.2)	(25.0)	(25.4)
	115.3	-192.0	6.2	128.2	-206.4	36.6	73.0	-123.8	-39.3	50.1
	3.800	-6.329	0.204	4.224	-6.804	1.207	2.405	-4.081	-1.295	1.652
N	3034	3034	3034	3034	3034	3034	3034	3034	3034	3034
X2	19.7	33.4	9.0	14.8	25.3	4.6	12.2	17.3	4.2	2.5
Gvalue	20.3	32.4	9.0	14.8	25.2	4.7	12.4	16.6	4.2	2.5
Gv/N	0.668	1.067	0.298	0.488	0.829	0.156	0.410	0.547	0.137	0.083
Prob	0.00015	0.00000	0.02892	0.00199	0.00001	0.19294	0.00605	0.00086	0.24430	0.47024

	81	82	83	84	85	86	87	88	89	90
a_O	416	668	754	390	666	765	377	685	720	419
	(13.7)	(22.0)	(24.9)	(12.9)	(22.0)	(25.2)	(12.4)	(22.6)	(23.7)	(13.8)
a_E	346.9	699.9	800.8	346.9	699.9	800.8	346.9	699.9	800.8	346.9
	(11.4)	(23.1)	(26.4)	(11.4)	(23.1)	(26.4)	(11.4)	(23.1)	(26.4)	(11.4)
	151.0	-62.3	-90.8	91.2	-66.1	-69.9	62.6	-29.5	-153.1	158.1
	4.977	-2.054	-2.992	3.007	-2.180	-2.305	2.064	-0.972	-5.047	5.212
t_O	680	405	874	644	456	920	686	447	940	627
	(22.4)	(13.3)	(28.8)	(21.2)	(15.0)	(30.3)	(22.6)	(14.7)	(31.0)	(20.7)
t_E	635.0	407.6	896.6	635.0	407.6	896.6	635.0	407.6	896.6	635.0
	(20.9)	(13.4)	(29.6)	(20.9)	(13.4)	(29.6)	(20.9)	(13.4)	(29.6)	(20.9)
	93.0	-5.3	-44.6	18.0	102.2	47.4	105.9	82.4	88.9	-16.0

	3.066	−0.174	−1.470	0.595	3.369	1.564	3.491	2.715	2.930	−0.527
g_O	919	1207	605	979	1177	600	922	1199	575	911
	(30.3)	(39.8)	(19.9)	(32.3)	(38.8)	(19.8)	(30.4)	(39.5)	(19.0)	(30.0)
g_E	954.2	1167.5	565.3	954.2	1167.5	565.3	954.2	1167.5	565.3	954.2
	(31.5)	(38.5)	(18.6)	(31.5)	(38.5)	(18.6)	(31.5)	(38.5)	(18.6)	(31.5)
	−69.1	80.2	82.1	50.2	19.0	71.4	−63.3	63.8	19.5	−84.4
	−2.278	2.645	2.705	1.655	0.626	2.355	−2.088	2.102	0.643	−2.783
c_O	1019	754	801	1021	735	749	1049	703	799	1077
	(33.6)	(24.9)	(26.4)	(33.7)	(24.2)	(24.7)	(34.6)	(23.2)	(26.3)	(35.5)
c_E	1097.8	758.9	771.3	1097.8	758.9	771.3	1097.8	758.9	771.3	1097.8
	(36.2)	(25.0)	(25.4)	(36.2)	(25.0)	(25.4)	(36.2)	(25.0)	(25.4)	(36.2)
	−151.8	−9.8	60.5	−148.1	−47.1	−44.0	−95.4	−107.6	56.3	−41.2
	−5.003	−0.323	1.993	−4.881	−1.551	−1.450	−3.144	−3.546	1.857	−1.358
N	3034	3034	3034	3034	3034	3034	3034	3034	3034	3034
X2	23.9	2.8	7.2	11.5	8.2	5.0	9.9	9.1	11.4	17.4
Gvalue	23.1	2.8	7.2	11.4	8.0	5.0	9.8	9.1	11.7	16.5
Gv/N	0.763	0.094	0.238	0.376	0.265	0.164	0.324	0.299	0.384	0.544
Prob	0.00004	0.41611	0.06550	0.00975	0.04543	0.17425	0.02019	0.02842	0.00867	0.00089

	91	92	93	94	95	96	97	98	99	100
a_O	681	752	391	663	770	398	672	748	369	729
	(22.4)	(24.8)	(12.9)	(21.9)	(25.4)	(13.1)	(22.2)	(24.8)	(12.2)	(24.2)
a_E	699.9	800.8	346.9	698.3	798.9	346.1	696.9	797.3	345.5	696.2
	(23.1)	(26.4)	(11.4)	(23.1)	(26.4)	(11.4)	(23.1)	(26.4)	(11.4)	(23.1)
	−37.3	−94.5	93.5	−68.8	−56.8	111.1	−48.9	−95.6	48.6	67.1
	−1.229	−3.115	3.081	−2.272	−1.876	3.671	−1.619	−3.163	1.610	2.223
t_O	418	890	709	476	919	700	464	903	689	409
	(13.8)	(29.3)	(23.4)	(15.7)	(30.4)	(23.1)	(15.4)	(29.9)	(22.8)	(13.6)
t_E	407.6	896.6	635.0	406.7	894.5	633.6	405.9	892.7	632.3	405.5
	(13.4)	(29.6)	(20.9)	(13.4)	(29.6)	(20.9)	(13.4)	(29.6)	(20.9)	(13.4)
	21.0	−13.1	156.2	149.8	49.6	139.6	124.1	20.6	118.3	7.0
	0.691	−0.432	5.149	4.948	1.640	4.611	4.109	0.683	3.916	0.233
g_O	1168	587	913	1129	563	870	1094	575	908	1169
	(38.5)	(19.3)	(30.1)	(37.3)	(18.6)	(28.7)	(36.2)	(19.0)	(30.1)	(38.7)
g_E	1167.5	565.3	954.2	1164.8	564.0	952.0	1162.5	562.9	950.1	1161.4
	(38.5)	(18.6)	(31.5)	(38.5)	(18.6)	(31.5)	(38.5)	(18.6)	(31.5)	(38.5)
	0.9	44.2	−80.6	−70.6	−2.0	−156.8	−132.9	24.5	−82.4	15.3
	0.031	1.456	−2.658	−2.331	−0.067	−5.179	−4.401	0.810	−2.726	0.507
c_O	767	805	1021	759	775	1059	791	795	1055	711
	(25.3)	(26.5)	(33.7)	(25.1)	(25.6)	(35.0)	(26.2)	(26.3)	(34.9)	(23.6)
c_E	758.9	771.3	1097.8	757.2	769.5	1095.3	755.7	768.0	1093.1	754.9
	(25.0)	(25.4)	(36.2)	(25.0)	(25.4)	(36.2)	(25.0)	(25.4)	(36.2)	(25.0)
	16.3	68.8	−148.1	3.7	10.9	−71.3	72.3	54.9	−74.8	−85.2
	0.536	2.268	−4.881	0.122	0.362	−2.356	2.394	1.817	−2.477	−2.823
N	3034	3034	3034	3027	3027	3027	3021	3021	3021	3018
X2	0.9	5.3	21.4	14.7	1.8	23.0	14.9	4.4	9.9	4.2
Gvalue	0.9	5.4	21.0	14.1	1.8	22.6	14.6	4.4	9.7	4.2
Gv/N	0.028	0.176	0.691	0.466	0.058	0.748	0.483	0.147	0.323	0.139
Prob	0.83458	0.14777	0.00011	0.00275	0.62257	0.00005	0.00220	0.21849	0.02088	0.24014

	101	102	103	104	105	106	107	108	109	110
a_O	767	389	705	728	381	672	791	377	670	730
	(25.4)	(12.9)	(23.4)	(24.2)	(12.7)	(22.3)	(26.3)	(12.5)	(22.3)	(24.3)
a_E	796.6	345.1	694.6	794.7	344.3	693.9	793.9	344.0	692.5	792.3
	(26.4)	(11.4)	(23.1)	(26.4)	(11.4)	(23.1)	(26.4)	(11.4)	(23.1)	(26.4)
	−58.0	93.1	21.0	−127.6	77.1	−43.1	−5.8	69.1	−44.3	−119.6
	−1.922	3.085	0.696	−4.239	2.562	−1.433	−0.193	2.298	−1.476	−3.985
t_O	867	654	425	920	667	432	848	681	447	896
	(28.7)	(21.7)	(14.1)	(30.6)	(22.2)	(14.4)	(28.2)	(22.6)	(14.9)	(29.8)
t_E	891.9	631.7	404.6	889.8	630.2	404.2	888.9	629.6	403.3	887.1
	(29.6)	(20.9)	(13.4)	(29.6)	(20.9)	(13.4)	(29.6)	(20.9)	(13.4)	(29.6)
	−49.0	45.4	41.9	61.4	75.7	57.6	−79.9	106.9	91.9	17.8
	−1.624	1.504	1.392	2.041	2.513	1.914	−2.656	3.554	3.060	0.594
g_O	589	932	1099	595	933	1155	594	894	1117	564
	(19.5)	(30.9)	(36.5)	(19.8)	(31.0)	(38.4)	(19.7)	(29.7)	(37.2)	(18.8)
g_E	562.3	949.2	1158.7	561.0	947.0	1157.5	560.5	946.0	1155.2	559.4
	(18.6)	(31.5)	(38.5)	(18.6)	(31.5)	(38.5)	(18.6)	(31.5)	(38.5)	(18.6)
	54.6	−34.1	−116.2	69.9	−27.8	−5.1	69.0	−101.2	−75.2	9.3
	1.808	−1.129	−3.861	2.323	−0.922	−0.168	2.294	−3.363	−2.504	0.311
c_O	795	1043	782	768	1030	749	775	1056	768	812
	(26.3)	(34.6)	(26.0)	(25.5)	(34.2)	(24.9)	(25.8)	(35.1)	(25.6)	(27.0)
c_E	767.3	1092.0	753.2	765.5	1089.5	752.4	764.7	1088.4	750.9	763.2
	(25.4)	(36.2)	(25.0)	(25.4)	(36.2)	(25.0)	(25.4)	(36.2)	(25.0)	(25.4)
	56.5	−95.8	58.8	5.1	−115.6	−6.8	20.7	−63.8	34.6	100.7
	1.871	−3.173	1.952	0.168	−3.840	−0.226	0.688	−2.121	1.152	3.354
N	3018	3018	3011	3011	3011	3008	3008	3008	3002	3002
X2	4.1	8.9	5.4	8.7	9.5	2.6	4.0	11.2	7.1	8.2
Gvalue	4.0	8.7	5.4	8.8	9.4	2.6	4.0	11.1	7.0	8.2
Gv/N	0.134	0.288	0.179	0.292	0.312	0.086	0.134	0.367	0.232	0.274
Prob	0.25664	0.03384	0.14536	0.03205	0.02447	0.45824	0.25867	0.01146	0.07293	0.04165

	111	112	113	114	115	116	117	118	119	120
a_O	370	640	806	349	711	802	353	654	763	383
	(12.3)	(21.4)	(26.9)	(11.6)	(23.8)	(26.8)	(11.8)	(21.9)	(25.5)	(12.8)
a_E	343.3	691.4	791.0	342.7	690.0	789.4	342.0	689.3	788.6	341.7
	(11.4)	(23.1)	(26.4)	(11.4)	(23.1)	(26.4)	(11.4)	(23.1)	(26.4)	(11.4)
	55.4	−98.8	30.3	12.7	42.7	25.3	22.3	−68.7	−50.4	87.4
	1.847	−3.297	1.010	0.423	1.426	0.847	0.745	−2.301	−1.688	2.926
t_O	646	427	872	691	420	852	663	398	867	639
	(21.5)	(14.2)	(29.1)	(23.1)	(14.0)	(28.5)	(22.2)	(13.3)	(29.0)	(21.4)
t_E	628.3	402.7	885.6	627.3	401.9	883.9	626.0	401.5	883.0	625.4
	(20.9)	(13.4)	(29.6)	(20.9)	(13.4)	(29.6)	(20.9)	(13.4)	(29.6)	(20.9)
	35.8	50.1	−27.1	133.7	37.1	−62.6	76.1	−6.9	−31.7	27.5
	1.193	1.671	−0.904	4.460	1.239	−2.092	2.543	−0.231	−1.060	0.919
g_O	905	1189	584	890	1096	586	896	1184	588	933
	(30.1)	(39.7)	(19.5)	(29.7)	(36.6)	(19.6)	(30.0)	(39.6)	(19.7)	(31.2)
g_E	944.2	1153.3	558.4	942.6	1151.0	557.3	940.7	1149.8	556.7	939.8
	(31.5)	(38.5)	(18.6)	(31.5)	(38.5)	(18.6)	(31.5)	(38.5)	(18.6)	(31.5)
	−76.7	72.5	52.3	−102.2	−107.3	58.8	−87.2	69.3	64.2	−13.5
	−2.554	2.419	1.745	−3.409	−3.588	1.967	−2.916	2.321	2.149	−0.450
c_O	1081	741	735	1067	764	751	1079	752	770	1033
	(36.0)	(24.7)	(24.5)	(35.6)	(25.5)	(25.1)	(36.1)	(25.2)	(25.8)	(34.6)
c_E	1086.2	749.7	761.9	1084.4	748.2	760.4	1082.2	747.4	759.6	1081.1
	(36.2)	(25.0)	(25.4)	(36.2)	(25.0)	(25.4)	(36.2)	(25.0)	(25.4)	(36.2)
	−10.4	−17.2	−52.9	−34.5	32.0	−18.7	−6.5	9.2	20.9	−94.1
	−0.347	−0.574	−1.764	−1.152	1.071	−0.624	−0.216	0.309	0.699	−3.150

N	3002	2997	2997	2997	2991	2991	2991	2988	2988	2988
X2	4.2	6.5	2.6	9.8	4.4	2.9	4.7	2.9	3.0	7.5
Gvalue	4.2	6.5	2.6	9.6	4.4	2.9	4.7	2.9	3.0	7.3
Gv/N	0.140	0.219	0.087	0.322	0.149	0.098	0.156	0.097	0.100	0.245
Prob	0.24170	0.08772	0.45579	0.02183	0.21747	0.40230	0.19879	0.40694	0.39213	0.06228

	121	122	123	124	125	126	127	128	129	130
a_O	680	771	389	667	783	383	644	781	380	647
	(22.8)	(25.8)	(13.0)	(22.4)	(26.2)	(12.8)	(21.7)	(26.3)	(12.8)	(21.8)
a_E	688.8	788.1	341.5	688.1	787.3	341.1	686.1	784.9	340.1	685.1
	(23.1)	(26.4)	(11.4)	(23.1)	(26.4)	(11.4)	(23.1)	(26.4)	(11.4)	(23.1)
	-17.5	-33.8	101.4	-41.6	-8.6	88.7	-81.5	-7.9	84.3	-74.1
	-0.588	-1.133	3.396	-1.395	-0.288	2.974	-2.740	-0.264	2.836	-2.496
t_O	417	860	689	444	820	661	435	862	675	413
	(14.0)	(28.8)	(23.1)	(14.9)	(27.5)	(22.2)	(14.6)	(29.0)	(22.7)	(13.9)
t_E	401.2	882.4	625.0	400.8	881.5	624.4	399.6	878.8	622.5	399.0
	(13.4)	(29.6)	(20.9)	(13.4)	(29.6)	(20.9)	(13.4)	(29.6)	(20.9)	(13.4)
	32.2	-44.2	134.4	90.9	-118.6	75.4	73.9	-33.4	109.3	28.4
	1.079	-1.481	4.499	3.047	-3.977	2.527	2.484	-1.122	3.677	0.956
g_O	1144	573	934	1155	600	880	1173	535	888	1174
	(38.3)	(19.2)	(31.3)	(38.7)	(20.1)	(29.5)	(39.4)	(18.0)	(29.9)	(39.5)
g_E	1149.1	556.4	939.1	1147.9	555.8	938.2	1144.4	554.1	935.3	1142.9
	(38.5)	(18.6)	(31.5)	(38.5)	(18.6)	(31.5)	(38.5)	(18.6)	(31.5)	(38.5)
	-10.1	33.7	-10.2	14.2	91.8	-112.7	57.8	-37.6	-92.3	63.0
	-0.338	1.130	-0.342	0.477	3.077	-3.777	1.944	-1.265	-3.102	2.122
c_O	745	782	974	717	780	1059	722	796	1031	736
	(24.9)	(26.2)	(32.6)	(24.0)	(26.1)	(35.5)	(24.3)	(26.8)	(34.7)	(24.8)
c_E	746.9	759.1	1080.4	746.2	758.4	1079.3	743.9	756.1	1076.1	742.9
	(25.0)	(25.4)	(36.2)	(25.0)	(25.4)	(36.2)	(25.0)	(25.4)	(36.2)	(25.0)
	-3.8	46.4	-202.0	-57.2	43.9	-40.3	-43.2	81.9	-88.3	-13.7
	-0.127	1.555	-6.765	-1.916	1.472	-1.351	-1.451	2.755	-2.967	-0.463
N	2986	2986	2986	2983	2983	2983	2974	2974	2974	2970
X2	0.8	2.1	23.7	6.5	8.4	11.3	7.1	3.1	13.4	3.5
Gvalue	0.8	2.1	23.5	6.4	8.5	11.1	7.0	3.1	13.2	3.5
Gv/N	0.025	0.071	0.789	0.213	0.283	0.373	0.237	0.104	0.443	0.119
Prob	0.86015	0.54751	0.00003	0.09567	0.03751	0.01108	0.07061	0.37849	0.00427	0.31453

	131	132	133	134	135	136	137	138	139	140
a_O	779	403	649	745	388	656	757	344	650	751
	(26.2)	(13.6)	(21.9)	(25.1)	(13.1)	(22.2)	(25.6)	(11.6)	(22.1)	(25.5)
a_E	783.9	339.6	683.8	782.3	338.9	681.7	779.9	337.9	679.1	777.0
	(26.4)	(11.4)	(23.1)	(26.4)	(11.4)	(23.1)	(26.4)	(11.4)	(23.1)	(26.4)
	-9.7	137.9	-67.7	-72.8	104.9	-50.4	-45.2	12.3	-57.0	-51.2
	-0.328	4.643	-2.285	-2.456	3.539	-1.705	-1.529	0.416	-1.937	-1.738
t_O	885	633	438	872	640	428	802	658	429	857
	(29.8)	(21.3)	(14.8)	(29.4)	(21.6)	(14.5)	(27.1)	(22.3)	(14.6)	(29.1)
t_E	877.7	621.6	398.2	875.9	620.4	397.0	873.2	618.5	395.6	870.0
	(29.6)	(20.9)	(13.4)	(29.6)	(20.9)	(13.4)	(29.6)	(20.9)	(13.4)	(29.6)
	14.7	22.9	83.4	-7.8	39.8	64.3	-136.5	81.5	69.6	-25.8
	0.496	0.772	2.812	-0.262	1.344	2.175	-4.619	2.757	2.365	-0.875
g_O	555	932	1136	565	910	1161	593	902	1132	595
	(18.7)	(31.4)	(38.3)	(19.1)	(30.7)	(39.3)	(20.1)	(30.5)	(38.5)	(20.2)
g_E	553.4	934.1	1140.6	552.3	932.2	1137.1	550.6	929.4	1132.9	548.6
	(18.6)	(31.5)	(38.5)	(18.6)	(31.5)	(38.5)	(18.6)	(31.5)	(38.5)	(18.6)
	3.2	-4.2	-9.2	25.7	-43.9	48.2	88.0	-53.9	-1.8	96.7
	0.108	-0.141	-0.310	0.868	-1.480	1.632	2.977	-1.825	-0.061	3.285
c_O	751	1002	741	782	1026	710	803	1051	733	741
	(25.3)	(33.7)	(25.0)	(26.4)	(34.6)	(24.0)	(27.2)	(35.6)	(24.9)	(25.2)
c_E	755.1	1074.6	741.4	753.5	1072.5	739.1	751.2	1069.2	736.4	748.4
	(25.4)	(36.2)	(25.0)	(25.4)	(36.2)	(25.0)	(25.4)	(36.2)	(25.0)	(25.4)
	-8.1	-140.2	-0.8	58.0	-90.9	-57.1	107.0	-36.1	-6.8	-14.8
	-0.272	-4.722	-0.027	1.957	-3.066	-1.933	3.621	-1.222	-0.230	-0.503
N	2970	2970	2964	2964	2964	2955	2955	2955	2944	2944
X2	0.1	16.9	5.8	3.2	10.3	5.0	13.3	3.7	4.1	5.1
Gvalue	0.1	16.4	5.7	3.2	10.0	5.0	13.3	3.7	4.0	5.0
Gv/N	0.004	0.552	0.191	0.107	0.336	0.169	0.451	0.125	0.137	0.169
Prob	0.98959	0.00095	0.12942	0.36497	0.01883	0.17203	0.00398	0.29514	0.25757	0.17350

	141	142	143	144	145	146	147	148	149	150
a_O	355	625	743	351	662	732	348	685	770	372
	(12.1)	(21.3)	(25.3)	(12.0)	(22.6)	(25.0)	(11.9)	(23.4)	(26.3)	(12.7)
a_E	336.7	677.3	774.9	335.7	676.4	773.9	335.3	674.8	772.0	334.5
	(11.4)	(23.1)	(26.4)	(11.4)	(23.1)	(26.4)	(11.4)	(23.1)	(26.4)	(11.4)
	37.7	-100.4	-62.5	31.2	-28.4	-81.4	25.9	20.6	-4.0	79.1
	1.279	-3.421	-2.128	1.063	-0.970	-2.776	0.884	0.705	-0.137	2.704
t_O	658	421	836	613	418	876	623	404	855	652
	(22.4)	(14.3)	(28.5)	(20.9)	(14.3)	(29.9)	(21.2)	(13.8)	(29.2)	(22.3)
t_E	616.2	394.5	867.6	614.5	393.9	866.4	613.7	393.0	864.4	612.2
	(20.9)	(13.4)	(29.6)	(20.9)	(13.4)	(29.6)	(20.9)	(13.4)	(29.6)	(20.9)
	86.4	54.8	-62.1	-3.1	49.6	19.2	18.8	22.3	-18.6	82.1
	2.934	1.866	-2.114	-0.104	1.690	0.656	0.640	0.762	-0.637	2.806
g_O	922	1096	555	961	1105	577	848	1138	548	922
	(31.3)	(37.3)	(18.9)	(32.7)	(37.7)	(19.7)	(28.9)	(38.9)	(18.7)	(31.5)
g_E	925.9	1129.8	547.1	923.4	1128.3	546.3	922.1	1125.6	545.0	919.9
	(31.5)	(38.5)	(18.6)	(31.5)	(38.5)	(18.6)	(31.5)	(38.5)	(18.6)	(31.5)
	-7.8	-66.6	16.0	76.7	-46.1	63.1	-142.1	25.0	6.0	4.1
	-0.265	-2.269	0.545	2.613	-1.572	2.151	-4.848	0.853	0.205	0.141
c_O	1009	794	802	1011	747	747	1113	698	752	979
	(34.3)	(27.0)	(27.3)	(34.4)	(25.5)	(25.5)	(38.0)	(23.9)	(25.7)	(33.5)
c_E	1065.2	734.4	746.4	1062.3	733.4	745.4	1060.9	731.6	743.6	1058.4
	(36.2)	(25.0)	(25.4)	(36.2)	(25.0)	(25.4)	(36.2)	(25.0)	(25.4)	(36.2)
	-109.4	123.9	115.2	-100.1	27.5	3.2	106.7	-65.7	16.9	-152.6
	-3.717	4.221	3.924	-3.411	0.936	0.110	3.641	-2.247	0.577	-5.217
N	2944	2936	2936	2936	2932	2932	2932	2925	2925	2925
X2	6.8	11.7	6.7	4.7	2.5	4.1	9.1	2.1	0.2	12.7
Gvalue	6.8	11.6	6.7	4.7	2.5	4.1	9.3	2.2	0.2	12.7
Gv/N	0.231	0.396	0.227	0.161	0.085	0.140	0.316	0.074	0.007	0.434
Prob	0.07879	0.00879	0.08375	0.19354	0.47851	0.25021	0.02599	0.53842	0.97467	0.00534

Bpar_12822
5UTR:

	50	49	48	47	46	45	44	43	42	41
a_O	753	843	703	742	754	673	734	748	695	716
	(18.0)	(20.2)	(16.8)	(17.8)	(18.0)	(16.1)	(17.6)	(17.9)	(16.6)	(17.1)
a_E	787.2	787.2	787.2	787.2	787.2	787.2	787.2	787.2	787.2	787.2
	(18.8)	(18.8)	(18.8)	(18.8)	(18.8)	(18.8)	(18.8)	(18.8)	(18.8)	(18.8)
	-66.9	115.4	-159.1	-87.8	-65.0	-211.0	-102.8	-76.5	-173.2	-135.8
	-1.602	2.763	-3.808	-2.101	-1.556	-5.050	-2.460	-1.830	-4.145	-3.250
t_O	662	758	708	720	830	736	720	767	717	707
	(15.8)	(18.1)	(16.9)	(17.2)	(19.9)	(17.6)	(17.2)	(18.4)	(17.2)	(16.9)
t_E	740.7	740.7	740.7	740.7	740.7	740.7	740.7	740.7	740.7	740.7
	(17.7)	(17.7)	(17.7)	(17.7)	(17.7)	(17.7)	(17.7)	(17.7)	(17.7)	(17.7)
	-148.8	34.9	-64.0	-40.9	188.9	-9.4	-40.9	53.5	-46.7	-65.9
	-3.561	0.836	-1.532	-0.978	4.521	-0.226	-0.978	1.280	-1.118	-1.577
g_O	1383	1297	1330	1320	1307	1371	1323	1293	1321	1407
	(33.1)	(31.0)	(31.8)	(31.6)	(31.3)	(32.8)	(31.7)	(30.9)	(31.6)	(33.7)
g_E	1287.3	1287.3	1287.3	1287.3	1287.3	1287.3	1287.3	1287.3	1287.3	1287.3
	(30.8)	(30.8)	(30.8)	(30.8)	(30.8)	(30.8)	(30.8)	(30.8)	(30.8)	(30.8)
	198.3	19.4	86.7	66.1	39.6	172.6	72.3	11.3	68.2	250.1
	4.745	0.464	2.076	1.583	0.948	4.132	1.730	0.272	1.632	5.986
c_O	1380	1280	1437	1396	1287	1398	1401	1370	1445	1348
	(33.0)	(30.6)	(34.4)	(33.4)	(30.8)	(33.5)	(33.5)	(32.8)	(34.6)	(32.3)
c_E	1362.7	1362.7	1362.7	1362.7	1362.7	1362.7	1362.7	1362.7	1362.7	1362.7
	(32.6)	(32.6)	(32.6)	(32.6)	(32.6)	(32.6)	(32.6)	(32.6)	(32.6)	(32.6)
	34.8	-160.3	152.6	67.4	-147.1	71.5	77.6	14.6	169.5	-29.3
	0.833	-3.837	3.651	1.613	-3.522	1.711	1.858	0.350	4.056	-0.700
N	4178	4178	4178	4178	4178	4178	4178	4178	4178	4178
X2	17.2	9.4	15.9	4.8	16.7	23.0	6.2	2.9	17.4	19.3
Gvalue	17.3	9.5	16.2	4.9	16.4	23.7	6.3	3.0	17.8	19.2
Gv/N	0.415	0.226	0.388	0.116	0.392	0.567	0.151	0.071	0.425	0.459
Prob	0.00060	0.02377	0.00103	0.18212	0.00096	0.00003	0.09737	0.39598	0.00049	0.00025

	40	39	38	37	36	35	34	33	32	31
a_O	836	718	751	754	665	739	828	691	743	794
	(20.0)	(17.2)	(18.0)	(18.0)	(15.9)	(17.7)	(19.8)	(16.5)	(17.8)	(19.0)
a_E	787.2	787.2	787.2	787.2	787.2	787.2	787.2	787.2	787.2	787.2
	(18.8)	(18.8)	(18.8)	(18.8)	(18.8)	(18.8)	(18.8)	(18.8)	(18.8)	(18.8)
	100.5	-132.2	-70.7	-65.0	-224.4	-93.4	83.6	-180.2	-85.9	13.6
	2.406	-3.163	-1.693	-1.556	-5.371	-2.236	2.002	-4.312	-2.056	0.326
t_O	763	735	704	829	752	699	823	738	629	780
	(18.3)	(17.6)	(16.9)	(19.8)	(18.0)	(16.7)	(19.7)	(17.7)	(15.1)	(18.7)
t_E	740.7	740.7	740.7	740.7	740.7	740.7	740.7	740.7	740.7	740.7
	(17.7)	(17.7)	(17.7)	(17.7)	(17.7)	(17.7)	(17.7)	(17.7)	(17.7)	(17.7)
	45.2	-11.4	-71.6	186.7	22.7	-81.1	173.4	-5.4	-205.7	80.6
	1.082	-0.273	-1.714	4.468	0.544	-1.940	4.149	-0.130	-4.923	1.929
g_O	1230	1292	1326	1226	1323	1305	1206	1350	1318	1208
	(29.4)	(30.9)	(31.7)	(29.3)	(31.7)	(31.2)	(28.9)	(32.3)	(31.5)	(28.9)
g_E	1287.3	1287.3	1287.3	1287.3	1287.3	1287.3	1287.3	1287.3	1287.3	1287.3
	(30.8)	(30.8)	(30.8)	(30.8)	(30.8)	(30.8)	(30.8)	(30.8)	(30.8)	(30.8)
	-112.1	9.3	78.5	-119.7	72.3	35.6	-157.4	128.3	62.0	-153.7
	-2.683	0.223	1.878	-2.865	1.730	0.851	-3.768	3.071	1.485	-3.678
c_O	1349	1433	1397	1369	1438	1435	1321	1399	1488	1396
	(32.3)	(34.3)	(33.4)	(32.8)	(34.4)	(34.3)	(31.6)	(33.5)	(35.6)	(33.4)
c_E	1362.7	1362.7	1362.7	1362.7	1362.7	1362.7	1362.7	1362.7	1362.7	1362.7
	(32.6)	(32.6)	(32.6)	(32.6)	(32.6)	(32.6)	(32.6)	(32.6)	(32.6)	(32.6)
	-27.3	144.1	69.4	12.6	154.7	148.3	-82.1	73.5	261.8	67.4
	-0.653	3.450	1.662	0.302	3.702	3.551	-1.966	1.760	6.265	1.613
N	4178	4178	4178	4178	4178	4178	4178	4178	4178	4178
X2	6.4	9.8	5.5	14.9	24.3	9.4	17.7	15.8	31.6	7.8
Gvalue	6.4	9.9	5.6	14.5	25.3	9.4	17.4	16.2	32.2	7.9
Gv/N	0.152	0.237	0.133	0.348	0.605	0.226	0.417	0.389	0.771	0.189
Prob	0.09551	0.01944	0.13567	0.00225	0.00001	0.02417	0.00058	0.00101	0.00000	0.04803

	30	29	28	27	26	25	24	23	22	21
a_O	685	695	801	691	748	756	745	761	844	758
	(16.4)	(16.6)	(19.2)	(16.5)	(17.9)	(18.1)	(17.8)	(18.2)	(20.2)	(18.1)
a_E	787.2	787.2	787.2	787.2	787.2	787.2	787.2	787.2	787.2	787.2
	(18.8)	(18.8)	(18.8)	(18.8)	(18.8)	(18.8)	(18.8)	(18.8)	(18.8)	(18.8)
	-190.6	-173.2	27.8	-180.2	-76.5	-61.2	-82.1	-51.6	117.6	-57.3
	-4.561	-4.145	0.665	-4.312	-1.830	-1.464	-1.966	-1.234	2.814	-1.372
t_O	737	722	870	751	711	847	749	798	863	760
	(17.6)	(17.3)	(20.8)	(18.0)	(17.0)	(20.3)	(17.9)	(19.1)	(20.7)	(18.2)
t_E	740.7	740.7	740.7	740.7	740.7	740.7	740.7	740.7	740.7	740.7
	(17.7)	(17.7)	(17.7)	(17.7)	(17.7)	(17.7)	(17.7)	(17.7)	(17.7)	(17.7)
	-7.4	-37.0	279.9	20.7	-58.3	227.1	16.6	118.9	263.7	39.0
	-0.178	-0.885	6.699	0.495	-1.394	5.436	0.398	2.845	6.312	0.934
g_O	1289	1304	1139	1277	1257	1161	1178	1154	1043	1125
	(30.9)	(31.2)	(27.3)	(30.6)	(30.1)	(27.8)	(28.2)	(27.6)	(25.0)	(26.9)
g_E	1287.3	1287.3	1287.3	1287.3	1287.3	1287.3	1287.3	1287.3	1287.3	1287.3
	(30.8)	(30.8)	(30.8)	(30.8)	(30.8)	(30.8)	(30.8)	(30.8)	(30.8)	(30.8)
	3.3	33.5	-278.9	-20.6	-60.0	-239.9	-209.1	-252.4	-439.1	-303.3
	0.080	0.803	-6.675	-0.493	-1.435	-5.741	-5.005	-6.040	-10.509	-7.259
c_O	1467	1457	1368	1459	1462	1414	1506	1465	1428	1535
	(35.1)	(34.9)	(32.7)	(34.9)	(35.0)	(33.8)	(36.0)	(35.1)	(34.2)	(36.7)
c_E	1362.7	1362.7	1362.7	1362.7	1362.7	1362.7	1362.7	1362.7	1362.7	1362.7
	(32.6)	(32.6)	(32.6)	(32.6)	(32.6)	(32.6)	(32.6)	(32.6)	(32.6)	(32.6)
	216.4	195.0	10.6	199.2	205.6	104.5	301.1	212.1	133.7	365.5
	5.179	4.666	0.254	4.769	4.922	2.501	7.208	5.076	3.199	8.748
N	4178	4178	4178	4178	4178	4178	4178	4178	4178	4178
X2	21.3	18.0	39.9	18.8	11.1	30.8	26.7	26.8	73.8	43.8
Gvalue	21.7	18.3	39.4	19.1	11.0	30.6	26.5	27.0	75.9	43.9
Gv/N	0.519	0.439	0.943	0.458	0.263	0.731	0.635	0.646	1.816	1.051
Prob	0.00008	0.00038	0.00000	0.00026	0.01182	0.00000	0.00001	0.00001	-0.00000	0.00000

	20	19	18	17	16	15	14	13	12	11
a_O	846	896	835	939	907	856	1011	1047	1045	1159
	(20.2)	(21.4)	(20.0)	(22.5)	(21.7)	(20.5)	(24.2)	(25.1)	(25.0)	(27.7)
a_E	787.2	787.2	787.2	787.2	787.2	787.2	787.2	787.2	787.2	787.2
	(18.8)	(18.8)	(18.8)	(18.8)	(18.8)	(18.8)	(18.8)	(18.8)	(18.8)	(18.8)
	121.8	231.9	98.4	331.1	256.9	143.4	505.9	597.2	592.0	896.6
	2.916	5.552	2.355	7.925	6.149	3.432	12.108	14.293	14.170	21.460
t_O	793	864	808	818	908	815	687	791	562	452
	(19.0)	(20.7)	(19.3)	(19.6)	(21.7)	(19.5)	(16.4)	(18.9)	(13.5)	(10.8)

```
t_E        740.7   740.7   740.7   740.7   740.7   740.7   740.7   740.7   740.7   740.7
         ( 17.7) ( 17.7) ( 17.7) ( 17.7) ( 17.7) ( 17.7) ( 17.7) ( 17.7) ( 17.7) ( 17.7)
           108.1   266.0   140.5   162.3   369.8   155.7  -103.5   103.9  -310.4  -446.5
           2.588   6.367   3.362   3.885   8.850   3.728  -2.476   2.486  -7.429 -10.688
g_O         1073     972     990    1017     941    1081    1125    1132    1454    1628
         ( 25.7) ( 23.3) ( 23.7) ( 24.3) ( 22.5) ( 25.9) ( 26.9) ( 27.1) ( 34.8) ( 39.0)
g_E       1287.3  1287.3  1287.3  1287.3  1287.3  1287.3  1287.3  1287.3  1287.3  1287.3
         ( 30.8) ( 30.8) ( 30.8) ( 30.8) ( 30.8) ( 30.8) ( 30.8) ( 30.8) ( 30.8) ( 30.8)
          -390.8  -546.2  -520.0  -479.5  -589.8  -377.7  -303.3  -291.1   354.0   764.4
          -9.354 -13.074 -12.446 -11.476 -14.117  -9.040  -7.259  -6.968   8.473  18.296
c_O         1466    1446    1545    1404    1422    1426    1355    1208    1117     939
         ( 35.1) ( 34.6) ( 37.0) ( 33.6) ( 34.0) ( 34.1) ( 32.4) ( 28.9) ( 26.7) ( 22.5)
c_E       1362.7  1362.7  1362.7  1362.7  1362.7  1362.7  1362.7  1362.7  1362.7  1362.7
         ( 32.6) ( 32.6) ( 32.6) ( 32.6) ( 32.6) ( 32.6) ( 32.6) ( 32.6) ( 32.6) ( 32.6)
           214.2   171.6   387.9    83.8   121.1   129.5   -15.4  -291.2  -444.2  -699.4
           5.127   4.107   9.285   2.006   2.899   3.099  -0.368  -6.969 -10.631 -16.740

N           4178    4178    4178    4178    4178    4178    4178    4178    4178    4178
X2          51.6   117.9   102.1    95.3   151.8    49.5    88.0   125.4   193.4   510.0
Gvalue      53.4   123.3   106.8    97.8   158.0    50.9    83.7   118.8   191.5   515.1
Gv/N       1.278   2.951   2.557   2.341   3.782   1.219   2.005   2.842   4.583  12.329
Prob     0.00000 0.00000 0.00000 0.00000 0.00000 0.00000 0.00000 0.00000 0.00000 0.00000
         -------------------------------------------------------------------------------
              10       9       8       7       6       5       4       3       2       1
         -------------------------------------------------------------------------------
a_O         1086    1024    1187    1146     999     992    1007    1024     788     575
         ( 26.0) ( 24.5) ( 28.4) ( 27.4) ( 23.9) ( 23.7) ( 24.1) ( 24.5) ( 18.9) ( 13.8)
a_E        787.2   787.2   787.2   787.2   787.2   787.2   787.2   787.2   787.2   787.2
         ( 18.8) ( 18.8) ( 18.8) ( 18.8) ( 18.8) ( 18.8) ( 18.8) ( 18.8) ( 18.8) ( 18.8)
           698.8   538.6   974.9   860.7   476.0   458.7   495.9   538.6     1.6  -361.3
          16.727  12.890  23.335  20.601  11.393  10.980  11.869  12.890   0.037  -8.647
t_O          502     457     505     708     694     699     692     581     806     487
         ( 12.0) ( 10.9) ( 12.1) ( 16.9) ( 16.6) ( 16.7) ( 16.6) ( 13.9) ( 19.3) ( 11.7)
t_E        740.7   740.7   740.7   740.7   740.7   740.7   740.7   740.7   740.7   740.7
         ( 17.7) ( 17.7) ( 17.7) ( 17.7) ( 17.7) ( 17.7) ( 17.7) ( 17.7) ( 17.7) ( 17.7)
          -390.6  -441.4  -386.9   -64.0   -90.4   -81.1   -94.2  -282.2   136.1  -408.5
          -9.349 -10.565  -9.261  -1.532  -2.165  -1.940  -2.254  -6.755   3.258  -9.777
g_O         1766    1950    1617    1318    1223     951     946    1507     795     596
         ( 42.3) ( 46.7) ( 38.7) ( 31.5) ( 29.3) ( 22.8) ( 22.6) ( 36.1) ( 19.0) ( 14.3)
g_E       1287.3  1287.3  1287.3  1287.3  1287.3  1287.3  1287.3  1287.3  1287.3  1287.3
         ( 30.8) ( 30.8) ( 30.8) ( 30.8) ( 30.8) ( 30.8) ( 30.8) ( 30.8) ( 30.8) ( 30.8)
          1116.6  1619.5   737.3    62.0  -125.4  -576.0  -582.9   474.8  -766.4  -918.0
          26.726  38.762  17.648   1.485  -3.002 -13.786 -13.952  11.365 -18.343 -21.971
c_O          824     747     869    1006    1262    1536    1533    1066    1789    2520
         ( 19.7) ( 17.9) ( 20.8) ( 24.1) ( 30.2) ( 36.8) ( 36.7) ( 25.5) ( 42.8) ( 60.3)
c_E       1362.7  1362.7  1362.7  1362.7  1362.7  1362.7  1362.7  1362.7  1362.7  1362.7
         ( 32.6) ( 32.6) ( 32.6) ( 32.6) ( 32.6) ( 32.6) ( 32.6) ( 32.6) ( 32.6) ( 32.6)
          -829.0  -898.1  -781.9  -610.6  -193.8   367.7   361.0  -523.5   973.9  3098.5
         -19.843 -21.497 -18.715 -14.615  -4.638   8.802   8.641 -12.531  23.309  74.162

N           4178    4178    4178    4178    4178    4178    4178    4178    4178    4178
X2         581.3   799.2   541.3   259.1    70.6   165.5   176.4   207.7   327.4  1498.2
Gvalue     595.8   818.5   543.5   248.1    66.4   169.4   179.8   207.6   345.2  1410.8
Gv/N      14.261  19.590  13.008   5.939   1.589   4.056   4.304   4.969   8.262  33.768
Prob     0.00000 0.00000 0.00000 0.00000 0.00000 0.00000 0.00000 0.00000 0.00000 0.00000
         -------------------------------------------------------------------------------

CDS_INITIAL:
         -------------------------------------------------------------------------------
               1       2       3       4       5       6       7       8       9      10
         -------------------------------------------------------------------------------
a_O         3410       0       0    1829    1248     703    1233    1213     767    1135
         ( 81.6) (  0.0) (  0.0) ( 43.8) ( 29.9) ( 16.8) ( 29.5) ( 29.0) ( 18.4) ( 27.2)
a_E        780.6  1002.0   202.9   780.6  1002.0   202.9   780.6  1002.0   202.9   780.6
         ( 18.7) ( 24.0) (  4.9) ( 18.7) ( 24.0) (  4.9) ( 18.7) ( 24.0) (  4.9) ( 18.7)
         10055.5     0.0     0.0  3114.7   547.9  1747.1  1127.3   463.5  2039.8   849.7
         240.677   0.000   0.000  74.549  13.114  41.817  26.982  11.094  48.823  20.338
t_O          318    4178       0     492     650     793     504     956     736     658
         (  7.6) (100.0) (  0.0) ( 11.8) ( 15.6) ( 19.0) ( 12.1) ( 22.9) ( 17.6) ( 15.7)
t_E        499.0  1191.5   265.8   499.0  1191.5   265.8   499.0  1191.5   265.8   499.0
         ( 11.9) ( 28.5) (  6.4) ( 11.9) ( 28.5) (  6.4) ( 11.9) ( 28.5) (  6.4) ( 11.9)
          -286.6 10483.9     0.0   -13.9  -787.8  1733.6    10.0  -421.0  1499.2   364.0
          -6.859 250.931   0.000  -0.333 -18.855  41.493   0.240 -10.076  35.883   8.712
g_O          450       0    4178     806     776    1040    1251     743    1112    1058
         ( 10.8) (  0.0) (100.0) ( 19.3) ( 18.6) ( 24.9) ( 29.9) ( 17.8) ( 26.6) ( 25.3)
g_E       1685.2   855.9  1703.2  1685.2   855.9  1703.2  1685.2   855.9  1703.2  1685.2
         ( 40.3) ( 20.5) ( 40.8) ( 40.3) ( 20.5) ( 40.8) ( 40.3) ( 20.5) ( 40.8) ( 40.3)
         -1188.4     0.0  7497.9 -1188.9  -152.1 -1026.1  -745.5  -210.2  -948.2  -985.0
         -28.443   0.000 179.461 -28.457  -3.641 -24.559 -17.842  -5.032 -22.696 -23.576
c_O            0       0       0    1051    1504    1642    1190    1266    1563    1327
         (  0.0) (  0.0) (  0.0) ( 25.2) ( 36.0) ( 39.3) ( 28.5) ( 30.3) ( 37.4) ( 31.8)
c_E       1213.2  1128.6  2006.1  1213.2  1128.6  2006.1  1213.2  1128.6  2006.1  1213.2
         ( 29.0) ( 27.0) ( 48.0) ( 29.0) ( 27.0) ( 48.0) ( 29.0) ( 27.0) ( 48.0) ( 29.0)
             0.0     0.0     0.0  -301.7   863.8  -657.6   -45.9   290.9  -780.1   238.0
           0.000   0.000   0.000  -7.220  20.674 -15.741  -1.099   6.963 -18.673   5.696

N           4178    4178    4178    4178    4178    4178    4178    4178    4178    4178
X2       11041.2 10472.7  6070.6  1888.6   438.8  2602.5   374.6   122.6  2703.1   455.7
Gvalue    8580.5 10483.9  7497.9  1610.1   471.8  1797.0   346.0   123.2  1810.6   466.7
Gv/N     205.375 250.931 179.461  38.539  11.292  43.011   8.281   2.949  43.338  11.170
Prob     1.00000 1.00000 1.00000 1.00000 0.00000 1.00000 0.00000 0.00000 1.00000 0.00000
         -------------------------------------------------------------------------------
              11      12      13      14      15      16      17      18      19      20
         -------------------------------------------------------------------------------
a_O          905     637    1072    1066     624     962     978     552    1003     957
         ( 21.7) ( 15.2) ( 25.7) ( 25.5) ( 14.9) ( 23.0) ( 23.4) ( 13.2) ( 24.0) ( 22.9)
a_E       1002.0   202.9   780.6  1002.0   202.9   780.6  1002.0   202.9   780.6  1002.0
         ( 24.0) (  4.9) ( 18.7) ( 24.0) (  4.9) ( 18.7) ( 24.0) (  4.9) ( 18.7) ( 24.0)
          -184.4  1457.5   680.1   131.9  1402.0   402.0   -47.5  1104.9   502.9   -88.0
          -4.413  34.885  16.279   3.158  33.557   9.623  -1.137  26.446  12.037  -2.107
t_O         1205     745     647    1193     735     711    1306     737     593    1264
         ( 28.8) ( 17.8) ( 15.5) ( 28.6) ( 17.6) ( 17.0) ( 31.3) ( 17.6) ( 14.2) ( 30.3)
t_E       1191.5   265.8   499.0  1191.5   265.8   499.0  1191.5   265.8   499.0  1191.5
         ( 28.5) (  6.4) ( 11.9) ( 28.5) (  6.4) ( 11.9) ( 28.5) (  6.4) ( 11.9) ( 28.5)
            27.2  1535.6   336.1     3.1  1495.1   503.4   239.8  1503.2   204.7   149.4
           0.652  36.755   8.044   0.074  35.786  12.050   5.738  35.979   4.899   3.576
g_O          669    1054    1181     651    1087    1168     643    1161    1262     678
         ( 16.0) ( 25.2) ( 28.3) ( 15.6) ( 26.0) ( 28.0) ( 15.4) ( 27.8) ( 30.2) ( 16.2)
g_E        855.9  1703.2  1685.2   855.9  1703.2  1685.2   855.9  1703.2  1685.2   855.9
         ( 20.5) ( 40.8) ( 40.3) ( 20.5) ( 40.8) ( 40.3) ( 20.5) ( 40.8) ( 40.3) ( 20.5)
          -329.7 -1011.7  -839.8  -356.3  -976.4  -856.4  -367.8  -889.9  -729.9  -316.0
          -7.890 -24.215 -20.099  -8.528 -23.369 -20.497  -8.804 -21.299 -17.470  -7.563
```

```
c_O        1399    1742    1278    1268    1732    1337    1251    1728    1320    1279
         ( 33.5) ( 41.7) ( 30.6) ( 30.3) ( 41.5) ( 32.0) ( 29.9) ( 41.4) ( 31.6) ( 30.6)
c_E      1128.6  2006.1  1213.2  1128.6  2006.1  1213.2  1128.6  2006.1  1213.2  1128.6
         ( 27.0) ( 48.0) ( 29.0) ( 27.0) ( 48.0) ( 29.0) ( 27.0) ( 48.0) ( 29.0) ( 27.0)
          601.0  -491.7   133.0   295.4  -508.9   259.9   257.6  -515.7   222.8   320.0
         14.384 -11.770   3.184   7.070 -12.179   6.220   6.167 -12.342   5.332   7.660

N          4178    4178    4178    4178    4178    4178    4178    4178    4178    4178
X2        115.2  2074.8   307.0    70.4  1962.5   303.6    77.8  1647.1   196.8    63.5
Gvalue    114.2  1489.7   309.5    74.1  1412.0   309.0    82.1  1202.6   200.4    65.4
Gv/N      2.733  35.656   7.407   1.773  33.795   7.395   1.964  28.783   4.797   1.566
Prob    0.00000 0.00000 0.00000 0.00000 0.00000 0.00000 0.00000 0.00000 0.00000 0.00000
        -------------------------------------------------------------------------------
             21      22      23      24      25      26      27      28      29      30
        -------------------------------------------------------------------------------
a_O         537     886     911     421     788     866     360     781     821     359
         ( 12.9) ( 21.2) ( 21.8) ( 10.1) ( 18.9) ( 20.7) (  8.6) ( 18.7) ( 19.7) (  8.6)
a_E       202.9   780.6  1002.0   202.9   780.6  1002.0   202.9   780.6  1002.0   202.9
         (  4.9) ( 18.7) ( 24.0) (  4.9) ( 18.7) ( 24.0) (  4.9) ( 18.7) ( 24.0) (  4.9)
         1045.3   224.4  -173.5   614.6    14.9  -252.7   412.8     0.8  -327.2   409.7
         25.019   5.372  -4.154  14.710   0.356  -6.049   9.881   0.019  -7.831   9.806
t_O         592     666    1229     590     658    1254     527     557    1228     461
         ( 14.2) ( 15.9) ( 29.4) ( 14.1) ( 15.7) ( 30.0) ( 12.6) ( 13.3) ( 29.4) ( 11.0)
t_E       265.8   499.0  1191.5   265.8   499.0  1191.5   265.8   499.0  1191.5   265.8
         (  6.4) ( 11.9) ( 28.5) (  6.4) ( 11.9) ( 28.5) (  6.4) ( 11.9) ( 28.5) (  6.4)
          948.1   384.5    76.2   940.9   364.0   128.3   721.4   122.5    74.2   507.7
         22.692   9.203   1.825  22.520   8.712   3.071  17.266   2.931   1.776  12.151
g_O        1175    1395     789    1339    1420     783    1365    1556     789    1420
         ( 28.1) ( 33.4) ( 18.9) ( 32.0) ( 34.0) ( 18.7) ( 32.7) ( 37.2) ( 18.9) ( 34.0)
g_E      1703.2  1685.2   855.9  1703.2  1685.2   855.9  1703.2  1685.2   855.9  1703.2
         ( 40.8) ( 40.3) ( 20.5) ( 40.8) ( 40.3) ( 20.5) ( 40.8) ( 40.3) ( 20.5) ( 40.8)
         -872.5  -527.3  -128.4  -644.3  -486.3  -139.4  -604.3  -248.2  -128.4  -516.5
        -20.882 -12.621  -3.074 -15.422 -11.639  -3.337 -14.465  -5.942  -3.074 -12.363
c_O        1874    1231    1249    1828    1312    1275    1926    1284    1340    1938
         ( 44.9) ( 29.5) ( 29.9) ( 43.8) ( 31.4) ( 30.5) ( 46.1) ( 30.7) ( 32.1) ( 46.4)
c_E      2006.1  1213.2  1128.6  2006.1  1213.2  1128.6  2006.1  1213.2  1128.6  2006.1
         ( 48.0) ( 29.0) ( 27.0) ( 48.0) ( 29.0) ( 27.0) ( 48.0) ( 29.0) ( 27.0) ( 48.0)
         -255.2    35.9   253.2  -339.8   205.5   311.0  -156.9   145.7   460.2  -133.8
         -6.109   0.859   6.061  -8.134   4.918   7.445  -3.755   3.487  11.014  -3.202

N          4178    4178    4178    4178    4178    4178    4178    4178    4178    4178
X2       1122.9   120.3    27.5   723.5   100.5    47.0   448.7    20.8    78.7   312.8
Gvalue    865.7   117.5    27.5   571.3    98.0    47.2   373.0    20.7    78.7   267.1
Gv/N     20.720   2.813   0.658  13.674   2.346   1.130   8.928   0.496   1.884   6.392
Prob    0.00000 0.00000 0.00000 0.00000 0.00000 0.00000 0.00000 0.00012 0.00000 0.00000
        -------------------------------------------------------------------------------
             31      32      33      34      35      36      37      38      39      40
        -------------------------------------------------------------------------------
a_O         771     797     373     737     756     322     720     794     302     663
         ( 18.5) ( 19.1) (  8.9) ( 17.6) ( 18.1) (  7.7) ( 17.2) ( 19.0) (  7.2) ( 15.9)
a_E       780.6  1002.0   202.9   780.6  1002.0   202.9   780.6  1002.0   202.9   780.6
         ( 18.7) ( 24.0) (  4.9) ( 18.7) ( 24.0) (  4.9) ( 18.7) ( 24.0) (  4.9) ( 18.7)
          -19.1  -364.9   454.2   -84.7  -426.0   297.4  -116.4  -369.5   240.2  -216.5
         -0.457  -8.734  10.871  -2.028 -10.196   7.119  -2.785  -8.845   5.749  -5.182
t_O         571    1212     452     503    1251     423     568    1222     457     556
         ( 13.7) ( 29.0) ( 10.8) ( 12.0) ( 29.9) ( 10.1) ( 13.6) ( 29.2) ( 10.9) ( 13.3)
t_E       499.0  1191.5   265.8   499.0  1191.5   265.8   499.0  1191.5   265.8   499.0
         ( 11.9) ( 28.5) (  6.4) ( 11.9) ( 28.5) (  6.4) ( 11.9) ( 28.5) (  6.4) ( 11.9)
          153.9    41.4   479.9     8.0   122.0   393.1   147.1    61.9   495.3   120.2
          3.683   0.992  11.487   0.192   2.920   9.408   3.521   1.480  11.855   2.878
g_O        1582     929    1411    1636     898    1441    1690     859    1517    1702
         ( 37.9) ( 22.2) ( 33.8) ( 39.2) ( 21.5) ( 34.5) ( 40.4) ( 20.6) ( 36.3) ( 40.7)
g_E      1685.2   855.9  1703.2  1685.2   855.9  1703.2  1685.2   855.9  1703.2  1685.2
         ( 40.3) ( 20.5) ( 40.8) ( 40.3) ( 20.5) ( 40.8) ( 40.3) ( 20.5) ( 40.8) ( 40.3)
         -200.0   152.3  -531.2   -97.0    86.2  -481.8     9.6     6.2  -351.3    33.8
         -4.786   3.644 -12.714  -2.321   2.064 -11.533   0.230   0.148  -8.409   0.808
c_O        1254    1240    1942    1302    1273    1992    1200    1303    1902    1257
         ( 30.0) ( 29.7) ( 46.5) ( 31.2) ( 30.5) ( 47.7) ( 28.7) ( 31.2) ( 45.5) ( 30.1)
c_E      1213.2  1128.6  2006.1  1213.2  1128.6  2006.1  1213.2  1128.6  2006.1  1213.2
         ( 29.0) ( 27.0) ( 48.0) ( 29.0) ( 27.0) ( 48.0) ( 29.0) ( 27.0) ( 48.0) ( 29.0)
           83.0   233.5  -126.1   184.0   306.6   -28.0   -26.2   374.5  -202.6    89.2
          1.986   5.588  -3.017   4.403   7.337  -0.671  -0.628   8.963  -4.850   2.135

N          4178    4178    4178    4178    4178    4178    4178    4178    4178    4178
X2         18.2    59.5   325.2    10.4    83.9   203.3    14.4    70.9   211.7    26.0
Gvalue     17.8    62.2   276.9    10.3    88.8   180.6    14.1    73.0   181.6    26.7
Gv/N      0.427   1.489   6.628   0.247   2.125   4.323   0.338   1.747   4.346   0.639
Prob    0.00047 0.00000 0.00000 0.01609 0.00000 0.00000 0.00275 0.00000 0.00000 0.00001
        -------------------------------------------------------------------------------
             41      42      43      44      45      46      47      48      49      50
        -------------------------------------------------------------------------------
a_O         806     340     758     812     331     706     825     287     733     767
         ( 19.3) (  8.1) ( 18.1) ( 19.4) (  7.9) ( 16.9) ( 19.7) (  6.9) ( 17.5) ( 18.4)
a_E      1002.0   202.9   780.6  1002.0   202.9   780.6  1002.0   202.9   780.6  1002.0
         ( 24.0) (  4.9) ( 18.7) ( 24.0) (  4.9) ( 18.7) ( 24.0) (  4.9) ( 18.7) ( 24.0)
         -350.9   351.0   -44.5  -341.5   324.0  -141.8  -320.8   199.0   -92.2  -410.0
         -8.400   8.402  -1.066  -8.174   7.754  -3.395  -7.678   4.764  -2.208  -9.814
t_O        1215     435     499    1214     384     538    1280     444     538    1267
         ( 29.1) ( 10.4) ( 11.9) ( 29.1) (  9.2) ( 12.9) ( 30.6) ( 10.6) ( 12.9) ( 30.3)
t_E      1191.5   265.8   499.0  1191.5   265.8   499.0  1191.5   265.8   499.0  1191.5
         ( 28.5) (  6.4) ( 11.9) ( 28.5) (  6.4) ( 11.9) ( 28.5) (  6.4) ( 11.9) ( 28.5)
           47.5   428.5    -0.0    45.5   282.5    80.9   183.5   455.6    80.9   155.8
          1.138  10.257  -0.001   1.089   6.762   1.937   4.392  10.905   1.937   3.728
g_O         860    1486    1618     915    1514    1664     838    1537    1708     879
         ( 20.6) ( 35.6) ( 38.7) ( 21.9) ( 36.2) ( 39.8) ( 20.1) ( 36.8) ( 40.9) ( 21.0)
g_E       855.9  1703.2  1685.2   855.9  1703.2  1685.2   855.9  1703.2  1685.2   855.9
         ( 20.5) ( 40.8) ( 40.3) ( 20.5) ( 40.8) ( 40.3) ( 20.5) ( 40.8) ( 40.3) ( 20.5)
            8.2  -405.5  -131.7   122.2  -356.6   -42.1   -35.4  -315.7    45.9    46.8
          0.196  -9.705  -3.152   2.924  -8.535  -1.009  -0.848  -7.556   1.099   1.120
c_O        1297    1917    1303    1237    1949    1270    1235    1910    1199    1265
         ( 31.0) ( 45.9) ( 31.2) ( 29.6) ( 46.6) ( 30.4) ( 29.6) ( 45.7) ( 28.7) ( 30.3)
c_E      1128.6  2006.1  1213.2  1128.6  2006.1  1213.2  1128.6  2006.1  1213.2  1128.6
         ( 27.0) ( 48.0) ( 29.0) ( 27.0) ( 48.0) ( 29.0) ( 27.0) ( 48.0) ( 29.0) ( 27.0)
          360.8  -174.1   186.1   226.9  -112.5   116.2   222.5  -187.4   -28.2   288.7
          8.635  -4.167   4.455   5.431  -2.692   2.782   5.327  -4.487  -0.675   6.909

N          4178    4178    4178    4178    4178    4178    4178    4178    4178    4178
X2         64.0   232.0    10.0    51.0   156.1    13.1    48.3   175.1     6.4    77.0
Gvalue     65.6   200.0     9.9    53.1   137.4    13.2    49.8   151.5     6.4    81.2
Gv/N      1.570   4.786   0.236   1.270   3.289   0.317   1.193   3.626   0.153   1.944
Prob    0.00000 0.00000 0.01972 0.00000 0.00000 0.00417 0.00000 0.00000 0.09344 0.00000
        -------------------------------------------------------------------------------
```

	51	52	53	54	55	56	57	58	59	60
a_O	292	696	799	263	724	874	290	735	797	240
	(7.0)	(16.7)	(19.1)	(6.3)	(17.3)	(20.9)	(6.9)	(17.6)	(19.1)	(5.7)
a_E	202.9	780.6	1002.0	202.9	780.6	1002.0	202.9	780.6	1002.0	202.9
	(4.9)	(18.7)	(24.0)	(4.9)	(18.7)	(24.0)	(4.9)	(18.7)	(24.0)	(4.9)
	212.6	−159.7	−361.8	136.5	−109.0	−239.0	207.1	−88.5	−364.9	80.6
	5.088	−3.822	−8.660	3.266	−2.609	−5.720	4.958	−2.118	−8.734	1.929
t_O	400	543	1281	371	502	1200	386	529	1196	363
	(9.6)	(13.0)	(30.7)	(8.9)	(12.0)	(28.7)	(9.2)	(12.7)	(28.6)	(8.7)
t_E	265.8	499.0	1191.5	265.8	499.0	1191.5	265.8	499.0	1191.5	265.8
	(6.4)	(11.9)	(28.5)	(6.4)	(11.9)	(28.5)	(6.4)	(11.9)	(28.5)	(6.4)
	327.0	91.7	185.6	247.4	6.0	17.1	288.0	61.7	9.1	226.2
	7.826	2.196	4.443	5.922	0.143	0.410	6.894	1.478	0.218	5.415
g_O	1541	1727	812	1547	1743	880	1587	1687	922	1600
	(36.9)	(41.3)	(19.4)	(37.0)	(41.7)	(21.1)	(38.0)	(40.4)	(22.1)	(38.3)
g_E	1703.2	1685.2	855.9	1703.2	1685.2	855.9	1703.2	1685.2	855.9	1703.2
	(40.8)	(40.3)	(20.5)	(40.8)	(40.3)	(20.5)	(40.8)	(40.3)	(20.5)	(40.8)
	−308.5	84.6	−85.5	−297.7	117.5	48.9	−224.3	3.6	137.2	−200.1
	−7.384	2.025	−2.047	−7.125	2.814	1.169	−5.370	0.086	3.283	−4.789
c_O	1945	1212	1286	1997	1209	1224	1915	1227	1263	1975
	(46.6)	(29.0)	(30.8)	(47.8)	(28.9)	(29.3)	(45.8)	(29.4)	(30.2)	(47.3)
c_E	2006.1	1213.2	1128.6	2006.1	1213.2	1128.6	2006.1	1213.2	1128.6	2006.1
	(48.0)	(29.0)	(27.0)	(48.0)	(29.0)	(27.0)	(48.0)	(29.0)	(27.0)	(48.0)
	−120.2	−2.4	335.8	−18.1	−8.4	198.7	−177.9	27.8	284.2	−61.6
	−2.878	−0.057	8.038	−0.433	−0.200	4.755	−4.259	0.665	6.803	−1.475
N	4178	4178	4178	4178	4178	4178	4178	4178	4178	4178
X2	124.2	14.1	72.1	73.8	6.1	25.2	103.8	4.6	63.1	49.1
Gvalue	110.8	14.3	74.1	68.1	6.2	25.7	92.9	4.6	65.6	45.1
Gv/N	2.652	0.343	1.774	1.630	0.148	0.615	2.223	0.111	1.569	1.080
Prob	0.00000	0.00251	0.00000	0.00000	0.10234	0.00001	0.00000	0.19993	0.00000	0.00000

	61	62	63	64	65	66	67	68	69	70
a_O	687	830	281	687	868	259	714	856	289	757
	(16.4)	(19.9)	(6.7)	(16.4)	(20.8)	(6.2)	(17.1)	(20.5)	(6.9)	(18.1)
a_E	780.6	1002.0	202.9	780.6	1002.0	202.9	780.6	1002.0	202.9	780.6
	(18.7)	(24.0)	(4.9)	(18.7)	(24.0)	(4.9)	(18.7)	(24.0)	(4.9)	(18.7)
	−175.5	−312.7	183.0	−175.5	−249.3	126.4	−127.3	−269.7	204.4	−46.5
	−4.200	−7.484	4.380	−4.200	−5.967	3.026	−3.048	−6.455	4.893	−1.112
t_O	499	1224	340	539	1184	348	532	1181	323	539
	(11.9)	(29.3)	(8.1)	(12.9)	(28.3)	(8.3)	(12.7)	(28.3)	(7.7)	(12.9)
t_E	499.0	1191.5	265.8	499.0	1191.5	265.8	499.0	1191.5	265.8	499.0
	(11.9)	(28.5)	(6.4)	(11.9)	(28.5)	(6.4)	(11.9)	(28.5)	(6.4)	(11.9)
	−0.0	66.0	167.4	83.1	−14.9	187.5	68.1	−20.8	125.9	83.1
	−0.001	1.579	4.007	1.989	−0.356	4.488	1.630	−0.499	3.013	1.989
g_O	1768	841	1675	1710	849	1667	1717	886	1611	1637
	(42.3)	(20.1)	(40.1)	(40.9)	(20.3)	(39.9)	(41.1)	(21.2)	(38.6)	(39.2)
g_E	1685.2	855.9	1703.2	1685.2	855.9	1703.2	1685.2	855.9	1703.2	1685.2
	(40.3)	(20.5)	(40.8)	(40.3)	(20.5)	(40.8)	(40.3)	(20.5)	(40.8)	(40.3)
	169.6	−29.6	−56.0	50.0	−13.8	−71.7	64.2	61.2	−179.4	−95.0
	4.059	−0.707	−1.340	1.196	−0.329	−1.716	1.536	1.465	−4.293	−2.274
c_O	1224	1283	1882	1242	1277	1904	1215	1255	1955	1245
	(29.3)	(30.7)	(45.0)	(29.7)	(30.6)	(45.6)	(29.1)	(30.0)	(46.8)	(29.8)
c_E	1213.2	1128.6	2006.1	1213.2	1128.6	2006.1	1213.2	1128.6	2006.1	1213.2
	(29.0)	(27.0)	(48.0)	(29.0)	(27.0)	(48.0)	(29.0)	(27.0)	(48.0)	(29.0)
	21.7	329.0	−240.3	58.3	315.5	−198.8	3.6	266.5	−100.8	64.5
	0.520	7.876	−5.751	1.396	7.552	−4.759	0.087	6.378	−2.413	1.543
N	4178	4178	4178	4178	4178	4178	4178	4178	4178	4178
X2	15.4	51.8	58.9	15.5	37.5	46.9	8.5	36.6	55.1	6.1
Gvalue	15.8	52.8	54.1	15.9	37.6	43.4	8.6	37.2	50.1	6.1
Gv/N	0.378	1.263	1.296	0.380	0.900	1.040	0.205	0.890	1.200	0.145
Prob	0.00125	0.00000	0.00000	0.00121	0.00000	0.00000	0.03537	0.00000	0.00000	0.10864

	71	72	73	74	75	76	77	78	79	80
a_O	905	254	774	912	280	685	949	233	761	863
	(21.7)	(6.1)	(18.5)	(21.8)	(6.7)	(16.4)	(22.7)	(5.6)	(18.2)	(20.7)
a_E	1002.0	202.9	780.6	1002.0	202.9	780.6	1002.0	202.9	780.6	1002.0
	(24.0)	(4.9)	(18.7)	(24.0)	(4.9)	(18.7)	(24.0)	(4.9)	(18.7)	(24.0)
	−184.4	114.1	−13.1	−171.7	180.4	−179.0	−103.2	64.5	−38.7	−257.8
	−4.413	2.731	−0.314	−4.110	4.317	−4.284	−2.470	1.543	−0.926	−6.171
t_O	1172	353	500	1202	322	515	1164	338	524	1239
	(28.1)	(8.4)	(12.0)	(28.8)	(7.7)	(12.3)	(27.9)	(8.1)	(12.5)	(29.7)
t_E	1191.5	265.8	499.0	1191.5	265.8	499.0	1191.5	265.8	499.0	1191.5
	(28.5)	(6.4)	(11.9)	(28.5)	(6.4)	(11.9)	(28.5)	(6.4)	(11.9)	(28.5)
	−38.6	200.3	2.0	21.2	123.5	32.5	−54.3	162.4	51.2	97.0
	−0.924	4.794	0.047	0.507	2.956	0.777	−1.299	3.888	1.226	2.321
g_O	884	1609	1659	807	1616	1664	843	1622	1642	845
	(21.2)	(38.5)	(39.7)	(19.3)	(38.7)	(39.8)	(20.2)	(38.8)	(39.3)	(20.2)
g_E	855.9	1703.2	1685.2	855.9	1703.2	1685.2	855.9	1703.2	1685.2	855.9
	(20.5)	(40.8)	(40.3)	(20.5)	(40.8)	(40.3)	(20.5)	(40.8)	(40.3)	(20.5)
	57.1	−183.1	−52.0	−95.0	−169.9	−42.1	−25.6	−158.5	−85.3	−21.7
	1.366	−4.384	−1.245	−2.273	−4.067	−1.009	−0.613	−3.794	−2.041	−0.519
c_O	1217	1962	1245	1257	1960	1314	1222	1985	1251	1231
	(29.1)	(47.0)	(29.8)	(30.1)	(46.9)	(31.5)	(29.2)	(47.5)	(29.9)	(29.5)
c_E	1128.6	2006.1	1213.2	1128.6	2006.1	1213.2	1128.6	2006.1	1213.2	1128.6
	(27.0)	(48.0)	(29.0)	(27.0)	(48.0)	(29.0)	(27.0)	(48.0)	(29.0)	(27.0)
	183.6	−87.1	64.5	270.9	−91.1	209.8	194.3	−41.9	76.8	213.8
	4.394	−2.086	1.543	6.484	−2.179	5.021	4.652	−1.003	1.838	5.118
N	4178	4178	4178	4178	4178	4178	4178	4178	4178	4178
X2	17.6	47.7	1.3	25.6	46.7	20.9	11.4	28.2	4.0	30.6
Gvalue	17.7	44.1	1.3	25.4	42.9	21.1	11.2	26.5	4.0	31.3
Gv/N	0.424	1.055	0.031	0.607	1.027	0.506	0.268	0.633	0.096	0.749
Prob	0.00051	0.00000	0.73043	0.00001	0.00000	0.00010	0.01060	0.00001	0.26030	0.00000

	81	82	83	84	85	86	87	88	89	90
a_O	282	708	936	235	732	964	264	750	946	247
	(6.7)	(16.9)	(22.4)	(5.6)	(17.5)	(23.1)	(6.3)	(18.0)	(22.6)	(5.9)
a_E	202.9	780.6	1002.0	202.9	780.6	1002.0	202.9	780.6	1002.0	202.9
	(4.9)	(18.7)	(24.0)	(4.9)	(18.7)	(24.0)	(4.9)	(18.7)	(24.0)	(4.9)
	185.7	−138.2	−127.6	69.0	−94.1	−74.6	139.0	−60.0	−108.9	97.1
	4.444	−3.308	−3.055	1.652	−2.252	−1.786	3.326	−1.436	−2.606	2.325
t_O	316	547	1136	308	486	1180	296	494	1150	315
	(7.6)	(13.1)	(27.2)	(7.4)	(11.6)	(28.2)	(7.1)	(11.8)	(27.5)	(7.5)
t_E	265.8	499.0	1191.5	265.8	499.0	1191.5	265.8	499.0	1191.5	265.8
	(6.4)	(11.9)	(28.5)	(6.4)	(11.9)	(28.5)	(6.4)	(11.9)	(28.5)	(6.4)
	109.3	100.4	−108.3	90.8	−25.7	−22.8	63.7	−10.0	−81.5	107.0

	2.617	2.404	-2.592	2.172	-0.615	-0.546	1.525	-0.239	-1.950	2.561
g_O	1659	1714	864	1653	1670	890	1656	1691	851	1676
	(39.7)	(41.0)	(20.7)	(39.6)	(40.0)	(21.3)	(39.6)	(40.5)	(20.4)	(40.1)
g_E	1703.2	1685.2	855.9	1703.2	1685.2	855.9	1703.2	1685.2	855.9	1703.2
	(40.8)	(40.3)	(20.5)	(40.8)	(40.3)	(20.5)	(40.8)	(40.3)	(20.5)	(40.8)
	-87.3	58.1	16.3	-99.0	-30.3	69.5	-93.1	11.6	-9.8	-54.0
	-2.089	1.390	0.389	-2.369	-0.725	1.664	-2.229	0.278	-0.234	-1.293
c_O	1921	1209	1242	1982	1290	1144	1962	1243	1231	1940
	(46.0)	(28.9)	(29.7)	(47.4)	(30.9)	(27.4)	(47.0)	(29.8)	(29.5)	(46.4)
c_E	2006.1	1213.2	1128.6	2006.1	1213.2	1128.6	2006.1	1213.2	1128.6	2006.1
	(48.0)	(29.0)	(27.0)	(48.0)	(29.0)	(27.0)	(48.0)	(29.0)	(27.0)	(48.0)
	-166.5	-8.4	237.8	-47.8	158.4	31.0	-87.1	60.4	213.8	-129.9
	-3.984	-0.200	5.693	-1.145	3.791	0.743	-2.086	1.445	5.118	-3.110
N	4178	4178	4178	4178	4178	4178	4178	4178	4178	4178
X2	45.1	11.9	18.4	13.5	8.4	3.1	24.1	2.0	13.9	21.3
Gvalue	41.2	11.9	18.2	13.0	8.3	3.1	22.4	2.0	13.7	20.2
Gv/N	0.986	0.286	0.435	0.311	0.199	0.075	0.536	0.048	0.328	0.483
Prob	0.00000	0.00756	0.00040	0.00467	0.03959	0.37311	0.00005	0.56984	0.00333	0.00016

	91	92	93	94	95	96	97	98	99	100
a_O	723	946	199	749	889	198	765	914	206	764
	(17.3)	(22.6)	(4.8)	(17.9)	(21.3)	(4.7)	(18.3)	(21.9)	(4.9)	(18.3)
a_E	780.6	1002.0	202.9	780.6	1002.0	202.9	780.6	1002.0	202.9	780.6
	(18.7)	(24.0)	(4.9)	(18.7)	(24.0)	(4.9)	(18.7)	(24.0)	(4.9)	(18.7)
	-110.8	-108.9	-7.7	-61.9	-212.8	-9.7	-30.9	-168.1	6.2	-32.8
	-2.653	-2.606	-0.185	-1.481	-5.094	-0.232	-0.739	-4.024	0.149	-0.786
t_O	458	1175	317	497	1245	298	475	1256	318	504
	(11.0)	(28.1)	(7.6)	(11.9)	(29.8)	(7.1)	(11.4)	(30.1)	(7.6)	(12.1)
t_E	499.0	1191.5	265.8	499.0	1191.5	265.8	499.0	1191.5	265.8	499.0
	(11.9)	(28.5)	(6.4)	(11.9)	(28.5)	(6.4)	(11.9)	(28.5)	(6.4)	(11.9)
	-78.6	-32.7	111.7	-4.0	109.4	68.1	-46.9	132.5	114.0	10.0
	-1.880	-0.783	2.673	-0.096	2.620	1.631	-1.121	3.172	2.729	0.240
g_O	1721	841	1651	1718	829	1684	1658	798	1659	1742
	(41.2)	(20.1)	(39.5)	(41.1)	(19.8)	(40.3)	(39.7)	(19.1)	(39.7)	(41.7)
g_E	1685.2	855.9	1703.2	1685.2	855.9	1703.2	1685.2	855.9	1703.2	1685.2
	(40.3)	(20.5)	(40.8)	(40.3)	(20.5)	(40.8)	(40.3)	(20.5)	(40.8)	(40.3)
	72.3	-29.6	-102.8	66.2	-53.0	-38.2	-54.0	-111.8	-87.3	115.5
	1.732	-0.707	-2.461	1.585	-1.268	-0.915	-1.292	-2.676	-2.089	2.764
c_O	1276	1216	2011	1214	1215	1998	1280	1210	1995	1168
	(30.5)	(29.1)	(48.1)	(29.1)	(29.1)	(47.8)	(30.6)	(29.0)	(47.8)	(28.0)
c_E	1213.2	1128.6	2006.1	1213.2	1128.6	2006.1	1213.2	1128.6	2006.1	1213.2
	(29.0)	(27.0)	(48.0)	(29.0)	(27.0)	(48.0)	(29.0)	(27.0)	(48.0)	(29.0)
	128.8	181.4	9.9	1.6	179.3	-16.1	137.2	168.6	-22.1	-88.7
	3.083	4.342	0.237	0.039	4.291	-0.385	3.285	4.034	-0.528	-2.122
N	4178	4178	4178	4178	4178	4178	4178	4178	4178	4178
X2	11.6	10.4	11.5	1.9	22.6	4.3	5.6	21.0	11.5	4.0
Gvalue	11.8	10.3	11.0	1.9	22.9	4.1	5.5	21.2	10.9	4.0
Gv/N	0.282	0.246	0.263	0.046	0.549	0.099	0.133	0.506	0.261	0.096
Prob	0.00818	0.01629	0.01179	0.58510	0.00004	0.24873	0.13605	0.00010	0.01226	0.26121

	101	102	103	104	105	106	107	108	109	110
a_O	978	217	713	954	208	817	933	212	731	965
	(23.4)	(5.2)	(17.1)	(22.8)	(5.0)	(19.6)	(22.3)	(5.1)	(17.5)	(23.1)
a_E	1002.0	202.9	780.6	1002.0	202.9	780.6	1002.0	202.9	780.6	1002.0
	(24.0)	(4.9)	(18.7)	(24.0)	(4.9)	(18.7)	(24.0)	(4.9)	(18.7)	(24.0)
	-47.5	29.2	-129.2	-93.7	10.3	74.5	-133.2	18.6	-96.0	-72.7
	-1.137	0.698	-3.091	-2.244	0.247	1.783	-3.188	0.445	-2.297	-1.740
t_O	1211	336	491	1247	305	502	1218	296	465	1238
	(29.0)	(8.0)	(11.8)	(29.8)	(7.3)	(12.0)	(29.2)	(7.1)	(11.1)	(29.6)
t_E	1191.5	265.8	499.0	1191.5	265.8	499.0	1191.5	265.8	499.0	1191.5
	(28.5)	(6.4)	(11.9)	(28.5)	(6.4)	(11.9)	(28.5)	(6.4)	(11.9)	(28.5)
	39.4	157.5	-15.9	113.6	83.9	6.0	53.7	63.7	-65.7	94.9
	0.943	3.769	-0.380	2.720	2.008	0.143	1.284	1.525	-1.571	2.271
g_O	796	1680	1729	828	1728	1683	785	1681	1736	844
	(19.1)	(40.2)	(41.4)	(19.8)	(41.4)	(40.3)	(18.8)	(40.2)	(41.6)	(20.2)
g_E	855.9	1703.2	1685.2	855.9	1703.2	1685.2	855.9	1703.2	1685.2	855.9
	(20.5)	(40.8)	(40.3)	(20.5)	(40.8)	(40.3)	(20.5)	(40.8)	(40.3)	(20.5)
	-115.5	-46.1	88.7	-54.9	49.9	-4.4	-135.8	-44.2	103.1	-23.7
	-2.765	-1.104	2.123	-1.314	1.194	-0.105	-3.250	-1.057	2.468	-0.566
c_O	1193	1945	1245	1149	1937	1176	1242	1989	1246	1131
	(28.6)	(46.6)	(29.8)	(27.5)	(46.4)	(28.1)	(29.7)	(47.6)	(29.8)	(27.1)
c_E	1128.6	2006.1	1213.2	1128.6	2006.1	1213.2	1128.6	2006.1	1213.2	1128.6
	(27.0)	(48.0)	(29.0)	(27.0)	(48.0)	(29.0)	(27.0)	(48.0)	(29.0)	(27.0)
	132.4	-120.2	64.5	41.2	-135.7	-73.2	237.8	-34.0	66.5	4.8
	3.170	-2.878	1.543	0.986	-3.248	-1.753	5.693	-0.813	1.592	0.115
N	4178	4178	4178	4178	4178	4178	4178	4178	4178	4178
X2	8.8	21.7	8.0	6.2	8.6	2.9	22.6	4.3	7.9	3.4
Gvalue	8.8	20.2	8.1	6.2	8.4	2.8	22.5	4.1	8.0	3.4
Gv/N	0.211	0.485	0.194	0.148	0.201	0.068	0.539	0.099	0.191	0.080
Prob	0.03203	0.00015	0.04369	0.10330	0.03834	0.41626	0.00005	0.24616	0.04625	0.34041

	111	112	113	114	115	116	117	118	119	120
a_O	242	761	952	216	734	950	208	724	1035	252
	(5.8)	(18.2)	(22.8)	(5.2)	(17.6)	(22.7)	(5.0)	(17.3)	(24.8)	(6.0)
a_E	202.9	780.6	1002.0	202.9	780.4	1001.8	202.9	780.4	1001.8	202.9
	(4.9)	(18.7)	(24.0)	(4.9)	(18.7)	(24.0)	(4.9)	(18.7)	(24.0)	(4.9)
	85.3	-38.7	-97.5	27.0	-90.0	-100.9	10.4	-108.6	67.5	109.3
	2.041	-0.926	-2.334	0.647	-2.155	-2.415	0.249	-2.601	1.616	2.618
t_O	322	491	1147	292	510	1234	292	485	1166	287
	(7.7)	(11.8)	(27.5)	(7.0)	(12.2)	(29.5)	(7.0)	(11.6)	(27.9)	(6.9)
t_E	265.8	499.0	1191.5	265.8	498.9	1191.2	265.7	498.9	1191.2	265.7
	(6.4)	(11.9)	(28.5)	(6.4)	(11.9)	(28.5)	(6.4)	(11.9)	(28.5)	(6.4)
	123.5	-15.9	-87.2	54.9	22.5	87.2	55.0	-27.4	-49.8	44.2
	2.956	-0.380	-2.088	1.314	0.538	2.087	1.317	-0.656	-1.193	1.058
g_O	1643	1726	847	1702	1759	835	1669	1692	847	1682
	(39.3)	(41.3)	(20.3)	(40.7)	(42.1)	(20.0)	(40.0)	(40.5)	(20.3)	(40.3)
g_E	1703.2	1685.2	855.9	1703.2	1684.8	855.7	1702.8	1684.8	855.7	1702.8
	(40.8)	(40.3)	(20.5)	(40.8)	(40.3)	(20.5)	(40.8)	(40.3)	(20.5)	(40.8)
	-118.3	82.6	-17.7	-2.5	151.6	-40.9	-67.0	14.4	-17.3	-41.4
	-2.832	1.976	-0.424	-0.059	3.630	-0.979	-1.603	0.345	-0.415	-0.991
c_O	1971	1200	1232	1968	1174	1158	2008	1276	1129	1956
	(47.2)	(28.7)	(29.5)	(47.1)	(28.1)	(27.7)	(48.1)	(30.5)	(27.0)	(46.8)
c_E	2006.1	1213.2	1128.6	2006.1	1212.9	1128.3	2005.6	1212.9	1128.3	2005.6
	(48.0)	(29.0)	(27.0)	(48.0)	(29.0)	(27.0)	(48.0)	(29.0)	(27.0)	(48.0)
	-69.5	-26.2	216.0	-75.4	-76.5	60.1	4.8	129.4	1.4	-97.9
	-1.664	-0.628	5.170	-1.805	-1.832	1.440	0.116	3.099	0.032	-2.344

```
N          4178    4178    4178    4178    4177    4177    4177    4177    4177    4177
X2         22.2     1.8    13.7     4.1     7.5     5.5     3.4     7.8     1.7    15.1
Gvalue     21.0     1.7    13.5     4.1     7.5     5.5     3.3     7.8     1.7    14.2
Gv/N      0.502   0.042   0.323   0.097   0.181   0.132   0.080   0.187   0.041   0.340
Prob    0.00011 0.62610 0.00365 0.25550 0.05645 0.13709 0.34465 0.04970 0.63391 0.00265
        -------------------------------------------------------------------------------
            121     122     123     124     125     126     127     128     129     130
        -------------------------------------------------------------------------------
a_O         669     980     199     740     980     211     704    1019     244     764
        ( 16.0) ( 23.5) (  4.8) ( 17.7) ( 23.5) (  5.1) ( 16.9) ( 24.4) (  5.8) ( 18.3)
a_E       780.4  1001.8   202.9   779.8  1001.1   202.7   779.5  1000.6   202.6   779.3
        ( 18.7) ( 24.0) (  4.9) ( 18.7) ( 24.0) (  4.9) ( 18.7) ( 24.0) (  4.9) ( 18.7)
         -206.1   -43.1    -7.6   -77.6   -41.7    16.9  -143.4    37.1    90.7   -30.3
         -4.934  -1.032  -0.183  -1.860  -0.999   0.405  -3.437   0.890   2.174  -0.726
t_O         497    1107     297     507    1174     287     494    1185     264     523
        ( 11.9) ( 26.5) (  7.1) ( 12.1) ( 28.1) (  6.9) ( 11.8) ( 28.4) (  6.3) ( 12.5)
t_E       498.9  1191.2   265.7   498.5  1190.3   265.6   498.3  1189.7   265.4   498.2
        ( 11.9) ( 28.5) (  6.4) ( 11.9) ( 28.5) (  6.4) ( 11.9) ( 28.5) (  6.4) ( 11.9)
           -3.8  -162.3    66.1    17.1   -32.4    44.6    -8.6    -9.5    -2.8    50.9
         -0.090  -3.885   1.581   0.409  -0.777   1.068  -0.205  -0.227  -0.068   1.219
g_O        1770     913    1681    1736     845    1719    1750     872    1648    1706
        ( 42.4) ( 21.9) ( 40.2) ( 41.6) ( 20.2) ( 41.2) ( 41.9) ( 20.9) ( 39.5) ( 40.9)
g_E      1684.8   855.7  1702.8  1683.6   855.1  1701.6  1682.8   854.7  1700.8  1682.4
        ( 40.3) ( 20.5) ( 40.8) ( 40.3) ( 20.5) ( 40.8) ( 40.3) ( 20.5) ( 40.8) ( 40.3)
          174.6   118.3   -43.4   106.4   -20.1    35.0   137.1    35.0  -103.9    47.6
          4.181   2.833  -1.038   2.550  -0.481   0.838   3.286   0.839  -2.491   1.140
c_O        1241    1177    2000    1191    1175    1957    1224    1096    2016    1178
        ( 29.7) ( 28.2) ( 47.9) ( 28.5) ( 28.2) ( 46.9) ( 29.3) ( 26.3) ( 48.3) ( 28.2)
c_E      1212.9  1128.3  2005.6  1212.0  1127.5  2004.1  1211.4  1127.0  2003.2  1211.2
        ( 29.0) ( 27.0) ( 48.0) ( 29.0) ( 27.0) ( 48.0) ( 29.0) ( 27.0) ( 48.0) ( 29.0)
           56.9    99.4   -11.1   -41.7    96.9   -93.2    25.2   -61.1    25.7   -65.4
          1.361   2.380  -0.267  -0.999   2.323  -2.232   0.605  -1.464   0.616  -1.568

N          4177    4177    4177    4174    4174    4174    4172    4172    4172    4171
X2         20.9    12.4     4.0     4.2     2.8     3.4    10.2     1.6    10.2     2.8
Gvalue     21.6    12.4     3.9     4.2     2.8     3.3    10.4     1.6     9.7     2.8
Gv/N      0.517   0.297   0.094   0.101   0.066   0.079   0.249   0.037   0.232   0.066
Prob    0.00008 0.00614 0.27129 0.24103 0.42947 0.34527 0.01565 0.66781 0.02153 0.42933
        -------------------------------------------------------------------------------
            131     132     133     134     135     136     137     138     139     140
        -------------------------------------------------------------------------------
a_O        1008     221     686    1003     226     708     968     202     738     919
        ( 24.2) (  5.3) ( 16.5) ( 24.1) (  5.4) ( 17.0) ( 23.2) (  4.8) ( 17.7) ( 22.1)
a_E      1000.4   202.6   778.7   999.6   202.4   778.4   999.2   202.3   778.4   999.2
        ( 24.0) (  4.9) ( 18.7) ( 24.0) (  4.9) ( 18.7) ( 24.0) (  4.9) ( 18.7) ( 24.0)
           15.3    38.5  -173.9     6.7    49.8  -134.1   -61.3    -0.6   -78.6  -153.7
          0.368   0.923  -4.173   0.162   1.195  -3.220  -1.472  -0.015  -1.886  -3.690
t_O        1110     268     552    1164     254     511    1138     292     527    1159
        ( 26.6) (  6.4) ( 13.2) ( 27.9) (  6.1) ( 12.3) ( 27.3) (  7.0) ( 12.7) ( 27.8)
t_E      1189.5   265.4   497.8  1188.6   265.2   497.6  1188.0   265.0   497.6  1188.0
        ( 28.5) (  6.4) ( 11.9) ( 28.5) (  6.4) ( 11.9) ( 28.5) (  6.4) ( 11.9) ( 28.5)
         -153.5     5.3   114.1   -48.7   -21.9    27.2   -97.9    56.6    60.5   -57.4
         -3.680   0.127   2.737  -1.169  -0.525   0.653  -2.351   1.358   1.453  -1.377
g_O         931    1694    1773     865    1688    1719     864    1657    1697     867
        ( 22.3) ( 40.6) ( 42.5) ( 20.8) ( 40.5) ( 41.3) ( 20.7) ( 39.8) ( 40.7) ( 20.8)
g_E       854.5  1700.4  1681.2   853.9  1699.2  1680.4   853.5  1698.3  1680.4   853.5
        ( 20.5) ( 40.8) ( 40.3) ( 20.5) ( 40.8) ( 40.3) ( 20.5) ( 40.8) ( 40.3) ( 20.5)
          159.7   -12.7   188.6    22.4   -22.2    78.2    21.2   -81.7    33.4    27.3
          3.829  -0.305   4.525   0.538  -0.533   1.876   0.510  -1.960   0.803   0.656
c_O        1122    1988    1157    1136    2000    1228    1196    2015    1204    1221
        ( 26.9) ( 47.7) ( 27.8) ( 27.3) ( 48.0) ( 29.5) ( 28.7) ( 48.4) ( 28.9) ( 29.3)
c_E      1126.7  2002.7  1210.3  1125.9  2001.3  1209.7  1125.4  2000.3  1209.7  1125.4
        ( 27.0) ( 48.0) ( 29.0) ( 27.0) ( 48.0) ( 29.0) ( 27.0) ( 48.0) ( 29.0) ( 27.0)
           -9.4   -29.3  -104.2    20.3    -2.5    36.9   145.6    29.5   -11.4   199.2
         -0.225  -0.702  -2.500   0.487  -0.060   0.885   3.496   0.708  -0.273   4.782

N          4171    4171    4168    4168    4168    4166    4166    4166    4166    4166
X2         12.2     1.8    24.3     0.8     3.3     7.9     7.6     3.9     4.0    15.5
Gvalue     12.2     1.8    24.5     0.8     3.2     8.1     7.6     3.8     4.0    15.4
Gv/N      0.292   0.043   0.588   0.018   0.077   0.194   0.182   0.091   0.097   0.371
Prob    0.00683 0.61765 0.00002 0.85912 0.36211 0.04447 0.05517 0.28669 0.25860 0.00147
        -------------------------------------------------------------------------------
            141     142     143     144     145     146     147     148     149     150
        -------------------------------------------------------------------------------
a_O         202     726     955     173     728     943     188     711    1005     220
        (  4.8) ( 17.4) ( 22.9) (  4.2) ( 17.5) ( 22.6) (  4.5) ( 17.1) ( 24.1) (  5.3)
a_E       202.3   778.4   999.2   202.3   778.2   998.9   202.3   778.0   998.7   202.2
        (  4.9) ( 18.7) ( 24.0) (  4.9) ( 18.7) ( 24.0) (  4.9) ( 18.7) ( 24.0) (  4.9)
           -0.6  -101.1   -86.3   -54.2   -97.0  -108.6   -27.5  -128.0    12.7    37.1
         -0.015  -2.427  -2.072  -1.300  -2.330  -2.609  -0.661  -3.074   0.305   0.890
t_O         271     549    1173     264     513    1175     261     526    1117     272
        (  6.5) ( 13.2) ( 28.2) (  6.3) ( 12.3) ( 28.2) (  6.3) ( 12.6) ( 26.8) (  6.5)
t_E       265.0   497.6  1188.0   265.0   497.5  1187.8   265.0   497.3  1187.5   264.9
        (  6.4) ( 11.9) ( 28.5) (  6.4) ( 11.9) ( 28.5) (  6.4) ( 11.9) ( 28.5) (  6.4)
           12.0   108.0   -29.9    -2.1    31.6   -25.4    -7.9    58.9  -136.7    14.4
          0.289   2.592  -0.717  -0.050   0.758  -0.609  -0.190   1.415  -3.282   0.345
g_O        1679    1639     928    1691    1739     891    1681    1637     890    1656
        ( 40.3) ( 39.3) ( 22.3) ( 40.6) ( 41.8) ( 21.4) ( 40.4) ( 39.3) ( 21.4) ( 39.8)
g_E      1698.3  1680.4   853.5  1698.3  1680.0   853.2  1697.9  1679.6   853.0  1697.5
        ( 40.8) ( 40.3) ( 20.5) ( 40.8) ( 40.3) ( 20.5) ( 40.8) ( 40.3) ( 20.5) ( 40.8)
          -38.5   -81.7   155.4   -14.6   120.1    77.2   -33.7   -84.0    75.5   -82.0
         -0.923  -1.961   3.731  -0.351   2.884   1.852  -0.809  -2.018   1.813  -1.970
c_O        2014    1252    1110    2038    1185    1156    2035    1290    1152    2016
        ( 48.3) ( 30.1) ( 26.6) ( 48.9) ( 28.5) ( 27.8) ( 48.9) ( 31.0) ( 27.7) ( 48.4)
c_E      2000.3  1209.7  1125.4  2000.3  1209.4  1125.1  1999.8  1209.1  1124.8  1999.3
        ( 48.0) ( 29.0) ( 27.0) ( 48.0) ( 29.0) ( 27.0) ( 48.0) ( 29.0) ( 27.0) ( 48.0)
           27.5    86.1   -30.5    76.1   -48.3    62.7    71.0   167.1    55.0    33.5
          0.660   2.066  -0.732   1.827  -1.160   1.505   1.704   4.012   1.322   0.804

N          4166    4166    4166    4166    4165    4165    4165    4164    4164    4164
X2          0.4    11.3     8.9     5.0     6.3     5.8     1.9    13.9     6.5     2.9
Gvalue      0.4    11.2     8.7     5.2     6.3     5.8     1.9    13.9     6.5     2.9
Gv/N      0.011   0.270   0.209   0.125   0.152   0.140   0.045   0.335   0.157   0.069
Prob    0.93015 0.01054 0.03337 0.15686 0.09646 0.12090 0.59848 0.00299 0.08819 0.41227
        -------------------------------------------------------------------------------
```

Bper_TOHAMA1
5UTR:

	50	49	48	47	46	45	44	43	42	41
a_O	576	833	582	786	792	739	596	624	524	579
	(16.8)	(24.2)	(16.9)	(22.9)	(23.1)	(21.5)	(17.3)	(18.2)	(15.3)	(16.9)
a_E	666.4	666.4	666.4	666.4	666.4	666.4	666.4	666.4	666.4	666.4
	(19.4)	(19.4)	(19.4)	(19.4)	(19.4)	(19.4)	(19.4)	(19.4)	(19.4)	(19.4)
	-167.9	371.8	-157.6	259.5	273.6	152.9	-133.1	-82.0	-251.9	-162.8
	-4.887	10.821	-4.587	7.553	7.962	4.449	-3.872	-2.387	-7.331	-4.737
t_O	565	619	517	539	625	584	593	823	571	537
	(16.4)	(18.0)	(15.0)	(15.7)	(18.2)	(17.0)	(17.3)	(24.0)	(16.6)	(15.6)
t_E	628.8	628.8	628.8	628.8	628.8	628.8	628.8	628.8	628.8	628.8
	(18.3)	(18.3)	(18.3)	(18.3)	(18.3)	(18.3)	(18.3)	(18.3)	(18.3)	(18.3)
	-120.9	-19.4	-202.4	-166.1	-7.6	-86.3	-69.5	443.0	-110.1	-169.5
	-3.519	-0.566	-5.891	-4.835	-0.221	-2.513	-2.023	12.893	-3.205	-4.933
g_O	1262	1016	1223	987	1007	1046	1023	975	1203	1276
	(36.7)	(29.6)	(35.6)	(28.7)	(29.3)	(30.4)	(29.8)	(28.4)	(35.0)	(37.1)
g_E	1035.5	1035.5	1035.5	1035.5	1035.5	1035.5	1035.5	1035.5	1035.5	1035.5
	(30.1)	(30.1)	(30.1)	(30.1)	(30.1)	(30.1)	(30.1)	(30.1)	(30.1)	(30.1)
	499.2	-38.7	407.0	-94.8	-56.3	21.0	-24.9	-117.5	360.6	532.9
	14.528	-1.127	11.844	-2.758	-1.638	0.612	-0.725	-3.419	10.496	15.509
c_O	1033	968	1114	1124	1012	1067	1224	1014	1138	1044
	(30.1)	(28.2)	(32.4)	(32.7)	(29.5)	(31.1)	(35.6)	(29.5)	(33.1)	(30.4)
c_E	1105.3	1105.3	1105.3	1105.3	1105.3	1105.3	1105.3	1105.3	1105.3	1105.3
	(32.2)	(32.2)	(32.2)	(32.2)	(32.2)	(32.2)	(32.2)	(32.2)	(32.2)	(32.2)
	-139.7	-256.8	17.5	37.8	-178.5	-75.2	249.8	-174.8	66.4	-119.1
	-4.067	-7.473	0.510	1.099	-5.194	-2.189	7.269	-5.087	1.932	-3.466
N	3436	3436	3436	3436	3436	3436	3436	3436	3436	3436
X2	73.0	59.2	64.6	36.9	32.4	12.5	22.4	73.8	63.8	84.1
Gvalue	70.7	56.9	64.5	36.4	31.2	12.4	22.3	68.7	65.0	81.5
Gv/N	2.056	1.656	1.876	1.059	0.909	0.360	0.648	2.000	1.892	2.372
Prob	0.00000	0.00000	0.00000	0.00000	0.00000	0.00625	0.00006	0.00000	0.00000	0.00000

	40	39	38	37	36	35	34	33	32	31
a_O	830	774	815	588	508	572	612	552	756	734
	(24.2)	(22.5)	(23.7)	(17.1)	(14.8)	(16.6)	(17.8)	(16.1)	(22.0)	(21.4)
a_E	666.4	666.4	666.4	666.4	666.4	666.4	666.4	666.4	666.4	666.4
	(19.4)	(19.4)	(19.4)	(19.4)	(19.4)	(19.4)	(19.4)	(19.4)	(19.4)	(19.4)
	364.5	231.8	328.2	-147.2	-275.7	-174.7	-104.2	-207.9	190.8	141.9
	10.608	6.745	9.551	-4.283	-8.024	-5.085	-3.032	-6.051	5.553	4.129
t_O	620	598	543	867	590	554	640	568	532	606
	(18.0)	(17.4)	(15.8)	(25.2)	(17.2)	(16.1)	(18.6)	(16.5)	(15.5)	(17.6)
t_E	628.8	628.8	628.8	628.8	628.8	628.8	628.8	628.8	628.8	628.8
	(18.3)	(18.3)	(18.3)	(18.3)	(18.3)	(18.3)	(18.3)	(18.3)	(18.3)	(18.3)
	-17.5	-60.1	-159.3	557.0	-75.2	-140.3	22.6	-115.5	-177.9	-44.8
	-0.509	-1.748	-4.637	16.211	-2.187	-4.084	0.658	-3.362	-5.177	-1.303
g_O	960	941	1012	943	1002	1213	910	1019	1012	938
	(27.9)	(27.4)	(29.5)	(27.4)	(29.2)	(35.3)	(26.5)	(29.7)	(29.5)	(27.3)
g_E	1035.5	1035.5	1035.5	1035.5	1035.5	1035.5	1035.5	1035.5	1035.5	1035.5
	(30.1)	(30.1)	(30.1)	(30.1)	(30.1)	(30.1)	(30.1)	(30.1)	(30.1)	(30.1)
	-145.4	-180.2	-46.5	-176.6	-66.0	383.7	-235.2	-32.8	-46.5	-185.6
	-4.233	-5.244	-1.355	-5.138	-1.920	11.168	-6.845	-0.955	-1.355	-5.401
c_O	1026	1123	1066	1038	1336	1097	1274	1297	1136	1158
	(29.9)	(32.7)	(31.0)	(30.2)	(38.9)	(31.9)	(37.1)	(37.7)	(33.1)	(33.7)
c_E	1105.3	1105.3	1105.3	1105.3	1105.3	1105.3	1105.3	1105.3	1105.3	1105.3
	(32.2)	(32.2)	(32.2)	(32.2)	(32.2)	(32.2)	(32.2)	(32.2)	(32.2)	(32.2)
	-152.7	35.7	-77.1	-130.4	506.6	-16.5	362.0	414.9	62.3	107.9
	-4.445	1.040	-2.245	-3.794	14.743	-0.480	10.535	12.076	1.813	3.141
N	3436	3436	3436	3436	3436	3436	3436	3436	3436	3436
X2	51.5	27.8	46.8	111.8	89.3	52.7	45.6	59.0	28.3	19.4
Gvalue	48.8	27.2	45.2	102.9	89.7	52.2	45.2	58.7	28.7	19.4
Gv/N	1.421	0.793	1.314	2.995	2.611	1.519	1.315	1.708	0.834	0.566
Prob	0.00000	0.00001	0.00000	0.00000	0.00000	0.00000	0.00000	0.00000	0.00000	0.00022

	30	29	28	27	26	25	24	23	22	21
a_O	532	538	584	518	568	565	764	576	647	601
	(15.5)	(15.7)	(17.0)	(15.1)	(16.5)	(16.4)	(22.2)	(16.8)	(18.8)	(17.5)
a_E	666.4	666.4	666.4	666.4	666.4	666.4	666.4	666.4	666.4	666.4
	(19.4)	(19.4)	(19.4)	(19.4)	(19.4)	(19.4)	(19.4)	(19.4)	(19.4)	(19.4)
	-239.6	-230.3	-154.1	-261.0	-181.5	-186.5	208.9	-167.9	-38.2	-124.1
	-6.974	-6.702	-4.486	-7.595	-5.281	-5.427	6.079	-4.887	-1.111	-3.612
t_O	596	607	719	605	575	693	578	615	885	830
	(17.3)	(17.7)	(20.9)	(17.6)	(16.7)	(20.2)	(16.8)	(17.9)	(25.8)	(24.2)
t_E	628.8	628.8	628.8	628.8	628.8	628.8	628.8	628.8	628.8	628.8
	(18.3)	(18.3)	(18.3)	(18.3)	(18.3)	(18.3)	(18.3)	(18.3)	(18.3)	(18.3)
	-63.9	-42.8	192.8	-46.7	-102.9	134.7	-97.4	-27.3	604.9	460.8
	-1.859	-1.247	5.610	-1.359	-2.994	3.921	-2.834	-0.794	17.606	13.412
g_O	972	971	868	947	892	866	975	1088	787	813
	(28.3)	(28.3)	(25.3)	(27.6)	(26.0)	(25.2)	(28.4)	(31.7)	(22.9)	(23.7)
g_E	1035.5	1035.5	1035.5	1035.5	1035.5	1035.5	1035.5	1035.5	1035.5	1035.5
	(30.1)	(30.1)	(30.1)	(30.1)	(30.1)	(30.1)	(30.1)	(30.1)	(30.1)	(30.1)
	-123.1	-125.0	-306.4	-169.3	-266.2	-309.7	-117.5	107.5	-432.0	-393.4
	-3.583	-3.637	-8.917	-4.927	-7.747	-9.013	-3.419	3.130	-12.572	-11.450
c_O	1336	1320	1265	1366	1401	1312	1119	1157	1117	1192
	(38.9)	(38.4)	(36.8)	(39.8)	(40.8)	(38.2)	(32.6)	(33.7)	(32.5)	(34.7)
c_E	1105.3	1105.3	1105.3	1105.3	1105.3	1105.3	1105.3	1105.3	1105.3	1105.3
	(32.2)	(32.2)	(32.2)	(32.2)	(32.2)	(32.2)	(32.2)	(32.2)	(32.2)	(32.2)
	506.6	468.7	341.5	578.6	664.3	449.9	27.6	105.8	23.6	180.1
	14.743	13.640	9.938	16.839	19.334	13.094	0.804	3.080	0.686	5.241
N	3436	3436	3436	3436	3436	3436	3436	3436	3436	3436
X2	80.9	71.2	73.3	103.0	118.1	88.4	22.1	17.6	164.7	125.4
Gvalue	80.0	70.6	73.7	101.7	113.8	88.5	21.6	18.2	158.3	123.4
Gv/N	2.328	2.055	2.146	2.959	3.312	2.575	0.630	0.528	4.608	3.591
Prob	0.00000	0.00000	0.00000	0.00000	0.00000	0.00000	0.00008	0.00041	0.00000	0.00000

	20	19	18	17	16	15	14	13	12	11
a_O	645	881	657	695	709	863	996	826	845	1077
	(18.8)	(25.6)	(19.1)	(20.2)	(20.6)	(25.1)	(29.0)	(24.0)	(24.6)	(31.3)
a_E	666.4	666.4	666.4	666.4	666.4	666.4	666.4	666.4	666.4	666.4
	(19.4)	(19.4)	(19.4)	(19.4)	(19.4)	(19.4)	(19.4)	(19.4)	(19.4)	(19.4)
	-42.1	491.9	-18.6	58.5	87.9	446.3	800.6	354.7	401.3	1034.1
	-1.224	14.317	-0.542	1.701	2.558	12.988	23.299	10.324	11.680	30.095
t_O	665	708	614	884	716	662	555	620	469	353
	(19.4)	(20.6)	(17.9)	(25.7)	(20.8)	(19.3)	(16.2)	(18.0)	(13.6)	(10.3)

```
t_E       628.8   628.8   628.8   628.8   628.8   628.8   628.8   628.8   628.8   628.8
        ( 18.3) ( 18.3) ( 18.3) ( 18.3) ( 18.3) ( 18.3) ( 18.3) ( 18.3) ( 18.3) ( 18.3)
           74.4   168.0   -29.2   602.3   186.0    68.1  -138.6   -17.5  -275.0  -407.6
          2.167   4.889  -0.851  17.528   5.412   1.983  -4.033  -0.509  -8.004 -11.863
g_O         792     725     779     723     686     839     846    1064    1293    1252
        ( 23.1) ( 21.1) ( 22.7) ( 21.0) ( 20.0) ( 24.4) ( 24.6) ( 31.0) ( 37.6) ( 36.4)
g_E      1035.5  1035.5  1035.5  1035.5  1035.5  1035.5  1035.5  1035.5  1035.5  1035.5
        ( 30.1) ( 30.1) ( 30.1) ( 30.1) ( 30.1) ( 30.1) ( 30.1) ( 30.1) ( 30.1) ( 30.1)
         -424.7  -516.9  -443.5  -519.5  -565.0  -353.2  -342.1    57.7   574.2   475.3
        -12.360 -15.045 -12.908 -15.119 -16.443 -10.278  -9.955   1.679  16.711  13.833
c_O        1334    1122    1386    1134    1325    1072    1039     926     829     754
        ( 38.8) ( 32.7) ( 40.3) ( 33.0) ( 38.6) ( 31.2) ( 30.2) ( 26.9) ( 24.1) ( 21.9)
c_E      1105.3  1105.3  1105.3  1105.3  1105.3  1105.3  1105.3  1105.3  1105.3  1105.3
        ( 32.2) ( 32.2) ( 32.2) ( 32.2) ( 32.2) ( 32.2) ( 32.2) ( 32.2) ( 32.2) ( 32.2)
          501.8    33.7   627.4    58.2   480.5   -65.5  -128.5  -327.8  -476.9  -576.8
         14.604   0.981  18.259   1.693  13.984  -1.908  -3.740  -9.539 -13.879 -16.786

N          3436    3436    3436    3436    3436    3436    3436    3436    3436    3436
X2        107.4   172.5   135.3   199.9   176.5    98.1   210.4    68.2   221.6   530.9
Gvalue    109.5   176.7   136.0   199.4   189.4    95.7   191.4    67.2   223.6   525.0
Gv/N      3.186   5.142   3.958   5.803   5.511   2.785   5.571   1.956   6.508  15.280
Prob    0.00000 0.00000 0.00000 0.00000 0.00000 0.00000 0.00000 0.00000 0.00000 0.00000
        -------------------------------------------------------------------------------
             10       9       8       7       6       5       4       3       2       1
        -------------------------------------------------------------------------------
a_O         865     833     912     876     763     722     746     816     558     434
        ( 25.2) ( 24.2) ( 26.5) ( 25.5) ( 22.2) ( 21.0) ( 21.7) ( 23.7) ( 16.2) ( 12.6)
a_E       666.4   666.4   666.4   666.4   666.4   666.4   666.4   666.4   666.4   666.4
        ( 19.4) ( 19.4) ( 19.4) ( 19.4) ( 19.4) ( 19.4) ( 19.4) ( 19.4) ( 19.4) ( 19.4)
          451.3   371.8   572.3   479.2   206.6   115.8   168.4   330.6  -198.1  -372.2
         13.135  10.821  16.657  13.946   6.013   3.369   4.901   9.621  -5.765 -10.833
t_O         383     346     422     554     551     599     565     460     663     376
        ( 11.1) ( 10.1) ( 12.3) ( 16.1) ( 16.0) ( 17.4) ( 16.4) ( 13.4) ( 19.3) ( 10.9)
t_E       628.8   628.8   628.8   628.8   628.8   628.8   628.8   628.8   628.8   628.8
        ( 18.3) ( 18.3) ( 18.3) ( 18.3) ( 18.3) ( 18.3) ( 18.3) ( 18.3) ( 18.3) ( 18.3)
         -379.8  -413.4  -336.6  -140.3  -145.6   -58.2  -120.9  -287.6    70.2  -386.7
        -11.053 -12.031  -9.796  -4.084  -4.236  -1.693  -3.519  -8.370   2.044 -11.254
g_O        1554    1467    1225    1037    1157     948     710    1104     788     639
        ( 45.2) ( 42.7) ( 35.7) ( 30.2) ( 33.7) ( 27.6) ( 20.7) ( 32.1) ( 22.9) ( 18.6)
g_E      1035.5  1035.5  1035.5  1035.5  1035.5  1035.5  1035.5  1035.5  1035.5  1035.5
        ( 30.1) ( 30.1) ( 30.1) ( 30.1) ( 30.1) ( 30.1) ( 30.1) ( 30.1) ( 30.1) ( 30.1)
         1261.6  1021.9   411.6     2.9   256.6  -167.5  -535.9   141.3  -430.5  -617.0
         36.716  29.741  11.980   0.085   7.469  -4.874 -15.597   4.114 -12.530 -17.956
c_O         634     790     877     969     965    1167    1415    1056    1427    1987
        ( 18.5) ( 23.0) ( 25.5) ( 28.2) ( 28.1) ( 34.0) ( 41.2) ( 30.7) ( 41.5) ( 57.8)
c_E      1105.3  1105.3  1105.3  1105.3  1105.3  1105.3  1105.3  1105.3  1105.3  1105.3
        ( 32.2) ( 32.2) ( 32.2) ( 32.2) ( 32.2) ( 32.2) ( 32.2) ( 32.2) ( 32.2) ( 32.2)
         -704.8  -530.6  -405.8  -255.0  -262.0   126.8   699.1   -96.3   729.1  2330.9
        -20.511 -15.442 -11.810  -7.422  -7.624   3.691  20.346  -2.804  21.220  67.836

N          3436    3436    3436    3436    3436    3436    3436    3436    3436    3436
X2        615.8   438.6   240.4    91.6    55.7    16.9   205.1    85.6   172.3  1037.9
Gvalue    628.3   449.7   241.6    86.8    55.8    17.0   210.7    88.0   170.7   955.0
Gv/N     18.287  13.089   7.031   2.525   1.623   0.493   6.131   2.561   4.969  27.793
Prob    0.00000 0.00000 0.00000 0.00000 0.00000 0.00072 0.00000 0.00000 0.00000 0.00000
        -------------------------------------------------------------------------------

CDS_INITIAL:
        -------------------------------------------------------------------------------
              1       2       3       4       5       6       7       8       9      10
        -------------------------------------------------------------------------------
a_O        2906       0       2    1623    1192     560    1193     970     570     917
        ( 84.6) (  0.0) (  0.1) ( 47.2) ( 34.7) ( 16.3) ( 34.7) ( 28.2) ( 16.6) ( 26.7)
a_E       645.5   839.8   173.9   645.5   839.8   173.9   645.5   839.8   173.9   645.5
        ( 18.8) ( 24.4) (  5.1) ( 18.8) ( 24.4) (  5.1) ( 18.8) ( 24.4) (  5.1) ( 18.8)
         8744.5     0.0   -17.9  2993.0   835.0  1310.1  1465.6   279.7  1353.6   644.0
        254.497   0.000  -0.520  87.107  24.302  38.127  42.655   8.140  39.395  18.742
t_O         180    3436       0     404     447     678     413     742     583     516
        (  5.2) (100.0) (  0.0) ( 11.8) ( 13.0) ( 19.7) ( 12.0) ( 21.6) ( 17.0) ( 15.0)
t_E       411.8   975.7   226.5   411.8   975.7   226.5   411.8   975.7   226.5   411.8
        ( 12.0) ( 28.4) (  6.6) ( 12.0) ( 28.4) (  6.6) ( 12.0) ( 28.4) (  6.6) ( 12.0)
         -297.9  8650.9     0.0   -15.4  -697.9  1486.7     2.4  -406.4  1102.4   232.8
         -8.670 251.772   0.000  -0.449 -20.311  43.269   0.071 -11.828  32.084   6.776
g_O         350       0    3434     610     609     730     941     534     834     787
        ( 10.2) (  0.0) ( 99.9) ( 17.8) ( 17.7) ( 21.2) ( 27.4) ( 15.5) ( 24.3) ( 22.9)
g_E      1375.5   697.7  1375.5  1375.5   697.7  1375.5  1375.5   697.7  1375.5  1375.5
        ( 40.0) ( 20.3) ( 40.0) ( 40.0) ( 20.3) ( 40.0) ( 40.0) ( 20.3) ( 40.0) ( 40.0)
         -958.0     0.0  6283.4  -992.0  -165.5  -925.0  -714.4  -285.5  -834.6  -878.8
        -27.882   0.000 182.869 -28.869  -4.818 -26.921 -20.791  -8.310 -24.290 -25.576
c_O           0       0       0     799    1188    1468     889    1190    1449    1216
        (  0.0) (  0.0) (  0.0) ( 23.3) ( 34.6) ( 42.7) ( 25.9) ( 34.6) ( 42.2) ( 35.4)
c_E      1003.3   922.8  1660.1  1003.3   922.8  1660.1  1003.3   922.8  1660.1  1003.3
        ( 29.2) ( 26.9) ( 48.3) ( 29.2) ( 26.9) ( 48.3) ( 29.2) ( 26.9) ( 48.3) ( 29.2)
            0.0     0.0     0.0  -363.9   600.2  -361.1  -215.1   605.2  -394.1   467.6
          0.000   0.000   0.000 -10.589  17.467 -10.508  -6.259  17.613 -11.471  13.609

N          3436    3436    3436    3436    3436    3436    3436    3436    3436    3436
X2       9815.1  8663.5  5136.9  1948.2   521.7  2082.8   614.7   191.9  1703.8   437.5
Gvalue   7488.6  8650.9  6265.5  1621.8   571.7  1510.7   538.6   192.9  1227.3   465.6
Gv/N    217.945 251.772 182.349  47.200  16.640  43.968  15.675   5.615  35.718  13.552
Prob    1.00000 1.00000 1.00000 1.00000 0.00000 1.00000 0.00000 0.00000 0.00000 0.00000
        -------------------------------------------------------------------------------
             11      12      13      14      15      16      17      18      19      20
        -------------------------------------------------------------------------------
a_O         958     486    1089    1012     472     763     968     437     769     717
        ( 27.9) ( 14.1) ( 31.7) ( 29.5) ( 13.7) ( 22.2) ( 28.2) ( 12.7) ( 22.4) ( 20.9)
a_E       839.8   173.9   645.5   839.8   173.9   645.5   839.8   173.9   645.5   839.8
        ( 24.4) (  5.1) ( 18.8) ( 24.4) (  5.1) ( 18.8) ( 24.4) (  5.1) ( 18.8) ( 24.4)
          252.4   999.2  1139.2   377.6   942.8   255.3   275.1   805.6   269.3  -226.6
          7.345  29.080  33.155  10.989  27.439   7.430   8.007  23.445   7.839  -6.596
t_O         944     812     487     967     600     567    1041     776     487    1033
        ( 27.5) ( 23.6) ( 14.2) ( 28.1) ( 17.5) ( 16.5) ( 30.3) ( 22.6) ( 14.2) ( 30.1)
t_E       975.7   226.5   411.8   975.7   226.5   411.8   975.7   226.5   411.8   975.7
        ( 28.4) (  6.6) ( 12.0) ( 28.4) (  6.6) ( 12.0) ( 28.4) (  6.6) ( 12.0) ( 28.4)
          -62.4  2073.5   163.4   -17.4  1169.0   362.7   134.8  1911.2   163.4   117.8
         -1.818  60.345   4.756  -0.507  34.023  10.557   3.922  55.621   4.756   3.428
g_O         473     770     888     491    1023     872     496     832    1168     509
        ( 13.8) ( 22.4) ( 25.8) ( 14.3) ( 29.8) ( 25.4) ( 14.4) ( 24.2) ( 34.0) ( 14.8)
g_E       697.7  1375.5  1375.5   697.7  1375.5  1375.5   697.7  1375.5  1375.5   697.7
        ( 20.3) ( 40.0) ( 40.0) ( 20.3) ( 40.0) ( 40.0) ( 20.3) ( 40.0) ( 40.0) ( 20.3)
         -367.7  -893.5  -777.1  -345.0  -605.8  -794.8  -338.4  -836.6  -381.9  -321.0
        -10.700 -26.005 -22.617 -10.040 -17.632 -23.132  -9.850 -24.348 -11.115  -9.341
```

c_O	1061	1368	972	966	1341	1234	931	1391	1012	1177
	(30.9)	(39.8)	(28.3)	(28.1)	(39.0)	(35.9)	(27.1)	(40.5)	(29.5)	(34.3)
c_E	922.8	1660.1	1003.3	922.8	1660.1	1003.3	922.8	1660.1	1003.3	922.8
	(26.9)	(48.3)	(29.2)	(26.9)	(48.3)	(29.2)	(26.9)	(48.3)	(29.2)	(26.9)
	296.1	-529.5	-61.6	88.3	-572.5	510.8	16.4	-492.0	17.5	572.7
	8.617	-15.410	-1.793	2.571	-16.662	14.866	0.478	-14.319	0.508	16.668
N	3436	3436	3436	3436	3436	3436	3436	3436	3436	3436
X2	110.7	2391.9	492.2	98.6	1278.9	317.2	82.3	1989.8	68.7	142.3
Gvalue	118.4	1649.6	463.9	103.6	933.5	334.0	87.9	1388.1	68.3	142.9
Gv/N	3.445	48.010	13.500	3.014	27.168	9.720	2.558	40.398	1.988	4.159
Prob	0.00000	1.00000	0.00000	0.00000	0.00000	0.00000	0.00000	0.00000	0.00000	0.00000

	21	22	23	24	25	26	27	28	29	30
a_O	418	693	716	525	639	694	302	780	619	285
	(12.2)	(20.2)	(20.8)	(15.3)	(18.6)	(20.2)	(8.8)	(22.7)	(18.0)	(8.3)
a_E	173.9	645.5	839.8	173.9	645.5	839.8	173.9	645.5	839.8	173.9
	(5.1)	(18.8)	(24.4)	(5.1)	(18.8)	(24.4)	(5.1)	(18.8)	(24.4)	(5.1)
	733.4	98.5	-228.3	1160.4	-12.9	-264.6	333.5	295.3	-377.6	281.7
	21.344	2.866	-6.645	33.772	-0.374	-7.702	9.707	8.596	-10.990	8.199
t_O	501	523	959	456	685	1180	405	447	951	336
	(14.6)	(15.2)	(27.9)	(13.3)	(19.9)	(34.3)	(11.8)	(13.0)	(27.7)	(9.8)
t_E	226.5	411.8	975.7	226.5	411.8	975.7	226.5	411.8	975.7	226.5
	(6.6)	(12.0)	(28.4)	(6.6)	(12.0)	(28.4)	(6.6)	(12.0)	(28.4)	(6.6)
	795.5	250.1	-33.2	638.2	697.2	448.6	470.7	73.4	-48.9	265.0
	23.151	7.278	-0.966	18.573	20.292	13.055	13.700	2.135	-1.422	7.713
g_O	841	1038	783	990	1110	575	1243	1226	641	1080
	(24.5)	(30.2)	(22.8)	(28.8)	(32.3)	(16.7)	(36.2)	(35.7)	(18.7)	(31.4)
g_E	1375.5	1375.5	697.7	1375.5	1375.5	697.7	1375.5	1375.5	697.7	1375.5
	(40.0)	(40.0)	(20.3)	(40.0)	(40.0)	(20.3)	(40.0)	(40.0)	(20.3)	(40.0)
	-827.6	-584.4	180.7	-651.2	-476.0	-222.4	-251.9	-282.0	-108.6	-522.5
	-24.085	-17.007	5.259	-18.953	-13.854	-6.472	-7.331	-8.208	-3.160	-15.206
c_O	1676	1182	978	1465	1002	987	1486	983	1225	1735
	(48.8)	(34.4)	(28.5)	(42.6)	(29.2)	(28.7)	(43.2)	(28.6)	(35.7)	(50.5)
c_E	1660.1	1003.3	922.8	1660.1	1003.3	922.8	1660.1	1003.3	922.8	1660.1
	(48.3)	(29.2)	(26.9)	(48.3)	(29.2)	(26.9)	(48.3)	(29.2)	(26.9)	(48.3)
	32.0	387.5	113.6	-366.3	-2.6	132.7	-329.3	-40.2	694.0	153.1
	0.930	11.277	3.306	-10.661	-0.076	3.863	-9.583	-1.170	20.198	4.457
N	3436	3436	3436	3436	3436	3436	3436	3436	3436	3436
X2	883.4	148.2	32.3	1072.7	232.6	94.1	266.1	47.7	162.2	190.9
Gvalue	733.2	151.7	32.8	781.1	205.7	94.3	223.1	46.5	158.9	177.4
Gv/N	21.339	4.415	0.954	22.731	5.988	2.744	6.493	1.353	4.625	5.163
Prob	0.00000	0.00000	0.00000	0.00000	0.00000	0.00000	0.00000	0.00000	0.00000	0.00000

	31	32	33	34	35	36	37	38	39	40
a_O	561	605	258	566	625	445	533	599	225	559
	(16.3)	(17.6)	(7.5)	(16.5)	(18.2)	(13.0)	(15.5)	(17.4)	(6.5)	(16.3)
a_E	645.5	839.8	173.9	645.5	839.8	173.9	645.5	839.8	173.9	645.5
	(18.8)	(24.4)	(5.1)	(18.8)	(24.4)	(5.1)	(18.8)	(24.4)	(5.1)	(18.8)
	-157.4	-396.8	203.7	-148.7	-369.2	836.4	-204.1	-404.8	116.0	-160.8
	-4.580	-11.547	5.928	-4.328	-10.746	24.344	-5.940	-11.780	3.377	-4.680
t_O	652	1168	348	404	1140	314	402	974	564	417
	(19.0)	(34.0)	(10.1)	(11.8)	(33.2)	(9.1)	(11.7)	(28.3)	(16.4)	(12.1)
t_E	411.8	975.7	226.5	411.8	975.7	226.5	411.8	975.7	226.5	411.8
	(12.0)	(28.4)	(6.6)	(12.0)	(28.4)	(6.6)	(12.0)	(28.4)	(6.6)	(12.0)
	599.3	420.1	298.9	-15.4	354.7	205.1	-19.3	-3.5	1029.1	10.5
	17.441	12.227	8.699	-0.449	10.324	5.970	-0.563	-0.102	29.950	0.306
g_O	1263	701	1076	1262	641	1132	1315	863	1131	1312
	(36.8)	(20.4)	(31.3)	(36.7)	(18.7)	(32.9)	(38.3)	(25.1)	(32.9)	(38.2)
g_E	1375.5	697.7	1375.5	1375.5	697.7	1375.5	1375.5	697.7	1375.5	1375.5
	(40.0)	(20.3)	(40.0)	(40.0)	(20.3)	(40.0)	(40.0)	(20.3)	(40.0)	(40.0)
	-215.4	6.7	-528.5	-217.3	-108.6	-441.2	-118.2	367.1	-442.8	-123.9
	-6.270	0.195	-15.382	-6.323	-3.160	-12.840	-3.440	10.684	-12.887	-3.607
c_O	960	962	1754	1204	1030	1545	1186	1000	1516	1148
	(27.9)	(28.0)	(51.0)	(35.0)	(30.0)	(45.0)	(34.5)	(29.1)	(44.1)	(33.4)
c_E	1003.3	922.8	1660.1	1003.3	922.8	1660.1	1003.3	922.8	1660.1	1003.3
	(29.2)	(26.9)	(48.3)	(29.2)	(26.9)	(48.3)	(29.2)	(26.9)	(48.3)	(29.2)
	-84.7	80.0	193.0	439.1	226.3	-222.0	396.8	160.6	-275.3	309.3
	-2.465	2.328	5.618	12.779	6.588	-6.462	11.549	4.675	-8.012	9.002
N	3436	3436	3436	3436	3436	3436	3436	3436	3436	3436
X2	162.3	105.2	176.4	59.4	99.6	507.8	55.8	114.7	573.9	35.4
Gvalue	141.7	110.0	167.1	57.7	103.3	378.4	55.2	119.5	427.0	35.1
Gv/N	4.125	3.203	4.862	1.679	3.005	11.013	1.606	3.477	12.428	1.022
Prob	0.00000	0.00000	0.00000	0.00000	0.00000	0.00000	0.00000	0.00000	0.00000	0.00000

	41	42	43	44	45	46	47	48	49	50
a_O	630	431	573	668	243	551	836	413	775	625
	(18.3)	(12.5)	(16.7)	(19.4)	(7.1)	(16.0)	(24.3)	(12.0)	(22.6)	(18.2)
a_E	839.8	173.9	645.5	839.8	173.9	645.5	839.8	173.9	645.5	839.8
	(24.4)	(5.1)	(18.8)	(24.4)	(5.1)	(18.8)	(24.4)	(5.1)	(18.8)	(24.4)
	-362.1	782.6	-136.5	-305.7	162.7	-174.4	-7.5	714.7	283.5	-369.2
	-10.539	22.776	-3.972	-8.898	4.736	-5.075	-0.219	20.799	8.250	-10.746
t_O	970	345	408	1171	317	389	1003	295	393	1209
	(28.2)	(10.0)	(11.9)	(34.1)	(9.2)	(11.3)	(29.2)	(8.6)	(11.4)	(35.2)
t_E	975.7	226.5	411.8	975.7	226.5	411.8	975.7	226.5	411.8	975.7
	(28.4)	(6.6)	(12.0)	(28.4)	(6.6)	(12.0)	(28.4)	(6.6)	(12.0)	(28.4)
	-11.5	290.4	-7.5	427.2	213.1	-44.3	55.3	155.9	-36.7	518.3
	-0.334	8.450	-0.219	12.433	6.203	-1.289	1.608	4.537	-1.068	15.084
g_O	858	1122	1263	673	1194	1517	644	1198	1338	638
	(25.0)	(32.7)	(36.8)	(19.6)	(34.7)	(44.2)	(18.7)	(34.9)	(38.9)	(18.6)
g_E	697.7	1375.5	1375.5	697.7	1375.5	1375.5	697.7	1375.5	1375.5	697.7
	(20.3)	(40.0)	(40.0)	(20.3)	(40.0)	(40.0)	(20.3)	(40.0)	(40.0)	(20.3)
	355.0	-457.2	-215.4	-48.4	-338.0	297.2	-103.1	-331.1	-73.9	-114.1
	10.331	-13.306	-6.270	-1.410	-9.837	8.649	-3.000	-9.637	-2.150	-3.320
c_O	978	1538	1192	924	1682	979	953	1530	930	964
	(28.5)	(44.8)	(34.7)	(26.9)	(49.0)	(28.5)	(27.7)	(44.5)	(27.1)	(28.1)
c_E	922.8	1660.1	1003.3	922.8	1660.1	1003.3	922.8	1660.1	1003.3	922.8
	(26.9)	(48.3)	(29.2)	(26.9)	(48.3)	(29.2)	(26.9)	(48.3)	(29.2)	(26.9)
	113.6	-235.0	410.8	2.4	44.1	-48.0	61.3	-249.7	-141.1	84.2
	3.306	-6.839	11.957	0.069	1.283	-1.397	1.785	-7.268	-4.107	2.450
N	3436	3436	3436	3436	3436	3436	3436	3436	3436	3436
X2	92.6	498.0	52.9	75.1	87.9	30.2	5.9	382.8	33.2	117.6
Gvalue	95.0	380.8	51.4	75.4	81.9	30.5	6.0	289.7	31.8	119.2
Gv/N	2.764	11.082	1.496	2.194	2.385	0.888	0.174	8.432	0.926	3.468
Prob	0.00000	0.00000	0.00000	0.00000	0.00000	0.00000	0.11234	0.00000	0.00000	0.00000

	51	52	53	54	55	56	57	58	59	60
a_O	262	553	615	207	547	861	200	567	845	398
	(7.6)	(16.1)	(17.9)	(6.0)	(15.9)	(25.1)	(5.8)	(16.5)	(24.6)	(11.6)
a_E	173.9	645.5	839.8	173.9	645.5	839.8	173.9	645.5	839.8	173.9
	(5.1)	(18.8)	(24.4)	(5.1)	(18.8)	(24.4)	(5.1)	(18.8)	(24.4)	(5.1)
	214.9	-171.0	-383.2	72.2	-181.1	43.0	56.0	-147.0	10.5	659.3
	6.254	-4.977	-11.151	2.102	-5.270	1.251	1.631	-4.278	0.305	19.187
t_O	294	407	1198	298	392	967	287	392	936	298
	(8.6)	(11.8)	(34.9)	(8.7)	(11.4)	(28.1)	(8.4)	(11.4)	(27.2)	(8.7)
t_E	226.5	411.8	975.7	226.5	411.8	975.7	226.5	411.8	975.7	226.5
	(6.6)	(12.0)	(28.4)	(6.6)	(12.0)	(28.4)	(6.6)	(12.0)	(28.4)	(6.6)
	153.4	-9.5	491.7	163.5	-38.6	-17.4	135.9	-38.6	-77.9	163.5
	4.464	-0.277	14.309	4.759	-1.123	-0.507	3.955	-1.123	-2.266	4.759
g_O	1419	1560	645	1164	1373	681	1443	1331	669	1236
	(41.3)	(45.4)	(18.8)	(33.9)	(40.0)	(19.8)	(42.0)	(38.7)	(19.5)	(36.0)
g_E	1375.5	1375.5	697.7	1375.5	1375.5	697.7	1375.5	1375.5	697.7	1375.5
	(40.0)	(40.0)	(20.3)	(40.0)	(40.0)	(20.3)	(40.0)	(40.0)	(20.3)	(40.0)
	88.3	392.8	-101.2	-388.7	-4.9	-32.9	138.2	-87.4	-56.1	-264.4
	2.569	11.433	-2.947	-11.314	-0.142	-0.958	4.021	-2.545	-1.634	-7.696
c_O	1461	916	978	1767	1124	927	1506	1146	986	1504
	(42.5)	(26.7)	(28.5)	(51.4)	(32.7)	(27.0)	(43.8)	(33.4)	(28.7)	(43.8)
c_E	1660.1	1003.3	922.8	1660.1	1003.3	922.8	1660.1	1003.3	922.8	1660.1
	(48.3)	(29.2)	(26.9)	(48.3)	(29.2)	(26.9)	(48.3)	(29.2)	(26.9)	(48.3)
	-373.3	-166.8	113.6	220.5	255.4	8.4	-293.4	304.8	130.6	-297.0
	-10.864	-4.854	3.306	6.419	7.432	0.244	-8.540	8.870	3.801	-8.645
N	3436	3436	3436	3436	3436	3436	3436	3436	3436	3436
X2	90.1	45.7	118.1	68.3	30.5	1.0	37.7	32.2	7.2	340.4
Gvalue	83.2	45.5	120.9	67.5	30.8	1.0	36.7	31.8	7.1	261.3
Gv/N	2.422	1.325	3.518	1.966	0.896	0.030	1.067	0.924	0.207	7.605
Prob	0.00000	0.00000	0.00000	0.00000	0.00000	0.79377	0.00000	0.00000	0.06884	0.00000

	61	62	63	64	65	66	67	68	69	70
a_O	525	627	207	698	650	185	543	717	197	575
	(15.3)	(18.2)	(6.0)	(20.3)	(18.9)	(5.4)	(15.8)	(20.9)	(5.7)	(16.7)
a_E	645.5	839.8	173.9	645.5	839.8	173.9	645.5	839.8	173.9	645.5
	(18.8)	(24.4)	(5.1)	(18.8)	(24.4)	(5.1)	(18.8)	(24.4)	(5.1)	(18.8)
	-216.9	-366.4	72.2	109.2	-333.0	23.0	-187.7	-226.6	49.2	-132.9
	-6.313	-10.663	2.102	3.179	-9.692	0.669	-5.464	-6.596	1.433	-3.869
t_O	589	1188	258	418	1110	266	387	884	253	405
	(17.1)	(34.6)	(7.5)	(12.2)	(32.3)	(7.7)	(11.3)	(25.7)	(7.4)	(11.8)
t_E	411.8	975.7	226.5	411.8	975.7	226.5	411.8	975.7	226.5	411.8
	(12.0)	(28.4)	(6.6)	(12.0)	(28.4)	(6.6)	(12.0)	(28.4)	(6.6)	(12.0)
	421.6	467.7	67.2	12.5	286.2	85.5	-48.0	-174.6	56.0	-13.5
	12.271	13.610	1.956	0.365	8.329	2.489	-1.398	-5.081	1.629	-0.391
g_O	1404	644	1456	1350	650	1264	1549	662	1275	1297
	(40.9)	(18.7)	(42.4)	(39.3)	(18.9)	(36.8)	(45.1)	(19.3)	(37.1)	(37.7)
g_E	1375.5	697.7	1375.5	1375.5	697.7	1375.5	1375.5	697.7	1375.5	1375.5
	(40.0)	(20.3)	(40.0)	(40.0)	(20.3)	(40.0)	(40.0)	(20.3)	(40.0)	(40.0)
	57.7	-103.1	165.5	-50.4	-92.0	-213.8	368.1	-69.5	-193.6	-152.3
	1.679	-3.000	4.817	-1.468	-2.677	-6.222	10.714	-2.022	-5.633	-4.434
c_O	918	977	1515	970	1026	1721	957	1173	1711	1159
	(26.7)	(28.4)	(44.1)	(28.2)	(29.9)	(50.1)	(27.9)	(34.1)	(49.8)	(33.7)
c_E	1003.3	922.8	1660.1	1003.3	922.8	1660.1	1003.3	922.8	1660.1	1003.3
	(29.2)	(26.9)	(48.3)	(29.2)	(26.9)	(48.3)	(29.2)	(26.9)	(48.3)	(29.2)
	-163.1	111.5	-277.1	-65.5	217.5	124.0	-90.4	562.8	103.4	334.4
	-4.748	3.244	-8.065	-1.906	6.330	3.609	-2.632	16.379	3.008	9.732
N	3436	3436	3436	3436	3436	3436	3436	3436	3436	3436
X2	106.6	107.4	28.1	5.9	76.1	18.9	41.8	96.2	15.1	36.4
Gvalue	99.3	109.6	27.8	5.8	78.7	18.7	41.9	92.1	15.0	35.7
Gv/N	2.890	3.191	0.810	0.170	2.290	0.545	1.220	2.680	0.437	1.038
Prob	0.00000	0.00000	0.00000	0.11916	0.00000	0.00031	0.00000	0.00000	0.00180	0.00000

	71	72	73	74	75	76	77	78	79	80
a_O	951	174	582	933	401	518	711	161	568	689
	(27.7)	(5.1)	(16.9)	(27.2)	(11.7)	(15.1)	(20.7)	(4.7)	(16.5)	(20.1)
a_E	839.8	173.9	645.5	839.8	173.9	645.5	839.8	173.9	645.5	839.8
	(24.4)	(5.1)	(18.8)	(24.4)	(5.1)	(18.8)	(24.4)	(5.1)	(18.8)	(24.4)
	236.6	0.3	-120.5	196.4	670.2	-227.9	-236.7	-24.7	-145.2	-272.7
	6.885	0.008	-3.506	5.717	19.507	-6.633	-6.889	-0.720	-4.227	-7.936
t_O	929	480	379	937	266	413	1154	460	594	975
	(27.0)	(14.0)	(11.0)	(27.3)	(7.7)	(12.0)	(33.6)	(13.4)	(17.3)	(28.4)
t_E	975.7	226.5	411.8	975.7	226.5	411.8	975.7	226.5	411.8	975.7
	(28.4)	(6.6)	(12.0)	(28.4)	(6.6)	(12.0)	(28.4)	(6.6)	(12.0)	(28.4)
	-91.2	721.0	-62.9	-75.9	85.5	2.4	387.3	651.8	435.3	-1.5
	-2.655	20.984	-1.830	-2.210	2.489	0.071	11.270	18.970	12.668	-0.043
g_O	644	1243	1298	609	1255	1498	638	1301	1311	845
	(18.7)	(36.2)	(37.8)	(17.7)	(36.5)	(43.6)	(18.6)	(37.9)	(38.2)	(24.6)
g_E	697.7	1375.5	1375.5	697.7	1375.5	1375.5	697.7	1375.5	1375.5	697.7
	(20.3)	(40.0)	(40.0)	(20.3)	(40.0)	(40.0)	(20.3)	(40.0)	(40.0)	(20.3)
	-103.1	-251.9	-150.5	-165.5	-230.2	255.7	-114.1	-145.0	-125.8	323.8
	-3.000	-7.331	-4.379	-4.818	-6.700	7.442	-3.320	-4.219	-3.662	9.424
c_O	912	1539	1177	957	1514	1007	933	1514	963	927
	(26.5)	(44.8)	(34.3)	(27.9)	(44.1)	(29.3)	(27.2)	(44.1)	(28.0)	(27.0)
c_E	922.8	1660.1	1003.3	922.8	1660.1	1003.3	922.8	1660.1	1003.3	922.8
	(26.9)	(48.3)	(29.2)	(26.9)	(48.3)	(29.2)	(26.9)	(48.3)	(29.2)	(26.9)
	-21.5	-233.1	375.9	69.6	-278.9	7.4	20.5	-278.9	-79.0	8.4
	-0.626	-6.785	10.939	2.026	-8.118	0.216	0.596	-8.118	-2.298	0.244
N	3436	3436	3436	3436	3436	3436	3436	3436	3436	3436
X2	21.2	305.3	43.3	24.4	327.1	36.1	57.5	258.6	94.6	58.2
Gvalue	20.8	236.3	42.1	24.6	246.6	37.6	57.0	203.1	85.2	58.0
Gv/N	0.604	6.876	1.224	0.715	7.178	1.095	1.657	5.912	2.481	1.688
Prob	0.00012	0.00000	0.00000	0.00002	0.00000	0.00000	0.00000	0.00000	0.00000	0.00000

	81	82	83	84	85	86	87	88	89	90
a_O	191	578	732	162	565	758	195	556	972	397
	(5.6)	(16.8)	(21.3)	(4.7)	(16.4)	(22.1)	(5.7)	(16.2)	(28.3)	(11.6)
a_E	173.9	645.5	839.8	173.9	645.5	839.8	173.9	645.5	839.8	173.9
	(5.1)	(18.8)	(24.4)	(5.1)	(18.8)	(24.4)	(5.1)	(18.8)	(24.4)	(5.1)
	35.9	-127.6	-201.1	-22.9	-150.5	-155.3	44.8	-165.9	284.3	655.6
	1.045	-3.714	-5.852	-0.666	-4.379	-4.520	1.303	-4.829	8.273	19.080
t_O	477	438	1081	235	388	901	442	397	916	237
	(13.9)	(12.7)	(31.5)	(6.8)	(11.3)	(26.2)	(12.9)	(11.6)	(26.7)	(6.9)
t_E	226.5	411.8	975.7	226.5	411.8	975.7	226.5	411.8	975.7	226.5
	(6.6)	(12.0)	(28.4)	(6.6)	(12.0)	(28.4)	(6.6)	(12.0)	(28.4)	(6.6)
	710.5	54.1	221.5	17.3	-46.2	-143.6	591.0	-29.0	-115.8	21.5

	20.679	1.574	6.446	0.504	−1.343	−4.180	17.201	−0.845	−3.369	0.625
g_O	1256	1514	678	1480	1335	656	1308	1505	639	1308
	(36.6)	(44.1)	(19.7)	(43.1)	(38.9)	(19.1)	(38.1)	(43.8)	(18.6)	(38.1)
g_E	1375.5	1375.5	697.7	1375.5	1375.5	697.7	1375.5	1375.5	697.7	1375.5
	(40.0)	(40.0)	(20.3)	(40.0)	(40.0)	(20.3)	(40.0)	(40.0)	(20.3)	(40.0)
	−228.4	290.6	−38.8	216.6	−79.7	−80.8	−131.7	270.9	−112.2	−131.7
	−6.647	8.458	−1.128	6.305	−2.320	−2.351	−3.833	7.885	−3.267	−3.833
c_O	1512	906	945	1559	1148	1121	1491	978	909	1494
	(44.0)	(26.4)	(27.5)	(45.4)	(33.4)	(32.6)	(43.4)	(28.5)	(26.5)	(43.5)
c_E	1660.1	1003.3	922.8	1660.1	1003.3	922.8	1660.1	1003.3	922.8	1660.1
	(48.3)	(29.2)	(26.9)	(48.3)	(29.2)	(26.9)	(48.3)	(29.2)	(26.9)	(48.3)
	−282.6	−184.8	44.9	−195.9	309.3	436.2	−320.4	−50.0	−27.4	−315.0
	−8.224	−5.380	1.306	−5.702	9.002	12.694	−9.323	−1.454	−0.798	−9.167
N	3436	3436	3436	3436	3436	3436	3436	3436	3436	3436
X2	302.3	32.1	26.3	15.2	33.5	58.7	228.1	25.8	29.6	306.8
Gvalue	235.5	32.2	26.5	15.2	33.0	56.5	183.7	26.0	28.8	230.4
Gv/N	6.853	0.938	0.772	0.441	0.961	1.643	5.346	0.758	0.839	6.705
Prob	0.00000	0.00000	0.00001	0.00168	0.00000	0.00000	0.00000	0.00001	0.00000	0.00000

	91	92	93	94	95	96	97	98	99	100
a_O	534	766	171	595	744	158	557	719	172	580
	(15.5)	(22.3)	(5.0)	(17.3)	(21.7)	(4.6)	(16.2)	(20.9)	(5.0)	(16.9)
a_E	645.5	839.8	173.9	645.5	839.8	173.9	645.5	839.8	173.9	645.5
	(18.8)	(24.4)	(5.1)	(18.8)	(24.4)	(5.1)	(18.8)	(24.4)	(5.1)	(18.8)
	−202.5	−140.9	−5.7	−96.9	−180.2	−30.2	−164.2	−223.3	−3.7	−124.1
	−5.892	−4.100	−0.165	−2.819	−5.244	−0.880	−4.779	−6.498	−0.108	−3.610
t_O	383	902	251	370	941	227	373	953	232	369
	(11.1)	(26.3)	(7.3)	(10.8)	(27.4)	(6.6)	(10.9)	(27.7)	(6.8)	(10.7)
t_E	411.8	975.7	226.5	411.8	975.7	226.5	411.8	975.7	226.5	411.8
	(12.0)	(28.4)	(6.6)	(12.0)	(28.4)	(6.6)	(12.0)	(28.4)	(6.6)	(12.0)
	−55.5	−141.8	51.6	−79.2	−68.2	1.0	−73.8	−45.0	11.1	−81.0
	−1.615	−4.126	1.501	−2.304	−1.986	0.029	−2.148	−1.308	0.324	−2.356
g_O	1546	638	1478	1520	619	1297	1319	825	1300	1598
	(45.0)	(18.6)	(43.0)	(44.2)	(18.0)	(37.7)	(38.4)	(24.0)	(37.8)	(46.5)
g_E	1375.5	697.7	1375.5	1375.5	697.7	1375.5	1375.5	697.7	1375.5	1375.5
	(40.0)	(20.3)	(40.0)	(40.0)	(20.3)	(40.0)	(40.0)	(20.3)	(40.0)	(40.0)
	361.4	−114.1	212.4	303.8	−148.1	−152.5	−110.6	276.6	−146.9	479.3
	10.519	−3.320	6.180	8.841	−4.310	−4.439	−3.217	8.051	−4.274	13.950
c_O	973	1130	1536	951	1132	1754	1187	939	1732	889
	(28.3)	(32.9)	(44.7)	(27.7)	(32.9)	(51.0)	(34.5)	(27.3)	(50.4)	(25.9)
c_E	1003.3	922.8	1660.1	1003.3	922.8	1660.1	1003.3	922.8	1660.1	1003.3
	(29.2)	(26.9)	(48.3)	(29.2)	(26.9)	(48.3)	(29.2)	(26.9)	(48.3)	(29.2)
	−59.7	457.7	−238.7	−101.8	462.5	193.0	399.1	32.6	146.9	−215.1
	−1.737	13.322	−6.946	−2.964	13.462	5.618	11.617	0.950	4.275	−6.259
N	3436	3436	3436	3436	3436	3436	3436	3436	3436	3436
X2	43.3	63.7	19.6	26.1	68.4	11.2	51.7	41.4	7.4	60.1
Gvalue	43.8	61.0	19.6	25.9	66.0	11.3	50.6	41.0	7.4	59.2
Gv/N	1.274	1.776	0.570	0.754	1.922	0.328	1.472	1.194	0.217	1.724
Prob	0.00000	0.00000	0.00021	0.00001	0.00000	0.01030	0.00000	0.00000	0.05886	0.00000

	101	102	103	104	105	106	107	108	109	110
a_O	727	162	578	970	150	562	756	175	572	761
	(21.2)	(4.7)	(16.8)	(28.2)	(4.4)	(16.4)	(22.0)	(5.1)	(16.6)	(22.1)
a_E	839.8	173.9	645.5	839.8	173.9	645.5	839.8	173.9	645.5	839.8
	(24.4)	(5.1)	(18.8)	(24.4)	(5.1)	(18.8)	(24.4)	(5.1)	(18.8)	(24.4)
	−209.7	−22.9	−127.6	279.7	−44.3	−155.6	−158.9	2.3	−138.2	−149.9
	−6.102	−0.666	−3.714	8.140	−1.289	−4.530	−4.624	0.067	−4.023	−4.363
t_O	953	235	565	917	450	400	944	246	381	1137
	(27.7)	(6.8)	(16.4)	(26.7)	(13.1)	(11.6)	(27.5)	(7.2)	(11.1)	(33.1)
t_E	975.7	226.5	411.8	975.7	226.5	411.8	975.7	226.5	411.8	975.7
	(28.4)	(6.6)	(12.0)	(28.4)	(6.6)	(12.0)	(28.4)	(6.6)	(12.0)	(28.4)
	−45.0	17.3	357.5	−113.9	617.9	−23.2	−62.4	40.6	−59.2	347.8
	−1.308	0.504	10.403	−3.314	17.982	−0.676	−1.818	1.183	−1.723	10.122
g_O	633	1312	1363	641	1342	1533	845	1477	1526	665
	(18.4)	(38.2)	(39.7)	(18.7)	(39.1)	(44.6)	(24.6)	(43.0)	(44.4)	(19.4)
g_E	697.7	1375.5	1375.5	697.7	1375.5	1375.5	697.7	1375.5	1375.5	697.7
	(20.3)	(40.0)	(40.0)	(20.3)	(40.0)	(40.0)	(20.3)	(40.0)	(40.0)	(20.3)
	−123.1	−124.1	−24.8	−108.6	−66.3	332.5	323.8	210.2	317.0	−63.8
	−3.584	−3.612	−0.721	−3.160	−1.929	9.677	9.424	6.118	9.226	−1.856
c_O	1123	1727	930	908	1494	941	891	1538	957	873
	(32.7)	(50.3)	(27.1)	(26.4)	(43.5)	(27.4)	(25.9)	(44.8)	(27.9)	(25.4)
c_E	922.8	1660.1	1003.3	922.8	1660.1	1003.3	922.8	1660.1	1003.3	922.8
	(26.9)	(48.3)	(29.2)	(26.9)	(48.3)	(29.2)	(26.9)	(48.3)	(29.2)	(26.9)
	440.9	136.5	−141.1	−29.4	−315.0	−120.7	−62.5	−235.0	−90.4	−96.9
	12.833	3.972	−4.107	−0.856	−9.167	−3.512	−1.820	−6.839	−2.632	−2.820
N	3436	3436	3436	3436	3436	3436	3436	3436	3436	3436
X2	65.1	6.8	69.5	28.6	241.3	33.0	41.6	18.1	29.3	38.3
Gvalue	63.2	6.8	63.9	27.8	192.3	33.0	39.9	18.2	29.1	37.2
Gv/N	1.839	0.197	1.861	0.809	5.597	0.960	1.162	0.528	0.848	1.083
Prob	0.00000	0.07905	0.00000	0.00000	0.00000	0.00000	0.00000	0.00041	0.00000	0.00000

	111	112	113	114	115	116	117	118	119	120
a_O	156	779	722	151	565	774	161	581	824	173
	(4.5)	(22.7)	(21.0)	(4.4)	(16.4)	(22.5)	(4.7)	(16.9)	(24.0)	(5.0)
a_E	173.9	645.5	839.8	173.9	645.3	839.5	173.8	645.3	839.5	173.8
	(5.1)	(18.8)	(24.4)	(5.1)	(18.8)	(24.4)	(5.1)	(18.8)	(24.4)	(5.1)
	−33.8	293.0	−218.2	−42.6	−150.1	−125.8	−24.6	−121.9	−30.8	−1.6
	−0.984	8.526	−6.350	−1.239	−4.370	−3.662	−0.718	−3.550	−0.896	−0.047
t_O	248	380	898	239	415	909	223	354	894	234
	(7.2)	(11.1)	(26.1)	(7.0)	(12.1)	(26.5)	(6.5)	(10.3)	(26.0)	(6.8)
t_E	226.5	411.8	975.7	226.5	411.7	975.5	226.4	411.7	975.5	226.4
	(6.6)	(12.0)	(28.4)	(6.6)	(12.0)	(28.4)	(6.6)	(12.0)	(28.4)	(6.6)
	45.0	−61.0	−149.1	25.7	6.7	−128.3	−6.8	−106.8	−155.9	15.4
	1.309	−1.777	−4.340	0.747	0.195	−3.735	−0.198	−3.110	−4.539	0.448
g_O	1278	1326	661	1314	1551	633	1497	1321	665	1462
	(37.2)	(38.6)	(19.2)	(38.2)	(45.2)	(18.4)	(43.6)	(38.5)	(19.4)	(42.6)
g_E	1375.5	1375.5	697.7	1375.5	1375.0	697.5	1375.1	1375.0	697.5	1375.1
	(40.0)	(40.0)	(20.3)	(40.0)	(40.0)	(20.3)	(40.0)	(40.0)	(20.3)	(40.0)
	−188.0	−97.1	−71.4	−120.3	373.5	−122.8	254.2	−105.9	−63.4	179.1
	−5.472	−2.826	−2.077	−3.501	10.874	−3.574	7.400	−3.084	−1.845	5.214
c_O	1754	951	1155	1732	904	1119	1554	1179	1052	1566
	(51.0)	(27.7)	(33.6)	(50.4)	(26.3)	(32.6)	(45.2)	(34.3)	(30.6)	(45.6)
c_E	1660.1	1003.3	922.8	1660.1	1003.0	922.6	1659.6	1003.0	922.6	1659.6
	(48.3)	(29.2)	(26.9)	(48.3)	(29.2)	(26.9)	(48.3)	(29.2)	(26.9)	(48.3)
	193.0	−101.8	518.4	146.9	−187.9	432.0	−204.4	381.2	276.3	−181.8
	5.618	−2.964	15.088	4.275	−5.470	12.577	−5.949	11.097	8.043	−5.294

N	3436	3436	3436	3436	3435	3435	3435	3435	3435	3435
X2	16.1	34.6	83.1	9.6	42.3	57.4	18.5	47.5	26.8	11.0
Gvalue	16.2	33.0	79.7	9.7	42.2	55.2	18.4	46.5	26.2	11.0
Gv/N	0.471	0.960	2.320	0.282	1.228	1.606	0.535	1.353	0.763	0.321
Prob	0.00104	0.00000	0.00000	0.02139	0.00000	0.00000	0.00037	0.00000	0.00001	0.01167

	121	122	123	124	125	126	127	128	129	130
a_O	759	801	173	567	760	173	559	774	171	846
	(22.1)	(23.3)	(5.0)	(16.5)	(22.2)	(5.0)	(16.3)	(22.6)	(5.0)	(24.7)
a_E	645.1	839.3	173.8	644.5	838.5	173.6	644.0	837.8	173.5	643.8
	(18.8)	(24.4)	(5.1)	(18.8)	(24.4)	(5.1)	(18.8)	(24.4)	(5.1)	(18.8)
	246.8	−74.8	−1.5	−145.3	−149.5	−1.2	−158.2	−122.6	−4.9	462.2
	7.188	−2.178	−0.044	−4.236	−4.357	−0.035	−4.615	−3.578	−0.142	13.487
t_O	411	836	430	384	1085	225	376	919	202	358
	(12.0)	(24.3)	(12.5)	(11.2)	(31.6)	(6.6)	(11.0)	(26.8)	(5.9)	(10.4)
t_E	411.5	975.2	226.4	411.2	974.3	226.2	410.8	973.5	226.0	410.7
	(12.0)	(28.4)	(6.6)	(12.0)	(28.4)	(6.6)	(12.0)	(28.4)	(6.6)	(12.0)
	−1.1	−257.5	551.8	−52.5	233.5	−2.3	−66.6	−105.8	−45.3	−98.3
	−0.032	−7.498	16.069	−1.531	6.805	−0.068	−1.943	−3.088	−1.322	−2.869
g_O	1353	680	1286	1541	642	1527	1340	872	1280	1319
	(39.4)	(19.8)	(37.4)	(44.9)	(18.7)	(44.5)	(39.1)	(25.4)	(37.3)	(38.5)
g_E	1374.6	697.3	1374.7	1373.4	696.6	1373.5	1372.2	696.0	1372.3	1371.8
	(40.0)	(20.3)	(40.0)	(40.0)	(20.3)	(40.0)	(40.0)	(20.3)	(40.0)	(40.0)
	−43.0	−34.1	−171.6	354.8	−104.9	323.5	−63.7	393.1	−178.3	−103.6
	−1.251	−0.992	−4.998	10.340	−3.057	9.427	−1.859	11.467	−5.202	−3.024
c_O	911	1117	1545	939	944	1506	1153	863	1775	904
	(26.5)	(32.5)	(45.0)	(27.4)	(27.5)	(43.9)	(33.6)	(25.2)	(51.8)	(26.4)
c_E	1002.7	922.3	1659.1	1001.8	921.5	1657.7	1001.0	920.7	1656.2	1000.7
	(29.2)	(26.9)	(48.3)	(29.2)	(26.9)	(48.3)	(29.2)	(26.9)	(48.3)	(29.2)
	−174.8	427.9	−220.2	−121.7	45.6	−289.0	326.1	−111.7	245.9	−183.7
	−5.090	12.461	−6.413	−3.546	1.329	−8.424	9.512	−3.257	7.172	−5.360
N	3434	3434	3434	3431	3431	3431	3428	3428	3428	3427
X2	28.8	63.1	196.8	35.5	24.8	31.0	38.0	56.0	17.3	81.7
Gvalue	28.0	61.6	158.4	35.2	24.7	30.9	37.5	52.9	17.4	76.5
Gv/N	0.816	1.793	4.614	1.027	0.719	0.900	1.095	1.544	0.506	2.234
Prob	0.00000	0.00000	0.00000	0.00000	0.00002	0.00000	0.00000	0.00000	0.00060	0.00000

	131	132	133	134	135	136	137	138	139	140
a_O	981	371	547	802	174	555	768	150	538	703
	(28.6)	(10.8)	(16.0)	(23.4)	(5.1)	(16.2)	(22.4)	(4.4)	(15.7)	(20.5)
a_E	837.6	173.4	643.2	836.8	173.3	643.0	836.6	173.2	643.0	836.6
	(24.4)	(5.1)	(18.8)	(24.4)	(5.1)	(18.8)	(24.4)	(5.1)	(18.8)	(24.4)
	310.1	564.4	−177.3	−68.2	1.5	−163.4	−131.4	−43.1	−191.9	−244.6
	9.050	16.468	−5.177	−1.992	0.044	−4.774	−3.839	−1.260	−5.605	−7.146
t_O	914	208	635	881	194	422	1102	211	398	907
	(26.7)	(6.1)	(18.5)	(25.7)	(5.7)	(12.3)	(32.2)	(6.2)	(11.6)	(26.5)
t_E	973.2	225.9	410.3	972.3	225.7	410.2	972.1	225.6	410.2	972.1
	(28.4)	(6.6)	(12.0)	(28.4)	(6.6)	(12.0)	(28.4)	(6.6)	(12.0)	(28.4)
	−114.7	−34.4	554.5	−173.8	−58.7	23.9	276.5	−28.3	−24.1	−125.7
	−3.347	−1.002	16.195	−5.076	−1.715	0.698	8.079	−0.827	−0.703	−3.671
g_O	693	1317	1348	861	1460	1305	668	1496	1564	882
	(20.2)	(38.4)	(39.4)	(25.1)	(42.6)	(38.1)	(19.5)	(43.7)	(45.7)	(25.8)
g_E	695.8	1371.9	1370.6	695.2	1370.7	1370.2	695.0	1370.3	1370.2	695.0
	(20.3)	(40.0)	(40.0)	(20.3)	(40.0)	(40.0)	(20.3)	(40.0)	(40.0)	(20.3)
	−5.7	−107.7	−44.9	368.3	184.2	−127.3	−53.0	262.5	413.7	420.3
	−0.165	−3.141	−1.312	10.755	5.380	−3.720	−1.548	7.669	12.086	12.278
c_O	839	1531	894	880	1596	1141	885	1566	923	931
	(24.5)	(44.7)	(26.1)	(25.7)	(46.6)	(33.3)	(25.9)	(45.7)	(27.0)	(27.2)
c_E	920.4	1655.7	999.8	919.6	1654.3	999.5	919.3	1653.8	999.5	919.3
	(26.9)	(48.3)	(29.2)	(26.9)	(48.3)	(29.2)	(26.9)	(48.3)	(29.2)	(26.9)
	−155.4	−239.9	−200.0	−77.5	−114.5	302.1	−67.4	−170.9	−147.0	23.5
	−4.534	−6.999	−5.841	−2.262	−3.345	8.827	−1.968	−4.992	−4.295	0.686
N	3427	3427	3424	3424	3424	3423	3423	3423	3423	3423
X2	35.4	238.2	149.0	51.3	12.3	35.5	25.3	20.2	50.8	76.1
Gvalue	34.4	182.5	132.4	48.8	12.5	35.3	24.8	20.2	50.7	73.5
Gv/N	1.003	5.325	3.866	1.425	0.364	1.031	0.724	0.589	1.482	2.147
Prob	0.00000	0.00000	0.00000	0.00000	0.00599	0.00000	0.00002	0.00016	0.00000	0.00000

	141	142	143	144	145	146	147	148	149	150
a_O	154	603	762	146	562	770	152	562	798	170
	(4.5)	(17.6)	(22.3)	(4.3)	(16.4)	(22.5)	(4.4)	(16.4)	(23.3)	(5.0)
a_E	173.2	642.6	836.1	173.1	642.5	835.9	173.0	642.5	835.9	173.0
	(5.1)	(18.8)	(24.4)	(5.1)	(18.8)	(24.4)	(5.1)	(18.8)	(24.4)	(5.1)
	−36.2	−76.8	−141.4	−49.7	−150.4	−126.4	−39.4	−150.4	−74.0	−6.0
	−1.057	−2.245	−4.134	−1.453	−4.397	−3.696	−1.153	−4.397	−2.163	−0.177
t_O	199	380	889	200	604	1132	205	425	1067	212
	(5.8)	(11.1)	(26.0)	(5.8)	(17.7)	(33.1)	(6.0)	(12.4)	(31.2)	(6.2)
t_E	225.6	410.0	971.5	225.5	409.9	971.2	225.4	409.9	971.2	225.4
	(6.6)	(12.0)	(28.4)	(6.6)	(12.0)	(28.4)	(6.6)	(12.0)	(28.4)	(6.6)
	−50.0	−57.7	−157.8	−48.0	468.4	346.9	−39.0	30.8	200.7	−26.1
	−1.461	−1.687	−4.612	−1.404	13.696	10.142	−1.140	0.901	5.870	−0.762
g_O	1307	1248	906	1328	1339	665	1261	1298	675	1480
	(38.2)	(36.5)	(26.5)	(38.8)	(39.2)	(19.4)	(36.9)	(38.0)	(19.7)	(43.3)
g_E	1370.3	1369.4	694.6	1369.5	1369.0	694.4	1369.1	1369.0	694.4	1369.1
	(40.0)	(40.0)	(20.3)	(40.0)	(40.0)	(20.3)	(40.0)	(40.0)	(20.3)	(40.0)
	−123.7	−231.8	481.4	−81.8	−59.4	−57.6	−207.5	−138.3	−38.3	230.5
	−3.614	−6.775	14.072	−2.391	−1.738	−1.683	−6.067	−4.045	−1.119	6.739
c_O	1763	1190	864	1747	915	853	1802	1135	880	1558
	(51.5)	(34.8)	(25.3)	(51.1)	(26.8)	(24.9)	(52.7)	(33.2)	(25.7)	(45.6)
c_E	1653.8	998.9	918.8	1652.8	998.6	918.5	1652.4	998.6	918.5	1652.4
	(48.3)	(29.2)	(26.9)	(48.3)	(29.2)	(26.9)	(48.3)	(29.2)	(26.9)	(48.3)
	225.4	416.6	−106.3	193.6	−160.1	−126.3	312.4	290.6	−75.4	−183.2
	6.586	12.177	−3.106	5.658	−4.680	−3.692	9.135	8.496	−2.205	−5.358
N	3423	3421	3421	3421	3420	3420	3420	3420	3420	3420
X2	15.4	52.0	81.2	13.8	109.7	37.7	26.5	32.9	13.3	15.2
Gvalue	15.5	50.3	76.0	14.0	98.5	36.6	26.5	32.7	13.1	15.1
Gv/N	0.453	1.469	2.220	0.410	2.881	1.072	0.775	0.955	0.382	0.442
Prob	0.00142	0.00000	0.00000	0.00288	0.00000	0.00000	0.00001	0.00000	0.00446	0.00172

Bpse_K96243
5UTR:

	50	49	48	47	46	45	44	43	42	41
a_O	889	959	935	889	949	908	1003	985	894	933
	(15.5)	(16.8)	(16.3)	(15.5)	(16.6)	(15.9)	(17.5)	(17.2)	(15.6)	(16.3)
a_E	1048.7	1048.7	1048.7	1048.7	1048.7	1048.7	1048.7	1048.7	1048.7	1048.7
	(18.3)	(18.3)	(18.3)	(18.3)	(18.3)	(18.3)	(18.3)	(18.3)	(18.3)	(18.3)
	-293.7	-171.4	-214.5	-293.7	-189.5	-261.5	-89.3	-123.4	-285.3	-218.0
	-5.132	-2.996	-3.749	-5.132	-3.312	-4.571	-1.560	-2.156	-4.986	-3.811
t_O	903	1003	950	882	1009	911	910	1051	953	911
	(15.8)	(17.5)	(16.6)	(15.4)	(17.6)	(15.9)	(15.9)	(18.4)	(16.7)	(15.9)
t_E	969.8	969.8	969.8	969.8	969.8	969.8	969.8	969.8	969.8	969.8
	(16.9)	(16.9)	(16.9)	(16.9)	(16.9)	(16.9)	(16.9)	(16.9)	(16.9)	(16.9)
	-128.9	67.5	-39.2	-167.4	79.9	-114.0	-115.9	169.0	-33.3	-114.0
	-2.253	1.179	-0.686	-2.926	1.397	-1.992	-2.025	2.953	-0.583	-1.992
g_O	1944	1850	1901	1907	1904	1874	1899	1712	1917	1901
	(34.0)	(32.3)	(33.2)	(33.3)	(33.3)	(32.8)	(33.2)	(29.9)	(33.5)	(33.2)
g_E	1804.6	1804.6	1804.6	1804.6	1804.6	1804.6	1804.6	1804.6	1804.6	1804.6
	(31.5)	(31.5)	(31.5)	(31.5)	(31.5)	(31.5)	(31.5)	(31.5)	(31.5)	(31.5)
	289.4	92.0	197.9	210.6	204.2	141.5	193.7	-180.3	231.7	197.9
	5.057	1.608	3.459	3.680	3.569	2.473	3.385	-3.151	4.050	3.459
c_O	1986	1910	1936	2044	1860	2029	1910	1974	1958	1977
	(34.7)	(33.4)	(33.8)	(35.7)	(32.5)	(35.5)	(33.4)	(34.5)	(34.2)	(34.6)
c_E	1899.0	1899.0	1899.0	1899.0	1899.0	1899.0	1899.0	1899.0	1899.0	1899.0
	(33.2)	(33.2)	(33.2)	(33.2)	(33.2)	(33.2)	(33.2)	(33.2)	(33.2)	(33.2)
	178.0	22.1	74.8	300.9	-77.1	268.8	22.1	153.0	119.9	159.2
	3.111	0.387	1.307	5.258	-1.348	4.697	0.387	2.674	2.095	2.783
N	5722	5722	5722	5722	5722	5722	5722	5722	5722	5722
X2	43.7	10.0	18.6	49.2	17.3	34.0	10.7	18.4	31.9	24.7
Gvalue	44.8	10.2	19.0	50.4	17.5	34.8	10.7	18.3	33.0	25.1
Gv/N	0.783	0.178	0.332	0.880	0.306	0.607	0.187	0.320	0.577	0.439
Prob	0.00000	0.01684	0.00028	0.00000	0.00055	0.00000	0.01343	0.00038	0.00000	0.00001

	40	39	38	37	36	35	34	33	32	31
a_O	950	879	1033	1047	881	984	1038	895	1044	1066
	(16.6)	(15.4)	(18.1)	(18.3)	(15.4)	(17.2)	(18.1)	(15.6)	(18.2)	(18.6)
a_E	1048.7	1048.7	1048.7	1048.7	1048.7	1048.7	1048.7	1048.7	1048.7	1048.7
	(18.3)	(18.3)	(18.3)	(18.3)	(18.3)	(18.3)	(18.3)	(18.3)	(18.3)	(18.3)
	-187.7	-310.2	-31.1	-3.3	-306.9	-125.2	-21.2	-283.6	-9.3	35.0
	-3.281	-5.422	-0.543	-0.058	-5.364	-2.189	-0.370	-4.956	-0.162	0.611
t_O	1111	928	925	1081	983	959	1089	989	939	1098
	(19.4)	(16.2)	(16.2)	(18.9)	(17.2)	(16.8)	(19.0)	(17.3)	(16.4)	(19.2)
t_E	969.8	969.8	969.8	969.8	969.8	969.8	969.8	969.8	969.8	969.8
	(16.9)	(16.9)	(16.9)	(16.9)	(16.9)	(16.9)	(16.9)	(16.9)	(16.9)	(16.9)
	302.0	-81.8	-87.5	234.6	26.5	-21.5	252.4	38.7	-60.7	272.6
	5.278	-1.430	-1.530	4.101	0.464	-0.376	4.412	0.677	-1.060	4.764
g_O	1706	1863	1748	1733	1831	1822	1659	1803	1779	1610
	(29.8)	(32.6)	(30.5)	(30.3)	(32.0)	(31.8)	(29.0)	(31.5)	(31.1)	(28.1)
g_E	1804.6	1804.6	1804.6	1804.6	1804.6	1804.6	1804.6	1804.6	1804.6	1804.6
	(31.5)	(31.5)	(31.5)	(31.5)	(31.5)	(31.5)	(31.5)	(31.5)	(31.5)	(31.5)
	-191.7	118.7	-111.3	-140.3	53.2	35.0	-279.1	-3.1	-50.8	-367.4
	-3.349	2.075	-1.946	-2.451	0.931	0.612	-4.877	-0.055	-0.887	-6.420
c_O	1955	2052	2016	1861	2027	1957	1936	2035	1960	1948
	(34.2)	(35.9)	(35.2)	(32.5)	(35.4)	(34.2)	(33.8)	(35.6)	(34.3)	(34.0)
c_E	1899.0	1899.0	1899.0	1899.0	1899.0	1899.0	1899.0	1899.0	1899.0	1899.0
	(33.2)	(33.2)	(33.2)	(33.2)	(33.2)	(33.2)	(33.2)	(33.2)	(33.2)	(33.2)
	113.7	318.1	241.1	-75.2	264.5	117.8	74.8	281.6	124.0	99.3
	1.987	5.559	4.214	-1.313	4.623	2.059	1.307	4.921	2.167	1.736
N	5722	5722	5722	5722	5722	5722	5722	5722	5722	5722
X2	36.9	43.5	11.3	16.3	36.0	6.0	27.2	32.6	3.3	39.5
Gvalue	36.3	44.8	11.2	15.9	37.4	6.1	27.0	33.6	3.3	39.6
Gv/N	0.635	0.783	0.196	0.278	0.653	0.107	0.471	0.587	0.058	0.691
Prob	0.00000	0.00000	0.01069	0.00117	0.00000	0.10613	0.00001	0.00000	0.34542	0.00000

	30	29	28	27	26	25	24	23	22	21
a_O	912	1021	1057	999	1018	1105	1029	1114	1173	1079
	(15.9)	(17.8)	(18.5)	(17.5)	(17.8)	(19.3)	(18.0)	(19.5)	(20.5)	(18.9)
a_E	1048.7	1048.7	1048.7	1048.7	1048.7	1048.7	1048.7	1048.7	1048.7	1048.7
	(18.3)	(18.3)	(18.3)	(18.3)	(18.3)	(18.3)	(18.3)	(18.3)	(18.3)	(18.3)
	-254.7	-54.6	16.8	-96.9	-60.4	115.7	-38.9	134.7	262.9	61.6
	-4.451	-0.954	0.293	-1.694	-1.055	2.022	-0.680	2.354	4.594	1.076
t_O	1017	1005	1118	1082	1031	1120	1046	981	1161	1136
	(17.8)	(17.6)	(19.5)	(18.9)	(18.0)	(19.6)	(18.3)	(17.1)	(20.3)	(19.9)
t_E	969.8	969.8	969.8	969.8	969.8	969.8	969.8	969.8	969.8	969.8
	(16.9)	(16.9)	(16.9)	(16.9)	(16.9)	(16.9)	(16.9)	(16.9)	(16.9)	(16.9)
	96.6	71.6	317.9	236.9	126.1	322.5	158.2	22.5	417.8	359.3
	1.689	1.252	5.556	4.140	2.204	5.636	2.765	0.393	7.301	6.280
g_O	1790	1738	1571	1672	1709	1525	1651	1612	1369	1445
	(31.3)	(30.4)	(27.5)	(29.2)	(29.9)	(26.7)	(28.9)	(28.2)	(23.9)	(25.3)
g_E	1804.6	1804.6	1804.6	1804.6	1804.6	1804.6	1804.6	1804.6	1804.6	1804.6
	(31.5)	(31.5)	(31.5)	(31.5)	(31.5)	(31.5)	(31.5)	(31.5)	(31.5)	(31.5)
	-29.0	-130.7	-435.5	-255.2	-186.0	-513.4	-293.7	-363.8	-756.4	-642.2
	-0.507	-2.283	-7.611	-4.459	-3.250	-8.972	-5.133	-6.358	-13.218	-11.223
c_O	2003	1958	1976	1969	1964	1972	1996	2015	2019	2062
	(35.0)	(34.2)	(34.5)	(34.4)	(34.3)	(34.5)	(34.9)	(35.2)	(35.3)	(36.0)
c_E	1899.0	1899.0	1899.0	1899.0	1899.0	1899.0	1899.0	1899.0	1899.0	1899.0
	(33.2)	(33.2)	(33.2)	(33.2)	(33.2)	(33.2)	(33.2)	(33.2)	(33.2)	(33.2)
	213.7	119.9	157.2	142.6	132.3	148.9	199.0	239.0	247.5	339.7
	3.734	2.095	2.747	2.493	2.312	2.601	3.477	4.177	4.326	5.937
N	5722	5722	5722	5722	5722	5722	5722	5722	5722	5722
X2	25.9	6.3	56.1	27.6	12.0	72.4	24.4	31.8	165.2	115.0
Gvalue	26.6	6.3	56.3	27.4	12.0	73.6	24.5	32.4	171.8	118.4
Gv/N	0.465	0.110	0.985	0.479	0.210	1.287	0.429	0.566	3.003	2.069
Prob	0.00001	0.09790	0.00000	0.00000	0.00724	0.00000	0.00002	0.00000	0.00000	0.00000

	20	19	18	17	16	15	14	13	12	11
a_O	1146	1171	1171	1258	1308	1311	1469	1506	1469	1575
	(20.0)	(20.5)	(20.5)	(22.0)	(22.9)	(22.9)	(25.7)	(26.3)	(25.7)	(27.5)
a_E	1048.7	1048.7	1048.7	1048.7	1048.7	1048.7	1048.7	1048.7	1048.7	1048.7
	(18.3)	(18.3)	(18.3)	(18.3)	(18.3)	(18.3)	(18.3)	(18.3)	(18.3)	(18.3)
	203.5	258.5	258.5	458.0	578.1	585.5	990.3	1090.2	990.3	1281.3
	3.556	4.517	4.517	8.004	10.104	10.232	17.308	19.053	17.308	22.392
t_O	1125	1181	1164	1128	1216	1077	970	945	739	590
	(19.7)	(20.6)	(20.3)	(19.7)	(21.3)	(18.8)	(17.0)	(16.5)	(12.9)	(10.3)

```
t_E      969.8   969.8   969.8   969.8   969.8   969.8   969.8   969.8   969.8   969.8
        ( 16.9) ( 16.9) ( 16.9) ( 16.9) ( 16.9) ( 16.9) ( 16.9) ( 16.9) ( 16.9) ( 16.9)
         334.0   465.3   424.9   340.9   550.1   225.8     0.4   -49.0  -401.7  -586.4
         5.836   8.132   7.425   5.957   9.615   3.946   0.006  -0.856  -7.021 -10.249
g_O       1483    1350    1464    1436    1338    1554    1547    1630    2008    2258
        ( 25.9) ( 23.6) ( 25.6) ( 25.1) ( 23.4) ( 27.2) ( 27.0) ( 28.5) ( 35.1) ( 39.5)
g_E     1804.6  1804.6  1804.6  1804.6  1804.6  1804.6  1804.6  1804.6  1804.6  1804.6
        ( 31.5) ( 31.5) ( 31.5) ( 31.5) ( 31.5) ( 31.5) ( 31.5) ( 31.5) ( 31.5) ( 31.5)
        -582.1  -783.6  -612.4  -656.1  -800.5  -464.6  -476.5  -331.7   429.0  1012.3
       -10.173 -13.694 -10.702 -11.467 -13.990  -8.120  -8.327  -5.797   7.497  17.691
c_O       1968    2020    1923    1900    1860    1780    1736    1641    1506    1299
        ( 34.4) ( 35.3) ( 33.6) ( 33.2) ( 32.5) ( 31.1) ( 30.3) ( 28.7) ( 26.3) ( 22.7)
c_E     1899.0  1899.0  1899.0  1899.0  1899.0  1899.0  1899.0  1899.0  1899.0  1899.0
        ( 33.2) ( 33.2) ( 33.2) ( 33.2) ( 33.2) ( 33.2) ( 33.2) ( 33.2) ( 33.2) ( 33.2)
         140.6   249.6    48.4     2.1   -77.1  -230.3  -311.5  -479.2  -698.3  -986.5
         2.456   4.363   0.846   0.036  -1.348  -4.025  -5.444  -8.374 -12.204 -17.240

N         5722    5722    5722    5722    5722    5722    5722    5722    5722    5722
X2        93.7   182.5   117.7   142.9   248.1   119.7   219.2   252.0   327.7   716.4
Gvalue    95.9   189.8   119.3   144.8   250.6   116.3   202.7   230.4   319.2   720.6
Gv/N     1.676   3.318   2.085   2.530   4.380   2.033   3.542   4.026   5.579  12.594
Prob   0.00000 0.00000 0.00000 0.00000 0.00000 0.00000 0.00000 0.00000 0.00000 0.00000
       -------------------------------------------------------------------------------
            10       9       8       7       6       5       4       3       2       1
       -------------------------------------------------------------------------------
a_O       1395    1464    1604    1513    1464    1394    1211    1524    1100     740
        ( 24.4) ( 25.6) ( 28.0) ( 26.4) ( 25.6) ( 24.4) ( 21.2) ( 26.6) ( 19.2) ( 12.9)
a_E     1048.7  1048.7  1048.7  1048.7  1048.7  1048.7  1048.7  1048.7  1048.7  1048.7
        ( 18.3) ( 18.3) ( 18.3) ( 18.3) ( 18.3) ( 18.3) ( 18.3) ( 18.3) ( 18.3) ( 18.3)
         796.2   977.0  1363.4  1109.3   977.0   793.7   348.6  1139.4   105.2  -515.9
        13.915  17.074  23.827  19.387  17.074  13.871   6.093  19.913   1.838  -9.017
t_O        638     587     690     911     870    1066     961     741    1059     532
        ( 11.1) ( 10.3) ( 12.1) ( 15.9) ( 15.2) ( 18.6) ( 16.8) ( 13.0) ( 18.5) (  9.3)
t_E      969.8   969.8   969.8   969.8   969.8   969.8   969.8   969.8   969.8   969.8
        ( 16.9) ( 16.9) ( 16.9) ( 16.9) ( 16.9) ( 16.9) ( 16.9) ( 16.9) ( 16.9) ( 16.9)
        -534.4  -589.4  -469.8  -114.0  -189.0   201.6   -17.6  -398.8   186.3  -638.9
        -9.339 -10.301  -8.210  -1.992  -3.303   3.523  -0.307  -6.970   3.256 -11.166
g_O       2580    2659    2195    1882    1749    1379    1430    1864    1162    1582
        ( 45.1) ( 46.5) ( 38.4) ( 32.9) ( 30.6) ( 24.1) ( 25.0) ( 32.6) ( 20.3) ( 27.6)
g_E     1804.6  1804.6  1804.6  1804.6  1804.6  1804.6  1804.6  1804.6  1804.6  1804.6
        ( 31.5) ( 31.5) ( 31.5) ( 31.5) ( 31.5) ( 31.5) ( 31.5) ( 31.5) ( 31.5) ( 31.5)
        1844.5  2061.4   859.8   158.1  -109.4  -741.8  -665.4   120.8 -1023.0  -416.5
        32.236  36.026  15.027   2.764  -1.912 -12.964 -11.628   2.111 -17.878  -7.279
c_O       1109    1012    1233    1416    1639    1883    2120    1593    2401    2868
        ( 19.4) ( 17.7) ( 21.5) ( 24.7) ( 28.6) ( 32.9) ( 37.0) ( 27.8) ( 42.0) ( 50.1)
c_E     1899.0  1899.0  1899.0  1899.0  1899.0  1899.0  1899.0  1899.0  1899.0  1899.0
        ( 33.2) ( 33.2) ( 33.2) ( 33.2) ( 33.2) ( 33.2) ( 33.2) ( 33.2) ( 33.2) ( 33.2)
       -1192.9 -1273.9 -1065.0  -831.1  -482.6   -31.8   466.9  -559.7  1126.4  2365.0
       -20.848 -22.263 -18.612 -14.525  -8.434  -0.555   8.159  -9.782  19.686  41.332

N         5722    5722    5722    5722    5722    5722    5722    5722    5722    5722
X2       889.7  1134.5   692.9   335.3   212.1   223.8   128.7   320.7   372.2   810.4
Gvalue   913.5  1175.1   688.5   322.3   196.0   221.7   132.6   301.7   395.0   793.7
Gv/N    15.964  20.536  12.032   5.633   3.425   3.874   2.317   5.272   6.903  13.871
Prob   0.00000 0.00000 0.00000 0.00000 0.00000 0.00000 0.00000 0.00000 0.00000 0.00000
       -------------------------------------------------------------------------------

CDS_INITIAL:
       -------------------------------------------------------------------------------
             1       2       3       4       5       6       7       8       9      10
       -------------------------------------------------------------------------------
a_O       4655       0       1    2657    1790    1032    1736    1735    1006    1635
        ( 81.4) (  0.0) (  0.0) ( 46.4) ( 31.3) ( 18.0) ( 30.3) ( 30.3) ( 17.6) ( 28.6)
a_E     1076.8  1358.5   276.4  1076.8  1358.5   276.4  1076.8  1358.5   276.4  1076.8
        ( 18.8) ( 23.7) (  4.8) ( 18.8) ( 23.7) (  4.8) ( 18.8) ( 23.7) (  4.8) ( 18.8)
       13629.2     0.0   -11.2  4799.5   987.4  2719.1  1658.1   848.8  2599.2  1365.6
       238.189   0.000  -0.196  83.878  17.257  47.520  28.978  14.834  45.426  23.867
t_O        388    5722       0     645     778    1009     727    1287     979     906
        (  6.8) (100.0) (  0.0) ( 11.3) ( 13.6) ( 17.6) ( 12.7) ( 22.5) ( 17.1) ( 15.8)
t_E      719.4  1580.6   401.7   719.4  1580.6   401.7   719.4  1580.6   401.7   719.4
        ( 12.6) ( 27.6) (  7.0) ( 12.6) ( 27.6) (  7.0) ( 12.6) ( 27.6) (  7.0) ( 12.6)
        -479.1 14723.2     0.0  -140.8 -1102.9  1858.8    15.3  -528.9  1744.4   417.9
        -8.373 257.309   0.000  -2.461 -19.275  32.485   0.267  -9.242  30.486   7.303
g_O        679       0    5721    1097    1130    1531    1703     971    1596    1496
        ( 11.9) (  0.0) (100.0) ( 19.2) ( 19.7) ( 26.8) ( 29.8) ( 17.0) ( 27.9) ( 26.1)
g_E     2336.3  1156.4  2463.1  2336.3  1156.4  2463.1  2336.3  1156.4  2463.1  2336.3
        ( 40.8) ( 20.2) ( 43.0) ( 40.8) ( 20.2) ( 43.0) ( 40.8) ( 20.2) ( 43.0) ( 40.8)
       -1678.1     0.0  9642.3 -1658.6   -52.1 -1456.0 -1076.8  -339.3 -1385.1 -1333.7
       -29.327   0.000 168.513 -28.987  -0.911 -25.446 -18.819  -5.929 -24.207 -23.308
c_O          0       0       0    1323    2024    2150    1556    1729    2141    1685
        (  0.0) (  0.0) (  0.0) ( 23.1) ( 35.4) ( 37.6) ( 27.2) ( 30.2) ( 37.4) ( 29.4)
c_E     1589.5  1626.6  2580.8  1589.5  1626.6  2580.8  1589.5  1626.6  2580.8  1589.5
        ( 27.8) ( 28.4) ( 45.1) ( 27.8) ( 28.4) ( 45.1) ( 27.8) ( 28.4) ( 45.1) ( 27.8)
           0.0     0.0     0.0  -485.6   884.9  -785.3   -66.3   211.2  -800.0   196.6
         0.000   0.000   0.000  -8.486  15.465 -13.725  -1.159   3.690 -13.981   3.436

N         5722    5722    5722    5722    5722    5722    5722    5722    5722    5722
X2     14807.7 14993.1  7565.9  3028.6   642.3  3408.5   576.0   195.0  3135.9   645.7
Gvalue 11472.0 14723.2  9631.1  2514.5   717.3  2336.5   530.2   191.9  2158.5   646.4
Gv/N   200.489 257.309 168.317  43.944  12.536  40.834   9.267   3.353  37.724  11.297
Prob   1.00000 1.00000 1.00000 1.00000 0.00000 1.00000 0.00000 0.00000 1.00000 0.00000
       -------------------------------------------------------------------------------
            11      12      13      14      15      16      17      18      19      20
       -------------------------------------------------------------------------------
a_O       1427     909    1503    1433     836    1420    1424     823    1343    1395
        ( 24.9) ( 15.9) ( 26.3) ( 25.0) ( 14.6) ( 24.8) ( 24.9) ( 14.4) ( 23.5) ( 24.4)
a_E     1358.5   276.4  1076.8  1358.5   276.4  1076.8  1358.5   276.4  1076.8  1358.5
        ( 23.7) (  4.8) ( 18.8) ( 23.7) (  4.8) ( 18.8) ( 23.7) (  4.8) ( 18.8) ( 23.7)
         140.4  2164.3  1002.3   153.0  1850.5   785.7   134.1  1795.9   593.3    73.9
         2.453  37.824  17.517   2.673  32.340  13.731   2.343  31.387  10.369   1.292
t_O       1544     936     869    1703     970     902    1737     919     838    1620
        ( 27.0) ( 16.4) ( 15.2) ( 29.8) ( 17.0) ( 15.8) ( 30.4) ( 16.1) ( 14.6) ( 28.3)
t_E     1580.6   401.7   719.4  1580.6   401.7   719.4  1580.6   401.7   719.4  1580.6
        ( 27.6) (  7.0) ( 12.6) ( 27.6) (  7.0) ( 12.6) ( 27.6) (  7.0) ( 12.6) ( 27.6)
         -72.3  1583.7   328.3   254.1  1710.5   408.0   327.9  1521.2   255.7    79.9
        -1.263  27.677   5.738   4.442  29.893   7.131   5.730  26.586   4.469   1.396
g_O        925    1554    1665     884    1602    1667     904    1633    1872     974
        ( 16.2) ( 27.2) ( 29.1) ( 15.4) ( 28.0) ( 29.1) ( 15.8) ( 28.5) ( 32.7) ( 17.0)
g_E     1156.4  2463.1  2336.3  1156.4  2463.1  2336.3  1156.4  2463.1  2336.3  1156.4
        ( 20.2) ( 43.0) ( 40.8) ( 20.2) ( 43.0) ( 40.8) ( 20.2) ( 43.0) ( 40.8) ( 20.2)
        -413.0 -1431.5 -1128.0  -474.8 -1378.3 -1125.3  -445.1 -1342.4  -829.5  -334.3
        -7.218 -25.018 -19.713  -8.298 -24.088 -19.667  -7.779 -23.460 -14.496  -5.843
```

c_O	1826	2323	1685	1702	2314	1733	1657	2347	1669	1733
	(31.9)	(40.6)	(29.4)	(29.7)	(40.4)	(30.3)	(29.0)	(41.0)	(29.2)	(30.3)
c_E	1626.6	2580.8	1589.5	1626.6	2580.8	1589.5	1626.6	2580.8	1589.5	1626.6
	(28.4)	(45.1)	(27.8)	(28.4)	(45.1)	(27.8)	(28.4)	(45.1)	(27.8)	(28.4)
	422.4	-489.0	196.6	154.3	-505.0	299.6	61.4	-445.8	162.9	219.7
	7.381	-8.545	3.436	2.697	-8.826	5.236	1.073	-7.790	2.847	3.839
N	5722	5722	5722	5722	5722	5722	5722	5722	5722	5722
X2	75.0	2519.9	398.4	81.2	2265.7	360.4	74.3	2048.2	181.6	37.7
Gvalue	77.5	1827.5	399.3	86.6	1677.6	368.0	78.3	1529.1	182.5	39.2
Gv/N	1.354	31.938	6.979	1.513	29.319	6.431	1.368	26.723	3.189	0.684
Prob	0.00000	1.00000	0.00000	0.00000	1.00000	0.00000	0.00000	1.00000	0.00000	0.00000

	21	22	23	24	25	26	27	28	29	30
a_O	709	1206	1269	583	1123	1177	526	1091	1182	489
	(12.4)	(21.1)	(22.2)	(10.2)	(19.6)	(20.6)	(9.2)	(19.1)	(20.7)	(8.5)
a_E	276.4	1076.8	1358.5	276.4	1076.8	1358.5	276.4	1076.8	1358.5	276.4
	(4.8)	(18.8)	(23.7)	(4.8)	(18.8)	(23.7)	(4.8)	(18.8)	(23.7)	(4.8)
	1335.7	273.3	-173.0	870.2	94.3	-337.6	676.9	28.5	-329.0	557.9
	23.344	4.776	-3.023	15.208	1.648	-5.900	11.830	0.499	-5.750	9.751
t_O	891	869	1566	748	804	1557	698	779	1588	681
	(15.6)	(15.2)	(27.4)	(13.1)	(14.1)	(27.2)	(12.2)	(13.6)	(27.8)	(11.9)
t_E	401.7	719.4	1580.6	401.7	719.4	1580.6	401.7	719.4	1580.6	401.7
	(7.0)	(12.6)	(27.6)	(7.0)	(12.6)	(27.6)	(7.0)	(12.6)	(27.6)	(7.0)
	1419.8	328.3	-29.0	930.2	178.8	-46.8	771.4	124.0	14.9	719.1
	24.812	5.738	-0.506	16.256	3.124	-0.817	13.482	2.167	0.261	12.567
g_O	1740	1866	1021	1920	2114	1133	2015	2088	1137	2009
	(30.4)	(32.6)	(17.8)	(33.6)	(36.9)	(19.8)	(35.2)	(36.5)	(19.9)	(35.1)
g_E	2463.1	2336.3	1156.4	2463.1	2336.3	1156.4	2463.1	2336.3	1156.4	2463.1
	(43.0)	(40.8)	(20.2)	(43.0)	(40.8)	(20.2)	(43.0)	(40.8)	(20.2)	(43.0)
	-1209.5	-838.8	-254.2	-956.6	-422.7	-46.2	-809.3	-469.2	-38.4	-818.8
	-21.137	-14.659	-4.443	-16.717	-7.387	-0.808	-14.143	-8.199	-0.671	-14.310
c_O	2382	1781	1866	2471	1681	1855	2483	1764	1815	2543
	(41.6)	(31.1)	(32.6)	(43.2)	(29.4)	(32.4)	(43.4)	(30.8)	(31.7)	(44.4)
c_E	2580.8	1589.5	1626.6	2580.8	1589.5	1626.6	2580.8	1589.5	1626.6	2580.8
	(45.1)	(27.8)	(28.4)	(45.1)	(27.8)	(28.4)	(45.1)	(27.8)	(28.4)	(45.1)
	-381.9	405.2	512.5	-214.9	188.2	487.5	-191.9	367.5	397.9	-75.1
	-6.674	7.081	8.956	-3.755	3.288	8.520	-3.353	6.422	6.954	-1.312
N	5722	5722	5722	5722	5722	5722	5722	5722	5722	5722
X2	1500.8	164.3	57.1	763.1	38.3	57.2	529.2	50.7	45.1	442.1
Gvalue	1164.2	168.0	56.3	629.0	38.5	56.9	447.2	50.9	45.4	383.1
Gv/N	20.345	2.936	0.984	10.992	0.674	0.995	7.815	0.889	0.793	6.695
Prob	0.00000	0.00000	0.00000	0.00000	0.00000	0.00000	0.00000	0.00000	0.00000	0.00000

	31	32	33	34	35	36	37	38	39	40
a_O	1011	1133	516	1012	1122	470	1003	1097	456	968
	(17.7)	(19.8)	(9.0)	(17.7)	(19.6)	(8.2)	(17.5)	(19.2)	(8.0)	(16.9)
a_E	1076.8	1358.5	276.4	1076.8	1358.5	276.4	1076.8	1358.5	276.4	1076.8
	(18.8)	(23.7)	(4.8)	(18.8)	(23.7)	(4.8)	(18.8)	(23.7)	(4.8)	(18.8)
	-127.5	-411.3	644.2	-125.7	-429.2	499.0	-142.5	-469.1	456.6	-206.3
	-2.229	-7.189	11.259	-2.196	-7.501	8.721	-2.490	-8.198	7.979	-3.605
t_O	780	1600	597	706	1565	572	759	1610	556	698
	(13.6)	(28.0)	(10.4)	(12.3)	(27.4)	(10.0)	(13.3)	(28.1)	(9.7)	(12.2)
t_E	719.4	1580.6	401.7	719.4	1580.6	401.7	719.4	1580.6	401.7	719.4
	(12.6)	(27.6)	(7.0)	(12.6)	(27.6)	(7.0)	(12.6)	(27.6)	(7.0)	(12.6)
	126.1	39.1	473.2	-26.6	-30.9	404.4	81.3	59.4	361.6	-42.2
	2.205	0.684	8.269	-0.464	-0.541	7.068	1.421	1.039	6.319	-0.737
g_O	2276	1125	2037	2341	1159	2095	2371	1151	2063	2373
	(39.8)	(19.7)	(35.6)	(40.9)	(20.3)	(36.6)	(41.4)	(20.1)	(36.1)	(41.5)
g_E	2336.3	1156.4	2463.1	2336.3	1156.4	2463.1	2336.3	1156.4	2463.1	2336.3
	(40.8)	(20.2)	(43.0)	(40.8)	(20.2)	(43.0)	(40.8)	(20.2)	(43.0)	(40.8)
	-119.0	-61.9	-773.9	9.5	5.3	-678.3	70.0	-10.7	-731.4	74.0
	-2.079	-1.081	-13.524	0.166	0.092	-11.854	1.223	-0.187	-12.782	1.294
c_O	1655	1864	2572	1663	1876	2585	1589	1864	2647	1683
	(28.9)	(32.6)	(44.9)	(29.1)	(32.8)	(45.2)	(27.8)	(32.6)	(46.3)	(29.4)
c_E	1589.5	1626.6	2580.8	1589.5	1626.6	2580.8	1589.5	1626.6	2580.8	1589.5
	(27.8)	(28.4)	(45.1)	(27.8)	(28.4)	(45.1)	(27.8)	(28.4)	(45.1)	(27.8)
	133.7	507.9	-17.6	150.3	535.3	8.4	-1.0	507.9	134.1	192.4
	2.336	8.877	-0.307	2.627	9.355	0.147	-0.018	8.877	2.343	3.362
N	5722	5722	5722	5722	5722	5722	5722	5722	5722	5722
X2	13.4	73.2	376.4	7.6	79.6	262.9	7.8	85.6	242.7	17.7
Gvalue	13.3	73.9	325.9	7.6	80.4	233.6	7.8	87.6	220.8	18.0
Gv/N	0.233	1.291	5.696	0.133	1.405	4.082	0.137	1.531	3.859	0.315
Prob	0.00402	0.00000	0.00000	0.05525	0.00000	0.00000	0.04956	0.00000	0.00000	0.00044

	41	42	43	44	45	46	47	48	49	50
a_O	1087	432	963	1193	433	1057	1131	417	985	1133
	(19.0)	(7.5)	(16.8)	(20.8)	(7.6)	(18.5)	(19.8)	(7.3)	(17.2)	(19.8)
a_E	1358.5	276.4	1076.8	1358.5	276.4	1076.8	1358.5	276.4	1076.8	1358.5
	(23.7)	(4.8)	(18.8)	(23.7)	(4.8)	(18.8)	(23.7)	(4.8)	(18.8)	(23.7)
	-484.7	385.8	-215.2	-310.0	388.7	-39.3	-414.6	343.0	-175.6	-411.3
	-8.472	6.743	-3.760	-5.418	6.794	-0.686	-7.246	5.994	-3.069	-7.189
t_O	1629	537	742	1551	528	731	1633	558	727	1622
	(28.5)	(9.4)	(13.0)	(27.1)	(9.2)	(12.8)	(28.5)	(9.8)	(12.7)	(28.3)
t_E	1580.6	401.7	719.4	1580.6	401.7	719.4	1580.6	401.7	719.4	1580.6
	(27.6)	(7.0)	(12.6)	(27.6)	(7.0)	(12.6)	(27.6)	(7.0)	(12.6)	(27.6)
	98.4	311.9	45.9	-58.5	288.8	23.4	106.6	366.9	15.3	84.0
	1.719	5.450	0.802	-1.023	5.047	0.408	1.863	6.412	0.267	1.468
g_O	1118	2109	2410	1195	2132	2342	1154	2165	2378	1166
	(19.5)	(36.9)	(42.1)	(20.9)	(37.3)	(40.9)	(20.2)	(37.8)	(41.6)	(20.4)
g_E	1156.4	2463.1	2336.3	1156.4	2463.1	2336.3	1156.4	2463.1	2336.3	1156.4
	(20.2)	(43.0)	(40.8)	(20.2)	(43.0)	(40.8)	(20.2)	(43.0)	(40.8)	(20.2)
	-75.4	-654.7	149.8	78.6	-615.6	11.5	-4.7	-558.6	84.2	19.4
	-1.318	-11.442	2.617	1.373	-10.758	0.201	-0.082	-9.763	1.472	0.338
c_O	1888	2644	1607	1783	2629	1592	1804	2582	1632	1801
	(33.0)	(46.2)	(28.1)	(31.2)	(45.9)	(27.8)	(31.5)	(45.1)	(28.5)	(31.5)
c_E	1626.6	2580.8	1589.5	1626.6	2580.8	1589.5	1626.6	2580.8	1589.5	1626.6
	(28.4)	(45.1)	(27.8)	(28.4)	(45.1)	(27.8)	(28.4)	(45.1)	(27.8)	(28.4)
	562.8	127.9	35.2	327.4	97.3	5.0	373.5	2.4	86.1	366.9
	9.835	2.236	0.615	5.722	1.700	0.087	6.528	0.042	1.505	6.412
N	5722	5722	5722	5722	5722	5722	5722	5722	5722	5722
X2	99.0	185.6	15.3	37.1	173.9	0.6	59.2	168.4	9.8	57.3
Gvalue	101.0	170.9	15.7	37.5	159.2	0.6	60.8	153.6	10.0	58.9
Gv/N	1.765	2.987	0.274	0.655	2.782	0.010	1.063	2.684	0.175	1.030
Prob	0.00000	0.00000	0.00132	0.00000	0.00000	0.90306	0.00000	0.00000	0.01848	0.00000

	51	52	53	54	55	56	57	58	59	60
a_O	380	1003	1115	395	1036	1161	360	1016	1127	360
	(6.6)	(17.5)	(19.5)	(6.9)	(18.1)	(20.3)	(6.3)	(17.8)	(19.7)	(6.3)
a_E	276.4	1076.8	1358.5	276.4	1076.8	1358.5	276.4	1076.8	1358.5	276.4
	(4.8)	(18.8)	(23.7)	(4.8)	(18.8)	(23.7)	(4.8)	(18.8)	(23.7)	(4.8)
	241.9	−142.5	−440.5	282.0	−80.1	−364.8	190.3	−118.1	−421.1	190.3
	4.228	−2.490	−7.699	4.929	−1.399	−6.376	3.325	−2.065	−7.360	3.325
t_O	533	745	1642	495	749	1619	520	767	1617	462
	(9.3)	(13.0)	(28.7)	(8.7)	(13.1)	(28.3)	(9.1)	(13.4)	(28.3)	(8.1)
t_E	401.7	719.4	1580.6	401.7	719.4	1580.6	401.7	719.4	1580.6	401.7
	(7.0)	(12.6)	(27.6)	(7.0)	(12.6)	(27.6)	(7.0)	(12.6)	(27.6)	(7.0)
	301.6	52.1	125.3	206.8	60.4	77.8	268.5	98.3	73.7	129.3
	5.270	0.910	2.189	3.615	1.055	1.360	4.693	1.717	1.289	2.260
g_O	2188	2330	1161	2206	2380	1160	2187	2356	1133	2275
	(38.2)	(40.7)	(20.3)	(38.6)	(41.6)	(20.3)	(38.2)	(41.2)	(19.8)	(39.8)
g_E	2463.1	2336.3	1156.4	2463.1	2336.3	1156.4	2463.1	2336.3	1156.4	2463.1
	(43.0)	(40.8)	(20.2)	(43.0)	(40.8)	(20.2)	(43.0)	(40.8)	(20.2)	(43.0)
	−518.3	−12.5	9.3	−486.4	88.3	7.3	−520.1	39.6	−46.2	−361.5
	−9.058	−0.219	0.163	−8.501	1.543	0.128	−9.089	0.693	−0.808	−6.318
c_O	2621	1644	1804	2626	1557	1782	2655	1583	1845	2625
	(45.8)	(28.7)	(31.5)	(45.9)	(27.2)	(31.1)	(46.4)	(27.7)	(32.2)	(45.9)
c_E	2580.8	1589.5	1626.6	2580.8	1589.5	1626.6	2580.8	1589.5	1626.6	2580.8
	(45.1)	(27.8)	(28.4)	(45.1)	(27.8)	(28.4)	(45.1)	(27.8)	(28.4)	(45.1)
	81.0	110.8	373.5	91.2	−64.3	325.3	150.5	−13.0	465.0	89.1
	1.416	1.937	6.528	1.593	−1.124	5.684	2.630	−0.227	8.126	1.558
N	5722	5722	5722	5722	5722	5722	5722	5722	5722	5722
X2	113.1	7.9	65.4	100.2	4.2	44.5	93.2	6.8	70.1	49.5
Gvalue	106.2	7.9	67.6	93.6	4.3	45.6	89.2	6.8	71.3	47.2
Gv/N	1.856	0.139	1.181	1.637	0.074	0.796	1.559	0.118	1.246	0.825
Prob	0.00000	0.04717	0.00000	0.00000	0.23560	0.00000	0.00000	0.07940	0.00000	0.00000

	61	62	63	64	65	66	67	68	69	70
a_O	949	1194	370	968	1173	381	944	1199	365	939
	(16.6)	(20.9)	(6.5)	(16.9)	(20.5)	(6.7)	(16.5)	(21.0)	(6.4)	(16.4)
a_E	1076.8	1358.5	276.4	1076.8	1358.5	276.4	1076.8	1358.5	276.4	1076.8
	(18.8)	(23.7)	(4.8)	(18.8)	(23.7)	(4.8)	(18.8)	(23.7)	(4.8)	(18.8)
	−239.8	−308.3	215.8	−206.3	−344.5	244.6	−248.5	−299.5	203.0	−257.2
	−4.191	−5.387	3.772	−3.605	−6.020	4.274	−4.344	−5.235	3.547	−4.495
t_O	725	1591	491	672	1558	493	725	1551	449	753
	(12.7)	(27.8)	(8.6)	(11.7)	(27.2)	(8.6)	(12.7)	(27.1)	(7.8)	(13.2)
t_E	719.4	1580.6	401.7	719.4	1580.6	401.7	719.4	1580.6	401.7	719.4
	(12.6)	(27.6)	(7.0)	(12.6)	(27.6)	(7.0)	(12.6)	(27.6)	(7.0)	(12.6)
	11.2	21.0	197.2	−91.6	−44.8	202.0	11.2	−58.5	100.0	68.7
	0.196	0.366	3.447	−1.601	−0.783	3.531	0.196	−1.023	1.748	1.201
g_O	2465	1105	2291	2442	1168	2299	2448	1164	2341	2354
	(43.1)	(19.3)	(40.0)	(42.7)	(20.4)	(40.2)	(42.8)	(20.3)	(40.9)	(41.1)
g_E	2336.3	1156.4	2463.1	2336.3	1156.4	2463.1	2336.3	1156.4	2463.1	2336.3
	(40.8)	(20.2)	(43.0)	(40.8)	(20.2)	(43.0)	(40.8)	(20.2)	(43.0)	(40.8)
	264.4	−100.4	−331.9	216.2	23.4	−317.1	228.7	15.3	−238.1	35.6
	4.621	−1.755	−5.801	3.778	0.409	−5.541	3.997	0.268	−4.161	0.622
c_O	1583	1832	2570	1640	1823	2549	1605	1808	2567	1676
	(27.7)	(32.0)	(44.9)	(28.7)	(31.9)	(44.5)	(28.0)	(31.6)	(44.9)	(29.3)
c_E	1589.5	1626.6	2580.8	1589.5	1626.6	2580.8	1589.5	1626.6	2580.8	1589.5
	(27.8)	(28.4)	(45.1)	(27.8)	(28.4)	(45.1)	(27.8)	(28.4)	(45.1)	(27.8)
	−13.0	435.8	−21.6	102.6	415.7	−63.2	31.1	382.4	−27.5	177.6
	−0.227	7.616	−0.377	1.793	7.264	−1.105	0.544	6.683	−0.481	3.104
N	5722	5722	5722	5722	5722	5722	5722	5722	5722	5722
X2	22.3	48.2	63.6	20.5	49.5	71.7	21.9	39.6	40.1	24.1
Gvalue	22.8	48.1	59.5	20.9	49.8	66.3	22.6	39.6	37.4	24.7
Gv/N	0.399	0.840	1.040	0.365	0.871	1.159	0.394	0.693	0.653	0.432
Prob	0.00004	0.00000	0.00000	0.00011	0.00000	0.00000	0.00005	0.00000	0.00000	0.00002

	71	72	73	74	75	76	77	78	79	80
a_O	1204	345	1014	1210	340	901	1228	358	964	1293
	(21.0)	(6.0)	(17.7)	(21.1)	(5.9)	(15.7)	(21.5)	(6.3)	(16.8)	(22.6)
a_E	1358.5	276.4	1076.8	1358.5	276.4	1076.8	1358.5	276.4	1076.8	1358.5
	(23.7)	(4.8)	(18.8)	(23.7)	(4.8)	(18.8)	(23.7)	(4.8)	(18.8)	(23.7)
	−290.8	153.0	−121.9	−280.2	140.8	−321.2	−248.1	185.2	−213.4	−127.8
	−5.081	2.673	−2.130	−4.896	2.461	−5.614	−4.335	3.237	−3.729	−2.234
t_O	1545	481	706	1566	479	751	1592	464	722	1555
	(27.0)	(8.4)	(12.3)	(27.4)	(8.4)	(13.1)	(27.8)	(8.1)	(12.6)	(27.2)
t_E	1580.6	401.7	719.4	1580.6	401.7	719.4	1580.6	401.7	719.4	1580.6
	(27.6)	(7.0)	(12.6)	(27.6)	(7.0)	(12.6)	(27.6)	(7.0)	(12.6)	(27.6)
	−70.3	173.4	−26.6	−29.0	168.7	64.5	23.0	133.9	5.2	−50.7
	−1.229	3.030	−0.464	−0.506	2.948	1.128	0.402	2.340	0.091	−0.886
g_O	1207	2308	2365	1215	2332	2353	1098	2384	2354	1193
	(21.1)	(40.3)	(41.3)	(21.2)	(40.8)	(41.1)	(19.2)	(41.7)	(41.1)	(20.8)
g_E	1156.4	2463.1	2336.3	1156.4	2463.1	2336.3	1156.4	2463.1	2336.3	1156.4
	(20.2)	(43.0)	(40.8)	(20.2)	(43.0)	(40.8)	(20.2)	(43.0)	(40.8)	(20.2)
	103.5	−300.3	57.8	120.2	−255.1	33.6	−113.7	−155.7	35.6	74.4
	1.808	−5.248	1.011	2.101	−4.459	0.587	−1.987	−2.721	0.622	1.301
c_O	1766	2588	1637	1731	2571	1717	1804	2516	1682	1681
	(30.9)	(45.2)	(28.6)	(30.3)	(44.9)	(30.0)	(31.5)	(44.0)	(29.4)	(29.4)
c_E	1626.6	2580.8	1589.5	1626.6	2580.8	1589.5	1626.6	2580.8	1589.5	1626.6
	(28.4)	(45.1)	(27.8)	(28.4)	(45.1)	(27.8)	(28.4)	(45.1)	(27.8)	(28.4)
	290.5	14.4	96.4	215.4	−19.6	265.0	373.5	−128.0	190.3	110.7
	5.076	0.252	1.685	3.765	−0.342	4.631	6.528	−2.236	3.325	1.934
N	5722	5722	5722	5722	5722	5722	5722	5722	5722	5722
X2	32.5	42.5	5.7	26.0	36.5	40.4	34.9	37.9	17.3	6.6
Gvalue	32.9	40.5	5.7	26.5	34.8	41.9	34.7	35.4	17.7	6.6
Gv/N	0.575	0.708	0.100	0.463	0.608	0.732	0.607	0.619	0.309	0.115
Prob	0.00000	0.00000	0.12456	0.00001	0.00000	0.00000	0.00000	0.00000	0.00051	0.08667

	81	82	83	84	85	86	87	88	89	90
a_O	333	947	1257	311	971	1279	308	1010	1268	332
	(5.8)	(16.6)	(22.0)	(5.4)	(17.0)	(22.4)	(5.4)	(17.7)	(22.2)	(5.8)
a_E	276.4	1076.8	1358.5	276.4	1076.8	1358.5	276.4	1076.8	1358.5	276.4
	(4.8)	(18.8)	(23.7)	(4.8)	(18.8)	(23.7)	(4.8)	(18.8)	(23.7)	(4.8)
	124.1	−243.3	−195.3	73.4	−200.9	−154.3	66.7	−129.4	−174.9	121.7
	2.168	−4.252	−3.412	1.282	−3.511	−2.696	1.165	−2.262	−3.056	2.127
t_O	451	738	1551	483	708	1553	415	723	1561	445
	(7.9)	(12.9)	(27.1)	(8.4)	(12.4)	(27.1)	(7.3)	(12.6)	(27.3)	(7.8)
t_E	401.7	719.4	1580.6	401.7	719.4	1580.6	401.7	719.4	1580.6	401.7
	(7.0)	(12.6)	(27.6)	(7.0)	(12.6)	(27.6)	(7.0)	(12.6)	(27.6)	(7.0)
	104.5	37.7	−58.5	178.1	−22.6	−54.6	27.1	7.2	−38.9	91.2

```
          1.826   0.658  -1.023   3.113  -0.396  -0.955   0.474   0.126  -0.679   1.594
g_O        2240    2428    1224    2296    2420    1162    2316    2387    1125    2375
        ( 39.1) ( 42.4) ( 21.4) ( 40.1) ( 42.3) ( 20.3) ( 40.5) ( 41.7) ( 19.7) ( 41.5)
g_E      2463.1  2336.3  1156.4  2463.1  2336.3  1156.4  2463.1  2336.3  1156.4  2463.1
        ( 43.0) ( 40.8) ( 20.2) ( 43.0) ( 40.8) ( 20.2) ( 43.0) ( 40.8) ( 20.2) ( 43.0)
         -425.4   187.0   139.2  -322.6   170.4    11.3  -285.3   102.6   -61.9  -173.1
         -7.434   3.269   2.432  -5.639   2.979   0.198  -4.986   1.792  -1.081  -3.024
c_O        2698    1609    1690    2632    1623    1728    2683    1602    1768    2570
        ( 47.2) ( 28.1) ( 29.5) ( 46.0) ( 28.4) ( 30.2) ( 46.9) ( 28.0) ( 30.9) ( 44.9)
c_E      2580.8  1589.5  1626.6  2580.8  1589.5  1626.6  2580.8  1589.5  1626.6  2580.8
        ( 45.1) ( 27.8) ( 28.4) ( 45.1) ( 27.8) ( 28.4) ( 45.1) ( 27.8) ( 28.4) ( 45.1)
          239.6    39.2   129.3   103.4    67.7   209.0   208.4    25.1   294.8   -21.6
          4.188   0.686   2.260   1.807   1.183   3.653   3.642   0.439   5.152  -0.377

N          5722    5722    5722    5722    5722    5722    5722    5722    5722    5722
X2         43.2    20.0    14.6    33.2    14.3    11.5    16.9     5.4    19.4    19.1
Gvalue     42.8    20.6    14.7    32.2    14.6    11.5    16.9     5.4    19.2    18.3
Gv/N      0.748   0.360   0.256   0.563   0.255   0.200   0.295   0.095   0.336   0.319
Prob    0.00000 0.00013 0.00213 0.00000 0.00219 0.00950 0.00075 0.14196 0.00025 0.00039
        -------------------------------------------------------------------------------
             91      92      93      94      95      96      97      98      99     100
        -------------------------------------------------------------------------------
a_O         986    1315     324    1029    1299     307    1059    1271     283    1005
        ( 17.2) ( 23.0) (  5.7) ( 18.0) ( 22.7) (  5.4) ( 18.5) ( 22.2) (  4.9) ( 17.6)
a_E      1076.8  1358.5   276.4  1076.6  1358.3   276.4  1076.6  1358.3   276.4  1076.4
        ( 18.8) ( 23.7) (  4.8) ( 18.8) ( 23.7) (  4.8) ( 18.8) ( 23.7) (  4.8) ( 18.8)
         -173.8   -85.6   103.0   -93.1  -115.9    64.6   -35.0  -168.8    13.4  -138.0
         -3.037  -1.496   1.799  -1.628  -2.026   1.129  -0.611  -2.951   0.235  -2.413
t_O         693    1494     428     746    1644     471     664    1625     438     715
        ( 12.1) ( 26.1) (  7.5) ( 13.0) ( 28.7) (  8.2) ( 11.6) ( 28.4) (  7.7) ( 12.5)
t_E       719.4  1580.6   401.7   719.3  1580.3   401.6   719.3  1580.3   401.6   719.2
        ( 12.6) ( 27.6) (  7.0) ( 12.6) ( 27.6) (  7.0) ( 12.6) ( 27.6) (  7.0) ( 12.6)
          -51.8  -168.3    54.4    54.4   130.0   150.2  -106.2    90.7    76.0    -8.3
         -0.906  -2.941   0.950   0.951   2.272   2.625  -1.856   1.585   1.329  -0.145
g_O        2392    1161    2352    2369    1125    2302    2443    1145    2320    2463
        ( 41.8) ( 20.3) ( 41.1) ( 41.4) ( 19.7) ( 40.2) ( 42.7) ( 20.0) ( 40.6) ( 43.1)
g_E      2336.3  1156.4  2463.1  2335.9  1156.2  2462.7  2335.9  1156.2  2462.7  2335.4
        ( 40.8) ( 20.2) ( 43.0) ( 40.8) ( 20.2) ( 43.0) ( 40.8) ( 20.2) ( 43.0) ( 40.8)
          112.8     9.3  -217.2    66.8   -61.5  -310.7   219.1   -22.2  -277.0   261.9
          1.971   0.163  -3.795   1.167  -1.074  -5.430   3.830  -0.388  -4.841   4.579
c_O        1651    1752    2618    1577    1653    2641    1555    1680    2680    1537
        ( 28.9) ( 30.6) ( 45.8) ( 27.6) ( 28.9) ( 46.2) ( 27.2) ( 29.4) ( 46.8) ( 26.9)
c_E      1589.5  1626.6  2580.8  1589.2  1626.3  2580.4  1589.2  1626.3  2580.4  1588.9
        ( 27.8) ( 28.4) ( 45.1) ( 27.8) ( 28.4) ( 45.1) ( 27.8) ( 28.4) ( 45.1) ( 27.8)
          125.3   260.3    74.9   -24.4    53.9   122.7   -67.7   109.2   203.1  -102.2
          2.191   4.549   1.309  -0.426   0.941   2.145  -1.183   1.908   3.550  -1.786

N          5722    5722    5722    5721    5721    5721    5721    5721    5721    5720
X2         12.3    15.8    15.5     3.7     6.4    27.3    10.2     8.8    15.6    13.4
Gvalue     12.5    15.7    15.1     3.7     6.4    26.8    10.2     8.8    15.6    13.4
Gv/N      0.219   0.274   0.263   0.064   0.113   0.468   0.179   0.155   0.273   0.235
Prob    0.00576 0.00131 0.00175 0.29793 0.09186 0.00001 0.01666 0.03137 0.00137 0.00379
        -------------------------------------------------------------------------------
            101     102     103     104     105     106     107     108     109     110
        -------------------------------------------------------------------------------
a_O        1321     298    1055    1290     319    1012    1338     316    1067    1327
        ( 23.1) (  5.2) ( 18.4) ( 22.6) (  5.6) ( 17.7) ( 23.4) (  5.5) ( 18.7) ( 23.2)
a_E      1358.0   276.3  1076.4  1358.0   276.3  1076.4  1358.0   276.3  1076.3  1357.8
        ( 23.7) (  4.8) ( 18.8) ( 23.7) (  4.8) ( 18.8) ( 23.7) (  4.8) ( 18.8) ( 23.7)
          -73.1    45.0   -42.5  -132.6    91.7  -125.0   -39.8    84.8   -18.4   -60.9
         -1.277   0.788  -0.742  -2.318   1.603  -2.185  -0.696   1.483  -0.322  -1.065
t_O        1630     441     686    1549     420     778    1562     435     717    1609
        ( 28.5) (  7.7) ( 12.0) ( 27.1) (  7.3) ( 13.6) ( 27.3) (  7.6) ( 12.5) ( 28.1)
t_E      1580.0   401.5   719.2  1580.0   401.5   719.2  1580.0   401.5   719.0  1579.7
        ( 27.6) (  7.0) ( 12.6) ( 27.6) (  7.0) ( 12.6) ( 27.6) (  7.0) ( 12.6) ( 27.6)
          101.6    82.7   -64.8   -61.4    37.8   122.4   -35.8    69.7    -4.1    59.1
          1.776   1.446  -1.132  -1.073   0.661   2.139  -0.626   1.218  -0.071   1.033
g_O        1104    2321    2433    1186    2319    2445    1150    2362    2318    1157
        ( 19.3) ( 40.6) ( 42.5) ( 20.7) ( 40.5) ( 42.7) ( 20.1) ( 41.3) ( 40.5) ( 20.2)
g_E      1156.0  2462.3  2335.4  1156.0  2462.3  2335.4  1156.0  2462.3  2335.0  1155.8
        ( 20.2) ( 43.0) ( 40.8) ( 20.2) ( 43.0) ( 40.8) ( 20.2) ( 43.0) ( 40.8) ( 20.2)
         -101.5  -274.3   199.1    60.9  -278.0   224.2   -11.9  -196.4   -34.0     2.5
         -1.775  -4.795   3.481   1.064  -4.861   3.919  -0.208  -3.433  -0.594   0.044
c_O        1665    2660    1546    1695    2662    1485    1670    2607    1617    1626
        ( 29.1) ( 46.5) ( 27.0) ( 29.6) ( 46.5) ( 26.0) ( 29.2) ( 45.6) ( 28.3) ( 28.4)
c_E      1626.0  2579.9  1588.9  1626.0  2579.9  1588.9  1626.0  2579.9  1588.7  1625.7
        ( 28.4) ( 45.1) ( 27.8) ( 28.4) ( 45.1) ( 27.8) ( 28.4) ( 45.1) ( 27.8) ( 28.4)
           78.9   162.7   -84.7   140.9   166.8  -200.9    89.2    54.5    57.2     0.6
          1.380   2.844  -1.481   2.463   2.916  -3.513   1.559   0.952   1.000   0.010

N          5720    5720    5720    5720    5720    5720    5720    5720    5719    5719
X2          5.9    16.2     7.2     7.7    18.4    20.6     1.7    12.9     0.7     1.2
Gvalue      5.9    16.1     7.2     7.7    18.2    20.6     1.7    12.6     0.7     1.2
Gv/N      0.103   0.282   0.125   0.135   0.318   0.361   0.030   0.220   0.012   0.022
Prob    0.11738 0.00106 0.06657 0.05168 0.00040 0.00013 0.63393 0.00562 0.87024 0.74226
        -------------------------------------------------------------------------------
            111     112     113     114     115     116     117     118     119     120
        -------------------------------------------------------------------------------
a_O         284     974    1321     316    1064    1367     307    1059    1374     327
        (  5.0) ( 17.0) ( 23.1) (  5.5) ( 18.6) ( 23.9) (  5.4) ( 18.5) ( 24.0) (  5.7)
a_E       276.3  1076.3  1357.8   276.3  1075.9  1357.3   276.2  1075.5  1356.9   276.1
        (  4.8) ( 18.8) ( 23.7) (  4.8) ( 18.8) ( 23.7) (  4.8) ( 18.8) ( 23.7) (  4.8)
           15.7  -194.5   -72.6    84.9   -23.6    19.4    65.0   -32.8    34.5   110.7
          0.274  -3.401  -1.269   1.485  -0.413   0.339   1.137  -0.573   0.604   1.938
t_O         428     752    1515     433     727    1566     461     729    1518     430
        (  7.5) ( 13.1) ( 26.5) (  7.6) ( 12.7) ( 27.4) (  8.1) ( 12.8) ( 26.6) (  7.5)
t_E       401.5   719.0  1579.7   401.5   718.8  1579.2   401.3   718.5  1578.6   401.2
        (  7.0) ( 12.6) ( 27.6) (  7.0) ( 12.6) ( 27.6) (  7.0) ( 12.6) ( 27.6) (  7.0)
           54.8    67.4  -126.8    65.5    16.5   -26.2   127.8    21.1  -118.9    59.7
          0.958   1.179  -2.216   1.145   0.289  -0.459   2.236   0.369  -2.080   1.044
g_O        2333    2464    1192    2326    2367    1104    2349    2339    1160    2383
        ( 40.8) ( 43.1) ( 20.8) ( 40.7) ( 41.4) ( 19.3) ( 41.1) ( 40.9) ( 20.3) ( 41.7)
g_E      2461.8  2335.0  1155.8  2461.8  2334.2  1155.3  2461.0  2333.4  1154.9  2460.1
        ( 43.0) ( 40.8) ( 20.2) ( 43.0) ( 40.8) ( 20.2) ( 43.0) ( 40.8) ( 20.2) ( 43.0)
         -250.8   264.9    73.6  -264.0    66.0  -100.4  -218.8    11.2    10.1  -151.8
         -4.385   4.632   1.287  -4.617   1.155  -1.756  -3.827   0.196   0.177  -2.656
c_O        2674    1529    1691    2644    1559    1680    2600    1588    1663    2575
        ( 46.8) ( 26.7) ( 29.6) ( 46.2) ( 27.3) ( 29.4) ( 45.5) ( 27.8) ( 29.1) ( 45.1)
c_E      2579.5  1588.7  1625.7  2579.5  1588.1  1625.2  2578.6  1587.6  1624.6  2577.6
        ( 45.1) ( 27.8) ( 28.4) ( 45.1) ( 27.8) ( 28.4) ( 45.1) ( 27.8) ( 28.4) ( 45.1)
          192.5  -117.1   133.1   130.7   -57.7   111.5    43.1     0.9    77.7    -5.3
          3.366  -2.047   2.328   2.285  -1.009   1.951   0.753   0.016   1.360  -0.093
```

N	5719	5719	5719	5719	5717	5717	5717	5715	5715	5715
X2	12.2	20.6	7.4	17.3	1.2	4.3	17.6	0.4	3.5	13.9
Gvalue	12.2	20.8	7.4	17.1	1.2	4.3	17.1	0.4	3.5	13.3
Gv/N	0.214	0.364	0.130	0.299	0.021	0.076	0.300	0.007	0.061	0.233
Prob	0.00667	0.00012	0.05998	0.00067	0.74812	0.22834	0.00066	0.93609	0.32112	0.00396

	121	122	123	124	125	126	127	128	129	130
a_O	1021	1373	284	1007	1384	275	1040	1350	296	1008
	(17.9)	(24.0)	(5.0)	(17.6)	(24.2)	(4.8)	(18.2)	(23.6)	(5.2)	(17.7)
a_E	1075.5	1356.9	276.1	1075.3	1356.6	276.0	1074.8	1355.9	275.9	1074.6
	(18.8)	(23.7)	(4.8)	(18.8)	(23.7)	(4.8)	(18.8)	(23.7)	(4.8)	(18.8)
	-106.2	32.5	16.1	-132.2	55.3	-2.0	-68.4	-11.8	41.7	-128.9
	-1.858	0.568	0.282	-2.314	0.968	-0.036	-1.197	-0.206	0.730	-2.258
t_O	687	1555	400	726	1537	458	669	1597	437	733
	(12.0)	(27.2)	(7.0)	(12.7)	(26.9)	(8.0)	(11.7)	(28.0)	(7.7)	(12.8)
t_E	718.5	1578.6	401.2	718.4	1578.3	401.1	718.0	1577.5	400.9	717.9
	(12.6)	(27.6)	(7.0)	(12.6)	(27.6)	(7.0)	(12.6)	(27.6)	(7.0)	(12.6)
	-61.7	-46.9	-2.3	15.3	-81.6	121.5	-94.6	39.2	75.4	30.5
	-1.079	-0.820	-0.041	0.267	-1.428	2.126	-1.657	0.687	1.320	0.534
g_O	2325	1220	2338	2441	1118	2436	2361	1166	2322	2405
	(40.7)	(21.3)	(40.9)	(42.7)	(19.6)	(42.6)	(41.3)	(20.4)	(40.7)	(42.1)
g_E	2333.4	1154.9	2460.1	2333.0	1154.7	2459.7	2331.8	1154.1	2458.4	2331.4
	(40.8)	(20.2)	(43.0)	(40.8)	(20.2)	(43.0)	(40.8)	(20.2)	(43.0)	(40.8)
	-16.8	133.7	-238.1	220.9	-72.3	-47.1	58.8	23.9	-265.1	149.6
	-0.294	2.340	-4.165	3.866	-1.265	-0.825	1.030	0.418	-4.641	2.619
c_O	1682	1567	2693	1540	1675	2545	1641	1598	2656	1564
	(29.4)	(27.4)	(47.1)	(27.0)	(29.3)	(44.5)	(28.7)	(28.0)	(46.5)	(27.4)
c_E	1587.6	1624.6	2577.6	1587.3	1624.3	2577.2	1586.4	1623.4	2575.8	1586.2
	(27.8)	(28.4)	(45.1)	(27.8)	(28.4)	(45.1)	(27.8)	(28.4)	(45.1)	(27.8)
	194.4	-113.1	235.8	-93.1	103.0	-64.0	111.0	-50.5	162.8	-44.0
	3.402	-1.979	4.126	-1.630	1.802	-1.120	1.943	-0.884	2.850	-0.771
N	5715	5715	5715	5714	5714	5714	5711	5711	5711	5710
X2	9.8	6.3	11.5	10.8	4.4	8.7	6.7	0.8	14.8	7.1
Gvalue	9.8	6.2	11.5	10.9	4.4	8.4	6.8	0.8	14.8	7.1
Gv/N	0.171	0.109	0.201	0.190	0.077	0.146	0.119	0.014	0.259	0.125
Prob	0.02077	0.10184	0.00939	0.01250	0.22237	0.03930	0.07917	0.85232	0.00202	0.06758

	131	132	133	134	135	136	137	138	139	140
a_O	1305	295	1030	1397	288	1029	1418	295	1040	1326
	(22.9)	(5.2)	(18.0)	(24.5)	(5.0)	(18.0)	(24.9)	(5.2)	(18.2)	(23.3)
a_E	1355.7	275.8	1074.4	1355.4	275.8	1073.6	1354.5	275.6	1073.2	1354.0
	(23.7)	(4.8)	(18.8)	(23.7)	(4.8)	(18.8)	(23.7)	(4.8)	(18.8)	(23.7)
	-99.4	39.7	-86.9	84.4	25.0	-87.4	130.0	40.2	-65.5	-55.4
	-1.741	0.694	-1.522	1.478	0.438	-1.531	2.278	0.704	-1.148	-0.972
t_O	1507	376	724	1525	419	704	1559	417	736	1579
	(26.4)	(6.6)	(12.7)	(26.7)	(7.3)	(12.3)	(27.3)	(7.3)	(12.9)	(27.7)
t_E	1577.2	400.8	717.8	1577.0	400.8	717.3	1575.9	400.5	717.0	1575.3
	(27.6)	(7.0)	(12.6)	(27.6)	(7.0)	(12.6)	(27.6)	(7.0)	(12.6)	(27.6)
	-137.3	-48.1	12.5	-102.2	37.3	-26.3	-33.5	33.7	38.5	7.4
	-2.405	-0.842	0.219	-1.790	0.654	-0.461	-0.588	0.591	0.674	0.130
g_O	1170	2339	2439	1166	2359	2421	1160	2407	2377	1197
	(20.5)	(41.0)	(42.7)	(20.4)	(41.3)	(42.4)	(20.3)	(42.2)	(41.7)	(21.0)
g_E	1153.9	2458.0	2331.0	1153.7	2457.5	2329.3	1152.9	2455.8	2328.5	1152.5
	(20.2)	(43.0)	(40.8)	(20.2)	(43.0)	(40.8)	(20.2)	(43.0)	(40.8)	(20.2)
	32.4	-232.1	221.0	24.7	-193.1	186.9	14.2	-96.6	98.0	90.7
	0.567	-4.064	3.871	0.432	-3.382	3.276	0.249	-1.694	1.718	1.590
c_O	1728	2700	1516	1621	2643	1551	1568	2586	1550	1601
	(30.3)	(47.3)	(26.6)	(28.4)	(46.3)	(27.2)	(27.5)	(45.3)	(27.2)	(28.1)
c_E	1623.2	2575.4	1585.9	1622.9	2574.9	1584.8	1621.7	2573.1	1584.2	1621.2
	(28.4)	(45.1)	(27.8)	(28.4)	(45.1)	(27.8)	(28.4)	(45.1)	(27.8)	(28.4)
	216.3	255.1	-136.7	-3.8	137.9	-66.8	-105.7	25.8	-67.7	-40.1
	3.788	4.468	-2.394	-0.066	2.415	-1.171	-1.852	0.452	-1.187	-0.703
N	5710	5710	5709	5709	5709	5705	5705	5705	5703	5703
X2	12.0	14.7	10.0	3.1	7.1	6.4	5.0	3.1	3.3	2.6
Gvalue	11.9	14.7	10.0	3.1	7.1	6.4	5.0	3.1	3.3	2.5
Gv/N	0.209	0.257	0.175	0.055	0.125	0.112	0.087	0.053	0.058	0.045
Prob	0.00757	0.00213	0.01881	0.37268	0.06757	0.09308	0.17486	0.38389	0.34955	0.46825

	141	142	143	144	145	146	147	148	149	150
a_O	285	1042	1357	315	1010	1259	284	1048	1309	256
	(5.0)	(18.3)	(23.8)	(5.5)	(17.7)	(22.1)	(5.0)	(18.4)	(23.0)	(4.5)
a_E	275.5	1072.5	1353.1	275.3	1072.3	1352.8	275.2	1071.9	1352.3	275.1
	(4.8)	(18.8)	(23.7)	(4.8)	(18.8)	(23.7)	(4.8)	(18.8)	(23.7)	(4.8)
	19.4	-60.1	7.9	84.9	-120.9	-181.0	17.8	-47.3	-85.3	-36.9
	0.339	-1.055	0.139	1.489	-2.122	-3.176	0.312	-0.831	-1.497	-0.648
t_O	433	737	1549	414	709	1672	378	718	1621	447
	(7.6)	(12.9)	(27.2)	(7.3)	(12.4)	(29.3)	(6.6)	(12.6)	(28.5)	(7.8)
t_E	400.3	716.5	1574.2	400.1	716.4	1573.9	400.0	716.1	1573.4	399.8
	(7.0)	(12.6)	(27.6)	(7.0)	(12.6)	(27.6)	(7.0)	(12.6)	(27.6)	(7.0)
	67.9	41.5	-50.0	28.4	-14.7	202.1	-42.7	3.7	96.7	99.7
	1.191	0.729	-0.877	0.498	-0.258	3.548	-0.750	0.065	1.697	1.750
g_O	2342	2362	1163	2379	2386	1105	2410	2310	1160	2333
	(41.1)	(41.4)	(20.4)	(41.7)	(41.9)	(19.4)	(42.3)	(40.6)	(20.4)	(41.0)
g_E	2454.9	2326.9	1151.7	2453.2	2326.5	1151.5	2452.8	2325.6	1151.1	2451.9
	(43.0)	(40.8)	(20.2)	(43.0)	(40.8)	(20.2)	(43.0)	(40.8)	(20.2)	(43.0)
	-220.6	70.8	22.7	-146.2	120.6	-91.1	-84.8	-31.2	17.9	-232.0
	-3.868	1.242	0.398	-2.565	2.116	-1.599	-1.489	-0.548	0.314	-4.073
c_O	2643	1558	1630	2591	1593	1662	2626	1620	1606	2660
	(46.3)	(27.3)	(28.6)	(45.5)	(28.0)	(29.2)	(46.1)	(28.4)	(28.2)	(46.7)
c_E	2572.2	1583.1	1620.0	2570.4	1582.8	1619.8	2570.0	1582.3	1619.2	2569.1
	(45.1)	(27.8)	(28.4)	(45.1)	(27.8)	(28.4)	(45.1)	(27.8)	(28.4)	(45.1)
	143.5	-49.8	20.0	41.3	20.4	85.6	113.2	76.3	-26.3	185.0
	2.515	-0.874	0.351	0.725	0.358	1.502	1.988	1.340	-0.461	3.248
N	5703	5699	5699	5699	5698	5698	5698	5696	5696	5696
X2	10.1	2.4	0.6	8.6	5.3	15.6	3.5	1.5	3.0	15.9
Gvalue	10.1	2.4	0.6	8.4	5.3	15.6	3.5	1.5	3.0	15.8
Gv/N	0.178	0.042	0.010	0.147	0.094	0.275	0.061	0.027	0.053	0.277
Prob	0.01751	0.49673	0.89906	0.03871	0.14822	0.00134	0.32473	0.67289	0.39038	0.00127

Bqui_TOULOUSE
5UTR:

	50	49	48	47	46	45	44	43	42	41
a_O	395	412	394	362	368	343	374	374	373	366
	(34.6)	(36.1)	(34.6)	(31.8)	(32.3)	(30.1)	(32.8)	(32.8)	(32.7)	(32.1)
a_E	382.3	382.3	382.3	382.3	382.3	382.3	382.3	382.3	382.3	382.3
	(33.5)	(33.5)	(33.5)	(33.5)	(33.5)	(33.5)	(33.5)	(33.5)	(33.5)	(33.5)
	25.9	61.7	23.8	-39.5	-28.0	-74.4	-16.4	-16.4	-18.3	-31.9
	2.268	5.412	2.087	-3.462	-2.458	-6.524	-1.437	-1.437	-1.608	-2.794
t_O	376	351	375	381	368	421	362	374	398	369
	(33.0)	(30.8)	(32.9)	(33.4)	(32.3)	(36.9)	(31.8)	(32.8)	(34.9)	(32.4)
t_E	369.7	369.7	369.7	369.7	369.7	369.7	369.7	369.7	369.7	369.7
	(32.4)	(32.4)	(32.4)	(32.4)	(32.4)	(32.4)	(32.4)	(32.4)	(32.4)	(32.4)
	12.7	-36.4	10.7	23.0	-3.4	109.4	-15.2	8.7	58.7	-1.4
	1.116	-3.195	0.938	2.014	-0.296	9.599	-1.335	0.761	5.152	-0.121
g_O	199	200	207	211	239	215	215	231	195	213
	(17.5)	(17.5)	(18.2)	(18.5)	(21.0)	(18.9)	(18.9)	(20.3)	(17.1)	(18.7)
g_E	219.5	219.5	219.5	219.5	219.5	219.5	219.5	219.5	219.5	219.5
	(19.3)	(19.3)	(19.3)	(19.3)	(19.3)	(19.3)	(19.3)	(19.3)	(19.3)	(19.3)
	-39.0	-37.2	-24.2	-16.6	40.7	-8.8	-8.8	23.7	-46.1	-12.7
	-3.418	-3.260	-2.124	-1.457	3.574	-0.776	-0.776	2.075	-4.044	-1.118
c_O	170	177	164	186	165	161	189	161	174	192
	(14.9)	(15.5)	(14.4)	(16.3)	(14.5)	(14.1)	(16.6)	(14.1)	(15.3)	(16.8)
c_E	168.6	168.6	168.6	168.6	168.6	168.6	168.6	168.6	168.6	168.6
	(14.8)	(14.8)	(14.8)	(14.8)	(14.8)	(14.8)	(14.8)	(14.8)	(14.8)	(14.8)
	2.9	17.3	-9.0	36.6	-7.0	-14.8	43.3	-14.8	11.1	50.0
	0.254	1.517	-0.789	3.213	-0.618	-1.296	3.795	-1.296	0.970	4.386
N	1140	1140	1140	1140	1140	1140	1140	1140	1140	1140
X2	2.5	5.4	1.3	3.6	2.4	11.6	2.9	1.2	5.3	4.1
Gvalue	2.5	5.4	1.3	3.5	2.3	11.4	2.8	1.2	5.4	4.0
Gv/N	0.220	0.475	0.112	0.308	0.203	1.003	0.247	0.103	0.469	0.352
Prob	0.47357	0.14409	0.73426	0.31891	0.51019	0.00961	0.42063	0.76012	0.14781	0.25980

	40	39	38	37	36	35	34	33	32	31
a_O	374	375	390	355	347	365	375	375	393	355
	(32.8)	(32.9)	(34.2)	(31.1)	(30.4)	(32.0)	(32.9)	(32.9)	(34.5)	(31.1)
a_E	382.3	382.3	382.3	382.3	382.3	382.3	382.3	382.3	382.3	382.3
	(33.5)	(33.5)	(33.5)	(33.5)	(33.5)	(33.5)	(33.5)	(33.5)	(33.5)	(33.5)
	-16.4	-14.4	15.6	-52.6	-67.2	-33.8	-14.4	-14.4	21.7	-52.6
	-1.437	-1.265	1.368	-4.611	-5.895	-2.962	-1.265	-1.265	1.907	-4.611
t_O	370	398	351	379	411	375	386	396	351	362
	(32.5)	(34.9)	(30.8)	(33.2)	(36.1)	(32.9)	(33.9)	(34.7)	(30.8)	(31.8)
t_E	369.7	369.7	369.7	369.7	369.7	369.7	369.7	369.7	369.7	369.7
	(32.4)	(32.4)	(32.4)	(32.4)	(32.4)	(32.4)	(32.4)	(32.4)	(32.4)	(32.4)
	0.6	58.7	-36.4	18.9	87.1	10.7	33.3	54.4	-36.4	-15.2
	0.054	5.152	-3.195	1.654	7.638	0.938	2.924	4.776	-3.195	-1.335
g_O	211	195	232	218	212	225	199	195	213	237
	(18.5)	(17.1)	(20.4)	(19.1)	(18.6)	(19.7)	(17.5)	(17.1)	(18.7)	(20.8)
g_E	219.5	219.5	219.5	219.5	219.5	219.5	219.5	219.5	219.5	219.5
	(19.3)	(19.3)	(19.3)	(19.3)	(19.3)	(19.3)	(19.3)	(19.3)	(19.3)	(19.3)
	-16.6	-46.1	25.8	-2.9	-14.7	11.2	-39.0	-46.1	-12.7	36.4
	-1.457	-4.044	2.260	-0.257	-1.288	0.982	-3.418	-4.044	-1.118	3.195
c_O	185	172	167	188	170	175	180	174	183	186
	(16.2)	(15.1)	(14.6)	(16.5)	(14.9)	(15.4)	(15.8)	(15.3)	(16.1)	(16.3)
c_E	168.6	168.6	168.6	168.6	168.6	168.6	168.6	168.6	168.6	168.6
	(14.8)	(14.8)	(14.8)	(14.8)	(14.8)	(14.8)	(14.8)	(14.8)	(14.8)	(14.8)
	34.4	6.9	-3.1	41.0	2.9	13.1	23.6	11.1	30.1	36.6
	3.021	0.610	-0.272	3.600	0.254	1.151	2.074	0.970	2.639	3.213
N	1140	1140	1140	1140	1140	1140	1140	1140	1140	1140
X2	2.1	5.1	1.8	4.4	8.1	1.2	3.5	4.9	2.7	5.3
Gvalue	2.1	5.2	1.8	4.4	8.1	1.3	3.6	5.0	2.7	5.3
Gv/N	0.181	0.453	0.161	0.386	0.709	0.110	0.314	0.437	0.233	0.462
Prob	0.55887	0.16047	0.60785	0.22161	0.04432	0.74099	0.31057	0.17338	0.44795	0.15336

	30	29	28	27	26	25	24	23	22	21
a_O	369	393	386	357	372	376	397	386	426	424
	(32.4)	(34.5)	(33.9)	(31.3)	(32.6)	(33.0)	(34.8)	(33.9)	(37.4)	(37.2)
a_E	382.3	382.3	382.3	382.3	382.3	382.3	382.3	382.3	382.3	382.3
	(33.5)	(33.5)	(33.5)	(33.5)	(33.5)	(33.5)	(33.5)	(33.5)	(33.5)	(33.5)
	-26.1	21.7	7.5	-48.9	-20.3	-12.5	30.0	7.5	92.3	87.8
	-2.289	1.907	0.656	-4.285	-1.779	-1.093	2.632	0.656	8.093	7.705
t_O	396	386	395	429	380	380	387	350	373	373
	(34.7)	(33.9)	(34.6)	(37.6)	(33.3)	(33.3)	(33.9)	(30.7)	(32.7)	(32.7)
t_E	369.7	369.7	369.7	369.7	369.7	369.7	369.7	369.7	369.7	369.7
	(32.4)	(32.4)	(32.4)	(32.4)	(32.4)	(32.4)	(32.4)	(32.4)	(32.4)	(32.4)
	54.4	33.3	52.3	127.7	20.9	20.9	35.4	-38.3	6.6	6.6
	4.776	2.924	4.589	11.199	1.834	1.834	3.107	-3.361	0.583	0.583
g_O	211	200	177	183	208	198	185	202	183	195
	(18.5)	(17.5)	(15.5)	(16.1)	(18.2)	(17.4)	(16.2)	(17.7)	(16.1)	(17.1)
g_E	219.5	219.5	219.5	219.5	219.5	219.5	219.5	219.5	219.5	219.5
	(19.3)	(19.3)	(19.3)	(19.3)	(19.3)	(19.3)	(19.3)	(19.3)	(19.3)	(19.3)
	-16.6	-37.2	-76.1	-66.5	-22.3	-40.8	-63.2	-33.5	-66.5	-46.1
	-1.457	-3.260	-6.678	-5.834	-1.959	-3.576	-5.545	-2.940	-5.834	-4.044
c_O	164	161	182	171	180	186	171	202	158	148
	(14.4)	(14.1)	(16.0)	(15.0)	(15.8)	(16.3)	(15.0)	(17.7)	(13.9)	(13.0)
c_E	168.6	168.6	168.6	168.6	168.6	168.6	168.6	168.6	168.6	168.6
	(14.8)	(14.8)	(14.8)	(14.8)	(14.8)	(14.8)	(14.8)	(14.8)	(14.8)	(14.8)
	-9.0	-14.8	27.9	4.9	23.6	36.6	4.9	73.1	-20.4	-38.5
	-0.789	-1.296	2.449	0.431	2.074	3.213	0.431	6.414	-1.793	-3.378
N	1140	1140	1140	1140	1140	1140	1140	1140	1140	1140
X2	2.8	3.1	11.1	17.3	1.9	4.3	6.8	9.1	11.8	9.8
Gvalue	2.8	3.1	11.6	17.2	1.9	4.3	7.1	8.8	12.0	9.9
Gv/N	0.241	0.275	1.016	1.510	0.170	0.378	0.624	0.769	1.048	0.866
Prob	0.43147	0.37171	0.00896	0.00064	0.58633	0.23024	0.06833	0.03255	0.00755	0.01963

	20	19	18	17	16	15	14	13	12	11
a_O	411	458	457	462	463	455	453	493	481	425
	(36.1)	(40.2)	(40.1)	(40.5)	(40.6)	(39.9)	(39.7)	(43.2)	(42.2)	(37.3)
a_E	382.3	382.3	382.3	382.3	382.3	382.3	382.3	382.3	382.3	382.3
	(33.5)	(33.5)	(33.5)	(33.5)	(33.5)	(33.5)	(33.5)	(33.5)	(33.5)	(33.5)
	59.5	165.5	163.2	175.0	177.4	158.5	153.8	250.8	221.0	90.0
	5.223	14.521	14.314	15.352	15.561	13.901	13.490	22.000	19.385	7.899
t_O	380	342	362	330	342	376	296	250	220	169
	(33.3)	(30.0)	(31.8)	(28.9)	(30.0)	(33.0)	(26.0)	(21.9)	(19.3)	(14.8)

```
t_E         369.7    369.7    369.7    369.7    369.7    369.7    369.7    369.7    369.7    369.7
         ( 32.4)  ( 32.4)  ( 32.4)  ( 32.4)  ( 32.4)  ( 32.4)  ( 32.4)  ( 32.4)  ( 32.4)  ( 32.4)
            20.9    -53.3    -15.2    -75.0    -53.3     12.7   -131.6   -195.6   -228.4   -264.6
           1.834   -4.671   -1.335   -6.575   -4.671    1.116  -11.544  -17.158  -20.033  -23.208
g_O          202      186      173      183      183      202      271      296      362      470
         ( 17.7)  ( 16.3)  ( 15.2)  ( 16.1)  ( 16.1)  ( 17.7)  ( 23.8)  ( 26.0)  ( 31.8)  ( 41.2)
g_E        219.5    219.5    219.5    219.5    219.5    219.5    219.5    219.5    219.5    219.5
         ( 19.3)  ( 19.3)  ( 19.3)  ( 19.3)  ( 19.3)  ( 19.3)  ( 19.3)  ( 19.3)  ( 19.3)  ( 19.3)
           -33.5    -61.6    -82.3    -66.5    -66.5    -33.5    114.3    177.1    362.3    715.8
          -2.940   -5.400   -7.221   -5.834   -5.834   -2.940   10.027   15.534   31.782   62.792
c_O          147      154      148      165      152      107      120      101       77       76
         ( 12.9)  ( 13.5)  ( 13.0)  ( 14.5)  ( 13.3)  (  9.4)  ( 10.5)  (  8.9)  (  6.8)  (  6.7)
c_E        168.6    168.6    168.6    168.6    168.6    168.6    168.6    168.6    168.6    168.6
         ( 14.8)  ( 14.8)  ( 14.8)  ( 14.8)  ( 14.8)  ( 14.8)  ( 14.8)  ( 14.8)  ( 14.8)  ( 14.8)
           -40.2    -27.8    -38.5     -7.0    -31.4    -97.3    -81.6   -103.5   -120.7   -121.1
          -3.530   -2.441   -3.378   -0.618   -2.758   -8.531   -7.154   -9.075  -10.584  -10.621

N           1140     1140     1140     1140     1140     1140     1140     1140     1140     1140
X2           6.6     23.4     27.1     27.0     26.8     37.8     53.9    124.6    228.4    450.5
Gvalue       6.7     22.9     27.1     26.5     26.2     40.4     54.9    128.8    234.3    420.2
Gv/N       0.588    2.009    2.380    2.325    2.298    3.547    4.819   11.301   20.549   36.861
Prob     0.08199  0.00004  0.00001  0.00001  0.00001  0.00000  0.00000  0.00000  0.00000  0.00000
         ------------------------------------------------------------------------------------------
              10        9        8        7        6        5        4        3        2        1
         ------------------------------------------------------------------------------------------
a_O          432      407      463      471      452      458      439      512      331      346
         ( 37.9)  ( 35.7)  ( 40.6)  ( 41.3)  ( 39.6)  ( 40.2)  ( 38.5)  ( 44.9)  ( 29.0)  ( 30.4)
a_E        382.3    382.3    382.3    382.3    382.3    382.3    382.3    382.3    382.3    382.3
         ( 33.5)  ( 33.5)  ( 33.5)  ( 33.5)  ( 33.5)  ( 33.5)  ( 33.5)  ( 33.5)  ( 33.5)  ( 33.5)
           105.6     51.0    177.4    196.6    151.4    165.5    121.5    299.2    -95.4    -69.0
           9.267    4.474   15.561   17.246   13.285   14.521   10.655   26.244   -8.364   -6.053
t_O          176      192      213      311      360      331      370      296      440      394
         ( 15.4)  ( 16.8)  ( 18.7)  ( 27.3)  ( 31.6)  ( 29.0)  ( 32.5)  ( 26.0)  ( 38.6)  ( 34.6)
t_E        369.7    369.7    369.7    369.7    369.7    369.7    369.7    369.7    369.7    369.7
         ( 32.4)  ( 32.4)  ( 32.4)  ( 32.4)  ( 32.4)  ( 32.4)  ( 32.4)  ( 32.4)  ( 32.4)  ( 32.4)
          -261.2   -251.6   -234.9   -107.5    -19.1    -73.2      0.6   -131.6    153.2     50.2
         -22.916  -22.069  -20.604   -9.432   -1.678   -6.419    0.054  -11.544   13.440    4.402
g_O          478      470      357      268      219      212      183      189      140      175
         ( 41.9)  ( 41.2)  ( 31.3)  ( 23.5)  ( 19.2)  ( 18.6)  ( 16.1)  ( 16.6)  ( 12.3)  ( 15.4)
g_E        219.5    219.5    219.5    219.5    219.5    219.5    219.5    219.5    219.5    219.5
         ( 19.3)  ( 19.3)  ( 19.3)  ( 19.3)  ( 19.3)  ( 19.3)  ( 19.3)  ( 19.3)  ( 19.3)  ( 19.3)
           744.1    715.8    347.4    107.1     -0.9    -14.7    -66.5    -56.5   -125.9    -79.3
          65.276   62.792   30.472    9.393   -0.082   -1.288   -5.834   -4.956  -11.042   -6.952
c_O           54       71      107       90      109      139      148      143      229      225
         (  4.7)  (  6.2)  (  9.4)  (  7.9)  (  9.6)  ( 12.2)  ( 13.0)  ( 12.5)  ( 20.1)  ( 19.7)
c_E        168.6    168.6    168.6    168.6    168.6    168.6    168.6    168.6    168.6    168.6
         ( 14.8)  ( 14.8)  ( 14.8)  ( 14.8)  ( 14.8)  ( 14.8)  ( 14.8)  ( 14.8)  ( 14.8)  ( 14.8)
          -122.9   -122.8    -97.3   -112.9    -95.0    -53.6    -38.5    -47.0    140.3    130.0
         -10.784  -10.770   -8.531   -9.908   -8.336   -4.702   -3.378   -4.126   12.311   11.400

N           1140     1140     1140     1140     1140     1140     1140     1140     1140     1140
X2         490.3    429.5    192.1     77.3     34.0     24.5     17.0     66.8     70.7     33.0
Gvalue     465.6    392.5    192.6     83.2     36.3     24.1     17.1     64.0     72.3     31.9
Gv/N      40.842   34.427   16.898    7.299    3.188    2.111    1.498    5.618    6.345    2.798
Prob     0.00000  0.00000  0.00000  0.00000  0.00000  0.00002  0.00068  0.00000  0.00000  0.00000
         ------------------------------------------------------------------------------------------

CDS_INITIAL:
         ------------------------------------------------------------------------------------------
               1        2        3        4        5        6        7        8        9       10
         ------------------------------------------------------------------------------------------
a_O         1019        0        1      540      387      413      462      502      403      448
         ( 89.4)  (  0.0)  (  0.1)  ( 47.4)  ( 33.9)  ( 36.2)  ( 40.5)  ( 44.0)  ( 35.4)  ( 39.3)
a_E        318.5    343.3    326.9    318.5    343.3    326.9    318.5    343.3    326.9    318.5
         ( 27.9)  ( 30.1)  ( 28.7)  ( 27.9)  ( 30.1)  ( 28.7)  ( 27.9)  ( 30.1)  ( 28.7)  ( 27.9)
          2369.9      0.0    -11.6    570.1     92.7    193.1    343.6    381.5    168.7    305.6
         207.889    0.000   -1.016   50.008    8.134   16.939   30.140   33.464   14.796   26.808
t_O           46     1140        0      164      256      424      202      310      436      242
         (  4.0)  (100.0)  (  0.0)  ( 14.4)  ( 22.5)  ( 37.2)  ( 17.7)  ( 27.2)  ( 38.2)  ( 21.2)
t_E        213.1    356.5    477.2    213.1    356.5    477.2    213.1    356.5    477.2    213.1
         ( 18.7)  ( 31.3)  ( 41.9)  ( 18.7)  ( 31.3)  ( 41.9)  ( 18.7)  ( 31.3)  ( 41.9)  ( 18.7)
          -141.1   2650.3      0.0    -85.9   -169.6   -100.2    -21.6    -86.7    -78.7     61.5
         -12.373  232.484    0.000   -7.537  -14.874   -8.788   -1.897   -7.603   -6.902    5.397
g_O           75        0     1138      274      158      136      283      142      171      233
         (  6.6)  (  0.0)  ( 99.8)  ( 24.0)  ( 13.9)  ( 11.9)  ( 24.8)  ( 12.5)  ( 15.0)  ( 20.4)
g_E        375.2    185.6    185.8    375.2    185.6    185.8    375.2    185.6    185.8    375.2
         ( 32.9)  ( 16.3)  ( 16.3)  ( 32.9)  ( 16.3)  ( 16.3)  ( 32.9)  ( 16.3)  ( 16.3)  ( 32.9)
          -241.5      0.0   4125.1   -172.2    -50.8    -84.8   -159.6    -76.0    -28.4   -222.0
         -21.183    0.000  361.854  -15.108   -4.460   -7.442  -14.000   -6.668   -2.488  -19.473
c_O            0        0        1      162      339      167      193      186      130      217
         (  0.0)  (  0.0)  (  0.1)  ( 14.2)  ( 29.7)  ( 14.6)  ( 16.9)  ( 16.3)  ( 11.4)  ( 19.0)
c_E        233.2    254.6    150.1    233.2    254.6    150.1    233.2    254.6    150.1    233.2
         ( 20.5)  ( 22.3)  ( 13.2)  ( 20.5)  ( 22.3)  ( 13.2)  ( 20.5)  ( 22.3)  ( 13.2)  ( 20.5)
             0.0      0.0    -10.0   -118.0    194.1     35.5    -73.0   -116.8    -37.4    -31.2
           0.000    0.000   -0.879  -10.351   17.027    3.118   -6.403  -10.245   -3.285   -2.737

N           1140     1140     1140     1140     1140     1140     1140     1140     1140     1140
X2        2144.8   2505.3   5830.7    214.3     66.0     43.8     94.8    108.1     25.1    111.6
Gvalue    1987.4   2650.3   4103.5    193.9     66.4     43.6     89.4    102.0     24.2    113.9
Gv/N     174.333  232.484  359.959   17.012    5.827    3.826    7.840    8.949    2.121    9.995
Prob     1.00000  1.00000  1.00000  0.00000  0.00000  0.00000  0.00000  0.00000  0.00002  0.00000
         ------------------------------------------------------------------------------------------
              11       12       13       14       15       16       17       18       19       20
         ------------------------------------------------------------------------------------------
a_O          407      411      422      395      351      410      375      359      422      377
         ( 35.7)  ( 36.1)  ( 37.0)  ( 34.6)  ( 30.8)  ( 36.0)  ( 32.9)  ( 31.5)  ( 37.0)  ( 33.1)
a_E        343.3    326.9    318.5    343.3    326.9    318.5    343.3    326.9    318.5    343.3
         ( 30.1)  ( 28.7)  ( 27.9)  ( 30.1)  ( 28.7)  ( 27.9)  ( 30.1)  ( 28.7)  ( 27.9)  ( 30.1)
           138.5    188.2    237.4    110.8     49.9    207.0     66.2     67.2    237.4     70.6
          12.152   16.507   20.826    9.720    4.379   18.159    5.809    5.899   20.826    6.192
t_O          375      432      251      395      469      256      395      468      237      396
         ( 32.9)  ( 37.9)  ( 22.0)  ( 34.6)  ( 41.1)  ( 22.5)  ( 34.6)  ( 41.1)  ( 20.8)  ( 34.7)
t_E        356.5    477.2    213.1    356.5    477.2    213.1    356.5    477.2    213.1    356.5
         ( 31.3)  ( 41.9)  ( 18.7)  ( 31.3)  ( 41.9)  ( 18.7)  ( 31.3)  ( 41.9)  ( 18.7)  ( 31.3)
            37.9    -85.9     82.1     81.0    -16.2     93.9     81.0    -18.2     50.4     83.2
           3.326   -7.537    7.206    7.105   -1.421    8.235    7.105   -1.593    4.418    7.298
g_O          130      154      260      136      157      245      143      135      251      139
         ( 11.4)  ( 13.5)  ( 22.8)  ( 11.9)  ( 13.8)  ( 21.5)  ( 12.5)  ( 11.8)  ( 22.0)  ( 12.2)
g_E        185.6    185.8    375.2    185.6    185.8    375.2    185.6    185.8    375.2    185.6
         ( 16.3)  ( 16.3)  ( 32.9)  ( 16.3)  ( 16.3)  ( 32.9)  ( 16.3)  ( 16.3)  ( 32.9)  ( 16.3)
           -92.5    -57.8   -190.7    -84.5    -52.9   -208.8    -74.5    -86.2   -201.8    -80.3
          -8.118   -5.069  -16.728   -7.416   -4.637  -18.318   -6.539   -7.562  -17.701   -7.048
```

c_O	228	143	207	214	163	229	227	178	230	228
	(20.0)	(12.5)	(18.2)	(18.8)	(14.3)	(20.1)	(19.9)	(15.6)	(20.2)	(20.0)
c_E	254.6	150.1	233.2	254.6	150.1	233.2	254.6	150.1	233.2	254.6
	(22.3)	(13.2)	(20.5)	(22.3)	(13.2)	(20.5)	(22.3)	(13.2)	(20.5)	(22.3)
	-50.3	-13.9	-49.3	-74.4	26.8	-8.3	-52.1	60.6	-6.3	-50.3
	-4.414	-1.222	-4.325	-6.523	2.350	-0.726	-4.570	5.315	-0.554	-4.414
N	1140	1140	1140	1140	1140	1140	1140	1140	1140	1140
X2	32.2	31.7	78.7	31.7	7.5	80.1	19.8	22.4	77.4	22.2
Gvalue	33.6	30.5	79.6	32.9	7.7	83.8	20.6	23.5	79.7	23.1
Gv/N	2.946	2.678	6.979	2.886	0.672	7.350	1.805	2.058	6.989	2.028
Prob	0.00000	0.00000	0.00000	0.00000	0.05370	0.00000	0.00013	0.00003	0.00000	0.00004

	21	22	23	24	25	26	27	28	29	30
a_O	358	372	369	348	368	339	318	324	332	314
	(31.4)	(32.6)	(32.4)	(30.5)	(32.3)	(29.7)	(27.9)	(28.4)	(29.1)	(27.5)
a_E	326.9	318.5	343.3	326.9	318.5	343.3	326.9	318.5	343.3	326.9
	(28.7)	(27.9)	(30.1)	(28.7)	(27.9)	(30.1)	(28.7)	(27.9)	(30.1)	(28.7)
	65.1	115.5	53.3	43.5	106.3	-8.6	-17.6	11.0	-22.2	-25.3
	5.707	10.128	4.672	3.818	9.321	-0.751	-1.541	0.969	-1.951	-2.219
t_O	457	243	391	474	232	374	471	242	373	498
	(40.1)	(21.3)	(34.3)	(41.6)	(20.4)	(32.8)	(41.3)	(21.2)	(32.7)	(43.7)
t_E	477.2	213.1	356.5	477.2	213.1	356.5	477.2	213.1	356.5	477.2
	(41.9)	(18.7)	(31.3)	(41.9)	(18.7)	(31.3)	(41.9)	(18.7)	(31.3)	(41.9)
	-39.5	63.8	72.2	-6.3	39.4	35.8	-12.3	61.5	33.7	42.6
	-3.463	5.595	6.334	-0.555	3.457	3.142	-1.076	5.397	2.959	3.733
g_O	149	291	137	173	304	173	181	337	184	153
	(13.1)	(25.5)	(12.0)	(15.2)	(26.7)	(15.2)	(15.9)	(29.6)	(16.1)	(13.4)
g_E	185.8	375.2	185.6	185.8	375.2	185.6	185.8	375.2	185.6	185.8
	(16.3)	(32.9)	(16.3)	(16.3)	(32.9)	(16.3)	(16.3)	(32.9)	(16.3)	(16.3)
	-65.7	-147.9	-83.2	-24.7	-127.9	-24.3	-9.4	-72.3	-3.1	-59.4
	-5.767	-12.972	-7.295	-2.164	-11.221	-2.130	-0.828	-6.346	-0.276	-5.211
c_O	176	234	243	145	236	254	170	237	251	175
	(15.4)	(20.5)	(21.3)	(12.7)	(20.7)	(22.3)	(14.9)	(20.8)	(22.0)	(15.4)
c_E	150.1	233.2	254.6	150.1	233.2	254.6	150.1	233.2	254.6	150.1
	(13.2)	(20.5)	(22.3)	(13.2)	(20.5)	(22.3)	(13.2)	(20.5)	(22.3)	(13.2)
	55.9	1.6	-22.7	-10.1	5.7	-1.2	42.2	7.7	-7.2	53.6
	4.907	0.145	-1.989	-0.886	0.498	-0.106	3.705	0.676	-0.628	4.704
N	1140	1140	1140	1140	1140	1140	1140	1140	1140	1140
X2	15.5	32.1	18.5	2.4	22.9	1.8	3.1	8.0	1.2	11.3
Gvalue	15.8	33.0	19.6	2.4	23.4	1.8	3.0	7.9	1.2	11.5
Gv/N	1.383	2.896	1.723	0.213	2.055	0.155	0.260	0.696	0.105	1.007
Prob	0.00126	0.00000	0.00020	0.48761	0.00003	0.62094	0.39669	0.04744	0.75482	0.00941

	31	32	33	34	35	36	37	38	39	40
a_O	350	289	323	329	327	340	347	328	342	337
	(30.7)	(25.4)	(28.3)	(28.9)	(28.7)	(29.8)	(30.4)	(28.8)	(30.0)	(29.6)
a_E	318.5	343.3	326.9	318.5	343.3	326.9	318.5	343.3	326.9	318.5
	(27.9)	(30.1)	(28.7)	(27.9)	(30.1)	(28.7)	(27.9)	(30.1)	(28.7)	(27.9)
	66.0	-99.5	-7.8	21.3	-31.8	26.7	59.4	-29.9	30.9	38.0
	5.786	-8.731	-0.681	1.867	-2.792	2.343	5.212	-2.625	2.709	3.333
t_O	243	398	474	228	378	477	260	390	457	222
	(21.3)	(34.9)	(41.6)	(20.0)	(33.2)	(41.8)	(22.8)	(34.2)	(40.1)	(19.5)
t_E	213.1	356.5	477.2	213.1	356.5	477.2	213.1	356.5	477.2	213.1
	(18.7)	(31.3)	(41.9)	(18.7)	(31.3)	(41.9)	(18.7)	(31.3)	(41.9)	(18.7)
	63.8	87.6	-6.3	30.8	44.2	-0.3	103.4	70.0	-39.5	18.1
	5.595	7.687	-0.555	2.701	3.881	-0.030	9.071	6.143	-3.463	1.592
g_O	320	189	163	364	183	165	327	178	182	348
	(28.1)	(16.6)	(14.3)	(31.9)	(16.1)	(14.5)	(28.7)	(15.6)	(16.0)	(30.5)
g_E	375.2	185.6	185.8	375.2	185.6	185.8	375.2	185.6	185.8	375.2
	(32.9)	(16.3)	(16.3)	(32.9)	(16.3)	(16.3)	(32.9)	(16.3)	(16.3)	(32.9)
	-101.8	6.9	-42.7	-22.0	-5.1	-39.1	-89.9	-14.8	-7.5	-52.4
	-8.932	0.606	-3.741	-1.933	-0.449	-3.434	-7.886	-1.302	-0.657	-4.592
c_O	227	264	180	219	252	158	206	244	159	233
	(19.9)	(23.2)	(15.8)	(19.2)	(22.1)	(13.9)	(18.1)	(21.4)	(13.9)	(20.4)
c_E	233.2	254.6	150.1	233.2	254.6	150.1	233.2	254.6	150.1	233.2
	(20.5)	(22.3)	(13.2)	(20.5)	(22.3)	(13.2)	(20.5)	(22.3)	(13.2)	(20.5)
	-12.2	19.1	65.3	-27.5	-5.2	16.1	-51.1	-20.8	18.2	-0.4
	-1.069	1.679	5.728	-2.410	-0.454	1.414	-4.479	-1.821	1.599	-0.031
N	1140	1140	1140	1140	1140	1140	1140	1140	1140	1140
X2	15.6	13.8	8.8	2.6	2.1	3.3	22.2	4.6	2.1	3.4
Gvalue	15.7	14.1	8.6	2.6	2.1	3.3	21.9	4.5	2.1	3.4
Gv/N	1.380	1.240	0.751	0.226	0.186	0.293	1.919	0.395	0.188	0.301
Prob	0.00129	0.00272	0.03570	0.46198	0.54790	0.34224	0.00007	0.21173	0.54348	0.32910

	41	42	43	44	45	46	47	48	49	50
a_O	330	318	312	303	304	340	305	339	340	297
	(28.9)	(27.9)	(27.4)	(26.6)	(26.7)	(29.8)	(26.8)	(29.7)	(29.8)	(26.1)
a_E	343.3	326.9	318.5	343.3	326.9	318.5	343.3	326.9	318.5	343.3
	(30.1)	(28.7)	(27.9)	(30.1)	(28.7)	(27.9)	(30.1)	(28.7)	(27.9)	(30.1)
	-26.1	-17.6	-12.9	-75.7	-44.2	44.4	-72.2	24.6	44.4	-86.1
	-2.289	-1.541	-1.133	-6.639	-3.874	3.892	-6.331	2.161	3.892	-7.550
t_O	395	475	236	385	481	226	375	485	211	377
	(34.6)	(41.7)	(20.7)	(33.8)	(42.2)	(19.8)	(32.9)	(42.5)	(18.5)	(33.1)
t_E	356.5	477.2	213.1	356.5	477.2	213.1	356.5	477.2	213.1	356.5
	(31.3)	(41.9)	(18.7)	(31.3)	(41.9)	(18.7)	(31.3)	(41.9)	(18.7)	(31.3)
	81.0	-4.3	48.2	59.2	7.7	26.5	37.9	15.8	-4.2	42.1
	7.105	-0.380	4.224	5.193	0.674	2.328	3.326	1.385	-0.368	3.696
g_O	176	175	369	202	214	337	210	171	380	212
	(15.4)	(15.4)	(32.4)	(17.7)	(18.8)	(29.6)	(18.4)	(15.0)	(33.3)	(18.6)
g_E	185.6	185.8	375.2	185.6	185.8	375.2	185.6	185.8	375.2	185.6
	(16.3)	(16.3)	(32.9)	(16.3)	(16.3)	(32.9)	(16.3)	(16.3)	(32.9)	(16.3)
	-18.7	-20.9	-12.3	34.3	60.5	-72.3	51.9	-28.4	9.7	56.4
	-1.636	-1.836	-1.076	3.005	5.309	-6.346	4.555	-2.488	0.850	4.951
c_O	239	172	223	250	141	237	250	145	209	254
	(21.0)	(15.1)	(19.6)	(21.9)	(12.4)	(20.8)	(21.9)	(12.7)	(18.3)	(22.3)
c_E	254.6	150.1	233.2	254.6	150.1	233.2	254.6	150.1	233.2	254.6
	(22.3)	(13.2)	(20.5)	(22.3)	(13.2)	(20.5)	(22.3)	(13.2)	(20.5)	(22.3)
	-30.2	46.8	-19.9	-9.1	-17.7	7.7	-9.1	-10.1	-45.8	-1.2
	-2.652	4.102	-1.746	-0.800	-1.554	0.676	-0.800	-0.886	-4.014	-0.106
N	1140	1140	1140	1140	1140	1140	1140	1140	1140	1140
X2	6.1	4.1	3.1	8.5	6.5	6.2	8.5	1.9	4.0	11.2
Gvalue	6.0	3.9	3.1	8.6	6.3	6.3	8.6	2.0	4.1	11.3
Gv/N	0.528	0.345	0.269	0.758	0.555	0.550	0.750	0.172	0.359	0.991
Prob	0.11086	0.26895	0.38202	0.03443	0.09678	0.09926	0.03590	0.58144	0.25118	0.01021

	51	52	53	54	55	56	57	58	59	60
a_O	353	351	290	297	335	294	320	301	328	326
	(31.0)	(30.8)	(25.4)	(26.1)	(29.4)	(25.8)	(28.1)	(26.4)	(28.8)	(28.6)
a_E	326.9	318.5	343.3	326.9	318.5	343.3	326.9	318.5	343.3	326.9
	(28.7)	(27.9)	(30.1)	(28.7)	(27.9)	(30.1)	(28.7)	(27.9)	(30.1)	(28.7)
	54.2	68.2	-97.9	-57.0	33.8	-91.2	-13.7	-34.1	-29.9	-1.8
	4.756	5.978	-8.585	-4.999	2.964	-7.997	-1.198	-2.989	-2.625	-0.159
t_O	454	247	413	481	213	390	485	227	366	483
	(39.8)	(21.7)	(36.2)	(42.2)	(18.7)	(34.2)	(42.5)	(19.9)	(32.1)	(42.4)
t_E	477.2	213.1	356.5	477.2	213.1	356.5	477.2	213.1	356.5	477.2
	(41.9)	(18.7)	(31.3)	(41.9)	(18.7)	(31.3)	(41.9)	(18.7)	(31.3)	(41.9)
	-45.2	72.9	121.5	7.7	-0.2	70.0	15.8	28.7	19.2	11.7
	-3.965	6.395	10.657	0.674	-0.019	6.143	1.385	2.515	1.687	1.029
g_O	175	327	188	202	346	196	185	370	178	182
	(15.4)	(28.7)	(16.5)	(17.7)	(30.4)	(17.2)	(16.2)	(32.5)	(15.6)	(16.0)
g_E	185.8	375.2	185.6	185.8	375.2	185.6	185.8	375.2	185.6	185.8
	(16.3)	(32.9)	(16.3)	(16.3)	(32.9)	(16.3)	(16.3)	(32.9)	(16.3)	(16.3)
	-20.9	-89.9	4.9	33.8	-56.0	21.4	-1.6	-10.3	-14.8	-7.5
	-1.836	-7.886	0.428	2.966	-4.916	1.879	-0.137	-0.903	-1.302	-0.657
c_O	158	215	249	160	246	260	150	242	268	149
	(13.9)	(18.9)	(21.8)	(14.0)	(21.6)	(22.8)	(13.2)	(21.2)	(23.5)	(13.1)
c_E	150.1	233.2	254.6	150.1	233.2	254.6	150.1	233.2	254.6	150.1
	(13.2)	(20.5)	(22.3)	(13.2)	(20.5)	(22.3)	(13.2)	(20.5)	(22.3)	(13.2)
	16.1	-34.9	-11.1	20.4	26.3	10.9	-0.3	18.0	27.5	-2.3
	1.414	-3.061	-0.972	1.785	2.310	0.957	-0.025	1.577	2.411	-0.199
N	1140	1140	1140	1140	1140	1140	1140	1140	1140	1140
X2	4.2	16.3	17.4	4.8	3.8	10.9	0.3	2.3	1.9	0.2
Gvalue	4.2	16.3	17.4	4.9	3.9	11.2	0.3	2.3	1.9	0.2
Gv/N	0.370	1.426	1.527	0.427	0.339	0.981	0.024	0.199	0.171	0.014
Prob	0.23903	0.00101	0.00058	0.18207	0.27676	0.01076	0.96410	0.51821	0.58287	0.98382

	61	62	63	64	65	66	67	68	69	70
a_O	298	291	294	305	281	336	293	348	340	326
	(26.1)	(25.5)	(25.8)	(26.8)	(24.6)	(29.5)	(25.7)	(30.5)	(29.8)	(28.6)
a_E	318.5	343.3	326.9	318.5	343.3	326.9	318.5	343.3	326.9	318.5
	(27.9)	(30.1)	(28.7)	(27.9)	(30.1)	(28.7)	(27.9)	(30.1)	(28.7)	(27.9)
	-39.7	-96.2	-62.4	-26.5	-112.6	18.4	-48.9	9.4	26.7	15.1
	-3.482	-8.439	-5.472	-2.322	-9.873	1.618	-4.294	0.829	2.343	1.326
t_O	228	387	470	242	395	459	246	355	445	208
	(20.0)	(33.9)	(41.2)	(21.2)	(34.6)	(40.3)	(21.6)	(31.1)	(39.0)	(18.2)
t_E	213.1	356.5	477.2	213.1	356.5	477.2	213.1	356.5	477.2	213.1
	(18.7)	(31.3)	(41.9)	(18.7)	(31.3)	(41.9)	(18.7)	(31.3)	(41.9)	(18.7)
	30.8	63.5	-14.2	61.5	81.0	-35.6	70.6	-3.0	-62.1	-10.1
	2.701	5.571	-1.249	5.397	7.105	-3.127	6.194	-0.264	-5.449	-0.886
g_O	354	210	204	362	191	207	355	180	199	385
	(31.1)	(18.4)	(17.9)	(31.8)	(16.8)	(18.2)	(31.1)	(15.8)	(17.5)	(33.8)
g_E	375.2	185.6	185.8	375.2	185.6	185.8	375.2	185.6	185.8	375.2
	(32.9)	(16.3)	(16.3)	(32.9)	(16.3)	(16.3)	(32.9)	(16.3)	(16.3)	(32.9)
	-41.2	51.9	38.2	-25.9	11.0	44.8	-39.3	-11.0	27.4	19.9
	-3.610	4.555	3.348	-2.272	0.965	3.927	-3.444	-0.964	2.399	1.744
c_O	260	252	172	231	273	138	246	257	156	221
	(22.8)	(22.1)	(15.1)	(20.3)	(23.9)	(12.1)	(21.6)	(22.5)	(13.7)	(19.4)
c_E	233.2	254.6	150.1	233.2	254.6	150.1	233.2	254.6	150.1	233.2
	(20.5)	(22.3)	(13.2)	(20.5)	(22.3)	(13.2)	(20.5)	(22.3)	(13.2)	(20.5)
	56.6	-5.2	46.8	-4.3	38.1	-23.3	26.3	4.8	11.9	-23.7
	4.967	-0.454	4.102	-0.380	3.341	-2.041	2.310	0.422	1.048	-2.080
N	1140	1140	1140	1140	1140	1140	1140	1140	1140	1140
X2	6.6	13.8	8.4	5.0	17.0	4.3	8.9	0.3	3.9	1.2
Gvalue	6.6	14.1	8.3	4.8	17.5	4.3	8.7	0.3	3.9	1.2
Gv/N	0.576	1.233	0.729	0.423	1.538	0.377	0.766	0.023	0.340	0.105
Prob	0.08725	0.00283	0.04008	0.18527	0.00055	0.23116	0.03304	0.96698	0.27453	0.75315

	71	72	73	74	75	76	77	78	79	80
a_O	317	289	311	309	296	307	290	293	292	305
	(27.8)	(25.4)	(27.3)	(27.1)	(26.0)	(26.9)	(25.4)	(25.7)	(25.6)	(26.8)
a_E	343.3	326.9	318.5	343.3	326.9	318.5	343.3	326.9	318.5	343.3
	(30.1)	(28.7)	(27.9)	(30.1)	(28.7)	(27.9)	(30.1)	(28.7)	(27.9)	(30.1)
	-50.5	-71.2	-14.9	-65.1	-58.8	-22.6	-97.9	-64.2	-50.8	-72.2
	-4.434	-6.249	-1.305	-5.708	-5.157	-1.985	-8.585	-5.628	-4.454	-6.331
t_O	400	473	239	362	482	251	390	499	221	375
	(35.1)	(41.5)	(21.0)	(31.8)	(42.3)	(22.0)	(34.2)	(43.8)	(19.4)	(32.9)
t_E	356.5	477.2	213.1	356.5	477.2	213.1	356.5	477.2	213.1	356.5
	(31.3)	(41.9)	(18.7)	(31.3)	(41.9)	(18.7)	(31.3)	(41.9)	(18.7)	(31.3)
	92.1	-8.3	54.8	11.1	9.7	82.1	70.0	44.6	16.1	37.9
	8.077	-0.729	4.807	0.970	0.851	7.206	6.143	3.916	1.409	3.326
g_O	186	211	355	212	187	356	178	193	373	197
	(16.3)	(18.5)	(31.1)	(18.6)	(16.4)	(31.2)	(15.6)	(16.9)	(32.7)	(17.3)
g_E	185.6	185.8	375.2	185.6	185.8	375.2	185.6	185.8	375.2	185.6
	(16.3)	(16.3)	(32.9)	(16.3)	(16.3)	(32.9)	(16.3)	(16.3)	(32.9)	(16.3)
	0.8	53.7	-39.3	56.4	2.4	-37.4	-14.8	14.7	-4.4	23.5
	0.074	4.712	-3.444	4.951	0.214	-3.278	-1.302	1.290	-0.382	2.064
c_O	237	167	235	257	175	226	282	155	254	263
	(20.8)	(14.6)	(20.6)	(22.5)	(15.4)	(19.8)	(24.7)	(13.6)	(22.3)	(23.1)
c_E	254.6	150.1	233.2	254.6	150.1	233.2	254.6	150.1	233.2	254.6
	(22.3)	(13.2)	(20.5)	(22.3)	(13.2)	(20.5)	(22.3)	(13.2)	(20.5)	(22.3)
	-34.0	35.5	3.7	4.8	53.6	-14.1	57.6	9.9	43.5	17.1
	-2.979	3.118	0.321	0.422	4.704	-1.240	5.056	0.866	3.812	1.497
N	1140	1140	1140	1140	1140	1140	1140	1140	1140	1140
X2	8.5	9.7	4.4	7.3	7.1	8.4	14.7	5.0	4.4	6.2
Gvalue	8.4	9.7	4.3	7.3	7.0	8.0	15.0	5.1	4.4	6.3
Gv/N	0.738	0.852	0.379	0.636	0.612	0.703	1.312	0.444	0.384	0.557
Prob	0.03812	0.02114	0.22848	0.06434	0.07250	0.04574	0.00186	0.16740	0.22317	0.09583

	81	82	83	84	85	86	87	88	89	90
a_O	304	300	325	327	336	353	294	289	308	329
	(26.7)	(26.3)	(28.5)	(28.7)	(29.5)	(31.0)	(25.8)	(25.4)	(27.0)	(28.9)
a_E	326.9	318.5	343.3	326.9	318.5	343.3	326.9	318.5	343.3	326.9
	(28.7)	(27.9)	(30.1)	(28.7)	(27.9)	(30.1)	(28.7)	(27.9)	(30.1)	(28.7)
	-44.2	-36.0	-35.6	0.2	35.9	19.7	-62.4	-56.2	-66.9	4.2
	-3.874	-3.154	-3.125	0.017	3.148	1.724	-5.472	-4.932	-5.864	0.369
t_O	503	240	354	474	220	378	499	226	360	460
	(44.1)	(21.1)	(31.1)	(41.6)	(19.3)	(33.2)	(43.8)	(19.8)	(31.6)	(40.4)
t_E	477.2	213.1	356.5	477.2	213.1	356.5	477.2	213.1	356.5	477.2
	(41.9)	(18.7)	(31.3)	(41.9)	(18.7)	(31.3)	(41.9)	(18.7)	(31.3)	(41.9)
	53.0	57.0	-5.0	-6.3	14.0	44.2	44.6	26.5	7.0	-33.7

	4.652	5.003	−0.439	−0.555	1.228	3.881	3.916	2.328	0.615	−2.958
g_O	191	358	195	184	363	167	203	390	199	198
	(16.8)	(31.4)	(17.1)	(16.1)	(31.8)	(14.6)	(17.8)	(34.2)	(17.5)	(17.4)
g_E	185.8	375.2	185.6	185.8	375.2	185.6	185.8	375.2	185.6	185.8
	(16.3)	(32.9)	(16.3)	(16.3)	(32.9)	(16.3)	(16.3)	(32.9)	(16.3)	(16.3)
	10.6	−33.6	19.3	−3.5	−24.0	−35.2	36.0	30.2	27.8	25.2
	0.928	−2.945	1.694	−0.311	−2.103	−3.091	3.156	2.650	2.438	2.212
c_O	142	242	266	155	221	242	144	235	273	153
	(12.5)	(21.2)	(23.3)	(13.6)	(19.4)	(21.2)	(12.6)	(20.6)	(23.9)	(13.4)
c_E	150.1	233.2	254.6	150.1	233.2	254.6	150.1	233.2	254.6	150.1
	(13.2)	(20.5)	(22.3)	(13.2)	(20.5)	(22.3)	(13.2)	(20.5)	(22.3)	(13.2)
	−15.8	18.0	23.3	9.9	−23.7	−24.6	−12.0	3.7	38.1	5.8
	−1.389	1.577	2.044	0.866	−2.080	−2.155	−1.055	0.321	3.341	0.506
N	1140	1140	1140	1140	1140	1140	1140	1140	1140	1140
X2	3.6	5.6	2.0	0.2	2.2	4.1	6.2	4.1	6.0	1.5
Gvalue	3.6	5.5	2.0	0.2	2.2	4.1	6.2	4.2	6.0	1.5
Gv/N	0.317	0.481	0.174	0.017	0.194	0.360	0.545	0.367	0.530	0.130
Prob	0.30654	0.13943	0.57555	0.97856	0.53011	0.25077	0.10157	0.24251	0.10954	0.68705

	91	92	93	94	95	96	97	98	99	100
a_O	310	312	324	299	334	314	335	302	309	311
	(27.2)	(27.4)	(28.4)	(26.2)	(29.3)	(27.5)	(29.4)	(26.5)	(27.1)	(27.3)
a_E	318.5	343.3	326.9	318.5	343.3	326.9	318.5	343.3	326.9	318.5
	(27.9)	(30.1)	(28.7)	(27.9)	(30.1)	(28.7)	(27.9)	(30.1)	(28.7)	(27.9)
	−16.8	−59.7	−5.8	−37.8	−18.4	−25.3	33.8	−77.4	−34.8	−14.9
	−1.476	−5.234	−0.507	−3.318	−1.610	−2.219	2.964	−6.792	−3.054	−1.305
t_O	214	377	459	257	376	509	218	373	484	221
	(18.8)	(33.1)	(40.3)	(22.5)	(33.0)	(44.6)	(19.1)	(32.7)	(42.5)	(19.4)
t_E	213.1	356.5	477.2	213.1	356.5	477.2	213.1	356.5	477.2	213.1
	(18.7)	(31.3)	(41.9)	(18.7)	(31.3)	(41.9)	(18.7)	(31.3)	(41.9)	(18.7)
	1.8	42.1	−35.6	96.3	40.0	65.7	9.9	33.7	13.8	16.1
	0.156	3.696	−3.127	8.443	3.511	5.766	0.868	2.959	1.207	1.409
g_O	382	191	197	361	176	186	359	199	193	358
	(33.5)	(16.8)	(17.3)	(31.7)	(15.4)	(16.3)	(31.5)	(17.5)	(16.9)	(31.4)
g_E	375.2	185.6	185.8	375.2	185.6	185.8	375.2	185.6	185.8	375.2
	(32.9)	(16.3)	(16.3)	(32.9)	(16.3)	(16.3)	(32.9)	(16.3)	(16.3)	(32.9)
	13.8	11.0	23.1	−27.8	−18.7	0.4	−31.7	27.8	14.7	−33.6
	1.206	0.965	2.026	−2.441	−1.636	0.038	−2.777	2.438	1.290	−2.945
c_O	234	260	160	223	254	131	228	266	154	250
	(20.5)	(22.8)	(14.0)	(19.6)	(22.3)	(11.5)	(20.0)	(23.3)	(13.5)	(21.9)
c_E	233.2	254.6	150.1	233.2	254.6	150.1	233.2	254.6	150.1	233.2
	(20.5)	(22.3)	(13.2)	(20.5)	(22.3)	(13.2)	(20.5)	(22.3)	(13.2)	(20.5)
	1.6	10.9	20.4	−19.9	−1.2	−35.7	−10.2	23.3	7.8	34.8
	0.145	0.957	1.785	−1.746	−0.106	−3.134	−0.898	2.044	0.686	3.055
N	1140	1140	1140	1140	1140	1140	1140	1140	1140	1140
X2	0.4	4.3	2.0	11.2	1.8	5.1	1.8	7.2	1.5	2.5
Gvalue	0.4	4.4	2.0	10.7	1.8	5.1	1.8	7.4	1.5	2.5
Gv/N	0.032	0.384	0.178	0.938	0.158	0.451	0.156	0.648	0.129	0.215
Prob	0.94839	0.22399	0.56732	0.01352	0.61369	0.16149	0.62074	0.06061	0.68887	0.48388

	101	102	103	104	105	106	107	108	109	110
a_O	333	325	315	323	315	299	338	339	335	308
	(29.2)	(28.5)	(27.6)	(28.3)	(27.6)	(26.2)	(29.6)	(29.7)	(29.4)	(27.0)
a_E	343.3	326.9	318.5	343.3	326.9	318.5	343.3	326.9	318.5	343.3
	(30.1)	(28.7)	(27.9)	(30.1)	(28.7)	(27.9)	(30.1)	(28.7)	(27.9)	(30.1)
	−20.3	−3.8	−7.0	−39.4	−23.4	−37.8	−10.5	24.6	33.8	−66.9
	−1.781	−0.333	−0.615	−3.455	−2.050	−3.318	−0.924	2.161	2.964	−5.864
t_O	362	481	228	376	477	205	360	458	233	356
	(31.8)	(42.2)	(20.0)	(33.0)	(41.8)	(18.0)	(31.6)	(40.2)	(20.4)	(31.2)
t_E	356.5	477.2	213.1	356.5	477.2	213.1	356.5	477.2	213.1	356.5
	(31.3)	(41.9)	(18.7)	(31.3)	(41.9)	(18.7)	(31.3)	(41.9)	(18.7)	(31.3)
	11.1	7.7	30.8	40.0	−0.3	−15.9	7.0	−37.6	41.6	−1.0
	0.970	0.674	2.701	3.511	−0.030	−1.395	0.615	−3.295	3.647	−0.090
g_O	205	188	379	190	189	376	186	207	349	193
	(18.0)	(16.5)	(33.2)	(16.7)	(16.6)	(33.0)	(16.3)	(18.2)	(30.6)	(16.9)
g_E	185.6	185.8	375.2	185.6	185.8	375.2	185.6	185.8	375.2	185.6
	(16.3)	(16.3)	(32.9)	(16.3)	(16.3)	(32.9)	(16.3)	(16.3)	(32.9)	(16.3)
	40.8	4.5	7.7	8.9	6.5	1.6	0.8	44.8	−50.5	15.1
	3.580	0.391	0.673	0.785	0.569	0.143	0.074	3.927	−4.430	1.328
c_O	240	146	218	251	159	260	256	136	223	283
	(21.1)	(12.8)	(19.1)	(22.0)	(13.9)	(22.8)	(22.5)	(11.9)	(19.6)	(24.8)
c_E	254.6	150.1	233.2	254.6	150.1	233.2	254.6	150.1	233.2	254.6
	(22.3)	(13.2)	(20.5)	(22.3)	(13.2)	(20.5)	(22.3)	(13.2)	(20.5)	(22.3)
	−28.4	−8.2	−29.3	−7.2	18.2	56.6	2.8	−26.9	−19.9	59.8
	−2.487	−0.716	−2.574	−0.628	1.599	4.967	0.246	−2.360	−1.746	5.250
N	1140	1140	1140	1140	1140	1140	1140	1140	1140	1140
X2	3.3	0.2	2.1	2.4	1.0	4.6	0.1	5.0	5.0	7.1
Gvalue	3.2	0.2	2.1	2.4	1.0	4.5	0.1	4.9	5.0	7.1
Gv/N	0.282	0.016	0.185	0.213	0.088	0.396	0.011	0.433	0.435	0.624
Prob	0.35953	0.98023	0.55095	0.48847	0.79953	0.21139	0.98866	0.17667	0.17465	0.06842

	111	112	113	114	115	116	117	118	119	120
a_O	332	300	338	350	302	332	333	317	336	320
	(29.1)	(26.3)	(29.6)	(30.7)	(26.5)	(29.1)	(29.2)	(27.8)	(29.5)	(28.1)
a_E	326.9	318.5	343.3	326.9	318.5	343.3	326.9	318.5	343.3	326.9
	(28.7)	(27.9)	(30.1)	(28.7)	(27.9)	(30.1)	(28.7)	(27.9)	(30.1)	(28.7)
	10.3	−36.0	−10.5	47.8	−32.2	−22.2	12.3	−3.0	−14.5	−13.7
	0.901	−3.154	−0.924	4.192	−2.823	−1.951	1.079	−0.267	−1.268	−1.198
t_O	474	220	369	452	230	346	499	202	332	510
	(41.6)	(19.3)	(32.4)	(39.6)	(20.2)	(30.4)	(43.8)	(17.7)	(29.1)	(44.7)
t_E	477.2	213.1	356.5	477.2	213.1	356.5	477.2	213.1	356.5	477.2
	(41.9)	(18.7)	(31.3)	(41.9)	(18.7)	(31.3)	(41.9)	(18.7)	(31.3)	(41.9)
	−6.3	14.0	25.4	−49.0	35.1	−20.7	44.6	−21.6	−47.3	67.9
	−0.555	1.228	2.229	−4.297	3.078	−1.817	3.916	−1.897	−4.149	5.953
g_O	170	382	187	180	369	183	176	415	203	184
	(14.9)	(33.5)	(16.4)	(15.8)	(32.4)	(16.1)	(15.4)	(36.4)	(17.8)	(16.1)
g_E	185.8	375.2	185.6	185.8	375.2	185.6	185.8	375.2	185.6	185.8
	(16.3)	(32.9)	(16.3)	(16.3)	(32.9)	(16.3)	(16.3)	(32.9)	(16.3)	(16.3)
	−30.2	13.8	2.9	−11.4	−12.3	−5.1	−19.0	83.7	36.4	−3.5
	−2.648	1.206	0.250	−0.999	−1.076	−0.449	−1.670	7.343	3.196	−0.311
c_O	164	238	246	158	239	279	132	206	269	126
	(14.4)	(20.9)	(21.6)	(13.9)	(21.0)	(24.5)	(11.6)	(18.1)	(23.6)	(11.1)
c_E	150.1	233.2	254.6	150.1	233.2	254.6	150.1	233.2	254.6	150.1
	(13.2)	(20.5)	(22.3)	(13.2)	(20.5)	(22.3)	(13.2)	(20.5)	(22.3)	(13.2)
	29.0	9.7	−16.9	16.1	11.8	51.1	−34.0	−51.1	29.6	−44.2
	2.540	0.855	−1.484	1.414	1.034	4.479	−2.982	−4.479	2.596	−3.875

N	1140	1140	1140	1140	1140	1140	1140	1140	1140	1140
X2	2.7	1.5	0.8	3.6	2.4	3.1	3.8	8.0	4.3	6.3
Gvalue	2.7	1.5	0.8	3.5	2.4	3.0	3.9	8.0	4.3	6.5
Gv/N	0.239	0.135	0.072	0.310	0.213	0.262	0.343	0.700	0.374	0.568
Prob	0.43644	0.67273	0.84471	0.31655	0.48919	0.39286	0.27169	0.04646	0.23399	0.09043

	121	122	123	124	125	126	127	128	129	130
a_O	293	332	326	319	338	318	325	338	312	307
	(25.7)	(29.1)	(28.6)	(28.0)	(29.6)	(27.9)	(28.5)	(29.7)	(27.4)	(27.0)
a_E	318.5	343.3	326.9	318.5	343.3	326.9	318.2	343.0	326.6	318.2
	(27.9)	(30.1)	(28.7)	(27.9)	(30.1)	(28.7)	(27.9)	(30.1)	(28.7)	(27.9)
	-48.9	-22.2	-1.8	0.9	-10.5	-17.6	13.6	-9.9	-28.6	-22.1
	-4.294	-1.951	-0.159	0.083	-0.924	-1.541	1.198	-0.873	-2.509	-1.940
t_O	258	337	485	242	373	497	222	342	481	219
	(22.6)	(29.6)	(42.5)	(21.2)	(32.7)	(43.6)	(19.5)	(30.0)	(42.2)	(19.2)
t_E	213.1	356.5	477.2	213.1	356.5	477.2	212.9	356.2	476.8	212.9
	(18.7)	(31.3)	(41.9)	(18.7)	(31.3)	(41.9)	(18.7)	(31.3)	(41.9)	(18.7)
	98.6	-37.9	15.8	61.5	33.7	40.5	18.5	-27.8	8.5	12.3
	8.652	-3.328	1.385	5.397	2.959	3.550	1.627	-2.443	0.749	1.082
g_O	360	196	178	374	184	173	378	184	198	391
	(31.6)	(17.2)	(15.6)	(32.8)	(16.1)	(15.2)	(33.2)	(16.2)	(17.4)	(34.3)
g_E	375.2	185.6	185.8	375.2	185.6	185.8	374.9	185.4	185.6	374.9
	(32.9)	(16.3)	(16.3)	(32.9)	(16.3)	(16.3)	(32.9)	(16.3)	(16.3)	(32.9)
	-29.7	21.4	-15.2	-2.4	-3.1	-24.7	6.3	-2.8	25.6	33.0
	-2.610	1.879	-1.336	-0.208	-0.276	-2.164	0.554	-0.248	2.245	2.895
c_O	229	275	151	205	245	152	214	275	148	222
	(20.1)	(24.1)	(13.2)	(18.0)	(21.5)	(13.3)	(18.8)	(24.1)	(13.0)	(19.5)
c_E	233.2	254.6	150.1	233.2	254.6	150.1	233.0	254.4	150.0	233.0
	(20.5)	(22.3)	(13.2)	(20.5)	(22.3)	(13.2)	(20.5)	(22.3)	(13.2)	(20.5)
	-8.3	42.4	1.7	-52.8	-18.8	3.7	-36.4	42.9	-4.0	-21.4
	-0.726	3.718	0.151	-4.632	-1.653	0.328	-3.192	3.764	-0.350	-1.881
N	1140	1140	1140	1140	1140	1140	1139	1139	1139	1139
X2	12.2	3.7	0.5	7.3	1.2	2.0	2.1	2.3	1.5	1.8
Gvalue	11.7	3.6	0.5	7.3	1.2	2.0	2.1	2.3	1.5	1.8
Gv/N	1.022	0.319	0.041	0.641	0.106	0.174	0.188	0.201	0.135	0.157
Prob	0.00866	0.30417	0.92632	0.06276	0.74966	0.57637	0.54407	0.51523	0.67379	0.61830

	131	132	133	134	135	136	137	138	139	140
a_O	319	323	289	327	316	297	343	284	317	347
	(28.0)	(28.4)	(25.4)	(28.7)	(27.7)	(26.1)	(30.1)	(25.0)	(27.9)	(30.5)
a_E	343.0	326.6	318.2	343.0	326.6	318.0	342.7	326.3	318.0	342.7
	(30.1)	(28.7)	(27.9)	(30.1)	(28.7)	(27.9)	(30.1)	(28.7)	(27.9)	(30.1)
	-46.3	-7.2	-55.7	-31.3	-20.9	-40.5	0.6	-78.9	-1.9	8.6
	-4.064	-0.632	-4.892	-2.744	-1.834	-3.561	0.052	-6.935	-0.170	0.760
t_O	364	474	254	380	479	209	334	508	222	354
	(32.0)	(41.6)	(22.3)	(33.4)	(42.1)	(18.4)	(29.3)	(44.6)	(19.5)	(31.1)
t_E	356.2	476.8	212.9	356.2	476.8	212.7	355.9	476.3	212.7	355.9
	(31.3)	(41.9)	(18.7)	(31.3)	(41.9)	(18.7)	(31.3)	(41.9)	(18.7)	(31.3)
	15.8	-5.5	89.6	49.2	4.5	-7.4	-42.4	65.4	18.9	-3.8
	1.385	-0.482	7.868	4.316	0.395	-0.651	-3.726	5.746	1.663	-0.330
g_O	190	184	363	175	179	415	185	193	366	183
	(16.7)	(16.2)	(31.9)	(15.4)	(15.7)	(36.5)	(16.3)	(17.0)	(32.2)	(16.1)
g_E	185.4	185.6	374.9	185.4	185.6	374.5	185.3	185.5	374.5	185.3
	(16.3)	(16.3)	(32.9)	(16.3)	(16.3)	(32.9)	(16.3)	(16.3)	(32.9)	(16.3)
	9.3	-3.2	-23.3	-20.2	-13.0	85.2	-0.5	15.4	-16.9	-4.5
	0.815	-0.283	-2.049	-1.777	-1.141	7.484	-0.044	1.352	-1.482	-0.394
c_O	266	158	233	257	165	217	276	153	233	254
	(23.4)	(13.9)	(20.5)	(22.6)	(14.5)	(19.1)	(24.3)	(13.4)	(20.5)	(22.3)
c_E	254.4	150.0	233.0	254.4	150.0	232.8	254.2	149.9	232.8	254.2
	(22.3)	(13.2)	(20.5)	(22.3)	(13.2)	(20.5)	(22.3)	(13.2)	(20.5)	(22.3)
	23.8	16.4	0.1	5.3	31.4	-30.4	45.5	6.3	0.5	-0.3
	2.086	1.440	0.005	0.462	2.760	-2.675	3.999	0.555	0.041	-0.028
N	1139	1139	1139	1139	1139	1138	1138	1138	1138	1138
X2	2.5	0.5	11.0	2.9	2.1	6.9	3.2	8.0	0.6	0.1
Gvalue	2.5	0.5	10.6	2.9	2.0	6.8	3.2	8.2	0.6	0.1
Gv/N	0.222	0.043	0.932	0.258	0.180	0.597	0.281	0.718	0.052	0.008
Prob	0.47067	0.92137	0.01402	0.40124	0.56227	0.07865	0.36171	0.04258	0.89724	0.99287

	141	142	143	144	145	146	147	148	149	150
a_O	311	336	321	325	341	338	326	312	345	296
	(27.3)	(29.5)	(28.2)	(28.6)	(30.0)	(29.8)	(28.7)	(27.5)	(30.4)	(26.1)
a_E	326.3	318.0	342.7	326.3	317.4	342.1	325.8	317.4	342.1	325.8
	(28.7)	(27.9)	(30.1)	(28.7)	(27.9)	(30.1)	(28.7)	(27.9)	(30.1)	(28.7)
	-29.9	37.1	-42.0	-2.7	48.9	-8.2	0.5	-10.7	5.8	-56.7
	-2.630	3.257	-3.691	-0.233	4.304	-0.718	0.043	-0.944	0.512	-4.992
t_O	479	203	350	484	210	362	465	204	359	481
	(42.1)	(17.8)	(30.8)	(42.5)	(18.5)	(31.9)	(40.9)	(18.0)	(31.6)	(42.3)
t_E	476.3	212.7	355.9	476.3	212.4	355.3	475.5	212.4	355.3	475.5
	(41.9)	(18.7)	(31.3)	(41.9)	(18.7)	(31.3)	(41.9)	(18.7)	(31.3)	(41.9)
	5.3	-19.0	-11.7	15.5	-4.7	13.6	-20.8	-16.4	7.5	11.1
	0.470	-1.671	-1.026	1.358	-0.414	1.198	-1.828	-1.443	0.662	0.974
g_O	183	356	210	181	364	189	188	369	179	198
	(16.1)	(31.3)	(18.5)	(15.9)	(32.0)	(16.6)	(16.5)	(32.5)	(15.8)	(17.4)
g_E	185.5	374.5	185.3	185.5	373.9	184.9	185.1	373.9	184.9	185.1
	(16.3)	(32.9)	(16.3)	(16.3)	(32.9)	(16.3)	(16.3)	(32.9)	(16.3)	(16.3)
	-4.9	-36.1	52.7	-8.8	-19.5	8.2	5.8	-9.7	-11.7	26.6
	-0.429	-3.174	4.628	-0.774	-1.714	0.725	0.509	-0.852	-1.027	2.343
c_O	165	243	257	148	221	247	157	251	253	161
	(14.5)	(21.4)	(22.6)	(13.0)	(19.5)	(21.7)	(13.8)	(22.1)	(22.3)	(14.2)
c_E	149.9	232.8	254.2	149.9	232.4	253.7	149.6	232.4	253.7	149.6
	(13.2)	(20.5)	(22.3)	(13.2)	(20.5)	(22.3)	(13.2)	(20.5)	(22.3)	(13.2)
	31.7	20.9	5.7	-3.7	-22.2	-13.2	15.1	38.7	-1.4	23.6
	2.788	1.837	0.502	-0.328	-1.950	-1.166	1.332	3.410	-0.125	2.079
N	1138	1138	1138	1138	1136	1136	1136	1136	1136	1136
X2	2.3	2.8	4.8	0.3	2.6	0.4	0.6	2.0	0.3	4.5
Gvalue	2.3	2.8	4.7	0.3	2.6	0.4	0.6	1.9	0.3	4.6
Gv/N	0.198	0.249	0.413	0.023	0.226	0.039	0.056	0.171	0.023	0.404
Prob	0.52091	0.41829	0.19497	0.96741	0.46359	0.93089	0.88799	0.58346	0.96777	0.20454

Bsui_1330
5UTR:

	50	49	48	47	46	45	44	43	42	41
a_O	759	807	710	761	826	766	806	847	766	772
	(23.2)	(24.7)	(21.7)	(23.3)	(25.2)	(23.4)	(24.6)	(25.9)	(23.4)	(23.6)
a_E	808.5	808.5	808.5	808.5	808.5	808.5	808.5	808.5	808.5	808.5
	(24.7)	(24.7)	(24.7)	(24.7)	(24.7)	(24.7)	(24.7)	(24.7)	(24.7)	(24.7)
	-95.9	-3.0	-184.5	-92.2	35.4	-82.7	-5.0	78.8	-82.7	-71.3
	-2.931	-0.092	-5.637	-2.816	1.080	-2.528	-0.153	2.407	-2.528	-2.180
t_O	789	757	842	722	772	766	738	761	755	778
	(24.1)	(23.1)	(25.7)	(22.1)	(23.6)	(23.4)	(22.5)	(23.3)	(23.1)	(23.8)
t_E	759.8	759.8	759.8	759.8	759.8	759.8	759.8	759.8	759.8	759.8
	(23.2)	(23.2)	(23.2)	(23.2)	(23.2)	(23.2)	(23.2)	(23.2)	(23.2)	(23.2)
	59.5	-5.6	172.9	-73.7	24.6	12.4	-43.0	2.4	-9.6	36.8
	1.817	-0.172	5.284	-2.253	0.750	0.379	-1.314	0.072	-0.294	1.124
g_O	922	834	881	914	823	867	906	830	875	887
	(28.2)	(25.5)	(26.9)	(27.9)	(25.1)	(26.5)	(27.7)	(25.4)	(26.7)	(27.1)
g_E	884.0	884.0	884.0	884.0	884.0	884.0	884.0	884.0	884.0	884.0
	(27.0)	(27.0)	(27.0)	(27.0)	(27.0)	(27.0)	(27.0)	(27.0)	(27.0)	(27.0)
	77.6	-97.1	-6.0	61.0	-117.7	-33.7	44.5	-104.6	-17.9	6.0
	2.371	-2.967	-0.183	1.864	-3.596	-1.029	1.361	-3.197	-0.547	0.184
c_O	803	875	840	876	852	874	823	835	877	836
	(24.5)	(26.7)	(25.7)	(26.8)	(26.0)	(26.7)	(25.1)	(25.5)	(26.8)	(25.5)
c_E	820.7	820.7	820.7	820.7	820.7	820.7	820.7	820.7	820.7	820.7
	(25.1)	(25.1)	(25.1)	(25.1)	(25.1)	(25.1)	(25.1)	(25.1)	(25.1)	(25.1)
	-35.0	112.2	39.1	114.3	63.8	110.1	4.7	28.9	116.4	30.9
	-1.068	3.427	1.195	3.492	1.951	3.362	0.143	0.883	3.558	0.945
N	3273	3273	3273	3273	3273	3273	3273	3273	3273	3273
X2	6.2	6.4	21.4	9.4	6.0	6.1	1.2	5.4	6.2	2.4
Gvalue	6.2	6.4	21.6	9.4	6.0	6.0	1.2	5.4	6.2	2.4
Gv/N	0.189	0.196	0.659	0.288	0.185	0.185	0.036	0.166	0.189	0.073
Prob	0.10245	0.09303	0.00008	0.02421	0.10870	0.10933	0.75537	0.14335	0.10323	0.49356

	40	39	38	37	36	35	34	33	32	31
a_O	787	748	763	741	675	724	784	714	798	810
	(24.0)	(22.9)	(23.3)	(22.6)	(20.6)	(22.1)	(24.0)	(21.8)	(24.4)	(24.7)
a_E	808.5	808.5	808.5	808.5	808.5	808.5	808.5	808.5	808.5	808.5
	(24.7)	(24.7)	(24.7)	(24.7)	(24.7)	(24.7)	(24.7)	(24.7)	(24.7)	(24.7)
	-42.4	-116.4	-88.4	-129.2	-243.6	-159.9	-48.3	-177.5	-20.9	3.0
	-1.297	-3.556	-2.701	-3.948	-7.444	-4.884	-1.475	-5.424	-0.638	0.091
t_O	801	794	730	806	789	770	825	819	769	773
	(24.5)	(24.3)	(22.3)	(24.6)	(24.1)	(23.5)	(25.2)	(25.0)	(23.5)	(23.6)
t_E	759.8	759.8	759.8	759.8	759.8	759.8	759.8	759.8	759.8	759.8
	(23.2)	(23.2)	(23.2)	(23.2)	(23.2)	(23.2)	(23.2)	(23.2)	(23.2)	(23.2)
	84.6	69.9	-58.5	95.1	59.5	20.5	135.8	122.9	18.5	26.6
	2.583	2.135	-1.786	2.906	1.817	0.626	4.149	3.754	0.564	0.812
g_O	818	854	914	832	867	909	861	894	863	811
	(25.0)	(26.1)	(27.9)	(25.4)	(26.5)	(27.8)	(26.3)	(27.3)	(26.4)	(24.8)
g_E	884.0	884.0	884.0	884.0	884.0	884.0	884.0	884.0	884.0	884.0
	(27.0)	(27.0)	(27.0)	(27.0)	(27.0)	(27.0)	(27.0)	(27.0)	(27.0)	(27.0)
	-126.9	-59.0	61.0	-100.9	-33.7	50.7	-45.4	20.1	-41.5	-139.8
	-3.879	-1.802	1.864	-3.082	-1.029	1.549	-1.387	0.615	-1.268	-4.271
c_O	867	877	866	894	942	870	803	846	843	879
	(26.5)	(26.8)	(26.5)	(27.3)	(28.8)	(26.6)	(24.5)	(25.8)	(25.8)	(26.9)
c_E	820.7	820.7	820.7	820.7	820.7	820.7	820.7	820.7	820.7	820.7
	(25.1)	(25.1)	(25.1)	(25.1)	(25.1)	(25.1)	(25.1)	(25.1)	(25.1)	(25.1)
	95.2	116.4	93.1	153.0	259.8	101.6	-35.0	51.4	45.3	120.7
	2.909	3.558	2.845	4.675	7.937	3.103	-1.068	1.571	1.383	3.688
N	3273	3273	3273	3273	3273	3273	3273	3273	3273	3273
X2	10.3	11.0	7.3	18.1	41.4	12.6	7.3	16.6	1.4	10.4
Gvalue	10.4	11.0	7.3	18.0	41.9	12.9	7.2	16.9	1.4	10.5
Gv/N	0.318	0.335	0.222	0.551	1.281	0.394	0.219	0.516	0.041	0.320
Prob	0.01551	0.01188	0.06398	0.00043	0.00000	0.00485	0.06651	0.00075	0.71666	0.01488

	30	29	28	27	26	25	24	23	22	21
a_O	743	788	805	790	840	846	827	898	880	831
	(22.7)	(24.1)	(24.6)	(24.1)	(25.7)	(25.8)	(25.3)	(27.4)	(26.9)	(25.4)
a_E	808.5	808.5	808.5	808.5	808.5	808.5	808.5	808.5	808.5	808.5
	(24.7)	(24.7)	(24.7)	(24.7)	(24.7)	(24.7)	(24.7)	(24.7)	(24.7)	(24.7)
	-125.6	-40.5	-7.0	-36.6	64.2	76.7	37.4	188.5	149.1	45.6
	-3.836	-1.237	-0.214	-1.118	1.961	2.343	1.143	5.760	4.556	1.393
t_O	802	737	787	768	679	695	751	692	801	785
	(24.5)	(22.5)	(24.0)	(23.5)	(20.7)	(21.2)	(22.9)	(21.1)	(24.5)	(24.0)
t_E	759.8	759.8	759.8	759.8	759.8	759.8	759.8	759.8	759.8	759.8
	(23.2)	(23.2)	(23.2)	(23.2)	(23.2)	(23.2)	(23.2)	(23.2)	(23.2)	(23.2)
	86.7	-44.9	55.3	16.4	-152.7	-123.9	-17.5	-129.4	84.6	51.2
	2.648	-1.373	1.690	0.503	-4.666	-3.787	-0.536	-3.953	2.583	1.564
g_O	859	896	842	839	904	840	828	846	771	817
	(26.2)	(27.4)	(25.7)	(25.6)	(27.6)	(25.7)	(25.3)	(25.8)	(23.6)	(25.0)
g_E	884.0	884.0	884.0	884.0	884.0	884.0	884.0	884.0	884.0	884.0
	(27.0)	(27.0)	(27.0)	(27.0)	(27.0)	(27.0)	(27.0)	(27.0)	(27.0)	(27.0)
	-49.3	24.2	-82.0	-87.7	40.4	-85.8	-108.4	-74.3	-210.9	-128.8
	-1.506	0.738	-2.504	-2.679	1.236	-2.621	-3.311	-2.271	-6.444	-3.935
c_O	869	852	839	876	850	892	867	837	821	840
	(26.6)	(26.0)	(25.6)	(26.8)	(26.0)	(27.3)	(26.5)	(25.6)	(25.1)	(25.7)
c_E	820.7	820.7	820.7	820.7	820.7	820.7	820.7	820.7	820.7	820.7
	(25.1)	(25.1)	(25.1)	(25.1)	(25.1)	(25.1)	(25.1)	(25.1)	(25.1)	(25.1)
	99.5	63.8	37.1	114.3	59.7	148.7	95.2	33.0	0.7	39.1
	3.039	1.951	1.132	3.492	1.824	4.543	2.909	1.008	0.020	1.195
N	3273	3273	3273	3273	3273	3273	3273	3273	3273	3273
X2	11.2	2.6	3.4	6.5	11.3	15.7	6.7	17.9	23.0	7.0
Gvalue	11.3	2.6	3.4	6.5	11.6	15.7	6.7	17.8	23.4	7.1
Gv/N	0.344	0.078	0.104	0.198	0.355	0.478	0.205	0.543	0.716	0.217
Prob	0.01039	0.46448	0.33257	0.08987	0.00883	0.00133	0.08156	0.00049	0.00003	0.06852

	20	19	18	17	16	15	14	13	12	11
a_O	929	944	885	927	986	957	990	1042	1037	993
	(28.4)	(28.8)	(27.0)	(28.3)	(30.1)	(29.2)	(30.2)	(31.8)	(31.7)	(30.3)
a_E	808.5	808.5	808.5	808.5	808.5	808.5	808.5	808.5	808.5	808.5
	(24.7)	(24.7)	(24.7)	(24.7)	(24.7)	(24.7)	(24.7)	(24.7)	(24.7)	(24.7)
	258.1	292.5	160.0	253.6	391.4	322.7	401.0	528.7	516.2	408.2
	7.886	8.937	4.888	7.747	11.958	9.860	12.251	16.154	15.772	12.472
t_O	733	782	803	719	734	762	622	600	553	426
	(22.4)	(23.9)	(24.5)	(22.0)	(22.4)	(23.3)	(19.0)	(18.3)	(16.9)	(13.0)

```
t_E        759.8   759.8   759.8   759.8   759.8   759.8   759.8   759.8   759.8   759.8
         ( 23.2) ( 23.2) ( 23.2) ( 23.2) ( 23.2) ( 23.2) ( 23.2) ( 23.2) ( 23.2) ( 23.2)
           -52.7    45.0    88.8   -79.4   -50.8     4.4  -249.0  -283.4  -351.4  -493.0
          -1.610   1.375   2.712  -2.426  -1.551   0.133  -7.607  -8.658 -10.736 -15.063
g_O          786     706     861     823     811     841    1003     961    1188    1380
         ( 24.0) ( 21.6) ( 26.3) ( 25.1) ( 24.8) ( 25.7) ( 30.6) ( 29.4) ( 36.3) ( 42.2)
g_E        884.0   884.0   884.0   884.0   884.0   884.0   884.0   884.0   884.0   884.0
         ( 27.0) ( 27.0) ( 27.0) ( 27.0) ( 27.0) ( 27.0) ( 27.0) ( 27.0) ( 27.0) ( 27.0)
          -184.7  -317.5   -45.4  -117.7  -139.8   -83.9   253.3   160.5   702.3  1229.3
          -5.643  -9.700  -1.387  -3.596  -4.271  -2.563   7.740   4.904  21.457  37.557
c_O          825     841     724     804     742     713     658     670     495     474
         ( 25.2) ( 25.7) ( 22.1) ( 24.6) ( 22.7) ( 21.8) ( 20.1) ( 20.5) ( 15.1) ( 14.5)
c_E        820.7   820.7   820.7   820.7   820.7   820.7   820.7   820.7   820.7   820.7
         ( 25.1) ( 25.1) ( 25.1) ( 25.1) ( 25.1) ( 25.1) ( 25.1) ( 25.1) ( 25.1) ( 25.1)
             8.7    41.2  -181.5   -33.0  -149.5  -200.6  -290.7  -271.8  -500.5  -520.4
           0.265   1.258  -5.545  -1.008  -4.569  -6.127  -8.883  -8.305 -15.292 -15.899

N           3273    3273    3273    3273    3273    3273    3273    3273    3273    3273
X2          29.8    59.7    21.7    24.1    53.4    43.5   114.0   135.4   354.6   613.5
Gvalue      29.4    61.2    21.9    23.5    51.3    42.7   114.6   134.0   366.6   624.1
Gv/N       0.898   1.870   0.669   0.717   1.567   1.303   3.502   4.096  11.200  19.067
Prob     0.00000 0.00000 0.00007 0.00003 0.00000 0.00000 0.00000 0.00000 0.00000 0.00000
         -------------------------------------------------------------------------------
              10       9       8       7       6       5       4       3       2       1
         -------------------------------------------------------------------------------
a_O          896     925    1070     981    1006     986     867    1014     806     838
         ( 27.4) ( 28.3) ( 32.7) ( 30.0) ( 30.7) ( 30.1) ( 26.5) ( 31.0) ( 24.6) ( 25.6)
a_E        808.5   808.5   808.5   808.5   808.5   808.5   808.5   808.5   808.5   808.5
         ( 24.7) ( 24.7) ( 24.7) ( 24.7) ( 24.7) ( 24.7) ( 24.7) ( 24.7) ( 24.7) ( 24.7)
           184.1   249.0   599.7   379.4   439.7   391.4   121.1   459.3    -5.0    60.0
           5.626   7.608  18.322  11.592  13.434  11.958   3.700  14.032  -0.153   1.834
t_O          431     452     496     705     681     693     793     604     817     609
         ( 13.2) ( 13.8) ( 15.2) ( 21.5) ( 20.8) ( 21.2) ( 24.2) ( 18.5) ( 25.0) ( 18.6)
t_E        759.8   759.8   759.8   759.8   759.8   759.8   759.8   759.8   759.8   759.8
         ( 23.2) ( 23.2) ( 23.2) ( 23.2) ( 23.2) ( 23.2) ( 23.2) ( 23.2) ( 23.2) ( 23.2)
          -488.7  -469.5  -423.1  -105.6  -149.2  -127.6    67.8  -277.2   118.6  -269.5
         -14.932 -14.346 -12.927  -3.226  -4.557  -3.898   2.071  -8.471   3.622  -8.234
g_O         1506    1507    1241    1020     923     867     785     905     686     652
         ( 46.0) ( 46.0) ( 37.9) ( 31.2) ( 28.2) ( 26.5) ( 24.0) ( 27.7) ( 21.0) ( 19.9)
g_E        884.0   884.0   884.0   884.0   884.0   884.0   884.0   884.0   884.0   884.0
         ( 27.0) ( 27.0) ( 27.0) ( 27.0) ( 27.0) ( 27.0) ( 27.0) ( 27.0) ( 27.0) ( 27.0)
          1604.7  1607.7   841.9   291.9    79.7   -33.7  -186.5    42.5  -347.9  -397.0
          49.027  49.121  25.724   8.919   2.435  -1.029  -5.697   1.298 -10.630 -12.128
c_O          440     389     466     567     663     727     828     750     964    1174
         ( 13.4) ( 11.9) ( 14.2) ( 17.3) ( 20.3) ( 22.2) ( 25.3) ( 22.9) ( 29.5) ( 35.9)
c_E        820.7   820.7   820.7   820.7   820.7   820.7   820.7   820.7   820.7   820.7
         ( 25.1) ( 25.1) ( 25.1) ( 25.1) ( 25.1) ( 25.1) ( 25.1) ( 25.1) ( 25.1) ( 25.1)
          -548.5  -580.8  -527.5  -419.3  -282.9  -176.2    14.7  -135.1   310.4   840.7
         -16.760 -17.745 -16.115 -12.811  -8.643  -5.384   0.450  -4.127   9.482  25.686

N           3273    3273    3273    3273    3273    3273    3273    3273    3273    3273
X2         766.0   807.6   473.6   140.1    88.4    55.9    16.8    90.8    73.7   244.0
Gvalue     751.5   806.4   491.1   146.4    87.3    53.9    17.2    89.4    76.0   234.3
Gv/N      22.961  24.638  15.003   4.474   2.669   1.647   0.524   2.733   2.322   7.158
Prob     0.00000 0.00000 0.00000 0.00000 0.00000 0.00000 0.00066 0.00000 -0.00000 0.00000
         -------------------------------------------------------------------------------

CDS_INITIAL:
         -------------------------------------------------------------------------------
               1       2       3       4       5       6       7       8       9      10
         -------------------------------------------------------------------------------
a_O         2627       0       0    1405     935     757    1018    1105     747    1003
         ( 80.3) (  0.0) (  0.0) ( 42.9) ( 28.6) ( 23.1) ( 31.1) ( 33.8) ( 22.8) ( 30.6)
a_E        756.1   860.0   375.2   756.1   860.0   375.2   756.1   860.0   375.2   756.1
         ( 23.1) ( 26.3) ( 11.5) ( 23.1) ( 26.3) ( 11.5) ( 23.1) ( 26.3) ( 11.5) ( 23.1)
          6543.4     0.0     0.0  1741.1   156.4  1062.6   605.5   554.0  1028.7   566.8
         199.921   0.000   0.000  53.196   4.777  32.467  18.501  16.926  31.431  17.318
t_O          313    3273       0     389     561     762     476     827     825     578
         (  9.6) (100.0) (  0.0) ( 11.9) ( 17.1) ( 23.3) ( 14.5) ( 25.3) ( 25.2) ( 17.7)
t_E        443.8   969.2   686.7   443.8   969.2   686.7   443.8   969.2   686.7   443.8
         ( 13.6) ( 29.6) ( 21.0) ( 13.6) ( 29.6) ( 21.0) ( 13.6) ( 29.6) ( 21.0) ( 13.6)
          -218.6  7966.6     0.0  -102.6  -613.4   158.7    66.6  -262.4   302.9   305.3
          -6.680 243.404   0.000  -3.135 -18.742   4.848   2.035  -8.017   9.253   9.328
g_O          333       0    3273     816     611     787    1051     578     797     904
         ( 10.2) (  0.0) (100.0) ( 24.9) ( 18.7) ( 24.0) ( 32.1) ( 17.7) ( 24.4) ( 27.6)
g_E       1256.7   611.2  1027.9  1256.7   611.2  1027.9  1256.7   611.2  1027.9  1256.7
         ( 38.4) ( 18.7) ( 31.4) ( 38.4) ( 18.7) ( 31.4) ( 38.4) ( 18.7) ( 31.4) ( 38.4)
          -884.5     0.0  7581.4  -704.7    -0.3  -420.4  -375.7   -64.5  -405.6  -595.6
         -27.025   0.000 231.634 -21.532  -0.010 -12.843 -11.479  -1.971 -12.391 -18.197
c_O            0       0       0     663    1166     967     728     763     904     788
         (  0.0) (  0.0) (  0.0) ( 20.3) ( 35.6) ( 29.5) ( 22.2) ( 23.3) ( 27.6) ( 24.1)
c_E        816.4   832.7  1183.2   816.4   832.7  1183.2   816.4   832.7  1183.2   816.4
         ( 24.9) ( 25.4) ( 36.2) ( 24.9) ( 25.4) ( 36.2) ( 24.9) ( 25.4) ( 36.2) ( 24.9)
             0.0     0.0     0.0  -275.9   785.2  -390.3  -166.8  -133.3  -486.6   -55.7
           0.000   0.000   0.000  -8.430  23.990 -11.924  -5.096  -4.073 -14.868  -1.703

N           3273    3273    3273    3273    3273    3273    3273    3273    3273    3273
X2        6163.2  7780.3  7148.6   747.0   311.9   492.7   136.3    98.3   514.0   221.1
Gvalue    5440.3  7966.6  7581.4   657.8   327.8   410.7   129.6    93.8   439.4   220.9
Gv/N     166.217 243.404 231.634  20.099  10.015  12.548   3.960   2.865  13.424   6.748
Prob     1.00000 1.00000 1.00000 0.00000 0.00000 0.00000 0.00000 0.00000 0.00000 0.00000
         -------------------------------------------------------------------------------
              11      12      13      14      15      16      17      18      19      20
         -------------------------------------------------------------------------------
a_O          925     741    1014     849     612     974     927     655     989     912
         ( 28.3) ( 22.6) ( 31.0) ( 25.9) ( 18.7) ( 29.8) ( 28.3) ( 20.0) ( 30.2) ( 27.9)
a_E        860.0   375.2   756.1   860.0   375.2   756.1   860.0   375.2   756.1   860.0
         ( 26.3) ( 11.5) ( 23.1) ( 26.3) ( 11.5) ( 23.1) ( 26.3) ( 11.5) ( 23.1) ( 26.3)
           134.8  1008.5   595.2   -21.9   598.8   493.3   139.1   729.9   531.1   107.1
           4.118  30.813  18.184  -0.668  18.296  15.072   4.250  22.299  16.227   3.272
t_O          956     850     558    1048     873     565    1044     831     524     985
         ( 29.2) ( 26.0) ( 17.0) ( 32.0) ( 26.7) ( 17.3) ( 31.9) ( 25.4) ( 16.0) ( 30.1)
t_E        969.2   686.7   443.8   969.2   686.7   443.8   969.2   686.7   443.8   969.2
         ( 29.6) ( 21.0) ( 13.6) ( 29.6) ( 21.0) ( 13.6) ( 29.6) ( 21.0) ( 13.6) ( 29.6)
           -26.2   362.8   255.5   163.9   419.2   272.7   155.3   317.1   174.0    31.9
          -0.799  11.084   7.805   5.008  12.809   8.333   4.745   9.689   5.316   0.975
g_O          530     785     909     523     785     941     500     743     892     549
         ( 16.2) ( 24.0) ( 27.8) ( 16.0) ( 24.0) ( 28.8) ( 15.3) ( 22.7) ( 27.3) ( 16.8)
g_E        611.2  1027.9  1256.7   611.2  1027.9  1256.7   611.2  1027.9  1256.7   611.2
         ( 18.7) ( 31.4) ( 38.4) ( 18.7) ( 31.4) ( 38.4) ( 18.7) ( 31.4) ( 38.4) ( 18.7)
          -151.0  -423.3  -588.8  -163.0  -423.3  -544.5  -200.8  -482.3  -611.5  -117.8
          -4.615 -12.933 -17.991  -4.979 -12.933 -16.635  -6.134 -14.737 -18.683  -3.599
```

c_O	862	897	792	853	1003	793	802	1044	868	827
	(26.3)	(27.4)	(24.2)	(26.1)	(30.6)	(24.2)	(24.5)	(31.9)	(26.5)	(25.3)
c_E	832.7	1183.2	816.4	832.7	1183.2	816.4	832.7	1183.2	816.4	832.7
	(25.4)	(36.2)	(24.9)	(25.4)	(36.2)	(24.9)	(25.4)	(36.2)	(24.9)	(25.4)
	59.7	-496.8	-48.0	41.2	-331.5	-46.1	-60.2	-261.4	106.5	-11.3
	1.824	-15.179	-1.466	1.258	-10.127	-1.407	-1.839	-7.986	3.253	-0.345
N	3273	3273	3273	3273	3273	3273	3273	3273	3273	3273
X2	16.9	522.1	214.3	19.8	284.9	175.8	32.3	334.3	195.3	9.8
Gvalue	17.3	451.2	213.8	20.3	263.3	175.5	33.4	303.2	200.1	9.9
Gv/N	0.528	13.785	6.532	0.619	8.045	5.363	1.022	9.265	6.113	0.303
Prob	0.00062	0.00000	0.00000	0.00015	0.00000	0.00000	0.00000	0.00000	0.00000	0.01918

	21	22	23	24	25	26	27	28	29	30
a_O	608	875	855	557	817	793	510	836	777	516
	(18.6)	(26.7)	(26.1)	(17.0)	(25.0)	(24.2)	(15.6)	(25.5)	(23.7)	(15.8)
a_E	375.2	756.1	860.0	375.2	756.1	860.0	375.2	756.1	860.0	375.2
	(11.5)	(23.1)	(26.3)	(11.5)	(23.1)	(26.3)	(11.5)	(23.1)	(26.3)	(11.5)
	586.9	255.6	-10.0	440.1	126.6	-128.6	313.1	167.9	-157.7	328.8
	17.933	7.809	-0.305	13.447	3.867	-3.930	9.565	5.131	-4.819	10.046
t_O	849	535	1005	834	478	988	802	515	999	795
	(25.9)	(16.3)	(30.7)	(25.5)	(14.6)	(30.2)	(24.5)	(15.7)	(30.5)	(24.3)
t_E	686.7	443.8	969.2	686.7	443.8	969.2	686.7	443.8	969.2	686.7
	(21.0)	(13.6)	(29.6)	(21.0)	(13.6)	(29.6)	(21.0)	(13.6)	(29.6)	(21.0)
	360.4	199.9	73.0	324.3	70.9	38.0	249.1	153.2	60.6	233.0
	11.010	6.107	2.229	9.907	2.166	1.162	7.610	4.680	1.850	7.117
g_O	761	1053	556	792	1123	607	862	1106	614	876
	(23.3)	(32.2)	(17.0)	(24.2)	(34.3)	(18.5)	(26.3)	(33.8)	(18.8)	(26.8)
g_E	1027.9	1256.7	611.2	1027.9	1256.7	611.2	1027.9	1256.7	611.2	1027.9
	(31.4)	(38.4)	(18.7)	(31.4)	(38.4)	(18.7)	(31.4)	(38.4)	(18.7)	(31.4)
	-457.6	-372.4	-105.2	-413.0	-252.6	-8.3	-303.5	-282.6	5.7	-280.2
	-13.981	-11.379	-3.214	-12.618	-7.719	-0.254	-9.272	-8.633	0.173	-8.561
c_O	1055	810	857	1090	855	885	1099	816	883	1086
	(32.2)	(24.7)	(26.2)	(33.3)	(26.1)	(27.0)	(33.6)	(24.9)	(27.0)	(33.2)
c_E	1183.2	816.4	832.7	1183.2	816.4	832.7	1183.2	816.4	832.7	1183.2
	(36.2)	(24.9)	(25.4)	(36.2)	(24.9)	(25.4)	(36.2)	(24.9)	(25.4)	(36.2)
	-242.0	-12.7	49.4	-178.9	79.1	107.9	-162.3	-0.7	103.7	-186.2
	-7.394	-0.387	1.509	-5.466	2.416	3.297	-4.958	-0.022	3.167	-5.689
N	3273	3273	3273	3273	3273	3273	3273	3273	3273	3273
X2	266.0	70.5	7.0	181.2	23.6	8.9	100.6	37.9	12.0	100.4
Gvalue	247.7	70.4	7.2	172.5	23.9	9.0	96.3	37.8	12.2	95.4
Gv/N	7.568	2.150	0.219	5.270	0.730	0.274	2.944	1.156	0.372	2.914
Prob	0.00000	0.00000	0.06640	0.00000	0.00003	0.02962	0.00000	0.00000	0.00677	0.00000

	31	32	33	34	35	36	37	38	39	40
a_O	813	777	479	803	742	452	735	732	499	733
	(24.8)	(23.7)	(14.6)	(24.5)	(22.7)	(13.8)	(22.5)	(22.4)	(15.2)	(22.4)
a_E	756.1	860.0	375.2	756.1	860.0	375.2	756.1	860.0	375.2	756.1
	(23.1)	(26.3)	(11.5)	(23.1)	(26.3)	(11.5)	(23.1)	(26.3)	(11.5)	(23.1)
	118.0	-157.7	234.0	96.6	-219.0	168.3	-41.6	-235.9	284.5	-45.5
	3.604	-4.819	7.148	2.953	-6.691	5.143	-1.272	-7.208	8.694	-1.390
t_O	490	1033	817	507	990	781	499	1010	755	469
	(15.0)	(31.6)	(25.0)	(15.5)	(30.2)	(23.9)	(15.2)	(30.9)	(23.1)	(14.3)
t_E	443.8	969.2	686.7	443.8	969.2	686.7	443.8	969.2	686.7	443.8
	(13.6)	(29.6)	(21.0)	(13.6)	(29.6)	(21.0)	(13.6)	(29.6)	(21.0)	(13.6)
	97.0	131.8	284.0	134.9	42.1	201.1	116.9	83.4	143.3	51.7
	2.963	4.026	8.677	4.122	1.286	6.144	3.572	2.547	4.378	1.580
g_O	1162	616	891	1185	666	921	1250	670	941	1251
	(35.5)	(18.8)	(27.2)	(36.2)	(20.3)	(28.1)	(38.2)	(20.5)	(28.8)	(38.2)
g_E	1256.7	611.2	1027.9	1256.7	611.2	1027.9	1256.7	611.2	1027.9	1256.7
	(38.4)	(18.7)	(31.4)	(38.4)	(18.7)	(31.4)	(38.4)	(18.7)	(31.4)	(38.4)
	-182.1	9.7	-254.7	-139.2	114.4	-202.3	-13.4	123.2	-166.3	-11.4
	-5.563	0.296	-7.783	-4.254	3.497	-6.181	-0.408	3.763	-5.080	-0.347
c_O	808	847	1086	778	875	1119	789	861	1078	820
	(24.7)	(25.9)	(33.2)	(23.8)	(26.7)	(34.2)	(24.1)	(26.3)	(32.9)	(25.1)
c_E	816.4	832.7	1183.2	816.4	832.7	1183.2	816.4	832.7	1183.2	816.4
	(24.9)	(25.4)	(36.2)	(24.9)	(25.4)	(36.2)	(24.9)	(25.4)	(36.2)	(24.9)
	-16.6	28.9	-186.2	-74.9	86.8	-124.9	-53.8	57.6	-200.8	7.3
	-0.508	0.884	-5.689	-2.288	2.652	-3.816	-1.644	1.761	-6.135	0.223
N	3273	3273	3273	3273	3273	3273	3273	3273	3273	3273
X2	16.3	12.5	79.7	17.8	23.7	43.3	8.4	27.4	64.3	2.2
Gvalue	16.2	12.7	77.0	17.4	24.3	42.2	8.1	28.2	60.8	2.2
Gv/N	0.496	0.387	2.353	0.533	0.743	1.290	0.249	0.862	1.857	0.066
Prob	0.00102	0.00539	0.00000	0.00057	0.00002	0.00000	0.04307	0.00000	0.00000	0.54068

	41	42	43	44	45	46	47	48	49	50
a_O	745	483	739	686	472	725	763	480	742	737
	(22.8)	(14.8)	(22.6)	(21.0)	(14.4)	(22.2)	(23.3)	(14.7)	(22.7)	(22.5)
a_E	860.0	375.2	756.1	860.0	375.2	756.1	860.0	375.2	756.1	860.0
	(26.3)	(11.5)	(23.1)	(26.3)	(11.5)	(23.1)	(26.3)	(11.5)	(23.1)	(26.3)
	-213.9	243.9	-33.8	-310.1	216.6	-60.9	-182.6	236.4	-27.9	-227.5
	-6.535	7.453	-1.033	-9.476	6.619	-1.861	-5.580	7.224	-0.854	-6.951
t_O	1007	723	525	999	779	457	966	783	486	966
	(30.8)	(22.1)	(16.0)	(30.5)	(23.8)	(14.0)	(29.5)	(23.9)	(14.8)	(29.5)
t_E	969.2	686.7	443.8	969.2	686.7	443.8	969.2	686.7	443.8	969.2
	(29.6)	(21.0)	(13.6)	(29.6)	(21.0)	(13.6)	(29.6)	(21.0)	(13.6)	(29.6)
	77.1	74.6	176.3	60.6	196.6	26.7	-6.3	205.6	88.2	-6.3
	2.356	2.279	5.388	1.850	6.006	0.816	-0.194	6.282	2.695	-0.194
g_O	633	942	1198	655	958	1241	692	886	1211	661
	(19.3)	(28.8)	(36.6)	(20.0)	(29.3)	(37.9)	(21.1)	(27.1)	(37.0)	(20.2)
g_E	611.2	1027.9	1256.7	611.2	1027.9	1256.7	611.2	1027.9	1256.7	611.2
	(18.7)	(31.4)	(38.4)	(18.7)	(31.4)	(38.4)	(18.7)	(31.4)	(38.4)	(18.7)
	44.4	-164.4	-114.6	90.7	-135.0	-31.2	171.9	-263.3	-89.7	103.6
	1.358	-5.024	-3.502	2.772	-4.124	-0.953	5.252	-8.044	-2.741	3.166
c_O	888	1125	811	933	1064	850	852	1124	834	909
	(27.1)	(34.4)	(24.8)	(28.5)	(32.5)	(26.0)	(26.0)	(34.3)	(25.5)	(27.8)
c_E	832.7	1183.2	816.4	832.7	1183.2	816.4	832.7	1183.2	816.4	832.7
	(25.4)	(36.2)	(24.9)	(25.4)	(36.2)	(24.9)	(25.4)	(36.2)	(24.9)	(25.4)
	114.3	-113.5	-10.7	212.3	-226.0	68.6	39.1	-115.4	35.7	159.5
	3.492	-3.468	-0.327	6.487	-6.905	2.097	1.195	-3.526	1.089	4.872
N	3273	3273	3273	3273	3273	3273	3273	3273	3273	3273
X2	21.3	42.9	18.0	51.4	54.2	3.3	22.1	65.3	6.3	28.7
Gvalue	21.9	40.6	17.2	53.5	52.3	3.2	22.1	63.4	6.2	29.3
Gv/N	0.670	1.239	0.526	1.634	1.597	0.099	0.675	1.936	0.190	0.894
Prob	0.00007	0.00000	0.00064	0.00000	0.00000	0.35489	0.00006	0.00000	0.10185	0.00000

	51	52	53	54	55	56	57	58	59	60
a_O	501	752	715	449	733	791	437	728	766	438
	(15.3)	(23.0)	(21.8)	(13.7)	(22.4)	(24.2)	(13.4)	(22.2)	(23.4)	(13.4)
a_E	375.2	756.1	860.0	375.2	756.1	860.0	375.2	756.1	860.0	375.2
	(11.5)	(23.1)	(26.3)	(11.5)	(23.1)	(26.3)	(11.5)	(23.1)	(26.3)	(11.5)
	289.7	-8.2	-264.0	161.2	-45.5	-132.3	133.2	-55.2	-177.3	135.5
	8.851	-0.250	-8.067	4.926	-1.390	-4.042	4.071	-1.685	-5.418	4.141
t_O	733	484	1017	676	483	986	742	511	992	729
	(22.4)	(14.8)	(31.1)	(20.7)	(14.8)	(30.1)	(22.7)	(15.6)	(30.3)	(22.3)
t_E	686.7	443.8	969.2	686.7	443.8	969.2	686.7	443.8	969.2	686.7
	(21.0)	(13.6)	(29.6)	(21.0)	(13.6)	(29.6)	(21.0)	(13.6)	(29.6)	(21.0)
	95.8	83.9	98.0	-21.1	81.7	33.9	115.0	144.0	46.2	87.3
	2.926	2.562	2.993	-0.646	2.496	1.037	3.515	4.400	1.411	2.666
g_O	955	1213	668	1044	1264	611	984	1220	644	1016
	(29.2)	(37.1)	(20.4)	(31.9)	(38.6)	(18.7)	(30.1)	(37.3)	(19.7)	(31.0)
g_E	1027.9	1256.7	611.2	1027.9	1256.7	611.2	1027.9	1256.7	611.2	1027.9
	(31.4)	(38.4)	(18.7)	(31.4)	(38.4)	(18.7)	(31.4)	(38.4)	(18.7)	(31.4)
	-140.5	-85.9	118.8	32.4	14.7	-0.3	-85.9	-72.3	67.4	-23.7
	-4.294	-2.623	3.629	0.990	0.448	-0.010	-2.625	-2.209	2.059	-0.724
c_O	1084	824	873	1104	793	885	1110	814	871	1090
	(33.1)	(25.2)	(26.7)	(33.7)	(24.2)	(27.0)	(33.9)	(24.9)	(26.6)	(33.3)
c_E	1183.2	816.4	832.7	1183.2	816.4	832.7	1183.2	816.4	832.7	1183.2
	(36.2)	(24.9)	(25.4)	(36.2)	(24.9)	(25.4)	(36.2)	(24.9)	(25.4)	(36.2)
	-189.9	15.3	82.6	-153.0	-46.1	107.9	-141.8	-4.7	78.4	-178.9
	-5.801	0.469	2.524	-4.675	-1.407	3.297	-4.333	-0.144	2.396	-5.466
N	3273	3273	3273	3273	3273	3273	3273	3273	3273	3273
X2	58.8	5.2	34.0	20.2	4.9	9.1	21.0	12.3	14.3	20.6
Gvalue	55.0	5.2	35.3	19.5	4.8	9.2	20.5	11.8	14.7	20.2
Gv/N	1.682	0.158	1.079	0.595	0.146	0.281	0.627	0.362	0.448	0.617
Prob	0.00000	0.16042	0.00000	0.00022	0.18789	0.02670	0.00013	0.00798	0.00212	0.00015

	61	62	63	64	65	66	67	68	69	70
a_O	680	774	457	721	724	455	720	769	439	730
	(20.8)	(23.6)	(14.0)	(22.0)	(22.1)	(13.9)	(22.0)	(23.5)	(13.4)	(22.3)
a_E	756.1	860.0	375.2	756.1	860.0	375.2	756.1	860.0	375.2	756.1
	(23.1)	(26.3)	(11.5)	(23.1)	(26.3)	(11.5)	(23.1)	(26.3)	(11.5)	(23.1)
	-144.3	-163.1	180.2	-68.6	-249.3	175.5	-70.5	-172.0	137.9	-51.3
	-4.408	-4.983	5.507	-2.095	-7.616	5.361	-2.153	-5.255	4.212	-1.567
t_O	490	1014	759	506	977	728	478	969	725	484
	(15.0)	(31.0)	(23.2)	(15.5)	(29.9)	(22.2)	(14.6)	(29.6)	(22.2)	(14.8)
t_E	443.8	969.2	686.7	443.8	969.2	686.7	443.8	969.2	686.7	443.8
	(13.6)	(29.6)	(21.0)	(13.6)	(29.6)	(21.0)	(13.6)	(29.6)	(21.0)	(13.6)
	97.0	91.7	152.1	132.7	15.7	85.1	70.9	-0.3	78.8	83.9
	2.963	2.802	4.646	4.053	0.480	2.601	2.166	-0.011	2.407	2.562
g_O	1264	615	1011	1219	637	1047	1217	585	1012	1296
	(38.6)	(18.8)	(30.9)	(37.2)	(19.5)	(32.0)	(37.2)	(17.9)	(30.9)	(39.6)
g_E	1256.7	611.2	1027.9	1256.7	611.2	1027.9	1256.7	611.2	1027.9	1256.7
	(38.4)	(18.7)	(31.4)	(38.4)	(18.7)	(31.4)	(38.4)	(18.7)	(31.4)	(38.4)
	14.7	7.7	-33.6	-74.2	52.7	38.5	-78.1	-51.2	-31.6	79.8
	0.448	0.235	-1.025	-2.269	1.611	1.177	-2.387	-1.564	-0.965	2.439
c_O	839	870	1046	827	935	1043	858	950	1097	763
	(25.6)	(26.6)	(32.0)	(25.3)	(28.6)	(31.9)	(26.2)	(29.0)	(33.5)	(23.3)
c_E	816.4	832.7	1183.2	816.4	832.7	1183.2	816.4	832.7	1183.2	816.4
	(24.9)	(25.4)	(36.2)	(24.9)	(25.4)	(36.2)	(24.9)	(25.4)	(36.2)	(24.9)
	45.9	76.3	-257.9	21.4	216.8	-263.1	85.4	250.5	-166.0	-103.2
	1.402	2.332	-7.879	0.654	6.623	-8.039	2.608	7.653	-5.072	-3.152
N	3273	3273	3273	3273	3273	3273	3273	3273	3273	3273
X2	13.1	12.4	41.6	11.6	35.2	36.4	7.7	27.3	19.5	9.3
Gvalue	13.2	12.6	40.9	11.3	36.0	36.0	7.7	26.9	19.1	9.2
Gv/N	0.404	0.386	1.249	0.344	1.099	1.099	0.234	0.823	0.583	0.282
Prob	0.00415	0.00554	0.00000	0.01039	0.00000	0.00000	0.05328	0.00001	0.00026	0.02644

	71	72	73	74	75	76	77	78	79	80
a_O	747	464	694	770	438	720	766	468	740	834
	(22.8)	(14.2)	(21.2)	(23.5)	(13.4)	(22.0)	(23.4)	(14.3)	(22.6)	(25.5)
a_E	860.0	375.2	756.1	860.0	375.2	756.1	860.0	375.2	756.1	860.0
	(26.3)	(11.5)	(23.1)	(26.3)	(11.5)	(23.1)	(26.3)	(11.5)	(23.1)	(26.3)
	-210.5	197.1	-119.0	-170.2	135.5	-70.5	-177.3	206.8	-31.9	-51.2
	-6.430	6.022	-3.635	-5.201	4.141	-2.153	-5.418	6.319	-0.974	-1.564
t_O	1030	762	468	935	743	476	1010	687	475	1004
	(31.5)	(23.3)	(14.3)	(28.6)	(22.7)	(14.5)	(30.9)	(21.0)	(14.5)	(30.7)
t_E	969.2	686.7	443.8	969.2	686.7	443.8	969.2	686.7	443.8	969.2
	(29.6)	(21.0)	(13.6)	(29.6)	(21.0)	(13.6)	(29.6)	(21.0)	(13.6)	(29.6)
	125.4	158.7	49.6	-67.1	117.2	66.6	83.4	0.7	64.5	70.9
	3.831	4.848	1.516	-2.051	3.581	2.035	2.547	0.021	1.970	2.166
g_O	618	965	1289	656	1013	1254	641	1016	1249	575
	(18.9)	(29.5)	(39.4)	(20.0)	(31.0)	(38.3)	(19.6)	(31.0)	(38.2)	(17.6)
g_E	611.2	1027.9	1256.7	611.2	1027.9	1256.7	611.2	1027.9	1256.7	611.2
	(18.7)	(31.4)	(38.4)	(18.7)	(31.4)	(38.4)	(18.7)	(31.4)	(38.4)	(18.7)
	13.7	-121.9	65.4	92.9	-29.6	-5.4	61.1	-23.7	-15.3	-70.2
	0.420	-3.725	1.999	2.838	-0.905	-0.165	1.867	-0.724	-0.469	-2.143
c_O	878	1082	822	912	1079	823	856	1102	809	860
	(26.8)	(33.1)	(25.1)	(27.9)	(33.0)	(25.1)	(26.2)	(33.7)	(24.7)	(26.3)
c_E	832.7	1183.2	816.4	832.7	1183.2	816.4	832.7	1183.2	816.4	832.7
	(25.4)	(36.2)	(24.9)	(25.4)	(36.2)	(24.9)	(25.4)	(36.2)	(24.9)	(25.4)
	93.1	-193.5	11.3	166.0	-199.0	13.3	47.3	-156.7	-14.7	55.6
	2.845	-5.912	0.346	5.072	-6.079	0.407	1.446	-4.788	-0.448	1.698
N	3273	3273	3273	3273	3273	3273	3273	3273	3273	3273
X2	21.2	41.8	7.3	21.5	24.5	4.1	14.1	28.7	2.6	5.1
Gvalue	21.8	40.4	7.4	21.5	24.1	4.1	14.4	27.1	2.6	5.1
Gv/N	0.666	1.233	0.226	0.658	0.738	0.125	0.441	0.828	0.079	0.156
Prob	0.00007	0.00000	0.06014	0.00008	0.00002	0.25207	0.00236	0.00001	0.45783	0.16443

	81	82	83	84	85	86	87	88	89	90
a_O	454	714	804	424	711	813	430	744	774	454
	(13.9)	(21.8)	(24.6)	(13.0)	(21.7)	(24.8)	(13.1)	(22.7)	(23.6)	(13.9)
a_E	375.2	756.1	860.0	375.2	756.1	860.0	375.2	756.1	860.0	375.2
	(11.5)	(23.1)	(26.3)	(11.5)	(23.1)	(26.3)	(11.5)	(23.1)	(26.3)	(11.5)
	173.1	-81.8	-108.3	103.7	-87.5	-91.4	117.2	-24.0	-163.1	173.1
	5.288	-2.500	-3.308	3.167	-2.672	-2.792	3.581	-0.734	-4.983	5.288
t_O	729	473	956	680	494	1000	743	478	1008	682
	(22.3)	(14.5)	(29.2)	(20.8)	(15.1)	(30.6)	(22.7)	(14.6)	(30.8)	(20.8)
t_E	686.7	443.8	969.2	686.7	443.8	969.2	686.7	443.8	969.2	686.7
	(21.0)	(13.6)	(29.6)	(21.0)	(13.6)	(29.6)	(21.0)	(13.6)	(29.6)	(21.0)
	87.3	60.2	-26.2	-13.2	105.8	62.6	117.2	70.9	79.2	-9.3

	2.666	1.839	-0.799	-0.405	3.232	1.913	3.581	2.166	2.419	-0.283
g_O	1008	1281	637	1076	1261	654	1005	1287	617	999
	(30.8)	(39.1)	(19.5)	(32.9)	(38.5)	(20.0)	(30.7)	(39.3)	(18.9)	(30.5)
g_E	1027.9	1256.7	611.2	1027.9	1256.7	611.2	1027.9	1256.7	611.2	1027.9
	(31.4)	(38.4)	(18.7)	(31.4)	(38.4)	(18.7)	(31.4)	(38.4)	(18.7)	(31.4)
	-39.4	49.1	52.7	98.4	8.6	88.6	-45.3	61.3	11.7	-57.0
	-1.205	1.499	1.611	3.006	0.263	2.707	-1.385	1.874	0.358	-1.742
c_O	1082	805	876	1093	807	806	1095	764	874	1138
	(33.1)	(24.6)	(26.8)	(33.4)	(24.7)	(24.6)	(33.5)	(23.3)	(26.7)	(34.8)
c_E	1183.2	816.4	832.7	1183.2	816.4	832.7	1183.2	816.4	832.7	1183.2
	(36.2)	(24.9)	(25.4)	(36.2)	(24.9)	(25.4)	(36.2)	(24.9)	(25.4)	(36.2)
	-193.5	-22.6	88.9	-173.4	-18.6	-52.5	-169.7	-101.3	84.7	-88.7
	-5.912	-0.689	2.716	-5.297	-0.569	-1.603	-5.184	-3.095	2.588	-2.709
N	3273	3273	3273	3273	3273	3273	3273	3273	3273	3273
X2	28.2	4.9	7.2	15.5	8.5	7.4	19.7	6.9	12.3	19.1
Gvalue	27.4	4.9	7.2	15.4	8.3	7.4	19.4	6.9	12.5	18.1
Gv/N	0.836	0.149	0.220	0.471	0.255	0.226	0.593	0.211	0.382	0.553
Prob	0.00000	0.17994	0.06572	0.00149	0.03956	0.06063	0.00023	0.07457	0.00582	0.00042

	91	92	93	94	95	96	97	98	99	100
a_O	748	828	441	710	834	434	724	812	427	777
	(22.9)	(25.3)	(13.5)	(21.8)	(25.6)	(13.3)	(22.3)	(25.0)	(13.1)	(24.0)
a_E	755.9	859.7	375.1	753.1	856.6	373.7	750.3	853.4	372.3	748.5
	(23.1)	(26.3)	(11.5)	(23.1)	(26.3)	(11.5)	(23.1)	(26.3)	(11.5)	(23.1)
	-15.7	-62.3	142.8	-83.7	-44.6	129.8	-51.7	-80.8	117.0	58.1
	-0.479	-1.904	4.363	-2.567	-1.367	3.981	-1.593	-2.488	3.601	1.793
t_O	477	952	763	516	988	768	503	960	743	467
	(14.6)	(29.1)	(23.3)	(15.8)	(30.3)	(23.6)	(15.5)	(29.6)	(22.9)	(14.4)
t_E	443.7	968.9	686.4	442.1	965.3	683.9	440.4	961.8	681.4	439.4
	(13.6)	(29.6)	(21.0)	(13.6)	(29.6)	(21.0)	(13.6)	(29.6)	(21.0)	(13.6)
	69.0	-33.5	161.3	159.6	45.9	178.1	133.6	-3.5	128.6	57.0
	2.110	-1.023	4.931	4.895	1.407	5.463	4.113	-0.109	3.959	1.759
g_O	1227	630	976	1230	616	935	1181	617	955	1240
	(37.5)	(19.3)	(29.8)	(37.7)	(18.9)	(28.7)	(36.4)	(19.0)	(29.4)	(38.3)
g_E	1256.3	611.0	1027.6	1251.7	608.7	1023.8	1247.1	606.5	1020.1	1244.0
	(38.4)	(18.7)	(31.4)	(38.4)	(18.7)	(31.4)	(38.4)	(18.7)	(31.4)	(38.4)
	-57.9	38.6	-100.6	-43.0	14.6	-169.7	-128.6	21.2	-125.9	-8.0
	-1.771	1.180	-3.074	-1.320	0.448	-5.206	-3.960	0.652	-3.876	-0.248
c_O	820	862	1092	804	822	1123	840	859	1123	756
	(25.1)	(26.3)	(33.4)	(24.7)	(25.2)	(34.4)	(25.9)	(26.4)	(34.6)	(23.3)
c_E	816.1	832.4	1182.9	813.1	829.4	1178.5	810.1	826.3	1174.2	808.1
	(24.9)	(25.4)	(36.2)	(24.9)	(25.4)	(36.2)	(24.9)	(25.4)	(36.2)	(24.9)
	7.8	60.2	-174.5	-18.1	-14.6	-108.4	60.8	66.7	-100.1	-100.8
	0.238	1.841	-5.334	-0.556	-0.449	-3.324	1.873	2.053	-3.082	-3.112
N	3272	3272	3272	3260	3260	3260	3248	3248	3248	3240
X2	3.3	3.1	29.7	15.3	1.3	30.4	14.4	3.5	20.0	6.2
Gvalue	3.2	3.1	29.0	14.7	1.3	29.8	14.1	3.5	19.6	6.2
Gv/N	0.099	0.095	0.886	0.452	0.039	0.913	0.434	0.108	0.602	0.192
Prob	0.35766	0.37533	0.00000	0.00207	0.73369	0.00000	0.00280	0.32001	0.00021	0.10107

	101	102	103	104	105	106	107	108	109	110
a_O	826	431	754	791	409	718	866	420	720	778
	(25.5)	(13.3)	(23.4)	(24.5)	(12.7)	(22.3)	(26.9)	(13.1)	(22.4)	(24.3)
a_E	851.3	371.4	745.2	847.6	369.8	743.4	845.5	368.9	741.1	842.9
	(26.3)	(11.5)	(23.1)	(26.3)	(11.5)	(23.1)	(26.3)	(11.5)	(23.1)	(26.3)
	-49.9	128.2	17.6	-109.4	82.4	-49.9	41.4	109.0	-41.6	-124.7
	-1.540	3.957	0.546	-3.392	2.553	-1.551	1.286	3.386	-1.296	-3.887
t_O	937	725	463	983	708	458	898	739	482	964
	(28.9)	(22.4)	(14.4)	(30.5)	(21.9)	(14.2)	(27.9)	(23.0)	(15.0)	(30.0)
t_E	959.4	679.7	437.5	955.3	676.8	436.4	952.9	675.1	435.0	949.9
	(29.6)	(21.0)	(13.6)	(29.6)	(21.0)	(13.6)	(29.6)	(21.0)	(13.6)	(29.6)
	-44.3	93.5	52.5	56.3	63.8	44.3	-106.5	133.6	98.9	28.4
	-1.367	2.885	1.629	1.745	1.979	1.377	-3.311	4.153	3.082	0.884
g_O	628	981	1173	633	992	1246	630	948	1175	595
	(19.4)	(30.3)	(36.4)	(19.6)	(30.8)	(38.7)	(19.6)	(29.5)	(36.6)	(18.5)
g_E	605.0	1017.6	1238.6	602.4	1013.2	1235.6	600.9	1010.6	1231.7	599.0
	(18.7)	(31.4)	(38.4)	(18.7)	(31.4)	(38.4)	(18.7)	(31.4)	(38.4)	(18.7)
	46.8	-71.8	-127.8	62.7	-41.9	20.9	59.6	-121.3	-110.8	-8.0
	1.446	-2.215	-3.960	1.945	-1.298	0.650	1.852	-3.770	-3.455	-0.250
c_O	849	1103	836	819	1117	796	824	1111	831	871
	(26.2)	(34.0)	(25.9)	(25.4)	(34.6)	(24.7)	(25.6)	(34.5)	(25.9)	(27.2)
c_E	824.3	1171.3	804.6	820.7	1166.2	802.6	818.7	1163.3	800.2	816.1
	(25.4)	(36.2)	(24.9)	(25.4)	(36.2)	(24.9)	(25.4)	(36.2)	(24.9)	(25.4)
	50.2	-132.5	63.9	-3.4	-96.3	-13.2	10.7	-102.3	62.9	113.4
	1.550	-4.090	1.982	-0.106	-2.986	-0.411	0.332	-3.178	1.960	3.534
N	3240	3240	3226	3226	3226	3218	3218	3218	3208	3208
X2	2.9	17.9	6.3	6.2	8.1	2.1	5.1	19.4	9.5	8.9
Gvalue	2.9	17.4	6.3	6.2	8.0	2.1	5.1	19.0	9.3	9.0
Gv/N	0.089	0.538	0.196	0.192	0.247	0.064	0.160	0.590	0.291	0.280
Prob	0.40951	0.00058	0.09728	0.10209	0.04642	0.55701	0.16206	0.00027	0.02515	0.02959

	111	112	113	114	115	116	117	118	119	120
a_O	401	697	850	388	744	842	383	716	801	411
	(12.5)	(21.8)	(26.6)	(12.1)	(23.3)	(26.4)	(12.0)	(22.5)	(25.2)	(12.9)
a_E	367.8	739.5	841.1	367.0	736.7	837.9	365.6	735.3	836.4	364.9
	(11.5)	(23.1)	(26.3)	(11.5)	(23.1)	(26.3)	(11.5)	(23.1)	(26.3)	(11.5)
	69.4	-82.5	17.9	43.3	14.7	8.2	35.7	-38.1	-69.2	97.8
	2.163	-2.576	0.560	1.352	0.460	0.256	1.118	-1.198	-2.174	3.073
t_O	708	482	938	741	460	903	697	435	926	684
	(22.1)	(15.1)	(29.3)	(23.1)	(14.4)	(28.3)	(21.9)	(13.7)	(29.1)	(21.5)
t_E	673.0	434.1	947.9	671.5	432.4	944.3	669.0	431.6	942.5	667.8
	(21.0)	(13.6)	(29.6)	(21.0)	(13.6)	(29.6)	(21.0)	(13.6)	(29.6)	(21.0)
	71.8	101.0	-19.6	145.8	56.8	-80.8	57.1	6.8	-32.8	32.8
	2.237	3.154	-0.612	4.556	1.782	-2.533	1.790	0.212	-1.029	1.032
g_O	960	1247	612	948	1162	642	955	1246	632	991
	(29.9)	(39.0)	(19.1)	(29.6)	(36.4)	(20.1)	(29.9)	(39.1)	(19.9)	(31.1)
g_E	1007.5	1229.1	597.7	1005.3	1224.4	595.5	1001.5	1222.1	594.4	999.7
	(31.4)	(38.4)	(18.7)	(31.4)	(38.4)	(18.7)	(31.4)	(38.4)	(18.7)	(31.4)
	-92.7	36.2	28.9	-111.3	-121.6	96.6	-90.9	48.2	77.6	-17.2
	-2.891	1.130	0.903	-3.476	-3.815	3.028	-2.850	1.514	2.438	-0.541
c_O	1139	775	801	1124	823	802	1154	786	824	1097
	(35.5)	(24.2)	(25.0)	(35.1)	(25.8)	(25.1)	(36.2)	(24.7)	(25.9)	(34.5)
c_E	1159.7	798.4	814.3	1157.2	795.4	811.3	1152.8	793.9	809.8	1150.7
	(36.2)	(24.9)	(25.4)	(36.2)	(24.9)	(25.4)	(36.2)	(24.9)	(25.4)	(36.2)
	-41.1	-46.1	-26.5	-65.4	56.1	-18.5	2.3	-15.8	28.7	-104.8
	-1.280	-1.441	-0.827	-2.044	1.760	-0.579	0.072	-0.495	0.902	-3.293

N	3208	3201	3201	3201	3189	3189	3189	3183	3183	3183
X2	7.4	8.7	0.8	12.6	6.0	5.6	4.2	1.1	4.4	8.8
Gvalue	7.4	8.5	0.8	12.4	6.0	5.5	4.2	1.1	4.4	8.6
Gv/N	0.229	0.267	0.024	0.388	0.187	0.173	0.131	0.034	0.138	0.270
Prob	0.06144	0.03594	0.86016	0.00607	0.11270	0.13850	0.24388	0.78188	0.22228	0.03503

	121	122	123	124	125	126	127	128	129	130
a_O	721	818	420	721	831	415	673	838	411	678
	(22.7)	(25.8)	(13.2)	(22.8)	(26.2)	(13.1)	(21.3)	(26.6)	(13.0)	(21.5)
a_E	733.5	834.2	364.0	731.8	832.4	363.2	729.1	829.3	361.8	727.2
	(23.1)	(26.3)	(11.5)	(23.1)	(26.3)	(11.5)	(23.1)	(26.3)	(11.5)	(23.1)
	-24.7	-32.2	120.3	-21.5	-2.8	110.7	-107.7	17.6	104.8	-95.0
	-0.779	-1.013	3.788	-0.680	-0.089	3.495	-3.413	0.557	3.321	-3.019
t_O	453	905	751	482	881	712	468	893	716	440
	(14.3)	(28.5)	(23.7)	(15.2)	(27.8)	(22.5)	(14.8)	(28.3)	(22.7)	(14.0)
t_E	430.5	940.2	666.1	429.6	938.1	664.6	428.0	934.5	662.1	426.9
	(13.6)	(29.6)	(21.0)	(13.6)	(29.6)	(21.0)	(13.6)	(29.6)	(21.0)	(13.6)
	46.1	-69.0	180.2	111.0	-110.6	98.0	83.7	-81.2	112.1	26.6
	1.451	-2.172	5.676	3.502	-3.492	3.095	2.652	-2.572	3.551	0.846
g_O	1215	617	969	1203	633	924	1239	589	956	1249
	(38.3)	(19.4)	(30.5)	(38.0)	(20.0)	(29.2)	(39.3)	(18.7)	(30.3)	(39.7)
g_E	1219.1	592.9	997.1	1216.4	591.6	994.9	1211.8	589.3	991.2	1208.7
	(38.4)	(18.7)	(31.4)	(38.4)	(18.7)	(31.4)	(38.4)	(18.7)	(31.4)	(38.4)
	-8.1	49.2	-55.5	-26.6	85.7	-136.7	55.1	-0.6	-69.1	81.9
	-0.256	1.551	-1.747	-0.840	2.706	-4.315	1.745	-0.020	-2.189	2.603
c_O	786	835	1035	762	823	1117	776	836	1073	781
	(24.8)	(26.3)	(32.6)	(24.1)	(26.0)	(35.3)	(24.6)	(26.5)	(34.0)	(24.8)
c_E	791.9	807.7	1147.8	790.2	805.9	1145.3	787.2	802.9	1140.9	785.2
	(24.9)	(25.4)	(36.2)	(24.9)	(25.4)	(36.2)	(24.9)	(25.4)	(36.2)	(24.9)
	-11.8	55.5	-214.1	-55.3	34.5	-55.8	-22.2	67.6	-131.7	-8.3
	-0.371	1.747	-6.744	-1.747	1.088	-1.762	-0.703	2.141	-4.173	-0.265
N	3175	3175	3175	3168	3168	3168	3156	3156	3156	3148
X2	1.4	3.5	31.3	7.7	6.7	16.5	8.8	3.3	16.4	5.1
[truncated: 898,427 more chars]
