# Supplementary material for: Comparative genomic analysis of translation initiation mechanisms for genes lacking the Shine–Dalgarno sequence in prokaryotes
Source: Nucleic Acids Res. 2017 Feb 21;45(7):3922–31. doi: 10.1093/nar/gkx124 (PMC5397173; doi:10.1093/nar/gkx124)
Supplement: Supplementary Data [file nar-02696-h-2016-File009.pdf › nar-02696-h-2016-File009.pdf]

Suuplementary Data - *G*-statistic for non-SD genes

Aaeo_VF5
5UTR:

	100	99	98	97	96	95	94	93	92	91
a_O	174	148	173	182	168	142	147	156	159	161
	(32.0)	(27.3)	(31.9)	(33.5)	(30.9)	(26.2)	(27.1)	(28.7)	(29.3)	(29.7)
a_E	172.2	172.2	172.2	172.2	172.2	172.2	172.2	172.2	172.2	172.2
	(31.7)	(31.7)	(31.7)	(31.7)	(31.7)	(31.7)	(31.7)	(31.7)	(31.7)	(31.7)
	3.7	-44.8	1.7	20.2	-8.2	-54.7	-46.4	-30.8	-25.3	-21.6
	0.681	-8.243	0.310	3.726	-1.514	-10.073	-8.554	-5.664	-4.657	-3.974
t_O	149	151	162	145	141	142	148	139	152	153
	(27.4)	(27.8)	(29.8)	(26.7)	(26.0)	(26.2)	(27.3)	(25.6)	(28.0)	(28.2)
t_E	167.3	167.3	167.3	167.3	167.3	167.3	167.3	167.3	167.3	167.3
	(30.8)	(30.8)	(30.8)	(30.8)	(30.8)	(30.8)	(30.8)	(30.8)	(30.8)	(30.8)
	-34.5	-30.9	-10.4	-41.4	-48.2	-46.5	-36.2	-51.5	-29.1	-27.3
	-6.348	-5.691	-1.910	-7.631	-8.873	-8.566	-6.672	-9.478	-5.359	-5.025
g_O	113	134	108	104	112	125	140	125	112	129
	(20.8)	(24.7)	(19.9)	(19.2)	(20.6)	(23.0)	(25.8)	(23.0)	(20.6)	(23.8)
g_E	98.7	98.7	98.7	98.7	98.7	98.7	98.7	98.7	98.7	98.7
	(18.2)	(18.2)	(18.2)	(18.2)	(18.2)	(18.2)	(18.2)	(18.2)	(18.2)	(18.2)
	30.6	82.0	19.5	10.9	28.4	59.1	98.0	59.1	28.4	69.2
	5.644	15.106	3.594	2.015	5.227	10.890	18.041	10.890	5.227	12.735
c_O	107	110	100	112	122	134	108	123	120	100
	(19.7)	(20.3)	(18.4)	(20.6)	(22.5)	(24.7)	(19.9)	(22.7)	(22.1)	(18.4)
c_E	104.9	104.9	104.9	104.9	104.9	104.9	104.9	104.9	104.9	104.9
	(19.3)	(19.3)	(19.3)	(19.3)	(19.3)	(19.3)	(19.3)	(19.3)	(19.3)	(19.3)
	4.2	10.4	-9.6	14.7	36.8	65.6	6.3	39.2	32.3	-9.6
	0.781	1.923	-1.762	2.702	6.786	12.084	1.159	7.211	5.944	-1.762
N	543	543	543	543	543	543	543	543	543	543
X2	4.1	17.9	1.3	4.3	8.8	24.2	23.3	16.4	6.4	11.5
Gvalue	4.1	16.8	1.3	4.4	8.8	23.5	21.6	16.1	6.3	10.7
Gv/N	0.759	3.095	0.232	0.812	1.627	4.335	3.973	2.959	1.155	1.974
Prob	0.24870	0.00077	0.73863	0.22037	0.03158	0.00003	0.00008	0.00110	0.09910	0.01335

	90	89	88	87	86	85	84	83	82	81
a_O	163	157	166	148	143	173	158	174	141	152
	(30.0)	(28.9)	(30.6)	(27.3)	(26.3)	(31.9)	(29.1)	(32.0)	(26.0)	(28.0)
a_E	172.2	172.2	172.2	172.2	172.2	172.2	172.2	172.2	172.2	172.2
	(31.7)	(31.7)	(31.7)	(31.7)	(31.7)	(31.7)	(31.7)	(31.7)	(31.7)	(31.7)
	-17.8	-28.9	-12.1	-44.8	-53.1	1.7	-27.1	3.7	-56.3	-37.9
	-3.282	-5.330	-2.228	-8.243	-9.775	0.310	-4.995	0.681	-10.369	-6.973
t_O	166	154	146	138	159	143	150	141	159	164
	(30.6)	(28.4)	(26.9)	(25.4)	(29.3)	(26.3)	(27.6)	(26.0)	(29.3)	(30.2)
t_E	167.3	167.3	167.3	167.3	167.3	167.3	167.3	167.3	167.3	167.3
	(30.8)	(30.8)	(30.8)	(30.8)	(30.8)	(30.8)	(30.8)	(30.8)	(30.8)	(30.8)
	-2.5	-25.5	-39.7	-53.1	-16.1	-44.8	-32.7	-48.2	-16.1	-6.5
	-0.466	-4.688	-7.314	-9.777	-2.969	-8.257	-6.021	-8.873	-2.969	-1.193
g_O	104	112	115	125	125	114	111	124	114	113
	(19.2)	(20.6)	(21.2)	(23.0)	(23.0)	(21.0)	(20.4)	(22.8)	(21.0)	(20.8)
g_E	98.7	98.7	98.7	98.7	98.7	98.7	98.7	98.7	98.7	98.7
	(18.2)	(18.2)	(18.2)	(18.2)	(18.2)	(18.2)	(18.2)	(18.2)	(18.2)	(18.2)
	10.9	28.4	35.2	59.1	59.1	32.9	26.1	56.7	32.9	30.6
	2.015	5.227	6.487	10.890	10.890	6.064	4.814	10.436	6.064	5.644
c_O	110	120	116	132	116	113	124	104	129	114
	(20.3)	(22.1)	(21.4)	(24.3)	(21.4)	(20.8)	(22.8)	(19.2)	(23.8)	(21.0)
c_E	104.9	104.9	104.9	104.9	104.9	104.9	104.9	104.9	104.9	104.9
	(19.3)	(19.3)	(19.3)	(19.3)	(19.3)	(19.3)	(19.3)	(19.3)	(19.3)	(19.3)
	10.4	32.3	23.3	60.7	23.3	16.8	41.5	-1.8	53.4	19.0
	1.923	5.944	4.297	11.172	4.297	3.096	7.640	-0.330	9.826	3.493
N	543	543	543	543	543	543	543	543	543	543
X2	1.0	6.4	6.8	22.5	13.5	6.5	8.0	10.7	14.0	5.3
Gvalue	1.0	6.3	6.8	22.0	13.3	6.6	7.8	10.4	13.9	5.3
Gv/N	0.190	1.153	1.243	4.042	2.444	1.213	1.438	1.915	2.551	0.972
Prob	0.79331	0.09968	0.08030	0.00007	0.00409	0.08631	0.05010	0.01548	0.00311	0.15255

	80	79	78	77	76	75	74	73	72	71
a_O	139	168	159	144	154	167	140	152	150	160
	(25.6)	(30.9)	(29.3)	(26.5)	(28.4)	(30.8)	(25.8)	(28.0)	(27.6)	(29.5)
a_E	172.2	172.2	172.2	172.2	172.2	172.2	172.2	172.2	172.2	172.2
	(31.7)	(31.7)	(31.7)	(31.7)	(31.7)	(31.7)	(31.7)	(31.7)	(31.7)	(31.7)
	-59.5	-8.2	-25.3	-51.4	-34.3	-10.2	-57.9	-37.9	-41.3	-23.4
	-10.954	-1.514	-4.657	-9.473	-6.323	-1.872	-10.663	-6.973	-7.613	-4.317
t_O	154	164	154	152	152	157	150	140	165	153
	(28.4)	(30.2)	(28.4)	(28.0)	(28.0)	(28.9)	(27.6)	(25.8)	(30.4)	(28.2)
t_E	167.3	167.3	167.3	167.3	167.3	167.3	167.3	167.3	167.3	167.3
	(30.8)	(30.8)	(30.8)	(30.8)	(30.8)	(30.8)	(30.8)	(30.8)	(30.8)	(30.8)
	-25.5	-6.5	-25.5	-29.1	-29.1	-19.9	-32.7	-49.8	-4.5	-27.3
	-4.688	-1.193	-4.688	-5.359	-5.359	-3.664	-6.021	-9.177	-0.830	-5.025
g_O	125	102	119	124	113	109	135	113	126	109
	(23.0)	(18.8)	(21.9)	(22.8)	(20.8)	(20.1)	(24.9)	(20.8)	(23.2)	(20.1)
g_E	98.7	98.7	98.7	98.7	98.7	98.7	98.7	98.7	98.7	98.7
	(18.2)	(18.2)	(18.2)	(18.2)	(18.2)	(18.2)	(18.2)	(18.2)	(18.2)	(18.2)
	59.1	6.8	44.6	56.7	30.6	21.7	84.6	30.6	61.6	21.7
	10.890	1.247	8.211	10.436	5.644	3.997	15.588	5.644	11.347	3.997
c_O	125	109	111	123	124	110	118	138	102	121
	(23.0)	(20.1)	(20.4)	(22.7)	(22.8)	(20.3)	(21.7)	(25.4)	(18.8)	(22.3)
c_E	104.9	104.9	104.9	104.9	104.9	104.9	104.9	104.9	104.9	104.9
	(19.3)	(19.3)	(19.3)	(19.3)	(19.3)	(19.3)	(19.3)	(19.3)	(19.3)	(19.3)
	43.8	8.4	12.5	39.2	41.5	10.4	27.8	75.7	-5.7	34.6
	8.071	1.539	2.311	7.211	7.640	1.923	5.115	13.940	-1.053	6.363
N	543	543	543	543	543	543	543	543	543	543
X2	18.3	0.4	6.6	15.6	8.9	2.1	22.8	19.3	10.5	5.6
Gvalue	18.0	0.4	6.4	15.3	8.7	2.1	21.8	18.6	10.0	5.5
Gv/N	3.319	0.080	1.177	2.815	1.602	0.385	4.019	3.434	1.851	1.019
Prob	0.00044	0.93292	0.09411	0.00159	0.03361	0.55400	0.00007	0.00032	0.01815	0.13671

	70	69	68	67	66	65	64	63	62	61
a_O	176	156	151	153	170	171	174	163	165	171
	(32.4)	(28.7)	(27.8)	(28.2)	(31.3)	(31.5)	(32.0)	(30.0)	(30.4)	(31.5)
a_E	172.2	172.2	172.2	172.2	172.2	172.2	172.2	172.2	172.2	172.2
	(31.7)	(31.7)	(31.7)	(31.7)	(31.7)	(31.7)	(31.7)	(31.7)	(31.7)	(31.7)
	7.8	-30.8	-39.6	-36.1	-4.3	-2.3	3.7	-17.8	-14.0	-2.3
	1.430	-5.664	-7.294	-6.649	-0.791	-0.426	0.681	-3.282	-2.582	-0.426
t_O	165	155	131	158	141	143	156	144	146	165
	(30.4)	(28.5)	(24.1)	(29.1)	(26.0)	(26.3)	(28.7)	(26.5)	(26.9)	(30.4)

t_E	167.3	167.3	167.3	167.3	167.3	167.3	167.3	167.3	167.3	167.3
	(30.8)	(30.8)	(30.8)	(30.8)	(30.8)	(30.8)	(30.8)	(30.8)	(30.8)	(30.8)
	-4.5	-23.6	-64.0	-18.0	-48.2	-44.8	-21.8	-43.1	-39.7	-4.5
	-0.830	-4.349	-11.793	-3.318	-8.873	-8.257	-4.008	-7.945	-7.314	-0.830
g_O	114	111	136	139	116	127	115	120	109	108
	(21.0)	(20.4)	(25.0)	(25.6)	(21.4)	(23.4)	(21.2)	(22.1)	(20.1)	(19.9)
g_E	98.7	98.7	98.7	98.7	98.7	98.7	98.7	98.7	98.7	98.7
	(18.2)	(18.2)	(18.2)	(18.2)	(18.2)	(18.2)	(18.2)	(18.2)	(18.2)	(18.2)
	32.9	26.1	87.3	95.3	37.5	64.1	35.2	47.0	21.7	19.5
	6.064	4.814	16.073	17.545	6.913	11.807	6.487	8.650	3.997	3.594
c_O	88	121	125	93	116	102	98	116	123	99
	(16.2)	(22.3)	(23.0)	(17.1)	(21.4)	(18.8)	(18.0)	(21.4)	(22.7)	(18.2)
c_E	104.9	104.9	104.9	104.9	104.9	104.9	104.9	104.9	104.9	104.9
	(19.3)	(19.3)	(19.3)	(19.3)	(19.3)	(19.3)	(19.3)	(19.3)	(19.3)	(19.3)
	-30.9	34.6	43.8	-22.4	23.3	-5.7	-13.3	23.3	39.2	-11.5
	-5.694	6.363	8.071	-4.124	4.297	-1.053	-2.456	4.297	7.211	-2.111
N	543	543	543	543	543	543	543	543	543	543
X2	5.2	6.4	28.4	20.5	8.4	11.7	3.9	9.5	7.2	1.3
Gvalue	5.3	6.3	27.5	18.8	8.4	11.2	3.8	9.3	7.1	1.2
Gv/N	0.970	1.165	5.058	3.453	1.547	2.071	0.705	1.720	1.314	0.227
Prob	0.15338	0.09689	0.00000	0.00031	0.03838	0.01047	0.28093	0.02509	0.06780	0.74518
	60	59	58	57	56	55	54	53	52	51
a_O	147	164	169	169	183	169	173	139	168	174
	(27.1)	(30.2)	(31.1)	(31.1)	(33.7)	(31.1)	(31.9)	(25.6)	(30.9)	(32.0)
a_E	172.2	172.2	172.2	172.2	172.2	172.2	172.2	172.2	172.2	172.2
	(31.7)	(31.7)	(31.7)	(31.7)	(31.7)	(31.7)	(31.7)	(31.7)	(31.7)	(31.7)
	-46.4	-15.9	-6.3	-6.3	22.3	-6.3	1.7	-59.5	-8.2	3.7
	-8.554	-2.933	-1.153	-1.153	4.116	-1.153	0.310	-10.954	-1.514	0.681
t_O	165	154	146	140	138	154	157	146	160	157
	(30.4)	(28.4)	(26.9)	(25.8)	(25.4)	(28.4)	(28.9)	(26.9)	(29.5)	(28.9)
t_E	167.3	167.3	167.3	167.3	167.3	167.3	167.3	167.3	167.3	167.3
	(30.8)	(30.8)	(30.8)	(30.8)	(30.8)	(30.8)	(30.8)	(30.8)	(30.8)	(30.8)
	-4.5	-25.5	-39.7	-49.8	-53.1	-25.5	-19.9	-39.7	-14.2	-19.9
	-0.830	-4.688	-7.314	-9.177	-9.777	-4.688	-3.664	-7.314	-2.619	-3.664
g_O	115	106	118	115	125	113	106	134	123	101
	(21.2)	(19.5)	(21.7)	(21.2)	(23.0)	(20.8)	(19.5)	(24.7)	(22.7)	(18.6)
g_E	98.7	98.7	98.7	98.7	98.7	98.7	98.7	98.7	98.7	98.7
	(18.2)	(18.2)	(18.2)	(18.2)	(18.2)	(18.2)	(18.2)	(18.2)	(18.2)	(18.2)
	35.2	15.2	42.2	35.2	59.1	30.6	15.2	82.0	54.2	4.7
	6.487	2.798	7.776	6.487	10.890	5.644	2.798	15.106	9.985	0.868
c_O	116	119	110	119	97	107	107	124	92	111
	(21.4)	(21.9)	(20.3)	(21.9)	(17.9)	(19.7)	(19.7)	(22.8)	(16.9)	(20.4)
c_E	104.9	104.9	104.9	104.9	104.9	104.9	104.9	104.9	104.9	104.9
	(19.3)	(19.3)	(19.3)	(19.3)	(19.3)	(19.3)	(19.3)	(19.3)	(19.3)	(19.3)
	23.3	30.0	10.4	30.0	-15.2	4.2	4.2	41.5	-24.1	12.5
	4.297	5.528	1.923	5.528	-2.797	0.781	0.781	7.640	-4.446	2.311
N	543	543	543	543	543	543	543	543	543	543
X2	7.6	3.9	6.8	9.1	13.4	3.2	1.2	25.2	8.0	1.1
Gvalue	7.6	3.8	6.7	9.1	13.2	3.2	1.2	24.3	7.6	1.1
Gv/N	1.400	0.704	1.232	1.685	2.431	0.584	0.225	4.478	1.406	0.196
Prob	0.05501	0.28135	0.08243	0.02739	0.00422	0.36629	0.74791	0.00002	0.05414	0.78526
	50	49	48	47	46	45	44	43	42	41
a_O	166	147	161	153	194	176	157	162	171	161
	(30.6)	(27.1)	(29.7)	(28.2)	(35.7)	(32.4)	(28.9)	(29.8)	(31.5)	(29.7)
a_E	172.2	172.2	172.2	172.2	172.2	172.2	172.2	172.2	172.2	172.2
	(31.7)	(31.7)	(31.7)	(31.7)	(31.7)	(31.7)	(31.7)	(31.7)	(31.7)	(31.7)
	-12.1	-46.4	-21.6	-36.1	46.3	7.8	-28.9	-19.7	-2.3	-21.6
	-2.228	-8.554	-3.974	-6.649	8.534	1.430	-5.330	-3.630	-0.426	-3.974
t_O	153	166	164	172	158	174	160	157	173	173
	(28.2)	(30.6)	(30.2)	(31.7)	(29.1)	(32.0)	(29.5)	(28.9)	(31.9)	(31.9)
t_E	167.3	167.3	167.3	167.3	167.3	167.3	167.3	167.3	167.3	167.3
	(30.8)	(30.8)	(30.8)	(30.8)	(30.8)	(30.8)	(30.8)	(30.8)	(30.8)	(30.8)
	-27.3	-2.5	-6.5	9.6	-18.0	13.7	-14.2	-19.9	11.7	11.7
	-5.025	-0.466	-1.193	1.767	-3.318	2.528	-2.619	-3.664	2.146	2.146
g_O	117	114	105	109	97	98	106	107	94	94
	(21.5)	(21.0)	(19.3)	(20.1)	(17.9)	(18.0)	(19.5)	(19.7)	(17.3)	(17.3)
g_E	98.7	98.7	98.7	98.7	98.7	98.7	98.7	98.7	98.7	98.7
	(18.2)	(18.2)	(18.2)	(18.2)	(18.2)	(18.2)	(18.2)	(18.2)	(18.2)	(18.2)
	39.9	32.9	13.1	21.7	-3.3	-1.3	15.2	17.3	-9.1	-9.1
	7.343	6.064	2.405	3.997	-0.610	-0.246	2.798	3.194	-1.679	-1.679
c_O	107	116	113	109	94	95	120	117	105	115
	(19.7)	(21.4)	(20.8)	(20.1)	(17.3)	(17.5)	(22.1)	(21.5)	(19.3)	(21.2)
c_E	104.9	104.9	104.9	104.9	104.9	104.9	104.9	104.9	104.9	104.9
	(19.3)	(19.3)	(19.3)	(19.3)	(19.3)	(19.3)	(19.3)	(19.3)	(19.3)	(19.3)
	4.2	23.3	16.8	8.4	-20.6	-18.8	32.3	25.5	0.2	21.1
	0.781	4.297	3.096	1.539	-3.799	-3.469	5.944	4.704	0.037	3.894
N	543	543	543	543	543	543	543	543	543	543
X2	4.9	7.2	1.8	3.5	4.4	1.3	4.4	3.3	0.4	2.1
Gvalue	4.7	7.3	1.8	3.6	4.4	1.3	4.3	3.3	0.4	2.1
Gv/N	0.871	1.341	0.334	0.654	0.808	0.243	0.793	0.605	0.079	0.387
Prob	0.19264	0.06342	0.61242	0.31402	0.22266	0.72392	0.23042	0.34976	0.93469	0.55172
	40	39	38	37	36	35	34	33	32	31
a_O	195	165	152	164	173	173	177	161	194	159
	(35.9)	(30.4)	(28.0)	(30.2)	(31.9)	(31.9)	(32.6)	(29.7)	(35.7)	(29.3)
a_E	172.2	172.2	172.2	172.2	172.2	172.2	172.2	172.2	172.2	172.2
	(31.7)	(31.7)	(31.7)	(31.7)	(31.7)	(31.7)	(31.7)	(31.7)	(31.7)	(31.7)
	48.6	-14.0	-37.9	-15.9	1.7	1.7	9.8	-21.6	46.3	-25.3
	8.947	-2.582	-6.973	-2.933	0.310	0.310	1.808	-3.974	8.534	-4.657
t_O	154	153	156	157	182	146	158	171	169	197
	(28.4)	(28.2)	(28.7)	(28.9)	(33.5)	(26.9)	(29.1)	(31.5)	(31.1)	(36.3)
t_E	167.3	167.3	167.3	167.3	167.3	167.3	167.3	167.3	167.3	167.3
	(30.8)	(30.8)	(30.8)	(30.8)	(30.8)	(30.8)	(30.8)	(30.8)	(30.8)	(30.8)
	-25.5	-27.3	-21.8	-19.9	30.7	-39.7	-18.0	7.5	3.5	64.5
	-4.688	-5.025	-4.008	-3.664	5.658	-7.314	-3.318	1.389	0.640	11.870
g_O	97	106	123	103	82	123	94	111	78	89
	(17.9)	(19.5)	(22.7)	(19.0)	(15.1)	(22.7)	(17.3)	(20.4)	(14.4)	(16.4)
g_E	98.7	98.7	98.7	98.7	98.7	98.7	98.7	98.7	98.7	98.7
	(18.2)	(18.2)	(18.2)	(18.2)	(18.2)	(18.2)	(18.2)	(18.2)	(18.2)	(18.2)
	-3.3	15.2	54.2	8.8	-30.4	54.2	-9.1	26.1	-36.7	-18.4
	-0.610	2.798	9.985	1.629	-5.589	9.985	-1.679	4.814	-6.753	-3.381
c_O	97	119	112	119	106	101	114	100	102	98
	(17.9)	(21.9)	(20.6)	(21.9)	(19.5)	(18.6)	(21.0)	(18.4)	(18.8)	(18.0)
c_E	104.9	104.9	104.9	104.9	104.9	104.9	104.9	104.9	104.9	104.9

```
        ( 19.3) ( 19.3) ( 19.3) ( 19.3) ( 19.3) ( 19.3) ( 19.3) ( 19.3) ( 19.3) ( 19.3)
          -15.2    30.0    14.7    30.0     2.2    -7.7    19.0    -9.6    -5.7   -13.3
         -2.797   5.528   2.702   5.528   0.407  -1.409   3.493  -1.762  -1.053  -2.456

N           543     543     543     543     543     543     543     543     543     543
X2          4.7     4.0     9.6     3.1     4.1     8.9     1.7     2.6     7.2     7.7
Gvalue      4.6     3.9     9.3     3.0     4.3     8.5     1.7     2.5     7.4     7.5
Gv/N      0.852   0.719   1.706   0.560   0.786   1.572   0.304   0.467   1.368   1.376
Prob    0.20141 0.27217 0.02597 0.38553 0.23412 0.03612 0.64801 0.46900 0.05944 0.05825
        -------------------------------------------------------------------------------
             30      29      28      27      26      25      24      23      22      21
        -------------------------------------------------------------------------------
a_O         171     175     166     179     194     177     193     181     187     174
        ( 31.5) ( 32.2) ( 30.6) ( 33.0) ( 35.7) ( 32.6) ( 35.5) ( 33.3) ( 34.4) ( 32.0)
a_E       172.2   172.2   172.2   172.2   172.2   172.2   172.2   172.2   172.2   172.2
        ( 31.7) ( 31.7) ( 31.7) ( 31.7) ( 31.7) ( 31.7) ( 31.7) ( 31.7) ( 31.7) ( 31.7)
           -2.3     5.7   -12.1    13.9    46.3     9.8    44.1    18.1    30.9     3.7
         -0.426   1.055  -2.228   2.569   8.534   1.808   8.123   3.338   5.695   0.681
t_O         166     172     183     154     161     160     177     148     182     169
        ( 30.6) ( 31.7) ( 33.7) ( 28.4) ( 29.7) ( 29.5) ( 32.6) ( 27.3) ( 33.5) ( 31.1)
t_E       167.3   167.3   167.3   167.3   167.3   167.3   167.3   167.3   167.3   167.3
        ( 30.8) ( 30.8) ( 30.8) ( 30.8) ( 30.8) ( 30.8) ( 30.8) ( 30.8) ( 30.8) ( 30.8)
           -2.5     9.6    32.9   -25.5   -12.3   -14.2    20.0   -36.2    30.7     3.5
         -0.466   1.767   6.058  -4.688  -2.266  -2.619   3.686  -6.672   5.658   0.640
g_O          88      89      88      87      83      96      75     100      87      96
        ( 16.2) ( 16.4) ( 16.2) ( 16.0) ( 15.3) ( 17.7) ( 13.8) ( 18.4) ( 16.0) ( 17.7)
g_E        98.7    98.7    98.7    98.7    98.7    98.7    98.7    98.7    98.7    98.7
        ( 18.2) ( 18.2) ( 18.2) ( 18.2) ( 18.2) ( 18.2) ( 18.2) ( 18.2) ( 18.2) ( 18.2)
          -20.1   -18.4   -20.1   -21.9   -28.7    -5.3   -41.1     2.7   -21.9    -5.3
         -3.709  -3.381  -3.709  -4.033  -5.287  -0.970  -7.577   0.493  -4.033  -0.970
c_O         118     107     106     123     105     110      98     114      87     104
        ( 21.7) ( 19.7) ( 19.5) ( 22.7) ( 19.3) ( 20.3) ( 18.0) ( 21.0) ( 16.0) ( 19.2)
c_E       104.9   104.9   104.9   104.9   104.9   104.9   104.9   104.9   104.9   104.9
        ( 19.3) ( 19.3) ( 19.3) ( 19.3) ( 19.3) ( 19.3) ( 19.3) ( 19.3) ( 19.3) ( 19.3)
           27.8     4.2     2.2    39.2     0.2    10.4   -13.3    19.0   -32.6    -1.8
          5.115   0.781   0.407   7.211   0.037   1.923  -2.456   3.493  -5.995  -0.330

N           543     543     543     543     543     543     543     543     543     543
X2          2.8     1.2     2.9     5.8     5.5     0.8     9.2     3.5     7.0     0.1
Gvalue      2.8     1.2     2.9     5.7     5.5     0.8     9.6     3.5     7.2     0.1
Gv/N      0.513   0.221   0.528   1.058   1.018   0.142   1.776   0.652   1.324   0.022
Prob    0.42562 0.75273 0.41253 0.12466 0.13683 0.85606 0.02187 0.31533 0.06616 0.98958
        -------------------------------------------------------------------------------
             20      19      18      17      16      15      14      13      12      11
        -------------------------------------------------------------------------------
a_O         193     203     173     182     177     173     206     249     302     270
        ( 35.5) ( 37.4) ( 31.9) ( 33.5) ( 32.6) ( 31.9) ( 37.9) ( 45.9) ( 55.6) ( 49.7)
a_E       172.2   172.2   172.2   172.2   172.2   172.2   172.2   172.2   172.2   172.2
        ( 31.7) ( 31.7) ( 31.7) ( 31.7) ( 31.7) ( 31.7) ( 31.7) ( 31.7) ( 31.7) ( 31.7)
           44.1    66.9     1.7    20.2     9.8     1.7    73.9   183.8   339.4   243.0
          8.123  12.321   0.310   3.726   1.808   0.310  13.616  33.845  62.514  44.751
t_O         181     185     202     192     224     250     222     205     165     175
        ( 33.3) ( 34.1) ( 37.2) ( 35.4) ( 41.3) ( 46.0) ( 40.9) ( 37.8) ( 30.4) ( 32.2)
t_E       167.3   167.3   167.3   167.3   167.3   167.3   167.3   167.3   167.3   167.3
        ( 30.8) ( 30.8) ( 30.8) ( 30.8) ( 30.8) ( 30.8) ( 30.8) ( 30.8) ( 30.8) ( 30.8)
           28.6    37.3    76.2    52.9   130.8   200.9   125.7    83.4    -4.5    15.8
          5.259   6.865  14.036   9.751  24.094  37.003  23.146  15.358  -0.830   2.912
g_O          81      61      65      80      55      46      48      32      29      32
        ( 14.9) ( 11.2) ( 12.0) ( 14.7) ( 10.1) (  8.5) (  8.8) (  5.9) (  5.3) (  5.9)
g_E        98.7    98.7    98.7    98.7    98.7    98.7    98.7    98.7    98.7    98.7
        ( 18.2) ( 18.2) ( 18.2) ( 18.2) ( 18.2) ( 18.2) ( 18.2) ( 18.2) ( 18.2) ( 18.2)
          -32.0   -58.7   -54.3   -33.6   -64.3   -70.2   -69.2   -72.1   -71.0   -72.1
         -5.887 -10.805  -9.993  -6.181 -11.840 -12.930 -12.740 -13.272 -13.079 -13.272
c_O          88      94     103      89      87      74      67      57      47      66
        ( 16.2) ( 17.3) ( 19.0) ( 16.4) ( 16.0) ( 13.6) ( 12.3) ( 10.5) (  8.7) ( 12.2)
c_E       104.9   104.9   104.9   104.9   104.9   104.9   104.9   104.9   104.9   104.9
        ( 19.3) ( 19.3) ( 19.3) ( 19.3) ( 19.3) ( 19.3) ( 19.3) ( 19.3) ( 19.3) ( 19.3)
          -30.9   -20.6    -3.8   -29.3   -32.6   -51.6   -60.1   -69.5   -75.5   -61.2
         -5.694  -3.799  -0.693  -5.388  -5.995  -9.511 -11.063 -12.806 -13.898 -11.264

N           543     543     543     543     543     543     543     543     543     543
X2          9.5    22.9    18.7    10.2    41.8    78.1    64.3   109.7   179.1   115.4
Gvalue      9.8    24.9    19.9    10.4    43.8    80.8    70.4   125.6   188.5   125.6
Gv/N      1.801   4.582   3.660   1.908   8.067  14.872  12.959  23.125  34.706  23.127
Prob    0.02055 0.00002 0.00018 0.01573 0.00000 0.00000 0.00000 0.00000 0.00000 0.00000
        -------------------------------------------------------------------------------
             10       9       8       7       6       5       4       3       2       1
        -------------------------------------------------------------------------------
a_O         254     201     196     232     204     190     192     185     133     120
        ( 46.8) ( 37.0) ( 36.1) ( 42.7) ( 37.6) ( 35.0) ( 35.4) ( 34.1) ( 24.5) ( 22.1)
a_E       172.2   172.2   172.2   172.2   172.2   172.2   172.2   172.2   172.2   172.2
        ( 31.7) ( 31.7) ( 31.7) ( 31.7) ( 31.7) ( 31.7) ( 31.7) ( 31.7) ( 31.7) ( 31.7)
          197.6    62.3    50.8   138.4    69.2    37.5    41.9    26.6   -68.6   -86.6
         36.384  11.466   9.363  25.491  12.751   6.900   7.713   4.901 -12.642 -15.953
t_O         204     258     265     216     226     220     196     137     206     269
        ( 37.6) ( 47.5) ( 48.8) ( 39.8) ( 41.6) ( 40.5) ( 36.1) ( 25.2) ( 37.9) ( 49.5)
t_E       167.3   167.3   167.3   167.3   167.3   167.3   167.3   167.3   167.3   167.3
        ( 30.8) ( 30.8) ( 30.8) ( 30.8) ( 30.8) ( 30.8) ( 30.8) ( 30.8) ( 30.8) ( 30.8)
           81.0   223.6   243.9   110.4   136.0   120.6    62.1   -54.7    85.8   255.6
         14.916  41.180  44.910  20.341  25.049  22.204  11.443 -10.073  15.802  47.073
g_O          21      25      30      16      40      54      59     105      98      69
        (  3.9) (  4.6) (  5.5) (  2.9) (  7.4) (  9.9) ( 10.9) ( 19.3) ( 18.0) ( 12.7)
g_E        98.7    98.7    98.7    98.7    98.7    98.7    98.7    98.7    98.7    98.7
        ( 18.2) ( 18.2) ( 18.2) ( 18.2) ( 18.2) ( 18.2) ( 18.2) ( 18.2) ( 18.2) ( 18.2)
          -65.0   -68.6   -71.4   -58.2   -72.2   -65.1   -60.7    13.1    -1.3   -49.4
        -11.968 -12.642 -13.156 -10.721 -13.302 -11.989 -11.175   2.405  -0.246  -9.090
c_O          64      59      52      79      73      79      96     116     106      85
        ( 11.8) ( 10.9) (  9.6) ( 14.5) ( 13.4) ( 14.5) ( 17.7) ( 21.4) ( 19.5) ( 15.7)
c_E       104.9   104.9   104.9   104.9   104.9   104.9   104.9   104.9   104.9   104.9
        ( 19.3) ( 19.3) ( 19.3) ( 19.3) ( 19.3) ( 19.3) ( 19.3) ( 19.3) ( 19.3) ( 19.3)
          -63.2   -67.9   -73.0   -44.8   -52.9   -44.8   -17.0    23.3     2.2   -35.8
        -11.648 -12.506 -13.441  -8.251  -9.748  -8.251  -3.135   4.297   0.407  -6.586

N           543     543     543     543     543     543     543     543     543     543
X2        124.1   129.1   134.9   110.7    71.1    45.1    23.9     8.0    17.9    90.4
Gvalue    150.3   149.3   150.3   145.8    80.1    48.1    26.3     8.3    18.0    83.9
Gv/N     27.684  27.499  27.677  26.860  14.750   8.864   4.846   1.530   3.321  15.444
Prob    0.00000 0.00000 0.00000 0.00000 0.00000 0.00000 0.00001 0.04003 0.00043 0.00000
        -------------------------------------------------------------------------------

CDS_INITIAL:
        -------------------------------------------------------------------------------
```

	1	2	3	4	5	6	7	8	9	10
a_O	448	0	0	249	233	223	245	208	197	188
	(82.5)	(0.0)	(0.0)	(45.9)	(42.9)	(41.1)	(45.1)	(38.3)	(36.3)	(34.6)
a_E	177.6	190.9	166.0	177.6	190.9	166.0	177.6	190.9	166.0	177.6
	(32.7)	(35.2)	(30.6)	(32.7)	(35.2)	(30.6)	(32.7)	(35.2)	(30.6)	(32.7)
	828.9	0.0	0.0	168.2	92.8	131.7	157.6	35.6	67.5	21.3
	152.648	0.000	0.000	30.976	17.095	24.253	29.017	6.565	12.430	3.929
t_O	34	543	1	68	128	127	89	195	102	107
	(6.3)	(100.0)	(0.2)	(12.5)	(23.6)	(23.4)	(16.4)	(35.9)	(18.8)	(19.7)
t_E	91.3	178.2	116.4	91.3	178.2	116.4	91.3	178.2	116.4	91.3
	(16.8)	(32.8)	(21.4)	(16.8)	(32.8)	(21.4)	(16.8)	(32.8)	(21.4)	(16.8)
	-67.2	1209.9	-9.5	-40.1	-84.7	22.2	-4.6	35.1	-26.9	33.9
	-12.373	222.812	-1.752	-7.386	-15.606	4.095	-0.845	6.461	-4.947	6.244
g_O	61	0	542	159	109	104	147	67	123	157
	(11.2)	(0.0)	(99.8)	(29.3)	(20.1)	(19.2)	(27.1)	(12.3)	(22.7)	(28.9)
g_E	187.7	81.3	129.1	187.7	81.3	129.1	187.7	81.3	129.1	187.7
	(34.6)	(15.0)	(23.8)	(34.6)	(15.0)	(23.8)	(34.6)	(15.0)	(23.8)	(34.6)
	-137.1	0.0	1555.1	-52.7	63.8	-45.0	-71.8	-26.0	-11.9	-56.0
	-25.251	0.000	286.386	-9.712	11.757	-8.286	-13.228	-4.783	-2.198	-10.322
c_O	0	0	0	67	73	89	62	73	121	91
	(0.0)	(0.0)	(0.0)	(12.3)	(13.4)	(16.4)	(11.4)	(13.4)	(22.3)	(16.8)
c_E	86.4	92.5	131.5	86.4	92.5	131.5	86.4	92.5	131.5	86.4
	(15.9)	(17.0)	(24.2)	(15.9)	(17.0)	(24.2)	(15.9)	(17.0)	(24.2)	(15.9)
	0.0	0.0	0.0	-34.0	-34.6	-69.5	-41.1	-34.6	-20.2	9.5
	0.000	0.000	0.000	-6.266	-6.373	-12.808	-7.569	-6.373	-3.724	1.752
N	543	543	543	543	543	543	543	543	543	543
X2	619.4	1111.4	1732.2	43.4	37.0	39.2	41.3	9.8	8.7	8.6
Gvalue	624.6	1209.9	1545.6	41.3	37.3	39.4	40.0	10.1	8.5	8.7
Gv/N	115.024	222.812	284.634	7.612	6.872	7.254	7.375	1.869	1.561	1.602
Prob	0.00000	0.00000	1.00000	0.00000	0.00000	0.00000	0.00000	0.01733	0.03712	0.03355

	11	12	13	14	15	16	17	18	19	20
a_O	147	168	200	185	177	195	164	186	198	172
	(27.1)	(30.9)	(36.8)	(34.1)	(32.6)	(35.9)	(30.2)	(34.3)	(36.5)	(31.7)
a_E	190.9	166.0	177.6	190.9	166.0	177.6	190.9	166.0	177.6	190.9
	(35.2)	(30.6)	(32.7)	(35.2)	(30.6)	(32.7)	(35.2)	(30.6)	(32.7)	(35.2)
	-76.9	4.1	47.4	-11.7	22.7	36.4	-49.8	42.4	43.0	-35.9
	-14.154	0.747	8.737	-2.146	4.189	6.701	-9.180	7.800	7.917	-6.611
t_O	263	141	113	221	126	108	230	123	102	222
	(48.4)	(26.0)	(20.8)	(40.7)	(23.2)	(19.9)	(42.4)	(22.7)	(18.8)	(40.9)
t_E	178.2	116.4	91.3	178.2	116.4	91.3	178.2	116.4	91.3	178.2
	(32.8)	(21.4)	(16.8)	(32.8)	(21.4)	(16.8)	(32.8)	(21.4)	(16.8)	(32.8)
	204.7	54.2	48.1	95.1	20.1	36.2	117.3	13.7	22.6	97.5
	37.692	9.977	8.865	17.510	3.695	6.672	21.605	2.516	4.154	17.959
g_O	63	131	149	69	117	163	74	112	153	75
	(11.6)	(24.1)	(27.4)	(12.7)	(21.5)	(30.0)	(13.6)	(20.6)	(28.2)	(13.8)
g_E	81.3	129.1	187.7	81.3	129.1	187.7	81.3	129.1	187.7	81.3
	(15.0)	(23.8)	(34.6)	(15.0)	(23.8)	(34.6)	(15.0)	(23.8)	(34.6)	(15.0)
	-32.2	3.8	-68.8	-22.7	-23.1	-46.0	-14.0	-31.9	-62.5	-12.2
	-5.926	0.700	-12.666	-4.178	-4.246	-8.464	-2.574	-5.866	-11.513	-2.238
c_O	70	103	81	68	123	77	75	122	90	74
	(12.9)	(19.0)	(14.9)	(12.5)	(22.7)	(14.2)	(13.8)	(22.5)	(16.6)	(13.6)
c_E	92.5	131.5	86.4	92.5	131.5	86.4	92.5	131.5	86.4	92.5
	(17.0)	(24.2)	(15.9)	(17.0)	(24.2)	(15.9)	(17.0)	(24.2)	(15.9)	(17.0)
	-39.1	-50.4	-10.4	-41.9	-16.5	-17.7	-31.5	-18.4	7.4	-33.1
	-7.193	-9.281	-1.914	-7.714	-3.043	-3.256	-5.801	-3.385	1.366	-6.090
N	543	543	543	543	543	543	543	543	543	543
X2	60.0	11.5	16.3	18.8	3.2	9.0	22.8	5.8	10.1	16.8
Gvalue	56.6	11.6	16.4	18.9	3.2	9.0	22.0	5.8	10.4	16.4
Gv/N	10.419	2.143	3.022	3.472	0.596	1.653	4.049	1.065	1.924	3.020
Prob	0.00000	0.00874	0.00093	0.00029	0.35678	0.02965	0.00007	0.12279	0.01512	0.00094

	21	22	23	24	25	26	27	28	29	30
a_O	165	181	166	172	173	152	173	181	177	172
	(30.4)	(33.3)	(30.6)	(31.7)	(31.9)	(28.0)	(31.9)	(33.3)	(32.6)	(31.7)
a_E	166.0	177.6	190.9	166.0	177.6	190.9	166.0	177.6	190.9	166.0
	(30.6)	(32.7)	(35.2)	(30.6)	(32.7)	(35.2)	(30.6)	(32.7)	(35.2)	(30.6)
	-2.0	6.8	-46.4	12.2	-9.1	-69.3	14.3	6.8	-26.8	12.2
	-0.361	1.253	-8.551	2.255	-1.683	-12.763	2.638	1.253	-4.935	2.255
t_O	127	109	195	108	102	190	129	99	185	137
	(23.4)	(20.1)	(35.9)	(19.9)	(18.8)	(35.0)	(23.8)	(18.2)	(34.1)	(25.2)
t_E	116.4	91.3	178.2	116.4	91.3	178.2	116.4	91.3	178.2	116.4
	(21.4)	(16.8)	(32.8)	(21.4)	(16.8)	(32.8)	(21.4)	(16.8)	(32.8)	(21.4)
	22.2	38.6	35.1	-16.1	22.6	24.3	26.6	16.0	13.8	44.8
	4.095	7.104	6.461	-2.964	4.154	4.477	4.901	2.943	2.542	8.242
g_O	124	157	93	114	162	104	105	174	96	115
	(22.8)	(28.9)	(17.1)	(21.0)	(29.8)	(19.2)	(19.3)	(32.0)	(17.7)	(21.2)
g_E	129.1	187.7	81.3	129.1	187.7	81.3	129.1	187.7	81.3	129.1
	(23.8)	(34.6)	(15.0)	(23.8)	(34.6)	(15.0)	(23.8)	(34.6)	(15.0)	(23.8)
	-10.0	-56.0	24.9	-28.4	-47.7	51.1	-43.4	-26.3	31.8	-26.6
	-1.846	-10.322	4.593	-5.227	-8.780	9.419	-7.995	-4.850	5.864	-4.903
c_O	127	96	89	149	106	97	136	89	85	119
	(23.4)	(17.7)	(16.4)	(27.4)	(19.5)	(17.9)	(25.0)	(16.4)	(15.7)	(21.9)
c_E	131.5	86.4	92.5	131.5	86.4	92.5	131.5	86.4	92.5	131.5
	(24.2)	(15.9)	(17.0)	(24.2)	(15.9)	(17.0)	(24.2)	(15.9)	(17.0)	(24.2)
	-8.9	20.3	-6.9	37.1	43.4	9.2	9.1	5.3	-14.4	-23.9
	-1.645	3.739	-1.274	6.837	7.997	1.687	1.668	0.985	-2.656	-4.393
N	543	543	543	543	543	543	543	543	543	543
X2	1.3	9.6	6.6	4.9	9.3	15.2	6.3	1.8	4.5	6.6
Gvalue	1.3	9.6	6.7	4.9	9.2	15.3	6.6	1.8	4.4	6.5
Gv/N	0.242	1.774	1.229	0.901	1.689	2.820	1.212	0.330	0.815	1.200
Prob	0.72548	0.02195	0.08314	0.17982	0.02713	0.00157	0.08649	0.61641	0.21914	0.08896

	31	32	33	34	35	36	37	38	39	40
a_O	148	157	173	187	186	185	174	177	181	171
	(27.3)	(28.9)	(31.9)	(34.4)	(34.3)	(34.1)	(32.0)	(32.6)	(33.3)	(31.5)
a_E	177.6	190.9	166.0	177.6	190.9	166.0	177.6	190.9	166.0	177.6
	(32.7)	(35.2)	(30.6)	(32.7)	(35.2)	(30.6)	(32.7)	(35.2)	(30.6)	(32.7)
	-54.0	-61.4	14.3	19.2	-9.7	40.1	-7.2	-26.8	31.4	-13.0
	-9.948	-11.311	2.638	3.540	-1.788	7.391	-1.324	-4.935	5.774	-2.396
t_O	114	209	114	103	191	113	106	193	118	93
	(21.0)	(38.5)	(21.0)	(19.0)	(35.2)	(20.8)	(19.5)	(35.5)	(21.7)	(17.1)
t_E	91.3	178.2	116.4	91.3	178.2	116.4	91.3	178.2	116.4	91.3
	(16.8)	(32.8)	(21.4)	(16.8)	(32.8)	(21.4)	(16.8)	(32.8)	(21.4)	(16.8)
	50.6	66.6	-4.7	24.8	26.4	-6.6	31.6	30.7	3.3	3.4

	9.313	12.262	−0.859	4.565	4.870	−1.218	5.819	5.662	0.610	0.623
g_O	178	85	132	182	90	111	173	93	113	186
	(32.8)	(15.7)	(24.3)	(33.5)	(16.6)	(20.4)	(31.9)	(17.1)	(20.8)	(34.3)
g_E	187.7	81.3	129.1	187.7	81.3	129.1	187.7	81.3	129.1	187.7
	(34.6)	(15.0)	(23.8)	(34.6)	(15.0)	(23.8)	(34.6)	(15.0)	(23.8)	(34.6)
	−18.9	7.5	5.8	−11.2	18.2	−33.6	−28.2	24.9	−30.1	−3.3
	−3.472	1.382	1.075	−2.060	3.358	−6.180	−5.190	4.593	−5.548	−0.616
c_O	103	92	124	71	76	134	90	80	131	93
	(19.0)	(16.9)	(22.8)	(13.1)	(14.0)	(24.7)	(16.6)	(14.7)	(24.1)	(17.1)
c_E	86.4	92.5	131.5	86.4	92.5	131.5	86.4	92.5	131.5	86.4
	(15.9)	(17.0)	(24.2)	(15.9)	(17.0)	(24.2)	(15.9)	(17.0)	(24.2)	(15.9)
	36.3	−1.1	−14.7	−27.8	−29.9	5.0	7.4	−23.3	−1.1	13.8
	6.682	−0.193	−2.698	−5.123	−5.508	0.912	1.366	−4.286	−0.201	2.535
N	543	543	543	543	543	543	543	543	543	543
X2	14.3	11.5	0.8	4.9	4.9	4.9	3.7	5.6	3.4	0.8
Gvalue	14.0	11.6	0.8	5.0	5.1	4.9	3.6	5.6	3.4	0.8
Gv/N	2.575	2.139	0.156	0.922	0.932	0.905	0.672	1.033	0.634	0.146
Prob	0.00293	0.00882	0.83846	0.17144	0.16740	0.17834	0.30224	0.13217	0.32797	0.85107

	41	42	43	44	45	46	47	48	49	50
a_O	173	175	170	162	171	164	184	176	178	192
	(31.9)	(32.2)	(31.3)	(29.8)	(31.5)	(30.2)	(33.9)	(32.4)	(32.8)	(35.4)
a_E	190.9	166.0	177.6	190.9	166.0	177.6	190.9	166.0	177.6	190.9
	(35.2)	(30.6)	(32.7)	(35.2)	(30.6)	(32.7)	(35.2)	(30.6)	(32.7)	(35.2)
	−34.1	18.5	−14.9	−53.2	10.2	−26.2	−13.6	20.6	0.7	2.2
	−6.280	3.409	−2.749	−9.801	1.875	−4.823	−2.502	3.798	0.136	0.399
t_O	166	114	119	195	127	98	186	113	95	160
	(30.6)	(21.0)	(21.9)	(35.9)	(23.4)	(18.0)	(34.3)	(20.8)	(17.5)	(29.5)
t_E	178.2	116.4	91.3	178.2	116.4	91.3	178.2	116.4	91.3	178.2
	(32.8)	(21.4)	(16.8)	(32.8)	(21.4)	(16.8)	(32.8)	(21.4)	(16.8)	(32.8)
	−23.6	−4.7	63.0	35.1	22.2	13.8	15.9	−6.6	7.5	−34.5
	−4.345	−0.859	11.603	6.461	4.095	2.547	2.925	−1.218	1.381	−6.357
g_O	110	120	162	85	113	192	77	120	182	81
	(20.3)	(22.1)	(29.8)	(15.7)	(20.8)	(35.4)	(14.2)	(22.1)	(33.5)	(14.9)
g_E	81.3	129.1	187.7	81.3	129.1	187.7	81.3	129.1	187.7	81.3
	(15.0)	(23.8)	(34.6)	(15.0)	(23.8)	(34.6)	(15.0)	(23.8)	(34.6)	(15.0)
	66.4	−17.6	−47.7	7.5	−30.1	8.7	−8.4	−17.6	−11.2	−0.7
	12.235	−3.235	−8.780	1.382	−5.548	1.609	−1.551	−3.235	−2.060	−0.121
c_O	94	134	92	101	132	89	96	134	88	110
	(17.3)	(24.7)	(16.9)	(18.6)	(24.3)	(16.4)	(17.7)	(24.7)	(16.2)	(20.3)
c_E	92.5	131.5	86.4	92.5	131.5	86.4	92.5	131.5	86.4	92.5
	(17.0)	(24.2)	(15.9)	(17.0)	(24.2)	(15.9)	(17.0)	(24.2)	(15.9)	(17.0)
	3.0	5.0	11.6	17.7	0.9	5.3	7.1	5.0	3.3	38.1
	0.547	0.912	2.141	3.260	0.167	0.985	1.303	0.912	0.607	7.008
N	543	543	543	543	543	543	543	543	543	543
X2	12.7	1.2	12.6	6.9	3.1	1.7	1.0	1.4	0.4	5.2
Gvalue	11.7	1.2	12.0	7.1	3.2	1.7	1.0	1.4	0.4	5.0
Gv/N	2.157	0.227	2.215	1.302	0.589	0.318	0.175	0.257	0.065	0.930
Prob	0.00844	0.74495	0.00729	0.06976	0.36219	0.63057	0.81296	0.70675	0.95012	0.16833

	51	52	53	54	55	56	57	58	59	60
a_O	151	156	172	158	165	177	167	163	168	196
	(27.8)	(28.7)	(31.7)	(29.1)	(30.4)	(32.6)	(30.8)	(30.0)	(30.9)	(36.1)
a_E	166.0	177.6	190.9	166.0	177.6	190.9	166.0	177.6	190.9	166.0
	(30.6)	(32.7)	(35.2)	(30.6)	(32.7)	(35.2)	(30.6)	(32.7)	(35.2)	(30.6)
	−28.6	−40.5	−35.9	−15.6	−24.3	−26.8	2.0	−28.0	−43.0	65.2
	−5.262	−7.461	−6.611	−2.869	−4.483	−4.935	0.375	−5.161	−7.913	12.000
t_O	107	124	177	118	105	190	106	96	187	110
	(19.7)	(22.8)	(32.6)	(21.7)	(19.3)	(35.0)	(19.5)	(17.7)	(34.4)	(20.3)
t_E	116.4	91.3	178.2	116.4	91.3	178.2	116.4	91.3	178.2	116.4
	(21.4)	(16.8)	(32.8)	(21.4)	(16.8)	(32.8)	(21.4)	(16.8)	(32.8)	(21.4)
	−17.9	75.9	−2.4	3.3	29.3	24.3	−19.8	9.6	18.0	−12.4
	−3.304	13.970	−0.450	0.610	5.397	4.477	−3.639	1.766	3.310	−2.276
g_O	150	165	90	119	177	76	130	189	84	115
	(27.6)	(30.4)	(16.6)	(21.9)	(32.6)	(14.0)	(23.9)	(34.8)	(15.5)	(21.2)
g_E	129.1	187.7	81.3	129.1	187.7	81.3	129.1	187.7	81.3	129.1
	(23.8)	(34.6)	(15.0)	(23.8)	(34.6)	(15.0)	(23.8)	(34.6)	(15.0)	(23.8)
	45.0	−42.5	18.2	−19.4	−20.7	−10.3	1.8	2.6	5.4	−26.6
	8.284	−7.827	3.358	−3.575	−3.819	−1.897	0.327	0.488	1.000	−4.903
c_O	135	98	104	148	96	100	140	95	104	122
	(24.9)	(18.0)	(19.2)	(27.3)	(17.7)	(18.4)	(25.8)	(17.5)	(19.2)	(22.5)
c_E	131.5	86.4	92.5	131.5	86.4	92.5	131.5	86.4	92.5	131.5
	(24.2)	(15.9)	(17.0)	(24.2)	(15.9)	(17.0)	(24.2)	(15.9)	(17.0)	(24.2)
	7.0	24.8	24.3	34.9	20.3	15.5	17.4	18.1	24.3	−18.4
	1.289	4.561	4.478	6.425	3.739	2.861	3.212	3.334	4.478	−3.385
N	543	543	543	543	543	543	543	543	543	543
X2	5.6	18.6	4.2	3.3	4.6	2.7	1.5	2.3	4.7	8.0
Gvalue	5.5	17.6	4.2	3.2	4.5	2.7	1.5	2.3	4.7	7.8
Gv/N	1.007	3.243	0.775	0.590	0.834	0.506	0.275	0.427	0.874	1.435
Prob	0.14054	0.00053	0.23979	0.36115	0.20966	0.43234	0.68352	0.50868	0.19125	0.05048

	61	62	63	64	65	66	67	68	69	70
a_O	160	187	149	165	167	174	185	195	163	169
	(29.5)	(34.4)	(27.4)	(30.4)	(30.8)	(32.0)	(34.1)	(35.9)	(30.0)	(31.1)
a_E	177.6	190.9	166.0	177.6	190.9	166.0	177.6	190.9	166.0	177.6
	(32.7)	(35.2)	(30.6)	(32.7)	(35.2)	(30.6)	(32.7)	(35.2)	(30.6)	(32.7)
	−33.5	−7.8	−32.2	−24.3	−44.7	16.4	15.0	8.2	−5.9	−16.8
	−6.160	−1.428	−5.924	−4.483	−8.233	3.023	2.770	1.519	−1.089	−3.100
t_O	111	174	125	98	178	115	103	179	107	93
	(20.4)	(32.0)	(23.0)	(18.0)	(32.8)	(21.2)	(19.0)	(33.0)	(19.7)	(17.1)
t_E	91.3	178.2	116.4	91.3	178.2	116.4	91.3	178.2	116.4	91.3
	(16.8)	(32.8)	(21.4)	(16.8)	(32.8)	(21.4)	(16.8)	(32.8)	(21.4)	(16.8)
	43.3	−8.3	17.9	13.8	−0.4	−2.7	24.8	1.6	−17.9	3.4
	7.978	−1.538	3.299	2.547	−0.083	−0.496	4.565	0.286	−3.304	0.623
g_O	170	82	124	178	82	126	160	75	128	190
	(31.3)	(15.1)	(22.8)	(32.8)	(15.1)	(23.2)	(29.5)	(13.8)	(23.6)	(35.0)
g_E	187.7	81.3	129.1	187.7	81.3	129.1	187.7	81.3	129.1	187.7
	(34.6)	(15.0)	(23.8)	(34.6)	(15.0)	(23.8)	(34.6)	(15.0)	(23.8)	(34.6)
	−33.6	1.3	−10.0	−18.9	1.3	−6.2	−51.1	−12.2	−2.2	4.7
	−6.195	0.248	−1.846	−3.472	0.248	−1.133	−9.403	−2.238	−0.408	0.860
c_O	102	100	145	102	116	128	95	94	145	91
	(18.8)	(18.4)	(26.7)	(18.8)	(21.4)	(23.6)	(17.5)	(17.3)	(26.7)	(16.8)
c_E	86.4	92.5	131.5	86.4	92.5	131.5	86.4	92.5	131.5	86.4
	(15.9)	(17.0)	(24.2)	(15.9)	(17.0)	(24.2)	(15.9)	(17.0)	(24.2)	(15.9)
	33.9	15.5	28.2	33.9	52.5	−7.0	18.1	3.0	28.2	9.5
	6.251	2.861	5.201	6.251	9.660	−1.288	3.334	0.547	5.201	1.752

N	543	543	543	543	543	543	543	543	543	543
X2	10.5	0.8	4.0	4.7	9.0	0.6	6.7	0.6	2.2	0.7
Gvalue	10.2	0.8	4.0	4.6	8.6	0.6	6.9	0.6	2.2	0.7
Gv/N	1.873	0.143	0.730	0.843	1.592	0.105	1.265	0.114	0.399	0.134
Prob	0.01719	0.85534	0.26539	0.20543	0.03444	0.90348	0.07617	0.89201	0.53823	0.86618

	71	72	73	74	75	76	77	78	79	80
a_O	172	177	175	193	179	157	198	172	173	182
	(31.7)	(32.6)	(32.2)	(35.5)	(33.0)	(28.9)	(36.5)	(31.7)	(31.9)	(33.5)
a_E	190.9	166.0	177.6	190.9	166.0	177.6	190.9	166.0	177.6	190.9
	(35.2)	(30.6)	(32.7)	(35.2)	(30.6)	(32.7)	(35.2)	(30.6)	(32.7)	(35.2)
	-35.9	22.7	-5.2	4.2	27.0	-38.8	14.4	12.2	-9.1	-17.4
	-6.611	4.189	-0.962	0.771	4.977	-7.139	2.656	2.255	-1.683	-3.207
t_O	196	122	105	186	124	89	179	121	86	191
	(36.1)	(22.5)	(19.3)	(34.3)	(22.8)	(16.4)	(33.0)	(22.3)	(15.8)	(35.2)
t_E	178.2	116.4	91.3	178.2	116.4	91.3	178.2	116.4	91.3	178.2
	(32.8)	(21.4)	(16.8)	(32.8)	(21.4)	(16.8)	(32.8)	(21.4)	(16.8)	(32.8)
	37.3	11.6	29.3	15.9	15.8	-4.6	1.6	9.5	-10.3	26.4
	6.863	2.129	5.397	2.925	2.906	-0.845	0.286	1.744	-1.902	4.870
g_O	82	107	165	79	111	186	75	110	191	80
	(15.1)	(19.7)	(30.4)	(14.5)	(20.4)	(34.3)	(13.8)	(20.3)	(35.2)	(14.7)
g_E	81.3	129.1	187.7	81.3	129.1	187.7	81.3	129.1	187.7	81.3
	(15.0)	(23.8)	(34.6)	(15.0)	(23.8)	(34.6)	(15.0)	(23.8)	(34.6)	(15.0)
	1.3	-40.2	-42.5	-4.6	-33.6	-3.3	-12.2	-35.2	6.7	-2.6
	0.248	-7.404	-7.827	-0.846	-6.180	-0.616	-2.238	-6.491	1.234	-0.486
c_O	93	137	98	85	129	111	91	140	93	90
	(17.1)	(25.2)	(18.0)	(15.7)	(23.8)	(20.4)	(16.8)	(25.8)	(17.1)	(16.6)
c_E	92.5	131.5	86.4	92.5	131.5	86.4	92.5	131.5	86.4	92.5
	(17.0)	(24.2)	(15.9)	(17.0)	(24.2)	(15.9)	(17.0)	(24.2)	(15.9)	(17.0)
	0.9	11.1	24.8	-14.4	-5.0	55.7	-3.0	17.4	13.8	-5.0
	0.175	2.050	4.561	-2.656	-0.929	10.259	-0.558	3.212	2.535	-0.918
N	543	543	543	543	543	543	543	543	543	543
X2	3.7	5.0	6.4	1.0	4.1	9.5	0.8	3.8	1.0	1.4
Gvalue	3.7	5.2	6.4	1.1	4.2	9.0	0.8	3.9	1.0	1.4
Gv/N	0.675	0.964	1.170	0.194	0.774	1.659	0.146	0.720	0.183	0.260
Prob	0.30006	0.15555	0.09570	0.78820	0.24013	0.02917	0.85088	0.27124	0.80280	0.70335

	81	82	83	84	85	86	87	88	89	90
a_O	185	171	193	181	183	200	177	171	191	176
	(34.1)	(31.5)	(35.5)	(33.3)	(33.7)	(36.8)	(32.6)	(31.5)	(35.2)	(32.4)
a_E	166.0	177.6	190.9	166.0	177.6	190.9	166.0	177.6	190.9	166.0
	(30.6)	(32.7)	(35.2)	(30.6)	(32.7)	(35.2)	(30.6)	(32.7)	(35.2)	(30.6)
	40.1	-13.0	4.2	31.4	10.9	18.6	22.7	-13.0	0.2	20.6
	7.391	-2.396	0.771	5.774	2.007	3.423	4.189	-2.396	0.030	3.798
t_O	134	104	169	102	83	173	123	91	190	111
	(24.7)	(19.2)	(31.1)	(18.8)	(15.3)	(31.9)	(22.7)	(16.8)	(35.0)	(20.4)
t_E	116.4	91.3	178.2	116.4	91.3	178.2	116.4	91.3	178.2	116.4
	(21.4)	(16.8)	(32.8)	(21.4)	(16.8)	(32.8)	(21.4)	(16.8)	(32.8)	(21.4)
	37.8	27.0	-18.0	-26.9	-15.9	-10.3	13.7	-0.6	24.3	-10.5
	6.968	4.979	-3.308	-4.947	-2.921	-1.896	2.516	-0.119	4.477	-1.927
g_O	109	180	79	127	194	89	122	190	85	106
	(20.1)	(33.1)	(14.5)	(23.4)	(35.7)	(16.4)	(22.5)	(35.0)	(15.7)	(19.5)
g_E	129.1	187.7	81.3	129.1	187.7	81.3	129.1	187.7	81.3	129.1
	(23.8)	(34.6)	(15.0)	(23.8)	(34.6)	(15.0)	(23.8)	(34.6)	(15.0)	(23.8)
	-36.9	-15.0	-4.6	-4.2	12.9	16.0	-13.8	4.7	7.5	-41.8
	-6.799	-2.770	-0.846	-0.772	2.367	2.954	-2.547	0.860	1.382	-7.701
c_O	115	88	102	133	83	81	121	91	77	150
	(21.2)	(16.2)	(18.8)	(24.5)	(15.3)	(14.9)	(22.3)	(16.8)	(14.2)	(27.6)
c_E	131.5	86.4	92.5	131.5	86.4	92.5	131.5	86.4	92.5	131.5
	(24.2)	(15.9)	(17.0)	(24.2)	(15.9)	(17.0)	(24.2)	(15.9)	(17.0)	(24.2)
	-30.9	3.3	19.9	2.9	-6.6	-21.6	-20.2	9.5	-28.3	39.4
	-5.694	0.607	3.662	0.538	-1.215	-3.969	-3.724	1.752	-5.210	7.253
N	543	543	543	543	543	543	543	543	543	543
X2	10.1	2.4	1.5	3.2	1.3	2.7	2.3	0.5	3.5	7.6
Gvalue	10.1	2.3	1.5	3.2	1.3	2.8	2.4	0.5	3.7	7.7
Gv/N	1.866	0.421	0.279	0.593	0.237	0.512	0.434	0.097	0.679	1.423
Prob	0.01746	0.51568	0.67896	0.35909	0.73226	0.42659	0.50184	0.91344	0.29700	0.05198

	91	92	93	94	95	96	97	98	99	100
a_O	183	176	180	189	191	174	194	206	169	164
	(33.7)	(32.4)	(33.1)	(34.8)	(35.2)	(32.0)	(35.7)	(37.9)	(31.1)	(30.2)
a_E	177.6	190.9	166.0	177.6	190.9	166.0	177.6	190.9	166.0	177.6
	(32.7)	(35.2)	(30.6)	(32.7)	(35.2)	(30.6)	(32.7)	(35.2)	(30.6)	(32.7)
	10.9	-28.6	29.2	23.5	0.2	16.4	34.2	31.3	6.1	-26.2
	2.007	-5.274	5.374	4.319	0.030	3.023	6.299	5.769	1.121	-4.823
t_O	102	172	112	82	170	120	80	176	123	104
	(18.8)	(31.7)	(20.6)	(15.1)	(31.3)	(22.1)	(14.7)	(32.4)	(22.7)	(19.2)
t_E	91.3	178.2	116.4	91.3	178.2	116.4	91.3	178.2	116.4	91.3
	(16.8)	(32.8)	(21.4)	(16.8)	(32.8)	(21.4)	(16.8)	(32.8)	(21.4)	(16.8)
	22.6	-12.2	-8.5	-17.7	-16.1	7.4	-21.2	-4.4	13.7	27.0
	4.154	-2.252	-1.574	-3.252	-2.958	1.363	-3.901	-0.814	2.516	4.979
g_O	171	88	105	183	77	118	175	70	119	187
	(31.5)	(16.2)	(19.3)	(33.7)	(14.2)	(21.7)	(32.2)	(12.9)	(21.9)	(34.4)
g_E	187.7	81.3	129.1	187.7	81.3	129.1	187.7	81.3	129.1	187.7
	(34.6)	(15.0)	(23.8)	(34.6)	(15.0)	(23.8)	(34.6)	(15.0)	(23.8)	(34.6)
	-31.8	13.9	-43.4	-9.2	-8.4	-21.2	-24.5	-21.0	-19.4	-1.4
	-5.862	2.555	-7.995	-1.702	-1.551	-3.912	-4.509	-3.868	-3.575	-0.250
c_O	87	107	146	89	105	131	94	91	132	88
	(16.0)	(19.7)	(26.9)	(16.4)	(19.3)	(24.1)	(17.3)	(16.8)	(24.3)	(16.2)
c_E	86.4	92.5	131.5	86.4	92.5	131.5	86.4	92.5	131.5	86.4
	(15.9)	(17.0)	(24.2)	(15.9)	(17.0)	(24.2)	(15.9)	(17.0)	(24.2)	(15.9)
	1.3	31.1	30.4	5.3	26.6	-1.1	15.9	-3.0	0.9	3.3
	0.234	5.728	5.606	0.985	4.891	-0.201	2.932	-0.558	0.167	0.607
N	543	543	543	543	543	543	543	543	543	543
X2	2.9	4.2	7.4	1.9	2.3	1.5	4.4	2.8	1.2	2.8
Gvalue	2.9	4.1	7.7	1.9	2.2	1.5	4.5	2.9	1.2	2.8
Gv/N	0.533	0.756	1.411	0.349	0.411	0.273	0.822	0.529	0.229	0.514
Prob	0.40794	0.25041	0.05351	0.59433	0.52588	0.68652	0.21568	0.41186	0.74290	0.42506

Abor_SK2
5UTR:

	100	99	98	97	96	95	94	93	92	91
a_O	89	86	92	96	95	93	98	87	92	101
	(23.1)	(22.3)	(23.8)	(24.9)	(24.6)	(24.1)	(25.4)	(22.5)	(23.8)	(26.2)
a_E	94.5	94.5	94.5	94.5	94.5	94.5	94.5	94.5	94.5	94.5
	(24.5)	(24.5)	(24.5)	(24.5)	(24.5)	(24.5)	(24.5)	(24.5)	(24.5)	(24.5)
	-10.8	-16.3	-5.0	2.9	0.9	-3.1	7.0	-14.5	-5.0	13.3
	-2.790	-4.223	-1.303	0.757	0.234	-0.796	1.820	-3.751	-1.303	3.453
t_O	95	104	92	95	87	79	84	95	82	97
	(24.6)	(26.9)	(23.8)	(24.6)	(22.5)	(20.5)	(21.8)	(24.6)	(21.2)	(25.1)
t_E	92.6	92.6	92.6	92.6	92.6	92.6	92.6	92.6	92.6	92.6
	(24.0)	(24.0)	(24.0)	(24.0)	(24.0)	(24.0)	(24.0)	(24.0)	(24.0)	(24.0)
	4.8	24.1	-1.2	4.8	-10.9	-25.1	-16.4	4.8	-20.0	9.0
	1.254	6.250	-0.315	1.254	-2.817	-6.506	-4.247	1.254	-5.170	2.328
g_O	99	98	102	93	96	116	100	105	110	88
	(25.6)	(25.4)	(26.4)	(24.1)	(24.9)	(30.1)	(25.9)	(27.2)	(28.5)	(22.8)
g_E	92.9	92.9	92.9	92.9	92.9	92.9	92.9	92.9	92.9	92.9
	(24.1)	(24.1)	(24.1)	(24.1)	(24.1)	(24.1)	(24.1)	(24.1)	(24.1)	(24.1)
	12.6	10.5	19.1	0.2	6.3	51.5	14.7	25.7	37.2	-9.5
	3.262	2.714	4.939	0.052	1.633	13.347	3.816	6.661	9.630	-2.471
c_O	103	98	100	102	108	98	104	99	102	100
	(26.7)	(25.4)	(25.9)	(26.4)	(28.0)	(25.4)	(26.9)	(25.6)	(26.4)	(25.9)
c_E	105.9	105.9	105.9	105.9	105.9	105.9	105.9	105.9	105.9	105.9
	(27.4)	(27.4)	(27.4)	(27.4)	(27.4)	(27.4)	(27.4)	(27.4)	(27.4)	(27.4)
	-5.8	-15.3	-11.5	-7.7	4.2	-15.3	-3.8	-13.4	-7.7	-11.5
	-1.502	-3.956	-2.990	-2.003	1.078	-3.956	-0.996	-3.475	-2.003	-2.990
N	386	386	386	386	386	386	386	386	386	386
X2	0.9	3.0	1.3	0.2	0.5	8.4	1.5	2.7	4.6	1.2
Gvalue	0.9	3.0	1.3	0.2	0.5	8.1	1.5	2.7	4.5	1.2
Gv/N	0.225	0.785	0.331	0.060	0.127	2.088	0.393	0.688	1.154	0.321
Prob	0.83316	0.38705	0.73472	0.97230	0.92083	0.04476	0.67860	0.44752	0.21651	0.74396

	90	89	88	87	86	85	84	83	82	81
a_O	81	89	92	91	82	87	110	99	107	74
	(21.0)	(23.1)	(23.8)	(23.6)	(21.2)	(22.5)	(28.5)	(25.6)	(27.7)	(19.2)
a_E	94.5	94.5	94.5	94.5	94.5	94.5	94.5	94.5	94.5	94.5
	(24.5)	(24.5)	(24.5)	(24.5)	(24.5)	(24.5)	(24.5)	(24.5)	(24.5)	(24.5)
	-25.1	-10.8	-5.0	-7.0	-23.4	-14.5	33.3	9.1	26.5	-36.3
	-6.492	-2.790	-1.303	-1.804	-6.051	-3.751	8.626	2.359	6.858	-9.396
t_O	94	91	108	97	103	85	75	75	83	106
	(24.4)	(23.6)	(28.0)	(25.1)	(26.7)	(22.0)	(19.4)	(19.4)	(21.5)	(27.5)
t_E	92.6	92.6	92.6	92.6	92.6	92.6	92.6	92.6	92.6	92.6
	(24.0)	(24.0)	(24.0)	(24.0)	(24.0)	(24.0)	(24.0)	(24.0)	(24.0)	(24.0)
	2.8	-3.2	33.2	9.0	21.9	-14.6	-31.6	-31.6	-18.2	28.6
	0.726	-0.827	8.603	2.328	5.675	-3.776	-8.196	-8.196	-4.711	7.417
g_O	113	99	100	93	107	108	91	112	100	111
	(29.3)	(25.6)	(25.9)	(24.1)	(27.7)	(28.0)	(23.6)	(29.0)	(25.9)	(28.8)
g_E	92.9	92.9	92.9	92.9	92.9	92.9	92.9	92.9	92.9	92.9
	(24.1)	(24.1)	(24.1)	(24.1)	(24.1)	(24.1)	(24.1)	(24.1)	(24.1)	(24.1)
	44.3	12.6	14.7	0.2	30.2	32.5	-3.8	41.9	14.7	39.5
	11.468	3.262	3.816	0.052	7.834	8.428	-0.974	10.850	3.816	10.238
c_O	98	107	86	105	94	106	110	100	96	95
	(25.4)	(27.7)	(22.3)	(27.2)	(24.4)	(27.5)	(28.5)	(25.9)	(24.9)	(24.6)
c_E	105.9	105.9	105.9	105.9	105.9	105.9	105.9	105.9	105.9	105.9
	(27.4)	(27.4)	(27.4)	(27.4)	(27.4)	(27.4)	(27.4)	(27.4)	(27.4)	(27.4)
	-15.3	2.1	-35.9	-1.9	-22.5	0.1	8.3	-11.5	-18.9	-20.7
	-3.956	0.552	-9.292	-0.485	-5.824	0.031	2.143	-2.990	-4.901	-5.365
N	386	386	386	386	386	386	386	386	386	386
X2	6.9	0.8	6.9	0.3	6.3	3.7	6.1	7.8	4.1	11.1
Gvalue	6.7	0.8	7.0	0.3	6.3	3.6	6.2	7.8	4.1	11.2
Gv/N	1.746	0.198	1.824	0.090	1.634	0.931	1.599	2.024	1.062	2.893
Prob	0.08072	0.85827	0.07067	0.95069	0.09756	0.30876	0.10346	0.05007	0.25108	0.01085

	80	79	78	77	76	75	74	73	72	71
a_O	96	99	87	89	102	106	89	90	87	97
	(24.9)	(25.6)	(22.5)	(23.1)	(26.4)	(27.5)	(23.1)	(23.3)	(22.5)	(25.1)
a_E	94.5	94.5	94.5	94.5	94.5	94.5	94.5	94.5	94.5	94.5
	(24.5)	(24.5)	(24.5)	(24.5)	(24.5)	(24.5)	(24.5)	(24.5)	(24.5)	(24.5)
	2.9	9.1	-14.5	-10.8	15.5	24.2	-10.8	-8.9	-14.5	5.0
	0.757	2.359	-3.751	-2.790	4.008	6.278	-2.790	-2.300	-3.751	1.286
t_O	93	99	108	79	91	70	86	91	88	95
	(24.1)	(25.6)	(28.0)	(20.5)	(23.6)	(18.1)	(22.3)	(23.6)	(22.8)	(24.6)
t_E	92.6	92.6	92.6	92.6	92.6	92.6	92.6	92.6	92.6	92.6
	(24.0)	(24.0)	(24.0)	(24.0)	(24.0)	(24.0)	(24.0)	(24.0)	(24.0)	(24.0)
	0.8	13.2	33.2	-25.1	-3.2	-39.2	-12.7	-3.2	-9.0	4.8
	0.202	3.423	8.603	-6.506	-0.827	-10.152	-3.300	-0.827	-2.328	1.254
g_O	92	93	97	102	84	105	96	102	99	98
	(23.8)	(24.1)	(25.1)	(26.4)	(21.8)	(27.2)	(24.9)	(26.4)	(25.6)	(25.4)
g_E	92.9	92.9	92.9	92.9	92.9	92.9	92.9	92.9	92.9	92.9
	(24.1)	(24.1)	(24.1)	(24.1)	(24.1)	(24.1)	(24.1)	(24.1)	(24.1)	(24.1)
	-1.8	0.2	8.4	19.1	-16.9	25.7	6.3	19.1	12.6	10.5
	-0.464	0.052	2.171	4.939	-4.383	6.661	1.633	4.939	3.262	2.714
c_O	105	95	94	116	109	105	115	103	112	96
	(27.2)	(24.6)	(24.4)	(30.1)	(28.2)	(27.2)	(29.8)	(26.7)	(29.0)	(24.9)
c_E	105.9	105.9	105.9	105.9	105.9	105.9	105.9	105.9	105.9	105.9
	(27.4)	(27.4)	(27.4)	(27.4)	(27.4)	(27.4)	(27.4)	(27.4)	(27.4)	(27.4)
	-1.9	-20.7	-22.5	21.0	6.2	-1.9	18.9	-5.8	12.5	-18.9
	-0.485	-5.365	-5.824	5.452	1.608	-0.485	4.890	-1.502	3.228	-4.901
N	386	386	386	386	386	386	386	386	386	386
X2	0.0	1.8	4.7	4.2	1.6	8.5	1.7	1.2	1.6	1.3
Gvalue	0.0	1.8	4.6	4.2	1.6	8.9	1.7	1.2	1.6	1.4
Gv/N	0.011	0.468	1.198	1.095	0.407	2.302	0.433	0.310	0.411	0.353
Prob	0.99783	0.61315	0.20151	0.23783	0.66641	0.03082	0.64324	0.75380	0.66274	0.71438

	70	69	68	67	66	65	64	63	62	61
a_O	96	104	92	107	98	93	97	97	92	101
	(24.9)	(26.9)	(23.8)	(27.7)	(25.4)	(24.1)	(25.1)	(25.1)	(23.8)	(26.2)
a_E	94.5	94.5	94.5	94.5	94.5	94.5	94.5	94.5	94.5	94.5
	(24.5)	(24.5)	(24.5)	(24.5)	(24.5)	(24.5)	(24.5)	(24.5)	(24.5)	(24.5)
	2.9	19.8	-5.0	26.5	7.0	-3.1	5.0	5.0	-5.0	13.3
	0.757	5.133	-1.303	6.858	1.820	-0.796	1.286	1.286	-1.303	3.453
t_O	88	89	77	77	91	84	90	90	72	105
	(22.8)	(23.1)	(19.9)	(19.9)	(23.6)	(21.8)	(23.3)	(23.3)	(18.7)	(27.2)

t_E	92.6	92.6	92.6	92.6	92.6	92.6	92.6	92.6	92.6	92.6
	(24.0)	(24.0)	(24.0)	(24.0)	(24.0)	(24.0)	(24.0)	(24.0)	(24.0)	(24.0)
	-9.0	-7.1	-28.4	-28.4	-3.2	-16.4	-5.1	-5.1	-36.2	26.4
	-2.328	-1.834	-7.365	-7.365	-0.827	-4.247	-1.333	-1.333	-9.391	6.831
g_O	91	106	106	84	97	107	92	97	118	95
	(23.6)	(27.5)	(27.5)	(21.8)	(25.1)	(27.7)	(23.8)	(25.1)	(30.6)	(24.6)
g_E	92.9	92.9	92.9	92.9	92.9	92.9	92.9	92.9	92.9	92.9
	(24.1)	(24.1)	(24.1)	(24.1)	(24.1)	(24.1)	(24.1)	(24.1)	(24.1)	(24.1)
	-3.8	28.0	28.0	-16.9	8.4	30.2	-1.8	8.4	56.4	4.2
	-0.974	7.245	7.245	-4.383	2.171	7.834	-0.464	2.171	14.622	1.100
c_O	111	87	111	118	100	102	107	102	104	85
	(28.8)	(22.5)	(28.8)	(30.6)	(25.9)	(26.4)	(27.7)	(26.4)	(26.9)	(22.0)
c_E	105.9	105.9	105.9	105.9	105.9	105.9	105.9	105.9	105.9	105.9
	(27.4)	(27.4)	(27.4)	(27.4)	(27.4)	(27.4)	(27.4)	(27.4)	(27.4)	(27.4)
	10.4	-34.3	10.4	25.4	-11.5	-7.7	2.1	-7.7	-3.8	-37.4
	2.683	-8.879	2.683	6.592	-2.990	-2.003	0.552	-2.003	-0.996	-9.699
N	386	386	386	386	386	386	386	386	386	386
X2	0.5	6.3	4.8	6.5	0.7	3.1	0.2	0.5	11.5	6.3
Gvalue	0.5	6.4	4.9	6.6	0.7	3.0	0.2	0.5	11.3	6.5
Gv/N	0.138	1.666	1.261	1.702	0.174	0.787	0.041	0.120	2.932	1.686
Prob	0.91168	0.09241	0.18186	0.08696	0.88016	0.38554	0.98428	0.92674	0.01013	0.08935

	60	59	58	57	56	55	54	53	52	51
a_O	98	82	97	95	87	100	73	101	112	86
	(25.4)	(21.2)	(25.1)	(24.6)	(22.5)	(25.9)	(18.9)	(26.2)	(29.0)	(22.3)
a_E	94.5	94.5	94.5	94.5	94.5	94.5	94.5	94.5	94.5	94.5
	(24.5)	(24.5)	(24.5)	(24.5)	(24.5)	(24.5)	(24.5)	(24.5)	(24.5)	(24.5)
	7.0	-23.4	5.0	0.9	-14.5	11.2	-37.8	13.3	37.9	-16.3
	1.820	-6.051	1.286	0.234	-3.751	2.904	-9.784	3.453	9.829	-4.223
t_O	87	105	111	87	103	100	103	90	81	96
	(22.5)	(27.2)	(28.8)	(22.5)	(26.7)	(25.9)	(26.7)	(23.3)	(21.0)	(24.9)
t_E	92.6	92.6	92.6	92.6	92.6	92.6	92.6	92.6	92.6	92.6
	(24.0)	(24.0)	(24.0)	(24.0)	(24.0)	(24.0)	(24.0)	(24.0)	(24.0)	(24.0)
	-10.9	26.4	40.2	-10.9	21.9	15.4	21.9	-5.1	-21.7	6.9
	-2.817	6.831	10.417	-2.817	5.675	3.978	5.675	-1.333	-5.622	1.788
g_O	104	105	93	95	96	100	103	105	95	89
	(26.9)	(27.2)	(24.1)	(24.6)	(24.9)	(25.9)	(26.7)	(27.2)	(24.6)	(23.1)
g_E	92.9	92.9	92.9	92.9	92.9	92.9	92.9	92.9	92.9	92.9
	(24.1)	(24.1)	(24.1)	(24.1)	(24.1)	(24.1)	(24.1)	(24.1)	(24.1)	(24.1)
	23.5	25.7	0.2	4.2	6.3	14.7	21.3	25.7	4.2	-7.6
	6.082	6.661	0.052	1.100	1.633	3.816	5.508	6.661	1.100	-1.978
c_O	97	94	85	109	100	86	107	90	98	115
	(25.1)	(24.4)	(22.0)	(28.2)	(25.9)	(22.3)	(27.7)	(23.3)	(25.4)	(29.8)
c_E	105.9	105.9	105.9	105.9	105.9	105.9	105.9	105.9	105.9	105.9
	(27.4)	(27.4)	(27.4)	(27.4)	(27.4)	(27.4)	(27.4)	(27.4)	(27.4)	(27.4)
	-17.1	-22.5	-37.4	6.2	-11.5	-35.9	2.1	-29.4	-15.3	18.9
	-4.431	-5.824	-9.699	1.608	-2.990	-9.292	0.552	-7.604	-3.956	4.890
N	386	386	386	386	386	386	386	386	386	386
X2	2.5	6.2	7.9	0.5	2.2	5.2	7.2	4.5	5.3	1.8
Gvalue	2.5	6.2	7.9	0.5	2.2	5.4	7.5	4.5	5.2	1.8
Gv/N	0.654	1.618	2.056	0.125	0.566	1.406	1.951	1.177	1.352	0.477
Prob	0.47092	0.10032	0.04733	0.92245	0.53476	0.14314	0.05681	0.20832	0.15658	0.60630

	50	49	48	47	46	45	44	43	42	41
a_O	89	94	83	95	77	77	91	97	78	87
	(23.1)	(24.4)	(21.5)	(24.6)	(19.9)	(19.9)	(23.6)	(25.1)	(20.2)	(22.5)
a_E	94.5	94.5	94.5	94.5	94.5	94.5	94.5	94.5	94.5	94.5
	(24.5)	(24.5)	(24.5)	(24.5)	(24.5)	(24.5)	(24.5)	(24.5)	(24.5)	(24.5)
	-10.8	-1.1	-21.6	0.9	-31.6	-31.6	-7.0	5.0	-30.0	-14.5
	-2.790	-0.284	-5.603	0.234	-8.192	-8.192	-1.804	1.286	-7.777	-3.751
t_O	86	106	92	90	105	119	82	88	110	98
	(22.3)	(27.5)	(23.8)	(23.3)	(27.2)	(30.8)	(21.2)	(22.8)	(28.5)	(25.4)
t_E	92.6	92.6	92.6	92.6	92.6	92.6	92.6	92.6	92.6	92.6
	(24.0)	(24.0)	(24.0)	(24.0)	(24.0)	(24.0)	(24.0)	(24.0)	(24.0)	(24.0)
	-12.7	28.6	-1.2	-5.1	26.4	59.7	-20.0	-9.0	37.9	11.1
	-3.300	7.417	-0.315	-1.333	6.831	15.459	-5.170	-2.328	9.808	2.872
g_O	103	88	104	91	95	79	115	108	80	101
	(26.7)	(22.8)	(26.9)	(23.6)	(24.6)	(20.5)	(29.8)	(28.0)	(20.7)	(26.2)
g_E	92.9	92.9	92.9	92.9	92.9	92.9	92.9	92.9	92.9	92.9
	(24.1)	(24.1)	(24.1)	(24.1)	(24.1)	(24.1)	(24.1)	(24.1)	(24.1)	(24.1)
	21.3	-9.5	23.5	-3.8	4.2	-25.6	49.1	32.5	-23.9	16.9
	5.508	-2.471	6.082	-0.974	1.100	-6.634	12.716	8.428	-6.197	4.375
c_O	108	98	107	110	109	111	98	93	118	100
	(28.0)	(25.4)	(27.7)	(28.5)	(28.2)	(28.8)	(25.4)	(24.1)	(30.6)	(25.9)
c_E	105.9	105.9	105.9	105.9	105.9	105.9	105.9	105.9	105.9	105.9
	(27.4)	(27.4)	(27.4)	(27.4)	(27.4)	(27.4)	(27.4)	(27.4)	(27.4)	(27.4)
	4.2	-15.3	2.1	8.3	6.2	10.4	-15.3	-24.2	25.4	-11.5
	1.078	-3.956	0.552	2.143	1.608	2.683	-3.956	-6.277	6.592	-2.990
N	386	386	386	386	386	386	386	386	386	386
X2	1.9	2.8	2.8	0.3	5.1	13.1	7.2	4.3	9.3	2.0
Gvalue	1.9	2.7	2.8	0.3	5.2	12.8	6.9	4.3	9.4	2.0
Gv/N	0.496	0.706	0.716	0.070	1.348	3.317	1.786	1.108	2.426	0.506
Prob	0.59005	0.43587	0.42960	0.96572	0.15751	0.00508	0.07534	0.23309	0.02481	0.58213

	40	39	38	37	36	35	34	33	32	31
a_O	98	76	100	91	87	100	77	84	81	84
	(25.4)	(19.7)	(25.9)	(23.6)	(22.5)	(25.9)	(19.9)	(21.8)	(21.0)	(21.8)
a_E	94.5	94.5	94.5	94.5	94.5	94.5	94.5	94.5	94.5	94.5
	(24.5)	(24.5)	(24.5)	(24.5)	(24.5)	(24.5)	(24.5)	(24.5)	(24.5)	(24.5)
	7.0	-33.2	11.2	-7.0	-14.5	11.2	-31.6	-19.9	-25.1	-19.9
	1.820	-8.600	2.904	-1.804	-3.751	2.904	-8.192	-5.149	-6.492	-5.149
t_O	102	107	89	93	110	100	95	84	99	104
	(26.4)	(27.7)	(23.1)	(24.1)	(28.5)	(25.9)	(24.6)	(21.8)	(25.6)	(26.9)
t_E	92.6	92.6	92.6	92.6	92.6	92.6	92.6	92.6	92.6	92.6
	(24.0)	(24.0)	(24.0)	(24.0)	(24.0)	(24.0)	(24.0)	(24.0)	(24.0)	(24.0)
	19.7	30.9	-7.1	0.8	37.9	15.4	4.8	-16.4	13.2	24.1
	5.104	8.007	-1.834	0.202	9.808	3.978	1.254	-4.247	3.423	6.250
g_O	87	89	91	90	87	85	107	101	93	93
	(22.5)	(23.1)	(23.6)	(23.3)	(22.5)	(22.0)	(27.7)	(26.2)	(24.1)	(24.1)
g_E	92.9	92.9	92.9	92.9	92.9	92.9	92.9	92.9	92.9	92.9
	(24.1)	(24.1)	(24.1)	(24.1)	(24.1)	(24.1)	(24.1)	(24.1)	(24.1)	(24.1)
	-11.4	-7.6	-3.8	-5.7	-11.4	-15.1	30.2	16.9	0.2	0.2
	-2.958	-1.978	-0.974	-1.479	-2.958	-3.914	7.834	4.375	0.052	0.052
c_O	99	114	106	112	102	101	107	117	113	105
	(25.6)	(29.5)	(27.5)	(29.0)	(26.4)	(26.2)	(27.7)	(30.3)	(29.3)	(27.2)
c_E	105.9	105.9	105.9	105.9	105.9	105.9	105.9	105.9	105.9	105.9

	(27.4)	(27.4)	(27.4)	(27.4)	(27.4)	(27.4)	(27.4)	(27.4)	(27.4)	(27.4)
	-13.4	16.7	0.1	12.5	-7.7	-9.6	2.1	23.2	14.6	-1.9
	-3.475	4.331	0.031	3.228	-2.003	-2.499	0.552	6.020	3.777	-0.485
N	386	386	386	386	386	386	386	386	386	386
X2	1.9	6.7	0.5	0.6	4.4	1.8	5.5	3.8	2.9	2.6
Gvalue	1.9	6.8	0.5	0.6	4.2	1.8	5.6	3.9	2.9	2.6
Gv/N	0.491	1.761	0.127	0.147	1.096	0.469	1.449	0.998	0.760	0.668
Prob	0.59478	0.07867	0.92108	0.90364	0.23776	0.61306	0.13328	0.27777	0.40200	0.46122

	30	29	28	27	26	25	24	23	22	21
a_O	86	97	89	77	88	83	83	91	95	97
	(22.3)	(25.1)	(23.1)	(19.9)	(22.8)	(21.5)	(21.5)	(23.6)	(24.6)	(25.1)
a_E	94.5	94.5	94.5	94.5	94.5	94.5	94.5	94.5	94.5	94.5
	(24.5)	(24.5)	(24.5)	(24.5)	(24.5)	(24.5)	(24.5)	(24.5)	(24.5)	(24.5)
	-16.3	5.0	-10.8	-31.6	-12.6	-21.6	-21.6	-7.0	0.9	5.0
	-4.223	1.286	-2.790	-8.192	-3.273	-5.603	-5.603	-1.804	0.234	1.286
t_O	110	95	112	108	88	112	103	98	100	87
	(28.5)	(24.6)	(29.0)	(28.0)	(22.8)	(29.0)	(26.7)	(25.4)	(25.9)	(22.5)
t_E	92.6	92.6	92.6	92.6	92.6	92.6	92.6	92.6	92.6	92.6
	(24.0)	(24.0)	(24.0)	(24.0)	(24.0)	(24.0)	(24.0)	(24.0)	(24.0)	(24.0)
	37.9	4.8	42.6	33.2	-9.0	42.6	21.9	11.1	15.4	-10.9
	9.808	1.254	11.032	8.603	-2.328	11.032	5.675	2.872	3.978	-2.817
g_O	84	100	90	76	85	75	92	84	90	84
	(21.8)	(25.9)	(23.3)	(19.7)	(22.0)	(19.4)	(23.8)	(21.8)	(23.3)	(21.8)
g_E	92.9	92.9	92.9	92.9	92.9	92.9	92.9	92.9	92.9	92.9
	(24.1)	(24.1)	(24.1)	(24.1)	(24.1)	(24.1)	(24.1)	(24.1)	(24.1)	(24.1)
	-16.9	14.7	-5.7	-30.5	-15.1	-32.1	-1.8	-16.9	-5.7	-16.9
	-4.383	3.816	-1.479	-7.907	-3.914	-8.317	-0.464	-4.383	-1.479	-4.383
c_O	106	94	95	125	125	116	108	113	101	118
	(27.5)	(24.4)	(24.6)	(32.4)	(32.4)	(30.1)	(28.0)	(29.3)	(26.2)	(30.6)
c_E	105.9	105.9	105.9	105.9	105.9	105.9	105.9	105.9	105.9	105.9
	(27.4)	(27.4)	(27.4)	(27.4)	(27.4)	(27.4)	(27.4)	(27.4)	(27.4)	(27.4)
	0.1	-22.5	-20.7	41.4	41.4	21.0	4.2	14.6	-9.6	25.4
	0.031	-5.824	-5.365	10.715	10.715	5.452	1.078	3.777	-2.499	6.592
N	386	386	386	386	386	386	386	386	386	386
X2	4.9	2.0	5.6	12.3	4.8	9.9	2.6	1.8	0.9	2.6
Gvalue	4.8	2.1	5.4	12.4	4.6	9.9	2.6	1.8	0.9	2.6
Gv/N	1.232	0.532	1.398	3.219	1.199	2.564	0.685	0.462	0.234	0.677
Prob	0.19047	0.56154	0.14491	0.00606	0.20099	0.01947	0.44964	0.61834	0.82488	0.45492

	20	19	18	17	16	15	14	13	12	11
a_O	108	107	100	92	107	101	109	117	118	137
	(28.0)	(27.7)	(25.9)	(23.8)	(27.7)	(26.2)	(28.2)	(30.3)	(30.6)	(35.5)
a_E	94.5	94.5	94.5	94.5	94.5	94.5	94.5	94.5	94.5	94.5
	(24.5)	(24.5)	(24.5)	(24.5)	(24.5)	(24.5)	(24.5)	(24.5)	(24.5)	(24.5)
	28.7	26.5	11.2	-5.0	26.5	13.3	31.0	49.9	52.3	101.6
	7.443	6.858	2.904	-1.303	6.858	3.453	8.032	12.915	13.546	26.325
t_O	82	98	88	91	107	98	87	79	84	61
	(21.2)	(25.4)	(22.8)	(23.6)	(27.7)	(25.4)	(22.5)	(20.5)	(21.8)	(15.8)
t_E	92.6	92.6	92.6	92.6	92.6	92.6	92.6	92.6	92.6	92.6
	(24.0)	(24.0)	(24.0)	(24.0)	(24.0)	(24.0)	(24.0)	(24.0)	(24.0)	(24.0)
	-20.0	11.1	-9.0	-3.2	30.9	11.1	-10.9	-25.1	-16.4	-50.9
	-5.170	2.872	-2.328	-0.827	8.007	2.872	-2.817	-6.506	-4.247	-13.196
g_O	89	71	63	80	65	61	92	67	79	77
	(23.1)	(18.4)	(16.3)	(20.7)	(16.8)	(15.8)	(23.8)	(17.4)	(20.5)	(19.9)
g_E	92.9	92.9	92.9	92.9	92.9	92.9	92.9	92.9	92.9	92.9
	(24.1)	(24.1)	(24.1)	(24.1)	(24.1)	(24.1)	(24.1)	(24.1)	(24.1)	(24.1)
	-7.6	-38.2	-48.9	-23.9	-46.4	-51.3	-1.8	-43.8	-25.6	-28.9
	-1.978	-9.890	-12.678	-6.197	-12.028	-13.295	-0.464	-11.346	-6.634	-7.489
c_O	107	110	135	123	107	126	98	123	105	111
	(27.7)	(28.5)	(35.0)	(31.9)	(27.7)	(32.6)	(25.4)	(31.9)	(27.2)	(28.8)
c_E	105.9	105.9	105.9	105.9	105.9	105.9	105.9	105.9	105.9	105.9
	(27.4)	(27.4)	(27.4)	(27.4)	(27.4)	(27.4)	(27.4)	(27.4)	(27.4)	(27.4)
	2.1	8.3	65.4	36.7	2.1	43.7	-15.3	36.7	-1.9	10.4
	0.552	2.143	16.956	9.516	0.552	11.321	-3.956	9.516	-0.485	2.683
N	386	386	386	386	386	386	386	386	386	386
X2	3.3	7.3	18.1	4.6	12.3	15.5	3.2	17.3	8.7	32.8
Gvalue	3.3	7.7	18.7	4.6	13.1	16.8	3.1	17.7	8.4	32.1
Gv/N	0.847	1.984	4.853	1.189	3.389	4.352	0.795	4.579	2.180	8.323
Prob	0.35184	0.05365	0.00031	0.20452	0.00446	0.00078	0.38096	0.00051	0.03819	0.00000

	10	9	8	7	6	5	4	3	2	1
a_O	115	98	123	117	104	117	114	100	50	98
	(29.8)	(25.4)	(31.9)	(30.3)	(26.9)	(30.3)	(29.5)	(25.9)	(13.0)	(25.4)
a_E	94.5	94.5	94.5	94.5	94.5	94.5	94.5	94.5	94.5	94.5
	(24.5)	(24.5)	(24.5)	(24.5)	(24.5)	(24.5)	(24.5)	(24.5)	(24.5)	(24.5)
	45.0	7.0	64.7	49.9	19.8	49.9	42.7	11.2	-63.7	7.0
	11.667	1.820	16.765	12.915	5.133	12.915	11.050	2.904	-16.505	1.820
t_O	67	94	74	92	99	78	72	81	100	104
	(17.4)	(24.4)	(19.2)	(23.8)	(25.6)	(20.2)	(18.7)	(21.0)	(25.9)	(26.9)
t_E	92.6	92.6	92.6	92.6	92.6	92.6	92.6	92.6	92.6	92.6
	(24.0)	(24.0)	(24.0)	(24.0)	(24.0)	(24.0)	(24.0)	(24.0)	(24.0)	(24.0)
	-43.4	2.8	-33.2	-1.2	13.2	-26.8	-36.2	-21.7	15.4	24.1
	-11.237	0.726	-8.601	-0.315	3.423	-6.939	-9.391	-5.622	3.978	6.250
g_O	94	89	93	55	89	66	86	108	83	49
	(24.4)	(23.1)	(24.1)	(14.2)	(23.1)	(17.1)	(22.3)	(28.0)	(21.5)	(12.7)
g_E	92.9	92.9	92.9	92.9	92.9	92.9	92.9	92.9	92.9	92.9
	(24.1)	(24.1)	(24.1)	(24.1)	(24.1)	(24.1)	(24.1)	(24.1)	(24.1)	(24.1)
	2.2	-7.6	0.2	-57.7	-7.6	-45.1	-13.3	32.5	-18.7	-62.7
	0.573	-1.978	0.052	-14.938	-1.978	-11.691	-3.439	8.428	-4.846	-16.241
c_O	110	105	96	122	94	125	114	97	153	135
	(28.5)	(27.2)	(24.9)	(31.6)	(24.4)	(32.4)	(29.5)	(25.1)	(39.6)	(35.0)
c_E	105.9	105.9	105.9	105.9	105.9	105.9	105.9	105.9	105.9	105.9
	(27.4)	(27.4)	(27.4)	(27.4)	(27.4)	(27.4)	(27.4)	(27.4)	(27.4)	(27.4)
	8.3	-1.9	-18.9	34.4	-22.5	41.4	16.7	-17.1	112.5	65.4
	2.143	-0.485	-4.901	8.922	-5.824	10.715	4.331	-4.431	29.139	16.956
N	386	386	386	386	386	386	386	386	386	386
X2	11.7	0.3	13.2	23.2	2.9	18.9	9.7	5.0	43.5	30.2
Gvalue	12.1	0.3	12.8	25.4	2.9	19.3	9.8	4.9	45.4	33.9
Gv/N	3.146	0.083	3.314	6.584	0.754	5.001	2.551	1.279	11.765	8.785
Prob	0.00690	0.95631	0.00510	0.00001	0.40555	0.00024	0.01992	0.17650	0.00000	0.00000

CDS_INITIAL:

	1	2	3	4	5	6	7	8	9	10
a_O	280	0	0	152	112	86	122	126	104	121
	(72.5)	(0.0)	(0.0)	(39.4)	(29.0)	(22.3)	(31.6)	(32.6)	(26.9)	(31.3)
a_E	86.9	108.4	61.7	86.9	108.4	61.7	86.9	108.4	61.7	86.9
	(22.5)	(28.1)	(16.0)	(22.5)	(28.1)	(16.0)	(22.5)	(28.1)	(16.0)	(22.5)
	655.4	0.0	0.0	170.1	7.3	57.2	82.9	37.9	108.7	80.2
	169.788	0.000	0.000	44.058	1.893	14.827	21.464	9.820	28.171	20.772
t_O	56	386	0	40	82	123	59	110	89	67
	(14.5)	(100.0)	(0.0)	(10.4)	(21.2)	(31.9)	(15.3)	(28.5)	(23.1)	(17.4)
t_E	58.3	114.5	85.1	58.3	114.5	85.1	58.3	114.5	85.1	58.3
	(15.1)	(29.7)	(22.1)	(15.1)	(29.7)	(22.1)	(15.1)	(29.7)	(22.1)	(15.1)
	-4.6	938.2	0.0	-30.2	-54.7	90.5	1.3	-8.8	7.9	18.5
	-1.191	243.057	0.000	-7.824	-14.184	23.456	0.340	-2.284	2.052	4.801
g_O	50	0	386	98	74	88	118	52	92	88
	(13.0)	(0.0)	(100.0)	(25.4)	(19.2)	(22.8)	(30.6)	(13.5)	(23.8)	(22.8)
g_E	137.3	74.1	115.0	137.3	74.1	115.0	137.3	74.1	115.0	137.3
	(35.6)	(19.2)	(29.8)	(35.6)	(19.2)	(29.8)	(35.6)	(19.2)	(29.8)	(35.6)
	-101.0	0.0	934.5	-66.1	-0.2	-47.2	-35.7	-36.8	-41.1	-78.3
	-26.165	0.000	242.096	-17.114	-0.047	-12.221	-9.251	-9.539	-10.657	-20.275
c_O	0	0	0	96	118	89	87	98	101	110
	(0.0)	(0.0)	(0.0)	(24.9)	(30.6)	(23.1)	(22.5)	(25.4)	(26.2)	(28.5)
c_E	103.5	89.0	124.2	103.5	89.0	124.2	103.5	89.0	124.2	103.5
	(26.8)	(23.1)	(32.2)	(26.8)	(23.1)	(32.2)	(26.8)	(23.1)	(32.2)	(26.8)
	0.0	0.0	0.0	-14.4	66.5	-59.3	-30.2	18.9	-41.7	13.4
	0.000	0.000	0.000	-3.743	17.239	-15.355	-7.829	4.887	-10.806	3.470
N	386	386	386	386	386	386	386	386	386	386
X2	588.4	915.3	909.1	66.4	18.8	42.8	19.5	10.5	38.2	32.8
Gvalue	549.8	938.2	934.5	59.4	18.9	41.3	18.2	11.1	33.8	33.8
Gv/N	142.432	243.057	242.096	15.377	4.902	10.707	4.724	2.884	8.759	8.769
Prob	0.00000	0.00000	0.00000	0.00000	0.00028	0.00000	0.00039	0.01104	0.00000	0.00000

	11	12	13	14	15	16	17	18	19	20
a_O	87	70	122	93	83	121	107	80	113	97
	(22.5)	(18.1)	(31.6)	(24.1)	(21.5)	(31.3)	(27.7)	(20.7)	(29.3)	(25.1)
a_E	108.4	61.7	86.9	108.4	61.7	86.9	108.4	61.7	86.9	108.4
	(28.1)	(16.0)	(22.5)	(28.1)	(16.0)	(22.5)	(28.1)	(16.0)	(22.5)	(28.1)
	-38.3	17.8	82.9	-28.5	49.3	80.2	-2.8	41.7	59.4	-21.6
	-9.915	4.602	21.464	-7.386	12.782	20.772	-0.723	10.794	15.394	-5.587
t_O	120	102	77	132	108	74	119	95	54	112
	(31.1)	(26.4)	(19.9)	(34.2)	(28.0)	(19.2)	(30.8)	(24.6)	(14.0)	(29.0)
t_E	114.5	85.1	58.3	114.5	85.1	58.3	114.5	85.1	58.3	114.5
	(29.7)	(22.1)	(15.1)	(29.7)	(22.1)	(15.1)	(29.7)	(22.1)	(15.1)	(29.7)
	11.3	36.9	42.7	37.6	51.4	35.2	9.2	20.9	-8.4	-4.9
	2.919	9.557	11.067	9.729	13.318	9.112	2.378	5.402	-2.166	-1.280
g_O	79	110	82	65	81	83	61	92	98	72
	(20.5)	(28.5)	(21.2)	(16.8)	(21.0)	(21.5)	(15.8)	(23.8)	(25.4)	(18.7)
g_E	74.1	115.0	137.3	74.1	115.0	137.3	74.1	115.0	137.3	74.1
	(19.2)	(29.8)	(35.6)	(19.2)	(29.8)	(35.6)	(19.2)	(29.8)	(35.6)	(19.2)
	10.1	-9.9	-84.5	-17.0	-56.8	-83.5	-23.7	-41.1	-66.1	-4.1
	2.627	-2.558	-21.893	-4.408	-14.727	-21.638	-6.144	-10.657	-17.114	-1.067
c_O	100	104	105	96	114	108	99	119	121	105
	(25.9)	(26.9)	(27.2)	(24.9)	(29.5)	(28.0)	(25.6)	(30.8)	(31.3)	(27.2)
c_E	89.0	124.2	103.5	89.0	124.2	103.5	89.0	124.2	103.5	89.0
	(23.1)	(32.2)	(26.8)	(23.1)	(32.2)	(26.8)	(23.1)	(32.2)	(26.8)	(23.1)
	23.3	-36.9	3.0	14.5	-19.5	9.2	21.1	-10.1	37.8	34.7
	6.033	-9.550	0.782	3.761	-5.045	2.380	5.457	-2.620	9.793	8.989
N	386	386	386	386	386	386	386	386	386	386
X2	6.2	8.0	42.4	6.5	24.4	39.3	3.6	11.4	22.4	4.2
Gvalue	6.4	7.9	44.1	6.5	24.4	41.0	3.7	11.3	22.8	4.1
Gv/N	1.663	2.051	11.421	1.697	6.327	10.627	0.968	2.919	5.907	1.055
Prob	0.09290	0.04775	0.00000	0.08776	0.00002	0.00000	0.29126	0.01037	0.00004	0.25361

	21	22	23	24	25	26	27	28	29	30
a_O	76	112	117	77	103	91	72	90	92	73
	(19.7)	(29.0)	(30.3)	(19.9)	(26.7)	(23.6)	(18.7)	(23.3)	(23.8)	(18.9)
a_E	61.7	86.9	108.4	61.7	86.9	108.4	61.7	86.9	108.4	61.7
	(16.0)	(22.5)	(28.1)	(16.0)	(22.5)	(28.1)	(16.0)	(22.5)	(28.1)	(16.0)
	31.8	56.9	17.9	34.2	35.1	-31.9	22.3	6.4	-30.2	24.7
	8.235	14.742	4.626	8.865	9.087	-8.252	5.784	1.648	-7.822	6.386
t_O	100	61	111	80	76	126	103	67	133	88
	(25.9)	(15.8)	(28.8)	(20.7)	(19.7)	(32.6)	(26.7)	(17.4)	(34.5)	(22.8)
t_E	85.1	58.3	114.5	85.1	58.3	114.5	85.1	58.3	114.5	85.1
	(22.1)	(15.1)	(29.7)	(22.1)	(15.1)	(29.7)	(22.1)	(15.1)	(29.7)	(22.1)
	32.2	5.4	-6.9	-9.9	40.2	24.1	39.3	18.5	39.8	5.8
	8.344	1.406	-1.784	-2.575	10.409	6.250	10.171	4.801	10.323	1.514
g_O	90	110	68	104	100	78	98	110	77	98
	(23.3)	(28.5)	(17.6)	(26.9)	(25.9)	(20.2)	(25.4)	(28.5)	(19.9)	(25.4)
g_E	115.0	137.3	74.1	115.0	137.3	74.1	115.0	137.3	74.1	115.0
	(29.8)	(35.6)	(19.2)	(29.8)	(35.6)	(19.2)	(29.8)	(35.6)	(19.2)	(29.8)
	-44.2	-48.7	-11.7	-21.0	-63.4	8.0	-31.4	-48.7	5.9	-31.4
	-11.451	-12.625	-3.022	-5.441	-16.416	2.078	-8.144	-12.625	1.537	-8.144
c_O	120	103	90	125	107	91	113	119	84	127
	(31.1)	(26.7)	(23.3)	(32.4)	(27.7)	(23.6)	(29.3)	(30.8)	(21.8)	(32.9)
c_E	124.2	103.5	89.0	124.2	103.5	89.0	124.2	103.5	89.0	124.2
	(32.2)	(26.8)	(23.1)	(32.2)	(26.8)	(23.1)	(32.2)	(26.8)	(23.1)	(32.2)
	-8.2	-1.0	2.0	1.7	7.1	4.0	-21.3	33.2	-9.7	5.7
	-2.122	-0.260	0.517	0.434	1.843	1.043	-5.517	8.603	-2.521	1.485
N	386	386	386	386	386	386	386	386	386	386
X2	11.5	12.8	1.3	5.2	18.6	4.2	9.0	9.1	5.9	4.8
Gvalue	11.6	12.6	1.3	5.0	19.0	4.3	8.9	9.4	5.9	4.8
Gv/N	3.006	3.263	0.336	1.283	4.922	1.120	2.294	2.427	1.518	1.241
Prob	0.00887	0.00560	0.72961	0.17531	0.00027	0.22863	0.03126	0.02478	0.11867	0.18780

	31	32	33	34	35	36	37	38	39	40
a_O	102	92	63	101	94	57	95	88	65	92
	(26.4)	(23.8)	(16.3)	(26.2)	(24.4)	(14.8)	(24.6)	(22.8)	(16.8)	(23.8)
a_E	86.9	108.4	61.7	86.9	108.4	61.7	86.9	108.4	61.7	86.9
	(22.5)	(28.1)	(16.0)	(22.5)	(28.1)	(16.0)	(22.5)	(28.1)	(16.0)	(22.5)
	32.7	-30.2	2.7	30.4	-26.8	-9.0	17.0	-36.7	6.9	10.5
	8.483	-7.822	0.703	7.884	-6.944	-2.320	4.401	-9.508	1.777	2.733
t_O	60	125	84	61	118	113	69	139	100	61
	(15.5)	(32.4)	(21.8)	(15.8)	(30.6)	(29.3)	(17.9)	(36.0)	(25.9)	(15.8)
t_E	58.3	114.5	85.1	58.3	114.5	85.1	58.3	114.5	85.1	58.3
	(15.1)	(29.7)	(22.1)	(15.1)	(29.7)	(22.1)	(15.1)	(29.7)	(22.1)	(15.1)
	3.4	21.9	-2.2	5.4	7.1	64.0	23.1	53.9	32.2	5.4

	0.869	5.684	-0.580	1.406	1.842	16.584	5.996	13.967	8.344	1.406
g_O	111	71	100	117	69	87	101	74	99	127
	(28.8)	(18.4)	(25.9)	(30.3)	(17.9)	(22.5)	(26.2)	(19.2)	(25.6)	(32.9)
g_E	137.3	74.1	115.0	137.3	74.1	115.0	137.3	74.1	115.0	137.3
	(35.6)	(19.2)	(29.8)	(35.6)	(19.2)	(29.8)	(35.6)	(19.2)	(29.8)	(35.6)
	-47.2	-6.0	-28.0	-37.4	-9.8	-48.6	-62.0	-0.2	-29.7	-19.8
	-12.220	-1.567	-7.264	-9.689	-2.545	-12.597	-16.059	-0.047	-7.707	-5.120
c_O	113	98	139	107	105	129	121	85	122	106
	(29.3)	(25.4)	(36.0)	(27.7)	(27.2)	(33.4)	(31.3)	(22.0)	(31.6)	(27.5)
c_E	103.5	89.0	124.2	103.5	89.0	124.2	103.5	89.0	124.2	103.5
	(26.8)	(23.1)	(32.2)	(26.8)	(23.1)	(32.2)	(26.8)	(23.1)	(32.2)	(26.8)
	19.8	18.9	31.4	7.1	34.7	9.9	37.8	-7.8	-4.3	5.1
	5.140	4.887	8.128	1.843	8.989	2.553	9.793	-2.029	-1.112	1.310
N	386	386	386	386	386	386	386	386	386	386
X2	8.6	4.5	3.8	5.5	5.2	16.5	15.2	9.3	5.1	1.3
Gvalue	8.8	4.6	3.8	5.6	5.2	16.3	15.9	9.2	5.0	1.3
Gv/N	2.272	1.182	0.987	1.443	1.343	4.220	4.130	2.382	1.302	0.328
Prob	0.03250	0.20671	0.28272	0.13441	0.15880	0.00099	0.00117	0.02680	0.16989	0.73756

	41	42	43	44	45	46	47	48	49	50
a_O	87	66	95	99	64	80	97	64	95	99
	(22.5)	(17.1)	(24.6)	(25.6)	(16.6)	(20.7)	(25.1)	(16.6)	(24.6)	(25.6)
a_E	108.4	61.7	86.9	108.4	61.7	86.9	108.4	61.7	86.9	108.4
	(28.1)	(16.0)	(22.5)	(28.1)	(16.0)	(22.5)	(28.1)	(16.0)	(22.5)	(28.1)
	-38.3	9.0	17.0	-18.0	4.8	-13.2	-21.6	4.8	17.0	-18.0
	-9.915	2.327	4.401	-4.655	1.236	-3.417	-5.587	1.236	4.401	-4.655
t_O	127	97	70	115	80	57	118	98	60	119
	(32.9)	(25.1)	(18.1)	(29.8)	(20.7)	(14.8)	(30.6)	(25.4)	(15.5)	(30.8)
t_E	114.5	85.1	58.3	114.5	85.1	58.3	114.5	85.1	58.3	114.5
	(29.7)	(22.1)	(15.1)	(29.7)	(22.1)	(15.1)	(29.7)	(22.1)	(15.1)	(29.7)
	26.3	25.3	25.5	1.0	-9.9	-2.7	7.1	27.6	3.4	9.2
	6.820	6.562	6.604	0.261	-2.575	-0.690	1.842	7.151	0.869	2.378
g_O	69	94	120	83	119	120	78	87	120	74
	(17.9)	(24.4)	(31.1)	(21.5)	(30.8)	(31.1)	(20.2)	(22.5)	(31.1)	(19.2)
g_E	74.1	115.0	137.3	74.1	115.0	137.3	74.1	115.0	137.3	74.1
	(19.2)	(29.8)	(35.6)	(19.2)	(29.8)	(35.6)	(19.2)	(29.8)	(35.6)	(19.2)
	-9.8	-38.0	-32.3	18.9	8.0	-32.3	8.0	-48.6	-32.3	-0.2
	-2.545	-9.842	-8.363	4.884	2.082	-8.363	2.078	-12.597	-8.363	-0.047
c_O	103	129	101	89	123	129	93	137	111	94
	(26.7)	(33.4)	(26.2)	(23.1)	(31.9)	(33.4)	(24.1)	(35.5)	(28.8)	(24.4)
c_E	89.0	124.2	103.5	89.0	124.2	103.5	89.0	124.2	103.5	89.0
	(23.1)	(32.2)	(26.8)	(23.1)	(32.2)	(26.8)	(23.1)	(32.2)	(26.8)	(23.1)
	30.1	9.9	-4.9	-0.0	-2.3	56.8	8.2	27.0	15.5	10.3
	7.792	2.553	-1.281	-0.004	-0.601	14.719	2.114	6.982	4.022	2.658
N	386	386	386	386	386	386	386	386	386	386
X2	8.1	6.0	5.3	1.9	0.5	9.0	1.7	10.2	3.5	1.3
Gvalue	8.3	6.2	5.3	1.9	0.5	8.7	1.7	10.7	3.6	1.3
Gv/N	2.151	1.601	1.362	0.485	0.142	2.250	0.448	2.772	0.929	0.334
Prob	0.04012	0.10323	0.15395	0.59902	0.90821	0.03381	0.63038	0.01346	0.30977	0.73143

	51	52	53	54	55	56	57	58	59	60
a_O	63	80	83	71	81	73	57	80	84	63
	(16.3)	(20.7)	(21.5)	(18.4)	(21.0)	(18.9)	(14.8)	(20.7)	(21.8)	(16.3)
a_E	61.7	86.9	108.4	61.7	86.9	108.4	61.7	86.9	108.4	61.7
	(16.0)	(22.5)	(28.1)	(16.0)	(22.5)	(28.1)	(16.0)	(22.5)	(28.1)	(16.0)
	2.7	-13.2	-44.3	20.0	-11.3	-57.7	-9.0	-13.2	-42.8	2.7
	0.703	-3.417	-11.484	5.190	-2.938	-14.956	-2.320	-3.417	-11.101	0.703
t_O	95	63	102	86	79	139	97	58	122	92
	(24.6)	(16.3)	(26.4)	(22.3)	(20.5)	(36.0)	(25.1)	(15.0)	(31.6)	(23.8)
t_E	85.1	58.3	114.5	85.1	58.3	114.5	85.1	58.3	114.5	85.1
	(22.1)	(15.1)	(29.7)	(22.1)	(15.1)	(29.7)	(22.1)	(15.1)	(29.7)	(22.1)
	20.9	9.7	-23.6	1.8	47.9	53.9	25.3	-0.7	15.5	14.3
	5.402	2.505	-6.108	0.455	12.404	13.967	6.562	-0.179	4.012	3.701
g_O	104	125	86	97	116	78	104	142	80	96
	(26.9)	(32.4)	(22.3)	(25.1)	(30.1)	(20.2)	(26.9)	(36.8)	(20.7)	(24.9)
g_E	115.0	137.3	74.1	115.0	137.3	74.1	115.0	137.3	74.1	115.0
	(29.8)	(35.6)	(19.2)	(29.8)	(35.6)	(19.2)	(29.8)	(35.6)	(19.2)	(29.8)
	-21.0	-23.4	25.6	-33.1	-39.1	8.0	-21.0	9.6	12.3	-34.8
	-5.441	-6.068	6.642	-8.577	-10.122	2.078	-5.441	2.489	3.181	-9.004
c_O	124	118	115	132	110	96	128	106	100	135
	(32.1)	(30.6)	(29.8)	(34.2)	(28.5)	(24.9)	(33.2)	(27.5)	(25.9)	(35.0)
c_E	124.2	103.5	89.0	124.2	103.5	89.0	124.2	103.5	89.0	124.2
	(32.2)	(26.8)	(23.1)	(32.2)	(26.8)	(23.1)	(32.2)	(26.8)	(23.1)	(32.2)
	-0.3	30.9	58.9	16.2	13.4	14.5	7.8	5.1	23.3	22.6
	-0.086	8.015	15.266	4.185	3.470	3.761	2.017	1.310	6.033	5.852
N	386	386	386	386	386	386	386	386	386	386
X2	2.2	4.0	16.8	4.8	11.4	17.6	3.2	0.8	7.8	4.7
Gvalue	2.2	4.0	16.7	4.8	10.9	18.7	3.2	0.8	8.2	4.8
Gv/N	0.578	1.035	4.316	1.252	2.814	4.850	0.818	0.202	2.126	1.252
Prob	0.52619	0.26202	0.00083	0.18433	0.01249	0.00031	0.36771	0.85389	0.04195	0.18446

	61	62	63	64	65	66	67	68	69	70
a_O	95	96	55	88	84	66	79	101	49	77
	(24.6)	(24.9)	(14.2)	(22.8)	(21.8)	(17.1)	(20.5)	(26.2)	(12.7)	(19.9)
a_E	86.9	108.4	61.7	86.9	108.4	61.7	86.9	108.4	61.7	86.9
	(22.5)	(28.1)	(16.0)	(22.5)	(28.1)	(16.0)	(22.5)	(28.1)	(16.0)	(22.5)
	17.0	-23.3	-12.6	2.3	-42.8	9.0	-15.0	-14.3	-22.5	-18.6
	4.401	-6.045	-3.257	0.587	-11.101	2.327	-3.889	-3.702	-5.834	-4.814
t_O	71	122	97	60	124	88	63	112	86	70
	(18.4)	(31.6)	(25.1)	(15.5)	(32.1)	(22.8)	(16.3)	(29.0)	(22.3)	(18.1)
t_E	58.3	114.5	85.1	58.3	114.5	85.1	58.3	114.5	85.1	58.3
	(15.1)	(29.7)	(22.1)	(15.1)	(29.7)	(22.1)	(15.1)	(29.7)	(22.1)	(15.1)
	27.9	15.5	25.3	3.4	19.8	5.8	9.7	-4.9	1.8	25.5
	7.221	4.012	6.562	0.869	5.123	1.514	2.505	-1.280	0.455	6.604
g_O	119	75	106	143	72	109	145	81	129	136
	(30.8)	(19.4)	(27.5)	(37.0)	(18.7)	(28.2)	(37.6)	(21.0)	(33.4)	(35.2)
g_E	137.3	74.1	115.0	137.3	74.1	115.0	137.3	74.1	115.0	137.3
	(35.6)	(19.2)	(29.8)	(35.6)	(19.2)	(29.8)	(35.6)	(19.2)	(29.8)	(35.6)
	-34.0	1.8	-17.4	11.7	-4.1	-11.8	15.9	14.4	29.5	-2.5
	-8.809	0.474	-4.499	3.026	-1.067	-3.050	4.112	3.742	7.650	-0.658
c_O	101	93	128	95	106	123	99	92	122	103
	(26.2)	(24.1)	(33.2)	(24.6)	(27.5)	(31.9)	(25.6)	(23.8)	(31.6)	(26.7)
c_E	103.5	89.0	124.2	103.5	89.0	124.2	103.5	89.0	124.2	103.5
	(26.8)	(23.1)	(32.2)	(26.8)	(23.1)	(32.2)	(26.8)	(23.1)	(32.2)	(26.8)
	-4.9	8.2	7.8	-16.3	37.0	-2.3	-8.8	6.1	-4.3	-1.0
	-1.281	2.114	2.017	-4.219	9.595	-0.601	-2.281	1.576	-1.112	-0.260

N	386	386	386	386	386	386	386	386	386	386
X2	6.0	2.1	3.2	1.0	9.6	0.7	1.7	1.3	4.3	3.5
Gvalue	5.9	2.1	3.2	1.0	9.8	0.7	1.7	1.3	4.5	3.4
Gv/N	1.532	0.556	0.824	0.263	2.550	0.189	0.446	0.336	1.158	0.873
Prob	0.11596	0.54272	0.36486	0.79771	0.01995	0.86626	0.63176	0.72976	0.21484	0.33826

	71	72	73	74	75	76	77	78	79	80
a_O	86	69	81	91	57	76	94	61	98	89
	(22.3)	(17.9)	(21.0)	(23.6)	(14.8)	(19.7)	(24.4)	(15.8)	(25.4)	(23.1)
a_E	108.4	61.7	86.9	108.4	61.7	86.9	108.4	61.7	86.9	108.4
	(28.1)	(16.0)	(22.5)	(28.1)	(16.0)	(22.5)	(28.1)	(16.0)	(22.5)	(28.1)
	−39.8	15.5	−11.3	−31.9	−9.0	−20.3	−26.8	−1.3	23.6	−35.1
	−10.317	4.022	−2.938	−8.252	−2.320	−5.266	−6.944	−0.339	6.119	−9.095
t_O	135	72	53	117	94	72	120	94	64	111
	(35.0)	(18.7)	(13.7)	(30.3)	(24.4)	(18.7)	(31.1)	(24.4)	(16.6)	(28.8)
t_E	114.5	85.1	58.3	114.5	85.1	58.3	114.5	85.1	58.3	114.5
	(29.7)	(22.1)	(15.1)	(29.7)	(22.1)	(15.1)	(29.7)	(22.1)	(15.1)	(29.7)
	44.5	−24.1	−10.2	5.1	18.6	30.3	11.3	18.6	11.8	−6.9
	11.522	−6.248	−2.639	1.311	4.829	7.844	2.919	4.829	3.067	−1.784
g_O	70	128	132	75	111	135	67	114	123	84
	(18.1)	(33.2)	(34.2)	(19.4)	(28.8)	(35.0)	(17.4)	(29.5)	(31.9)	(21.8)
g_E	74.1	115.0	137.3	74.1	115.0	137.3	74.1	115.0	137.3	74.1
	(19.2)	(29.8)	(35.6)	(19.2)	(29.8)	(35.6)	(19.2)	(29.8)	(35.6)	(19.2)
	−7.9	27.3	−10.3	1.8	−8.0	−4.5	−13.5	−2.1	−27.0	21.1
	−2.060	7.075	−2.681	0.474	−2.061	−1.170	−3.492	−0.541	−6.999	5.464
c_O	95	117	120	103	124	103	105	117	101	102
	(24.6)	(30.3)	(31.1)	(26.7)	(32.1)	(26.7)	(27.2)	(30.3)	(26.2)	(26.4)
c_E	89.0	124.2	103.5	89.0	124.2	103.5	89.0	124.2	103.5	89.0
	(23.1)	(32.2)	(26.8)	(23.1)	(32.2)	(26.8)	(23.1)	(32.2)	(26.8)	(23.1)
	12.4	−13.9	35.5	30.1	−0.3	−1.0	34.7	−13.9	−4.9	27.8
	3.207	−3.604	9.196	7.792	−0.086	−0.260	8.989	−3.604	−1.281	7.200
N	386	386	386	386	386	386	386	386	386	386
X2	8.9	4.8	3.7	5.1	1.4	4.6	5.7	1.4	3.5	6.8
Gvalue	9.1	4.8	3.6	5.1	1.4	4.4	5.7	1.3	3.5	6.9
Gv/N	2.353	1.245	0.937	1.325	0.363	1.149	1.472	0.345	0.906	1.785
Prob	0.02822	0.18656	0.30581	0.16356	0.70553	0.21829	0.12817	0.72136	0.32095	0.07549

	81	82	83	84	85	86	87	88	89	90
a_O	52	88	96	50	79	101	62	85	115	67
	(13.5)	(22.8)	(24.9)	(13.0)	(20.5)	(26.2)	(16.1)	(22.0)	(29.8)	(17.4)
a_E	61.7	86.9	108.4	61.7	86.9	108.4	61.7	86.9	108.4	61.7
	(16.0)	(22.5)	(28.1)	(16.0)	(22.5)	(28.1)	(16.0)	(22.5)	(28.1)	(16.0)
	−17.7	2.3	−23.3	−21.0	−15.0	−14.3	0.7	−3.7	13.6	11.1
	−4.590	0.587	−6.045	−5.430	−3.889	−3.702	0.177	−0.961	3.519	2.884
t_O	88	59	101	100	68	124	99	58	113	76
	(22.8)	(15.3)	(26.2)	(25.9)	(17.6)	(32.1)	(25.6)	(15.0)	(29.3)	(19.7)
t_E	85.1	58.3	114.5	85.1	58.3	114.5	85.1	58.3	114.5	85.1
	(22.1)	(15.1)	(29.7)	(22.1)	(15.1)	(29.7)	(22.1)	(15.1)	(29.7)	(22.1)
	5.8	1.3	−25.3	32.2	20.8	19.8	29.9	−0.7	−3.0	−17.2
	1.514	0.340	−6.564	8.344	5.394	5.123	7.745	−0.179	−0.771	−4.466
g_O	111	141	79	101	135	59	103	138	74	107
	(28.8)	(36.5)	(20.5)	(26.2)	(35.0)	(15.3)	(26.7)	(35.8)	(19.2)	(27.7)
g_E	115.0	137.3	74.1	115.0	137.3	74.1	115.0	137.3	74.1	115.0
	(29.8)	(35.6)	(19.2)	(29.8)	(35.6)	(19.2)	(29.8)	(35.6)	(19.2)	(29.8)
	−8.0	7.5	10.1	−26.3	−4.5	−26.9	−22.8	1.5	−0.2	−15.5
	−2.061	1.955	2.627	−6.816	−1.170	−6.962	−5.904	0.376	−0.047	−4.021
c_O	135	98	110	135	104	102	122	105	84	136
	(35.0)	(25.4)	(28.5)	(35.0)	(26.9)	(26.4)	(31.6)	(27.2)	(21.8)	(35.2)
c_E	124.2	103.5	89.0	124.2	103.5	89.0	124.2	103.5	89.0	124.2
	(32.2)	(26.8)	(23.1)	(32.2)	(26.8)	(23.1)	(32.2)	(26.8)	(23.1)	(32.2)
	22.6	−10.7	46.6	22.6	1.0	27.8	−4.3	3.0	−9.7	24.8
	5.852	−2.774	12.069	5.852	0.259	7.200	−1.112	0.782	−2.521	6.415
N	386	386	386	386	386	386	386	386	386	386
X2	2.7	0.4	8.3	7.5	2.4	6.3	3.6	0.1	0.7	3.1
Gvalue	2.8	0.4	8.1	7.5	2.3	6.4	3.5	0.1	0.7	3.1
Gv/N	0.714	0.109	2.087	1.950	0.594	1.659	0.906	0.018	0.181	0.812
Prob	0.43047	0.93614	0.04489	0.05688	0.51398	0.09361	0.32133	0.99536	0.87312	0.37115

	91	92	93	94	95	96	97	98	99	100
a_O	77	104	64	88	96	64	80	110	59	70
	(19.9)	(26.9)	(16.6)	(22.8)	(24.9)	(16.6)	(20.7)	(28.5)	(15.3)	(18.1)
a_E	86.9	108.4	61.7	86.9	108.4	61.7	86.9	108.4	61.7	86.9
	(22.5)	(28.1)	(16.0)	(22.5)	(28.1)	(16.0)	(22.5)	(28.1)	(16.0)	(22.5)
	−18.6	−8.6	4.8	2.3	−23.3	4.8	−13.2	3.2	−5.2	−30.2
	−4.814	−2.235	1.236	0.587	−6.045	1.236	−3.417	0.833	−1.347	−7.833
t_O	70	122	100	56	133	83	58	116	92	59
	(18.1)	(31.6)	(25.9)	(14.5)	(34.5)	(21.5)	(15.0)	(30.1)	(23.8)	(15.3)
t_E	58.3	114.5	85.1	58.3	114.5	85.1	58.3	114.5	85.1	58.3
	(15.1)	(29.7)	(22.1)	(15.1)	(29.7)	(22.1)	(15.1)	(29.7)	(22.1)	(15.1)
	25.5	15.5	32.2	−4.6	39.8	−4.2	−0.7	3.0	14.3	1.3
	6.604	4.012	8.344	−1.191	10.323	−1.088	−0.179	0.784	3.701	0.340
g_O	147	73	112	143	76	112	128	82	121	160
	(38.1)	(18.9)	(29.0)	(37.0)	(19.7)	(29.0)	(33.2)	(21.2)	(31.3)	(41.5)
g_E	137.3	74.1	115.0	137.3	74.1	115.0	137.3	74.1	115.0	137.3
	(35.6)	(19.2)	(29.8)	(35.6)	(19.2)	(29.8)	(35.6)	(19.2)	(29.8)	(35.6)
	20.1	−2.2	−6.0	11.7	3.9	−6.0	−17.9	16.6	12.2	49.0
	5.212	−0.561	−1.559	3.026	1.002	−1.559	−4.640	4.310	3.162	12.698
c_O	92	87	110	99	81	127	120	78	114	97
	(23.8)	(22.5)	(28.5)	(25.6)	(21.0)	(32.9)	(31.1)	(20.2)	(29.5)	(25.1)
c_E	103.5	89.0	124.2	103.5	89.0	124.2	103.5	89.0	124.2	103.5
	(26.8)	(23.1)	(32.2)	(26.8)	(23.1)	(32.2)	(26.8)	(23.1)	(32.2)	(26.8)
	−21.7	−4.0	−26.7	−8.8	−15.3	5.7	35.5	−20.6	−19.5	−12.6
	−5.616	−1.029	−6.904	−2.281	−3.957	1.485	9.196	−5.336	−5.045	−3.261
N	386	386	386	386	386	386	386	386	386	386
X2	5.4	0.7	4.4	0.5	5.2	0.3	3.8	2.2	1.8	7.5
Gvalue	5.4	0.7	4.3	0.5	5.1	0.3	3.7	2.3	1.8	7.5
Gv/N	1.387	0.188	1.117	0.141	1.324	0.074	0.959	0.591	0.470	1.945
Prob	0.14756	0.86744	0.22987	0.90906	0.16393	0.96243	0.29541	0.51631	0.61156	0.05737

Acin_ADP1
5UTR:

	100	99	98	97	96	95	94	93	92	91
a_O	246	223	256	241	247	260	248	249	232	264
	(32.8)	(29.7)	(34.1)	(32.1)	(32.9)	(34.7)	(33.1)	(33.2)	(30.9)	(35.2)
a_E	254.6	254.6	254.6	254.6	254.6	254.6	254.6	254.6	254.6	254.6
	(33.9)	(33.9)	(33.9)	(33.9)	(33.9)	(33.9)	(33.9)	(33.9)	(33.9)	(33.9)
	-16.9	-59.1	2.8	-26.5	-15.0	10.9	-13.1	-11.1	-43.2	19.1
	-2.259	-7.885	0.369	-3.533	-2.001	1.450	-1.742	-1.482	-5.756	2.547
t_O	254	260	246	263	259	248	253	257	265	252
	(33.9)	(34.7)	(32.8)	(35.1)	(34.5)	(33.1)	(33.7)	(34.3)	(35.3)	(33.6)
t_E	256.0	256.0	256.0	256.0	256.0	256.0	256.0	256.0	256.0	256.0
	(34.1)	(34.1)	(34.1)	(34.1)	(34.1)	(34.1)	(34.1)	(34.1)	(34.1)	(34.1)
	-4.0	8.0	-19.7	14.1	6.0	-15.8	-6.0	1.9	18.3	-8.0
	-0.539	1.067	-2.622	1.884	0.797	-2.107	-0.803	0.259	2.433	-1.066
g_O	126	144	134	123	123	126	142	129	145	123
	(16.8)	(19.2)	(17.9)	(16.4)	(16.4)	(16.8)	(18.9)	(17.2)	(19.3)	(16.4)
g_E	117.4	117.4	117.4	117.4	117.4	117.4	117.4	117.4	117.4	117.4
	(15.7)	(15.7)	(15.7)	(15.7)	(15.7)	(15.7)	(15.7)	(15.7)	(15.7)	(15.7)
	17.9	58.9	35.5	11.5	11.5	17.9	54.1	24.4	61.3	11.5
	2.381	7.849	4.732	1.534	1.534	2.381	7.210	3.247	8.171	1.534
c_O	124	123	114	123	121	116	107	115	108	111
	(16.5)	(16.4)	(15.2)	(16.4)	(16.1)	(15.5)	(14.3)	(15.3)	(14.4)	(14.8)
c_E	122.0	122.0	122.0	122.0	122.0	122.0	122.0	122.0	122.0	122.0
	(16.3)	(16.3)	(16.3)	(16.3)	(16.3)	(16.3)	(16.3)	(16.3)	(16.3)	(16.3)
	4.1	2.1	-15.4	2.1	-1.9	-11.6	-28.0	-13.5	-26.3	-20.9
	0.546	0.276	-2.054	0.276	-0.258	-1.552	-3.736	-1.805	-3.503	-2.790
N	750	750	750	750	750	750	750	750	750	750
X2	1.0	10.0	3.3	1.2	0.5	1.3	7.2	1.7	10.4	1.7
Gvalue	1.0	9.8	3.2	1.2	0.5	1.3	7.0	1.6	10.1	1.7
Gv/N	0.128	1.306	0.425	0.160	0.072	0.171	0.928	0.220	1.345	0.225
Prob	0.81021	0.02038	0.36360	0.75216	0.91063	0.73327	0.07306	0.64836	0.01782	0.63968

	90	89	88	87	86	85	84	83	82	81
a_O	261	267	261	251	251	248	232	250	232	262
	(34.8)	(35.6)	(34.8)	(33.5)	(33.5)	(33.1)	(30.9)	(33.3)	(30.9)	(34.9)
a_E	254.6	254.6	254.6	254.6	254.6	254.6	254.6	254.6	254.6	254.6
	(33.9)	(33.9)	(33.9)	(33.9)	(33.9)	(33.9)	(33.9)	(33.9)	(33.9)	(33.9)
	12.9	25.4	12.9	-7.2	-7.2	-13.1	-43.2	-9.2	-43.2	15.0
	1.722	3.380	1.722	-0.958	-0.958	-1.742	-5.756	-1.221	-5.756	1.996
t_O	228	255	250	243	248	250	290	267	264	243
	(30.4)	(34.0)	(33.3)	(32.4)	(33.1)	(33.3)	(38.7)	(35.6)	(35.2)	(32.4)
t_E	256.0	256.0	256.0	256.0	256.0	256.0	256.0	256.0	256.0	256.0
	(34.1)	(34.1)	(34.1)	(34.1)	(34.1)	(34.1)	(34.1)	(34.1)	(34.1)	(34.1)
	-52.9	-2.1	-11.9	-25.4	-15.8	-11.9	72.3	22.4	16.2	-25.4
	-7.050	-0.274	-1.589	-3.385	-2.107	-1.589	9.635	2.987	2.158	-3.385
g_O	142	117	123	108	126	131	109	118	121	123
	(18.9)	(15.6)	(16.4)	(14.4)	(16.8)	(17.5)	(14.5)	(15.7)	(16.1)	(16.4)
g_E	117.4	117.4	117.4	117.4	117.4	117.4	117.4	117.4	117.4	117.4
	(15.7)	(15.7)	(15.7)	(15.7)	(15.7)	(15.7)	(15.7)	(15.7)	(15.7)	(15.7)
	54.1	-0.8	11.5	-18.0	17.9	28.8	-16.1	1.2	7.4	11.5
	7.210	-0.101	1.534	-2.399	2.381	3.835	-2.153	0.166	0.980	1.534
c_O	119	111	116	148	125	121	119	115	133	122
	(15.9)	(14.8)	(15.5)	(19.7)	(16.7)	(16.1)	(15.9)	(15.3)	(17.7)	(16.3)
c_E	122.0	122.0	122.0	122.0	122.0	122.0	122.0	122.0	122.0	122.0
	(16.3)	(16.3)	(16.3)	(16.3)	(16.3)	(16.3)	(16.3)	(16.3)	(16.3)	(16.3)
	-5.9	-20.9	-11.6	57.3	6.1	-1.9	-5.9	-13.5	23.0	0.1
	-0.782	-2.790	-1.552	7.634	0.818	-0.258	-0.782	-1.805	3.070	0.008
N	750	750	750	750	750	750	750	750	750	750
X2	8.5	1.6	0.9	7.0	1.0	1.9	7.2	1.0	3.4	1.1
Gvalue	8.3	1.6	0.9	6.7	1.0	1.8	7.1	1.0	3.4	1.2
Gv/N	1.101	0.215	0.115	0.893	0.133	0.246	0.944	0.128	0.453	0.154
Prob	0.04102	0.65590	0.83418	0.08231	0.80150	0.60474	0.06946	0.81161	0.33442	0.76467

	80	79	78	77	76	75	74	73	72	71
a_O	247	241	246	236	232	248	270	282	263	257
	(32.9)	(32.1)	(32.8)	(31.5)	(30.9)	(33.1)	(36.0)	(37.6)	(35.1)	(34.3)
a_E	254.6	254.6	254.6	254.6	254.6	254.6	254.6	254.6	254.6	254.6
	(33.9)	(33.9)	(33.9)	(33.9)	(33.9)	(33.9)	(33.9)	(33.9)	(33.9)	(33.9)
	-15.0	-26.5	-16.9	-35.8	-43.2	-13.1	31.7	57.6	17.0	4.8
	-2.001	-3.533	-2.259	-4.779	-5.756	-1.742	4.223	7.681	2.271	0.638
t_O	280	256	258	256	267	251	257	219	246	273
	(37.3)	(34.1)	(34.4)	(34.1)	(35.6)	(33.5)	(34.3)	(29.2)	(32.8)	(36.4)
t_E	256.0	256.0	256.0	256.0	256.0	256.0	256.0	256.0	256.0	256.0
	(34.1)	(34.1)	(34.1)	(34.1)	(34.1)	(34.1)	(34.1)	(34.1)	(34.1)	(34.1)
	50.1	-0.1	4.0	-0.1	22.4	-10.0	1.9	-68.4	-19.7	35.0
	6.682	-0.008	0.527	-0.008	2.987	-1.328	0.259	-9.123	-2.622	4.672
g_O	99	135	125	124	142	120	119	127	123	123
	(13.2)	(18.0)	(16.7)	(16.5)	(18.9)	(16.0)	(15.9)	(16.9)	(16.4)	(16.4)
g_E	117.4	117.4	117.4	117.4	117.4	117.4	117.4	117.4	117.4	117.4
	(15.7)	(15.7)	(15.7)	(15.7)	(15.7)	(15.7)	(15.7)	(15.7)	(15.7)	(15.7)
	-33.7	37.8	15.7	13.6	54.1	5.3	3.3	20.0	11.5	11.5
	-4.496	5.035	2.097	1.814	7.210	0.706	0.435	2.668	1.534	1.534
c_O	124	118	121	134	109	131	104	122	118	97
	(16.5)	(15.7)	(16.1)	(17.9)	(14.5)	(17.5)	(13.9)	(16.3)	(15.7)	(12.9)
c_E	122.0	122.0	122.0	122.0	122.0	122.0	122.0	122.0	122.0	122.0
	(16.3)	(16.3)	(16.3)	(16.3)	(16.3)	(16.3)	(16.3)	(16.3)	(16.3)	(16.3)
	4.1	-7.8	-1.9	25.2	-24.5	18.7	-33.2	0.1	-7.8	-44.4
	0.546	-1.041	-0.258	3.361	-3.268	2.495	-4.420	0.008	-1.041	-5.925
N	750	750	750	750	750	750	750	750	750	750
X2	5.4	3.5	0.8	2.9	9.0	1.0	3.6	9.1	1.1	6.5
Gvalue	5.5	3.4	0.8	2.9	8.8	1.0	3.7	9.2	1.1	6.9
Gv/N	0.731	0.453	0.107	0.388	1.174	0.131	0.497	1.233	0.142	0.919
Prob	0.13970	0.33474	0.84891	0.40540	0.03203	0.80518	0.29275	0.02619	0.78516	0.07551

	70	69	68	67	66	65	64	63	62	61
a_O	253	265	217	255	237	244	250	249	286	239
	(33.7)	(35.3)	(28.9)	(34.0)	(31.6)	(32.5)	(33.3)	(33.2)	(38.1)	(31.9)
a_E	254.6	254.6	254.6	254.6	254.6	254.6	254.6	254.6	254.6	254.6
	(33.9)	(33.9)	(33.9)	(33.9)	(33.9)	(33.9)	(33.9)	(33.9)	(33.9)	(33.9)
	-3.2	21.2	-69.4	0.8	-34.0	-20.8	-9.2	-11.1	66.5	-30.3
	-0.431	2.824	-9.251	0.101	-4.532	-2.772	-1.221	-1.482	8.864	-4.035
t_O	245	253	245	256	253	240	255	260	238	278
	(32.7)	(33.7)	(32.7)	(34.1)	(33.7)	(32.0)	(34.0)	(34.7)	(31.7)	(37.1)

```
t_E       256.0   256.0   256.0   256.0   256.0   256.0   256.0   256.0   256.0   256.0
        ( 34.1) ( 34.1) ( 34.1) ( 34.1) ( 34.1) ( 34.1) ( 34.1) ( 34.1) ( 34.1) ( 34.1)
          -21.6    -6.0   -21.6    -0.1    -6.0   -31.0    -2.1     8.0   -34.8    45.8
         -2.877  -0.803  -2.877  -0.008  -0.803  -4.138  -0.274   1.067  -4.635   6.103
g_O         130     117     146     119     140     130     128     111     109     106
        ( 17.3) ( 15.6) ( 19.5) ( 15.9) ( 18.7) ( 17.3) ( 17.1) ( 14.8) ( 14.5) ( 14.1)
g_E       117.4   117.4   117.4   117.4   117.4   117.4   117.4   117.4   117.4   117.4
        ( 15.7) ( 15.7) ( 15.7) ( 15.7) ( 15.7) ( 15.7) ( 15.7) ( 15.7) ( 15.7) ( 15.7)
           26.6    -0.8    63.7     3.3    49.3    26.6    22.2   -12.4   -16.1   -21.6
          3.540  -0.101   8.495   0.435   6.579   3.540   2.956  -1.654  -2.153  -2.883
c_O         122     115     142     120     120     136     117     130     117     127
        ( 16.3) ( 15.3) ( 18.9) ( 16.0) ( 16.0) ( 18.1) ( 15.6) ( 17.3) ( 15.6) ( 16.9)
c_E       122.0   122.0   122.0   122.0   122.0   122.0   122.0   122.0   122.0   122.0
        ( 16.3) ( 16.3) ( 16.3) ( 16.3) ( 16.3) ( 16.3) ( 16.3) ( 16.3) ( 16.3) ( 16.3)
            0.1   -13.5    43.2    -3.9    -3.9    29.6    -9.7    16.6    -9.7    10.3
          0.008  -1.805   5.758  -0.521  -0.521   3.949  -1.298   2.210  -1.298   1.369

N           750     750     750     750     750     750     750     750     750     750
X2          1.8     0.9    16.3     0.1     5.6     4.4     1.3     1.1     5.9     4.2
Gvalue      1.8     0.9    15.9     0.1     5.4     4.3     1.2     1.1     5.8     4.2
Gv/N      0.240   0.115   2.124   0.007   0.723   0.579   0.164   0.141   0.778   0.554
Prob    0.61425 0.83486 0.00117 0.99664 0.14352 0.22697 0.74655 0.78749 0.11984 0.24496
        --------------------------------------------------------------------------------
             60      59      58      57      56      55      54      53      52      51
        --------------------------------------------------------------------------------
a_O         233     242     250     224     252     256     244     233     233     253
        ( 31.1) ( 32.3) ( 33.3) ( 29.9) ( 33.6) ( 34.1) ( 32.5) ( 31.1) ( 31.1) ( 33.7)
a_E       254.6   254.6   254.6   254.6   254.6   254.6   254.6   254.6   254.6   254.6
        ( 33.9) ( 33.9) ( 33.9) ( 33.9) ( 33.9) ( 33.9) ( 33.9) ( 33.9) ( 33.9) ( 33.9)
          -41.3   -24.6    -9.2   -57.4    -5.2     2.8   -20.8   -41.3   -41.3    -3.2
         -5.513  -3.281  -1.221  -7.653  -0.695   0.369  -2.772  -5.513  -5.513  -0.431
t_O         263     260     262     269     261     259     260     271     291     261
        ( 35.1) ( 34.7) ( 34.9) ( 35.9) ( 34.8) ( 34.5) ( 34.7) ( 36.1) ( 38.8) ( 34.8)
t_E       256.0   256.0   256.0   256.0   256.0   256.0   256.0   256.0   256.0   256.0
        ( 34.1) ( 34.1) ( 34.1) ( 34.1) ( 34.1) ( 34.1) ( 34.1) ( 34.1) ( 34.1) ( 34.1)
           14.1     8.0    12.1    26.6    10.0     6.0     8.0    30.8    74.5    10.0
          1.884   1.067   1.610   3.545   1.338   0.797   1.067   4.106   9.935   1.338
g_O         128     125     125     136     116     120     117     116     114     117
        ( 17.1) ( 16.7) ( 16.7) ( 18.1) ( 15.5) ( 16.0) ( 15.6) ( 15.5) ( 15.2) ( 15.6)
g_E       117.4   117.4   117.4   117.4   117.4   117.4   117.4   117.4   117.4   117.4
        ( 15.7) ( 15.7) ( 15.7) ( 15.7) ( 15.7) ( 15.7) ( 15.7) ( 15.7) ( 15.7) ( 15.7)
           22.2    15.7    15.7    40.0    -2.7     5.3    -0.8    -2.7    -6.7    -0.8
          2.956   2.097   2.097   5.340  -0.366   0.706  -0.101  -0.366  -0.888  -0.101
c_O         126     123     113     121     121     115     129     130     112     119
        ( 16.8) ( 16.4) ( 15.1) ( 16.1) ( 16.1) ( 15.3) ( 17.2) ( 17.3) ( 14.9) ( 15.9)
c_E       122.0   122.0   122.0   122.0   122.0   122.0   122.0   122.0   122.0   122.0
        ( 16.3) ( 16.3) ( 16.3) ( 16.3) ( 16.3) ( 16.3) ( 16.3) ( 16.3) ( 16.3) ( 16.3)
            8.2     2.1   -17.3    -1.9    -1.9   -13.5    14.5    16.6   -19.1    -5.9
          1.092   0.276  -2.302  -0.258  -0.258  -1.805   1.928   2.210  -2.547  -0.782

N           750     750     750     750     750     750     750     750     750     750
X2          3.1     1.2     1.4     7.3     0.1     0.5     0.9     3.3     7.5     0.2
Gvalue      3.1     1.2     1.4     7.3     0.1     0.5     0.9     3.3     7.4     0.2
Gv/N      0.419   0.159   0.184   0.974   0.020   0.067   0.121   0.438   0.987   0.024
Prob    0.37011 0.75533 0.70947 0.06286 0.98566 0.91758 0.82317 0.35009 0.06021 0.98070
        --------------------------------------------------------------------------------
             50      49      48      47      46      45      44      43      42      41
        --------------------------------------------------------------------------------
a_O         230     229     238     239     258     254     233     250     266     268
        ( 30.7) ( 30.5) ( 31.7) ( 31.9) ( 34.4) ( 33.9) ( 31.1) ( 33.3) ( 35.5) ( 35.7)
a_E       254.6   254.6   254.6   254.6   254.6   254.6   254.6   254.6   254.6   254.6
        ( 33.9) ( 33.9) ( 33.9) ( 33.9) ( 33.9) ( 33.9) ( 33.9) ( 33.9) ( 33.9) ( 33.9)
          -46.8   -48.6   -32.1   -30.3     6.8    -1.2   -41.3    -9.2    23.3    27.5
         -6.237  -6.476  -4.284  -4.035   0.907  -0.165  -5.513  -1.221   3.101   3.660
t_O         276     266     274     269     241     251     260     244     256     274
        ( 36.8) ( 35.5) ( 36.5) ( 35.9) ( 32.1) ( 33.5) ( 34.7) ( 32.5) ( 34.1) ( 36.5)
t_E       256.0   256.0   256.0   256.0   256.0   256.0   256.0   256.0   256.0   256.0
        ( 34.1) ( 34.1) ( 34.1) ( 34.1) ( 34.1) ( 34.1) ( 34.1) ( 34.1) ( 34.1) ( 34.1)
           41.5    20.3    37.2    26.6   -29.2   -10.0     8.0   -23.5    -0.1    37.2
          5.528   2.710   4.956   3.545  -3.888  -1.328   1.067  -3.131  -0.008   4.956
g_O         123     133     126     114     126     111     136     131     104      95
        ( 16.4) ( 17.7) ( 16.8) ( 15.2) ( 16.8) ( 14.8) ( 18.1) ( 17.5) ( 13.9) ( 12.7)
g_E       117.4   117.4   117.4   117.4   117.4   117.4   117.4   117.4   117.4   117.4
        ( 15.7) ( 15.7) ( 15.7) ( 15.7) ( 15.7) ( 15.7) ( 15.7) ( 15.7) ( 15.7) ( 15.7)
           11.5    33.2    17.9    -6.7    17.9   -12.4    40.0    28.8   -25.2   -40.2
          1.534   4.431   2.381  -0.888   2.381  -1.654   5.340   3.835  -3.356  -5.359
c_O         121     122     112     128     125     134     121     125     124     113
        ( 16.1) ( 16.3) ( 14.9) ( 17.1) ( 16.7) ( 17.9) ( 16.1) ( 16.7) ( 16.5) ( 15.1)
c_E       122.0   122.0   122.0   122.0   122.0   122.0   122.0   122.0   122.0   122.0
        ( 16.3) ( 16.3) ( 16.3) ( 16.3) ( 16.3) ( 16.3) ( 16.3) ( 16.3) ( 16.3) ( 16.3)
           -1.9     0.1   -19.1    12.4     6.1    25.2    -1.9     6.1     4.1   -17.3
         -0.258   0.008  -2.547   1.647   0.818   3.361  -0.258   0.818   0.546  -2.302

N           750     750     750     750     750     750     750     750     750     750
X2          4.2     5.0     3.8     2.0     1.6     1.6     4.9     2.3     2.1     6.9
Gvalue      4.3     5.0     3.8     2.0     1.6     1.6     4.8     2.3     2.1     7.2
Gv/N      0.567   0.673   0.506   0.269   0.218   0.214   0.636   0.301   0.283   0.956
Prob    0.23546 0.16858 0.28410 0.56904 0.65091 0.65858 0.18961 0.52103 0.54758 0.06674
        --------------------------------------------------------------------------------
             40      39      38      37      36      35      34      33      32      31
        --------------------------------------------------------------------------------
a_O         264     268     234     258     227     266     262     235     255     237
        ( 35.2) ( 35.7) ( 31.2) ( 34.4) ( 30.3) ( 35.5) ( 34.9) ( 31.3) ( 34.0) ( 31.6)
a_E       254.6   254.6   254.6   254.6   254.6   254.6   254.6   254.6   254.6   254.6
        ( 33.9) ( 33.9) ( 33.9) ( 33.9) ( 33.9) ( 33.9) ( 33.9) ( 33.9) ( 33.9) ( 33.9)
           19.1    27.5   -39.5     6.8   -52.1    23.3    15.0   -37.7     0.8   -34.0
          2.547   3.660  -5.270   0.907  -6.951   3.101   1.996  -5.025   0.101  -4.532
t_O         267     251     243     264     282     246     274     276     237     263
        ( 35.6) ( 33.5) ( 32.4) ( 35.2) ( 37.6) ( 32.8) ( 36.5) ( 36.8) ( 31.6) ( 35.1)
t_E       256.0   256.0   256.0   256.0   256.0   256.0   256.0   256.0   256.0   256.0
        ( 34.1) ( 34.1) ( 34.1) ( 34.1) ( 34.1) ( 34.1) ( 34.1) ( 34.1) ( 34.1) ( 34.1)
           22.4   -10.0   -25.4    16.2    54.5   -19.7    37.2    41.5   -36.6    14.1
          2.987  -1.328  -3.385   2.158   7.265  -2.622   4.956   5.528  -4.881   1.884
g_O         111     114     148     119     121     125     102     119     136     111
        ( 14.8) ( 15.2) ( 19.7) ( 15.9) ( 16.1) ( 16.7) ( 13.6) ( 15.9) ( 18.1) ( 14.8)
g_E       117.4   117.4   117.4   117.4   117.4   117.4   117.4   117.4   117.4   117.4
        ( 15.7) ( 15.7) ( 15.7) ( 15.7) ( 15.7) ( 15.7) ( 15.7) ( 15.7) ( 15.7) ( 15.7)
          -12.4    -6.7    68.6     3.3     7.4    15.7   -28.7     3.3    40.0   -12.4
         -1.654  -0.888   9.148   0.435   0.980   2.097  -3.820   0.435   5.340  -1.654
c_O         108     117     125     109     120     113     112     120     122     139
        ( 14.4) ( 15.6) ( 16.7) ( 14.5) ( 16.0) ( 15.1) ( 14.9) ( 16.0) ( 16.3) ( 18.5)
c_E       122.0   122.0   122.0   122.0   122.0   122.0   122.0   122.0   122.0   122.0
```

```
          ( 16.3) ( 16.3) ( 16.3) ( 16.3) ( 16.3) ( 16.3) ( 16.3) ( 16.3) ( 16.3) ( 16.3)
            -26.3    -9.7     6.1   -24.5    -3.9   -17.3   -19.1    -3.9     0.1    36.3
           -3.503  -1.298   0.818  -3.268  -0.521  -2.302  -2.547  -0.521   0.008   4.845

N             750     750     750     750     750     750     750     750     750     750
X2            2.8     1.1    10.4     1.7     5.8     2.1     4.3     3.1     4.4     4.1
Gvalue        2.8     1.1     9.8     1.7     5.8     2.1     4.4     3.1     4.3     4.1
Gv/N        0.376   0.146   1.312   0.232   0.774   0.275   0.586   0.417   0.568   0.542
Prob      0.41973 0.77848 0.02000 0.62734 0.12162 0.56002 0.22211 0.37273 0.23470 0.25463
          ------------------------------------------------------------------------------
               30      29      28      27      26      25      24      23      22      21
          ------------------------------------------------------------------------------
a_O           247     265     258     256     257     270     256     264     259     246
          ( 32.9) ( 35.3) ( 34.4) ( 34.1) ( 34.3) ( 36.0) ( 34.1) ( 35.2) ( 34.5) ( 32.8)
a_E         254.6   254.6   254.6   254.6   254.6   254.6   254.6   254.6   254.6   254.6
          ( 33.9) ( 33.9) ( 33.9) ( 33.9) ( 33.9) ( 33.9) ( 33.9) ( 33.9) ( 33.9) ( 33.9)
            -15.0    21.2     6.8     2.8     4.8    31.7     2.8    19.1     8.8   -16.9
           -2.001   2.824   0.907   0.369   0.638   4.223   0.369   2.547   1.178  -2.259
t_O           272     244     273     233     238     257     250     256     287     264
          ( 36.3) ( 32.5) ( 36.4) ( 31.1) ( 31.7) ( 34.3) ( 33.3) ( 34.1) ( 38.3) ( 35.2)
t_E         256.0   256.0   256.0   256.0   256.0   256.0   256.0   256.0   256.0   256.0
          ( 34.1) ( 34.1) ( 34.1) ( 34.1) ( 34.1) ( 34.1) ( 34.1) ( 34.1) ( 34.1) ( 34.1)
             32.9   -23.5    35.0   -43.9   -34.8     1.9   -11.9    -0.1    65.5    16.2
            4.389  -3.131   4.672  -5.856  -4.635   0.259  -1.589  -0.008   8.739   2.158
g_O           105     125      82     127     133     105     115     103      89     121
          ( 14.0) ( 16.7) ( 10.9) ( 16.9) ( 17.7) ( 14.0) ( 15.3) ( 13.7) ( 11.9) ( 16.1)
g_E         117.4   117.4   117.4   117.4   117.4   117.4   117.4   117.4   117.4   117.4
          ( 15.7) ( 15.7) ( 15.7) ( 15.7) ( 15.7) ( 15.7) ( 15.7) ( 15.7) ( 15.7) ( 15.7)
            -23.4    15.7   -58.8    20.0    33.2   -23.4    -4.7   -26.9   -49.3     7.4
           -3.121   2.097  -7.844   2.668   4.431  -3.121  -0.628  -3.590  -6.569   0.980
c_O           126     116     137     134     122     118     129     127     115     119
          ( 16.8) ( 15.5) ( 18.3) ( 17.9) ( 16.3) ( 15.7) ( 17.2) ( 16.9) ( 15.3) ( 15.9)
c_E         122.0   122.0   122.0   122.0   122.0   122.0   122.0   122.0   122.0   122.0
          ( 16.3) ( 16.3) ( 16.3) ( 16.3) ( 16.3) ( 16.3) ( 16.3) ( 16.3) ( 16.3) ( 16.3)
              8.2   -11.6    31.8    25.2     0.1    -7.8    14.5    10.3   -13.5    -5.9
            1.092  -1.552   4.245   3.361   0.008  -1.041   1.928   1.369  -1.805  -0.782

N             750     750     750     750     750     750     750     750     750     750
X2            2.7     1.8    13.7     4.1     3.4     2.4     0.6     2.3    11.1     0.7
Gvalue        2.7     1.8    14.9     4.1     3.3     2.4     0.6     2.4    11.6     0.7
Gv/N        0.359   0.236   1.981   0.541   0.442   0.320   0.080   0.318   1.544   0.097
Prob      0.44154 0.62078 0.00194 0.25494 0.34558 0.49375 0.89716 0.49655 0.00898 0.86751
          ------------------------------------------------------------------------------
               20      19      18      17      16      15      14      13      12      11
          ------------------------------------------------------------------------------
a_O           275     274     256     279     282     276     276     276     250     299
          ( 36.7) ( 36.5) ( 34.1) ( 37.2) ( 37.6) ( 36.8) ( 36.8) ( 36.8) ( 33.3) ( 39.9)
a_E         254.6   254.6   254.6   254.6   254.6   254.6   254.6   254.6   254.6   254.6
          ( 33.9) ( 33.9) ( 33.9) ( 33.9) ( 33.9) ( 33.9) ( 33.9) ( 33.9) ( 33.9) ( 33.9)
             42.3    40.2     2.8    51.0    57.6    44.5    44.5    44.5    -9.2    96.1
            5.647   5.360   0.369   6.803   7.681   5.934   5.934   5.934  -1.221  12.811
t_O           238     287     292     244     272     255     260     256     258     215
          ( 31.7) ( 38.3) ( 38.9) ( 32.5) ( 36.3) ( 34.0) ( 34.7) ( 34.1) ( 34.4) ( 28.7)
t_E         256.0   256.0   256.0   256.0   256.0   256.0   256.0   256.0   256.0   256.0
          ( 34.1) ( 34.1) ( 34.1) ( 34.1) ( 34.1) ( 34.1) ( 34.1) ( 34.1) ( 34.1) ( 34.1)
            -34.8    65.5    76.8   -23.5    32.9    -2.1     8.0    -0.1     4.0   -75.1
           -4.635   8.739  10.236  -3.131   4.389  -0.274   1.067  -0.008   0.527 -10.014
g_O           111      68      83      77      65      96      85      90     120     103
          ( 14.8) (  9.1) ( 11.1) ( 10.3) (  8.7) ( 12.8) ( 11.3) ( 12.0) ( 16.0) ( 13.7)
g_E         117.4   117.4   117.4   117.4   117.4   117.4   117.4   117.4   117.4   117.4
          ( 15.7) ( 15.7) ( 15.7) ( 15.7) ( 15.7) ( 15.7) ( 15.7) ( 15.7) ( 15.7) ( 15.7)
            -12.4   -74.2   -57.5   -64.9   -76.8   -38.6   -54.9   -47.8     5.3   -26.9
           -1.654  -9.899  -7.671  -8.657 -10.245  -5.147  -7.316  -6.375   0.706  -3.590
c_O           126     121     119     150     131     123     129     128     122     133
          ( 16.8) ( 16.1) ( 15.9) ( 20.0) ( 17.5) ( 16.4) ( 17.2) ( 17.1) ( 16.3) ( 17.7)
c_E         122.0   122.0   122.0   122.0   122.0   122.0   122.0   122.0   122.0   122.0
          ( 16.3) ( 16.3) ( 16.3) ( 16.3) ( 16.3) ( 16.3) ( 16.3) ( 16.3) ( 16.3) ( 16.3)
              8.2    -1.9    -5.9    62.1    18.7     2.1    14.5    12.4     0.1    23.0
            1.092  -0.258  -0.782   8.274   2.495   0.276   1.928   1.647   0.008   3.070

N             750     750     750     750     750     750     750     750     750     750
X2            3.4    26.0    15.2    23.2    28.0     5.7    11.2     8.5     0.2    17.1
Gvalue        3.4    29.6    16.1    24.7    32.4     5.9    12.1     9.0     0.2    17.1
Gv/N        0.450   3.942   2.152   3.289   4.320   0.789   1.613   1.199   0.021   2.278
Prob      0.33735 0.00000 0.00106 0.00002 0.00000 0.11583 0.00706 0.02941 0.98414 0.00068
          ------------------------------------------------------------------------------
               10       9       8       7       6       5       4       3       2       1
          ------------------------------------------------------------------------------
a_O           291     273     276     291     311     287     282     273     240     228
          ( 38.8) ( 36.4) ( 36.8) ( 38.8) ( 41.5) ( 38.3) ( 37.6) ( 36.4) ( 32.0) ( 30.4)
a_E         254.6   254.6   254.6   254.6   254.6   254.6   254.6   254.6   254.6   254.6
          ( 33.9) ( 33.9) ( 33.9) ( 33.9) ( 33.9) ( 33.9) ( 33.9) ( 33.9) ( 33.9) ( 33.9)
             77.7    38.1    44.5    77.7   124.4    68.7    57.6    38.1   -28.4   -50.4
           10.364   5.074   5.934  10.364  16.588   9.162   7.681   5.074  -3.785  -6.714
t_O           218     248     208     248     235     241     236     215     237     314
          ( 29.1) ( 33.1) ( 27.7) ( 33.1) ( 31.3) ( 32.1) ( 31.5) ( 28.7) ( 31.6) ( 41.9)
t_E         256.0   256.0   256.0   256.0   256.0   256.0   256.0   256.0   256.0   256.0
          ( 34.1) ( 34.1) ( 34.1) ( 34.1) ( 34.1) ( 34.1) ( 34.1) ( 34.1) ( 34.1) ( 34.1)
            -70.1   -15.8   -86.4   -15.8   -40.3   -29.2   -38.5   -75.1   -36.6   128.2
           -9.348  -2.107 -11.524  -2.107  -5.371  -3.888  -5.127 -10.014  -4.881  17.090
g_O           134     118     123      83      99      92     104     149     133      75
          ( 17.9) ( 15.7) ( 16.4) ( 11.1) ( 13.2) ( 12.3) ( 13.9) ( 19.9) ( 17.7) ( 10.0)
g_E         117.4   117.4   117.4   117.4   117.4   117.4   117.4   117.4   117.4   117.4
          ( 15.7) ( 15.7) ( 15.7) ( 15.7) ( 15.7) ( 15.7) ( 15.7) ( 15.7) ( 15.7) ( 15.7)
             35.5     1.2    11.5   -57.5   -33.7   -44.8   -25.2    71.1    33.2   -67.2
            4.732   0.166   1.534  -7.671  -4.496  -5.977  -3.356   9.478   4.431  -8.959
c_O           107     111     143     128     105     130     128     113     140     133
          ( 14.3) ( 14.8) ( 19.1) ( 17.1) ( 14.0) ( 17.3) ( 17.1) ( 15.1) ( 18.7) ( 17.7)
c_E         122.0   122.0   122.0   122.0   122.0   122.0   122.0   122.0   122.0   122.0
          ( 16.3) ( 16.3) ( 16.3) ( 16.3) ( 16.3) ( 16.3) ( 16.3) ( 16.3) ( 16.3) ( 16.3)
            -28.0   -20.9    45.5    12.4   -31.5    16.6    12.4   -17.3    38.6    23.0
           -3.736  -2.790   6.066   1.647  -4.195   2.210   1.647  -2.302   5.147   3.070

N             750     750     750     750     750     750     750     750     750     750
X2           15.0     2.6    14.7    15.8    19.5    11.0     6.3    17.1     7.0    32.2
Gvalue       15.1     2.6    15.1    16.7    18.9    11.3     6.3    16.8     6.8    33.7
Gv/N        2.011   0.343   2.011   2.232   2.527   1.507   0.844   2.236   0.912   4.488
Prob      0.00175 0.46253 0.00175 0.00080 0.00028 0.01019 0.09646 0.00079 0.07712 0.00000
          ------------------------------------------------------------------------------

CDS_INITIAL:
          ------------------------------------------------------------------------------
```

	1	2	3	4	5	6	7	8	9	10
a_O	457	0	0	347	240	229	309	281	238	311
	(60.9)	(0.0)	(0.0)	(46.3)	(32.0)	(30.5)	(41.2)	(37.5)	(31.7)	(41.5)
a_E	208.6	244.1	213.7	208.6	244.1	213.7	208.6	244.1	213.7	208.6
	(27.8)	(32.5)	(28.5)	(27.8)	(32.5)	(28.5)	(27.8)	(32.5)	(28.5)	(27.8)
	716.6	0.0	0.0	353.0	-8.1	31.8	242.7	79.1	51.4	248.3
	95.552	0.000	0.000	47.073	-1.086	4.236	32.361	10.546	6.849	33.106
t_O	203	750	0	136	229	283	157	243	265	177
	(27.1)	(100.0)	(0.0)	(18.1)	(30.5)	(37.7)	(20.9)	(32.4)	(35.3)	(23.6)
t_E	146.8	231.5	283.1	146.8	231.5	283.1	146.8	231.5	283.1	146.8
	(19.6)	(30.9)	(37.7)	(19.6)	(30.9)	(37.7)	(19.6)	(30.9)	(37.7)	(19.6)
	131.5	1763.3	0.0	-20.9	-5.0	-0.2	21.0	23.6	-35.0	66.1
	17.529	235.100	0.000	-2.783	-0.663	-0.030	2.799	3.142	-4.672	8.815
g_O	90	0	750	126	122	123	165	88	145	121
	(12.0)	(0.0)	(100.0)	(16.8)	(16.3)	(16.4)	(22.0)	(11.7)	(19.3)	(16.1)
g_E	232.6	114.4	137.7	232.6	114.4	137.7	232.6	114.4	137.7	232.6
	(31.0)	(15.3)	(18.4)	(31.0)	(15.3)	(18.4)	(31.0)	(15.3)	(18.4)	(31.0)
	-170.9	0.0	2542.6	-154.5	15.6	-27.8	-113.3	-46.2	15.0	-158.1
	-22.788	0.000	339.008	-20.597	2.085	-3.701	-15.107	-6.162	1.999	-21.086
c_O	0	0	0	141	159	115	119	138	102	141
	(0.0)	(0.0)	(0.0)	(18.8)	(21.2)	(15.3)	(15.9)	(18.4)	(13.6)	(18.8)
c_E	161.9	160.0	115.5	161.9	160.0	115.5	161.9	160.0	115.5	161.9
	(21.6)	(21.3)	(15.4)	(21.6)	(21.3)	(15.4)	(21.6)	(21.3)	(15.4)	(21.6)
	0.0	0.0	0.0	-39.0	-1.9	-1.1	-73.3	-40.8	-25.4	-39.0
	0.000	0.000	0.000	-5.201	-0.258	-0.144	-9.773	-5.436	-3.390	-5.201
N	750	750	750	750	750	750	750	750	750	750
X2	566.4	1679.8	3335.2	144.1	0.6	2.7	80.0	15.3	5.9	112.7
Gvalue	677.2	1763.3	2542.6	138.7	0.6	2.7	77.1	15.7	5.9	117.2
Gv/N	90.294	235.100	339.008	18.491	0.079	0.361	10.280	2.090	0.786	15.632
Prob	0.00000	1.00000	1.00000	0.00000	0.89798	0.43917	0.00000	0.00132	0.11697	0.00000

	11	12	13	14	15	16	17	18	19	20
a_O	232	240	290	250	215	272	243	217	270	248
	(30.9)	(32.0)	(38.7)	(33.3)	(28.7)	(36.3)	(32.4)	(28.9)	(36.0)	(33.1)
a_E	244.1	213.7	208.6	244.1	213.7	208.6	244.1	213.7	208.6	244.1
	(32.5)	(28.5)	(27.8)	(32.5)	(28.5)	(27.8)	(32.5)	(28.5)	(27.8)	(32.5)
	-23.6	55.8	191.0	11.9	2.7	144.3	-2.2	6.7	139.2	7.8
	-3.147	7.442	25.464	1.590	0.360	19.235	-0.295	0.899	18.562	1.046
t_O	282	280	172	261	271	177	275	276	179	249
	(37.6)	(37.3)	(22.9)	(34.8)	(36.1)	(23.6)	(36.7)	(36.8)	(23.9)	(33.2)
t_E	231.5	283.1	146.8	231.5	283.1	146.8	231.5	283.1	146.8	231.5
	(30.9)	(37.7)	(19.6)	(30.9)	(37.7)	(19.6)	(30.9)	(37.7)	(19.6)	(30.9)
	111.3	-6.2	54.4	62.6	-23.7	66.1	94.7	-14.0	70.9	36.3
	14.840	-0.825	7.251	8.348	-3.160	8.815	12.628	-1.872	9.450	4.839
g_O	79	116	139	84	133	141	84	129	139	86
	(10.5)	(15.5)	(18.5)	(11.2)	(17.7)	(18.8)	(11.2)	(17.2)	(18.5)	(11.5)
g_E	114.4	137.7	232.6	114.4	137.7	232.6	114.4	137.7	232.6	114.4
	(15.3)	(18.4)	(31.0)	(15.3)	(18.4)	(31.0)	(15.3)	(18.4)	(31.0)	(15.3)
	-58.5	-39.8	-143.1	-51.9	-9.2	-141.1	-51.9	-16.8	-143.1	-49.1
	-7.805	-5.303	-19.082	-6.924	-1.230	-18.820	-6.924	-2.244	-19.082	-6.549
c_O	157	114	149	155	131	160	148	128	162	167
	(20.9)	(15.2)	(19.9)	(20.7)	(17.5)	(21.3)	(19.7)	(17.1)	(21.6)	(22.3)
c_E	160.0	115.5	161.9	160.0	115.5	161.9	160.0	115.5	161.9	160.0
	(21.3)	(15.4)	(21.6)	(21.3)	(15.4)	(21.6)	(21.3)	(15.4)	(21.6)	(21.3)
	-5.9	-3.1	-24.8	-9.8	32.9	-3.8	-23.0	26.2	0.2	14.4
	-0.784	-0.408	-3.304	-1.304	4.387	-0.509	-3.069	3.496	0.022	1.916
N	750	750	750	750	750	750	750	750	750	750
X2	22.6	6.7	74.7	12.1	2.8	61.5	17.2	2.1	62.7	8.8
Gvalue	23.3	6.8	77.5	12.8	2.7	65.4	17.6	2.1	67.1	9.4
Gv/N	3.103	0.905	10.329	1.710	0.357	8.721	2.340	0.279	8.952	1.252
Prob	0.00004	0.07888	0.00000	0.00503	0.44398	0.00000	0.00054	0.55376	0.00000	0.02454

	21	22	23	24	25	26	27	28	29	30
a_O	242	255	261	226	248	241	246	223	230	231
	(32.3)	(34.0)	(34.8)	(30.1)	(33.1)	(32.1)	(32.8)	(29.7)	(30.7)	(30.8)
a_E	213.7	208.6	244.1	213.7	208.6	244.1	213.7	208.6	244.1	213.7
	(28.5)	(27.8)	(32.5)	(28.5)	(27.8)	(32.5)	(28.5)	(27.8)	(32.5)	(28.5)
	60.3	102.3	34.9	25.4	85.7	-6.2	69.4	29.7	-27.4	36.1
	8.039	13.644	4.657	3.385	11.429	-0.824	9.248	3.958	-3.651	4.808
t_O	278	165	246	279	174	262	263	169	262	255
	(37.1)	(22.0)	(32.8)	(37.2)	(23.2)	(34.9)	(35.1)	(22.5)	(34.9)	(34.0)
t_E	283.1	146.8	231.5	283.1	146.8	231.5	283.1	146.8	231.5	283.1
	(37.7)	(19.6)	(30.9)	(37.7)	(19.6)	(30.9)	(37.7)	(19.6)	(30.9)	(37.7)
	-10.1	38.5	29.9	-8.2	59.0	64.9	-38.8	47.5	64.9	-53.3
	-1.351	5.128	3.986	-1.088	7.872	8.648	-5.168	6.332	8.648	-7.111
g_O	114	170	84	134	162	88	121	192	90	154
	(15.2)	(22.7)	(11.2)	(17.9)	(21.6)	(11.7)	(16.1)	(25.6)	(12.0)	(20.5)
g_E	137.7	232.6	114.4	137.7	232.6	114.4	137.7	232.6	114.4	137.7
	(18.4)	(31.0)	(15.3)	(18.4)	(31.0)	(15.3)	(18.4)	(31.0)	(15.3)	(18.4)
	-43.1	-106.6	-51.9	-7.3	-117.2	-46.2	-31.3	-73.6	-43.2	34.5
	-5.741	-14.212	-6.924	-0.972	-15.625	-6.162	-4.170	-9.820	-5.763	4.596
c_O	116	160	159	111	166	159	120	166	168	110
	(15.5)	(21.3)	(21.2)	(14.8)	(22.1)	(21.2)	(16.0)	(22.1)	(22.4)	(14.7)
c_E	115.5	161.9	160.0	115.5	161.9	160.0	115.5	161.9	160.0	115.5
	(15.4)	(21.6)	(21.3)	(15.4)	(21.6)	(21.3)	(15.4)	(21.6)	(21.3)	(15.4)
	0.9	-3.8	-1.9	-8.9	8.3	-1.9	9.1	8.3	16.5	-10.8
	0.123	-0.509	-0.258	-1.187	1.102	-0.258	1.212	1.102	2.195	-1.441
N	750	750	750	750	750	750	750	750	750	750
X2	7.9	29.4	10.2	1.1	34.0	10.2	8.5	11.5	10.5	6.4
Gvalue	8.0	30.4	11.0	1.0	35.8	10.5	8.4	11.8	10.7	6.4
Gv/N	1.071	4.052	1.461	0.139	4.778	1.405	1.122	1.572	1.428	0.852
Prob	0.04534	0.00000	0.01195	0.79139	0.00000	0.01453	0.03823	0.00814	0.01339	0.09413

	31	32	33	34	35	36	37	38	39	40
a_O	241	239	217	224	235	246	238	202	207	215
	(32.1)	(31.9)	(28.9)	(29.9)	(31.3)	(32.8)	(31.7)	(26.9)	(27.6)	(28.7)
a_E	208.6	244.1	213.7	208.6	244.1	213.7	208.6	244.1	213.7	208.6
	(27.8)	(32.5)	(28.5)	(27.8)	(32.5)	(28.5)	(27.8)	(32.5)	(28.5)	(27.8)
	69.5	-10.1	6.7	31.8	-17.9	69.4	62.7	-76.5	-13.1	12.9
	9.266	-1.348	0.899	4.243	-2.383	9.248	8.356	-10.199	-1.747	1.722
t_O	160	265	286	169	247	260	163	246	286	162
	(21.3)	(35.3)	(38.1)	(22.5)	(32.9)	(34.7)	(21.7)	(32.8)	(38.1)	(21.6)
t_E	146.8	231.5	283.1	146.8	231.5	283.1	146.8	231.5	283.1	146.8
	(19.6)	(30.9)	(37.7)	(19.6)	(30.9)	(37.7)	(19.6)	(30.9)	(37.7)	(19.6)
	27.4	71.6	5.8	47.5	32.0	-44.3	34.0	29.9	5.8	31.8

	3.660	9.551	0.774	6.332	4.269	-5.904	4.536	3.986	0.774	4.242
g_O	169	112	133	190	102	113	200	119	138	210
	(22.5)	(14.9)	(17.7)	(25.3)	(13.6)	(15.1)	(26.7)	(15.9)	(18.4)	(28.0)
g_E	232.6	114.4	137.7	232.6	114.4	137.7	232.6	114.4	137.7	232.6
	(31.0)	(15.3)	(18.4)	(31.0)	(15.3)	(18.4)	(31.0)	(15.3)	(18.4)	(31.0)
	-108.0	-4.8	-9.2	-76.9	-23.4	-44.7	-60.4	9.3	0.6	-42.9
	-14.394	-0.640	-1.230	-10.248	-3.127	-5.956	-8.052	1.244	0.082	-5.722
c_O	180	134	114	167	166	131	149	183	119	163
	(24.0)	(17.9)	(15.2)	(22.3)	(22.1)	(17.5)	(19.9)	(24.4)	(15.9)	(21.7)
c_E	161.9	160.0	115.5	161.9	160.0	115.5	161.9	160.0	115.5	161.9
	(21.6)	(21.3)	(15.4)	(21.6)	(21.3)	(15.4)	(21.6)	(21.3)	(15.4)	(21.6)
	38.1	-47.5	-3.1	10.3	12.3	32.9	-24.8	49.2	7.0	2.2
	5.081	-6.330	-0.408	1.376	1.638	4.387	-3.304	6.564	0.936	0.289
N	750	750	750	750	750	750	750	750	750	750
X2	25.6	9.2	0.3	12.4	3.0	13.3	11.5	11.7	0.3	4.0
Gvalue	27.1	9.3	0.3	12.8	3.0	13.3	11.5	12.0	0.3	4.0
Gv/N	3.614	1.234	0.035	1.703	0.398	1.775	1.536	1.595	0.046	0.531
Prob	0.00001	0.02610	0.96666	0.00516	0.39391	0.00401	0.00922	0.00752	0.95193	0.26357

	41	42	43	44	45	46	47	48	49	50
a_O	215	223	216	217	207	235	214	208	206	210
	(28.7)	(29.7)	(28.8)	(28.9)	(27.6)	(31.3)	(28.5)	(27.7)	(27.5)	(28.0)
a_E	244.1	213.7	208.6	244.1	213.7	208.6	244.1	213.7	208.6	244.1
	(32.5)	(28.5)	(27.8)	(32.5)	(28.5)	(27.8)	(32.5)	(28.5)	(27.8)	(32.5)
	-54.6	19.1	15.0	-51.1	-13.1	55.9	-56.3	-11.2	-5.2	-63.2
	-7.280	2.546	1.997	-6.812	-1.747	7.456	-7.512	-1.488	-0.700	-8.428
t_O	276	310	186	254	292	146	255	304	162	244
	(36.8)	(41.3)	(24.8)	(33.9)	(38.9)	(19.5)	(34.0)	(40.5)	(21.6)	(32.5)
t_E	231.5	283.1	146.8	231.5	283.1	146.8	231.5	283.1	146.8	231.5
	(30.9)	(37.7)	(19.6)	(30.9)	(37.7)	(19.6)	(30.9)	(37.7)	(19.6)	(30.9)
	97.1	56.3	87.9	47.1	18.1	-1.7	49.3	43.3	31.8	25.7
	12.941	7.500	11.723	6.283	2.407	-0.226	6.575	5.771	4.242	3.422
g_O	98	108	202	96	133	217	115	117	220	111
	(13.1)	(14.4)	(26.9)	(12.8)	(17.7)	(28.9)	(15.3)	(15.6)	(29.3)	(14.8)
g_E	114.4	137.7	232.6	114.4	137.7	232.6	114.4	137.7	232.6	114.4
	(15.3)	(18.4)	(31.0)	(15.3)	(18.4)	(31.0)	(15.3)	(18.4)	(31.0)	(15.3)
	-30.4	-52.5	-57.0	-33.7	-9.2	-30.1	1.2	-38.1	-24.5	-6.7
	-4.049	-6.996	-7.596	-4.495	-1.230	-4.015	0.154	-5.081	-3.265	-0.900
c_O	161	109	146	183	118	152	166	121	162	185
	(21.5)	(14.5)	(19.5)	(24.4)	(15.7)	(20.3)	(22.1)	(16.1)	(21.6)	(24.7)
c_E	160.0	115.5	161.9	160.0	115.5	161.9	160.0	115.5	161.9	160.0
	(21.3)	(15.4)	(21.6)	(21.3)	(15.4)	(21.6)	(21.3)	(15.4)	(21.6)	(21.3)
	2.1	-12.7	-30.2	49.2	5.0	-19.2	12.3	11.2	0.2	53.8
	0.276	-1.694	-4.029	6.564	0.663	-2.562	1.638	1.490	0.022	7.172
N	750	750	750	750	750	750	750	750	750	750
X2	14.4	9.7	16.3	11.5	0.7	5.0	6.3	5.1	2.3	9.5
Gvalue	14.2	10.2	15.7	11.6	0.7	4.9	6.4	5.2	2.2	9.5
Gv/N	1.888	1.357	2.094	1.541	0.093	0.652	0.855	0.692	0.299	1.266
Prob	0.00270	0.01711	0.00130	0.00907	0.87333	0.17979	0.09316	0.15852	0.52408	0.02335

	51	52	53	54	55	56	57	58	59	60
a_O	244	215	223	244	217	212	218	191	221	231
	(32.5)	(28.7)	(29.7)	(32.5)	(28.9)	(28.3)	(29.1)	(25.5)	(29.5)	(30.8)
a_E	213.7	208.6	244.1	213.7	208.6	244.1	213.7	208.6	244.1	213.7
	(28.5)	(27.8)	(32.5)	(28.5)	(27.8)	(32.5)	(28.5)	(27.8)	(32.5)	(28.5)
	64.8	12.9	-40.3	64.8	17.1	-59.8	8.8	-33.7	-44.0	36.1
	8.641	1.722	-5.378	8.641	2.273	-7.973	1.170	-4.499	-5.861	4.808
t_O	262	169	251	265	194	260	287	158	249	289
	(34.9)	(22.5)	(33.5)	(35.3)	(25.9)	(34.7)	(38.3)	(21.1)	(33.2)	(38.5)
t_E	283.1	146.8	231.5	283.1	146.8	231.5	283.1	146.8	231.5	283.1
	(37.7)	(19.6)	(30.9)	(37.7)	(19.6)	(30.9)	(37.7)	(19.6)	(30.9)	(37.7)
	-40.6	47.5	40.6	-35.0	108.0	60.4	7.8	23.1	36.3	11.9
	-5.414	6.332	5.414	-4.672	14.406	8.050	1.044	3.084	4.839	1.586
g_O	119	207	114	127	214	106	145	236	123	119
	(15.9)	(27.6)	(15.2)	(16.9)	(28.5)	(14.1)	(19.3)	(31.5)	(16.4)	(15.9)
g_E	137.7	232.6	114.4	137.7	232.6	114.4	137.7	232.6	114.4	137.7
	(18.4)	(31.0)	(15.3)	(18.4)	(31.0)	(15.3)	(18.4)	(31.0)	(15.3)	(18.4)
	-34.7	-48.3	-0.8	-20.5	-35.7	-16.2	15.0	6.9	17.8	-34.7
	-4.630	-6.435	-0.113	-2.738	-4.754	-2.162	1.999	0.915	2.370	-4.630
c_O	125	159	162	114	125	172	100	165	157	111
	(16.7)	(21.2)	(21.6)	(15.2)	(16.7)	(22.9)	(13.3)	(22.0)	(20.9)	(14.8)
c_E	115.5	161.9	160.0	115.5	161.9	160.0	115.5	161.9	160.0	115.5
	(15.4)	(21.6)	(21.3)	(15.4)	(21.6)	(21.3)	(15.4)	(21.6)	(21.3)	(15.4)
	19.7	-5.8	4.1	-3.1	-64.7	24.9	-28.9	6.2	-5.9	-8.9
	2.623	-0.771	0.545	-0.408	-8.626	3.326	-3.852	0.829	-0.784	-1.187
N	750	750	750	750	750	750	750	750	750	750
X2	9.2	6.4	3.5	6.3	25.4	9.3	2.6	2.4	4.2	4.2
Gvalue	9.1	6.4	3.5	6.2	24.7	9.3	2.7	2.5	4.2	4.3
Gv/N	1.220	0.847	0.467	0.824	3.298	1.242	0.362	0.329	0.564	0.578
Prob	0.02737	0.09554	0.31998	0.10331	0.00002	0.02539	0.43807	0.48084	0.23732	0.22747

	61	62	63	64	65	66	67	68	69	70
a_O	219	209	198	193	204	218	193	221	210	216
	(29.2)	(27.9)	(26.4)	(25.7)	(27.2)	(29.1)	(25.7)	(29.5)	(28.0)	(28.8)
a_E	208.6	244.1	213.7	208.6	244.1	213.7	208.6	244.1	213.7	208.6
	(27.8)	(32.5)	(28.5)	(27.8)	(32.5)	(28.5)	(27.8)	(32.5)	(28.5)	(27.8)
	21.2	-64.9	-30.1	-30.1	-73.2	8.8	-30.1	-44.0	-7.2	15.0
	2.830	-8.654	-4.018	-4.010	-9.764	1.170	-4.010	-5.861	-0.966	1.997
t_O	161	247	263	166	251	286	152	227	305	153
	(21.5)	(32.9)	(35.1)	(22.1)	(33.5)	(38.1)	(20.3)	(30.3)	(40.7)	(20.4)
t_E	146.8	231.5	283.1	146.8	231.5	283.1	146.8	231.5	283.1	146.8
	(19.6)	(30.9)	(37.7)	(19.6)	(30.9)	(37.7)	(19.6)	(30.9)	(37.7)	(19.6)
	29.6	32.0	-38.8	40.7	40.6	5.8	10.5	-8.9	45.4	12.6
	3.950	4.269	-5.168	5.427	5.414	0.774	1.398	-1.188	6.057	1.674
g_O	209	120	162	232	108	127	234	144	118	222
	(27.9)	(16.0)	(21.6)	(30.9)	(14.4)	(16.9)	(31.2)	(19.2)	(15.7)	(29.6)
g_E	232.6	114.4	137.7	232.6	114.4	137.7	232.6	114.4	137.7	232.6
	(31.0)	(15.3)	(18.4)	(31.0)	(15.3)	(18.4)	(31.0)	(15.3)	(18.4)	(31.0)
	-44.7	11.4	52.7	-1.2	-12.5	-20.5	2.8	66.2	-36.4	-20.7
	-5.961	1.522	7.023	-0.158	-1.664	-2.738	0.376	8.828	-4.857	-2.759
c_O	161	174	127	159	187	119	171	158	117	159
	(21.5)	(23.2)	(16.9)	(21.2)	(24.9)	(15.9)	(22.8)	(21.1)	(15.6)	(21.2)
c_E	161.9	160.0	115.5	161.9	160.0	115.5	161.9	160.0	115.5	161.9
	(21.6)	(21.3)	(15.4)	(21.6)	(21.3)	(15.4)	(21.6)	(21.3)	(15.4)	(21.6)
	-1.8	29.3	24.0	-5.8	58.4	7.0	18.7	-3.9	2.9	-5.8
	-0.244	3.901	3.203	-0.771	7.786	0.936	2.488	-0.522	0.392	-0.771

N	750	750	750	750	750	750	750	750	750	750
X2	4.3	7.6	8.0	3.7	13.2	1.1	1.9	9.9	4.6	1.1
Gvalue	4.3	7.8	7.8	3.7	13.3	1.1	1.9	9.4	4.7	1.1
Gv/N	0.575	1.039	1.040	0.487	1.771	0.143	0.252	1.258	0.626	0.140
Prob	0.22972	0.05057	0.05035	0.30146	0.00407	0.78369	0.59554	0.02407	0.19571	0.78827

	71	72	73	74	75	76	77	78	79	80
a_O	227	210	204	229	185	218	230	220	211	217
	(30.3)	(28.0)	(27.2)	(30.5)	(24.7)	(29.1)	(30.7)	(29.3)	(28.1)	(28.9)
a_E	244.1	213.7	208.6	244.1	213.7	208.6	244.1	213.7	208.6	244.1
	(32.5)	(28.5)	(27.8)	(32.5)	(28.5)	(27.8)	(32.5)	(28.5)	(27.8)	(32.5)
	-33.0	-7.2	-9.2	-29.3	-53.3	19.1	-27.4	12.9	4.7	-51.1
	-4.398	-0.966	-1.223	-3.901	-7.104	2.551	-3.651	1.717	0.633	-6.812
t_O	240	287	147	264	314	165	236	285	158	241
	(32.0)	(38.3)	(19.6)	(35.2)	(41.9)	(22.0)	(31.5)	(38.0)	(21.1)	(32.1)
t_E	231.5	283.1	146.8	231.5	283.1	146.8	231.5	283.1	146.8	231.5
	(30.9)	(37.7)	(19.6)	(30.9)	(37.7)	(19.6)	(30.9)	(37.7)	(19.6)	(30.9)
	17.3	7.8	0.3	69.4	65.0	38.5	9.1	3.8	23.1	19.4
	2.308	1.044	0.040	9.249	8.671	5.128	1.212	0.505	3.084	2.585
g_O	113	146	223	104	130	207	114	134	203	107
	(15.1)	(19.5)	(29.7)	(13.9)	(17.3)	(27.6)	(15.2)	(17.9)	(27.1)	(14.3)
g_E	114.4	137.7	232.6	114.4	137.7	232.6	114.4	137.7	232.6	114.4
	(15.3)	(18.4)	(31.0)	(15.3)	(18.4)	(31.0)	(15.3)	(18.4)	(31.0)	(15.3)
	-2.8	17.1	-18.8	-19.9	-14.9	-48.3	-0.8	-7.3	-55.2	-14.4
	-0.378	2.281	-2.505	-2.649	-1.993	-6.435	-0.113	-0.972	-7.367	-1.914
c_O	170	107	176	153	121	160	170	111	178	185
	(22.7)	(14.3)	(23.5)	(20.4)	(16.1)	(21.3)	(22.7)	(14.8)	(23.7)	(24.7)
c_E	160.0	115.5	161.9	160.0	115.5	161.9	160.0	115.5	161.9	160.0
	(21.3)	(15.4)	(21.6)	(21.3)	(15.4)	(21.6)	(21.3)	(15.4)	(21.6)	(21.3)
	20.7	-16.4	29.4	-13.6	11.2	-3.8	20.7	-8.9	33.7	53.8
	2.757	-2.191	3.914	-1.817	1.490	-0.509	2.757	-1.187	4.495	7.172
N	750	750	750	750	750	750	750	750	750	750
X2	2.2	1.2	1.7	6.8	7.9	5.5	1.5	0.5	6.2	7.8
Gvalue	2.2	1.3	1.7	6.6	8.0	5.5	1.5	0.5	6.3	7.7
Gv/N	0.289	0.167	0.226	0.881	1.063	0.736	0.205	0.064	0.845	1.031
Prob	0.53776	0.73991	0.63786	0.08556	0.04657	0.13753	0.67390	0.92327	0.09639	0.05187

	81	82	83	84	85	86	87	88	89	90
a_O	240	208	228	221	205	249	216	182	236	232
	(32.0)	(27.7)	(30.4)	(29.5)	(27.3)	(33.2)	(28.8)	(24.3)	(31.5)	(30.9)
a_E	213.7	208.6	244.1	213.7	208.6	244.1	213.7	208.6	244.1	213.7
	(28.5)	(27.8)	(32.5)	(28.5)	(27.8)	(32.5)	(28.5)	(27.8)	(32.5)	(28.5)
	55.8	-1.3	-31.1	14.9	-7.2	9.9	4.7	-49.7	-15.9	38.2
	7.442	-0.170	-4.151	1.992	-0.962	1.318	0.629	-6.630	-2.126	5.096
t_O	254	144	248	266	132	232	295	153	221	292
	(33.9)	(19.2)	(33.1)	(35.5)	(17.6)	(30.9)	(39.3)	(20.4)	(29.5)	(38.9)
t_E	283.1	146.8	231.5	283.1	146.8	231.5	283.1	146.8	231.5	283.1
	(37.7)	(19.6)	(30.9)	(37.7)	(19.6)	(30.9)	(37.7)	(19.6)	(30.9)	(37.7)
	-55.1	-5.6	34.2	-33.2	-28.1	1.0	24.3	12.6	-20.5	18.1
	-7.350	-0.752	4.554	-4.422	-3.752	0.134	3.236	1.674	-2.735	2.407
g_O	123	220	115	157	238	105	126	253	122	118
	(16.4)	(29.3)	(15.3)	(20.9)	(31.7)	(14.0)	(16.8)	(33.7)	(16.3)	(15.7)
g_E	137.7	232.6	114.4	137.7	232.6	114.4	137.7	232.6	114.4	137.7
	(18.4)	(31.0)	(15.3)	(18.4)	(31.0)	(15.3)	(18.4)	(31.0)	(15.3)	(18.4)
	-27.8	-24.5	1.2	41.2	10.9	-18.1	-22.4	42.6	15.6	-36.4
	-3.701	-3.265	0.154	5.494	1.459	-2.407	-2.982	5.674	2.085	-4.857
c_O	133	178	159	106	175	164	113	162	171	108
	(17.7)	(23.7)	(21.2)	(14.1)	(23.3)	(21.9)	(15.1)	(21.6)	(22.8)	(14.4)
c_E	115.5	161.9	160.0	115.5	161.9	160.0	115.5	161.9	160.0	115.5
	(15.4)	(21.6)	(21.3)	(15.4)	(21.6)	(21.3)	(15.4)	(21.6)	(21.3)	(15.4)
	37.4	33.7	-1.9	-18.3	27.2	8.2	-5.0	0.2	22.8	-14.6
	4.991	4.495	-0.258	-2.436	3.626	1.088	-0.670	0.022	3.041	-1.944
N	750	750	750	750	750	750	750	750	750	750
X2	10.4	2.3	2.2	4.8	2.7	1.0	1.6	5.4	2.0	5.2
Gvalue	10.4	2.3	2.2	4.7	2.8	1.0	1.6	5.6	2.0	5.3
Gv/N	1.382	0.306	0.299	0.627	0.370	0.133	0.213	0.740	0.265	0.703
Prob	0.01570	0.51276	0.52311	0.19476	0.42800	0.80179	0.66013	0.13563	0.57441	0.15288

	91	92	93	94	95	96	97	98	99	100
a_O	189	238	219	201	239	235	208	246	205	207
	(25.2)	(31.7)	(29.2)	(26.8)	(31.9)	(31.3)	(27.7)	(32.8)	(27.3)	(27.6)
a_E	208.6	244.1	213.7	208.6	244.1	213.7	208.6	244.1	213.7	208.6
	(27.8)	(32.5)	(28.5)	(27.8)	(32.5)	(28.5)	(27.8)	(32.5)	(28.5)	(27.8)
	-37.4	-12.1	10.8	-15.0	-10.1	44.8	-1.3	3.8	-17.0	-3.3
	-4.983	-1.608	1.443	-2.000	-1.348	5.967	-0.170	0.506	-2.260	-0.436
t_O	157	246	298	142	229	267	149	221	301	130
	(20.9)	(32.8)	(39.7)	(18.9)	(30.5)	(35.6)	(19.9)	(29.5)	(40.1)	(17.3)
t_E	146.8	231.5	283.1	146.8	231.5	283.1	146.8	231.5	283.1	146.8
	(19.6)	(30.9)	(37.7)	(19.6)	(30.9)	(37.7)	(19.6)	(30.9)	(37.7)	(19.6)
	21.0	29.9	30.5	-9.5	-5.0	-31.3	4.3	-20.5	36.9	-31.7
	2.799	3.986	4.073	-1.271	-0.663	-4.172	0.578	-2.735	4.918	-4.225
g_O	240	100	132	231	107	134	224	107	143	239
	(32.0)	(13.3)	(17.6)	(30.8)	(14.3)	(17.9)	(29.9)	(14.3)	(19.1)	(31.9)
g_E	232.6	114.4	137.7	232.6	114.4	137.7	232.6	114.4	137.7	232.6
	(31.0)	(15.3)	(18.4)	(31.0)	(15.3)	(18.4)	(31.0)	(15.3)	(18.4)	(31.0)
	15.0	-26.9	-11.1	-3.2	-14.4	-7.3	-16.9	-14.4	10.8	13.0
	2.006	-3.593	-1.486	-0.423	-1.914	-0.972	-2.249	-1.914	1.442	1.732
c_O	164	166	101	176	175	114	169	176	101	174
	(21.9)	(22.1)	(13.5)	(23.5)	(23.3)	(15.2)	(22.5)	(23.5)	(13.5)	(23.2)
c_E	161.9	160.0	115.5	161.9	160.0	115.5	161.9	160.0	115.5	161.9
	(21.6)	(21.3)	(15.4)	(21.6)	(21.3)	(15.4)	(21.6)	(21.3)	(15.4)	(21.6)
	4.2	12.3	-27.2	29.4	31.4	-3.1	14.5	33.6	-27.2	25.0
	0.559	1.638	-3.622	3.914	4.191	-0.408	1.929	4.482	-3.622	3.339
N	750	750	750	750	750	750	750	750	750	750
X2	2.8	3.1	3.0	1.7	2.0	3.2	0.7	2.6	3.5	3.0
Gvalue	2.9	3.2	3.1	1.6	2.0	3.1	0.7	2.5	3.6	3.1
Gv/N	0.381	0.423	0.407	0.220	0.266	0.416	0.088	0.339	0.477	0.411
Prob	0.41438	0.36631	0.38356	0.64888	0.57294	0.37345	0.88256	0.46701	0.31100	0.37958

Adeh_2CPC
5UTR:

	100	99	98	97	96	95	94	93	92	91
a_O	62	64	82	71	61	78	62	71	74	70
	(11.0)	(11.4)	(14.6)	(12.6)	(10.8)	(13.9)	(11.0)	(12.6)	(13.1)	(12.4)
a_E	66.1	66.1	66.1	66.1	66.1	66.1	66.1	66.1	66.1	66.1
	(11.7)	(11.7)	(11.7)	(11.7)	(11.7)	(11.7)	(11.7)	(11.7)	(11.7)	(11.7)
	-8.0	-4.2	35.3	10.1	-9.9	25.8	-8.0	10.1	16.6	8.0
	-1.420	-0.744	6.266	1.792	-1.750	4.574	-1.420	1.792	2.956	1.414
t_O	70	72	65	75	74	69	70	75	69	54
	(12.4)	(12.8)	(11.5)	(13.3)	(13.1)	(12.3)	(12.4)	(13.3)	(12.3)	(9.6)
t_E	72.5	72.5	72.5	72.5	72.5	72.5	72.5	72.5	72.5	72.5
	(12.9)	(12.9)	(12.9)	(12.9)	(12.9)	(12.9)	(12.9)	(12.9)	(12.9)	(12.9)
	-5.0	-1.1	-14.2	5.0	3.0	-6.9	-5.0	5.0	-6.9	-31.9
	-0.883	-0.188	-2.531	0.892	0.527	-1.223	-0.883	0.892	-1.223	-5.659
g_O	226	240	202	209	231	190	212	206	208	245
	(40.1)	(42.6)	(35.9)	(37.1)	(41.0)	(33.7)	(37.7)	(36.6)	(36.9)	(43.5)
g_E	201.7	201.7	201.7	201.7	201.7	201.7	201.7	201.7	201.7	201.7
	(35.8)	(35.8)	(35.8)	(35.8)	(35.8)	(35.8)	(35.8)	(35.8)	(35.8)	(35.8)
	51.5	83.5	0.7	14.9	62.7	-22.7	21.2	8.8	12.9	95.4
	9.145	14.835	0.117	2.651	11.143	-4.023	3.762	1.555	2.284	16.939
c_O	205	187	214	208	197	226	219	211	212	194
	(36.4)	(33.2)	(38.0)	(36.9)	(35.0)	(40.1)	(38.9)	(37.5)	(37.7)	(34.5)
c_E	222.7	222.7	222.7	222.7	222.7	222.7	222.7	222.7	222.7	222.7
	(39.6)	(39.6)	(39.6)	(39.6)	(39.6)	(39.6)	(39.6)	(39.6)	(39.6)	(39.6)
	-33.9	-65.3	-17.0	-28.4	-48.3	6.7	-7.3	-22.7	-20.8	-53.5
	-6.021	-11.597	-3.019	-5.036	-8.572	1.192	-1.293	-4.035	-3.698	-9.499
N	563	563	563	563	563	563	563	563	563	563
X2	4.7	13.1	4.9	1.7	7.7	3.0	0.9	1.1	1.8	18.0
Gvalue	4.6	13.0	4.7	1.7	7.6	2.9	0.9	1.1	1.8	18.0
Gv/N	0.820	2.306	0.833	0.299	1.348	0.520	0.166	0.204	0.318	3.195
Prob	0.20205	0.00467	0.19606	0.64027	0.05527	0.40311	0.81724	0.76545	0.61658	0.00044

	90	89	88	87	86	85	84	83	82	81
a_O	55	62	63	63	61	62	59	76	59	61
	(9.8)	(11.0)	(11.2)	(11.2)	(10.8)	(11.0)	(10.5)	(13.5)	(10.5)	(10.8)
a_E	66.1	66.1	66.1	66.1	66.1	66.1	66.1	66.1	66.1	66.1
	(11.7)	(11.7)	(11.7)	(11.7)	(11.7)	(11.7)	(11.7)	(11.7)	(11.7)	(11.7)
	-20.3	-8.0	-6.1	-6.1	-9.9	-8.0	-13.5	21.1	-13.5	-9.9
	-3.601	-1.420	-1.085	-1.085	-1.750	-1.420	-2.391	3.756	-2.391	-1.750
t_O	86	68	69	71	76	57	76	71	65	78
	(15.3)	(12.1)	(12.3)	(12.6)	(13.5)	(10.1)	(13.5)	(12.6)	(11.5)	(13.9)
t_E	72.5	72.5	72.5	72.5	72.5	72.5	72.5	72.5	72.5	72.5
	(12.9)	(12.9)	(12.9)	(12.9)	(12.9)	(12.9)	(12.9)	(12.9)	(12.9)	(12.9)
	29.3	-8.8	-6.9	-3.0	7.1	-27.5	7.1	-3.0	-14.2	11.3
	5.204	-1.558	-1.223	-0.538	1.262	-4.879	1.262	-0.538	-2.531	2.015
g_O	201	226	208	239	219	223	231	204	229	218
	(35.7)	(40.1)	(36.9)	(42.5)	(38.9)	(39.6)	(41.0)	(36.2)	(40.7)	(38.7)
g_E	201.7	201.7	201.7	201.7	201.7	201.7	201.7	201.7	201.7	201.7
	(35.8)	(35.8)	(35.8)	(35.8)	(35.8)	(35.8)	(35.8)	(35.8)	(35.8)	(35.8)
	-1.3	51.5	12.9	81.2	36.1	44.8	62.7	4.7	58.2	33.9
	-0.238	9.145	2.284	14.419	6.414	7.965	11.143	0.832	10.339	6.030
c_O	221	207	223	190	207	221	197	212	210	206
	(39.3)	(36.8)	(39.6)	(33.7)	(36.8)	(39.3)	(35.0)	(37.7)	(37.3)	(36.6)
c_E	222.7	222.7	222.7	222.7	222.7	222.7	222.7	222.7	222.7	222.7
	(39.6)	(39.6)	(39.6)	(39.6)	(39.6)	(39.6)	(39.6)	(39.6)	(39.6)	(39.6)
	-3.3	-30.2	0.7	-60.3	-30.2	-3.3	-48.3	-20.8	-24.6	-32.1
	-0.591	-5.366	0.117	-10.709	-5.366	-0.591	-8.572	-3.698	-4.370	-5.694
N	563	563	563	563	563	563	563	563	563	563
X2	4.4	4.6	0.5	11.9	3.2	5.9	8.2	2.0	6.0	3.4
Gvalue	4.4	4.5	0.5	11.7	3.1	6.0	8.1	2.0	5.9	3.4
Gv/N	0.775	0.800	0.093	2.087	0.559	1.074	1.441	0.352	1.046	0.600
Prob	0.22487	0.21177	0.91396	0.00830	0.36908	0.10929	0.04371	0.57577	0.11708	0.33672

	80	79	78	77	76	75	74	73	72	71
a_O	57	62	63	72	71	67	66	61	66	54
	(10.1)	(11.0)	(11.2)	(12.8)	(12.6)	(11.9)	(11.7)	(10.8)	(11.7)	(9.6)
a_E	66.1	66.1	66.1	66.1	66.1	66.1	66.1	66.1	66.1	66.1
	(11.7)	(11.7)	(11.7)	(11.7)	(11.7)	(11.7)	(11.7)	(11.7)	(11.7)	(11.7)
	-16.9	-8.0	-6.1	12.2	10.1	1.8	-0.3	-9.9	-0.3	-21.9
	-3.008	-1.420	-1.085	2.175	1.792	0.311	-0.046	-1.750	-0.046	-3.887
t_O	67	76	66	78	72	60	61	61	60	79
	(11.9)	(13.5)	(11.7)	(13.9)	(12.8)	(10.7)	(10.8)	(10.8)	(10.7)	(14.0)
t_E	72.5	72.5	72.5	72.5	72.5	72.5	72.5	72.5	72.5	72.5
	(12.9)	(12.9)	(12.9)	(12.9)	(12.9)	(12.9)	(12.9)	(12.9)	(12.9)	(12.9)
	-10.6	7.1	-12.5	11.3	-1.1	-22.8	-21.1	-21.1	-22.8	13.5
	-1.888	1.262	-2.212	2.015	-0.188	-4.042	-3.752	-3.752	-4.042	2.398
g_O	215	225	221	201	197	215	230	209	228	229
	(38.2)	(40.0)	(39.3)	(35.7)	(35.0)	(38.2)	(40.9)	(37.1)	(40.5)	(40.7)
g_E	201.7	201.7	201.7	201.7	201.7	201.7	201.7	201.7	201.7	201.7
	(35.8)	(35.8)	(35.8)	(35.8)	(35.8)	(35.8)	(35.8)	(35.8)	(35.8)	(35.8)
	27.5	49.3	40.5	-1.3	-9.2	27.5	60.5	14.9	56.0	58.2
	4.889	8.750	7.186	-0.238	-1.640	4.889	10.740	2.651	9.939	10.339
c_O	224	200	213	212	223	221	206	232	209	201
	(39.8)	(35.5)	(37.8)	(37.7)	(39.6)	(39.3)	(36.6)	(41.2)	(37.1)	(35.7)
c_E	222.7	222.7	222.7	222.7	222.7	222.7	222.7	222.7	222.7	222.7
	(39.6)	(39.6)	(39.6)	(39.6)	(39.6)	(39.6)	(39.6)	(39.6)	(39.6)	(39.6)
	2.7	-42.9	-18.9	-20.8	0.7	-3.3	-32.1	19.0	-26.5	-41.2
	0.474	-7.629	-3.359	-3.698	0.117	-0.591	-5.694	3.383	-4.704	-7.311
N	563	563	563	563	563	563	563	563	563	563
X2	2.6	5.4	3.0	1.4	0.5	3.1	7.1	2.9	6.4	8.6
Gvalue	2.6	5.4	3.0	1.4	0.5	3.2	7.0	3.0	6.5	8.7
Gv/N	0.466	0.962	0.529	0.254	0.082	0.566	1.248	0.532	1.147	1.539
Prob	0.45297	0.14363	0.39482	0.69835	0.92683	0.36360	0.07112	0.39223	0.09143	0.03412

	70	69	68	67	66	65	64	63	62	61
a_O	65	73	68	61	69	77	65	64	66	56
	(11.5)	(13.0)	(12.1)	(10.8)	(12.3)	(13.7)	(11.5)	(11.4)	(11.7)	(9.9)
a_E	66.1	66.1	66.1	66.1	66.1	66.1	66.1	66.1	66.1	66.1
	(11.7)	(11.7)	(11.7)	(11.7)	(11.7)	(11.7)	(11.7)	(11.7)	(11.7)	(11.7)
	-2.2	14.4	3.8	-9.9	5.9	23.4	-2.2	-4.2	-0.3	-18.6
	-0.398	2.563	0.674	-1.750	1.041	4.163	-0.398	-0.744	-0.046	-3.308
t_O	61	62	69	71	74	73	68	76	65	66
	(10.8)	(11.0)	(12.3)	(12.6)	(13.1)	(13.0)	(12.1)	(13.5)	(11.5)	(11.7)

t_E	72.5	72.5	72.5	72.5	72.5	72.5	72.5	72.5	72.5	72.5
	(12.9)	(12.9)	(12.9)	(12.9)	(12.9)	(12.9)	(12.9)	(12.9)	(12.9)	(12.9)
	-21.1	-19.5	-6.9	-3.0	3.0	0.9	-8.8	7.1	-14.2	-12.5
	-3.752	-3.455	-1.223	-0.538	0.527	0.168	-1.558	1.262	-2.531	-2.212
g_O	192	214	213	206	217	206	206	213	207	200
	(34.1)	(38.0)	(37.8)	(36.6)	(38.5)	(36.6)	(36.6)	(37.8)	(36.8)	(35.5)
g_E	201.7	201.7	201.7	201.7	201.7	201.7	201.7	201.7	201.7	201.7
	(35.8)	(35.8)	(35.8)	(35.8)	(35.8)	(35.8)	(35.8)	(35.8)	(35.8)	(35.8)
	-18.9	25.4	23.3	8.8	31.8	8.8	8.8	23.3	10.8	-3.3
	-3.351	4.511	4.136	1.555	5.648	1.555	1.555	4.136	1.918	-0.591
c_O	245	214	213	225	203	207	224	210	225	241
	(43.5)	(38.0)	(37.8)	(40.0)	(36.1)	(36.8)	(39.8)	(37.3)	(40.0)	(42.8)
c_E	222.7	222.7	222.7	222.7	222.7	222.7	222.7	222.7	222.7	222.7
	(39.6)	(39.6)	(39.6)	(39.6)	(39.6)	(39.6)	(39.6)	(39.6)	(39.6)	(39.6)
	46.8	-17.0	-18.9	4.7	-37.5	-30.2	2.7	-24.6	4.7	38.1
	8.318	-3.019	-3.359	0.832	-6.669	-5.366	0.474	-4.370	0.832	6.772
N	563	563	563	563	563	563	563	563	563	563
X2	4.6	3.3	1.3	0.5	3.1	3.0	0.4	1.6	0.9	3.7
Gvalue	4.6	3.4	1.3	0.6	3.1	2.9	0.4	1.6	1.0	3.7
Gv/N	0.817	0.600	0.227	0.099	0.547	0.519	0.073	0.283	0.173	0.662
Prob	0.20378	0.33669	0.73433	0.90603	0.37940	0.40395	0.93845	0.66104	0.80742	0.29249

	60	59	58	57	56	55	54	53	52	51
a_O	47	70	63	63	63	67	48	64	71	60
	(8.3)	(12.4)	(11.2)	(11.2)	(11.2)	(11.9)	(8.5)	(11.4)	(12.6)	(10.7)
a_E	66.1	66.1	66.1	66.1	66.1	66.1	66.1	66.1	66.1	66.1
	(11.7)	(11.7)	(11.7)	(11.7)	(11.7)	(11.7)	(11.7)	(11.7)	(11.7)	(11.7)
	-32.1	8.0	-6.1	-6.1	-6.1	1.8	-30.8	-4.2	10.1	-11.7
	-5.701	1.414	-1.085	-1.085	-1.085	0.311	-5.464	-0.744	1.792	-2.073
t_O	78	74	89	70	60	67	71	75	68	56
	(13.9)	(13.1)	(15.8)	(12.4)	(10.7)	(11.9)	(12.6)	(13.3)	(12.1)	(9.9)
t_E	72.5	72.5	72.5	72.5	72.5	72.5	72.5	72.5	72.5	72.5
	(12.9)	(12.9)	(12.9)	(12.9)	(12.9)	(12.9)	(12.9)	(12.9)	(12.9)	(12.9)
	11.3	3.0	36.4	-5.0	-22.8	-10.6	-3.0	5.0	-8.8	-29.0
	2.015	0.527	6.470	-0.883	-4.042	-1.888	-0.538	0.892	-1.558	-5.145
g_O	212	213	194	225	206	213	221	213	198	217
	(37.7)	(37.8)	(34.5)	(40.0)	(36.6)	(37.8)	(39.3)	(37.8)	(35.2)	(38.5)
g_E	201.7	201.7	201.7	201.7	201.7	201.7	201.7	201.7	201.7	201.7
	(35.8)	(35.8)	(35.8)	(35.8)	(35.8)	(35.8)	(35.8)	(35.8)	(35.8)	(35.8)
	21.2	23.3	-15.0	49.3	8.8	23.3	40.5	23.3	-7.3	31.8
	3.762	4.136	-2.672	8.750	1.555	4.136	7.186	4.136	-1.292	5.648
c_O	226	206	217	205	234	216	223	211	226	230
	(40.1)	(36.6)	(38.5)	(36.4)	(41.6)	(38.4)	(39.6)	(37.5)	(40.1)	(40.9)
c_E	222.7	222.7	222.7	222.7	222.7	222.7	222.7	222.7	222.7	222.7
	(39.6)	(39.6)	(39.6)	(39.6)	(39.6)	(39.6)	(39.6)	(39.6)	(39.6)	(39.6)
	6.7	-32.1	-11.2	-33.9	23.2	-13.1	0.7	-22.7	6.7	14.9
	1.192	-5.694	-1.988	-6.021	4.126	-2.334	0.117	-4.035	1.192	2.646
N	563	563	563	563	563	563	563	563	563	563
X2	6.5	2.1	4.3	4.3	3.0	1.3	6.9	1.4	0.8	5.7
Gvalue	7.1	2.2	4.1	4.3	3.1	1.3	7.3	1.4	0.8	6.1
Gv/N	1.267	0.383	0.724	0.760	0.553	0.226	1.302	0.249	0.134	1.075
Prob	0.06775	0.54046	0.25328	0.23266	0.37481	0.73605	0.06212	0.70552	0.85998	0.10903

	50	49	48	47	46	45	44	43	42	41
a_O	62	67	63	63	77	70	76	67	56	71
	(11.0)	(11.9)	(11.2)	(11.2)	(13.7)	(12.4)	(13.5)	(11.9)	(9.9)	(12.6)
a_E	66.1	66.1	66.1	66.1	66.1	66.1	66.1	66.1	66.1	66.1
	(11.7)	(11.7)	(11.7)	(11.7)	(11.7)	(11.7)	(11.7)	(11.7)	(11.7)	(11.7)
	-8.0	1.8	-6.1	-6.1	23.4	8.0	21.1	1.8	-18.6	10.1
	-1.420	0.311	-1.085	-1.085	4.163	1.414	3.756	0.311	-3.308	1.792
t_O	63	63	62	62	76	73	66	78	68	53
	(11.2)	(11.2)	(11.0)	(11.0)	(13.5)	(13.0)	(11.7)	(13.9)	(12.1)	(9.4)
t_E	72.5	72.5	72.5	72.5	72.5	72.5	72.5	72.5	72.5	72.5
	(12.9)	(12.9)	(12.9)	(12.9)	(12.9)	(12.9)	(12.9)	(12.9)	(12.9)	(12.9)
	-17.7	-17.7	-19.5	-19.5	7.1	0.9	-12.5	11.3	-8.8	-33.3
	-3.153	-3.153	-3.455	-3.455	1.262	0.168	-2.212	2.015	-1.558	-5.906
g_O	194	212	206	198	195	198	220	217	206	240
	(34.5)	(37.7)	(36.6)	(35.2)	(34.6)	(35.2)	(39.1)	(38.5)	(36.6)	(42.6)
g_E	201.7	201.7	201.7	201.7	201.7	201.7	201.7	201.7	201.7	201.7
	(35.8)	(35.8)	(35.8)	(35.8)	(35.8)	(35.8)	(35.8)	(35.8)	(35.8)	(35.8)
	-15.0	21.2	8.8	-7.3	-13.1	-7.3	38.3	31.8	8.8	83.5
	-2.672	3.762	1.555	-1.292	-2.330	-1.292	6.799	5.648	1.555	14.835
c_O	244	221	232	240	215	222	201	201	233	199
	(43.3)	(39.3)	(41.2)	(42.6)	(38.2)	(39.4)	(35.7)	(35.7)	(41.4)	(35.3)
c_E	222.7	222.7	222.7	222.7	222.7	222.7	222.7	222.7	222.7	222.7
	(39.6)	(39.6)	(39.6)	(39.6)	(39.6)	(39.6)	(39.6)	(39.6)	(39.6)	(39.6)
	44.6	-3.3	19.0	36.0	-15.1	-1.3	-41.2	-41.2	21.1	-44.7
	7.929	-0.591	3.383	6.390	-2.677	-0.238	-7.311	-7.311	3.753	-7.945
N	563	563	563	563	563	563	563	563	563	563
X2	3.8	1.8	2.2	3.1	2.4	0.3	5.8	3.7	2.4	15.4
Gvalue	3.9	1.9	2.2	3.1	2.3	0.3	5.8	3.7	2.5	15.6
Gv/N	0.684	0.330	0.397	0.558	0.417	0.052	1.032	0.663	0.442	2.776
Prob	0.27803	0.60299	0.52468	0.37033	0.50302	0.96107	0.12124	0.29194	0.47692	0.00135

	40	39	38	37	36	35	34	33	32	31
a_O	75	66	65	59	62	72	74	76	82	58
	(13.3)	(11.7)	(11.5)	(10.5)	(11.0)	(12.8)	(13.1)	(13.5)	(14.6)	(10.3)
a_E	66.1	66.1	66.1	66.1	66.1	66.1	66.1	66.1	66.1	66.1
	(11.7)	(11.7)	(11.7)	(11.7)	(11.7)	(11.7)	(11.7)	(11.7)	(11.7)	(11.7)
	18.9	-0.3	-2.2	-13.5	-8.0	12.2	16.6	21.1	35.3	-15.2
	3.353	-0.046	-0.398	-2.391	-1.420	2.175	2.956	3.756	6.266	-2.703
t_O	73	83	102	91	109	121	79	48	67	43
	(13.0)	(14.7)	(18.1)	(16.2)	(19.4)	(21.5)	(14.0)	(8.5)	(11.9)	(7.6)
t_E	72.5	72.5	72.5	72.5	72.5	72.5	72.5	72.5	72.5	72.5
	(12.9)	(12.9)	(12.9)	(12.9)	(12.9)	(12.9)	(12.9)	(12.9)	(12.9)	(12.9)
	0.9	22.4	69.6	41.3	88.8	123.9	13.5	-39.6	-10.6	-45.0
	0.168	3.976	12.355	7.334	15.773	21.999	2.398	-7.039	-1.888	-7.986
g_O	218	197	204	217	194	182	217	212	171	202
	(38.7)	(35.0)	(36.2)	(38.5)	(34.5)	(32.3)	(38.5)	(37.7)	(30.4)	(35.9)
g_E	201.7	201.7	201.7	201.7	201.7	201.7	201.7	201.7	201.7	201.7
	(35.8)	(35.8)	(35.8)	(35.8)	(35.8)	(35.8)	(35.8)	(35.8)	(35.8)	(35.8)
	33.9	-9.2	4.7	31.8	-15.0	-37.4	31.8	21.2	-56.4	0.7
	6.030	-1.640	0.832	5.648	-2.672	-6.635	5.648	3.762	-10.021	0.117
c_O	197	217	192	196	198	188	193	227	243	260
	(35.0)	(38.5)	(34.1)	(34.8)	(35.2)	(33.4)	(34.3)	(40.3)	(43.2)	(46.2)
c_E	222.7	222.7	222.7	222.7	222.7	222.7	222.7	222.7	222.7	222.7

```
        ( 39.6) ( 39.6) ( 39.6) ( 39.6) ( 39.6) ( 39.6) ( 39.6) ( 39.6) ( 39.6) ( 39.6)
          -48.3   -11.2   -56.9   -50.0   -46.5   -63.6   -55.2     8.7    42.5    80.6
         -8.572  -1.988 -10.108  -8.883  -8.259 -11.303  -9.804   1.553   7.542  14.315

N           563     563     563     563     563     563     563     563     563     563
X2          5.5     1.8    16.2     9.8    21.6    40.2     6.6    10.4    10.8    19.3
Gvalue      5.5     1.7    15.1     9.6    19.3    35.1     6.7    11.4    10.7    21.1
Gv/N      0.979   0.302   2.682   1.708   3.421   6.236   1.197   2.032   1.899   3.744
Prob    0.13799 0.63730 0.00174 0.02216 0.00024 0.00000 0.08063 0.00957 0.01352 0.00010
        -------------------------------------------------------------------------------
             30      29      28      27      26      25      24      23      22      21
        -------------------------------------------------------------------------------
a_O          57      67      55      47      48      53      58      57      59      52
        ( 10.1) ( 11.9) (  9.8) (  8.3) (  8.5) (  9.4) ( 10.3) ( 10.1) ( 10.5) (  9.2)
a_E        66.1    66.1    66.1    66.1    66.1    66.1    66.1    66.1    66.1    66.1
        ( 11.7) ( 11.7) ( 11.7) ( 11.7) ( 11.7) ( 11.7) ( 11.7) ( 11.7) ( 11.7) ( 11.7)
          -16.9     1.8   -20.3   -32.1   -30.8   -23.5   -15.2   -16.9   -13.5   -25.0
         -3.008   0.311  -3.601  -5.701  -5.464  -4.167  -2.703  -3.008  -2.391  -4.440
t_O          51      59      51      53      50      65      53      52      57      79
        (  9.1) ( 10.5) (  9.1) (  9.4) (  8.9) ( 11.5) (  9.4) (  9.2) ( 10.1) ( 14.0)
t_E        72.5    72.5    72.5    72.5    72.5    72.5    72.5    72.5    72.5    72.5
        ( 12.9) ( 12.9) ( 12.9) ( 12.9) ( 12.9) ( 12.9) ( 12.9) ( 12.9) ( 12.9) ( 12.9)
          -35.9   -24.4   -35.9   -33.3   -37.2   -14.2   -33.3   -34.6   -27.5    13.5
         -6.380  -4.327  -6.380  -5.906  -6.607  -2.531  -5.906  -6.147  -4.879   2.398
g_O         211     212     242     221     227     225     224     223     206     222
        ( 37.5) ( 37.7) ( 43.0) ( 39.3) ( 40.3) ( 40.0) ( 39.8) ( 39.6) ( 36.6) ( 39.4)
g_E       201.7   201.7   201.7   201.7   201.7   201.7   201.7   201.7   201.7   201.7
        ( 35.8) ( 35.8) ( 35.8) ( 35.8) ( 35.8) ( 35.8) ( 35.8) ( 35.8) ( 35.8) ( 35.8)
           19.1    21.2    88.2    40.5    53.7    49.3    47.0    44.8     8.8    42.6
          3.390   3.762  15.672   7.186   9.541   8.750   8.356   7.965   1.555   7.574
c_O         244     225     215     242     238     220     228     231     241     210
        ( 43.3) ( 40.0) ( 38.2) ( 43.0) ( 42.3) ( 39.1) ( 40.5) ( 41.0) ( 42.8) ( 37.3)
c_E       222.7   222.7   222.7   222.7   222.7   222.7   222.7   222.7   222.7   222.7
        ( 39.6) ( 39.6) ( 39.6) ( 39.6) ( 39.6) ( 39.6) ( 39.6) ( 39.6) ( 39.6) ( 39.6)
           44.6     4.7   -15.1    40.3    31.7    -5.3    10.8    17.0    38.1   -24.6
          7.929   0.832  -2.677   7.157   5.629  -0.943   1.916   3.014   6.772  -4.370

N           563     563     563     563     563     563     563     563     563     563
X2         10.1     3.1    16.6    14.3    16.2     6.1     8.9     9.6     5.7     6.4
Gvalue     10.9     3.3    17.0    15.4    17.4     6.2     9.4    10.3     6.0     6.5
Gv/N      1.930   0.578   3.014   2.735   3.099   1.109   1.663   1.823   1.057   1.162
Prob    0.01247 0.35418 0.00072 0.00151 0.00057 0.10042 0.02484 0.01645 0.11401 0.08809
        -------------------------------------------------------------------------------
             20      19      18      17      16      15      14      13      12      11
        -------------------------------------------------------------------------------
a_O          67      60      62      58      57      77     101     101     115     155
        ( 11.9) ( 10.7) ( 11.0) ( 10.3) ( 10.1) ( 13.7) ( 17.9) ( 17.9) ( 20.4) ( 27.5)
a_E        66.1    66.1    66.1    66.1    66.1    66.1    66.1    66.1    66.1    66.1
        ( 11.7) ( 11.7) ( 11.7) ( 11.7) ( 11.7) ( 11.7) ( 11.7) ( 11.7) ( 11.7) ( 11.7)
            1.8   -11.7    -8.0   -15.2   -16.9    23.4    85.5    85.5   127.3   264.1
          0.311  -2.073  -1.420  -2.703  -3.008   4.163  15.195  15.195  22.604  46.902
t_O          79      84      63      96     107     104     107     124     116      94
        ( 14.0) ( 14.9) ( 11.2) ( 17.1) ( 19.0) ( 18.5) ( 19.0) ( 22.0) ( 20.6) ( 16.7)
t_E        72.5    72.5    72.5    72.5    72.5    72.5    72.5    72.5    72.5    72.5
        ( 12.9) ( 12.9) ( 12.9) ( 12.9) ( 12.9) ( 12.9) ( 12.9) ( 12.9) ( 12.9) ( 12.9)
           13.5    24.7   -17.7    53.8    83.2    75.0    83.2   133.0   108.9    48.7
          2.398   4.381  -3.153   9.561  14.780  13.315  14.780  23.623  19.351   8.659
g_O         167     162     171     172     158     145     145     120     117     116
        ( 29.7) ( 28.8) ( 30.4) ( 30.6) ( 28.1) ( 25.8) ( 25.8) ( 21.3) ( 20.8) ( 20.6)
g_E       201.7   201.7   201.7   201.7   201.7   201.7   201.7   201.7   201.7   201.7
        ( 35.8) ( 35.8) ( 35.8) ( 35.8) ( 35.8) ( 35.8) ( 35.8) ( 35.8) ( 35.8) ( 35.8)
          -63.0   -71.0   -56.4   -54.7   -77.1   -95.7   -95.7  -124.6  -127.4  -128.3
        -11.191 -12.605 -10.021  -9.724 -13.697 -16.993 -16.993 -22.130 -22.629 -22.790
c_O         250     257     267     237     241     237     210     218     215     198
        ( 44.4) ( 45.6) ( 47.4) ( 42.1) ( 42.8) ( 42.1) ( 37.3) ( 38.7) ( 38.2) ( 35.2)
c_E       222.7   222.7   222.7   222.7   222.7   222.7   222.7   222.7   222.7   222.7
        ( 39.6) ( 39.6) ( 39.6) ( 39.6) ( 39.6) ( 39.6) ( 39.6) ( 39.6) ( 39.6) ( 39.6)
           57.9    73.7    97.0    29.6    38.1    29.6   -24.6    -9.2   -15.1   -46.5
         10.282  13.091  17.221   5.251   6.772   5.251  -4.370  -1.641  -2.677  -8.259

N           563     563     563     563     563     563     563     563     563     563
X2          9.9    15.5    15.0    13.9    28.6    32.3    51.4    88.1    98.0   164.9
Gvalue     10.1    15.7    14.8    13.4    27.3    32.3    48.5    84.7    93.7   138.0
Gv/N      1.800   2.793   2.626   2.385   4.846   5.735   8.611  15.046  16.648  24.512
Prob    0.01748 0.00129 0.00201 0.00379 0.00001 0.00000 0.00000 0.00000 0.00000 0.00000
        -------------------------------------------------------------------------------
             10       9       8       7       6       5       4       3       2       1
        -------------------------------------------------------------------------------
a_O          82      84      81      49      57      63      48      56      60      31
        ( 14.6) ( 14.9) ( 14.4) (  8.7) ( 10.1) ( 11.2) (  8.5) (  9.9) ( 10.7) (  5.5)
a_E        66.1    66.1    66.1    66.1    66.1    66.1    66.1    66.1    66.1    66.1
        ( 11.7) ( 11.7) ( 11.7) ( 11.7) ( 11.7) ( 11.7) ( 11.7) ( 11.7) ( 11.7) ( 11.7)
           35.3    40.2    32.9   -29.4   -16.9    -6.1   -30.8   -18.6   -11.7   -47.0
          6.266   7.138   5.836  -5.219  -3.008  -1.085  -5.464  -3.308  -2.073  -8.343
t_O         118     100     100     114      58      68      66      45      54      23
        ( 21.0) ( 17.8) ( 17.8) ( 20.2) ( 10.3) ( 12.1) ( 11.7) (  8.0) (  9.6) (  4.1)
t_E        72.5    72.5    72.5    72.5    72.5    72.5    72.5    72.5    72.5    72.5
        ( 12.9) ( 12.9) ( 12.9) ( 12.9) ( 12.9) ( 12.9) ( 12.9) ( 12.9) ( 12.9) ( 12.9)
          114.9    64.2    64.2   103.1   -25.9    -8.8   -12.5   -43.0   -31.9   -52.8
         20.401  11.409  11.409  18.313  -4.606  -1.558  -2.212  -7.631  -5.659  -9.384
g_O         145     144     136     134     177     161     217     218     173     153
        ( 25.8) ( 25.6) ( 24.2) ( 23.8) ( 31.4) ( 28.6) ( 38.5) ( 38.7) ( 30.7) ( 27.2)
g_E       201.7   201.7   201.7   201.7   201.7   201.7   201.7   201.7   201.7   201.7
        ( 35.8) ( 35.8) ( 35.8) ( 35.8) ( 35.8) ( 35.8) ( 35.8) ( 35.8) ( 35.8) ( 35.8)
          -95.7   -97.0  -107.2  -109.6   -46.2   -72.5    31.8    33.9   -53.1   -84.5
        -16.993 -17.230 -19.034 -19.459  -8.204 -12.882   5.648   6.030  -9.424 -15.012
c_O         218     235     246     266     271     271     232     244     276     356
        ( 38.7) ( 41.7) ( 43.7) ( 47.2) ( 48.1) ( 48.1) ( 41.2) ( 43.3) ( 49.0) ( 63.2)
c_E       222.7   222.7   222.7   222.7   222.7   222.7   222.7   222.7   222.7   222.7
        ( 39.6) ( 39.6) ( 39.6) ( 39.6) ( 39.6) ( 39.6) ( 39.6) ( 39.6) ( 39.6) ( 39.6)
           -9.2    25.3    49.0    94.6   106.5   106.5    19.0    44.6   118.5   334.1
         -1.641   4.499   8.707  16.802  18.910  18.910   3.383   7.929  21.051  59.343

N           563     563     563     563     563     563     563     563     563     563
X2         48.3    32.4    37.6    59.3    17.7    19.1     7.1    15.4    22.2   144.1
Gvalue     45.2    32.7    39.0    58.8    17.4    19.1     7.6    17.0    21.9   149.8
Gv/N      8.032   5.816   6.919  10.436   3.091   3.385   1.355   3.021   3.895  26.604
Prob    0.00000 0.00000 0.00000 0.00000 0.00058 0.00027 0.05435 0.00070 0.00007 0.00000
        -------------------------------------------------------------------------------

CDS_INITIAL:
        -------------------------------------------------------------------------------
```

	1	2	3	4	5	6	7	8	9	10
a_O	372	0	0	171	101	51	100	99	49	88
	(66.1)	(0.0)	(0.0)	(30.4)	(17.9)	(9.1)	(17.8)	(17.6)	(8.7)	(15.6)
a_E	69.9	113.9	7.4	69.9	113.9	7.4	69.9	113.9	7.4	69.9
	(12.4)	(20.2)	(1.3)	(12.4)	(20.2)	(1.3)	(12.4)	(20.2)	(1.3)	(12.4)
	1243.8	0.0	0.0	305.9	-24.2	197.2	71.6	-27.7	185.5	40.5
	220.915	0.000	0.000	54.336	-4.300	35.019	12.717	-4.918	32.949	7.195
t_O	12	563	0	39	77	38	52	98	33	57
	(2.1)	(100.0)	(0.0)	(6.9)	(13.7)	(6.7)	(9.2)	(17.4)	(5.9)	(10.1)
t_E	55.8	148.9	10.1	55.8	148.9	10.1	55.8	148.9	10.1	55.8
	(9.9)	(26.5)	(1.8)	(9.9)	(26.5)	(1.8)	(9.9)	(26.5)	(1.8)	(9.9)
	-36.9	1497.4	0.0	-28.0	-101.6	101.0	-7.4	-82.0	78.4	2.4
	-6.554	265.970	0.000	-4.971	-18.043	17.937	-1.313	-14.568	13.923	0.419
g_O	179	0	563	162	137	161	186	147	177	189
	(31.8)	(0.0)	(100.0)	(28.8)	(24.3)	(28.6)	(33.0)	(26.1)	(31.4)	(33.6)
g_E	254.5	129.2	259.5	254.5	129.2	259.5	254.5	129.2	259.5	254.5
	(45.2)	(22.9)	(46.1)	(45.2)	(22.9)	(46.1)	(45.2)	(22.9)	(46.1)	(45.2)
	-126.0	0.0	871.9	-146.4	16.1	-153.8	-116.7	38.0	-135.5	-112.5
	-22.379	0.000	154.875	-25.997	2.864	-27.310	-20.720	6.752	-24.067	-19.980
c_O	0	0	0	191	248	313	225	219	304	229
	(0.0)	(0.0)	(0.0)	(33.9)	(44.0)	(55.6)	(40.0)	(38.9)	(54.0)	(40.7)
c_E	182.8	171.0	286.0	182.8	171.0	286.0	182.8	171.0	286.0	182.8
	(32.5)	(30.4)	(50.8)	(32.5)	(30.4)	(50.8)	(32.5)	(30.4)	(50.8)	(32.5)
	0.0	0.0	0.0	16.9	184.3	56.4	93.6	108.2	37.1	103.3
	0.000	0.000	0.000	2.995	32.728	10.023	16.622	19.227	6.585	18.351
N	563	563	563	563	563	563	563	563	563	563
X2	1545.0	1565.4	658.3	185.3	71.3	375.3	41.4	35.3	314.3	33.3
Gvalue	1080.9	1497.4	871.9	148.4	74.6	200.8	41.1	36.6	165.5	33.7
Gv/N	191.982	265.970	154.875	26.364	13.249	35.670	7.306	6.492	29.390	5.986
Prob	0.00000	1.00000	0.00000	0.00000	-0.00000	0.00000	0.00000	0.00000	0.00000	0.00000

	11	12	13	14	15	16	17	18	19	20
a_O	85	29	87	98	40	81	104	21	94	103
	(15.1)	(5.2)	(15.5)	(17.4)	(7.1)	(14.4)	(18.5)	(3.7)	(16.7)	(18.3)
a_E	113.9	7.4	69.9	113.9	7.4	69.9	113.9	7.4	69.9	113.9
	(20.2)	(1.3)	(12.4)	(20.2)	(1.3)	(12.4)	(20.2)	(1.3)	(12.4)	(20.2)
	-49.7	79.4	38.1	-29.4	135.2	23.9	-18.8	43.9	55.7	-20.6
	-8.827	14.097	6.760	-5.222	24.014	4.238	-3.346	7.800	9.888	-3.668
t_O	128	36	46	143	30	63	149	40	75	155
	(22.7)	(6.4)	(8.2)	(25.4)	(5.3)	(11.2)	(26.5)	(7.1)	(13.3)	(27.5)
t_E	148.9	10.1	55.8	148.9	10.1	55.8	148.9	10.1	55.8	148.9
	(26.5)	(1.8)	(9.9)	(26.5)	(1.8)	(9.9)	(26.5)	(1.8)	(9.9)	(26.5)
	-38.8	91.8	-17.8	-11.6	65.5	15.2	0.2	110.4	44.3	12.4
	-6.884	16.302	-3.165	-2.062	11.642	2.703	0.027	19.610	7.864	2.202
g_O	121	153	199	126	182	197	120	199	181	101
	(21.5)	(27.2)	(35.3)	(22.4)	(32.3)	(35.0)	(21.3)	(35.3)	(32.1)	(17.9)
g_E	129.2	259.5	254.5	129.2	259.5	254.5	129.2	259.5	254.5	129.2
	(22.9)	(46.1)	(45.2)	(22.9)	(46.1)	(45.2)	(22.9)	(46.1)	(45.2)	(22.9)
	-15.8	-161.7	-97.9	-6.3	-129.2	-100.9	-17.7	-105.7	-123.4	-49.7
	-2.809	-28.723	-17.392	-1.113	-22.945	-17.924	-3.139	-18.776	-21.915	-8.827
c_O	229	345	231	196	311	222	190	303	213	204
	(40.7)	(61.3)	(41.0)	(34.8)	(55.2)	(39.4)	(33.7)	(53.8)	(37.8)	(36.2)
c_E	171.0	286.0	182.8	171.0	286.0	182.8	171.0	286.0	182.8	171.0
	(30.4)	(50.8)	(32.5)	(30.4)	(50.8)	(32.5)	(30.4)	(50.8)	(32.5)	(30.4)
	133.6	129.4	108.2	53.4	52.1	86.4	39.9	35.0	65.2	71.9
	23.737	22.978	19.225	9.482	9.251	15.342	7.093	6.208	11.589	12.768
N	563	563	563	563	563	563	563	563	563	563
X2	30.4	186.1	30.8	6.2	209.0	24.1	3.6	129.3	41.1	13.8
Gvalue	29.4	138.8	30.6	6.1	123.6	24.5	3.6	83.6	41.8	13.9
Gv/N	5.217	24.654	5.428	1.086	21.961	4.359	0.635	14.843	7.425	2.476
Prob	0.00000	0.00000	0.00000	0.10628	0.00000	0.00002	0.31135	0.00000	0.00000	0.00299

	21	22	23	24	25	26	27	28	29	30
a_O	23	68	77	16	82	97	22	60	88	19
	(4.1)	(12.1)	(13.7)	(2.8)	(14.6)	(17.2)	(3.9)	(10.7)	(15.6)	(3.4)
a_E	7.4	69.9	113.9	7.4	69.9	113.9	7.4	69.9	113.9	7.4
	(1.3)	(12.4)	(20.2)	(1.3)	(12.4)	(20.2)	(1.3)	(12.4)	(20.2)	(1.3)
	52.3	-3.8	-60.2	24.8	26.2	-31.1	48.1	-18.3	-45.3	35.9
	9.286	-0.669	-10.700	4.397	4.647	-5.522	8.535	-3.258	-8.054	6.382
t_O	28	61	162	23	53	147	33	62	148	31
	(5.0)	(10.8)	(28.8)	(4.1)	(9.4)	(26.1)	(5.9)	(11.0)	(26.3)	(5.5)
t_E	10.1	55.8	148.9	10.1	55.8	148.9	10.1	55.8	148.9	10.1
	(1.8)	(9.9)	(26.5)	(1.8)	(9.9)	(26.5)	(1.8)	(9.9)	(26.5)	(1.8)
	57.3	10.8	27.3	38.0	-5.5	-3.8	78.4	13.0	-1.8	69.8
	10.179	1.918	4.844	6.754	-0.980	-0.679	13.923	2.308	-0.327	12.391
g_O	176	208	106	178	227	121	199	233	119	192
	(31.3)	(36.9)	(18.8)	(31.6)	(40.3)	(21.5)	(35.3)	(41.4)	(21.1)	(34.1)
g_E	259.5	254.5	129.2	259.5	254.5	129.2	259.5	254.5	129.2	259.5
	(46.1)	(45.2)	(22.9)	(46.1)	(45.2)	(22.9)	(46.1)	(45.2)	(22.9)	(46.1)
	-136.7	-83.9	-41.9	-134.3	-51.9	-15.8	-105.7	-41.1	-19.5	-115.7
	-24.285	-14.910	-7.444	-23.846	-9.223	-2.809	-18.776	-7.308	-3.467	-20.558
c_O	336	226	218	346	201	198	309	208	208	321
	(59.7)	(40.1)	(38.7)	(61.5)	(35.7)	(35.2)	(54.9)	(36.9)	(36.9)	(57.0)
c_E	286.0	182.8	171.0	286.0	182.8	171.0	286.0	182.8	171.0	286.0
	(50.8)	(32.5)	(30.4)	(50.8)	(32.5)	(30.4)	(50.8)	(32.5)	(30.4)	(50.8)
	108.2	96.0	105.8	131.7	38.3	57.9	47.8	53.8	81.4	74.1
	19.224	17.052	18.784	23.401	6.795	10.293	8.484	9.561	14.453	13.158
N	563	563	563	563	563	563	563	563	563	563
X2	100.6	19.3	30.1	64.9	7.0	7.3	97.2	7.4	14.7	83.7
Gvalue	81.1	19.1	30.9	60.3	7.0	7.2	68.5	7.3	14.7	64.0
Gv/N	14.405	3.392	5.484	10.706	1.239	1.283	12.166	1.304	2.605	11.372
Prob	0.00000	0.00026	0.00000	0.00000	0.07264	0.06511	0.00000	0.06177	0.00212	0.00000

	31	32	33	34	35	36	37	38	39	40
a_O	57	82	23	80	100	13	66	92	13	67
	(10.1)	(14.6)	(4.1)	(14.2)	(17.8)	(2.3)	(11.7)	(16.3)	(2.3)	(11.9)
a_E	69.9	113.9	7.4	69.9	113.9	7.4	69.9	113.9	7.4	69.9
	(12.4)	(20.2)	(1.3)	(12.4)	(20.2)	(1.3)	(12.4)	(20.2)	(1.3)	(12.4)
	-23.3	-53.8	52.3	21.6	-26.0	14.7	-7.6	-39.2	14.7	-5.7
	-4.133	-9.562	9.286	3.832	-4.611	2.614	-1.349	-6.967	2.614	-1.011
t_O	59	154	28	57	158	31	68	149	20	56
	(10.5)	(27.4)	(5.0)	(10.1)	(28.1)	(5.5)	(12.1)	(26.5)	(3.6)	(9.9)
t_E	55.8	148.9	10.1	55.8	148.9	10.1	55.8	148.9	10.1	55.8
	(9.9)	(26.5)	(1.8)	(9.9)	(26.5)	(1.8)	(9.9)	(26.5)	(1.8)	(9.9)
	6.5	10.3	57.3	2.4	18.7	69.8	26.8	0.2	27.5	0.3

	1.157	1.834	10.179	0.419	3.321	12.391	4.763	0.027	4.880	0.060
g_O	239	124	205	220	112	210	243	110	215	248
	(42.5)	(22.0)	(36.4)	(39.1)	(19.9)	(37.3)	(43.2)	(19.5)	(38.2)	(44.0)
g_E	254.5	129.2	259.5	254.5	129.2	259.5	254.5	129.2	259.5	254.5
	(45.2)	(22.9)	(46.1)	(45.2)	(22.9)	(46.1)	(45.2)	(22.9)	(46.1)	(45.2)
	-30.0	-10.1	-96.7	-64.1	-32.0	-89.0	-22.5	-35.3	-81.0	-12.8
	-5.337	-1.800	-17.179	-11.387	-5.675	-15.800	-3.994	-6.278	-14.379	-2.282
c_O	208	203	307	206	193	309	186	212	315	192
	(36.9)	(36.1)	(54.5)	(36.6)	(34.3)	(54.9)	(33.0)	(37.7)	(56.0)	(34.1)
c_E	182.8	171.0	286.0	182.8	171.0	286.0	182.8	171.0	286.0	182.8
	(32.5)	(30.4)	(50.8)	(32.5)	(30.4)	(50.8)	(32.5)	(30.4)	(50.8)	(32.5)
	53.8	69.5	43.5	49.3	46.6	47.8	6.6	91.0	60.8	19.0
	9.561	12.351	7.720	8.762	8.279	8.484	1.164	16.166	10.800	3.366
N	563	563	563	563	563	563	563	563	563	563
X2	7.0	15.3	78.0	9.1	7.3	59.1	3.4	16.8	24.7	0.8
Gvalue	7.0	15.9	56.3	9.2	7.4	43.3	3.3	16.6	22.0	0.8
Gv/N	1.248	2.824	10.007	1.627	1.314	7.688	0.584	2.948	3.916	0.133
Prob	0.07114	0.00119	0.00000	0.02723	0.06024	0.00000	0.34956	0.00086	0.00006	0.86112

	41	42	43	44	45	46	47	48	49	50
a_O	90	17	68	83	14	60	91	19	54	100
	(16.0)	(3.0)	(12.1)	(14.7)	(2.5)	(10.7)	(16.2)	(3.4)	(9.6)	(17.8)
a_E	113.9	7.4	69.9	113.9	7.4	69.9	113.9	7.4	69.9	113.9
	(20.2)	(1.3)	(12.4)	(20.2)	(1.3)	(12.4)	(20.2)	(1.3)	(12.4)	(20.2)
	-42.3	28.4	-3.8	-52.5	17.9	-18.3	-40.8	35.9	-27.9	-26.0
	-7.518	5.038	-0.669	-9.321	3.184	-3.258	-7.245	6.382	-4.953	-4.611
t_O	145	24	47	161	26	51	128	26	66	122
	(25.8)	(4.3)	(8.3)	(28.6)	(4.6)	(9.1)	(22.7)	(4.6)	(11.7)	(21.7)
t_E	148.9	10.1	55.8	148.9	10.1	55.8	148.9	10.1	55.8	148.9
	(26.5)	(1.8)	(9.9)	(26.5)	(1.8)	(9.9)	(26.5)	(1.8)	(9.9)	(26.5)
	-7.7	41.7	-16.2	25.1	49.4	-9.2	-38.8	49.4	22.1	-48.7
	-1.375	7.411	-2.875	4.460	8.768	-1.640	-6.884	8.768	3.923	-8.642
g_O	142	212	244	122	217	257	130	202	253	120
	(25.2)	(37.7)	(43.3)	(21.7)	(38.5)	(45.6)	(23.1)	(35.9)	(44.9)	(21.3)
g_E	129.2	259.5	254.5	129.2	259.5	254.5	129.2	259.5	254.5	129.2
	(22.9)	(46.1)	(45.2)	(22.9)	(46.1)	(45.2)	(22.9)	(46.1)	(45.2)	(22.9)
	26.9	-85.8	-20.6	-13.9	-77.7	5.0	1.7	-101.3	-3.0	-17.7
	4.776	-15.237	-3.654	-2.475	-13.799	0.890	0.295	-17.985	-0.534	-3.139
c_O	186	310	204	197	306	195	214	316	190	221
	(33.0)	(55.1)	(36.2)	(35.0)	(54.4)	(34.6)	(38.0)	(56.1)	(33.7)	(39.3)
c_E	171.0	286.0	182.8	171.0	286.0	182.8	171.0	286.0	182.8	171.0
	(30.4)	(50.8)	(32.5)	(30.4)	(50.8)	(32.5)	(30.4)	(50.8)	(32.5)	(30.4)
	31.2	49.9	44.9	55.7	41.3	25.3	95.9	63.0	14.8	113.3
	5.538	8.867	7.970	9.886	7.341	4.493	17.032	11.190	2.625	20.116
N	563	563	563	563	563	563	563	563	563	563
X2	7.7	42.6	4.4	13.7	39.5	2.7	18.3	59.4	5.8	21.8
Gvalue	8.0	34.2	4.3	14.4	30.9	2.7	18.0	47.0	6.0	21.0
Gv/N	1.421	6.079	0.772	2.550	5.493	0.486	3.198	8.355	1.061	3.723
Prob	0.04601	0.00000	0.22615	0.00246	0.00000	0.43452	0.00044	0.00000	0.11297	0.00011

	51	52	53	54	55	56	57	58	59	60
a_O	14	65	92	17	74	90	16	71	102	14
	(2.5)	(11.5)	(16.3)	(3.0)	(13.1)	(16.0)	(2.8)	(12.6)	(18.1)	(2.5)
a_E	7.4	69.9	113.9	7.4	69.9	113.9	7.4	69.9	113.9	7.4
	(1.3)	(12.4)	(20.2)	(1.3)	(12.4)	(20.2)	(1.3)	(12.4)	(20.2)	(1.3)
	17.9	-9.5	-39.2	28.4	8.4	-42.3	24.8	2.2	-22.4	17.9
	3.184	-1.681	-6.967	5.038	1.495	-7.518	4.397	0.391	-3.986	3.184
t_O	20	67	142	20	61	146	20	59	141	18
	(3.6)	(11.9)	(25.2)	(3.6)	(10.8)	(25.9)	(3.6)	(10.5)	(25.0)	(3.2)
t_E	10.1	55.8	148.9	10.1	55.8	148.9	10.1	55.8	148.9	10.1
	(1.8)	(9.9)	(26.5)	(1.8)	(9.9)	(26.5)	(1.8)	(9.9)	(26.5)	(1.8)
	27.5	24.4	-13.5	27.5	10.8	-5.8	27.5	6.5	-15.4	20.9
	4.880	4.340	-2.401	4.880	1.918	-1.028	4.880	1.157	-2.738	3.719
g_O	221	238	121	224	251	132	215	233	124	257
	(39.3)	(42.3)	(21.5)	(39.8)	(44.6)	(23.4)	(38.2)	(41.4)	(22.0)	(45.6)
g_E	259.5	254.5	129.2	259.5	254.5	129.2	259.5	254.5	129.2	259.5
	(46.1)	(45.2)	(22.9)	(46.1)	(45.2)	(22.9)	(46.1)	(45.2)	(22.9)	(46.1)
	-71.0	-31.9	-15.8	-66.0	-7.0	5.7	-81.0	-41.1	-10.1	-5.1
	-12.619	-5.669	-2.809	-11.718	-1.237	1.016	-14.379	-7.308	-1.800	-0.897
c_O	308	193	208	302	177	195	312	200	196	274
	(54.7)	(34.3)	(36.9)	(53.6)	(31.4)	(34.6)	(55.4)	(35.5)	(34.8)	(48.7)
c_E	286.0	182.8	171.0	286.0	182.8	171.0	286.0	182.8	171.0	286.0
	(50.8)	(32.5)	(30.4)	(50.8)	(32.5)	(30.4)	(50.8)	(32.5)	(30.4)	(50.8)
	45.6	21.1	81.4	32.8	-11.3	51.1	54.3	36.1	53.4	-23.5
	8.101	3.740	14.453	5.833	-2.011	9.079	9.637	6.407	9.482	-4.178
N	563	563	563	563	563	563	563	563	563	563
X2	23.2	4.2	13.0	28.1	0.9	8.5	29.9	3.6	5.5	12.7
Gvalue	20.0	4.1	12.8	22.7	0.9	8.7	25.5	3.6	5.4	10.3
Gv/N	3.546	0.730	2.276	4.034	0.165	1.549	4.536	0.647	0.958	1.827
Prob	0.00017	0.24984	0.00506	0.00005	0.81797	0.03328	0.00001	0.30268	0.14506	0.01630

	61	62	63	64	65	66	67	68	69	70
a_O	71	103	14	60	87	13	61	113	10	60
	(12.6)	(18.3)	(2.5)	(10.7)	(15.5)	(2.3)	(10.8)	(20.1)	(1.8)	(10.7)
a_E	69.9	113.9	7.4	69.9	113.9	7.4	69.9	113.9	7.4	69.9
	(12.4)	(20.2)	(1.3)	(12.4)	(20.2)	(1.3)	(12.4)	(20.2)	(1.3)	(12.4)
	2.2	-20.6	17.9	-18.3	-46.8	14.7	-16.6	-1.7	6.1	-18.3
	0.391	-3.668	3.184	-3.258	-8.316	2.614	-2.954	-0.304	1.079	-3.258
t_O	59	151	24	44	143	16	47	144	19	52
	(10.5)	(26.8)	(4.3)	(7.8)	(25.4)	(2.8)	(8.3)	(25.6)	(3.4)	(9.2)
t_E	55.8	148.9	10.1	55.8	148.9	10.1	55.8	148.9	10.1	55.8
	(9.9)	(26.5)	(1.8)	(9.9)	(26.5)	(1.8)	(9.9)	(26.5)	(1.8)	(9.9)
	6.5	4.2	41.7	-21.0	-11.6	14.8	-16.2	-9.7	24.2	-7.4
	1.157	0.743	7.411	-3.722	-2.062	2.636	-2.875	-1.720	4.290	-1.313
g_O	252	113	214	273	133	245	269	118	238	253
	(44.8)	(20.1)	(38.0)	(48.5)	(23.6)	(43.5)	(47.8)	(21.0)	(42.3)	(44.9)
g_E	254.5	129.2	259.5	254.5	129.2	259.5	254.5	129.2	259.5	254.5
	(45.2)	(22.9)	(46.1)	(45.2)	(22.9)	(46.1)	(45.2)	(22.9)	(46.1)	(45.2)
	-5.0	-30.2	-82.6	38.3	7.8	-28.2	29.8	-21.3	-41.2	-3.0
	-0.886	-5.369	-14.667	6.803	1.380	-5.017	5.292	-3.792	-7.325	-0.534
c_O	181	196	311	186	200	289	186	188	296	198
	(32.1)	(34.8)	(55.2)	(33.0)	(35.5)	(51.3)	(33.0)	(33.4)	(52.6)	(35.2)
c_E	182.8	171.0	286.0	182.8	171.0	286.0	182.8	171.0	286.0	182.8
	(32.5)	(30.4)	(50.8)	(32.5)	(30.4)	(50.8)	(32.5)	(30.4)	(50.8)	(32.5)
	-3.5	53.4	52.1	6.6	62.6	6.0	6.6	35.5	20.3	31.7
	-0.620	9.482	9.251	1.164	11.111	1.065	1.164	6.312	3.607	5.636

N	563	563	563	563	563	563	563	563	563	563
X2	0.2	6.7	35.4	5.3	11.6	8.6	3.4	2.8	11.0	2.9
Gvalue	0.2	6.7	29.2	5.6	11.9	7.3	3.5	2.8	9.3	3.0
Gv/N	0.042	1.188	5.179	0.986	2.114	1.298	0.627	0.496	1.651	0.531
Prob	0.97173	0.08249	0.00000	0.13559	0.00773	0.06277	0.31673	0.42453	0.02559	0.39287

	71	72	73	74	75	76	77	78	79	80
a_O	103	11	73	104	14	63	102	12	64	127
	(18.3)	(2.0)	(13.0)	(18.5)	(2.5)	(11.2)	(18.1)	(2.1)	(11.4)	(22.6)
a_E	113.9	7.4	69.9	113.9	7.4	69.9	113.9	7.4	69.9	113.9
	(20.2)	(1.3)	(12.4)	(20.2)	(1.3)	(12.4)	(20.2)	(1.3)	(12.4)	(20.2)
	-20.6	8.8	6.3	-18.8	17.9	-13.1	-22.4	11.7	-11.3	27.7
	-3.668	1.559	1.122	-3.346	3.184	-2.329	-3.986	2.072	-2.007	4.927
t_O	125	20	46	155	14	49	138	15	46	131
	(22.2)	(3.6)	(8.2)	(27.5)	(2.5)	(8.7)	(24.5)	(2.7)	(8.2)	(23.3)
t_E	148.9	10.1	55.8	148.9	10.1	55.8	148.9	10.1	55.8	148.9
	(26.5)	(1.8)	(9.9)	(26.5)	(1.8)	(9.9)	(26.5)	(1.8)	(9.9)	(26.5)
	-43.8	27.5	-17.8	12.4	9.2	-12.8	-21.0	12.0	-17.8	-33.6
	-7.776	4.880	-3.165	2.202	1.642	-2.272	-3.734	2.127	-3.165	-5.967
g_O	148	226	243	117	244	260	125	230	273	143
	(26.3)	(40.1)	(43.2)	(20.8)	(43.3)	(46.2)	(22.2)	(40.9)	(48.5)	(25.4)
g_E	129.2	259.5	254.5	129.2	259.5	254.5	129.2	259.5	254.5	129.2
	(22.9)	(46.1)	(45.2)	(22.9)	(46.1)	(45.2)	(22.9)	(46.1)	(45.2)	(22.9)
	40.3	-62.5	-22.5	-23.2	-30.1	11.1	-8.2	-55.6	38.3	29.1
	7.154	-11.109	-3.994	-4.113	-5.351	1.972	-1.458	-9.872	6.803	5.167
c_O	187	306	201	187	291	191	198	306	180	162
	(33.2)	(54.4)	(35.7)	(33.2)	(51.7)	(33.9)	(35.2)	(54.4)	(32.0)	(28.8)
c_E	171.0	286.0	182.8	171.0	286.0	182.8	171.0	286.0	182.8	171.0
	(30.4)	(50.8)	(32.5)	(30.4)	(50.8)	(32.5)	(30.4)	(50.8)	(32.5)	(30.4)
	33.4	41.3	38.3	33.4	10.0	16.9	57.9	41.3	-5.5	-17.6
	5.924	7.341	6.795	5.924	1.785	2.995	10.293	7.341	-0.971	-3.127
N	563	563	563	563	563	563	563	563	563	563
X2	9.1	17.3	4.2	3.7	8.5	2.0	6.4	10.1	3.6	5.6
Gvalue	9.2	15.0	4.3	3.8	7.1	2.1	6.3	9.4	3.7	5.6
Gv/N	1.634	2.671	0.758	0.667	1.260	0.366	1.115	1.668	0.659	1.000
Prob	0.02673	0.00178	0.23374	0.28943	0.06898	0.55944	0.09881	0.02454	0.29441	0.13119

	81	82	83	84	85	86	87	88	89	90
a_O	16	61	118	17	65	96	9	76	93	13
	(2.8)	(10.8)	(21.0)	(3.0)	(11.5)	(17.1)	(1.6)	(13.5)	(16.5)	(2.3)
a_E	7.4	69.9	113.9	7.4	69.9	113.9	7.4	69.9	113.9	7.4
	(1.3)	(12.4)	(20.2)	(1.3)	(12.4)	(20.2)	(1.3)	(12.4)	(20.2)	(1.3)
	24.8	-16.6	8.4	28.4	-9.5	-32.8	3.6	12.7	-37.6	14.7
	4.397	-2.954	1.497	5.038	-1.681	-5.819	0.634	2.256	-6.686	2.614
t_O	10	59	144	10	50	138	9	53	141	12
	(1.8)	(10.5)	(25.6)	(1.8)	(8.9)	(24.5)	(1.6)	(9.4)	(25.0)	(2.1)
t_E	10.1	55.8	148.9	10.1	55.8	148.9	10.1	55.8	148.9	10.1
	(1.8)	(9.9)	(26.5)	(1.8)	(9.9)	(26.5)	(1.8)	(9.9)	(26.5)	(1.8)
	-0.1	6.5	-9.7	-0.1	-11.0	-21.0	-2.0	-5.5	-15.4	4.2
	-0.022	1.157	-1.720	-0.022	-1.959	-3.734	-0.357	-0.980	-2.738	0.751
g_O	248	256	120	265	250	131	240	249	129	238
	(44.0)	(45.5)	(21.3)	(47.1)	(44.4)	(23.3)	(42.6)	(44.2)	(22.9)	(42.3)
g_E	259.5	254.5	129.2	259.5	254.5	129.2	259.5	254.5	129.2	259.5
	(46.1)	(45.2)	(22.9)	(46.1)	(45.2)	(22.9)	(46.1)	(45.2)	(22.9)	(46.1)
	-22.6	3.0	-17.7	11.0	-8.9	3.7	-37.6	-10.9	-0.3	-41.2
	-4.006	0.532	-3.139	1.961	-1.587	0.654	-6.673	-1.935	-0.061	-7.325
c_O	289	187	181	271	198	198	305	185	200	300
	(51.3)	(33.2)	(32.1)	(48.1)	(35.2)	(35.2)	(54.2)	(32.9)	(35.5)	(53.3)
c_E	286.0	182.8	171.0	286.0	182.8	171.0	286.0	182.8	171.0	286.0
	(50.8)	(32.5)	(30.4)	(50.8)	(32.5)	(30.4)	(50.8)	(32.5)	(30.4)	(50.8)
	6.0	8.6	20.5	-29.2	31.7	57.9	39.2	4.5	62.6	28.6
	1.065	1.526	3.637	-5.193	5.636	10.293	6.962	0.803	11.111	5.086
N	563	563	563	563	563	563	563	563	563	563
X2	10.6	1.4	1.5	13.4	2.3	7.9	3.2	0.8	9.1	7.1
Gvalue	8.1	1.5	1.5	10.0	2.3	7.8	3.2	0.8	9.2	6.3
Gv/N	1.434	0.261	0.275	1.784	0.409	1.394	0.567	0.144	1.626	1.126
Prob	0.04455	0.68925	0.67107	0.01818	0.51199	0.04925	0.36326	0.84714	0.02731	0.09612

	91	92	93	94	95	96	97	98	99	100
a_O	57	118	10	63	109	9	54	128	6	69
	(10.1)	(21.0)	(1.8)	(11.2)	(19.4)	(1.6)	(9.6)	(22.7)	(1.1)	(12.3)
a_E	69.9	113.9	7.4	69.9	113.9	7.4	69.9	113.9	7.4	69.9
	(12.4)	(20.2)	(1.3)	(12.4)	(20.2)	(1.3)	(12.4)	(20.2)	(1.3)	(12.4)
	-23.3	8.4	6.1	-13.1	-9.5	3.6	-27.9	30.0	-2.5	-1.8
	-4.133	1.497	1.079	-2.329	-1.689	0.634	-4.953	5.323	-0.442	-0.320
t_O	54	158	9	52	154	15	54	144	18	55
	(9.6)	(28.1)	(1.6)	(9.2)	(27.4)	(2.7)	(9.6)	(25.6)	(3.2)	(9.8)
t_E	55.8	148.9	10.1	55.8	148.9	10.1	55.8	148.9	10.1	55.8
	(9.9)	(26.5)	(1.8)	(9.9)	(26.5)	(1.8)	(9.9)	(26.5)	(1.8)	(9.9)
	-3.6	18.7	-2.0	-7.4	10.3	12.0	-3.6	-9.7	20.9	-1.7
	-0.640	3.321	-0.357	-1.313	1.834	2.127	-0.640	-1.720	3.719	-0.293
g_O	268	115	260	254	124	257	262	127	235	267
	(47.6)	(20.4)	(46.2)	(45.1)	(22.0)	(45.6)	(46.5)	(22.6)	(41.7)	(47.4)
g_E	254.5	129.2	259.5	254.5	129.2	259.5	254.5	129.2	259.5	254.5
	(45.2)	(22.9)	(46.1)	(45.2)	(22.9)	(46.1)	(45.2)	(22.9)	(46.1)	(45.2)
	27.7	-26.7	0.9	-1.0	-10.1	-5.1	15.2	-4.3	-46.7	25.6
	4.918	-4.747	0.164	-0.180	-1.800	-0.897	2.701	-0.765	-8.291	4.545
c_O	184	172	284	194	176	282	193	164	304	172
	(32.7)	(30.6)	(50.4)	(34.5)	(31.3)	(50.1)	(34.3)	(29.1)	(54.0)	(30.6)
c_E	182.8	171.0	286.0	182.8	171.0	286.0	182.8	171.0	286.0	182.8
	(32.5)	(30.4)	(50.8)	(32.5)	(30.4)	(50.8)	(32.5)	(30.4)	(50.8)	(32.5)
	2.5	1.9	-4.0	23.2	10.0	-8.0	21.1	-13.8	37.1	-20.9
	0.444	0.340	-0.714	4.116	1.785	-1.417	3.740	-2.451	6.585	-3.705
N	563	563	563	563	563	563	563	563	563	563
X2	3.2	2.3	1.1	1.6	0.7	2.9	4.5	2.2	10.0	1.3
Gvalue	3.3	2.3	1.0	1.7	0.7	2.5	4.8	2.2	8.8	1.3
Gv/N	0.589	0.411	0.172	0.294	0.130	0.447	0.848	0.388	1.570	0.226
Prob	0.34519	0.51036	0.80925	0.64702	0.86528	0.47253	0.18910	0.53528	0.03148	0.73548

Aehr_HLHE1
5UTR:

	100	99	98	97	96	95	94	93	92	91
a_O	34	34	39	38	39	35	36	44	43	35
	(16.0)	(16.0)	(18.3)	(17.8)	(18.3)	(16.4)	(16.9)	(20.7)	(20.2)	(16.4)
a_E	40.4	40.4	40.4	40.4	40.4	40.4	40.4	40.4	40.4	40.4
	(18.9)	(18.9)	(18.9)	(18.9)	(18.9)	(18.9)	(18.9)	(18.9)	(18.9)	(18.9)
	−11.7	−11.7	−2.7	−4.6	−2.7	−10.0	−8.2	7.6	5.4	−10.0
	−5.474	−5.474	−1.255	−2.150	−1.255	−4.683	−3.864	3.568	2.558	−4.683
t_O	38	25	28	40	35	42	42	32	31	38
	(17.8)	(11.7)	(13.1)	(18.8)	(16.4)	(19.7)	(19.7)	(15.0)	(14.6)	(17.8)
t_E	37.1	37.1	37.1	37.1	37.1	37.1	37.1	37.1	37.1	37.1
	(17.4)	(17.4)	(17.4)	(17.4)	(17.4)	(17.4)	(17.4)	(17.4)	(17.4)	(17.4)
	1.8	−19.8	−15.8	6.0	−4.1	10.4	10.4	−9.5	−11.2	1.8
	0.836	−9.279	−7.413	2.807	−1.933	4.871	4.871	−4.460	−5.244	0.836
g_O	68	77	68	68	66	77	62	75	78	72
	(31.9)	(36.2)	(31.9)	(31.9)	(31.0)	(36.2)	(29.1)	(35.2)	(36.6)	(33.8)
g_E	62.9	62.9	62.9	62.9	62.9	62.9	62.9	62.9	62.9	62.9
	(29.5)	(29.5)	(29.5)	(29.5)	(29.5)	(29.5)	(29.5)	(29.5)	(29.5)	(29.5)
	10.6	31.1	10.6	10.6	6.3	31.1	−1.8	26.3	33.5	19.4
	4.958	14.600	4.958	4.958	2.962	14.600	−0.858	12.368	15.735	9.113
c_O	73	77	78	67	73	59	73	62	61	68
	(34.3)	(36.2)	(36.6)	(31.5)	(34.3)	(27.7)	(34.3)	(29.1)	(28.6)	(31.9)
c_E	72.6	72.6	72.6	72.6	72.6	72.6	72.6	72.6	72.6	72.6
	(34.1)	(34.1)	(34.1)	(34.1)	(34.1)	(34.1)	(34.1)	(34.1)	(34.1)	(34.1)
	0.8	9.1	11.2	−10.8	0.8	−24.5	0.8	−19.6	−21.2	−8.9
	0.377	4.254	5.254	−5.050	0.377	−11.491	0.377	−9.188	−9.971	−4.179
N	213	213	213	213	213	213	213	213	213	213
X2	1.4	8.4	3.1	1.2	0.3	7.1	1.1	4.9	6.6	2.3
Gvalue	1.5	8.7	3.3	1.2	0.3	7.0	1.1	4.9	6.6	2.3
Gv/N	0.696	4.101	1.544	0.564	0.150	3.297	0.526	2.288	3.078	1.087
Prob	0.68646	0.03302	0.34922	0.75260	0.95615	0.07116	0.77229	0.18137	0.08751	0.50953

	90	89	88	87	86	85	84	83	82	81
a_O	34	34	40	33	34	45	46	40	37	41
	(16.0)	(16.0)	(18.8)	(15.5)	(16.0)	(21.1)	(21.6)	(18.8)	(17.4)	(19.2)
a_E	40.4	40.4	40.4	40.4	40.4	40.4	40.4	40.4	40.4	40.4
	(18.9)	(18.9)	(18.9)	(18.9)	(18.9)	(18.9)	(18.9)	(18.9)	(18.9)	(18.9)
	−11.7	−11.7	−0.7	−13.3	−11.7	9.8	12.0	−0.7	−6.4	1.3
	−5.474	−5.474	−0.337	−6.238	−5.474	4.598	5.650	−0.337	−3.020	0.606
t_O	37	37	33	38	33	37	31	34	35	35
	(17.4)	(17.4)	(15.5)	(17.8)	(15.5)	(17.4)	(14.6)	(16.0)	(16.4)	(16.4)
t_E	37.1	37.1	37.1	37.1	37.1	37.1	37.1	37.1	37.1	37.1
	(17.4)	(17.4)	(17.4)	(17.4)	(17.4)	(17.4)	(17.4)	(17.4)	(17.4)	(17.4)
	−0.2	−0.2	−7.8	1.8	−7.8	−0.2	−11.2	−6.0	−4.1	−4.1
	−0.112	−0.112	−3.645	0.836	−3.645	−0.112	−5.244	−2.803	−1.933	−1.933
g_O	65	68	69	68	70	68	66	66	61	61
	(30.5)	(31.9)	(32.4)	(31.9)	(32.9)	(31.9)	(31.0)	(31.0)	(28.6)	(28.6)
g_E	62.9	62.9	62.9	62.9	62.9	62.9	62.9	62.9	62.9	62.9
	(29.5)	(29.5)	(29.5)	(29.5)	(29.5)	(29.5)	(29.5)	(29.5)	(29.5)	(29.5)
	4.2	10.6	12.7	10.6	14.9	10.6	6.3	6.3	−3.8	−3.8
	1.985	4.958	5.976	4.958	7.009	4.958	2.962	2.962	−1.775	−1.775
c_O	77	74	71	74	76	63	70	73	80	76
	(36.2)	(34.7)	(33.3)	(34.7)	(35.7)	(29.6)	(32.9)	(34.3)	(37.6)	(35.7)
c_E	72.6	72.6	72.6	72.6	72.6	72.6	72.6	72.6	72.6	72.6
	(34.1)	(34.1)	(34.1)	(34.1)	(34.1)	(34.1)	(34.1)	(34.1)	(34.1)	(34.1)
	9.1	2.8	−3.2	2.8	7.0	−17.9	−5.1	0.8	15.5	7.0
	4.254	1.327	−1.486	1.327	3.266	−8.390	−2.397	0.377	7.291	3.266
N	213	213	213	213	213	213	213	213	213	213
X2	1.3	1.4	1.1	1.8	2.4	2.2	2.0	0.4	1.2	0.3
Gvalue	1.4	1.5	1.1	1.9	2.5	2.2	2.1	0.4	1.2	0.3
Gv/N	0.652	0.698	0.509	0.882	1.155	1.053	0.970	0.199	0.564	0.164
Prob	0.70805	0.68546	0.78108	0.59786	0.48260	0.52345	0.55883	0.93530	0.75292	0.95046

	80	79	78	77	76	75	74	73	72	71
a_O	36	41	31	38	41	35	39	33	37	35
	(16.9)	(19.2)	(14.6)	(17.8)	(19.2)	(16.4)	(18.3)	(15.5)	(17.4)	(16.4)
a_E	40.4	40.4	40.4	40.4	40.4	40.4	40.4	40.4	40.4	40.4
	(18.9)	(18.9)	(18.9)	(18.9)	(18.9)	(18.9)	(18.9)	(18.9)	(18.9)	(18.9)
	−8.2	1.3	−16.4	−4.6	1.3	−10.0	−2.7	−13.3	−6.4	−10.0
	−3.864	0.606	−7.680	−2.150	0.606	−4.683	−1.255	−6.238	−3.020	−4.683
t_O	37	39	34	23	32	45	38	42	37	36
	(17.4)	(18.3)	(16.0)	(10.8)	(15.0)	(21.1)	(17.8)	(19.7)	(17.4)	(16.9)
t_E	37.1	37.1	37.1	37.1	37.1	37.1	37.1	37.1	37.1	37.1
	(17.4)	(17.4)	(17.4)	(17.4)	(17.4)	(17.4)	(17.4)	(17.4)	(17.4)	(17.4)
	−0.2	3.9	−6.0	−22.0	−9.5	17.3	1.8	10.4	−0.2	−2.2
	−0.112	1.809	−2.803	−10.337	−4.460	8.134	0.836	4.871	−0.112	−1.036
g_O	66	67	71	76	68	61	68	71	63	67
	(31.0)	(31.5)	(33.3)	(35.7)	(31.9)	(28.6)	(31.9)	(33.3)	(29.6)	(31.5)
g_E	62.9	62.9	62.9	62.9	62.9	62.9	62.9	62.9	62.9	62.9
	(29.5)	(29.5)	(29.5)	(29.5)	(29.5)	(29.5)	(29.5)	(29.5)	(29.5)	(29.5)
	6.3	8.4	17.2	28.7	10.6	−3.8	10.6	17.2	0.2	8.4
	2.962	3.953	8.054	13.478	4.958	−1.775	4.958	8.054	0.075	3.953
c_O	74	66	77	76	72	72	68	67	76	75
	(34.7)	(31.0)	(36.2)	(35.7)	(33.8)	(33.8)	(31.9)	(31.5)	(35.7)	(35.2)
c_E	72.6	72.6	72.6	72.6	72.6	72.6	72.6	72.6	72.6	72.6
	(34.1)	(34.1)	(34.1)	(34.1)	(34.1)	(34.1)	(34.1)	(34.1)	(34.1)	(34.1)
	2.8	−12.6	9.1	7.0	−1.2	−1.2	−8.9	−10.8	7.0	4.9
	1.327	−5.907	4.254	3.266	−0.561	−0.561	−4.179	−5.050	3.266	2.290
N	213	213	213	213	213	213	213	213	213	213
X2	0.6	1.0	3.7	8.4	1.1	2.4	0.8	3.5	0.4	1.1
Gvalue	0.7	1.0	3.9	9.1	1.2	2.4	0.8	3.5	0.4	1.1
Gv/N	0.312	0.461	1.826	4.257	0.543	1.115	0.359	1.637	0.209	0.525
Prob	0.88152	0.80565	0.27377	0.02841	0.76364	0.49823	0.85796	0.32250	0.93077	0.77291

	70	69	68	67	66	65	64	63	62	61
a_O	37	34	39	36	29	39	38	40	31	32
	(17.4)	(16.0)	(18.3)	(16.9)	(13.6)	(18.3)	(17.8)	(18.8)	(14.6)	(15.0)
a_E	40.4	40.4	40.4	40.4	40.4	40.4	40.4	40.4	40.4	40.4
	(18.9)	(18.9)	(18.9)	(18.9)	(18.9)	(18.9)	(18.9)	(18.9)	(18.9)	(18.9)
	−6.4	−11.7	−2.7	−8.2	−19.2	−2.7	−4.6	−0.7	−16.4	−14.9
	−3.020	−5.474	−1.255	−3.864	−9.001	−1.255	−2.150	−0.337	−7.680	−6.974
t_O	42	47	40	31	38	40	49	40	34	39
	(19.7)	(22.1)	(18.8)	(14.6)	(17.8)	(18.8)	(23.0)	(18.8)	(16.0)	(18.3)

```
t_E        37.1     37.1     37.1     37.1     37.1     37.1     37.1     37.1     37.1     37.1
        ( 17.4)  ( 17.4)  ( 17.4)  ( 17.4)  ( 17.4)  ( 17.4)  ( 17.4)  ( 17.4)  ( 17.4)  ( 17.4)
           10.4     22.2      6.0    -11.2      1.8      6.0     27.2      6.0     -6.0      3.9
          4.871   10.415    2.807   -5.244    0.836    2.807   12.775    2.807   -2.803    1.809
g_O          68       58       67       78       78       70       59       78       76       75
        ( 31.9)  ( 27.2)  ( 31.5)  ( 36.6)  ( 36.6)  ( 32.9)  ( 27.7)  ( 36.6)  ( 35.7)  ( 35.2)
g_E        62.9     62.9     62.9     62.9     62.9     62.9     62.9     62.9     62.9     62.9
        ( 29.5)  ( 29.5)  ( 29.5)  ( 29.5)  ( 29.5)  ( 29.5)  ( 29.5)  ( 29.5)  ( 29.5)  ( 29.5)
           10.6     -9.4      8.4     33.5     33.5     14.9     -7.6     33.5     28.7     26.3
          4.958   -4.434    3.953   15.735   15.735    7.009   -3.564   15.735   13.478   12.368
c_O          66       74       67       68       68       64       67       55       72       67
        ( 31.0)  ( 34.7)  ( 31.5)  ( 31.9)  ( 31.9)  ( 30.0)  ( 31.5)  ( 25.8)  ( 33.8)  ( 31.5)
c_E        72.6     72.6     72.6     72.6     72.6     72.6     72.6     72.6     72.6     72.6
        ( 34.1)  ( 34.1)  ( 34.1)  ( 34.1)  ( 34.1)  ( 34.1)  ( 34.1)  ( 34.1)  ( 34.1)  ( 34.1)
          -12.6      2.8    -10.8     -8.9     -8.9    -16.1    -10.8    -30.5     -1.2    -10.8
         -5.907    1.327   -5.050   -4.179   -4.179   -7.577   -5.050  -14.338   -0.561   -5.050

N           213      213      213      213      213      213      213      213      213      213
X2          1.9      4.0      1.0      5.4      7.1      2.1      4.6      8.1      5.2      4.6
Gvalue      1.9      3.9      1.0      5.2      7.2      2.1      4.3      8.2      5.2      4.6
Gv/N      0.902    1.833    0.454    2.447    3.391    0.983    2.012    3.867    2.434    2.153
Prob    0.58884  0.27195  0.80929  0.15691  0.06513  0.55310  0.23230  0.04135  0.15880  0.20473
        ------------------------------------------------------------------------------------------
             60       59       58       57       56       55       54       53       52       51
        ------------------------------------------------------------------------------------------
a_O          33       33       44       43       47       33       32       33       54       32
        ( 15.5)  ( 15.5)  ( 20.7)  ( 20.2)  ( 22.1)  ( 15.5)  ( 15.0)  ( 15.5)  ( 25.4)  ( 15.0)
a_E        40.4     40.4     40.4     40.4     40.4     40.4     40.4     40.4     40.4     40.4
        ( 18.9)  ( 18.9)  ( 18.9)  ( 18.9)  ( 18.9)  ( 18.9)  ( 18.9)  ( 18.9)  ( 18.9)  ( 18.9)
          -13.3    -13.3      7.6      5.4     14.3    -13.3    -14.9    -13.3     31.4    -14.9
         -6.238   -6.238    3.568    2.558    6.722   -6.238   -6.974   -6.238   14.762   -6.974
t_O          34       34       45       41       28       47       38       32       41       33
        ( 16.0)  ( 16.0)  ( 21.1)  ( 19.2)  ( 13.1)  ( 22.1)  ( 17.8)  ( 15.0)  ( 19.2)  ( 15.5)
t_E        37.1     37.1     37.1     37.1     37.1     37.1     37.1     37.1     37.1     37.1
        ( 17.4)  ( 17.4)  ( 17.4)  ( 17.4)  ( 17.4)  ( 17.4)  ( 17.4)  ( 17.4)  ( 17.4)  ( 17.4)
           -6.0     -6.0     17.3      8.2    -15.8     22.2      1.8     -9.5      8.2     -7.8
         -2.803   -2.803    8.134    3.827   -7.413   10.415    0.836   -4.460    3.827   -3.645
g_O          69       74       59       71       73       62       60       85       51       63
        ( 32.4)  ( 34.7)  ( 27.7)  ( 33.3)  ( 34.3)  ( 29.1)  ( 28.2)  ( 39.9)  ( 23.9)  ( 29.6)
g_E        62.9     62.9     62.9     62.9     62.9     62.9     62.9     62.9     62.9     62.9
        ( 29.5)  ( 29.5)  ( 29.5)  ( 29.5)  ( 29.5)  ( 29.5)  ( 29.5)  ( 29.5)  ( 29.5)  ( 29.5)
           12.7     24.0     -7.6     17.2     21.7     -1.8     -5.7     51.1    -21.4      0.2
          5.976   11.270   -3.564    8.054   10.185   -0.858   -2.677   24.006  -10.058    0.075
c_O          77       72       65       58       65       71       83       63       67       85
        ( 36.2)  ( 33.8)  ( 30.5)  ( 27.2)  ( 30.5)  ( 33.3)  ( 39.0)  ( 29.6)  ( 31.5)  ( 39.9)
c_E        72.6     72.6     72.6     72.6     72.6     72.6     72.6     72.6     72.6     72.6
        ( 34.1)  ( 34.1)  ( 34.1)  ( 34.1)  ( 34.1)  ( 34.1)  ( 34.1)  ( 34.1)  ( 34.1)  ( 34.1)
            9.1     -1.2    -14.4    -26.0    -14.4     -3.2     22.2    -17.9    -10.8     26.8
          4.254   -0.561   -6.749  -12.227   -6.749   -1.486   10.434   -8.390   -5.050   12.585

N           213      213      213      213      213      213      213      213      213      213
X2          2.5      3.6      3.0      4.6      5.7      4.0      3.4     11.1      7.7      4.3
Gvalue      2.5      3.6      3.0      4.7      5.8      3.9      3.4     10.5      7.4      4.3
Gv/N      1.189    1.668    1.389    2.212    2.745    1.833    1.618    4.919    3.481    2.041
Prob    0.46937  0.31398  0.39802  0.19410  0.11927  0.27198  0.32769  0.01492  0.05977  0.22631
        ------------------------------------------------------------------------------------------
             50       49       48       47       46       45       44       43       42       41
        ------------------------------------------------------------------------------------------
a_O          45       41       30       33       43       45       30       48       38       46
        ( 21.1)  ( 19.2)  ( 14.1)  ( 15.5)  ( 20.2)  ( 21.1)  ( 14.1)  ( 22.5)  ( 17.8)  ( 21.6)
a_E        40.4     40.4     40.4     40.4     40.4     40.4     40.4     40.4     40.4     40.4
        ( 18.9)  ( 18.9)  ( 18.9)  ( 18.9)  ( 18.9)  ( 18.9)  ( 18.9)  ( 18.9)  ( 18.9)  ( 18.9)
            9.8      1.3    -17.8    -13.3      5.4      9.8    -17.8     16.6     -4.6     12.0
          4.598    0.606   -8.356   -6.238    2.558    4.598   -8.356    7.813   -2.150    5.650
t_O          35       37       42       40       32       28       28       39       49       36
        ( 16.4)  ( 17.4)  ( 19.7)  ( 18.8)  ( 15.0)  ( 13.1)  ( 13.1)  ( 18.3)  ( 23.0)  ( 16.9)
t_E        37.1     37.1     37.1     37.1     37.1     37.1     37.1     37.1     37.1     37.1
        ( 17.4)  ( 17.4)  ( 17.4)  ( 17.4)  ( 17.4)  ( 17.4)  ( 17.4)  ( 17.4)  ( 17.4)  ( 17.4)
           -4.1     -0.2     10.4      6.0     -9.5    -15.8    -15.8      3.9     27.2     -2.2
         -1.933   -0.112    4.871    2.807   -4.460   -7.413   -7.413    1.809   12.775   -1.036
g_O          72       71       70       77       66       57       68       71       59       60
        ( 33.8)  ( 33.3)  ( 32.9)  ( 36.2)  ( 31.0)  ( 26.8)  ( 31.9)  ( 33.3)  ( 27.7)  ( 28.2)
g_E        62.9     62.9     62.9     62.9     62.9     62.9     62.9     62.9     62.9     62.9
        ( 29.5)  ( 29.5)  ( 29.5)  ( 29.5)  ( 29.5)  ( 29.5)  ( 29.5)  ( 29.5)  ( 29.5)  ( 29.5)
           19.4     17.2     14.9     31.1      6.3    -11.3     10.6     17.2     -7.6     -5.7
          9.113    8.054    7.009   14.600    2.962   -5.289    4.958    8.054   -3.564   -2.677
c_O          61       64       71       63       72       83       87       55       67       71
        ( 28.6)  ( 30.0)  ( 33.3)  ( 29.6)  ( 33.8)  ( 39.0)  ( 40.8)  ( 25.8)  ( 31.5)  ( 33.3)
c_E        72.6     72.6     72.6     72.6     72.6     72.6     72.6     72.6     72.6     72.6
        ( 34.1)  ( 34.1)  ( 34.1)  ( 34.1)  ( 34.1)  ( 34.1)  ( 34.1)  ( 34.1)  ( 34.1)  ( 34.1)
          -21.2    -16.1     -3.2    -17.9     -1.2     22.2     31.5    -30.5    -10.8     -3.2
         -9.971   -7.577   -1.486   -8.390   -0.561   10.434   14.781  -14.338   -5.050   -1.486

N           213      213      213      213      213      213      213      213      213      213
X2          3.8      2.1      4.1      6.0      1.0      4.8      8.2      6.8      4.6      1.0
Gvalue      3.8      2.1      4.3      5.9      1.1      5.0      8.5      7.1      4.3      1.0
Gv/N      1.807    0.971    2.038    2.779    0.499    2.330    3.970    3.339    2.012    0.451
Prob    0.27813  0.55844  0.22696  0.11565  0.78589  0.17449  0.03747  0.06839  0.23230  0.81066
        ------------------------------------------------------------------------------------------
             40       39       38       37       36       35       34       33       32       31
        ------------------------------------------------------------------------------------------
a_O          47       29       42       43       41       43       42       21       41       51
        ( 22.1)  ( 13.6)  ( 19.7)  ( 20.2)  ( 19.2)  ( 20.2)  ( 19.7)  (  9.9)  ( 19.2)  ( 23.9)
a_E        40.4     40.4     40.4     40.4     40.4     40.4     40.4     40.4     40.4     40.4
        ( 18.9)  ( 18.9)  ( 18.9)  ( 18.9)  ( 18.9)  ( 18.9)  ( 18.9)  ( 18.9)  ( 18.9)  ( 18.9)
           14.3    -19.2      3.3      5.4      1.3      5.4      3.3    -27.4      1.3     23.9
          6.722   -9.001    1.571    2.558    0.606    2.558    1.571  -12.882    0.606   11.205
t_O          45       50       37       35       40       30       45       41       40       35
        ( 21.1)  ( 23.5)  ( 17.4)  ( 16.4)  ( 18.8)  ( 14.1)  ( 21.1)  ( 19.2)  ( 18.8)  ( 16.4)
t_E        37.1     37.1     37.1     37.1     37.1     37.1     37.1     37.1     37.1     37.1
        ( 17.4)  ( 17.4)  ( 17.4)  ( 17.4)  ( 17.4)  ( 17.4)  ( 17.4)  ( 17.4)  ( 17.4)  ( 17.4)
           17.3     29.8     -0.2     -4.1      6.0    -12.8     17.3      8.2      6.0     -4.1
          8.134   13.984   -0.112   -1.933    2.807   -5.999    8.134    3.827    2.807   -1.933
g_O          47       60       57       73       62       76       50       76       62       60
        ( 22.1)  ( 28.2)  ( 26.8)  ( 34.3)  ( 29.1)  ( 35.7)  ( 23.5)  ( 35.7)  ( 29.1)  ( 28.2)
g_E        62.9     62.9     62.9     62.9     62.9     62.9     62.9     62.9     62.9     62.9
        ( 29.5)  ( 29.5)  ( 29.5)  ( 29.5)  ( 29.5)  ( 29.5)  ( 29.5)  ( 29.5)  ( 29.5)  ( 29.5)
          -27.4     -5.7    -11.3     21.7     -1.8     28.7    -23.0     28.7     -1.8     -5.7
        -12.874   -2.677   -5.289   10.185   -0.858   13.478  -10.791   13.478   -0.858   -2.677
c_O          74       74       77       62       70       64       76       75       70       67
        ( 34.7)  ( 34.7)  ( 36.2)  ( 29.1)  ( 32.9)  ( 30.0)  ( 35.7)  ( 35.2)  ( 32.9)  ( 31.5)
c_E        72.6     72.6     72.6     72.6     72.6     72.6     72.6     72.6     72.6     72.6
```

```
        ( 34.1) ( 34.1) ( 34.1) ( 34.1) ( 34.1) ( 34.1) ( 34.1) ( 34.1) ( 34.1) ( 34.1)
             2.8     2.8     9.1   -19.6    -5.1   -16.1     7.0     4.9    -5.1   -10.8
           1.327   1.327   4.254  -9.188  -2.397  -7.577   3.266   2.290  -2.397  -5.050

N            213     213     213     213     213     213     213     213     213     213
X2           6.8     7.8     0.9     3.5     0.3     5.3     4.6    12.5     0.3     3.5
Gvalue       7.0     7.7     0.9     3.5     0.3     5.2     4.6    14.3     0.3     3.3
Gv/N       3.309   3.634   0.424   1.623   0.158   2.461   2.180   6.713   0.158   1.545
Prob     0.07038 0.05171 0.82472 0.32646 0.95317 0.15496 0.19980 0.00252 0.95317 0.34886
         -------------------------------------------------------------------------------
              30      29      28      27      26      25      24      23      22      21
         -------------------------------------------------------------------------------
a_O           38      28      39      43      41      52      27      47      49      49
        ( 17.8) ( 13.1) ( 18.3) ( 20.2) ( 19.2) ( 24.4) ( 12.7) ( 22.1) ( 23.0) ( 23.0)
a_E         40.4    40.4    40.4    40.4    40.4    40.4    40.4    40.4    40.4    40.4
        ( 18.9) ( 18.9) ( 18.9) ( 18.9) ( 18.9) ( 18.9) ( 18.9) ( 18.9) ( 18.9) ( 18.9)
            -4.6   -20.5    -2.7     5.4     1.3    26.4   -21.7    14.3    19.0    19.0
          -2.150  -9.613  -1.255   2.558   0.606  12.373 -10.192   6.722   8.925   8.925
t_O           34      34      46      35      32      34      37      29      31      40
        ( 16.0) ( 16.0) ( 21.6) ( 16.4) ( 15.0) ( 16.0) ( 17.4) ( 13.6) ( 14.6) ( 18.8)
t_E         37.1    37.1    37.1    37.1    37.1    37.1    37.1    37.1    37.1    37.1
        ( 17.4) ( 17.4) ( 17.4) ( 17.4) ( 17.4) ( 17.4) ( 17.4) ( 17.4) ( 17.4) ( 17.4)
            -6.0    -6.0    19.7    -4.1    -9.5    -6.0    -0.2   -14.3   -11.2     6.0
          -2.803  -2.803   9.264  -1.933  -4.460  -2.803  -0.112  -6.722  -5.244   2.807
g_O           73      57      68      64      58      49      63      57      49      49
        ( 34.3) ( 26.8) ( 31.9) ( 30.0) ( 27.2) ( 23.0) ( 29.6) ( 26.8) ( 23.0) ( 23.0)
g_E         62.9    62.9    62.9    62.9    62.9    62.9    62.9    62.9    62.9    62.9
        ( 29.5) ( 29.5) ( 29.5) ( 29.5) ( 29.5) ( 29.5) ( 29.5) ( 29.5) ( 29.5) ( 29.5)
            21.7   -11.3    10.6     2.2    -9.4   -24.5     0.2   -11.3   -24.5   -24.5
          10.185  -5.289   4.958   1.023  -4.434 -11.504   0.075  -5.289 -11.504 -11.504
c_O           68      94      60      71      82      78      86      80      84      75
        ( 31.9) ( 44.1) ( 28.2) ( 33.3) ( 38.5) ( 36.6) ( 40.4) ( 37.6) ( 39.4) ( 35.2)
c_E         72.6    72.6    72.6    72.6    72.6    72.6    72.6    72.6    72.6    72.6
        ( 34.1) ( 34.1) ( 34.1) ( 34.1) ( 34.1) ( 34.1) ( 34.1) ( 34.1) ( 34.1) ( 34.1)
            -8.9    48.6   -22.9    -3.2    20.0    11.2    29.1    15.5    24.5     4.9
          -4.179  22.801 -10.739  -1.486   9.375   5.254  13.678   7.291  11.504   2.290

N            213     213     213     213     213     213     213     213     213     213
X2           2.3    10.9     4.8     0.3     2.3     7.1     6.9     4.2     7.7     5.2
Gvalue       2.2    10.9     4.7     0.3     2.3     7.1     7.3     4.3     7.8     5.4
Gv/N       1.053   5.097   2.227   0.163   1.086   3.320   3.449   2.002   3.680   2.517
Prob     0.52345 0.01253 0.19152 0.95107 0.50983 0.06965 0.06165 0.23430 0.04947 0.14712
         -------------------------------------------------------------------------------
              20      19      18      17      16      15      14      13      12      11
         -------------------------------------------------------------------------------
a_O           52      51      46      44      43      40      55      48      52      73
        ( 24.4) ( 23.9) ( 21.6) ( 20.7) ( 20.2) ( 18.8) ( 25.8) ( 22.5) ( 24.4) ( 34.3)
a_E         40.4    40.4    40.4    40.4    40.4    40.4    40.4    40.4    40.4    40.4
        ( 18.9) ( 18.9) ( 18.9) ( 18.9) ( 18.9) ( 18.9) ( 18.9) ( 18.9) ( 18.9) ( 18.9)
            26.4    23.9    12.0     7.6     5.4    -0.7    34.0    16.6    26.4    86.5
          12.373  11.205   5.650   3.568   2.558  -0.337  15.983   7.813  12.373  40.621
t_O           34      52      37      32      36      40      38      35      39      33
        ( 16.0) ( 24.4) ( 17.4) ( 15.0) ( 16.9) ( 18.8) ( 17.8) ( 16.4) ( 18.3) ( 15.5)
t_E         37.1    37.1    37.1    37.1    37.1    37.1    37.1    37.1    37.1    37.1
        ( 17.4) ( 17.4) ( 17.4) ( 17.4) ( 17.4) ( 17.4) ( 17.4) ( 17.4) ( 17.4) ( 17.4)
            -6.0    35.1    -0.2    -9.5    -2.2     6.0     1.8    -4.1     3.9    -7.8
          -2.803  16.459  -0.112  -4.460  -1.036   2.807   0.836  -1.933   1.809  -3.645
g_O           54      30      44      60      41      47      50      50      58      39
        ( 25.4) ( 14.1) ( 20.7) ( 28.2) ( 19.2) ( 22.1) ( 23.5) ( 23.5) ( 27.2) ( 18.3)
g_E         62.9    62.9    62.9    62.9    62.9    62.9    62.9    62.9    62.9    62.9
        ( 29.5) ( 29.5) ( 29.5) ( 29.5) ( 29.5) ( 29.5) ( 29.5) ( 29.5) ( 29.5) ( 29.5)
           -16.5   -44.4   -31.5    -5.7   -35.1   -27.4   -23.0   -23.0    -9.4   -37.3
          -7.752 -20.864 -14.777  -2.677 -16.488 -12.874 -10.791 -10.791  -4.434 -17.515
c_O           73      80      86      77      93      86      70      80      64      68
        ( 34.3) ( 37.6) ( 40.4) ( 36.2) ( 43.7) ( 40.4) ( 32.9) ( 37.6) ( 30.0) ( 31.9)
c_E         72.6    72.6    72.6    72.6    72.6    72.6    72.6    72.6    72.6    72.6
        ( 34.1) ( 34.1) ( 34.1) ( 34.1) ( 34.1) ( 34.1) ( 34.1) ( 34.1) ( 34.1) ( 34.1)
             0.8    15.5    29.1     9.1    46.1    29.1    -5.1    15.5   -16.1    -8.9
           0.377   7.291  13.678   4.254  21.624  13.678  -2.397   7.291  -7.577  -4.179

N            213     213     213     213     213     213     213     213     213     213
X2           4.9    26.7     9.0     1.4    13.6     6.7     8.1     5.0     4.9    36.2
Gvalue       4.7    30.0     9.5     1.5    14.2     7.0     7.7     5.1     4.6    32.5
Gv/N       2.195  14.091   4.438   0.685   6.659   3.274   3.632   2.381   2.171  15.281
Prob     0.19719 0.00000 0.02384 0.69175 0.00267 0.07275 0.05181 0.16660 0.20146 0.00000
         -------------------------------------------------------------------------------
              10       9       8       7       6       5       4       3       2       1
         -------------------------------------------------------------------------------
a_O           40      66      59      46      35      46      48      49      26      39
        ( 18.8) ( 31.0) ( 27.7) ( 21.6) ( 16.4) ( 21.6) ( 22.5) ( 23.0) ( 12.2) ( 18.3)
a_E         40.4    40.4    40.4    40.4    40.4    40.4    40.4    40.4    40.4    40.4
        ( 18.9) ( 18.9) ( 18.9) ( 18.9) ( 18.9) ( 18.9) ( 18.9) ( 18.9) ( 18.9) ( 18.9)
            -0.7    64.9    44.8    12.0   -10.0    12.0    16.6    19.0   -22.9    -2.7
          -0.337  30.479  21.035   5.650  -4.683   5.650   7.813   8.925 -10.736  -1.255
t_O           39      40      44      45      41      36      28      30      45      35
        ( 18.3) ( 18.8) ( 20.7) ( 21.1) ( 19.2) ( 16.9) ( 13.1) ( 14.1) ( 21.1) ( 16.4)
t_E         37.1    37.1    37.1    37.1    37.1    37.1    37.1    37.1    37.1    37.1
        ( 17.4) ( 17.4) ( 17.4) ( 17.4) ( 17.4) ( 17.4) ( 17.4) ( 17.4) ( 17.4) ( 17.4)
             3.9     6.0    15.0    17.3     8.2    -2.2   -15.8   -12.8    17.3    -4.1
           1.809   2.807   7.025   8.134   3.827  -1.036  -7.413  -5.999   8.134  -1.933
g_O           48      43      42      38      50      59      69      69      53      42
        ( 22.5) ( 20.2) ( 19.7) ( 17.8) ( 23.5) ( 27.7) ( 32.4) ( 32.4) ( 24.9) ( 19.7)
g_E         62.9    62.9    62.9    62.9    62.9    62.9    62.9    62.9    62.9    62.9
        ( 29.5) ( 29.5) ( 29.5) ( 29.5) ( 29.5) ( 29.5) ( 29.5) ( 29.5) ( 29.5) ( 29.5)
           -26.0   -32.7   -34.0   -38.3   -23.0    -7.6    12.7    12.7   -18.2   -34.0
         -12.199 -15.370 -15.940 -17.993 -10.791  -3.564   5.976   5.976  -8.538 -15.940
c_O           86      64      68      84      87      72      68      65      89      97
        ( 40.4) ( 30.0) ( 31.9) ( 39.4) ( 40.8) ( 33.8) ( 31.9) ( 30.5) ( 41.8) ( 45.5)
c_E         72.6    72.6    72.6    72.6    72.6    72.6    72.6    72.6    72.6    72.6
        ( 34.1) ( 34.1) ( 34.1) ( 34.1) ( 34.1) ( 34.1) ( 34.1) ( 34.1) ( 34.1) ( 34.1)
            29.1   -16.1    -8.9    24.5    31.5    -1.2    -8.9   -14.4    36.3    56.2
          13.678  -7.577  -4.179  11.504  14.781  -0.561  -4.179  -6.749  17.020  26.390

N            213     213     213     213     213     213     213     213     213     213
X2           6.1    23.8    17.1    14.1     6.6     1.1     4.6     4.6    12.1    15.3
Gvalue       6.3    22.0    16.9    15.5     6.7     1.0     4.7     4.6    12.5    15.5
Gv/N       2.952  10.339   7.940   7.295   3.135   0.489   2.198   2.154   5.881   7.262
Prob     0.09845 0.00006 0.00074 0.00141 0.08291 0.79100 0.19673 0.20466 0.00578 0.00146
         -------------------------------------------------------------------------------

CDS_INITIAL:
         -------------------------------------------------------------------------------
```

	1	2	3	4	5	6	7	8	9	10
a_O	180	0	0	86	54	44	54	63	46	61
	(84.5)	(0.0)	(0.0)	(40.4)	(25.4)	(20.7)	(25.4)	(29.6)	(21.6)	(28.6)
a_E	36.2	52.6	11.6	36.2	52.6	11.6	36.2	52.6	11.6	36.2
	(17.0)	(24.7)	(5.4)	(17.0)	(24.7)	(5.4)	(17.0)	(24.7)	(5.4)	(17.0)
	577.0	0.0	0.0	148.7	2.9	117.3	43.1	22.8	126.7	63.5
	270.905	0.000	0.000	69.789	1.373	55.073	20.225	10.720	59.497	29.828
t_O	8	213	0	15	33	34	23	39	27	27
	(3.8)	(100.0)	(0.0)	(7.0)	(15.5)	(16.0)	(10.8)	(18.3)	(12.7)	(12.7)
t_E	25.0	60.5	18.6	25.0	60.5	18.6	25.0	60.5	18.6	25.0
	(11.7)	(28.4)	(8.7)	(11.7)	(28.4)	(8.7)	(11.7)	(28.4)	(8.7)	(11.7)
	-18.2	535.9	0.0	-15.3	-40.1	41.2	-3.8	-34.3	20.2	4.2
	-8.557	251.592	0.000	-7.191	-18.803	19.330	-1.795	-16.104	9.506	1.957
g_O	25	0	213	54	52	41	74	44	72	56
	(11.7)	(0.0)	(100.0)	(25.4)	(24.4)	(19.2)	(34.7)	(20.7)	(33.8)	(26.3)
g_E	84.1	48.1	86.1	84.1	48.1	86.1	84.1	48.1	86.1	84.1
	(39.5)	(22.6)	(40.4)	(39.5)	(22.6)	(40.4)	(39.5)	(22.6)	(40.4)	(39.5)
	-60.7	0.0	385.9	-47.9	8.1	-60.8	-19.0	-7.8	-25.8	-45.6
	-28.489	0.000	181.160	-22.488	3.825	-28.562	-8.924	-3.665	-12.090	-21.408
c_O	0	0	0	58	74	94	62	67	68	69
	(0.0)	(0.0)	(0.0)	(27.2)	(34.7)	(44.1)	(29.1)	(31.5)	(31.9)	(32.4)
c_E	67.6	51.8	96.7	67.6	51.8	96.7	67.6	51.8	96.7	67.6
	(31.7)	(24.3)	(45.4)	(31.7)	(24.3)	(45.4)	(31.7)	(24.3)	(45.4)	(31.7)
	0.0	0.0	0.0	-17.8	52.7	-5.4	-10.8	34.4	-47.9	2.8
	0.000	0.000	0.000	-8.363	24.758	-2.538	-5.058	16.165	-22.509	1.302
N	213	213	213	213	213	213	213	213	213	213
X2	691.1	536.4	313.9	84.5	22.4	127.0	10.6	14.5	116.7	26.5
Gvalue	498.1	535.9	385.9	67.6	23.8	92.2	9.5	15.2	73.3	24.9
Gv/N	233.859	251.592	181.160	31.746	11.153	43.303	4.448	7.115	34.403	11.679
Prob	0.00000	0.00000	0.00000	0.00000	0.00003	0.00000	0.02360	0.00169	0.00000	0.00002

	11	12	13	14	15	16	17	18	19	20
a_O	48	35	48	48	28	59	46	32	54	50
	(22.5)	(16.4)	(22.5)	(22.5)	(13.1)	(27.7)	(21.6)	(15.0)	(25.4)	(23.5)
a_E	52.6	11.6	36.2	52.6	11.6	36.2	52.6	11.6	36.2	52.6
	(24.7)	(5.4)	(17.0)	(24.7)	(5.4)	(17.0)	(24.7)	(5.4)	(17.0)	(24.7)
	-8.7	77.3	27.0	-8.7	49.3	57.5	-12.3	64.9	43.1	-5.0
	-4.088	36.288	12.669	-4.088	23.163	27.004	-5.756	30.485	20.225	-2.342
t_O	47	42	29	52	28	22	56	27	32	56
	(22.1)	(19.7)	(13.6)	(24.4)	(13.1)	(10.3)	(26.3)	(12.7)	(15.0)	(26.3)
t_E	60.5	18.6	25.0	60.5	18.6	25.0	60.5	18.6	25.0	60.5
	(28.4)	(8.7)	(11.7)	(28.4)	(8.7)	(11.7)	(28.4)	(8.7)	(11.7)	(28.4)
	-23.8	68.6	8.6	-15.8	23.0	-5.6	-8.7	20.2	15.8	-8.7
	-11.173	32.211	4.048	-7.426	10.814	-2.636	-4.100	9.506	7.425	-4.100
g_O	56	50	74	41	62	68	46	43	66	44
	(26.3)	(23.5)	(34.7)	(19.2)	(29.1)	(31.9)	(21.6)	(20.2)	(31.0)	(20.7)
g_E	48.1	86.1	84.1	48.1	86.1	84.1	48.1	86.1	84.1	48.1
	(22.6)	(40.4)	(39.5)	(22.6)	(40.4)	(39.5)	(22.6)	(40.4)	(39.5)	(22.6)
	17.1	-54.3	-19.0	-13.1	-40.7	-29.0	-4.1	-59.7	-32.1	-7.8
	8.016	-25.515	-8.924	-6.134	-19.116	-13.599	-1.912	-28.032	-15.049	-3.665
c_O	62	86	62	72	95	64	65	111	61	63
	(29.1)	(40.4)	(29.1)	(33.8)	(44.6)	(30.0)	(30.5)	(52.1)	(28.6)	(29.6)
c_E	51.8	96.7	67.6	51.8	96.7	67.6	51.8	96.7	67.6	51.8
	(24.3)	(45.4)	(31.7)	(24.3)	(45.4)	(31.7)	(24.3)	(45.4)	(31.7)	(24.3)
	22.2	-20.2	-10.8	47.4	-3.5	-7.1	29.5	30.5	-12.6	24.6
	10.443	-9.504	-5.058	22.237	-1.621	-3.313	13.832	14.329	-5.908	11.558
N	213	213	213	213	213	213	213	213	213	213
X2	6.7	93.1	6.2	10.5	34.8	17.9	4.6	63.4	15.2	3.2
Gvalue	6.8	71.3	5.8	9.8	28.2	15.9	4.4	56.0	14.3	3.1
Gv/N	3.197	33.480	2.736	4.588	13.241	7.456	2.064	26.287	6.693	1.450
Prob	0.07821	0.00000	0.12030	0.02060	0.00000	0.00120	0.22178	0.00000	0.00258	0.37815

	21	22	23	24	25	26	27	28	29	30
a_O	36	54	56	28	44	52	18	43	44	28
	(16.9)	(25.4)	(26.3)	(13.1)	(20.7)	(24.4)	(8.5)	(20.2)	(20.7)	(13.1)
a_E	11.6	36.2	52.6	11.6	36.2	52.6	11.6	36.2	52.6	11.6
	(5.4)	(17.0)	(24.7)	(5.4)	(17.0)	(24.7)	(5.4)	(17.0)	(24.7)	(5.4)
	81.5	43.1	7.1	49.3	17.1	-1.1	15.8	14.7	-15.6	49.3
	38.277	20.225	3.336	23.163	8.019	-0.521	7.423	6.908	-7.343	23.163
t_O	21	25	58	20	28	50	22	24	61	19
	(9.9)	(11.7)	(27.2)	(9.4)	(13.1)	(23.5)	(10.3)	(11.3)	(28.6)	(8.9)
t_E	18.6	25.0	60.5	18.6	25.0	60.5	18.6	25.0	60.5	18.6
	(8.7)	(11.7)	(28.4)	(8.7)	(11.7)	(28.4)	(8.7)	(11.7)	(28.4)	(8.7)
	5.2	0.0	-5.0	3.0	6.4	-19.1	7.5	-1.9	0.9	0.9
	2.438	0.006	-2.336	1.406	2.986	-8.982	3.515	-0.914	0.432	0.420
g_O	58	74	36	69	65	46	73	68	61	73
	(27.2)	(34.7)	(16.9)	(32.4)	(30.5)	(21.6)	(34.3)	(31.9)	(28.6)	(34.3)
g_E	86.1	84.1	48.1	86.1	84.1	48.1	86.1	84.1	48.1	86.1
	(40.4)	(39.5)	(22.6)	(40.4)	(39.5)	(22.6)	(40.4)	(39.5)	(22.6)	(40.4)
	-45.8	-19.0	-20.8	-30.6	-33.6	-4.1	-24.1	-29.0	29.0	-24.1
	-21.514	-8.924	-9.782	-14.343	-15.753	-1.912	-11.312	-13.599	13.630	-11.312
c_O	98	60	63	96	76	65	100	78	47	93
	(46.0)	(28.2)	(29.6)	(45.1)	(35.7)	(30.5)	(46.9)	(36.6)	(22.1)	(43.7)
c_E	96.7	67.6	51.8	96.7	67.6	51.8	96.7	67.6	51.8	96.7
	(45.4)	(31.7)	(24.3)	(45.4)	(31.7)	(24.3)	(45.4)	(31.7)	(24.3)	(45.4)
	2.5	-14.4	24.6	-1.5	17.7	29.5	6.6	22.3	-9.2	-7.3
	1.189	-6.742	11.558	-0.694	8.329	13.832	3.110	10.451	-4.307	-3.445
N	213	213	213	213	213	213	213	213	213	213
X2	60.8	10.8	5.8	26.7	7.4	5.3	6.3	6.0	5.3	25.3
Gvalue	43.4	9.7	5.9	20.3	7.6	5.2	5.8	6.1	5.1	18.8
Gv/N	20.389	4.565	2.776	9.532	3.581	2.418	2.736	2.846	2.412	8.827
Prob	0.00000	0.02106	0.11593	0.00015	0.05437	0.16113	0.12027	0.10867	0.16198	0.00030

	31	32	33	34	35	36	37	38	39	40
a_O	38	43	18	45	45	14	38	52	12	37
	(17.8)	(20.2)	(8.5)	(21.1)	(21.1)	(6.6)	(17.8)	(24.4)	(5.6)	(17.4)
a_E	36.2	52.6	11.6	36.2	52.6	11.6	36.2	52.6	11.6	36.2
	(17.0)	(24.7)	(5.4)	(17.0)	(24.7)	(5.4)	(17.0)	(24.7)	(5.4)	(17.0)
	3.6	-17.3	15.8	19.5	-14.0	5.3	3.6	-1.1	0.8	1.5
	1.694	-8.104	7.423	9.150	-6.560	2.470	1.694	-0.521	0.380	0.723
t_O	31	59	25	34	58	26	31	56	21	40
	(14.6)	(27.7)	(11.7)	(16.0)	(27.2)	(12.2)	(14.6)	(26.3)	(9.9)	(18.8)
t_E	25.0	60.5	18.6	25.0	60.5	18.6	25.0	60.5	18.6	25.0
	(11.7)	(28.4)	(8.7)	(11.7)	(28.4)	(8.7)	(11.7)	(28.4)	(8.7)	(11.7)
	13.4	-3.0	14.9	20.9	-5.0	17.5	13.4	-8.7	5.2	37.6

	6.269	−1.429	6.995	9.824	−2.336	8.232	6.269	−4.100	2.438	17.662
g_O	69	53	85	68	61	64	66	57	81	66
	(32.4)	(24.9)	(39.9)	(31.9)	(28.6)	(30.0)	(31.0)	(26.8)	(38.0)	(31.0)
g_E	84.1	48.1	86.1	84.1	48.1	86.1	84.1	48.1	86.1	84.1
	(39.5)	(22.6)	(40.4)	(39.5)	(22.6)	(40.4)	(39.5)	(22.6)	(40.4)	(39.5)
	−27.4	10.3	−2.2	−29.0	29.0	−38.0	−32.1	19.4	−9.9	−32.1
	−12.853	4.846	−1.025	−13.599	13.630	−17.824	−15.049	9.106	−4.643	−15.049
c_O	75	58	85	66	49	109	78	48	99	70
	(35.2)	(27.2)	(39.9)	(31.0)	(23.0)	(51.2)	(36.6)	(22.5)	(46.5)	(32.9)
c_E	67.6	51.8	96.7	67.6	51.8	96.7	67.6	51.8	96.7	67.6
	(31.7)	(24.3)	(45.4)	(31.7)	(24.3)	(45.4)	(31.7)	(24.3)	(45.4)	(31.7)
	15.5	13.1	−22.0	−3.2	−5.5	26.0	22.3	−7.3	4.6	4.8
	7.287	6.137	−10.327	−1.510	−2.573	12.210	10.451	−3.450	2.145	2.266
N	213	213	213	213	213	213	213	213	213	213
X2	5.1	3.0	7.2	8.5	4.8	10.7	7.0	2.3	0.7	13.0
Gvalue	5.1	3.1	6.5	8.2	4.6	10.8	7.2	2.2	0.7	11.9
Gv/N	2.397	1.451	3.066	3.866	2.161	5.088	3.365	1.035	0.320	5.602
Prob	0.16429	0.37793	0.08846	0.04140	0.20327	0.01264	0.06677	0.53109	0.87742	0.00762

	41	42	43	44	45	46	47	48	49	50
a_O	39	16	36	39	10	30	52	15	38	40
	(18.3)	(7.5)	(16.9)	(18.3)	(4.7)	(14.1)	(24.4)	(7.0)	(17.8)	(18.8)
a_E	52.6	11.6	36.2	52.6	11.6	36.2	52.6	11.6	36.2	52.6
	(24.7)	(5.4)	(17.0)	(24.7)	(5.4)	(17.0)	(24.7)	(5.4)	(17.0)	(24.7)
	−23.3	10.3	−0.5	−23.3	−3.0	−11.3	−1.1	7.7	3.6	−21.8
	−10.926	4.829	−0.223	−10.926	−1.395	−5.321	−0.521	3.618	1.694	−10.255
t_O	73	20	32	67	22	37	60	33	29	58
	(34.3)	(9.4)	(15.0)	(31.5)	(10.3)	(17.4)	(28.2)	(15.5)	(13.6)	(27.2)
t_E	60.5	18.6	25.0	60.5	18.6	25.0	60.5	18.6	25.0	60.5
	(28.4)	(8.7)	(11.7)	(28.4)	(8.7)	(11.7)	(28.4)	(8.7)	(11.7)	(28.4)
	27.3	3.0	15.8	13.6	7.5	29.0	−1.1	38.0	8.6	−5.0
	12.827	1.406	7.425	6.377	3.515	13.629	−0.506	17.836	4.048	−2.336
g_O	55	60	78	53	72	85	44	72	83	52
	(25.8)	(28.2)	(36.6)	(24.9)	(33.8)	(39.9)	(20.7)	(33.8)	(39.0)	(24.4)
g_E	48.1	86.1	84.1	48.1	86.1	84.1	48.1	86.1	84.1	48.1
	(22.6)	(40.4)	(39.5)	(22.6)	(40.4)	(39.5)	(22.6)	(40.4)	(39.5)	(22.6)
	14.8	−43.3	−11.8	10.3	−25.8	1.7	−7.8	−25.8	−2.3	8.1
	6.942	−20.346	−5.550	4.846	−12.090	0.811	−3.665	−12.090	−1.064	3.825
c_O	46	117	67	54	109	61	57	93	63	63
	(21.6)	(54.9)	(31.5)	(25.4)	(51.2)	(28.6)	(26.8)	(43.7)	(29.6)	(29.6)
c_E	51.8	96.7	67.6	51.8	96.7	67.6	51.8	96.7	67.6	51.8
	(24.3)	(45.4)	(31.7)	(24.3)	(45.4)	(31.7)	(24.3)	(45.4)	(31.7)	(24.3)
	−11.0	44.5	−1.2	4.5	26.0	−12.6	10.9	−7.3	−8.9	24.6
	−5.145	20.887	−0.586	2.091	12.210	−5.908	5.101	−3.445	−4.193	11.558
N	213	213	213	213	213	213	213	213	213	213
X2	7.7	13.9	2.4	4.8	4.7	7.5	0.9	14.7	1.1	5.8
Gvalue	7.9	14.4	2.3	5.1	4.8	6.8	0.9	12.6	1.0	5.9
Gv/N	3.699	6.775	1.065	2.388	2.240	3.211	0.408	5.920	0.486	2.792
Prob	0.04860	0.00237	0.51842	0.16553	0.18927	0.07722	0.83284	0.00556	0.79295	0.11418

	51	52	53	54	55	56	57	58	59	60
a_O	22	38	36	9	40	47	17	42	44	9
	(10.3)	(17.8)	(16.9)	(4.2)	(18.8)	(22.1)	(8.0)	(19.7)	(20.7)	(4.2)
a_E	11.6	36.2	52.6	11.6	36.2	52.6	11.6	36.2	52.6	11.6
	(5.4)	(17.0)	(24.7)	(5.4)	(17.0)	(24.7)	(5.4)	(17.0)	(24.7)	(5.4)
	28.2	3.6	−27.2	−4.6	7.9	−10.5	13.0	12.4	−15.6	−4.6
	13.218	1.694	−12.791	−2.146	3.710	−4.932	6.098	5.820	−7.343	−2.146
t_O	23	25	69	24	24	62	23	32	65	19
	(10.8)	(11.7)	(32.4)	(11.3)	(11.3)	(29.1)	(10.8)	(15.0)	(30.5)	(8.9)
t_E	18.6	25.0	60.5	18.6	25.0	60.5	18.6	25.0	60.5	18.6
	(8.7)	(11.7)	(28.4)	(8.7)	(11.7)	(28.4)	(8.7)	(11.7)	(28.4)	(8.7)
	9.9	0.0	18.0	12.3	−1.9	3.0	9.9	15.8	9.2	0.9
	4.635	0.006	8.473	5.795	−0.914	1.386	4.635	7.425	4.337	0.420
g_O	79	67	46	82	86	46	79	75	51	83
	(37.1)	(31.5)	(21.6)	(38.5)	(40.4)	(21.6)	(37.1)	(35.2)	(23.9)	(39.0)
g_E	86.1	84.1	48.1	86.1	84.1	48.1	86.1	84.1	48.1	86.1
	(40.4)	(39.5)	(22.6)	(40.4)	(39.5)	(22.6)	(40.4)	(39.5)	(22.6)	(40.4)
	−13.6	−30.5	−4.1	−8.0	3.8	−4.1	−13.6	−17.3	6.0	−6.1
	−6.383	−14.331	−1.912	−3.755	1.765	−1.912	−6.383	−8.099	2.821	−2.856
c_O	89	83	62	98	63	58	94	64	53	102
	(41.8)	(39.0)	(29.1)	(46.0)	(29.6)	(27.2)	(44.1)	(30.0)	(24.9)	(47.9)
c_E	96.7	67.6	51.8	96.7	67.6	51.8	96.7	67.6	51.8	96.7
	(45.4)	(31.7)	(24.3)	(45.4)	(31.7)	(24.3)	(45.4)	(31.7)	(24.3)	(45.4)
	−14.8	34.0	22.2	2.5	−8.9	13.1	−5.4	−7.1	2.4	10.8
	−6.970	15.963	10.443	1.189	−4.193	6.137	−2.538	−3.313	1.122	5.069
N	213	213	213	213	213	213	213	213	213	213
X2	11.6	7.1	8.5	2.4	0.8	1.5	4.2	4.1	1.9	1.0
Gvalue	9.6	7.1	9.0	2.3	0.8	1.4	3.9	3.9	2.0	1.0
Gv/N	4.500	3.332	4.213	1.083	0.367	0.679	1.813	1.832	0.937	0.487
Prob	0.02245	0.06887	0.02964	0.51121	0.85360	0.69487	0.27684	0.27214	0.57308	0.79233

	61	62	63	64	65	66	67	68	69	70
a_O	35	39	12	42	46	13	32	52	20	37
	(16.4)	(18.3)	(5.6)	(19.7)	(21.6)	(6.1)	(15.0)	(24.4)	(9.4)	(17.4)
a_E	36.2	52.6	11.6	36.2	52.6	11.6	36.2	52.6	11.6	36.2
	(17.0)	(24.7)	(5.4)	(17.0)	(24.7)	(5.4)	(17.0)	(24.7)	(5.4)	(17.0)
	−2.4	−23.3	0.8	12.4	−12.3	3.0	−8.0	−1.1	21.8	1.5
	−1.142	−10.926	0.380	5.820	−5.756	1.389	−3.737	−0.521	10.227	0.723
t_O	19	83	24	22	59	22	14	62	19	28
	(8.9)	(39.0)	(11.3)	(10.3)	(27.7)	(10.3)	(6.6)	(29.1)	(8.9)	(13.1)
t_E	25.0	60.5	18.6	25.0	60.5	18.6	25.0	60.5	18.6	25.0
	(11.7)	(28.4)	(8.7)	(11.7)	(28.4)	(8.7)	(11.7)	(28.4)	(8.7)	(11.7)
	−10.4	52.4	12.3	−5.6	−3.0	7.5	−16.2	3.0	0.9	6.4
	−4.892	24.589	5.795	−2.636	−1.429	3.515	−7.619	1.386	0.420	2.986
g_O	89	44	90	87	45	84	99	40	95	79
	(41.8)	(20.7)	(42.3)	(40.8)	(21.1)	(39.4)	(46.5)	(18.8)	(44.6)	(37.1)
g_E	84.1	48.1	86.1	84.1	48.1	86.1	84.1	48.1	86.1	84.1
	(39.5)	(22.6)	(40.4)	(39.5)	(22.6)	(40.4)	(39.5)	(22.6)	(40.4)	(39.5)
	10.0	−7.8	8.0	5.8	−6.0	−4.1	32.2	−14.7	18.7	−10.0
	4.692	−3.665	3.745	2.730	−2.799	−1.946	15.117	−6.912	8.776	−4.677
c_O	70	47	87	62	63	94	68	59	79	69
	(32.9)	(22.1)	(40.8)	(29.1)	(29.6)	(44.1)	(31.9)	(27.7)	(37.1)	(32.4)
c_E	67.6	51.8	96.7	67.6	51.8	96.7	67.6	51.8	96.7	67.6
	(31.7)	(24.3)	(45.4)	(31.7)	(24.3)	(45.4)	(31.7)	(24.3)	(45.4)	(31.7)
	4.8	−9.2	−18.5	−10.8	24.6	−5.4	0.7	15.3	−32.0	2.8
	2.266	−4.307	−8.670	−5.058	11.558	−2.538	0.351	7.190	−15.028	1.302

N	213	213	213	213	213	213	213	213	213	213
X2	1.8	12.6	2.8	1.8	3.5	0.9	8.0	2.4	10.3	0.7
Gvalue	2.0	12.1	2.7	1.8	3.4	0.9	8.8	2.4	9.4	0.7
Gv/N	0.924	5.691	1.250	0.856	1.574	0.420	4.113	1.143	4.394	0.334
Prob	0.57889	0.00698	0.44658	0.60994	0.34049	0.82677	0.03266	0.48719	0.02487	0.87034

	71	72	73	74	75	76	77	78	79	80
a_O	55	18	34	44	13	48	55	11	33	50
	(25.8)	(8.5)	(16.0)	(20.7)	(6.1)	(22.5)	(25.8)	(5.2)	(15.5)	(23.5)
a_E	52.6	11.6	36.2	52.6	11.6	36.2	52.6	11.6	36.2	52.6
	(24.7)	(5.4)	(17.0)	(24.7)	(5.4)	(17.0)	(24.7)	(5.4)	(17.0)	(24.7)
	5.0	15.8	-4.3	-15.6	3.0	27.0	5.0	-1.2	-6.2	-5.0
	2.346	7.423	-2.035	-7.343	1.389	12.669	2.346	-0.550	-2.900	-2.342
t_O	58	21	27	70	21	25	52	17	35	59
	(27.2)	(9.9)	(12.7)	(32.9)	(9.9)	(11.7)	(24.4)	(8.0)	(16.4)	(27.7)
t_E	60.5	18.6	25.0	60.5	18.6	25.0	60.5	18.6	25.0	60.5
	(28.4)	(8.7)	(11.7)	(28.4)	(8.7)	(11.7)	(28.4)	(8.7)	(11.7)	(28.4)
	-5.0	5.2	4.2	20.3	5.2	0.0	-15.8	-3.0	23.6	-3.0
	-2.336	2.438	1.957	9.541	2.438	0.006	-7.426	-1.400	11.066	-1.429
g_O	46	84	85	44	83	86	43	78	69	48
	(21.6)	(39.4)	(39.9)	(20.7)	(39.0)	(40.4)	(20.2)	(36.6)	(32.4)	(22.5)
g_E	48.1	86.1	84.1	48.1	86.1	84.1	48.1	86.1	84.1	48.1
	(22.6)	(40.4)	(39.5)	(22.6)	(40.4)	(39.5)	(22.6)	(40.4)	(39.5)	(22.6)
	-4.1	-4.1	1.7	-7.8	-6.1	3.8	-9.6	-15.4	-27.4	-0.2
	-1.912	-1.946	0.811	-3.665	-2.856	1.765	-4.510	-7.235	-12.853	-0.077
c_O	54	90	67	55	96	54	63	107	76	56
	(25.4)	(42.3)	(31.5)	(25.8)	(45.1)	(25.4)	(29.6)	(50.2)	(35.7)	(26.3)
c_E	51.8	96.7	67.6	51.8	96.7	67.6	51.8	96.7	67.6	51.8
	(24.3)	(45.4)	(31.7)	(24.3)	(45.4)	(31.7)	(24.3)	(45.4)	(31.7)	(24.3)
	4.5	-13.0	-1.2	6.6	-1.5	-24.3	24.6	21.6	17.7	8.7
	2.091	-6.104	-0.586	3.077	-0.694	-11.410	11.558	10.126	8.329	4.081
N	213	213	213	213	213	213	213	213	213	213
X2	0.4	4.4	0.3	3.4	0.6	6.6	4.3	2.0	8.1	0.5
Gvalue	0.4	3.9	0.3	3.4	0.6	6.5	4.2	2.0	7.8	0.5
Gv/N	0.189	1.810	0.147	1.611	0.276	3.030	1.968	0.941	3.642	0.232
Prob	0.93982	0.27744	0.95765	0.32990	0.89898	0.09151	0.24158	0.57149	0.05131	0.92000

	81	82	83	84	85	86	87	88	89	90
a_O	22	30	45	14	35	45	10	33	57	19
	(10.3)	(14.1)	(21.1)	(6.6)	(16.4)	(21.1)	(4.7)	(15.5)	(26.8)	(8.9)
a_E	11.6	36.2	52.6	11.6	36.2	52.6	11.6	36.2	52.6	11.6
	(5.4)	(17.0)	(24.7)	(5.4)	(17.0)	(24.7)	(5.4)	(17.0)	(24.7)	(5.4)
	28.2	-11.3	-14.0	5.3	-2.4	-14.0	-3.0	-6.2	9.2	18.7
	13.218	-5.321	-6.560	2.470	-1.142	-6.560	-1.395	-2.900	4.343	8.800
t_O	21	30	71	24	26	57	19	21	62	14
	(9.9)	(14.1)	(33.3)	(11.3)	(12.2)	(26.8)	(8.9)	(9.9)	(29.1)	(6.6)
t_E	18.6	25.0	60.5	18.6	25.0	60.5	18.6	25.0	60.5	18.6
	(8.7)	(11.7)	(28.4)	(8.7)	(11.7)	(28.4)	(8.7)	(11.7)	(28.4)	(8.7)
	5.2	11.0	22.6	12.3	2.1	-6.9	0.9	-7.3	3.0	-7.9
	2.438	5.143	10.623	5.795	0.963	-3.226	0.420	-3.433	1.386	-3.705
g_O	84	72	50	88	78	57	97	90	37	93
	(39.4)	(33.8)	(23.5)	(41.3)	(36.6)	(26.8)	(45.5)	(42.3)	(17.4)	(43.7)
g_E	86.1	84.1	48.1	86.1	84.1	48.1	86.1	84.1	48.1	86.1
	(40.4)	(39.5)	(22.6)	(40.4)	(39.5)	(22.6)	(40.4)	(39.5)	(22.6)	(40.4)
	-4.1	-22.4	3.9	3.8	-11.8	19.4	23.1	12.1	-19.4	14.3
	-1.946	-10.535	1.836	1.805	-5.550	9.106	10.858	5.689	-9.102	6.733
c_O	86	81	47	87	74	54	87	69	57	87
	(40.4)	(38.0)	(22.1)	(40.8)	(34.7)	(25.4)	(40.8)	(32.4)	(26.8)	(40.8)
c_E	96.7	67.6	51.8	96.7	67.6	51.8	96.7	67.6	51.8	96.7
	(45.4)	(31.7)	(24.3)	(45.4)	(31.7)	(24.3)	(45.4)	(31.7)	(24.3)	(45.4)
	-20.2	29.2	-9.2	-18.5	13.3	4.5	-18.5	2.8	10.9	-18.5
	-9.504	13.723	-4.307	-8.670	6.257	2.091	-8.670	1.302	5.101	-8.670
N	213	213	213	213	213	213	213	213	213	213
X2	10.9	6.5	3.4	3.1	1.1	3.0	2.6	1.4	3.5	7.4
Gvalue	9.0	6.4	3.4	3.0	1.1	3.0	2.6	1.4	3.7	6.7
Gv/N	4.205	3.010	1.592	1.400	0.528	1.411	1.213	0.657	1.727	3.158
Prob	0.02986	0.09323	0.33505	0.39448	0.77111	0.39083	0.46035	0.70552	0.29832	0.08113

	91	92	93	94	95	96	97	98	99	100
a_O	30	56	12	37	52	9	33	58	16	33
	(14.1)	(26.3)	(5.6)	(17.4)	(24.4)	(4.2)	(15.5)	(27.2)	(7.5)	(15.5)
a_E	36.2	52.6	11.6	36.2	52.6	11.6	36.2	52.6	11.6	36.2
	(17.0)	(24.7)	(5.4)	(17.0)	(24.7)	(5.4)	(17.0)	(24.7)	(5.4)	(17.0)
	-11.3	7.1	0.8	1.5	-1.1	-4.6	-6.2	11.4	10.3	-6.2
	-5.321	3.336	0.380	0.723	-0.521	-2.146	-2.900	5.366	4.829	-2.900
t_O	27	63	26	19	71	21	20	62	17	15
	(12.7)	(29.6)	(12.2)	(8.9)	(33.3)	(9.9)	(9.4)	(29.1)	(8.0)	(7.0)
t_E	25.0	60.5	18.6	25.0	60.5	18.6	25.0	60.5	18.6	25.0
	(11.7)	(28.4)	(8.7)	(11.7)	(28.4)	(8.7)	(11.7)	(28.4)	(8.7)	(11.7)
	4.2	5.0	17.5	-10.4	22.6	5.2	-8.9	3.0	-3.0	-15.3
	1.957	2.355	8.232	-4.892	10.623	2.438	-4.186	1.386	-1.400	-7.191
g_O	95	35	79	77	38	91	89	48	93	88
	(44.6)	(16.4)	(37.1)	(36.2)	(17.8)	(42.7)	(41.8)	(22.5)	(43.7)	(41.3)
g_E	84.1	48.1	86.1	84.1	48.1	86.1	84.1	48.1	86.1	84.1
	(39.5)	(22.6)	(40.4)	(39.5)	(22.6)	(40.4)	(39.5)	(22.6)	(40.4)	(39.5)
	23.1	-22.2	-13.6	-13.7	-17.9	10.1	10.0	-0.2	14.3	7.9
	10.828	-10.436	-6.383	-6.412	-8.397	4.731	4.692	-0.077	6.733	3.705
c_O	61	59	96	80	52	92	71	45	87	77
	(28.6)	(27.7)	(45.1)	(37.6)	(24.4)	(43.2)	(33.3)	(21.1)	(40.8)	(36.2)
c_E	67.6	51.8	96.7	67.6	51.8	96.7	67.6	51.8	96.7	67.6
	(31.7)	(24.3)	(45.4)	(31.7)	(24.3)	(45.4)	(31.7)	(24.3)	(45.4)	(31.7)
	-12.6	15.3	-1.5	26.9	0.4	-9.2	6.9	-12.7	-18.5	20.0
	-5.908	7.190	-0.694	12.621	0.171	-4.341	3.244	-5.961	-8.670	9.384
N	213	213	213	213	213	213	213	213	213	213
X2	3.3	4.9	3.6	4.3	3.9	1.4	1.7	1.5	3.3	5.8
Gvalue	3.3	5.2	3.3	4.3	4.0	1.5	1.8	1.5	3.2	6.4
Gv/N	1.556	2.444	1.536	2.040	1.877	0.681	0.850	0.713	1.492	2.998
Prob	0.34560	0.15730	0.35161	0.22651	0.26179	0.69359	0.61255	0.67780	0.36491	0.09428

Aful_DSM4304
5UTR:

	100	99	98	97	96	95	94	93	92	91
a_O	249	224	252	224	238	226	250	219	221	226
	(29.5)	(26.5)	(29.9)	(26.5)	(28.2)	(26.8)	(29.6)	(25.9)	(26.2)	(26.8)
a_E	260.2	260.2	260.2	260.2	260.2	260.2	260.2	260.2	260.2	260.2
	(30.8)	(30.8)	(30.8)	(30.8)	(30.8)	(30.8)	(30.8)	(30.8)	(30.8)	(30.8)
	-21.9	-67.1	-16.1	-67.1	-42.4	-63.7	-20.0	-75.5	-72.1	-63.7
	-2.592	-7.948	-1.908	-7.948	-5.025	-7.543	-2.365	-8.942	-8.547	-7.543
t_O	232	221	238	237	220	209	235	241	234	223
	(27.5)	(26.2)	(28.2)	(28.1)	(26.1)	(24.8)	(27.8)	(28.6)	(27.7)	(26.4)
t_E	251.2	251.2	251.2	251.2	251.2	251.2	251.2	251.2	251.2	251.2
	(29.8)	(29.8)	(29.8)	(29.8)	(29.8)	(29.8)	(29.8)	(29.8)	(29.8)	(29.8)
	-37.0	-56.7	-25.8	-27.7	-58.4	-76.9	-31.4	-20.1	-33.3	-53.2
	-4.380	-6.716	-3.053	-3.277	-6.922	-9.116	-3.721	-2.376	-3.942	-6.301
g_O	196	233	176	186	189	212	172	198	219	202
	(23.2)	(27.6)	(20.9)	(22.0)	(22.4)	(25.1)	(20.4)	(23.5)	(25.9)	(23.9)
g_E	163.4	163.4	163.4	163.4	163.4	163.4	163.4	163.4	163.4	163.4
	(19.4)	(19.4)	(19.4)	(19.4)	(19.4)	(19.4)	(19.4)	(19.4)	(19.4)	(19.4)
	71.2	165.2	26.1	48.1	54.9	110.3	17.6	76.0	128.2	85.6
	8.438	19.578	3.088	5.699	6.508	13.069	2.081	9.000	15.186	10.139
c_O	167	166	178	197	197	197	187	186	170	193
	(19.8)	(19.7)	(21.1)	(23.3)	(23.3)	(23.3)	(22.2)	(22.0)	(20.1)	(22.9)
c_E	169.1	169.1	169.1	169.1	169.1	169.1	169.1	169.1	169.1	169.1
	(20.0)	(20.0)	(20.0)	(20.0)	(20.0)	(20.0)	(20.0)	(20.0)	(20.0)	(20.0)
	-4.3	-6.2	18.2	60.1	60.1	60.1	37.5	35.3	1.7	50.9
	-0.504	-0.737	2.154	7.118	7.118	7.118	4.448	4.188	0.204	6.035
N	844	844	844	844	844	844	844	844	844	844
X2	8.5	38.3	2.4	13.5	14.4	30.6	3.8	15.9	26.0	20.1
Gvalue	8.1	35.3	2.4	13.4	14.2	29.8	3.7	15.8	24.5	19.7
Gv/N	0.962	4.177	0.281	1.592	1.678	3.528	0.443	1.870	2.901	2.331
Prob	0.04357	0.00000	0.49965	0.00377	0.00269	0.00000	0.29091	0.00126	0.00002	0.00020

	90	89	88	87	86	85	84	83	82	81
a_O	212	242	247	253	243	244	213	244	211	223
	(25.1)	(28.7)	(29.3)	(30.0)	(28.8)	(28.9)	(25.2)	(28.9)	(25.0)	(26.4)
a_E	260.2	260.2	260.2	260.2	260.2	260.2	260.2	260.2	260.2	260.2
	(30.8)	(30.8)	(30.8)	(30.8)	(30.8)	(30.8)	(30.8)	(30.8)	(30.8)	(30.8)
	-86.8	-35.1	-25.7	-14.2	-33.2	-31.3	-85.2	-31.3	-88.4	-68.8
	-10.288	-4.154	-3.043	-1.678	-3.934	-3.712	-10.099	-3.712	-10.476	-8.149
t_O	237	225	227	215	240	219	232	241	252	233
	(28.1)	(26.7)	(26.9)	(25.5)	(28.4)	(25.9)	(27.5)	(28.6)	(29.9)	(27.6)
t_E	251.2	251.2	251.2	251.2	251.2	251.2	251.2	251.2	251.2	251.2
	(29.8)	(29.8)	(29.8)	(29.8)	(29.8)	(29.8)	(29.8)	(29.8)	(29.8)	(29.8)
	-27.7	-49.6	-46.1	-67.0	-22.0	-60.2	-37.0	-20.1	1.5	-35.1
	-3.277	-5.881	-5.458	-7.936	-2.603	-7.127	-4.380	-2.376	0.180	-4.161
g_O	213	210	188	194	205	186	203	195	188	206
	(25.2)	(24.9)	(22.3)	(23.0)	(24.3)	(22.0)	(24.1)	(23.1)	(22.3)	(24.4)
g_E	163.4	163.4	163.4	163.4	163.4	163.4	163.4	163.4	163.4	163.4
	(19.4)	(19.4)	(19.4)	(19.4)	(19.4)	(19.4)	(19.4)	(19.4)	(19.4)	(19.4)
	112.8	105.3	52.6	66.5	92.9	48.1	88.0	68.9	52.6	95.3
	13.368	12.474	6.237	7.880	11.006	5.699	10.427	8.158	6.237	11.297
c_O	182	167	182	182	156	195	196	164	193	182
	(21.6)	(19.8)	(21.6)	(21.6)	(18.5)	(23.1)	(23.2)	(19.4)	(22.9)	(21.6)
c_E	169.1	169.1	169.1	169.1	169.1	169.1	169.1	169.1	169.1	169.1
	(20.0)	(20.0)	(20.0)	(20.0)	(20.0)	(20.0)	(20.0)	(20.0)	(20.0)	(20.0)
	26.7	-4.3	26.7	26.7	-25.2	55.5	57.8	-10.1	50.9	26.7
	3.160	-0.504	3.160	3.160	-2.990	6.574	6.846	-1.199	6.035	3.160
N	844	844	844	844	844	844	844	844	844	844
X2	25.7	17.3	7.7	12.1	13.2	12.2	23.9	7.7	16.4	18.7
Gvalue	25.0	16.3	7.6	12.0	12.5	12.1	23.6	7.3	16.7	18.1
Gv/N	2.963	1.935	0.897	1.427	1.480	1.434	2.794	0.870	1.977	2.148
Prob	0.00002	0.00097	0.05580	0.00725	0.00588	0.00705	0.00003	0.06169	0.00082	0.00041

	80	79	78	77	76	75	74	73	72	71
a_O	238	240	242	252	247	228	243	248	212	215
	(28.2)	(28.4)	(28.7)	(29.9)	(29.3)	(27.0)	(28.8)	(29.4)	(25.1)	(25.5)
a_E	260.2	260.2	260.2	260.2	260.2	260.2	260.2	260.2	260.2	260.2
	(30.8)	(30.8)	(30.8)	(30.8)	(30.8)	(30.8)	(30.8)	(30.8)	(30.8)	(30.8)
	-42.4	-38.8	-35.1	-16.1	-25.7	-60.2	-33.2	-23.8	-86.8	-82.0
	-5.025	-4.592	-4.154	-1.908	-3.043	-7.133	-3.934	-2.818	-10.288	-9.718
t_O	248	254	244	208	246	254	233	238	249	247
	(29.4)	(30.1)	(28.9)	(24.6)	(29.1)	(30.1)	(27.6)	(28.2)	(29.5)	(29.3)
t_E	251.2	251.2	251.2	251.2	251.2	251.2	251.2	251.2	251.2	251.2
	(29.8)	(29.8)	(29.8)	(29.8)	(29.8)	(29.8)	(29.8)	(29.8)	(29.8)	(29.8)
	-6.4	5.6	-14.3	-78.6	-10.4	5.6	-35.1	-25.8	-4.5	-8.4
	-0.763	0.658	-1.691	-9.309	-1.229	0.658	-4.161	-3.053	-0.528	-0.996
g_O	180	179	187	204	181	186	190	180	203	204
	(21.3)	(21.2)	(22.2)	(24.2)	(21.4)	(22.0)	(22.5)	(21.3)	(24.1)	(24.2)
g_E	163.4	163.4	163.4	163.4	163.4	163.4	163.4	163.4	163.4	163.4
	(19.4)	(19.4)	(19.4)	(19.4)	(19.4)	(19.4)	(19.4)	(19.4)	(19.4)	(19.4)
	34.7	32.6	50.4	90.4	36.9	48.1	57.2	34.7	88.0	90.4
	4.117	3.857	5.967	10.716	4.377	5.699	6.780	4.117	10.427	10.716
c_O	178	171	171	180	170	176	178	178	180	178
	(21.1)	(20.3)	(20.3)	(21.3)	(20.1)	(20.9)	(21.1)	(21.1)	(21.3)	(21.1)
c_E	169.1	169.1	169.1	169.1	169.1	169.1	169.1	169.1	169.1	169.1
	(20.0)	(20.0)	(20.0)	(20.0)	(20.0)	(20.0)	(20.0)	(20.0)	(20.0)	(20.0)
	18.2	3.7	3.7	22.4	1.7	14.0	18.2	18.2	22.4	18.2
	2.154	0.443	0.443	2.654	0.204	1.658	2.154	2.154	2.654	2.154
N	844	844	844	844	844	844	844	844	844	844
X2	4.1	3.1	4.9	18.5	2.7	7.4	7.2	3.4	19.2	18.4
Gvalue	4.1	3.1	4.8	18.2	2.6	7.4	7.1	3.4	19.1	18.2
Gv/N	0.482	0.367	0.566	2.154	0.310	0.881	0.838	0.399	2.265	2.156
Prob	0.25410	0.37730	0.18896	0.00040	0.45467	0.05913	0.06959	0.33805	0.00026	0.00040

	70	69	68	67	66	65	64	63	62	61
a_O	243	233	253	255	240	265	256	268	245	207
	(28.8)	(27.6)	(30.0)	(30.2)	(28.4)	(31.4)	(30.3)	(31.8)	(29.0)	(24.5)
a_E	260.2	260.2	260.2	260.2	260.2	260.2	260.2	260.2	260.2	260.2
	(30.8)	(30.8)	(30.8)	(30.8)	(30.8)	(30.8)	(30.8)	(30.8)	(30.8)	(30.8)
	-33.2	-51.4	-14.2	-10.3	-38.8	9.7	-8.3	15.9	-29.5	-94.7
	-3.934	-6.092	-1.678	-1.215	-4.592	1.153	-0.983	1.881	-3.490	-11.216
t_O	249	262	231	242	254	269	227	241	240	258
	(29.5)	(31.0)	(27.4)	(28.7)	(30.1)	(31.9)	(26.9)	(28.6)	(28.4)	(30.6)

t_E	251.2	251.2	251.2	251.2	251.2	251.2	251.2	251.2	251.2	251.2
	(29.8)	(29.8)	(29.8)	(29.8)	(29.8)	(29.8)	(29.8)	(29.8)	(29.8)	(29.8)
	-4.5	22.0	-38.8	-18.1	5.6	36.7	-46.1	-20.1	-22.0	13.7
	-0.528	2.604	-4.598	-2.149	0.658	4.354	-5.458	-2.376	-2.603	1.623
g_O	179	177	192	166	190	179	177	185	188	193
	(21.2)	(21.0)	(22.7)	(19.7)	(22.5)	(21.2)	(21.0)	(21.9)	(22.3)	(22.9)
g_E	163.4	163.4	163.4	163.4	163.4	163.4	163.4	163.4	163.4	163.4
	(19.4)	(19.4)	(19.4)	(19.4)	(19.4)	(19.4)	(19.4)	(19.4)	(19.4)	(19.4)
	32.6	28.2	61.8	5.2	57.2	32.6	28.2	45.8	52.6	64.2
	3.857	3.343	7.327	0.611	6.780	3.857	3.343	5.432	6.237	7.603
c_O	173	172	168	181	160	131	184	150	171	186
	(20.5)	(20.4)	(19.9)	(21.4)	(19.0)	(15.5)	(21.8)	(17.8)	(20.3)	(22.0)
c_E	169.1	169.1	169.1	169.1	169.1	169.1	169.1	169.1	169.1	169.1
	(20.0)	(20.0)	(20.0)	(20.0)	(20.0)	(20.0)	(20.0)	(20.0)	(20.0)	(20.0)
	7.8	5.8	-2.3	24.5	-17.8	-66.9	31.0	-36.0	3.7	35.3
	0.925	0.683	-0.269	2.907	-2.106	-7.932	3.672	-4.269	0.443	4.188
N	844	844	844	844	844	844	844	844	844	844
X2	2.7	4.5	6.8	1.3	6.4	11.4	4.8	5.7	5.1	18.1
Gvalue	2.7	4.5	6.6	1.3	6.2	12.1	4.8	5.6	5.0	18.6
Gv/N	0.320	0.538	0.783	0.154	0.739	1.432	0.575	0.668	0.587	2.198
Prob	0.43952	0.20863	0.08554	0.72899	0.10049	0.00710	0.18317	0.13078	0.17528	0.00034

	60	59	58	57	56	55	54	53	52	51
a_O	228	256	246	235	259	226	211	232	243	237
	(27.0)	(30.3)	(29.1)	(27.8)	(30.7)	(26.8)	(25.0)	(27.5)	(28.8)	(28.1)
a_E	260.2	260.2	260.2	260.2	260.2	260.2	260.2	260.2	260.2	260.2
	(30.8)	(30.8)	(30.8)	(30.8)	(30.8)	(30.8)	(30.8)	(30.8)	(30.8)	(30.8)
	-60.2	-8.3	-27.6	-47.8	-2.4	-63.7	-88.4	-53.2	-33.2	-44.2
	-7.133	-0.983	-3.267	-5.668	-0.279	-7.543	-10.476	-6.302	-3.934	-5.241
t_O	259	245	266	268	250	261	283	244	259	261
	(30.7)	(29.0)	(31.5)	(31.8)	(29.6)	(30.9)	(33.5)	(28.9)	(30.7)	(30.9)
t_E	251.2	251.2	251.2	251.2	251.2	251.2	251.2	251.2	251.2	251.2
	(29.8)	(29.8)	(29.8)	(29.8)	(29.8)	(29.8)	(29.8)	(29.8)	(29.8)	(29.8)
	15.8	-12.3	30.4	34.6	-2.5	19.9	67.4	-14.3	15.8	19.9
	1.867	-1.460	3.598	4.101	-0.293	2.357	7.983	-1.691	1.867	2.357
g_O	181	183	162	189	195	183	181	184	191	182
	(21.4)	(21.7)	(19.2)	(22.4)	(23.1)	(21.7)	(21.4)	(21.8)	(22.6)	(21.6)
g_E	163.4	163.4	163.4	163.4	163.4	163.4	163.4	163.4	163.4	163.4
	(19.4)	(19.4)	(19.4)	(19.4)	(19.4)	(19.4)	(19.4)	(19.4)	(19.4)	(19.4)
	36.9	41.4	-2.9	54.9	68.9	41.4	36.9	43.6	59.5	39.2
	4.377	4.902	-0.340	6.508	8.158	4.902	4.377	5.166	7.053	4.639
c_O	176	160	170	152	140	174	169	184	151	164
	(20.9)	(19.0)	(20.1)	(18.0)	(16.6)	(20.6)	(20.0)	(21.8)	(17.9)	(19.4)
c_E	169.1	169.1	169.1	169.1	169.1	169.1	169.1	169.1	169.1	169.1
	(20.0)	(20.0)	(20.0)	(20.0)	(20.0)	(20.0)	(20.0)	(20.0)	(20.0)	(20.0)
	14.0	-17.8	1.7	-32.5	-52.9	9.9	-0.3	31.0	-34.3	-10.1
	1.658	-2.106	0.204	-3.848	-6.273	1.168	-0.033	3.672	-4.059	-1.199
N	844	844	844	844	844	844	844	844	844	844
X2	6.4	3.1	1.7	9.3	11.1	7.3	15.2	7.2	8.0	4.7
Gvalue	6.5	3.0	1.7	9.2	11.1	7.5	15.6	7.1	7.8	4.7
Gv/N	0.769	0.353	0.196	1.092	1.313	0.885	1.851	0.845	0.927	0.556
Prob	0.09008	0.39479	0.64699	0.02655	0.01128	0.05843	0.00135	0.06779	0.04982	0.19566

	50	49	48	47	46	45	44	43	42	41
a_O	250	241	245	298	262	285	272	279	261	271
	(29.6)	(28.6)	(29.0)	(35.3)	(31.0)	(33.8)	(32.2)	(33.1)	(30.9)	(32.1)
a_E	260.2	260.2	260.2	260.2	260.2	260.2	260.2	260.2	260.2	260.2
	(30.8)	(30.8)	(30.8)	(30.8)	(30.8)	(30.8)	(30.8)	(30.8)	(30.8)	(30.8)
	-20.0	-36.9	-29.5	80.9	3.7	51.9	24.2	39.0	1.6	22.1
	-2.365	-4.373	-3.490	9.584	0.433	6.154	2.864	4.617	0.195	2.617
t_O	273	277	272	216	250	230	231	238	248	245
	(32.3)	(32.8)	(32.2)	(25.6)	(29.6)	(27.3)	(27.4)	(28.2)	(29.4)	(29.0)
t_E	251.2	251.2	251.2	251.2	251.2	251.2	251.2	251.2	251.2	251.2
	(29.8)	(29.8)	(29.8)	(29.8)	(29.8)	(29.8)	(29.8)	(29.8)	(29.8)	(29.8)
	45.4	54.1	43.2	-65.3	-2.5	-40.6	-38.8	-25.8	-6.4	-12.3
	5.374	6.407	5.117	-7.736	-0.293	-4.814	-4.598	-3.053	-0.763	-1.460
g_O	176	148	172	182	173	165	186	149	167	179
	(20.9)	(17.5)	(20.4)	(21.6)	(20.5)	(19.5)	(22.0)	(17.7)	(19.8)	(21.2)
g_E	163.4	163.4	163.4	163.4	163.4	163.4	163.4	163.4	163.4	163.4
	(19.4)	(19.4)	(19.4)	(19.4)	(19.4)	(19.4)	(19.4)	(19.4)	(19.4)	(19.4)
	26.1	-29.4	17.6	39.2	19.7	3.1	48.1	-27.6	7.2	32.6
	3.088	-3.480	2.081	4.639	2.330	0.371	5.699	-3.266	0.853	3.857
c_O	145	178	155	148	159	164	155	178	168	149
	(17.2)	(21.1)	(18.4)	(17.5)	(18.8)	(19.4)	(18.4)	(21.1)	(19.9)	(17.7)
c_E	169.1	169.1	169.1	169.1	169.1	169.1	169.1	169.1	169.1	169.1
	(20.0)	(20.0)	(20.0)	(20.0)	(20.0)	(20.0)	(20.0)	(20.0)	(20.0)	(20.0)
	-44.7	18.2	-27.1	-39.5	-19.7	-10.1	-27.1	18.2	-2.3	-37.8
	-5.291	2.154	-3.207	-4.682	-2.329	-1.199	-3.207	2.154	-0.269	-4.476
N	844	844	844	844	844	844	844	844	844	844
X2	6.7	6.0	4.2	15.2	1.2	4.3	6.5	3.8	0.1	4.5
Gvalue	6.8	6.0	4.2	15.2	1.2	4.3	6.4	3.8	0.1	4.5
Gv/N	0.806	0.707	0.501	1.805	0.141	0.511	0.758	0.452	0.015	0.537
Prob	0.07857	0.11316	0.23762	0.00163	0.75592	0.22921	0.09363	0.28258	0.98811	0.20910

	40	39	38	37	36	35	34	33	32	31
a_O	296	274	279	267	282	364	377	431	461	378
	(35.1)	(32.5)	(33.1)	(31.6)	(33.4)	(43.1)	(44.7)	(51.1)	(54.6)	(44.8)
a_E	260.2	260.2	260.2	260.2	260.2	260.2	260.2	260.2	260.2	260.2
	(30.8)	(30.8)	(30.8)	(30.8)	(30.8)	(30.8)	(30.8)	(30.8)	(30.8)	(30.8)
	76.4	28.4	39.0	13.8	45.4	244.4	279.6	435.1	527.4	282.4
	9.047	3.360	4.617	1.637	5.382	28.963	33.132	51.550	62.489	33.458
t_O	246	259	216	196	140	131	165	196	148	171
	(29.1)	(30.7)	(25.6)	(23.2)	(16.6)	(15.5)	(19.5)	(23.2)	(17.5)	(20.3)
t_E	251.2	251.2	251.2	251.2	251.2	251.2	251.2	251.2	251.2	251.2
	(29.8)	(29.8)	(29.8)	(29.8)	(29.8)	(29.8)	(29.8)	(29.8)	(29.8)	(29.8)
	-10.4	15.8	-65.3	-97.3	-163.7	-170.6	-138.8	-97.3	-156.6	-131.6
	-1.229	1.867	-7.736	-11.532	-19.400	-20.215	-16.440	-11.532	-18.559	-15.590
g_O	150	148	193	212	213	206	164	102	141	153
	(17.8)	(17.5)	(22.9)	(25.1)	(25.2)	(24.4)	(19.4)	(12.1)	(16.7)	(18.1)
g_E	163.4	163.4	163.4	163.4	163.4	163.4	163.4	163.4	163.4	163.4
	(19.4)	(19.4)	(19.4)	(19.4)	(19.4)	(19.4)	(19.4)	(19.4)	(19.4)	(19.4)
	-25.7	-29.4	64.2	110.3	112.8	95.3	1.1	-96.2	-41.6	-20.2
	-3.050	-3.480	7.603	13.069	13.368	11.297	0.133	-11.396	-4.935	-2.393
c_O	152	163	156	169	209	143	138	115	94	142
	(18.0)	(19.3)	(18.5)	(20.0)	(24.8)	(16.9)	(16.4)	(13.6)	(11.1)	(16.8)
c_E	169.1	169.1	169.1	169.1	169.1	169.1	169.1	169.1	169.1	169.1

```
         ( 20.0) ( 20.0) ( 20.0) ( 20.0) ( 20.0) ( 20.0) ( 20.0) ( 20.0) ( 20.0) ( 20.0)
           -32.5   -12.1   -25.2    -0.3    88.5   -48.0   -56.2   -88.7  -110.4   -49.7
          -3.848  -1.428  -2.990  -0.033  10.480  -5.689  -6.654 -10.513 -13.085  -5.885

N            844     844     844     844     844     844     844     844     844     844
X2           7.9     2.7    12.7    26.8    75.5   114.1    87.8   164.7   233.9    84.0
Gvalue       7.8     2.7    12.6    26.5    83.0   121.2    85.8   152.8   218.7    80.9
Gv/N       0.920   0.319   1.495   3.140   9.830  14.356  10.172  18.109  25.910   9.589
Prob     0.05112 0.44174 0.00553 0.00001 0.00000 0.00000 0.00000 0.00000 0.00000 0.00000
         -------------------------------------------------------------------------------
              30      29      28      27      26      25      24      23      22      21
         -------------------------------------------------------------------------------
a_O          269     226     201     207     318     331     334     343     324     263
         ( 31.9) ( 26.8) ( 23.8) ( 24.5) ( 37.7) ( 39.2) ( 39.6) ( 40.6) ( 38.4) ( 31.2)
a_E        260.2   260.2   260.2   260.2   260.2   260.2   260.2   260.2   260.2   260.2
         ( 30.8) ( 30.8) ( 30.8) ( 30.8) ( 30.8) ( 30.8) ( 30.8) ( 30.8) ( 30.8) ( 30.8)
            17.9   -63.7  -103.7   -94.7   127.6   159.4   166.8   189.6   142.2     5.7
           2.125  -7.543 -12.292 -11.216  15.122  18.883  19.768  22.462  16.843   0.672
t_O          258     324     455     440     375     383     315     289     260     279
         ( 30.6) ( 38.4) ( 53.9) ( 52.1) ( 44.4) ( 45.4) ( 37.3) ( 34.2) ( 30.8) ( 33.1)
t_E        251.2   251.2   251.2   251.2   251.2   251.2   251.2   251.2   251.2   251.2
         ( 29.8) ( 29.8) ( 29.8) ( 29.8) ( 29.8) ( 29.8) ( 29.8) ( 29.8) ( 29.8) ( 29.8)
            13.7   164.8   540.4   493.1   300.4   323.0   142.5    80.9    17.8    58.5
           1.623  19.527  64.033  58.427  35.591  38.266  16.882   9.589   2.112   6.929
g_O          150     148      98     108      73      64     110     117     112     122
         ( 17.8) ( 17.5) ( 11.6) ( 12.8) (  8.6) (  7.6) ( 13.0) ( 13.9) ( 13.3) ( 14.5)
g_E        163.4   163.4   163.4   163.4   163.4   163.4   163.4   163.4   163.4   163.4
         ( 19.4) ( 19.4) ( 19.4) ( 19.4) ( 19.4) ( 19.4) ( 19.4) ( 19.4) ( 19.4) ( 19.4)
           -25.7   -29.4  -100.2   -89.5  -117.7  -120.0   -87.1   -78.2   -84.7   -71.4
          -3.050  -3.480 -11.878 -10.603 -13.942 -14.219 -10.321  -9.268 -10.031  -8.454
c_O          167     146      90      89      78      66      85      95     148     180
         ( 19.8) ( 17.3) ( 10.7) ( 10.5) (  9.2) (  7.8) ( 10.1) ( 11.3) ( 17.5) ( 21.3)
c_E        169.1   169.1   169.1   169.1   169.1   169.1   169.1   169.1   169.1   169.1
         ( 20.0) ( 20.0) ( 20.0) ( 20.0) ( 20.0) ( 20.0) ( 20.0) ( 20.0) ( 20.0) ( 20.0)
            -4.3   -43.0  -113.6  -114.3  -120.7  -124.2  -117.0  -109.6   -39.5    22.4
          -0.504  -5.090 -13.456 -13.542 -14.306 -14.718 -13.859 -12.986  -4.682   2.654

N            844     844     844     844     844     844     844     844     844     844
X2           1.6    30.2   241.9   209.5   173.0   211.8    96.5    77.7    34.8    14.3
Gvalue       1.6    28.8   222.9   194.7   189.6   238.1   105.2    82.7    35.8    15.2
Gv/N       0.194   3.414  26.408  23.066  22.464  28.212  12.470   9.797   4.241   1.801
Prob     0.65037 0.00000 0.00000 0.00000 0.00000 0.00000 0.00000 0.00000 0.00000 0.00165
         -------------------------------------------------------------------------------
              20      19      18      17      16      15      14      13      12      11
         -------------------------------------------------------------------------------
a_O          227     202     225     243     296     273     271     263     268     305
         ( 26.9) ( 23.9) ( 26.7) ( 28.8) ( 35.1) ( 32.3) ( 32.1) ( 31.2) ( 31.8) ( 36.1)
a_E        260.2   260.2   260.2   260.2   260.2   260.2   260.2   260.2   260.2   260.2
         ( 30.8) ( 30.8) ( 30.8) ( 30.8) ( 30.8) ( 30.8) ( 30.8) ( 30.8) ( 30.8) ( 30.8)
           -61.9  -102.3   -65.4   -33.2    76.4    26.3    22.1     5.7    15.9    97.0
          -7.338 -12.116  -7.746  -3.934   9.047   3.112   2.617   0.672   1.881  11.487
t_O          269     307     299     271     244     254     237     241     228     225
         ( 31.9) ( 36.4) ( 35.4) ( 32.1) ( 28.9) ( 30.1) ( 28.1) ( 28.6) ( 27.0) ( 26.7)
t_E        251.2   251.2   251.2   251.2   251.2   251.2   251.2   251.2   251.2   251.2
         ( 29.8) ( 29.8) ( 29.8) ( 29.8) ( 29.8) ( 29.8) ( 29.8) ( 29.8) ( 29.8) ( 29.8)
            36.7   123.1   104.1    41.0   -14.3     5.6   -27.7   -20.1   -44.3   -49.6
           4.354  14.582  12.331   4.862  -1.691   0.658  -3.277  -2.376  -5.244  -5.881
g_O          142     110      95     101     103     107     138     105      87     100
         ( 16.8) ( 13.0) ( 11.3) ( 12.0) ( 12.2) ( 12.7) ( 16.4) ( 12.4) ( 10.3) ( 11.8)
g_E        163.4   163.4   163.4   163.4   163.4   163.4   163.4   163.4   163.4   163.4
         ( 19.4) ( 19.4) ( 19.4) ( 19.4) ( 19.4) ( 19.4) ( 19.4) ( 19.4) ( 19.4) ( 19.4)
           -39.9   -87.1  -103.1   -97.2   -95.1   -90.7   -46.7   -92.9  -109.7   -98.3
          -4.732 -10.321 -12.214 -11.520 -11.269 -10.741  -5.533 -11.010 -12.999 -11.642
c_O          206     225     225     229     201     210     198     235     261     214
         ( 24.4) ( 26.7) ( 26.7) ( 27.1) ( 23.8) ( 24.9) ( 23.5) ( 27.8) ( 30.9) ( 25.4)
c_E        169.1   169.1   169.1   169.1   169.1   169.1   169.1   169.1   169.1   169.1
         ( 20.0) ( 20.0) ( 20.0) ( 20.0) ( 20.0) ( 20.0) ( 20.0) ( 20.0) ( 20.0) ( 20.0)
            81.2   128.4   128.4   138.8    69.4    90.9    62.4   154.6   226.4   100.7
           9.624  15.215  15.215  16.442   8.220  10.768   7.392  18.313  26.829  11.930

N            844     844     844     844     844     844     844     844     844     844
X2          16.3    61.3    60.9    47.7    33.5    30.0    10.1    47.0    88.0    47.0
Gvalue      16.1    62.1    64.0    49.4    36.4    32.0    10.1    47.3    88.3    49.7
Gv/N       1.908   7.360   7.586   5.851   4.307   3.796   1.199   5.599  10.467   5.894
Prob     0.00108 0.00000 0.00000 0.00000 0.00000 0.00000 0.01761 0.00000 0.00000 0.00000
         -------------------------------------------------------------------------------
              10       9       8       7       6       5       4       3       2       1
         -------------------------------------------------------------------------------
a_O          406     351     240     269     245     270     266     256     216     173
         ( 48.1) ( 41.6) ( 28.4) ( 31.9) ( 29.0) ( 32.0) ( 31.5) ( 30.3) ( 25.6) ( 20.5)
a_E        260.2   260.2   260.2   260.2   260.2   260.2   260.2   260.2   260.2   260.2
         ( 30.8) ( 30.8) ( 30.8) ( 30.8) ( 30.8) ( 30.8) ( 30.8) ( 30.8) ( 30.8) ( 30.8)
           361.3   210.2   -38.8    17.9   -29.5    20.0    11.8    -8.3   -80.4  -141.2
          42.811  24.904  -4.592   2.125  -3.490   2.370   1.394  -0.983  -9.525 -16.729
t_O          230     235     272     354     343     259     256     276     269     228
         ( 27.3) ( 27.8) ( 32.2) ( 41.9) ( 40.6) ( 30.7) ( 30.3) ( 32.7) ( 31.9) ( 27.0)
t_E        251.2   251.2   251.2   251.2   251.2   251.2   251.2   251.2   251.2   251.2
         ( 29.8) ( 29.8) ( 29.8) ( 29.8) ( 29.8) ( 29.8) ( 29.8) ( 29.8) ( 29.8) ( 29.8)
           -40.6   -31.4    43.2   242.8   213.6    15.8     9.6    51.9    36.7   -44.3
          -4.814  -3.721   5.117  28.764  25.304   1.867   1.139   6.147   4.354  -5.244
g_O           65      84     112      62     114     136     148     190     178     164
         (  7.7) ( 10.0) ( 13.3) (  7.3) ( 13.5) ( 16.1) ( 17.5) ( 22.5) ( 21.1) ( 19.4)
g_E        163.4   163.4   163.4   163.4   163.4   163.4   163.4   163.4   163.4   163.4
         ( 19.4) ( 19.4) ( 19.4) ( 19.4) ( 19.4) ( 19.4) ( 19.4) ( 19.4) ( 19.4) ( 19.4)
          -119.9  -111.8   -84.7  -120.2   -82.1   -50.0   -29.4    57.2    30.4     1.1
         -14.202 -13.249 -10.031 -14.241  -9.732  -5.923  -3.480   6.780   3.600   0.133
c_O          143     174     220     159     142     179     174     122     181     279
         ( 16.9) ( 20.6) ( 26.1) ( 18.8) ( 16.8) ( 21.2) ( 20.6) ( 14.5) ( 21.4) ( 33.1)
c_E        169.1   169.1   169.1   169.1   169.1   169.1   169.1   169.1   169.1   169.1
         ( 20.0) ( 20.0) ( 20.0) ( 20.0) ( 20.0) ( 20.0) ( 20.0) ( 20.0) ( 20.0) ( 20.0)
           -48.0     9.9   115.7   -19.7   -49.7    20.3     9.9   -79.7    24.5   279.3
          -5.689   1.168  13.706  -2.329  -5.885   2.403   1.168  -9.445   2.907  33.089

N            844     844     844     844     844     844     844     844     844     844
X2         146.9    71.5    34.8   105.9    53.7     5.8     1.8    20.0    10.9   102.7
Gvalue     152.8    76.8    35.5   120.8    52.3     6.1     1.9    21.1    11.3    94.9
Gv/N      18.105   9.101   4.201  14.318   6.197   0.718   0.221   2.499   1.335  11.248
Prob     0.00000 0.00000 0.00000 0.00000 0.00000 0.10891 0.60111 0.00010 0.01037 0.00000
         -------------------------------------------------------------------------------

CDS_INITIAL:
         -------------------------------------------------------------------------------
```

	1	2	3	4	5	6	7	8	9	10
a_O	609	0	0	329	335	264	298	272	242	306
	(72.2)	(0.0)	(0.0)	(39.0)	(39.7)	(31.3)	(35.3)	(32.2)	(28.7)	(36.3)
a_E	266.8	263.7	164.9	266.8	263.7	164.9	266.8	263.7	164.9	266.8
	(31.6)	(31.2)	(19.5)	(31.6)	(31.2)	(19.5)	(31.6)	(31.2)	(19.5)	(31.6)
	1005.2	0.0	0.0	137.9	160.5	248.4	65.9	17.0	185.6	83.9
	119.101	0.000	0.000	16.336	19.012	29.429	7.808	2.009	21.987	9.939
t_O	11	844	0	79	229	185	133	312	209	150
	(1.3)	(100.0)	(0.0)	(9.4)	(27.1)	(21.9)	(15.8)	(37.0)	(24.8)	(17.8)
t_E	129.8	274.5	185.2	129.8	274.5	185.2	129.8	274.5	185.2	129.8
	(15.4)	(32.5)	(21.9)	(15.4)	(32.5)	(21.9)	(15.4)	(32.5)	(21.9)	(15.4)
	-54.3	1895.7	0.0	-78.5	-83.1	-0.3	6.5	79.8	50.6	43.4
	-6.434	224.608	0.000	-9.296	-9.843	-0.040	0.766	9.456	5.995	5.140
g_O	224	0	844	270	153	234	271	153	209	260
	(26.5)	(0.0)	(100.0)	(32.0)	(18.1)	(27.7)	(32.1)	(18.1)	(24.8)	(30.8)
g_E	316.3	148.5	259.3	316.3	148.5	259.3	316.3	148.5	259.3	316.3
	(37.5)	(17.6)	(30.7)	(37.5)	(17.6)	(30.7)	(37.5)	(17.6)	(30.7)	(37.5)
	-154.6	0.0	1992.3	-85.5	9.2	-48.0	-83.9	9.2	-90.1	-102.0
	-18.323	0.000	236.055	-10.135	1.088	-5.687	-9.935	1.088	-10.675	-12.085
c_O	0	0	0	166	127	161	142	107	184	128
	(0.0)	(0.0)	(0.0)	(19.7)	(15.0)	(19.1)	(16.8)	(12.7)	(21.8)	(15.2)
c_E	131.0	157.3	234.6	131.0	157.3	234.6	131.0	157.3	234.6	131.0
	(15.5)	(18.6)	(27.8)	(15.5)	(18.6)	(27.8)	(15.5)	(18.6)	(27.8)	(15.5)
	0.0	0.0	0.0	78.5	-54.4	-121.3	22.8	-82.5	-89.4	-6.0
	0.000	0.000	0.000	9.301	-6.445	-14.368	2.701	-9.775	-10.598	-0.713
N	844	844	844	844	844	844	844	844	844	844
X2	705.6	1750.6	1903.5	50.5	32.8	85.1	11.1	21.6	59.8	19.0
Gvalue	796.3	1895.7	1992.3	52.4	32.2	78.8	11.3	23.4	56.6	19.2
Gv/N	94.345	224.608	236.055	6.205	3.813	9.335	1.341	2.778	6.710	2.281
Prob	0.00000	1.00000	1.00000	0.00000	0.00000	0.00000	0.01013	0.00003	0.00000	0.00024

	11	12	13	14	15	16	17	18	19	20
a_O	216	213	293	256	190	293	241	190	283	257
	(25.6)	(25.2)	(34.7)	(30.3)	(22.5)	(34.7)	(28.6)	(22.5)	(33.5)	(30.5)
a_E	263.7	164.9	266.8	263.7	164.9	266.8	263.7	164.9	266.8	263.7
	(31.2)	(19.5)	(31.6)	(31.2)	(19.5)	(31.6)	(31.2)	(19.5)	(31.6)	(31.2)
	-86.1	109.0	54.9	-15.1	53.8	54.9	-43.3	53.8	33.4	-13.1
	-10.204	12.910	6.503	-1.787	6.371	6.503	-5.130	6.371	3.952	-1.556
t_O	354	185	140	308	220	130	350	213	124	315
	(41.9)	(21.9)	(16.6)	(36.5)	(26.1)	(15.4)	(41.5)	(25.2)	(14.7)	(37.3)
t_E	274.5	185.2	129.8	274.5	185.2	129.8	274.5	185.2	129.8	274.5
	(32.5)	(21.9)	(15.4)	(32.5)	(21.9)	(15.4)	(32.5)	(21.9)	(15.4)	(32.5)
	180.0	-0.3	21.2	70.8	75.8	0.4	170.0	59.6	-11.3	86.6
	21.323	-0.040	2.509	8.393	8.985	0.046	20.139	7.067	-1.344	10.261
g_O	134	264	289	144	232	292	118	227	281	141
	(15.9)	(31.3)	(34.2)	(17.1)	(27.5)	(34.6)	(14.0)	(26.9)	(33.3)	(16.7)
g_E	148.5	259.3	316.3	148.5	259.3	316.3	148.5	259.3	316.3	148.5
	(17.6)	(30.7)	(37.5)	(17.6)	(30.7)	(37.5)	(17.6)	(30.7)	(37.5)	(17.6)
	-27.5	9.5	-52.3	-8.8	-51.6	-46.8	-54.2	-60.3	-66.6	-14.6
	-3.258	1.131	-6.191	-1.045	-6.110	-5.541	-6.424	-7.150	-7.889	-1.726
c_O	140	182	122	136	202	129	135	214	156	131
	(16.6)	(21.6)	(14.5)	(16.1)	(23.9)	(15.3)	(16.0)	(25.4)	(18.5)	(15.5)
c_E	157.3	234.6	131.0	157.3	234.6	131.0	157.3	234.6	131.0	157.3
	(18.6)	(27.8)	(15.5)	(18.6)	(27.8)	(15.5)	(18.6)	(27.8)	(15.5)	(18.6)
	-32.7	-92.5	-17.5	-39.6	-60.5	-4.1	-41.3	-39.4	54.4	-48.0
	-3.871	-10.954	-2.068	-4.695	-7.167	-0.481	-4.896	-4.666	6.444	-5.685
N	844	844	844	844	844	844	844	844	844	844
X2	34.9	25.9	6.4	7.3	17.8	4.5	32.1	13.8	9.9	10.9
Gvalue	33.7	25.7	6.3	7.3	17.5	4.4	31.1	13.7	9.8	10.9
Gv/N	3.990	3.046	0.752	0.866	2.079	0.527	3.689	1.621	1.162	1.293
Prob	0.00000	0.00001	0.09592	0.06256	0.00055	0.21703	0.00000	0.00337	0.02027	0.01219

	21	22	23	24	25	26	27	28	29	30
a_O	197	270	243	201	270	256	202	249	241	191
	(23.3)	(32.0)	(28.8)	(23.8)	(32.0)	(30.3)	(23.9)	(29.5)	(28.6)	(22.6)
a_E	164.9	266.8	263.7	164.9	266.8	263.7	164.9	266.8	263.7	164.9
	(19.5)	(31.6)	(31.2)	(19.5)	(31.6)	(31.2)	(19.5)	(31.6)	(31.2)	(19.5)
	70.0	6.4	-39.6	79.5	6.4	-15.1	81.9	-34.4	-43.3	56.1
	8.294	0.762	-4.697	9.420	0.762	-1.787	9.705	-4.075	-5.130	6.642
t_O	198	160	299	177	139	285	175	139	285	206
	(23.5)	(19.0)	(35.4)	(21.0)	(16.5)	(33.8)	(20.7)	(16.5)	(33.8)	(24.4)
t_E	185.2	129.8	274.5	185.2	129.8	274.5	185.2	129.8	274.5	185.2
	(21.9)	(15.4)	(32.5)	(21.9)	(15.4)	(32.5)	(21.9)	(15.4)	(32.5)	(21.9)
	26.5	66.9	51.0	-16.0	19.0	21.3	-19.8	19.0	21.3	43.9
	3.143	7.930	6.046	-1.893	2.254	2.524	-2.343	2.254	2.524	5.204
g_O	218	287	159	247	293	160	226	315	161	215
	(25.8)	(34.0)	(18.8)	(29.3)	(34.7)	(19.0)	(26.8)	(37.3)	(19.1)	(25.5)
g_E	259.3	316.3	148.5	259.3	316.3	148.5	259.3	316.3	148.5	259.3
	(30.7)	(37.5)	(17.6)	(30.7)	(37.5)	(17.6)	(30.7)	(37.5)	(17.6)	(30.7)
	-75.6	-55.9	21.8	-24.0	-44.9	23.9	-62.1	-2.7	26.1	-80.5
	-8.957	-6.621	2.580	-2.838	-5.323	2.834	-7.355	-0.318	3.089	-9.539
c_O	231	127	143	219	142	143	241	141	157	232
	(27.4)	(15.0)	(16.9)	(25.9)	(16.8)	(16.9)	(28.6)	(16.7)	(18.6)	(27.5)
c_E	234.6	131.0	157.3	234.6	131.0	157.3	234.6	131.0	157.3	234.6
	(27.8)	(15.5)	(18.6)	(27.8)	(15.5)	(18.6)	(27.8)	(15.5)	(18.6)	(27.8)
	-7.2	-8.0	-27.3	-30.2	22.8	-27.3	12.9	20.6	-0.7	-5.2
	-0.853	-0.944	-3.236	-3.577	2.701	-3.236	1.531	2.446	-0.078	-0.619
N	844	844	844	844	844	844	844	844	844	844
X2	13.8	9.9	5.8	9.9	3.3	2.8	13.3	2.6	3.4	14.1
Gvalue	13.7	9.5	5.9	9.4	3.3	2.8	13.0	2.6	3.4	14.2
Gv/N	1.628	1.127	0.694	1.113	0.395	0.336	1.537	0.308	0.406	1.687
Prob	0.00328	0.02323	0.11896	0.02453	0.34312	0.41762	0.00469	0.45827	0.33046	0.00260

	31	32	33	34	35	36	37	38	39	40
a_O	278	258	193	253	234	211	285	262	204	269
	(32.9)	(30.6)	(22.9)	(30.0)	(27.7)	(25.0)	(33.8)	(31.0)	(24.2)	(31.9)
a_E	266.8	263.7	164.9	266.8	263.7	164.9	266.8	263.7	164.9	266.8
	(31.6)	(31.2)	(19.5)	(31.6)	(31.2)	(19.5)	(31.6)	(31.2)	(19.5)	(31.6)
	22.9	-11.2	60.7	-26.9	-55.8	104.0	37.6	-3.3	86.7	4.4
	2.708	-1.325	7.188	-3.185	-6.616	12.317	4.455	-0.390	10.277	0.522
t_O	146	304	193	131	280	178	136	279	185	134
	(17.3)	(36.0)	(22.9)	(15.5)	(33.2)	(21.1)	(16.1)	(33.1)	(21.9)	(15.9)
t_E	129.8	274.5	185.2	129.8	274.5	185.2	129.8	274.5	185.2	129.8
	(15.4)	(32.5)	(21.9)	(15.4)	(32.5)	(21.9)	(15.4)	(32.5)	(21.9)	(15.4)
	34.3	62.0	16.0	2.4	11.0	-14.1	12.7	9.0	-0.3	8.5

	4.068	7.342	1.894	0.285	1.306	−1.666	1.503	1.064	−0.040	1.010
g_O	283	146	230	317	170	247	295	156	231	294
	(33.5)	(17.3)	(27.3)	(37.6)	(20.1)	(29.3)	(35.0)	(18.5)	(27.4)	(34.8)
g_E	316.3	148.5	259.3	316.3	148.5	259.3	316.3	148.5	259.3	316.3
	(37.5)	(17.6)	(30.7)	(37.5)	(17.6)	(30.7)	(37.5)	(17.6)	(30.7)	(37.5)
	−63.0	−4.9	−55.1	1.3	46.0	−24.0	−41.2	15.4	−53.3	−43.1
	−7.470	−0.582	−6.529	0.155	5.453	−2.838	−4.883	1.827	−6.320	−5.103
c_O	137	136	228	143	160	208	128	147	224	147
	(16.2)	(16.1)	(27.0)	(16.9)	(19.0)	(24.6)	(15.2)	(17.4)	(26.5)	(17.4)
c_E	131.0	157.3	234.6	131.0	157.3	234.6	131.0	157.3	234.6	131.0
	(15.5)	(18.6)	(27.8)	(15.5)	(18.6)	(27.8)	(15.5)	(18.6)	(27.8)	(15.5)
	12.2	−39.6	−13.1	25.0	5.4	−50.1	−6.0	−20.0	−20.8	33.8
	1.443	−4.695	−1.548	2.958	0.639	−5.937	−0.713	−2.365	−2.460	4.002
N	844	844	844	844	844	844	844	844	844	844
X2	6.3	6.2	8.6	1.8	6.6	16.7	3.0	1.1	12.8	3.7
Gvalue	6.3	6.2	8.5	1.8	6.6	15.8	3.0	1.2	12.3	3.6
Gv/N	0.748	0.740	1.005	0.213	0.782	1.875	0.361	0.136	1.456	0.431
Prob	0.09718	0.10014	0.03708	0.61535	0.08581	0.00123	0.38402	0.76489	0.00645	0.30341

	41	42	43	44	45	46	47	48	49	50
a_O	250	194	283	251	188	255	235	193	259	255
	(29.6)	(23.0)	(33.5)	(29.7)	(22.3)	(30.2)	(27.8)	(22.9)	(30.7)	(30.2)
a_E	263.7	164.9	266.8	263.7	164.9	266.8	263.7	164.9	266.8	263.7
	(31.2)	(19.5)	(31.6)	(31.2)	(19.5)	(31.6)	(31.2)	(19.5)	(31.6)	(31.2)
	−26.6	63.0	33.4	−24.7	49.2	−23.1	−54.1	60.7	−15.4	−17.0
	−3.150	7.463	3.952	−2.925	5.832	−2.735	−6.407	7.188	−1.822	−2.016
t_O	279	192	144	278	214	119	305	197	124	289
	(33.1)	(22.7)	(17.1)	(32.9)	(25.4)	(14.1)	(36.1)	(23.3)	(14.7)	(34.2)
t_E	274.5	185.2	129.8	274.5	185.2	129.8	274.5	185.2	129.8	274.5
	(32.5)	(21.9)	(15.4)	(32.5)	(21.9)	(15.4)	(32.5)	(21.9)	(15.4)	(32.5)
	9.0	13.9	29.9	7.0	61.9	−20.7	64.2	24.4	−11.3	29.7
	1.064	1.648	3.541	0.824	7.338	−2.451	7.603	2.891	−1.344	3.514
g_O	153	247	272	155	249	311	146	254	307	136
	(18.1)	(29.3)	(32.2)	(18.4)	(29.5)	(36.8)	(17.3)	(30.1)	(36.4)	(16.1)
g_E	148.5	259.3	316.3	148.5	259.3	316.3	148.5	259.3	316.3	148.5
	(17.6)	(30.7)	(37.5)	(17.6)	(30.7)	(37.5)	(17.6)	(30.7)	(37.5)	(17.6)
	9.2	−24.0	−82.2	13.3	−20.1	−10.6	−4.9	−10.4	−18.4	−23.9
	1.088	−2.838	−9.735	1.579	−2.385	−1.256	−0.582	−1.236	−2.181	−2.829
c_O	162	211	145	160	193	159	158	200	154	164
	(19.2)	(25.0)	(17.2)	(19.0)	(22.9)	(18.8)	(18.7)	(23.7)	(18.2)	(19.4)
c_E	157.3	234.6	131.0	157.3	234.6	131.0	157.3	234.6	131.0	157.3
	(18.6)	(27.8)	(15.5)	(18.6)	(27.8)	(15.5)	(18.6)	(27.8)	(15.5)	(18.6)
	9.5	−44.8	29.3	5.4	−75.4	61.5	1.3	−63.9	49.7	13.6
	1.123	−5.307	3.477	0.639	−8.932	7.285	0.160	−7.568	5.890	1.614
N	844	844	844	844	844	844	844	844	844	844
X2	1.1	8.3	10.2	1.0	15.5	7.5	6.5	10.8	4.8	2.4
Gvalue	1.1	8.1	10.4	1.0	15.6	7.1	6.5	10.8	4.6	2.4
Gv/N	0.126	0.966	1.235	0.117	1.853	0.844	0.775	1.275	0.542	0.284
Prob	0.78548	0.04303	0.01526	0.80414	0.00135	0.06800	0.08819	0.01311	0.20547	0.49486

	51	52	53	54	55	56	57	58	59	60
a_O	189	298	255	171	289	239	180	291	237	215
	(22.4)	(35.3)	(30.2)	(20.3)	(34.2)	(28.3)	(21.3)	(34.5)	(28.1)	(25.5)
a_E	164.9	266.8	263.7	164.9	266.8	263.7	164.9	266.8	263.7	164.9
	(19.5)	(31.6)	(31.2)	(19.5)	(31.6)	(31.2)	(19.5)	(31.6)	(31.2)	(19.5)
	51.5	65.9	−17.0	12.4	46.2	−46.9	31.5	50.5	−50.5	114.0
	6.101	7.808	−2.016	1.464	5.472	−5.560	3.729	5.986	−5.985	13.507
t_O	175	126	280	194	126	289	206	112	267	183
	(20.7)	(14.9)	(33.2)	(23.0)	(14.9)	(34.2)	(24.4)	(13.3)	(31.6)	(21.7)
t_E	185.2	129.8	274.5	185.2	129.8	274.5	185.2	129.8	274.5	185.2
	(21.9)	(15.4)	(32.5)	(21.9)	(15.4)	(32.5)	(21.9)	(15.4)	(32.5)	(21.9)
	−19.8	−7.5	11.0	18.1	−7.5	29.7	43.9	−33.0	−14.9	−4.3
	−2.343	−0.888	1.306	2.141	−0.888	3.514	5.204	−3.915	−1.763	−0.511
g_O	245	276	143	278	290	161	244	328	162	232
	(29.0)	(32.7)	(16.9)	(32.9)	(34.4)	(19.1)	(28.9)	(38.9)	(19.2)	(27.5)
g_E	259.3	316.3	148.5	259.3	316.3	148.5	259.3	316.3	148.5	259.3
	(30.7)	(37.5)	(17.6)	(30.7)	(37.5)	(17.6)	(30.7)	(37.5)	(17.6)	(30.7)
	−27.7	−75.3	−10.7	38.8	−50.4	26.1	−29.6	23.7	28.2	−51.6
	−3.287	−8.923	−1.274	4.595	−5.975	3.089	−3.510	2.812	3.346	−6.110
c_O	235	144	166	201	139	155	214	113	178	214
	(27.8)	(17.1)	(19.7)	(23.8)	(16.5)	(18.4)	(25.4)	(13.4)	(21.1)	(25.4)
c_E	234.6	131.0	157.3	234.6	131.0	157.3	234.6	131.0	157.3	234.6
	(27.8)	(15.5)	(18.6)	(27.8)	(15.5)	(18.6)	(27.8)	(15.5)	(18.6)	(27.8)
	0.7	27.1	17.8	−62.2	16.4	−4.6	−39.4	−33.5	43.9	−39.4
	0.088	3.217	2.111	−7.368	1.941	−0.547	−4.666	−3.967	5.207	−4.666
N	844	844	844	844	844	844	844	844	844	844
X2	4.9	10.2	1.1	6.8	4.6	4.2	6.4	7.6	6.8	19.9
Gvalue	4.7	10.2	1.1	7.0	4.6	4.2	6.4	7.7	6.8	18.7
Gv/N	0.560	1.214	0.127	0.832	0.550	0.497	0.756	0.915	0.806	2.219
Prob	0.19321	0.01659	0.78460	0.07113	0.20008	0.24156	0.09438	0.05206	0.07857	0.00031

	61	62	63	64	65	66	67	68	69	70
a_O	282	257	191	277	227	201	266	265	186	273
	(33.4)	(30.5)	(22.6)	(32.8)	(26.9)	(23.8)	(31.5)	(31.4)	(22.0)	(32.3)
a_E	266.8	263.7	164.9	266.8	263.7	164.9	266.8	263.7	164.9	266.8
	(31.6)	(31.2)	(19.5)	(31.6)	(31.2)	(19.5)	(31.6)	(31.2)	(19.5)	(31.6)
	31.2	−13.1	56.1	20.8	−68.0	79.5	−1.6	2.7	44.7	12.5
	3.701	−1.556	6.642	2.461	−8.052	9.420	−0.191	0.320	5.299	1.485
t_O	145	268	199	130	311	183	145	269	177	137
	(17.2)	(31.8)	(23.6)	(15.4)	(36.8)	(21.7)	(17.2)	(31.9)	(21.0)	(16.2)
t_E	129.8	274.5	185.2	129.8	274.5	185.2	129.8	274.5	185.2	129.8
	(15.4)	(32.5)	(21.9)	(15.4)	(32.5)	(21.9)	(15.4)	(32.5)	(21.9)	(15.4)
	32.1	−12.9	28.7	0.4	77.6	−4.3	32.1	−11.0	−16.0	14.8
	3.804	−1.532	3.397	0.046	9.189	−0.511	3.804	−1.300	−1.893	1.752
g_O	299	150	237	295	135	237	301	145	248	314
	(35.4)	(17.8)	(28.1)	(35.0)	(16.0)	(28.1)	(35.7)	(17.2)	(29.4)	(37.2)
g_E	316.3	148.5	259.3	316.3	148.5	259.3	316.3	148.5	259.3	316.3
	(37.5)	(17.6)	(30.7)	(37.5)	(17.6)	(30.7)	(37.5)	(17.6)	(30.7)	(37.5)
	−33.7	3.1	−42.6	−41.2	−25.7	−42.6	−29.9	−6.9	−22.0	−4.7
	−3.995	0.363	−5.044	−4.883	−3.044	−5.044	−3.547	−0.814	−2.612	−0.554
c_O	118	169	217	142	171	223	132	165	233	120
	(14.0)	(20.0)	(25.7)	(16.8)	(20.3)	(26.4)	(15.6)	(19.5)	(27.6)	(14.2)
c_E	131.0	157.3	234.6	131.0	157.3	234.6	131.0	157.3	234.6	131.0
	(15.5)	(18.6)	(27.8)	(15.5)	(18.6)	(27.8)	(15.5)	(18.6)	(27.8)	(15.5)
	−24.7	24.2	−33.9	22.8	28.5	−22.7	1.9	15.7	−3.2	−21.1
	−2.932	2.866	−4.016	2.701	3.377	−2.686	0.227	1.862	−0.384	−2.504

N	844	844	844	844	844	844	844	844	844	844
X2	4.9	1.2	8.4	2.7	12.3	10.4	2.5	0.6	3.6	1.5
Gvalue	4.9	1.2	8.3	2.7	12.4	10.0	2.5	0.6	3.5	1.5
Gv/N	0.578	0.141	0.978	0.326	1.470	1.179	0.294	0.068	0.410	0.179
Prob	0.18114	0.75603	0.04100	0.43181	0.00611	0.01899	0.47905	0.90330	0.32621	0.67992

	71	72	73	74	75	76	77	78	79	80
a_O	273	177	260	247	172	256	270	173	267	256
	(32.3)	(21.0)	(30.8)	(29.3)	(20.4)	(30.3)	(32.0)	(20.5)	(31.7)	(30.4)
a_E	263.7	164.9	266.8	263.7	164.9	266.8	263.7	164.9	266.5	263.3
	(31.2)	(19.5)	(31.6)	(31.2)	(19.5)	(31.6)	(31.2)	(19.5)	(31.6)	(31.2)
	19.0	25.0	-13.4	-32.2	14.4	-21.2	12.8	16.5	1.0	-14.5
	2.254	2.962	-1.592	-3.819	1.711	-2.508	1.522	1.958	0.121	-1.717
t_O	287	189	132	288	195	145	285	214	136	283
	(34.0)	(22.4)	(15.6)	(34.1)	(23.1)	(17.2)	(33.8)	(25.4)	(16.1)	(33.6)
t_E	274.5	185.2	129.8	274.5	185.2	129.8	274.5	185.2	129.7	274.2
	(32.5)	(21.9)	(15.4)	(32.5)	(21.9)	(15.4)	(32.5)	(21.9)	(15.4)	(32.5)
	25.5	7.7	4.4	27.6	20.2	32.1	21.3	61.9	13.0	17.8
	3.018	0.917	0.525	3.266	2.390	3.804	2.524	7.338	1.543	2.116
g_O	149	268	310	149	244	324	140	248	293	148
	(17.7)	(31.8)	(36.7)	(17.7)	(28.9)	(38.4)	(16.6)	(29.4)	(34.8)	(17.6)
g_E	148.5	259.3	316.3	148.5	259.3	316.3	148.5	259.3	316.0	148.3
	(17.6)	(30.7)	(37.5)	(17.6)	(30.7)	(37.5)	(17.6)	(30.7)	(37.5)	(17.6)
	1.0	17.7	-12.6	1.0	-29.6	15.5	-16.5	-22.0	-44.2	-0.6
	0.124	2.103	-1.488	0.124	-3.510	1.836	-1.950	-2.612	-5.247	-0.071
c_O	135	210	142	160	233	119	149	209	147	156
	(16.0)	(24.9)	(16.8)	(19.0)	(27.6)	(14.1)	(17.7)	(24.8)	(17.4)	(18.5)
c_E	157.3	234.6	131.0	157.3	234.6	131.0	157.3	234.6	130.9	157.1
	(18.6)	(27.8)	(15.5)	(18.6)	(27.8)	(15.5)	(18.6)	(27.8)	(15.5)	(18.6)
	-41.3	-46.6	22.8	5.4	-3.2	-22.9	-16.2	-48.3	34.1	-2.3
	-4.896	-5.518	2.701	0.639	-0.384	-2.719	-1.920	-5.728	4.048	-0.270

N	844	844	844	844	844	844	844	844	843	843
X2	4.1	3.8	1.3	1.8	1.7	3.5	1.5	8.2	4.0	0.5
Gvalue	4.2	3.9	1.2	1.8	1.7	3.5	1.5	8.1	3.9	0.5
Gv/N	0.500	0.464	0.146	0.210	0.206	0.413	0.176	0.956	0.466	0.059
Prob	0.23902	0.27112	0.74550	0.62136	0.62795	0.32292	0.68525	0.04469	0.26969	0.92028

	81	82	83	84	85	86	87	88	89	90
a_O	170	238	244	168	283	267	183	238	251	186
	(20.2)	(28.2)	(28.9)	(19.9)	(33.6)	(31.7)	(21.7)	(28.3)	(29.8)	(22.1)
a_E	164.7	266.5	263.3	164.7	266.5	263.3	164.7	266.2	263.0	164.5
	(19.5)	(31.6)	(31.2)	(19.5)	(31.6)	(31.2)	(19.5)	(31.6)	(31.2)	(19.5)
	10.7	-53.8	-37.2	6.6	34.0	7.4	38.5	-53.3	-23.5	45.6
	1.269	-6.384	-4.416	0.782	4.036	0.874	4.565	-6.325	-2.790	5.416
t_O	208	141	302	216	129	281	212	126	302	218
	(24.7)	(16.7)	(35.8)	(25.6)	(15.3)	(33.3)	(25.1)	(15.0)	(35.9)	(25.9)
t_E	185.0	129.7	274.2	185.0	129.7	274.2	185.0	129.5	273.9	184.7
	(21.9)	(15.4)	(32.5)	(21.9)	(15.4)	(32.5)	(21.9)	(15.4)	(32.5)	(21.9)
	48.9	23.7	58.3	67.0	-1.3	13.7	57.9	-6.9	59.0	72.2
	5.796	2.807	6.914	7.953	-0.154	1.629	6.865	-0.819	7.008	8.574
g_O	245	331	147	257	309	158	234	323	138	239
	(29.1)	(39.3)	(17.4)	(30.5)	(36.7)	(18.7)	(27.8)	(38.4)	(16.4)	(28.4)
g_E	259.0	316.0	148.3	259.0	316.0	148.3	259.0	315.6	148.1	258.7
	(30.7)	(37.5)	(17.6)	(30.7)	(37.5)	(17.6)	(30.7)	(37.5)	(17.6)	(30.7)
	-27.2	30.8	-2.6	-3.9	-13.8	20.0	-47.4	15.0	-19.5	-37.8
	-3.222	3.649	-0.307	-0.464	-1.635	2.375	-5.628	1.779	-2.321	-4.487
c_O	220	133	150	202	122	137	214	155	151	199
	(26.1)	(15.8)	(17.8)	(24.0)	(14.5)	(16.3)	(25.4)	(18.4)	(17.9)	(23.6)
c_E	234.3	130.9	157.1	234.3	130.9	157.1	234.3	130.7	157.0	234.1
	(27.8)	(15.5)	(18.6)	(27.8)	(15.5)	(18.6)	(27.8)	(15.5)	(18.6)	(27.8)
	-27.8	4.3	-14.0	-60.0	-17.2	-37.6	-38.9	52.8	-11.7	-64.6
	-3.298	0.505	-1.655	-7.119	-2.036	-4.458	-4.612	6.268	-1.387	-7.673

N	843	843	843	843	843	843	843	842	842	842
X2	4.7	4.8	4.6	9.8	1.8	3.4	10.2	7.8	4.4	15.5
Gvalue	4.6	4.9	4.5	9.7	1.8	3.5	10.0	7.6	4.3	15.4
Gv/N	0.545	0.577	0.536	1.152	0.211	0.420	1.191	0.904	0.509	1.831
Prob	0.20436	0.18205	0.21038	0.02121	0.61959	0.31593	0.01824	0.05482	0.23217	0.00149

	91	92	93	94	95	96	97	98	99	100
a_O	261	251	192	282	278	170	270	280	193	268
	(31.0)	(29.8)	(22.8)	(33.5)	(33.0)	(20.2)	(32.1)	(33.3)	(22.9)	(31.8)
a_E	266.2	263.0	164.5	266.2	263.0	164.5	266.2	263.0	164.5	266.2
	(31.6)	(31.2)	(19.5)	(31.6)	(31.2)	(19.5)	(31.6)	(31.2)	(19.5)	(31.6)
	-10.2	-23.5	59.3	32.6	30.8	11.1	7.7	35.0	61.6	3.7
	-1.217	-2.790	7.039	3.869	3.656	1.318	0.916	4.159	7.314	0.435
t_O	120	263	194	132	251	208	140	275	191	133
	(14.3)	(31.2)	(23.0)	(15.7)	(29.8)	(24.7)	(16.6)	(32.7)	(22.7)	(15.8)
t_E	129.5	273.9	184.7	129.5	273.9	184.7	129.5	273.9	184.7	129.5
	(15.4)	(32.5)	(21.9)	(15.4)	(32.5)	(21.9)	(15.4)	(32.5)	(21.9)	(15.4)
	-18.3	-21.3	19.0	5.1	-43.8	49.4	21.8	2.2	12.7	7.1
	-2.171	-2.535	2.256	0.600	-5.204	5.861	2.593	0.263	1.514	0.843
g_O	340	153	234	297	150	249	298	155	238	306
	(40.4)	(18.2)	(27.8)	(35.3)	(17.8)	(29.6)	(35.4)	(18.4)	(28.3)	(36.3)
g_E	315.6	148.1	258.7	315.6	148.1	258.7	315.6	148.1	258.7	315.6
	(37.5)	(17.6)	(30.7)	(37.5)	(17.6)	(30.7)	(37.5)	(17.6)	(30.7)	(37.5)
	50.6	9.9	-46.9	-36.1	3.8	-18.9	-34.2	14.1	-39.6	-18.9
	6.015	1.177	-5.568	-4.284	0.448	-2.251	-4.061	1.670	-4.705	-2.244
c_O	121	175	222	131	163	215	134	132	220	135
	(14.4)	(20.8)	(26.4)	(15.6)	(19.4)	(25.5)	(15.9)	(15.7)	(26.1)	(16.0)
c_E	130.7	157.0	234.1	130.7	157.0	234.1	130.7	157.0	234.1	130.7
	(15.5)	(18.6)	(27.8)	(15.5)	(18.6)	(27.8)	(15.5)	(18.6)	(27.8)	(15.5)
	-18.7	38.1	-23.5	0.5	12.3	-36.5	6.6	-45.7	-27.3	8.7
	-2.224	4.524	-2.792	0.063	1.463	-4.340	0.785	-5.429	-3.240	1.029

N	842	842	842	842	842	842	842	842	842	842
X2	3.4	3.2	8.0	2.1	3.0	5.0	2.0	5.4	7.6	0.5
Gvalue	3.4	3.2	7.9	2.1	3.1	5.0	2.0	5.6	7.4	0.5
Gv/N	0.404	0.375	0.934	0.248	0.363	0.589	0.233	0.664	0.882	0.064
Prob	0.33406	0.36822	0.04884	0.55405	0.38257	0.17497	0.57968	0.13349	0.05938	0.91028

Amar_MARIES
5UTR:

	100	99	98	97	96	95	94	93	92	91
a_O	78	64	83	68	61	70	57	64	56	75
	(28.9)	(23.7)	(30.7)	(25.2)	(22.6)	(25.9)	(21.1)	(23.7)	(20.7)	(27.8)
a_E	68.6	68.6	68.6	68.6	68.6	68.6	68.6	68.6	68.6	68.6
	(25.4)	(25.4)	(25.4)	(25.4)	(25.4)	(25.4)	(25.4)	(25.4)	(25.4)	(25.4)
	20.0	-8.9	31.6	-1.2	-14.3	2.8	-21.1	-8.9	-22.7	13.4
	7.420	-3.291	11.715	-0.442	-5.306	1.048	-7.821	-3.291	-8.418	4.955
t_O	68	83	65	69	88	80	67	83	75	70
	(25.2)	(30.7)	(24.1)	(25.6)	(32.6)	(29.6)	(24.8)	(30.7)	(27.8)	(25.9)
t_E	77.0	77.0	77.0	77.0	77.0	77.0	77.0	77.0	77.0	77.0
	(28.5)	(28.5)	(28.5)	(28.5)	(28.5)	(28.5)	(28.5)	(28.5)	(28.5)	(28.5)
	-16.9	12.5	-22.0	-15.1	23.5	6.2	-18.6	12.5	-3.9	-13.3
	-6.248	4.629	-8.145	-5.594	8.721	2.280	-6.891	4.629	-1.448	-4.929
g_O	69	67	74	65	53	64	73	69	66	62
	(25.6)	(24.8)	(27.4)	(24.1)	(19.6)	(23.7)	(27.0)	(25.6)	(24.4)	(23.0)
g_E	64.5	64.5	64.5	64.5	64.5	64.5	64.5	64.5	64.5	64.5
	(23.9)	(23.9)	(23.9)	(23.9)	(23.9)	(23.9)	(23.9)	(23.9)	(23.9)	(23.9)
	9.2	5.0	20.2	0.9	-20.9	-1.1	18.0	9.2	3.0	-5.0
	3.415	1.857	7.498	0.342	-7.734	-0.398	6.661	3.415	1.094	-1.844
c_O	55	56	48	68	68	56	73	54	73	63
	(20.4)	(20.7)	(17.8)	(25.2)	(25.2)	(20.7)	(27.0)	(20.0)	(27.0)	(23.3)
c_E	59.9	59.9	59.9	59.9	59.9	59.9	59.9	59.9	59.9	59.9
	(22.2)	(22.2)	(22.2)	(22.2)	(22.2)	(22.2)	(22.2)	(22.2)	(22.2)	(22.2)
	-9.4	-7.5	-21.2	17.3	17.3	-7.5	28.9	-11.2	28.9	6.4
	-3.463	-2.779	-7.863	6.405	6.405	-2.779	10.713	-4.134	10.713	2.370
N	270	270	270	270	270	270	270	270	270	270
X2	3.0	1.1	8.6	1.9	5.6	0.4	7.2	1.7	5.3	1.5
Gvalue	3.0	1.1	8.7	1.9	5.6	0.4	7.2	1.7	5.2	1.5
Gv/N	1.124	0.416	3.205	0.711	2.087	0.151	2.661	0.620	1.941	0.553
Prob	0.38640	0.77124	0.03426	0.58909	0.13076	0.93882	0.06624	0.64291	0.15505	0.68380

	90	89	88	87	86	85	84	83	82	81
a_O	71	72	78	61	66	74	58	72	77	72
	(26.3)	(26.7)	(28.9)	(22.6)	(24.4)	(27.4)	(21.5)	(26.7)	(28.5)	(26.7)
a_E	68.6	68.6	68.6	68.6	68.6	68.6	68.6	68.6	68.6	68.6
	(25.4)	(25.4)	(25.4)	(25.4)	(25.4)	(25.4)	(25.4)	(25.4)	(25.4)	(25.4)
	4.9	7.0	20.0	-14.3	-5.1	11.2	-19.5	7.0	17.8	7.0
	1.809	2.580	7.420	-5.306	-1.889	4.153	-7.211	2.580	6.589	2.580
t_O	63	66	70	83	65	72	85	67	65	73
	(23.3)	(24.4)	(25.9)	(30.7)	(24.1)	(26.7)	(31.5)	(24.8)	(24.1)	(27.0)
t_E	77.0	77.0	77.0	77.0	77.0	77.0	77.0	77.0	77.0	77.0
	(28.5)	(28.5)	(28.5)	(28.5)	(28.5)	(28.5)	(28.5)	(28.5)	(28.5)	(28.5)
	-25.3	-20.3	-13.3	12.5	-22.0	-9.6	16.8	-18.6	-22.0	-7.8
	-9.353	-7.524	-4.929	4.629	-8.145	-3.567	6.240	-6.891	-8.145	-2.871
g_O	65	69	61	71	66	70	70	68	70	68
	(24.1)	(25.6)	(22.6)	(26.3)	(24.4)	(25.9)	(25.9)	(25.2)	(25.9)	(25.2)
g_E	64.5	64.5	64.5	64.5	64.5	64.5	64.5	64.5	64.5	64.5
	(23.9)	(23.9)	(23.9)	(23.9)	(23.9)	(23.9)	(23.9)	(23.9)	(23.9)	(23.9)
	0.9	9.2	-6.9	13.5	3.0	11.4	11.4	7.1	11.4	7.1
	0.342	3.415	-2.549	5.017	1.094	4.211	4.211	2.630	4.211	2.630
c_O	71	63	61	55	73	54	57	63	58	57
	(26.3)	(23.3)	(22.6)	(20.4)	(27.0)	(20.0)	(21.1)	(23.3)	(21.5)	(21.1)
c_E	59.9	59.9	59.9	59.9	59.9	59.9	59.9	59.9	59.9	59.9
	(22.2)	(22.2)	(22.2)	(22.2)	(22.2)	(22.2)	(22.2)	(22.2)	(22.2)	(22.2)
	24.2	6.4	2.3	-9.4	28.9	-11.2	-5.6	6.4	-3.7	-5.6
	8.958	2.370	0.837	-3.463	10.713	-4.134	-2.081	2.370	-1.370	-2.081
N	270	270	270	270	270	270	270	270	270	270
X2	4.7	2.2	2.1	2.4	4.9	1.8	3.1	1.8	3.4	0.7
Gvalue	4.7	2.3	2.1	2.4	4.8	1.8	3.1	1.9	3.5	0.7
Gv/N	1.756	0.842	0.779	0.877	1.773	0.663	1.158	0.689	1.284	0.259
Prob	0.19166	0.51765	0.55100	0.49945	0.18807	0.61703	0.37237	0.60163	0.32501	0.87361

	80	79	78	77	76	75	74	73	72	71
a_O	61	66	60	63	76	68	70	73	64	72
	(22.6)	(24.4)	(22.2)	(23.3)	(28.1)	(25.2)	(25.9)	(27.0)	(23.7)	(26.7)
a_E	68.6	68.6	68.6	68.6	68.6	68.6	68.6	68.6	68.6	68.6
	(25.4)	(25.4)	(25.4)	(25.4)	(25.4)	(25.4)	(25.4)	(25.4)	(25.4)	(25.4)
	-14.3	-5.1	-16.1	-10.7	15.6	-1.2	2.8	9.1	-8.9	7.0
	-5.306	-1.889	-5.953	-3.974	5.767	-0.442	1.048	3.362	-3.291	2.580
t_O	68	71	75	67	75	85	80	79	71	75
	(25.2)	(26.3)	(27.8)	(24.8)	(27.8)	(31.5)	(29.6)	(29.3)	(26.3)	(27.8)
t_E	77.0	77.0	77.0	77.0	77.0	77.0	77.0	77.0	77.0	77.0
	(28.5)	(28.5)	(28.5)	(28.5)	(28.5)	(28.5)	(28.5)	(28.5)	(28.5)	(28.5)
	-16.9	-11.5	-3.9	-18.6	-3.9	16.8	6.2	4.1	-11.5	-3.9
	-6.248	-4.253	-1.448	-6.891	-1.448	6.240	2.280	1.516	-4.253	-1.448
g_O	76	73	68	73	64	57	62	60	73	66
	(28.1)	(27.0)	(25.2)	(27.0)	(23.7)	(21.1)	(23.0)	(22.2)	(27.0)	(24.4)
g_E	64.5	64.5	64.5	64.5	64.5	64.5	64.5	64.5	64.5	64.5
	(23.9)	(23.9)	(23.9)	(23.9)	(23.9)	(23.9)	(23.9)	(23.9)	(23.9)	(23.9)
	24.8	18.0	7.1	18.0	-1.1	-14.2	-5.0	-8.8	18.0	3.0
	9.202	6.661	2.630	6.661	-0.398	-5.245	-1.844	-3.242	6.661	1.094
c_O	65	60	67	67	55	60	58	58	62	57
	(24.1)	(22.2)	(24.8)	(24.8)	(20.4)	(22.2)	(21.5)	(21.5)	(23.0)	(21.1)
c_E	59.9	59.9	59.9	59.9	59.9	59.9	59.9	59.9	59.9	59.9
	(22.2)	(22.2)	(22.2)	(22.2)	(22.2)	(22.2)	(22.2)	(22.2)	(22.2)	(22.2)
	10.7	0.2	15.1	15.1	-9.4	0.2	-3.7	-3.7	4.3	-5.6
	3.950	0.089	5.576	5.576	-3.463	0.089	-1.370	-1.370	1.598	-2.081
N	270	270	270	270	270	270	270	270	270	270
X2	4.4	1.7	2.2	3.7	1.3	1.7	0.3	0.7	2.0	0.4
Gvalue	4.3	1.6	2.2	3.7	1.2	1.7	0.3	0.7	1.9	0.4
Gv/N	1.598	0.608	0.805	1.371	0.458	0.641	0.113	0.265	0.715	0.145
Prob	0.22933	0.65023	0.53693	0.29547	0.74441	0.63011	0.95884	0.86951	0.58700	0.94215

	70	69	68	67	66	65	64	63	62	61
a_O	71	80	65	87	71	73	71	62	76	64
	(26.3)	(29.6)	(24.1)	(32.2)	(26.3)	(27.0)	(26.3)	(23.0)	(28.1)	(23.7)
a_E	68.6	68.6	68.6	68.6	68.6	68.6	68.6	68.6	68.6	68.6
	(25.4)	(25.4)	(25.4)	(25.4)	(25.4)	(25.4)	(25.4)	(25.4)	(25.4)	(25.4)
	4.9	24.6	-7.0	41.3	4.9	9.1	4.9	-12.5	15.6	-8.9
	1.809	9.110	-2.595	15.313	1.809	3.362	1.809	-4.646	5.767	-3.291
t_O	70	64	79	63	73	79	71	74	70	76
	(25.9)	(23.7)	(29.3)	(23.3)	(27.0)	(29.3)	(26.3)	(27.4)	(25.9)	(28.1)

t_E	77.0	77.0	77.0	77.0	77.0	77.0	77.0	77.0	77.0	77.0
	(28.5)	(28.5)	(28.5)	(28.5)	(28.5)	(28.5)	(28.5)	(28.5)	(28.5)	(28.5)
	-13.3	-23.6	4.1	-25.3	-7.8	4.1	-11.5	-5.8	-13.3	-1.9
	-4.929	-8.754	1.516	-9.353	-2.871	1.516	-4.253	-2.164	-4.929	-0.721
g_O	71	71	58	64	66	64	74	76	64	67
	(26.3)	(26.3)	(21.5)	(23.7)	(24.4)	(23.7)	(27.4)	(28.1)	(23.7)	(24.8)
g_E	64.5	64.5	64.5	64.5	64.5	64.5	64.5	64.5	64.5	64.5
	(23.9)	(23.9)	(23.9)	(23.9)	(23.9)	(23.9)	(23.9)	(23.9)	(23.9)	(23.9)
	13.5	13.5	-12.4	-1.1	3.0	-1.1	20.2	24.8	-1.1	5.0
	5.017	5.017	-4.590	-0.398	1.094	-0.398	7.498	9.202	-0.398	1.857
c_O	58	55	68	56	60	54	54	58	60	63
	(21.5)	(20.4)	(25.2)	(20.7)	(22.2)	(20.0)	(20.0)	(21.5)	(22.2)	(23.3)
c_E	59.9	59.9	59.9	59.9	59.9	59.9	59.9	59.9	59.9	59.9
	(22.2)	(22.2)	(22.2)	(22.2)	(22.2)	(22.2)	(22.2)	(22.2)	(22.2)	(22.2)
	-3.7	-9.4	17.3	-7.5	0.2	-11.2	-11.2	-3.7	0.2	6.4
	-1.370	-3.463	6.405	-2.779	0.089	-4.134	-4.134	-1.370	0.089	2.370
N	270	270	270	270	270	270	270	270	270	270
X2	1.4	5.1	2.0	7.7	0.3	0.9	2.5	2.8	1.4	0.6
Gvalue	1.4	5.2	2.0	7.5	0.3	0.9	2.5	2.8	1.4	0.6
Gv/N	0.527	1.910	0.735	2.783	0.121	0.345	0.919	1.021	0.529	0.215
Prob	0.70047	0.16075	0.57539	0.05718	0.95518	0.81801	0.47878	0.43062	0.69882	0.90088

	60	59	58	57	56	55	54	53	52	51
a_O	84	64	74	64	72	84	64	80	70	57
	(31.1)	(23.7)	(27.4)	(23.7)	(26.7)	(31.1)	(23.7)	(29.6)	(25.9)	(21.1)
a_E	68.6	68.6	68.6	68.6	68.6	68.6	68.6	68.6	68.6	68.6
	(25.4)	(25.4)	(25.4)	(25.4)	(25.4)	(25.4)	(25.4)	(25.4)	(25.4)	(25.4)
	34.0	-8.9	11.2	-8.9	7.0	34.0	-8.9	24.6	2.8	-21.1
	12.602	-3.291	4.153	-3.291	2.580	12.602	-3.291	9.110	1.048	-7.821
t_O	70	78	65	74	88	74	79	69	74	80
	(25.9)	(28.9)	(24.1)	(27.4)	(32.6)	(27.4)	(29.3)	(25.6)	(27.4)	(29.6)
t_E	77.0	77.0	77.0	77.0	77.0	77.0	77.0	77.0	77.0	77.0
	(28.5)	(28.5)	(28.5)	(28.5)	(28.5)	(28.5)	(28.5)	(28.5)	(28.5)	(28.5)
	-13.3	2.1	-22.0	-5.8	23.5	-5.8	4.1	-15.1	-5.8	6.2
	-4.929	0.761	-8.145	-2.164	8.721	-2.164	1.516	-5.594	-2.164	2.280
g_O	62	71	62	66	55	55	58	57	65	73
	(23.0)	(26.3)	(23.0)	(24.4)	(20.4)	(20.4)	(21.5)	(21.1)	(24.1)	(27.0)
g_E	64.5	64.5	64.5	64.5	64.5	64.5	64.5	64.5	64.5	64.5
	(23.9)	(23.9)	(23.9)	(23.9)	(23.9)	(23.9)	(23.9)	(23.9)	(23.9)	(23.9)
	-5.0	13.5	-5.0	3.0	-17.6	-17.6	-12.4	-14.2	0.9	18.0
	-1.844	5.017	-1.844	1.094	-6.517	-6.517	-4.590	-5.245	0.342	6.661
c_O	54	57	69	66	55	57	69	64	61	60
	(20.0)	(21.1)	(25.6)	(24.4)	(20.4)	(21.1)	(25.6)	(23.7)	(22.6)	(22.2)
c_E	59.9	59.9	59.9	59.9	59.9	59.9	59.9	59.9	59.9	59.9
	(22.2)	(22.2)	(22.2)	(22.2)	(22.2)	(22.2)	(22.2)	(22.2)	(22.2)	(22.2)
	-11.2	-5.6	19.6	12.8	-9.4	-5.6	19.6	8.5	2.3	0.2
	-4.134	-2.081	7.246	4.757	-3.463	-2.081	7.246	3.155	0.837	0.089
N	270	270	270	270	270	270	270	270	270	270
X2	4.8	1.1	3.8	1.1	3.6	5.1	2.4	3.9	0.2	3.2
Gvalue	4.6	1.1	3.8	1.1	3.6	5.0	2.4	3.8	0.2	3.3
Gv/N	1.695	0.406	1.411	0.396	1.321	1.840	0.881	1.426	0.063	1.209
Prob	0.20565	0.77807	0.28290	0.78421	0.31214	0.17423	0.49777	0.27820	0.98238	0.35281

	50	49	48	47	46	45	44	43	42	41
a_O	73	65	72	64	57	63	70	68	63	71
	(27.0)	(24.1)	(26.7)	(23.7)	(21.1)	(23.3)	(25.9)	(25.2)	(23.3)	(26.3)
a_E	68.6	68.6	68.6	68.6	68.6	68.6	68.6	68.6	68.6	68.6
	(25.4)	(25.4)	(25.4)	(25.4)	(25.4)	(25.4)	(25.4)	(25.4)	(25.4)	(25.4)
	9.1	-7.0	7.0	-8.9	-21.1	-10.7	2.8	-1.2	-10.7	4.9
	3.362	-2.595	2.580	-3.291	-7.821	-3.974	1.048	-0.442	-3.974	1.809
t_O	80	76	79	77	81	62	57	80	78	92
	(29.6)	(28.1)	(29.3)	(28.5)	(30.0)	(23.0)	(21.1)	(29.6)	(28.9)	(34.1)
t_E	77.0	77.0	77.0	77.0	77.0	77.0	77.0	77.0	77.0	77.0
	(28.5)	(28.5)	(28.5)	(28.5)	(28.5)	(28.5)	(28.5)	(28.5)	(28.5)	(28.5)
	6.2	-1.9	4.1	0.0	8.2	-26.8	-34.3	6.2	2.1	32.8
	2.280	-0.721	1.516	0.015	3.054	-9.939	-12.688	2.280	0.761	12.147
g_O	59	62	63	60	73	81	63	61	84	46
	(21.9)	(23.0)	(23.3)	(22.2)	(27.0)	(30.0)	(23.3)	(22.6)	(31.1)	(17.0)
g_E	64.5	64.5	64.5	64.5	64.5	64.5	64.5	64.5	64.5	64.5
	(23.9)	(23.9)	(23.9)	(23.9)	(23.9)	(23.9)	(23.9)	(23.9)	(23.9)	(23.9)
	-10.6	-5.0	-3.0	-8.8	18.0	36.8	-3.0	-6.9	44.3	-31.2
	-3.922	-1.844	-1.127	-3.242	6.661	13.630	-1.127	-2.549	16.398	-11.539
c_O	58	67	56	69	59	64	80	61	45	61
	(21.5)	(24.8)	(20.7)	(25.6)	(21.9)	(23.7)	(29.6)	(22.6)	(16.7)	(22.6)
c_E	59.9	59.9	59.9	59.9	59.9	59.9	59.9	59.9	59.9	59.9
	(22.2)	(22.2)	(22.2)	(22.2)	(22.2)	(22.2)	(22.2)	(22.2)	(22.2)	(22.2)
	-3.7	15.1	-7.5	19.6	-1.7	8.5	46.3	2.3	-25.7	2.3
	-1.370	5.576	-2.779	7.246	-0.647	3.155	17.166	0.837	-9.523	0.837
N	270	270	270	270	270	270	270	270	270	270
X2	0.9	1.1	0.5	2.0	3.3	7.9	12.0	0.3	10.0	8.4
Gvalue	0.9	1.1	0.5	2.0	3.4	7.8	11.9	0.3	9.9	8.8
Gv/N	0.349	0.415	0.190	0.728	1.246	2.871	4.399	0.126	3.661	3.254
Prob	0.81510	0.77199	0.91616	0.57946	0.33866	0.05141	0.00781	0.95217	0.01956	0.03229

	40	39	38	37	36	35	34	33	32	31
a_O	76	71	48	85	62	86	80	55	78	77
	(28.1)	(26.3)	(17.8)	(31.5)	(23.0)	(31.9)	(29.6)	(20.4)	(28.9)	(28.5)
a_E	68.6	68.6	68.6	68.6	68.6	68.6	68.6	68.6	68.6	68.6
	(25.4)	(25.4)	(25.4)	(25.4)	(25.4)	(25.4)	(25.4)	(25.4)	(25.4)	(25.4)
	15.6	4.9	-34.3	36.4	-12.5	38.9	24.6	-24.3	20.0	17.8
	5.767	1.809	-12.697	13.497	-4.646	14.401	9.110	-9.002	7.420	6.589
t_O	81	72	81	72	84	75	71	82	73	74
	(30.0)	(26.7)	(30.0)	(26.7)	(31.1)	(27.8)	(26.3)	(30.4)	(27.0)	(27.4)
t_E	77.0	77.0	77.0	77.0	77.0	77.0	77.0	77.0	77.0	77.0
	(28.5)	(28.5)	(28.5)	(28.5)	(28.5)	(28.5)	(28.5)	(28.5)	(28.5)	(28.5)
	8.2	-9.6	8.2	-9.6	14.7	-3.9	-11.5	10.4	-7.8	-5.8
	3.054	-3.567	3.054	-3.567	5.430	-1.448	-4.253	3.837	-2.871	-2.164
g_O	57	69	76	66	68	62	68	76	70	63
	(21.1)	(25.6)	(28.1)	(24.4)	(25.2)	(23.0)	(25.2)	(28.1)	(25.9)	(23.3)
g_E	64.5	64.5	64.5	64.5	64.5	64.5	64.5	64.5	64.5	64.5
	(23.9)	(23.9)	(23.9)	(23.9)	(23.9)	(23.9)	(23.9)	(23.9)	(23.9)	(23.9)
	-14.2	9.2	24.8	3.0	7.1	-5.0	7.1	24.8	11.4	-3.0
	-5.245	3.415	9.202	1.094	2.630	-1.844	2.630	9.202	4.211	-1.127
c_O	56	58	65	47	56	47	51	57	49	56
	(20.7)	(21.5)	(24.1)	(17.4)	(20.7)	(17.4)	(18.9)	(21.1)	(18.1)	(20.7)
c_E	59.9	59.9	59.9	59.9	59.9	59.9	59.9	59.9	59.9	59.9

```
        ( 22.2) ( 22.2) ( 22.2) ( 22.2) ( 22.2) ( 22.2) ( 22.2) ( 22.2) ( 22.2) ( 22.2)
           -7.5    -3.7    10.7   -22.8    -7.5   -22.8   -16.4    -5.6   -19.7    -7.5
         -2.779  -1.370   3.950  -8.432  -2.779  -8.432  -6.064  -2.081  -7.278  -2.779

N           270     270     270     270     270     270     270     270     270     270
X2          2.1     0.8     8.9     7.0     1.7     7.3     3.9     5.2     3.9     1.4
Gvalue      2.2     0.8     9.5     7.0     1.7     7.2     3.8     5.3     4.0     1.4
Gv/N      0.797   0.286   3.509   2.591   0.636   2.677   1.424   1.956   1.482   0.518
Prob    0.54152 0.85583 0.02359 0.07200 0.63311 0.06498 0.27880 0.15241 0.26140 0.70555
        -------------------------------------------------------------------------------
             30      29      28      27      26      25      24      23      22      21
        -------------------------------------------------------------------------------
a_O          58      75      60      63      71      70      75      66      73      65
        ( 21.5) ( 27.8) ( 22.2) ( 23.3) ( 26.3) ( 25.9) ( 27.8) ( 24.4) ( 27.0) ( 24.1)
a_E        68.6    68.6    68.6    68.6    68.6    68.6    68.6    68.6    68.6    68.6
        ( 25.4) ( 25.4) ( 25.4) ( 25.4) ( 25.4) ( 25.4) ( 25.4) ( 25.4) ( 25.4) ( 25.4)
          -19.5    13.4   -16.1   -10.7     4.9     2.8    13.4    -5.1     9.1    -7.0
         -7.211   4.955  -5.953  -3.974   1.809   1.048   4.955  -1.889   3.362  -2.595
t_O          91      65      67      77      76      75      80      71      67      89
        ( 33.7) ( 24.1) ( 24.8) ( 28.5) ( 28.1) ( 27.8) ( 29.6) ( 26.3) ( 24.8) ( 33.0)
t_E        77.0    77.0    77.0    77.0    77.0    77.0    77.0    77.0    77.0    77.0
        ( 28.5) ( 28.5) ( 28.5) ( 28.5) ( 28.5) ( 28.5) ( 28.5) ( 28.5) ( 28.5) ( 28.5)
           30.5   -22.0   -18.6     0.0    -1.9    -3.9     6.2   -11.5   -18.6    25.8
         11.278  -8.145  -6.891   0.015  -0.721  -1.448   2.280  -4.253  -6.891   9.565
g_O          69      70      75      67      80      74      59      83      77      59
        ( 25.6) ( 25.9) ( 27.8) ( 24.8) ( 29.6) ( 27.4) ( 21.9) ( 30.7) ( 28.5) ( 21.9)
g_E        64.5    64.5    64.5    64.5    64.5    64.5    64.5    64.5    64.5    64.5
        ( 23.9) ( 23.9) ( 23.9) ( 23.9) ( 23.9) ( 23.9) ( 23.9) ( 23.9) ( 23.9) ( 23.9)
            9.2    11.4    22.5     5.0    34.4    20.2   -10.6    41.8    27.2   -10.6
          3.415   4.211   8.345   1.857  12.725   7.498  -3.922  15.466  10.068  -3.922
c_O          52      60      68      63      43      51      56      50      53      57
        ( 19.3) ( 22.2) ( 25.2) ( 23.3) ( 15.9) ( 18.9) ( 20.7) ( 18.5) ( 19.6) ( 21.1)
c_E        59.9    59.9    59.9    59.9    59.9    59.9    59.9    59.9    59.9    59.9
        ( 22.2) ( 22.2) ( 22.2) ( 22.2) ( 22.2) ( 22.2) ( 22.2) ( 22.2) ( 22.2) ( 22.2)
          -14.7     0.2    17.3     6.4   -28.5   -16.4    -7.5   -18.0   -12.9    -5.6
         -5.435   0.089   6.405   2.370 -10.548  -6.064  -2.779  -6.679  -4.792  -2.081

N           270     270     270     270     270     270     270     270     270     270
X2          5.5     2.9     5.2     0.7     8.6     2.8     1.4     7.5     4.8     2.7
Gvalue      5.5     3.0     5.1     0.7     8.8     2.8     1.4     7.1     4.7     2.6
Gv/N      2.047   1.111   1.905   0.268   3.265   1.033   0.534   2.646   1.747   0.966
Prob    0.13699 0.39187 0.16149 0.86789 0.03184 0.42507 0.69547 0.06747 0.19375 0.45591
        -------------------------------------------------------------------------------
             20      19      18      17      16      15      14      13      12      11
        -------------------------------------------------------------------------------
a_O          60      65      52      67      58      68      59      81      71      72
        ( 22.2) ( 24.1) ( 19.3) ( 24.8) ( 21.5) ( 25.2) ( 21.9) ( 30.0) ( 26.3) ( 26.7)
a_E        68.6    68.6    68.6    68.6    68.6    68.6    68.6    68.6    68.6    68.6
        ( 25.4) ( 25.4) ( 25.4) ( 25.4) ( 25.4) ( 25.4) ( 25.4) ( 25.4) ( 25.4) ( 25.4)
          -16.1    -7.0   -28.8    -3.2   -19.5    -1.2   -17.8    26.9     4.9     7.0
         -5.953  -2.595 -10.672  -1.171  -7.211  -0.442  -6.589   9.969   1.809   2.580
t_O          94      94     117      86      94      82      95      81      93      73
        ( 34.8) ( 34.8) ( 43.3) ( 31.9) ( 34.8) ( 30.4) ( 35.2) ( 30.0) ( 34.4) ( 27.0)
t_E        77.0    77.0    77.0    77.0    77.0    77.0    77.0    77.0    77.0    77.0
        ( 28.5) ( 28.5) ( 28.5) ( 28.5) ( 28.5) ( 28.5) ( 28.5) ( 28.5) ( 28.5) ( 28.5)
           37.6    37.6    98.0    19.1    37.6    10.4    40.0     8.2    35.2    -7.8
         13.908  13.908  36.281   7.058  13.908   3.837  14.801   3.054  13.024  -2.871
g_O          61      49      40      56      57      51      44      38      49      60
        ( 22.6) ( 18.1) ( 14.8) ( 20.7) ( 21.1) ( 18.9) ( 16.3) ( 14.1) ( 18.1) ( 22.2)
g_E        64.5    64.5    64.5    64.5    64.5    64.5    64.5    64.5    64.5    64.5
        ( 23.9) ( 23.9) ( 23.9) ( 23.9) ( 23.9) ( 23.9) ( 23.9) ( 23.9) ( 23.9) ( 23.9)
           -6.9   -27.0   -38.3   -15.9   -14.2   -24.0   -33.7   -40.3   -27.0    -8.8
         -2.549  -9.998 -14.175  -5.888  -5.245  -8.895 -12.486 -14.910  -9.998  -3.242
c_O          55      62      61      61      61      69      72      70      57      65
        ( 20.4) ( 23.0) ( 22.6) ( 22.6) ( 22.6) ( 25.6) ( 26.7) ( 25.9) ( 21.1) ( 24.1)
c_E        59.9    59.9    59.9    59.9    59.9    59.9    59.9    59.9    59.9    59.9
        ( 22.2) ( 22.2) ( 22.2) ( 22.2) ( 22.2) ( 22.2) ( 22.2) ( 22.2) ( 22.2) ( 22.2)
           -9.4     4.3     2.3     2.3     2.3    19.6    26.5    21.9    -5.6    10.7
         -3.463   1.598   0.837   0.837   0.837   7.246   9.831   8.097  -2.081   3.950

N           270     270     270     270     270     270     270     270     270     270
X2          5.4     7.8    34.2     2.2     6.3     4.6    14.6    15.1     7.3     1.1
Gvalue      5.2     7.9    33.1     2.3     6.2     4.7    15.0    16.8     7.4     1.1
Gv/N      1.943   2.913  12.272   0.837   2.289   1.745   5.557   6.210   2.753   0.418
Prob    0.15465 0.04891 0.00000 0.52027 0.10315 0.19412 0.00181 0.00079 0.05932 0.77028
        -------------------------------------------------------------------------------
             10       9       8       7       6       5       4       3       2       1
        -------------------------------------------------------------------------------
a_O          67      72      74      74      50      67      63      61      71      65
        ( 24.8) ( 26.7) ( 27.4) ( 27.4) ( 18.5) ( 24.8) ( 23.3) ( 22.6) ( 26.3) ( 24.1)
a_E        68.6    68.6    68.6    68.6    68.6    68.6    68.6    68.6    68.6    68.6
        ( 25.4) ( 25.4) ( 25.4) ( 25.4) ( 25.4) ( 25.4) ( 25.4) ( 25.4) ( 25.4) ( 25.4)
           -3.2     7.0    11.2    11.2   -31.6    -3.2   -10.7   -14.3     4.9    -7.0
         -1.171   2.580   4.153   4.153 -11.714  -1.171  -3.974  -5.306   1.809  -2.595
t_O          89      87      79      90      81      71     100      77      58      99
        ( 33.0) ( 32.2) ( 29.3) ( 33.3) ( 30.0) ( 26.3) ( 37.0) ( 28.5) ( 21.5) ( 36.7)
t_E        77.0    77.0    77.0    77.0    77.0    77.0    77.0    77.0    77.0    77.0
        ( 28.5) ( 28.5) ( 28.5) ( 28.5) ( 28.5) ( 28.5) ( 28.5) ( 28.5) ( 28.5) ( 28.5)
           25.8    21.3     4.1    28.1     8.2   -11.5    52.3     0.0   -32.8    49.8
          9.565   7.886   1.516  10.418   3.054  -4.253  19.380   0.015 -12.163  18.449
g_O          44      43      62      49      64      64      60      83      79      50
        ( 16.3) ( 15.9) ( 23.0) ( 18.1) ( 23.7) ( 23.7) ( 22.2) ( 30.7) ( 29.3) ( 18.5)
g_E        64.5    64.5    64.5    64.5    64.5    64.5    64.5    64.5    64.5    64.5
        ( 23.9) ( 23.9) ( 23.9) ( 23.9) ( 23.9) ( 23.9) ( 23.9) ( 23.9) ( 23.9) ( 23.9)
          -33.7   -34.9    -5.0   -27.0    -1.1    -1.1    -8.8    41.8    31.9   -25.5
        -12.486 -12.935  -1.844  -9.998  -0.398  -0.398  -3.242  15.466  11.830  -9.454
c_O          70      68      55      57      75      68      47      49      62      56
        ( 25.9) ( 25.2) ( 20.4) ( 21.1) ( 27.8) ( 25.2) ( 17.4) ( 18.1) ( 23.0) ( 20.7)
c_E        59.9    59.9    59.9    59.9    59.9    59.9    59.9    59.9    59.9    59.9
        ( 22.2) ( 22.2) ( 22.2) ( 22.2) ( 22.2) ( 22.2) ( 22.2) ( 22.2) ( 22.2) ( 22.2)
           21.9    17.3    -9.4    -5.6    33.8    17.3   -22.8   -19.7     4.3    -7.5
          8.097   6.405  -3.463  -2.081  12.508   6.405  -8.432  -7.278   1.598  -2.779

N           270     270     270     270     270     270     270     270     270     270
X2         10.2     9.8     1.0     6.5     9.1     1.6    10.4     8.1     8.1    10.0
Gvalue     10.8    10.6     1.0     6.7     9.3     1.6    10.1     7.8     8.3     9.8
Gv/N      4.005   3.936   0.362   2.492   3.450   0.583   3.732   2.897   3.074   3.620
Prob    0.01278 0.01392 0.80678 0.08112 0.02537 0.66538 0.01793 0.04984 0.04022 0.02058
        -------------------------------------------------------------------------------

CDS_INITIAL:
        -------------------------------------------------------------------------------
```

	1	2	3	4	5	6	7	8	9	10
a_O	114	0	0	56	59	60	77	62	54	86
	(42.2)	(0.0)	(0.0)	(20.7)	(21.9)	(22.2)	(28.5)	(23.0)	(20.0)	(31.9)
a_E	74.6	76.4	58.1	74.6	76.4	58.1	74.6	76.4	58.1	74.6
	(27.6)	(28.3)	(21.5)	(27.6)	(28.3)	(21.5)	(27.6)	(28.3)	(21.5)	(27.6)
	96.7	0.0	0.0	−32.1	−30.5	3.8	4.9	−25.9	−7.9	24.4
	35.800	0.000	0.000	−11.901	−11.286	1.418	1.800	−9.582	−2.939	9.052
t_O	75	270	0	70	74	72	49	77	85	65
	(27.8)	(100.0)	(0.0)	(25.9)	(27.4)	(26.7)	(18.1)	(28.5)	(31.5)	(24.1)
t_E	47.0	81.3	69.3	47.0	81.3	69.3	47.0	81.3	69.3	47.0
	(17.4)	(30.1)	(25.7)	(17.4)	(30.1)	(25.7)	(17.4)	(30.1)	(25.7)	(17.4)
	70.2	648.4	0.0	55.8	−13.8	5.5	4.1	−8.3	34.7	42.2
	25.994	240.159	0.000	20.684	−5.128	2.034	1.533	−3.070	12.852	15.638
g_O	81	0	270	75	76	78	86	74	72	74
	(30.0)	(0.0)	(100.0)	(27.8)	(28.1)	(28.9)	(31.9)	(27.4)	(26.7)	(27.4)
g_E	98.2	52.3	76.6	98.2	52.3	76.6	98.2	52.3	76.6	98.2
	(36.4)	(19.4)	(28.4)	(36.4)	(19.4)	(28.4)	(36.4)	(19.4)	(28.4)	(36.4)
	−31.2	0.0	680.5	−40.4	56.7	2.9	−22.8	51.3	−8.9	−41.8
	−11.541	0.000	252.053	−14.962	20.998	1.072	−8.438	18.984	−3.279	−15.498
c_O	0	0	0	69	61	60	58	57	59	45
	(0.0)	(0.0)	(0.0)	(25.6)	(22.6)	(22.2)	(21.5)	(21.1)	(21.9)	(16.7)
c_E	50.2	60.0	66.0	50.2	60.0	66.0	50.2	60.0	66.0	50.2
	(18.6)	(22.2)	(24.4)	(18.6)	(22.2)	(24.4)	(18.6)	(22.2)	(24.4)	(18.6)
	0.0	0.0	0.0	43.8	2.0	−11.5	16.7	−5.9	−13.2	−9.9
	0.000	0.000	0.000	16.220	0.732	−4.244	6.173	−2.180	−4.907	−3.670
N	270	270	270	270	270	270	270	270	270	270
X2	90.8	627.1	682.1	28.4	15.3	0.7	2.9	12.0	4.9	15.2
Gvalue	135.7	648.4	680.5	27.1	14.4	0.8	2.9	11.2	4.7	14.9
Gv/N	50.253	240.159	252.053	10.040	5.316	0.280	1.067	4.153	1.727	5.522
Prob	0.00000	0.00000	0.00000	0.00001	0.00246	0.86013	0.41021	0.01063	0.19832	0.00190

	11	12	13	14	15	16	17	18	19	20
a_O	61	68	69	69	55	84	67	63	69	60
	(22.6)	(25.2)	(25.6)	(25.6)	(20.4)	(31.1)	(24.8)	(23.3)	(25.6)	(22.2)
a_E	76.4	58.1	74.6	76.4	58.1	74.6	76.4	58.1	74.6	76.4
	(28.3)	(21.5)	(27.6)	(28.3)	(21.5)	(27.6)	(28.3)	(21.5)	(27.6)	(28.3)
	−27.4	21.4	−10.8	−14.0	−6.1	19.9	−17.6	10.2	−10.8	−29.0
	−10.162	7.911	−3.994	−5.196	−2.246	7.377	−6.505	3.765	−3.994	−10.730
t_O	98	84	64	92	80	59	105	75	55	98
	(36.3)	(31.1)	(23.7)	(34.1)	(29.6)	(21.9)	(38.9)	(27.8)	(20.4)	(36.3)
t_E	81.3	69.3	47.0	81.3	69.3	47.0	81.3	69.3	47.0	81.3
	(30.1)	(25.7)	(17.4)	(30.1)	(25.7)	(17.4)	(30.1)	(25.7)	(17.4)	(30.1)
	36.7	32.3	39.6	22.8	23.0	26.9	53.8	11.8	17.4	36.7
	13.600	11.964	14.662	8.461	8.503	9.962	19.937	4.387	6.426	13.600
g_O	58	75	85	53	78	78	56	76	95	57
	(21.5)	(27.8)	(31.5)	(19.6)	(28.9)	(28.9)	(20.7)	(28.1)	(35.2)	(21.1)
g_E	52.3	76.6	98.2	52.3	76.6	98.2	52.3	76.6	98.2	52.3
	(19.4)	(28.4)	(36.4)	(19.4)	(28.4)	(36.4)	(19.4)	(28.4)	(36.4)	(19.4)
	11.9	−3.1	−24.5	1.3	2.9	−35.9	7.6	−1.1	−6.3	9.7
	4.412	−1.148	−9.076	0.493	1.072	−13.294	2.804	−0.418	−2.317	3.602
c_O	53	43	52	56	57	49	42	56	51	55
	(19.6)	(15.9)	(19.3)	(20.7)	(21.1)	(18.1)	(15.6)	(20.7)	(18.9)	(20.4)
c_E	60.0	66.0	50.2	60.0	66.0	50.2	60.0	66.0	50.2	60.0
	(22.2)	(24.4)	(18.6)	(22.2)	(24.4)	(18.6)	(22.2)	(24.4)	(18.6)	(22.2)
	−13.2	−36.9	3.6	−7.8	−16.7	−2.4	−30.0	−18.4	1.5	−9.6
	−4.883	−13.653	1.328	−2.876	−6.197	−0.905	−11.107	−6.823	0.569	−3.558
N	270	270	270	270	270	270	270	270	270	270
X2	8.0	12.8	8.4	2.4	3.1	8.4	13.8	2.4	1.9	7.8
Gvalue	8.0	13.7	7.9	2.4	3.1	8.5	13.9	2.5	1.8	7.9
Gv/N	2.967	5.075	2.920	0.883	1.133	3.140	5.130	0.911	0.684	2.913
Prob	0.04579	0.00334	0.04845	0.49680	0.38271	0.03712	0.00312	0.48247	0.60467	0.04886

	21	22	23	24	25	26	27	28	29	30
a_O	51	75	66	56	76	82	62	85	56	58
	(18.9)	(27.8)	(24.4)	(20.7)	(28.1)	(30.4)	(23.0)	(31.5)	(20.7)	(21.5)
a_E	58.1	74.6	76.4	58.1	74.6	76.4	58.1	74.6	76.4	58.1
	(21.5)	(27.6)	(28.3)	(21.5)	(27.6)	(28.3)	(21.5)	(27.6)	(28.3)	(21.5)
	−13.3	0.8	−19.3	−4.2	2.8	11.6	8.0	22.2	−34.8	−0.2
	−4.935	0.291	−7.143	−1.539	1.040	4.310	2.971	8.210	−12.876	−0.086
t_O	86	49	85	87	49	79	80	45	87	78
	(31.9)	(18.1)	(31.5)	(32.2)	(18.1)	(29.3)	(29.6)	(16.7)	(32.2)	(28.9)
t_E	69.3	47.0	81.3	69.3	47.0	81.3	69.3	47.0	81.3	69.3
	(25.7)	(17.4)	(30.1)	(25.7)	(17.4)	(30.1)	(25.7)	(17.4)	(30.1)	(25.7)
	37.1	4.1	7.7	39.6	4.1	−4.5	23.0	−3.9	11.9	18.4
	13.748	1.533	2.835	14.653	1.533	−1.649	8.503	−1.431	4.400	6.828
g_O	79	95	69	76	101	66	74	85	66	75
	(29.3)	(35.2)	(25.6)	(28.1)	(37.4)	(24.4)	(27.4)	(31.5)	(24.4)	(27.8)
g_E	76.6	98.2	52.3	76.6	98.2	52.3	76.6	98.2	52.3	76.6
	(28.4)	(36.4)	(19.4)	(28.4)	(36.4)	(19.4)	(28.4)	(36.4)	(19.4)	(28.4)
	4.9	−6.3	38.1	−1.1	5.7	30.6	−5.0	−24.5	30.6	−3.1
	1.831	−2.317	14.125	−0.418	2.118	11.338	−1.869	−9.076	11.338	−1.148
c_O	54	51	50	51	44	43	54	55	61	59
	(20.0)	(18.9)	(18.5)	(18.9)	(16.3)	(15.9)	(20.0)	(20.4)	(22.6)	(21.9)
c_E	66.0	50.2	60.0	66.0	50.2	60.0	66.0	50.2	60.0	66.0
	(24.4)	(18.6)	(22.2)	(24.4)	(18.6)	(22.2)	(24.4)	(18.6)	(22.2)	(24.4)
	−21.7	1.5	−18.3	−26.3	−11.7	−28.7	−21.7	10.0	2.0	−13.2
	−8.034	0.569	−6.765	−9.747	−4.321	−10.622	−8.034	3.690	0.732	−4.907
N	270	270	270	270	270	270	270	270	270	270
X2	7.2	0.2	8.6	8.0	1.0	8.9	4.2	3.8	9.4	1.9
Gvalue	7.0	0.2	8.2	8.0	1.0	9.1	4.2	3.8	9.7	1.9
Gv/N	2.611	0.076	3.052	2.950	0.371	3.377	1.572	1.393	3.594	0.686
Prob	0.07032	0.97697	0.04129	0.04675	0.80105	0.02777	0.23631	0.28847	0.02126	0.60353

	31	32	33	34	35	36	37	38	39	40
a_O	81	58	66	90	57	54	78	64	51	71
	(30.0)	(21.5)	(24.4)	(33.3)	(21.1)	(20.0)	(28.9)	(23.7)	(18.9)	(26.3)
a_E	74.6	76.4	58.1	74.6	76.4	58.1	74.6	76.4	58.1	74.6
	(27.6)	(28.3)	(21.5)	(27.6)	(28.3)	(21.5)	(27.6)	(28.3)	(21.5)	(27.6)
	13.3	−31.9	16.8	33.8	−33.4	−7.9	6.9	−22.6	−13.3	−7.0
	4.932	−11.829	6.219	12.504	−12.359	−2.939	2.569	−8.386	−4.935	−2.607
t_O	49	97	69	55	87	72	63	80	80	58
	(18.1)	(35.9)	(25.6)	(20.4)	(32.2)	(26.7)	(23.3)	(29.6)	(29.6)	(21.5)
t_E	47.0	81.3	69.3	47.0	81.3	69.3	47.0	81.3	69.3	47.0
	(17.4)	(30.1)	(25.7)	(17.4)	(30.1)	(25.7)	(17.4)	(30.1)	(25.7)	(17.4)
	4.1	34.4	−0.6	17.4	11.9	5.5	37.0	−2.5	23.0	24.5

	1.533	12.724	-0.226	6.426	4.400	2.034	13.698	-0.924	8.503	9.059
g_O	88	73	80	83	70	76	84	70	80	88
	(32.6)	(27.0)	(29.6)	(30.7)	(25.9)	(28.1)	(31.1)	(25.9)	(29.6)	(32.6)
g_E	98.2	52.3	76.6	98.2	52.3	76.6	98.2	52.3	76.6	98.2
	(36.4)	(19.4)	(28.4)	(36.4)	(19.4)	(28.4)	(36.4)	(19.4)	(28.4)	(36.4)
	-19.3	48.6	7.0	-27.9	40.7	-1.1	-26.2	40.7	7.0	-19.3
	-7.136	17.991	2.600	-10.327	15.076	-0.418	-9.706	15.076	2.600	-7.136
c_O	52	42	55	42	56	68	45	56	59	53
	(19.3)	(15.6)	(20.4)	(15.6)	(20.7)	(25.2)	(16.7)	(20.7)	(21.9)	(19.6)
c_E	50.2	60.0	66.0	50.2	60.0	66.0	50.2	60.0	66.0	50.2
	(18.6)	(22.2)	(24.4)	(18.6)	(22.2)	(24.4)	(18.6)	(22.2)	(24.4)	(18.6)
	3.6	-30.0	-20.1	-15.0	-7.8	4.0	-9.9	-7.8	-13.2	5.7
	1.328	-11.107	-7.435	-5.572	-2.876	1.495	-3.670	-2.876	-4.907	2.102
N	270	270	270	270	270	270	270	270	270	270
X2	1.8	21.0	3.1	8.2	11.6	0.5	8.2	8.3	3.4	4.0
Gvalue	1.8	21.0	3.1	8.2	11.5	0.5	7.8	7.8	3.4	3.8
Gv/N	0.657	7.780	1.158	3.032	4.242	0.172	2.891	2.890	1.261	1.417
Prob	0.62063	0.00011	0.37267	0.04232	0.00951	0.92637	0.05019	0.05023	0.33331	0.28075

	41	42	43	44	45	46	47	48	49	50
a_O	51	41	69	65	63	81	70	56	86	62
	(18.9)	(15.2)	(25.6)	(24.1)	(23.3)	(30.0)	(25.9)	(20.7)	(31.9)	(23.0)
a_E	76.4	58.1	74.6	76.4	58.1	74.6	76.4	58.1	74.6	76.4
	(28.3)	(21.5)	(27.6)	(28.3)	(21.5)	(27.6)	(28.3)	(21.5)	(27.6)	(28.3)
	-41.2	-28.6	-10.8	-21.0	10.2	13.3	-12.2	-4.2	24.4	-25.9
	-15.260	-10.596	-3.994	-7.770	3.765	4.932	-4.525	-1.539	9.052	-9.582
t_O	84	88	63	82	81	52	80	81	46	96
	(31.1)	(32.6)	(23.3)	(30.4)	(30.0)	(19.3)	(29.6)	(30.0)	(17.0)	(35.6)
t_E	81.3	69.3	47.0	81.3	69.3	47.0	81.3	69.3	47.0	81.3
	(30.1)	(25.7)	(17.4)	(30.1)	(25.7)	(17.4)	(30.1)	(25.7)	(17.4)	(30.1)
	5.6	42.0	37.0	1.5	25.3	10.6	-2.5	25.3	-1.9	32.0
	2.065	15.567	13.698	0.552	9.355	3.915	-0.924	9.355	-0.714	11.856
g_O	81	75	87	60	69	85	71	74	90	58
	(30.0)	(27.8)	(32.2)	(22.2)	(25.6)	(31.5)	(26.3)	(27.4)	(33.3)	(21.5)
g_E	52.3	76.6	98.2	52.3	76.6	98.2	52.3	76.6	98.2	52.3
	(19.4)	(28.4)	(36.4)	(19.4)	(28.4)	(36.4)	(19.4)	(28.4)	(36.4)	(19.4)
	70.7	-3.1	-21.0	16.4	-14.4	-24.5	43.3	-5.0	-15.7	11.9
	26.202	-1.148	-7.791	6.071	-5.318	-9.076	16.037	-1.869	-5.800	4.412
c_O	54	66	51	63	57	52	49	59	48	54
	(20.0)	(24.4)	(18.9)	(23.3)	(21.1)	(19.3)	(18.1)	(21.9)	(17.8)	(20.0)
c_E	60.0	66.0	50.2	60.0	66.0	50.2	60.0	66.0	50.2	60.0
	(22.2)	(24.4)	(18.6)	(22.2)	(24.4)	(18.6)	(22.2)	(24.4)	(18.6)	(22.2)
	-11.4	-0.0	1.5	6.1	-16.7	3.6	-19.9	-13.2	-4.4	-11.4
	-4.228	-0.008	0.569	2.262	-6.197	1.328	-7.363	-4.907	-1.620	-4.228
N	270	270	270	270	270	270	270	270	270	270
X2	24.8	10.1	7.2	3.0	4.4	2.9	9.2	2.9	2.5	6.6
Gvalue	23.7	10.3	6.7	3.0	4.3	3.0	8.7	2.8	2.5	6.6
Gv/N	8.780	3.814	2.482	1.115	1.605	1.099	3.225	1.040	0.918	2.459
Prob	0.00003	0.01619	0.08201	0.39003	0.22759	0.39665	0.03344	0.42214	0.47894	0.08434

	51	52	53	54	55	56	57	58	59	60
a_O	58	60	67	64	60	66	66	77	65	65
	(21.5)	(22.2)	(24.8)	(23.7)	(22.2)	(24.4)	(24.4)	(28.5)	(24.1)	(24.1)
a_E	58.1	74.6	76.4	58.1	74.6	76.4	58.1	74.6	76.4	58.1
	(21.5)	(27.6)	(28.3)	(21.5)	(27.6)	(28.3)	(21.5)	(27.6)	(28.3)	(21.5)
	-0.2	-26.1	-17.6	12.3	-26.1	-19.3	16.8	4.9	-21.0	14.6
	-0.086	-9.685	-6.505	4.572	-9.685	-7.143	6.219	1.800	-7.770	5.390
t_O	72	49	81	71	53	75	78	46	95	70
	(26.7)	(18.1)	(30.0)	(26.3)	(19.6)	(27.8)	(28.9)	(17.0)	(35.2)	(25.9)
t_E	69.3	47.0	81.3	69.3	47.0	81.3	69.3	47.0	81.3	69.3
	(25.7)	(17.4)	(30.1)	(25.7)	(17.4)	(30.1)	(25.7)	(17.4)	(30.1)	(25.7)
	5.5	4.1	-0.5	3.4	12.8	-12.0	18.4	-1.9	29.7	1.4
	2.034	1.533	-0.191	1.270	4.738	-4.452	6.828	-0.714	10.995	0.517
g_O	84	113	63	72	112	67	71	104	57	82
	(31.1)	(41.9)	(23.3)	(26.7)	(41.5)	(24.8)	(26.3)	(38.5)	(21.1)	(30.4)
g_E	76.6	98.2	52.3	76.6	98.2	52.3	76.6	98.2	52.3	76.6
	(28.4)	(36.4)	(19.4)	(28.4)	(36.4)	(19.4)	(28.4)	(36.4)	(19.4)	(28.4)
	15.6	31.8	23.4	-8.9	29.5	33.1	-10.7	12.0	9.7	11.2
	5.766	11.767	8.652	-3.279	10.926	12.256	-3.970	4.436	3.602	4.165
c_O	56	48	59	63	45	62	55	43	53	53
	(20.7)	(17.8)	(21.9)	(23.3)	(16.7)	(23.0)	(20.4)	(15.9)	(19.6)	(19.6)
c_E	66.0	50.2	60.0	66.0	50.2	60.0	66.0	50.2	60.0	66.0
	(24.4)	(18.6)	(22.2)	(24.4)	(18.6)	(22.2)	(24.4)	(18.6)	(22.2)	(24.4)
	-18.4	-4.4	-2.0	-5.9	-9.9	4.0	-20.1	-13.4	-13.2	-23.3
	-6.823	-1.620	-0.749	-2.179	-3.670	1.491	-7.435	-4.955	-4.883	-8.619
N	270	270	270	270	270	270	270	270	270	270
X2	2.3	5.3	3.3	1.0	6.1	6.1	4.4	1.5	5.3	3.8
Gvalue	2.4	5.4	3.3	1.0	6.2	5.8	4.4	1.5	5.2	3.9
Gv/N	0.891	1.995	1.207	0.383	2.309	2.151	1.643	0.567	1.944	1.452
Prob	0.49279	0.14554	0.35342	0.79279	0.10069	0.12128	0.21816	0.67507	0.15444	0.27017

	61	62	63	64	65	66	67	68	69	70
a_O	67	54	39	65	58	41	83	71	44	74
	(24.8)	(20.0)	(14.4)	(24.1)	(21.5)	(15.2)	(30.7)	(26.3)	(16.3)	(27.4)
a_E	74.6	76.4	58.1	74.6	76.4	58.1	74.6	76.4	58.1	74.6
	(27.6)	(28.3)	(21.5)	(27.6)	(28.3)	(21.5)	(27.6)	(28.3)	(21.5)	(27.6)
	-14.4	-37.5	-31.1	-17.9	-31.9	-28.6	17.7	-10.4	-24.5	-1.2
	-5.338	-13.871	-11.523	-6.638	-11.829	-10.596	6.553	-3.844	-9.069	-0.449
t_O	55	93	81	58	81	71	44	81	73	48
	(20.4)	(34.4)	(30.0)	(21.5)	(30.0)	(26.3)	(16.3)	(30.0)	(27.0)	(17.8)
t_E	47.0	81.3	69.3	47.0	81.3	69.3	47.0	81.3	69.3	47.0
	(17.4)	(30.1)	(25.7)	(17.4)	(30.1)	(25.7)	(17.4)	(30.1)	(25.7)	(17.4)
	17.4	25.1	25.3	24.5	-0.5	3.4	-5.8	-0.5	7.6	2.1
	6.426	9.298	9.355	9.059	-0.191	1.270	-2.132	-0.191	2.808	0.768
g_O	86	66	88	96	71	86	95	53	86	85
	(31.9)	(24.4)	(32.6)	(35.6)	(26.3)	(31.9)	(35.2)	(19.6)	(31.9)	(31.5)
g_E	98.2	52.3	76.6	98.2	52.3	76.6	98.2	52.3	76.6	98.2
	(36.4)	(19.4)	(28.4)	(36.4)	(19.4)	(28.4)	(36.4)	(19.4)	(28.4)	(36.4)
	-22.8	30.6	24.5	-4.3	43.3	20.0	-6.3	1.3	20.0	-24.5
	-8.438	11.338	9.072	-1.597	16.037	7.402	-2.317	0.493	7.402	-9.076
c_O	62	57	62	51	60	72	48	65	67	63
	(23.0)	(21.1)	(23.0)	(18.9)	(22.2)	(26.7)	(17.8)	(24.1)	(24.8)	(23.3)
c_E	50.2	60.0	66.0	50.2	60.0	66.0	50.2	60.0	66.0	50.2
	(18.6)	(22.2)	(24.4)	(18.6)	(22.2)	(24.4)	(18.6)	(22.2)	(24.4)	(18.6)
	26.1	-5.9	-7.8	1.5	-0.0	12.5	-4.4	10.4	2.0	28.5
	9.662	-2.180	-2.879	0.569	-0.015	4.632	-1.620	3.838	0.738	10.564

N	270	270	270	270	270	270	270	270	270	270
X2	6.4	12.0	10.2	3.9	11.1	6.8	1.3	0.8	4.8	5.0
Gvalue	6.2	12.4	10.9	3.8	10.8	7.3	1.3	0.8	5.1	4.9
Gv/N	2.312	4.585	4.025	1.393	4.003	2.708	0.484	0.296	1.878	1.807
Prob	0.10042	0.00619	0.01246	0.28847	0.01280	0.06262	0.72722	0.84945	0.16662	0.18083

	71	72	73	74	75	76	77	78	79	80
a_O	76	44	65	65	49	68	59	60	61	60
	(28.1)	(16.3)	(24.1)	(24.1)	(18.1)	(25.2)	(21.9)	(22.2)	(22.6)	(22.2)
a_E	76.4	58.1	74.6	76.4	58.1	74.6	76.4	58.1	74.6	76.4
	(28.3)	(21.5)	(27.6)	(28.3)	(21.5)	(27.6)	(28.3)	(21.5)	(27.6)	(28.3)
	-0.8	-24.5	-17.9	-21.0	-16.7	-12.6	-30.5	3.8	-24.6	-29.0
	-0.283	-9.069	-6.638	-7.770	-6.193	-4.672	-11.286	1.418	-9.099	-10.730
t_O	84	66	57	88	67	48	92	66	64	93
	(31.1)	(24.4)	(21.1)	(32.6)	(24.8)	(17.8)	(34.1)	(24.4)	(23.7)	(34.4)
t_E	81.3	69.3	47.0	81.3	69.3	47.0	81.3	69.3	47.0	81.3
	(30.1)	(25.7)	(17.4)	(30.1)	(25.7)	(17.4)	(30.1)	(25.7)	(17.4)	(30.1)
	5.6	-6.5	22.1	14.0	-4.5	2.1	22.8	-6.5	39.6	25.1
	2.065	-2.389	8.168	5.196	-1.679	0.768	8.461	-2.389	14.662	9.298
g_O	53	89	101	59	78	97	56	77	96	53
	(19.6)	(33.0)	(37.4)	(21.9)	(28.9)	(35.9)	(20.7)	(28.5)	(35.6)	(19.6)
g_E	52.3	76.6	98.2	52.3	76.6	98.2	52.3	76.6	98.2	52.3
	(19.4)	(28.4)	(36.4)	(19.4)	(28.4)	(36.4)	(19.4)	(28.4)	(36.4)	(19.4)
	1.3	26.8	5.7	14.1	2.9	-2.3	7.6	0.9	-4.3	1.3
	0.493	9.921	2.118	5.235	1.072	-0.869	2.804	0.322	-1.597	0.493
c_O	57	71	47	58	76	57	63	67	49	64
	(21.1)	(26.3)	(17.4)	(21.5)	(28.1)	(21.1)	(23.3)	(24.8)	(18.1)	(23.7)
c_E	60.0	66.0	50.2	60.0	66.0	50.2	60.0	66.0	50.2	60.0
	(22.2)	(24.4)	(18.6)	(22.2)	(24.4)	(18.6)	(22.2)	(24.4)	(18.6)	(22.2)
	-5.9	10.3	-6.3	-4.0	21.4	14.4	6.1	2.0	-2.4	8.2
	-2.180	3.832	-2.319	-1.471	7.933	5.332	2.262	0.738	-0.905	3.044
N	270	270	270	270	270	270	270	270	270	270
X2	0.3	6.0	3.7	3.2	3.0	1.5	5.8	0.2	8.7	5.5
Gvalue	0.3	6.2	3.6	3.2	3.1	1.5	6.1	0.2	8.3	5.7
Gv/N	0.095	2.293	1.330	1.191	1.132	0.560	2.242	0.088	3.061	2.105
Prob	0.96802	0.10262	0.30931	0.35967	0.38305	0.67953	0.10905	0.97126	0.04084	0.12806

	81	82	83	84	85	86	87	88	89	90
a_O	46	75	59	55	54	57	61	62	62	47
	(17.0)	(27.8)	(21.9)	(20.4)	(20.0)	(21.1)	(22.6)	(23.0)	(23.0)	(17.4)
a_E	58.1	74.6	76.4	58.1	74.6	76.4	58.1	74.6	76.4	58.1
	(21.5)	(27.6)	(28.3)	(21.5)	(27.6)	(28.3)	(21.5)	(27.6)	(28.3)	(21.5)
	-21.5	0.8	-30.5	-6.1	-34.9	-33.4	5.9	-23.0	-25.9	-20.0
	-7.967	0.291	-11.286	-2.246	-12.931	-12.359	2.188	-8.502	-9.582	-7.391
t_O	78	63	90	67	52	95	68	47	88	85
	(28.9)	(23.3)	(33.3)	(24.8)	(19.3)	(35.2)	(25.2)	(17.4)	(32.6)	(31.5)
t_E	69.3	47.0	81.3	69.3	47.0	81.3	69.3	47.0	81.3	69.3
	(25.7)	(17.4)	(30.1)	(25.7)	(17.4)	(30.1)	(25.7)	(17.4)	(30.1)	(25.7)
	18.4	37.0	18.4	-4.5	10.6	29.7	-2.6	0.1	14.0	34.7
	6.828	13.698	6.812	-1.679	3.915	10.995	-0.958	0.019	5.196	12.852
g_O	77	87	60	80	107	55	69	107	62	71
	(28.5)	(32.2)	(22.2)	(29.6)	(39.6)	(20.4)	(25.6)	(39.6)	(23.0)	(26.3)
g_E	76.6	98.2	52.3	76.6	98.2	52.3	76.6	98.2	52.3	76.6
	(28.4)	(36.4)	(19.4)	(28.4)	(36.4)	(19.4)	(28.4)	(36.4)	(19.4)	(28.4)
	0.9	-21.0	16.4	7.0	18.4	5.5	-14.4	18.4	21.0	-10.7
	0.322	-7.791	6.071	2.600	6.818	2.020	-5.318	6.818	7.779	-3.970
c_O	69	45	61	68	57	63	72	54	58	67
	(25.6)	(16.7)	(22.6)	(25.2)	(21.1)	(23.3)	(26.7)	(20.0)	(21.5)	(24.8)
c_E	66.0	50.2	60.0	66.0	50.2	60.0	66.0	50.2	60.0	66.0
	(24.4)	(18.6)	(22.2)	(24.4)	(18.6)	(22.2)	(24.4)	(18.6)	(22.2)	(24.4)
	6.1	-9.9	2.0	4.0	14.4	6.1	12.5	7.8	-4.0	2.0
	2.263	-3.670	0.732	1.495	5.332	2.262	4.632	2.889	-1.471	0.738
N	270	270	270	270	270	270	270	270	270	270
X2	3.8	7.3	6.0	0.5	7.9	7.5	1.5	3.2	5.1	6.1
Gvalue	3.9	6.8	6.3	0.5	8.5	7.9	1.5	3.3	5.2	6.0
Gv/N	1.447	2.528	2.330	0.170	3.135	2.918	0.543	1.225	1.923	2.229
Prob	0.27178	0.07764	0.09829	0.92783	0.03732	0.04857	0.68997	0.34672	0.15823	0.11071

	91	92	93	94	95	96	97	98	99	100
a_O	78	73	56	67	62	47	68	65	46	63
	(28.9)	(27.0)	(20.7)	(24.8)	(23.0)	(17.4)	(25.2)	(24.1)	(17.0)	(23.3)
a_E	74.6	76.4	58.1	74.6	76.4	58.1	74.6	76.4	58.1	74.6
	(27.6)	(28.3)	(21.5)	(27.6)	(28.3)	(21.5)	(27.6)	(28.3)	(21.5)	(27.6)
	6.9	-6.6	-4.2	-14.4	-25.9	-20.0	-12.6	-21.0	-21.5	-21.3
	2.569	-2.450	-1.539	-5.338	-9.582	-7.391	-4.672	-7.770	-7.967	-7.892
t_O	55	92	73	52	97	67	68	85	75	50
	(20.4)	(34.1)	(27.0)	(19.3)	(35.9)	(24.8)	(25.2)	(31.5)	(27.8)	(18.5)
t_E	47.0	81.3	69.3	47.0	81.3	69.3	47.0	81.3	69.3	47.0
	(17.4)	(30.1)	(25.7)	(17.4)	(30.1)	(25.7)	(17.4)	(30.1)	(25.7)	(17.4)
	17.4	22.8	7.6	10.6	34.4	-4.5	50.3	7.7	11.8	6.2
	6.426	8.461	2.808	3.915	12.724	-1.679	18.633	2.835	4.387	2.312
g_O	97	56	87	107	61	86	90	48	90	99
	(35.9)	(20.7)	(32.2)	(39.6)	(22.6)	(31.9)	(33.3)	(17.8)	(33.3)	(36.7)
g_E	98.2	52.3	76.6	98.2	52.3	76.6	98.2	52.3	76.6	98.2
	(36.4)	(19.4)	(28.4)	(36.4)	(19.4)	(28.4)	(36.4)	(19.4)	(28.4)	(36.4)
	-2.3	7.6	22.2	18.4	18.7	20.0	-15.7	-8.3	29.1	1.6
	-0.869	2.804	8.233	6.818	6.919	7.402	-5.800	-3.077	10.777	0.610
c_O	40	49	54	44	50	70	44	72	59	58
	(14.8)	(18.1)	(20.0)	(16.3)	(18.5)	(25.9)	(16.3)	(26.7)	(21.9)	(21.5)
c_E	50.2	60.0	66.0	50.2	60.0	66.0	50.2	60.0	66.0	50.2
	(18.6)	(22.2)	(24.4)	(18.6)	(22.2)	(24.4)	(18.6)	(22.2)	(24.4)	(18.6)
	-18.2	-19.9	-21.7	-11.7	-18.3	8.2	-11.7	26.2	-13.2	16.7
	-6.752	-7.363	-8.034	-4.321	-6.765	3.042	-4.321	9.706	-4.907	6.173
N	270	270	270	270	270	270	270	270	270	270
X2	3.6	3.8	3.9	2.9	8.9	3.6	11.5	4.6	6.1	3.2
Gvalue	3.7	3.9	4.0	2.9	8.9	3.7	10.4	4.6	6.2	3.2
Gv/N	1.374	1.453	1.468	1.075	3.297	1.373	3.841	1.694	2.289	1.203
Prob	0.29455	0.26982	0.26533	0.40707	0.03064	0.29479	0.01567	0.20579	0.10314	0.35501

Apha_HZ
5UTR:

	100	99	98	97	96	95	94	93	92	91
a_O	103	127	107	108	115	117	99	122	103	112
	(26.7)	(32.9)	(27.7)	(28.0)	(29.8)	(30.3)	(25.6)	(31.6)	(26.7)	(29.0)
a_E	112.3	112.3	112.3	112.3	112.3	112.3	112.3	112.3	112.3	112.3
	(29.1)	(29.1)	(29.1)	(29.1)	(29.1)	(29.1)	(29.1)	(29.1)	(29.1)	(29.1)
	-17.8	31.3	-10.3	-8.4	5.5	9.6	-25.0	20.2	-17.8	-0.6
	-4.612	8.096	-2.679	-2.183	1.417	2.487	-6.465	5.239	-4.612	-0.154
t_O	134	118	117	130	118	122	113	130	120	129
	(34.7)	(30.6)	(30.3)	(33.7)	(30.6)	(31.6)	(29.3)	(33.7)	(31.1)	(33.4)
t_E	125.3	125.3	125.3	125.3	125.3	125.3	125.3	125.3	125.3	125.3
	(32.5)	(32.5)	(32.5)	(32.5)	(32.5)	(32.5)	(32.5)	(32.5)	(32.5)	(32.5)
	18.0	-14.1	-16.0	9.6	-14.1	-6.5	-23.3	9.6	-10.3	7.6
	4.674	-3.659	-4.144	2.493	-3.659	-1.675	-6.039	2.493	-2.676	1.958
g_O	77	71	86	60	74	87	93	74	89	71
	(19.9)	(18.4)	(22.3)	(15.5)	(19.2)	(22.5)	(24.1)	(19.2)	(23.1)	(18.4)
g_E	78.9	78.9	78.9	78.9	78.9	78.9	78.9	78.9	78.9	78.9
	(20.5)	(20.5)	(20.5)	(20.5)	(20.5)	(20.5)	(20.5)	(20.5)	(20.5)	(20.5)
	-3.8	-15.0	14.7	-32.9	-9.6	16.9	30.5	-9.6	21.4	-15.0
	-0.992	-3.899	3.818	-8.528	-2.477	4.384	7.900	-2.477	5.532	-3.899
c_O	72	70	76	88	79	60	81	60	74	74
	(18.7)	(18.1)	(19.7)	(22.8)	(20.5)	(15.5)	(21.0)	(15.5)	(19.2)	(19.2)
c_E	69.5	69.5	69.5	69.5	69.5	69.5	69.5	69.5	69.5	69.5
	(18.0)	(18.0)	(18.0)	(18.0)	(18.0)	(18.0)	(18.0)	(18.0)	(18.0)	(18.0)
	5.1	1.0	13.6	41.6	20.3	-17.6	24.8	-17.6	9.3	9.3
	1.325	0.266	3.527	10.769	5.251	-4.564	6.434	-4.564	2.412	2.412
N	386	386	386	386	386	386	386	386	386	386
X2	1.5	3.1	2.0	9.8	2.1	2.4	7.2	2.6	2.6	1.2
Gvalue	1.5	3.1	2.0	9.8	2.1	2.4	7.1	2.7	2.5	1.2
Gv/N	0.395	0.805	0.523	2.550	0.533	0.631	1.830	0.691	0.657	0.317
Prob	0.67689	0.37536	0.56849	0.01994	0.56055	0.48691	0.06992	0.44611	0.46892	0.74735

	90	89	88	87	86	85	84	83	82	81
a_O	112	119	124	121	121	104	107	115	118	104
	(29.0)	(30.8)	(32.1)	(31.3)	(31.3)	(26.9)	(27.7)	(29.8)	(30.6)	(26.9)
a_E	112.3	112.3	112.3	112.3	112.3	112.3	112.3	112.3	112.3	112.3
	(29.1)	(29.1)	(29.1)	(29.1)	(29.1)	(29.1)	(29.1)	(29.1)	(29.1)	(29.1)
	-0.6	13.8	24.6	18.1	18.1	-16.0	-10.3	5.5	11.7	-16.0
	-0.154	3.575	6.369	4.680	4.680	-4.136	-2.679	1.417	3.029	-4.136
t_O	135	105	113	123	109	120	132	123	119	134
	(35.0)	(27.2)	(29.3)	(31.9)	(28.2)	(31.1)	(34.2)	(31.9)	(30.8)	(34.7)
t_E	125.3	125.3	125.3	125.3	125.3	125.3	125.3	125.3	125.3	125.3
	(32.5)	(32.5)	(32.5)	(32.5)	(32.5)	(32.5)	(32.5)	(32.5)	(32.5)	(32.5)
	20.2	-37.1	-23.3	-4.5	-30.3	-10.3	13.8	-4.5	-12.2	18.0
	5.229	-9.606	-6.039	-1.169	-7.860	-2.676	3.575	-1.169	-3.169	4.674
g_O	80	100	76	75	82	89	71	76	82	79
	(20.7)	(25.9)	(19.7)	(19.4)	(21.2)	(23.1)	(18.4)	(19.7)	(21.2)	(20.5)
g_E	78.9	78.9	78.9	78.9	78.9	78.9	78.9	78.9	78.9	78.9
	(20.5)	(20.5)	(20.5)	(20.5)	(20.5)	(20.5)	(20.5)	(20.5)	(20.5)	(20.5)
	2.1	47.3	-5.8	-7.7	6.2	21.4	-15.0	-5.8	6.2	0.1
	0.554	12.254	-1.494	-1.989	1.617	5.532	-3.899	-1.494	1.617	0.032
c_O	59	62	73	67	74	73	76	72	67	69
	(15.3)	(16.1)	(18.9)	(17.4)	(19.2)	(18.9)	(19.7)	(18.7)	(17.4)	(17.9)
c_E	69.5	69.5	69.5	69.5	69.5	69.5	69.5	69.5	69.5	69.5
	(18.0)	(18.0)	(18.0)	(18.0)	(18.0)	(18.0)	(18.0)	(18.0)	(18.0)	(18.0)
	-19.3	-14.1	7.2	-4.9	9.3	7.2	13.6	5.1	-4.9	-1.0
	-5.002	-3.663	1.865	-1.266	2.412	1.865	3.527	1.325	-1.266	-0.252
N	386	386	386	386	386	386	386	386	386	386
X2	2.4	10.1	2.7	1.0	3.2	2.3	2.0	0.3	0.8	1.2
Gvalue	2.4	9.9	2.7	1.0	3.3	2.3	2.0	0.3	0.8	1.2
Gv/N	0.627	2.560	0.702	0.256	0.848	0.586	0.525	0.079	0.210	0.318
Prob	0.48989	0.01959	0.43869	0.80384	0.35119	0.52022	0.56685	0.95878	0.84656	0.74678

	80	79	78	77	76	75	74	73	72	71
a_O	120	95	116	116	114	110	114	118	117	128
	(31.1)	(24.6)	(30.1)	(30.1)	(29.5)	(28.5)	(29.5)	(30.6)	(30.3)	(33.2)
a_E	112.3	112.3	112.3	112.3	112.3	112.3	112.3	112.3	112.3	112.3
	(29.1)	(29.1)	(29.1)	(29.1)	(29.1)	(29.1)	(29.1)	(29.1)	(29.1)	(29.1)
	15.9	-31.8	7.5	7.5	3.4	-4.5	3.4	11.7	9.6	33.5
	4.125	-8.234	1.950	1.950	0.889	-1.178	0.889	3.029	2.487	8.680
t_O	118	119	115	116	131	124	112	119	117	113
	(30.6)	(30.8)	(29.8)	(30.1)	(33.9)	(32.1)	(29.0)	(30.8)	(30.3)	(29.3)
t_E	125.3	125.3	125.3	125.3	125.3	125.3	125.3	125.3	125.3	125.3
	(32.5)	(32.5)	(32.5)	(32.5)	(32.5)	(32.5)	(32.5)	(32.5)	(32.5)	(32.5)
	-14.1	-12.2	-19.7	-17.8	11.7	-2.5	-25.1	-12.2	-16.0	-23.3
	-3.659	-3.169	-5.100	-4.624	3.032	-0.658	-6.501	-3.169	-4.144	-6.039
g_O	85	87	81	90	64	76	79	73	88	73
	(22.0)	(22.5)	(21.0)	(23.3)	(16.6)	(19.7)	(20.5)	(18.9)	(22.8)	(18.9)
g_E	78.9	78.9	78.9	78.9	78.9	78.9	78.9	78.9	78.9	78.9
	(20.5)	(20.5)	(20.5)	(20.5)	(20.5)	(20.5)	(20.5)	(20.5)	(20.5)	(20.5)
	12.6	16.9	4.2	23.6	-26.9	-5.8	0.1	-11.4	19.1	-11.4
	3.259	4.384	1.082	6.116	-6.956	-1.494	0.032	-2.958	4.955	-2.958
c_O	63	85	74	64	77	76	81	76	64	72
	(16.3)	(22.0)	(19.2)	(16.6)	(19.9)	(19.7)	(21.0)	(19.7)	(16.6)	(18.7)
c_E	69.5	69.5	69.5	69.5	69.5	69.5	69.5	69.5	69.5	69.5
	(18.0)	(18.0)	(18.0)	(18.0)	(18.0)	(18.0)	(18.0)	(18.0)	(18.0)	(18.0)
	-12.4	34.3	9.3	-10.5	15.8	13.6	24.8	13.6	-10.5	5.1
	-3.200	8.874	2.412	-2.728	4.095	3.527	6.434	3.527	-2.728	1.325
N	386	386	386	386	386	386	386	386	386	386
X2	2.0	7.3	1.3	2.8	3.9	0.8	3.3	1.7	2.2	3.9
Gvalue	2.0	7.2	1.3	2.8	4.1	0.8	3.3	1.7	2.2	3.9
Gv/N	0.525	1.855	0.344	0.713	1.060	0.198	0.854	0.429	0.570	1.008
Prob	0.56673	0.06698	0.72248	0.43127	0.25173	0.85827	0.34833	0.64698	0.53165	0.27331

	70	69	68	67	66	65	64	63	62	61
a_O	106	106	122	103	132	113	107	107	123	105
	(27.5)	(27.5)	(31.6)	(26.7)	(34.2)	(29.3)	(27.7)	(27.7)	(31.9)	(27.2)
a_E	112.3	112.3	112.3	112.3	112.3	112.3	112.3	112.3	112.3	112.3
	(29.1)	(29.1)	(29.1)	(29.1)	(29.1)	(29.1)	(29.1)	(29.1)	(29.1)	(29.1)
	-12.2	-12.2	20.2	-17.8	42.7	1.4	-10.3	-10.3	22.4	-14.1
	-3.170	-3.170	5.239	-4.612	11.056	0.365	-2.679	-2.679	5.802	-3.655
t_O	130	131	120	120	124	109	130	139	122	125
	(33.7)	(33.9)	(31.1)	(31.1)	(32.1)	(28.2)	(33.7)	(36.0)	(31.6)	(32.4)

t_E	125.3	125.3	125.3	125.3	125.3	125.3	125.3	125.3	125.3	125.3
	(32.5)	(32.5)	(32.5)	(32.5)	(32.5)	(32.5)	(32.5)	(32.5)	(32.5)	(32.5)
	9.6	11.7	-10.3	-10.3	-2.5	-30.3	9.6	28.9	-6.5	-0.6
	2.493	3.032	-2.676	-2.676	-0.658	-7.860	2.493	7.486	-1.675	-0.143
g_O	78	84	83	90	71	91	83	65	87	79
	(20.2)	(21.8)	(21.5)	(23.3)	(18.4)	(23.6)	(21.5)	(16.8)	(22.5)	(20.5)
g_E	78.9	78.9	78.9	78.9	78.9	78.9	78.9	78.9	78.9	78.9
	(20.5)	(20.5)	(20.5)	(20.5)	(20.5)	(20.5)	(20.5)	(20.5)	(20.5)	(20.5)
	-1.9	10.4	8.3	23.6	-15.0	25.9	8.3	-25.3	16.9	0.1
	-0.483	2.705	2.158	6.116	-3.899	6.705	2.158	-6.543	4.384	0.032
c_O	72	65	61	73	59	73	66	75	54	77
	(18.7)	(16.8)	(15.8)	(18.9)	(15.3)	(18.9)	(17.1)	(19.4)	(14.0)	(19.9)
c_E	69.5	69.5	69.5	69.5	69.5	69.5	69.5	69.5	69.5	69.5
	(18.0)	(18.0)	(18.0)	(18.0)	(18.0)	(18.0)	(18.0)	(18.0)	(18.0)	(18.0)
	5.1	-8.7	-15.9	7.2	-19.3	7.2	-6.8	11.4	-27.2	15.8
	1.325	-2.249	-4.118	1.865	-5.002	1.865	-1.761	2.966	-7.056	4.095
N	386	386	386	386	386	386	386	386	386	386
X2	0.6	1.2	2.3	2.7	5.9	4.1	0.8	4.7	5.4	1.3
Gvalue	0.6	1.2	2.3	2.7	5.8	4.1	0.8	4.8	5.6	1.3
Gv/N	0.165	0.319	0.603	0.693	1.497	1.075	0.211	1.231	1.454	0.329
Prob	0.88798	0.74541	0.50717	0.44460	0.12284	0.24596	0.84626	0.19094	0.13198	0.73634

	60	59	58	57	56	55	54	53	52	51
a_O	113	114	95	108	97	110	97	130	104	98
	(29.3)	(29.5)	(24.6)	(28.0)	(25.1)	(28.5)	(25.1)	(33.7)	(26.9)	(25.4)
a_E	112.3	112.3	112.3	112.3	112.3	112.3	112.3	112.3	112.3	112.3
	(29.1)	(29.1)	(29.1)	(29.1)	(29.1)	(29.1)	(29.1)	(29.1)	(29.1)	(29.1)
	1.4	3.4	-31.8	-8.4	-28.4	-4.5	-28.4	38.1	-16.0	-26.7
	0.365	0.889	-8.234	-2.183	-7.360	-1.178	-7.360	9.860	-4.136	-6.915
t_O	108	130	124	133	124	131	139	113	136	122
	(28.0)	(33.7)	(32.1)	(34.5)	(32.1)	(33.9)	(36.0)	(29.3)	(35.2)	(31.6)
t_E	125.3	125.3	125.3	125.3	125.3	125.3	125.3	125.3	125.3	125.3
	(32.5)	(32.5)	(32.5)	(32.5)	(32.5)	(32.5)	(32.5)	(32.5)	(32.5)	(32.5)
	-32.1	9.6	-2.5	15.9	-2.5	11.7	28.9	-23.3	22.3	-6.5
	-8.304	2.493	-0.658	4.123	-0.658	3.032	7.486	-6.039	5.787	-1.675
g_O	82	79	91	87	97	67	82	71	97	89
	(21.2)	(20.5)	(23.6)	(22.5)	(25.1)	(17.4)	(21.2)	(18.4)	(25.1)	(23.1)
g_E	78.9	78.9	78.9	78.9	78.9	78.9	78.9	78.9	78.9	78.9
	(20.5)	(20.5)	(20.5)	(20.5)	(20.5)	(20.5)	(20.5)	(20.5)	(20.5)	(20.5)
	6.2	0.1	25.9	16.9	40.0	-22.0	6.2	-15.0	40.0	21.4
	1.617	0.032	6.705	4.384	10.356	-5.692	1.617	-3.899	10.356	5.532
c_O	83	63	76	58	68	78	68	72	49	77
	(21.5)	(16.3)	(19.7)	(15.0)	(17.6)	(20.2)	(17.6)	(18.7)	(12.7)	(19.9)
c_E	69.5	69.5	69.5	69.5	69.5	69.5	69.5	69.5	69.5	69.5
	(18.0)	(18.0)	(18.0)	(18.0)	(18.0)	(18.0)	(18.0)	(18.0)	(18.0)	(18.0)
	29.5	-12.4	13.6	-21.0	-2.9	18.0	-2.9	5.1	-34.2	15.8
	7.641	-3.200	3.527	-5.431	-0.763	4.670	-0.763	1.325	-8.869	4.095
N	386	386	386	386	386	386	386	386	386	386
X2	5.1	0.8	5.1	3.4	6.3	3.2	3.7	4.9	11.7	4.0
Gvalue	5.1	0.8	5.2	3.4	6.1	3.2	3.8	4.8	12.1	4.0
Gv/N	1.320	0.214	1.340	0.892	1.575	0.832	0.981	1.247	3.138	1.038
Prob	0.16505	0.84295	0.15952	0.32816	0.10778	0.36017	0.28552	0.18584	0.00701	0.26094

	50	49	48	47	46	45	44	43	42	41
a_O	94	109	111	108	121	114	114	121	115	120
	(24.4)	(28.2)	(28.8)	(28.0)	(31.3)	(29.5)	(29.5)	(31.3)	(29.8)	(31.1)
a_E	112.3	112.3	112.3	112.3	112.3	112.3	112.3	112.3	112.3	112.3
	(29.1)	(29.1)	(29.1)	(29.1)	(29.1)	(29.1)	(29.1)	(29.1)	(29.1)	(29.1)
	-33.4	-6.5	-2.6	-8.4	18.1	3.4	3.4	18.1	5.5	15.9
	-8.662	-1.683	-0.668	-2.183	4.680	0.889	0.889	4.680	1.417	4.125
t_O	131	132	128	136	124	117	108	118	116	121
	(33.9)	(34.2)	(33.2)	(35.2)	(32.1)	(30.3)	(28.0)	(30.6)	(30.1)	(31.3)
t_E	125.3	125.3	125.3	125.3	125.3	125.3	125.3	125.3	125.3	125.3
	(32.5)	(32.5)	(32.5)	(32.5)	(32.5)	(32.5)	(32.5)	(32.5)	(32.5)	(32.5)
	11.7	13.8	5.5	22.3	-2.5	-16.0	-32.1	-14.1	-17.8	-8.4
	3.032	3.575	1.426	5.787	-0.658	-4.144	-8.304	-3.659	-4.624	-2.178
g_O	85	83	75	74	76	84	101	81	92	76
	(22.0)	(21.5)	(19.4)	(19.2)	(19.7)	(21.8)	(26.2)	(21.0)	(23.8)	(19.7)
g_E	78.9	78.9	78.9	78.9	78.9	78.9	78.9	78.9	78.9	78.9
	(20.5)	(20.5)	(20.5)	(20.5)	(20.5)	(20.5)	(20.5)	(20.5)	(20.5)	(20.5)
	12.6	8.3	-7.7	-9.6	-5.8	10.4	49.8	4.2	28.2	-5.8
	3.259	2.158	-1.989	-2.477	-1.494	2.705	12.898	1.082	7.299	-1.494
c_O	76	62	72	68	65	71	63	66	63	69
	(19.7)	(16.1)	(18.7)	(17.6)	(16.8)	(18.4)	(16.3)	(17.1)	(16.3)	(17.9)
c_E	69.5	69.5	69.5	69.5	69.5	69.5	69.5	69.5	69.5	69.5
	(18.0)	(18.0)	(18.0)	(18.0)	(18.0)	(18.0)	(18.0)	(18.0)	(18.0)	(18.0)
	13.6	-14.1	5.1	-2.9	-8.7	3.1	-12.4	-6.8	-12.4	-1.0
	3.527	-3.663	1.325	-0.763	-2.249	0.792	-3.200	-1.761	-3.200	-0.252
N	386	386	386	386	386	386	386	386	386	386
X2	4.3	1.5	0.4	1.4	1.1	0.9	9.2	1.3	3.5	0.8
Gvalue	4.5	1.5	0.4	1.4	1.1	0.9	8.8	1.3	3.4	0.8
Gv/N	1.156	0.387	0.094	0.364	0.279	0.242	2.283	0.342	0.893	0.202
Prob	0.21576	0.68333	0.94771	0.70390	0.78243	0.81685	0.03190	0.72432	0.32785	0.85459

	40	39	38	37	36	35	34	33	32	31
a_O	119	117	122	118	95	108	113	109	112	104
	(30.8)	(30.3)	(31.6)	(30.6)	(24.6)	(28.0)	(29.3)	(28.2)	(29.0)	(26.9)
a_E	112.3	112.3	112.3	112.3	112.3	112.3	112.3	112.3	112.3	112.3
	(29.1)	(29.1)	(29.1)	(29.1)	(29.1)	(29.1)	(29.1)	(29.1)	(29.1)	(29.1)
	13.8	9.6	20.2	11.7	-31.8	-8.4	1.4	-6.5	-0.6	-16.0
	3.575	2.487	5.239	3.029	-8.234	-2.183	0.365	-1.683	-0.154	-4.136
t_O	123	128	117	123	127	125	124	124	121	128
	(31.9)	(33.2)	(30.3)	(31.9)	(32.9)	(32.4)	(32.1)	(32.1)	(31.3)	(33.2)
t_E	125.3	125.3	125.3	125.3	125.3	125.3	125.3	125.3	125.3	125.3
	(32.5)	(32.5)	(32.5)	(32.5)	(32.5)	(32.5)	(32.5)	(32.5)	(32.5)	(32.5)
	-4.5	5.5	-16.0	-4.5	3.5	-0.6	-2.5	-2.5	-8.4	5.5
	-1.169	1.426	-4.144	-1.169	0.899	-0.143	-0.658	-0.658	-2.178	1.426
g_O	87	76	93	83	86	90	86	82	74	87
	(22.5)	(19.7)	(24.1)	(21.5)	(22.3)	(23.3)	(22.3)	(21.2)	(19.2)	(22.5)
g_E	78.9	78.9	78.9	78.9	78.9	78.9	78.9	78.9	78.9	78.9
	(20.5)	(20.5)	(20.5)	(20.5)	(20.5)	(20.5)	(20.5)	(20.5)	(20.5)	(20.5)
	16.9	-5.8	30.5	8.3	14.7	23.6	14.7	6.2	-9.6	16.9
	4.384	-1.494	7.900	2.158	3.818	6.116	3.818	1.617	-2.477	4.384
c_O	57	65	54	62	78	63	63	71	79	67
	(14.8)	(16.8)	(14.0)	(16.1)	(20.2)	(16.3)	(16.3)	(18.4)	(20.5)	(17.4)
c_E	69.5	69.5	69.5	69.5	69.5	69.5	69.5	69.5	69.5	69.5

```
       ( 18.0) ( 18.0) ( 18.0) ( 18.0) ( 18.0) ( 18.0) ( 18.0) ( 18.0) ( 18.0) ( 18.0)
         -22.6    -8.7   -27.2   -14.1    18.0   -12.4   -12.4     3.1    20.3    -4.9
        -5.851  -2.249  -7.056  -3.663   4.670  -3.200  -3.200   0.792   5.251  -1.266

N          386     386     386     386     386     386     386     386     386     386
X2         3.5     0.7     7.3     1.3     4.4     2.3     1.3     0.3     1.8     1.6
Gvalue     3.6     0.7     7.5     1.4     4.5     2.3     1.3     0.3     1.7     1.6
Gv/N     0.939   0.171   1.939   0.355   1.154   0.589   0.326   0.068   0.443   0.408
Prob   0.30515 0.88255 0.05796 0.71268 0.21656 0.51728 0.73942 0.96723 0.63463 0.66521
       ---------------------------------------------------------------------------------
            30      29      28      27      26      25      24      23      22      21
       ---------------------------------------------------------------------------------
a_O        109     104     122     108     116     128     115     119     114     100
       ( 28.2) ( 26.9) ( 31.6) ( 28.0) ( 30.1) ( 33.2) ( 29.8) ( 30.8) ( 29.5) ( 25.9)
a_E      112.3   112.3   112.3   112.3   112.3   112.3   112.3   112.3   112.3   112.3
       ( 29.1) ( 29.1) ( 29.1) ( 29.1) ( 29.1) ( 29.1) ( 29.1) ( 29.1) ( 29.1) ( 29.1)
          -6.5   -16.0    20.2    -8.4     7.5    33.5     5.5    13.8     3.4   -23.2
        -1.683  -4.136   5.239  -2.183   1.950   8.680   1.417   3.575   0.889  -6.009
t_O        132     115     124     113     102     114     124     138     132     123
       ( 34.2) ( 29.8) ( 32.1) ( 29.3) ( 26.4) ( 29.5) ( 32.1) ( 35.8) ( 34.2) ( 31.9)
t_E      125.3   125.3   125.3   125.3   125.3   125.3   125.3   125.3   125.3   125.3
       ( 32.5) ( 32.5) ( 32.5) ( 32.5) ( 32.5) ( 32.5) ( 32.5) ( 32.5) ( 32.5) ( 32.5)
          13.8   -19.7    -2.5   -23.3   -41.9   -21.5    -2.5    26.7    13.8    -4.5
         3.575  -5.100  -0.658  -6.039 -10.863  -5.572  -0.658   6.916   3.575  -1.169
g_O         86      88      82      94     104      83      86      64      78      98
       ( 22.3) ( 22.8) ( 21.2) ( 24.4) ( 26.9) ( 21.5) ( 22.3) ( 16.6) ( 20.2) ( 25.4)
g_E       78.9    78.9    78.9    78.9    78.9    78.9    78.9    78.9    78.9    78.9
       ( 20.5) ( 20.5) ( 20.5) ( 20.5) ( 20.5) ( 20.5) ( 20.5) ( 20.5) ( 20.5) ( 20.5)
          14.7    19.1     6.2    32.8    57.4     8.3    14.7   -26.9    -1.9    42.4
         3.818   4.955   1.617   8.505  14.858   2.158   3.818  -6.956  -0.483  10.983
c_O         59      79      58      71      64      61      61      65      62      65
       ( 15.3) ( 20.5) ( 15.0) ( 18.4) ( 16.6) ( 15.8) ( 15.8) ( 16.8) ( 16.1) ( 16.8)
c_E       69.5    69.5    69.5    69.5    69.5    69.5    69.5    69.5    69.5    69.5
       ( 18.0) ( 18.0) ( 18.0) ( 18.0) ( 18.0) ( 18.0) ( 18.0) ( 18.0) ( 18.0) ( 18.0)
         -19.3    20.3   -21.0     3.1   -10.5   -15.9   -15.9    -8.7   -14.1    -8.7
        -5.002   5.251  -5.431   0.792  -2.728  -4.118  -4.118  -2.249  -3.663  -2.249

N          386     386     386     386     386     386     386     386     386     386
X2         2.7     3.8     2.9     4.3    12.8     4.5     1.7     4.8     1.2     6.3
Gvalue     2.7     3.7     3.0     4.2    12.4     4.4     1.8     5.0     1.2     6.0
Gv/N     0.709   0.970   0.767   1.075   3.216   1.149   0.459   1.286   0.318   1.557
Prob   0.43425 0.29037 0.39797 0.24566 0.00609 0.21821 0.62078 0.17450 0.74605 0.11120
       ---------------------------------------------------------------------------------
            20      19      18      17      16      15      14      13      12      11
       ---------------------------------------------------------------------------------
a_O        102     124      93     113     122     121     125     110     104     115
       ( 26.4) ( 32.1) ( 24.1) ( 29.3) ( 31.6) ( 31.3) ( 32.4) ( 28.5) ( 26.9) ( 29.8)
a_E      112.3   112.3   112.3   112.3   112.3   112.3   112.3   112.3   112.3   112.3
       ( 29.1) ( 29.1) ( 29.1) ( 29.1) ( 29.1) ( 29.1) ( 29.1) ( 29.1) ( 29.1) ( 29.1)
         -19.6    24.6   -35.1     1.4    20.2    18.1    26.8    -4.5   -16.0     5.5
        -5.083   6.369  -9.085   0.365   5.239   4.680   6.941  -1.178  -4.136   1.417
t_O        133     136     148     153     148     133     106     144     133     116
       ( 34.5) ( 35.2) ( 38.3) ( 39.6) ( 38.3) ( 34.5) ( 27.5) ( 37.3) ( 34.5) ( 30.1)
t_E      125.3   125.3   125.3   125.3   125.3   125.3   125.3   125.3   125.3   125.3
       ( 32.5) ( 32.5) ( 32.5) ( 32.5) ( 32.5) ( 32.5) ( 32.5) ( 32.5) ( 32.5) ( 32.5)
          15.9    22.3    49.3    61.2    49.3    15.9   -35.4    40.1    15.9   -17.8
         4.123   5.787  12.782  15.848  12.782   4.123  -9.177  10.392   4.123  -4.624
g_O         74      61      65      63      50      65      65      52      71      67
       ( 19.2) ( 15.8) ( 16.8) ( 16.3) ( 13.0) ( 16.8) ( 16.8) ( 13.5) ( 18.4) ( 17.4)
g_E       78.9    78.9    78.9    78.9    78.9    78.9    78.9    78.9    78.9    78.9
       ( 20.5) ( 20.5) ( 20.5) ( 20.5) ( 20.5) ( 20.5) ( 20.5) ( 20.5) ( 20.5) ( 20.5)
          -9.6   -31.5   -25.3   -28.4   -45.7   -25.3   -25.3   -43.4   -15.0   -22.0
        -2.477  -8.148  -6.543  -7.362 -11.830  -6.543  -6.543 -11.247  -3.899  -5.692
c_O         77      65      80      57      66      67      90      80      78      88
       ( 19.9) ( 16.8) ( 20.7) ( 14.8) ( 17.1) ( 17.4) ( 23.3) ( 20.7) ( 20.2) ( 22.8)
c_E       69.5    69.5    69.5    69.5    69.5    69.5    69.5    69.5    69.5    69.5
       ( 18.0) ( 18.0) ( 18.0) ( 18.0) ( 18.0) ( 18.0) ( 18.0) ( 18.0) ( 18.0) ( 18.0)
          15.8    -8.7    22.5   -22.6    -6.8    -4.9    46.6    22.5    18.0    41.6
         4.095  -2.249   5.839  -5.851  -1.761  -1.266  12.062   5.839   4.670  10.769

N          386     386     386     386     386     386     386     386     386     386
X2         2.5     6.5    11.5    11.6    15.7     3.7    12.9    13.6     2.9     7.5
Gvalue     2.5     6.8    11.6    11.6    17.1     3.8    12.7    14.7     2.9     7.2
Gv/N     0.658   1.760   2.993   3.001   4.430   0.993   3.283   3.807   0.758   1.870
Prob   0.46794 0.07876 0.00908 0.00896 0.00067 0.27988 0.00541 0.00210 0.40345 0.06529
       ---------------------------------------------------------------------------------
            10       9       8       7       6       5       4       3       2       1
       ---------------------------------------------------------------------------------
a_O        127      96     107     109     112     125     111     106     121     112
       ( 32.9) ( 24.9) ( 27.7) ( 28.2) ( 29.0) ( 32.4) ( 28.8) ( 27.5) ( 31.3) ( 29.0)
a_E      112.3   112.3   112.3   112.3   112.3   112.3   112.3   112.3   112.3   112.3
       ( 29.1) ( 29.1) ( 29.1) ( 29.1) ( 29.1) ( 29.1) ( 29.1) ( 29.1) ( 29.1) ( 29.1)
          31.3   -30.1   -10.3    -6.5    -0.6    26.8    -2.6   -12.2    18.1    -0.6
         8.096  -7.799  -2.679  -1.683  -0.154   6.941  -0.668  -3.170   4.680  -0.154
t_O        122     146     136     152     137     118     140     126     119     155
       ( 31.6) ( 37.8) ( 35.2) ( 39.4) ( 35.5) ( 30.6) ( 36.3) ( 32.6) ( 30.8) ( 40.2)
t_E      125.3   125.3   125.3   125.3   125.3   125.3   125.3   125.3   125.3   125.3
       ( 32.5) ( 32.5) ( 32.5) ( 32.5) ( 32.5) ( 32.5) ( 32.5) ( 32.5) ( 32.5) ( 32.5)
          -6.5    44.7    22.3    58.8    24.5   -14.1    31.1     1.5   -12.2    66.0
        -1.675  11.580   5.787  15.228   6.350  -3.659   8.060   0.376  -3.169  17.098
g_O         55      69      68      50      77      68      85      91      67      56
       ( 14.2) ( 17.9) ( 17.6) ( 13.0) ( 19.9) ( 17.6) ( 22.0) ( 23.6) ( 17.4) ( 14.5)
g_E       78.9    78.9    78.9    78.9    78.9    78.9    78.9    78.9    78.9    78.9
       ( 20.5) ( 20.5) ( 20.5) ( 20.5) ( 20.5) ( 20.5) ( 20.5) ( 20.5) ( 20.5) ( 20.5)
         -39.7   -18.6   -20.3   -45.7    -3.8   -20.3    12.6    25.9   -22.0   -38.5
       -10.297  -4.811  -5.255 -11.830  -0.992  -5.255   3.259   6.705  -5.692  -9.961
c_O         82      75      75      75      60      75      50      63      79      63
       ( 21.2) ( 19.4) ( 19.4) ( 19.4) ( 15.5) ( 19.4) ( 13.0) ( 16.3) ( 20.5) ( 16.3)
c_E       69.5    69.5    69.5    69.5    69.5    69.5    69.5    69.5    69.5    69.5
       ( 18.0) ( 18.0) ( 18.0) ( 18.0) ( 18.0) ( 18.0) ( 18.0) ( 18.0) ( 18.0) ( 18.0)
          27.2    11.4    11.4    11.4   -17.6    11.4   -32.9   -12.4    20.3   -12.4
         7.034   2.966   2.966   2.966  -4.564   2.966  -8.527  -3.200   5.251  -3.200

N          386     386     386     386     386     386     386     386     386     386
X2        11.5     7.5     3.1    16.8     2.4     3.8     7.7     2.8     4.1    14.3
Gvalue    12.2     7.5     3.2    18.1     2.5     3.8     8.2     2.7     4.1    14.6
Gv/N     3.158   1.937   0.820   4.681   0.640   0.993   2.124   0.711   1.069   3.783
Prob   0.00676 0.05820 0.36708 0.00043 0.48037 0.27999 0.04208 0.43255 0.24798 0.00219
       ---------------------------------------------------------------------------------

CDS_INITIAL:
       ---------------------------------------------------------------------------------
```

	1	2	3	4	5	6	7	8	9	10
a_O	212	0	1	105	131	117	118	134	102	132
	(54.9)	(0.0)	(0.3)	(27.2)	(33.9)	(30.3)	(30.6)	(34.7)	(26.4)	(34.2)
a_E	115.6	112.4	103.2	115.6	112.4	103.2	115.6	112.4	103.2	115.6
	(30.0)	(29.1)	(26.7)	(30.0)	(29.1)	(26.7)	(30.0)	(29.1)	(26.7)	(30.0)
	257.0	0.0	-9.3	-20.3	40.1	29.4	4.7	47.1	-2.4	34.9
	66.569	0.000	-2.402	-5.255	10.400	7.609	1.230	12.210	-0.617	9.045
t_O	83	386	0	73	127	121	97	117	157	90
	(21.5)	(100.0)	(0.0)	(18.9)	(32.9)	(31.3)	(25.1)	(30.3)	(40.7)	(23.3)
t_E	77.3	123.4	136.2	77.3	123.4	136.2	77.3	123.4	136.2	77.3
	(20.0)	(32.0)	(35.3)	(20.0)	(32.0)	(35.3)	(20.0)	(32.0)	(35.3)	(20.0)
	11.8	880.7	0.0	-8.4	7.4	-28.7	44.0	-12.4	44.5	27.4
	3.054	228.157	0.000	-2.170	1.917	-7.440	11.403	-3.205	11.534	7.087
g_O	91	0	385	107	62	90	109	70	71	112
	(23.6)	(0.0)	(99.7)	(27.7)	(16.1)	(23.3)	(28.2)	(18.1)	(18.4)	(29.0)
g_E	130.0	69.0	87.2	130.0	69.0	87.2	130.0	69.0	87.2	130.0
	(33.7)	(17.9)	(22.6)	(33.7)	(17.9)	(22.6)	(33.7)	(17.9)	(22.6)	(33.7)
	-64.9	0.0	1143.2	-41.7	-13.3	5.6	-38.4	2.0	-29.2	-33.4
	-16.815	0.000	296.158	-10.791	-3.443	1.455	-9.947	0.514	-7.575	-8.645
c_O	0	0	0	101	66	58	62	65	56	52
	(0.0)	(0.0)	(0.0)	(26.2)	(17.1)	(15.0)	(16.1)	(16.8)	(14.5)	(13.5)
c_E	63.0	81.2	59.3	63.0	81.2	59.3	63.0	81.2	59.3	63.0
	(16.3)	(21.0)	(15.4)	(16.3)	(21.0)	(15.4)	(16.3)	(21.0)	(15.4)	(16.3)
	0.0	0.0	0.0	95.2	-27.4	-2.6	-2.1	-29.0	-6.5	-20.0
	0.000	0.000	0.000	24.659	-7.105	-0.677	-0.539	-7.512	-1.672	-5.191
N	386	386	386	386	386	386	386	386	386	386
X2	155.4	821.9	1313.2	28.1	6.8	3.7	8.5	7.7	6.4	8.8
Gvalue	203.8	880.7	1133.9	24.9	6.8	3.7	8.3	7.7	6.4	8.9
Gv/N	52.808	228.157	293.756	6.443	1.769	0.948	2.148	2.007	1.670	2.296
Prob	0.00000	0.00000	0.00000	0.00002	0.07764	0.30081	0.04038	0.05155	0.09188	0.03119

	11	12	13	14	15	16	17	18	19	20
a_O	110	111	120	107	111	118	130	114	122	103
	(28.5)	(28.8)	(31.1)	(27.7)	(28.8)	(30.6)	(33.7)	(29.5)	(31.6)	(26.7)
a_E	112.4	103.2	115.6	112.4	103.2	115.6	112.4	103.2	115.6	112.4
	(29.1)	(26.7)	(30.0)	(29.1)	(26.7)	(30.0)	(29.1)	(26.7)	(30.0)	(29.1)
	-4.7	16.2	8.9	-10.5	16.2	4.7	37.8	22.7	13.0	-18.0
	-1.225	4.191	2.296	-2.725	4.191	1.230	9.804	5.880	3.379	-4.656
t_O	137	143	80	120	128	89	133	147	91	141
	(35.5)	(37.0)	(20.7)	(31.1)	(33.2)	(23.1)	(34.5)	(38.1)	(23.6)	(36.5)
t_E	123.4	136.2	77.3	123.4	136.2	77.3	123.4	136.2	77.3	123.4
	(32.0)	(35.3)	(20.0)	(32.0)	(35.3)	(20.0)	(32.0)	(35.3)	(20.0)	(32.0)
	28.8	13.8	5.5	-6.6	-16.0	25.1	20.0	22.3	29.7	37.7
	7.448	3.585	1.418	-1.714	-4.140	6.493	5.189	5.787	7.687	9.768
g_O	67	77	122	72	89	112	57	72	104	77
	(17.4)	(19.9)	(31.6)	(18.7)	(23.1)	(29.0)	(14.8)	(18.7)	(26.9)	(19.9)
g_E	69.0	87.2	130.0	69.0	87.2	130.0	69.0	87.2	130.0	69.0
	(17.9)	(22.6)	(33.7)	(17.9)	(22.6)	(33.7)	(17.9)	(22.6)	(33.7)	(17.9)
	-4.0	-19.2	-15.5	6.1	3.6	-33.4	-21.8	-27.6	-46.4	16.9
	-1.028	-4.979	-4.011	1.580	0.924	-8.645	-5.649	-7.160	-12.021	4.368
c_O	72	55	64	87	58	67	66	53	69	65
	(18.7)	(14.2)	(16.6)	(22.5)	(15.0)	(17.4)	(17.1)	(13.7)	(17.9)	(16.8)
c_E	81.2	59.3	63.0	81.2	59.3	63.0	81.2	59.3	63.0	81.2
	(21.0)	(15.4)	(16.3)	(21.0)	(15.4)	(16.3)	(21.0)	(15.4)	(16.3)	(21.0)
	-17.4	-8.3	1.9	11.9	-2.6	8.1	-27.4	-11.9	12.4	-29.0
	-4.505	-2.156	0.497	3.087	-0.677	2.110	-7.105	-3.094	3.225	-7.512
N	386	386	386	386	386	386	386	386	386	386
X2	2.7	2.4	0.8	0.9	1.2	4.6	8.5	5.3	8.5	7.5
Gvalue	2.7	2.5	0.8	0.9	1.1	4.6	8.6	5.5	8.8	7.6
Gv/N	0.689	0.642	0.200	0.228	0.298	1.189	2.239	1.412	2.270	1.968
Prob	0.44686	0.47919	0.85656	0.83003	0.76504	0.20451	0.03444	0.14165	0.03261	0.05509

	21	22	23	24	25	26	27	28	29	30
a_O	104	111	112	99	121	95	96	115	106	104
	(26.9)	(28.8)	(29.0)	(25.6)	(31.3)	(24.6)	(24.9)	(29.8)	(27.5)	(26.9)
a_E	103.2	115.6	112.4	103.2	115.6	112.4	103.2	115.6	112.4	103.2
	(26.7)	(30.0)	(29.1)	(26.7)	(30.0)	(29.1)	(26.7)	(30.0)	(29.1)	(26.7)
	1.6	-9.1	-0.8	-8.2	10.9	-31.9	-13.9	-1.3	-12.4	1.6
	0.417	-2.360	-0.202	-2.131	2.836	-8.275	-3.597	-0.335	-3.215	0.417
t_O	137	89	131	142	94	138	141	78	138	141
	(35.5)	(23.1)	(33.9)	(36.8)	(24.4)	(35.8)	(36.5)	(20.2)	(35.8)	(36.5)
t_E	136.2	77.3	123.4	136.2	77.3	123.4	136.2	77.3	123.4	136.2
	(35.3)	(20.0)	(32.0)	(35.3)	(20.0)	(32.0)	(35.3)	(20.0)	(32.0)	(35.3)
	1.5	25.1	15.8	11.7	36.7	31.0	9.7	1.4	31.0	9.7
	0.392	6.493	4.082	3.044	9.520	8.023	2.506	0.359	8.023	2.506
g_O	92	124	65	73	105	80	82	129	63	90
	(23.8)	(32.1)	(16.8)	(18.9)	(27.2)	(20.7)	(21.2)	(33.4)	(16.3)	(23.3)
g_E	87.2	130.0	69.0	87.2	130.0	69.0	87.2	130.0	69.0	87.2
	(22.6)	(33.7)	(17.9)	(22.6)	(33.7)	(17.9)	(22.6)	(33.7)	(17.9)	(22.6)
	9.8	-11.7	-7.8	-26.0	-44.8	23.6	-10.1	-2.0	-11.5	5.6
	2.535	-3.032	-2.018	-6.738	-11.616	6.123	-2.629	-0.512	-2.976	1.455
c_O	53	62	78	72	66	73	67	64	79	51
	(13.7)	(16.1)	(20.2)	(18.7)	(17.1)	(18.9)	(17.4)	(16.6)	(20.5)	(13.2)
c_E	59.3	63.0	81.2	59.3	63.0	81.2	59.3	63.0	81.2	59.3
	(15.4)	(16.3)	(21.0)	(15.4)	(16.3)	(21.0)	(15.4)	(16.3)	(21.0)	(15.4)
	-11.9	-2.1	-6.4	27.9	6.0	-15.6	16.3	1.9	-4.4	-15.4
	-3.094	-0.539	-1.646	7.226	1.564	-4.046	4.225	0.497	-1.145	-3.994
N	386	386	386	386	386	386	386	386	386	386
X2	0.9	2.2	0.8	5.4	8.8	7.0	2.0	0.0	2.7	1.4
Gvalue	1.0	2.2	0.8	5.4	8.9	7.0	2.0	0.0	2.6	1.5
Gv/N	0.250	0.563	0.216	1.401	2.304	1.825	0.506	0.008	0.686	0.384
Prob	0.80972	0.53744	0.84103	0.14421	0.03072	0.07050	0.58233	0.99852	0.44929	0.68617

	31	32	33	34	35	36	37	38	39	40
a_O	114	95	103	103	111	98	119	87	103	102
	(29.5)	(24.6)	(26.7)	(26.7)	(28.8)	(25.4)	(30.8)	(22.5)	(26.7)	(26.4)
a_E	115.6	112.4	103.2	115.6	112.4	103.2	115.6	112.4	103.2	115.6
	(30.0)	(29.1)	(26.7)	(30.0)	(29.1)	(26.7)	(30.0)	(29.1)	(26.7)	(30.0)
	-3.3	-31.9	-0.4	-23.9	-2.8	-10.1	6.8	-44.6	-0.4	-25.6
	-0.848	-8.275	-0.103	-6.182	-0.716	-2.625	1.761	-11.543	-0.103	-6.637
t_O	89	139	137	86	132	146	75	155	146	89
	(23.1)	(36.0)	(35.5)	(22.3)	(34.2)	(37.8)	(19.4)	(40.2)	(37.8)	(23.1)
t_E	77.3	123.4	136.2	77.3	123.4	136.2	77.3	123.4	136.2	77.3
	(20.0)	(32.0)	(35.3)	(20.0)	(32.0)	(35.3)	(20.0)	(32.0)	(35.3)	(20.0)
	25.1	33.2	1.5	18.3	17.9	20.2	-4.6	70.8	20.2	25.1

	6.493	8.601	0.392	4.747	4.634	5.231	−1.179	18.341	5.231	6.493
g_O	109	73	77	131	62	86	130	67	84	122
	(28.2)	(18.9)	(19.9)	(33.9)	(16.1)	(22.3)	(33.7)	(17.4)	(21.8)	(31.6)
g_E	130.0	69.0	87.2	130.0	69.0	87.2	130.0	69.0	87.2	130.0
	(33.7)	(17.9)	(22.6)	(33.7)	(17.9)	(22.6)	(33.7)	(17.9)	(22.6)	(33.7)
	−38.4	8.2	−19.2	2.0	−13.3	−2.5	0.0	−4.0	−6.3	−15.5
	−9.947	2.124	−4.979	0.524	−3.443	−0.635	0.004	−1.028	−1.645	−4.011
c_O	74	79	69	66	81	56	62	77	53	73
	(19.2)	(20.5)	(17.9)	(17.1)	(21.0)	(14.5)	(16.1)	(19.9)	(13.7)	(18.9)
c_E	63.0	81.2	59.3	63.0	81.2	59.3	63.0	81.2	59.3	63.0
	(16.3)	(21.0)	(15.4)	(16.3)	(21.0)	(15.4)	(16.3)	(21.0)	(15.4)	(16.3)
	23.7	−4.4	20.9	6.0	−0.5	−6.5	−2.1	−8.3	−11.9	21.4
	6.141	−1.145	5.403	1.564	−0.125	−1.672	−0.539	−2.139	−3.094	5.543
N	386	386	386	386	386	386	386	386	386	386
X2	7.1	5.0	2.8	2.5	1.3	1.2	0.2	14.1	1.5	5.4
Gvalue	7.1	5.0	2.8	2.5	1.3	1.2	0.2	14.0	1.5	5.4
Gv/N	1.839	1.305	0.714	0.654	0.349	0.299	0.048	3.630	0.389	1.388
Prob	0.06884	0.16920	0.43098	0.47119	0.71757	0.76361	0.98022	0.00289	0.68154	0.14733

	41	42	43	44	45	46	47	48	49	50
a_O	117	104	133	113	121	93	91	91	103	96
	(30.3)	(26.9)	(34.5)	(29.3)	(31.3)	(24.1)	(23.6)	(23.6)	(26.7)	(24.9)
a_E	112.4	103.2	115.6	112.4	103.2	115.6	112.4	103.2	115.6	112.4
	(29.1)	(26.7)	(30.0)	(29.1)	(26.7)	(30.0)	(29.1)	(26.7)	(30.0)	(29.1)
	9.4	1.6	37.2	1.2	38.5	−40.5	−38.4	−22.9	−23.9	−30.3
	2.437	0.417	9.633	0.317	9.977	−10.503	−9.954	−5.931	−6.182	−7.841
t_O	122	137	72	116	139	94	128	140	91	123
	(31.6)	(35.5)	(18.7)	(30.1)	(36.0)	(24.4)	(33.2)	(36.3)	(23.6)	(31.9)
t_E	123.4	136.2	77.3	123.4	136.2	77.3	123.4	136.2	77.3	123.4
	(32.0)	(35.3)	(20.0)	(32.0)	(35.3)	(20.0)	(32.0)	(35.3)	(20.0)	(32.0)
	−2.7	1.5	−10.2	−14.3	5.6	36.7	9.5	7.6	29.7	−0.7
	−0.697	0.392	−2.655	−3.694	1.442	9.520	2.453	1.972	7.687	−0.183
g_O	69	80	124	73	74	137	77	90	128	84
	(17.9)	(20.7)	(32.1)	(18.9)	(19.2)	(35.5)	(19.9)	(23.3)	(33.2)	(21.8)
g_E	69.0	87.2	130.0	69.0	87.2	130.0	69.0	87.2	130.0	69.0
	(17.9)	(22.6)	(33.7)	(17.9)	(22.6)	(33.7)	(17.9)	(22.6)	(33.7)	(17.9)
	−0.0	−13.9	−11.7	8.2	−24.4	14.4	16.9	5.6	−4.0	33.0
	−0.007	−3.589	−3.032	2.124	−6.309	3.727	4.368	1.455	−1.024	8.552
c_O	78	65	57	84	52	62	90	65	64	83
	(20.2)	(16.8)	(14.8)	(21.8)	(13.5)	(16.1)	(23.3)	(16.8)	(16.6)	(21.5)
c_E	81.2	59.3	63.0	81.2	59.3	63.0	81.2	59.3	63.0	81.2
	(21.0)	(15.4)	(16.3)	(21.0)	(15.4)	(16.3)	(21.0)	(15.4)	(16.3)	(21.0)
	−6.4	11.9	−11.5	5.6	−13.7	−2.1	18.4	11.9	1.9	3.6
	−1.646	3.079	−2.979	1.453	−3.549	−0.539	4.774	3.079	0.497	0.921
N	386	386	386	386	386	386	386	386	386	386
X2	0.3	1.2	3.8	0.8	6.0	8.4	6.1	2.2	3.9	5.7
Gvalue	0.3	1.2	3.7	0.8	6.0	8.5	6.3	2.2	3.8	5.6
Gv/N	0.086	0.299	0.968	0.199	1.561	2.206	1.641	0.575	0.978	1.450
Prob	0.95380	0.76388	0.29145	0.85685	0.11042	0.03649	0.09651	0.52829	0.28688	0.13302

	51	52	53	54	55	56	57	58	59	60
a_O	95	112	109	108	111	101	90	101	91	94
	(24.6)	(29.0)	(28.2)	(28.0)	(28.8)	(26.2)	(23.3)	(26.2)	(23.6)	(24.4)
a_E	103.2	115.6	112.4	103.2	115.6	112.4	103.2	115.6	112.4	103.2
	(26.7)	(30.0)	(29.1)	(26.7)	(30.0)	(29.1)	(26.7)	(30.0)	(29.1)	(26.7)
	−15.7	−7.2	−6.7	9.8	−9.1	−21.6	−24.6	−27.4	−38.4	−17.6
	−4.075	−1.860	−1.730	2.545	−2.360	−5.592	−6.381	−7.088	−9.954	−4.547
t_O	145	67	134	135	66	145	135	90	150	149
	(37.6)	(17.4)	(34.7)	(35.0)	(17.1)	(37.6)	(35.0)	(23.3)	(38.9)	(38.6)
t_E	136.2	77.3	123.4	136.2	77.3	123.4	136.2	77.3	123.4	136.2
	(35.3)	(20.0)	(32.0)	(35.3)	(20.0)	(32.0)	(35.3)	(20.0)	(32.0)	(35.3)
	18.1	−19.2	22.2	−2.5	−20.9	46.9	−2.5	27.4	58.7	26.7
	4.679	−4.969	5.748	−0.642	−5.409	12.147	−0.642	7.087	15.201	6.909
g_O	86	141	72	88	134	68	86	138	68	85
	(22.3)	(36.5)	(18.7)	(22.8)	(34.7)	(17.6)	(22.3)	(35.8)	(17.6)	(22.0)
g_E	87.2	130.0	69.0	87.2	130.0	69.0	87.2	130.0	69.0	87.2
	(22.6)	(33.7)	(17.9)	(22.6)	(33.7)	(17.9)	(22.6)	(33.7)	(17.9)	(22.6)
	−2.5	22.9	6.1	1.5	8.1	−2.0	−2.5	16.5	−2.0	−4.4
	−0.635	5.938	1.580	0.398	2.108	−0.522	−0.635	4.274	−0.522	−1.143
c_O	60	66	71	55	75	72	75	57	77	58
	(15.5)	(17.1)	(18.4)	(14.2)	(19.4)	(18.7)	(19.4)	(14.8)	(19.9)	(15.0)
c_E	59.3	63.0	81.2	59.3	63.0	81.2	59.3	63.0	81.2	59.3
	(15.4)	(16.3)	(21.0)	(15.4)	(16.3)	(21.0)	(15.4)	(16.3)	(21.0)	(15.4)
	1.4	6.0	−19.1	−8.3	26.0	−17.4	35.2	−11.5	−8.3	−2.6
	0.354	1.564	−4.957	−2.156	6.745	−4.505	9.113	−2.979	−2.139	−0.677
N	386	386	386	386	386	386	386	386	386	386
X2	1.2	2.6	2.4	0.6	4.2	6.0	5.9	5.0	10.1	2.1
Gvalue	1.2	2.6	2.5	0.6	4.2	5.9	5.6	5.0	10.0	2.1
Gv/N	0.323	0.674	0.641	0.145	1.085	1.528	1.454	1.295	2.585	0.542
Prob	0.74211	0.45753	0.48012	0.90533	0.24186	0.11666	0.13196	0.17180	0.01874	0.55367

	61	62	63	64	65	66	67	68	69	70
a_O	114	100	87	115	98	110	108	109	96	121
	(29.5)	(25.9)	(22.5)	(29.8)	(25.4)	(28.5)	(28.0)	(28.2)	(24.9)	(31.3)
a_E	115.6	112.4	103.2	115.6	112.4	103.2	115.6	112.4	103.2	115.6
	(30.0)	(29.1)	(26.7)	(30.0)	(29.1)	(26.7)	(30.0)	(29.1)	(26.7)	(30.0)
	−3.3	−23.4	−29.7	−1.3	−26.9	14.0	−14.8	−6.7	−13.9	10.9
	−0.848	−6.052	−7.697	−0.335	−6.957	3.638	−3.829	−1.730	−3.597	2.836
t_O	83	141	135	76	134	123	79	112	147	78
	(21.5)	(36.5)	(35.0)	(19.7)	(34.7)	(31.9)	(20.5)	(29.0)	(38.1)	(20.2)
t_E	77.3	123.4	136.2	77.3	123.4	136.2	77.3	123.4	136.2	77.3
	(20.0)	(32.0)	(35.3)	(20.0)	(32.0)	(35.3)	(20.0)	(32.0)	(35.3)	(20.0)
	11.8	37.7	−2.5	−2.6	22.2	−25.2	3.4	−21.6	22.3	1.4
	3.054	9.768	−0.642	−0.673	5.748	−6.518	0.885	−5.603	5.787	0.359
g_O	129	75	102	122	73	101	134	70	79	121
	(33.4)	(19.4)	(26.4)	(31.6)	(18.9)	(26.2)	(34.7)	(18.1)	(20.5)	(31.3)
g_E	130.0	69.0	87.2	130.0	69.0	87.2	130.0	69.0	87.2	130.0
	(33.7)	(17.9)	(22.6)	(33.7)	(17.9)	(22.6)	(33.7)	(17.9)	(22.6)	(33.7)
	−2.0	12.5	31.9	−15.5	8.2	29.6	8.1	2.0	−15.7	−17.3
	−0.512	3.232	8.264	−4.011	2.124	7.667	2.108	0.514	−4.059	−4.494
c_O	60	70	62	73	81	52	65	95	64	66
	(15.5)	(18.1)	(16.1)	(18.9)	(21.0)	(13.5)	(16.8)	(24.6)	(16.6)	(17.1)
c_E	63.0	81.2	59.3	63.0	81.2	59.3	63.0	81.2	59.3	63.0
	(16.3)	(21.0)	(15.4)	(16.3)	(21.0)	(15.4)	(16.3)	(21.0)	(15.4)	(16.3)
	−5.9	−20.9	5.5	21.4	−0.5	−13.7	4.0	29.7	9.7	6.0
	−1.541	−5.402	1.419	5.543	−0.125	−3.549	1.027	7.701	2.517	1.564

N	386	386	386	386	386	386	386	386	386	386
X2	0.6	6.0	5.2	2.1	3.0	4.8	0.7	3.5	2.5	1.0
Gvalue	0.6	6.0	5.2	2.0	3.0	4.8	0.7	3.4	2.5	1.0
Gv/N	0.153	1.546	1.344	0.524	0.789	1.238	0.191	0.882	0.649	0.265
Prob	0.89874	0.11314	0.15863	0.56789	0.38445	0.18867	0.86466	0.33338	0.47449	0.79569

	71	72	73	74	75	76	77	78	79	80
a_O	101	108	105	101	93	133	103	110	120	103
	(26.2)	(28.0)	(27.2)	(26.2)	(24.1)	(34.5)	(26.7)	(28.5)	(31.1)	(26.7)
a_E	112.4	103.2	115.6	112.4	103.2	115.6	112.4	103.2	115.6	112.4
	(29.1)	(26.7)	(30.0)	(29.1)	(26.7)	(30.0)	(29.1)	(26.7)	(30.0)	(29.1)
	-21.6	9.8	-20.3	-21.6	-19.4	37.2	-18.0	14.0	8.9	-18.0
	-5.592	2.545	-5.255	-5.592	-5.014	9.633	-4.656	3.638	2.296	-4.656
t_O	129	141	85	114	149	72	142	130	88	137
	(33.4)	(36.5)	(22.0)	(29.5)	(38.6)	(18.7)	(36.8)	(33.7)	(22.8)	(35.5)
t_E	123.4	136.2	77.3	123.4	136.2	77.3	123.4	136.2	77.3	123.4
	(32.0)	(35.3)	(20.0)	(32.0)	(35.3)	(20.0)	(32.0)	(35.3)	(20.0)	(32.0)
	11.5	9.7	16.1	-18.0	26.7	-10.2	40.0	-12.2	22.8	28.8
	2.992	2.506	4.176	-4.658	6.909	-2.655	10.358	-3.160	5.905	7.448
g_O	74	83	125	88	78	115	71	84	121	70
	(19.2)	(21.5)	(32.4)	(22.8)	(20.2)	(29.8)	(18.4)	(21.8)	(31.3)	(18.1)
g_E	69.0	87.2	130.0	69.0	87.2	130.0	69.0	87.2	130.0	69.0
	(17.9)	(22.6)	(33.7)	(17.9)	(22.6)	(33.7)	(17.9)	(22.6)	(33.7)	(17.9)
	10.3	-8.3	-9.8	42.8	-17.5	-28.2	4.0	-6.3	-17.3	2.0
	2.674	-2.140	-2.536	11.081	-4.522	-7.302	1.043	-1.645	-4.494	0.514
c_O	82	54	71	83	66	66	70	62	57	76
	(21.2)	(14.0)	(18.4)	(21.5)	(17.1)	(17.1)	(18.1)	(16.1)	(14.8)	(19.7)
c_E	81.2	59.3	63.0	81.2	59.3	63.0	81.2	59.3	63.0	81.2
	(21.0)	(15.4)	(16.3)	(21.0)	(15.4)	(16.3)	(21.0)	(15.4)	(16.3)	(21.0)
	1.5	-10.2	16.9	3.6	14.1	6.0	-20.9	5.5	-11.5	-10.1
	0.395	-2.630	4.369	0.921	3.648	1.564	-5.402	1.419	-2.979	-2.626
N	386	386	386	386	386	386	386	386	386	386
X2	1.8	1.1	2.9	7.1	3.9	4.8	5.2	1.0	2.8	2.6
Gvalue	1.8	1.1	2.9	6.8	3.9	4.8	5.2	1.0	2.8	2.6
Gv/N	0.469	0.281	0.754	1.752	1.021	1.241	1.343	0.251	0.729	0.680
Prob	0.61297	0.78060	0.40568	0.07989	0.26796	0.18774	0.15885	0.80849	0.42141	0.45326

	81	82	83	84	85	86	87	88	89	90
a_O	96	101	98	82	92	87	107	119	109	103
	(24.9)	(26.2)	(25.4)	(21.2)	(23.8)	(22.5)	(27.7)	(30.8)	(28.2)	(26.7)
a_E	103.2	115.6	112.4	103.2	115.6	112.4	103.2	115.6	112.4	103.2
	(26.7)	(30.0)	(29.1)	(26.7)	(30.0)	(29.1)	(26.7)	(30.0)	(29.1)	(26.7)
	-13.9	-27.4	-26.9	-37.7	-42.1	-44.6	7.7	6.8	-6.7	-0.4
	-3.597	-7.088	-6.957	-9.769	-10.905	-11.543	2.006	1.761	-1.730	-0.103
t_O	145	98	123	149	97	149	123	95	116	134
	(37.6)	(25.4)	(31.9)	(38.6)	(25.1)	(38.6)	(31.9)	(24.6)	(30.1)	(34.7)
t_E	136.2	77.3	123.4	136.2	77.3	123.4	136.2	77.3	123.4	136.2
	(35.3)	(20.0)	(32.0)	(35.3)	(20.0)	(32.0)	(35.3)	(20.0)	(32.0)	(35.3)
	18.1	46.5	-0.7	26.7	44.0	56.3	-25.2	39.1	-14.3	-4.5
	4.679	12.041	-0.183	6.909	11.403	14.583	-6.518	10.142	-3.694	-1.154
g_O	88	127	84	88	125	83	85	112	90	93
	(22.8)	(32.9)	(21.8)	(22.8)	(32.4)	(21.5)	(22.0)	(29.0)	(23.3)	(24.1)
g_E	87.2	130.0	69.0	87.2	130.0	69.0	87.2	130.0	69.0	87.2
	(22.6)	(33.7)	(17.9)	(22.6)	(33.7)	(17.9)	(22.6)	(33.7)	(17.9)	(22.6)
	1.5	-5.9	33.0	1.5	-9.8	30.6	-4.4	-33.4	47.8	11.9
	0.398	-1.532	8.552	0.398	-2.536	7.936	-1.143	-8.645	12.381	3.084
c_O	57	60	81	67	72	67	71	60	71	56
	(14.8)	(15.5)	(21.0)	(17.4)	(18.7)	(17.4)	(18.4)	(15.5)	(18.4)	(14.5)
c_E	59.3	63.0	81.2	59.3	63.0	81.2	59.3	63.0	81.2	59.3
	(15.4)	(16.3)	(21.0)	(15.4)	(16.3)	(21.0)	(15.4)	(16.3)	(21.0)	(15.4)
	-4.6	-5.9	-0.5	16.3	19.1	-25.8	25.5	-5.9	-19.1	-6.5
	-1.179	-1.541	-0.125	4.225	4.953	-6.691	6.611	-1.541	-4.957	-1.672
N	386	386	386	386	386	386	386	386	386	386
X2	1.2	7.6	5.1	6.5	11.3	16.4	3.8	6.8	8.2	0.6
Gvalue	1.2	7.3	5.0	6.8	11.2	16.5	3.7	6.6	7.7	0.6
Gv/N	0.302	1.880	1.287	1.763	2.914	4.285	0.956	1.718	1.999	0.155
Prob	0.76168	0.06409	0.17404	0.07833	0.01045	0.00088	0.29705	0.08467	0.05222	0.89643

	91	92	93	94	95	96	97	98	99	100
a_O	123	120	112	131	102	117	112	111	94	102
	(31.9)	(31.1)	(29.0)	(34.4)	(26.8)	(30.7)	(29.9)	(29.6)	(25.1)	(27.8)
a_E	115.6	112.4	103.2	114.2	110.9	101.9	112.4	109.2	100.3	110.0
	(30.0)	(29.1)	(26.7)	(30.0)	(29.1)	(26.7)	(30.0)	(29.1)	(26.7)	(30.0)
	15.2	15.7	18.3	36.1	-17.1	32.4	-0.7	3.7	-12.1	-15.3
	3.927	4.073	4.750	9.468	-4.496	8.510	-0.188	0.974	-3.231	-4.175
t_O	83	113	137	87	129	119	86	124	131	87
	(21.5)	(29.3)	(35.5)	(22.8)	(33.9)	(31.2)	(22.9)	(33.1)	(34.9)	(23.7)
t_E	77.3	123.4	136.2	76.3	121.8	134.5	75.1	119.8	132.4	73.5
	(20.0)	(32.0)	(35.3)	(20.0)	(32.0)	(35.3)	(20.0)	(32.0)	(35.3)	(20.0)
	11.8	-19.8	1.5	22.8	14.9	-29.1	23.3	8.5	-2.7	29.3
	3.054	-5.133	0.392	5.988	3.914	-7.639	6.212	2.258	-0.723	7.992
g_O	130	73	75	108	90	84	128	67	78	107
	(33.7)	(18.9)	(19.4)	(28.3)	(23.6)	(22.0)	(34.1)	(17.9)	(20.8)	(29.2)
g_E	130.0	69.0	87.2	128.3	68.1	86.1	126.3	67.0	84.7	123.6
	(33.7)	(17.9)	(22.6)	(33.7)	(17.9)	(22.6)	(33.7)	(17.9)	(22.6)	(33.7)
	0.0	8.2	-22.7	-37.2	50.1	-4.2	3.4	-0.1	-12.9	-30.9
	0.004	2.124	-5.872	-9.769	13.159	-1.091	0.919	-0.025	-3.452	-8.407
c_O	50	80	62	55	60	61	49	73	72	71
	(13.0)	(20.7)	(16.1)	(14.4)	(15.7)	(16.0)	(13.1)	(19.5)	(19.2)	(19.3)
c_E	63.0	81.2	59.3	62.2	80.2	58.6	61.3	78.9	57.6	59.9
	(16.3)	(21.0)	(15.4)	(16.3)	(21.0)	(15.4)	(16.3)	(21.0)	(15.4)	(16.3)
	-23.2	-2.5	5.5	-13.6	-34.8	5.0	-21.9	-11.4	32.1	24.0
	-6.007	-0.638	1.419	-3.567	-9.135	1.311	-5.832	-3.039	8.548	6.549
N	386	386	386	381	381	381	375	375	375	367
X2	3.6	1.6	2.6	8.0	13.3	4.2	4.1	0.6	4.5	7.3
Gvalue	3.8	1.6	2.7	8.1	13.1	4.2	4.2	0.6	4.3	7.2
Gv/N	0.978	0.426	0.688	2.121	3.442	1.090	1.111	0.168	1.142	1.958
Prob	0.28676	0.64963	0.44778	0.04440	0.00440	0.24539	0.24414	0.88965	0.23253	0.06616

Atum_C58_CEREON
5UTR:

	100	99	98	97	96	95	94	93	92	91
a_O	288	279	283	283	291	268	243	270	265	275
	(23.3)	(22.6)	(22.9)	(22.9)	(23.5)	(21.7)	(19.6)	(21.8)	(21.4)	(22.2)
a_E	287.8	287.8	287.8	287.8	287.8	287.8	287.8	287.8	287.8	287.8
	(23.3)	(23.3)	(23.3)	(23.3)	(23.3)	(23.3)	(23.3)	(23.3)	(23.3)	(23.3)
	0.5	-17.2	-9.4	-9.4	6.5	-38.1	-82.1	-34.4	-43.7	-24.9
	0.040	-1.393	-0.762	-0.762	0.528	-3.081	-6.641	-2.779	-3.529	-2.015
t_O	263	281	253	285	248	283	297	281	280	282
	(21.3)	(22.7)	(20.5)	(23.0)	(20.0)	(22.9)	(24.0)	(22.7)	(22.6)	(22.8)
t_E	285.5	285.5	285.5	285.5	285.5	285.5	285.5	285.5	285.5	285.5
	(23.1)	(23.1)	(23.1)	(23.1)	(23.1)	(23.1)	(23.1)	(23.1)	(23.1)	(23.1)
	-43.2	-9.0	-61.2	-1.1	-69.9	-5.0	23.4	-9.0	-11.0	-7.0
	-3.495	-0.727	-4.948	-0.086	-5.650	-0.407	1.891	-0.727	-0.885	-0.567
g_O	336	342	378	345	337	357	374	318	353	341
	(27.2)	(27.6)	(30.6)	(27.9)	(27.2)	(28.9)	(30.2)	(25.7)	(28.5)	(27.6)
g_E	316.1	316.1	316.1	316.1	316.1	316.1	316.1	316.1	316.1	316.1
	(25.6)	(25.6)	(25.6)	(25.6)	(25.6)	(25.6)	(25.6)	(25.6)	(25.6)	(25.6)
	41.1	54.0	135.3	60.5	43.2	87.0	125.9	3.9	78.0	51.8
	3.324	4.362	10.937	4.887	3.495	7.031	10.178	0.315	6.309	4.187
c_O	350	335	323	324	361	329	323	368	339	339
	(28.3)	(27.1)	(26.1)	(26.2)	(29.2)	(26.6)	(26.1)	(29.7)	(27.4)	(27.4)
c_E	347.7	347.7	347.7	347.7	347.7	347.7	347.7	347.7	347.7	347.7
	(28.1)	(28.1)	(28.1)	(28.1)	(28.1)	(28.1)	(28.1)	(28.1)	(28.1)	(28.1)
	4.7	-24.9	-47.5	-45.7	27.2	-36.3	-47.5	41.8	-17.1	-17.1
	0.380	-2.009	-3.842	-3.692	2.198	-2.935	-3.842	3.383	-1.383	-1.383
N	1237	1237	1237	1237	1237	1237	1237	1237	1237	1237
X2	3.1	2.9	17.7	4.3	6.9	7.7	19.8	2.4	6.4	2.8
Gvalue	3.1	2.9	17.1	4.3	7.1	7.5	19.6	2.4	6.3	2.8
Gv/N	0.249	0.233	1.386	0.348	0.571	0.608	1.586	0.192	0.512	0.223
Prob	0.38025	0.41037	0.00066	0.23080	0.06988	0.05710	0.00020	0.49929	0.09654	0.43129

	90	89	88	87	86	85	84	83	82	81
a_O	285	304	295	277	277	284	275	275	269	273
	(23.0)	(24.6)	(23.8)	(22.4)	(22.4)	(23.0)	(22.2)	(22.2)	(21.7)	(22.1)
a_E	287.8	287.8	287.8	287.8	287.8	287.8	287.8	287.8	287.8	287.8
	(23.3)	(23.3)	(23.3)	(23.3)	(23.3)	(23.3)	(23.3)	(23.3)	(23.3)	(23.3)
	-5.5	33.4	14.7	-21.1	-21.1	-7.5	-24.9	-24.9	-36.3	-28.7
	-0.442	2.700	1.187	-1.705	-1.705	-0.602	-2.015	-2.015	-2.931	-2.323
t_O	272	260	265	287	269	280	302	273	306	295
	(22.0)	(21.0)	(21.4)	(23.2)	(21.7)	(22.6)	(24.4)	(22.1)	(24.7)	(23.8)
t_E	285.5	285.5	285.5	285.5	285.5	285.5	285.5	285.5	285.5	285.5
	(23.1)	(23.1)	(23.1)	(23.1)	(23.1)	(23.1)	(23.1)	(23.1)	(23.1)	(23.1)
	-26.4	-48.7	-39.5	2.9	-32.1	-11.0	33.9	-24.5	42.4	19.3
	-2.135	-3.937	-3.197	0.238	-2.594	-0.885	2.738	-1.981	3.426	1.556
g_O	364	356	335	328	354	319	315	329	309	356
	(29.4)	(28.8)	(27.1)	(26.5)	(28.6)	(25.8)	(25.5)	(26.6)	(25.0)	(28.8)
g_E	316.1	316.1	316.1	316.1	316.1	316.1	316.1	316.1	316.1	316.1
	(25.6)	(25.6)	(25.6)	(25.6)	(25.6)	(25.6)	(25.6)	(25.6)	(25.6)	(25.6)
	102.8	84.7	39.0	24.3	80.3	5.9	-2.1	26.4	-14.0	84.7
	8.311	6.849	3.152	1.966	6.488	0.478	-0.171	2.134	-1.129	6.849
c_O	316	317	342	345	337	354	345	360	353	313
	(25.5)	(25.6)	(27.6)	(27.9)	(27.2)	(28.6)	(27.9)	(29.1)	(28.5)	(25.3)
c_E	347.7	347.7	347.7	347.7	347.7	347.7	347.7	347.7	347.7	347.7
	(28.1)	(28.1)	(28.1)	(28.1)	(28.1)	(28.1)	(28.1)	(28.1)	(28.1)	(28.1)
	-60.3	-58.5	-11.2	-5.3	-21.0	12.8	-5.3	25.1	10.8	-65.7
	-4.878	-4.732	-0.908	-0.428	-1.697	1.034	-0.428	2.030	0.870	-5.315
N	1237	1237	1237	1237	1237	1237	1237	1237	1237	1237
X2	10.8	11.0	2.9	0.9	6.2	0.3	1.5	2.1	2.9	9.6
Gvalue	10.6	10.9	2.9	0.9	6.1	0.3	1.5	2.1	2.9	9.5
Gv/N	0.855	0.880	0.234	0.071	0.493	0.024	0.124	0.169	0.236	0.768
Prob	0.01421	0.01234	0.40736	0.83021	0.10707	0.96020	0.67543	0.55451	0.40359	0.02330

	80	79	78	77	76	75	74	73	72	71
a_O	240	284	249	269	264	269	287	307	276	313
	(19.4)	(23.0)	(20.1)	(21.7)	(21.3)	(21.7)	(23.2)	(24.8)	(22.3)	(25.3)
a_E	287.8	287.8	287.8	287.8	287.8	287.8	287.8	287.8	287.8	287.8
	(23.3)	(23.3)	(23.3)	(23.3)	(23.3)	(23.3)	(23.3)	(23.3)	(23.3)	(23.3)
	-87.1	-7.5	-72.0	-36.3	-45.5	-36.3	-1.5	39.8	-23.0	52.7
	-7.041	-0.602	-5.823	-2.931	-3.677	-2.931	-0.121	3.214	-1.860	4.257
t_O	290	285	314	293	311	296	276	267	262	260
	(23.4)	(23.0)	(25.4)	(23.7)	(25.1)	(23.9)	(22.3)	(21.6)	(21.2)	(21.0)
t_E	285.5	285.5	285.5	285.5	285.5	285.5	285.5	285.5	285.5	285.5
	(23.1)	(23.1)	(23.1)	(23.1)	(23.1)	(23.1)	(23.1)	(23.1)	(23.1)	(23.1)
	9.0	-1.1	59.7	15.1	53.1	21.3	-18.7	-35.8	-45.1	-48.7
	0.728	-0.086	4.825	1.223	4.296	1.723	-1.515	-2.897	-3.643	-3.937
g_O	343	340	332	344	330	327	341	329	340	327
	(27.7)	(27.5)	(26.8)	(27.8)	(26.7)	(26.4)	(27.6)	(26.6)	(27.5)	(26.4)
g_E	316.1	316.1	316.1	316.1	316.1	316.1	316.1	316.1	316.1	316.1
	(25.6)	(25.6)	(25.6)	(25.6)	(25.6)	(25.6)	(25.6)	(25.6)	(25.6)	(25.6)
	56.1	49.6	32.7	58.3	28.5	22.3	51.8	26.4	49.6	22.3
	4.536	4.014	2.641	4.711	2.303	1.799	4.187	2.134	4.014	1.799
c_O	364	328	342	331	332	345	333	334	359	337
	(29.4)	(26.5)	(27.6)	(26.8)	(26.8)	(27.9)	(26.9)	(27.0)	(29.0)	(27.2)
c_E	347.7	347.7	347.7	347.7	347.7	347.7	347.7	347.7	347.7	347.7
	(28.1)	(28.1)	(28.1)	(28.1)	(28.1)	(28.1)	(28.1)	(28.1)	(28.1)	(28.1)
	33.4	-38.2	-11.2	-32.5	-30.6	-5.3	-28.7	-26.8	23.0	-21.0
	2.703	-3.087	-0.908	-2.628	-2.474	-0.428	-2.320	-2.165	1.863	-1.697
N	1237	1237	1237	1237	1237	1237	1237	1237	1237	1237
X2	11.1	3.0	9.0	4.7	5.6	2.0	2.9	3.6	4.6	5.2
Gvalue	11.5	3.0	9.1	4.7	5.5	2.0	2.9	3.6	4.6	5.2
Gv/N	0.927	0.239	0.736	0.376	0.448	0.164	0.232	0.287	0.373	0.421
Prob	0.00947	0.39899	0.02797	0.19891	0.13591	0.56759	0.41229	0.31357	0.20215	0.15690

	70	69	68	67	66	65	64	63	62	61
a_O	264	272	282	312	272	248	293	300	268	310
	(21.3)	(22.0)	(22.8)	(25.2)	(22.0)	(20.0)	(23.7)	(24.3)	(21.7)	(25.1)
a_E	287.8	287.8	287.8	287.8	287.8	287.8	287.8	287.8	287.8	287.8
	(23.3)	(23.3)	(23.3)	(23.3)	(23.3)	(23.3)	(23.3)	(23.3)	(23.3)	(23.3)
	-45.5	-30.6	-11.4	50.5	-30.6	-73.7	10.6	25.0	-38.1	46.2
	-3.677	-2.475	-0.920	4.082	-2.475	-5.961	0.857	2.022	-3.081	3.733
t_O	269	274	243	263	306	301	300	285	285	275
	(21.7)	(22.2)	(19.6)	(21.3)	(24.7)	(24.3)	(24.3)	(23.0)	(23.0)	(22.2)

```
t_E       285.5   285.5   285.5   285.5   285.5   285.5   285.5   285.5   285.5   285.5
        ( 23.1) ( 23.1) ( 23.1) ( 23.1) ( 23.1) ( 23.1) ( 23.1) ( 23.1) ( 23.1) ( 23.1)
          -32.1   -22.6   -78.4   -43.2    42.4    31.8    29.7    -1.1    -1.1   -20.7
         -2.594  -1.826  -6.337  -3.495   3.426   2.568   2.398  -0.086  -0.086  -1.671
g_O         352     339     341     309     332     348     319     324     320     309
        ( 28.5) ( 27.4) ( 27.6) ( 25.0) ( 26.8) ( 28.1) ( 25.8) ( 26.2) ( 25.9) ( 25.0)
g_E       316.1   316.1   316.1   316.1   316.1   316.1   316.1   316.1   316.1   316.1
        ( 25.6) ( 25.6) ( 25.6) ( 25.6) ( 25.6) ( 25.6) ( 25.6) ( 25.6) ( 25.6) ( 25.6)
           75.8    47.5    51.8   -14.0    32.7    67.0     5.9    16.1     7.9   -14.0
          6.129   3.840   4.187  -1.129   2.641   5.417   0.478   1.300   0.641  -1.129
c_O         352     352     371     353     327     340     325     328     364     343
        ( 28.5) ( 28.5) ( 30.0) ( 28.5) ( 26.4) ( 27.5) ( 26.3) ( 26.5) ( 29.4) ( 27.7)
c_E       347.7   347.7   347.7   347.7   347.7   347.7   347.7   347.7   347.7   347.7
        ( 28.1) ( 28.1) ( 28.1) ( 28.1) ( 28.1) ( 28.1) ( 28.1) ( 28.1) ( 28.1) ( 28.1)
            8.7     8.7    48.2    10.8   -40.1   -15.1   -43.8   -38.2    33.4    -9.3
          0.706   0.706   3.898   0.870  -3.239  -1.225  -3.542  -3.087   2.703  -0.748

N          1237    1237    1237    1237    1237    1237    1237    1237    1237    1237
X2          7.1     3.0    10.0     4.1     4.4     9.7     2.3     1.8     2.2     2.3
Gvalue      7.0     3.0    10.2     4.1     4.4     9.9     2.4     1.8     2.2     2.3
Gv/N      0.565   0.245   0.828   0.328   0.352   0.799   0.190   0.149   0.177   0.185
Prob    0.07233 0.38697 0.01662 0.25553 0.22555 0.01961 0.50229 0.60489 0.53317 0.51394
        -------------------------------------------------------------------------------
             60      59      58      57      56      55      54      53      52      51
        -------------------------------------------------------------------------------
a_O         300     288     285     310     278     271     277     270     301     285
        ( 24.3) ( 23.3) ( 23.0) ( 25.1) ( 22.5) ( 21.9) ( 22.4) ( 21.8) ( 24.3) ( 23.0)
a_E       287.8   287.8   287.8   287.8   287.8   287.8   287.8   287.8   287.8   287.8
        ( 23.3) ( 23.3) ( 23.3) ( 23.3) ( 23.3) ( 23.3) ( 23.3) ( 23.3) ( 23.3) ( 23.3)
           25.0     0.5    -5.5    46.2   -19.2   -32.5   -21.1   -34.4    27.1    -5.5
          2.022   0.040  -0.442   3.733  -1.549  -2.628  -1.705  -2.779   2.191  -0.442
t_O         275     259     286     281     247     288     279     287     266     285
        ( 22.2) ( 20.9) ( 23.1) ( 22.7) ( 20.0) ( 23.3) ( 22.6) ( 23.2) ( 21.5) ( 23.0)
t_E       285.5   285.5   285.5   285.5   285.5   285.5   285.5   285.5   285.5   285.5
        ( 23.1) ( 23.1) ( 23.1) ( 23.1) ( 23.1) ( 23.1) ( 23.1) ( 23.1) ( 23.1) ( 23.1)
          -20.7   -50.5     0.9    -9.0   -71.6     5.0   -12.9     2.9   -37.7    -1.1
         -1.671  -4.084   0.076  -0.727  -5.789   0.401  -1.044   0.238  -3.047  -0.086
g_O         331     332     327     304     384     326     343     334     318     356
        ( 26.8) ( 26.8) ( 26.4) ( 24.6) ( 31.0) ( 26.4) ( 27.7) ( 27.0) ( 25.7) ( 28.8)
g_E       316.1   316.1   316.1   316.1   316.1   316.1   316.1   316.1   316.1   316.1
        ( 25.6) ( 25.6) ( 25.6) ( 25.6) ( 25.6) ( 25.6) ( 25.6) ( 25.6) ( 25.6) ( 25.6)
           30.6    32.7    22.3   -23.7   149.5    20.2    56.1    36.9     3.9    84.7
          2.472   2.641   1.799  -1.912  12.089   1.632   4.536   2.981   0.315   6.849
c_O         331     358     339     342     328     352     338     346     352     311
        ( 26.8) ( 28.9) ( 27.4) ( 27.6) ( 26.5) ( 28.5) ( 27.3) ( 28.0) ( 28.5) ( 25.1)
c_E       347.7   347.7   347.7   347.7   347.7   347.7   347.7   347.7   347.7   347.7
        ( 28.1) ( 28.1) ( 28.1) ( 28.1) ( 28.1) ( 28.1) ( 28.1) ( 28.1) ( 28.1) ( 28.1)
          -32.5    21.0   -17.1   -11.2   -38.2     8.7   -19.0    -3.3     8.7   -69.3
         -2.628   1.696  -1.383  -0.908  -3.087   0.706  -1.540  -0.268   0.706  -5.603

N          1237    1237    1237    1237    1237    1237    1237    1237    1237    1237
X2          2.4     3.6     0.6     2.3    21.2     1.4     3.1     2.1     2.0     8.9
Gvalue      2.4     3.6     0.6     2.3    20.6     1.4     3.1     2.1     2.0     8.9
Gv/N      0.195   0.294   0.050   0.187   1.663   0.111   0.248   0.172   0.164   0.718
Prob    0.49100 0.30298 0.89206 0.51084 0.00013 0.71043 0.38219 0.54522 0.56534 0.03088
        -------------------------------------------------------------------------------
             50      49      48      47      46      45      44      43      42      41
        -------------------------------------------------------------------------------
a_O         294     298     297     292     318     260     279     271     270     279
        ( 23.8) ( 24.1) ( 24.0) ( 23.6) ( 25.7) ( 21.0) ( 22.6) ( 21.9) ( 21.8) ( 22.6)
a_E       287.8   287.8   287.8   287.8   287.8   287.8   287.8   287.8   287.8   287.8
        ( 23.3) ( 23.3) ( 23.3) ( 23.3) ( 23.3) ( 23.3) ( 23.3) ( 23.3) ( 23.3) ( 23.3)
           12.6    20.9    18.8     8.6    63.6   -52.7   -17.2   -32.5   -34.4   -17.2
          1.021   1.686   1.519   0.692   5.139  -4.263  -1.393  -2.628  -2.779  -1.393
t_O         248     275     277     264     284     293     294     302     310     253
        ( 20.0) ( 22.2) ( 22.4) ( 21.3) ( 23.0) ( 23.7) ( 23.8) ( 24.4) ( 25.1) ( 20.5)
t_E       285.5   285.5   285.5   285.5   285.5   285.5   285.5   285.5   285.5   285.5
        ( 23.1) ( 23.1) ( 23.1) ( 23.1) ( 23.1) ( 23.1) ( 23.1) ( 23.1) ( 23.1) ( 23.1)
          -69.9   -20.7   -16.8   -41.4    -3.1    15.1    17.2    33.9    51.0   -61.2
         -5.650  -1.671  -1.358  -3.346  -0.247   1.223   1.390   2.738   4.121  -4.948
g_O         341     297     321     344     299     339     338     332     326     352
        ( 27.6) ( 24.0) ( 25.9) ( 27.8) ( 24.2) ( 27.4) ( 27.3) ( 26.8) ( 26.4) ( 28.5)
g_E       316.1   316.1   316.1   316.1   316.1   316.1   316.1   316.1   316.1   316.1
        ( 25.6) ( 25.6) ( 25.6) ( 25.6) ( 25.6) ( 25.6) ( 25.6) ( 25.6) ( 25.6) ( 25.6)
           51.8   -36.9    10.0    58.3   -33.2    47.5    45.4    32.7    20.2    75.8
          4.187  -2.987   0.805   4.711  -2.682   3.840   3.668   2.641   1.632   6.129
c_O         354     367     342     337     336     345     326     332     331     353
        ( 28.6) ( 29.7) ( 27.6) ( 27.2) ( 27.2) ( 27.9) ( 26.4) ( 26.8) ( 26.8) ( 28.5)
c_E       347.7   347.7   347.7   347.7   347.7   347.7   347.7   347.7   347.7   347.7
        ( 28.1) ( 28.1) ( 28.1) ( 28.1) ( 28.1) ( 28.1) ( 28.1) ( 28.1) ( 28.1) ( 28.1)
           12.8    39.7   -11.2   -21.0   -22.9    -5.3   -41.9   -30.6   -32.5    10.8
          1.034   3.212  -0.908  -1.697  -1.853  -0.428  -3.391  -2.474  -2.628   0.870

N          1237    1237    1237    1237    1237    1237    1237    1237    1237    1237
X2          7.2     3.0     0.7     4.5     4.5     4.6     3.4     3.4     4.3     8.1
Gvalue      7.3     3.0     0.7     4.5     4.4     4.6     3.4     3.4     4.3     8.1
Gv/N      0.593   0.241   0.058   0.360   0.357   0.372   0.274   0.278   0.346   0.659
Prob    0.06203 0.39414 0.86823 0.21600 0.21993 0.20295 0.33599 0.32946 0.23291 0.04309
        -------------------------------------------------------------------------------
             40      39      38      37      36      35      34      33      32      31
        -------------------------------------------------------------------------------
a_O         295     292     291     314     290     253     252     283     272     290
        ( 23.8) ( 23.6) ( 23.5) ( 25.4) ( 23.4) ( 20.5) ( 20.4) ( 22.9) ( 22.0) ( 23.4)
a_E       287.8   287.8   287.8   287.8   287.8   287.8   287.8   287.8   287.8   287.8
        ( 23.3) ( 23.3) ( 23.3) ( 23.3) ( 23.3) ( 23.3) ( 23.3) ( 23.3) ( 23.3) ( 23.3)
           14.7     8.6     6.5    54.8     4.5   -65.1   -66.9    -9.4   -30.6     4.5
          1.187   0.692   0.528   4.432   0.365  -5.265  -5.405  -0.762  -2.475   0.365
t_O         324     285     256     282     284     305     321     301     303     294
        ( 26.2) ( 23.0) ( 20.7) ( 22.8) ( 23.0) ( 24.7) ( 25.9) ( 24.3) ( 24.5) ( 23.8)
t_E       285.5   285.5   285.5   285.5   285.5   285.5   285.5   285.5   285.5   285.5
        ( 23.1) ( 23.1) ( 23.1) ( 23.1) ( 23.1) ( 23.1) ( 23.1) ( 23.1) ( 23.1) ( 23.1)
           81.9    -1.1   -55.9    -7.0    -3.1    40.2    75.2    31.8    36.0    17.2
          6.621  -0.086  -4.519  -0.567  -0.247   3.253   6.077   2.568   2.909   1.390
g_O         283     322     356     315     334     342     334     321     300     308
        ( 22.9) ( 26.0) ( 28.8) ( 25.5) ( 27.0) ( 27.6) ( 27.0) ( 25.9) ( 24.3) ( 24.9)
g_E       316.1   316.1   316.1   316.1   316.1   316.1   316.1   316.1   316.1   316.1
        ( 25.6) ( 25.6) ( 25.6) ( 25.6) ( 25.6) ( 25.6) ( 25.6) ( 25.6) ( 25.6) ( 25.6)
          -62.5    12.0    84.7    -2.1    36.9    54.0    36.9    10.0   -31.3   -15.9
         -5.055   0.969   6.849  -0.171   2.981   4.362   2.981   0.805  -2.529  -1.286
c_O         335     338     334     326     329     337     330     332     362     345
        ( 27.1) ( 27.3) ( 27.0) ( 26.4) ( 26.6) ( 27.2) ( 26.7) ( 26.8) ( 29.3) ( 27.9)
c_E       347.7   347.7   347.7   347.7   347.7   347.7   347.7   347.7   347.7   347.7
```

	(28.1)	(28.1)	(28.1)	(28.1)	(28.1)	(28.1)	(28.1)	(28.1)	(28.1)	(28.1)
	-24.9	-19.0	-26.8	-41.9	-36.3	-21.0	-34.4	-30.6	29.3	-5.3
	-2.009	-1.540	-2.165	-3.391	-2.935	-1.697	-2.782	-2.474	2.366	-0.428
N	1237	1237	1237	1237	1237	1237	1237	1237	1237	1237
X2	9.3	0.4	8.7	3.8	2.0	8.0	10.8	1.7	3.3	0.5
Gvalue	9.2	0.4	8.6	3.8	2.0	8.1	10.8	1.7	3.3	0.5
Gv/N	0.744	0.036	0.695	0.303	0.165	0.653	0.872	0.137	0.270	0.040
Prob	0.02677	0.93073	0.03523	0.28966	0.56306	0.04442	0.01295	0.63798	0.34215	0.92019

	30	29	28	27	26	25	24	23	22	21
a_O	288	288	328	275	270	319	298	319	289	271
	(23.3)	(23.3)	(26.5)	(22.2)	(21.8)	(25.8)	(24.1)	(25.8)	(23.4)	(21.9)
a_E	287.8	287.8	287.8	287.8	287.8	287.8	287.8	287.8	287.8	287.8
	(23.3)	(23.3)	(23.3)	(23.3)	(23.3)	(23.3)	(23.3)	(23.3)	(23.3)	(23.3)
	0.5	0.5	85.9	-24.9	-34.4	65.8	20.9	65.8	2.5	-32.5
	0.040	0.040	6.943	-2.015	-2.779	5.317	1.686	5.317	0.203	-2.628
t_O	288	271	300	296	277	294	297	273	304	322
	(23.3)	(21.9)	(24.3)	(23.9)	(22.4)	(23.8)	(24.0)	(22.1)	(24.6)	(26.0)
t_E	285.5	285.5	285.5	285.5	285.5	285.5	285.5	285.5	285.5	285.5
	(23.1)	(23.1)	(23.1)	(23.1)	(23.1)	(23.1)	(23.1)	(23.1)	(23.1)	(23.1)
	5.0	-28.3	29.7	21.3	-16.8	17.2	23.4	-24.5	38.1	77.4
	0.401	-2.288	2.398	1.723	-1.358	1.390	1.891	-1.981	3.081	6.258
g_O	332	350	278	314	347	290	292	289	288	347
	(26.8)	(28.3)	(22.5)	(25.4)	(28.1)	(23.4)	(23.6)	(23.4)	(23.3)	(28.1)
g_E	316.1	316.1	316.1	316.1	316.1	316.1	316.1	316.1	316.1	316.1
	(25.6)	(25.6)	(25.6)	(25.6)	(25.6)	(25.6)	(25.6)	(25.6)	(25.6)	(25.6)
	32.7	71.4	-71.3	-4.1	64.8	-49.9	-46.2	-51.7	-53.6	64.8
	2.641	5.772	-5.767	-0.332	5.240	-4.035	-3.738	-4.182	-4.329	5.240
c_O	329	328	331	352	343	334	350	356	356	297
	(26.6)	(26.5)	(26.8)	(28.5)	(27.7)	(27.0)	(28.3)	(28.8)	(28.8)	(24.0)
c_E	347.7	347.7	347.7	347.7	347.7	347.7	347.7	347.7	347.7	347.7
	(28.1)	(28.1)	(28.1)	(28.1)	(28.1)	(28.1)	(28.1)	(28.1)	(28.1)	(28.1)
	-36.3	-38.2	-32.5	8.7	-9.3	-26.8	4.7	16.9	16.9	-93.6
	-2.935	-3.087	-2.628	0.706	-0.748	-2.165	0.380	1.364	1.364	-7.563
N	1237	1237	1237	1237	1237	1237	1237	1237	1237	1237
X2	1.8	5.5	11.7	1.0	4.4	6.3	2.7	6.5	3.9	16.0
Gvalue	1.8	5.4	11.7	1.0	4.4	6.3	2.7	6.4	3.9	16.2
Gv/N	0.148	0.437	0.946	0.082	0.354	0.508	0.219	0.519	0.319	1.307
Prob	0.60795	0.14433	0.00850	0.79636	0.22392	0.09874	0.43833	0.09289	0.26779	0.00105

	20	19	18	17	16	15	14	13	12	11
a_O	302	320	290	308	327	281	299	304	303	298
	(24.4)	(25.9)	(23.4)	(24.9)	(26.4)	(22.7)	(24.2)	(24.6)	(24.5)	(24.1)
a_E	287.8	287.8	287.8	287.8	287.8	287.8	287.8	287.8	287.8	287.8
	(23.3)	(23.3)	(23.3)	(23.3)	(23.3)	(23.3)	(23.3)	(23.3)	(23.3)	(23.3)
	29.2	68.0	4.5	41.9	83.6	-13.3	22.9	33.4	31.3	20.9
	2.360	5.496	0.365	3.387	6.760	-1.078	1.854	2.700	2.530	1.686
t_O	298	345	355	288	303	340	273	299	310	275
	(24.1)	(27.9)	(28.7)	(23.3)	(24.5)	(27.5)	(22.1)	(24.2)	(25.1)	(22.2)
t_E	285.5	285.5	285.5	285.5	285.5	285.5	285.5	285.5	285.5	285.5
	(23.1)	(23.1)	(23.1)	(23.1)	(23.1)	(23.1)	(23.1)	(23.1)	(23.1)	(23.1)
	25.5	130.5	154.6	5.0	36.0	118.7	-24.5	27.6	51.0	-20.7
	2.060	10.553	12.499	0.401	2.909	9.598	-1.981	2.228	4.121	-1.671
g_O	314	215	223	256	207	257	270	202	244	254
	(25.4)	(17.4)	(18.0)	(20.7)	(16.7)	(20.8)	(21.8)	(16.3)	(19.7)	(20.5)
g_E	316.1	316.1	316.1	316.1	316.1	316.1	316.1	316.1	316.1	316.1
	(25.6)	(25.6)	(25.6)	(25.6)	(25.6)	(25.6)	(25.6)	(25.6)	(25.6)	(25.6)
	-4.1	-165.7	-155.5	-107.9	-175.2	-106.3	-85.1	-180.9	-126.3	-111.0
	-0.332	-13.393	-12.575	-8.723	-14.164	-8.595	-6.876	-14.621	-10.208	-8.977
c_O	323	357	369	385	400	359	395	432	380	410
	(26.1)	(28.9)	(29.8)	(31.1)	(32.3)	(29.0)	(31.9)	(34.9)	(30.7)	(33.1)
c_E	347.7	347.7	347.7	347.7	347.7	347.7	347.7	347.7	347.7	347.7
	(28.1)	(28.1)	(28.1)	(28.1)	(28.1)	(28.1)	(28.1)	(28.1)	(28.1)	(28.1)
	-47.5	18.9	44.0	78.6	112.2	23.0	100.9	187.7	67.6	135.2
	-3.842	1.530	3.554	6.350	9.070	1.863	8.153	15.171	5.465	10.933
N	1237	1237	1237	1237	1237	1237	1237	1237	1237	1237
X2	3.0	48.6	45.6	16.9	51.9	22.0	14.1	63.2	22.3	24.1
Gvalue	3.0	51.8	47.6	17.5	56.6	22.1	14.2	67.8	23.6	24.4
Gv/N	0.245	4.186	3.844	1.415	4.575	1.787	1.150	5.479	1.908	1.972
Prob	0.38600	0.00000	0.00000	0.00056	0.00000	0.00006	0.00261	0.00000	0.00003	0.00002

	10	9	8	7	6	5	4	3	2	1
a_O	332	304	315	329	309	323	295	303	281	314
	(26.8)	(24.6)	(25.5)	(26.6)	(25.0)	(26.1)	(23.8)	(24.5)	(22.7)	(25.4)
a_E	287.8	287.8	287.8	287.8	287.8	287.8	287.8	287.8	287.8	287.8
	(23.3)	(23.3)	(23.3)	(23.3)	(23.3)	(23.3)	(23.3)	(23.3)	(23.3)	(23.3)
	95.0	33.4	57.0	88.1	44.0	74.7	14.7	31.3	-13.3	54.8
	7.678	2.700	4.608	7.126	3.560	6.035	1.187	2.530	-1.078	4.432
t_O	290	322	296	297	293	264	284	272	244	258
	(23.4)	(26.0)	(23.9)	(24.0)	(23.7)	(21.3)	(23.0)	(22.0)	(19.7)	(20.9)
t_E	285.5	285.5	285.5	285.5	285.5	285.5	285.5	285.5	285.5	285.5
	(23.1)	(23.1)	(23.1)	(23.1)	(23.1)	(23.1)	(23.1)	(23.1)	(23.1)	(23.1)
	9.0	77.4	21.3	23.4	15.1	-41.4	-3.1	-26.4	-76.7	-52.3
	0.728	6.258	1.723	1.891	1.223	-3.346	-0.247	-2.135	-6.201	-4.229
g_O	220	228	242	208	229	289	328	338	336	268
	(17.8)	(18.4)	(19.6)	(16.8)	(18.5)	(23.4)	(26.5)	(27.3)	(27.2)	(21.7)
g_E	316.1	316.1	316.1	316.1	316.1	316.1	316.1	316.1	316.1	316.1
	(25.6)	(25.6)	(25.6)	(25.6)	(25.6)	(25.6)	(25.6)	(25.6)	(25.6)	(25.6)
	-159.4	-148.9	-129.2	-174.1	-147.6	-51.7	24.3	45.4	41.1	-88.4
	-12.887	-12.039	-10.447	-14.070	-11.930	-4.182	1.966	3.668	3.324	-7.147
c_O	395	383	384	403	406	361	330	324	376	397
	(31.9)	(31.0)	(31.0)	(32.6)	(32.8)	(29.2)	(26.7)	(26.2)	(30.4)	(32.1)
c_E	347.7	347.7	347.7	347.7	347.7	347.7	347.7	347.7	347.7	347.7
	(28.1)	(28.1)	(28.1)	(28.1)	(28.1)	(28.1)	(28.1)	(28.1)	(28.1)	(28.1)
	100.9	74.2	76.4	119.1	126.0	27.2	-34.4	-45.7	58.9	105.4
	8.153	5.995	6.172	9.625	10.183	2.198	-2.782	-3.692	4.764	8.518
N	1237	1237	1237	1237	1237	1237	1237	1237	1237	1237
X2	42.5	33.7	24.1	52.1	35.5	8.8	1.5	4.6	9.8	19.4
Gvalue	45.4	36.0	25.4	56.5	37.6	8.7	1.5	4.6	10.0	19.5
Gv/N	3.672	2.914	2.057	4.571	3.036	0.704	0.125	0.370	0.808	1.574
Prob	0.00000	0.00000	0.00001	0.00000	0.00000	0.03342	0.67135	0.20503	0.01858	0.00022

CDS_INITIAL:

	1	2	3	4	5	6	7	8	9	10
a_O	530	0	0	428	306	288	337	329	258	384
	(42.8)	(0.0)	(0.0)	(34.6)	(24.7)	(23.3)	(27.2)	(26.6)	(20.9)	(31.0)
a_E	284.6	318.1	134.6	284.6	318.1	134.6	284.6	318.1	134.6	284.6
	(23.0)	(25.7)	(10.9)	(23.0)	(25.7)	(10.9)	(23.0)	(25.7)	(10.9)	(23.0)
	659.0	0.0	0.0	349.2	-23.7	438.0	113.8	22.2	335.6	230.0
	53.270	0.000	0.000	28.226	-1.914	35.405	9.201	1.797	27.128	18.590
t_O	401	1237	0	164	268	256	204	329	279	207
	(32.4)	(100.0)	(0.0)	(13.3)	(21.7)	(20.7)	(16.5)	(26.6)	(22.6)	(16.7)
t_E	170.2	362.5	224.3	170.2	362.5	224.3	170.2	362.5	224.3	170.2
	(13.8)	(29.3)	(18.1)	(13.8)	(29.3)	(18.1)	(13.8)	(29.3)	(18.1)	(13.8)
	687.5	3036.9	0.0	-12.1	-161.8	67.7	74.0	-63.7	121.7	81.1
	55.578	245.505	0.000	-0.977	-13.083	5.469	5.983	-5.152	9.841	6.559
g_O	306	0	1236	304	269	388	390	278	351	377
	(24.7)	(0.0)	(99.9)	(24.6)	(21.7)	(31.4)	(31.5)	(22.5)	(28.4)	(30.5)
g_E	473.9	234.1	396.1	473.9	234.1	396.1	473.9	234.1	396.1	473.9
	(38.3)	(18.9)	(32.0)	(38.3)	(18.9)	(32.0)	(38.3)	(18.9)	(32.0)	(38.3)
	-267.7	0.0	2813.2	-269.9	74.8	-16.0	-151.9	95.6	-84.8	-172.4
	-21.639	0.000	227.420	-21.819	6.044	-1.293	-12.284	7.726	-6.857	-13.941
c_O	0	0	1	341	394	305	306	301	349	269
	(0.0)	(0.0)	(0.1)	(27.6)	(31.9)	(24.7)	(24.7)	(24.3)	(28.2)	(21.7)
c_E	308.3	322.4	482.0	308.3	322.4	482.0	308.3	322.4	482.0	308.3
	(24.9)	(26.1)	(39.0)	(24.9)	(26.1)	(39.0)	(24.9)	(26.1)	(39.0)	(24.9)
	0.0	0.0	-12.4	68.7	158.1	-279.1	-4.6	-41.3	-225.3	-73.4
	0.000	0.000	-0.999	5.554	12.782	-22.564	-0.374	-3.338	-18.215	-5.934
N	1237	1237	1237	1237	1237	1237	1237	1237	1237	1237
X2	892.5	2984.6	2620.0	136.8	46.2	244.3	31.2	13.1	168.2	67.5
Gvalue	1078.8	3036.9	2800.8	135.9	47.4	210.5	31.2	12.8	147.2	65.2
Gv/N	87.209	245.505	226.421	10.984	3.829	17.017	2.526	1.032	11.898	5.274
Prob	0.00000	1.00000	1.00000	0.00000	0.00000	0.00000	0.00000	0.00516	0.00000	0.00000

	11	12	13	14	15	16	17	18	19	20
a_O	304	253	360	291	240	366	306	247	369	313
	(24.6)	(20.5)	(29.1)	(23.5)	(19.4)	(29.6)	(24.7)	(20.0)	(29.8)	(25.3)
a_E	318.1	134.6	284.6	318.1	134.6	284.6	318.1	134.6	284.6	318.1
	(25.7)	(10.9)	(23.0)	(25.7)	(10.9)	(23.0)	(25.7)	(10.9)	(23.0)	(25.7)
	-27.5	319.2	169.1	-51.8	277.5	184.0	-23.7	299.7	191.6	-10.1
	-2.224	25.802	13.671	-4.185	22.429	14.877	-1.914	24.232	15.486	-0.813
t_O	323	255	221	322	270	216	378	259	226	313
	(26.1)	(20.6)	(17.9)	(26.0)	(21.8)	(17.5)	(30.6)	(20.9)	(18.3)	(25.3)
t_E	362.5	224.3	170.2	362.5	224.3	170.2	362.5	224.3	170.2	362.5
	(29.3)	(18.1)	(13.8)	(29.3)	(18.1)	(13.8)	(29.3)	(18.1)	(13.8)	(29.3)
	-74.5	65.4	115.6	-76.2	100.1	103.1	31.7	74.5	128.3	-91.8
	-6.020	5.286	9.341	-6.162	8.092	8.331	2.565	6.021	10.370	-7.425
g_O	278	351	345	267	360	362	243	383	346	261
	(22.5)	(28.4)	(27.9)	(21.6)	(29.1)	(29.3)	(19.6)	(31.0)	(28.0)	(21.1)
g_E	234.1	396.1	473.9	234.1	396.1	473.9	234.1	396.1	473.9	234.1
	(18.9)	(32.0)	(38.3)	(18.9)	(32.0)	(38.3)	(18.9)	(32.0)	(38.3)	(18.9)
	95.6	-84.8	-219.0	70.2	-68.8	-195.0	18.1	-25.7	-217.6	56.8
	7.726	-6.857	-17.705	5.677	-5.559	-15.762	1.466	-2.079	-17.595	4.590
c_O	332	378	311	357	367	293	310	348	296	350
	(26.8)	(30.6)	(25.1)	(28.9)	(29.7)	(23.7)	(25.1)	(28.1)	(23.9)	(28.3)
c_E	322.4	482.0	308.3	322.4	482.0	308.3	322.4	482.0	308.3	322.4
	(26.1)	(39.0)	(24.9)	(26.1)	(39.0)	(24.9)	(26.1)	(39.0)	(24.9)	(26.1)
	19.5	-183.7	5.4	72.9	-200.0	-29.9	-24.3	-226.7	-24.1	57.6
	1.580	-14.850	0.435	5.890	-16.170	-2.415	-1.961	-18.324	-1.952	4.654
N	1237	1237	1237	1237	1237	1237	1237	1237	1237	1237
X2	13.4	135.8	70.2	15.2	122.5	62.8	1.9	136.8	78.3	12.3
Gvalue	13.1	116.0	71.0	15.1	108.8	62.2	1.9	121.8	78.1	12.4
Gv/N	1.062	9.382	5.742	1.219	8.793	5.031	0.156	9.849	6.310	1.006
Prob	0.00434	0.00000	0.00000	0.00175	0.00000	0.00000	0.58582	0.00000	0.00000	0.00601

	21	22	23	24	25	26	27	28	29	30
a_O	265	313	310	227	332	307	244	327	303	253
	(21.4)	(25.3)	(25.1)	(18.4)	(26.8)	(24.8)	(19.7)	(26.4)	(24.5)	(20.5)
a_E	134.6	284.6	318.1	134.6	284.6	318.1	134.6	284.6	318.1	134.6
	(10.9)	(23.0)	(25.7)	(10.9)	(23.0)	(25.7)	(10.9)	(23.0)	(25.7)	(10.9)
	358.9	59.5	-15.9	237.1	102.2	-21.8	290.1	90.7	-29.4	319.2
	29.011	4.807	-1.288	19.171	8.262	-1.759	23.455	7.335	-2.378	25.802
t_O	293	234	325	271	198	322	285	216	318	266
	(23.7)	(18.9)	(26.3)	(21.9)	(16.0)	(26.0)	(23.0)	(17.5)	(25.7)	(21.5)
t_E	224.3	170.2	362.5	224.3	170.2	362.5	224.3	170.2	362.5	224.3
	(18.1)	(13.8)	(29.3)	(18.1)	(13.8)	(29.3)	(18.1)	(13.8)	(29.3)	(18.1)
	156.5	149.1	-70.9	102.5	60.0	-76.2	136.5	103.1	-83.2	90.7
	12.654	12.053	-5.732	8.284	4.851	-6.162	11.033	8.331	-6.728	7.331
g_O	350	378	243	369	387	272	336	392	285	376
	(28.3)	(30.6)	(19.6)	(29.8)	(31.3)	(22.0)	(27.2)	(31.7)	(23.0)	(30.4)
g_E	396.1	473.9	234.1	396.1	473.9	234.1	396.1	473.9	234.1	396.1
	(32.0)	(38.3)	(18.9)	(32.0)	(38.3)	(18.9)	(32.0)	(38.3)	(18.9)	(32.0)
	-86.6	-170.9	18.1	-52.3	-156.8	81.6	-110.5	-148.7	112.1	-39.1
	-6.999	-13.816	1.466	-4.225	-12.672	6.599	-8.937	-12.022	9.066	-3.163
c_O	329	312	359	370	320	336	372	302	331	342
	(26.6)	(25.2)	(29.0)	(29.9)	(25.9)	(27.2)	(30.1)	(24.4)	(26.8)	(27.6)
c_E	482.0	308.3	322.4	482.0	308.3	322.4	482.0	308.3	322.4	482.0
	(39.0)	(24.9)	(26.1)	(39.0)	(24.9)	(26.1)	(39.0)	(24.9)	(26.1)	(39.0)
	-251.2	7.4	77.3	-195.6	23.8	27.8	-192.7	-12.5	17.5	-234.7
	-20.310	0.598	6.247	-15.815	1.923	2.250	-15.576	-1.012	1.414	-18.970
N	1237	1237	1237	1237	1237	1237	1237	1237	1237	1237
X2	201.2	46.2	8.6	100.9	28.8	11.6	139.4	32.9	17.5	153.5
Gvalue	177.6	45.1	8.6	91.7	29.2	11.5	123.4	32.6	17.0	136.1
Gv/N	14.357	3.642	0.693	7.415	2.364	0.928	9.976	2.632	1.373	11.000
Prob	0.00000	0.00000	0.03564	0.00000	0.00000	0.00938	0.00000	0.00000	0.00071	0.00000

	31	32	33	34	35	36	37	38	39	40
a_O	339	300	221	324	308	219	345	323	197	315
	(27.4)	(24.3)	(17.9)	(26.2)	(24.9)	(17.7)	(27.9)	(26.1)	(15.9)	(25.5)
a_E	284.6	318.1	134.6	284.6	318.1	134.6	284.6	318.1	134.6	284.6
	(23.0)	(25.7)	(10.9)	(23.0)	(25.7)	(10.9)	(23.0)	(25.7)	(10.9)	(23.0)
	118.5	-35.1	219.0	83.9	-19.8	213.1	132.7	9.9	150.0	63.8
	9.579	-2.837	17.707	6.785	-1.602	17.225	10.728	0.803	12.122	5.162
t_O	194	348	255	194	337	279	214	358	298	205
	(15.7)	(28.1)	(20.6)	(15.7)	(27.2)	(22.6)	(17.3)	(28.9)	(24.1)	(16.6)
t_E	170.2	362.5	224.3	170.2	362.5	224.3	170.2	362.5	224.3	170.2
	(13.8)	(29.3)	(18.1)	(13.8)	(29.3)	(18.1)	(13.8)	(29.3)	(18.1)	(13.8)
	50.9	-28.3	65.4	50.9	-49.1	121.7	98.1	-8.9	169.3	76.4

```
         4.113  -2.291   5.286   4.113  -3.969   9.841   7.932  -0.717  13.686   6.174
g_O        368     257     403     363     267     354     364     253     352     403
        ( 29.7) ( 20.8) ( 32.6) ( 29.3) ( 21.6) ( 28.6) ( 29.4) ( 20.5) ( 28.5) ( 32.6)
g_E      473.9   234.1   396.1   473.9   234.1   396.1   473.9   234.1   396.1   473.9
        ( 38.3) ( 18.9) ( 32.0) ( 38.3) ( 18.9) ( 32.0) ( 38.3) ( 18.9) ( 32.0) ( 38.3)
        -186.1    48.0    14.0  -193.5    70.2   -79.5  -192.0    39.3   -83.1  -130.6
       -15.045   3.878   1.129 -15.644   5.677  -6.429 -15.525   3.176  -6.715 -10.557
c_O        336     332     358     356     325     385     314     303     390     314
        ( 27.2) ( 26.8) ( 28.9) ( 28.8) ( 26.3) ( 31.1) ( 25.4) ( 24.5) ( 31.5) ( 25.4)
c_E      308.3   322.4   482.0   308.3   322.4   482.0   308.3   322.4   482.0   308.3
        ( 24.9) ( 26.1) ( 39.0) ( 24.9) ( 26.1) ( 39.0) ( 24.9) ( 26.1) ( 39.0) ( 24.9)
          57.8    19.5  -212.9   102.4     5.3  -173.0    11.5   -37.5  -165.1    11.5
         4.670   1.580 -17.210   8.276   0.427 -13.982   0.926  -3.036 -13.350   0.926

N         1237    1237    1237    1237    1237    1237    1237    1237    1237    1237
X2        39.9     4.1    91.6    42.1     6.8    90.2    49.7     2.8    75.5    21.1
Gvalue    41.0     4.1    85.5    43.7     6.6    82.3    50.2     2.8    71.0    21.1
Gv/N     3.317   0.330   6.911   3.530   0.533   6.655   4.061   0.227   5.743   1.706
Prob   0.00000 0.25219 0.00000 0.00000 0.08590 0.00000 0.00000 0.42258 0.00000 0.00010
       -------------------------------------------------------------------------------
            41      42      43      44      45      46      47      48      49      50
       -------------------------------------------------------------------------------
a_O        304     198     343     302     216     337     306     226     336     321
        ( 24.6) ( 16.0) ( 27.7) ( 24.4) ( 17.5) ( 27.2) ( 24.7) ( 18.3) ( 27.2) ( 25.9)
a_E      318.1   134.6   284.6   318.1   134.6   284.6   318.1   134.6   284.6   318.1
        ( 25.7) ( 10.9) ( 23.0) ( 25.7) ( 10.9) ( 23.0) ( 25.7) ( 10.9) ( 23.0) ( 25.7)
         -27.5   152.7   127.9   -31.3   204.2   113.8   -23.7   234.1   111.5     5.9
        -2.224  12.346  10.343  -2.532  16.507   9.201  -1.914  18.925   9.012   0.476
t_O        362     285     173     346     262     182     344     267     191     318
        ( 29.3) ( 23.0) ( 14.0) ( 28.0) ( 21.2) ( 14.7) ( 27.8) ( 21.6) ( 15.4) ( 25.7)
t_E      362.5   224.3   170.2   362.5   224.3   170.2   362.5   224.3   170.2   362.5
        ( 29.3) ( 18.1) ( 13.8) ( 29.3) ( 18.1) ( 13.8) ( 29.3) ( 18.1) ( 13.8) ( 29.3)
          -0.9   136.5     5.7   -32.2    81.4    24.5   -36.0    93.0    44.1   -83.2
        -0.075  11.033   0.463  -2.600   6.579   1.980  -2.908   7.520   3.568  -6.728
g_O        247     360     409     258     362     393     260     362     402     271
        ( 20.0) ( 29.1) ( 33.1) ( 20.9) ( 29.3) ( 31.8) ( 21.0) ( 29.3) ( 32.5) ( 21.9)
g_E      234.1   396.1   473.9   234.1   396.1   473.9   234.1   396.1   473.9   234.1
        ( 18.9) ( 32.0) ( 38.3) ( 18.9) ( 32.0) ( 38.3) ( 18.9) ( 32.0) ( 38.3) ( 18.9)
          26.5   -68.8  -120.4    50.2   -65.1  -147.1    54.6   -65.1  -132.3    79.3
         2.142  -5.559  -9.737   4.055  -5.266 -11.891   4.411  -5.266 -10.692   6.414
c_O        324     394     312     331     397     325     327     382     308     327
        ( 26.2) ( 31.9) ( 25.2) ( 26.8) ( 32.1) ( 26.3) ( 26.4) ( 30.9) ( 24.9) ( 26.4)
c_E      322.4   482.0   308.3   322.4   482.0   308.3   322.4   482.0   308.3   322.4
        ( 26.1) ( 39.0) ( 24.9) ( 26.1) ( 39.0) ( 24.9) ( 26.1) ( 39.0) ( 24.9) ( 26.1)
           3.3  -158.8     7.4    17.5  -154.0    34.2     9.3  -177.6    -0.6     9.3
         0.264 -12.837   0.598   1.414 -12.448   2.768   0.754 -14.357  -0.052   0.754

N         1237    1237    1237    1237    1237    1237    1237    1237    1237    1237
X2         1.3    65.6    20.9     4.2    73.4    25.2     4.3    93.8    22.7    11.4
Gvalue     1.3    61.6    20.6     4.2    66.4    25.4     4.3    84.4    22.7    11.3
Gv/N     0.108   4.983   1.668   0.338   5.371   2.057   0.344   6.822   1.836   0.915
Prob   0.71987 0.00000 0.00013 0.24310 0.00000 0.00001 0.23544 0.00000 0.00005 0.01012
       -------------------------------------------------------------------------------
            51      52      53      54      55      56      57      58      59      60
       -------------------------------------------------------------------------------
a_O        218     340     294     204     318     280     219     335     295     192
        ( 17.6) ( 27.5) ( 23.8) ( 16.5) ( 25.7) ( 22.6) ( 17.7) ( 27.1) ( 23.8) ( 15.5)
a_E      134.6   284.6   318.1   134.6   284.6   318.1   134.6   284.6   318.1   134.6
        ( 10.9) ( 23.0) ( 25.7) ( 10.9) ( 23.0) ( 25.7) ( 10.9) ( 23.0) ( 25.7) ( 10.9)
         210.1   120.9   -46.3   169.5    70.5   -71.4   213.1   109.1   -44.4   136.3
        16.985   9.770  -3.741  13.705   5.698  -5.771  17.225   8.824  -3.592  11.016
t_O        285     179     339     232     186     375     267     195     373     291
        ( 23.0) ( 14.5) ( 27.4) ( 18.8) ( 15.0) ( 30.3) ( 21.6) ( 15.8) ( 30.2) ( 23.5)
t_E      224.3   170.2   362.5   224.3   170.2   362.5   224.3   170.2   362.5   224.3
        ( 18.1) ( 13.8) ( 29.3) ( 18.1) ( 13.8) ( 29.3) ( 18.1) ( 13.8) ( 29.3) ( 18.1)
         136.5    18.1   -45.4    15.6    33.1    25.5    93.0    53.1    21.4   151.5
        11.033   1.466  -3.668   1.264   2.677   2.062   7.520   4.296   1.728  12.246
g_O        348     404     256     404     415     253     361     405     263     362
        ( 28.1) ( 32.7) ( 20.7) ( 32.7) ( 33.5) ( 20.5) ( 29.2) ( 32.7) ( 21.3) ( 29.3)
g_E      396.1   473.9   234.1   396.1   473.9   234.1   396.1   473.9   234.1   396.1
        ( 32.0) ( 38.3) ( 18.9) ( 32.0) ( 38.3) ( 18.9) ( 32.0) ( 38.3) ( 18.9) ( 32.0)
         -90.1  -128.9    45.8    16.0  -110.1    39.3   -67.0  -127.2    61.2   -65.1
        -7.281 -10.421   3.702   1.293  -8.902   3.176  -5.413 -10.285   4.950  -5.266
c_O        386     314     348     397     318     329     390     302     306     392
        ( 31.2) ( 25.4) ( 28.1) ( 32.1) ( 25.7) ( 26.6) ( 31.5) ( 24.4) ( 24.7) ( 31.7)
c_E      482.0   308.3   322.4   482.0   308.3   322.4   482.0   308.3   322.4   482.0
        ( 39.0) ( 24.9) ( 26.1) ( 39.0) ( 24.9) ( 26.1) ( 39.0) ( 24.9) ( 26.1) ( 39.0)
        -171.4    11.5    53.2  -154.0    19.7    13.4  -165.1   -12.5   -31.9  -162.0
       -13.857   0.926   4.305 -12.448   1.589   1.083 -13.350  -1.012  -2.578 -13.095

N         1237    1237    1237    1237    1237    1237    1237    1237    1237    1237
X2        93.0    21.6     7.4    51.1    13.0     6.7    81.6    22.7     6.4    64.0
Gvalue    85.1    21.5     7.4    47.2    13.1     6.8    74.0    22.6     6.3    60.6
Gv/N     6.880   1.741   0.598   3.813   1.062   0.550   5.981   1.823   0.509   4.902
Prob   0.00000 0.00008 0.06029 0.00000 0.00435 0.07850 -0.00000 0.00005 0.09813 0.00000
       -------------------------------------------------------------------------------
            61      62      63      64      65      66      67      68      69      70
       -------------------------------------------------------------------------------
a_O        339     303     227     326     295     202     304     289     193     284
        ( 27.4) ( 24.5) ( 18.4) ( 26.4) ( 23.8) ( 16.3) ( 24.6) ( 23.4) ( 15.6) ( 23.0)
a_E      284.6   318.1   134.6   284.6   318.1   134.6   284.6   318.1   134.6   284.6
        ( 23.0) ( 25.7) ( 10.9) ( 23.0) ( 25.7) ( 10.9) ( 23.0) ( 25.7) ( 10.9) ( 23.0)
         118.5   -29.4   237.1    88.5   -44.4   163.9    40.0   -55.4   139.0    -1.3
         9.579  -2.378  19.171   7.151  -3.592  13.248   3.234  -4.479  11.236  -0.103
t_O        194     353     249     186     353     253     195     353     290     226
        ( 15.7) ( 28.5) ( 20.1) ( 15.0) ( 28.5) ( 20.5) ( 15.8) ( 28.5) ( 23.4) ( 18.3)
t_E      170.2   362.5   224.3   170.2   362.5   224.3   170.2   362.5   224.3   170.2
        ( 13.8) ( 29.3) ( 18.1) ( 13.8) ( 29.3) ( 18.1) ( 13.8) ( 29.3) ( 18.1) ( 13.8)
          50.9   -18.7    52.0    33.1   -18.7    60.9    53.1   -18.7   149.0   128.3
         4.113  -1.510   4.203   2.677  -1.510   4.923   4.296  -1.510  12.042  10.370
g_O        389     229     382     426     239     362     426     260     360     425
        ( 31.4) ( 18.5) ( 30.9) ( 34.4) ( 19.3) ( 29.3) ( 34.4) ( 21.0) ( 29.1) ( 34.4)
g_E      473.9   234.1   396.1   473.9   234.1   396.1   473.9   234.1   396.1   473.9
        ( 38.3) ( 18.9) ( 32.0) ( 38.3) ( 18.9) ( 32.0) ( 38.3) ( 18.9) ( 32.0) ( 38.3)
        -153.6   -10.1   -27.7   -90.7     9.9   -65.1   -90.7    54.6   -68.8   -92.5
       -12.414  -0.815  -2.236  -7.336   0.801  -5.266  -7.336   4.411  -5.559  -7.481
c_O        315     352     379     299     350     420     312     335     394     302
        ( 25.5) ( 28.5) ( 30.6) ( 24.2) ( 28.3) ( 34.0) ( 25.2) ( 27.1) ( 31.9) ( 24.4)
c_E      308.3   322.4   482.0   308.3   322.4   482.0   308.3   322.4   482.0   308.3
        ( 24.9) ( 26.1) ( 39.0) ( 24.9) ( 26.1) ( 39.0) ( 24.9) ( 26.1) ( 39.0) ( 24.9)
          13.5    61.9  -182.2   -18.4    57.6  -115.6     7.4    25.8  -158.8   -12.5
         1.091   5.005 -14.727  -1.484   4.654  -9.345   0.598   2.082 -12.837  -1.012
```

N	1237	1237	1237	1237	1237	1237	1237	1237	1237	1237
X2	29.1	3.8	88.6	12.6	4.4	48.3	9.8	6.3	63.9	23.5
Gvalue	29.3	3.7	79.3	12.5	4.4	44.0	9.8	6.2	60.4	22.0
Gv/N	2.370	0.302	6.411	1.008	0.353	3.560	0.792	0.505	4.882	1.775
Prob	0.00000	0.29207	0.00000	0.00595	0.22418	0.00000	0.02032	0.10026	0.00000	0.00007

	71	72	73	74	75	76	77	78	79	80
a_O	295	168	307	292	166	285	263	178	293	284
	(23.8)	(13.6)	(24.8)	(23.6)	(13.4)	(23.0)	(21.3)	(14.4)	(23.7)	(23.0)
a_E	318.1	134.6	284.6	318.1	134.6	284.6	318.1	134.6	284.6	318.1
	(25.7)	(10.9)	(23.0)	(25.7)	(10.9)	(23.0)	(25.7)	(10.9)	(23.0)	(25.7)
	-44.4	74.4	46.4	-49.9	69.5	0.7	-100.0	99.4	17.0	-64.4
	-3.592	6.012	3.754	-4.038	5.619	0.058	-8.084	8.034	1.371	-5.203
t_O	369	234	201	376	270	205	354	253	182	384
	(29.8)	(18.9)	(16.2)	(30.4)	(21.8)	(16.6)	(28.6)	(20.5)	(14.7)	(31.0)
t_E	362.5	224.3	170.2	362.5	224.3	170.2	362.5	224.3	170.2	362.5
	(29.3)	(18.1)	(13.8)	(29.3)	(18.1)	(13.8)	(29.3)	(18.1)	(13.8)	(29.3)
	13.2	19.8	67.0	27.6	100.1	76.4	-16.7	60.9	24.5	44.3
	1.067	1.599	5.413	2.229	8.092	6.174	-1.352	4.923	1.980	3.584
g_O	256	386	425	253	396	425	244	397	444	227
	(20.7)	(31.2)	(34.4)	(20.5)	(32.0)	(34.4)	(19.7)	(32.1)	(35.9)	(18.4)
g_E	234.1	396.1	473.9	234.1	396.1	473.9	234.1	396.1	473.9	234.1
	(18.9)	(32.0)	(38.3)	(18.9)	(32.0)	(38.3)	(18.9)	(32.0)	(38.3)	(18.9)
	45.8	-19.9	-92.5	39.3	-0.2	-92.5	20.2	1.8	-57.8	-14.0
	3.702	-1.609	-7.481	3.176	-0.013	-7.481	1.634	0.149	-4.675	-1.130
c_O	317	449	304	316	405	322	376	409	318	342
	(25.6)	(36.3)	(24.6)	(25.5)	(32.7)	(26.0)	(30.4)	(33.1)	(25.7)	(27.6)
c_E	322.4	482.0	308.3	322.4	482.0	308.3	322.4	482.0	308.3	322.4
	(26.1)	(39.0)	(24.9)	(26.1)	(39.0)	(24.9)	(26.1)	(39.0)	(24.9)	(26.1)
	-10.6	-63.6	-8.6	-12.6	-140.9	28.0	115.7	-134.3	19.7	40.4
	-0.861	-5.143	-0.694	-1.019	-11.393	2.260	9.356	-10.855	1.589	3.269
N	1237	1237	1237	1237	1237	1237	1237	1237	1237	1237
X2	3.9	11.2	12.4	4.3	28.9	12.8	19.1	28.7	3.3	6.3
Gvalue	3.9	10.6	12.3	4.3	28.5	12.5	19.2	27.8	3.3	6.4
Gv/N	0.316	0.860	0.993	0.348	2.306	1.012	1.553	2.251	0.265	0.520
Prob	0.27127	0.01387	0.00649	0.22980	0.00000	0.00581	0.00025	0.00000	0.35123	0.09238

	81	82	83	84	85	86	87	88	89	90
a_O	190	320	319	162	312	266	181	311	249	169
	(15.4)	(25.9)	(25.8)	(13.1)	(25.2)	(21.5)	(14.6)	(25.1)	(20.1)	(13.7)
a_E	134.6	284.6	318.1	134.6	284.6	318.1	134.6	284.6	318.1	134.6
	(10.9)	(23.0)	(25.7)	(10.9)	(23.0)	(25.7)	(10.9)	(23.0)	(25.7)	(10.9)
	130.9	74.9	1.9	59.9	57.3	-95.1	107.1	55.1	-121.9	76.8
	10.580	6.058	0.150	4.845	4.630	-7.689	8.659	4.453	-9.856	6.210
t_O	250	200	348	241	199	365	246	192	390	257
	(20.2)	(16.2)	(28.1)	(19.5)	(16.1)	(29.5)	(19.9)	(15.5)	(31.5)	(20.8)
t_E	224.3	170.2	362.5	224.3	170.2	362.5	224.3	170.2	362.5	224.3
	(18.1)	(13.8)	(29.3)	(18.1)	(13.8)	(29.3)	(18.1)	(13.8)	(29.3)	(18.1)
	54.2	64.6	-28.3	34.6	62.3	5.1	45.4	46.4	57.1	69.9
	4.382	5.225	-2.291	2.796	5.038	0.412	3.671	3.749	4.617	5.652
g_O	366	414	228	397	424	222	418	421	247	379
	(29.6)	(33.5)	(18.4)	(32.1)	(34.3)	(17.9)	(33.8)	(34.0)	(20.0)	(30.6)
g_E	396.1	473.9	234.1	396.1	473.9	234.1	396.1	473.9	234.1	396.1
	(32.0)	(38.3)	(18.9)	(32.0)	(38.3)	(18.9)	(32.0)	(38.3)	(18.9)	(32.0)
	-57.8	-111.9	-12.0	1.8	-94.3	-23.6	45.0	-99.6	26.5	-33.4
	-4.674	-9.042	-0.973	0.149	-7.624	-1.905	3.640	-8.054	2.142	-2.701
c_O	431	303	342	437	302	384	392	313	351	432
	(34.8)	(24.5)	(27.6)	(35.3)	(24.4)	(31.0)	(31.7)	(25.3)	(28.4)	(34.9)
c_E	482.0	308.3	322.4	482.0	308.3	322.4	482.0	308.3	322.4	482.0
	(39.0)	(24.9)	(26.1)	(39.0)	(24.9)	(26.1)	(39.0)	(24.9)	(26.1)	(39.0)
	-96.3	-10.6	40.4	-85.6	-12.5	134.4	-162.0	9.4	59.7	-94.6
	-7.788	-0.853	3.269	-6.920	-1.012	10.862	-13.095	0.762	4.829	-7.644
N	1237	1237	1237	1237	1237	1237	1237	1237	1237	1237
X2	33.4	17.3	1.9	11.0	12.9	21.0	36.1	11.2	20.3	19.4
Gvalue	30.9	17.2	1.9	10.8	12.8	20.8	35.6	11.3	21.4	18.8
Gv/N	2.500	1.388	0.155	0.870	1.031	1.680	2.875	0.911	1.733	1.517
Prob	0.00000	0.00065	0.58924	0.01308	0.00519	0.00012	0.00000	0.01038	0.00009	0.00031

	91	92	93	94	95	96	97	98	99	100
a_O	280	302	170	306	280	172	321	310	177	312
	(22.6)	(24.4)	(13.7)	(24.8)	(22.7)	(13.9)	(26.0)	(25.1)	(14.3)	(25.3)
a_E	284.6	318.1	134.6	284.4	317.8	134.5	284.2	317.6	134.4	284.2
	(23.0)	(25.7)	(10.9)	(23.0)	(25.7)	(10.9)	(23.0)	(25.7)	(10.9)	(23.0)
	-9.2	-31.3	79.3	44.8	-70.9	84.5	78.2	-14.9	97.4	58.3
	-0.744	-2.532	6.409	3.623	-5.739	6.838	6.333	-1.209	7.887	4.719
t_O	188	368	261	181	363	223	192	364	242	188
	(15.2)	(29.7)	(21.1)	(14.6)	(29.4)	(18.0)	(15.5)	(29.5)	(19.6)	(15.2)
t_E	170.2	362.5	224.3	170.0	362.2	224.1	169.9	361.9	224.0	169.9
	(13.8)	(29.3)	(18.1)	(13.8)	(29.3)	(18.1)	(13.8)	(29.3)	(18.1)	(13.8)
	37.5	11.2	79.1	22.7	1.7	-2.3	47.0	4.3	37.5	38.1
	3.031	0.902	6.392	1.833	0.135	-0.183	3.805	0.345	3.038	3.085
g_O	450	206	361	466	230	383	431	224	378	456
	(36.4)	(16.7)	(29.2)	(37.7)	(18.6)	(31.0)	(34.9)	(18.1)	(30.6)	(36.9)
g_E	473.9	234.1	396.1	473.5	233.9	395.8	473.1	233.7	395.4	473.1
	(38.3)	(18.9)	(32.0)	(38.3)	(18.9)	(32.0)	(38.3)	(18.9)	(32.0)	(38.3)
	-46.5	-52.7	-67.0	-14.9	-7.8	-25.1	-80.4	-19.0	-34.1	-33.6
	-3.762	-4.259	-5.413	-1.203	-0.627	-2.031	-6.507	-1.541	-2.761	-2.721
c_O	319	361	445	283	363	458	291	337	438	279
	(25.8)	(29.2)	(36.0)	(22.9)	(29.4)	(37.1)	(23.6)	(27.3)	(35.5)	(22.6)
c_E	308.3	322.4	482.0	308.1	322.1	481.6	307.8	321.8	481.2	307.8
	(24.9)	(26.1)	(39.0)	(24.9)	(26.1)	(39.0)	(24.9)	(26.1)	(39.0)	(24.9)
	21.7	81.7	-71.0	-48.0	86.8	-46.0	-32.7	31.0	-82.4	-54.9
	1.756	6.606	-5.741	-3.887	7.020	-3.720	-2.649	2.511	-6.670	-4.442
N	1237	1237	1237	1236	1236	1236	1235	1235	1235	1235
X2	3.5	8.9	21.2	4.5	9.8	12.0	12.3	1.3	19.6	8.0
Gvalue	3.5	8.9	20.4	4.5	9.7	11.2	12.1	1.3	18.4	7.9
Gv/N	0.281	0.718	1.647	0.365	0.788	0.904	0.983	0.106	1.494	0.641
Prob	0.32411	0.03091	0.00014	0.21133	0.02090	0.01081	0.00692	0.72768	0.00036	0.04771

Avar_ATCC29413
5UTR:

	100	99	98	97	96	95	94	93	92	91
a_O	674	661	714	727	715	680	692	717	729	760
	(30.4)	(29.8)	(32.2)	(32.8)	(32.2)	(30.6)	(31.2)	(32.3)	(32.9)	(34.2)
a_E	741.0	741.0	741.0	741.0	741.0	741.0	741.0	741.0	741.0	741.0
	(33.4)	(33.4)	(33.4)	(33.4)	(33.4)	(33.4)	(33.4)	(33.4)	(33.4)	(33.4)
	-127.8	-151.1	-53.1	-27.8	-51.1	-116.9	-94.8	-47.3	-23.9	38.4
	-5.760	-6.809	-2.392	-1.253	-2.305	-5.268	-4.270	-2.131	-1.076	1.731
t_O	670	720	667	661	680	668	694	667	675	667
	(30.2)	(32.4)	(30.1)	(29.8)	(30.6)	(30.1)	(31.3)	(30.1)	(30.4)	(30.1)
t_E	691.8	691.8	691.8	691.8	691.8	691.8	691.8	691.8	691.8	691.8
	(31.2)	(31.2)	(31.2)	(31.2)	(31.2)	(31.2)	(31.2)	(31.2)	(31.2)	(31.2)
	-43.0	57.5	-48.8	-60.3	-23.5	-46.8	4.3	-48.8	-33.3	-48.8
	-1.937	2.589	-2.198	-2.716	-1.058	-2.111	0.195	-2.198	-1.499	-2.198
g_O	399	440	442	426	426	430	440	432	407	380
	(18.0)	(19.8)	(19.9)	(19.2)	(19.2)	(19.4)	(19.8)	(19.5)	(18.3)	(17.1)
g_E	361.7	361.7	361.7	361.7	361.7	361.7	361.7	361.7	361.7	361.7
	(16.3)	(16.3)	(16.3)	(16.3)	(16.3)	(16.3)	(16.3)	(16.3)	(16.3)	(16.3)
	78.4	172.5	177.3	139.5	139.5	148.9	172.5	153.6	96.1	37.6
	3.534	7.776	7.992	6.287	6.287	6.708	7.776	6.920	4.333	1.694
c_O	476	398	396	405	398	441	393	403	408	412
	(21.5)	(17.9)	(17.8)	(18.3)	(17.9)	(19.9)	(17.7)	(18.2)	(18.4)	(18.6)
c_E	424.5	424.5	424.5	424.5	424.5	424.5	424.5	424.5	424.5	424.5
	(19.1)	(19.1)	(19.1)	(19.1)	(19.1)	(19.1)	(19.1)	(19.1)	(19.1)	(19.1)
	109.1	-51.3	-55.0	-38.0	-51.3	33.7	-60.5	-41.8	-32.3	-24.6
	4.916	-2.310	-2.478	-1.714	-2.310	1.519	-2.728	-1.885	-1.455	-1.107
N	2219	2219	2219	2219	2219	2219	2219	2219	2219	2219
X2	16.9	28.4	21.6	14.0	14.2	19.4	22.6	16.4	6.9	2.7
Gvalue	16.7	27.7	20.5	13.4	13.6	18.8	21.6	15.7	6.7	2.7
Gv/N	0.753	1.246	0.924	0.604	0.615	0.848	0.973	0.706	0.303	0.120
Prob	0.00081	0.00000	0.00013	0.00386	0.00344	0.00030	0.00008	0.00133	0.08145	0.44562

	90	89	88	87	86	85	84	83	82	81
a_O	719	685	762	731	734	724	688	713	732	726
	(32.4)	(30.9)	(34.3)	(32.9)	(33.1)	(32.6)	(31.0)	(32.1)	(33.0)	(32.7)
a_E	741.0	741.0	741.0	741.0	741.0	741.0	741.0	741.0	741.0	741.0
	(33.4)	(33.4)	(33.4)	(33.4)	(33.4)	(33.4)	(33.4)	(33.4)	(33.4)	(33.4)
	-43.4	-107.7	42.5	-19.9	-14.0	-33.7	-102.2	-55.0	-18.0	-29.8
	-1.956	-4.855	1.916	-0.898	-0.631	-1.518	-4.605	-2.479	-0.809	-1.341
t_O	644	671	624	721	700	680	694	659	675	681
	(29.0)	(30.2)	(28.1)	(32.5)	(31.5)	(30.6)	(31.3)	(29.7)	(30.4)	(30.7)
t_E	691.8	691.8	691.8	691.8	691.8	691.8	691.8	691.8	691.8	691.8
	(31.2)	(31.2)	(31.2)	(31.2)	(31.2)	(31.2)	(31.2)	(31.2)	(31.2)	(31.2)
	-92.3	-41.0	-128.8	59.5	16.4	-23.5	4.3	-64.1	-33.3	-21.5
	-4.159	-1.849	-5.804	2.683	0.740	-1.058	0.195	-2.888	-1.499	-0.969
g_O	455	433	427	385	396	408	433	450	422	417
	(20.5)	(19.5)	(19.2)	(17.4)	(17.8)	(18.4)	(19.5)	(20.3)	(19.0)	(18.8)
g_E	361.7	361.7	361.7	361.7	361.7	361.7	361.7	361.7	361.7	361.7
	(16.3)	(16.3)	(16.3)	(16.3)	(16.3)	(16.3)	(16.3)	(16.3)	(16.3)	(16.3)
	208.9	155.9	141.8	48.2	71.8	98.4	155.9	196.7	130.2	118.8
	9.416	7.026	6.392	2.170	3.238	4.434	7.026	8.864	5.869	5.352
c_O	401	430	406	382	389	407	404	397	390	395
	(18.1)	(19.4)	(18.3)	(17.2)	(17.5)	(18.3)	(18.2)	(17.9)	(17.6)	(17.8)
c_E	424.5	424.5	424.5	424.5	424.5	424.5	424.5	424.5	424.5	424.5
	(19.1)	(19.1)	(19.1)	(19.1)	(19.1)	(19.1)	(19.1)	(19.1)	(19.1)	(19.1)
	-45.6	11.1	-36.1	-80.5	-67.9	-34.2	-39.9	-53.1	-66.1	-56.8
	-2.056	0.502	-1.628	-3.629	-3.059	-1.542	-1.800	-2.394	-2.977	-2.562
N	2219	2219	2219	2219	2219	2219	2219	2219	2219	2219
X2	29.4	19.0	19.9	7.1	6.4	7.3	18.9	26.0	13.4	11.0
Gvalue	27.6	18.3	19.4	7.2	6.4	7.0	18.1	24.5	13.0	10.6
Gv/N	1.245	0.824	0.876	0.325	0.287	0.317	0.817	1.103	0.584	0.479
Prob	0.00000	0.00038	0.00022	0.06514	0.09472	0.07105	0.00041	0.00002	0.00474	0.01384

	80	79	78	77	76	75	74	73	72	71
a_O	732	714	658	681	691	743	696	729	726	702
	(33.0)	(32.2)	(29.7)	(30.7)	(31.1)	(33.5)	(31.4)	(32.9)	(32.7)	(31.6)
a_E	741.0	741.0	741.0	741.0	741.0	741.0	741.0	741.0	741.0	741.0
	(33.4)	(33.4)	(33.4)	(33.4)	(33.4)	(33.4)	(33.4)	(33.4)	(33.4)	(33.4)
	-18.0	-53.1	-156.4	-115.1	-96.6	3.9	-87.3	-23.9	-29.8	-76.0
	-0.809	-2.392	-7.048	-5.186	-4.354	0.177	-3.933	-1.076	-1.341	-3.424
t_O	685	706	695	666	699	644	641	707	662	695
	(30.9)	(31.8)	(31.3)	(30.0)	(31.5)	(29.0)	(28.9)	(31.9)	(29.8)	(31.3)
t_E	691.8	691.8	691.8	691.8	691.8	691.8	691.8	691.8	691.8	691.8
	(31.2)	(31.2)	(31.2)	(31.2)	(31.2)	(31.2)	(31.2)	(31.2)	(31.2)	(31.2)
	-13.6	28.6	6.3	-50.7	14.4	-92.3	-97.8	30.7	-58.4	6.3
	-0.613	1.290	0.286	-2.285	0.649	-4.159	-4.409	1.382	-2.630	0.286
g_O	435	397	448	452	425	418	449	399	436	408
	(19.6)	(17.9)	(20.2)	(20.4)	(19.2)	(18.8)	(20.2)	(18.0)	(19.6)	(18.4)
g_E	361.7	361.7	361.7	361.7	361.7	361.7	361.7	361.7	361.7	361.7
	(16.3)	(16.3)	(16.3)	(16.3)	(16.3)	(16.3)	(16.3)	(16.3)	(16.3)	(16.3)
	160.6	74.0	191.8	201.6	137.2	121.0	194.3	78.4	163.0	98.4
	7.239	3.336	8.645	9.084	6.182	5.455	8.754	3.534	7.346	4.434
c_O	367	402	418	420	404	414	433	384	395	414
	(16.5)	(18.1)	(18.8)	(18.9)	(18.2)	(18.7)	(19.5)	(17.3)	(17.8)	(18.7)
c_E	424.5	424.5	424.5	424.5	424.5	424.5	424.5	424.5	424.5	424.5
	(19.1)	(19.1)	(19.1)	(19.1)	(19.1)	(19.1)	(19.1)	(19.1)	(19.1)	(19.1)
	-106.8	-43.7	-12.8	-8.9	-39.9	-20.7	17.2	-76.9	-56.8	-20.7
	-4.812	-1.970	-0.579	-0.401	-1.800	-0.932	0.777	-3.468	-2.562	-0.932
N	2219	2219	2219	2219	2219	2219	2219	2219	2219	2219
X2	22.8	5.9	30.0	28.4	15.5	12.3	27.7	8.2	18.9	8.3
Gvalue	22.3	5.8	28.9	26.9	15.0	12.0	26.4	8.2	18.0	8.1
Gv/N	1.005	0.263	1.304	1.213	0.677	0.541	1.188	0.371	0.813	0.364
Prob	0.00006	0.11946	0.00000	0.00001	0.00179	0.00736	0.00001	0.04125	0.00043	0.04463

	70	69	68	67	66	65	64	63	62	61
a_O	709	696	715	751	713	711	736	730	715	702
	(32.0)	(31.4)	(32.2)	(33.8)	(32.1)	(32.0)	(33.2)	(32.9)	(32.2)	(31.6)
a_E	741.0	741.0	741.0	741.0	741.0	741.0	741.0	741.0	741.0	741.0
	(33.4)	(33.4)	(33.4)	(33.4)	(33.4)	(33.4)	(33.4)	(33.4)	(33.4)	(33.4)
	-62.7	-87.3	-51.1	20.1	-55.0	-58.8	-10.0	-21.9	-51.1	-76.0
	-2.824	-3.933	-2.305	0.904	-2.479	-2.652	-0.452	-0.987	-2.305	-3.424
t_O	699	667	647	655	679	675	648	650	645	694
	(31.5)	(30.1)	(29.2)	(29.5)	(30.6)	(30.4)	(29.2)	(29.3)	(29.1)	(31.3)

t_E	691.8	691.8	691.8	691.8	691.8	691.8	691.8	691.8	691.8	691.8
	(31.2)	(31.2)	(31.2)	(31.2)	(31.2)	(31.2)	(31.2)	(31.2)	(31.2)	(31.2)
	14.4	-48.8	-86.7	-71.7	-25.4	-33.3	-84.8	-81.1	-90.4	4.3
	0.649	-2.198	-3.907	-3.230	-1.146	-1.499	-3.823	-3.654	-4.075	0.195
g_O	438	411	419	376	397	421	389	415	436	406
	(19.7)	(18.5)	(18.9)	(16.9)	(17.9)	(19.0)	(17.5)	(18.7)	(19.6)	(18.3)
g_E	361.7	361.7	361.7	361.7	361.7	361.7	361.7	361.7	361.7	361.7
	(16.3)	(16.3)	(16.3)	(16.3)	(16.3)	(16.3)	(16.3)	(16.3)	(16.3)	(16.3)
	167.8	105.1	123.3	29.2	74.0	127.9	56.7	114.2	163.0	93.9
	7.561	4.738	5.558	1.318	3.336	5.765	2.555	5.146	7.346	4.232
c_O	373	445	438	437	430	412	446	424	423	417
	(16.8)	(20.1)	(19.7)	(19.7)	(19.4)	(18.6)	(20.1)	(19.1)	(19.1)	(18.8)
c_E	424.5	424.5	424.5	424.5	424.5	424.5	424.5	424.5	424.5	424.5
	(19.1)	(19.1)	(19.1)	(19.1)	(19.1)	(19.1)	(19.1)	(19.1)	(19.1)	(19.1)
	-96.4	42.0	27.5	25.4	11.1	-24.6	44.1	-0.9	-2.9	-14.8
	-4.346	1.895	1.239	1.146	0.502	-1.107	1.989	-0.042	-0.132	-0.667
N	2219	2219	2219	2219	2219	2219	2219	2219	2219	2219
X2	23.8	11.4	13.3	3.0	4.8	11.7	6.0	10.6	19.4	7.6
Gvalue	23.1	11.1	13.0	3.1	4.7	11.3	6.0	10.3	18.5	7.5
Gv/N	1.040	0.501	0.584	0.138	0.213	0.507	0.269	0.462	0.834	0.336
Prob	0.00004	0.01110	0.00470	0.38273	0.19249	0.01039	0.11339	0.01652	0.00035	0.05861

	60	59	58	57	56	55	54	53	52	51
a_O	721	687	679	737	777	733	719	698	763	723
	(32.5)	(31.0)	(30.6)	(33.2)	(35.0)	(33.0)	(32.4)	(31.5)	(34.4)	(32.6)
a_E	741.0	741.0	741.0	741.0	741.0	741.0	741.0	741.0	741.0	741.0
	(33.4)	(33.4)	(33.4)	(33.4)	(33.4)	(33.4)	(33.4)	(33.4)	(33.4)	(33.4)
	-39.5	-104.0	-118.7	-8.1	73.6	-16.0	-43.4	-83.5	44.6	-35.6
	-1.781	-4.688	-5.351	-0.363	3.319	-0.720	-1.956	-3.764	2.009	-1.606
t_O	660	666	699	641	641	675	688	684	650	730
	(29.7)	(30.0)	(31.5)	(28.9)	(28.9)	(30.4)	(31.0)	(30.8)	(29.3)	(32.9)
t_E	691.8	691.8	691.8	691.8	691.8	691.8	691.8	691.8	691.8	691.8
	(31.2)	(31.2)	(31.2)	(31.2)	(31.2)	(31.2)	(31.2)	(31.2)	(31.2)	(31.2)
	-62.2	-50.7	14.4	-97.8	-97.8	-33.3	-7.7	-15.6	-81.1	78.4
	-2.802	-2.285	0.649	-4.409	-4.409	-1.499	-0.345	-0.702	-3.654	3.533
g_O	411	404	401	412	398	416	393	426	364	367
	(18.5)	(18.2)	(18.1)	(18.6)	(17.9)	(18.7)	(17.7)	(19.2)	(16.4)	(16.5)
g_E	361.7	361.7	361.7	361.7	361.7	361.7	361.7	361.7	361.7	361.7
	(16.3)	(16.3)	(16.3)	(16.3)	(16.3)	(16.3)	(16.3)	(16.3)	(16.3)	(16.3)
	105.1	89.5	82.8	107.4	76.2	116.5	65.3	139.5	4.7	10.8
	4.738	4.031	3.732	4.839	3.435	5.249	2.944	6.287	0.212	0.485
c_O	427	462	440	429	403	395	419	411	442	399
	(19.2)	(20.8)	(19.8)	(19.3)	(18.2)	(17.8)	(18.9)	(18.5)	(19.9)	(18.0)
c_E	424.5	424.5	424.5	424.5	424.5	424.5	424.5	424.5	424.5	424.5
	(19.1)	(19.1)	(19.1)	(19.1)	(19.1)	(19.1)	(19.1)	(19.1)	(19.1)	(19.1)
	5.1	78.3	31.6	9.1	-41.8	-56.8	-10.9	-26.5	35.8	-49.4
	0.229	3.528	1.425	0.411	-1.885	-2.562	-0.490	-1.194	1.612	-2.225
N	2219	2219	2219	2219	2219	2219	2219	2219	2219	2219
X2	8.8	13.2	10.1	10.8	10.2	10.7	3.5	14.5	3.9	4.2
Gvalue	8.5	13.0	10.1	10.6	10.2	10.4	3.4	13.9	4.0	4.1
Gv/N	0.383	0.587	0.456	0.478	0.459	0.468	0.153	0.626	0.178	0.187
Prob	0.03682	0.00460	0.01763	0.01407	0.01703	0.01560	0.33466	0.00305	0.26618	0.24590

	50	49	48	47	46	45	44	43	42	41
a_O	769	720	672	729	734	709	717	720	675	706
	(34.7)	(32.4)	(30.3)	(32.9)	(33.1)	(32.0)	(32.3)	(32.4)	(30.4)	(31.8)
a_E	741.0	741.0	741.0	741.0	741.0	741.0	741.0	741.0	741.0	741.0
	(33.4)	(33.4)	(33.4)	(33.4)	(33.4)	(33.4)	(33.4)	(33.4)	(33.4)	(33.4)
	57.0	-41.5	-131.4	-23.9	-14.0	-62.7	-47.3	-41.5	-126.0	-68.4
	2.567	-1.869	-5.923	-1.076	-0.631	-2.824	-2.131	-1.869	-5.678	-3.082
t_O	673	678	699	707	675	708	722	688	709	689
	(30.3)	(30.6)	(31.5)	(31.9)	(30.4)	(31.9)	(32.5)	(31.0)	(32.0)	(31.1)
t_E	691.8	691.8	691.8	691.8	691.8	691.8	691.8	691.8	691.8	691.8
	(31.2)	(31.2)	(31.2)	(31.2)	(31.2)	(31.2)	(31.2)	(31.2)	(31.2)	(31.2)
	-37.2	-27.4	14.4	30.7	-33.3	32.7	61.6	-7.7	34.7	-5.7
	-1.674	-1.235	0.649	1.382	-1.499	1.474	2.777	-0.345	1.566	-0.255
g_O	380	373	386	380	386	389	369	392	405	410
	(17.1)	(16.8)	(17.4)	(17.1)	(17.4)	(17.5)	(16.6)	(17.7)	(18.3)	(18.5)
g_E	361.7	361.7	361.7	361.7	361.7	361.7	361.7	361.7	361.7	361.7
	(16.3)	(16.3)	(16.3)	(16.3)	(16.3)	(16.3)	(16.3)	(16.3)	(16.3)	(16.3)
	37.6	23.0	50.3	37.6	50.3	56.7	14.8	63.2	91.7	102.9
	1.694	1.038	2.266	1.694	2.266	2.555	0.668	2.846	4.132	4.636
c_O	397	448	462	403	424	413	411	419	430	414
	(17.9)	(20.2)	(20.8)	(18.2)	(19.1)	(18.6)	(18.5)	(18.9)	(19.4)	(18.7)
c_E	424.5	424.5	424.5	424.5	424.5	424.5	424.5	424.5	424.5	424.5
	(19.1)	(19.1)	(19.1)	(19.1)	(19.1)	(19.1)	(19.1)	(19.1)	(19.1)	(19.1)
	-53.1	48.3	78.3	-41.8	-0.9	-22.6	-26.5	-10.9	11.1	-20.7
	-2.394	2.179	3.528	-1.885	-0.042	-1.020	-1.194	-0.490	0.502	-0.932
N	2219	2219	2219	2219	2219	2219	2219	2219	2219	2219
X2	4.3	2.5	11.5	2.5	2.1	4.1	2.7	3.2	11.6	8.4
Gvalue	4.3	2.5	11.5	2.5	2.1	4.1	2.7	3.2	11.6	8.1
Gv/N	0.193	0.113	0.520	0.115	0.094	0.185	0.120	0.143	0.521	0.367
Prob	0.23163	0.47265	0.00913	0.46705	0.55527	0.24987	0.44628	0.36583	0.00905	0.04307

	40	39	38	37	36	35	34	33	32	31
a_O	706	759	732	732	754	760	757	676	764	757
	(31.8)	(34.2)	(33.0)	(33.0)	(34.0)	(34.2)	(34.1)	(30.5)	(34.4)	(34.1)
a_E	741.0	741.0	741.0	741.0	741.0	741.0	741.0	741.0	741.0	741.0
	(33.4)	(33.4)	(33.4)	(33.4)	(33.4)	(33.4)	(33.4)	(33.4)	(33.4)	(33.4)
	-68.4	36.4	-18.0	-18.0	26.2	38.4	32.3	-124.2	46.6	32.3
	-3.082	1.639	-0.809	-0.809	1.179	1.731	1.454	-5.597	2.101	1.454
t_O	694	713	675	679	747	721	731	770	693	698
	(31.3)	(32.1)	(30.4)	(30.6)	(33.7)	(32.5)	(32.9)	(34.7)	(31.2)	(31.5)
t_E	691.8	691.8	691.8	691.8	691.8	691.8	691.8	691.8	691.8	691.8
	(31.2)	(31.2)	(31.2)	(31.2)	(31.2)	(31.2)	(31.2)	(31.2)	(31.2)	(31.2)
	4.3	43.0	-33.3	-25.4	114.6	59.5	80.5	164.8	2.3	12.4
	0.195	1.936	-1.499	-1.146	5.165	2.683	3.628	7.429	0.105	0.558
g_O	402	366	376	369	337	341	341	387	344	320
	(18.1)	(16.5)	(16.9)	(16.6)	(15.2)	(15.4)	(15.4)	(17.4)	(15.5)	(14.4)
g_E	361.7	361.7	361.7	361.7	361.7	361.7	361.7	361.7	361.7	361.7
	(16.3)	(16.3)	(16.3)	(16.3)	(16.3)	(16.3)	(16.3)	(16.3)	(16.3)	(16.3)
	85.0	8.7	29.2	14.8	-47.6	-40.1	-40.1	52.4	-34.4	-78.3
	3.832	0.394	1.318	0.668	-2.145	-1.808	-1.808	2.362	-1.552	-3.530
c_O	417	381	436	439	381	397	390	386	418	444
	(18.8)	(17.2)	(19.6)	(19.8)	(17.2)	(17.9)	(17.6)	(17.4)	(18.8)	(20.0)
c_E	424.5	424.5	424.5	424.5	424.5	424.5	424.5	424.5	424.5	424.5

```
        ( 19.1) ( 19.1) ( 19.1) ( 19.1) ( 19.1) ( 19.1) ( 19.1) ( 19.1) ( 19.1) ( 19.1)
          -14.8   -82.3    23.4    29.6   -82.3   -53.1   -66.1   -73.3   -12.8    39.9
         -0.667  -3.710   1.053   1.332  -3.710  -2.394  -2.977  -3.305  -0.579   1.800

N          2219    2219    2219    2219    2219    2219    2219    2219    2219    2219
X2          6.3     5.6     1.4     1.0    10.8     4.7     6.5    19.8     1.7     6.1
Gvalue      6.2     5.7     1.4     1.0    10.8     4.7     6.6    19.7     1.7     6.3
Gv/N      0.278   0.259   0.063   0.045   0.489   0.212   0.297   0.889   0.076   0.283
Prob    0.10392 0.12516 0.70656 0.80360 0.01260 0.19399 0.08585 0.00019 0.64095 0.09894
        -------------------------------------------------------------------------------
             30      29      28      27      26      25      24      23      22      21
        -------------------------------------------------------------------------------
a_O         730     815     747     728     754     813     755     761     816     809
        ( 32.9) ( 36.7) ( 33.7) ( 32.8) ( 34.0) ( 36.6) ( 34.0) ( 34.3) ( 36.8) ( 36.5)
a_E       741.0   741.0   741.0   741.0   741.0   741.0   741.0   741.0   741.0   741.0
        ( 33.4) ( 33.4) ( 33.4) ( 33.4) ( 33.4) ( 33.4) ( 33.4) ( 33.4) ( 33.4) ( 33.4)
          -21.9   155.1    12.0   -25.8    26.2   150.7    28.2    40.5   157.3   142.0
         -0.987   6.989   0.540  -1.165   1.179   6.791   1.270   1.823   7.087   6.398
t_O         741     672     713     727     701     676     720     678     678     673
        ( 33.4) ( 30.3) ( 32.1) ( 32.8) ( 31.6) ( 30.5) ( 32.4) ( 30.6) ( 30.6) ( 30.3)
t_E       691.8   691.8   691.8   691.8   691.8   691.8   691.8   691.8   691.8   691.8
        ( 31.2) ( 31.2) ( 31.2) ( 31.2) ( 31.2) ( 31.2) ( 31.2) ( 31.2) ( 31.2) ( 31.2)
          101.7   -39.1    43.0    72.1    18.4   -31.3    57.5   -27.4   -27.4   -37.2
          4.585  -1.762   1.936   3.248   0.831  -1.411   2.589  -1.235  -1.235  -1.674
g_O         342     321     336     335     344     311     283     334     296     304
        ( 15.4) ( 14.5) ( 15.1) ( 15.1) ( 15.5) ( 14.0) ( 12.8) ( 15.1) ( 13.3) ( 13.7)
g_E       361.7   361.7   361.7   361.7   361.7   361.7   361.7   361.7   361.7   361.7
        ( 16.3) ( 16.3) ( 16.3) ( 16.3) ( 16.3) ( 16.3) ( 16.3) ( 16.3) ( 16.3) ( 16.3)
          -38.2   -76.6   -49.5   -51.3   -34.4   -93.9  -138.8   -53.1  -118.6  -105.6
         -1.723  -3.450  -2.229  -2.312  -1.552  -4.230  -6.256  -2.395  -5.345  -4.759
c_O         406     411     423     429     420     419     461     446     429     433
        ( 18.3) ( 18.5) ( 19.1) ( 19.3) ( 18.9) ( 18.9) ( 20.8) ( 20.1) ( 19.3) ( 19.5)
c_E       424.5   424.5   424.5   424.5   424.5   424.5   424.5   424.5   424.5   424.5
        ( 19.1) ( 19.1) ( 19.1) ( 19.1) ( 19.1) ( 19.1) ( 19.1) ( 19.1) ( 19.1) ( 19.1)
          -36.1   -26.5    -2.9     9.1    -8.9   -10.9    76.1    44.1     9.1    17.2
         -1.628  -1.194  -0.132   0.411  -0.401  -0.490   3.430   1.989   0.411   0.777

N          2219    2219    2219    2219    2219    2219    2219    2219    2219    2219
X2          5.5    12.9     2.5     4.0     1.3    14.5    21.7     4.0    19.8    16.1
Gvalue      5.5    12.9     2.6     4.1     1.3    14.7    23.0     4.1    20.4    16.5
Gv/N      0.247   0.582   0.115   0.183   0.057   0.661   1.034   0.183   0.919   0.742
Prob    0.13967 0.00484 0.46475 0.25593 0.73625 0.00213 0.00004 0.25536 0.00014 0.00091
        -------------------------------------------------------------------------------
             20      19      18      17      16      15      14      13      12      11
        -------------------------------------------------------------------------------
a_O         782     807     791     785     751     779     815     758     752     856
        ( 35.2) ( 36.4) ( 35.6) ( 35.4) ( 33.8) ( 35.1) ( 36.7) ( 34.2) ( 33.9) ( 38.6)
a_E       741.0   741.0   741.0   741.0   741.0   741.0   741.0   741.0   741.0   741.0
        ( 33.4) ( 33.4) ( 33.4) ( 33.4) ( 33.4) ( 33.4) ( 33.4) ( 33.4) ( 33.4) ( 33.4)
           84.2   137.6   103.2    90.5    20.1    77.8   155.1    34.3    22.1   246.9
          3.792   6.202   4.652   4.078   0.904   3.508   6.989   1.546   0.995  11.127
t_O         693     739     722     721     789     767     703     785     732     634
        ( 31.2) ( 33.3) ( 32.5) ( 32.5) ( 35.6) ( 34.6) ( 31.7) ( 35.4) ( 33.0) ( 28.6)
t_E       691.8   691.8   691.8   691.8   691.8   691.8   691.8   691.8   691.8   691.8
        ( 31.2) ( 31.2) ( 31.2) ( 31.2) ( 31.2) ( 31.2) ( 31.2) ( 31.2) ( 31.2) ( 31.2)
            2.3    97.5    61.6    59.5   207.4   158.2    22.5   198.3    82.6  -110.7
          0.105   4.393   2.777   2.683   9.345   7.130   1.014   8.938   3.723  -4.989
g_O         293     249     228     216     191     227     225     200     280     249
        ( 13.2) ( 11.2) ( 10.3) (  9.7) (  8.6) ( 10.2) ( 10.1) (  9.0) ( 12.6) ( 11.2)
g_E       361.7   361.7   361.7   361.7   361.7   361.7   361.7   361.7   361.7   361.7
        ( 16.3) ( 16.3) ( 16.3) ( 16.3) ( 16.3) ( 16.3) ( 16.3) ( 16.3) ( 16.3) ( 16.3)
         -123.4  -185.9  -210.4  -222.7  -243.9  -211.5  -213.6  -237.0  -143.3  -185.9
         -5.560  -8.377  -9.481 -10.034 -10.990  -9.529  -9.625 -10.678  -6.458  -8.377
c_O         451     424     478     497     488     446     476     476     455     480
        ( 20.3) ( 19.1) ( 21.5) ( 22.4) ( 22.0) ( 20.1) ( 21.5) ( 21.5) ( 20.5) ( 21.6)
c_E       424.5   424.5   424.5   424.5   424.5   424.5   424.5   424.5   424.5   424.5
        ( 19.1) ( 19.1) ( 19.1) ( 19.1) ( 19.1) ( 19.1) ( 19.1) ( 19.1) ( 19.1) ( 19.1)
           54.7    -0.9   113.5   156.8   136.1    44.1   109.1   109.1    63.2   118.0
          2.465  -0.042   5.117   7.067   6.135   1.989   4.916   4.916   2.849   5.319

N          2219    2219    2219    2219    2219    2219    2219    2219    2219    2219
X2         17.0    44.2    60.8    74.9   103.8    61.3    65.5    91.4    23.1    65.0
Gvalue     17.8    48.3    68.0    84.2   119.7    68.7    73.1   104.8    24.6    68.4
Gv/N      0.802   2.176   3.065   3.793   5.394   3.098   3.294   4.722   1.109   3.081
Prob    0.00048 0.00000 0.00000 0.00000 0.00000 0.00000 0.00000 0.00000 0.00002 0.00000
        -------------------------------------------------------------------------------
             10       9       8       7       6       5       4       3       2       1
        -------------------------------------------------------------------------------
a_O         833     778     907     911     913     831    1020     857     581     550
        ( 37.5) ( 35.1) ( 40.9) ( 41.1) ( 41.1) ( 37.4) ( 46.0) ( 38.6) ( 26.2) ( 24.8)
a_E       741.0   741.0   741.0   741.0   741.0   741.0   741.0   741.0   741.0   741.0
        ( 33.4) ( 33.4) ( 33.4) ( 33.4) ( 33.4) ( 33.4) ( 33.4) ( 33.4) ( 33.4) ( 33.4)
          194.9    75.7   366.6   376.2   381.1   190.4   651.8   249.2  -282.7  -327.9
          8.783   3.413  16.521  16.955  17.173   8.582  29.373  11.230 -12.741 -14.779
t_O         667     726     655     717     704     724     554     541     835    1017
        ( 30.1) ( 32.7) ( 29.5) ( 32.3) ( 31.7) ( 32.6) ( 25.0) ( 24.4) ( 37.6) ( 45.8)
t_E       691.8   691.8   691.8   691.8   691.8   691.8   691.8   691.8   691.8   691.8
        ( 31.2) ( 31.2) ( 31.2) ( 31.2) ( 31.2) ( 31.2) ( 31.2) ( 31.2) ( 31.2) ( 31.2)
          -48.8    70.0   -71.7    51.2    24.5    65.8  -246.2  -266.1   314.1   783.6
         -2.198   3.154  -3.230   2.309   1.106   2.965 -11.094 -11.992  14.155  35.314
g_O         226     250     226     187     198     263     284     317     188     195
        ( 10.2) ( 11.3) ( 10.2) (  8.4) (  8.9) ( 11.9) ( 12.8) ( 14.3) (  8.5) (  8.8)
g_E       361.7   361.7   361.7   361.7   361.7   361.7   361.7   361.7   361.7   361.7
        ( 16.3) ( 16.3) ( 16.3) ( 16.3) ( 16.3) ( 16.3) ( 16.3) ( 16.3) ( 16.3) ( 16.3)
         -212.5  -184.6  -212.5  -246.7  -238.6  -167.6  -137.3   -83.6  -246.0  -240.9
         -9.577  -8.320  -9.577 -11.117 -10.751  -7.551  -6.187  -3.766 -11.086 -10.856
c_O         493     465     431     404     404     401     361     504     615     457
        ( 22.2) ( 21.0) ( 19.4) ( 18.2) ( 18.2) ( 18.1) ( 16.3) ( 22.7) ( 27.7) ( 20.6)
c_E       424.5   424.5   424.5   424.5   424.5   424.5   424.5   424.5   424.5   424.5
        ( 19.1) ( 19.1) ( 19.1) ( 19.1) ( 19.1) ( 19.1) ( 19.1) ( 19.1) ( 19.1) ( 19.1)
          147.6    84.8    13.2   -39.9   -39.9   -45.6  -116.9   173.1   456.1    67.5
          6.651   3.822   0.593  -1.800  -1.800  -2.056  -5.270   7.801  20.553   3.042

N          2219    2219    2219    2219    2219    2219    2219    2219    2219    2219
X2         74.3    41.9    90.1   125.2   115.2    40.6   158.6    71.4   233.1   281.4
Gvalue     81.2    45.9    95.6   140.8   127.1    43.1   151.4    72.7   241.4   282.3
Gv/N      3.659   2.070   4.307   6.347   5.728   1.941   6.822   3.274  10.881  12.721
Prob    0.00000 0.00000 0.00000 0.00000 0.00000 0.00000 0.00000 0.00000 0.00000 0.00000
        -------------------------------------------------------------------------------

CDS_INITIAL:
        -------------------------------------------------------------------------------
```

	1	2	3	4	5	6	7	8	9	10
a_O	2036	0	0	1065	774	758	974	877	774	819
	(91.8)	(0.0)	(0.0)	(48.0)	(34.9)	(34.2)	(43.9)	(39.5)	(34.9)	(36.9)
a_E	615.3	680.2	682.8	615.3	680.2	682.8	615.3	680.2	682.8	615.3
	(27.7)	(30.7)	(30.8)	(27.7)	(30.7)	(30.8)	(27.7)	(30.7)	(30.8)	(27.7)
	4872.5	0.0	0.0	1168.5	200.0	158.3	894.6	445.7	194.0	468.4
	219.582	0.000	0.000	52.658	9.011	7.134	40.317	20.086	8.742	21.107
t_O	57	2219	0	327	490	841	385	595	754	451
	(2.6)	(100.0)	(0.0)	(14.7)	(22.1)	(37.9)	(17.4)	(26.8)	(34.0)	(20.3)
t_E	434.4	677.2	756.0	434.4	677.2	756.0	434.4	677.2	756.0	434.4
	(19.6)	(30.5)	(34.1)	(19.6)	(30.5)	(34.1)	(19.6)	(30.5)	(34.1)	(19.6)
	-231.5	5267.4	0.0	-185.7	-317.0	179.3	-92.9	-153.9	-3.9	33.9
	-10.433	237.379	0.000	-8.369	-14.288	8.081	-4.188	-6.937	-0.176	1.526
g_O	126	0	2219	398	277	270	415	234	253	419
	(5.7)	(0.0)	(100.0)	(17.9)	(12.5)	(12.2)	(18.7)	(10.5)	(11.4)	(18.9)
g_E	710.0	367.9	372.8	710.0	367.9	372.8	710.0	367.9	372.8	710.0
	(32.0)	(16.6)	(16.8)	(32.0)	(16.6)	(16.8)	(32.0)	(16.6)	(16.8)	(32.0)
	-435.7	0.0	7916.7	-460.7	-157.1	-174.2	-445.7	-211.7	-196.1	-442.0
	-19.635	0.000	356.768	-20.763	-7.082	-7.849	-20.085	-9.541	-8.838	-19.917
c_O	0	0	0	429	678	350	445	513	438	530
	(0.0)	(0.0)	(0.0)	(19.3)	(30.6)	(15.8)	(20.1)	(23.1)	(19.7)	(23.9)
c_E	459.3	493.8	407.4	459.3	493.8	407.4	459.3	493.8	407.4	459.3
	(20.7)	(22.3)	(18.4)	(20.7)	(22.3)	(18.4)	(20.7)	(22.3)	(18.4)	(20.7)
	0.0	0.0	0.0	-58.5	430.0	-106.4	-28.1	39.2	63.4	151.8
	0.000	0.000	0.000	-2.639	19.376	-4.793	-1.268	1.766	2.856	6.840
N	2219	2219	2219	2219	2219	2219	2219	2219	2219	2219
X2	4547.6	5052.4	10990.0	494.3	155.8	54.3	337.7	116.4	53.0	198.2
Gvalue	4205.3	5267.4	7916.7	463.5	155.7	57.1	327.9	119.3	57.3	212.0
Gv/N	189.513	237.379	356.768	20.887	7.017	2.573	14.776	5.374	2.584	9.556
Prob	1.00000	1.00000	1.00000	0.00000	0.00000	0.00000	0.00000	0.00000	0.00000	0.00000

	11	12	13	14	15	16	17	18	19	20
a_O	744	782	782	713	772	768	699	728	695	682
	(33.5)	(35.2)	(35.2)	(32.1)	(34.8)	(34.6)	(31.5)	(32.8)	(31.3)	(30.7)
a_E	680.2	682.8	615.3	680.2	682.8	615.3	680.2	682.8	615.3	680.2
	(30.7)	(30.8)	(27.7)	(30.7)	(30.8)	(27.7)	(30.7)	(30.8)	(27.7)	(30.7)
	133.4	212.1	374.9	67.1	189.5	340.4	38.1	93.2	169.2	3.6
	6.011	9.557	16.895	3.025	8.539	15.342	1.717	4.202	7.627	0.161
t_O	630	722	498	755	773	488	743	778	501	762
	(28.4)	(32.5)	(22.4)	(34.0)	(34.8)	(22.0)	(33.5)	(35.1)	(22.6)	(34.3)
t_E	677.2	756.0	434.4	677.2	756.0	434.4	677.2	756.0	434.4	677.2
	(30.5)	(34.1)	(19.6)	(30.5)	(34.1)	(19.6)	(30.5)	(34.1)	(19.6)	(30.5)
	-91.0	-66.4	136.1	164.3	34.5	113.6	137.9	44.7	143.0	179.9
	-4.099	-2.991	6.135	7.404	1.554	5.119	6.213	2.016	6.443	8.106
g_O	246	263	445	229	290	465	272	284	504	281
	(11.1)	(11.9)	(20.1)	(10.3)	(13.1)	(21.0)	(12.3)	(12.8)	(22.7)	(12.7)
g_E	367.9	372.8	710.0	367.9	372.8	710.0	367.9	372.8	710.0	367.9
	(16.6)	(16.8)	(32.0)	(16.6)	(16.8)	(32.0)	(16.6)	(16.8)	(32.0)	(16.6)
	-198.0	-183.5	-415.8	-217.1	-145.6	-393.6	-164.2	-154.5	-345.4	-151.4
	-8.921	-8.269	-18.738	-9.782	-6.563	-17.738	-7.401	-6.962	-15.567	-6.821
c_O	599	452	494	522	384	498	505	429	519	494
	(27.0)	(20.4)	(22.3)	(23.5)	(17.3)	(22.4)	(22.8)	(19.3)	(23.4)	(22.3)
c_E	493.8	407.4	459.3	493.8	407.4	459.3	493.8	407.4	459.3	493.8
	(22.3)	(18.4)	(20.7)	(22.3)	(18.4)	(20.7)	(22.3)	(18.4)	(20.7)	(22.3)
	231.4	93.8	72.0	58.0	-45.5	80.6	22.7	44.3	126.9	0.5
	10.430	4.229	3.243	2.616	-2.050	3.631	1.023	1.994	5.717	0.021
N	2219	2219	2219	2219	2219	2219	2219	2219	2219	2219
X2	72.1	53.1	156.0	64.6	31.8	132.3	32.2	25.9	88.1	31.1
Gvalue	75.9	56.1	167.2	72.4	32.8	141.0	34.5	27.7	93.6	32.6
Gv/N	3.420	2.527	7.535	3.262	1.480	6.355	1.553	1.250	4.220	1.467
Prob	0.00000	0.00000	0.00000	0.00000	0.00000	0.00000	0.00000	0.00000	0.00000	0.00000

	21	22	23	24	25	26	27	28	29	30
a_O	695	731	708	741	724	691	675	673	665	740
	(31.3)	(32.9)	(31.9)	(33.4)	(32.6)	(31.1)	(30.4)	(30.3)	(30.0)	(33.3)
a_E	682.8	615.3	680.2	682.8	615.3	680.2	682.8	615.3	680.2	682.8
	(30.8)	(27.7)	(30.7)	(30.8)	(27.7)	(30.7)	(30.8)	(27.7)	(30.7)	(30.8)
	24.5	251.8	56.7	121.1	235.5	21.7	-15.6	120.6	-30.1	119.0
	1.106	11.350	2.555	5.459	10.613	0.980	-0.702	5.435	-1.356	5.362
t_O	763	443	657	733	440	702	767	463	705	731
	(34.4)	(20.0)	(29.6)	(33.0)	(19.8)	(31.6)	(34.6)	(20.9)	(31.8)	(32.9)
t_E	756.0	434.4	677.2	756.0	434.4	677.2	756.0	434.4	677.2	756.0
	(34.1)	(19.6)	(30.5)	(34.1)	(19.6)	(30.5)	(34.1)	(19.6)	(30.5)	(34.1)
	14.2	17.4	-39.7	-45.2	11.3	50.6	22.3	59.1	56.8	-49.1
	0.638	0.785	-1.790	-2.037	0.510	2.279	1.003	2.663	2.560	-2.212
g_O	318	506	318	298	533	310	311	600	351	314
	(14.3)	(22.8)	(14.3)	(13.4)	(24.0)	(14.0)	(14.0)	(27.0)	(15.8)	(14.2)
g_E	372.8	710.0	367.9	372.8	710.0	367.9	372.8	710.0	367.9	372.8
	(16.8)	(32.0)	(16.6)	(16.8)	(32.0)	(16.6)	(16.8)	(32.0)	(16.6)	(16.8)
	-101.1	-342.8	-92.6	-133.4	-305.7	-106.1	-112.7	-202.0	-32.9	-107.8
	-4.555	-15.448	-4.174	-6.013	-13.775	-4.781	-5.079	-9.103	-1.484	-4.856
c_O	443	539	536	447	522	516	466	483	498	434
	(20.0)	(24.3)	(24.2)	(20.1)	(23.5)	(23.3)	(21.0)	(21.8)	(22.4)	(19.6)
c_E	407.4	459.3	493.8	407.4	459.3	493.8	407.4	459.3	493.8	407.4
	(18.4)	(20.7)	(22.3)	(18.4)	(20.7)	(22.3)	(18.4)	(20.7)	(22.3)	(18.4)
	74.2	172.5	88.0	82.9	133.6	45.4	125.2	48.6	8.5	54.8
	3.342	7.774	3.964	3.734	6.021	2.048	5.641	2.191	0.383	2.471
N	2219	2219	2219	2219	2219	2219	2219	2219	2219	2219
X2	11.4	94.4	12.1	24.5	72.0	11.2	18.9	25.6	2.3	16.6
Gvalue	11.8	99.0	12.3	25.4	74.8	11.7	19.1	26.3	2.3	17.0
Gv/N	0.531	4.460	0.555	1.143	3.369	0.526	0.863	1.185	0.103	0.765
Prob	0.00820	0.00000	0.00635	0.00001	0.00000	0.00859	0.00025	0.00001	0.51412	0.00071

	31	32	33	34	35	36	37	38	39	40
a_O	676	644	663	675	647	683	677	628	677	624
	(30.5)	(29.0)	(29.9)	(30.4)	(29.2)	(30.8)	(30.5)	(28.3)	(30.5)	(28.1)
a_E	615.3	680.2	682.8	615.3	680.2	682.8	615.3	680.2	682.8	615.3
	(27.7)	(30.7)	(30.8)	(27.7)	(30.7)	(30.8)	(27.7)	(30.7)	(30.8)	(27.7)
	127.1	-70.5	-39.1	125.0	-64.8	0.3	129.3	-100.3	-11.6	17.5
	5.730	-3.175	-1.762	5.631	-2.919	0.015	5.828	-4.520	-0.524	0.787
t_O	459	709	833	421	707	782	473	742	786	473
	(20.7)	(32.0)	(37.5)	(19.0)	(31.9)	(35.2)	(21.3)	(33.4)	(35.4)	(21.3)
t_E	434.4	677.2	756.0	434.4	677.2	756.0	434.4	677.2	756.0	434.4
	(19.6)	(30.5)	(34.1)	(19.6)	(30.5)	(34.1)	(19.6)	(30.5)	(34.1)	(19.6)
	50.6	65.1	161.7	-26.3	61.0	53.0	80.6	135.7	61.3	80.6

	2.281	2.936	7.287	-1.187	2.747	2.388	3.631	6.115	2.761	3.631
g_O	615	308	313	635	341	336	624	329	324	663
	(27.7)	(13.9)	(14.1)	(28.6)	(15.4)	(15.1)	(28.1)	(14.8)	(14.6)	(29.9)
g_E	710.0	367.9	372.8	710.0	367.9	372.8	710.0	367.9	372.8	710.0
	(32.0)	(16.6)	(16.8)	(32.0)	(16.6)	(16.8)	(32.0)	(16.6)	(16.8)	(32.0)
	-176.7	-109.4	-109.4	-141.8	-51.7	-69.8	-161.1	-73.4	-90.9	-90.8
	-7.962	-4.930	-4.930	-6.389	-2.330	-3.145	-7.261	-3.310	-4.095	-4.092
c_O	469	558	410	488	524	418	445	520	432	459
	(21.1)	(25.1)	(18.5)	(22.0)	(23.6)	(18.8)	(20.1)	(23.4)	(19.5)	(20.7)
c_E	459.3	493.8	407.4	459.3	493.8	407.4	459.3	493.8	407.4	459.3
	(20.7)	(22.3)	(18.4)	(20.7)	(22.3)	(18.4)	(20.7)	(22.3)	(18.4)	(20.7)
	19.6	136.5	5.2	59.2	62.3	21.4	-28.1	53.8	50.6	-0.6
	0.884	6.150	0.232	2.666	2.806	0.965	-1.268	2.426	2.280	-0.027
N	2219	2219	2219	2219	2219	2219	2219	2219	2219	2219
X2	20.3	21.5	18.0	15.9	6.7	4.8	20.5	15.7	9.1	6.7
Gvalue	20.7	21.8	18.3	16.0	6.8	4.9	20.6	15.8	9.4	6.6
Gv/N	0.932	0.981	0.826	0.721	0.305	0.221	0.930	0.710	0.422	0.299
Prob	0.00012	0.00007	0.00037	0.00113	0.07968	0.17833	0.00013	0.00127	0.02486	0.08431

	41	42	43	44	45	46	47	48	49	50
a_O	640	677	595	620	685	596	608	660	613	586
	(28.8)	(30.5)	(26.8)	(27.9)	(30.9)	(26.9)	(27.4)	(29.7)	(27.6)	(26.4)
a_E	680.2	682.8	615.3	680.2	682.8	615.3	680.2	682.8	615.3	680.2
	(30.7)	(30.8)	(27.7)	(30.7)	(30.8)	(27.7)	(30.7)	(30.8)	(27.7)	(30.7)
	-78.0	-11.6	-40.0	-114.9	4.3	-38.0	-136.5	-44.9	-4.6	-174.7
	-3.515	-0.524	-1.801	-5.179	0.195	-1.714	-6.150	-2.024	-0.209	-7.874
t_O	691	753	481	687	742	451	710	768	480	749
	(31.1)	(33.9)	(21.7)	(31.0)	(33.4)	(20.3)	(32.0)	(34.6)	(21.6)	(33.8)
t_E	677.2	756.0	434.4	677.2	756.0	434.4	677.2	756.0	434.4	677.2
	(30.5)	(34.1)	(19.6)	(30.5)	(34.1)	(19.6)	(30.5)	(34.1)	(19.6)	(30.5)
	27.9	-5.9	98.1	19.8	-27.6	33.9	67.2	24.3	95.9	151.0
	1.260	-0.266	4.420	0.893	-1.246	1.526	3.030	1.094	4.320	6.806
g_O	346	344	655	382	367	682	378	350	674	378
	(15.6)	(15.5)	(29.5)	(17.2)	(16.5)	(30.7)	(17.0)	(15.8)	(30.4)	(17.0)
g_E	367.9	372.8	710.0	367.9	372.8	710.0	367.9	372.8	710.0	367.9
	(16.6)	(16.8)	(32.0)	(16.6)	(16.8)	(32.0)	(16.6)	(16.8)	(32.0)	(16.6)
	-42.4	-55.3	-105.6	28.8	-11.5	-54.9	20.6	-44.1	-70.1	20.6
	-1.910	-2.491	-4.760	1.299	-0.516	-2.473	0.927	-1.989	-3.161	0.927
c_O	542	445	488	530	425	490	523	441	452	506
	(24.4)	(20.1)	(22.0)	(23.9)	(19.2)	(22.1)	(23.6)	(19.9)	(20.4)	(22.8)
c_E	493.8	407.4	459.3	493.8	407.4	459.3	493.8	407.4	459.3	493.8
	(22.3)	(18.4)	(20.7)	(22.3)	(18.4)	(20.7)	(22.3)	(18.4)	(20.7)	(22.3)
	101.0	78.5	59.2	75.1	35.9	63.4	60.2	69.8	-14.5	24.8
	4.553	3.537	2.666	3.382	1.617	2.858	2.711	3.147	-0.652	1.116
N	2219	2219	2219	2219	2219	2219	2219	2219	2219	2219
X2	8.7	5.7	11.7	8.7	1.1	4.4	11.3	5.1	6.7	21.3
Gvalue	8.6	5.7	11.6	8.8	1.1	4.4	11.5	5.1	6.6	21.6
Gv/N	0.387	0.257	0.525	0.395	0.050	0.197	0.518	0.229	0.298	0.975
Prob	0.03519	0.12692	0.00871	0.03252	0.77642	0.22434	0.00933	0.16627	0.08529	0.00008

	51	52	53	54	55	56	57	58	59	60
a_O	698	607	626	656	601	614	641	607	643	674
	(31.5)	(27.4)	(28.2)	(29.6)	(27.1)	(27.7)	(28.9)	(27.4)	(29.0)	(30.4)
a_E	682.8	615.3	680.2	682.8	615.3	680.2	682.8	615.3	680.2	682.8
	(30.8)	(27.7)	(30.7)	(30.8)	(27.7)	(30.7)	(30.8)	(27.7)	(30.7)	(30.8)
	30.7	-16.5	-104.0	-52.6	-28.3	-125.8	-81.1	-16.5	-72.3	-17.6
	1.382	-0.745	-4.686	-2.371	-1.276	-5.667	-3.653	-0.745	-3.260	-0.791
t_O	722	437	722	743	452	725	757	471	689	732
	(32.5)	(19.7)	(32.5)	(33.5)	(20.4)	(32.7)	(34.1)	(21.2)	(31.1)	(33.0)
t_E	756.0	434.4	677.2	756.0	434.4	677.2	756.0	434.4	677.2	756.0
	(34.1)	(19.6)	(30.5)	(34.1)	(19.6)	(30.5)	(34.1)	(19.6)	(30.5)	(34.1)
	-66.4	5.3	92.6	-25.7	35.9	99.0	2.1	76.2	23.9	-47.1
	-2.991	0.237	4.172	-1.158	1.620	4.460	0.094	3.436	1.076	-2.124
g_O	341	681	349	390	674	370	370	670	373	385
	(15.4)	(30.7)	(15.7)	(17.6)	(30.4)	(16.7)	(16.7)	(30.2)	(16.8)	(17.4)
g_E	372.8	710.0	367.9	372.8	710.0	367.9	372.8	710.0	367.9	372.8
	(16.8)	(32.0)	(16.6)	(16.8)	(32.0)	(16.6)	(16.8)	(32.0)	(16.6)	(16.8)
	-60.8	-56.8	-36.7	35.2	-70.1	4.3	-5.5	-77.7	10.4	24.8
	-2.738	-2.559	-1.655	1.588	-3.161	0.194	-0.249	-3.501	0.467	1.120
c_O	458	494	522	430	492	510	451	471	514	428
	(20.6)	(22.3)	(23.5)	(19.4)	(22.2)	(23.0)	(20.3)	(21.2)	(23.2)	(19.3)
c_E	407.4	459.3	493.8	407.4	459.3	493.8	407.4	459.3	493.8	407.4
	(18.4)	(20.7)	(22.3)	(18.4)	(20.7)	(22.3)	(18.4)	(20.7)	(22.3)	(18.4)
	107.2	72.0	58.0	46.4	67.7	33.0	91.6	23.7	41.3	42.2
	4.829	3.243	2.616	2.089	3.050	1.486	4.130	1.068	1.860	1.900
N	2219	2219	2219	2219	2219	2219	2219	2219	2219	2219
X2	10.8	3.9	9.9	3.3	5.2	10.4	7.2	5.8	3.1	2.3
Gvalue	10.7	3.9	9.9	3.3	5.2	10.5	7.1	5.7	3.2	2.3
Gv/N	0.482	0.175	0.447	0.149	0.233	0.474	0.322	0.257	0.143	0.104
Prob	0.01345	0.27381	0.01933	0.34712	0.15954	0.01471	0.06755	0.12664	0.36633	0.51254

	61	62	63	64	65	66	67	68	69	70
a_O	595	646	712	630	626	675	616	633	671	588
	(26.8)	(29.1)	(32.1)	(28.4)	(28.2)	(30.4)	(27.8)	(28.5)	(30.2)	(26.5)
a_E	615.3	680.2	682.8	615.3	680.2	682.8	615.3	680.2	682.8	615.3
	(27.7)	(30.7)	(30.8)	(27.7)	(30.7)	(30.8)	(27.7)	(30.7)	(30.8)	(27.7)
	-40.0	-66.7	59.6	29.7	-104.0	-15.6	1.3	-91.1	-23.5	-53.4
	-1.801	-3.005	2.684	1.338	-4.686	-0.702	0.061	-4.104	-1.058	-2.407
t_O	435	718	701	457	724	759	430	671	706	439
	(19.6)	(32.4)	(31.6)	(20.6)	(32.6)	(34.2)	(19.4)	(30.2)	(31.8)	(19.8)
t_E	434.4	677.2	756.0	434.4	677.2	756.0	434.4	677.2	756.0	434.4
	(19.6)	(30.5)	(34.1)	(19.6)	(30.5)	(34.1)	(19.6)	(30.5)	(34.1)	(19.6)
	1.2	84.1	-105.8	46.4	96.8	6.1	-8.7	-12.3	-96.5	9.3
	0.056	3.789	-4.768	2.091	4.364	0.275	-0.393	-0.553	-4.350	0.419
g_O	716	361	399	646	340	410	717	388	431	694
	(32.3)	(16.3)	(18.0)	(29.1)	(15.3)	(18.5)	(32.3)	(17.5)	(19.4)	(31.3)
g_E	710.0	367.9	372.8	710.0	367.9	372.8	710.0	367.9	372.8	710.0
	(32.0)	(16.6)	(16.8)	(32.0)	(16.6)	(16.8)	(32.0)	(16.6)	(16.8)	(32.0)
	12.1	-13.6	54.3	-122.0	-53.5	78.1	14.1	41.4	125.1	-31.6
	0.543	-0.612	2.445	-5.500	-2.413	3.517	0.634	1.865	5.638	-1.425
c_O	473	494	407	486	529	375	456	527	411	498
	(21.3)	(22.3)	(18.3)	(21.9)	(23.8)	(16.9)	(20.5)	(23.7)	(18.5)	(22.4)
c_E	459.3	493.8	407.4	459.3	493.8	407.4	459.3	493.8	407.4	459.3
	(20.7)	(22.3)	(18.4)	(20.7)	(22.3)	(18.4)	(20.7)	(22.3)	(18.4)	(20.7)
	27.8	0.5	-0.9	54.9	72.9	-62.2	-6.6	68.6	7.2	80.6
	1.253	0.021	-0.039	2.475	3.286	-2.804	-0.296	3.094	0.323	3.631

N	2219	2219	2219	2219	2219	2219	2219	2219	2219	2219
X2	1.1	4.3	7.1	8.8	12.2	6.4	0.1	6.7	12.6	4.9
Gvalue	1.1	4.3	7.1	9.0	12.2	6.4	0.1	6.7	12.3	4.8
Gv/N	0.051	0.193	0.321	0.405	0.551	0.286	0.006	0.301	0.553	0.217
Prob	0.76857	0.23151	0.06796	0.02958	0.00665	0.09555	0.98697	0.08271	0.00652	0.18566

	71	72	73	74	75	76	77	78	79	80
a_O	607	680	573	627	665	603	639	714	627	649
	(27.4)	(30.6)	(25.8)	(28.3)	(30.0)	(27.2)	(28.8)	(32.2)	(28.3)	(29.2)
a_E	680.2	682.8	615.3	680.2	682.8	615.3	680.2	682.8	615.3	680.2
	(30.7)	(30.8)	(27.7)	(30.7)	(30.8)	(27.7)	(30.7)	(30.8)	(27.7)	(30.7)
	-138.2	-5.7	-81.7	-102.1	-35.2	-24.4	-79.9	63.7	23.6	-61.0
	-6.230	-0.255	-3.681	-4.603	-1.587	-1.100	-3.600	2.872	1.062	-2.748
t_O	694	775	465	697	767	426	693	725	453	717
	(31.3)	(34.9)	(21.0)	(31.4)	(34.6)	(19.2)	(31.2)	(32.7)	(20.4)	(32.3)
t_E	677.2	756.0	434.4	677.2	756.0	434.4	677.2	756.0	434.4	677.2
	(30.5)	(34.1)	(19.6)	(30.5)	(34.1)	(19.6)	(30.5)	(34.1)	(19.6)	(30.5)
	34.1	38.6	63.3	40.2	22.3	-16.6	32.0	-60.6	38.0	82.0
	1.536	1.738	2.855	1.814	1.003	-0.748	1.444	-2.732	1.714	3.694
g_O	361	369	718	381	383	717	386	386	694	335
	(16.3)	(16.6)	(32.4)	(17.2)	(17.3)	(32.3)	(17.4)	(17.4)	(31.3)	(15.1)
g_E	367.9	372.8	710.0	367.9	372.8	710.0	367.9	372.8	710.0	367.9
	(16.6)	(16.8)	(32.0)	(16.6)	(16.8)	(32.0)	(16.6)	(16.8)	(32.0)	(16.6)
	-13.6	-7.5	16.1	26.8	20.7	14.1	37.2	26.9	-31.6	-62.7
	-0.612	-0.338	0.725	1.206	0.934	0.634	1.675	1.213	-1.425	-2.825
c_O	557	395	463	514	404	473	501	394	445	518
	(25.1)	(17.8)	(20.9)	(23.2)	(18.2)	(21.3)	(22.6)	(17.8)	(20.1)	(23.3)
c_E	493.8	407.4	459.3	493.8	407.4	459.3	493.8	407.4	459.3	493.8
	(22.3)	(18.4)	(20.7)	(22.3)	(18.4)	(20.7)	(22.3)	(18.4)	(20.7)	(22.3)
	134.2	-24.5	7.4	41.3	-6.8	27.8	14.6	-26.4	-28.1	49.6
	6.049	-1.103	0.335	1.860	-0.308	1.253	0.656	-1.191	-1.268	2.236

N	2219	2219	2219	2219	2219	2219	2219	2219	2219	2219
X2	16.5	0.9	5.2	6.0	0.9	0.9	3.9	3.6	1.8	7.9
Gvalue	16.5	0.9	5.2	6.1	0.9	0.9	3.9	3.6	1.8	7.9
Gv/N	0.743	0.041	0.235	0.276	0.042	0.040	0.176	0.162	0.082	0.358
Prob	0.00090	0.82317	0.15708	0.10532	0.81631	0.82904	0.27219	0.30832	0.61062	0.04709

	81	82	83	84	85	86	87	88	89	90
a_O	703	578	650	684	600	628	669	591	664	680
	(31.7)	(26.0)	(29.3)	(30.8)	(27.0)	(28.3)	(30.1)	(26.6)	(29.9)	(30.7)
a_E	682.8	615.3	680.2	682.8	615.3	680.2	682.8	615.0	679.9	682.5
	(30.8)	(27.7)	(30.7)	(30.8)	(27.7)	(30.7)	(30.8)	(27.7)	(30.7)	(30.8)
	40.9	-72.3	-59.1	2.3	-30.3	-100.3	-27.4	-47.1	-31.4	-5.1
	1.844	-3.260	-2.662	0.105	-1.364	-4.520	-1.235	-2.126	-1.417	-0.228
t_O	740	447	712	774	487	701	798	413	705	736
	(33.3)	(20.1)	(32.1)	(34.9)	(21.9)	(31.6)	(36.0)	(18.6)	(31.8)	(33.2)
t_E	756.0	434.4	677.2	756.0	434.4	677.2	756.0	434.2	676.9	755.6
	(34.1)	(19.6)	(30.5)	(34.1)	(19.6)	(30.5)	(34.1)	(19.6)	(30.5)	(34.1)
	-31.6	25.6	71.4	36.5	111.4	48.5	86.4	-41.3	57.4	-38.7
	-1.423	1.154	3.219	1.646	5.019	2.186	3.893	-1.863	2.589	-1.745
g_O	392	716	353	392	706	363	395	712	376	415
	(17.7)	(32.3)	(15.9)	(17.7)	(31.8)	(16.4)	(17.8)	(32.1)	(17.0)	(18.7)
g_E	372.8	710.0	367.9	372.8	710.0	367.9	372.8	709.7	367.7	372.6
	(16.8)	(32.0)	(16.6)	(16.8)	(32.0)	(16.6)	(16.8)	(32.0)	(16.6)	(16.8)
	39.4	12.1	-29.1	39.4	-8.0	-9.6	45.8	4.7	16.8	89.4
	1.777	0.543	-1.311	1.777	-0.359	-0.434	2.062	0.210	0.758	4.032
c_O	384	478	504	369	426	527	357	502	473	387
	(17.3)	(21.5)	(22.7)	(16.6)	(19.2)	(23.7)	(16.1)	(22.6)	(21.3)	(17.4)
c_E	407.4	459.3	493.8	407.4	459.3	493.8	407.4	459.1	493.5	407.2
	(18.4)	(20.7)	(22.3)	(18.4)	(20.7)	(22.3)	(18.4)	(20.7)	(22.3)	(18.4)
	-45.5	38.2	20.7	-73.1	-64.1	68.6	-94.3	89.7	-40.2	-39.5
	-2.050	1.720	0.931	-3.295	-2.890	3.094	-4.252	4.045	-1.814	-1.780

N	2219	2219	2219	2219	2219	2219	2219	2218	2218	2218
X2	3.3	3.4	3.9	5.0	9.2	7.1	10.2	6.0	2.6	6.3
Gvalue	3.3	3.5	3.9	5.1	9.0	7.2	10.4	5.9	2.6	6.2
Gv/N	0.148	0.157	0.178	0.232	0.406	0.324	0.468	0.266	0.116	0.279
Prob	0.35074	0.32380	0.26775	0.16118	0.02912	0.06593	0.01548	0.11644	0.46044	0.10240

	91	92	93	94	95	96	97	98	99	100
a_O	545	662	689	584	690	670	586	665	676	593
	(24.6)	(29.8)	(31.1)	(26.3)	(31.1)	(30.2)	(26.4)	(30.0)	(30.5)	(26.7)
a_E	615.0	679.9	682.5	615.0	679.9	682.5	615.0	679.9	682.5	615.0
	(27.7)	(30.7)	(30.8)	(27.7)	(30.7)	(30.8)	(27.7)	(30.7)	(30.8)	(27.7)
	-131.8	-35.3	13.0	-60.5	20.3	-24.8	-56.7	-29.5	-13.0	-43.3
	-5.942	-1.593	0.586	-2.728	0.917	-1.119	-2.556	-1.329	-0.586	-1.952
t_O	474	712	758	442	663	741	434	691	794	462
	(21.4)	(32.1)	(34.2)	(19.9)	(29.9)	(33.4)	(19.6)	(31.2)	(35.8)	(20.8)
t_E	434.2	676.9	755.6	434.2	676.9	755.6	434.2	676.9	755.6	434.2
	(19.6)	(30.5)	(34.1)	(19.6)	(30.5)	(34.1)	(19.6)	(30.5)	(34.1)	(19.6)
	83.2	72.1	4.8	15.8	-27.4	-28.9	-0.4	28.6	78.7	57.4
	3.750	3.249	0.216	0.711	-1.237	-1.305	-0.017	1.288	3.548	2.587
g_O	720	348	397	708	383	416	705	377	378	717
	(32.5)	(15.7)	(17.9)	(31.9)	(17.3)	(18.8)	(31.8)	(17.0)	(17.0)	(32.3)
g_E	709.7	367.7	372.6	709.7	367.7	372.6	709.7	367.7	372.6	709.7
	(32.0)	(16.6)	(16.8)	(32.0)	(16.6)	(16.8)	(32.0)	(16.6)	(16.8)	(32.0)
	20.8	-38.3	50.4	-3.3	31.3	91.7	-9.3	18.9	10.9	14.7
	0.938	-1.727	2.270	-0.151	1.409	4.132	-0.420	0.850	0.490	0.664
c_O	479	496	374	484	482	391	493	485	370	446
	(21.6)	(22.4)	(16.9)	(21.8)	(21.7)	(17.6)	(22.2)	(21.9)	(16.7)	(20.1)
c_E	459.1	493.5	407.2	459.1	493.5	407.2	459.1	493.5	407.2	459.1
	(20.7)	(22.3)	(18.4)	(20.7)	(22.3)	(18.4)	(20.7)	(22.3)	(18.4)	(20.7)
	40.7	4.9	-63.7	51.1	-22.8	-31.8	70.3	-16.9	-71.0	-25.8
	1.834	0.222	-2.872	2.306	-1.029	-1.436	3.168	-0.764	-3.200	-1.163

N	2218	2218	2218	2218	2218	2218	2218	2218	2218	2218
X2	12.6	3.4	4.4	3.1	1.3	6.2	3.9	1.0	5.5	3.0
Gvalue	12.8	3.4	4.4	3.1	1.3	6.0	3.9	1.0	5.6	3.0
Gv/N	0.579	0.151	0.199	0.138	0.060	0.272	0.174	0.045	0.251	0.135
Prob	0.00499	0.34011	0.21921	0.38155	0.72049	0.10962	0.27581	0.79995	0.13427	0.39206

Ayel_PHYTOPLASMA
5UTR:

	100	99	98	97	96	95	94	93	92	91
a_O	66	69	87	83	81	68	76	75	71	67
	(37.9)	(39.7)	(50.0)	(47.7)	(46.6)	(39.1)	(43.7)	(43.1)	(40.8)	(38.5)
a_E	75.6	75.6	75.6	75.6	75.6	75.6	75.6	75.6	75.6	75.6
	(43.4)	(43.4)	(43.4)	(43.4)	(43.4)	(43.4)	(43.4)	(43.4)	(43.4)	(43.4)
	-17.9	-12.6	24.5	15.5	11.2	-14.4	0.8	-1.2	-8.9	-16.1
	-10.282	-7.224	14.072	8.934	6.448	-8.260	0.484	-0.664	-5.102	-9.280
t_O	61	60	53	55	49	58	55	49	57	64
	(35.1)	(34.5)	(30.5)	(31.6)	(28.2)	(33.3)	(31.6)	(28.2)	(32.8)	(36.8)
t_E	58.5	58.5	58.5	58.5	58.5	58.5	58.5	58.5	58.5	58.5
	(33.6)	(33.6)	(33.6)	(33.6)	(33.6)	(33.6)	(33.6)	(33.6)	(33.6)	(33.6)
	5.1	3.0	-10.5	-6.8	-17.4	-1.0	-6.8	-17.4	-3.0	11.5
	2.910	1.722	-6.036	-3.922	-10.000	-0.595	-3.922	-10.000	-1.724	6.585
g_O	26	19	19	19	18	20	21	25	24	17
	(14.9)	(10.9)	(10.9)	(10.9)	(10.3)	(11.5)	(12.1)	(14.4)	(13.8)	(9.8)
g_E	17.0	17.0	17.0	17.0	17.0	17.0	17.0	17.0	17.0	17.0
	(9.8)	(9.8)	(9.8)	(9.8)	(9.8)	(9.8)	(9.8)	(9.8)	(9.8)	(9.8)
	22.0	4.2	4.2	4.2	2.0	6.4	8.8	19.2	16.5	-0.1
	12.645	2.391	2.391	2.391	1.146	3.696	5.058	11.032	9.464	-0.034
c_O	21	26	15	17	26	28	22	25	22	26
	(12.1)	(14.9)	(8.6)	(9.8)	(14.9)	(16.1)	(12.6)	(14.4)	(12.6)	(14.9)
c_E	22.9	22.9	22.9	22.9	22.9	22.9	22.9	22.9	22.9	22.9
	(13.1)	(13.1)	(13.1)	(13.1)	(13.1)	(13.1)	(13.1)	(13.1)	(13.1)	(13.1)
	-3.6	6.7	-12.7	-10.1	6.7	11.3	-1.7	4.5	-1.7	6.7
	-2.059	3.833	-7.272	-5.796	3.833	6.513	-0.981	2.559	-0.981	3.833
N	174	174	174	174	174	174	174	174	174	174
X2	6.2	1.3	5.2	2.7	2.4	2.4	1.2	5.5	3.2	1.9
Gvalue	5.6	1.3	5.5	2.8	2.5	2.4	1.1	5.1	2.9	1.9
Gv/N	3.214	0.722	3.155	1.607	1.428	1.353	0.640	2.927	1.658	1.104
Prob	0.13322	0.73937	0.13931	0.42406	0.47814	0.50206	0.77396	0.16517	0.40980	0.58894

	90	89	88	87	86	85	84	83	82	81
a_O	71	71	61	75	73	72	70	76	84	72
	(40.8)	(40.8)	(35.1)	(43.1)	(42.0)	(41.4)	(40.2)	(43.7)	(48.3)	(41.4)
a_E	75.6	75.6	75.6	75.6	75.6	75.6	75.6	75.6	75.6	75.6
	(43.4)	(43.4)	(43.4)	(43.4)	(43.4)	(43.4)	(43.4)	(43.4)	(43.4)	(43.4)
	-8.9	-8.9	-26.1	-1.2	-5.1	-7.0	-10.7	0.8	17.7	-7.0
	-5.102	-5.102	-15.027	-0.664	-2.914	-4.016	-6.171	0.484	10.198	-4.016
t_O	60	50	66	54	64	53	64	56	42	55
	(34.5)	(28.7)	(37.9)	(31.0)	(36.8)	(30.5)	(36.8)	(32.2)	(24.1)	(31.6)
t_E	58.5	58.5	58.5	58.5	58.5	58.5	58.5	58.5	58.5	58.5
	(33.6)	(33.6)	(33.6)	(33.6)	(33.6)	(33.6)	(33.6)	(33.6)	(33.6)	(33.6)
	3.0	-15.7	15.9	-8.7	11.5	-10.5	11.5	-4.9	-27.9	-6.8
	1.722	-9.043	9.125	-4.989	6.585	-6.036	6.585	-2.833	-16.013	-3.922
g_O	21	29	20	21	17	25	19	17	21	19
	(12.1)	(16.7)	(11.5)	(12.1)	(9.8)	(14.4)	(10.9)	(9.8)	(12.1)	(10.9)
g_E	17.0	17.0	17.0	17.0	17.0	17.0	17.0	17.0	17.0	17.0
	(9.8)	(9.8)	(9.8)	(9.8)	(9.8)	(9.8)	(9.8)	(9.8)	(9.8)	(9.8)
	8.8	30.9	6.4	8.8	-0.1	19.2	4.2	-0.1	8.8	4.2
	5.058	17.744	3.696	5.058	-0.034	11.032	2.391	-0.034	5.058	2.391
c_O	22	24	27	24	20	24	21	25	27	28
	(12.6)	(13.8)	(15.5)	(13.8)	(11.5)	(13.8)	(12.1)	(14.4)	(15.5)	(16.1)
c_E	22.9	22.9	22.9	22.9	22.9	22.9	22.9	22.9	22.9	22.9
	(13.1)	(13.1)	(13.1)	(13.1)	(13.1)	(13.1)	(13.1)	(13.1)	(13.1)	(13.1)
	-1.7	2.3	9.0	2.3	-5.4	2.3	-3.6	4.5	9.0	11.3
	-0.981	1.330	5.152	1.330	-3.083	1.330	-2.059	2.559	5.152	6.513
N	174	174	174	174	174	174	174	174	174	174
X2	1.3	10.0	5.0	1.3	1.0	4.5	1.3	0.3	7.3	1.8
Gvalue	1.2	8.6	5.1	1.3	1.0	4.0	1.3	0.3	7.6	1.7
Gv/N	0.698	4.930	2.946	0.735	0.554	2.310	0.746	0.175	4.395	0.966
Prob	0.74943	0.03546	0.16281	0.73417	0.81014	0.25930	0.72980	0.95909	0.05388	0.64112

	80	79	78	77	76	75	74	73	72	71
a_O	73	66	75	74	74	74	73	85	73	78
	(42.0)	(37.9)	(43.1)	(42.5)	(42.5)	(42.5)	(42.0)	(48.9)	(42.0)	(44.8)
a_E	75.6	75.6	75.6	75.6	75.6	75.6	75.6	75.6	75.6	75.6
	(43.4)	(43.4)	(43.4)	(43.4)	(43.4)	(43.4)	(43.4)	(43.4)	(43.4)	(43.4)
	-5.1	-17.9	-1.2	-3.1	-3.1	-3.1	-5.1	20.0	-5.1	4.9
	-2.914	-10.282	-0.664	-1.797	-1.797	-1.797	-2.914	11.476	-2.914	2.826
t_O	66	54	63	51	61	57	63	57	57	55
	(37.9)	(31.0)	(36.2)	(29.3)	(35.1)	(32.8)	(36.2)	(32.8)	(32.8)	(31.6)
t_E	58.5	58.5	58.5	58.5	58.5	58.5	58.5	58.5	58.5	58.5
	(33.6)	(33.6)	(33.6)	(33.6)	(33.6)	(33.6)	(33.6)	(33.6)	(33.6)	(33.6)
	15.9	-8.7	9.3	-14.0	5.1	-3.0	9.3	-3.0	-3.0	-6.8
	9.125	-4.989	5.342	-8.063	2.910	-1.724	5.342	-1.724	-1.724	-3.922
g_O	17	26	16	19	15	17	16	15	17	24
	(9.8)	(14.9)	(9.2)	(10.9)	(8.6)	(9.8)	(9.2)	(8.6)	(9.8)	(13.8)
g_E	17.0	17.0	17.0	17.0	17.0	17.0	17.0	17.0	17.0	17.0
	(9.8)	(9.8)	(9.8)	(9.8)	(9.8)	(9.8)	(9.8)	(9.8)	(9.8)	(9.8)
	-0.1	22.0	-2.0	4.2	-3.8	-0.1	-2.0	-3.8	-0.1	16.5
	-0.034	12.645	-1.147	2.391	-2.188	-0.034	-1.147	-2.188	-0.034	9.464
c_O	18	28	20	30	24	26	22	17	27	17
	(10.3)	(16.1)	(11.5)	(17.2)	(13.8)	(14.9)	(12.6)	(9.8)	(15.5)	(9.8)
c_E	22.9	22.9	22.9	22.9	22.9	22.9	22.9	22.9	22.9	22.9
	(13.1)	(13.1)	(13.1)	(13.1)	(13.1)	(13.1)	(13.1)	(13.1)	(13.1)	(13.1)
	-8.6	11.3	-5.4	16.3	2.3	6.7	-1.7	-10.1	9.0	-10.1
	-4.954	6.513	-3.083	9.358	1.330	3.833	-0.981	-5.796	5.152	-5.796
N	174	174	174	174	174	174	174	174	174	174
X2	2.1	7.4	0.8	3.5	0.4	0.5	0.5	3.0	0.9	4.6
Gvalue	2.1	6.8	0.8	3.3	0.4	0.5	0.5	3.1	0.8	4.5
Gv/N	1.222	3.887	0.448	1.888	0.255	0.278	0.299	1.767	0.479	2.572
Prob	0.54656	0.07985	0.85452	0.34963	0.93100	0.92256	0.91431	0.38016	0.84143	0.21447

	70	69	68	67	66	65	64	63	62	61
a_O	65	68	71	66	81	70	66	85	77	78
	(37.4)	(39.1)	(40.8)	(37.9)	(46.6)	(40.2)	(37.9)	(48.9)	(44.3)	(44.8)
a_E	75.6	75.6	75.6	75.6	75.6	75.6	75.6	75.6	75.6	75.6
	(43.4)	(43.4)	(43.4)	(43.4)	(43.4)	(43.4)	(43.4)	(43.4)	(43.4)	(43.4)
	-19.6	-14.4	-8.9	-17.9	11.2	-10.7	-17.9	20.0	2.9	4.9
	-11.267	-8.260	-5.102	-10.282	6.448	-6.171	-10.282	11.476	1.647	2.826
t_O	61	58	60	72	56	54	71	51	62	56
	(35.1)	(33.3)	(34.5)	(41.4)	(32.2)	(31.0)	(40.8)	(29.3)	(35.6)	(32.2)

t_E	58.5	58.5	58.5	58.5	58.5	58.5	58.5	58.5	58.5	58.5
	(33.6)	(33.6)	(33.6)	(33.6)	(33.6)	(33.6)	(33.6)	(33.6)	(33.6)	(33.6)
	5.1	-1.0	3.0	29.9	-4.9	-8.7	27.5	-14.0	7.2	-4.9
	2.910	-0.595	1.722	17.156	-2.833	-4.989	15.776	-8.063	4.117	-2.833
g_O	24	24	20	13	9	24	16	16	13	15
	(13.8)	(13.8)	(11.5)	(7.5)	(5.2)	(13.8)	(9.2)	(9.2)	(7.5)	(8.6)
g_E	17.0	17.0	17.0	17.0	17.0	17.0	17.0	17.0	17.0	17.0
	(9.8)	(9.8)	(9.8)	(9.8)	(9.8)	(9.8)	(9.8)	(9.8)	(9.8)	(9.8)
	16.5	16.5	6.4	-7.0	-11.5	16.5	-2.0	-2.0	-7.0	-3.8
	9.464	9.464	3.696	-4.035	-6.597	9.464	-1.147	-1.147	-4.035	-2.188
c_O	24	24	23	23	28	26	21	22	22	25
	(13.8)	(13.8)	(13.2)	(13.2)	(16.1)	(14.9)	(12.1)	(12.6)	(12.6)	(14.4)
c_E	22.9	22.9	22.9	22.9	22.9	22.9	22.9	22.9	22.9	22.9
	(13.1)	(13.1)	(13.1)	(13.1)	(13.1)	(13.1)	(13.1)	(13.1)	(13.1)	(13.1)
	2.3	2.3	0.3	0.3	11.3	6.7	-3.6	-1.7	-1.7	4.5
	1.330	1.330	0.150	0.150	6.513	3.833	-2.059	-0.981	-0.981	2.559
N	174	174	174	174	174	174	174	174	174	174
X2	4.5	3.7	0.8	5.3	5.4	4.0	4.1	2.2	1.2	0.6
Gvalue	4.2	3.4	0.8	5.2	6.1	3.7	4.0	2.2	1.3	0.6
Gv/N	2.438	1.939	0.466	2.989	3.531	2.137	2.287	1.285	0.748	0.363
Prob	0.23652	0.33744	0.84674	0.15772	0.10483	0.29348	0.26361	0.52492	0.72859	0.88919

	60	59	58	57	56	55	54	53	52	51
a_O	86	79	73	66	78	73	76	79	82	73
	(49.4)	(45.4)	(42.0)	(37.9)	(44.8)	(42.0)	(43.7)	(45.4)	(47.1)	(42.0)
a_E	75.6	75.6	75.6	75.6	75.6	75.6	75.6	75.6	75.6	75.6
	(43.4)	(43.4)	(43.4)	(43.4)	(43.4)	(43.4)	(43.4)	(43.4)	(43.4)	(43.4)
	22.2	7.0	-5.1	-17.9	4.9	-5.1	0.8	7.0	13.4	-5.1
	12.767	4.019	-2.914	-10.282	2.826	-2.914	0.484	4.019	7.684	-2.914
t_O	55	49	67	55	50	66	67	59	57	65
	(31.6)	(28.2)	(38.5)	(31.6)	(28.7)	(37.9)	(38.5)	(33.9)	(32.8)	(37.4)
t_E	58.5	58.5	58.5	58.5	58.5	58.5	58.5	58.5	58.5	58.5
	(33.6)	(33.6)	(33.6)	(33.6)	(33.6)	(33.6)	(33.6)	(33.6)	(33.6)	(33.6)
	-6.8	-17.4	18.1	-6.8	-15.7	15.9	18.1	1.0	-3.0	13.7
	-3.922	-10.000	10.422	-3.922	-9.043	9.125	10.422	0.554	-1.724	7.846
g_O	14	22	17	30	22	20	8	12	21	16
	(8.0)	(12.6)	(9.8)	(17.2)	(12.6)	(11.5)	(4.6)	(6.9)	(12.1)	(9.2)
g_E	17.0	17.0	17.0	17.0	17.0	17.0	17.0	17.0	17.0	17.0
	(9.8)	(9.8)	(9.8)	(9.8)	(9.8)	(9.8)	(9.8)	(9.8)	(9.8)	(9.8)
	-5.5	11.3	-0.1	34.0	11.3	6.4	-12.1	-8.4	8.8	-2.0
	-3.153	6.475	-0.034	19.525	6.475	3.696	-6.947	-4.829	5.058	-1.147
c_O	19	24	17	23	24	15	23	24	14	20
	(10.9)	(13.8)	(9.8)	(13.2)	(13.8)	(8.6)	(13.2)	(13.8)	(8.0)	(11.5)
c_E	22.9	22.9	22.9	22.9	22.9	22.9	22.9	22.9	22.9	22.9
	(13.1)	(13.1)	(13.1)	(13.1)	(13.1)	(13.1)	(13.1)	(13.1)	(13.1)	(13.1)
	-7.0	2.3	-10.1	0.3	2.3	-12.7	0.3	2.3	-13.7	-5.4
	-4.049	1.330	-5.796	0.150	1.330	-7.272	0.150	1.330	-7.897	-3.083
N	174	174	174	174	174	174	174	174	174	174
X2	2.8	3.2	2.8	11.3	2.8	4.3	6.0	1.7	5.0	1.2
Gvalue	2.9	3.2	2.9	9.5	2.8	4.6	7.1	1.9	5.4	1.2
Gv/N	1.644	1.824	1.677	5.471	1.588	2.634	4.108	1.075	3.121	0.702
Prob	0.41365	0.36548	0.40448	0.02313	0.42947	0.20493	0.06733	0.59990	0.14290	0.74789

	50	49	48	47	46	45	44	43	42	41
a_O	76	85	74	67	83	71	86	80	76	71
	(43.7)	(48.9)	(42.5)	(38.5)	(47.7)	(40.8)	(49.4)	(46.0)	(43.7)	(40.8)
a_E	75.6	75.6	75.6	75.6	75.6	75.6	75.6	75.6	75.6	75.6
	(43.4)	(43.4)	(43.4)	(43.4)	(43.4)	(43.4)	(43.4)	(43.4)	(43.4)	(43.4)
	0.8	20.0	-3.1	-16.1	15.5	-8.9	22.2	9.1	0.8	-8.9
	0.484	11.476	-1.797	-9.280	8.934	-5.102	12.767	5.226	0.484	-5.102
t_O	47	45	62	58	55	56	56	48	66	67
	(27.0)	(25.9)	(35.6)	(33.3)	(31.6)	(32.2)	(32.2)	(27.6)	(37.9)	(38.5)
t_E	58.5	58.5	58.5	58.5	58.5	58.5	58.5	58.5	58.5	58.5
	(33.6)	(33.6)	(33.6)	(33.6)	(33.6)	(33.6)	(33.6)	(33.6)	(33.6)	(33.6)
	-20.6	-23.6	7.2	-1.0	-6.8	-4.9	-4.9	-19.0	15.9	18.1
	-11.843	-13.588	4.117	-0.595	-3.922	-2.833	-2.833	-10.933	9.125	10.422
g_O	18	17	14	17	17	23	12	19	9	11
	(10.3)	(9.8)	(8.0)	(9.8)	(9.8)	(13.2)	(6.9)	(10.9)	(5.2)	(6.3)
g_E	17.0	17.0	17.0	17.0	17.0	17.0	17.0	17.0	17.0	17.0
	(9.8)	(9.8)	(9.8)	(9.8)	(9.8)	(9.8)	(9.8)	(9.8)	(9.8)	(9.8)
	2.0	-0.1	-5.5	-0.1	-0.1	13.8	-8.4	4.2	-11.5	-9.6
	1.146	-0.034	-3.153	-0.034	-0.034	7.945	-4.829	2.391	-6.597	-5.526
c_O	33	27	24	32	19	24	20	27	23	25
	(19.0)	(15.5)	(13.8)	(18.4)	(10.9)	(13.8)	(11.5)	(15.5)	(13.2)	(14.4)
c_E	22.9	22.9	22.9	22.9	22.9	22.9	22.9	22.9	22.9	22.9
	(13.1)	(13.1)	(13.1)	(13.1)	(13.1)	(13.1)	(13.1)	(13.1)	(13.1)	(13.1)
	24.2	9.0	2.3	21.5	-7.0	2.3	-5.4	9.0	0.3	4.5
	13.909	5.152	1.330	12.355	-4.049	1.330	-3.083	5.152	0.150	2.559
N	174	174	174	174	174	174	174	174	174	174
X2	6.8	5.0	0.8	4.6	1.6	2.5	3.4	3.1	4.7	3.8
Gvalue	6.4	5.2	0.9	4.3	1.6	2.3	3.5	3.2	5.5	4.1
Gv/N	3.696	3.005	0.497	2.446	0.929	1.340	2.023	1.835	3.162	2.353
Prob	0.09243	0.15576	0.83376	0.23511	0.65550	0.50638	0.31825	0.36272	0.13856	0.25155

	40	39	38	37	36	35	34	33	32	31
a_O	84	71	72	71	75	67	73	72	66	78
	(48.3)	(40.8)	(41.4)	(40.8)	(43.1)	(38.5)	(42.0)	(41.4)	(37.9)	(44.8)
a_E	75.6	75.6	75.6	75.6	75.6	75.6	75.6	75.6	75.6	75.6
	(43.4)	(43.4)	(43.4)	(43.4)	(43.4)	(43.4)	(43.4)	(43.4)	(43.4)	(43.4)
	17.7	-8.9	-7.0	-8.9	-1.2	-16.1	-5.1	-7.0	-17.9	4.9
	10.198	-5.102	-4.016	-5.102	-0.664	-9.280	-2.914	-4.016	-10.282	2.826
t_O	52	55	65	68	64	64	64	67	60	53
	(29.9)	(31.6)	(37.4)	(39.1)	(36.8)	(36.8)	(36.8)	(38.5)	(34.5)	(30.5)
t_E	58.5	58.5	58.5	58.5	58.5	58.5	58.5	58.5	58.5	58.5
	(33.6)	(33.6)	(33.6)	(33.6)	(33.6)	(33.6)	(33.6)	(33.6)	(33.6)	(33.6)
	-12.3	-6.8	13.7	20.4	11.5	11.5	11.5	18.1	3.0	-10.5
	-7.060	-3.922	7.846	11.735	6.585	6.585	6.585	10.422	1.722	-6.036
g_O	18	20	16	16	13	19	9	19	21	15
	(10.3)	(11.5)	(9.2)	(9.2)	(7.5)	(10.9)	(5.2)	(10.9)	(12.1)	(8.6)
g_E	17.0	17.0	17.0	17.0	17.0	17.0	17.0	17.0	17.0	17.0
	(9.8)	(9.8)	(9.8)	(9.8)	(9.8)	(9.8)	(9.8)	(9.8)	(9.8)	(9.8)
	2.0	6.4	-2.0	-2.0	-7.0	4.2	-11.5	4.2	8.8	-3.8
	1.146	3.696	-1.147	-1.147	-4.035	2.391	-6.597	2.391	5.058	-2.188
c_O	20	28	21	19	22	24	28	16	27	28
	(11.5)	(16.1)	(12.1)	(10.9)	(12.6)	(13.8)	(16.1)	(9.2)	(15.5)	(16.1)
c_E	22.9	22.9	22.9	22.9	22.9	22.9	22.9	22.9	22.9	22.9

```
        ( 13.1) ( 13.1) ( 13.1) ( 13.1) ( 13.1) ( 13.1) ( 13.1) ( 13.1) ( 13.1) ( 13.1)
           -5.4    11.3    -3.6    -7.0    -1.7     2.3    11.3   -11.4     9.0    11.3
         -3.083   6.513  -2.059  -4.049  -0.981   1.330   6.513  -6.570   5.152   6.513

N           174     174     174     174     174     174     174     174     174     174
X2          2.1     2.2     1.1     2.5     1.5     1.8     5.5     3.7     2.9     2.0
Gvalue      2.1     2.1     1.1     2.5     1.6     1.8     6.2     3.9     2.9     1.9
Gv/N      1.201   1.186   0.624   1.437   0.905   1.026   3.587   2.226   1.650   1.115
Prob    0.55383 0.55946 0.78055 0.47509 0.66504 0.61810 0.10047 0.27543 0.41182 0.58496
        -------------------------------------------------------------------------------
             30      29      28      27      26      25      24      23      22      21
        -------------------------------------------------------------------------------
a_O          79      92      81      86      87      75      79      75      76      94
        ( 45.4) ( 52.9) ( 46.6) ( 49.4) ( 50.0) ( 43.1) ( 45.4) ( 43.1) ( 43.7) ( 54.0)
a_E        75.6    75.6    75.6    75.6    75.6    75.6    75.6    75.6    75.6    75.6
        ( 43.4) ( 43.4) ( 43.4) ( 43.4) ( 43.4) ( 43.4) ( 43.4) ( 43.4) ( 43.4) ( 43.4)
            7.0    36.2    11.2    22.2    24.5    -1.2     7.0    -1.2     0.8    41.0
          4.019  20.790   6.448  12.767  14.072  -0.664   4.019  -0.664   0.484  23.565
t_O          63      52      50      46      51      59      63      64      54      49
        ( 36.2) ( 29.9) ( 28.7) ( 26.4) ( 29.3) ( 33.9) ( 36.2) ( 36.8) ( 31.0) ( 28.2)
t_E        58.5    58.5    58.5    58.5    58.5    58.5    58.5    58.5    58.5    58.5
        ( 33.6) ( 33.6) ( 33.6) ( 33.6) ( 33.6) ( 33.6) ( 33.6) ( 33.6) ( 33.6) ( 33.6)
            9.3   -12.3   -15.7   -22.1   -14.0     1.0     9.3    11.5    -8.7   -17.4
          5.342  -7.060  -9.043 -12.728  -8.063   0.554   5.342   6.585  -4.989 -10.000
g_O          19      17      13      19      14      16      17      13      16      10
        ( 10.9) (  9.8) (  7.5) ( 10.9) (  8.0) (  9.2) (  9.8) (  7.5) (  9.2) (  5.7)
g_E        17.0    17.0    17.0    17.0    17.0    17.0    17.0    17.0    17.0    17.0
        (  9.8) (  9.8) (  9.8) (  9.8) (  9.8) (  9.8) (  9.8) (  9.8) (  9.8) (  9.8)
            4.2    -0.1    -7.0     4.2    -5.5    -2.0    -0.1    -7.0    -2.0   -10.6
          2.391  -0.034  -4.035   2.391  -3.153  -1.147  -0.034  -4.035  -1.147  -6.119
c_O          13      13      30      23      22      24      15      22      28      21
        (  7.5) (  7.5) ( 17.2) ( 13.2) ( 12.6) ( 13.8) (  8.6) ( 12.6) ( 16.1) ( 12.1)
c_E        22.9    22.9    22.9    22.9    22.9    22.9    22.9    22.9    22.9    22.9
        ( 13.1) ( 13.1) ( 13.1) ( 13.1) ( 13.1) ( 13.1) ( 13.1) ( 13.1) ( 13.1) ( 13.1)
          -14.7   -14.7    16.3     0.3    -1.7     2.3   -12.7    -1.7    11.3    -3.6
         -8.441  -8.441   9.358   0.150  -0.981   1.330  -7.272  -0.981   6.513  -2.059

N           174     174     174     174     174     174     174     174     174     174
X2          5.0     8.6     4.8     4.3     3.3     0.1     3.2     1.5     1.6     9.1
Gvalue      5.8     9.1     4.7     4.5     3.3     0.1     3.6     1.6     1.5     9.4
Gv/N      3.310   5.254   2.728   2.579   1.875   0.073   2.054   0.905   0.861   5.387
Prob    0.12390 0.02746 0.19131 0.21335 0.35282 0.98842 0.31132 0.66504 0.68283 0.02472
        -------------------------------------------------------------------------------
             20      19      18      17      16      15      14      13      12      11
        -------------------------------------------------------------------------------
a_O          64      86      73      73      86      76      75      88      81      75
        ( 36.8) ( 49.4) ( 42.0) ( 42.0) ( 49.4) ( 43.7) ( 43.1) ( 50.6) ( 46.6) ( 43.1)
a_E        75.6    75.6    75.6    75.6    75.6    75.6    75.6    75.6    75.6    75.6
        ( 43.4) ( 43.4) ( 43.4) ( 43.4) ( 43.4) ( 43.4) ( 43.4) ( 43.4) ( 43.4) ( 43.4)
          -21.3    22.2    -5.1    -5.1    22.2     0.8    -1.2    26.8    11.2    -1.2
        -12.234  12.767  -2.914  -2.914  12.767   0.484  -0.664  15.389   6.448  -0.664
t_O          71      62      59      62      54      56      52      52      59      55
        ( 40.8) ( 35.6) ( 33.9) ( 35.6) ( 31.0) ( 32.2) ( 29.9) ( 29.9) ( 33.9) ( 31.6)
t_E        58.5    58.5    58.5    58.5    58.5    58.5    58.5    58.5    58.5    58.5
        ( 33.6) ( 33.6) ( 33.6) ( 33.6) ( 33.6) ( 33.6) ( 33.6) ( 33.6) ( 33.6) ( 33.6)
           27.5     7.2     1.0     7.2    -8.7    -4.9   -12.3   -12.3     1.0    -6.8
         15.776   4.117   0.554   4.117  -4.989  -2.833  -7.060  -7.060   0.554  -3.922
g_O          23       6      25      13       8      21      10       5      14      16
        ( 13.2) (  3.4) ( 14.4) (  7.5) (  4.6) ( 12.1) (  5.7) (  2.9) (  8.0) (  9.2)
g_E        17.0    17.0    17.0    17.0    17.0    17.0    17.0    17.0    17.0    17.0
        (  9.8) (  9.8) (  9.8) (  9.8) (  9.8) (  9.8) (  9.8) (  9.8) (  9.8) (  9.8)
           13.8   -12.5    19.2    -7.0   -12.1     8.8   -10.6   -12.3    -5.5    -2.0
          7.945  -7.195  11.032  -4.035  -6.947   5.058  -6.119  -7.043  -3.153  -1.147
c_O          16      20      17      26      26      21      37      29      20      28
        (  9.2) ( 11.5) (  9.8) ( 14.9) ( 14.9) ( 12.1) ( 21.3) ( 16.7) ( 11.5) ( 16.1)
c_E        22.9    22.9    22.9    22.9    22.9    22.9    22.9    22.9    22.9    22.9
        ( 13.1) ( 13.1) ( 13.1) ( 13.1) ( 13.1) ( 13.1) ( 13.1) ( 13.1) ( 13.1) ( 13.1)
          -11.4    -5.4   -10.1     6.7     6.7    -3.6    35.6    13.8    -5.4    11.3
         -6.570  -3.083  -5.796   3.833   3.833  -2.059  20.460   7.916  -3.083   6.513

N           174     174     174     174     174     174     174     174     174     174
X2          8.6     9.1     5.3     1.7     7.0     1.2    12.4    12.9     1.3     1.4
Gvalue      8.6    11.5     5.0     1.7     8.1     1.1    11.5    16.0     1.3     1.4
Gv/N      4.917   6.607   2.875   1.001   4.664   0.650   6.616   9.201   0.767   0.780
Prob    0.03583 0.00933 0.17157 0.62777 0.04370 0.76969 0.00925 0.00113 0.72104 0.71556
        -------------------------------------------------------------------------------
             10       9       8       7       6       5       4       3       2       1
        -------------------------------------------------------------------------------
a_O          81      94      73      69      64      69      77      79      68      80
        ( 46.6) ( 54.0) ( 42.0) ( 39.7) ( 36.8) ( 39.7) ( 44.3) ( 45.4) ( 39.1) ( 46.0)
a_E        75.6    75.6    75.6    75.6    75.6    75.6    75.6    75.6    75.6    75.6
        ( 43.4) ( 43.4) ( 43.4) ( 43.4) ( 43.4) ( 43.4) ( 43.4) ( 43.4) ( 43.4) ( 43.4)
           11.2    41.0    -5.1   -12.6   -21.3   -12.6     2.9     7.0   -14.4     9.1
          6.448  23.565  -2.914  -7.224 -12.234  -7.224   1.647   4.019  -8.260   5.226
t_O          67      53      72      79      63      59      63      56      71      64
        ( 38.5) ( 30.5) ( 41.4) ( 45.4) ( 36.2) ( 33.9) ( 36.2) ( 32.2) ( 40.8) ( 36.8)
t_E        58.5    58.5    58.5    58.5    58.5    58.5    58.5    58.5    58.5    58.5
        ( 33.6) ( 33.6) ( 33.6) ( 33.6) ( 33.6) ( 33.6) ( 33.6) ( 33.6) ( 33.6) ( 33.6)
           18.1   -10.5    29.9    47.4     9.3     1.0     9.3    -4.9    27.5    11.5
         10.422  -6.036  17.156  27.249   5.342   0.554   5.342  -2.833  15.776   6.585
g_O           5       8      11       5      21      20      12      17      16      15
        (  2.9) (  4.6) (  6.3) (  2.9) ( 12.1) ( 11.5) (  6.9) (  9.8) (  9.2) (  8.6)
g_E        17.0    17.0    17.0    17.0    17.0    17.0    17.0    17.0    17.0    17.0
        (  9.8) (  9.8) (  9.8) (  9.8) (  9.8) (  9.8) (  9.8) (  9.8) (  9.8) (  9.8)
          -12.3   -12.1    -9.6   -12.3     8.8     6.4    -8.4    -0.1    -2.0    -3.8
         -7.043  -6.947  -5.526  -7.043   5.058   3.696  -4.829  -0.034  -1.147  -2.188
c_O          21      19      18      21      26      26      22      22      19      15
        ( 12.1) ( 10.9) ( 10.3) ( 12.1) ( 14.9) ( 14.9) ( 12.6) ( 12.6) ( 10.9) (  8.6)
c_E        22.9    22.9    22.9    22.9    22.9    22.9    22.9    22.9    22.9    22.9
        ( 13.1) ( 13.1) ( 13.1) ( 13.1) ( 13.1) ( 13.1) ( 13.1) ( 13.1) ( 13.1) ( 13.1)
           -3.6    -7.0    -8.6    -3.6     6.7     6.7    -1.7    -1.7    -7.0   -12.7
         -2.059  -4.049  -4.954  -2.059   3.833   3.833  -0.981  -0.981  -4.049  -7.272

N           174     174     174     174     174     174     174     174     174     174
X2         10.3    10.5     6.4    16.4     3.5     1.5     1.9     0.3     4.1     3.7
Gvalue     13.5    11.4     6.5    19.0     3.5     1.5     2.1     0.3     4.0     4.1
Gv/N      7.767   6.533   3.761  10.922   1.999   0.859   1.180   0.170   2.320   2.351
Prob    0.00365 0.00989 0.08795 0.00027 0.32362 0.68355 0.56150 0.96075 0.25760 0.25187
        -------------------------------------------------------------------------------

CDS_INITIAL:
        -------------------------------------------------------------------------------
```

	1	2	3	4	5	6	7	8	9	10
a_O	92	0	0	81	74	83	80	73	72	79
	(52.9)	(0.0)	(0.0)	(46.6)	(42.5)	(47.7)	(46.0)	(42.0)	(41.4)	(45.4)
a_E	67.0	68.6	68.1	67.0	68.6	68.1	67.0	68.6	68.1	67.0
	(38.5)	(39.4)	(39.1)	(38.5)	(39.4)	(39.1)	(38.5)	(39.4)	(39.1)	(38.5)
	58.3	0.0	0.0	30.7	11.3	33.0	28.4	9.1	8.1	26.0
	33.521	0.000	0.000	17.657	6.469	18.938	16.297	5.240	4.662	14.951
t_O	65	174	0	31	58	56	35	57	67	49
	(37.4)	(100.0)	(0.0)	(17.8)	(33.3)	(32.2)	(20.1)	(32.8)	(38.5)	(28.2)
t_E	42.1	59.2	71.1	42.1	59.2	71.1	42.1	59.2	71.1	42.1
	(24.2)	(34.0)	(40.8)	(24.2)	(34.0)	(40.8)	(24.2)	(34.0)	(40.8)	(24.2)
	56.6	375.0	0.0	-18.9	-2.4	-26.7	-12.9	-4.4	-7.9	15.0
	32.526	215.509	0.000	-10.870	-1.404	-15.340	-7.390	-2.520	-4.541	8.605
g_O	17	0	174	36	17	22	34	12	16	26
	(9.8)	(0.0)	(100.0)	(20.7)	(9.8)	(12.6)	(19.5)	(6.9)	(9.2)	(14.9)
g_E	39.3	16.9	13.0	39.3	16.9	13.0	39.3	16.9	13.0	39.3
	(22.6)	(9.7)	(7.5)	(22.6)	(9.7)	(7.5)	(22.6)	(9.7)	(7.5)	(22.6)
	-28.5	0.0	903.7	-6.2	0.2	23.3	-9.8	-8.2	6.7	-21.4
	-16.351	0.000	519.366	-3.579	0.116	13.372	-5.614	-4.722	3.869	-12.310
c_O	0	0	0	26	25	13	25	32	19	20
	(0.0)	(0.0)	(0.0)	(14.9)	(14.4)	(7.5)	(14.4)	(18.4)	(10.9)	(11.5)
c_E	25.7	29.3	21.9	25.7	29.3	21.9	25.7	29.3	21.9	25.7
	(14.8)	(16.8)	(12.6)	(14.8)	(16.8)	(12.6)	(14.8)	(16.8)	(12.6)	(14.8)
	0.0	0.0	0.0	0.6	-7.9	-13.6	-1.3	5.7	-5.4	-10.0
	0.000	0.000	0.000	0.367	-4.546	-7.799	-0.775	3.260	-3.111	-5.749
N	174	174	174	174	174	174	174	174	174	174
X2	60.1	337.1	2161.3	6.1	1.1	16.4	4.4	2.0	1.6	9.0
Gvalue	86.5	375.0	903.7	6.2	1.1	16.0	4.4	2.2	1.5	9.6
Gv/N	49.696	215.509	519.366	3.575	0.635	9.171	2.519	1.259	0.878	5.496
Prob	0.00000	0.00000	0.00000	0.10136	0.77610	0.00116	0.22304	0.53391	0.67595	0.02266

	11	12	13	14	15	16	17	18	19	20
a_O	63	59	77	66	81	88	83	71	73	77
	(36.2)	(33.9)	(44.3)	(37.9)	(46.6)	(50.6)	(47.7)	(40.8)	(42.0)	(44.3)
a_E	68.6	68.1	67.0	68.6	68.1	67.0	68.6	68.1	67.0	68.6
	(39.4)	(39.1)	(38.5)	(39.4)	(39.1)	(38.5)	(39.4)	(39.1)	(38.5)	(39.4)
	-10.7	-16.9	21.4	-5.1	28.2	48.0	31.7	6.0	12.5	17.8
	-6.146	-9.684	12.303	-2.910	16.211	27.567	18.205	3.456	7.188	10.248
t_O	78	70	45	66	56	44	63	73	32	57
	(44.8)	(40.2)	(25.9)	(37.9)	(32.2)	(25.3)	(36.2)	(42.0)	(18.4)	(32.8)
t_E	59.2	71.1	42.1	59.2	71.1	42.1	59.2	71.1	42.1	59.2
	(34.0)	(40.8)	(24.2)	(34.0)	(40.8)	(24.2)	(34.0)	(40.8)	(24.2)	(34.0)
	42.9	-2.1	6.1	14.3	-26.7	4.0	7.8	3.9	-17.5	-4.4
	24.673	-1.220	3.498	8.204	-15.340	2.284	4.463	2.248	-10.052	-2.520
g_O	8	13	25	10	27	22	7	18	37	18
	(4.6)	(7.5)	(14.4)	(5.7)	(15.5)	(12.6)	(4.0)	(10.3)	(21.3)	(10.3)
g_E	16.9	13.0	39.3	16.9	13.0	39.3	16.9	13.0	39.3	16.9
	(9.7)	(7.5)	(22.6)	(9.7)	(7.5)	(22.6)	(9.7)	(7.5)	(22.6)	(9.7)
	-12.0	0.1	-22.6	-10.5	39.6	-25.5	-12.3	11.8	-4.4	2.3
	-6.876	0.041	-12.964	-6.031	22.767	-14.641	-7.091	6.789	-2.513	1.306
c_O	25	32	27	32	10	20	21	12	32	22
	(14.4)	(18.4)	(15.5)	(18.4)	(5.7)	(11.5)	(12.1)	(6.9)	(18.4)	(12.6)
c_E	29.3	21.9	25.7	29.3	21.9	25.7	29.3	21.9	25.7	29.3
	(16.8)	(12.6)	(14.8)	(16.8)	(12.6)	(14.8)	(16.8)	(12.6)	(14.8)	(16.8)
	-7.9	24.2	2.7	5.7	-15.7	-10.0	-14.0	-14.4	14.1	-12.6
	-4.546	13.934	1.552	3.260	-9.015	-5.749	-8.028	-8.303	8.089	-7.233
N	174	174	174	174	174	174	174	174	174	174
X2	11.7	5.9	6.9	3.9	27.3	15.5	11.4	6.6	4.6	3.0
Gvalue	12.4	5.3	7.6	4.4	25.4	16.5	13.1	7.3	4.7	3.1
Gv/N	7.104	3.070	4.389	2.524	14.623	9.461	7.549	4.190	2.710	1.801
Prob	0.00624	0.14839	0.05414	0.22213	0.00001	0.00091	0.00435	0.06319	0.19380	0.37141

	21	22	23	24	25	26	27	28	29	30
a_O	75	83	74	86	81	60	78	89	88	77
	(43.1)	(47.7)	(42.5)	(49.4)	(46.6)	(34.5)	(44.8)	(51.1)	(50.6)	(44.3)
a_E	68.1	67.0	68.6	68.1	67.0	68.6	68.1	67.0	68.6	68.1
	(39.1)	(38.5)	(39.4)	(39.1)	(38.5)	(39.4)	(39.1)	(38.5)	(39.4)	(39.1)
	14.6	35.5	11.3	40.3	30.7	-16.0	21.3	50.5	43.9	19.0
	8.375	20.420	6.469	23.132	17.657	-9.219	12.227	29.036	25.219	10.928
t_O	60	42	70	57	42	64	55	32	55	52
	(34.5)	(24.1)	(40.2)	(32.8)	(24.1)	(36.8)	(31.6)	(18.4)	(31.6)	(29.9)
t_E	71.1	42.1	59.2	71.1	42.1	59.2	71.1	42.1	59.2	71.1
	(40.8)	(24.2)	(34.0)	(40.8)	(24.2)	(34.0)	(40.8)	(24.2)	(34.0)	(40.8)
	-20.3	-0.1	23.4	-25.1	-0.1	9.9	-28.2	-17.5	-8.2	-32.5
	-11.677	-0.066	13.436	-14.454	-0.066	5.692	-16.205	-10.052	-4.689	-18.673
g_O	21	29	12	13	29	20	13	28	16	23
	(12.1)	(16.7)	(6.9)	(7.5)	(16.7)	(11.5)	(7.5)	(16.1)	(9.2)	(13.2)
g_E	13.0	39.3	16.9	13.0	39.3	16.9	13.0	39.3	16.9	13.0
	(7.5)	(22.6)	(9.7)	(7.5)	(22.6)	(9.7)	(7.5)	(22.6)	(9.7)	(7.5)
	20.3	-17.6	-8.2	0.1	-17.6	6.7	0.1	-18.9	-1.7	26.4
	11.642	-10.091	-4.722	0.041	-10.091	3.873	0.041	-10.872	-1.005	15.155
c_O	18	20	18	18	22	30	28	25	15	22
	(10.3)	(11.5)	(10.3)	(10.3)	(12.6)	(17.2)	(16.1)	(14.4)	(8.6)	(12.6)
c_E	21.9	25.7	29.3	21.9	25.7	29.3	21.9	25.7	29.3	21.9
	(12.6)	(14.8)	(16.8)	(12.6)	(14.8)	(16.8)	(12.6)	(14.8)	(16.8)	(12.6)
	-7.1	-10.0	-17.5	-7.1	-6.8	1.4	13.7	-1.3	-20.1	0.2
	-4.066	-5.749	-10.070	-4.066	-3.914	0.831	7.895	-0.775	-11.535	0.105
N	174	174	174	174	174	174	174	174	174	174
X2	8.1	7.8	8.2	8.2	6.1	2.0	6.8	12.9	12.8	14.1
Gvalue	7.4	7.9	8.9	8.1	6.2	2.0	6.9	12.8	13.9	13.1
Gv/N	4.274	4.514	5.112	4.653	3.586	1.178	3.957	7.337	7.989	7.514
Prob	0.05923	0.04911	0.03071	0.04407	0.10048	0.56223	0.07564	0.00517	0.00304	0.00448

	31	32	33	34	35	36	37	38	39	40
a_O	78	76	77	87	64	63	83	76	72	87
	(44.8)	(43.7)	(44.3)	(50.0)	(36.8)	(36.2)	(47.7)	(43.7)	(41.4)	(50.0)
a_E	67.0	68.6	68.1	67.0	68.6	68.1	67.0	68.6	68.1	67.0
	(38.5)	(39.4)	(39.1)	(38.5)	(39.4)	(39.1)	(38.5)	(39.4)	(39.1)	(38.5)
	23.7	15.6	19.0	45.4	-8.8	-9.7	35.5	15.6	8.1	45.4
	13.620	8.973	10.928	26.111	-5.085	-5.590	20.420	8.973	4.662	26.111
t_O	44	59	57	46	63	75	35	53	67	41
	(25.3)	(33.9)	(32.8)	(26.4)	(36.2)	(43.1)	(20.1)	(30.5)	(38.5)	(23.6)
t_E	42.1	59.2	71.1	42.1	59.2	71.1	42.1	59.2	71.1	42.1
	(24.2)	(34.0)	(40.8)	(24.2)	(34.0)	(40.8)	(24.2)	(34.0)	(40.8)	(24.2)
	4.0	-0.5	-25.1	8.2	7.8	8.1	-12.9	-11.8	-7.9	-2.1

	2.284	−0.269	−14.454	4.738	4.463	4.640	−7.390	−6.775	−4.541	−1.200
g_O	28	12	18	23	16	12	29	15	16	18
	(16.1)	(6.9)	(10.3)	(13.2)	(9.2)	(6.9)	(16.7)	(8.6)	(9.2)	(10.3)
g_E	39.3	16.9	13.0	39.3	16.9	13.0	39.3	16.9	13.0	39.3
	(22.6)	(9.7)	(7.5)	(22.6)	(9.7)	(7.5)	(22.6)	(9.7)	(7.5)	(22.6)
	−18.9	−8.2	11.8	−24.6	−1.7	−1.9	−17.6	−3.6	6.7	−28.1
	−10.872	−4.722	6.789	−14.131	−1.005	−1.066	−10.091	−2.055	3.869	−16.131
c_O	24	27	22	18	31	24	27	30	19	28
	(13.8)	(15.5)	(12.6)	(10.3)	(17.8)	(13.8)	(15.5)	(17.2)	(10.9)	(16.1)
c_E	25.7	29.3	21.9	25.7	29.3	21.9	25.7	29.3	21.9	25.7
	(14.8)	(16.8)	(12.6)	(14.8)	(16.8)	(12.6)	(14.8)	(16.8)	(12.6)	(14.8)
	−3.3	−4.4	0.2	−12.8	3.5	4.4	2.7	1.4	−5.4	4.8
	−1.870	−2.522	0.105	−7.354	2.027	2.514	1.552	0.831	−3.111	2.780
N	174	174	174	174	174	174	174	174	174	174
X2	5.2	2.4	5.9	15.4	0.7	0.9	7.7	1.7	1.6	17.7
Gvalue	5.5	2.5	5.9	16.3	0.7	0.9	7.8	1.7	1.5	20.1
Gv/N	3.161	1.460	3.368	9.363	0.399	0.498	4.492	0.974	0.878	11.560
Prob	0.13858	0.46794	0.11863	0.00099	0.87447	0.83367	0.04998	0.63808	0.67595	0.00016

	41	42	43	44	45	46	47	48	49	50
a_O	80	75	83	78	75	80	76	83	67	74
	(46.0)	(43.1)	(47.7)	(44.8)	(43.1)	(46.0)	(43.7)	(47.7)	(38.5)	(42.5)
a_E	68.6	68.1	67.0	68.6	68.1	67.0	68.6	68.1	67.0	68.6
	(39.4)	(39.1)	(38.5)	(39.4)	(39.1)	(38.5)	(39.4)	(39.1)	(38.5)	(39.4)
	24.6	14.6	35.5	20.1	14.6	28.4	15.6	33.0	−0.0	11.3
	14.162	8.375	20.420	11.538	8.375	16.297	8.973	18.938	−0.008	6.469
t_O	63	59	42	45	68	39	54	59	52	61
	(36.2)	(33.9)	(24.1)	(25.9)	(39.1)	(22.4)	(31.0)	(33.9)	(29.9)	(35.1)
t_E	59.2	71.1	42.1	59.2	71.1	42.1	59.2	71.1	42.1	59.2
	(34.0)	(40.8)	(24.2)	(34.0)	(40.8)	(24.2)	(34.0)	(40.8)	(24.2)	(34.0)
	7.8	−22.0	−0.1	−24.7	−6.0	−5.9	−10.0	−22.0	22.1	3.6
	4.463	−12.622	−0.066	−14.216	−3.451	−3.383	−5.743	−12.622	12.684	2.059
g_O	15	16	25	17	8	29	13	8	26	9
	(8.6)	(9.2)	(14.4)	(9.8)	(4.6)	(16.7)	(7.5)	(4.6)	(14.9)	(5.2)
g_E	16.9	13.0	39.3	16.9	13.0	39.3	16.9	13.0	39.3	16.9
	(9.7)	(7.5)	(22.6)	(9.7)	(7.5)	(22.6)	(9.7)	(7.5)	(22.6)	(9.7)
	−3.6	6.7	−22.6	0.2	−7.7	−17.6	−6.8	−7.7	−21.4	−11.3
	−2.055	3.869	−12.964	0.116	−4.439	−10.091	−3.919	−4.439	−12.310	−6.518
c_O	16	24	24	34	23	26	31	24	29	30
	(9.2)	(13.8)	(13.8)	(19.5)	(13.2)	(14.9)	(17.8)	(13.8)	(16.7)	(17.2)
c_E	29.3	21.9	25.7	29.3	21.9	25.7	29.3	21.9	25.7	29.3
	(16.8)	(12.6)	(14.8)	(16.8)	(12.6)	(14.8)	(16.8)	(12.6)	(14.8)	(16.8)
	−19.3	4.4	−3.3	10.2	2.2	0.6	3.5	4.4	7.0	1.4
	−11.117	2.514	−1.870	5.833	1.284	0.367	2.027	2.514	4.049	0.831
N	174	174	174	174	174	174	174	174	174	174
X2	8.4	3.7	9.1	5.5	2.8	5.4	2.3	7.4	7.3	4.2
Gvalue	9.5	3.7	9.6	5.7	3.1	5.5	2.3	7.6	7.7	4.9
Gv/N	5.452	2.136	5.521	3.272	1.769	3.189	1.338	4.391	4.414	2.841
Prob	0.02347	0.29372	0.02223	0.12752	0.37972	0.13569	0.50707	0.05408	0.05309	0.17593

	51	52	53	54	55	56	57	58	59	60
a_O	74	66	74	75	74	76	61	66	67	67
	(42.5)	(37.9)	(42.5)	(43.1)	(42.5)	(43.7)	(35.1)	(37.9)	(38.5)	(38.5)
a_E	68.1	67.0	68.6	68.1	67.0	68.6	68.1	67.0	68.6	68.1
	(39.1)	(38.5)	(39.4)	(39.1)	(38.5)	(39.4)	(39.1)	(38.5)	(39.4)	(39.1)
	12.4	−2.0	11.3	14.6	14.7	15.6	−13.4	−2.0	−3.1	−2.1
	7.122	−1.149	6.469	8.375	8.443	8.973	−7.675	−1.149	−1.796	−1.205
t_O	68	51	66	64	48	68	78	52	60	70
	(39.1)	(29.3)	(37.9)	(36.8)	(27.6)	(39.1)	(44.8)	(29.9)	(34.5)	(40.2)
t_E	71.1	42.1	59.2	71.1	42.1	59.2	71.1	42.1	59.2	71.1
	(40.8)	(24.2)	(34.0)	(40.8)	(24.2)	(34.0)	(40.8)	(24.2)	(34.0)	(40.8)
	−6.0	19.7	14.3	−13.4	12.7	18.8	14.5	22.1	1.5	−2.1
	−3.451	11.301	8.204	−7.708	7.292	10.786	8.342	12.684	0.885	−1.220
g_O	18	33	13	16	28	6	8	31	23	12
	(10.3)	(19.0)	(7.5)	(9.2)	(16.1)	(3.4)	(4.6)	(17.8)	(13.2)	(6.9)
g_E	13.0	39.3	16.9	13.0	39.3	16.9	13.0	39.3	16.9	13.0
	(7.5)	(22.6)	(9.7)	(7.5)	(22.6)	(9.7)	(7.5)	(22.6)	(9.7)	(7.5)
	11.8	−11.5	−6.8	6.7	−18.9	−12.4	−7.7	−14.6	14.2	−1.9
	6.789	−6.581	−3.919	3.869	−10.872	−7.141	−4.439	−8.410	8.149	−1.066
c_O	14	24	21	19	24	24	27	25	24	25
	(8.0)	(13.8)	(12.1)	(10.9)	(13.8)	(13.8)	(15.5)	(14.4)	(13.8)	(14.4)
c_E	21.9	25.7	29.3	21.9	25.7	29.3	21.9	25.7	29.3	21.9
	(12.6)	(14.8)	(16.8)	(12.6)	(14.8)	(16.8)	(12.6)	(14.8)	(16.8)	(12.6)
	−12.5	−3.3	−14.0	−5.4	−3.3	−9.6	11.3	−1.3	−9.6	6.6
	−7.207	−1.870	−8.028	−3.111	−1.870	−5.491	6.484	−0.775	−5.491	3.792
N	174	174	174	174	174	174	174	174	174	174
X2	5.5	3.0	4.4	2.5	4.9	10.1	4.5	4.1	3.2	0.5
Gvalue	5.7	3.0	4.7	2.5	5.2	12.4	4.7	4.1	3.0	0.5
Gv/N	3.253	1.702	2.726	1.425	2.994	7.127	2.712	2.350	1.748	0.301
Prob	0.12935	0.39769	0.19159	0.47911	0.15714	0.00613	0.19361	0.25198	0.38538	0.91378

	61	62	63	64	65	66	67	68	69	70
a_O	76	64	75	58	69	79	68	57	63	62
	(43.7)	(36.8)	(43.1)	(33.3)	(39.7)	(45.4)	(39.1)	(32.8)	(36.2)	(35.6)
a_E	67.0	68.6	68.1	67.0	68.6	68.1	67.0	68.6	68.1	67.0
	(38.5)	(39.4)	(39.1)	(38.5)	(39.4)	(39.1)	(38.5)	(39.4)	(39.1)	(38.5)
	19.1	−8.8	14.6	−16.7	0.8	23.6	2.0	−21.1	−9.7	−9.6
	11.001	−5.085	8.375	−9.624	0.483	13.540	1.150	−12.118	−5.590	−5.535
t_O	46	67	65	56	60	61	49	68	73	55
	(26.4)	(38.5)	(37.4)	(32.2)	(34.5)	(35.1)	(28.2)	(39.1)	(42.0)	(31.6)
t_E	42.1	59.2	71.1	42.1	59.2	71.1	42.1	59.2	71.1	42.1
	(24.2)	(34.0)	(40.8)	(24.2)	(34.0)	(40.8)	(24.2)	(34.0)	(40.8)	(24.2)
	8.2	16.5	−11.6	32.1	1.5	−18.6	15.0	18.8	3.9	29.5
	4.738	9.487	−6.670	18.430	0.885	−10.713	8.605	10.786	2.248	16.961
g_O	30	14	10	35	15	16	38	18	14	32
	(17.2)	(8.0)	(5.7)	(20.1)	(8.6)	(9.2)	(21.8)	(10.3)	(8.0)	(18.4)
g_E	39.3	16.9	13.0	39.3	16.9	13.0	39.3	16.9	13.0	39.3
	(22.6)	(9.7)	(7.5)	(22.6)	(9.7)	(7.5)	(22.6)	(9.7)	(7.5)	(22.6)
	−16.1	−5.3	−5.2	−8.0	−3.6	6.7	−2.5	2.3	2.2	−13.1
	−9.270	−3.028	−2.984	−4.613	−2.055	3.869	−1.416	1.306	1.236	−7.514
c_O	22	29	24	25	30	18	19	31	24	25
	(12.6)	(16.7)	(13.8)	(14.4)	(17.2)	(10.3)	(10.9)	(17.8)	(13.8)	(14.4)
c_E	25.7	29.3	21.9	25.7	29.3	21.9	25.7	29.3	21.9	25.7
	(14.8)	(16.8)	(12.6)	(14.8)	(16.8)	(12.6)	(14.8)	(16.8)	(12.6)	(14.8)
	−6.8	−0.6	4.4	−1.3	1.4	−7.1	−11.5	3.5	4.4	−1.3
	−3.914	−0.327	2.514	−0.775	0.831	−4.066	−6.582	2.027	2.514	−0.775

N	174	174	174	174	174	174	174	174	174	174
X2	4.3	1.8	2.1	6.3	0.2	4.6	2.9	3.4	0.7	5.7
Gvalue	4.4	1.8	2.1	5.9	0.3	4.6	3.1	3.5	0.7	5.5
Gv/N	2.555	1.046	1.235	3.418	0.145	2.630	1.756	2.001	0.409	3.138
Prob	0.21714	0.61049	0.54201	0.11418	0.96887	0.20562	0.38308	0.32309	0.87054	0.14100

	71	72	73	74	75	76	77	78	79	80
a_O	72	71	67	72	67	74	74	71	64	63
	(41.4)	(40.8)	(38.5)	(41.4)	(38.5)	(42.5)	(42.5)	(40.8)	(36.8)	(36.2)
a_E	68.6	68.1	67.0	68.6	68.1	67.0	68.6	68.1	67.0	68.6
	(39.4)	(39.1)	(38.5)	(39.4)	(39.1)	(38.5)	(39.4)	(39.1)	(38.5)	(39.4)
	7.0	6.0	-0.0	7.0	-2.1	14.7	11.3	6.0	-5.9	-10.7
	4.026	3.456	-0.008	4.026	-1.205	8.443	6.469	3.456	-3.378	-6.146
t_O	65	73	46	59	76	45	68	74	47	64
	(37.4)	(42.0)	(26.4)	(33.9)	(43.7)	(25.9)	(39.1)	(42.5)	(27.0)	(36.8)
t_E	59.2	71.1	42.1	59.2	71.1	42.1	59.2	71.1	42.1	59.2
	(34.0)	(40.8)	(24.2)	(34.0)	(40.8)	(24.2)	(34.0)	(40.8)	(24.2)	(34.0)
	12.1	3.9	8.2	-0.5	10.2	6.1	18.8	6.0	10.4	9.9
	6.939	2.248	4.738	-0.269	5.859	3.498	10.786	3.436	6.003	5.692
g_O	11	11	33	15	11	30	15	13	35	20
	(6.3)	(6.3)	(19.0)	(8.6)	(6.3)	(17.2)	(8.6)	(7.5)	(20.1)	(11.5)
g_E	16.9	13.0	39.3	16.9	13.0	39.3	16.9	13.0	39.3	16.9
	(9.7)	(7.5)	(22.6)	(9.7)	(7.5)	(22.6)	(9.7)	(7.5)	(22.6)	(9.7)
	-9.4	-3.6	-11.5	-3.6	-3.6	-16.1	-3.6	0.1	-8.0	6.7
	-5.429	-2.078	-6.581	-2.055	-2.078	-9.270	-2.055	0.041	-4.613	3.873
c_O	26	19	28	28	20	25	17	16	28	27
	(14.9)	(10.9)	(16.1)	(16.1)	(11.5)	(14.4)	(9.8)	(9.2)	(16.1)	(15.5)
c_E	29.3	21.9	25.7	29.3	21.9	25.7	29.3	21.9	25.7	29.3
	(16.8)	(12.6)	(14.8)	(16.8)	(12.6)	(14.8)	(16.8)	(12.6)	(14.8)	(16.8)
	-6.2	-5.4	4.8	-2.5	-3.6	-1.3	-18.5	-10.1	4.8	-4.4
	-3.556	-3.111	2.780	-1.445	-2.096	-0.775	-10.628	-5.781	2.780	-2.522
N	174	174	174	174	174	174	174	174	174	174
X2	3.2	0.9	1.6	0.4	0.8	3.1	7.1	1.8	1.4	1.6
Gvalue	3.4	0.9	1.6	0.4	0.8	3.3	8.0	2.0	1.4	1.6
Gv/N	1.981	0.515	0.928	0.257	0.481	1.897	4.572	1.152	0.792	0.897
Prob	0.32773	0.82636	0.65598	0.93021	0.84077	0.34748	0.04694	0.57136	0.71077	0.66832

	81	82	83	84	85	86	87	88	89	90
a_O	72	60	66	65	64	78	71	63	75	78
	(41.4)	(34.5)	(37.9)	(37.4)	(36.8)	(44.8)	(40.8)	(36.2)	(43.1)	(44.8)
a_E	68.1	67.0	68.6	68.1	67.0	68.6	68.1	67.0	68.6	68.1
	(39.1)	(38.5)	(39.4)	(39.1)	(38.5)	(39.4)	(39.1)	(38.5)	(39.4)	(39.1)
	8.1	-13.3	-5.1	-6.0	-5.9	20.1	6.0	-7.8	13.4	21.3
	4.662	-7.617	-2.910	-3.433	-3.378	11.538	3.456	-4.465	7.713	12.227
t_O	58	51	65	81	60	59	69	50	55	74
	(33.3)	(29.3)	(37.4)	(46.6)	(34.5)	(33.9)	(39.7)	(28.7)	(31.6)	(42.5)
t_E	71.1	42.1	59.2	71.1	42.1	59.2	71.1	42.1	59.2	71.1
	(40.8)	(24.2)	(34.0)	(40.8)	(24.2)	(34.0)	(40.8)	(24.2)	(34.0)	(40.8)
	-23.6	19.7	12.1	21.2	42.6	-0.5	-4.1	17.3	-8.2	6.0
	-13.548	11.301	6.939	12.177	24.504	-0.269	-2.344	9.942	-4.689	3.436
g_O	14	39	17	8	28	8	10	35	14	4
	(8.0)	(22.4)	(9.8)	(4.6)	(16.1)	(4.6)	(5.7)	(20.1)	(8.0)	(2.3)
g_E	13.0	39.3	16.9	13.0	39.3	16.9	13.0	39.3	16.9	13.0
	(7.5)	(22.6)	(9.7)	(7.5)	(22.6)	(9.7)	(7.5)	(22.6)	(9.7)	(7.5)
	2.2	-0.5	0.2	-7.7	-18.9	-12.0	-5.2	-8.0	-5.3	-9.4
	1.236	-0.289	0.116	-4.439	-10.872	-6.876	-2.984	-4.613	-3.028	-5.407
c_O	30	24	26	20	22	29	24	26	30	18
	(17.2)	(13.8)	(14.9)	(11.5)	(12.6)	(16.7)	(13.8)	(14.9)	(17.2)	(10.3)
c_E	21.9	25.7	29.3	21.9	25.7	29.3	21.9	25.7	29.3	21.9
	(12.6)	(14.8)	(16.8)	(12.6)	(14.8)	(16.8)	(12.6)	(14.8)	(16.8)	(12.6)
	18.9	-3.3	-6.2	-3.6	-6.8	-0.6	4.4	0.6	1.4	-7.1
	10.838	-1.870	-3.556	-2.096	-3.914	-0.327	2.514	0.367	0.831	-4.066
N	174	174	174	174	174	174	174	174	174	174
X2	5.7	2.7	1.0	3.6	11.5	6.0	1.1	2.2	1.4	8.5
Gvalue	5.5	2.7	1.0	3.8	11.0	7.1	1.1	2.1	1.4	10.8
Gv/N	3.188	1.525	0.590	2.208	6.340	4.066	0.642	1.230	0.827	6.190
Prob	0.13587	0.44818	0.79498	0.27898	0.01155	0.06955	0.77313	0.54380	0.69644	0.01303

	91	92	93	94	95	96	97	98	99	100
a_O	68	72	59	78	82	66	60	72	81	66
	(39.1)	(41.4)	(33.9)	(44.8)	(47.1)	(37.9)	(34.5)	(41.4)	(46.6)	(37.9)
a_E	67.0	68.6	68.1	67.0	68.6	68.1	67.0	68.6	68.1	67.0
	(38.5)	(39.4)	(39.1)	(38.5)	(39.4)	(39.1)	(38.5)	(39.4)	(39.1)	(38.5)
	2.0	7.0	-16.9	23.7	29.3	-4.0	-13.3	7.0	28.2	-2.0
	1.150	4.026	-9.684	13.620	16.844	-2.327	-7.617	4.026	16.211	-1.149
t_O	50	60	69	37	50	78	47	60	67	47
	(28.7)	(34.5)	(39.7)	(21.3)	(28.7)	(44.8)	(27.0)	(34.5)	(38.5)	(27.0)
t_E	42.1	59.2	71.1	42.1	59.2	71.1	42.1	59.2	71.1	42.1
	(24.2)	(34.0)	(40.8)	(24.2)	(34.0)	(40.8)	(24.2)	(34.0)	(40.8)	(24.2)
	17.3	1.5	-4.1	-9.5	-16.9	14.5	10.4	1.5	-7.9	10.4
	9.942	0.885	-2.344	-5.449	-9.741	8.342	6.003	0.885	-4.541	6.003
g_O	31	14	17	31	13	7	31	17	11	38
	(17.8)	(8.0)	(9.8)	(17.8)	(7.5)	(4.0)	(17.8)	(9.8)	(6.3)	(21.8)
g_E	39.3	16.9	13.0	39.3	16.9	13.0	39.3	16.9	13.0	39.3
	(22.6)	(9.7)	(7.5)	(22.6)	(9.7)	(7.5)	(22.6)	(9.7)	(7.5)	(22.6)
	-14.6	-5.3	9.2	-14.6	-6.8	-8.6	-14.6	0.2	-3.6	-2.5
	-8.410	-3.028	5.295	-8.410	-3.919	-4.959	-8.410	0.116	-2.078	-1.416
c_O	25	28	29	28	29	23	36	25	15	23
	(14.4)	(16.1)	(16.7)	(16.1)	(16.7)	(13.2)	(20.7)	(14.4)	(8.6)	(13.2)
c_E	25.7	29.3	21.9	25.7	29.3	21.9	25.7	29.3	21.9	25.7
	(14.8)	(16.8)	(12.6)	(14.8)	(16.8)	(12.6)	(14.8)	(16.8)	(12.6)	(14.8)
	-1.3	-2.5	16.3	4.8	-0.6	2.2	24.3	-7.9	-11.4	-5.1
	-0.775	-1.445	9.346	2.780	-0.327	1.284	13.973	-4.546	-6.532	-2.917
N	174	174	174	174	174	174	174	174	174	174
X2	3.3	0.7	4.8	4.4	5.0	3.5	7.2	0.8	5.2	0.9
Gvalue	3.3	0.8	4.5	4.4	5.0	4.1	6.9	0.8	5.3	0.9
Gv/N	1.907	0.439	2.613	2.541	2.857	2.340	3.948	0.482	3.059	0.520
Prob	0.34517	0.85823	0.20812	0.21948	0.17391	0.25381	0.07616	0.84028	0.14959	0.82409

Azoa_EBN1
5UTR:

	100	99	98	97	96	95	94	93	92	91
a_O	107	97	124	127	98	128	138	112	122	121
	(17.8)	(16.1)	(20.6)	(21.1)	(16.3)	(21.3)	(22.9)	(18.6)	(20.3)	(20.1)
a_E	116.3	116.3	116.3	116.3	116.3	116.3	116.3	116.3	116.3	116.3
	(19.3)	(19.3)	(19.3)	(19.3)	(19.3)	(19.3)	(19.3)	(19.3)	(19.3)	(19.3)
	-17.8	-35.2	15.9	22.4	-33.5	24.6	47.3	-8.4	11.7	9.6
	-2.957	-5.842	2.648	3.721	-5.569	4.084	7.851	-1.395	1.946	1.600
t_O	114	107	115	118	115	105	130	103	91	110
	(18.9)	(17.8)	(19.1)	(19.6)	(19.1)	(17.4)	(21.6)	(17.1)	(15.1)	(18.3)
t_E	113.7	113.7	113.7	113.7	113.7	113.7	113.7	113.7	113.7	113.7
	(18.9)	(18.9)	(18.9)	(18.9)	(18.9)	(18.9)	(18.9)	(18.9)	(18.9)	(18.9)
	0.7	-12.9	2.7	8.9	2.7	-16.6	34.9	-20.3	-40.5	-7.2
	0.116	-2.143	0.451	1.472	0.451	-2.762	5.805	-3.367	-6.720	-1.193
g_O	186	194	193	174	187	197	160	186	196	167
	(30.9)	(32.2)	(32.1)	(28.9)	(31.1)	(32.7)	(26.6)	(30.9)	(32.6)	(27.7)
g_E	171.6	171.6	171.6	171.6	171.6	171.6	171.6	171.6	171.6	171.6
	(28.5)	(28.5)	(28.5)	(28.5)	(28.5)	(28.5)	(28.5)	(28.5)	(28.5)	(28.5)
	30.0	47.6	45.4	4.8	32.1	54.4	-22.4	30.0	52.1	-9.1
	4.979	7.908	7.536	0.803	5.339	9.034	-3.721	4.979	8.657	-1.508
c_O	195	204	170	183	202	172	174	201	193	204
	(32.4)	(33.9)	(28.2)	(30.4)	(33.6)	(28.6)	(28.9)	(33.4)	(32.1)	(33.9)
c_E	200.5	200.5	200.5	200.5	200.5	200.5	200.5	200.5	200.5	200.5
	(33.3)	(33.3)	(33.3)	(33.3)	(33.3)	(33.3)	(33.3)	(33.3)	(33.3)	(33.3)
	-10.8	7.1	-56.1	-33.4	3.1	-52.7	-49.3	1.1	-14.7	7.1
	-1.792	1.183	-9.311	-5.543	0.510	-8.753	-8.186	0.176	-2.435	1.183
N	602	602	602	602	602	602	602	602	602	602
X2	2.1	6.6	7.8	2.7	4.3	9.6	10.7	2.4	8.5	0.5
Gvalue	2.1	6.7	8.0	2.7	4.4	9.7	10.5	2.4	8.7	0.5
Gv/N	0.347	1.105	1.323	0.453	0.732	1.604	1.750	0.393	1.449	0.082
Prob	0.55418	0.08382	0.04669	0.43587	0.22069	0.02173	0.01453	0.49970	0.03322	0.92026

	90	89	88	87	86	85	84	83	82	81
a_O	110	106	106	105	122	117	81	102	139	103
	(18.3)	(17.6)	(17.6)	(17.4)	(20.3)	(19.4)	(13.5)	(16.9)	(23.1)	(17.1)
a_E	116.3	116.3	116.3	116.3	116.3	116.3	116.3	116.3	116.3	116.3
	(19.3)	(19.3)	(19.3)	(19.3)	(19.3)	(19.3)	(19.3)	(19.3)	(19.3)	(19.3)
	-12.2	-19.6	-19.6	-21.4	11.7	1.4	-58.6	-26.7	49.6	-25.0
	-2.029	-3.260	-3.260	-3.560	1.946	0.240	-9.729	-4.440	8.242	-4.150
t_O	103	104	119	100	101	123	114	94	116	98
	(17.1)	(17.3)	(19.8)	(16.6)	(16.8)	(20.4)	(18.9)	(15.6)	(19.3)	(16.3)
t_E	113.7	113.7	113.7	113.7	113.7	113.7	113.7	113.7	113.7	113.7
	(18.9)	(18.9)	(18.9)	(18.9)	(18.9)	(18.9)	(18.9)	(18.9)	(18.9)	(18.9)
	-20.3	-18.5	10.9	-25.6	-23.8	19.4	0.7	-35.7	4.7	-29.0
	-3.367	-3.066	1.819	-4.251	-3.960	3.231	0.116	-5.928	0.789	-4.824
g_O	215	199	168	207	203	170	193	206	181	214
	(35.7)	(33.1)	(27.9)	(34.4)	(33.7)	(28.2)	(32.1)	(34.2)	(30.1)	(35.5)
g_E	171.6	171.6	171.6	171.6	171.6	171.6	171.6	171.6	171.6	171.6
	(28.5)	(28.5)	(28.5)	(28.5)	(28.5)	(28.5)	(28.5)	(28.5)	(28.5)	(28.5)
	97.0	59.0	-7.1	77.6	68.2	-3.2	45.4	75.3	19.3	94.5
	16.105	9.794	-1.183	12.898	11.333	-0.529	7.536	12.504	3.207	15.699
c_O	174	193	209	190	176	192	214	200	166	187
	(28.9)	(32.1)	(34.7)	(31.6)	(29.2)	(31.9)	(35.5)	(33.2)	(27.6)	(31.1)
c_E	200.5	200.5	200.5	200.5	200.5	200.5	200.5	200.5	200.5	200.5
	(33.3)	(33.3)	(33.3)	(33.3)	(33.3)	(33.3)	(33.3)	(33.3)	(33.3)	(33.3)
	-49.3	-14.7	17.4	-20.4	-45.8	-16.6	28.0	-0.9	-62.6	-26.0
	-8.186	-2.435	2.893	-3.386	-7.612	-2.754	4.643	-0.156	-10.405	-4.321
N	602	602	602	602	602	602	602	602	602	602
X2	15.8	6.4	1.6	10.6	10.4	1.1	14.3	12.0	10.9	15.1
Gvalue	15.2	6.2	1.6	10.2	10.3	1.1	15.4	11.9	11.0	14.5
Gv/N	2.523	1.033	0.269	1.702	1.708	0.188	2.566	1.980	1.832	2.404
Prob	0.00166	0.10132	0.65513	0.01660	0.01632	0.76950	0.00147	0.00766	0.01157	0.00233

	80	79	78	77	76	75	74	73	72	71
a_O	116	111	96	121	142	107	120	131	110	116
	(19.3)	(18.4)	(15.9)	(20.1)	(23.6)	(17.8)	(19.9)	(21.8)	(18.3)	(19.3)
a_E	116.3	116.3	116.3	116.3	116.3	116.3	116.3	116.3	116.3	116.3
	(19.3)	(19.3)	(19.3)	(19.3)	(19.3)	(19.3)	(19.3)	(19.3)	(19.3)	(19.3)
	-0.6	-10.3	-36.8	9.6	56.8	-17.8	7.6	31.2	-12.2	-0.6
	-0.093	-1.714	-6.113	1.600	9.427	-2.957	1.255	5.188	-2.029	-0.093
t_O	99	117	119	89	102	86	104	127	112	100
	(16.4)	(19.4)	(19.8)	(14.8)	(16.9)	(14.3)	(17.3)	(21.1)	(18.6)	(16.6)
t_E	113.7	113.7	113.7	113.7	113.7	113.7	113.7	113.7	113.7	113.7
	(18.9)	(18.9)	(18.9)	(18.9)	(18.9)	(18.9)	(18.9)	(18.9)	(18.9)	(18.9)
	-27.3	6.8	10.9	-43.5	-22.1	-47.9	-18.5	28.2	-3.3	-25.6
	-4.539	1.129	1.819	-7.229	-3.665	-7.965	-3.066	4.686	-0.544	-4.251
g_O	191	170	184	192	176	195	179	161	179	206
	(31.7)	(28.2)	(30.6)	(31.9)	(29.2)	(32.4)	(29.7)	(26.7)	(29.7)	(34.2)
g_E	171.6	171.6	171.6	171.6	171.6	171.6	171.6	171.6	171.6	171.6
	(28.5)	(28.5)	(28.5)	(28.5)	(28.5)	(28.5)	(28.5)	(28.5)	(28.5)	(28.5)
	40.9	-3.2	25.7	43.1	8.9	49.9	15.1	-20.5	15.1	75.3
	6.797	-0.529	4.265	7.165	1.480	8.282	2.511	-3.411	2.511	12.504
c_O	196	204	203	200	182	214	199	183	201	180
	(32.6)	(33.9)	(33.7)	(33.2)	(30.2)	(35.5)	(33.1)	(30.4)	(33.4)	(29.9)
c_E	200.5	200.5	200.5	200.5	200.5	200.5	200.5	200.5	200.5	200.5
	(33.3)	(33.3)	(33.3)	(33.3)	(33.3)	(33.3)	(33.3)	(33.3)	(33.3)	(33.3)
	-8.8	7.1	5.1	-0.9	-35.2	28.0	-2.9	-33.4	1.1	-38.8
	-1.468	1.183	0.846	-0.156	-5.844	4.643	-0.487	-5.543	0.176	-6.441
N	602	602	602	602	602	602	602	602	602	602
X2	4.2	0.4	4.7	8.0	8.7	11.6	1.3	5.6	0.7	10.6
Gvalue	4.2	0.4	4.9	8.3	8.4	12.1	1.3	5.5	0.7	10.4
Gv/N	0.696	0.069	0.817	1.380	1.398	2.003	0.214	0.920	0.114	1.720
Prob	0.24153	0.93650	0.17793	0.04010	0.03815	0.00718	0.73227	0.13645	0.87660	0.01580

	70	69	68	67	66	65	64	63	62	61
a_O	120	104	122	135	99	98	120	108	119	131
	(19.9)	(17.3)	(20.3)	(22.4)	(16.4)	(16.3)	(19.9)	(17.9)	(19.8)	(21.8)
a_E	116.3	116.3	116.3	116.3	116.3	116.3	116.3	116.3	116.3	116.3
	(19.3)	(19.3)	(19.3)	(19.3)	(19.3)	(19.3)	(19.3)	(19.3)	(19.3)	(19.3)
	7.6	-23.2	11.7	40.3	-31.9	-33.5	7.6	-16.0	5.5	31.2
	1.255	-3.856	1.946	6.695	-5.291	-5.569	1.255	-2.650	0.914	5.188
t_O	119	103	96	116	102	110	126	97	94	126
	(19.8)	(17.1)	(15.9)	(19.3)	(16.9)	(18.3)	(20.9)	(16.1)	(15.6)	(20.9)

t_E	113.7	113.7	113.7	113.7	113.7	113.7	113.7	113.7	113.7	113.7
	(18.9)	(18.9)	(18.9)	(18.9)	(18.9)	(18.9)	(18.9)	(18.9)	(18.9)	(18.9)
	10.9	-20.3	-32.4	4.7	-22.1	-7.2	26.0	-30.7	-35.7	26.0
	1.819	-3.367	-5.383	0.789	-3.665	-1.193	4.318	-5.105	-5.928	4.318
g_O	186	177	171	184	183	208	168	197	200	149
	(30.9)	(29.4)	(28.4)	(30.6)	(30.4)	(34.6)	(27.9)	(32.7)	(33.2)	(24.8)
g_E	171.6	171.6	171.6	171.6	171.6	171.6	171.6	171.6	171.6	171.6
	(28.5)	(28.5)	(28.5)	(28.5)	(28.5)	(28.5)	(28.5)	(28.5)	(28.5)	(28.5)
	30.0	11.0	-1.2	25.7	23.5	80.0	-7.1	54.4	61.3	-42.1
	4.979	1.822	-0.199	4.265	3.910	13.293	-1.183	9.034	10.176	-6.991
c_O	177	218	213	167	218	186	188	200	189	196
	(29.4)	(36.2)	(35.4)	(27.7)	(36.2)	(30.9)	(31.2)	(33.2)	(31.4)	(32.6)
c_E	200.5	200.5	200.5	200.5	200.5	200.5	200.5	200.5	200.5	200.5
	(33.3)	(33.3)	(33.3)	(33.3)	(33.3)	(33.3)	(33.3)	(33.3)	(33.3)	(33.3)
	-44.1	36.6	25.8	-61.0	36.6	-27.9	-24.1	-0.9	-22.3	-8.8
	-7.322	6.071	4.290	-10.135	6.071	-4.629	-4.011	-0.156	-3.699	-1.468
N	602	602	602	602	602	602	602	602	602	602
X2	4.3	4.0	3.8	9.5	6.1	11.8	2.3	6.8	8.8	6.3
Gvalue	4.4	4.0	3.9	9.7	6.2	11.5	2.3	6.8	8.8	6.3
Gv/N	0.731	0.670	0.655	1.614	1.026	1.903	0.379	1.123	1.463	1.047
Prob	0.22107	0.25781	0.26786	0.02115	0.10345	0.00951	0.51598	0.07995	0.03200	0.09780

	60	59	58	57	56	55	54	53	52	51
a_O	90	97	124	99	127	121	106	123	129	100
	(15.0)	(16.1)	(20.6)	(16.4)	(21.1)	(20.1)	(17.6)	(20.4)	(21.4)	(16.6)
a_E	116.3	116.3	116.3	116.3	116.3	116.3	116.3	116.3	116.3	116.3
	(19.3)	(19.3)	(19.3)	(19.3)	(19.3)	(19.3)	(19.3)	(19.3)	(19.3)	(19.3)
	-46.1	-35.2	15.9	-31.9	22.4	9.6	-19.6	13.8	26.8	-30.2
	-7.660	-5.842	2.648	-5.291	3.721	1.600	-3.260	2.296	4.449	-5.011
t_O	115	113	126	115	118	114	101	101	122	104
	(19.1)	(18.8)	(20.9)	(19.1)	(19.6)	(18.9)	(16.8)	(16.8)	(20.3)	(17.3)
t_E	113.7	113.7	113.7	113.7	113.7	113.7	113.7	113.7	113.7	113.7
	(18.9)	(18.9)	(18.9)	(18.9)	(18.9)	(18.9)	(18.9)	(18.9)	(18.9)	(18.9)
	2.7	-1.3	26.0	2.7	8.9	0.7	-23.8	-23.8	17.3	-18.5
	0.451	-0.215	4.318	0.451	1.472	0.116	-3.960	-3.960	2.874	-3.066
g_O	198	187	160	199	175	193	205	176	154	188
	(32.9)	(31.1)	(26.6)	(33.1)	(29.1)	(32.1)	(34.1)	(29.2)	(25.6)	(31.2)
g_E	171.6	171.6	171.6	171.6	171.6	171.6	171.6	171.6	171.6	171.6
	(28.5)	(28.5)	(28.5)	(28.5)	(28.5)	(28.5)	(28.5)	(28.5)	(28.5)	(28.5)
	56.7	32.1	-22.4	59.0	6.9	45.4	72.9	8.9	-33.3	34.3
	9.413	5.339	-3.721	9.794	1.141	7.536	12.112	1.480	-5.537	5.701
c_O	199	205	192	189	182	174	190	202	197	210
	(33.1)	(34.1)	(31.9)	(31.4)	(30.2)	(28.9)	(31.6)	(33.6)	(32.7)	(34.9)
c_E	200.5	200.5	200.5	200.5	200.5	200.5	200.5	200.5	200.5	200.5
	(33.3)	(33.3)	(33.3)	(33.3)	(33.3)	(33.3)	(33.3)	(33.3)	(33.3)	(33.3)
	-2.9	9.2	-16.6	-22.3	-35.2	-49.3	-20.4	3.1	-6.9	19.5
	-0.487	1.522	-2.754	-3.699	-5.844	-8.186	-3.386	0.510	-1.143	3.240
N	602	602	602	602	602	602	602	602	602	602
X2	10.0	4.7	3.0	7.6	2.9	6.4	9.4	1.9	3.9	5.1
Gvalue	10.3	4.8	3.0	7.5	2.9	6.4	9.1	2.0	3.9	5.2
Gv/N	1.718	0.804	0.492	1.254	0.490	1.066	1.507	0.327	0.643	0.864
Prob	0.01588	0.18409	0.39738	0.05629	0.39985	0.09310	0.02834	0.57908	0.27552	0.15751

	50	49	48	47	46	45	44	43	42	41
a_O	96	125	103	112	104	91	124	108	94	116
	(15.9)	(20.8)	(17.1)	(18.6)	(17.3)	(15.1)	(20.6)	(17.9)	(15.6)	(19.3)
a_E	116.3	116.3	116.3	116.3	116.3	116.3	116.3	116.3	116.3	116.3
	(19.3)	(19.3)	(19.3)	(19.3)	(19.3)	(19.3)	(19.3)	(19.3)	(19.3)	(19.3)
	-36.8	18.1	-25.0	-8.4	-23.2	-44.6	15.9	-16.0	-40.0	-0.6
	-6.113	3.003	-4.150	-1.395	-3.856	-7.411	2.648	-2.650	-6.643	-0.093
t_O	109	105	109	122	141	110	116	147	128	92
	(18.1)	(17.4)	(18.1)	(20.3)	(23.4)	(18.3)	(19.3)	(24.4)	(21.3)	(15.3)
t_E	113.7	113.7	113.7	113.7	113.7	113.7	113.7	113.7	113.7	113.7
	(18.9)	(18.9)	(18.9)	(18.9)	(18.9)	(18.9)	(18.9)	(18.9)	(18.9)	(18.9)
	-9.1	-16.6	-9.1	17.3	60.8	-7.2	4.7	75.6	30.4	-38.9
	-1.513	-2.762	-1.513	2.874	10.101	-1.193	0.789	12.566	5.056	-6.459
g_O	197	171	197	181	171	185	175	176	182	190
	(32.7)	(28.4)	(32.7)	(30.1)	(28.4)	(30.7)	(29.1)	(29.2)	(30.2)	(31.6)
g_E	171.6	171.6	171.6	171.6	171.6	171.6	171.6	171.6	171.6	171.6
	(28.5)	(28.5)	(28.5)	(28.5)	(28.5)	(28.5)	(28.5)	(28.5)	(28.5)	(28.5)
	54.4	-1.2	54.4	19.3	-1.2	27.8	6.9	8.9	21.4	38.7
	9.034	-0.199	9.034	3.207	-0.199	4.621	1.141	1.480	3.558	6.430
c_O	200	201	193	187	186	216	187	171	198	204
	(33.2)	(33.4)	(32.1)	(31.1)	(30.9)	(35.9)	(31.1)	(28.4)	(32.9)	(33.9)
c_E	200.5	200.5	200.5	200.5	200.5	200.5	200.5	200.5	200.5	200.5
	(33.3)	(33.3)	(33.3)	(33.3)	(33.3)	(33.3)	(33.3)	(33.3)	(33.3)	(33.3)
	-0.9	1.1	-14.7	-26.0	-27.9	32.2	-26.0	-54.4	-4.9	7.1
	-0.156	0.176	-2.435	-4.321	-4.629	5.354	-4.321	-9.033	-0.816	1.183
N	602	602	602	602	602	602	602	602	602	602
X2	7.5	1.3	5.7	2.2	8.9	7.9	1.5	14.8	6.7	6.2
Gvalue	7.5	1.3	5.6	2.2	8.5	8.3	1.5	14.2	7.0	6.4
Gv/N	1.253	0.219	0.937	0.364	1.416	1.371	0.256	2.363	1.156	1.060
Prob	0.05645	0.72501	0.13053	0.53386	0.03629	0.04101	0.67243	0.00261	0.07319	0.09440

	40	39	38	37	36	35	34	33	32	31
a_O	129	106	131	123	108	131	120	107	128	132
	(21.4)	(17.6)	(21.8)	(20.4)	(17.9)	(21.8)	(19.9)	(17.8)	(21.3)	(21.9)
a_E	116.3	116.3	116.3	116.3	116.3	116.3	116.3	116.3	116.3	116.3
	(19.3)	(19.3)	(19.3)	(19.3)	(19.3)	(19.3)	(19.3)	(19.3)	(19.3)	(19.3)
	26.8	-19.6	31.2	13.8	-16.0	31.2	7.6	-17.8	24.6	33.5
	4.449	-3.260	5.188	2.296	-2.650	5.188	1.255	-2.957	4.084	5.561
t_O	119	111	112	136	113	121	137	123	128	166
	(19.8)	(18.4)	(18.6)	(22.6)	(18.8)	(20.1)	(22.8)	(20.4)	(21.3)	(27.6)
t_E	113.7	113.7	113.7	113.7	113.7	113.7	113.7	113.7	113.7	113.7
	(18.9)	(18.9)	(18.9)	(18.9)	(18.9)	(18.9)	(18.9)	(18.9)	(18.9)	(18.9)
	10.9	-5.2	-3.3	48.8	-1.3	15.2	51.2	19.4	30.4	125.8
	1.819	-0.870	-0.544	8.112	-0.215	2.519	8.505	3.231	5.056	20.894
g_O	156	193	165	170	170	171	155	184	154	134
	(25.9)	(32.1)	(27.4)	(28.2)	(28.2)	(28.4)	(25.7)	(30.6)	(25.6)	(22.3)
g_E	171.6	171.6	171.6	171.6	171.6	171.6	171.6	171.6	171.6	171.6
	(28.5)	(28.5)	(28.5)	(28.5)	(28.5)	(28.5)	(28.5)	(28.5)	(28.5)	(28.5)
	-29.7	45.4	-12.9	-3.2	-3.2	-1.2	-31.5	25.7	-33.3	-66.3
	-4.940	7.536	-2.150	-0.529	-0.529	-0.199	-5.239	4.265	-5.537	-11.011
c_O	198	192	194	173	211	179	190	188	192	170
	(32.9)	(31.9)	(32.2)	(28.7)	(35.0)	(29.7)	(31.6)	(31.2)	(31.9)	(28.2)
c_E	200.5	200.5	200.5	200.5	200.5	200.5	200.5	200.5	200.5	200.5

```
         ( 33.3) ( 33.3) ( 33.3) ( 33.3) ( 33.3) ( 33.3) ( 33.3) ( 33.3) ( 33.3) ( 33.3)
            -4.9   -16.6   -12.7   -51.0    21.6   -40.6   -20.4   -24.1   -16.6   -56.1
          -0.816  -2.754  -2.114  -8.470   3.589  -6.737  -3.386  -4.011  -2.754  -9.311

N            602     602     602     602     602     602     602     602     602     602
X2           3.1     4.0     2.4     8.6     1.2     4.6     7.1     3.2     5.2    39.1
Gvalue       3.1     3.9     2.3     8.5     1.2     4.6     6.8     3.2     5.1    36.9
Gv/N       0.512   0.652   0.379   1.408   0.194   0.771   1.135   0.528   0.850   6.133
Prob     0.37869 0.26952 0.51603 0.03711 0.76106 0.19987 0.07740 0.36505 0.16344 0.00000
         -------------------------------------------------------------------------------
              30      29      28      27      26      25      24      23      22      21
         -------------------------------------------------------------------------------
a_O           93     112     119      89     123     140      89     109     121     109
         ( 15.4) ( 18.6) ( 19.8) ( 14.8) ( 20.4) ( 23.3) ( 14.8) ( 18.1) ( 20.1) ( 18.1)
a_E        116.3   116.3   116.3   116.3   116.3   116.3   116.3   116.3   116.3   116.3
         ( 19.3) ( 19.3) ( 19.3) ( 19.3) ( 19.3) ( 19.3) ( 19.3) ( 19.3) ( 19.3) ( 19.3)
           -41.6    -8.4     5.5   -47.6    13.8    52.0   -47.6   -14.1     9.6   -14.1
          -6.902  -1.395   0.914  -7.905   2.296   8.634  -7.905  -2.341   1.600  -2.341
t_O          128     125     134     105     125     115     111     116     144     127
         ( 21.3) ( 20.8) ( 22.3) ( 17.4) ( 20.8) ( 19.1) ( 18.4) ( 19.3) ( 23.9) ( 21.1)
t_E        113.7   113.7   113.7   113.7   113.7   113.7   113.7   113.7   113.7   113.7
         ( 18.9) ( 18.9) ( 18.9) ( 18.9) ( 18.9) ( 18.9) ( 18.9) ( 18.9) ( 18.9) ( 18.9)
            30.4    23.8    44.1   -16.6    23.8     2.7    -5.2     4.7    68.2    28.2
           5.056   3.953   7.333  -2.762   3.953   0.451  -0.870   0.789  11.323   4.686
g_O          164     158     138     176     143     132     172     190     141     140
         ( 27.2) ( 26.2) ( 22.9) ( 29.2) ( 23.8) ( 21.9) ( 28.6) ( 31.6) ( 23.4) ( 23.3)
g_E        171.6   171.6   171.6   171.6   171.6   171.6   171.6   171.6   171.6   171.6
         ( 28.5) ( 28.5) ( 28.5) ( 28.5) ( 28.5) ( 28.5) ( 28.5) ( 28.5) ( 28.5) ( 28.5)
           -14.9   -26.1   -60.1     8.9   -52.1   -69.3     0.8    38.7   -55.4   -57.0
          -2.468  -4.334  -9.991   1.480  -8.662 -11.506   0.133   6.430  -9.200  -9.466
c_O          217     207     211     232     211     215     230     187     196     226
         ( 36.0) ( 34.4) ( 35.0) ( 38.5) ( 35.0) ( 35.7) ( 38.2) ( 31.1) ( 32.6) ( 37.5)
c_E        200.5   200.5   200.5   200.5   200.5   200.5   200.5   200.5   200.5   200.5
         ( 33.3) ( 33.3) ( 33.3) ( 33.3) ( 33.3) ( 33.3) ( 33.3) ( 33.3) ( 33.3) ( 33.3)
            34.4    13.3    21.6    67.8    21.6    30.1    63.2   -26.0    -8.8    54.2
           5.712   2.204   3.589  11.259   3.589   4.998  10.500  -4.321  -1.468   9.000

N            602     602     602     602     602     602     602     602     602     602
X2           8.2     2.6    10.8    12.1     6.8    15.0    10.8     3.4    13.9    11.1
Gvalue       8.4     2.6    11.1    12.5     7.1    15.5    11.2     3.3    13.6    11.3
Gv/N       1.398   0.428   1.845   2.072   1.176   2.578   1.858   0.556   2.254   1.879
Prob     0.03815 0.46189 0.01116 0.00592 0.06944 0.00142 0.01078 0.34130 0.00355 0.01016
         -------------------------------------------------------------------------------
              20      19      18      17      16      15      14      13      12      11
         -------------------------------------------------------------------------------
a_O          127     134      95     135     127      92     123     127     122     153
         ( 21.1) ( 22.3) ( 15.8) ( 22.4) ( 21.1) ( 15.3) ( 20.4) ( 21.1) ( 20.3) ( 25.4)
a_E        116.3   116.3   116.3   116.3   116.3   116.3   116.3   116.3   116.3   116.3
         ( 19.3) ( 19.3) ( 19.3) ( 19.3) ( 19.3) ( 19.3) ( 19.3) ( 19.3) ( 19.3) ( 19.3)
            22.4    38.0   -38.4    40.3    22.4   -43.1    13.8    22.4    11.7    84.0
           3.721   6.314  -6.379   6.695   3.721  -7.159   2.296   3.721   1.946  13.950
t_O          133     142     124     112     144     124     116     143     101      94
         ( 22.1) ( 23.6) ( 20.6) ( 18.6) ( 23.9) ( 20.6) ( 19.3) ( 23.8) ( 16.8) ( 15.6)
t_E        113.7   113.7   113.7   113.7   113.7   113.7   113.7   113.7   113.7   113.7
         ( 18.9) ( 18.9) ( 18.9) ( 18.9) ( 18.9) ( 18.9) ( 18.9) ( 18.9) ( 18.9) ( 18.9)
            41.8    63.2    21.6    -3.3    68.2    21.6     4.7    65.7   -23.8   -35.7
           6.947  10.506   3.591  -0.544  11.323   3.591   0.789  10.914  -3.960  -5.928
g_O          140     129     138     122     127     153     131     120     161     146
         ( 23.3) ( 21.4) ( 22.9) ( 20.3) ( 21.1) ( 25.4) ( 21.8) ( 19.9) ( 26.7) ( 24.3)
g_E        171.6   171.6   171.6   171.6   171.6   171.6   171.6   171.6   171.6   171.6
         ( 28.5) ( 28.5) ( 28.5) ( 28.5) ( 28.5) ( 28.5) ( 28.5) ( 28.5) ( 28.5) ( 28.5)
           -57.0   -73.6   -60.1   -83.2   -76.4   -35.1   -70.7   -85.8   -20.5   -47.2
          -9.466 -12.229  -9.991 -13.827 -12.699  -5.832 -11.749 -14.259  -3.411  -7.836
c_O          202     197     245     233     204     233     232     212     218     209
         ( 33.6) ( 32.7) ( 40.7) ( 38.7) ( 33.9) ( 38.7) ( 38.5) ( 35.2) ( 36.2) ( 34.7)
c_E        200.5   200.5   200.5   200.5   200.5   200.5   200.5   200.5   200.5   200.5
         ( 33.3) ( 33.3) ( 33.3) ( 33.3) ( 33.3) ( 33.3) ( 33.3) ( 33.3) ( 33.3) ( 33.3)
             3.1    -6.9    98.3    70.1     7.1    70.1    67.8    23.7    36.6    17.4
           0.510  -1.143  16.327  11.640   1.183  11.640  11.259   3.939   6.071   2.893

N            602     602     602     602     602     602     602     602     602     602
X2          10.1    20.4    21.3    22.7    20.7    13.3    15.0    24.7     3.9    19.2
Gvalue      10.3    20.8    21.4    23.9    21.2    13.5    15.6    26.0     3.9    18.5
Gv/N       1.712   3.448   3.548   3.964   3.528   2.240   2.594   4.314   0.648   3.078
Prob     0.01613 0.00012 0.00009 0.00003 0.00009 0.00369 0.00136 0.00001 0.27258 0.00034
         -------------------------------------------------------------------------------
              10       9       8       7       6       5       4       3       2       1
         -------------------------------------------------------------------------------
a_O          131     122     146     131     128     144     117     140     124     111
         ( 21.8) ( 20.3) ( 24.3) ( 21.8) ( 21.3) ( 23.9) ( 19.4) ( 23.3) ( 20.6) ( 18.4)
a_E        116.3   116.3   116.3   116.3   116.3   116.3   116.3   116.3   116.3   116.3
         ( 19.3) ( 19.3) ( 19.3) ( 19.3) ( 19.3) ( 19.3) ( 19.3) ( 19.3) ( 19.3) ( 19.3)
            31.2    11.7    66.5    31.2    24.6    61.6     1.4    52.0    15.9   -10.3
           5.188   1.946  11.040   5.188   4.084  10.229   0.240   8.634   2.648  -1.714
t_O           99      81     106     129     116     104     101      79     121      68
         ( 16.4) ( 13.5) ( 17.6) ( 21.4) ( 19.3) ( 17.3) ( 16.8) ( 13.1) ( 20.1) ( 11.3)
t_E        113.7   113.7   113.7   113.7   113.7   113.7   113.7   113.7   113.7   113.7
         ( 18.9) ( 18.9) ( 18.9) ( 18.9) ( 18.9) ( 18.9) ( 18.9) ( 18.9) ( 18.9) ( 18.9)
           -27.3   -54.9   -14.8    32.7     4.7   -18.5   -23.8   -57.5    15.2   -69.9
          -4.539  -9.114  -2.454   5.430   0.789  -3.066  -3.960  -9.545   2.519 -11.603
g_O          159     156     152     113     154     118     176     172     124     151
         ( 26.4) ( 25.9) ( 25.2) ( 18.8) ( 25.6) ( 19.6) ( 29.2) ( 28.6) ( 20.6) ( 25.1)
g_E        171.6   171.6   171.6   171.6   171.6   171.6   171.6   171.6   171.6   171.6
         ( 28.5) ( 28.5) ( 28.5) ( 28.5) ( 28.5) ( 28.5) ( 28.5) ( 28.5) ( 28.5) ( 28.5)
           -24.3   -29.7   -36.9   -94.4   -33.3   -88.4     8.9     0.8   -80.6   -38.6
          -4.028  -4.940  -6.125 -15.684  -5.537 -14.681   1.480   0.133 -13.384  -6.416
c_O          213     243     198     229     204     236     208     211     233     272
         ( 35.4) ( 40.4) ( 32.9) ( 38.0) ( 33.9) ( 39.2) ( 34.6) ( 35.0) ( 38.7) ( 45.2)
c_E        200.5   200.5   200.5   200.5   200.5   200.5   200.5   200.5   200.5   200.5
         ( 33.3) ( 33.3) ( 33.3) ( 33.3) ( 33.3) ( 33.3) ( 33.3) ( 33.3) ( 33.3) ( 33.3)
            25.8    93.5    -4.9    60.9     7.1    77.0    15.3    21.6    70.1   166.0
           4.290  15.532  -0.816  10.123   1.183  12.793   2.548   3.589  11.640  27.574

N            602     602     602     602     602     602     602     602     602     602
X2           5.5    20.1    10.4    28.0     3.1    30.5     1.8    16.0    19.5    46.6
Gvalue       5.5    20.6     9.9    30.4     3.1    31.8     1.9    16.9    20.6    47.2
Gv/N       0.910   3.425   1.646   5.056   0.519   5.276   0.309   2.811   3.424   7.841
Prob     0.13980 0.00013 0.01937 0.00000 0.37294 0.00000 0.60217 0.00073 0.00013 0.00000
         -------------------------------------------------------------------------------

CDS_INITIAL:
         -------------------------------------------------------------------------------
```

	1	2	3	4	5	6	7	8	9	10
a_O	447	0	0	246	171	109	140	155	105	143
	(74.3)	(0.0)	(0.0)	(40.9)	(28.4)	(18.1)	(23.3)	(25.7)	(17.4)	(23.8)
a_E	114.5	146.5	53.1	114.5	146.5	53.1	114.5	146.5	53.1	114.5
	(19.0)	(24.3)	(8.8)	(19.0)	(24.3)	(8.8)	(19.0)	(24.3)	(8.8)	(19.0)
	1217.6	0.0	0.0	376.3	52.9	156.8	56.3	17.5	143.2	63.6
	202.260	0.000	0.000	62.501	8.789	26.052	9.351	2.908	23.792	10.559
t_O	56	602	0	64	114	115	87	144	120	96
	(9.3)	(100.0)	(0.0)	(10.6)	(18.9)	(19.1)	(14.5)	(23.9)	(19.9)	(15.9)
t_E	75.5	171.7	61.4	75.5	171.7	61.4	75.5	171.7	61.4	75.5
	(12.5)	(28.5)	(10.2)	(12.5)	(28.5)	(10.2)	(12.5)	(28.5)	(10.2)	(12.5)
	-33.5	1510.5	0.0	-21.2	-93.4	144.5	24.6	-50.7	161.0	46.1
	-5.565	250.913	0.000	-3.521	-15.509	23.999	4.088	-8.414	26.740	7.651
g_O	99	0	602	104	105	149	168	121	139	153
	(16.4)	(0.0)	(100.0)	(17.3)	(17.4)	(24.8)	(27.9)	(20.1)	(23.1)	(25.4)
g_E	233.0	126.8	219.2	233.0	126.8	219.2	233.0	126.8	219.2	233.0
	(38.7)	(21.1)	(36.4)	(38.7)	(21.1)	(36.4)	(38.7)	(21.1)	(36.4)	(38.7)
	-169.5	0.0	1216.1	-167.8	-39.6	-115.1	-109.9	-11.3	-126.7	-128.7
	-28.153	0.000	202.017	-27.873	-6.576	-19.119	-18.259	-1.877	-21.044	-21.382
c_O	0	0	0	188	212	229	207	182	238	210
	(0.0)	(0.0)	(0.0)	(31.2)	(35.2)	(38.0)	(34.4)	(30.2)	(39.5)	(34.9)
c_E	179.0	157.0	268.3	179.0	157.0	268.3	179.0	157.0	268.3	179.0
	(29.7)	(26.1)	(44.6)	(29.7)	(26.1)	(44.6)	(29.7)	(26.1)	(44.6)	(29.7)
	0.0	0.0	0.0	18.5	127.2	-72.6	60.3	53.7	-57.1	67.2
	0.000	0.000	0.000	3.078	21.137	-12.053	10.010	8.920	-9.479	11.159
N	602	602	602	602	602	602	602	602	602	602
X2	1226.6	1508.8	1051.0	224.7	46.5	134.0	30.0	9.2	139.6	45.5
Gvalue	1014.6	1510.5	1216.1	205.8	47.2	113.7	31.3	9.3	120.5	48.1
Gv/N	168.541	250.913	202.017	34.185	7.841	18.880	5.191	1.537	20.009	7.987
Prob	0.00000	1.00000	0.00000	0.00000	0.00000	0.00000	0.00000	0.02610	0.00000	0.00000

	11	12	13	14	15	16	17	18	19	20
a_O	138	79	136	140	101	126	130	73	142	150
	(22.9)	(13.1)	(22.6)	(23.3)	(16.8)	(20.9)	(21.6)	(12.1)	(23.6)	(24.9)
a_E	146.5	53.1	114.5	146.5	53.1	114.5	146.5	53.1	114.5	146.5
	(24.3)	(8.8)	(19.0)	(24.3)	(8.8)	(19.0)	(24.3)	(8.8)	(19.0)	(24.3)
	-16.5	62.8	46.8	-12.7	129.9	24.1	-31.0	46.5	61.1	7.1
	-2.737	10.433	7.774	-2.107	21.582	4.006	-5.158	7.725	10.154	1.180
t_O	182	112	98	171	104	91	179	99	82	154
	(30.2)	(18.6)	(16.3)	(28.4)	(17.3)	(15.1)	(29.7)	(16.4)	(13.6)	(25.6)
t_E	171.7	61.4	75.5	171.7	61.4	75.5	171.7	61.4	75.5	171.7
	(28.5)	(10.2)	(12.5)	(28.5)	(10.2)	(12.5)	(28.5)	(10.2)	(12.5)	(28.5)
	21.2	134.8	51.1	-1.4	109.7	33.9	14.9	94.7	13.5	-33.5
	3.526	22.390	8.482	-0.229	18.230	5.635	2.479	15.733	2.241	-5.563
g_O	108	166	160	91	138	169	107	149	183	114
	(17.9)	(27.6)	(26.6)	(15.1)	(22.9)	(28.1)	(17.8)	(24.8)	(30.4)	(18.9)
g_E	126.8	219.2	233.0	126.8	219.2	233.0	126.8	219.2	233.0	126.8
	(21.1)	(36.4)	(38.7)	(21.1)	(36.4)	(38.7)	(21.1)	(36.4)	(38.7)	(21.1)
	-34.6	-92.4	-120.3	-60.4	-127.8	-108.6	-36.3	-115.1	-88.4	-24.2
	-5.753	-15.342	-19.983	-10.026	-21.224	-18.034	-6.031	-19.119	-14.689	-4.025
c_O	174	245	208	200	259	216	186	281	195	184
	(28.9)	(40.7)	(34.6)	(33.2)	(43.0)	(35.9)	(30.9)	(46.7)	(32.4)	(30.6)
c_E	157.0	268.3	179.0	157.0	268.3	179.0	157.0	268.3	179.0	157.0
	(26.1)	(44.6)	(29.7)	(26.1)	(44.6)	(29.7)	(26.1)	(44.6)	(29.7)	(26.1)
	35.7	-44.5	62.6	96.7	-18.3	81.3	63.0	26.0	33.5	58.3
	5.929	-7.398	10.391	16.069	-3.039	13.499	10.460	4.314	5.561	9.686
N	602	602	602	602	602	602	602	602	602	602
X2	5.7	69.4	38.3	22.1	103.3	29.6	10.6	53.7	19.3	7.8
Gvalue	5.8	60.7	40.1	22.3	93.6	30.7	10.5	52.1	19.7	7.7
Gv/N	0.965	10.083	6.665	3.707	15.550	5.106	1.751	8.653	3.267	1.278
Prob	0.12124	0.00000	0.00000	0.00006	0.00000	0.00000	0.01450	0.00000	0.00020	0.05283

	21	22	23	24	25	26	27	28	29	30
a_O	91	123	125	74	115	121	58	101	118	67
	(15.1)	(20.4)	(20.8)	(12.3)	(19.1)	(20.1)	(9.6)	(16.8)	(19.6)	(11.1)
a_E	53.1	114.5	146.5	53.1	114.5	146.5	53.1	114.5	146.5	53.1
	(8.8)	(19.0)	(24.3)	(8.8)	(19.0)	(24.3)	(8.8)	(19.0)	(24.3)	(8.8)
	98.1	17.6	-39.7	49.2	1.0	-46.3	10.3	-25.3	-51.0	31.2
	16.293	2.926	-6.588	8.165	0.166	-7.685	1.706	-4.210	-8.478	5.181
t_O	105	70	179	106	105	157	101	94	179	83
	(17.4)	(11.6)	(29.7)	(17.6)	(17.4)	(26.1)	(16.8)	(15.6)	(29.7)	(13.8)
t_E	61.4	75.5	171.7	61.4	75.5	171.7	61.4	75.5	171.7	61.4
	(10.2)	(12.5)	(28.5)	(10.2)	(12.5)	(28.5)	(10.2)	(12.5)	(28.5)	(10.2)
	112.8	-10.6	14.9	115.9	69.2	-28.1	100.7	41.1	14.9	50.1
	18.739	-1.767	2.479	19.251	11.494	-4.666	16.722	6.834	2.479	8.329
g_O	145	191	127	169	178	107	171	206	134	177
	(24.1)	(31.7)	(21.1)	(28.1)	(29.6)	(17.8)	(28.4)	(34.2)	(22.3)	(29.4)
g_E	219.2	233.0	126.8	219.2	233.0	126.8	219.2	233.0	126.8	219.2
	(36.4)	(38.7)	(21.1)	(36.4)	(38.7)	(21.1)	(36.4)	(38.7)	(21.1)	(36.4)
	-119.9	-76.0	0.4	-88.0	-95.9	-36.3	-85.0	-50.8	14.8	-75.8
	-19.916	-12.616	0.072	-14.613	-15.926	-6.031	-14.118	-8.433	2.465	-12.585
c_O	261	218	171	253	204	217	272	201	171	275
	(43.4)	(36.2)	(28.4)	(42.0)	(33.9)	(36.0)	(45.2)	(33.4)	(28.4)	(45.7)
c_E	268.3	179.0	157.0	268.3	179.0	157.0	268.3	179.0	157.0	268.3
	(44.6)	(29.7)	(26.1)	(44.6)	(29.7)	(26.1)	(44.6)	(29.7)	(26.1)	(44.6)
	-14.4	86.0	29.1	-29.7	53.4	140.4	7.4	46.7	29.1	13.5
	-2.396	14.292	4.839	-4.939	8.875	23.316	1.234	7.756	4.839	2.250
N	602	602	602	602	602	602	602	602	602	602
X2	83.5	17.1	4.7	53.1	28.0	31.7	36.7	12.0	7.5	19.6
Gvalue	76.6	17.1	4.8	47.3	27.7	29.7	33.4	11.7	7.9	19.1
Gv/N	12.720	2.834	0.803	7.865	4.609	4.935	5.544	1.947	1.305	3.175
Prob	0.00000	0.00069	0.18449	0.00000	0.00000	0.00000	0.00000	0.00841	0.04909	0.00026

	31	32	33	34	35	36	37	38	39	40
a_O	115	125	65	120	129	63	113	133	70	106
	(19.1)	(20.8)	(10.8)	(19.9)	(21.4)	(10.5)	(18.8)	(22.1)	(11.6)	(17.6)
a_E	114.5	146.5	53.1	114.5	146.5	53.1	114.5	146.5	53.1	114.5
	(19.0)	(24.3)	(8.8)	(19.0)	(24.3)	(8.8)	(19.0)	(24.3)	(8.8)	(19.0)
	1.0	-39.7	26.3	11.3	-32.8	21.6	-3.0	-25.7	38.7	-16.4
	0.166	-6.588	4.372	1.870	-5.449	3.583	-0.496	-4.269	6.432	-2.717
t_O	77	193	89	82	182	93	99	164	82	115
	(12.8)	(32.1)	(14.8)	(13.6)	(30.2)	(15.4)	(16.4)	(27.2)	(13.6)	(19.1)
t_E	75.5	171.7	61.4	75.5	171.7	61.4	75.5	171.7	61.4	75.5
	(12.5)	(28.5)	(10.2)	(12.5)	(28.5)	(10.2)	(12.5)	(28.5)	(10.2)	(12.5)
	3.0	45.2	66.2	13.5	21.2	77.3	53.6	-15.0	47.6	96.7

	0.495	7.502	10.995	2.241	3.526	12.848	8.902	-2.497	7.899	16.065
g_O	220	124	182	211	134	190	201	112	178	189
	(36.5)	(20.6)	(30.2)	(35.0)	(22.3)	(31.6)	(33.4)	(18.6)	(29.6)	(31.4)
g_E	233.0	126.8	219.2	233.0	126.8	219.2	233.0	126.8	219.2	233.0
	(38.7)	(21.1)	(36.4)	(38.7)	(21.1)	(36.4)	(38.7)	(21.1)	(36.4)	(38.7)
	-25.3	-5.5	-67.8	-41.9	14.8	-54.4	-59.4	-27.8	-74.2	-79.1
	-4.200	-0.914	-11.257	-6.957	2.465	-9.036	-9.869	-4.613	-12.323	-13.145
c_O	190	160	266	189	157	256	189	193	272	192
	(31.6)	(26.6)	(44.2)	(31.4)	(26.1)	(42.5)	(31.4)	(32.1)	(45.2)	(31.9)
c_E	179.0	157.0	268.3	179.0	157.0	268.3	179.0	157.0	268.3	179.0
	(29.7)	(26.1)	(44.6)	(29.7)	(26.1)	(44.6)	(29.7)	(26.1)	(44.6)	(29.7)
	22.7	6.0	-4.6	20.6	-0.1	-24.0	20.6	79.6	7.4	27.0
	3.779	0.994	-0.764	3.427	-0.012	-3.995	3.427	13.222	1.234	4.486
N	602	602	602	602	602	602	602	602	602	602
X2	1.4	5.9	21.5	3.5	3.1	22.6	12.3	11.5	20.1	30.5
Gvalue	1.4	6.0	20.1	3.5	3.2	20.5	11.8	11.1	19.5	28.2
Gv/N	0.239	0.993	3.346	0.582	0.529	3.400	1.965	1.843	3.241	4.689
Prob	0.69643	0.11270	0.00016	0.32059	0.36372	0.00014	0.00800	0.01121	0.00021	0.00000

	41	42	43	44	45	46	47	48	49	50
a_O	115	53	115	119	60	96	118	64	122	120
	(19.1)	(8.8)	(19.1)	(19.8)	(10.0)	(15.9)	(19.6)	(10.6)	(20.3)	(19.9)
a_E	146.5	53.1	114.5	146.5	53.1	114.5	146.5	53.1	114.5	146.5
	(24.3)	(8.8)	(19.0)	(24.3)	(8.8)	(19.0)	(24.3)	(8.8)	(19.0)	(24.3)
	-55.7	-0.2	1.0	-49.5	14.7	-33.8	-51.0	23.9	15.5	-47.9
	-9.247	-0.029	0.166	-8.216	2.440	-5.621	-8.478	3.975	2.571	-7.952
t_O	185	87	76	187	83	92	170	78	80	188
	(30.7)	(14.5)	(12.6)	(31.1)	(13.8)	(15.3)	(28.2)	(13.0)	(13.3)	(31.2)
t_E	171.7	61.4	75.5	171.7	61.4	75.5	171.7	61.4	75.5	171.7
	(28.5)	(10.2)	(12.5)	(28.5)	(10.2)	(12.5)	(28.5)	(10.2)	(12.5)	(28.5)
	27.6	60.7	1.0	31.9	50.1	36.3	-3.4	37.4	9.2	34.1
	4.589	10.091	0.158	5.307	8.329	6.031	-0.559	6.218	1.530	5.668
g_O	119	210	206	134	181	216	128	201	216	124
	(19.8)	(34.9)	(34.2)	(22.3)	(30.1)	(35.9)	(21.3)	(33.4)	(35.9)	(20.6)
g_E	126.8	219.2	233.0	126.8	219.2	233.0	126.8	219.2	233.0	126.8
	(21.1)	(36.4)	(38.7)	(21.1)	(36.4)	(38.7)	(21.1)	(36.4)	(38.7)	(21.1)
	-15.1	-18.1	-50.8	14.8	-69.4	-32.8	2.4	-34.9	-32.8	-5.5
	-2.505	-3.005	-8.433	2.465	-11.526	-5.441	0.406	-5.801	-5.441	-0.914
c_O	183	252	205	162	278	198	186	259	184	170
	(30.4)	(41.9)	(34.1)	(26.9)	(46.2)	(32.9)	(30.9)	(43.0)	(30.6)	(28.2)
c_E	157.0	268.3	179.0	157.0	268.3	179.0	157.0	268.3	179.0	157.0
	(26.1)	(44.6)	(29.7)	(26.1)	(44.6)	(29.7)	(26.1)	(44.6)	(29.7)	(26.1)
	56.0	-31.6	55.7	10.1	19.7	40.0	63.0	-18.3	10.2	27.0
	9.302	-5.251	9.252	1.675	3.276	6.651	10.460	-3.039	1.698	4.480
N	602	602	602	602	602	602	602	602	602	602
X2	12.6	12.1	6.9	7.1	15.6	9.9	10.9	8.6	2.1	7.5
Gvalue	12.9	10.9	6.9	7.4	15.2	9.8	11.0	8.1	2.2	7.7
Gv/N	2.140	1.807	1.143	1.229	2.520	1.620	1.829	1.353	0.358	1.281
Prob	0.00490	0.01240	0.07573	0.06018	0.00168	0.02078	0.01168	0.04312	0.54086	0.05231

	51	52	53	54	55	56	57	58	59	60
a_O	60	129	112	68	120	118	57	103	121	58
	(10.0)	(21.4)	(18.6)	(11.3)	(19.9)	(19.6)	(9.5)	(17.1)	(20.1)	(9.6)
a_E	53.1	114.5	146.5	53.1	114.5	146.5	53.1	114.5	146.5	53.1
	(8.8)	(19.0)	(24.3)	(8.8)	(19.0)	(24.3)	(8.8)	(19.0)	(24.3)	(8.8)
	14.7	30.8	-60.1	33.7	11.3	-51.0	8.1	-21.8	-46.3	10.3
	2.440	5.110	-9.989	5.593	1.870	-8.478	1.347	-3.622	-7.685	1.706
t_O	81	73	162	93	76	201	86	95	189	79
	(13.5)	(12.1)	(26.9)	(15.4)	(12.6)	(33.4)	(14.3)	(15.8)	(31.4)	(13.1)
t_E	61.4	75.5	171.7	61.4	75.5	171.7	61.4	75.5	171.7	61.4
	(10.2)	(12.5)	(28.5)	(10.2)	(12.5)	(28.5)	(10.2)	(12.5)	(28.5)	(10.2)
	45.0	-5.0	-18.8	77.3	1.0	63.4	58.1	43.6	36.3	39.9
	7.472	-0.825	-3.127	12.848	0.158	10.525	9.645	7.241	6.031	6.632
g_O	203	209	158	204	190	129	208	240	126	210
	(33.7)	(34.7)	(26.2)	(33.9)	(31.6)	(21.4)	(34.6)	(39.9)	(20.9)	(34.9)
g_E	219.2	233.0	126.8	219.2	233.0	126.8	219.2	233.0	126.8	219.2
	(36.4)	(38.7)	(21.1)	(36.4)	(38.7)	(21.1)	(36.4)	(38.7)	(21.1)	(36.4)
	-31.2	-45.5	69.6	-29.4	-77.5	4.5	-21.9	14.2	-1.6	-18.1
	-5.191	-7.552	11.554	-4.883	-12.882	0.743	-3.637	2.356	-0.259	-3.005
c_O	258	191	170	237	216	154	251	164	166	255
	(42.9)	(31.7)	(28.2)	(39.4)	(35.9)	(25.6)	(41.7)	(27.2)	(27.6)	(42.4)
c_E	268.3	179.0	157.0	268.3	179.0	157.0	268.3	179.0	157.0	268.3
	(44.6)	(29.7)	(26.1)	(44.6)	(29.7)	(26.1)	(44.6)	(29.7)	(26.1)	(44.6)
	-20.2	24.9	27.0	-58.8	81.3	-6.0	-33.5	-28.6	18.4	-26.0
	-3.359	4.132	4.480	-9.770	13.499	-0.999	-5.562	-4.756	3.061	-4.311
N	602	602	602	602	602	602	602	602	602	602
X2	8.8	5.2	17.4	25.2	15.9	10.6	11.9	7.6	6.7	6.6
Gvalue	8.2	5.2	17.6	22.8	15.9	10.8	10.8	7.3	6.9	6.2
Gv/N	1.363	0.865	2.918	3.787	2.646	1.790	1.793	1.217	1.148	1.022
Prob	0.04197	0.15738	0.00054	0.00004	0.00117	0.01299	0.01289	0.06212	0.07470	0.10442

	61	62	63	64	65	66	67	68	69	70
a_O	96	133	60	91	116	52	103	131	59	117
	(15.9)	(22.1)	(10.0)	(15.1)	(19.3)	(8.6)	(17.1)	(21.8)	(9.8)	(19.4)
a_E	114.5	146.5	53.1	114.5	146.5	53.1	114.5	146.5	53.1	114.5
	(19.0)	(24.3)	(8.8)	(19.0)	(24.3)	(8.8)	(19.0)	(24.3)	(8.8)	(19.0)
	-33.8	-25.7	14.7	-41.8	-54.1	-2.2	-21.8	-29.3	12.5	5.1
	-5.621	-4.269	2.440	-6.945	-8.993	-0.357	-3.622	-4.864	2.070	0.839
t_O	76	167	92	89	172	81	75	170	77	89
	(12.6)	(27.7)	(15.3)	(14.8)	(28.6)	(13.5)	(12.5)	(28.2)	(12.8)	(14.8)
t_E	75.5	171.7	61.4	75.5	171.7	61.4	75.5	171.7	61.4	75.5
	(12.5)	(28.5)	(10.2)	(12.5)	(28.5)	(10.2)	(12.5)	(28.5)	(10.2)	(12.5)
	1.0	-9.3	74.5	29.2	0.6	45.0	-1.0	-3.4	35.0	29.2
	0.158	-1.537	12.379	4.854	0.103	7.472	-0.174	-0.559	5.808	4.854
g_O	225	123	192	232	124	221	218	138	217	222
	(37.4)	(20.4)	(31.9)	(38.5)	(20.6)	(36.7)	(36.2)	(22.9)	(36.0)	(36.9)
g_E	233.0	126.8	219.2	233.0	126.8	219.2	233.0	126.8	219.2	233.0
	(38.7)	(21.1)	(36.4)	(38.7)	(21.1)	(36.4)	(38.7)	(21.1)	(36.4)	(38.7)
	-15.7	-7.5	-50.9	-2.0	-5.5	3.5	-29.0	23.4	-4.5	-21.5
	-2.616	-1.238	-8.463	-0.336	-0.914	0.587	-4.824	3.887	-0.741	-3.571
c_O	205	179	258	190	190	248	206	163	249	174
	(34.1)	(29.7)	(42.9)	(31.6)	(31.6)	(41.2)	(34.2)	(27.1)	(41.4)	(28.9)
c_E	179.0	157.0	268.3	179.0	157.0	268.3	179.0	157.0	268.3	179.0
	(29.7)	(26.1)	(44.6)	(29.7)	(26.1)	(44.6)	(29.7)	(26.1)	(44.6)	(29.7)
	55.7	46.9	-20.2	22.7	72.4	-39.0	58.0	12.1	-37.2	-9.8
	9.252	7.785	-3.359	3.779	12.028	-6.486	9.630	2.018	-6.179	-1.625

N	602	602	602	602	602	602	602	602	602	602
X2	7.1	4.6	20.0	7.9	13.3	7.9	6.2	2.9	6.1	3.1
Gvalue	7.1	4.5	18.0	8.1	13.4	7.3	6.1	2.9	5.8	3.0
Gv/N	1.173	0.741	2.997	1.352	2.223	1.216	1.010	0.482	0.958	0.497
Prob	0.06988	0.21560	0.00043	0.04326	0.00388	0.06238	0.10766	0.40675	0.12347	0.39244

	71	72	73	74	75	76	77	78	79	80
a_O	121	49	115	151	59	99	130	56	118	119
	(20.1)	(8.1)	(19.1)	(25.1)	(9.8)	(16.4)	(21.6)	(9.3)	(19.6)	(19.8)
a_E	146.5	53.1	114.5	146.5	53.1	114.5	146.5	53.1	114.5	146.5
	(24.3)	(8.8)	(19.0)	(24.3)	(8.8)	(19.0)	(24.3)	(8.8)	(19.0)	(24.3)
	-46.3	-7.9	1.0	9.2	12.5	-28.8	-31.0	6.0	7.1	-49.5
	-7.685	-1.304	0.166	1.521	2.070	-4.784	-5.158	0.994	1.180	-8.216
t_O	176	84	91	165	85	96	176	75	61	187
	(29.2)	(14.0)	(15.1)	(27.4)	(14.1)	(15.9)	(29.2)	(12.5)	(10.1)	(31.1)
t_E	171.7	61.4	75.5	171.7	61.4	75.5	171.7	61.4	75.5	171.7
	(28.5)	(10.2)	(12.5)	(28.5)	(10.2)	(12.5)	(28.5)	(10.2)	(12.5)	(28.5)
	8.7	52.8	33.9	-13.1	55.4	46.1	8.7	30.1	-26.1	31.9
	1.450	8.764	5.635	-2.179	9.203	7.651	1.450	5.001	-4.328	5.307
g_O	137	212	206	136	192	221	121	207	230	127
	(22.8)	(35.2)	(34.2)	(22.6)	(31.9)	(36.7)	(20.1)	(34.4)	(38.2)	(21.1)
g_E	126.8	219.2	233.0	126.8	219.2	233.0	126.8	219.2	233.0	126.8
	(21.1)	(36.4)	(38.7)	(21.1)	(36.4)	(38.7)	(21.1)	(36.4)	(38.7)	(21.1)
	21.2	-14.2	-50.8	19.1	-50.9	-23.4	-11.3	-23.8	-6.0	0.4
	3.528	-2.366	-8.433	3.171	-8.463	-3.886	-1.877	-3.951	-0.995	0.072
c_O	168	257	190	150	266	186	175	264	193	169
	(27.9)	(42.7)	(31.6)	(24.9)	(44.2)	(30.9)	(29.1)	(43.9)	(32.1)	(28.1)
c_E	157.0	268.3	179.0	157.0	268.3	179.0	157.0	268.3	179.0	157.0
	(26.1)	(44.6)	(29.7)	(26.1)	(44.6)	(29.7)	(26.1)	(44.6)	(29.7)	(26.1)
	22.7	-22.1	22.7	-13.8	-4.6	14.4	37.9	-8.6	29.2	24.8
	3.766	-3.677	3.779	-2.285	-0.764	2.384	6.297	-1.421	4.843	4.122
N	602	602	602	602	602	602	602	602	602	602
X2	6.1	9.4	7.0	1.4	13.2	8.5	4.3	3.9	4.0	7.4
Gvalue	6.4	8.5	6.9	1.4	12.3	8.2	4.3	3.8	4.2	7.7
Gv/N	1.059	1.417	1.147	0.229	2.045	1.364	0.712	0.623	0.699	1.284
Prob	0.09473	0.03624	0.07502	0.71112	0.00639	0.04183	0.23223	0.28948	0.23960	0.05191

	81	82	83	84	85	86	87	88	89	90
a_O	70	130	124	59	101	125	63	107	143	68
	(11.6)	(21.6)	(20.6)	(9.8)	(16.8)	(20.8)	(10.5)	(17.8)	(23.8)	(11.3)
a_E	53.1	114.5	146.5	53.1	114.5	146.5	53.1	114.5	146.5	53.1
	(8.8)	(19.0)	(24.3)	(8.8)	(19.0)	(24.3)	(8.8)	(19.0)	(24.3)	(8.8)
	38.7	33.0	-41.3	12.5	-25.3	-39.7	21.6	-14.5	-6.9	33.7
	6.432	5.483	-6.866	2.070	-4.210	-6.588	3.583	-2.409	-1.145	5.593
t_O	83	90	177	74	83	174	69	75	175	84
	(13.8)	(15.0)	(29.4)	(12.3)	(13.8)	(28.9)	(11.5)	(12.5)	(29.1)	(14.0)
t_E	61.4	75.5	171.7	61.4	75.5	171.7	61.4	75.5	171.7	61.4
	(10.2)	(12.5)	(28.5)	(10.2)	(12.5)	(28.5)	(10.2)	(12.5)	(28.5)	(10.2)
	50.1	31.6	10.8	27.7	15.7	4.6	16.2	-1.0	6.7	52.8
	8.329	5.243	1.791	4.605	2.603	0.772	2.690	-0.174	1.110	8.764
g_O	207	194	128	218	228	140	215	238	107	222
	(34.4)	(32.2)	(21.3)	(36.2)	(37.9)	(23.3)	(35.7)	(39.5)	(17.8)	(36.9)
g_E	219.2	233.0	126.8	219.2	233.0	126.8	219.2	233.0	126.8	219.2
	(36.4)	(38.7)	(21.1)	(36.4)	(38.7)	(21.1)	(36.4)	(38.7)	(21.1)	(36.4)
	-23.8	-71.1	2.4	-2.5	-9.9	27.8	-8.4	10.1	-36.3	5.6
	-3.951	-11.810	0.406	-0.411	-1.648	4.612	-1.395	1.674	-6.031	0.922
c_O	242	188	173	251	190	163	255	182	177	228
	(40.2)	(31.2)	(28.7)	(41.7)	(31.6)	(27.1)	(42.4)	(30.2)	(29.4)	(37.9)
c_E	268.3	179.0	157.0	268.3	179.0	157.0	268.3	179.0	157.0	268.3
	(44.6)	(29.7)	(26.1)	(44.6)	(29.7)	(26.1)	(44.6)	(29.7)	(26.1)	(44.6)
	-50.0	18.5	33.5	-33.5	22.7	12.1	-26.0	6.1	42.4	-74.2
	-8.298	3.078	5.564	-5.562	3.779	2.018	-4.311	1.018	7.037	-12.332
N	602	602	602	602	602	602	602	602	602	602
X2	16.3	11.9	5.3	4.4	3.1	4.8	3.5	0.7	5.8	18.6
Gvalue	15.1	12.0	5.4	4.2	3.2	4.9	3.4	0.7	5.8	17.7
Gv/N	2.512	1.994	0.895	0.702	0.523	0.815	0.567	0.110	0.971	2.948
Prob	0.00171	0.00738	0.14545	0.23814	0.36889	0.17883	0.33214	0.88176	0.11940	0.00050

	91	92	93	94	95	96	97	98	99	100
a_O	99	125	60	112	130	61	105	112	44	100
	(16.4)	(20.8)	(10.0)	(18.6)	(21.6)	(10.1)	(17.4)	(18.6)	(7.3)	(16.6)
a_E	114.5	146.5	53.1	114.5	146.5	53.1	114.5	146.5	53.1	114.5
	(19.0)	(24.3)	(8.8)	(19.0)	(24.3)	(8.8)	(19.0)	(24.3)	(8.8)	(19.0)
	-28.8	-39.7	14.7	-4.9	-31.0	17.0	-18.2	-60.1	-16.5	-27.1
	-4.784	-6.588	2.440	-0.822	-5.158	2.816	-3.022	-9.989	-2.744	-4.499
t_O	83	179	71	98	180	68	90	187	79	71
	(13.8)	(29.7)	(11.8)	(16.3)	(29.9)	(11.3)	(15.0)	(31.1)	(13.1)	(11.8)
t_E	75.5	171.7	61.4	75.5	171.7	61.4	75.5	171.7	61.4	75.5
	(12.5)	(28.5)	(10.2)	(12.5)	(28.5)	(10.2)	(12.5)	(28.5)	(10.2)	(12.5)
	15.7	14.9	20.7	51.1	17.0	14.0	31.6	31.9	39.9	-8.8
	2.603	2.479	3.442	8.482	2.826	2.321	5.243	5.307	6.632	-1.457
g_O	225	140	220	230	129	220	215	138	209	229
	(37.4)	(23.3)	(36.5)	(38.2)	(21.4)	(36.5)	(35.7)	(22.9)	(34.7)	(38.0)
g_E	233.0	126.8	219.2	233.0	126.8	219.2	233.0	126.8	219.2	233.0
	(38.7)	(21.1)	(36.4)	(38.7)	(21.1)	(36.4)	(38.7)	(21.1)	(36.4)	(38.7)
	-15.7	27.8	1.5	-6.0	4.5	1.5	-34.6	23.4	-20.0	-8.0
	-2.616	4.612	0.253	-0.995	0.743	0.253	-5.747	3.887	-3.322	-1.322
c_O	195	158	251	162	163	253	192	165	270	202
	(32.4)	(26.2)	(41.7)	(26.9)	(27.1)	(42.0)	(31.9)	(27.4)	(44.9)	(33.6)
c_E	179.0	157.0	268.3	179.0	157.0	268.3	179.0	157.0	268.3	179.0
	(29.7)	(26.1)	(44.6)	(29.7)	(26.1)	(44.6)	(29.7)	(26.1)	(44.6)	(29.7)
	33.5	1.9	-33.5	-32.3	12.1	-29.7	27.0	16.3	3.4	48.9
	5.561	0.321	-5.562	-5.359	2.018	-4.939	4.486	2.711	0.563	8.127
N	602	602	602	602	602	602	602	602	602	602
X2	4.6	4.8	3.5	8.4	2.5	2.8	5.9	10.9	7.1	5.1
Gvalue	4.6	5.0	3.4	7.9	2.6	2.7	5.8	11.5	6.8	5.1
Gv/N	0.763	0.825	0.573	1.306	0.430	0.451	0.960	1.916	1.129	0.849
Prob	0.20421	0.17445	0.32751	0.04891	0.45961	0.43817	0.12275	0.00917	0.07877	0.16377

Babo_BIOVAR1_9941
5UTR:

	100	99	98	97	96	95	94	93	92	91
a_O	97	96	87	95	76	93	100	86	97	89
	(26.1)	(25.8)	(23.4)	(25.5)	(20.4)	(25.0)	(26.9)	(23.1)	(26.1)	(23.9)
a_E	91.9	91.9	91.9	91.9	91.9	91.9	91.9	91.9	91.9	91.9
	(24.7)	(24.7)	(24.7)	(24.7)	(24.7)	(24.7)	(24.7)	(24.7)	(24.7)	(24.7)
	10.4	8.3	-9.6	6.2	-28.9	2.1	16.8	-11.5	10.4	-5.8
	2.794	2.230	-2.583	1.672	-7.780	0.573	4.518	-3.088	2.794	-1.555
t_O	85	82	85	88	102	77	98	100	85	89
	(22.8)	(22.0)	(22.8)	(23.7)	(27.4)	(20.7)	(26.3)	(26.9)	(22.8)	(23.9)
t_E	92.1	92.1	92.1	92.1	92.1	92.1	92.1	92.1	92.1	92.1
	(24.7)	(24.7)	(24.7)	(24.7)	(24.7)	(24.7)	(24.7)	(24.7)	(24.7)	(24.7)
	-13.6	-19.0	-13.6	-7.9	20.9	-27.5	12.3	16.5	-13.6	-6.0
	-3.646	-5.102	-3.646	-2.134	5.623	-7.395	3.294	4.448	-3.646	-1.618
g_O	114	100	107	80	99	96	88	92	104	95
	(30.6)	(26.9)	(28.8)	(21.5)	(26.6)	(25.8)	(23.7)	(24.7)	(28.0)	(25.5)
g_E	91.2	91.2	91.2	91.2	91.2	91.2	91.2	91.2	91.2	91.2
	(24.5)	(24.5)	(24.5)	(24.5)	(24.5)	(24.5)	(24.5)	(24.5)	(24.5)	(24.5)
	50.9	18.4	34.2	-21.0	16.2	9.8	-6.3	1.6	27.3	7.7
	13.670	4.947	9.185	-5.640	4.362	2.642	-1.695	0.427	7.337	2.079
c_O	76	94	93	109	95	106	86	94	86	99
	(20.4)	(25.3)	(25.0)	(29.3)	(25.5)	(28.5)	(23.1)	(25.3)	(23.1)	(26.6)
c_E	96.8	96.8	96.8	96.8	96.8	96.8	96.8	96.8	96.8	96.8
	(26.0)	(26.0)	(26.0)	(26.0)	(26.0)	(26.0)	(26.0)	(26.0)	(26.0)	(26.0)
	-36.8	-5.5	-7.4	25.9	-3.5	19.3	-20.3	-5.5	-20.3	4.5
	-9.880	-1.478	-1.997	6.962	-0.953	5.180	-5.465	-1.478	-5.465	1.202
N	372	372	372	372	372	372	372	372	372	372
X2	11.0	2.2	3.7	3.2	4.5	3.6	2.4	1.2	3.8	0.4
Gvalue	10.9	2.2	3.6	3.2	4.7	3.7	2.4	1.1	3.8	0.4
Gv/N	2.937	0.597	0.958	0.860	1.252	1.000	0.652	0.308	1.020	0.108
Prob	0.01213	0.52787	0.31247	0.36184	0.19881	0.29339	0.48870	0.76592	0.28451	0.93956

	90	89	88	87	86	85	84	83	82	81
a_O	98	77	83	91	88	102	75	78	91	96
	(26.3)	(20.7)	(22.3)	(24.5)	(23.7)	(27.4)	(20.2)	(21.0)	(24.5)	(25.8)
a_E	91.9	91.9	91.9	91.9	91.9	91.9	91.9	91.9	91.9	91.9
	(24.7)	(24.7)	(24.7)	(24.7)	(24.7)	(24.7)	(24.7)	(24.7)	(24.7)	(24.7)
	12.5	-27.3	-17.0	-1.9	-7.7	21.2	-30.5	-25.7	-1.9	8.3
	3.363	-7.341	-4.565	-0.503	-2.072	5.694	-8.212	-6.895	-0.503	2.230
t_O	81	102	88	82	90	84	106	114	85	87
	(21.8)	(27.4)	(23.7)	(22.0)	(24.2)	(22.6)	(28.5)	(30.6)	(22.8)	(23.4)
t_E	92.1	92.1	92.1	92.1	92.1	92.1	92.1	92.1	92.1	92.1
	(24.7)	(24.7)	(24.7)	(24.7)	(24.7)	(24.7)	(24.7)	(24.7)	(24.7)	(24.7)
	-20.7	20.9	-7.9	-19.0	-4.1	-15.4	29.9	48.7	-13.6	-9.8
	-5.574	5.623	-2.134	-5.102	-1.095	-4.138	8.035	13.101	-3.646	-2.644
g_O	94	93	102	104	96	87	95	102	107	90
	(25.3)	(25.0)	(27.4)	(28.0)	(25.8)	(23.4)	(25.5)	(27.4)	(28.8)	(24.2)
g_E	91.2	91.2	91.2	91.2	91.2	91.2	91.2	91.2	91.2	91.2
	(24.5)	(24.5)	(24.5)	(24.5)	(24.5)	(24.5)	(24.5)	(24.5)	(24.5)	(24.5)
	5.7	3.6	22.8	27.3	9.8	-8.2	7.7	22.8	34.2	-2.4
	1.523	0.972	6.131	7.337	2.642	-2.210	2.079	6.131	9.185	-0.646
c_O	99	100	99	95	98	99	96	78	89	99
	(26.6)	(26.9)	(26.6)	(25.5)	(26.3)	(26.6)	(25.8)	(21.0)	(23.9)	(26.6)
c_E	96.8	96.8	96.8	96.8	96.8	96.8	96.8	96.8	96.8	96.8
	(26.0)	(26.0)	(26.0)	(26.0)	(26.0)	(26.0)	(26.0)	(26.0)	(26.0)	(26.0)
	4.5	6.5	4.5	-3.5	2.4	4.5	-1.6	-33.7	-14.9	4.5
	1.202	1.754	1.202	-0.953	0.655	1.202	-0.423	-9.051	-4.015	1.202
N	372	372	372	372	372	372	372	372	372	372
X2	1.9	3.6	2.4	2.9	0.5	2.1	5.4	12.3	3.9	0.5
Gvalue	1.9	3.7	2.4	2.9	0.5	2.0	5.5	12.2	3.8	0.5
Gv/N	0.514	1.007	0.634	0.780	0.129	0.548	1.480	3.286	1.021	0.141
Prob	0.59111	0.29001	0.50119	0.40734	0.92325	0.56469	0.13826	0.00665	0.28410	0.91308

	80	79	78	77	76	75	74	73	72	71
a_O	96	87	91	95	92	102	71	93	97	95
	(25.8)	(23.4)	(24.5)	(25.5)	(24.7)	(27.4)	(19.1)	(25.0)	(26.1)	(25.5)
a_E	91.9	91.9	91.9	91.9	91.9	91.9	91.9	91.9	91.9	91.9
	(24.7)	(24.7)	(24.7)	(24.7)	(24.7)	(24.7)	(24.7)	(24.7)	(24.7)	(24.7)
	8.3	-9.6	-1.9	6.2	0.1	21.2	-36.7	2.1	10.4	6.2
	2.230	-2.583	-0.503	1.672	0.032	5.694	-9.866	0.573	2.794	1.672
t_O	78	80	96	92	79	81	82	95	92	109
	(21.0)	(21.5)	(25.8)	(24.7)	(21.2)	(21.8)	(22.0)	(25.5)	(24.7)	(29.3)
t_E	92.1	92.1	92.1	92.1	92.1	92.1	92.1	92.1	92.1	92.1
	(24.7)	(24.7)	(24.7)	(24.7)	(24.7)	(24.7)	(24.7)	(24.7)	(24.7)	(24.7)
	-25.9	-22.5	8.0	-0.1	-24.2	-20.7	-19.0	6.0	-0.1	36.8
	-6.950	-6.039	2.163	-0.032	-6.498	-5.574	-5.102	1.606	-0.032	9.898
g_O	96	104	91	101	89	95	113	95	88	81
	(25.8)	(28.0)	(24.5)	(27.2)	(23.9)	(25.5)	(30.4)	(25.5)	(23.7)	(21.8)
g_E	91.2	91.2	91.2	91.2	91.2	91.2	91.2	91.2	91.2	91.2
	(24.5)	(24.5)	(24.5)	(24.5)	(24.5)	(24.5)	(24.5)	(24.5)	(24.5)	(24.5)
	9.8	27.3	-0.4	20.6	-4.4	7.7	48.4	7.7	-6.3	-19.2
	2.642	7.337	-0.113	5.536	-1.174	2.079	13.015	2.079	-1.695	-5.170
c_O	102	101	94	84	112	94	106	89	95	87
	(27.4)	(27.2)	(25.3)	(22.6)	(30.1)	(25.3)	(28.5)	(23.9)	(25.5)	(23.4)
c_E	96.8	96.8	96.8	96.8	96.8	96.8	96.8	96.8	96.8	96.8
	(26.0)	(26.0)	(26.0)	(26.0)	(26.0)	(26.0)	(26.0)	(26.0)	(26.0)	(26.0)
	10.7	8.6	-5.5	-23.8	32.7	-5.5	19.3	-14.9	-3.5	-18.6
	2.875	2.312	-1.478	-6.401	8.789	-1.478	5.180	-4.015	-0.953	-4.988
N	372	372	372	372	372	372	372	372	372	372
X2	2.9	3.8	0.3	2.8	4.3	2.7	12.0	0.9	0.4	5.4
Gvalue	3.0	3.8	0.3	2.9	4.3	2.7	12.0	0.9	0.4	5.3
Gv/N	0.797	1.027	0.069	0.776	1.149	0.722	3.227	0.243	0.113	1.413
Prob	0.39700	0.28160	0.96779	0.40958	0.23325	0.44285	0.00737	0.82424	0.93583	0.15398

	70	69	68	67	66	65	64	63	62	61
a_O	93	88	82	88	87	102	93	94	84	95
	(25.0)	(23.7)	(22.0)	(23.7)	(23.4)	(27.4)	(25.0)	(25.3)	(22.6)	(25.5)
a_E	91.9	91.9	91.9	91.9	91.9	91.9	91.9	91.9	91.9	91.9
	(24.7)	(24.7)	(24.7)	(24.7)	(24.7)	(24.7)	(24.7)	(24.7)	(24.7)	(24.7)
	2.1	-7.7	-18.8	-7.7	-9.6	21.2	2.1	4.2	-15.2	6.2
	0.573	-2.072	-5.044	-2.072	-2.583	5.694	0.573	1.120	-4.079	1.672
t_O	108	95	81	94	89	76	95	90	86	79
	(29.0)	(25.5)	(21.8)	(25.3)	(23.9)	(20.4)	(25.5)	(24.2)	(23.1)	(21.2)

t_E	92.1	92.1	92.1	92.1	92.1	92.1	92.1	92.1	92.1	92.1
	(24.7)	(24.7)	(24.7)	(24.7)	(24.7)	(24.7)	(24.7)	(24.7)	(24.7)	(24.7)
	34.5	6.0	-20.7	3.9	-6.0	-29.1	6.0	-4.1	-11.7	-24.2
	9.272	1.606	-5.574	1.054	-1.618	-7.833	1.606	-1.095	-3.148	-6.498
g_O	90	93	102	83	100	89	96	104	102	97
	(24.2)	(25.0)	(27.4)	(22.3)	(26.9)	(23.9)	(25.8)	(28.0)	(27.4)	(26.1)
g_E	91.2	91.2	91.2	91.2	91.2	91.2	91.2	91.2	91.2	91.2
	(24.5)	(24.5)	(24.5)	(24.5)	(24.5)	(24.5)	(24.5)	(24.5)	(24.5)	(24.5)
	-2.4	3.6	22.8	-15.7	18.4	-4.4	9.8	27.3	22.8	11.9
	-0.646	0.972	6.131	-4.209	4.947	-1.174	2.642	7.337	6.131	3.210
c_O	81	96	107	107	96	105	88	84	100	101
	(21.8)	(25.8)	(28.8)	(28.8)	(25.8)	(28.2)	(23.7)	(22.6)	(26.9)	(27.2)
c_E	96.8	96.8	96.8	96.8	96.8	96.8	96.8	96.8	96.8	96.8
	(26.0)	(26.0)	(26.0)	(26.0)	(26.0)	(26.0)	(26.0)	(26.0)	(26.0)	(26.0)
	-28.9	-1.6	21.5	21.5	-1.6	17.1	-16.8	-23.8	6.5	8.6
	-7.756	-0.423	5.769	5.769	-0.423	4.596	-4.504	-6.401	1.754	2.312
N	372	372	372	372	372	372	372	372	372	372
X2	5.4	0.3	4.8	2.0	1.2	4.7	1.2	3.6	2.5	2.5
Gvalue	5.4	0.3	4.8	2.0	1.2	4.8	1.2	3.6	2.4	2.6
Gv/N	1.444	0.082	1.283	0.542	0.323	1.284	0.316	0.962	0.658	0.696
Prob	0.14661	0.95897	0.18936	0.56927	0.75282	0.18906	0.75878	0.31088	0.48467	0.45952

	60	59	58	57	56	55	54	53	52	51
a_O	91	78	78	84	75	114	87	80	100	81
	(24.5)	(21.0)	(21.0)	(22.6)	(20.2)	(30.6)	(23.4)	(21.5)	(26.9)	(21.8)
a_E	91.9	91.9	91.9	91.9	91.9	91.9	91.9	91.9	91.9	91.9
	(24.7)	(24.7)	(24.7)	(24.7)	(24.7)	(24.7)	(24.7)	(24.7)	(24.7)	(24.7)
	-1.9	-25.7	-25.7	-15.2	-30.5	49.0	-9.6	-22.3	16.8	-20.5
	-0.503	-6.895	-6.895	-4.079	-8.212	13.181	-2.583	-5.983	4.518	-5.517
t_O	91	114	111	88	72	64	108	92	96	97
	(24.5)	(30.6)	(29.8)	(23.7)	(19.4)	(17.2)	(29.0)	(24.7)	(25.8)	(26.1)
t_E	92.1	92.1	92.1	92.1	92.1	92.1	92.1	92.1	92.1	92.1
	(24.7)	(24.7)	(24.7)	(24.7)	(24.7)	(24.7)	(24.7)	(24.7)	(24.7)	(24.7)
	-2.1	48.7	41.5	-7.9	-35.4	-46.5	34.5	-0.1	8.0	10.1
	-0.567	13.101	11.165	-2.134	-9.514	-12.510	9.272	-0.032	2.163	2.726
g_O	89	86	103	101	104	91	84	91	91	96
	(23.9)	(23.1)	(27.7)	(27.2)	(28.0)	(24.5)	(22.6)	(24.5)	(24.5)	(25.8)
g_E	91.2	91.2	91.2	91.2	91.2	91.2	91.2	91.2	91.2	91.2
	(24.5)	(24.5)	(24.5)	(24.5)	(24.5)	(24.5)	(24.5)	(24.5)	(24.5)	(24.5)
	-4.4	-10.1	25.0	20.6	27.3	-0.4	-13.8	-0.4	-0.4	9.8
	-1.174	-2.720	6.732	5.536	7.337	-0.113	-3.719	-0.113	-0.113	2.642
c_O	101	94	80	99	121	103	93	109	85	98
	(27.2)	(25.3)	(21.5)	(26.6)	(32.5)	(27.7)	(25.0)	(29.3)	(22.8)	(26.3)
c_E	96.8	96.8	96.8	96.8	96.8	96.8	96.8	96.8	96.8	96.8
	(26.0)	(26.0)	(26.0)	(26.0)	(26.0)	(26.0)	(26.0)	(26.0)	(26.0)	(26.0)
	8.6	-5.5	-30.5	4.5	54.0	12.8	-7.4	25.9	-22.1	2.4
	2.312	-1.478	-8.194	1.202	14.523	3.444	-1.997	6.962	-5.936	0.655
N	372	372	372	372	372	372	372	372	372	372
X2	0.3	7.7	10.4	2.0	15.3	14.2	3.7	3.1	2.3	1.8
Gvalue	0.3	7.5	10.4	2.0	15.4	14.9	3.6	3.1	2.4	1.9
Gv/N	0.069	2.008	2.807	0.525	4.135	4.003	0.973	0.834	0.632	0.505
Prob	0.96801	0.05831	0.01515	0.58216	0.00152	0.00191	0.30560	0.37612	0.50266	0.59779

	50	49	48	47	46	45	44	43	42	41
a_O	89	100	85	93	96	104	88	86	72	75
	(23.9)	(26.9)	(22.8)	(25.0)	(25.8)	(28.0)	(23.7)	(23.1)	(19.4)	(20.2)
a_E	91.9	91.9	91.9	91.9	91.9	91.9	91.9	91.9	91.9	91.9
	(24.7)	(24.7)	(24.7)	(24.7)	(24.7)	(24.7)	(24.7)	(24.7)	(24.7)	(24.7)
	-5.8	16.8	-13.3	2.1	8.3	25.6	-7.7	-11.5	-35.2	-30.5
	-1.555	4.518	-3.587	0.573	2.230	6.892	-2.072	-3.088	-9.463	-8.212
t_O	85	73	92	92	92	98	95	97	90	101
	(22.8)	(19.6)	(24.7)	(24.7)	(24.7)	(26.3)	(25.5)	(26.1)	(24.2)	(27.2)
t_E	92.1	92.1	92.1	92.1	92.1	92.1	92.1	92.1	92.1	92.1
	(24.7)	(24.7)	(24.7)	(24.7)	(24.7)	(24.7)	(24.7)	(24.7)	(24.7)	(24.7)
	-13.6	-33.9	-0.1	-0.1	-0.1	12.3	6.0	10.1	-4.1	18.7
	-3.646	-9.105	-0.032	-0.032	-0.032	3.294	1.606	2.726	-1.095	5.033
g_O	100	97	99	95	92	98	108	87	110	101
	(26.9)	(26.1)	(26.6)	(25.5)	(24.7)	(26.3)	(29.0)	(23.4)	(29.6)	(27.2)
g_E	91.2	91.2	91.2	91.2	91.2	91.2	91.2	91.2	91.2	91.2
	(24.5)	(24.5)	(24.5)	(24.5)	(24.5)	(24.5)	(24.5)	(24.5)	(24.5)	(24.5)
	18.4	11.9	16.2	7.7	1.6	14.1	36.5	-8.2	41.2	20.6
	4.947	3.210	4.362	2.079	0.427	3.783	9.811	-2.210	11.078	5.536
c_O	98	102	96	92	92	72	81	102	100	95
	(26.3)	(27.4)	(25.8)	(24.7)	(24.7)	(19.4)	(21.8)	(27.4)	(26.9)	(25.5)
c_E	96.8	96.8	96.8	96.8	96.8	96.8	96.8	96.8	96.8	96.8
	(26.0)	(26.0)	(26.0)	(26.0)	(26.0)	(26.0)	(26.0)	(26.0)	(26.0)	(26.0)
	2.4	10.7	-1.6	-9.3	-9.3	-42.6	-28.9	10.7	6.5	-3.5
	0.655	2.875	-0.423	-2.510	-2.510	-11.453	-7.756	2.875	1.754	-0.953
N	372	372	372	372	372	372	372	372	372	372
X2	1.5	5.3	1.2	0.4	0.4	8.8	5.9	1.1	8.3	5.1
Gvalue	1.5	5.6	1.2	0.4	0.4	9.4	5.9	1.1	8.5	5.2
Gv/N	0.400	1.498	0.320	0.110	0.114	2.516	1.589	0.303	2.274	1.404
Prob	0.68526	0.13433	0.75511	0.93846	0.93511	0.02488	0.11609	0.77087	0.03744	0.15621

	40	39	38	37	36	35	34	33	32	31
a_O	101	95	95	94	81	88	89	68	87	114
	(27.2)	(25.5)	(25.5)	(25.3)	(21.8)	(23.7)	(23.9)	(18.3)	(23.4)	(30.6)
a_E	91.9	91.9	91.9	91.9	91.9	91.9	91.9	91.9	91.9	91.9
	(24.7)	(24.7)	(24.7)	(24.7)	(24.7)	(24.7)	(24.7)	(24.7)	(24.7)	(24.7)
	19.0	6.2	6.2	4.2	-20.5	-7.7	-5.8	-41.0	-9.6	49.0
	5.103	1.672	1.672	1.120	-5.517	-2.072	-1.555	-11.027	-2.583	13.181
t_O	87	94	95	103	100	119	116	115	93	77
	(23.4)	(25.3)	(25.5)	(27.7)	(26.9)	(32.0)	(31.2)	(30.9)	(25.0)	(20.7)
t_E	92.1	92.1	92.1	92.1	92.1	92.1	92.1	92.1	92.1	92.1
	(24.7)	(24.7)	(24.7)	(24.7)	(24.7)	(24.7)	(24.7)	(24.7)	(24.7)	(24.7)
	-9.8	3.9	6.0	23.1	16.5	61.1	53.6	51.2	1.9	-27.5
	-2.644	1.054	1.606	6.218	4.448	16.422	14.416	13.756	0.508	-7.395
g_O	94	86	95	90	99	73	83	105	102	83
	(25.3)	(23.1)	(25.5)	(24.2)	(26.6)	(19.6)	(22.3)	(28.2)	(27.4)	(22.3)
g_E	91.2	91.2	91.2	91.2	91.2	91.2	91.2	91.2	91.2	91.2
	(24.5)	(24.5)	(24.5)	(24.5)	(24.5)	(24.5)	(24.5)	(24.5)	(24.5)	(24.5)
	5.7	-10.1	7.7	-2.4	16.2	-32.5	-15.7	29.6	22.8	-15.7
	1.523	-2.720	2.079	-0.646	4.362	-8.741	-4.209	7.948	6.131	-4.209
c_O	90	97	87	85	92	92	84	84	90	98
	(24.2)	(26.1)	(23.4)	(22.8)	(24.7)	(24.7)	(22.6)	(22.6)	(24.2)	(26.3)
c_E	96.8	96.8	96.8	96.8	96.8	96.8	96.8	96.8	96.8	96.8

```
        ( 26.0) ( 26.0) ( 26.0) ( 26.0) ( 26.0) ( 26.0) ( 26.0) ( 26.0) ( 26.0) ( 26.0)
          -13.1     0.4   -18.6   -22.1    -9.3    -9.3   -23.8   -23.8   -13.1     2.4
         -3.519   0.113  -4.988  -5.936  -2.510  -2.510  -6.401  -6.401  -3.519   0.655

N           372     372     372     372     372     372     372     372     372     372
X2          1.7     0.4     1.3     2.8     2.9    11.9     8.7    15.7     2.0     8.5
Gvalue      1.7     0.4     1.4     2.8     2.9    11.5     8.4    15.9     2.0     8.3
Gv/N      0.463   0.120   0.369   0.756   0.782   3.099   2.251   4.277   0.537   2.232
Prob    0.63237 0.93075 0.71155 0.42159 0.40558 0.00919 0.03889 0.00118 0.57310 0.04017
        -------------------------------------------------------------------------------
             30      29      28      27      26      25      24      23      22      21
        -------------------------------------------------------------------------------
a_O         100      97      95      79      96      95      89      87      95      78
        ( 26.9) ( 26.1) ( 25.5) ( 21.2) ( 25.8) ( 25.5) ( 23.9) ( 23.4) ( 25.5) ( 21.0)
a_E        91.9    91.9    91.9    91.9    91.9    91.9    91.9    91.9    91.9    91.9
        ( 24.7) ( 24.7) ( 24.7) ( 24.7) ( 24.7) ( 24.7) ( 24.7) ( 24.7) ( 24.7) ( 24.7)
           16.8    10.4     6.2   -24.0     8.3     6.2    -5.8    -9.6     6.2   -25.7
          4.518   2.794   1.672  -6.443   2.230   1.672  -1.555  -2.583   1.672  -6.895
t_O          85      75     100     104      72      71      97      84     108     104
        ( 22.8) ( 20.2) ( 26.9) ( 28.0) ( 19.4) ( 19.1) ( 26.1) ( 22.6) ( 29.0) ( 28.0)
t_E        92.1    92.1    92.1    92.1    92.1    92.1    92.1    92.1    92.1    92.1
        ( 24.7) ( 24.7) ( 24.7) ( 24.7) ( 24.7) ( 24.7) ( 24.7) ( 24.7) ( 24.7) ( 24.7)
          -13.6   -30.7    16.5    25.4   -35.4   -36.9    10.1   -15.4    34.5    25.4
         -3.646  -8.264   4.448   6.819  -9.514  -9.916   2.726  -4.138   9.272   6.819
g_O          93      99      84      90      94      99      97      95      76      97
        ( 25.0) ( 26.6) ( 22.6) ( 24.2) ( 25.3) ( 26.6) ( 26.1) ( 25.5) ( 20.4) ( 26.1)
g_E        91.2    91.2    91.2    91.2    91.2    91.2    91.2    91.2    91.2    91.2
        ( 24.5) ( 24.5) ( 24.5) ( 24.5) ( 24.5) ( 24.5) ( 24.5) ( 24.5) ( 24.5) ( 24.5)
            3.6    16.2   -13.8    -2.4     5.7    16.2    11.9     7.7   -27.7    11.9
          0.972   4.362  -3.719  -0.646   1.523   4.362   3.210   2.079  -7.454   3.210
c_O          94     101      93      99     110     107      89     106      93      93
        ( 25.3) ( 27.2) ( 25.0) ( 26.6) ( 29.6) ( 28.8) ( 23.9) ( 28.5) ( 25.0) ( 25.0)
c_E        96.8    96.8    96.8    96.8    96.8    96.8    96.8    96.8    96.8    96.8
        ( 26.0) ( 26.0) ( 26.0) ( 26.0) ( 26.0) ( 26.0) ( 26.0) ( 26.0) ( 26.0) ( 26.0)
           -5.5     8.6    -7.4     4.5    28.1    21.5   -14.9    19.3    -7.4    -7.4
         -1.478   2.312  -1.997   1.202   7.566   5.769  -4.015   5.180  -1.997  -1.997

N           372     372     372     372     372     372     372     372     372     372
X2          1.4     4.3     1.5     3.4     6.4     6.7     1.4     2.0     5.5     4.2
Gvalue      1.4     4.5     1.5     3.5     6.7     7.0     1.4     2.0     5.6     4.2
Gv/N      0.365   1.204   0.404   0.931   1.805   1.888   0.366   0.538   1.493   1.136
Prob    0.71524 0.21423 0.68167 0.32531 0.08153 0.07117 0.71494 0.57185 0.13540 0.23813
        -------------------------------------------------------------------------------
             20      19      18      17      16      15      14      13      12      11
        -------------------------------------------------------------------------------
a_O          98     117      80      89     110     115     101      88      93     118
        ( 26.3) ( 31.5) ( 21.5) ( 23.9) ( 29.6) ( 30.9) ( 27.2) ( 23.7) ( 25.0) ( 31.7)
a_E        91.9    91.9    91.9    91.9    91.9    91.9    91.9    91.9    91.9    91.9
        ( 24.7) ( 24.7) ( 24.7) ( 24.7) ( 24.7) ( 24.7) ( 24.7) ( 24.7) ( 24.7) ( 24.7)
           12.5    56.4   -22.3    -5.8    39.5    51.5    19.0    -7.7     2.1    58.9
          3.363  15.162  -5.983  -1.555  10.606  13.837   5.103  -2.072   0.573  15.832
t_O          94      99     109      87      89      91      98     104     113      98
        ( 25.3) ( 26.6) ( 29.3) ( 23.4) ( 23.9) ( 24.5) ( 26.3) ( 28.0) ( 30.4) ( 26.3)
t_E        92.1    92.1    92.1    92.1    92.1    92.1    92.1    92.1    92.1    92.1
        ( 24.7) ( 24.7) ( 24.7) ( 24.7) ( 24.7) ( 24.7) ( 24.7) ( 24.7) ( 24.7) ( 24.7)
            3.9    14.4    36.8    -9.8    -6.0    -2.1    12.3    25.4    46.3    12.3
          1.054   3.868   9.898  -2.644  -1.618  -0.567   3.294   6.819  12.451   3.294
g_O          86      48      77      82      69      65      79      57      77      57
        ( 23.1) ( 12.9) ( 20.7) ( 22.0) ( 18.5) ( 17.5) ( 21.2) ( 15.3) ( 20.7) ( 15.3)
g_E        91.2    91.2    91.2    91.2    91.2    91.2    91.2    91.2    91.2    91.2
        ( 24.5) ( 24.5) ( 24.5) ( 24.5) ( 24.5) ( 24.5) ( 24.5) ( 24.5) ( 24.5) ( 24.5)
          -10.1   -61.6   -26.1   -17.5   -38.5   -44.0   -22.7   -53.6   -26.1   -53.6
         -2.720 -16.567  -7.011  -4.693 -10.352 -11.839  -6.104 -14.407  -7.011 -14.407
c_O          94     108     106     114     104     101      94     123      89      99
        ( 25.3) ( 29.0) ( 28.5) ( 30.6) ( 28.0) ( 27.2) ( 25.3) ( 33.1) ( 23.9) ( 26.6)
c_E        96.8    96.8    96.8    96.8    96.8    96.8    96.8    96.8    96.8    96.8
        ( 26.0) ( 26.0) ( 26.0) ( 26.0) ( 26.0) ( 26.0) ( 26.0) ( 26.0) ( 26.0) ( 26.0)
           -5.5    23.7    19.3    37.3    14.9     8.6    -5.5    59.0   -14.9     4.5
         -1.478   6.363   5.180  10.030   4.017   2.312  -1.478  15.847  -4.015   1.202

N           372     372     372     372     372     372     372     372     372     372
X2          0.8    29.1     7.8     4.4     9.6    13.5     3.0    21.6     7.6    20.7
Gvalue      0.8    32.8     7.8     4.2     9.9    13.9     3.0    23.0     7.4    22.0
Gv/N      0.219   8.827   2.084   1.138   2.654   3.743   0.816   6.187   1.998   5.921
Prob    0.84560 0.00000 0.05141 0.23722 0.01968 0.00301 0.38640 0.00004 0.05929 0.00006
        -------------------------------------------------------------------------------
             10       9       8       7       6       5       4       3       2       1
        -------------------------------------------------------------------------------
a_O         104     116     101     104      99     103      82      95      90      92
        ( 28.0) ( 31.2) ( 27.2) ( 28.0) ( 26.6) ( 27.7) ( 22.0) ( 25.5) ( 24.2) ( 24.7)
a_E        91.9    91.9    91.9    91.9    91.9    91.9    91.9    91.9    91.9    91.9
        ( 24.7) ( 24.7) ( 24.7) ( 24.7) ( 24.7) ( 24.7) ( 24.7) ( 24.7) ( 24.7) ( 24.7)
           25.6    53.9    19.0    25.6    14.6    23.4   -18.8     6.2    -3.8     0.1
          6.892  14.497   5.103   6.892   3.938   6.290  -5.044   1.672  -1.032   0.032
t_O          88      94      94     114      94      90      90      74      81      71
        ( 23.7) ( 25.3) ( 25.3) ( 30.6) ( 25.3) ( 24.2) ( 24.2) ( 19.9) ( 21.8) ( 19.1)
t_E        92.1    92.1    92.1    92.1    92.1    92.1    92.1    92.1    92.1    92.1
        ( 24.7) ( 24.7) ( 24.7) ( 24.7) ( 24.7) ( 24.7) ( 24.7) ( 24.7) ( 24.7) ( 24.7)
           -7.9     3.9     3.9    48.7     3.9    -4.1    -4.1   -32.3   -20.7   -36.9
         -2.134   1.054   1.054  13.101   1.054  -1.095  -1.095  -8.688  -5.574  -9.916
g_O          75      74      83      54      74      82     104     102      92      73
        ( 20.2) ( 19.9) ( 22.3) ( 14.5) ( 19.9) ( 22.0) ( 28.0) ( 27.4) ( 24.7) ( 19.6)
g_E        91.2    91.2    91.2    91.2    91.2    91.2    91.2    91.2    91.2    91.2
        ( 24.5) ( 24.5) ( 24.5) ( 24.5) ( 24.5) ( 24.5) ( 24.5) ( 24.5) ( 24.5) ( 24.5)
          -29.4   -30.9   -15.7   -56.6   -30.9   -17.5    27.3    22.8     1.6   -32.5
         -7.890  -8.319  -4.209 -15.218  -8.319  -4.693   7.337   6.131   0.427  -8.741
c_O         105      88      94     100     105      97      96     101     109     136
        ( 28.2) ( 23.7) ( 25.3) ( 26.9) ( 28.2) ( 26.1) ( 25.8) ( 27.2) ( 29.3) ( 36.6)
c_E        96.8    96.8    96.8    96.8    96.8    96.8    96.8    96.8    96.8    96.8
        ( 26.0) ( 26.0) ( 26.0) ( 26.0) ( 26.0) ( 26.0) ( 26.0) ( 26.0) ( 26.0) ( 26.0)
           17.1   -16.8    -5.5     6.5    17.1     0.4    -1.6     8.6    25.9    92.5
          4.596  -4.504  -1.478   1.754   4.596   0.113  -0.423   2.312   6.962  24.868

N           372     372     372     372     372     372     372     372     372     372
X2          5.3    10.4     1.8    22.1     4.5     2.3     2.9     5.1     2.9    24.3
Gvalue      5.4    10.1     1.7    24.3     4.7     2.3     2.9     5.3     2.9    23.2
Gv/N      1.464   2.728   0.470   6.529   1.269   0.616   0.775   1.428   0.783   6.244
Prob    0.14198 0.01736 0.62613 0.00002 0.19347 0.51444 0.40992 0.15042 0.40527 0.00004
        -------------------------------------------------------------------------------

CDS_INITIAL:
        -------------------------------------------------------------------------------
```

	1	2	3	4	5	6	7	8	9	10
a_O	248	0	0	141	101	95	119	115	89	99
	(66.7)	(0.0)	(0.0)	(37.9)	(27.2)	(25.5)	(32.0)	(30.9)	(23.9)	(26.6)
a_E	85.6	95.9	47.5	85.6	95.9	47.5	85.6	95.9	47.5	85.6
	(23.0)	(25.8)	(12.8)	(23.0)	(25.8)	(12.8)	(23.0)	(25.8)	(12.8)	(23.0)
	527.9	0.0	0.0	140.9	10.4	131.7	78.5	41.7	111.7	28.9
	141.910	0.000	0.000	37.877	2.799	35.397	21.114	11.213	30.040	7.772
t_O	73	372	0	44	89	79	59	88	87	58
	(19.6)	(100.0)	(0.0)	(11.8)	(23.9)	(21.2)	(15.9)	(23.7)	(23.4)	(15.6)
t_E	51.8	107.5	79.2	51.8	107.5	79.2	51.8	107.5	79.2	51.8
	(13.9)	(28.9)	(21.3)	(13.9)	(28.9)	(21.3)	(13.9)	(28.9)	(21.3)	(13.9)
	50.0	923.3	0.0	−14.4	−33.7	−0.5	15.3	−35.3	16.3	13.0
	13.435	248.191	0.000	−3.879	−9.058	−0.124	4.104	−9.491	4.375	3.502
g_O	51	0	372	95	62	90	103	70	101	98
	(13.7)	(0.0)	(100.0)	(25.5)	(16.7)	(24.2)	(27.7)	(18.8)	(27.2)	(26.3)
g_E	138.0	73.1	116.1	138.0	73.1	116.1	138.0	73.1	116.1	138.0
	(37.1)	(19.7)	(31.2)	(37.1)	(19.7)	(31.2)	(37.1)	(19.7)	(31.2)	(37.1)
	−101.5	0.0	866.5	−70.9	−20.5	−45.8	−60.3	−6.1	−28.1	−67.1
	−27.294	0.000	232.919	−19.071	−5.507	−12.314	−16.200	−1.650	−7.558	−18.035
c_O	0	0	0	92	120	108	91	99	95	117
	(0.0)	(0.0)	(0.0)	(24.7)	(32.3)	(29.0)	(24.5)	(26.6)	(25.5)	(31.5)
c_E	96.6	95.4	129.2	96.6	95.4	129.2	96.6	95.4	129.2	96.6
	(26.0)	(25.6)	(34.7)	(26.0)	(25.6)	(34.7)	(26.0)	(25.6)	(34.7)	(26.0)
	0.0	0.0	0.0	−9.0	55.1	−38.7	−10.9	7.4	−58.4	44.8
	0.000	0.000	0.000	−2.417	14.809	−10.399	−2.926	1.978	−15.697	12.047
N	372	372	372	372	372	372	372	372	372	372
X2	468.6	914.7	820.1	50.7	11.5	56.8	23.3	7.6	48.0	18.7
Gvalue	476.3	923.3	866.5	46.5	11.3	46.7	22.7	7.6	41.5	19.7
Gv/N	128.050	248.191	232.919	12.510	3.043	12.561	6.093	2.050	11.160	5.285
Prob	0.00000	0.00000	0.00000	0.00000	0.01012	0.00000	0.00005	0.05441	0.00000	0.00020

	11	12	13	14	15	16	17	18	19	20
a_O	102	82	129	92	74	106	83	75	108	112
	(27.4)	(22.0)	(34.7)	(24.7)	(19.9)	(28.5)	(22.3)	(20.2)	(29.0)	(30.1)
a_E	95.9	47.5	85.6	95.9	47.5	85.6	95.9	47.5	85.6	95.9
	(25.8)	(12.8)	(23.0)	(25.8)	(12.8)	(23.0)	(25.8)	(12.8)	(23.0)	(25.8)
	12.5	89.5	106.0	−7.7	65.6	45.4	−24.0	68.5	50.3	34.7
	3.367	24.066	28.484	−2.067	17.634	12.215	−6.459	18.414	13.530	9.329
t_O	90	94	60	103	110	67	114	89	57	99
	(24.2)	(25.3)	(16.1)	(27.7)	(29.6)	(18.0)	(30.6)	(23.9)	(15.3)	(26.6)
t_E	107.5	79.2	51.8	107.5	79.2	51.8	107.5	79.2	51.8	107.5
	(28.9)	(21.3)	(13.9)	(28.9)	(21.3)	(13.9)	(28.9)	(21.3)	(13.9)	(28.9)
	−32.1	32.1	17.5	−8.9	72.2	34.4	13.3	20.7	10.8	−16.4
	−8.619	8.638	4.716	−2.393	19.404	9.241	3.571	5.563	2.908	−4.408
g_O	89	100	105	68	82	101	66	87	100	72
	(23.9)	(26.9)	(28.2)	(18.3)	(22.0)	(27.2)	(17.7)	(23.4)	(26.9)	(19.4)
g_E	73.1	116.1	138.0	73.1	116.1	138.0	73.1	116.1	138.0	73.1
	(19.7)	(31.2)	(37.1)	(19.7)	(31.2)	(37.1)	(19.7)	(31.2)	(37.1)	(19.7)
	34.9	−29.8	−57.4	−9.9	−57.0	−63.1	−13.6	−50.2	−64.4	−2.3
	9.392	−8.018	−15.429	−2.663	−15.324	−16.950	−3.644	−13.489	−17.317	−0.607
c_O	91	96	78	109	106	98	109	121	107	89
	(24.5)	(25.8)	(21.0)	(29.3)	(28.5)	(26.3)	(29.3)	(32.5)	(28.8)	(23.9)
c_E	95.4	129.2	96.6	95.4	129.2	96.6	95.4	129.2	96.6	95.4
	(25.6)	(34.7)	(26.0)	(25.6)	(34.7)	(26.0)	(25.6)	(34.7)	(26.0)	(25.6)
	−8.6	−57.0	−33.4	29.1	−41.9	2.8	29.1	−15.8	21.9	−12.3
	−2.304	−15.322	−8.972	7.817	−11.271	0.754	7.817	−4.256	5.877	−3.317
N	372	372	372	372	372	372	372	372	372	372
X2	6.9	38.6	34.8	2.7	40.9	19.3	4.8	24.9	18.0	3.8
Gvalue	6.8	34.8	32.7	2.6	38.9	19.6	4.8	23.2	18.6	3.7
Gv/N	1.836	9.364	8.799	0.695	10.444	5.259	1.285	6.231	4.999	0.997
Prob	0.07751	0.00000	0.00000	0.46030	0.00000	0.00021	0.18847	0.00004	0.00033	0.29476

	21	22	23	24	25	26	27	28	29	30
a_O	70	98	108	75	87	90	68	94	99	76
	(18.8)	(26.3)	(29.0)	(20.2)	(23.4)	(24.2)	(18.3)	(25.3)	(26.6)	(20.4)
a_E	47.5	85.6	95.9	47.5	85.6	95.9	47.5	85.6	95.9	47.5
	(12.8)	(23.0)	(25.8)	(12.8)	(23.0)	(25.8)	(12.8)	(23.0)	(25.8)	(12.8)
	54.3	26.6	25.6	68.5	2.9	−11.5	48.8	17.7	6.2	71.4
	14.589	7.158	6.884	18.414	0.786	−3.086	13.113	4.760	1.679	19.200
t_O	106	75	104	85	66	101	100	60	113	93
	(28.5)	(20.2)	(28.0)	(22.8)	(17.7)	(27.2)	(26.9)	(16.1)	(30.4)	(25.0)
t_E	79.2	51.8	107.5	79.2	51.8	107.5	79.2	51.8	107.5	79.2
	(21.3)	(13.9)	(28.9)	(21.3)	(13.9)	(28.9)	(21.3)	(13.9)	(28.9)	(21.3)
	61.7	55.4	−7.0	11.9	31.9	−12.7	46.6	17.5	11.2	29.8
	16.588	14.893	−1.876	3.212	8.570	−3.411	12.516	4.716	3.004	8.011
g_O	96	100	66	89	107	81	94	121	65	99
	(25.8)	(26.9)	(17.7)	(23.9)	(28.8)	(21.8)	(25.3)	(32.5)	(17.5)	(26.6)
g_E	116.1	138.0	73.1	116.1	138.0	73.1	116.1	138.0	73.1	116.1
	(31.2)	(37.1)	(19.7)	(31.2)	(37.1)	(19.7)	(31.2)	(37.1)	(19.7)	(31.2)
	−36.5	−64.4	−13.6	−47.3	−54.5	16.5	−39.7	−31.8	−15.3	−31.5
	−9.804	−17.317	−3.644	−12.712	−14.637	4.446	−10.664	−8.553	−4.122	−8.473
c_O	100	99	94	123	112	100	110	97	95	104
	(26.9)	(26.6)	(25.3)	(33.1)	(30.1)	(26.9)	(29.6)	(26.1)	(25.5)	(28.0)
c_E	129.2	96.6	95.4	129.2	96.6	95.4	129.2	96.6	95.4	129.2
	(34.7)	(26.0)	(25.6)	(34.7)	(26.0)	(25.6)	(34.7)	(26.0)	(25.6)	(34.7)
	−51.2	4.8	−2.8	−12.1	33.1	9.4	−35.4	0.8	−0.8	−45.1
	−13.766	1.302	−0.741	−3.242	8.902	2.539	−9.506	0.211	−0.208	−12.124
N	372	372	372	372	372	372	372	372	372	372
X2	29.8	22.7	2.4	22.9	13.3	1.8	21.3	4.2	1.3	26.9
Gvalue	28.3	22.5	2.3	21.1	13.5	1.8	20.3	4.2	1.3	24.6
Gv/N	7.607	6.036	0.623	5.671	3.620	0.488	5.459	1.134	0.353	6.615
Prob	0.00000	0.00005	0.50893	0.00010	0.00373	0.61127	0.00015	0.23882	0.72631	0.00002

	31	32	33	34	35	36	37	38	39	40
a_O	108	88	68	81	85	63	82	82	59	83
	(29.0)	(23.7)	(18.3)	(21.8)	(22.8)	(16.9)	(22.0)	(22.0)	(15.9)	(22.3)
a_E	85.6	95.9	47.5	85.6	95.9	47.5	85.6	95.9	47.5	85.6
	(23.0)	(25.8)	(12.8)	(23.0)	(25.8)	(12.8)	(23.0)	(25.8)	(12.8)	(23.0)
	50.3	−15.2	48.8	−8.9	−20.6	35.6	−7.0	−25.7	25.6	−5.0
	13.530	−4.080	13.113	−2.380	−5.526	9.562	−1.869	−6.915	6.874	−1.351
t_O	60	124	100	70	113	85	68	121	95	60
	(16.1)	(33.3)	(26.9)	(18.8)	(30.4)	(22.8)	(18.3)	(32.5)	(25.5)	(16.1)
t_E	51.8	107.5	79.2	51.8	107.5	79.2	51.8	107.5	79.2	51.8
	(13.9)	(28.9)	(21.3)	(13.9)	(28.9)	(21.3)	(13.9)	(28.9)	(21.3)	(13.9)
	17.5	35.3	46.6	42.0	11.2	11.9	36.9	28.5	34.5	17.5

	4.716	9.489	12.516	11.303	3.004	3.212	9.921	7.667	9.270	4.716
g_O	111	69	99	131	73	106	134	91	107	127
	(29.8)	(18.5)	(26.6)	(35.2)	(19.6)	(28.5)	(36.0)	(24.5)	(28.8)	(34.1)
g_E	138.0	73.1	116.1	138.0	73.1	116.1	138.0	73.1	116.1	138.0
	(37.1)	(19.7)	(31.2)	(37.1)	(19.7)	(31.2)	(37.1)	(19.7)	(31.2)	(37.1)
	-48.3	-8.0	-31.5	-13.6	-0.3	-19.3	-7.9	39.8	-17.4	-21.1
	-12.994	-2.161	-8.473	-3.667	-0.074	-5.178	-2.120	10.691	-4.687	-5.673
c_O	93	91	105	90	101	118	88	78	111	102
	(25.0)	(24.5)	(28.2)	(24.2)	(27.2)	(31.7)	(23.7)	(21.0)	(29.8)	(27.4)
c_E	96.6	95.4	129.2	96.6	95.4	129.2	96.6	95.4	129.2	96.6
	(26.0)	(25.6)	(34.7)	(26.0)	(25.6)	(34.7)	(26.0)	(25.6)	(34.7)	(26.0)
	-7.1	-8.6	-43.5	-12.8	11.5	-21.4	-16.4	-31.4	-33.7	11.1
	-1.903	-2.304	-11.700	-3.428	3.104	-5.743	-4.415	-8.439	-9.052	2.978
N	372	372	372	372	372	372	372	372	372	372
X2	12.6	3.6	21.3	7.4	1.9	7.3	6.1	11.2	9.2	2.5
Gvalue	12.5	3.5	20.3	6.8	1.9	6.9	5.6	11.2	8.9	2.5
Gv/N	3.349	0.945	5.456	1.827	0.508	1.852	1.516	3.003	2.405	0.671
Prob	0.00597	0.31886	0.00015	0.07863	0.59541	0.07551	0.13045	0.01084	0.02999	0.47604

	41	42	43	44	45	46	47	48	49	50
a_O	91	60	80	93	65	101	97	68	89	81
	(24.5)	(16.1)	(21.5)	(25.0)	(17.5)	(27.2)	(26.1)	(18.3)	(23.9)	(21.8)
a_E	95.9	47.5	85.6	95.9	47.5	85.6	95.9	47.5	85.6	95.9
	(25.8)	(12.8)	(23.0)	(25.8)	(12.8)	(23.0)	(25.8)	(12.8)	(23.0)	(25.8)
	-9.6	28.0	-10.7	-5.8	40.8	33.5	2.2	48.8	7.0	-27.4
	-2.579	7.533	-2.885	-1.549	10.958	9.015	0.581	13.113	1.891	-7.365
t_O	118	93	67	105	97	53	114	100	62	97
	(31.7)	(25.0)	(18.0)	(28.2)	(26.1)	(14.2)	(30.6)	(26.9)	(16.7)	(26.1)
t_E	107.5	79.2	51.8	107.5	79.2	51.8	107.5	79.2	51.8	107.5
	(28.9)	(21.3)	(13.9)	(28.9)	(21.3)	(13.9)	(28.9)	(21.3)	(13.9)	(28.9)
	21.9	29.8	34.4	-5.0	39.3	2.3	13.3	46.6	22.2	-20.0
	5.884	8.011	9.241	-1.354	10.552	0.631	3.571	12.516	5.966	-5.383
g_O	58	99	124	83	102	111	79	78	119	96
	(15.6)	(26.6)	(33.3)	(22.3)	(27.4)	(29.8)	(21.2)	(21.0)	(32.0)	(25.8)
g_E	73.1	116.1	138.0	73.1	116.1	138.0	73.1	116.1	138.0	73.1
	(19.7)	(31.2)	(37.1)	(19.7)	(31.2)	(37.1)	(19.7)	(31.2)	(37.1)	(19.7)
	-26.9	-31.5	-26.5	21.0	-26.4	-48.3	12.2	-62.0	-35.3	52.2
	-7.231	-8.473	-7.133	5.645	-7.092	-12.994	3.275	-16.673	-9.478	14.039
c_O	105	120	101	91	108	107	82	126	102	98
	(28.2)	(32.3)	(27.2)	(24.5)	(29.0)	(28.8)	(22.0)	(33.9)	(27.4)	(26.3)
c_E	95.4	129.2	96.6	95.4	129.2	96.6	95.4	129.2	96.6	95.4
	(25.6)	(34.7)	(26.0)	(25.6)	(34.7)	(26.0)	(25.6)	(34.7)	(26.0)	(25.6)
	20.2	-17.7	9.0	-8.6	-38.7	21.9	-24.8	-6.3	11.1	5.3
	5.420	-4.756	2.414	-2.304	-10.399	5.877	-6.667	-1.689	2.978	1.424
N	372	372	372	372	372	372	372	372	372	372
X2	5.4	8.8	6.4	1.7	15.6	9.2	2.7	26.9	5.0	10.6
Gvalue	5.6	8.6	6.1	1.6	15.0	9.4	2.8	27.0	5.1	10.1
Gv/N	1.493	2.315	1.638	0.438	4.019	2.529	0.759	7.267	1.358	2.714
Prob	0.13542	0.03492	0.10723	0.65265	0.00186	0.02434	0.41975	0.00001	0.16809	0.01778

	51	52	53	54	55	56	57	58	59	60
a_O	74	87	93	69	85	84	63	80	73	68
	(19.9)	(23.4)	(25.0)	(18.5)	(22.8)	(22.6)	(16.9)	(21.5)	(19.6)	(18.3)
a_E	47.5	85.6	95.9	47.5	85.6	95.9	47.5	85.6	95.9	47.5
	(12.8)	(23.0)	(25.8)	(12.8)	(23.0)	(25.8)	(12.8)	(23.0)	(25.8)	(12.8)
	65.6	2.9	-5.8	51.5	-1.1	-22.3	35.6	-10.7	-39.9	48.8
	17.634	0.786	-1.549	13.847	-0.295	-5.996	9.562	-2.885	-10.719	13.113
t_O	82	55	103	85	64	107	91	78	104	86
	(22.0)	(14.8)	(27.7)	(22.8)	(17.2)	(28.8)	(24.5)	(21.0)	(28.0)	(23.1)
t_E	79.2	51.8	107.5	79.2	51.8	107.5	79.2	51.8	107.5	79.2
	(21.3)	(13.9)	(28.9)	(21.3)	(13.9)	(28.9)	(21.3)	(13.9)	(28.9)	(21.3)
	5.6	6.5	-8.9	11.9	27.0	-1.1	25.2	63.7	-7.0	14.1
	1.514	1.750	-2.393	3.212	7.251	-0.294	6.776	17.133	-1.876	3.790
g_O	100	132	82	106	124	79	98	125	86	112
	(26.9)	(35.5)	(22.0)	(28.5)	(33.3)	(21.2)	(26.3)	(33.6)	(23.1)	(30.1)
g_E	116.1	138.0	73.1	116.1	138.0	73.1	116.1	138.0	73.1	116.1
	(31.2)	(37.1)	(19.7)	(31.2)	(37.1)	(19.7)	(31.2)	(37.1)	(19.7)	(31.2)
	-29.8	-11.7	18.8	-19.3	-26.5	12.2	-33.2	-24.7	27.9	-8.0
	-8.018	-3.156	5.042	-5.178	-7.133	3.275	-8.922	-6.650	7.490	-2.156
c_O	116	98	94	112	99	102	120	89	109	106
	(31.2)	(26.3)	(25.3)	(30.1)	(26.6)	(27.4)	(32.3)	(23.9)	(29.3)	(28.5)
c_E	129.2	96.6	95.4	129.2	96.6	95.4	129.2	96.6	95.4	129.2
	(34.7)	(26.0)	(25.6)	(34.7)	(26.0)	(25.6)	(34.7)	(26.0)	(25.6)	(34.7)
	-25.0	2.8	-2.8	-32.0	4.8	13.7	-17.7	-14.6	29.1	-41.9
	-6.712	0.754	-0.741	-8.594	1.302	3.676	-4.756	-3.925	7.817	-11.271
N	372	372	372	372	372	372	372	372	372	372
X2	18.4	0.5	1.4	13.3	4.3	2.4	10.3	15.4	9.8	13.7
Gvalue	16.4	0.5	1.3	12.2	4.2	2.5	9.9	13.7	10.1	12.9
Gv/N	4.418	0.134	0.360	3.287	1.125	0.660	2.659	3.673	2.713	3.476
Prob	0.00092	0.91928	0.72001	0.00664	0.24204	0.48311	0.01951	0.00340	0.01780	0.00479

	61	62	63	64	65	66	67	68	69	70
a_O	72	84	57	87	87	59	89	93	44	98
	(19.4)	(22.6)	(15.3)	(23.4)	(23.4)	(15.9)	(23.9)	(25.0)	(11.8)	(26.3)
a_E	85.6	95.9	47.5	85.6	95.9	47.5	85.6	95.9	47.5	85.6
	(23.0)	(25.8)	(12.8)	(23.0)	(25.8)	(12.8)	(23.0)	(25.8)	(12.8)	(23.0)
	-24.8	-22.3	20.8	2.9	-17.0	25.6	7.0	-5.8	-6.7	26.6
	-6.675	-5.996	5.584	0.786	-4.568	6.874	1.891	-1.549	-1.813	7.158
t_O	65	127	85	56	99	84	67	104	83	60
	(17.5)	(34.1)	(22.8)	(15.1)	(26.6)	(22.6)	(18.0)	(28.0)	(22.3)	(16.1)
t_E	51.8	107.5	79.2	51.8	107.5	79.2	51.8	107.5	79.2	51.8
	(13.9)	(28.9)	(21.3)	(13.9)	(28.9)	(21.3)	(13.9)	(28.9)	(21.3)	(13.9)
	29.4	42.2	11.9	8.6	-16.4	9.8	34.4	-7.0	7.7	17.5
	7.906	11.351	3.212	2.324	-4.408	2.639	9.241	-1.876	2.074	4.716
g_O	128	70	117	133	77	121	108	73	121	123
	(34.4)	(18.8)	(31.5)	(35.8)	(20.7)	(32.5)	(29.0)	(19.6)	(32.5)	(33.1)
g_E	138.0	73.1	116.1	138.0	73.1	116.1	138.0	73.1	116.1	138.0
	(37.1)	(19.7)	(31.2)	(37.1)	(19.7)	(31.2)	(37.1)	(19.7)	(31.2)	(37.1)
	-19.3	-6.1	1.8	-9.8	7.9	10.0	-52.9	-0.3	10.0	-28.3
	-5.178	-1.650	0.495	-2.640	2.130	2.699	-14.234	-0.074	2.699	-7.610
c_O	107	91	113	96	109	108	108	102	124	91
	(28.8)	(24.5)	(30.4)	(25.8)	(29.3)	(29.0)	(29.0)	(27.4)	(33.3)	(24.5)
c_E	96.6	95.4	129.2	96.6	95.4	129.2	96.6	95.4	129.2	96.6
	(26.0)	(25.6)	(34.7)	(26.0)	(25.6)	(34.7)	(26.0)	(25.6)	(34.7)	(26.0)
	21.9	-8.6	-30.2	-1.2	29.1	-38.7	24.1	13.7	-10.2	-10.9
	5.877	-2.304	-8.130	-0.326	7.817	-10.399	6.472	3.676	-2.729	-2.926

N	372	372	372	372	372	372	372	372	372	372
X2	7.3	5.3	4.4	0.5	3.7	6.7	12.4	0.7	0.9	5.1
Gvalue	7.2	5.2	4.3	0.5	3.6	6.7	12.5	0.7	0.9	5.0
Gv/N	1.931	1.401	1.161	0.144	0.971	1.814	3.371	0.177	0.231	1.338
Prob	0.06632	0.15683	0.22917	0.91066	0.30646	0.08038	0.00574	0.88328	0.83561	0.17347

	71	72	73	74	75	76	77	78	79	80
a_O	91	69	84	80	52	75	90	62	89	95
	(24.5)	(18.5)	(22.6)	(21.5)	(14.0)	(20.2)	(24.2)	(16.7)	(23.9)	(25.5)
a_E	95.9	47.5	85.6	95.9	47.5	85.6	95.9	47.5	85.6	95.9
	(25.8)	(12.8)	(23.0)	(25.8)	(12.8)	(23.0)	(25.8)	(12.8)	(23.0)	(25.8)
	-9.6	51.5	-3.1	-29.0	9.4	-19.7	-11.5	33.0	7.0	-1.8
	-2.579	13.847	-0.826	-7.809	2.528	-5.307	-3.086	8.877	1.891	-0.496
t_O	116	81	65	95	92	65	114	90	74	104
	(31.2)	(21.8)	(17.5)	(25.5)	(24.7)	(17.5)	(30.6)	(24.2)	(19.9)	(28.0)
t_E	107.5	79.2	51.8	107.5	79.2	51.8	107.5	79.2	51.8	107.5
	(28.9)	(21.3)	(13.9)	(28.9)	(21.3)	(13.9)	(28.9)	(21.3)	(13.9)	(28.9)
	17.6	3.6	29.4	-23.6	27.5	29.4	13.3	22.9	52.7	-7.0
	4.718	0.961	7.906	-6.337	7.391	7.906	3.571	6.166	14.160	-1.876
g_O	70	110	128	87	112	136	87	108	116	69
	(18.8)	(29.6)	(34.4)	(23.4)	(30.1)	(36.6)	(23.4)	(29.0)	(31.2)	(18.5)
g_E	73.1	116.1	138.0	73.1	116.1	138.0	73.1	116.1	138.0	73.1
	(19.7)	(31.2)	(37.1)	(19.7)	(31.2)	(37.1)	(19.7)	(31.2)	(37.1)	(19.7)
	-6.1	-11.8	-5.178	30.2	-8.0	-4.0	30.2	-15.6	-40.3	-8.0
	-1.650	-3.183	-5.178	8.118	-2.156	-1.069	8.118	-4.191	-10.832	-2.161
c_O	95	112	95	110	116	96	81	112	93	104
	(25.5)	(30.1)	(25.5)	(29.6)	(31.2)	(25.8)	(21.8)	(30.1)	(25.0)	(28.0)
c_E	95.4	129.2	96.6	95.4	129.2	96.6	95.4	129.2	96.6	95.4
	(25.6)	(34.7)	(26.0)	(25.6)	(34.7)	(26.0)	(25.6)	(34.7)	(26.0)	(25.6)
	-0.8	-32.0	-3.2	31.4	-25.0	-1.2	-26.5	-32.0	-7.1	18.0
	-0.208	-8.594	-0.857	8.429	-6.712	-0.326	-7.120	-8.594	-1.903	4.833
N	372	372	372	372	372	372	372	372	372	372
X2	1.1	12.4	4.1	9.0	4.0	4.7	5.6	8.7	13.3	1.1
Gvalue	1.0	11.3	3.9	8.9	3.9	4.5	5.5	8.4	12.3	1.1
Gv/N	0.280	3.032	1.045	2.402	1.050	1.205	1.483	2.259	3.317	0.301
Prob	0.79084	0.01031	0.27382	0.03015	0.27175	0.21401	0.13761	0.03838	0.00631	0.77196

	81	82	83	84	85	86	87	88	89	90
a_O	63	74	89	59	84	98	51	93	83	67
	(16.9)	(19.9)	(23.9)	(15.9)	(22.6)	(26.3)	(13.7)	(25.0)	(22.3)	(18.0)
a_E	47.5	85.6	95.9	47.5	85.6	95.9	47.5	85.6	95.9	47.5
	(12.8)	(23.0)	(25.8)	(12.8)	(23.0)	(25.8)	(12.8)	(23.0)	(25.8)	(12.8)
	35.6	-21.5	-13.3	25.6	-3.1	4.2	7.2	15.5	-24.0	46.1
	9.562	-5.770	-3.586	6.874	-0.826	1.127	1.947	4.175	-6.459	12.386
t_O	99	66	109	85	62	95	73	51	105	63
	(26.6)	(17.7)	(29.3)	(22.8)	(16.7)	(25.5)	(19.6)	(13.7)	(28.2)	(16.9)
t_E	79.2	51.8	107.5	79.2	51.8	107.5	79.2	51.8	107.5	79.2
	(21.3)	(13.9)	(28.9)	(21.3)	(13.9)	(28.9)	(21.3)	(13.9)	(28.9)	(21.3)
	44.1	31.9	2.9	11.9	22.2	-23.6	-12.0	-1.7	-5.0	-28.9
	11.856	8.570	0.786	3.212	5.966	-6.337	-3.215	-0.448	-1.354	-7.764
g_O	95	122	72	123	132	84	125	121	78	119
	(25.5)	(32.8)	(19.4)	(33.1)	(35.5)	(22.6)	(33.6)	(32.5)	(21.0)	(32.0)
g_E	116.1	138.0	73.1	116.1	138.0	73.1	116.1	138.0	73.1	116.1
	(31.2)	(37.1)	(19.7)	(31.2)	(37.1)	(19.7)	(31.2)	(37.1)	(19.7)	(31.2)
	-38.1	-30.1	-2.3	14.2	-11.7	23.3	18.5	-31.8	10.0	5.9
	-10.237	-8.084	-0.607	3.828	-3.156	6.253	4.974	-8.553	2.699	1.588
c_O	115	110	102	105	94	95	123	107	106	123
	(30.9)	(29.6)	(27.4)	(28.2)	(25.3)	(25.5)	(33.1)	(28.8)	(28.5)	(33.1)
c_E	129.2	96.6	95.4	129.2	96.6	95.4	129.2	96.6	95.4	129.2
	(34.7)	(26.0)	(25.6)	(34.7)	(26.0)	(25.6)	(34.7)	(26.0)	(25.6)	(34.7)
	-26.7	28.6	13.7	-43.5	-5.1	-0.8	-12.1	21.9	22.4	-12.1
	-7.190	7.677	3.676	-11.700	-1.383	-0.208	-3.242	5.877	6.012	-3.242
N	372	372	372	372	372	372	372	372	372	372
X2	15.4	9.1	1.0	8.1	2.4	3.1	1.7	3.9	3.3	11.7
Gvalue	14.8	8.9	1.0	8.2	2.2	3.1	1.7	3.9	3.3	11.0
Gv/N	3.991	2.393	0.268	2.213	0.601	0.836	0.463	1.051	0.898	2.968
Prob	0.00195	0.03065	0.80173	0.04141	0.52468	0.37505	0.63177	0.27129	0.34182	0.01152

	91	92	93	94	95	96	97	98	99	100
a_O	100	101	62	89	89	64	81	98	58	94
	(26.9)	(27.2)	(16.7)	(24.4)	(24.4)	(17.5)	(22.3)	(27.0)	(16.0)	(26.0)
a_E	85.6	95.9	47.5	83.9	94.1	46.6	83.5	93.6	46.4	83.0
	(23.0)	(25.8)	(12.8)	(23.0)	(25.8)	(12.8)	(23.0)	(25.8)	(12.8)	(23.0)
	31.2	10.4	33.0	10.4	-10.0	40.6	-4.9	9.0	26.0	23.4
	8.391	2.799	8.877	2.854	-2.728	11.118	-1.346	2.477	7.161	6.468
t_O	63	93	92	66	101	85	71	97	80	50
	(16.9)	(25.0)	(24.7)	(18.1)	(27.7)	(23.3)	(19.6)	(26.7)	(22.0)	(13.9)
t_E	51.8	107.5	79.2	50.9	105.5	77.7	50.6	104.9	77.3	50.3
	(13.9)	(28.9)	(21.3)	(13.9)	(28.9)	(21.3)	(13.9)	(28.9)	(21.3)	(13.9)
	24.6	-27.0	27.5	34.4	-8.9	15.2	48.1	-15.3	5.5	-0.6
	6.604	-7.267	7.391	9.421	-2.425	4.158	13.262	-4.208	1.505	-0.169
g_O	119	74	108	113	85	102	108	71	113	133
	(32.0)	(19.9)	(29.0)	(31.0)	(23.3)	(27.9)	(29.8)	(19.6)	(31.1)	(36.8)
g_E	138.0	73.1	116.1	135.4	71.8	113.9	134.7	71.4	113.3	133.9
	(37.1)	(19.7)	(31.2)	(37.1)	(19.7)	(31.2)	(37.1)	(19.7)	(31.2)	(37.1)
	-35.3	1.7	-15.6	-40.9	28.8	-22.5	-47.7	-0.7	-0.5	-1.8
	-9.478	0.466	-4.191	-11.200	7.885	-6.167	-13.129	-0.202	-0.151	-0.509
c_O	90	104	110	97	90	114	103	97	112	84
	(24.2)	(28.0)	(29.6)	(26.6)	(24.7)	(31.2)	(28.4)	(26.7)	(30.9)	(23.3)
c_E	96.6	95.4	129.2	94.8	93.6	126.8	94.3	93.1	126.1	93.8
	(26.0)	(25.6)	(34.7)	(26.0)	(25.6)	(34.7)	(26.0)	(25.6)	(34.7)	(26.0)
	-12.8	18.0	-35.4	4.5	-7.0	-24.2	18.2	8.0	-26.5	-18.5
	-3.428	4.833	-9.506	1.225	-1.930	-6.623	5.026	2.205	-7.296	-5.111
N	372	372	372	365	365	365	363	363	363	361
X2	7.9	3.0	9.9	8.6	3.1	9.7	14.4	1.0	4.6	2.5
Gvalue	7.8	3.1	9.6	8.4	2.9	9.1	13.8	1.0	4.4	2.5
Gv/N	2.088	0.831	2.571	2.300	0.801	2.487	3.812	0.271	1.220	0.679
Prob	0.05104	0.37761	0.02267	0.03852	0.40336	0.02827	0.00314	0.80479	0.21872	0.48412

Bafz_PKO
5UTR:

	100	99	98	97	96	95	94	93	92	91
a_O	81	95	86	94	95	87	87	77	89	84
	(36.5)	(42.8)	(38.7)	(42.3)	(42.8)	(39.2)	(39.2)	(34.7)	(40.1)	(37.8)
a_E	91.5	91.5	91.5	91.5	91.5	91.5	91.5	91.5	91.5	91.5
	(41.2)	(41.2)	(41.2)	(41.2)	(41.2)	(41.2)	(41.2)	(41.2)	(41.2)	(41.2)
	-19.7	7.2	-10.6	5.1	7.2	-8.7	-8.7	-26.5	-4.9	-14.3
	-8.879	3.231	-4.786	2.301	3.231	-3.936	-3.936	-11.953	-2.204	-6.455
t_O	76	81	76	80	77	81	76	80	77	82
	(34.2)	(36.5)	(34.2)	(36.0)	(34.7)	(36.5)	(34.2)	(36.0)	(34.7)	(36.9)
t_E	80.1	80.1	80.1	80.1	80.1	80.1	80.1	80.1	80.1	80.1
	(36.1)	(36.1)	(36.1)	(36.1)	(36.1)	(36.1)	(36.1)	(36.1)	(36.1)	(36.1)
	-8.0	1.8	-8.0	-0.2	-6.1	1.8	-8.0	-0.2	-6.1	3.8
	-3.615	0.797	-3.615	-0.108	-2.755	0.797	-3.615	-0.108	-2.755	1.713
g_O	34	24	27	22	25	38	26	31	28	30
	(15.3)	(10.8)	(12.2)	(9.9)	(11.3)	(17.1)	(11.7)	(14.0)	(12.6)	(13.5)
g_E	27.3	27.3	27.3	27.3	27.3	27.3	27.3	27.3	27.3	27.3
	(12.3)	(12.3)	(12.3)	(12.3)	(12.3)	(12.3)	(12.3)	(12.3)	(12.3)	(12.3)
	14.8	-6.2	-0.7	-9.5	-4.5	25.0	-2.6	7.8	1.4	5.6
	6.689	-2.809	-0.295	-4.300	-2.007	11.284	-1.169	3.519	0.611	2.519
c_O	31	22	33	26	25	16	33	34	28	26
	(14.0)	(9.9)	(14.9)	(11.7)	(11.3)	(7.2)	(14.9)	(15.3)	(12.6)	(11.7)
c_E	23.1	23.1	23.1	23.1	23.1	23.1	23.1	23.1	23.1	23.1
	(10.4)	(10.4)	(10.4)	(10.4)	(10.4)	(10.4)	(10.4)	(10.4)	(10.4)	(10.4)
	18.3	-2.1	23.6	6.2	4.0	-11.7	23.6	26.4	10.8	6.2
	8.251	-0.941	10.642	2.801	1.810	-5.275	10.642	11.879	4.885	2.801
N	222	222	222	222	222	222	222	222	222	222
X2	5.8	0.6	4.8	1.5	0.6	6.6	4.8	8.0	1.3	1.3
Gvalue	5.4	0.6	4.3	1.5	0.6	6.4	4.3	7.4	1.2	1.3
Gv/N	2.447	0.278	1.946	0.694	0.279	2.870	1.924	3.337	0.537	0.578
Prob	0.14273	0.89251	0.22883	0.67289	0.89218	0.09484	0.23367	0.05997	0.75472	0.73322

	90	89	88	87	86	85	84	83	82	81
a_O	87	81	101	74	94	82	77	84	97	93
	(39.2)	(36.5)	(45.5)	(33.3)	(42.3)	(36.9)	(34.7)	(37.8)	(43.7)	(41.9)
a_E	91.5	91.5	91.5	91.5	91.5	91.5	91.5	91.5	91.5	91.5
	(41.2)	(41.2)	(41.2)	(41.2)	(41.2)	(41.2)	(41.2)	(41.2)	(41.2)	(41.2)
	-8.7	-19.7	20.0	-31.4	5.1	-17.9	-26.5	-14.3	11.4	3.1
	-3.936	-8.879	9.008	-14.137	2.301	-8.082	-11.953	-6.455	5.120	1.381
t_O	89	88	67	92	73	86	82	77	71	68
	(40.1)	(39.6)	(30.2)	(41.4)	(32.9)	(38.7)	(36.9)	(34.7)	(32.0)	(30.6)
t_E	80.1	80.1	80.1	80.1	80.1	80.1	80.1	80.1	80.1	80.1
	(36.1)	(36.1)	(36.1)	(36.1)	(36.1)	(36.1)	(36.1)	(36.1)	(36.1)	(36.1)
	18.7	16.5	-24.0	25.4	-13.6	12.2	3.8	-6.1	-17.2	-22.3
	8.428	7.437	-10.794	11.460	-6.121	5.487	1.713	-2.755	-7.730	-10.048
g_O	22	33	31	26	24	26	34	37	30	36
	(9.9)	(14.9)	(14.0)	(11.7)	(10.8)	(11.7)	(15.3)	(16.7)	(13.5)	(16.2)
g_E	27.3	27.3	27.3	27.3	27.3	27.3	27.3	27.3	27.3	27.3
	(12.3)	(12.3)	(12.3)	(12.3)	(12.3)	(12.3)	(12.3)	(12.3)	(12.3)	(12.3)
	-9.5	12.4	7.8	-2.6	-6.2	-2.6	14.8	22.4	5.6	19.8
	-4.300	5.605	3.519	-1.169	-2.809	-1.169	6.689	10.098	2.519	8.936
c_O	24	20	23	30	31	28	29	24	24	25
	(10.8)	(9.0)	(10.4)	(13.5)	(14.0)	(12.6)	(13.1)	(10.8)	(10.8)	(11.3)
c_E	23.1	23.1	23.1	23.1	23.1	23.1	23.1	23.1	23.1	23.1
	(10.4)	(10.4)	(10.4)	(10.4)	(10.4)	(10.4)	(10.4)	(10.4)	(10.4)	(10.4)
	1.9	-5.7	-0.1	15.8	18.3	10.8	13.3	1.9	1.9	4.0
	0.855	-2.573	-0.063	7.099	8.251	4.885	5.977	0.855	0.855	1.810
N	222	222	222	222	222	222	222	222	222	222
X2	2.3	3.6	3.6	7.2	3.8	2.5	5.5	4.2	1.7	4.8
Gvalue	2.3	3.5	3.7	7.2	3.6	2.5	5.4	3.9	1.7	4.6
Gv/N	1.047	1.590	1.670	3.253	1.623	1.122	2.426	1.742	0.764	2.079
Prob	0.50788	0.31683	0.29492	0.06515	0.30770	0.47693	0.14568	0.27629	0.63777	0.20232

	80	79	78	77	76	75	74	73	72	71
a_O	86	90	85	91	98	99	96	87	91	82
	(38.7)	(40.5)	(38.3)	(41.0)	(44.1)	(44.6)	(43.2)	(39.2)	(41.0)	(36.9)
a_E	91.5	91.5	91.5	91.5	91.5	91.5	91.5	91.5	91.5	91.5
	(41.2)	(41.2)	(41.2)	(41.2)	(41.2)	(41.2)	(41.2)	(41.2)	(41.2)	(41.2)
	-10.6	-2.9	-12.5	-1.0	13.5	15.6	9.3	-8.7	-1.0	-17.9
	-4.786	-1.322	-5.626	-0.431	6.078	7.046	4.171	-3.936	-0.431	-8.082
t_O	94	86	80	74	79	76	73	86	73	86
	(42.3)	(38.7)	(36.0)	(33.3)	(35.6)	(34.2)	(32.9)	(38.7)	(32.9)	(38.7)
t_E	80.1	80.1	80.1	80.1	80.1	80.1	80.1	80.1	80.1	80.1
	(36.1)	(36.1)	(36.1)	(36.1)	(36.1)	(36.1)	(36.1)	(36.1)	(36.1)	(36.1)
	30.0	12.2	-0.2	-11.8	-2.2	-8.0	-13.6	12.2	-13.6	12.2
	13.530	5.487	-0.108	-5.297	-1.002	-3.615	-6.121	5.487	-6.121	5.487
g_O	23	26	34	29	26	30	34	29	27	32
	(10.4)	(11.7)	(15.3)	(13.1)	(11.7)	(13.5)	(15.3)	(13.1)	(12.2)	(14.4)
g_E	27.3	27.3	27.3	27.3	27.3	27.3	27.3	27.3	27.3	27.3
	(12.3)	(12.3)	(12.3)	(12.3)	(12.3)	(12.3)	(12.3)	(12.3)	(12.3)	(12.3)
	-7.9	-2.6	14.8	3.4	-2.6	5.6	14.8	3.4	-0.7	10.1
	-3.574	-1.169	6.689	1.550	-1.169	2.519	6.689	1.550	-0.295	4.548
c_O	19	20	23	28	19	17	19	20	31	22
	(8.6)	(9.0)	(10.4)	(12.6)	(8.6)	(7.7)	(8.6)	(9.0)	(14.0)	(9.9)
c_E	23.1	23.1	23.1	23.1	23.1	23.1	23.1	23.1	23.1	23.1
	(10.4)	(10.4)	(10.4)	(10.4)	(10.4)	(10.4)	(10.4)	(10.4)	(10.4)	(10.4)
	-7.4	-5.7	-0.1	10.8	-7.4	-10.4	-7.4	-5.7	18.3	-2.1
	-3.322	-2.573	-0.063	4.885	-3.322	-4.676	-3.322	-2.573	8.251	-0.941
N	222	222	222	222	222	222	222	222	222	222
X2	4.1	0.9	2.1	1.6	1.3	2.7	3.2	1.2	3.4	2.3
Gvalue	4.1	0.9	2.0	1.6	1.3	2.8	3.1	1.2	3.1	2.2
Gv/N	1.848	0.423	0.892	0.706	0.586	1.274	1.417	0.528	1.404	1.012
Prob	0.25071	0.81596	0.57653	0.66666	0.72914	0.41869	0.36965	0.75963	0.37393	0.52294

	70	69	68	67	66	65	64	63	62	61
a_O	87	81	95	100	71	86	91	83	96	99
	(39.2)	(36.5)	(42.8)	(45.0)	(32.0)	(38.7)	(41.0)	(37.4)	(43.2)	(44.6)
a_E	91.5	91.5	91.5	91.5	91.5	91.5	91.5	91.5	91.5	91.5
	(41.2)	(41.2)	(41.2)	(41.2)	(41.2)	(41.2)	(41.2)	(41.2)	(41.2)	(41.2)
	-8.7	-19.7	7.2	17.8	-36.0	-10.6	-1.0	-16.1	9.3	15.6
	-3.936	-8.879	3.231	8.023	-16.211	-4.786	-0.431	-7.274	4.171	7.046
t_O	88	95	72	68	81	82	73	76	74	77
	(39.6)	(42.8)	(32.4)	(30.6)	(36.5)	(36.9)	(32.9)	(34.2)	(33.3)	(34.7)

t_E	80.1	80.1	80.1	80.1	80.1	80.1	80.1	80.1	80.1	80.1
	(36.1)	(36.1)	(36.1)	(36.1)	(36.1)	(36.1)	(36.1)	(36.1)	(36.1)	(36.1)
	16.5	32.4	-15.4	-22.3	1.8	3.8	-13.6	-8.0	-11.8	-6.1
	7.437	14.580	-6.931	-10.048	0.797	1.713	-6.121	-3.615	-5.297	-2.755
g_O	26	19	24	39	42	31	28	30	22	32
	(11.7)	(8.6)	(10.8)	(17.6)	(18.9)	(14.0)	(12.6)	(13.5)	(9.9)	(14.4)
g_E	27.3	27.3	27.3	27.3	27.3	27.3	27.3	27.3	27.3	27.3
	(12.3)	(12.3)	(12.3)	(12.3)	(12.3)	(12.3)	(12.3)	(12.3)	(12.3)	(12.3)
	-2.6	-13.8	-6.2	27.7	36.1	7.8	1.4	5.6	-9.5	10.1
	-1.169	-6.223	-2.809	12.493	16.258	3.519	0.611	2.519	-4.300	4.548
c_O	21	27	31	15	28	23	30	33	30	14
	(9.5)	(12.2)	(14.0)	(6.8)	(12.6)	(10.4)	(13.5)	(14.9)	(13.5)	(6.3)
c_E	23.1	23.1	23.1	23.1	23.1	23.1	23.1	23.1	23.1	23.1
	(10.4)	(10.4)	(10.4)	(10.4)	(10.4)	(10.4)	(10.4)	(10.4)	(10.4)	(10.4)
	-3.9	8.5	18.3	-12.9	10.8	-0.1	15.8	23.6	15.8	-14.0
	-1.779	3.826	8.251	-5.817	4.885	-0.063	7.099	10.642	7.099	-6.300
N	222	222	222	222	222	222	222	222	222	222
X2	1.2	7.2	4.1	10.4	13.5	0.9	2.7	5.5	3.8	5.1
Gvalue	1.2	7.3	3.9	10.3	12.7	0.9	2.6	5.0	3.7	5.6
Gv/N	0.555	3.304	1.742	4.650	5.730	0.383	1.158	2.273	1.673	2.539
Prob	0.74553	0.06193	0.27614	0.01600	0.00528	0.83720	0.46259	0.16844	0.29405	0.13074

	60	59	58	57	56	55	54	53	52	51
a_O	88	100	95	82	92	87	101	97	95	95
	(39.6)	(45.0)	(42.8)	(36.9)	(41.4)	(39.2)	(45.5)	(43.7)	(42.8)	(42.8)
a_E	91.5	91.5	91.5	91.5	91.5	91.5	91.5	91.5	91.5	91.5
	(41.2)	(41.2)	(41.2)	(41.2)	(41.2)	(41.2)	(41.2)	(41.2)	(41.2)	(41.2)
	-6.8	17.8	7.2	-17.9	1.0	-8.7	20.0	11.4	7.2	7.2
	-3.075	8.023	3.231	-8.082	0.470	-3.936	9.008	5.120	3.231	3.231
t_O	88	68	77	87	81	84	70	77	67	68
	(39.6)	(30.6)	(34.7)	(39.2)	(36.5)	(37.8)	(31.5)	(34.7)	(30.2)	(30.6)
t_E	80.1	80.1	80.1	80.1	80.1	80.1	80.1	80.1	80.1	80.1
	(36.1)	(36.1)	(36.1)	(36.1)	(36.1)	(36.1)	(36.1)	(36.1)	(36.1)	(36.1)
	16.5	-22.3	-6.1	14.3	1.8	7.9	-18.9	-6.1	-24.0	-22.3
	7.437	-10.048	-2.755	6.457	0.797	3.579	-8.515	-2.755	-10.794	-10.048
g_O	21	28	25	28	27	24	21	24	30	37
	(9.5)	(12.6)	(11.3)	(12.6)	(12.2)	(10.8)	(9.5)	(10.8)	(13.5)	(16.7)
g_E	27.3	27.3	27.3	27.3	27.3	27.3	27.3	27.3	27.3	27.3
	(12.3)	(12.3)	(12.3)	(12.3)	(12.3)	(12.3)	(12.3)	(12.3)	(12.3)	(12.3)
	-11.1	1.4	-4.5	1.4	-0.7	-6.2	-11.1	-6.2	5.6	22.4
	-4.984	0.611	-2.007	0.611	-0.295	-2.809	-4.984	-2.809	2.519	10.098
c_O	25	26	25	25	22	27	30	24	30	22
	(11.3)	(11.7)	(11.3)	(11.3)	(9.9)	(12.2)	(13.5)	(10.8)	(13.5)	(9.9)
c_E	23.1	23.1	23.1	23.1	23.1	23.1	23.1	23.1	23.1	23.1
	(10.4)	(10.4)	(10.4)	(10.4)	(10.4)	(10.4)	(10.4)	(10.4)	(10.4)	(10.4)
	4.0	6.2	4.0	4.0	-2.1	8.5	15.8	1.9	15.8	-2.1
	1.810	2.801	1.810	1.810	-0.941	3.826	7.099	0.855	7.099	-0.941
N	222	222	222	222	222	222	222	222	222	222
X2	2.5	3.0	0.6	1.8	0.1	1.5	5.8	0.9	4.6	5.4
Gvalue	2.6	3.1	0.6	1.8	0.1	1.5	5.8	0.9	4.6	5.2
Gv/N	1.188	1.386	0.279	0.796	0.030	0.660	2.607	0.410	2.055	2.340
Prob	0.45111	0.37988	0.89218	0.62231	0.99548	0.69021	0.12238	0.82302	0.20676	0.15808

	50	49	48	47	46	45	44	43	42	41
a_O	78	88	89	86	101	86	88	97	82	95
	(35.1)	(39.6)	(40.1)	(38.7)	(45.5)	(38.7)	(39.6)	(43.7)	(36.9)	(42.8)
a_E	91.5	91.5	91.5	91.5	91.5	91.5	91.5	91.5	91.5	91.5
	(41.2)	(41.2)	(41.2)	(41.2)	(41.2)	(41.2)	(41.2)	(41.2)	(41.2)	(41.2)
	-24.9	-6.8	-4.9	-10.6	20.0	-10.6	-6.8	11.4	-17.9	7.2
	-11.202	-3.075	-2.204	-4.786	9.008	-4.786	-3.075	5.120	-8.082	3.231
t_O	87	84	80	81	69	84	79	87	84	82
	(39.2)	(37.8)	(36.0)	(36.5)	(31.1)	(37.8)	(35.6)	(39.2)	(37.8)	(36.9)
t_E	80.1	80.1	80.1	80.1	80.1	80.1	80.1	80.1	80.1	80.1
	(36.1)	(36.1)	(36.1)	(36.1)	(36.1)	(36.1)	(36.1)	(36.1)	(36.1)	(36.1)
	14.3	7.9	-0.2	1.8	-20.6	7.9	-2.2	14.3	7.9	3.8
	6.457	3.579	-0.108	0.797	-9.288	3.579	-1.002	6.457	3.579	1.713
g_O	24	28	27	32	27	30	29	21	36	29
	(10.8)	(12.6)	(12.2)	(14.4)	(12.2)	(13.5)	(13.1)	(9.5)	(16.2)	(13.1)
g_E	27.3	27.3	27.3	27.3	27.3	27.3	27.3	27.3	27.3	27.3
	(12.3)	(12.3)	(12.3)	(12.3)	(12.3)	(12.3)	(12.3)	(12.3)	(12.3)	(12.3)
	-6.2	1.4	-0.7	10.1	-0.7	5.6	3.4	-11.1	19.8	3.4
	-2.809	0.611	-0.295	4.548	-0.295	2.519	1.550	-4.984	8.936	1.550
c_O	33	22	26	23	25	22	26	17	20	16
	(14.9)	(9.9)	(11.7)	(10.4)	(11.3)	(9.9)	(11.7)	(7.7)	(9.0)	(7.2)
c_E	23.1	23.1	23.1	23.1	23.1	23.1	23.1	23.1	23.1	23.1
	(10.4)	(10.4)	(10.4)	(10.4)	(10.4)	(10.4)	(10.4)	(10.4)	(10.4)	(10.4)
	23.6	-2.1	6.2	-0.1	4.0	-2.1	6.2	-10.4	-5.7	-11.7
	10.642	-0.941	2.801	-0.063	1.810	-0.941	2.801	-4.676	-2.573	-5.275
N	222	222	222	222	222	222	222	222	222	222
X2	7.3	0.4	0.4	1.1	2.7	0.8	0.6	4.0	4.3	2.4
Gvalue	6.9	0.4	0.4	1.1	2.7	0.8	0.6	4.3	4.1	2.7
Gv/N	3.088	0.174	0.193	0.496	1.234	0.371	0.273	1.917	1.860	1.220
Prob	0.07663	0.94317	0.93412	0.77685	0.43358	0.84393	0.89479	0.23523	0.24781	0.43898

	40	39	38	37	36	35	34	33	32	31
a_O	86	83	89	91	86	105	86	96	102	94
	(38.7)	(37.4)	(40.1)	(41.0)	(38.7)	(47.3)	(38.7)	(43.2)	(45.9)	(42.3)
a_E	91.5	91.5	91.5	91.5	91.5	91.5	91.5	91.5	91.5	91.5
	(41.2)	(41.2)	(41.2)	(41.2)	(41.2)	(41.2)	(41.2)	(41.2)	(41.2)	(41.2)
	-10.6	-16.1	-4.9	-1.0	-10.6	28.9	-10.6	9.3	22.2	5.1
	-4.786	-7.274	-2.204	-0.431	-4.786	13.039	-4.786	4.171	10.003	2.301
t_O	88	91	87	80	76	77	81	71	76	78
	(39.6)	(41.0)	(39.2)	(36.0)	(34.2)	(34.7)	(36.5)	(32.0)	(34.2)	(35.1)
t_E	80.1	80.1	80.1	80.1	80.1	80.1	80.1	80.1	80.1	80.1
	(36.1)	(36.1)	(36.1)	(36.1)	(36.1)	(36.1)	(36.1)	(36.1)	(36.1)	(36.1)
	16.5	23.2	14.3	-0.2	-8.0	-6.1	1.8	-17.2	-8.0	-4.2
	7.437	10.439	6.457	-0.108	-3.615	-2.755	0.797	-7.730	-3.615	-1.884
g_O	25	28	24	26	38	28	31	33	23	28
	(11.3)	(12.6)	(10.8)	(11.7)	(17.1)	(12.6)	(14.0)	(14.9)	(10.4)	(12.6)
g_E	27.3	27.3	27.3	27.3	27.3	27.3	27.3	27.3	27.3	27.3
	(12.3)	(12.3)	(12.3)	(12.3)	(12.3)	(12.3)	(12.3)	(12.3)	(12.3)	(12.3)
	-4.5	1.4	-6.2	-2.6	25.0	1.4	7.8	12.4	-7.9	1.4
	-2.007	0.611	-2.809	-1.169	11.284	0.611	3.519	5.605	-3.574	0.611
c_O	23	20	22	25	22	12	24	22	21	22
	(10.4)	(9.0)	(9.9)	(11.3)	(9.9)	(5.4)	(10.8)	(9.9)	(9.5)	(9.9)
c_E	23.1	23.1	23.1	23.1	23.1	23.1	23.1	23.1	23.1	23.1

```
        ( 10.4) ( 10.4) ( 10.4) ( 10.4) ( 10.4) ( 10.4) ( 10.4) ( 10.4) ( 10.4) ( 10.4)
           -0.1    -5.7    -2.1     4.0    -2.1   -15.7     1.9    -2.1    -3.9    -2.1
         -0.063  -2.573  -0.941   1.810  -0.941  -7.066   0.855  -0.941  -1.779  -0.941

N           222     222     222     222     222     222     222     222     222     222
X2          1.3     2.7     1.1     0.2     4.8     7.4     0.9     2.5     2.3     0.2
Gvalue      1.3     2.7     1.1     0.2     4.3     8.5     0.9     2.5     2.3     0.2
Gv/N      0.581   1.203   0.503   0.102   1.942   3.828   0.385   1.105   1.035   0.087
Prob    0.73137 0.44525 0.77318 0.97335 0.22981 0.03675 0.83656 0.48392 0.51281 0.97886
        -------------------------------------------------------------------------------
             30      29      28      27      26      25      24      23      22      21
        -------------------------------------------------------------------------------
a_O          82      91      92      96      89      88     100     104      95     100
        ( 36.9) ( 41.0) ( 41.4) ( 43.2) ( 40.1) ( 39.6) ( 45.0) ( 46.8) ( 42.8) ( 45.0)
a_E        91.5    91.5    91.5    91.5    91.5    91.5    91.5    91.5    91.5    91.5
        ( 41.2) ( 41.2) ( 41.2) ( 41.2) ( 41.2) ( 41.2) ( 41.2) ( 41.2) ( 41.2) ( 41.2)
          -17.9    -1.0     1.0     9.3    -4.9    -6.8    17.8    26.7     7.2    17.8
         -8.082  -0.431   0.470   4.171  -2.204  -3.075   8.023  12.018   3.231   8.023
t_O          90      78      85      89      82      85      80      75      69      77
        ( 40.5) ( 35.1) ( 38.3) ( 40.1) ( 36.9) ( 38.3) ( 36.0) ( 33.8) ( 31.1) ( 34.7)
t_E        80.1    80.1    80.1    80.1    80.1    80.1    80.1    80.1    80.1    80.1
        ( 36.1) ( 36.1) ( 36.1) ( 36.1) ( 36.1) ( 36.1) ( 36.1) ( 36.1) ( 36.1) ( 36.1)
           20.9    -4.2    10.1    18.7     3.8    10.1    -0.2    -9.9   -20.6    -6.1
          9.428  -1.884   4.528   8.428   1.713   4.528  -0.108  -4.462  -9.288  -2.755
g_O          22      29      29      25      27      18      24      24      37      30
        (  9.9) ( 13.1) ( 13.1) ( 11.3) ( 12.2) (  8.1) ( 10.8) ( 10.8) ( 16.7) ( 13.5)
g_E        27.3    27.3    27.3    27.3    27.3    27.3    27.3    27.3    27.3    27.3
        ( 12.3) ( 12.3) ( 12.3) ( 12.3) ( 12.3) ( 12.3) ( 12.3) ( 12.3) ( 12.3) ( 12.3)
           -9.5     3.4     3.4    -4.5    -0.7   -15.0    -6.2    -6.2    22.4     5.6
         -4.300   1.550   1.550  -2.007  -0.295  -6.772  -2.809  -2.809  10.098   2.519
c_O          28      24      16      12      24      31      18      19      21      15
        ( 12.6) ( 10.8) (  7.2) (  5.4) ( 10.8) ( 14.0) (  8.1) (  8.6) (  9.5) (  6.8)
c_E        23.1    23.1    23.1    23.1    23.1    23.1    23.1    23.1    23.1    23.1
        ( 10.4) ( 10.4) ( 10.4) ( 10.4) ( 10.4) ( 10.4) ( 10.4) ( 10.4) ( 10.4) ( 10.4)
           10.8     1.9   -11.7   -15.7     1.9    18.3    -8.9    -7.4    -3.9   -12.9
          4.885   0.855  -5.275  -7.066   0.855   8.251  -4.024  -3.322  -1.779  -5.817

N           222     222     222     222     222     222     222     222     222     222
X2          4.3     0.2     2.6     6.7     0.2     6.3     2.3     3.2     5.3     4.0
Gvalue      4.3     0.2     2.8     7.8     0.2     6.5     2.4     3.2     5.0     4.4
Gv/N      1.932   0.088   1.272   3.526   0.069   2.932   1.081   1.424   2.262   1.969
Prob    0.23185 0.97821 0.41954 0.04973 0.98485 0.08929 0.49370 0.36726 0.17016 0.22406
        -------------------------------------------------------------------------------
             20      19      18      17      16      15      14      13      12      11
        -------------------------------------------------------------------------------
a_O         106     103      94      98      94      91     103     100     102     103
        ( 47.7) ( 46.4) ( 42.3) ( 44.1) ( 42.3) ( 41.0) ( 46.4) ( 45.0) ( 45.9) ( 46.4)
a_E        91.5    91.5    91.5    91.5    91.5    91.5    91.5    91.5    91.5    91.5
        ( 41.2) ( 41.2) ( 41.2) ( 41.2) ( 41.2) ( 41.2) ( 41.2) ( 41.2) ( 41.2) ( 41.2)
           31.2    24.4     5.1    13.5     5.1    -1.0    24.4    17.8    22.2    24.4
         14.068  11.006   2.301   6.078   2.301  -0.431  11.006   8.023  10.003  11.006
t_O          63      80      84      83      80      81      78      87      84      82
        ( 28.4) ( 36.0) ( 37.8) ( 37.4) ( 36.0) ( 36.5) ( 35.1) ( 39.2) ( 37.8) ( 36.9)
t_E        80.1    80.1    80.1    80.1    80.1    80.1    80.1    80.1    80.1    80.1
        ( 36.1) ( 36.1) ( 36.1) ( 36.1) ( 36.1) ( 36.1) ( 36.1) ( 36.1) ( 36.1) ( 36.1)
          -30.3    -0.2     7.9     5.9    -0.2     1.8    -4.2    14.3     7.9     3.8
        -13.644  -0.108   3.579   2.641  -0.108   0.797  -1.884   6.457   3.579   1.713
g_O          32      22      26      19      20      22      22      16      24      20
        ( 14.4) (  9.9) ( 11.7) (  8.6) (  9.0) (  9.9) (  9.9) (  7.2) ( 10.8) (  9.0)
g_E        27.3    27.3    27.3    27.3    27.3    27.3    27.3    27.3    27.3    27.3
        ( 12.3) ( 12.3) ( 12.3) ( 12.3) ( 12.3) ( 12.3) ( 12.3) ( 12.3) ( 12.3) ( 12.3)
           10.1    -9.5    -2.6   -13.8   -12.5    -9.5    -9.5   -17.1    -6.2   -12.5
          4.548  -4.300  -1.169  -6.223  -5.626  -4.300  -4.300  -7.717  -2.809  -5.626
c_O          21      17      18      22      28      28      19      19      12      17
        (  9.5) (  7.7) (  8.1) (  9.9) ( 12.6) ( 12.6) (  8.6) (  8.6) (  5.4) (  7.7)
c_E        23.1    23.1    23.1    23.1    23.1    23.1    23.1    23.1    23.1    23.1
        ( 10.4) ( 10.4) ( 10.4) ( 10.4) ( 10.4) ( 10.4) ( 10.4) ( 10.4) ( 10.4) ( 10.4)
           -3.9   -10.4    -8.9    -2.1    10.8    10.8    -7.4    -7.4   -15.7   -10.4
         -1.779  -4.676  -4.024  -0.941   4.885   4.885  -3.322  -3.322  -7.066  -4.676

N           222     222     222     222     222     222     222     222     222     222
X2          6.9     4.1     1.4     3.2     3.1     2.1     3.3     6.8     7.1     5.1
Gvalue      7.1     4.3     1.5     3.5     3.2     2.1     3.3     7.6     8.2     5.4
Gv/N      3.194   1.922   0.687   1.555   1.452   0.951   1.500   3.440   3.706   2.417
Prob    0.06909 0.23402 0.67635 0.32704 0.35830 0.54943 0.34364 0.05416 0.04154 0.14687
        -------------------------------------------------------------------------------
             10       9       8       7       6       5       4       3       2       1
        -------------------------------------------------------------------------------
a_O          85      99      80     105      99     106      92      94      93      85
        ( 38.3) ( 44.6) ( 36.0) ( 47.3) ( 44.6) ( 47.7) ( 41.4) ( 42.3) ( 41.9) ( 38.3)
a_E        91.5    91.5    91.5    91.5    91.5    91.5    91.5    91.5    91.5    91.5
        ( 41.2) ( 41.2) ( 41.2) ( 41.2) ( 41.2) ( 41.2) ( 41.2) ( 41.2) ( 41.2) ( 41.2)
          -12.5    15.6   -21.5    28.9    15.6    31.2     1.0     5.1     3.1   -12.5
         -5.626   7.046  -9.664  13.039   7.046  14.068   0.470   2.301   1.381  -5.626
t_O          92      69     100      86      73      83      81      79      76     100
        ( 41.4) ( 31.1) ( 45.0) ( 38.7) ( 32.9) ( 37.4) ( 36.5) ( 35.6) ( 34.2) ( 45.0)
t_E        80.1    80.1    80.1    80.1    80.1    80.1    80.1    80.1    80.1    80.1
        ( 36.1) ( 36.1) ( 36.1) ( 36.1) ( 36.1) ( 36.1) ( 36.1) ( 36.1) ( 36.1) ( 36.1)
           25.4   -20.6    44.3    12.2   -13.6     5.9     1.8    -2.2    -8.0    44.3
         11.460  -9.288  19.968   5.487  -6.121   2.641   0.797  -1.002  -3.615  19.968
g_O          24      29      23      23      23      18      31      27      29      19
        ( 10.8) ( 13.1) ( 10.4) ( 10.4) ( 10.4) (  8.1) ( 14.0) ( 12.2) ( 13.1) (  8.6)
g_E        27.3    27.3    27.3    27.3    27.3    27.3    27.3    27.3    27.3    27.3
        ( 12.3) ( 12.3) ( 12.3) ( 12.3) ( 12.3) ( 12.3) ( 12.3) ( 12.3) ( 12.3) ( 12.3)
           -6.2     3.4    -7.9    -7.9    -7.9   -15.0     7.8    -0.7     3.4   -13.8
         -2.809   1.550  -3.574  -3.574  -3.574  -6.772   3.519  -0.295   1.550  -6.223
c_O          21      25      19       8      27      15      18      22      24      18
        (  9.5) ( 11.3) (  8.6) (  3.6) ( 12.2) (  6.8) (  8.1) (  9.9) ( 10.8) (  8.1)
c_E        23.1    23.1    23.1    23.1    23.1    23.1    23.1    23.1    23.1    23.1
        ( 10.4) ( 10.4) ( 10.4) ( 10.4) ( 10.4) ( 10.4) ( 10.4) ( 10.4) ( 10.4) ( 10.4)
           -3.9     4.0    -7.4   -16.9     8.5   -12.9    -8.9    -2.1     1.9    -8.9
         -1.779   1.810  -3.322  -7.633   3.826  -5.817  -4.024  -0.941   0.855  -4.024

N           222     222     222     222     222     222     222     222     222     222
X2          2.8     2.4     7.8    13.0     2.6     8.4     1.6     0.1     0.4     9.0
Gvalue      2.8     2.5     7.6    16.2     2.6     9.1     1.7     0.1     0.4     9.1
Gv/N      1.246   1.117   3.407   7.319   1.178   4.120   0.762   0.063   0.170   4.095
Prob    0.42921 0.47905 0.05594 0.00101 0.45503 0.02742 0.63896 0.98679 0.94480 0.02811
        -------------------------------------------------------------------------------

CDS_INITIAL:
        -------------------------------------------------------------------------------
```

	1	2	3	4	5	6	7	8	9	10
a_O	163	0	0	118	111	98	119	111	100	108
	(73.4)	(0.0)	(0.0)	(53.2)	(50.0)	(44.1)	(53.6)	(50.0)	(45.0)	(48.6)
a_E	93.2	87.7	84.2	93.2	87.7	84.2	93.2	87.7	84.2	93.2
	(42.0)	(39.5)	(37.9)	(42.0)	(39.5)	(37.9)	(42.0)	(39.5)	(37.9)	(42.0)
	182.2	0.0	0.0	55.7	52.4	29.8	58.2	52.4	34.5	31.8
	82.085	0.000	0.000	25.080	23.599	13.441	26.197	23.599	15.536	14.338
t_O	46	222	0	36	71	90	49	75	88	48
	(20.7)	(100.0)	(0.0)	(16.2)	(32.0)	(40.5)	(22.1)	(33.8)	(39.6)	(21.6)
t_E	52.7	78.1	95.1	52.7	78.1	95.1	52.7	78.1	95.1	52.7
	(23.7)	(35.2)	(42.8)	(23.7)	(35.2)	(42.8)	(23.7)	(35.2)	(42.8)	(23.7)
	-12.5	464.1	0.0	-27.4	-13.5	-9.9	-7.1	-6.0	-13.7	-9.0
	-5.627	209.037	0.000	-12.354	-6.064	-4.472	-3.205	-2.703	-6.154	-4.032
g_O	13	0	222	31	17	13	41	16	25	40
	(5.9)	(0.0)	(100.0)	(14.0)	(7.7)	(5.9)	(18.5)	(7.2)	(11.3)	(18.0)
g_E	52.9	23.3	22.0	52.9	23.3	22.0	52.9	23.3	22.0	52.9
	(23.8)	(10.5)	(9.9)	(23.8)	(10.5)	(9.9)	(23.8)	(10.5)	(9.9)	(23.8)
	-36.5	0.0	1025.6	-33.2	-10.8	-13.7	-20.9	-12.1	6.3	-22.4
	-16.444	0.000	461.994	-14.943	-4.847	-6.181	-9.437	-5.436	2.842	-10.096
c_O	0	0	0	37	23	21	13	20	9	26
	(0.0)	(0.0)	(0.0)	(16.7)	(10.4)	(9.5)	(5.9)	(9.0)	(4.1)	(11.7)
c_E	23.2	32.9	20.7	23.2	32.9	20.7	23.2	32.9	20.7	23.2
	(10.4)	(14.8)	(9.3)	(10.4)	(14.8)	(9.3)	(10.4)	(14.8)	(9.3)	(10.4)
	0.0	0.0	0.0	34.6	-16.5	0.6	-15.0	-20.0	-15.0	6.0
	0.000	0.000	0.000	15.597	-7.445	0.272	-6.770	-8.992	-6.753	2.696
N	222	222	222	222	222	222	222	222	222	222
X2	106.4	409.4	2014.5	29.2	11.6	6.3	14.6	13.7	10.5	6.3
Gvalue	133.2	464.1	1025.6	29.7	11.6	6.8	15.1	14.4	12.1	6.5
Gv/N	60.013	209.037	461.994	13.379	5.243	3.061	6.785	6.468	5.470	2.906
Prob	0.00000	0.00000	0.00000	0.00000	0.00873	0.07872	0.00176	0.00245	0.00691	0.09160

	11	12	13	14	15	16	17	18	19	20
a_O	105	108	102	102	89	92	74	77	98	88
	(47.3)	(48.6)	(45.9)	(45.9)	(40.1)	(41.4)	(33.3)	(34.7)	(44.1)	(39.6)
a_E	87.7	84.2	93.2	87.7	84.2	93.2	87.7	84.2	93.2	87.7
	(39.5)	(37.9)	(42.0)	(39.5)	(37.9)	(42.0)	(39.5)	(37.9)	(42.0)	(39.5)
	37.9	53.9	18.4	30.9	10.0	-2.4	-25.1	-13.7	9.8	0.7
	17.067	24.266	8.289	13.915	4.483	-1.076	-11.298	-6.168	4.432	0.301
t_O	78	76	65	92	94	60	96	106	62	91
	(35.1)	(34.2)	(29.3)	(41.4)	(42.3)	(27.0)	(43.2)	(47.7)	(27.9)	(41.0)
t_E	78.1	95.1	52.7	78.1	95.1	52.7	78.1	95.1	52.7	78.1
	(35.2)	(42.8)	(23.7)	(35.2)	(42.8)	(23.7)	(35.2)	(42.8)	(23.7)	(35.2)
	-0.1	-34.1	27.3	30.2	-2.2	15.6	39.7	23.0	20.2	27.9
	-0.055	-15.352	12.295	13.617	-0.988	7.022	17.890	10.359	9.088	12.573
g_O	20	16	37	10	17	44	14	20	34	18
	(9.0)	(7.2)	(16.7)	(4.5)	(7.7)	(19.8)	(6.3)	(9.0)	(15.3)	(8.1)
g_E	23.3	22.0	52.9	23.3	22.0	52.9	23.3	22.0	52.9	23.3
	(10.5)	(9.9)	(23.8)	(10.5)	(9.9)	(23.8)	(10.5)	(9.9)	(23.8)	(10.5)
	-6.2	-10.2	-26.5	-16.9	-8.8	-16.3	-14.3	-3.9	-30.1	-9.3
	-2.774	-4.614	-11.938	-7.632	-3.974	-7.328	-6.441	-1.747	-13.560	-4.206
c_O	19	22	18	18	22	26	38	19	28	25
	(8.6)	(9.9)	(8.1)	(8.1)	(9.9)	(11.7)	(17.1)	(8.6)	(12.6)	(11.3)
c_E	32.9	20.7	23.2	32.9	20.7	23.2	32.9	20.7	23.2	32.9
	(14.8)	(9.3)	(10.4)	(14.8)	(9.3)	(10.4)	(14.8)	(9.3)	(10.4)	(14.8)
	-20.9	2.7	-9.1	-21.8	2.7	6.0	10.9	-3.3	10.6	-13.8
	-9.420	1.207	-4.097	-9.801	1.207	2.696	4.889	-1.467	4.773	-6.214
N	222	222	222	222	222	222	222	222	222	222
X2	9.8	12.3	9.7	19.2	1.5	2.9	10.8	2.2	9.7	5.3
Gvalue	10.7	12.2	10.1	22.4	1.6	2.9	11.2	2.2	10.5	5.4
Gv/N	4.817	5.507	4.549	10.100	0.728	1.314	5.040	0.977	4.733	2.455
Prob	0.01350	0.00665	0.01774	0.00005	0.65566	0.40442	0.01075	0.53814	0.01472	0.14168

	21	22	23	24	25	26	27	28	29	30
a_O	90	97	85	91	87	76	82	104	74	96
	(40.5)	(43.7)	(38.3)	(41.0)	(39.2)	(34.2)	(36.9)	(46.8)	(33.3)	(43.2)
a_E	84.2	93.2	87.7	84.2	93.2	87.7	84.2	93.2	87.7	84.2
	(37.9)	(42.0)	(39.5)	(37.9)	(42.0)	(39.5)	(37.9)	(42.0)	(39.5)	(37.9)
	12.1	7.7	-5.3	14.2	-12.0	-21.7	-4.3	22.8	-25.1	25.3
	5.439	3.491	-2.365	6.406	-5.397	-9.778	-1.921	10.271	-11.298	11.384
t_O	89	54	90	93	61	102	95	60	102	79
	(40.1)	(24.3)	(40.5)	(41.9)	(27.5)	(45.9)	(42.8)	(27.0)	(45.9)	(35.6)
t_E	95.1	52.7	78.1	95.1	52.7	78.1	95.1	52.7	78.1	95.1
	(42.8)	(23.7)	(35.2)	(42.8)	(23.7)	(35.2)	(42.8)	(23.7)	(35.2)	(42.8)
	-11.8	2.7	25.6	-4.2	17.9	54.6	-0.2	15.6	54.6	-29.3
	-5.318	1.194	11.539	-1.873	8.048	24.579	-0.093	7.022	24.579	-13.203
g_O	23	51	14	19	48	13	27	33	23	25
	(10.4)	(23.0)	(6.3)	(8.6)	(21.6)	(5.9)	(12.2)	(14.9)	(10.4)	(11.3)
g_E	22.0	52.9	23.3	22.0	52.9	23.3	22.0	52.9	23.3	22.0
	(9.9)	(23.8)	(10.5)	(9.9)	(23.8)	(10.5)	(9.9)	(23.8)	(10.5)	(9.9)
	2.0	-3.8	-14.3	-5.6	-9.4	-15.2	11.0	-31.2	-0.7	6.3
	0.887	-1.711	-6.441	-2.538	-4.232	-6.849	4.941	-14.049	-0.295	2.842
c_O	20	20	33	19	26	31	18	25	23	22
	(9.0)	(9.0)	(14.9)	(8.6)	(11.7)	(14.0)	(8.1)	(11.3)	(10.4)	(9.9)
c_E	20.7	23.2	32.9	20.7	23.2	32.9	20.7	23.2	32.9	20.7
	(9.3)	(10.4)	(14.8)	(9.3)	(10.4)	(14.8)	(9.3)	(10.4)	(14.8)	(9.3)
	-1.4	-5.9	0.1	-3.3	6.0	-3.8	-5.0	3.8	-16.5	2.7
	-0.620	-2.654	0.051	-1.467	2.696	-1.698	-2.266	1.709	-7.445	1.207
N	222	222	222	222	222	222	222	222	222	222
X2	0.9	0.7	5.6	1.2	2.5	13.6	1.5	9.9	12.5	4.9
Gvalue	0.9	0.7	6.2	1.2	2.5	13.9	1.5	11.0	12.3	4.9
Gv/N	0.388	0.321	2.785	0.527	1.115	6.255	0.661	4.953	5.542	2.229
Prob	0.83463	0.87027	0.10309	0.75999	0.47978	0.00306	0.68993	0.01174	0.00641	0.17556

	31	32	33	34	35	36	37	38	39	40
a_O	104	82	92	86	85	86	101	79	73	81
	(46.8)	(36.9)	(41.4)	(38.7)	(38.3)	(38.7)	(45.5)	(35.6)	(32.9)	(36.5)
a_E	93.2	87.7	84.2	93.2	87.7	84.2	93.2	87.7	84.2	93.2
	(42.0)	(39.5)	(37.9)	(42.0)	(39.5)	(37.9)	(42.0)	(39.5)	(37.9)	(42.0)
	22.8	-11.0	16.4	-13.8	-5.3	3.7	16.2	-16.4	-20.8	-22.7
	10.271	-4.936	7.382	-6.231	-2.365	1.675	7.312	-7.408	-9.356	-10.239
t_O	52	106	91	68	94	98	57	96	113	72
	(23.4)	(47.7)	(41.0)	(30.6)	(42.3)	(44.1)	(25.7)	(43.2)	(50.9)	(32.4)
t_E	52.7	78.1	95.1	52.7	78.1	95.1	52.7	78.1	95.1	52.7
	(23.7)	(35.2)	(42.8)	(23.7)	(35.2)	(42.8)	(23.7)	(35.2)	(42.8)	(23.7)
	-1.4	64.9	-8.0	34.7	34.9	5.9	9.0	39.7	39.0	45.0

	-0.618	29.217	-3.615	15.626	15.735	2.649	4.037	17.890	17.554	20.253
g_O	43	15	18	45	18	21	39	11	18	48
	(19.4)	(6.8)	(8.1)	(20.3)	(8.1)	(9.5)	(17.6)	(5.0)	(8.1)	(21.6)
g_E	52.9	23.3	22.0	52.9	23.3	22.0	52.9	23.3	22.0	52.9
	(23.8)	(10.5)	(9.9)	(23.8)	(10.5)	(9.9)	(23.8)	(10.5)	(9.9)	(23.8)
	-17.9	-13.2	-7.3	-14.6	-9.3	-2.0	-23.8	-16.5	-7.3	-9.4
	-8.052	-5.968	-3.281	-6.584	-4.206	-0.912	-10.734	-7.450	-3.281	-4.232
c_O	23	19	21	23	25	17	25	36	18	21
	(10.4)	(8.6)	(9.5)	(10.4)	(11.3)	(7.7)	(11.3)	(16.2)	(8.1)	(9.5)
c_E	23.2	32.9	20.7	23.2	32.9	20.7	23.2	32.9	20.7	23.2
	(10.4)	(14.8)	(9.3)	(10.4)	(14.8)	(9.3)	(10.4)	(14.8)	(9.3)	(10.4)
	-0.3	-20.9	0.6	-0.3	-13.8	-6.7	3.8	6.4	-5.0	-4.1
	-0.156	-9.420	0.272	-0.156	-6.214	-3.016	1.709	2.878	-2.266	-1.863
N	222	222	222	222	222	222	222	222	222	222
X2	3.1	19.2	1.7	6.2	6.5	0.8	4.8	11.8	5.9	9.3
Gvalue	3.2	19.7	1.7	5.9	6.5	0.9	5.2	13.1	5.9	8.7
Gv/N	1.446	8.892	0.758	2.656	2.950	0.397	2.324	5.910	2.650	3.919
Prob	0.36048	0.00019	0.64100	0.11672	0.08777	0.82988	0.16051	0.00439	0.11747	0.03356

	41	42	43	44	45	46	47	48	49	50
a_O	80	83	94	88	77	88	82	76	93	76
	(36.0)	(37.4)	(42.3)	(39.6)	(34.7)	(39.6)	(36.9)	(34.2)	(41.9)	(34.2)
a_E	87.7	84.2	93.2	87.7	84.2	93.2	87.7	84.2	93.2	87.7
	(39.5)	(37.9)	(42.0)	(39.5)	(37.9)	(42.0)	(39.5)	(37.9)	(42.0)	(39.5)
	-14.6	-2.3	1.6	0.7	-13.7	-10.1	-11.0	-15.5	-0.4	-21.7
	-6.596	-1.038	0.722	0.301	-6.168	-4.553	-4.936	-6.983	-0.182	-9.778
t_O	92	106	65	86	101	66	85	108	59	82
	(41.4)	(47.7)	(29.3)	(38.7)	(45.5)	(29.7)	(38.3)	(48.6)	(26.6)	(36.9)
t_E	78.1	95.1	52.7	78.1	95.1	52.7	78.1	95.1	52.7	78.1
	(35.2)	(42.8)	(23.7)	(35.2)	(42.8)	(23.7)	(35.2)	(42.8)	(23.7)	(35.2)
	30.2	23.0	27.3	16.7	12.2	29.7	14.5	27.5	13.3	8.1
	13.617	10.359	12.295	7.504	5.474	13.392	6.521	12.374	6.012	3.637
g_O	21	19	37	20	23	46	16	21	46	31
	(9.5)	(8.6)	(16.7)	(9.0)	(10.4)	(20.7)	(7.2)	(9.5)	(20.7)	(14.0)
g_E	23.3	22.0	52.9	23.3	22.0	52.9	23.3	22.0	52.9	23.3
	(10.5)	(9.9)	(23.8)	(10.5)	(9.9)	(23.8)	(10.5)	(9.9)	(23.8)	(10.5)
	-4.4	-5.6	-26.5	-6.2	2.0	-12.9	-12.1	-2.0	-12.9	17.6
	-1.990	-2.538	-11.938	-2.774	0.887	-5.819	-5.436	-0.912	-5.819	7.939
c_O	29	14	26	28	21	22	39	17	24	33
	(13.1)	(6.3)	(11.7)	(12.6)	(9.5)	(9.9)	(17.6)	(7.7)	(10.8)	(14.9)
c_E	32.9	20.7	23.2	32.9	20.7	23.2	32.9	20.7	23.2	32.9
	(14.8)	(9.3)	(10.4)	(14.8)	(9.3)	(10.4)	(14.8)	(9.3)	(10.4)	(14.8)
	-7.4	-11.0	6.0	-9.1	0.6	-2.3	13.2	-6.7	1.7	0.1
	-3.331	-4.932	2.696	-4.101	0.272	-1.030	5.930	-3.016	0.758	0.051
N	222	222	222	222	222	222	222	222	222	222
X2	3.9	3.9	8.0	2.0	1.0	4.6	4.4	3.3	1.7	4.3
Gvalue	3.8	4.1	8.4	2.1	1.0	4.4	4.6	3.2	1.7	4.1
Gv/N	1.701	1.851	3.775	0.930	0.465	1.990	2.079	1.463	0.769	1.849
Prob	0.28664	0.24998	0.03878	0.55923	0.79364	0.21979	0.20221	0.35504	0.63524	0.25027

	51	52	53	54	55	56	57	58	59	60
a_O	85	91	82	81	82	89	101	95	86	74
	(38.3)	(41.0)	(36.9)	(36.5)	(36.9)	(40.1)	(45.5)	(42.8)	(38.7)	(33.3)
a_E	84.2	93.2	87.7	84.2	93.2	87.7	84.2	93.2	87.7	84.2
	(37.9)	(42.0)	(39.5)	(37.9)	(42.0)	(39.5)	(37.9)	(42.0)	(39.5)	(37.9)
	1.7	-4.4	-11.0	-6.2	-21.0	2.7	36.8	3.6	-3.3	-19.0
	0.760	-1.960	-4.936	-2.793	-9.459	1.210	16.596	1.636	-1.487	-8.577
t_O	93	64	83	89	65	82	87	65	89	104
	(41.9)	(28.8)	(37.4)	(40.1)	(29.3)	(36.9)	(39.2)	(29.3)	(40.1)	(46.8)
t_E	95.1	52.7	78.1	95.1	52.7	78.1	95.1	52.7	78.1	95.1
	(42.8)	(23.7)	(35.2)	(42.8)	(23.7)	(35.2)	(42.8)	(23.7)	(35.2)	(42.8)
	-4.2	24.9	10.2	-11.8	27.3	8.1	-15.5	27.3	23.3	18.6
	-1.873	11.212	4.587	-5.318	12.295	3.637	-6.980	12.295	10.515	8.379
g_O	19	47	24	29	57	16	16	47	15	23
	(8.6)	(21.2)	(10.8)	(13.1)	(25.7)	(7.2)	(7.2)	(21.2)	(6.8)	(10.4)
g_E	22.0	52.9	23.3	22.0	52.9	23.3	22.0	52.9	23.3	22.0
	(9.9)	(23.8)	(10.5)	(9.9)	(23.8)	(10.5)	(9.9)	(23.8)	(10.5)	(9.9)
	-5.6	-11.2	1.4	15.9	8.4	-12.1	-10.2	-11.2	-13.2	2.0
	-2.538	-5.035	0.613	7.174	3.800	-5.436	-4.614	-5.035	-5.968	0.887
c_O	25	20	33	23	18	35	18	15	32	21
	(11.3)	(9.0)	(14.9)	(10.4)	(8.1)	(15.8)	(8.1)	(6.8)	(14.4)	(9.5)
c_E	20.7	23.2	32.9	20.7	23.2	32.9	20.7	23.2	32.9	20.7
	(9.3)	(10.4)	(14.8)	(9.3)	(10.4)	(14.8)	(9.3)	(10.4)	(14.8)	(9.3)
	9.4	-5.9	0.1	4.8	-9.1	4.2	-5.0	-13.0	-1.9	0.6
	4.251	-2.654	0.051	2.183	-4.097	1.910	-2.266	-5.878	-0.837	0.272
N	222	222	222	222	222	222	222	222	222	222
X2	1.4	3.6	0.7	3.0	5.7	2.7	6.1	6.5	4.6	2.1
Gvalue	1.3	3.5	0.7	2.8	5.6	2.9	6.1	6.7	4.9	2.1
Gv/N	0.600	1.563	0.315	1.246	2.538	1.321	2.736	3.018	2.223	0.961
Prob	0.72168	0.32463	0.87327	0.42904	0.13078	0.40217	0.10808	0.08213	0.17669	0.54534

	61	62	63	64	65	66	67	68	69	70
a_O	102	80	83	82	99	77	93	87	87	96
	(45.9)	(36.0)	(37.4)	(36.9)	(44.6)	(34.7)	(41.9)	(39.2)	(39.2)	(43.4)
a_E	93.2	87.7	84.2	93.2	87.7	84.2	93.2	87.7	84.2	92.8
	(42.0)	(39.5)	(37.9)	(42.0)	(39.5)	(37.9)	(42.0)	(39.5)	(37.9)	(42.0)
	18.4	-14.6	-2.3	-21.0	24.1	-13.7	-0.4	-1.3	5.8	6.5
	8.289	-6.596	-1.038	-9.459	10.844	-6.168	-0.182	-0.598	2.601	2.962
t_O	51	86	101	54	67	106	60	86	96	49
	(23.0)	(38.7)	(45.5)	(24.3)	(30.2)	(47.7)	(27.0)	(38.7)	(43.2)	(22.2)
t_E	52.7	78.1	95.1	52.7	78.1	95.1	52.7	78.1	95.1	52.5
	(23.7)	(35.2)	(42.8)	(23.7)	(35.2)	(42.8)	(23.7)	(35.2)	(42.8)	(23.7)
	-3.3	16.7	12.2	2.7	-20.5	23.0	15.6	16.7	1.8	-6.7
	-1.498	7.504	5.474	1.194	-9.223	10.359	7.022	7.504	0.812	-3.020
g_O	37	21	21	62	18	19	44	15	16	53
	(16.7)	(9.5)	(9.5)	(27.9)	(8.1)	(8.6)	(19.8)	(6.8)	(7.2)	(24.0)
g_E	52.9	23.3	22.0	52.9	23.3	22.0	52.9	23.3	22.0	52.7
	(23.8)	(10.5)	(9.9)	(23.8)	(10.5)	(9.9)	(23.8)	(10.5)	(9.9)	(23.8)
	-26.5	-4.4	-2.0	19.6	-9.3	-5.6	-16.3	-13.2	-10.2	0.6
	-11.938	-1.990	-0.912	8.830	-4.206	-2.538	-7.328	-5.968	-4.614	0.276
c_O	32	35	17	24	38	20	25	34	23	23
	(14.4)	(15.8)	(7.7)	(10.8)	(17.1)	(9.0)	(11.3)	(15.3)	(10.4)	(10.4)
c_E	23.2	32.9	20.7	23.2	32.9	20.7	23.2	32.9	20.7	23.1
	(10.4)	(14.8)	(9.3)	(10.4)	(14.8)	(9.3)	(10.4)	(14.8)	(9.3)	(10.4)
	20.7	4.2	-6.7	1.7	10.9	-1.4	3.8	2.1	4.8	-0.1
	9.304	1.910	-3.016	0.758	4.889	-0.620	1.709	0.967	2.183	-0.062

N	222	222	222	222	222	222	222	222	222	221
X2	9.0	1.8	1.1	3.0	5.0	2.3	2.7	3.8	2.0	0.3
Gvalue	9.2	1.8	1.1	2.9	5.1	2.3	2.7	4.2	2.2	0.3
Gv/N	4.157	0.828	0.508	1.323	2.304	1.033	1.222	1.905	0.982	0.156
Prob	0.02640	0.60652	0.77016	0.40160	0.16358	0.51370	0.43823	0.23778	0.53603	0.95140

	71	72	73	74	75	76	77	78	79	80
a_O	102	79	91	92	84	83	92	89	90	85
	(46.2)	(35.7)	(41.2)	(41.6)	(38.0)	(37.6)	(41.6)	(40.3)	(40.9)	(38.6)
a_E	87.3	83.8	92.8	87.3	83.8	92.8	87.3	83.8	92.4	86.9
	(39.5)	(37.9)	(42.0)	(39.5)	(37.9)	(42.0)	(39.5)	(37.9)	(42.0)	(39.5)
	31.8	-9.3	-3.5	9.7	0.4	-18.5	9.7	10.8	-4.7	-3.7
	14.395	-4.201	-1.597	4.393	0.198	-8.368	4.393	4.867	-2.120	-1.688
t_O	74	109	56	86	100	56	78	98	57	78
	(33.5)	(49.3)	(25.3)	(38.9)	(45.2)	(25.3)	(35.3)	(44.3)	(25.9)	(35.5)
t_E	77.7	94.7	52.5	77.7	94.7	52.5	77.7	94.7	52.2	77.4
	(35.2)	(42.8)	(23.7)	(35.2)	(42.8)	(23.7)	(35.2)	(42.8)	(23.7)	(35.2)
	-7.2	30.7	7.3	17.4	10.9	7.3	0.6	6.8	10.0	1.3
	-3.275	13.899	3.316	7.890	4.953	3.316	0.263	3.062	4.543	0.586
g_O	16	16	44	16	16	55	18	15	51	23
	(7.2)	(7.2)	(19.9)	(7.2)	(7.2)	(24.9)	(8.1)	(6.8)	(23.2)	(10.5)
g_E	23.2	21.9	52.7	23.2	21.9	52.7	23.2	21.9	52.5	23.1
	(10.5)	(9.9)	(23.8)	(10.5)	(9.9)	(23.8)	(10.5)	(9.9)	(23.8)	(10.5)
	-11.9	-10.1	-15.9	-11.9	-10.1	4.7	-9.2	-11.4	-2.9	-0.2
	-5.395	-4.570	-7.181	-5.395	-4.570	2.130	-4.151	-5.160	-1.307	-0.108
c_O	29	17	30	27	21	27	33	19	22	34
	(13.1)	(7.7)	(13.6)	(12.2)	(9.5)	(12.2)	(14.9)	(8.6)	(10.0)	(15.5)
c_E	32.8	20.6	23.1	32.8	20.6	23.1	32.8	20.6	23.0	32.6
	(14.8)	(9.3)	(10.4)	(14.8)	(9.3)	(10.4)	(14.8)	(9.3)	(10.4)	(14.8)
	-7.1	-6.5	15.8	-10.5	0.8	8.5	0.4	-3.1	-1.9	2.8
	-3.227	-2.960	7.132	-4.751	0.359	3.845	0.186	-1.396	-0.858	1.256
N	221	221	221	221	221	221	221	221	220	220
X2	5.3	4.7	3.8	4.4	1.9	2.0	1.4	2.8	0.6	0.1
Gvalue	5.5	4.8	3.7	4.7	2.1	2.0	1.5	3.0	0.6	0.1
Gv/N	2.497	2.168	1.670	2.136	0.940	0.922	0.692	1.372	0.258	0.046
Prob	0.13753	0.18773	0.29685	0.19336	0.55645	0.56451	0.67562	0.38654	0.90361	0.99158

	81	82	83	84	85	86	87	88	89	90
a_O	82	91	87	91	87	97	85	95	65	88
	(37.3)	(41.4)	(39.5)	(41.4)	(39.5)	(44.1)	(38.6)	(43.8)	(30.0)	(40.6)
a_E	83.4	92.4	86.9	83.4	92.4	86.9	83.4	91.1	85.7	82.3
	(37.9)	(42.0)	(39.5)	(37.9)	(42.0)	(39.5)	(37.9)	(42.0)	(39.5)	(37.9)
	-2.8	-2.7	0.2	15.9	-10.4	21.4	3.2	8.0	-35.9	11.9
	-1.264	-1.229	0.112	7.212	-4.730	9.719	1.466	3.668	-16.557	5.466
t_O	95	58	81	91	50	66	96	64	87	95
	(43.2)	(26.4)	(36.8)	(41.4)	(22.7)	(30.0)	(43.6)	(29.5)	(40.1)	(43.8)
t_E	94.2	52.2	77.4	94.2	52.2	77.4	94.2	51.5	76.3	93.0
	(42.8)	(23.7)	(35.2)	(42.8)	(23.7)	(35.2)	(42.8)	(23.7)	(35.2)	(42.8)
	1.5	12.2	7.5	-6.4	-4.3	-21.0	3.5	27.8	22.8	4.1
	0.688	5.540	3.388	-2.900	-1.971	-9.527	1.609	12.814	10.520	1.900
g_O	23	53	21	22	61	22	14	43	34	17
	(10.5)	(24.1)	(9.5)	(10.0)	(27.7)	(10.0)	(6.4)	(19.8)	(15.7)	(7.8)
g_E	21.8	52.5	23.1	21.8	52.5	23.1	21.8	51.7	22.8	21.5
	(9.9)	(23.8)	(10.5)	(9.9)	(23.8)	(10.5)	(9.9)	(23.8)	(10.5)	(9.9)
	2.4	1.1	-4.0	0.3	18.4	-2.2	-12.4	-15.9	27.2	-8.0
	1.084	0.496	-1.835	0.148	8.366	-0.992	-5.659	-7.335	12.517	-3.709
c_O	20	18	31	16	22	35	25	15	31	17
	(9.1)	(8.2)	(14.1)	(7.3)	(10.0)	(15.9)	(11.4)	(6.9)	(14.3)	(7.8)
c_E	20.5	23.0	32.6	20.5	23.0	32.6	20.5	22.7	32.2	20.2
	(9.3)	(10.4)	(14.8)	(9.3)	(10.4)	(14.8)	(9.3)	(10.4)	(14.8)	(9.3)
	-1.0	-8.8	-3.2	-8.0	-1.9	4.9	9.9	-12.4	-2.4	-5.9
	-0.461	-3.986	-1.458	-3.614	-0.858	2.215	4.495	-5.698	-1.086	-2.728
N	220	220	220	220	220	220	220	217	217	217
X2	0.1	1.7	0.4	1.8	1.8	3.1	3.9	7.3	12.0	1.9
Gvalue	0.1	1.8	0.5	1.9	1.8	3.1	4.2	7.5	11.7	2.0
Gv/N	0.047	0.820	0.206	0.846	0.807	1.415	1.912	3.448	5.394	0.928
Prob	0.99142	0.61399	0.92888	0.60165	0.62036	0.37455	0.23996	0.05800	0.00847	0.56942

	91	92	93	94	95	96	97	98	99	100
a_O	89	83	82	75	90	78	96	94	77	87
	(41.4)	(38.6)	(38.1)	(35.2)	(42.3)	(36.6)	(45.1)	(44.1)	(36.2)	(40.8)
a_E	90.3	84.9	81.5	89.4	84.1	80.7	89.4	84.1	80.7	89.4
	(42.0)	(39.5)	(37.9)	(42.0)	(39.5)	(37.9)	(42.0)	(39.5)	(37.9)	(42.0)
	-2.5	-3.8	1.0	-26.4	12.2	-5.4	13.6	20.9	-7.3	-4.8
	-1.167	-1.750	0.460	-12.387	5.717	-2.536	6.397	9.810	-3.437	-2.244
t_O	51	77	98	62	73	90	56	70	98	64
	(23.7)	(35.8)	(45.6)	(29.1)	(34.3)	(42.3)	(26.3)	(32.9)	(46.0)	(30.0)
t_E	51.0	75.6	92.1	50.6	74.9	91.2	50.6	74.9	91.2	50.6
	(23.7)	(35.2)	(42.8)	(23.7)	(35.2)	(42.8)	(23.7)	(35.2)	(42.8)	(23.7)
	-0.1	2.8	12.2	25.3	-3.7	-2.5	11.5	-9.5	14.0	30.2
	-0.027	1.315	5.656	11.881	-1.758	-1.163	5.379	-4.444	6.570	14.172
g_O	51	18	21	49	24	22	38	22	19	39
	(23.7)	(8.4)	(9.8)	(23.0)	(11.3)	(10.3)	(17.8)	(10.3)	(8.9)	(18.3)
g_E	51.3	22.6	21.3	50.8	22.4	21.1	50.8	22.4	21.1	50.8
	(23.8)	(10.5)	(9.9)	(23.8)	(10.5)	(9.9)	(23.8)	(10.5)	(9.9)	(23.8)
	-0.5	-8.2	-0.7	-3.5	3.3	1.7	-22.0	-0.8	-4.1	-20.6
	-0.246	-3.806	-0.315	-1.649	1.571	0.820	-10.350	-0.357	-1.907	-9.672
c_O	24	37	14	27	26	23	23	27	19	23
	(11.2)	(17.2)	(6.5)	(12.7)	(12.2)	(10.8)	(10.8)	(12.7)	(8.9)	(10.8)
c_E	22.4	31.9	20.0	22.2	31.6	19.9	22.2	31.6	19.9	22.2
	(10.4)	(14.8)	(9.3)	(10.4)	(14.8)	(9.3)	(10.4)	(14.8)	(9.3)	(10.4)
	3.2	11.0	-10.1	10.5	-10.2	6.8	1.6	-8.5	-1.7	1.6
	1.498	5.100	-4.676	4.924	-4.768	3.169	0.732	-3.994	-0.790	0.732
N	215	215	215	213	213	213	213	213	213	213
X2	0.1	1.8	2.2	6.0	1.6	0.6	4.3	2.2	0.9	6.4
Gvalue	0.1	1.8	2.4	5.9	1.6	0.6	4.6	2.2	0.9	6.4
Gv/N	0.058	0.859	1.125	2.769	0.763	0.290	2.158	1.014	0.435	2.988
Prob	0.98870	0.60473	0.48999	0.11672	0.65379	0.89231	0.20392	0.53986	0.81879	0.09517

Bant_STERNE
5UTR:

	100	99	98	97	96	95	94	93	92	91
a_O	155	155	162	142	164	146	145	152	153	156
	(35.9)	(35.9)	(37.5)	(32.9)	(38.0)	(33.8)	(33.6)	(35.2)	(35.4)	(36.1)
a_E	157.6	157.6	157.6	157.6	157.6	157.6	157.6	157.6	157.6	157.6
	(36.5)	(36.5)	(36.5)	(36.5)	(36.5)	(36.5)	(36.5)	(36.5)	(36.5)	(36.5)
	-5.2	-5.2	8.9	-29.6	13.0	-22.3	-24.2	-11.0	-9.1	-3.2
	-1.198	-1.198	2.060	-6.857	3.018	-5.172	-5.598	-2.550	-2.103	-0.742
t_O	135	140	136	151	144	157	146	164	131	152
	(31.2)	(32.4)	(31.5)	(35.0)	(33.3)	(36.3)	(33.8)	(38.0)	(30.3)	(35.2)
t_E	140.6	140.6	140.6	140.6	140.6	140.6	140.6	140.6	140.6	140.6
	(32.5)	(32.5)	(32.5)	(32.5)	(32.5)	(32.5)	(32.5)	(32.5)	(32.5)	(32.5)
	-11.0	-1.2	-9.1	21.5	6.9	34.6	11.0	50.5	-18.5	23.7
	-2.545	-0.282	-2.099	4.984	1.588	8.014	2.543	11.683	-4.293	5.481
g_O	72	84	72	78	63	74	75	64	78	65
	(16.7)	(19.4)	(16.7)	(18.1)	(14.6)	(17.1)	(17.4)	(14.8)	(18.1)	(15.0)
g_E	74.2	74.2	74.2	74.2	74.2	74.2	74.2	74.2	74.2	74.2
	(17.2)	(17.2)	(17.2)	(17.2)	(17.2)	(17.2)	(17.2)	(17.2)	(17.2)	(17.2)
	-4.4	20.8	-4.4	7.7	-20.7	-0.5	1.5	-19.0	7.7	-17.3
	-1.017	4.809	-1.017	1.789	-4.784	-0.106	0.358	-4.394	1.789	-3.996
c_O	70	53	62	61	61	55	66	52	70	59
	(16.2)	(12.3)	(14.4)	(14.1)	(14.1)	(12.7)	(15.3)	(12.0)	(16.2)	(13.7)
c_E	59.6	59.6	59.6	59.6	59.6	59.6	59.6	59.6	59.6	59.6
	(13.8)	(13.8)	(13.8)	(13.8)	(13.8)	(13.8)	(13.8)	(13.8)	(13.8)	(13.8)
	22.6	-12.4	5.0	2.9	2.9	-8.7	13.6	-14.1	22.6	-1.1
	5.240	-2.859	1.157	0.679	0.679	-2.024	3.142	-3.264	5.240	-0.253
N	432	432	432	432	432	432	432	432	432	432
X2	2.2	2.1	0.4	2.5	2.1	3.1	1.9	6.5	2.8	2.1
Gvalue	2.1	2.0	0.4	2.6	2.2	3.1	1.9	6.4	2.7	2.1
Gv/N	0.480	0.469	0.102	0.596	0.501	0.712	0.445	1.475	0.632	0.490
Prob	0.55738	0.56673	0.93163	0.46231	0.53917	0.38022	0.58842	0.09479	0.43485	0.54813

	90	89	88	87	86	85	84	83	82	81
a_O	162	138	143	145	143	148	152	152	144	151
	(37.5)	(31.9)	(33.1)	(33.6)	(33.1)	(34.3)	(35.2)	(35.2)	(33.3)	(35.0)
a_E	157.6	157.6	157.6	157.6	157.6	157.6	157.6	157.6	157.6	157.6
	(36.5)	(36.5)	(36.5)	(36.5)	(36.5)	(36.5)	(36.5)	(36.5)	(36.5)	(36.5)
	8.9	-36.7	-27.8	-24.2	-27.8	-18.6	-11.0	-11.0	-26.0	-12.9
	2.060	-8.489	-6.440	-5.598	-6.440	-4.311	-2.550	-2.550	-6.021	-2.995
t_O	144	129	142	140	129	136	137	126	145	151
	(33.3)	(29.9)	(32.9)	(32.4)	(29.9)	(31.5)	(31.7)	(29.2)	(33.6)	(35.0)
t_E	140.6	140.6	140.6	140.6	140.6	140.6	140.6	140.6	140.6	140.6
	(32.5)	(32.5)	(32.5)	(32.5)	(32.5)	(32.5)	(32.5)	(32.5)	(32.5)	(32.5)
	6.9	-22.2	2.8	-1.2	-22.2	-9.1	-7.1	-27.6	8.9	21.5
	1.588	-5.147	0.647	-0.282	-5.147	-2.099	-1.650	-6.400	2.064	4.984
g_O	77	106	81	86	92	81	86	92	87	71
	(17.8)	(24.5)	(18.8)	(19.9)	(21.3)	(18.8)	(19.9)	(21.3)	(20.1)	(16.4)
g_E	74.2	74.2	74.2	74.2	74.2	74.2	74.2	74.2	74.2	74.2
	(17.2)	(17.2)	(17.2)	(17.2)	(17.2)	(17.2)	(17.2)	(17.2)	(17.2)	(17.2)
	5.6	75.5	14.1	25.3	39.5	14.1	25.3	39.5	27.6	-6.3
	1.306	17.484	3.273	5.860	9.141	3.273	5.860	9.141	6.394	-1.462
c_O	49	59	66	61	68	67	57	62	56	59
	(11.3)	(13.7)	(15.3)	(14.1)	(15.7)	(15.5)	(13.2)	(14.4)	(13.0)	(13.7)
c_E	59.6	59.6	59.6	59.6	59.6	59.6	59.6	59.6	59.6	59.6
	(13.8)	(13.8)	(13.8)	(13.8)	(13.8)	(13.8)	(13.8)	(13.8)	(13.8)	(13.8)
	-19.1	-1.1	13.6	2.9	18.0	15.8	-5.0	5.0	-6.9	-1.1
	-4.424	-0.253	3.142	0.679	4.177	3.656	-1.155	1.157	-1.594	-0.253
N	432	432	432	432	432	432	432	432	432	432
X2	2.2	17.0	2.7	2.9	7.8	2.3	2.3	6.1	3.7	1.2
Gvalue	2.3	15.5	2.7	2.8	7.5	2.2	2.2	5.8	3.6	1.2
Gv/N	0.531	3.595	0.622	0.660	1.732	0.520	0.505	1.348	0.843	0.273
Prob	0.51353	0.00142	0.44260	0.41544	0.05806	0.52295	0.53567	0.12043	0.30269	0.75819

	80	79	78	77	76	75	74	73	72	71
a_O	155	157	166	153	160	140	148	160	157	148
	(35.9)	(36.3)	(38.4)	(35.4)	(37.0)	(32.4)	(34.3)	(37.0)	(36.3)	(34.3)
a_E	157.6	157.6	157.6	157.6	157.6	157.6	157.6	157.6	157.6	157.6
	(36.5)	(36.5)	(36.5)	(36.5)	(36.5)	(36.5)	(36.5)	(36.5)	(36.5)	(36.5)
	-5.2	-1.2	17.2	-9.1	4.8	-33.2	-18.6	4.8	-1.2	-18.6
	-1.198	-0.282	3.986	-2.103	1.115	-7.679	-4.311	1.115	-0.282	-4.311
t_O	139	153	138	128	126	161	138	133	150	140
	(32.2)	(35.4)	(31.9)	(29.6)	(29.2)	(37.3)	(31.9)	(30.8)	(34.7)	(32.4)
t_E	140.6	140.6	140.6	140.6	140.6	140.6	140.6	140.6	140.6	140.6
	(32.5)	(32.5)	(32.5)	(32.5)	(32.5)	(32.5)	(32.5)	(32.5)	(32.5)	(32.5)
	-3.2	25.8	-5.2	-24.1	-27.6	43.6	-5.2	-14.8	19.4	-1.2
	-0.741	5.982	-1.197	-5.568	-6.400	10.093	-1.197	-3.426	4.489	-0.282
g_O	70	63	77	87	84	73	84	82	63	75
	(16.2)	(14.6)	(17.8)	(20.1)	(19.4)	(16.9)	(19.4)	(19.0)	(14.6)	(17.4)
g_E	74.2	74.2	74.2	74.2	74.2	74.2	74.2	74.2	74.2	74.2
	(17.2)	(17.2)	(17.2)	(17.2)	(17.2)	(17.2)	(17.2)	(17.2)	(17.2)	(17.2)
	-8.2	-20.7	5.6	27.6	20.8	-2.4	20.8	16.3	-20.7	1.5
	-1.901	-4.784	1.306	6.394	4.809	-0.565	4.809	3.779	-4.784	0.358
c_O	68	59	51	64	62	58	62	57	62	69
	(15.7)	(13.7)	(11.8)	(14.8)	(14.4)	(13.4)	(14.4)	(13.2)	(14.4)	(16.0)
c_E	59.6	59.6	59.6	59.6	59.6	59.6	59.6	59.6	59.6	59.6
	(13.8)	(13.8)	(13.8)	(13.8)	(13.8)	(13.8)	(13.8)	(13.8)	(13.8)	(13.8)
	18.0	-1.1	-15.8	9.2	5.0	-3.1	5.0	-5.0	5.0	20.3
	4.177	-0.253	-3.660	2.135	1.157	-0.708	1.157	-1.155	1.157	4.705
N	432	432	432	432	432	432	432	432	432	432
X2	1.5	2.8	1.8	3.8	2.9	5.0	2.0	1.4	2.4	2.1
Gvalue	1.5	2.9	1.9	3.7	2.9	4.9	2.0	1.4	2.5	2.0
Gv/N	0.337	0.662	0.435	0.858	0.681	1.141	0.458	0.313	0.580	0.471
Prob	0.69298	0.41368	0.59753	0.29480	0.40066	0.17701	0.57670	0.71666	0.47400	0.56520

	70	69	68	67	66	65	64	63	62	61
a_O	162	162	161	159	161	161	145	159	151	152
	(37.5)	(37.5)	(37.3)	(36.8)	(37.3)	(37.3)	(33.6)	(36.8)	(35.0)	(35.2)
a_E	157.6	157.6	157.6	157.6	157.6	157.6	157.6	157.6	157.6	157.6
	(36.5)	(36.5)	(36.5)	(36.5)	(36.5)	(36.5)	(36.5)	(36.5)	(36.5)	(36.5)
	8.9	8.9	6.9	2.8	6.9	6.9	-24.2	2.8	-12.9	-11.0
	2.060	2.060	1.586	0.646	1.586	1.586	-5.598	0.646	-2.995	-2.550
t_O	139	137	136	145	131	129	139	146	130	144
	(32.2)	(31.7)	(31.5)	(33.6)	(30.3)	(29.9)	(32.2)	(33.8)	(30.1)	(33.3)

t_E	140.6	140.6	140.6	140.6	140.6	140.6	140.6	140.6	140.6	140.6
	(32.5)	(32.5)	(32.5)	(32.5)	(32.5)	(32.5)	(32.5)	(32.5)	(32.5)	(32.5)
	-3.2	-7.1	-9.1	8.9	-18.5	-22.2	-3.2	11.0	-20.4	6.9
	-0.741	-1.650	-2.099	2.064	-4.293	-5.147	-0.741	2.543	-4.722	1.588
g_O	72	79	84	65	81	77	84	76	88	77
	(16.7)	(18.3)	(19.4)	(15.0)	(18.8)	(17.8)	(19.4)	(17.6)	(20.4)	(17.8)
g_E	74.2	74.2	74.2	74.2	74.2	74.2	74.2	74.2	74.2	74.2
	(17.2)	(17.2)	(17.2)	(17.2)	(17.2)	(17.2)	(17.2)	(17.2)	(17.2)	(17.2)
	-4.4	9.8	20.8	-17.3	14.1	5.6	20.8	3.6	29.9	5.6
	-1.017	2.278	4.809	-3.996	3.273	1.306	4.809	0.829	6.933	1.306
c_O	59	54	51	63	59	65	64	51	63	59
	(13.7)	(12.5)	(11.8)	(14.6)	(13.7)	(15.0)	(14.8)	(11.8)	(14.6)	(13.7)
c_E	59.6	59.6	59.6	59.6	59.6	59.6	59.6	59.6	59.6	59.6
	(13.8)	(13.8)	(13.8)	(13.8)	(13.8)	(13.8)	(13.8)	(13.8)	(13.8)	(13.8)
	-1.1	-10.6	-15.8	7.1	-1.1	11.4	9.2	-15.8	7.1	-1.1
	-0.253	-2.446	-3.660	1.643	-0.253	2.635	2.135	-3.660	1.643	-0.253
N	432	432	432	432	432	432	432	432	432	432
X2	0.2	1.0	2.7	1.5	1.4	1.6	2.6	1.5	3.8	0.4
Gvalue	0.2	1.0	2.7	1.5	1.3	1.6	2.6	1.5	3.7	0.4
Gv/N	0.049	0.243	0.636	0.357	0.312	0.381	0.605	0.359	0.858	0.090
Prob	0.97556	0.78942	0.43195	0.67251	0.71747	0.64926	0.45523	0.67099	0.29471	0.94222

	60	59	58	57	56	55	54	53	52	51
a_O	132	148	149	151	150	160	160	171	167	164
	(30.6)	(34.3)	(34.5)	(35.0)	(34.7)	(37.0)	(37.0)	(39.6)	(38.7)	(38.0)
a_E	157.6	157.6	157.6	157.6	157.6	157.6	157.6	157.6	157.6	157.6
	(36.5)	(36.5)	(36.5)	(36.5)	(36.5)	(36.5)	(36.5)	(36.5)	(36.5)	(36.5)
	-46.8	-18.6	-16.7	-12.9	-14.8	4.8	4.8	27.9	19.3	13.0
	-10.836	-4.311	-3.875	-2.995	-3.437	1.115	1.115	6.455	4.474	3.018
t_O	146	161	153	142	136	152	150	145	144	137
	(33.8)	(37.3)	(35.4)	(32.9)	(31.5)	(35.2)	(34.7)	(33.6)	(33.3)	(31.7)
t_E	140.6	140.6	140.6	140.6	140.6	140.6	140.6	140.6	140.6	140.6
	(32.5)	(32.5)	(32.5)	(32.5)	(32.5)	(32.5)	(32.5)	(32.5)	(32.5)	(32.5)
	11.0	43.6	25.8	2.8	-9.1	23.7	19.4	8.9	6.9	-7.1
	2.543	10.093	5.982	0.647	-2.099	5.481	4.489	2.064	1.588	-1.650
g_O	81	67	71	75	82	68	69	63	74	74
	(18.8)	(15.5)	(16.4)	(17.4)	(19.0)	(15.7)	(16.0)	(14.6)	(17.1)	(17.1)
g_E	74.2	74.2	74.2	74.2	74.2	74.2	74.2	74.2	74.2	74.2
	(17.2)	(17.2)	(17.2)	(17.2)	(17.2)	(17.2)	(17.2)	(17.2)	(17.2)	(17.2)
	14.1	-13.7	-6.3	1.5	16.3	-11.9	-10.1	-20.7	-0.5	-0.5
	3.273	-3.179	-1.462	0.358	3.779	-2.760	-2.334	-4.784	-0.106	-0.106
c_O	73	56	59	64	64	52	53	53	47	57
	(16.9)	(13.0)	(13.7)	(14.8)	(14.8)	(12.0)	(12.3)	(12.3)	(10.9)	(13.2)
c_E	59.6	59.6	59.6	59.6	59.6	59.6	59.6	59.6	59.6	59.6
	(13.8)	(13.8)	(13.8)	(13.8)	(13.8)	(13.8)	(13.8)	(13.8)	(13.8)	(13.8)
	29.7	-6.9	-1.1	9.2	9.2	-14.1	-12.4	-12.4	-22.2	-5.0
	6.882	-1.594	-0.253	2.135	2.135	-3.264	-2.859	-2.859	-5.150	-1.155
N	432	432	432	432	432	432	432	432	432	432
X2	8.0	4.5	1.7	0.6	1.7	2.4	1.8	3.7	3.3	0.5
Gvalue	8.0	4.4	1.7	0.6	1.6	2.5	1.8	3.8	3.5	0.5
Gv/N	1.862	1.011	0.391	0.145	0.379	0.573	0.411	0.876	0.806	0.107
Prob	0.04514	0.22457	0.63962	0.89014	0.65098	0.48013	0.62028	0.28593	0.32290	0.92745

	50	49	48	47	46	45	44	43	42	41
a_O	149	151	166	170	158	156	154	162	162	170
	(34.5)	(35.0)	(38.4)	(39.4)	(36.6)	(36.1)	(35.6)	(37.5)	(37.5)	(39.4)
a_E	157.6	157.6	157.6	157.6	157.6	157.6	157.6	157.6	157.6	157.6
	(36.5)	(36.5)	(36.5)	(36.5)	(36.5)	(36.5)	(36.5)	(36.5)	(36.5)	(36.5)
	-16.7	-12.9	17.2	25.7	0.8	-3.2	-7.1	8.9	8.9	25.7
	-3.875	-2.995	3.986	5.956	0.181	-0.742	-1.652	2.060	2.060	5.956
t_O	124	147	134	128	145	141	143	121	127	126
	(28.7)	(34.0)	(31.0)	(29.6)	(33.6)	(32.6)	(33.1)	(28.0)	(29.4)	(29.2)
t_E	140.6	140.6	140.6	140.6	140.6	140.6	140.6	140.6	140.6	140.6
	(32.5)	(32.5)	(32.5)	(32.5)	(32.5)	(32.5)	(32.5)	(32.5)	(32.5)	(32.5)
	-31.2	13.1	-12.9	-24.1	8.9	0.8	4.8	-36.3	-25.9	-27.6
	-7.217	3.025	-2.987	-5.568	2.064	0.181	1.116	-8.414	-5.986	-6.400
g_O	93	74	71	78	68	76	75	82	76	82
	(21.5)	(17.1)	(16.4)	(18.1)	(15.7)	(17.6)	(17.4)	(19.0)	(17.6)	(19.0)
g_E	74.2	74.2	74.2	74.2	74.2	74.2	74.2	74.2	74.2	74.2
	(17.2)	(17.2)	(17.2)	(17.2)	(17.2)	(17.2)	(17.2)	(17.2)	(17.2)	(17.2)
	41.9	-0.5	-6.3	7.7	-11.9	3.6	1.5	16.3	3.6	16.3
	9.706	-0.106	-1.462	1.789	-2.760	0.829	0.358	3.779	0.829	3.779
c_O	66	60	61	56	61	59	60	67	67	54
	(15.3)	(13.9)	(14.1)	(13.0)	(14.1)	(13.7)	(13.9)	(15.5)	(15.5)	(12.5)
c_E	59.6	59.6	59.6	59.6	59.6	59.6	59.6	59.6	59.6	59.6
	(13.8)	(13.8)	(13.8)	(13.8)	(13.8)	(13.8)	(13.8)	(13.8)	(13.8)	(13.8)
	13.6	0.9	2.9	-6.9	2.9	-1.1	0.9	15.8	15.8	-10.6
	3.142	0.209	0.679	-1.594	0.679	-0.253	0.209	3.656	3.656	-2.446
N	432	432	432	432	432	432	432	432	432	432
X2	7.9	0.6	0.9	2.5	0.7	0.1	0.1	4.6	2.4	3.8
Gvalue	7.6	0.6	0.9	2.5	0.7	0.1	0.1	4.7	2.4	3.8
Gv/N	1.757	0.132	0.216	0.583	0.164	0.015	0.031	1.082	0.560	0.890
Prob	0.05533	0.90295	0.81763	0.47169	0.87090	0.99572	0.98732	0.19723	0.48983	0.27887

	40	39	38	37	36	35	34	33	32	31
a_O	163	167	160	145	162	164	162	157	161	156
	(37.7)	(38.7)	(37.0)	(33.6)	(37.5)	(38.0)	(37.5)	(36.3)	(37.3)	(36.1)
a_E	157.6	157.6	157.6	157.6	157.6	157.6	157.6	157.6	157.6	157.6
	(36.5)	(36.5)	(36.5)	(36.5)	(36.5)	(36.5)	(36.5)	(36.5)	(36.5)	(36.5)
	11.0	19.3	4.8	-24.2	8.9	13.0	8.9	-1.2	6.9	-3.2
	2.538	4.474	1.115	-5.598	2.060	3.018	2.060	-0.282	1.586	-0.742
t_O	152	127	138	138	133	119	144	136	129	135
	(35.2)	(29.4)	(31.9)	(31.9)	(30.8)	(27.5)	(33.3)	(31.5)	(29.9)	(31.2)
t_E	140.6	140.6	140.6	140.6	140.6	140.6	140.6	140.6	140.6	140.6
	(32.5)	(32.5)	(32.5)	(32.5)	(32.5)	(32.5)	(32.5)	(32.5)	(32.5)	(32.5)
	23.7	-25.9	-5.2	-5.2	-14.8	-39.7	6.9	-9.1	-22.2	-11.0
	5.481	-5.986	-1.197	-1.197	-3.426	-9.193	1.588	-2.099	-5.147	-2.545
g_O	65	78	83	82	80	93	82	87	76	73
	(15.0)	(18.1)	(19.2)	(19.0)	(18.5)	(21.5)	(19.0)	(20.1)	(17.6)	(16.9)
g_E	74.2	74.2	74.2	74.2	74.2	74.2	74.2	74.2	74.2	74.2
	(17.2)	(17.2)	(17.2)	(17.2)	(17.2)	(17.2)	(17.2)	(17.2)	(17.2)	(17.2)
	-17.3	7.7	18.5	16.3	12.0	41.9	16.3	27.6	3.6	-2.4
	-3.996	1.789	4.291	3.779	2.773	9.706	3.779	6.394	0.829	-0.565
c_O	52	60	51	67	57	56	44	52	66	68
	(12.0)	(13.9)	(11.8)	(15.5)	(13.2)	(13.0)	(10.2)	(12.0)	(15.3)	(15.7)
c_E	59.6	59.6	59.6	59.6	59.6	59.6	59.6	59.6	59.6	59.6

```
        ( 13.8) ( 13.8) ( 13.8) ( 13.8) ( 13.8) ( 13.8) ( 13.8) ( 13.8) ( 13.8) ( 13.8)
          -14.1     0.9   -15.8    15.8    -5.0    -6.9   -26.6   -14.1    13.6    18.0
         -3.264   0.209  -3.660   3.656  -1.155  -1.594  -6.165  -3.264   3.142   4.177

N           432     432     432     432     432     432     432     432     432     432
X2          3.2     2.1     2.3     2.8     1.1     8.5     5.1     3.3     1.8     1.5
Gvalue      3.3     2.1     2.4     2.8     1.1     8.4     5.5     3.2     1.8     1.4
Gv/N      0.759   0.487   0.549   0.641   0.252   1.937   1.263   0.749   0.411   0.326
Prob    0.35046 0.55143 0.49861 0.42889 0.77979 0.03900 0.14121 0.35660 0.62037 0.70322
        --------------------------------------------------------------------------------
             30      29      28      27      26      25      24      23      22      21
        --------------------------------------------------------------------------------
a_O         174     164     143     172     187     188     163     162     166     153
        ( 40.3) ( 38.0) ( 33.1) ( 39.8) ( 43.3) ( 43.5) ( 37.7) ( 37.5) ( 38.4) ( 35.4)
a_E       157.6   157.6   157.6   157.6   157.6   157.6   157.6   157.6   157.6   157.6
        ( 36.5) ( 36.5) ( 36.5) ( 36.5) ( 36.5) ( 36.5) ( 36.5) ( 36.5) ( 36.5) ( 36.5)
           34.4    13.0   -27.8    30.1    63.9    66.3    11.0     8.9    17.2    -9.1
          7.969   3.018  -6.440   6.957  14.803  15.346   2.538   2.060   3.986  -2.103
t_O         131     127     167     145     125     137     128     140     146     149
        ( 30.3) ( 29.4) ( 38.7) ( 33.6) ( 28.9) ( 31.7) ( 29.6) ( 32.4) ( 33.8) ( 34.5)
t_E       140.6   140.6   140.6   140.6   140.6   140.6   140.6   140.6   140.6   140.6
        ( 32.5) ( 32.5) ( 32.5) ( 32.5) ( 32.5) ( 32.5) ( 32.5) ( 32.5) ( 32.5) ( 32.5)
          -18.5   -25.9    57.4     8.9   -29.4    -7.1   -24.1    -1.2    11.0    17.3
         -4.293  -5.986  13.298   2.064  -6.810  -1.650  -5.568  -0.282   2.543   3.998
g_O          69      87      68      64      71      63      72      74      65      78
        ( 16.0) ( 20.1) ( 15.7) ( 14.8) ( 16.4) ( 14.6) ( 16.7) ( 17.1) ( 15.0) ( 18.1)
g_E        74.2    74.2    74.2    74.2    74.2    74.2    74.2    74.2    74.2    74.2
        ( 17.2) ( 17.2) ( 17.2) ( 17.2) ( 17.2) ( 17.2) ( 17.2) ( 17.2) ( 17.2) ( 17.2)
          -10.1    27.6   -11.9   -19.0    -6.3   -20.7    -4.4    -0.5   -17.3     7.7
         -2.334   6.394  -2.760  -4.394  -1.462  -4.784  -1.017  -0.106  -3.996   1.789
c_O          58      54      54      51      49      44      69      56      55      52
        ( 13.4) ( 12.5) ( 12.5) ( 11.8) ( 11.3) ( 10.2) ( 16.0) ( 13.0) ( 12.7) ( 12.0)
c_E        59.6    59.6    59.6    59.6    59.6    59.6    59.6    59.6    59.6    59.6
        ( 13.8) ( 13.8) ( 13.8) ( 13.8) ( 13.8) ( 13.8) ( 13.8) ( 13.8) ( 13.8) ( 13.8)
           -3.1   -10.6   -10.6   -15.8   -19.1   -26.6    20.3    -6.9    -8.7   -14.1
         -0.708  -2.446  -2.446  -3.660  -4.424  -6.165   4.705  -1.594  -2.024  -3.264

N           432     432     432     432     432     432     432     432     432     432
X2          2.8     4.3     7.3     4.1     9.2    11.7     2.9     0.3     2.1     1.8
Gvalue      2.7     4.2     7.1     4.2     9.1    11.9     2.8     0.3     2.2     1.8
Gv/N      0.634   0.980   1.653   0.968   2.107   2.748   0.658   0.079   0.509   0.420
Prob    0.43369 0.23743 0.06758 0.24250 0.02796 0.00784 0.41659 0.95224 0.53230 0.61148
        --------------------------------------------------------------------------------
             20      19      18      17      16      15      14      13      12      11
        --------------------------------------------------------------------------------
a_O         155     180     151     140     170     162     163     181     141     143
        ( 35.9) ( 41.7) ( 35.0) ( 32.4) ( 39.4) ( 37.5) ( 37.7) ( 41.9) ( 32.6) ( 33.1)
a_E       157.6   157.6   157.6   157.6   157.6   157.6   157.6   157.6   157.6   157.6
        ( 36.5) ( 36.5) ( 36.5) ( 36.5) ( 36.5) ( 36.5) ( 36.5) ( 36.5) ( 36.5) ( 36.5)
           -5.2    47.8   -12.9   -33.2    25.7     8.9    11.0    50.1   -31.4   -27.8
         -1.198  11.069  -2.995  -7.679   5.956   2.060   2.538  11.595  -7.270  -6.440
t_O         149     155     157     162     158     152     144     130     160     149
        ( 34.5) ( 35.9) ( 36.3) ( 37.5) ( 36.6) ( 35.2) ( 33.3) ( 30.1) ( 37.0) ( 34.5)
t_E       140.6   140.6   140.6   140.6   140.6   140.6   140.6   140.6   140.6   140.6
        ( 32.5) ( 32.5) ( 32.5) ( 32.5) ( 32.5) ( 32.5) ( 32.5) ( 32.5) ( 32.5) ( 32.5)
           17.3    30.2    34.6    45.9    36.8    23.7     6.9   -20.4    41.3    17.3
          3.998   6.992   8.014  10.620   8.529   5.481   1.588  -4.722   9.569   3.998
g_O          69      44      54      60      38      53      60      51      48      54
        ( 16.0) ( 10.2) ( 12.5) ( 13.9) (  8.8) ( 12.3) ( 13.9) ( 11.8) ( 11.1) ( 12.5)
g_E        74.2    74.2    74.2    74.2    74.2    74.2    74.2    74.2    74.2    74.2
        ( 17.2) ( 17.2) ( 17.2) ( 17.2) ( 17.2) ( 17.2) ( 17.2) ( 17.2) ( 17.2) ( 17.2)
          -10.1   -46.0   -34.4   -25.5   -50.9   -35.7   -25.5   -38.3   -41.9   -34.4
         -2.334 -10.653  -7.955  -5.912 -11.780  -8.266  -5.912  -8.862  -9.688  -7.955
c_O          59      53      70      70      66      65      65      70      83      86
        ( 13.7) ( 12.3) ( 16.2) ( 16.2) ( 15.3) ( 15.0) ( 15.0) ( 16.2) ( 19.2) ( 19.9)
c_E        59.6    59.6    59.6    59.6    59.6    59.6    59.6    59.6    59.6    59.6
        ( 13.8) ( 13.8) ( 13.8) ( 13.8) ( 13.8) ( 13.8) ( 13.8) ( 13.8) ( 13.8) ( 13.8)
           -1.1   -12.4    22.6    22.6    13.6    11.4    11.4    22.6    55.1    63.2
         -0.253  -2.859   5.240   5.240   3.142   2.635   2.635   5.240  12.758  14.633

N           432     432     432     432     432     432     432     432     432     432
X2          0.9    17.7     9.5     9.8    21.5     7.6     3.5    13.4    22.9    19.1
Gvalue      0.9    19.7    10.0     9.8    25.3     8.3     3.7    14.0    23.2    18.3
Gv/N      0.212   4.549   2.304   2.269   5.848   1.911   0.849   3.251   5.370   4.236
Prob    0.82133 0.00020 0.01897 0.02033 0.00001 0.04102 0.29953 0.00285 0.00004 0.00038
        --------------------------------------------------------------------------------
             10       9       8       7       6       5       4       3       2       1
        --------------------------------------------------------------------------------
a_O         170     157     169     185     149     177     156     127     187     154
        ( 39.4) ( 36.3) ( 39.1) ( 42.8) ( 34.5) ( 41.0) ( 36.1) ( 29.4) ( 43.3) ( 35.6)
a_E       157.6   157.6   157.6   157.6   157.6   157.6   157.6   157.6   157.6   157.6
        ( 36.5) ( 36.5) ( 36.5) ( 36.5) ( 36.5) ( 36.5) ( 36.5) ( 36.5) ( 36.5) ( 36.5)
           25.7    -1.2    23.6    59.3   -16.7    41.1    -3.2   -54.8    63.9    -7.1
          5.956  -0.282   5.459  13.724  -3.875   9.508  -0.742 -12.696  14.803  -1.652
t_O         165     144     143     151     151     143     134     116      96     137
        ( 38.2) ( 33.3) ( 33.1) ( 35.0) ( 35.0) ( 33.1) ( 31.0) ( 26.9) ( 22.2) ( 31.7)
t_E       140.6   140.6   140.6   140.6   140.6   140.6   140.6   140.6   140.6   140.6
        ( 32.5) ( 32.5) ( 32.5) ( 32.5) ( 32.5) ( 32.5) ( 32.5) ( 32.5) ( 32.5) ( 32.5)
           52.8     6.9     4.8    21.5    21.5     4.8   -12.9   -44.6   -73.3    -7.1
         12.219   1.588   1.116   4.984   4.984   1.116  -2.987 -10.333 -16.962  -1.650
g_O          39      61      59      36      84      63      92     124      96     104
        (  9.0) ( 14.1) ( 13.7) (  8.3) ( 19.4) ( 14.6) ( 21.3) ( 28.7) ( 22.2) ( 24.1)
g_E        74.2    74.2    74.2    74.2    74.2    74.2    74.2    74.2    74.2    74.2
        ( 17.2) ( 17.2) ( 17.2) ( 17.2) ( 17.2) ( 17.2) ( 17.2) ( 17.2) ( 17.2) ( 17.2)
          -50.2   -23.9   -27.1   -52.1    20.8   -20.7    39.5   127.3    49.4    70.1
        -11.621  -5.544  -6.272 -12.061   4.809  -4.784   9.141  29.456  11.430  16.237
c_O          58      70      61      60      48      49      50      65      53      37
        ( 13.4) ( 16.2) ( 14.1) ( 13.9) ( 11.1) ( 11.3) ( 11.6) ( 15.0) ( 12.3) (  8.6)
c_E        59.6    59.6    59.6    59.6    59.6    59.6    59.6    59.6    59.6    59.6
        ( 13.8) ( 13.8) ( 13.8) ( 13.8) ( 13.8) ( 13.8) ( 13.8) ( 13.8) ( 13.8) ( 13.8)
           -3.1    22.6     2.9     0.9   -20.7   -19.1   -17.5    11.4   -12.4   -35.2
         -0.708   5.240   0.679   0.209  -4.791  -4.424  -4.046   2.635  -2.859  -8.152

N           432     432     432     432     432     432     432     432     432     432
X2         22.0     4.3     4.0    25.2     4.8     6.0     6.1    44.1    26.7    20.7
Gvalue     25.3     4.3     4.2    29.6     4.9     6.1     5.9    39.2    27.7    20.7
Gv/N      5.846   1.002   0.982   6.856   1.126   1.416   1.366   9.063   6.412   4.783
Prob    0.00001 0.22791 0.23640 0.00000 0.18214 0.10611 0.11643 0.00000 0.00000 0.00012
        --------------------------------------------------------------------------------

CDS_INITIAL:
        --------------------------------------------------------------------------------
```

	1	2	3	4	5	6	7	8	9	10
a_O	237	0	0	186	193	152	170	158	155	170
	(54.9)	(0.0)	(0.0)	(43.1)	(44.7)	(35.2)	(39.4)	(36.6)	(35.9)	(39.4)
a_E	143.8	145.2	161.0	143.8	145.2	161.0	143.8	145.2	161.0	143.8
	(33.3)	(33.6)	(37.3)	(33.3)	(33.6)	(37.3)	(33.3)	(33.6)	(37.3)	(33.3)
	237.0	0.0	0.0	95.8	109.7	-17.5	57.0	26.6	-11.8	57.0
	54.854	0.000	0.000	22.184	25.399	-4.061	13.196	6.156	-2.738	13.196
t_O	91	432	0	89	126	137	99	138	143	103
	(21.1)	(100.0)	(0.0)	(20.6)	(29.2)	(31.7)	(22.9)	(31.9)	(33.1)	(23.8)
t_E	90.4	142.4	156.4	90.4	142.4	156.4	90.4	142.4	156.4	90.4
	(20.9)	(33.0)	(36.2)	(20.9)	(33.0)	(36.2)	(20.9)	(33.0)	(36.2)	(20.9)
	1.1	959.1	0.0	-2.9	-30.8	-36.3	17.9	-8.6	-25.6	26.8
	0.256	222.007	0.000	-0.665	-7.123	-8.396	4.141	-1.989	-5.926	6.197
g_O	104	0	432	112	74	83	108	86	88	115
	(24.1)	(0.0)	(100.0)	(25.9)	(17.1)	(19.2)	(25.0)	(19.9)	(20.4)	(26.6)
g_E	132.1	61.2	68.5	132.1	61.2	68.5	132.1	61.2	68.5	132.1
	(30.6)	(14.2)	(15.8)	(30.6)	(14.2)	(15.8)	(30.6)	(14.2)	(15.8)	(30.6)
	-49.7	0.0	1591.5	-37.0	28.0	32.0	-43.5	58.4	44.2	-31.9
	-11.512	0.000	368.413	-8.555	6.485	7.396	-10.068	13.521	10.225	-7.377
c_O	0	0	0	45	39	60	55	50	46	44
	(0.0)	(0.0)	(0.0)	(10.4)	(9.0)	(13.9)	(12.7)	(11.6)	(10.6)	(10.2)
c_E	65.7	83.2	46.1	65.7	83.2	46.1	65.7	83.2	46.1	65.7
	(15.2)	(19.2)	(10.7)	(15.2)	(19.2)	(10.7)	(15.2)	(19.2)	(10.7)	(15.2)
	0.0	0.0	0.0	-34.1	-59.1	31.6	-19.6	-50.9	-0.2	-35.3
	0.000	0.000	0.000	-7.885	-13.670	7.313	-4.527	-11.774	-0.052	-8.168
N	432	432	432	432	432	432	432	432	432	432
X2	132.2	878.9	2293.7	22.0	43.7	10.2	11.7	24.5	6.9	15.9
Gvalue	188.3	959.1	1591.5	21.9	47.9	9.7	11.8	25.5	6.5	16.6
Gv/N	43.598	222.007	368.413	5.078	11.091	2.252	2.741	5.913	1.508	3.848
Prob	0.00000	0.00000	1.00000	0.00007	0.00000	0.02101	0.00794	0.00001	0.08901	0.00084

	11	12	13	14	15	16	17	18	19	20
a_O	123	145	183	154	136	174	147	157	169	163
	(28.5)	(33.6)	(42.4)	(35.6)	(31.5)	(40.3)	(34.0)	(36.3)	(39.1)	(37.7)
a_E	145.2	161.0	143.8	145.2	161.0	143.8	145.2	161.0	143.8	145.2
	(33.6)	(37.3)	(33.3)	(33.6)	(37.3)	(33.3)	(33.6)	(37.3)	(33.3)	(33.6)
	-40.9	-30.4	88.3	18.0	-45.9	66.4	3.5	-8.0	54.7	37.6
	-9.467	-7.039	20.449	4.172	-10.636	15.380	0.816	-1.842	12.657	8.702
t_O	186	130	90	148	155	103	152	142	111	152
	(43.1)	(30.1)	(20.8)	(34.3)	(35.9)	(23.8)	(35.2)	(32.9)	(25.7)	(35.2)
t_E	142.4	156.4	90.4	142.4	156.4	90.4	142.4	156.4	90.4	142.4
	(33.0)	(36.2)	(20.9)	(33.0)	(36.2)	(20.9)	(33.0)	(36.2)	(20.9)	(33.0)
	99.5	-48.1	-0.9	11.5	-2.8	26.8	19.9	-27.4	45.5	19.9
	23.022	-11.124	-0.207	2.660	-0.641	6.197	4.609	-6.346	10.522	4.609
g_O	61	110	99	66	99	102	67	91	98	65
	(14.1)	(25.5)	(22.9)	(15.3)	(22.9)	(23.6)	(15.5)	(21.1)	(22.7)	(15.0)
g_E	61.2	68.5	132.1	61.2	68.5	132.1	61.2	68.5	132.1	61.2
	(14.2)	(15.8)	(30.6)	(14.2)	(15.8)	(30.6)	(14.2)	(15.8)	(30.6)	(14.2)
	-0.5	104.3	-57.1	9.9	73.0	-52.7	12.1	51.8	-58.5	7.8
	-0.110	24.145	-13.217	2.288	16.901	-12.208	2.790	11.986	-13.544	1.794
c_O	62	47	60	64	42	53	66	42	54	52
	(14.4)	(10.9)	(13.9)	(14.8)	(9.7)	(12.3)	(15.3)	(9.7)	(12.5)	(12.0)
c_E	83.2	46.1	65.7	83.2	46.1	65.7	83.2	46.1	65.7	83.2
	(19.2)	(10.7)	(15.2)	(19.2)	(10.7)	(15.2)	(19.2)	(10.7)	(15.2)	(19.2)
	-36.4	1.8	-10.9	-33.5	-7.8	-22.8	-30.5	-7.8	-21.2	-48.8
	-8.425	0.415	-2.522	-7.756	-1.816	-5.272	-7.058	-1.816	-4.904	-11.301
N	432	432	432	432	432	432	432	432	432	432
X2	22.2	31.3	19.5	5.5	17.9	17.4	4.8	9.2	20.0	14.7
Gvalue	21.7	27.6	19.4	5.9	16.4	17.7	5.0	8.6	20.4	16.4
Gv/N	5.020	6.398	4.502	1.364	3.808	4.097	1.156	1.982	4.731	3.804
Prob	0.00008	0.00000	0.00022	0.11692	0.00092	0.00051	0.17219	0.03574	0.00014	0.00092

	21	22	23	24	25	26	27	28	29	30
a_O	152	166	160	165	155	155	165	165	157	154
	(35.2)	(38.4)	(37.0)	(38.2)	(35.9)	(35.9)	(38.2)	(38.2)	(36.3)	(35.6)
a_E	161.0	143.8	145.2	161.0	143.8	145.2	161.0	143.8	145.2	161.0
	(37.3)	(33.3)	(33.6)	(37.3)	(33.3)	(33.6)	(37.3)	(33.3)	(33.6)	(37.3)
	-17.5	47.8	31.0	8.0	23.3	20.1	8.0	45.5	24.4	-13.7
	-4.061	11.056	7.166	1.861	5.403	4.664	1.861	10.528	5.656	-3.182
t_O	166	106	141	135	106	155	149	85	151	147
	(38.4)	(24.5)	(32.6)	(31.2)	(24.5)	(35.9)	(34.5)	(19.7)	(35.0)	(34.0)
t_E	156.4	90.4	142.4	156.4	90.4	142.4	156.4	90.4	142.4	156.4
	(36.2)	(20.9)	(33.0)	(36.2)	(20.9)	(33.0)	(36.2)	(20.9)	(33.0)	(36.2)
	19.8	33.6	-2.7	-39.7	33.6	26.4	-14.4	-10.6	17.8	-18.2
	4.582	7.786	-0.629	-9.193	7.786	6.102	-3.340	-2.445	4.117	-4.215
g_O	69	112	65	86	111	72	86	114	51	88
	(16.0)	(25.9)	(15.0)	(19.9)	(25.7)	(16.7)	(19.9)	(26.4)	(11.8)	(20.4)
g_E	68.5	132.1	61.2	68.5	132.1	61.2	68.5	132.1	61.2	68.5
	(15.8)	(30.6)	(14.2)	(15.8)	(30.6)	(14.2)	(15.8)	(30.6)	(14.2)	(15.8)
	1.1	-37.0	7.8	39.2	-38.6	23.3	39.2	-33.6	-18.7	44.2
	0.247	-8.555	1.794	9.077	-8.940	5.397	9.077	-7.774	-4.319	10.225
c_O	45	48	66	46	60	50	32	68	73	43
	(10.4)	(11.1)	(15.3)	(10.6)	(13.9)	(11.6)	(7.4)	(15.7)	(16.9)	(10.0)
c_E	46.1	65.7	83.2	46.1	65.7	83.2	46.1	65.7	83.2	46.1
	(10.7)	(15.2)	(19.2)	(10.7)	(15.2)	(19.2)	(10.7)	(15.2)	(19.2)	(10.7)
	-2.2	-30.1	-30.5	-0.2	-10.9	-50.9	-23.4	4.7	-19.0	-6.0
	-0.509	-6.976	-7.058	-0.052	-2.522	-11.774	-5.412	1.082	-4.400	-1.391
N	432	432	432	432	432	432	432	432	432	432
X2	1.1	13.9	5.3	7.5	7.4	16.9	9.3	6.0	4.4	6.7
Gvalue	1.1	14.3	5.5	7.3	7.5	19.0	9.4	6.0	4.5	6.2
Gv/N	0.261	3.310	1.273	1.693	1.727	4.389	2.186	1.391	1.053	1.437
Prob	0.77085	0.00252	0.13867	0.06249	0.05853	0.00028	0.02394	0.11119	0.20799	0.10188

	31	32	33	34	35	36	37	38	39	40
a_O	163	148	163	160	143	145	164	133	151	151
	(37.7)	(34.3)	(37.7)	(37.0)	(33.1)	(33.6)	(38.0)	(30.8)	(35.0)	(35.0)
a_E	143.8	145.2	161.0	143.8	145.2	161.0	143.8	145.2	161.0	143.8
	(33.3)	(33.6)	(37.3)	(33.3)	(33.6)	(37.3)	(33.3)	(33.6)	(37.3)	(33.3)
	41.0	5.6	4.0	34.3	-4.5	-30.4	43.2	-23.4	-19.4	14.8
	9.480	1.286	0.918	7.929	-1.032	-7.039	10.002	-5.424	-4.495	3.436
t_O	107	154	146	116	147	165	95	150	155	97
	(24.8)	(35.6)	(33.8)	(26.9)	(34.0)	(38.2)	(22.0)	(34.7)	(35.9)	(22.5)
t_E	90.4	142.4	156.4	90.4	142.4	156.4	90.4	142.4	156.4	90.4
	(20.9)	(33.0)	(36.2)	(20.9)	(33.0)	(36.2)	(20.9)	(33.0)	(36.2)	(20.9)
	36.0	24.2	-20.1	57.7	9.4	17.7	9.3	15.7	-2.8	13.6

	8.325	5.601	−4.647	13.362	2.181	4.093	2.159	3.628	−0.641	3.140
g_O	100	65	80	99	65	69	117	82	76	113
	(23.1)	(15.0)	(18.5)	(22.9)	(15.0)	(16.0)	(27.1)	(19.0)	(17.6)	(26.2)
g_E	132.1	61.2	68.5	132.1	61.2	68.5	132.1	61.2	68.5	132.1
	(30.6)	(14.2)	(15.8)	(30.6)	(14.2)	(15.8)	(30.6)	(14.2)	(15.8)	(30.6)
	−55.7	7.8	24.9	−57.1	7.8	1.1	−28.4	47.9	15.9	−35.3
	−12.885	1.794	5.765	−13.217	1.794	0.247	−6.572	11.084	3.672	−8.167
c_O	62	65	43	57	77	53	56	67	50	71
	(14.4)	(15.0)	(10.0)	(13.2)	(17.8)	(12.3)	(13.0)	(15.5)	(11.6)	(16.4)
c_E	65.7	83.2	46.1	65.7	83.2	46.1	65.7	83.2	46.1	65.7
	(15.2)	(19.2)	(10.7)	(15.2)	(19.2)	(10.7)	(15.2)	(19.2)	(10.7)	(15.2)
	−7.2	−32.0	−6.0	−16.2	−11.8	14.8	−17.9	−28.9	8.1	11.0
	−1.665	−7.411	−1.391	−3.750	−2.740	3.416	−4.143	−6.699	1.874	2.549
N	432	432	432	432	432	432	432	432	432	432
X2	13.6	5.2	2.9	18.5	0.9	3.1	6.2	11.6	1.8	4.0
Gvalue	14.1	5.5	2.8	18.7	0.9	3.1	6.3	11.2	1.8	4.1
Gv/N	3.254	1.271	0.645	4.325	0.203	0.718	1.448	2.589	0.410	0.959
Prob	0.00283	0.13916	0.42565	0.00032	0.83085	0.37618	0.09990	0.01077	0.62167	0.24661

	41	42	43	44	45	46	47	48	49	50
a_O	128	165	147	139	159	150	151	168	149	136
	(29.6)	(38.2)	(34.0)	(32.2)	(36.8)	(34.7)	(35.0)	(38.9)	(34.5)	(31.5)
a_E	145.2	161.0	143.8	145.2	161.0	143.8	145.2	161.0	143.8	145.2
	(33.6)	(37.3)	(33.3)	(33.6)	(37.3)	(33.3)	(33.6)	(37.3)	(33.3)	(33.6)
	−32.4	8.0	6.6	−12.2	−4.0	12.8	11.7	14.2	10.7	−17.9
	−7.491	1.861	1.518	−2.829	−0.933	2.952	2.715	3.296	2.471	−4.142
t_O	167	161	101	156	163	101	133	144	106	159
	(38.7)	(37.3)	(23.4)	(36.1)	(37.7)	(23.4)	(30.8)	(33.3)	(24.5)	(36.8)
t_E	142.4	156.4	90.4	142.4	156.4	90.4	142.4	156.4	90.4	142.4
	(33.0)	(36.2)	(20.9)	(33.0)	(36.2)	(20.9)	(33.0)	(36.2)	(20.9)	(33.0)
	53.3	9.4	22.3	28.5	13.5	22.3	−18.1	−23.8	33.6	35.1
	12.340	2.165	5.159	6.606	3.123	5.159	−4.190	−5.503	7.786	8.135
g_O	68	74	119	59	72	104	74	73	111	71
	(15.7)	(17.1)	(27.5)	(13.7)	(16.7)	(24.1)	(17.1)	(16.9)	(25.7)	(16.4)
g_E	61.2	68.5	132.1	61.2	68.5	132.1	61.2	68.5	132.1	61.2
	(14.2)	(15.8)	(30.6)	(14.2)	(15.8)	(30.6)	(14.2)	(15.8)	(30.6)	(14.2)
	14.2	11.5	−24.8	−4.4	7.2	−49.7	28.0	9.4	−38.6	21.0
	3.298	2.662	−5.750	−1.017	1.677	−11.512	6.485	2.166	−8.940	4.862
c_O	69	32	65	78	38	77	74	47	66	66
	(16.0)	(7.4)	(15.0)	(18.1)	(8.8)	(17.8)	(17.1)	(10.9)	(15.3)	(15.3)
c_E	83.2	46.1	65.7	83.2	46.1	65.7	83.2	46.1	65.7	83.2
	(19.2)	(10.7)	(15.2)	(19.2)	(10.7)	(15.2)	(19.2)	(10.7)	(15.2)	(19.2)
	−25.7	−23.4	−1.4	−10.0	−14.7	24.4	−17.3	1.8	0.6	−30.5
	−5.959	−5.412	−0.324	−2.309	−3.404	5.656	−3.994	0.415	0.138	−7.058
N	432	432	432	432	432	432	432	432	432	432
X2	9.5	5.0	2.6	2.0	1.9	9.4	4.5	1.6	6.2	7.6
Gvalue	9.4	5.5	2.6	1.9	2.0	9.7	4.4	1.6	6.3	7.8
Gv/N	2.187	1.275	0.604	0.451	0.463	2.255	1.017	0.374	1.455	1.797
Prob	0.02388	0.13806	0.45602	0.58337	0.57254	0.02089	0.22201	0.65556	0.09851	0.05117

	51	52	53	54	55	56	57	58	59	60
a_O	146	165	129	151	153	169	160	158	151	174
	(33.8)	(38.2)	(29.9)	(35.0)	(35.4)	(39.1)	(37.0)	(36.6)	(35.0)	(40.3)
a_E	161.0	143.8	145.2	161.0	143.8	145.2	161.0	143.8	145.2	161.0
	(37.3)	(33.3)	(33.6)	(37.3)	(33.3)	(33.6)	(37.3)	(33.3)	(33.6)	(37.3)
	−28.6	45.5	−30.6	−19.4	19.1	51.2	−2.1	29.9	11.7	27.0
	−6.623	10.528	−7.084	−4.495	4.414	11.851	−0.475	6.910	2.715	6.241
t_O	162	105	156	151	88	138	144	89	164	136
	(37.5)	(24.3)	(36.1)	(35.0)	(20.4)	(31.9)	(33.3)	(20.6)	(38.0)	(31.5)
t_E	156.4	90.4	142.4	156.4	90.4	142.4	156.4	90.4	142.4	156.4
	(36.2)	(20.9)	(33.0)	(36.2)	(20.9)	(33.0)	(36.2)	(20.9)	(33.0)	(36.2)
	11.4	31.3	28.5	−10.6	−4.8	−8.6	−23.8	−2.9	46.4	−38.0
	2.643	7.252	6.606	−2.452	−1.118	−1.989	−5.503	−0.665	10.742	−8.796
g_O	62	109	72	88	126	66	90	118	50	84
	(14.4)	(25.2)	(16.7)	(20.4)	(29.2)	(15.3)	(20.8)	(27.3)	(11.6)	(19.4)
g_E	68.5	132.1	61.2	68.5	132.1	61.2	68.5	132.1	61.2	68.5
	(15.8)	(30.6)	(14.2)	(15.8)	(30.6)	(14.2)	(15.8)	(30.6)	(14.2)	(15.8)
	−12.3	−41.9	23.3	44.2	−11.9	9.9	49.2	−26.6	−20.3	34.3
	−2.848	−9.696	5.397	10.225	−2.754	2.288	11.394	−6.163	−4.693	7.951
c_O	62	53	75	42	65	59	38	67	67	38
	(14.4)	(12.3)	(17.4)	(9.7)	(15.0)	(13.7)	(8.8)	(15.5)	(15.5)	(8.8)
c_E	46.1	65.7	83.2	46.1	65.7	83.2	46.1	65.7	83.2	46.1
	(10.7)	(15.2)	(19.2)	(10.7)	(15.2)	(19.2)	(10.7)	(15.2)	(19.2)	(10.7)
	36.7	−22.8	−15.5	−7.8	−1.4	−40.5	−14.7	2.6	−28.9	−14.7
	8.498	−5.272	−3.582	−1.816	−0.324	−9.372	−3.404	0.606	−6.699	−3.404
N	432	432	432	432	432	432	432	432	432	432
X2	7.7	12.0	5.8	6.7	0.9	11.4	9.2	3.0	8.7	8.7
Gvalue	7.2	12.1	5.8	6.3	0.9	12.0	8.7	3.0	8.9	8.6
Gv/N	1.670	2.812	1.336	1.461	0.218	2.777	2.012	0.689	2.065	1.991
Prob	0.06539	0.00690	0.12328	0.09742	0.81536	0.00739	0.03370	0.39546	0.03035	0.03506

	61	62	63	64	65	66	67	68	69	70
a_O	133	136	173	137	151	151	142	145	167	152
	(30.8)	(31.5)	(40.0)	(31.7)	(35.0)	(35.0)	(32.9)	(33.6)	(38.7)	(35.2)
a_E	143.8	145.2	161.0	143.8	145.2	161.0	143.8	145.2	161.0	143.8
	(33.3)	(33.6)	(37.3)	(33.3)	(33.6)	(37.3)	(33.3)	(33.6)	(37.3)	(33.3)
	−20.7	−17.9	24.8	−13.2	11.7	−19.4	−3.5	−0.5	12.2	16.9
	−4.789	−4.142	5.743	−3.054	2.715	−4.495	−0.809	−0.114	2.815	3.923
t_O	103	146	149	105	144	158	101	149	159	96
	(23.8)	(33.8)	(34.5)	(24.3)	(33.3)	(36.6)	(23.4)	(34.5)	(36.8)	(22.2)
t_E	90.4	142.4	156.4	90.4	142.4	156.4	90.4	142.4	156.4	90.4
	(20.9)	(33.0)	(36.2)	(20.9)	(33.0)	(36.2)	(20.9)	(33.0)	(36.2)	(20.9)
	26.8	7.4	−14.4	31.3	3.3	3.2	22.3	13.6	5.3	11.4
	6.197	1.705	−3.340	7.252	0.762	0.749	5.159	3.143	1.218	2.647
g_O	136	67	69	139	62	79	128	66	62	125
	(31.5)	(15.5)	(16.0)	(32.2)	(14.4)	(18.3)	(29.6)	(15.3)	(14.4)	(28.9)
g_E	132.1	61.2	68.5	132.1	61.2	68.5	132.1	61.2	68.5	132.1
	(30.6)	(14.2)	(15.8)	(30.6)	(14.2)	(15.8)	(30.6)	(14.2)	(15.8)	(30.6)
	7.9	12.1	1.1	14.2	1.5	22.6	−8.1	9.9	−12.3	−13.8
	1.836	2.790	0.247	3.281	0.355	5.233	−1.865	2.288	−2.848	−3.193
c_O	60	83	41	51	75	44	61	72	44	59
	(13.9)	(19.2)	(9.5)	(11.8)	(17.4)	(10.2)	(14.1)	(16.7)	(10.2)	(13.7)
c_E	65.7	83.2	46.1	65.7	83.2	46.1	65.7	83.2	46.1	65.7
	(15.2)	(19.2)	(10.7)	(15.2)	(19.2)	(10.7)	(15.2)	(19.2)	(10.7)	(15.2)
	−10.9	−0.3	−9.6	−25.8	−15.5	−4.1	−9.1	−20.7	−4.1	−12.7
	−2.522	−0.070	−2.230	−5.981	−3.582	−0.955	−2.097	−4.800	−0.955	−2.939

N	432	432	432	432	432	432	432	432	432	432
X2	3.2	1.2	1.8	6.3	1.1	2.4	1.7	2.2	1.0	1.9
Gvalue	3.1	1.2	1.8	6.5	1.1	2.3	1.7	2.2	1.0	1.9
Gv/N	0.721	0.282	0.421	1.498	0.250	0.531	0.389	0.517	0.229	0.438
Prob	0.37399	0.74822	0.61130	0.09085	0.78201	0.51334	0.64134	0.52542	0.80332	0.59474

	71	72	73	74	75	76	77	78	79	80
a_O	144	164	137	131	155	149	149	146	138	142
	(33.3)	(38.0)	(31.7)	(30.3)	(35.9)	(34.5)	(34.5)	(33.8)	(31.9)	(32.9)
a_E	145.2	161.0	143.8	145.2	161.0	143.8	145.2	161.0	143.8	145.2
	(33.6)	(37.3)	(33.3)	(33.6)	(37.3)	(33.3)	(33.6)	(37.3)	(33.3)	(33.6)
	−2.5	6.0	−13.2	−27.0	−11.8	10.7	7.6	−28.6	−11.3	−6.4
	−0.575	1.388	−3.054	−6.261	−2.738	2.471	1.760	−6.623	−2.611	−1.486
t_O	137	165	110	160	167	108	159	163	109	144
	(31.7)	(38.2)	(25.5)	(37.0)	(38.7)	(25.0)	(36.8)	(37.7)	(25.2)	(33.3)
t_E	142.4	156.4	90.4	142.4	156.4	90.4	142.4	156.4	90.4	142.4
	(33.0)	(36.2)	(20.9)	(33.0)	(36.2)	(20.9)	(33.0)	(36.2)	(20.9)	(33.0)
	−10.5	17.7	43.1	37.4	21.9	38.3	35.1	13.5	40.7	3.3
	−2.436	4.093	9.966	8.651	5.074	8.868	8.135	3.123	9.415	0.762
g_O	73	63	140	67	62	113	56	73	114	69
	(16.9)	(14.6)	(32.4)	(15.5)	(14.4)	(26.2)	(13.0)	(16.9)	(26.4)	(16.0)
g_E	61.2	68.5	132.1	61.2	68.5	132.1	61.2	68.5	132.1	61.2
	(14.2)	(15.8)	(30.6)	(14.2)	(15.8)	(30.6)	(14.2)	(15.8)	(30.6)	(14.2)
	25.7	−10.5	16.3	12.1	−12.3	−35.3	−10.0	9.4	−33.6	16.5
	5.938	−2.427	3.769	2.790	−2.848	−8.167	−2.318	2.166	−7.774	3.812
c_O	78	40	45	74	48	62	68	50	71	77
	(18.1)	(9.3)	(10.4)	(17.1)	(11.1)	(14.4)	(15.7)	(11.6)	(16.4)	(17.8)
c_E	83.2	46.1	65.7	83.2	46.1	65.7	83.2	46.1	65.7	83.2
	(19.2)	(10.7)	(15.2)	(19.2)	(10.7)	(15.2)	(19.2)	(10.7)	(15.2)	(19.2)
	−10.0	−11.4	−34.1	−17.3	3.9	−7.2	−27.4	8.1	11.0	−11.8
	−2.309	−2.633	−7.885	−3.994	0.892	−1.665	−6.333	1.874	2.549	−2.740
N	432	432	432	432	432	432	432	432	432	432
X2	2.8	1.8	11.5	5.1	1.6	6.6	5.2	2.3	6.9	1.5
Gvalue	2.7	1.8	12.1	5.1	1.6	6.5	5.4	2.3	6.8	1.5
Gv/N	0.618	0.421	2.796	1.185	0.380	1.507	1.244	0.541	1.578	0.348
Prob	0.44561	0.61119	0.00711	0.16340	0.65037	0.08930	0.14629	0.50558	0.07794	0.68149

	81	82	83	84	85	86	87	88	89	90
a_O	156	139	134	169	159	137	155	145	131	144
	(36.1)	(32.2)	(31.0)	(39.1)	(36.8)	(31.7)	(35.9)	(33.6)	(30.3)	(33.3)
a_E	161.0	143.8	145.2	161.0	143.8	145.2	161.0	143.8	145.2	161.0
	(37.3)	(33.3)	(33.6)	(37.3)	(33.3)	(33.6)	(37.3)	(33.3)	(33.6)	(37.3)
	−9.9	−9.4	−21.6	16.3	32.0	−16.0	−11.8	2.5	−27.0	−32.2
	−2.291	−2.166	−5.000	3.780	7.418	−3.708	−2.738	0.578	−6.261	−7.451
t_O	168	105	151	155	92	169	168	95	162	158
	(38.9)	(24.3)	(35.0)	(35.9)	(21.3)	(39.1)	(38.9)	(22.0)	(37.5)	(36.6)
t_E	156.4	90.4	142.4	156.4	90.4	142.4	156.4	90.4	142.4	156.4
	(36.2)	(20.9)	(33.0)	(36.2)	(20.9)	(33.0)	(36.2)	(20.9)	(33.0)	(36.2)
	24.1	31.3	17.8	−2.8	3.1	58.0	24.1	9.3	41.9	3.2
	5.569	7.252	4.117	−0.641	0.724	13.419	5.569	2.159	9.691	0.749
g_O	56	137	54	61	119	48	64	129	62	79
	(13.0)	(31.7)	(12.5)	(14.1)	(27.5)	(11.1)	(14.8)	(29.9)	(14.4)	(18.3)
g_E	68.5	132.1	61.2	68.5	132.1	61.2	68.5	132.1	61.2	68.5
	(15.8)	(30.6)	(14.2)	(15.8)	(30.6)	(14.2)	(15.8)	(30.6)	(14.2)	(15.8)
	−22.5	10.0	−13.6	−14.1	−24.8	−23.4	−8.6	−6.1	1.5	22.6
	−5.211	2.314	−3.144	−3.261	−5.750	−5.412	−1.999	−1.414	0.355	5.233
c_O	52	51	93	47	62	78	45	63	77	51
	(12.0)	(11.8)	(21.5)	(10.9)	(14.4)	(18.1)	(10.4)	(14.6)	(17.8)	(11.8)
c_E	46.1	65.7	83.2	46.1	65.7	83.2	46.1	65.7	83.2	46.1
	(10.7)	(15.2)	(19.2)	(10.7)	(15.2)	(19.2)	(10.7)	(15.2)	(19.2)	(10.7)
	12.5	−25.8	20.8	1.8	−7.2	−10.0	−2.2	−5.3	−11.8	10.3
	2.893	−5.981	4.820	0.415	−1.665	−2.309	−0.509	−1.225	−2.740	2.379
N	432	432	432	432	432	432	432	432	432	432
X2	4.0	6.0	3.4	1.2	3.1	8.6	1.4	0.4	4.6	4.0
Gvalue	4.1	6.1	3.4	1.3	3.1	8.6	1.4	0.4	4.5	3.9
Gv/N	0.959	1.419	0.792	0.292	0.728	1.990	0.323	0.098	1.045	0.909
Prob	0.24635	0.10539	0.33100	0.73783	0.37000	0.03518	0.70674	0.93573	0.21110	0.26934

	91	92	93	94	95	96	97	98	99	100
a_O	130	135	164	154	131	160	150	135	160	152
	(30.1)	(31.2)	(38.0)	(35.6)	(30.3)	(37.0)	(34.7)	(31.2)	(37.0)	(35.2)
a_E	143.8	145.2	161.0	143.8	145.2	161.0	143.8	145.2	161.0	143.8
	(33.3)	(33.6)	(37.3)	(33.3)	(33.6)	(37.3)	(33.3)	(33.6)	(37.3)	(33.3)
	−26.2	−19.8	6.0	21.2	−27.0	−2.1	12.8	−19.8	−2.1	16.9
	−6.054	−4.573	1.388	4.907	−6.261	−0.475	2.952	−4.573	−0.475	3.923
t_O	97	153	154	97	143	161	88	153	155	90
	(22.5)	(35.4)	(35.6)	(22.5)	(33.1)	(37.3)	(20.4)	(35.4)	(35.9)	(20.8)
t_E	90.4	142.4	156.4	90.4	142.4	156.4	90.4	142.4	156.4	90.4
	(20.9)	(33.0)	(36.2)	(20.9)	(33.0)	(36.2)	(20.9)	(33.0)	(36.2)	(20.9)
	13.6	22.0	−4.7	13.6	1.3	9.4	−4.8	22.0	−2.8	−0.9
	3.140	5.103	−1.099	3.140	0.295	2.165	−1.118	5.103	−0.641	−0.207
g_O	131	61	63	125	66	65	134	67	67	120
	(30.3)	(14.1)	(14.6)	(28.9)	(15.3)	(15.0)	(31.0)	(15.5)	(15.5)	(27.8)
g_E	132.1	61.2	68.5	132.1	61.2	68.5	132.1	61.2	68.5	132.1
	(30.6)	(14.2)	(15.8)	(30.6)	(14.2)	(15.8)	(30.6)	(14.2)	(15.8)	(30.6)
	−2.2	−0.5	−10.5	−13.8	9.9	−6.8	3.8	12.1	−2.9	−23.0
	−0.503	−0.110	−2.427	−3.193	2.288	−1.564	0.890	2.790	−0.672	−5.333
c_O	74	83	51	56	92	46	60	77	50	70
	(17.1)	(19.2)	(11.8)	(13.0)	(21.3)	(10.6)	(13.9)	(17.8)	(11.6)	(16.2)
c_E	65.7	83.2	46.1	65.7	83.2	46.1	65.7	83.2	46.1	65.7
	(15.2)	(19.2)	(10.7)	(15.2)	(19.2)	(10.7)	(15.2)	(19.2)	(10.7)	(15.2)
	17.6	−0.3	10.3	−17.9	18.6	−0.2	−10.9	−11.8	8.1	8.9
	4.074	−0.070	2.379	−4.143	4.307	−0.052	−2.522	−2.740	1.874	2.053
N	432	432	432	432	432	432	432	432	432	432
X2	2.8	1.5	1.0	3.0	2.7	0.3	0.9	2.5	0.4	1.9
Gvalue	2.8	1.5	1.0	3.1	2.7	0.3	0.9	2.5	0.4	1.9
Gv/N	0.657	0.351	0.241	0.711	0.630	0.074	0.202	0.581	0.086	0.436
Prob	0.41704	0.67830	0.79141	0.38045	0.43692	0.95622	0.83229	0.47372	0.94644	0.59695

Baph_BP
5UTR:

	100	99	98	97	96	95	94	93	92	91
a_O	60	65	58	64	56	69	63	64	56	65
	(40.0)	(43.3)	(38.7)	(42.7)	(37.3)	(46.0)	(42.0)	(42.7)	(37.3)	(43.3)
a_E	66.4	66.4	66.4	66.4	66.4	66.4	66.4	66.4	66.4	66.4
	(44.3)	(44.3)	(44.3)	(44.3)	(44.3)	(44.3)	(44.3)	(44.3)	(44.3)	(44.3)
	-12.2	-2.8	-15.8	-4.8	-19.1	5.2	-6.7	-4.8	-19.1	-2.8
	-8.156	-1.899	-10.506	-3.193	-12.764	3.478	-4.466	-3.193	-12.764	-1.899
t_O	62	56	65	59	61	50	63	57	67	47
	(41.3)	(37.3)	(43.3)	(39.3)	(40.7)	(33.3)	(42.0)	(38.0)	(44.7)	(31.3)
t_E	58.2	58.2	58.2	58.2	58.2	58.2	58.2	58.2	58.2	58.2
	(38.8)	(38.8)	(38.8)	(38.8)	(38.8)	(38.8)	(38.8)	(38.8)	(38.8)	(38.8)
	7.8	-4.3	14.3	1.6	5.7	-15.2	10.0	-2.4	18.8	-20.1
	5.214	-2.890	9.562	1.060	3.808	-10.136	6.643	-1.596	12.563	-13.405
g_O	16	17	14	13	13	16	13	12	13	18
	(10.7)	(11.3)	(9.3)	(8.7)	(8.7)	(10.7)	(8.7)	(8.0)	(8.7)	(12.0)
g_E	11.9	11.9	11.9	11.9	11.9	11.9	11.9	11.9	11.9	11.9
	(7.9)	(7.9)	(7.9)	(7.9)	(7.9)	(7.9)	(7.9)	(7.9)	(7.9)	(7.9)
	9.5	12.1	4.6	2.3	2.3	9.5	2.3	0.2	2.3	14.9
	6.316	8.085	3.034	1.532	1.532	6.316	1.532	0.134	1.532	9.932
c_O	12	12	13	14	20	15	11	17	14	20
	(8.0)	(8.0)	(8.7)	(9.3)	(13.3)	(10.0)	(7.3)	(11.3)	(9.3)	(13.3)
c_E	13.5	13.5	13.5	13.5	13.5	13.5	13.5	13.5	13.5	13.5
	(9.0)	(9.0)	(9.0)	(9.0)	(9.0)	(9.0)	(9.0)	(9.0)	(9.0)	(9.0)
	-2.7	-2.7	-0.9	1.1	15.9	3.3	-4.4	8.0	1.1	15.9
	-1.825	-1.825	-0.590	0.748	10.580	2.181	-2.949	5.309	0.748	10.580
N	150	150	150	150	150	150	150	150	150	150
X2	2.4	2.5	2.2	0.2	5.1	2.8	1.1	1.1	3.1	8.5
Gvalue	2.3	2.2	2.2	0.2	4.7	2.8	1.1	1.0	3.1	7.8
Gv/N	1.549	1.470	1.500	0.148	3.156	1.840	0.760	0.654	2.080	5.208
Prob	0.50815	0.53084	0.52229	0.97392	0.19232	0.43017	0.76746	0.80588	0.37351	0.05006

	90	89	88	87	86	85	84	83	82	81
a_O	61	69	54	67	68	67	78	68	64	70
	(40.7)	(46.0)	(36.0)	(44.7)	(45.3)	(44.7)	(52.0)	(45.3)	(42.7)	(46.7)
a_E	66.4	66.4	66.4	66.4	66.4	66.4	66.4	66.4	66.4	66.4
	(44.3)	(44.3)	(44.3)	(44.3)	(44.3)	(44.3)	(44.3)	(44.3)	(44.3)	(44.3)
	-10.4	5.2	-22.4	1.1	3.2	1.1	25.0	3.2	-4.8	7.3
	-6.948	3.478	-14.927	0.750	2.104	0.750	16.683	2.104	-3.193	4.872
t_O	66	50	61	53	51	60	43	60	63	55
	(44.0)	(33.3)	(40.7)	(35.3)	(34.0)	(40.0)	(28.7)	(40.0)	(42.0)	(36.7)
t_E	58.2	58.2	58.2	58.2	58.2	58.2	58.2	58.2	58.2	58.2
	(38.8)	(38.8)	(38.8)	(38.8)	(38.8)	(38.8)	(38.8)	(38.8)	(38.8)	(38.8)
	16.6	-15.2	5.7	-9.9	-13.5	3.6	-26.0	3.6	10.0	-6.2
	11.053	-10.136	3.808	-6.626	-8.992	2.423	-17.364	2.423	6.643	-4.160
g_O	11	18	17	16	19	12	17	10	13	17
	(7.3)	(12.0)	(11.3)	(10.7)	(12.7)	(8.0)	(11.3)	(6.7)	(8.7)	(11.3)
g_E	11.9	11.9	11.9	11.9	11.9	11.9	11.9	11.9	11.9	11.9
	(7.9)	(7.9)	(7.9)	(7.9)	(7.9)	(7.9)	(7.9)	(7.9)	(7.9)	(7.9)
	-1.7	14.9	12.1	9.5	17.8	0.2	12.1	-3.5	2.3	12.1
	-1.153	9.932	8.085	6.316	11.853	0.134	8.085	-2.319	1.532	8.085
c_O	12	13	18	14	12	11	12	12	10	8
	(8.0)	(8.7)	(12.0)	(9.3)	(8.0)	(7.3)	(8.0)	(8.0)	(6.7)	(5.3)
c_E	13.5	13.5	13.5	13.5	13.5	13.5	13.5	13.5	13.5	13.5
	(9.0)	(9.0)	(9.0)	(9.0)	(9.0)	(9.0)	(9.0)	(9.0)	(9.0)	(9.0)
	-2.7	-0.9	10.5	1.1	-2.7	-4.4	-2.7	-2.7	-5.9	-8.3
	-1.825	-0.590	6.993	0.748	-1.825	-2.949	-1.825	-1.825	-3.952	-5.542
N	150	150	150	150	150	150	150	150	150	150
X2	1.7	4.4	6.2	1.9	5.3	0.5	8.3	0.6	1.5	4.8
Gvalue	1.7	4.0	5.9	1.8	4.7	0.5	8.4	0.6	1.5	4.9
Gv/N	1.126	2.685	3.959	1.188	3.141	0.357	5.578	0.383	1.030	3.255
Prob	0.63936	0.25854	0.11463	0.61899	0.19421	0.91087	0.03900	0.90235	0.67185	0.18063

	80	79	78	77	76	75	74	73	72	71
a_O	70	60	62	63	65	61	66	71	72	78
	(46.7)	(40.0)	(41.3)	(42.0)	(43.3)	(40.7)	(44.0)	(47.3)	(48.0)	(52.0)
a_E	66.4	66.4	66.4	66.4	66.4	66.4	66.4	66.4	66.4	66.4
	(44.3)	(44.3)	(44.3)	(44.3)	(44.3)	(44.3)	(44.3)	(44.3)	(44.3)	(44.3)
	7.3	-12.2	-8.6	-6.7	-2.8	-10.4	-0.9	9.4	11.6	25.0
	4.872	-8.156	-5.718	-4.466	-1.899	-6.948	-0.585	6.284	7.715	16.683
t_O	51	61	63	62	60	66	62	49	54	50
	(34.0)	(40.7)	(42.0)	(41.3)	(40.0)	(44.0)	(41.3)	(32.7)	(36.0)	(33.3)
t_E	58.2	58.2	58.2	58.2	58.2	58.2	58.2	58.2	58.2	58.2
	(38.8)	(38.8)	(38.8)	(38.8)	(38.8)	(38.8)	(38.8)	(38.8)	(38.8)	(38.8)
	-13.5	5.7	10.0	7.8	3.6	16.6	7.8	-16.9	-8.1	-15.2
	-8.992	3.808	6.643	5.214	2.423	11.053	5.214	-11.253	-5.405	-10.136
g_O	14	14	11	13	12	15	6	13	11	8
	(9.3)	(9.3)	(7.3)	(8.7)	(8.0)	(10.0)	(4.0)	(8.7)	(7.3)	(5.3)
g_E	11.9	11.9	11.9	11.9	11.9	11.9	11.9	11.9	11.9	11.9
	(7.9)	(7.9)	(7.9)	(7.9)	(7.9)	(7.9)	(7.9)	(7.9)	(7.9)	(7.9)
	4.6	4.6	-1.7	2.3	0.2	6.9	-8.2	2.3	-1.7	-6.4
	3.034	3.034	-1.153	1.532	0.134	4.630	-5.478	1.532	-1.153	-4.236
c_O	15	15	14	12	13	8	16	17	13	14
	(10.0)	(10.0)	(9.3)	(8.0)	(8.7)	(5.3)	(10.7)	(11.3)	(8.7)	(9.3)
c_E	13.5	13.5	13.5	13.5	13.5	13.5	13.5	13.5	13.5	13.5
	(9.0)	(9.0)	(9.0)	(9.0)	(9.0)	(9.0)	(9.0)	(9.0)	(9.0)	(9.0)
	3.3	3.3	1.1	-2.7	-0.9	-8.3	5.6	8.0	-0.9	1.1
	2.181	2.181	0.748	-1.825	-0.590	-5.542	3.704	5.309	-0.590	0.748
N	150	150	150	150	150	150	150	150	150	150
X2	1.6	1.3	0.8	0.7	0.1	4.5	3.7	2.8	0.9	4.5
Gvalue	1.6	1.3	0.8	0.7	0.1	4.8	4.3	2.8	0.9	4.6
Gv/N	1.095	0.866	0.520	0.456	0.068	3.193	2.855	1.873	0.567	3.059
Prob	0.64980	0.72920	0.85440	0.87701	0.99160	0.18786	0.23252	0.42194	0.83747	0.20448

	70	69	68	67	66	65	64	63	62	61
a_O	65	65	62	72	56	65	68	69	60	60
	(43.3)	(43.3)	(41.3)	(48.0)	(37.3)	(43.3)	(45.3)	(46.0)	(40.0)	(40.0)
a_E	66.4	66.4	66.4	66.4	66.4	66.4	66.4	66.4	66.4	66.4
	(44.3)	(44.3)	(44.3)	(44.3)	(44.3)	(44.3)	(44.3)	(44.3)	(44.3)	(44.3)
	-2.8	-2.8	-8.6	11.6	-19.1	-2.8	3.2	5.2	-12.2	-12.2
	-1.899	-1.899	-5.718	7.715	-12.764	-1.899	2.104	3.478	-8.156	-8.156
t_O	56	63	65	51	65	59	54	56	61	63
	(37.3)	(42.0)	(43.3)	(34.0)	(43.3)	(39.3)	(36.0)	(37.3)	(40.7)	(42.0)

t_E	58.2	58.2	58.2	58.2	58.2	58.2	58.2	58.2	58.2	58.2
	(38.8)	(38.8)	(38.8)	(38.8)	(38.8)	(38.8)	(38.8)	(38.8)	(38.8)	(38.8)
	-4.3	10.0	14.3	-13.5	14.3	1.6	-8.1	-4.3	5.7	10.0
	-2.890	6.643	9.562	-8.992	9.562	1.060	-5.405	-2.890	3.808	6.643
g_O	17	14	13	11	15	8	12	13	15	10
	(11.3)	(9.3)	(8.7)	(7.3)	(10.0)	(5.3)	(8.0)	(8.7)	(10.0)	(6.7)
g_E	11.9	11.9	11.9	11.9	11.9	11.9	11.9	11.9	11.9	11.9
	(7.9)	(7.9)	(7.9)	(7.9)	(7.9)	(7.9)	(7.9)	(7.9)	(7.9)	(7.9)
	12.1	4.6	2.3	-1.7	6.9	-6.4	0.2	2.3	6.9	-3.5
	8.085	3.034	1.532	-1.153	4.630	-4.236	0.134	1.532	4.630	-2.319
c_O	12	8	10	16	14	18	16	12	14	17
	(8.0)	(5.3)	(6.7)	(10.7)	(9.3)	(12.0)	(10.7)	(8.0)	(9.3)	(11.3)
c_E	13.5	13.5	13.5	13.5	13.5	13.5	13.5	13.5	13.5	13.5
	(9.0)	(9.0)	(9.0)	(9.0)	(9.0)	(9.0)	(9.0)	(9.0)	(9.0)	(9.0)
	-2.7	-8.3	-5.9	5.6	1.1	10.5	5.6	-2.7	1.1	8.0
	-1.825	-5.542	-3.952	3.704	0.748	6.993	3.704	-1.825	0.748	5.309
N	150	150	150	150	150	150	150	150	150	150
X2	2.5	3.0	2.1	1.9	3.3	2.9	0.8	0.4	1.6	2.3
Gvalue	2.2	3.4	2.1	1.9	3.3	2.9	0.8	0.4	1.5	2.2
Gv/N	1.470	2.235	1.425	1.274	2.176	1.919	0.537	0.296	1.030	1.476
Prob	0.53084	0.34031	0.54441	0.59117	0.35264	0.41071	0.84832	0.93115	0.67201	0.52918

	60	59	58	57	56	55	54	53	52	51
a_O	53	73	57	64	51	61	69	62	55	62
	(35.3)	(48.7)	(38.0)	(42.7)	(34.0)	(40.7)	(46.0)	(41.3)	(36.7)	(41.3)
a_E	66.4	66.4	66.4	66.4	66.4	66.4	66.4	66.4	66.4	66.4
	(44.3)	(44.3)	(44.3)	(44.3)	(44.3)	(44.3)	(44.3)	(44.3)	(44.3)	(44.3)
	-24.0	13.7	-17.5	-4.8	-27.0	-10.4	5.2	-8.6	-20.8	-8.6
	-15.971	9.165	-11.647	-3.193	-17.984	-6.948	3.478	-5.718	-13.858	-5.718
t_O	71	46	59	62	76	59	65	65	65	66
	(47.3)	(30.7)	(39.3)	(41.3)	(50.7)	(39.3)	(43.3)	(43.3)	(43.3)	(44.0)
t_E	58.2	58.2	58.2	58.2	58.2	58.2	58.2	58.2	58.2	58.2
	(38.8)	(38.8)	(38.8)	(38.8)	(38.8)	(38.8)	(38.8)	(38.8)	(38.8)	(38.8)
	28.2	-21.7	1.6	7.8	40.5	1.6	14.3	14.3	14.3	16.6
	18.803	-14.439	1.060	5.214	27.023	1.060	9.562	9.562	9.562	11.053
g_O	12	17	16	10	14	15	6	12	12	12
	(8.0)	(11.3)	(10.7)	(6.7)	(9.3)	(10.0)	(4.0)	(8.0)	(8.0)	(8.0)
g_E	11.9	11.9	11.9	11.9	11.9	11.9	11.9	11.9	11.9	11.9
	(7.9)	(7.9)	(7.9)	(7.9)	(7.9)	(7.9)	(7.9)	(7.9)	(7.9)	(7.9)
	0.2	12.1	9.5	-3.5	4.6	6.9	-8.2	0.2	0.2	0.2
	0.134	8.085	6.316	-2.319	3.034	4.630	-5.478	0.134	0.134	0.134
c_O	14	14	18	14	9	15	10	11	18	10
	(9.3)	(9.3)	(12.0)	(9.3)	(6.0)	(10.0)	(6.7)	(7.3)	(12.0)	(6.7)
c_E	13.5	13.5	13.5	13.5	13.5	13.5	13.5	13.5	13.5	13.5
	(9.0)	(9.0)	(9.0)	(9.0)	(9.0)	(9.0)	(9.0)	(9.0)	(9.0)	(9.0)
	1.1	1.1	10.5	1.1	-7.2	3.3	-5.9	-4.4	10.5	-5.9
	0.748	0.748	6.993	0.748	-4.821	2.181	-3.952	-2.949	6.993	-3.952
N	150	150	150	150	150	150	150	150	150	150
X2	5.6	5.4	4.3	0.7	10.9	1.4	4.7	1.5	4.3	2.2
Gvalue	5.6	5.3	4.1	0.7	10.9	1.4	5.4	1.5	4.2	2.3
Gv/N	3.714	3.559	2.723	0.450	7.252	0.924	3.610	1.029	2.832	1.517
Prob	0.13447	0.14864	0.25252	0.87897	0.01241	0.70876	0.14381	0.67228	0.23594	0.51725

	50	49	48	47	46	45	44	43	42	41
a_O	68	70	67	67	54	53	64	53	66	61
	(45.3)	(46.7)	(44.7)	(44.7)	(36.0)	(35.3)	(42.7)	(35.3)	(44.0)	(40.7)
a_E	66.4	66.4	66.4	66.4	66.4	66.4	66.4	66.4	66.4	66.4
	(44.3)	(44.3)	(44.3)	(44.3)	(44.3)	(44.3)	(44.3)	(44.3)	(44.3)	(44.3)
	3.2	7.3	1.1	1.1	-22.4	-24.0	-4.8	-24.0	-0.9	-10.4
	2.104	4.872	0.750	0.750	-14.927	-15.971	-3.193	-15.971	-0.585	-6.948
t_O	55	53	58	65	61	63	61	61	49	62
	(36.7)	(35.3)	(38.7)	(43.3)	(40.7)	(42.0)	(40.7)	(40.7)	(32.7)	(41.3)
t_E	58.2	58.2	58.2	58.2	58.2	58.2	58.2	58.2	58.2	58.2
	(38.8)	(38.8)	(38.8)	(38.8)	(38.8)	(38.8)	(38.8)	(38.8)	(38.8)	(38.8)
	-6.2	-9.9	-0.4	14.3	5.7	10.0	5.7	5.7	-16.9	7.8
	-4.160	-6.626	-0.279	9.562	3.808	6.643	3.808	3.808	-11.253	5.214
g_O	14	16	9	9	15	15	12	19	15	13
	(9.3)	(10.7)	(6.0)	(6.0)	(10.0)	(10.0)	(8.0)	(12.7)	(10.0)	(8.7)
g_E	11.9	11.9	11.9	11.9	11.9	11.9	11.9	11.9	11.9	11.9
	(7.9)	(7.9)	(7.9)	(7.9)	(7.9)	(7.9)	(7.9)	(7.9)	(7.9)	(7.9)
	4.6	9.5	-5.0	-5.0	6.9	6.9	0.2	17.8	6.9	2.3
	3.034	6.316	-3.352	-3.352	4.630	4.630	0.134	11.853	4.630	1.532
c_O	13	11	16	9	20	19	13	17	20	14
	(8.7)	(7.3)	(10.7)	(6.0)	(13.3)	(12.7)	(8.7)	(11.3)	(13.3)	(9.3)
c_E	13.5	13.5	13.5	13.5	13.5	13.5	13.5	13.5	13.5	13.5
	(9.0)	(9.0)	(9.0)	(9.0)	(9.0)	(9.0)	(9.0)	(9.0)	(9.0)	(9.0)
	-0.9	-4.4	5.6	-7.2	15.9	13.1	-0.9	8.0	15.9	1.1
	-0.590	-2.949	3.704	-4.821	10.580	8.752	-0.590	5.309	10.580	0.748
N	150	150	150	150	150	150	150	150	150	150
X2	0.6	2.5	1.2	3.0	6.5	6.2	0.2	8.0	5.5	0.8
Gvalue	0.6	2.4	1.2	3.2	6.1	6.1	0.2	7.5	5.1	0.8
Gv/N	0.388	1.612	0.822	2.139	4.091	4.053	0.159	4.999	3.373	0.547
Prob	0.90043	0.49028	0.74502	0.36060	0.10513	0.10779	0.97116	0.05758	0.16751	0.84455

	40	39	38	37	36	35	34	33	32	31
a_O	58	76	81	80	68	65	57	71	68	56
	(38.7)	(50.7)	(54.0)	(53.3)	(45.3)	(43.3)	(38.0)	(47.3)	(45.3)	(37.3)
a_E	66.4	66.4	66.4	66.4	66.4	66.4	66.4	66.4	66.4	66.4
	(44.3)	(44.3)	(44.3)	(44.3)	(44.3)	(44.3)	(44.3)	(44.3)	(44.3)	(44.3)
	-15.8	20.4	32.1	29.7	3.2	-2.8	-17.5	9.4	3.2	-19.1
	-10.506	13.623	21.400	19.811	2.104	-1.899	-11.647	6.284	2.104	-12.764
t_O	65	52	50	49	65	65	72	60	55	72
	(43.3)	(34.7)	(33.3)	(32.7)	(43.3)	(43.3)	(48.0)	(40.0)	(36.7)	(48.0)
t_E	58.2	58.2	58.2	58.2	58.2	58.2	58.2	58.2	58.2	58.2
	(38.8)	(38.8)	(38.8)	(38.8)	(38.8)	(38.8)	(38.8)	(38.8)	(38.8)	(38.8)
	14.3	-11.7	-15.2	-16.9	14.3	14.3	30.6	3.6	-6.2	30.6
	9.562	-7.822	-10.136	-11.253	9.562	9.562	20.410	2.423	-4.160	20.410
g_O	13	11	7	12	4	7	7	10	15	11
	(8.7)	(7.3)	(4.7)	(8.0)	(2.7)	(4.7)	(4.7)	(6.7)	(10.0)	(7.3)
g_E	11.9	11.9	11.9	11.9	11.9	11.9	11.9	11.9	11.9	11.9
	(7.9)	(7.9)	(7.9)	(7.9)	(7.9)	(7.9)	(7.9)	(7.9)	(7.9)	(7.9)
	2.3	-1.7	-7.4	0.2	-8.7	-7.4	-7.4	-3.5	6.9	-1.7
	1.532	-1.153	-4.953	0.134	-5.815	-4.953	-4.953	-2.319	4.630	-1.153
c_O	14	11	12	9	13	13	14	9	12	11
	(9.3)	(7.3)	(8.0)	(6.0)	(8.7)	(8.7)	(9.3)	(6.0)	(8.0)	(7.3)
c_E	13.5	13.5	13.5	13.5	13.5	13.5	13.5	13.5	13.5	13.5

	(9.0)	(9.0)	(9.0)	(9.0)	(9.0)	(9.0)	(9.0)	(9.0)	(9.0)	(9.0)
	1.1	-4.4	-2.7	-7.2	-0.9	-0.9	1.1	-7.2	-2.7	-4.4
	0.748	-2.949	-1.825	-4.821	-0.590	-0.590	0.748	-4.821	-1.825	-2.949
N	150	150	150	150	150	150	150	150	150	150
X2	2.0	2.6	6.5	5.7	6.1	2.9	6.6	2.1	1.2	5.4
Gvalue	2.0	2.5	6.7	5.8	7.9	3.2	6.8	2.3	1.1	5.3
Gv/N	1.336	1.698	4.487	3.871	5.262	2.121	4.559	1.567	0.750	3.544
Prob	0.57148	0.46679	0.08101	0.12142	0.04829	0.36458	0.07722	0.50303	0.77121	0.15010

	30	29	28	27	26	25	24	23	22	21
a_O	74	69	67	70	74	54	64	66	58	70
	(49.3)	(46.0)	(44.7)	(46.7)	(49.3)	(36.0)	(42.7)	(44.0)	(38.7)	(46.7)
a_E	66.4	66.4	66.4	66.4	66.4	66.4	66.4	66.4	66.4	66.4
	(44.3)	(44.3)	(44.3)	(44.3)	(44.3)	(44.3)	(44.3)	(44.3)	(44.3)	(44.3)
	15.9	5.2	1.1	7.3	15.9	-22.4	-4.8	-0.9	-15.8	7.3
	10.633	3.478	0.750	4.872	10.633	-14.927	-3.193	-0.585	-10.506	4.872
t_O	59	58	61	56	50	72	68	51	70	66
	(39.3)	(38.7)	(40.7)	(37.3)	(33.3)	(48.0)	(45.3)	(34.0)	(46.7)	(44.0)
t_E	58.2	58.2	58.2	58.2	58.2	58.2	58.2	58.2	58.2	58.2
	(38.8)	(38.8)	(38.8)	(38.8)	(38.8)	(38.8)	(38.8)	(38.8)	(38.8)	(38.8)
	1.6	-0.4	5.7	-4.3	-15.2	30.6	21.1	-13.5	25.8	16.6
	1.060	-0.279	3.808	-2.890	-10.136	20.410	14.094	-8.992	17.214	11.053
g_O	7	12	10	9	8	5	7	14	10	10
	(4.7)	(8.0)	(6.7)	(6.0)	(5.3)	(3.3)	(4.7)	(9.3)	(6.7)	(6.7)
g_E	11.9	11.9	11.9	11.9	11.9	11.9	11.9	11.9	11.9	11.9
	(7.9)	(7.9)	(7.9)	(7.9)	(7.9)	(7.9)	(7.9)	(7.9)	(7.9)	(7.9)
	-7.4	0.2	-3.5	-5.0	-6.4	-8.7	-7.4	4.6	-3.5	-3.5
	-4.953	0.134	-2.319	-3.352	-4.236	-5.781	-4.953	3.034	-2.319	-2.319
c_O	10	11	12	15	18	19	11	19	12	4
	(6.7)	(7.3)	(8.0)	(10.0)	(12.0)	(12.7)	(7.3)	(12.7)	(8.0)	(2.7)
c_E	13.5	13.5	13.5	13.5	13.5	13.5	13.5	13.5	13.5	13.5
	(9.0)	(9.0)	(9.0)	(9.0)	(9.0)	(9.0)	(9.0)	(9.0)	(9.0)	(9.0)
	-5.9	-4.4	-2.7	3.3	10.5	13.1	-4.4	13.1	-2.7	-9.7
	-3.952	-2.949	-1.825	2.181	6.993	8.752	-2.949	8.752	-1.825	-6.468
N	150	150	150	150	150	150	150	150	150	150
X2	3.8	0.5	0.6	1.2	4.8	11.9	4.2	3.6	3.9	8.2
Gvalue	4.2	0.6	0.6	1.2	4.9	12.7	4.5	3.3	3.8	10.7
Gv/N	2.789	0.383	0.413	0.811	3.255	8.455	3.000	2.209	2.563	7.137
Prob	0.24234	0.90210	0.89195	0.74895	0.18061	0.00538	0.21235	0.34579	0.27867	0.01343

	20	19	18	17	16	15	14	13	12	11
a_O	63	65	76	79	72	71	70	82	80	73
	(42.0)	(43.3)	(50.7)	(52.7)	(48.0)	(47.3)	(46.7)	(54.7)	(53.3)	(48.7)
a_E	66.4	66.4	66.4	66.4	66.4	66.4	66.4	66.4	66.4	66.4
	(44.3)	(44.3)	(44.3)	(44.3)	(44.3)	(44.3)	(44.3)	(44.3)	(44.3)	(44.3)
	-6.7	-2.8	20.4	27.4	11.6	9.4	7.3	34.5	29.7	13.7
	-4.466	-1.899	13.623	18.238	7.715	6.284	4.872	23.006	19.811	9.165
t_O	60	65	59	50	51	62	51	43	49	49
	(40.0)	(43.3)	(39.3)	(33.3)	(34.0)	(41.3)	(34.0)	(28.7)	(32.7)	(32.7)
t_E	58.2	58.2	58.2	58.2	58.2	58.2	58.2	58.2	58.2	58.2
	(38.8)	(38.8)	(38.8)	(38.8)	(38.8)	(38.8)	(38.8)	(38.8)	(38.8)	(38.8)
	3.6	14.3	1.6	-15.2	-13.5	7.8	-13.5	-26.0	-16.9	-16.9
	2.423	9.562	1.060	-10.136	-8.992	5.214	-8.992	-17.364	-11.253	-11.253
g_O	9	8	6	7	12	7	14	13	12	17
	(6.0)	(5.3)	(4.0)	(4.7)	(8.0)	(4.7)	(9.3)	(8.7)	(8.0)	(11.3)
g_E	11.9	11.9	11.9	11.9	11.9	11.9	11.9	11.9	11.9	11.9
	(7.9)	(7.9)	(7.9)	(7.9)	(7.9)	(7.9)	(7.9)	(7.9)	(7.9)	(7.9)
	-5.0	-6.4	-8.2	-7.4	0.2	-7.4	4.6	2.3	0.2	12.1
	-3.352	-4.236	-5.478	-4.953	0.134	-4.953	3.034	1.532	0.134	8.085
c_O	18	12	9	14	15	10	15	12	9	11
	(12.0)	(8.0)	(6.0)	(9.3)	(10.0)	(6.7)	(10.0)	(8.0)	(6.0)	(7.3)
c_E	13.5	13.5	13.5	13.5	13.5	13.5	13.5	13.5	13.5	13.5
	(9.0)	(9.0)	(9.0)	(9.0)	(9.0)	(9.0)	(9.0)	(9.0)	(9.0)	(9.0)
	10.5	-2.7	-7.2	1.1	3.3	-5.9	3.3	-2.7	-7.2	-4.4
	6.993	-1.825	-4.821	0.748	2.181	-3.952	2.181	-1.825	-4.821	-2.949
N	150	150	150	150	150	150	150	150	150	150
X2	2.5	2.3	5.8	5.6	1.5	3.5	1.6	7.9	5.7	4.7
Gvalue	2.4	2.4	6.6	5.8	1.6	3.9	1.6	8.0	5.8	4.6
Gv/N	1.599	1.602	4.384	3.898	1.039	2.594	1.095	5.349	3.871	3.048
Prob	0.49396	0.49306	0.08672	0.11927	0.66891	0.27348	0.64980	0.04552	0.12142	0.20602

	10	9	8	7	6	5	4	3	2	1
a_O	75	78	76	81	83	89	74	93	57	35
	(50.0)	(52.0)	(50.7)	(54.0)	(55.3)	(59.3)	(49.3)	(62.0)	(38.0)	(23.3)
a_E	66.4	66.4	66.4	66.4	66.4	66.4	66.4	66.4	66.4	66.4
	(44.3)	(44.3)	(44.3)	(44.3)	(44.3)	(44.3)	(44.3)	(44.3)	(44.3)	(44.3)
	18.2	25.0	20.4	32.1	36.9	52.0	15.9	62.6	-17.5	-44.9
	12.119	16.683	13.623	21.400	24.628	34.691	10.633	41.701	-11.647	-29.911
t_O	46	41	52	45	48	46	53	40	64	89
	(30.7)	(27.3)	(34.7)	(30.0)	(32.0)	(30.7)	(35.3)	(26.7)	(42.7)	(59.3)
t_E	58.2	58.2	58.2	58.2	58.2	58.2	58.2	58.2	58.2	58.2
	(38.8)	(38.8)	(38.8)	(38.8)	(38.8)	(38.8)	(38.8)	(38.8)	(38.8)	(38.8)
	-21.7	-28.7	-11.7	-23.2	-18.5	-21.7	-9.9	-30.0	12.1	75.6
	-14.439	-19.160	-7.822	-15.444	-12.343	-14.439	-6.626	-20.009	8.092	50.383
g_O	17	20	13	7	7	2	7	7	7	6
	(11.3)	(13.3)	(8.7)	(4.7)	(4.7)	(1.3)	(4.7)	(4.7)	(4.7)	(4.0)
g_E	11.9	11.9	11.9	11.9	11.9	11.9	11.9	11.9	11.9	11.9
	(7.9)	(7.9)	(7.9)	(7.9)	(7.9)	(7.9)	(7.9)	(7.9)	(7.9)	(7.9)
	12.1	20.8	2.3	-7.4	-7.4	-7.1	-7.4	-7.4	-7.4	-8.2
	8.085	13.845	1.532	-4.953	-4.953	-4.756	-4.953	-4.953	-4.953	-5.478
c_O	12	11	9	17	12	13	16	10	22	20
	(8.0)	(7.3)	(6.0)	(11.3)	(8.0)	(8.7)	(10.7)	(6.7)	(14.7)	(13.3)
c_E	13.5	13.5	13.5	13.5	13.5	13.5	13.5	13.5	13.5	13.5
	(9.0)	(9.0)	(9.0)	(9.0)	(9.0)	(9.0)	(9.0)	(9.0)	(9.0)	(9.0)
	-2.7	-4.4	-7.2	8.0	-2.7	-0.9	5.6	-5.9	21.7	15.9
	-1.825	-2.949	-4.821	5.309	-1.825	-0.590	3.704	-3.952	14.434	10.580
N	150	150	150	150	150	150	150	150	150	150
X2	6.0	13.1	3.6	9.1	8.1	18.5	3.8	19.2	9.4	37.3
Gvalue	5.9	12.6	3.8	9.5	8.3	22.4	4.1	19.2	8.9	38.4
Gv/N	3.940	8.419	2.512	6.313	5.507	14.906	2.758	12.787	5.926	25.574
Prob	0.11611	0.00551	0.28757	0.02365	0.04091	0.00005	0.24705	0.00025	0.03080	0.00000

CDS_INITIAL:

```
               1       2       3       4       5       6       7       8       9      10
        --------------------------------------------------------------------------------
a_O          149       0       0      93      72      72      97      71      73      88
         ( 99.3) (  0.0) (  0.0) ( 62.0) ( 48.0) ( 48.0) ( 64.7) ( 47.3) ( 48.7) ( 58.7)
a_E         62.4    53.9    64.3    62.4    53.9    64.3    62.4    53.9    64.3    62.4
         ( 41.6) ( 36.0) ( 42.9) ( 41.6) ( 36.0) ( 42.9) ( 41.6) ( 36.0) ( 42.9) ( 41.6)
           259.2     0.0     0.0    74.1    41.6    16.3    85.4    39.0    18.5    60.4
         172.773   0.000   0.000  49.391  27.713  10.857  56.962  26.004  12.350  40.252
t_O            1     150       2      29      41      55      25      56      61      24
         (  0.7) (100.0) (  1.3) ( 19.3) ( 27.3) ( 36.7) ( 16.7) ( 37.3) ( 40.7) ( 16.0)
t_E         37.8    52.0    62.9    37.8    52.0    62.9    37.8    52.0    62.9    37.8
         ( 25.2) ( 34.6) ( 41.9) ( 25.2) ( 34.6) ( 41.9) ( 25.2) ( 34.6) ( 41.9) ( 25.2)
            -7.3   318.0   -13.8   -15.4   -19.4   -14.7   -20.7     8.4    -3.7   -21.8
          -4.844 211.977  -9.195 -10.283 -12.966  -9.825 -13.812   5.570  -2.476 -14.566
g_O            0       0     148      15      11       6      14       9       9      21
         (  0.0) (  0.0) ( 98.7) ( 10.0) (  7.3) (  4.0) (  9.3) (  6.0) (  6.0) ( 14.0)
g_E         31.0    18.5    12.0    31.0    18.5    12.0    31.0    18.5    12.0    31.0
         ( 20.7) ( 12.4) (  8.0) ( 20.7) ( 12.4) (  8.0) ( 20.7) ( 12.4) (  8.0) ( 20.7)
             0.0     0.0   744.6   -21.8   -11.5    -8.3   -22.3   -13.0    -5.1   -16.4
           0.000   0.000 496.388 -14.549  -7.652  -5.520 -14.867  -8.669  -3.414 -10.947
c_O            0       0       0      13      26      17      14      14       7      17
         (  0.0) (  0.0) (  0.0) (  8.7) ( 17.3) ( 11.3) (  9.3) (  9.3) (  4.7) ( 11.3)
c_E         18.7    25.5    10.9    18.7    25.5    10.9    18.7    25.5    10.9    18.7
         ( 12.4) ( 17.0) (  7.2) ( 12.4) ( 17.0) (  7.2) ( 12.4) ( 17.0) (  7.2) ( 12.4)
             0.0     0.0     0.0    -9.4     0.9    15.3    -8.1   -16.8    -6.1    -3.2
           0.000   0.000   0.000  -6.278   0.612  10.175  -5.377 -11.226  -4.092  -2.129

N            150     150     150     150     150     150     150     150     150     150
X2         205.6   282.9  1681.2    27.0    11.4     8.4    34.0    15.8     3.3    18.9
Gvalue     251.9   318.0   730.8    27.4    11.6     8.5    34.4    17.5     3.6    18.9
Gv/N     167.929 211.977 487.193  18.282   7.707   5.687  22.906  11.680   2.368  12.610
Prob     0.00000 0.00000 0.00000 0.00000 0.00905 0.03624 0.00000 0.00055 0.31400 0.00028
        --------------------------------------------------------------------------------
              11      12      13      14      15      16      17      18      19      20
        --------------------------------------------------------------------------------
a_O           66      70      79      66      60      77      51      78      75      74
         ( 44.0) ( 46.7) ( 52.7) ( 44.0) ( 40.0) ( 51.3) ( 34.0) ( 52.0) ( 50.0) ( 49.3)
a_E         53.9    64.3    62.4    53.9    64.3    62.4    53.9    64.3    62.4    53.9
         ( 36.0) ( 42.9) ( 41.6) ( 36.0) ( 42.9) ( 41.6) ( 36.0) ( 42.9) ( 41.6) ( 36.0)
            26.6    11.9    37.2    26.6    -8.3    32.3    -5.7    30.1    27.5    46.8
          17.746   7.926  24.771  17.746  -5.538  21.511  -3.819  20.086  18.321  31.186
t_O           54      66      37      54      72      35      70      46      34      55
         ( 36.0) ( 44.0) ( 24.7) ( 36.0) ( 48.0) ( 23.3) ( 46.7) ( 30.7) ( 22.7) ( 36.7)
t_E         52.0    62.9    37.8    52.0    62.9    37.8    52.0    62.9    37.8    52.0
         ( 34.6) ( 41.9) ( 25.2) ( 34.6) ( 41.9) ( 25.2) ( 34.6) ( 41.9) ( 25.2) ( 34.6)
             4.1     6.4    -1.7     4.1    19.5    -5.5    41.7   -28.8    -7.3     6.2
           2.753   4.254  -1.101   2.753  12.994  -3.634  27.790 -19.177  -4.845   4.149
g_O           11       5      18      13       2      21      13      16      26       9
         (  7.3) (  3.3) ( 12.0) (  8.7) (  1.3) ( 14.0) (  8.7) ( 10.7) ( 17.3) (  6.0)
g_E         18.5    12.0    31.0    18.5    12.0    31.0    18.5    12.0    31.0    18.5
         ( 12.4) (  8.0) ( 20.7) ( 12.4) (  8.0) ( 20.7) ( 12.4) (  8.0) ( 20.7) ( 12.4)
           -11.5    -8.7   -19.6    -9.2    -7.2   -16.4    -9.2     9.3    -9.2   -13.0
          -7.652  -5.815 -13.083  -6.148  -4.770 -10.947  -6.148   6.205  -6.150  -8.669
c_O           19       9      16      17      16      17      16      10      15      12
         ( 12.7) (  6.0) ( 10.7) ( 11.3) ( 10.7) ( 11.3) ( 10.7) (  6.7) ( 10.0) (  8.0)
c_E         25.5    10.9    18.7    25.5    10.9    18.7    25.5    10.9    18.7    25.5
         ( 17.0) (  7.2) ( 12.4) ( 17.0) (  7.2) ( 12.4) ( 17.0) (  7.2) ( 12.4) ( 17.0)
           -11.2    -3.4    -4.9   -13.8    12.4    -3.2   -15.0    -1.6    -6.6   -18.1
          -7.498  -2.245  -3.297  -9.230   8.283  -2.129  -9.981  -1.090  -4.381 -12.088

N            150     150     150     150     150     150     150     150     150     150
X2           7.5     5.0    10.3     7.3    12.3     7.0    11.6     8.9     4.5    19.7
Gvalue       8.0     6.2    10.9     7.7    16.5     7.2    11.8     9.0     4.4    21.9
Gv/N       5.349   4.119   7.291   5.121  10.969   4.801   7.842   6.024   2.945  14.578
Prob     0.04554 0.10321 0.01208 0.05307 0.00092 0.06575 0.00824 0.02882 0.21980 0.00007
        --------------------------------------------------------------------------------
              21      22      23      24      25      26      27      28      29      30
        --------------------------------------------------------------------------------
a_O           64      82      63      62      64      57      59      75      53      76
         ( 42.7) ( 54.7) ( 42.0) ( 41.3) ( 42.7) ( 38.0) ( 39.3) ( 50.0) ( 35.3) ( 50.7)
a_E         64.3    62.4    53.9    64.3    62.4    53.9    64.3    62.4    53.9    64.3
         ( 42.9) ( 41.6) ( 36.0) ( 42.9) ( 41.6) ( 36.0) ( 42.9) ( 41.6) ( 36.0) ( 42.9)
            -0.6    44.7    19.5    -4.5     3.1     6.3   -10.2    27.5    -1.9    25.4
          -0.400  29.786  13.032  -3.012   2.099   4.185  -6.768  18.321  -1.251  16.939
t_O           61      33      53      74      46      54      62      34      58      55
         ( 40.7) ( 22.0) ( 35.3) ( 49.3) ( 30.7) ( 36.0) ( 41.3) ( 22.7) ( 38.7) ( 36.7)
t_E         62.9    37.8    52.0    62.9    37.8    52.0    62.9    37.8    52.0    62.9
         ( 41.9) ( 25.2) ( 34.6) ( 41.9) ( 25.2) ( 34.6) ( 41.9) ( 25.2) ( 34.6) ( 41.9)
            -3.7    -9.0     2.1    24.1    18.0     4.1    -1.8    -7.3    12.7   -14.7
          -2.476  -6.016   1.381  16.058  11.985   2.753  -1.172  -4.845   8.483  -9.825
g_O           10      23      14       6      20      13      11      23      11       7
         (  6.7) ( 15.3) (  9.3) (  4.0) ( 13.3) (  8.7) (  7.3) ( 15.3) (  7.3) (  4.7)
g_E         12.0    31.0    18.5    12.0    31.0    18.5    12.0    31.0    18.5    12.0
         (  8.0) ( 20.7) ( 12.4) (  8.0) ( 20.7) ( 12.4) (  8.0) ( 20.7) ( 12.4) (  8.0)
            -3.6   -13.8    -7.9    -8.3   -17.6    -9.2    -1.8   -13.8   -11.5    -7.5
          -2.389  -9.200  -5.237  -5.520 -11.727  -6.148  -1.230  -9.200  -7.652  -5.001
c_O           15      12      20       8      20      26      18      18      28      12
         ( 10.0) (  8.0) ( 13.3) (  5.3) ( 13.3) ( 17.3) ( 12.0) ( 12.0) ( 18.7) (  8.0)
c_E         10.9    18.7    25.5    10.9    18.7    25.5    10.9    18.7    25.5    10.9
         (  7.2) ( 12.4) ( 17.0) (  7.2) ( 12.4) ( 17.0) (  7.2) ( 12.4) ( 17.0) (  7.2)
             9.7   -10.6    -9.8    -4.9     2.7     0.9    18.2    -1.3     5.1     2.4
           6.475  -7.075  -6.525  -3.252   1.830   0.612  12.145  -0.882   3.426   1.610

N            150     150     150     150     150     150     150     150     150     150
X2           2.0    11.2     3.9     5.8     5.8     1.9     5.2     5.0     4.0     5.3
Gvalue       1.8    11.2     4.0     6.4     6.3     2.1     4.5     5.1     4.5     5.6
Gv/N       1.210   7.495   2.650   4.274   4.187   1.402   2.975   3.394   3.006   3.722
Prob     0.61171 0.01048 0.26413 0.09325 0.09871 0.55132 0.21562 0.16525 0.21146 0.13376
        --------------------------------------------------------------------------------
              31      32      33      34      35      36      37      38      39      40
        --------------------------------------------------------------------------------
a_O           70      58      71      55      52      60      65      44      62      62
         ( 46.7) ( 38.7) ( 47.3) ( 36.7) ( 34.7) ( 40.0) ( 43.3) ( 29.3) ( 41.3) ( 41.3)
a_E         62.4    53.9    64.3    62.4    53.9    64.3    62.4    53.9    64.3    62.4
         ( 41.6) ( 36.0) ( 42.9) ( 41.6) ( 36.0) ( 42.9) ( 41.6) ( 36.0) ( 42.9) ( 41.6)
            16.0     8.4    14.1   -14.0    -3.8    -8.3     5.2   -17.9    -4.5    -0.9
          10.660   5.603   9.382  -9.310  -2.548  -5.538   3.476 -11.956  -3.012  -0.591
t_O           35      50      63      44      53      70      44      61      62      36
         ( 23.3) ( 33.3) ( 42.0) ( 29.3) ( 35.3) ( 46.7) ( 29.3) ( 40.7) ( 41.3) ( 24.0)
t_E         37.8    52.0    62.9    37.8    52.0    62.9    37.8    52.0    62.9    37.8
         ( 25.2) ( 34.6) ( 41.9) ( 25.2) ( 34.6) ( 41.9) ( 25.2) ( 34.6) ( 41.9) ( 25.2)
            -5.5    -3.9     0.2    13.3     2.1    15.0    13.3    19.5    -1.8    -3.6
```

	-3.634	-2.582	0.153	8.856	1.381	10.003	8.856	13.023	-1.172	-2.386
g_O	27	19	9	32	17	12	23	19	12	31
	(18.0)	(12.7)	(6.0)	(21.3)	(11.3)	(8.0)	(15.3)	(12.7)	(8.0)	(20.7)
g_E	31.0	18.5	12.0	31.0	18.5	12.0	31.0	18.5	12.0	31.0
	(20.7)	(12.4)	(8.0)	(20.7)	(12.4)	(8.0)	(20.7)	(12.4)	(8.0)	(20.7)
	-7.5	0.9	-5.1	1.9	-2.9	0.1	-13.8	0.9	0.1	-0.1
	-5.028	0.628	-3.414	1.290	-1.959	0.051	-9.200	0.628	0.051	-0.062
c_O	18	23	7	19	28	8	18	26	14	21
	(12.0)	(15.3)	(4.7)	(12.7)	(18.7)	(5.3)	(12.0)	(17.3)	(9.3)	(14.0)
c_E	18.7	25.5	10.9	18.7	25.5	10.9	18.7	25.5	10.9	18.7
	(12.4)	(17.0)	(7.2)	(12.4)	(17.0)	(7.2)	(12.4)	(17.0)	(7.2)	(12.4)
	-1.3	-4.8	-6.1	0.7	5.1	-4.9	-1.3	0.9	7.1	4.9
	-0.882	-3.218	-4.092	0.439	3.426	-3.252	-0.882	0.612	4.755	3.287
N	150	150	150	150	150	150	150	150	150	150
X2	1.7	0.6	2.8	1.9	0.5	1.8	3.2	3.4	1.0	0.4
Gvalue	1.7	0.6	3.0	1.9	0.5	1.9	3.4	3.5	0.9	0.4
Gv/N	1.116	0.431	2.029	1.276	0.300	1.264	2.250	2.308	0.621	0.248
Prob	0.64277	0.88556	0.38497	0.59047	0.92958	0.59431	0.33729	0.32576	0.81769	0.94593

	41	42	43	44	45	46	47	48	49	50
a_O	47	73	66	49	70	63	42	64	55	46
	(31.3)	(48.7)	(44.0)	(32.7)	(46.7)	(42.0)	(28.0)	(42.7)	(36.7)	(30.7)
a_E	53.9	64.3	62.4	53.9	64.3	62.4	53.9	64.3	62.4	53.9
	(36.0)	(42.9)	(41.6)	(36.0)	(42.9)	(41.6)	(36.0)	(42.9)	(41.6)	(36.0)
	-13.0	18.5	7.3	-9.4	11.9	1.1	-21.0	-0.6	-14.0	-14.7
	-8.638	12.350	4.873	-6.283	7.926	0.744	-14.018	-0.400	-9.310	-9.773
t_O	62	54	36	50	55	34	51	57	52	64
	(41.3)	(36.0)	(24.0)	(33.3)	(36.7)	(22.7)	(34.0)	(38.0)	(34.7)	(42.7)
t_E	52.0	62.9	37.8	52.0	62.9	37.8	52.0	62.9	37.8	52.0
	(34.6)	(41.9)	(25.2)	(34.6)	(41.9)	(25.2)	(34.6)	(41.9)	(25.2)	(34.6)
	21.9	-16.5	-3.6	-3.9	-14.7	-7.3	-1.9	-11.2	33.1	26.6
	14.581	-10.968	-2.386	-2.582	-9.825	-4.845	-1.287	-7.468	22.049	17.761
g_O	22	13	30	28	12	37	24	14	30	12
	(14.7)	(8.7)	(20.0)	(18.7)	(8.0)	(24.7)	(16.0)	(9.3)	(20.0)	(8.0)
g_E	18.5	12.0	31.0	18.5	12.0	31.0	18.5	12.0	31.0	18.5
	(12.4)	(8.0)	(20.7)	(12.4)	(8.0)	(20.7)	(12.4)	(8.0)	(20.7)	(12.4)
	7.5	2.2	-2.1	23.1	0.1	13.0	12.4	4.4	-2.1	-10.4
	5.028	1.442	-1.372	15.403	0.051	8.654	8.269	2.937	-1.372	-6.956
c_O	19	10	18	23	13	16	33	15	13	28
	(12.7)	(6.7)	(12.0)	(15.3)	(8.7)	(10.7)	(22.0)	(10.0)	(8.7)	(18.7)
c_E	25.5	10.9	18.7	25.5	10.9	18.7	25.5	10.9	18.7	25.5
	(17.0)	(7.2)	(12.4)	(17.0)	(7.2)	(12.4)	(17.0)	(7.2)	(12.4)	(17.0)
	-11.2	-1.6	-1.3	-4.8	4.7	-4.9	16.9	9.7	-9.4	5.1
	-7.498	-1.090	-0.882	-3.218	3.131	-3.297	11.267	6.475	-6.278	3.426
N	150	150	150	150	150	150	150	150	150	150
X2	5.2	2.6	0.4	5.6	1.9	1.9	6.5	2.5	8.0	6.5
Gvalue	5.2	2.6	0.3	5.0	1.9	1.9	6.3	2.3	7.6	6.7
Gv/N	3.472	1.735	0.233	3.320	1.282	1.257	4.232	1.543	5.090	4.458
Prob	0.15715	0.45704	0.95048	0.17332	0.58840	0.59665	0.09586	0.50972	0.05419	0.08258

	51	52	53	54	55	56	57	58	59	60
a_O	71	53	52	72	58	49	66	59	59	69
	(47.3)	(35.3)	(34.7)	(48.0)	(38.7)	(32.7)	(44.0)	(39.3)	(39.3)	(46.0)
a_E	64.3	62.4	53.9	64.3	62.4	53.9	64.3	62.4	53.9	64.3
	(42.9)	(41.6)	(36.0)	(42.9)	(41.6)	(36.0)	(42.9)	(41.6)	(36.0)	(42.9)
	14.1	-17.4	-3.8	16.3	-8.6	-9.4	3.4	-6.7	10.6	9.7
	9.382	-11.589	-2.548	10.857	-5.710	-6.283	2.295	-4.464	7.044	6.489
t_O	53	43	52	56	46	57	59	45	50	55
	(35.3)	(28.7)	(34.7)	(37.3)	(30.7)	(38.0)	(39.3)	(30.0)	(33.3)	(36.7)
t_E	62.9	37.8	52.0	62.9	37.8	52.0	62.9	37.8	52.0	62.9
	(41.9)	(25.2)	(34.6)	(41.9)	(25.2)	(34.6)	(41.9)	(25.2)	(34.6)	(41.9)
	-18.1	11.0	0.1	-13.0	18.0	10.5	-7.5	15.6	-3.9	-14.7
	-12.086	7.337	0.034	-8.659	11.985	7.015	-5.017	10.406	-2.582	-9.825
g_O	15	42	26	16	28	18	11	33	19	14
	(10.0)	(28.0)	(17.3)	(10.7)	(18.7)	(12.0)	(7.3)	(22.0)	(12.7)	(9.3)
g_E	12.0	31.0	18.5	12.0	31.0	18.5	12.0	31.0	18.5	12.0
	(8.0)	(20.7)	(12.4)	(8.0)	(20.7)	(12.4)	(8.0)	(20.7)	(12.4)	(8.0)
	6.8	25.4	17.6	9.3	-5.8	-1.1	-1.8	4.0	0.9	4.4
	4.526	16.922	11.733	6.205	-3.856	-0.702	-1.230	2.685	0.628	2.937
c_O	11	12	20	6	18	26	14	13	22	12
	(7.3)	(8.0)	(13.3)	(4.0)	(12.0)	(17.3)	(9.3)	(8.7)	(14.7)	(8.0)
c_E	10.9	18.7	25.5	10.9	18.7	25.5	10.9	18.7	25.5	10.9
	(7.2)	(12.4)	(17.0)	(7.2)	(12.4)	(17.0)	(7.2)	(12.4)	(17.0)	(7.2)
	0.3	-10.6	-9.8	-7.1	-1.3	0.9	7.1	-9.4	-6.6	2.4
	0.199	-7.075	-6.525	-4.740	-0.882	0.612	4.755	-6.278	-4.382	1.610
N	150	150	150	150	150	150	150	150	150	150
X2	3.0	8.4	4.3	5.2	2.4	1.0	1.3	3.4	1.1	1.8
Gvalue	3.0	8.4	4.0	5.5	2.3	1.0	1.2	3.5	1.1	1.8
Gv/N	2.022	5.595	2.694	3.663	1.537	0.642	0.804	2.349	0.709	1.210
Prob	0.38658	0.03856	0.25701	0.13899	0.51145	0.81027	0.75174	0.31770	0.78597	0.61172

	61	62	63	64	65	66	67	68	69	70
a_O	62	50	78	57	59	69	58	55	61	60
	(41.3)	(33.3)	(52.0)	(38.0)	(39.3)	(46.0)	(38.7)	(36.7)	(40.7)	(40.0)
a_E	62.4	53.9	64.3	62.4	53.9	64.3	62.4	53.9	64.3	62.4
	(41.6)	(36.0)	(42.9)	(41.6)	(36.0)	(42.9)	(41.6)	(36.0)	(42.9)	(41.6)
	-0.9	-7.6	30.1	-10.4	10.6	9.7	-8.6	2.1	-6.4	-4.8
	-0.591	-5.065	20.086	-6.934	7.044	6.489	-5.710	1.418	-4.286	-3.195
t_O	33	53	57	41	52	61	37	50	65	45
	(22.0)	(35.3)	(38.0)	(27.3)	(34.7)	(40.7)	(24.7)	(33.3)	(43.3)	(30.0)
t_E	37.8	52.0	62.9	37.8	52.0	62.9	37.8	52.0	62.9	37.8
	(25.2)	(34.6)	(41.9)	(25.2)	(34.6)	(41.9)	(25.2)	(34.6)	(41.9)	(25.2)
	-9.0	2.1	-11.2	6.6	0.1	-3.7	-1.7	-3.9	4.3	15.6
	-6.016	1.381	-7.468	4.392	0.034	-2.476	-1.101	-2.582	2.866	10.406
g_O	34	24	10	33	17	10	34	21	16	31
	(22.7)	(16.0)	(6.7)	(22.0)	(11.3)	(6.7)	(22.7)	(14.0)	(10.7)	(20.7)
g_E	31.0	18.5	12.0	31.0	18.5	12.0	31.0	18.5	12.0	31.0
	(20.7)	(12.4)	(8.0)	(20.7)	(12.4)	(8.0)	(20.7)	(12.4)	(8.0)	(20.7)
	6.2	12.4	-3.6	4.0	-2.9	-3.6	6.2	5.2	9.3	-0.1
	4.119	8.269	-2.389	2.685	-1.959	-2.389	4.119	3.497	6.205	-0.062
c_O	21	23	5	19	22	10	21	24	8	14
	(14.0)	(15.3)	(3.3)	(12.7)	(14.7)	(6.7)	(14.0)	(16.0)	(5.3)	(9.3)
c_E	18.7	25.5	10.9	18.7	25.5	10.9	18.7	25.5	10.9	18.7
	(12.4)	(17.0)	(7.2)	(12.4)	(17.0)	(7.2)	(12.4)	(17.0)	(7.2)	(12.4)
	4.9	-4.8	-7.7	0.7	-6.6	-1.6	4.9	-3.0	-4.9	-8.1
	3.287	-3.218	-5.166	0.439	-4.382	-1.090	3.287	-1.996	-3.252	-5.377

N	150	150	150	150	150	150	150	150	150	150
X2	1.2	2.2	6.9	0.9	1.1	0.8	0.9	0.5	2.4	2.6
Gvalue	1.2	2.1	7.6	0.9	1.1	0.8	0.9	0.5	2.3	2.7
Gv/N	0.800	1.368	5.063	0.582	0.738	0.535	0.596	0.337	1.533	1.772
Prob	0.75300	0.56177	0.05516	0.83195	0.77554	0.84897	0.82698	0.91756	0.51264	0.44751

	71	72	73	74	75	76	77	78	79	80
a_O	52	63	54	44	71	58	60	62	59	48
	(34.7)	(42.0)	(36.0)	(29.3)	(47.3)	(38.7)	(40.0)	(41.3)	(39.3)	(32.0)
a_E	53.9	64.3	62.4	53.9	64.3	62.4	53.9	64.3	62.4	53.9
	(36.0)	(42.9)	(41.6)	(36.0)	(42.9)	(41.6)	(36.0)	(42.9)	(41.6)	(36.0)
	-3.8	-2.6	-15.7	-17.9	14.1	-8.6	12.8	-4.5	-6.7	-11.2
	-2.548	-1.717	-10.461	-11.956	9.382	-5.710	8.508	-3.012	-4.464	-7.475
t_O	54	67	37	58	63	38	48	60	37	56
	(36.0)	(44.7)	(24.7)	(38.7)	(42.0)	(25.3)	(32.0)	(40.0)	(24.7)	(37.3)
t_E	52.0	62.9	37.8	52.0	62.9	37.8	52.0	62.9	37.8	52.0
	(34.6)	(41.9)	(25.2)	(34.6)	(41.9)	(25.2)	(34.6)	(41.9)	(25.2)	(34.6)
	4.1	8.5	-1.7	12.7	0.2	0.3	-7.6	-5.6	-1.7	8.4
	2.753	5.662	-1.101	8.483	0.153	0.221	-5.091	-3.758	-1.101	5.570
g_O	18	10	37	18	9	30	17	13	27	16
	(12.0)	(6.7)	(24.7)	(12.0)	(6.0)	(20.0)	(11.3)	(8.7)	(18.0)	(10.7)
g_E	18.5	12.0	31.0	18.5	12.0	31.0	18.5	12.0	31.0	18.5
	(12.4)	(8.0)	(20.7)	(12.4)	(8.0)	(20.7)	(12.4)	(8.0)	(20.7)	(12.4)
	-1.1	-3.6	13.0	-1.1	-5.1	-2.1	-2.9	2.2	-7.5	-4.7
	-0.702	-2.389	8.654	-0.702	-3.414	-1.372	-1.959	1.442	-5.028	-3.137
c_O	26	10	22	30	7	24	25	15	27	30
	(17.3)	(6.7)	(14.7)	(20.0)	(4.7)	(16.0)	(16.7)	(10.0)	(18.0)	(20.0)
c_E	25.5	10.9	18.7	25.5	10.9	18.7	25.5	10.9	18.7	25.5
	(17.0)	(7.2)	(12.4)	(17.0)	(7.2)	(12.4)	(17.0)	(7.2)	(12.4)	(17.0)
	0.9	-1.6	7.2	9.6	-6.1	12.0	-1.1	9.7	19.9	9.6
	0.612	-1.090	4.808	6.431	-4.092	8.030	-0.718	6.475	13.274	6.431
N	150	150	150	150	150	150	150	150	150	150
X2	0.2	0.7	2.9	3.3	2.8	1.9	1.1	1.9	4.4	2.1
Gvalue	0.2	0.7	2.9	3.4	3.0	1.8	1.1	1.7	4.0	2.1
Gv/N	0.115	0.466	1.901	2.255	2.029	1.169	0.740	1.147	2.682	1.389
Prob	0.98188	0.87329	0.41519	0.33634	0.38497	0.62523	0.77476	0.63234	0.25905	0.55514

	81	82	83	84	85	86	87	88	89	90
a_O	71	63	56	61	60	59	70	66	60	64
	(47.3)	(42.0)	(37.3)	(40.7)	(40.0)	(39.3)	(46.7)	(44.0)	(40.0)	(42.7)
a_E	64.3	62.4	53.9	64.3	62.4	53.9	64.3	62.4	53.9	64.3
	(42.9)	(41.6)	(36.0)	(42.9)	(41.6)	(36.0)	(42.9)	(41.6)	(36.0)	(42.9)
	14.1	1.1	4.2	-6.4	-4.8	10.6	11.9	7.3	12.8	-0.6
	9.382	0.744	2.790	-4.286	-3.195	7.044	7.926	4.873	8.508	-0.400
t_O	56	33	53	64	34	51	59	42	43	56
	(37.3)	(22.0)	(35.3)	(42.7)	(22.7)	(34.0)	(39.3)	(28.0)	(28.7)	(37.3)
t_E	62.9	37.8	52.0	62.9	37.8	52.0	62.9	37.8	52.0	62.9
	(41.9)	(25.2)	(34.6)	(41.9)	(25.2)	(34.6)	(41.9)	(25.2)	(34.6)	(41.9)
	-13.0	-9.0	2.1	2.2	-7.3	-1.9	-7.5	8.8	-16.3	-13.0
	-8.659	-6.016	1.381	1.499	-4.845	-1.287	-5.017	5.849	-10.868	-8.659
g_O	8	33	14	16	33	13	14	24	20	13
	(5.3)	(22.0)	(9.3)	(10.7)	(22.0)	(8.7)	(9.3)	(16.0)	(13.3)	(8.7)
g_E	12.0	31.0	18.5	12.0	31.0	18.5	12.0	31.0	18.5	12.0
	(8.0)	(20.7)	(12.4)	(8.0)	(20.7)	(12.4)	(8.0)	(20.7)	(12.4)	(8.0)
	-6.4	4.0	-7.9	9.3	4.0	-9.2	4.4	-12.4	3.0	2.2
	-4.291	2.685	-5.237	6.205	2.685	-6.148	2.937	-8.238	2.029	1.442
c_O	15	21	27	9	23	27	7	18	27	17
	(10.0)	(14.0)	(18.0)	(6.0)	(15.3)	(18.0)	(4.7)	(12.0)	(18.0)	(11.3)
c_E	10.9	18.7	25.5	10.9	18.7	25.5	10.9	18.7	25.5	10.9
	(7.2)	(12.4)	(17.0)	(7.2)	(12.4)	(17.0)	(7.2)	(12.4)	(17.0)	(7.2)
	9.7	4.9	3.0	-3.4	9.6	3.0	-6.1	-1.3	3.0	15.3
	6.475	3.287	1.995	-2.245	6.390	1.995	-4.092	-0.882	1.995	10.175
N	150	150	150	150	150	150	150	150	150	150
X2	4.3	1.0	1.3	1.9	1.6	2.2	2.5	2.3	2.4	4.3
Gvalue	4.4	1.0	1.4	1.8	1.6	2.4	2.6	2.4	2.5	3.8
Gv/N	2.907	0.700	0.928	1.173	1.035	1.604	1.754	1.601	1.665	2.559
Prob	0.22507	0.78922	0.70751	0.62389	0.67017	0.49247	0.45215	0.49322	0.47585	0.27951

	91	92	93	94	95	96	97	98	99	100
a_O	68	53	59	57	48	72	53	61	65	68
	(45.3)	(35.3)	(39.3)	(38.0)	(32.0)	(48.0)	(35.3)	(40.7)	(43.3)	(45.3)
a_E	62.4	53.9	64.3	62.4	53.9	64.3	62.4	53.9	64.3	62.4
	(41.6)	(36.0)	(42.9)	(41.6)	(36.0)	(42.9)	(41.6)	(36.0)	(42.9)	(41.6)
	11.6	-1.9	-10.2	-10.4	-11.2	16.3	-17.4	15.0	1.4	11.6
	7.727	-1.251	-6.768	-6.934	-7.475	10.857	-11.589	9.994	0.937	7.727
t_O	43	52	70	42	68	58	40	38	59	30
	(28.7)	(34.7)	(46.7)	(28.0)	(45.3)	(38.7)	(26.7)	(25.3)	(39.3)	(20.0)
t_E	37.8	52.0	62.9	37.8	52.0	62.9	37.8	52.0	62.9	37.8
	(25.2)	(34.6)	(41.9)	(25.2)	(34.6)	(41.9)	(25.2)	(34.6)	(41.9)	(25.2)
	11.0	0.1	15.0	8.8	36.6	-9.4	4.5	-23.8	-7.5	-13.9
	7.337	0.034	10.003	5.849	24.367	-6.254	2.968	-15.867	-5.017	-9.281
g_O	24	22	8	33	19	10	34	21	18	34
	(16.0)	(14.7)	(5.3)	(22.0)	(12.7)	(6.7)	(22.7)	(14.0)	(12.0)	(22.7)
g_E	31.0	18.5	12.0	31.0	18.5	12.0	31.0	18.5	12.0	31.0
	(20.7)	(12.4)	(8.0)	(20.7)	(12.4)	(8.0)	(20.7)	(12.4)	(8.0)	(20.7)
	-12.4	7.5	-6.4	4.0	0.9	-3.6	6.2	5.2	14.7	6.2
	-8.238	5.028	-4.291	2.685	0.628	-2.389	4.119	3.497	9.807	4.119
c_O	15	23	13	18	15	10	23	30	8	18
	(10.0)	(15.3)	(8.7)	(12.0)	(10.0)	(6.7)	(15.3)	(20.0)	(5.3)	(12.0)
c_E	18.7	25.5	10.9	18.7	25.5	10.9	18.7	25.5	10.9	18.7
	(12.4)	(17.0)	(7.2)	(12.4)	(17.0)	(7.2)	(12.4)	(17.0)	(7.2)	(12.4)
	-6.6	-4.8	4.7	-1.3	-16.0	-1.6	9.6	9.6	-4.9	-1.3
	-4.381	-3.218	3.131	-0.882	-10.648	-1.090	6.390	6.431	-3.252	-0.882
N	150	150	150	150	150	150	150	150	150	150
X2	3.5	0.9	3.0	1.1	10.0	1.7	2.8	5.8	4.0	2.4
Gvalue	3.7	0.9	3.1	1.1	10.3	1.7	2.8	6.1	3.7	2.5
Gv/N	2.445	0.593	2.075	0.718	6.874	1.124	1.889	4.055	2.475	1.683
Prob	0.29973	0.82783	0.37458	0.78268	0.01610	0.63993	0.41804	0.10767	0.29417	0.47081

Bavi_197N
5UTR:

	100	99	98	97	96	95	94	93	92	91
a_O	166	143	155	185	143	160	156	152	144	150
	(23.1)	(19.9)	(21.5)	(25.7)	(19.9)	(22.2)	(21.7)	(21.1)	(20.0)	(20.8)
a_E	152.7	152.7	152.7	152.7	152.7	152.7	152.7	152.7	152.7	152.7
	(21.2)	(21.2)	(21.2)	(21.2)	(21.2)	(21.2)	(21.2)	(21.2)	(21.2)	(21.2)
	27.8	-18.7	4.7	71.1	-18.7	15.0	6.8	-1.3	-16.8	-5.3
	3.866	-2.594	0.658	9.877	-2.594	2.090	0.941	-0.180	-2.333	-0.730
t_O	142	140	148	142	141	161	153	150	152	150
	(19.7)	(19.4)	(20.6)	(19.7)	(19.6)	(22.4)	(21.2)	(20.8)	(21.1)	(20.8)
t_E	162.1	162.1	162.1	162.1	162.1	162.1	162.1	162.1	162.1	162.1
	(22.5)	(22.5)	(22.5)	(22.5)	(22.5)	(22.5)	(22.5)	(22.5)	(22.5)	(22.5)
	-37.6	-41.1	-27.0	-37.6	-39.4	-2.2	-17.7	-23.3	-19.6	-23.3
	-5.227	-5.705	-3.746	-5.227	-5.467	-0.310	-2.461	-3.238	-2.721	-3.238
g_O	208	244	206	181	214	201	200	213	219	201
	(28.9)	(33.9)	(28.6)	(25.1)	(29.7)	(27.9)	(27.8)	(29.6)	(30.4)	(27.9)
g_E	185.4	185.4	185.4	185.4	185.4	185.4	185.4	185.4	185.4	185.4
	(25.7)	(25.7)	(25.7)	(25.7)	(25.7)	(25.7)	(25.7)	(25.7)	(25.7)	(25.7)
	47.9	134.1	43.5	-8.6	61.5	32.5	30.4	59.2	73.0	32.5
	6.655	18.626	6.038	-1.199	8.538	4.520	4.220	8.221	10.142	4.520
c_O	204	193	211	212	222	198	211	205	205	219
	(28.3)	(26.8)	(29.3)	(29.4)	(30.8)	(27.5)	(29.3)	(28.5)	(28.5)	(30.4)
c_E	219.9	219.9	219.9	219.9	219.9	219.9	219.9	219.9	219.9	219.9
	(30.5)	(30.5)	(30.5)	(30.5)	(30.5)	(30.5)	(30.5)	(30.5)	(30.5)	(30.5)
	-30.5	-50.3	-17.4	-15.4	4.3	-41.5	-17.4	-28.7	-28.7	-1.7
	-4.243	-6.986	-2.411	-2.144	0.597	-5.760	-2.411	-3.985	-3.985	-0.238
N	720	720	720	720	720	720	720	720	720	720
X2	7.6	25.5	3.9	9.7	7.8	3.9	2.1	6.0	8.2	2.3
Gvalue	7.6	24.1	3.9	9.4	7.7	3.9	2.1	5.9	7.9	2.3
Gv/N	1.052	3.342	0.539	1.307	1.074	0.540	0.289	0.818	1.102	0.314
Prob	0.05575	0.00002	0.27469	0.02428	0.05185	0.27384	0.55530	0.11720	0.04737	0.51993

	90	89	88	87	86	85	84	83	82	81
a_O	126	133	149	127	140	163	125	141	135	137
	(17.5)	(18.5)	(20.7)	(17.6)	(19.4)	(22.6)	(17.4)	(19.6)	(18.8)	(19.0)
a_E	152.7	152.7	152.7	152.7	152.7	152.7	152.7	152.7	152.7	152.7
	(21.2)	(21.2)	(21.2)	(21.2)	(21.2)	(21.2)	(21.2)	(21.2)	(21.2)	(21.2)
	-48.4	-36.7	-7.2	-46.7	-24.2	21.4	-50.0	-22.4	-33.2	-29.6
	-6.715	-5.091	-1.002	-6.490	-3.364	2.970	-6.939	-3.109	-4.608	-4.116
t_O	144	158	136	149	163	174	146	151	156	148
	(20.0)	(21.9)	(18.9)	(20.7)	(22.6)	(24.2)	(20.3)	(21.0)	(21.7)	(20.6)
t_E	162.1	162.1	162.1	162.1	162.1	162.1	162.1	162.1	162.1	162.1
	(22.5)	(22.5)	(22.5)	(22.5)	(22.5)	(22.5)	(22.5)	(22.5)	(22.5)	(22.5)
	-34.1	-8.1	-47.8	-25.1	1.8	24.6	-30.6	-21.5	-12.0	-27.0
	-4.741	-1.130	-6.637	-3.493	0.245	3.418	-4.247	-2.980	-1.668	-3.746
g_O	219	207	205	218	199	178	226	218	212	223
	(30.4)	(28.7)	(28.5)	(30.3)	(27.6)	(24.7)	(31.4)	(30.3)	(29.4)	(31.0)
g_E	185.4	185.4	185.4	185.4	185.4	185.4	185.4	185.4	185.4	185.4
	(25.7)	(25.7)	(25.7)	(25.7)	(25.7)	(25.7)	(25.7)	(25.7)	(25.7)	(25.7)
	73.0	45.7	41.3	70.7	28.2	-14.4	89.6	70.7	56.9	82.4
	10.142	6.346	5.732	9.819	3.922	-2.006	12.441	9.819	7.905	11.448
c_O	231	222	230	226	218	205	223	210	217	212
	(32.1)	(30.8)	(31.9)	(31.4)	(30.3)	(28.5)	(31.0)	(29.2)	(30.1)	(29.4)
c_E	219.9	219.9	219.9	219.9	219.9	219.9	219.9	219.9	219.9	219.9
	(30.5)	(30.5)	(30.5)	(30.5)	(30.5)	(30.5)	(30.5)	(30.5)	(30.5)	(30.5)
	22.8	4.3	20.7	12.4	-3.7	-28.7	6.3	-19.3	-5.7	-15.4
	3.172	0.597	2.881	1.729	-0.514	-3.985	0.878	-2.677	-0.789	-2.144
N	720	720	720	720	720	720	720	720	720	720
X2	13.3	5.2	6.8	11.3	2.1	2.9	15.6	7.8	6.1	10.8
Gvalue	13.4	5.2	7.0	11.3	2.1	2.9	15.4	7.6	6.1	10.4
Gv/N	1.857	0.723	0.974	1.565	0.289	0.397	2.134	1.052	0.840	1.442
Prob	0.00390	0.15752	0.07151	0.01036	0.55647	0.41352	0.00153	0.05564	0.10919	0.01558

	80	79	78	77	76	75	74	73	72	71
a_O	145	160	143	143	150	135	150	171	160	154
	(20.1)	(22.2)	(19.9)	(19.9)	(20.8)	(18.8)	(20.8)	(23.8)	(22.2)	(21.4)
a_E	152.7	152.7	152.7	152.7	152.7	152.7	152.7	152.7	152.7	152.7
	(21.2)	(21.2)	(21.2)	(21.2)	(21.2)	(21.2)	(21.2)	(21.2)	(21.2)	(21.2)
	-14.9	15.0	-18.7	-18.7	-5.3	-33.2	-5.3	38.8	15.0	2.7
	-2.071	2.090	-2.594	-2.594	-0.730	-4.608	-0.730	5.392	2.090	0.377
t_O	152	158	154	154	168	146	162	178	150	141
	(21.1)	(21.9)	(21.4)	(21.4)	(23.3)	(20.3)	(22.5)	(24.7)	(20.8)	(19.6)
t_E	162.1	162.1	162.1	162.1	162.1	162.1	162.1	162.1	162.1	162.1
	(22.5)	(22.5)	(22.5)	(22.5)	(22.5)	(22.5)	(22.5)	(22.5)	(22.5)	(22.5)
	-19.6	-8.1	-15.8	-15.8	12.0	-30.6	-0.2	33.3	-23.3	-39.4
	-2.721	-1.130	-2.198	-2.198	1.663	-4.247	-0.033	4.620	-3.238	-5.467
g_O	207	203	210	214	189	219	188	171	203	213
	(28.7)	(28.2)	(29.2)	(29.7)	(26.2)	(30.4)	(26.1)	(23.8)	(28.2)	(29.6)
g_E	185.4	185.4	185.4	185.4	185.4	185.4	185.4	185.4	185.4	185.4
	(25.7)	(25.7)	(25.7)	(25.7)	(25.7)	(25.7)	(25.7)	(25.7)	(25.7)	(25.7)
	45.7	36.9	52.4	61.5	7.3	73.0	5.3	-27.6	36.9	59.2
	6.346	5.123	7.277	8.538	1.018	10.142	0.736	-3.833	5.123	8.221
c_O	216	199	213	209	213	220	220	200	207	212
	(30.0)	(27.6)	(29.6)	(29.0)	(29.6)	(30.6)	(30.6)	(27.8)	(28.7)	(29.4)
c_E	219.9	219.9	219.9	219.9	219.9	219.9	219.9	219.9	219.9	219.9
	(30.5)	(30.5)	(30.5)	(30.5)	(30.5)	(30.5)	(30.5)	(30.5)	(30.5)	(30.5)
	-7.7	-39.7	-13.5	-21.2	-13.5	0.3	0.3	-37.9	-25.0	-15.4
	-1.063	-5.510	-1.876	-2.941	-1.876	0.039	0.039	-5.260	-3.466	-2.144
N	720	720	720	720	720	720	720	720	720	720
X2	3.6	4.1	4.5	6.0	0.5	9.7	0.1	6.7	3.7	7.2
Gvalue	3.5	4.1	4.4	5.8	0.5	9.5	0.1	6.6	3.7	7.1
Gv/N	0.491	0.573	0.610	0.805	0.076	1.326	0.012	0.920	0.510	0.987
Prob	0.31631	0.24830	0.22233	0.12214	0.90914	0.02285	0.99373	0.08490	0.29923	0.06868

	70	69	68	67	66	65	64	63	62	61
a_O	155	145	156	154	153	156	161	148	153	146
	(21.5)	(20.1)	(21.7)	(21.4)	(21.2)	(21.7)	(22.4)	(20.6)	(21.2)	(20.3)
a_E	152.7	152.7	152.7	152.7	152.7	152.7	152.7	152.7	152.7	152.7
	(21.2)	(21.2)	(21.2)	(21.2)	(21.2)	(21.2)	(21.2)	(21.2)	(21.2)	(21.2)
	4.7	-14.9	6.8	2.7	0.7	6.8	17.1	-9.2	0.7	-13.0
	0.658	-2.071	0.941	0.377	0.097	0.941	2.382	-1.272	0.097	-1.806
t_O	154	156	147	148	141	146	170	163	154	169
	(21.4)	(21.7)	(20.4)	(20.6)	(19.6)	(20.3)	(23.6)	(22.6)	(21.4)	(23.5)

t_E	162.1	162.1	162.1	162.1	162.1	162.1	162.1	162.1	162.1	162.1
	(22.5)	(22.5)	(22.5)	(22.5)	(22.5)	(22.5)	(22.5)	(22.5)	(22.5)	(22.5)
	-15.8	-12.0	-28.8	-27.0	-39.4	-30.6	16.1	1.8	-15.8	14.0
	-2.198	-1.668	-3.998	-3.746	-5.467	-4.247	2.241	0.245	-2.198	1.951
g_O	208	204	192	213	194	204	187	185	207	192
	(28.9)	(28.3)	(26.7)	(29.6)	(26.9)	(28.3)	(26.0)	(25.7)	(28.7)	(26.7)
g_E	185.4	185.4	185.4	185.4	185.4	185.4	185.4	185.4	185.4	185.4
	(25.7)	(25.7)	(25.7)	(25.7)	(25.7)	(25.7)	(25.7)	(25.7)	(25.7)	(25.7)
	47.9	39.1	13.5	59.2	17.7	39.1	3.3	-0.7	45.7	13.5
	6.655	5.427	1.874	8.221	2.452	5.427	0.455	-0.103	6.346	1.874
c_O	203	215	225	205	232	214	202	224	206	213
	(28.2)	(29.9)	(31.2)	(28.5)	(32.2)	(29.7)	(28.1)	(31.1)	(28.6)	(29.6)
c_E	219.9	219.9	219.9	219.9	219.9	219.9	219.9	219.9	219.9	219.9
	(30.5)	(30.5)	(30.5)	(30.5)	(30.5)	(30.5)	(30.5)	(30.5)	(30.5)	(30.5)
	-32.4	-9.6	10.4	-28.7	24.9	-11.6	-34.2	8.4	-26.8	-13.5
	-4.499	-1.335	1.444	-3.985	3.464	-1.606	-4.754	1.161	-3.726	-1.876
N	720	720	720	720	720	720	720	720	720	720
X2	4.5	2.6	1.8	6.4	3.8	3.7	2.3	0.2	3.8	1.0
Gvalue	4.4	2.5	1.9	6.2	3.9	3.7	2.3	0.2	3.7	1.0
Gv/N	0.616	0.353	0.261	0.866	0.546	0.514	0.324	0.031	0.519	0.143
Prob	0.21829	0.46726	0.59710	0.10074	0.26867	0.29550	0.50648	0.97327	0.29115	0.79337
	60	59	58	57	56	55	54	53	52	51
a_O	164	152	154	160	149	148	130	145	162	146
	(22.8)	(21.1)	(21.4)	(22.2)	(20.7)	(20.6)	(18.1)	(20.1)	(22.5)	(20.3)
a_E	152.7	152.7	152.7	152.7	152.7	152.7	152.7	152.7	152.7	152.7
	(21.2)	(21.2)	(21.2)	(21.2)	(21.2)	(21.2)	(21.2)	(21.2)	(21.2)	(21.2)
	23.5	-1.3	2.7	15.0	-7.2	-9.2	-41.8	-14.9	19.3	-13.0
	3.267	-0.180	0.377	2.090	-1.002	-1.272	-5.800	-2.071	2.675	-1.806
t_O	159	170	152	154	162	170	144	148	147	160
	(22.1)	(23.6)	(21.1)	(21.4)	(22.5)	(23.6)	(20.0)	(20.6)	(20.4)	(22.2)
t_E	162.1	162.1	162.1	162.1	162.1	162.1	162.1	162.1	162.1	162.1
	(22.5)	(22.5)	(22.5)	(22.5)	(22.5)	(22.5)	(22.5)	(22.5)	(22.5)	(22.5)
	-6.2	16.1	-19.6	-15.8	-0.2	16.1	-34.1	-27.0	-28.8	-4.2
	-0.858	2.241	-2.721	-2.198	-0.033	2.241	-4.741	-3.746	-3.998	-0.585
g_O	203	202	215	180	208	203	204	201	197	216
	(28.2)	(28.1)	(29.9)	(25.0)	(28.9)	(28.2)	(28.3)	(27.9)	(27.4)	(30.0)
g_E	185.4	185.4	185.4	185.4	185.4	185.4	185.4	185.4	185.4	185.4
	(25.7)	(25.7)	(25.7)	(25.7)	(25.7)	(25.7)	(25.7)	(25.7)	(25.7)	(25.7)
	36.9	34.7	63.8	-10.6	47.9	36.9	39.1	32.5	24.0	66.1
	5.123	4.821	8.856	-1.470	6.655	5.123	5.427	4.520	3.330	9.175
c_O	194	196	199	226	201	199	242	226	214	198
	(26.9)	(27.2)	(27.6)	(31.4)	(27.9)	(27.6)	(33.6)	(31.4)	(29.7)	(27.5)
c_E	219.9	219.9	219.9	219.9	219.9	219.9	219.9	219.9	219.9	219.9
	(30.5)	(30.5)	(30.5)	(30.5)	(30.5)	(30.5)	(30.5)	(30.5)	(30.5)	(30.5)
	-48.6	-45.0	-39.7	12.4	-36.1	-39.7	46.4	12.4	-11.6	-41.5
	-6.743	-6.254	-5.510	1.729	-5.007	-5.510	6.450	1.729	-1.606	-5.760
N	720	720	720	720	720	720	720	720	720	720
X2	5.6	4.5	7.4	1.1	4.5	4.2	9.5	3.1	2.9	7.6
Gvalue	5.7	4.5	7.2	1.1	4.4	4.2	9.6	3.1	2.9	7.4
Gv/N	0.789	0.627	1.001	0.151	0.613	0.582	1.336	0.432	0.401	1.024
Prob	0.12834	0.21074	0.06566	0.77973	0.22038	0.24157	0.02212	0.37508	0.40890	0.06087
	50	49	48	47	46	45	44	43	42	41
a_O	155	138	146	150	153	153	150	177	133	157
	(21.5)	(19.2)	(20.3)	(20.8)	(21.2)	(21.2)	(20.8)	(24.6)	(18.5)	(21.8)
a_E	152.7	152.7	152.7	152.7	152.7	152.7	152.7	152.7	152.7	152.7
	(21.2)	(21.2)	(21.2)	(21.2)	(21.2)	(21.2)	(21.2)	(21.2)	(21.2)	(21.2)
	4.7	-27.8	-13.0	-5.3	0.7	0.7	-5.3	52.4	-36.7	8.8
	0.658	-3.868	-1.806	-0.730	0.097	0.097	-0.730	7.277	-5.091	1.225
t_O	133	179	159	140	178	151	141	141	173	160
	(18.5)	(24.9)	(22.1)	(19.4)	(24.7)	(21.0)	(19.6)	(19.6)	(24.0)	(22.2)
t_E	162.1	162.1	162.1	162.1	162.1	162.1	162.1	162.1	162.1	162.1
	(22.5)	(22.5)	(22.5)	(22.5)	(22.5)	(22.5)	(22.5)	(22.5)	(22.5)	(22.5)
	-52.7	35.5	-6.2	-41.1	33.3	-21.5	-39.4	-39.4	22.5	-4.2
	-7.315	4.925	-0.858	-5.705	4.620	-2.980	-5.467	-5.467	3.121	-0.585
g_O	211	198	198	198	191	212	206	195	196	198
	(29.3)	(27.5)	(27.5)	(27.5)	(26.5)	(29.4)	(28.6)	(27.1)	(27.2)	(27.5)
g_E	185.4	185.4	185.4	185.4	185.4	185.4	185.4	185.4	185.4	185.4
	(25.7)	(25.7)	(25.7)	(25.7)	(25.7)	(25.7)	(25.7)	(25.7)	(25.7)	(25.7)
	54.7	26.1	26.1	26.1	11.4	56.9	43.5	19.8	21.9	26.1
	7.590	3.625	3.625	3.625	1.587	7.905	6.038	2.743	3.036	3.625
c_O	221	205	217	232	198	204	223	207	218	205
	(30.7)	(28.5)	(30.1)	(32.2)	(27.5)	(28.3)	(31.0)	(28.7)	(30.3)	(28.5)
c_E	219.9	219.9	219.9	219.9	219.9	219.9	219.9	219.9	219.9	219.9
	(30.5)	(30.5)	(30.5)	(30.5)	(30.5)	(30.5)	(30.5)	(30.5)	(30.5)	(30.5)
	2.3	-28.7	-5.7	24.9	-41.5	-30.5	6.3	-25.0	-3.7	-28.7
	0.317	-3.985	-0.789	3.464	-5.760	-4.243	0.878	-3.466	-0.514	-3.985
N	720	720	720	720	720	720	720	720	720	720
X2	8.8	5.0	1.2	4.6	3.9	5.7	5.1	7.9	3.9	2.0
Gvalue	9.0	5.0	1.2	4.7	3.9	5.6	5.2	7.8	4.0	2.0
Gv/N	1.251	0.698	0.171	0.654	0.545	0.779	0.720	1.088	0.552	0.281
Prob	0.02918	0.17018	0.74500	0.19416	0.26952	0.13227	0.15871	0.04963	0.26429	0.56825
	40	39	38	37	36	35	34	33	32	31
a_O	172	142	145	178	132	158	170	170	154	149
	(23.9)	(19.7)	(20.1)	(24.7)	(18.3)	(21.9)	(23.6)	(23.6)	(21.4)	(20.7)
a_E	152.7	152.7	152.7	152.7	152.7	152.7	152.7	152.7	152.7	152.7
	(21.2)	(21.2)	(21.2)	(21.2)	(21.2)	(21.2)	(21.2)	(21.2)	(21.2)	(21.2)
	41.1	-20.5	-14.9	54.7	-38.4	10.9	36.6	36.6	2.7	-7.2
	5.702	-2.853	-2.071	7.596	-5.329	1.512	5.084	5.084	0.377	-1.002
t_O	176	160	144	152	180	159	170	170	152	190
	(24.4)	(22.2)	(20.0)	(21.1)	(25.0)	(22.1)	(23.6)	(23.6)	(21.1)	(26.4)
t_E	162.1	162.1	162.1	162.1	162.1	162.1	162.1	162.1	162.1	162.1
	(22.5)	(22.5)	(22.5)	(22.5)	(22.5)	(22.5)	(22.5)	(22.5)	(22.5)	(22.5)
	28.9	-4.2	-34.1	-19.6	37.7	-6.2	16.1	16.1	-19.6	60.3
	4.016	-0.585	-4.741	-2.721	5.231	-0.858	2.241	2.241	-2.721	8.375
g_O	190	203	213	178	186	185	166	187	201	179
	(26.4)	(28.2)	(29.6)	(24.7)	(25.8)	(25.7)	(23.1)	(26.0)	(27.9)	(24.9)
g_E	185.4	185.4	185.4	185.4	185.4	185.4	185.4	185.4	185.4	185.4
	(25.7)	(25.7)	(25.7)	(25.7)	(25.7)	(25.7)	(25.7)	(25.7)	(25.7)	(25.7)
	9.4	36.9	59.2	-14.4	1.3	-0.7	-36.6	3.3	32.5	-12.5
	1.302	5.123	8.221	-2.006	0.175	-0.103	-5.089	0.455	4.520	-1.739
c_O	182	215	218	212	222	218	214	193	213	202
	(25.3)	(29.9)	(30.3)	(29.4)	(30.8)	(30.3)	(29.7)	(26.8)	(29.6)	(28.1)
c_E	219.9	219.9	219.9	219.9	219.9	219.9	219.9	219.9	219.9	219.9

```
        ( 30.5) ( 30.5) ( 30.5) ( 30.5) ( 30.5) ( 30.5) ( 30.5) ( 30.5) ( 30.5) ( 30.5)
          -68.8    -9.6    -3.7   -15.4     4.3    -3.7   -11.6   -50.3   -13.5   -34.2
         -9.554  -1.335  -0.514  -2.144   0.597  -0.514  -1.606  -6.986  -1.876  -4.754

N           720     720     720     720     720     720     720     720     720     720
X2         10.3     2.6     6.5     5.4     4.8     0.3     4.5     5.7     2.2     6.6
Gvalue     10.6     2.5     6.4     5.2     4.9     0.3     4.5     5.7     2.2     6.3
Gv/N      1.466   0.350   0.894   0.725   0.674   0.036   0.630   0.794   0.299   0.881
Prob    0.01439 0.47115 0.09211 0.15631 0.18274 0.96695 0.20925 0.12625 0.54070 0.09609
        --------------------------------------------------------------------------------
             30      29      28      27      26      25      24      23      22      21
        --------------------------------------------------------------------------------
a_O         132     160     152     128     154     148     143     152     176     140
        ( 18.3) ( 22.2) ( 21.1) ( 17.8) ( 21.4) ( 20.6) ( 19.9) ( 21.1) ( 24.4) ( 19.4)
a_E       152.7   152.7   152.7   152.7   152.7   152.7   152.7   152.7   152.7   152.7
        ( 21.2) ( 21.2) ( 21.2) ( 21.2) ( 21.2) ( 21.2) ( 21.2) ( 21.2) ( 21.2) ( 21.2)
          -38.4    15.0    -1.3   -45.1     2.7    -9.2   -18.7    -1.3    50.1   -24.2
         -5.329   2.090  -0.180  -6.262   0.377  -1.272  -2.594  -0.180   6.959  -3.364
t_O         168     179     200     178     159     191     173     162     173     169
        ( 23.3) ( 24.9) ( 27.8) ( 24.7) ( 22.1) ( 26.5) ( 24.0) ( 22.5) ( 24.0) ( 23.5)
t_E       162.1   162.1   162.1   162.1   162.1   162.1   162.1   162.1   162.1   162.1
        ( 22.5) ( 22.5) ( 22.5) ( 22.5) ( 22.5) ( 22.5) ( 22.5) ( 22.5) ( 22.5) ( 22.5)
           12.0    35.5    84.0    33.3    -6.2    62.6    22.5    -0.2    22.5    14.0
          1.663   4.925  11.666   4.620  -0.858   8.698   3.121  -0.033   3.121   1.951
g_O         196     176     173     184     185     157     172     184     147     182
        ( 27.2) ( 24.4) ( 24.0) ( 25.6) ( 25.7) ( 21.8) ( 23.9) ( 25.6) ( 20.4) ( 25.3)
g_E       185.4   185.4   185.4   185.4   185.4   185.4   185.4   185.4   185.4   185.4
        ( 25.7) ( 25.7) ( 25.7) ( 25.7) ( 25.7) ( 25.7) ( 25.7) ( 25.7) ( 25.7) ( 25.7)
           21.9   -18.3   -23.9    -2.7    -0.7   -52.2   -25.8    -2.7   -68.2    -6.7
          3.036  -2.536  -3.319  -0.379  -0.103  -7.244  -3.577  -0.379  -9.470  -0.928
c_O         224     205     195     230     222     224     232     222     224     229
        ( 31.1) ( 28.5) ( 27.1) ( 31.9) ( 30.8) ( 31.1) ( 32.2) ( 30.8) ( 31.1) ( 31.8)
c_E       219.9   219.9   219.9   219.9   219.9   219.9   219.9   219.9   219.9   219.9
        ( 30.5) ( 30.5) ( 30.5) ( 30.5) ( 30.5) ( 30.5) ( 30.5) ( 30.5) ( 30.5) ( 30.5)
            8.4   -28.7   -46.8    20.7     4.3     8.4    24.9     4.3     8.4    18.7
          1.161  -3.985  -6.500   2.881   0.597   1.161   3.464   0.597   1.161   2.591

N           720     720     720     720     720     720     720     720     720     720
X2          3.7     3.6    12.5     6.0     0.1     9.7     3.0     0.0    12.3     1.8
Gvalue      3.8     3.6    12.0     6.2     0.1     9.7     3.0     0.0    12.7     1.8
Gv/N      0.530   0.494   1.667   0.860   0.013   1.343   0.414   0.005   1.771   0.250
Prob    0.28213 0.31338 0.00737 0.10264 0.99257 0.02162 0.39404 0.99837 0.00521 0.61431
        --------------------------------------------------------------------------------
             20      19      18      17      16      15      14      13      12      11
        --------------------------------------------------------------------------------
a_O         151     146     145     153     144     149     157     171     171     191
        ( 21.0) ( 20.3) ( 20.1) ( 21.2) ( 20.0) ( 20.7) ( 21.8) ( 23.8) ( 23.8) ( 26.5)
a_E       152.7   152.7   152.7   152.7   152.7   152.7   152.7   152.7   152.7   152.7
        ( 21.2) ( 21.2) ( 21.2) ( 21.2) ( 21.2) ( 21.2) ( 21.2) ( 21.2) ( 21.2) ( 21.2)
           -3.3   -13.0   -14.9     0.7   -16.8    -7.2     8.8    38.8    38.8    85.6
         -0.456  -1.806  -2.071   0.097  -2.333  -1.002   1.225   5.392   5.392  11.891
t_O         203     212     195     183     214     205     188     205     195     161
        ( 28.2) ( 29.4) ( 27.1) ( 25.4) ( 29.7) ( 28.5) ( 26.1) ( 28.5) ( 27.1) ( 22.4)
t_E       162.1   162.1   162.1   162.1   162.1   162.1   162.1   162.1   162.1   162.1
        ( 22.5) ( 22.5) ( 22.5) ( 22.5) ( 22.5) ( 22.5) ( 22.5) ( 22.5) ( 22.5) ( 22.5)
           91.3   113.7    72.0    44.3   118.8    96.2    55.7    96.2    72.0    -2.2
         12.680  15.797  10.003   6.158  16.504  13.363   7.734  13.363  10.003  -0.310
g_O         147     131     110     128     131     129     133     113     141     153
        ( 20.4) ( 18.2) ( 15.3) ( 17.8) ( 18.2) ( 17.9) ( 18.5) ( 15.7) ( 19.6) ( 21.2)
g_E       185.4   185.4   185.4   185.4   185.4   185.4   185.4   185.4   185.4   185.4
        ( 25.7) ( 25.7) ( 25.7) ( 25.7) ( 25.7) ( 25.7) ( 25.7) ( 25.7) ( 25.7) ( 25.7)
          -68.2   -91.0  -114.8   -94.8   -91.0   -93.5   -88.3  -111.9   -77.2   -58.7
         -9.470 -12.633 -15.946 -13.167 -12.633 -12.991 -12.266 -15.536 -10.716  -8.156
c_O         219     231     270     256     231     237     242     231     213     215
        ( 30.4) ( 32.1) ( 37.5) ( 35.6) ( 32.1) ( 32.9) ( 33.6) ( 32.1) ( 29.6) ( 29.9)
c_E       219.9   219.9   219.9   219.9   219.9   219.9   219.9   219.9   219.9   219.9
        ( 30.5) ( 30.5) ( 30.5) ( 30.5) ( 30.5) ( 30.5) ( 30.5) ( 30.5) ( 30.5) ( 30.5)
           -1.7    22.8   110.9    77.9    22.8    35.6    46.4    22.8   -13.5    -9.6
         -0.238   3.172  15.407  10.822   3.172   4.942   6.450   3.172  -1.876  -1.335

N           720     720     720     720     720     720     720     720     720     720
X2         18.3    32.1    49.1    26.4    33.6    29.9    21.3    42.4    19.7    15.4
Gvalue     18.1    32.6    53.2    28.2    33.9    31.1    22.6    46.0    20.2    15.0
Gv/N      2.516   4.529   7.393   3.911   4.710   4.313   3.144   6.390   2.803   2.090
Prob    0.00042 0.00000 0.00000 0.00000 0.00000 0.00000 0.00005 0.00000 0.00016 0.00178
        --------------------------------------------------------------------------------
             10       9       8       7       6       5       4       3       2       1
        --------------------------------------------------------------------------------
a_O         186     188     183     180     189     150     164     199     114      89
        ( 25.8) ( 26.1) ( 25.4) ( 25.0) ( 26.2) ( 20.8) ( 22.8) ( 27.6) ( 15.8) ( 12.4)
a_E       152.7   152.7   152.7   152.7   152.7   152.7   152.7   152.7   152.7   152.7
        ( 21.2) ( 21.2) ( 21.2) ( 21.2) ( 21.2) ( 21.2) ( 21.2) ( 21.2) ( 21.2) ( 21.2)
           73.5    78.3    66.4    59.3    80.7    -5.3    23.5   105.5   -66.6   -96.0
         10.209  10.878   9.218   8.240  11.214  -0.730   3.267  14.657  -9.245 -13.338
t_O         171     163     167     197     146     167     141     128     194     136
        ( 23.8) ( 22.6) ( 23.2) ( 27.4) ( 20.3) ( 23.2) ( 19.6) ( 17.8) ( 26.9) ( 18.9)
t_E       162.1   162.1   162.1   162.1   162.1   162.1   162.1   162.1   162.1   162.1
        ( 22.5) ( 22.5) ( 22.5) ( 22.5) ( 22.5) ( 22.5) ( 22.5) ( 22.5) ( 22.5) ( 22.5)
           18.2     1.8     9.9    76.8   -30.6     9.9   -39.4   -60.5    69.7   -47.8
          2.533   0.245   1.376  10.664  -4.247   1.376  -5.467  -8.402   9.674  -6.637
g_O         128     145     156     106     137     139     144     196     110      89
        ( 17.8) ( 20.1) ( 21.7) ( 14.7) ( 19.0) ( 19.3) ( 20.0) ( 27.2) ( 15.3) ( 12.4)
g_E       185.4   185.4   185.4   185.4   185.4   185.4   185.4   185.4   185.4   185.4
        ( 25.7) ( 25.7) ( 25.7) ( 25.7) ( 25.7) ( 25.7) ( 25.7) ( 25.7) ( 25.7) ( 25.7)
          -94.8   -71.2   -53.8  -118.5   -82.9   -80.0   -72.7    21.9  -114.8  -130.6
        -13.167  -9.893  -7.475 -16.457 -11.507 -11.115 -10.102   3.036 -15.946 -18.139
c_O         235     224     214     237     248     264     271     197     302     406
        ( 32.6) ( 31.1) ( 29.7) ( 32.9) ( 34.4) ( 36.7) ( 37.6) ( 27.4) ( 41.9) ( 56.4)
c_E       219.9   219.9   219.9   219.9   219.9   219.9   219.9   219.9   219.9   219.9
        ( 30.5) ( 30.5) ( 30.5) ( 30.5) ( 30.5) ( 30.5) ( 30.5) ( 30.5) ( 30.5) ( 30.5)
           31.3     8.4   -11.6    35.6    59.7    96.6   113.3   -43.3   191.7   498.1
          4.347   1.161  -1.606   4.942   8.297  13.417  15.743  -6.008  26.629  69.174

N           720     720     720     720     720     720     720     720     720     720
X2         26.6    17.1    11.0    47.7    26.5    20.7    24.7    24.2    77.4   238.4
Gvalue     28.2    17.2    10.9    53.2    27.0    21.2    24.8    23.6    80.0   223.6
Gv/N      3.922   2.390   1.513   7.389   3.756   2.948   3.441   3.283  11.112  31.060
Prob    0.00000 0.00064 0.01232 0.00000 0.00001 0.00009 0.00002 0.00003 0.00000 0.00000
        --------------------------------------------------------------------------------

CDS_INITIAL:
        --------------------------------------------------------------------------------
```

	1	2	3	4	5	6	7	8	9	10
a_O	655	0	0	319	207	120	224	236	153	185
	(91.0)	(0.0)	(0.0)	(44.3)	(28.7)	(16.7)	(31.1)	(32.8)	(21.2)	(25.7)
a_E	145.2	177.6	69.3	145.2	177.6	69.3	145.2	177.6	69.3	145.2
	(20.2)	(24.7)	(9.6)	(20.2)	(24.7)	(9.6)	(20.2)	(24.7)	(9.6)	(20.2)
	1973.8	0.0	0.0	502.3	63.3	131.8	194.3	134.1	242.4	89.7
	274.134	0.000	0.000	69.759	8.794	18.305	26.986	18.621	33.664	12.457
t_O	65	720	0	114	122	187	106	169	149	129
	(9.0)	(100.0)	(0.0)	(15.8)	(16.9)	(26.0)	(14.7)	(23.5)	(20.7)	(17.9)
t_E	96.6	209.4	113.3	96.6	209.4	113.3	96.6	209.4	113.3	96.6
	(13.4)	(29.1)	(15.7)	(13.4)	(29.1)	(15.7)	(13.4)	(29.1)	(15.7)	(13.4)
	-51.4	1778.3	0.0	37.9	-131.8	187.5	19.8	-72.5	81.7	74.7
	-7.145	246.990	0.000	5.259	-18.309	26.046	2.748	-10.065	11.351	10.381
g_O	0	0	720	115	122	122	179	105	143	169
	(0.0)	(0.0)	(100.0)	(16.0)	(16.9)	(16.9)	(24.9)	(14.6)	(19.9)	(23.5)
g_E	274.3	145.4	243.1	274.3	145.4	243.1	274.3	145.4	243.1	274.3
	(38.1)	(20.2)	(33.8)	(38.1)	(20.2)	(33.8)	(38.1)	(20.2)	(33.8)	(38.1)
	0.0	0.0	1563.3	-200.0	-42.9	-168.3	-152.8	-68.4	-151.8	-163.7
	0.000	0.000	217.126	-27.773	-5.954	-23.370	-21.229	-9.501	-21.084	-22.742
c_O	0	0	0	172	269	291	211	210	275	237
	(0.0)	(0.0)	(0.0)	(23.9)	(37.4)	(40.4)	(29.3)	(29.2)	(38.2)	(32.9)
c_E	203.9	187.5	294.3	203.9	187.5	294.3	203.9	187.5	294.3	203.9
	(28.3)	(26.0)	(40.9)	(28.3)	(26.0)	(40.9)	(28.3)	(26.0)	(40.9)	(28.3)
	0.0	0.0	0.0	-58.6	194.2	-6.6	14.4	47.6	-37.3	71.2
	0.000	0.000	0.000	-8.137	26.966	-0.914	1.995	6.608	-5.184	9.891
N	720	720	720	720	720	720	720	720	720	720
X2	2279.0	1755.5	1412.1	308.8	80.5	145.5	77.1	40.9	154.9	67.6
Gvalue	1922.3	1778.3	1563.3	281.6	82.8	144.5	75.6	40.8	135.0	71.9
Gv/N	266.989	246.990	217.126	39.108	11.496	20.067	10.500	5.662	18.748	9.988
Prob	1.00000	1.00000	1.00000	0.00000	0.00000	0.00000	-0.00000	0.00000	0.00000	0.00000

	11	12	13	14	15	16	17	18	19	20
a_O	178	103	168	175	124	179	203	94	188	168
	(24.7)	(14.3)	(23.3)	(24.3)	(17.2)	(24.9)	(28.2)	(13.1)	(26.1)	(23.3)
a_E	177.6	69.3	145.2	177.6	69.3	145.2	177.6	69.3	145.2	177.6
	(24.7)	(9.6)	(20.2)	(24.7)	(9.6)	(20.2)	(24.7)	(9.6)	(20.2)	(24.7)
	0.7	81.7	49.1	-5.2	144.3	75.0	54.2	57.3	97.2	-18.8
	0.099	11.341	6.814	-0.729	20.045	10.414	7.523	7.963	13.499	-2.605
t_O	225	180	125	220	183	130	218	192	127	231
	(31.2)	(25.0)	(17.4)	(30.6)	(25.4)	(18.1)	(30.3)	(26.7)	(17.6)	(32.1)
t_E	209.4	113.3	96.6	209.4	113.3	96.6	209.4	113.3	96.6	209.4
	(29.1)	(15.7)	(13.4)	(29.1)	(15.7)	(13.4)	(29.1)	(15.7)	(13.4)	(29.1)
	32.3	166.8	64.6	21.7	175.6	77.3	17.5	202.7	69.6	45.3
	4.488	23.163	8.965	3.014	24.389	10.740	2.434	28.149	9.669	6.296
g_O	78	130	182	86	135	165	91	124	195	94
	(10.8)	(18.1)	(25.3)	(11.9)	(18.8)	(22.9)	(12.6)	(17.2)	(27.1)	(13.1)
g_E	145.4	243.1	274.3	145.4	243.1	274.3	145.4	243.1	274.3	145.4
	(20.2)	(33.8)	(38.1)	(20.2)	(33.8)	(38.1)	(20.2)	(33.8)	(38.1)	(20.2)
	-97.2	-162.8	-149.4	-90.4	-158.9	-167.8	-85.3	-167.0	-133.1	-82.0
	-13.499	-22.609	-20.745	-12.551	-22.063	-23.301	-11.852	-23.193	-18.489	-11.396
c_O	239	307	245	239	278	246	208	310	210	227
	(33.2)	(42.6)	(34.0)	(33.2)	(38.6)	(34.2)	(28.9)	(43.1)	(29.2)	(31.5)
c_E	187.5	294.3	203.9	187.5	294.3	203.9	187.5	294.3	203.9	187.5
	(26.0)	(40.9)	(28.3)	(26.0)	(40.9)	(28.3)	(26.0)	(40.9)	(28.3)	(26.0)
	116.0	25.9	89.9	116.0	-31.7	92.3	43.1	32.2	12.3	86.8
	16.108	3.600	12.484	16.108	-4.403	12.814	5.992	4.472	1.709	12.051
N	720	720	720	720	720	720	720	720	720	720
X2	46.6	108.9	51.3	39.0	135.1	71.7	26.6	122.8	45.4	29.3
Gvalue	51.8	111.6	54.1	42.1	129.4	76.8	29.5	125.2	46.0	31.3
Gv/N	7.196	15.495	7.519	5.843	17.968	10.667	4.098	17.392	6.388	4.346
Prob	0.00000	0.00000	0.00000	0.00000	0.00000	0.00000	0.00000	0.00000	0.00000	0.00000

	21	22	23	24	25	26	27	28	29	30
a_O	103	145	172	97	145	171	83	156	162	101
	(14.3)	(20.1)	(23.9)	(13.5)	(20.1)	(23.8)	(11.5)	(21.7)	(22.5)	(14.0)
a_E	69.3	145.2	177.6	69.3	145.2	177.6	69.3	145.2	177.6	69.3
	(9.6)	(20.2)	(24.7)	(9.6)	(20.2)	(24.7)	(9.6)	(20.2)	(24.7)	(9.6)
	81.7	-0.4	-11.1	65.3	-0.4	-13.0	30.0	22.4	-29.9	76.1
	11.341	-0.049	-1.543	9.063	-0.049	-1.811	4.162	3.116	-4.149	10.571
t_O	156	126	207	146	124	188	144	110	215	128
	(21.7)	(17.5)	(28.7)	(20.3)	(17.2)	(26.1)	(20.0)	(15.3)	(29.9)	(17.8)
t_E	113.3	96.6	209.4	113.3	96.6	209.4	113.3	96.6	209.4	113.3
	(15.7)	(13.4)	(29.1)	(15.7)	(13.4)	(29.1)	(15.7)	(13.4)	(29.1)	(15.7)
	99.9	67.1	-4.8	74.1	62.0	-40.6	69.2	28.7	11.3	31.3
	13.874	9.316	-0.666	10.298	8.617	-5.633	9.605	3.984	1.573	4.350
g_O	154	201	125	171	236	125	189	223	138	187
	(21.4)	(27.9)	(17.4)	(23.8)	(32.8)	(17.4)	(26.2)	(31.0)	(19.2)	(26.0)
g_E	243.1	274.3	145.4	243.1	274.3	145.4	243.1	274.3	145.4	243.1
	(33.8)	(38.1)	(20.2)	(33.8)	(38.1)	(20.2)	(33.8)	(38.1)	(20.2)	(33.8)
	-140.7	-125.0	-37.9	-120.4	-71.0	-37.9	-95.2	-92.4	-14.5	-98.2
	-19.535	-17.366	-5.257	-16.718	-9.867	-5.257	-13.223	-12.833	-2.011	-13.636
c_O	307	248	216	306	215	236	304	231	205	304
	(42.6)	(34.4)	(30.0)	(42.5)	(29.9)	(32.8)	(42.2)	(32.1)	(28.5)	(42.2)
c_E	294.3	203.9	187.5	294.3	203.9	187.5	294.3	203.9	187.5	294.3
	(40.9)	(28.3)	(26.0)	(40.9)	(28.3)	(26.0)	(40.9)	(28.3)	(26.0)	(40.9)
	25.9	97.0	61.1	23.8	22.7	108.6	19.7	57.6	36.6	19.7
	3.600	13.476	8.487	3.311	3.155	15.078	2.735	7.995	5.078	2.735
N	720	720	720	720	720	720	720	720	720	720
X2	65.7	38.1	7.4	42.4	13.8	17.8	23.4	15.9	3.5	29.7
Gvalue	66.8	38.7	7.3	42.9	13.4	17.1	23.6	16.3	3.5	28.9
Gv/N	9.279	5.377	1.021	5.954	1.856	2.377	3.278	2.262	0.491	4.020
Prob	0.00000	0.00000	0.06155	0.00000	0.00391	0.00067	0.00003	0.00099	0.31593	0.00000

	31	32	33	34	35	36	37	38	39	40
a_O	157	152	84	149	133	75	139	169	70	124
	(21.8)	(21.1)	(11.7)	(20.7)	(18.5)	(10.4)	(19.3)	(23.5)	(9.7)	(17.2)
a_E	145.2	177.6	69.3	145.2	177.6	69.3	145.2	177.6	69.3	145.2
	(20.2)	(24.7)	(9.6)	(20.2)	(24.7)	(9.6)	(20.2)	(24.7)	(9.6)	(20.2)
	24.6	-47.4	32.3	7.7	-77.0	11.9	-12.1	-16.9	1.4	-39.1
	3.415	-6.583	4.491	1.076	-10.693	1.649	-1.678	-2.342	0.197	-5.431
t_O	100	224	150	104	184	113	110	201	138	129
	(13.9)	(31.1)	(20.8)	(14.4)	(25.6)	(15.7)	(15.3)	(27.9)	(19.2)	(17.9)
t_E	96.6	209.4	113.3	96.6	209.4	113.3	96.6	209.4	113.3	96.6
	(13.4)	(29.1)	(15.7)	(13.4)	(29.1)	(15.7)	(13.4)	(29.1)	(15.7)	(13.4)
	7.0	30.2	84.3	15.5	-47.6	-0.5	28.7	-16.5	54.5	74.7

	0.974	4.190	11.706	2.146	-6.612	-0.072	3.984	-2.289	7.573	10.381
g_O	230	134	184	247	160	231	236	137	213	247
	(31.9)	(18.6)	(25.6)	(34.3)	(22.2)	(32.1)	(32.8)	(19.0)	(29.6)	(34.3)
g_E	274.3	145.4	243.1	274.3	145.4	243.1	274.3	145.4	243.1	274.3
	(38.1)	(20.2)	(33.8)	(38.1)	(20.2)	(33.8)	(38.1)	(20.2)	(33.8)	(38.1)
	-81.1	-21.9	-102.6	-51.8	30.5	-23.7	-71.0	-16.4	-56.4	-51.8
	-11.261	-3.048	-14.244	-7.201	4.242	-3.286	-9.867	-2.274	-7.830	-7.201
c_O	233	210	302	220	243	301	235	213	299	220
	(32.4)	(29.2)	(41.9)	(30.6)	(33.8)	(41.8)	(32.6)	(29.6)	(41.5)	(30.6)
c_E	203.9	187.5	294.3	203.9	187.5	294.3	203.9	187.5	294.3	203.9
	(28.3)	(26.0)	(40.9)	(28.3)	(26.0)	(40.9)	(28.3)	(26.0)	(40.9)	(28.3)
	62.1	47.6	15.6	33.4	126.0	13.5	66.6	54.3	9.5	33.4
	8.623	6.608	2.164	4.633	17.498	1.879	9.255	7.541	1.313	4.633
N	720	720	720	720	720	720	720	720	720	720
X2	12.4	8.3	29.6	4.7	32.2	1.2	12.2	4.7	9.2	18.0
Gvalue	12.6	8.4	29.6	4.7	31.9	1.2	12.2	4.6	9.0	17.2
Gv/N	1.750	1.168	4.117	0.654	4.435	0.170	1.693	0.637	1.254	2.383
Prob	0.00558	0.03831	0.00000	0.19420	0.00000	0.74693	0.00676	0.20466	0.02890	0.00066

	41	42	43	44	45	46	47	48	49	50
a_O	146	78	143	144	76	133	185	76	135	150
	(20.3)	(10.8)	(19.9)	(20.0)	(10.6)	(18.5)	(25.7)	(10.6)	(18.8)	(20.8)
a_E	177.6	69.3	145.2	177.6	69.3	145.2	177.6	69.3	145.2	177.6
	(24.7)	(9.6)	(20.2)	(24.7)	(9.6)	(20.2)	(24.7)	(9.6)	(20.2)	(24.7)
	-57.3	18.5	-4.3	-60.5	14.0	-23.3	15.0	14.0	-19.6	-50.7
	-7.956	2.565	-0.600	-8.399	1.951	-3.236	2.085	1.951	-2.725	-7.048
t_O	228	129	89	215	113	95	212	131	108	238
	(31.7)	(17.9)	(12.4)	(29.9)	(15.7)	(13.2)	(29.4)	(18.2)	(15.0)	(33.1)
t_E	209.4	113.3	96.6	209.4	113.3	96.6	209.4	113.3	96.6	209.4
	(29.1)	(15.7)	(13.4)	(29.1)	(15.7)	(13.4)	(29.1)	(15.7)	(13.4)	(29.1)
	38.8	33.6	-14.5	11.3	-0.5	-3.1	5.2	38.1	24.2	60.9
	5.386	4.663	-2.014	1.573	-0.072	-0.428	0.724	5.295	3.361	8.460
g_O	140	208	243	162	228	240	159	219	277	144
	(19.4)	(28.9)	(33.8)	(22.5)	(31.7)	(33.3)	(22.1)	(30.4)	(38.5)	(20.0)
g_E	145.4	243.1	274.3	145.4	243.1	274.3	145.4	243.1	274.3	145.4
	(20.2)	(33.8)	(38.1)	(20.2)	(33.8)	(38.1)	(20.2)	(33.8)	(38.1)	(20.2)
	-10.7	-64.9	-58.9	35.0	-29.3	-64.2	28.4	-45.8	5.4	-2.9
	-1.481	-9.018	-8.186	4.854	-4.071	-8.913	3.939	-6.360	0.745	-0.396
c_O	206	305	245	199	303	252	164	294	200	188
	(28.6)	(42.4)	(34.0)	(27.6)	(42.1)	(35.0)	(22.8)	(40.8)	(27.8)	(26.1)
c_E	187.5	294.3	203.9	187.5	294.3	203.9	187.5	294.3	203.9	187.5
	(26.0)	(40.9)	(28.3)	(26.0)	(40.9)	(28.3)	(26.0)	(40.9)	(28.3)	(26.0)
	38.7	21.8	89.9	23.7	17.6	106.7	-43.9	-0.6	-7.8	1.0
	5.381	3.023	12.484	3.288	2.449	14.813	-6.103	-0.086	-1.083	0.136
N	720	720	720	720	720	720	720	720	720	720
X2	9.3	8.7	12.5	9.1	1.8	16.7	4.5	5.8	2.2	8.2
Gvalue	9.6	8.9	12.1	9.5	1.8	16.1	4.6	5.8	2.1	8.3
Gv/N	1.331	1.232	1.684	1.316	0.256	2.235	0.644	0.799	0.297	1.152
Prob	0.02249	0.03109	0.00696	0.02360	0.60488	0.00109	0.20019	0.12428	0.54408	0.04027

	51	52	53	54	55	56	57	58	59	60
a_O	76	145	152	77	135	165	94	133	165	73
	(10.6)	(20.1)	(21.1)	(10.7)	(18.8)	(22.9)	(13.1)	(18.5)	(22.9)	(10.1)
a_E	69.3	145.2	177.6	69.3	145.2	177.6	69.3	145.2	177.6	69.3
	(9.6)	(20.2)	(24.7)	(9.6)	(20.2)	(24.7)	(9.6)	(20.2)	(24.7)	(9.6)
	14.0	-0.4	-47.4	16.2	-19.6	-24.4	57.3	-23.3	-24.4	7.6
	1.951	-0.049	-6.583	2.256	-2.725	-3.384	7.963	-3.236	-3.384	1.057
t_O	118	106	222	114	113	226	126	109	232	125
	(16.4)	(14.7)	(30.8)	(15.8)	(15.7)	(31.4)	(17.5)	(15.1)	(32.2)	(17.4)
t_E	113.3	96.6	209.4	113.3	96.6	209.4	113.3	96.6	209.4	113.3
	(15.7)	(13.4)	(29.1)	(15.7)	(13.4)	(29.1)	(15.7)	(13.4)	(29.1)	(15.7)
	9.7	19.8	25.9	1.5	35.5	34.5	26.9	26.4	47.5	24.7
	1.344	2.748	3.600	0.206	4.937	4.786	3.731	3.671	6.602	3.424
g_O	230	244	162	218	264	140	242	259	143	207
	(31.9)	(33.9)	(22.5)	(30.3)	(36.7)	(19.4)	(33.6)	(36.0)	(19.9)	(28.7)
g_E	243.1	274.3	145.4	243.1	274.3	145.4	243.1	274.3	145.4	243.1
	(33.8)	(38.1)	(20.2)	(33.8)	(38.1)	(20.2)	(33.8)	(38.1)	(20.2)	(33.8)
	-25.5	-57.2	35.0	-47.6	-20.3	-10.7	-2.3	-29.8	-4.8	-66.6
	-3.549	-7.942	4.854	-6.608	-2.815	-1.481	-0.315	-4.138	-0.670	-9.252
c_O	296	225	184	311	208	189	258	219	180	315
	(41.1)	(31.2)	(25.6)	(43.2)	(28.9)	(26.2)	(35.8)	(30.4)	(25.0)	(43.8)
c_E	294.3	203.9	187.5	294.3	203.9	187.5	294.3	203.9	187.5	294.3
	(40.9)	(28.3)	(26.0)	(40.9)	(28.3)	(26.0)	(40.9)	(28.3)	(26.0)	(40.9)
	3.4	44.2	-7.0	34.3	8.2	3.0	-67.9	31.2	-14.7	42.8
	0.471	6.143	-0.966	4.765	1.140	0.416	-9.437	4.335	-2.044	5.945
N	720	720	720	720	720	720	720	720	720	720
X2	1.6	6.5	6.4	4.4	4.0	2.4	14.7	4.6	3.7	8.2
Gvalue	1.6	6.5	6.5	4.5	3.9	2.4	14.0	4.5	3.6	8.5
Gv/N	0.216	0.901	0.906	0.619	0.536	0.336	1.942	0.632	0.503	1.174
Prob	0.66909	0.09029	0.08881	0.21641	0.27707	0.48986	0.00293	0.20790	0.30535	0.03754

	61	62	63	64	65	66	67	68	69	70
a_O	145	145	63	123	159	70	121	161	70	119
	(20.1)	(20.1)	(8.8)	(17.1)	(22.1)	(9.7)	(16.8)	(22.4)	(9.7)	(16.5)
a_E	145.2	177.6	69.3	145.2	177.6	69.3	145.2	177.6	69.3	145.2
	(20.2)	(24.7)	(9.6)	(20.2)	(24.7)	(9.6)	(20.2)	(24.7)	(9.6)	(20.2)
	-0.4	-58.9	-12.0	-40.8	-35.3	1.4	-44.1	-31.7	1.4	-47.3
	-0.049	-8.178	-1.666	-5.663	-4.897	0.197	-6.122	-4.400	0.197	-6.572
t_O	90	210	105	98	218	108	90	208	125	103
	(12.5)	(29.2)	(14.6)	(13.6)	(30.3)	(15.0)	(12.5)	(28.9)	(17.4)	(14.3)
t_E	96.6	209.4	113.3	96.6	209.4	113.3	96.6	209.4	113.3	96.6
	(13.4)	(29.1)	(15.7)	(13.4)	(29.1)	(15.7)	(13.4)	(29.1)	(15.7)	(13.4)
	-12.7	1.2	-15.9	2.9	17.5	-10.3	-12.7	-2.8	24.7	13.3
	-1.758	0.164	-2.209	0.404	2.434	-1.427	-1.758	-0.391	3.424	1.849
g_O	291	157	247	274	152	252	288	147	209	279
	(40.4)	(21.8)	(34.3)	(38.1)	(21.1)	(35.0)	(40.0)	(20.4)	(29.0)	(38.8)
g_E	274.3	145.4	243.1	274.3	145.4	243.1	274.3	145.4	243.1	274.3
	(38.1)	(20.2)	(33.8)	(38.1)	(20.2)	(33.8)	(38.1)	(20.2)	(33.8)	(38.1)
	34.3	24.0	7.8	-0.7	13.4	18.0	28.0	3.1	-63.2	9.4
	4.768	3.337	1.082	-0.092	1.864	2.506	3.890	0.437	-8.783	1.308
c_O	194	208	305	225	191	290	221	204	316	219
	(26.9)	(28.9)	(42.4)	(31.2)	(26.5)	(40.3)	(30.7)	(28.3)	(43.9)	(30.4)
c_E	203.9	187.5	294.3	203.9	187.5	294.3	203.9	187.5	294.3	203.9
	(28.3)	(26.0)	(40.9)	(28.3)	(26.0)	(40.9)	(28.3)	(26.0)	(40.9)	(28.3)
	-19.4	43.1	21.8	44.2	7.0	-8.6	35.5	34.4	44.9	31.2
	-2.692	5.992	3.023	6.143	0.978	-1.188	4.933	4.776	6.242	4.335

N	720	720	720	720	720	720	720	720	720	720
X2	1.9	9.2	1.6	5.6	2.7	0.6	6.6	3.0	7.6	6.3
Gvalue	1.9	9.5	1.7	5.7	2.7	0.6	6.8	3.0	7.8	6.6
Gv/N	0.270	1.314	0.229	0.792	0.380	0.089	0.942	0.423	1.080	0.919
Prob	0.58480	0.02372	0.64764	0.12713	0.43459	0.88765	0.07908	0.38452	0.05081	0.08512

	71	72	73	74	75	76	77	78	79	80
a_O	172	74	127	163	80	128	149	70	133	156
	(23.9)	(10.3)	(17.6)	(22.6)	(11.1)	(17.8)	(20.7)	(9.7)	(18.5)	(21.7)
a_E	177.6	69.3	145.2	177.6	69.3	145.2	177.6	69.3	145.2	177.6
	(24.7)	(9.6)	(20.2)	(24.7)	(9.6)	(20.2)	(24.7)	(9.6)	(20.2)	(24.7)
	−11.1	9.7	−34.0	−28.0	23.0	−32.2	−52.4	1.4	−23.3	−40.5
	−1.543	1.351	−4.719	−3.896	3.193	−4.477	−7.278	0.197	−3.236	−5.630
t_O	205	129	99	206	118	98	211	106	108	208
	(28.5)	(17.9)	(13.8)	(28.6)	(16.4)	(13.6)	(29.3)	(14.7)	(15.0)	(28.9)
t_E	209.4	113.3	96.6	209.4	113.3	96.6	209.4	113.3	96.6	209.4
	(29.1)	(15.7)	(13.4)	(29.1)	(15.7)	(13.4)	(29.1)	(15.7)	(13.4)	(29.1)
	−8.7	33.6	5.0	−6.8	9.7	2.9	3.2	−14.0	24.2	−2.8
	−1.212	4.663	0.688	−0.940	1.344	0.404	0.443	−1.951	3.361	−0.391
g_O	155	235	303	141	231	288	134	238	276	138
	(21.5)	(32.6)	(42.1)	(19.6)	(32.1)	(40.0)	(18.6)	(33.1)	(38.3)	(19.2)
g_E	145.4	243.1	274.3	145.4	243.1	274.3	145.4	243.1	274.3	145.4
	(20.2)	(33.8)	(38.1)	(20.2)	(33.8)	(38.1)	(20.2)	(33.8)	(38.1)	(20.2)
	19.7	−16.0	60.2	−8.7	−23.7	28.0	−21.9	−10.2	3.3	−14.5
	2.743	−2.222	8.366	−1.213	−3.286	3.890	−3.048	−1.412	0.465	−2.011
c_O	188	282	191	210	291	206	226	306	203	218
	(26.1)	(39.2)	(26.5)	(29.2)	(40.4)	(28.6)	(31.4)	(42.5)	(28.2)	(30.3)
c_E	187.5	294.3	203.9	187.5	294.3	203.9	187.5	294.3	203.9	187.5
	(26.0)	(40.9)	(28.3)	(26.0)	(40.9)	(28.3)	(26.0)	(40.9)	(28.3)	(26.0)
	1.0	−24.1	−25.0	47.6	−6.6	4.1	84.4	23.8	−1.9	65.7
	0.136	−3.347	−3.477	6.608	−0.914	0.576	11.721	3.311	−0.260	9.123
N	720	720	720	720	720	720	720	720	720	720
X2	0.9	3.3	6.2	4.1	2.5	2.8	13.4	1.0	2.4	8.0
Gvalue	0.9	3.2	6.2	4.0	2.4	2.8	13.2	1.1	2.4	7.9
Gv/N	0.124	0.445	0.857	0.560	0.337	0.393	1.838	0.146	0.329	1.091
Prob	0.82736	0.36141	0.10346	0.25824	0.48911	0.41866	0.00416	0.78903	0.49897	0.04907

	81	82	83	84	85	86	87	88	89	90
a_O	81	107	171	77	134	160	78	112	159	60
	(11.2)	(14.9)	(23.8)	(10.7)	(18.6)	(22.2)	(10.8)	(15.6)	(22.1)	(8.3)
a_E	69.3	145.2	177.6	69.3	145.2	177.6	69.3	145.2	177.6	69.3
	(9.6)	(20.2)	(24.7)	(9.6)	(20.2)	(24.7)	(9.6)	(20.2)	(24.7)	(9.6)
	25.3	−65.3	−13.0	16.2	−21.5	−33.5	18.5	−58.1	−35.3	−17.3
	3.512	−9.069	−1.811	2.256	−2.982	−4.649	2.565	−8.072	−4.897	−2.400
t_O	129	107	211	121	109	210	109	99	203	108
	(17.9)	(14.9)	(29.3)	(16.8)	(15.1)	(29.2)	(15.1)	(13.8)	(28.2)	(15.0)
t_E	113.3	96.6	209.4	113.3	96.6	209.4	113.3	96.6	209.4	113.3
	(15.7)	(13.4)	(29.1)	(15.7)	(13.4)	(29.1)	(15.7)	(13.4)	(29.1)	(15.7)
	33.6	22.0	3.2	16.0	26.4	1.2	−8.4	5.0	−12.6	−10.3
	4.663	3.053	0.443	2.222	3.671	0.164	−1.161	0.688	−1.753	−1.427
g_O	237	303	139	232	266	159	239	284	164	254
	(32.9)	(42.1)	(19.3)	(32.2)	(36.9)	(22.1)	(33.2)	(39.4)	(22.8)	(35.3)
g_E	243.1	274.3	145.4	243.1	274.3	145.4	243.1	274.3	145.4	243.1
	(33.8)	(38.1)	(20.2)	(33.8)	(38.1)	(20.2)	(33.8)	(38.1)	(20.2)	(33.8)
	−12.1	60.2	−12.6	−21.8	−16.4	28.4	−8.2	19.7	39.4	22.2
	−1.683	8.366	−1.747	−3.021	−2.279	3.939	−1.139	2.732	5.473	3.084
c_O	273	203	199	290	211	191	294	225	194	298
	(37.9)	(28.2)	(27.6)	(40.3)	(29.3)	(26.5)	(40.8)	(31.2)	(26.9)	(41.4)
c_E	294.3	203.9	187.5	294.3	203.9	187.5	294.3	203.9	187.5	294.3
	(40.9)	(28.3)	(26.0)	(40.9)	(28.3)	(26.0)	(40.9)	(28.3)	(26.0)	(40.9)
	−41.0	−1.9	23.7	−8.6	14.4	7.0	−0.6	44.2	13.2	7.4
	−5.700	−0.260	3.288	−1.188	1.995	0.978	−0.086	6.143	1.834	1.031
N	720	720	720	720	720	720	720	720	720	720
X2	5.9	14.2	1.2	2.0	3.0	3.1	1.3	10.2	4.7	2.0
Gvalue	5.7	15.0	1.2	1.9	2.9	3.1	1.3	10.7	4.7	2.1
Gv/N	0.792	2.090	0.173	0.267	0.406	0.432	0.178	1.491	0.656	0.289
Prob	0.12686	0.00178	0.74276	0.58796	0.40411	0.37532	0.73284	0.01323	0.19309	0.55624

	91	92	93	94	95	96	97	98	99	100
a_O	142	176	69	135	143	66	133	165	73	146
	(19.7)	(24.4)	(9.6)	(18.8)	(19.9)	(9.2)	(18.5)	(22.9)	(10.1)	(20.3)
a_E	145.2	177.6	69.3	145.2	177.6	69.3	145.2	177.6	69.3	145.2
	(20.2)	(24.7)	(9.6)	(20.2)	(24.7)	(9.6)	(20.2)	(24.7)	(9.6)	(20.2)
	−6.3	−3.3	−0.6	−19.6	−62.0	−6.4	−23.3	−24.4	7.6	1.7
	−0.872	−0.455	−0.081	−2.725	−8.617	−0.893	−3.236	−3.384	1.057	0.230
t_O	90	208	125	103	221	107	89	221	120	96
	(12.5)	(28.9)	(17.4)	(14.3)	(30.7)	(14.9)	(12.4)	(30.7)	(16.7)	(13.3)
t_E	96.6	209.4	113.3	96.6	209.4	113.3	96.6	209.4	113.3	96.6
	(13.4)	(29.1)	(15.7)	(13.4)	(29.1)	(15.7)	(13.4)	(29.1)	(15.7)	(13.4)
	−12.7	−2.8	24.7	13.3	23.8	−12.2	−14.5	23.8	13.9	−1.1
	−1.758	−0.391	3.424	1.849	3.307	−1.690	−2.014	3.307	1.927	−0.154
g_O	269	132	249	273	133	224	281	151	223	267
	(37.4)	(18.3)	(34.6)	(37.9)	(18.5)	(31.1)	(39.0)	(21.0)	(31.0)	(37.1)
g_E	274.3	145.4	243.1	274.3	145.4	243.1	274.3	145.4	243.1	274.3
	(38.1)	(20.2)	(33.8)	(38.1)	(20.2)	(33.8)	(38.1)	(20.2)	(33.8)	(38.1)
	−10.6	−25.6	11.9	−2.7	−23.8	−36.7	13.5	11.3	−38.6	−14.5
	−1.467	−3.554	1.648	−0.369	−3.302	−5.101	1.875	1.575	−5.355	−2.009
c_O	219	204	277	209	223	323	217	183	304	211
	(30.4)	(28.3)	(38.5)	(29.0)	(31.0)	(44.9)	(30.1)	(25.4)	(42.2)	(29.3)
c_E	203.9	187.5	294.3	203.9	187.5	294.3	203.9	187.5	294.3	203.9
	(28.3)	(26.0)	(40.9)	(28.3)	(26.0)	(40.9)	(28.3)	(26.0)	(40.9)	(28.3)
	31.2	34.4	−33.6	10.3	77.3	60.1	26.9	−8.9	19.7	14.4
	4.335	4.776	−4.664	1.424	10.737	8.346	3.742	−1.238	2.735	1.995
N	720	720	720	720	720	720	720	720	720	720
X2	1.7	2.7	2.4	1.3	15.2	4.8	2.6	1.9	2.6	0.4
Gvalue	1.7	2.7	2.4	1.3	15.3	4.8	2.6	1.9	2.6	0.4
Gv/N	0.238	0.377	0.327	0.178	2.125	0.662	0.366	0.260	0.364	0.062
Prob	0.63342	0.43764	0.50170	0.73327	0.00158	0.18951	0.45081	0.59946	0.45409	0.93028

Bbac_HD100
5UTR:

	100	99	98	97	96	95	94	93	92	91
a_O	158	171	180	170	164	172	135	147	161	163
	(25.9)	(28.0)	(29.5)	(27.8)	(26.8)	(28.2)	(22.1)	(24.1)	(26.4)	(26.7)
a_E	167.4	167.4	167.4	167.4	167.4	167.4	167.4	167.4	167.4	167.4
	(27.4)	(27.4)	(27.4)	(27.4)	(27.4)	(27.4)	(27.4)	(27.4)	(27.4)	(27.4)
	-18.3	7.3	26.1	5.2	-6.8	9.3	-58.1	-38.2	-12.6	-8.7
	-2.992	1.188	4.272	0.854	-1.105	1.523	-9.508	-6.256	-2.058	-1.424
t_O	147	164	144	153	163	148	167	175	143	168
	(24.1)	(26.8)	(23.6)	(25.0)	(26.7)	(24.2)	(27.3)	(28.6)	(23.4)	(27.5)
t_E	167.5	167.5	167.5	167.5	167.5	167.5	167.5	167.5	167.5	167.5
	(27.4)	(27.4)	(27.4)	(27.4)	(27.4)	(27.4)	(27.4)	(27.4)	(27.4)	(27.4)
	-38.4	-6.9	-43.6	-27.7	-8.9	-36.7	-1.0	15.3	-45.2	1.0
	-6.285	-1.137	-7.128	-4.538	-1.456	-5.999	-0.167	2.506	-7.405	0.161
g_O	152	129	138	139	138	133	152	138	153	150
	(24.9)	(21.1)	(22.6)	(22.7)	(22.6)	(21.8)	(24.9)	(22.6)	(25.0)	(24.5)
g_E	131.8	131.8	131.8	131.8	131.8	131.8	131.8	131.8	131.8	131.8
	(21.6)	(21.6)	(21.6)	(21.6)	(21.6)	(21.6)	(21.6)	(21.6)	(21.6)	(21.6)
	43.3	-5.6	12.6	14.7	12.6	2.4	43.3	12.6	45.6	38.8
	7.087	-0.913	2.070	2.413	2.070	0.388	7.087	2.070	7.462	6.344
c_O	154	147	149	149	146	158	157	151	154	130
	(25.2)	(24.1)	(24.4)	(24.4)	(23.9)	(25.9)	(25.7)	(24.7)	(25.2)	(21.3)
c_E	144.3	144.3	144.3	144.3	144.3	144.3	144.3	144.3	144.3	144.3
	(23.6)	(23.6)	(23.6)	(23.6)	(23.6)	(23.6)	(23.6)	(23.6)	(23.6)	(23.6)
	20.1	5.5	9.6	9.6	3.5	28.7	26.6	13.8	20.1	-27.1
	3.294	0.905	1.577	1.577	0.573	4.705	4.349	2.257	3.294	-4.429
N	611	611	611	611	611	611	611	611	611	611
X2	6.8	0.3	4.7	1.8	0.5	3.7	10.5	3.4	7.9	4.0
Gvalue	6.7	0.3	4.8	1.9	0.5	3.8	10.8	3.5	7.9	4.0
Gv/N	1.104	0.043	0.790	0.307	0.082	0.617	1.761	0.576	1.293	0.651
Prob	0.08046	0.96683	0.18474	0.59919	0.91918	0.28736	0.01309	0.31798	0.04810	0.26401

	90	89	88	87	86	85	84	83	82	81
a_O	165	161	160	151	158	162	168	157	158	164
	(27.0)	(26.4)	(26.2)	(24.7)	(25.9)	(26.5)	(27.5)	(25.7)	(25.9)	(26.8)
a_E	167.4	167.4	167.4	167.4	167.4	167.4	167.4	167.4	167.4	167.4
	(27.4)	(27.4)	(27.4)	(27.4)	(27.4)	(27.4)	(27.4)	(27.4)	(27.4)	(27.4)
	-4.8	-12.6	-14.5	-31.2	-18.3	-10.6	1.2	-20.2	-18.3	-6.8
	-0.783	-2.058	-2.371	-5.099	-2.992	-1.742	0.193	-3.299	-2.992	-1.105
t_O	138	142	169	183	151	153	172	150	149	159
	(22.6)	(23.2)	(27.7)	(30.0)	(24.7)	(25.0)	(28.2)	(24.5)	(24.4)	(26.0)
t_E	167.5	167.5	167.5	167.5	167.5	167.5	167.5	167.5	167.5	167.5
	(27.4)	(27.4)	(27.4)	(27.4)	(27.4)	(27.4)	(27.4)	(27.4)	(27.4)	(27.4)
	-53.5	-46.9	3.0	32.4	-31.3	-27.7	9.1	-33.1	-34.9	-16.6
	-8.754	-7.679	0.490	5.298	-5.129	-4.538	1.489	-5.421	-5.711	-2.714
g_O	153	148	119	130	158	151	139	155	143	153
	(25.0)	(24.2)	(19.5)	(21.3)	(25.9)	(24.7)	(22.7)	(25.4)	(23.4)	(25.0)
g_E	131.8	131.8	131.8	131.8	131.8	131.8	131.8	131.8	131.8	131.8
	(21.6)	(21.6)	(21.6)	(21.6)	(21.6)	(21.6)	(21.6)	(21.6)	(21.6)	(21.6)
	45.6	34.3	-24.4	-3.6	57.2	41.0	14.7	50.2	23.3	45.6
	7.462	5.609	-3.985	-0.592	9.369	6.714	2.413	8.219	3.811	7.462
c_O	155	160	163	147	144	145	132	149	161	135
	(25.4)	(26.2)	(26.7)	(24.1)	(23.6)	(23.7)	(21.6)	(24.4)	(26.4)	(22.1)
c_E	144.3	144.3	144.3	144.3	144.3	144.3	144.3	144.3	144.3	144.3
	(23.6)	(23.6)	(23.6)	(23.6)	(23.6)	(23.6)	(23.6)	(23.6)	(23.6)	(23.6)
	22.3	33.1	39.8	5.5	-0.5	1.5	-23.4	9.6	35.4	-17.9
	3.643	5.424	6.516	0.905	-0.085	0.243	-3.838	1.577	5.786	-2.932
N	611	611	611	611	611	611	611	611	611	611
X2	9.4	7.8	4.0	3.1	7.4	4.2	1.6	6.7	5.5	4.5
Gvalue	9.6	7.9	4.0	3.1	7.1	4.1	1.6	6.6	5.5	4.4
Gv/N	1.569	1.295	0.650	0.512	1.163	0.678	0.258	1.075	0.893	0.712
Prob	0.02246	0.04781	0.26463	0.37187	0.06851	0.24676	0.66432	0.08698	0.14116	0.22593

	80	79	78	77	76	75	74	73	72	71
a_O	170	160	160	157	160	158	157	167	153	156
	(27.8)	(26.2)	(26.2)	(25.7)	(26.2)	(25.9)	(25.7)	(27.3)	(25.0)	(25.5)
a_E	167.4	167.4	167.4	167.4	167.4	167.4	167.4	167.4	167.4	167.4
	(27.4)	(27.4)	(27.4)	(27.4)	(27.4)	(27.4)	(27.4)	(27.4)	(27.4)	(27.4)
	5.2	-14.5	-14.5	-20.2	-14.5	-18.3	-20.2	-0.8	-27.5	-22.0
	0.854	-2.371	-2.371	-3.299	-2.371	-2.992	-3.299	-0.134	-4.508	-3.605
t_O	143	155	170	172	150	169	142	146	150	155
	(23.4)	(25.4)	(27.8)	(28.2)	(24.5)	(27.7)	(23.2)	(23.9)	(24.5)	(25.4)
t_E	167.5	167.5	167.5	167.5	167.5	167.5	167.5	167.5	167.5	167.5
	(27.4)	(27.4)	(27.4)	(27.4)	(27.4)	(27.4)	(27.4)	(27.4)	(27.4)	(27.4)
	-45.2	-24.1	5.0	9.1	-33.1	3.0	-46.9	-40.1	-33.1	-24.1
	-7.405	-3.938	0.821	1.489	-5.421	0.490	-7.679	-6.568	-5.421	-3.938
g_O	157	149	132	139	142	136	163	157	147	155
	(25.7)	(24.4)	(21.6)	(22.7)	(23.2)	(22.3)	(26.7)	(25.7)	(24.1)	(25.4)
g_E	131.8	131.8	131.8	131.8	131.8	131.8	131.8	131.8	131.8	131.8
	(21.6)	(21.6)	(21.6)	(21.6)	(21.6)	(21.6)	(21.6)	(21.6)	(21.6)	(21.6)
	54.9	36.5	0.4	14.7	21.1	8.5	69.2	54.9	32.0	50.2
	8.984	5.975	0.059	2.413	3.458	1.390	11.328	8.984	5.245	8.219
c_O	141	147	149	143	159	148	149	141	161	145
	(23.1)	(24.1)	(24.4)	(23.4)	(26.0)	(24.2)	(24.4)	(23.1)	(26.4)	(23.7)
c_E	144.3	144.3	144.3	144.3	144.3	144.3	144.3	144.3	144.3	144.3
	(23.6)	(23.6)	(23.6)	(23.6)	(23.6)	(23.6)	(23.6)	(23.6)	(23.6)	(23.6)
	-6.4	5.5	9.6	-2.5	30.9	7.6	9.6	-6.4	35.4	1.5
	-1.055	0.905	1.577	-0.411	5.063	1.240	1.577	-1.055	5.786	0.243
N	611	611	611	611	611	611	611	611	611	611
X2	8.5	3.6	0.5	1.2	4.5	0.8	12.1	7.6	6.8	5.8
Gvalue	8.4	3.5	0.5	1.2	4.5	0.8	11.8	7.5	6.7	5.6
Gv/N	1.378	0.571	0.086	0.192	0.729	0.128	1.926	1.226	1.102	0.919
Prob	0.03810	0.32196	0.91357	0.75885	0.21638	0.85433	0.00822	0.05772	0.08097	0.13195

	70	69	68	67	66	65	64	63	62	61
a_O	160	149	155	157	144	186	155	154	174	183
	(26.2)	(24.4)	(25.4)	(25.7)	(23.6)	(30.4)	(25.4)	(25.2)	(28.5)	(30.0)
a_E	167.4	167.4	167.4	167.4	167.4	167.4	167.4	167.4	167.4	167.4
	(27.4)	(27.4)	(27.4)	(27.4)	(27.4)	(27.4)	(27.4)	(27.4)	(27.4)	(27.4)
	-14.5	-34.7	-23.9	-20.2	-43.4	39.2	-23.9	-25.7	13.4	32.6
	-2.371	-5.682	-3.908	-3.299	-7.100	6.411	-3.908	-4.209	2.199	5.334
t_O	163	159	150	155	177	140	163	185	170	174
	(26.7)	(26.0)	(24.5)	(25.4)	(29.0)	(22.9)	(26.7)	(30.3)	(27.8)	(28.5)

t_E	167.5	167.5	167.5	167.5	167.5	167.5	167.5	167.5	167.5	167.5
	(27.4)	(27.4)	(27.4)	(27.4)	(27.4)	(27.4)	(27.4)	(27.4)	(27.4)	(27.4)
	-8.9	-16.6	-33.1	-24.1	19.5	-50.2	-8.9	36.7	5.0	13.2
	-1.456	-2.714	-5.421	-3.938	3.193	-8.221	-1.456	6.014	0.821	2.165
g_O	127	142	154	143	137	134	145	129	140	126
	(20.8)	(23.2)	(25.2)	(23.4)	(22.4)	(21.9)	(23.7)	(21.1)	(22.9)	(20.6)
g_E	131.8	131.8	131.8	131.8	131.8	131.8	131.8	131.8	131.8	131.8
	(21.6)	(21.6)	(21.6)	(21.6)	(21.6)	(21.6)	(21.6)	(21.6)	(21.6)	(21.6)
	-9.5	21.1	47.9	23.3	10.6	4.4	27.6	-5.6	16.9	-11.4
	-1.549	3.458	7.839	3.811	1.728	0.719	4.523	-0.913	2.759	-1.862
c_O	161	161	152	156	153	151	148	143	127	128
	(26.4)	(26.4)	(24.9)	(25.5)	(25.0)	(24.7)	(24.2)	(23.4)	(20.8)	(20.9)
c_E	144.3	144.3	144.3	144.3	144.3	144.3	144.3	144.3	144.3	144.3
	(23.6)	(23.6)	(23.6)	(23.6)	(23.6)	(23.6)	(23.6)	(23.6)	(23.6)	(23.6)
	35.4	35.4	15.9	24.4	18.0	13.8	7.6	-2.5	-32.4	-30.6
	5.786	5.786	2.600	3.995	2.946	2.257	1.240	-0.411	-5.297	-5.011
N	611	611	611	611	611	611	611	611	611	611
X2	2.6	5.2	6.9	3.5	4.5	6.9	2.5	3.0	2.9	3.8
Gvalue	2.5	5.2	6.8	3.5	4.7	7.1	2.4	2.9	2.9	3.8
Gv/N	0.410	0.848	1.111	0.568	0.767	1.166	0.399	0.481	0.482	0.626
Prob	0.47432	0.15901	0.07897	0.32433	0.19632	0.06800	0.48657	0.40072	0.40049	0.28116

	60	59	58	57	56	55	54	53	52	51
a_O	164	192	149	173	167	174	179	174	155	147
	(26.8)	(31.4)	(24.4)	(28.3)	(27.3)	(28.5)	(29.3)	(28.5)	(25.4)	(24.1)
a_E	167.4	167.4	167.4	167.4	167.4	167.4	167.4	167.4	167.4	167.4
	(27.4)	(27.4)	(27.4)	(27.4)	(27.4)	(27.4)	(27.4)	(27.4)	(27.4)	(27.4)
	-6.8	52.6	-34.7	11.4	-0.8	13.4	24.0	13.4	-23.9	-38.2
	-1.105	8.613	-5.682	1.860	-0.134	2.199	3.922	2.199	-3.908	-6.256
t_O	161	148	184	155	153	151	144	161	172	203
	(26.4)	(24.2)	(30.1)	(25.4)	(25.0)	(24.7)	(23.6)	(26.4)	(28.2)	(33.2)
t_E	167.5	167.5	167.5	167.5	167.5	167.5	167.5	167.5	167.5	167.5
	(27.4)	(27.4)	(27.4)	(27.4)	(27.4)	(27.4)	(27.4)	(27.4)	(27.4)	(27.4)
	-12.8	-36.7	34.6	-24.1	-27.7	-31.3	-43.6	-12.8	9.1	78.0
	-2.089	-5.999	5.655	-3.938	-4.538	-5.129	-7.128	-2.089	1.489	12.769
g_O	143	135	141	129	136	136	134	131	133	128
	(23.4)	(22.1)	(23.1)	(21.1)	(22.3)	(22.3)	(21.9)	(21.4)	(21.8)	(20.9)
g_E	131.8	131.8	131.8	131.8	131.8	131.8	131.8	131.8	131.8	131.8
	(21.6)	(21.6)	(21.6)	(21.6)	(21.6)	(21.6)	(21.6)	(21.6)	(21.6)	(21.6)
	23.3	6.4	19.0	-5.6	8.5	8.5	4.4	-1.6	2.4	-7.5
	3.811	1.053	3.107	-0.913	1.390	1.390	0.719	-0.268	0.388	-1.232
c_O	143	136	137	154	155	150	154	145	151	133
	(23.4)	(22.3)	(22.4)	(25.2)	(25.4)	(24.5)	(25.2)	(23.7)	(24.7)	(21.8)
c_E	144.3	144.3	144.3	144.3	144.3	144.3	144.3	144.3	144.3	144.3
	(23.6)	(23.6)	(23.6)	(23.6)	(23.6)	(23.6)	(23.6)	(23.6)	(23.6)	(23.6)
	-2.5	-16.0	-14.1	20.1	22.3	11.7	20.1	1.5	13.8	-21.6
	-0.411	-2.625	-2.316	3.294	3.643	1.916	3.294	0.243	2.257	-3.538
N	611	611	611	611	611	611	611	611	611	611
X2	1.3	6.4	4.7	1.8	2.2	2.2	4.8	0.5	1.4	11.0
Gvalue	1.3	6.4	4.7	1.8	2.2	2.3	4.9	0.5	1.4	10.6
Gv/N	0.206	1.043	0.765	0.302	0.361	0.376	0.807	0.085	0.226	1.743
Prob	0.73870	0.09487	0.19743	0.60474	0.53048	0.51325	0.17704	0.91418	0.70941	0.01379

	50	49	48	47	46	45	44	43	42	41
a_O	152	173	179	178	167	156	158	178	176	165
	(24.9)	(28.3)	(29.3)	(29.1)	(27.3)	(25.5)	(25.9)	(29.1)	(28.8)	(27.0)
a_E	167.4	167.4	167.4	167.4	167.4	167.4	167.4	167.4	167.4	167.4
	(27.4)	(27.4)	(27.4)	(27.4)	(27.4)	(27.4)	(27.4)	(27.4)	(27.4)	(27.4)
	-29.4	11.4	24.0	21.8	-0.8	-22.0	-18.3	21.8	17.6	-4.8
	-4.805	1.860	3.922	3.574	-0.134	-3.605	-2.992	3.574	2.883	-0.783
t_O	166	153	138	155	161	187	174	194	189	192
	(27.2)	(25.0)	(22.6)	(25.4)	(26.4)	(30.6)	(28.5)	(31.8)	(30.9)	(31.4)
t_E	167.5	167.5	167.5	167.5	167.5	167.5	167.5	167.5	167.5	167.5
	(27.4)	(27.4)	(27.4)	(27.4)	(27.4)	(27.4)	(27.4)	(27.4)	(27.4)	(27.4)
	-3.0	-27.7	-53.5	-24.1	-12.8	41.2	13.2	57.0	45.6	52.4
	-0.492	-4.538	-8.754	-3.938	-2.089	6.737	2.165	9.323	7.467	8.576
g_O	145	135	133	123	127	138	126	128	125	138
	(23.7)	(22.1)	(21.8)	(20.1)	(20.8)	(22.6)	(20.6)	(20.9)	(20.5)	(22.6)
g_E	131.8	131.8	131.8	131.8	131.8	131.8	131.8	131.8	131.8	131.8
	(21.6)	(21.6)	(21.6)	(21.6)	(21.6)	(21.6)	(21.6)	(21.6)	(21.6)	(21.6)
	27.6	6.4	2.4	-17.0	-9.5	12.6	-11.4	-7.5	-13.3	12.6
	4.523	1.053	0.388	-2.788	-1.549	2.070	-1.862	-1.232	-2.174	2.070
c_O	148	150	161	155	156	130	153	111	121	116
	(24.2)	(24.5)	(26.4)	(25.4)	(25.5)	(21.3)	(25.0)	(18.2)	(19.8)	(19.0)
c_E	144.3	144.3	144.3	144.3	144.3	144.3	144.3	144.3	144.3	144.3
	(23.6)	(23.6)	(23.6)	(23.6)	(23.6)	(23.6)	(23.6)	(23.6)	(23.6)	(23.6)
	7.6	11.7	35.4	22.3	24.4	-27.1	18.0	-58.2	-42.6	-50.6
	1.240	1.916	5.786	3.643	3.995	-4.429	2.946	-9.523	-6.964	-8.279
N	611	611	611	611	611	611	611	611	611	611
X2	2.8	1.7	8.0	3.0	1.4	4.7	1.6	12.6	7.3	9.4
Gvalue	2.8	1.8	8.2	3.0	1.4	4.7	1.6	13.1	7.4	9.7
Gv/N	0.466	0.291	1.342	0.491	0.224	0.773	0.257	2.142	1.213	1.584
Prob	0.41536	0.61909	0.04204	0.39180	0.71346	0.19314	0.66682	0.00445	0.05996	0.02153

	40	39	38	37	36	35	34	33	32	31
a_O	159	165	162	145	159	147	154	153	183	166
	(26.0)	(27.0)	(26.5)	(23.7)	(26.0)	(24.1)	(25.2)	(25.0)	(30.0)	(27.2)
a_E	167.4	167.4	167.4	167.4	167.4	167.4	167.4	167.4	167.4	167.4
	(27.4)	(27.4)	(27.4)	(27.4)	(27.4)	(27.4)	(27.4)	(27.4)	(27.4)	(27.4)
	-16.4	-4.8	-10.6	-41.7	-16.4	-38.2	-25.7	-27.5	32.6	-2.8
	-2.683	-0.783	-1.742	-6.821	-2.683	-6.256	-4.209	-4.508	5.334	-0.460
t_O	202	166	165	178	191	218	212	165	155	171
	(33.1)	(27.2)	(27.0)	(29.1)	(31.3)	(35.7)	(34.7)	(27.0)	(25.4)	(28.0)
t_E	167.5	167.5	167.5	167.5	167.5	167.5	167.5	167.5	167.5	167.5
	(27.4)	(27.4)	(27.4)	(27.4)	(27.4)	(27.4)	(27.4)	(27.4)	(27.4)	(27.4)
	75.6	-3.0	-5.0	21.6	50.1	114.9	99.9	-5.0	-24.1	7.1
	12.380	-0.492	-0.815	3.539	8.205	18.800	16.345	-0.815	-3.938	1.154
g_O	123	150	158	144	121	129	116	157	126	122
	(20.1)	(24.5)	(25.9)	(23.6)	(19.8)	(21.1)	(19.0)	(25.7)	(20.6)	(20.0)
g_E	131.8	131.8	131.8	131.8	131.8	131.8	131.8	131.8	131.8	131.8
	(21.6)	(21.6)	(21.6)	(21.6)	(21.6)	(21.6)	(21.6)	(21.6)	(21.6)	(21.6)
	-17.0	38.8	57.2	25.5	-20.7	-5.6	-29.7	54.9	-11.4	-18.9
	-2.788	6.344	9.369	4.166	-3.392	-0.913	-4.854	8.984	-1.862	-3.092
c_O	127	130	126	144	140	117	129	136	147	152
	(20.8)	(21.3)	(20.6)	(23.6)	(22.9)	(19.1)	(21.1)	(22.3)	(24.1)	(24.9)
c_E	144.3	144.3	144.3	144.3	144.3	144.3	144.3	144.3	144.3	144.3

```
        ( 23.6) ( 23.6) ( 23.6) ( 23.6) ( 23.6) ( 23.6) ( 23.6) ( 23.6) ( 23.6) ( 23.6)
          -32.4   -27.1   -34.1    -0.5    -8.4   -49.0   -28.8   -16.0     5.5    15.9
         -5.297  -4.429  -5.582  -0.085  -1.374  -8.021  -4.721  -2.625   0.905   2.600

N           611     611     611     611     611     611     611     611     611     611
X2         10.2     4.0     7.7     4.8     4.7    22.9    16.4     6.6     2.7     1.2
Gvalue      9.8     3.9     7.5     4.9     4.6    22.1    15.6     6.3     2.7     1.2
Gv/N      1.611   0.639   1.230   0.799   0.756   3.609   2.561   1.036   0.439   0.203
Prob    0.01993 0.27175 0.05715 0.18081 0.20181 0.00006 0.00134 0.09672 0.44369 0.74281
        --------------------------------------------------------------------------------
             30      29      28      27      26      25      24      23      22      21
        --------------------------------------------------------------------------------
a_O         189     159     153     167     140     165     170     175     167     162
        ( 30.9) ( 26.0) ( 25.0) ( 27.3) ( 22.9) ( 27.0) ( 27.8) ( 28.6) ( 27.3) ( 26.5)
a_E       167.4   167.4   167.4   167.4   167.4   167.4   167.4   167.4   167.4   167.4
        ( 27.4) ( 27.4) ( 27.4) ( 27.4) ( 27.4) ( 27.4) ( 27.4) ( 27.4) ( 27.4) ( 27.4)
           45.9   -16.4   -27.5    -0.8   -50.1    -4.8     5.2    15.5    -0.8   -10.6
          7.504  -2.683  -4.508  -0.134  -8.194  -0.783   0.854   2.540  -0.134  -1.742
t_O         176     169     193     179     189     174     160     146     152     170
        ( 28.8) ( 27.7) ( 31.6) ( 29.3) ( 30.9) ( 28.5) ( 26.2) ( 23.9) ( 24.9) ( 27.8)
t_E       167.5   167.5   167.5   167.5   167.5   167.5   167.5   167.5   167.5   167.5
        ( 27.4) ( 27.4) ( 27.4) ( 27.4) ( 27.4) ( 27.4) ( 27.4) ( 27.4) ( 27.4) ( 27.4)
           17.4     3.0    54.7    23.8    45.6    13.2   -14.7   -40.1   -29.5     5.0
          2.848   0.490   8.949   3.887   7.467   2.165  -2.402  -6.568  -4.834   0.821
g_O         135     139     130     117     130     121     127     155     151     143
        ( 22.1) ( 22.7) ( 21.3) ( 19.1) ( 21.3) ( 19.8) ( 20.8) ( 25.4) ( 24.7) ( 23.4)
g_E       131.8   131.8   131.8   131.8   131.8   131.8   131.8   131.8   131.8   131.8
        ( 21.6) ( 21.6) ( 21.6) ( 21.6) ( 21.6) ( 21.6) ( 21.6) ( 21.6) ( 21.6) ( 21.6)
            6.4    14.7    -3.6   -27.9    -3.6   -20.7    -9.5    50.2    41.0    23.3
          1.053   2.413  -0.592  -4.568  -0.592  -3.392  -1.549   8.219   6.714   3.811
c_O         111     144     135     148     152     151     154     135     141     136
        ( 18.2) ( 23.6) ( 22.1) ( 24.2) ( 24.9) ( 24.7) ( 25.2) ( 22.1) ( 23.1) ( 22.3)
c_E       144.3   144.3   144.3   144.3   144.3   144.3   144.3   144.3   144.3   144.3
        ( 23.6) ( 23.6) ( 23.6) ( 23.6) ( 23.6) ( 23.6) ( 23.6) ( 23.6) ( 23.6) ( 23.6)
          -58.2    -0.5   -17.9     7.6    15.9    13.8    20.1   -17.9    -6.4   -16.0
         -9.523  -0.085  -2.932   1.240   2.600   2.257   3.294  -2.932  -1.055  -2.625

N           611     611     611     611     611     611     611     611     611     611
X2         11.0     0.8     5.7     2.6     7.7     1.5     1.2     7.8     4.3     1.6
Gvalue     11.5     0.8     5.6     2.6     7.8     1.5     1.2     7.7     4.2     1.6
Gv/N      1.883   0.135   0.918   0.426   1.282   0.247   0.197   1.259   0.691   0.265
Prob    0.00927 0.84286 0.13243 0.45745 0.04956 0.68072 0.75218 0.05284 0.23844 0.65525
        --------------------------------------------------------------------------------
             20      19      18      17      16      15      14      13      12      11
        --------------------------------------------------------------------------------
a_O         188     177     168     158     191     174     176     172     159     274
        ( 30.8) ( 29.0) ( 27.5) ( 25.9) ( 31.3) ( 28.5) ( 28.8) ( 28.2) ( 26.0) ( 44.8)
a_E       167.4   167.4   167.4   167.4   167.4   167.4   167.4   167.4   167.4   167.4
        ( 27.4) ( 27.4) ( 27.4) ( 27.4) ( 27.4) ( 27.4) ( 27.4) ( 27.4) ( 27.4) ( 27.4)
           43.6    19.7     1.2   -18.3    50.4    13.4    17.6     9.3   -16.4   270.0
          7.138   3.227   0.193  -2.992   8.242   2.199   2.883   1.523  -2.683  44.188
t_O         156     169     187     202     189     194     183     193     213     136
        ( 25.5) ( 27.7) ( 30.6) ( 33.1) ( 30.9) ( 31.8) ( 30.0) ( 31.6) ( 34.9) ( 22.3)
t_E       167.5   167.5   167.5   167.5   167.5   167.5   167.5   167.5   167.5   167.5
        ( 27.4) ( 27.4) ( 27.4) ( 27.4) ( 27.4) ( 27.4) ( 27.4) ( 27.4) ( 27.4) ( 27.4)
          -22.2     3.0    41.2    75.6    45.6    57.0    32.4    54.7   102.3   -56.7
         -3.635   0.490   6.737  12.380   7.467   9.323   5.298   8.949  16.751  -9.277
g_O         124     105      82      95      93     102     111      88     101      79
        ( 20.3) ( 17.2) ( 13.4) ( 15.5) ( 15.2) ( 16.7) ( 18.2) ( 14.4) ( 16.5) ( 12.9)
g_E       131.8   131.8   131.8   131.8   131.8   131.8   131.8   131.8   131.8   131.8
        ( 21.6) ( 21.6) ( 21.6) ( 21.6) ( 21.6) ( 21.6) ( 21.6) ( 21.6) ( 21.6) ( 21.6)
          -15.2   -47.8   -77.9   -62.2   -64.9   -52.3   -38.2   -71.1   -53.8   -80.9
         -2.482  -7.818 -12.742 -10.186 -10.619  -8.563  -6.246 -11.640  -8.805 -13.240
c_O         143     160     174     156     138     141     141     158     138     122
        ( 23.4) ( 26.2) ( 28.5) ( 25.5) ( 22.6) ( 23.1) ( 23.1) ( 25.9) ( 22.6) ( 20.0)
c_E       144.3   144.3   144.3   144.3   144.3   144.3   144.3   144.3   144.3   144.3
        ( 23.6) ( 23.6) ( 23.6) ( 23.6) ( 23.6) ( 23.6) ( 23.6) ( 23.6) ( 23.6) ( 23.6)
           -2.5    33.1    65.2    24.4   -12.2    -6.4    -6.4    28.7   -12.2   -40.9
         -0.411   5.424  10.676   3.995  -2.004  -1.055  -1.055   4.705  -2.004  -6.693

N           611     611     611     611     611     611     611     611     611     611
X2          3.8     7.7    27.2    18.9    17.8    11.3     5.2    19.9    20.3    98.4
Gvalue      3.7     8.1    29.7    19.5    18.9    11.6     5.4    21.6    19.9    91.5
Gv/N      0.610   1.322   4.864   3.197   3.086   1.904   0.880   3.536   3.259  14.979
Prob    0.29231 0.04438 0.00000 0.00021 0.00029 0.00874 0.14635 0.00008 0.00018 0.00000
        --------------------------------------------------------------------------------
             10       9       8       7       6       5       4       3       2       1
        --------------------------------------------------------------------------------
a_O         191     215     203     172     201     199     193     189     168     137
        ( 31.3) ( 35.2) ( 33.2) ( 28.2) ( 32.9) ( 32.6) ( 31.6) ( 30.9) ( 27.5) ( 22.4)
a_E       167.4   167.4   167.4   167.4   167.4   167.4   167.4   167.4   167.4   167.4
        ( 27.4) ( 27.4) ( 27.4) ( 27.4) ( 27.4) ( 27.4) ( 27.4) ( 27.4) ( 27.4) ( 27.4)
           50.4   107.6    78.3     9.3    73.5    68.8    54.9    45.9     1.2   -54.9
          8.242  17.608  12.809   1.523  12.031  11.260   8.986   7.504   0.193  -8.990
t_O         190     165     167     226     181     153     153     149     152     195
        ( 31.1) ( 27.0) ( 27.3) ( 37.0) ( 29.6) ( 25.0) ( 25.0) ( 24.4) ( 24.9) ( 31.9)
t_E       167.5   167.5   167.5   167.5   167.5   167.5   167.5   167.5   167.5   167.5
        ( 27.4) ( 27.4) ( 27.4) ( 27.4) ( 27.4) ( 27.4) ( 27.4) ( 27.4) ( 27.4) ( 27.4)
           47.9    -5.0    -1.0   135.4    28.0   -27.7   -27.7   -34.9   -29.5    59.3
          7.835  -0.815  -0.167  22.156   4.589  -4.538  -4.538  -5.711  -4.834   9.699
g_O          98     104      99      81     100     145     125     154     128      85
        ( 16.0) ( 17.0) ( 16.2) ( 13.3) ( 16.4) ( 23.7) ( 20.5) ( 25.2) ( 20.9) ( 13.9)
g_E       131.8   131.8   131.8   131.8   131.8   131.8   131.8   131.8   131.8   131.8
        ( 21.6) ( 21.6) ( 21.6) ( 21.6) ( 21.6) ( 21.6) ( 21.6) ( 21.6) ( 21.6) ( 21.6)
          -58.1   -49.3   -56.7   -78.9   -55.3    27.6   -13.3    47.9    -7.5   -74.6
         -9.510  -8.070  -9.278 -12.912  -9.043   4.523  -2.174   7.839  -1.232 -12.208
c_O         132     127     142     132     129     114     140     119     163     194
        ( 21.6) ( 20.8) ( 23.2) ( 21.6) ( 21.1) ( 18.7) ( 22.9) ( 19.5) ( 26.7) ( 31.8)
c_E       144.3   144.3   144.3   144.3   144.3   144.3   144.3   144.3   144.3   144.3
        ( 23.6) ( 23.6) ( 23.6) ( 23.6) ( 23.6) ( 23.6) ( 23.6) ( 23.6) ( 23.6) ( 23.6)
          -23.4   -32.4    -4.5   -23.4   -28.8   -53.7    -8.4   -45.8    39.8   114.9
         -3.838  -5.297  -0.734  -3.838  -4.721  -8.785  -1.374  -7.498   6.516  18.812

N           611     611     611     611     611     611     611     611     611     611
X2         16.1    21.5    15.8    41.2    17.1    14.9     5.6    13.0     4.0    43.8
Gvalue     16.7    20.9    16.1    42.3    17.4    15.0     5.5    13.0     3.9    44.7
Gv/N      2.729   3.425   2.630   6.929   2.856   2.460   0.901   2.135   0.644   7.313
Prob    0.00082 0.00011 0.00110 0.00000 0.00057 0.00179 0.13820 0.00455 0.26892 0.00000
        --------------------------------------------------------------------------------

CDS_INITIAL:
        --------------------------------------------------------------------------------
```

	1	2	3	4	5	6	7	8	9	10
a_O	370	0	0	260	213	188	232	221	194	221
	(60.6)	(0.0)	(0.0)	(42.6)	(34.9)	(30.8)	(38.0)	(36.2)	(31.8)	(36.2)
a_E	165.9	187.0	101.8	165.9	187.0	101.8	165.9	187.0	101.8	165.9
	(27.2)	(30.6)	(16.7)	(27.2)	(30.6)	(16.7)	(27.2)	(30.6)	(16.7)	(27.2)
	593.5	0.0	0.0	233.6	55.4	230.5	155.5	73.8	250.1	126.7
	97.132	0.000	0.000	38.228	9.065	37.730	25.458	12.073	40.930	20.737
t_O	144	611	0	104	127	159	108	169	144	137
	(23.6)	(100.0)	(0.0)	(17.0)	(20.8)	(26.0)	(17.7)	(27.7)	(23.6)	(22.4)
t_E	107.2	180.4	150.0	107.2	180.4	150.0	107.2	180.4	150.0	107.2
	(17.5)	(29.5)	(24.6)	(17.5)	(29.5)	(24.6)	(17.5)	(29.5)	(24.6)	(17.5)
	85.0	1490.6	0.0	-6.3	-89.2	18.4	1.6	-22.1	-11.8	67.3
	13.919	243.967	0.000	-1.025	-14.595	3.017	0.269	-3.616	-1.938	11.008
g_O	97	0	611	131	122	130	145	91	150	125
	(15.9)	(0.0)	(100.0)	(21.4)	(20.0)	(21.3)	(23.7)	(14.9)	(24.5)	(20.5)
g_E	205.7	100.8	174.6	205.7	100.8	174.6	205.7	100.8	174.6	205.7
	(33.7)	(16.5)	(28.6)	(33.7)	(16.5)	(28.6)	(33.7)	(16.5)	(28.6)	(33.7)
	-145.8	0.0	1530.7	-118.2	46.7	-76.7	-101.4	-18.6	-45.5	-124.5
	-23.861	0.000	250.528	-19.340	7.636	-12.550	-16.588	-3.037	-7.455	-20.373
c_O	0	0	0	116	149	134	126	130	123	128
	(0.0)	(0.0)	(0.0)	(19.0)	(24.4)	(21.9)	(20.6)	(21.3)	(20.1)	(20.9)
c_E	132.2	142.8	184.5	132.2	142.8	184.5	132.2	142.8	184.5	132.2
	(21.6)	(23.4)	(30.2)	(21.6)	(23.4)	(30.2)	(21.6)	(23.4)	(30.2)	(21.6)
	0.0	0.0	0.0	-30.4	12.7	-85.8	-12.2	-24.4	-99.8	-8.3
	0.000	0.000	0.000	-4.975	2.078	-14.035	-1.994	-3.992	-16.332	-1.366
N	611	611	611	611	611	611	611	611	611	611
X2	453.3	1458.2	1527.2	82.5	24.2	98.7	44.5	9.0	107.6	58.4
Gvalue	532.7	1490.6	1530.7	78.7	25.6	86.5	43.7	8.7	92.9	61.1
Gv/N	87.190	243.967	250.528	12.887	4.184	14.163	7.146	1.428	15.205	10.007
Prob	0.00000	1.00000	1.00000	0.00000	0.00001	0.00000	0.00000	0.03319	0.00000	0.00000

	11	12	13	14	15	16	17	18	19	20
a_O	165	153	192	156	142	185	170	130	197	152
	(27.0)	(25.0)	(31.4)	(25.5)	(23.2)	(30.3)	(27.8)	(21.3)	(32.2)	(24.9)
a_E	187.0	101.8	165.9	187.0	101.8	165.9	187.0	101.8	165.9	187.0
	(30.6)	(16.7)	(27.2)	(30.6)	(16.7)	(27.2)	(30.6)	(16.7)	(27.2)	(30.6)
	-41.4	124.6	56.1	-56.6	94.4	40.3	-32.5	63.5	67.6	-63.0
	-6.769	20.389	9.175	-9.264	15.455	6.592	-5.313	10.392	11.072	-10.319
t_O	227	169	145	221	171	146	221	190	151	248
	(37.2)	(27.7)	(23.7)	(36.2)	(28.0)	(23.9)	(36.2)	(31.1)	(24.7)	(40.6)
t_E	180.4	150.0	107.2	180.4	150.0	107.2	180.4	150.0	107.2	180.4
	(29.5)	(24.6)	(17.5)	(29.5)	(24.6)	(17.5)	(29.5)	(24.6)	(17.5)	(29.5)
	104.3	40.2	87.6	89.7	44.7	90.3	89.7	89.7	103.5	157.8
	17.067	6.581	14.344	14.678	7.318	14.772	14.678	14.684	16.942	25.828
g_O	102	151	131	80	164	142	98	155	130	82
	(16.7)	(24.7)	(21.4)	(13.1)	(26.8)	(23.2)	(16.0)	(25.4)	(21.3)	(13.4)
g_E	100.8	174.6	205.7	100.8	174.6	205.7	100.8	174.6	205.7	100.8
	(16.5)	(28.6)	(33.7)	(16.5)	(28.6)	(33.7)	(16.5)	(28.6)	(33.7)	(16.5)
	2.5	-43.8	-118.2	-36.9	-20.5	-105.2	-5.5	-36.9	-119.3	-33.8
	0.406	-7.176	-19.340	-6.044	-3.360	-17.216	-0.893	-6.039	-19.519	-5.532
c_O	117	138	143	154	134	138	122	136	133	129
	(19.1)	(22.6)	(23.4)	(25.2)	(21.9)	(22.6)	(20.0)	(22.3)	(21.8)	(21.1)
c_E	142.8	184.5	132.2	142.8	184.5	132.2	142.8	184.5	132.2	142.8
	(23.4)	(30.2)	(21.6)	(23.4)	(30.2)	(21.6)	(23.4)	(30.2)	(21.6)	(23.4)
	-46.6	-80.2	22.4	23.3	-85.8	11.8	-38.4	-83.0	1.5	-26.2
	-7.628	-13.125	3.662	3.812	-14.035	1.926	-6.282	-13.585	0.249	-4.287
N	611	611	611	611	611	611	611	611	611	611
X2	19.3	43.0	45.4	19.4	33.3	36.2	13.8	33.4	51.6	36.7
Gvalue	18.8	40.7	47.9	19.4	32.9	37.1	13.4	33.3	53.4	34.8
Gv/N	3.076	6.669	7.841	3.182	5.378	6.073	2.189	5.451	8.745	5.690
Prob	0.00030	0.00000	0.00000	0.00022	0.00000	0.00000	0.00389	0.00000	0.00000	0.00000

	21	22	23	24	25	26	27	28	29	30
a_O	143	172	138	148	172	138	156	212	156	127
	(23.4)	(28.2)	(22.6)	(24.2)	(28.2)	(22.6)	(25.5)	(34.7)	(25.5)	(20.8)
a_E	101.8	165.9	187.0	101.8	165.9	187.0	101.8	165.9	187.0	101.8
	(16.7)	(27.2)	(30.6)	(16.7)	(27.2)	(30.6)	(16.7)	(27.2)	(30.6)	(16.7)
	97.1	12.4	-83.9	110.7	12.4	-83.9	133.1	103.9	-56.6	56.1
	15.892	2.026	-13.733	18.113	2.026	-13.733	21.780	17.007	-9.264	9.182
t_O	180	119	220	169	149	237	178	141	235	181
	(29.5)	(19.5)	(36.0)	(27.7)	(24.4)	(38.8)	(29.1)	(23.1)	(38.5)	(29.6)
t_E	150.0	107.2	180.4	150.0	107.2	180.4	150.0	107.2	180.4	150.0
	(24.6)	(17.5)	(29.5)	(24.6)	(17.5)	(29.5)	(24.6)	(17.5)	(29.5)	(24.6)
	65.5	24.9	87.3	40.2	98.2	129.3	60.8	77.3	124.2	67.9
	10.725	4.075	14.285	6.581	16.067	21.163	9.955	12.658	20.332	11.113
g_O	130	171	107	137	141	103	128	134	92	148
	(21.3)	(28.0)	(17.5)	(22.4)	(23.1)	(16.9)	(20.9)	(21.9)	(15.1)	(24.2)
g_E	174.6	205.7	100.8	174.6	205.7	100.8	174.6	205.7	100.8	174.6
	(28.6)	(33.7)	(16.5)	(28.6)	(33.7)	(16.5)	(28.6)	(33.7)	(16.5)	(28.6)
	-76.7	-63.1	12.8	-66.4	-106.4	4.5	-79.5	-114.8	-16.7	-48.9
	-12.550	-10.330	2.102	-10.874	-17.421	0.739	-13.006	-18.790	-2.741	-8.005
c_O	158	149	146	157	149	133	149	124	128	155
	(25.9)	(24.4)	(23.9)	(25.7)	(24.4)	(21.8)	(24.4)	(20.3)	(20.9)	(25.4)
c_E	184.5	132.2	142.8	184.5	132.2	142.8	184.5	132.2	142.8	184.5
	(30.2)	(21.6)	(23.4)	(30.2)	(21.6)	(23.4)	(30.2)	(21.6)	(23.4)	(30.2)
	-49.1	35.6	6.5	-50.7	35.6	-18.9	-63.7	-16.0	-28.0	-54.1
	-8.028	5.820	1.064	-8.303	5.820	-3.091	-10.431	-2.611	-4.580	-8.848
N	611	611	611	611	611	611	611	611	611	611
X2	37.8	9.5	22.0	35.5	39.0	31.3	53.3	49.0	24.0	21.4
Gvalue	36.9	9.7	22.7	33.7	39.7	31.0	50.7	50.5	22.9	21.0
Gv/N	6.040	1.590	3.717	5.517	6.492	5.078	8.298	8.263	3.747	3.441
Prob	0.00000	0.02112	0.00005	0.00000	0.00000	0.00000	0.00000	0.00000	0.00004	0.00010

	31	32	33	34	35	36	37	38	39	40
a_O	168	149	127	168	136	119	178	132	146	179
	(27.5)	(24.4)	(20.8)	(27.5)	(22.3)	(19.5)	(29.1)	(21.6)	(23.9)	(29.3)
a_E	165.9	187.0	101.8	165.9	187.0	101.8	165.9	187.0	101.8	165.9
	(27.2)	(30.6)	(16.7)	(27.2)	(30.6)	(16.7)	(27.2)	(30.6)	(16.7)	(27.2)
	4.2	-67.7	56.1	4.2	-86.7	37.1	25.0	-92.0	105.2	27.2
	0.685	-11.087	9.182	0.685	-14.184	6.069	4.095	-15.057	17.218	4.446
t_O	147	238	177	154	247	174	131	255	184	140
	(24.1)	(39.0)	(29.0)	(25.2)	(40.4)	(28.5)	(21.4)	(41.7)	(30.1)	(22.9)
t_E	107.2	180.4	150.0	107.2	180.4	150.0	107.2	180.4	150.0	107.2
	(17.5)	(29.5)	(24.6)	(17.5)	(29.5)	(24.6)	(17.5)	(29.5)	(24.6)	(17.5)
	92.9	131.9	58.5	111.6	155.2	51.5	52.6	176.5	75.1	74.8

	15.201	21.580	9.573	18.270	25.397	8.437	8.605	28.880	12.287	12.242
g_O	157	88	159	169	94	164	171	84	151	163
	(25.7)	(14.4)	(26.0)	(27.7)	(15.4)	(26.8)	(28.0)	(13.7)	(24.7)	(26.7)
g_E	205.7	100.8	174.6	205.7	100.8	174.6	205.7	100.8	174.6	205.7
	(33.7)	(16.5)	(28.6)	(33.7)	(16.5)	(28.6)	(33.7)	(16.5)	(28.6)	(33.7)
	-84.8	-23.8	-29.8	-66.4	-13.1	-20.5	-63.1	-30.6	-43.8	-75.8
	-13.874	-3.903	-4.869	-10.860	-2.139	-3.360	-10.330	-5.004	-7.176	-12.404
c_O	139	136	148	120	134	154	131	140	130	129
	(22.7)	(22.3)	(24.2)	(19.6)	(21.9)	(25.2)	(21.4)	(22.9)	(21.3)	(21.1)
c_E	132.2	142.8	184.5	132.2	142.8	184.5	132.2	142.8	184.5	132.2
	(21.6)	(23.4)	(30.2)	(21.6)	(23.4)	(30.2)	(21.6)	(23.4)	(30.2)	(21.6)
	13.9	-13.2	-65.3	-23.3	-17.0	-55.7	-2.5	-5.5	-91.1	-6.4
	2.268	-2.167	-10.687	-3.815	-2.785	-9.117	-0.404	-0.903	-14.906	-1.048
N	611	611	611	611	611	611	611	611	611	611
X2	26.7	28.1	19.7	28.1	39.5	12.4	12.0	49.9	46.1	20.0
Gvalue	26.2	27.0	19.5	26.1	38.4	12.4	12.0	48.4	45.4	19.8
Gv/N	4.280	4.423	3.198	4.280	6.289	2.028	1.966	7.917	7.424	3.237
Prob	0.00001	0.00001	0.00021	0.00001	0.00000	0.00615	0.00735	0.00000	0.00000	0.00019

	41	42	43	44	45	46	47	48	49	50
a_O	144	140	190	150	121	157	150	131	161	140
	(23.6)	(22.9)	(31.1)	(24.5)	(19.8)	(25.7)	(24.5)	(21.4)	(26.4)	(22.9)
a_E	187.0	101.8	165.9	187.0	101.8	165.9	187.0	101.8	165.9	187.0
	(30.6)	(16.7)	(27.2)	(30.6)	(16.7)	(27.2)	(30.6)	(16.7)	(27.2)	(30.6)
	-75.3	89.1	51.5	-66.2	41.7	-17.4	-66.2	66.0	-9.7	-81.1
	-12.324	14.587	8.428	-10.833	6.831	-2.840	-10.833	10.800	-1.586	-13.273
t_O	233	171	133	226	177	137	218	167	127	240
	(38.1)	(28.0)	(21.8)	(37.0)	(29.0)	(22.4)	(35.7)	(27.3)	(20.8)	(39.3)
t_E	180.4	150.0	107.2	180.4	150.0	107.2	180.4	150.0	107.2	180.4
	(29.5)	(24.6)	(17.5)	(29.5)	(24.6)	(17.5)	(29.5)	(24.6)	(17.5)	(29.5)
	119.2	44.7	57.4	101.8	58.5	67.3	82.5	35.8	43.1	137.0
	19.507	7.318	9.396	16.665	9.573	11.008	13.503	5.853	7.054	22.419
g_O	96	158	162	97	173	185	99	155	175	87
	(15.7)	(25.9)	(26.5)	(15.9)	(28.3)	(30.3)	(16.2)	(25.4)	(28.6)	(14.2)
g_E	100.8	174.6	205.7	100.8	174.6	205.7	100.8	174.6	205.7	100.8
	(16.5)	(28.6)	(33.7)	(16.5)	(28.6)	(33.7)	(16.5)	(28.6)	(33.7)	(16.5)
	-9.3	-31.6	-77.3	-7.4	-3.2	-39.2	-3.5	-36.9	-56.5	-25.6
	-1.523	-5.165	-12.654	-1.210	-0.519	-6.411	-0.574	-6.039	-9.247	-4.184
c_O	138	142	126	138	140	132	144	158	148	144
	(22.6)	(23.2)	(20.6)	(22.6)	(22.9)	(21.6)	(23.6)	(25.9)	(24.2)	(23.6)
c_E	142.8	184.5	132.2	142.8	184.5	132.2	142.8	184.5	132.2	142.8
	(23.4)	(30.2)	(21.6)	(23.4)	(30.2)	(21.6)	(23.4)	(30.2)	(21.6)	(23.4)
	-9.4	-74.4	-12.2	-9.4	-77.3	-0.5	2.4	-49.1	33.3	2.4
	-1.540	-12.178	-1.994	-1.540	-12.656	-0.079	0.399	-8.028	5.454	0.399
N	611	611	611	611	611	611	611	611	611	611
X2	25.6	28.6	19.3	19.2	19.2	10.9	15.2	16.3	10.3	33.4
Gvalue	25.2	27.9	19.4	18.8	19.7	10.3	15.2	15.8	10.2	32.8
Gv/N	4.120	4.563	3.177	3.082	3.228	1.679	2.496	2.586	1.674	5.362
Prob	0.00001	0.00000	0.00022	0.00030	0.00019	0.01651	0.00162	0.00125	0.01671	0.00000

	51	52	53	54	55	56	57	58	59	60
a_O	124	166	129	138	175	143	119	146	140	113
	(20.3)	(27.2)	(21.1)	(22.6)	(28.6)	(23.4)	(19.5)	(23.9)	(22.9)	(18.5)
a_E	101.8	165.9	187.0	101.8	165.9	187.0	101.8	165.9	187.0	101.8
	(16.7)	(27.2)	(30.6)	(16.7)	(27.2)	(30.6)	(16.7)	(27.2)	(30.6)	(16.7)
	48.8	0.2	-95.8	83.9	18.6	-76.8	37.1	-37.3	-81.1	23.5
	7.994	0.026	-15.685	13.729	3.052	-12.565	6.069	-6.112	-13.273	3.849
t_O	167	145	219	153	130	213	172	142	221	157
	(27.3)	(23.7)	(35.8)	(25.0)	(21.3)	(34.9)	(28.2)	(23.2)	(36.2)	(25.7)
t_E	150.0	107.2	180.4	150.0	107.2	180.4	150.0	107.2	180.4	150.0
	(24.6)	(17.5)	(29.5)	(24.6)	(17.5)	(29.5)	(24.6)	(17.5)	(29.5)	(24.6)
	35.8	87.6	84.9	6.0	50.2	70.7	47.0	79.9	89.7	14.2
	5.853	14.344	13.893	0.977	8.214	11.576	7.689	13.076	14.678	2.329
g_O	159	174	103	155	173	103	156	190	98	172
	(26.0)	(28.5)	(16.9)	(25.4)	(28.3)	(16.9)	(25.5)	(31.1)	(16.0)	(28.2)
g_E	174.6	205.7	100.8	174.6	205.7	100.8	174.6	205.7	100.8	174.6
	(28.6)	(33.7)	(16.5)	(28.6)	(33.7)	(16.5)	(28.6)	(33.7)	(16.5)	(28.6)
	-29.8	-58.2	4.5	-36.9	-59.8	4.5	-35.1	-30.1	-5.5	-5.1
	-4.869	-9.521	0.739	-6.039	-9.793	0.739	-5.750	-4.925	-0.893	-0.843
c_O	161	126	160	165	133	152	164	133	152	169
	(26.4)	(20.6)	(26.2)	(27.0)	(21.8)	(24.9)	(26.8)	(21.8)	(24.9)	(27.7)
c_E	184.5	132.2	142.8	184.5	132.2	142.8	184.5	132.2	142.8	184.5
	(30.2)	(21.6)	(23.4)	(30.2)	(21.6)	(23.4)	(30.2)	(21.6)	(23.4)	(30.2)
	-43.9	-12.2	36.4	-36.9	1.5	19.0	-38.7	1.5	19.0	-29.7
	-7.189	-1.994	5.962	-6.042	0.249	3.112	-6.332	0.249	3.112	-4.864
N	611	611	611	611	611	611	611	611	611	611
X2	11.1	18.5	28.4	17.2	10.5	16.9	10.4	14.9	21.6	2.9
Gvalue	10.9	17.4	30.0	16.0	10.5	17.5	10.2	14.0	22.1	2.9
Gv/N	1.789	2.856	4.908	2.625	1.722	2.861	1.676	2.287	3.623	0.472
Prob	0.01211	0.00057	0.00000	0.00111	0.01460	0.00056	0.01664	0.00294	0.00006	0.40984

	61	62	63	64	65	66	67	68	69	70
a_O	152	149	123	164	145	120	139	160	109	160
	(24.9)	(24.4)	(20.1)	(26.8)	(23.7)	(19.6)	(22.7)	(26.2)	(17.8)	(26.2)
a_E	165.9	187.0	101.8	165.9	187.0	101.8	165.9	187.0	101.8	165.9
	(27.2)	(30.6)	(16.7)	(27.2)	(30.6)	(16.7)	(27.2)	(30.6)	(16.7)	(27.2)
	-26.6	-67.7	46.5	-3.8	-73.8	39.4	-49.2	-50.0	14.8	-11.6
	-4.360	-11.087	7.604	-0.625	-12.081	6.449	-8.055	-8.176	2.427	-1.903
t_O	124	209	168	107	211	147	134	194	172	126
	(20.3)	(34.2)	(27.5)	(17.5)	(34.5)	(24.1)	(21.9)	(31.8)	(28.2)	(20.6)
t_E	107.2	180.4	150.0	107.2	180.4	150.0	107.2	180.4	150.0	107.2
	(17.5)	(29.5)	(24.6)	(17.5)	(29.5)	(24.6)	(17.5)	(29.5)	(24.6)	(17.5)
	36.2	61.5	38.0	-0.4	66.1	-6.0	59.9	28.2	47.0	40.8
	5.917	10.061	6.216	-0.059	10.815	-0.986	9.796	4.610	7.689	6.672
g_O	194	105	184	193	101	177	208	116	166	193
	(31.8)	(17.2)	(30.1)	(31.6)	(16.5)	(29.0)	(34.0)	(19.0)	(27.2)	(31.6)
g_E	205.7	100.8	174.6	205.7	100.8	174.6	205.7	100.8	174.6	205.7
	(33.7)	(16.5)	(28.6)	(33.7)	(16.5)	(28.6)	(33.7)	(16.5)	(28.6)	(33.7)
	-22.6	8.6	19.3	-24.5	0.5	4.8	4.7	32.7	-16.8	-24.5
	-3.706	1.414	3.161	-4.014	0.076	0.793	0.771	5.345	-2.742	-4.014
c_O	141	148	136	147	154	167	130	141	164	132
	(23.1)	(24.2)	(22.3)	(24.1)	(25.2)	(27.3)	(21.3)	(23.1)	(26.8)	(21.6)
c_E	132.2	142.8	184.5	132.2	142.8	184.5	132.2	142.8	184.5	132.2
	(21.6)	(23.4)	(30.2)	(21.6)	(23.4)	(30.2)	(21.6)	(23.4)	(30.2)	(21.6)
	18.1	10.6	-83.0	31.1	23.3	-33.3	-4.4	-3.5	-38.7	-0.5
	2.960	1.738	-13.585	5.091	3.812	-5.457	-0.727	-0.581	-6.332	-0.079

N	611	611	611	611	611	611	611	611	611	611
X2	5.0	12.6	19.8	2.4	15.5	5.0	11.1	7.3	6.4	4.3
Gvalue	5.0	13.0	20.7	2.4	16.0	4.9	10.9	7.3	6.4	4.1
Gv/N	0.811	2.126	3.396	0.394	2.622	0.799	1.784	1.199	1.042	0.677
Prob	0.17513	0.00466	0.00012	0.49222	0.00112	0.18078	0.01227	0.06223	0.09519	0.24710

	71	72	73	74	75	76	77	78	79	80
a_O	152	105	158	145	126	154	158	117	152	163
	(24.9)	(17.2)	(25.9)	(23.7)	(20.6)	(25.2)	(25.9)	(19.1)	(24.9)	(26.7)
a_E	187.0	101.8	165.9	187.0	101.8	165.9	187.0	101.8	165.9	187.0
	(30.6)	(16.7)	(27.2)	(30.6)	(16.7)	(27.2)	(30.6)	(16.7)	(27.2)	(30.6)
	-63.0	6.4	-15.5	-73.8	53.7	-23.0	-53.3	32.5	-26.6	-44.8
	-10.319	1.053	-2.530	-12.081	8.783	-3.758	-8.724	5.318	-4.360	-7.338
t_O	221	167	107	206	147	114	198	162	112	195
	(36.2)	(27.3)	(17.5)	(33.7)	(24.1)	(18.7)	(32.4)	(26.5)	(18.3)	(31.9)
t_E	180.4	150.0	107.2	180.4	150.0	107.2	180.4	150.0	107.2	180.4
	(29.5)	(24.6)	(17.5)	(29.5)	(24.6)	(17.5)	(29.5)	(24.6)	(17.5)	(29.5)
	89.7	35.8	-0.4	54.6	-6.0	14.1	36.8	24.8	9.9	30.3
	14.678	5.853	-0.059	8.942	-0.986	2.302	6.028	4.066	1.613	4.962
g_O	86	172	210	100	165	205	103	171	217	100
	(14.1)	(28.2)	(34.4)	(16.4)	(27.0)	(33.6)	(16.9)	(28.0)	(35.5)	(16.4)
g_E	100.8	174.6	205.7	100.8	174.6	205.7	100.8	174.6	205.7	100.8
	(16.5)	(28.6)	(33.7)	(16.5)	(28.6)	(33.7)	(16.5)	(28.6)	(33.7)	(16.5)
	-27.3	-5.1	8.8	-1.5	-18.6	-1.3	4.5	-7.1	23.3	-1.5
	-4.461	-0.843	1.436	-0.250	-3.052	-0.215	0.739	-1.164	3.813	-0.250
c_O	152	167	136	160	173	138	152	161	130	153
	(24.9)	(27.3)	(22.3)	(26.2)	(28.3)	(22.6)	(24.9)	(26.4)	(21.3)	(25.0)
c_E	142.8	184.5	132.2	142.8	184.5	132.2	142.8	184.5	132.2	142.8
	(23.4)	(30.2)	(21.6)	(23.4)	(30.2)	(21.6)	(23.4)	(30.2)	(21.6)	(23.4)
	19.0	-33.3	7.6	36.4	-22.3	11.8	19.0	-43.9	-4.4	21.1
	3.112	-5.457	1.248	5.962	-3.654	1.926	3.112	-7.189	-0.727	3.461
N	611	611	611	611	611	611	611	611	611	611
X2	18.4	3.7	0.6	15.2	7.0	1.5	6.9	6.3	2.0	5.0
Gvalue	18.4	3.7	0.6	15.7	6.7	1.6	7.1	6.3	2.1	5.1
Gv/N	3.010	0.606	0.095	2.572	1.091	0.254	1.154	1.030	0.338	0.834
Prob	0.00037	0.29514	0.90068	0.00130	0.08339	0.67031	0.07027	0.09813	0.55845	0.16480

	81	82	83	84	85	86	87	88	89	90
a_O	128	153	154	85	175	165	108	160	188	98
	(20.9)	(25.0)	(25.2)	(13.9)	(28.6)	(27.0)	(17.7)	(26.2)	(30.8)	(16.0)
a_E	101.8	165.9	187.0	101.8	165.9	187.0	101.8	165.9	187.0	101.8
	(16.7)	(27.2)	(30.6)	(16.7)	(27.2)	(30.6)	(16.7)	(27.2)	(30.6)	(16.7)
	58.5	-24.8	-59.9	-30.7	18.6	-41.4	12.7	-11.6	1.9	-7.5
	9.582	-4.060	-9.796	-5.027	3.052	-6.769	2.079	-1.903	0.318	-1.230
t_O	169	113	193	182	113	197	175	114	202	177
	(27.7)	(18.5)	(31.6)	(29.8)	(18.5)	(32.2)	(28.6)	(18.7)	(33.1)	(29.0)
t_E	150.0	107.2	180.4	150.0	107.2	180.4	150.0	107.2	180.4	150.0
	(24.6)	(17.5)	(29.5)	(24.6)	(17.5)	(29.5)	(24.6)	(17.5)	(29.5)	(24.6)
	40.2	11.9	26.0	70.3	11.9	34.6	53.9	14.1	45.7	58.5
	6.581	1.956	4.260	11.503	1.956	5.671	8.813	2.302	7.472	9.573
g_O	143	193	93	172	201	83	167	208	90	167
	(23.4)	(31.6)	(15.2)	(28.2)	(32.9)	(13.6)	(27.3)	(34.0)	(14.7)	(27.3)
g_E	174.6	205.7	100.8	174.6	205.7	100.8	174.6	205.7	100.8	174.6
	(28.6)	(33.7)	(16.5)	(28.6)	(33.7)	(16.5)	(28.6)	(33.7)	(16.5)	(28.6)
	-57.1	-24.5	-14.9	-5.1	-9.2	-32.2	-14.9	4.7	-20.3	-14.9
	-9.344	-4.014	-2.442	-0.843	-1.508	-5.270	-2.431	0.771	-3.329	-2.431
c_O	171	152	171	172	122	166	161	129	131	169
	(28.0)	(24.9)	(28.0)	(28.2)	(20.0)	(27.2)	(26.4)	(21.1)	(21.4)	(27.7)
c_E	184.5	132.2	142.8	184.5	132.2	142.8	184.5	132.2	142.8	184.5
	(30.2)	(21.6)	(23.4)	(30.2)	(21.6)	(23.4)	(30.2)	(21.6)	(23.4)	(30.2)
	-26.0	42.3	61.7	-24.2	-19.7	50.0	-43.9	-6.4	-22.6	-29.7
	-4.263	6.929	10.093	-3.959	-3.219	8.186	-7.189	-1.048	-3.694	-4.864
N	611	611	611	611	611	611	611	611	611	611
X2	15.8	5.1	12.9	10.5	1.7	11.0	7.9	0.8	4.7	6.6
Gvalue	15.6	5.0	12.9	10.2	1.7	11.1	7.8	0.7	4.7	6.4
Gv/N	2.558	0.811	2.115	1.674	0.281	1.817	1.273	0.122	0.767	1.048
Prob	0.00135	0.17513	0.00480	0.01673	0.63268	0.01118	0.05087	0.86248	0.19641	0.09357

	91	92	93	94	95	96	97	98	99	100
a_O	153	168	119	165	177	105	185	179	108	161
	(25.0)	(27.5)	(19.5)	(27.0)	(29.0)	(17.2)	(30.3)	(29.3)	(17.7)	(26.4)
a_E	165.9	187.0	101.8	165.9	187.0	101.8	165.6	186.7	101.7	165.6
	(27.2)	(30.6)	(16.7)	(27.2)	(30.6)	(16.7)	(27.2)	(30.6)	(16.7)	(27.2)
	-24.8	-36.1	37.1	-1.8	-19.5	6.4	40.9	-15.1	13.1	-9.2
	-4.060	-5.901	6.069	-0.300	-3.194	1.053	6.702	-2.480	2.140	-1.502
t_O	125	171	158	123	193	147	95	186	147	129
	(20.5)	(28.0)	(25.9)	(20.1)	(31.6)	(24.1)	(15.6)	(30.5)	(24.1)	(21.1)
t_E	107.2	180.4	150.0	107.2	180.4	150.0	107.0	180.1	149.8	107.0
	(17.5)	(29.5)	(24.6)	(17.5)	(29.5)	(24.6)	(17.5)	(29.5)	(24.6)	(17.5)
	38.5	-18.3	16.3	33.9	26.0	-6.0	-22.6	11.9	-5.5	48.2
	6.293	-3.000	2.672	5.543	4.260	-0.986	-3.706	1.959	-0.909	7.907
g_O	205	85	158	186	88	169	199	88	170	178
	(33.6)	(13.9)	(25.9)	(30.4)	(14.4)	(27.7)	(32.6)	(14.4)	(27.9)	(29.2)
g_E	205.7	100.8	174.6	205.7	100.8	174.6	205.3	100.6	174.3	205.3
	(33.7)	(16.5)	(28.6)	(33.7)	(16.5)	(28.6)	(33.7)	(16.5)	(28.6)	(33.7)
	-1.3	-28.9	-31.6	-37.4	-23.8	-11.0	-12.4	-23.6	-8.5	-50.8
	-0.215	-4.735	-5.165	-6.117	-3.903	-1.801	-2.041	-3.862	-1.395	-8.334
c_O	128	187	176	137	153	190	131	157	185	142
	(20.9)	(30.6)	(28.8)	(22.4)	(25.0)	(31.1)	(21.5)	(25.7)	(30.3)	(23.3)
c_E	132.2	142.8	184.5	132.2	142.8	184.5	132.0	142.6	184.2	132.0
	(21.6)	(23.4)	(30.2)	(21.6)	(23.4)	(30.2)	(21.6)	(23.4)	(30.2)	(21.6)
	-8.3	100.9	-16.7	9.7	21.1	11.1	-2.0	30.3	1.5	20.7
	-1.366	16.513	-2.727	1.586	3.461	1.817	-0.334	4.970	0.253	3.391
N	611	611	611	611	611	611	610	610	610	610
X2	4.1	18.6	5.3	4.4	3.8	0.5	3.8	3.6	0.6	9.0
Gvalue	4.0	17.6	5.2	4.3	3.8	0.5	3.8	3.6	0.5	8.9
Gv/N	0.652	2.877	0.849	0.711	0.624	0.082	0.620	0.587	0.090	1.462
Prob	0.26323	0.00054	0.15848	0.22659	0.28264	0.91859	0.28594	0.31046	0.90779	0.03043

Bbro_RB50
5UTR:

	100	99	98	97	96	95	94	93	92	91
a_O	232	193	197	234	171	197	201	167	199	233
	(21.1)	(17.5)	(17.9)	(21.3)	(15.5)	(17.9)	(18.3)	(15.2)	(18.1)	(21.2)
a_E	202.4	202.4	202.4	202.4	202.4	202.4	202.4	202.4	202.4	202.4
	(18.4)	(18.4)	(18.4)	(18.4)	(18.4)	(18.4)	(18.4)	(18.4)	(18.4)	(18.4)
	63.3	-18.4	-10.7	67.9	-57.7	-10.7	-2.8	-64.2	-6.8	65.6
	5.755	-1.671	-0.970	6.170	-5.243	-0.970	-0.255	-5.839	-0.615	5.962
t_O	214	198	189	208	189	171	216	184	177	228
	(19.5)	(18.0)	(17.2)	(18.9)	(17.2)	(15.5)	(19.6)	(16.7)	(16.1)	(20.7)
t_E	203.8	203.8	203.8	203.8	203.8	203.8	203.8	203.8	203.8	203.8
	(18.5)	(18.5)	(18.5)	(18.5)	(18.5)	(18.5)	(18.5)	(18.5)	(18.5)	(18.5)
	20.9	-11.4	-28.5	8.5	-28.5	-60.0	25.2	-37.6	-49.9	51.2
	1.904	-1.036	-2.587	0.775	-2.587	-5.453	2.287	-3.416	-4.534	4.656
g_O	328	331	382	320	350	357	346	350	361	313
	(29.8)	(30.1)	(34.7)	(29.1)	(31.8)	(32.5)	(31.5)	(31.8)	(32.8)	(28.5)
g_E	318.7	318.7	318.7	318.7	318.7	318.7	318.7	318.7	318.7	318.7
	(29.0)	(29.0)	(29.0)	(29.0)	(29.0)	(29.0)	(29.0)	(29.0)	(29.0)	(29.0)
	18.8	25.0	138.4	2.6	65.5	81.0	56.8	65.5	89.9	-11.3
	1.712	2.275	12.579	0.233	5.958	7.362	5.166	5.958	8.176	-1.031
c_O	326	378	332	338	390	375	337	399	363	326
	(29.6)	(34.4)	(30.2)	(30.7)	(35.5)	(34.1)	(30.6)	(36.3)	(33.0)	(29.6)
c_E	375.1	375.1	375.1	375.1	375.1	375.1	375.1	375.1	375.1	375.1
	(34.1)	(34.1)	(34.1)	(34.1)	(34.1)	(34.1)	(34.1)	(34.1)	(34.1)	(34.1)
	-91.5	5.8	-81.0	-70.4	30.4	-0.2	-72.2	49.3	-23.8	-91.5
	-8.314	0.531	-7.366	-6.399	2.764	-0.016	-6.561	4.483	-2.162	-8.314
N	1100	1100	1100	1100	1100	1100	1100	1100	1100	1100
X2	11.5	1.1	18.7	8.7	9.6	10.0	6.9	12.7	9.6	14.0
Gvalue	11.6	1.1	18.2	8.6	9.8	10.2	7.0	13.0	9.5	14.0
Gv/N	1.057	0.100	1.655	0.780	0.891	0.923	0.637	1.186	0.865	1.273
Prob	0.00878	0.77717	0.00040	0.03545	0.02029	0.01733	0.07172	0.00454	0.02320	0.00290

	90	89	88	87	86	85	84	83	82	81
a_O	161	191	197	164	175	221	209	191	211	160
	(14.6)	(17.4)	(17.9)	(14.9)	(15.9)	(20.1)	(19.0)	(17.4)	(19.2)	(14.5)
a_E	202.4	202.4	202.4	202.4	202.4	202.4	202.4	202.4	202.4	202.4
	(18.4)	(18.4)	(18.4)	(18.4)	(18.4)	(18.4)	(18.4)	(18.4)	(18.4)	(18.4)
	-73.7	-22.2	-10.7	-69.0	-50.9	38.8	13.4	-22.2	17.5	-75.2
	-6.700	-2.015	-0.970	-6.275	-4.630	3.531	1.217	-2.015	1.594	-6.840
t_O	189	155	213	188	182	202	163	170	208	202
	(17.2)	(14.1)	(19.4)	(17.1)	(16.5)	(18.4)	(14.8)	(15.5)	(18.9)	(18.4)
t_E	203.8	203.8	203.8	203.8	203.8	203.8	203.8	203.8	203.8	203.8
	(18.5)	(18.5)	(18.5)	(18.5)	(18.5)	(18.5)	(18.5)	(18.5)	(18.5)	(18.5)
	-28.5	-84.8	18.9	-30.3	-41.1	-3.5	-72.8	-61.6	8.5	-3.5
	-2.587	-7.711	1.714	-2.755	-3.740	-0.322	-6.618	-5.602	0.775	-0.322
g_O	375	371	353	370	384	344	353	377	337	362
	(34.1)	(33.7)	(32.1)	(33.6)	(34.9)	(31.3)	(32.1)	(34.3)	(30.6)	(32.9)
g_E	318.7	318.7	318.7	318.7	318.7	318.7	318.7	318.7	318.7	318.7
	(29.0)	(29.0)	(29.0)	(29.0)	(29.0)	(29.0)	(29.0)	(29.0)	(29.0)	(29.0)
	122.0	112.7	72.1	110.4	143.1	52.5	72.1	126.6	37.6	92.2
	11.087	10.246	6.556	10.036	13.009	4.774	6.556	11.511	3.417	8.381
c_O	375	383	337	378	359	333	375	362	344	376
	(34.1)	(34.8)	(30.6)	(34.4)	(32.6)	(30.3)	(34.1)	(32.9)	(31.3)	(34.2)
c_E	375.1	375.1	375.1	375.1	375.1	375.1	375.1	375.1	375.1	375.1
	(34.1)	(34.1)	(34.1)	(34.1)	(34.1)	(34.1)	(34.1)	(34.1)	(34.1)	(34.1)
	-0.2	16.0	-72.2	5.8	-31.5	-79.3	-0.2	-25.7	-59.5	1.8
	-0.016	1.453	-6.561	0.531	-2.862	-7.206	-0.016	-2.338	-5.412	0.166
N	1100	1100	1100	1100	1100	1100	1100	1100	1100	1100
X2	19.5	21.1	8.1	16.8	20.1	8.5	12.1	17.4	4.1	14.8
Gvalue	19.6	21.7	8.1	16.9	19.5	8.5	12.5	17.1	4.1	15.2
Gv/N	1.783	1.973	0.739	1.538	1.777	0.776	1.140	1.556	0.375	1.384
Prob	0.00020	0.00008	0.04351	0.00073	0.00021	0.03612	0.00574	0.00067	0.24812	0.00163

	80	79	78	77	76	75	74	73	72	71
a_O	202	220	176	188	222	186	209	238	196	220
	(18.4)	(20.0)	(16.0)	(17.1)	(20.2)	(16.9)	(19.0)	(21.6)	(17.8)	(20.0)
a_E	202.4	202.4	202.4	202.4	202.4	202.4	202.4	202.4	202.4	202.4
	(18.4)	(18.4)	(18.4)	(18.4)	(18.4)	(18.4)	(18.4)	(18.4)	(18.4)	(18.4)
	-0.8	36.7	-49.2	-27.8	41.0	-31.5	13.4	77.1	-12.6	36.7
	-0.074	3.333	-4.474	-2.524	3.729	-2.859	1.217	7.009	-1.147	3.333
t_O	184	215	213	202	213	194	179	195	181	181
	(16.7)	(19.5)	(19.4)	(18.4)	(19.4)	(17.6)	(16.3)	(17.7)	(16.5)	(16.5)
t_E	203.8	203.8	203.8	203.8	203.8	203.8	203.8	203.8	203.8	203.8
	(18.5)	(18.5)	(18.5)	(18.5)	(18.5)	(18.5)	(18.5)	(18.5)	(18.5)	(18.5)
	-37.6	23.0	18.9	-3.5	18.9	-19.1	-46.4	-17.2	-42.9	-42.9
	-3.416	2.095	1.714	-0.322	1.714	-1.735	-4.220	-1.561	-3.901	-3.901
g_O	372	333	343	360	330	347	383	350	324	347
	(33.8)	(30.3)	(31.2)	(32.7)	(30.0)	(31.5)	(34.8)	(31.8)	(29.5)	(31.5)
g_E	318.7	318.7	318.7	318.7	318.7	318.7	318.7	318.7	318.7	318.7
	(29.0)	(29.0)	(29.0)	(29.0)	(29.0)	(29.0)	(29.0)	(29.0)	(29.0)	(29.0)
	115.0	29.2	50.4	87.7	23.0	59.0	140.7	65.5	10.6	59.0
	10.455	2.654	4.579	7.972	2.087	5.363	12.794	5.958	0.968	5.363
c_O	342	332	368	350	335	373	329	317	399	352
	(31.1)	(30.2)	(33.5)	(31.8)	(30.5)	(33.9)	(29.9)	(28.8)	(36.3)	(32.0)
c_E	375.1	375.1	375.1	375.1	375.1	375.1	375.1	375.1	375.1	375.1
	(34.1)	(34.1)	(34.1)	(34.1)	(34.1)	(34.1)	(34.1)	(34.1)	(34.1)	(34.1)
	-63.2	-81.0	-14.0	-48.5	-75.7	-4.2	-86.3	-106.7	49.3	-44.7
	-5.743	-7.366	-1.277	-4.406	-6.885	-0.379	-7.843	-9.698	4.483	-4.066
N	1100	1100	1100	1100	1100	1100	1100	1100	1100	1100
X2	13.7	7.7	5.8	8.1	7.0	4.3	21.9	18.7	4.4	8.0
Gvalue	13.4	7.9	6.0	7.9	7.1	4.3	21.4	18.8	4.4	8.0
Gv/N	1.222	0.716	0.542	0.719	0.644	0.390	1.949	1.707	0.403	0.729
Prob	0.00377	0.04867	0.11374	0.04784	0.06910	0.23134	0.00009	0.00030	0.21844	0.04554

	70	69	68	67	66	65	64	63	62	61
a_O	208	197	197	226	196	179	234	200	215	194
	(18.9)	(17.9)	(17.9)	(20.5)	(17.8)	(16.3)	(21.3)	(18.2)	(19.5)	(17.6)
a_E	202.4	202.4	202.4	202.4	202.4	202.4	202.4	202.4	202.4	202.4
	(18.4)	(18.4)	(18.4)	(18.4)	(18.4)	(18.4)	(18.4)	(18.4)	(18.4)	(18.4)
	11.3	-10.7	-10.7	49.8	-12.6	-44.0	67.9	-4.8	25.9	-16.5
	1.030	-0.970	-0.970	4.530	-1.147	-4.000	6.170	-0.436	2.359	-1.497
t_O	213	191	179	225	184	190	202	212	206	235
	(19.4)	(17.4)	(16.3)	(20.5)	(16.7)	(17.3)	(18.4)	(19.3)	(18.7)	(21.4)

```
t_E        203.8   203.8   203.8   203.8   203.8   203.8   203.8   203.8   203.8   203.8
         ( 18.5) ( 18.5) ( 18.5) ( 18.5) ( 18.5) ( 18.5) ( 18.5) ( 18.5) ( 18.5) ( 18.5)
            18.9   -24.7   -46.4    44.6   -37.6   -26.6    -3.5    16.8     4.5    67.0
           1.714  -2.249  -4.220   4.052  -3.416  -2.419  -0.322   1.524   0.406   6.091
g_O          328     355     370     329     336     365     347     310     320     344
         ( 29.8) ( 32.3) ( 33.6) ( 29.9) ( 30.5) ( 33.2) ( 31.5) ( 28.2) ( 29.1) ( 31.3)
g_E        318.7   318.7   318.7   318.7   318.7   318.7   318.7   318.7   318.7   318.7
         ( 29.0) ( 29.0) ( 29.0) ( 29.0) ( 29.0) ( 29.0) ( 29.0) ( 29.0) ( 29.0) ( 29.0)
            18.8    76.5   110.4    20.9    35.5    99.0    59.0   -17.2     2.6    52.5
           1.712   6.958  10.036   1.899   3.225   8.998   5.363  -1.564   0.233   4.774
c_O          351     357     354     320     384     366     317     378     359     327
         ( 31.9) ( 32.5) ( 32.2) ( 29.1) ( 34.9) ( 33.3) ( 28.8) ( 34.4) ( 32.6) ( 29.7)
c_E        375.1   375.1   375.1   375.1   375.1   375.1   375.1   375.1   375.1   375.1
         ( 34.1) ( 34.1) ( 34.1) ( 34.1) ( 34.1) ( 34.1) ( 34.1) ( 34.1) ( 34.1) ( 34.1)
           -46.6   -35.3   -41.0  -101.7    18.0   -18.0  -106.7     5.8   -31.5   -89.7
          -4.236  -3.208  -3.725  -9.242   1.639  -1.633  -9.698   0.531  -2.862  -8.158

N           1100    1100    1100    1100    1100    1100    1100    1100    1100    1100
X2           2.4     5.9    12.6    13.4     3.3    10.6    16.5     0.6     1.5    13.3
Gvalue       2.4     5.8    12.3    13.6     3.3    10.4    16.6     0.6     1.5    13.3
Gv/N       0.219   0.530   1.122   1.239   0.302   0.946   1.513   0.056   0.136   1.210
Prob     0.49122 0.12003 0.00631 0.00345 0.34475 0.01538 0.00084 0.89200 0.68299 0.00401
         --------------------------------------------------------------------------------
              60      59      58      57      56      55      54      53      52      51
         --------------------------------------------------------------------------------
a_O          178     190     227     165     196     217     181     184     217     178
         ( 16.2) ( 17.3) ( 20.6) ( 15.0) ( 17.8) ( 19.7) ( 16.5) ( 16.7) ( 19.7) ( 16.2)
a_E        202.4   202.4   202.4   202.4   202.4   202.4   202.4   202.4   202.4   202.4
         ( 18.4) ( 18.4) ( 18.4) ( 18.4) ( 18.4) ( 18.4) ( 18.4) ( 18.4) ( 18.4) ( 18.4)
           -45.8   -24.0    52.1   -67.4   -12.6    30.2   -40.5   -35.1    30.2   -45.8
          -4.159  -2.186   4.732  -6.130  -1.147   2.746  -3.679  -3.190   2.746  -4.159
t_O          213     192     235     211     187     219     206     175     213     179
         ( 19.4) ( 17.5) ( 21.4) ( 19.2) ( 17.0) ( 19.9) ( 18.7) ( 15.9) ( 19.4) ( 16.3)
t_E        203.8   203.8   203.8   203.8   203.8   203.8   203.8   203.8   203.8   203.8
         ( 18.5) ( 18.5) ( 18.5) ( 18.5) ( 18.5) ( 18.5) ( 18.5) ( 18.5) ( 18.5) ( 18.5)
            18.9   -22.9    67.0    14.7   -32.1    31.5     4.5   -53.3    18.9   -46.4
           1.714  -2.079   6.091   1.336  -2.922   2.868   0.406  -4.844   1.714  -4.220
g_O          340     331     304     352     377     301     346     369     323     367
         ( 30.9) ( 30.1) ( 27.6) ( 32.0) ( 34.3) ( 27.4) ( 31.5) ( 33.5) ( 29.4) ( 33.4)
g_E        318.7   318.7   318.7   318.7   318.7   318.7   318.7   318.7   318.7   318.7
         ( 29.0) ( 29.0) ( 29.0) ( 29.0) ( 29.0) ( 29.0) ( 29.0) ( 29.0) ( 29.0) ( 29.0)
            44.0    25.0   -28.7    69.9   126.6   -34.4    56.8   108.1     8.6   103.5
           3.995   2.275  -2.614   6.356  11.511  -3.131   5.166   9.828   0.783   9.412
c_O          369     387     334     372     340     363     367     372     347     376
         ( 33.5) ( 35.2) ( 30.4) ( 33.8) ( 30.9) ( 33.0) ( 33.4) ( 33.8) ( 31.5) ( 34.2)
c_E        375.1   375.1   375.1   375.1   375.1   375.1   375.1   375.1   375.1   375.1
         ( 34.1) ( 34.1) ( 34.1) ( 34.1) ( 34.1) ( 34.1) ( 34.1) ( 34.1) ( 34.1) ( 34.1)
           -12.1    24.2   -77.5    -6.2   -66.8   -23.8   -16.0    -6.2   -54.0     1.8
          -1.098   2.199  -7.046  -0.559  -6.072  -2.162  -1.455  -0.559  -4.911   0.166

N           1100    1100    1100    1100    1100    1100    1100    1100    1100    1100
X2           4.9     2.3    13.0    10.7    15.5     3.6     4.8    13.7     3.6    13.3
Gvalue       5.0     2.3    12.8    11.0    15.1     3.5     4.8    13.6     3.7    13.2
Gv/N       0.452   0.210   1.163   1.002   1.371   0.321   0.438   1.234   0.332   1.199
Prob     0.17398 0.51007 0.00510 0.01160 0.00175 0.31630 0.18547 0.00355 0.30137 0.00425
         --------------------------------------------------------------------------------
              50      49      48      47      46      45      44      43      42      41
         --------------------------------------------------------------------------------
a_O          208     212     187     198     181     213     205     209     189     201
         ( 18.9) ( 19.3) ( 17.0) ( 18.0) ( 16.5) ( 19.4) ( 18.6) ( 19.0) ( 17.2) ( 18.3)
a_E        202.4   202.4   202.4   202.4   202.4   202.4   202.4   202.4   202.4   202.4
         ( 18.4) ( 18.4) ( 18.4) ( 18.4) ( 18.4) ( 18.4) ( 18.4) ( 18.4) ( 18.4) ( 18.4)
            11.3    19.6   -29.6    -8.7   -40.5    21.7     5.2    13.4   -25.9    -2.8
           1.030   1.784  -2.692  -0.793  -3.679   1.975   0.474   1.217  -2.356  -0.255
t_O          193     228     192     193     222     191     191     202     205     193
         ( 17.5) ( 20.7) ( 17.5) ( 17.5) ( 20.2) ( 17.4) ( 17.4) ( 18.4) ( 18.6) ( 17.5)
t_E        203.8   203.8   203.8   203.8   203.8   203.8   203.8   203.8   203.8   203.8
         ( 18.5) ( 18.5) ( 18.5) ( 18.5) ( 18.5) ( 18.5) ( 18.5) ( 18.5) ( 18.5) ( 18.5)
           -21.0    51.2   -22.9   -21.0    38.0   -24.7   -24.7    -3.5     2.4   -21.0
          -1.907   4.656  -2.079  -1.907   3.457  -2.249  -2.249  -0.322   0.222  -1.907
g_O          336     347     319     344     352     338     331     313     319     328
         ( 30.5) ( 31.5) ( 29.0) ( 31.3) ( 32.0) ( 30.7) ( 30.1) ( 28.5) ( 29.0) ( 29.8)
g_E        318.7   318.7   318.7   318.7   318.7   318.7   318.7   318.7   318.7   318.7
         ( 29.0) ( 29.0) ( 29.0) ( 29.0) ( 29.0) ( 29.0) ( 29.0) ( 29.0) ( 29.0) ( 29.0)
            35.5    59.0     0.6    52.5    69.9    39.7    25.0   -11.3     0.6    18.8
           3.225   5.363   0.051   4.774   6.356   3.609   2.275  -1.031   0.051   1.712
c_O          363     313     402     365     345     358     373     376     387     378
         ( 33.0) ( 28.5) ( 36.5) ( 33.2) ( 31.4) ( 32.5) ( 33.9) ( 34.2) ( 35.2) ( 34.4)
c_E        375.1   375.1   375.1   375.1   375.1   375.1   375.1   375.1   375.1   375.1
         ( 34.1) ( 34.1) ( 34.1) ( 34.1) ( 34.1) ( 34.1) ( 34.1) ( 34.1) ( 34.1) ( 34.1)
           -23.8  -113.3    55.7   -19.9   -57.7   -33.4    -4.2     1.8    24.2     5.8
          -2.162 -10.298   5.064  -1.810  -5.245  -3.035  -0.379   0.166   2.199   0.531

N           1100    1100    1100    1100    1100    1100    1100    1100    1100    1100
X2           2.1    16.1     3.8     2.9     9.8     3.3     1.3     0.3     1.3     0.9
Gvalue       2.0    16.6     3.8     2.9     9.8     3.3     1.3     0.3     1.3     0.9
Gv/N       0.186   1.505   0.344   0.264   0.888   0.300   0.121   0.030   0.117   0.080
Prob     0.56253 0.00087 0.28565 0.40644 0.02060 0.34797 0.72187 0.95364 0.73139 0.83011
         --------------------------------------------------------------------------------
              40      39      38      37      36      35      34      33      32      31
         --------------------------------------------------------------------------------
a_O          230     197     185     219     198     217     244     172     196     215
         ( 20.9) ( 17.9) ( 16.8) ( 19.9) ( 18.0) ( 19.7) ( 22.2) ( 15.6) ( 17.8) ( 19.5)
a_E        202.4   202.4   202.4   202.4   202.4   202.4   202.4   202.4   202.4   202.4
         ( 18.4) ( 18.4) ( 18.4) ( 18.4) ( 18.4) ( 18.4) ( 18.4) ( 18.4) ( 18.4) ( 18.4)
            58.8   -10.7   -33.3    34.5    -8.7    30.2    91.2   -56.0   -12.6    25.9
           5.344  -0.970  -3.025   3.137  -0.793   2.746   8.290  -5.091  -1.147   2.359
t_O          226     211     188     199     189     211     243     224     191     234
         ( 20.5) ( 19.2) ( 17.1) ( 18.1) ( 17.2) ( 19.2) ( 22.1) ( 20.4) ( 17.4) ( 21.3)
t_E        203.8   203.8   203.8   203.8   203.8   203.8   203.8   203.8   203.8   203.8
         ( 18.5) ( 18.5) ( 18.5) ( 18.5) ( 18.5) ( 18.5) ( 18.5) ( 18.5) ( 18.5) ( 18.5)
            46.8    14.7   -30.3    -9.4   -28.5    14.7    85.5    42.4   -24.7    64.7
           4.253   1.336  -2.755  -0.859  -2.587   1.336   7.777   3.853  -2.249   5.883
g_O          314     328     348     312     350     303     269     328     341     301
         ( 28.5) ( 29.8) ( 31.6) ( 28.4) ( 31.8) ( 27.5) ( 24.5) ( 29.8) ( 31.0) ( 27.4)
g_E        318.7   318.7   318.7   318.7   318.7   318.7   318.7   318.7   318.7   318.7
         ( 29.0) ( 29.0) ( 29.0) ( 29.0) ( 29.0) ( 29.0) ( 29.0) ( 29.0) ( 29.0) ( 29.0)
            -9.4    18.8    61.2   -13.3    65.5   -30.7   -91.2    18.8    46.1   -34.4
          -0.852   1.712   5.561  -1.209   5.958  -2.787  -8.295   1.712   4.189  -3.131
c_O          330     364     379     370     363     369     344     376     372     350
         ( 30.0) ( 33.1) ( 34.5) ( 33.6) ( 33.0) ( 33.5) ( 31.3) ( 34.2) ( 33.8) ( 31.8)
c_E        375.1   375.1   375.1   375.1   375.1   375.1   375.1   375.1   375.1   375.1
```

```
        ( 34.1) ( 34.1) ( 34.1) ( 34.1) ( 34.1) ( 34.1) ( 34.1) ( 34.1) ( 34.1) ( 34.1)
          -84.5   -21.8     7.9   -10.1   -23.8   -12.1   -59.5     1.8    -6.2   -48.5
         -7.684  -1.986   0.715  -0.919  -2.162  -1.098  -5.412   0.166  -0.559  -4.406

N          1100    1100    1100    1100    1100    1100    1100    1100    1100    1100
X2         11.7     1.0     5.5     1.7     4.6     2.2    26.4     6.8     2.6     7.9
Gvalue     11.7     1.0     5.4     1.6     4.6     2.2    26.0     7.0     2.6     7.8
Gv/N      1.060   0.091   0.495   0.150   0.415   0.197   2.361   0.639   0.234   0.706
Prob    0.00864 0.80185 0.14173 0.64830 0.20667 0.53829 0.00001 0.07097 0.46244 0.05116
        -------------------------------------------------------------------------------
             30      29      28      27      26      25      24      23      22      21
        -------------------------------------------------------------------------------
a_O         193     171     203     188     189     182     180     193     202     200
        ( 17.5) ( 15.5) ( 18.5) ( 17.1) ( 17.2) ( 16.5) ( 16.4) ( 17.5) ( 18.4) ( 18.2)
a_E       202.4   202.4   202.4   202.4   202.4   202.4   202.4   202.4   202.4   202.4
        ( 18.4) ( 18.4) ( 18.4) ( 18.4) ( 18.4) ( 18.4) ( 18.4) ( 18.4) ( 18.4) ( 18.4)
          -18.4   -57.7     1.2   -27.8   -25.9   -38.7   -42.2   -18.4    -0.8    -4.8
         -1.671  -5.243   0.107  -2.524  -2.356  -3.517  -3.840  -1.671  -0.074  -0.436
t_O         210     217     230     185     213     230     197     205     246     220
        ( 19.1) ( 19.7) ( 20.9) ( 16.8) ( 19.4) ( 20.9) ( 17.9) ( 18.6) ( 22.4) ( 20.0)
t_E       203.8   203.8   203.8   203.8   203.8   203.8   203.8   203.8   203.8   203.8
        ( 18.5) ( 18.5) ( 18.5) ( 18.5) ( 18.5) ( 18.5) ( 18.5) ( 18.5) ( 18.5) ( 18.5)
           12.6    27.3    55.7   -35.8    18.9    55.7   -13.3     2.4    92.6    33.7
          1.148   2.480   5.062  -3.252   1.714   5.062  -1.212   0.222   8.422   3.063
g_O         306     342     290     353     311     297     326     282     275     308
        ( 27.8) ( 31.1) ( 26.4) ( 32.1) ( 28.3) ( 27.0) ( 29.6) ( 25.6) ( 25.0) ( 28.0)
g_E       318.7   318.7   318.7   318.7   318.7   318.7   318.7   318.7   318.7   318.7
        ( 29.0) ( 29.0) ( 29.0) ( 29.0) ( 29.0) ( 29.0) ( 29.0) ( 29.0) ( 29.0) ( 29.0)
          -24.9    48.2   -54.8    72.1   -15.3   -41.9    14.7   -69.0   -81.1   -21.1
         -2.266   4.384  -4.979   6.556  -1.386  -3.811   1.339  -6.276  -7.377  -1.916
c_O         391     370     377     374     387     391     397     420     377     372
        ( 35.5) ( 33.6) ( 34.3) ( 34.0) ( 35.2) ( 35.5) ( 36.1) ( 38.2) ( 34.3) ( 33.8)
c_E       375.1   375.1   375.1   375.1   375.1   375.1   375.1   375.1   375.1   375.1
        ( 34.1) ( 34.1) ( 34.1) ( 34.1) ( 34.1) ( 34.1) ( 34.1) ( 34.1) ( 34.1) ( 34.1)
           32.5   -10.1     3.8    -2.2    24.2    32.5    45.1    95.0     3.8    -6.2
          2.953  -0.919   0.348  -0.198   2.199   2.953   4.098   8.636   0.348  -0.559

N          1100    1100    1100    1100    1100    1100    1100    1100    1100    1100
X2          1.8     7.5     6.0     6.4     1.9     7.6     4.2    10.1    14.8     1.7
Gvalue      1.8     7.7     5.9     6.4     1.9     7.5     4.2    10.0    14.5     1.7
Gv/N      0.165   0.702   0.538   0.582   0.171   0.686   0.384   0.912   1.318   0.152
Prob    0.61224 0.05223 0.11564 0.09360 0.59715 0.05631 0.23798 0.01832 0.00230 0.64204
        -------------------------------------------------------------------------------
             20      19      18      17      16      15      14      13      12      11
        -------------------------------------------------------------------------------
a_O         201     192     178     228     198     182     223     221     231     277
        ( 18.3) ( 17.5) ( 16.2) ( 20.7) ( 18.0) ( 16.5) ( 20.3) ( 20.1) ( 21.0) ( 25.2)
a_E       202.4   202.4   202.4   202.4   202.4   202.4   202.4   202.4   202.4   202.4
        ( 18.4) ( 18.4) ( 18.4) ( 18.4) ( 18.4) ( 18.4) ( 18.4) ( 18.4) ( 18.4) ( 18.4)
           -2.8   -20.3   -45.8    54.3    -8.7   -38.7    43.2    38.8    61.0   173.8
         -0.255  -1.843  -4.159   4.935  -0.793  -3.517   3.928   3.531   5.549  15.800
t_O         239     228     209     235     256     221     220     239     226     202
        ( 21.7) ( 20.7) ( 19.0) ( 21.4) ( 23.3) ( 20.1) ( 20.0) ( 21.7) ( 20.5) ( 18.4)
t_E       203.8   203.8   203.8   203.8   203.8   203.8   203.8   203.8   203.8   203.8
        ( 18.5) ( 18.5) ( 18.5) ( 18.5) ( 18.5) ( 18.5) ( 18.5) ( 18.5) ( 18.5) ( 18.5)
           76.2    51.2    10.6    67.0   116.8    35.9    33.7    76.2    46.8    -3.5
          6.928   4.656   0.961   6.091  10.619   3.260   3.063   6.928   4.253  -0.322
g_O         267     229     221     231     228     265     228     211     275     229
        ( 24.3) ( 20.8) ( 20.1) ( 21.0) ( 20.7) ( 24.1) ( 20.7) ( 19.2) ( 25.0) ( 20.8)
g_E       318.7   318.7   318.7   318.7   318.7   318.7   318.7   318.7   318.7   318.7
        ( 29.0) ( 29.0) ( 29.0) ( 29.0) ( 29.0) ( 29.0) ( 29.0) ( 29.0) ( 29.0) ( 29.0)
          -94.6  -151.4  -161.8  -148.7  -152.7   -97.8  -152.7  -174.1   -81.1  -151.4
         -8.596 -13.765 -14.713 -13.520 -13.886  -8.894 -13.886 -15.823  -7.377 -13.765
c_O         393     451     492     406     418     432     429     429     368     392
        ( 35.7) ( 41.0) ( 44.7) ( 36.9) ( 38.0) ( 39.3) ( 39.0) ( 39.0) ( 33.5) ( 35.6)
c_E       375.1   375.1   375.1   375.1   375.1   375.1   375.1   375.1   375.1   375.1
        ( 34.1) ( 34.1) ( 34.1) ( 34.1) ( 34.1) ( 34.1) ( 34.1) ( 34.1) ( 34.1) ( 34.1)
           36.7   166.2   267.0    64.3    90.6   122.0   115.2   115.2   -14.0    34.6
          3.333  15.113  24.270   5.845   8.232  11.095  10.475  10.475  -1.277   3.143

N          1100    1100    1100    1100    1100    1100    1100    1100    1100    1100
X2         15.3    44.0    69.5    34.7    44.2    21.2    37.0    51.9    12.6    53.5
Gvalue     15.5    45.8    70.0    36.9    45.9    21.4    39.4    56.2    12.6    53.4
Gv/N      1.409   4.160   6.360   3.352   4.172   1.944   3.580   5.110   1.148   4.856
Prob    0.00143 0.00000 0.00000 0.00000 0.00000 0.00009 0.00000 0.00000 0.00552 0.00000
        -------------------------------------------------------------------------------
             10       9       8       7       6       5       4       3       2       1
        -------------------------------------------------------------------------------
a_O         235     230     275     235     227     223     209     243     168     148
        ( 21.4) ( 20.9) ( 25.0) ( 21.4) ( 20.6) ( 20.3) ( 19.0) ( 22.1) ( 15.3) ( 13.5)
a_E       202.4   202.4   202.4   202.4   202.4   202.4   202.4   202.4   202.4   202.4
        ( 18.4) ( 18.4) ( 18.4) ( 18.4) ( 18.4) ( 18.4) ( 18.4) ( 18.4) ( 18.4) ( 18.4)
           70.2    58.8   168.6    70.2    52.1    43.2    13.4    88.8   -62.6   -92.7
          6.379   5.344  15.324   6.379   4.732   3.928   1.217   8.075  -5.692  -8.425
t_O         224     204     207     246     204     201     180     147     201     140
        ( 20.4) ( 18.5) ( 18.8) ( 22.4) ( 18.5) ( 18.3) ( 16.4) ( 13.4) ( 18.3) ( 12.7)
t_E       203.8   203.8   203.8   203.8   203.8   203.8   203.8   203.8   203.8   203.8
        ( 18.5) ( 18.5) ( 18.5) ( 18.5) ( 18.5) ( 18.5) ( 18.5) ( 18.5) ( 18.5) ( 18.5)
           42.4     0.4     6.5    92.6     0.4    -5.5   -44.7   -96.0    -5.5  -105.1
          3.853   0.040   0.590   8.422   0.040  -0.502  -4.061  -8.729  -0.502  -9.556
g_O         235     296     237     223     261     215     277     417     201     148
        ( 21.4) ( 26.9) ( 21.5) ( 20.3) ( 23.7) ( 19.5) ( 25.2) ( 37.9) ( 18.3) ( 13.5)
g_E       318.7   318.7   318.7   318.7   318.7   318.7   318.7   318.7   318.7   318.7
        ( 29.0) ( 29.0) ( 29.0) ( 29.0) ( 29.0) ( 29.0) ( 29.0) ( 29.0) ( 29.0) ( 29.0)
         -143.2   -43.8  -140.4  -159.3  -104.3  -169.3   -77.7   224.2  -185.3  -227.1
        -13.020  -3.980 -12.766 -14.480  -9.481 -15.389  -7.066  20.378 -16.848 -20.642
c_O         406     370     381     396     408     461     434     293     530     664
        ( 36.9) ( 33.6) ( 34.6) ( 36.0) ( 37.1) ( 41.9) ( 39.5) ( 26.6) ( 48.2) ( 60.4)
c_E       375.1   375.1   375.1   375.1   375.1   375.1   375.1   375.1   375.1   375.1
        ( 34.1) ( 34.1) ( 34.1) ( 34.1) ( 34.1) ( 34.1) ( 34.1) ( 34.1) ( 34.1) ( 34.1)
           64.3   -10.1    11.9    43.0    68.6   190.1   126.6  -144.7   366.5   758.4
          5.845  -0.919   1.083   3.906   6.239  17.286  11.511 -13.158  33.314  68.949

N          1100    1100    1100    1100    1100    1100    1100    1100    1100    1100
X2         31.8     5.4    47.1    43.9    16.3    55.6    17.7    72.2   113.3   348.6
Gvalue     33.6     5.3    46.5    46.5    16.8    58.6    17.6    72.2   113.0   333.6
Gv/N      3.057   0.485   4.231   4.226   1.530   5.323   1.602   6.565  10.273  30.327
Prob    0.00000 0.14918 0.00000 0.00000 0.00077 0.00000 0.00053 0.00000 0.00000 0.00000
        -------------------------------------------------------------------------------

CDS_INITIAL:
        -------------------------------------------------------------------------------
```

	1	2	3	4	5	6	7	8	9	10
a_O	901	0	0	446	282	172	302	269	182	288
	(81.9)	(0.0)	(0.0)	(40.5)	(25.6)	(15.6)	(27.5)	(24.5)	(16.5)	(26.2)
a_E	197.8	261.5	56.5	197.8	261.5	56.5	197.8	261.5	56.5	197.8
	(18.0)	(23.8)	(5.1)	(18.0)	(23.8)	(5.1)	(18.0)	(23.8)	(5.1)	(18.0)
	2732.0	0.0	0.0	725.1	42.5	383.1	255.5	15.1	425.9	216.3
	248.362	0.000	0.000	65.918	3.861	34.827	23.227	1.375	38.722	19.665
t_O	77	1100	0	154	180	192	131	256	175	158
	(7.0)	(100.0)	(0.0)	(14.0)	(16.4)	(17.5)	(11.9)	(23.3)	(15.9)	(14.4)
t_E	129.2	310.6	69.1	129.2	310.6	69.1	129.2	310.6	69.1	129.2
	(11.7)	(28.2)	(6.3)	(11.7)	(28.2)	(6.3)	(11.7)	(28.2)	(6.3)	(11.7)
	-79.7	2782.0	0.0	54.2	-196.4	392.2	3.7	-99.0	325.0	63.7
	-7.242	252.905	0.000	4.924	-17.855	35.657	0.336	-9.000	29.550	5.789
g_O	122	0	1100	215	160	247	345	202	277	286
	(11.1)	(0.0)	(100.0)	(19.5)	(14.5)	(22.5)	(31.4)	(18.4)	(25.2)	(26.0)
g_E	441.3	228.9	441.1	441.3	228.9	441.1	441.3	228.9	441.1	441.3
	(40.1)	(20.8)	(40.1)	(40.1)	(20.8)	(40.1)	(40.1)	(20.8)	(40.1)	(40.1)
	-313.7	0.0	2010.1	-309.2	-114.6	-286.5	-169.8	-50.5	-257.8	-248.0
	-28.517	0.000	182.737	-28.106	-10.415	-26.047	-15.437	-4.588	-23.437	-22.550
c_O	0	0	0	285	478	489	322	373	466	368
	(0.0)	(0.0)	(0.0)	(25.9)	(43.5)	(44.5)	(29.3)	(33.9)	(42.4)	(33.5)
c_E	331.7	299.0	533.2	331.7	299.0	533.2	331.7	299.0	533.2	331.7
	(30.2)	(27.2)	(48.5)	(30.2)	(27.2)	(48.5)	(30.2)	(27.2)	(48.5)	(30.2)
	0.0	0.0	0.0	-86.6	448.6	-84.7	-19.2	165.1	-125.6	76.3
	0.000	0.000	0.000	-7.869	40.785	-7.700	-1.745	15.005	-11.420	6.941
N	1100	1100	1100	1100	1100	1100	1100	1100	1100	1100
X2	3083.1	2795.6	1642.8	438.7	184.5	543.8	76.2	31.3	510.6	106.1
Gvalue	2338.6	2782.0	2010.1	383.5	180.1	404.1	70.2	30.7	367.6	108.3
Gv/N	212.603	252.905	182.737	34.867	16.375	36.737	6.381	2.791	33.414	9.844
Prob	1.00000	1.00000	1.00000	0.00000	0.00000	0.00000	0.00000	0.00000	0.00000	0.00000

	11	12	13	14	15	16	17	18	19	20
a_O	244	158	238	260	157	247	244	154	264	238
	(22.2)	(14.4)	(21.6)	(23.6)	(14.3)	(22.5)	(22.2)	(14.0)	(24.0)	(21.6)
a_E	261.5	56.5	197.8	261.5	56.5	197.8	261.5	56.5	197.8	261.5
	(23.8)	(5.1)	(18.0)	(23.8)	(5.1)	(18.0)	(23.8)	(5.1)	(18.0)	(23.8)
	-33.9	325.1	88.0	-3.1	321.0	109.7	-33.9	309.0	152.3	-44.9
	-3.081	29.553	7.999	-0.280	29.185	9.968	-3.081	28.087	13.849	-4.082
t_O	270	197	177	265	192	172	337	192	158	315
	(24.5)	(17.9)	(16.1)	(24.1)	(17.5)	(15.6)	(30.6)	(17.5)	(14.4)	(28.6)
t_E	310.6	69.1	129.2	310.6	69.1	129.2	310.6	69.1	129.2	310.6
	(28.2)	(6.3)	(11.7)	(28.2)	(6.3)	(11.7)	(28.2)	(6.3)	(11.7)	(28.2)
	-75.7	412.6	111.5	-84.2	392.2	98.5	55.0	392.2	63.7	8.8
	-6.878	37.506	10.139	-7.652	35.657	8.957	4.996	35.657	5.789	0.804
g_O	174	254	311	176	264	305	155	250	296	165
	(15.8)	(23.1)	(28.3)	(16.0)	(24.0)	(27.7)	(14.1)	(22.7)	(26.9)	(15.0)
g_E	228.9	441.1	441.3	228.9	441.1	441.3	228.9	441.1	441.3	228.9
	(20.8)	(40.1)	(40.1)	(20.8)	(40.1)	(40.1)	(20.8)	(40.1)	(40.1)	(20.8)
	-95.4	-280.4	-217.6	-92.5	-271.1	-225.3	-120.8	-284.0	-236.4	-108.0
	-8.673	-25.495	-19.782	-8.407	-24.645	-20.481	-10.984	-25.815	-21.489	-9.817
c_O	412	491	374	399	487	376	364	504	382	382
	(37.5)	(44.6)	(34.0)	(36.3)	(44.3)	(34.2)	(33.1)	(45.8)	(34.7)	(34.7)
c_E	299.0	533.2	331.7	299.0	533.2	331.7	299.0	533.2	331.7	299.0
	(27.2)	(48.5)	(30.2)	(27.2)	(48.5)	(30.2)	(27.2)	(48.5)	(30.2)	(27.2)
	264.3	-81.0	89.7	230.3	-88.3	94.2	143.3	-56.8	107.8	187.2
	24.023	-7.367	8.153	20.939	-8.031	8.562	13.026	-5.167	9.798	17.023
N	1100	1100	1100	1100	1100	1100	1100	1100	1100	1100
X2	62.4	501.7	69.7	52.4	472.4	74.4	41.4	471.2	84.0	43.1
Gvalue	59.3	376.2	71.6	50.6	353.8	77.1	43.5	360.4	87.4	43.2
Gv/N	5.391	34.198	6.509	4.600	32.165	7.006	3.958	32.762	7.947	3.927
Prob	0.00000	0.00000	0.00000	0.00000	0.00000	0.00000	0.00000	0.00000	0.00000	0.00000

	21	22	23	24	25	26	27	28	29	30
a_O	130	222	222	122	218	241	107	217	196	104
	(11.8)	(20.2)	(20.2)	(11.1)	(19.8)	(21.9)	(9.7)	(19.7)	(17.8)	(9.5)
a_E	56.5	197.8	261.5	56.5	197.8	261.5	56.5	197.8	261.5	56.5
	(5.1)	(18.0)	(23.8)	(5.1)	(18.0)	(23.8)	(5.1)	(18.0)	(23.8)	(5.1)
	216.8	51.2	-72.8	187.9	42.3	-39.4	136.7	40.1	-113.1	127.0
	19.705	4.652	-6.617	17.084	3.848	-3.585	12.431	3.649	-10.281	11.545
t_O	162	188	305	166	175	309	157	153	315	123
	(14.7)	(17.1)	(27.7)	(15.1)	(15.9)	(28.1)	(14.3)	(13.9)	(28.6)	(11.2)
t_E	69.1	129.2	310.6	69.1	129.2	310.6	69.1	129.2	310.6	69.1
	(6.3)	(11.7)	(28.2)	(6.3)	(11.7)	(28.2)	(6.3)	(11.7)	(28.2)	(6.3)
	275.9	141.1	-11.1	290.8	106.3	-3.2	257.5	51.8	8.8	141.7
	25.081	12.830	-1.011	26.436	9.663	-0.292	23.412	4.711	0.804	12.884
g_O	292	351	208	311	342	200	289	365	223	342
	(26.5)	(31.9)	(18.9)	(28.3)	(31.1)	(18.2)	(26.3)	(33.2)	(20.3)	(31.1)
g_E	441.1	441.3	228.9	441.1	441.3	228.9	441.1	441.3	228.9	441.1
	(40.1)	(40.1)	(20.8)	(40.1)	(40.1)	(20.8)	(40.1)	(40.1)	(20.8)	(40.1)
	-241.0	-160.7	-39.8	-217.4	-174.3	-54.0	-244.5	-138.5	-11.6	-174.1
	-21.907	-14.605	-3.618	-19.768	-15.846	-4.905	-22.224	-12.592	-1.055	-15.830
c_O	516	339	365	501	365	350	547	365	366	531
	(46.9)	(30.8)	(33.2)	(45.5)	(33.2)	(31.8)	(49.7)	(33.2)	(33.3)	(48.3)
c_E	533.2	331.7	299.0	533.2	331.7	299.0	533.2	331.7	299.0	533.2
	(48.5)	(30.2)	(27.2)	(48.5)	(30.2)	(27.2)	(48.5)	(30.2)	(27.2)	(48.5)
	-33.9	14.7	145.7	-62.5	69.7	110.3	27.9	69.7	148.1	-4.5
	-3.083	1.334	13.244	-5.680	6.341	10.029	2.534	6.341	13.462	-0.406
N	1100	1100	1100	1100	1100	1100	1100	1100	1100	1100
X2	271.4	48.4	22.6	252.1	44.0	14.0	209.7	22.8	31.7	104.2
Gvalue	217.8	46.3	22.0	198.8	44.1	13.7	177.7	23.2	32.2	90.1
Gv/N	19.797	4.212	1.999	18.072	4.006	1.248	16.153	2.108	2.930	8.193
Prob	0.00000	0.00000	0.00007	0.00000	0.00000	0.00330	0.00000	0.00004	0.00000	0.00000

	31	32	33	34	35	36	37	38	39	40
a_O	218	204	104	192	204	77	186	208	90	178
	(19.8)	(18.5)	(9.5)	(17.5)	(18.5)	(7.0)	(16.9)	(18.9)	(8.2)	(16.2)
a_E	197.8	261.5	56.5	197.8	261.5	56.5	197.8	261.5	56.5	197.8
	(18.0)	(23.8)	(5.1)	(18.0)	(23.8)	(5.1)	(18.0)	(23.8)	(5.1)	(18.0)
	42.3	-101.4	127.0	-11.5	-101.4	47.7	-22.9	-95.3	83.9	-37.6
	3.848	-9.217	11.545	-1.045	-9.217	4.339	-2.086	-8.663	7.625	-3.419
t_O	167	321	133	150	305	131	140	315	127	173
	(15.2)	(29.2)	(12.1)	(13.6)	(27.7)	(11.9)	(12.7)	(28.6)	(11.5)	(15.7)
t_E	129.2	310.6	69.1	129.2	310.6	69.1	129.2	310.6	69.1	129.2
	(11.7)	(28.2)	(6.3)	(11.7)	(28.2)	(6.3)	(11.7)	(28.2)	(6.3)	(11.7)
	85.8	21.1	174.0	44.9	-11.1	167.4	22.6	8.8	154.5	101.1

	7.801	1.920	15.821	4.079	−1.011	15.223	2.051	0.804	14.042	9.191
g_O	370	231	351	392	228	331	434	201	376	411
	(33.6)	(21.0)	(31.9)	(35.6)	(20.7)	(30.1)	(39.5)	(18.3)	(34.2)	(37.4)
g_E	441.3	228.9	441.1	441.3	228.9	441.1	441.3	228.9	441.1	441.3
	(40.1)	(20.8)	(40.1)	(40.1)	(20.8)	(40.1)	(40.1)	(20.8)	(40.1)	(40.1)
	−130.3	4.3	−160.5	−92.8	−1.8	−190.2	−14.4	−52.2	−120.2	−58.4
	−11.849	0.387	−14.589	−8.437	−0.160	−17.288	−1.310	−4.747	−10.924	−5.309
c_O	345	344	512	366	363	561	340	376	507	338
	(31.4)	(31.3)	(46.5)	(33.3)	(33.0)	(51.0)	(30.9)	(34.2)	(46.1)	(30.7)
c_E	331.7	299.0	533.2	331.7	299.0	533.2	331.7	299.0	533.2	331.7
	(30.2)	(27.2)	(48.5)	(30.2)	(27.2)	(48.5)	(30.2)	(27.2)	(48.5)	(30.2)
	27.0	96.5	−41.6	71.9	140.9	56.9	16.7	172.4	−51.2	12.6
	2.458	8.776	−3.783	6.540	12.809	5.177	1.520	15.673	−4.651	1.149
N	1100	1100	1100	1100	1100	1100	1100	1100	1100	1100
X2	25.2	19.8	118.2	12.6	26.5	91.8	1.9	34.3	79.2	19.1
Gvalue	24.8	20.5	98.9	12.5	26.6	82.0	1.9	33.7	67.0	17.7
Gv/N	2.257	1.867	8.994	1.137	2.422	7.451	0.176	3.067	6.091	1.612
Prob	0.00002	0.00013	0.00000	0.00584	0.00001	0.00000	0.58683	0.00000	0.00000	0.00050

	41	42	43	44	45	46	47	48	49	50
a_O	191	80	202	230	100	174	218	81	189	226
	(17.4)	(7.3)	(18.4)	(20.9)	(9.1)	(15.8)	(19.8)	(7.4)	(17.2)	(20.5)
a_E	261.5	56.5	197.8	261.5	56.5	197.8	261.5	56.5	197.8	261.5
	(23.8)	(5.1)	(18.0)	(23.8)	(5.1)	(18.0)	(23.8)	(5.1)	(18.0)	(23.8)
	−120.1	55.7	8.4	−59.1	114.3	−44.7	−79.4	58.4	−17.3	−66.0
	−10.916	5.064	0.766	−5.375	10.388	−4.061	−7.218	5.311	−1.570	−6.002
t_O	329	122	132	315	115	146	319	101	145	318
	(29.9)	(11.1)	(12.0)	(28.6)	(10.5)	(13.3)	(29.0)	(9.2)	(13.2)	(28.9)
t_E	310.6	69.1	129.2	310.6	69.1	129.2	310.6	69.1	129.2	310.6
	(28.2)	(6.3)	(11.7)	(28.2)	(6.3)	(11.7)	(28.2)	(6.3)	(11.7)	(28.2)
	37.8	138.6	5.7	8.8	117.0	35.8	17.0	76.6	33.5	15.0
	3.441	12.598	0.521	0.804	10.640	3.252	1.546	6.960	3.049	1.359
g_O	252	335	409	222	382	413	223	383	433	224
	(22.9)	(30.5)	(37.2)	(20.2)	(34.7)	(37.5)	(20.3)	(34.8)	(39.4)	(20.4)
g_E	228.9	441.1	441.3	228.9	441.1	441.3	228.9	441.1	441.3	228.9
	(20.8)	(40.1)	(40.1)	(20.8)	(40.1)	(40.1)	(20.8)	(40.1)	(40.1)	(20.8)
	48.5	−184.4	−62.1	−13.5	−110.0	−54.7	−11.6	−108.3	−16.4	−9.7
	4.409	−16.765	−5.646	−1.232	−9.999	−4.970	−1.055	−9.843	−1.488	−0.878
c_O	328	563	357	333	503	367	340	535	333	332
	(29.8)	(51.2)	(32.5)	(30.3)	(45.7)	(33.4)	(30.9)	(48.6)	(30.3)	(30.2)
c_E	299.0	533.2	331.7	299.0	533.2	331.7	299.0	533.2	331.7	299.0
	(27.2)	(48.5)	(30.2)	(27.2)	(48.5)	(30.2)	(27.2)	(48.5)	(30.2)	(27.2)
	60.8	61.2	52.4	71.8	−58.7	74.1	87.5	3.5	2.5	69.6
	5.527	5.560	4.763	6.528	−5.339	6.740	7.951	0.321	0.229	6.326
N	1100	1100	1100	1100	1100	1100	1100	1100	1100	1100
X2	25.3	77.4	4.4	7.9	73.6	10.6	13.3	33.0	2.5	8.8
Gvalue	27.1	71.0	4.4	8.0	62.6	10.6	13.5	30.2	2.4	8.9
Gv/N	2.461	6.457	0.404	0.725	5.690	0.961	1.223	2.749	0.221	0.806
Prob	0.00001	0.00000	0.21735	0.04656	0.00000	0.01429	0.00375	0.00000	0.48853	0.03110

	51	52	53	54	55	56	57	58	59	60
a_O	78	192	203	69	200	219	70	194	219	57
	(7.1)	(17.5)	(18.5)	(6.3)	(18.2)	(19.9)	(6.4)	(17.6)	(19.9)	(5.2)
a_E	56.5	197.8	261.5	56.5	197.8	261.5	56.5	197.8	261.5	56.5
	(5.1)	(18.0)	(23.8)	(5.1)	(18.0)	(23.8)	(5.1)	(18.0)	(23.8)	(5.1)
	50.4	−11.5	−102.9	27.6	4.4	−77.8	30.1	−7.6	−77.8	1.0
	4.579	−1.045	−9.353	2.512	0.396	−7.069	2.732	−0.690	−7.069	0.095
t_O	124	158	304	98	140	310	105	143	315	98
	(11.3)	(14.4)	(27.6)	(8.9)	(12.7)	(28.2)	(9.5)	(13.0)	(28.6)	(8.9)
t_E	69.1	129.2	310.6	69.1	129.2	310.6	69.1	129.2	310.6	69.1
	(6.3)	(11.7)	(28.2)	(6.3)	(11.7)	(28.2)	(6.3)	(11.7)	(28.2)	(6.3)
	144.9	63.7	−13.1	68.4	22.6	−1.2	87.8	29.1	8.8	68.4
	13.171	5.789	−1.189	6.216	2.051	−0.111	7.978	2.646	0.804	6.216
g_O	387	416	238	393	432	237	401	423	238	402
	(35.2)	(37.8)	(21.6)	(35.7)	(39.3)	(21.5)	(36.5)	(38.5)	(21.6)	(36.5)
g_E	441.1	441.3	228.9	441.1	441.3	228.9	441.1	441.3	228.9	441.1
	(40.1)	(40.1)	(20.8)	(40.1)	(40.1)	(20.8)	(40.1)	(40.1)	(20.8)	(40.1)
	−101.4	−49.1	18.6	−90.8	−18.3	16.5	−76.5	−35.8	18.6	−74.7
	−9.215	−4.459	1.691	−8.258	−1.666	1.502	−6.957	−3.251	1.691	−6.792
c_O	511	334	355	540	328	334	524	340	328	543
	(46.5)	(30.4)	(32.3)	(49.1)	(29.8)	(30.4)	(47.6)	(30.9)	(29.8)	(49.4)
c_E	533.2	331.7	299.0	533.2	331.7	299.0	533.2	331.7	299.0	533.2
	(48.5)	(30.2)	(27.2)	(48.5)	(30.2)	(27.2)	(48.5)	(30.2)	(27.2)	(48.5)
	−43.5	4.5	122.0	13.6	−7.4	74.0	−18.3	16.7	60.8	19.7
	−3.958	0.412	11.088	1.237	−0.676	6.729	−1.665	1.520	5.527	1.791
N	1100	1100	1100	1100	1100	1100	1100	1100	1100	1100
X2	59.3	8.1	24.1	20.2	1.2	11.3	25.7	2.5	10.2	15.7
Gvalue	50.4	7.7	24.6	18.8	1.1	11.6	23.0	2.5	10.5	14.4
Gv/N	4.578	0.697	2.237	1.708	0.104	1.052	2.088	0.225	0.953	1.311
Prob	0.00000	0.05336	0.00002	0.00030	0.76588	0.00901	0.00004	0.47946	0.01488	0.00239

	61	62	63	64	65	66	67	68	69	70
a_O	188	231	86	176	218	86	166	226	69	212
	(17.1)	(21.0)	(7.8)	(16.0)	(19.8)	(7.8)	(15.1)	(20.5)	(6.3)	(19.3)
a_E	197.8	261.5	56.5	197.8	261.5	56.5	197.8	261.5	56.5	197.8
	(18.0)	(23.8)	(5.1)	(18.0)	(23.8)	(5.1)	(18.0)	(23.8)	(5.1)	(18.0)
	−19.2	−57.4	72.3	−41.2	−79.4	72.3	−58.2	−66.0	27.6	29.3
	−1.743	−5.216	6.575	−3.742	−7.218	6.575	−5.295	−6.002	2.512	2.666
t_O	125	307	90	144	315	94	149	328	107	126
	(11.4)	(27.9)	(8.2)	(13.1)	(28.6)	(8.5)	(13.5)	(29.8)	(9.7)	(11.5)
t_E	129.2	310.6	69.1	129.2	310.6	69.1	129.2	310.6	69.1	129.2
	(11.7)	(28.2)	(6.3)	(11.7)	(28.2)	(6.3)	(11.7)	(28.2)	(6.3)	(11.7)
	−8.2	−7.2	47.5	31.3	8.8	57.8	42.6	35.7	93.5	−6.3
	−0.745	−0.653	4.316	2.847	0.804	5.251	3.870	3.249	8.497	−0.568
g_O	445	216	419	427	233	390	443	228	396	432
	(40.5)	(19.6)	(38.1)	(38.8)	(21.2)	(35.5)	(40.3)	(20.7)	(36.0)	(39.3)
g_E	441.3	228.9	441.1	441.3	228.9	441.1	441.3	228.9	441.1	441.3
	(40.1)	(20.8)	(40.1)	(40.1)	(20.8)	(40.1)	(40.1)	(20.8)	(40.1)	(40.1)
	7.5	−25.0	−43.2	−28.1	8.3	−96.1	3.5	−1.8	−85.5	−18.3
	0.682	−2.274	−3.924	−2.551	0.756	−8.738	0.317	−0.160	−7.774	−1.666
c_O	342	346	505	353	334	530	342	318	528	330
	(31.1)	(31.5)	(45.9)	(32.1)	(30.4)	(48.2)	(31.1)	(28.9)	(48.0)	(30.0)
c_E	331.7	299.0	533.2	331.7	299.0	533.2	331.7	299.0	533.2	331.7
	(30.2)	(27.2)	(48.5)	(30.2)	(27.2)	(48.5)	(30.2)	(27.2)	(48.5)	(30.2)
	20.8	101.1	−55.0	43.9	74.0	−6.5	20.8	39.3	−10.4	−3.5
	1.894	9.192	−4.996	3.987	6.729	−0.587	1.894	3.569	−0.948	−0.316

N	1100	1100	1100	1100	1100	1100	1100	1100	1100	1100
X2	1.0	11.7	24.3	5.9	11.5	30.3	8.5	7.0	28.2	1.3
Gvalue	1.0	11.5	21.7	5.9	11.8	27.5	8.6	7.2	25.2	1.3
Gv/N	0.089	1.049	1.971	0.541	1.071	2.500	0.786	0.656	2.288	0.116
Prob	0.80648	0.00916	0.00008	0.11427	0.00818	0.00000	0.03443	0.06544	0.00001	0.73526

	71	72	73	74	75	76	77	78	79	80
a_O	266	73	191	255	74	187	248	70	205	215
	(24.2)	(6.6)	(17.4)	(23.2)	(6.7)	(17.0)	(22.5)	(6.4)	(18.6)	(19.5)
a_E	261.5	56.5	197.8	261.5	56.5	197.8	261.5	56.5	197.8	261.5
	(23.8)	(5.1)	(18.0)	(23.8)	(5.1)	(18.0)	(23.8)	(5.1)	(18.0)	(23.8)
	9.0	37.5	−13.4	−12.9	40.0	−21.1	−26.4	30.1	14.6	−84.3
	0.817	3.406	−1.221	−1.175	3.636	−1.915	−2.398	2.732	1.326	−7.661
t_O	316	105	129	292	84	142	318	89	140	347
	(28.7)	(9.5)	(11.7)	(26.5)	(7.6)	(12.9)	(28.9)	(8.1)	(12.7)	(31.5)
t_E	310.6	69.1	129.2	310.6	69.1	129.2	310.6	69.1	129.2	310.6
	(28.2)	(6.3)	(11.7)	(28.2)	(6.3)	(11.7)	(28.2)	(6.3)	(11.7)	(28.2)
	10.9	87.8	−0.3	−36.1	32.7	26.9	15.0	45.0	22.6	76.9
	0.988	7.978	−0.030	−3.280	2.974	2.446	1.359	4.087	2.051	6.990
g_O	223	420	442	219	401	443	229	411	408	232
	(20.3)	(38.2)	(40.2)	(19.9)	(36.5)	(40.3)	(20.8)	(37.4)	(37.1)	(21.1)
g_E	228.9	441.1	441.3	228.9	441.1	441.3	228.9	441.1	441.3	228.9
	(20.8)	(40.1)	(40.1)	(20.8)	(40.1)	(40.1)	(20.8)	(40.1)	(40.1)	(20.8)
	−11.6	−41.3	1.5	−19.3	−76.5	3.5	0.2	−58.2	−64.0	6.3
	−1.055	−3.752	0.134	−1.757	−6.957	0.317	0.022	−5.290	−5.814	0.571
c_O	295	502	338	334	541	328	305	530	347	306
	(26.8)	(45.6)	(30.7)	(30.4)	(49.2)	(29.8)	(27.7)	(48.2)	(31.5)	(27.8)
c_E	299.0	533.2	331.7	299.0	533.2	331.7	299.0	533.2	331.7	299.0
	(27.2)	(48.5)	(30.2)	(27.2)	(48.5)	(30.2)	(27.2)	(48.5)	(30.2)	(27.2)
	−7.9	−60.6	12.6	74.0	15.6	−7.4	12.2	−6.5	31.2	14.2
	−0.716	−5.510	1.149	6.729	1.422	−0.676	1.108	−0.587	2.837	1.294
N	1100	1100	1100	1100	1100	1100	1100	1100	1100	1100
X2	0.4	26.3	0.4	5.8	12.4	1.9	1.0	11.0	4.4	12.8
Gvalue	0.4	23.3	0.4	5.7	11.8	1.9	1.0	10.4	4.4	13.1
Gv/N	0.034	2.122	0.033	0.517	1.074	0.172	0.092	0.942	0.400	1.194
Prob	0.94564	0.00003	0.94886	0.12772	0.00803	0.59581	0.79898	0.01572	0.22088	0.00435

	81	82	83	84	85	86	87	88	89	90
a_O	81	170	237	62	184	238	75	197	246	63
	(7.4)	(15.5)	(21.5)	(5.6)	(16.7)	(21.6)	(6.8)	(17.9)	(22.4)	(5.7)
a_E	56.5	197.8	261.5	56.5	197.8	261.5	56.5	197.8	261.5	56.5
	(5.1)	(18.0)	(23.8)	(5.1)	(18.0)	(23.8)	(5.1)	(18.0)	(23.8)	(5.1)
	58.4	−51.6	−46.7	11.6	−26.7	−44.9	42.5	−1.7	−30.1	13.8
	5.311	−4.687	−4.246	1.052	−2.425	−4.082	3.868	−0.151	−2.741	1.252
t_O	77	138	312	77	121	315	83	125	321	68
	(7.0)	(12.5)	(28.4)	(7.0)	(11.0)	(28.6)	(7.5)	(11.4)	(29.2)	(6.2)
t_E	69.1	129.2	310.6	69.1	129.2	310.6	69.1	129.2	310.6	69.1
	(6.3)	(11.7)	(28.2)	(6.3)	(11.7)	(28.2)	(6.3)	(11.7)	(28.2)	(6.3)
	16.6	18.3	2.8	16.6	−15.8	8.8	30.3	−8.2	21.1	−2.3
	1.508	1.660	0.253	1.508	−1.436	0.804	2.758	−0.745	1.920	−0.205
g_O	415	437	227	440	441	232	418	444	221	437
	(37.7)	(39.7)	(20.6)	(40.0)	(40.1)	(21.1)	(38.0)	(40.4)	(20.1)	(39.7)
g_E	441.1	441.3	228.9	441.1	441.3	228.9	441.1	441.3	228.9	441.1
	(40.1)	(40.1)	(20.8)	(40.1)	(40.1)	(20.8)	(40.1)	(40.1)	(20.8)	(40.1)
	−50.7	−8.5	−3.7	−2.3	−0.5	6.3	−45.1	5.5	−15.5	−8.3
	−4.611	−0.771	−0.340	−0.209	−0.048	0.571	−4.096	0.499	−1.408	−0.751
c_O	527	355	324	521	354	315	524	334	312	532
	(47.9)	(32.3)	(29.5)	(47.4)	(32.2)	(28.6)	(47.6)	(30.4)	(28.4)	(48.4)
c_E	533.2	331.7	299.0	533.2	331.7	299.0	533.2	331.7	299.0	533.2
	(48.5)	(30.2)	(27.2)	(48.5)	(30.2)	(27.2)	(48.5)	(30.2)	(27.2)	(48.5)
	−12.4	48.1	52.1	−24.2	46.0	32.9	−18.3	4.5	26.6	−2.5
	−1.127	4.374	4.737	−2.199	4.180	2.992	−1.665	0.412	2.421	−0.225
N	1100	1100	1100	1100	1100	1100	1100	1100	1100	1100
X2	13.2	6.2	4.4	1.7	3.0	3.1	10.2	0.2	2.1	0.8
Gvalue	11.9	6.3	4.4	1.7	3.0	3.1	9.5	0.2	2.1	0.8
Gv/N	1.081	0.576	0.404	0.152	0.271	0.285	0.865	0.016	0.193	0.072
Prob	0.00777	0.09619	0.21763	0.64330	0.39482	0.37123	0.02323	0.98205	0.54766	0.85267

	91	92	93	94	95	96	97	98	99	100
a_O	195	241	51	197	231	52	196	240	60	191
	(17.7)	(21.9)	(4.6)	(17.9)	(21.0)	(4.7)	(17.8)	(21.8)	(5.5)	(17.4)
a_E	197.8	261.5	56.5	197.8	261.5	56.5	197.7	261.3	56.4	197.7
	(18.0)	(23.8)	(5.1)	(18.0)	(23.8)	(5.1)	(18.0)	(23.8)	(5.1)	(18.0)
	−5.6	−39.4	−10.4	−1.7	−57.4	−8.6	−3.3	−40.8	7.4	−13.1
	−0.511	−3.585	−0.946	−0.151	−5.216	−0.781	−0.300	−3.715	0.671	−1.190
t_O	120	325	72	116	303	86	116	296	71	127
	(10.9)	(29.5)	(6.5)	(10.5)	(27.5)	(7.8)	(10.6)	(26.9)	(6.5)	(11.6)
t_E	129.2	310.6	69.1	129.2	310.6	69.1	129.0	310.3	69.1	129.0
	(11.7)	(28.2)	(6.3)	(11.7)	(28.2)	(6.3)	(11.7)	(28.2)	(6.3)	(11.7)
	−17.7	29.4	5.8	−24.9	−15.0	37.5	−24.7	−28.0	3.9	−4.1
	−1.606	2.676	0.531	−2.267	−1.367	3.413	−2.250	−2.546	0.355	−0.370
g_O	456	209	436	456	218	430	438	235	417	432
	(41.5)	(19.0)	(39.6)	(41.5)	(19.8)	(39.1)	(39.9)	(21.4)	(37.9)	(39.3)
g_E	441.3	228.9	441.1	441.3	228.9	441.1	440.9	228.7	440.7	440.9
	(40.1)	(20.8)	(40.1)	(40.1)	(20.8)	(40.1)	(40.1)	(20.8)	(40.1)	(40.1)
	30.0	−38.0	−10.2	30.0	−21.2	−22.0	−5.7	12.8	−46.2	−17.5
	2.724	−3.453	−0.931	2.724	−1.930	−2.001	−0.519	1.168	−4.203	−1.596
c_O	329	325	541	331	348	532	349	328	551	349
	(29.9)	(29.5)	(49.2)	(30.1)	(31.6)	(48.4)	(31.8)	(29.8)	(50.1)	(31.8)
c_E	331.7	299.0	533.2	331.7	299.0	533.2	331.4	298.7	532.8	331.4
	(30.2)	(27.2)	(48.5)	(30.2)	(27.2)	(48.5)	(30.2)	(27.2)	(48.5)	(30.2)
	−5.5	54.3	15.6	−1.5	105.7	−2.5	36.0	61.4	37.1	36.0
	−0.496	4.934	1.422	−0.134	9.609	−0.225	3.279	5.587	3.377	3.279
N	1100	1100	1100	1100	1100	1100	1099	1099	1099	1099
X2	1.2	6.3	0.8	1.8	12.3	4.8	2.3	5.4	2.2	1.4
Gvalue	1.2	6.3	0.8	1.9	12.1	4.5	2.3	5.4	2.2	1.4
Gv/N	0.111	0.572	0.076	0.171	1.097	0.406	0.210	0.493	0.200	0.123
Prob	0.74907	0.09801	0.84009	0.59732	0.00717	0.21532	0.51006	0.14364	0.53272	0.71660

Bbur_B31
5UTR:

	100	99	98	97	96	95	94	93	92	91
a_O	168	145	177	182	197	175	182	176	168	170
	(38.1)	(32.9)	(40.1)	(41.3)	(44.7)	(39.7)	(41.3)	(39.9)	(38.1)	(38.5)
a_E	179.9	179.9	179.9	179.9	179.9	179.9	179.9	179.9	179.9	179.9
	(40.8)	(40.8)	(40.8)	(40.8)	(40.8)	(40.8)	(40.8)	(40.8)	(40.8)	(40.8)
	-22.9	-62.5	-5.7	4.3	35.8	-9.6	4.3	-7.7	-22.9	-19.2
	-5.203	-14.173	-1.293	0.970	8.125	-2.180	0.970	-1.738	-5.203	-4.353
t_O	159	176	164	148	134	160	154	159	173	159
	(36.1)	(39.9)	(37.2)	(33.6)	(30.4)	(36.3)	(34.9)	(36.1)	(39.2)	(36.1)
t_E	162.1	162.1	162.1	162.1	162.1	162.1	162.1	162.1	162.1	162.1
	(36.8)	(36.8)	(36.8)	(36.8)	(36.8)	(36.8)	(36.8)	(36.8)	(36.8)	(36.8)
	-6.1	29.0	3.9	-26.9	-51.0	-4.1	-15.7	-6.1	22.6	-6.1
	-1.381	6.580	0.879	-6.097	-11.559	-0.934	-3.569	-1.381	5.119	-1.381
g_O	74	58	53	59	49	68	67	47	50	56
	(16.8)	(13.2)	(12.0)	(13.4)	(11.1)	(15.4)	(15.2)	(10.7)	(11.3)	(12.7)
g_E	52.1	52.1	52.1	52.1	52.1	52.1	52.1	52.1	52.1	52.1
	(11.8)	(11.8)	(11.8)	(11.8)	(11.8)	(11.8)	(11.8)	(11.8)	(11.8)	(11.8)
	51.8	12.4	1.7	14.6	-6.1	36.1	33.6	-9.8	-4.2	8.0
	11.750	2.801	0.393	3.307	-1.381	8.189	7.619	-2.213	-0.951	1.813
c_O	40	62	47	52	61	38	38	59	50	56
	(9.1)	(14.1)	(10.7)	(11.8)	(13.8)	(8.6)	(8.6)	(13.4)	(11.3)	(12.7)
c_E	46.9	46.9	46.9	46.9	46.9	46.9	46.9	46.9	46.9	46.9
	(10.6)	(10.6)	(10.6)	(10.6)	(10.6)	(10.6)	(10.6)	(10.6)	(10.6)	(10.6)
	-12.7	34.6	0.2	10.7	32.0	-16.0	-16.0	27.1	6.4	19.8
	-2.891	7.842	0.040	2.429	7.265	-3.631	-3.631	6.135	1.446	4.498
N	441	441	441	441	441	441	441	441	441	441
X2	11.0	13.5	0.1	2.7	10.9	6.7	6.4	3.8	1.8	2.6
Gvalue	10.0	13.4	0.1	2.7	10.8	6.4	6.1	3.5	1.8	2.5
Gv/N	2.275	3.050	0.019	0.608	2.451	1.444	1.389	0.804	0.411	0.578
Prob	0.01830	0.00376	0.99377	0.44324	0.01282	0.09498	0.10561	0.31500	0.61265	0.46689

	90	89	88	87	86	85	84	83	82	81
a_O	164	170	176	166	171	181	180	152	178	168
	(37.2)	(38.5)	(39.9)	(37.6)	(38.8)	(41.0)	(40.8)	(34.5)	(40.4)	(38.1)
a_E	179.9	179.9	179.9	179.9	179.9	179.9	179.9	179.9	179.9	179.9
	(40.8)	(40.8)	(40.8)	(40.8)	(40.8)	(40.8)	(40.8)	(40.8)	(40.8)	(40.8)
	-30.3	-19.2	-7.7	-26.6	-17.3	2.3	0.3	-51.2	-3.7	-22.9
	-6.872	-4.353	-1.738	-6.043	-3.924	0.512	0.057	-11.607	-0.845	-5.203
t_O	167	161	153	152	177	165	149	175	161	166
	(37.9)	(36.5)	(34.7)	(34.5)	(40.1)	(37.4)	(33.8)	(39.7)	(36.5)	(37.6)
t_E	162.1	162.1	162.1	162.1	162.1	162.1	162.1	162.1	162.1	162.1
	(36.8)	(36.8)	(36.8)	(36.8)	(36.8)	(36.8)	(36.8)	(36.8)	(36.8)	(36.8)
	10.0	-2.1	-17.6	-19.5	31.2	5.9	-25.1	26.9	-2.1	7.9
	2.268	-0.485	-3.998	-4.424	7.072	1.339	-5.683	6.090	-0.485	1.802
g_O	61	59	70	54	53	54	52	55	56	64
	(13.8)	(13.4)	(15.9)	(12.2)	(12.0)	(12.2)	(11.8)	(12.5)	(12.7)	(14.5)
g_E	52.1	52.1	52.1	52.1	52.1	52.1	52.1	52.1	52.1	52.1
	(11.8)	(11.8)	(11.8)	(11.8)	(11.8)	(11.8)	(11.8)	(11.8)	(11.8)	(11.8)
	19.1	14.6	41.2	3.8	1.7	3.8	-0.3	5.9	8.0	26.2
	4.341	3.307	9.350	0.858	0.393	0.858	-0.064	1.331	1.813	5.948
c_O	49	51	42	69	40	41	60	59	46	43
	(11.1)	(11.6)	(9.5)	(15.6)	(9.1)	(9.3)	(13.6)	(13.4)	(10.4)	(9.8)
c_E	46.9	46.9	46.9	46.9	46.9	46.9	46.9	46.9	46.9	46.9
	(10.6)	(10.6)	(10.6)	(10.6)	(10.6)	(10.6)	(10.6)	(10.6)	(10.6)	(10.6)
	4.3	8.5	-9.3	53.2	-12.7	-11.0	29.5	27.1	-1.8	-7.5
	0.968	1.933	-2.106	12.074	-2.891	-2.504	6.696	6.135	-0.409	-1.698
N	441	441	441	441	441	441	441	441	441	441
X2	3.1	1.8	7.2	12.2	2.8	0.9	4.7	8.6	0.3	3.9
Gvalue	3.1	1.8	6.7	10.9	2.9	0.9	4.4	8.6	0.3	3.7
Gv/N	0.705	0.402	1.509	2.466	0.650	0.205	1.006	1.950	0.073	0.849
Prob	0.37491	0.62128	0.08383	0.01243	0.41279	0.82463	0.21792	0.03514	0.95559	0.29038

	80	79	78	77	76	75	74	73	72	71
a_O	158	181	194	189	176	173	157	173	197	177
	(35.8)	(41.0)	(44.0)	(42.9)	(39.9)	(39.2)	(35.6)	(39.2)	(44.7)	(40.1)
a_E	179.9	179.9	179.9	179.9	179.9	179.9	179.9	179.9	179.9	179.9
	(40.8)	(40.8)	(40.8)	(40.8)	(40.8)	(40.8)	(40.8)	(40.8)	(40.8)	(40.8)
	-41.0	2.3	29.3	18.7	-7.7	-13.5	-42.7	-13.5	35.8	-5.7
	-9.291	0.512	6.652	4.242	-1.738	-3.057	-9.684	-3.057	8.125	-1.293
t_O	171	156	149	156	168	155	175	155	148	171
	(38.8)	(35.4)	(33.8)	(35.4)	(38.1)	(35.1)	(39.7)	(35.1)	(33.6)	(38.8)
t_E	162.1	162.1	162.1	162.1	162.1	162.1	162.1	162.1	162.1	162.1
	(36.8)	(36.8)	(36.8)	(36.8)	(36.8)	(36.8)	(36.8)	(36.8)	(36.8)	(36.8)
	18.3	-11.9	-25.1	-11.9	12.1	-13.8	26.9	-13.8	-26.9	18.3
	4.158	-2.702	-5.683	-2.702	2.736	-3.137	6.090	-3.137	-6.097	4.158
g_O	54	56	47	50	57	57	56	66	52	44
	(12.2)	(12.7)	(10.7)	(11.3)	(12.9)	(12.9)	(12.7)	(15.0)	(11.8)	(10.0)
g_E	52.1	52.1	52.1	52.1	52.1	52.1	52.1	52.1	52.1	52.1
	(11.8)	(11.8)	(11.8)	(11.8)	(11.8)	(11.8)	(11.8)	(11.8)	(11.8)	(11.8)
	3.8	8.0	-9.8	-4.2	10.2	10.2	8.0	31.1	-0.3	-14.9
	0.858	1.813	-2.213	-0.951	2.303	2.303	1.813	7.055	-0.064	-3.388
c_O	58	48	51	46	40	56	53	47	44	49
	(13.2)	(10.9)	(11.6)	(10.4)	(9.1)	(12.7)	(12.0)	(10.7)	(10.0)	(11.1)
c_E	46.9	46.9	46.9	46.9	46.9	46.9	46.9	46.9	46.9	46.9
	(10.6)	(10.6)	(10.6)	(10.6)	(10.6)	(10.6)	(10.6)	(10.6)	(10.6)	(10.6)
	24.6	2.2	8.5	-1.8	-12.7	19.8	12.9	0.2	-5.6	4.3
	5.581	0.500	1.933	-0.409	-2.891	4.498	2.933	0.040	-1.278	0.968
N	441	441	441	441	441	441	441	441	441	441
X2	5.8	0.5	3.0	0.8	1.8	2.8	5.0	4.3	3.0	1.9
Gvalue	5.8	0.5	3.0	0.8	1.8	2.7	5.1	4.0	3.0	2.0
Gv/N	1.306	0.123	0.689	0.180	0.410	0.607	1.152	0.901	0.686	0.445
Prob	0.12392	0.90970	0.38604	0.85118	0.61283	0.44434	0.16588	0.26425	0.38777	0.57998

	70	69	68	67	66	65	64	63	62	61
a_O	184	150	178	201	165	185	182	154	180	179
	(41.7)	(34.0)	(40.4)	(45.6)	(37.4)	(42.0)	(41.3)	(34.9)	(40.8)	(40.6)
a_E	179.9	179.9	179.9	179.9	179.9	179.9	179.9	179.9	179.9	179.9
	(40.8)	(40.8)	(40.8)	(40.8)	(40.8)	(40.8)	(40.8)	(40.8)	(40.8)	(40.8)
	8.3	-54.5	-3.7	44.6	-28.5	10.4	4.3	-47.8	0.3	-1.7
	1.892	-12.355	-0.845	10.123	-6.459	2.357	0.970	-10.847	0.057	-0.395
t_O	155	182	166	137	155	167	144	170	151	173
	(35.1)	(41.3)	(37.6)	(31.1)	(35.1)	(37.9)	(32.7)	(38.5)	(34.2)	(39.2)

t_E	162.1	162.1	162.1	162.1	162.1	162.1	162.1	162.1	162.1	162.1
	(36.8)	(36.8)	(36.8)	(36.8)	(36.8)	(36.8)	(36.8)	(36.8)	(36.8)	(36.8)
	-13.8	42.2	7.9	-46.1	-13.8	10.0	-34.1	16.2	-21.4	22.6
	-3.137	9.571	1.802	-10.442	-3.137	2.268	-7.722	3.681	-4.846	5.119
g_O	53	53	55	59	55	42	59	65	51	50
	(12.0)	(12.0)	(12.5)	(13.4)	(12.5)	(9.5)	(13.4)	(14.7)	(11.6)	(11.3)
g_E	52.1	52.1	52.1	52.1	52.1	52.1	52.1	52.1	52.1	52.1
	(11.8)	(11.8)	(11.8)	(11.8)	(11.8)	(11.8)	(11.8)	(11.8)	(11.8)	(11.8)
	1.7	1.7	5.9	14.6	5.9	-18.2	14.6	28.7	-2.3	-4.2
	0.393	0.393	1.331	3.307	1.331	-4.120	3.307	6.498	-0.512	-0.951
c_O	49	56	42	44	66	47	56	52	59	39
	(11.1)	(12.7)	(9.5)	(10.0)	(15.0)	(10.7)	(12.7)	(11.8)	(13.4)	(8.8)
c_E	46.9	46.9	46.9	46.9	46.9	46.9	46.9	46.9	46.9	46.9
	(10.6)	(10.6)	(10.6)	(10.6)	(10.6)	(10.6)	(10.6)	(10.6)	(10.6)	(10.6)
	4.3	19.8	-9.3	-5.6	45.1	0.2	19.8	10.7	27.1	-14.4
	0.968	4.498	-2.106	-1.278	10.219	0.040	4.498	2.429	6.135	-3.267
N	441	441	441	441	441	441	441	441	441	441
X2	0.5	9.2	0.8	7.4	9.5	2.3	4.7	7.8	3.9	2.2
Gvalue	0.5	9.3	0.8	7.5	8.6	2.4	4.6	7.8	3.7	2.2
Gv/N	0.116	2.106	0.182	1.709	1.955	0.546	1.053	1.761	0.834	0.506
Prob	0.91603	0.02570	0.84923	0.05666	0.03480	0.49224	0.19996	0.05107	0.29848	0.52603
	60	59	58	57	56	55	54	53	52	51
a_O	175	171	182	158	193	161	160	163	184	205
	(39.7)	(38.8)	(41.3)	(35.8)	(43.8)	(36.5)	(36.3)	(37.0)	(41.7)	(46.5)
a_E	179.9	179.9	179.9	179.9	179.9	179.9	179.9	179.9	179.9	179.9
	(40.8)	(40.8)	(40.8)	(40.8)	(40.8)	(40.8)	(40.8)	(40.8)	(40.8)	(40.8)
	-9.6	-17.3	4.3	-41.0	27.2	-35.7	-37.5	-32.1	8.3	53.6
	-2.180	-3.924	0.970	-9.291	6.165	-8.094	-8.496	-7.282	1.892	12.156
t_O	167	182	173	176	156	155	181	171	142	143
	(37.9)	(41.3)	(39.2)	(39.9)	(35.4)	(35.1)	(41.0)	(38.8)	(32.2)	(32.4)
t_E	162.1	162.1	162.1	162.1	162.1	162.1	162.1	162.1	162.1	162.1
	(36.8)	(36.8)	(36.8)	(36.8)	(36.8)	(36.8)	(36.8)	(36.8)	(36.8)	(36.8)
	10.0	42.2	22.6	29.0	-11.9	-13.8	40.0	18.3	-37.6	-35.8
	2.268	9.571	5.119	6.580	-2.702	-3.137	9.066	4.158	-8.515	-8.120
g_O	50	51	50	55	58	69	54	55	59	48
	(11.3)	(11.6)	(11.3)	(12.5)	(13.2)	(15.6)	(12.2)	(12.5)	(13.4)	(10.9)
g_E	52.1	52.1	52.1	52.1	52.1	52.1	52.1	52.1	52.1	52.1
	(11.8)	(11.8)	(11.8)	(11.8)	(11.8)	(11.8)	(11.8)	(11.8)	(11.8)	(11.8)
	-4.2	-2.3	-4.2	5.9	12.4	38.7	3.8	5.9	14.6	-7.9
	-0.951	-0.512	-0.951	1.331	2.801	8.767	0.858	1.331	3.307	-1.801
c_O	49	37	36	52	34	56	46	52	56	45
	(11.1)	(8.4)	(8.2)	(11.8)	(7.7)	(12.7)	(10.4)	(11.8)	(12.7)	(10.2)
c_E	46.9	46.9	46.9	46.9	46.9	46.9	46.9	46.9	46.9	46.9
	(10.6)	(10.6)	(10.6)	(10.6)	(10.6)	(10.6)	(10.6)	(10.6)	(10.6)	(10.6)
	4.3	-17.6	-19.1	10.7	-21.9	19.8	-1.8	10.7	19.8	-3.7
	0.968	-3.983	-4.322	2.429	-4.963	4.498	-0.409	2.429	4.498	-0.849
N	441	441	441	441	441	441	441	441	441	441
X2	0.5	5.0	3.4	4.6	5.4	9.5	4.5	2.8	5.2	6.2
Gvalue	0.5	5.1	3.6	4.6	5.7	9.0	4.5	2.8	5.2	6.1
Gv/N	0.105	1.153	0.815	1.049	1.300	2.033	1.019	0.636	1.182	1.386
Prob	0.92708	0.16569	0.30852	0.20134	0.12528	0.02973	0.21286	0.42271	0.15692	0.10628
	50	49	48	47	46	45	44	43	42	41
a_O	174	187	187	178	188	167	185	176	158	163
	(39.5)	(42.4)	(42.4)	(40.4)	(42.6)	(37.9)	(42.0)	(39.9)	(35.8)	(37.0)
a_E	179.9	179.9	179.9	179.9	179.9	179.9	179.9	179.9	179.9	179.9
	(40.8)	(40.8)	(40.8)	(40.8)	(40.8)	(40.8)	(40.8)	(40.8)	(40.8)	(40.8)
	-11.6	14.5	14.5	-3.7	16.6	-24.8	10.4	-7.7	-41.0	-32.1
	-2.620	3.295	3.295	-0.845	3.767	-5.624	2.357	-1.738	-9.291	-7.282
t_O	181	155	153	154	148	166	151	159	181	169
	(41.0)	(35.1)	(34.7)	(34.9)	(33.6)	(37.6)	(34.2)	(36.1)	(41.0)	(38.3)
t_E	162.1	162.1	162.1	162.1	162.1	162.1	162.1	162.1	162.1	162.1
	(36.8)	(36.8)	(36.8)	(36.8)	(36.8)	(36.8)	(36.8)	(36.8)	(36.8)	(36.8)
	40.0	-13.8	-17.6	-15.7	-26.9	7.9	-21.4	-6.1	40.0	14.1
	9.066	-3.137	-3.998	-3.569	-6.097	1.802	-4.846	-1.381	9.066	3.207
g_O	30	58	54	55	50	55	61	44	52	60
	(6.8)	(13.2)	(12.2)	(12.5)	(11.3)	(12.5)	(13.8)	(10.0)	(11.8)	(13.6)
g_E	52.1	52.1	52.1	52.1	52.1	52.1	52.1	52.1	52.1	52.1
	(11.8)	(11.8)	(11.8)	(11.8)	(11.8)	(11.8)	(11.8)	(11.8)	(11.8)	(11.8)
	-33.2	12.4	3.8	5.9	-4.2	5.9	19.1	-14.9	-0.3	16.8
	-7.521	2.801	0.858	1.331	-0.951	1.331	4.341	-3.388	-0.064	3.820
c_O	56	41	47	54	55	53	44	62	50	49
	(12.7)	(9.3)	(10.7)	(12.2)	(12.5)	(12.0)	(10.0)	(14.1)	(11.3)	(11.1)
c_E	46.9	46.9	46.9	46.9	46.9	46.9	46.9	46.9	46.9	46.9
	(10.6)	(10.6)	(10.6)	(10.6)	(10.6)	(10.6)	(10.6)	(10.6)	(10.6)	(10.6)
	19.8	-11.0	0.2	15.2	17.5	12.9	-5.6	34.6	6.4	4.3
	4.498	-2.504	0.040	3.446	3.968	2.933	-1.278	7.842	1.446	0.968
N	441	441	441	441	441	441	441	441	441	441
X2	13.6	2.0	0.9	1.6	3.1	2.0	2.6	6.3	5.1	3.2
Gvalue	15.1	2.0	0.9	1.6	3.0	2.0	2.5	5.9	5.1	3.1
Gv/N	3.423	0.455	0.195	0.364	0.687	0.442	0.574	1.335	1.157	0.714
Prob	0.00174	0.57133	0.83457	0.65857	0.38690	0.58269	0.46987	0.11713	0.16439	0.36943
	40	39	38	37	36	35	34	33	32	31
a_O	167	178	169	188	186	173	179	172	177	202
	(37.9)	(40.4)	(38.3)	(42.6)	(42.2)	(39.2)	(40.6)	(39.0)	(40.1)	(45.8)
a_E	179.9	179.9	179.9	179.9	179.9	179.9	179.9	179.9	179.9	179.9
	(40.8)	(40.8)	(40.8)	(40.8)	(40.8)	(40.8)	(40.8)	(40.8)	(40.8)	(40.8)
	-24.8	-3.7	-21.1	16.6	12.5	-13.5	-1.7	-15.4	-5.7	46.9
	-5.624	-0.845	-4.779	3.767	2.825	-3.057	-0.395	-3.492	-1.293	10.628
t_O	190	154	165	144	161	173	170	166	149	165
	(43.1)	(34.9)	(37.4)	(32.7)	(36.5)	(39.2)	(38.5)	(37.6)	(33.8)	(37.4)
t_E	162.1	162.1	162.1	162.1	162.1	162.1	162.1	162.1	162.1	162.1
	(36.8)	(36.8)	(36.8)	(36.8)	(36.8)	(36.8)	(36.8)	(36.8)	(36.8)	(36.8)
	60.4	-15.7	5.9	-34.1	-2.1	22.6	16.2	7.9	-25.1	5.9
	13.698	-3.569	1.339	-7.722	-0.485	5.119	3.681	1.802	-5.683	1.339
g_O	54	66	56	47	50	59	52	55	56	39
	(12.2)	(15.0)	(12.7)	(10.7)	(11.3)	(13.4)	(11.8)	(12.5)	(12.7)	(8.8)
g_E	52.1	52.1	52.1	52.1	52.1	52.1	52.1	52.1	52.1	52.1
	(11.8)	(11.8)	(11.8)	(11.8)	(11.8)	(11.8)	(11.8)	(11.8)	(11.8)	(11.8)
	3.8	31.1	8.0	-9.8	-4.2	14.6	-0.3	5.9	8.0	-22.7
	0.858	7.055	1.813	-2.213	-0.951	3.307	-0.064	1.331	1.813	-5.136
c_O	30	43	51	62	44	36	40	48	59	35
	(6.8)	(9.8)	(11.6)	(14.1)	(10.0)	(8.2)	(9.1)	(10.9)	(13.4)	(7.9)
c_E	46.9	46.9	46.9	46.9	46.9	46.9	46.9	46.9	46.9	46.9

```
        ( 10.6) ( 10.6) ( 10.6) ( 10.6) ( 10.6) ( 10.6) ( 10.6) ( 10.6) ( 10.6) ( 10.6)
          -26.8    -7.5     8.5    34.6    -5.6   -19.1   -12.7     2.2    27.1   -20.5
         -6.082  -1.698   1.933   7.842  -1.278  -4.322  -2.891   0.500   6.135  -4.649

N           441     441     441     441     441     441     441     441     441     441
X2         11.9     4.4     1.4     7.7     0.5     4.4     1.4     0.6     4.5     9.1
Gvalue     12.6     4.2     1.3     7.4     0.5     4.6     1.5     0.6     4.3     9.6
Gv/N      2.849   0.943   0.306   1.674   0.110   1.046   0.331   0.141   0.972   2.181
Prob    0.00568 0.24481 0.71757 0.06060 0.92172 0.20243 0.69193 0.89109 0.23212 0.02209
        --------------------------------------------------------------------------------
             30      29      28      27      26      25      24      23      22      21
        --------------------------------------------------------------------------------
a_O         196     171     193     195     157     176     206     202     185     207
        ( 44.4) ( 38.8) ( 43.8) ( 44.2) ( 35.6) ( 39.9) ( 46.7) ( 45.8) ( 42.0) ( 46.9)
a_E       179.9   179.9   179.9   179.9   179.9   179.9   179.9   179.9   179.9   179.9
        ( 40.8) ( 40.8) ( 40.8) ( 40.8) ( 40.8) ( 40.8) ( 40.8) ( 40.8) ( 40.8) ( 40.8)
           33.7   -17.3    27.2    31.5   -42.7    -7.7    55.9    46.9    10.4    58.2
          7.632  -3.924   6.165   7.140  -9.684  -1.738  12.670  10.628   2.357  13.186
t_O         162     183     162     159     171     161     161     136     172     152
        ( 36.7) ( 41.5) ( 36.7) ( 36.1) ( 38.8) ( 36.5) ( 36.5) ( 30.8) ( 39.0) ( 34.5)
t_E       162.1   162.1   162.1   162.1   162.1   162.1   162.1   162.1   162.1   162.1
        ( 36.8) ( 36.8) ( 36.8) ( 36.8) ( 36.8) ( 36.8) ( 36.8) ( 36.8) ( 36.8) ( 36.8)
           -0.1    44.4    -0.1    -6.1    18.3    -2.1    -2.1   -47.7    20.4   -19.5
         -0.033  10.078  -0.033  -1.381   4.158  -0.485  -0.485 -10.818   4.637  -4.424
g_O          45      49      45      51      59      54      46      59      45      55
        ( 10.2) ( 11.1) ( 10.2) ( 11.6) ( 13.4) ( 12.2) ( 10.4) ( 13.4) ( 10.2) ( 12.5)
g_E        52.1    52.1    52.1    52.1    52.1    52.1    52.1    52.1    52.1    52.1
        ( 11.8) ( 11.8) ( 11.8) ( 11.8) ( 11.8) ( 11.8) ( 11.8) ( 11.8) ( 11.8) ( 11.8)
          -13.3    -6.1   -13.3    -2.3    14.6     3.8   -11.5    14.6   -13.3     5.9
         -3.006  -1.381  -3.006  -0.512   3.307   0.858  -2.614   3.307  -3.006   1.331
c_O          38      38      41      36      54      50      28      44      39      27
        (  8.6) (  8.6) (  9.3) (  8.2) ( 12.2) ( 11.3) (  6.3) ( 10.0) (  8.8) (  6.1)
c_E        46.9    46.9    46.9    46.9    46.9    46.9    46.9    46.9    46.9    46.9
        ( 10.6) ( 10.6) ( 10.6) ( 10.6) ( 10.6) ( 10.6) ( 10.6) ( 10.6) ( 10.6) ( 10.6)
          -16.0   -16.0   -11.0   -19.1    15.2     6.4   -28.9    -5.6   -14.4   -29.8
         -3.631  -3.631  -2.504  -4.322   3.446   1.446  -6.553  -1.278  -3.267  -6.764

N           441     441     441     441     441     441     441     441     441     441
X2          4.1     5.0     2.7     3.9     5.4     0.4    12.1     8.0     3.1    13.3
Gvalue      4.2     5.0     2.7     4.1     5.4     0.4    13.3     8.1     3.2    14.7
Gv/N      0.962   1.143   0.621   0.926   1.227   0.081   3.018   1.838   0.722   3.330
Prob    0.23651 0.16872 0.43353 0.25266 0.14415 0.94923 0.00402 0.04388 0.36429 0.00211
        --------------------------------------------------------------------------------
             20      19      18      17      16      15      14      13      12      11
        --------------------------------------------------------------------------------
a_O         182     193     179     186     198     196     193     201     172     194
        ( 41.3) ( 43.8) ( 40.6) ( 42.2) ( 44.9) ( 44.4) ( 43.8) ( 45.6) ( 39.0) ( 44.0)
a_E       179.9   179.9   179.9   179.9   179.9   179.9   179.9   179.9   179.9   179.9
        ( 40.8) ( 40.8) ( 40.8) ( 40.8) ( 40.8) ( 40.8) ( 40.8) ( 40.8) ( 40.8) ( 40.8)
            4.3    27.2    -1.7    12.5    38.0    33.7    27.2    44.6   -15.4    29.3
          0.970   6.165  -0.395   2.825   8.621   7.632   6.165  10.123  -3.492   6.652
t_O         180     157     187     166     163     156     150     156     198     159
        ( 40.8) ( 35.6) ( 42.4) ( 37.6) ( 37.0) ( 35.4) ( 34.0) ( 35.4) ( 44.9) ( 36.1)
t_E       162.1   162.1   162.1   162.1   162.1   162.1   162.1   162.1   162.1   162.1
        ( 36.8) ( 36.8) ( 36.8) ( 36.8) ( 36.8) ( 36.8) ( 36.8) ( 36.8) ( 36.8) ( 36.8)
           37.8   -10.0    53.5     7.9     1.9   -11.9   -23.2   -11.9    79.3    -6.1
          8.564  -2.265  12.132   1.802   0.421  -2.702  -5.266  -2.702  17.979  -1.381
g_O          48      46      32      45      33      44      49      35      35      47
        ( 10.9) ( 10.4) (  7.3) ( 10.2) (  7.5) ( 10.0) ( 11.1) (  7.9) (  7.9) ( 10.7)
g_E        52.1    52.1    52.1    52.1    52.1    52.1    52.1    52.1    52.1    52.1
        ( 11.8) ( 11.8) ( 11.8) ( 11.8) ( 11.8) ( 11.8) ( 11.8) ( 11.8) ( 11.8) ( 11.8)
           -7.9   -11.5   -31.2   -13.3   -30.2   -14.9    -6.1   -27.9   -27.9    -9.8
         -1.801  -2.614  -7.085  -3.006  -6.846  -3.388  -1.381  -6.327  -6.327  -2.213
c_O          31      45      43      44      47      45      49      49      36      41
        (  7.0) ( 10.2) (  9.8) ( 10.0) ( 10.7) ( 10.2) ( 11.1) ( 11.1) (  8.2) (  9.3)
c_E        46.9    46.9    46.9    46.9    46.9    46.9    46.9    46.9    46.9    46.9
        ( 10.6) ( 10.6) ( 10.6) ( 10.6) ( 10.6) ( 10.6) ( 10.6) ( 10.6) ( 10.6) ( 10.6)
          -25.7    -3.7    -7.5    -5.6     0.2    -3.7     4.3     4.3   -19.1   -11.0
         -5.824  -0.849  -1.698  -1.278   0.040  -0.849   0.968   0.968  -4.322  -2.504

N           441     441     441     441     441     441     441     441     441     441
X2          7.7     1.9    11.9     1.5     8.9     3.0     2.1     8.4    16.5     2.4
Gvalue      8.4     1.9    13.0     1.5     9.9     3.1     2.1     9.1    16.9     2.4
Gv/N      1.908   0.437   2.954   0.343   2.237   0.693   0.486   2.062   3.838   0.554
Prob    0.03819 0.58742 0.00458 0.67969 0.01975 0.38295 0.54330 0.02810 0.00073 0.48572
        --------------------------------------------------------------------------------
             10       9       8       7       6       5       4       3       2       1
        --------------------------------------------------------------------------------
a_O         177     157     210     203     205     224     213     197     184     150
        ( 40.1) ( 35.7) ( 47.6) ( 46.0) ( 46.5) ( 50.8) ( 48.3) ( 44.7) ( 41.7) ( 34.0)
a_E       179.9   179.5   179.9   179.9   179.9   179.9   179.9   179.9   179.9   179.9
        ( 40.8) ( 40.8) ( 40.8) ( 40.8) ( 40.8) ( 40.8) ( 40.8) ( 40.8) ( 40.8) ( 40.8)
           -5.7   -42.0    65.0    49.1    53.6    98.3    72.0    35.8     8.3   -54.5
         -1.293  -9.544  14.748  11.135  12.156  22.287  16.329   8.125   1.892 -12.355
t_O         165     169     167     161     163     134     142     138     164     197
        ( 37.4) ( 38.4) ( 37.9) ( 36.5) ( 37.0) ( 30.4) ( 32.2) ( 31.3) ( 37.2) ( 44.7)
t_E       162.1   161.7   162.1   162.1   162.1   162.1   162.1   162.1   162.1   162.1
        ( 36.8) ( 36.8) ( 36.8) ( 36.8) ( 36.8) ( 36.8) ( 36.8) ( 36.8) ( 36.8) ( 36.8)
            5.9    14.9    10.0    -2.1     1.9   -51.0   -37.6   -44.4     3.9    76.9
          1.339   3.389   2.268  -0.485   0.421 -11.559  -8.515 -10.064   0.879  17.435
g_O          35      55      29      32      37      42      48      58      62      52
        (  7.9) ( 12.5) (  6.6) (  7.3) (  8.4) (  9.5) ( 10.9) ( 13.2) ( 14.1) ( 11.8)
g_E        52.1    52.0    52.1    52.1    52.1    52.1    52.1    52.1    52.1    52.1
        ( 11.8) ( 11.8) ( 11.8) ( 11.8) ( 11.8) ( 11.8) ( 11.8) ( 11.8) ( 11.8) ( 11.8)
          -27.9     6.1   -34.0   -31.2   -25.4   -18.2    -7.9    12.4    21.5    -0.3
         -6.327   1.391  -7.716  -7.085  -5.756  -4.120  -1.801   2.801   4.869  -0.064
c_O          64      59      35      45      36      41      38      48      31      42
        ( 14.5) ( 13.4) (  7.9) ( 10.2) (  8.2) (  9.3) (  8.6) ( 10.9) (  7.0) (  9.5)
c_E        46.9    46.8    46.9    46.9    46.9    46.9    46.9    46.9    46.9    46.9
        ( 10.6) ( 10.6) ( 10.6) ( 10.6) ( 10.6) ( 10.6) ( 10.6) ( 10.6) ( 10.6) ( 10.6)
           39.8    27.3   -20.5    -3.7   -19.1   -11.0   -16.0     2.2   -25.7    -9.3
          9.016   6.210  -4.649  -0.849  -4.322  -2.504  -3.631   0.500  -5.824  -2.106

N           441     440     441     441     441     441     441     441     441     441
X2         12.0     6.5    18.5    10.8    10.4    18.4    10.6     5.9     7.4    13.0
Gvalue     12.1     6.4    20.5    12.0    11.0    18.1    10.5     6.0     8.0    12.8
Gv/N      2.735   1.446   4.650   2.716   2.499   4.104   2.381   1.362   1.817   2.910
Prob    0.00718 0.09527 0.00013 0.00747 0.01161 0.00042 0.01475 0.11120 0.04579 0.00501
        --------------------------------------------------------------------------------

CDS_INITIAL:
        --------------------------------------------------------------------------------
```

	1	2	3	4	5	6	7	8	9	10
a_O	219	0	0	192	190	173	219	184	160	191
	(49.7)	(0.0)	(0.0)	(43.5)	(43.1)	(39.2)	(49.7)	(41.7)	(36.3)	(43.3)
a_E	179.0	170.3	163.4	179.0	170.3	163.4	179.0	170.3	163.4	179.0
	(40.6)	(38.6)	(37.0)	(40.6)	(38.6)	(37.0)	(40.6)	(38.6)	(37.0)	(40.6)
	88.2	0.0	0.0	26.8	41.5	19.8	88.2	28.4	-6.7	24.7
	20.011	0.000	0.000	6.087	9.420	4.491	20.011	6.445	-1.515	5.603
t_O	165	441	0	85	135	186	107	167	159	112
	(37.4)	(100.0)	(0.0)	(19.3)	(30.6)	(42.2)	(24.3)	(37.9)	(36.1)	(25.4)
t_E	104.7	154.1	186.0	104.7	154.1	186.0	104.7	154.1	186.0	104.7
	(23.7)	(34.9)	(42.2)	(23.7)	(34.9)	(42.2)	(23.7)	(34.9)	(42.2)	(23.7)
	150.2	927.6	0.0	-35.4	-35.7	-0.1	4.7	26.9	-50.0	15.2
	34.054	210.333	0.000	-8.026	-8.088	-0.022	1.066	6.106	-11.328	3.436
g_O	57	0	441	94	63	56	84	60	93	91
	(12.9)	(0.0)	(100.0)	(21.3)	(14.3)	(12.7)	(19.0)	(13.6)	(21.1)	(20.6)
g_E	110.0	49.8	48.7	110.0	49.8	48.7	110.0	49.8	48.7	110.0
	(24.9)	(11.3)	(11.0)	(24.9)	(11.3)	(11.0)	(24.9)	(11.3)	(11.0)	(24.9)
	-75.0	0.0	1943.3	-29.6	29.7	15.6	-45.3	22.4	120.3	-34.5
	-16.997	0.000	440.662	-6.704	6.727	3.546	-10.276	5.079	27.283	-7.829
c_O	0	0	0	70	53	26	31	30	29	47
	(0.0)	(0.0)	(0.0)	(15.9)	(12.0)	(5.9)	(7.0)	(6.8)	(6.6)	(10.7)
c_E	47.3	66.8	42.9	47.3	66.8	42.9	47.3	66.8	42.9	47.3
	(10.7)	(15.2)	(9.7)	(10.7)	(15.2)	(9.7)	(10.7)	(15.2)	(9.7)	(10.7)
	0.0	0.0	0.0	54.9	-24.6	-26.0	-26.2	-48.1	-22.7	-0.6
	0.000	0.000	0.000	12.457	-5.571	-5.898	-5.934	-10.896	-5.142	-0.127
N	441	441	441	441	441	441	441	441	441	441
X2	116.5	821.3	3552.2	17.9	11.0	8.3	20.7	24.6	48.8	4.6
Gvalue	163.5	927.6	1943.3	16.8	11.0	9.3	21.5	29.7	41.0	4.8
Gv/N	37.068	210.333	440.662	3.814	2.487	2.118	4.867	6.732	9.298	1.083
Prob	0.00000	0.00000	1.00000	0.00077	0.01190	0.02511	0.00008	0.00000	0.00000	0.18891

	11	12	13	14	15	16	17	18	19	20
a_O	155	162	207	177	175	197	156	176	215	154
	(35.1)	(36.7)	(46.9)	(40.1)	(39.7)	(44.7)	(35.4)	(39.9)	(48.8)	(34.9)
a_E	170.3	163.4	179.0	170.3	163.4	179.0	170.3	163.4	179.0	170.3
	(38.6)	(37.0)	(40.6)	(38.6)	(37.0)	(40.6)	(38.6)	(37.0)	(40.6)	(38.6)
	-29.2	-2.7	60.1	13.6	24.1	37.7	-27.4	26.2	78.7	-31.0
	-6.627	-0.621	13.624	3.086	5.456	8.542	-6.215	5.942	17.848	-7.037
t_O	183	184	129	178	171	116	187	159	118	183
	(41.5)	(41.7)	(29.3)	(40.4)	(38.8)	(26.3)	(42.4)	(36.1)	(26.8)	(41.5)
t_E	154.1	186.0	104.7	154.1	186.0	104.7	154.1	186.0	104.7	154.1
	(34.9)	(42.2)	(23.7)	(34.9)	(42.2)	(23.7)	(34.9)	(42.2)	(23.7)	(34.9)
	63.0	-4.1	53.9	51.4	-28.8	23.8	72.5	-50.0	28.3	63.0
	14.284	-0.923	12.224	11.657	-6.541	5.404	16.430	-11.328	6.412	14.284
g_O	59	68	63	47	66	76	47	68	75	52
	(13.4)	(15.4)	(14.3)	(10.7)	(15.0)	(17.2)	(10.7)	(15.4)	(17.0)	(11.8)
g_E	49.8	48.7	110.0	49.8	48.7	110.0	49.8	48.7	110.0	49.8
	(11.3)	(11.0)	(24.9)	(11.3)	(11.0)	(24.9)	(11.3)	(11.0)	(24.9)	(11.3)
	20.0	45.4	-70.2	-5.4	40.1	-56.2	-5.4	45.4	-57.5	4.5
	4.544	10.293	-15.926	-1.227	9.097	-12.747	-1.227	10.293	-13.029	1.027
c_O	44	27	42	39	29	52	51	38	33	52
	(10.0)	(6.1)	(9.5)	(8.8)	(6.6)	(11.8)	(11.6)	(8.6)	(7.5)	(11.8)
c_E	66.8	42.9	47.3	66.8	42.9	47.3	66.8	42.9	47.3	66.8
	(15.2)	(9.7)	(10.7)	(15.2)	(9.7)	(10.7)	(15.2)	(9.7)	(10.7)	(15.2)
	-36.8	-25.0	-9.9	-42.0	-22.7	9.9	-27.6	-9.2	-23.7	-26.1
	-8.339	-5.663	-2.256	-9.525	-5.142	2.244	-6.251	-2.080	-5.382	-5.915
N	441	441	441	441	441	441	441	441	441	441
X2	16.3	13.6	30.7	15.7	12.7	14.0	12.1	13.1	24.4	10.4
Gvalue	17.0	13.6	33.8	17.6	12.7	15.2	12.1	12.5	25.8	10.4
Gv/N	3.862	3.086	7.667	3.991	2.870	3.444	2.737	2.826	5.849	2.358
Prob	0.00070	0.00349	0.00000	0.00053	0.00545	0.00166	0.00715	0.00595	0.00001	0.01545

	21	22	23	24	25	26	27	28	29	30
a_O	159	186	175	154	171	140	169	180	132	184
	(36.1)	(42.2)	(39.7)	(34.9)	(38.8)	(31.7)	(38.3)	(40.8)	(29.9)	(41.7)
a_E	163.4	179.0	170.3	163.4	179.0	170.3	163.4	179.0	170.3	163.4
	(37.0)	(40.6)	(38.6)	(37.0)	(40.6)	(38.6)	(37.0)	(40.6)	(38.6)	(37.0)
	-8.6	14.2	9.5	-18.2	-15.7	-54.9	11.4	1.9	-67.3	43.8
	-1.957	3.218	2.149	-4.127	-3.562	-12.448	2.595	0.438	-15.260	9.921
t_O	175	122	177	200	141	191	189	137	213	175
	(39.7)	(27.7)	(40.1)	(45.4)	(32.0)	(43.3)	(42.9)	(31.1)	(48.3)	(39.7)
t_E	186.0	104.7	154.1	186.0	104.7	154.1	186.0	104.7	154.1	186.0
	(42.2)	(23.7)	(34.9)	(42.2)	(23.7)	(34.9)	(42.2)	(23.7)	(34.9)	(42.2)
	-21.4	37.4	49.1	28.9	84.0	82.1	6.0	73.7	138.0	-21.4
	-4.858	8.474	11.139	6.559	19.049	18.615	1.350	16.721	31.290	-4.858
g_O	68	85	37	62	84	65	49	90	41	45
	(15.4)	(19.3)	(8.4)	(14.1)	(19.0)	(14.7)	(11.1)	(20.4)	(9.3)	(10.2)
g_E	48.7	110.0	49.8	48.7	110.0	49.8	48.7	110.0	49.8	48.7
	(11.0)	(24.9)	(11.3)	(11.0)	(24.9)	(11.3)	(11.0)	(24.9)	(11.3)	(11.0)
	45.4	-43.8	-22.0	29.9	-45.3	34.7	0.6	-36.1	-15.9	-7.1
	10.293	-9.942	-4.980	6.788	-10.276	7.861	0.135	-8.194	-3.610	-1.614
c_O	39	48	52	25	45	45	34	34	55	37
	(8.8)	(10.9)	(11.8)	(5.7)	(10.2)	(10.2)	(7.7)	(7.7)	(12.5)	(8.4)
c_E	42.9	47.3	66.8	42.9	47.3	66.8	42.9	47.3	66.8	42.9
	(9.7)	(10.7)	(15.2)	(9.7)	(10.7)	(15.2)	(9.7)	(10.7)	(15.2)	(9.7)
	-7.4	1.5	-26.1	-27.0	-4.4	-35.6	-15.8	-22.4	-21.4	-10.9
	-1.675	0.329	-5.915	-6.116	-1.009	-8.070	-3.576	-5.084	-4.858	-2.473
N	441	441	441	441	441	441	441	441	441	441
X2	8.8	8.8	10.1	12.7	19.2	26.0	2.1	17.4	34.8	4.3
Gvalue	7.9	9.2	10.6	13.7	18.5	26.3	2.2	17.1	33.4	4.3
Gv/N	1.802	2.080	2.393	3.104	4.203	5.958	0.503	3.881	7.563	0.976
Prob	0.04711	0.02709	0.01440	0.00336	0.00034	0.00001	0.52826	0.00067	0.00000	0.23049

	31	32	33	34	35	36	37	38	39	40
a_O	192	164	173	198	166	180	182	157	157	181
	(43.5)	(37.2)	(39.2)	(44.9)	(37.6)	(40.8)	(41.3)	(35.6)	(35.6)	(41.0)
a_E	179.0	170.3	163.4	179.0	170.3	163.4	179.0	170.3	163.4	179.0
	(40.6)	(38.6)	(37.0)	(40.6)	(38.6)	(37.0)	(40.6)	(38.6)	(37.0)	(40.6)
	26.8	-12.4	19.8	39.9	-8.5	34.9	6.0	-25.6	-12.5	3.9
	6.087	-2.814	4.491	9.040	-1.936	7.911	1.355	-5.800	-2.834	0.895
t_O	120	189	166	127	198	181	126	187	195	117
	(27.2)	(42.9)	(37.6)	(28.8)	(44.9)	(41.0)	(28.6)	(42.4)	(44.2)	(26.5)
t_E	104.7	154.1	186.0	104.7	154.1	186.0	104.7	154.1	186.0	104.7
	(23.7)	(34.9)	(42.2)	(23.7)	(34.9)	(42.2)	(23.7)	(34.9)	(42.2)	(23.7)
	32.8	77.3	-37.9	49.1	99.4	-10.0	46.7	72.5	18.3	26.0

	7.436	17.517	-8.583	11.135	22.529	-2.258	10.596	16.430	4.156	5.906
g_O	89	46	67	79	35	51	93	40	56	89
	(20.2)	(10.4)	(15.2)	(17.9)	(7.9)	(11.6)	(21.1)	(9.1)	(12.7)	(20.2)
g_E	110.0	49.8	48.7	110.0	49.8	48.7	110.0	49.8	48.7	110.0
	(24.9)	(11.3)	(11.0)	(24.9)	(11.3)	(11.0)	(24.9)	(11.3)	(11.0)	(24.9)
	-37.7	-7.3	42.7	-52.3	-24.7	4.7	-31.2	-17.5	15.6	-37.7
	-8.554	-1.649	9.692	-11.863	-5.593	1.066	-7.084	-3.970	3.546	-8.554
c_O	40	42	35	37	42	29	40	57	33	54
	(9.1)	(9.5)	(7.9)	(8.4)	(9.5)	(6.6)	(9.1)	(12.9)	(7.5)	(12.2)
c_E	47.3	66.8	42.9	47.3	66.8	42.9	47.3	66.8	42.9	47.3
	(10.7)	(15.2)	(9.7)	(10.7)	(15.2)	(9.7)	(10.7)	(15.2)	(9.7)	(10.7)
	-13.4	-39.0	-14.2	-18.1	-39.0	-22.7	-13.4	-18.1	-17.3	14.4
	-3.033	-8.846	-3.221	-4.114	-8.846	-5.142	-3.033	-4.111	-3.918	3.254
N	441	441	441	441	441	441	441	441	441	441
X2	8.3	17.7	11.0	17.7	26.3	6.4	8.1	11.5	4.0	6.4
Gvalue	8.5	18.6	10.5	18.5	27.1	7.0	8.1	11.2	4.2	6.6
Gv/N	1.936	4.208	2.378	4.198	6.154	1.577	1.833	2.549	0.950	1.503
Prob	0.03613	0.00034	0.01484	0.00034	0.00001	0.07336	0.04429	0.01049	0.24157	0.08481

	41	42	43	44	45	46	47	48	49	50
a_O	155	145	183	150	143	191	143	181	179	146
	(35.1)	(32.9)	(41.5)	(34.0)	(32.4)	(43.3)	(32.4)	(41.0)	(40.6)	(33.1)
a_E	170.3	163.4	179.0	170.3	163.4	179.0	170.3	163.4	179.0	170.3
	(38.6)	(37.0)	(40.6)	(38.6)	(37.0)	(40.6)	(38.6)	(37.0)	(40.6)	(38.6)
	-29.2	-34.6	8.0	-38.1	-38.1	24.7	-50.0	37.1	-0.1	-45.0
	-6.627	-7.846	1.817	-8.644	-8.638	5.603	-11.340	8.410	-0.017	-10.203
t_O	189	189	130	182	205	132	179	185	117	168
	(42.9)	(42.9)	(29.5)	(41.3)	(46.5)	(29.9)	(40.6)	(42.0)	(26.5)	(38.1)
t_E	154.1	186.0	104.7	154.1	186.0	104.7	154.1	186.0	104.7	154.1
	(34.9)	(42.2)	(23.7)	(34.9)	(42.2)	(23.7)	(34.9)	(42.2)	(23.7)	(34.9)
	77.3	6.0	56.3	60.7	39.8	61.2	53.7	-2.1	26.0	29.1
	17.517	1.350	12.774	13.753	9.019	13.885	12.177	-0.474	5.906	6.597
g_O	49	65	79	35	56	73	40	48	96	65
	(11.1)	(14.7)	(17.9)	(7.9)	(12.7)	(16.6)	(9.1)	(10.9)	(21.8)	(14.7)
g_E	49.8	48.7	110.0	49.8	48.7	110.0	49.8	48.7	110.0	49.8
	(11.3)	(11.0)	(24.9)	(11.3)	(11.0)	(24.9)	(11.3)	(11.0)	(24.9)	(11.3)
	-1.6	37.5	-52.3	-24.7	15.6	-59.9	-17.5	-1.4	-26.2	34.7
	-0.353	8.509	-11.863	-5.593	3.546	-13.577	-3.970	-0.316	-5.930	7.861
c_O	48	42	49	74	37	45	79	27	49	62
	(10.9)	(9.5)	(11.1)	(16.8)	(8.4)	(10.2)	(17.9)	(6.1)	(11.1)	(14.1)
c_E	66.8	42.9	47.3	66.8	42.9	47.3	66.8	42.9	47.3	66.8
	(15.2)	(9.7)	(10.7)	(15.2)	(9.7)	(10.7)	(15.2)	(9.7)	(10.7)	(15.2)
	-31.8	-1.7	3.5	15.1	-10.9	-4.4	26.4	-25.0	3.5	-9.3
	-7.203	-0.393	0.794	3.423	-2.473	-1.009	5.996	-5.663	0.794	-2.107
N	441	441	441	441	441	441	441	441	441	441
X2	14.6	7.6	15.0	12.7	6.4	20.5	12.6	7.8	3.3	9.7
Gvalue	14.7	7.1	15.5	13.0	6.4	21.6	12.6	8.6	3.3	9.5
Gv/N	3.334	1.620	3.523	2.939	1.453	4.902	2.864	1.957	0.754	2.148
Prob	0.00209	0.06746	0.00141	0.00472	0.09332	0.00008	0.00551	0.03463	0.34434	0.02364

	51	52	53	54	55	56	57	58	59	60
a_O	152	163	142	148	167	150	175	191	153	140
	(34.5)	(37.0)	(32.2)	(33.6)	(37.9)	(34.0)	(39.7)	(43.3)	(34.7)	(31.7)
a_E	163.4	179.0	170.3	163.4	179.0	170.3	163.4	179.0	170.3	163.4
	(37.0)	(40.6)	(38.6)	(37.0)	(40.6)	(38.6)	(37.0)	(40.6)	(38.6)	(37.0)
	-21.9	-30.6	-51.7	-29.3	-23.2	-38.1	24.1	24.7	-32.8	-43.2
	-4.975	-6.937	-11.713	-6.634	-5.271	-8.644	5.456	5.603	-7.443	-9.803
t_O	184	144	183	185	132	157	154	109	163	196
	(41.7)	(32.7)	(41.5)	(42.0)	(29.9)	(35.6)	(34.9)	(24.7)	(37.0)	(44.4)
t_E	186.0	104.7	154.1	186.0	104.7	154.1	186.0	104.7	154.1	186.0
	(42.2)	(23.7)	(34.9)	(42.2)	(23.7)	(34.9)	(42.2)	(23.7)	(34.9)	(42.2)
	-4.1	91.9	63.0	-2.1	61.2	5.9	-58.2	8.8	18.4	20.4
	-0.923	20.830	14.284	-0.474	13.885	1.343	-13.203	2.001	4.167	4.632
g_O	61	80	48	58	101	51	53	93	54	47
	(13.8)	(18.1)	(10.9)	(13.2)	(22.9)	(11.6)	(12.0)	(21.1)	(12.2)	(10.7)
g_E	48.7	110.0	49.8	48.7	110.0	49.8	48.7	110.0	49.8	48.7
	(11.0)	(24.9)	(11.3)	(11.0)	(24.9)	(11.3)	(11.0)	(24.9)	(11.3)	(11.0)
	27.5	-51.0	-3.5	20.3	-17.3	2.5	9.0	-31.2	8.8	-3.3
	6.228	-11.556	-0.795	4.595	-3.913	0.558	2.032	-7.084	1.991	-0.759
c_O	44	54	68	50	41	83	59	48	71	58
	(10.0)	(12.2)	(15.4)	(11.3)	(9.3)	(18.8)	(13.4)	(10.9)	(16.1)	(13.2)
c_E	42.9	47.3	66.8	42.9	47.3	66.8	42.9	47.3	66.8	42.9
	(9.7)	(10.7)	(15.2)	(9.7)	(10.7)	(15.2)	(9.7)	(10.7)	(15.2)	(9.7)
	2.3	14.4	2.4	15.4	-11.7	36.0	37.7	1.5	8.6	35.1
	0.517	3.254	0.537	3.486	-2.650	8.159	8.542	0.329	1.951	7.948
N	441	441	441	441	441	441	441	441	441	441
X2	3.9	25.4	10.2	4.4	9.5	6.4	12.8	3.6	2.9	9.3
Gvalue	3.7	24.7	10.2	4.3	9.0	6.2	12.5	3.7	2.9	8.9
Gv/N	0.847	5.591	2.314	0.974	2.051	1.416	2.827	0.849	0.666	2.018
Prob	0.29151	0.00002	0.01691	0.23128	0.02872	0.10026	0.00595	0.29024	0.40154	0.03066

	61	62	63	64	65	66	67	68	69	70
a_O	192	147	155	175	175	149	194	177	174	179
	(43.5)	(33.3)	(35.1)	(39.7)	(39.7)	(33.8)	(44.0)	(40.1)	(39.5)	(40.6)
a_E	179.0	170.3	163.4	179.0	170.3	163.4	179.0	170.3	163.4	179.0
	(40.6)	(38.6)	(37.0)	(40.6)	(38.6)	(37.0)	(40.6)	(38.6)	(37.0)	(40.6)
	26.8	-43.3	-16.3	-8.0	9.5	-27.4	31.1	13.6	21.9	-0.1
	6.087	-9.818	-3.699	-1.810	2.149	-6.224	7.062	3.086	4.972	-0.017
t_O	113	178	193	107	148	197	107	163	161	110
	(25.6)	(40.4)	(43.8)	(24.3)	(33.6)	(44.7)	(24.3)	(37.0)	(36.5)	(24.9)
t_E	104.7	154.1	186.0	104.7	154.1	186.0	104.7	154.1	186.0	104.7
	(23.7)	(34.9)	(42.2)	(23.7)	(34.9)	(42.2)	(23.7)	(34.9)	(42.2)	(23.7)
	17.3	51.4	14.2	4.7	-11.9	22.5	4.7	18.4	-46.6	10.9
	3.922	11.657	3.211	1.066	-2.696	5.110	1.066	4.167	-10.558	2.475
g_O	90	44	51	112	42	39	90	47	59	100
	(20.4)	(10.0)	(11.6)	(25.4)	(9.5)	(8.8)	(20.4)	(10.7)	(13.4)	(22.7)
g_E	110.0	49.8	48.7	110.0	49.8	48.7	110.0	49.8	48.7	110.0
	(24.9)	(11.3)	(11.0)	(24.9)	(11.3)	(11.0)	(24.9)	(11.3)	(11.0)	(24.9)
	-36.1	-10.9	4.7	4.0	-14.3	-17.3	-36.1	-5.4	22.6	-19.1
	-8.194	-2.465	1.066	0.912	-3.239	-3.930	-8.194	-1.227	5.132	-4.326
c_O	46	72	42	47	76	56	50	54	47	52
	(10.4)	(16.3)	(9.5)	(10.7)	(17.2)	(12.7)	(11.3)	(12.2)	(10.7)	(11.8)
c_E	47.3	66.8	42.9	47.3	66.8	42.9	47.3	66.8	42.9	47.3
	(10.7)	(15.2)	(9.7)	(10.7)	(15.2)	(9.7)	(10.7)	(15.2)	(9.7)	(10.7)
	-2.5	10.7	-1.7	-0.6	19.6	29.9	5.6	-23.0	8.6	9.9
	-0.573	2.435	-0.393	-0.127	4.434	6.783	1.268	-5.219	1.958	2.244

N	441	441	441	441	441	441	441	441	441	441
X2	5.3	8.0	0.8	0.2	2.8	7.9	5.1	3.4	6.6	1.7
Gvalue	5.5	8.0	0.8	0.2	2.9	7.7	5.3	3.6	6.6	1.7
Gv/N	1.242	1.810	0.186	0.041	0.649	1.740	1.203	0.808	1.504	0.377
Prob	0.13990	0.04641	0.84513	0.98067	0.41356	0.05327	0.15084	0.31288	0.08453	0.64541

	71	72	73	74	75	76	77	78	79	80
a_O	170	176	181	153	164	202	186	166	170	193
	(38.5)	(39.9)	(41.0)	(34.7)	(37.2)	(45.8)	(42.2)	(37.6)	(38.5)	(43.8)
a_E	170.3	163.4	179.0	170.3	163.4	179.0	170.3	163.4	179.0	170.3
	(38.6)	(37.0)	(40.6)	(38.6)	(37.0)	(40.6)	(38.6)	(37.0)	(40.6)	(38.6)
	-0.6	26.2	3.9	-32.8	1.3	48.8	32.8	5.3	-17.6	48.2
	-0.147	5.942	0.895	-7.443	0.284	11.055	7.427	1.200	-3.993	10.940
t_O	164	195	117	174	190	105	151	184	117	138
	(37.2)	(44.2)	(26.5)	(39.5)	(43.1)	(23.8)	(34.2)	(41.7)	(26.5)	(31.3)
t_E	154.1	186.0	104.7	154.1	186.0	104.7	154.1	186.0	104.7	154.1
	(34.9)	(42.2)	(23.7)	(34.9)	(42.2)	(23.7)	(34.9)	(42.2)	(23.7)	(34.9)
	20.5	18.3	26.0	42.3	8.0	0.7	-6.1	-4.1	26.0	-30.4
	4.648	4.156	5.906	9.602	1.811	0.148	-1.376	-0.923	5.906	-6.892
g_O	37	37	92	52	44	102	47	52	112	50
	(8.4)	(8.4)	(20.9)	(11.8)	(10.0)	(23.1)	(10.7)	(11.8)	(25.4)	(11.3)
g_E	49.8	48.7	110.0	49.8	48.7	110.0	49.8	48.7	110.0	49.8
	(11.3)	(11.0)	(24.9)	(11.3)	(11.0)	(24.9)	(11.3)	(11.0)	(24.9)	(11.3)
	-22.0	-20.3	-32.9	4.5	-8.9	-15.4	-5.4	6.8	4.0	0.4
	-4.980	-4.611	-7.459	1.027	-2.026	-3.496	-1.227	1.545	0.912	0.098
c_O	70	33	51	62	43	32	57	39	42	60
	(15.9)	(7.5)	(11.6)	(14.1)	(9.8)	(7.3)	(12.9)	(8.8)	(9.5)	(13.6)
c_E	66.8	42.9	47.3	66.8	42.9	47.3	66.8	42.9	47.3	66.8
	(15.2)	(9.7)	(10.7)	(15.2)	(9.7)	(10.7)	(15.2)	(9.7)	(10.7)	(15.2)
	6.5	-17.3	7.7	-9.3	0.3	-25.0	-18.1	-7.4	-9.9	-12.9
	1.473	-3.918	1.752	-2.107	0.057	-5.665	-4.111	-1.675	-2.256	-2.932
N	441	441	441	441	441	441	441	441	441	441
X2	4.1	6.5	4.7	4.8	0.5	8.5	3.1	0.6	2.5	5.4
Gvalue	4.4	6.9	4.8	4.8	0.6	9.0	3.1	0.6	2.5	5.4
Gv/N	0.994	1.568	1.095	1.078	0.126	2.042	0.712	0.146	0.569	1.214
Prob	0.22294	0.07459	0.18488	0.19071	0.90652	0.02925	0.37031	0.88624	0.47344	0.14771

	81	82	83	84	85	86	87	88	89	90
a_O	162	184	147	187	195	179	178	162	144	142
	(36.7)	(41.7)	(33.3)	(42.4)	(44.2)	(40.6)	(40.4)	(36.7)	(32.7)	(32.2)
a_E	163.4	179.0	170.3	163.4	179.0	170.3	163.4	179.0	170.3	163.4
	(37.0)	(40.6)	(38.6)	(37.0)	(40.6)	(38.6)	(37.0)	(40.6)	(38.6)	(37.0)
	-2.7	10.1	-43.3	50.5	33.3	17.8	30.5	-32.4	-48.4	-39.8
	-0.621	2.282	-9.818	11.454	7.553	4.033	6.921	-7.347	-10.964	-9.030
t_O	204	107	179	191	104	155	175	114	170	210
	(46.3)	(24.3)	(40.6)	(43.3)	(23.6)	(35.1)	(39.7)	(25.9)	(38.5)	(47.6)
t_E	186.0	104.7	154.1	186.0	104.7	154.1	186.0	104.7	154.1	186.0
	(42.2)	(23.7)	(34.9)	(42.2)	(23.7)	(34.9)	(42.2)	(23.7)	(34.9)	(42.2)
	37.6	4.7	53.7	10.0	-1.3	1.9	-21.4	19.5	33.5	50.9
	8.522	1.066	12.177	2.276	-0.305	0.425	-4.858	4.412	7.588	11.534
g_O	41	91	49	33	100	55	43	100	52	48
	(9.3)	(20.6)	(11.1)	(7.5)	(22.7)	(12.5)	(9.8)	(22.7)	(11.8)	(10.9)
g_E	48.7	110.0	49.8	48.7	110.0	49.8	48.7	110.0	49.8	48.7
	(11.0)	(24.9)	(11.3)	(11.0)	(24.9)	(11.3)	(11.0)	(24.9)	(11.3)	(11.0)
	-14.1	-34.5	-1.6	-25.7	-19.1	11.0	-10.7	-19.1	4.5	-1.4
	-3.201	-7.829	-0.353	-5.825	-4.326	2.485	-2.429	-4.326	1.027	-0.316
c_O	34	59	66	30	42	52	45	65	75	41
	(7.7)	(13.4)	(15.0)	(6.8)	(9.5)	(11.8)	(10.2)	(14.7)	(17.0)	(9.3)
c_E	42.9	47.3	66.8	42.9	47.3	66.8	42.9	47.3	66.8	42.9
	(9.7)	(10.7)	(15.2)	(9.7)	(10.7)	(15.2)	(9.7)	(10.7)	(15.2)	(9.7)
	-15.8	26.1	-1.6	-21.4	-9.9	-26.1	4.4	41.4	17.3	-3.7
	-3.576	5.925	-0.372	-4.858	-2.256	-5.915	0.987	9.383	3.925	-0.831
N	441	441	441	441	441	441	441	441	441	441
X2	4.8	6.4	7.3	12.5	2.9	4.3	2.7	10.0	6.8	6.0
Gvalue	5.0	6.4	7.2	13.4	2.9	4.5	2.7	9.4	6.9	6.0
Gv/N	1.124	1.445	1.634	3.046	0.667	1.028	0.621	2.123	1.576	1.356
Prob	0.17492	0.09492	0.06558	0.00379	0.40105	0.20946	0.43341	0.02486	0.07355	0.11260

	91	92	93	94	95	96	97	98	99	100
a_O	159	166	173	176	197	149	151	178	158	171
	(36.1)	(37.6)	(39.2)	(40.6)	(45.4)	(34.3)	(35.4)	(41.8)	(37.1)	(40.9)
a_E	179.0	170.3	163.4	176.2	167.6	160.8	172.9	164.5	157.8	169.7
	(40.6)	(38.6)	(37.0)	(40.6)	(38.6)	(37.0)	(40.6)	(38.6)	(37.0)	(40.6)
	-37.7	-8.5	19.8	-0.4	63.6	-22.7	-41.0	28.0	0.4	2.6
	-8.558	-1.936	4.491	-0.090	14.661	-5.225	-9.621	6.575	0.086	0.625
t_O	119	158	171	128	150	196	137	153	178	116
	(27.0)	(35.8)	(38.8)	(29.5)	(34.6)	(45.2)	(32.2)	(35.9)	(41.8)	(27.8)
t_E	104.7	154.1	186.0	103.0	151.6	183.1	101.1	148.8	179.7	99.2
	(23.7)	(34.9)	(42.2)	(23.7)	(34.9)	(42.2)	(23.7)	(34.9)	(42.2)	(23.7)
	30.5	8.0	-28.8	55.6	-3.2	26.7	83.2	8.5	-3.4	36.3
	6.922	1.807	-6.541	12.810	-0.743	6.152	19.535	1.987	-0.803	8.675
g_O	99	48	46	73	46	58	97	40	51	78
	(22.4)	(10.9)	(10.4)	(16.8)	(10.6)	(13.4)	(22.8)	(9.4)	(12.0)	(18.7)
g_E	110.0	49.8	48.7	108.3	49.0	47.9	106.3	48.1	47.0	104.3
	(24.9)	(11.3)	(11.0)	(24.9)	(11.3)	(11.0)	(24.9)	(11.3)	(11.0)	(24.9)
	-20.9	-3.5	-5.3	-57.5	-5.8	22.1	-17.7	-14.7	8.2	-45.3
	-4.734	-0.795	-1.191	-13.257	-1.337	5.097	-4.155	-3.460	1.932	-10.833
c_O	64	69	51	57	41	31	41	55	39	53
	(14.5)	(15.6)	(11.6)	(13.1)	(9.4)	(7.1)	(9.6)	(12.9)	(9.2)	(12.7)
c_E	47.3	66.8	42.9	46.5	65.8	42.2	45.7	64.6	41.4	44.8
	(10.7)	(15.2)	(9.7)	(10.7)	(15.2)	(9.7)	(10.7)	(15.2)	(9.7)	(10.7)
	38.8	4.4	17.7	23.1	-38.7	-19.1	-8.8	-17.6	-4.7	17.8
	8.788	1.002	4.014	5.331	-8.928	-4.404	-2.077	-4.135	-1.101	4.254
N	441	441	441	434	434	434	426	426	426	418
X2	11.2	0.3	3.5	19.9	14.7	6.9	16.8	4.0	0.5	11.0
Gvalue	10.7	0.3	3.4	20.8	15.9	7.0	15.7	4.1	0.5	11.4
Gv/N	2.419	0.078	0.774	4.794	3.654	1.620	3.683	0.968	0.114	2.720
Prob	0.01368	0.95133	0.33247	0.00012	0.00121	0.07095	0.00131	0.24844	0.92242	0.00988

Bcen_AU1054
5UTR:

	100	99	98	97	96	95	94	93	92	91
a_O	250	230	257	228	232	244	267	235	218	261
	(18.3)	(16.8)	(18.8)	(16.7)	(16.9)	(17.8)	(19.5)	(17.2)	(15.9)	(19.1)
a_E	247.1	247.1	247.1	247.1	247.1	247.1	247.1	247.1	247.1	247.1
	(18.0)	(18.0)	(18.0)	(18.0)	(18.0)	(18.0)	(18.0)	(18.0)	(18.0)	(18.0)
	5.9	-33.0	20.2	-36.7	-29.2	-6.1	41.4	-23.6	-54.6	28.6
	0.428	-2.408	1.476	-2.678	-2.136	-0.449	3.023	-1.722	-3.989	2.088
t_O	284	243	238	267	252	224	260	245	222	209
	(20.7)	(17.8)	(17.4)	(19.5)	(18.4)	(16.4)	(19.0)	(17.9)	(16.2)	(15.3)
t_E	257.9	257.9	257.9	257.9	257.9	257.9	257.9	257.9	257.9	257.9
	(18.8)	(18.8)	(18.8)	(18.8)	(18.8)	(18.8)	(18.8)	(18.8)	(18.8)	(18.8)
	54.7	-29.0	-38.3	18.5	-11.7	-63.2	4.2	-25.2	-66.6	-87.9
	3.997	-2.115	-2.795	1.350	-0.855	-4.614	0.305	-1.839	-4.864	-6.422
g_O	414	423	421	435	411	452	392	462	453	428
	(30.2)	(30.9)	(30.8)	(31.8)	(30.0)	(33.0)	(28.6)	(33.7)	(33.1)	(31.3)
g_E	391.2	391.2	391.2	391.2	391.2	391.2	391.2	391.2	391.2	391.2
	(28.6)	(28.6)	(28.6)	(28.6)	(28.6)	(28.6)	(28.6)	(28.6)	(28.6)	(28.6)
	46.8	66.0	61.7	92.2	40.5	130.5	1.5	153.6	132.8	76.8
	3.418	4.822	4.507	6.736	2.957	9.531	0.110	11.219	9.698	5.613
c_O	421	473	453	439	474	449	450	427	476	471
	(30.8)	(34.6)	(33.1)	(32.1)	(34.6)	(32.8)	(32.9)	(31.2)	(34.8)	(34.4)
c_E	472.7	472.7	472.7	472.7	472.7	472.7	472.7	472.7	472.7	472.7
	(34.5)	(34.5)	(34.5)	(34.5)	(34.5)	(34.5)	(34.5)	(34.5)	(34.5)	(34.5)
	-97.6	0.5	-38.6	-65.0	2.5	-46.3	-44.4	-86.9	6.5	-3.5
	-7.129	0.038	-2.823	-4.749	0.184	-3.380	-3.241	-6.348	0.478	-0.254
N	1369	1369	1369	1369	1369	1369	1369	1369	1369	1369
X2	9.7	4.6	5.0	9.1	2.1	15.1	2.7	18.5	18.2	13.5
Gvalue	9.8	4.6	5.0	9.0	2.1	14.9	2.7	17.9	18.1	14.1
Gv/N	0.713	0.336	0.366	0.659	0.151	1.088	0.197	1.309	1.323	1.026
Prob	0.02066	0.20353	0.17071	0.02906	0.55968	0.00190	0.44144	0.00046	0.00042	0.00284

	90	89	88	87	86	85	84	83	82	81
a_O	263	222	267	248	238	241	224	236	257	211
	(19.2)	(16.2)	(19.5)	(18.1)	(17.4)	(17.6)	(16.4)	(17.2)	(18.8)	(15.4)
a_E	247.1	247.1	247.1	247.1	247.1	247.1	247.1	247.1	247.1	247.1
	(18.0)	(18.0)	(18.0)	(18.0)	(18.0)	(18.0)	(18.0)	(18.0)	(18.0)	(18.0)
	32.8	-47.5	41.4	1.8	-17.8	-12.0	-44.0	-21.7	20.2	-66.6
	2.398	-3.473	3.023	0.133	-1.303	-0.879	-3.211	-1.583	1.476	-4.867
t_O	256	218	258	241	214	261	245	232	254	250
	(18.7)	(15.9)	(18.8)	(17.6)	(15.6)	(19.1)	(17.9)	(16.9)	(18.6)	(18.3)
t_E	257.9	257.9	257.9	257.9	257.9	257.9	257.9	257.9	257.9	257.9
	(18.8)	(18.8)	(18.8)	(18.8)	(18.8)	(18.8)	(18.8)	(18.8)	(18.8)	(18.8)
	-3.8	-73.3	0.2	-32.7	-79.9	6.2	-25.2	-49.1	-7.8	-15.6
	-0.279	-5.355	0.012	-2.389	-5.836	0.453	-1.839	-3.590	-0.568	-1.139
g_O	411	484	415	468	446	427	440	443	419	454
	(30.0)	(35.4)	(30.3)	(34.2)	(32.6)	(31.2)	(32.1)	(32.4)	(30.6)	(33.2)
g_E	391.2	391.2	391.2	391.2	391.2	391.2	391.2	391.2	391.2	391.2
	(28.6)	(28.6)	(28.6)	(28.6)	(28.6)	(28.6)	(28.6)	(28.6)	(28.6)	(28.6)
	40.5	205.9	48.9	167.7	116.8	74.7	103.3	110.1	57.4	135.1
	2.957	15.042	3.573	12.247	8.534	5.454	7.548	8.040	4.195	9.866
c_O	439	445	429	412	471	440	460	458	439	454
	(32.1)	(32.5)	(31.3)	(30.1)	(34.4)	(32.1)	(33.6)	(33.5)	(32.1)	(33.2)
c_E	472.7	472.7	472.7	472.7	472.7	472.7	472.7	472.7	472.7	472.7
	(34.5)	(34.5)	(34.5)	(34.5)	(34.5)	(34.5)	(34.5)	(34.5)	(34.5)	(34.5)
	-65.0	-53.8	-83.3	-113.3	-3.5	-63.2	-25.1	-29.0	-65.0	-36.7
	-4.749	-3.931	-6.085	-8.277	-0.254	-4.613	-1.836	-2.119	-4.749	-2.683
N	1369	1369	1369	1369	1369	1369	1369	1369	1369	1369
X2	4.4	32.3	7.1	24.0	15.5	5.7	9.2	10.4	4.8	16.3
Gvalue	4.5	31.3	7.2	23.5	15.6	5.7	9.1	10.2	4.8	16.1
Gv/N	0.326	2.283	0.523	1.714	1.141	0.415	0.662	0.747	0.354	1.177
Prob	0.21538	0.00000	0.06714	0.00003	0.00136	0.12820	0.02838	0.01672	0.18367	0.00108

	80	79	78	77	76	75	74	73	72	71
a_O	235	275	239	230	238	246	262	251	204	256
	(17.2)	(20.1)	(17.5)	(16.8)	(17.4)	(18.0)	(19.1)	(18.3)	(14.9)	(18.7)
a_E	247.1	247.1	247.1	247.1	247.1	247.1	247.1	247.1	247.1	247.1
	(18.0)	(18.0)	(18.0)	(18.0)	(18.0)	(18.0)	(18.0)	(18.0)	(18.0)	(18.0)
	-23.6	58.9	-15.9	-33.0	-17.8	-2.2	30.7	7.9	-78.2	18.1
	-1.722	4.299	-1.162	-2.408	-1.303	-0.159	2.243	0.576	-5.711	1.325
t_O	237	264	252	230	246	226	232	273	257	233
	(17.3)	(19.3)	(18.4)	(16.8)	(18.0)	(16.5)	(16.9)	(19.9)	(18.8)	(17.0)
t_E	257.9	257.9	257.9	257.9	257.9	257.9	257.9	257.9	257.9	257.9
	(18.8)	(18.8)	(18.8)	(18.8)	(18.8)	(18.8)	(18.8)	(18.8)	(18.8)	(18.8)
	-40.1	12.3	-11.7	-52.7	-23.3	-59.7	-49.1	31.0	-1.8	-47.4
	-2.929	0.899	-0.855	-3.850	-1.701	-4.362	-3.590	2.266	-0.134	-3.459
g_O	435	405	421	472	445	449	425	411	459	422
	(31.8)	(29.6)	(30.8)	(34.5)	(32.5)	(32.8)	(31.0)	(30.0)	(33.5)	(30.8)
g_E	391.2	391.2	391.2	391.2	391.2	391.2	391.2	391.2	391.2	391.2
	(28.6)	(28.6)	(28.6)	(28.6)	(28.6)	(28.6)	(28.6)	(28.6)	(28.6)	(28.6)
	92.2	28.0	61.7	177.1	114.6	123.6	70.3	40.5	146.6	63.9
	6.736	2.044	4.507	12.938	8.369	9.031	5.137	2.957	10.709	4.664
c_O	462	425	457	437	440	448	450	434	449	458
	(33.7)	(31.0)	(33.4)	(31.9)	(32.1)	(32.7)	(32.9)	(31.7)	(32.8)	(33.5)
c_E	472.7	472.7	472.7	472.7	472.7	472.7	472.7	472.7	472.7	472.7
	(34.5)	(34.5)	(34.5)	(34.5)	(34.5)	(34.5)	(34.5)	(34.5)	(34.5)	(34.5)
	-21.2	-90.5	-31.0	-68.7	-63.2	-48.2	-44.4	-74.2	-46.3	-29.0
	-1.551	-6.610	-2.261	-5.019	-4.613	-3.518	-3.241	-5.421	-3.380	-2.119
N	1369	1369	1369	1369	1369	1369	1369	1369	1369	1369
X2	7.4	8.6	3.2	23.6	10.5	13.8	7.5	5.1	20.4	5.6
Gvalue	7.3	8.7	3.1	22.7	10.3	13.6	7.5	5.2	20.3	5.6
Gv/N	0.534	0.632	0.229	1.661	0.751	0.992	0.549	0.378	1.484	0.411
Prob	0.06261	0.03429	0.37030	0.00005	0.01628	0.00354	0.05700	0.15961	0.00015	0.13123

	70	69	68	67	66	65	64	63	62	61
a_O	278	244	249	241	235	273	230	243	226	258
	(20.3)	(17.8)	(18.2)	(17.6)	(17.2)	(19.9)	(16.8)	(17.8)	(16.5)	(18.8)
a_E	247.1	247.1	247.1	247.1	247.1	247.1	247.1	247.1	247.1	247.1
	(18.0)	(18.0)	(18.0)	(18.0)	(18.0)	(18.0)	(18.0)	(18.0)	(18.0)	(18.0)
	65.5	-6.1	3.8	-12.0	-23.6	54.4	-33.0	-8.1	-40.3	22.3
	4.787	-0.449	0.280	-0.879	-1.722	3.977	-2.408	-0.593	-2.946	1.629
t_O	241	247	238	265	265	248	265	253	241	265
	(17.6)	(18.0)	(17.4)	(19.4)	(19.4)	(18.1)	(19.4)	(18.5)	(17.6)	(19.4)

```
t_E       257.9   257.9   257.9   257.9   257.9   257.9   257.9   257.9   257.9   257.9
        ( 18.8) ( 18.8) ( 18.8) ( 18.8) ( 18.8) ( 18.8) ( 18.8) ( 18.8) ( 18.8) ( 18.8)
          -32.7   -21.4   -38.3    14.4    14.4   -19.5    14.4    -9.7   -32.7    14.4
         -2.389  -1.561  -2.795   1.048   1.048  -1.421   1.048  -0.712  -2.389   1.048
g_O         409     434     428     401     402     417     425     420     442     419
        ( 29.9) ( 31.7) ( 31.3) ( 29.3) ( 29.4) ( 30.5) ( 31.0) ( 30.7) ( 32.3) ( 30.6)
g_E       391.2   391.2   391.2   391.2   391.2   391.2   391.2   391.2   391.2   391.2
        ( 28.6) ( 28.6) ( 28.6) ( 28.6) ( 28.6) ( 28.6) ( 28.6) ( 28.6) ( 28.6) ( 28.6)
           36.3    90.0    76.8    19.7    21.8    53.2    70.3    59.6   107.8    57.4
          2.651   6.575   5.613   1.442   1.592   3.883   5.137   4.351   7.875   4.195
c_O         441     444     454     462     467     431     449     453     460     427
        ( 32.2) ( 32.4) ( 33.2) ( 33.7) ( 34.1) ( 31.5) ( 32.8) ( 33.1) ( 33.6) ( 31.2)
c_E       472.7   472.7   472.7   472.7   472.7   472.7   472.7   472.7   472.7   472.7
        ( 34.5) ( 34.5) ( 34.5) ( 34.5) ( 34.5) ( 34.5) ( 34.5) ( 34.5) ( 34.5) ( 34.5)
          -61.3   -55.7   -36.7   -21.2   -11.4   -79.7   -46.3   -38.6   -25.1   -86.9
         -4.478  -4.068  -2.683  -1.551  -0.833  -5.820  -3.380  -2.823  -1.836  -6.348

N          1369    1369    1369    1369    1369    1369    1369    1369    1369    1369
X2          7.9     6.9     5.7     0.8     1.2     8.5     5.5     3.1     9.8     7.1
Gvalue      7.8     6.8     5.7     0.8     1.2     8.5     5.4     3.1     9.7     7.2
Gv/N      0.571   0.497   0.416   0.061   0.084   0.619   0.398   0.224   0.705   0.523
Prob    0.04980 0.07851 0.12738 0.84209 0.76340 0.03722 0.14187 0.38228 0.02178 0.06678
        ------------------------------------------------------------------------------
             60      59      58      57      56      55      54      53      52      51
        ------------------------------------------------------------------------------
a_O         234     240     249     226     250     269     224     254     249     239
        ( 17.1) ( 17.5) ( 18.2) ( 16.5) ( 18.3) ( 19.6) ( 16.4) ( 18.6) ( 18.2) ( 17.5)
a_E       247.1   247.1   247.1   247.1   247.1   247.1   247.1   247.1   247.1   247.1
        ( 18.0) ( 18.0) ( 18.0) ( 18.0) ( 18.0) ( 18.0) ( 18.0) ( 18.0) ( 18.0) ( 18.0)
          -25.5   -14.0     3.8   -40.3     5.9    45.7   -44.0    14.0     3.8   -15.9
         -1.861  -1.021   0.280  -2.946   0.428   3.339  -3.211   1.023   0.280  -1.162
t_O         251     263     276     239     234     272     239     225     256     233
        ( 18.3) ( 19.2) ( 20.2) ( 17.5) ( 17.1) ( 19.9) ( 17.5) ( 16.4) ( 18.7) ( 17.0)
t_E       257.9   257.9   257.9   257.9   257.9   257.9   257.9   257.9   257.9   257.9
        ( 18.8) ( 18.8) ( 18.8) ( 18.8) ( 18.8) ( 18.8) ( 18.8) ( 18.8) ( 18.8) ( 18.8)
          -13.7    10.3    37.4   -36.4   -45.5    28.9   -36.4   -61.4    -3.8   -47.4
         -0.997   0.749   2.732  -2.660  -3.327   2.112  -2.660  -4.488  -0.279  -3.459
g_O         407     401     426     426     434     403     423     438     388     443
        ( 29.7) ( 29.3) ( 31.1) ( 31.1) ( 31.7) ( 29.4) ( 30.9) ( 32.0) ( 28.3) ( 32.4)
g_E       391.2   391.2   391.2   391.2   391.2   391.2   391.2   391.2   391.2   391.2
        ( 28.6) ( 28.6) ( 28.6) ( 28.6) ( 28.6) ( 28.6) ( 28.6) ( 28.6) ( 28.6) ( 28.6)
           32.1    19.7    72.5    72.5    90.0    23.8    66.0    98.9    -6.5   110.1
          2.347   1.442   5.296   5.296   6.575   1.742   4.822   7.222  -0.473   8.040
c_O         477     465     418     478     451     425     483     452     476     454
        ( 34.8) ( 34.0) ( 30.5) ( 34.9) ( 32.9) ( 31.0) ( 35.3) ( 33.0) ( 34.8) ( 33.2)
c_E       472.7   472.7   472.7   472.7   472.7   472.7   472.7   472.7   472.7   472.7
        ( 34.5) ( 34.5) ( 34.5) ( 34.5) ( 34.5) ( 34.5) ( 34.5) ( 34.5) ( 34.5) ( 34.5)
            8.6   -15.4  -102.9    10.6   -42.5   -90.5    20.7   -40.6     6.5   -36.7
          0.625  -1.121  -7.515   0.773  -3.102  -6.610   1.515  -2.962   0.478  -2.683

N          1369    1369    1369    1369    1369    1369    1369    1369    1369    1369
X2          1.6     0.7    10.7     6.3     7.9     7.9     6.3    10.9     0.1    10.3
Gvalue      1.6     0.7    10.9     6.3     7.8     8.0     6.4    10.9     0.1    10.1
Gv/N      0.114   0.049   0.793   0.463   0.573   0.583   0.466   0.795   0.006   0.736
Prob    0.66910 0.87948 0.01256 0.09645 0.04922 0.04634 0.09443 0.01237 0.99428 0.01797
        ------------------------------------------------------------------------------
             50      49      48      47      46      45      44      43      42      41
        ------------------------------------------------------------------------------
a_O         233     269     240     253     221     229     253     238     217     234
        ( 17.0) ( 19.6) ( 17.5) ( 18.5) ( 16.1) ( 16.7) ( 18.5) ( 17.4) ( 15.9) ( 17.1)
a_E       247.1   247.1   247.1   247.1   247.1   247.1   247.1   247.1   247.1   247.1
        ( 18.0) ( 18.0) ( 18.0) ( 18.0) ( 18.0) ( 18.0) ( 18.0) ( 18.0) ( 18.0) ( 18.0)
          -27.4    45.7   -14.0    12.0   -49.3   -34.8    12.0   -17.8   -56.4   -25.5
         -1.999   3.339  -1.021   0.874  -3.603  -2.544   0.874  -1.303  -4.117  -1.861
t_O         227     243     267     245     246     257     249     294     287     250
        ( 16.6) ( 17.8) ( 19.5) ( 17.9) ( 18.0) ( 18.8) ( 18.2) ( 21.5) ( 21.0) ( 18.3)
t_E       257.9   257.9   257.9   257.9   257.9   257.9   257.9   257.9   257.9   257.9
        ( 18.8) ( 18.8) ( 18.8) ( 18.8) ( 18.8) ( 18.8) ( 18.8) ( 18.8) ( 18.8) ( 18.8)
          -58.0   -29.0    18.5   -25.2   -23.3    -1.8   -17.5    77.0    61.3   -15.6
         -4.235  -2.115   1.350  -1.839  -1.701  -0.134  -1.280   5.624   4.479  -1.139
g_O         410     428     405     414     420     443     422     387     412     435
        ( 29.9) ( 31.3) ( 29.6) ( 30.2) ( 30.7) ( 32.4) ( 30.8) ( 28.3) ( 30.1) ( 31.8)
g_E       391.2   391.2   391.2   391.2   391.2   391.2   391.2   391.2   391.2   391.2
        ( 28.6) ( 28.6) ( 28.6) ( 28.6) ( 28.6) ( 28.6) ( 28.6) ( 28.6) ( 28.6) ( 28.6)
           38.4    76.8    28.0    46.8    59.6   110.1    63.9    -8.5    42.6    92.2
          2.804   5.613   2.044   3.418   4.351   8.040   4.664  -0.618   3.110   6.736
c_O         499     429     457     457     482     440     445     450     453     450
        ( 36.4) ( 31.3) ( 33.4) ( 33.4) ( 35.2) ( 32.1) ( 32.5) ( 32.9) ( 33.1) ( 32.9)
c_E       472.7   472.7   472.7   472.7   472.7   472.7   472.7   472.7   472.7   472.7
        ( 34.5) ( 34.5) ( 34.5) ( 34.5) ( 34.5) ( 34.5) ( 34.5) ( 34.5) ( 34.5) ( 34.5)
           54.0   -83.3   -31.0   -31.0    18.7   -63.2   -53.8   -44.4   -38.6   -44.4
          3.941  -6.085  -2.261  -2.261   1.366  -4.613  -3.931  -3.241  -2.823  -3.241

N          1369    1369    1369    1369    1369    1369    1369    1369    1369    1369
X2          6.9    10.3     1.5     2.6     5.6    10.4     4.5     6.5     8.9     6.9
Gvalue      7.0    10.3     1.5     2.6     5.7    10.2     4.5     6.3     8.9     6.8
Gv/N      0.511   0.752   0.112   0.192   0.413   0.748   0.326   0.462   0.650   0.495
Prob    0.07188 0.01622 0.67559 0.45287 0.12939 0.01660 0.21509 0.09688 0.03062 0.07915
        ------------------------------------------------------------------------------
             40      39      38      37      36      35      34      33      32      31
        ------------------------------------------------------------------------------
a_O         235     267     259     255     232     262     238     230     277     248
        ( 17.2) ( 19.5) ( 18.9) ( 18.6) ( 16.9) ( 19.1) ( 17.4) ( 16.8) ( 20.2) ( 18.1)
a_E       247.1   247.1   247.1   247.1   247.1   247.1   247.1   247.1   247.1   247.1
        ( 18.0) ( 18.0) ( 18.0) ( 18.0) ( 18.0) ( 18.0) ( 18.0) ( 18.0) ( 18.0) ( 18.0)
          -23.6    41.4    24.4    16.1   -29.2    30.7   -17.8   -33.0    63.3     1.8
         -1.722   3.023   1.781   1.174  -2.136   2.243  -1.303  -2.408   4.624   0.133
t_O         290     252     266     281     282     274     292     264     229     298
        ( 21.2) ( 18.4) ( 19.4) ( 20.5) ( 20.6) ( 20.0) ( 21.3) ( 19.3) ( 16.7) ( 21.8)
t_E       257.9   257.9   257.9   257.9   257.9   257.9   257.9   257.9   257.9   257.9
        ( 18.8) ( 18.8) ( 18.8) ( 18.8) ( 18.8) ( 18.8) ( 18.8) ( 18.8) ( 18.8) ( 18.8)
           68.0   -11.7    16.4    48.2    50.3    33.1    72.5    12.3   -54.5    86.1
          4.967  -0.855   1.199   3.518   3.677   2.421   5.294   0.899  -3.979   6.288
g_O         397     417     404     375     409     408     382     404     394     356
        ( 29.0) ( 30.5) ( 29.5) ( 27.4) ( 29.9) ( 29.8) ( 27.9) ( 29.5) ( 28.8) ( 26.0)
g_E       391.2   391.2   391.2   391.2   391.2   391.2   391.2   391.2   391.2   391.2
        ( 28.6) ( 28.6) ( 28.6) ( 28.6) ( 28.6) ( 28.6) ( 28.6) ( 28.6) ( 28.6) ( 28.6)
           11.6    53.2    25.9   -31.8    36.3    34.2   -18.3    25.9     5.5   -67.2
          0.846   3.883   1.893  -2.324   2.651   2.499  -1.335   1.893   0.403  -4.910
c_O         447     433     440     458     446     425     457     471     469     467
        ( 32.7) ( 31.6) ( 32.1) ( 33.5) ( 32.6) ( 31.0) ( 33.4) ( 34.4) ( 34.3) ( 34.1)
c_E       472.7   472.7   472.7   472.7   472.7   472.7   472.7   472.7   472.7   472.7
```

```
        ( 34.5) ( 34.5) ( 34.5) ( 34.5) ( 34.5) ( 34.5) ( 34.5) ( 34.5) ( 34.5) ( 34.5)
          -50.1   -76.0   -63.2   -29.0   -51.9   -90.5   -31.0    -3.5    -7.5   -11.4
         -3.656  -5.555  -4.613  -2.119  -3.794  -6.610  -2.261  -0.254  -0.544  -0.833

N          1369    1369    1369    1369    1369    1369    1369    1369    1369    1369
X2          6.1     6.8     3.5     3.5     5.5     7.4     5.6     1.7     6.9     9.5
Gvalue      5.9     6.8     3.5     3.4     5.5     7.6     5.4     1.8     6.9     9.3
Gv/N      0.434   0.496   0.259   0.249   0.399   0.552   0.395   0.129   0.504   0.678
Prob    0.11420 0.07866 0.31455 0.33319 0.14121 0.05595 0.14437 0.62150 0.07506 0.02582
        --------------------------------------------------------------------------------
             30      29      28      27      26      25      24      23      22      21
        --------------------------------------------------------------------------------
a_O         220     257     234     210     233     221     232     246     253     235
        ( 16.1) ( 18.8) ( 17.1) ( 15.3) ( 17.0) ( 16.1) ( 16.9) ( 18.0) ( 18.5) ( 17.2)
a_E       247.1   247.1   247.1   247.1   247.1   247.1   247.1   247.1   247.1   247.1
        ( 18.0) ( 18.0) ( 18.0) ( 18.0) ( 18.0) ( 18.0) ( 18.0) ( 18.0) ( 18.0) ( 18.0)
          -51.1    20.2   -25.5   -68.3   -27.4   -49.3   -29.2    -2.2    12.0   -23.6
         -3.732   1.476  -1.861  -4.990  -1.999  -3.603  -2.136  -0.159   0.874  -1.722
t_O         250     251     307     301     269     298     257     283     296     277
        ( 18.3) ( 18.3) ( 22.4) ( 22.0) ( 19.6) ( 21.8) ( 18.8) ( 20.7) ( 21.6) ( 20.2)
t_E       257.9   257.9   257.9   257.9   257.9   257.9   257.9   257.9   257.9   257.9
        ( 18.8) ( 18.8) ( 18.8) ( 18.8) ( 18.8) ( 18.8) ( 18.8) ( 18.8) ( 18.8) ( 18.8)
          -15.6   -13.7   107.0    93.0    22.6    86.1    -1.8    52.5    81.5    39.5
         -1.139  -0.997   7.813   6.792   1.653   6.288  -0.134   3.837   5.955   2.888
g_O         384     377     382     380     389     346     409     302     316     354
        ( 28.0) ( 27.5) ( 27.9) ( 27.8) ( 28.4) ( 25.3) ( 29.9) ( 22.1) ( 23.1) ( 25.9)
g_E       391.2   391.2   391.2   391.2   391.2   391.2   391.2   391.2   391.2   391.2
        ( 28.6) ( 28.6) ( 28.6) ( 28.6) ( 28.6) ( 28.6) ( 28.6) ( 28.6) ( 28.6) ( 28.6)
          -14.4   -28.0   -18.3   -22.2    -4.5   -85.1    36.3  -156.4  -135.0   -70.8
         -1.049  -2.043  -1.335  -1.620  -0.328  -6.213   2.651 -11.423  -9.861  -5.174
c_O         515     484     446     478     478     504     471     538     504     503
        ( 37.6) ( 35.4) ( 32.6) ( 34.9) ( 34.9) ( 36.8) ( 34.4) ( 39.3) ( 36.8) ( 36.7)
c_E       472.7   472.7   472.7   472.7   472.7   472.7   472.7   472.7   472.7   472.7
        ( 34.5) ( 34.5) ( 34.5) ( 34.5) ( 34.5) ( 34.5) ( 34.5) ( 34.5) ( 34.5) ( 34.5)
           88.2    22.8   -51.9    10.6    10.6    64.5    -3.5   139.1    64.5    62.4
          6.442   1.664  -3.794   0.773   0.773   4.715  -0.254  10.164   4.715   4.559

N          1369    1369    1369    1369    1369    1369    1369    1369    1369    1369
X2          7.1     1.4    11.8    13.1     1.4    16.3     1.7    31.8    22.3     7.5
Gvalue      7.1     1.4    11.3    13.1     1.4    16.3     1.7    33.1    23.0     7.5
Gv/N      0.521   0.100   0.823   0.955   0.099   1.187   0.127   2.418   1.682   0.551
Prob    0.06769 0.71232 0.01037 0.00447 0.71499 0.00100 0.62720 0.00000 0.00004 0.05652
        --------------------------------------------------------------------------------
             20      19      18      17      16      15      14      13      12      11
        --------------------------------------------------------------------------------
a_O         266     250     230     282     221     249     279     264     262     280
        ( 19.4) ( 18.3) ( 16.8) ( 20.6) ( 16.1) ( 18.2) ( 20.4) ( 19.3) ( 19.1) ( 20.5)
a_E       247.1   247.1   247.1   247.1   247.1   247.1   247.1   247.1   247.1   247.1
        ( 18.0) ( 18.0) ( 18.0) ( 18.0) ( 18.0) ( 18.0) ( 18.0) ( 18.0) ( 18.0) ( 18.0)
           39.2     5.9   -33.0    74.5   -49.3     3.8    67.8    35.0    30.7    70.0
          2.866   0.428  -2.408   5.444  -3.603   0.280   4.951   2.553   2.243   5.115
t_O         303     330     327     297     331     296     273     305     287     273
        ( 22.1) ( 24.1) ( 23.9) ( 21.7) ( 24.2) ( 21.6) ( 19.9) ( 22.3) ( 21.0) ( 19.9)
t_E       257.9   257.9   257.9   257.9   257.9   257.9   257.9   257.9   257.9   257.9
        ( 18.8) ( 18.8) ( 18.8) ( 18.8) ( 18.8) ( 18.8) ( 18.8) ( 18.8) ( 18.8) ( 18.8)
           97.6   162.7   155.2    83.8   165.1    81.5    31.0   102.3    61.3    31.0
          7.130  11.881  11.337   6.121  12.063   5.955   2.266   7.471   4.479   2.266
g_O         296     279     272     272     278     288     292     297     290     306
        ( 21.6) ( 20.4) ( 19.9) ( 19.9) ( 20.3) ( 21.0) ( 21.3) ( 21.7) ( 21.2) ( 22.4)
g_E       391.2   391.2   391.2   391.2   391.2   391.2   391.2   391.2   391.2   391.2
        ( 28.6) ( 28.6) ( 28.6) ( 28.6) ( 28.6) ( 28.6) ( 28.6) ( 28.6) ( 28.6) ( 28.6)
         -165.2  -188.7  -197.8  -197.8  -190.0  -176.5  -170.9  -163.7  -173.7  -150.4
        -12.064 -13.782 -14.446 -14.446 -13.879 -12.891 -12.482 -11.959 -12.687 -10.987
c_O         504     510     540     518     539     536     525     503     530     510
        ( 36.8) ( 37.3) ( 39.4) ( 37.8) ( 39.4) ( 39.2) ( 38.3) ( 36.7) ( 38.7) ( 37.3)
c_E       472.7   472.7   472.7   472.7   472.7   472.7   472.7   472.7   472.7   472.7
        ( 34.5) ( 34.5) ( 34.5) ( 34.5) ( 34.5) ( 34.5) ( 34.5) ( 34.5) ( 34.5) ( 34.5)
           64.5    77.4   143.7    94.7   141.4   134.6   110.1    62.4   121.2    77.4
          4.715   5.652  10.494   6.919  10.329   9.834   8.042   4.559   8.853   5.652

N          1369    1369    1369    1369    1369    1369    1369    1369    1369    1369
X2         34.6    55.3    65.6    51.5    65.5    41.3    36.0    34.4    37.3    26.8
Gvalue     36.2    57.2    68.1    55.3    67.2    43.5    38.0    35.9    39.5    28.0
Gv/N      2.647   4.179   4.977   4.039   4.911   3.178   2.777   2.624   2.887   2.047
Prob    0.00000 0.00000 0.00000 0.00000 0.00000 0.00000 0.00000 0.00000 0.00000 0.00000
        --------------------------------------------------------------------------------
             10       9       8       7       6       5       4       3       2       1
        --------------------------------------------------------------------------------
a_O         267     263     324     310     325     260     289     323     203     155
        ( 19.5) ( 19.2) ( 23.7) ( 22.6) ( 23.7) ( 19.0) ( 21.1) ( 23.6) ( 14.8) ( 11.3)
a_E       247.1   247.1   247.1   247.1   247.1   247.1   247.1   247.1   247.1   247.1
        ( 18.0) ( 18.0) ( 18.0) ( 18.0) ( 18.0) ( 18.0) ( 18.0) ( 18.0) ( 18.0) ( 18.0)
           41.4    32.8   175.6   140.6   178.1    26.5    90.6   173.1   -79.8  -144.6
          3.023   2.398  12.827  10.272  13.013   1.934   6.615  12.642  -5.829 -10.560
t_O         245     247     252     267     249     248     237     233     258     113
        ( 17.9) ( 18.0) ( 18.4) ( 19.5) ( 18.2) ( 18.1) ( 17.3) ( 17.0) ( 18.8) (  8.3)
t_E       257.9   257.9   257.9   257.9   257.9   257.9   257.9   257.9   257.9   257.9
        ( 18.8) ( 18.8) ( 18.8) ( 18.8) ( 18.8) ( 18.8) ( 18.8) ( 18.8) ( 18.8) ( 18.8)
          -25.2   -21.4   -11.7    18.5   -17.5   -19.5   -40.1   -47.4     0.2  -186.5
         -1.839  -1.561  -0.855   1.350  -1.280  -1.421  -2.929  -3.459   0.012 -13.624
g_O         323     362     272     268     292     277     308     379     262     323
        ( 23.6) ( 26.4) ( 19.9) ( 19.6) ( 21.3) ( 20.2) ( 22.5) ( 27.7) ( 19.1) ( 23.6)
g_E       391.2   391.2   391.2   391.2   391.2   391.2   391.2   391.2   391.2   391.2
        ( 28.6) ( 28.6) ( 28.6) ( 28.6) ( 28.6) ( 28.6) ( 28.6) ( 28.6) ( 28.6) ( 28.6)
         -123.8   -56.3  -197.8  -202.8  -170.9  -191.3  -147.4   -24.1  -210.1  -123.8
         -9.046  -4.109 -14.446 -14.814 -12.482 -13.975 -10.765  -1.761 -15.349  -9.046
c_O         534     497     521     524     503     584     535     434     646     778
        ( 39.0) ( 36.3) ( 38.1) ( 38.3) ( 36.7) ( 42.7) ( 39.1) ( 31.7) ( 47.2) ( 56.8)
c_E       472.7   472.7   472.7   472.7   472.7   472.7   472.7   472.7   472.7   472.7
        ( 34.5) ( 34.5) ( 34.5) ( 34.5) ( 34.5) ( 34.5) ( 34.5) ( 34.5) ( 34.5) ( 34.5)
          130.1    49.7   101.3   107.9    62.4   246.9   132.4   -74.2   403.4   775.2
          9.506   3.634   7.399   7.881   4.559  18.032   9.670  -5.421  29.469  56.623

N          1369    1369    1369    1369    1369    1369    1369    1369    1369    1369
X2         22.1     4.9    65.3    60.7    52.0    60.6    34.7    29.3   114.1   324.8
Gvalue     22.5     4.9    67.4    64.2    52.2    62.6    35.5    27.4   113.7   320.3
Gv/N      1.644   0.361   4.925   4.689   3.810   4.571   2.591   2.000   8.303  23.394
Prob    0.00005 0.17627 0.00000 0.00000 0.00000 0.00000 0.00000 0.00000 0.00000 0.00000
        --------------------------------------------------------------------------------

CDS_INITIAL:
        --------------------------------------------------------------------------------
```

	1	2	3	4	5	6	7	8	9	10
a_O	1193	0	0	583	402	249	405	393	230	372
	(87.1)	(0.0)	(0.0)	(42.6)	(29.4)	(18.2)	(29.6)	(28.7)	(16.8)	(27.2)
a_E	258.0	324.2	81.5	258.0	324.2	81.5	258.0	324.2	81.5	258.0
	(18.8)	(23.7)	(6.0)	(18.8)	(23.7)	(6.0)	(18.8)	(23.7)	(6.0)	(18.8)
	3653.9	0.0	0.0	950.7	173.0	556.1	365.4	151.4	477.2	272.4
	266.905	0.000	0.000	69.446	12.640	40.624	26.688	11.057	34.857	19.895
t_O	58	1369	0	179	178	220	173	265	254	214
	(4.2)	(100.0)	(0.0)	(13.1)	(13.0)	(16.1)	(12.6)	(19.4)	(18.6)	(15.6)
t_E	171.0	387.0	101.9	171.0	387.0	101.9	171.0	387.0	101.9	171.0
	(12.5)	(28.3)	(7.4)	(12.5)	(28.3)	(7.4)	(12.5)	(28.3)	(7.4)	(12.5)
	-125.4	3459.5	0.0	16.4	-276.5	338.8	4.1	-200.7	464.1	96.0
	-9.161	252.700	0.000	1.198	-20.194	24.747	0.296	-14.658	33.904	7.015
g_O	118	0	1369	213	208	364	392	225	342	327
	(8.6)	(0.0)	(100.0)	(15.6)	(15.2)	(26.6)	(28.6)	(16.4)	(25.0)	(23.9)
g_E	545.5	270.0	568.5	545.5	270.0	568.5	545.5	270.0	568.5	545.5
	(39.8)	(19.7)	(41.5)	(39.8)	(19.7)	(41.5)	(39.8)	(19.7)	(41.5)	(39.8)
	-361.3	0.0	2406.2	-400.6	-108.5	-324.6	-259.0	-82.0	-347.6	-334.6
	-26.391	0.000	175.764	-29.260	-7.924	-23.710	-18.918	-5.989	-25.392	-24.443
c_O	0	0	0	394	581	536	399	486	543	456
	(0.0)	(0.0)	(0.0)	(28.8)	(42.4)	(39.2)	(29.1)	(35.5)	(39.7)	(33.3)
c_E	394.6	387.9	617.1	394.6	387.9	617.1	394.6	387.9	617.1	394.6
	(28.8)	(28.3)	(45.1)	(28.8)	(28.3)	(45.1)	(28.8)	(28.3)	(45.1)	(28.8)
	0.0	0.0	0.0	-1.2	469.4	-151.1	8.9	219.1	-139.0	131.9
	0.000	0.000	0.000	-0.087	34.290	-11.035	0.647	16.006	-10.150	9.635
N	1369	1369	1369	1369	1369	1369	1369	1369	1369	1369
X2	4193.4	3474.2	1927.6	612.5	241.9	565.4	127.1	85.4	596.9	158.3
Gvalue	3167.2	3459.5	2406.2	565.3	257.5	419.3	119.3	87.8	454.8	165.7
Gv/N	231.353	252.700	175.764	41.296	18.812	30.626	8.712	6.416	33.219	12.102
Prob	1.00000	1.00000	1.00000	0.00000	0.00000	0.00000	0.00000	0.00000	0.00000	0.00000

	11	12	13	14	15	16	17	18	19	20
a_O	295	187	351	294	183	331	296	185	292	308
	(21.5)	(13.7)	(25.6)	(21.5)	(13.4)	(24.2)	(21.6)	(13.5)	(21.3)	(22.5)
a_E	324.2	81.5	258.0	324.2	81.5	258.0	324.2	81.5	258.0	324.2
	(23.7)	(6.0)	(18.8)	(23.7)	(6.0)	(18.8)	(23.7)	(6.0)	(18.8)	(23.7)
	-55.6	310.6	216.2	-57.4	296.0	165.0	-53.8	303.3	72.4	-31.5
	-4.062	22.686	15.792	-4.194	21.623	12.055	-3.930	22.153	5.287	-2.301
t_O	358	256	199	364	233	222	361	267	216	379
	(26.2)	(18.7)	(14.5)	(26.6)	(17.0)	(16.2)	(26.4)	(19.5)	(15.8)	(27.7)
t_E	387.0	101.9	171.0	387.0	101.9	171.0	387.0	101.9	171.0	387.0
	(28.3)	(7.4)	(12.5)	(28.3)	(7.4)	(12.5)	(28.3)	(7.4)	(12.5)	(28.3)
	-55.7	471.8	60.4	-44.5	385.6	115.9	-50.2	514.6	101.0	-15.8
	-4.069	34.464	4.411	-3.254	28.163	8.468	-3.663	37.586	7.374	-1.152
g_O	196	349	355	203	378	375	204	339	401	208
	(14.3)	(25.5)	(25.9)	(14.8)	(27.6)	(27.4)	(14.9)	(24.8)	(29.3)	(15.2)
g_E	270.0	568.5	545.5	270.0	568.5	545.5	270.0	568.5	545.5	270.0
	(19.7)	(41.5)	(39.8)	(19.7)	(41.5)	(39.8)	(19.7)	(41.5)	(39.8)	(19.7)
	-125.5	-340.6	-304.9	-115.7	-308.5	-281.0	-114.3	-350.5	-246.7	-108.5
	-9.168	-24.878	-22.275	-8.455	-22.538	-20.527	-8.350	-25.605	-18.023	-7.924
c_O	520	577	464	508	575	441	508	578	460	474
	(38.0)	(42.1)	(33.9)	(37.1)	(42.0)	(32.2)	(37.1)	(42.2)	(33.6)	(34.6)
c_E	387.9	617.1	394.6	387.9	617.1	394.6	387.9	617.1	394.6	387.9
	(28.3)	(45.1)	(28.8)	(28.3)	(45.1)	(28.8)	(28.3)	(45.1)	(28.8)	(28.3)
	304.8	-77.6	150.4	274.0	-81.3	98.1	274.0	-75.7	141.1	190.0
	22.263	-5.666	10.982	20.017	-5.938	7.163	20.017	-5.530	10.306	13.880
N	1369	1369	1369	1369	1369	1369	1369	1369	1369	1369
X2	70.0	457.1	116.8	58.0	361.9	94.6	57.5	494.2	65.4	34.3
Gvalue	67.9	364.2	122.0	56.3	291.7	98.0	55.8	391.6	67.7	34.3
Gv/N	4.963	26.606	8.911	4.114	21.310	7.159	4.074	28.604	4.944	2.504
Prob	0.00000	0.00000	0.00000	0.00000	0.00000	0.00000	0.00000	0.00000	0.00000	0.00000

	21	22	23	24	25	26	27	28	29	30
a_O	152	284	306	138	228	263	144	241	270	116
	(11.1)	(20.7)	(22.4)	(10.1)	(16.7)	(19.2)	(10.5)	(17.6)	(19.7)	(8.5)
a_E	81.5	258.0	324.2	81.5	258.0	324.2	81.5	258.0	324.2	81.5
	(6.0)	(18.8)	(23.7)	(6.0)	(18.8)	(23.7)	(6.0)	(18.8)	(23.7)	(6.0)
	189.4	54.6	-35.3	145.3	-56.3	-110.0	163.9	-32.8	-98.7	81.9
	13.838	3.989	-2.577	10.616	-4.113	-8.033	11.972	-2.395	-7.211	5.980
t_O	226	203	371	215	226	367	196	210	359	159
	(16.5)	(14.8)	(27.1)	(15.7)	(16.5)	(26.8)	(14.3)	(15.3)	(26.2)	(11.6)
t_E	101.9	171.0	387.0	101.9	171.0	387.0	101.9	171.0	387.0	101.9
	(7.4)	(12.5)	(28.3)	(7.4)	(12.5)	(28.3)	(7.4)	(12.5)	(28.3)	(7.4)
	360.2	69.7	-31.3	321.2	126.1	-38.9	256.5	86.3	-53.9	141.6
	26.310	5.090	-2.284	23.462	9.210	-2.840	18.739	6.305	-3.934	10.342
g_O	370	409	218	395	482	274	386	484	259	442
	(27.0)	(29.9)	(15.9)	(28.9)	(35.2)	(20.0)	(28.2)	(35.4)	(18.9)	(32.3)
g_E	568.5	545.5	270.0	568.5	545.5	270.0	568.5	545.5	270.0	568.5
	(41.5)	(39.8)	(19.7)	(41.5)	(39.8)	(19.7)	(41.5)	(39.8)	(19.7)	(41.5)
	-317.8	-235.5	-93.2	-287.7	-119.2	8.1	-298.9	-115.7	-21.5	-222.5
	-23.217	-17.202	-6.809	-21.013	-8.708	0.594	-21.834	-8.452	-1.569	-16.253
c_O	621	473	474	621	433	465	643	434	481	652
	(45.4)	(34.6)	(34.6)	(45.4)	(31.6)	(34.0)	(47.0)	(31.7)	(35.1)	(47.6)
c_E	617.1	394.6	387.9	617.1	394.6	387.9	617.1	394.6	387.9	617.1
	(45.1)	(28.8)	(28.3)	(45.1)	(28.8)	(28.3)	(45.1)	(28.8)	(28.3)	(45.1)
	7.8	171.4	190.0	7.8	80.4	168.6	52.8	82.6	206.9	71.7
	0.569	12.523	13.880	0.569	5.875	12.314	3.860	6.034	15.115	5.238
N	1369	1369	1369	1369	1369	1369	1369	1369	1369	1369
X2	281.6	58.3	30.8	217.8	32.3	27.9	194.6	20.9	33.9	76.8
Gvalue	239.6	60.2	30.3	186.7	31.0	27.8	174.4	20.4	32.9	72.6
Gv/N	17.500	4.400	2.210	13.634	2.264	2.034	12.738	1.493	2.401	5.307
Prob	0.00000	0.00000	0.00000	0.00000	0.00000	0.00000	0.00000	0.00014	0.00000	0.00000

	31	32	33	34	35	36	37	38	39	40
a_O	244	263	142	266	270	112	248	275	116	228
	(17.8)	(19.2)	(10.4)	(19.4)	(19.7)	(8.2)	(18.1)	(20.1)	(8.5)	(16.7)
a_E	258.0	324.2	81.5	258.0	324.2	81.5	258.0	324.2	81.5	258.0
	(18.8)	(23.7)	(6.0)	(18.8)	(23.7)	(6.0)	(18.8)	(23.7)	(6.0)	(18.8)
	-27.2	-110.0	157.7	16.3	-98.7	71.2	-19.5	-90.5	81.9	-56.3
	-1.984	-8.033	11.516	1.192	-7.211	5.200	-1.427	-6.607	5.980	-4.113
t_O	201	381	170	210	385	146	173	370	174	168
	(14.7)	(27.8)	(12.4)	(15.3)	(28.1)	(10.7)	(12.6)	(27.0)	(12.7)	(12.3)
t_E	171.0	387.0	101.9	171.0	387.0	101.9	171.0	387.0	101.9	171.0
	(12.5)	(28.3)	(7.4)	(12.5)	(28.3)	(7.4)	(12.5)	(28.3)	(7.4)	(12.5)
	65.0	-11.8	174.1	86.3	-3.9	105.1	4.1	-33.2	186.3	-5.9

	4.749	−0.865	12.719	6.305	−0.287	7.677	0.296	−2.424	13.610	−0.432
g_O	483	275	434	464	266	428	540	274	460	515
	(35.3)	(20.1)	(31.7)	(33.9)	(19.4)	(31.3)	(39.4)	(20.0)	(33.6)	(37.6)
g_E	545.5	270.0	568.5	545.5	270.0	568.5	545.5	270.0	568.5	545.5
	(39.8)	(19.7)	(41.5)	(39.8)	(19.7)	(41.5)	(39.8)	(19.7)	(41.5)	(39.8)
	−117.5	10.2	−234.3	−150.1	−7.9	−243.0	−10.8	8.1	−194.8	−59.2
	−8.580	0.742	−17.117	−10.963	−0.575	−17.751	−0.792	0.594	−14.233	−4.322
c_O	441	450	623	429	448	683	408	450	619	458
	(32.2)	(32.9)	(45.5)	(31.3)	(32.7)	(49.9)	(29.8)	(32.9)	(45.2)	(33.5)
c_E	394.6	387.9	617.1	394.6	387.9	617.1	394.6	387.9	617.1	394.6
	(28.8)	(28.3)	(45.1)	(28.8)	(28.3)	(45.1)	(28.8)	(28.3)	(45.1)	(28.8)
	98.1	133.6	11.8	71.7	129.0	138.6	27.3	133.6	3.8	136.5
	7.163	9.761	0.864	5.239	9.426	10.121	1.991	9.761	0.276	9.970
N	1369	1369	1369	1369	1369	1369	1369	1369	1369	1369
X2	18.6	21.7	122.3	24.3	18.4	72.3	0.9	18.2	86.4	15.4
Gvalue	18.4	22.0	109.3	24.3	18.5	71.8	0.9	18.1	77.1	15.1
Gv/N	1.348	1.605	7.981	1.773	1.354	5.248	0.067	1.324	5.633	1.102
Prob	0.00036	0.00007	0.00000	0.00002	0.00034	0.00000	0.82101	0.00041	0.00000	0.00174

	41	42	43	44	45	46	47	48	49	50
a_O	265	118	238	278	120	238	255	131	224	303
	(19.4)	(8.6)	(17.4)	(20.3)	(8.8)	(17.4)	(18.6)	(9.6)	(16.4)	(22.1)
a_E	324.2	81.5	258.0	324.2	81.5	258.0	324.2	81.5	258.0	324.2
	(23.7)	(6.0)	(18.8)	(23.7)	(6.0)	(18.8)	(23.7)	(6.0)	(18.8)	(23.7)
	−106.8	87.3	−38.3	−85.4	92.8	−38.3	−122.4	124.3	−63.2	−40.9
	−7.801	6.378	−2.801	−6.239	6.781	−2.801	−8.940	9.081	−4.620	−2.988
t_O	394	149	183	380	165	161	385	136	191	381
	(28.8)	(10.9)	(13.4)	(27.8)	(12.1)	(11.8)	(28.1)	(9.9)	(14.0)	(27.8)
t_E	387.0	101.9	171.0	387.0	101.9	171.0	387.0	101.9	171.0	387.0
	(28.3)	(7.4)	(12.5)	(28.3)	(7.4)	(12.5)	(28.3)	(7.4)	(12.5)	(28.3)
	14.2	113.3	24.9	−13.8	159.2	−19.4	−3.9	78.6	42.3	−11.8
	1.037	8.278	1.815	−1.009	11.625	−1.415	−0.287	5.742	3.089	−0.865
g_O	254	449	537	265	469	536	265	458	530	259
	(18.6)	(32.8)	(39.2)	(19.4)	(34.3)	(39.2)	(19.4)	(33.5)	(38.7)	(18.9)
g_E	270.0	568.5	545.5	270.0	568.5	545.5	270.0	568.5	545.5	270.0
	(19.7)	(41.5)	(39.8)	(19.7)	(41.5)	(39.8)	(19.7)	(41.5)	(39.8)	(19.7)
	−31.0	−211.9	−16.8	−9.8	−180.5	−18.7	−9.8	−198.0	−30.5	−21.5
	−2.262	−15.480	−1.225	−0.719	−13.184	−1.369	−0.719	−14.462	−2.225	−1.569
c_O	456	653	411	446	615	434	464	644	424	426
	(33.3)	(47.7)	(30.0)	(32.6)	(44.9)	(31.7)	(33.9)	(47.0)	(31.0)	(31.1)
c_E	387.9	617.1	394.6	387.9	617.1	394.6	387.9	617.1	394.6	387.9
	(28.3)	(45.1)	(28.8)	(28.3)	(45.1)	(28.8)	(28.3)	(45.1)	(28.8)	(28.3)
	147.5	73.8	33.5	124.5	−4.2	82.6	166.2	54.9	60.9	79.8
	10.774	5.392	2.445	9.093	−0.309	6.034	12.142	4.012	4.451	5.829
N	1369	1369	1369	1369	1369	1369	1369	1369	1369	1369
X2	23.8	65.4	3.2	15.5	74.7	6.2	29.8	64.1	9.4	5.7
Gvalue	23.9	62.5	3.2	15.4	67.3	6.2	30.1	59.9	9.5	5.6
Gv/N	1.747	4.568	0.235	1.127	4.914	0.450	2.197	4.372	0.695	0.408
Prob	0.00003	0.00000	0.35955	0.00149	0.00000	0.10427	0.00000	0.00000	0.02314	0.13394

	51	52	53	54	55	56	57	58	59	60
a_O	108	270	253	101	233	295	121	238	286	104
	(7.9)	(19.7)	(18.5)	(7.4)	(17.0)	(21.5)	(8.8)	(17.4)	(20.9)	(7.6)
a_E	81.5	258.0	324.2	81.5	258.0	324.2	81.5	258.0	324.2	81.5
	(6.0)	(18.8)	(23.7)	(6.0)	(18.8)	(23.7)	(6.0)	(18.8)	(23.7)	(6.0)
	60.8	24.6	−125.4	43.3	−47.4	−55.6	95.6	−38.3	−71.6	50.7
	4.440	1.799	−9.161	3.164	−3.465	−4.062	6.984	−2.801	−5.233	3.702
t_O	149	160	399	149	181	394	153	156	376	134
	(10.9)	(11.7)	(29.1)	(10.9)	(13.2)	(28.8)	(11.2)	(11.4)	(27.5)	(9.8)
t_E	101.9	171.0	387.0	101.9	171.0	387.0	101.9	171.0	387.0	101.9
	(7.4)	(12.5)	(28.3)	(7.4)	(12.5)	(28.3)	(7.4)	(12.5)	(28.3)	(7.4)
	113.3	−21.3	24.4	113.3	20.6	14.2	124.5	−28.6	−21.6	73.5
	8.278	−1.552	1.785	8.278	1.505	1.037	9.092	−2.091	−1.579	5.367
g_O	469	505	274	498	554	266	453	560	279	489
	(34.3)	(36.9)	(20.0)	(36.4)	(40.5)	(19.4)	(33.1)	(40.9)	(20.4)	(35.7)
g_E	568.5	545.5	270.0	568.5	545.5	270.0	568.5	545.5	270.0	568.5
	(41.5)	(39.8)	(19.7)	(41.5)	(39.8)	(19.7)	(41.5)	(39.8)	(19.7)	(41.5)
	−180.5	−77.8	8.1	−131.9	17.2	−7.9	−205.8	29.5	18.4	−147.3
	−13.184	−5.685	0.594	−9.634	1.259	−0.575	−15.031	2.154	1.342	−10.762
c_O	643	434	443	621	401	414	642	415	428	642
	(47.0)	(31.7)	(32.4)	(45.4)	(29.3)	(30.2)	(46.9)	(30.3)	(31.3)	(46.9)
c_E	617.1	394.6	387.9	617.1	394.6	387.9	617.1	394.6	387.9	617.1
	(45.1)	(28.8)	(28.3)	(45.1)	(28.8)	(28.3)	(45.1)	(28.8)	(28.3)	(45.1)
	52.8	82.6	117.7	7.8	12.9	53.9	50.8	41.8	84.2	50.8
	3.860	6.034	8.595	0.569	0.943	3.937	3.708	3.056	6.150	3.708
N	1369	1369	1369	1369	1369	1369	1369	1369	1369	1369
X2	48.9	8.2	23.9	35.2	3.2	4.6	69.3	4.3	9.2	28.5
Gvalue	46.5	8.2	24.8	32.5	3.3	4.6	65.1	4.4	9.3	27.6
Gv/N	3.394	0.596	1.813	2.377	0.242	0.337	4.753	0.319	0.679	2.015
Prob	0.00000	0.04280	0.00002	0.00000	0.34627	0.20289	0.00000	0.22493	0.02556	0.00000

	61	62	63	64	65	66	67	68	69	70
a_O	237	257	105	236	286	104	249	286	99	220
	(17.3)	(18.8)	(7.7)	(17.2)	(20.9)	(7.6)	(18.2)	(20.9)	(7.2)	(16.1)
a_E	258.0	324.2	81.5	258.0	324.2	81.5	258.0	324.2	81.5	258.0
	(18.8)	(23.7)	(6.0)	(18.8)	(23.7)	(6.0)	(18.8)	(23.7)	(6.0)	(18.8)
	−40.2	−119.3	53.2	−42.0	−71.6	50.7	−17.6	−71.6	38.5	−70.0
	−2.935	−8.716	3.885	−3.068	−5.233	3.702	−1.287	−5.233	2.812	−5.117
t_O	180	416	140	163	368	150	181	375	137	174
	(13.1)	(30.4)	(10.2)	(11.9)	(26.9)	(11.0)	(13.2)	(27.4)	(10.0)	(12.7)
t_E	171.0	387.0	101.9	171.0	387.0	101.9	171.0	387.0	101.9	171.0
	(12.5)	(28.3)	(7.4)	(12.5)	(28.3)	(7.4)	(12.5)	(28.3)	(7.4)	(12.5)
	18.5	60.2	89.0	−15.6	−37.0	116.1	20.6	−23.6	81.2	6.1
	1.351	4.397	6.503	−1.139	−2.702	8.480	1.505	−1.721	5.931	0.444
g_O	536	274	502	559	275	474	513	278	500	549
	(39.2)	(20.0)	(36.7)	(40.8)	(20.1)	(34.6)	(37.5)	(20.3)	(36.5)	(40.1)
g_E	545.5	270.0	568.5	545.5	270.0	568.5	545.5	270.0	568.5	545.5
	(39.8)	(19.7)	(41.5)	(39.8)	(19.7)	(41.5)	(39.8)	(19.7)	(41.5)	(39.8)
	−18.7	8.1	−124.9	27.4	10.2	−172.4	−62.9	16.3	−128.4	7.1
	−1.369	0.594	−9.124	2.004	0.742	−12.590	−4.597	1.191	−9.380	0.520
c_O	416	422	622	411	440	641	426	430	633	426
	(30.4)	(30.8)	(45.4)	(30.0)	(32.1)	(46.8)	(31.1)	(31.4)	(46.2)	(31.1)
c_E	394.6	387.9	617.1	394.6	387.9	617.1	394.6	387.9	617.1	394.6
	(28.8)	(28.3)	(45.1)	(28.8)	(28.3)	(45.1)	(28.8)	(28.3)	(45.1)	(28.8)
	43.9	71.1	9.8	33.5	110.9	48.7	65.2	88.6	32.2	65.2
	3.210	5.193	0.716	2.445	8.100	3.556	4.765	6.471	2.350	4.765

N	1369	1369	1369	1369	1369	1369	1369	1369	1369	1369
X2	3.5	19.1	28.9	3.3	12.5	45.6	5.3	9.7	24.5	8.2
Gvalue	3.5	20.1	27.1	3.3	12.4	43.1	5.3	9.7	23.4	8.4
Gv/N	0.257	1.467	1.980	0.242	0.907	3.148	0.387	0.709	1.713	0.613
Prob	0.31767	0.00016	0.00001	0.34612	0.00608	0.00000	0.15139	0.02129	0.00003	0.03853

	71	72	73	74	75	76	77	78	79	80
a_O	301	121	237	247	87	234	279	81	250	309
	(22.0)	(8.8)	(17.3)	(18.0)	(6.4)	(17.1)	(20.4)	(5.9)	(18.3)	(22.6)
a_E	324.2	81.5	258.0	324.2	81.5	258.0	324.2	81.5	258.0	324.2
	(23.7)	(6.0)	(18.8)	(23.7)	(6.0)	(18.8)	(23.7)	(6.0)	(18.8)	(23.7)
	−44.6	95.6	−40.2	−134.3	11.3	−45.6	−83.7	−1.0	−15.7	−29.6
	−3.259	6.984	−2.935	−9.809	0.829	−3.333	−6.115	−0.074	−1.145	−2.162
t_O	371	114	168	415	129	193	382	147	175	382
	(27.1)	(8.3)	(12.3)	(30.3)	(9.4)	(14.1)	(27.9)	(10.7)	(12.8)	(27.9)
t_E	387.0	101.9	171.0	387.0	101.9	171.0	387.0	101.9	171.0	387.0
	(28.3)	(7.4)	(12.5)	(28.3)	(7.4)	(12.5)	(28.3)	(7.4)	(12.5)	(28.3)
	−31.3	25.7	−5.9	58.1	60.9	46.7	−9.9	107.8	8.1	−9.9
	−2.284	1.874	−0.432	4.240	4.450	3.415	−0.721	7.876	0.593	−0.721
g_O	292	507	541	273	517	515	270	507	543	257
	(21.3)	(37.0)	(39.5)	(19.9)	(37.8)	(37.6)	(19.7)	(37.0)	(39.7)	(18.8)
g_E	270.0	568.5	545.5	270.0	568.5	545.5	270.0	568.5	545.5	270.0
	(19.7)	(41.5)	(39.8)	(19.7)	(41.5)	(39.8)	(19.7)	(41.5)	(39.8)	(19.7)
	45.8	−116.1	−8.9	6.1	−98.2	−59.2	0.1	−116.1	−4.9	−25.3
	3.347	−8.481	−0.647	0.446	−7.173	−4.322	0.005	−8.481	−0.357	−1.848
c_O	405	627	423	434	636	427	438	634	401	421
	(29.6)	(45.8)	(30.9)	(31.7)	(46.5)	(31.2)	(32.0)	(46.3)	(29.3)	(30.8)
c_E	387.9	617.1	394.6	387.9	617.1	394.6	387.9	617.1	394.6	387.9
	(28.3)	(45.1)	(28.8)	(28.3)	(45.1)	(28.8)	(28.3)	(45.1)	(28.8)	(28.3)
	34.9	19.9	58.8	97.5	38.3	67.4	106.4	34.2	12.9	68.9
	2.551	1.455	4.295	7.119	2.801	4.923	7.771	2.500	0.943	5.035
N	1369	1369	1369	1369	1369	1369	1369	1369	1369	1369
X2	4.9	27.4	3.8	25.9	12.8	9.4	12.8	27.1	0.5	4.2
Gvalue	4.9	25.1	3.8	27.3	12.4	9.3	12.9	24.9	0.5	4.2
Gv/N	0.355	1.832	0.280	1.995	0.906	0.682	0.941	1.821	0.033	0.304
Prob	0.18258	0.00001	0.27954	0.00001	0.00611	0.02508	0.00491	0.00002	0.92839	0.24439

	81	82	83	84	85	86	87	88	89	90
a_O	103	226	283	93	262	313	100	242	314	99
	(7.5)	(16.5)	(20.7)	(6.8)	(19.1)	(22.9)	(7.3)	(17.7)	(22.9)	(7.2)
a_E	81.5	258.0	324.2	81.5	258.0	324.2	81.5	258.0	324.2	81.5
	(6.0)	(18.8)	(23.7)	(6.0)	(18.8)	(23.7)	(6.0)	(18.8)	(23.7)	(6.0)
	48.2	−59.8	−76.9	24.5	8.1	−21.9	40.9	−30.9	−20.0	38.5
	3.521	−4.368	−5.614	1.792	0.594	−1.602	2.987	−2.259	−1.461	2.812
t_O	150	192	395	137	173	370	122	175	387	155
	(11.0)	(14.0)	(28.9)	(10.0)	(12.6)	(27.0)	(8.9)	(12.8)	(28.3)	(11.3)
t_E	101.9	171.0	387.0	101.9	171.0	387.0	101.9	171.0	387.0	101.9
	(7.4)	(12.5)	(28.3)	(7.4)	(12.5)	(28.3)	(7.4)	(12.5)	(28.3)	(7.4)
	116.1	44.5	16.2	81.2	4.1	−33.2	44.0	8.1	0.1	130.1
	8.480	3.251	1.186	5.931	0.296	−2.424	3.214	0.593	0.005	9.505
g_O	504	552	284	476	543	277	532	539	272	470
	(36.8)	(40.3)	(20.7)	(34.8)	(39.7)	(20.2)	(38.9)	(39.4)	(19.9)	(34.3)
g_E	568.5	545.5	270.0	568.5	545.5	270.0	568.5	545.5	270.0	568.5
	(41.5)	(39.8)	(19.7)	(41.5)	(39.8)	(19.7)	(41.5)	(39.8)	(19.7)	(41.5)
	−121.4	13.2	28.8	−169.1	−4.9	14.2	−70.6	−12.8	4.1	−178.9
	−8.868	0.963	2.103	−12.350	−0.357	1.041	−5.158	−0.937	0.298	−13.065
c_O	612	399	407	663	391	409	615	413	396	645
	(44.7)	(29.1)	(29.7)	(48.4)	(28.6)	(29.9)	(44.9)	(30.2)	(28.9)	(47.1)
c_E	617.1	394.6	387.9	617.1	394.6	387.9	617.1	394.6	387.9	617.1
	(45.1)	(28.8)	(28.3)	(45.1)	(28.8)	(28.3)	(45.1)	(28.8)	(28.3)	(45.1)
	−10.2	8.9	39.1	95.1	−7.2	43.3	−4.2	37.6	16.3	57.0
	−0.744	0.647	2.857	6.946	−0.523	3.164	−0.309	2.750	1.194	4.164
N	1369	1369	1369	1369	1369	1369	1369	1369	1369	1369
X2	35.8	6.7	7.1	32.2	0.1	2.5	10.5	2.0	0.5	49.8
Gvalue	32.7	6.7	7.3	31.7	0.1	2.5	10.1	2.0	0.5	46.8
Gv/N	2.389	0.493	0.531	2.319	0.010	0.179	0.734	0.148	0.037	3.416
Prob	0.00000	0.08047	0.06378	0.00000	0.98796	0.48407	0.01812	0.56740	0.91788	0.00000

	91	92	93	94	95	96	97	98	99	100
a_O	233	299	99	242	271	84	234	299	88	247
	(17.0)	(21.8)	(7.2)	(17.7)	(19.8)	(6.1)	(17.1)	(21.8)	(6.4)	(18.1)
a_E	258.0	324.2	81.5	258.0	324.2	81.5	258.0	324.2	81.5	257.8
	(18.8)	(23.7)	(6.0)	(18.8)	(23.7)	(6.0)	(18.8)	(23.7)	(6.0)	(18.8)
	−47.4	−48.3	38.5	−30.9	−97.1	5.1	−45.6	−48.3	13.5	−21.1
	−3.465	−3.529	2.812	−2.259	−7.091	0.369	−3.333	−3.529	0.985	−1.542
t_O	164	384	130	192	394	121	175	386	123	172
	(12.0)	(28.0)	(9.5)	(14.0)	(28.8)	(8.8)	(12.8)	(28.2)	(9.0)	(12.6)
t_E	171.0	387.0	101.9	171.0	387.0	101.9	171.0	387.0	101.9	170.9
	(12.5)	(28.3)	(7.4)	(12.5)	(28.3)	(7.4)	(12.5)	(28.3)	(7.4)	(12.5)
	−13.7	−5.9	63.4	44.5	14.2	41.7	8.1	−1.9	46.4	2.3
	−1.000	−0.432	4.631	3.251	1.037	3.043	0.593	−0.141	3.388	0.167
g_O	562	274	529	547	248	524	561	278	462	597
	(41.1)	(20.0)	(38.6)	(40.0)	(18.1)	(38.3)	(41.0)	(20.3)	(33.7)	(43.6)
g_E	545.5	270.0	568.5	545.5	270.0	568.5	545.5	270.0	568.5	545.1
	(39.8)	(19.7)	(41.5)	(39.8)	(19.7)	(41.5)	(39.8)	(19.7)	(41.5)	(39.8)
	33.6	8.1	−76.2	3.1	−42.1	−85.4	31.5	16.3	−191.7	108.7
	2.454	0.594	−5.566	0.227	−3.075	−6.241	2.304	1.191	−14.002	7.946
c_O	410	412	611	388	456	640	399	406	696	352
	(29.9)	(30.1)	(44.6)	(28.3)	(33.3)	(46.7)	(29.1)	(29.7)	(50.8)	(25.7)
c_E	394.6	387.9	617.1	394.6	387.9	617.1	394.6	387.9	617.1	394.3
	(28.8)	(28.3)	(45.1)	(28.8)	(28.3)	(45.1)	(28.8)	(28.3)	(45.1)	(28.8)
	31.4	49.6	−12.2	−13.1	147.5	46.6	8.9	37.0	167.4	−79.9
	2.293	3.627	−0.889	−0.956	10.774	3.404	0.647	2.704	12.231	−5.841
N	1369	1369	1369	1369	1369	1369	1369	1369	1369	1368
X2	3.8	3.5	14.3	3.7	22.6	8.0	2.8	3.0	34.9	9.9
Gvalue	3.9	3.6	13.5	3.6	22.5	7.9	2.9	3.1	35.6	10.0
Gv/N	0.283	0.260	0.988	0.263	1.644	0.576	0.210	0.225	2.602	0.729
Prob	0.27495	0.31382	0.00363	0.30712	0.00005	0.04855	0.41047	0.38018	0.00000	0.01876

Bcer_ATCC14579
5UTR:

	100	99	98	97	96	95	94	93	92	91
a_O	115	102	110	104	122	113	107	111	120	107
	(37.7)	(33.4)	(36.1)	(34.1)	(40.0)	(37.0)	(35.1)	(36.4)	(39.3)	(35.1)
a_E	113.1	113.1	113.1	113.1	113.1	113.1	113.1	113.1	113.1	113.1
	(37.1)	(37.1)	(37.1)	(37.1)	(37.1)	(37.1)	(37.1)	(37.1)	(37.1)	(37.1)
	3.9	-21.0	-6.1	-17.4	18.5	-0.1	-11.8	-4.1	14.3	-11.8
	1.276	-6.891	-1.986	-5.702	6.081	-0.046	-3.872	-1.345	4.681	-3.872
t_O	100	109	100	106	93	97	112	93	99	99
	(32.8)	(35.7)	(32.8)	(34.8)	(30.5)	(31.8)	(36.7)	(30.5)	(32.5)	(32.5)
t_E	98.1	98.1	98.1	98.1	98.1	98.1	98.1	98.1	98.1	98.1
	(32.2)	(32.2)	(32.2)	(32.2)	(32.2)	(32.2)	(32.2)	(32.2)	(32.2)	(32.2)
	3.9	23.0	3.9	16.4	-9.9	-2.2	29.7	-9.9	1.8	1.8
	1.265	7.538	1.265	5.391	-3.250	-0.711	9.739	-3.250	0.599	0.599
g_O	51	50	56	57	54	47	50	61	52	58
	(16.7)	(16.4)	(18.4)	(18.7)	(17.7)	(15.4)	(16.4)	(20.0)	(17.0)	(19.0)
g_E	53.2	53.2	53.2	53.2	53.2	53.2	53.2	53.2	53.2	53.2
	(17.5)	(17.5)	(17.5)	(17.5)	(17.5)	(17.5)	(17.5)	(17.5)	(17.5)	(17.5)
	-4.4	-6.3	5.7	7.8	1.6	-11.7	-6.3	16.6	-2.4	10.0
	-1.431	-2.052	1.863	2.558	0.509	-3.836	-2.052	5.450	-0.797	3.264
c_O	39	44	39	38	36	48	36	40	34	41
	(12.8)	(14.4)	(12.8)	(12.5)	(11.8)	(15.7)	(11.8)	(13.1)	(11.1)	(13.4)
c_E	40.6	40.6	40.6	40.6	40.6	40.6	40.6	40.6	40.6	40.6
	(13.3)	(13.3)	(13.3)	(13.3)	(13.3)	(13.3)	(13.3)	(13.3)	(13.3)	(13.3)
	-3.2	7.1	-3.2	-5.0	-8.7	16.0	-8.7	-1.2	-12.1	0.8
	-1.035	2.313	-1.035	-1.655	-2.844	5.262	-2.844	-0.397	-3.961	0.257
N	305	305	305	305	305	305	305	305	305	305
X2	0.2	2.8	0.3	1.8	1.5	2.1	3.0	1.4	1.5	0.8
Gvalue	0.2	2.8	0.3	1.8	1.5	2.0	3.0	1.4	1.6	0.8
Gv/N	0.075	0.907	0.107	0.591	0.496	0.669	0.971	0.459	0.522	0.249
Prob	0.97275	0.42891	0.95478	0.61459	0.67959	0.56384	0.39757	0.70580	0.66100	0.85919

	90	89	88	87	86	85	84	83	82	81
a_O	103	100	109	109	109	107	116	116	120	111
	(33.8)	(32.8)	(35.7)	(35.7)	(35.7)	(35.1)	(38.0)	(38.0)	(39.3)	(36.4)
a_E	113.1	113.1	113.1	113.1	113.1	113.1	113.1	113.1	113.1	113.1
	(37.1)	(37.1)	(37.1)	(37.1)	(37.1)	(37.1)	(37.1)	(37.1)	(37.1)	(37.1)
	-19.2	-24.6	-8.0	-8.0	-8.0	-11.8	5.9	5.9	14.3	-4.1
	-6.300	-8.055	-2.620	-2.620	-2.620	-3.872	1.946	1.946	4.681	-1.345
t_O	103	106	112	106	93	92	90	100	95	98
	(33.8)	(34.8)	(36.7)	(34.8)	(30.5)	(30.2)	(29.5)	(32.8)	(31.1)	(32.1)
t_E	98.1	98.1	98.1	98.1	98.1	98.1	98.1	98.1	98.1	98.1
	(32.2)	(32.2)	(32.2)	(32.2)	(32.2)	(32.2)	(32.2)	(32.2)	(32.2)	(32.2)
	10.1	16.4	29.7	16.4	-9.9	-11.8	-15.5	3.9	-6.1	-0.2
	3.299	5.391	9.739	5.391	-3.250	-3.867	-5.080	1.265	-1.994	-0.059
g_O	58	64	49	49	56	54	52	47	58	53
	(19.0)	(21.0)	(16.1)	(16.1)	(18.4)	(17.7)	(17.0)	(15.4)	(19.0)	(17.4)
g_E	53.2	53.2	53.2	53.2	53.2	53.2	53.2	53.2	53.2	53.2
	(17.5)	(17.5)	(17.5)	(17.5)	(17.5)	(17.5)	(17.5)	(17.5)	(17.5)	(17.5)
	10.0	23.6	-8.1	-8.1	5.7	1.6	-2.4	-11.7	10.0	-0.5
	3.264	7.733	-2.661	-2.661	1.863	0.509	-0.797	-3.836	3.264	-0.150
c_O	41	35	35	41	47	52	47	42	32	43
	(13.4)	(11.5)	(11.5)	(13.4)	(15.4)	(17.0)	(15.4)	(13.8)	(10.5)	(14.1)
c_E	40.6	40.6	40.6	40.6	40.6	40.6	40.6	40.6	40.6	40.6
	(13.3)	(13.3)	(13.3)	(13.3)	(13.3)	(13.3)	(13.3)	(13.3)	(13.3)	(13.3)
	0.8	-10.4	-10.4	0.8	13.7	25.7	13.7	2.8	-15.2	4.9
	0.257	-3.412	-3.412	0.257	4.504	8.430	4.504	0.927	-5.000	1.612
N	305	305	305	305	305	305	305	305	305	305
X2	1.6	5.1	3.2	1.1	1.6	3.9	1.8	0.9	2.8	0.2
Gvalue	1.6	5.1	3.2	1.1	1.5	3.7	1.7	0.9	2.9	0.2
Gv/N	0.520	1.657	1.047	0.367	0.497	1.200	0.573	0.301	0.951	0.058
Prob	0.66270	0.16797	0.36289	0.77253	0.67873	0.30048	0.62658	0.82092	0.40729	0.98119

	80	79	78	77	76	75	74	73	72	71
a_O	90	109	108	114	107	125	100	105	103	109
	(29.5)	(35.7)	(35.4)	(37.4)	(35.1)	(41.0)	(32.8)	(34.4)	(33.8)	(35.7)
a_E	113.1	113.1	113.1	113.1	113.1	113.1	113.1	113.1	113.1	113.1
	(37.1)	(37.1)	(37.1)	(37.1)	(37.1)	(37.1)	(37.1)	(37.1)	(37.1)	(37.1)
	-41.1	-8.0	-9.9	1.9	-11.8	25.1	-24.6	-15.5	-19.2	-8.0
	-13.467	-2.620	-3.249	0.612	-3.872	8.222	-8.055	-5.098	-6.300	-2.620
t_O	108	96	104	103	109	81	103	93	100	104
	(35.4)	(31.5)	(34.1)	(33.8)	(35.7)	(26.6)	(33.8)	(30.5)	(32.8)	(34.1)
t_E	98.1	98.1	98.1	98.1	98.1	98.1	98.1	98.1	98.1	98.1
	(32.2)	(32.2)	(32.2)	(32.2)	(32.2)	(32.2)	(32.2)	(32.2)	(32.2)	(32.2)
	20.8	-4.1	12.2	10.1	23.0	-31.0	10.1	-9.9	3.9	12.2
	6.816	-1.356	3.990	3.299	7.538	-10.168	3.299	-3.250	1.265	3.990
g_O	54	53	61	50	51	46	47	56	61	45
	(17.7)	(17.4)	(20.0)	(16.4)	(16.7)	(15.1)	(15.4)	(18.4)	(20.0)	(14.8)
g_E	53.2	53.2	53.2	53.2	53.2	53.2	53.2	53.2	53.2	53.2
	(17.5)	(17.5)	(17.5)	(17.5)	(17.5)	(17.5)	(17.5)	(17.5)	(17.5)	(17.5)
	1.6	-0.5	16.6	-6.3	-4.4	-13.4	-11.7	5.7	16.6	-15.1
	0.509	-0.150	5.450	-2.052	-1.431	-4.403	-3.836	1.863	5.450	-4.956
c_O	53	47	32	38	38	53	55	51	41	47
	(17.4)	(15.4)	(10.5)	(12.5)	(12.5)	(17.4)	(18.0)	(16.7)	(13.4)	(15.4)
c_E	40.6	40.6	40.6	40.6	40.6	40.6	40.6	40.6	40.6	40.6
	(13.3)	(13.3)	(13.3)	(13.3)	(13.3)	(13.3)	(13.3)	(13.3)	(13.3)	(13.3)
	28.2	13.7	-15.2	-5.0	-5.0	28.2	33.4	23.2	0.8	13.7
	9.254	4.504	-5.000	-1.655	-1.655	9.254	10.939	7.619	0.257	4.504
N	305	305	305	305	305	305	305	305	305	305
X2	9.5	1.2	3.5	0.6	1.8	9.0	7.6	3.6	2.1	2.8
Gvalue	9.5	1.2	3.6	0.6	1.8	8.9	7.2	3.5	2.0	2.8
Gv/N	3.111	0.377	1.191	0.204	0.580	2.905	2.347	1.134	0.672	0.917
Prob	0.02344	0.76486	0.30394	0.89162	0.62173	0.03122	0.06700	0.32632	0.56248	0.42390

	70	69	68	67	66	65	64	63	62	61
a_O	115	110	105	94	103	117	118	111	113	108
	(37.7)	(36.1)	(34.4)	(30.8)	(33.8)	(38.4)	(38.7)	(36.4)	(37.0)	(35.4)
a_E	113.1	113.1	113.1	113.1	113.1	113.1	113.1	113.1	113.1	113.1
	(37.1)	(37.1)	(37.1)	(37.1)	(37.1)	(37.1)	(37.1)	(37.1)	(37.1)	(37.1)
	3.9	-6.1	-15.5	-34.7	-19.2	8.0	10.1	-4.1	-0.1	-9.9
	1.276	-1.986	-5.098	-11.386	-6.300	2.621	3.302	-1.345	-0.046	-3.249
t_O	95	100	94	123	98	101	103	101	92	103
	(31.1)	(32.8)	(30.8)	(40.3)	(32.1)	(33.1)	(33.8)	(33.1)	(30.2)	(33.8)

t_E	98.1	98.1	98.1	98.1	98.1	98.1	98.1	98.1	98.1	98.1
	(32.2)	(32.2)	(32.2)	(32.2)	(32.2)	(32.2)	(32.2)	(32.2)	(32.2)	(32.2)
	-6.1	3.9	-8.0	55.7	-0.2	5.9	10.1	5.9	-11.8	10.1
	-1.994	1.265	-2.625	18.252	-0.059	1.936	3.299	1.936	-3.867	3.299
g_O	57	59	55	43	56	47	50	51	61	56
	(18.7)	(19.3)	(18.0)	(14.1)	(18.4)	(15.4)	(16.4)	(16.7)	(20.0)	(18.4)
g_E	53.2	53.2	53.2	53.2	53.2	53.2	53.2	53.2	53.2	53.2
	(17.5)	(17.5)	(17.5)	(17.5)	(17.5)	(17.5)	(17.5)	(17.5)	(17.5)	(17.5)
	7.8	12.1	3.6	-18.4	5.7	-11.7	-6.3	-4.4	16.6	5.7
	2.558	3.982	1.180	-6.018	1.863	-3.836	-2.052	-1.431	5.450	1.863
c_O	38	36	51	45	48	40	34	42	39	38
	(12.5)	(11.8)	(16.7)	(14.8)	(15.7)	(13.1)	(11.1)	(13.8)	(12.8)	(12.5)
c_E	40.6	40.6	40.6	40.6	40.6	40.6	40.6	40.6	40.6	40.6
	(13.3)	(13.3)	(13.3)	(13.3)	(13.3)	(13.3)	(13.3)	(13.3)	(13.3)	(13.3)
	-5.0	-8.7	23.2	9.2	16.0	-1.2	-12.1	2.8	-3.2	-5.0
	-1.655	-2.844	7.619	3.029	5.262	-0.397	-3.961	0.927	-1.035	-1.655
N	305	305	305	305	305	305	305	305	305	305
X2	0.6	1.3	3.5	12.0	2.4	1.0	1.7	0.3	1.6	0.8
Gvalue	0.6	1.3	3.3	11.8	2.3	1.0	1.8	0.3	1.5	0.8
Gv/N	0.185	0.416	1.075	3.878	0.766	0.324	0.588	0.087	0.503	0.258
Prob	0.90473	0.73641	0.35070	0.00800	0.50558	0.80388	0.61639	0.96640	0.67453	0.85287

	60	59	58	57	56	55	54	53	52	51
a_O	84	102	110	103	113	107	113	116	118	122
	(27.5)	(33.4)	(36.1)	(33.8)	(37.0)	(35.1)	(37.0)	(38.0)	(38.7)	(40.0)
a_E	113.1	113.1	113.1	113.1	113.1	113.1	113.1	113.1	113.1	113.1
	(37.1)	(37.1)	(37.1)	(37.1)	(37.1)	(37.1)	(37.1)	(37.1)	(37.1)	(37.1)
	-49.9	-21.0	-6.1	-19.2	-0.1	-11.8	-0.1	5.9	10.1	18.5
	-16.370	-6.891	-1.986	-6.300	-0.046	-3.872	-0.046	1.946	3.302	6.081
t_O	122	105	91	108	93	111	98	90	88	98
	(40.0)	(34.4)	(29.8)	(35.4)	(30.5)	(36.4)	(32.1)	(29.5)	(28.9)	(32.1)
t_E	98.1	98.1	98.1	98.1	98.1	98.1	98.1	98.1	98.1	98.1
	(32.2)	(32.2)	(32.2)	(32.2)	(32.2)	(32.2)	(32.2)	(32.2)	(32.2)	(32.2)
	53.2	14.3	-13.7	20.8	-9.9	27.4	-0.2	-15.5	-19.1	-0.2
	17.451	4.687	-4.477	6.816	-3.250	9.000	-0.059	-5.080	-6.264	-0.059
g_O	58	56	63	53	55	50	50	51	54	49
	(19.0)	(18.4)	(20.7)	(17.4)	(18.0)	(16.4)	(16.4)	(16.7)	(17.7)	(16.1)
g_E	53.2	53.2	53.2	53.2	53.2	53.2	53.2	53.2	53.2	53.2
	(17.5)	(17.5)	(17.5)	(17.5)	(17.5)	(17.5)	(17.5)	(17.5)	(17.5)	(17.5)
	10.0	5.7	21.2	-0.5	3.6	-6.3	-6.3	-4.4	1.6	-8.1
	3.264	1.863	6.962	-0.150	1.180	-2.052	-2.052	-1.431	0.509	-2.661
c_O	41	42	41	41	44	37	44	48	45	36
	(13.4)	(13.8)	(13.4)	(13.4)	(14.4)	(12.1)	(14.4)	(15.7)	(14.8)	(11.8)
c_E	40.6	40.6	40.6	40.6	40.6	40.6	40.6	40.6	40.6	40.6
	(13.3)	(13.3)	(13.3)	(13.3)	(13.3)	(13.3)	(13.3)	(13.3)	(13.3)	(13.3)
	0.8	2.8	0.8	0.8	7.1	-6.9	7.1	16.0	9.2	-8.7
	0.257	0.927	0.257	0.257	2.313	-2.259	2.313	5.262	3.029	-2.844
N	305	305	305	305	305	305	305	305	305	305
X2	13.7	1.8	2.4	1.9	0.6	2.5	0.5	2.2	1.7	1.6
Gvalue	14.0	1.8	2.3	1.9	0.6	2.5	0.5	2.1	1.8	1.6
Gv/N	4.602	0.585	0.756	0.622	0.198	0.817	0.156	0.697	0.576	0.517
Prob	0.00286	0.61810	0.51143	0.59373	0.89586	0.47675	0.92421	0.54661	0.62439	0.66456

	50	49	48	47	46	45	44	43	42	41
a_O	104	104	118	116	119	107	123	122	119	110
	(34.1)	(34.1)	(38.7)	(38.0)	(39.0)	(35.1)	(40.3)	(40.0)	(39.0)	(36.1)
a_E	113.1	113.1	113.1	113.1	113.1	113.1	113.1	113.1	113.1	113.1
	(37.1)	(37.1)	(37.1)	(37.1)	(37.1)	(37.1)	(37.1)	(37.1)	(37.1)	(37.1)
	-17.4	-17.4	10.1	5.9	12.2	-11.8	20.7	18.5	12.2	-6.1
	-5.702	-5.702	3.302	1.946	3.989	-3.872	6.789	6.081	3.989	-1.986
t_O	91	106	93	99	103	98	90	88	96	107
	(29.8)	(34.8)	(30.5)	(32.5)	(33.8)	(32.1)	(29.5)	(28.9)	(31.5)	(35.1)
t_E	98.1	98.1	98.1	98.1	98.1	98.1	98.1	98.1	98.1	98.1
	(32.2)	(32.2)	(32.2)	(32.2)	(32.2)	(32.2)	(32.2)	(32.2)	(32.2)	(32.2)
	-13.7	16.4	-9.9	1.8	10.1	-0.2	-15.5	-19.1	-4.1	18.6
	-4.477	5.391	-3.250	0.599	3.299	-0.059	-5.080	-6.264	-1.356	6.100
g_O	65	50	50	53	46	57	50	51	55	48
	(21.3)	(16.4)	(16.4)	(17.4)	(15.1)	(18.7)	(16.4)	(16.7)	(18.0)	(15.7)
g_E	53.2	53.2	53.2	53.2	53.2	53.2	53.2	53.2	53.2	53.2
	(17.5)	(17.5)	(17.5)	(17.5)	(17.5)	(17.5)	(17.5)	(17.5)	(17.5)	(17.5)
	26.0	-6.3	-6.3	-0.5	-13.4	7.8	-6.3	-4.4	3.6	-9.9
	8.515	-2.052	-2.052	-0.150	-4.403	2.558	-2.052	-1.431	1.180	-3.255
c_O	45	45	44	37	37	43	42	44	35	40
	(14.8)	(14.8)	(14.4)	(12.1)	(12.1)	(14.1)	(13.8)	(14.4)	(11.5)	(13.1)
c_E	40.6	40.6	40.6	40.6	40.6	40.6	40.6	40.6	40.6	40.6
	(13.3)	(13.3)	(13.3)	(13.3)	(13.3)	(13.3)	(13.3)	(13.3)	(13.3)	(13.3)
	9.2	9.2	7.1	-6.9	-6.9	4.9	2.8	7.1	-10.4	-1.2
	3.029	3.029	2.313	-2.259	-2.259	1.612	0.927	2.313	-3.412	-0.397
N	305	305	305	305	305	305	305	305	305	305
X2	4.3	2.0	1.0	0.4	1.9	0.7	1.8	2.1	1.2	1.4
Gvalue	4.2	2.0	1.0	0.4	1.9	0.7	1.8	2.1	1.2	1.4
Gv/N	1.364	0.665	0.314	0.136	0.626	0.240	0.584	0.699	0.401	0.463
Prob	0.24460	0.56669	0.81184	0.93701	0.59170	0.86593	0.61906	0.54526	0.74770	0.70301

	40	39	38	37	36	35	34	33	32	31
a_O	113	118	106	129	124	136	130	112	113	131
	(37.0)	(38.7)	(34.8)	(42.3)	(40.7)	(44.6)	(42.6)	(36.7)	(37.0)	(43.0)
a_E	113.1	113.1	113.1	113.1	113.1	113.1	113.1	113.1	113.1	113.1
	(37.1)	(37.1)	(37.1)	(37.1)	(37.1)	(37.1)	(37.1)	(37.1)	(37.1)	(37.1)
	-0.1	10.1	-13.7	34.0	22.9	50.2	36.3	-2.1	-0.1	38.6
	-0.046	3.302	-4.488	11.149	7.503	16.467	11.894	-0.698	-0.046	12.644
t_O	95	95	104	99	96	79	86	98	82	95
	(31.1)	(31.1)	(34.1)	(32.5)	(31.5)	(25.9)	(28.2)	(32.1)	(26.9)	(31.1)
t_E	98.1	98.1	98.1	98.1	98.1	98.1	98.1	98.1	98.1	98.1
	(32.2)	(32.2)	(32.2)	(32.2)	(32.2)	(32.2)	(32.2)	(32.2)	(32.2)	(32.2)
	-6.1	-6.1	12.2	1.8	-4.1	-34.2	-22.6	-0.2	-29.4	-6.1
	-1.994	-1.994	3.990	0.599	-1.356	-11.212	-7.418	-0.059	-9.634	-1.994
g_O	56	53	50	46	49	53	63	54	65	51
	(18.4)	(17.4)	(16.4)	(15.1)	(16.1)	(17.4)	(20.7)	(17.7)	(21.3)	(16.7)
g_E	53.2	53.2	53.2	53.2	53.2	53.2	53.2	53.2	53.2	53.2
	(17.5)	(17.5)	(17.5)	(17.5)	(17.5)	(17.5)	(17.5)	(17.5)	(17.5)	(17.5)
	5.7	-0.5	-6.3	-13.4	-8.1	-0.5	21.2	1.6	26.0	-4.4
	1.863	-0.150	-2.052	-4.403	-2.661	-0.150	6.962	0.509	8.515	-1.431
c_O	41	39	45	31	36	37	26	41	45	28
	(13.4)	(12.8)	(14.8)	(10.2)	(11.8)	(12.1)	(8.5)	(13.4)	(14.8)	(9.2)
c_E	40.6	40.6	40.6	40.6	40.6	40.6	40.6	40.6	40.6	40.6

	(13.3)	(13.3)	(13.3)	(13.3)	(13.3)	(13.3)	(13.3)	(13.3)	(13.3)	(13.3)
	0.8	-3.2	9.2	-16.7	-8.7	-6.9	-23.2	0.8	9.2	-20.8
	0.257	-1.035	3.029	-5.489	-2.844	-2.259	-7.603	0.257	3.029	-6.827
N	305	305	305	305	305	305	305	305	305	305
X2	0.2	0.4	1.5	5.5	2.0	8.7	11.1	0.0	5.7	6.9
Gvalue	0.2	0.4	1.5	5.7	2.0	8.7	11.7	0.0	5.7	7.3
Gv/N	0.080	0.123	0.478	1.856	0.642	2.846	3.835	0.008	1.864	2.392
Prob	0.97022	0.94516	0.69173	0.12925	0.58103	0.03388	0.00849	0.99895	0.12801	0.06305

	30	29	28	27	26	25	24	23	22	21
a_O	103	140	113	122	126	113	106	109	104	101
	(33.8)	(45.9)	(37.0)	(40.0)	(41.3)	(37.0)	(34.8)	(35.7)	(34.1)	(33.1)
a_E	113.1	113.1	113.1	113.1	113.1	113.1	113.1	113.1	113.1	113.1
	(37.1)	(37.1)	(37.1)	(37.1)	(37.1)	(37.1)	(37.1)	(37.1)	(37.1)	(37.1)
	-19.2	59.8	-0.1	18.5	27.3	-0.1	-13.7	-8.0	-17.4	-22.8
	-6.300	19.612	-0.046	6.081	8.946	-0.046	-4.488	-2.620	-5.702	-7.476
t_O	117	79	94	97	80	79	90	90	116	84
	(38.4)	(25.9)	(30.8)	(31.8)	(26.2)	(25.9)	(29.5)	(29.5)	(38.0)	(27.5)
t_E	98.1	98.1	98.1	98.1	98.1	98.1	98.1	98.1	98.1	98.1
	(32.2)	(32.2)	(32.2)	(32.2)	(32.2)	(32.2)	(32.2)	(32.2)	(32.2)	(32.2)
	41.3	-34.2	-8.0	-2.2	-32.6	-34.2	-15.5	-15.5	38.9	-26.1
	13.525	-11.212	-2.625	-0.711	-10.694	-11.212	-5.080	-5.080	12.757	-8.541
g_O	43	51	56	52	62	76	69	76	56	87
	(14.1)	(16.7)	(18.4)	(17.0)	(20.3)	(24.9)	(22.6)	(24.9)	(18.4)	(28.5)
g_E	53.2	53.2	53.2	53.2	53.2	53.2	53.2	53.2	53.2	53.2
	(17.5)	(17.5)	(17.5)	(17.5)	(17.5)	(17.5)	(17.5)	(17.5)	(17.5)	(17.5)
	-18.4	-4.4	5.7	-2.4	18.9	54.1	35.8	54.1	5.7	85.5
	-6.018	-1.431	1.863	-0.797	6.200	17.747	11.741	17.747	1.863	28.027
c_O	42	35	42	34	37	37	40	30	29	33
	(13.8)	(11.5)	(13.8)	(11.1)	(12.1)	(12.1)	(13.1)	(9.8)	(9.5)	(10.8)
c_E	40.6	40.6	40.6	40.6	40.6	40.6	40.6	40.6	40.6	40.6
	(13.3)	(13.3)	(13.3)	(13.3)	(13.3)	(13.3)	(13.3)	(13.3)	(13.3)	(13.3)
	2.8	-10.4	2.8	-12.1	-6.9	-6.9	-1.2	-18.2	-19.5	-13.7
	0.927	-3.412	0.927	-3.961	-2.259	-2.259	-0.397	-5.957	-6.403	-4.490
N	305	305	305	305	305	305	305	305	305	305
X2	6.6	11.0	0.4	1.8	6.6	13.8	5.8	13.3	7.5	26.2
Gvalue	6.5	10.8	0.4	1.9	6.7	12.9	5.4	12.5	7.7	22.9
Gv/N	2.134	3.557	0.119	0.612	2.194	4.230	1.776	4.090	2.514	7.519
Prob	0.08931	0.01257	0.94802	0.60031	0.08245	0.00485	0.14375	0.00592	0.05341	0.00004

	20	19	18	17	16	15	14	13	12	11
a_O	118	125	127	111	117	116	124	126	131	109
	(38.7)	(41.0)	(41.6)	(36.4)	(38.4)	(38.0)	(40.7)	(41.3)	(43.0)	(35.7)
a_E	113.1	113.1	113.1	113.1	113.1	113.1	113.1	113.1	113.1	113.1
	(37.1)	(37.1)	(37.1)	(37.1)	(37.1)	(37.1)	(37.1)	(37.1)	(37.1)	(37.1)
	10.1	25.1	29.5	-4.1	8.0	5.9	22.9	27.3	38.6	-8.0
	3.302	8.222	9.675	-1.345	2.621	1.946	7.503	8.946	12.644	-2.620
t_O	86	100	105	123	115	102	97	97	80	117
	(28.2)	(32.8)	(34.4)	(40.3)	(37.7)	(33.4)	(31.8)	(31.8)	(26.2)	(38.4)
t_E	98.1	98.1	98.1	98.1	98.1	98.1	98.1	98.1	98.1	98.1
	(32.2)	(32.2)	(32.2)	(32.2)	(32.2)	(32.2)	(32.2)	(32.2)	(32.2)	(32.2)
	-22.6	3.9	14.3	55.7	36.6	8.0	-2.2	-2.2	-32.6	41.3
	-7.418	1.265	4.687	18.252	11.994	2.614	-0.711	-0.711	-10.694	13.525
g_O	52	46	38	35	39	41	41	40	37	32
	(17.0)	(15.1)	(12.5)	(11.5)	(12.8)	(13.4)	(13.4)	(13.1)	(12.1)	(10.5)
g_E	53.2	53.2	53.2	53.2	53.2	53.2	53.2	53.2	53.2	53.2
	(17.5)	(17.5)	(17.5)	(17.5)	(17.5)	(17.5)	(17.5)	(17.5)	(17.5)	(17.5)
	-2.4	-13.4	-25.6	-29.3	-24.3	-21.4	-21.4	-22.9	-26.9	-32.6
	-0.797	-4.403	-8.398	-9.623	-7.955	-7.018	-7.018	-7.495	-8.824	-10.678
c_O	49	34	35	36	34	46	43	42	57	47
	(16.1)	(11.1)	(11.5)	(11.8)	(11.1)	(15.1)	(14.1)	(13.8)	(18.7)	(15.4)
c_E	40.6	40.6	40.6	40.6	40.6	40.6	40.6	40.6	40.6	40.6
	(13.3)	(13.3)	(13.3)	(13.3)	(13.3)	(13.3)	(13.3)	(13.3)	(13.3)	(13.3)
	18.4	-12.1	-10.4	-8.7	-12.1	11.5	4.9	2.8	38.7	13.7
	6.034	-3.961	-3.412	-2.844	-3.961	3.759	1.612	0.927	12.672	4.504
N	305	305	305	305	305	305	305	305	305	305
X2	3.5	3.4	7.3	13.1	7.9	3.8	4.0	4.8	17.7	13.3
Gvalue	3.4	3.4	7.8	13.5	8.2	4.0	4.2	5.1	17.7	14.4
Gv/N	1.122	1.122	2.552	4.440	2.699	1.301	1.386	1.667	5.798	4.730
Prob	0.33116	0.33090	0.05069	0.00360	0.04144	0.26487	0.23786	0.16568	0.00051	0.00238

	10	9	8	7	6	5	4	3	2	1
a_O	111	116	102	128	129	116	114	108	118	107
	(36.4)	(38.0)	(33.4)	(42.0)	(42.3)	(38.0)	(37.4)	(35.4)	(38.7)	(35.1)
a_E	113.1	113.1	113.1	113.1	113.1	113.1	113.1	113.1	113.1	113.1
	(37.1)	(37.1)	(37.1)	(37.1)	(37.1)	(37.1)	(37.1)	(37.1)	(37.1)	(37.1)
	-4.1	5.9	-21.0	31.7	34.0	5.9	1.9	-9.9	10.1	-11.8
	-1.345	1.946	-6.891	10.410	11.149	1.946	0.612	-3.249	3.302	-3.872
t_O	110	97	119	104	89	95	89	62	81	97
	(36.1)	(31.8)	(39.0)	(34.1)	(29.2)	(31.1)	(29.2)	(20.3)	(26.6)	(31.8)
t_E	98.1	98.1	98.1	98.1	98.1	98.1	98.1	98.1	98.1	98.1
	(32.2)	(32.2)	(32.2)	(32.2)	(32.2)	(32.2)	(32.2)	(32.2)	(32.2)	(32.2)
	25.2	-2.2	46.0	12.2	-17.3	-6.1	-17.3	-56.9	-31.0	-2.2
	8.266	-0.711	15.079	3.990	-5.676	-1.994	-5.676	-18.651	-10.168	-0.711
g_O	46	43	40	33	46	46	74	99	61	67
	(15.1)	(14.1)	(13.1)	(10.8)	(15.1)	(15.1)	(24.3)	(32.5)	(20.0)	(22.0)
g_E	53.2	53.2	53.2	53.2	53.2	53.2	53.2	53.2	53.2	53.2
	(17.5)	(17.5)	(17.5)	(17.5)	(17.5)	(17.5)	(17.5)	(17.5)	(17.5)	(17.5)
	-13.4	-18.4	-22.9	-31.6	-13.4	-13.4	48.8	122.9	16.6	30.8
	-4.403	-6.018	-7.495	-10.346	-4.403	-4.403	15.986	40.281	5.450	10.108
c_O	38	49	44	40	41	48	28	36	45	34
	(12.5)	(16.1)	(14.4)	(13.1)	(13.4)	(15.7)	(9.2)	(11.8)	(14.8)	(11.1)
c_E	40.6	40.6	40.6	40.6	40.6	40.6	40.6	40.6	40.6	40.6
	(13.3)	(13.3)	(13.3)	(13.3)	(13.3)	(13.3)	(13.3)	(13.3)	(13.3)	(13.3)
	-5.0	18.4	7.1	-1.2	0.8	16.0	-20.8	-8.7	9.2	-12.1
	-1.655	6.034	2.313	-0.397	0.257	5.262	-6.827	-2.844	3.029	-3.961
N	305	305	305	305	305	305	305	305	305	305
X2	2.6	3.8	9.1	10.0	4.1	2.5	12.9	53.4	4.8	5.0
Gvalue	2.6	3.8	9.2	11.2	4.0	2.5	12.5	47.4	4.9	4.8
Gv/N	0.862	1.252	3.006	3.657	1.328	0.811	4.096	15.537	1.613	1.565
Prob	0.45221	0.28178	0.02714	0.01093	0.25623	0.48013	0.00587	0.00000	0.17773	0.18919

CDS_INITIAL:

	1	2	3	4	5	6	7	8	9	10
a_O	157	0	0	136	115	123	104	99	102	118
	(51.5)	(0.0)	(0.0)	(44.6)	(37.7)	(40.3)	(34.1)	(32.5)	(33.4)	(38.7)
a_E	99.6	103.5	115.9	99.6	103.5	115.9	99.6	103.5	115.9	99.6
	(32.7)	(33.9)	(38.0)	(32.7)	(33.9)	(38.0)	(32.7)	(33.9)	(38.0)	(32.7)
	142.7	0.0	0.0	84.6	24.2	14.6	8.9	-8.8	-26.1	39.9
	46.802	0.000	0.000	27.736	7.939	4.772	2.915	-2.891	-8.565	13.080
t_O	81	305	1	59	107	96	69	106	110	75
	(26.6)	(100.0)	(0.3)	(19.3)	(35.1)	(31.5)	(22.6)	(34.8)	(36.1)	(24.6)
t_E	63.1	99.3	109.5	63.1	99.3	109.5	63.1	99.3	109.5	63.1
	(20.7)	(32.6)	(35.9)	(20.7)	(32.6)	(35.9)	(20.7)	(32.6)	(35.9)	(20.7)
	40.4	684.2	-9.4	-8.0	15.9	-25.2	12.3	13.7	1.1	25.9
	13.245	224.343	-3.079	-2.613	5.208	-8.256	4.028	4.507	0.359	8.479
g_O	67	0	304	58	47	44	94	47	65	76
	(22.0)	(0.0)	(99.7)	(19.0)	(15.4)	(14.4)	(30.8)	(15.4)	(21.3)	(24.9)
g_E	96.2	43.1	48.8	96.2	43.1	48.8	96.2	43.1	48.8	96.2
	(31.5)	(14.1)	(16.0)	(31.5)	(14.1)	(16.0)	(31.5)	(14.1)	(16.0)	(31.5)
	-48.4	0.0	1112.3	-58.6	8.1	-9.1	-4.3	8.1	37.3	-35.8
	-15.876	0.000	364.699	-19.229	2.666	-2.982	-1.402	2.666	12.226	-11.727
c_O	0	0	0	52	36	42	38	53	28	36
	(0.0)	(0.0)	(0.0)	(17.0)	(11.8)	(13.8)	(12.5)	(17.4)	(9.2)	(11.8)
c_E	46.1	59.0	30.8	46.1	59.0	30.8	46.1	59.0	30.8	46.1
	(15.1)	(19.4)	(10.1)	(15.1)	(19.4)	(10.1)	(15.1)	(19.4)	(10.1)	(15.1)
	0.0	0.0	0.0	12.6	-35.6	26.0	-14.6	-11.4	-5.4	-17.8
	0.000	0.000	0.000	4.131	-11.679	8.522	-4.797	-3.752	-1.763	-5.821
N	305	305	305	305	305	305	305	305	305	305
X2	93.0	631.4	1589.1	29.4	11.2	6.6	2.2	1.6	7.3	12.0
Gvalue	134.7	684.2	1102.9	30.6	12.6	6.3	2.3	1.6	6.9	12.2
Gv/N	44.171	224.343	361.620	10.025	4.135	2.056	0.744	0.530	2.258	4.012
Prob	0.00000	0.00000	0.00000	0.00000	0.00556	0.09912	0.51820	0.65539	0.07558	0.00662

	11	12	13	14	15	16	17	18	19	20
a_O	99	99	116	99	108	124	104	118	128	100
	(32.5)	(32.5)	(38.0)	(32.5)	(35.4)	(40.7)	(34.1)	(38.7)	(42.0)	(32.8)
a_E	103.5	115.9	99.6	103.5	115.9	99.6	103.5	115.9	99.6	103.5
	(33.9)	(38.0)	(32.7)	(33.9)	(38.0)	(32.7)	(33.9)	(38.0)	(32.7)	(33.9)
	-8.8	-31.3	35.3	-8.8	-15.3	54.2	1.0	4.2	64.1	-6.9
	-2.891	-10.251	11.558	-2.891	-5.020	17.778	0.323	1.367	21.016	-2.261
t_O	115	111	59	100	116	69	111	96	65	113
	(37.7)	(36.4)	(19.3)	(32.8)	(38.0)	(22.6)	(36.4)	(31.5)	(21.3)	(37.0)
t_E	99.3	109.5	63.1	99.3	109.5	63.1	99.3	109.5	63.1	99.3
	(32.6)	(35.9)	(20.7)	(32.6)	(35.9)	(20.7)	(32.6)	(35.9)	(20.7)	(32.6)
	33.7	3.1	-8.0	1.3	13.5	12.3	24.6	-25.2	3.8	29.1
	11.035	1.021	-2.613	0.431	4.419	4.028	8.074	-8.256	1.249	9.543
g_O	45	66	88	50	53	75	44	66	78	53
	(14.8)	(21.6)	(28.9)	(16.4)	(17.4)	(24.6)	(14.4)	(21.6)	(25.6)	(17.4)
g_E	43.1	48.8	96.2	43.1	48.8	96.2	43.1	48.8	96.2	43.1
	(14.1)	(16.0)	(31.5)	(14.1)	(16.0)	(31.5)	(14.1)	(16.0)	(31.5)	(14.1)
	3.9	39.9	-15.6	14.8	8.8	-37.3	1.8	39.9	-32.7	21.9
	1.269	13.075	-5.119	4.865	2.876	-12.224	0.593	13.075	-10.707	7.182
c_O	46	29	42	56	28	37	46	25	34	39
	(15.1)	(9.5)	(13.8)	(18.4)	(9.2)	(12.1)	(15.1)	(8.2)	(11.1)	(12.8)
c_E	59.0	30.8	46.1	59.0	30.8	46.1	59.0	30.8	46.1	59.0
	(19.4)	(10.1)	(15.1)	(19.4)	(10.1)	(15.1)	(19.4)	(10.1)	(15.1)	(19.4)
	-23.0	-3.5	-7.8	-5.9	-5.4	-16.2	-23.0	-10.5	-20.7	-32.3
	-7.529	-1.159	-2.545	-1.942	-1.763	-5.318	-7.529	-3.432	-6.772	-10.605
N	305	305	305	305	305	305	305	305	305	305
X2	5.6	8.7	4.0	1.5	1.6	12.9	4.3	8.9	14.7	11.1
Gvalue	5.7	8.2	3.9	1.4	1.6	13.0	4.5	8.4	14.6	11.8
Gv/N	1.885	2.687	1.281	0.463	0.511	4.264	1.461	2.754	4.787	3.859
Prob	0.12454	0.04213	0.27174	0.70286	0.66857	0.00462	0.21612	0.03844	0.00219	0.00821

	21	22	23	24	25	26	27	28	29	30
a_O	106	114	90	110	93	96	107	103	99	109
	(34.8)	(37.4)	(29.5)	(36.1)	(30.5)	(31.5)	(35.1)	(33.8)	(32.5)	(35.7)
a_E	115.9	99.6	103.5	115.9	99.6	103.5	115.9	99.6	103.5	115.9
	(38.0)	(32.7)	(33.9)	(38.0)	(32.7)	(33.9)	(38.0)	(32.7)	(33.9)	(38.0)
	-19.0	30.7	-25.2	-11.6	-12.8	-14.5	-17.2	6.8	-8.8	-13.4
	-6.227	10.059	-8.253	-3.790	-4.210	-4.741	-5.627	2.235	-2.891	-4.408
t_O	106	63	106	108	91	130	116	69	105	114
	(34.8)	(20.7)	(34.8)	(35.4)	(29.8)	(42.6)	(38.0)	(22.6)	(34.4)	(37.4)
t_E	109.5	63.1	99.3	109.5	63.1	99.3	109.5	63.1	99.3	109.5
	(35.9)	(20.7)	(32.6)	(35.9)	(20.7)	(32.6)	(35.9)	(20.7)	(32.6)	(35.9)
	-6.8	-0.2	13.7	-2.9	66.6	69.9	13.5	12.3	11.6	9.3
	-2.228	-0.080	4.507	-0.947	21.827	22.926	4.419	4.028	3.812	3.043
g_O	55	83	52	50	82	26	48	83	38	48
	(18.0)	(27.2)	(17.0)	(16.4)	(26.9)	(8.5)	(15.7)	(27.2)	(12.5)	(15.7)
g_E	48.8	96.2	43.1	48.8	96.2	43.1	48.8	96.2	43.1	48.8
	(16.0)	(31.5)	(14.1)	(16.0)	(31.5)	(14.1)	(16.0)	(31.5)	(14.1)	(16.0)
	13.2	-24.4	19.5	2.4	-26.1	-26.3	-1.6	-24.4	-9.6	-1.6
	4.321	-8.012	6.397	0.803	-8.567	-8.619	-0.514	-8.012	-3.141	-0.514
c_O	38	45	57	37	39	53	34	50	63	34
	(12.5)	(14.8)	(18.7)	(12.1)	(12.8)	(17.4)	(11.1)	(16.4)	(20.7)	(11.1)
c_E	30.8	46.1	59.0	30.8	46.1	59.0	30.8	46.1	59.0	30.8
	(10.1)	(15.1)	(19.4)	(10.1)	(15.1)	(19.4)	(10.1)	(15.1)	(19.4)	(10.1)
	15.9	-2.1	-4.0	13.5	-13.0	-11.4	6.7	8.2	8.2	6.7
	5.217	-0.691	-1.315	4.432	-4.259	-3.752	2.188	2.686	2.681	2.188
N	305	305	305	305	305	305	305	305	305	305
X2	3.4	3.9	4.1	1.6	15.9	17.4	1.4	2.8	1.4	0.9
Gvalue	3.3	3.9	4.1	1.5	14.6	17.7	1.4	2.9	1.4	0.9
Gv/N	1.082	1.275	1.336	0.499	4.791	5.814	0.466	0.937	0.461	0.308
Prob	0.34756	0.27362	0.25364	0.67740	0.00218	0.00050	0.70069	0.41384	0.70438	0.81591

	31	32	33	34	35	36	37	38	39	40
a_O	116	95	124	111	100	117	104	86	104	98
	(38.0)	(31.1)	(40.7)	(36.4)	(32.8)	(38.4)	(34.1)	(28.2)	(34.1)	(32.1)
a_E	99.6	103.5	115.9	99.6	103.5	115.9	99.6	103.5	115.9	99.6
	(32.7)	(33.9)	(38.0)	(32.7)	(33.9)	(38.0)	(32.7)	(33.9)	(38.0)	(32.7)
	35.3	-16.3	16.7	24.0	-6.9	2.1	8.9	-31.9	-22.6	-3.3
	11.558	-5.343	5.469	7.853	-2.261	0.702	2.915	-10.450	-7.408	-1.071
t_O	71	97	114	71	109	114	78	116	115	81
	(23.3)	(31.8)	(37.4)	(23.3)	(35.7)	(37.4)	(25.6)	(38.0)	(37.7)	(26.6)
t_E	63.1	99.3	109.5	63.1	99.3	109.5	63.1	99.3	109.5	63.1
	(20.7)	(32.6)	(35.9)	(20.7)	(32.6)	(35.9)	(20.7)	(32.6)	(35.9)	(20.7)
	16.7	-4.6	9.3	16.7	20.2	9.3	33.0	36.0	11.4	40.4

	5.475	-1.519	3.043	5.475	6.629	3.043	10.824	11.790	3.728	13.245
g_O	72	44	33	62	38	39	71	48	60	79
	(23.6)	(14.4)	(10.8)	(20.3)	(12.5)	(12.8)	(23.3)	(15.7)	(19.7)	(25.9)
g_E	96.2	43.1	48.8	96.2	43.1	48.8	96.2	43.1	48.8	96.2
	(31.5)	(14.1)	(16.0)	(31.5)	(14.1)	(16.0)	(31.5)	(14.1)	(16.0)	(31.5)
	-41.7	1.8	-25.8	-54.4	-9.6	-17.5	-43.1	10.3	24.8	-31.1
	-13.662	0.593	-8.462	-17.844	-3.141	-5.728	-14.124	3.385	8.137	-10.184
c_O	46	69	34	61	58	35	52	55	26	47
	(15.1)	(22.6)	(11.1)	(20.0)	(19.0)	(11.5)	(17.0)	(18.0)	(8.5)	(15.4)
c_E	46.1	59.0	30.8	46.1	59.0	30.8	46.1	59.0	30.8	46.1
	(15.1)	(19.4)	(10.1)	(15.1)	(19.4)	(10.1)	(15.1)	(19.4)	(10.1)	(15.1)
	-0.1	21.5	6.7	34.3	-2.1	8.9	12.6	-7.8	-8.8	1.9
	-0.044	7.052	2.188	11.231	-0.677	2.917	4.131	-2.557	-2.901	0.618
N	305	305	305	305	305	305	305	305	305	305
X2	9.7	2.5	6.2	19.3	1.7	2.7	11.0	6.6	4.8	8.2
Gvalue	10.1	2.4	6.8	20.5	1.7	2.8	11.4	6.6	4.7	8.0
Gv/N	3.327	0.782	2.238	6.715	0.550	0.934	3.747	2.168	1.556	2.608
Prob	0.01735	0.49622	0.07769	0.00013	0.64181	0.41543	0.00962	0.08537	0.19148	0.04700

	41	42	43	44	45	46	47	48	49	50
a_O	99	111	98	94	110	114	97	114	107	87
	(32.5)	(36.4)	(32.1)	(30.8)	(36.1)	(37.4)	(31.8)	(37.4)	(35.1)	(28.5)
a_E	103.5	115.9	99.6	103.5	115.9	99.6	103.5	115.9	99.6	103.5
	(33.9)	(38.0)	(32.7)	(33.9)	(38.0)	(32.7)	(33.9)	(38.0)	(32.7)	(33.9)
	-8.8	-9.7	-3.3	-18.1	-11.6	30.7	-12.6	-3.8	15.2	-30.2
	-2.891	-3.166	-1.071	-5.939	-3.790	10.059	-4.131	-1.258	4.995	-9.912
t_O	108	115	68	97	116	70	112	116	65	121
	(35.4)	(37.7)	(22.3)	(31.8)	(38.0)	(23.0)	(36.7)	(38.0)	(21.3)	(39.7)
t_E	99.3	109.5	63.1	99.3	109.5	63.1	99.3	109.5	63.1	99.3
	(32.6)	(35.9)	(20.7)	(32.6)	(35.9)	(20.7)	(32.6)	(35.9)	(20.7)	(32.6)
	18.0	11.4	10.1	-4.6	13.5	14.5	26.9	13.5	3.8	47.7
	5.916	3.728	3.319	-1.519	4.419	4.747	8.806	4.419	1.249	15.646
g_O	43	40	97	56	45	81	39	44	81	46
	(14.1)	(13.1)	(31.8)	(18.4)	(14.8)	(26.6)	(12.8)	(14.4)	(26.6)	(15.1)
g_E	43.1	48.8	96.2	43.1	48.8	96.2	43.1	48.8	96.2	43.1
	(14.1)	(16.0)	(31.5)	(14.1)	(16.0)	(31.5)	(14.1)	(16.0)	(31.5)	(14.1)
	-0.2	-15.9	1.7	29.3	-7.3	-27.8	-7.8	-9.1	-27.8	6.0
	-0.069	-5.211	0.551	9.610	-2.386	-9.114	-2.559	-2.982	-9.114	1.961
c_O	55	39	42	58	34	40	57	31	52	51
	(18.0)	(12.8)	(13.8)	(19.0)	(11.1)	(13.1)	(18.7)	(10.2)	(17.0)	(16.7)
c_E	59.0	30.8	46.1	59.0	30.8	46.1	59.0	30.8	46.1	59.0
	(19.4)	(10.1)	(15.1)	(19.4)	(10.1)	(15.1)	(19.4)	(10.1)	(15.1)	(19.4)
	-7.8	18.4	-7.8	-2.1	6.7	-11.3	-4.0	0.4	12.6	-14.9
	-2.557	6.018	-2.545	-0.677	2.188	-3.704	-1.315	0.117	4.131	-4.896
N	305	305	305	305	305	305	305	305	305	305
X2	1.2	4.2	0.8	4.8	1.3	6.0	2.5	0.9	3.8	8.6
Gvalue	1.2	4.2	0.8	4.5	1.3	6.1	2.4	0.9	3.8	8.5
Gv/N	0.399	1.370	0.253	1.475	0.430	1.988	0.800	0.296	1.261	2.798
Prob	0.74922	0.24288	0.85610	0.21251	0.72619	0.10863	0.48602	0.82446	0.27861	0.03616

	51	52	53	54	55	56	57	58	59	60
a_O	123	99	98	124	97	114	113	109	95	116
	(40.3)	(32.5)	(32.1)	(40.7)	(31.8)	(37.4)	(37.0)	(35.7)	(31.1)	(38.0)
a_E	115.9	99.6	103.5	115.9	99.6	103.5	115.9	99.6	103.5	115.9
	(38.0)	(32.7)	(33.9)	(38.0)	(32.7)	(33.9)	(38.0)	(32.7)	(33.9)	(38.0)
	14.6	-1.3	-10.7	16.7	-5.2	22.0	-5.8	19.6	-16.3	0.1
	4.772	-0.423	-3.514	5.469	-1.713	7.217	-1.899	6.412	-5.343	0.043
t_O	116	80	104	93	66	92	112	69	100	110
	(38.0)	(26.2)	(34.1)	(30.5)	(21.6)	(30.2)	(36.7)	(22.6)	(32.8)	(36.1)
t_E	109.5	63.1	99.3	109.5	63.1	99.3	109.5	63.1	99.3	109.5
	(35.9)	(20.7)	(32.6)	(35.9)	(20.7)	(32.6)	(35.9)	(20.7)	(32.6)	(35.9)
	13.5	37.9	9.5	-30.3	5.9	-14.1	5.2	12.3	1.3	1.1
	4.419	12.430	3.123	-9.934	1.929	-4.634	1.689	4.028	0.431	0.359
g_O	34	80	34	53	99	36	51	87	45	46
	(11.1)	(26.2)	(11.1)	(17.4)	(32.5)	(11.8)	(16.7)	(28.5)	(14.8)	(15.1)
g_E	48.8	96.2	43.1	48.8	96.2	43.1	48.8	96.2	43.1	48.8
	(16.0)	(31.5)	(14.1)	(16.0)	(31.5)	(14.1)	(16.0)	(31.5)	(14.1)	(16.0)
	-24.6	-29.4	-16.1	8.8	5.8	-13.0	4.5	-17.4	3.9	-5.4
	-8.052	-9.653	-5.290	2.876	1.888	-4.252	1.481	-5.713	1.269	-1.776
c_O	32	46	69	35	43	63	29	40	65	33
	(10.5)	(15.1)	(22.6)	(11.5)	(14.1)	(20.7)	(9.5)	(13.1)	(21.3)	(10.8)
c_E	30.8	46.1	59.0	30.8	46.1	59.0	30.8	46.1	59.0	30.8
	(10.1)	(15.1)	(19.4)	(10.1)	(15.1)	(19.4)	(10.1)	(15.1)	(19.4)	(10.1)
	2.4	-0.1	21.5	8.9	-5.9	8.2	-3.5	-11.3	12.5	4.5
	0.787	-0.044	7.052	2.917	-1.942	2.681	-1.159	-3.704	4.098	1.477
N	305	305	305	305	305	305	305	305	305	305
X2	5.4	7.2	4.1	4.0	0.5	3.0	0.3	3.1	1.4	0.3
Gvalue	5.9	7.0	4.2	4.1	0.5	3.1	0.3	3.1	1.4	0.3
Gv/N	1.925	2.310	1.371	1.328	0.161	1.012	0.112	1.023	0.455	0.104
Prob	0.11802	0.07051	0.24258	0.25595	0.92067	0.37837	0.95191	0.37331	0.70838	0.95701

	61	62	63	64	65	66	67	68	69	70
a_O	101	110	119	86	99	114	113	105	109	86
	(33.1)	(36.1)	(39.0)	(28.2)	(32.5)	(37.4)	(37.0)	(34.4)	(35.7)	(28.2)
a_E	99.6	103.5	115.9	99.6	103.5	115.9	99.6	103.5	115.9	99.6
	(32.7)	(33.9)	(38.0)	(32.7)	(33.9)	(38.0)	(32.7)	(33.9)	(38.0)	(32.7)
	2.7	13.4	6.2	-25.3	-8.8	-3.8	28.4	3.0	-13.4	-25.3
	0.893	4.388	2.037	-8.306	-2.891	-1.258	9.318	0.985	-4.408	-8.306
t_O	69	98	102	70	103	108	71	96	111	79
	(22.6)	(32.1)	(33.4)	(23.0)	(33.8)	(35.4)	(23.3)	(31.5)	(36.4)	(25.9)
t_E	63.1	99.3	109.5	63.1	99.3	109.5	63.1	99.3	109.5	63.1
	(20.7)	(32.6)	(35.9)	(20.7)	(32.6)	(35.9)	(20.7)	(32.6)	(35.9)	(20.7)
	12.3	-2.7	-14.4	14.5	7.4	-2.9	16.7	-6.6	3.1	35.4
	4.028	-0.876	-4.717	4.747	2.440	-0.947	5.475	-2.156	1.021	11.623
g_O	95	37	46	94	44	50	86	45	48	104
	(31.1)	(12.1)	(15.1)	(30.8)	(14.4)	(16.4)	(28.2)	(14.8)	(15.7)	(34.1)
g_E	96.2	43.1	48.8	96.2	43.1	48.8	96.2	43.1	48.8	96.2
	(31.5)	(14.1)	(16.0)	(31.5)	(14.1)	(16.0)	(31.5)	(14.1)	(16.0)	(31.5)
	-2.3	-11.3	-5.4	-4.3	1.8	2.4	-19.2	3.9	-1.6	16.3
	-0.758	-3.705	-1.776	-1.402	0.593	0.803	-6.299	1.269	-0.514	5.343
c_O	40	60	38	55	59	33	35	59	37	36
	(13.1)	(19.7)	(12.5)	(18.0)	(19.3)	(10.8)	(11.5)	(19.3)	(12.1)	(11.8)
c_E	46.1	59.0	30.8	46.1	59.0	30.8	46.1	59.0	30.8	46.1
	(15.1)	(19.4)	(10.1)	(15.1)	(19.4)	(10.1)	(15.1)	(19.4)	(10.1)	(15.1)
	-11.3	1.9	15.9	19.5	-0.1	4.5	-19.2	-0.1	13.5	-17.8
	-3.704	0.634	5.217	6.392	-0.027	1.477	-6.306	-0.027	4.432	-5.821

N	305	305	305	305	305	305	305	305	305	305
X2	1.4	1.3	2.4	4.4	0.3	0.2	6.5	0.2	1.7	8.7
Gvalue	1.4	1.3	2.3	4.4	0.4	0.2	6.7	0.2	1.6	8.7
Gv/N	0.459	0.440	0.760	1.431	0.115	0.076	2.188	0.071	0.531	2.839
Prob	0.70556	0.71935	0.50914	0.22480	0.95024	0.97227	0.08304	0.97473	0.65467	0.03419

	71	72	73	74	75	76	77	78	79	80
a_O	98	108	100	101	110	105	97	110	104	101
	(32.1)	(35.4)	(32.8)	(33.1)	(36.1)	(34.4)	(31.8)	(36.1)	(34.1)	(33.1)
a_E	103.5	115.9	99.6	103.5	115.9	99.6	103.5	115.9	99.6	103.5
	(33.9)	(38.0)	(32.7)	(33.9)	(38.0)	(32.7)	(33.9)	(38.0)	(32.7)	(33.9)
	-10.7	-15.3	0.7	-5.0	-11.6	11.0	-12.6	-11.6	8.9	-5.0
	-3.514	-5.020	0.231	-1.625	-3.790	3.602	-4.131	-3.790	2.915	-1.625
t_O	103	126	62	100	110	71	106	115	68	102
	(33.8)	(41.3)	(20.3)	(32.8)	(36.1)	(23.3)	(34.8)	(37.7)	(22.3)	(33.4)
t_E	99.3	109.5	63.1	99.3	109.5	63.1	99.3	109.5	63.1	99.3
	(32.6)	(35.9)	(20.7)	(32.6)	(35.9)	(20.7)	(32.6)	(35.9)	(20.7)	(32.6)
	7.4	35.5	-2.2	1.3	1.1	16.7	13.7	11.4	10.1	5.4
	2.440	11.632	-0.730	0.431	0.359	5.475	4.507	3.728	3.319	1.764
g_O	45	31	102	50	52	88	44	42	80	45
	(14.8)	(10.2)	(33.4)	(16.4)	(17.0)	(28.9)	(14.4)	(13.8)	(26.2)	(14.8)
g_E	43.1	48.8	96.2	43.1	48.8	96.2	43.1	48.8	96.2	43.1
	(14.1)	(16.0)	(31.5)	(14.1)	(16.0)	(31.5)	(14.1)	(16.0)	(31.5)	(14.1)
	3.9	-28.1	12.0	14.8	6.6	-15.6	1.8	-12.6	-29.4	3.9
	1.269	-9.220	3.942	4.865	2.172	-5.119	0.593	-4.127	-9.653	1.269
c_O	59	40	41	54	33	41	58	38	53	57
	(19.3)	(13.1)	(13.4)	(17.7)	(10.8)	(13.4)	(19.0)	(12.5)	(17.4)	(18.7)
c_E	59.0	30.8	46.1	59.0	30.8	46.1	59.0	30.8	46.1	59.0
	(19.4)	(10.1)	(15.1)	(19.4)	(10.1)	(15.1)	(19.4)	(10.1)	(15.1)	(19.4)
	-0.1	20.9	-9.6	-9.6	4.5	-9.6	-2.1	15.9	14.9	-4.0
	-0.027	6.837	-3.133	-3.161	1.477	-3.133	-0.677	5.217	4.873	-1.315
N	305	305	305	305	305	305	305	305	305	305
X2	0.5	12.3	0.9	1.6	0.7	2.5	0.9	3.2	4.3	0.3
Gvalue	0.5	12.9	0.9	1.6	0.7	2.5	0.9	3.1	4.4	0.3
Gv/N	0.168	4.229	0.311	0.511	0.219	0.826	0.292	1.027	1.453	0.093
Prob	0.91592	0.00486	0.81393	0.66912	0.88051	0.47182	0.82755	0.37159	0.21837	0.96280

	81	82	83	84	85	86	87	88	89	90
a_O	105	111	105	117	107	103	116	106	97	98
	(34.4)	(36.4)	(34.4)	(38.4)	(35.1)	(33.8)	(38.0)	(34.8)	(31.8)	(32.1)
a_E	115.9	99.6	103.5	115.9	99.6	103.5	115.9	99.6	103.5	115.9
	(38.0)	(32.7)	(33.9)	(38.0)	(32.7)	(33.9)	(38.0)	(32.7)	(33.9)	(38.0)
	-20.8	24.0	3.0	2.1	15.2	-1.0	0.1	13.1	-12.6	-32.9
	-6.821	7.853	0.985	0.702	4.995	-0.333	0.043	4.296	-4.131	-10.800
t_O	120	65	94	106	83	100	119	70	104	112
	(39.3)	(21.3)	(30.8)	(34.8)	(27.2)	(32.8)	(39.0)	(23.0)	(34.1)	(36.7)
t_E	109.5	63.1	99.3	109.5	63.1	99.3	109.5	63.1	99.3	109.5
	(35.9)	(20.7)	(32.6)	(35.9)	(20.7)	(32.6)	(35.9)	(20.7)	(32.6)	(35.9)
	22.1	3.8	-10.4	-6.8	45.4	1.3	19.9	14.5	9.5	5.2
	7.239	1.249	-3.409	-2.228	14.900	0.431	6.526	4.747	3.123	1.689
g_O	48	82	40	50	72	48	39	84	52	53
	(15.7)	(26.9)	(13.1)	(16.4)	(23.6)	(15.7)	(12.8)	(27.5)	(17.0)	(17.4)
g_E	48.8	96.2	43.1	48.8	96.2	43.1	48.8	96.2	43.1	48.8
	(16.0)	(31.5)	(14.1)	(16.0)	(31.5)	(14.1)	(16.0)	(31.5)	(14.1)	(16.0)
	-1.6	-26.1	-6.0	2.4	-41.7	10.3	-17.5	-22.7	19.5	8.8
	-0.514	-8.567	-1.961	0.803	-13.662	3.385	-5.728	-7.449	6.397	2.876
c_O	32	47	66	32	43	54	31	45	52	42
	(10.5)	(15.4)	(21.6)	(10.5)	(14.1)	(17.7)	(10.2)	(14.8)	(17.0)	(13.8)
c_E	30.8	46.1	59.0	30.8	46.1	59.0	30.8	46.1	59.0	30.8
	(10.1)	(15.1)	(19.4)	(10.1)	(15.1)	(19.4)	(10.1)	(15.1)	(19.4)	(10.1)
	2.4	1.9	14.7	2.4	-5.9	-9.6	0.4	-2.1	-13.2	26.0
	0.787	0.618	4.822	0.787	-1.942	-3.161	0.117	-0.691	-4.330	8.522
N	305	305	305	305	305	305	305	305	305	305
X2	2.1	3.5	1.4	0.2	13.1	1.0	2.8	2.7	3.3	7.3
Gvalue	2.1	3.5	1.3	0.2	13.1	1.0	2.9	2.8	3.2	7.0
Gv/N	0.691	1.153	0.437	0.064	4.290	0.323	0.958	0.903	1.059	2.288
Prob	0.55035	0.31850	0.72127	0.97854	0.00446	0.80463	0.40388	0.43136	0.35764	0.07259

	91	92	93	94	95	96	97	98	99	100
a_O	108	112	127	112	115	100	101	90	94	98
	(35.4)	(36.7)	(41.6)	(36.7)	(37.7)	(32.8)	(33.3)	(29.7)	(31.0)	(32.3)
a_E	99.6	103.5	115.9	99.6	103.5	115.9	99.0	102.8	115.2	99.0
	(32.7)	(33.9)	(38.0)	(32.7)	(33.9)	(38.0)	(32.7)	(33.9)	(38.0)	(32.7)
	17.4	17.7	23.2	26.2	24.2	-29.6	4.1	-24.0	-38.2	-2.0
	5.700	5.791	7.592	8.582	7.939	-9.695	1.337	-7.917	-12.605	-0.653
t_O	60	100	108	64	100	120	67	106	122	77
	(19.7)	(32.8)	(35.4)	(21.0)	(32.8)	(39.3)	(22.1)	(35.0)	(40.3)	(25.4)
t_E	63.1	99.3	109.5	63.1	99.3	109.5	62.7	98.7	108.7	62.7
	(20.7)	(32.6)	(35.9)	(20.7)	(32.6)	(35.9)	(20.7)	(32.6)	(35.9)	(20.7)
	-6.1	1.3	-2.9	1.8	1.3	22.1	8.9	15.1	28.1	31.6
	-1.996	0.431	-0.947	0.579	0.431	7.239	2.927	4.997	9.269	10.434
g_O	90	39	43	93	35	48	95	48	47	82
	(29.5)	(12.8)	(14.1)	(30.5)	(11.5)	(15.7)	(31.4)	(15.8)	(15.5)	(27.1)
g_E	96.2	43.1	48.8	96.2	43.1	48.8	95.5	42.8	48.5	95.5
	(31.5)	(14.1)	(16.0)	(31.5)	(14.1)	(16.0)	(31.5)	(14.1)	(16.0)	(31.5)
	-11.9	-7.8	-10.9	-6.2	-14.6	-1.6	-1.1	11.0	-2.9	-25.1
	-3.909	-2.559	-3.562	-2.040	-4.781	-0.514	-0.350	3.616	-0.956	-8.267
c_O	47	54	27	36	55	37	40	59	40	46
	(15.4)	(17.7)	(8.9)	(11.8)	(18.0)	(12.1)	(13.2)	(19.5)	(13.2)	(15.2)
c_E	46.1	59.0	30.8	46.1	59.0	30.8	45.8	58.7	30.6	45.8
	(15.1)	(19.4)	(10.1)	(15.1)	(19.4)	(10.1)	(15.1)	(19.4)	(10.1)	(15.1)
	1.9	-9.6	-7.1	-17.8	-7.8	13.5	-10.8	0.7	21.4	0.5
	0.618	-3.161	-2.344	-5.821	-2.557	4.432	-3.555	0.229	7.055	0.156
N	305	305	305	305	305	305	303	303	303	303
X2	1.3	1.5	2.2	3.8	3.1	4.5	1.1	2.8	8.4	5.2
Gvalue	1.3	1.5	2.3	4.0	3.1	4.5	1.1	2.8	8.4	5.1
Gv/N	0.413	0.502	0.739	1.301	1.032	1.462	0.359	0.925	2.764	1.670
Prob	0.73844	0.67539	0.52129	0.26481	0.36933	0.21599	0.77968	0.42283	0.03886	0.16746

Bcic_HC
5UTR:

	100	99	98	97	96	95	94	93	92	91
a_O	58	75	69	76	75	65	70	83	68	69
	(31.5)	(40.8)	(37.5)	(41.3)	(40.8)	(35.3)	(38.0)	(45.1)	(37.0)	(37.5)
a_E	72.1	72.1	72.1	72.1	72.1	72.1	72.1	72.1	72.1	72.1
	(39.2)	(39.2)	(39.2)	(39.2)	(39.2)	(39.2)	(39.2)	(39.2)	(39.2)	(39.2)
	-25.3	5.8	-6.1	7.9	5.8	-13.5	-4.2	23.3	-8.0	-6.1
	-13.752	3.172	-3.335	4.308	3.172	-7.361	-2.289	12.654	-4.366	-3.335
t_O	63	53	63	61	66	65	59	53	57	65
	(34.2)	(28.8)	(34.2)	(33.2)	(35.9)	(35.3)	(32.1)	(28.8)	(31.0)	(35.3)
t_E	65.1	65.1	65.1	65.1	65.1	65.1	65.1	65.1	65.1	65.1
	(35.4)	(35.4)	(35.4)	(35.4)	(35.4)	(35.4)	(35.4)	(35.4)	(35.4)	(35.4)
	-4.1	-21.7	-4.1	-7.9	1.9	-0.1	-11.5	-21.7	-15.1	-0.1
	-2.211	-11.817	-2.211	-4.280	1.021	-0.073	-6.277	-11.817	-8.201	-0.073
g_O	31	31	28	22	25	20	35	20	31	23
	(16.8)	(16.8)	(15.2)	(12.0)	(13.6)	(10.9)	(19.0)	(10.9)	(16.8)	(12.5)
g_E	23.2	23.2	23.2	23.2	23.2	23.2	23.2	23.2	23.2	23.2
	(12.6)	(12.6)	(12.6)	(12.6)	(12.6)	(12.6)	(12.6)	(12.6)	(12.6)	(12.6)
	18.0	18.0	10.5	-2.3	3.8	-5.9	28.8	-5.9	18.0	-0.4
	9.777	9.777	5.733	-1.262	2.039	-3.220	15.656	-3.220	9.777	-0.208
c_O	32	25	24	25	18	34	20	28	28	27
	(17.4)	(13.6)	(13.0)	(13.6)	(9.8)	(18.5)	(10.9)	(15.2)	(15.2)	(14.7)
c_E	23.6	23.6	23.6	23.6	23.6	23.6	23.6	23.6	23.6	23.6
	(12.8)	(12.8)	(12.8)	(12.8)	(12.8)	(12.8)	(12.8)	(12.8)	(12.8)	(12.8)
	19.5	2.9	0.8	2.9	-9.8	24.8	-6.6	9.6	9.6	7.3
	10.587	1.563	0.436	1.563	-5.302	13.489	-3.601	5.200	5.200	3.947
N	184	184	184	184	184	184	184	184	184	184
X2	8.5	5.1	1.2	0.6	1.6	5.7	7.2	5.1	4.7	0.6
Gvalue	8.1	5.0	1.1	0.6	1.7	5.2	6.4	5.2	4.4	0.6
Gv/N	4.401	2.695	0.623	0.330	0.931	2.836	3.489	2.817	2.410	0.330
Prob	0.04402	0.17484	0.76609	0.89496	0.63417	0.15655	0.09288	0.15883	0.21827	0.89476

	90	89	88	87	86	85	84	83	82	81
a_O	76	69	72	68	79	62	74	70	66	70
	(41.3)	(37.5)	(39.1)	(37.0)	(42.9)	(33.7)	(40.2)	(38.0)	(35.9)	(38.0)
a_E	72.1	72.1	72.1	72.1	72.1	72.1	72.1	72.1	72.1	72.1
	(39.2)	(39.2)	(39.2)	(39.2)	(39.2)	(39.2)	(39.2)	(39.2)	(39.2)	(39.2)
	7.9	-6.1	-0.3	-8.0	14.4	-18.8	3.8	-4.2	-11.7	-4.2
	4.308	-3.335	-0.150	-4.366	7.803	-10.206	2.050	-2.289	-6.379	-2.289
t_O	57	59	62	74	57	62	62	68	72	64
	(31.0)	(32.1)	(33.7)	(40.2)	(31.0)	(33.7)	(33.7)	(37.0)	(39.1)	(34.8)
t_E	65.1	65.1	65.1	65.1	65.1	65.1	65.1	65.1	65.1	65.1
	(35.4)	(35.4)	(35.4)	(35.4)	(35.4)	(35.4)	(35.4)	(35.4)	(35.4)	(35.4)
	-15.1	-11.5	-6.0	19.0	-15.1	-6.0	-6.0	6.0	14.6	-2.1
	-8.201	-6.277	-3.254	10.348	-8.201	-3.254	-3.254	3.259	7.924	-1.150
g_O	32	32	22	19	30	34	24	24	22	20
	(17.4)	(17.4)	(12.0)	(10.3)	(16.3)	(18.5)	(13.0)	(13.0)	(12.0)	(10.9)
g_E	23.2	23.2	23.2	23.2	23.2	23.2	23.2	23.2	23.2	23.2
	(12.6)	(12.6)	(12.6)	(12.6)	(12.6)	(12.6)	(12.6)	(12.6)	(12.6)	(12.6)
	20.6	20.6	-2.3	-7.6	15.4	26.0	1.6	1.6	-2.3	-5.9
	11.197	11.197	-1.262	-4.118	8.392	14.137	0.893	0.893	-1.262	-3.220
c_O	19	24	28	23	18	26	24	22	24	30
	(10.3)	(13.0)	(15.2)	(12.5)	(9.8)	(14.1)	(13.0)	(12.0)	(13.0)	(16.3)
c_E	23.6	23.6	23.6	23.6	23.6	23.6	23.6	23.6	23.6	23.6
	(12.8)	(12.8)	(12.8)	(12.8)	(12.8)	(12.8)	(12.8)	(12.8)	(12.8)	(12.8)
	-8.2	0.8	9.6	-1.2	-9.8	5.0	0.8	-3.1	0.8	14.4
	-4.480	0.436	5.200	-0.647	-5.302	2.734	0.436	-1.681	0.436	7.821
N	184	184	184	184	184	184	184	184	184	184
X2	5.4	4.1	1.0	2.2	5.0	6.8	0.2	0.3	1.3	2.3
Gvalue	5.2	3.7	1.0	2.2	5.0	6.3	0.2	0.3	1.3	2.1
Gv/N	2.824	2.020	0.534	1.217	2.692	3.411	0.124	0.181	0.718	1.162
Prob	0.15794	0.29377	0.80555	0.52420	0.17519	0.09892	0.97278	0.95363	0.72426	0.54419

	80	79	78	77	76	75	74	73	72	71
a_O	73	64	65	54	62	58	64	91	60	63
	(39.7)	(34.8)	(35.3)	(29.3)	(33.7)	(31.5)	(35.0)	(49.5)	(32.6)	(34.2)
a_E	72.1	72.1	72.1	72.1	72.1	72.1	71.7	72.1	72.1	72.1
	(39.2)	(39.2)	(39.2)	(39.2)	(39.2)	(39.2)	(39.2)	(39.2)	(39.2)	(39.2)
	1.7	-15.3	-13.5	-31.3	-18.8	-25.3	-14.6	42.3	-22.1	-17.1
	0.943	-8.327	-7.361	-16.998	-10.206	-13.752	-7.991	22.976	-12.015	-9.275
t_O	52	66	70	74	58	70	64	54	73	70
	(28.3)	(35.9)	(38.0)	(40.2)	(31.5)	(38.0)	(35.0)	(29.3)	(39.7)	(38.0)
t_E	65.1	65.1	65.1	65.1	65.1	65.1	64.7	65.1	65.1	65.1
	(35.4)	(35.4)	(35.4)	(35.4)	(35.4)	(35.4)	(35.4)	(35.4)	(35.4)	(35.4)
	-23.3	1.9	10.2	19.0	-13.3	10.2	-1.4	-20.1	16.8	10.2
	-12.671	1.021	5.560	10.348	-7.248	5.560	-0.775	-10.943	9.128	5.560
g_O	30	32	26	30	38	32	31	21	31	22
	(16.3)	(17.4)	(14.1)	(16.3)	(20.7)	(17.4)	(16.9)	(11.4)	(16.8)	(12.0)
g_E	23.2	23.2	23.2	23.2	23.2	23.2	23.1	23.2	23.2	23.2
	(12.6)	(12.6)	(12.6)	(12.6)	(12.6)	(12.6)	(12.6)	(12.6)	(12.6)	(12.6)
	15.4	20.6	5.9	15.4	37.5	20.6	18.3	-4.2	18.0	-2.3
	8.392	11.197	3.229	8.392	20.394	11.197	10.015	-2.267	9.777	-1.262
c_O	29	22	23	26	26	24	24	18	20	29
	(15.8)	(12.0)	(12.5)	(14.1)	(14.1)	(13.0)	(13.1)	(9.8)	(10.9)	(15.8)
c_E	23.6	23.6	23.6	23.6	23.6	23.6	23.5	23.6	23.6	23.6
	(12.8)	(12.8)	(12.8)	(12.8)	(12.8)	(12.8)	(12.8)	(12.8)	(12.8)	(12.8)
	11.9	-3.1	-1.2	5.0	5.0	0.8	1.1	-9.8	-6.6	11.9
	6.492	-1.681	-0.647	2.734	2.734	0.436	0.581	-5.302	-3.601	6.492
N	184	184	184	184	184	184	183	184	184	184
X2	5.9	4.4	1.4	8.0	11.9	6.5	3.6	8.4	6.2	2.8
Gvalue	5.8	4.1	1.4	8.2	10.4	6.3	3.3	8.2	6.1	2.8
Gv/N	3.156	2.210	0.782	4.476	5.674	3.440	1.830	4.464	3.290	1.514
Prob	0.12139	0.25439	0.69663	0.04138	0.01518	0.09659	0.34098	0.04179	0.10907	0.42570

	70	69	68	67	66	65	64	63	62	61
a_O	71	71	68	48	68	72	66	78	63	76
	(38.6)	(38.6)	(37.0)	(26.1)	(37.0)	(39.1)	(35.9)	(42.4)	(34.2)	(41.3)
a_E	72.1	72.1	72.1	72.1	72.1	72.1	72.1	72.1	72.1	72.1
	(39.2)	(39.2)	(39.2)	(39.2)	(39.2)	(39.2)	(39.2)	(39.2)	(39.2)	(39.2)
	-2.3	-2.3	-8.0	-39.1	-8.0	-0.3	-11.7	12.2	-17.1	7.9
	-1.227	-1.227	-4.366	-21.254	-4.366	-0.150	-6.379	6.624	-9.275	4.308
t_O	54	61	66	72	65	65	65	59	65	56
	(29.3)	(33.2)	(35.9)	(39.1)	(35.3)	(35.3)	(35.3)	(32.1)	(35.3)	(30.4)

t_E	65.1	65.1	65.1	65.1	65.1	65.1	65.1	65.1	65.1	65.1
	(35.4)	(35.4)	(35.4)	(35.4)	(35.4)	(35.4)	(35.4)	(35.4)	(35.4)	(35.4)
	-20.1	-7.9	1.9	14.6	-0.1	-0.1	-0.1	-11.5	-0.1	-16.8
	-10.943	-4.280	1.021	7.924	-0.073	-0.073	-0.073	-6.277	-0.073	-9.135
g_O	28	19	26	33	23	24	23	28	25	28
	(15.2)	(10.3)	(14.1)	(17.9)	(12.5)	(13.0)	(12.5)	(15.2)	(13.6)	(15.2)
g_E	23.2	23.2	23.2	23.2	23.2	23.2	23.2	23.2	23.2	23.2
	(12.6)	(12.6)	(12.6)	(12.6)	(12.6)	(12.6)	(12.6)	(12.6)	(12.6)	(12.6)
	10.5	-7.6	5.9	23.3	-0.4	1.6	-0.4	10.5	3.8	10.5
	5.733	-4.118	3.229	12.650	-0.208	0.893	-0.208	5.733	2.039	5.733
c_O	31	33	24	31	28	23	30	19	31	24
	(16.8)	(17.9)	(13.0)	(16.8)	(15.2)	(12.5)	(16.3)	(10.3)	(16.8)	(13.0)
c_E	23.6	23.6	23.6	23.6	23.6	23.6	23.6	23.6	23.6	23.6
	(12.8)	(12.8)	(12.8)	(12.8)	(12.8)	(12.8)	(12.8)	(12.8)	(12.8)	(12.8)
	16.9	22.1	0.8	16.9	9.6	-1.2	14.4	-8.2	16.9	0.8
	9.187	12.022	0.436	9.187	5.200	-0.647	7.821	-4.480	9.187	0.436
N	184	184	184	184	184	184	184	184	184	184
X2	5.2	4.8	0.6	15.3	1.1	0.0	2.3	2.9	3.6	2.5
Gvalue	5.1	4.4	0.6	15.7	1.0	0.0	2.1	2.9	3.5	2.5
Gv/N	2.750	2.397	0.320	8.506	0.552	0.024	1.160	1.600	1.878	1.343
Prob	0.16749	0.22036	0.89897	0.00134	0.79729	0.99761	0.54486	0.40027	0.32659	0.48064

	60	59	58	57	56	55	54	53	52	51
a_O	63	71	62	71	68	76	82	66	69	70
	(34.2)	(38.6)	(33.7)	(38.6)	(37.0)	(41.3)	(44.6)	(35.9)	(37.5)	(38.0)
a_E	72.1	72.1	72.1	72.1	72.1	72.1	72.1	72.1	72.1	72.1
	(39.2)	(39.2)	(39.2)	(39.2)	(39.2)	(39.2)	(39.2)	(39.2)	(39.2)	(39.2)
	-17.1	-2.3	-18.8	-2.3	-8.0	7.9	21.0	-11.7	-6.1	-4.2
	-9.275	-1.227	-10.206	-1.227	-4.366	4.308	11.421	-6.379	-3.335	-2.289
t_O	67	64	64	69	59	68	60	79	65	69
	(36.4)	(34.8)	(34.8)	(37.5)	(32.1)	(37.0)	(32.6)	(42.9)	(35.3)	(37.5)
t_E	65.1	65.1	65.1	65.1	65.1	65.1	65.1	65.1	65.1	65.1
	(35.4)	(35.4)	(35.4)	(35.4)	(35.4)	(35.4)	(35.4)	(35.4)	(35.4)	(35.4)
	3.9	-2.1	-2.1	8.1	-11.5	6.0	-9.7	30.7	-0.1	8.1
	2.132	-1.150	-1.150	4.402	-6.277	3.259	-5.287	16.661	-0.073	4.402
g_O	28	22	29	22	25	16	23	18	32	21
	(15.2)	(12.0)	(15.8)	(12.0)	(13.6)	(8.7)	(12.5)	(9.8)	(17.4)	(11.4)
g_E	23.2	23.2	23.2	23.2	23.2	23.2	23.2	23.2	23.2	23.2
	(12.6)	(12.6)	(12.6)	(12.6)	(12.6)	(12.6)	(12.6)	(12.6)	(12.6)	(12.6)
	10.5	-2.3	13.0	-2.3	3.8	-11.9	-0.4	-9.1	20.6	-4.2
	5.733	-1.262	7.044	-1.262	2.039	-6.456	-0.208	-4.959	11.197	-2.267
c_O	26	27	29	22	32	24	19	21	18	24
	(14.1)	(14.7)	(15.8)	(12.0)	(17.4)	(13.0)	(10.3)	(11.4)	(9.8)	(13.0)
c_E	23.6	23.6	23.6	23.6	23.6	23.6	23.6	23.6	23.6	23.6
	(12.8)	(12.8)	(12.8)	(12.8)	(12.8)	(12.8)	(12.8)	(12.8)	(12.8)	(12.8)
	5.0	7.3	11.9	-3.1	19.5	0.8	-8.2	-4.9	-9.8	0.8
	2.734	3.947	6.492	-1.681	10.587	0.436	-4.480	-2.667	-5.302	0.436
N	184	184	184	184	184	184	184	184	184	184
X2	2.5	0.6	4.1	0.4	3.9	2.6	2.6	5.0	4.8	0.5
Gvalue	2.4	0.6	4.0	0.4	3.6	2.8	2.7	4.9	4.6	0.5
Gv/N	1.324	0.307	2.179	0.231	1.983	1.546	1.446	2.656	2.487	0.281
Prob	0.48692	0.90436	0.26039	0.93506	0.30194	0.41608	0.44709	0.18025	0.20567	0.91495

	50	49	48	47	46	45	44	43	42	41
a_O	61	72	70	68	79	60	72	77	73	69
	(33.2)	(39.1)	(38.0)	(37.0)	(42.9)	(32.6)	(39.1)	(41.8)	(39.7)	(37.5)
a_E	72.1	72.1	72.1	72.1	72.1	72.1	72.1	72.1	72.1	72.1
	(39.2)	(39.2)	(39.2)	(39.2)	(39.2)	(39.2)	(39.2)	(39.2)	(39.2)	(39.2)
	-20.5	-0.3	-4.2	-8.0	14.4	-22.1	-0.3	10.0	1.7	-6.1
	-11.120	-0.150	-2.289	-4.366	7.803	-12.015	-0.150	5.459	0.943	-3.335
t_O	75	66	68	62	61	78	70	56	77	67
	(40.8)	(35.9)	(37.0)	(33.7)	(33.2)	(42.4)	(38.0)	(30.4)	(41.8)	(36.4)
t_E	65.1	65.1	65.1	65.1	65.1	65.1	65.1	65.1	65.1	65.1
	(35.4)	(35.4)	(35.4)	(35.4)	(35.4)	(35.4)	(35.4)	(35.4)	(35.4)	(35.4)
	21.3	1.9	6.0	-6.0	-7.9	28.3	10.2	-16.8	25.9	3.9
	11.582	1.021	3.259	-3.254	-4.280	15.370	5.560	-9.135	14.093	2.132
g_O	30	25	22	28	21	22	24	29	16	29
	(16.3)	(13.6)	(12.0)	(15.2)	(11.4)	(12.0)	(13.0)	(15.8)	(8.7)	(15.8)
g_E	23.2	23.2	23.2	23.2	23.2	23.2	23.2	23.2	23.2	23.2
	(12.6)	(12.6)	(12.6)	(12.6)	(12.6)	(12.6)	(12.6)	(12.6)	(12.6)	(12.6)
	15.4	3.8	-2.3	10.5	-4.2	-2.3	1.6	13.0	-11.9	13.0
	8.392	2.039	-1.262	5.733	-2.267	-1.262	0.893	7.044	-6.456	7.044
c_O	18	21	24	26	23	24	18	22	18	19
	(9.8)	(11.4)	(13.0)	(14.1)	(12.5)	(13.0)	(9.8)	(12.0)	(9.8)	(10.3)
c_E	23.6	23.6	23.6	23.6	23.6	23.6	23.6	23.6	23.6	23.6
	(12.8)	(12.8)	(12.8)	(12.8)	(12.8)	(12.8)	(12.8)	(12.8)	(12.8)	(12.8)
	-9.8	-4.9	0.8	5.0	-1.2	0.8	-9.8	-3.1	-9.8	-8.2
	-5.302	-2.667	0.436	2.734	-0.647	0.436	-5.302	-1.681	-5.302	-4.480
N	184	184	184	184	184	184	184	184	184	184
X2	6.6	0.4	0.3	1.6	1.1	4.7	1.7	3.2	5.8	2.5
Gvalue	6.5	0.4	0.3	1.6	1.1	4.7	1.8	3.1	6.0	2.5
Gv/N	3.553	0.244	0.143	0.847	0.610	2.528	1.001	1.687	3.278	1.361
Prob	0.08821	0.92993	0.96678	0.66881	0.77169	0.19912	0.60570	0.37577	0.11011	0.47461

	40	39	38	37	36	35	34	33	32	31
a_O	80	76	67	59	79	77	68	68	84	74
	(43.5)	(41.3)	(36.4)	(32.1)	(42.9)	(41.8)	(37.0)	(37.0)	(45.7)	(40.2)
a_E	72.1	72.1	72.1	72.1	72.1	72.1	72.1	72.1	72.1	72.1
	(39.2)	(39.2)	(39.2)	(39.2)	(39.2)	(39.2)	(39.2)	(39.2)	(39.2)	(39.2)
	16.6	7.9	-9.9	-23.7	14.4	10.0	-8.0	-8.0	25.6	3.8
	8.995	4.308	-5.381	-12.893	7.803	5.459	-4.366	-4.366	13.900	2.050
t_O	59	66	66	74	71	69	67	67	56	59
	(32.1)	(35.9)	(35.9)	(40.2)	(38.6)	(37.5)	(36.4)	(36.4)	(30.4)	(32.1)
t_E	65.1	65.1	65.1	65.1	65.1	65.1	65.1	65.1	65.1	65.1
	(35.4)	(35.4)	(35.4)	(35.4)	(35.4)	(35.4)	(35.4)	(35.4)	(35.4)	(35.4)
	-11.5	1.9	1.9	19.0	12.4	8.1	3.9	3.9	-16.8	-11.5
	-6.277	1.021	1.021	10.348	6.734	4.402	2.132	2.132	-9.135	-6.277
g_O	27	22	24	20	14	20	24	19	18	21
	(14.7)	(12.0)	(13.0)	(10.9)	(7.6)	(10.9)	(13.0)	(10.3)	(9.8)	(11.4)
g_E	23.2	23.2	23.2	23.2	23.2	23.2	23.2	23.2	23.2	23.2
	(12.6)	(12.6)	(12.6)	(12.6)	(12.6)	(12.6)	(12.6)	(12.6)	(12.6)	(12.6)
	8.2	-2.3	1.6	-5.9	-14.1	-5.9	1.6	-7.6	-9.1	-4.2
	4.461	-1.262	0.893	-3.220	-7.681	-3.220	0.893	-4.118	-4.959	-2.267
c_O	18	20	27	31	20	18	25	30	26	30
	(9.8)	(10.9)	(14.7)	(16.8)	(10.9)	(9.8)	(13.6)	(16.3)	(14.1)	(16.3)
c_E	23.6	23.6	23.6	23.6	23.6	23.6	23.6	23.6	23.6	23.6

	(12.8)	(12.8)	(12.8)	(12.8)	(12.8)	(12.8)	(12.8)	(12.8)	(12.8)	(12.8)
	-9.8	-6.6	7.3	16.9	-6.6	-9.8	2.9	14.4	5.0	14.4
	-5.302	-3.601	3.947	9.187	-3.601	-5.302	1.563	7.821	2.734	7.821
N	184	184	184	184	184	184	184	184	184	184
X2	3.4	0.8	0.9	6.4	5.4	2.3	0.4	2.8	4.6	2.6
Gvalue	3.5	0.9	0.9	6.3	6.0	2.5	0.4	2.7	4.7	2.4
Gv/N	1.878	0.467	0.480	3.422	3.255	1.339	0.222	1.469	2.541	1.327
Prob	0.32669	0.83529	0.82949	0.09806	0.11210	0.48174	0.93862	0.43977	0.19724	0.48594

	30	29	28	27	26	25	24	23	22	21
a_O	69	74	78	66	68	74	82	70	67	78
	(37.5)	(40.2)	(42.4)	(35.9)	(37.0)	(40.2)	(44.6)	(38.0)	(36.4)	(42.4)
a_E	72.1	72.1	72.1	72.1	72.1	72.1	72.1	72.1	72.1	72.1
	(39.2)	(39.2)	(39.2)	(39.2)	(39.2)	(39.2)	(39.2)	(39.2)	(39.2)	(39.2)
	-6.1	3.8	12.2	-11.7	-8.0	3.8	21.0	-4.2	-9.9	12.2
	-3.335	2.050	6.624	-6.379	-4.366	2.050	11.421	-2.289	-5.381	6.624
t_O	76	69	68	78	73	71	55	71	71	64
	(41.3)	(37.5)	(37.0)	(42.4)	(39.7)	(38.6)	(29.9)	(38.6)	(38.6)	(34.8)
t_E	65.1	65.1	65.1	65.1	65.1	65.1	65.1	65.1	65.1	65.1
	(35.4)	(35.4)	(35.4)	(35.4)	(35.4)	(35.4)	(35.4)	(35.4)	(35.4)	(35.4)
	23.6	8.1	6.0	28.3	16.8	12.4	-18.5	12.4	12.4	-2.1
	12.830	4.402	3.259	15.370	9.128	6.734	-10.049	6.734	6.734	-1.150
g_O	20	20	20	14	23	19	19	17	22	20
	(10.9)	(10.9)	(10.9)	(7.6)	(12.5)	(10.3)	(10.3)	(9.2)	(12.0)	(10.9)
g_E	23.2	23.2	23.2	23.2	23.2	23.2	23.2	23.2	23.2	23.2
	(12.6)	(12.6)	(12.6)	(12.6)	(12.6)	(12.6)	(12.6)	(12.6)	(12.6)	(12.6)
	-5.9	-5.9	-5.9	-14.1	-0.4	-7.6	-7.6	-10.6	-2.3	-5.9
	-3.220	-3.220	-3.220	-7.681	-0.208	-4.118	-4.118	-5.740	-1.262	-3.220
c_O	19	21	18	26	20	20	28	26	24	22
	(10.3)	(11.4)	(9.8)	(14.1)	(10.9)	(10.9)	(15.2)	(14.1)	(13.0)	(12.0)
c_E	23.6	23.6	23.6	23.6	23.6	23.6	23.6	23.6	23.6	23.6
	(12.8)	(12.8)	(12.8)	(12.8)	(12.8)	(12.8)	(12.8)	(12.8)	(12.8)	(12.8)
	-8.2	-4.9	-9.8	5.0	-6.6	-6.6	9.6	5.0	0.8	-3.1
	-4.480	-2.667	-5.302	2.734	-3.601	-3.601	5.200	2.734	0.436	-1.681
N	184	184	184	184	184	184	184	184	184	184
X2	3.3	1.0	2.4	7.0	1.8	1.9	4.5	2.5	1.0	1.0
Gvalue	3.3	1.0	2.5	7.4	1.8	2.0	4.5	2.6	1.0	1.1
Gv/N	1.796	0.565	1.361	4.044	0.953	1.066	2.455	1.440	0.527	0.573
Prob	0.34709	0.79157	0.47437	0.05911	0.62501	0.58048	0.21084	0.44897	0.80870	0.78817

	20	19	18	17	16	15	14	13	12	11
a_O	69	85	92	87	81	77	87	75	85	83
	(37.5)	(46.2)	(50.0)	(47.3)	(44.0)	(41.8)	(47.3)	(40.8)	(46.4)	(45.1)
a_E	72.1	72.1	72.1	72.1	72.1	72.1	72.1	72.1	71.7	72.1
	(39.2)	(39.2)	(39.2)	(39.2)	(39.2)	(39.2)	(39.2)	(39.2)	(39.2)	(39.2)
	-6.1	27.9	44.8	32.6	18.8	10.0	32.6	5.8	28.8	23.3
	-3.335	15.159	24.321	17.715	10.202	5.459	17.715	3.172	15.748	12.654
t_O	69	68	60	69	61	72	62	67	50	52
	(37.5)	(37.0)	(32.6)	(37.5)	(33.2)	(39.1)	(33.7)	(36.4)	(27.3)	(28.3)
t_E	65.1	65.1	65.1	65.1	65.1	65.1	65.1	65.1	64.7	65.1
	(35.4)	(35.4)	(35.4)	(35.4)	(35.4)	(35.4)	(35.4)	(35.4)	(35.4)	(35.4)
	8.1	6.0	-9.7	8.1	-7.9	14.6	-6.0	3.9	-25.8	-23.3
	4.402	3.259	-5.287	4.402	-4.280	7.924	-3.254	2.132	-14.095	-12.671
g_O	23	13	11	7	16	11	16	25	27	30
	(12.5)	(7.1)	(6.0)	(3.8)	(8.7)	(6.0)	(8.7)	(13.6)	(14.8)	(16.3)
g_E	23.2	23.2	23.2	23.2	23.2	23.2	23.2	23.2	23.1	23.2
	(12.6)	(12.6)	(12.6)	(12.6)	(12.6)	(12.6)	(12.6)	(12.6)	(12.6)	(12.6)
	-0.4	-15.1	-16.4	-16.8	-11.9	-16.4	-11.9	3.8	8.5	15.4
	-0.208	-8.180	-8.919	-9.115	-6.456	-8.919	-6.456	2.039	4.646	8.392
c_O	23	18	21	21	26	24	19	17	21	19
	(12.5)	(9.8)	(11.4)	(11.4)	(14.1)	(13.0)	(10.3)	(9.2)	(11.5)	(10.3)
c_E	23.6	23.6	23.6	23.6	23.6	23.6	23.6	23.6	23.5	23.6
	(12.8)	(12.8)	(12.8)	(12.8)	(12.8)	(12.8)	(12.8)	(12.8)	(12.8)	(12.8)
	-1.2	-9.8	-4.9	-4.9	5.0	0.8	-8.2	-11.2	-4.7	-8.2
	-0.647	-5.302	-2.667	-2.667	2.734	0.436	-4.480	-6.063	-2.556	-4.480
N	184	184	184	184	184	184	184	184	183	184
X2	0.4	8.2	12.6	14.9	3.8	7.5	6.3	2.2	6.7	7.2
Gvalue	0.4	9.1	13.7	19.0	4.0	9.0	6.5	2.4	6.8	7.2
Gv/N	0.211	4.936	7.448	10.335	2.200	4.900	3.525	1.280	3.742	3.896
Prob	0.94257	0.02822	0.00334	0.00027	0.25639	0.02909	0.09024	0.50214	0.07688	0.06672

	10	9	8	7	6	5	4	3	2	1
a_O	85	88	72	84	90	82	82	80	66	57
	(46.2)	(47.8)	(39.1)	(45.7)	(48.9)	(44.6)	(44.6)	(43.5)	(35.9)	(31.0)
a_E	72.1	72.1	72.1	72.1	72.1	72.1	72.1	72.1	72.1	72.1
	(39.2)	(39.2)	(39.2)	(39.2)	(39.2)	(39.2)	(39.2)	(39.2)	(39.2)	(39.2)
	27.9	35.0	-0.3	25.6	39.8	21.0	21.0	16.6	-11.7	-26.9
	15.159	19.012	-0.150	13.900	21.642	11.421	11.421	8.995	-6.379	-14.592
t_O	62	49	64	62	65	67	77	59	75	81
	(33.7)	(26.6)	(34.8)	(33.7)	(35.3)	(36.4)	(41.8)	(32.1)	(40.8)	(44.0)
t_E	65.1	65.1	65.1	65.1	65.1	65.1	65.1	65.1	65.1	65.1
	(35.4)	(35.4)	(35.4)	(35.4)	(35.4)	(35.4)	(35.4)	(35.4)	(35.4)	(35.4)
	-6.0	-27.8	-2.1	-6.0	-0.1	3.9	25.9	-11.5	21.3	35.5
	-3.254	-15.105	-1.150	-3.254	-0.073	2.132	14.093	-6.277	11.582	19.284
g_O	26	26	17	17	13	16	14	23	15	13
	(14.1)	(14.1)	(9.2)	(9.2)	(7.1)	(8.7)	(7.6)	(12.5)	(8.2)	(7.1)
g_E	23.2	23.2	23.2	23.2	23.2	23.2	23.2	23.2	23.2	23.2
	(12.6)	(12.6)	(12.6)	(12.6)	(12.6)	(12.6)	(12.6)	(12.6)	(12.6)	(12.6)
	5.9	5.9	-10.6	-10.6	-15.1	-11.9	-14.1	-0.4	-13.1	-15.1
	3.229	3.229	-5.740	-5.740	-8.180	-6.456	-7.681	-0.208	-7.105	-8.180
c_O	11	21	31	21	16	19	11	22	28	33
	(6.0)	(11.4)	(16.8)	(11.4)	(8.7)	(10.3)	(6.0)	(12.0)	(15.2)	(17.9)
c_E	23.6	23.6	23.6	23.6	23.6	23.6	23.6	23.6	23.6	23.6
	(12.8)	(12.8)	(12.8)	(12.8)	(12.8)	(12.8)	(12.8)	(12.8)	(12.8)	(12.8)
	-16.8	-4.9	16.9	-4.9	-12.4	-8.2	-16.8	-3.1	9.6	22.1
	-9.128	-2.667	9.187	-2.667	-6.761	-4.480	-9.128	-1.681	5.200	12.022
N	184	184	184	184	184	184	184	184	184	184
X2	9.5	8.1	4.0	4.0	11.4	4.5	13.9	1.5	5.8	15.3
Gvalue	11.1	8.2	4.0	4.1	12.2	4.8	16.0	1.5	6.1	15.7
Gv/N	6.006	4.469	2.147	2.240	6.628	2.617	8.705	0.829	3.297	8.534
Prob	0.01145	0.04161	0.26689	0.24871	0.00674	0.18584	0.00112	0.67663	0.10841	0.00131

CDS_INITIAL:

	1	2	3	4	5	6	7	8	9	10
a_O	161	0	0	100	66	61	85	89	85	82
	(87.5)	(0.0)	(0.0)	(54.3)	(35.9)	(33.2)	(46.2)	(48.4)	(46.2)	(44.6)
a_E	60.2	58.0	67.3	60.2	58.0	67.3	60.2	58.0	67.3	60.2
	(32.7)	(31.5)	(36.6)	(32.7)	(31.5)	(36.6)	(32.7)	(31.5)	(36.6)	(32.7)
	316.7	0.0	0.0	101.5	17.0	−12.0	58.6	76.2	39.7	50.7
	172.145	0.000	0.000	55.158	9.266	−6.514	31.869	41.418	21.576	27.541
t_O	9	184	0	31	59	91	35	51	61	44
	(4.9)	(100.0)	(0.0)	(16.8)	(32.1)	(49.5)	(19.0)	(27.7)	(33.2)	(23.9)
t_E	37.0	61.3	80.1	37.0	61.3	80.1	37.0	61.3	80.1	37.0
	(20.1)	(33.3)	(43.5)	(20.1)	(33.3)	(43.5)	(20.1)	(33.3)	(43.5)	(20.1)
	−25.5	404.5	0.0	−11.0	−4.5	23.3	−3.9	−18.8	−33.2	15.2
	−13.833	219.832	0.000	−5.972	−2.452	12.653	−2.126	−10.197	−18.039	8.272
g_O	14	0	184	30	20	17	31	24	21	18
	(7.6)	(0.0)	(100.0)	(16.3)	(10.9)	(9.2)	(16.8)	(13.0)	(11.4)	(9.8)
g_E	49.9	29.5	20.1	49.9	29.5	20.1	49.9	29.5	20.1	49.9
	(27.1)	(16.0)	(10.9)	(27.1)	(16.0)	(10.9)	(27.1)	(16.0)	(10.9)	(27.1)
	−35.6	0.0	814.5	−30.5	−15.5	−5.7	−29.5	−9.8	1.8	−36.7
	−19.330	0.000	442.666	−16.569	−8.424	−3.112	−16.016	−5.352	0.980	−19.936
c_O	0	0	0	23	39	15	33	20	17	40
	(0.0)	(0.0)	(0.0)	(12.5)	(21.2)	(8.2)	(17.9)	(10.9)	(9.2)	(21.7)
c_E	36.9	35.2	16.5	36.9	35.2	16.5	36.9	35.2	16.5	36.9
	(20.1)	(19.1)	(9.0)	(20.1)	(19.1)	(9.0)	(20.1)	(19.1)	(9.0)	(20.1)
	0.0	0.0	0.0	−21.8	7.9	−2.9	−7.4	−22.6	1.0	6.4
	0.000	0.000	0.000	−11.833	4.308	−1.566	−4.028	−12.309	0.538	3.481
N	184	184	184	184	184	184	184	184	184	184
X2	252.7	368.3	1498.9	40.4	4.6	2.7	17.9	25.9	9.3	29.8
Gvalue	255.7	404.5	814.5	38.2	5.0	2.7	17.8	24.9	9.3	35.6
Gv/N	138.983	219.832	442.666	20.784	2.698	1.462	9.699	13.559	5.055	19.360
Prob	0.00000	0.00000	0.00000	0.00000	0.17449	0.44203	0.00047	0.00002	0.02554	0.00000

	11	12	13	14	15	16	17	18	19	20
a_O	70	78	85	63	61	76	62	77	70	62
	(38.0)	(42.4)	(46.2)	(34.2)	(33.2)	(41.3)	(33.7)	(41.8)	(38.0)	(33.7)
a_E	58.0	67.3	60.2	58.0	67.3	60.2	58.0	67.3	60.2	58.0
	(31.5)	(36.6)	(32.7)	(31.5)	(36.6)	(32.7)	(31.5)	(36.6)	(32.7)	(31.5)
	26.3	23.0	58.6	10.4	−12.0	35.4	8.3	20.7	21.1	8.3
	14.304	12.513	31.869	5.659	−6.514	19.249	4.491	11.272	11.472	4.491
t_O	66	78	41	65	89	47	72	83	44	65
	(35.9)	(42.4)	(22.3)	(35.3)	(48.4)	(25.5)	(39.1)	(45.1)	(23.9)	(35.3)
t_E	61.3	80.1	37.0	61.3	80.1	37.0	61.3	80.1	37.0	61.3
	(33.3)	(43.5)	(20.1)	(33.3)	(43.5)	(20.1)	(33.3)	(43.5)	(20.1)	(33.3)
	9.8	−4.1	8.4	7.6	18.8	22.5	23.2	6.0	15.2	7.6
	5.300	−2.224	4.561	4.141	10.225	12.206	12.592	3.239	8.272	4.141
g_O	19	13	22	26	11	29	18	10	35	19
	(10.3)	(7.1)	(12.0)	(14.1)	(6.0)	(15.8)	(9.8)	(5.4)	(19.0)	(10.3)
g_E	29.5	20.1	49.9	29.5	20.1	49.9	29.5	20.1	49.9	29.5
	(16.0)	(10.9)	(27.1)	(16.0)	(10.9)	(27.1)	(16.0)	(10.9)	(27.1)	(16.0)
	−16.7	−11.4	−36.0	−6.5	−13.3	−31.4	−17.7	−14.0	−24.8	−16.7
	−9.062	−6.170	−19.567	−3.536	−7.218	−17.085	−9.643	−7.598	−13.466	−9.062
c_O	29	15	36	30	23	32	32	14	35	38
	(15.8)	(8.2)	(19.6)	(16.3)	(12.5)	(17.4)	(17.4)	(7.6)	(19.0)	(20.7)
c_E	35.2	16.5	36.9	35.2	16.5	36.9	35.2	16.5	36.9	35.2
	(19.1)	(9.0)	(20.1)	(19.1)	(9.0)	(20.1)	(19.1)	(9.0)	(20.1)	(19.1)
	−11.3	−2.9	−1.8	−9.6	15.2	−9.2	−6.2	−4.6	−3.7	5.7
	−6.136	−1.566	−0.990	−5.242	8.285	−4.976	−3.346	−2.511	−2.034	3.124
N	184	184	184	184	184	184	184	184	184	184
X2	7.7	4.4	26.2	1.8	8.3	16.2	6.9	7.0	7.4	4.4
Gvalue	8.1	4.7	29.2	1.9	8.8	17.3	7.5	8.1	7.8	5.0
Gv/N	4.407	2.553	15.874	1.022	4.778	9.394	4.093	4.402	4.245	2.694
Prob	0.04382	0.19530	0.00000	0.59754	0.03219	0.00062	0.05675	0.04399	0.05008	0.17491

	21	22	23	24	25	26	27	28	29	30
a_O	77	71	53	74	83	57	67	72	62	74
	(41.8)	(38.6)	(28.8)	(40.2)	(45.1)	(31.0)	(36.4)	(39.1)	(33.7)	(40.2)
a_E	67.3	60.2	58.0	67.3	60.2	58.0	67.3	60.2	58.0	67.3
	(36.6)	(32.7)	(31.5)	(36.6)	(32.7)	(31.5)	(36.6)	(32.7)	(31.5)	(36.6)
	20.7	23.4	−9.6	14.1	53.3	−2.0	−0.6	25.8	8.3	14.1
	11.272	12.731	−5.197	7.637	28.971	−1.081	−0.323	14.005	4.491	7.637
t_O	77	35	70	75	32	67	83	36	77	70
	(41.8)	(19.0)	(38.0)	(40.8)	(17.4)	(36.4)	(45.1)	(19.6)	(41.8)	(38.0)
t_E	80.1	37.0	61.3	80.1	37.0	61.3	80.1	37.0	61.3	80.1
	(43.5)	(20.1)	(33.3)	(43.5)	(20.1)	(33.3)	(43.5)	(20.1)	(33.3)	(43.5)
	−6.0	−3.9	18.6	−9.8	−9.3	11.9	6.0	−2.0	35.1	−18.8
	−3.275	−2.126	10.098	−5.335	−5.060	6.476	3.239	−1.084	19.085	−10.229
g_O	14	39	26	18	32	26	14	43	16	21
	(7.6)	(21.2)	(14.1)	(9.8)	(17.4)	(14.1)	(7.6)	(23.4)	(8.7)	(11.4)
g_E	20.1	49.9	29.5	20.1	49.9	29.5	20.1	49.9	29.5	20.1
	(10.9)	(27.1)	(16.0)	(10.9)	(27.1)	(16.0)	(10.9)	(27.1)	(16.0)	(10.9)
	−10.2	−19.2	−6.5	−4.0	−28.4	−6.5	−10.2	−12.7	−19.5	1.8
	−5.517	−10.417	−3.536	−2.176	−15.428	−3.536	−5.517	−6.922	−10.620	0.980
c_O	16	39	35	17	37	34	20	33	29	19
	(8.7)	(21.2)	(19.0)	(9.2)	(20.1)	(18.5)	(10.9)	(17.9)	(15.8)	(10.3)
c_E	16.5	36.9	35.2	16.5	36.9	35.2	16.5	36.9	35.2	16.5
	(9.0)	(20.1)	(19.1)	(9.0)	(20.1)	(19.1)	(9.0)	(20.1)	(19.1)	(9.0)
	−1.0	4.3	−0.5	1.0	0.2	−2.4	7.7	−7.4	−11.3	5.3
	−0.548	2.321	−0.251	0.538	0.085	−1.315	4.166	−4.028	−6.136	2.899
N	184	184	184	184	184	184	184	184	184	184
X2	3.4	4.5	2.1	1.2	15.7	1.0	2.7	3.7	11.6	2.3
Gvalue	3.6	4.6	2.1	1.2	15.8	1.0	2.9	3.6	12.6	2.4
Gv/N	1.933	2.509	1.115	0.663	8.567	0.543	1.566	1.970	6.821	1.286
Prob	0.31359	0.20211	0.56190	0.74814	0.00127	0.80125	0.41032	0.30484	0.00572	0.50004

	31	32	33	34	35	36	37	38	39	40
a_O	58	48	62	64	55	68	66	42	71	56
	(31.5)	(26.1)	(33.7)	(34.8)	(29.9)	(37.0)	(35.9)	(22.8)	(38.6)	(30.4)
a_E	60.2	58.0	67.3	60.2	58.0	67.3	60.2	58.0	67.3	60.2
	(32.7)	(31.5)	(36.6)	(32.7)	(31.5)	(36.6)	(32.7)	(31.5)	(36.6)	(32.7)
	−4.3	−18.2	−10.2	7.8	−5.8	1.4	12.1	−27.1	7.6	−8.1
	−2.350	−9.876	−5.525	4.255	−3.178	0.768	6.595	−14.738	4.133	−4.405
t_O	37	75	88	38	61	77	32	60	73	43
	(20.1)	(40.8)	(47.8)	(20.7)	(33.2)	(41.8)	(17.4)	(32.6)	(39.7)	(23.4)
t_E	37.0	61.3	80.1	37.0	61.3	80.1	37.0	61.3	80.1	37.0
	(20.1)	(33.3)	(43.5)	(20.1)	(33.3)	(43.5)	(20.1)	(33.3)	(43.5)	(20.1)
	−0.0	30.3	16.6	2.0	−0.6	−6.0	−9.3	−2.6	−13.5	12.9

	-0.012	16.444	9.030	1.089	-0.325	-3.275	-5.060	-1.398	-7.338	7.010
g_O	48	34	19	42	44	21	45	36	18	48
	(26.1)	(18.5)	(10.3)	(22.8)	(23.9)	(11.4)	(24.5)	(19.6)	(9.8)	(26.1)
g_E	49.9	29.5	20.1	49.9	29.5	20.1	49.9	29.5	20.1	49.9
	(27.1)	(16.0)	(10.9)	(27.1)	(16.0)	(10.9)	(27.1)	(16.0)	(10.9)	(27.1)
	-3.7	9.7	-2.2	-14.4	35.3	1.8	-9.2	14.4	-4.0	-3.7
	-1.988	5.290	-1.181	-7.835	19.176	0.980	-5.020	7.838	-2.176	-1.988
c_O	41	27	15	40	24	18	41	46	22	37
	(22.3)	(14.7)	(8.2)	(21.7)	(13.0)	(9.8)	(22.3)	(25.0)	(12.0)	(20.1)
c_E	36.9	35.2	16.5	36.9	35.2	16.5	36.9	35.2	16.5	36.9
	(20.1)	(19.1)	(9.0)	(20.1)	(19.1)	(9.0)	(20.1)	(19.1)	(9.0)	(20.1)
	8.6	-14.4	-2.9	6.4	-18.4	3.1	8.6	24.5	12.6	0.2
	4.669	-7.810	-1.566	3.481	-10.014	1.688	4.669	13.335	6.862	0.085
N	184	184	184	184	184	184	184	184	184	184
X2	0.6	7.4	1.4	1.8	10.9	0.3	2.2	9.2	2.9	1.3
Gvalue	0.6	7.4	1.4	1.8	10.4	0.3	2.2	9.3	2.7	1.3
Gv/N	0.319	4.048	0.758	0.990	5.659	0.160	1.184	5.037	1.481	0.702
Prob	0.89943	0.05891	0.70678	0.61023	0.01537	0.96094	0.53631	0.02593	0.43595	0.73107

	41	42	43	44	45	46	47	48	49	50
a_O	52	69	55	56	58	65	56	67	56	50
	(28.3)	(37.5)	(29.9)	(30.4)	(31.5)	(35.3)	(30.4)	(36.4)	(30.4)	(27.2)
a_E	58.0	67.3	60.2	58.0	67.3	60.2	58.0	67.3	60.2	58.0
	(31.5)	(36.6)	(32.7)	(31.5)	(36.6)	(32.7)	(31.5)	(36.6)	(32.7)	(31.5)
	-11.4	3.4	-9.9	-3.9	-17.2	10.0	-3.9	-0.6	-8.1	-14.8
	-6.175	1.874	-5.403	-2.139	-9.373	5.417	-2.139	-0.323	-4.405	-8.069
t_O	69	78	47	62	81	40	61	79	37	65
	(37.5)	(42.4)	(25.5)	(33.7)	(44.0)	(21.7)	(33.2)	(42.9)	(20.1)	(35.3)
t_E	61.3	80.1	37.0	61.3	80.1	37.0	61.3	80.1	37.0	61.3
	(33.3)	(43.5)	(20.1)	(33.3)	(43.5)	(20.1)	(33.3)	(43.5)	(20.1)	(33.3)
	16.3	-4.1	22.5	1.4	1.9	6.2	-0.6	-2.1	-0.0	7.6
	8.875	-2.224	12.206	0.766	1.014	3.376	-0.325	-1.158	-0.012	4.141
g_O	32	20	45	36	22	49	38	18	44	33
	(17.4)	(10.9)	(24.5)	(19.6)	(12.0)	(26.6)	(20.7)	(9.8)	(23.9)	(17.9)
g_E	29.5	20.1	49.9	29.5	20.1	49.9	29.5	20.1	49.9	29.5
	(16.0)	(10.9)	(27.1)	(16.0)	(10.9)	(27.1)	(16.0)	(10.9)	(27.1)	(16.0)
	5.3	-0.2	-9.2	14.4	3.9	-1.7	19.3	-4.0	-11.0	7.5
	2.870	-0.128	-5.020	7.838	2.139	-0.931	10.506	-2.176	-5.984	4.063
c_O	31	17	37	30	23	30	29	20	47	36
	(16.8)	(9.2)	(20.1)	(16.3)	(12.5)	(16.3)	(15.8)	(10.9)	(25.5)	(19.6)
c_E	35.2	16.5	36.9	35.2	16.5	36.9	35.2	16.5	36.9	35.2
	(19.1)	(9.0)	(20.1)	(19.1)	(9.0)	(20.1)	(19.1)	(9.0)	(20.1)	(19.1)
	-7.9	1.0	0.2	-9.6	15.2	-12.5	-11.3	7.7	22.7	1.6
	-4.311	0.538	0.085	-5.242	8.285	-6.770	-6.136	4.166	12.329	0.844
N	184	184	184	184	184	184	184	184	184	184
X2	2.3	0.1	3.6	2.3	4.0	1.9	3.6	1.0	3.7	1.8
Gvalue	2.3	0.1	3.4	2.2	3.8	2.0	3.5	0.9	3.5	1.8
Gv/N	1.258	0.061	1.867	1.222	2.064	1.092	1.906	0.509	1.929	0.980
Prob	0.50966	0.99038	0.32922	0.52240	0.28408	0.57032	0.31976	0.81655	0.31449	0.61440

	51	52	53	54	55	56	57	58	59	60
a_O	75	61	70	65	56	53	59	48	49	70
	(40.8)	(33.2)	(38.0)	(35.3)	(30.4)	(28.8)	(32.1)	(26.1)	(26.6)	(38.0)
a_E	67.3	60.2	58.0	67.3	60.2	58.0	67.3	60.2	58.0	67.3
	(36.6)	(32.7)	(31.5)	(36.6)	(32.7)	(31.5)	(36.6)	(32.7)	(31.5)	(36.6)
	16.3	1.6	26.3	-4.5	-8.1	-9.6	-15.5	-21.7	-16.5	5.5
	8.834	0.872	14.304	-2.454	-4.405	-5.197	-8.439	-11.818	-8.984	2.996
t_O	74	42	60	87	37	66	91	48	57	70
	(40.2)	(22.8)	(32.6)	(47.3)	(20.1)	(35.9)	(49.5)	(26.1)	(31.0)	(38.0)
t_E	80.1	37.0	61.3	80.1	37.0	61.3	80.1	37.0	61.3	80.1
	(43.5)	(20.1)	(33.3)	(43.5)	(20.1)	(33.3)	(43.5)	(20.1)	(33.3)	(43.5)
	-11.7	10.6	-2.6	14.4	-0.0	9.8	23.3	25.0	-8.3	-18.8
	-6.344	5.773	-1.398	7.846	-0.012	5.300	12.653	13.564	-4.506	-10.229
g_O	20	51	22	18	52	27	19	48	40	20
	(10.9)	(27.7)	(12.0)	(9.8)	(28.3)	(14.7)	(10.3)	(26.1)	(21.7)	(10.9)
g_E	20.1	49.9	29.5	20.1	49.9	29.5	20.1	49.9	29.5	20.1
	(10.9)	(27.1)	(16.0)	(10.9)	(27.1)	(16.0)	(10.9)	(27.1)	(16.0)	(10.9)
	-0.2	2.3	-12.9	-4.0	4.4	-4.7	-2.2	-3.7	24.5	-0.2
	-0.128	1.249	-6.987	-2.176	2.371	-2.565	-1.181	-1.988	13.289	-0.128
c_O	15	30	32	14	39	38	15	40	38	24
	(8.2)	(16.3)	(17.4)	(7.6)	(21.2)	(20.7)	(8.2)	(21.7)	(20.7)	(13.0)
c_E	16.5	36.9	35.2	16.5	36.9	35.2	16.5	36.9	35.2	16.5
	(9.0)	(20.1)	(19.1)	(9.0)	(20.1)	(19.1)	(9.0)	(20.1)	(19.1)	(9.0)
	-2.9	-12.5	-6.2	-4.6	4.3	5.7	-2.9	6.4	5.7	18.0
	-1.566	-6.770	-3.346	-2.511	2.321	3.124	-1.566	3.481	3.124	9.756
N	184	184	184	184	184	184	184	184	184	184
X2	1.5	2.0	4.7	1.3	0.5	1.2	2.7	6.1	5.7	4.8
Gvalue	1.5	2.1	4.7	1.3	0.5	1.2	2.7	6.0	5.4	4.4
Gv/N	0.797	1.124	2.573	0.705	0.275	0.663	1.468	3.240	2.924	2.395
Prob	0.69014	0.55848	0.19227	0.72998	0.91768	0.74810	0.43993	0.11354	0.14596	0.22083

	61	62	63	64	65	66	67	68	69	70
a_O	48	45	64	70	49	55	55	59	75	61
	(26.1)	(24.5)	(34.8)	(38.0)	(26.6)	(29.9)	(29.9)	(32.1)	(40.8)	(33.2)
a_E	60.2	58.0	67.3	60.2	58.0	67.3	60.2	58.0	67.3	60.2
	(32.7)	(31.5)	(36.6)	(32.7)	(31.5)	(36.6)	(32.7)	(31.5)	(36.6)	(32.7)
	-21.7	-22.8	-6.4	21.1	-16.5	-22.2	-9.9	2.0	16.3	1.6
	-11.818	-12.416	-3.495	11.472	-8.984	-12.064	-5.403	1.093	8.834	0.872
t_O	57	76	82	48	65	87	37	59	82	27
	(31.0)	(41.3)	(44.6)	(26.1)	(35.3)	(47.3)	(20.1)	(32.1)	(44.6)	(14.7)
t_E	37.0	61.3	80.1	37.0	61.3	80.1	37.0	61.3	80.1	37.0
	(20.1)	(33.3)	(43.5)	(20.1)	(33.3)	(43.5)	(20.1)	(33.3)	(43.5)	(20.1)
	49.2	32.7	3.9	25.0	7.6	14.4	-0.0	-4.5	3.9	-17.0
	26.755	17.758	2.120	13.564	4.141	7.846	-0.012	-2.452	2.120	-9.256
g_O	46	34	20	39	27	18	52	26	20	49
	(25.0)	(18.5)	(10.9)	(21.2)	(14.7)	(9.8)	(28.3)	(14.1)	(10.9)	(26.6)
g_E	49.9	29.5	20.1	49.9	29.5	20.1	49.9	29.5	20.1	49.9
	(27.1)	(16.0)	(10.9)	(27.1)	(16.0)	(10.9)	(27.1)	(16.0)	(10.9)	(27.1)
	-7.4	9.7	-0.2	-19.2	-4.7	-4.0	4.4	-6.5	-0.2	-1.7
	-4.033	5.290	-0.128	-10.417	-2.565	-2.176	2.371	-3.536	-0.128	-0.931
c_O	33	29	18	27	43	24	40	40	7	47
	(17.9)	(15.8)	(9.8)	(14.7)	(23.4)	(13.0)	(21.7)	(21.7)	(3.8)	(25.5)
c_E	36.9	35.2	16.5	36.9	35.2	16.5	36.9	35.2	16.5	36.9
	(20.1)	(19.1)	(9.0)	(20.1)	(19.1)	(9.0)	(20.1)	(19.1)	(9.0)	(20.1)
	-7.4	-11.3	3.1	-16.9	17.1	18.0	6.4	10.2	-12.0	22.7
	-4.028	-6.136	1.688	-9.185	9.313	9.756	3.481	5.519	-6.530	12.329

N	184	184	184	184	184	184	184	184	184	184
X2	14.0	8.2	0.3	9.9	3.5	6.5	0.8	1.2	6.4	5.5
Gvalue	12.7	8.3	0.3	10.0	3.5	6.2	0.8	1.1	7.9	5.5
Gv/N	6.875	4.496	0.185	5.434	1.906	3.362	0.436	0.623	4.297	3.015
Prob	0.00546	0.04071	0.95210	0.01858	0.31990	0.10288	0.84871	0.76571	0.04800	0.13584

	71	72	73	74	75	76	77	78	79	80
a_O	53	59	58	44	72	52	55	77	67	55
	(28.8)	(32.1)	(31.5)	(23.9)	(39.1)	(28.3)	(29.9)	(41.8)	(36.4)	(29.9)
a_E	58.0	67.3	60.2	58.0	67.3	60.2	58.0	67.3	60.2	58.0
	(31.5)	(36.6)	(32.7)	(31.5)	(36.6)	(32.7)	(31.5)	(36.6)	(32.7)	(31.5)
	-9.6	-15.5	-4.3	-24.3	9.7	-15.2	-5.8	20.7	14.3	-5.8
	-5.197	-8.439	-2.350	-13.215	5.286	-8.279	-3.178	11.272	7.791	-3.178
t_O	63	79	46	76	74	41	60	75	35	71
	(34.2)	(42.9)	(25.0)	(41.3)	(40.2)	(22.3)	(32.6)	(40.8)	(19.0)	(38.6)
t_E	61.3	80.1	37.0	61.3	80.1	37.0	61.3	80.1	37.0	61.3
	(33.3)	(43.5)	(20.1)	(33.3)	(43.5)	(20.1)	(33.3)	(43.5)	(20.1)	(33.3)
	3.4	-2.1	20.0	32.7	-11.7	8.4	-2.6	-9.8	-3.9	20.9
	1.874	-1.158	10.871	17.758	-6.344	4.561	-1.398	-5.335	-2.126	11.337
g_O	36	26	44	20	24	54	34	19	43	23
	(19.6)	(14.1)	(23.9)	(10.9)	(13.0)	(29.3)	(18.5)	(10.3)	(23.4)	(12.5)
g_E	29.5	20.1	49.9	29.5	20.1	49.9	29.5	20.1	49.9	29.5
	(16.0)	(10.9)	(27.1)	(16.0)	(10.9)	(27.1)	(16.0)	(10.9)	(27.1)	(16.0)
	14.4	13.3	-11.0	-15.5	8.5	8.6	9.7	-2.2	-12.7	-11.4
	7.838	7.249	-5.984	-8.424	4.603	4.677	5.290	-1.181	-6.922	-6.193
c_O	32	20	36	44	14	37	35	13	39	35
	(17.4)	(10.9)	(19.6)	(23.9)	(7.6)	(20.1)	(19.0)	(7.1)	(21.2)	(19.0)
c_E	35.2	16.5	36.9	35.2	16.5	36.9	35.2	16.5	36.9	35.2
	(19.1)	(9.0)	(20.1)	(19.1)	(9.0)	(20.1)	(19.1)	(9.0)	(20.1)	(19.1)
	-6.2	7.7	-1.8	19.6	-4.6	0.2	-0.5	-6.2	4.3	-0.5
	-3.346	4.166	-0.990	10.629	-2.511	0.085	-0.251	-3.379	2.321	-0.251

N	184	184	184	184	184	184	184	184	184	184
X2	2.2	3.5	3.0	12.1	1.9	1.9	0.9	2.5	1.9	3.1
Gvalue	2.1	3.3	2.8	12.4	1.9	1.9	0.9	2.5	2.0	3.2
Gv/N	1.168	1.818	1.548	6.748	1.034	1.044	0.463	1.377	1.064	1.715
Prob	0.54194	0.34139	0.41562	0.00608	0.59296	0.58886	0.83699	0.46915	0.58123	0.36828

	81	82	83	84	85	86	87	88	89	90
a_O	70	59	63	77	50	48	75	65	59	72
	(38.3)	(32.1)	(34.2)	(41.8)	(27.2)	(26.1)	(40.8)	(35.3)	(32.1)	(39.1)
a_E	66.9	60.2	58.0	67.3	60.2	58.0	67.3	60.2	58.0	67.3
	(36.6)	(32.7)	(31.5)	(36.6)	(32.7)	(31.5)	(36.6)	(32.7)	(31.5)	(36.6)
	6.3	-2.4	10.4	20.7	-18.6	-18.2	16.3	10.0	2.0	9.7
	3.429	-1.294	5.659	11.272	-10.092	-9.876	8.834	5.417	1.093	5.286
t_O	79	34	57	84	45	71	77	38	66	79
	(43.2)	(18.5)	(31.0)	(45.7)	(24.5)	(38.6)	(41.8)	(20.7)	(35.9)	(42.9)
t_E	79.6	37.0	61.3	80.1	37.0	61.3	80.1	37.0	61.3	80.1
	(43.5)	(20.1)	(33.3)	(43.5)	(20.1)	(33.3)	(43.5)	(20.1)	(33.3)	(43.5)
	-1.3	-5.8	-8.3	8.0	17.6	20.9	-6.0	2.0	9.8	-2.1
	-0.694	-3.136	-4.506	4.372	9.560	11.337	-3.275	1.089	5.300	-1.158
g_O	20	45	27	15	48	21	13	44	29	13
	(10.9)	(24.5)	(14.7)	(8.2)	(26.1)	(11.4)	(7.1)	(23.9)	(15.8)	(7.1)
g_E	20.0	49.9	29.5	20.1	49.9	29.5	20.1	49.9	29.5	20.1
	(10.9)	(27.1)	(16.0)	(10.9)	(27.1)	(16.0)	(10.9)	(27.1)	(16.0)	(10.9)
	-0.0	-9.2	-4.7	-8.8	-3.7	-14.2	-11.4	-11.0	-0.9	-11.4
	-0.009	-5.020	-2.565	-4.786	-1.988	-7.731	-6.170	-5.984	-0.502	-6.170
c_O	14	46	37	8	41	44	19	37	30	20
	(7.7)	(25.0)	(20.1)	(4.3)	(22.3)	(23.9)	(10.3)	(20.1)	(16.3)	(10.9)
c_E	16.4	36.9	35.2	16.5	36.9	35.2	16.5	36.9	35.2	16.5
	(9.0)	(20.1)	(19.1)	(9.0)	(20.1)	(19.1)	(9.0)	(20.1)	(19.1)	(9.0)
	-4.5	20.2	3.6	-11.6	8.6	19.6	5.3	0.2	-9.6	7.7
	-2.442	10.992	1.970	-6.301	4.669	10.629	2.899	0.085	-5.242	4.166

N	183	184	184	184	184	184	184	184	184	184
X2	0.5	3.0	1.0	7.3	4.0	7.9	3.9	1.1	1.2	3.6
Gvalue	0.5	2.8	1.0	8.4	4.0	8.0	4.2	1.1	1.2	3.9
Gv/N	0.284	1.541	0.558	4.557	2.149	4.359	2.288	0.607	0.649	2.124
Prob	0.91449	0.41769	0.79468	0.03870	0.26654	0.04559	0.23975	0.77292	0.75435	0.27157

	91	92	93	94	95	96	97	98	99	100
a_O	50	53	73	66	51	67	51	61	60	42
	(27.2)	(28.8)	(39.7)	(35.9)	(27.7)	(36.4)	(27.9)	(33.3)	(32.8)	(23.1)
a_E	60.2	58.0	67.3	60.2	58.0	67.3	59.9	57.7	66.9	59.5
	(32.7)	(31.5)	(36.6)	(32.7)	(31.5)	(36.6)	(32.7)	(31.5)	(36.6)	(32.7)
	-18.6	-9.6	11.9	12.1	-13.1	-0.6	-16.4	6.8	-13.1	-29.3
	-10.092	-5.197	6.454	6.595	-7.133	-0.323	-8.943	3.722	-7.169	-16.113
t_O	46	60	71	42	67	81	44	58	86	43
	(25.0)	(32.6)	(38.6)	(22.8)	(36.4)	(44.0)	(24.0)	(31.7)	(47.0)	(23.6)
t_E	37.0	61.3	80.1	37.0	61.3	80.1	36.8	61.0	79.6	36.6
	(20.1)	(33.3)	(43.5)	(20.1)	(33.3)	(43.5)	(20.1)	(33.3)	(43.5)	(20.1)
	20.0	-2.6	-17.1	10.6	11.9	1.9	15.7	-5.8	13.2	13.8
	10.871	-1.398	-9.281	5.773	6.476	1.014	8.580	-3.162	7.224	7.603
g_O	48	26	26	50	28	23	53	37	21	58
	(26.1)	(14.1)	(14.1)	(27.2)	(15.2)	(12.5)	(29.0)	(20.2)	(11.5)	(31.9)
g_E	49.9	29.5	20.1	49.9	29.5	20.1	49.6	29.3	20.0	49.3
	(27.1)	(16.0)	(10.9)	(27.1)	(16.0)	(10.9)	(27.1)	(16.0)	(10.9)	(27.1)
	-3.7	-6.5	13.3	0.3	-2.9	6.2	7.0	17.3	2.0	18.8
	-1.988	-3.536	7.249	0.148	-1.553	3.347	3.849	9.428	1.110	10.330
c_O	40	45	14	26	38	13	35	27	16	39
	(21.7)	(24.5)	(7.6)	(14.1)	(20.7)	(7.1)	(19.1)	(14.8)	(8.7)	(21.4)
c_E	36.9	35.2	16.5	36.9	35.2	16.5	36.7	35.0	16.4	36.5
	(20.1)	(19.1)	(9.0)	(20.1)	(19.1)	(9.0)	(20.1)	(19.1)	(9.0)	(20.1)
	6.4	22.0	-4.6	-18.2	5.7	-6.2	-3.4	-14.1	-0.8	5.1
	3.481	11.970	-2.511	-9.911	3.124	-3.379	-1.836	-7.691	-0.455	2.815

N	184	184	184	184	184	184	183	183	183	182
X2	4.2	3.6	3.6	4.5	1.7	1.2	3.0	4.2	1.3	8.0
Gvalue	4.2	3.4	3.5	4.8	1.7	1.2	3.0	4.2	1.3	8.4
Gv/N	2.273	1.840	1.911	2.605	0.914	0.659	1.649	2.296	0.710	4.635
Prob	0.24253	0.33600	0.31875	0.18763	0.64086	0.74991	0.38890	0.24050	0.72946	0.03781

Bcla_KSMK16
5UTR:

	100	99	98	97	96	95	94	93	92	91
a_O	29	38	35	27	38	24	26	33	29	39
	(25.9)	(33.9)	(31.2)	(24.1)	(33.9)	(21.4)	(23.2)	(29.5)	(25.9)	(34.8)
a_E	34.7	34.7	34.7	34.7	34.7	34.7	34.7	34.7	34.7	34.7
	(31.0)	(31.0)	(31.0)	(31.0)	(31.0)	(31.0)	(31.0)	(31.0)	(31.0)	(31.0)
	-10.5	6.8	0.5	-13.6	6.8	-17.7	-15.1	-3.4	-10.5	9.0
	-9.337	6.106	0.484	-12.139	6.106	-15.838	-13.441	-3.011	-9.337	8.076
t_O	31	33	36	28	30	36	35	32	31	39
	(27.7)	(29.5)	(32.1)	(25.0)	(26.8)	(32.1)	(31.2)	(28.6)	(27.7)	(34.8)
t_E	33.0	33.0	33.0	33.0	33.0	33.0	33.0	33.0	33.0	33.0
	(29.4)	(29.4)	(29.4)	(29.4)	(29.4)	(29.4)	(29.4)	(29.4)	(29.4)	(29.4)
	-3.8	0.1	6.3	-9.2	-5.7	6.3	4.2	-1.9	-3.8	13.1
	-3.411	0.054	5.652	-8.170	-5.057	5.652	3.734	-1.706	-3.411	11.697
g_O	26	22	13	25	21	37	21	25	26	13
	(23.2)	(19.6)	(11.6)	(22.3)	(18.8)	(33.0)	(18.8)	(22.3)	(23.2)	(11.6)
g_E	22.6	22.6	22.6	22.6	22.6	22.6	22.6	22.6	22.6	22.6
	(20.2)	(20.2)	(20.2)	(20.2)	(20.2)	(20.2)	(20.2)	(20.2)	(20.2)	(20.2)
	7.4	-1.1	-14.3	5.1	-3.0	36.6	-3.0	5.1	7.4	-14.3
	6.568	-1.005	-12.807	4.565	-2.704	32.659	-2.704	4.565	6.568	-12.807
c_O	26	19	28	32	23	15	30	22	26	21
	(23.2)	(17.0)	(25.0)	(28.6)	(20.5)	(13.4)	(26.8)	(19.6)	(23.2)	(18.8)
c_E	21.7	21.7	21.7	21.7	21.7	21.7	21.7	21.7	21.7	21.7
	(19.4)	(19.4)	(19.4)	(19.4)	(19.4)	(19.4)	(19.4)	(19.4)	(19.4)	(19.4)
	9.3	-5.1	14.2	24.8	2.6	-11.1	19.4	0.5	9.3	-1.4
	8.329	-4.555	12.676	22.117	2.333	-9.928	17.277	0.485	8.329	-1.281
N	112	112	112	112	112	112	112	112	112	112
X2	2.4	0.7	6.1	7.6	0.8	14.9	5.6	0.4	2.4	5.7
Gvalue	2.4	0.7	6.7	7.1	0.8	14.1	5.5	0.4	2.4	6.4
Gv/N	2.150	0.600	6.005	6.373	0.678	12.545	4.866	0.333	2.150	5.685
Prob	0.49217	0.87988	0.08118	0.06763	0.85918	0.00284	0.14165	0.94586	0.49217	0.09505

	90	89	88	87	86	85	84	83	82	81
a_O	26	31	37	35	29	27	31	38	30	36
	(23.2)	(27.7)	(33.0)	(31.2)	(25.9)	(24.1)	(27.7)	(33.9)	(26.8)	(32.1)
a_E	34.7	34.7	34.7	34.7	34.7	34.7	34.7	34.7	34.7	34.7
	(31.0)	(31.0)	(31.0)	(31.0)	(31.0)	(31.0)	(31.0)	(31.0)	(31.0)	(31.0)
	-15.1	-7.0	4.7	0.5	-10.5	-13.6	-7.0	6.8	-8.8	2.6
	-13.441	-6.289	4.183	0.484	-9.337	-12.139	-6.289	6.106	-7.843	2.309
t_O	40	39	28	38	33	41	34	33	34	29
	(35.7)	(34.8)	(25.0)	(33.9)	(29.5)	(36.6)	(30.4)	(29.5)	(30.4)	(25.9)
t_E	33.0	33.0	33.0	33.0	33.0	33.0	33.0	33.0	33.0	33.0
	(29.4)	(29.4)	(29.4)	(29.4)	(29.4)	(29.4)	(29.4)	(29.4)	(29.4)	(29.4)
	15.5	13.1	-9.2	10.8	0.1	17.9	2.1	0.1	2.1	-7.4
	13.806	11.697	-8.170	9.635	0.054	15.959	1.868	0.054	1.868	-6.644
g_O	23	21	19	22	28	22	22	20	23	28
	(20.5)	(18.8)	(17.0)	(19.6)	(25.0)	(19.6)	(19.6)	(17.9)	(20.5)	(25.0)
g_E	22.6	22.6	22.6	22.6	22.6	22.6	22.6	22.6	22.6	22.6
	(20.2)	(20.2)	(20.2)	(20.2)	(20.2)	(20.2)	(20.2)	(20.2)	(20.2)	(20.2)
	0.9	-3.0	-6.5	-1.1	12.1	-1.1	-1.1	-4.8	0.9	12.1
	0.775	-2.704	-5.842	-1.005	10.779	-1.005	-1.005	-4.317	0.775	10.779
c_O	23	21	28	17	22	22	25	21	25	19
	(20.5)	(18.8)	(25.0)	(15.2)	(19.6)	(19.6)	(22.3)	(18.8)	(22.3)	(17.0)
c_E	21.7	21.7	21.7	21.7	21.7	21.7	21.7	21.7	21.7	21.7
	(19.4)	(19.4)	(19.4)	(19.4)	(19.4)	(19.4)	(19.4)	(19.4)	(19.4)	(19.4)
	2.6	-1.4	14.2	-8.3	0.5	0.5	7.0	-1.4	7.0	-5.1
	2.333	-1.281	12.676	-7.452	0.485	0.485	6.258	-1.281	6.258	-4.555
N	112	112	112	112	112	112	112	112	112	112
X2	3.8	1.6	3.3	1.8	2.3	3.7	0.9	0.6	1.2	2.2
Gvalue	3.9	1.6	3.2	1.9	2.2	3.7	0.9	0.6	1.2	2.1
Gv/N	3.472	1.423	2.847	1.662	1.980	3.300	0.831	0.561	1.058	1.889
Prob	0.27369	0.66085	0.36341	0.60167	0.52838	0.29618	0.81788	0.89001	0.75668	0.54882

	80	79	78	77	76	75	74	73	72	71
a_O	35	27	36	29	45	30	26	38	30	39
	(31.2)	(24.1)	(32.1)	(25.9)	(40.2)	(26.8)	(23.2)	(33.9)	(26.8)	(34.8)
a_E	34.7	34.7	34.7	34.7	34.7	34.7	34.7	34.7	34.7	34.7
	(31.0)	(31.0)	(31.0)	(31.0)	(31.0)	(31.0)	(31.0)	(31.0)	(31.0)	(31.0)
	0.5	-13.6	2.6	-10.5	23.3	-8.8	-15.1	6.8	-8.8	9.0
	0.484	-12.139	2.309	-9.337	20.817	-7.843	-13.441	6.106	-7.843	8.076
t_O	24	38	39	33	29	41	43	30	44	43
	(21.4)	(33.9)	(34.8)	(29.5)	(25.9)	(36.6)	(38.4)	(26.8)	(39.3)	(38.4)
t_E	33.0	33.0	33.0	33.0	33.0	33.0	33.0	33.0	33.0	33.0
	(29.4)	(29.4)	(29.4)	(29.4)	(29.4)	(29.4)	(29.4)	(29.4)	(29.4)	(29.4)
	-15.2	10.8	13.1	0.1	-7.4	17.9	22.8	-5.7	25.4	22.8
	-13.609	9.635	11.697	0.054	-6.644	15.959	20.394	-5.057	22.675	20.394
g_O	28	19	18	30	20	17	11	24	15	17
	(25.0)	(17.0)	(16.1)	(26.8)	(17.9)	(15.2)	(9.8)	(21.4)	(13.4)	(15.2)
g_E	22.6	22.6	22.6	22.6	22.6	22.6	22.6	22.6	22.6	22.6
	(20.2)	(20.2)	(20.2)	(20.2)	(20.2)	(20.2)	(20.2)	(20.2)	(20.2)	(20.2)
	12.1	-6.5	-8.1	17.1	-4.8	-9.6	-15.8	2.9	-12.3	-9.6
	10.779	-5.842	-7.272	15.245	-4.317	-8.603	-14.118	2.633	-10.944	-8.603
c_O	25	28	19	20	18	24	32	20	23	13
	(22.3)	(25.0)	(17.0)	(17.9)	(16.1)	(21.4)	(28.6)	(17.9)	(20.5)	(11.6)
c_E	21.7	21.7	21.7	21.7	21.7	21.7	21.7	21.7	21.7	21.7
	(19.4)	(19.4)	(19.4)	(19.4)	(19.4)	(19.4)	(19.4)	(19.4)	(19.4)	(19.4)
	7.0	14.2	-5.1	-3.3	-6.8	4.8	24.8	-3.3	2.6	-13.4
	6.258	12.676	-4.555	-2.963	-6.053	4.258	22.117	-2.963	2.333	-11.926
N	112	112	112	112	112	112	112	112	112	112
X2	4.2	4.9	2.4	3.5	4.4	4.2	16.0	0.8	6.9	8.5
Gvalue	4.4	4.8	2.4	3.4	4.3	4.2	16.7	0.8	7.0	8.9
Gv/N	3.912	4.330	2.179	2.998	3.802	3.770	14.952	0.719	6.221	7.940
Prob	0.22309	0.18317	0.48616	0.33962	0.23486	0.23838	0.00080	0.84829	0.07295	0.03074

	70	69	68	67	66	65	64	63	62	61
a_O	37	33	47	40	33	26	35	34	41	30
	(33.0)	(29.5)	(42.0)	(35.7)	(29.5)	(23.2)	(31.2)	(30.4)	(36.6)	(26.8)
a_E	34.7	34.7	34.7	34.7	34.7	34.7	34.7	34.7	34.7	34.7
	(31.0)	(31.0)	(31.0)	(31.0)	(31.0)	(31.0)	(31.0)	(31.0)	(31.0)	(31.0)
	4.7	-3.4	28.4	11.3	-3.4	-15.1	0.5	-1.4	13.6	-8.8
	4.183	-3.011	25.392	10.091	-3.011	-13.441	0.484	-1.290	12.151	-7.843
t_O	37	31	30	27	38	42	29	37	28	38
	(33.0)	(27.7)	(26.8)	(24.1)	(33.9)	(37.5)	(25.9)	(33.0)	(25.0)	(33.9)

t_E	33.0	33.0	33.0	33.0	33.0	33.0	33.0	33.0	33.0	33.0
	(29.4)	(29.4)	(29.4)	(29.4)	(29.4)	(29.4)	(29.4)	(29.4)	(29.4)	(29.4)
	8.5	-3.8	-5.7	-10.8	10.8	20.3	-7.4	8.5	-9.2	10.8
	7.619	-3.411	-5.057	-9.631	9.635	18.155	-6.644	7.619	-8.170	9.635
g_O	21	25	16	21	23	22	24	21	25	24
	(18.8)	(22.3)	(14.3)	(18.8)	(20.5)	(19.6)	(21.4)	(18.8)	(22.3)	(21.4)
g_E	22.6	22.6	22.6	22.6	22.6	22.6	22.6	22.6	22.6	22.6
	(20.2)	(20.2)	(20.2)	(20.2)	(20.2)	(20.2)	(20.2)	(20.2)	(20.2)	(20.2)
	-3.0	5.1	-11.0	-3.0	0.9	-1.1	2.9	-3.0	5.1	2.9
	-2.704	4.565	-9.830	-2.704	0.775	-1.005	2.633	-2.704	4.565	2.633
c_O	17	23	19	24	18	22	24	20	18	20
	(15.2)	(20.5)	(17.0)	(21.4)	(16.1)	(19.6)	(21.4)	(17.9)	(16.1)	(17.9)
c_E	21.7	21.7	21.7	21.7	21.7	21.7	21.7	21.7	21.7	21.7
	(19.4)	(19.4)	(19.4)	(19.4)	(19.4)	(19.4)	(19.4)	(19.4)	(19.4)	(19.4)
	-8.3	2.6	-5.1	4.8	-6.8	0.5	4.8	-3.3	-6.8	-3.3
	-7.452	2.333	-4.555	4.258	-6.053	0.485	4.258	-2.963	-6.053	-2.963
N	112	112	112	112	112	112	112	112	112	112
X2	1.8	0.5	6.9	2.2	1.5	4.7	0.8	0.8	2.8	1.6
Gvalue	1.8	0.5	6.7	2.3	1.5	4.7	0.8	0.7	2.8	1.6
Gv/N	1.647	0.476	5.950	2.014	1.346	4.194	0.731	0.663	2.493	1.462
Prob	0.60532	0.91152	0.08340	0.52099	0.68059	0.19534	0.84501	0.86317	0.42474	0.65103

	60	59	58	57	56	55	54	53	52	51
a_O	30	26	29	32	39	35	33	41	43	38
	(26.8)	(23.2)	(25.9)	(28.6)	(34.8)	(31.2)	(29.5)	(36.6)	(38.4)	(33.9)
a_E	34.7	34.7	34.7	34.7	34.7	34.7	34.7	34.7	34.7	34.7
	(31.0)	(31.0)	(31.0)	(31.0)	(31.0)	(31.0)	(31.0)	(31.0)	(31.0)	(31.0)
	-8.8	-15.1	-10.5	-5.2	9.0	0.5	-3.4	13.6	18.4	6.8
	-7.843	-13.441	-9.337	-4.678	8.076	0.484	-3.011	12.151	16.401	6.106
t_O	31	32	38	44	34	38	36	31	29	35
	(27.7)	(28.6)	(33.9)	(39.3)	(30.4)	(33.9)	(32.1)	(27.7)	(25.9)	(31.2)
t_E	33.0	33.0	33.0	33.0	33.0	33.0	33.0	33.0	33.0	33.0
	(29.4)	(29.4)	(29.4)	(29.4)	(29.4)	(29.4)	(29.4)	(29.4)	(29.4)	(29.4)
	-3.8	-1.9	10.8	25.4	2.1	10.8	6.3	-3.8	-7.4	4.2
	-3.411	-1.706	9.635	22.675	1.868	9.635	5.652	-3.411	-6.644	3.734
g_O	27	29	24	25	17	15	22	22	24	20
	(24.1)	(25.9)	(21.4)	(22.3)	(15.2)	(13.4)	(19.6)	(19.6)	(21.4)	(17.9)
g_E	22.6	22.6	22.6	22.6	22.6	22.6	22.6	22.6	22.6	22.6
	(20.2)	(20.2)	(20.2)	(20.2)	(20.2)	(20.2)	(20.2)	(20.2)	(20.2)	(20.2)
	9.7	14.5	2.9	5.1	-9.6	-12.3	-1.1	-1.1	2.9	-4.8
	8.641	12.981	2.633	4.565	-8.603	-10.944	-1.005	-1.005	2.633	-4.317
c_O	24	25	21	11	22	24	21	18	16	19
	(21.4)	(22.3)	(18.8)	(9.8)	(19.6)	(21.4)	(18.8)	(16.1)	(14.3)	(17.0)
c_E	21.7	21.7	21.7	21.7	21.7	21.7	21.7	21.7	21.7	21.7
	(19.4)	(19.4)	(19.4)	(19.4)	(19.4)	(19.4)	(19.4)	(19.4)	(19.4)	(19.4)
	4.8	7.0	-1.4	-15.0	0.5	4.8	-1.4	-6.8	-9.8	-5.1
	4.258	6.258	-1.281	-13.373	0.485	4.258	-1.281	-6.053	-8.746	-4.555
N	112	112	112	112	112	112	112	112	112	112
X2	1.9	4.5	1.8	9.5	1.9	3.5	0.4	1.9	4.0	1.1
Gvalue	1.8	4.6	1.8	10.3	2.0	3.8	0.4	1.9	4.1	1.1
Gv/N	1.645	4.092	1.649	9.189	1.825	3.433	0.355	1.683	3.644	0.968
Prob	0.60570	0.20504	0.60481	0.01624	0.56332	0.27866	0.94081	0.59673	0.25284	0.78096

	50	49	48	47	46	45	44	43	42	41
a_O	25	32	37	35	46	37	34	44	33	43
	(22.3)	(28.6)	(33.0)	(31.2)	(41.1)	(33.0)	(30.4)	(39.3)	(29.5)	(38.4)
a_E	34.7	34.7	34.7	34.7	34.7	34.7	34.7	34.7	34.7	34.7
	(31.0)	(31.0)	(31.0)	(31.0)	(31.0)	(31.0)	(31.0)	(31.0)	(31.0)	(31.0)
	-16.4	-5.2	4.7	0.5	25.9	4.7	-1.4	20.8	-3.4	18.4
	-14.675	-4.678	4.183	0.484	23.085	4.183	-1.290	18.589	-3.011	16.401
t_O	38	30	36	28	33	34	34	19	29	25
	(33.9)	(26.8)	(32.1)	(25.0)	(29.5)	(30.4)	(30.4)	(17.0)	(25.9)	(22.3)
t_E	33.0	33.0	33.0	33.0	33.0	33.0	33.0	33.0	33.0	33.0
	(29.4)	(29.4)	(29.4)	(29.4)	(29.4)	(29.4)	(29.4)	(29.4)	(29.4)	(29.4)
	10.8	-5.7	6.3	-9.2	0.1	2.1	2.1	-20.9	-7.4	-13.8
	9.635	-5.057	5.652	-8.170	0.054	1.868	1.868	-18.700	-6.644	-12.354
g_O	30	30	26	28	15	22	29	22	27	25
	(26.8)	(26.8)	(23.2)	(25.0)	(13.4)	(19.6)	(25.9)	(19.6)	(24.1)	(22.3)
g_E	22.6	22.6	22.6	22.6	22.6	22.6	22.6	22.6	22.6	22.6
	(20.2)	(20.2)	(20.2)	(20.2)	(20.2)	(20.2)	(20.2)	(20.2)	(20.2)	(20.2)
	17.1	17.1	7.4	12.1	-12.3	-1.1	14.5	-1.1	9.7	5.1
	15.245	15.245	6.568	10.779	-10.944	-1.005	12.981	-1.005	8.641	4.565
c_O	19	20	13	21	18	19	15	27	23	19
	(17.0)	(17.9)	(11.6)	(18.8)	(16.1)	(17.0)	(13.4)	(24.1)	(20.5)	(17.0)
c_E	21.7	21.7	21.7	21.7	21.7	21.7	21.7	21.7	21.7	21.7
	(19.4)	(19.4)	(19.4)	(19.4)	(19.4)	(19.4)	(19.4)	(19.4)	(19.4)	(19.4)
	-5.1	-3.3	-13.4	-1.4	-6.8	-5.1	-11.1	11.7	2.6	-5.1
	-4.555	-2.963	-11.926	-1.281	-6.053	-4.555	-9.928	10.469	2.333	-4.555
N	112	112	112	112	112	112	112	112	112	112
X2	6.3	3.1	4.5	2.1	6.8	0.5	4.0	9.7	1.5	4.5
Gvalue	6.3	2.9	5.0	2.0	6.9	0.5	4.1	10.5	1.5	4.5
Gv/N	5.650	2.547	4.478	1.812	6.142	0.491	3.631	9.353	1.318	4.057
Prob	0.09672	0.41493	0.17071	0.56631	0.07586	0.90779	0.25431	0.01493	0.68769	0.20838

	40	39	38	37	36	35	34	33	32	31
a_O	38	40	39	38	35	42	36	33	34	36
	(33.9)	(35.7)	(34.8)	(33.9)	(31.2)	(37.5)	(32.1)	(29.5)	(30.4)	(32.1)
a_E	34.7	34.7	34.7	34.7	34.7	34.7	34.7	34.7	34.7	34.7
	(31.0)	(31.0)	(31.0)	(31.0)	(31.0)	(31.0)	(31.0)	(31.0)	(31.0)	(31.0)
	6.8	11.3	9.0	6.8	0.5	16.0	2.6	-3.4	-1.4	2.6
	6.106	10.091	8.076	6.106	0.484	14.255	2.309	-3.011	-1.290	2.309
t_O	28	27	34	30	36	32	41	36	32	26
	(25.0)	(24.1)	(30.4)	(26.8)	(32.1)	(28.6)	(36.6)	(32.1)	(28.6)	(23.2)
t_E	33.0	33.0	33.0	33.0	33.0	33.0	33.0	33.0	33.0	33.0
	(29.4)	(29.4)	(29.4)	(29.4)	(29.4)	(29.4)	(29.4)	(29.4)	(29.4)	(29.4)
	-9.2	-10.8	2.1	-5.7	6.3	-1.9	17.9	6.3	-1.9	-12.4
	-8.170	-9.631	1.868	-5.057	5.652	-1.706	15.959	5.652	-1.706	-11.027
g_O	22	24	23	23	16	22	17	23	26	26
	(19.6)	(21.4)	(20.5)	(20.5)	(14.3)	(19.6)	(15.2)	(20.5)	(23.2)	(23.2)
g_E	22.6	22.6	22.6	22.6	22.6	22.6	22.6	22.6	22.6	22.6
	(20.2)	(20.2)	(20.2)	(20.2)	(20.2)	(20.2)	(20.2)	(20.2)	(20.2)	(20.2)
	-1.1	2.9	0.9	0.9	-11.0	-1.1	-9.6	0.9	7.4	7.4
	-1.005	2.633	0.775	0.775	-9.830	-1.005	-8.603	0.775	6.568	6.568
c_O	24	21	16	21	25	16	18	20	20	24
	(21.4)	(18.8)	(14.3)	(18.8)	(22.3)	(14.3)	(16.1)	(17.9)	(17.9)	(21.4)
c_E	21.7	21.7	21.7	21.7	21.7	21.7	21.7	21.7	21.7	21.7

```
        ( 19.4) ( 19.4) ( 19.4) ( 19.4) ( 19.4) ( 19.4) ( 19.4) ( 19.4) ( 19.4) ( 19.4)
            4.8    -1.4    -9.8    -1.4     7.0    -9.8    -6.8    -3.3    -3.3     4.8
          4.258  -1.281  -8.746  -1.281   6.258  -8.746  -6.053  -2.963  -2.963   4.258

N           112     112     112     112     112     112     112     112     112     112
X2          1.3     2.0     2.1     0.6     2.7     3.1     4.0     0.5     0.7     2.3
Gvalue      1.3     2.0     2.2     0.6     2.9     3.1     4.0     0.5     0.7     2.4
Gv/N      1.190   1.811   1.973   0.542   2.565   2.798   3.611   0.453   0.609   2.109
Prob    0.72145 0.56651 0.53011 0.89470 0.41171 0.37150 0.25672 0.91721 0.87731 0.50079
        ------------------------------------------------------------------------------
             30      29      28      27      26      25      24      23      22      21
        ------------------------------------------------------------------------------
a_O          33      41      34      34      32      30      37      30      31      30
        ( 29.5) ( 36.6) ( 30.4) ( 30.4) ( 28.6) ( 26.8) ( 33.0) ( 26.8) ( 27.7) ( 26.8)
a_E        34.7    34.7    34.7    34.7    34.7    34.7    34.7    34.7    34.7    34.7
        ( 31.0) ( 31.0) ( 31.0) ( 31.0) ( 31.0) ( 31.0) ( 31.0) ( 31.0) ( 31.0) ( 31.0)
           -3.4    13.6    -1.4    -1.4    -5.2    -8.8     4.7    -8.8    -7.0    -8.8
         -3.011  12.151  -1.290  -1.290  -4.678  -7.843   4.183  -7.843  -6.289  -7.843
t_O          29      27      31      28      35      36      28      32      37      39
        ( 25.9) ( 24.1) ( 27.7) ( 25.0) ( 31.2) ( 32.1) ( 25.0) ( 28.6) ( 33.0) ( 34.8)
t_E        33.0    33.0    33.0    33.0    33.0    33.0    33.0    33.0    33.0    33.0
        ( 29.4) ( 29.4) ( 29.4) ( 29.4) ( 29.4) ( 29.4) ( 29.4) ( 29.4) ( 29.4) ( 29.4)
           -7.4   -10.8    -3.8    -9.2     4.2     6.3    -9.2    -1.9     8.5    13.1
         -6.644  -9.631  -3.411  -8.170   3.734   5.652  -8.170  -1.706   7.619  11.697
g_O          25      26      23      32      18      27      29      31      22      17
        ( 22.3) ( 23.2) ( 20.5) ( 28.6) ( 16.1) ( 24.1) ( 25.9) ( 27.7) ( 19.6) ( 15.2)
g_E        22.6    22.6    22.6    22.6    22.6    22.6    22.6    22.6    22.6    22.6
        ( 20.2) ( 20.2) ( 20.2) ( 20.2) ( 20.2) ( 20.2) ( 20.2) ( 20.2) ( 20.2) ( 20.2)
            5.1     7.4     0.9    22.3    -8.1     9.7    14.5    19.7    -1.1    -9.6
          4.565   6.568   0.775  19.949  -7.272   8.641  12.981  17.568  -1.005  -8.603
c_O          25      18      24      18      27      19      18      19      22      26
        ( 22.3) ( 16.1) ( 21.4) ( 16.1) ( 24.1) ( 17.0) ( 16.1) ( 17.0) ( 19.6) ( 23.2)
c_E        21.7    21.7    21.7    21.7    21.7    21.7    21.7    21.7    21.7    21.7
        ( 19.4) ( 19.4) ( 19.4) ( 19.4) ( 19.4) ( 19.4) ( 19.4) ( 19.4) ( 19.4) ( 19.4)
            7.0    -6.8     4.8    -6.8    11.7    -5.1    -6.8    -5.1     0.5     9.3
          6.258  -6.053   4.258  -6.053  10.469  -4.555  -6.053  -4.555   0.485   8.329

N           112     112     112     112     112     112     112     112     112     112
X2          1.3     3.4     0.4     5.3     2.5     2.1     3.4     4.2     0.9     4.0
Gvalue      1.3     3.4     0.4     5.0     2.5     2.1     3.3     3.9     0.9     4.0
Gv/N      1.168   3.035   0.333   4.437   2.253   1.895   2.942   3.464   0.810   3.580
Prob    0.72725 0.33405 0.94576 0.17407 0.47103 0.54750 0.34838 0.27478 0.82367 0.26040
        ------------------------------------------------------------------------------
             20      19      18      17      16      15      14      13      12      11
        ------------------------------------------------------------------------------
a_O          42      33      38      31      34      40      38      36      44      37
        ( 37.5) ( 29.5) ( 33.9) ( 27.7) ( 30.4) ( 35.7) ( 33.9) ( 32.1) ( 39.3) ( 33.0)
a_E        34.7    34.7    34.7    34.7    34.7    34.7    34.7    34.7    34.7    34.7
        ( 31.0) ( 31.0) ( 31.0) ( 31.0) ( 31.0) ( 31.0) ( 31.0) ( 31.0) ( 31.0) ( 31.0)
           16.0    -3.4     6.8    -7.0    -1.4    11.3     6.8     2.6    20.8     4.7
         14.255  -3.011   6.106  -6.289  -1.290  10.091   6.106   2.309  18.589   4.183
t_O          24      37      37      34      33      29      31      32      26      41
        ( 21.4) ( 33.0) ( 33.0) ( 30.4) ( 29.5) ( 25.9) ( 27.7) ( 28.6) ( 23.2) ( 36.6)
t_E        33.0    33.0    33.0    33.0    33.0    33.0    33.0    33.0    33.0    33.0
        ( 29.4) ( 29.4) ( 29.4) ( 29.4) ( 29.4) ( 29.4) ( 29.4) ( 29.4) ( 29.4) ( 29.4)
          -15.2     8.5     8.5     2.1     0.1    -7.4    -3.8    -1.9   -12.4    17.9
        -13.609   7.619   7.619   1.868   0.054  -6.644  -3.411  -1.706 -11.027  15.959
g_O          25      19      13      27      18      19      16      23      17      13
        ( 22.3) ( 17.0) ( 11.6) ( 24.1) ( 16.1) ( 17.0) ( 14.3) ( 20.5) ( 15.2) ( 11.6)
g_E        22.6    22.6    22.6    22.6    22.6    22.6    22.6    22.6    22.6    22.6
        ( 20.2) ( 20.2) ( 20.2) ( 20.2) ( 20.2) ( 20.2) ( 20.2) ( 20.2) ( 20.2) ( 20.2)
            5.1    -6.5   -14.3     9.7    -8.1    -6.5   -11.0     0.9    -9.6   -14.3
          4.565  -5.842 -12.807   8.641  -7.272  -5.842  -9.830   0.775  -8.603 -12.807
c_O          21      23      24      20      27      24      27      21      25      21
        ( 18.8) ( 20.5) ( 21.4) ( 17.9) ( 24.1) ( 21.4) ( 24.1) ( 18.8) ( 22.3) ( 18.8)
c_E        21.7    21.7    21.7    21.7    21.7    21.7    21.7    21.7    21.7    21.7
        ( 19.4) ( 19.4) ( 19.4) ( 19.4) ( 19.4) ( 19.4) ( 19.4) ( 19.4) ( 19.4) ( 19.4)
           -1.4     2.6     4.8    -3.3    11.7     4.8    11.7    -1.4     7.0    -1.4
         -1.281   2.333   4.258  -2.963  10.469   4.258  10.469  -1.281   6.258  -1.281

N           112     112     112     112     112     112     112     112     112     112
X2          4.2     1.2     5.1     1.4     2.2     2.1     3.6     0.1     5.8     6.2
Gvalue      4.4     1.2     5.8     1.4     2.2     2.1     3.7     0.1     5.8     6.8
Gv/N      3.929   1.099   5.177   1.256   1.961   1.863   3.335   0.096   5.217   6.054
Prob    0.22130 0.74553 0.12186 0.70395 0.53269 0.55458 0.29148 0.99090 0.11952 0.07924
        ------------------------------------------------------------------------------
             10       9       8       7       6       5       4       3       2       1
        ------------------------------------------------------------------------------
a_O          32      35      29      31      44      49      36      34      36      30
        ( 28.6) ( 31.2) ( 25.9) ( 27.7) ( 39.3) ( 43.8) ( 32.1) ( 30.4) ( 32.1) ( 26.8)
a_E        34.7    34.7    34.7    34.7    34.7    34.7    34.7    34.7    34.7    34.7
        ( 31.0) ( 31.0) ( 31.0) ( 31.0) ( 31.0) ( 31.0) ( 31.0) ( 31.0) ( 31.0) ( 31.0)
           -5.2     0.5   -10.5    -7.0    20.8    33.7     2.6    -1.4     2.6    -8.8
         -4.678   0.484  -9.337  -6.289  18.589  30.119   2.309  -1.290   2.309  -7.843
t_O          42      35      30      39      19      17      28      25      26      33
        ( 37.5) ( 31.2) ( 26.8) ( 34.8) ( 17.0) ( 15.2) ( 25.0) ( 22.3) ( 23.2) ( 29.5)
t_E        33.0    33.0    33.0    33.0    33.0    33.0    33.0    33.0    33.0    33.0
        ( 29.4) ( 29.4) ( 29.4) ( 29.4) ( 29.4) ( 29.4) ( 29.4) ( 29.4) ( 29.4) ( 29.4)
           20.3     4.2    -5.7    13.1   -20.9   -22.5    -9.2   -13.8   -12.4     0.1
         18.155   3.734  -5.057  11.697 -18.700 -20.108  -8.170 -12.354 -11.027   0.054
g_O          12      19      23       9      25      22      30      33      31      34
        ( 10.7) ( 17.0) ( 20.5) (  8.0) ( 22.3) ( 19.6) ( 26.8) ( 29.5) ( 27.7) ( 30.4)
g_E        22.6    22.6    22.6    22.6    22.6    22.6    22.6    22.6    22.6    22.6
        ( 20.2) ( 20.2) ( 20.2) ( 20.2) ( 20.2) ( 20.2) ( 20.2) ( 20.2) ( 20.2) ( 20.2)
          -15.2    -6.5     0.9   -16.5     5.1    -1.1    17.1    25.1    19.7    27.9
        -13.537  -5.842   0.775 -14.776   4.565  -1.005  15.245  22.386  17.568  24.877
c_O          26      23      30      33      24      24      18      20      19      15
        ( 23.2) ( 20.5) ( 26.8) ( 29.5) ( 21.4) ( 21.4) ( 16.1) ( 17.9) ( 17.0) ( 13.4)
c_E        21.7    21.7    21.7    21.7    21.7    21.7    21.7    21.7    21.7    21.7
        ( 19.4) ( 19.4) ( 19.4) ( 19.4) ( 19.4) ( 19.4) ( 19.4) ( 19.4) ( 19.4) ( 19.4)
            9.3     2.6    19.4    27.6     4.8     4.8    -6.8    -3.3    -5.1   -11.1
          8.329   2.333  17.277  24.621   4.258   4.258  -6.053  -2.963  -4.555  -9.928

N           112     112     112     112     112     112     112     112     112     112
X2          8.5     0.8     4.4    15.5     8.9    13.9     3.9     6.9     5.0     8.5
Gvalue      9.3     0.8     4.1    17.1     9.8    14.9     3.7     6.5     4.8     8.0
Gv/N      8.270   0.709   3.658  15.253   8.712  13.264   3.331   5.780   4.295   7.159
Prob    0.02600 0.85079 0.25124 0.00068 0.02074 0.00194 0.29204 0.09072 0.18619 0.04563
        ------------------------------------------------------------------------------

CDS_INITIAL:
        ------------------------------------------------------------------------------
```

	1	2	3	4	5	6	7	8	9	10
a_O	47	0	0	41	30	25	28	36	45	35
	(42.0)	(0.0)	(0.0)	(36.6)	(26.8)	(22.3)	(25.0)	(32.1)	(40.2)	(31.2)
a_E	30.6	34.5	30.6	30.6	34.5	30.6	30.6	34.5	30.6	30.6
	(27.3)	(30.8)	(27.3)	(27.3)	(30.8)	(27.3)	(27.3)	(30.8)	(27.3)	(27.3)
	40.5	0.0	0.0	24.1	-8.4	-10.0	-4.9	3.0	34.8	9.5
	36.123	0.000	0.000	21.512	-7.506	-8.962	-4.377	2.713	31.101	8.475
t_O	33	112	0	25	38	45	17	23	22	25
	(29.5)	(100.0)	(0.0)	(22.3)	(33.9)	(40.2)	(15.2)	(20.5)	(19.6)	(22.3)
t_E	22.1	36.7	33.4	22.1	36.7	33.4	22.1	36.7	33.4	22.1
	(19.8)	(32.7)	(29.9)	(19.8)	(32.7)	(29.9)	(19.8)	(32.7)	(29.9)	(19.8)
	26.3	250.2	0.0	6.1	2.7	26.7	-9.0	-21.4	-18.4	6.1
	23.511	223.359	0.000	5.417	2.435	23.850	-8.024	-19.148	-16.454	5.417
g_O	32	0	112	26	24	26	41	25	23	33
	(28.6)	(0.0)	(100.0)	(23.2)	(21.4)	(23.2)	(36.6)	(22.3)	(20.5)	(29.5)
g_E	37.0	17.6	25.7	37.0	17.6	25.7	37.0	17.6	25.7	37.0
	(33.0)	(15.7)	(22.9)	(33.0)	(15.7)	(22.9)	(33.0)	(15.7)	(22.9)	(33.0)
	-9.3	0.0	330.0	-18.3	14.8	0.7	8.5	17.5	-5.0	-7.5
	-8.272	0.000	294.671	-16.362	13.246	0.601	7.546	15.620	-4.504	-6.717
c_O	0	0	0	20	20	16	26	28	22	19
	(0.0)	(0.0)	(0.0)	(17.9)	(17.9)	(14.3)	(23.2)	(25.0)	(19.6)	(17.0)
c_E	22.3	23.2	22.3	22.3	23.2	22.3	22.3	23.2	22.3	22.3
	(19.9)	(20.7)	(19.9)	(19.9)	(20.7)	(19.9)	(19.9)	(20.7)	(19.9)	(19.9)
	0.0	0.0	0.0	-4.4	-6.0	-10.7	8.0	10.5	-0.7	-6.1
	0.000	0.000	0.000	-3.905	-5.313	-9.528	7.105	9.385	-0.590	-5.450
N	112	112	112	112	112	112	112	112	112	112
X2	37.1	230.2	376.8	7.4	3.4	6.8	2.5	9.2	11.0	1.9
Gvalue	57.5	250.2	330.0	7.5	3.2	6.7	2.5	9.6	10.7	1.9
Gv/N	51.362	223.359	294.671	6.663	2.861	5.962	2.250	8.571	9.554	1.725
Prob	0.00000	0.00000	0.00000	0.05852	0.36112	0.08294	0.47159	0.02230	0.01346	0.58659

	11	12	13	14	15	16	17	18	19	20
a_O	33	22	29	45	31	41	31	33	25	38
	(29.5)	(19.6)	(25.9)	(40.2)	(27.7)	(36.6)	(27.7)	(29.5)	(22.3)	(33.9)
a_E	34.5	30.6	30.6	34.5	30.6	30.6	34.5	30.6	30.6	34.5
	(30.8)	(27.3)	(27.3)	(30.8)	(27.3)	(27.3)	(30.8)	(27.3)	(27.3)	(30.8)
	-3.0	-14.5	-3.0	23.9	0.9	24.1	-6.7	5.1	-10.0	7.3
	-2.640	-12.909	-2.716	21.323	0.795	21.512	-5.941	4.531	-8.967	6.533
t_O	46	33	28	34	39	26	37	36	27	36
	(41.1)	(29.5)	(25.0)	(30.4)	(34.8)	(23.2)	(33.0)	(32.1)	(24.1)	(32.1)
t_E	36.7	33.4	22.1	36.7	33.4	22.1	36.7	33.4	22.1	36.7
	(32.7)	(29.9)	(19.8)	(32.7)	(29.9)	(19.8)	(32.7)	(29.9)	(19.8)	(32.7)
	20.9	-0.9	13.1	-5.1	12.0	8.3	0.7	5.3	10.7	-1.3
	18.641	-0.787	11.734	-4.574	10.704	7.455	0.609	4.735	9.561	-1.169
g_O	11	34	32	20	27	30	18	26	35	20
	(9.8)	(30.4)	(28.6)	(17.9)	(24.1)	(26.8)	(16.1)	(23.2)	(31.2)	(17.9)
g_E	17.6	25.7	37.0	17.6	25.7	37.0	17.6	25.7	37.0	17.6
	(15.7)	(22.9)	(33.0)	(15.7)	(22.9)	(33.0)	(15.7)	(22.9)	(33.0)	(15.7)
	-10.4	19.1	-9.3	5.1	2.7	-12.6	0.8	0.7	-3.9	5.1
	-9.254	17.074	-8.272	4.527	2.444	-11.213	0.687	0.601	-3.447	4.527
c_O	22	23	23	13	15	15	26	17	25	18
	(19.6)	(20.5)	(20.5)	(11.6)	(13.4)	(13.4)	(23.2)	(15.2)	(22.3)	(16.1)
c_E	23.2	22.3	22.3	23.2	22.3	22.3	23.2	22.3	22.3	23.2
	(20.7)	(19.9)	(19.9)	(20.7)	(19.9)	(19.9)	(20.7)	(19.9)	(19.9)	(20.7)
	-2.4	1.4	1.4	-15.1	-11.9	-11.9	5.9	-9.3	5.7	-9.1
	-2.100	1.209	1.250	-13.454	-10.661	-10.634	5.274	-8.283	5.081	-8.168
N	112	112	112	112	112	112	112	112	112	112
X2	5.0	5.1	2.3	8.2	3.4	8.0	0.7	1.7	2.5	1.9
Gvalue	5.2	5.1	2.2	8.8	3.7	8.0	0.7	1.8	2.5	1.9
Gv/N	4.647	4.587	1.995	7.821	3.282	7.120	0.629	1.584	2.228	1.722
Prob	0.15738	0.16200	0.52515	0.03266	0.29864	0.04654	0.87210	0.62058	0.47617	0.58732

	21	22	23	24	25	26	27	28	29	30
a_O	32	42	30	38	35	39	31	41	30	32
	(28.6)	(37.5)	(26.8)	(33.9)	(31.2)	(34.8)	(27.7)	(36.6)	(26.8)	(28.6)
a_E	30.6	30.6	34.5	30.6	30.6	34.5	30.6	30.6	34.5	30.6
	(27.3)	(27.3)	(30.8)	(27.3)	(27.3)	(30.8)	(27.3)	(27.3)	(30.8)	(27.3)
	3.0	26.7	-8.4	16.6	9.5	9.5	0.9	24.1	-8.4	3.0
	2.635	23.844	-7.506	14.790	8.475	8.514	0.795	21.512	-7.506	2.635
t_O	35	18	38	34	21	37	29	28	32	43
	(31.2)	(16.1)	(33.9)	(30.4)	(18.8)	(33.0)	(25.9)	(25.0)	(28.6)	(38.4)
t_E	33.4	22.1	36.7	33.4	22.1	36.7	33.4	22.1	36.7	33.4
	(29.9)	(19.8)	(32.7)	(29.9)	(19.8)	(32.7)	(29.9)	(19.8)	(32.7)	(29.9)
	3.2	-7.5	2.7	1.1	-2.2	0.7	-8.3	13.1	-8.7	21.6
	2.843	-6.659	2.435	1.002	-1.988	0.609	-7.383	11.734	-7.770	19.299
g_O	23	32	24	16	32	19	29	18	23	27
	(20.5)	(28.6)	(21.4)	(14.3)	(28.6)	(17.0)	(25.9)	(16.1)	(20.5)	(24.1)
g_E	25.7	37.0	17.6	25.7	37.0	17.6	25.7	37.0	17.6	25.7
	(22.9)	(33.0)	(15.7)	(22.9)	(33.0)	(15.7)	(22.9)	(33.0)	(15.7)	(22.9)
	-5.0	-9.3	14.8	-15.1	-9.3	2.9	7.1	-25.9	12.3	2.7
	-4.504	-8.272	13.246	-13.502	-8.272	2.560	6.326	-23.147	10.946	2.444
c_O	22	20	20	24	24	17	23	25	27	10
	(19.6)	(17.9)	(17.9)	(21.4)	(21.4)	(15.2)	(20.5)	(22.3)	(24.1)	(8.9)
c_E	22.3	22.3	23.2	22.3	22.3	23.2	22.3	22.3	23.2	22.3
	(19.9)	(19.9)	(20.7)	(19.9)	(19.9)	(20.7)	(19.9)	(19.9)	(20.7)	(19.9)
	-0.7	-4.4	-6.0	3.5	3.5	-10.6	1.4	5.7	8.2	-16.1
	-0.590	-3.905	-5.313	3.085	3.128	-9.450	1.209	5.081	7.297	-14.348
N	112	112	112	112	112	112	112	112	112	112
X2	0.4	6.0	3.4	5.6	1.5	2.4	1.0	15.2	3.4	9.7
Gvalue	0.4	5.6	3.2	6.0	1.5	2.5	1.1	17.0	3.3	11.2
Gv/N	0.384	5.009	2.861	5.376	1.343	2.233	0.947	15.180	2.967	10.030
Prob	0.93393	0.13222	0.36112	0.11061	0.68124	0.47515	0.78670	0.00071	0.34448	0.01053

	31	32	33	34	35	36	37	38	39	40
a_O	39	34	47	39	34	32	37	34	31	38
	(34.8)	(30.4)	(42.0)	(34.8)	(30.4)	(28.6)	(33.0)	(30.4)	(27.7)	(33.9)
a_E	30.6	34.5	30.6	30.6	34.5	30.6	30.6	34.5	30.6	30.6
	(27.3)	(30.8)	(27.3)	(27.3)	(30.8)	(27.3)	(27.3)	(30.8)	(27.3)	(27.3)
	19.0	-1.0	40.5	19.0	-1.0	3.0	14.1	-1.0	0.9	16.6
	16.980	-0.908	36.133	16.980	-0.908	2.635	12.631	-0.908	0.795	14.782
t_O	27	42	32	21	40	35	26	46	42	24
	(24.1)	(37.5)	(28.6)	(18.8)	(35.7)	(31.2)	(23.2)	(41.1)	(37.5)	(21.4)
t_E	22.1	36.7	33.4	22.1	36.7	33.4	22.1	36.7	33.4	22.1
	(19.8)	(32.7)	(29.9)	(19.8)	(32.7)	(29.9)	(19.8)	(32.7)	(29.9)	(19.8)
	10.7	11.4	-2.8	-2.2	7.0	3.2	8.3	20.9	19.1	3.9

	9.561	10.197	-2.521	-1.988	6.227	2.843	7.455	18.641	17.086	3.451
g_O	27	15	18	29	15	24	27	17	16	33
	(24.1)	(13.4)	(16.1)	(25.9)	(13.4)	(21.4)	(24.1)	(15.2)	(14.3)	(29.5)
g_E	37.0	17.6	25.7	37.0	17.6	25.7	37.0	17.6	25.7	37.0
	(33.0)	(15.7)	(22.9)	(33.0)	(15.7)	(22.9)	(33.0)	(15.7)	(22.9)	(33.0)
	-17.0	-4.8	-12.8	-14.1	-4.8	-3.2	-17.0	-1.2	-15.1	-7.5
	-15.171	-4.311	-11.403	-12.595	-4.311	-2.875	-15.171	-1.086	-13.502	-6.717
c_O	19	21	15	23	23	21	22	15	23	17
	(17.0)	(18.8)	(13.4)	(20.5)	(20.5)	(18.8)	(19.6)	(13.4)	(20.5)	(15.2)
c_E	22.3	23.2	22.3	22.3	23.2	22.3	22.3	23.2	22.3	22.3
	(19.9)	(20.7)	(19.9)	(19.9)	(20.7)	(19.9)	(19.9)	(20.7)	(19.9)	(19.9)
	-6.1	-4.2	-11.9	1.4	-0.4	-2.6	-0.6	-13.1	1.4	-9.2
	-5.450	-3.749	-10.661	1.250	-0.370	-2.308	-0.551	-11.691	1.209	-8.253
N	112	112	112	112	112	112	112	112	112	112
X2	6.6	1.4	13.6	4.1	0.7	0.3	4.7	5.3	5.9	3.7
Gvalue	6.6	1.4	12.9	4.1	0.7	0.3	4.9	5.6	6.3	3.7
Gv/N	5.920	1.230	11.547	3.648	0.638	0.295	4.364	4.957	5.588	3.263
Prob	0.08465	0.71086	0.00478	0.25240	0.86965	0.95429	0.18022	0.13557	0.09970	0.30125

	41	42	43	44	45	46	47	48	49	50
a_O	33	33	33	40	41	38	32	39	36	38
	(29.5)	(29.5)	(29.5)	(35.7)	(36.6)	(33.9)	(28.6)	(34.8)	(32.1)	(33.9)
a_E	34.5	30.6	30.6	34.5	30.6	30.6	34.5	30.6	30.6	34.5
	(30.8)	(27.3)	(27.3)	(30.8)	(27.3)	(27.3)	(30.8)	(27.3)	(27.3)	(30.8)
	-3.0	5.1	5.1	11.8	24.1	16.6	-4.8	19.0	11.8	7.3
	-2.640	4.531	4.523	10.540	21.521	14.782	-4.319	16.988	10.528	6.533
t_O	37	30	21	26	40	24	35	31	20	30
	(33.0)	(26.8)	(18.8)	(23.2)	(35.7)	(21.4)	(31.2)	(27.7)	(17.9)	(26.8)
t_E	36.7	33.4	22.1	36.7	33.4	22.1	36.7	33.4	22.1	36.7
	(32.7)	(29.9)	(19.8)	(32.7)	(29.9)	(19.8)	(32.7)	(29.9)	(19.8)	(32.7)
	0.7	-6.5	-2.2	-17.9	14.3	3.9	-3.2	-4.7	-4.1	-12.0
	0.609	-5.821	-1.988	-15.953	12.787	3.451	-2.897	-4.200	-3.635	-10.741
g_O	17	23	32	20	17	34	22	26	32	21
	(15.2)	(20.5)	(28.6)	(17.9)	(15.2)	(30.4)	(19.6)	(23.2)	(28.6)	(18.8)
g_E	17.6	25.7	37.0	17.6	25.7	37.0	17.6	25.7	37.0	17.6
	(15.7)	(22.9)	(33.0)	(15.7)	(22.9)	(33.0)	(15.7)	(22.9)	(33.0)	(15.7)
	-1.2	-5.0	-9.3	5.1	-14.0	-5.7	9.8	0.7	-9.3	7.4
	-1.086	-4.504	-8.272	4.527	-12.505	-5.109	8.724	0.601	-8.272	6.583
c_O	25	26	26	26	14	16	23	16	24	23
	(22.3)	(23.2)	(23.2)	(23.2)	(12.5)	(14.3)	(20.5)	(14.3)	(21.4)	(20.5)
c_E	23.2	22.3	22.3	23.2	22.3	22.3	23.2	22.3	22.3	23.2
	(20.7)	(19.9)	(19.9)	(20.7)	(19.9)	(19.9)	(20.7)	(19.9)	(19.9)	(20.7)
	3.7	7.9	8.0	5.9	-13.1	-10.6	-0.4	-10.7	3.5	-0.4
	3.321	7.059	7.105	5.274	-11.675	-9.499	-0.370	-9.528	3.128	-0.370
N	112	112	112	112	112	112	112	112	112	112
X2	0.2	1.4	1.5	4.6	10.9	4.0	1.3	4.3	2.0	2.2
Gvalue	0.2	1.4	1.5	4.9	11.3	4.1	1.3	4.3	2.0	2.2
Gv/N	0.203	1.264	1.369	4.388	10.128	3.625	1.138	3.862	1.749	2.004
Prob	0.97306	0.70176	0.67472	0.17815	0.01001	0.25502	0.73520	0.22843	0.58109	0.52319

	51	52	53	54	55	56	57	58	59	60
a_O	29	38	36	24	29	27	25	30	34	26
	(25.9)	(33.9)	(32.1)	(21.4)	(25.9)	(24.1)	(22.3)	(26.8)	(30.4)	(23.2)
a_E	30.6	30.6	34.5	30.6	30.6	34.5	30.6	30.6	34.5	30.6
	(27.3)	(27.3)	(30.8)	(27.3)	(27.3)	(30.8)	(27.3)	(27.3)	(30.8)	(27.3)
	-3.0	16.6	3.0	-11.6	-3.0	-13.3	-10.0	-1.1	-1.0	-8.4
	-2.710	14.782	2.713	-10.353	-2.716	-11.835	-8.962	-0.994	-0.908	-7.500
t_O	31	12	34	32	28	43	39	32	43	33
	(27.7)	(10.7)	(30.4)	(28.6)	(25.0)	(38.4)	(34.8)	(28.6)	(38.4)	(29.5)
t_E	33.4	22.1	36.7	33.4	22.1	36.7	33.4	22.1	36.7	33.4
	(29.9)	(19.8)	(32.7)	(29.9)	(19.8)	(32.7)	(29.9)	(19.8)	(32.7)	(29.9)
	-4.7	-14.7	-5.1	-2.8	13.1	13.7	12.0	23.6	13.7	-0.9
	-4.200	-13.128	-4.574	-2.521	11.734	12.247	10.704	21.041	12.247	-0.787
g_O	31	34	17	33	37	18	31	34	19	23
	(27.7)	(30.4)	(15.2)	(29.5)	(33.0)	(16.1)	(27.7)	(30.4)	(17.0)	(20.5)
g_E	25.7	37.0	17.6	25.7	37.0	17.6	25.7	37.0	17.6	25.7
	(22.9)	(33.0)	(15.7)	(22.9)	(33.0)	(15.7)	(22.9)	(33.0)	(15.7)	(22.9)
	11.7	-5.7	-1.2	16.6	0.0	0.8	11.7	-5.7	2.9	-5.0
	10.454	-5.109	-1.086	14.812	0.028	0.687	10.454	-5.109	2.560	-4.504
c_O	21	28	25	23	18	24	17	16	16	30
	(18.8)	(25.0)	(22.3)	(20.5)	(16.1)	(21.4)	(15.2)	(14.3)	(14.3)	(26.8)
c_E	22.3	22.3	23.2	22.3	22.3	23.2	22.3	22.3	23.2	22.3
	(19.9)	(19.9)	(20.7)	(19.9)	(19.9)	(20.7)	(19.9)	(19.9)	(20.7)	(19.9)
	-2.6	12.7	3.7	1.4	-7.7	1.6	-9.3	-10.6	-11.9	17.7
	-2.308	11.357	3.321	1.209	-6.901	1.438	-8.283	-9.499	-10.626	15.811
N	112	112	112	112	112	112	112	112	112	112
X2	1.4	8.1	0.4	3.6	2.5	2.8	4.3	6.4	3.5	3.6
Gvalue	1.4	8.9	0.4	3.5	2.4	2.8	4.4	6.1	3.7	3.4
Gv/N	1.236	7.903	0.373	3.147	2.144	2.537	3.913	5.439	3.273	3.021
Prob	0.70925	0.03134	0.93647	0.31763	0.49330	0.41670	0.22302	0.10723	0.29986	0.33623

	61	62	63	64	65	66	67	68	69	70
a_O	35	38	22	26	35	27	30	34	33	27
	(31.2)	(33.9)	(19.6)	(23.2)	(31.2)	(24.1)	(26.8)	(30.4)	(29.5)	(24.1)
a_E	30.6	34.5	30.6	30.6	34.5	30.6	30.6	34.5	30.6	30.6
	(27.3)	(30.8)	(27.3)	(27.3)	(30.8)	(27.3)	(27.3)	(30.8)	(27.3)	(27.3)
	9.5	7.3	-14.5	-8.4	1.0	-6.7	-1.1	-1.0	5.1	-6.7
	8.475	6.533	-12.909	-7.505	0.877	-5.968	-0.994	-0.908	4.531	-5.974
t_O	31	36	40	29	38	37	23	40	39	21
	(27.7)	(32.1)	(35.7)	(25.9)	(33.9)	(33.0)	(20.5)	(35.7)	(34.8)	(18.8)
t_E	22.1	36.7	33.4	22.1	36.7	33.4	22.1	36.7	33.4	22.1
	(19.8)	(32.7)	(29.9)	(19.8)	(32.7)	(29.9)	(19.8)	(32.7)	(29.9)	(19.8)
	20.9	-1.3	14.3	15.6	2.7	7.5	1.7	7.0	12.0	-2.2
	18.626	-1.169	12.787	13.970	2.435	6.677	1.559	6.227	10.704	-1.988
g_O	28	17	26	33	16	29	43	17	26	42
	(25.0)	(15.2)	(23.2)	(29.5)	(14.3)	(25.9)	(38.4)	(15.2)	(23.2)	(37.5)
g_E	37.0	17.6	25.7	37.0	17.6	25.7	37.0	17.6	25.7	37.0
	(33.0)	(15.7)	(22.9)	(33.0)	(15.7)	(22.9)	(33.0)	(15.7)	(22.9)	(33.0)
	-15.6	-1.2	0.7	-7.5	-3.1	7.1	13.0	-1.2	0.7	10.7
	-13.915	-1.086	0.601	-6.717	-2.754	6.326	11.572	-1.086	0.601	9.538
c_O	18	21	24	24	23	19	16	21	14	22
	(16.1)	(18.8)	(21.4)	(21.4)	(20.5)	(17.0)	(14.3)	(18.8)	(12.5)	(19.6)
c_E	22.3	23.2	22.3	22.3	23.2	22.3	22.3	23.2	22.3	22.3
	(19.9)	(20.7)	(19.9)	(19.9)	(20.7)	(19.9)	(19.9)	(20.7)	(19.9)	(19.9)
	-7.7	-4.2	3.5	3.5	-0.4	-6.1	-10.6	-4.2	-13.1	-0.6
	-6.901	-3.749	3.085	3.128	-0.370	-5.484	-9.499	-3.749	-11.675	-0.551

N	112	112	112	112	112	112	112	112	112	112
X2	7.2	0.6	3.8	3.4	0.2	1.7	2.8	0.5	4.2	1.2
Gvalue	7.0	0.6	4.0	3.2	0.2	1.7	3.0	0.5	4.7	1.1
Gv/N	6.285	0.529	3.565	2.876	0.188	1.551	2.638	0.484	4.161	1.025
Prob	0.07066	0.89821	0.26224	0.35882	0.97585	0.62883	0.39870	0.90956	0.19844	0.76552

	71	72	73	74	75	76	77	78	79	80
a_O	33	33	33	38	39	36	27	37	43	34
	(29.5)	(29.5)	(29.5)	(33.9)	(34.8)	(32.1)	(24.1)	(33.0)	(38.4)	(30.4)
a_E	34.5	30.6	30.6	34.5	30.6	30.6	34.5	30.6	30.6	34.5
	(30.8)	(27.3)	(27.3)	(30.8)	(27.3)	(27.3)	(30.8)	(27.3)	(27.3)	(30.8)
	−3.0	5.1	5.1	7.3	19.0	11.8	−13.3	14.2	29.4	−1.0
	−2.640	4.531	4.523	6.533	16.988	10.528	−11.835	12.639	26.219	−0.908
t_O	35	38	22	39	27	27	44	27	21	39
	(31.2)	(33.9)	(19.6)	(34.8)	(24.1)	(24.1)	(39.3)	(24.1)	(18.8)	(34.8)
t_E	36.7	33.4	22.1	36.7	33.4	22.1	36.7	33.4	22.1	36.7
	(32.7)	(29.9)	(19.8)	(32.7)	(29.9)	(19.8)	(32.7)	(29.9)	(19.8)	(32.7)
	−3.2	9.7	−0.3	4.8	−11.6	10.7	16.1	−11.6	−2.2	4.8
	−2.897	8.667	−0.255	4.308	−10.319	9.561	14.338	−10.319	−1.988	4.308
g_O	22	25	33	12	29	27	21	31	31	23
	(19.6)	(22.3)	(29.5)	(10.7)	(25.9)	(24.1)	(18.8)	(27.7)	(27.7)	(20.5)
g_E	17.6	25.7	37.0	17.6	25.7	37.0	17.6	25.7	37.0	17.6
	(15.7)	(22.9)	(33.0)	(15.7)	(22.9)	(33.0)	(15.7)	(22.9)	(33.0)	(15.7)
	9.8	−1.3	−7.5	−9.2	7.1	−17.0	7.4	11.7	−10.9	12.3
	8.724	−1.173	−6.717	−8.230	6.326	−15.171	6.583	10.454	−9.771	10.946
c_O	22	16	24	23	17	22	20	17	17	16
	(19.6)	(14.3)	(21.4)	(20.5)	(15.2)	(19.6)	(17.9)	(15.2)	(15.2)	(14.3)
c_E	23.2	22.3	22.3	23.2	22.3	22.3	23.2	22.3	22.3	23.2
	(20.7)	(19.9)	(19.9)	(20.7)	(19.9)	(19.9)	(20.7)	(19.9)	(19.9)	(20.7)
	−2.4	−10.7	3.5	−0.4	−9.3	−0.6	−6.0	−9.3	−9.2	−11.9
	−2.100	−9.528	3.128	−0.370	−8.283	−0.551	−5.313	−8.283	−8.253	−10.626
N	112	112	112	112	112	112	112	112	112	112
X2	1.3	2.6	0.8	2.3	5.3	4.7	4.2	5.0	7.4	4.0
Gvalue	1.2	2.8	0.8	2.5	5.3	4.9	4.2	5.0	7.0	4.2
Gv/N	1.086	2.497	0.679	2.241	4.712	4.367	3.772	4.491	6.207	3.720
Prob	0.74907	0.42405	0.85880	0.47356	0.15257	0.17991	0.23817	0.16964	0.07344	0.24405

	81	82	83	84	85	86	87	88	89	90
a_O	36	34	36	29	30	31	32	29	32	32
	(32.1)	(30.4)	(32.1)	(25.9)	(26.8)	(27.7)	(28.6)	(25.9)	(28.6)	(28.6)
a_E	30.6	30.6	34.5	30.6	30.6	34.5	30.6	30.6	34.5	30.6
	(27.3)	(27.3)	(30.8)	(27.3)	(27.3)	(30.8)	(27.3)	(27.3)	(30.8)	(27.3)
	11.8	7.2	3.0	−3.0	−1.1	−6.7	3.0	−3.0	−4.8	3.0
	10.536	6.473	2.713	−2.710	−0.994	−5.941	2.635	−2.716	−4.319	2.635
t_O	22	24	39	37	26	32	28	26	41	30
	(19.6)	(21.4)	(34.8)	(33.0)	(23.2)	(28.6)	(25.0)	(23.2)	(36.6)	(26.8)
t_E	33.4	22.1	36.7	33.4	22.1	36.7	33.4	22.1	36.7	33.4
	(29.9)	(19.8)	(32.7)	(29.9)	(19.8)	(32.7)	(29.9)	(19.8)	(32.7)	(29.9)
	−18.4	3.9	4.8	7.5	8.3	−8.7	−9.9	8.3	9.2	−6.5
	−16.454	3.451	4.308	6.677	7.455	−7.770	−8.883	7.455	8.190	−5.821
g_O	36	29	19	23	36	19	23	36	17	30
	(32.1)	(25.9)	(17.0)	(20.5)	(32.1)	(17.0)	(20.5)	(32.1)	(15.2)	(26.8)
g_E	25.7	37.0	17.6	25.7	37.0	17.6	25.7	37.0	17.6	25.7
	(22.9)	(33.0)	(15.7)	(22.9)	(33.0)	(15.7)	(22.9)	(33.0)	(15.7)	(22.9)
	24.4	−14.1	2.9	−5.0	−1.9	2.9	−5.0	−1.9	−1.2	9.4
	21.753	−12.595	2.560	−4.504	−1.735	2.560	−4.504	−1.735	−1.086	8.360
c_O	18	25	18	23	20	30	29	21	22	20
	(16.1)	(22.3)	(16.1)	(20.5)	(17.9)	(26.8)	(25.9)	(18.8)	(19.6)	(17.9)
c_E	22.3	22.3	23.2	22.3	22.3	23.2	22.3	22.3	23.2	22.3
	(19.9)	(19.9)	(20.7)	(19.9)	(19.9)	(20.7)	(19.9)	(19.9)	(20.7)	(19.9)
	−7.8	5.7	−9.1	1.4	−4.4	15.4	15.2	−2.5	−2.4	−4.4
	−6.933	5.081	−8.168	1.209	−3.905	13.752	13.528	−2.270	−2.100	−3.940
N	112	112	112	112	112	112	112	112	112	112
X2	9.9	2.6	1.5	0.8	0.9	3.0	3.2	0.9	0.8	1.4
Gvalue	10.0	2.7	1.6	0.8	0.9	2.9	3.1	0.8	0.8	1.4
Gv/N	8.902	2.411	1.413	0.672	0.822	2.601	2.777	0.734	0.686	1.233
Prob	0.01882	0.44027	0.66338	0.86069	0.82042	0.40522	0.37501	0.84418	0.85711	0.70997

	91	92	93	94	95	96	97	98	99	100
a_O	37	33	33	28	36	28	39	31	30	32
	(33.0)	(29.5)	(29.5)	(25.0)	(32.1)	(25.0)	(34.8)	(27.7)	(26.8)	(28.6)
a_E	30.6	34.5	30.6	30.6	34.5	30.6	30.6	34.5	30.6	30.6
	(27.3)	(30.8)	(27.3)	(27.3)	(30.8)	(27.3)	(27.3)	(30.8)	(27.3)	(27.3)
	14.1	−3.0	5.1	−4.9	3.0	−4.9	19.0	−6.7	−1.1	2.9
	12.631	−2.640	4.531	−4.377	2.713	−4.371	16.980	−5.941	−0.987	2.628
t_O	19	39	36	22	34	28	25	28	32	29
	(17.0)	(34.8)	(32.1)	(19.6)	(30.4)	(25.0)	(22.3)	(25.0)	(28.6)	(25.9)
t_E	22.1	36.7	33.4	22.1	36.7	33.4	22.1	36.7	33.4	22.1
	(19.8)	(32.7)	(29.9)	(19.8)	(32.7)	(29.9)	(19.8)	(32.7)	(29.9)	(19.8)
	−5.8	4.8	5.3	−0.3	−5.1	−9.9	6.1	−15.1	−2.8	15.6
	−5.194	4.308	4.735	−0.255	−4.574	−8.883	5.417	−13.475	−2.521	13.970
g_O	31	14	22	32	16	24	25	24	25	32
	(27.7)	(12.5)	(19.6)	(28.6)	(14.3)	(21.4)	(22.3)	(21.4)	(22.3)	(28.6)
g_E	37.0	17.6	25.7	37.0	17.6	25.7	37.0	17.6	25.7	37.0
	(33.0)	(15.7)	(22.9)	(33.0)	(15.7)	(22.9)	(33.0)	(15.7)	(22.9)	(33.0)
	−10.9	−6.4	−6.8	−9.3	−3.1	−3.2	−19.6	14.8	−1.3	−9.3
	−9.771	−5.748	−6.054	−8.272	−2.754	−2.875	−17.483	13.246	−1.173	−8.272
c_O	25	26	21	30	26	32	23	29	25	19
	(22.3)	(23.2)	(18.8)	(26.8)	(23.2)	(28.6)	(20.5)	(25.9)	(22.3)	(17.0)
c_E	22.3	23.2	22.3	22.3	23.2	22.3	22.3	23.2	22.3	22.3
	(19.9)	(20.7)	(19.9)	(19.9)	(20.7)	(19.9)	(19.9)	(20.7)	(19.9)	(19.9)
	5.7	5.9	−2.6	17.8	5.9	23.0	1.4	12.9	5.6	−6.1
	5.081	5.274	−2.308	15.864	5.274	20.553	1.250	11.538	5.036	−5.450
N	112	112	112	112	112	112	112	112	112	112
X2	3.1	1.3	1.0	3.5	0.7	5.4	6.6	6.2	0.4	3.4
Gvalue	3.1	1.3	1.0	3.3	0.7	5.0	6.9	6.0	0.4	3.2
Gv/N	2.746	1.194	0.904	2.960	0.659	4.423	6.164	5.367	0.355	2.876
Prob	0.38004	0.72039	0.79827	0.34550	0.86425	0.17518	0.07504	0.11106	0.94078	0.35876

Bflo
5UTR:

	100	99	98	97	96	95	94	93	92	91
a_O	83	78	81	64	79	73	86	84	89	82
	(41.1)	(38.6)	(40.1)	(31.7)	(39.1)	(36.1)	(42.6)	(41.6)	(44.1)	(40.6)
a_E	85.8	85.8	85.8	85.8	85.8	85.8	85.8	85.8	85.8	85.8
	(42.5)	(42.5)	(42.5)	(42.5)	(42.5)	(42.5)	(42.5)	(42.5)	(42.5)	(42.5)
	-5.5	-14.8	-9.3	-37.5	-13.0	-23.6	0.4	-3.5	6.6	-7.4
	-2.707	-7.343	-4.598	-18.560	-6.440	-11.660	0.218	-1.744	3.247	-3.659
t_O	79	78	74	87	75	85	79	81	82	75
	(39.1)	(38.6)	(36.6)	(43.1)	(37.1)	(42.1)	(39.1)	(40.1)	(40.6)	(37.1)
t_E	81.0	81.0	81.0	81.0	81.0	81.0	81.0	81.0	81.0	81.0
	(40.1)	(40.1)	(40.1)	(40.1)	(40.1)	(40.1)	(40.1)	(40.1)	(40.1)	(40.1)
	-4.0	-6.0	-13.4	12.3	-11.6	8.1	-4.0	-0.1	1.9	-11.6
	-1.994	-2.953	-6.658	6.113	-5.752	4.015	-1.994	-0.040	0.956	-5.752
g_O	22	25	22	24	29	23	18	23	18	25
	(10.9)	(12.4)	(10.9)	(11.9)	(14.4)	(11.4)	(8.9)	(11.4)	(8.9)	(12.4)
g_E	18.5	18.5	18.5	18.5	18.5	18.5	18.5	18.5	18.5	18.5
	(9.2)	(9.2)	(9.2)	(9.2)	(9.2)	(9.2)	(9.2)	(9.2)	(9.2)	(9.2)
	7.5	14.9	7.5	12.4	25.9	9.9	-1.1	9.9	-1.1	14.9
	3.727	7.400	3.727	6.134	12.845	4.909	-0.527	4.909	-0.527	7.400
c_O	18	21	25	27	19	21	19	14	13	20
	(8.9)	(10.4)	(12.4)	(13.4)	(9.4)	(10.4)	(9.4)	(6.9)	(6.4)	(9.9)
c_E	16.6	16.6	16.6	16.6	16.6	16.6	16.6	16.6	16.6	16.6
	(8.2)	(8.2)	(8.2)	(8.2)	(8.2)	(8.2)	(8.2)	(8.2)	(8.2)	(8.2)
	2.8	9.8	20.4	26.1	5.0	9.8	5.0	-4.8	-6.4	7.4
	1.400	4.839	10.076	12.939	2.495	4.839	2.495	-2.395	-3.177	3.642
N	202	202	202	202	202	202	202	202	202	202
X2	0.9	4.2	5.7	14.0	7.2	4.3	0.4	1.5	0.9	3.5
Gvalue	0.9	3.9	5.1	13.4	6.4	4.2	0.4	1.5	1.0	3.3
Gv/N	0.426	1.943	2.546	6.626	3.148	2.102	0.192	0.731	0.499	1.631
Prob	0.83506	0.26971	0.16157	0.00388	0.09537	0.23603	0.94268	0.68780	0.79926	0.34833

	90	89	88	87	86	85	84	83	82	81
a_O	89	84	80	71	85	80	77	88	87	91
	(44.1)	(41.6)	(39.6)	(35.1)	(42.1)	(39.6)	(38.1)	(43.6)	(43.1)	(45.0)
a_E	85.8	85.8	85.8	85.8	85.8	85.8	85.8	85.8	85.8	85.8
	(42.5)	(42.5)	(42.5)	(42.5)	(42.5)	(42.5)	(42.5)	(42.5)	(42.5)	(42.5)
	6.6	-3.5	-11.2	-26.9	-1.6	-11.2	-16.6	4.5	2.5	10.8
	3.247	-1.744	-5.525	-13.294	-0.769	-5.525	-8.232	2.226	1.216	5.322
t_O	76	84	72	79	72	74	89	73	84	87
	(37.6)	(41.6)	(35.6)	(39.1)	(35.6)	(36.6)	(44.1)	(36.1)	(41.6)	(43.1)
t_E	81.0	81.0	81.0	81.0	81.0	81.0	81.0	81.0	81.0	81.0
	(40.1)	(40.1)	(40.1)	(40.1)	(40.1)	(40.1)	(40.1)	(40.1)	(40.1)	(40.1)
	-9.8	6.0	-17.0	-4.0	-17.0	-13.4	16.7	-15.3	6.0	12.3
	-4.832	2.984	-8.432	-1.994	-8.432	-6.658	8.256	-7.552	2.984	6.113
g_O	24	17	27	26	22	25	20	24	17	8
	(11.9)	(8.4)	(13.4)	(12.9)	(10.9)	(12.4)	(9.9)	(11.9)	(8.4)	(4.0)
g_E	18.5	18.5	18.5	18.5	18.5	18.5	18.5	18.5	18.5	18.5
	(9.2)	(9.2)	(9.2)	(9.2)	(9.2)	(9.2)	(9.2)	(9.2)	(9.2)	(9.2)
	12.4	-2.9	20.3	17.6	7.5	14.9	3.0	12.4	-2.9	-13.4
	6.134	-1.460	10.049	8.705	3.727	7.400	1.501	6.134	-1.460	-6.657
c_O	13	17	23	26	23	23	16	17	14	16
	(6.4)	(8.4)	(11.4)	(12.9)	(11.4)	(11.4)	(7.9)	(8.4)	(6.9)	(7.9)
c_E	16.6	16.6	16.6	16.6	16.6	16.6	16.6	16.6	16.6	16.6
	(8.2)	(8.2)	(8.2)	(8.2)	(8.2)	(8.2)	(8.2)	(8.2)	(8.2)	(8.2)
	-6.4	0.7	14.9	23.2	14.9	14.9	-1.3	0.7	-4.8	-1.3
	-3.177	0.360	7.371	11.489	7.371	7.371	-0.621	0.360	-2.395	-0.621
N	202	202	202	202	202	202	202	202	202	202
X2	2.8	0.3	7.7	10.9	4.1	5.7	1.8	2.5	0.7	6.8
Gvalue	2.8	0.3	7.0	9.9	3.8	5.2	1.8	2.4	0.7	8.4
Gv/N	1.372	0.140	3.463	4.906	1.898	2.587	0.904	1.168	0.346	4.157
Prob	0.42828	0.96313	0.07205	0.01934	0.27999	0.15602	0.60941	0.50112	0.87352	0.03849

	80	79	78	77	76	75	74	73	72	71
a_O	89	79	81	78	85	87	101	83	82	92
	(44.1)	(39.1)	(40.1)	(38.6)	(42.1)	(43.1)	(50.0)	(41.1)	(40.6)	(45.5)
a_E	85.8	85.8	85.8	85.8	85.8	85.8	85.8	85.8	85.8	85.8
	(42.5)	(42.5)	(42.5)	(42.5)	(42.5)	(42.5)	(42.5)	(42.5)	(42.5)	(42.5)
	6.6	-13.0	-9.3	-14.8	-1.6	2.5	33.0	-5.5	-7.4	12.9
	3.247	-6.440	-4.598	-7.343	-0.769	1.216	16.333	-2.707	-3.659	6.376
t_O	79	78	82	74	84	74	63	84	85	78
	(39.1)	(38.6)	(40.6)	(36.6)	(41.6)	(36.6)	(31.2)	(41.6)	(42.1)	(38.6)
t_E	81.0	81.0	81.0	81.0	81.0	81.0	81.0	81.0	81.0	81.0
	(40.1)	(40.1)	(40.1)	(40.1)	(40.1)	(40.1)	(40.1)	(40.1)	(40.1)	(40.1)
	-4.0	-6.0	1.9	-13.4	6.0	-13.4	-31.7	6.0	8.1	-6.0
	-1.994	-2.953	0.956	-6.658	2.984	-6.658	-15.707	2.984	4.015	-2.953
g_O	15	29	23	29	18	28	22	19	27	18
	(7.4)	(14.4)	(11.4)	(14.4)	(8.9)	(13.9)	(10.9)	(9.4)	(13.4)	(8.9)
g_E	18.5	18.5	18.5	18.5	18.5	18.5	18.5	18.5	18.5	18.5
	(9.2)	(9.2)	(9.2)	(9.2)	(9.2)	(9.2)	(9.2)	(9.2)	(9.2)	(9.2)
	-6.4	25.9	9.9	25.9	-1.1	23.1	7.5	0.9	20.3	-1.1
	-3.147	12.845	4.909	12.845	-0.527	11.429	3.727	0.461	10.049	-0.527
c_O	19	16	16	21	15	13	16	16	8	14
	(9.4)	(7.9)	(7.9)	(10.4)	(7.4)	(6.4)	(7.9)	(7.9)	(4.0)	(6.9)
c_E	16.6	16.6	16.6	16.6	16.6	16.6	16.6	16.6	16.6	16.6
	(8.2)	(8.2)	(8.2)	(8.2)	(8.2)	(8.2)	(8.2)	(8.2)	(8.2)	(8.2)
	5.0	-1.3	-1.3	9.8	-3.1	-6.4	-1.3	-1.3	-11.7	-4.8
	2.495	-0.621	-0.621	4.839	-1.541	-3.177	-0.621	-0.621	-5.801	-2.395
N	202	202	202	202	202	202	202	202	202	202
X2	1.2	6.6	1.4	8.4	0.3	6.3	7.4	0.2	8.7	1.0
Gvalue	1.2	5.7	1.3	7.4	0.3	5.7	7.5	0.2	9.3	1.0
Gv/N	0.601	2.831	0.645	3.683	0.147	2.810	3.732	0.116	4.604	0.502
Prob	0.74948	0.12615	0.72827	0.05914	0.96058	0.12846	0.05655	0.97189	0.02555	0.79767

	70	69	68	67	66	65	64	63	62	61
a_O	86	88	87	86	90	85	70	76	88	74
	(42.6)	(43.6)	(43.1)	(42.6)	(44.6)	(42.1)	(34.7)	(37.6)	(43.6)	(36.6)
a_E	85.8	85.8	85.8	85.8	85.8	85.8	85.8	85.8	85.8	85.8
	(42.5)	(42.5)	(42.5)	(42.5)	(42.5)	(42.5)	(42.5)	(42.5)	(42.5)	(42.5)
	0.4	4.5	2.5	0.4	8.6	-1.6	-28.5	-18.4	4.5	-21.9
	0.218	2.226	1.216	0.218	4.279	-0.769	-14.089	-9.109	2.226	-10.823
t_O	79	83	75	83	77	78	90	90	69	90
	(39.1)	(41.1)	(37.1)	(41.1)	(38.1)	(38.6)	(44.6)	(44.6)	(34.2)	(44.6)

t_E	81.0	81.0	81.0	81.0	81.0	81.0	81.0	81.0	81.0	81.0
	(40.1)	(40.1)	(40.1)	(40.1)	(40.1)	(40.1)	(40.1)	(40.1)	(40.1)	(40.1)
	-4.0	4.0	-11.6	4.0	-7.9	-6.0	18.9	18.9	-22.2	18.9
	-1.994	1.964	-5.752	1.964	-3.899	-2.953	9.345	9.345	-10.988	9.345
g_O	20	18	25	17	20	18	20	18	28	16
	(9.9)	(8.9)	(12.4)	(8.4)	(9.9)	(8.9)	(9.9)	(8.9)	(13.9)	(7.9)
g_E	18.5	18.5	18.5	18.5	18.5	18.5	18.5	18.5	18.5	18.5
	(9.2)	(9.2)	(9.2)	(9.2)	(9.2)	(9.2)	(9.2)	(9.2)	(9.2)	(9.2)
	3.0	-1.1	14.9	-2.9	3.0	-1.1	3.0	-1.1	23.1	-4.7
	1.501	-0.527	7.400	-1.460	1.501	-0.527	1.501	-0.527	11.429	-2.334
c_O	17	13	15	16	15	21	22	18	17	22
	(8.4)	(6.4)	(7.4)	(7.9)	(7.4)	(10.4)	(10.9)	(8.9)	(8.4)	(10.9)
c_E	16.6	16.6	16.6	16.6	16.6	16.6	16.6	16.6	16.6	16.6
	(8.2)	(8.2)	(8.2)	(8.2)	(8.2)	(8.2)	(8.2)	(8.2)	(8.2)	(8.2)
	0.7	-6.4	-3.1	-1.3	-3.1	9.8	12.3	2.8	0.7	12.3
	0.360	-3.177	-1.541	-0.621	-1.541	4.839	6.082	1.400	0.360	6.082
N	202	202	202	202	202	202	202	202	202	202
X2	0.2	0.9	2.9	0.2	0.7	1.3	5.7	2.2	6.7	4.7
Gvalue	0.2	1.0	2.7	0.2	0.7	1.2	5.7	2.2	6.1	4.6
Gv/N	0.085	0.486	1.324	0.101	0.341	0.590	2.838	1.109	3.028	2.270
Prob	0.98195	0.80573	0.44474	0.97692	0.87593	0.75482	0.12531	0.52408	0.10607	0.20486

	60	59	58	57	56	55	54	53	52	51
a_O	88	82	83	89	86	82	72	78	74	82
	(43.6)	(40.6)	(41.1)	(44.1)	(42.6)	(40.6)	(35.6)	(38.6)	(36.6)	(40.6)
a_E	85.8	85.8	85.8	85.8	85.8	85.8	85.8	85.8	85.8	85.8
	(42.5)	(42.5)	(42.5)	(42.5)	(42.5)	(42.5)	(42.5)	(42.5)	(42.5)	(42.5)
	4.5	-7.4	-5.5	6.6	0.4	-7.4	-25.2	-14.8	-21.9	-7.4
	2.226	-3.659	-2.707	3.247	0.218	-3.659	-12.484	-7.343	-10.823	-3.659
t_O	81	80	86	79	79	84	91	90	91	85
	(40.1)	(39.6)	(42.6)	(39.1)	(39.1)	(41.6)	(45.0)	(44.6)	(45.0)	(42.1)
t_E	81.0	81.0	81.0	81.0	81.0	81.0	81.0	81.0	81.0	81.0
	(40.1)	(40.1)	(40.1)	(40.1)	(40.1)	(40.1)	(40.1)	(40.1)	(40.1)	(40.1)
	-0.1	-2.1	10.2	-4.0	-4.0	6.0	21.1	18.9	21.1	8.1
	-0.040	-1.023	5.058	-1.994	-1.994	2.984	10.444	9.345	10.444	4.015
g_O	19	27	14	18	19	19	23	16	14	19
	(9.4)	(13.4)	(6.9)	(8.9)	(9.4)	(9.4)	(11.4)	(7.9)	(6.9)	(9.4)
g_E	18.5	18.5	18.5	18.5	18.5	18.5	18.5	18.5	18.5	18.5
	(9.2)	(9.2)	(9.2)	(9.2)	(9.2)	(9.2)	(9.2)	(9.2)	(9.2)	(9.2)
	0.9	20.3	-7.9	-1.1	0.9	0.9	9.9	-4.7	-7.9	0.9
	0.461	10.049	-3.893	-0.527	0.461	0.461	4.909	-2.334	-3.893	0.461
c_O	14	13	19	16	18	17	16	18	23	16
	(6.9)	(6.4)	(9.4)	(7.9)	(8.9)	(8.4)	(7.9)	(8.9)	(11.4)	(7.9)
c_E	16.6	16.6	16.6	16.6	16.6	16.6	16.6	16.6	16.6	16.6
	(8.2)	(8.2)	(8.2)	(8.2)	(8.2)	(8.2)	(8.2)	(8.2)	(8.2)	(8.2)
	-4.8	-6.4	5.0	-1.3	2.8	0.7	-1.3	2.8	14.9	-1.3
	-2.395	-3.177	2.495	-0.621	1.400	0.360	-0.621	1.400	7.371	-0.621
N	202	202	202	202	202	202	202	202	202	202
X2	0.5	4.8	1.8	0.2	0.2	0.3	4.5	2.2	6.4	0.4
Gvalue	0.5	4.4	1.9	0.2	0.2	0.3	4.5	2.2	6.3	0.4
Gv/N	0.253	2.190	0.953	0.105	0.085	0.146	2.248	1.068	3.099	0.196
Prob	0.91641	0.21926	0.58830	0.97563	0.98199	0.96098	0.20871	0.54041	0.09966	0.94112

	50	49	48	47	46	45	44	43	42	41
a_O	86	90	94	94	88	81	95	76	85	85
	(42.6)	(44.6)	(46.5)	(46.5)	(43.6)	(40.1)	(47.0)	(37.6)	(42.1)	(42.1)
a_E	85.8	85.8	85.8	85.8	85.8	85.8	85.8	85.8	85.8	85.8
	(42.5)	(42.5)	(42.5)	(42.5)	(42.5)	(42.5)	(42.5)	(42.5)	(42.5)	(42.5)
	0.4	8.6	17.2	17.2	4.5	-9.3	19.4	-18.4	-1.6	-1.6
	0.218	4.279	8.517	8.517	2.226	-4.598	9.603	-9.109	-0.769	-0.769
t_O	89	82	75	69	81	92	71	86	89	81
	(44.1)	(40.6)	(37.1)	(34.2)	(40.1)	(45.5)	(35.1)	(42.6)	(44.1)	(40.1)
t_E	81.0	81.0	81.0	81.0	81.0	81.0	81.0	81.0	81.0	81.0
	(40.1)	(40.1)	(40.1)	(40.1)	(40.1)	(40.1)	(40.1)	(40.1)	(40.1)	(40.1)
	16.7	1.9	-11.6	-22.2	-0.1	23.3	-18.8	10.2	16.7	-0.1
	8.256	0.956	-5.752	-10.988	-0.040	11.554	-9.298	5.058	8.256	-0.040
g_O	16	13	16	19	18	12	18	21	10	19
	(7.9)	(6.4)	(7.9)	(9.4)	(8.9)	(5.9)	(8.9)	(10.4)	(5.0)	(9.4)
g_E	18.5	18.5	18.5	18.5	18.5	18.5	18.5	18.5	18.5	18.5
	(9.2)	(9.2)	(9.2)	(9.2)	(9.2)	(9.2)	(9.2)	(9.2)	(9.2)	(9.2)
	-4.7	-9.2	-4.7	0.9	-1.1	-10.4	-1.1	5.2	-12.3	0.9
	-2.334	-4.569	-2.334	0.461	-0.527	-5.169	-0.527	2.591	-6.112	0.461
c_O	11	17	17	20	15	17	18	19	18	17
	(5.4)	(8.4)	(8.4)	(9.9)	(7.4)	(8.4)	(8.9)	(9.4)	(8.9)	(8.4)
c_E	16.6	16.6	16.6	16.6	16.6	16.6	16.6	16.6	16.6	16.6
	(8.2)	(8.2)	(8.2)	(8.2)	(8.2)	(8.2)	(8.2)	(8.2)	(8.2)	(8.2)
	-9.1	0.7	0.7	7.4	-3.1	0.7	2.8	5.0	2.8	0.7
	-4.508	0.360	0.360	3.642	-1.541	0.360	1.400	2.495	1.400	0.360
N	202	202	202	202	202	202	202	202	202	202
X2	3.0	1.9	1.6	3.3	0.2	4.1	2.4	2.1	4.8	0.0
Gvalue	3.3	2.1	1.6	3.3	0.2	4.3	2.4	2.1	5.6	0.0
Gv/N	1.632	1.027	0.791	1.632	0.119	2.148	1.178	1.035	2.775	0.013
Prob	0.34807	0.55722	0.65979	0.34813	0.97087	0.22718	0.49735	0.55387	0.13244	0.99888

	40	39	38	37	36	35	34	33	32	31
a_O	91	83	86	86	77	86	76	69	81	92
	(45.0)	(41.1)	(42.6)	(42.6)	(38.1)	(42.6)	(37.6)	(34.2)	(40.1)	(45.5)
a_E	85.8	85.8	85.8	85.8	85.8	85.8	85.8	85.8	85.8	85.8
	(42.5)	(42.5)	(42.5)	(42.5)	(42.5)	(42.5)	(42.5)	(42.5)	(42.5)	(42.5)
	10.8	-5.5	0.4	0.4	-16.6	0.4	-18.4	-30.0	-9.3	12.9
	5.322	-2.707	0.218	0.218	-8.232	0.218	-9.109	-14.871	-4.598	6.376
t_O	79	88	77	80	90	77	100	90	82	78
	(39.1)	(43.6)	(38.1)	(39.6)	(44.6)	(38.1)	(49.5)	(44.6)	(40.6)	(38.6)
t_E	81.0	81.0	81.0	81.0	81.0	81.0	81.0	81.0	81.0	81.0
	(40.1)	(40.1)	(40.1)	(40.1)	(40.1)	(40.1)	(40.1)	(40.1)	(40.1)	(40.1)
	-4.0	14.5	-7.9	-2.1	18.9	-7.9	42.0	18.9	1.9	-6.0
	-1.994	7.179	-3.899	-1.023	9.345	-3.899	20.815	9.345	0.956	-2.953
g_O	18	19	19	18	17	23	8	20	22	18
	(8.9)	(9.4)	(9.4)	(8.9)	(8.4)	(11.4)	(4.0)	(9.9)	(10.9)	(8.9)
g_E	18.5	18.5	18.5	18.5	18.5	18.5	18.5	18.5	18.5	18.5
	(9.2)	(9.2)	(9.2)	(9.2)	(9.2)	(9.2)	(9.2)	(9.2)	(9.2)	(9.2)
	-1.1	0.9	0.9	-1.1	-2.9	9.9	-13.4	3.0	7.5	-1.1
	-0.527	0.461	0.461	-0.527	-1.460	4.909	-6.657	1.501	3.727	-0.527
c_O	14	12	20	18	18	16	18	23	17	14
	(6.9)	(5.9)	(9.9)	(8.9)	(8.9)	(7.9)	(8.9)	(11.4)	(8.4)	(6.9)
c_E	16.6	16.6	16.6	16.6	16.6	16.6	16.6	16.6	16.6	16.6

```
        (  8.2) (  8.2) (  8.2) (  8.2) (  8.2) (  8.2) (  8.2) (  8.2) (  8.2) (  8.2)
           -4.8    -7.8     7.4     2.8     2.8    -1.3     2.8    14.9     0.7    -4.8
         -2.395  -3.884   3.642   1.400   1.400  -0.621   1.400   7.371   0.360  -2.395

N           202     202     202     202     202     202     202     202     202     202
X2          0.8     2.0     0.9     0.1     2.1     1.3    11.7     6.8     0.9     1.0
Gvalue      0.8     2.1     0.9     0.1     2.1     1.2    13.0     6.8     0.9     1.0
Gv/N      0.407   1.049   0.423   0.068   1.053   0.607   6.448   3.345   0.445   0.502
Prob    0.84419 0.54826 0.83660 0.98690 0.54649 0.74671 0.00458 0.08003 0.82556 0.79767
        -------------------------------------------------------------------------------
             30      29      28      27      26      25      24      23      22      21
        -------------------------------------------------------------------------------
a_O          67      93      81      93      88      82      82     106      76      84
        ( 33.2) ( 46.0) ( 40.1) ( 46.0) ( 43.6) ( 40.6) ( 40.6) ( 52.5) ( 37.6) ( 41.6)
a_E        85.8    85.8    85.8    85.8    85.8    85.8    85.8    85.8    85.8    85.8
        ( 42.5) ( 42.5) ( 42.5) ( 42.5) ( 42.5) ( 42.5) ( 42.5) ( 42.5) ( 42.5) ( 42.5)
          -33.1    15.0    -9.3    15.0     4.5    -7.4    -7.4    44.9   -18.4    -3.5
        -16.391   7.441  -4.598   7.441   2.226  -3.659  -3.659  22.213  -9.109  -1.744
t_O         103      84      84      82      76      84      81      64      91      87
        ( 51.0) ( 41.6) ( 41.6) ( 40.6) ( 37.6) ( 41.6) ( 40.1) ( 31.7) ( 45.0) ( 43.1)
t_E        81.0    81.0    81.0    81.0    81.0    81.0    81.0    81.0    81.0    81.0
        ( 40.1) ( 40.1) ( 40.1) ( 40.1) ( 40.1) ( 40.1) ( 40.1) ( 40.1) ( 40.1) ( 40.1)
           49.4     6.0     6.0     1.9    -9.8     6.0    -0.1   -30.2    21.1    12.3
         24.453   2.984   2.984   0.956  -4.832   2.984  -0.040 -14.958  10.444   6.113
g_O          18      16      20      18      20      22      24      15      16      13
        (  8.9) (  7.9) (  9.9) (  8.9) (  9.9) ( 10.9) ( 11.9) (  7.4) (  7.9) (  6.4)
g_E        18.5    18.5    18.5    18.5    18.5    18.5    18.5    18.5    18.5    18.5
        (  9.2) (  9.2) (  9.2) (  9.2) (  9.2) (  9.2) (  9.2) (  9.2) (  9.2) (  9.2)
           -1.1    -4.7     3.0    -1.1     3.0     7.5    12.4    -6.4    -4.7    -9.2
         -0.527  -2.334   1.501  -0.527   1.501   3.727   6.134  -3.147  -2.334  -4.569
c_O          14       9      17       9      18      14      15      17      19      18
        (  6.9) (  4.5) (  8.4) (  4.5) (  8.9) (  6.9) (  7.4) (  8.4) (  9.4) (  8.9)
c_E        16.6    16.6    16.6    16.6    16.6    16.6    16.6    16.6    16.6    16.6
        (  8.2) (  8.2) (  8.2) (  8.2) (  8.2) (  8.2) (  8.2) (  8.2) (  8.2) (  8.2)
           -4.8   -11.1     0.7   -11.1     2.8    -4.8    -3.1     0.7     5.0     2.8
         -2.395  -5.476   0.360  -5.476   1.400  -2.395  -1.541   0.360   2.495   1.400

N           202     202     202     202     202     202     202     202     202     202
X2         10.5     4.6     0.5     4.1     0.6     1.3     1.9     9.0     3.0     2.2
Gvalue     10.4     5.3     0.5     4.8     0.6     1.3     1.8     9.0     3.0     2.4
Gv/N      5.141   2.614   0.247   2.394   0.296   0.657   0.894   4.468   1.496   1.200
Prob    0.01557 0.15236 0.91931 0.18421 0.89702 0.72255 0.61358 0.02895 0.38827 0.48920
        -------------------------------------------------------------------------------
             20      19      18      17      16      15      14      13      12      11
        -------------------------------------------------------------------------------
a_O          97      95      82      94      84     111      94     100      97      99
        ( 48.0) ( 47.0) ( 40.6) ( 46.5) ( 41.6) ( 55.0) ( 46.5) ( 49.5) ( 48.0) ( 49.0)
a_E        85.8    85.8    85.8    85.8    85.8    85.8    85.8    85.8    85.8    85.8
        ( 42.5) ( 42.5) ( 42.5) ( 42.5) ( 42.5) ( 42.5) ( 42.5) ( 42.5) ( 42.5) ( 42.5)
           23.8    19.4    -7.4    17.2    -3.5    57.2    17.2    30.7    23.8    28.4
         11.806   9.603  -3.659   8.517  -1.744  28.326   8.517  15.187  11.806  14.050
t_O          77      82      82      88      80      66      80      78      71      75
        ( 38.1) ( 40.6) ( 40.6) ( 43.6) ( 39.6) ( 32.7) ( 39.6) ( 38.6) ( 35.1) ( 37.1)
t_E        81.0    81.0    81.0    81.0    81.0    81.0    81.0    81.0    81.0    81.0
        ( 40.1) ( 40.1) ( 40.1) ( 40.1) ( 40.1) ( 40.1) ( 40.1) ( 40.1) ( 40.1) ( 40.1)
           -7.9     1.9     1.9    14.5    -2.1   -27.1    -2.1    -6.0   -18.8   -11.6
         -3.899   0.956   0.956   7.179  -1.023 -13.415  -1.023  -2.953  -9.298  -5.752
g_O          12       8      18      11      19      10      15      16      22      14
        (  5.9) (  4.0) (  8.9) (  5.4) (  9.4) (  5.0) (  7.4) (  7.9) ( 10.9) (  6.9)
g_E        18.5    18.5    18.5    18.5    18.5    18.5    18.5    18.5    18.5    18.5
        (  9.2) (  9.2) (  9.2) (  9.2) (  9.2) (  9.2) (  9.2) (  9.2) (  9.2) (  9.2)
          -10.4   -13.4    -1.1   -11.5     0.9   -12.3    -6.4    -4.7     7.5    -7.9
         -5.169  -6.657  -0.527  -5.686   0.461  -6.112  -3.147  -2.334   3.727  -3.893
c_O          16      17      20       9      19      15      13       8      12      14
        (  7.9) (  8.4) (  9.9) (  4.5) (  9.4) (  7.4) (  6.4) (  4.0) (  5.9) (  6.9)
c_E        16.6    16.6    16.6    16.6    16.6    16.6    16.6    16.6    16.6    16.6
        (  8.2) (  8.2) (  8.2) (  8.2) (  8.2) (  8.2) (  8.2) (  8.2) (  8.2) (  8.2)
           -1.3     0.7     7.4   -11.1     5.0    -3.1    -6.4   -11.7    -7.8    -4.8
         -0.621   0.360   3.642  -5.476   2.495  -1.541  -3.177  -5.801  -3.884  -2.395

N           202     202     202     202     202     202     202     202     202     202
X2          4.0     7.0     0.9     8.0     0.4    14.3     2.3     7.3     4.7     4.0
Gvalue      4.3     8.6     0.8     9.2     0.4    14.7     2.4     8.3     4.7     4.1
Gv/N      2.117   4.262   0.412   4.534   0.189   7.258   1.169   4.099   2.351   2.010
Prob    0.23310 0.03497 0.84151 0.02727 0.94397 0.00213 0.50069 0.04057 0.19110 0.25501
        -------------------------------------------------------------------------------
             10       9       8       7       6       5       4       3       2       1
        -------------------------------------------------------------------------------
a_O          99      94     100      94     105      96      92     114      86      58
        ( 49.0) ( 46.5) ( 49.5) ( 46.5) ( 52.0) ( 47.5) ( 45.5) ( 56.4) ( 42.6) ( 28.7)
a_E        85.8    85.8    85.8    85.8    85.8    85.8    85.8    85.8    85.8    85.8
        ( 42.5) ( 42.5) ( 42.5) ( 42.5) ( 42.5) ( 42.5) ( 42.5) ( 42.5) ( 42.5) ( 42.5)
           28.4    17.2    30.7    17.2    42.5    21.6    12.9    64.8     0.4   -45.4
         14.050   8.517  15.187   8.517  21.018  10.699   6.376  32.102   0.218 -22.473
t_O          74      78      71      87      69      84      76      65      87     116
        ( 36.6) ( 38.6) ( 35.1) ( 43.1) ( 34.2) ( 41.6) ( 37.6) ( 32.2) ( 43.1) ( 57.4)
t_E        81.0    81.0    81.0    81.0    81.0    81.0    81.0    81.0    81.0    81.0
        ( 40.1) ( 40.1) ( 40.1) ( 40.1) ( 40.1) ( 40.1) ( 40.1) ( 40.1) ( 40.1) ( 40.1)
          -13.4    -6.0   -18.8    12.3   -22.2     6.0    -9.8   -28.7    12.3    83.2
         -6.658  -2.953  -9.298   6.113 -10.988   2.984  -4.832 -14.194   6.113  41.191
g_O          14      16      20       7      13      11      16       8      14       9
        (  6.9) (  7.9) (  9.9) (  3.5) (  6.4) (  5.4) (  7.9) (  4.0) (  6.9) (  4.5)
g_E        18.5    18.5    18.5    18.5    18.5    18.5    18.5    18.5    18.5    18.5
        (  9.2) (  9.2) (  9.2) (  9.2) (  9.2) (  9.2) (  9.2) (  9.2) (  9.2) (  9.2)
           -7.9    -4.7     3.0   -13.6    -9.2   -11.5    -4.7   -13.4    -7.9   -13.0
         -3.893  -2.334   1.501  -6.751  -4.569  -5.686  -2.334  -6.657  -3.893  -6.440
c_O          15      14      11      14      15      11      18      15      15      19
        (  7.4) (  6.9) (  5.4) (  6.9) (  7.4) (  5.4) (  8.9) (  7.4) (  7.4) (  9.4)
c_E        16.6    16.6    16.6    16.6    16.6    16.6    16.6    16.6    16.6    16.6
        (  8.2) (  8.2) (  8.2) (  8.2) (  8.2) (  8.2) (  8.2) (  8.2) (  8.2) (  8.2)
           -3.1    -4.8    -9.1    -4.8    -3.1    -9.1     2.8    -3.1    -3.1     5.0
         -1.541  -2.395  -4.508  -2.395  -1.541  -4.508   1.400  -1.541  -1.541   2.495

N           202     202     202     202     202     202     202     202     202     202
X2          3.9     1.7     5.6     8.8     7.9     6.3     1.2    18.6     1.7    29.3
Gvalue      4.0     1.7     5.8    11.1     7.9     7.0     1.2    19.6     1.8    29.8
Gv/N      1.957   0.835   2.882   5.484   3.920   3.489   0.611   9.710   0.897  14.773
Prob    0.26659 0.63979 0.12063 0.01131 0.04772 0.07039 0.74487 0.00020 0.61248 0.00000
        -------------------------------------------------------------------------------

CDS_INITIAL:
        -------------------------------------------------------------------------------
```

	1	2	3	4	5	6	7	8	9	10
a_O	188	0	0	119	93	77	117	82	86	101
	(93.1)	(0.0)	(0.0)	(58.9)	(46.0)	(38.1)	(57.9)	(40.6)	(42.6)	(50.0)
a_E	76.2	70.2	80.9	76.2	70.2	80.9	76.2	70.2	80.9	76.2
	(37.7)	(34.8)	(40.1)	(37.7)	(34.8)	(40.1)	(37.7)	(34.8)	(40.1)	(37.7)
	339.5	0.0	0.0	106.1	52.3	-7.7	100.3	25.5	10.4	56.9
	168.092	0.000	0.000	52.516	25.901	-3.807	49.670	12.617	5.160	28.172
t_O	7	202	0	44	70	97	50	81	84	55
	(3.5)	(100.0)	(0.0)	(21.8)	(34.7)	(48.0)	(24.8)	(40.1)	(41.6)	(27.2)
t_E	51.9	69.6	89.0	51.9	69.6	89.0	51.9	69.6	89.0	51.9
	(25.7)	(34.5)	(44.1)	(25.7)	(34.5)	(44.1)	(25.7)	(34.5)	(44.1)	(25.7)
	-28.0	430.3	0.0	-14.5	0.7	16.6	-3.7	24.5	-9.8	6.4
	-13.883	213.013	0.000	-7.179	0.367	8.233	-1.830	12.130	-4.837	3.178
g_O	7	0	202	15	15	10	20	15	16	23
	(3.5)	(0.0)	(100.0)	(7.4)	(7.4)	(5.0)	(9.9)	(7.4)	(7.9)	(11.4)
g_E	44.7	26.4	18.6	44.7	26.4	18.6	44.7	26.4	18.6	44.7
	(22.1)	(13.1)	(9.2)	(22.1)	(13.1)	(9.2)	(22.1)	(13.1)	(9.2)	(22.1)
	-26.0	0.0	962.9	-32.8	-17.0	-12.4	-32.2	-17.0	-4.9	-30.6
	-12.850	0.000	476.686	-16.216	-8.405	-6.161	-15.925	-8.405	-2.412	-15.131
c_O	0	0	0	24	24	18	15	24	16	23
	(0.0)	(0.0)	(0.0)	(11.9)	(11.9)	(8.9)	(7.4)	(11.9)	(7.9)	(11.4)
c_E	29.2	35.8	13.4	29.2	35.8	13.4	29.2	35.8	13.4	29.2
	(14.5)	(17.7)	(6.6)	(14.5)	(17.7)	(6.6)	(14.5)	(17.7)	(6.6)	(14.5)
	0.0	0.0	0.0	-9.4	-19.1	10.6	-20.0	-19.1	5.7	-11.0
	0.000	0.000	0.000	-4.674	-9.473	5.266	-9.902	-9.473	2.815	-5.449
N	202	202	202	202	202	202	202	202	202	202
X2	263.9	384.0	1988.1	45.9	16.2	6.5	42.5	12.6	1.5	20.1
Gvalue	285.5	430.3	962.9	49.4	16.9	7.1	44.5	13.9	1.5	21.8
Gv/N	141.360	213.013	476.686	24.447	8.390	3.531	22.015	6.869	0.726	10.771
Prob	0.00000	0.00000	0.00000	0.00000	0.00072	0.06777	0.00000	0.00308	0.69005	0.00007

	11	12	13	14	15	16	17	18	19	20
a_O	81	82	95	77	73	92	62	90	87	67
	(40.1)	(40.6)	(47.0)	(38.1)	(36.1)	(45.5)	(30.7)	(44.6)	(43.1)	(33.2)
a_E	70.2	80.9	76.2	70.2	80.9	76.2	70.2	80.9	76.2	70.2
	(34.8)	(40.1)	(37.7)	(34.8)	(40.1)	(37.7)	(34.8)	(40.1)	(37.7)	(34.8)
	23.2	2.1	41.9	14.2	-15.1	34.7	-15.4	19.1	23.1	-6.2
	11.479	1.053	20.738	7.052	-7.465	17.161	-7.623	9.451	11.414	-3.092
t_O	90	92	52	83	101	50	90	81	50	87
	(44.6)	(45.5)	(25.7)	(41.1)	(50.0)	(24.8)	(44.6)	(40.1)	(24.8)	(43.1)
t_E	69.6	89.0	51.9	69.6	89.0	51.9	69.6	89.0	51.9	69.6
	(34.5)	(44.1)	(25.7)	(34.5)	(44.1)	(25.7)	(34.5)	(44.1)	(25.7)	(34.5)
	46.2	6.0	0.2	29.2	25.5	-3.7	46.2	-15.3	-3.7	38.8
	22.866	2.988	0.117	14.434	12.614	-1.830	22.866	-7.581	-1.830	19.184
g_O	9	15	27	16	18	31	23	21	42	25
	(4.5)	(7.4)	(13.4)	(7.9)	(8.9)	(15.3)	(11.4)	(10.4)	(20.8)	(12.4)
g_E	26.4	18.6	44.7	26.4	18.6	44.7	26.4	18.6	44.7	26.4
	(13.1)	(9.2)	(22.1)	(13.1)	(9.2)	(22.1)	(13.1)	(9.2)	(22.1)	(13.1)
	-19.4	-6.5	-27.2	-16.0	-1.2	-22.7	-6.4	5.0	-5.2	-2.8
	-9.595	-3.220	-13.476	-7.943	-0.614	-11.232	-3.154	2.489	-2.589	-1.365
c_O	22	13	28	26	10	29	27	10	23	23
	(10.9)	(6.4)	(13.9)	(12.9)	(5.0)	(14.4)	(13.4)	(5.0)	(11.4)	(11.4)
c_E	35.8	13.4	29.2	35.8	13.4	29.2	35.8	13.4	29.2	35.8
	(17.7)	(6.6)	(14.5)	(17.7)	(6.6)	(14.5)	(17.7)	(6.6)	(14.5)	(17.7)
	-21.4	-0.8	-2.4	-16.6	-5.8	-0.4	-15.2	-5.8	-11.0	-20.3
	-10.579	-0.385	-1.180	-8.202	-2.894	-0.214	-7.508	-2.894	-5.449	-10.047
N	202	202	202	202	202	202	202	202	202	202
X2	24.4	0.8	11.7	10.0	3.3	7.5	9.5	2.9	3.1	9.1
Gvalue	28.6	0.9	12.5	10.8	3.3	7.8	9.3	3.0	3.1	9.5
Gv/N	14.172	0.437	6.200	5.340	1.640	3.885	4.581	1.464	1.548	4.679
Prob	0.00000	0.82972	0.00579	0.01293	0.34576	0.04927	0.02610	0.39813	0.37260	0.02384

	21	22	23	24	25	26	27	28	29	30
a_O	79	71	58	83	78	64	82	82	57	89
	(39.1)	(35.1)	(28.7)	(41.1)	(38.6)	(31.7)	(40.6)	(40.6)	(28.2)	(44.1)
a_E	80.9	76.2	70.2	80.9	76.2	70.2	80.9	76.2	70.2	80.9
	(40.1)	(37.7)	(34.8)	(40.1)	(37.7)	(34.8)	(40.1)	(37.7)	(34.8)	(40.1)
	-3.8	-10.0	-22.1	4.2	3.6	-11.8	2.1	12.0	-23.7	16.9
	-1.900	-4.971	-10.961	2.062	1.800	-5.857	1.053	5.953	-11.753	8.362
t_O	92	65	82	94	53	76	100	50	88	81
	(45.5)	(32.2)	(40.6)	(46.5)	(26.2)	(37.6)	(49.5)	(24.8)	(43.6)	(40.1)
t_E	89.0	51.9	69.6	89.0	51.9	69.6	89.0	51.9	69.6	89.0
	(44.1)	(25.7)	(34.5)	(44.1)	(25.7)	(34.5)	(44.1)	(25.7)	(34.5)	(44.1)
	6.0	29.3	26.8	10.2	2.3	13.3	23.2	-3.7	41.2	-15.3
	2.988	14.506	13.276	5.055	1.118	6.587	11.504	-1.830	20.400	-7.581
g_O	21	41	23	13	39	25	11	40	19	19
	(10.4)	(20.3)	(11.4)	(6.4)	(19.3)	(12.4)	(5.4)	(19.8)	(9.4)	(9.4)
g_E	18.6	44.7	26.4	18.6	44.7	26.4	18.6	44.7	26.4	18.6
	(9.2)	(22.1)	(13.1)	(9.2)	(22.1)	(13.1)	(9.2)	(22.1)	(13.1)	(9.2)
	5.0	-7.1	-6.4	-9.4	-10.6	-2.8	-11.6	-8.9	-12.5	0.7
	2.489	-3.505	-3.154	-4.632	-5.265	-1.365	-5.739	-4.398	-6.200	0.369
c_O	10	25	39	12	32	37	9	30	38	13
	(5.0)	(12.4)	(19.3)	(5.9)	(15.8)	(18.3)	(4.5)	(14.9)	(18.8)	(6.4)
c_E	13.4	29.2	35.8	13.4	29.2	35.8	13.4	29.2	35.8	13.4
	(6.6)	(14.5)	(17.7)	(6.6)	(14.5)	(17.7)	(6.6)	(14.5)	(17.7)	(6.6)
	-5.8	-7.8	6.8	-2.6	5.8	2.5	-7.2	1.6	4.6	-0.8
	-2.894	-3.859	3.354	-1.307	2.882	1.254	-3.544	0.785	2.291	-0.385
N	202	202	202	202	202	202	202	202	202	202
X2	1.3	4.6	5.1	2.2	1.1	1.2	5.9	1.0	9.6	1.5
Gvalue	1.4	4.4	5.1	2.4	1.1	1.2	6.6	1.0	9.6	1.5
Gv/N	0.682	2.171	2.515	1.178	0.536	0.619	3.275	0.511	4.738	0.764
Prob	0.71064	0.22265	0.16603	0.49735	0.78136	0.74107	0.08524	0.79346	0.02260	0.67241

	31	32	33	34	35	36	37	38	39	40
a_O	84	67	86	78	58	92	69	71	93	74
	(41.6)	(33.2)	(42.6)	(38.6)	(28.7)	(45.5)	(34.2)	(35.1)	(46.0)	(36.6)
a_E	76.2	70.2	80.9	76.2	70.2	80.9	76.2	70.2	80.9	76.2
	(37.7)	(34.8)	(40.1)	(37.7)	(34.8)	(40.1)	(37.7)	(34.8)	(40.1)	(37.7)
	16.4	-6.2	10.4	3.6	-22.1	23.6	-13.7	1.6	25.8	-4.3
	8.102	-3.092	5.160	1.800	-10.961	11.663	-6.783	0.799	12.786	-2.149
t_O	51	84	87	62	77	88	58	66	88	56
	(25.2)	(41.6)	(43.1)	(30.7)	(38.1)	(43.6)	(28.7)	(32.7)	(43.6)	(27.7)
t_E	51.9	69.6	89.0	51.9	69.6	89.0	51.9	69.6	89.0	51.9
	(25.7)	(34.5)	(44.1)	(25.7)	(34.5)	(44.1)	(25.7)	(34.5)	(44.1)	(25.7)
	-1.7	31.5	-4.0	22.1	15.5	-2.0	12.9	-7.1	-2.0	8.6

	-0.866	15.604	-1.988	10.936	7.670	-1.015	6.401	-3.499	-1.015	4.235
g_O	44	23	17	34	32	14	48	29	11	40
	(21.8)	(11.4)	(8.4)	(16.8)	(15.8)	(6.9)	(23.8)	(14.4)	(5.4)	(19.8)
g_E	44.7	26.4	18.6	44.7	26.4	18.6	44.7	26.4	18.6	44.7
	(22.1)	(13.1)	(9.2)	(22.1)	(13.1)	(9.2)	(22.1)	(13.1)	(9.2)	(22.1)
	-1.4	-6.4	-3.1	-18.6	12.3	-8.0	6.8	5.4	-11.6	-8.9
	-0.685	-3.154	-1.542	-9.209	6.075	-3.961	3.388	2.679	-5.739	-4.398
c_O	23	28	12	28	35	8	27	36	10	32
	(11.4)	(13.9)	(5.9)	(13.9)	(17.3)	(4.0)	(13.4)	(17.8)	(5.0)	(15.8)
c_E	29.2	35.8	13.4	29.2	35.8	13.4	29.2	35.8	13.4	29.2
	(14.5)	(17.7)	(6.6)	(14.5)	(17.7)	(6.6)	(14.5)	(17.7)	(6.6)	(14.5)
	-11.0	-13.7	-2.6	-2.4	-1.5	-8.2	-4.3	0.5	-5.8	5.8
	-5.449	-6.778	-1.307	-1.180	-0.740	-4.083	-2.110	0.243	-2.894	2.882
N	202	202	202	202	202	202	202	202	202	202
X2	2.1	5.2	0.7	4.6	4.1	4.8	1.8	0.5	5.8	1.1
Gvalue	2.2	5.2	0.7	4.7	4.1	5.3	1.8	0.4	6.3	1.2
Gv/N	1.102	2.579	0.324	2.348	2.044	2.605	0.895	0.222	3.138	0.570
Prob	0.52678	0.15709	0.88396	0.19164	0.24791	0.15362	0.61303	0.93012	0.09625	0.76460

	41	42	43	44	45	46	47	48	49	50
a_O	64	81	75	55	77	81	61	98	83	56
	(31.7)	(40.1)	(37.1)	(27.2)	(38.1)	(40.1)	(30.2)	(48.5)	(41.1)	(27.7)
a_E	70.2	80.9	76.2	70.2	80.9	76.2	70.2	80.9	76.2	70.2
	(34.8)	(40.1)	(37.7)	(34.8)	(40.1)	(37.7)	(34.8)	(40.1)	(37.7)	(34.8)
	-11.8	0.1	-2.4	-26.8	-7.7	9.9	-17.1	37.5	14.2	-25.3
	-5.857	0.056	-1.181	-13.286	-3.807	4.896	-8.482	18.554	7.022	-12.528
t_O	75	91	55	78	81	57	82	75	51	65
	(37.1)	(45.0)	(27.2)	(38.6)	(40.1)	(28.2)	(40.6)	(37.1)	(25.2)	(32.2)
t_E	69.6	89.0	51.9	69.6	89.0	51.9	69.6	89.0	51.9	69.6
	(34.5)	(44.1)	(25.7)	(34.5)	(44.1)	(25.7)	(34.5)	(44.1)	(25.7)	(34.5)
	11.1	4.0	6.4	17.7	-15.3	10.7	26.8	-25.7	-1.7	-8.9
	5.516	1.971	3.178	8.766	-7.581	5.309	13.276	-12.735	-0.866	-4.429
g_O	26	19	43	39	22	43	23	19	41	34
	(12.9)	(9.4)	(21.3)	(19.3)	(10.9)	(21.3)	(11.4)	(9.4)	(20.3)	(16.8)
g_E	26.4	18.6	44.7	26.4	18.6	44.7	26.4	18.6	44.7	26.4
	(13.1)	(9.2)	(22.1)	(13.1)	(9.2)	(22.1)	(13.1)	(9.2)	(22.1)	(13.1)
	-0.8	0.7	-3.3	30.4	7.3	-3.3	-6.4	0.7	-7.1	17.2
	-0.410	0.369	-1.649	15.042	3.620	-1.649	-3.154	0.369	-3.505	8.495
c_O	37	11	29	30	22	21	36	10	27	47
	(18.3)	(5.4)	(14.4)	(14.9)	(10.9)	(10.4)	(17.8)	(5.0)	(13.4)	(23.3)
c_E	35.8	13.4	29.2	35.8	13.4	29.2	35.8	13.4	29.2	35.8
	(17.7)	(6.6)	(14.5)	(17.7)	(6.6)	(14.5)	(17.7)	(6.6)	(14.5)	(17.7)
	2.5	-4.3	-0.4	-10.5	21.8	-13.9	0.5	-5.8	-4.3	25.7
	1.254	-2.145	-0.214	-5.213	10.807	-6.866	0.243	-2.894	-2.110	12.725
N	202	202	202	202	202	202	202	202	202	202
X2	1.0	0.5	0.3	11.2	7.1	3.2	3.8	6.7	1.1	8.9
Gvalue	1.0	0.5	0.3	10.7	6.1	3.4	3.8	6.7	1.1	8.6
Gv/N	0.504	0.251	0.134	5.309	3.039	1.690	1.883	3.294	0.540	4.263
Prob	0.79708	0.91738	0.96562	0.01331	0.10504	0.33199	0.28351	0.08377	0.77911	0.03493

	51	52	53	54	55	56	57	58	59	60
a_O	77	73	67	73	81	65	81	81	55	79
	(38.1)	(36.1)	(33.2)	(36.1)	(40.1)	(32.2)	(40.1)	(40.1)	(27.2)	(39.1)
a_E	80.9	76.2	70.2	80.9	76.2	70.2	80.9	76.2	70.2	80.9
	(40.1)	(37.7)	(34.8)	(40.1)	(37.7)	(34.8)	(40.1)	(37.7)	(34.8)	(40.1)
	-7.7	-6.3	-6.2	-15.1	9.9	-10.0	0.1	9.9	-26.8	-3.8
	-3.807	-3.103	-3.092	-7.465	4.896	-4.950	0.056	4.896	-13.286	-1.900
t_O	94	59	70	91	51	83	83	49	88	93
	(46.5)	(29.2)	(34.7)	(45.0)	(25.2)	(41.1)	(41.1)	(24.3)	(43.6)	(46.0)
t_E	89.0	51.9	69.6	89.0	51.9	69.6	89.0	51.9	69.6	89.0
	(44.1)	(25.7)	(34.5)	(44.1)	(25.7)	(34.5)	(44.1)	(25.7)	(34.5)	(44.1)
	10.2	15.2	0.7	4.0	-1.7	29.2	-11.6	-5.6	41.2	8.1
	5.055	7.510	0.367	1.971	-0.866	14.434	-5.764	-2.773	20.400	4.016
g_O	22	41	29	23	45	27	18	42	23	20
	(10.9)	(20.3)	(14.4)	(11.4)	(22.3)	(13.4)	(8.9)	(20.8)	(11.4)	(9.9)
g_E	18.6	44.7	26.4	18.6	44.7	26.4	18.6	44.7	26.4	18.6
	(9.2)	(22.1)	(13.1)	(9.2)	(22.1)	(13.1)	(9.2)	(22.1)	(13.1)	(9.2)
	7.3	-7.1	5.4	9.7	0.6	1.2	-1.2	-5.2	-6.4	2.8
	3.620	-3.505	2.679	4.797	0.300	0.584	-0.614	-2.589	-3.154	1.404
c_O	9	29	36	15	25	27	20	30	36	10
	(4.5)	(14.4)	(17.8)	(7.4)	(12.4)	(13.4)	(9.9)	(14.9)	(17.8)	(5.0)
c_E	13.4	29.2	35.8	13.4	29.2	35.8	13.4	29.2	35.8	13.4
	(6.6)	(14.5)	(17.7)	(6.6)	(14.5)	(17.7)	(6.6)	(14.5)	(17.7)	(6.6)
	-7.2	-0.4	0.5	3.4	-7.8	-15.2	16.0	1.6	0.5	-5.8
	-3.544	-0.214	0.243	1.681	-3.859	-7.508	7.938	0.785	0.243	-2.894
N	202	202	202	202	202	202	202	202	202	202
X2	2.5	1.4	0.4	2.0	0.9	5.1	3.7	0.6	8.6	1.2
Gvalue	2.7	1.4	0.4	2.0	1.0	5.2	3.3	0.6	8.5	1.3
Gv/N	1.324	0.687	0.196	0.984	0.472	2.559	1.616	0.320	4.203	0.626
Prob	0.44445	0.70847	0.94102	0.57507	0.81254	0.15987	0.35273	0.88582	0.03690	0.73777

	61	62	63	64	65	66	67	68	69	70
a_O	80	67	74	70	65	84	75	52	88	74
	(39.6)	(33.2)	(36.6)	(34.7)	(32.2)	(41.6)	(37.1)	(25.7)	(43.6)	(36.6)
a_E	76.2	70.2	80.9	76.2	70.2	80.9	76.2	70.2	80.9	76.2
	(37.7)	(34.8)	(40.1)	(37.7)	(34.8)	(40.1)	(37.7)	(34.8)	(40.1)	(37.7)
	7.8	-6.2	-13.3	-11.9	-10.0	6.2	-2.4	-31.2	14.7	-4.3
	3.852	-3.092	-6.571	-5.884	-4.950	3.083	-1.181	-15.449	7.283	-2.149
t_O	54	70	93	56	73	91	50	80	81	56
	(26.7)	(34.7)	(46.0)	(27.7)	(36.1)	(45.0)	(24.8)	(39.6)	(40.1)	(27.7)
t_E	51.9	69.6	89.0	51.9	69.6	89.0	51.9	69.6	89.0	51.9
	(25.7)	(34.5)	(44.1)	(25.7)	(34.5)	(44.1)	(25.7)	(34.5)	(44.1)	(25.7)
	4.3	0.7	8.1	8.6	6.9	4.0	-3.7	22.2	-15.3	8.6
	2.139	0.367	4.016	4.235	3.416	1.971	-1.830	10.996	-7.581	4.235
g_O	45	34	22	47	27	13	45	28	23	51
	(22.3)	(16.8)	(10.9)	(23.3)	(13.4)	(6.4)	(22.3)	(13.9)	(11.4)	(25.2)
g_E	44.7	26.4	18.6	44.7	26.4	18.6	44.7	26.4	18.6	44.7
	(22.1)	(13.1)	(9.2)	(22.1)	(13.1)	(9.2)	(22.1)	(13.1)	(9.2)	(22.1)
	0.6	17.2	7.3	4.7	1.2	-9.4	0.6	3.3	9.7	13.5
	0.300	8.495	3.620	2.337	0.584	-4.632	0.300	1.613	4.797	6.660
c_O	23	31	13	29	37	14	32	42	10	21
	(11.4)	(15.3)	(6.4)	(14.4)	(18.3)	(6.9)	(15.8)	(20.8)	(5.0)	(10.4)
c_E	29.2	35.8	13.4	29.2	35.8	13.4	29.2	35.8	13.4	29.2
	(14.5)	(17.7)	(6.6)	(14.5)	(17.7)	(6.6)	(14.5)	(17.7)	(6.6)	(14.5)
	-11.0	-8.8	-0.8	-0.4	2.5	1.2	5.8	13.5	-5.8	-13.9
	-5.449	-4.380	-0.385	-0.214	1.254	0.612	2.882	6.694	-2.894	-6.866

N	202	202	202	202	202	202	202	202	202	202
X2	1.6	3.0	1.4	1.0	0.6	1.9	0.4	7.4	3.2	3.6
Gvalue	1.7	2.8	1.4	1.0	0.6	2.1	0.3	7.8	3.2	3.8
Gv/N	0.842	1.389	0.681	0.473	0.302	1.034	0.172	3.854	1.605	1.880
Prob	0.63653	0.42245	0.71142	0.81193	0.89396	0.55405	0.95091	0.05065	0.35579	0.28425

	71	72	73	74	75	76	77	78	79	80
a_O	64	86	75	56	77	72	62	71	75	70
	(31.7)	(42.6)	(37.1)	(27.7)	(38.1)	(35.6)	(30.7)	(35.1)	(37.1)	(34.7)
a_E	70.2	80.9	76.2	70.2	80.9	76.2	70.2	80.9	76.2	70.2
	(34.8)	(40.1)	(37.7)	(34.8)	(40.1)	(37.7)	(34.8)	(40.1)	(37.7)	(34.8)
	-11.8	10.4	-2.4	-25.3	-7.7	-8.2	-15.4	-18.6	-2.4	-0.4
	-5.857	5.160	-1.181	-12.528	-3.807	-4.044	-7.623	-9.214	-1.181	-0.195
t_O	79	84	60	79	91	64	76	93	51	65
	(39.1)	(41.6)	(29.7)	(39.1)	(45.0)	(31.7)	(37.6)	(46.0)	(25.2)	(32.2)
t_E	69.6	89.0	51.9	69.6	89.0	51.9	69.6	89.0	51.9	69.6
	(34.5)	(44.1)	(25.7)	(34.5)	(44.1)	(25.7)	(34.5)	(44.1)	(25.7)	(34.5)
	19.9	-9.8	17.4	19.9	4.0	26.9	13.3	8.1	-1.7	-8.9
	9.875	-4.837	8.636	9.875	1.971	13.301	6.587	4.016	-0.866	-4.429
g_O	31	19	42	30	22	43	27	24	52	30
	(15.3)	(9.4)	(20.8)	(14.9)	(10.9)	(21.3)	(13.4)	(11.9)	(25.7)	(14.9)
g_E	26.4	18.6	44.7	26.4	18.6	44.7	26.4	18.6	44.7	26.4
	(13.1)	(9.2)	(22.1)	(13.1)	(9.2)	(22.1)	(13.1)	(9.2)	(22.1)	(13.1)
	9.9	0.7	-5.2	7.6	7.3	-3.3	1.2	12.2	15.7	7.6
	4.910	0.369	-2.589	3.778	3.620	-1.649	0.584	6.017	7.791	3.778
c_O	28	13	25	37	12	23	37	14	24	37
	(13.9)	(6.4)	(12.4)	(18.3)	(5.9)	(11.4)	(18.3)	(6.9)	(11.9)	(18.3)
c_E	35.8	13.4	29.2	35.8	13.4	29.2	35.8	13.4	29.2	35.8
	(17.7)	(6.6)	(14.5)	(17.7)	(6.6)	(14.5)	(17.7)	(6.6)	(14.5)	(17.7)
	-13.7	-0.8	-7.8	2.5	-2.6	-11.0	2.5	1.2	-9.4	2.5
	-6.778	-0.385	-3.859	1.254	-1.307	-5.449	1.254	0.612	-4.674	1.254

N	202	202	202	202	202	202	202	202	202	202
X2	4.3	0.6	2.1	4.7	1.0	4.4	1.6	3.0	2.2	0.8
Gvalue	4.3	0.6	2.0	4.8	1.0	4.4	1.6	2.9	2.2	0.8
Gv/N	2.150	0.306	1.007	2.378	0.478	2.160	0.801	1.432	1.069	0.408
Prob	0.22669	0.89222	0.56533	0.18678	0.80983	0.22491	0.65529	0.40845	0.53993	0.84377

	81	82	83	84	85	86	87	88	89	90
a_O	77	62	56	71	66	64	79	65	71	91
	(38.1)	(30.7)	(27.7)	(35.1)	(32.7)	(31.7)	(39.1)	(32.2)	(35.1)	(45.0)
a_E	80.9	76.2	70.2	80.9	76.2	70.2	80.9	76.2	70.2	80.9
	(40.1)	(37.7)	(34.8)	(40.1)	(37.7)	(34.8)	(40.1)	(37.7)	(34.8)	(40.1)
	-7.7	-25.6	-25.3	-18.6	-19.0	-11.8	-3.8	-20.7	1.6	21.3
	-3.807	-12.662	-12.528	-9.214	-9.393	-5.857	-1.900	-10.233	0.799	10.552
t_O	94	63	62	94	58	84	78	60	68	83
	(46.5)	(31.2)	(30.7)	(46.5)	(28.7)	(41.6)	(38.6)	(29.7)	(33.7)	(41.1)
t_E	89.0	51.9	69.6	89.0	51.9	69.6	89.0	51.9	69.6	89.0
	(44.1)	(25.7)	(34.5)	(44.1)	(25.7)	(34.5)	(44.1)	(25.7)	(34.5)	(44.1)
	10.2	24.5	-14.4	10.2	12.9	31.5	-20.6	17.4	-3.2	-11.6
	5.055	12.111	-7.125	5.055	6.401	15.604	-10.215	8.636	-1.595	-5.764
g_O	17	43	30	24	48	25	33	49	22	18
	(8.4)	(21.3)	(14.9)	(11.9)	(23.8)	(12.4)	(16.3)	(24.3)	(10.9)	(8.9)
g_E	18.6	44.7	26.4	18.6	44.7	26.4	18.6	44.7	26.4	18.6
	(9.2)	(22.1)	(13.1)	(9.2)	(22.1)	(13.1)	(9.2)	(22.1)	(13.1)	(9.2)
	-3.1	-3.3	7.6	12.2	6.8	-2.8	37.7	9.0	-8.1	-1.2
	-1.542	-1.649	3.778	6.017	3.388	-1.365	18.678	4.458	-3.985	-0.614
c_O	14	34	54	13	30	29	12	28	41	10
	(6.9)	(16.8)	(26.7)	(6.4)	(14.9)	(14.4)	(5.9)	(13.9)	(20.3)	(5.0)
c_E	13.4	29.2	35.8	13.4	29.2	35.8	13.4	29.2	35.8	13.4
	(6.6)	(14.5)	(17.7)	(6.6)	(14.5)	(17.7)	(6.6)	(14.5)	(17.7)	(6.6)
	1.2	10.3	44.5	-0.8	1.6	-12.1	-2.6	-2.4	11.2	-5.8
	0.612	5.103	22.043	-0.385	0.785	-6.013	-1.307	-1.180	5.556	-2.894

N	202	202	202	202	202	202	202	202	202	202
X2	0.6	5.9	13.5	3.1	2.4	4.9	12.6	3.4	1.6	2.5
Gvalue	0.6	5.9	12.5	3.0	2.4	4.8	10.6	3.4	1.6	2.6
Gv/N	0.318	2.904	6.168	1.473	1.181	2.370	5.256	1.681	0.775	1.280
Prob	0.88682	0.11832	0.00597	0.39540	0.49645	0.18809	0.01399	0.33456	0.66727	0.46020

	91	92	93	94	95	96	97	98	99	100
a_O	68	53	82	75	58	72	80	65	82	81
	(33.7)	(26.2)	(40.6)	(37.1)	(28.7)	(35.6)	(39.6)	(32.2)	(40.6)	(40.1)
a_E	76.2	70.2	80.9	76.2	70.2	80.9	76.2	70.2	80.9	76.2
	(37.7)	(34.8)	(40.1)	(37.7)	(34.8)	(40.1)	(37.7)	(34.8)	(40.1)	(37.7)
	-15.5	-29.8	2.1	-2.4	-22.1	-16.9	7.8	-10.0	2.1	9.9
	-7.668	-14.747	1.053	-1.181	-10.961	-8.346	3.852	-4.950	1.053	4.896
t_O	61	93	83	50	81	105	56	72	82	54
	(30.2)	(46.0)	(41.1)	(24.8)	(40.1)	(52.0)	(27.7)	(35.6)	(40.6)	(26.7)
t_E	51.9	69.6	89.0	51.9	69.6	89.0	51.9	69.6	89.0	51.9
	(25.7)	(34.5)	(44.1)	(25.7)	(34.5)	(44.1)	(25.7)	(34.5)	(44.1)	(25.7)
	19.8	53.8	-11.6	-3.7	24.5	34.6	8.6	4.8	-13.5	4.3
	9.778	26.648	-5.764	-1.830	12.130	17.151	4.235	2.386	-6.679	2.139
g_O	45	15	18	43	30	16	38	24	20	36
	(22.3)	(7.4)	(8.9)	(21.3)	(14.9)	(7.9)	(18.8)	(11.9)	(9.9)	(17.8)
g_E	44.7	26.4	18.6	44.7	26.4	18.6	44.7	26.4	18.6	44.7
	(22.1)	(13.1)	(9.2)	(22.1)	(13.1)	(9.2)	(22.1)	(13.1)	(9.2)	(22.1)
	0.6	-17.0	-1.2	-3.3	7.6	-4.9	-12.3	-4.6	2.8	-15.6
	0.300	-8.405	-0.614	-1.649	3.778	-2.412	-6.108	-2.280	1.404	-7.713
c_O	28	41	19	34	33	9	28	41	18	31
	(13.9)	(20.3)	(9.4)	(16.8)	(16.3)	(4.5)	(13.9)	(20.3)	(8.9)	(15.3)
c_E	29.2	35.8	13.4	29.2	35.8	13.4	29.2	35.8	13.4	29.2
	(14.5)	(17.7)	(6.6)	(14.5)	(17.7)	(6.6)	(14.5)	(17.7)	(6.6)	(14.5)
	-2.4	11.2	13.3	10.3	-5.3	-7.2	-2.4	11.2	10.6	3.7
	-1.180	5.556	6.576	5.103	-2.620	-3.544	-1.180	5.556	5.266	1.818

N	202	202	202	202	202	202	202	202	202	202
X2	2.5	17.8	2.8	0.9	4.7	5.7	1.6	1.5	2.3	2.2
Gvalue	2.5	18.3	2.5	0.9	4.7	5.8	1.6	1.4	2.1	2.3
Gv/N	1.231	9.052	1.251	0.444	2.327	2.850	0.799	0.711	1.045	1.140
Prob	0.47786	0.00038	0.47051	0.82618	0.19510	0.12409	0.65623	0.69692	0.54990	0.51211

5UTR:

	100	99	98	97	96	95	94	93	92	91
a_O	902	864	873	891	898	923	905	905	928	935
	(32.1)	(30.7)	(31.1)	(31.7)	(31.9)	(32.8)	(32.2)	(32.2)	(33.0)	(33.3)
a_E	995.8	995.8	995.8	995.8	995.8	995.8	995.8	995.8	995.8	995.8
	(35.4)	(35.4)	(35.4)	(35.4)	(35.4)	(35.4)	(35.4)	(35.4)	(35.4)	(35.4)
	-178.5	-245.3	-229.8	-198.2	-185.7	-140.2	-173.1	-173.1	-130.9	-117.8
	-6.350	-8.728	-8.175	-7.050	-6.605	-4.986	-6.157	-6.157	-4.656	-4.191
t_O	826	842	831	880	848	804	861	830	827	896
	(29.4)	(30.0)	(29.6)	(31.3)	(30.2)	(28.6)	(30.6)	(29.5)	(29.4)	(31.9)
t_E	870.6	870.6	870.6	870.6	870.6	870.6	870.6	870.6	870.6	870.6
	(31.0)	(31.0)	(31.0)	(31.0)	(31.0)	(31.0)	(31.0)	(31.0)	(31.0)	(31.0)
	-86.9	-56.3	-77.4	18.8	-44.7	-128.0	-19.2	-79.3	-85.0	51.5
	-3.093	-2.004	-2.755	0.670	-1.589	-4.555	-0.682	-2.823	-3.026	1.831
g_O	581	560	602	532	582	598	544	588	544	504
	(20.7)	(19.9)	(21.4)	(18.9)	(20.7)	(21.3)	(19.4)	(20.9)	(19.4)	(17.9)
g_E	475.1	475.1	475.1	475.1	475.1	475.1	475.1	475.1	475.1	475.1
	(16.9)	(16.9)	(16.9)	(16.9)	(16.9)	(16.9)	(16.9)	(16.9)	(16.9)	(16.9)
	233.8	184.1	285.0	120.3	236.2	275.1	147.3	250.7	147.3	59.5
	8.316	6.548	10.137	4.279	8.401	9.786	5.239	8.917	5.239	2.115
c_O	502	545	505	508	483	486	501	488	512	476
	(17.9)	(19.4)	(18.0)	(18.1)	(17.2)	(17.3)	(17.8)	(17.4)	(18.2)	(16.9)
c_E	469.4	469.4	469.4	469.4	469.4	469.4	469.4	469.4	469.4	469.4
	(16.7)	(16.7)	(16.7)	(16.7)	(16.7)	(16.7)	(16.7)	(16.7)	(16.7)	(16.7)
	67.4	162.7	73.8	80.2	27.5	33.7	65.2	37.9	88.9	13.2
	2.396	5.788	2.624	2.854	0.979	1.200	2.320	1.347	3.162	0.471
N	2811	2811	2811	2811	2811	2811	2811	2811	2811	2811
X2	37.0	45.7	53.5	21.1	34.6	42.8	20.5	37.7	20.6	6.3
Gvalue	35.7	45.1	51.5	21.2	33.3	40.6	20.3	36.1	20.2	6.3
Gv/N	1.269	1.605	1.831	0.753	1.186	1.445	0.721	1.285	0.720	0.225
Prob	0.00000	0.00000	0.00000	0.00010	0.00000	0.00000	0.00015	0.00000	0.00015	0.09643

	90	89	88	87	86	85	84	83	82	81
a_O	879	943	862	884	876	926	885	884	883	919
	(31.3)	(33.5)	(30.7)	(31.4)	(31.2)	(32.9)	(31.5)	(31.4)	(31.4)	(32.7)
a_E	995.8	995.8	995.8	995.8	995.8	995.8	995.8	995.8	995.8	995.8
	(35.4)	(35.4)	(35.4)	(35.4)	(35.4)	(35.4)	(35.4)	(35.4)	(35.4)	(35.4)
	-219.3	-102.8	-248.8	-210.6	-224.6	-134.6	-208.8	-210.6	-212.3	-147.5
	-7.803	-3.656	-8.850	-7.491	-7.989	-4.788	-7.428	-7.491	-7.553	-5.248
t_O	856	801	869	835	865	884	877	875	872	798
	(30.5)	(28.5)	(30.9)	(29.7)	(30.8)	(31.4)	(31.2)	(31.1)	(31.0)	(28.4)
t_E	870.6	870.6	870.6	870.6	870.6	870.6	870.6	870.6	870.6	870.6
	(31.0)	(31.0)	(31.0)	(31.0)	(31.0)	(31.0)	(31.0)	(31.0)	(31.0)	(31.0)
	-29.0	-133.5	-3.3	-69.8	-11.2	26.9	12.8	8.7	2.7	-139.0
	-1.033	-4.751	-0.116	-2.483	-0.400	0.958	0.454	0.311	0.097	-4.946
g_O	565	554	569	550	574	536	569	543	555	579
	(20.1)	(19.7)	(20.2)	(19.6)	(20.4)	(19.1)	(20.2)	(19.3)	(19.7)	(20.6)
g_E	475.1	475.1	475.1	475.1	475.1	475.1	475.1	475.1	475.1	475.1
	(16.9)	(16.9)	(16.9)	(16.9)	(16.9)	(16.9)	(16.9)	(16.9)	(16.9)	(16.9)
	195.8	170.2	205.2	161.0	217.0	129.2	205.2	145.0	172.5	229.0
	6.964	6.054	7.299	5.726	7.720	4.597	7.299	5.159	6.136	8.145
c_O	511	513	511	542	496	465	480	509	501	515
	(18.2)	(18.2)	(18.2)	(19.3)	(17.6)	(16.5)	(17.1)	(18.1)	(17.8)	(18.3)
c_E	469.4	469.4	469.4	469.4	469.4	469.4	469.4	469.4	469.4	469.4
	(16.7)	(16.7)	(16.7)	(16.7)	(16.7)	(16.7)	(16.7)	(16.7)	(16.7)	(16.7)
	86.7	91.1	86.7	155.8	54.6	-8.8	21.4	82.4	65.2	95.4
	3.085	3.240	3.085	5.543	1.943	-0.314	0.761	2.931	2.320	3.395
N	2811	2811	2811	2811	2811	2811	2811	2811	2811	2811
X2	34.6	25.5	40.2	37.0	36.5	12.9	31.2	25.6	28.3	39.1
Gvalue	34.1	24.9	39.9	36.4	35.8	12.7	30.5	25.6	28.1	37.8
Gv/N	1.214	0.887	1.418	1.296	1.274	0.453	1.086	0.910	1.000	1.345
Prob	0.00000	0.00002	0.00000	0.00000	0.00000	0.00523	0.00000	0.00001	0.00000	0.00000

	80	79	78	77	76	75	74	73	72	71
a_O	900	927	891	936	937	899	947	937	932	890
	(32.0)	(33.0)	(31.7)	(33.3)	(33.3)	(32.0)	(33.7)	(33.3)	(33.2)	(31.7)
a_E	995.8	995.8	995.8	995.8	995.8	995.8	995.8	995.8	995.8	995.8
	(35.4)	(35.4)	(35.4)	(35.4)	(35.4)	(35.4)	(35.4)	(35.4)	(35.4)	(35.4)
	-182.1	-132.7	-198.2	-115.9	-114.1	-183.9	-95.2	-114.1	-123.4	-199.9
	-6.478	-4.722	-7.050	-4.125	-4.058	-6.541	-3.386	-4.058	-4.391	-7.113
t_O	878	816	846	865	844	860	838	835	861	870
	(31.2)	(29.0)	(30.1)	(30.8)	(30.0)	(30.6)	(29.8)	(29.7)	(30.6)	(30.9)
t_E	870.6	870.6	870.6	870.6	870.6	870.6	870.6	870.6	870.6	870.6
	(31.0)	(31.0)	(31.0)	(31.0)	(31.0)	(31.0)	(31.0)	(31.0)	(31.0)	(31.0)
	14.8	-105.8	-48.6	-11.2	-52.5	-21.1	-64.0	-69.8	-19.2	-1.3
	0.526	-3.763	-1.728	-0.400	-1.866	-0.752	-2.278	-2.483	-0.682	-0.045
g_O	536	589	572	546	537	546	569	536	522	563
	(19.1)	(21.0)	(20.3)	(19.4)	(19.1)	(19.4)	(20.2)	(19.1)	(18.6)	(20.0)
g_E	475.1	475.1	475.1	475.1	475.1	475.1	475.1	475.1	475.1	475.1
	(16.9)	(16.9)	(16.9)	(16.9)	(16.9)	(16.9)	(16.9)	(16.9)	(16.9)	(16.9)
	129.2	253.1	212.3	151.8	131.5	151.8	205.2	129.2	98.2	191.1
	4.597	9.003	7.552	5.401	4.677	5.401	7.299	4.597	3.494	6.797
c_O	497	479	502	464	493	506	457	503	496	488
	(17.7)	(17.0)	(17.9)	(16.5)	(17.5)	(18.0)	(16.3)	(17.9)	(17.6)	(17.4)
c_E	469.4	469.4	469.4	469.4	469.4	469.4	469.4	469.4	469.4	469.4
	(16.7)	(16.7)	(16.7)	(16.7)	(16.7)	(16.7)	(16.7)	(16.7)	(16.7)	(16.7)
	56.7	19.3	67.4	-10.8	48.3	75.9	-24.5	69.5	54.6	37.9
	2.018	0.688	2.396	-0.384	1.719	2.701	-0.872	2.472	1.943	1.347
N	2811	2811	2811	2811	2811	2811	2811	2811	2811	2811
X2	18.7	35.7	33.7	14.3	13.5	23.0	22.5	15.1	10.3	28.2
Gvalue	18.7	33.9	32.9	13.8	13.3	22.7	21.4	14.9	10.2	27.7
Gv/N	0.664	1.206	1.170	0.493	0.472	0.808	0.763	0.528	0.364	0.986
Prob	0.00032	0.00000	0.00000	0.00312	0.00411	0.00005	0.00009	0.00195	0.01662	0.00000

	70	69	68	67	66	65	64	63	62	61
a_O	871	931	930	962	869	922	917	889	899	876
	(31.0)	(33.1)	(33.1)	(34.2)	(30.9)	(32.8)	(32.6)	(31.6)	(32.0)	(31.2)
a_E	995.8	995.8	995.8	995.8	995.8	995.8	995.8	995.8	995.8	995.8
	(35.4)	(35.4)	(35.4)	(35.4)	(35.4)	(35.4)	(35.4)	(35.4)	(35.4)	(35.4)
	-233.3	-125.3	-127.2	-66.5	-236.7	-142.0	-151.2	-201.7	-183.9	-224.6
	-8.299	-4.458	-4.524	-2.364	-8.422	-5.052	-5.379	-7.176	-6.541	-7.989
t_O	936	835	859	803	877	848	837	873	837	891
	(33.3)	(29.7)	(30.6)	(28.6)	(31.2)	(30.2)	(29.8)	(31.1)	(29.8)	(31.7)
t_E	870.6	870.6	870.6	870.6	870.6	870.6	870.6	870.6	870.6	870.6

	(31.0)	(31.0)	(31.0)	(31.0)	(31.0)	(31.0)	(31.0)	(31.0)	(31.0)	(31.0)
	135.5	-69.8	-23.1	-129.9	12.8	-44.7	-66.0	4.7	-66.0	41.2
	4.821	-2.483	-0.822	-4.620	0.454	-1.589	-2.346	0.168	-2.346	1.466
g_O	542	582	548	589	593	530	555	563	563	525
	(19.3)	(20.7)	(19.5)	(21.0)	(21.1)	(18.9)	(19.7)	(20.0)	(20.0)	(18.7)
g_E	475.1	475.1	475.1	475.1	475.1	475.1	475.1	475.1	475.1	475.1
	(16.9)	(16.9)	(16.9)	(16.9)	(16.9)	(16.9)	(16.9)	(16.9)	(16.9)	(16.9)
	142.7	236.2	156.4	253.1	262.8	115.8	172.5	191.1	191.1	104.8
	5.078	8.401	5.563	9.003	9.350	4.121	6.136	6.797	6.797	3.728
c_O	462	463	474	457	472	511	502	486	512	519
	(16.4)	(16.5)	(16.9)	(16.3)	(16.8)	(18.2)	(17.9)	(17.3)	(18.2)	(18.5)
c_E	469.4	469.4	469.4	469.4	469.4	469.4	469.4	469.4	469.4	469.4
	(16.7)	(16.7)	(16.7)	(16.7)	(16.7)	(16.7)	(16.7)	(16.7)	(16.7)	(16.7)
	-14.7	-12.8	9.2	-24.5	5.2	86.7	67.4	33.7	88.9	104.2
	-0.524	-0.454	0.327	-0.872	0.183	3.085	2.396	1.200	3.162	3.707
N	2811	2811	2811	2811	2811	2811	2811	2811	2811	2811
X2	30.1	29.8	15.7	34.0	45.5	16.1	23.2	28.3	30.8	25.4
Gvalue	30.2	28.3	15.3	32.2	44.0	15.9	22.7	27.8	30.1	25.6
Gv/N	1.076	1.007	0.544	1.147	1.566	0.565	0.806	0.989	1.072	0.912
Prob	0.00000	0.00000	0.00158	0.00000	0.00000	0.00120	0.00005	0.00000	0.00000	0.00001

	60	59	58	57	56	55	54	53	52	51
a_O	851	867	884	877	918	898	862	863	879	911
	(30.3)	(30.8)	(31.4)	(31.2)	(32.7)	(31.9)	(30.7)	(30.7)	(31.3)	(32.4)
a_E	995.8	995.8	995.8	995.8	995.8	995.8	995.8	995.8	995.8	995.8
	(35.4)	(35.4)	(35.4)	(35.4)	(35.4)	(35.4)	(35.4)	(35.4)	(35.4)	(35.4)
	-267.5	-240.2	-210.6	-222.8	-149.4	-185.7	-248.8	-247.1	-219.3	-162.2
	-9.515	-8.544	-7.491	-7.927	-5.314	-6.605	-8.850	-8.789	-7.803	-5.769
t_O	929	882	826	878	845	882	904	877	892	866
	(33.0)	(31.4)	(29.4)	(31.2)	(30.1)	(31.4)	(32.2)	(31.2)	(31.7)	(30.8)
t_E	870.6	870.6	870.6	870.6	870.6	870.6	870.6	870.6	870.6	870.6
	(31.0)	(31.0)	(31.0)	(31.0)	(31.0)	(31.0)	(31.0)	(31.0)	(31.0)	(31.0)
	120.6	22.9	-86.9	14.8	-50.5	22.9	68.0	12.8	43.2	-9.2
	4.289	0.814	-3.093	0.526	-1.797	0.814	2.419	0.454	1.538	-0.329
g_O	544	572	564	548	530	532	540	574	546	526
	(19.4)	(20.3)	(20.1)	(19.5)	(18.9)	(18.9)	(19.2)	(20.4)	(19.4)	(18.7)
g_E	475.1	475.1	475.1	475.1	475.1	475.1	475.1	475.1	475.1	475.1
	(16.9)	(16.9)	(16.9)	(16.9)	(16.9)	(16.9)	(16.9)	(16.9)	(16.9)	(16.9)
	147.3	212.3	193.4	156.4	115.8	120.3	138.2	217.0	151.8	107.0
	5.239	7.552	6.881	5.563	4.121	4.279	4.917	7.720	5.401	3.807
c_O	487	490	537	508	518	499	505	497	494	508
	(17.3)	(17.4)	(19.1)	(18.1)	(18.4)	(17.8)	(18.0)	(17.7)	(17.6)	(18.1)
c_E	469.4	469.4	469.4	469.4	469.4	469.4	469.4	469.4	469.4	469.4
	(16.7)	(16.7)	(16.7)	(16.7)	(16.7)	(16.7)	(16.7)	(16.7)	(16.7)	(16.7)
	35.8	42.0	144.4	80.2	102.0	61.0	73.8	56.7	50.4	80.2
	1.273	1.495	5.138	2.854	3.629	2.169	2.624	2.018	1.793	2.854
N	2811	2811	2811	2811	2811	2811	2811	2811	2811	2811
X2	35.6	37.5	41.2	28.6	18.2	18.4	30.8	40.0	26.1	15.9
Gvalue	36.2	37.0	40.3	28.6	18.0	18.5	31.2	39.5	26.1	15.8
Gv/N	1.287	1.316	1.435	1.016	0.639	0.657	1.110	1.404	0.930	0.562
Prob	0.00000	0.00000	0.00000	0.00000	0.00045	0.00035	0.00000	0.00000	0.00001	0.00124

	50	49	48	47	46	45	44	43	42	41
a_O	882	829	918	899	886	894	898	937	926	951
	(31.4)	(29.5)	(32.7)	(32.0)	(31.5)	(31.8)	(31.9)	(33.3)	(32.9)	(33.8)
a_E	995.8	995.8	995.8	995.8	995.8	995.8	995.8	995.8	995.8	995.8
	(35.4)	(35.4)	(35.4)	(35.4)	(35.4)	(35.4)	(35.4)	(35.4)	(35.4)	(35.4)
	-214.1	-304.0	-149.4	-183.9	-207.0	-192.8	-185.7	-114.1	-134.6	-87.6
	-7.616	-10.813	-5.314	-6.541	-7.365	-6.860	-6.605	-4.058	-4.788	-3.115
t_O	879	918	828	884	888	872	866	859	869	850
	(31.3)	(32.7)	(29.5)	(31.4)	(31.6)	(31.0)	(30.8)	(30.6)	(30.9)	(30.2)
t_E	870.6	870.6	870.6	870.6	870.6	870.6	870.6	870.6	870.6	870.6
	(31.0)	(31.0)	(31.0)	(31.0)	(31.0)	(31.0)	(31.0)	(31.0)	(31.0)	(31.0)
	16.8	97.3	-83.2	26.9	35.1	2.7	-9.2	-23.1	-3.3	-40.8
	0.598	3.460	-2.958	0.958	1.248	0.097	-0.329	-0.822	-0.116	-1.451
g_O	533	563	543	526	531	541	561	538	540	548
	(19.0)	(20.0)	(19.3)	(18.7)	(18.9)	(19.2)	(20.0)	(19.1)	(19.2)	(19.5)
g_E	475.1	475.1	475.1	475.1	475.1	475.1	475.1	475.1	475.1	475.1
	(16.9)	(16.9)	(16.9)	(16.9)	(16.9)	(16.9)	(16.9)	(16.9)	(16.9)	(16.9)
	122.5	191.1	145.0	107.0	118.1	140.5	186.4	133.7	138.2	156.4
	4.359	6.797	5.159	3.807	4.200	4.998	6.631	4.757	4.917	5.563
c_O	517	501	522	502	506	504	486	477	476	462
	(18.4)	(17.8)	(18.6)	(17.9)	(18.0)	(17.9)	(17.3)	(17.0)	(16.9)	(16.4)
c_E	469.4	469.4	469.4	469.4	469.4	469.4	469.4	469.4	469.4	469.4
	(16.7)	(16.7)	(16.7)	(16.7)	(16.7)	(16.7)	(16.7)	(16.7)	(16.7)	(16.7)
	99.8	65.2	110.8	67.4	75.9	71.6	33.7	15.3	13.2	-14.7
	3.551	2.320	3.942	2.396	2.701	2.548	1.200	0.543	0.471	-0.524
N	2811	2811	2811	2811	2811	2811	2811	2811	2811	2811
X2	25.0	48.9	23.8	17.3	21.9	22.1	25.7	12.1	13.8	13.8
Gvalue	25.1	49.6	23.3	17.4	22.0	22.0	25.2	11.8	13.6	13.3
Gv/N	0.891	1.764	0.829	0.619	0.784	0.783	0.897	0.420	0.483	0.473
Prob	0.00002	0.00000	0.00003	0.00058	0.00006	0.00006	0.00001	0.00812	0.00353	0.00402

	40	39	38	37	36	35	34	33	32	31
a_O	941	933	952	956	929	987	980	977	999	994
	(33.5)	(33.2)	(33.9)	(34.0)	(33.0)	(35.1)	(34.9)	(34.8)	(35.5)	(35.4)
a_E	995.8	995.8	995.8	995.8	995.8	995.8	995.8	995.8	995.8	995.8
	(35.4)	(35.4)	(35.4)	(35.4)	(35.4)	(35.4)	(35.4)	(35.4)	(35.4)	(35.4)
	-106.5	-121.6	-85.7	-78.0	-129.0	-17.5	-31.4	-37.3	6.4	-3.6
	-3.790	-4.325	-3.047	-2.775	-4.590	-0.624	-1.116	-1.325	0.228	-0.128
t_O	871	890	918	888	904	845	936	949	890	934
	(31.0)	(31.7)	(32.7)	(31.6)	(32.2)	(30.1)	(33.3)	(33.8)	(31.7)	(33.2)
t_E	870.6	870.6	870.6	870.6	870.6	870.6	870.6	870.6	870.6	870.6
	(31.0)	(31.0)	(31.0)	(31.0)	(31.0)	(31.0)	(31.0)	(31.0)	(31.0)	(31.0)
	0.7	39.2	97.3	35.1	68.0	-50.5	135.5	163.6	39.2	131.2
	0.026	1.393	3.460	1.248	2.419	-1.797	4.821	5.819	1.393	4.668
g_O	551	544	514	496	506	512	464	468	459	405
	(19.6)	(19.4)	(18.3)	(17.6)	(18.0)	(18.2)	(16.5)	(16.6)	(16.3)	(14.4)
g_E	475.1	475.1	475.1	475.1	475.1	475.1	475.1	475.1	475.1	475.1
	(16.9)	(16.9)	(16.9)	(16.9)	(16.9)	(16.9)	(16.9)	(16.9)	(16.9)	(16.9)
	163.3	147.3	80.8	42.6	63.7	76.5	-22.0	-14.1	-31.7	-129.4
	5.808	5.239	2.876	1.517	2.266	2.723	-0.782	-0.503	-1.128	-4.602
c_O	448	444	427	471	472	467	431	417	463	478
	(15.9)	(15.8)	(15.2)	(16.8)	(16.8)	(16.6)	(15.3)	(14.8)	(16.5)	(17.0)
c_E	469.4	469.4	469.4	469.4	469.4	469.4	469.4	469.4	469.4	469.4
	(16.7)	(16.7)	(16.7)	(16.7)	(16.7)	(16.7)	(16.7)	(16.7)	(16.7)	(16.7)

	-41.9	-49.5	-80.9	3.1	5.2	-4.8	-73.6	-98.8	-12.8	17.3
	-1.489	-1.759	-2.878	0.112	0.183	-0.172	-2.619	-3.514	-0.454	0.615

N	2811	2811	2811	2811	2811	2811	2811	2811	2811	2811
X2	16.1	15.8	11.5	2.9	7.8	3.7	8.6	13.4	1.1	15.1
Gvalue	15.6	15.4	11.5	2.9	7.8	3.6	8.5	13.4	1.1	15.6
Gv/N	0.554	0.548	0.411	0.102	0.278	0.130	0.304	0.477	0.038	0.554
Prob	0.00138	0.00150	0.00914	0.41278	0.04975	0.30266	0.03609	0.00384	0.78192	0.00139

	30	29	28	27	26	25	24	23	22	21
a_O	997	1001	934	981	973	1017	975	1081	1076	1138
	(35.5)	(35.6)	(33.2)	(34.9)	(34.6)	(36.2)	(34.7)	(38.5)	(38.3)	(40.5)
a_E	995.8	995.8	995.8	995.8	995.8	995.8	995.8	995.8	995.8	995.8
	(35.4)	(35.4)	(35.4)	(35.4)	(35.4)	(35.4)	(35.4)	(35.4)	(35.4)	(35.4)
	2.4	10.4	-119.7	-29.4	-45.1	42.8	-41.2	177.5	166.7	303.8
	0.085	0.370	-4.258	-1.046	-1.604	1.524	-1.465	6.314	5.930	10.807
t_O	959	927	993	967	939	989	999	924	942	902
	(34.1)	(33.0)	(35.3)	(34.4)	(33.4)	(35.2)	(35.5)	(32.9)	(33.5)	(32.1)
t_E	870.6	870.6	870.6	870.6	870.6	870.6	870.6	870.6	870.6	870.6
	(31.0)	(31.0)	(31.0)	(31.0)	(31.0)	(31.0)	(31.0)	(31.0)	(31.0)	(31.0)
	185.4	116.3	261.2	203.0	142.0	252.1	274.8	109.9	148.4	63.8
	6.596	4.137	9.291	7.222	5.050	8.970	9.775	3.911	5.280	2.271
g_O	402	455	453	462	446	400	425	408	379	350
	(14.3)	(16.2)	(16.1)	(16.4)	(15.9)	(14.2)	(15.1)	(14.5)	(13.5)	(12.5)
g_E	475.1	475.1	475.1	475.1	475.1	475.1	475.1	475.1	475.1	475.1
	(16.9)	(16.9)	(16.9)	(16.9)	(16.9)	(16.9)	(16.9)	(16.9)	(16.9)	(16.9)
	-134.4	-39.4	-43.2	-25.9	-56.4	-137.7	-94.8	-124.3	-171.3	-214.0
	-4.780	-1.401	-1.537	-0.921	-2.008	-4.898	-3.371	-4.422	-6.096	-7.611
c_O	453	428	431	401	453	405	412	398	414	421
	(16.1)	(15.2)	(15.3)	(14.3)	(16.1)	(14.4)	(14.7)	(14.2)	(14.7)	(15.0)
c_E	469.4	469.4	469.4	469.4	469.4	469.4	469.4	469.4	469.4	469.4
	(16.7)	(16.7)	(16.7)	(16.7)	(16.7)	(16.7)	(16.7)	(16.7)	(16.7)	(16.7)
	-32.3	-79.1	-73.6	-126.4	-32.3	-119.6	-107.5	-131.4	-104.0	-91.7
	-1.148	-2.814	-2.619	-4.495	-1.148	-4.254	-3.825	-4.674	-3.701	-3.261

N	2811	2811	2811	2811	2811	2811	2811	2811	2811	2811
X2	20.8	8.2	25.2	21.2	8.3	37.3	31.7	30.9	38.3	59.4
Gvalue	21.1	8.2	24.6	21.4	8.2	37.7	31.3	31.7	39.7	62.0
Gv/N	0.752	0.293	0.877	0.761	0.290	1.341	1.114	1.129	1.413	2.205
Prob	0.00010	0.04143	0.00002	0.00009	0.04274	0.00000	0.00000	0.00000	0.00000	0.00000

	20	19	18	17	16	15	14	13	12	11
a_O	1222	1206	1162	1164	1167	1247	1518	1719	1610	1470
	(43.5)	(42.9)	(41.3)	(41.4)	(41.5)	(44.4)	(54.0)	(61.2)	(57.3)	(52.3)
a_E	995.8	995.8	995.8	995.8	995.8	995.8	995.8	995.8	995.8	995.8
	(35.4)	(35.4)	(35.4)	(35.4)	(35.4)	(35.4)	(35.4)	(35.4)	(35.4)	(35.4)
	500.3	461.9	358.7	363.3	370.3	561.0	1280.0	1877.0	1547.0	1145.0
	17.797	16.433	12.761	12.925	13.172	19.958	45.534	66.772	55.034	40.734
t_O	807	859	878	931	994	1037	817	662	673	719
	(28.7)	(30.6)	(31.2)	(33.1)	(35.4)	(36.9)	(29.1)	(23.6)	(23.9)	(25.6)
t_E	870.6	870.6	870.6	870.6	870.6	870.6	870.6	870.6	870.6	870.6
	(31.0)	(31.0)	(31.0)	(31.0)	(31.0)	(31.0)	(31.0)	(31.0)	(31.0)	(31.0)
	-122.5	-23.1	14.8	124.8	263.4	362.7	-103.9	-362.7	-346.6	-275.2
	-4.358	-0.822	0.526	4.440	9.372	12.902	-3.696	-12.904	-12.329	-9.789
g_O	292	216	213	206	164	166	168	144	179	207
	(10.4)	(7.7)	(7.6)	(7.3)	(5.8)	(5.9)	(6.0)	(5.1)	(6.4)	(7.4)
g_E	475.1	475.1	475.1	475.1	475.1	475.1	475.1	475.1	475.1	475.1
	(16.9)	(16.9)	(16.9)	(16.9)	(16.9)	(16.9)	(16.9)	(16.9)	(16.9)	(16.9)
	-284.3	-340.5	-341.8	-344.3	-348.9	-349.1	-349.3	-343.8	-349.5	-344.0
	-10.114	-12.115	-12.159	-12.249	-12.412	-12.420	-12.427	-12.231	-12.433	-12.237
c_O	490	530	558	510	486	361	308	286	349	415
	(17.4)	(18.9)	(19.9)	(18.1)	(17.3)	(12.8)	(11.0)	(10.2)	(12.4)	(14.8)
c_E	469.4	469.4	469.4	469.4	469.4	469.4	469.4	469.4	469.4	469.4
	(16.7)	(16.7)	(16.7)	(16.7)	(16.7)	(16.7)	(16.7)	(16.7)	(16.7)	(16.7)
	42.0	128.6	192.9	84.6	33.7	-189.6	-259.6	-283.4	-206.9	-102.3
	1.495	4.576	6.862	3.008	1.200	-6.746	-9.235	-10.083	-7.361	-3.639

N	2811	2811	2811	2811	2811	2811	2811	2811	2811	2811
X2	127.5	193.7	189.1	188.5	251.2	321.3	531.2	877.7	639.1	409.8
Gvalue	135.5	226.9	224.6	228.4	318.5	384.9	567.2	887.0	644.0	423.6
Gv/N	4.820	8.072	7.990	8.124	11.331	13.693	20.176	31.554	22.911	15.069
Prob	0.00000	0.00000	0.00000	0.00000	0.00000	0.00000	0.00000	0.00000	0.00000	0.00000

	10	9	8	7	6	5	4	3	2	1
a_O	1289	1230	1233	1257	1421	1342	1236	1404	1045	760
	(45.9)	(43.8)	(43.9)	(44.7)	(50.6)	(47.7)	(44.0)	(49.9)	(37.2)	(27.0)
a_E	995.8	995.8	995.8	995.8	995.8	995.8	995.8	995.8	995.8	995.8
	(35.4)	(35.4)	(35.4)	(35.4)	(35.4)	(35.4)	(35.4)	(35.4)	(35.4)	(35.4)
	665.3	519.6	526.9	585.6	1010.5	800.8	534.2	964.6	100.8	-410.8
	23.668	18.484	18.743	20.832	35.948	28.488	19.002	34.316	3.585	-14.612
t_O	852	874	891	930	748	701	729	521	913	1382
	(30.3)	(31.1)	(31.7)	(33.1)	(26.6)	(24.9)	(25.9)	(18.5)	(32.5)	(49.2)
t_E	870.6	870.6	870.6	870.6	870.6	870.6	870.6	870.6	870.6	870.6
	(31.0)	(31.0)	(31.0)	(31.0)	(31.0)	(31.0)	(31.0)	(31.0)	(31.0)	(31.0)
	-36.9	6.7	41.2	122.7	-227.1	-303.8	-258.9	-535.0	86.8	1277.1
	-1.312	0.240	1.466	4.364	-8.080	-10.809	-9.209	-19.034	3.086	45.434
g_O	195	263	293	254	296	347	436	537	365	238
	(6.9)	(9.4)	(10.4)	(9.0)	(10.5)	(12.3)	(15.5)	(19.1)	(13.0)	(8.5)
g_E	475.1	475.1	475.1	475.1	475.1	475.1	475.1	475.1	475.1	475.1
	(16.9)	(16.9)	(16.9)	(16.9)	(16.9)	(16.9)	(16.9)	(16.9)	(16.9)	(16.9)
	-347.3	-311.1	-283.3	-318.1	-280.1	-218.1	-74.9	131.5	-192.5	-329.1
	-12.356	-11.067	-10.078	-11.318	-9.966	-7.759	-2.666	4.677	-6.848	-11.706
c_O	475	444	394	370	346	421	410	349	488	431
	(16.9)	(15.8)	(14.0)	(13.2)	(12.3)	(15.0)	(14.6)	(12.4)	(17.4)	(15.3)
c_E	469.4	469.4	469.4	469.4	469.4	469.4	469.4	469.4	469.4	469.4
	(16.7)	(16.7)	(16.7)	(16.7)	(16.7)	(16.7)	(16.7)	(16.7)	(16.7)	(16.7)
	11.2	-49.5	-138.0	-176.1	-211.1	-91.7	-111.0	-206.9	37.9	-73.6
	0.399	-1.759	-4.910	-6.266	-7.510	-3.261	-3.949	-7.361	1.347	-2.619

N	2811	2811	2811	2811	2811	2811	2811	2811	2811	2811
X2	251.9	151.2	138.9	196.5	298.8	193.0	91.7	346.7	30.8	477.7
Gvalue	292.3	165.8	146.8	214.0	292.1	187.2	89.4	354.1	32.9	463.7
Gv/N	10.399	5.898	5.221	7.613	10.392	6.659	3.179	12.598	1.171	16.496
Prob	0.00000	0.00000	0.00000	0.00000	0.00000	0.00000	0.00000	0.00000	0.00000	0.00000

CDS_INITIAL:

	1	2	3	4	5	6	7	8	9	10

a_O	2589	0	0	1782	1642	1442	1534	1351	1314	1333
	(92.1)	(0.0)	(0.0)	(63.4)	(58.4)	(51.3)	(54.6)	(48.1)	(46.7)	(47.4)
a_E	844.6	937.4	684.1	844.6	937.4	684.1	844.6	937.4	684.1	844.6
	(30.0)	(33.3)	(24.3)	(30.0)	(33.3)	(24.3)	(30.0)	(33.3)	(24.3)	(30.0)
	5800.3	0.0	0.0	2661.1	1841.0	2150.6	1831.0	987.6	1715.4	1216.6
	206.344	0.000	0.000	94.666	65.492	76.507	65.136	35.134	61.025	43.281
t_O	142	2811	0	239	480	703	382	677	606	584
	(5.1)	(100.0)	(0.0)	(8.5)	(17.1)	(25.0)	(13.6)	(24.1)	(21.6)	(20.8)
t_E	539.7	844.3	843.2	539.7	844.3	843.2	539.7	844.3	843.2	539.7
	(19.2)	(30.0)	(30.0)	(19.2)	(30.0)	(30.0)	(19.2)	(30.0)	(30.0)	(19.2)
	-379.2	6762.0	0.0	-389.3	-542.1	-255.7	-264.0	-299.0	-400.4	92.2
	-13.489	240.556	0.000	-13.851	-19.286	-9.097	-9.393	-10.637	-14.243	3.278
g_O	80	0	2811	583	311	369	475	315	516	486
	(2.8)	(0.0)	(100.0)	(20.7)	(11.1)	(13.1)	(16.9)	(11.2)	(18.4)	(17.3)
g_E	906.2	455.3	648.3	906.2	455.3	648.3	906.2	455.3	648.3	906.2
	(32.2)	(16.2)	(23.1)	(32.2)	(16.2)	(23.1)	(32.2)	(16.2)	(23.1)	(32.2)
	-388.4	0.0	8247.1	-514.4	-237.0	-415.9	-613.7	-232.0	-235.6	-605.7
	-13.816	0.000	293.387	-18.298	-8.433	-14.796	-21.832	-8.255	-8.380	-21.546
c_O	0	0	0	207	378	297	420	468	375	408
	(0.0)	(0.0)	(0.0)	(7.4)	(13.4)	(10.6)	(14.9)	(16.6)	(13.3)	(14.5)
c_E	520.5	574.1	635.4	520.5	574.1	635.4	520.5	574.1	635.4	520.5
	(18.5)	(20.4)	(22.6)	(18.5)	(20.4)	(22.6)	(18.5)	(20.4)	(22.6)	(18.5)
	0.0	0.0	0.0	-381.7	-315.9	-451.7	-180.2	-191.2	-395.5	-198.7
	0.000	0.000	0.000	-13.580	-11.237	-16.071	-6.410	-6.801	-14.069	-7.068
N	2811	2811	2811	2811	2811	2811	2811	2811	2811	2811
X2	5169.8	6547.9	9377.3	1512.1	799.5	1163.6	833.5	278.5	780.5	505.3
Gvalue	5032.8	6762.0	8247.1	1375.6	745.9	1027.2	773.1	265.4	684.0	504.4
Gv/N	179.039	240.556	293.387	48.938	26.535	36.544	27.502	9.441	24.333	17.945
Prob	1.00000	1.00000	1.00000	0.00000	0.00000	0.00000	0.00000	0.00000	0.00000	0.00000

	11	12	13	14	15	16	17	18	19	20
a_O	1102	1262	1267	1034	1106	1072	906	1093	1146	835
	(39.2)	(44.9)	(45.1)	(36.8)	(39.3)	(38.1)	(32.2)	(38.9)	(40.8)	(29.7)
a_E	937.4	684.1	844.6	937.4	684.1	844.6	937.4	684.1	844.6	937.4
	(33.3)	(24.3)	(30.0)	(33.3)	(24.3)	(30.0)	(33.3)	(24.3)	(30.0)	(33.3)
	356.6	1545.6	1027.7	202.9	1062.7	511.2	-61.7	1024.4	699.5	-193.1
	12.686	54.985	36.560	7.218	37.805	18.186	-2.195	36.441	24.884	-6.871
t_O	1069	829	623	1137	914	775	1202	878	687	1208
	(38.0)	(29.5)	(22.2)	(40.4)	(32.5)	(27.6)	(42.8)	(31.2)	(24.4)	(43.0)
t_E	844.3	843.2	539.7	844.3	843.2	539.7	844.3	843.2	539.7	844.3
	(30.0)	(30.0)	(19.2)	(30.0)	(30.0)	(19.2)	(30.0)	(30.0)	(19.2)	(30.0)
	504.5	-28.2	178.9	676.8	147.4	560.9	849.2	71.0	331.6	865.4
	17.947	-1.003	6.363	24.078	5.242	19.953	30.209	2.525	11.796	30.787
g_O	250	335	530	268	390	548	289	372	504	281
	(8.9)	(11.9)	(18.9)	(9.5)	(13.9)	(19.5)	(10.3)	(13.2)	(17.9)	(10.0)
g_E	455.3	648.3	906.2	455.3	648.3	906.2	455.3	648.3	906.2	455.3
	(16.2)	(23.1)	(32.2)	(16.2)	(23.1)	(32.2)	(16.2)	(23.1)	(32.2)	(16.2)
	-299.7	-442.4	-568.6	-284.0	-396.4	-551.3	-262.7	-413.3	-591.4	-271.2
	-10.662	-15.737	-20.228	-10.104	-14.102	-19.613	-9.345	-14.702	-21.040	-9.647
c_O	390	385	391	372	401	416	414	468	474	487
	(13.9)	(13.7)	(13.9)	(13.2)	(14.3)	(14.8)	(14.7)	(16.6)	(16.9)	(17.3)
c_E	574.1	635.4	520.5	574.1	635.4	520.5	574.1	635.4	520.5	574.1
	(20.4)	(22.6)	(18.5)	(20.4)	(22.6)	(18.5)	(20.4)	(22.6)	(18.5)	(20.4)
	-301.5	-385.8	-223.7	-322.8	-369.1	-186.4	-270.6	-286.2	-88.7	-160.2
	-10.727	-13.724	-7.957	-11.482	-13.132	-6.632	-9.628	-10.182	-3.155	-5.698
N	2811	2811	2811	2811	2811	2811	2811	2811	2811	2811
X2	240.3	738.5	412.6	259.6	455.5	326.4	257.9	407.7	330.5	247.8
Gvalue	259.9	689.3	414.3	272.9	444.5	334.4	254.2	395.9	351.0	240.9
Gv/N	9.244	24.522	14.737	9.709	15.812	11.894	9.042	14.083	12.486	8.571
Prob	0.00000	0.00000	0.00000	0.00000	0.00000	0.00000	0.00000	0.00000	0.00000	0.00000

	21	22	23	24	25	26	27	28	29	30
a_O	1033	978	753	898	897	728	800	817	673	797
	(36.7)	(34.8)	(26.8)	(31.9)	(31.9)	(25.9)	(28.5)	(29.1)	(23.9)	(28.4)
a_E	684.1	844.6	937.4	684.1	844.6	937.4	684.1	844.6	937.4	684.1
	(24.3)	(30.0)	(33.3)	(24.3)	(30.0)	(33.3)	(24.3)	(30.0)	(33.3)	(24.3)
	851.5	286.9	-329.8	488.7	108.0	-368.1	250.4	-54.3	-446.0	243.5
	30.291	10.206	-11.734	17.384	3.843	-13.093	8.909	-1.930	-15.866	8.663
t_O	815	699	1194	850	672	1089	866	698	1184	858
	(29.0)	(24.9)	(42.5)	(30.2)	(23.9)	(38.7)	(30.8)	(24.8)	(42.1)	(30.5)
t_E	843.2	539.7	844.3	843.2	539.7	844.3	843.2	539.7	844.3	843.2
	(30.0)	(19.2)	(30.0)	(30.0)	(19.2)	(30.0)	(30.0)	(19.2)	(30.0)	(30.0)
	-55.5	361.6	827.6	13.6	294.7	554.3	46.2	359.1	800.7	29.8
	-1.974	12.863	29.440	0.485	10.483	19.719	1.643	12.774	28.485	1.061
g_O	461	625	356	503	715	408	567	770	430	554
	(16.4)	(22.2)	(12.7)	(17.9)	(25.4)	(14.5)	(20.2)	(27.4)	(15.3)	(19.7)
g_E	648.3	906.2	455.3	648.3	906.2	455.3	648.3	906.2	455.3	648.3
	(23.1)	(32.2)	(16.2)	(23.1)	(32.2)	(16.2)	(23.1)	(32.2)	(16.2)	(23.1)
	-314.4	-464.5	-175.1	-255.3	-339.0	-89.5	-152.0	-250.9	-49.1	-174.2
	-11.183	-16.523	-6.230	-9.082	-12.058	-3.182	-5.406	-8.926	-1.747	-6.196
c_O	502	509	508	560	527	586	578	526	524	602
	(17.9)	(18.1)	(18.1)	(19.9)	(18.7)	(20.8)	(20.6)	(18.7)	(18.6)	(21.4)
c_E	635.4	520.5	574.1	635.4	520.5	574.1	635.4	520.5	574.1	635.4
	(22.6)	(18.5)	(20.4)	(22.6)	(18.5)	(20.4)	(22.6)	(18.5)	(20.4)	(22.6)
	-236.6	-22.7	-124.2	-141.5	13.1	24.1	-109.4	11.1	-95.6	-65.0
	-8.416	-0.808	-4.418	-5.032	0.467	0.859	-3.893	0.395	-3.401	-2.312
N	2811	2811	2811	2811	2811	2811	2811	2811	2811	2811
X2	261.0	155.6	210.3	108.5	76.1	122.8	35.6	67.9	217.0	34.4
Gvalue	245.0	161.3	198.4	105.5	76.9	120.9	35.2	65.0	210.0	34.2
Gv/N	8.717	5.739	7.058	3.754	2.735	4.303	1.253	2.313	7.471	1.216
Prob	0.00000	0.00000	0.00000	0.00000	-0.00000	0.00000	0.00000	0.00000	0.00000	0.00000

	31	32	33	34	35	36	37	38	39	40
a_O	817	647	750	770	620	697	783	626	708	820
	(29.1)	(23.0)	(26.7)	(27.4)	(22.1)	(24.8)	(27.9)	(22.3)	(25.2)	(29.2)
a_E	844.6	937.4	684.1	844.6	937.4	684.1	844.6	937.4	684.1	844.6
	(30.0)	(33.3)	(24.3)	(30.0)	(33.3)	(24.3)	(30.0)	(33.3)	(24.3)	(30.0)
	-54.3	-479.7	138.0	-142.4	-512.6	26.1	-118.6	-505.5	48.7	-48.4
	-1.930	-17.066	4.909	-5.065	-18.234	0.927	-4.218	-17.982	1.731	-1.723
t_O	685	1197	791	630	1163	864	632	1111	834	626
	(24.4)	(42.6)	(28.1)	(22.4)	(41.4)	(30.7)	(22.5)	(39.5)	(29.7)	(22.3)
t_E	539.7	844.3	843.2	539.7	844.3	843.2	539.7	844.3	843.2	539.7
	(19.2)	(30.0)	(30.0)	(19.2)	(30.0)	(30.0)	(19.2)	(30.0)	(30.0)	(19.2)
	326.6	835.7	-101.1	194.9	744.9	42.1	199.6	609.9	-18.3	185.7
	11.620	29.728	-3.598	6.935	26.499	1.497	7.100	21.699	-0.652	6.607

g_O	759	419	601	820	439	629	843	466	640	863
	(27.0)	(14.9)	(21.4)	(29.2)	(15.6)	(22.4)	(30.0)	(16.6)	(22.8)	(30.7)
g_E	906.2	455.3	648.3	906.2	455.3	648.3	906.2	455.3	648.3	906.2
	(32.2)	(16.2)	(23.1)	(32.2)	(16.2)	(23.1)	(32.2)	(16.2)	(23.1)	(32.2)
	-269.2	-69.6	-91.1	-164.0	-32.0	-38.0	-122.0	21.7	-16.5	-84.4
	-9.575	-2.475	-3.240	-5.835	-1.137	-1.353	-4.339	0.772	-0.587	-3.002
c_O	550	548	669	591	589	621	553	608	629	502
	(19.6)	(19.5)	(23.8)	(21.0)	(21.0)	(22.1)	(19.7)	(21.6)	(22.4)	(17.9)
c_E	520.5	574.1	635.4	520.5	574.1	635.4	520.5	574.1	635.4	520.5
	(18.5)	(20.4)	(22.6)	(18.5)	(20.4)	(22.6)	(18.5)	(20.4)	(22.6)	(18.5)
	60.7	-50.9	69.0	150.2	30.3	-28.5	67.0	69.9	-12.7	-36.3
	2.159	-1.811	2.453	5.343	1.077	-1.012	2.385	2.486	-0.453	-1.291
N	2811	2811	2811	2811	2811	2811	2811	2811	2811	2811
X2	65.6	241.4	14.8	39.5	228.7	1.7	26.7	189.9	1.1	17.2
Gvalue	63.9	235.4	14.7	38.8	230.7	1.7	26.1	196.1	1.1	16.6
Gv/N	2.274	8.376	0.525	1.379	8.205	0.059	0.928	6.974	0.039	0.591
Prob	0.00000	0.00000	0.00205	0.00000	0.00000	0.64597	0.00001	0.00000	0.77736	0.00085

	41	42	43	44	45	46	47	48	49	50
a_O	660	711	782	701	749	776	702	763	803	681
	(23.5)	(25.3)	(27.8)	(24.9)	(26.6)	(27.6)	(25.0)	(27.1)	(28.6)	(24.2)
a_E	937.4	684.1	844.6	937.4	684.1	844.6	937.4	684.1	844.6	937.4
	(33.3)	(24.3)	(30.0)	(33.3)	(24.3)	(30.0)	(33.3)	(24.3)	(30.0)	(33.3)
	-463.1	54.9	-120.4	-407.4	135.8	-131.4	-406.0	166.6	-81.1	-435.2
	-16.475	1.952	-4.283	-14.493	4.831	-4.676	-14.442	5.927	-2.884	-15.482
t_O	1120	849	589	1038	823	637	1006	835	663	1039
	(39.8)	(30.2)	(21.0)	(36.9)	(29.3)	(22.7)	(35.8)	(29.7)	(23.6)	(37.0)
t_E	844.3	843.2	539.7	844.3	843.2	539.7	844.3	843.2	539.7	844.3
	(30.0)	(30.0)	(19.2)	(30.0)	(30.0)	(19.2)	(30.0)	(30.0)	(19.2)	(30.0)
	633.0	11.6	103.0	428.8	-39.9	211.2	352.6	-16.4	272.9	431.2
	22.517	0.413	3.664	15.253	-1.421	7.513	12.542	-0.582	9.707	15.339
g_O	443	609	881	469	648	891	472	630	839	499
	(15.8)	(21.7)	(31.3)	(16.7)	(23.1)	(31.7)	(16.8)	(22.4)	(29.8)	(17.8)
g_E	455.3	648.3	906.2	455.3	648.3	906.2	455.3	648.3	906.2	455.3
	(16.2)	(23.1)	(32.2)	(16.2)	(23.1)	(32.2)	(16.2)	(23.1)	(32.2)	(16.2)
	-24.2	-76.2	-49.8	27.9	-0.6	-30.2	34.1	-36.1	-129.4	91.5
	-0.861	-2.710	-1.771	0.991	-0.022	-1.076	1.212	-1.284	-4.602	3.256
c_O	588	642	559	603	591	507	631	583	506	592
	(20.9)	(22.8)	(19.9)	(21.5)	(21.0)	(18.0)	(22.4)	(20.7)	(18.0)	(21.1)
c_E	574.1	635.4	520.5	574.1	635.4	520.5	574.1	635.4	520.5	574.1
	(20.4)	(22.6)	(18.5)	(20.4)	(22.6)	(18.5)	(20.4)	(22.6)	(18.5)	(20.4)
	28.2	13.3	79.8	59.3	-85.6	-26.6	119.4	-100.3	-28.6	36.5
	1.004	0.473	2.840	2.111	-3.046	-0.946	4.247	-3.570	-1.016	1.297
N	2811	2811	2811	2811	2811	2811	2811	2811	2811	2811
X2	172.8	3.6	12.7	105.9	9.7	23.7	96.3	14.0	35.6	119.8
Gvalue	173.9	3.6	12.6	108.6	9.6	22.9	100.0	13.8	33.8	124.0
Gv/N	6.185	0.128	0.449	3.863	0.343	0.815	3.558	0.492	1.204	4.411
Prob	0.00000	0.30980	0.00553	0.00000	0.02190	0.00004	0.00000	0.00316	0.00000	0.00000

	51	52	53	54	55	56	57	58	59	60
a_O	714	796	723	696	713	751	722	758	801	734
	(25.4)	(28.3)	(25.7)	(24.8)	(25.4)	(26.7)	(25.7)	(27.0)	(28.5)	(26.1)
a_E	684.1	844.6	937.4	684.1	844.6	937.4	684.1	844.6	937.4	684.1
	(24.3)	(30.0)	(33.3)	(24.3)	(30.0)	(33.3)	(24.3)	(30.0)	(33.3)	(24.3)
	61.1	-94.3	-375.5	24.0	-241.5	-333.0	77.9	-164.0	-251.9	103.4
	2.174	-3.355	-13.358	0.855	-8.591	-11.845	2.771	-5.833	-8.960	3.678
t_O	848	643	951	866	654	902	861	658	869	847
	(30.2)	(22.9)	(33.8)	(30.8)	(23.3)	(32.1)	(30.6)	(23.4)	(30.9)	(30.1)
t_E	843.2	539.7	844.3	843.2	539.7	844.3	843.2	539.7	844.3	843.2
	(30.0)	(19.2)	(30.0)	(30.0)	(19.2)	(30.0)	(30.0)	(19.2)	(30.0)	(30.0)
	9.6	225.2	226.3	46.2	251.3	119.2	35.9	260.8	50.1	7.6
	0.341	8.013	8.052	1.643	8.939	4.242	1.279	9.279	1.783	0.270
g_O	620	881	501	599	936	521	623	887	482	634
	(22.1)	(31.3)	(17.8)	(21.3)	(33.3)	(18.5)	(22.2)	(31.6)	(17.1)	(22.6)
g_E	648.3	906.2	455.3	648.3	906.2	455.3	648.3	906.2	455.3	648.3
	(23.1)	(32.2)	(16.2)	(23.1)	(32.2)	(16.2)	(23.1)	(32.2)	(16.2)	(23.1)
	-55.4	-49.8	95.9	-94.8	60.5	140.5	-49.6	-38.1	55.0	-28.3
	-1.969	-1.771	3.412	-3.371	2.151	4.999	-1.765	-1.355	1.957	-1.006
c_O	629	491	636	650	508	637	605	508	659	596
	(22.4)	(17.5)	(22.6)	(23.1)	(18.1)	(22.7)	(21.5)	(18.1)	(23.4)	(21.2)
c_E	635.4	520.5	574.1	635.4	520.5	574.1	635.4	520.5	574.1	635.4
	(22.6)	(18.5)	(20.4)	(22.6)	(18.5)	(20.4)	(22.6)	(18.5)	(20.4)	(22.6)
	-12.7	-57.3	130.4	29.5	-24.7	132.6	-59.3	-24.7	181.9	-76.3
	-0.453	-2.037	4.637	1.051	-0.877	4.716	-2.110	-0.877	6.471	-2.714
N	2811	2811	2811	2811	2811	2811	2811	2811	2811	2811
X2	2.6	24.9	73.8	4.9	46.0	57.4	4.9	35.5	34.7	6.4
Gvalue	2.6	23.9	77.1	5.0	45.6	59.4	4.9	34.1	35.1	6.4
Gv/N	0.094	0.850	2.743	0.178	1.622	2.112	0.175	1.214	1.250	0.227
Prob	0.45136	0.00003	0.00000	0.17193	0.00000	0.00000	0.17819	0.00000	0.00000	0.09428

	61	62	63	64	65	66	67	68	69	70
a_O	818	844	700	824	874	738	880	951	739	797
	(29.1)	(30.0)	(24.9)	(29.3)	(31.1)	(26.3)	(31.3)	(33.8)	(26.3)	(28.4)
a_E	844.6	937.4	684.1	844.6	937.4	684.1	844.6	937.4	684.1	844.6
	(30.0)	(33.3)	(24.3)	(30.0)	(33.3)	(24.3)	(30.0)	(33.3)	(24.3)	(30.0)
	-52.3	-177.1	32.2	-40.7	-122.4	112.0	72.3	27.4	114.1	-92.4
	-1.861	-6.301	1.145	-1.446	-4.353	3.983	2.572	0.976	4.060	-3.288
t_O	585	866	845	566	902	808	545	836	792	559
	(20.8)	(30.8)	(30.1)	(20.1)	(32.1)	(28.7)	(19.4)	(29.7)	(28.2)	(19.9)
t_E	539.7	844.3	843.2	539.7	844.3	843.2	539.7	844.3	843.2	539.7
	(19.2)	(30.0)	(30.0)	(19.2)	(30.0)	(30.0)	(19.2)	(30.0)	(30.0)	(19.2)
	94.3	43.9	3.6	53.9	119.2	-68.9	10.7	-16.5	-99.3	39.3
	3.355	1.563	0.127	1.917	4.242	-2.453	0.379	-0.588	-3.531	1.398
g_O	885	474	667	885	414	627	882	412	625	906
	(31.5)	(16.9)	(23.7)	(31.5)	(14.7)	(22.3)	(31.4)	(14.7)	(22.2)	(32.2)
g_E	906.2	455.3	648.3	906.2	455.3	648.3	906.2	455.3	648.3	906.2
	(32.2)	(16.2)	(23.1)	(32.2)	(16.2)	(23.1)	(32.2)	(16.2)	(23.1)	(32.2)
	-42.0	38.2	37.9	-42.0	-78.7	-41.9	-47.8	-82.3	-45.8	-0.5
	-1.494	1.360	1.349	-1.494	-2.799	-1.491	-1.702	-2.927	-1.628	-0.018
c_O	523	627	599	536	621	638	504	612	655	549
	(18.6)	(22.3)	(21.3)	(19.1)	(22.1)	(22.7)	(17.9)	(21.8)	(23.3)	(19.5)
c_E	520.5	574.1	635.4	520.5	574.1	635.4	520.5	574.1	635.4	520.5
	(18.5)	(20.4)	(22.6)	(18.5)	(20.4)	(22.6)	(18.5)	(20.4)	(22.6)	(18.5)
	5.1	110.6	-70.7	31.5	97.6	5.2	-32.4	78.4	39.8	58.6
	0.180	3.936	-2.514	1.121	3.473	0.186	-1.154	2.787	1.416	2.084

N	2811	2811	2811	2811	2811	2811	2811	2811	2811	2811
X2	5.2	15.5	3.0	2.7	15.8	6.4	2.7	6.9	9.0	4.9
Gvalue	5.1	15.7	3.0	2.7	15.8	6.4	2.7	7.0	8.9	5.0
Gv/N	0.180	0.558	0.108	0.097	0.563	0.226	0.096	0.248	0.317	0.176
Prob	0.16739	0.00132	0.38694	0.43550	0.00122	0.09570	0.44040	0.07242	0.03041	0.17549

	71	72	73	74	75	76	77	78	79	80
a_O	899	765	831	924	768	847	912	741	853	919
	(32.0)	(27.2)	(29.6)	(32.9)	(27.3)	(30.1)	(32.4)	(26.4)	(30.3)	(32.7)
a_E	937.4	684.1	844.6	937.4	684.1	844.6	937.4	684.1	844.6	937.4
	(33.3)	(24.3)	(30.0)	(33.3)	(24.3)	(30.0)	(33.3)	(24.3)	(30.0)	(33.3)
	−75.2	171.0	−26.9	−26.6	177.7	4.8	−50.1	118.4	16.9	−36.4
	−2.674	6.085	−0.958	−0.945	6.322	0.172	−1.781	4.213	0.602	−1.294
t_O	885	822	579	840	870	567	886	814	536	830
	(31.5)	(29.2)	(20.6)	(29.9)	(30.9)	(20.2)	(31.5)	(29.0)	(19.1)	(29.5)
t_E	844.3	843.2	539.7	844.3	843.2	539.7	844.3	843.2	539.7	844.3
	(30.0)	(30.0)	(19.2)	(30.0)	(30.0)	(19.2)	(30.0)	(30.0)	(19.2)	(30.0)
	83.3	−41.9	81.4	−8.6	54.4	56.0	85.4	−57.4	−7.4	−28.4
	2.964	−1.490	2.896	−0.305	1.936	1.991	3.039	−2.042	−0.262	−1.009
g_O	428	612	928	439	617	909	429	617	912	415
	(15.2)	(21.8)	(33.0)	(15.6)	(21.9)	(32.3)	(15.3)	(21.9)	(32.4)	(14.8)
g_E	455.3	648.3	906.2	455.3	648.3	906.2	455.3	648.3	906.2	455.3
	(16.2)	(23.1)	(32.2)	(16.2)	(23.1)	(32.2)	(16.2)	(23.1)	(32.2)	(16.2)
	−52.9	−70.5	44.0	−32.0	−61.1	5.5	−51.0	−61.1	11.5	−76.9
	−1.881	−2.509	1.566	−1.137	−2.173	0.196	−1.814	−2.173	0.411	−2.735
c_O	599	612	473	608	556	488	584	639	510	647
	(21.3)	(21.8)	(16.8)	(21.6)	(19.8)	(17.4)	(20.8)	(22.7)	(18.1)	(23.0)
c_E	574.1	635.4	520.5	574.1	635.4	520.5	574.1	635.4	520.5	574.1
	(20.4)	(22.6)	(18.5)	(20.4)	(22.6)	(18.5)	(20.4)	(22.6)	(18.5)	(20.4)
	51.0	−45.9	−90.5	69.9	−148.4	−62.9	20.1	7.2	−20.7	154.8
	1.813	−1.633	−3.219	2.486	−5.280	−2.237	0.714	0.257	−0.738	5.507
N	2811	2811	2811	2811	2811	2811	2811	2811	2811	2811
X2	6.3	13.0	7.9	2.8	22.6	3.4	4.4	7.3	0.4	13.4
Gvalue	6.3	12.7	8.0	2.8	22.6	3.4	4.4	7.2	0.4	13.2
Gv/N	0.223	0.452	0.285	0.099	0.805	0.123	0.158	0.256	0.013	0.469
Prob	0.09963	0.00534	0.04596	0.42819	0.00005	0.32804	0.21802	0.06608	0.94881	0.00426

	81	82	83	84	85	86	87	88	89	90
a_O	713	845	911	718	838	951	715	784	949	672
	(25.4)	(30.1)	(32.4)	(25.5)	(29.8)	(33.8)	(25.4)	(27.9)	(33.8)	(23.9)
a_E	684.1	844.6	937.4	684.1	844.6	937.4	684.1	844.6	937.4	684.1
	(24.3)	(30.0)	(33.3)	(24.3)	(30.0)	(33.3)	(24.3)	(30.0)	(33.3)	(24.3)
	59.0	0.8	−52.0	69.5	−13.1	27.4	63.2	−116.7	23.4	−24.0
	2.100	0.030	−1.850	2.472	−0.466	0.976	2.248	−4.152	0.832	−0.852
t_O	860	553	885	863	578	867	848	565	810	876
	(30.6)	(19.7)	(31.5)	(30.7)	(20.6)	(30.8)	(30.2)	(20.1)	(28.8)	(31.2)
t_E	843.2	539.7	844.3	843.2	539.7	844.3	843.2	539.7	844.3	843.2
	(30.0)	(19.2)	(30.0)	(30.0)	(19.2)	(30.0)	(30.0)	(19.2)	(30.0)	(30.0)
	33.9	26.9	83.3	40.0	79.3	46.0	9.6	51.8	−67.2	66.8
	1.206	0.958	2.964	1.424	2.820	1.636	0.341	1.842	−2.390	2.377
g_O	599	909	442	642	895	435	641	943	452	611
	(21.3)	(32.3)	(15.7)	(22.8)	(31.8)	(15.5)	(22.8)	(33.5)	(16.1)	(21.7)
g_E	648.3	906.2	455.3	648.3	906.2	455.3	648.3	906.2	455.3	648.3
	(23.1)	(32.2)	(16.2)	(23.1)	(32.2)	(16.2)	(23.1)	(32.2)	(16.2)	(23.1)
	−94.8	5.5	−26.1	−12.5	−22.4	−39.6	−14.5	75.0	−6.5	−72.4
	−3.371	0.196	−0.930	−0.446	−0.795	−1.410	−0.517	2.667	−0.232	−2.576
c_O	639	504	573	588	500	558	607	519	600	652
	(22.7)	(17.9)	(20.4)	(20.9)	(17.8)	(19.9)	(21.6)	(18.5)	(21.3)	(23.2)
c_E	635.4	520.5	574.1	635.4	520.5	574.1	635.4	520.5	574.1	635.4
	(22.6)	(18.5)	(20.4)	(22.6)	(18.5)	(20.4)	(22.6)	(18.5)	(20.4)	(22.6)
	7.2	−32.4	−2.1	−91.2	−40.1	−31.6	−55.5	−3.0	53.1	33.6
	0.257	−1.154	−0.075	−3.243	−1.428	−1.126	−1.974	−0.105	1.887	1.197
N	2811	2811	2811	2811	2811	2811	2811	2811	2811	2811
X2	5.3	0.9	3.1	5.7	3.7	2.2	2.8	7.0	2.7	4.1
Gvalue	5.4	0.9	3.1	5.8	3.7	2.2	2.8	7.1	2.7	4.1
Gv/N	0.192	0.031	0.109	0.206	0.130	0.077	0.099	0.252	0.097	0.146
Prob	0.14437	0.83475	0.38058	0.12162	0.30000	0.53769	0.42777	0.06888	0.43415	0.25164

	91	92	93	94	95	96	97	98	99	100
a_O	813	936	697	826	879	728	836	913	659	819
	(28.9)	(33.3)	(24.8)	(29.4)	(31.3)	(25.9)	(29.8)	(32.5)	(23.5)	(29.2)
a_E	844.6	937.4	684.1	844.0	936.7	683.6	844.0	936.7	683.6	844.0
	(30.0)	(33.3)	(24.3)	(30.0)	(33.3)	(24.3)	(30.0)	(33.3)	(24.3)	(30.0)
	−62.0	−2.7	26.1	−35.6	−111.8	91.6	−15.9	−46.8	−48.3	−49.2
	−2.204	−0.098	0.927	−1.266	−3.980	3.262	−0.565	−1.666	−1.720	−1.752
t_O	537	790	855	531	910	833	555	870	886	512
	(19.1)	(28.1)	(30.4)	(18.9)	(32.4)	(29.7)	(19.8)	(31.0)	(31.5)	(18.2)
t_E	539.7	844.3	843.2	539.3	843.7	842.6	539.3	843.7	842.6	539.3
	(19.2)	(30.0)	(30.0)	(19.2)	(30.0)	(30.0)	(19.2)	(30.0)	(30.0)	(19.2)
	−5.4	−105.0	23.7	−16.5	137.7	−19.1	31.8	53.4	89.0	−53.2
	−0.191	−3.737	0.844	−0.587	4.901	−0.681	1.133	1.901	3.167	−1.894
g_O	954	470	617	937	442	660	870	430	670	956
	(33.9)	(16.7)	(21.9)	(33.4)	(15.7)	(23.5)	(31.0)	(15.3)	(23.9)	(34.0)
g_E	906.2	455.3	648.3	905.6	454.9	647.8	905.6	454.9	647.8	905.6
	(32.2)	(16.2)	(23.1)	(32.2)	(16.2)	(23.1)	(32.2)	(16.2)	(23.1)	(32.2)
	98.0	29.9	−61.1	63.9	−25.5	24.5	−69.8	−48.5	45.1	103.5
	3.486	1.065	−2.173	2.274	−0.909	0.874	−2.484	−1.727	1.604	3.686
c_O	507	615	642	515	578	588	548	596	594	522
	(18.0)	(21.9)	(22.8)	(18.3)	(20.6)	(20.9)	(19.5)	(21.2)	(21.1)	(18.6)
c_E	520.5	574.1	635.4	520.1	573.6	634.9	520.1	573.6	634.9	520.1
	(18.5)	(20.4)	(22.6)	(18.5)	(20.4)	(22.6)	(18.5)	(20.4)	(22.6)	(18.5)
	−26.6	84.8	13.3	−10.2	8.7	−90.3	57.3	45.6	−79.2	3.8
	−0.946	3.015	0.473	−0.362	0.311	−3.215	2.038	1.623	−2.819	0.135
N	2811	2811	2811	2809	2809	2809	2809	2809	2809	2809
X2	4.1	6.9	2.0	1.6	9.2	6.7	3.4	3.7	6.5	4.9
Gvalue	4.0	6.9	2.0	1.6	9.1	6.7	3.4	3.7	6.5	4.9
Gv/N	0.144	0.245	0.072	0.058	0.324	0.239	0.122	0.131	0.233	0.175
Prob	0.25775	0.07523	0.57010	0.65013	0.02782	0.08140	0.33184	0.29933	0.08809	0.17817

Bgar_PBI
5UTR:

	100	99	98	97	96	95	94	93	92	91
a_O	56	58	70	66	68	66	65	66	62	62
	(33.7)	(34.9)	(42.2)	(39.8)	(41.0)	(39.8)	(39.2)	(39.8)	(37.3)	(37.3)
a_E	68.3	68.3	68.3	68.3	68.3	68.3	68.3	68.3	68.3	68.3
	(41.2)	(41.2)	(41.2)	(41.2)	(41.2)	(41.2)	(41.2)	(41.2)	(41.2)	(41.2)
	-22.3	-19.0	3.4	-4.5	-0.6	-4.5	-6.5	-4.5	-12.0	-12.0
	-13.407	-11.433	2.061	-2.736	-0.373	-2.736	-3.890	-2.736	-7.240	-7.240
t_O	77	69	60	58	58	55	62	67	72	68
	(46.4)	(41.6)	(36.1)	(34.9)	(34.9)	(33.1)	(37.3)	(40.4)	(43.4)	(41.0)
t_E	61.0	61.0	61.0	61.0	61.0	61.0	61.0	61.0	61.0	61.0
	(36.7)	(36.7)	(36.7)	(36.7)	(36.7)	(36.7)	(36.7)	(36.7)	(36.7)	(36.7)
	35.9	17.0	-2.0	-5.8	-5.8	-11.4	2.0	12.6	23.9	14.8
	21.625	10.258	-1.183	-3.513	-3.513	-6.850	1.227	7.587	14.396	8.914
g_O	18	19	16	19	20	27	15	12	14	21
	(10.8)	(11.4)	(9.6)	(11.4)	(12.0)	(16.3)	(9.0)	(7.2)	(8.4)	(12.7)
g_E	19.7	19.7	19.7	19.7	19.7	19.7	19.7	19.7	19.7	19.7
	(11.9)	(11.9)	(11.9)	(11.9)	(11.9)	(11.9)	(11.9)	(11.9)	(11.9)	(11.9)
	-3.2	-1.4	-6.6	-1.4	0.6	17.0	-8.2	-11.9	-9.5	2.7
	-1.946	-0.817	-4.000	-0.817	0.376	10.271	-4.917	-7.160	-5.753	1.630
c_O	15	20	20	23	20	18	24	21	18	15
	(9.0)	(12.0)	(12.0)	(13.9)	(12.0)	(10.8)	(14.5)	(12.7)	(10.8)	(9.0)
c_E	17.0	17.0	17.0	17.0	17.0	17.0	17.0	17.0	17.0	17.0
	(10.2)	(10.2)	(10.2)	(10.2)	(10.2)	(10.2)	(10.2)	(10.2)	(10.2)	(10.2)
	-3.8	6.5	6.5	13.9	6.5	2.0	16.5	8.9	2.0	-3.8
	-2.273	3.902	3.902	8.360	3.902	1.227	9.954	5.331	1.227	-2.273
N	166	166	166	166	166	166	166	166	166	166
X2	6.8	3.2	1.3	2.4	0.7	3.4	4.2	4.6	4.3	1.7
Gvalue	6.6	3.2	1.3	2.2	0.7	3.2	3.9	5.0	4.4	1.7
Gv/N	3.999	1.910	0.780	1.295	0.393	1.912	2.375	3.023	2.630	1.031
Prob	0.08435	0.36596	0.73052	0.54179	0.88431	0.36567	0.26780	0.17048	0.22452	0.63450

	90	89	88	87	86	85	84	83	82	81
a_O	64	64	59	54	68	65	66	53	66	66
	(38.6)	(38.6)	(35.5)	(32.5)	(41.0)	(39.2)	(39.8)	(31.9)	(39.8)	(39.8)
a_E	68.3	68.3	68.3	68.3	68.3	68.3	68.3	68.3	68.3	68.3
	(41.2)	(41.2)	(41.2)	(41.2)	(41.2)	(41.2)	(41.2)	(41.2)	(41.2)	(41.2)
	-8.3	-8.3	-17.3	-25.4	-0.6	-6.5	-4.5	-26.9	-4.5	-4.5
	-5.025	-5.025	-10.415	-15.294	-0.373	-3.890	-2.736	-16.204	-2.736	-2.736
t_O	61	64	63	74	69	62	56	65	58	57
	(36.7)	(38.6)	(38.0)	(44.6)	(41.6)	(37.3)	(33.7)	(39.2)	(34.9)	(34.3)
t_E	61.0	61.0	61.0	61.0	61.0	61.0	61.0	61.0	61.0	61.0
	(36.7)	(36.7)	(36.7)	(36.7)	(36.7)	(36.7)	(36.7)	(36.7)	(36.7)	(36.7)
	0.0	6.2	4.1	28.6	17.0	2.0	-9.6	8.3	-5.8	-7.7
	0.012	3.715	2.461	17.239	10.258	1.227	-5.759	4.987	-3.513	-4.646
g_O	20	24	27	16	12	17	23	23	25	23
	(12.0)	(14.5)	(16.3)	(9.6)	(7.2)	(10.2)	(13.9)	(13.9)	(15.1)	(13.9)
g_E	19.7	19.7	19.7	19.7	19.7	19.7	19.7	19.7	19.7	19.7
	(11.9)	(11.9)	(11.9)	(11.9)	(11.9)	(11.9)	(11.9)	(11.9)	(11.9)	(11.9)
	0.6	9.5	17.0	-6.6	-11.9	-5.0	7.1	7.1	11.9	7.1
	0.376	5.724	10.271	-4.000	-7.160	-3.009	4.306	4.306	7.192	4.306
c_O	21	14	17	22	17	22	21	25	17	20
	(12.7)	(8.4)	(10.2)	(13.3)	(10.2)	(13.3)	(12.7)	(15.1)	(10.2)	(12.0)
c_E	17.0	17.0	17.0	17.0	17.0	17.0	17.0	17.0	17.0	17.0
	(10.2)	(10.2)	(10.2)	(10.2)	(10.2)	(10.2)	(10.2)	(10.2)	(10.2)	(10.2)
	8.9	-5.5	-0.0	11.3	-0.0	11.3	8.9	19.3	-0.0	6.5
	5.331	-3.285	-0.012	6.818	-0.012	6.818	5.331	11.599	-0.012	3.902
N	166	166	166	166	166	166	166	166	166	166
X2	1.2	1.9	4.0	7.9	4.1	2.0	2.0	8.0	1.7	1.4
Gvalue	1.2	1.9	3.8	7.9	4.5	1.9	1.9	7.8	1.5	1.4
Gv/N	0.695	1.128	2.305	4.763	2.714	1.147	1.143	4.687	0.932	0.826
Prob	0.76430	0.59930	0.28093	0.04798	0.21182	0.59262	0.59410	0.05078	0.67163	0.71239

	80	79	78	77	76	75	74	73	72	71
a_O	66	65	69	82	70	76	76	71	80	70
	(39.8)	(39.2)	(41.6)	(49.4)	(42.2)	(45.8)	(45.8)	(42.8)	(48.2)	(42.2)
a_E	68.3	68.3	68.3	68.3	68.3	68.3	68.3	68.3	68.3	68.3
	(41.2)	(41.2)	(41.2)	(41.2)	(41.2)	(41.2)	(41.2)	(41.2)	(41.2)	(41.2)
	-4.5	-6.5	1.4	30.0	3.4	16.2	16.2	5.5	25.3	3.4
	-2.736	-3.890	0.836	18.046	2.061	9.768	9.768	3.304	15.226	2.061
t_O	65	66	58	47	49	56	56	55	45	55
	(39.2)	(39.8)	(34.9)	(28.3)	(29.5)	(33.7)	(33.7)	(33.1)	(27.1)	(33.1)
t_E	61.0	61.0	61.0	61.0	61.0	61.0	61.0	61.0	61.0	61.0
	(36.7)	(36.7)	(36.7)	(36.7)	(36.7)	(36.7)	(36.7)	(36.7)	(36.7)	(36.7)
	8.3	10.4	-5.8	-24.5	-21.5	-9.6	-9.6	-11.4	-27.4	-11.4
	4.987	6.278	-3.513	-14.755	-12.922	-5.759	-5.759	-6.850	-16.484	-6.850
g_O	25	22	26	12	25	20	23	22	20	28
	(15.1)	(13.3)	(15.7)	(7.2)	(15.1)	(12.0)	(13.9)	(13.3)	(12.0)	(16.9)
g_E	19.7	19.7	19.7	19.7	19.7	19.7	19.7	19.7	19.7	19.7
	(11.9)	(11.9)	(11.9)	(11.9)	(11.9)	(11.9)	(11.9)	(11.9)	(11.9)	(11.9)
	11.9	4.9	14.5	-11.9	11.9	0.6	7.1	4.9	0.6	19.7
	7.192	2.940	8.708	-7.160	7.192	0.376	4.306	2.940	0.376	11.878
c_O	10	13	13	25	22	14	11	18	21	13
	(6.0)	(7.8)	(7.8)	(15.1)	(13.3)	(8.4)	(6.6)	(10.8)	(12.7)	(7.8)
c_E	17.0	17.0	17.0	17.0	17.0	17.0	17.0	17.0	17.0	17.0
	(10.2)	(10.2)	(10.2)	(10.2)	(10.2)	(10.2)	(10.2)	(10.2)	(10.2)	(10.2)
	-10.6	-7.0	-7.0	19.3	11.3	-5.5	-9.6	2.0	8.9	-7.0
	-6.400	-4.211	-4.211	11.599	6.818	-3.285	-5.777	1.227	5.331	-4.211
N	166	166	166	166	166	166	166	166	166	166
X2	4.7	1.8	3.1	12.7	5.3	1.8	4.0	1.0	7.1	5.1
Gvalue	5.1	1.9	3.0	12.8	5.2	1.8	4.2	1.0	7.4	4.8
Gv/N	3.043	1.117	1.820	7.731	3.149	1.100	2.538	0.621	4.449	2.878
Prob	0.16810	0.60313	0.38839	0.00501	0.15590	0.60911	0.23941	0.79384	0.06056	0.18886

	70	69	68	67	66	65	64	63	62	61
a_O	77	62	63	82	59	63	64	67	59	69
	(46.4)	(37.3)	(38.0)	(49.4)	(35.5)	(38.0)	(38.6)	(40.4)	(35.5)	(41.6)
a_E	68.3	68.3	68.3	68.3	68.3	68.3	68.3	68.3	68.3	68.3
	(41.2)	(41.2)	(41.2)	(41.2)	(41.2)	(41.2)	(41.2)	(41.2)	(41.2)	(41.2)
	18.4	-12.0	-10.2	30.0	-17.3	-10.2	-8.3	-2.6	-17.3	1.4
	11.109	-7.240	-6.142	18.046	-10.415	-6.142	-5.025	-1.563	-10.415	0.836
t_O	60	77	61	53	56	59	57	57	65	69
	(36.1)	(46.4)	(36.7)	(31.9)	(33.7)	(35.5)	(34.3)	(34.3)	(39.2)	(41.6)

t_E	61.0	61.0	61.0	61.0	61.0	61.0	61.0	61.0	61.0	61.0
	(36.7)	(36.7)	(36.7)	(36.7)	(36.7)	(36.7)	(36.7)	(36.7)	(36.7)	(36.7)
	-2.0	35.9	0.0	-14.9	-9.6	-3.9	-7.7	-7.7	8.3	17.0
	-1.183	21.625	0.012	-8.966	-5.759	-2.358	-4.646	-4.646	4.987	10.258
g_O	15	9	24	14	29	24	24	14	16	16
	(9.0)	(5.4)	(14.5)	(8.4)	(17.5)	(14.5)	(14.5)	(8.4)	(9.6)	(9.6)
g_E	19.7	19.7	19.7	19.7	19.7	19.7	19.7	19.7	19.7	19.7
	(11.9)	(11.9)	(11.9)	(11.9)	(11.9)	(11.9)	(11.9)	(11.9)	(11.9)	(11.9)
	-8.2	-14.1	9.5	-9.5	22.5	9.5	9.5	-9.5	-6.6	-6.6
	-4.917	-8.489	5.724	-5.753	13.528	5.724	5.724	-5.753	-4.000	-4.000
c_O	14	18	18	17	22	20	21	28	26	12
	(8.4)	(10.8)	(10.8)	(10.2)	(13.3)	(12.0)	(12.7)	(16.9)	(15.7)	(7.2)
c_E	17.0	17.0	17.0	17.0	17.0	17.0	17.0	17.0	17.0	17.0
	(10.2)	(10.2)	(10.2)	(10.2)	(10.2)	(10.2)	(10.2)	(10.2)	(10.2)	(10.2)
	-5.5	2.0	2.0	-0.0	11.3	6.5	8.9	27.9	22.1	-8.4
	-3.285	1.227	1.227	-0.012	6.818	3.902	5.331	16.814	13.291	-5.044
N	166	166	166	166	166	166	166	166	166	166
X2	2.8	10.6	1.4	5.4	7.5	1.9	2.4	9.0	7.0	3.2
Gvalue	2.9	11.8	1.4	5.5	6.9	1.9	2.3	8.1	6.4	3.4
Gv/N	1.725	7.122	0.820	3.315	4.172	1.125	1.383	4.851	3.862	2.049
Prob	0.41325	0.00801	0.71454	0.13846	0.07429	0.60024	0.51323	0.04492	0.09322	0.33376

	60	59	58	57	56	55	54	53	52	51
a_O	59	67	65	66	71	64	74	76	67	71
	(35.5)	(40.4)	(39.2)	(39.8)	(42.8)	(38.6)	(44.6)	(45.8)	(40.4)	(42.8)
a_E	68.3	68.3	68.3	68.3	68.3	68.3	68.3	68.3	68.3	68.3
	(41.2)	(41.2)	(41.2)	(41.2)	(41.2)	(41.2)	(41.2)	(41.2)	(41.2)	(41.2)
	-17.3	-2.6	-6.5	-4.5	5.5	-8.3	11.8	16.2	-2.6	5.5
	-10.415	-1.563	-3.890	-2.736	3.304	-5.025	7.133	9.768	-1.563	3.304
t_O	74	57	60	65	45	60	59	56	55	53
	(44.6)	(34.3)	(36.1)	(39.2)	(27.1)	(36.1)	(35.5)	(33.7)	(33.1)	(31.9)
t_E	61.0	61.0	61.0	61.0	61.0	61.0	61.0	61.0	61.0	61.0
	(36.7)	(36.7)	(36.7)	(36.7)	(36.7)	(36.7)	(36.7)	(36.7)	(36.7)	(36.7)
	28.6	-7.7	-2.0	8.3	-27.4	-2.0	-3.9	-9.6	-11.4	-14.9
	17.239	-4.646	-1.183	4.987	-16.484	-1.183	-2.358	-5.759	-6.850	-8.966
g_O	16	18	27	21	30	20	16	18	24	17
	(9.6)	(10.8)	(16.3)	(12.7)	(18.1)	(12.0)	(9.6)	(10.8)	(14.5)	(10.2)
g_E	19.7	19.7	19.7	19.7	19.7	19.7	19.7	19.7	19.7	19.7
	(11.9)	(11.9)	(11.9)	(11.9)	(11.9)	(11.9)	(11.9)	(11.9)	(11.9)	(11.9)
	-6.6	-3.2	17.0	2.7	25.3	0.6	-6.6	-3.2	9.5	-5.0
	-4.000	-1.946	10.271	1.630	15.220	0.376	-4.000	-1.946	5.724	-3.009
c_O	17	24	14	14	20	22	17	16	20	25
	(10.2)	(14.5)	(8.4)	(8.4)	(12.0)	(13.3)	(10.2)	(9.6)	(12.0)	(15.1)
c_E	17.0	17.0	17.0	17.0	17.0	17.0	17.0	17.0	17.0	17.0
	(10.2)	(10.2)	(10.2)	(10.2)	(10.2)	(10.2)	(10.2)	(10.2)	(10.2)	(10.2)
	-0.0	16.5	-5.5	-5.5	6.5	11.3	-0.0	-2.0	6.5	19.3
	-0.012	9.954	-3.285	-3.285	3.902	6.818	-0.012	-1.180	3.902	11.599
N	166	166	166	166	166	166	166	166	166	166
X2	4.7	3.3	3.4	1.0	10.2	1.8	1.2	1.5	2.1	5.3
Gvalue	4.7	3.0	3.2	1.0	9.9	1.6	1.3	1.5	2.0	4.9
Gv/N	2.811	1.799	1.913	0.596	5.941	0.986	0.763	0.883	1.212	2.927
Prob	0.19790	0.39383	0.36534	0.80379	0.01977	0.65093	0.73716	0.69028	0.56985	0.18238

	50	49	48	47	46	45	44	43	42	41
a_O	63	77	64	65	79	69	64	71	66	68
	(38.0)	(46.4)	(38.6)	(39.2)	(47.6)	(41.6)	(38.6)	(42.8)	(39.8)	(41.0)
a_E	68.3	68.3	68.3	68.3	68.3	68.3	68.3	68.3	68.3	68.3
	(41.2)	(41.2)	(41.2)	(41.2)	(41.2)	(41.2)	(41.2)	(41.2)	(41.2)	(41.2)
	-10.2	18.4	-8.3	-6.5	23.0	1.4	-8.3	5.5	-4.5	-0.6
	-6.142	11.109	-5.025	-3.890	13.838	0.836	-5.025	3.304	-2.736	-0.373
t_O	60	54	64	61	49	62	65	59	66	67
	(36.1)	(32.5)	(38.6)	(36.7)	(29.5)	(37.3)	(39.2)	(35.5)	(39.8)	(40.4)
t_E	61.0	61.0	61.0	61.0	61.0	61.0	61.0	61.0	61.0	61.0
	(36.7)	(36.7)	(36.7)	(36.7)	(36.7)	(36.7)	(36.7)	(36.7)	(36.7)	(36.7)
	-2.0	-13.1	6.2	0.0	-21.5	2.0	8.3	-3.9	10.4	12.6
	-1.183	-7.920	3.715	0.012	-12.922	1.227	4.987	-2.358	6.278	7.587
g_O	15	16	19	23	23	17	24	18	18	19
	(9.0)	(9.6)	(11.4)	(13.9)	(13.9)	(10.2)	(14.5)	(10.8)	(10.8)	(11.4)
g_E	19.7	19.7	19.7	19.7	19.7	19.7	19.7	19.7	19.7	19.7
	(11.9)	(11.9)	(11.9)	(11.9)	(11.9)	(11.9)	(11.9)	(11.9)	(11.9)	(11.9)
	-8.2	-6.6	-1.4	7.1	7.1	-5.0	9.5	-3.2	-3.2	-1.4
	-4.917	-4.000	-0.817	4.306	4.306	-3.009	5.724	-1.946	-1.946	-0.817
c_O	28	19	19	17	15	18	13	18	16	12
	(16.9)	(11.4)	(11.4)	(10.2)	(9.0)	(10.8)	(7.8)	(10.8)	(9.6)	(7.2)
c_E	17.0	17.0	17.0	17.0	17.0	17.0	17.0	17.0	17.0	17.0
	(10.2)	(10.2)	(10.2)	(10.2)	(10.2)	(10.2)	(10.2)	(10.2)	(10.2)	(10.2)
	27.9	4.2	4.2	-0.0	-3.8	2.0	-7.0	2.0	-2.0	-8.4
	16.814	2.533	2.533	-0.012	-2.273	1.227	-4.211	1.227	-1.180	-5.044
N	166	166	166	166	166	166	166	166	166	166
X2	8.6	2.8	0.7	0.7	4.8	0.4	2.4	0.4	0.7	2.1
Gvalue	7.6	2.9	0.7	0.7	4.9	0.5	2.4	0.4	0.7	2.2
Gv/N	4.572	1.722	0.405	0.416	2.949	0.280	1.474	0.227	0.416	1.353
Prob	0.05532	0.41396	0.87960	0.87539	0.17959	0.92638	0.48494	0.94513	0.87548	0.52293

	40	39	38	37	36	35	34	33	32	31
a_O	72	66	64	67	67	65	60	75	74	59
	(43.4)	(39.8)	(38.6)	(40.4)	(40.4)	(39.2)	(36.1)	(45.2)	(44.6)	(35.5)
a_E	68.3	68.3	68.3	68.3	68.3	68.3	68.3	68.3	68.3	68.3
	(41.2)	(41.2)	(41.2)	(41.2)	(41.2)	(41.2)	(41.2)	(41.2)	(41.2)	(41.2)
	7.6	-4.5	-8.3	-2.6	-2.6	-6.5	-15.6	14.0	11.8	-17.3
	4.564	-2.736	-5.025	-1.563	-1.563	-3.890	-9.377	8.443	7.133	-10.415
t_O	59	61	70	59	61	65	65	55	54	64
	(35.5)	(36.7)	(42.2)	(35.5)	(36.7)	(39.2)	(39.2)	(33.1)	(32.5)	(38.6)
t_E	61.0	61.0	61.0	61.0	61.0	61.0	61.0	61.0	61.0	61.0
	(36.7)	(36.7)	(36.7)	(36.7)	(36.7)	(36.7)	(36.7)	(36.7)	(36.7)	(36.7)
	-3.9	0.0	19.3	-3.9	0.0	8.3	8.3	-11.4	-13.1	6.2
	-2.358	0.012	11.620	-2.358	0.012	4.987	4.987	-6.850	-7.920	3.715
g_O	20	18	17	18	16	26	24	22	21	30
	(12.0)	(10.8)	(10.2)	(10.8)	(9.6)	(15.7)	(14.5)	(13.3)	(12.7)	(18.1)
g_E	19.7	19.7	19.7	19.7	19.7	19.7	19.7	19.7	19.7	19.7
	(11.9)	(11.9)	(11.9)	(11.9)	(11.9)	(11.9)	(11.9)	(11.9)	(11.9)	(11.9)
	0.6	-3.2	-5.0	-3.2	-6.6	14.5	9.5	4.9	2.7	25.3
	0.376	-1.946	-3.009	-1.946	-4.000	8.708	5.724	2.940	1.630	15.220
c_O	15	21	15	22	22	10	17	14	17	13
	(9.0)	(12.7)	(9.0)	(13.3)	(13.3)	(6.0)	(10.2)	(8.4)	(10.2)	(7.8)
c_E	17.0	17.0	17.0	17.0	17.0	17.0	17.0	17.0	17.0	17.0

```
        ( 10.2) ( 10.2) ( 10.2) ( 10.2) ( 10.2) ( 10.2) ( 10.2) ( 10.2) ( 10.2) ( 10.2)
           -3.8     8.9    -3.8    11.3    11.3   -10.6    -0.0    -5.5    -0.0    -7.0
         -2.273   5.331  -2.273   6.818   6.818  -6.400  -0.012  -3.285  -0.012  -4.211

N           166     166     166     166     166     166     166     166     166     166
X2          0.5     1.2     2.2     1.7     2.2     5.3     2.2     2.0     1.4     7.8
Gvalue      0.5     1.1     2.2     1.6     2.1     5.7     2.2     2.1     1.4     7.2
Gv/N      0.310   0.662   1.314   0.951   1.267   3.405   1.322   1.248   0.831   4.308
Prob    0.91585 0.77739 0.53575 0.66418 0.55127 0.12982 0.53314 0.55770 0.71018 0.06721
        -------------------------------------------------------------------------------
             30      29      28      27      26      25      24      23      22      21
        -------------------------------------------------------------------------------
a_O          64      66      73      75      75      72      69      80      65      60
        ( 38.6) ( 39.8) ( 44.0) ( 45.2) ( 45.2) ( 43.4) ( 41.6) ( 48.2) ( 39.2) ( 36.1)
a_E        68.3    68.3    68.3    68.3    68.3    68.3    68.3    68.3    68.3    68.3
        ( 41.2) ( 41.2) ( 41.2) ( 41.2) ( 41.2) ( 41.2) ( 41.2) ( 41.2) ( 41.2) ( 41.2)
           -8.3    -4.5     9.7    14.0    14.0     7.6     1.4    25.3    -6.5   -15.6
         -5.025  -2.736   5.840   8.443   8.443   4.564   0.836  15.226  -3.890  -9.377
t_O          64      63      54      65      59      64      64      54      54      73
        ( 38.6) ( 38.0) ( 32.5) ( 39.2) ( 35.5) ( 38.6) ( 38.6) ( 32.5) ( 32.5) ( 44.0)
t_E        61.0    61.0    61.0    61.0    61.0    61.0    61.0    61.0    61.0    61.0
        ( 36.7) ( 36.7) ( 36.7) ( 36.7) ( 36.7) ( 36.7) ( 36.7) ( 36.7) ( 36.7) ( 36.7)
            6.2     4.1   -13.1     8.3    -3.9     6.2     6.2   -13.1   -13.1    26.2
          3.715   2.461  -7.920   4.987  -2.358   3.715   3.715  -7.920  -7.920  15.809
g_O          18      25      21      14      16      18      17      17      31      22
        ( 10.8) ( 15.1) ( 12.7) (  8.4) (  9.6) ( 10.8) ( 10.2) ( 10.2) ( 18.7) ( 13.3)
g_E        19.7    19.7    19.7    19.7    19.7    19.7    19.7    19.7    19.7    19.7
        ( 11.9) ( 11.9) ( 11.9) ( 11.9) ( 11.9) ( 11.9) ( 11.9) ( 11.9) ( 11.9) ( 11.9)
           -3.2    11.9     2.7    -9.5    -6.6    -3.2    -5.0    -5.0    28.1     4.9
         -1.946   7.192   1.630  -5.753  -4.000  -1.946  -3.009  -3.009  16.952   2.940
c_O          20      12      18      12      16      12      16      15      16      11
        ( 12.0) (  7.2) ( 10.8) (  7.2) (  9.6) (  7.2) (  9.6) (  9.0) (  9.6) (  6.6)
c_E        17.0    17.0    17.0    17.0    17.0    17.0    17.0    17.0    17.0    17.0
        ( 10.2) ( 10.2) ( 10.2) ( 10.2) ( 10.2) ( 10.2) ( 10.2) ( 10.2) ( 10.2) ( 10.2)
            6.5    -8.4     2.0    -8.4    -2.0    -8.4    -2.0    -3.8    -2.0    -9.6
          3.902  -5.044   1.227  -5.044  -1.180  -5.044  -1.180  -2.273  -1.180  -5.777

N           166     166     166     166     166     166     166     166     166     166
X2          1.1     3.1     1.3     4.0     1.5     2.0     0.6     3.4     7.5     5.8
Gvalue      1.1     3.1     1.3     4.4     1.5     2.1     0.6     3.4     6.6     6.0
Gv/N      0.645   1.873   0.777   2.632   0.904   1.288   0.361   2.025   3.963   3.596
Prob    0.78417 0.37508 0.73144 0.22420 0.68206 0.54428 0.89648 0.33913 0.08663 0.11312
        -------------------------------------------------------------------------------
             20      19      18      17      16      15      14      13      12      11
        -------------------------------------------------------------------------------
a_O          56      72      82      74      75      64      73      72      72      78
        ( 33.7) ( 43.4) ( 49.4) ( 44.6) ( 45.2) ( 38.6) ( 44.0) ( 43.4) ( 43.4) ( 47.0)
a_E        68.3    68.3    68.3    68.3    68.3    68.3    68.3    68.3    68.3    68.3
        ( 41.2) ( 41.2) ( 41.2) ( 41.2) ( 41.2) ( 41.2) ( 41.2) ( 41.2) ( 41.2) ( 41.2)
          -22.3     7.6    30.0    11.8    14.0    -8.3     9.7     7.6     7.6    20.7
        -13.407   4.564  18.046   7.133   8.443  -5.025   5.840   4.564   4.564  12.466
t_O          66      61      61      65      61      69      55      59      65      61
        ( 39.8) ( 36.7) ( 36.7) ( 39.2) ( 36.7) ( 41.6) ( 33.1) ( 35.5) ( 39.2) ( 36.7)
t_E        61.0    61.0    61.0    61.0    61.0    61.0    61.0    61.0    61.0    61.0
        ( 36.7) ( 36.7) ( 36.7) ( 36.7) ( 36.7) ( 36.7) ( 36.7) ( 36.7) ( 36.7) ( 36.7)
           10.4     0.0     0.0     8.3     0.0    17.0   -11.4    -3.9     8.3     0.0
          6.278   0.012   0.012   4.987   0.012  10.258  -6.850  -2.358   4.987   0.012
g_O          30      20      12      12      14      17      24      17      15      18
        ( 18.1) ( 12.0) (  7.2) (  7.2) (  8.4) ( 10.2) ( 14.5) ( 10.2) (  9.0) ( 10.8)
g_E        19.7    19.7    19.7    19.7    19.7    19.7    19.7    19.7    19.7    19.7
        ( 11.9) ( 11.9) ( 11.9) ( 11.9) ( 11.9) ( 11.9) ( 11.9) ( 11.9) ( 11.9) ( 11.9)
           25.3     0.6   -11.9   -11.9    -9.5    -5.0     9.5    -5.0    -8.2    -3.2
         15.220   0.376  -7.160  -7.160  -5.753  -3.009   5.724  -3.009  -4.917  -1.946
c_O          14      13      11      15      16      16      14      18      14       9
        (  8.4) (  7.8) (  6.6) (  9.0) (  9.6) (  9.6) (  8.4) ( 10.8) (  8.4) (  5.4)
c_E        17.0    17.0    17.0    17.0    17.0    17.0    17.0    17.0    17.0    17.0
        ( 10.2) ( 10.2) ( 10.2) ( 10.2) ( 10.2) ( 10.2) ( 10.2) ( 10.2) ( 10.2) ( 10.2)
           -5.5    -7.0    -9.6    -3.8    -2.0    -2.0    -5.5     2.0    -5.5   -11.5
         -3.285  -4.211  -5.777  -2.273  -1.180  -1.180  -3.285   1.227  -3.285  -6.903

N           166     166     166     166     166     166     166     166     166     166
X2          8.6     1.1     7.9     4.0     2.4     1.8     2.4     0.7     2.1     5.3
Gvalue      8.0     1.2     8.5     4.5     2.5     1.7     2.4     0.7     2.2     6.0
Gv/N      4.806   0.741   5.122   2.688   1.522   1.044   1.429   0.424   1.349   3.629
Prob    0.04647 0.74569 0.03670 0.21571 0.47052 0.62957 0.49889 0.87238 0.52426 0.11041
        -------------------------------------------------------------------------------
             10       9       8       7       6       5       4       3       2       1
        -------------------------------------------------------------------------------
a_O          72      70      80      70      73      74      71      72      82      61
        ( 43.4) ( 42.2) ( 48.2) ( 42.2) ( 44.0) ( 44.6) ( 42.8) ( 43.4) ( 49.4) ( 36.7)
a_E        68.3    68.3    68.3    68.3    68.3    68.3    68.3    68.3    68.3    68.3
        ( 41.2) ( 41.2) ( 41.2) ( 41.2) ( 41.2) ( 41.2) ( 41.2) ( 41.2) ( 41.2) ( 41.2)
            7.6     3.4    25.3     3.4     9.7    11.8     5.5     7.6    30.0   -13.8
          4.564   2.061  15.226   2.061   5.840   7.133   3.304   4.564  18.046  -8.318
t_O          58      56      57      64      58      70      55      62      57      80
        ( 34.9) ( 33.7) ( 34.3) ( 38.6) ( 34.9) ( 42.2) ( 33.1) ( 37.3) ( 34.3) ( 48.2)
t_E        61.0    61.0    61.0    61.0    61.0    61.0    61.0    61.0    61.0    61.0
        ( 36.7) ( 36.7) ( 36.7) ( 36.7) ( 36.7) ( 36.7) ( 36.7) ( 36.7) ( 36.7) ( 36.7)
           -5.8    -9.6    -7.7     6.2    -5.8    19.3   -11.4     2.0    -7.7    43.4
         -3.513  -5.759  -4.646   3.715  -3.513  11.620  -6.850   1.227  -4.646  26.151
g_O          21      26      14      23      14       9      23      19      16      16
        ( 12.7) ( 15.7) (  8.4) ( 13.9) (  8.4) (  5.4) ( 13.9) ( 11.4) (  9.6) (  9.6)
g_E        19.7    19.7    19.7    19.7    19.7    19.7    19.7    19.7    19.7    19.7
        ( 11.9) ( 11.9) ( 11.9) ( 11.9) ( 11.9) ( 11.9) ( 11.9) ( 11.9) ( 11.9) ( 11.9)
            2.7    14.5    -9.5     7.1    -9.5   -14.1     7.1    -1.4    -6.6    -6.6
          1.630   8.708  -5.753   4.306  -5.753  -8.489   4.306  -0.817  -4.000  -4.000
c_O          15      14      15       9      21      13      17      13      11       9
        (  9.0) (  8.4) (  9.0) (  5.4) ( 12.7) (  7.8) ( 10.2) (  7.8) (  6.6) (  5.4)
c_E        17.0    17.0    17.0    17.0    17.0    17.0    17.0    17.0    17.0    17.0
        ( 10.2) ( 10.2) ( 10.2) ( 10.2) ( 10.2) ( 10.2) ( 10.2) ( 10.2) ( 10.2) ( 10.2)
           -3.8    -5.5    -3.8   -11.5     8.9    -7.0    -0.0    -7.0    -9.6   -11.5
         -2.273  -3.285  -2.273  -6.903   5.331  -4.211  -0.012  -4.211  -5.777  -6.903

N           166     166     166     166     166     166     166     166     166     166
X2          0.7     3.0     4.1     4.5     3.0     8.6     1.3     1.2     5.8    11.2
Gvalue      0.7     2.9     4.2     5.3     3.2    10.0     1.2     1.3     6.0    11.5
Gv/N      0.408   1.725   2.554   3.179   1.906   6.054   0.747   0.763   3.622   6.930
Prob    0.87845 0.41311 0.23667 0.15261 0.36692 0.01815 0.74324 0.73704 0.11098 0.00929
        -------------------------------------------------------------------------------

CDS_INITIAL:
        -------------------------------------------------------------------------------
```

	1	2	3	4	5	6	7	8	9	10
a_O	134	0	0	96	83	69	88	78	73	78
	(80.7)	(0.0)	(0.0)	(57.8)	(50.0)	(41.6)	(53.0)	(47.0)	(44.0)	(47.0)
a_E	69.2	64.8	62.5	69.2	64.8	62.5	69.2	64.8	62.5	69.2
	(41.7)	(39.0)	(37.6)	(41.7)	(39.0)	(37.6)	(41.7)	(39.0)	(37.6)	(41.7)
	177.0	0.0	0.0	62.8	41.2	13.7	42.2	29.0	22.7	18.6
	106.640	0.000	0.000	37.827	24.822	8.267	25.449	17.488	13.703	11.221
t_O	23	166	0	25	58	73	41	65	61	40
	(13.9)	(100.0)	(0.0)	(15.1)	(34.9)	(44.0)	(24.7)	(39.2)	(36.7)	(24.1)
t_E	39.7	58.4	71.0	39.7	58.4	71.0	39.7	58.4	71.0	39.7
	(23.9)	(35.2)	(42.7)	(23.9)	(35.2)	(42.7)	(23.9)	(35.2)	(42.7)	(23.9)
	-25.1	346.8	0.0	-23.1	-0.8	4.2	2.6	13.9	-18.4	0.6
	-15.136	208.943	0.000	-13.941	-0.477	2.503	1.574	8.388	-11.107	0.346
g_O	9	0	166	19	12	13	32	10	23	33
	(5.4)	(0.0)	(100.0)	(11.4)	(7.2)	(7.8)	(19.3)	(6.0)	(13.9)	(19.9)
g_E	39.7	17.9	16.5	39.7	17.9	16.5	39.7	17.9	16.5	39.7
	(23.9)	(10.8)	(10.0)	(23.9)	(10.8)	(10.0)	(23.9)	(10.8)	(10.0)	(23.9)
	-26.7	0.0	766.0	-28.0	-9.7	-6.2	-13.8	-11.7	15.2	-12.2
	-16.091	0.000	461.418	-16.866	-5.817	-3.758	-8.307	-7.044	9.161	-7.343
c_O	0	0	0	26	13	11	5	13	9	15
	(0.0)	(0.0)	(0.0)	(15.7)	(7.8)	(6.6)	(3.0)	(7.8)	(5.4)	(9.0)
c_E	17.4	24.9	16.1	17.4	24.9	16.1	17.4	24.9	16.1	17.4
	(10.5)	(15.0)	(9.7)	(10.5)	(15.0)	(9.7)	(10.5)	(15.0)	(9.7)	(10.5)
	0.0	0.0	0.0	21.0	-16.9	-8.3	-12.5	-16.9	-10.4	-4.4
	0.000	0.000	0.000	12.634	-10.181	-5.011	-7.502	-10.181	-6.276	-2.652
N	166	166	166	166	166	166	166	166	166	166
X2	108.8	305.9	1501.5	30.9	12.8	3.1	15.4	12.7	8.8	2.6
Gvalue	125.2	346.8	766.0	32.6	13.9	3.3	18.6	14.4	9.1	2.6
Gv/N	75.413	208.943	461.418	19.654	8.346	2.001	11.214	8.651	5.481	1.572
Prob	0.00000	0.00000	0.00000	0.00000	0.00311	0.34458	0.00033	0.00245	0.02800	0.45594

	11	12	13	14	15	16	17	18	19	20
a_O	62	73	80	66	70	68	58	70	85	71
	(37.3)	(44.0)	(48.2)	(39.8)	(42.2)	(41.0)	(34.9)	(42.2)	(51.2)	(42.8)
a_E	64.8	62.5	69.2	64.8	62.5	69.2	64.8	62.5	69.2	64.8
	(39.0)	(37.6)	(41.7)	(39.0)	(37.6)	(41.7)	(39.0)	(37.6)	(41.7)	(39.0)
	-5.4	22.7	23.2	2.5	15.9	-2.4	-12.8	15.9	34.9	13.1
	-3.249	13.703	13.949	1.513	9.601	-1.458	-7.699	9.601	21.029	7.875
t_O	70	62	42	71	71	46	72	62	43	67
	(42.2)	(37.3)	(25.3)	(42.8)	(42.8)	(27.7)	(43.4)	(37.3)	(25.9)	(40.4)
t_E	58.4	71.0	39.7	58.4	71.0	39.7	58.4	71.0	39.7	58.4
	(35.2)	(42.7)	(23.9)	(35.2)	(42.7)	(23.9)	(35.2)	(42.7)	(23.9)	(35.2)
	25.4	-16.7	4.7	27.7	0.1	13.5	30.2	-16.7	6.8	18.4
	15.284	-10.074	2.832	16.715	0.058	8.143	18.164	-10.074	4.118	11.093
g_O	20	17	26	10	14	30	13	22	23	16
	(12.0)	(10.2)	(15.7)	(6.0)	(8.4)	(18.1)	(7.8)	(13.3)	(13.9)	(9.6)
g_E	17.9	16.5	39.7	17.9	16.5	39.7	17.9	16.5	39.7	17.9
	(10.8)	(10.0)	(23.9)	(10.8)	(10.0)	(23.9)	(10.8)	(10.0)	(23.9)	(10.8)
	4.3	1.0	-22.0	-11.7	-4.6	-16.8	-8.4	12.6	-25.1	-3.7
	2.614	0.580	-13.254	-7.044	-2.797	-10.121	-5.048	7.585	-15.122	-2.211
c_O	14	14	18	19	11	22	23	12	15	12
	(8.4)	(8.4)	(10.8)	(11.4)	(6.6)	(13.3)	(13.9)	(7.2)	(9.0)	(7.2)
c_E	24.9	16.1	17.4	24.9	16.1	17.4	24.9	16.1	17.4	24.9
	(15.0)	(9.7)	(10.5)	(15.0)	(9.7)	(10.5)	(15.0)	(9.7)	(10.5)	(15.0)
	-16.1	-3.8	1.3	-10.3	-8.3	10.4	-3.7	-7.0	-4.4	-17.5
	-9.714	-2.310	0.772	-6.193	-5.011	6.262	-2.202	-4.209	-2.652	-10.555
N	166	166	166	166	166	166	166	166	166	166
X2	7.4	3.2	6.6	7.7	2.9	4.6	5.4	4.9	11.2	8.8
Gvalue	8.2	3.2	7.1	8.3	3.1	4.7	5.3	4.8	12.2	10.3
Gv/N	4.935	1.899	4.299	4.992	1.851	2.827	3.214	2.902	7.374	6.202
Prob	0.04221	0.36878	0.06769	0.04045	0.38066	0.19575	0.14881	0.18562	0.00660	0.01622

	21	22	23	24	25	26	27	28	29	30
a_O	57	68	61	65	75	63	72	76	53	70
	(34.3)	(41.0)	(36.7)	(39.2)	(45.2)	(38.0)	(43.4)	(45.8)	(31.9)	(42.2)
a_E	62.5	69.2	64.8	62.5	69.2	64.8	62.5	69.2	64.8	62.5
	(37.6)	(41.7)	(39.0)	(37.6)	(41.7)	(39.0)	(37.6)	(41.7)	(39.0)	(37.6)
	-10.4	-2.4	-7.3	5.2	12.0	-3.5	20.4	14.2	-21.2	15.9
	-6.291	-1.458	-4.391	3.111	7.245	-2.086	12.319	8.555	-12.792	9.601
t_O	73	46	66	71	42	71	67	46	83	64
	(44.0)	(27.7)	(39.8)	(42.8)	(25.3)	(42.8)	(40.4)	(27.7)	(50.0)	(38.6)
t_E	71.0	39.7	58.4	71.0	39.7	58.4	71.0	39.7	58.4	71.0
	(42.7)	(23.9)	(35.2)	(42.7)	(23.9)	(35.2)	(42.7)	(23.9)	(35.2)	(42.7)
	4.2	13.5	16.2	0.1	4.7	27.7	-7.7	13.5	58.4	-13.2
	2.503	8.143	9.732	0.058	2.832	16.715	-4.626	8.143	35.157	-7.951
g_O	15	41	10	15	28	16	17	28	12	15
	(9.0)	(24.7)	(6.0)	(9.0)	(16.9)	(9.6)	(10.2)	(16.9)	(7.2)	(9.0)
g_E	16.5	39.7	17.9	16.5	39.7	17.9	16.5	39.7	17.9	16.5
	(10.0)	(23.9)	(10.8)	(10.0)	(23.9)	(10.8)	(10.0)	(23.9)	(10.8)	(10.0)
	-2.9	2.7	-11.7	-2.9	-19.5	-3.7	1.0	-19.5	-9.7	-2.9
	-1.750	1.599	-7.044	-1.750	-11.773	-2.211	0.580	-11.773	-5.817	-1.750
c_O	21	11	29	15	21	16	10	16	18	17
	(12.7)	(6.6)	(17.5)	(9.0)	(12.7)	(9.6)	(6.0)	(9.6)	(10.8)	(10.2)
c_E	16.1	17.4	24.9	16.1	17.4	24.9	16.1	17.4	24.9	16.1
	(9.7)	(10.5)	(15.0)	(9.7)	(10.5)	(15.0)	(9.7)	(10.5)	(15.0)	(9.7)
	11.3	-10.1	8.8	-2.0	8.0	-14.2	-9.5	-2.6	-11.7	1.9
	6.794	-6.055	5.322	-1.228	4.801	-8.528	-5.704	-1.585	-7.039	1.172
N	166	166	166	166	166	166	166	166	166	166
X2	2.2	3.4	5.4	0.3	4.8	6.2	4.0	5.2	16.4	1.8
Gvalue	2.1	3.7	6.0	0.3	5.2	6.5	4.3	5.5	15.8	1.8
Gv/N	1.256	2.229	3.618	0.191	3.104	3.891	2.569	3.340	9.508	1.071
Prob	0.55505	0.29570	0.11130	0.95671	0.16090	0.09132	0.23429	0.13598	0.00126	0.61972

	31	32	33	34	35	36	37	38	39	40
a_O	76	74	71	70	59	66	78	59	61	73
	(45.8)	(44.6)	(42.8)	(42.2)	(35.5)	(39.8)	(47.0)	(35.5)	(36.7)	(44.0)
a_E	69.2	64.8	62.5	69.2	64.8	62.5	69.2	64.8	62.5	69.2
	(41.7)	(39.0)	(37.6)	(41.7)	(39.0)	(37.6)	(41.7)	(39.0)	(37.6)	(41.7)
	14.2	19.7	18.2	1.6	-11.0	7.3	18.6	-11.0	-2.9	7.8
	8.555	11.897	10.951	0.944	-6.617	4.373	11.221	-6.617	-1.748	4.675
t_O	42	67	64	47	76	72	46	75	76	42
	(25.3)	(40.4)	(38.6)	(28.3)	(45.8)	(43.4)	(27.7)	(45.2)	(45.8)	(25.3)
t_E	39.7	58.4	71.0	39.7	58.4	71.0	39.7	58.4	71.0	39.7
	(23.9)	(35.2)	(42.7)	(23.9)	(35.2)	(42.7)	(23.9)	(35.2)	(42.7)	(23.9)
	4.7	18.4	-13.2	15.8	40.0	2.1	13.5	37.5	10.4	4.7

	2.832	11.093	-7.951	9.538	24.124	1.273	8.143	22.610	6.294	2.832
g_O	36	9	17	36	12	11	21	9	16	29
	(21.7)	(5.4)	(10.2)	(21.7)	(7.2)	(6.6)	(12.7)	(5.4)	(9.6)	(17.5)
g_E	39.7	17.9	16.5	39.7	17.9	16.5	39.7	17.9	16.5	39.7
	(23.9)	(10.8)	(10.0)	(23.9)	(10.8)	(10.0)	(23.9)	(10.8)	(10.0)	(23.9)
	-7.0	-12.4	1.0	-7.0	-9.7	-9.0	-26.7	-12.4	-1.0	-18.2
	-4.237	-7.482	0.580	-4.237	-5.817	-5.394	-16.109	-7.482	-0.623	-10.968
c_O	12	16	14	13	19	17	21	23	13	22
	(7.2)	(9.6)	(8.4)	(7.8)	(11.4)	(10.2)	(12.7)	(13.9)	(7.8)	(13.3)
c_E	17.4	24.9	16.1	17.4	24.9	16.1	17.4	24.9	16.1	17.4
	(10.5)	(15.0)	(9.7)	(10.5)	(15.0)	(9.7)	(10.5)	(15.0)	(9.7)	(10.5)
	-8.9	-14.2	-3.8	-7.5	-10.3	1.9	8.0	-3.7	-5.5	10.4
	-5.348	-8.528	-2.310	-4.540	-6.193	1.172	4.801	-2.202	-3.306	6.262
N	166	166	166	166	166	166	166	166	166	166
X2	2.8	10.2	2.1	2.8	9.2	2.1	11.7	9.8	1.0	4.5
Gvalue	3.0	11.6	2.1	2.8	9.1	2.4	13.4	10.5	1.0	4.7
Gv/N	1.802	6.980	1.270	1.705	5.497	1.423	8.056	6.308	0.617	2.801
Prob	0.39291	0.00894	0.55021	0.41840	0.02767	0.50061	0.00389	0.01495	0.79528	0.19929

	41	42	43	44	45	46	47	48	49	50
a_O	58	77	60	58	55	68	51	59	67	60
	(34.9)	(46.4)	(36.1)	(34.9)	(33.1)	(41.0)	(30.7)	(35.5)	(40.4)	(36.1)
a_E	64.8	62.5	69.2	64.8	62.5	69.2	64.8	62.5	69.2	64.8
	(39.0)	(37.6)	(41.7)	(39.0)	(37.6)	(41.7)	(39.0)	(37.6)	(41.7)	(39.0)
	-12.8	32.2	-17.2	-12.8	-14.0	-2.4	-24.4	-6.7	-4.4	-9.2
	-7.699	19.403	-10.335	-7.699	-8.437	-1.458	-14.673	-4.061	-2.633	-5.514
t_O	74	61	54	75	79	47	74	71	50	63
	(44.6)	(36.7)	(32.5)	(45.2)	(47.6)	(28.3)	(44.6)	(42.8)	(30.1)	(38.0)
t_E	58.4	71.0	39.7	58.4	71.0	39.7	58.4	71.0	39.7	58.4
	(35.2)	(42.7)	(23.9)	(35.2)	(42.7)	(23.9)	(35.2)	(42.7)	(23.9)	(35.2)
	35.0	-18.4	33.2	37.5	17.0	15.8	35.0	0.1	23.0	9.6
	21.112	-11.107	19.992	22.610	10.227	9.538	21.112	0.058	13.875	5.758
g_O	13	12	34	12	17	30	12	20	28	19
	(7.8)	(7.2)	(20.5)	(7.2)	(10.2)	(18.1)	(7.2)	(12.0)	(16.9)	(11.4)
g_E	17.9	16.5	39.7	17.9	16.5	39.7	17.9	16.5	39.7	17.9
	(10.8)	(10.0)	(23.9)	(10.8)	(10.0)	(23.9)	(10.8)	(10.0)	(23.9)	(10.8)
	-8.4	-7.7	-10.5	-9.7	1.0	-16.8	-9.7	7.6	-19.5	2.2
	-5.048	-4.626	-6.343	-5.817	0.580	-10.121	-5.817	4.598	-11.773	1.309
c_O	21	16	18	21	15	21	29	16	21	24
	(12.7)	(9.6)	(10.8)	(12.7)	(9.0)	(12.7)	(17.5)	(9.6)	(12.7)	(14.5)
c_E	24.9	16.1	17.4	24.9	16.1	17.4	24.9	16.1	17.4	24.9
	(15.0)	(9.7)	(10.5)	(15.0)	(9.7)	(10.5)	(15.0)	(9.7)	(10.5)	(15.0)
	-7.2	-0.1	1.3	-7.2	-2.0	8.0	8.8	-0.1	8.0	-1.8
	-4.312	-0.066	0.772	-4.312	-1.228	4.801	5.322	-0.066	4.801	-1.067
N	166	166	166	166	166	166	166	166	166	166
X2	6.8	6.0	7.2	8.0	1.9	4.5	9.7	0.9	6.9	0.8
Gvalue	6.7	6.0	6.8	7.9	1.9	4.6	9.9	0.9	7.1	0.8
Gv/N	4.051	3.604	4.086	4.781	1.142	2.760	5.944	0.530	4.269	0.485
Prob	0.08119	0.11247	0.07916	0.04735	0.59440	0.20512	0.01974	0.83011	0.06919	0.84809

	51	52	53	54	55	56	57	58	59	60
a_O	71	68	55	45	63	61	60	62	51	56
	(42.8)	(41.2)	(33.3)	(27.3)	(38.2)	(37.0)	(36.4)	(37.8)	(31.1)	(34.1)
a_E	62.5	68.8	64.4	62.1	68.8	64.4	62.1	68.4	64.0	61.7
	(37.6)	(41.7)	(39.0)	(37.6)	(41.7)	(39.0)	(37.6)	(41.7)	(39.0)	(37.6)
	18.2	-1.6	-17.3	-29.0	-11.1	-6.6	-4.1	-12.2	-23.1	-10.9
	10.951	-0.969	-10.483	-17.561	-6.730	-3.971	-2.493	-7.414	-14.098	-6.637
t_O	63	51	67	84	54	65	71	45	64	73
	(38.0)	(30.9)	(40.6)	(50.9)	(32.7)	(39.4)	(43.0)	(27.4)	(39.0)	(44.5)
t_E	71.0	39.5	58.0	70.5	39.5	58.0	70.5	39.2	57.7	70.1
	(42.7)	(23.9)	(35.2)	(42.7)	(23.9)	(35.2)	(42.7)	(23.9)	(35.2)	(42.7)
	-15.0	26.1	19.2	29.4	33.8	14.7	1.0	12.3	13.3	5.9
	-9.022	15.836	11.651	17.804	20.508	8.915	0.579	7.523	8.096	3.613
g_O	12	29	17	16	35	18	15	36	16	17
	(7.2)	(17.6)	(10.3)	(9.7)	(21.2)	(10.9)	(9.1)	(22.0)	(9.8)	(10.4)
g_E	16.5	39.5	17.8	16.4	39.5	17.8	16.4	39.2	17.7	16.3
	(10.0)	(23.9)	(10.8)	(10.0)	(23.9)	(10.8)	(10.0)	(23.9)	(10.8)	(10.0)
	-7.7	-17.9	-1.6	-0.8	-8.4	0.3	-2.7	-6.2	-3.3	1.4
	-4.626	-10.822	-0.989	-0.509	-5.083	0.200	-1.651	-3.756	-2.001	0.838
c_O	20	17	26	20	13	21	19	21	33	18
	(12.0)	(10.3)	(15.8)	(12.1)	(7.9)	(12.7)	(11.5)	(12.8)	(20.1)	(11.0)
c_E	16.1	17.3	24.8	16.0	17.3	24.8	16.0	17.2	24.6	15.9
	(9.7)	(10.5)	(15.0)	(9.7)	(10.5)	(15.0)	(9.7)	(10.5)	(15.0)	(9.7)
	8.8	-0.5	2.6	9.0	-7.4	-6.9	6.6	8.5	19.4	4.6
	5.295	-0.320	1.550	5.473	-4.472	-4.185	4.018	5.169	11.818	2.777
N	166	165	165	165	165	165	165	164	164	164
X2	4.3	6.1	2.8	8.3	7.4	1.6	0.8	2.6	6.4	1.0
Gvalue	4.3	6.1	2.9	8.6	7.0	1.6	0.7	2.5	6.3	1.0
Gv/N	2.597	3.725	1.728	5.207	4.224	0.959	0.453	1.522	3.815	0.591
Prob	0.22973	0.10474	0.41516	0.03525	0.07289	0.66338	0.86181	0.47595	0.09978	0.80880

	61	62	63	64	65	66	67	68	69	70
a_O	62	53	59	51	54	58	72	61	68	68
	(37.8)	(32.3)	(36.0)	(31.3)	(33.1)	(35.6)	(44.4)	(37.7)	(42.0)	(42.0)
a_E	68.4	64.0	61.7	68.0	63.6	61.3	67.6	63.2	61.0	67.6
	(41.7)	(39.0)	(37.6)	(41.7)	(39.0)	(37.6)	(41.7)	(39.0)	(37.6)	(41.7)
	-12.2	-20.0	-5.3	-29.3	-17.6	-6.5	9.2	-4.3	14.9	0.9
	-7.414	-12.165	-3.238	-17.975	-10.827	-3.984	5.667	-2.663	9.171	0.554
t_O	43	66	76	52	70	81	42	68	69	42
	(26.2)	(40.2)	(46.3)	(31.9)	(42.9)	(49.7)	(25.9)	(42.0)	(42.6)	(25.9)
t_E	39.2	57.7	70.1	39.0	57.3	69.7	38.8	57.0	69.2	38.8
	(23.9)	(35.2)	(42.7)	(23.9)	(35.2)	(42.7)	(23.9)	(35.2)	(42.7)	(23.9)
	7.9	17.8	12.3	29.9	27.9	24.4	6.7	24.0	-0.5	6.7
	4.804	10.826	7.494	18.361	17.131	14.977	4.167	14.828	-0.298	4.167
g_O	35	18	18	44	12	12	36	10	13	35
	(21.3)	(11.0)	(11.0)	(27.0)	(7.4)	(7.4)	(22.2)	(6.2)	(8.0)	(21.6)
g_E	39.2	17.7	16.3	39.0	17.6	16.2	38.7	17.5	16.1	38.7
	(23.9)	(10.8)	(10.0)	(23.9)	(10.8)	(10.0)	(23.9)	(10.8)	(10.0)	(23.9)
	-8.0	0.5	3.5	10.7	-9.2	-7.2	-5.3	-11.2	-5.6	-7.1
	-4.855	0.334	2.142	6.545	-5.656	-4.443	-3.257	-6.917	-3.460	-4.384
c_O	24	27	11	16	27	12	12	23	12	17
	(14.6)	(16.5)	(6.7)	(9.8)	(16.6)	(7.4)	(7.4)	(14.2)	(7.4)	(10.5)
c_E	17.2	24.6	15.9	17.1	24.5	15.8	17.0	24.3	15.7	17.0
	(10.5)	(15.0)	(9.7)	(10.5)	(15.0)	(9.7)	(10.5)	(15.0)	(9.7)	(10.5)
	16.1	5.0	-8.1	-2.0	5.4	-6.5	-8.3	-2.5	-6.4	0.1
	9.816	3.062	-4.910	-1.256	3.283	-4.018	-5.118	-1.564	-3.951	0.059

N	164	164	164	163	163	163	162	162	162	162
X2	4.1	3.3	2.3	9.3	6.3	4.0	2.2	5.5	2.3	0.6
Gvalue	3.9	3.4	2.4	9.3	6.4	4.1	2.4	6.0	2.4	0.6
Gv/N	2.353	2.057	1.489	5.675	3.932	2.532	1.458	3.684	1.462	0.395
Prob	0.27720	0.33751	0.48588	0.02613	0.09329	0.24803	0.50086	0.11318	0.49958	0.88722

	71	72	73	74	75	76	77	78	79	80
a_O	58	50	69	62	62	65	61	62	62	62
	(35.8)	(30.9)	(42.6)	(38.3)	(38.3)	(40.1)	(37.7)	(38.3)	(38.3)	(38.3)
a_E	63.2	61.0	67.6	63.2	61.0	67.6	63.2	61.0	67.6	63.2
	(39.0)	(37.6)	(41.7)	(39.0)	(37.6)	(41.7)	(39.0)	(37.6)	(41.7)	(39.0)
	-10.0	-19.8	2.9	-2.4	2.1	-5.0	-4.3	2.1	-10.6	-2.4
	-6.143	-12.237	1.805	-1.462	1.291	-3.092	-2.663	1.291	-6.566	-1.462
t_O	57	84	39	65	70	51	64	75	52	63
	(35.2)	(51.9)	(24.1)	(40.1)	(43.2)	(31.5)	(39.5)	(46.3)	(32.1)	(38.9)
t_E	57.0	69.2	38.8	57.0	69.2	38.8	57.0	69.2	38.8	57.0
	(35.2)	(42.7)	(23.9)	(35.2)	(42.7)	(23.9)	(35.2)	(42.7)	(23.9)	(35.2)
	0.0	32.5	0.5	17.1	1.5	28.0	14.8	12.0	30.6	12.6
	0.012	20.037	0.301	10.553	0.941	17.284	9.166	7.397	18.870	7.797
g_O	18	13	34	21	15	34	13	14	32	13
	(11.1)	(8.0)	(21.0)	(13.0)	(9.3)	(21.0)	(8.0)	(8.6)	(19.8)	(8.0)
g_E	17.5	16.1	38.7	17.5	16.1	38.7	17.5	16.1	38.7	17.5
	(10.8)	(10.0)	(23.9)	(10.8)	(10.0)	(23.9)	(10.8)	(10.0)	(23.9)	(10.8)
	1.0	-5.6	-8.9	7.6	-2.2	-8.9	-7.7	-4.0	-12.2	-7.7
	0.611	-3.460	-5.476	4.709	-1.342	-5.476	-4.782	-2.445	-7.549	-4.782
c_O	29	15	20	14	15	12	24	11	16	24
	(17.9)	(9.3)	(12.3)	(8.6)	(9.3)	(7.4)	(14.8)	(6.8)	(9.9)	(14.8)
c_E	24.3	15.7	17.0	24.3	15.7	17.0	24.3	15.7	17.0	24.3
	(15.0)	(9.7)	(10.5)	(15.0)	(9.7)	(10.5)	(15.0)	(9.7)	(10.5)	(15.0)
	10.2	-1.3	6.6	-15.4	-1.3	-8.3	-0.6	-7.8	-1.8	-0.6
	6.327	-0.807	4.082	-9.532	-0.807	-5.118	-0.371	-4.804	-1.142	-0.371
N	162	162	162	162	162	162	162	162	162	162
X2	1.3	5.8	1.2	6.2	0.1	6.0	2.1	2.2	6.2	1.8
Gvalue	1.3	5.7	1.2	6.9	0.1	5.8	2.2	2.3	5.9	1.9
Gv/N	0.807	3.533	0.713	4.268	0.084	3.598	1.350	1.439	3.613	1.183
Prob	0.72738	0.12586	0.76389	0.07468	0.98728	0.12021	0.53444	0.50654	0.11896	0.58989

	81	82	83	84	85	86	87	88	89	90
a_O	56	54	56	65	74	75	52	63	49	64
	(34.6)	(33.3)	(34.6)	(40.1)	(45.7)	(46.3)	(32.1)	(39.1)	(30.4)	(39.8)
a_E	61.0	67.6	63.2	61.0	67.6	63.2	61.0	67.1	62.8	60.6
	(37.6)	(41.7)	(39.0)	(37.6)	(41.7)	(39.0)	(37.6)	(41.7)	(39.0)	(37.6)
	-9.5	-24.2	-13.5	8.3	13.5	25.7	-16.5	-8.0	-24.3	7.0
	-5.871	-14.929	-8.357	5.145	8.327	15.857	-10.209	-4.976	-15.109	4.357
t_O	76	55	61	71	33	43	71	44	61	74
	(46.9)	(34.0)	(37.7)	(43.8)	(20.4)	(26.5)	(43.8)	(27.3)	(37.9)	(46.0)
t_E	69.2	38.8	57.0	69.2	38.8	57.0	69.2	38.5	56.6	68.8
	(42.7)	(23.9)	(35.2)	(42.7)	(23.9)	(35.2)	(42.7)	(23.9)	(35.2)	(42.7)
	14.2	38.5	8.3	3.6	-10.6	-24.2	3.6	11.7	9.1	10.8
	8.738	23.767	5.120	2.198	-6.551	-14.953	2.198	7.273	5.621	6.679
g_O	13	35	15	7	41	21	18	36	19	9
	(8.0)	(21.6)	(9.3)	(4.3)	(25.3)	(13.0)	(11.1)	(22.4)	(11.8)	(5.6)
g_E	16.1	38.7	17.5	16.1	38.7	17.5	16.1	38.5	17.4	16.0
	(10.0)	(23.9)	(10.8)	(10.0)	(23.9)	(10.8)	(10.0)	(23.9)	(10.8)	(10.0)
	-5.6	-7.1	-4.6	-11.7	4.7	7.6	4.0	-4.8	3.3	-10.4
	-3.460	-4.384	-2.867	-7.213	2.873	4.709	2.441	-3.001	2.071	-6.452
c_O	17	18	30	19	14	23	21	18	32	14
	(10.5)	(11.1)	(18.5)	(11.7)	(8.6)	(14.2)	(13.0)	(11.2)	(19.9)	(8.7)
c_E	15.7	17.0	24.3	15.7	17.0	24.3	15.7	16.8	24.2	15.6
	(9.7)	(10.5)	(15.0)	(9.7)	(10.5)	(15.0)	(9.7)	(10.5)	(15.0)	(9.7)
	2.8	2.2	12.6	7.3	-5.4	-2.5	12.3	2.4	18.0	-3.0
	1.713	1.333	7.801	4.523	-3.307	-1.564	7.594	1.480	11.184	-1.850
N	162	162	162	162	162	162	162	161	161	161
X2	1.8	10.0	2.8	6.2	2.1	6.4	3.4	1.3	6.1	3.8
Gvalue	1.8	9.4	2.7	7.5	2.2	6.6	3.3	1.2	6.1	4.4
Gv/N	1.120	5.787	1.697	4.654	1.342	4.049	2.024	0.776	3.768	2.734
Prob	0.61174	0.02471	0.43197	0.05656	0.53708	0.08736	0.35052	0.74133	0.10844	0.22121

	91	92	93	94	95	96	97	98	99	100
a_O	57	62	65	59	70	56	72	71	53	59
	(35.4)	(38.5)	(40.4)	(36.9)	(43.8)	(35.0)	(45.0)	(44.4)	(33.1)	(36.9)
a_E	67.1	62.8	60.6	66.7	62.4	60.2	66.7	62.4	60.2	66.7
	(41.7)	(39.0)	(37.6)	(41.7)	(39.0)	(37.6)	(41.7)	(39.0)	(37.6)	(41.7)
	-18.7	-1.6	9.1	-14.5	16.1	-8.1	11.0	18.3	-13.5	-14.5
	-11.589	-0.994	5.677	-9.068	10.035	-5.075	6.856	11.437	-8.450	-9.068
t_O	42	56	66	43	54	70	35	59	73	47
	(26.1)	(34.8)	(41.0)	(26.9)	(33.8)	(43.8)	(21.9)	(36.9)	(45.6)	(29.4)
t_E	38.5	56.6	68.8	38.3	56.3	68.4	38.3	56.3	68.4	38.3
	(23.9)	(35.2)	(42.7)	(23.9)	(35.2)	(42.7)	(23.9)	(35.2)	(42.7)	(23.9)
	7.3	-1.3	-5.5	10.0	-4.5	3.3	-6.3	5.6	9.5	19.3
	4.516	-0.789	-3.424	6.252	-2.800	2.040	-3.917	3.472	5.956	12.059
g_O	44	18	13	42	18	18	36	13	16	37
	(27.3)	(11.2)	(8.1)	(26.2)	(11.2)	(11.2)	(22.5)	(8.1)	(10.0)	(23.1)
g_E	38.5	17.4	16.0	38.3	17.3	15.9	38.3	17.3	15.9	38.3
	(23.9)	(10.8)	(10.0)	(23.9)	(10.8)	(10.0)	(23.9)	(10.8)	(10.0)	(23.9)
	11.8	1.2	-5.4	7.8	1.4	4.4	-4.4	-7.4	0.1	-2.5
	7.301	0.753	-3.381	4.897	0.898	2.751	-2.739	-4.639	0.090	-1.548
c_O	18	25	17	16	18	16	17	17	18	17
	(11.2)	(15.5)	(10.6)	(10.0)	(11.2)	(10.0)	(10.6)	(10.6)	(11.2)	(10.6)
c_E	16.8	24.2	15.6	16.7	24.0	15.5	16.7	24.0	15.5	16.7
	(10.5)	(15.0)	(9.7)	(10.5)	(15.0)	(9.7)	(10.5)	(15.0)	(9.7)	(10.5)
	2.4	1.7	3.0	-1.5	-10.4	1.1	0.5	-11.7	5.4	0.5
	1.480	1.071	1.854	-0.908	-6.475	0.668	0.324	-7.330	3.402	0.324
N	161	161	161	160	160	160	160	160	160	160
X2	2.7	0.1	1.1	1.9	2.5	0.6	0.8	4.4	1.6	2.9
Gvalue	2.7	0.1	1.2	1.9	2.7	0.6	0.8	4.7	1.6	2.8
Gv/N	1.707	0.042	0.727	1.173	1.659	0.385	0.523	2.940	0.998	1.767
Prob	0.43217	0.99546	0.76028	0.59827	0.44816	0.89288	0.84069	0.19482	0.66022	0.41910

5UTR:

	100	99	98	97	96	95	94	93	92	91
a_O	59	70	67	58	55	70	52	68	70	62
	(29.1)	(34.5)	(33.0)	(28.6)	(27.1)	(34.5)	(25.6)	(33.5)	(34.5)	(30.5)
a_E	69.1	69.1	69.1	69.1	69.1	69.1	69.1	69.1	69.1	69.1
	(34.0)	(34.0)	(34.0)	(34.0)	(34.0)	(34.0)	(34.0)	(34.0)	(34.0)	(34.0)
	-18.7	1.8	-4.2	-20.3	-25.1	1.8	-29.6	-2.2	1.8	-13.5
	-9.194	0.882	-2.047	-10.015	-12.375	0.882	-14.573	-1.085	0.882	-6.632
t_O	65	65	74	66	69	64	74	64	59	61
	(32.0)	(32.0)	(36.5)	(32.5)	(34.0)	(31.5)	(36.5)	(31.5)	(29.1)	(30.0)
t_E	65.6	65.6	65.6	65.6	65.6	65.6	65.6	65.6	65.6	65.6
	(32.3)	(32.3)	(32.3)	(32.3)	(32.3)	(32.3)	(32.3)	(32.3)	(32.3)	(32.3)
	-1.2	-1.2	17.8	0.8	7.0	-3.2	17.8	-3.2	-12.5	-8.9
	-0.598	-0.598	8.773	0.385	3.425	-1.567	8.773	-1.567	-6.173	-4.378
g_O	37	45	28	41	42	37	43	37	36	42
	(18.2)	(22.2)	(13.8)	(20.2)	(20.7)	(18.2)	(21.2)	(18.2)	(17.7)	(20.7)
g_E	34.3	34.3	34.3	34.3	34.3	34.3	34.3	34.3	34.3	34.3
	(16.9)	(16.9)	(16.9)	(16.9)	(16.9)	(16.9)	(16.9)	(16.9)	(16.9)	(16.9)
	5.5	24.3	-11.4	14.5	16.9	5.5	19.3	5.5	3.4	16.9
	2.720	11.986	-5.631	7.160	8.332	2.720	9.527	2.720	1.674	8.332
c_O	42	23	34	38	37	32	34	34	38	38
	(20.7)	(11.3)	(16.7)	(18.7)	(18.2)	(15.8)	(16.7)	(16.7)	(18.7)	(18.7)
c_E	33.9	33.9	33.9	33.9	33.9	33.9	33.9	33.9	33.9	33.9
	(16.7)	(16.7)	(16.7)	(16.7)	(16.7)	(16.7)	(16.7)	(16.7)	(16.7)	(16.7)
	17.9	-17.9	0.1	8.6	6.4	-3.8	0.1	0.1	8.6	8.6
	8.817	-8.817	0.059	4.230	3.147	-1.856	0.059	0.059	4.230	4.230
N	203	203	203	203	203	203	203	203	203	203
X2	3.6	6.9	2.3	3.6	5.0	0.4	7.5	0.3	1.2	3.2
Gvalue	3.5	7.0	2.3	3.6	5.1	0.4	7.7	0.3	1.2	3.2
Gv/N	1.745	3.453	1.155	1.761	2.529	0.180	3.787	0.127	0.614	1.552
Prob	0.31538	0.07158	0.50393	0.31112	0.16224	0.94733	0.05294	0.96758	0.74169	0.36889

	90	89	88	87	86	85	84	83	82	81
a_O	61	61	71	69	71	73	75	80	57	64
	(30.0)	(30.0)	(35.0)	(34.0)	(35.0)	(36.0)	(36.9)	(39.4)	(28.1)	(31.5)
a_E	69.1	69.1	69.1	69.1	69.1	69.1	69.1	69.1	69.1	69.1
	(34.0)	(34.0)	(34.0)	(34.0)	(34.0)	(34.0)	(34.0)	(34.0)	(34.0)	(34.0)
	-15.2	-15.2	3.8	-0.2	3.8	8.0	12.3	23.4	-22.0	-9.8
	-7.502	-7.502	1.887	-0.108	1.887	3.938	6.043	11.533	-10.819	-4.844
t_O	76	54	63	52	60	60	71	59	52	73
	(37.4)	(26.6)	(31.0)	(25.6)	(29.6)	(29.6)	(35.0)	(29.1)	(25.6)	(36.0)
t_E	65.6	65.6	65.6	65.6	65.6	65.6	65.6	65.6	65.6	65.6
	(32.3)	(32.3)	(32.3)	(32.3)	(32.3)	(32.3)	(32.3)	(32.3)	(32.3)	(32.3)
	22.3	-21.0	-5.1	-24.2	-10.7	-10.7	11.2	-12.5	-24.2	15.6
	11.007	-10.361	-2.520	-11.911	-5.284	-5.284	5.523	-6.173	-11.911	7.676
g_O	37	37	31	43	31	33	27	43	46	37
	(18.2)	(18.2)	(15.3)	(21.2)	(15.3)	(16.3)	(13.3)	(21.2)	(22.7)	(18.2)
g_E	34.3	34.3	34.3	34.3	34.3	34.3	34.3	34.3	34.3	34.3
	(16.9)	(16.9)	(16.9)	(16.9)	(16.9)	(16.9)	(16.9)	(16.9)	(16.9)	(16.9)
	5.5	5.5	-6.3	19.3	-6.3	-2.6	-13.0	19.3	26.9	5.5
	2.720	2.720	-3.125	9.527	-3.125	-1.294	-6.397	9.527	13.248	2.720
c_O	29	51	38	39	41	37	30	21	48	29
	(14.3)	(25.1)	(18.7)	(19.2)	(20.2)	(18.2)	(14.8)	(10.3)	(23.6)	(14.3)
c_E	33.9	33.9	33.9	33.9	33.9	33.9	33.9	33.9	33.9	33.9
	(16.7)	(16.7)	(16.7)	(16.7)	(16.7)	(16.7)	(16.7)	(16.7)	(16.7)	(16.7)
	-9.1	41.5	8.6	10.8	15.5	6.4	-7.4	-20.2	33.3	-9.1
	-4.494	20.462	4.230	5.340	7.634	3.147	-3.647	-9.933	16.391	-4.494
N	203	203	203	203	203	203	203	203	203	203
X2	3.5	11.8	1.0	5.8	2.3	1.0	3.0	9.5	14.7	2.1
Gvalue	3.5	10.8	1.0	5.8	2.3	1.0	3.1	10.1	14.0	2.1
Gv/N	1.731	5.319	0.473	2.848	1.112	0.507	1.522	4.955	6.911	1.058
Prob	0.31899	0.01287	0.81098	0.12273	0.52075	0.79403	0.37795	0.01807	0.00287	0.54228

	80	79	78	77	76	75	74	73	72	71
a_O	63	65	60	53	68	60	56	69	79	66
	(31.0)	(32.0)	(29.6)	(26.1)	(33.5)	(29.6)	(27.6)	(34.0)	(38.9)	(32.5)
a_E	69.1	69.1	69.1	69.1	69.1	69.1	69.1	69.1	69.1	69.1
	(34.0)	(34.0)	(34.0)	(34.0)	(34.0)	(34.0)	(34.0)	(34.0)	(34.0)	(34.0)
	-11.7	-8.0	-17.0	-28.1	-2.2	-17.0	-23.6	-0.2	21.1	-6.1
	-5.745	-3.926	-8.356	-13.859	-1.085	-8.356	-11.605	-0.108	10.410	-2.994
t_O	64	75	72	74	74	70	77	58	63	72
	(31.5)	(36.9)	(35.5)	(36.5)	(36.5)	(34.5)	(37.9)	(28.6)	(31.0)	(35.5)
t_E	65.6	65.6	65.6	65.6	65.6	65.6	65.6	65.6	65.6	65.6
	(32.3)	(32.3)	(32.3)	(32.3)	(32.3)	(32.3)	(32.3)	(32.3)	(32.3)	(32.3)
	-3.2	20.1	13.4	17.8	17.8	9.1	24.7	-14.3	-5.1	13.4
	-1.567	9.884	6.593	8.773	8.773	4.467	12.144	-7.045	-2.520	6.593
g_O	40	38	39	44	29	37	34	36	36	32
	(19.7)	(18.7)	(19.2)	(21.7)	(14.3)	(18.2)	(16.7)	(17.7)	(17.7)	(15.8)
g_E	34.3	34.3	34.3	34.3	34.3	34.3	34.3	34.3	34.3	34.3
	(16.9)	(16.9)	(16.9)	(16.9)	(16.9)	(16.9)	(16.9)	(16.9)	(16.9)	(16.9)
	12.2	7.7	9.9	21.8	-9.8	5.5	-0.7	3.4	3.4	-4.5
	6.013	3.792	4.889	10.745	-4.829	2.720	-0.333	1.674	1.674	-2.225
c_O	36	25	32	32	32	36	36	40	25	33
	(17.7)	(12.3)	(15.8)	(15.8)	(15.8)	(17.7)	(17.7)	(19.7)	(12.3)	(16.3)
c_E	33.9	33.9	33.9	33.9	33.9	33.9	33.9	33.9	33.9	33.9
	(16.7)	(16.7)	(16.7)	(16.7)	(16.7)	(16.7)	(16.7)	(16.7)	(16.7)	(16.7)
	4.2	-15.3	-3.8	-3.8	-3.8	4.2	4.2	13.1	-15.3	-1.9
	2.090	-7.530	-1.856	-1.856	-1.856	2.090	2.090	6.474	-7.530	-0.913
N	203	203	203	203	203	203	203	203	203	203
X2	1.6	4.3	2.6	7.7	2.0	1.8	4.6	2.0	4.0	0.9
Gvalue	1.6	4.5	2.6	7.7	2.0	1.9	4.7	2.0	4.1	0.9
Gv/N	0.791	2.219	1.270	3.804	1.004	0.920	2.295	0.996	2.035	0.460
Prob	0.65831	0.21190	0.46116	0.05210	0.56456	0.60018	0.19855	0.56808	0.24771	0.81706

	70	69	68	67	66	65	64	63	62	61
a_O	69	75	76	62	63	70	69	70	58	58
	(34.0)	(36.9)	(37.4)	(30.5)	(31.0)	(34.5)	(34.0)	(34.5)	(28.6)	(28.6)
a_E	69.1	69.1	69.1	69.1	69.1	69.1	69.1	69.1	69.1	69.1
	(34.0)	(34.0)	(34.0)	(34.0)	(34.0)	(34.0)	(34.0)	(34.0)	(34.0)	(34.0)
	-0.2	12.3	14.4	-13.5	-11.7	1.8	-0.2	1.8	-20.3	-20.3
	-0.108	6.043	7.116	-6.632	-5.745	0.882	-0.108	0.882	-10.015	-10.015
t_O	68	56	53	67	61	61	67	66	75	59
	(33.5)	(27.6)	(26.1)	(33.0)	(30.0)	(30.0)	(33.0)	(32.5)	(36.9)	(29.1)
t_E	65.6	65.6	65.6	65.6	65.6	65.6	65.6	65.6	65.6	65.6

	(32.3)	(32.3)	(32.3)	(32.3)	(32.3)	(32.3)	(32.3)	(32.3)	(32.3)	(32.3)
	4.9	-17.7	-22.6	2.8	-8.9	-8.9	2.8	0.8	20.1	-12.5
	2.397	-8.738	-11.145	1.384	-4.378	-4.378	1.384	0.385	9.884	-6.173
g_O	38	31	38	37	42	33	37	34	39	48
	(18.7)	(15.3)	(18.7)	(18.2)	(20.7)	(16.3)	(18.2)	(16.7)	(19.2)	(23.6)
g_E	34.3	34.3	34.3	34.3	34.3	34.3	34.3	34.3	34.3	34.3
	(16.9)	(16.9)	(16.9)	(16.9)	(16.9)	(16.9)	(16.9)	(16.9)	(16.9)	(16.9)
	7.7	-6.3	7.7	5.5	16.9	-2.6	5.5	-0.7	9.9	32.1
	3.792	-3.125	3.792	2.720	8.332	-1.294	2.720	-0.333	4.889	15.837
c_O	28	41	36	37	37	39	30	33	31	38
	(13.8)	(20.2)	(17.7)	(18.2)	(18.2)	(19.2)	(14.8)	(16.3)	(15.3)	(18.7)
c_E	33.9	33.9	33.9	33.9	33.9	33.9	33.9	33.9	33.9	33.9
	(16.7)	(16.7)	(16.7)	(16.7)	(16.7)	(16.7)	(16.7)	(16.7)	(16.7)	(16.7)
	-10.8	15.5	4.2	6.4	6.4	10.8	-7.4	-1.9	-5.6	8.6
	-5.307	7.634	2.090	3.147	3.147	5.340	-3.647	-0.913	-2.767	4.230
N	203	203	203	203	203	203	203	203	203	203
X2	1.5	3.7	3.6	1.2	2.8	1.1	0.7	0.0	4.0	8.4
Gvalue	1.6	3.7	3.8	1.3	2.8	1.1	0.7	0.0	4.0	7.9
Gv/N	0.773	1.814	1.852	0.619	1.355	0.550	0.348	0.021	1.991	3.880
Prob	0.66637	0.29788	0.28852	0.73960	0.43168	0.77330	0.87167	0.99763	0.25695	0.04863
	60	59	58	57	56	55	54	53	52	51
a_O	70	82	65	62	60	62	67	73	79	72
	(34.5)	(40.4)	(32.0)	(30.5)	(29.6)	(30.5)	(33.0)	(36.0)	(38.9)	(35.5)
a_E	69.1	69.1	69.1	69.1	69.1	69.1	69.1	69.1	69.1	69.1
	(34.0)	(34.0)	(34.0)	(34.0)	(34.0)	(34.0)	(34.0)	(34.0)	(34.0)	(34.0)
	1.8	28.0	-8.0	-13.5	-17.0	-13.5	-4.2	8.0	21.1	5.9
	0.882	13.816	-3.926	-6.632	-8.356	-6.632	-2.047	3.938	10.410	2.906
t_O	75	58	63	64	70	54	74	63	67	63
	(36.9)	(28.6)	(31.0)	(31.5)	(34.5)	(26.6)	(36.5)	(31.0)	(33.0)	(31.0)
t_E	65.6	65.6	65.6	65.6	65.6	65.6	65.6	65.6	65.6	65.6
	(32.3)	(32.3)	(32.3)	(32.3)	(32.3)	(32.3)	(32.3)	(32.3)	(32.3)	(32.3)
	20.1	-14.3	-5.1	-3.2	9.1	-21.0	17.8	-5.1	2.8	-5.1
	9.884	-7.045	-2.520	-1.567	4.467	-10.361	8.773	-2.520	1.384	-2.520
g_O	26	25	29	42	38	46	31	31	33	34
	(12.8)	(12.3)	(14.3)	(20.7)	(18.7)	(22.7)	(15.3)	(15.3)	(16.3)	(16.7)
g_E	34.3	34.3	34.3	34.3	34.3	34.3	34.3	34.3	34.3	34.3
	(16.9)	(16.9)	(16.9)	(16.9)	(16.9)	(16.9)	(16.9)	(16.9)	(16.9)	(16.9)
	-14.5	-15.9	-9.8	16.9	7.7	26.9	-6.3	-6.3	-2.6	-0.7
	-7.127	-7.819	-4.829	8.332	3.792	13.248	-3.125	-3.125	-1.294	-0.333
c_O	32	38	46	35	35	41	31	36	24	34
	(15.8)	(18.7)	(22.7)	(17.2)	(17.2)	(20.2)	(15.3)	(17.7)	(11.8)	(16.7)
c_E	33.9	33.9	33.9	33.9	33.9	33.9	33.9	33.9	33.9	33.9
	(16.7)	(16.7)	(16.7)	(16.7)	(16.7)	(16.7)	(16.7)	(16.7)	(16.7)	(16.7)
	-3.8	8.6	28.0	2.2	2.2	15.5	-5.6	4.2	-16.6	0.1
	-1.856	4.230	13.779	1.060	1.060	7.634	-2.767	2.090	-8.194	0.059
N	203	203	203	203	203	203	203	203	203	203
X2	3.5	6.3	5.5	2.5	1.9	8.2	1.7	0.8	4.4	0.2
Gvalue	3.6	6.5	5.1	2.4	2.0	7.9	1.7	0.8	4.7	0.2
Gv/N	1.784	3.183	2.504	1.194	0.963	3.890	0.834	0.384	2.306	0.112
Prob	0.30536	0.09118	0.16574	0.48906	0.58190	0.04821	0.63843	0.85455	0.19674	0.97296
	50	49	48	47	46	45	44	43	42	41
a_O	74	61	71	78	66	71	70	66	64	71
	(36.5)	(30.0)	(35.0)	(38.4)	(32.5)	(35.0)	(34.5)	(32.5)	(31.5)	(35.0)
a_E	69.1	69.1	69.1	69.1	69.1	69.1	69.1	69.1	69.1	69.1
	(34.0)	(34.0)	(34.0)	(34.0)	(34.0)	(34.0)	(34.0)	(34.0)	(34.0)	(34.0)
	10.1	-15.2	3.8	18.9	-6.1	3.8	1.8	-6.1	-9.8	3.8
	4.984	-7.502	1.887	9.299	-2.994	1.887	0.882	-2.994	-4.844	1.887
t_O	66	66	55	55	63	59	67	65	73	62
	(32.5)	(32.5)	(27.1)	(27.1)	(31.0)	(29.1)	(33.0)	(32.0)	(36.0)	(30.5)
t_E	65.6	65.6	65.6	65.6	65.6	65.6	65.6	65.6	65.6	65.6
	(32.3)	(32.3)	(32.3)	(32.3)	(32.3)	(32.3)	(32.3)	(32.3)	(32.3)	(32.3)
	0.8	0.8	-19.4	-19.4	-5.1	-12.5	2.8	-1.2	15.6	-7.0
	0.385	0.385	-9.558	-9.558	-2.520	-6.173	1.384	-0.598	7.676	-3.457
g_O	35	36	43	35	43	45	31	41	35	42
	(17.2)	(17.7)	(21.2)	(17.2)	(21.2)	(22.2)	(15.3)	(20.2)	(17.2)	(20.7)
g_E	34.3	34.3	34.3	34.3	34.3	34.3	34.3	34.3	34.3	34.3
	(16.9)	(16.9)	(16.9)	(16.9)	(16.9)	(16.9)	(16.9)	(16.9)	(16.9)	(16.9)
	1.3	3.4	19.3	1.3	19.3	24.3	-6.3	14.5	1.3	16.9
	0.656	1.674	9.527	0.656	9.527	11.986	-3.125	7.160	0.656	8.332
c_O	28	40	34	35	31	28	35	31	31	28
	(13.8)	(19.7)	(16.7)	(17.2)	(15.3)	(13.8)	(17.2)	(15.3)	(15.3)	(13.8)
c_E	33.9	33.9	33.9	33.9	33.9	33.9	33.9	33.9	33.9	33.9
	(16.7)	(16.7)	(16.7)	(16.7)	(16.7)	(16.7)	(16.7)	(16.7)	(16.7)	(16.7)
	-10.8	13.1	0.1	2.2	-5.6	-10.8	2.2	-5.6	-5.6	-10.8
	-5.307	6.474	0.059	1.060	-2.767	-5.307	1.060	-2.767	-2.767	-5.307
N	203	203	203	203	203	203	203	203	203	203
X2	1.4	2.1	4.0	2.9	2.7	5.1	0.4	1.7	1.5	3.0
Gvalue	1.5	2.1	3.9	3.0	2.5	4.9	0.4	1.6	1.5	3.0
Gv/N	0.719	1.032	1.915	1.458	1.246	2.393	0.202	0.801	0.722	1.455
Prob	0.69172	0.55285	0.27377	0.39793	0.46986	0.18245	0.93830	0.65361	0.69031	0.39879
	40	39	38	37	36	35	34	33	32	31
a_O	69	66	59	54	65	58	64	69	60	71
	(34.0)	(32.5)	(29.1)	(26.6)	(32.0)	(28.6)	(31.5)	(34.0)	(29.6)	(35.0)
a_E	69.1	69.1	69.1	69.1	69.1	69.1	69.1	69.1	69.1	69.1
	(34.0)	(34.0)	(34.0)	(34.0)	(34.0)	(34.0)	(34.0)	(34.0)	(34.0)	(34.0)
	-0.2	-6.1	-18.7	-26.6	-8.0	-20.3	-9.8	-0.2	-17.0	3.8
	-0.108	-2.994	-9.194	-13.126	-3.926	-10.015	-4.844	-0.108	-8.356	1.887
t_O	75	69	65	75	75	78	85	56	70	59
	(36.9)	(34.0)	(32.0)	(36.9)	(36.9)	(38.4)	(41.9)	(27.6)	(34.5)	(29.1)
t_E	65.6	65.6	65.6	65.6	65.6	65.6	65.6	65.6	65.6	65.6
	(32.3)	(32.3)	(32.3)	(32.3)	(32.3)	(32.3)	(32.3)	(32.3)	(32.3)	(32.3)
	20.1	7.0	-1.2	20.1	20.1	27.0	44.0	-17.7	9.1	-12.5
	9.884	3.425	-0.598	9.884	9.884	13.293	21.683	-8.738	4.467	-6.173
g_O	31	40	39	28	24	41	24	50	28	38
	(15.3)	(19.7)	(19.2)	(13.8)	(11.8)	(20.2)	(11.8)	(24.6)	(13.8)	(18.7)
g_E	34.3	34.3	34.3	34.3	34.3	34.3	34.3	34.3	34.3	34.3
	(16.9)	(16.9)	(16.9)	(16.9)	(16.9)	(16.9)	(16.9)	(16.9)	(16.9)	(16.9)
	-6.3	12.2	9.9	-11.4	-17.2	14.5	-17.2	37.6	-11.4	7.7
	-3.125	6.013	4.889	-5.631	-8.471	7.160	-8.471	18.508	-5.631	3.792
c_O	28	28	40	46	39	26	30	28	45	35
	(13.8)	(13.8)	(19.7)	(22.7)	(19.2)	(12.8)	(14.8)	(13.8)	(22.2)	(17.2)
c_E	33.9	33.9	33.9	33.9	33.9	33.9	33.9	33.9	33.9	33.9
	(16.7)	(16.7)	(16.7)	(16.7)	(16.7)	(16.7)	(16.7)	(16.7)	(16.7)	(16.7)

	-10.8	-10.8	13.1	28.0	10.8	-13.9	-7.4	-10.8	25.4	2.2
	-5.307	-5.307	6.474	13.779	5.340	-6.827	-3.647	-5.307	12.505	1.060
N	203	203	203	203	203	203	203	203	203	203
X2	2.7	2.3	3.2	10.1	5.5	7.3	9.7	9.6	6.3	1.1
Gvalue	2.7	2.3	3.2	10.0	5.7	7.3	9.6	8.8	6.1	1.2
Gv/N	1.343	1.136	1.572	4.907	2.826	3.612	4.721	4.354	2.986	0.567
Prob	0.43578	0.51138	0.36311	0.01890	0.12516	0.06201	0.02245	0.03150	0.10868	0.76492

	30	29	28	27	26	25	24	23	22	21
a_O	65	63	58	72	73	81	73	71	87	80
	(32.0)	(31.0)	(28.6)	(35.5)	(36.0)	(39.9)	(36.0)	(35.0)	(42.9)	(39.4)
a_E	69.1	69.1	69.1	69.1	69.1	69.1	69.1	69.1	69.1	69.1
	(34.0)	(34.0)	(34.0)	(34.0)	(34.0)	(34.0)	(34.0)	(34.0)	(34.0)	(34.0)
	-8.0	-11.7	-20.3	5.9	8.0	25.7	8.0	3.8	40.1	23.4
	-3.926	-5.745	-10.015	2.906	3.938	12.669	3.938	1.887	19.732	11.533
t_O	66	69	90	70	59	57	62	57	52	45
	(32.5)	(34.0)	(44.3)	(34.5)	(29.1)	(28.1)	(30.5)	(28.1)	(25.6)	(22.2)
t_E	65.6	65.6	65.6	65.6	65.6	65.6	65.6	65.6	65.6	65.6
	(32.3)	(32.3)	(32.3)	(32.3)	(32.3)	(32.3)	(32.3)	(32.3)	(32.3)	(32.3)
	0.8	7.0	56.9	9.1	-12.5	-16.0	-7.0	-16.0	-24.2	-33.9
	0.385	3.425	28.027	4.467	-6.173	-7.900	-3.457	-7.900	-11.911	-16.717
g_O	38	33	25	34	33	28	35	34	32	38
	(18.7)	(16.3)	(12.3)	(16.7)	(16.3)	(13.8)	(17.2)	(16.7)	(15.8)	(18.7)
g_E	34.3	34.3	34.3	34.3	34.3	34.3	34.3	34.3	34.3	34.3
	(16.9)	(16.9)	(16.9)	(16.9)	(16.9)	(16.9)	(16.9)	(16.9)	(16.9)	(16.9)
	7.7	-2.6	-15.9	-0.7	-2.6	-11.4	1.3	-0.7	-4.5	7.7
	3.792	-1.294	-7.819	-0.333	-1.294	-5.631	0.656	-0.333	-2.225	3.792
c_O	34	38	30	27	38	37	33	41	32	40
	(16.7)	(18.7)	(14.8)	(13.3)	(18.7)	(18.2)	(16.3)	(20.2)	(15.8)	(19.7)
c_E	33.9	33.9	33.9	33.9	33.9	33.9	33.9	33.9	33.9	33.9
	(16.7)	(16.7)	(16.7)	(16.7)	(16.7)	(16.7)	(16.7)	(16.7)	(16.7)	(16.7)
	0.1	8.6	-7.4	-12.4	8.6	6.4	-1.9	15.5	-3.8	13.1
	0.059	4.230	-3.647	-6.085	4.230	3.147	-0.913	7.634	-1.856	6.474
N	203	203	203	203	203	203	203	203	203	203
X2	0.6	1.3	13.9	1.8	1.4	4.6	0.5	2.7	7.7	9.7
Gvalue	0.6	1.2	13.3	1.9	1.4	4.6	0.5	2.6	7.6	10.3
Gv/N	0.310	0.615	6.546	0.954	0.702	2.285	0.225	1.287	3.741	5.082
Prob	0.88982	0.74117	0.00405	0.58557	0.69973	0.20028	0.92840	0.45511	0.05519	0.01606

	20	19	18	17	16	15	14	13	12	11
a_O	87	71	72	64	81	67	72	66	79	85
	(42.9)	(35.0)	(35.5)	(31.5)	(39.9)	(33.0)	(35.5)	(32.5)	(38.9)	(41.9)
a_E	69.1	69.1	69.1	69.1	69.1	69.1	69.1	69.1	69.1	69.1
	(34.0)	(34.0)	(34.0)	(34.0)	(34.0)	(34.0)	(34.0)	(34.0)	(34.0)	(34.0)
	40.1	3.8	5.9	-9.8	25.7	-4.2	5.9	-6.1	21.1	35.2
	19.732	1.887	2.906	-4.844	12.669	-2.047	2.906	-2.994	10.410	17.331
t_O	60	75	75	72	80	72	69	78	60	65
	(29.6)	(36.9)	(36.9)	(35.5)	(39.4)	(35.5)	(34.0)	(38.4)	(29.6)	(32.0)
t_E	65.6	65.6	65.6	65.6	65.6	65.6	65.6	65.6	65.6	65.6
	(32.3)	(32.3)	(32.3)	(32.3)	(32.3)	(32.3)	(32.3)	(32.3)	(32.3)	(32.3)
	-10.7	20.1	20.1	13.4	31.7	13.4	7.0	27.0	-10.7	-1.2
	-5.284	9.884	9.884	6.593	15.629	6.593	3.425	13.293	-5.284	-0.598
g_O	26	30	28	25	19	27	25	27	35	24
	(12.8)	(14.8)	(13.8)	(12.3)	(9.4)	(13.3)	(12.3)	(13.3)	(17.2)	(11.8)
g_E	34.3	34.3	34.3	34.3	34.3	34.3	34.3	34.3	34.3	34.3
	(16.9)	(16.9)	(16.9)	(16.9)	(16.9)	(16.9)	(16.9)	(16.9)	(16.9)	(16.9)
	-14.5	-8.1	-11.4	-15.9	-22.5	-13.0	-15.9	-13.0	1.3	-17.2
	-7.127	-3.994	-5.631	-7.819	-11.079	-6.397	-7.819	-6.397	0.656	-8.471
c_O	30	27	28	42	23	37	37	32	29	29
	(14.8)	(13.3)	(13.8)	(20.7)	(11.3)	(18.2)	(18.2)	(15.8)	(14.3)	(14.3)
c_E	33.9	33.9	33.9	33.9	33.9	33.9	33.9	33.9	33.9	33.9
	(16.7)	(16.7)	(16.7)	(16.7)	(16.7)	(16.7)	(16.7)	(16.7)	(16.7)	(16.7)
	-7.4	-12.4	-10.8	17.9	-17.9	6.4	6.4	-3.8	-9.1	-9.1
	-3.647	-6.085	-5.307	8.817	-8.817	3.147	3.147	-1.856	-4.494	-4.494
N	203	203	203	203	203	203	203	203	203	203
X2	7.6	3.4	3.7	5.5	15.6	2.5	3.1	4.2	2.6	7.5
Gvalue	7.5	3.4	3.8	5.6	17.1	2.6	3.4	4.2	2.6	7.6
Gv/N	3.675	1.692	1.852	2.747	8.402	1.296	1.659	2.047	1.288	3.767
Prob	0.05861	0.32922	0.28864	0.13410	0.00069	0.45217	0.33834	0.24525	0.45478	0.05388

	10	9	8	7	6	5	4	3	2	1
a_O	96	80	78	102	97	83	63	80	60	64
	(47.3)	(39.4)	(38.4)	(50.2)	(47.8)	(40.9)	(31.0)	(39.4)	(29.6)	(31.5)
a_E	69.1	69.1	69.1	69.1	69.1	69.1	69.1	69.1	69.1	69.1
	(34.0)	(34.0)	(34.0)	(34.0)	(34.0)	(34.0)	(34.0)	(34.0)	(34.0)	(34.0)
	63.1	23.4	18.9	79.4	65.8	30.4	-11.7	23.4	-17.0	-9.8
	31.084	11.533	9.299	39.119	32.398	14.976	-5.745	11.533	-8.356	-4.844
t_O	47	67	56	56	55	58	78	63	81	65
	(23.2)	(33.0)	(27.6)	(27.6)	(27.1)	(28.6)	(38.4)	(31.0)	(39.9)	(32.0)
t_E	65.6	65.6	65.6	65.6	65.6	65.6	65.6	65.6	65.6	65.6
	(32.3)	(32.3)	(32.3)	(32.3)	(32.3)	(32.3)	(32.3)	(32.3)	(32.3)	(32.3)
	-31.4	2.8	-17.7	-17.7	-19.4	-14.3	27.0	-5.1	34.1	-1.2
	-15.447	1.384	-8.738	-8.738	-9.558	-7.045	13.293	-2.520	16.816	-0.598
g_O	31	32	33	16	27	30	28	32	24	26
	(15.3)	(15.8)	(16.3)	(7.9)	(13.3)	(14.8)	(13.8)	(15.8)	(11.8)	(12.8)
g_E	34.3	34.3	34.3	34.3	34.3	34.3	34.3	34.3	34.3	34.3
	(16.9)	(16.9)	(16.9)	(16.9)	(16.9)	(16.9)	(16.9)	(16.9)	(16.9)	(16.9)
	-6.3	-4.5	-2.6	-24.4	-13.0	-8.1	-11.4	-4.5	-17.2	-14.5
	-3.125	-2.225	-1.294	-12.039	-6.397	-3.994	-5.631	-2.225	-8.471	-7.127
c_O	29	24	36	29	24	32	34	28	38	48
	(14.3)	(11.8)	(17.7)	(14.3)	(11.8)	(15.8)	(16.7)	(13.8)	(18.7)	(23.6)
c_E	33.9	33.9	33.9	33.9	33.9	33.9	33.9	33.9	33.9	33.9
	(16.7)	(16.7)	(16.7)	(16.7)	(16.7)	(16.7)	(16.7)	(16.7)	(16.7)	(16.7)
	-9.1	-16.6	4.2	-9.1	-16.6	-3.8	0.1	-10.8	8.6	33.3
	-4.494	-8.194	2.090	-4.494	-8.194	-1.856	0.059	-5.307	4.230	16.391
N	203	203	203	203	203	203	203	203	203	203
X2	16.8	4.8	2.7	27.6	17.5	4.3	4.1	3.0	8.4	8.2
Gvalue	16.3	5.1	2.8	28.1	16.7	4.2	4.0	3.0	8.6	7.8
Gv/N	8.018	2.498	1.357	13.848	8.249	2.082	1.976	1.481	4.219	3.823
Prob	0.00100	0.16668	0.43098	0.00000	0.00080	0.23801	0.26017	0.39055	0.03567	0.05123

CDS_INITIAL:

	1	2	3	4	5	6	7	8	9	10

a_O	170	0	0	95	76	65	81	95	60	76
	(83.7)	(0.0)	(0.0)	(46.8)	(37.4)	(32.0)	(39.9)	(46.8)	(29.6)	(37.4)
a_E	58.7	63.4	59.6	58.7	63.4	59.6	58.7	63.4	59.6	58.7
	(28.9)	(31.2)	(29.3)	(28.9)	(31.2)	(29.3)	(28.9)	(31.2)	(29.3)	(28.9)
	361.3	0.0	0.0	91.4	27.5	11.4	52.1	76.7	0.9	39.2
	177.998	0.000	0.000	45.004	13.529	5.602	25.649	37.797	0.439	19.295
t_O	25	203	0	26	52	78	32	52	73	51
	(12.3)	(100.0)	(0.0)	(12.8)	(25.6)	(38.4)	(15.8)	(25.6)	(36.0)	(25.1)
t_E	38.2	60.9	81.5	38.2	60.9	81.5	38.2	60.9	81.5	38.2
	(18.8)	(30.0)	(40.2)	(18.8)	(30.0)	(40.2)	(18.8)	(30.0)	(40.2)	(18.8)
	-21.2	488.7	0.0	-20.0	-16.5	-6.9	-11.4	-16.5	-16.1	29.4
	-10.461	240.722	0.000	-9.875	-8.113	-3.387	-5.607	-8.113	-7.935	14.483
g_O	8	0	203	37	23	26	51	27	41	35
	(3.9)	(0.0)	(100.0)	(18.2)	(11.3)	(12.8)	(25.1)	(13.3)	(20.2)	(17.2)
g_E	63.2	34.0	31.7	63.2	34.0	31.7	63.2	34.0	31.7	63.2
	(31.1)	(16.7)	(15.6)	(31.1)	(16.7)	(15.6)	(31.1)	(16.7)	(15.6)	(31.1)
	-33.1	0.0	753.9	-39.6	-18.0	-10.3	-21.9	-12.4	21.1	-41.4
	-16.293	0.000	371.396	-19.529	-8.851	-5.075	-10.794	-6.125	10.395	-20.390
c_O	0	0	0	45	52	34	39	29	29	41
	(0.0)	(0.0)	(0.0)	(22.2)	(25.6)	(16.7)	(19.2)	(14.3)	(14.3)	(20.2)
c_E	42.8	44.6	30.2	42.8	44.6	30.2	42.8	44.6	30.2	42.8
	(21.1)	(22.0)	(14.9)	(21.1)	(22.0)	(14.9)	(21.1)	(22.0)	(14.9)	(21.1)
	0.0	0.0	0.0	4.5	15.8	8.0	-7.3	-25.0	-2.4	-3.5
	0.000	0.000	0.000	2.208	7.807	3.934	-3.585	-12.330	-1.189	-1.748
N	203	203	203	203	203	203	203	203	203	203
X2	306.4	473.4	1097.1	37.3	8.6	2.1	12.2	23.9	3.7	22.0
Gvalue	307.0	488.7	753.9	36.2	8.9	2.2	11.5	22.8	3.5	23.6
Gv/N	151.244	240.722	371.396	17.808	4.373	1.074	5.663	11.229	1.711	11.639
Prob	0.00000	0.00000	0.00000	0.00000	0.03098	0.53596	0.00933	0.00004	0.32427	0.00003

	11	12	13	14	15	16	17	18	19	20
a_O	79	55	69	64	63	80	72	63	82	79
	(38.9)	(27.1)	(34.0)	(31.5)	(31.0)	(39.4)	(35.5)	(31.0)	(40.4)	(38.9)
a_E	63.4	59.6	58.7	63.4	59.6	58.7	63.4	59.6	58.7	63.4
	(31.2)	(29.3)	(28.9)	(31.2)	(29.3)	(28.9)	(31.2)	(29.3)	(28.9)	(31.2)
	34.7	-8.8	22.2	1.1	7.1	49.4	18.2	7.1	54.7	34.7
	17.076	-4.312	10.949	0.557	3.490	24.353	8.982	3.490	26.957	17.076
t_O	61	96	48	73	83	48	67	74	39	65
	(30.0)	(47.3)	(23.6)	(36.0)	(40.9)	(23.6)	(33.0)	(36.5)	(19.2)	(32.0)
t_E	60.9	81.5	38.2	60.9	81.5	38.2	60.9	81.5	38.2	60.9
	(30.0)	(40.2)	(18.8)	(30.0)	(40.2)	(18.8)	(30.0)	(40.2)	(18.8)	(30.0)
	0.2	31.4	21.9	26.4	3.0	21.9	12.7	-14.3	1.6	8.4
	0.077	15.470	10.764	13.008	1.476	10.764	6.277	-7.052	0.768	4.149
g_O	23	36	38	25	20	32	31	29	34	27
	(11.3)	(17.7)	(18.7)	(12.3)	(9.9)	(15.8)	(15.3)	(14.3)	(16.7)	(13.3)
g_E	34.0	31.7	63.2	34.0	31.7	63.2	34.0	31.7	63.2	34.0
	(16.7)	(15.6)	(31.1)	(16.7)	(15.6)	(31.1)	(16.7)	(15.6)	(31.1)	(16.7)
	-18.0	9.2	-38.7	-15.4	-18.4	-43.6	-5.7	-5.2	-42.2	-12.4
	-8.851	4.515	-19.059	-7.567	-9.074	-21.467	-2.813	-2.541	-20.778	-6.125
c_O	40	16	48	41	37	43	33	37	48	32
	(19.7)	(7.9)	(23.6)	(20.2)	(18.2)	(21.2)	(16.3)	(18.2)	(23.6)	(15.8)
c_E	44.6	30.2	42.8	44.6	30.2	42.8	44.6	30.2	42.8	44.6
	(22.0)	(14.9)	(21.1)	(22.0)	(14.9)	(21.1)	(22.0)	(14.9)	(21.1)	(22.0)
	-8.8	-20.4	11.0	-7.0	14.9	0.4	-20.0	14.9	11.0	-21.3
	-4.334	-10.031	5.408	-3.445	7.364	0.184	-9.830	7.364	5.408	-10.502
N	203	203	203	203	203	203	203	203	203	203
X2	7.9	10.2	15.0	5.1	6.1	25.6	5.1	2.6	23.4	9.1
Gvalue	8.1	11.5	16.4	5.2	6.6	28.1	5.3	2.6	25.1	9.3
Gv/N	3.968	5.642	8.062	2.553	3.256	13.834	2.616	1.261	12.354	4.598
Prob	0.04488	0.00951	0.00095	0.15887	0.08543	0.00000	0.15042	0.46454	0.00001	0.02516

	21	22	23	24	25	26	27	28	29	30
a_O	70	73	71	69	69	70	55	53	58	50
	(34.5)	(36.0)	(35.0)	(34.0)	(34.0)	(34.5)	(27.1)	(26.1)	(28.6)	(24.6)
a_E	59.6	58.7	63.4	59.6	58.7	63.4	59.6	58.7	63.4	59.6
	(29.3)	(28.9)	(31.2)	(29.3)	(28.9)	(31.2)	(29.3)	(28.9)	(31.2)	(29.3)
	22.6	31.7	16.0	20.3	22.2	13.8	-8.8	-10.9	-10.4	-17.5
	11.144	15.637	7.879	10.006	10.949	6.789	-4.312	-5.366	-5.120	-8.615
t_O	80	45	62	78	35	65	90	49	68	80
	(39.4)	(22.2)	(30.5)	(38.4)	(17.2)	(32.0)	(44.3)	(24.1)	(33.5)	(39.4)
t_E	81.5	38.2	60.9	81.5	38.2	60.9	81.5	38.2	60.9	81.5
	(40.2)	(18.8)	(30.0)	(40.2)	(18.8)	(30.0)	(40.2)	(18.8)	(30.0)	(40.2)
	-3.0	14.7	2.2	-6.9	-6.2	8.4	17.8	24.3	14.9	-3.0
	-1.479	7.230	1.071	-3.387	-3.043	4.149	8.780	11.984	7.364	-1.479
g_O	27	39	28	29	59	25	28	58	34	29
	(13.3)	(19.2)	(13.8)	(14.3)	(29.1)	(12.3)	(13.8)	(28.6)	(16.7)	(14.3)
g_E	31.7	63.2	34.0	31.7	63.2	34.0	31.7	63.2	34.0	31.7
	(15.6)	(31.1)	(16.7)	(15.6)	(31.1)	(16.7)	(15.6)	(31.1)	(16.7)	(15.6)
	-8.7	-37.7	-10.9	-5.2	-8.2	-15.4	-6.9	-10.0	0.0	-5.2
	-4.266	-18.562	-5.349	-2.541	-4.018	-7.567	-3.421	-4.926	0.009	-2.541
c_O	26	46	42	27	40	43	30	43	43	44
	(12.8)	(22.7)	(20.7)	(13.3)	(19.7)	(21.2)	(14.8)	(21.2)	(21.2)	(21.7)
c_E	30.2	42.8	44.6	30.2	42.8	44.6	30.2	42.8	44.6	30.2
	(14.9)	(21.1)	(22.0)	(14.9)	(21.1)	(22.0)	(14.9)	(21.1)	(22.0)	(14.9)
	-7.8	6.6	-5.1	-6.1	-5.4	-3.2	-0.5	0.4	-3.2	33.0
	-3.863	3.253	-2.532	-3.008	-2.679	-1.595	-0.228	0.184	-1.595	16.268
N	203	203	203	203	203	203	203	203	203	203
X2	3.1	14.2	2.1	2.2	2.5	3.4	1.7	4.0	1.3	8.1
Gvalue	3.1	15.3	2.2	2.2	2.5	3.6	1.7	3.8	1.3	7.4
Gv/N	1.535	7.558	1.070	1.070	1.210	1.777	0.819	1.876	0.657	3.634
Prob	0.37395	0.00155	0.53762	0.53729	0.48334	0.30721	0.64528	0.28294	0.72111	0.06083

	31	32	33	34	35	36	37	38	39	40
a_O	67	64	55	53	55	50	63	50	69	78
	(33.0)	(31.5)	(27.1)	(26.1)	(27.1)	(24.6)	(31.0)	(24.6)	(34.0)	(38.4)
a_E	58.7	63.4	59.6	58.7	63.4	59.6	58.7	63.4	59.6	58.7
	(28.9)	(31.2)	(29.3)	(28.9)	(31.2)	(29.3)	(28.9)	(31.2)	(29.3)	(28.9)
	17.6	1.1	-8.8	-10.9	-15.7	-17.5	8.8	-23.8	20.3	44.3
	8.690	0.557	-4.312	-5.366	-7.733	-8.615	4.350	-11.725	10.006	21.799
t_O	53	76	83	63	71	85	55	67	76	40
	(26.1)	(37.4)	(40.9)	(31.0)	(35.0)	(41.9)	(27.1)	(33.0)	(37.4)	(19.7)
t_E	38.2	60.9	81.5	38.2	60.9	81.5	38.2	60.9	81.5	38.2
	(18.8)	(30.0)	(40.2)	(18.8)	(30.0)	(40.2)	(18.8)	(30.0)	(40.2)	(18.8)
	34.6	33.6	3.0	62.9	21.7	7.1	40.0	12.7	-10.6	3.6
	17.059	16.558	1.476	31.006	10.708	3.506	19.710	6.277	-5.245	1.785

```
g_O          48      31      34      49      27      33      43      44      28      48
        ( 23.6) ( 15.3) ( 16.7) ( 24.1) ( 13.3) ( 16.3) ( 21.2) ( 21.7) ( 13.8) ( 23.6)
g_E        63.2    34.0    31.7    63.2    34.0    31.7    63.2    34.0    31.7    63.2
        ( 31.1) ( 16.7) ( 15.6) ( 31.1) ( 16.7) ( 15.6) ( 31.1) ( 16.7) ( 15.6) ( 31.1)
          -26.4    -5.7     4.8   -25.0   -12.4     2.7   -33.1    22.7    -6.9   -26.4
        -13.026  -2.813   2.349 -12.302  -6.125   1.310 -16.330  11.188  -3.421 -13.026
c_O          35      32      31      38      50      35      42      42      30      37
        ( 17.2) ( 15.8) ( 15.3) ( 18.7) ( 24.6) ( 17.2) ( 20.7) ( 20.7) ( 14.8) ( 18.2)
c_E        42.8    44.6    30.2    42.8    44.6    30.2    42.8    44.6    30.2    42.8
        ( 21.1) ( 22.0) ( 14.9) ( 21.1) ( 22.0) ( 14.9) ( 21.1) ( 22.0) ( 14.9) ( 21.1)
          -14.1   -21.3     1.6    -9.1    11.3    10.3    -1.6    -5.1    -0.5   -10.8
         -6.948 -10.502   0.766  -4.465   5.575   5.050  -0.794  -2.532  -0.228  -5.320

N           203     203     203     203     203     203     203     203     203     203
X2         12.0     7.6     0.6    20.4     4.9     2.5    14.2     6.6     2.3    10.9
Gvalue     11.7     7.7     0.6    18.0     4.9     2.5    14.1     6.5     2.3    10.6
Gv/N      5.775   3.800   0.279   8.873   2.425   1.250   6.937   3.209   1.112   5.237
Prob    0.00840 0.05232 0.90387 0.00044 0.17756 0.46857 0.00279 0.08913 0.52082 0.01389
        -------------------------------------------------------------------------------
             41      42      43      44      45      46      47      48      49      50
        -------------------------------------------------------------------------------
a_O          70      61      58      58      61      61      50      71      73      69
        ( 34.5) ( 30.0) ( 28.6) ( 28.6) ( 30.0) ( 30.0) ( 24.6) ( 35.0) ( 36.0) ( 34.0)
a_E        63.4    59.6    58.7    63.4    59.6    58.7    63.4    59.6    58.7    63.4
        ( 31.2) ( 29.3) ( 28.9) ( 31.2) ( 29.3) ( 28.9) ( 31.2) ( 29.3) ( 28.9) ( 31.2)
           13.8     2.9    -1.5   -10.4     2.9     4.6   -23.8    25.0    31.7    11.6
          6.789   1.440  -0.720  -5.120   1.440   2.273 -11.725  12.295  15.637   5.714
t_O          60      80      43      76      76      51      77      69      39      53
        ( 29.6) ( 39.4) ( 21.2) ( 37.4) ( 37.4) ( 25.1) ( 37.9) ( 34.0) ( 19.2) ( 26.1)
t_E        60.9    81.5    38.2    60.9    81.5    38.2    60.9    81.5    38.2    60.9
        ( 30.0) ( 40.2) ( 18.8) ( 30.0) ( 40.2) ( 18.8) ( 30.0) ( 40.2) ( 18.8) ( 30.0)
           -1.8    -3.0    10.1    33.6   -10.6    29.4    36.1   -23.0     1.6   -14.8
         -0.902  -1.479   4.983  16.558  -5.245  14.483  17.768 -11.331   0.768  -7.274
g_O          32      30      57      28      37      49      36      33      58      35
        ( 15.8) ( 14.8) ( 28.1) ( 13.8) ( 18.2) ( 24.1) ( 17.7) ( 16.3) ( 28.6) ( 17.2)
g_E        34.0    31.7    63.2    34.0    31.7    63.2    34.0    31.7    63.2    34.0
        ( 16.7) ( 15.6) ( 31.1) ( 16.7) ( 15.6) ( 31.1) ( 16.7) ( 15.6) ( 31.1) ( 16.7)
           -3.9    -3.3   -11.8   -10.9    11.4   -25.0     4.1     2.7   -10.0     2.0
         -1.903  -1.626  -5.818  -5.349   5.639 -12.302   2.037   1.310  -4.926   1.009
c_O          41      32      45      41      29      42      40      30      33      46
        ( 20.2) ( 15.8) ( 22.2) ( 20.2) ( 14.3) ( 20.7) ( 19.7) ( 14.8) ( 16.3) ( 22.7)
c_E        44.6    30.2    42.8    44.6    30.2    42.8    44.6    30.2    42.8    44.6
        ( 22.0) ( 14.9) ( 21.1) ( 22.0) ( 14.9) ( 21.1) ( 22.0) ( 14.9) ( 21.1) ( 22.0)
           -7.0     3.6     4.5    -7.0    -2.4    -1.6    -8.8    -0.5   -17.2     2.7
         -3.445   1.792   2.208  -3.445  -1.189  -0.794  -4.334  -0.228  -8.464   1.350

N           203     203     203     203     203     203     203     203     203     203
X2          1.1     0.3     1.3     5.6     1.3     7.6     7.7     4.2     6.2     1.6
Gvalue      1.1     0.3     1.3     5.4     1.3     7.4     7.6     4.2     6.1     1.6
Gv/N      0.540   0.127   0.653   2.644   0.645   3.660   3.745   2.046   3.013   0.799
Prob    0.77792 0.96790 0.72327 0.14676 0.72700 0.05938 0.05498 0.24539 0.10606 0.65437
        -------------------------------------------------------------------------------
             51      52      53      54      55      56      57      58      59      60
        -------------------------------------------------------------------------------
a_O          80      61      65      63      58      62      59      58      60      61
        ( 39.4) ( 30.0) ( 32.0) ( 31.0) ( 28.6) ( 30.5) ( 29.1) ( 28.6) ( 29.6) ( 30.0)
a_E        59.6    58.7    63.4    59.6    58.7    63.4    59.6    58.7    63.4    59.6
        ( 29.3) ( 28.9) ( 31.2) ( 29.3) ( 28.9) ( 31.2) ( 29.3) ( 28.9) ( 31.2) ( 29.3)
           47.2     4.6     3.2     7.1    -1.5    -2.8    -1.1    -1.5    -6.7     2.9
         23.260   2.273   1.559   3.490  -0.720  -1.400  -0.545  -0.720  -3.293   1.440
t_O          63      44      57      81      41      51      84      56      56      69
        ( 31.0) ( 21.7) ( 28.1) ( 39.9) ( 20.2) ( 25.1) ( 41.4) ( 27.6) ( 27.6) ( 34.0)
t_E        81.5    38.2    60.9    81.5    38.2    60.9    81.5    38.2    60.9    81.5
        ( 40.2) ( 18.8) ( 30.0) ( 40.2) ( 18.8) ( 30.0) ( 40.2) ( 18.8) ( 30.0) ( 40.2)
          -32.5    12.4    -7.6    -1.0     5.7   -18.1     5.0    42.8    -9.4   -23.0
        -15.992   6.095  -3.737  -0.506   2.827  -8.932   2.485  21.063  -4.648 -11.331
g_O          31      50      30      33      50      42      29      54      35      47
        ( 15.3) ( 24.6) ( 14.8) ( 16.3) ( 24.6) ( 20.7) ( 14.3) ( 26.6) ( 17.2) ( 23.2)
g_E        31.7    63.2    34.0    31.7    63.2    34.0    31.7    63.2    34.0    31.7
        ( 15.6) ( 31.1) ( 16.7) ( 15.6) ( 31.1) ( 16.7) ( 15.6) ( 31.1) ( 16.7) ( 15.6)
           -1.4   -23.5    -7.5     2.7   -23.5    17.8    -5.2   -17.0     2.0    37.0
         -0.679 -11.558  -3.692   1.310 -11.558   8.755  -2.541  -8.388   1.009  18.241
c_O          29      48      51      26      54      48      31      35      52      26
        ( 14.3) ( 23.6) ( 25.1) ( 12.8) ( 26.6) ( 23.6) ( 15.3) ( 17.2) ( 25.6) ( 12.8)
c_E        30.2    42.8    44.6    30.2    42.8    44.6    30.2    42.8    44.6    30.2
        ( 14.9) ( 21.1) ( 22.0) ( 14.9) ( 21.1) ( 22.0) ( 14.9) ( 21.1) ( 22.0) ( 14.9)
           -2.4    11.0    13.6    -7.8    25.1     6.9     1.6   -14.1    15.8    -7.8
         -1.189   5.408   6.682  -3.863  12.350   3.422   0.766  -6.948   7.807  -3.863

N           203     203     203     203     203     203     203     203     203     203
X2         11.3     4.4     1.7     0.8     5.9     3.8     0.3    11.0     1.8     9.9
Gvalue     11.0     4.5     1.6     0.9     5.9     3.7     0.3    10.2     1.8     9.1
Gv/N      5.400   2.218   0.812   0.430   2.898   1.844   0.165   5.006   0.875   4.487
Prob    0.01193 0.21210 0.64867 0.83174 0.11741 0.29047 0.95316 0.01724 0.62001 0.02789
        -------------------------------------------------------------------------------
             61      62      63      64      65      66      67      68      69      70
        -------------------------------------------------------------------------------
a_O          63      56      67      63      61      69      50      49      72      69
        ( 31.0) ( 27.6) ( 33.0) ( 31.0) ( 30.0) ( 34.0) ( 24.6) ( 24.1) ( 35.5) ( 34.0)
a_E        58.7    63.4    59.6    58.7    63.4    59.6    58.7    63.4    59.6    58.7
        ( 28.9) ( 31.2) ( 29.3) ( 28.9) ( 31.2) ( 29.3) ( 28.9) ( 31.2) ( 29.3) ( 28.9)
            8.8   -14.0    15.8     8.8    -4.8    20.3   -16.1   -25.3    27.3    22.2
          4.350  -6.880   7.775   4.350  -2.354  10.006  -7.932 -12.466  13.461  10.949
t_O          44      59      90      42      72      74      40      79      71      49
        ( 21.7) ( 29.1) ( 44.3) ( 20.7) ( 35.5) ( 36.5) ( 19.7) ( 38.9) ( 35.0) ( 24.1)
t_E        38.2    60.9    81.5    38.2    60.9    81.5    38.2    60.9    81.5    38.2
        ( 18.8) ( 30.0) ( 40.2) ( 18.8) ( 30.0) ( 40.2) ( 18.8) ( 30.0) ( 40.2) ( 18.8)
           12.4    -3.8    17.8     7.9    24.1   -14.3     3.6    41.1   -19.6    24.3
          6.095  -1.864   8.780   3.893  11.851  -7.052   1.785  20.225  -9.661  11.984
g_O          51      37      27      53      26      37      65      28      25      45
        ( 25.1) ( 18.2) ( 13.3) ( 26.1) ( 12.8) ( 18.2) ( 32.0) ( 13.8) ( 12.3) ( 22.2)
g_E        63.2    34.0    31.7    63.2    34.0    31.7    63.2    34.0    31.7    63.2
        ( 31.1) ( 16.7) ( 15.6) ( 31.1) ( 16.7) ( 15.6) ( 31.1) ( 16.7) ( 15.6) ( 31.1)
          -21.9     6.3    -8.7   -18.7   -13.9    11.4     3.6   -10.9   -11.9   -30.6
        -10.794   3.092  -4.266  -9.209  -6.865   5.639   1.776  -5.349  -5.846 -15.074
c_O          45      51      19      45      44      23      48      47      35      40
        ( 22.2) ( 25.1) (  9.4) ( 22.2) ( 21.7) ( 11.3) ( 23.6) ( 23.2) ( 17.2) ( 19.7)
c_E        42.8    44.6    30.2    42.8    44.6    30.2    42.8    44.6    30.2    42.8
        ( 21.1) ( 22.0) ( 14.9) ( 21.1) ( 22.0) ( 14.9) ( 21.1) ( 22.0) ( 14.9) ( 21.1)
            4.5    13.6   -17.6     4.5    -1.3   -12.6    11.0     4.8    10.3    -5.4
          2.208   6.682  -8.695   2.208  -0.636  -6.196   5.408   2.375   5.050  -2.679
```

N	203	203	203	203	203	203	203	203	203	203
X2	3.7	2.1	6.7	2.4	4.0	4.8	2.1	9.8	6.1	10.3
Gvalue	3.8	2.1	7.3	2.5	4.1	4.9	2.1	9.7	6.1	10.5
Gv/N	1.859	1.030	3.594	1.243	1.996	2.398	1.036	4.785	3.003	5.180
Prob	0.28688	0.55366	0.06304	0.47126	0.25583	0.18170	0.55117	0.02116	0.10700	0.01465

	71	72	73	74	75	76	77	78	79	80
a_O	63	54	53	70	56	52	55	65	55	46
	(31.0)	(26.6)	(26.1)	(34.5)	(27.6)	(25.6)	(27.1)	(32.0)	(27.1)	(22.7)
a_E	63.4	59.6	58.7	63.4	59.6	58.7	63.4	59.6	58.7	63.4
	(31.2)	(29.3)	(28.9)	(31.2)	(29.3)	(28.9)	(31.2)	(29.3)	(28.9)	(31.2)
	-0.9	-10.6	-10.9	13.8	-6.9	-12.7	-15.7	11.4	-7.2	-29.6
	-0.429	-5.210	-5.366	6.789	-3.396	-6.240	-7.733	5.602	-3.561	-14.566
t_O	74	86	44	52	95	48	63	78	39	74
	(36.5)	(42.4)	(21.7)	(25.6)	(46.8)	(23.6)	(31.0)	(38.4)	(19.2)	(36.5)
t_E	60.9	81.5	38.2	60.9	81.5	38.2	60.9	81.5	38.2	60.9
	(30.0)	(40.2)	(18.8)	(30.0)	(40.2)	(18.8)	(30.0)	(40.2)	(18.8)	(30.0)
	28.8	9.2	12.4	-16.5	29.1	21.9	4.2	-6.9	1.6	28.8
	14.178	4.538	6.095	-8.113	14.329	10.764	2.082	-3.387	0.768	14.178
g_O	30	37	59	31	25	56	26	33	49	45
	(14.8)	(18.2)	(29.1)	(15.3)	(12.3)	(27.6)	(12.8)	(16.3)	(24.1)	(22.2)
g_E	34.0	31.7	63.2	34.0	31.7	63.2	34.0	31.7	63.2	34.0
	(16.7)	(15.6)	(31.1)	(16.7)	(15.6)	(31.1)	(16.7)	(15.6)	(31.1)	(16.7)
	-7.5	11.4	-8.2	-5.7	-11.9	-13.6	-13.9	2.7	-25.0	25.3
	-3.692	5.639	-4.018	-2.813	-5.846	-6.693	-6.865	1.310	-12.302	12.439
c_O	36	26	47	50	27	47	59	27	60	38
	(17.7)	(12.8)	(23.2)	(24.6)	(13.3)	(23.2)	(29.1)	(13.3)	(29.6)	(18.7)
c_E	44.6	30.2	42.8	44.6	30.2	42.8	44.6	30.2	42.8	44.6
	(22.0)	(14.9)	(21.1)	(22.0)	(14.9)	(21.1)	(22.0)	(14.9)	(21.1)	(22.0)
	-15.5	-7.8	8.8	11.3	-6.1	8.8	32.9	-6.1	40.5	-12.3
	-7.637	-3.863	4.320	5.575	-3.008	4.320	16.200	-3.008	19.950	-6.037
N	203	203	203	203	203	203	203	203	203	203
X2	5.0	2.2	2.1	2.9	4.2	4.5	7.7	1.0	10.4	12.2
Gvalue	4.9	2.2	2.1	2.9	4.2	4.4	7.5	1.0	9.9	12.2
Gv/N	2.420	1.104	1.032	1.439	2.078	2.151	3.683	0.516	4.855	6.013
Prob	0.17832	0.52387	0.55295	0.40406	0.23877	0.22445	0.05817	0.78953	0.01984	0.00671

	81	82	83	84	85	86	87	88	89	90
a_O	56	64	68	62	47	54	53	60	60	63
	(27.6)	(31.5)	(33.5)	(30.5)	(23.2)	(26.6)	(26.1)	(29.6)	(29.6)	(31.0)
a_E	59.6	58.7	63.4	59.6	58.7	63.4	59.6	58.7	63.4	59.6
	(29.3)	(28.9)	(31.2)	(29.3)	(28.9)	(31.2)	(29.3)	(28.9)	(31.2)	(29.3)
	-6.9	11.0	9.4	5.0	-21.0	-17.4	-12.4	2.6	-6.7	7.1
	-3.396	5.412	4.653	2.457	-10.321	-8.569	-6.089	1.259	-3.293	3.490
t_O	88	48	59	79	49	73	84	44	52	80
	(43.3)	(23.6)	(29.1)	(38.9)	(24.1)	(36.0)	(41.4)	(21.7)	(25.6)	(39.4)
t_E	81.5	38.2	60.9	81.5	38.2	60.9	81.5	38.2	60.9	81.5
	(40.2)	(18.8)	(30.0)	(40.2)	(18.8)	(30.0)	(40.2)	(18.8)	(30.0)	(40.2)
	13.5	21.9	-3.8	-5.0	24.3	26.4	5.0	12.4	-16.5	-3.0
	6.637	10.764	-1.864	-2.439	11.984	13.008	2.485	6.095	-8.113	-1.479
g_O	31	48	28	29	58	38	40	58	40	35
	(15.3)	(23.6)	(13.8)	(14.3)	(28.6)	(18.7)	(19.7)	(28.6)	(19.7)	(17.2)
g_E	31.7	63.2	34.0	31.7	63.2	34.0	31.7	63.2	34.0	31.7
	(15.6)	(31.1)	(16.7)	(15.6)	(31.1)	(16.7)	(15.6)	(31.1)	(16.7)	(15.6)
	-1.4	-26.4	-10.9	-5.2	-10.0	8.5	18.6	-10.0	13.0	6.9
	-0.679	-13.026	-5.349	-2.541	-4.926	4.174	9.169	-4.926	6.415	3.418
c_O	28	43	48	33	49	38	26	41	51	25
	(13.8)	(21.2)	(23.6)	(16.3)	(24.1)	(18.7)	(12.8)	(20.2)	(25.1)	(12.3)
c_E	30.2	42.8	44.6	30.2	42.8	44.6	30.2	42.8	44.6	30.2
	(14.9)	(21.1)	(22.0)	(14.9)	(21.1)	(22.0)	(14.9)	(21.1)	(22.0)	(14.9)
	-4.3	0.4	6.9	5.8	13.2	-12.3	-7.8	-3.5	13.6	-9.5
	-2.116	0.184	3.422	2.848	6.516	-6.037	-3.863	-1.748	6.682	-4.681
N	203	203	203	203	203	203	203	203	203	203
X2	0.9	6.6	1.7	0.7	6.7	5.3	3.6	1.4	3.5	1.5
Gvalue	0.9	6.8	1.8	0.7	6.6	5.2	3.5	1.4	3.4	1.5
Gv/N	0.445	3.334	0.863	0.325	3.252	2.576	1.701	0.679	1.691	0.749
Prob	0.82450	0.07966	0.62559	0.88265	0.08578	0.15582	0.32685	0.71043	0.32954	0.67779

	91	92	93	94	95	96	97	98	99	100
a_O	64	53	65	54	77	60	66	60	66	50
	(31.5)	(26.1)	(32.0)	(26.6)	(37.9)	(29.6)	(32.5)	(29.6)	(32.5)	(24.6)
a_E	58.7	63.4	59.6	58.7	63.4	59.6	58.7	63.4	59.6	58.7
	(28.9)	(31.2)	(29.3)	(28.9)	(31.2)	(29.3)	(28.9)	(31.2)	(29.3)	(28.9)
	11.0	-19.1	11.4	-9.1	29.8	0.9	15.4	-6.7	13.6	-16.1
	5.412	-9.386	5.602	-4.472	14.699	0.439	7.582	-3.293	6.681	-7.932
t_O	38	63	75	50	60	86	37	67	79	44
	(18.7)	(31.0)	(36.9)	(24.6)	(29.6)	(42.4)	(18.2)	(33.0)	(38.9)	(21.7)
t_E	38.2	60.9	81.5	38.2	60.9	81.5	38.2	60.9	81.5	38.2
	(18.8)	(30.0)	(40.2)	(18.8)	(30.0)	(40.2)	(18.8)	(30.0)	(40.2)	(18.8)
	-0.5	4.2	-12.5	26.8	-1.8	9.2	-2.4	12.7	-5.0	12.4
	-0.225	2.082	-6.155	13.223	-0.902	4.538	-1.191	6.277	-2.439	6.095
g_O	54	36	29	46	23	35	56	37	29	66
	(26.6)	(17.7)	(14.3)	(22.7)	(11.3)	(17.2)	(27.6)	(18.2)	(14.3)	(32.5)
g_E	63.2	34.0	31.7	63.2	34.0	31.7	63.2	34.0	31.7	63.2
	(31.1)	(16.7)	(15.6)	(31.1)	(16.7)	(15.6)	(31.1)	(16.7)	(15.6)	(31.1)
	-17.0	4.1	-5.2	-29.3	-18.0	6.9	-13.6	6.3	-5.2	5.7
	-8.388	2.037	-2.541	-14.412	-8.851	3.418	-6.693	3.092	-2.541	2.796
c_O	47	51	34	53	43	22	44	39	29	43
	(23.2)	(25.1)	(16.7)	(26.1)	(21.2)	(10.8)	(21.7)	(19.2)	(14.3)	(21.2)
c_E	42.8	44.6	30.2	42.8	44.6	30.2	42.8	44.6	30.2	42.8
	(21.1)	(22.0)	(14.9)	(21.1)	(22.0)	(14.9)	(21.1)	(22.0)	(14.9)	(21.1)
	8.8	13.6	8.0	22.6	-3.2	-14.0	2.4	-10.6	-2.4	0.4
	4.320	6.682	3.934	11.145	-1.595	-6.890	1.185	-5.198	-1.189	0.184
N	203	203	203	203	203	203	203	203	203	203
X2	2.2	2.8	1.7	11.1	6.5	2.8	1.8	1.8	1.1	2.3
Gvalue	2.3	2.9	1.7	11.1	6.8	3.1	1.8	1.8	1.0	2.3
Gv/N	1.119	1.414	0.840	5.484	3.351	1.506	0.884	0.878	0.512	1.143
Prob	0.51786	0.41216	0.63564	0.01103	0.07846	0.38294	0.61615	0.61869	0.79179	0.50860

5UTR:

	100	99	98	97	96	95	94	93	92	91
a_O	337	324	328	354	309	342	335	302	325	346
	(19.8)	(19.1)	(19.3)	(20.8)	(18.2)	(20.1)	(19.7)	(17.8)	(19.1)	(20.4)
a_E	341.0	341.0	341.0	341.0	341.0	341.0	341.0	341.0	341.0	341.0
	(20.1)	(20.1)	(20.1)	(20.1)	(20.1)	(20.1)	(20.1)	(20.1)	(20.1)	(20.1)
	-7.9	-33.1	-25.5	26.5	-60.9	2.0	-11.9	-73.3	-31.2	10.1
	-0.466	-1.949	-1.499	1.563	-3.584	0.121	-0.698	-4.318	-1.837	0.596
t_O	375	338	337	333	315	298	339	363	335	333
	(22.1)	(19.9)	(19.8)	(19.6)	(18.6)	(17.6)	(20.0)	(21.4)	(19.7)	(19.6)
t_E	338.6	338.6	338.6	338.6	338.6	338.6	338.6	338.6	338.6	338.6
	(19.9)	(19.9)	(19.9)	(19.9)	(19.9)	(19.9)	(19.9)	(19.9)	(19.9)	(19.9)
	76.6	-1.2	-3.2	-11.1	-45.5	-76.1	0.8	50.5	-7.2	-11.1
	4.509	-0.072	-0.189	-0.655	-2.681	-4.484	0.046	2.974	-0.423	-0.655
g_O	474	484	536	484	538	545	485	489	523	519
	(27.9)	(28.5)	(31.6)	(28.5)	(31.7)	(32.1)	(28.6)	(28.8)	(30.8)	(30.6)
g_E	484.3	484.3	484.3	484.3	484.3	484.3	484.3	484.3	484.3	484.3
	(28.5)	(28.5)	(28.5)	(28.5)	(28.5)	(28.5)	(28.5)	(28.5)	(28.5)	(28.5)
	-20.3	-0.5	108.8	-0.5	113.2	128.8	1.5	9.5	80.5	71.9
	-1.196	-0.031	6.408	-0.031	6.668	7.584	0.086	0.560	4.740	4.234
c_O	512	552	497	527	536	513	539	544	515	500
	(30.2)	(32.5)	(29.3)	(31.0)	(31.6)	(30.2)	(31.7)	(32.0)	(30.3)	(29.4)
c_E	534.1	534.1	534.1	534.1	534.1	534.1	534.1	534.1	534.1	534.1
	(31.5)	(31.5)	(31.5)	(31.5)	(31.5)	(31.5)	(31.5)	(31.5)	(31.5)	(31.5)
	-43.4	36.3	-71.6	-14.2	3.7	-41.4	9.8	19.9	-37.6	-66.1
	-2.554	2.138	-4.220	-0.836	0.219	-2.441	0.574	1.171	-2.214	-3.891
N	1698	1698	1698	1698	1698	1698	1698	1698	1698	1698
X2	5.1	1.4	8.6	0.7	10.6	13.3	0.2	6.4	4.6	4.8
Gvalue	5.0	1.5	8.5	0.7	10.5	13.2	0.2	6.6	4.5	4.8
Gv/N	0.293	0.085	0.500	0.040	0.621	0.780	0.009	0.387	0.266	0.285
Prob	0.17362	0.69358	0.03685	0.87773	0.01448	0.00413	0.98509	0.08660	0.21118	0.18455

	90	89	88	87	86	85	84	83	82	81
a_O	314	327	332	340	316	343	325	292	317	303
	(18.5)	(19.3)	(19.6)	(20.0)	(18.6)	(20.2)	(19.1)	(17.2)	(18.7)	(17.8)
a_E	341.0	341.0	341.0	341.0	341.0	341.0	341.0	341.0	341.0	341.0
	(20.1)	(20.1)	(20.1)	(20.1)	(20.1)	(20.1)	(20.1)	(20.1)	(20.1)	(20.1)
	-51.8	-27.4	-17.7	-2.0	-48.1	4.1	-31.2	-90.6	-46.2	-71.6
	-3.048	-1.612	-1.043	-0.115	-2.832	0.239	-1.837	-5.333	-2.723	-4.214
t_O	316	312	360	328	340	365	332	340	309	352
	(18.6)	(18.4)	(21.2)	(19.3)	(20.0)	(21.5)	(19.6)	(20.0)	(18.2)	(20.7)
t_E	338.6	338.6	338.6	338.6	338.6	338.6	338.6	338.6	338.6	338.6
	(19.9)	(19.9)	(19.9)	(19.9)	(19.9)	(19.9)	(19.9)	(19.9)	(19.9)	(19.9)
	-43.7	-51.1	44.1	-20.9	2.8	54.8	-13.1	2.8	-56.5	27.3
	-2.572	-3.008	2.598	-1.230	0.164	3.227	-0.771	0.164	-3.330	1.608
g_O	540	547	477	497	549	470	477	561	513	492
	(31.8)	(32.2)	(28.1)	(29.3)	(32.3)	(27.7)	(28.1)	(33.0)	(30.2)	(29.0)
g_E	484.3	484.3	484.3	484.3	484.3	484.3	484.3	484.3	484.3	484.3
	(28.5)	(28.5)	(28.5)	(28.5)	(28.5)	(28.5)	(28.5)	(28.5)	(28.5)	(28.5)
	117.6	133.3	-14.4	25.8	137.8	-28.1	-14.4	165.0	59.1	15.6
	6.929	7.848	-0.850	1.519	8.113	-1.655	-0.850	9.719	3.483	0.918
c_O	528	512	529	533	493	520	564	505	559	551
	(31.1)	(30.2)	(31.2)	(31.4)	(29.0)	(30.6)	(33.2)	(29.7)	(32.9)	(32.4)
c_E	534.1	534.1	534.1	534.1	534.1	534.1	534.1	534.1	534.1	534.1
	(31.5)	(31.5)	(31.5)	(31.5)	(31.5)	(31.5)	(31.5)	(31.5)	(31.5)	(31.5)
	-12.2	-43.4	-10.2	-2.3	-79.0	-27.9	61.3	-56.7	50.8	34.2
	-0.720	-2.554	-0.603	-0.135	-4.655	-1.644	3.613	-3.338	2.994	2.016
N	1698	1698	1698	1698	1698	1698	1698	1698	1698	1698
X2	10.1	11.7	1.7	0.7	13.7	2.9	2.7	20.8	7.1	5.4
Gvalue	10.0	11.5	1.7	0.7	13.4	2.8	2.6	20.6	7.2	5.6
Gv/N	0.588	0.675	0.101	0.040	0.791	0.166	0.155	1.212	0.424	0.328
Prob	0.01865	0.00950	0.63211	0.87953	0.00380	0.42020	0.45081	0.00013	0.06562	0.13461

	80	79	78	77	76	75	74	73	72	71
a_O	326	350	359	334	346	328	349	340	307	347
	(19.2)	(20.6)	(21.1)	(19.7)	(20.4)	(19.3)	(20.6)	(20.0)	(18.1)	(20.4)
a_E	341.0	341.0	341.0	341.0	341.0	341.0	341.0	341.0	341.0	341.0
	(20.1)	(20.1)	(20.1)	(20.1)	(20.1)	(20.1)	(20.1)	(20.1)	(20.1)	(20.1)
	-29.3	18.3	37.0	-13.8	10.1	-25.5	16.2	-2.0	-64.5	12.1
	-1.725	1.077	2.178	-0.813	0.596	-1.499	0.956	-0.115	-3.796	0.716
t_O	281	331	306	321	330	314	314	357	334	330
	(16.5)	(19.5)	(18.0)	(18.9)	(19.4)	(18.5)	(18.5)	(21.0)	(19.7)	(19.4)
t_E	338.6	338.6	338.6	338.6	338.6	338.6	338.6	338.6	338.6	338.6
	(19.9)	(19.9)	(19.9)	(19.9)	(19.9)	(19.9)	(19.9)	(19.9)	(19.9)	(19.9)
	-104.8	-15.0	-62.0	-34.3	-17.0	-47.4	-47.4	37.8	-9.2	-17.0
	-6.172	-0.886	-3.650	-2.019	-1.001	-2.790	-2.790	2.224	-0.539	-1.001
g_O	552	512	470	521	493	532	536	490	517	515
	(32.5)	(30.2)	(27.7)	(30.7)	(29.0)	(31.3)	(31.6)	(28.9)	(30.4)	(30.3)
g_E	484.3	484.3	484.3	484.3	484.3	484.3	484.3	484.3	484.3	484.3
	(28.5)	(28.5)	(28.5)	(28.5)	(28.5)	(28.5)	(28.5)	(28.5)	(28.5)	(28.5)
	144.5	57.0	-28.1	76.2	17.6	100.0	108.8	11.5	67.6	63.4
	8.512	3.358	-1.655	4.487	1.038	5.891	6.408	0.679	3.983	3.732
c_O	539	505	563	522	529	524	499	511	540	506
	(31.7)	(29.7)	(33.2)	(30.7)	(31.2)	(30.9)	(29.4)	(30.1)	(31.8)	(29.8)
c_E	534.1	534.1	534.1	534.1	534.1	534.1	534.1	534.1	534.1	534.1
	(31.5)	(31.5)	(31.5)	(31.5)	(31.5)	(31.5)	(31.5)	(31.5)	(31.5)	(31.5)
	9.8	-56.7	59.2	-24.0	-10.2	-20.1	-67.9	-45.3	11.8	-54.8
	0.574	-3.338	3.489	-1.414	-0.603	-1.184	-4.001	-2.666	0.693	-3.226
N	1698	1698	1698	1698	1698	1698	1698	1698	1698	1698
X2	20.0	3.6	6.1	4.1	0.5	7.2	9.8	2.1	5.7	3.8
Gvalue	20.2	3.6	6.1	4.1	0.5	7.1	9.7	2.1	5.8	3.7
Gv/N	1.189	0.211	0.361	0.240	0.029	0.417	0.573	0.122	0.341	0.221
Prob	0.00016	0.30955	0.10508	0.25375	0.91876	0.06923	0.02105	0.55808	0.12200	0.29014

	70	69	68	67	66	65	64	63	62	61
a_O	329	312	336	343	318	353	355	339	322	351
	(19.4)	(18.4)	(19.8)	(20.2)	(18.7)	(20.8)	(20.9)	(20.0)	(19.0)	(20.7)
a_E	341.0	341.0	341.0	341.0	341.0	341.0	341.0	341.0	341.0	341.0
	(20.1)	(20.1)	(20.1)	(20.1)	(20.1)	(20.1)	(20.1)	(20.1)	(20.1)	(20.1)
	-23.5	-55.4	-9.9	4.1	-44.4	24.5	28.6	-3.9	-36.9	20.3
	-1.386	-3.264	-0.582	0.239	-2.613	1.441	1.685	-0.232	-2.172	1.198
t_O	364	333	327	394	326	310	363	327	333	341
	(21.4)	(19.6)	(19.3)	(23.2)	(19.2)	(18.3)	(21.4)	(19.3)	(19.6)	(20.1)
t_E	338.6	338.6	338.6	338.6	338.6	338.6	338.6	338.6	338.6	338.6

	(19.9)	(19.9)	(19.9)	(19.9)	(19.9)	(19.9)	(19.9)	(19.9)	(19.9)	(19.9)
	52.6	-11.1	-22.8	119.4	-24.7	-54.7	50.5	-22.8	-11.1	4.8
	3.100	-0.655	-1.344	7.031	-1.457	-3.223	2.974	-1.344	-0.655	0.283
g_O	501	509	553	477	490	498	498	524	534	487
	(29.5)	(30.0)	(32.6)	(28.1)	(28.9)	(29.3)	(29.3)	(30.9)	(31.4)	(28.7)
g_E	484.3	484.3	484.3	484.3	484.3	484.3	484.3	484.3	484.3	484.3
	(28.5)	(28.5)	(28.5)	(28.5)	(28.5)	(28.5)	(28.5)	(28.5)	(28.5)	(28.5)
	34.0	50.7	146.8	-14.4	11.5	27.9	27.9	82.6	104.4	5.5
	2.005	2.986	8.645	-0.850	0.679	1.640	1.640	4.867	6.149	0.323
c_O	504	544	482	484	564	537	482	508	509	519
	(29.7)	(32.0)	(28.4)	(28.5)	(33.2)	(31.6)	(28.4)	(29.9)	(30.0)	(30.6)
c_E	534.1	534.1	534.1	534.1	534.1	534.1	534.1	534.1	534.1	534.1
	(31.5)	(31.5)	(31.5)	(31.5)	(31.5)	(31.5)	(31.5)	(31.5)	(31.5)	(31.5)
	-58.6	19.9	-99.0	-95.4	61.3	5.7	-99.0	-51.0	-49.1	-29.9
	-3.449	1.171	-5.832	-5.620	3.613	0.337	-5.832	-3.003	-2.891	-1.759
N	1698	1698	1698	1698	1698	1698	1698	1698	1698	1698
X2	4.6	4.0	15.3	13.9	3.8	3.2	7.8	4.9	7.4	0.8
Gvalue	4.6	4.1	15.1	13.6	3.8	3.3	7.9	4.9	7.3	0.8
Gv/N	0.270	0.239	0.887	0.800	0.222	0.195	0.467	0.288	0.431	0.045
Prob	0.20440	0.25592	0.00176	0.00352	0.28790	0.34652	0.04735	0.18006	0.06254	0.85958

	60	59	58	57	56	55	54	53	52	51
a_O	310	355	337	293	370	389	335	364	349	333
	(18.3)	(20.9)	(19.8)	(17.3)	(21.8)	(22.9)	(19.7)	(21.4)	(20.6)	(19.6)
a_E	341.0	341.0	341.0	341.0	341.0	341.0	341.0	341.0	341.0	341.0
	(20.1)	(20.1)	(20.1)	(20.1)	(20.1)	(20.1)	(20.1)	(20.1)	(20.1)	(20.1)
	-59.1	28.6	-7.9	-88.9	60.4	102.5	-11.9	47.6	16.2	-15.8
	-3.478	1.685	-0.466	-5.233	3.560	6.037	-0.698	2.801	0.956	-0.929
t_O	349	330	375	353	359	336	315	351	347	319
	(20.6)	(19.4)	(22.1)	(20.8)	(21.1)	(19.8)	(18.6)	(20.7)	(20.4)	(18.8)
t_E	338.6	338.6	338.6	338.6	338.6	338.6	338.6	338.6	338.6	338.6
	(19.9)	(19.9)	(19.9)	(19.9)	(19.9)	(19.9)	(19.9)	(19.9)	(19.9)	(19.9)
	21.1	-17.0	76.6	29.4	42.0	-5.2	-45.5	25.2	17.0	-38.1
	1.243	-1.001	4.509	1.731	2.473	-0.306	-2.681	1.486	1.001	-2.241
g_O	507	516	493	493	498	486	518	512	461	487
	(29.9)	(30.4)	(29.0)	(29.0)	(29.3)	(28.6)	(30.5)	(30.2)	(27.1)	(28.7)
g_E	484.3	484.3	484.3	484.3	484.3	484.3	484.3	484.3	484.3	484.3
	(28.5)	(28.5)	(28.5)	(28.5)	(28.5)	(28.5)	(28.5)	(28.5)	(28.5)	(28.5)
	46.5	65.5	17.6	17.6	27.9	3.5	69.8	57.0	-45.4	5.5
	2.739	3.858	1.038	1.038	1.640	0.204	4.109	3.358	-2.674	0.323
c_O	532	497	493	559	471	487	530	471	541	559
	(31.3)	(29.3)	(29.0)	(32.9)	(27.7)	(28.7)	(31.2)	(27.7)	(31.9)	(32.9)
c_E	534.1	534.1	534.1	534.1	534.1	534.1	534.1	534.1	534.1	534.1
	(31.5)	(31.5)	(31.5)	(31.5)	(31.5)	(31.5)	(31.5)	(31.5)	(31.5)	(31.5)
	-4.3	-71.6	-79.0	50.8	-118.5	-90.0	-8.3	-118.5	13.8	50.8
	-0.252	-4.220	-4.655	2.994	-6.980	-5.301	-0.487	-6.980	0.812	2.994
N	1698	1698	1698	1698	1698	1698	1698	1698	1698	1698
X2	4.2	5.5	7.3	8.7	11.6	11.0	4.1	11.1	1.6	2.5
Gvalue	4.3	5.5	7.2	9.0	11.8	10.8	4.1	11.3	1.6	2.5
Gv/N	0.252	0.322	0.426	0.529	0.693	0.635	0.243	0.666	0.095	0.147
Prob	0.23288	0.14042	0.06475	0.02942	0.00821	0.01296	0.24876	0.01018	0.65546	0.47522

	50	49	48	47	46	45	44	43	42	41
a_O	329	322	316	364	341	319	357	344	329	371
	(19.4)	(19.0)	(18.6)	(21.4)	(20.1)	(18.8)	(21.0)	(20.3)	(19.4)	(21.8)
a_E	341.0	341.0	341.0	341.0	341.0	341.0	341.0	341.0	341.0	341.0
	(20.1)	(20.1)	(20.1)	(20.1)	(20.1)	(20.1)	(20.1)	(20.1)	(20.1)	(20.1)
	-23.5	-36.9	-48.1	47.6	0.0	-42.5	32.8	6.1	-23.5	62.6
	-1.386	-2.172	-2.832	2.801	0.003	-2.503	1.931	0.358	-1.386	3.687
t_O	342	339	347	314	369	376	347	369	323	305
	(20.1)	(20.0)	(20.4)	(18.5)	(21.7)	(22.1)	(20.4)	(21.7)	(19.0)	(18.0)
t_E	338.6	338.6	338.6	338.6	338.6	338.6	338.6	338.6	338.6	338.6
	(19.9)	(19.9)	(19.9)	(19.9)	(19.9)	(19.9)	(19.9)	(19.9)	(19.9)	(19.9)
	6.8	0.8	17.0	-47.4	63.4	78.8	17.0	63.4	-30.5	-63.8
	0.402	0.046	1.001	-2.790	3.736	4.639	1.001	3.736	-1.795	-3.755
g_O	536	489	484	518	476	496	474	472	524	485
	(31.6)	(28.8)	(28.5)	(30.5)	(28.0)	(29.2)	(27.9)	(27.8)	(30.9)	(28.6)
g_E	484.3	484.3	484.3	484.3	484.3	484.3	484.3	484.3	484.3	484.3
	(28.5)	(28.5)	(28.5)	(28.5)	(28.5)	(28.5)	(28.5)	(28.5)	(28.5)	(28.5)
	108.8	9.5	-0.5	69.8	-16.4	23.7	-20.3	-24.2	82.6	1.5
	6.408	0.560	-0.031	4.109	-0.965	1.399	-1.196	-1.426	4.867	0.086
c_O	491	548	551	502	512	507	520	513	522	537
	(28.9)	(32.3)	(32.4)	(29.6)	(30.2)	(29.9)	(30.6)	(30.2)	(30.7)	(31.6)
c_E	534.1	534.1	534.1	534.1	534.1	534.1	534.1	534.1	534.1	534.1
	(31.5)	(31.5)	(31.5)	(31.5)	(31.5)	(31.5)	(31.5)	(31.5)	(31.5)	(31.5)
	-82.7	28.1	34.2	-62.3	-43.4	-52.9	-27.9	-41.4	-24.0	5.7
	-4.871	1.653	2.016	-3.670	-2.554	-3.115	-1.644	-2.441	-1.414	0.337
N	1698	1698	1698	1698	1698	1698	1698	1698	1698	1698
X2	9.5	1.5	2.6	7.6	3.8	7.2	1.6	3.9	4.7	6.0
Gvalue	9.4	1.5	2.6	7.6	3.7	7.1	1.5	3.8	4.6	6.0
Gv/N	0.553	0.087	0.154	0.449	0.219	0.419	0.091	0.226	0.271	0.356
Prob	0.02462	0.68708	0.45595	0.05442	0.29299	0.06821	0.67195	0.27946	0.20275	0.10981

	40	39	38	37	36	35	34	33	32	31
a_O	346	315	337	357	317	340	331	319	391	335
	(20.4)	(18.6)	(19.8)	(21.0)	(18.7)	(20.0)	(19.5)	(18.8)	(23.0)	(19.7)
a_E	341.0	341.0	341.0	341.0	341.0	341.0	341.0	341.0	341.0	341.0
	(20.1)	(20.1)	(20.1)	(20.1)	(20.1)	(20.1)	(20.1)	(20.1)	(20.1)	(20.1)
	10.1	-49.9	-7.9	32.8	-46.2	-2.0	-19.7	-42.5	107.0	-11.9
	0.596	-2.940	-0.466	1.931	-2.723	-0.115	-1.158	-2.503	6.304	-0.698
t_O	369	339	302	340	374	343	400	337	282	372
	(21.7)	(20.0)	(17.8)	(20.0)	(22.0)	(20.2)	(23.6)	(19.8)	(16.6)	(21.9)
t_E	338.6	338.6	338.6	338.6	338.6	338.6	338.6	338.6	338.6	338.6
	(19.9)	(19.9)	(19.9)	(19.9)	(19.9)	(19.9)	(19.9)	(19.9)	(19.9)	(19.9)
	63.4	0.8	-69.1	2.8	74.4	8.8	133.3	-3.2	-103.2	70.0
	3.736	0.046	-4.070	0.164	4.379	0.521	7.850	-0.189	-6.076	4.121
g_O	450	524	508	496	486	513	476	521	477	476
	(26.5)	(30.9)	(29.9)	(29.2)	(28.6)	(30.2)	(28.0)	(30.7)	(28.1)	(28.0)
g_E	484.3	484.3	484.3	484.3	484.3	484.3	484.3	484.3	484.3	484.3
	(28.5)	(28.5)	(28.5)	(28.5)	(28.5)	(28.5)	(28.5)	(28.5)	(28.5)	(28.5)
	-66.1	82.6	48.6	23.7	3.5	59.1	-16.4	76.2	-14.4	-16.4
	-3.890	4.867	2.863	1.399	0.204	3.483	-0.965	4.487	-0.850	-0.965
c_O	533	520	551	505	521	502	491	521	548	515
	(31.4)	(30.6)	(32.4)	(29.7)	(30.7)	(29.6)	(28.9)	(30.7)	(32.3)	(30.3)
c_E	534.1	534.1	534.1	534.1	534.1	534.1	534.1	534.1	534.1	534.1
	(31.5)	(31.5)	(31.5)	(31.5)	(31.5)	(31.5)	(31.5)	(31.5)	(31.5)	(31.5)

```
           -2.3   -27.9    34.2   -56.7   -26.0   -62.3   -82.7   -26.0    28.1   -37.6
         -0.135  -1.644   2.016  -3.338  -1.529  -3.670  -4.871  -1.529   1.653  -2.214

N          1698    1698    1698    1698    1698    1698    1698    1698    1698    1698
X2          5.2     5.6     5.7     2.6     5.7     3.7    15.0     4.5    17.3     4.2
Gvalue      5.2     5.6     5.8     2.6     5.6     3.7    14.5     4.5    17.5     4.1
Gv/N      0.307   0.329   0.343   0.156   0.332   0.218   0.856   0.265   1.031   0.243
Prob    0.15706 0.13369 0.12039 0.44892 0.13071 0.29497 0.00226 0.21226 0.00056 0.24758
        -------------------------------------------------------------------------------
             30      29      28      27      26      25      24      23      22      21
        -------------------------------------------------------------------------------
a_O         310     335     326     338     373     382     326     345     358     322
        ( 18.3) ( 19.7) ( 19.2) ( 19.9) ( 22.0) ( 22.5) ( 19.2) ( 20.3) ( 21.1) ( 19.0)
a_E       341.0   341.0   341.0   341.0   341.0   341.0   341.0   341.0   341.0   341.0
        ( 20.1) ( 20.1) ( 20.1) ( 20.1) ( 20.1) ( 20.1) ( 20.1) ( 20.1) ( 20.1) ( 20.1)
          -59.1   -11.9   -29.3    -5.9    67.0    86.8   -29.3     8.1    34.9   -36.9
         -3.478  -0.698  -1.725  -0.349   3.944   5.111  -1.725   0.477   2.054  -2.172
t_O         355     294     368     323     322     325     321     318     355     321
        ( 20.9) ( 17.3) ( 21.7) ( 19.0) ( 19.0) ( 19.1) ( 18.9) ( 18.7) ( 20.9) ( 18.9)
t_E       338.6   338.6   338.6   338.6   338.6   338.6   338.6   338.6   338.6   338.6
        ( 19.9) ( 19.9) ( 19.9) ( 19.9) ( 19.9) ( 19.9) ( 19.9) ( 19.9) ( 19.9) ( 19.9)
           33.6   -83.1    61.3   -30.5   -32.4   -26.7   -34.3   -39.9    33.6   -34.3
          1.977  -4.892   3.608  -1.795  -1.907  -1.570  -2.019  -2.352   1.977  -2.019
g_O         491     542     484     481     483     478     500     500     425     504
        ( 28.9) ( 31.9) ( 28.5) ( 28.3) ( 28.4) ( 28.2) ( 29.4) ( 29.4) ( 25.0) ( 29.7)
g_E       484.3   484.3   484.3   484.3   484.3   484.3   484.3   484.3   484.3   484.3
        ( 28.5) ( 28.5) ( 28.5) ( 28.5) ( 28.5) ( 28.5) ( 28.5) ( 28.5) ( 28.5) ( 28.5)
           13.6   122.1    -0.5    -6.5    -2.5   -12.5    32.0    32.0  -111.0    40.3
          0.799   7.190  -0.031  -0.384  -0.149  -0.733   1.883   1.883  -6.535   2.371
c_O         542     527     520     556     520     513     551     535     560     551
        ( 31.9) ( 31.0) ( 30.6) ( 32.7) ( 30.6) ( 30.2) ( 32.4) ( 31.5) ( 33.0) ( 32.4)
c_E       534.1   534.1   534.1   534.1   534.1   534.1   534.1   534.1   534.1   534.1
        ( 31.5) ( 31.5) ( 31.5) ( 31.5) ( 31.5) ( 31.5) ( 31.5) ( 31.5) ( 31.5) ( 31.5)
           15.8   -14.2   -27.9    44.6   -27.9   -41.4    34.2     1.7    52.9    34.2
          0.932  -0.836  -1.644   2.626  -1.644  -2.441   2.016   0.101   3.118   2.016

N          1698    1698    1698    1698    1698    1698    1698    1698    1698    1698
X2          3.8    13.0     3.6     1.7     4.2     6.4     2.6     1.8    10.1     3.3
Gvalue      3.9    13.0     3.5     1.7     4.1     6.2     2.6     1.8    10.4     3.3
Gv/N      0.229   0.764   0.208   0.098   0.243   0.367   0.155   0.108   0.614   0.196
Prob    0.27328 0.00468 0.31734 0.64563 0.24808 0.10082 0.45197 0.60740 0.01533 0.34377
        -------------------------------------------------------------------------------
             20      19      18      17      16      15      14      13      12      11
        -------------------------------------------------------------------------------
a_O         377     368     322     345     333     320     346     372     341     439
        ( 22.2) ( 21.7) ( 19.0) ( 20.3) ( 19.6) ( 18.8) ( 20.4) ( 21.9) ( 20.1) ( 25.9)
a_E       341.0   341.0   341.0   341.0   341.0   341.0   341.0   341.0   341.0   341.0
        ( 20.1) ( 20.1) ( 20.1) ( 20.1) ( 20.1) ( 20.1) ( 20.1) ( 20.1) ( 20.1) ( 20.1)
           75.7    56.1   -36.9     8.1   -15.8   -40.6    10.1    64.8     0.0   221.9
          4.459   3.306  -2.172   0.477  -0.929  -2.393   0.596   3.815   0.003  13.066
t_O         343     380     358     377     377     354     336     375     390     333
        ( 20.2) ( 22.4) ( 21.1) ( 22.2) ( 22.2) ( 20.8) ( 19.8) ( 22.1) ( 23.0) ( 19.6)
t_E       338.6   338.6   338.6   338.6   338.6   338.6   338.6   338.6   338.6   338.6
        ( 19.9) ( 19.9) ( 19.9) ( 19.9) ( 19.9) ( 19.9) ( 19.9) ( 19.9) ( 19.9) ( 19.9)
            8.8    87.6    39.9    81.0    81.0    31.5    -5.2    76.6   110.2   -11.1
          0.521   5.162   2.348   4.769   4.769   1.854  -0.306   4.509   6.491  -0.655
g_O         437     371     383     387     379     396     379     356     391     403
        ( 25.7) ( 21.8) ( 22.6) ( 22.8) ( 22.3) ( 23.3) ( 22.3) ( 21.0) ( 23.0) ( 23.7)
g_E       484.3   484.3   484.3   484.3   484.3   484.3   484.3   484.3   484.3   484.3
        ( 28.5) ( 28.5) ( 28.5) ( 28.5) ( 28.5) ( 28.5) ( 28.5) ( 28.5) ( 28.5) ( 28.5)
          -89.8  -197.7  -179.7  -173.5  -185.8  -159.4  -185.8  -219.1  -167.3  -148.1
         -5.286 -11.643 -10.583 -10.220 -10.941  -9.386 -10.941 -12.903  -9.852  -8.720
c_O         541     579     635     589     609     628     637     595     576     523
        ( 31.9) ( 34.1) ( 37.4) ( 34.7) ( 35.9) ( 37.0) ( 37.5) ( 35.0) ( 33.9) ( 30.8)
c_E       534.1   534.1   534.1   534.1   534.1   534.1   534.1   534.1   534.1   534.1
        ( 31.5) ( 31.5) ( 31.5) ( 31.5) ( 31.5) ( 31.5) ( 31.5) ( 31.5) ( 31.5) ( 31.5)
           13.8    93.4   219.7   115.2   159.7   203.3   224.3   128.4    86.9   -22.1
          0.812   5.499  12.936   6.782   9.407  11.973  13.213   7.561   5.118  -1.299

N          1698    1698    1698    1698    1698    1698    1698    1698    1698    1698
X2          8.6    37.5    42.4    29.6    37.9    34.6    42.8    47.6    29.0    42.1
Gvalue      8.6    39.5    42.9    30.7    39.2    34.8    43.5    50.6    29.9    40.6
Gv/N      0.506   2.324   2.529   1.807   2.307   2.048   2.561   2.983   1.759   2.392
Prob    0.03521 0.00000 0.00000 0.00000 0.00000 0.00000 0.00000 0.00000 0.00000 0.00000
        -------------------------------------------------------------------------------
             10       9       8       7       6       5       4       3       2       1
        -------------------------------------------------------------------------------
a_O         413     356     394     347     346     377     368     330     360     359
        ( 24.3) ( 21.0) ( 23.2) ( 20.4) ( 20.4) ( 22.2) ( 21.7) ( 19.4) ( 21.2) ( 21.1)
a_E       341.0   341.0   341.0   341.0   341.0   341.0   341.0   341.0   341.0   341.0
        ( 20.1) ( 20.1) ( 20.1) ( 20.1) ( 20.1) ( 20.1) ( 20.1) ( 20.1) ( 20.1) ( 20.1)
          158.3    30.7   113.9    12.1    10.1    75.7    56.1   -21.6    39.1    37.0
          9.322   1.808   6.707   0.716   0.596   4.459   3.306  -1.272   2.302   2.178
t_O         360     371     320     378     355     312     314     317     314     216
        ( 21.2) ( 21.8) ( 18.8) ( 22.3) ( 20.9) ( 18.4) ( 18.5) ( 18.7) ( 18.5) ( 12.7)
t_E       338.6   338.6   338.6   338.6   338.6   338.6   338.6   338.6   338.6   338.6
        ( 19.9) ( 19.9) ( 19.9) ( 19.9) ( 19.9) ( 19.9) ( 19.9) ( 19.9) ( 19.9) ( 19.9)
           44.1    67.8   -36.2    83.2    33.6   -51.1   -47.4   -41.8   -47.4  -194.2
          2.598   3.992  -2.130   4.900   1.977  -3.008  -2.790  -2.462  -2.790 -11.438
g_O         382     408     435     312     433     453     481     588     446     458
        ( 22.5) ( 24.0) ( 25.6) ( 18.4) ( 25.5) ( 26.7) ( 28.3) ( 34.6) ( 26.3) ( 27.0)
g_E       484.3   484.3   484.3   484.3   484.3   484.3   484.3   484.3   484.3   484.3
        ( 28.5) ( 28.5) ( 28.5) ( 28.5) ( 28.5) ( 28.5) ( 28.5) ( 28.5) ( 28.5) ( 28.5)
         -181.2  -139.8   -93.3  -274.3   -96.9   -60.5    -6.5   228.3   -73.4   -51.1
        -10.673  -8.235  -5.497 -16.156  -5.707  -3.561  -0.384  13.442  -4.324  -3.008
c_O         543     563     549     661     564     556     535     463     578     665
        ( 32.0) ( 33.2) ( 32.3) ( 38.9) ( 33.2) ( 32.7) ( 31.5) ( 27.3) ( 34.0) ( 39.2)
c_E       534.1   534.1   534.1   534.1   534.1   534.1   534.1   534.1   534.1   534.1
        ( 31.5) ( 31.5) ( 31.5) ( 31.5) ( 31.5) ( 31.5) ( 31.5) ( 31.5) ( 31.5) ( 31.5)
           17.9    59.2    30.1   281.7    61.3    44.6     1.7  -132.4    91.2   291.4
          1.051   3.489   1.774  16.590   3.613   2.626   0.101  -7.795   5.372  17.163

N          1698    1698    1698    1698    1698    1698    1698    1698    1698    1698
X2         38.3    17.3    14.7    96.1     8.0     8.8     4.0    33.4     9.5    78.8
Gvalue     39.0    17.9    14.5   102.7     8.1     8.8     3.9    32.5     9.5    83.1
Gv/N      2.298   1.053   0.853   6.049   0.478   0.517   0.232   1.913   0.559   4.895
Prob    0.00000 0.00046 0.00231 0.00000 0.04354 0.03248 0.26760 0.00000 0.02346 0.00000
        -------------------------------------------------------------------------------

CDS_INITIAL:
        -------------------------------------------------------------------------------
              1       2       3       4       5       6       7       8       9      10
```

a_O	1077	0	0	576	346	299	426	433	322	443
	(63.4)	(0.0)	(0.0)	(33.9)	(20.4)	(17.6)	(25.1)	(25.5)	(19.0)	(26.1)
a_E	367.9	413.2	124.5	367.9	413.2	124.5	367.9	413.2	124.5	367.9
	(21.7)	(24.3)	(7.3)	(21.7)	(24.3)	(7.3)	(21.7)	(24.3)	(7.3)	(21.7)
	2313.9	0.0	0.0	516.5	-122.9	523.7	125.0	40.5	611.7	164.7
	136.270	0.000	0.000	30.421	-7.237	30.843	7.362	2.383	36.026	9.698
t_O	2	1698	0	212	327	261	237	370	298	278
	(0.1)	(100.0)	(0.0)	(12.5)	(19.3)	(15.4)	(14.0)	(21.8)	(17.6)	(16.4)
t_E	224.5	479.9	190.1	224.5	479.9	190.1	224.5	479.9	190.1	224.5
	(13.2)	(28.3)	(11.2)	(13.2)	(28.3)	(11.2)	(13.2)	(28.3)	(11.2)	(13.2)
	-18.9	4291.3	0.0	-24.3	-250.9	165.5	25.7	-192.5	268.0	118.8
	-1.112	252.725	0.000	-1.432	-14.776	9.749	1.511	-11.334	15.785	6.997
g_O	619	0	1698	432	420	555	542	414	546	482
	(36.5)	(0.0)	(100.0)	(25.4)	(24.7)	(32.7)	(31.9)	(24.4)	(32.2)	(28.4)
g_E	654.0	342.8	623.1	654.0	342.8	623.1	654.0	342.8	623.1	654.0
	(38.5)	(20.2)	(36.7)	(38.5)	(20.2)	(36.7)	(38.5)	(20.2)	(36.7)	(38.5)
	-68.1	0.0	3404.3	-358.3	170.5	-128.5	-203.6	156.2	-144.3	-294.2
	-4.008	0.000	200.492	-21.099	10.043	-7.569	-11.990	9.198	-8.497	-17.323
c_O	0	0	0	478	605	583	493	481	532	495
	(0.0)	(0.0)	(0.0)	(28.2)	(35.6)	(34.3)	(29.0)	(28.3)	(31.3)	(29.2)
c_E	451.6	462.0	760.3	451.6	462.0	760.3	451.6	462.0	760.3	451.6
	(26.6)	(27.2)	(44.8)	(26.6)	(27.2)	(44.8)	(26.6)	(27.2)	(44.8)	(26.6)
	0.0	0.0	0.0	54.2	326.2	-309.5	86.4	38.7	-379.9	90.8
	0.000	0.000	0.000	3.194	19.211	-18.230	5.088	2.279	-22.371	5.345
N	1698	1698	1698	1698	1698	1698	1698	1698	1698	1698
X2	2041.0	4309.9	2929.0	195.3	121.3	319.6	32.8	41.7	452.4	77.5
Gvalue	2226.9	4291.3	3404.3	188.2	123.0	251.2	33.5	42.9	355.6	80.1
Gv/N	131.150	252.725	200.492	11.084	7.242	14.794	1.971	2.526	20.942	4.716
Prob	1.00000	1.00000	1.00000	0.00000	0.00000	0.00000	0.00000	0.00000	0.00000	0.00000

	11	12	13	14	15	16	17	18	19	20
a_O	340	240	371	355	258	429	373	303	421	420
	(20.0)	(14.1)	(21.8)	(20.9)	(15.2)	(25.3)	(22.0)	(17.8)	(24.8)	(24.7)
a_E	413.2	124.5	367.9	413.2	124.5	367.9	413.2	124.5	367.9	413.2
	(24.3)	(7.3)	(21.7)	(24.3)	(7.3)	(21.7)	(24.3)	(7.3)	(21.7)	(24.3)
	-132.6	314.9	6.3	-107.8	375.8	131.9	-76.4	538.8	113.6	13.6
	-7.812	18.543	0.371	-6.351	22.132	7.768	-4.500	31.730	6.690	0.804
t_O	423	314	293	444	289	245	413	311	256	422
	(24.9)	(18.5)	(17.3)	(26.1)	(17.0)	(14.4)	(24.3)	(18.3)	(15.1)	(24.9)
t_E	479.9	190.1	224.5	479.9	190.1	224.5	479.9	190.1	224.5	479.9
	(28.3)	(11.2)	(13.2)	(28.3)	(11.2)	(13.2)	(28.3)	(11.2)	(13.2)	(28.3)
	-106.8	315.3	156.0	-69.0	242.2	42.8	-124.0	306.3	67.2	-108.5
	-6.288	18.567	9.188	-4.066	14.264	2.520	-7.303	18.038	3.957	-6.391
g_O	422	539	538	375	515	544	385	448	556	364
	(24.9)	(31.7)	(31.7)	(22.1)	(30.3)	(32.0)	(22.7)	(26.4)	(32.7)	(21.4)
g_E	342.8	623.1	654.0	342.8	623.1	654.0	342.8	623.1	654.0	342.8
	(20.2)	(36.7)	(38.5)	(20.2)	(36.7)	(38.5)	(20.2)	(36.7)	(38.5)	(20.2)
	175.3	-156.3	-210.1	67.3	-196.3	-200.3	89.3	-295.6	-180.5	43.6
	10.327	-9.208	-12.371	3.961	-11.561	-11.798	5.260	-17.411	-10.630	2.568
c_O	513	605	496	524	636	480	527	636	465	492
	(30.2)	(35.6)	(29.2)	(30.9)	(37.5)	(28.3)	(31.0)	(37.5)	(27.4)	(29.0)
c_E	462.0	760.3	451.6	462.0	760.3	451.6	462.0	760.3	451.6	462.0
	(27.2)	(44.8)	(26.6)	(27.2)	(44.8)	(26.6)	(27.2)	(44.8)	(26.6)	(27.2)
	107.4	-276.4	92.9	131.9	-227.0	58.5	138.7	-227.0	27.1	61.8
	6.323	-16.278	5.474	7.768	-13.369	3.443	8.167	-13.369	1.597	3.642
N	1698	1698	1698	1698	1698	1698	1698	1698	1698	1698
X2	43.6	230.9	45.8	22.2	233.6	32.3	27.6	402.2	27.2	10.3
Gvalue	43.3	197.4	45.2	22.3	194.7	32.8	27.6	322.4	27.4	10.6
Gv/N	2.550	11.624	2.662	1.311	11.466	1.933	1.623	18.987	1.615	0.623
Prob	0.00000	0.00000	0.00000	0.00006	0.00000	0.00000	0.00000	0.00000	0.00000	0.01423

	21	22	23	24	25	26	27	28	29	30
a_O	273	401	415	242	396	345	257	403	387	233
	(16.1)	(23.6)	(24.4)	(14.3)	(23.3)	(20.3)	(15.1)	(23.7)	(22.8)	(13.7)
a_E	124.5	367.9	413.2	124.5	367.9	413.2	124.5	367.9	413.2	124.5
	(7.3)	(21.7)	(24.3)	(7.3)	(21.7)	(24.3)	(7.3)	(21.7)	(24.3)	(7.3)
	428.5	69.2	3.5	321.5	58.4	-124.5	372.3	73.5	-50.8	291.9
	25.235	4.073	0.209	18.934	3.437	-7.333	21.928	4.330	-2.989	17.190
t_O	310	239	419	309	266	418	278	240	436	295
	(18.3)	(14.1)	(24.7)	(18.2)	(15.7)	(24.6)	(16.4)	(14.1)	(25.7)	(17.4)
t_E	190.1	224.5	479.9	190.1	224.5	479.9	190.1	224.5	479.9	190.1
	(11.2)	(13.2)	(28.3)	(11.2)	(13.2)	(28.3)	(11.2)	(13.2)	(28.3)	(11.2)
	303.3	29.9	-113.7	300.3	90.2	-115.5	211.4	32.0	-83.7	259.4
	17.862	1.760	-6.698	17.687	5.313	-6.799	12.451	1.886	-4.927	15.274
g_O	500	557	380	498	562	399	517	558	374	527
	(29.4)	(32.8)	(22.4)	(29.3)	(33.1)	(23.5)	(30.4)	(32.9)	(22.0)	(31.0)
g_E	623.1	654.0	342.8	623.1	654.0	342.8	623.1	654.0	342.8	623.1
	(36.7)	(38.5)	(20.2)	(36.7)	(38.5)	(20.2)	(36.7)	(38.5)	(20.2)	(36.7)
	-220.1	-178.8	78.2	-223.3	-170.4	121.1	-193.1	-177.1	65.1	-176.6
	-12.965	-10.531	4.607	-13.148	-10.034	7.130	-11.369	-10.432	3.833	-10.400
c_O	615	501	484	649	474	536	646	497	501	643
	(36.2)	(29.5)	(28.5)	(38.2)	(27.9)	(31.6)	(38.0)	(29.3)	(29.5)	(37.9)
c_E	760.3	451.6	462.0	760.3	451.6	462.0	760.3	451.6	462.0	760.3
	(44.8)	(26.6)	(27.2)	(44.8)	(26.6)	(27.2)	(44.8)	(26.6)	(27.2)	(44.8)
	-260.8	103.9	45.0	-205.4	45.8	159.2	-210.4	95.1	81.1	-215.4
	-15.360	6.121	2.648	-12.095	2.698	9.375	-12.392	5.603	4.778	-12.687
N	1698	1698	1698	1698	1698	1698	1698	1698	1698	1698
X2	304.7	23.7	12.8	226.6	23.9	40.3	216.8	23.1	11.8	185.3
Gvalue	250.8	24.2	13.0	193.2	24.0	40.3	180.3	23.5	11.8	159.2
Gv/N	14.773	1.424	0.766	11.378	1.414	2.373	10.617	1.386	0.695	9.377
Prob	0.00000	0.00002	0.00463	0.00000	0.00002	0.00000	0.00000	0.00003	0.00812	0.00000

	31	32	33	34	35	36	37	38	39	40
a_O	398	384	232	361	373	246	419	391	240	396
	(23.4)	(22.6)	(13.7)	(21.3)	(22.0)	(14.5)	(24.7)	(23.0)	(14.1)	(23.3)
a_E	367.9	413.2	124.5	367.9	413.2	124.5	367.9	413.2	124.5	367.9
	(21.7)	(24.3)	(7.3)	(21.7)	(24.3)	(7.3)	(21.7)	(24.3)	(7.3)	(21.7)
	62.7	-56.3	288.6	-13.6	-76.4	334.9	109.1	-43.2	314.9	58.4
	3.691	-3.318	16.999	-0.801	-4.500	19.722	6.423	-2.547	18.543	3.437
t_O	238	444	281	244	446	250	235	444	288	235
	(14.0)	(26.1)	(16.5)	(14.4)	(26.3)	(14.7)	(13.8)	(26.1)	(17.0)	(13.8)
t_E	224.5	479.9	190.1	224.5	479.9	190.1	224.5	479.9	190.1	224.5
	(13.2)	(28.3)	(11.2)	(13.2)	(28.3)	(11.2)	(13.2)	(28.3)	(11.2)	(13.2)
	27.8	-69.0	219.7	40.6	-65.4	137.0	21.5	-69.0	239.4	21.5
	1.635	-4.066	12.940	2.392	-3.849	8.071	1.264	-4.066	14.097	1.264

g_O	586	400	571	579	386	527	555	409	539	579
	(34.5)	(23.6)	(33.6)	(34.1)	(22.7)	(31.0)	(32.7)	(24.1)	(31.7)	(34.1)
g_E	654.0	342.8	623.1	654.0	342.8	623.1	654.0	342.8	623.1	654.0
	(38.5)	(20.2)	(36.7)	(38.5)	(20.2)	(36.7)	(38.5)	(20.2)	(36.7)	(38.5)
	-128.6	123.4	-99.8	-141.0	91.6	-176.6	-182.2	144.3	-156.3	-141.0
	-7.576	7.266	-5.875	-8.305	5.392	-10.400	-10.728	8.501	-9.208	-8.305
c_O	476	470	614	514	493	675	489	454	631	488
	(28.0)	(27.7)	(36.2)	(30.3)	(29.0)	(39.8)	(28.8)	(26.7)	(37.2)	(28.7)
c_E	451.6	462.0	760.3	451.6	462.0	760.3	451.6	462.0	760.3	451.6
	(26.6)	(27.2)	(44.8)	(26.6)	(27.2)	(44.8)	(26.6)	(27.2)	(44.8)	(26.6)
	50.0	16.1	-262.4	133.0	64.0	-160.6	77.7	-15.9	-235.2	75.6
	2.945	0.946	-15.452	7.831	3.767	-9.457	4.578	-0.938	-13.850	4.451
N	1698	1698	1698	1698	1698	1698	1698	1698	1698	1698
X2	11.7	14.4	168.7	19.0	13.8	161.7	25.7	16.8	190.8	14.2
Gvalue	11.8	14.1	146.2	19.0	13.8	134.7	26.1	16.1	162.7	14.4
Gv/N	0.696	0.828	8.611	1.117	0.810	7.936	1.537	0.950	9.582	0.847
Prob	0.00805	0.00283	0.00000	0.00028	0.00326	0.00000	0.00001	0.00106	0.00000	0.00243

	41	42	43	44	45	46	47	48	49	50
a_O	362	241	367	338	229	386	361	229	397	365
	(21.3)	(14.2)	(21.6)	(19.9)	(13.5)	(22.7)	(21.3)	(13.5)	(23.4)	(21.5)
a_E	413.2	124.5	367.9	413.2	124.5	367.9	413.2	124.5	367.9	413.2
	(24.3)	(7.3)	(21.7)	(24.3)	(7.3)	(21.7)	(24.3)	(7.3)	(21.7)	(24.3)
	-95.8	318.2	-1.7	-135.8	278.9	37.1	-97.6	278.9	60.5	-90.6
	-5.644	18.738	-0.102	-8.001	16.428	2.188	-5.746	16.428	3.564	-5.336
t_O	439	280	260	475	303	241	453	231	254	481
	(25.9)	(16.5)	(15.3)	(28.0)	(17.8)	(14.2)	(26.7)	(13.6)	(15.0)	(28.3)
t_E	479.9	190.1	224.5	479.9	190.1	224.5	479.9	190.1	224.5	479.9
	(28.3)	(11.2)	(13.2)	(28.3)	(11.2)	(13.2)	(28.3)	(11.2)	(13.2)	(28.3)
	-78.2	216.9	76.3	-9.8	282.6	34.2	-52.3	90.1	62.7	2.2
	-4.606	12.777	4.494	-0.574	16.643	2.012	-3.078	5.307	3.692	0.129
g_O	405	509	591	374	566	625	380	586	591	375
	(23.9)	(30.0)	(34.8)	(22.0)	(33.3)	(36.8)	(22.4)	(34.5)	(34.8)	(22.1)
g_E	342.8	623.1	654.0	342.8	623.1	654.0	342.8	623.1	654.0	342.8
	(20.2)	(36.7)	(38.5)	(20.2)	(36.7)	(38.5)	(20.2)	(36.7)	(38.5)	(20.2)
	135.0	-205.9	-119.7	65.1	-108.8	-56.7	78.2	-72.0	-119.7	67.3
	7.949	-12.128	-7.049	3.833	-6.410	-3.337	4.607	-4.240	-7.049	3.961
c_O	492	668	480	511	600	446	504	652	456	477
	(29.0)	(39.3)	(28.3)	(30.1)	(35.3)	(26.3)	(29.7)	(38.4)	(26.9)	(28.1)
c_E	462.0	760.3	451.6	462.0	760.3	451.6	462.0	760.3	451.6	462.0
	(27.2)	(44.8)	(26.6)	(27.2)	(44.8)	(26.6)	(27.2)	(44.8)	(26.6)	(27.2)
	61.8	-172.8	58.5	103.0	-284.1	-11.2	87.6	-200.3	8.8	30.4
	3.642	-10.179	3.443	6.063	-16.730	-0.660	5.161	-11.797	0.516	1.791
N	1698	1698	1698	1698	1698	1698	1698	1698	1698	1698
X2	23.1	183.5	13.5	21.8	193.7	3.5	16.0	114.0	12.3	9.1
Gvalue	22.8	156.3	13.4	22.4	168.6	3.4	16.0	96.7	12.3	9.3
Gv/N	1.341	9.207	0.786	1.321	9.931	0.202	0.944	5.697	0.723	0.546
Prob	0.00005	0.00000	0.00393	0.00005	0.00000	0.32911	0.00112	0.00000	0.00650	0.02589

	51	52	53	54	55	56	57	58	59	60
a_O	218	390	375	211	386	348	188	337	364	197
	(12.8)	(23.0)	(22.1)	(12.4)	(22.7)	(20.5)	(11.1)	(19.8)	(21.4)	(11.6)
a_E	124.5	367.9	413.2	124.5	367.9	413.2	124.5	367.9	413.2	124.5
	(7.3)	(21.7)	(24.3)	(7.3)	(21.7)	(24.3)	(7.3)	(21.7)	(24.3)	(7.3)
	244.1	45.6	-72.8	222.5	37.1	-119.6	154.8	-59.1	-92.3	180.7
	14.375	2.684	-4.288	13.102	2.188	-7.042	9.118	-3.479	-5.439	10.640
t_O	250	192	480	269	236	520	255	238	454	298
	(14.7)	(11.3)	(28.3)	(15.8)	(13.9)	(30.6)	(15.0)	(14.0)	(26.7)	(17.6)
t_E	190.1	224.5	479.9	190.1	224.5	479.9	190.1	224.5	479.9	190.1
	(11.2)	(13.2)	(28.3)	(11.2)	(13.2)	(28.3)	(11.2)	(13.2)	(28.3)	(11.2)
	137.0	-60.1	0.2	186.9	23.6	83.5	149.9	27.8	-50.4	268.0
	8.071	-3.538	0.012	11.005	1.387	4.915	8.827	1.635	-2.967	15.785
g_O	558	633	337	582	602	350	607	651	365	579
	(32.9)	(37.3)	(19.8)	(34.3)	(35.5)	(20.6)	(35.7)	(38.3)	(21.5)	(34.1)
g_E	623.1	654.0	342.8	623.1	654.0	342.8	623.1	654.0	342.8	623.1
	(36.7)	(38.5)	(20.2)	(36.7)	(38.5)	(20.2)	(36.7)	(38.5)	(20.2)	(36.7)
	-123.2	-41.3	-11.6	-79.5	-99.7	14.5	-31.8	-6.0	45.7	-85.1
	-7.255	-2.431	-0.681	-4.681	-5.873	0.853	-1.875	-0.351	2.693	-5.009
c_O	672	483	506	636	474	480	648	472	515	624
	(39.6)	(28.4)	(29.8)	(37.5)	(27.9)	(28.3)	(38.2)	(27.8)	(30.3)	(36.7)
c_E	760.3	451.6	462.0	760.3	451.6	462.0	760.3	451.6	462.0	760.3
	(44.8)	(26.6)	(27.2)	(44.8)	(26.6)	(27.2)	(44.8)	(26.6)	(27.2)	(44.8)
	-165.9	64.9	92.0	-227.0	45.8	36.6	-207.1	41.6	111.8	-246.5
	-9.767	3.819	5.418	-13.369	2.698	2.157	-12.194	2.452	6.583	-14.517
N	1698	1698	1698	1698	1698	1698	1698	1698	1698	1698
X2	106.1	8.9	7.8	115.8	6.7	14.5	71.5	4.3	14.8	131.0
Gvalue	92.1	9.1	7.8	102.9	6.8	15.0	65.8	4.4	14.8	117.1
Gv/N	5.422	0.534	0.460	6.057	0.400	0.883	3.876	0.258	0.871	6.899
Prob	0.00000	0.02839	0.05014	0.00000	0.07880	0.00183	0.00000	0.22344	0.00200	0.00000

	61	62	63	64	65	66	67	68	69	70
a_O	367	356	190	379	329	208	383	374	202	349
	(21.6)	(21.0)	(11.2)	(22.3)	(19.4)	(12.2)	(22.6)	(22.0)	(11.9)	(20.6)
a_E	367.9	413.2	124.5	367.9	413.2	124.5	367.9	413.2	124.5	367.9
	(21.7)	(24.3)	(7.3)	(21.7)	(24.3)	(7.3)	(21.7)	(24.3)	(7.3)	(21.7)
	-1.7	-106.1	160.5	22.6	-150.0	213.3	30.9	-74.6	195.4	-36.7
	-0.102	-6.251	9.452	1.331	-8.833	12.565	1.819	-4.394	11.506	-2.164
t_O	231	458	274	192	491	251	215	444	268	224
	(13.6)	(27.0)	(16.1)	(11.3)	(28.9)	(14.8)	(12.7)	(26.1)	(15.8)	(13.2)
t_E	224.5	479.9	190.1	224.5	479.9	190.1	224.5	479.9	190.1	224.5
	(13.2)	(28.3)	(11.2)	(13.2)	(28.3)	(11.2)	(13.2)	(28.3)	(11.2)	(13.2)
	13.2	-42.8	200.4	-60.1	22.5	139.6	-18.6	-69.0	184.2	-1.0
	0.775	-2.520	11.804	-3.538	1.322	8.221	-1.096	-4.066	10.846	-0.060
g_O	608	382	560	628	393	573	615	380	569	680
	(35.8)	(22.5)	(33.0)	(37.0)	(23.1)	(33.7)	(36.2)	(22.4)	(33.5)	(40.0)
g_E	654.0	342.8	623.1	654.0	342.8	623.1	654.0	342.8	623.1	654.0
	(38.5)	(20.2)	(36.7)	(38.5)	(20.2)	(36.7)	(38.5)	(20.2)	(36.7)	(38.5)
	-88.7	82.6	-119.6	-50.9	107.3	-96.1	-75.6	78.2	-103.4	53.1
	-5.221	4.867	-7.045	-2.999	6.321	-5.660	-4.452	4.607	-6.090	3.125
c_O	492	502	674	499	485	666	485	500	659	445
	(29.0)	(29.6)	(39.7)	(29.4)	(28.6)	(39.2)	(28.6)	(29.4)	(38.8)	(26.2)
c_E	451.6	462.0	760.3	451.6	462.0	760.3	451.6	462.0	760.3	451.6
	(26.6)	(27.2)	(44.8)	(26.6)	(27.2)	(44.8)	(26.6)	(27.2)	(44.8)	(26.6)
	84.2	83.3	-162.3	99.5	47.1	-176.3	69.1	79.0	-188.4	-13.2
	4.960	4.906	-9.561	5.861	2.771	-10.384	4.071	4.651	-11.095	-0.776

N	1698	1698	1698	1698	1698	1698	1698	1698	1698	1698
X2	7.0	16.9	87.6	11.0	25.9	91.2	5.8	13.6	98.3	2.1
Gvalue	7.0	17.0	79.0	11.1	26.9	80.5	5.8	13.5	87.7	2.1
Gv/N	0.412	1.002	4.650	0.656	1.582	4.742	0.342	0.797	5.167	0.124
Prob	0.07178	0.00070	0.00000	0.01102	0.00001	0.00000	0.12174	0.00362	0.00000	0.55086

	71	72	73	74	75	76	77	78	79	80
a_O	354	187	379	392	203	348	367	159	348	373
	(20.8)	(11.0)	(22.3)	(23.1)	(12.0)	(20.5)	(21.6)	(9.4)	(20.5)	(22.0)
a_E	413.2	124.5	367.9	413.2	124.5	367.9	413.2	124.5	367.9	413.2
	(24.3)	(7.3)	(21.7)	(24.3)	(7.3)	(21.7)	(24.3)	(7.3)	(21.7)	(24.3)
	-109.5	152.0	22.6	-41.4	198.3	-38.6	-87.1	77.7	-38.6	-76.4
	-6.451	8.952	1.331	-2.435	11.681	-2.276	-5.129	4.574	-2.276	-4.500
t_O	478	260	234	485	219	258	462	257	237	483
	(28.2)	(15.3)	(13.8)	(28.6)	(12.9)	(15.2)	(27.2)	(15.1)	(14.0)	(28.4)
t_E	479.9	190.1	224.5	479.9	190.1	224.5	479.9	190.1	224.5	479.9
	(28.3)	(11.2)	(13.2)	(28.3)	(11.2)	(13.2)	(28.3)	(11.2)	(13.2)	(28.3)
	-3.8	162.9	19.4	10.2	62.1	71.7	-35.1	155.1	25.7	6.2
	-0.224	9.594	1.141	0.604	3.655	4.225	-2.069	9.132	1.511	0.366
g_O	359	560	609	339	607	607	377	613	646	347
	(21.1)	(33.0)	(35.9)	(20.0)	(35.7)	(35.7)	(22.2)	(36.1)	(38.0)	(20.4)
g_E	342.8	623.1	654.0	342.8	623.1	654.0	342.8	623.1	654.0	342.8
	(20.2)	(36.7)	(38.5)	(20.2)	(36.7)	(38.5)	(20.2)	(36.7)	(38.5)	(20.2)
	33.1	-119.6	-86.8	-7.6	-31.8	-90.5	71.6	-20.1	-15.9	8.4
	1.948	-7.045	-5.112	-0.449	-1.875	-5.330	4.218	-1.183	-0.935	0.494
c_O	507	691	476	482	669	485	492	669	467	495
	(29.9)	(40.7)	(28.0)	(28.4)	(39.4)	(28.6)	(29.0)	(39.4)	(27.5)	(29.2)
c_E	462.0	760.3	451.6	462.0	760.3	451.6	462.0	760.3	451.6	462.0
	(27.2)	(44.8)	(26.6)	(27.2)	(44.8)	(26.6)	(27.2)	(44.8)	(26.6)	(27.2)
	94.2	-132.0	50.0	40.8	-171.1	69.1	61.8	-171.1	31.2	68.2
	5.546	-7.774	2.945	2.402	-10.076	4.071	3.642	-10.076	1.840	4.018
N	1698	1698	1698	1698	1698	1698	1698	1698	1698	1698
X2	13.6	69.8	5.1	2.1	65.2	11.9	11.2	44.2	2.4	6.3
Gvalue	13.9	63.3	5.2	2.1	57.5	11.7	11.3	41.5	2.4	6.4
Gv/N	0.820	3.727	0.306	0.121	3.385	0.690	0.663	2.447	0.141	0.378
Prob	0.00301	0.00000	0.15856	0.56052	0.00000	0.00840	0.01044	0.00000	0.49566	0.09291

	81	82	83	84	85	86	87	88	89	90
a_O	170	353	321	165	355	360	186	352	361	188
	(10.0)	(20.8)	(18.9)	(9.7)	(20.9)	(21.2)	(11.0)	(20.7)	(21.3)	(11.1)
a_E	124.5	367.9	413.2	124.5	367.9	413.2	124.5	367.9	413.2	124.5
	(7.3)	(21.7)	(24.3)	(7.3)	(21.7)	(24.3)	(7.3)	(21.7)	(24.3)	(7.3)
	105.8	-29.1	-162.1	92.8	-25.3	-99.3	149.2	-31.0	-97.6	154.8
	6.230	-1.715	-9.549	5.466	-1.489	-5.847	8.787	-1.828	-5.746	9.118
t_O	219	234	486	251	238	452	213	227	458	246
	(12.9)	(13.8)	(28.6)	(14.8)	(14.0)	(26.6)	(12.5)	(13.4)	(27.0)	(14.5)
t_E	190.1	224.5	479.9	190.1	224.5	479.9	190.1	224.5	479.9	190.1
	(11.2)	(13.2)	(28.3)	(11.2)	(13.2)	(28.3)	(11.2)	(13.2)	(28.3)	(11.2)
	62.1	19.4	12.3	139.6	27.8	-54.1	48.5	5.0	-42.8	126.9
	3.655	1.141	0.723	8.221	1.635	-3.189	2.858	0.295	-2.520	7.474
g_O	613	651	362	590	637	387	616	640	361	594
	(36.1)	(38.3)	(21.3)	(34.7)	(37.5)	(22.8)	(36.3)	(37.7)	(21.3)	(35.0)
g_E	623.1	654.0	342.8	623.1	654.0	342.8	623.1	654.0	342.8	623.1
	(36.7)	(38.5)	(20.2)	(36.7)	(38.5)	(20.2)	(36.7)	(38.5)	(20.2)	(36.7)
	-20.1	-6.0	39.4	-64.5	-33.5	93.8	-14.2	-27.7	37.3	-56.9
	-1.183	-0.351	2.319	-3.796	-1.974	5.524	-0.834	-1.629	2.195	-3.349
c_O	696	460	529	692	468	499	683	479	518	670
	(41.0)	(27.1)	(31.2)	(40.8)	(27.6)	(29.4)	(40.2)	(28.2)	(30.5)	(39.5)
c_E	760.3	451.6	462.0	760.3	451.6	462.0	760.3	451.6	462.0	760.3
	(44.8)	(26.6)	(27.2)	(44.8)	(26.6)	(27.2)	(44.8)	(26.6)	(27.2)	(44.8)
	-122.9	16.9	143.2	-130.2	33.3	76.8	-146.4	56.3	118.5	-169.4
	-7.240	0.994	8.434	-7.668	1.962	4.524	-8.621	3.319	6.976	-9.974
N	1698	1698	1698	1698	1698	1698	1698	1698	1698	1698
X2	26.6	1.2	31.4	40.6	2.3	17.1	41.0	2.7	15.3	60.9
Gvalue	24.8	1.2	32.7	37.8	2.3	17.2	37.2	2.6	15.4	55.5
Gv/N	1.462	0.069	1.927	2.223	0.134	1.011	2.189	0.156	0.906	3.269
Prob	0.00002	0.75977	0.00000	0.00000	0.51629	0.00065	0.00000	0.44913	0.00152	0.00000

	91	92	93	94	95	96	97	98	99	100
a_O	355	375	194	357	392	160	351	328	167	345
	(20.9)	(22.1)	(11.4)	(21.0)	(23.1)	(9.4)	(20.7)	(19.3)	(9.8)	(20.3)
a_E	367.9	413.2	124.5	367.6	413.0	124.5	367.6	413.0	124.5	367.6
	(21.7)	(24.3)	(7.3)	(21.7)	(24.3)	(7.3)	(21.7)	(24.3)	(7.3)	(21.7)
	-25.3	-72.8	172.0	-21.0	-40.9	80.3	-32.5	-151.1	98.2	-43.9
	-1.489	-4.288	10.127	-1.237	-2.409	4.735	-1.917	-8.907	5.785	-2.585
t_O	246	509	245	244	460	236	233	464	240	238
	(14.5)	(30.0)	(14.4)	(14.4)	(27.1)	(13.9)	(13.7)	(27.3)	(14.1)	(14.0)
t_E	224.5	479.9	190.1	224.4	479.6	190.0	224.4	479.6	190.0	224.4
	(13.2)	(28.3)	(11.2)	(13.2)	(28.3)	(11.2)	(13.2)	(28.3)	(11.2)	(13.2)
	45.0	59.9	124.4	40.9	-38.4	102.4	17.6	-30.7	112.2	28.0
	2.648	3.529	7.326	2.411	-2.264	6.037	1.035	-1.811	6.614	1.653
g_O	637	339	578	607	355	632	635	421	583	699
	(37.5)	(20.0)	(34.0)	(35.8)	(20.9)	(37.2)	(37.4)	(24.8)	(34.4)	(41.2)
g_E	654.0	342.8	623.1	653.6	342.6	622.8	653.6	342.6	622.8	653.6
	(38.5)	(20.2)	(36.7)	(38.5)	(20.2)	(36.7)	(38.5)	(20.2)	(36.7)	(38.5)
	-33.5	-7.6	-86.9	-89.8	25.2	18.6	-36.7	173.4	-76.9	93.9
	-1.974	-0.449	-5.118	-5.291	1.484	1.097	-2.160	10.220	-4.533	5.533
c_O	460	475	681	489	490	669	478	484	707	415
	(27.1)	(28.0)	(40.1)	(28.8)	(28.9)	(39.4)	(28.2)	(28.5)	(41.7)	(24.5)
c_E	451.6	462.0	760.3	451.4	461.8	759.8	451.4	461.8	759.8	451.4
	(26.6)	(27.2)	(44.8)	(26.6)	(27.2)	(44.8)	(26.6)	(27.2)	(44.8)	(26.6)
	16.9	26.3	-150.0	78.3	58.2	-170.3	54.8	45.5	-101.9	-69.7
	0.994	1.549	-8.831	4.615	3.428	-10.036	3.229	2.683	-6.003	-4.109
N	1698	1698	1698	1697	1697	1697	1697	1697	1697	1697
X2	3.1	5.7	66.1	8.5	4.0	32.3	3.2	37.0	33.9	8.3
Gvalue	3.0	5.8	59.5	8.4	4.0	31.1	3.2	37.1	31.6	8.3
Gv/N	0.179	0.341	3.504	0.497	0.238	1.833	0.187	2.186	1.863	0.491
Prob	0.38430	0.12257	0.00000	0.03781	0.25795	0.00000	0.36649	0.00000	0.00000	0.03964

```
5UTR:
          -------------------------------------------------------------------------------
              100       99       98       97       96       95       94       93       92       91
          -------------------------------------------------------------------------------
a_O            36       50       52       48       48       42       50       48       48       54
          ( 16.1) ( 22.3) ( 23.2) ( 21.4) ( 21.4) ( 18.8) ( 22.3) ( 21.4) ( 21.4) ( 24.1)
a_E          53.1     53.1     53.1     53.1     53.1     53.1     53.1     53.1     53.1     53.1
          ( 23.7) ( 23.7) ( 23.7) ( 23.7) ( 23.7) ( 23.7) ( 23.7) ( 23.7) ( 23.7) ( 23.7)
            -28.0     -6.0     -2.2     -9.7     -9.7    -19.7     -6.0     -9.7     -9.7      1.8
          -12.499   -2.694   -0.981   -4.336   -4.336   -8.801   -2.694   -4.336   -4.336    0.801
t_O            49       40       54       57       52       55       57       54       45       43
          ( 21.9) ( 17.9) ( 24.1) ( 25.4) ( 23.2) ( 24.6) ( 25.4) ( 24.1) ( 20.1) ( 19.2)
t_E          49.5     49.5     49.5     49.5     49.5     49.5     49.5     49.5     49.5     49.5
          ( 22.1) ( 22.1) ( 22.1) ( 22.1) ( 22.1) ( 22.1) ( 22.1) ( 22.1) ( 22.1) ( 22.1)
             -1.0    -17.0      9.4     16.1      5.1     11.6     16.1      9.4     -8.6    -12.1
           -0.444   -7.610    4.195    7.180    2.288    5.174    7.180    4.195   -3.829   -5.405
g_O            67       69       56       49       57       64       54       57       60       56
          ( 29.9) ( 30.8) ( 25.0) ( 21.9) ( 25.4) ( 28.6) ( 24.1) ( 25.4) ( 26.8) ( 25.0)
g_E          57.3     57.3     57.3     57.3     57.3     57.3     57.3     57.3     57.3     57.3
          ( 25.6) ( 25.6) ( 25.6) ( 25.6) ( 25.6) ( 25.6) ( 25.6) ( 25.6) ( 25.6) ( 25.6)
             21.1     25.7     -2.5    -15.3     -0.5     14.2     -6.3     -0.5      5.6     -2.5
            9.397   11.490   -1.113   -6.815   -0.232    6.359   -2.826   -0.232    2.504   -1.113
c_O            72       65       62       70       67       63       63       65       71       71
          ( 32.1) ( 29.0) ( 27.7) ( 31.2) ( 29.9) ( 28.1) ( 28.1) ( 29.0) ( 31.7) ( 31.7)
c_E          64.1     64.1     64.1     64.1     64.1     64.1     64.1     64.1     64.1     64.1
          ( 28.6) ( 28.6) ( 28.6) ( 28.6) ( 28.6) ( 28.6) ( 28.6) ( 28.6) ( 28.6) ( 28.6)
             16.7      1.8     -4.2     12.3      5.9     -2.2     -2.2      1.8     14.5     14.5
            7.441    0.782   -1.870    5.474    2.619   -1.000   -1.000    0.782    6.451    6.451

N             224      224      224      224      224      224      224      224      224      224
X2            8.1      4.4      0.5      3.4      0.7      3.7      1.5      0.9      1.8      1.6
Gvalue        8.7      4.4      0.5      3.4      0.8      3.9      1.5      0.9      1.8      1.6
Gv/N        3.896    1.968    0.232    1.503    0.339    1.732    0.660    0.410    0.790    0.735
Prob      0.03315  0.22068  0.91446  0.33854  0.85897  0.27481  0.68736  0.82099  0.62140  0.64871
          -------------------------------------------------------------------------------
               90       89       88       87       86       85       84       83       82       81
          -------------------------------------------------------------------------------
a_O            51       43       44       52       48       47       56       52       56       47
          ( 22.8) ( 19.2) ( 19.6) ( 23.2) ( 21.4) ( 21.0) ( 25.0) ( 23.2) ( 25.0) ( 21.0)
a_E          53.1     53.1     53.1     53.1     53.1     53.1     53.1     53.1     53.1     53.1
          ( 23.7) ( 23.7) ( 23.7) ( 23.7) ( 23.7) ( 23.7) ( 23.7) ( 23.7) ( 23.7) ( 23.7)
             -4.1    -18.2    -16.6     -2.2     -9.7    -11.5      5.9     -2.2      5.9    -11.5
           -1.846   -8.107   -7.393   -0.981   -4.336   -5.129    2.649   -0.981    2.649   -5.129
t_O            56       53       43       49       60       46       47       34       52       50
          ( 25.0) ( 23.7) ( 19.2) ( 21.9) ( 26.8) ( 20.5) ( 21.0) ( 15.2) ( 23.2) ( 22.3)
t_E          49.5     49.5     49.5     49.5     49.5     49.5     49.5     49.5     49.5     49.5
          ( 22.1) ( 22.1) ( 22.1) ( 22.1) ( 22.1) ( 22.1) ( 22.1) ( 22.1) ( 22.1) ( 22.1)
             13.8      7.2    -12.1     -1.0     23.1     -6.7     -4.9    -25.5      5.1      1.0
            6.169    3.233   -5.405   -0.444   10.306   -3.012   -2.175  -11.403    2.288    0.449
g_O            51       63       67       60       58       65       64       66       52       72
          ( 22.8) ( 28.1) ( 29.9) ( 26.8) ( 25.9) ( 29.0) ( 28.6) ( 29.5) ( 23.2) ( 32.1)
g_E          57.3     57.3     57.3     57.3     57.3     57.3     57.3     57.3     57.3     57.3
          ( 25.6) ( 25.6) ( 25.6) ( 25.6) ( 25.6) ( 25.6) ( 25.6) ( 25.6) ( 25.6) ( 25.6)
            -11.8     12.0     21.1      5.6      1.5     16.5     14.2     18.8    -10.0     33.0
           -5.272    5.374    9.397    2.504    0.665    7.358    6.359    8.371   -4.474   14.726
c_O            66       65       70       63       58       66       57       72       64       55
          ( 29.5) ( 29.0) ( 31.2) ( 28.1) ( 25.9) ( 29.5) ( 25.4) ( 32.1) ( 28.6) ( 24.6)
c_E          64.1     64.1     64.1     64.1     64.1     64.1     64.1     64.1     64.1     64.1
          ( 28.6) ( 28.6) ( 28.6) ( 28.6) ( 28.6) ( 28.6) ( 28.6) ( 28.6) ( 28.6) ( 28.6)
              3.8      1.8     12.3     -2.2    -11.7      3.8    -13.4     16.7     -0.3    -16.9
            1.694    0.782    5.474   -1.000   -5.203    1.694   -5.998    7.441   -0.116   -7.542

N             224      224      224      224      224      224      224      224      224      224
X2            1.7      2.8      4.6      0.2      3.3      2.1      1.9      7.2      0.8      5.8
Gvalue        1.7      2.9      4.6      0.2      3.2      2.0      1.9      7.7      0.8      5.6
Gv/N        0.745    1.281    2.074    0.079    1.432    0.911    0.835    3.429    0.347    2.504
Prob      0.64403  0.41203  0.19965  0.98113  0.36066  0.56392  0.59967  0.05308  0.85481  0.13232
          -------------------------------------------------------------------------------
               80       79       78       77       76       75       74       73       72       71
          -------------------------------------------------------------------------------
a_O            55       49       53       47       51       51       58       51       62       59
          ( 24.6) ( 21.9) ( 23.7) ( 21.0) ( 22.8) ( 22.8) ( 25.9) ( 22.8) ( 27.7) ( 26.3)
a_E          53.1     53.1     53.1     53.1     53.1     53.1     53.1     53.1     53.1     53.1
          ( 23.7) ( 23.7) ( 23.7) ( 23.7) ( 23.7) ( 23.7) ( 23.7) ( 23.7) ( 23.7) ( 23.7)
              3.8     -7.9     -0.2    -11.5     -4.1     -4.1     10.2     -4.1     19.2     12.4
            1.717   -3.524   -0.098   -5.129   -1.846   -1.846    4.561   -1.846    8.568    5.540
t_O            45       65       51       53       50       36       44       40       46       47
          ( 20.1) ( 29.0) ( 22.8) ( 23.7) ( 22.3) ( 16.1) ( 19.6) ( 17.9) ( 20.5) ( 21.0)
t_E          49.5     49.5     49.5     49.5     49.5     49.5     49.5     49.5     49.5     49.5
          ( 22.1) ( 22.1) ( 22.1) ( 22.1) ( 22.1) ( 22.1) ( 22.1) ( 22.1) ( 22.1) ( 22.1)
             -8.6     35.4      3.0      7.2      1.0    -22.9    -10.4    -17.0     -6.7     -4.9
           -3.829   15.810    1.359    3.233    0.449  -10.236   -4.627   -7.610   -3.012   -2.175
g_O            62       49       54       67       64       64       56       55       56       58
          ( 27.7) ( 21.9) ( 24.1) ( 29.9) ( 28.6) ( 28.6) ( 25.0) ( 24.6) ( 25.0) ( 25.9)
g_E          57.3     57.3     57.3     57.3     57.3     57.3     57.3     57.3     57.3     57.3
          ( 25.6) ( 25.6) ( 25.6) ( 25.6) ( 25.6) ( 25.6) ( 25.6) ( 25.6) ( 25.6) ( 25.6)
              9.9    -15.3     -6.3     21.1     14.2     14.2     -2.5     -4.4     -2.5      1.5
            4.403   -6.815   -2.826    9.397    6.359    6.359   -1.113   -1.978   -1.113    0.665
c_O            62       61       66       57       59       73       66       78       60       60
          ( 27.7) ( 27.2) ( 29.5) ( 25.4) ( 26.3) ( 32.6) ( 29.5) ( 34.8) ( 26.8) ( 26.8)
c_E          64.1     64.1     64.1     64.1     64.1     64.1     64.1     64.1     64.1     64.1
          ( 28.6) ( 28.6) ( 28.6) ( 28.6) ( 28.6) ( 28.6) ( 28.6) ( 28.6) ( 28.6) ( 28.6)
             -4.2     -6.1      3.8    -13.4     -9.8     18.9      3.8     30.5     -8.0     -8.0
           -1.870   -2.725    1.694   -5.998   -4.392    8.444    1.694   13.636   -3.566   -3.566

N             224      224      224      224      224      224      224      224      224      224
X2            0.9      6.5      0.3      3.4      1.3      5.8      1.1      5.0      2.0      1.1
Gvalue        0.9      6.1      0.3      3.4      1.3      6.1      1.2      4.9      2.0      1.0
Gv/N        0.421    2.745    0.129    1.503    0.570    2.721    0.515    2.202    0.877    0.464
Prob      0.81526  0.10457  0.96218  0.33838  0.73491  0.10712  0.76404  0.17683  0.57976  0.79154
          -------------------------------------------------------------------------------
               70       69       68       67       66       65       64       63       62       61
          -------------------------------------------------------------------------------
a_O            46       54       43       56       53       52       53       54       56       39
          ( 20.5) ( 24.1) ( 19.2) ( 25.0) ( 23.7) ( 23.2) ( 23.7) ( 24.1) ( 25.0) ( 17.4)
a_E          53.1     53.1     53.1     53.1     53.1     53.1     53.1     53.1     53.1     53.1
          ( 23.7) ( 23.7) ( 23.7) ( 23.7) ( 23.7) ( 23.7) ( 23.7) ( 23.7) ( 23.7) ( 23.7)
            -13.2      1.8    -18.2      5.9     -0.2     -2.2     -0.2      1.8      5.9    -24.1
           -5.903    0.801   -8.107    2.649   -0.098   -0.981   -0.098    0.801    2.649  -10.753
t_O            51       47       53       51       52       43       44       47       40       65
          ( 22.8) ( 21.0) ( 23.7) ( 22.8) ( 23.2) ( 19.2) ( 19.6) ( 21.0) ( 17.9) ( 29.0)
t_E          49.5     49.5     49.5     49.5     49.5     49.5     49.5     49.5     49.5     49.5
```

	(22.1)	(22.1)	(22.1)	(22.1)	(22.1)	(22.1)	(22.1)	(22.1)	(22.1)	(22.1)
	3.0	-4.9	7.2	3.0	5.1	-12.1	-10.4	-4.9	-17.0	35.4
	1.359	-2.175	3.233	1.359	2.288	-5.405	-4.627	-2.175	-7.610	15.810
g_O	59	43	65	49	51	67	66	54	62	59
	(26.3)	(19.2)	(29.0)	(21.9)	(22.8)	(29.9)	(29.5)	(24.1)	(27.7)	(26.3)
g_E	57.3	57.3	57.3	57.3	57.3	57.3	57.3	57.3	57.3	57.3
	(25.6)	(25.6)	(25.6)	(25.6)	(25.6)	(25.6)	(25.6)	(25.6)	(25.6)	(25.6)
	3.5	-24.6	16.5	-15.3	-11.8	21.1	18.8	-6.3	9.9	3.5
	1.577	-10.996	7.358	-6.815	-5.272	9.397	8.371	-2.826	4.403	1.577
c_O	68	80	63	68	68	62	61	69	66	61
	(30.4)	(35.7)	(28.1)	(30.4)	(30.4)	(27.7)	(27.2)	(30.8)	(29.5)	(27.2)
c_E	64.1	64.1	64.1	64.1	64.1	64.1	64.1	64.1	64.1	64.1
	(28.6)	(28.6)	(28.6)	(28.6)	(28.6)	(28.6)	(28.6)	(28.6)	(28.6)	(28.6)
	8.0	35.4	-2.2	8.0	8.0	-4.2	-6.1	10.1	3.8	-6.1
	3.558	15.794	-1.000	3.558	3.558	-1.870	-2.725	4.509	1.694	-2.725
N	224	224	224	224	224	224	224	224	224	224
X2	1.3	7.6	3.2	1.6	1.0	2.6	2.1	0.7	2.4	8.8
Gvalue	1.3	7.7	3.3	1.7	1.1	2.6	2.1	0.7	2.5	8.8
Gv/N	0.591	3.425	1.484	0.751	0.475	1.142	0.920	0.310	1.135	3.908
Prob	0.72350	0.05332	0.34436	0.64098	0.78573	0.46480	0.55973	0.87478	0.46757	0.03273

	60	59	58	57	56	55	54	53	52	51
a_O	48	49	50	44	49	55	40	59	58	60
	(21.4)	(21.9)	(22.3)	(19.6)	(21.9)	(24.6)	(17.9)	(26.3)	(25.9)	(26.8)
a_E	53.1	53.1	53.1	53.1	53.1	53.1	53.1	53.1	53.1	53.1
	(23.7)	(23.7)	(23.7)	(23.7)	(23.7)	(23.7)	(23.7)	(23.7)	(23.7)	(23.7)
	-9.7	-7.9	-6.0	-16.6	-7.9	3.8	-22.7	12.4	10.2	14.6
	-4.336	-3.524	-2.694	-7.393	-3.524	1.717	-10.124	5.540	4.561	6.535
t_O	55	55	60	52	60	42	56	54	47	45
	(24.6)	(24.6)	(26.8)	(23.2)	(26.8)	(18.8)	(25.0)	(24.1)	(21.0)	(20.1)
t_E	49.5	49.5	49.5	49.5	49.5	49.5	49.5	49.5	49.5	49.5
	(22.1)	(22.1)	(22.1)	(22.1)	(22.1)	(22.1)	(22.1)	(22.1)	(22.1)	(22.1)
	11.6	11.6	23.1	5.1	23.1	-13.8	13.8	9.4	-4.9	-8.6
	5.174	5.174	10.306	2.288	10.306	-6.161	6.169	4.195	-2.175	-3.829
g_O	68	54	51	62	69	48	70	52	57	62
	(30.4)	(24.1)	(22.8)	(27.7)	(30.8)	(21.4)	(31.2)	(23.2)	(25.4)	(27.7)
g_E	57.3	57.3	57.3	57.3	57.3	57.3	57.3	57.3	57.3	57.3
	(25.6)	(25.6)	(25.6)	(25.6)	(25.6)	(25.6)	(25.6)	(25.6)	(25.6)	(25.6)
	23.4	-6.3	-11.8	9.9	25.7	-16.9	28.1	-10.0	-0.5	9.9
	10.437	-2.826	-5.272	4.403	11.490	-7.560	12.556	-4.474	-0.232	4.403
c_O	53	66	63	66	46	79	58	59	62	57
	(23.7)	(29.5)	(28.1)	(29.5)	(20.5)	(35.3)	(25.9)	(26.3)	(27.7)	(25.4)
c_E	64.1	64.1	64.1	64.1	64.1	64.1	64.1	64.1	64.1	64.1
	(28.6)	(28.6)	(28.6)	(28.6)	(28.6)	(28.6)	(28.6)	(28.6)	(28.6)	(28.6)
	-20.2	3.8	-2.2	3.8	-30.6	32.9	-11.7	-9.8	-4.2	-13.4
	-9.020	1.694	-1.000	1.694	-13.647	14.709	-5.203	-4.392	-1.870	-5.998
N	224	224	224	224	224	224	224	224	224	224
X2	5.0	1.2	3.1	2.1	10.1	6.1	7.5	2.0	0.6	2.5
Gvalue	5.1	1.2	3.0	2.2	10.4	6.1	7.6	1.9	0.6	2.5
Gv/N	2.255	0.518	1.340	0.991	4.625	2.705	3.397	0.870	0.285	1.110
Prob	0.16808	0.76274	0.39144	0.52788	0.01574	0.10877	0.05479	0.58326	0.88764	0.47793

	50	49	48	47	46	45	44	43	42	41
a_O	59	39	33	45	59	47	49	56	42	54
	(26.3)	(17.4)	(14.7)	(20.1)	(26.3)	(21.0)	(21.9)	(25.0)	(18.8)	(24.1)
a_E	53.1	53.1	53.1	53.1	53.1	53.1	53.1	53.1	53.1	53.1
	(23.7)	(23.7)	(23.7)	(23.7)	(23.7)	(23.7)	(23.7)	(23.7)	(23.7)	(23.7)
	12.4	-24.1	-31.4	-14.9	12.4	-11.5	-7.9	5.9	-19.7	1.8
	5.540	-10.753	-14.021	-6.658	5.540	-5.129	-3.524	2.649	-8.801	0.801
t_O	53	58	46	44	49	61	50	50	50	59
	(23.7)	(25.9)	(20.5)	(19.6)	(21.9)	(27.2)	(22.3)	(22.3)	(22.3)	(26.3)
t_E	49.5	49.5	49.5	49.5	49.5	49.5	49.5	49.5	49.5	49.5
	(22.1)	(22.1)	(22.1)	(22.1)	(22.1)	(22.1)	(22.1)	(22.1)	(22.1)	(22.1)
	7.2	18.4	-6.7	-10.4	-1.0	25.5	1.0	1.0	1.0	20.7
	3.233	8.206	-3.012	-4.627	-0.444	11.378	0.449	0.449	0.449	9.249
g_O	52	58	69	69	54	55	70	49	66	54
	(23.2)	(25.9)	(30.8)	(30.8)	(24.1)	(24.6)	(31.2)	(21.9)	(29.5)	(24.1)
g_E	57.3	57.3	57.3	57.3	57.3	57.3	57.3	57.3	57.3	57.3
	(25.6)	(25.6)	(25.6)	(25.6)	(25.6)	(25.6)	(25.6)	(25.6)	(25.6)	(25.6)
	-10.0	1.5	25.7	25.7	-6.3	-4.4	28.1	-15.3	18.8	-6.3
	-4.474	0.665	11.490	11.490	-2.826	-1.978	12.556	-6.815	8.371	-2.826
c_O	60	69	76	66	62	61	55	69	66	57
	(26.8)	(30.8)	(33.9)	(29.5)	(27.7)	(27.2)	(24.6)	(30.8)	(29.5)	(25.4)
c_E	64.1	64.1	64.1	64.1	64.1	64.1	64.1	64.1	64.1	64.1
	(28.6)	(28.6)	(28.6)	(28.6)	(28.6)	(28.6)	(28.6)	(28.6)	(28.6)	(28.6)
	-8.0	10.1	25.8	3.8	-4.2	-6.1	-16.9	10.1	3.8	-13.4
	-3.566	4.509	11.524	1.694	-1.870	-2.725	-7.542	4.509	1.694	-5.998
N	224	224	224	224	224	224	224	224	224	224
X2	1.6	5.6	12.5	4.3	0.9	3.6	4.5	1.7	3.7	2.8
Gvalue	1.6	5.9	13.4	4.3	0.9	3.5	4.3	1.8	3.8	2.7
Gv/N	0.733	2.628	5.981	1.899	0.400	1.546	1.939	0.792	1.712	1.225
Prob	0.64974	0.11728	0.00385	0.23537	0.82635	0.32557	0.22673	0.62068	0.27978	0.43271

	40	39	38	37	36	35	34	33	32	31
a_O	54	43	50	53	44	53	49	45	59	56
	(24.1)	(19.2)	(22.3)	(23.7)	(19.6)	(23.7)	(21.9)	(20.1)	(26.3)	(25.0)
a_E	53.1	53.1	53.1	53.1	53.1	53.1	53.1	53.1	53.1	53.1
	(23.7)	(23.7)	(23.7)	(23.7)	(23.7)	(23.7)	(23.7)	(23.7)	(23.7)	(23.7)
	1.8	-18.2	-6.0	-0.2	-16.6	-0.2	-7.9	-14.9	12.4	5.9
	0.801	-8.107	-2.694	-0.098	-7.393	-0.098	-3.524	-6.658	5.540	2.649
t_O	55	51	43	48	52	38	52	56	49	47
	(24.6)	(22.8)	(19.2)	(21.4)	(23.2)	(17.0)	(23.2)	(25.0)	(21.9)	(21.0)
t_E	49.5	49.5	49.5	49.5	49.5	49.5	49.5	49.5	49.5	49.5
	(22.1)	(22.1)	(22.1)	(22.1)	(22.1)	(22.1)	(22.1)	(22.1)	(22.1)	(22.1)
	11.6	3.0	-12.1	-3.0	5.1	-20.1	5.1	13.8	-1.0	-4.9
	5.174	1.359	-5.405	-1.319	2.288	-8.970	2.288	6.169	-0.444	-2.175
g_O	43	68	57	58	67	67	61	55	61	54
	(19.2)	(30.4)	(25.4)	(25.9)	(29.9)	(29.9)	(27.2)	(24.6)	(27.2)	(24.1)
g_E	57.3	57.3	57.3	57.3	57.3	57.3	57.3	57.3	57.3	57.3
	(25.6)	(25.6)	(25.6)	(25.6)	(25.6)	(25.6)	(25.6)	(25.6)	(25.6)	(25.6)
	-24.6	23.4	-0.5	1.5	21.1	21.1	7.7	-4.4	7.7	-6.3
	-10.996	10.437	-0.232	0.665	9.397	9.397	3.446	-1.978	3.446	-2.826
c_O	72	62	74	65	61	66	62	68	55	67
	(32.1)	(27.7)	(33.0)	(29.0)	(27.2)	(29.5)	(27.7)	(30.4)	(24.6)	(29.9)
c_E	64.1	64.1	64.1	64.1	64.1	64.1	64.1	64.1	64.1	64.1
	(28.6)	(28.6)	(28.6)	(28.6)	(28.6)	(28.6)	(28.6)	(28.6)	(28.6)	(28.6)

	16.7	-4.2	21.2	1.8	-6.1	3.8	-4.2	8.0	-16.9	5.9
	7.441	-1.870	9.458	0.782	-2.725	1.694	-1.870	3.558	-7.542	2.619
N	224	224	224	224	224	224	224	224	224	224
X2	5.1	4.1	2.6	0.1	3.5	4.4	0.8	2.4	2.2	0.6
Gvalue	5.4	4.1	2.5	0.1	3.5	4.5	0.8	2.4	2.2	0.6
Gv/N	2.421	1.819	1.128	0.030	1.567	2.023	0.340	1.091	1.000	0.267
Prob	0.14335	0.25343	0.47041	0.99544	0.31945	0.20955	0.85866	0.48539	0.52395	0.89672

	30	29	28	27	26	25	24	23	22	21
a_O	57	56	63	45	59	56	48	53	55	60
	(25.4)	(25.0)	(28.1)	(20.1)	(26.3)	(25.0)	(21.4)	(23.7)	(24.6)	(26.8)
a_E	53.1	53.1	53.1	53.1	53.1	53.1	53.1	53.1	53.1	53.1
	(23.7)	(23.7)	(23.7)	(23.7)	(23.7)	(23.7)	(23.7)	(23.7)	(23.7)	(23.7)
	8.1	5.9	21.5	-14.9	12.4	5.9	-9.7	-0.2	3.8	14.6
	3.597	2.649	9.606	-6.658	5.540	2.649	-4.336	-0.098	1.717	6.535
t_O	48	54	51	38	51	43	46	50	41	54
	(21.4)	(24.1)	(22.8)	(17.0)	(22.8)	(19.2)	(20.5)	(22.3)	(18.3)	(24.1)
t_E	49.5	49.5	49.5	49.5	49.5	49.5	49.5	49.5	49.5	49.5
	(22.1)	(22.1)	(22.1)	(22.1)	(22.1)	(22.1)	(22.1)	(22.1)	(22.1)	(22.1)
	-3.0	9.4	3.0	-20.1	3.0	-12.1	-6.7	1.0	-15.4	9.4
	-1.319	4.195	1.359	-8.970	1.359	-5.405	-3.012	0.449	-6.897	4.195
g_O	55	60	48	72	58	57	59	59	56	57
	(24.6)	(26.8)	(21.4)	(32.1)	(25.9)	(25.4)	(26.3)	(26.3)	(25.0)	(25.4)
g_E	57.3	57.3	57.3	57.3	57.3	57.3	57.3	57.3	57.3	57.3
	(25.6)	(25.6)	(25.6)	(25.6)	(25.6)	(25.6)	(25.6)	(25.6)	(25.6)	(25.6)
	-4.4	5.6	-16.9	33.0	1.5	-0.5	3.5	3.5	-2.5	-0.5
	-1.978	2.504	-7.560	14.726	0.665	-0.232	1.577	1.577	-1.113	-0.232
c_O	64	54	62	69	56	68	71	62	72	53
	(28.6)	(24.1)	(27.7)	(30.8)	(25.0)	(30.4)	(31.7)	(27.7)	(32.1)	(23.7)
c_E	64.1	64.1	64.1	64.1	64.1	64.1	64.1	64.1	64.1	64.1
	(28.6)	(28.6)	(28.6)	(28.6)	(28.6)	(28.6)	(28.6)	(28.6)	(28.6)	(28.6)
	-0.3	-18.6	-4.2	10.1	-15.2	8.0	14.5	-4.2	16.7	-20.2
	-0.116	-8.289	-1.870	4.509	-6.778	3.558	6.451	-1.870	7.441	-9.020
N	224	224	224	224	224	224	224	224	224	224
X2	0.4	2.3	3.5	8.1	1.7	1.2	1.5	0.1	2.5	3.2
Gvalue	0.4	2.4	3.4	8.1	1.8	1.3	1.5	0.1	2.6	3.3
Gv/N	0.185	1.059	1.535	3.607	0.787	0.571	0.681	0.058	1.149	1.478
Prob	0.93717	0.49877	0.32876	0.04440	0.62323	0.73432	0.67649	0.98812	0.46205	0.34623

	20	19	18	17	16	15	14	13	12	11
a_O	55	65	59	55	56	58	58	62	74	89
	(24.6)	(29.0)	(26.3)	(24.6)	(25.0)	(25.9)	(25.9)	(27.7)	(33.0)	(39.7)
a_E	53.1	53.1	53.1	53.1	53.1	53.1	53.1	53.1	53.1	53.1
	(23.7)	(23.7)	(23.7)	(23.7)	(23.7)	(23.7)	(23.7)	(23.7)	(23.7)	(23.7)
	3.8	26.3	12.4	3.8	5.9	10.2	10.2	19.2	49.1	91.9
	1.717	11.724	5.540	1.717	2.649	4.561	4.561	8.568	21.916	41.025
t_O	58	38	42	44	46	54	57	56	48	43
	(25.9)	(17.0)	(18.8)	(19.6)	(20.5)	(24.1)	(25.4)	(25.0)	(21.4)	(19.2)
t_E	49.5	49.5	49.5	49.5	49.5	49.5	49.5	49.5	49.5	49.5
	(22.1)	(22.1)	(22.1)	(22.1)	(22.1)	(22.1)	(22.1)	(22.1)	(22.1)	(22.1)
	18.4	-20.1	-13.8	-10.4	-6.7	9.4	16.1	13.8	-3.0	-12.1
	8.206	-8.970	-6.161	-4.627	-3.012	4.195	7.180	6.169	-1.319	-5.405
g_O	51	54	40	58	60	34	50	34	42	39
	(22.8)	(24.1)	(17.9)	(25.9)	(26.8)	(15.2)	(22.3)	(15.2)	(18.8)	(17.4)
g_E	57.3	57.3	57.3	57.3	57.3	57.3	57.3	57.3	57.3	57.3
	(25.6)	(25.6)	(25.6)	(25.6)	(25.6)	(25.6)	(25.6)	(25.6)	(25.6)	(25.6)
	-11.8	-6.3	-28.7	1.5	5.6	-35.4	-13.6	-35.4	-26.0	-30.0
	-5.272	-2.826	-12.812	0.665	2.504	-15.823	-6.053	-15.823	-11.622	-13.373
c_O	60	67	83	67	62	78	59	72	60	53
	(26.8)	(29.9)	(37.1)	(29.9)	(27.7)	(34.8)	(26.3)	(32.1)	(26.8)	(23.7)
c_E	64.1	64.1	64.1	64.1	64.1	64.1	64.1	64.1	64.1	64.1
	(28.6)	(28.6)	(28.6)	(28.6)	(28.6)	(28.6)	(28.6)	(28.6)	(28.6)	(28.6)
	-8.0	5.9	42.8	5.9	-4.2	30.5	-9.8	16.7	-8.0	-20.2
	-3.566	2.619	19.114	2.619	-1.870	13.636	-4.392	7.441	-3.566	-9.020
N	224	224	224	224	224	224	224	224	224	224
X2	2.5	5.6	12.5	0.8	0.6	13.3	2.9	12.8	12.6	32.9
Gvalue	2.4	5.7	12.7	0.8	0.6	14.7	2.9	14.2	12.1	29.6
Gv/N	1.086	2.547	5.682	0.374	0.272	6.569	1.296	6.354	5.408	13.227
Prob	0.48776	0.12686	0.00527	0.84044	0.89446	0.00208	0.40669	0.00260	0.00700	0.00000

	10	9	8	7	6	5	4	3	2	1
a_O	72	73	64	64	69	55	69	60	37	50
	(32.1)	(32.6)	(28.6)	(28.6)	(30.8)	(24.6)	(30.8)	(26.8)	(16.5)	(22.3)
a_E	53.1	53.1	53.1	53.1	53.1	53.1	53.1	53.1	53.1	53.1
	(23.7)	(23.7)	(23.7)	(23.7)	(23.7)	(23.7)	(23.7)	(23.7)	(23.7)	(23.7)
	43.8	46.4	23.9	23.9	36.1	3.8	36.1	14.6	-26.7	-6.0
	19.562	20.733	10.658	10.658	16.125	1.717	16.125	6.535	-11.941	-2.694
t_O	35	54	48	51	50	53	37	48	45	64
	(15.6)	(24.1)	(21.4)	(22.8)	(22.3)	(23.7)	(16.5)	(21.4)	(20.1)	(28.6)
t_E	49.5	49.5	49.5	49.5	49.5	49.5	49.5	49.5	49.5	49.5
	(22.1)	(22.1)	(22.1)	(22.1)	(22.1)	(22.1)	(22.1)	(22.1)	(22.1)	(22.1)
	-24.3	9.4	-3.0	3.0	1.0	7.2	-21.5	-3.0	-8.6	32.9
	-10.832	4.195	-1.319	1.359	0.449	3.233	-9.615	-1.319	-3.829	14.681
g_O	45	52	49	39	53	61	55	72	73	37
	(20.1)	(23.2)	(21.9)	(17.4)	(23.7)	(27.2)	(24.6)	(32.1)	(32.6)	(16.5)
g_E	57.3	57.3	57.3	57.3	57.3	57.3	57.3	57.3	57.3	57.3
	(25.6)	(25.6)	(25.6)	(25.6)	(25.6)	(25.6)	(25.6)	(25.6)	(25.6)	(25.6)
	-21.7	-10.0	-15.3	-30.0	-8.2	7.7	-4.4	33.0	35.5	-32.3
	-9.681	-4.474	-6.815	-13.373	-3.658	3.446	-1.978	14.726	15.829	-14.426
c_O	72	45	63	70	52	55	63	44	69	73
	(32.1)	(20.1)	(28.1)	(31.2)	(23.2)	(24.6)	(28.1)	(19.6)	(30.8)	(32.6)
c_E	64.1	64.1	64.1	64.1	64.1	64.1	64.1	64.1	64.1	64.1
	(28.6)	(28.6)	(28.6)	(28.6)	(28.6)	(28.6)	(28.6)	(28.6)	(28.6)	(28.6)
	16.7	-31.9	-2.2	12.3	-21.8	-16.9	-2.2	-33.2	10.1	18.9
	7.441	-14.233	-1.000	5.474	-9.735	-7.542	-1.000	-14.800	4.509	8.444
N	224	224	224	224	224	224	224	224	224	224
X2	14.6	14.0	3.5	8.6	7.4	1.9	8.0	11.1	10.0	12.8
Gvalue	14.5	13.9	3.4	9.2	7.1	1.9	7.9	11.5	10.2	13.4
Gv/N	6.491	6.221	1.524	4.119	3.181	0.854	3.532	5.142	4.568	6.004
Prob	0.00226	0.00299	0.33214	0.02644	0.06802	0.59050	0.04785	0.00923	0.01669	0.00376

CDS_INITIAL:

	1	2	3	4	5	6	7	8	9	10

a_O	122	0	0	69	44	54	57	70	44	64
	(54.5)	(0.0)	(0.0)	(30.8)	(19.6)	(24.1)	(25.4)	(31.2)	(19.6)	(28.6)
a_E	51.3	61.3	24.8	51.3	61.3	24.8	51.3	61.3	24.8	51.3
	(22.9)	(27.4)	(11.1)	(22.9)	(27.4)	(11.1)	(22.9)	(27.4)	(11.1)	(22.9)
	211.5	0.0	0.0	41.0	-29.2	84.1	12.1	18.5	50.5	28.4
	94.425	0.000	0.000	18.294	-13.049	37.541	5.389	8.259	22.544	12.670
t_O	35	224	0	28	44	65	44	60	52	40
	(15.6)	(100.0)	(0.0)	(12.5)	(19.6)	(29.0)	(19.6)	(26.8)	(23.2)	(17.9)
t_E	33.4	60.9	35.2	33.4	60.9	35.2	33.4	60.9	35.2	33.4
	(14.9)	(27.2)	(15.7)	(14.9)	(27.2)	(15.7)	(14.9)	(27.2)	(15.7)	(14.9)
	3.3	583.3	0.0	-9.9	-28.6	79.7	24.3	-1.8	40.5	14.4
	1.461	260.385	0.000	-4.410	-12.789	35.558	10.826	-0.824	18.086	6.438
g_O	67	0	224	70	55	58	76	32	66	57
	(29.9)	(0.0)	(100.0)	(31.2)	(24.6)	(25.9)	(33.9)	(14.3)	(29.5)	(25.4)
g_E	84.8	42.1	70.2	84.8	42.1	70.2	84.8	42.1	70.2	84.8
	(37.8)	(18.8)	(31.3)	(37.8)	(18.8)	(31.3)	(37.8)	(18.8)	(31.3)	(37.8)
	-31.5	0.0	520.1	-26.8	29.4	-22.1	-16.6	-17.6	-8.1	-45.3
	-14.080	0.000	232.181	-11.973	13.104	-9.855	-7.419	-7.850	-3.600	-20.205
c_O	0	0	0	57	81	47	47	62	62	63
	(0.0)	(0.0)	(0.0)	(25.4)	(36.2)	(21.0)	(21.0)	(27.7)	(27.7)	(28.1)
c_E	54.5	59.6	93.8	54.5	59.6	93.8	54.5	59.6	93.8	54.5
	(24.4)	(26.6)	(41.9)	(24.4)	(26.6)	(41.9)	(24.4)	(26.6)	(41.9)	(24.4)
	0.0	0.0	0.0	5.0	49.7	-65.0	-14.0	4.9	-51.4	18.2
	0.000	0.000	0.000	2.241	22.167	-29.012	-6.247	2.170	-22.938	8.106
N	224	224	224	224	224	224	224	224	224	224
X2	155.9	599.5	491.2	9.7	21.2	85.1	6.0	3.8	33.9	14.9
Gvalue	183.2	583.3	520.1	9.3	21.1	76.7	5.7	3.9	31.6	15.7
Gv/N	81.806	260.385	232.181	4.151	9.433	34.232	2.549	1.755	14.092	7.009
Prob	0.00000	0.00000	0.00000	0.02557	0.00010	0.00000	0.12662	0.26896	0.00000	0.00131

	11	12	13	14	15	16	17	18	19	20
a_O	50	33	64	62	52	61	57	45	51	57
	(22.3)	(14.7)	(28.6)	(27.7)	(23.2)	(27.2)	(25.4)	(20.1)	(22.8)	(25.4)
a_E	61.3	24.8	51.3	61.3	24.8	51.3	61.3	24.8	51.3	61.3
	(27.4)	(11.1)	(22.9)	(27.4)	(11.1)	(22.9)	(27.4)	(11.1)	(22.9)	(27.4)
	-20.4	18.9	28.4	1.3	77.1	21.2	-8.4	53.7	-0.5	-8.4
	-9.122	8.431	12.670	0.597	34.399	9.461	-3.730	23.959	-0.243	-3.730
t_O	62	45	33	56	46	34	56	56	40	61
	(27.7)	(20.1)	(14.7)	(25.0)	(20.5)	(15.2)	(25.0)	(25.0)	(17.9)	(27.2)
t_E	60.9	35.2	33.4	60.9	35.2	33.4	60.9	35.2	33.4	60.9
	(27.2)	(15.7)	(14.9)	(27.2)	(15.7)	(14.9)	(27.2)	(15.7)	(14.9)	(27.2)
	2.2	22.0	-0.8	-9.4	24.6	1.2	-9.4	51.9	14.4	0.1
	0.964	9.843	-0.357	-4.218	10.964	0.539	-4.218	23.183	6.438	0.063
g_O	41	73	65	45	50	73	46	55	69	44
	(18.3)	(32.6)	(29.0)	(20.1)	(22.3)	(32.6)	(20.5)	(24.6)	(30.8)	(19.6)
g_E	42.1	70.2	84.8	42.1	70.2	84.8	42.1	70.2	84.8	42.1
	(18.8)	(31.3)	(37.8)	(18.8)	(31.3)	(37.8)	(18.8)	(31.3)	(37.8)	(18.8)
	-2.2	5.8	-34.5	6.0	-33.9	-21.8	8.1	-26.8	-28.4	3.8
	-0.985	2.589	-15.419	2.659	-15.121	-9.751	3.621	-11.953	-12.689	1.717
c_O	71	73	62	61	76	56	65	68	64	62
	(31.7)	(32.6)	(27.7)	(27.2)	(33.9)	(25.0)	(29.0)	(30.4)	(28.6)	(27.7)
c_E	59.6	93.8	54.5	59.6	93.8	54.5	59.6	93.8	54.5	59.6
	(26.6)	(41.9)	(24.4)	(26.6)	(41.9)	(24.4)	(26.6)	(41.9)	(24.4)	(26.6)
	24.8	-36.7	15.9	2.8	-32.0	2.9	11.2	-43.8	20.5	4.9
	11.077	-16.363	7.092	1.249	-14.303	1.317	5.017	-19.550	9.135	2.170
N	224	224	224	224	224	224	224	224	224	224
X2	4.3	10.2	8.8	0.6	42.4	3.5	1.5	39.1	5.9	0.5
Gvalue	4.3	10.1	8.9	0.6	35.7	3.5	1.5	35.0	5.9	0.5
Gv/N	1.934	4.500	3.986	0.287	15.939	1.565	0.689	15.639	2.642	0.219
Prob	0.22769	0.01790	0.03025	0.88678	0.00000	0.31996	0.67234	0.00000	0.11571	0.92091

	21	22	23	24	25	26	27	28	29	30
a_O	57	58	66	35	68	59	41	49	66	41
	(25.4)	(25.9)	(29.5)	(15.6)	(30.4)	(26.3)	(18.3)	(21.9)	(29.5)	(18.3)
a_E	24.8	51.3	61.3	24.8	51.3	61.3	24.8	51.3	61.3	24.8
	(11.1)	(22.9)	(27.4)	(11.1)	(22.9)	(27.4)	(11.1)	(22.9)	(27.4)	(11.1)
	94.9	14.3	9.7	24.1	38.4	-4.6	41.3	-4.4	9.7	41.3
	42.379	6.384	4.320	10.781	17.142	-2.045	18.422	-1.984	4.320	18.422
t_O	43	49	61	37	30	53	49	39	45	53
	(19.2)	(21.9)	(27.2)	(16.5)	(13.4)	(23.7)	(21.9)	(17.4)	(20.1)	(23.7)
t_E	35.2	33.4	60.9	35.2	33.4	60.9	35.2	33.4	60.9	35.2
	(15.7)	(14.9)	(27.2)	(15.7)	(14.9)	(27.2)	(15.7)	(14.9)	(27.2)	(15.7)
	17.2	37.6	0.1	3.6	-6.4	-14.8	32.4	12.1	-27.3	43.3
	7.660	16.766	0.063	1.626	-2.877	-6.598	14.443	5.396	-12.176	19.336
g_O	55	59	47	67	70	41	71	72	55	47
	(24.6)	(26.3)	(21.0)	(29.9)	(31.2)	(18.3)	(31.7)	(32.1)	(24.6)	(21.0)
g_E	70.2	84.8	42.1	70.2	84.8	42.1	70.2	84.8	42.1	70.2
	(31.3)	(37.8)	(18.8)	(31.3)	(37.8)	(18.8)	(31.3)	(37.8)	(18.8)	(31.3)
	-26.8	-42.8	10.3	-6.2	-26.8	-2.2	1.7	-23.5	29.4	-37.7
	-11.953	-19.097	4.602	-2.755	-11.973	-0.985	0.757	-10.504	13.104	-16.811
c_O	69	58	50	85	56	71	63	64	58	83
	(30.8)	(25.9)	(22.3)	(37.9)	(25.0)	(31.7)	(28.1)	(28.6)	(25.9)	(37.1)
c_E	93.8	54.5	59.6	93.8	54.5	59.6	93.8	54.5	59.6	93.8
	(41.9)	(24.4)	(26.6)	(41.9)	(24.4)	(26.6)	(41.9)	(24.4)	(26.6)	(41.9)
	-42.4	7.1	-17.6	-16.8	2.9	24.8	-50.2	20.5	-3.2	-20.4
	-18.938	3.181	-7.853	-7.502	1.317	11.077	-22.408	9.135	-1.424	-9.090
N	224	224	224	224	224	224	224	224	224	224
X2	53.4	16.2	2.5	5.3	8.4	3.3	26.1	4.6	8.5	28.5
Gvalue	42.9	16.2	2.5	4.8	8.1	3.2	25.1	4.6	8.6	26.6
Gv/N	19.147	7.233	1.131	2.150	3.609	1.450	11.213	2.043	3.823	11.856
Prob	0.00000	0.00103	0.46927	0.18573	0.04432	0.35509	0.00001	0.20566	0.03568	0.00001

	31	32	33	34	35	36	37	38	39	40
a_O	62	51	37	59	47	26	48	61	44	54
	(27.7)	(22.8)	(16.5)	(26.3)	(21.0)	(11.6)	(21.4)	(27.2)	(19.6)	(24.1)
a_E	51.3	61.3	24.8	51.3	61.3	24.8	51.3	61.3	24.8	51.3
	(22.9)	(27.4)	(11.1)	(22.9)	(27.4)	(11.1)	(22.9)	(27.4)	(11.1)	(22.9)
	23.6	-18.8	29.6	16.6	-25.0	2.5	-6.3	-0.7	50.5	5.6
	10.516	-8.402	13.233	7.395	-11.171	1.108	-2.827	-0.298	22.544	2.498
t_O	38	74	54	35	54	55	31	50	36	38
	(17.0)	(33.0)	(24.1)	(15.6)	(24.1)	(24.6)	(13.8)	(22.3)	(16.1)	(17.0)
t_E	33.4	60.9	35.2	33.4	60.9	35.2	33.4	60.9	35.2	33.4
	(14.9)	(27.2)	(15.7)	(14.9)	(27.2)	(15.7)	(14.9)	(27.2)	(15.7)	(14.9)
	9.8	28.8	46.1	3.3	-13.0	49.0	-4.6	-19.8	1.6	9.8
	4.376	12.841	20.602	1.461	-5.821	21.884	-2.065	-8.826	0.702	4.376

g_O	64	45	71	72	50	70	79	46	73	79
	(28.6)	(20.1)	(31.7)	(32.1)	(22.3)	(31.2)	(35.3)	(20.5)	(32.6)	(35.3)
g_E	84.8	42.1	70.2	84.8	42.1	70.2	84.8	42.1	70.2	84.8
	(37.8)	(18.8)	(31.3)	(37.8)	(18.8)	(31.3)	(37.8)	(18.8)	(31.3)	(37.8)
	-36.0	6.0	1.7	-23.5	17.2	-0.3	-11.2	8.1	5.8	-11.2
	-16.068	2.659	0.757	-10.504	7.658	-0.140	-4.981	3.621	2.589	-4.981
c_O	60	54	62	58	73	73	66	67	71	53
	(26.8)	(24.1)	(27.7)	(25.9)	(32.6)	(32.6)	(29.5)	(29.9)	(31.7)	(23.7)
c_E	54.5	59.6	93.8	54.5	59.6	93.8	54.5	59.6	93.8	54.5
	(24.4)	(26.6)	(41.9)	(24.4)	(26.6)	(41.9)	(24.4)	(26.6)	(41.9)	(24.4)
	11.4	-10.7	-51.4	7.1	29.6	-36.7	25.2	15.6	-39.6	-3.0
	5.107	-4.771	-22.938	3.181	13.200	-16.363	11.234	6.984	-17.676	-1.360
N	224	224	224	224	224	224	224	224	224	224
X2	8.5	5.3	26.8	3.4	8.6	15.8	3.2	3.2	20.6	1.2
Gvalue	8.8	5.2	26.1	3.4	8.7	14.5	3.0	3.3	18.3	1.2
Gv/N	3.931	2.326	11.653	1.532	3.866	6.489	1.360	1.481	8.158	0.534
Prob	0.03198	0.15703	0.00001	0.32980	0.03418	0.00226	0.38443	0.34523	0.00039	0.75398

	41	42	43	44	45	46	47	48	49	50
a_O	58	40	45	57	31	51	62	36	66	56
	(25.9)	(17.9)	(20.1)	(25.4)	(13.8)	(22.8)	(27.7)	(16.1)	(29.5)	(25.0)
a_E	61.3	24.8	51.3	61.3	24.8	51.3	61.3	24.8	51.3	61.3
	(27.4)	(11.1)	(22.9)	(27.4)	(11.1)	(22.9)	(27.4)	(11.1)	(22.9)	(27.4)
	-6.5	38.3	-11.7	-8.4	13.9	-0.5	1.3	26.9	33.3	-10.2
	-2.895	17.090	-5.243	-3.730	6.190	-0.243	0.597	11.995	14.879	-4.550
t_O	65	49	40	62	37	32	57	52	25	54
	(29.0)	(21.9)	(17.9)	(27.7)	(16.5)	(14.3)	(25.4)	(23.2)	(11.2)	(24.1)
t_E	60.9	35.2	33.4	60.9	35.2	33.4	60.9	35.2	33.4	60.9
	(27.2)	(15.7)	(14.9)	(27.2)	(15.7)	(14.9)	(27.2)	(15.7)	(14.9)	(27.2)
	8.4	32.4	14.4	2.2	3.6	-2.7	-7.6	40.5	-14.5	-13.0
	3.753	14.443	6.438	0.964	1.626	-1.225	-3.393	18.086	-6.467	-5.821
g_O	46	67	68	43	73	80	46	42	76	52
	(20.5)	(29.9)	(30.4)	(19.2)	(32.6)	(35.7)	(20.5)	(18.8)	(33.9)	(23.2)
g_E	42.1	70.2	84.8	42.1	70.2	84.8	42.1	70.2	84.8	42.1
	(18.8)	(31.3)	(37.8)	(18.8)	(31.3)	(37.8)	(18.8)	(31.3)	(37.8)	(18.8)
	8.1	-6.2	-30.0	1.8	5.8	-9.3	8.1	-43.1	-16.6	21.9
	3.621	-2.755	-13.391	0.795	2.589	-4.146	3.621	-19.240	-7.419	9.785
c_O	55	68	71	62	83	61	59	94	57	62
	(24.6)	(30.4)	(31.7)	(27.7)	(37.1)	(27.2)	(26.3)	(42.0)	(25.4)	(27.7)
c_E	59.6	93.8	54.5	59.6	93.8	54.5	59.6	93.8	54.5	59.6
	(26.6)	(41.9)	(24.4)	(26.6)	(41.9)	(24.4)	(26.6)	(41.9)	(24.4)	(26.6)
	-8.9	-43.8	37.4	4.9	-20.4	13.6	-1.2	0.3	5.0	4.9
	-3.958	-19.550	16.714	2.170	-9.090	6.092	-0.548	0.150	2.241	2.170
N	224	224	224	224	224	224	224	224	224	224
X2	1.2	22.0	10.4	0.4	3.0	1.1	0.6	24.4	7.4	3.7
Gvalue	1.2	20.7	10.1	0.4	2.9	1.1	0.6	24.6	7.2	3.5
Gv/N	0.520	9.229	4.518	0.199	1.315	0.478	0.277	10.991	3.233	1.584
Prob	0.76139	0.00012	0.01757	0.93082	0.40026	0.78399	0.89187	0.00002	0.06454	0.31464

	51	52	53	54	55	56	57	58	59	60
a_O	32	50	60	37	55	58	39	60	62	37
	(14.3)	(22.3)	(26.8)	(16.5)	(24.6)	(25.9)	(17.4)	(26.8)	(27.7)	(16.5)
a_E	24.8	51.3	61.3	24.8	51.3	61.3	24.8	51.3	61.3	24.8
	(11.1)	(22.9)	(27.4)	(11.1)	(22.9)	(27.4)	(11.1)	(22.9)	(27.4)	(11.1)
	16.3	-2.5	-2.6	29.6	7.7	-6.5	35.4	18.9	1.3	29.6
	7.297	-1.122	-1.179	13.233	3.446	-2.895	15.782	8.420	0.597	13.233
t_O	43	39	63	44	30	60	45	44	60	43
	(19.2)	(17.4)	(28.1)	(19.6)	(13.4)	(26.8)	(20.1)	(19.6)	(26.8)	(19.2)
t_E	35.2	33.4	60.9	35.2	33.4	60.9	35.2	33.4	60.9	35.2
	(15.7)	(14.9)	(27.2)	(15.7)	(14.9)	(27.2)	(15.7)	(14.9)	(27.2)	(15.7)
	17.2	12.1	4.2	19.6	-6.4	-1.8	22.0	24.3	-1.8	17.2
	7.660	5.396	1.880	8.741	-2.877	-0.824	9.843	10.826	-0.824	7.660
g_O	65	77	37	70	81	50	52	63	45	68
	(29.0)	(34.4)	(16.5)	(31.2)	(36.2)	(22.3)	(23.2)	(28.1)	(20.1)	(30.4)
g_E	70.2	84.8	42.1	70.2	84.8	42.1	70.2	84.8	42.1	70.2
	(31.3)	(37.8)	(18.8)	(31.3)	(37.8)	(18.8)	(31.3)	(37.8)	(18.8)	(31.3)
	-9.9	-14.8	-9.6	-0.3	-7.4	17.2	-31.1	-37.4	6.0	-4.2
	-4.431	-6.618	-4.280	-0.140	-3.299	7.658	-13.905	-16.702	2.659	-1.896
c_O	84	58	64	73	58	56	88	57	57	76
	(37.5)	(25.9)	(28.6)	(32.6)	(25.9)	(25.0)	(39.3)	(25.4)	(25.4)	(33.9)
c_E	93.8	54.5	59.6	93.8	54.5	59.6	93.8	54.5	59.6	93.8
	(41.9)	(24.4)	(26.6)	(41.9)	(24.4)	(26.6)	(41.9)	(24.4)	(26.6)	(41.9)
	-18.6	7.1	9.1	-36.7	7.1	-7.0	-11.3	5.0	-5.1	-32.0
	-8.302	3.181	4.054	-16.363	3.181	-3.129	-5.042	2.241	-2.284	-14.303
N	224	224	224	224	224	224	224	224	224	224
X2	5.2	1.9	1.0	12.8	1.0	1.9	15.9	10.6	0.3	11.2
Gvalue	5.0	1.9	1.1	12.3	1.0	1.8	15.0	10.7	0.3	10.5
Gv/N	2.223	0.836	0.474	5.471	0.450	0.810	6.677	4.785	0.148	4.694
Prob	0.17323	0.59917	0.78616	0.00656	0.79908	0.61199	0.00185	0.01335	0.95414	0.01466

	61	62	63	64	65	66	67	68	69	70
a_O	49	60	30	48	57	25	59	62	42	58
	(21.9)	(26.8)	(13.4)	(21.4)	(25.4)	(11.2)	(26.3)	(27.7)	(18.8)	(25.9)
a_E	51.3	61.3	24.8	51.3	61.3	24.8	51.3	61.3	24.8	51.3
	(22.9)	(27.4)	(11.1)	(22.9)	(27.4)	(11.1)	(22.9)	(27.4)	(11.1)	(22.9)
	-4.4	-2.6	11.5	-6.3	-8.4	0.4	16.6	1.3	44.3	14.3
	-1.984	-1.179	5.112	-2.827	-3.730	0.190	7.395	0.597	19.775	6.384
t_O	36	52	37	35	62	53	32	56	38	31
	(16.1)	(23.2)	(16.5)	(15.6)	(27.7)	(23.7)	(14.3)	(25.0)	(17.0)	(13.8)
t_E	33.4	60.9	35.2	33.4	60.9	35.2	33.4	60.9	35.2	33.4
	(14.9)	(27.2)	(15.7)	(14.9)	(27.2)	(15.7)	(14.9)	(27.2)	(15.7)	(14.9)
	5.4	-16.5	3.6	3.3	2.2	43.3	-2.7	-9.4	5.8	-4.6
	2.408	-7.358	1.626	1.461	0.964	19.336	-1.225	-4.218	2.575	-2.065
g_O	74	56	69	80	54	74	77	47	59	84
	(33.0)	(25.0)	(30.8)	(35.7)	(24.1)	(33.0)	(34.4)	(21.0)	(26.3)	(37.5)
g_E	84.8	42.1	70.2	84.8	42.1	70.2	84.8	42.1	70.2	84.8
	(37.8)	(18.8)	(31.3)	(37.8)	(18.8)	(31.3)	(37.8)	(18.8)	(31.3)	(37.8)
	-20.1	31.9	-2.3	-9.3	26.8	7.9	-14.8	10.3	-20.4	-1.6
	-8.986	14.243	-1.025	-4.146	11.981	3.523	-6.618	4.602	-9.124	-0.694
c_O	65	56	88	61	51	72	56	59	85	51
	(29.0)	(25.0)	(39.3)	(27.2)	(22.8)	(32.1)	(25.0)	(26.3)	(37.9)	(22.8)
c_E	54.5	59.6	93.8	54.5	59.6	93.8	54.5	59.6	93.8	54.5
	(24.4)	(26.6)	(41.9)	(24.4)	(26.6)	(41.9)	(24.4)	(26.6)	(41.9)	(24.4)
	22.8	-7.0	-11.3	13.6	-15.9	-38.1	2.9	-1.2	-16.8	-6.9
	10.177	-3.129	-5.042	6.092	-7.109	-17.026	1.317	-0.548	-7.502	-3.060

N	224	224	224	224	224	224	224	224	224	224
X2	3.7	6.1	1.6	1.3	4.9	14.3	2.0	1.0	14.8	1.3
Gvalue	3.6	5.8	1.5	1.3	4.7	13.5	1.9	1.0	12.8	1.3
Gv/N	1.616	2.577	0.671	0.580	2.106	6.024	0.868	0.432	5.723	0.565
Prob	0.30561	0.12320	0.68134	0.72935	0.19370	0.00368	0.58388	0.80882	0.00504	0.73721

	71	72	73	74	75	76	77	78	79	80
a_O	75	31	60	65	37	48	59	33	62	68
	(33.5)	(13.8)	(26.8)	(29.0)	(16.5)	(21.4)	(26.3)	(14.7)	(27.7)	(30.4)
a_E	61.3	24.8	51.3	61.3	24.8	51.3	61.3	24.8	51.3	61.3
	(27.4)	(11.1)	(22.9)	(27.4)	(11.1)	(22.9)	(27.4)	(11.1)	(22.9)	(27.4)
	30.2	13.9	18.9	7.5	29.6	-6.3	-4.6	18.9	23.6	14.0
	13.469	6.190	8.420	3.368	13.233	-2.827	-2.045	8.431	10.516	6.263
t_O	53	39	23	65	30	27	45	38	32	50
	(23.7)	(17.4)	(10.3)	(29.0)	(13.4)	(12.1)	(20.1)	(17.0)	(14.3)	(22.3)
t_E	60.9	35.2	33.4	60.9	35.2	33.4	60.9	35.2	33.4	60.9
	(27.2)	(15.7)	(14.9)	(27.2)	(15.7)	(14.9)	(27.2)	(15.7)	(14.9)	(27.2)
	-14.8	7.9	-17.2	8.4	-9.6	-11.5	-27.3	5.8	-2.7	-19.8
	-6.598	3.547	-7.662	3.753	-4.299	-5.129	-12.176	2.575	-1.225	-8.826
g_O	49	65	78	38	72	86	45	64	72	42
	(21.9)	(29.0)	(34.8)	(17.0)	(32.1)	(38.4)	(20.1)	(28.6)	(32.1)	(18.8)
g_E	42.1	70.2	84.8	42.1	70.2	84.8	42.1	70.2	84.8	42.1
	(18.8)	(31.3)	(37.8)	(18.8)	(31.3)	(37.8)	(18.8)	(31.3)	(37.8)	(18.8)
	14.8	-9.9	-13.0	-7.8	3.7	2.5	6.0	-11.8	-23.5	-0.2
	6.621	-4.431	-5.805	-3.491	1.667	1.097	2.659	-5.249	-10.504	-0.106
c_O	47	89	63	56	85	63	75	89	58	64
	(21.0)	(39.7)	(28.1)	(25.0)	(37.9)	(28.1)	(33.5)	(39.7)	(25.9)	(28.6)
c_E	59.6	93.8	54.5	59.6	93.8	54.5	59.6	93.8	54.5	59.6
	(26.6)	(41.9)	(24.4)	(26.6)	(41.9)	(24.4)	(26.6)	(41.9)	(24.4)	(26.6)
	-22.4	-9.4	18.2	-7.0	-16.8	18.2	34.4	-9.4	7.1	9.1
	-9.979	-4.201	8.106	-3.129	-7.502	8.106	15.372	-4.201	3.181	4.054
N	224	224	224	224	224	224	224	224	224	224
X2	7.9	2.6	6.6	1.1	7.7	2.8	8.4	3.7	4.4	3.0
Gvalue	7.9	2.5	6.9	1.1	6.9	2.8	8.5	3.5	4.4	3.1
Gv/N	3.513	1.105	3.059	0.501	3.098	1.247	3.809	1.556	1.968	1.386
Prob	0.04879	0.47994	0.07673	0.77192	0.07383	0.42472	0.03619	0.32264	0.22066	0.37587

	81	82	83	84	85	86	87	88	89	90
a_O	29	51	53	35	51	57	33	56	58	39
	(12.9)	(22.8)	(23.7)	(15.6)	(22.8)	(25.4)	(14.7)	(25.0)	(25.9)	(17.4)
a_E	24.8	51.3	61.3	24.8	51.3	61.3	24.8	51.3	61.3	24.8
	(11.1)	(22.9)	(27.4)	(11.1)	(22.9)	(27.4)	(11.1)	(22.9)	(27.4)	(11.1)
	9.1	-0.5	-15.5	24.1	-0.5	-8.4	18.9	9.9	-6.5	35.4
	4.064	-0.243	-6.912	10.781	-0.243	-3.730	8.431	4.409	-2.895	15.782
t_O	43	48	53	45	39	51	40	33	60	37
	(19.2)	(21.4)	(23.7)	(20.1)	(17.4)	(22.8)	(17.9)	(14.7)	(26.8)	(16.5)
t_E	35.2	33.4	60.9	35.2	33.4	60.9	35.2	33.4	60.9	35.2
	(15.7)	(14.9)	(27.2)	(15.7)	(14.9)	(27.2)	(15.7)	(14.9)	(27.2)	(15.7)
	17.2	34.8	-14.8	22.0	12.1	-18.1	10.2	-0.8	-1.8	3.6
	7.660	15.540	-6.598	9.843	5.396	-8.101	4.542	-0.357	-0.824	1.626
g_O	69	74	53	55	68	51	75	77	44	58
	(30.8)	(33.0)	(23.7)	(24.6)	(30.4)	(22.8)	(33.5)	(34.4)	(19.6)	(25.9)
g_E	70.2	84.8	42.1	70.2	84.8	42.1	70.2	84.8	42.1	70.2
	(31.3)	(37.8)	(18.8)	(31.3)	(37.8)	(18.8)	(31.3)	(37.8)	(18.8)	(31.3)
	-2.3	-20.1	24.4	-26.8	-30.0	19.5	10.0	-14.8	3.8	-22.1
	-1.025	-8.986	10.875	-11.953	-13.391	8.713	4.470	-6.618	1.717	-9.855
c_O	83	51	65	89	66	65	76	58	62	90
	(37.1)	(22.8)	(29.0)	(39.7)	(29.5)	(29.0)	(33.9)	(25.9)	(27.7)	(40.2)
c_E	93.8	54.5	59.6	93.8	54.5	59.6	93.8	54.5	59.6	93.8
	(41.9)	(24.4)	(26.6)	(41.9)	(24.4)	(26.6)	(41.9)	(24.4)	(26.6)	(41.9)
	-20.4	-6.9	11.2	-9.4	25.2	11.2	-32.0	7.1	4.9	-7.5
	-9.090	-3.060	5.017	-4.201	11.234	5.017	-14.303	3.181	2.170	-3.351
N	224	224	224	224	224	224	224	224	224	224
X2	3.7	8.0	5.5	10.4	6.7	4.3	7.1	1.4	0.4	10.5
Gvalue	3.6	7.3	5.3	10.0	6.7	4.3	7.0	1.4	0.4	9.4
Gv/N	1.608	3.251	2.382	4.469	2.995	1.899	3.141	0.616	0.168	4.202
Prob	0.30772	0.06342	0.14878	0.01847	0.08176	0.23536	0.07076	0.71042	0.94519	0.02427

	91	92	93	94	95	96	97	98	99	100
a_O	57	61	39	44	67	35	48	47	25	52
	(25.4)	(27.2)	(17.4)	(19.6)	(29.9)	(15.6)	(21.4)	(21.0)	(11.2)	(23.2)
a_E	51.3	61.3	24.8	51.3	61.3	24.8	51.3	61.3	24.8	51.3
	(22.9)	(27.4)	(11.1)	(22.9)	(27.4)	(11.1)	(22.9)	(27.4)	(11.1)	(22.9)
	12.1	-0.7	35.4	-13.5	11.8	24.1	-6.3	-25.0	0.4	1.5
	5.389	-0.298	15.782	-6.010	5.285	10.781	-2.827	-11.171	0.190	0.654
t_O	33	62	42	33	53	44	43	73	39	36
	(14.7)	(27.7)	(18.8)	(14.7)	(23.7)	(19.6)	(19.2)	(32.6)	(17.4)	(16.1)
t_E	33.4	60.9	35.2	33.4	60.9	35.2	33.4	60.9	35.2	33.4
	(14.9)	(27.2)	(15.7)	(14.9)	(27.2)	(15.7)	(14.9)	(27.2)	(15.7)	(14.9)
	-0.8	2.2	14.8	-0.8	-14.8	19.6	21.7	26.4	7.9	5.4
	-0.357	0.964	6.599	-0.357	-6.598	8.741	9.698	11.780	3.547	2.408
g_O	84	39	72	89	44	59	85	43	65	95
	(37.5)	(17.4)	(32.1)	(39.7)	(19.6)	(26.3)	(37.9)	(19.2)	(29.0)	(42.4)
g_E	84.8	42.1	70.2	84.8	42.1	70.2	84.8	42.1	70.2	84.8
	(37.8)	(18.8)	(31.3)	(37.8)	(18.8)	(31.3)	(37.8)	(18.8)	(31.3)	(37.8)
	-1.6	-6.0	3.7	8.6	3.8	-20.4	0.4	1.8	-9.9	21.6
	-0.694	-2.679	1.667	3.860	1.717	-9.124	0.196	0.795	-4.431	9.654
c_O	50	62	71	58	60	86	48	61	95	41
	(22.3)	(27.7)	(31.7)	(25.9)	(26.8)	(38.4)	(21.4)	(27.2)	(42.4)	(18.3)
c_E	54.5	59.6	93.8	54.5	59.6	93.8	54.5	59.6	93.8	54.5
	(24.4)	(26.6)	(41.9)	(24.4)	(26.6)	(41.9)	(24.4)	(26.6)	(41.9)	(24.4)
	-8.7	4.9	-39.6	7.1	0.8	-15.0	-12.3	2.8	2.4	-23.4
	-3.884	2.170	-17.676	3.181	0.343	-6.693	-5.478	1.249	1.049	-10.449
N	224	224	224	224	224	224	224	224	224	224
X2	1.0	0.3	15.1	1.5	1.6	8.8	3.8	5.8	0.8	4.8
Gvalue	1.0	0.4	14.3	1.5	1.7	8.3	3.6	5.9	0.8	5.1
Gv/N	0.455	0.157	6.372	0.674	0.747	3.705	1.589	2.654	0.356	2.266
Prob	0.79670	0.95009	0.00256	0.67994	0.64294	0.04020	0.31316	0.11435	0.85032	0.16638

5UTR:

	100	99	98	97	96	95	94	93	92	91
a_O	217	231	214	216	298	211	229	227	312	312
	(15.0)	(15.9)	(14.8)	(14.9)	(20.6)	(14.6)	(15.8)	(15.7)	(21.5)	(21.5)
a_E	257.0	257.0	257.0	257.0	257.0	257.0	257.0	257.0	257.0	257.0
	(17.7)	(17.7)	(17.7)	(17.7)	(17.7)	(17.7)	(17.7)	(17.7)	(17.7)	(17.7)
	-73.4	-49.3	-78.4	-75.1	88.2	-83.2	-52.8	-56.4	121.0	121.0
	-5.068	-3.402	-5.409	-5.183	6.087	-5.745	-3.647	-3.890	8.350	8.350
t_O	208	213	201	223	211	208	307	221	194	212
	(14.4)	(14.7)	(13.9)	(15.4)	(14.6)	(14.4)	(21.2)	(15.3)	(13.4)	(14.6)
t_E	250.5	250.5	250.5	250.5	250.5	250.5	250.5	250.5	250.5	250.5
	(17.3)	(17.3)	(17.3)	(17.3)	(17.3)	(17.3)	(17.3)	(17.3)	(17.3)	(17.3)
	-77.3	-69.1	-88.5	-51.9	-72.4	-77.3	124.9	-55.4	-99.2	-70.8
	-5.338	-4.768	-6.108	-3.579	-4.998	-5.338	8.618	-3.822	-6.844	-4.883
g_O	463	419	481	525	465	483	441	532	473	447
	(32.0)	(28.9)	(33.2)	(36.2)	(32.1)	(33.3)	(30.4)	(36.7)	(32.6)	(30.8)
g_E	444.0	444.0	444.0	444.0	444.0	444.0	444.0	444.0	444.0	444.0
	(30.6)	(30.6)	(30.6)	(30.6)	(30.6)	(30.6)	(30.6)	(30.6)	(30.6)	(30.6)
	38.9	-48.5	77.1	176.0	43.1	81.4	-5.9	192.5	59.9	6.1
	2.684	-3.346	5.320	12.150	2.972	5.619	-0.407	13.284	4.137	0.421
c_O	561	586	553	485	475	547	472	469	470	478
	(38.7)	(40.4)	(38.2)	(33.5)	(32.8)	(37.8)	(32.6)	(32.4)	(32.4)	(33.0)
c_E	497.5	497.5	497.5	497.5	497.5	497.5	497.5	497.5	497.5	497.5
	(34.3)	(34.3)	(34.3)	(34.3)	(34.3)	(34.3)	(34.3)	(34.3)	(34.3)	(34.3)
	134.7	191.8	116.9	-24.7	-44.0	103.7	-49.7	-55.4	-53.5	-38.3
	9.297	13.238	8.068	-1.708	-3.038	7.157	-3.432	-3.823	-3.693	-2.642
N	1449	1449	1449	1449	1449	1449	1449	1449	1449	1449
X2	22.4	25.4	26.3	24.7	14.8	23.8	17.1	26.1	27.9	18.5
Gvalue	22.8	24.9	27.1	24.3	14.8	24.5	16.4	25.3	28.2	18.0
Gv/N	1.575	1.722	1.871	1.680	1.023	1.693	1.132	1.749	1.949	1.246
Prob	0.00004	0.00002	0.00001	0.00002	0.00198	0.00002	0.00094	0.00001	0.00000	0.00043

	90	89	88	87	86	85	84	83	82	81
a_O	237	324	298	300	205	192	242	226	246	242
	(16.4)	(22.4)	(20.6)	(20.7)	(14.1)	(13.3)	(16.7)	(15.6)	(17.0)	(16.7)
a_E	257.0	257.0	257.0	257.0	257.0	257.0	257.0	257.0	257.0	257.0
	(17.7)	(17.7)	(17.7)	(17.7)	(17.7)	(17.7)	(17.7)	(17.7)	(17.7)	(17.7)
	-38.4	150.1	88.2	92.8	-92.7	-112.0	-29.1	-58.1	-21.5	-29.1
	-2.651	10.359	6.087	6.404	-6.398	-7.728	-2.010	-4.011	-1.487	-2.010
t_O	198	215	210	232	224	306	218	214	314	195
	(13.7)	(14.8)	(14.5)	(16.0)	(15.5)	(21.1)	(15.0)	(14.8)	(21.7)	(13.5)
t_E	250.5	250.5	250.5	250.5	250.5	250.5	250.5	250.5	250.5	250.5
	(17.3)	(17.3)	(17.3)	(17.3)	(17.3)	(17.3)	(17.3)	(17.3)	(17.3)	(17.3)
	-93.1	-65.7	-74.1	-35.6	-50.1	122.5	-60.6	-67.4	141.9	-97.7
	-6.428	-4.535	-5.112	-2.457	-3.457	8.453	-4.181	-4.652	9.792	-6.741
g_O	517	470	443	469	455	482	525	490	418	567
	(35.7)	(32.4)	(30.6)	(32.4)	(31.4)	(33.3)	(36.2)	(33.8)	(28.8)	(39.1)
g_E	444.0	444.0	444.0	444.0	444.0	444.0	444.0	444.0	444.0	444.0
	(30.6)	(30.6)	(30.6)	(30.6)	(30.6)	(30.6)	(30.6)	(30.6)	(30.6)	(30.6)
	157.5	53.6	-1.9	51.5	22.4	79.3	176.0	96.7	-50.4	277.4
	10.869	3.698	-0.132	3.552	1.543	5.469	12.150	6.673	-3.476	19.145
c_O	497	440	498	448	565	469	464	519	471	445
	(34.3)	(30.4)	(34.4)	(30.9)	(39.0)	(32.4)	(32.0)	(35.8)	(32.5)	(30.7)
c_E	497.5	497.5	497.5	497.5	497.5	497.5	497.5	497.5	497.5	497.5
	(34.3)	(34.3)	(34.3)	(34.3)	(34.3)	(34.3)	(34.3)	(34.3)	(34.3)	(34.3)
	-1.1	-108.1	0.9	-94.0	143.7	-55.4	-64.7	43.9	-51.6	-99.3
	-0.073	-7.463	0.065	-6.484	9.917	-3.823	-4.468	3.026	-3.562	-6.854
N	1449	1449	1449	1449	1449	1449	1449	1449	1449	1449
X2	24.6	30.7	13.1	14.9	22.8	33.6	22.1	14.8	19.5	52.8
Gvalue	24.9	29.8	13.2	14.7	23.3	34.4	21.6	15.0	18.4	51.3
Gv/N	1.716	2.058	0.908	1.015	1.605	2.371	1.490	1.037	1.267	3.540
Prob	0.00002	0.00000	0.00432	0.00208	0.00004	0.00000	0.00008	0.00179	0.00037	0.00000

	80	79	78	77	76	75	74	73	72	71
a_O	264	240	228	214	331	356	234	244	318	307
	(18.2)	(16.6)	(15.7)	(14.8)	(22.8)	(24.6)	(16.1)	(16.8)	(21.9)	(21.2)
a_E	257.0	257.0	257.0	257.0	257.0	257.0	257.0	257.0	257.0	257.0
	(17.7)	(17.7)	(17.7)	(17.7)	(17.7)	(17.7)	(17.7)	(17.7)	(17.7)	(17.7)
	14.2	-32.9	-54.6	-78.4	167.5	232.0	-43.9	-25.4	135.4	109.1
	0.978	-2.268	-3.769	-5.409	11.559	16.010	-3.029	-1.749	9.346	7.531
t_O	207	237	232	229	202	224	228	209	216	218
	(14.3)	(16.4)	(16.0)	(15.8)	(13.9)	(15.5)	(15.7)	(14.4)	(14.9)	(15.0)
t_E	250.5	250.5	250.5	250.5	250.5	250.5	250.5	250.5	250.5	250.5
	(17.3)	(17.3)	(17.3)	(17.3)	(17.3)	(17.3)	(17.3)	(17.3)	(17.3)	(17.3)
	-79.0	-26.3	-35.6	-41.1	-86.9	-50.1	-42.9	-75.7	-64.0	-60.6
	-5.450	-1.812	-2.457	-2.836	-6.000	-3.457	-2.962	-5.225	-4.418	-4.181
g_O	513	442	430	544	477	447	523	420	442	464
	(35.4)	(30.5)	(29.7)	(37.5)	(32.9)	(30.8)	(36.1)	(29.0)	(30.5)	(32.0)
g_E	444.0	444.0	444.0	444.0	444.0	444.0	444.0	444.0	444.0	444.0
	(30.6)	(30.6)	(30.6)	(30.6)	(30.6)	(30.6)	(30.6)	(30.6)	(30.6)	(30.6)
	148.3	-3.9	-27.5	221.1	68.5	6.1	171.4	-46.6	-3.9	41.0
	10.235	-0.270	-1.896	15.259	4.726	0.421	11.828	-3.216	-0.270	2.828
c_O	465	530	559	462	439	422	464	576	473	460
	(32.1)	(36.6)	(38.6)	(31.9)	(30.3)	(29.1)	(32.0)	(39.8)	(32.6)	(31.7)
c_E	497.5	497.5	497.5	497.5	497.5	497.5	497.5	497.5	497.5	497.5
	(34.3)	(34.3)	(34.3)	(34.3)	(34.3)	(34.3)	(34.3)	(34.3)	(34.3)	(34.3)
	-62.9	67.0	130.2	-68.5	-109.9	-139.0	-64.7	168.7	-47.8	-72.2
	-4.340	4.625	8.988	-4.725	-7.584	-9.590	-4.468	11.643	-3.301	-4.980
N	1449	1449	1449	1449	1449	1449	1449	1449	1449	1449
X2	20.6	4.0	12.7	34.1	40.0	52.4	20.4	21.2	20.4	17.7
Gvalue	20.6	4.0	12.5	33.2	39.1	49.0	19.8	21.0	19.7	17.4
Gv/N	1.423	0.274	0.866	2.288	2.701	3.383	1.368	1.453	1.358	1.198
Prob	0.00013	0.26412	0.00572	0.00000	0.00000	0.00000	0.00018	0.00010	0.00020	0.00060

	70	69	68	67	66	65	64	63	62	61
a_O	225	215	218	234	225	251	325	227	313	216
	(15.5)	(14.8)	(15.0)	(16.1)	(15.5)	(17.3)	(22.4)	(15.7)	(21.6)	(14.9)
a_E	257.0	257.0	257.0	257.0	257.0	257.0	257.0	257.0	257.0	257.0
	(17.7)	(17.7)	(17.7)	(17.7)	(17.7)	(17.7)	(17.7)	(17.7)	(17.7)	(17.7)
	-59.9	-76.7	-71.8	-43.9	-59.9	-11.9	152.6	-56.4	123.4	-75.1
	-4.131	-5.296	-4.953	-3.029	-4.131	-0.820	10.529	-3.890	8.515	-5.183
t_O	312	212	205	328	240	216	245	302	222	314
	(21.5)	(14.6)	(14.1)	(22.6)	(16.6)	(14.9)	(16.9)	(20.8)	(15.3)	(21.7)
t_E	250.5	250.5	250.5	250.5	250.5	250.5	250.5	250.5	250.5	250.5

	(17.3)	(17.3)	(17.3)	(17.3)	(17.3)	(17.3)	(17.3)	(17.3)	(17.3)	(17.3)
	137.0	-70.8	-82.2	176.8	-20.6	-64.0	-10.9	112.9	-53.6	141.9
	9.454	-4.883	-5.672	12.203	-1.418	-4.418	-0.751	7.794	-3.701	9.792
g_O	443	527	460	422	452	508	451	452	446	465
	(30.6)	(36.4)	(31.7)	(29.1)	(31.2)	(35.1)	(31.1)	(31.2)	(30.8)	(32.1)
g_E	444.0	444.0	444.0	444.0	444.0	444.0	444.0	444.0	444.0	444.0
	(30.6)	(30.6)	(30.6)	(30.6)	(30.6)	(30.6)	(30.6)	(30.6)	(30.6)	(30.6)
	-1.9	180.7	32.7	-42.8	16.2	136.9	14.2	16.2	4.1	43.1
	-0.132	12.472	2.253	-2.955	1.120	9.448	0.979	1.120	0.282	2.972
c_O	469	495	566	465	532	474	428	468	468	454
	(32.4)	(34.2)	(39.1)	(32.1)	(36.7)	(32.7)	(29.5)	(32.3)	(32.3)	(31.3)
c_E	497.5	497.5	497.5	497.5	497.5	497.5	497.5	497.5	497.5	497.5
	(34.3)	(34.3)	(34.3)	(34.3)	(34.3)	(34.3)	(34.3)	(34.3)	(34.3)	(34.3)
	-55.4	-5.0	146.0	-62.9	71.3	-45.9	-128.9	-57.3	-57.3	-83.1
	-3.823	-0.348	10.073	-4.340	4.919	-3.170	-8.893	-3.952	-3.952	-5.737
N	1449	1449	1449	1449	1449	1449	1449	1449	1449	1449
X2	20.7	28.3	24.2	29.3	7.0	15.2	27.9	16.0	17.2	27.4
Gvalue	19.8	28.2	24.7	27.2	7.1	15.1	27.0	15.5	16.6	26.7
Gv/N	1.368	1.945	1.701	1.879	0.489	1.041	1.865	1.070	1.143	1.844
Prob	0.00018	0.00000	0.00002	0.00001	0.06910	0.00175	0.00001	0.00143	0.00087	0.00001

	60	59	58	57	56	55	54	53	52	51
a_O	200	239	328	317	229	238	239	257	196	230
	(13.8)	(16.5)	(22.6)	(21.9)	(15.8)	(16.4)	(16.5)	(17.7)	(13.5)	(15.9)
a_E	257.0	257.0	257.0	257.0	257.0	257.0	257.0	257.0	257.0	257.0
	(17.7)	(17.7)	(17.7)	(17.7)	(17.7)	(17.7)	(17.7)	(17.7)	(17.7)	(17.7)
	-100.3	-34.7	160.0	133.0	-52.8	-36.6	-34.7	-0.0	-106.2	-51.1
	-6.923	-2.397	11.042	9.179	-3.647	-2.524	-2.397	-0.001	-7.331	-3.525
t_O	234	211	226	228	238	249	211	310	337	229
	(16.1)	(14.6)	(15.6)	(15.7)	(16.4)	(17.2)	(14.6)	(21.4)	(23.3)	(15.8)
t_E	250.5	250.5	250.5	250.5	250.5	250.5	250.5	250.5	250.5	250.5
	(17.3)	(17.3)	(17.3)	(17.3)	(17.3)	(17.3)	(17.3)	(17.3)	(17.3)	(17.3)
	-31.9	-72.4	-46.5	-42.9	-24.4	-3.0	-72.4	132.1	199.9	-41.1
	-2.201	-4.998	-3.211	-2.962	-1.682	-0.206	-4.998	9.119	13.797	-2.836
g_O	511	533	413	462	518	416	544	460	486	510
	(35.3)	(36.8)	(28.5)	(31.9)	(35.7)	(28.7)	(37.5)	(31.7)	(33.5)	(35.2)
g_E	444.0	444.0	444.0	444.0	444.0	444.0	444.0	444.0	444.0	444.0
	(30.6)	(30.6)	(30.6)	(30.6)	(30.6)	(30.6)	(30.6)	(30.6)	(30.6)	(30.6)
	143.7	194.9	-59.7	36.8	159.8	-54.1	221.1	32.7	87.9	141.4
	9.919	13.447	-4.121	2.540	11.028	-3.735	15.259	2.253	6.069	9.762
c_O	504	466	482	442	464	546	455	422	430	480
	(34.8)	(32.2)	(33.3)	(30.5)	(32.0)	(37.7)	(31.4)	(29.1)	(29.7)	(33.1)
c_E	497.5	497.5	497.5	497.5	497.5	497.5	497.5	497.5	497.5	497.5
	(34.3)	(34.3)	(34.3)	(34.3)	(34.3)	(34.3)	(34.3)	(34.3)	(34.3)	(34.3)
	13.0	-61.0	-30.6	-104.6	-64.7	101.5	-81.3	-139.0	-125.4	-34.4
	0.899	-4.211	-2.110	-7.220	-4.468	7.006	-5.612	-9.590	-8.658	-2.376
N	1449	1449	1449	1449	1449	1449	1449	1449	1449	1449
X2	23.9	27.3	24.6	23.0	18.3	7.9	33.7	26.2	57.5	15.1
Gvalue	24.5	26.7	23.2	22.3	17.8	7.8	32.6	25.8	56.2	14.8
Gv/N	1.694	1.842	1.601	1.537	1.231	0.540	2.252	1.780	3.877	1.024
Prob	0.00002	0.00001	0.00004	0.00006	0.00048	0.04975	0.00000	0.00001	0.00000	0.00196

	50	49	48	47	46	45	44	43	42	41
a_O	313	281	213	201	224	228	273	176	254	269
	(21.6)	(19.4)	(14.7)	(13.9)	(15.5)	(15.7)	(18.8)	(12.1)	(17.5)	(18.6)
a_E	257.0	257.0	257.0	257.0	257.0	257.0	257.0	257.0	257.0	257.0
	(17.7)	(17.7)	(17.7)	(17.7)	(17.7)	(17.7)	(17.7)	(17.7)	(17.7)	(17.7)
	123.4	50.2	-80.0	-98.8	-61.6	-54.6	33.0	-133.3	-6.0	24.5
	8.515	3.461	-5.522	-6.820	-4.250	-3.769	2.274	-9.198	-0.413	1.693
t_O	257	271	219	212	342	319	220	324	228	207
	(17.7)	(18.7)	(15.1)	(14.6)	(23.6)	(22.0)	(15.2)	(22.4)	(15.7)	(14.3)
t_E	250.5	250.5	250.5	250.5	250.5	250.5	250.5	250.5	250.5	250.5
	(17.3)	(17.3)	(17.3)	(17.3)	(17.3)	(17.3)	(17.3)	(17.3)	(17.3)	(17.3)
	13.2	42.6	-58.9	-70.8	213.0	154.2	-57.1	166.7	-42.9	-79.0
	0.909	2.942	-4.062	-4.883	14.697	10.644	-3.942	11.506	-2.962	-5.450
g_O	430	451	520	542	454	443	515	414	457	498
	(29.7)	(31.1)	(35.9)	(37.4)	(31.3)	(30.6)	(35.5)	(28.6)	(31.5)	(34.4)
g_E	444.0	444.0	444.0	444.0	444.0	444.0	444.0	444.0	444.0	444.0
	(30.6)	(30.6)	(30.6)	(30.6)	(30.6)	(30.6)	(30.6)	(30.6)	(30.6)	(30.6)
	-27.5	14.2	164.4	216.3	20.3	-1.9	152.9	-57.9	26.5	114.4
	-1.896	0.979	11.347	14.927	1.401	-0.132	10.551	-3.992	1.826	7.896
c_O	449	446	497	494	429	459	441	535	510	475
	(31.0)	(30.8)	(34.3)	(34.1)	(29.6)	(31.7)	(30.4)	(36.9)	(35.2)	(32.8)
c_E	497.5	497.5	497.5	497.5	497.5	497.5	497.5	497.5	497.5	497.5
	(34.3)	(34.3)	(34.3)	(34.3)	(34.3)	(34.3)	(34.3)	(34.3)	(34.3)	(34.3)
	-92.2	-97.5	-1.1	-7.0	-127.2	-74.0	-106.4	77.7	25.2	-44.0
	-6.361	-6.731	-0.073	-0.486	-8.775	-5.107	-7.342	5.362	1.743	-3.038
N	1449	1449	1449	1449	1449	1449	1449	1449	1449	1449
X2	17.5	9.4	24.5	39.8	47.3	25.0	22.5	51.9	2.8	15.7
Gvalue	16.9	9.4	24.5	39.7	44.5	23.7	22.3	53.3	2.8	15.9
Gv/N	1.167	0.652	1.690	2.739	3.073	1.635	1.541	3.677	0.194	1.101
Prob	0.00074	0.02388	0.00002	0.00000	0.00000	0.00003	0.00006	0.00000	0.42202	0.00116

	40	39	38	37	36	35	34	33	32	31
a_O	257	212	292	303	205	218	213	292	328	328
	(17.7)	(14.6)	(20.2)	(20.9)	(14.1)	(15.0)	(14.7)	(20.2)	(22.6)	(22.6)
a_E	257.0	257.0	257.0	257.0	257.0	257.0	257.0	257.0	257.0	257.0
	(17.7)	(17.7)	(17.7)	(17.7)	(17.7)	(17.7)	(17.7)	(17.7)	(17.7)	(17.7)
	-0.0	-81.6	74.5	99.8	-92.7	-71.8	-80.0	74.5	160.0	160.0
	-0.001	-5.634	5.144	6.885	-6.398	-4.953	-5.522	5.144	11.042	11.042
t_O	353	217	227	237	279	240	269	245	239	263
	(24.4)	(15.0)	(15.7)	(16.4)	(19.3)	(16.6)	(18.6)	(16.9)	(16.5)	(18.2)
t_E	250.5	250.5	250.5	250.5	250.5	250.5	250.5	250.5	250.5	250.5
	(17.3)	(17.3)	(17.3)	(17.3)	(17.3)	(17.3)	(17.3)	(17.3)	(17.3)	(17.3)
	242.2	-62.3	-44.7	-26.3	60.1	-20.6	38.3	-10.9	-22.5	25.6
	16.713	-4.300	-3.086	-1.812	4.149	-1.418	2.646	-0.751	-1.550	1.768
g_O	382	475	418	413	528	480	497	393	392	385
	(26.4)	(32.8)	(28.8)	(28.5)	(36.4)	(33.1)	(34.3)	(27.1)	(27.1)	(26.6)
g_E	444.0	444.0	444.0	444.0	444.0	444.0	444.0	444.0	444.0	444.0
	(30.6)	(30.6)	(30.6)	(30.6)	(30.6)	(30.6)	(30.6)	(30.6)	(30.6)	(30.6)
	-114.8	64.2	-50.4	-59.7	183.1	74.9	112.2	-95.8	-97.6	-109.7
	-7.925	4.431	-3.476	-4.121	12.634	5.171	7.742	-6.614	-6.735	-7.572
c_O	457	545	512	496	437	511	470	519	490	473
	(31.5)	(37.6)	(35.3)	(34.2)	(30.2)	(35.3)	(32.4)	(35.8)	(33.8)	(32.6)
c_E	497.5	497.5	497.5	497.5	497.5	497.5	497.5	497.5	497.5	497.5
	(34.3)	(34.3)	(34.3)	(34.3)	(34.3)	(34.3)	(34.3)	(34.3)	(34.3)	(34.3)

```
          -77.7    99.3    29.4    -3.1  -113.4    27.3   -53.5    43.9   -14.9   -47.8
         -5.360   6.855   2.026  -0.211  -7.825   1.884  -3.693   3.026  -1.031  -3.301

N          1449    1449    1449    1449    1449    1449    1449    1449    1449    1449
X2         53.9    19.1     8.9    11.1    37.0     9.7    16.8    11.7    26.3    29.3
Gvalue     49.6    19.6     8.8    10.7    37.1     9.9    17.0    11.7    25.0    28.1
Gv/N      3.426   1.352   0.608   0.741   2.561   0.683   1.173   0.806   1.725   1.937
Prob    0.00000 0.00021 0.03202 0.01325 0.00000 0.01941 0.00071 0.00855 0.00002 0.00000
        -------------------------------------------------------------------------------
             30      29      28      27      26      25      24      23      22      21
        -------------------------------------------------------------------------------
a_O         264     255     209     207     223     217     301     232     279     235
        ( 18.2) ( 17.6) ( 14.4) ( 14.3) ( 15.4) ( 15.0) ( 20.8) ( 16.0) ( 19.3) ( 16.2)
a_E       257.0   257.0   257.0   257.0   257.0   257.0   257.0   257.0   257.0   257.0
        ( 17.7) ( 17.7) ( 17.7) ( 17.7) ( 17.7) ( 17.7) ( 17.7) ( 17.7) ( 17.7) ( 17.7)
           14.2    -4.0   -86.4   -89.6   -63.3   -73.4    95.1   -47.5    45.8   -42.1
          0.978  -0.276  -5.965  -6.183  -4.369  -5.068   6.564  -3.278   3.161  -2.904
t_O         234     232     315     252     348     285     241     245     250     292
        ( 16.1) ( 16.0) ( 21.7) ( 17.4) ( 24.0) ( 19.7) ( 16.6) ( 16.9) ( 17.3) ( 20.2)
t_E       250.5   250.5   250.5   250.5   250.5   250.5   250.5   250.5   250.5   250.5
        ( 17.3) ( 17.3) ( 17.3) ( 17.3) ( 17.3) ( 17.3) ( 17.3) ( 17.3) ( 17.3) ( 17.3)
          -31.9   -35.6   144.3     3.0   228.8    73.5   -18.6   -10.9    -1.0    89.5
         -2.201  -2.457   9.961   0.208  15.791   5.076  -1.286  -0.751  -0.069   6.178
g_O         475     439     367     436     427     493     446     501     364     361
        ( 32.8) ( 30.3) ( 25.3) ( 30.1) ( 29.5) ( 34.0) ( 30.8) ( 34.6) ( 25.1) ( 24.9)
g_E       444.0   444.0   444.0   444.0   444.0   444.0   444.0   444.0   444.0   444.0
        ( 30.6) ( 30.6) ( 30.6) ( 30.6) ( 30.6) ( 30.6) ( 30.6) ( 30.6) ( 30.6) ( 30.6)
           64.2    -9.9  -139.7   -15.8   -33.3   103.3     4.1   121.1  -144.6  -149.4
          4.431  -0.681  -9.643  -1.089  -2.296   7.130   0.282   8.358  -9.977 -10.307
c_O         476     523     558     554     451     454     461     471     556     561
        ( 32.9) ( 36.1) ( 38.5) ( 38.2) ( 31.1) ( 31.3) ( 31.8) ( 32.5) ( 38.4) ( 38.7)
c_E       497.5   497.5   497.5   497.5   497.5   497.5   497.5   497.5   497.5   497.5
        ( 34.3) ( 34.3) ( 34.3) ( 34.3) ( 34.3) ( 34.3) ( 34.3) ( 34.3) ( 34.3) ( 34.3)
          -42.1    52.2   128.0   119.1   -88.6   -83.1   -70.3   -51.6   123.6   134.7
         -2.906   3.604   8.834   8.221  -6.112  -5.737  -4.852  -3.562   8.527   9.297

N          1449    1449    1449    1449    1449    1449    1449    1449    1449    1449
X2          4.4     2.7    46.3    16.3    47.4    20.2    10.6    11.3    23.2    32.4
Gvalue      4.4     2.8    46.2    16.8    43.7    20.3    10.3    11.1    23.8    32.8
Gv/N      0.301   0.190   3.187   1.157   3.014   1.400   0.708   0.767   1.643   2.264
Prob    0.22450 0.43100 0.00000 0.00079 0.00000 0.00015 0.01650 0.01113 0.00003 0.00000
        -------------------------------------------------------------------------------
             20      19      18      17      16      15      14      13      12      11
        -------------------------------------------------------------------------------
a_O         229     218     233     313     285     241     244     247     328     346
        ( 15.8) ( 15.0) ( 16.1) ( 21.6) ( 19.7) ( 16.6) ( 16.8) ( 17.0) ( 22.6) ( 23.9)
a_E       257.0   257.0   257.0   257.0   257.0   257.0   257.0   257.0   257.0   257.0
        ( 17.7) ( 17.7) ( 17.7) ( 17.7) ( 17.7) ( 17.7) ( 17.7) ( 17.7) ( 17.7) ( 17.7)
          -52.8   -71.8   -45.7   123.4    58.9   -31.0   -25.4   -19.6   160.0   205.7
         -3.647  -4.953  -3.154   8.515   4.066  -2.139  -1.749  -1.354  11.042  14.199
t_O         311     333     293     317     261     286     327     301     292     271
        ( 21.5) ( 23.0) ( 20.2) ( 21.9) ( 18.0) ( 19.7) ( 22.6) ( 20.8) ( 20.2) ( 18.7)
t_E       250.5   250.5   250.5   250.5   250.5   250.5   250.5   250.5   250.5   250.5
        ( 17.3) ( 17.3) ( 17.3) ( 17.3) ( 17.3) ( 17.3) ( 17.3) ( 17.3) ( 17.3) ( 17.3)
          134.6   189.6    91.8   149.3    21.4    75.8   174.3   110.6    89.5    42.6
          9.286  13.085   6.338  10.302   1.479   5.232  12.028   7.630   6.178   2.942
g_O         336     406     314     299     376     438     302     304     341     350
        ( 23.2) ( 28.0) ( 21.7) ( 20.6) ( 25.9) ( 30.2) ( 20.8) ( 21.0) ( 23.5) ( 24.2)
g_E       444.0   444.0   444.0   444.0   444.0   444.0   444.0   444.0   444.0   444.0
        ( 30.6) ( 30.6) ( 30.6) ( 30.6) ( 30.6) ( 30.6) ( 30.6) ( 30.6) ( 30.6) ( 30.6)
         -187.2   -72.6  -217.5  -236.4  -124.9   -11.8  -232.7  -230.3  -179.9  -166.5
        -12.922  -5.009 -15.011 -16.314  -8.623  -0.817 -16.061 -15.891 -12.419 -11.488
c_O         573     492     609     520     527     484     576     597     488     482
        ( 39.5) ( 34.0) ( 42.0) ( 35.9) ( 36.4) ( 33.4) ( 39.8) ( 41.2) ( 33.7) ( 33.3)
c_E       497.5   497.5   497.5   497.5   497.5   497.5   497.5   497.5   497.5   497.5
        ( 34.3) ( 34.3) ( 34.3) ( 34.3) ( 34.3) ( 34.3) ( 34.3) ( 34.3) ( 34.3) ( 34.3)
          161.8   -11.0   246.2    45.9    60.7   -26.7   168.7   217.6   -18.9   -30.6
         11.170  -0.759  16.993   3.170   4.186  -1.842  11.643  15.019  -1.303  -2.110

N          1449    1449    1449    1449    1449    1449    1449    1449    1449    1449
X2         55.4    36.4    72.5    78.2    15.6     6.5    81.8    74.6    50.5    52.9
Gvalue     56.3    34.2    74.9    82.2    16.1     6.3    84.9    78.3    50.7    51.4
Gv/N      3.887   2.364   5.166   5.673   1.109   0.433   5.861   5.404   3.499   3.544
Prob    0.00000 0.00000 0.00000 0.00000 0.00110 0.09879 0.00000 0.00000 0.00000 0.00000
        -------------------------------------------------------------------------------
             10       9       8       7       6       5       4       3       2       1
        -------------------------------------------------------------------------------
a_O         352     312     311     241     269     358     309     291     202     233
        ( 24.3) ( 21.5) ( 21.5) ( 16.6) ( 18.6) ( 24.7) ( 21.3) ( 20.1) ( 13.9) ( 16.1)
a_E       257.0   257.0   257.0   257.0   257.0   257.0   257.0   257.0   257.0   257.0
        ( 17.7) ( 17.7) ( 17.7) ( 17.7) ( 17.7) ( 17.7) ( 17.7) ( 17.7) ( 17.7) ( 17.7)
          221.4   121.0   118.6   -31.0    24.5   237.3   113.9    72.3   -97.3   -45.7
         15.281   8.350   8.185  -2.139   1.693  16.376   7.857   4.989  -6.715  -3.154
t_O         250     235     257     299     243     252     230     216     286     133
        ( 17.3) ( 16.2) ( 17.7) ( 20.6) ( 16.8) ( 17.4) ( 15.9) ( 14.9) ( 19.7) (  9.2)
t_E       250.5   250.5   250.5   250.5   250.5   250.5   250.5   250.5   250.5   250.5
        ( 17.3) ( 17.3) ( 17.3) ( 17.3) ( 17.3) ( 17.3) ( 17.3) ( 17.3) ( 17.3) ( 17.3)
           -1.0   -30.0    13.2   105.8   -14.8     3.0   -39.3   -64.0    75.8  -168.4
         -0.069  -2.072   0.909   7.304  -1.020   0.208  -2.710  -4.418   5.232 -11.622
g_O         336     362     424     307     451     345     321     540     290     412
        ( 23.2) ( 25.0) ( 29.3) ( 21.2) ( 31.1) ( 23.8) ( 22.2) ( 37.3) ( 20.0) ( 28.4)
g_E       444.0   444.0   444.0   444.0   444.0   444.0   444.0   444.0   444.0   444.0
        ( 30.6) ( 30.6) ( 30.6) ( 30.6) ( 30.6) ( 30.6) ( 30.6) ( 30.6) ( 30.6) ( 30.6)
         -187.2  -147.8   -39.0  -226.5    14.2  -174.0  -208.2   211.5  -247.0   -61.6
        -12.922 -10.197  -2.692 -15.631   0.979 -12.009 -14.368  14.596 -17.046  -4.249
c_O         511     540     457     602     486     494     589     402     671     671
        ( 35.3) ( 37.3) ( 31.5) ( 41.5) ( 33.5) ( 34.1) ( 40.6) ( 27.7) ( 46.3) ( 46.3)
c_E       497.5   497.5   497.5   497.5   497.5   497.5   497.5   497.5   497.5   497.5
        ( 34.3) ( 34.3) ( 34.3) ( 34.3) ( 34.3) ( 34.3) ( 34.3) ( 34.3) ( 34.3) ( 34.3)
           27.3    88.5   -77.7   229.5   -22.8    -7.0   198.8  -171.4   401.4   401.4
          1.884   6.105  -5.360  15.837  -1.573  -0.486  13.721 -11.830  27.703  27.703

N          1449    1449    1449    1449    1449    1449    1449    1449    1449    1449
X2         61.7    31.5    15.7    74.6     1.2    61.8    63.1    48.4   130.7   120.1
Gvalue     60.5    31.7    15.1    77.8     1.2    59.3    65.2    48.4   132.9   125.7
Gv/N      4.174   2.186   1.042   5.371   0.080   4.090   4.499   3.338   9.173   8.678
Prob    0.00000 0.00000 0.00174 0.00000 0.76309 0.00000 0.00000 0.00000 0.00000 0.00000
        -------------------------------------------------------------------------------

CDS_INITIAL:
        -------------------------------------------------------------------------------
              1       2       3       4       5       6       7       8       9      10
```

a_O	977	0	0	473	311	205	355	381	196	429
	(67.4)	(0.0)	(0.0)	(32.6)	(21.5)	(14.1)	(24.5)	(26.3)	(13.5)	(29.6)
a_E	260.3	344.5	77.9	260.3	344.5	77.9	260.3	344.5	77.9	260.3
	(18.0)	(23.8)	(5.4)	(18.0)	(23.8)	(5.4)	(18.0)	(23.8)	(5.4)	(18.0)
	2584.1	0.0	0.0	564.8	-63.7	396.5	220.2	76.7	361.5	428.5
	178.338	0.000	0.000	38.982	-4.396	27.361	15.195	5.290	24.945	29.574
t_O	147	1449	0	258	340	213	166	286	214	187
	(10.1)	(100.0)	(0.0)	(17.8)	(23.5)	(14.7)	(11.5)	(19.7)	(14.8)	(12.9)
t_E	182.8	389.4	110.3	182.8	389.4	110.3	182.8	389.4	110.3	182.8
	(12.6)	(26.9)	(7.6)	(12.6)	(26.9)	(7.6)	(12.6)	(26.9)	(7.6)	(12.6)
	-64.1	3807.8	0.0	177.7	-92.3	280.4	-32.1	-176.6	283.8	8.4
	-4.426	262.785	0.000	12.264	-6.371	19.354	-2.213	-12.187	19.583	0.581
g_O	325	0	1449	210	298	480	525	258	467	416
	(22.4)	(0.0)	(100.0)	(14.5)	(20.6)	(33.1)	(36.2)	(17.8)	(32.2)	(28.7)
g_E	578.3	303.4	614.9	578.3	303.4	614.9	578.3	303.4	614.9	578.3
	(39.9)	(20.9)	(42.4)	(39.9)	(20.9)	(42.4)	(39.9)	(20.9)	(42.4)	(39.9)
	-374.6	0.0	2484.2	-425.5	-10.8	-237.7	-101.6	-83.7	-256.9	-274.1
	-25.854	0.000	171.439	-29.364	-0.744	-16.407	-7.013	-5.776	-17.733	-18.919
c_O	0	0	0	508	500	551	403	524	572	417
	(0.0)	(0.0)	(0.0)	(35.1)	(34.5)	(38.0)	(27.8)	(36.2)	(39.5)	(28.8)
c_E	427.5	411.6	645.9	427.5	411.6	645.9	427.5	411.6	645.9	427.5
	(29.5)	(28.4)	(44.6)	(29.5)	(28.4)	(44.6)	(29.5)	(28.4)	(44.6)	(29.5)
	0.0	0.0	0.0	175.4	194.6	-175.1	-47.5	253.1	-139.0	-20.7
	0.000	0.000	0.000	12.102	13.429	-12.084	-3.279	17.464	-9.592	-1.428
N	1449	1449	1449	1449	1449	1449	1449	1449	1449	1449
X2	2518.2	3942.4	1965.6	454.4	28.6	346.3	42.3	68.8	320.4	155.2
Gvalue	2145.4	3807.8	2484.2	492.4	27.8	264.0	39.0	69.4	249.3	142.1
Gv/N	148.058	262.785	171.439	33.983	1.918	18.223	2.690	4.791	17.203	9.809
Prob	1.00000	1.00000	1.00000	0.00000	0.00000	0.00000	0.00000	0.00000	0.00000	0.00000

	11	12	13	14	15	16	17	18	19	20
a_O	314	196	298	276	156	324	381	173	343	371
	(21.7)	(13.5)	(20.6)	(19.0)	(10.8)	(22.4)	(26.3)	(11.9)	(23.7)	(25.6)
a_E	344.5	77.9	260.3	344.5	77.9	260.3	344.5	77.9	260.3	344.5
	(23.8)	(5.4)	(18.0)	(23.8)	(5.4)	(18.0)	(23.8)	(5.4)	(18.0)	(23.8)
	-58.3	361.5	80.5	-122.4	216.5	141.7	76.7	275.8	189.1	54.9
	-4.022	24.945	5.556	-8.449	14.939	9.782	5.290	19.037	13.054	3.790
t_O	387	297	199	332	217	209	363	200	193	327
	(26.7)	(20.5)	(13.7)	(22.9)	(15.0)	(14.4)	(25.1)	(13.8)	(13.3)	(22.6)
t_E	389.4	110.3	182.8	389.4	110.3	182.8	389.4	110.3	182.8	389.4
	(26.9)	(7.6)	(12.6)	(26.9)	(7.6)	(12.6)	(26.9)	(7.6)	(12.6)	(26.9)
	-4.9	588.5	33.7	-106.0	293.8	55.9	-51.0	238.1	20.9	-114.3
	-0.335	40.614	2.327	-7.312	20.275	3.858	-3.522	16.434	1.441	-7.887
g_O	250	405	376	342	462	491	253	487	439	239
	(17.3)	(28.0)	(25.9)	(23.6)	(31.9)	(33.9)	(17.5)	(33.6)	(30.3)	(16.5)
g_E	303.4	614.9	578.3	303.4	614.9	578.3	303.4	614.9	578.3	303.4
	(20.9)	(42.4)	(39.9)	(20.9)	(42.4)	(39.9)	(20.9)	(42.4)	(39.9)	(20.9)
	-96.9	-338.2	-323.8	81.8	-264.1	-160.8	-92.0	-227.1	-242.0	-114.1
	-6.684	-23.341	-22.347	5.648	-18.229	-11.096	-6.348	-15.673	-16.704	-7.875
c_O	498	551	576	499	614	425	452	589	474	512
	(34.4)	(38.0)	(39.8)	(34.4)	(42.4)	(29.3)	(31.2)	(40.6)	(32.7)	(35.3)
c_E	411.6	645.9	427.5	411.6	645.9	427.5	411.6	645.9	427.5	411.6
	(28.4)	(44.6)	(29.5)	(28.4)	(44.6)	(29.5)	(28.4)	(44.6)	(29.5)	(28.4)
	189.8	-175.1	343.5	192.2	-62.2	-4.9	84.7	-108.6	97.9	223.5
	13.099	-12.084	23.709	13.264	-4.291	-0.340	5.843	-7.496	6.760	15.427
N	1449	1449	1449	1449	1449	1449	1449	1449	1449	1449
X2	30.3	580.5	129.3	45.6	221.0	32.5	18.0	220.5	65.4	50.2
Gvalue	29.8	436.6	134.0	45.6	183.9	31.9	18.3	178.3	65.9	50.1
Gv/N	2.057	30.134	9.246	3.150	12.693	2.204	1.263	12.302	4.550	3.455
Prob	0.00000	0.00000	0.00000	0.00000	0.00000	0.00000	0.00038	0.00000	0.00000	0.00000

	21	22	23	24	25	26	27	28	29	30
a_O	169	290	274	135	321	395	110	313	288	121
	(11.7)	(20.0)	(18.9)	(9.3)	(22.2)	(27.3)	(7.6)	(21.6)	(19.9)	(8.4)
a_E	77.9	260.3	344.5	77.9	260.3	344.5	77.9	260.3	344.5	77.9
	(5.4)	(18.0)	(23.8)	(5.4)	(18.0)	(23.8)	(5.4)	(18.0)	(23.8)	(5.4)
	261.6	62.6	-125.5	148.3	134.5	108.0	75.8	115.3	-103.2	106.4
	18.051	4.318	-8.663	10.234	9.279	7.452	5.230	7.958	-7.125	7.344
t_O	202	181	388	175	189	313	212	183	399	179
	(13.9)	(12.5)	(26.8)	(12.1)	(13.0)	(21.6)	(14.6)	(12.6)	(27.5)	(12.4)
t_E	110.3	182.8	389.4	110.3	182.8	389.4	110.3	182.8	389.4	110.3
	(7.6)	(12.6)	(26.9)	(7.6)	(12.6)	(26.9)	(7.6)	(12.6)	(26.9)	(7.6)
	244.5	-3.7	-2.9	161.6	12.5	-136.8	277.1	0.3	19.4	173.4
	16.876	-0.252	-0.198	11.155	0.865	-9.440	19.125	0.023	1.336	11.968
g_O	472	528	279	455	442	291	483	462	303	438
	(32.6)	(36.4)	(19.3)	(31.4)	(30.5)	(20.1)	(33.3)	(31.9)	(20.9)	(30.2)
g_E	614.9	578.3	303.4	614.9	578.3	303.4	614.9	578.3	303.4	614.9
	(42.4)	(39.9)	(20.9)	(42.4)	(39.9)	(20.9)	(42.4)	(39.9)	(20.9)	(42.4)
	-249.6	-96.2	-46.8	-274.0	-237.7	-24.4	-233.2	-207.5	-0.9	-297.2
	-17.229	-6.638	-3.233	-18.912	-16.403	-1.681	-16.095	-14.323	-0.060	-20.507
c_O	606	450	508	684	497	450	644	491	459	711
	(41.8)	(31.1)	(35.1)	(47.2)	(34.3)	(31.1)	(44.4)	(33.9)	(31.7)	(49.1)
c_E	645.9	427.5	411.6	645.9	427.5	411.6	645.9	427.5	411.6	645.9
	(44.6)	(29.5)	(28.4)	(44.6)	(29.5)	(28.4)	(44.6)	(29.5)	(28.4)	(44.6)
	-77.3	46.2	213.8	78.4	149.8	80.3	-3.8	136.1	100.1	136.6
	-5.332	3.190	14.756	5.412	10.338	5.542	-0.261	9.390	6.907	9.425
N	1449	1449	1449	1449	1449	1449	1449	1449	1449	1449
X2	218.3	9.0	39.0	123.6	57.8	26.5	135.3	43.5	15.0	124.1
Gvalue	179.2	9.0	38.6	114.3	59.1	27.1	115.9	44.2	15.3	119.3
Gv/N	12.366	0.618	2.662	7.889	4.079	1.873	8.000	3.047	1.058	8.230
Prob	0.00000	0.02985	0.00000	0.00000	0.00000	0.00001	0.00000	0.00000	0.00156	0.00000

	31	32	33	34	35	36	37	38	39	40
a_O	202	284	167	220	325	115	216	275	109	253
	(13.9)	(19.6)	(11.5)	(15.2)	(22.4)	(7.9)	(14.9)	(19.0)	(7.5)	(17.5)
a_E	260.3	344.5	77.9	260.3	344.5	77.9	260.3	344.5	77.9	260.3
	(18.0)	(23.8)	(5.4)	(18.0)	(23.8)	(5.4)	(18.0)	(23.8)	(5.4)	(18.0)
	-102.5	-109.8	254.5	-74.1	-37.9	89.4	-80.7	-124.0	73.1	-14.5
	-7.075	-7.574	17.563	-5.113	-2.618	6.173	-5.567	-8.557	5.045	-0.999
t_O	291	348	147	177	391	139	193	322	133	159
	(20.1)	(24.0)	(10.1)	(12.2)	(27.0)	(9.6)	(13.3)	(22.2)	(9.2)	(11.0)
t_E	182.8	389.4	110.3	182.8	389.4	110.3	182.8	389.4	110.3	182.8
	(12.6)	(26.9)	(7.6)	(12.6)	(26.9)	(7.6)	(12.6)	(26.9)	(7.6)	(12.6)
	270.5	-78.3	84.5	-11.5	3.1	64.4	20.9	-122.5	49.8	-44.4
	18.667	-5.404	5.832	-0.792	0.216	4.441	1.441	-8.451	3.439	-3.065

g_O	552	262	434	585	284	546	551	397	499	531
	(38.1)	(18.1)	(30.0)	(40.4)	(19.6)	(37.7)	(38.0)	(27.4)	(34.4)	(36.6)
g_E	578.3	303.4	614.9	578.3	303.4	614.9	578.3	303.4	614.9	578.3
	(39.9)	(20.9)	(42.4)	(39.9)	(20.9)	(42.4)	(39.9)	(20.9)	(42.4)	(39.9)
	-51.5	-76.9	-302.4	13.4	-37.6	-129.7	-53.4	213.4	-208.4	-90.7
	-3.553	-5.310	-20.870	0.923	-2.595	-8.954	-3.684	14.727	-14.383	-6.260
c_O	404	555	701	467	449	649	489	455	708	506
	(27.9)	(38.3)	(48.4)	(32.2)	(31.0)	(44.8)	(33.7)	(31.4)	(48.9)	(34.9)
c_E	427.5	411.6	645.9	427.5	411.6	645.9	427.5	411.6	645.9	427.5
	(29.5)	(28.4)	(44.6)	(29.5)	(28.4)	(44.6)	(29.5)	(28.4)	(44.6)	(29.5)
	-45.6	331.8	114.8	82.6	78.1	6.2	131.5	91.2	130.0	170.7
	-3.149	22.900	7.922	5.701	5.391	0.430	9.076	6.297	8.972	11.779

N	1449	1449	1449	1449	1449	1449	1449	1449	1449	1449
X2	79.6	70.7	171.9	10.2	5.8	32.8	18.3	59.1	44.9	21.6
Gvalue	70.9	66.8	151.4	10.4	5.7	30.3	18.4	58.2	44.5	21.1
Gv/N	4.890	4.612	10.448	0.719	0.394	2.090	1.266	4.017	3.073	1.454
Prob	0.00000	0.00000	0.00000	0.01535	0.12663	0.00000	0.00037	0.00000	0.00000	0.00010

	41	42	43	44	45	46	47	48	49	50
a_O	230	118	219	248	117	245	262	97	207	254
	(15.9)	(8.1)	(15.1)	(17.1)	(8.1)	(16.9)	(18.1)	(6.7)	(14.3)	(17.5)
a_E	344.5	77.9	260.3	344.5	77.9	260.3	344.5	77.9	260.2	344.3
	(23.8)	(5.4)	(18.0)	(23.8)	(5.4)	(18.0)	(23.8)	(5.4)	(18.0)	(23.8)
	-185.9	97.9	-75.7	-163.1	95.0	-29.8	-143.5	42.4	-94.6	-154.5
	-12.829	6.753	-5.227	-11.254	6.559	-2.054	-9.903	2.928	-6.536	-10.671
t_O	405	134	188	388	149	267	342	145	179	370
	(28.0)	(9.2)	(13.0)	(26.8)	(10.3)	(18.4)	(23.6)	(10.0)	(12.4)	(25.6)
t_E	389.4	110.3	182.8	389.4	110.3	182.8	389.4	110.3	182.7	389.2
	(26.9)	(7.6)	(12.6)	(26.9)	(7.6)	(12.6)	(26.9)	(7.6)	(12.6)	(26.9)
	31.7	52.2	10.5	-2.9	89.7	202.2	-88.8	79.4	-7.3	-37.4
	2.190	3.604	0.723	-0.198	6.190	13.955	-6.132	5.479	-0.507	-2.581
g_O	372	492	626	281	511	501	391	505	526	377
	(25.7)	(34.0)	(43.2)	(19.4)	(35.3)	(34.6)	(27.0)	(34.9)	(36.3)	(26.0)
g_E	303.4	614.9	578.3	303.4	614.9	578.3	303.4	614.9	577.9	303.2
	(20.9)	(42.4)	(39.9)	(20.9)	(42.4)	(39.9)	(20.9)	(42.4)	(39.9)	(20.9)
	151.6	-219.4	99.1	-43.2	-189.1	-143.9	198.3	-198.8	-99.1	164.2
	10.460	-15.141	6.841	-2.979	-13.053	-9.928	13.683	-13.723	-6.843	11.339
c_O	442	705	416	532	672	436	454	702	536	447
	(30.5)	(48.7)	(28.7)	(36.7)	(46.4)	(30.1)	(31.3)	(48.4)	(37.0)	(30.9)
c_E	411.6	645.9	427.5	411.6	645.9	427.5	411.6	645.9	427.2	411.3
	(28.4)	(44.6)	(29.5)	(28.4)	(44.6)	(29.5)	(28.4)	(44.6)	(29.5)	(28.4)
	63.0	123.5	-22.6	273.0	53.3	17.2	89.0	117.0	243.3	74.4
	4.349	8.521	-1.562	18.843	3.676	1.189	6.145	8.072	16.801	5.138

N	1449	1449	1449	1449	1449	1449	1449	1449	1448	1448
X2	56.4	55.7	10.9	63.9	51.8	50.2	55.2	40.1	43.3	45.7
Gvalue	60.4	54.2	11.2	63.9	48.8	45.8	55.0	39.9	42.2	46.7
Gv/N	4.170	3.737	0.774	4.412	3.371	3.161	3.793	2.755	2.915	3.225
Prob	0.00000	0.00000	0.01058	0.00000	0.00000	0.00000	0.00000	0.00000	0.00000	0.00000

	51	52	53	54	55	56	57	58	59	60
a_O	116	216	308	111	200	302	146	233	262	98
	(8.0)	(14.9)	(21.3)	(7.7)	(13.8)	(20.9)	(10.1)	(16.1)	(18.1)	(6.8)
a_E	77.9	260.2	344.3	77.9	260.2	344.3	77.9	260.2	344.3	77.9
	(5.4)	(18.0)	(23.8)	(5.4)	(18.0)	(23.8)	(5.4)	(18.0)	(23.8)	(5.4)
	92.4	-80.4	-68.6	78.6	-105.2	-79.2	183.4	-51.4	-143.1	45.0
	6.381	-5.550	-4.739	5.430	-7.265	-5.468	12.669	-3.549	-9.885	3.108
t_O	150	188	434	213	177	425	134	169	358	120
	(10.4)	(13.0)	(30.0)	(14.7)	(12.2)	(29.4)	(9.3)	(11.7)	(24.7)	(8.3)
t_E	110.2	182.7	389.2	110.2	182.7	389.2	110.2	182.7	389.2	110.2
	(7.6)	(12.6)	(26.9)	(7.6)	(12.6)	(26.9)	(7.6)	(12.6)	(26.9)	(7.6)
	92.5	10.7	94.6	280.7	-11.2	74.9	52.4	-26.4	-59.8	20.4
	6.388	0.741	6.536	19.388	-0.776	5.170	3.619	-1.821	-4.128	1.412
g_O	563	533	305	532	653	305	449	620	376	599
	(38.9)	(36.8)	(21.1)	(36.7)	(45.1)	(21.1)	(31.0)	(42.8)	(26.0)	(41.4)
g_E	614.5	577.9	303.2	614.5	577.9	303.2	614.5	577.9	303.2	614.5
	(42.4)	(39.9)	(20.9)	(42.4)	(39.9)	(20.9)	(42.4)	(39.9)	(20.9)	(42.4)
	-98.5	-86.3	3.6	-153.3	159.5	3.6	-281.7	87.1	161.8	-30.5
	-6.801	-5.961	0.246	-10.589	11.012	0.246	-19.456	6.014	11.171	-2.108
c_O	619	511	401	592	418	416	719	426	452	631
	(42.7)	(35.3)	(27.7)	(40.9)	(28.9)	(28.7)	(49.7)	(29.4)	(31.2)	(43.6)
c_E	645.4	427.2	411.3	645.4	427.2	411.3	645.4	427.2	411.3	645.4
	(44.6)	(29.5)	(28.4)	(44.6)	(29.5)	(28.4)	(44.6)	(29.5)	(28.4)	(44.6)
	-51.8	183.1	-20.4	-102.3	-18.2	9.4	155.2	-2.4	85.3	-28.6
	-3.577	12.646	-1.406	-7.068	-1.254	0.652	10.718	-0.162	5.890	-1.973

N	1448	1448	1448	1448	1448	1448	1448	1448	1448	1448
X2	38.4	27.6	9.3	125.5	24.0	8.6	117.6	6.9	43.7	6.8
Gvalue	34.6	27.2	9.2	103.7	24.9	8.7	109.3	7.0	44.1	6.4
Gv/N	2.391	1.876	0.636	7.161	1.717	0.600	7.550	0.483	3.048	0.439
Prob	0.00000	0.00001	0.02655	0.00000	0.00002	0.03372	0.00000	0.07231	0.00000	0.09544

	61	62	63	64	65	66	67	68	69	70
a_O	218	244	100	201	238	106	268	276	94	284
	(15.1)	(16.9)	(6.9)	(13.9)	(16.4)	(7.3)	(18.5)	(19.1)	(6.5)	(19.6)
a_E	260.2	344.3	77.9	260.2	344.3	77.9	260.2	344.3	77.9	260.2
	(18.0)	(23.8)	(5.4)	(18.0)	(23.8)	(5.4)	(18.0)	(23.8)	(5.4)	(18.0)
	-77.1	-168.0	50.0	-103.7	-175.8	65.3	15.9	-122.1	35.3	49.8
	-5.324	-11.605	3.451	-7.163	-12.138	4.511	1.098	-8.429	2.440	3.438
t_O	171	455	215	253	341	206	173	336	148	198
	(11.8)	(31.4)	(14.8)	(17.5)	(23.5)	(14.2)	(11.9)	(23.2)	(10.2)	(13.7)
t_E	182.7	389.2	110.2	182.7	389.2	110.2	182.7	389.2	110.2	182.7
	(12.6)	(26.9)	(7.6)	(12.6)	(26.9)	(7.6)	(12.6)	(26.9)	(7.6)	(12.6)
	-22.6	142.2	287.4	164.7	-90.1	257.7	-18.9	-98.7	87.3	31.8
	-1.564	9.822	19.847	11.374	-6.223	17.800	-1.305	-6.818	6.029	2.198
g_O	539	319	550	529	310	547	512	415	525	531
	(37.2)	(22.0)	(38.0)	(36.5)	(21.4)	(37.8)	(35.4)	(28.7)	(36.3)	(36.7)
g_E	577.9	303.2	614.5	577.9	303.2	614.5	577.9	303.2	614.5	577.9
	(39.9)	(20.9)	(42.4)	(39.9)	(20.9)	(42.4)	(39.9)	(20.9)	(42.4)	(39.9)
	-75.2	32.4	-121.9	-93.6	13.7	-127.2	-124.1	260.5	-165.2	-90.0
	-5.194	2.234	-8.419	-6.466	0.946	-8.786	-8.568	17.987	-11.410	-6.214
c_O	520	430	583	465	559	589	495	421	681	435
	(35.9)	(29.7)	(40.3)	(32.1)	(38.6)	(40.7)	(34.2)	(29.1)	(47.0)	(30.0)
c_E	427.2	411.3	645.4	427.2	411.3	645.4	427.2	411.3	645.4	427.2
	(29.5)	(28.4)	(44.6)	(29.5)	(28.4)	(44.6)	(29.5)	(28.4)	(44.6)	(29.5)
	204.5	38.2	-118.6	78.9	343.0	-107.8	145.9	19.6	73.0	15.8
	14.123	2.640	-8.194	5.449	23.689	-7.445	10.075	1.355	5.044	1.090

N	1448	1448	1448	1448	1448	1448	1448	1448	1448	1448
X2	30.4	42.0	118.7	48.0	92.0	105.8	19.0	62.2	31.3	7.4
Gvalue	29.5	44.8	96.8	46.3	90.8	88.0	18.8	59.3	30.4	7.4
Gv/N	2.040	3.091	6.685	3.194	6.274	6.079	1.300	4.095	2.103	0.513
Prob	0.00000	0.00000	0.00000	0.00000	0.00000	0.00000	0.00030	0.00000	0.00000	0.05940

	71	72	73	74	75	76	77	78	79	80
a_O	291	111	220	301	107	240	260	113	236	362
	(20.1)	(7.7)	(15.2)	(20.8)	(7.4)	(16.6)	(18.0)	(7.8)	(16.3)	(25.0)
a_E	344.3	77.9	260.2	344.3	77.9	260.2	344.3	77.9	260.2	344.3
	(23.8)	(5.4)	(18.0)	(23.8)	(5.4)	(18.0)	(23.8)	(5.4)	(18.0)	(23.8)
	-97.9	78.6	-73.8	-80.9	67.9	-38.7	-146.0	84.1	-46.0	36.3
	-6.760	5.430	-5.096	-5.587	4.692	-2.674	-10.085	5.807	-3.178	2.507
t_O	363	137	166	446	111	157	463	144	165	374
	(25.1)	(9.5)	(11.5)	(30.8)	(7.7)	(10.8)	(32.0)	(9.9)	(11.4)	(25.8)
t_E	389.2	110.2	182.7	389.2	110.2	182.7	389.2	110.2	182.7	389.2
	(26.9)	(7.6)	(12.6)	(26.9)	(7.6)	(12.6)	(26.9)	(7.6)	(12.6)	(26.9)
	-50.5	59.6	-31.8	121.6	1.6	-47.6	160.9	77.0	-33.6	-29.7
	-3.490	4.119	-2.199	8.397	0.111	-3.288	11.109	5.321	-2.323	-2.054
g_O	368	491	612	302	600	497	332	587	524	287
	(25.4)	(33.9)	(42.3)	(20.9)	(41.4)	(34.3)	(22.9)	(40.5)	(36.2)	(19.8)
g_E	303.2	614.5	577.9	303.2	614.5	577.9	303.2	614.5	577.9	303.2
	(20.9)	(42.4)	(39.9)	(20.9)	(42.4)	(39.9)	(20.9)	(42.4)	(39.9)	(20.9)
	142.5	-220.3	70.1	-2.4	-28.6	-150.0	60.2	-53.7	-102.7	-31.6
	9.841	-15.211	4.839	-0.169	-1.974	-10.358	4.157	-3.707	-7.092	-2.180
c_O	426	709	450	399	630	554	393	604	523	425
	(29.4)	(49.0)	(31.1)	(27.6)	(43.5)	(38.3)	(27.1)	(41.7)	(36.1)	(29.4)
c_E	411.3	645.4	427.2	411.3	645.4	427.2	411.3	645.4	427.2	411.3
	(28.4)	(44.6)	(29.5)	(28.4)	(44.6)	(29.5)	(28.4)	(44.6)	(29.5)	(28.4)
	29.9	133.2	46.8	-24.2	-30.5	288.0	-35.8	-80.2	211.7	27.8
	2.065	9.197	3.235	-1.674	-2.108	19.892	-2.471	-5.537	14.620	1.923

N	1448	1448	1448	1448	1448	1448	1448	1448	1448	1448
X2	24.4	51.7	11.0	14.1	11.6	54.2	38.2	30.1	30.5	2.8
Gvalue	24.0	51.2	11.3	14.0	10.4	51.7	39.2	27.3	29.3	2.8
Gv/N	1.656	3.535	0.780	0.966	0.722	3.571	2.710	1.884	2.026	0.195
Prob	0.00003	0.00000	0.01026	0.00292	0.01511	0.00000	0.00000	0.00001	0.00000	0.41871

	81	82	83	84	85	86	87	88	89	90
a_O	120	207	371	128	211	384	102	251	293	113
	(8.3)	(14.3)	(25.6)	(8.8)	(14.6)	(26.5)	(7.0)	(17.3)	(20.2)	(7.8)
a_E	77.9	260.2	344.3	77.9	260.2	344.3	77.9	260.2	344.3	77.9
	(5.4)	(18.0)	(23.8)	(5.4)	(18.0)	(23.8)	(5.4)	(18.0)	(23.8)	(5.4)
	103.7	-94.6	55.4	127.1	-88.4	83.8	55.0	-18.0	-94.5	84.1
	7.163	-6.536	3.828	8.781	-6.104	5.788	3.799	-1.243	-6.529	5.807
t_O	196	258	326	117	158	302	115	185	345	122
	(13.5)	(17.8)	(22.5)	(8.1)	(10.9)	(20.9)	(7.9)	(12.8)	(23.8)	(8.4)
t_E	110.2	182.7	389.2	110.2	182.7	389.2	110.2	182.7	389.2	110.2
	(7.6)	(12.6)	(26.9)	(7.6)	(12.6)	(26.9)	(7.6)	(12.6)	(26.9)	(7.6)
	225.7	178.1	-115.5	14.0	-45.9	-153.2	9.8	4.6	-83.1	24.8
	15.588	12.297	-7.975	0.968	-3.171	-10.578	0.677	0.319	-5.741	1.714
g_O	535	525	334	524	635	310	537	612	295	593
	(36.9)	(36.3)	(23.1)	(36.2)	(43.9)	(21.4)	(37.1)	(42.3)	(20.4)	(41.0)
g_E	614.5	577.9	303.2	614.5	577.9	303.2	614.5	577.9	303.2	614.5
	(42.4)	(39.9)	(20.9)	(42.4)	(39.9)	(20.9)	(42.4)	(39.9)	(20.9)	(42.4)
	-148.2	-100.9	64.6	-166.9	119.6	13.7	-144.7	70.1	-16.2	-42.2
	-10.233	-6.968	4.459	-11.526	8.257	0.946	-9.994	4.839	-1.121	-2.912
c_O	597	458	417	679	444	452	694	400	515	620
	(41.2)	(31.6)	(28.8)	(46.9)	(30.7)	(31.2)	(47.9)	(27.6)	(35.6)	(42.8)
c_E	645.4	427.2	411.3	645.4	427.2	411.3	645.4	427.2	411.3	645.4
	(44.6)	(29.5)	(28.4)	(44.6)	(29.5)	(28.4)	(44.6)	(29.5)	(28.4)	(44.6)
	-93.2	63.8	11.5	68.8	34.3	85.3	100.7	-52.6	231.6	-49.9
	-6.434	4.407	0.792	4.753	2.369	5.890	6.953	-3.632	15.993	-3.444

N	1448	1448	1448	1448	1448	1448	1448	1448	1448	1448
X2	103.5	49.0	15.5	47.7	18.9	28.3	21.1	4.1	39.0	18.8
Gvalue	88.1	46.3	16.0	43.1	19.6	29.6	20.8	4.1	37.7	16.9
Gv/N	6.084	3.200	1.103	2.976	1.350	2.046	1.434	0.282	2.602	1.165
Prob	0.00000	0.00000	0.00115	0.00000	0.00021	0.00000	0.00012	0.25190	0.00000	0.00075

	91	92	93	94	95	96	97	98	99	100
a_O	302	392	106	243	274	88	235	339	106	219
	(20.9)	(27.1)	(7.3)	(16.8)	(19.0)	(6.1)	(16.3)	(23.5)	(7.3)	(15.2)
a_E	260.2	344.3	77.9	259.4	343.3	77.7	259.4	343.3	77.7	259.4
	(18.0)	(23.8)	(5.4)	(18.0)	(23.8)	(5.4)	(18.0)	(23.8)	(5.4)	(18.0)
	90.1	101.7	65.3	-31.8	-123.6	22.0	-46.5	-8.6	65.9	-74.2
	6.220	7.025	4.511	-2.204	-8.562	1.520	-3.221	-0.598	4.564	-5.141
t_O	182	323	152	186	361	132	154	347	112	170
	(12.6)	(22.3)	(10.5)	(12.9)	(25.0)	(9.1)	(10.7)	(24.0)	(7.8)	(11.8)
t_E	182.7	389.2	110.2	182.2	388.1	109.9	182.2	388.1	109.9	182.2
	(12.6)	(26.9)	(7.6)	(12.6)	(26.9)	(7.6)	(12.6)	(26.9)	(7.6)	(12.6)
	-1.4	-120.4	97.8	7.7	-52.3	48.4	-51.8	-77.7	4.2	-23.6
	-0.098	-8.314	6.751	0.531	-3.618	3.351	-3.587	-5.379	0.294	-1.632
g_O	586	301	617	546	279	640	641	307	546	555
	(40.5)	(20.8)	(42.6)	(37.8)	(19.3)	(44.3)	(44.4)	(21.3)	(37.8)	(38.4)
g_E	577.9	303.2	614.5	576.4	302.4	612.8	576.4	302.4	612.8	576.4
	(39.9)	(20.9)	(42.4)	(39.9)	(20.9)	(42.4)	(39.9)	(20.9)	(42.4)	(39.9)
	16.2	-4.4	5.1	-59.1	-44.9	55.7	136.3	9.3	-126.0	-41.9
	1.120	-0.306	0.352	-4.091	-3.111	3.855	9.438	0.643	-8.724	-2.902
c_O	378	432	573	469	530	584	414	451	680	500
	(26.1)	(29.8)	(39.6)	(32.5)	(36.7)	(40.4)	(28.7)	(31.2)	(47.1)	(34.6)
c_E	427.2	411.3	645.4	426.0	410.2	643.7	426.0	410.2	643.7	426.0
	(29.5)	(28.4)	(44.6)	(29.5)	(28.4)	(44.6)	(29.5)	(28.4)	(44.6)	(29.5)
	-92.5	42.4	-136.4	90.2	271.7	-113.6	-23.7	85.6	74.7	160.2
	-6.385	2.929	-9.422	6.247	18.815	-7.868	-1.638	5.928	5.172	11.093

N	1448	1448	1448	1444	1444	1444	1444	1444	1444	1444
X2	12.5	18.9	34.1	7.1	52.7	12.6	14.3	8.5	19.7	20.8
Gvalue	12.4	19.3	31.7	7.0	50.9	12.4	14.3	8.6	18.9	20.5
Gv/N	0.856	1.334	2.191	0.483	3.523	0.858	0.992	0.594	1.307	1.417
Prob	0.00613	0.00024	0.00000	0.07276	0.00000	0.00615	0.00249	0.03552	0.00029	0.00014

5UTR:

	100	99	98	97	96	95	94	93	92	91
a_O	100	110	92	101	89	94	103	94	94	97
	(25.1)	(27.6)	(23.1)	(25.3)	(22.3)	(23.6)	(25.8)	(23.6)	(23.6)	(24.3)
a_E	99.0	99.0	99.0	99.0	99.0	99.0	99.0	99.0	99.0	99.0
	(24.8)	(24.8)	(24.8)	(24.8)	(24.8)	(24.8)	(24.8)	(24.8)	(24.8)	(24.8)
	1.9	23.1	−13.6	4.0	−19.0	−9.8	8.1	−9.8	−9.8	−4.0
	0.484	5.787	−3.400	0.992	−4.768	−2.461	2.024	−2.461	−2.461	−1.012
t_O	89	83	88	97	98	91	108	106	100	102
	(22.3)	(20.8)	(22.1)	(24.3)	(24.6)	(22.8)	(27.1)	(26.6)	(25.1)	(25.6)
t_E	97.8	97.8	97.8	97.8	97.8	97.8	97.8	97.8	97.8	97.8
	(24.5)	(24.5)	(24.5)	(24.5)	(24.5)	(24.5)	(24.5)	(24.5)	(24.5)	(24.5)
	−16.7	−27.2	−18.5	−1.5	0.5	−13.0	21.5	17.2	4.6	8.7
	−4.184	−6.805	−4.635	−0.374	0.125	−3.264	5.398	4.305	1.141	2.176
g_O	117	109	115	92	105	100	95	106	111	103
	(29.3)	(27.3)	(28.8)	(23.1)	(26.3)	(25.1)	(23.8)	(26.6)	(27.8)	(25.8)
g_E	98.9	98.9	98.9	98.9	98.9	98.9	98.9	98.9	98.9	98.9
	(24.8)	(24.8)	(24.8)	(24.8)	(24.8)	(24.8)	(24.8)	(24.8)	(24.8)	(24.8)
	39.4	21.3	34.8	−13.2	12.7	2.3	−7.6	14.8	25.7	8.5
	9.880	5.335	8.717	−3.316	3.171	0.575	−1.897	3.705	6.444	2.118
c_O	93	97	104	109	107	114	93	93	94	97
	(23.3)	(24.3)	(26.1)	(27.3)	(26.8)	(28.6)	(23.3)	(23.3)	(23.6)	(24.3)
c_E	103.3	103.3	103.3	103.3	103.3	103.3	103.3	103.3	103.3	103.3
	(25.9)	(25.9)	(25.9)	(25.9)	(25.9)	(25.9)	(25.9)	(25.9)	(25.9)	(25.9)
	−19.6	−12.3	1.3	11.6	7.4	22.4	−19.6	−19.6	−17.8	−12.3
	−4.919	−3.083	0.327	2.908	1.862	5.604	−4.919	−4.919	−4.468	−3.083
N	399	399	399	399	399	399	399	399	399	399
X2	5.2	4.9	4.1	0.8	1.5	1.8	2.4	2.5	2.6	0.8
Gvalue	5.0	4.9	4.0	0.8	1.6	1.8	2.4	2.5	2.6	0.8
Gv/N	1.261	1.234	1.009	0.209	0.390	0.454	0.607	0.630	0.656	0.199
Prob	0.16949	0.17760	0.25871	0.84105	0.66946	0.61214	0.48973	0.47257	0.45430	0.85093

	90	89	88	87	86	85	84	83	82	81
a_O	101	95	82	95	91	112	85	79	93	100
	(25.3)	(23.8)	(20.6)	(23.8)	(22.8)	(28.1)	(21.3)	(19.8)	(23.3)	(25.1)
a_E	99.0	99.0	99.0	99.0	99.0	99.0	99.0	99.0	99.0	99.0
	(24.8)	(24.8)	(24.8)	(24.8)	(24.8)	(24.8)	(24.8)	(24.8)	(24.8)	(24.8)
	4.0	−7.9	−31.0	−7.9	−15.4	27.5	−26.0	−35.7	−11.7	1.9
	0.992	−1.983	−7.760	−1.983	−3.862	6.904	−6.513	−8.952	−2.933	0.484
t_O	93	95	102	95	89	85	112	129	88	102
	(23.3)	(23.8)	(25.6)	(23.8)	(22.3)	(21.3)	(28.1)	(32.3)	(22.1)	(25.6)
t_E	97.8	97.8	97.8	97.8	97.8	97.8	97.8	97.8	97.8	97.8
	(24.5)	(24.5)	(24.5)	(24.5)	(24.5)	(24.5)	(24.5)	(24.5)	(24.5)	(24.5)
	−9.3	−5.4	8.7	−5.4	−16.7	−23.8	30.5	71.6	−18.5	8.7
	−2.322	−1.359	2.176	−1.359	−4.184	−5.955	7.640	17.937	−4.635	2.176
g_O	103	105	110	110	118	94	94	101	117	101
	(25.8)	(26.3)	(27.6)	(27.6)	(29.6)	(23.6)	(23.6)	(25.3)	(29.3)	(25.3)
g_E	98.9	98.9	98.9	98.9	98.9	98.9	98.9	98.9	98.9	98.9
	(24.8)	(24.8)	(24.8)	(24.8)	(24.8)	(24.8)	(24.8)	(24.8)	(24.8)	(24.8)
	8.5	12.7	23.5	23.5	41.8	−9.5	−9.5	4.3	39.4	4.3
	2.118	3.171	5.887	5.887	10.468	−2.375	−2.375	1.084	9.880	1.084
c_O	102	104	105	99	101	108	108	90	101	96
	(25.6)	(26.1)	(26.3)	(24.8)	(25.3)	(27.1)	(27.1)	(22.6)	(25.3)	(24.1)
c_E	103.3	103.3	103.3	103.3	103.3	103.3	103.3	103.3	103.3	103.3
	(25.9)	(25.9)	(25.9)	(25.9)	(25.9)	(25.9)	(25.9)	(25.9)	(25.9)	(25.9)
	−2.7	1.3	3.3	−8.5	−4.6	9.5	9.5	−24.9	−4.6	−14.2
	−0.672	0.327	0.834	−2.134	−1.164	2.382	2.382	−6.240	−1.164	−3.550
N	399	399	399	399	399	399	399	399	399	399
X2	0.5	0.6	4.4	1.7	5.2	3.8	4.5	15.8	4.7	0.8
Gvalue	0.5	0.6	4.5	1.6	5.0	3.8	4.5	15.3	4.6	0.8
Gv/N	0.116	0.156	1.137	0.411	1.258	0.956	1.134	3.829	1.147	0.194
Prob	0.92718	0.89116	0.20918	0.65001	0.17034	0.28203	0.21016	0.00159	0.20541	0.85591

	80	79	78	77	76	75	74	73	72	71
a_O	105	96	100	96	98	103	85	96	108	94
	(26.3)	(24.1)	(25.1)	(24.1)	(24.6)	(25.8)	(21.3)	(24.1)	(27.1)	(23.6)
a_E	99.0	99.0	99.0	99.0	99.0	99.0	99.0	99.0	99.0	99.0
	(24.8)	(24.8)	(24.8)	(24.8)	(24.8)	(24.8)	(24.8)	(24.8)	(24.8)	(24.8)
	12.3	−6.0	1.9	−6.0	−2.1	8.1	−26.0	−6.0	18.7	−9.8
	3.076	−1.500	0.484	−1.500	−0.519	2.024	−6.513	−1.500	4.689	−2.461
t_O	80	93	107	95	86	87	83	105	93	115
	(20.1)	(23.3)	(26.8)	(23.8)	(21.6)	(21.8)	(20.8)	(26.3)	(23.3)	(28.8)
t_E	97.8	97.8	97.8	97.8	97.8	97.8	97.8	97.8	97.8	97.8
	(24.5)	(24.5)	(24.5)	(24.5)	(24.5)	(24.5)	(24.5)	(24.5)	(24.5)	(24.5)
	−32.1	−9.3	19.3	−5.4	−22.0	−20.3	−27.2	15.0	−9.3	37.4
	−8.036	−2.322	4.849	−1.359	−5.521	−5.081	−6.805	3.766	−2.322	9.368
g_O	103	105	94	109	100	112	123	102	102	86
	(25.8)	(26.3)	(23.6)	(27.3)	(25.1)	(28.1)	(30.8)	(25.6)	(25.6)	(21.6)
g_E	98.9	98.9	98.9	98.9	98.9	98.9	98.9	98.9	98.9	98.9
	(24.8)	(24.8)	(24.8)	(24.8)	(24.8)	(24.8)	(24.8)	(24.8)	(24.8)	(24.8)
	8.5	12.7	−9.5	21.3	2.3	28.0	53.7	6.4	6.4	−24.0
	2.118	3.171	−2.375	5.335	0.575	7.006	13.470	1.599	1.599	−6.007
c_O	111	105	98	99	115	97	108	96	96	104
	(27.8)	(26.3)	(24.6)	(24.8)	(28.8)	(24.3)	(27.1)	(24.1)	(24.1)	(26.1)
c_E	103.3	103.3	103.3	103.3	103.3	103.3	103.3	103.3	103.3	103.3
	(25.9)	(25.9)	(25.9)	(25.9)	(25.9)	(25.9)	(25.9)	(25.9)	(25.9)	(25.9)
	15.9	3.3	−10.4	−8.5	24.6	−12.3	9.5	−14.2	−14.2	1.3
	3.973	0.834	−2.611	−2.134	6.157	−3.083	2.382	−3.550	−3.550	0.327
N	399	399	399	399	399	399	399	399	399	399
X2	4.3	0.7	1.4	1.4	2.7	3.5	10.3	1.3	1.7	5.0
Gvalue	4.5	0.7	1.4	1.4	2.8	3.5	10.1	1.3	1.7	4.9
Gv/N	1.131	0.183	0.347	0.342	0.693	0.866	2.534	0.314	0.415	1.227
Prob	0.21108	0.86641	0.70952	0.71392	0.42959	0.32642	0.01765	0.74022	0.64673	0.17966

	70	69	68	67	66	65	64	63	62	61
a_O	93	94	94	88	99	109	104	104	93	94
	(23.3)	(23.6)	(23.6)	(22.1)	(24.8)	(27.3)	(26.1)	(26.1)	(23.3)	(23.6)
a_E	99.0	99.0	99.0	99.0	99.0	99.0	99.0	99.0	99.0	99.0
	(24.8)	(24.8)	(24.8)	(24.8)	(24.8)	(24.8)	(24.8)	(24.8)	(24.8)	(24.8)
	−11.7	−9.8	−9.8	−20.8	−0.1	20.9	10.2	10.2	−11.7	−9.8
	−2.933	−2.461	−2.461	−5.213	−0.020	5.236	2.547	2.547	−2.933	−2.461
t_O	114	100	90	89	101	84	103	92	91	85
	(28.6)	(25.1)	(22.6)	(22.3)	(25.3)	(21.1)	(25.8)	(23.1)	(22.8)	(21.3)
t_E	97.8	97.8	97.8	97.8	97.8	97.8	97.8	97.8	97.8	97.8

```
        ( 24.5) ( 24.5) ( 24.5) ( 24.5) ( 24.5) ( 24.5) ( 24.5) ( 24.5) ( 24.5) ( 24.5)
           35.1     4.6   -14.9   -16.7     6.6   -25.5    10.8   -11.2   -13.0   -23.8
          8.788   1.141  -3.726  -4.184   1.656  -6.383   2.701  -2.796  -3.264  -5.955
g_O          95      93     110     102      97      97     104     107     121     101
        ( 23.8) ( 23.3) ( 27.6) ( 25.6) ( 24.3) ( 24.3) ( 26.1) ( 26.8) ( 30.3) ( 25.3)
g_E        98.9    98.9    98.9    98.9    98.9    98.9    98.9    98.9    98.9    98.9
        ( 24.8) ( 24.8) ( 24.8) ( 24.8) ( 24.8) ( 24.8) ( 24.8) ( 24.8) ( 24.8) ( 24.8)
           -7.6   -11.4    23.5     6.4    -3.7    -3.7    10.5    16.9    48.9     4.3
         -1.897  -2.849   5.887   1.599  -0.924  -0.924   2.642   4.244  12.257   1.084
c_O          97     112     105     120     102     109      88      96      94     119
        ( 24.3) ( 28.1) ( 26.3) ( 30.1) ( 25.6) ( 27.3) ( 22.1) ( 24.1) ( 23.6) ( 29.8)
c_E       103.3   103.3   103.3   103.3   103.3   103.3   103.3   103.3   103.3   103.3
        ( 25.9) ( 25.9) ( 25.9) ( 25.9) ( 25.9) ( 25.9) ( 25.9) ( 25.9) ( 25.9) ( 25.9)
          -12.3    18.0     3.3    35.8    -2.7    11.6   -28.3   -14.2   -17.8    33.6
         -3.083   4.512   0.834   8.985  -0.672   2.908  -7.092  -3.550  -4.468   8.411

N           399     399     399     399     399     399     399     399     399     399
X2          3.6     1.4     2.2     4.8     0.2     3.3     3.1     1.8     6.6     4.3
Gvalue      3.5     1.4     2.1     4.7     0.2     3.3     3.2     1.8     6.4     4.3
Gv/N      0.875   0.344   0.534   1.187   0.040   0.837   0.799   0.446   1.592   1.079
Prob    0.32203 0.71226 0.54604 0.19231 0.98380 0.34210 0.36382 0.61983 0.09574 0.23027
        --------------------------------------------------------------------------------
             60      59      58      57      56      55      54      53      52      51
        --------------------------------------------------------------------------------
a_O          98      91      97      90      88     127      95      93     114      84
        ( 24.6) ( 22.8) ( 24.3) ( 22.6) ( 22.1) ( 31.8) ( 23.8) ( 23.3) ( 28.6) ( 21.1)
a_E        99.0    99.0    99.0    99.0    99.0    99.0    99.0    99.0    99.0    99.0
        ( 24.8) ( 24.8) ( 24.8) ( 24.8) ( 24.8) ( 24.8) ( 24.8) ( 24.8) ( 24.8) ( 24.8)
           -2.1   -15.4    -4.0   -17.2   -20.8    63.2    -7.9   -11.7    32.1   -27.7
         -0.519  -3.862  -1.012  -4.318  -5.213  15.830  -1.983  -2.933   8.039  -6.935
t_O          90     110      97      95      86      79     111      96      93     110
        ( 22.6) ( 27.6) ( 24.3) ( 23.8) ( 21.6) ( 19.8) ( 27.8) ( 24.1) ( 23.3) ( 27.6)
t_E        97.8    97.8    97.8    97.8    97.8    97.8    97.8    97.8    97.8    97.8
        ( 24.5) ( 24.5) ( 24.5) ( 24.5) ( 24.5) ( 24.5) ( 24.5) ( 24.5) ( 24.5) ( 24.5)
          -14.9    26.0    -1.5    -5.4   -22.0   -33.6    28.2    -3.5    -9.3    26.0
         -3.726   6.510  -0.374  -1.359  -5.521  -8.433   7.073  -0.869  -2.322   6.510
g_O          91      93     113     112     101      94      98      96      99     102
        ( 22.8) ( 23.3) ( 28.3) ( 28.1) ( 25.3) ( 23.6) ( 24.6) ( 24.1) ( 24.8) ( 25.6)
g_E        98.9    98.9    98.9    98.9    98.9    98.9    98.9    98.9    98.9    98.9
        ( 24.8) ( 24.8) ( 24.8) ( 24.8) ( 24.8) ( 24.8) ( 24.8) ( 24.8) ( 24.8) ( 24.8)
          -15.1   -11.4    30.2    28.0     4.3    -9.5    -1.7    -5.6     0.3     6.4
         -3.779  -2.849   7.572   7.006   1.084  -2.375  -0.429  -1.413   0.070   1.599
c_O         120     105      92     102     124      99      95     114      93     103
        ( 30.1) ( 26.3) ( 23.1) ( 25.6) ( 31.1) ( 24.8) ( 23.8) ( 28.6) ( 23.3) ( 25.8)
c_E       103.3   103.3   103.3   103.3   103.3   103.3   103.3   103.3   103.3   103.3
        ( 25.9) ( 25.9) ( 25.9) ( 25.9) ( 25.9) ( 25.9) ( 25.9) ( 25.9) ( 25.9) ( 25.9)
           35.8     3.3   -21.4    -2.7    45.2    -8.5   -16.0    22.4   -19.6    -0.7
          8.985   0.834  -5.365  -0.672  11.322  -2.134  -4.012   5.604  -4.919  -0.175

N           399     399     399     399     399     399     399     399     399     399
X2          3.9     2.6     3.3     2.7     6.8    11.9     2.6     1.6     3.5     3.9
Gvalue      3.8     2.5     3.3     2.6     6.7    11.5     2.6     1.6     3.5     4.0
Gv/N      0.961   0.633   0.821   0.657   1.673   2.887   0.649   0.389   0.868   0.998
Prob    0.28002 0.47052 0.35110 0.45379 0.08307 0.00922 0.45957 0.67015 0.32577 0.26321
        --------------------------------------------------------------------------------
             50      49      48      47      46      45      44      43      42      41
        --------------------------------------------------------------------------------
a_O         103     105      96      96     104     114      97     101      82      80
        ( 25.8) ( 26.3) ( 24.1) ( 24.1) ( 26.1) ( 28.6) ( 24.3) ( 25.3) ( 20.6) ( 20.1)
a_E        99.0    99.0    99.0    99.0    99.0    99.0    99.0    99.0    99.0    99.0
        ( 24.8) ( 24.8) ( 24.8) ( 24.8) ( 24.8) ( 24.8) ( 24.8) ( 24.8) ( 24.8) ( 24.8)
            8.1    12.3    -6.0    -6.0    10.2    32.1    -4.0     4.0   -31.0   -34.2
          2.024   3.076  -1.500  -1.500   2.547   8.039  -1.012   0.992  -7.760  -8.561
t_O          86      84     105      96      89      99     103      92      99     111
        ( 21.6) ( 21.1) ( 26.3) ( 24.1) ( 22.3) ( 24.8) ( 25.8) ( 23.1) ( 24.8) ( 27.8)
t_E        97.8    97.8    97.8    97.8    97.8    97.8    97.8    97.8    97.8    97.8
        ( 24.5) ( 24.5) ( 24.5) ( 24.5) ( 24.5) ( 24.5) ( 24.5) ( 24.5) ( 24.5) ( 24.5)
          -22.0   -25.5    15.0    -3.5   -16.7     2.5    10.8   -11.2     2.5    28.2
         -5.521  -6.383   3.766  -0.869  -4.184   0.631   2.701  -2.796   0.631   7.073
g_O         113     101     102     105     101     110     115      95     111     110
        ( 28.3) ( 25.3) ( 25.6) ( 26.3) ( 25.3) ( 27.6) ( 28.8) ( 23.8) ( 27.8) ( 27.6)
g_E        98.9    98.9    98.9    98.9    98.9    98.9    98.9    98.9    98.9    98.9
        ( 24.8) ( 24.8) ( 24.8) ( 24.8) ( 24.8) ( 24.8) ( 24.8) ( 24.8) ( 24.8) ( 24.8)
           30.2     4.3     6.4    12.7     4.3    23.5    34.8    -7.6    25.7    23.5
          7.572   1.084   1.599   3.171   1.084   5.887   8.717  -1.897   6.444   5.887
c_O          97     109      96     102     105      76      84     111     107      98
        ( 24.3) ( 27.3) ( 24.1) ( 25.6) ( 26.3) ( 19.0) ( 21.1) ( 27.8) ( 26.8) ( 24.6)
c_E       103.3   103.3   103.3   103.3   103.3   103.3   103.3   103.3   103.3   103.3
        ( 25.9) ( 25.9) ( 25.9) ( 25.9) ( 25.9) ( 25.9) ( 25.9) ( 25.9) ( 25.9) ( 25.9)
          -12.3    11.6   -14.2    -2.7     3.3   -46.7   -34.8    15.9     7.4   -10.4
         -3.083   2.908  -3.550  -0.672   0.834 -11.710  -8.729   3.973   1.862  -2.611

N           399     399     399     399     399     399     399     399     399     399
X2          4.0     2.6     1.3     0.5     1.1    10.8     6.6     1.1     4.6     7.0
Gvalue      4.0     2.7     1.3     0.5     1.1    11.4     6.7     1.1     4.7     7.1
Gv/N      0.992   0.685   0.314   0.130   0.282   2.846   1.678   0.273   1.176   1.788
Prob    0.26585 0.43467 0.74022 0.91510 0.77122 0.00994 0.08230 0.77971 0.19574 0.06778
        --------------------------------------------------------------------------------
             40      39      38      37      36      35      34      33      32      31
        --------------------------------------------------------------------------------
a_O         109     106     105     102      84      84      95      75      95     111
        ( 27.3) ( 26.6) ( 26.3) ( 25.6) ( 21.1) ( 21.1) ( 23.8) ( 18.8) ( 23.8) ( 27.8)
a_E        99.0    99.0    99.0    99.0    99.0    99.0    99.0    99.0    99.0    99.0
        ( 24.8) ( 24.8) ( 24.8) ( 24.8) ( 24.8) ( 24.8) ( 24.8) ( 24.8) ( 24.8) ( 24.8)
           20.9    14.4    12.3     6.0   -27.7   -27.7    -7.9   -41.7    -7.9    25.3
          5.236   3.609   3.076   1.506  -6.935  -6.935  -1.983 -10.452  -1.983   6.343
t_O          98     105      95     118     109     127     117     110      84      84
        ( 24.6) ( 26.3) ( 23.8) ( 29.6) ( 27.3) ( 31.8) ( 29.3) ( 27.6) ( 21.1) ( 21.1)
t_E        97.8    97.8    97.8    97.8    97.8    97.8    97.8    97.8    97.8    97.8
        ( 24.5) ( 24.5) ( 24.5) ( 24.5) ( 24.5) ( 24.5) ( 24.5) ( 24.5) ( 24.5) ( 24.5)
            0.5    15.0    -5.4    44.4    23.7    66.5    42.1    26.0   -25.5   -25.5
          0.125   3.766  -1.359  11.136   5.952  16.664  10.542   6.510  -6.383  -6.383
g_O          98      90     102      93     105      85      99     122     127      99
        ( 24.6) ( 22.6) ( 25.6) ( 23.3) ( 26.3) ( 21.3) ( 24.8) ( 30.6) ( 31.8) ( 24.8)
g_E        98.9    98.9    98.9    98.9    98.9    98.9    98.9    98.9    98.9    98.9
        ( 24.8) ( 24.8) ( 24.8) ( 24.8) ( 24.8) ( 24.8) ( 24.8) ( 24.8) ( 24.8) ( 24.8)
           -1.7   -16.9     6.4   -11.4    12.7   -25.7     0.3    51.3    63.6     0.3
         -0.429  -4.236   1.599  -2.849   3.171  -6.436   0.070  12.861  15.945   0.070
c_O          94      98      97      86     101     103      88      92      93     105
        ( 23.6) ( 24.6) ( 24.3) ( 21.6) ( 25.3) ( 25.8) ( 22.1) ( 23.1) ( 23.3) ( 26.3)
c_E       103.3   103.3   103.3   103.3   103.3   103.3   103.3   103.3   103.3   103.3
        ( 25.9) ( 25.9) ( 25.9) ( 25.9) ( 25.9) ( 25.9) ( 25.9) ( 25.9) ( 25.9) ( 25.9)
```

	-17.8	-10.4	-12.3	-31.6	-4.6	-0.7	-28.3	-21.4	-19.6	3.3
	-4.468	-2.611	-3.083	-7.922	-1.164	-0.175	-7.092	-5.365	-4.919	0.834
N	399	399	399	399	399	399	399	399	399	399
X2	1.9	2.1	0.9	7.5	4.0	13.0	6.2	14.0	11.1	3.4
Gvalue	1.9	2.1	0.9	7.5	4.1	12.4	6.1	14.2	10.6	3.4
Gv/N	0.464	0.527	0.232	1.871	1.024	3.118	1.537	3.554	2.660	0.864
Prob	0.60403	0.55117	0.81894	0.05847	0.25248	0.00601	0.10531	0.00267	0.01400	0.32762

	30	29	28	27	26	25	24	23	22	21
a_O	102	100	111	100	111	100	107	94	103	85
	(25.6)	(25.1)	(27.8)	(25.1)	(27.8)	(25.1)	(26.8)	(23.6)	(25.8)	(21.3)
a_E	99.0	99.0	99.0	99.0	99.0	99.0	99.0	99.0	99.0	99.0
	(24.8)	(24.8)	(24.8)	(24.8)	(24.8)	(24.8)	(24.8)	(24.8)	(24.8)	(24.8)
	6.0	1.9	25.3	1.9	25.3	1.9	16.5	-9.8	8.1	-26.0
	1.506	0.484	6.343	0.484	6.343	0.484	4.146	-2.461	2.024	-6.513
t_O	91	81	102	108	81	73	95	90	113	109
	(22.8)	(20.3)	(25.6)	(27.1)	(20.3)	(18.3)	(23.8)	(22.6)	(28.3)	(27.3)
t_E	97.8	97.8	97.8	97.8	97.8	97.8	97.8	97.8	97.8	97.8
	(24.5)	(24.5)	(24.5)	(24.5)	(24.5)	(24.5)	(24.5)	(24.5)	(24.5)	(24.5)
	-13.0	-30.5	8.7	21.5	-30.5	-42.6	-5.4	-14.9	32.8	23.7
	-3.264	-7.632	2.176	5.398	-7.632	-10.683	-1.359	-3.726	8.212	5.952
g_O	105	113	86	92	102	114	100	108	92	106
	(26.3)	(28.3)	(21.6)	(23.1)	(25.6)	(28.6)	(25.1)	(27.1)	(23.1)	(26.6)
g_E	98.9	98.9	98.9	98.9	98.9	98.9	98.9	98.9	98.9	98.9
	(24.8)	(24.8)	(24.8)	(24.8)	(24.8)	(24.8)	(24.8)	(24.8)	(24.8)	(24.8)
	12.7	30.2	-24.0	-13.2	6.4	32.5	2.3	19.1	-13.2	14.8
	3.171	7.572	-6.007	-3.316	1.599	8.142	0.575	4.787	-3.316	3.705
c_O	101	105	100	99	105	112	97	107	91	99
	(25.3)	(26.3)	(25.1)	(24.8)	(26.3)	(28.1)	(24.3)	(26.8)	(22.8)	(24.8)
c_E	103.3	103.3	103.3	103.3	103.3	103.3	103.3	103.3	103.3	103.3
	(25.9)	(25.9)	(25.9)	(25.9)	(25.9)	(25.9)	(25.9)	(25.9)	(25.9)	(25.9)
	-4.6	3.3	-6.6	-8.5	3.3	18.0	-12.3	7.4	-23.2	-8.5
	-1.164	0.834	-1.652	-2.134	0.834	4.512	-3.083	1.862	-5.805	-2.134
N	399	399	399	399	399	399	399	399	399	399
X2	1.0	4.9	3.4	1.7	4.4	9.3	1.1	1.8	4.5	4.0
Gvalue	1.0	5.0	3.4	1.7	4.6	9.8	1.1	1.8	4.4	4.0
Gv/N	0.249	1.258	0.860	0.431	1.144	2.455	0.279	0.461	1.114	1.010
Prob	0.80310	0.17051	0.32970	0.63216	0.20664	0.02037	0.77397	0.60630	0.21713	0.25837

	20	19	18	17	16	15	14	13	12	11
a_O	106	121	93	97	120	112	106	95	97	122
	(26.6)	(30.3)	(23.3)	(24.3)	(30.1)	(28.1)	(26.6)	(23.8)	(24.3)	(30.6)
a_E	99.0	99.0	99.0	99.0	99.0	99.0	99.0	99.0	99.0	99.0
	(24.8)	(24.8)	(24.8)	(24.8)	(24.8)	(24.8)	(24.8)	(24.8)	(24.8)	(24.8)
	14.4	48.5	-11.7	-4.0	46.1	27.5	14.4	-7.9	-4.0	50.9
	3.609	12.147	-2.933	-1.012	11.547	6.904	3.609	-1.983	-1.012	12.750
t_O	100	105	111	95	98	100	100	111	114	112
	(25.1)	(26.3)	(27.8)	(23.8)	(24.6)	(25.1)	(25.1)	(27.8)	(28.6)	(28.1)
t_E	97.8	97.8	97.8	97.8	97.8	97.8	97.8	97.8	97.8	97.8
	(24.5)	(24.5)	(24.5)	(24.5)	(24.5)	(24.5)	(24.5)	(24.5)	(24.5)	(24.5)
	4.6	15.0	28.2	-5.4	0.5	4.6	4.6	28.2	35.1	30.5
	1.141	3.766	7.073	-1.359	0.125	1.141	1.141	7.073	8.788	7.640
g_O	91	55	85	81	66	75	87	52	88	69
	(22.8)	(13.8)	(21.3)	(20.3)	(16.5)	(18.8)	(21.8)	(13.0)	(22.1)	(17.3)
g_E	98.9	98.9	98.9	98.9	98.9	98.9	98.9	98.9	98.9	98.9
	(24.8)	(24.8)	(24.8)	(24.8)	(24.8)	(24.8)	(24.8)	(24.8)	(24.8)	(24.8)
	-15.1	-64.5	-25.7	-32.3	-53.3	-41.4	-22.2	-66.8	-20.5	-49.6
	-3.779	-16.166	-6.436	-8.090	-13.367	-10.384	-5.573	-16.746	-5.133	-12.437
c_O	102	118	110	126	115	112	106	141	100	96
	(25.6)	(29.6)	(27.6)	(31.6)	(28.8)	(28.1)	(26.6)	(35.3)	(25.1)	(24.1)
c_E	103.3	103.3	103.3	103.3	103.3	103.3	103.3	103.3	103.3	103.3
	(25.9)	(25.9)	(25.9)	(25.9)	(25.9)	(25.9)	(25.9)	(25.9)	(25.9)	(25.9)
	-2.7	31.3	13.7	49.9	24.6	18.0	5.4	87.6	-6.6	-14.2
	-0.672	7.841	3.438	12.515	6.157	4.512	1.345	21.955	-1.652	-3.550
N	399	399	399	399	399	399	399	399	399	399
X2	1.2	26.9	4.5	8.3	16.7	8.2	2.0	37.9	4.0	16.9
Gvalue	1.2	30.3	4.6	8.2	17.8	8.7	2.1	41.1	4.0	17.6
Gv/N	0.298	7.587	1.142	2.055	4.462	2.173	0.521	10.299	0.991	4.403
Prob	0.75557	0.00000	0.20735	0.04210	0.00048	0.03402	0.55593	0.00000	0.26643	0.00054

	10	9	8	7	6	5	4	3	2	1
a_O	120	130	105	102	105	106	91	97	91	94
	(30.1)	(32.6)	(26.3)	(25.6)	(26.3)	(26.6)	(22.8)	(24.3)	(22.8)	(23.6)
a_E	99.0	99.0	99.0	99.0	99.0	99.0	99.0	99.0	99.0	99.0
	(24.8)	(24.8)	(24.8)	(24.8)	(24.8)	(24.8)	(24.8)	(24.8)	(24.8)	(24.8)
	46.1	70.7	12.3	6.0	12.3	14.4	-15.4	-4.0	-15.4	-9.8
	11.547	17.725	3.076	1.506	3.076	3.609	-3.862	-1.012	-3.862	-2.461
t_O	90	101	98	118	106	99	95	83	91	82
	(22.6)	(25.3)	(24.6)	(29.6)	(26.6)	(24.8)	(23.8)	(20.8)	(22.8)	(20.6)
t_E	97.8	97.8	97.8	97.8	97.8	97.8	97.8	97.8	97.8	97.8
	(24.5)	(24.5)	(24.5)	(24.5)	(24.5)	(24.5)	(24.5)	(24.5)	(24.5)	(24.5)
	-14.9	6.6	0.5	44.4	17.2	2.5	-5.4	-27.2	-13.0	-28.8
	-3.726	1.656	0.125	11.136	4.305	0.631	-1.359	-6.805	-3.264	-7.222
g_O	80	70	94	58	74	83	106	118	103	82
	(20.1)	(17.5)	(23.6)	(14.5)	(18.5)	(20.8)	(26.6)	(29.6)	(25.8)	(20.6)
g_E	98.9	98.9	98.9	98.9	98.9	98.9	98.9	98.9	98.9	98.9
	(24.8)	(24.8)	(24.8)	(24.8)	(24.8)	(24.8)	(24.8)	(24.8)	(24.8)	(24.8)
	-33.9	-48.3	-9.5	-61.9	-42.9	-29.0	14.8	41.8	8.5	-30.7
	-8.488	-12.113	-2.375	-15.503	-10.744	-7.275	3.705	10.468	2.118	-7.686
c_O	109	98	102	121	114	111	107	101	114	141
	(27.3)	(24.6)	(25.6)	(30.3)	(28.6)	(27.8)	(26.8)	(25.3)	(28.6)	(35.3)
c_E	103.3	103.3	103.3	103.3	103.3	103.3	103.3	103.3	103.3	103.3
	(25.9)	(25.9)	(25.9)	(25.9)	(25.9)	(25.9)	(25.9)	(25.9)	(25.9)	(25.9)
	11.6	-10.4	-2.7	38.2	22.4	15.9	7.4	-4.6	22.4	87.6
	2.908	-2.611	-0.672	9.563	5.604	3.973	1.862	-1.164	5.604	21.955
N	399	399	399	399	399	399	399	399	399	399
X2	9.0	18.5	0.6	24.2	8.4	3.6	1.4	6.0	2.4	19.4
Gvalue	8.9	18.6	0.6	26.7	8.9	3.7	1.4	5.9	2.4	18.3
Gv/N	2.240	4.657	0.154	6.701	2.242	0.937	0.346	1.486	0.597	4.587
Prob	0.03012	0.00033	0.89346	0.00001	0.03004	0.29103	0.71016	0.11505	0.49720	0.00038

CDS_INITIAL:

	1	2	3	4	5	6	7	8	9	10

```
          ------------------------------------------------------------------------------------------
a_O           262        0        0      141       97       97      117      122       98      104
          ( 65.7) (  0.0) (  0.0) ( 35.3) ( 24.3) ( 24.3) ( 29.3) ( 30.6) ( 24.6) ( 26.1)
a_E          91.1    103.1     51.5     91.1    103.1     51.5     91.1    103.1     51.5     91.1
          ( 22.8) ( 25.9) ( 12.9) ( 22.8) ( 25.9) ( 12.9) ( 22.8) ( 25.9) ( 12.9) ( 22.8)
            553.7      0.0      0.0    123.2    -11.9    122.9     58.6     41.0    126.2     27.6
          138.761    0.000    0.000   30.886   -2.987   30.800   14.686   10.267   31.621    6.915
t_O            77      399        0       48       96       89       62       99       90       68
          ( 19.3) (100.0) (  0.0) ( 12.0) ( 24.1) ( 22.3) ( 15.5) ( 24.8) ( 22.6) ( 17.0)
t_E          55.8    116.1     84.2     55.8    116.1     84.2     55.8    116.1     84.2     55.8
          ( 14.0) ( 29.1) ( 21.1) ( 14.0) ( 29.1) ( 21.1) ( 14.0) ( 29.1) ( 21.1) ( 14.0)
             49.6    985.3      0.0    -14.4    -36.5     10.0     13.1    -31.5     12.1     26.9
           12.432  246.941    0.000   -3.621   -9.138    2.497    3.276   -7.897    3.029    6.742
g_O            60        0      399      109       79       91      109       74      107      110
          ( 15.0) (  0.0) (100.0) ( 27.3) ( 19.8) ( 22.8) ( 27.3) ( 18.5) ( 26.8) ( 27.6)
g_E         147.6     77.8    124.4    147.6     77.8    124.4    147.6     77.8    124.4    147.6
          ( 37.0) ( 19.5) ( 31.2) ( 37.0) ( 19.5) ( 31.2) ( 37.0) ( 19.5) ( 31.2) ( 37.0)
           -108.0      0.0    930.2    -66.1      2.4    -56.9    -66.1     -7.4    -32.2    -64.7
          -27.071    0.000  233.124  -16.561    0.611  -14.254  -16.561   -1.853   -8.073  -16.209
c_O             0        0        0      101      127      122      111      104      104      117
          (  0.0) (  0.0) (  0.0) ( 25.3) ( 31.8) ( 30.6) ( 27.8) ( 26.1) ( 26.1) ( 29.3)
c_E         104.5    102.0    139.0    104.5    102.0    139.0    104.5    102.0    139.0    104.5
          ( 26.2) ( 25.6) ( 34.8) ( 26.2) ( 25.6) ( 34.8) ( 26.2) ( 25.6) ( 34.8) ( 26.2)
              0.0      0.0      0.0     -6.9     55.7    -31.8     13.3      4.1    -60.3     26.4
            0.000    0.000    0.000   -1.739   13.963   -7.970    3.341    1.018  -15.116    6.609

N             399      399      399      399      399      399      399      399      399      399
X2          485.3    972.5    881.0     38.7     10.0     51.6     18.6      6.2     53.7     15.6
Gvalue      495.2    985.3    930.2     35.8      9.8     44.2     18.9      6.1     45.7     16.2
Gv/N      124.122  246.941  233.124    8.965    2.449   11.074    4.744    1.535   11.463    4.057
Prob      0.00000  0.00000  0.00000  0.00000  0.02062  0.00000  0.00028  0.10561  0.00000  0.00104
          ------------------------------------------------------------------------------------------
               11       12       13       14       15       16       17       18       19       20
          ------------------------------------------------------------------------------------------
a_O           106       86      129      103       78      115       98       84      120      120
          ( 26.6) ( 21.6) ( 32.3) ( 25.8) ( 19.5) ( 28.8) ( 24.6) ( 21.1) ( 30.1) ( 30.1)
a_E         103.1     51.5     91.1    103.1     51.5     91.1    103.1     51.5     91.1    103.1
          ( 25.9) ( 12.9) ( 22.8) ( 25.9) ( 12.9) ( 22.8) ( 25.9) ( 12.9) ( 22.8) ( 25.9)
              5.8     88.3     89.8     -0.3     64.8     53.6    -10.0     82.2     66.2     36.3
            1.451   22.119   22.506   -0.073   16.244   13.442   -2.514   20.613   16.586    9.104
t_O           102       93       78      117      112       78      120       87       67       98
          ( 25.6) ( 23.3) ( 19.5) ( 29.3) ( 28.1) ( 19.5) ( 30.1) ( 21.8) ( 16.8) ( 24.6)
t_E         116.1     84.2     55.8    116.1     84.2     55.8    116.1     84.2     55.8    116.1
          ( 29.1) ( 21.1) ( 14.0) ( 29.1) ( 21.1) ( 14.0) ( 29.1) ( 21.1) ( 14.0) ( 29.1)
            -26.4     18.6     52.3      1.9     64.0     52.3      8.0      5.8     24.5    -33.2
           -6.610    4.659   13.098    0.464   16.047   13.098    1.999    1.450    6.145   -8.316
g_O            89      118      106       79       97      108       68       94      107       78
          ( 22.3) ( 29.6) ( 26.6) ( 19.8) ( 24.3) ( 27.1) ( 17.0) ( 23.6) ( 26.8) ( 19.5)
g_E          77.8    124.4    147.6     77.8    124.4    147.6     77.8    124.4    147.6     77.8
          ( 19.5) ( 31.2) ( 37.0) ( 19.5) ( 31.2) ( 37.0) ( 19.5) ( 31.2) ( 37.0) ( 19.5)
             24.0    -12.4    -70.2      2.4    -48.2    -67.5    -18.3    -52.6    -68.8      0.4
            6.006   -3.115  -17.588    0.611  -12.089  -16.908   -4.584  -13.195  -17.250    0.106
c_O           102      102       86      100      112       98      113      134      105      103
          ( 25.6) ( 25.6) ( 21.6) ( 25.1) ( 28.1) ( 24.6) ( 28.3) ( 33.6) ( 26.3) ( 25.8)
c_E         102.0    139.0    104.5    102.0    139.0    104.5    102.0    139.0    104.5    102.0
          ( 25.6) ( 34.8) ( 26.2) ( 25.6) ( 34.8) ( 26.2) ( 25.6) ( 34.8) ( 26.2) ( 25.6)
              0.0    -63.1    -33.6     -3.9    -48.3    -12.6     23.2     -9.8      0.9      2.0
            0.006  -15.818   -8.412   -0.987  -12.118   -3.169    5.808   -2.452    0.236    0.510

N             399      399      399      399      399      399      399      399      399      399
X2            3.4     34.2     39.6      0.1     34.1     26.1      2.8     28.2     22.6      5.6
Gvalue        3.4     31.3     38.3      0.1     32.3     25.8      2.8     25.6     22.8      5.6
Gv/N        0.853    7.845    9.605    0.016    8.084    6.463    0.709    6.416    5.717    1.403
Prob      0.33368  0.00000  0.00000  0.99565  0.00000  0.00001  0.41895  0.00001  0.00004  0.13278
          ------------------------------------------------------------------------------------------
               21       22       23       24       25       26       27       28       29       30
          ------------------------------------------------------------------------------------------
a_O            75      113      124       82       86      101       73      110      102       79
          ( 18.8) ( 28.3) ( 31.1) ( 20.6) ( 21.6) ( 25.3) ( 18.3) ( 27.6) ( 25.6) ( 19.8)
a_E          51.5     91.1    103.1     51.5     91.1    103.1     51.5     91.1    103.1     51.5
          ( 12.9) ( 22.8) ( 25.9) ( 12.9) ( 22.8) ( 25.9) ( 12.9) ( 22.8) ( 25.9) ( 12.9)
             56.4     48.7     45.7     76.3     -9.9     -4.2     51.0     41.5     -2.3     67.7
           14.144   12.214   11.446   19.132   -2.475   -1.064   12.778   10.406   -0.571   16.956
t_O           101       71      105       94       71      102      111       73      115       95
          ( 25.3) ( 17.8) ( 26.3) ( 23.6) ( 17.8) ( 25.6) ( 27.8) ( 18.3) ( 28.8) ( 23.8)
t_E          84.2     55.8    116.1     84.2     55.8    116.1     84.2     55.8    116.1     84.2
          ( 21.1) ( 14.0) ( 29.1) ( 21.1) ( 14.0) ( 29.1) ( 21.1) ( 14.0) ( 29.1) ( 21.1)
             36.9     34.2    -21.1     20.8     34.2    -26.4     61.5     39.2     -2.1     23.0
            9.237    8.576   -5.279    5.213    8.576   -6.610   15.405    9.834   -0.537    5.772
g_O           106      111       69       98      114       91       89      116       80       97
          ( 26.6) ( 27.8) ( 17.3) ( 24.6) ( 28.6) ( 22.8) ( 22.3) ( 29.1) ( 20.1) ( 24.3)
g_E         124.4    147.6     77.8    124.4    147.6     77.8    124.4    147.6     77.8    124.4
          ( 31.2) ( 37.0) ( 19.5) ( 31.2) ( 37.0) ( 19.5) ( 31.2) ( 37.0) ( 19.5) ( 31.2)
            -33.9    -63.3    -16.5    -46.7    -58.9     28.5    -59.6    -55.9      4.5    -48.2
           -8.496  -15.853   -4.147  -11.710  -14.757    7.155  -14.932  -14.005    1.124  -12.089
c_O           117      104      101      125      128      105      126      100      102      128
          ( 29.3) ( 26.1) ( 25.3) ( 31.3) ( 32.1) ( 26.3) ( 31.6) ( 25.1) ( 25.6) ( 32.1)
c_E         139.0    104.5    102.0    139.0    104.5    102.0    139.0    104.5    102.0    139.0
          ( 34.8) ( 26.2) ( 25.6) ( 34.8) ( 26.2) ( 25.6) ( 34.8) ( 26.2) ( 25.6) ( 34.8)
            -40.3     -1.1     -2.0    -26.5     51.9      6.1    -24.7     -8.9      0.0    -21.1
          -10.097   -0.265   -0.493   -6.644   12.996    1.532   -6.194   -2.221    0.006   -5.281

N             399      399      399      399      399      399      399      399      399      399
X2           20.3     18.5      6.3     26.2     17.3      4.1     28.8     16.2      0.1     23.0
Gvalue       19.1     18.6      6.1     23.9     17.3      4.0     28.2     16.0      0.1     21.4
Gv/N        4.788    4.672    1.527    5.992    4.340    1.013    7.058    4.014    0.021    5.358
Prob      0.00026  0.00032  0.10718  0.00003  0.00061  0.25718  0.00000  0.00113  0.99357  0.00009
          ------------------------------------------------------------------------------------------
               31       32       33       34       35       36       37       38       39       40
          ------------------------------------------------------------------------------------------
a_O           119       97       79       96       91       62       82       87       59       90
          ( 29.8) ( 24.3) ( 19.8) ( 24.1) ( 22.8) ( 15.5) ( 20.6) ( 21.8) ( 14.8) ( 22.6)
a_E          91.1    103.1     51.5     91.1    103.1     51.5     91.1    103.1     51.5     91.1
          ( 22.8) ( 25.9) ( 12.9) ( 22.8) ( 25.9) ( 12.9) ( 22.8) ( 25.9) ( 12.9) ( 22.8)
             63.6    -11.9     67.7     10.1    -22.8     23.1    -17.2    -29.6     16.1     -2.1
           15.949   -2.987   16.956    2.531   -5.714    5.777   -4.317   -7.424    4.031   -0.539
t_O            69      125       97       69      124       96       70      126       94       61
          ( 17.3) ( 31.3) ( 24.3) ( 17.3) ( 31.1) ( 24.1) ( 17.5) ( 31.6) ( 23.6) ( 15.3)
t_E          55.8    116.1     84.2     55.8    116.1     84.2     55.8    116.1     84.2     55.8
          ( 14.0) ( 29.1) ( 21.1) ( 14.0) ( 29.1) ( 21.1) ( 14.0) ( 29.1) ( 21.1) ( 14.0)
             29.3     18.5     27.6     29.3     16.4     25.3     31.8     20.7     20.8     10.9
            7.346    4.640    6.907    7.346    4.104    6.337    7.957    5.181    5.213    2.726
```

g_O	117	81	111	131	86	115	144	94	110	137
	(29.3)	(20.3)	(27.8)	(32.8)	(21.6)	(28.8)	(36.1)	(23.6)	(27.6)	(34.3)
g_E	147.6	77.8	124.4	147.6	77.8	124.4	147.6	77.8	124.4	147.6
	(37.0)	(19.5)	(31.2)	(37.0)	(19.5)	(31.2)	(37.0)	(19.5)	(31.2)	(37.0)
	-54.4	6.6	-25.3	-31.2	17.3	-18.0	-7.1	35.6	-27.0	-20.4
	-13.622	1.642	-6.332	-7.831	4.325	-4.520	-1.778	8.919	-6.774	-5.114
c_O	94	96	112	103	98	126	103	92	136	111
	(23.6)	(24.1)	(28.1)	(25.8)	(24.6)	(31.6)	(25.8)	(23.1)	(34.1)	(27.8)
c_E	104.5	102.0	139.0	104.5	102.0	139.0	104.5	102.0	139.0	104.5
	(26.2)	(25.6)	(34.8)	(26.2)	(25.6)	(34.8)	(26.2)	(25.6)	(34.8)	(26.2)
	-20.0	-11.6	-48.3	-3.0	-7.8	-24.7	-3.0	-19.0	-5.9	13.3
	-5.003	-2.912	-12.118	-0.762	-1.959	-6.194	-0.762	-4.753	-1.479	3.341
N	399	399	399	399	399	399	399	399	399	399
X2	19.1	1.5	23.3	5.3	3.0	5.7	4.6	7.7	4.0	1.7
Gvalue	18.6	1.5	21.6	5.1	3.0	5.6	4.4	7.7	4.0	1.7
Gv/N	4.669	0.384	5.413	1.285	0.755	1.400	1.100	1.923	0.991	0.415
Prob	0.00033	0.67492	0.00008	0.16277	0.38944	0.13348	0.22226	0.05329	0.26668	0.64695

	41	42	43	44	45	46	47	48	49	50
a_O	100	62	91	89	60	95	88	78	95	95
	(25.1)	(15.5)	(22.8)	(22.3)	(15.0)	(23.8)	(22.1)	(19.5)	(23.8)	(23.8)
a_E	103.1	51.5	91.1	103.1	51.5	91.1	103.1	51.5	91.1	103.1
	(25.9)	(12.9)	(22.8)	(25.9)	(12.9)	(22.8)	(25.9)	(12.9)	(22.8)	(25.9)
	-6.2	23.1	-0.2	-26.3	18.4	8.0	-27.9	64.8	8.0	-15.6
	-1.552	5.777	-0.041	-6.580	4.604	2.006	-7.005	16.244	2.006	-3.917
t_O	124	98	79	115	99	54	132	105	78	103
	(31.1)	(24.6)	(19.8)	(28.8)	(24.8)	(13.5)	(33.1)	(26.3)	(19.5)	(25.8)
t_E	116.1	84.2	55.8	116.1	84.2	55.8	116.1	84.2	55.8	116.1
	(29.1)	(21.1)	(14.0)	(29.1)	(21.1)	(14.0)	(29.1)	(21.1)	(14.0)	(29.1)
	16.4	29.9	54.9	-2.1	32.2	-3.5	33.9	46.5	52.3	-24.6
	4.104	7.482	13.770	-0.537	8.062	-0.886	8.505	11.647	13.098	-6.171
g_O	67	113	133	92	121	129	91	84	121	90
	(16.8)	(28.3)	(33.3)	(23.1)	(30.3)	(32.3)	(22.8)	(21.1)	(30.3)	(22.6)
g_E	77.8	124.4	147.6	77.8	124.4	147.6	77.8	124.4	147.6	77.8
	(19.5)	(31.2)	(37.0)	(19.5)	(31.2)	(37.0)	(19.5)	(31.2)	(37.0)	(19.5)
	-20.0	-21.7	-27.7	30.9	-6.7	-34.7	28.5	-65.9	-48.1	26.2
	-5.015	-5.435	-6.940	7.737	-1.671	-8.706	7.155	-16.527	-12.049	6.578
c_O	108	126	96	103	119	121	88	132	105	111
	(27.1)	(31.6)	(24.1)	(25.8)	(29.8)	(30.3)	(22.1)	(33.1)	(26.3)	(27.8)
c_E	102.0	139.0	104.5	102.0	139.0	104.5	102.0	139.0	104.5	102.0
	(25.6)	(34.8)	(26.2)	(25.6)	(34.8)	(26.2)	(25.6)	(34.8)	(26.2)	(25.6)
	12.4	-24.7	-16.3	2.0	-36.9	35.4	-26.0	-13.6	0.9	18.8
	3.101	-6.194	-4.097	0.510	-9.259	8.874	-6.507	-3.411	0.236	4.711
N	399	399	399	399	399	399	399	399	399	399
X2	2.5	6.7	11.8	4.6	7.0	5.2	8.6	32.3	13.8	4.8
Gvalue	2.5	6.5	10.7	4.5	6.9	5.1	8.6	31.7	13.1	4.8
Gv/N	0.638	1.630	2.693	1.129	1.736	1.288	2.148	7.953	3.291	1.201
Prob	0.46733	0.08948	0.01319	0.21175	0.07421	0.16181	0.03557	0.00000	0.00437	0.18782

	51	52	53	54	55	56	57	58	59	60
a_O	79	97	100	71	84	90	68	77	81	70
	(19.8)	(24.3)	(25.1)	(17.8)	(21.1)	(22.6)	(17.0)	(19.3)	(20.3)	(17.5)
a_E	51.5	91.1	103.1	51.5	91.1	103.1	51.5	91.1	103.1	51.5
	(12.9)	(22.8)	(25.9)	(12.9)	(22.8)	(25.9)	(12.9)	(22.8)	(25.9)	(12.9)
	67.7	12.2	-6.2	45.6	-13.6	-24.5	37.8	-25.9	-39.2	43.0
	16.956	3.061	-1.552	11.439	-3.408	-6.150	9.485	-6.482	-9.813	10.781
t_O	87	64	105	86	74	113	92	78	100	93
	(21.8)	(16.0)	(26.3)	(21.6)	(18.5)	(28.3)	(23.1)	(19.5)	(25.1)	(23.3)
t_E	84.2	55.8	116.1	84.2	55.8	116.1	84.2	55.8	116.1	84.2
	(21.1)	(14.0)	(29.1)	(21.1)	(14.0)	(29.1)	(21.1)	(14.0)	(29.1)	(21.1)
	5.8	17.6	-21.1	3.7	41.8	-6.1	16.4	52.3	-29.8	18.6
	1.450	4.401	-5.279	0.935	10.473	-1.522	4.110	13.098	-7.473	4.659
g_O	102	132	88	120	142	90	112	145	87	125
	(25.6)	(33.1)	(22.1)	(30.1)	(35.6)	(22.6)	(28.1)	(36.3)	(21.8)	(31.3)
g_E	124.4	147.6	77.8	124.4	147.6	77.8	124.4	147.6	77.8	124.4
	(31.2)	(37.0)	(19.5)	(31.2)	(37.0)	(19.5)	(31.2)	(37.0)	(19.5)	(31.2)
	-40.5	-29.5	21.7	-8.6	-11.0	26.2	-23.5	-5.1	19.5	1.2
	-10.142	-7.387	5.440	-2.156	-2.749	6.578	-5.886	-1.288	4.880	0.312
c_O	131	106	106	122	99	106	127	99	131	111
	(32.8)	(26.6)	(26.6)	(30.6)	(24.8)	(26.6)	(31.8)	(24.8)	(32.8)	(27.8)
c_E	139.0	104.5	102.0	139.0	104.5	102.0	139.0	104.5	102.0	139.0
	(34.8)	(26.2)	(25.6)	(34.8)	(26.2)	(25.6)	(34.8)	(26.2)	(25.6)	(34.8)
	-15.5	3.0	8.2	-31.8	-10.8	8.2	-22.9	-10.8	65.6	-49.9
	-3.884	0.742	2.050	-7.970	-2.698	2.050	-5.740	-2.698	16.439	-12.509
N	399	399	399	399	399	399	399	399	399	399
X2	19.3	3.3	2.7	9.7	7.0	3.8	8.3	11.4	16.3	13.2
Gvalue	17.5	3.3	2.6	9.0	6.5	3.8	7.9	10.5	16.1	12.9
Gv/N	4.380	0.816	0.659	2.248	1.619	0.956	1.969	2.630	4.033	3.242
Prob	0.00056	0.35372	0.45221	0.02969	0.09128	0.28238	0.04904	0.01479	0.00109	0.00477

	61	62	63	64	65	66	67	68	69	70
a_O	83	98	62	94	85	81	101	98	46	108
	(20.8)	(24.6)	(15.5)	(23.6)	(21.3)	(20.3)	(25.3)	(24.6)	(11.5)	(27.1)
a_E	91.1	103.1	51.5	91.1	103.1	51.5	91.1	103.1	51.5	91.1
	(22.8)	(25.9)	(12.9)	(22.8)	(25.9)	(12.9)	(22.8)	(25.9)	(12.9)	(22.8)
	-15.4	-10.0	23.1	5.9	-32.9	73.4	20.9	-10.0	-10.4	36.8
	-3.865	-2.514	5.777	1.486	-8.244	18.401	5.233	-2.514	-2.596	9.224
t_O	63	127	87	67	105	86	73	113	94	64
	(15.8)	(31.8)	(21.8)	(16.8)	(26.3)	(21.6)	(18.3)	(28.3)	(23.6)	(16.0)
t_E	55.8	116.1	84.2	55.8	116.1	84.2	55.8	116.1	84.2	55.8
	(14.0)	(29.1)	(21.1)	(14.0)	(29.1)	(21.1)	(14.0)	(29.1)	(21.1)	(14.0)
	15.3	22.8	5.8	24.5	-21.1	3.7	39.2	-6.1	20.8	17.6
	3.835	5.725	1.450	6.145	-5.279	0.935	9.834	-1.522	5.213	4.401
g_O	135	77	130	137	83	118	104	76	123	135
	(33.8)	(19.3)	(32.6)	(34.3)	(20.8)	(29.6)	(26.1)	(19.0)	(30.8)	(33.8)
g_E	147.6	77.8	124.4	147.6	77.8	124.4	147.6	77.8	124.4	147.6
	(37.0)	(19.5)	(31.2)	(37.0)	(19.5)	(31.2)	(37.0)	(19.5)	(31.2)	(37.0)
	-24.1	-1.6	11.5	-20.4	10.8	-12.4	-72.8	-3.5	-2.7	-24.1
	-6.035	-0.394	2.880	-5.114	2.697	-3.115	-18.249	-0.887	-0.688	-6.035
c_O	118	97	120	101	126	114	121	112	136	92
	(29.6)	(24.3)	(30.1)	(25.3)	(31.6)	(28.6)	(30.3)	(28.1)	(34.1)	(23.1)
c_E	104.5	102.0	139.0	104.5	102.0	139.0	104.5	102.0	139.0	104.5
	(26.2)	(25.6)	(34.8)	(26.2)	(25.6)	(34.8)	(26.2)	(25.6)	(34.8)	(26.2)
	28.6	-9.7	-35.2	-6.9	53.3	-45.2	35.4	21.0	-5.9	-23.5
	7.169	-2.438	-8.833	-1.739	13.353	-11.323	8.874	5.257	-1.479	-5.888

N	399	399	399	399	399	399	399	399	399	399
X2	4.5	1.5	5.1	3.2	10.3	21.8	21.9	1.4	1.8	6.9
Gvalue	4.4	1.5	5.1	3.1	10.1	19.5	22.7	1.3	1.8	6.8
Gv/N	1.104	0.379	1.273	0.778	2.528	4.898	5.693	0.335	0.450	1.701
Prob	0.22107	0.67902	0.16601	0.37575	0.01784	0.00021	0.00005	0.72035	0.61603	0.07898

	71	72	73	74	75	76	77	78	79	80
a_O	99	74	90	86	49	83	98	70	88	102
	(24.8)	(18.5)	(22.6)	(21.6)	(12.3)	(20.8)	(24.6)	(17.5)	(22.1)	(25.6)
a_E	103.1	51.5	91.1	103.1	51.5	91.1	103.1	51.5	91.1	103.1
	(25.9)	(12.9)	(22.8)	(25.9)	(12.9)	(22.8)	(25.9)	(12.9)	(22.8)	(25.9)
	-8.1	53.7	-2.1	-31.3	-4.8	-15.4	-10.0	43.0	-6.1	-2.3
	-2.035	13.458	-0.539	-7.837	-1.214	-3.865	-2.514	10.781	-1.518	-0.571
t_O	127	88	64	98	93	58	111	98	86	111
	(31.8)	(22.1)	(16.0)	(24.6)	(23.3)	(14.5)	(27.8)	(24.6)	(21.6)	(27.8)
t_E	116.1	84.2	55.8	116.1	84.2	55.8	116.1	84.2	55.8	116.1
	(29.1)	(21.1)	(14.0)	(29.1)	(21.1)	(14.0)	(29.1)	(21.1)	(14.0)	(29.1)
	22.8	7.9	17.6	-33.2	18.6	4.5	-9.9	29.9	74.4	-9.9
	5.725	1.971	4.401	-8.316	4.659	1.126	-2.489	7.482	18.650	-2.489
g_O	75	118	133	98	122	153	90	125	128	71
	(18.8)	(29.6)	(33.3)	(24.6)	(30.6)	(38.3)	(22.6)	(31.3)	(32.1)	(17.8)
g_E	77.8	124.4	147.6	77.8	124.4	147.6	77.8	124.4	147.6	77.8
	(19.5)	(31.2)	(37.0)	(19.5)	(31.2)	(37.0)	(19.5)	(31.2)	(37.0)	(19.5)
	-5.5	-12.4	-27.7	45.3	-4.7	11.0	26.2	1.2	-36.5	-13.0
	-1.373	-3.115	-6.940	11.345	-1.182	2.760	6.578	0.312	-9.138	-3.250
c_O	98	119	112	117	135	105	100	106	97	115
	(24.6)	(29.8)	(28.1)	(29.3)	(33.8)	(26.3)	(25.1)	(26.6)	(24.3)	(28.8)
c_E	102.0	139.0	104.5	102.0	139.0	104.5	102.0	139.0	104.5	102.0
	(25.6)	(34.8)	(26.2)	(25.6)	(34.8)	(26.2)	(25.6)	(34.8)	(26.2)	(25.6)
	-7.8	-36.9	15.5	32.1	-7.8	0.9	-3.9	-57.4	-14.5	27.6
	-1.959	-9.259	3.875	8.053	-1.967	0.236	-0.987	-14.394	-3.635	6.922
N	399	399	399	399	399	399	399	399	399	399
X2	1.5	13.2	3.2	13.1	1.2	1.0	2.4	16.8	19.6	2.5
Gvalue	1.4	12.2	3.2	13.0	1.2	1.0	2.3	16.7	17.4	2.4
Gv/N	0.357	3.055	0.797	3.246	0.296	0.256	0.589	4.180	4.359	0.612
Prob	0.69965	0.00676	0.36497	0.00474	0.75745	0.79569	0.50320	0.00082	0.00059	0.48577

	81	82	83	84	85	86	87	88	89	90
a_O	60	83	99	60	97	105	56	90	90	57
	(15.0)	(20.8)	(24.8)	(15.0)	(24.3)	(26.3)	(14.0)	(22.6)	(22.6)	(14.3)
a_E	51.5	91.1	103.1	51.5	91.1	103.1	51.5	91.1	103.1	51.5
	(12.9)	(22.8)	(25.9)	(12.9)	(22.8)	(25.9)	(12.9)	(22.8)	(25.9)	(12.9)
	18.4	-15.4	-8.1	18.4	12.2	3.7	9.4	-2.1	-24.5	11.6
	4.604	-3.865	-2.035	4.604	3.061	0.938	2.361	-0.539	-6.150	2.909
t_O	107	66	118	92	66	104	78	66	119	79
	(26.8)	(16.5)	(29.6)	(23.1)	(16.5)	(26.1)	(19.5)	(16.5)	(29.8)	(19.8)
t_E	84.2	55.8	116.1	84.2	55.8	116.1	84.2	55.8	116.1	84.2
	(21.1)	(14.0)	(29.1)	(21.1)	(14.0)	(29.1)	(21.1)	(14.0)	(29.1)	(21.1)
	51.4	22.2	3.9	16.4	22.2	-22.9	-11.8	22.2	5.9	-10.0
	12.881	5.556	0.972	4.110	5.556	-5.727	-2.969	5.556	1.483	-2.503
g_O	109	139	73	123	146	85	129	122	81	123
	(27.3)	(34.8)	(18.3)	(30.8)	(36.6)	(21.3)	(32.3)	(30.6)	(20.3)	(30.8)
g_E	124.4	147.6	77.8	124.4	147.6	77.8	124.4	147.6	77.8	124.4
	(31.2)	(37.0)	(19.5)	(31.2)	(37.0)	(19.5)	(31.2)	(37.0)	(19.5)	(31.2)
	-28.8	-16.7	-9.3	-2.7	-3.2	15.1	9.4	-46.5	6.6	-2.7
	-7.212	-4.179	-2.325	-0.688	-0.794	3.777	2.358	-11.645	1.642	-0.688
c_O	123	111	109	124	90	105	136	121	109	140
	(30.8)	(27.8)	(27.3)	(31.1)	(22.6)	(26.3)	(34.1)	(30.3)	(27.3)	(35.1)
c_E	139.0	104.5	102.0	139.0	104.5	102.0	139.0	104.5	102.0	139.0
	(34.8)	(26.2)	(25.6)	(34.8)	(26.2)	(25.6)	(34.8)	(26.2)	(25.6)	(34.8)
	-30.1	13.3	14.5	-28.3	-26.9	6.1	-5.9	35.4	14.5	2.0
	-7.532	3.341	3.633	-7.090	-6.752	1.532	-1.479	8.874	3.633	0.512
N	399	399	399	399	399	399	399	399	399	399
X2	11.4	3.5	1.0	3.8	4.3	2.0	1.1	8.9	2.4	0.9
Gvalue	10.9	3.4	1.0	3.7	4.3	2.1	1.1	9.0	2.4	0.9
Gv/N	2.742	0.853	0.244	0.937	1.072	0.519	0.271	2.246	0.608	0.230
Prob	0.01205	0.33351	0.80776	0.29109	0.23317	0.55751	0.78172	0.02980	0.48873	0.82146

	91	92	93	94	95	96	97	98	99	100
a_O	99	99	60	88	93	71	81	101	61	108
	(24.8)	(24.8)	(15.0)	(22.2)	(23.5)	(17.9)	(20.6)	(25.6)	(15.5)	(27.5)
a_E	91.1	103.1	51.5	90.4	102.4	51.1	89.9	101.9	50.8	89.7
	(22.8)	(25.9)	(12.9)	(22.8)	(25.9)	(12.9)	(22.8)	(25.9)	(12.9)	(22.8)
	16.5	-8.1	18.4	-4.7	-17.9	46.7	-17.0	-1.7	22.2	40.1
	4.137	-2.035	4.604	-1.194	-4.509	11.797	-4.305	-0.431	5.643	10.197
t_O	62	111	104	79	108	91	77	112	89	52
	(15.5)	(27.8)	(26.1)	(19.9)	(27.3)	(23.0)	(19.5)	(28.4)	(22.6)	(13.2)
t_E	55.8	116.1	84.2	55.4	115.2	83.5	55.1	114.6	83.1	55.0
	(14.0)	(29.1)	(21.1)	(14.0)	(29.1)	(21.1)	(14.0)	(29.1)	(21.1)	(14.0)
	13.1	-9.9	44.0	56.1	-13.9	15.6	51.5	-5.2	12.2	-5.8
	3.276	-2.489	11.038	14.176	-3.522	3.941	13.082	-1.316	3.099	-1.464
g_O	133	79	112	121	99	106	115	83	121	149
	(33.3)	(19.8)	(28.1)	(30.6)	(25.0)	(26.8)	(29.2)	(21.1)	(30.7)	(37.9)
g_E	147.6	77.8	124.4	146.5	77.2	123.4	145.7	76.8	122.8	145.4
	(37.0)	(19.5)	(31.2)	(37.0)	(19.5)	(31.2)	(37.0)	(19.5)	(31.2)	(37.0)
	-27.7	2.4	-23.5	-46.2	49.2	-32.3	-54.5	12.9	-3.6	7.3
	-6.940	0.611	-5.886	-11.679	12.433	-8.156	-13.830	3.263	-0.918	1.869
c_O	105	110	123	108	96	128	121	98	123	84
	(26.3)	(27.6)	(30.8)	(27.3)	(24.2)	(32.3)	(30.7)	(24.9)	(31.2)	(21.4)
c_E	104.5	102.0	139.0	103.7	101.2	137.9	103.2	100.7	137.2	103.0
	(26.2)	(25.6)	(34.8)	(26.2)	(25.6)	(34.8)	(26.2)	(25.6)	(34.8)	(26.2)
	0.9	16.6	-30.1	8.7	-10.2	-19.1	38.5	-5.3	-26.9	-34.2
	0.236	4.170	-7.532	2.193	-2.568	-4.834	9.761	-1.357	-6.840	-8.700
N	399	399	399	396	396	396	394	394	394	393
X2	2.8	1.0	9.2	14.7	7.7	11.6	19.1	0.6	4.0	7.5
Gvalue	2.8	1.0	8.9	13.8	7.3	10.9	18.6	0.6	3.9	7.5
Gv/N	0.709	0.257	2.224	3.495	1.834	2.748	4.709	0.159	0.984	1.903
Prob	0.41863	0.79483	0.03100	0.00313	0.06396	0.01239	0.00034	0.89036	0.27514	0.05814

5UTR:

	100	99	98	97	96	95	94	93	92	91
a_O	207	178	185	208	160	175	197	168	181	191
	(20.4)	(17.5)	(18.2)	(20.5)	(15.7)	(17.2)	(19.4)	(16.5)	(17.8)	(18.8)
a_E	189.3	189.3	189.3	189.3	189.3	189.3	189.3	189.3	189.3	189.3
	(18.6)	(18.6)	(18.6)	(18.6)	(18.6)	(18.6)	(18.6)	(18.6)	(18.6)	(18.6)
	37.0	-21.9	-8.5	39.2	-53.8	-27.5	15.7	-40.1	-16.2	3.4
	3.644	-2.155	-0.835	3.859	-5.295	-2.704	1.548	-3.946	-1.596	0.338
t_O	188	166	179	195	174	180	208	167	169	214
	(18.5)	(16.3)	(17.6)	(19.2)	(17.1)	(17.7)	(20.5)	(16.4)	(16.6)	(21.1)
t_E	189.1	189.1	189.1	189.1	189.1	189.1	189.1	189.1	189.1	189.1
	(18.6)	(18.6)	(18.6)	(18.6)	(18.6)	(18.6)	(18.6)	(18.6)	(18.6)	(18.6)
	-2.1	-43.2	-19.6	12.1	-28.9	-17.7	39.7	-41.4	-37.9	53.0
	-0.208	-4.251	-1.927	1.187	-2.843	-1.740	3.909	-4.079	-3.731	5.220
g_O	284	321	345	293	338	316	297	330	326	298
	(28.0)	(31.6)	(34.0)	(28.8)	(33.3)	(31.1)	(29.2)	(32.5)	(32.1)	(29.3)
g_E	293.4	293.4	293.4	293.4	293.4	293.4	293.4	293.4	293.4	293.4
	(28.9)	(28.9)	(28.9)	(28.9)	(28.9)	(28.9)	(28.9)	(28.9)	(28.9)	(28.9)
	-18.5	57.7	111.7	-0.8	95.6	46.9	7.2	77.5	68.7	9.2
	-1.824	5.677	10.998	-0.083	9.411	4.612	0.709	7.632	6.757	0.909
c_O	337	351	307	320	344	345	314	351	340	313
	(33.2)	(34.5)	(30.2)	(31.5)	(33.9)	(34.0)	(30.9)	(34.5)	(33.5)	(30.8)
c_E	344.2	344.2	344.2	344.2	344.2	344.2	344.2	344.2	344.2	344.2
	(33.9)	(33.9)	(33.9)	(33.9)	(33.9)	(33.9)	(33.9)	(33.9)	(33.9)	(33.9)
	-14.3	13.7	-70.3	-46.7	-0.5	1.5	-57.7	13.7	-8.4	-59.5
	-1.408	1.346	-6.917	-4.598	-0.045	0.152	-5.681	1.346	-0.828	-5.860
N	1016	1016	1016	1016	1016	1016	1016	1016	1016	1016
X2	2.1	6.2	13.7	3.7	12.5	3.3	4.9	9.7	6.2	6.2
Gvalue	2.1	6.3	13.4	3.7	12.5	3.2	4.9	9.7	6.1	6.2
Gv/N	0.204	0.617	1.319	0.366	1.228	0.319	0.485	0.953	0.602	0.607
Prob	0.55757	0.09924	0.00384	0.29290	0.00593	0.35541	0.17726	0.02145	0.10595	0.10391

	90	89	88	87	86	85	84	83	82	81
a_O	150	165	179	145	154	204	194	178	194	163
	(14.8)	(16.2)	(17.6)	(14.3)	(15.2)	(20.1)	(19.1)	(17.5)	(19.1)	(16.0)
a_E	189.3	189.3	189.3	189.3	189.3	189.3	189.3	189.3	189.3	189.3
	(18.6)	(18.6)	(18.6)	(18.6)	(18.6)	(18.6)	(18.6)	(18.6)	(18.6)	(18.6)
	-69.8	-45.3	-20.0	-77.3	-63.5	30.5	9.5	-21.9	9.5	-48.7
	-6.869	-4.461	-1.970	-7.608	-6.255	3.005	0.939	-2.155	0.939	-4.798
t_O	174	145	189	175	175	177	164	182	201	190
	(17.1)	(14.3)	(18.6)	(17.2)	(17.2)	(17.4)	(16.1)	(17.9)	(19.8)	(18.7)
t_E	189.1	189.1	189.1	189.1	189.1	189.1	189.1	189.1	189.1	189.1
	(18.6)	(18.6)	(18.6)	(18.6)	(18.6)	(18.6)	(18.6)	(18.6)	(18.6)	(18.6)
	-28.9	-76.9	-0.1	-27.0	-27.0	-23.3	-46.6	-13.9	24.6	1.9
	-2.843	-7.573	-0.012	-2.662	-2.662	-2.297	-4.591	-1.363	2.423	0.185
g_O	340	365	323	355	362	321	308	333	312	325
	(33.5)	(35.9)	(31.8)	(34.9)	(35.6)	(31.6)	(30.3)	(32.8)	(30.7)	(32.0)
g_E	293.4	293.4	293.4	293.4	293.4	293.4	293.4	293.4	293.4	293.4
	(28.9)	(28.9)	(28.9)	(28.9)	(28.9)	(28.9)	(28.9)	(28.9)	(28.9)	(28.9)
	100.2	159.4	62.0	135.3	152.1	57.7	29.9	84.3	38.3	66.4
	9.861	15.684	6.107	13.313	14.967	5.677	2.940	8.295	3.771	6.540
c_O	352	341	325	341	325	314	350	323	309	338
	(34.6)	(33.6)	(32.0)	(33.6)	(32.0)	(30.9)	(34.4)	(31.8)	(30.4)	(33.3)
c_E	344.2	344.2	344.2	344.2	344.2	344.2	344.2	344.2	344.2	344.2
	(33.9)	(33.9)	(33.9)	(33.9)	(33.9)	(33.9)	(33.9)	(33.9)	(33.9)	(33.9)
	15.7	-6.4	-37.4	-6.4	-37.4	-57.7	11.6	-41.1	-66.7	-12.3
	1.547	-0.633	-3.678	-0.633	-3.678	-5.681	1.145	-4.048	-6.567	-1.215
N	1016	1016	1016	1016	1016	1016	1016	1016	1016	1016
X2	16.9	30.9	4.6	24.4	24.7	7.2	4.3	7.6	5.7	7.2
Gvalue	17.2	30.7	4.6	24.5	24.1	7.2	4.4	7.4	5.7	7.2
Gv/N	1.695	3.017	0.448	2.410	2.373	0.704	0.433	0.729	0.565	0.712
Prob	0.00064	0.00000	0.20777	0.00002	0.00002	0.06722	0.22099	0.06004	0.12482	0.06479

	80	79	78	77	76	75	74	73	72	71
a_O	194	208	171	167	208	173	206	207	182	204
	(19.1)	(20.5)	(16.8)	(16.4)	(20.5)	(17.0)	(20.3)	(20.4)	(17.9)	(20.1)
a_E	189.3	189.3	189.3	189.3	189.3	189.3	189.3	189.3	189.3	189.3
	(18.6)	(18.6)	(18.6)	(18.6)	(18.6)	(18.6)	(18.6)	(18.6)	(18.6)	(18.6)
	9.5	39.2	-34.8	-41.8	39.2	-31.1	34.9	37.0	-14.3	30.5
	0.939	3.859	-3.421	-4.119	3.859	-3.065	3.430	3.644	-1.407	3.005
t_O	177	191	191	170	191	183	169	179	158	165
	(17.4)	(18.8)	(18.8)	(16.7)	(18.8)	(18.0)	(16.6)	(17.6)	(15.6)	(16.2)
t_E	189.1	189.1	189.1	189.1	189.1	189.1	189.1	189.1	189.1	189.1
	(18.6)	(18.6)	(18.6)	(18.6)	(18.6)	(18.6)	(18.6)	(18.6)	(18.6)	(18.6)
	-23.3	3.9	3.9	-36.1	3.9	-11.9	-37.9	-19.6	-56.7	-44.9
	-2.297	0.384	0.384	-3.556	0.384	-1.174	-3.731	-1.927	-5.582	-4.421
g_O	325	321	323	339	283	328	348	329	301	309
	(32.0)	(31.6)	(31.8)	(33.4)	(27.9)	(32.3)	(34.3)	(32.4)	(29.6)	(30.4)
g_E	293.4	293.4	293.4	293.4	293.4	293.4	293.4	293.4	293.4	293.4
	(28.9)	(28.9)	(28.9)	(28.9)	(28.9)	(28.9)	(28.9)	(28.9)	(28.9)	(28.9)
	66.4	57.7	62.0	97.9	-20.5	73.1	118.7	75.3	15.4	32.0
	6.540	5.677	6.107	9.636	-2.014	7.193	11.687	7.412	1.511	3.147
c_O	320	296	331	340	334	332	293	301	375	338
	(31.5)	(29.1)	(32.6)	(33.5)	(32.9)	(32.7)	(28.8)	(29.6)	(36.9)	(33.3)
c_E	344.2	344.2	344.2	344.2	344.2	344.2	344.2	344.2	344.2	344.2
	(33.9)	(33.9)	(33.9)	(33.9)	(33.9)	(33.9)	(33.9)	(33.9)	(33.9)	(33.9)
	-46.7	-89.4	-25.9	-8.4	-20.2	-24.0	-94.4	-80.8	64.2	-12.3
	-4.598	-8.796	-2.554	-0.828	-1.984	-2.364	-9.294	-7.952	6.320	-1.215
N	1016	1016	1016	1016	1016	1016	1016	1016	1016	1016
X2	6.0	11.2	5.3	11.7	2.5	6.1	21.4	11.9	8.3	5.1
Gvalue	5.9	11.4	5.2	11.5	2.5	6.0	21.3	12.0	8.6	5.2
Gv/N	0.584	1.124	0.517	1.133	0.245	0.591	2.092	1.179	0.842	0.516
Prob	0.11494	0.00964	0.15447	0.00924	0.47662	0.11141	0.00009	0.00747	0.03578	0.15492

	70	69	68	67	66	65	64	63	62	61
a_O	199	173	178	210	184	176	198	190	201	185
	(19.6)	(17.0)	(17.5)	(20.7)	(18.1)	(17.3)	(19.5)	(18.7)	(19.8)	(18.2)
a_E	189.3	189.3	189.3	189.3	189.3	189.3	189.3	189.3	189.3	189.3
	(18.6)	(18.6)	(18.6)	(18.6)	(18.6)	(18.6)	(18.6)	(18.6)	(18.6)	(18.6)
	19.9	-31.1	-21.9	43.6	-10.4	-25.6	17.8	1.4	24.1	-8.5
	1.960	-3.065	-2.155	4.292	-1.027	-2.522	1.753	0.140	2.375	-0.835
t_O	200	175	152	185	178	177	211	187	192	220
	(19.7)	(17.2)	(15.0)	(18.2)	(17.5)	(17.4)	(20.8)	(18.4)	(18.9)	(21.7)
t_E	189.1	189.1	189.1	189.1	189.1	189.1	189.1	189.1	189.1	189.1

```
        ( 18.6) ( 18.6) ( 18.6) ( 18.6) ( 18.6) ( 18.6) ( 18.6) ( 18.6) ( 18.6) ( 18.6)
          22.5   -27.0   -66.3    -8.0   -21.5   -23.3    46.3    -4.1     5.9    66.7
         2.215  -2.662  -6.528  -0.791  -2.112  -2.297   4.560  -0.403   0.583   6.564
g_O        296     341     357     305     309     334     325     277     298     308
        ( 29.1) ( 33.6) ( 35.1) ( 30.0) ( 30.4) ( 32.9) ( 32.0) ( 27.3) ( 29.3) ( 30.3)
g_E      293.4   293.4   293.4   293.4   293.4   293.4   293.4   293.4   293.4   293.4
        ( 28.9) ( 28.9) ( 28.9) ( 28.9) ( 28.9) ( 28.9) ( 28.9) ( 28.9) ( 28.9) ( 28.9)
           5.2   102.5   140.0    23.6    32.0    86.5    66.4   -31.9     9.2    29.9
         0.510  10.088  13.783   2.324   3.147   8.517   6.540  -3.140   0.909   2.940
c_O        321     327     329     316     345     329     282     362     325     303
        ( 31.6) ( 32.2) ( 32.4) ( 31.1) ( 34.0) ( 32.4) ( 27.8) ( 35.6) ( 32.0) ( 29.8)
c_E      344.2   344.2   344.2   344.2   344.2   344.2   344.2   344.2   344.2   344.2
        ( 33.9) ( 33.9) ( 33.9) ( 33.9) ( 33.9) ( 33.9) ( 33.9) ( 33.9) ( 33.9) ( 33.9)
         -44.9   -33.6   -29.8   -54.1     1.5   -29.8  -112.5    36.4   -37.4   -77.3
        -4.415  -3.305  -2.931  -5.323   0.152  -2.931 -11.069   3.587  -3.678  -7.609

N         1016    1016    1016    1016    1016    1016    1016    1016    1016    1016
X2         2.7    11.0    22.4     5.1     1.6     8.0    17.6     1.9     1.9    10.8
Gvalue     2.7    10.7    22.0     5.1     1.6     7.8    18.1     1.9     1.9    10.8
Gv/N     0.269   1.055   2.169   0.503   0.160   0.767   1.784   0.183   0.189   1.060
Prob   0.43385 0.01332 0.00006 0.16409 0.65397 0.05042 0.00041 0.60118 0.58889 0.01306
       -------------------------------------------------------------------------------
            60      59      58      57      56      55      54      53      52      51
       -------------------------------------------------------------------------------
a_O        170     192     206     141     200     205     165     186     197     173
        ( 16.7) ( 18.9) ( 20.3) ( 13.9) ( 19.7) ( 20.2) ( 16.2) ( 18.3) ( 19.4) ( 17.0)
a_E      189.3   189.3   189.3   189.3   189.3   189.3   189.3   189.3   189.3   189.3
        ( 18.6) ( 18.6) ( 18.6) ( 18.6) ( 18.6) ( 18.6) ( 18.6) ( 18.6) ( 18.6) ( 18.6)
         -36.5     5.5    34.9   -83.1    22.0    32.7   -45.3    -6.5    15.7   -31.1
        -3.597   0.537   3.430  -8.175   2.167   3.217  -4.461  -0.642   1.548  -3.065
t_O        193     179     210     194     185     201     194     162     199     168
        ( 19.0) ( 17.6) ( 20.7) ( 19.1) ( 18.2) ( 19.8) ( 19.1) ( 15.9) ( 19.6) ( 16.5)
t_E      189.1   189.1   189.1   189.1   189.1   189.1   189.1   189.1   189.1   189.1
        ( 18.6) ( 18.6) ( 18.6) ( 18.6) ( 18.6) ( 18.6) ( 18.6) ( 18.6) ( 18.6) ( 18.6)
           8.0   -19.6    44.1    10.0    -8.0    24.6    10.0   -50.0    20.4   -39.7
         0.784  -1.927   4.342   0.985  -0.791   2.423   0.985  -4.926   2.007  -3.906
g_O        323     299     287     341     333     274     310     336     286     335
        ( 31.8) ( 29.4) ( 28.2) ( 33.6) ( 32.8) ( 27.0) ( 30.5) ( 33.1) ( 28.1) ( 33.0)
g_E      293.4   293.4   293.4   293.4   293.4   293.4   293.4   293.4   293.4   293.4
        ( 28.9) ( 28.9) ( 28.9) ( 28.9) ( 28.9) ( 28.9) ( 28.9) ( 28.9) ( 28.9) ( 28.9)
          62.0    11.3   -12.7   102.5    84.3   -37.5    34.1    91.1   -14.7    88.8
         6.107   1.109  -1.250  10.088   8.295  -3.693   3.354   8.963  -1.442   8.739
c_O        330     346     313     340     298     336     347     332     334     340
        ( 32.5) ( 34.1) ( 30.8) ( 33.5) ( 29.3) ( 33.1) ( 34.2) ( 32.7) ( 32.9) ( 33.5)
c_E      344.2   344.2   344.2   344.2   344.2   344.2   344.2   344.2   344.2   344.2
        ( 33.9) ( 33.9) ( 33.9) ( 33.9) ( 33.9) ( 33.9) ( 33.9) ( 33.9) ( 33.9) ( 33.9)
         -27.9     3.5   -59.5    -8.4   -86.0   -16.3     5.6   -24.0   -20.2    -8.4
        -2.742   0.349  -5.860  -0.828  -8.460  -1.601   0.547  -2.364  -1.984  -0.828

N         1016    1016    1016    1016    1016    1016    1016    1016    1016    1016
X2         5.6     0.7     6.8    20.2    12.2     3.5     4.2    10.5     1.3     9.7
Gvalue     5.6     0.7     6.7    21.0    12.3     3.5     4.3    10.5     1.3     9.6
Gv/N     0.551   0.069   0.663   2.070   1.211   0.347   0.426   1.030   0.130   0.942
Prob   0.13273 0.87363 0.08080 0.00010 0.00641 0.31807 0.22802 0.01497 0.72442 0.02263
       -------------------------------------------------------------------------------
            50      49      48      47      46      45      44      43      42      41
       -------------------------------------------------------------------------------
a_O        199     205     176     201     181     197     186     196     183     203
        ( 19.6) ( 20.2) ( 17.3) ( 19.8) ( 17.8) ( 19.4) ( 18.3) ( 19.3) ( 18.0) ( 20.0)
a_E      189.3   189.3   189.3   189.3   189.3   189.3   189.3   189.3   189.3   189.3
        ( 18.6) ( 18.6) ( 18.6) ( 18.6) ( 18.6) ( 18.6) ( 18.6) ( 18.6) ( 18.6) ( 18.6)
          19.9    32.7   -25.6    24.1   -16.2    15.7    -6.5    13.7   -12.4    28.4
         1.960   3.217  -2.522   2.375  -1.596   1.548  -0.642   1.344  -1.217   2.794
t_O        168     204     173     173     214     177     177     179     188     173
        ( 16.5) ( 20.1) ( 17.0) ( 17.0) ( 21.1) ( 17.4) ( 17.4) ( 17.6) ( 18.5) ( 17.0)
t_E      189.1   189.1   189.1   189.1   189.1   189.1   189.1   189.1   189.1   189.1
        ( 18.6) ( 18.6) ( 18.6) ( 18.6) ( 18.6) ( 18.6) ( 18.6) ( 18.6) ( 18.6) ( 18.6)
         -39.7    31.0   -30.7   -30.7    53.0   -23.3   -23.3   -19.6    -2.1   -30.7
        -3.906   3.054  -3.023  -3.023   5.220  -2.297  -2.297  -1.927  -0.208  -3.023
g_O        314     296     301     306     321     330     298     303     287     316
        ( 30.9) ( 29.1) ( 29.6) ( 30.1) ( 31.6) ( 32.5) ( 29.3) ( 29.8) ( 28.2) ( 31.1)
g_E      293.4   293.4   293.4   293.4   293.4   293.4   293.4   293.4   293.4   293.4
        ( 28.9) ( 28.9) ( 28.9) ( 28.9) ( 28.9) ( 28.9) ( 28.9) ( 28.9) ( 28.9) ( 28.9)
          42.6     5.2    15.4    25.7    57.7    77.5     9.2    19.5   -12.7    46.9
         4.190   0.510   1.511   2.529   5.677   7.632   0.909   1.916  -1.250   4.612
c_O        335     311     366     336     300     312     355     338     358     324
        ( 33.0) ( 30.6) ( 36.0) ( 33.1) ( 29.5) ( 30.7) ( 34.9) ( 33.3) ( 35.2) ( 31.9)
c_E      344.2   344.2   344.2   344.2   344.2   344.2   344.2   344.2   344.2   344.2
        ( 33.9) ( 33.9) ( 33.9) ( 33.9) ( 33.9) ( 33.9) ( 33.9) ( 33.9) ( 33.9) ( 33.9)
         -18.2   -63.1    44.9   -16.3   -82.5   -61.3    21.9   -12.3    28.1   -39.2
        -1.792  -6.215   4.418  -1.601  -8.122  -6.038   2.153  -1.215   2.764  -3.863

N         1016    1016    1016    1016    1016    1016    1016    1016    1016    1016
X2         4.5     5.7     3.9     2.8    11.9     8.7     1.2     1.2     0.9     5.3
Gvalue     4.6     5.8     3.9     2.8    12.0     8.6     1.2     1.2     0.9     5.3
Gv/N     0.452   0.567   0.384   0.280   1.179   0.846   0.123   0.118   0.089   0.520
Prob   0.20452 0.12395 0.27212 0.41620 0.00745 0.03520 0.74144 0.75228 0.82483 0.15227
       -------------------------------------------------------------------------------
            40      39      38      37      36      35      34      33      32      31
       -------------------------------------------------------------------------------
a_O        212     188     177     201     185     200     232     162     200     208
        ( 20.9) ( 18.5) ( 17.4) ( 19.8) ( 18.2) ( 19.7) ( 22.8) ( 15.9) ( 19.7) ( 20.5)
a_E      189.3   189.3   189.3   189.3   189.3   189.3   189.3   189.3   189.3   189.3
        ( 18.6) ( 18.6) ( 18.6) ( 18.6) ( 18.6) ( 18.6) ( 18.6) ( 18.6) ( 18.6) ( 18.6)
          48.0    -2.6   -23.8    24.1    -8.5    22.0    94.4   -50.4    22.0    39.2
         4.729  -0.253  -2.339   2.375  -0.835   2.167   9.292  -4.965   2.167   3.859
t_O        199     194     178     202     181     188     226     205     160     216
        ( 19.6) ( 19.1) ( 17.5) ( 19.9) ( 17.8) ( 18.5) ( 22.2) ( 20.2) ( 15.7) ( 21.3)
t_E      189.1   189.1   189.1   189.1   189.1   189.1   189.1   189.1   189.1   189.1
        ( 18.6) ( 18.6) ( 18.6) ( 18.6) ( 18.6) ( 18.6) ( 18.6) ( 18.6) ( 18.6) ( 18.6)
          20.4    10.0   -21.5    26.7   -15.8    -2.1    80.7    33.2   -53.4    57.5
         2.007   0.985  -2.112   2.632  -1.552  -0.208   7.940   3.267  -5.256   5.664
g_O        285     292     322     260     314     292     259     298     304     278
        ( 28.1) ( 28.7) ( 31.7) ( 25.6) ( 30.9) ( 28.7) ( 25.5) ( 29.3) ( 29.9) ( 27.4)
g_E      293.4   293.4   293.4   293.4   293.4   293.4   293.4   293.4   293.4   293.4
        ( 28.9) ( 28.9) ( 28.9) ( 28.9) ( 28.9) ( 28.9) ( 28.9) ( 28.9) ( 28.9) ( 28.9)
         -16.6    -2.8    59.9   -62.9    42.6    -2.8   -64.6     9.2    21.5   -30.0
        -1.633  -0.279   5.891  -6.189   4.190  -0.279  -6.362   0.909   2.120  -2.954
c_O        320     342     339     353     336     336     299     351     352     314
        ( 31.5) ( 33.7) ( 33.4) ( 34.7) ( 33.1) ( 33.1) ( 29.4) ( 34.5) ( 34.6) ( 30.9)
c_E      344.2   344.2   344.2   344.2   344.2   344.2   344.2   344.2   344.2   344.2
        ( 33.9) ( 33.9) ( 33.9) ( 33.9) ( 33.9) ( 33.9) ( 33.9) ( 33.9) ( 33.9) ( 33.9)
```

	-46.7	-4.4	-10.4	17.8	-16.3	-16.3	-84.2	13.7	15.7	-57.7
	-4.598	-0.438	-1.022	1.748	-1.601	-1.601	-8.291	1.346	1.547	-5.681
N	1016	1016	1016	1016	1016	1016	1016	1016	1016	1016
X2	5.2	0.2	4.3	5.6	2.1	0.8	26.8	5.5	5.6	9.2
Gvalue	5.1	0.2	4.3	5.8	2.1	0.8	26.2	5.6	5.9	9.0
Gv/N	0.505	0.016	0.419	0.567	0.202	0.079	2.579	0.556	0.577	0.888
Prob	0.16277	0.98405	0.23539	0.12403	0.56091	0.84802	0.00001	0.12997	0.11858	0.02902

	30	29	28	27	26	25	24	23	22	21
a_O	171	177	188	183	177	173	159	175	179	181
	(16.8)	(17.4)	(18.5)	(18.0)	(17.4)	(17.0)	(15.6)	(17.2)	(17.6)	(17.8)
a_E	189.3	189.3	189.3	189.3	189.3	189.3	189.3	189.3	189.3	189.3
	(18.6)	(18.6)	(18.6)	(18.6)	(18.6)	(18.6)	(18.6)	(18.6)	(18.6)	(18.6)
	-34.8	-23.8	-2.6	-12.4	-23.8	-31.1	-55.5	-27.5	-20.0	-16.2
	-3.421	-2.339	-0.253	-1.217	-2.339	-3.065	-5.458	-2.704	-1.970	-1.596
t_O	209	182	221	176	203	220	201	198	221	204
	(20.6)	(17.9)	(21.8)	(17.3)	(20.0)	(21.7)	(19.8)	(19.5)	(21.8)	(20.1)
t_E	189.1	189.1	189.1	189.1	189.1	189.1	189.1	189.1	189.1	189.1
	(18.6)	(18.6)	(18.6)	(18.6)	(18.6)	(18.6)	(18.6)	(18.6)	(18.6)	(18.6)
	41.9	-13.9	69.0	-25.2	28.9	66.7	24.6	18.3	69.0	31.0
	4.125	-1.363	6.791	-2.480	2.843	6.564	2.423	1.801	6.791	3.054
g_O	282	326	269	317	292	265	299	274	255	293
	(27.8)	(32.1)	(26.5)	(31.2)	(28.7)	(26.1)	(29.4)	(27.0)	(25.1)	(28.8)
g_E	293.4	293.4	293.4	293.4	293.4	293.4	293.4	293.4	293.4	293.4
	(28.9)	(28.9)	(28.9)	(28.9)	(28.9)	(28.9)	(28.9)	(28.9)	(28.9)	(28.9)
	-22.4	68.7	-46.7	49.0	-2.8	-54.0	11.3	-37.5	-71.6	-0.8
	-2.204	6.757	-4.601	4.823	-0.279	-5.314	1.109	-3.693	-7.045	-0.083
c_O	354	331	338	340	344	358	357	369	361	338
	(34.8)	(32.6)	(33.3)	(33.5)	(33.9)	(35.2)	(35.1)	(36.3)	(35.5)	(33.3)
c_E	344.2	344.2	344.2	344.2	344.2	344.2	344.2	344.2	344.2	344.2
	(33.9)	(33.9)	(33.9)	(33.9)	(33.9)	(33.9)	(33.9)	(33.9)	(33.9)	(33.9)
	19.8	-25.9	-12.3	-8.4	-0.5	28.1	26.0	51.3	34.3	-12.3
	1.950	-2.554	-1.215	-0.828	-0.045	2.764	2.560	5.047	3.380	-1.215
N	1016	1016	1016	1016	1016	1016	1016	1016	1016	1016
X2	4.6	5.2	7.5	3.1	1.8	9.8	6.2	4.6	11.8	1.7
Gvalue	4.6	5.1	7.3	3.0	1.8	9.6	6.4	4.6	11.8	1.6
Gv/N	0.451	0.501	0.721	0.299	0.180	0.949	0.634	0.451	1.157	0.161
Prob	0.20484	0.16543	0.06212	0.38651	0.60923	0.02187	0.09204	0.20535	0.00827	0.65189

	20	19	18	17	16	15	14	13	12	11
a_O	193	190	180	215	182	156	197	208	223	262
	(19.0)	(18.7)	(17.7)	(21.2)	(17.9)	(15.4)	(19.4)	(20.5)	(21.9)	(25.8)
a_E	189.3	189.3	189.3	189.3	189.3	189.3	189.3	189.3	189.3	189.3
	(18.6)	(18.6)	(18.6)	(18.6)	(18.6)	(18.6)	(18.6)	(18.6)	(18.6)	(18.6)
	7.5	1.4	-18.1	54.8	-14.3	-60.3	15.7	39.2	73.1	170.3
	0.737	0.140	-1.783	5.390	-1.407	-5.940	1.548	3.859	7.194	16.765
t_O	226	227	197	212	223	212	207	224	202	182
	(22.2)	(22.3)	(19.4)	(20.9)	(21.9)	(20.9)	(20.4)	(22.0)	(19.9)	(17.9)
t_E	189.1	189.1	189.1	189.1	189.1	189.1	189.1	189.1	189.1	189.1
	(18.6)	(18.6)	(18.6)	(18.6)	(18.6)	(18.6)	(18.6)	(18.6)	(18.6)	(18.6)
	80.7	83.0	16.2	48.6	73.6	48.6	37.5	76.0	26.7	-13.9
	7.940	8.172	1.595	4.779	7.248	4.779	3.694	7.478	2.632	-1.363
g_O	248	205	209	224	227	234	221	200	240	225
	(24.4)	(20.2)	(20.6)	(22.0)	(22.3)	(23.0)	(21.8)	(19.7)	(23.6)	(22.1)
g_E	293.4	293.4	293.4	293.4	293.4	293.4	293.4	293.4	293.4	293.4
	(28.9)	(28.9)	(28.9)	(28.9)	(28.9)	(28.9)	(28.9)	(28.9)	(28.9)	(28.9)
	-83.4	-147.0	-141.8	-120.9	-116.5	-105.9	-125.3	-153.3	-96.5	-119.5
	-8.210	-14.471	-13.958	-11.904	-11.469	-10.423	-12.331	-15.090	-9.494	-11.760
c_O	349	394	430	365	384	414	391	384	351	347
	(34.4)	(38.8)	(42.3)	(35.9)	(37.8)	(40.7)	(38.5)	(37.8)	(34.5)	(34.2)
c_E	344.2	344.2	344.2	344.2	344.2	344.2	344.2	344.2	344.2	344.2
	(33.9)	(33.9)	(33.9)	(33.9)	(33.9)	(33.9)	(33.9)	(33.9)	(33.9)	(33.9)
	9.6	106.4	191.3	42.8	84.0	152.8	99.6	84.0	13.7	5.6
	0.945	10.474	18.832	4.210	8.265	15.041	9.806	8.265	1.346	0.547
N	1016	1016	1016	1016	1016	1016	1016	1016	1016	1016
X2	14.4	41.5	46.4	24.0	26.0	34.8	26.2	42.6	16.7	44.2
Gvalue	14.4	43.8	47.6	25.1	26.8	35.1	27.6	45.8	17.1	42.6
Gv/N	1.413	4.315	4.686	2.475	2.637	3.457	2.717	4.511	1.678	4.190
Prob	0.00246	0.00000	0.00000	0.00001	0.00001	0.00000	0.00000	0.00000	0.00069	0.00000

	10	9	8	7	6	5	4	3	2	1
a_O	217	209	251	208	213	215	193	228	165	144
	(21.4)	(20.6)	(24.7)	(20.5)	(21.0)	(21.2)	(19.0)	(22.4)	(16.2)	(14.2)
a_E	189.3	189.3	189.3	189.3	189.3	189.3	189.3	189.3	189.3	189.3
	(18.6)	(18.6)	(18.6)	(18.6)	(18.6)	(18.6)	(18.6)	(18.6)	(18.6)	(18.6)
	59.3	41.4	141.7	39.2	50.3	54.8	7.5	84.8	-45.3	-78.8
	5.836	4.075	13.942	3.859	4.948	5.390	0.737	8.351	-4.461	-7.752
t_O	211	189	186	242	186	183	170	137	188	144
	(20.8)	(18.6)	(18.3)	(23.8)	(18.3)	(18.0)	(16.7)	(13.5)	(18.5)	(14.2)
t_E	189.1	189.1	189.1	189.1	189.1	189.1	189.1	189.1	189.1	189.1
	(18.6)	(18.6)	(18.6)	(18.6)	(18.6)	(18.6)	(18.6)	(18.6)	(18.6)	(18.6)
	46.3	-0.1	-6.1	119.5	-6.1	-11.9	-36.1	-88.3	-2.1	-78.4
	4.560	-0.012	-0.597	11.760	-0.597	-1.174	-3.556	-8.686	-0.208	-7.717
g_O	209	274	216	193	246	197	257	378	183	136
	(20.6)	(27.0)	(21.3)	(19.0)	(24.2)	(19.4)	(25.3)	(37.2)	(18.0)	(13.4)
g_E	293.4	293.4	293.4	293.4	293.4	293.4	293.4	293.4	293.4	293.4
	(28.9)	(28.9)	(28.9)	(28.9)	(28.9)	(28.9)	(28.9)	(28.9)	(28.9)	(28.9)
	-141.8	-37.5	-132.3	-161.7	-86.7	-157.0	-68.1	191.5	-172.8	-209.2
	-13.958	-3.693	-13.025	-15.915	-8.536	-15.450	-6.705	18.847	-17.007	-20.586
c_O	379	344	363	373	371	421	396	273	480	592
	(37.3)	(33.9)	(35.7)	(36.7)	(36.5)	(41.4)	(39.0)	(26.9)	(47.2)	(58.3)
c_E	344.2	344.2	344.2	344.2	344.2	344.2	344.2	344.2	344.2	344.2
	(33.9)	(33.9)	(33.9)	(33.9)	(33.9)	(33.9)	(33.9)	(33.9)	(33.9)	(33.9)
	72.9	-0.5	38.5	59.9	55.6	169.5	111.0	-126.6	319.2	642.0
	7.179	-0.045	3.794	5.894	5.469	16.684	10.922	-12.459	31.415	63.185
N	1016	1016	1016	1016	1016	1016	1016	1016	1016	1016
X2	34.4	3.3	41.6	53.4	12.8	52.5	14.3	61.4	98.2	284.4
Gvalue	36.7	3.3	41.8	56.9	13.0	55.4	14.2	61.5	98.9	275.6
Gv/N	3.617	0.325	4.113	5.598	1.284	5.451	1.398	6.053	9.739	27.130
Prob	0.00000	0.34773	0.00000	0.00000	0.00454	0.00000	0.00264	0.00000	0.00000	0.00000

CDS_INITIAL:

	1	2	3	4	5	6	7	8	9	10

```
            ---------------------------------------------------------------------------------
a_O           769       0       0     397     257     143     269     246     161     263
            ( 75.7) (  0.0) (  0.0) ( 39.1) ( 25.3) ( 14.1) ( 26.5) ( 24.2) ( 15.8) ( 25.9)
a_E         182.9   241.4    51.7   182.9   241.4    51.7   182.9   241.4    51.7   182.9
            ( 18.0) ( 23.8) (  5.1) ( 18.0) ( 23.8) (  5.1) ( 18.0) ( 23.8) (  5.1) ( 18.0)
           2208.4     0.0     0.0   615.1    32.3   291.1   207.4     9.4   366.0   190.9
          217.364   0.000   0.000  60.546   3.175  28.655  20.414   0.921  36.020  18.791
t_O           118    1016       0     134     168     183     124     242     175     156
            ( 11.6) (100.0) (  0.0) ( 13.2) ( 16.5) ( 18.0) ( 12.2) ( 23.8) ( 17.2) ( 15.4)
t_E         119.3   287.7    64.5   119.3   287.7    64.5   119.3   287.7    64.5   119.3
            ( 11.7) ( 28.3) (  6.3) ( 11.7) ( 28.3) (  6.3) ( 11.7) ( 28.3) (  6.3) ( 11.7)
             -2.6  2563.8     0.0    31.2  -180.7   381.7     9.6   -83.7   349.4    83.7
           -0.253 252.345   0.000   3.066 -17.790  37.570   0.944  -8.240  34.387   8.238
g_O           129       0    1016     194     170     243     315     194     265     268
            ( 12.7) (  0.0) (100.0) ( 19.1) ( 16.7) ( 23.9) ( 31.0) ( 19.1) ( 26.1) ( 26.4)
g_E         407.7   210.0   408.5   407.7   210.0   408.5   407.7   210.0   408.5   407.7
            ( 40.1) ( 20.7) ( 40.2) ( 40.1) ( 20.7) ( 40.2) ( 40.1) ( 20.7) ( 40.2) ( 40.1)
           -296.9     0.0  1851.4  -288.2   -71.9  -252.4  -162.5   -30.8  -229.4  -224.9
          -29.222   0.000 182.226 -28.364  -7.076 -24.847 -15.999  -3.032 -22.575 -22.136
c_O             0       0       0     291     421     447     308     334     415     329
            (  0.0) (  0.0) (  0.0) ( 28.6) ( 41.4) ( 44.0) ( 30.3) ( 32.9) ( 40.8) ( 32.4)
c_E         306.0   276.9   491.3   306.0   276.9   491.3   306.0   276.9   491.3   306.0
            ( 30.1) ( 27.3) ( 48.4) ( 30.1) ( 27.3) ( 48.4) ( 30.1) ( 27.3) ( 48.4) ( 30.1)
              0.0     0.0     0.0   -29.3   352.8   -84.5     3.9   125.2  -140.1    47.6
            0.000   0.000   0.000  -2.886  34.720  -8.321   0.387  12.325 -13.793   4.685

N            1016    1016    1016    1016    1016    1016    1016    1016    1016    1016
X2         2374.0  2572.0  1510.9   365.0   133.4   450.2    61.8    20.3   482.9    95.9
Gvalue     1908.9  2563.8  1851.4   328.8   132.4   335.9    58.4    20.1   345.8    97.3
Gv/N      187.888 252.345 182.226  32.362  13.029  33.057   5.747   1.974  34.038   9.578
Prob      1.00000 1.00000 1.00000 0.00000 0.00000 0.00000 0.00000 0.00017 0.00000 0.00000
            ---------------------------------------------------------------------------------
               11      12      13      14      15      16      17      18      19      20
            ---------------------------------------------------------------------------------
a_O           228     138     219     231     143     231     229     132     249     219
            ( 22.4) ( 13.6) ( 21.6) ( 22.7) ( 14.1) ( 22.7) ( 22.5) ( 13.0) ( 24.5) ( 21.6)
a_E         241.4    51.7   182.9   241.4    51.7   182.9   241.4    51.7   182.9   241.4
            ( 23.8) (  5.1) ( 18.0) ( 23.8) (  5.1) ( 18.0) ( 23.8) (  5.1) ( 18.0) ( 23.8)
            -26.0   271.1    78.8   -20.3   291.1   107.8   -24.1   247.6   153.5   -42.6
           -2.557  26.686   7.755  -1.996  28.655  10.605  -2.371  24.371  15.110  -4.192
t_O           270     192     171     235     175     165     302     188     146     288
            ( 26.6) ( 18.9) ( 16.8) ( 23.1) ( 17.2) ( 16.2) ( 29.7) ( 18.5) ( 14.4) ( 28.3)
t_E         287.7    64.5   119.3   287.7    64.5   119.3   287.7    64.5   119.3   287.7
            ( 28.3) (  6.3) ( 11.7) ( 28.3) (  6.3) ( 11.7) ( 28.3) (  6.3) ( 11.7) ( 28.3)
            -34.3   418.9   123.1   -95.1   349.4   107.0    29.3   402.3    59.0     0.6
           -3.374  41.232  12.120  -9.359  34.387  10.535   2.885  39.594   5.806   0.060
g_O           153     247     281     184     236     281     148     248     279     150
            ( 15.1) ( 24.3) ( 27.7) ( 18.1) ( 23.2) ( 27.7) ( 14.6) ( 24.4) ( 27.5) ( 14.8)
g_E         210.0   408.5   407.7   210.0   408.5   407.7   210.0   408.5   407.7   210.0
            ( 20.7) ( 40.2) ( 40.1) ( 20.7) ( 40.2) ( 40.1) ( 20.7) ( 40.2) ( 40.1) ( 20.7)
            -96.9  -248.5  -209.2   -48.7  -259.0  -209.2  -103.6  -247.5  -211.7  -101.0
           -9.542 -24.462 -20.590  -4.792 -25.489 -20.590 -10.198 -24.364 -20.836  -9.939
c_O           365     439     345     366     462     339     337     448     342     359
            ( 35.9) ( 43.2) ( 34.0) ( 36.0) ( 45.5) ( 33.4) ( 33.2) ( 44.1) ( 33.7) ( 35.3)
c_E         276.9   491.3   306.0   276.9   491.3   306.0   276.9   491.3   306.0   276.9
            ( 27.3) ( 48.4) ( 30.1) ( 27.3) ( 48.4) ( 30.1) ( 27.3) ( 48.4) ( 30.1) ( 27.3)
            201.6   -98.9    82.7   204.2   -56.9    69.4   132.4   -82.7    76.0   186.4
           19.846  -9.733   8.138  20.098  -5.598   6.826  13.029  -8.142   7.480  18.349

N            1016    1016    1016    1016    1016    1016    1016    1016    1016    1016
X2           45.3   465.7    73.9    42.0   425.4    73.1    32.7   428.3    74.7    43.6
Gvalue       44.4   342.6    75.4    40.1   324.7    74.9    34.0   319.6    76.8    43.5
Gv/N        4.373  33.723   7.424   3.950  31.955   7.377   3.345  31.458   7.560   4.277
Prob      0.00000 0.00000 -0.00000 0.00000 0.00000 0.00000 0.00000 0.00000 0.00000 0.00000
            ---------------------------------------------------------------------------------
               21      22      23      24      25      26      27      28      29      30
            ---------------------------------------------------------------------------------
a_O           125     219     212     101     208     226      93     213     184      99
            ( 12.3) ( 21.6) ( 20.9) (  9.9) ( 20.5) ( 22.2) (  9.2) ( 21.0) ( 18.1) (  9.7)
a_E          51.7   182.9   241.4    51.7   182.9   241.4    51.7   182.9   241.4    51.7
            (  5.1) ( 18.0) ( 23.8) (  5.1) ( 18.0) ( 23.8) (  5.1) ( 18.0) ( 23.8) (  5.1)
            220.9    78.8   -55.0   135.4    53.4   -29.7   109.3    64.8   -99.9   128.7
           21.738   7.755  -5.414  13.326   5.255  -2.927  10.759   6.377  -9.830  12.672
t_O           145     168     270     161     166     278     148     150     289     115
            ( 14.3) ( 16.5) ( 26.6) ( 15.8) ( 16.3) ( 27.4) ( 14.6) ( 14.8) ( 28.4) ( 11.3)
t_E          64.5   119.3   287.7    64.5   119.3   287.7    64.5   119.3   287.7    64.5
            (  6.3) ( 11.7) ( 28.3) (  6.3) ( 11.7) ( 28.3) (  6.3) ( 11.7) ( 28.3) (  6.3)
            234.9   115.0   -34.3   294.6   109.7   -19.1   245.9    68.7     2.6   133.0
           23.125  11.322  -3.374  28.994  10.796  -1.876  24.200   6.763   0.257  13.093
g_O           268     320     198     295     323     171     278     336     202     314
            ( 26.4) ( 31.5) ( 19.5) ( 29.0) ( 31.8) ( 16.8) ( 27.4) ( 33.1) ( 19.9) ( 30.9)
g_E         408.5   407.7   210.0   408.5   407.7   210.0   408.5   407.7   210.0   408.5
            ( 40.2) ( 40.1) ( 20.7) ( 40.2) ( 40.1) ( 20.7) ( 40.2) ( 40.1) ( 20.7) ( 40.2)
           -225.9  -155.0   -23.4  -192.1  -150.5   -70.3  -214.0  -130.0   -15.7  -165.2
          -22.237 -15.261  -2.299 -18.903 -14.810  -6.920 -21.062 -12.797  -1.550 -16.263
c_O           478     309     336     459     319     341     497     317     341     488
            ( 47.0) ( 30.4) ( 33.1) ( 45.2) ( 31.4) ( 33.6) ( 48.9) ( 31.2) ( 33.6) ( 48.0)
c_E         491.3   306.0   276.9   491.3   306.0   276.9   491.3   306.0   276.9   491.3
            ( 48.4) ( 30.1) ( 27.3) ( 48.4) ( 30.1) ( 27.3) ( 48.4) ( 30.1) ( 27.3) ( 48.4)
            -26.3     6.0   130.0   -62.5    26.5   142.0    11.4    22.3   142.0    -6.6
           -2.589   0.586  12.794  -6.151   2.605  13.976   1.122   2.196  13.976  -0.654

N            1016    1016    1016    1016    1016    1016    1016    1016    1016    1016
X2          253.2    45.9    18.0   225.2    39.9    23.4   182.9    25.8    28.8   104.8
Gvalue      203.6    44.7    17.3   175.4    39.1    22.9   152.6    25.8    29.0    89.9
Gv/N       20.037   4.402   1.707  17.265   3.846   2.253  15.019   2.540   2.853   8.848
Prob      0.00000 0.00000 0.00060 0.00000 0.00000 0.00004 0.00000 0.00001 0.00000 0.00000
            ---------------------------------------------------------------------------------
               31      32      33      34      35      36      37      38      39      40
            ---------------------------------------------------------------------------------
a_O           210     192     102     190     187      79     178     189      79     162
            ( 20.7) ( 18.9) ( 10.0) ( 18.7) ( 18.4) (  7.8) ( 17.5) ( 18.6) (  7.8) ( 15.9)
a_E         182.9   241.4    51.7   182.9   241.4    51.7   182.9   241.4    51.7   182.9
            ( 18.0) ( 23.8) (  5.1) ( 18.0) ( 23.8) (  5.1) ( 18.0) ( 23.8) (  5.1) ( 18.0)
             57.9   -87.9   138.7    14.4   -95.4    67.1    -9.8   -92.4    67.1   -39.4
            5.701  -8.649  13.655   1.415  -9.395   6.602  -0.960  -9.099   6.602  -3.878
t_O           151     285     128     140     281     124     128     291     123     153
            ( 14.9) ( 28.1) ( 12.6) ( 13.8) ( 27.7) ( 12.2) ( 12.6) ( 28.6) ( 12.1) ( 15.1)
t_E         119.3   287.7    64.5   119.3   287.7    64.5   119.3   287.7    64.5   119.3
            ( 11.7) ( 28.3) (  6.3) ( 11.7) ( 28.3) (  6.3) ( 11.7) ( 28.3) (  6.3) ( 11.7)
             71.2    -5.4   175.5    44.8   -13.2   162.1    18.0     6.6   158.8    76.1
            7.006  -0.528  17.272   4.411  -1.302  15.957   1.775   0.654  15.632   7.495
```

g_O	339	217	336	354	228	301	393	181	338	388
	(33.4)	(21.4)	(33.1)	(34.8)	(22.4)	(29.6)	(38.7)	(17.8)	(33.3)	(38.2)
g_E	407.7	210.0	408.5	407.7	210.0	408.5	407.7	210.0	408.5	407.7
	(40.1)	(20.7)	(40.2)	(40.1)	(20.7)	(40.2)	(40.1)	(20.7)	(40.2)	(40.1)
	-125.1	14.2	-131.3	-100.0	37.4	-183.8	-28.9	-53.8	-128.1	-38.5
	-12.318	1.395	-12.923	-9.846	3.685	-18.095	-2.845	-5.300	-12.605	-3.787
c_O	316	322	450	332	320	512	317	355	476	313
	(31.1)	(31.7)	(44.3)	(32.7)	(31.5)	(50.4)	(31.2)	(34.9)	(46.9)	(30.8)
c_E	306.0	276.9	491.3	306.0	276.9	491.3	306.0	276.9	491.3	306.0
	(30.1)	(27.3)	(48.4)	(30.1)	(27.3)	(48.4)	(30.1)	(27.3)	(48.4)	(30.1)
	20.2	97.2	-79.1	54.1	92.6	42.2	22.3	176.4	-30.2	14.1
	1.993	9.563	-7.784	5.321	9.111	4.153	2.196	17.361	-2.971	1.386
N	1016	1016	1016	1016	1016	1016	1016	1016	1016	1016
X2	24.3	17.7	127.9	13.1	20.6	98.5	1.7	37.4	80.2	13.0
Gvalue	24.2	18.1	103.8	13.2	21.3	87.5	1.7	36.7	67.6	12.4
Gv/N	2.382	1.781	10.219	1.302	2.099	8.617	0.165	3.616	6.658	1.216
Prob	0.00002	0.00042	0.00000	0.00417	0.00009	0.00000	0.64108	0.00000	0.00000	0.00625

	41	42	43	44	45	46	47	48	49	50
a_O	190	80	183	211	98	153	207	81	183	203
	(18.7)	(7.9)	(18.0)	(20.8)	(9.6)	(15.1)	(20.4)	(8.0)	(18.0)	(20.0)
a_E	241.4	51.7	182.9	241.4	51.7	182.9	241.4	51.7	182.9	241.4
	(23.8)	(5.1)	(18.0)	(23.8)	(5.1)	(18.0)	(23.8)	(5.1)	(18.0)	(23.8)
	-90.9	69.9	0.1	-56.7	125.5	-54.7	-63.6	72.8	0.1	-70.3
	-8.950	6.884	0.011	-5.585	12.348	-5.384	-6.259	7.168	0.011	-6.918
t_O	292	121	117	272	115	144	300	102	139	284
	(28.7)	(11.9)	(11.5)	(26.8)	(11.3)	(14.2)	(29.5)	(10.0)	(13.7)	(28.0)
t_E	287.7	64.5	119.3	287.7	64.5	119.3	287.7	64.5	119.3	287.7
	(28.3)	(6.3)	(11.7)	(28.3)	(6.3)	(11.7)	(28.3)	(6.3)	(11.7)	(28.3)
	8.7	152.3	-4.5	-30.5	133.0	54.2	25.1	93.5	42.5	-7.3
	0.854	14.987	-0.447	-3.004	13.093	5.335	2.473	9.204	4.183	-0.723
g_O	239	312	374	221	344	377	212	357	383	224
	(23.5)	(30.7)	(36.8)	(21.8)	(33.9)	(37.1)	(20.9)	(35.1)	(37.7)	(22.0)
g_E	210.0	408.5	407.7	210.0	408.5	407.7	210.0	408.5	407.7	210.0
	(20.7)	(40.2)	(40.1)	(20.7)	(40.2)	(40.1)	(20.7)	(40.2)	(40.1)	(20.7)
	61.8	-168.2	-64.6	22.5	-118.2	-59.1	4.0	-96.2	-47.9	28.8
	6.079	-16.552	-6.356	2.215	-11.637	-5.814	0.390	-9.470	-4.716	2.840
c_O	295	503	342	312	459	342	297	476	311	305
	(29.0)	(49.5)	(33.7)	(30.7)	(45.2)	(33.7)	(29.2)	(46.9)	(30.6)	(30.0)
c_E	276.9	491.3	306.0	276.9	491.3	306.0	276.9	491.3	306.0	276.9
	(27.3)	(48.4)	(30.1)	(27.3)	(48.4)	(30.1)	(27.3)	(48.4)	(30.1)	(27.3)
	37.3	23.6	76.0	74.5	-62.5	76.0	41.6	-30.2	10.0	58.9
	3.675	2.324	7.480	7.328	-6.151	7.480	4.095	-2.971	0.985	5.802
N	1016	1016	1016	1016	1016	1016	1016	1016	1016	1016
X2	16.2	88.1	7.1	9.7	93.4	16.6	6.9	45.4	4.8	9.9
Gvalue	16.8	77.7	7.0	9.7	77.8	16.4	7.1	39.9	4.7	10.2
Gv/N	1.658	7.643	0.687	0.955	7.653	1.618	0.699	3.931	0.463	1.001
Prob	0.00076	0.00000	0.07244	0.02132	0.00000	0.00092	0.06862	0.00000	0.19517	0.01721

	51	52	53	54	55	56	57	58	59	60
a_O	74	184	184	71	181	215	69	178	218	55
	(7.3)	(18.1)	(18.1)	(7.0)	(17.8)	(21.2)	(6.8)	(17.5)	(21.5)	(5.4)
a_E	51.7	182.9	241.4	51.7	182.9	241.4	51.7	182.9	241.4	51.7
	(5.1)	(18.0)	(23.8)	(5.1)	(18.0)	(23.8)	(5.1)	(18.0)	(23.8)	(5.1)
	53.2	2.1	-99.9	45.1	-3.9	-49.7	39.9	-9.8	-44.4	6.9
	5.232	0.208	-9.830	4.442	-0.381	-4.896	3.928	-0.960	-4.370	0.676
t_O	109	144	294	101	129	293	116	125	276	104
	(10.7)	(14.2)	(28.9)	(9.9)	(12.7)	(28.8)	(11.4)	(12.3)	(27.2)	(10.2)
t_E	64.5	119.3	287.7	64.5	119.3	287.7	64.5	119.3	287.7	64.5
	(6.3)	(11.7)	(28.3)	(6.3)	(11.7)	(28.3)	(6.3)	(11.7)	(28.3)	(6.3)
	114.4	54.2	12.7	90.6	20.2	10.7	136.2	11.7	-22.9	99.4
	11.260	5.335	1.255	8.918	1.986	1.054	13.404	1.150	-2.255	9.782
g_O	357	372	204	348	393	214	353	399	214	359
	(35.1)	(36.6)	(20.1)	(34.3)	(38.7)	(21.1)	(34.7)	(39.3)	(21.1)	(35.3)
g_E	408.5	407.7	210.0	408.5	407.7	210.0	408.5	407.7	210.0	408.5
	(40.2)	(40.1)	(20.7)	(40.2)	(40.1)	(20.7)	(40.2)	(40.1)	(20.7)	(40.2)
	-96.2	-68.2	-11.9	-111.6	-28.9	8.0	-103.1	-17.3	8.0	-92.7
	-9.470	-6.714	-1.170	-10.981	-2.845	0.789	-10.147	-1.698	0.789	-9.129
c_O	476	316	334	496	313	294	478	314	308	498
	(46.9)	(31.1)	(32.9)	(48.8)	(30.8)	(28.9)	(47.0)	(30.9)	(30.3)	(49.0)
c_E	491.3	306.0	276.9	491.3	306.0	276.9	491.3	306.0	276.9	491.3
	(48.4)	(30.1)	(27.3)	(48.4)	(30.1)	(27.3)	(48.4)	(30.1)	(27.3)	(48.4)
	-30.2	20.2	125.2	9.4	14.1	35.2	-26.3	16.1	65.6	13.4
	-2.971	1.993	12.325	0.923	1.386	3.466	-2.589	1.587	6.452	1.321
N	1016	1016	1016	1016	1016	1016	1016	1016	1016	1016
X2	47.3	8.6	25.7	36.9	1.5	4.1	54.8	0.8	6.3	30.5
Gvalue	41.2	8.3	26.2	33.5	1.5	4.2	46.7	0.8	6.3	26.9
Gv/N	4.051	0.822	2.580	3.302	0.146	0.413	4.597	0.078	0.616	2.651
Prob	0.00000	0.03934	0.00001	0.00000	0.68573	0.24093	0.00000	0.85023	0.09949	0.00001

	61	62	63	64	65	66	67	68	69	70
a_O	184	215	91	169	195	75	172	220	76	193
	(18.1)	(21.2)	(9.0)	(16.6)	(19.2)	(7.4)	(16.9)	(21.7)	(7.5)	(19.0)
a_E	182.9	241.4	51.7	182.9	241.4	51.7	182.9	241.4	51.7	182.9
	(18.0)	(23.8)	(5.1)	(18.0)	(23.8)	(5.1)	(18.0)	(23.8)	(5.1)	(18.0)
	2.1	-49.7	103.0	-26.8	-83.2	55.9	-21.2	-40.8	58.6	20.7
	0.208	-4.896	10.139	-2.638	-8.189	5.501	-2.089	-4.014	5.773	2.033
t_O	115	272	89	123	273	105	124	292	107	134
	(11.3)	(26.8)	(8.8)	(12.1)	(26.9)	(10.3)	(12.2)	(28.7)	(10.5)	(13.2)
t_E	119.3	287.7	64.5	119.3	287.7	64.5	119.3	287.7	64.5	119.3
	(11.7)	(28.3)	(6.3)	(11.7)	(28.3)	(6.3)	(11.7)	(28.3)	(6.3)	(11.7)
	-8.4	-30.5	57.3	7.5	-28.6	102.4	9.6	8.7	108.3	31.2
	-0.830	-3.004	5.643	0.741	-2.818	10.074	0.944	0.854	10.663	3.066
g_O	393	201	379	396	222	356	397	219	338	388
	(38.7)	(19.8)	(37.3)	(39.0)	(21.9)	(35.0)	(39.1)	(21.6)	(33.3)	(38.2)
g_E	407.7	210.0	408.5	407.7	210.0	408.5	407.7	210.0	408.5	407.7
	(40.1)	(20.7)	(40.2)	(40.1)	(20.7)	(40.2)	(40.1)	(20.7)	(40.2)	(40.1)
	-28.9	-17.7	-56.8	-23.1	24.6	-97.9	-21.2	18.3	-128.1	-38.5
	-2.845	-1.739	-5.593	-2.274	2.422	-9.641	-2.082	1.803	-12.605	-3.787
c_O	324	328	457	328	326	480	323	285	495	301
	(31.9)	(32.3)	(45.0)	(32.3)	(32.1)	(47.2)	(31.8)	(28.1)	(48.7)	(29.6)
c_E	306.0	276.9	491.3	306.0	276.9	491.3	306.0	276.9	491.3	306.0
	(30.1)	(27.3)	(48.4)	(30.1)	(27.3)	(48.4)	(30.1)	(27.3)	(48.4)	(30.1)
	37.0	111.1	-66.2	45.5	106.4	-22.4	34.8	16.4	7.4	-10.0
	3.638	10.933	-6.517	4.475	10.474	-2.205	3.430	1.616	0.724	-0.984

N	1016	1016	1016	1016	1016	1016	1016	1016	1016	1016
X2	1.7	13.6	43.8	3.1	19.0	43.0	2.1	2.6	51.7	3.4
Gvalue	1.7	13.2	37.3	3.1	19.2	37.9	2.1	2.6	46.3	3.3
Gv/N	0.171	1.295	3.672	0.304	1.890	3.729	0.203	0.258	4.555	0.329
Prob	0.62924	0.00432	0.00000	0.37871	0.00025	0.00000	0.56022	0.45292	0.00000	0.34234

	71	72	73	74	75	76	77	78	79	80
a_O	241	66	191	229	75	180	238	63	196	201
	(23.7)	(6.5)	(18.8)	(22.5)	(7.4)	(17.7)	(23.4)	(6.2)	(19.3)	(19.8)
a_E	241.4	51.7	182.9	241.4	51.7	182.9	241.4	51.7	182.9	241.4
	(23.8)	(5.1)	(18.0)	(23.8)	(5.1)	(18.0)	(23.8)	(5.1)	(18.0)	(23.8)
	-0.7	32.3	16.5	-24.1	55.9	-5.8	-6.7	25.0	27.0	-73.6
	-0.072	3.180	1.620	-2.371	5.501	-0.575	-0.658	2.459	2.659	-7.241
t_O	299	90	123	274	76	122	275	85	135	330
	(29.4)	(8.9)	(12.1)	(27.0)	(7.5)	(12.0)	(27.1)	(8.4)	(13.3)	(32.5)
t_E	287.7	64.5	119.3	287.7	64.5	119.3	287.7	64.5	119.3	287.7
	(28.3)	(6.3)	(11.7)	(28.3)	(6.3)	(11.7)	(28.3)	(6.3)	(11.7)	(28.3)
	23.0	60.0	7.5	-26.7	25.0	5.5	-24.8	46.9	33.4	90.5
	2.269	5.904	0.741	-2.631	2.456	0.539	-2.443	4.620	3.287	8.912
g_O	200	406	385	195	358	385	220	367	374	196
	(19.7)	(40.0)	(37.9)	(19.2)	(35.2)	(37.9)	(21.7)	(36.1)	(36.8)	(19.3)
g_E	210.0	408.5	407.7	210.0	408.5	407.7	210.0	408.5	407.7	210.0
	(20.7)	(40.2)	(40.1)	(20.7)	(40.2)	(40.1)	(20.7)	(40.2)	(40.1)	(20.7)
	-19.6	-5.0	-44.2	-29.0	-94.5	-44.2	20.4	-78.6	-64.6	-27.1
	-1.926	-0.491	-4.346	-2.850	-9.300	-4.346	2.009	-7.740	-6.356	-2.667
c_O	276	454	317	318	507	329	283	501	311	289
	(27.2)	(44.7)	(31.2)	(31.3)	(49.9)	(32.4)	(27.9)	(49.3)	(30.6)	(28.4)
c_E	276.9	491.3	306.0	276.9	491.3	306.0	276.9	491.3	306.0	276.9
	(27.3)	(48.4)	(30.1)	(27.3)	(48.4)	(30.1)	(27.3)	(48.4)	(30.1)	(27.3)
	-1.8	-71.8	22.3	88.0	31.8	47.6	12.3	19.5	10.0	24.7
	-0.178	-7.063	2.196	8.662	3.133	4.685	1.212	1.921	0.985	2.432
N	1016	1016	1016	1016	1016	1016	1016	1016	1016	1016
X2	0.9	16.9	2.1	8.5	19.3	3.1	1.2	13.4	5.9	14.4
Gvalue	0.9	15.5	2.1	8.2	18.2	3.1	1.2	12.8	5.8	14.6
Gv/N	0.091	1.530	0.211	0.810	1.790	0.303	0.120	1.260	0.575	1.435
Prob	0.81847	0.00140	0.54350	0.04156	0.00040	0.37942	0.74944	0.00509	0.11957	0.00221

	81	82	83	84	85	86	87	88	89	90
a_O	75	168	215	61	174	226	76	186	235	64
	(7.4)	(16.5)	(21.2)	(6.0)	(17.1)	(22.2)	(7.5)	(18.3)	(23.1)	(6.3)
a_E	51.7	182.9	241.4	51.7	182.9	241.4	51.7	182.9	241.4	51.7
	(5.1)	(18.0)	(23.8)	(5.1)	(18.0)	(23.8)	(5.1)	(18.0)	(23.8)	(5.1)
	55.9	-28.6	-49.7	20.3	-17.4	-29.7	58.6	6.2	-12.6	27.4
	5.501	-2.819	-4.896	1.993	-1.717	-2.927	5.773	0.606	-1.237	2.696
t_O	87	126	291	73	115	282	78	124	284	65
	(8.6)	(12.4)	(28.6)	(7.2)	(11.3)	(27.8)	(7.7)	(12.2)	(28.0)	(6.4)
t_E	64.5	119.3	287.7	64.5	119.3	287.7	64.5	119.3	287.7	64.5
	(6.3)	(11.7)	(28.3)	(6.3)	(11.7)	(28.3)	(6.3)	(11.7)	(28.3)	(6.3)
	52.1	13.8	6.6	18.1	-8.4	-11.3	29.7	9.6	-7.3	1.0
	5.127	1.356	0.654	1.780	-0.830	-1.110	2.920	0.944	-0.723	0.100
g_O	389	388	205	382	395	206	398	391	213	396
	(38.3)	(38.2)	(20.2)	(37.6)	(38.9)	(20.3)	(39.2)	(38.5)	(21.0)	(39.0)
g_E	408.5	407.7	210.0	408.5	407.7	210.0	408.5	407.7	210.0	408.5
	(40.2)	(40.1)	(20.7)	(40.2)	(40.1)	(20.7)	(40.2)	(40.1)	(20.7)	(40.2)
	-38.1	-38.5	-9.9	-51.2	-25.0	-8.0	-20.7	-32.7	6.0	-24.6
	-3.746	-3.787	-0.978	-5.044	-2.465	-0.786	-2.041	-3.223	0.589	-2.423
c_O	465	334	305	500	332	302	464	315	284	491
	(45.8)	(32.9)	(30.0)	(49.2)	(32.7)	(29.7)	(45.7)	(31.0)	(28.0)	(48.3)
c_E	491.3	306.0	276.9	491.3	306.0	276.9	491.3	306.0	276.9	491.3
	(48.4)	(30.1)	(27.3)	(48.4)	(30.1)	(27.3)	(48.4)	(30.1)	(27.3)	(48.4)
	-51.2	58.4	58.9	17.5	54.1	52.4	-53.1	18.2	14.4	-0.7
	-5.042	5.748	5.802	1.721	5.321	5.157	-5.228	1.790	1.414	-0.066
N	1016	1016	1016	1016	1016	1016	1016	1016	1016	1016
X2	20.7	5.1	5.9	4.7	3.2	3.4	16.1	1.2	0.4	3.3
Gvalue	18.7	5.1	5.9	4.6	3.1	3.4	14.5	1.2	0.4	3.1
Gv/N	1.839	0.500	0.582	0.450	0.310	0.335	1.423	0.117	0.043	0.307
Prob	0.00032	0.16634	0.11612	0.20554	0.36982	0.33406	0.00234	0.75561	0.93205	0.37300

	91	92	93	94	95	96	97	98	99	100
a_O	185	225	48	182	218	49	165	220	59	185
	(18.2)	(22.1)	(4.7)	(17.9)	(21.5)	(4.8)	(16.2)	(21.7)	(5.8)	(18.2)
a_E	182.9	241.4	51.7	182.9	241.4	51.7	182.9	241.4	51.7	182.9
	(18.0)	(23.8)	(5.1)	(18.0)	(23.8)	(5.1)	(18.0)	(23.8)	(5.1)	(18.0)
	4.1	-31.6	-7.1	-1.9	-44.4	-5.2	-34.1	-40.8	15.7	4.1
	0.407	-3.110	-0.696	-0.186	-4.370	-0.512	-3.353	-4.014	1.541	0.407
t_O	105	281	67	113	293	88	117	275	71	112
	(10.3)	(27.7)	(6.6)	(11.1)	(28.8)	(8.7)	(11.5)	(27.1)	(7.0)	(11.0)
t_E	119.3	287.7	64.5	119.3	287.7	64.5	119.3	287.7	64.5	119.3
	(11.7)	(28.3)	(6.3)	(11.7)	(28.3)	(6.3)	(11.7)	(28.3)	(6.3)	(11.7)
	-26.8	-13.2	5.1	-12.3	10.7	54.7	-4.5	-24.8	13.6	-14.1
	-2.638	-1.302	0.503	-1.206	1.054	5.383	-0.447	-2.443	1.343	-1.391
g_O	417	199	394	419	191	394	410	212	378	408
	(41.0)	(19.6)	(38.8)	(41.2)	(18.8)	(38.8)	(40.4)	(20.9)	(37.2)	(40.2)
g_E	407.7	210.0	408.5	407.7	210.0	408.5	407.7	210.0	408.5	407.7
	(40.1)	(20.7)	(40.2)	(40.1)	(20.7)	(40.2)	(40.1)	(20.7)	(40.2)	(40.1)
	18.8	-21.5	-28.5	22.9	-36.3	-28.5	4.6	4.0	-58.7	0.6
	1.847	-2.113	-2.804	2.251	-3.571	-2.804	0.450	0.390	-5.774	0.055
c_O	309	311	507	302	314	485	324	309	508	311
	(30.4)	(30.6)	(49.9)	(29.7)	(30.9)	(47.7)	(31.9)	(30.4)	(50.0)	(30.6)
c_E	306.0	276.9	491.3	306.0	276.9	491.3	306.0	276.9	491.3	306.0
	(30.1)	(27.3)	(48.4)	(30.1)	(27.3)	(48.4)	(30.1)	(27.3)	(48.4)	(30.1)
	6.0	72.2	31.8	-8.0	78.9	-12.6	37.0	67.8	33.9	10.0
	0.586	7.108	3.133	-0.790	7.770	-1.239	3.638	6.670	3.336	0.985
N	1016	1016	1016	1016	1016	1016	1016	1016	1016	1016
X2	2.0	6.0	1.4	0.7	9.1	9.3	2.9	6.2	4.5	0.5
Gvalue	2.0	5.9	1.4	0.7	9.0	8.4	2.9	6.1	4.5	0.6
Gv/N	0.201	0.582	0.136	0.069	0.883	0.829	0.287	0.602	0.445	0.055
Prob	0.56276	0.11569	0.71055	0.87186	0.02962	0.03798	0.40536	0.10589	0.21010	0.90585

5UTR:

	100	99	98	97	96	95	94	93	92	91
a_O	161	153	150	171	127	155	155	138	146	160
	(20.2)	(19.2)	(18.8)	(21.4)	(15.9)	(19.4)	(19.4)	(17.3)	(18.3)	(20.1)
a_E	149.0	149.0	149.0	149.0	149.0	149.0	149.0	149.0	149.0	149.0
	(18.7)	(18.7)	(18.7)	(18.7)	(18.7)	(18.7)	(18.7)	(18.7)	(18.7)	(18.7)
	24.9	8.1	2.0	47.1	-40.6	12.2	12.2	-21.2	-6.0	22.8
	3.123	1.013	0.249	5.899	-5.087	1.531	1.531	-2.655	-0.747	2.854
t_O	147	143	127	143	143	116	156	140	146	162
	(18.4)	(17.9)	(15.9)	(17.9)	(17.9)	(14.5)	(19.5)	(17.5)	(18.3)	(20.3)
t_E	150.9	150.9	150.9	150.9	150.9	150.9	150.9	150.9	150.9	150.9
	(18.9)	(18.9)	(18.9)	(18.9)	(18.9)	(18.9)	(18.9)	(18.9)	(18.9)	(18.9)
	-7.6	-15.3	-43.7	-15.3	-15.3	-61.0	10.4	-20.9	-9.6	23.1
	-0.957	-1.920	-5.482	-1.920	-1.920	-7.641	1.307	-2.624	-1.201	2.890
g_O	235	224	267	242	270	258	243	276	253	237
	(29.4)	(28.1)	(33.5)	(30.3)	(33.8)	(32.3)	(30.5)	(34.6)	(31.7)	(29.7)
g_E	226.7	226.7	226.7	226.7	226.7	226.7	226.7	226.7	226.7	226.7
	(28.4)	(28.4)	(28.4)	(28.4)	(28.4)	(28.4)	(28.4)	(28.4)	(28.4)	(28.4)
	16.9	-5.3	87.4	31.7	94.4	66.8	33.8	108.7	55.6	21.1
	2.123	-0.668	10.955	3.967	11.834	8.369	4.234	13.617	6.965	2.644
c_O	255	278	254	242	258	269	244	244	253	239
	(32.0)	(34.8)	(31.8)	(30.3)	(32.3)	(33.7)	(30.6)	(30.6)	(31.7)	(29.9)
c_E	271.4	271.4	271.4	271.4	271.4	271.4	271.4	271.4	271.4	271.4
	(34.0)	(34.0)	(34.0)	(34.0)	(34.0)	(34.0)	(34.0)	(34.0)	(34.0)	(34.0)
	-31.9	13.3	-33.7	-55.6	-26.2	-4.9	-52.0	-52.0	-35.6	-60.8
	-3.993	1.664	-4.227	-6.963	-3.284	-0.609	-6.517	-6.517	-4.461	-7.624
N	798	798	798	798	798	798	798	798	798	798
X2	2.4	0.7	12.1	7.9	12.6	12.6	4.4	15.1	4.5	6.0
Gvalue	2.4	0.7	11.9	7.8	12.3	13.2	4.4	14.5	4.4	6.1
Gv/N	0.296	0.089	1.494	0.983	1.543	1.650	0.555	1.822	0.557	0.764
Prob	0.50147	0.87018	0.00764	0.04939	0.00638	0.00429	0.21859	0.00226	0.21712	0.10698

	90	89	88	87	86	85	84	83	82	81
a_O	138	133	152	134	140	152	149	145	168	139
	(17.3)	(16.7)	(19.0)	(16.8)	(17.5)	(19.0)	(18.7)	(18.2)	(21.1)	(17.4)
a_E	149.0	149.0	149.0	149.0	149.0	149.0	149.0	149.0	149.0	149.0
	(18.7)	(18.7)	(18.7)	(18.7)	(18.7)	(18.7)	(18.7)	(18.7)	(18.7)	(18.7)
	-21.2	-30.2	6.0	-28.5	-17.5	6.0	-0.0	-7.9	40.3	-19.3
	-2.655	-3.789	0.757	-3.566	-2.188	0.757	-0.003	-0.991	5.051	-2.423
t_O	126	128	151	128	133	157	135	128	143	156
	(15.8)	(16.0)	(18.9)	(16.0)	(16.7)	(19.7)	(16.9)	(16.0)	(17.9)	(19.5)
t_E	150.9	150.9	150.9	150.9	150.9	150.9	150.9	150.9	150.9	150.9
	(18.9)	(18.9)	(18.9)	(18.9)	(18.9)	(18.9)	(18.9)	(18.9)	(18.9)	(18.9)
	-45.4	-42.1	0.3	-42.1	-33.5	12.5	-30.0	-42.1	-15.3	10.4
	-5.689	-5.274	0.033	-5.274	-4.202	1.567	-3.761	-5.274	-1.920	1.307
g_O	256	248	254	280	263	254	260	262	250	249
	(32.1)	(31.1)	(31.8)	(35.1)	(33.0)	(31.8)	(32.6)	(32.8)	(31.3)	(31.2)
g_E	226.7	226.7	226.7	226.7	226.7	226.7	226.7	226.7	226.7	226.7
	(28.4)	(28.4)	(28.4)	(28.4)	(28.4)	(28.4)	(28.4)	(28.4)	(28.4)	(28.4)
	62.3	44.6	57.8	118.3	78.2	57.8	71.3	75.9	49.0	46.8
	7.804	5.587	7.244	14.825	9.796	7.244	8.937	9.509	6.135	5.861
c_O	278	289	241	256	262	235	254	263	237	254
	(34.8)	(36.2)	(30.2)	(32.1)	(32.8)	(29.4)	(31.8)	(33.0)	(29.7)	(31.8)
c_E	271.4	271.4	271.4	271.4	271.4	271.4	271.4	271.4	271.4	271.4
	(34.0)	(34.0)	(34.0)	(34.0)	(34.0)	(34.0)	(34.0)	(34.0)	(34.0)	(34.0)
	13.3	36.2	-57.3	-30.0	-18.5	-67.8	-33.7	-16.6	-64.3	-33.7
	1.664	4.540	-7.184	-3.757	-2.324	-8.490	-4.227	-2.082	-8.059	-4.227
N	798	798	798	798	798	798	798	798	798	798
X2	8.9	8.3	6.8	18.4	8.8	8.5	7.7	9.3	9.6	4.2
Gvalue	9.0	8.5	6.8	17.8	8.6	8.6	7.6	9.3	9.6	4.1
Gv/N	1.125	1.065	0.849	2.228	1.081	1.078	0.946	1.161	1.207	0.518
Prob	0.02961	0.03674	0.07938	0.00049	0.03471	0.03511	0.05627	0.02592	0.02200	0.24727

	80	79	78	77	76	75	74	73	72	71
a_O	140	157	136	125	169	137	154	169	139	152
	(17.5)	(19.7)	(17.0)	(15.7)	(21.2)	(17.2)	(19.3)	(21.2)	(17.4)	(19.0)
a_E	149.0	149.0	149.0	149.0	149.0	149.0	149.0	149.0	149.0	149.0
	(18.7)	(18.7)	(18.7)	(18.7)	(18.7)	(18.7)	(18.7)	(18.7)	(18.7)	(18.7)
	-17.5	16.4	-24.8	-43.9	42.5	-23.0	10.1	42.5	-19.3	6.0
	-2.188	2.055	-3.114	-5.504	5.332	-2.885	1.271	5.332	-2.423	0.757
t_O	148	139	143	143	146	143	132	149	147	144
	(18.5)	(17.4)	(17.9)	(17.9)	(18.3)	(17.9)	(16.5)	(18.7)	(18.4)	(18.0)
t_E	150.9	150.9	150.9	150.9	150.9	150.9	150.9	150.9	150.9	150.9
	(18.9)	(18.9)	(18.9)	(18.9)	(18.9)	(18.9)	(18.9)	(18.9)	(18.9)	(18.9)
	-5.7	-22.8	-15.3	-15.3	-9.6	-15.3	-35.3	-3.7	-7.6	-13.4
	-0.712	-2.855	-1.920	-1.920	-1.201	-1.920	-4.420	-0.466	-0.957	-1.682
g_O	266	258	234	276	232	253	259	242	238	252
	(33.3)	(32.3)	(29.3)	(34.6)	(29.1)	(31.7)	(32.5)	(30.3)	(29.8)	(31.6)
g_E	226.7	226.7	226.7	226.7	226.7	226.7	226.7	226.7	226.7	226.7
	(28.4)	(28.4)	(28.4)	(28.4)	(28.4)	(28.4)	(28.4)	(28.4)	(28.4)	(28.4)
	85.1	66.8	14.9	108.7	10.8	55.6	69.0	31.7	23.2	53.4
	10.664	8.369	1.864	13.617	1.349	6.965	8.652	3.967	2.907	6.688
c_O	244	244	285	254	251	265	253	238	274	250
	(30.6)	(30.6)	(35.7)	(31.8)	(31.5)	(33.2)	(31.7)	(29.8)	(34.3)	(31.3)
c_E	271.4	271.4	271.4	271.4	271.4	271.4	271.4	271.4	271.4	271.4
	(34.0)	(34.0)	(34.0)	(34.0)	(34.0)	(34.0)	(34.0)	(34.0)	(34.0)	(34.0)
	-52.0	-52.0	27.8	-33.7	-39.3	-12.7	-35.6	-62.6	5.1	-41.1
	-6.517	-6.517	3.482	-4.227	-4.925	-1.595	-4.461	-7.842	0.645	-5.155
N	798	798	798	798	798	798	798	798	798	798
X2	10.2	8.5	2.5	16.1	4.5	4.6	8.4	7.9	1.4	4.9
Gvalue	9.9	8.4	2.5	15.7	4.4	4.5	8.3	7.9	1.4	4.8
Gv/N	1.246	1.052	0.312	1.966	0.555	0.565	1.042	0.991	0.171	0.607
Prob	0.01908	0.03854	0.47735	0.00132	0.21849	0.21130	0.03991	0.04801	0.71297	0.18346

	70	69	68	67	66	65	64	63	62	61
a_O	159	157	133	158	151	149	152	162	149	139
	(19.9)	(19.7)	(16.7)	(19.8)	(18.9)	(18.7)	(19.0)	(20.3)	(18.7)	(17.4)
a_E	149.0	149.0	149.0	149.0	149.0	149.0	149.0	149.0	149.0	149.0
	(18.7)	(18.7)	(18.7)	(18.7)	(18.7)	(18.7)	(18.7)	(18.7)	(18.7)	(18.7)
	20.6	16.4	-30.2	18.5	4.0	-0.0	6.0	27.1	-0.0	-19.3
	2.586	2.055	-3.789	2.320	0.502	-0.003	0.757	3.394	-0.003	-2.423
t_O	150	141	131	148	156	152	157	147	135	175
	(18.8)	(17.7)	(16.4)	(18.5)	(19.5)	(19.0)	(19.7)	(18.4)	(16.9)	(21.9)
t_E	150.9	150.9	150.9	150.9	150.9	150.9	150.9	150.9	150.9	150.9

	(18.9)	(18.9)	(18.9)	(18.9)	(18.9)	(18.9)	(18.9)	(18.9)	(18.9)	(18.9)
	-1.7	-19.1	-37.0	-5.7	10.4	2.3	12.5	-7.6	-30.0	51.9
	-0.217	-2.391	-4.637	-0.712	1.307	0.284	1.567	-0.957	-3.761	6.507
g_O	252	243	275	236	237	234	250	233	222	232
	(31.6)	(30.5)	(34.5)	(29.6)	(29.7)	(29.3)	(31.3)	(29.2)	(27.8)	(29.1)
g_E	226.7	226.7	226.7	226.7	226.7	226.7	226.7	226.7	226.7	226.7
	(28.4)	(28.4)	(28.4)	(28.4)	(28.4)	(28.4)	(28.4)	(28.4)	(28.4)	(28.4)
	53.4	33.8	106.3	19.0	21.1	14.9	49.0	12.8	-9.3	10.8
	6.688	4.234	13.318	2.383	2.644	1.864	6.135	1.606	-1.161	1.349
c_O	237	257	259	256	254	263	239	256	292	252
	(29.7)	(32.2)	(32.5)	(32.1)	(31.8)	(33.0)	(29.9)	(32.1)	(36.6)	(31.6)
c_E	271.4	271.4	271.4	271.4	271.4	271.4	271.4	271.4	271.4	271.4
	(34.0)	(34.0)	(34.0)	(34.0)	(34.0)	(34.0)	(34.0)	(34.0)	(34.0)	(34.0)
	-64.3	-28.1	-24.3	-30.0	-33.7	-16.6	-60.8	-30.0	42.6	-37.5
	-8.059	-3.521	-3.045	-3.757	-4.227	-2.082	-7.624	-3.757	5.343	-4.693
N	798	798	798	798	798	798	798	798	798	798
X2	7.9	3.0	15.2	1.9	1.8	0.5	6.6	2.3	3.3	6.0
Gvalue	8.0	3.0	14.7	1.9	1.8	0.5	6.7	2.3	3.3	5.9
Gv/N	0.997	0.377	1.847	0.233	0.226	0.064	0.836	0.285	0.420	0.740
Prob	0.04693	0.38991	0.00205	0.60193	0.61338	0.91724	0.08329	0.51810	0.34103	0.11621

	60	59	58	57	56	55	54	53	52	51
a_O	136	155	164	135	141	161	141	152	165	145
	(17.0)	(19.4)	(20.6)	(16.9)	(17.7)	(20.2)	(17.7)	(19.0)	(20.7)	(18.2)
a_E	149.0	149.0	149.0	149.0	149.0	149.0	149.0	149.0	149.0	149.0
	(18.7)	(18.7)	(18.7)	(18.7)	(18.7)	(18.7)	(18.7)	(18.7)	(18.7)	(18.7)
	-24.8	12.2	31.4	-26.7	-15.6	24.9	-15.6	6.0	33.6	-7.9
	-3.114	1.531	3.940	-3.341	-1.953	3.123	-1.953	0.757	4.215	-0.991
t_O	150	138	161	155	144	163	145	127	162	134
	(18.8)	(17.3)	(20.2)	(19.4)	(18.0)	(20.4)	(18.2)	(15.9)	(20.3)	(16.8)
t_E	150.9	150.9	150.9	150.9	150.9	150.9	150.9	150.9	150.9	150.9
	(18.9)	(18.9)	(18.9)	(18.9)	(18.9)	(18.9)	(18.9)	(18.9)	(18.9)	(18.9)
	-1.7	-24.6	20.9	8.4	-13.4	25.2	-11.5	-43.7	23.1	-31.8
	-0.217	-3.084	2.622	1.049	-1.682	3.159	-1.442	-5.482	2.890	-3.982
g_O	256	237	226	252	270	209	246	242	216	266
	(32.1)	(29.7)	(28.3)	(31.6)	(33.8)	(26.2)	(30.8)	(30.3)	(27.1)	(33.3)
g_E	226.7	226.7	226.7	226.7	226.7	226.7	226.7	226.7	226.7	226.7
	(28.4)	(28.4)	(28.4)	(28.4)	(28.4)	(28.4)	(28.4)	(28.4)	(28.4)	(28.4)
	62.3	21.1	-1.4	53.4	94.4	-33.9	40.2	31.7	-20.8	85.1
	7.804	2.644	-0.170	6.688	11.834	-4.254	5.043	3.967	-2.613	10.664
c_O	256	268	247	256	243	265	266	277	255	253
	(32.1)	(33.6)	(31.0)	(32.1)	(30.5)	(33.2)	(33.3)	(34.7)	(32.0)	(31.7)
c_E	271.4	271.4	271.4	271.4	271.4	271.4	271.4	271.4	271.4	271.4
	(34.0)	(34.0)	(34.0)	(34.0)	(34.0)	(34.0)	(34.0)	(34.0)	(34.0)	(34.0)
	-30.0	-6.8	-46.6	-30.0	-53.8	-12.7	-10.8	11.2	-31.9	-35.6
	-3.757	-0.857	-5.841	-3.757	-6.741	-1.595	-1.350	1.408	-3.993	-4.461
N	798	798	798	798	798	798	798	798	798	798
X2	5.8	1.9	4.4	5.1	12.0	3.5	2.4	5.0	4.0	10.1
Gvalue	5.7	1.9	4.4	5.1	11.6	3.5	2.4	5.2	4.0	9.8
Gv/N	0.715	0.235	0.551	0.639	1.459	0.434	0.298	0.649	0.500	1.229
Prob	0.12663	0.59880	0.22166	0.16487	0.00871	0.32595	0.49710	0.15917	0.26288	0.02026

	50	49	48	47	46	45	44	43	42	41
a_O	151	156	147	147	133	162	156	171	135	142
	(18.9)	(19.5)	(18.4)	(18.4)	(16.7)	(20.3)	(19.5)	(21.4)	(16.9)	(17.8)
a_E	149.0	149.0	149.0	149.0	149.0	149.0	149.0	149.0	149.0	149.0
	(18.7)	(18.7)	(18.7)	(18.7)	(18.7)	(18.7)	(18.7)	(18.7)	(18.7)	(18.7)
	4.0	14.3	-4.0	-4.0	-30.2	27.1	14.3	47.1	-26.7	-13.7
	0.502	1.792	-0.500	-0.500	-3.789	3.394	1.792	5.899	-3.341	-1.715
t_O	149	170	140	134	153	147	156	154	157	142
	(18.7)	(21.3)	(17.5)	(16.8)	(19.2)	(18.4)	(19.5)	(19.3)	(19.7)	(17.8)
t_E	150.9	150.9	150.9	150.9	150.9	150.9	150.9	150.9	150.9	150.9
	(18.9)	(18.9)	(18.9)	(18.9)	(18.9)	(18.9)	(18.9)	(18.9)	(18.9)	(18.9)
	-3.7	40.6	-20.9	-31.8	4.3	-7.6	10.4	6.3	12.5	-17.2
	-0.466	5.086	-2.624	-3.982	0.538	-0.957	1.307	0.793	1.567	-2.156
g_O	237	243	233	247	257	238	237	232	227	259
	(29.7)	(30.5)	(29.2)	(31.0)	(32.2)	(29.8)	(29.7)	(29.1)	(28.4)	(32.5)
g_E	226.7	226.7	226.7	226.7	226.7	226.7	226.7	226.7	226.7	226.7
	(28.4)	(28.4)	(28.4)	(28.4)	(28.4)	(28.4)	(28.4)	(28.4)	(28.4)	(28.4)
	21.1	33.8	12.8	42.4	64.5	23.2	21.1	10.8	0.6	69.0
	2.644	4.234	1.606	5.314	8.086	2.907	2.644	1.349	0.080	8.652
c_O	261	229	278	270	255	251	249	241	279	255
	(32.7)	(28.7)	(34.8)	(33.8)	(32.0)	(31.5)	(31.2)	(30.2)	(35.0)	(32.0)
c_E	271.4	271.4	271.4	271.4	271.4	271.4	271.4	271.4	271.4	271.4
	(34.0)	(34.0)	(34.0)	(34.0)	(34.0)	(34.0)	(34.0)	(34.0)	(34.0)	(34.0)
	-20.5	-77.9	13.3	-2.9	-31.9	-39.3	-43.0	-57.3	15.3	-31.9
	-2.566	-9.758	1.664	-0.360	-3.993	-4.925	-5.385	-7.184	1.921	-3.993
N	798	798	798	798	798	798	798	798	798	798
X2	0.9	10.6	1.1	3.7	6.8	3.3	2.8	6.8	1.8	6.5
Gvalue	0.9	10.8	1.2	3.8	6.7	3.3	2.9	6.8	1.8	6.3
Gv/N	0.115	1.355	0.146	0.472	0.842	0.418	0.359	0.856	0.227	0.788
Prob	0.82075	0.01279	0.76218	0.28796	0.08147	0.34260	0.41264	0.07739	0.61159	0.09845

	40	39	38	37	36	35	34	33	32	31
a_O	169	159	141	156	143	150	172	150	145	161
	(21.2)	(19.9)	(17.7)	(19.5)	(17.9)	(18.8)	(21.6)	(18.8)	(18.2)	(20.2)
a_E	149.0	149.0	149.0	149.0	149.0	149.0	149.0	149.0	149.0	149.0
	(18.7)	(18.7)	(18.7)	(18.7)	(18.7)	(18.7)	(18.7)	(18.7)	(18.7)	(18.7)
	42.5	20.6	-15.6	14.3	-11.8	2.0	49.4	2.0	-7.9	24.9
	5.332	2.586	-1.953	1.792	-1.475	0.249	6.185	0.249	-0.991	3.123
t_O	165	161	141	143	151	148	184	167	134	160
	(20.7)	(20.2)	(17.7)	(17.9)	(18.9)	(18.5)	(23.1)	(20.9)	(16.8)	(20.1)
t_E	150.9	150.9	150.9	150.9	150.9	150.9	150.9	150.9	150.9	150.9
	(18.9)	(18.9)	(18.9)	(18.9)	(18.9)	(18.9)	(18.9)	(18.9)	(18.9)	(18.9)
	29.5	20.9	-19.1	-15.3	0.3	-5.7	73.1	33.9	-31.8	18.8
	3.702	2.622	-2.391	-1.920	0.033	-0.712	9.155	4.251	-3.982	2.356
g_O	216	211	249	219	236	217	190	220	237	215
	(27.1)	(26.4)	(31.2)	(27.4)	(29.6)	(27.2)	(23.8)	(27.6)	(29.7)	(26.9)
g_E	226.7	226.7	226.7	226.7	226.7	226.7	226.7	226.7	226.7	226.7
	(28.4)	(28.4)	(28.4)	(28.4)	(28.4)	(28.4)	(28.4)	(28.4)	(28.4)	(28.4)
	-20.8	-30.2	46.8	-15.1	19.0	-18.9	-67.1	-13.2	21.1	-22.7
	-2.613	-3.791	5.861	-1.892	2.383	-2.374	-8.405	-1.649	2.644	-2.851
c_O	248	267	267	280	268	283	252	261	282	262
	(31.1)	(33.5)	(33.5)	(35.1)	(33.6)	(35.5)	(31.6)	(32.7)	(35.3)	(32.8)
c_E	271.4	271.4	271.4	271.4	271.4	271.4	271.4	271.4	271.4	271.4
	(34.0)	(34.0)	(34.0)	(34.0)	(34.0)	(34.0)	(34.0)	(34.0)	(34.0)	(34.0)

	-44.8	-8.8	-8.8	17.4	-6.8	23.6	-37.5	-20.5	21.5	-18.5
	-5.613	-1.104	-1.104	2.179	-0.857	2.958	-4.693	-2.566	2.697	-2.324
N	798	798	798	798	798	798	798	798	798	798
X2	6.5	2.5	3.3	1.3	0.7	1.0	18.1	2.3	2.9	2.4
Gvalue	6.4	2.5	3.3	1.3	0.7	1.0	17.9	2.3	2.9	2.4
Gv/N	0.808	0.314	0.414	0.159	0.084	0.121	2.241	0.286	0.368	0.304
Prob	0.09168	0.47452	0.34754	0.73601	0.88078	0.80939	0.00047	0.51669	0.40124	0.48878

	30	29	28	27	26	25	24	23	22	21
a_O	137	117	144	131	136	139	117	124	127	145
	(17.2)	(14.7)	(18.0)	(16.4)	(17.0)	(17.4)	(14.7)	(15.5)	(15.9)	(18.2)
a_E	149.0	149.0	149.0	149.0	149.0	149.0	149.0	149.0	149.0	149.0
	(18.7)	(18.7)	(18.7)	(18.7)	(18.7)	(18.7)	(18.7)	(18.7)	(18.7)	(18.7)
	-23.0	-56.6	-9.8	-33.7	-24.8	-19.3	-56.6	-45.6	-40.6	-7.9
	-2.885	-7.092	-1.234	-4.229	-3.114	-2.423	-7.092	-5.710	-5.087	-0.991
t_O	164	168	184	141	163	174	159	154	174	157
	(20.6)	(21.1)	(23.1)	(17.7)	(20.4)	(21.8)	(19.9)	(19.3)	(21.8)	(19.7)
t_E	150.9	150.9	150.9	150.9	150.9	150.9	150.9	150.9	150.9	150.9
	(18.9)	(18.9)	(18.9)	(18.9)	(18.9)	(18.9)	(18.9)	(18.9)	(18.9)	(18.9)
	27.4	36.1	73.1	-19.1	25.2	49.6	16.7	6.3	49.6	12.5
	3.430	4.528	9.155	-2.391	3.159	6.220	2.092	0.793	6.220	1.567
g_O	224	241	217	233	211	214	249	214	194	209
	(28.1)	(30.2)	(27.2)	(29.2)	(26.4)	(26.8)	(31.2)	(26.8)	(24.3)	(26.2)
g_E	226.7	226.7	226.7	226.7	226.7	226.7	226.7	226.7	226.7	226.7
	(28.4)	(28.4)	(28.4)	(28.4)	(28.4)	(28.4)	(28.4)	(28.4)	(28.4)	(28.4)
	-5.3	29.5	-18.9	12.8	-30.2	-24.6	46.8	-24.6	-60.4	-33.9
	-0.668	3.700	-2.374	1.606	-3.791	-3.087	5.861	-3.087	-7.569	-4.254
c_O	273	272	253	293	288	271	273	306	303	287
	(34.2)	(34.1)	(31.7)	(36.7)	(36.1)	(34.0)	(34.2)	(38.3)	(38.0)	(36.0)
c_E	271.4	271.4	271.4	271.4	271.4	271.4	271.4	271.4	271.4	271.4
	(34.0)	(34.0)	(34.0)	(34.0)	(34.0)	(34.0)	(34.0)	(34.0)	(34.0)	(34.0)
	3.1	1.1	-35.6	44.8	34.1	-0.9	3.1	73.3	66.7	32.0
	0.392	0.140	-4.461	5.613	4.274	-0.110	0.392	9.191	8.353	4.009
N	798	798	798	798	798	798	798	798	798	798
X2	2.2	9.7	9.1	4.7	4.2	4.9	9.5	9.4	15.2	2.6
Gvalue	2.1	10.2	8.7	4.8	4.2	4.8	10.0	9.5	15.3	2.6
Gv/N	0.269	1.277	1.086	0.598	0.529	0.600	1.253	1.186	1.916	0.332
Prob	0.54251	0.01700	0.03408	0.18912	0.23852	0.18790	0.01858	0.02369	0.00158	0.44943

	20	19	18	17	16	15	14	13	12	11
a_O	144	141	126	164	136	132	164	166	178	191
	(18.0)	(17.7)	(15.8)	(20.6)	(17.0)	(16.5)	(20.6)	(20.8)	(22.3)	(23.9)
a_E	149.0	149.0	149.0	149.0	149.0	149.0	149.0	149.0	149.0	149.0
	(18.7)	(18.7)	(18.7)	(18.7)	(18.7)	(18.7)	(18.7)	(18.7)	(18.7)	(18.7)
	-9.8	-15.6	-42.3	31.4	-24.8	-32.0	31.4	35.8	63.3	94.8
	-1.234	-1.953	-5.297	3.940	-3.114	-4.010	3.940	4.492	7.931	11.884
t_O	179	177	159	174	182	163	172	184	172	143
	(22.4)	(22.2)	(19.9)	(21.8)	(22.8)	(20.4)	(21.6)	(23.1)	(21.6)	(17.9)
t_E	150.9	150.9	150.9	150.9	150.9	150.9	150.9	150.9	150.9	150.9
	(18.9)	(18.9)	(18.9)	(18.9)	(18.9)	(18.9)	(18.9)	(18.9)	(18.9)	(18.9)
	61.2	56.5	16.7	49.6	68.3	25.2	45.1	73.1	45.1	-15.3
	7.670	7.086	2.092	6.220	8.557	3.159	5.650	9.155	5.650	-1.920
g_O	188	164	164	154	162	183	157	156	189	156
	(23.6)	(20.6)	(20.6)	(19.3)	(20.3)	(22.9)	(19.7)	(19.5)	(23.7)	(19.5)
g_E	226.7	226.7	226.7	226.7	226.7	226.7	226.7	226.7	226.7	226.7
	(28.4)	(28.4)	(28.4)	(28.4)	(28.4)	(28.4)	(28.4)	(28.4)	(28.4)	(28.4)
	-70.3	-106.2	-106.2	-119.1	-108.8	-78.3	-115.3	-116.6	-68.7	-116.6
	-8.816	-13.304	-13.304	-14.921	-13.640	-9.817	-14.452	-14.610	-8.611	-14.610
c_O	287	316	349	306	318	320	305	292	259	308
	(36.0)	(39.6)	(43.7)	(38.3)	(39.8)	(40.1)	(38.2)	(36.6)	(32.5)	(38.6)
c_E	271.4	271.4	271.4	271.4	271.4	271.4	271.4	271.4	271.4	271.4
	(34.0)	(34.0)	(34.0)	(34.0)	(34.0)	(34.0)	(34.0)	(34.0)	(34.0)	(34.0)
	32.0	96.1	175.4	73.3	100.7	105.3	71.1	42.6	-24.3	77.8
	4.009	12.038	21.984	9.191	12.617	13.199	8.911	5.343	-3.045	9.754
N	798	798	798	798	798	798	798	798	798	798
X2	12.9	29.6	43.5	32.8	34.0	20.0	30.0	32.8	15.4	39.2
Gvalue	13.0	30.9	43.7	35.4	35.3	20.2	32.3	35.0	15.4	40.8
Gv/N	1.630	3.868	5.474	4.430	4.420	2.531	4.049	4.380	1.925	5.108
Prob	0.00463	0.00000	0.00000	0.00000	0.00000	0.00015	0.00000	0.00000	0.00154	0.00000

	10	9	8	7	6	5	4	3	2	1
a_O	177	184	189	166	162	149	147	175	105	94
	(22.2)	(23.1)	(23.7)	(20.8)	(20.3)	(18.7)	(18.4)	(21.9)	(13.2)	(11.8)
a_E	149.0	149.0	149.0	149.0	149.0	149.0	149.0	149.0	149.0	149.0
	(18.7)	(18.7)	(18.7)	(18.7)	(18.7)	(18.7)	(18.7)	(18.7)	(18.7)	(18.7)
	60.9	77.6	89.9	35.8	27.1	-0.0	-4.0	56.3	-73.5	-86.6
	7.636	9.727	11.261	4.492	3.394	-0.003	-0.500	7.051	-9.212	-10.854
t_O	165	149	152	176	147	158	135	120	155	118
	(20.7)	(18.7)	(19.0)	(22.1)	(18.4)	(19.8)	(16.9)	(15.0)	(19.4)	(14.8)
t_E	150.9	150.9	150.9	150.9	150.9	150.9	150.9	150.9	150.9	150.9
	(18.9)	(18.9)	(18.9)	(18.9)	(18.9)	(18.9)	(18.9)	(18.9)	(18.9)	(18.9)
	29.5	-3.7	2.3	54.2	-7.6	14.6	-30.0	-54.9	8.4	-58.0
	3.702	-0.466	0.284	6.796	-0.957	1.829	-3.761	-6.885	1.049	-7.267
g_O	164	200	164	153	186	150	205	285	132	105
	(20.6)	(25.1)	(20.6)	(19.2)	(23.3)	(18.8)	(25.7)	(35.7)	(16.5)	(13.2)
g_E	226.7	226.7	226.7	226.7	226.7	226.7	226.7	226.7	226.7	226.7
	(28.4)	(28.4)	(28.4)	(28.4)	(28.4)	(28.4)	(28.4)	(28.4)	(28.4)	(28.4)
	-106.2	-50.1	-106.2	-120.3	-73.6	-123.9	-41.2	130.5	-142.8	-161.6
	-13.304	-6.277	-13.304	-15.074	-9.220	-15.523	-5.165	16.354	-17.889	-20.252
c_O	292	265	293	303	303	341	311	218	406	481
	(36.6)	(33.2)	(36.7)	(38.0)	(38.0)	(42.7)	(39.0)	(27.3)	(50.9)	(60.3)
c_E	271.4	271.4	271.4	271.4	271.4	271.4	271.4	271.4	271.4	271.4
	(34.0)	(34.0)	(34.0)	(34.0)	(34.0)	(34.0)	(34.0)	(34.0)	(34.0)	(34.0)
	42.6	-12.7	44.8	66.7	66.7	155.6	84.6	-95.6	326.9	550.4
	5.343	-1.595	5.613	8.353	8.353	19.498	10.605	-11.979	40.968	68.971
N	798	798	798	798	798	798	798	798	798	798
X2	25.5	11.5	29.8	33.7	12.2	44.1	9.5	36.4	119.4	254.6
Gvalue	27.0	11.1	30.8	36.4	12.5	46.3	9.4	36.2	119.0	244.2
Gv/N	3.378	1.389	3.854	4.567	1.569	5.801	1.179	4.541	14.916	30.597
Prob	0.00001	0.01126	0.00000	0.00000	0.00581	0.00000	0.02436	0.00000	0.00000	0.00000

CDS_INITIAL:

	1	2	3	4	5	6	7	8	9	10

a_O	647	0	1	315	216	113	228	215	138	208
	(81.1)	(0.0)	(0.1)	(39.5)	(27.1)	(14.2)	(28.6)	(26.9)	(17.3)	(26.1)
a_E	145.1	190.9	40.7	145.1	190.9	40.7	145.1	190.9	40.7	145.1
	(18.2)	(23.9)	(5.1)	(18.2)	(23.9)	(5.1)	(18.2)	(23.9)	(5.1)	(18.2)
	1934.2	0.0	-7.4	488.2	53.4	231.0	206.0	51.2	337.2	149.7
	242.382	0.000	-0.929	61.182	6.694	28.944	25.814	6.413	42.260	18.764
t_O	57	798	0	121	107	156	89	177	145	128
	(7.1)	(100.0)	(0.0)	(15.2)	(13.4)	(19.5)	(11.2)	(22.2)	(18.2)	(16.0)
t_E	92.7	226.3	49.8	92.7	226.3	49.8	92.7	226.3	49.8	92.7
	(11.6)	(28.4)	(6.2)	(11.6)	(28.4)	(6.2)	(11.6)	(28.4)	(6.2)	(11.6)
	-55.5	2011.1	0.0	64.5	-160.3	356.2	-7.3	-87.0	309.9	82.6
	-6.949	252.014	0.000	8.077	-20.092	44.641	-0.911	-10.908	38.836	10.348
g_O	94	0	797	135	124	170	242	135	189	192
	(11.8)	(0.0)	(99.9)	(16.9)	(15.5)	(21.3)	(30.3)	(16.9)	(23.7)	(24.1)
g_E	321.0	164.8	319.0	321.0	164.8	319.0	321.0	164.8	319.0	321.0
	(40.2)	(20.7)	(40.0)	(40.2)	(20.7)	(40.0)	(40.2)	(20.7)	(40.0)	(40.2)
	-230.9	0.0	1459.5	-233.8	-70.6	-214.0	-136.7	-53.9	-197.9	-197.3
	-28.931	0.000	182.892	-29.303	-8.845	-26.819	-17.128	-6.754	-24.797	-24.726
c_O	0	0	0	227	351	359	239	271	326	270
	(0.0)	(0.0)	(0.0)	(28.4)	(44.0)	(45.0)	(29.9)	(34.0)	(40.9)	(33.8)
c_E	239.2	216.0	388.5	239.2	216.0	388.5	239.2	216.0	388.5	239.2
	(30.0)	(27.1)	(48.7)	(30.0)	(27.1)	(48.7)	(30.0)	(27.1)	(48.7)	(30.0)
	0.0	0.0	0.0	-23.8	341.0	-56.7	-0.4	123.1	-114.4	65.4
	0.000	0.000	0.000	-2.979	42.727	-7.108	-0.051	15.420	-14.333	8.196
N	798	798	798	798	798	798	798	798	798	798
X2	2149.0	2015.5	1193.2	315.8	160.8	427.0	66.9	33.2	478.0	96.5
Gvalue	1647.9	2011.1	1452.1	295.1	163.5	316.5	61.6	33.3	334.9	100.4
Gv/N	206.502	252.014	181.963	36.978	20.485	39.658	7.726	4.171	41.965	12.582
Prob	1.00000	1.00000	0.00000	0.00000	0.00000	0.00000	0.00000	0.00000	0.00000	0.00000

	11	12	13	14	15	16	17	18	19	20
a_O	176	107	175	185	102	178	192	109	200	165
	(22.1)	(13.4)	(21.9)	(23.2)	(12.8)	(22.3)	(24.1)	(13.7)	(25.1)	(20.7)
a_E	190.9	40.7	145.1	190.9	40.7	145.1	190.9	40.7	145.1	190.9
	(23.9)	(5.1)	(18.2)	(23.9)	(5.1)	(18.2)	(23.9)	(5.1)	(18.2)	(23.9)
	-28.6	207.0	65.5	-11.6	187.6	72.7	2.3	214.9	128.3	-48.1
	-3.579	25.944	8.210	-1.450	23.508	9.109	0.282	26.935	16.076	-6.024
t_O	216	152	126	202	137	135	233	137	105	238
	(27.1)	(19.0)	(15.8)	(25.3)	(17.2)	(16.9)	(29.2)	(17.2)	(13.2)	(29.8)
t_E	226.3	49.8	92.7	226.3	49.8	92.7	226.3	49.8	92.7	226.3
	(28.4)	(6.2)	(11.6)	(28.4)	(6.2)	(11.6)	(28.4)	(6.2)	(11.6)	(28.4)
	-20.2	339.2	77.3	-46.0	277.3	101.5	13.5	277.3	26.1	23.9
	-2.531	42.507	9.689	-5.760	34.744	12.715	1.694	34.744	3.276	2.996
g_O	108	179	222	124	194	207	125	183	213	111
	(13.5)	(22.4)	(27.8)	(15.5)	(24.3)	(25.9)	(15.7)	(22.9)	(26.7)	(13.9)
g_E	164.8	319.0	321.0	164.8	319.0	321.0	164.8	319.0	321.0	164.8
	(20.7)	(40.0)	(40.2)	(20.7)	(40.0)	(40.2)	(20.7)	(40.0)	(40.2)	(20.7)
	-91.3	-206.9	-163.7	-70.6	-193.0	-181.6	-69.1	-203.4	-174.7	-87.8
	-11.443	-25.924	-20.512	-8.845	-24.184	-22.755	-8.665	-25.490	-21.889	-10.999
c_O	298	360	275	287	365	278	248	369	280	284
	(37.3)	(45.1)	(34.5)	(36.0)	(45.7)	(34.8)	(31.1)	(46.2)	(35.1)	(35.6)
c_E	216.0	388.5	239.2	216.0	388.5	239.2	216.0	388.5	239.2	216.0
	(27.1)	(48.7)	(30.0)	(27.1)	(48.7)	(30.0)	(27.1)	(48.7)	(30.0)	(27.1)
	191.9	-54.9	76.7	163.2	-45.6	83.6	68.6	-38.0	88.2	155.6
	24.050	-6.877	9.612	20.457	-5.711	10.473	8.599	-4.766	11.051	19.495
N	798	798	798	798	798	798	798	798	798	798
X2	52.4	381.5	54.0	36.3	295.6	73.5	14.6	326.5	65.7	43.1
Gvalue	51.8	284.5	55.9	35.1	226.3	76.1	15.2	250.8	67.9	43.6
Gv/N	6.496	35.649	7.000	4.402	28.358	9.543	1.910	31.424	8.515	5.468
Prob	0.00000	0.00000	0.00000	0.00000	0.00000	-0.00000	0.00162	0.00000	0.00000	0.00000

	21	22	23	24	25	26	27	28	29	30
a_O	95	177	180	88	180	180	75	154	144	82
	(11.9)	(22.2)	(22.6)	(11.0)	(22.6)	(22.6)	(9.4)	(19.3)	(18.0)	(10.3)
a_E	40.7	145.1	190.9	40.7	145.1	190.9	40.7	145.1	190.9	40.7
	(5.1)	(18.2)	(23.9)	(5.1)	(18.2)	(23.9)	(5.1)	(18.2)	(23.9)	(5.1)
	161.2	70.3	-21.1	135.9	77.5	-21.1	91.8	18.3	-81.2	115.0
	20.202	8.808	-2.647	17.025	9.716	-2.647	11.506	2.291	-10.171	14.413
t_O	133	130	226	116	116	228	103	118	242	91
	(16.7)	(16.3)	(28.3)	(14.5)	(14.5)	(28.6)	(12.9)	(14.8)	(30.3)	(11.4)
t_E	49.8	92.7	226.3	49.8	92.7	226.3	49.8	92.7	226.3	49.8
	(6.2)	(11.6)	(28.4)	(6.2)	(11.6)	(28.4)	(6.2)	(11.6)	(28.4)	(6.2)
	261.3	87.9	-0.7	196.2	52.0	3.3	149.7	56.9	32.4	109.7
	32.742	11.015	-0.085	24.581	6.516	0.418	18.758	7.134	4.058	13.748
g_O	190	261	128	238	244	118	222	273	152	237
	(23.8)	(32.7)	(16.0)	(29.8)	(30.6)	(14.8)	(27.8)	(34.2)	(19.0)	(29.7)
g_E	319.0	321.0	164.8	319.0	321.0	164.8	319.0	321.0	164.8	319.0
	(40.0)	(40.2)	(20.7)	(40.0)	(40.2)	(20.7)	(40.0)	(40.2)	(20.7)	(40.0)
	-196.9	-108.0	-64.7	-139.5	-133.8	-78.9	-161.0	-88.4	-24.6	-140.9
	-24.677	-13.528	-8.112	-17.476	-16.766	-9.884	-20.173	-11.075	-3.086	-17.652
c_O	380	230	264	356	258	272	398	253	260	388
	(47.6)	(28.8)	(33.1)	(44.6)	(32.3)	(34.1)	(49.9)	(31.7)	(32.6)	(48.6)
c_E	388.5	239.2	216.0	388.5	239.2	216.0	388.5	239.2	216.0	388.5
	(48.7)	(30.0)	(27.1)	(48.7)	(30.0)	(27.1)	(48.7)	(30.0)	(27.1)	(48.7)
	-16.8	-18.0	106.1	-62.2	39.0	125.5	19.2	28.4	96.5	-1.0
	-2.110	-2.261	13.290	-7.798	4.892	15.728	2.406	3.556	12.094	-0.128
N	798	798	798	798	798	798	798	798	798	798
X2	263.9	33.6	19.5	166.4	34.2	28.5	115.5	15.4	22.6	97.2
Gvalue	208.7	32.2	19.5	130.3	34.8	28.9	99.7	15.2	23.1	82.8
Gv/N	26.157	4.034	2.447	16.333	4.358	3.615	12.497	1.906	2.895	10.380
Prob	0.00000	0.00000	0.00021	0.00000	0.00000	0.00000	0.00000	0.00164	0.00004	0.00000

	31	32	33	34	35	36	37	38	39	40
a_O	153	144	62	151	155	59	121	138	52	133
	(19.2)	(18.0)	(7.8)	(18.9)	(19.4)	(7.4)	(15.2)	(17.3)	(6.5)	(16.7)
a_E	145.1	190.9	40.7	145.1	190.9	40.7	145.1	190.9	40.7	145.1
	(18.2)	(23.9)	(5.1)	(18.2)	(23.9)	(5.1)	(18.2)	(23.9)	(5.1)	(18.2)
	16.2	-81.2	52.3	12.0	-64.5	43.9	-44.0	-89.5	25.6	-23.2
	2.026	-10.171	6.553	1.502	-8.088	5.503	-5.513	-11.219	3.204	-2.908
t_O	123	229	101	109	204	93	99	242	90	125
	(15.4)	(28.7)	(12.7)	(13.7)	(25.6)	(11.7)	(12.4)	(30.3)	(11.3)	(15.7)
t_E	92.7	226.3	49.8	92.7	226.3	49.8	92.7	226.3	49.8	92.7
	(11.6)	(28.4)	(6.2)	(11.6)	(28.4)	(6.2)	(11.6)	(28.4)	(6.2)	(11.6)
	69.5	5.4	142.8	35.3	-42.4	116.2	13.0	32.4	106.5	74.7
	8.716	0.671	17.897	4.422	-5.313	14.556	1.629	4.058	13.347	9.363

g_O	282	182	255	281	166	243	310	158	270	299
	(35.3)	(22.8)	(32.0)	(35.2)	(20.8)	(30.5)	(38.8)	(19.8)	(33.8)	(37.5)
g_E	321.0	164.8	319.0	321.0	164.8	319.0	321.0	164.8	319.0	321.0
	(40.2)	(20.7)	(40.0)	(40.2)	(20.7)	(40.0)	(40.2)	(20.7)	(40.0)	(40.2)
	-73.0	36.1	-114.2	-74.7	2.4	-132.3	-21.6	-13.4	-90.1	-42.4
	-9.147	4.521	-14.315	-9.365	0.295	-16.577	-2.701	-1.675	-11.289	-5.312
c_O	240	243	380	257	273	403	268	260	386	241
	(30.1)	(30.5)	(47.6)	(32.2)	(34.2)	(50.5)	(33.6)	(32.6)	(48.4)	(30.2)
c_E	239.2	216.0	388.5	239.2	216.0	388.5	239.2	216.0	388.5	239.2
	(30.0)	(27.1)	(48.7)	(30.0)	(27.1)	(48.7)	(30.0)	(27.1)	(48.7)	(30.0)
	1.6	57.3	-16.8	36.9	128.0	29.5	60.9	96.5	-5.0	3.6
	0.200	7.185	-2.110	4.623	16.037	3.698	7.636	12.094	-0.628	0.452
N	798	798	798	798	798	798	798	798	798	798
X2	15.1	16.7	76.9	9.4	24.0	64.4	8.3	25.0	43.1	13.8
Gvalue	14.3	17.6	64.0	9.4	23.4	57.3	8.4	26.0	37.0	12.7
Gv/N	1.795	2.206	8.026	1.182	2.931	7.180	1.051	3.258	4.635	1.595
Prob	0.00250	0.00053	0.00000	0.02406	0.00003	0.00000	0.03868	0.00001	0.00000	0.00527

	41	42	43	44	45	46	47	48	49	50
a_O	147	45	143	160	65	129	165	65	129	163
	(18.4)	(5.6)	(17.9)	(20.1)	(8.1)	(16.2)	(20.7)	(8.1)	(16.2)	(20.4)
a_E	190.9	40.7	145.1	190.9	40.7	145.1	190.9	40.7	145.1	190.9
	(23.9)	(5.1)	(18.2)	(23.9)	(5.1)	(18.2)	(23.9)	(5.1)	(18.2)	(23.9)
	-76.8	9.1	-4.2	-56.5	61.0	-30.4	-48.1	61.0	-30.4	-51.5
	-9.623	1.142	-0.529	-7.076	7.640	-3.808	-6.024	7.640	-3.808	-6.450
t_O	251	81	95	229	88	101	230	62	103	235
	(31.5)	(10.2)	(11.9)	(28.7)	(11.0)	(12.7)	(28.8)	(7.8)	(12.9)	(29.4)
t_E	226.3	49.8	92.7	226.3	49.8	92.7	226.3	49.8	92.7	226.3
	(28.4)	(6.2)	(11.6)	(28.4)	(6.2)	(11.6)	(28.4)	(6.2)	(11.6)	(28.4)
	51.9	78.8	4.6	5.4	100.2	17.3	7.4	27.2	21.7	17.6
	6.506	9.874	0.581	0.671	12.555	2.168	0.925	3.404	2.717	2.211
g_O	163	250	282	181	297	306	169	286	301	176
	(20.4)	(31.3)	(35.3)	(22.7)	(37.2)	(38.3)	(21.2)	(35.8)	(37.7)	(22.1)
g_E	164.8	319.0	321.0	164.8	319.0	321.0	164.8	319.0	321.0	164.8
	(20.7)	(40.0)	(40.2)	(20.7)	(40.0)	(40.2)	(20.7)	(40.0)	(40.2)	(20.7)
	-3.6	-121.9	-73.0	33.9	-42.5	-29.2	8.5	-62.5	-38.7	23.1
	-0.455	-15.275	-9.147	4.246	-5.323	-3.662	1.059	-7.831	-4.845	2.893
c_O	237	422	278	228	348	262	234	385	265	224
	(29.7)	(52.9)	(34.8)	(28.6)	(43.6)	(32.8)	(29.3)	(48.2)	(33.2)	(28.1)
c_E	216.0	388.5	239.2	216.0	388.5	239.2	216.0	388.5	239.2	216.0
	(27.1)	(48.7)	(30.0)	(27.1)	(48.7)	(30.0)	(27.1)	(48.7)	(30.0)	(27.1)
	44.1	69.8	83.6	24.7	-76.6	47.7	37.6	-7.0	54.3	16.4
	5.523	8.744	10.473	3.101	-9.605	5.978	4.706	-0.876	6.803	2.053
N	798	798	798	798	798	798	798	798	798	798
X2	14.8	37.8	11.1	7.3	49.6	5.4	5.2	21.0	7.0	5.5
Gvalue	15.6	35.8	11.0	7.5	42.0	5.4	5.3	18.6	6.9	5.6
Gv/N	1.950	4.485	1.378	0.942	5.267	0.676	0.665	2.336	0.867	0.708
Prob	0.00139	0.00000	0.01173	0.05715	0.00000	0.14486	0.15053	0.00032	0.07449	0.12998

	51	52	53	54	55	56	57	58	59	60
a_O	68	147	154	50	131	152	53	133	162	37
	(8.5)	(18.4)	(19.3)	(6.3)	(16.4)	(19.0)	(6.6)	(16.7)	(20.3)	(4.6)
a_E	40.7	145.1	190.9	40.7	145.1	190.9	40.7	145.1	190.9	40.7
	(5.1)	(18.2)	(23.9)	(5.1)	(18.2)	(23.9)	(5.1)	(18.2)	(23.9)	(5.1)
	69.9	3.8	-66.1	20.7	-26.8	-69.2	28.1	-23.2	-53.1	-7.0
	8.762	0.473	-8.286	2.589	-3.362	-8.676	3.519	-2.908	-6.660	-0.876
t_O	73	114	217	77	102	240	69	94	232	88
	(9.1)	(14.3)	(27.2)	(9.6)	(12.8)	(30.1)	(8.6)	(11.8)	(29.1)	(11.0)
t_E	49.8	92.7	226.3	49.8	92.7	226.3	49.8	92.7	226.3	49.8
	(6.2)	(11.6)	(28.4)	(6.2)	(11.6)	(28.4)	(6.2)	(11.6)	(28.4)	(6.2)
	55.8	47.1	-18.3	67.1	19.5	28.1	45.0	2.6	11.5	100.2
	6.996	5.907	-2.292	8.409	2.442	3.525	5.638	0.326	1.436	12.555
g_O	294	291	178	268	314	184	294	314	173	279
	(36.8)	(36.5)	(22.3)	(33.6)	(39.3)	(23.1)	(36.8)	(39.3)	(21.7)	(35.0)
g_E	319.0	321.0	164.8	319.0	321.0	164.8	319.0	321.0	164.8	319.0
	(40.0)	(40.2)	(20.7)	(40.0)	(40.2)	(20.7)	(40.0)	(40.2)	(20.7)	(40.0)
	-48.0	-57.0	27.4	-93.4	-13.8	40.5	-48.0	-13.8	16.7	-74.8
	-6.018	-7.148	3.430	-11.705	-1.726	5.075	-6.018	-1.726	2.099	-9.372
c_O	363	246	249	403	251	222	382	257	231	394
	(45.5)	(30.8)	(31.2)	(50.5)	(31.5)	(27.8)	(47.9)	(32.2)	(28.9)	(49.4)
c_E	388.5	239.2	216.0	388.5	239.2	216.0	388.5	239.2	216.0	388.5
	(48.7)	(30.0)	(27.1)	(48.7)	(30.0)	(27.1)	(48.7)	(30.0)	(27.1)	(48.7)
	-49.3	13.8	70.9	29.5	24.2	12.3	-12.9	36.9	31.1	11.1
	-6.180	1.728	8.885	3.698	3.029	1.535	-1.619	4.623	3.898	1.385
N	798	798	798	798	798	798	798	798	798	798
X2	32.8	7.9	13.6	25.7	3.0	11.1	13.2	2.5	6.0	34.7
Gvalue	28.4	7.7	13.9	23.9	3.0	11.6	12.1	2.5	6.2	29.5
Gv/N	3.561	0.960	1.738	2.991	0.382	1.459	1.521	0.314	0.773	3.691
Prob	0.00000	0.05364	0.00309	0.00003	0.38427	0.00871	0.00694	0.47434	0.10360	0.00000

	61	62	63	64	65	66	67	68	69	70
a_O	142	153	60	119	154	49	119	176	54	143
	(17.8)	(19.2)	(7.5)	(14.9)	(19.3)	(6.1)	(14.9)	(22.1)	(6.8)	(17.9)
a_E	145.1	190.9	40.7	145.1	190.9	40.7	145.1	190.9	40.7	145.1
	(18.2)	(23.9)	(5.1)	(18.2)	(23.9)	(5.1)	(18.2)	(23.9)	(5.1)	(18.2)
	-6.2	-67.7	46.7	-47.2	-66.1	18.3	-47.2	-28.6	30.6	-4.2
	-0.775	-8.482	5.849	-5.919	-8.286	2.290	-5.919	-3.579	3.838	-0.529
t_O	88	219	61	108	213	62	104	230	78	95
	(11.0)	(27.4)	(7.6)	(13.5)	(26.7)	(7.8)	(13.0)	(28.8)	(9.8)	(11.9)
t_E	92.7	226.3	49.8	92.7	226.3	49.8	92.7	226.3	49.8	92.7
	(11.6)	(28.4)	(6.2)	(11.6)	(28.4)	(6.2)	(11.6)	(28.4)	(6.2)	(11.6)
	-9.2	-14.4	24.7	33.0	-25.9	27.2	23.9	7.4	70.0	4.6
	-1.150	-1.809	3.100	4.132	-3.243	3.404	2.996	0.925	8.770	0.581
g_O	333	171	293	322	172	298	325	165	288	309
	(41.7)	(21.4)	(36.7)	(40.4)	(21.6)	(37.3)	(40.7)	(20.7)	(36.1)	(38.7)
g_E	321.0	164.8	319.0	321.0	164.8	319.0	321.0	164.8	319.0	321.0
	(40.2)	(20.7)	(40.0)	(40.2)	(20.7)	(40.0)	(40.2)	(20.7)	(40.0)	(40.2)
	24.5	12.6	-49.9	2.1	14.7	-40.6	8.1	0.3	-58.9	-23.5
	3.072	1.576	-6.247	0.260	1.837	-5.090	1.018	0.044	-7.383	-2.942
c_O	235	255	384	249	259	389	250	227	378	251
	(29.4)	(32.0)	(48.1)	(31.2)	(32.5)	(48.7)	(31.3)	(28.4)	(47.4)	(31.5)
c_E	239.2	216.0	388.5	239.2	216.0	388.5	239.2	216.0	388.5	239.2
	(30.0)	(27.1)	(48.7)	(30.0)	(27.1)	(48.7)	(30.0)	(27.1)	(48.7)	(30.0)
	-8.3	84.8	-9.0	20.0	94.1	1.0	22.1	22.6	-20.7	24.2
	-1.044	10.621	-1.124	2.505	11.798	0.122	2.767	2.837	-2.599	3.029

N	798	798	798	798	798	798	798	798	798	798
X2	0.8	15.0	13.9	7.6	16.8	6.1	6.6	1.8	23.6	1.1
Gvalue	0.8	15.2	12.6	7.8	16.8	5.8	6.9	1.8	21.0	1.1
Gv/N	0.104	1.906	1.577	0.978	2.106	0.725	0.861	0.226	2.626	0.139
Prob	0.84202	0.00165	0.00562	0.05018	0.00078	0.12240	0.07626	0.61384	0.00011	0.77425

	71	72	73	74	75	76	77	78	79	80
a_O	203	46	132	178	42	136	183	46	132	151
	(25.4)	(5.8)	(16.5)	(22.3)	(5.3)	(17.0)	(22.9)	(5.8)	(16.5)	(18.9)
a_E	190.9	40.7	145.1	190.9	40.7	145.1	190.9	40.7	145.1	190.9
	(23.9)	(5.1)	(18.2)	(23.9)	(5.1)	(18.2)	(23.9)	(5.1)	(18.2)	(23.9)
	25.0	11.3	-25.0	-24.9	2.7	-17.7	-15.4	11.3	-25.0	-70.8
	3.133	1.421	-3.136	-3.116	0.340	-2.213	-1.933	1.421	-3.136	-8.869
t_O	244	79	91	226	63	102	237	60	107	261
	(30.6)	(9.9)	(11.4)	(28.3)	(7.9)	(12.8)	(29.7)	(7.5)	(13.4)	(32.7)
t_E	226.3	49.8	92.7	226.3	49.8	92.7	226.3	49.8	92.7	226.3
	(28.4)	(6.2)	(11.6)	(28.4)	(6.2)	(11.6)	(28.4)	(6.2)	(11.6)	(28.4)
	36.7	72.9	-3.4	-0.7	29.6	19.5	21.8	22.4	30.7	74.4
	4.594	9.135	-0.424	-0.085	3.711	2.442	2.734	2.801	3.845	9.320
g_O	142	314	330	156	281	321	160	314	299	148
	(17.8)	(39.3)	(41.4)	(19.5)	(35.2)	(40.2)	(20.1)	(39.3)	(37.5)	(18.5)
g_E	164.8	319.0	321.0	164.8	319.0	321.0	164.8	319.0	321.0	164.8
	(20.7)	(40.0)	(40.2)	(20.7)	(40.0)	(40.2)	(20.7)	(40.0)	(40.2)	(20.7)
	-42.3	-10.0	18.3	-17.2	-71.3	0.1	-9.5	-10.0	-42.4	-31.9
	-5.305	-1.248	2.296	-2.152	-8.937	0.009	-1.192	-1.248	-5.312	-3.994
c_O	209	359	245	238	412	239	218	378	260	238
	(26.2)	(45.0)	(30.7)	(29.8)	(51.6)	(29.9)	(27.3)	(47.4)	(32.6)	(29.8)
c_E	216.0	388.5	239.2	216.0	388.5	239.2	216.0	388.5	239.2	216.0
	(27.1)	(48.7)	(30.0)	(27.1)	(48.7)	(30.0)	(27.1)	(48.7)	(30.0)	(27.1)
	-13.7	-56.7	11.7	46.3	48.4	-0.4	4.1	-20.7	43.4	46.3
	-1.715	-7.108	1.471	5.797	6.061	-0.051	0.514	-2.599	5.433	5.797
N	798	798	798	798	798	798	798	798	798	798
X2	5.5	20.1	1.6	3.6	9.5	1.5	1.0	3.2	6.7	17.6
Gvalue	5.6	17.6	1.6	3.5	9.4	1.5	1.0	3.0	6.6	18.0
Gv/N	0.707	2.200	0.206	0.445	1.176	0.186	0.124	0.375	0.829	2.255
Prob	0.13037	0.00054	0.64865	0.31464	0.02463	0.68507	0.80466	0.39231	0.08508	0.00044

	81	82	83	84	85	86	87	88	89	90
a_O	59	144	172	34	126	179	55	133	183	46
	(7.4)	(18.0)	(21.6)	(4.3)	(15.8)	(22.4)	(6.9)	(16.7)	(22.9)	(5.8)
a_E	40.7	145.1	190.9	40.7	145.1	190.9	40.7	145.1	190.9	40.7
	(5.1)	(18.2)	(23.9)	(5.1)	(18.2)	(23.9)	(5.1)	(18.2)	(23.9)	(5.1)
	43.9	-2.2	-35.8	-12.2	-35.6	-23.0	33.2	-23.2	-15.4	11.3
	5.503	-0.281	-4.489	-1.526	-4.463	-2.882	4.162	-2.908	-1.933	1.421
t_O	61	106	222	58	96	222	61	95	236	52
	(7.6)	(13.3)	(27.8)	(7.3)	(12.0)	(27.8)	(7.6)	(11.9)	(29.6)	(6.5)
t_E	49.8	92.7	226.3	49.8	92.7	226.3	49.8	92.7	226.3	49.8
	(6.2)	(11.6)	(28.4)	(6.2)	(11.6)	(28.4)	(6.2)	(11.6)	(28.4)	(6.2)
	24.7	28.4	-8.6	17.7	6.7	-8.6	24.7	4.6	19.7	4.5
	3.100	3.559	-1.077	2.215	0.839	-1.077	3.100	0.581	2.472	0.563
g_O	303	307	160	322	339	155	315	319	167	318
	(38.0)	(38.5)	(20.1)	(40.4)	(42.5)	(19.4)	(39.5)	(40.0)	(20.9)	(39.8)
g_E	319.0	321.0	164.8	319.0	321.0	164.8	319.0	321.0	164.8	319.0
	(40.0)	(40.2)	(20.7)	(40.0)	(40.2)	(20.7)	(40.0)	(40.2)	(20.7)	(40.0)
	-31.2	-27.3	-9.5	6.0	37.1	-19.1	-8.0	-3.9	4.4	-2.0
	-3.912	-3.423	-1.192	0.751	4.645	-2.388	-1.001	-0.491	0.548	-0.255
c_O	375	241	244	384	237	242	367	251	212	382
	(47.0)	(30.2)	(30.6)	(48.1)	(29.7)	(30.3)	(46.0)	(31.5)	(26.6)	(47.9)
c_E	388.5	239.2	216.0	388.5	239.2	216.0	388.5	239.2	216.0	388.5
	(48.7)	(30.0)	(27.1)	(48.7)	(30.0)	(27.1)	(48.7)	(30.0)	(27.1)	(48.7)
	-26.6	3.6	59.6	-9.0	-4.4	55.1	-41.8	24.2	-7.8	-12.9
	-3.327	0.452	7.466	-1.124	-0.549	6.905	-5.240	3.029	-0.983	-1.619
N	798	798	798	798	798	798	798	798	798	798
X2	12.1	2.5	5.7	2.5	3.7	4.5	8.8	1.7	0.8	0.9
Gvalue	10.9	2.5	5.7	2.5	3.8	4.5	8.2	1.7	0.8	0.9
Gv/N	1.364	0.308	0.708	0.316	0.472	0.559	1.022	0.211	0.105	0.110
Prob	0.01236	0.48269	0.12987	0.47191	0.28743	0.21610	0.04287	0.64066	0.84044	0.83037

	91	92	93	94	95	96	97	98	99	100
a_O	133	175	42	130	169	41	128	180	47	146
	(16.7)	(21.9)	(5.3)	(16.3)	(21.2)	(5.1)	(16.0)	(22.6)	(5.9)	(18.3)
a_E	145.1	190.9	40.7	145.1	190.9	40.7	145.1	190.9	40.7	145.1
	(18.2)	(23.9)	(5.1)	(18.2)	(23.9)	(5.1)	(18.2)	(23.9)	(5.1)	(18.2)
	-23.2	-30.4	2.7	-28.6	-41.1	0.7	-32.1	-21.1	13.6	1.8
	-2.908	-3.809	0.340	-3.586	-5.156	0.084	-4.028	-2.647	1.705	0.220
t_O	96	215	56	91	219	59	90	211	49	80
	(12.0)	(26.9)	(7.0)	(11.4)	(27.4)	(7.4)	(11.3)	(26.4)	(6.1)	(10.0)
t_E	92.7	226.3	49.8	92.7	226.3	49.8	92.7	226.3	49.8	92.7
	(11.6)	(28.4)	(6.2)	(11.6)	(28.4)	(6.2)	(11.6)	(28.4)	(6.2)	(11.6)
	6.7	-22.1	13.1	-3.4	-14.4	20.0	-5.3	-29.6	-1.6	-23.6
	0.839	-2.770	1.646	-0.424	-1.809	2.506	-0.669	-3.711	-0.200	-2.956
g_O	314	170	317	314	169	315	319	170	311	313
	(39.3)	(21.3)	(39.7)	(39.3)	(21.2)	(39.5)	(40.0)	(21.3)	(39.0)	(39.2)
g_E	321.0	164.8	319.0	321.0	164.8	319.0	321.0	164.8	319.0	321.0
	(40.2)	(20.7)	(40.0)	(40.2)	(20.7)	(40.0)	(40.2)	(20.7)	(40.0)	(40.2)
	-13.8	10.5	-4.0	-13.8	8.5	-8.0	-3.9	10.5	-15.8	-15.7
	-1.726	1.317	-0.504	-1.726	1.059	-1.001	-0.491	1.317	-1.984	-1.971
c_O	255	238	383	263	241	383	261	237	391	259
	(32.0)	(29.8)	(48.0)	(33.0)	(30.2)	(48.0)	(32.7)	(29.7)	(49.0)	(32.5)
c_E	239.2	216.0	388.5	239.2	216.0	388.5	239.2	216.0	388.5	239.2
	(30.0)	(27.1)	(48.7)	(30.0)	(27.1)	(48.7)	(30.0)	(27.1)	(48.7)	(30.0)
	32.6	46.3	-10.9	49.9	52.9	-10.9	45.5	44.1	5.0	41.2
	4.087	5.797	-1.372	6.252	6.627	-1.372	5.705	5.523	0.625	5.162
N	798	798	798	798	798	798	798	798	798	798
X2	2.3	4.3	0.9	4.1	5.8	1.8	4.1	3.9	1.2	3.6
Gvalue	2.3	4.3	0.9	4.1	5.8	1.7	4.1	3.8	1.2	3.6
Gv/N	0.292	0.536	0.110	0.515	0.721	0.217	0.517	0.482	0.147	0.455
Prob	0.50654	0.23333	0.83132	0.24973	0.12432	0.62927	0.24818	0.27889	0.75993	0.30460

5UTR:

	100	99	98	97	96	95	94	93	92	91
a_O	237	230	245	247	240	243	237	239	218	250
	(16.6)	(16.1)	(17.2)	(17.3)	(16.8)	(17.0)	(16.6)	(16.8)	(15.3)	(17.5)
a_E	248.9	248.9	248.9	248.9	248.9	248.9	248.9	248.9	248.9	248.9
	(17.5)	(17.5)	(17.5)	(17.5)	(17.5)	(17.5)	(17.5)	(17.5)	(17.5)	(17.5)
	-23.3	-36.4	-7.8	-3.8	-17.5	-11.7	-23.3	-19.4	-57.8	2.2
	-1.631	-2.550	-0.545	-0.268	-1.228	-0.820	-1.631	-1.363	-4.055	0.152
t_O	242	225	215	259	226	218	255	214	214	244
	(17.0)	(15.8)	(15.1)	(18.2)	(15.8)	(15.3)	(17.9)	(15.0)	(15.0)	(17.1)
t_E	251.9	251.9	251.9	251.9	251.9	251.9	251.9	251.9	251.9	251.9
	(17.7)	(17.7)	(17.7)	(17.7)	(17.7)	(17.7)	(17.7)	(17.7)	(17.7)	(17.7)
	-19.5	-50.9	-68.2	14.3	-49.1	-63.1	6.1	-69.9	-69.9	-15.6
	-1.368	-3.570	-4.782	1.002	-3.445	-4.425	0.430	-4.900	-4.900	-1.097
g_O	474	476	466	461	499	455	454	469	489	433
	(33.2)	(33.4)	(32.7)	(32.3)	(35.0)	(31.9)	(31.8)	(32.9)	(34.3)	(30.4)
g_E	423.6	423.6	423.6	423.6	423.6	423.6	423.6	423.6	423.6	423.6
	(29.7)	(29.7)	(29.7)	(29.7)	(29.7)	(29.7)	(29.7)	(29.7)	(29.7)	(29.7)
	106.5	111.0	88.9	78.0	163.5	65.1	62.9	95.5	140.4	19.0
	7.472	7.785	6.233	5.469	11.463	4.562	4.412	6.696	9.845	1.331
c_O	473	495	500	459	461	510	480	504	505	499
	(33.2)	(34.7)	(35.1)	(32.2)	(32.3)	(35.8)	(33.7)	(35.3)	(35.4)	(35.0)
c_E	501.5	501.5	501.5	501.5	501.5	501.5	501.5	501.5	501.5	501.5
	(35.2)	(35.2)	(35.2)	(35.2)	(35.2)	(35.2)	(35.2)	(35.2)	(35.2)	(35.2)
	-55.4	-13.0	-3.0	-81.3	-77.7	17.1	-42.1	5.0	7.0	-5.0
	-3.884	-0.908	-0.213	-5.703	-5.447	1.199	-2.953	0.349	0.490	-0.353
N	1426	1426	1426	1426	1426	1426	1426	1426	1426	1426
X2	8.6	10.9	9.7	7.1	19.7	7.2	3.7	11.0	19.7	0.5
Gvalue	8.4	10.8	9.9	7.1	19.1	7.4	3.7	11.1	19.7	0.5
Gv/N	0.589	0.756	0.693	0.500	1.343	0.515	0.258	0.781	1.380	0.033
Prob	0.03840	0.01298	0.01962	0.06790	0.00026	0.06153	0.29771	0.01100	0.00020	0.92380

	90	89	88	87	86	85	84	83	82	81
a_O	235	263	247	228	233	247	261	232	244	240
	(16.5)	(18.4)	(17.3)	(16.0)	(16.3)	(17.3)	(18.3)	(16.3)	(17.1)	(16.8)
a_E	248.9	248.9	248.9	248.9	248.9	248.9	248.9	248.9	248.9	248.9
	(17.5)	(17.5)	(17.5)	(17.5)	(17.5)	(17.5)	(17.5)	(17.5)	(17.5)	(17.5)
	-27.0	28.9	-3.8	-40.0	-30.8	-3.8	24.7	-32.7	-9.7	-17.5
	-1.897	2.030	-0.268	-2.807	-2.160	-0.268	1.735	-2.291	-0.683	-1.228
t_O	218	200	255	250	236	268	243	233	244	215
	(15.3)	(14.0)	(17.9)	(17.5)	(16.5)	(18.8)	(17.0)	(16.3)	(17.1)	(15.1)
t_E	251.9	251.9	251.9	251.9	251.9	251.9	251.9	251.9	251.9	251.9
	(17.7)	(17.7)	(17.7)	(17.7)	(17.7)	(17.7)	(17.7)	(17.7)	(17.7)	(17.7)
	-63.1	-92.4	6.1	-3.9	-30.9	33.1	-17.6	-36.4	-15.6	-68.2
	-4.425	-6.477	0.430	-0.272	-2.165	2.321	-1.233	-2.555	-1.097	-4.782
g_O	466	458	436	451	462	455	436	487	435	503
	(32.7)	(32.1)	(30.6)	(31.6)	(32.4)	(31.9)	(30.6)	(34.2)	(30.5)	(35.3)
g_E	423.6	423.6	423.6	423.6	423.6	423.6	423.6	423.6	423.6	423.6
	(29.7)	(29.7)	(29.7)	(29.7)	(29.7)	(29.7)	(29.7)	(29.7)	(29.7)	(29.7)
	88.9	71.5	25.1	56.5	80.2	65.1	25.1	135.8	23.1	172.8
	6.233	5.014	1.763	3.963	5.621	4.562	1.763	9.525	1.619	12.118
c_O	507	505	488	497	495	456	486	474	503	468
	(35.6)	(35.4)	(34.2)	(34.9)	(34.7)	(32.0)	(34.1)	(33.2)	(35.3)	(32.8)
c_E	501.5	501.5	501.5	501.5	501.5	501.5	501.5	501.5	501.5	501.5
	(35.2)	(35.2)	(35.2)	(35.2)	(35.2)	(35.2)	(35.2)	(35.2)	(35.2)	(35.2)
	11.0	7.0	-26.7	-9.0	-13.0	-86.8	-30.6	-53.5	3.0	-64.7
	0.773	0.490	-1.870	-0.631	-0.908	-6.085	-2.143	-3.752	0.208	-4.541
N	1426	1426	1426	1426	1426	1426	1426	1426	1426	1426
X2	9.7	14.3	0.8	3.6	5.6	7.5	1.7	13.6	0.7	22.9
Gvalue	9.8	15.1	0.8	3.6	5.5	7.5	1.7	13.2	0.7	22.3
Gv/N	0.684	1.056	0.055	0.252	0.388	0.529	0.122	0.927	0.046	1.567
Prob	0.02076	0.00177	0.85464	0.30807	0.13650	0.05630	0.62742	0.00418	0.88272	0.00006

	80	79	78	77	76	75	74	73	72	71
a_O	240	234	233	208	267	290	254	243	239	269
	(16.8)	(16.4)	(16.3)	(14.6)	(18.7)	(20.3)	(17.8)	(17.0)	(16.8)	(18.9)
a_E	248.9	248.9	248.9	248.9	248.9	248.9	248.9	248.9	248.9	248.9
	(17.5)	(17.5)	(17.5)	(17.5)	(17.5)	(17.5)	(17.5)	(17.5)	(17.5)	(17.5)
	-17.5	-28.9	-30.8	-74.7	37.4	88.6	10.3	-11.7	-19.4	41.7
	-1.228	-2.029	-2.160	-5.239	2.626	6.213	0.720	-0.820	-1.363	2.927
t_O	229	270	257	244	229	254	259	222	245	241
	(16.1)	(18.9)	(18.0)	(17.1)	(16.1)	(17.8)	(18.2)	(15.6)	(17.2)	(16.9)
t_E	251.9	251.9	251.9	251.9	251.9	251.9	251.9	251.9	251.9	251.9
	(17.7)	(17.7)	(17.7)	(17.7)	(17.7)	(17.7)	(17.7)	(17.7)	(17.7)	(17.7)
	-43.7	37.4	10.2	-15.6	-43.7	4.1	14.3	-56.2	-13.7	-21.4
	-3.068	2.620	0.715	-1.097	-3.068	0.289	1.002	-3.940	-0.961	-1.502
g_O	457	443	464	493	478	440	432	453	474	445
	(32.0)	(31.1)	(32.5)	(34.6)	(33.5)	(30.9)	(30.3)	(31.8)	(33.2)	(31.2)
g_E	423.6	423.6	423.6	423.6	423.6	423.6	423.6	423.6	423.6	423.6
	(29.7)	(29.7)	(29.7)	(29.7)	(29.7)	(29.7)	(29.7)	(29.7)	(29.7)	(29.7)
	69.3	39.7	84.5	149.6	115.5	33.4	16.9	60.8	106.5	43.8
	4.863	2.781	5.927	10.489	8.098	2.343	1.188	4.262	7.472	3.074
c_O	500	479	472	481	452	442	481	508	468	471
	(35.1)	(33.6)	(33.1)	(33.7)	(31.7)	(31.0)	(33.7)	(35.6)	(32.8)	(33.0)
c_E	501.5	501.5	501.5	501.5	501.5	501.5	501.5	501.5	501.5	501.5
	(35.2)	(35.2)	(35.2)	(35.2)	(35.2)	(35.2)	(35.2)	(35.2)	(35.2)	(35.2)
	-3.0	-44.0	-57.3	-40.2	-94.0	-111.7	-40.2	13.0	-64.7	-59.1
	-0.213	-3.086	-4.016	-2.818	-6.591	-7.832	-2.818	0.915	-4.541	-4.148
N	1426	1426	1426	1426	1426	1426	1426	1426	1426	1426
X2	5.0	4.1	6.7	19.2	15.3	14.5	1.3	5.8	8.8	5.0
Gvalue	5.1	4.1	6.6	19.0	15.2	14.4	1.3	5.9	8.7	5.0
Gv/N	0.354	0.286	0.466	1.334	1.066	1.013	0.092	0.416	0.607	0.352
Prob	0.16814	0.25327	0.08400	0.00027	0.00165	0.00237	0.72562	0.11511	0.03423	0.17038

	70	69	68	67	66	65	64	63	62	61
a_O	234	250	215	247	251	239	256	242	234	232
	(16.4)	(17.5)	(15.1)	(17.3)	(17.6)	(16.8)	(18.0)	(17.0)	(16.4)	(16.3)
a_E	248.9	248.9	248.9	248.9	248.9	248.9	248.9	248.9	248.9	248.9
	(17.5)	(17.5)	(17.5)	(17.5)	(17.5)	(17.5)	(17.5)	(17.5)	(17.5)	(17.5)
	-28.9	2.2	-63.0	-3.8	4.2	-19.4	14.4	-13.6	-28.9	-32.7
	-2.029	0.152	-4.417	-0.268	0.293	-1.363	1.007	-0.957	-2.029	-2.291
t_O	260	237	257	272	254	254	267	244	224	264
	(18.2)	(16.6)	(18.0)	(19.1)	(17.8)	(17.8)	(18.7)	(17.1)	(15.7)	(18.5)
t_E	251.9	251.9	251.9	251.9	251.9	251.9	251.9	251.9	251.9	251.9

	(17.7)	(17.7)	(17.7)	(17.7)	(17.7)	(17.7)	(17.7)	(17.7)	(17.7)	(17.7)
	16.4	-29.0	10.2	41.7	4.1	4.1	31.0	-15.6	-52.7	24.7
	1.147	-2.033	0.715	2.921	0.289	0.289	2.173	-1.097	-3.694	1.730
g_O	473	446	482	428	444	447	463	466	447	471
	(33.2)	(31.3)	(33.8)	(30.0)	(31.1)	(31.3)	(32.5)	(32.7)	(31.3)	(33.0)
g_E	423.6	423.6	423.6	423.6	423.6	423.6	423.6	423.6	423.6	423.6
	(29.7)	(29.7)	(29.7)	(29.7)	(29.7)	(29.7)	(29.7)	(29.7)	(29.7)	(29.7)
	104.3	45.9	124.5	8.8	41.7	48.0	82.3	88.9	48.0	99.9
	7.316	3.222	8.729	0.619	2.927	3.369	5.774	6.233	3.369	7.005
c_O	459	493	472	479	477	486	440	474	521	459
	(32.2)	(34.6)	(33.1)	(33.6)	(33.5)	(34.1)	(30.9)	(33.2)	(36.5)	(32.2)
c_E	501.5	501.5	501.5	501.5	501.5	501.5	501.5	501.5	501.5	501.5
	(35.2)	(35.2)	(35.2)	(35.2)	(35.2)	(35.2)	(35.2)	(35.2)	(35.2)	(35.2)
	-81.3	-16.9	-57.3	-44.0	-47.8	-30.6	-115.2	-53.5	39.7	-81.3
	-5.703	-1.185	-4.016	-3.086	-3.354	-2.143	-8.076	-3.752	2.785	-5.703
N	1426	1426	1426	1426	1426	1426	1426	1426	1426	1426
X2	10.5	2.2	14.5	2.7	2.2	2.2	12.3	6.2	6.0	10.6
Gvalue	10.4	2.2	14.4	2.6	2.2	2.2	12.5	6.1	6.2	10.6
Gv/N	0.731	0.156	1.011	0.185	0.156	0.152	0.877	0.427	0.431	0.741
Prob	0.01527	0.52831	0.00238	0.45022	0.52824	0.53768	0.00582	0.10716	0.10453	0.01429

	60	59	58	57	56	55	54	53	52	51
a_O	215	252	261	235	243	265	242	238	200	232
	(15.1)	(17.7)	(18.3)	(16.5)	(17.0)	(18.6)	(17.0)	(16.7)	(14.0)	(16.3)
a_E	248.9	248.9	248.9	248.9	248.9	248.9	248.9	248.9	248.9	248.9
	(17.5)	(17.5)	(17.5)	(17.5)	(17.5)	(17.5)	(17.5)	(17.5)	(17.5)	(17.5)
	-63.0	6.2	24.7	-27.0	-11.7	33.2	-13.6	-21.4	-87.5	-32.7
	-4.417	0.435	1.735	-1.897	-0.820	2.327	-0.957	-1.497	-6.138	-2.291
t_O	253	226	247	239	234	266	232	222	274	235
	(17.7)	(15.8)	(17.3)	(16.8)	(16.4)	(18.7)	(16.3)	(15.6)	(19.2)	(16.5)
t_E	251.9	251.9	251.9	251.9	251.9	251.9	251.9	251.9	251.9	251.9
	(17.7)	(17.7)	(17.7)	(17.7)	(17.7)	(17.7)	(17.7)	(17.7)	(17.7)	(17.7)
	2.1	-49.1	-9.8	-25.2	-34.6	28.9	-38.3	-56.2	46.0	-32.7
	0.148	-3.445	-0.687	-1.769	-2.426	2.024	-2.684	-3.940	3.224	-2.295
g_O	477	439	427	477	466	421	476	470	469	473
	(33.5)	(30.8)	(29.9)	(33.5)	(32.7)	(29.5)	(33.4)	(33.0)	(32.9)	(33.2)
g_E	423.6	423.6	423.6	423.6	423.6	423.6	423.6	423.6	423.6	423.6
	(29.7)	(29.7)	(29.7)	(29.7)	(29.7)	(29.7)	(29.7)	(29.7)	(29.7)	(29.7)
	113.2	31.3	6.8	113.2	88.9	-5.2	111.0	97.7	95.5	104.3
	7.941	2.197	0.477	7.941	6.233	-0.365	7.785	6.850	6.696	7.316
c_O	481	509	491	475	483	474	476	496	483	486
	(33.7)	(35.7)	(34.4)	(33.3)	(33.9)	(33.2)	(33.4)	(34.8)	(33.9)	(34.1)
c_E	501.5	501.5	501.5	501.5	501.5	501.5	501.5	501.5	501.5	501.5
	(35.2)	(35.2)	(35.2)	(35.2)	(35.2)	(35.2)	(35.2)	(35.2)	(35.2)	(35.2)
	-40.2	15.1	-20.8	-51.6	-36.3	-53.5	-49.7	-11.0	-36.3	-30.6
	-2.818	1.057	-1.460	-3.619	-2.549	-3.752	-3.487	-0.770	-2.549	-2.143
N	1426	1426	1426	1426	1426	1426	1426	1426	1426	1426
X2	12.2	3.4	0.9	9.6	6.3	3.3	9.5	9.2	17.1	8.5
Gvalue	12.2	3.5	0.9	9.4	6.3	3.3	9.4	9.2	17.6	8.4
Gv/N	0.853	0.243	0.065	0.656	0.438	0.234	0.657	0.642	1.233	0.587
Prob	0.00683	0.32454	0.81959	0.02485	0.09998	0.34197	0.02480	0.02721	0.00054	0.03885

	50	49	48	47	46	45	44	43	42	41
a_O	222	233	260	235	243	233	262	223	243	239
	(15.6)	(16.3)	(18.2)	(16.5)	(17.0)	(16.3)	(18.4)	(15.6)	(17.0)	(16.8)
a_E	248.9	248.9	248.9	248.9	248.9	248.9	248.9	248.9	248.9	248.9
	(17.5)	(17.5)	(17.5)	(17.5)	(17.5)	(17.5)	(17.5)	(17.5)	(17.5)	(17.5)
	-50.8	-30.8	22.6	-27.0	-11.7	-30.8	26.8	-49.0	-11.7	-19.4
	-3.564	-2.160	1.588	-1.897	-0.820	-2.160	1.882	-3.439	-0.820	-1.363
t_O	236	236	256	214	244	260	256	243	259	223
	(16.5)	(16.5)	(18.0)	(15.0)	(17.1)	(18.2)	(18.0)	(17.0)	(18.2)	(15.6)
t_E	251.9	251.9	251.9	251.9	251.9	251.9	251.9	251.9	251.9	251.9
	(17.7)	(17.7)	(17.7)	(17.7)	(17.7)	(17.7)	(17.7)	(17.7)	(17.7)	(17.7)
	-30.9	-30.9	8.2	-69.9	-15.6	16.4	8.2	-17.6	14.3	-54.4
	-2.165	-2.165	0.573	-4.900	-1.097	1.147	0.573	-1.233	1.002	-3.818
g_O	453	457	457	434	463	451	438	411	475	429
	(31.8)	(32.0)	(32.0)	(30.4)	(32.5)	(31.6)	(30.7)	(28.8)	(33.3)	(30.1)
g_E	423.6	423.6	423.6	423.6	423.6	423.6	423.6	423.6	423.6	423.6
	(29.7)	(29.7)	(29.7)	(29.7)	(29.7)	(29.7)	(29.7)	(29.7)	(29.7)	(29.7)
	60.8	69.3	69.3	21.0	82.3	56.5	29.3	-24.8	108.8	10.8
	4.262	4.863	4.863	1.475	5.774	3.963	2.052	-1.742	7.628	0.761
c_O	515	500	453	543	476	482	470	549	449	535
	(36.1)	(35.1)	(31.8)	(38.1)	(33.4)	(33.8)	(33.0)	(38.5)	(31.5)	(37.5)
c_E	501.5	501.5	501.5	501.5	501.5	501.5	501.5	501.5	501.5	501.5
	(35.2)	(35.2)	(35.2)	(35.2)	(35.2)	(35.2)	(35.2)	(35.2)	(35.2)	(35.2)
	27.3	-3.0	-92.2	86.3	-49.7	-38.3	-61.0	99.3	-99.3	69.1
	1.916	-0.213	-6.465	6.052	-3.487	-2.684	-4.279	6.965	-6.966	4.849
N	1426	1426	1426	1426	1426	1426	1426	1426	1426	1426
X2	6.3	4.7	7.9	10.2	5.4	3.8	3.2	7.9	12.1	6.0
Gvalue	6.4	4.6	8.0	10.4	5.3	3.8	3.2	7.9	12.0	6.1
Gv/N	0.449	0.326	0.559	0.730	0.370	0.266	0.228	0.551	0.844	0.429
Prob	0.09342	0.19994	0.04665	0.01536	0.15300	0.28401	0.35501	0.04903	0.00726	0.10603

	40	39	38	37	36	35	34	33	32	31
a_O	252	238	245	272	226	239	240	223	252	255
	(17.7)	(16.7)	(17.2)	(19.1)	(15.8)	(16.8)	(16.8)	(15.6)	(17.7)	(17.9)
a_E	248.9	248.9	248.9	248.9	248.9	248.9	248.9	248.9	248.9	248.9
	(17.5)	(17.5)	(17.5)	(17.5)	(17.5)	(17.5)	(17.5)	(17.5)	(17.5)	(17.5)
	6.2	-21.4	-7.8	48.2	-43.7	-19.4	-17.5	-49.0	6.2	12.3
	0.435	-1.497	-0.545	3.383	-3.062	-1.363	-1.228	-3.439	0.435	0.863
t_O	291	249	264	260	254	259	295	281	262	291
	(20.4)	(17.5)	(18.5)	(18.2)	(17.8)	(18.2)	(20.7)	(19.7)	(18.4)	(20.4)
t_E	251.9	251.9	251.9	251.9	251.9	251.9	251.9	251.9	251.9	251.9
	(17.7)	(17.7)	(17.7)	(17.7)	(17.7)	(17.7)	(17.7)	(17.7)	(17.7)	(17.7)
	83.9	-5.9	24.7	16.4	4.1	14.3	93.1	61.3	20.5	83.9
	5.881	-0.411	1.730	1.147	0.289	1.002	6.527	4.301	1.437	5.881
g_O	419	433	394	419	453	412	386	433	397	377
	(29.4)	(30.4)	(27.6)	(29.4)	(31.8)	(28.9)	(27.1)	(30.4)	(27.8)	(26.4)
g_E	423.6	423.6	423.6	423.6	423.6	423.6	423.6	423.6	423.6	423.6
	(29.7)	(29.7)	(29.7)	(29.7)	(29.7)	(29.7)	(29.7)	(29.7)	(29.7)	(29.7)
	-9.2	19.0	-57.1	-9.2	60.8	-22.9	-71.8	19.0	-51.5	-87.9
	-0.643	1.331	-4.004	-0.643	4.262	-1.606	-5.033	1.331	-3.612	-6.164
c_O	464	506	523	475	493	516	505	489	515	503
	(32.5)	(35.5)	(36.7)	(33.3)	(34.6)	(36.2)	(35.4)	(34.3)	(36.1)	(35.3)
c_E	501.5	501.5	501.5	501.5	501.5	501.5	501.5	501.5	501.5	501.5
	(35.2)	(35.2)	(35.2)	(35.2)	(35.2)	(35.2)	(35.2)	(35.2)	(35.2)	(35.2)

	-72.2	9.0	43.9	-51.6	-16.9	29.4	7.0	-24.7	27.3	3.0
	-5.060	0.631	3.076	-3.619	-1.185	2.060	0.490	-1.734	1.916	0.208
N	1426	1426	1426	1426	1426	1426	1426	1426	1426	1426
X2	8.9	0.8	3.6	3.8	4.3	1.3	11.0	6.6	2.5	11.3
Gvalue	8.7	0.8	3.7	3.8	4.3	1.3	10.8	6.5	2.5	11.2
Gv/N	0.612	0.054	0.256	0.267	0.304	0.093	0.755	0.459	0.175	0.788
Prob	0.03311	0.85724	0.30106	0.28280	0.22758	0.72169	0.01310	0.08781	0.47521	0.01049

	30	29	28	27	26	25	24	23	22	21
a_O	236	265	257	250	228	248	237	276	266	242
	(16.5)	(18.6)	(18.0)	(17.5)	(16.0)	(17.4)	(16.6)	(19.4)	(18.7)	(17.0)
a_E	248.9	248.9	248.9	248.9	248.9	248.9	248.9	248.9	248.9	248.9
	(17.5)	(17.5)	(17.5)	(17.5)	(17.5)	(17.5)	(17.5)	(17.5)	(17.5)	(17.5)
	-25.2	33.2	16.4	2.2	-40.0	-1.8	-23.3	57.0	35.3	-13.6
	-1.764	2.327	1.151	0.152	-2.807	-0.129	-1.631	3.998	2.476	-0.957
t_O	250	250	296	284	296	317	262	248	283	274
	(17.5)	(17.5)	(20.8)	(19.9)	(20.8)	(22.2)	(18.4)	(17.4)	(19.8)	(19.2)
t_E	251.9	251.9	251.9	251.9	251.9	251.9	251.9	251.9	251.9	251.9
	(17.7)	(17.7)	(17.7)	(17.7)	(17.7)	(17.7)	(17.7)	(17.7)	(17.7)	(17.7)
	-3.9	-3.9	95.4	68.0	95.4	145.6	20.5	-7.8	65.8	46.0
	-0.272	-0.272	6.689	4.770	6.689	10.211	1.437	-0.550	4.613	3.224
g_O	433	411	366	408	405	372	423	365	366	382
	(30.4)	(28.8)	(25.7)	(28.6)	(28.4)	(26.1)	(29.7)	(25.6)	(25.7)	(26.8)
g_E	423.6	423.6	423.6	423.6	423.6	423.6	423.6	423.6	423.6	423.6
	(29.7)	(29.7)	(29.7)	(29.7)	(29.7)	(29.7)	(29.7)	(29.7)	(29.7)	(29.7)
	19.0	-24.8	-107.0	-30.6	-36.4	-96.7	-1.2	-108.7	-107.0	-79.0
	1.331	-1.742	-7.504	-2.148	-2.552	-6.778	-0.085	-7.623	-7.504	-5.539
c_O	507	500	507	484	497	489	504	537	511	528
	(35.6)	(35.1)	(35.6)	(33.9)	(34.9)	(34.3)	(35.3)	(37.7)	(35.8)	(37.0)
c_E	501.5	501.5	501.5	501.5	501.5	501.5	501.5	501.5	501.5	501.5
	(35.2)	(35.2)	(35.2)	(35.2)	(35.2)	(35.2)	(35.2)	(35.2)	(35.2)	(35.2)
	11.0	-3.0	11.0	-34.4	-9.0	-24.7	5.0	73.4	19.1	54.3
	0.773	-0.213	0.773	-2.414	-0.631	-1.734	0.349	5.148	1.342	3.810
N	1426	1426	1426	1426	1426	1426	1426	1426	1426	1426
X2	1.0	1.4	15.9	5.3	10.3	23.4	1.0	13.6	13.0	7.6
Gvalue	1.0	1.4	15.8	5.1	10.0	22.4	1.0	13.9	13.2	7.7
Gv/N	0.068	0.099	1.110	0.359	0.699	1.570	0.069	0.973	0.927	0.538
Prob	0.80998	0.70179	0.00123	0.16317	0.01884	0.00005	0.80393	0.00309	0.00419	0.05330

	20	19	18	17	16	15	14	13	12	11
a_O	253	241	257	256	230	276	284	282	283	311
	(17.7)	(16.9)	(18.0)	(18.0)	(16.1)	(19.4)	(19.9)	(19.8)	(19.8)	(21.8)
a_E	248.9	248.9	248.9	248.9	248.9	248.9	248.9	248.9	248.9	248.9
	(17.5)	(17.5)	(17.5)	(17.5)	(17.5)	(17.5)	(17.5)	(17.5)	(17.5)	(17.5)
	8.2	-15.6	16.4	14.4	-36.4	57.0	74.9	70.4	72.6	138.5
	0.577	-1.093	1.151	1.007	-2.550	3.998	5.252	4.935	5.093	9.712
t_O	279	302	280	294	319	288	294	281	259	235
	(19.6)	(21.2)	(19.6)	(20.6)	(22.4)	(20.2)	(20.6)	(19.7)	(18.2)	(16.5)
t_E	251.9	251.9	251.9	251.9	251.9	251.9	251.9	251.9	251.9	251.9
	(17.7)	(17.7)	(17.7)	(17.7)	(17.7)	(17.7)	(17.7)	(17.7)	(17.7)	(17.7)
	56.9	109.4	59.1	90.8	150.5	77.0	90.8	61.3	14.3	-32.7
	3.991	7.675	4.145	6.364	10.557	5.402	6.364	4.301	1.002	-2.295
g_O	332	317	336	304	350	359	310	316	359	330
	(23.3)	(22.2)	(23.6)	(21.3)	(24.5)	(25.2)	(21.7)	(22.2)	(25.2)	(23.1)
g_E	423.6	423.6	423.6	423.6	423.6	423.6	423.6	423.6	423.6	423.6
	(29.7)	(29.7)	(29.7)	(29.7)	(29.7)	(29.7)	(29.7)	(29.7)	(29.7)	(29.7)
	-161.8	-183.8	-155.7	-201.7	-133.6	-118.8	-193.6	-185.2	-118.8	-164.8
	-11.347	-12.889	-10.919	-14.146	-9.370	-8.333	-13.576	-12.989	-8.333	-11.558
c_O	562	566	553	572	527	503	538	547	525	550
	(39.4)	(39.7)	(38.8)	(40.1)	(37.0)	(35.3)	(37.7)	(38.4)	(36.8)	(38.6)
c_E	501.5	501.5	501.5	501.5	501.5	501.5	501.5	501.5	501.5	501.5
	(35.2)	(35.2)	(35.2)	(35.2)	(35.2)	(35.2)	(35.2)	(35.2)	(35.2)	(35.2)
	128.0	136.9	108.1	150.4	52.2	3.0	75.6	95.0	48.0	101.5
	8.975	9.601	7.579	10.549	3.663	0.208	5.298	6.660	3.369	7.118
N	1426	1426	1426	1426	1426	1426	1426	1426	1426	1426
X2	30.1	45.3	26.8	50.9	33.4	18.0	45.1	39.2	15.8	42.0
Gvalue	31.3	47.0	27.9	53.8	32.8	18.2	47.6	41.4	16.1	42.4
Gv/N	2.195	3.294	1.957	3.774	2.300	1.275	3.338	2.906	1.132	2.977
Prob	0.00000	0.00000	0.00000	0.00000	0.00000	0.00040	0.00000	0.00000	0.00106	0.00000

	10	9	8	7	6	5	4	3	2	1
a_O	299	313	307	293	308	311	244	348	225	155
	(21.0)	(21.9)	(21.5)	(20.5)	(21.6)	(21.8)	(17.1)	(24.4)	(15.8)	(10.9)
a_E	248.9	248.9	248.9	248.9	248.9	248.9	248.9	248.9	248.9	248.9
	(17.5)	(17.5)	(17.5)	(17.5)	(17.5)	(17.5)	(17.5)	(17.5)	(17.5)	(17.5)
	109.6	143.4	128.8	95.5	131.2	138.5	-9.7	233.2	-45.5	-146.8
	7.687	10.056	9.030	6.700	9.200	9.712	-0.683	16.354	-3.188	-10.298
t_O	244	239	261	273	247	276	252	196	252	146
	(17.1)	(16.8)	(18.3)	(19.1)	(17.3)	(19.4)	(17.7)	(13.7)	(17.7)	(10.2)
t_E	251.9	251.9	251.9	251.9	251.9	251.9	251.9	251.9	251.9	251.9
	(17.7)	(17.7)	(17.7)	(17.7)	(17.7)	(17.7)	(17.7)	(17.7)	(17.7)	(17.7)
	-15.6	-25.2	18.4	43.8	-9.8	50.3	0.1	-98.4	0.1	-159.3
	-1.097	-1.769	1.292	3.072	-0.687	3.529	0.007	-6.903	0.007	-11.173
g_O	369	388	342	303	349	285	374	460	283	367
	(25.9)	(27.2)	(24.0)	(21.2)	(24.5)	(20.0)	(26.2)	(32.3)	(19.8)	(25.7)
g_E	423.6	423.6	423.6	423.6	423.6	423.6	423.6	423.6	423.6	423.6
	(29.7)	(29.7)	(29.7)	(29.7)	(29.7)	(29.7)	(29.7)	(29.7)	(29.7)	(29.7)
	-101.9	-68.1	-146.4	-203.1	-135.2	-225.9	-93.2	75.8	-228.3	-105.3
	-7.143	-4.778	-10.265	-14.240	-9.483	-15.842	-6.534	5.317	-16.010	-7.384
c_O	514	486	516	557	522	554	556	422	666	758
	(36.0)	(34.1)	(36.2)	(39.1)	(36.6)	(38.8)	(39.0)	(29.6)	(46.7)	(53.2)
c_E	501.5	501.5	501.5	501.5	501.5	501.5	501.5	501.5	501.5	501.5
	(35.2)	(35.2)	(35.2)	(35.2)	(35.2)	(35.2)	(35.2)	(35.2)	(35.2)	(35.2)
	25.3	-30.6	29.4	116.9	41.8	110.3	114.7	-145.7	377.8	626.2
	1.772	-2.143	2.060	8.197	2.930	7.733	8.042	-10.218	26.495	43.911
N	1426	1426	1426	1426	1426	1426	1426	1426	1426	1426
X2	17.7	20.6	30.0	50.0	28.1	68.6	11.8	67.6	102.9	218.7
Gvalue	17.4	19.5	30.2	53.2	27.9	73.2	11.9	64.9	104.1	214.7
Gv/N	1.219	1.366	2.117	3.729	1.959	5.132	0.832	4.550	7.303	15.056
Prob	0.00059	0.00022	0.00000	0.00000	0.00000	0.00000	0.00786	0.00000	0.00000	0.00000

CDS_INITIAL:

	1	2	3	4	5	6	7	8	9	10

a_O	1072	0	1	582	382	243	409	394	242	393
	(75.2)	(0.0)	(0.1)	(40.8)	(26.8)	(17.0)	(28.7)	(27.6)	(17.0)	(27.6)
a_E	261.1	332.6	69.0	261.1	332.6	69.0	261.1	332.6	69.0	261.1
	(18.3)	(23.3)	(4.8)	(18.3)	(23.3)	(4.8)	(18.3)	(23.3)	(4.8)	(18.3)
	3027.9	0.0	-8.5	932.9	105.7	612.1	367.0	133.4	607.6	321.3
	212.332	0.000	-0.594	65.419	7.414	42.922	25.738	9.356	42.606	22.531
t_O	135	1426	0	184	222	209	178	328	231	223
	(9.5)	(100.0)	(0.0)	(12.9)	(15.6)	(14.7)	(12.5)	(23.0)	(16.2)	(15.6)
t_E	178.1	395.1	96.1	178.1	395.1	96.1	178.1	395.1	96.1	178.1
	(12.5)	(27.7)	(6.7)	(12.5)	(27.7)	(6.7)	(12.5)	(27.7)	(6.7)	(12.5)
	-74.8	3660.7	0.0	12.0	-255.9	324.6	-0.2	-122.0	405.0	100.2
	-5.247	256.713	0.000	0.840	-17.946	22.762	-0.015	-8.559	28.400	7.030
g_O	219	0	1425	243	249	367	450	251	362	408
	(15.4)	(0.0)	(99.9)	(17.0)	(17.5)	(25.7)	(31.6)	(17.6)	(25.4)	(28.6)
g_E	579.6	287.0	608.5	579.6	287.0	608.5	579.6	287.0	608.5	579.6
	(40.6)	(20.1)	(42.7)	(40.6)	(20.1)	(42.7)	(40.6)	(20.1)	(42.7)	(40.6)
	-426.3	0.0	2425.1	-422.4	-70.8	-371.1	-227.7	-67.3	-376.0	-286.4
	-29.893	0.000	170.065	-29.625	-4.962	-26.027	-15.971	-4.720	-26.369	-20.087
c_O	0	0	0	417	573	607	389	453	591	402
	(0.0)	(0.0)	(0.0)	(29.2)	(40.2)	(42.6)	(27.3)	(31.8)	(41.4)	(28.2)
c_E	407.2	411.3	652.4	407.2	411.3	652.4	407.2	411.3	652.4	407.2
	(28.6)	(28.8)	(45.7)	(28.6)	(28.8)	(45.7)	(28.6)	(28.8)	(45.7)	(28.6)
	0.0	0.0	0.0	19.9	380.0	-87.5	-35.5	87.5	-116.8	-10.3
	0.000	0.000	0.000	1.394	26.650	-6.139	-2.492	6.138	-8.191	-0.722
N	1426	1426	1426	1426	1426	1426	1426	1426	1426	1426
X2	3159.8	3721.1	1911.1	590.2	151.8	670.6	113.5	31.5	728.9	128.8
Gvalue	2526.8	3660.7	2416.7	542.3	159.1	478.0	103.5	31.6	519.7	124.8
Gv/N	177.192	256.713	169.471	38.028	11.155	33.519	7.260	2.215	36.446	8.753
Prob	1.00000	1.00000	1.00000	0.00000	0.00000	0.00000	0.00000	0.00000	0.00000	0.00000

	11	12	13	14	15	16	17	18	19	20
a_O	347	204	359	320	189	330	338	191	332	339
	(24.3)	(14.3)	(25.2)	(22.4)	(13.3)	(23.1)	(23.7)	(13.4)	(23.3)	(23.8)
a_E	332.6	69.0	261.1	332.6	69.0	261.1	332.6	69.0	261.1	332.6
	(23.3)	(4.8)	(18.3)	(23.3)	(4.8)	(18.3)	(23.3)	(4.8)	(18.3)	(23.3)
	29.3	442.5	228.5	-24.8	381.1	154.5	10.8	389.1	159.4	12.9
	2.058	31.028	16.026	-1.738	26.722	10.833	0.759	27.287	11.180	0.902
t_O	339	211	227	389	239	228	381	220	212	376
	(23.8)	(14.8)	(15.9)	(27.3)	(16.8)	(16.0)	(26.7)	(15.4)	(14.9)	(26.4)
t_E	395.1	96.1	178.1	395.1	96.1	178.1	395.1	96.1	178.1	395.1
	(27.7)	(6.7)	(12.5)	(27.7)	(6.7)	(12.5)	(27.7)	(6.7)	(12.5)	(27.7)
	-103.8	331.7	110.1	-12.0	435.3	112.6	-27.6	364.2	73.9	-37.2
	-7.277	23.262	7.722	-0.845	30.525	7.897	-1.938	25.543	5.179	-2.609
g_O	248	381	397	214	413	420	221	403	437	222
	(17.4)	(26.7)	(27.8)	(15.0)	(29.0)	(29.5)	(15.5)	(28.3)	(30.6)	(15.6)
g_E	287.0	608.5	579.6	287.0	608.5	579.6	287.0	608.5	579.6	287.0
	(20.1)	(42.7)	(40.6)	(20.1)	(42.7)	(40.6)	(20.1)	(42.7)	(40.6)	(20.1)
	-72.5	-356.8	-300.4	-125.6	-320.1	-270.5	-115.5	-332.1	-246.8	-114.0
	-5.082	-25.019	-21.067	-8.811	-22.449	-18.970	-8.102	-23.291	-17.306	-7.998
c_O	492	630	443	503	585	448	486	612	445	489
	(34.5)	(44.2)	(31.1)	(35.3)	(41.0)	(31.4)	(34.1)	(42.9)	(31.2)	(34.3)
c_E	411.3	652.4	407.2	411.3	652.4	407.2	411.3	652.4	407.2	411.3
	(28.8)	(45.7)	(28.6)	(28.8)	(45.7)	(28.6)	(28.8)	(45.7)	(28.6)	(28.8)
	176.3	-44.0	74.7	202.5	-127.6	85.6	162.3	-78.2	79.0	169.3
	12.366	-3.085	5.239	14.202	-8.945	6.003	11.379	-5.485	5.543	11.871
N	1426	1426	1426	1426	1426	1426	1426	1426	1426	1426
X2	29.7	487.4	110.8	39.6	491.0	80.2	29.3	447.4	64.3	30.5
Gvalue	29.4	373.4	112.9	40.1	368.7	82.2	29.9	343.0	65.5	30.9
Gv/N	2.064	26.186	7.920	2.809	25.853	5.762	2.098	24.054	4.596	2.166
Prob	0.00000	0.00000	0.00000	0.00000	0.00000	0.00000	0.00000	0.00000	0.00000	0.00000

	21	22	23	24	25	26	27	28	29	30
a_O	188	318	320	143	268	303	123	257	269	135
	(13.2)	(22.3)	(22.4)	(10.0)	(18.8)	(21.2)	(8.6)	(18.0)	(18.9)	(9.5)
a_E	69.0	261.1	332.6	69.0	261.1	332.6	69.0	261.1	332.6	69.0
	(4.8)	(18.3)	(23.3)	(4.8)	(18.3)	(23.3)	(4.8)	(18.3)	(23.3)	(4.8)
	377.0	125.3	-24.8	208.5	13.9	-56.5	142.3	-8.2	-114.2	181.3
	26.441	8.787	-1.738	14.625	0.975	-3.965	9.980	-0.575	-8.011	12.717
t_O	208	214	349	181	207	363	213	201	381	174
	(14.6)	(15.0)	(24.5)	(12.7)	(14.5)	(25.5)	(14.9)	(14.1)	(26.7)	(12.2)
t_E	96.1	178.1	395.1	96.1	178.1	395.1	96.1	178.1	395.1	96.1
	(6.7)	(12.5)	(27.7)	(6.7)	(12.5)	(27.7)	(6.7)	(12.5)	(27.7)	(6.7)
	321.0	78.6	-86.5	229.0	62.2	-61.5	338.9	48.6	-27.6	206.4
	22.513	5.510	-6.069	16.061	4.364	-4.310	23.764	3.408	-1.938	14.477
g_O	405	429	242	456	476	274	478	506	279	471
	(28.4)	(30.1)	(17.0)	(32.0)	(33.4)	(19.2)	(33.5)	(35.5)	(19.6)	(33.0)
g_E	608.5	579.6	287.0	608.5	579.6	287.0	608.5	579.6	287.0	608.5
	(42.7)	(40.6)	(20.1)	(42.7)	(40.6)	(20.1)	(42.7)	(40.6)	(20.1)	(42.7)
	-329.8	-258.1	-82.6	-263.1	-187.4	-25.4	-230.8	-137.4	-15.8	-241.3
	-23.125	-18.101	-5.791	-18.452	-13.144	-1.784	-16.183	-9.635	-1.109	-16.921
c_O	625	465	515	646	475	486	612	462	497	646
	(43.8)	(32.6)	(36.1)	(45.3)	(33.3)	(34.1)	(42.9)	(32.4)	(34.9)	(45.3)
c_E	652.4	407.2	411.3	652.4	407.2	411.3	652.4	407.2	411.3	652.4
	(45.7)	(28.6)	(28.8)	(45.7)	(28.6)	(28.8)	(45.7)	(28.6)	(28.8)	(45.7)
	-53.6	123.5	231.6	-12.7	146.4	162.3	-78.2	116.7	188.2	-12.7
	-3.759	8.660	16.244	-0.891	10.263	11.379	-5.485	8.184	13.196	-0.891
N	1426	1426	1426	1426	1426	1426	1426	1426	1426	1426
X2	404.8	66.9	39.1	192.7	34.7	19.4	214.9	19.7	30.8	157.4
Gvalue	314.7	69.2	37.7	161.7	35.1	18.8	172.2	19.7	30.5	133.8
Gv/N	22.070	4.856	2.647	11.343	2.459	1.320	12.076	1.383	2.139	9.382
Prob	0.00000	0.00000	0.00000	0.00000	0.00000	0.00030	0.00000	0.00019	0.00000	0.00000

	31	32	33	34	35	36	37	38	39	40
a_O	249	281	149	250	281	123	265	287	122	253
	(17.5)	(19.7)	(10.4)	(17.5)	(19.7)	(8.6)	(18.6)	(20.1)	(8.6)	(17.7)
a_E	261.1	332.6	69.0	261.1	332.6	69.0	261.1	332.6	69.0	261.1
	(18.3)	(23.3)	(4.8)	(18.3)	(23.3)	(4.8)	(18.3)	(23.3)	(4.8)	(18.3)
	-23.7	-94.8	229.5	-21.8	-94.8	142.3	7.8	-84.7	139.2	-16.0
	-1.662	-6.648	16.097	-1.528	-6.648	9.980	0.546	-5.939	9.759	-1.123
t_O	215	395	156	163	376	138	216	396	148	180
	(15.1)	(27.7)	(10.9)	(11.4)	(26.4)	(9.7)	(15.1)	(27.8)	(10.4)	(12.6)
t_E	178.1	395.1	96.1	178.1	395.1	96.1	178.1	395.1	96.1	178.1
	(12.5)	(27.7)	(6.7)	(12.5)	(27.7)	(6.7)	(12.5)	(27.7)	(6.7)	(12.5)
	80.9	-0.1	151.0	-28.9	-37.2	99.8	83.3	1.9	127.7	3.8
	5.676	-0.010	10.591	-2.027	-2.609	6.996	5.843	0.131	8.955	0.267

g_O	525	245	464	560	281	496	574	277	475	565
	(36.8)	(17.2)	(32.5)	(39.3)	(19.7)	(34.8)	(40.3)	(19.4)	(33.3)	(39.6)
g_E	579.6	287.0	608.5	579.6	287.0	608.5	579.6	287.0	608.5	579.6
	(40.6)	(20.1)	(42.7)	(40.6)	(20.1)	(42.7)	(40.6)	(20.1)	(42.7)	(40.6)
	-103.8	-77.6	-251.6	-38.5	-11.9	-202.8	-11.1	-19.7	-235.3	-28.8
	-7.282	-5.439	-17.644	-2.699	-0.835	-14.221	-0.778	-1.380	-16.501	-2.019
c_O	437	505	657	453	488	669	371	466	681	428
	(30.6)	(35.4)	(46.1)	(31.8)	(34.2)	(46.9)	(26.0)	(32.7)	(47.8)	(30.0)
c_E	407.2	411.3	652.4	407.2	411.3	652.4	407.2	411.3	652.4	407.2
	(28.6)	(28.8)	(45.7)	(28.6)	(28.8)	(45.7)	(28.6)	(28.8)	(45.7)	(28.6)
	61.8	207.3	9.3	96.6	166.9	33.6	-69.0	116.4	58.5	42.7
	4.332	14.540	0.649	6.775	11.706	2.360	-4.842	8.164	4.100	2.993
N	1426	1426	1426	1426	1426	1426	1426	1426	1426	1426
X2	15.5	35.5	164.5	7.6	23.4	81.8	11.4	13.9	99.3	1.7
Gvalue	15.2	34.8	138.2	7.4	23.0	72.9	11.0	13.9	90.0	1.7
Gv/N	1.064	2.443	9.694	0.522	1.615	5.114	0.769	0.975	6.313	0.119
Prob	0.00167	0.00000	0.00000	0.05904	0.00004	0.00000	0.01190	0.00304	0.00000	0.63856

	41	42	43	44	45	46	47	48	49	50
a_O	271	102	240	299	107	272	278	105	240	288
	(19.0)	(7.2)	(16.8)	(21.0)	(7.5)	(19.1)	(19.5)	(7.4)	(16.8)	(20.2)
a_E	332.6	69.0	261.1	332.6	69.0	261.1	332.6	69.0	261.1	332.6
	(23.3)	(4.8)	(18.3)	(23.3)	(4.8)	(18.3)	(23.3)	(4.8)	(18.3)	(23.3)
	-111.1	79.8	-40.5	-63.7	94.0	22.2	-99.8	88.3	-40.5	-83.0
	-7.789	5.598	-2.841	-4.470	6.591	1.555	-6.995	6.190	-2.841	-5.820
t_O	419	136	185	414	141	186	390	138	186	388
	(29.4)	(9.5)	(13.0)	(29.0)	(9.9)	(13.0)	(27.3)	(9.7)	(13.0)	(27.2)
t_E	395.1	96.1	178.1	395.1	96.1	178.1	395.1	96.1	178.1	395.1
	(27.7)	(6.7)	(12.5)	(27.7)	(6.7)	(12.5)	(27.7)	(6.7)	(12.5)	(27.7)
	49.3	94.3	14.0	38.8	108.0	16.1	-10.1	99.8	16.1	-14.0
	3.456	6.616	0.985	2.718	7.573	1.131	-0.706	6.996	1.131	-0.983
g_O	262	507	569	274	508	548	264	512	561	288
	(18.4)	(35.6)	(39.9)	(19.2)	(35.6)	(38.4)	(18.5)	(35.9)	(39.3)	(20.2)
g_E	287.0	608.5	579.6	287.0	608.5	579.6	287.0	608.5	579.6	287.0
	(20.1)	(42.7)	(40.6)	(20.1)	(42.7)	(40.6)	(20.1)	(42.7)	(40.6)	(20.1)
	-47.8	-185.0	-21.0	-25.4	-183.4	-61.4	-44.1	-176.8	-36.6	2.0
	-3.351	-12.977	-1.470	-1.784	-12.862	-4.306	-3.095	-12.400	-2.563	0.138
c_O	474	681	432	439	670	420	494	671	439	462
	(33.2)	(47.8)	(30.3)	(30.8)	(47.0)	(29.5)	(34.6)	(47.1)	(30.8)	(32.4)
c_E	411.3	652.4	407.2	411.3	652.4	407.2	411.3	652.4	407.2	411.3
	(28.8)	(45.7)	(28.6)	(28.8)	(45.7)	(28.6)	(28.8)	(45.7)	(28.6)	(28.8)
	134.6	58.5	51.1	57.3	35.7	26.0	181.1	37.8	66.1	107.5
	9.436	4.100	3.585	4.016	2.504	1.826	12.697	2.648	4.633	7.535
N	1426	1426	1426	1426	1426	1426	1426	1426	1426	1426
X2	24.6	50.5	3.7	6.8	59.0	2.9	27.5	52.9	5.1	12.4
Gvalue	25.0	47.6	3.7	6.8	54.3	2.9	27.1	49.0	5.1	12.4
Gv/N	1.752	3.337	0.259	0.480	3.805	0.206	1.900	3.433	0.360	0.871
Prob	0.00002	0.00000	0.29585	0.07714	0.00000	0.40057	0.00001	0.00000	0.16257	0.00608

	51	52	53	54	55	56	57	58	59	60
a_O	92	254	268	104	272	281	87	255	304	98
	(6.5)	(17.8)	(18.8)	(7.3)	(19.1)	(19.7)	(6.1)	(17.9)	(21.3)	(6.9)
a_E	69.0	261.1	332.6	69.0	261.1	332.6	69.0	261.1	332.6	69.0
	(4.8)	(18.3)	(23.3)	(4.8)	(18.3)	(23.3)	(4.8)	(18.3)	(23.3)	(4.8)
	53.0	-14.1	-115.8	85.4	22.2	-94.8	40.4	-12.1	-54.7	68.9
	3.718	-0.987	-8.121	5.991	1.555	-6.648	2.834	-0.850	-3.838	4.829
t_O	156	188	450	109	179	398	134	187	393	114
	(10.9)	(13.2)	(31.6)	(7.6)	(12.6)	(27.9)	(9.4)	(13.1)	(27.6)	(8.0)
t_E	96.1	178.1	395.1	96.1	178.1	395.1	96.1	178.1	395.1	96.1
	(6.7)	(12.5)	(27.7)	(6.7)	(12.5)	(27.7)	(6.7)	(12.5)	(27.7)	(6.7)
	151.0	20.3	117.2	27.4	1.8	5.9	89.0	18.2	-4.1	38.8
	10.591	1.425	8.216	1.919	0.125	0.412	6.240	1.278	-0.290	2.724
g_O	516	541	282	532	576	272	511	572	276	559
	(36.2)	(37.9)	(19.8)	(37.3)	(40.4)	(19.1)	(35.8)	(40.1)	(19.4)	(39.2)
g_E	608.5	579.6	287.0	608.5	579.6	287.0	608.5	579.6	287.0	608.5
	(42.7)	(40.6)	(20.1)	(42.7)	(40.6)	(20.1)	(42.7)	(40.6)	(20.1)	(42.7)
	-170.2	-74.5	-9.9	-143.0	-7.1	-29.2	-178.5	-15.1	-21.6	-94.9
	-11.934	-5.226	-0.698	-10.025	-0.500	-2.050	-12.516	-1.056	-1.515	-6.653
c_O	662	443	426	681	399	475	694	412	453	655
	(46.4)	(31.1)	(29.9)	(47.8)	(28.0)	(33.3)	(48.7)	(28.9)	(31.8)	(45.9)
c_E	652.4	407.2	411.3	652.4	407.2	411.3	652.4	407.2	411.3	652.4
	(45.7)	(28.6)	(28.8)	(45.7)	(28.6)	(28.8)	(45.7)	(28.6)	(28.8)	(45.7)
	19.4	74.7	30.0	58.5	-16.2	136.8	85.8	9.7	87.5	5.2
	1.358	5.239	2.101	4.100	-1.136	9.596	6.019	0.680	6.138	0.367
N	1426	1426	1426	1426	1426	1426	1426	1426	1426	1426
X2	59.2	6.5	20.8	30.4	0.6	18.7	37.9	0.7	7.1	19.6
Gvalue	53.2	6.4	21.4	28.3	0.6	18.7	36.7	0.7	7.1	18.1
Gv/N	3.733	0.451	1.499	1.985	0.045	1.310	2.577	0.052	0.496	1.268
Prob	0.00000	0.09266	0.00009	0.00000	0.88768	0.00032	0.00000	0.86419	0.06960	0.00042

	61	62	63	64	65	66	67	68	69	70
a_O	263	294	112	251	279	107	233	289	100	213
	(18.4)	(20.6)	(7.9)	(17.6)	(19.6)	(7.5)	(16.3)	(20.3)	(7.0)	(14.9)
a_E	261.1	332.6	69.0	261.1	332.6	69.0	261.1	332.6	69.0	261.1
	(18.3)	(23.3)	(4.8)	(18.3)	(23.3)	(4.8)	(18.3)	(23.3)	(4.8)	(18.3)
	3.7	-72.6	108.6	-19.9	-98.1	94.0	-53.1	-81.3	74.3	-86.8
	0.263	-5.091	7.616	-1.393	-6.880	6.591	-3.725	-5.699	5.211	-6.087
t_O	171	383	104	166	418	118	171	384	119	207
	(12.0)	(26.9)	(7.3)	(11.6)	(29.3)	(8.3)	(12.0)	(26.9)	(8.3)	(14.5)
t_E	178.1	395.1	96.1	178.1	395.1	96.1	178.1	395.1	96.1	178.1
	(12.5)	(27.7)	(6.7)	(12.5)	(27.7)	(6.7)	(12.5)	(27.7)	(6.7)	(12.5)
	-13.9	-23.8	16.3	-23.4	47.2	48.3	-13.9	-21.8	50.8	62.2
	-0.977	-1.667	1.146	-1.639	3.308	3.391	-0.977	-1.531	3.560	4.364
g_O	570	273	561	582	291	527	592	295	565	562
	(40.0)	(19.1)	(39.3)	(40.8)	(20.4)	(37.0)	(41.5)	(20.7)	(39.6)	(39.4)
g_E	579.6	287.0	608.5	579.6	287.0	608.5	579.6	287.0	608.5	579.6
	(40.6)	(20.1)	(42.7)	(40.6)	(20.1)	(42.7)	(40.6)	(20.1)	(42.7)	(40.6)
	-19.0	-27.3	-91.2	4.9	8.0	-151.6	25.1	16.2	-83.8	-34.6
	-1.332	-1.917	-6.396	0.341	0.562	-10.629	1.761	1.135	-5.878	-2.427
c_O	422	476	649	427	438	674	430	458	642	444
	(29.6)	(33.4)	(45.5)	(29.9)	(30.7)	(47.3)	(30.2)	(32.1)	(45.0)	(31.1)
c_E	407.2	411.3	652.4	407.2	411.3	652.4	407.2	411.3	652.4	407.2
	(28.6)	(28.8)	(45.7)	(28.6)	(28.8)	(45.7)	(28.6)	(28.8)	(45.7)	(28.6)
	30.2	139.1	-6.8	40.6	55.1	43.9	46.9	98.6	-20.6	76.9
	2.116	9.756	-0.474	2.846	3.867	3.081	3.289	6.911	-1.445	5.391

N	1426	1426	1426	1426	1426	1426	1426	1426	1426	1426
X2	1.0	15.7	31.2	2.2	11.8	37.6	4.9	11.6	22.7	17.4
Gvalue	1.0	15.4	27.0	2.2	12.2	34.7	5.0	11.6	20.6	17.7
Gv/N	0.070	1.082	1.893	0.154	0.857	2.433	0.347	0.816	1.448	1.241
Prob	0.80294	0.00148	0.00001	0.53181	0.00669	0.00000	0.17520	0.00872	0.00012	0.00051

	71	72	73	74	75	76	77	78	79	80
a_O	296	98	231	297	94	222	294	98	226	317
	(20.8)	(6.9)	(16.2)	(20.8)	(6.6)	(15.6)	(20.6)	(6.9)	(15.8)	(22.2)
a_E	332.6	69.0	261.1	332.6	69.0	261.1	332.6	69.0	261.1	332.6
	(23.3)	(4.8)	(18.3)	(23.3)	(4.8)	(18.3)	(23.3)	(4.8)	(18.3)	(23.3)
	-69.1	68.9	-56.6	-67.3	58.2	-72.1	-72.6	68.9	-65.3	-30.5
	-4.844	4.829	-3.973	-4.720	4.082	-5.055	-5.091	4.829	-4.580	-2.140
t_O	402	133	167	400	110	197	398	119	193	395
	(28.2)	(9.3)	(11.7)	(28.1)	(7.7)	(13.8)	(27.9)	(8.3)	(13.5)	(27.7)
t_E	395.1	96.1	178.1	395.1	96.1	178.1	395.1	96.1	178.1	395.1
	(27.7)	(6.7)	(12.5)	(27.7)	(6.7)	(12.5)	(27.7)	(6.7)	(12.5)	(27.7)
	14.0	86.3	-21.5	9.9	29.6	39.7	5.9	50.8	31.0	-0.1
	0.980	6.054	-1.508	0.696	2.078	2.785	0.412	3.560	2.173	-0.010
g_O	279	532	617	304	558	559	277	569	576	273
	(19.6)	(37.3)	(43.3)	(21.3)	(39.1)	(39.2)	(19.4)	(39.9)	(40.4)	(19.1)
g_E	287.0	608.5	579.6	287.0	608.5	579.6	287.0	608.5	579.6	287.0
	(20.1)	(42.7)	(40.6)	(20.1)	(42.7)	(40.6)	(20.1)	(42.7)	(40.6)	(20.1)
	-15.8	-143.0	77.2	34.9	-96.7	-40.4	-19.7	-76.4	-7.1	-27.3
	-1.109	-10.025	5.415	2.451	-6.781	-2.834	-1.380	-5.357	-0.500	-1.917
c_O	449	663	411	425	664	448	457	640	431	441
	(31.5)	(46.5)	(28.8)	(29.8)	(46.6)	(31.4)	(32.0)	(44.9)	(30.2)	(30.9)
c_E	411.3	652.4	407.2	411.3	652.4	407.2	411.3	652.4	407.2	411.3
	(28.8)	(45.7)	(28.6)	(28.8)	(45.7)	(28.6)	(28.8)	(45.7)	(28.6)	(28.8)
	78.8	21.4	7.7	27.9	23.4	85.6	96.3	-24.5	49.0	61.5
	5.526	1.501	0.538	1.956	1.643	6.003	6.756	-1.721	3.437	4.315
N	1426	1426	1426	1426	1426	1426	1426	1426	1426	1426
X2	7.8	36.1	6.6	5.3	15.5	12.7	9.9	20.5	7.4	3.6
Gvalue	7.9	33.6	6.7	5.5	14.6	12.8	10.0	18.7	7.6	3.5
Gv/N	0.554	2.358	0.472	0.383	1.022	0.899	0.698	1.312	0.530	0.248
Prob	0.04821	0.00000	0.08106	0.14103	0.00222	0.00504	0.01899	0.00031	0.05615	0.31561

	81	82	83	84	85	86	87	88	89	90
a_O	101	255	296	91	230	306	87	244	336	90
	(7.1)	(17.9)	(20.8)	(6.4)	(16.1)	(21.5)	(6.1)	(17.1)	(23.6)	(6.3)
a_E	69.0	261.1	332.6	69.0	261.1	332.6	69.0	261.1	332.6	69.0
	(4.8)	(18.3)	(23.3)	(4.8)	(18.3)	(23.3)	(4.8)	(18.3)	(23.3)	(4.8)
	77.1	-12.1	-69.1	50.5	-58.4	-51.1	40.4	-33.1	6.8	47.9
	5.404	-0.850	-4.844	3.538	-4.095	-3.581	2.834	-2.322	0.475	3.360
t_O	113	203	401	122	178	367	103	198	394	105
	(7.9)	(14.2)	(28.1)	(8.6)	(12.5)	(25.7)	(7.2)	(13.9)	(27.6)	(7.4)
t_E	96.1	178.1	395.1	96.1	178.1	395.1	96.1	178.1	395.1	96.1
	(6.7)	(12.5)	(27.7)	(6.7)	(12.5)	(27.7)	(6.7)	(12.5)	(27.7)	(6.7)
	36.5	53.1	11.9	58.1	-0.2	-54.1	14.2	41.9	-2.1	18.5
	2.561	3.724	0.838	4.076	-0.015	-3.794	0.996	2.940	-0.150	1.298
g_O	548	556	301	551	603	277	579	554	269	582
	(38.4)	(39.0)	(21.1)	(38.6)	(42.3)	(19.4)	(40.6)	(38.8)	(18.9)	(40.8)
g_E	608.5	579.6	287.0	608.5	579.6	287.0	608.5	579.6	287.0	608.5
	(42.7)	(40.6)	(20.1)	(42.7)	(40.6)	(20.1)	(42.7)	(40.6)	(20.1)	(42.7)
	-114.8	-46.2	28.6	-109.4	47.8	-19.7	-57.6	-50.0	-34.9	-51.8
	-8.049	-3.239	2.008	-7.671	3.351	-1.380	-4.036	-3.507	-2.446	-3.635
c_O	664	412	428	662	415	476	657	430	427	649
	(46.6)	(28.9)	(30.0)	(46.4)	(29.1)	(33.4)	(46.1)	(30.2)	(29.9)	(45.5)
c_E	652.4	407.2	411.3	652.4	407.2	411.3	652.4	407.2	411.3	652.4
	(45.7)	(28.6)	(28.8)	(45.7)	(28.6)	(28.8)	(45.7)	(28.6)	(28.8)	(45.7)
	23.4	9.7	34.1	19.4	15.8	139.1	9.3	46.9	32.0	-6.8
	1.643	0.680	2.392	1.358	1.107	9.756	0.649	3.289	2.246	-0.474
N	1426	1426	1426	1426	1426	1426	1426	1426	1426	1426
X2	24.1	4.6	5.5	19.6	4.8	14.7	6.7	5.8	1.8	8.4
Gvalue	22.2	4.5	5.6	18.5	5.0	14.3	6.3	5.7	1.8	7.8
Gv/N	1.558	0.316	0.394	1.301	0.347	1.001	0.443	0.399	0.125	0.549
Prob	0.00006	0.21217	0.13161	0.00034	0.17531	0.00255	0.09726	0.12748	0.61805	0.04972

	91	92	93	94	95	96	97	98	99	100
a_O	256	304	79	260	327	82	268	311	79	251
	(18.0)	(21.3)	(5.5)	(18.2)	(22.9)	(5.8)	(18.8)	(21.8)	(5.5)	(17.6)
a_E	261.1	332.6	69.0	261.1	332.6	69.0	261.1	332.6	69.0	261.1
	(18.3)	(23.3)	(4.8)	(18.3)	(23.3)	(4.8)	(18.3)	(23.3)	(4.8)	(18.3)
	-10.2	-54.7	21.5	-2.3	-11.2	28.4	13.9	-41.8	21.5	-19.9
	-0.713	-3.838	1.505	-0.159	-0.783	1.990	0.975	-2.933	1.505	-1.393
t_O	183	368	112	202	391	119	176	413	110	197
	(12.8)	(25.8)	(7.9)	(14.2)	(27.4)	(8.3)	(12.3)	(29.0)	(7.7)	(13.8)
t_E	178.1	395.1	96.1	178.1	395.1	96.1	178.1	395.1	96.1	178.1
	(12.5)	(27.7)	(6.7)	(12.5)	(27.7)	(6.7)	(12.5)	(27.7)	(6.7)	(12.5)
	9.9	-52.2	34.2	50.9	-8.1	50.8	-4.2	36.7	29.6	39.7
	0.695	-3.663	2.398	3.566	-0.568	3.560	-0.294	2.571	2.078	2.785
g_O	584	304	576	583	274	567	564	273	574	584
	(41.0)	(21.3)	(40.4)	(40.9)	(19.2)	(39.8)	(39.6)	(19.1)	(40.3)	(41.0)
g_E	579.6	287.0	608.5	579.6	287.0	608.5	579.6	287.0	608.5	579.6
	(40.6)	(20.1)	(42.7)	(40.6)	(20.1)	(42.7)	(40.6)	(20.1)	(42.7)	(40.6)
	8.9	34.9	-63.2	6.9	-25.4	-80.1	-30.7	-27.3	-67.0	8.9
	0.623	2.451	-4.435	0.482	-1.784	-5.618	-2.155	-1.917	-4.699	0.623
c_O	403	450	659	381	434	658	418	429	663	394
	(28.3)	(31.6)	(46.2)	(26.7)	(30.4)	(46.1)	(29.3)	(30.1)	(46.5)	(27.6)
c_E	407.2	411.3	652.4	407.2	411.3	652.4	407.2	411.3	652.4	407.2
	(28.6)	(28.8)	(45.7)	(28.6)	(28.8)	(45.7)	(28.6)	(28.8)	(45.7)	(28.6)
	-8.3	81.0	13.3	-50.6	46.7	11.3	21.9	36.2	21.4	-25.9
	-0.583	5.678	0.932	-3.551	3.273	0.791	1.538	2.538	1.501	-1.818
N	1426	1426	1426	1426	1426	1426	1426	1426	1426	1426
X2	0.3	9.0	5.9	4.9	2.0	10.8	0.9	3.7	5.6	2.9
Gvalue	0.3	9.0	5.7	4.8	2.0	10.3	0.9	3.7	5.5	2.8
Gv/N	0.022	0.628	0.400	0.338	0.138	0.723	0.064	0.259	0.383	0.196
Prob	0.95787	0.02985	0.12655	0.18579	0.57865	0.01607	0.82265	0.29711	0.14059	0.42354

5UTR:

	100	99	98	97	96	95	94	93	92	91
a_O	53	59	50	51	53	39	45	52	59	47
	(32.7)	(36.4)	(30.9)	(31.5)	(32.7)	(24.1)	(27.8)	(32.1)	(36.4)	(29.0)
a_E	54.6	54.6	54.6	54.6	54.6	54.6	54.6	54.6	54.6	54.6
	(33.7)	(33.7)	(33.7)	(33.7)	(33.7)	(33.7)	(33.7)	(33.7)	(33.7)	(33.7)
	-3.1	9.2	-8.8	-6.9	-3.1	-26.2	-17.4	-5.1	9.2	-14.1
	-1.934	5.659	-5.421	-4.283	-1.934	-16.192	-10.733	-3.120	5.659	-8.686
t_O	49	48	53	54	61	67	57	57	55	60
	(30.2)	(29.6)	(32.7)	(33.3)	(37.7)	(41.4)	(35.2)	(35.2)	(34.0)	(37.0)
t_E	53.7	53.7	53.7	53.7	53.7	53.7	53.7	53.7	53.7	53.7
	(33.1)	(33.1)	(33.1)	(33.1)	(33.1)	(33.1)	(33.1)	(33.1)	(33.1)	(33.1)
	-9.0	-10.8	-1.4	0.6	15.6	29.7	6.8	6.8	2.6	13.3
	-5.541	-6.650	-0.859	0.371	9.599	18.303	4.197	4.197	1.624	8.217
g_O	24	32	30	24	22	28	22	24	23	28
	(14.8)	(19.8)	(18.5)	(14.8)	(13.6)	(17.3)	(13.6)	(14.8)	(14.2)	(17.3)
g_E	26.3	26.3	26.3	26.3	26.3	26.3	26.3	26.3	26.3	26.3
	(16.2)	(16.2)	(16.2)	(16.2)	(16.2)	(16.2)	(16.2)	(16.2)	(16.2)	(16.2)
	-4.4	12.6	7.9	-4.4	-7.8	3.6	-7.8	-4.4	-6.1	3.6
	-2.689	7.780	4.903	-2.689	-4.828	2.191	-4.828	-2.689	-3.785	2.191
c_O	36	23	29	33	26	28	38	29	25	27
	(22.2)	(14.2)	(17.9)	(20.4)	(16.0)	(17.3)	(23.5)	(17.9)	(15.4)	(16.7)
c_E	27.4	27.4	27.4	27.4	27.4	27.4	27.4	27.4	27.4	27.4
	(16.9)	(16.9)	(16.9)	(16.9)	(16.9)	(16.9)	(16.9)	(16.9)	(16.9)	(16.9)
	19.6	-8.1	3.2	12.2	-2.8	1.2	24.8	3.2	-4.6	-0.9
	12.084	-5.002	1.993	7.532	-1.719	0.711	15.291	1.993	-2.863	-0.527
N	162	162	162	162	162	162	162	162	162	162
X2	3.3	2.9	1.0	1.6	1.8	7.9	6.7	0.6	1.0	1.9
Gvalue	3.1	2.9	1.0	1.5	1.8	8.1	6.4	0.6	1.0	1.9
Gv/N	1.920	1.787	0.616	0.931	1.118	5.014	3.927	0.380	0.634	1.195
Prob	0.37498	0.40802	0.80175	0.68035	0.61251	0.04354	0.09526	0.89285	0.79454	0.58566

	90	89	88	87	86	85	84	83	82	81
a_O	48	54	42	50	50	57	54	53	55	51
	(29.6)	(33.3)	(25.9)	(30.9)	(30.9)	(35.2)	(33.3)	(32.7)	(34.0)	(31.5)
a_E	54.6	54.6	54.6	54.6	54.6	54.6	54.6	54.6	54.6	54.6
	(33.7)	(33.7)	(33.7)	(33.7)	(33.7)	(33.7)	(33.7)	(33.7)	(33.7)	(33.7)
	-12.4	-1.2	-22.0	-8.8	-8.8	4.9	-1.2	-3.1	0.8	-6.9
	-7.624	-0.724	-13.595	-5.421	-5.421	3.040	-0.724	-1.934	0.508	-4.283
t_O	56	55	54	58	48	55	54	54	53	54
	(34.6)	(34.0)	(33.3)	(35.8)	(29.6)	(34.0)	(33.3)	(33.3)	(32.7)	(33.3)
t_E	53.7	53.7	53.7	53.7	53.7	53.7	53.7	53.7	53.7	53.7
	(33.1)	(33.1)	(33.1)	(33.1)	(33.1)	(33.1)	(33.1)	(33.1)	(33.1)	(33.1)
	4.7	2.6	0.6	8.9	-10.8	2.6	0.6	0.6	-1.4	0.6
	2.899	1.624	0.371	5.516	-6.650	1.624	0.371	0.371	-0.859	0.371
g_O	28	18	34	26	31	22	28	30	27	28
	(17.3)	(11.1)	(21.0)	(16.0)	(19.1)	(13.6)	(17.3)	(18.5)	(16.7)	(17.3)
g_E	26.3	26.3	26.3	26.3	26.3	26.3	26.3	26.3	26.3	26.3
	(16.2)	(16.2)	(16.2)	(16.2)	(16.2)	(16.2)	(16.2)	(16.2)	(16.2)	(16.2)
	3.6	-13.6	17.5	-0.6	10.2	-7.8	3.6	7.9	1.5	3.6
	2.191	-8.410	10.811	-0.344	6.322	-4.828	2.191	4.903	0.901	2.191
c_O	30	35	32	28	33	28	26	25	27	29
	(18.5)	(21.6)	(19.8)	(17.3)	(20.4)	(17.3)	(16.0)	(15.4)	(16.7)	(17.9)
c_E	27.4	27.4	27.4	27.4	27.4	27.4	27.4	27.4	27.4	27.4
	(16.9)	(16.9)	(16.9)	(16.9)	(16.9)	(16.9)	(16.9)	(16.9)	(16.9)	(16.9)
	5.4	17.1	9.9	1.2	12.2	1.2	-2.8	-4.6	-0.9	3.2
	3.317	10.531	6.088	0.711	7.532	0.711	-1.719	-2.863	-0.527	1.993
N	162	162	162	162	162	162	162	162	162	162
X2	1.2	4.7	5.9	0.7	3.0	0.8	0.2	0.8	0.0	0.4
Gvalue	1.3	4.9	6.0	0.7	2.9	0.9	0.2	0.8	0.0	0.4
Gv/N	0.784	3.021	3.676	0.461	1.782	0.547	0.120	0.478	0.024	0.273
Prob	0.73614	0.17975	0.11385	0.86199	0.40931	0.82877	0.97853	0.85574	0.99801	0.93154

	80	79	78	77	76	75	74	73	72	71
a_O	50	52	36	51	46	48	57	50	48	54
	(30.9)	(32.1)	(22.2)	(31.5)	(28.4)	(29.6)	(35.2)	(30.9)	(29.6)	(33.3)
a_E	54.6	54.6	54.6	54.6	54.6	54.6	54.6	54.6	54.6	54.6
	(33.7)	(33.7)	(33.7)	(33.7)	(33.7)	(33.7)	(33.7)	(33.7)	(33.7)	(33.7)
	-8.8	-5.1	-30.0	-6.9	-15.8	-12.4	4.9	-8.8	-12.4	-1.2
	-5.421	-3.120	-18.504	-4.283	-9.723	-7.624	3.040	-5.421	-7.624	-0.724
t_O	48	48	52	53	51	59	53	63	49	53
	(29.6)	(29.6)	(32.1)	(32.7)	(31.5)	(36.4)	(32.7)	(38.9)	(30.2)	(32.7)
t_E	53.7	53.7	53.7	53.7	53.7	53.7	53.7	53.7	53.7	53.7
	(33.1)	(33.1)	(33.1)	(33.1)	(33.1)	(33.1)	(33.1)	(33.1)	(33.1)	(33.1)
	-10.8	-10.8	-3.3	-1.4	-5.3	11.1	-1.4	20.1	-9.0	-1.4
	-6.650	-6.650	-2.065	-0.859	-3.248	6.856	-0.859	12.423	-5.541	-0.859
g_O	37	35	43	32	30	30	25	28	34	23
	(22.8)	(21.6)	(26.5)	(19.8)	(18.5)	(18.5)	(15.4)	(17.3)	(21.0)	(14.2)
g_E	26.3	26.3	26.3	26.3	26.3	26.3	26.3	26.3	26.3	26.3
	(16.2)	(16.2)	(16.2)	(16.2)	(16.2)	(16.2)	(16.2)	(16.2)	(16.2)	(16.2)
	25.3	20.1	42.3	12.6	7.9	7.9	-2.5	3.6	17.5	-6.1
	15.627	12.381	26.139	7.780	4.903	4.903	-1.541	2.191	10.811	-3.785
c_O	27	27	31	26	35	25	27	21	31	32
	(16.7)	(16.7)	(19.1)	(16.0)	(21.6)	(15.4)	(16.7)	(13.0)	(19.1)	(19.8)
c_E	27.4	27.4	27.4	27.4	27.4	27.4	27.4	27.4	27.4	27.4
	(16.9)	(16.9)	(16.9)	(16.9)	(16.9)	(16.9)	(16.9)	(16.9)	(16.9)	(16.9)
	-0.9	-0.9	7.6	-2.8	17.1	-4.6	-0.9	-11.2	7.6	9.9
	-0.527	-0.527	4.683	-1.719	10.531	-2.863	-0.527	-6.925	4.683	6.088
N	162	162	162	162	162	162	162	162	162	162
X2	5.4	3.6	17.5	1.6	4.1	2.1	0.2	3.6	3.9	1.2
Gvalue	4.9	3.4	16.6	1.5	4.0	2.1	0.2	3.7	3.8	1.2
Gv/N	3.029	2.085	10.253	0.920	2.463	1.273	0.114	2.268	2.329	0.719
Prob	0.17868	0.33706	0.00085	0.68461	0.26255	0.55972	0.98010	0.29894	0.28707	0.76131

	70	69	68	67	66	65	64	63	62	61
a_O	56	56	46	50	56	60	55	56	52	52
	(34.6)	(34.6)	(28.4)	(30.9)	(34.6)	(37.0)	(34.0)	(34.6)	(32.1)	(32.1)
a_E	54.6	54.6	54.6	54.6	54.6	54.6	54.6	54.6	54.6	54.6
	(33.7)	(33.7)	(33.7)	(33.7)	(33.7)	(33.7)	(33.7)	(33.7)	(33.7)	(33.7)
	2.9	2.9	-15.8	-8.8	2.9	11.3	0.8	2.9	-5.1	-5.1
	1.763	1.763	-9.723	-5.421	1.763	7.000	0.508	1.763	-3.120	-3.120
t_O	55	57	64	57	51	53	45	49	59	48
	(34.0)	(35.2)	(39.5)	(35.2)	(31.5)	(32.7)	(27.8)	(30.2)	(36.4)	(29.6)
t_E	53.7	53.7	53.7	53.7	53.7	53.7	53.7	53.7	53.7	53.7

	(33.1)	(33.1)	(33.1)	(33.1)	(33.1)	(33.1)	(33.1)	(33.1)	(33.1)	(33.1)
	2.6	6.8	22.5	6.8	-5.3	-1.4	-15.9	-9.0	11.1	-10.8
	1.624	4.197	13.864	4.197	-3.248	-0.859	-9.819	-5.541	6.856	-6.650
g_O	23	15	31	25	28	21	26	30	25	39
	(14.2)	(9.3)	(19.1)	(15.4)	(17.3)	(13.0)	(16.0)	(18.5)	(15.4)	(24.1)
g_E	26.3	26.3	26.3	26.3	26.3	26.3	26.3	26.3	26.3	26.3
	(16.2)	(16.2)	(16.2)	(16.2)	(16.2)	(16.2)	(16.2)	(16.2)	(16.2)	(16.2)
	-6.1	-16.8	10.2	-2.5	3.6	-9.4	-0.6	7.9	-2.5	30.8
	-3.785	-10.384	6.322	-1.541	2.191	-5.815	-0.344	4.903	-1.541	19.007
c_O	28	34	21	30	27	28	36	27	26	23
	(17.3)	(21.0)	(13.0)	(18.5)	(16.7)	(17.3)	(22.2)	(16.7)	(16.0)	(14.2)
c_E	27.4	27.4	27.4	27.4	27.4	27.4	27.4	27.4	27.4	27.4
	(16.9)	(16.9)	(16.9)	(16.9)	(16.9)	(16.9)	(16.9)	(16.9)	(16.9)	(16.9)
	1.2	14.6	-11.2	5.4	-0.9	1.2	19.6	-0.9	-2.8	-8.1
	0.711	9.013	-6.925	3.317	-0.527	0.711	12.084	-0.527	-1.719	-5.002
N	162	162	162	162	162	162	162	162	162	162
X2	0.5	6.7	5.7	0.9	0.3	1.6	4.1	1.0	0.8	7.6
Gvalue	0.5	7.4	5.7	0.9	0.3	1.7	3.9	1.0	0.8	6.9
Gv/N	0.313	4.588	3.538	0.551	0.180	1.037	2.428	0.599	0.476	4.235
Prob	0.91743	0.05930	0.12545	0.82712	0.96168	0.64135	0.26868	0.80847	0.85643	0.07647

	60	59	58	57	56	55	54	53	52	51
a_O	58	56	52	51	51	55	58	54	51	51
	(35.8)	(34.6)	(32.1)	(31.5)	(31.5)	(34.0)	(35.8)	(33.3)	(31.5)	(31.5)
a_E	54.6	54.6	54.6	54.6	54.6	54.6	54.6	54.6	54.6	54.6
	(33.7)	(33.7)	(33.7)	(33.7)	(33.7)	(33.7)	(33.7)	(33.7)	(33.7)	(33.7)
	7.0	2.9	-5.1	-6.9	-6.9	0.8	7.0	-1.2	-6.9	-6.9
	4.339	1.763	-3.120	-4.283	-4.283	0.508	4.339	-0.724	-4.283	-4.283
t_O	57	50	48	47	62	61	51	56	62	55
	(35.2)	(30.9)	(29.6)	(29.0)	(38.3)	(37.7)	(31.5)	(34.6)	(38.3)	(34.0)
t_E	53.7	53.7	53.7	53.7	53.7	53.7	53.7	53.7	53.7	53.7
	(33.1)	(33.1)	(33.1)	(33.1)	(33.1)	(33.1)	(33.1)	(33.1)	(33.1)	(33.1)
	6.8	-7.1	-10.8	-12.5	17.8	15.6	-5.3	4.7	17.8	2.6
	4.197	-4.407	-6.650	-7.733	11.001	9.599	-3.248	2.899	11.001	1.624
g_O	26	32	26	35	26	19	27	21	25	28
	(16.0)	(19.8)	(16.0)	(21.6)	(16.0)	(11.7)	(16.7)	(13.0)	(15.4)	(17.3)
g_E	26.3	26.3	26.3	26.3	26.3	26.3	26.3	26.3	26.3	26.3
	(16.2)	(16.2)	(16.2)	(16.2)	(16.2)	(16.2)	(16.2)	(16.2)	(16.2)	(16.2)
	-0.6	12.6	-0.6	20.1	-0.6	-12.3	1.5	-9.4	-2.5	3.6
	-0.344	7.780	-0.344	12.381	-0.344	-7.609	0.901	-5.815	-1.541	2.191
c_O	21	24	36	29	23	27	26	31	24	28
	(13.0)	(14.8)	(22.2)	(17.9)	(14.2)	(16.7)	(16.0)	(19.1)	(14.8)	(17.3)
c_E	27.4	27.4	27.4	27.4	27.4	27.4	27.4	27.4	27.4	27.4
	(16.9)	(16.9)	(16.9)	(16.9)	(16.9)	(16.9)	(16.9)	(16.9)	(16.9)	(16.9)
	-11.2	-6.4	19.6	3.2	-8.1	-0.9	-2.8	7.6	-6.4	1.2
	-6.925	-3.958	12.084	1.993	-5.002	-0.527	-1.719	4.683	-3.958	0.711
N	162	162	162	162	162	162	162	162	162	162
X2	1.9	2.0	3.4	4.1	2.2	3.0	0.4	1.6	2.0	0.4
Gvalue	2.1	1.9	3.2	3.8	2.2	3.2	0.4	1.7	2.0	0.4
Gv/N	1.266	1.178	1.970	2.358	1.372	1.972	0.273	1.043	1.219	0.244
Prob	0.56176	0.59162	0.36310	0.28151	0.52737	0.36266	0.93139	0.63933	0.57776	0.94135

	50	49	48	47	46	45	44	43	42	41
a_O	54	56	56	59	56	53	48	62	54	49
	(33.3)	(34.6)	(34.6)	(36.4)	(34.6)	(32.7)	(29.6)	(38.3)	(33.3)	(30.2)
a_E	54.6	54.6	54.6	54.6	54.6	54.6	54.6	54.6	54.6	54.6
	(33.7)	(33.7)	(33.7)	(33.7)	(33.7)	(33.7)	(33.7)	(33.7)	(33.7)	(33.7)
	-1.2	2.9	2.9	9.2	2.9	-3.1	-12.4	15.8	-1.2	-10.6
	-0.724	1.763	1.763	5.659	1.763	-1.934	-7.624	9.743	-0.724	-6.535
t_O	60	52	44	53	52	61	51	50	59	44
	(37.0)	(32.1)	(27.2)	(32.7)	(32.1)	(37.7)	(31.5)	(30.9)	(36.4)	(27.2)
t_E	53.7	53.7	53.7	53.7	53.7	53.7	53.7	53.7	53.7	53.7
	(33.1)	(33.1)	(33.1)	(33.1)	(33.1)	(33.1)	(33.1)	(33.1)	(33.1)	(33.1)
	13.3	-3.3	-17.5	-1.4	-3.3	15.6	-5.3	-7.1	11.1	-17.5
	8.217	-2.065	-10.822	-0.859	-2.065	9.599	-3.248	-4.407	6.856	-10.822
g_O	25	26	30	22	36	26	28	29	26	30
	(15.4)	(16.0)	(18.5)	(13.6)	(22.2)	(16.0)	(17.3)	(17.9)	(16.0)	(18.5)
g_E	26.3	26.3	26.3	26.3	26.3	26.3	26.3	26.3	26.3	26.3
	(16.2)	(16.2)	(16.2)	(16.2)	(16.2)	(16.2)	(16.2)	(16.2)	(16.2)	(16.2)
	-2.5	-0.6	7.9	-7.8	22.7	-0.6	3.6	5.7	-0.6	7.9
	-1.541	-0.344	4.903	-4.828	13.987	-0.344	2.191	3.526	-0.344	4.903
c_O	23	28	32	28	18	22	35	21	23	39
	(14.2)	(17.3)	(19.8)	(17.3)	(11.1)	(13.6)	(21.6)	(13.0)	(14.2)	(24.1)
c_E	27.4	27.4	27.4	27.4	27.4	27.4	27.4	27.4	27.4	27.4
	(16.9)	(16.9)	(16.9)	(16.9)	(16.9)	(16.9)	(16.9)	(16.9)	(16.9)	(16.9)
	-8.1	1.2	9.9	1.2	-15.2	-9.7	17.1	-11.2	-8.1	27.5
	-5.002	0.711	6.088	0.711	-9.361	-5.991	10.531	-6.925	-5.002	16.945
N	162	162	162	162	162	162	162	162	162	162
X2	1.5	0.1	3.1	1.1	6.9	2.1	3.1	3.0	1.2	7.7
Gvalue	1.5	0.1	3.1	1.1	7.0	2.2	3.0	3.1	1.3	7.3
Gv/N	0.950	0.065	1.932	0.683	4.324	1.330	1.850	1.937	0.786	4.491
Prob	0.67330	0.99120	0.37200	0.77556	0.07177	0.54110	0.39199	0.37089	0.73544	0.06364

	40	39	38	37	36	35	34	33	32	31
a_O	63	45	56	47	51	41	55	54	49	53
	(38.9)	(27.8)	(34.6)	(29.0)	(31.5)	(25.3)	(34.0)	(33.3)	(30.2)	(32.7)
a_E	54.6	54.6	54.6	54.6	54.6	54.6	54.6	54.6	54.6	54.6
	(33.7)	(33.7)	(33.7)	(33.7)	(33.7)	(33.7)	(33.7)	(33.7)	(33.7)	(33.7)
	18.1	-17.4	2.9	-14.1	-6.9	-23.5	0.8	-1.2	-10.6	-3.1
	11.144	-10.733	1.763	-8.686	-4.283	-14.491	0.508	-0.724	-6.535	-1.934
t_O	47	59	46	55	64	67	70	53	60	45
	(29.0)	(36.4)	(28.4)	(34.0)	(39.5)	(41.4)	(43.2)	(32.7)	(37.0)	(27.8)
t_E	53.7	53.7	53.7	53.7	53.7	53.7	53.7	53.7	53.7	53.7
	(33.1)	(33.1)	(33.1)	(33.1)	(33.1)	(33.1)	(33.1)	(33.1)	(33.1)	(33.1)
	-12.5	11.1	-14.2	2.6	22.5	29.7	37.1	-1.4	13.3	-15.9
	-7.733	6.856	-8.789	1.624	13.864	18.303	22.908	-0.859	8.217	-9.819
g_O	31	31	37	25	22	32	15	25	24	30
	(19.1)	(19.1)	(22.8)	(15.4)	(13.6)	(19.8)	(9.3)	(15.4)	(14.8)	(18.5)
g_E	26.3	26.3	26.3	26.3	26.3	26.3	26.3	26.3	26.3	26.3
	(16.2)	(16.2)	(16.2)	(16.2)	(16.2)	(16.2)	(16.2)	(16.2)	(16.2)	(16.2)
	10.2	10.2	25.3	-2.5	-7.8	12.6	-16.8	-2.5	-4.4	7.9
	6.322	6.322	15.627	-1.541	-4.828	7.780	-10.384	-1.541	-2.689	4.903
c_O	21	27	23	35	25	22	22	30	29	34
	(13.0)	(16.7)	(14.2)	(21.6)	(15.4)	(13.6)	(13.6)	(18.5)	(17.9)	(21.0)
c_E	27.4	27.4	27.4	27.4	27.4	27.4	27.4	27.4	27.4	27.4
	(16.9)	(16.9)	(16.9)	(16.9)	(16.9)	(16.9)	(16.9)	(16.9)	(16.9)	(16.9)

	-11.2	-0.9	-8.1	17.1	-4.6	-9.7	-9.7	5.4	3.2	14.6
	-6.925	-0.527	-5.002	10.531	-2.863	-5.991	-5.991	3.317	1.993	9.013
N	162	162	162	162	162	162	162	162	162	162
X2	4.5	3.1	6.2	3.2	3.1	9.0	10.9	0.3	1.6	3.6
Gvalue	4.5	3.1	5.8	3.1	3.1	9.1	11.4	0.3	1.6	3.5
Gv/N	2.808	1.918	3.599	1.927	1.890	5.601	7.041	0.193	0.986	2.163
Prob	0.20795	0.37533	0.12015	0.37316	0.38219	0.02833	0.00972	0.95765	0.66012	0.32028

	30	29	28	27	26	25	24	23	22	21
a_O	51	49	53	54	54	46	60	48	61	71
	(31.5)	(30.2)	(32.7)	(33.3)	(33.3)	(28.4)	(37.0)	(29.6)	(37.7)	(43.8)
a_E	54.6	54.6	54.6	54.6	54.6	54.6	54.6	54.6	54.6	54.6
	(33.7)	(33.7)	(33.7)	(33.7)	(33.7)	(33.7)	(33.7)	(33.7)	(33.7)	(33.7)
	-6.9	-10.6	-3.1	-1.2	-1.2	-15.8	11.3	-12.4	13.5	37.3
	-4.283	-6.535	-1.934	-0.724	-0.724	-9.723	7.000	-7.624	8.361	23.038
t_O	54	61	65	62	52	57	48	43	47	47
	(33.3)	(37.7)	(40.1)	(38.3)	(32.1)	(35.2)	(29.6)	(26.5)	(29.0)	(29.0)
t_E	53.7	53.7	53.7	53.7	53.7	53.7	53.7	53.7	53.7	53.7
	(33.1)	(33.1)	(33.1)	(33.1)	(33.1)	(33.1)	(33.1)	(33.1)	(33.1)	(33.1)
	0.6	15.6	24.8	17.8	-3.3	6.8	-10.8	-19.1	-12.5	-12.5
	0.371	9.599	15.325	11.001	-2.065	4.197	-6.650	-11.796	-7.733	-7.733
g_O	33	27	20	25	30	32	29	29	26	26
	(20.4)	(16.7)	(12.3)	(15.4)	(18.5)	(19.8)	(17.9)	(17.9)	(16.0)	(16.0)
g_E	26.3	26.3	26.3	26.3	26.3	26.3	26.3	26.3	26.3	26.3
	(16.2)	(16.2)	(16.2)	(16.2)	(16.2)	(16.2)	(16.2)	(16.2)	(16.2)	(16.2)
	15.0	1.5	-10.9	-2.5	7.9	12.6	5.7	5.7	-0.6	-0.6
	9.277	0.901	-6.743	-1.541	4.903	7.780	3.526	3.526	-0.344	-0.344
c_O	24	25	24	21	26	27	25	42	28	18
	(14.8)	(15.4)	(14.8)	(13.0)	(16.0)	(16.7)	(15.4)	(25.9)	(17.3)	(11.1)
c_E	27.4	27.4	27.4	27.4	27.4	27.4	27.4	27.4	27.4	27.4
	(16.9)	(16.9)	(16.9)	(16.9)	(16.9)	(16.9)	(16.9)	(16.9)	(16.9)	(16.9)
	-6.4	-4.6	-6.4	-11.2	-2.8	-0.9	-4.6	35.8	1.2	-15.2
	-3.958	-2.863	-3.958	-6.925	-1.719	-0.527	-2.863	22.091	0.711	-9.361
N	162	162	162	162	162	162	162	162	162	162
X2	2.4	1.8	4.4	2.9	0.7	2.8	1.6	10.9	1.6	9.0
Gvalue	2.3	1.8	4.4	2.9	0.6	2.8	1.6	10.0	1.6	9.1
Gv/N	1.407	1.102	2.690	1.810	0.395	1.727	1.013	6.196	0.995	5.600
Prob	0.51650	0.61827	0.22528	0.40216	0.88721	0.42389	0.65010	0.01824	0.65653	0.02835

	20	19	18	17	16	15	14	13	12	11
a_O	60	67	68	58	62	63	53	61	65	64
	(37.0)	(41.4)	(42.0)	(35.8)	(38.3)	(38.9)	(32.7)	(37.7)	(40.1)	(39.5)
a_E	54.6	54.6	54.6	54.6	54.6	54.6	54.6	54.6	54.6	54.6
	(33.7)	(33.7)	(33.7)	(33.7)	(33.7)	(33.7)	(33.7)	(33.7)	(33.7)	(33.7)
	11.3	27.4	29.9	7.0	15.8	18.1	-3.1	13.5	22.7	20.4
	7.000	16.944	18.440	4.339	9.743	11.144	-1.934	8.361	14.006	12.566
t_O	61	52	44	61	53	65	66	51	41	46
	(37.7)	(32.1)	(27.2)	(37.7)	(32.7)	(40.1)	(40.7)	(31.5)	(25.3)	(28.4)
t_E	53.7	53.7	53.7	53.7	53.7	53.7	53.7	53.7	53.7	53.7
	(33.1)	(33.1)	(33.1)	(33.1)	(33.1)	(33.1)	(33.1)	(33.1)	(33.1)	(33.1)
	15.6	-3.3	-17.5	15.6	-1.4	24.8	27.2	-5.3	-22.1	-14.2
	9.599	-2.065	-10.822	9.599	-0.859	15.325	16.805	-3.248	-13.659	-8.789
g_O	19	12	20	11	19	13	18	22	29	28
	(11.7)	(7.4)	(12.3)	(6.8)	(11.7)	(8.0)	(11.1)	(13.6)	(17.9)	(17.3)
g_E	26.3	26.3	26.3	26.3	26.3	26.3	26.3	26.3	26.3	26.3
	(16.2)	(16.2)	(16.2)	(16.2)	(16.2)	(16.2)	(16.2)	(16.2)	(16.2)	(16.2)
	-12.3	-18.8	-10.9	-19.2	-12.3	-18.3	-13.6	-7.8	5.7	3.6
	-7.609	-11.613	-6.743	-11.827	-7.609	-11.296	-8.410	-4.828	3.526	2.191
c_O	22	31	30	32	28	21	25	28	27	24
	(13.6)	(19.1)	(18.5)	(19.8)	(17.3)	(13.0)	(15.4)	(17.3)	(16.7)	(14.8)
c_E	27.4	27.4	27.4	27.4	27.4	27.4	27.4	27.4	27.4	27.4
	(16.9)	(16.9)	(16.9)	(16.9)	(16.9)	(16.9)	(16.9)	(16.9)	(16.9)	(16.9)
	-9.7	7.6	5.4	9.9	1.2	-11.2	-4.6	1.2	-0.9	-6.4
	-5.991	4.683	3.317	6.088	0.711	-6.925	-2.863	0.711	-0.527	-3.958
N	162	162	162	162	162	162	162	162	162	162
X2	4.6	11.1	6.8	10.9	3.0	11.9	5.7	1.6	5.3	3.3
Gvalue	4.9	12.9	6.8	13.3	3.2	13.4	5.8	1.6	5.4	3.3
Gv/N	2.998	7.948	4.193	8.198	1.986	8.248	3.598	0.996	3.347	2.009
Prob	0.18255	0.00491	0.07882	0.00407	0.35921	0.00392	0.12024	0.65645	0.14339	0.35391

	10	9	8	7	6	5	4	3	2	1
a_O	66	69	68	68	71	76	50	73	50	49
	(40.7)	(42.6)	(42.0)	(42.0)	(43.8)	(46.9)	(30.9)	(45.1)	(30.9)	(30.2)
a_E	54.6	54.6	54.6	54.6	54.6	54.6	54.6	54.6	54.6	54.6
	(33.7)	(33.7)	(33.7)	(33.7)	(33.7)	(33.7)	(33.7)	(33.7)	(33.7)	(33.7)
	25.1	32.3	29.9	29.9	37.3	50.3	-8.8	42.4	-8.8	-10.6
	15.466	19.955	18.440	18.440	23.038	31.046	-5.421	26.191	-5.421	-6.535
t_O	44	45	40	50	54	47	52	43	61	48
	(27.2)	(27.8)	(24.7)	(30.9)	(33.3)	(29.0)	(32.1)	(26.5)	(37.7)	(29.6)
t_E	53.7	53.7	53.7	53.7	53.7	53.7	53.7	53.7	53.7	53.7
	(33.1)	(33.1)	(33.1)	(33.1)	(33.1)	(33.1)	(33.1)	(33.1)	(33.1)	(33.1)
	-17.5	-15.9	-23.6	-7.1	0.6	-12.5	-3.3	-19.1	15.6	-10.8
	-10.822	-9.819	-14.545	-4.407	0.371	-7.733	-2.065	-11.796	9.599	-6.650
g_O	34	27	31	23	22	18	31	22	12	16
	(21.0)	(16.7)	(19.1)	(14.2)	(13.6)	(11.1)	(19.1)	(13.6)	(7.4)	(9.9)
g_E	26.3	26.3	26.3	26.3	26.3	26.3	26.3	26.3	26.3	26.3
	(16.2)	(16.2)	(16.2)	(16.2)	(16.2)	(16.2)	(16.2)	(16.2)	(16.2)	(16.2)
	17.5	1.5	10.2	-6.1	-7.8	-13.6	10.2	-7.8	-18.8	-15.9
	10.811	0.901	6.322	-3.785	-4.828	-8.410	6.322	-4.828	-11.613	-9.802
c_O	18	21	23	21	15	21	29	24	39	49
	(11.1)	(13.0)	(14.2)	(13.0)	(9.3)	(13.0)	(17.9)	(14.8)	(24.1)	(30.2)
c_E	27.4	27.4	27.4	27.4	27.4	27.4	27.4	27.4	27.4	27.4
	(16.9)	(16.9)	(16.9)	(16.9)	(16.9)	(16.9)	(16.9)	(16.9)	(16.9)	(16.9)
	-15.2	-11.2	-8.1	-11.2	-18.1	-11.2	3.2	-6.4	27.5	56.9
	-9.361	-6.925	-5.002	-6.925	-11.178	-6.925	1.993	-3.958	16.945	35.097
N	162	162	162	162	162	162	162	162	162	162
X2	9.6	6.7	8.4	5.5	11.3	13.3	1.4	9.5	14.0	22.2
Gvalue	9.9	6.7	8.4	5.4	12.0	12.9	1.3	9.1	15.4	19.6
Gv/N	6.093	4.111	5.216	3.323	7.404	7.978	0.828	5.608	9.509	12.111
Prob	0.01970	0.08354	0.03759	0.14580	0.00740	0.00480	0.71944	0.02818	0.00150	0.00020

CDS_INITIAL:

	1	2	3	4	5	6	7	8	9	10

a_O	142	0	1	91	65	59	74	65	54	50
	(87.7)	(0.0)	(0.6)	(56.2)	(40.1)	(36.4)	(45.7)	(40.1)	(33.3)	(30.9)
a_E	45.1	48.4	47.3	45.1	48.4	47.3	45.1	48.4	47.3	45.1
	(27.8)	(29.9)	(29.2)	(27.8)	(29.9)	(29.2)	(27.8)	(29.9)	(29.2)	(27.8)
	326.0	0.0	-7.7	127.9	38.3	26.0	73.4	38.3	14.2	10.4
	201.231	0.000	-4.762	78.968	23.667	16.058	45.323	23.667	8.794	6.424
t_O	10	162	0	18	42	52	25	46	62	46
	(6.2)	(100.0)	(0.0)	(11.1)	(25.9)	(32.1)	(15.4)	(28.4)	(38.3)	(28.4)
t_E	31.7	50.9	66.2	31.7	50.9	66.2	31.7	50.9	66.2	31.7
	(19.6)	(31.4)	(40.9)	(19.6)	(31.4)	(40.9)	(19.6)	(31.4)	(40.9)	(19.6)
	-23.1	375.4	0.0	-20.4	-16.1	-25.2	-11.9	-9.2	-8.2	34.1
	-14.261	231.729	0.000	-12.608	-9.918	-15.540	-7.373	-5.697	-5.066	21.063
g_O	10	0	161	29	17	23	32	24	24	28
	(6.2)	(0.0)	(99.4)	(17.9)	(10.5)	(14.2)	(19.8)	(14.8)	(14.8)	(17.3)
g_E	50.2	26.5	25.3	50.2	26.5	25.3	50.2	26.5	25.3	50.2
	(31.0)	(16.3)	(15.6)	(31.0)	(16.3)	(15.6)	(31.0)	(16.3)	(15.6)	(31.0)
	-32.3	0.0	596.5	-31.8	-15.1	-4.3	-28.8	-4.7	-2.4	-32.7
	-19.913	0.000	368.226	-19.627	-9.300	-2.650	-17.769	-2.912	-1.505	-20.164
c_O	0	0	0	24	38	28	31	27	22	38
	(0.0)	(0.0)	(0.0)	(14.8)	(23.5)	(17.3)	(19.1)	(16.7)	(13.6)	(23.5)
c_E	35.0	36.3	23.2	35.0	36.3	23.2	35.0	36.3	23.2	35.0
	(21.6)	(22.4)	(14.3)	(21.6)	(22.4)	(14.3)	(21.6)	(22.4)	(14.3)	(21.6)
	0.0	0.0	0.0	-18.1	3.5	10.6	-7.6	-15.9	-2.3	6.2
	0.000	0.000	0.000	-11.197	2.185	6.529	-4.668	-9.839	-1.420	3.829
N	162	162	162	162	162	162	162	162	162	162
X2	290.7	354.1	864.6	65.2	10.7	7.1	27.1	8.8	1.3	17.0
Gvalue	270.6	375.4	588.8	57.6	10.7	7.1	25.1	8.5	1.3	18.1
Gv/N	167.057	231.729	363.464	35.535	6.635	4.396	15.514	5.220	0.803	11.152
Prob	0.00000	0.00000	0.00000	0.00000	0.01317	0.06810	0.00001	0.03745	0.72881	0.00043

	11	12	13	14	15	16	17	18	19	20
a_O	55	52	61	57	46	63	57	50	76	52
	(34.0)	(32.1)	(37.7)	(35.2)	(28.4)	(38.9)	(35.2)	(30.9)	(46.9)	(32.1)
a_E	48.4	47.3	45.1	48.4	47.3	45.1	48.4	47.3	45.1	48.4
	(29.9)	(29.2)	(27.8)	(29.9)	(29.2)	(27.8)	(29.9)	(29.2)	(27.8)	(29.9)
	14.1	9.8	37.0	18.6	-2.6	42.2	18.6	5.5	79.5	7.5
	8.683	6.045	22.812	11.512	-1.615	26.069	11.512	3.392	49.051	4.609
t_O	65	59	41	52	67	36	51	60	37	61
	(40.1)	(36.4)	(25.3)	(32.1)	(41.4)	(22.2)	(31.5)	(37.0)	(22.8)	(37.7)
t_E	50.9	66.2	31.7	50.9	66.2	31.7	50.9	66.2	31.7	50.9
	(31.4)	(40.9)	(19.6)	(31.4)	(40.9)	(19.6)	(31.4)	(40.9)	(19.6)	(31.4)
	31.9	-13.7	21.0	2.3	1.5	9.1	0.3	-11.9	11.3	22.2
	19.696	-8.433	12.949	1.431	0.941	5.590	0.181	-7.331	6.997	13.700
g_O	10	29	24	23	22	25	30	22	24	18
	(6.2)	(17.9)	(14.8)	(14.2)	(13.6)	(15.4)	(18.5)	(13.6)	(14.8)	(11.1)
g_E	26.5	25.3	50.2	26.5	25.3	50.2	26.5	25.3	50.2	26.5
	(16.3)	(15.6)	(31.0)	(16.3)	(15.6)	(31.0)	(16.3)	(15.6)	(31.0)	(16.3)
	-19.5	8.0	-35.4	-6.5	-6.1	-34.8	7.5	-6.1	-35.4	-13.9
	-12.021	4.957	-21.851	-3.999	-3.743	-21.501	4.625	-3.743	-21.851	-8.577
c_O	32	22	36	30	27	38	24	30	25	31
	(19.8)	(13.6)	(22.2)	(18.5)	(16.7)	(23.5)	(14.8)	(18.5)	(15.4)	(19.1)
c_E	36.3	23.2	35.0	36.3	23.2	35.0	36.3	23.2	35.0	36.3
	(22.4)	(14.3)	(21.6)	(22.4)	(14.3)	(21.6)	(22.4)	(14.3)	(21.6)	(22.4)
	-8.0	-2.3	2.0	-11.4	8.2	6.2	-19.8	15.5	-16.9	-9.7
	-4.949	-1.420	1.225	-7.030	5.084	3.829	-12.236	9.551	-10.404	-6.009
N	162	162	162	162	162	162	162	162	162	162
X2	15.6	1.9	22.0	3.1	1.1	20.6	6.1	3.2	38.6	5.8
Gvalue	18.5	1.9	24.5	3.1	1.1	22.7	6.6	3.0	38.5	6.0
Gv/N	11.408	1.149	15.135	1.915	0.667	13.987	4.083	1.869	23.793	3.723
Prob	0.00035	0.60163	0.00002	0.37614	0.78165	0.00005	0.08526	0.38742	0.00000	0.11008

	21	22	23	24	25	26	27	28	29	30
a_O	60	58	52	48	46	45	51	49	49	50
	(37.0)	(35.8)	(32.1)	(29.6)	(28.4)	(27.8)	(31.5)	(30.2)	(30.2)	(30.9)
a_E	47.3	45.1	48.4	47.3	45.1	48.4	47.3	45.1	48.4	47.3
	(29.2)	(27.8)	(29.9)	(29.2)	(27.8)	(29.9)	(29.2)	(27.8)	(29.9)	(29.2)
	28.5	29.3	7.5	1.4	1.9	-6.6	7.6	8.2	1.2	5.5
	17.575	18.079	4.609	0.837	1.175	-4.044	4.706	5.073	0.748	3.392
t_O	64	36	62	71	31	54	62	34	49	74
	(39.5)	(22.2)	(38.3)	(43.8)	(19.1)	(33.3)	(38.3)	(21.0)	(30.2)	(45.7)
t_E	66.2	31.7	50.9	66.2	31.7	50.9	66.2	31.7	50.9	66.2
	(40.9)	(19.6)	(31.4)	(40.9)	(19.6)	(31.4)	(40.9)	(19.6)	(31.4)	(40.9)
	-4.4	9.1	24.6	9.8	-1.5	6.5	-8.2	4.7	-3.6	16.4
	-2.721	5.590	15.170	6.080	-0.909	4.002	-5.066	2.880	-2.246	10.118
g_O	16	26	12	21	47	25	22	46	26	21
	(9.9)	(16.0)	(7.4)	(13.0)	(29.0)	(15.4)	(13.6)	(28.4)	(16.0)	(13.0)
g_E	25.3	50.2	26.5	25.3	50.2	26.5	25.3	50.2	26.5	25.3
	(15.6)	(31.0)	(16.3)	(15.6)	(31.0)	(16.3)	(15.6)	(31.0)	(16.3)	(15.6)
	-14.6	-34.2	-19.0	-7.7	-6.1	-2.9	-6.1	-8.0	-0.9	-7.7
	-9.012	-21.102	-11.725	-4.779	-3.793	-1.773	-3.743	-4.933	-0.585	-4.779
c_O	22	42	36	22	38	38	27	33	38	17
	(13.6)	(25.9)	(22.2)	(13.6)	(23.5)	(23.5)	(16.7)	(20.4)	(23.5)	(10.5)
c_E	23.2	35.0	36.3	23.2	35.0	36.3	23.2	35.0	36.3	23.2
	(14.3)	(21.6)	(22.4)	(14.3)	(21.6)	(22.4)	(14.3)	(21.6)	(22.4)	(14.3)
	-2.3	15.3	-0.5	-2.3	6.2	3.5	8.2	-3.9	3.5	-10.5
	-1.420	9.422	-0.333	-1.420	3.829	2.185	5.084	-2.422	2.185	-6.508
N	162	162	162	162	162	162	162	162	162	162
X2	6.9	17.3	10.6	1.1	0.5	0.6	1.6	1.0	0.2	3.4
Gvalue	7.2	19.4	12.5	1.2	0.5	0.6	1.6	1.0	0.2	3.6
Gv/N	4.422	11.989	7.721	0.718	0.302	0.371	0.982	0.598	0.102	2.222
Prob	0.06684	0.00022	0.00583	0.76167	0.92133	0.89634	0.66155	0.80898	0.98292	0.30797

	31	32	33	34	35	36	37	38	39	40
a_O	59	40	46	43	40	47	55	48	55	43
	(36.4)	(24.7)	(28.4)	(26.5)	(24.7)	(29.0)	(34.0)	(29.6)	(34.0)	(26.5)
a_E	45.1	48.4	47.3	45.1	48.4	47.3	45.1	48.4	47.3	45.1
	(27.8)	(29.9)	(29.2)	(27.8)	(29.9)	(29.2)	(27.8)	(29.9)	(29.2)	(27.8)
	31.8	-15.2	-2.6	-4.0	-15.2	-0.7	21.9	-0.8	16.5	-4.0
	19.636	-9.411	-1.615	-2.482	-9.411	-0.402	13.538	-0.489	10.202	-2.482
t_O	42	66	77	43	63	63	37	45	60	29
	(25.9)	(40.7)	(47.5)	(26.5)	(38.9)	(38.9)	(22.8)	(27.8)	(37.0)	(17.9)
t_E	31.7	50.9	66.2	31.7	50.9	66.2	31.7	50.9	66.2	31.7
	(19.6)	(31.4)	(40.9)	(19.6)	(31.4)	(40.9)	(19.6)	(31.4)	(40.9)	(19.6)
	23.5	34.4	23.2	26.1	27.0	-6.3	11.3	-11.0	-11.9	-5.2
	14.515	21.243	14.306	16.109	16.659	-3.903	6.997	-6.794	-7.331	-3.238

```
g_O         36      24      21      45      23      25      40      34      22      44
        ( 22.2) ( 14.8) ( 13.0) ( 27.8) ( 14.2) ( 15.4) ( 24.7) ( 21.0) ( 13.6) ( 27.2)
g_E       50.2    26.5    25.3    50.2    26.5    25.3    50.2    26.5    25.3    50.2
        ( 31.0) ( 16.3) ( 15.6) ( 31.0) ( 16.3) ( 15.6) ( 31.0) ( 16.3) ( 15.6) ( 31.0)
         -23.9    -4.7    -7.7    -9.8    -6.5    -0.5   -18.1    17.0    -6.1   -11.6
       -14.755  -2.912  -4.779  -6.047  -3.999  -0.307 -11.192  10.496  -3.743  -7.134
c_O         25      32      18      31      36      27      30      35      25      46
        ( 15.4) ( 19.8) ( 11.1) ( 19.1) ( 22.2) ( 16.7) ( 18.5) ( 21.6) ( 15.4) ( 28.4)
c_E       35.0    36.3    23.2    35.0    36.3    23.2    35.0    36.3    23.2    35.0
        ( 21.6) ( 22.4) ( 14.3) ( 21.6) ( 22.4) ( 14.3) ( 21.6) ( 22.4) ( 14.3) ( 21.6)
         -16.9    -8.0    -9.1    -7.6    -0.5     8.2    -9.3    -2.5     3.8    25.1
       -10.404  -4.949  -5.621  -4.668  -0.333   5.084  -5.732  -1.541   2.332  15.485

N          162     162     162     162     162     162     162     162     162     162
X2        14.5     6.7     3.7     5.1     4.8     0.8     5.8     2.9     2.4     4.5
Gvalue    14.6     6.4     3.7     4.7     4.7     0.8     5.8     2.7     2.4     4.3
Gv/N     8.991   3.971   2.291   2.912   2.916   0.471   3.611   1.672   1.461   2.631
Prob   0.00223 0.09234 0.29432 0.19375 0.19313 0.85821 0.11917 0.43876 0.49997 0.23446
       -------------------------------------------------------------------------------
            41      42      43      44      45      46      47      48      49      50
       -------------------------------------------------------------------------------
a_O         54      46      50      46      47      63      44      51      53      48
        ( 33.3) ( 28.4) ( 30.9) ( 28.4) ( 29.0) ( 38.9) ( 27.2) ( 31.5) ( 32.7) ( 29.6)
a_E       48.4    47.3    45.1    48.4    47.3    45.1    48.4    47.3    45.1    48.4
        ( 29.9) ( 29.2) ( 27.8) ( 29.9) ( 29.2) ( 27.8) ( 29.9) ( 29.2) ( 27.8) ( 29.9)
          11.8    -2.6    10.4    -4.7    -0.7    42.2    -8.4     7.6    17.2    -0.8
         7.302  -1.615   6.424  -2.886  -0.402  26.069  -5.175   4.706  10.622  -0.489
t_O         45      69      35      59      63      33      53      66      29      46
        ( 27.8) ( 42.6) ( 21.6) ( 36.4) ( 38.9) ( 20.4) ( 32.7) ( 40.7) ( 17.9) ( 28.4)
t_E       50.9    66.2    31.7    50.9    66.2    31.7    50.9    66.2    31.7    50.9
        ( 31.4) ( 40.9) ( 19.6) ( 31.4) ( 40.9) ( 19.6) ( 31.4) ( 40.9) ( 19.6) ( 31.4)
         -11.0     5.6     6.8    17.5    -6.3     2.6     4.4    -0.5    -5.2    -9.2
        -6.794   3.475   4.217  10.823  -3.903   1.579   2.705  -0.298  -3.238  -5.697
g_O         24      27      41      22      36      40      30      27      55      29
        ( 14.8) ( 16.7) ( 25.3) ( 13.6) ( 22.2) ( 24.7) ( 18.5) ( 16.7) ( 34.0) ( 17.9)
g_E       26.5    25.3    50.2    26.5    25.3    50.2    26.5    25.3    50.2    26.5
        ( 16.3) ( 15.6) ( 31.0) ( 16.3) ( 15.6) ( 31.0) ( 16.3) ( 15.6) ( 31.0) ( 16.3)
          -4.7     3.6   -16.6    -8.2    25.5   -18.1     7.5     3.6    10.1     5.3
        -2.912   2.233 -10.222  -5.032  15.764 -11.192   4.625   2.233   6.235   3.257
c_O         39      20      36      35      16      26      35      18      25      39
        ( 24.1) ( 12.3) ( 22.2) ( 21.6) (  9.9) ( 16.0) ( 21.6) ( 11.1) ( 15.4) ( 24.1)
c_E       36.3    23.2    35.0    36.3    23.2    35.0    36.3    23.2    35.0    36.3
        ( 22.4) ( 14.3) ( 21.6) ( 22.4) ( 14.3) ( 21.6) ( 22.4) ( 14.3) ( 21.6) ( 22.4)
           5.7    -5.9     2.0    -2.5   -11.9   -15.5    -2.5    -9.1   -16.9     5.7
         3.493  -3.644   1.225  -1.541  -7.323  -9.561  -1.541  -5.621 -10.404   3.493

N          162     162     162     162     162     162     162     162     162     162
X2         1.8     0.7     2.6     2.2     7.0    11.6     1.0     1.6     5.0     0.9
Gvalue     1.8     0.7     2.7     2.2     6.7    11.2     1.0     1.7     5.2     0.9
Gv/N     1.090   0.449   1.644   1.364   4.135   6.896   0.615   1.020   3.214   0.565
Prob   0.62237 0.86677 0.44646 0.52991 0.08213 0.01084 0.80230 0.64750 0.15724 0.82172
       -------------------------------------------------------------------------------
            51      52      53      54      55      56      57      58      59      60
       -------------------------------------------------------------------------------
a_O         53      54      43      50      45      44      50      41      46      47
        ( 32.7) ( 33.3) ( 26.5) ( 30.9) ( 27.8) ( 27.2) ( 30.9) ( 25.3) ( 28.4) ( 29.0)
a_E       47.3    45.1    48.4    47.3    45.1    48.4    47.3    45.1    48.4    47.3
        ( 29.2) ( 27.8) ( 29.9) ( 29.2) ( 27.8) ( 29.9) ( 29.2) ( 27.8) ( 29.9) ( 29.2)
          12.0    19.6   -10.2     5.5    -0.1    -8.4     5.5    -7.7    -4.7    -0.7
         7.408  12.068  -6.278   3.392  -0.072  -5.175   3.392  -4.778  -2.886  -0.402
t_O         55      36      63      58      38      52      69      41      55      62
        ( 34.0) ( 22.2) ( 38.9) ( 35.8) ( 23.5) ( 32.1) ( 42.6) ( 25.3) ( 34.0) ( 38.3)
t_E       66.2    31.7    50.9    66.2    31.7    50.9    66.2    31.7    50.9    66.2
        ( 40.9) ( 19.6) ( 31.4) ( 40.9) ( 19.6) ( 31.4) ( 40.9) ( 19.6) ( 31.4) ( 40.9)
         -20.5     9.1    27.0   -15.4    13.7     2.3     5.6    21.0     8.6    -8.2
       -12.628   5.590  16.659  -9.514   8.437   1.431   3.475  12.949   5.322  -5.066
g_O         28      44      24      28      41      29      21      41      25      28
        ( 17.3) ( 27.2) ( 14.8) ( 17.3) ( 25.3) ( 17.9) ( 13.0) ( 25.3) ( 15.4) ( 17.3)
g_E       25.3    50.2    26.5    25.3    50.2    26.5    25.3    50.2    26.5    25.3
        ( 15.6) ( 31.0) ( 16.3) ( 15.6) ( 31.0) ( 16.3) ( 15.6) ( 31.0) ( 16.3) ( 15.6)
           5.8   -11.6    -4.7     5.8   -16.6     5.3    -7.7   -16.6    -2.9     5.8
         3.573  -7.134  -2.912   3.573 -10.222   3.257  -4.779 -10.222  -1.773   3.573
c_O         26      28      32      26      38      37      22      39      36      25
        ( 16.0) ( 17.3) ( 19.8) ( 16.0) ( 23.5) ( 22.8) ( 13.6) ( 24.1) ( 22.2) ( 15.4)
c_E       23.2    35.0    36.3    23.2    35.0    36.3    23.2    35.0    36.3    23.2
        ( 14.3) ( 21.6) ( 22.4) ( 14.3) ( 21.6) ( 22.4) ( 14.3) ( 21.6) ( 22.4) ( 14.3)
           6.0   -12.5    -8.0     6.0     6.2     1.5    -2.3     8.4    -0.5     3.8
         3.684  -7.735  -4.949   3.684   3.829   0.910  -1.420   5.181  -0.333   2.332

N          162     162     162     162     162     162     162     162     162     162
X2         3.2     4.5     4.2     1.8     3.2     0.7     1.0     5.2     0.5     0.7
Gvalue     3.3     4.5     4.1     1.8     3.2     0.7     1.1     5.1     0.5     0.7
Gv/N     2.037   2.790   2.521   1.135   1.972   0.423   0.668   3.131   0.331   0.437
Prob   0.34774 0.21055 0.25259 0.60670 0.36246 0.87662 0.78142 0.16663 0.91087 0.87127
       -------------------------------------------------------------------------------
            61      62      63      64      65      66      67      68      69      70
       -------------------------------------------------------------------------------
a_O         46      32      41      43      39      48      47      45      57      39
        ( 28.4) ( 19.8) ( 25.3) ( 26.5) ( 24.1) ( 29.6) ( 29.0) ( 27.8) ( 35.2) ( 24.1)
a_E       45.1    48.4    47.3    45.1    48.4    47.3    45.1    48.4    47.3    45.1
        ( 27.8) ( 29.9) ( 29.2) ( 27.8) ( 29.9) ( 29.2) ( 27.8) ( 29.9) ( 29.2) ( 27.8)
           1.9   -26.5   -11.8    -4.0   -16.8     1.4     4.0    -6.6    21.2   -11.3
         1.175 -16.344  -7.264  -2.482 -10.395   0.837   2.448  -4.044  13.087  -6.952
t_O         29      53      65      38      65      62      34      52      51      45
        ( 17.9) ( 32.7) ( 40.1) ( 23.5) ( 40.1) ( 38.3) ( 21.0) ( 32.1) ( 31.5) ( 27.8)
t_E       31.7    50.9    66.2    31.7    50.9    66.2    31.7    50.9    66.2    31.7
        ( 19.6) ( 31.4) ( 40.9) ( 19.6) ( 31.4) ( 40.9) ( 19.6) ( 31.4) ( 40.9) ( 19.6)
          -5.2     4.4    -2.5    13.7    31.9    -8.2     4.7     2.3   -26.7    31.4
        -3.238   2.705  -1.519   8.437  19.696  -5.066   2.880   1.431 -16.464  19.384
g_O         46      35      23      47      23      29      46      28      25      50
        ( 28.4) ( 21.6) ( 14.2) ( 29.0) ( 14.2) ( 17.9) ( 28.4) ( 17.3) ( 15.4) ( 30.9)
g_E       50.2    26.5    25.3    50.2    26.5    25.3    50.2    26.5    25.3    50.2
        ( 31.0) ( 16.3) ( 15.6) ( 31.0) ( 16.3) ( 15.6) ( 31.0) ( 16.3) ( 15.6) ( 31.0)
          -8.0    19.5    -4.3    -6.1    -6.5     8.0    -8.0     3.1    -0.5    -0.3
        -4.933  12.057  -2.650  -3.793  -3.999   4.957  -4.933   1.932  -0.307  -0.215
c_O         41      42      33      34      35      23      35      37      29      28
        ( 25.3) ( 25.9) ( 20.4) ( 21.0) ( 21.6) ( 14.2) ( 21.6) ( 22.8) ( 17.9) ( 17.3)
c_E       35.0    36.3    23.2    35.0    36.3    23.2    35.0    36.3    23.2    35.0
        ( 21.6) ( 22.4) ( 14.3) ( 21.6) ( 22.4) ( 14.3) ( 21.6) ( 22.4) ( 14.3) ( 21.6)
          12.9    12.3    23.3    -2.0    -2.5    -0.4    -0.0     1.5    13.0   -12.5
         7.978   7.605  14.389  -1.243  -1.541  -0.222  -0.027   0.910   8.019  -7.735
```

N	162	162	162	162	162	162	162	162	162	162
X2	1.6	9.3	5.2	1.6	6.3	0.8	0.6	0.4	6.9	7.8
Gvalue	1.6	9.8	4.8	1.5	6.1	0.8	0.6	0.4	7.0	7.3
Gv/N	0.981	6.022	2.956	0.919	3.761	0.506	0.368	0.229	4.334	4.482
Prob	0.66199	0.02076	0.18798	0.68473	0.10716	0.84474	0.89726	0.94628	0.07122	0.06405

	71	72	73	74	75	76	77	78	79	80
a_O	39	46	47	46	43	42	35	50	41	41
	(24.1)	(28.4)	(29.0)	(28.4)	(26.5)	(25.9)	(21.6)	(30.9)	(25.3)	(25.3)
a_E	48.4	47.3	45.1	48.4	47.3	45.1	48.4	47.3	45.1	48.4
	(29.9)	(29.2)	(27.8)	(29.9)	(29.2)	(27.8)	(29.9)	(29.2)	(27.8)	(29.9)
	-16.8	-2.6	4.0	-4.7	-8.2	-5.9	-22.7	5.5	-7.7	-13.6
	-10.395	-1.615	2.448	-2.886	-5.090	-3.645	-14.005	3.392	-4.778	-8.397
t_O	53	64	40	49	70	39	60	61	33	57
	(32.7)	(39.5)	(24.7)	(30.2)	(43.2)	(24.1)	(37.0)	(37.7)	(20.4)	(35.2)
t_E	50.9	66.2	31.7	50.9	66.2	31.7	50.9	66.2	31.7	50.9
	(31.4)	(40.9)	(19.6)	(31.4)	(40.9)	(19.6)	(31.4)	(40.9)	(19.6)	(31.4)
	4.4	-4.4	18.5	-3.6	7.7	16.1	19.8	-10.1	2.6	13.0
	2.705	-2.721	11.414	-2.246	4.769	9.910	12.251	-6.209	1.579	8.029
g_O	31	32	44	23	26	45	22	30	38	29
	(19.1)	(19.8)	(27.2)	(14.2)	(16.0)	(27.8)	(13.6)	(18.5)	(23.5)	(17.9)
g_E	26.5	25.3	50.2	26.5	25.3	50.2	26.5	25.3	50.2	26.5
	(16.3)	(15.6)	(31.0)	(16.3)	(15.6)	(31.0)	(16.3)	(15.6)	(31.0)	(16.3)
	9.8	15.2	-11.6	-6.5	1.5	-9.8	-8.2	10.3	-21.1	5.3
	6.034	9.359	-7.134	-3.999	0.939	-6.047	-5.032	6.384	-13.038	3.257
c_O	39	20	31	44	23	36	45	21	50	35
	(24.1)	(12.3)	(19.1)	(27.2)	(14.2)	(22.2)	(27.8)	(13.0)	(30.9)	(21.6)
c_E	36.3	23.2	35.0	36.3	23.2	35.0	36.3	23.2	35.0	36.3
	(22.4)	(14.3)	(21.6)	(22.4)	(14.3)	(21.6)	(22.4)	(14.3)	(21.6)	(22.4)
	5.7	-5.9	-7.6	17.0	-0.4	2.0	19.4	-4.1	35.6	-2.5
	3.493	-3.644	-4.668	10.494	-0.222	1.225	11.981	-2.562	21.979	-1.541

N	162	162	162	162	162	162	162	162	162	162
X2	2.9	2.4	3.5	2.3	0.6	2.4	8.2	1.7	9.8	2.2
Gvalue	3.0	2.2	3.3	2.2	0.6	2.3	8.4	1.6	9.3	2.2
Gv/N	1.838	1.379	2.060	1.363	0.396	1.443	5.196	1.005	5.742	1.349
Prob	0.39511	0.52522	0.34245	0.53022	0.88699	0.50549	0.03814	0.65294	0.02553	0.53473

	81	82	83	84	85	86	87	88	89	90
a_O	46	51	49	46	53	42	32	37	51	47
	(28.4)	(31.5)	(30.2)	(28.4)	(32.7)	(25.9)	(19.8)	(22.8)	(31.5)	(29.0)
a_E	47.3	45.1	48.4	47.3	45.1	48.4	47.3	45.1	48.4	47.3
	(29.2)	(27.8)	(29.9)	(29.2)	(27.8)	(29.9)	(29.2)	(27.8)	(29.9)	(29.2)
	-2.6	12.6	1.2	-2.6	17.2	-11.9	-25.0	-14.6	5.3	-0.7
	-1.615	7.799	0.748	-1.615	10.622	-7.352	-15.460	-9.001	3.297	-0.402
t_O	70	33	56	64	26	59	70	30	49	68
	(43.2)	(20.4)	(34.6)	(39.5)	(16.0)	(36.4)	(43.2)	(18.5)	(30.2)	(42.0)
t_E	66.2	31.7	50.9	66.2	31.7	50.9	66.2	31.7	50.9	66.2
	(40.9)	(19.6)	(31.4)	(40.9)	(19.6)	(31.4)	(40.9)	(19.6)	(31.4)	(40.9)
	7.7	2.6	10.8	-4.4	-10.4	17.5	7.7	-3.4	-3.6	3.6
	4.769	1.579	6.665	-2.721	-6.409	10.823	4.769	-2.094	-2.246	2.199
g_O	28	38	24	23	51	29	34	55	27	22
	(17.3)	(23.5)	(14.8)	(14.2)	(31.5)	(17.9)	(21.0)	(34.0)	(16.7)	(13.6)
g_E	25.3	50.2	26.5	25.3	50.2	26.5	25.3	50.2	26.5	25.3
	(15.6)	(31.0)	(16.3)	(15.6)	(31.0)	(16.3)	(15.6)	(31.0)	(16.3)	(15.6)
	5.8	-21.1	-4.7	-4.3	1.7	5.3	20.2	10.1	1.1	-6.1
	3.573	-13.038	-2.912	-2.650	1.027	3.257	12.489	6.235	0.651	-3.743
c_O	18	40	33	29	32	32	26	40	35	25
	(11.1)	(24.7)	(20.4)	(17.9)	(19.8)	(19.8)	(16.0)	(24.7)	(21.6)	(15.4)
c_E	23.2	35.0	36.3	23.2	35.0	36.3	23.2	35.0	36.3	23.2
	(14.3)	(21.6)	(22.4)	(14.3)	(21.6)	(22.4)	(14.3)	(21.6)	(22.4)	(14.3)
	-9.1	10.6	-6.2	13.0	-5.8	-8.0	6.0	10.6	-2.5	3.8
	-5.621	6.564	-3.850	8.019	-3.565	-4.949	3.684	6.564	-1.541	2.332

N	162	162	162	162	162	162	162	162	162	162
X2	1.7	4.5	1.1	1.8	2.7	2.9	8.6	2.7	0.3	0.6
Gvalue	1.8	4.7	1.1	1.7	2.7	2.9	8.9	2.8	0.3	0.6
Gv/N	1.106	2.903	0.651	1.033	1.676	1.780	5.481	1.704	0.161	0.386
Prob	0.61684	0.19483	0.78788	0.64294	0.43768	0.41004	0.03094	0.43018	0.96717	0.89062

	91	92	93	94	95	96	97	98	99	100
a_O	44	36	41	42	50	40	54	54	52	42
	(27.2)	(22.2)	(25.3)	(25.9)	(30.9)	(24.7)	(33.3)	(33.3)	(32.1)	(25.9)
a_E	45.1	48.4	47.3	45.1	48.4	47.3	45.1	48.4	47.3	45.1
	(27.8)	(29.9)	(29.2)	(27.8)	(29.9)	(29.2)	(27.8)	(29.9)	(29.2)	(27.8)
	-2.1	-21.3	-11.8	-5.9	3.3	-13.5	19.6	11.8	9.8	-5.9
	-1.291	-13.153	-7.264	-3.645	2.010	-8.306	12.068	7.302	6.045	-3.645
t_O	42	51	67	43	55	76	37	51	64	32
	(25.9)	(31.5)	(41.4)	(26.5)	(34.0)	(46.9)	(22.8)	(31.5)	(39.5)	(19.8)
t_E	31.7	50.9	66.2	31.7	50.9	66.2	31.7	50.9	66.2	31.7
	(19.6)	(31.4)	(40.9)	(19.6)	(31.4)	(40.9)	(19.6)	(31.4)	(40.9)	(19.6)
	23.5	0.3	1.5	26.1	8.6	20.9	11.3	0.3	-4.4	0.5
	14.515	0.181	0.941	16.109	5.322	12.894	6.997	0.181	-2.721	0.316
g_O	43	33	23	40	25	29	35	35	25	50
	(26.5)	(20.4)	(14.2)	(24.7)	(15.4)	(17.9)	(21.6)	(21.6)	(15.4)	(30.9)
g_E	50.2	26.5	25.3	50.2	26.5	25.3	50.2	26.5	25.3	50.2
	(31.0)	(16.3)	(15.6)	(31.0)	(16.3)	(15.6)	(31.0)	(16.3)	(15.6)	(31.0)
	-13.3	14.5	-4.3	-18.1	-2.9	8.0	-25.2	19.5	-0.5	-0.3
	-8.192	8.971	-2.650	-11.192	-1.773	4.957	-15.563	12.057	-0.307	-0.215
c_O	33	42	31	37	32	17	36	22	21	38
	(20.4)	(25.9)	(19.1)	(22.8)	(19.8)	(10.5)	(22.2)	(13.6)	(13.0)	(23.5)
c_E	35.0	36.3	23.2	35.0	36.3	23.2	35.0	36.3	23.2	35.0
	(21.6)	(22.4)	(14.3)	(21.6)	(22.4)	(14.3)	(21.6)	(22.4)	(14.3)	(21.6)
	-3.9	12.3	18.0	4.1	-8.0	-10.5	2.0	-22.0	-4.1	6.2
	-2.422	7.605	11.124	2.510	-4.949	-6.508	1.225	-13.579	-2.562	3.829

N	162	162	162	162	162	162	162	162	162	162
X2	4.5	5.7	3.7	6.4	1.0	4.8	7.3	9.0	0.7	0.5
Gvalue	4.2	5.8	3.5	6.1	1.0	4.9	7.7	9.7	0.7	0.5
Gv/N	2.609	3.604	2.151	3.783	0.611	3.036	4.727	5.961	0.456	0.285
Prob	0.23797	0.11975	0.32279	0.10551	0.80377	0.17787	0.05363	0.02173	0.86421	0.92726

Bsui_1330
5UTR:

	100	99	98	97	96	95	94	93	92	91
a_O	112	120	99	103	100	103	107	103	114	112
	(25.2)	(27.0)	(22.3)	(23.2)	(22.5)	(23.2)	(24.1)	(23.2)	(25.7)	(25.2)
a_E	109.5	109.5	109.5	109.5	109.5	109.5	109.5	109.5	109.5	109.5
	(24.7)	(24.7)	(24.7)	(24.7)	(24.7)	(24.7)	(24.7)	(24.7)	(24.7)	(24.7)
	5.0	21.9	-20.0	-12.7	-18.2	-12.7	-5.0	-12.7	9.1	5.0
	1.125	4.935	-4.508	-2.852	-4.100	-2.852	-1.126	-2.852	2.054	1.125
t_O	100	97	105	103	110	103	110	113	99	106
	(22.5)	(21.8)	(23.6)	(23.2)	(24.8)	(23.2)	(24.8)	(25.5)	(22.3)	(23.9)
t_E	108.4	108.4	108.4	108.4	108.4	108.4	108.4	108.4	108.4	108.4
	(24.4)	(24.4)	(24.4)	(24.4)	(24.4)	(24.4)	(24.4)	(24.4)	(24.4)	(24.4)
	-16.1	-21.5	-6.7	-10.5	3.3	-10.5	3.3	9.4	-17.9	-4.7
	-3.625	-4.847	-1.499	-2.362	0.735	-2.362	0.735	2.125	-4.037	-1.060
g_O	130	116	121	114	116	114	117	118	125	114
	(29.3)	(26.1)	(27.3)	(25.7)	(26.1)	(25.7)	(26.4)	(26.6)	(28.2)	(25.7)
g_E	109.3	109.3	109.3	109.3	109.3	109.3	109.3	109.3	109.3	109.3
	(24.6)	(24.6)	(24.6)	(24.6)	(24.6)	(24.6)	(24.6)	(24.6)	(24.6)	(24.6)
	45.1	13.8	24.6	9.6	13.8	9.6	15.9	18.1	33.6	9.6
	10.156	3.109	5.543	2.162	3.109	2.162	3.588	4.071	7.557	2.162
c_O	102	111	119	124	118	124	110	110	106	112
	(23.0)	(25.0)	(26.8)	(27.9)	(26.6)	(27.9)	(24.8)	(24.8)	(23.9)	(25.2)
c_E	116.8	116.8	116.8	116.8	116.8	116.8	116.8	116.8	116.8	116.8
	(26.3)	(26.3)	(26.3)	(26.3)	(26.3)	(26.3)	(26.3)	(26.3)	(26.3)	(26.3)
	-27.6	-11.3	4.5	14.9	2.4	14.9	-13.2	-13.2	-20.6	-9.4
	-6.221	-2.542	1.005	3.346	0.548	3.346	-2.968	-2.968	-4.629	-2.113
N	444	444	444	444	444	444	444	444	444	444
X2	6.5	2.9	2.4	1.3	1.3	1.3	1.0	1.7	4.2	0.5
Gvalue	6.4	2.9	2.4	1.3	1.3	1.3	1.0	1.7	4.2	0.5
Gv/N	1.435	0.654	0.542	0.294	0.291	0.294	0.229	0.376	0.946	0.114
Prob	0.09485	0.40668	0.49280	0.72810	0.73068	0.72810	0.79743	0.64386	0.24069	0.91748

	90	89	88	87	86	85	84	83	82	81
a_O	111	105	99	102	102	119	87	90	106	107
	(25.0)	(23.6)	(22.3)	(23.0)	(23.0)	(26.8)	(19.6)	(20.3)	(23.9)	(24.1)
a_E	109.5	109.5	109.5	109.5	109.5	109.5	109.5	109.5	109.5	109.5
	(24.7)	(24.7)	(24.7)	(24.7)	(24.7)	(24.7)	(24.7)	(24.7)	(24.7)	(24.7)
	3.0	-8.9	-20.0	-14.5	-14.5	19.7	-40.1	-35.3	-6.9	-5.0
	0.667	-1.998	-4.508	-3.273	-3.273	4.445	-9.025	-7.962	-1.564	-1.126
t_O	101	110	110	105	96	105	118	133	103	115
	(22.7)	(24.8)	(24.8)	(23.6)	(21.6)	(23.6)	(26.6)	(30.0)	(23.2)	(25.9)
t_E	108.4	108.4	108.4	108.4	108.4	108.4	108.4	108.4	108.4	108.4
	(24.4)	(24.4)	(24.4)	(24.4)	(24.4)	(24.4)	(24.4)	(24.4)	(24.4)	(24.4)
	-14.2	3.3	3.3	-6.7	-23.3	-6.7	20.1	54.5	-10.5	13.6
	-3.208	0.735	0.735	-1.499	-5.245	-1.499	4.520	12.264	-2.362	3.071
g_O	119	105	115	132	125	103	117	118	117	119
	(26.8)	(23.6)	(25.9)	(29.7)	(28.2)	(23.2)	(26.4)	(26.6)	(26.4)	(26.8)
g_E	109.3	109.3	109.3	109.3	109.3	109.3	109.3	109.3	109.3	109.3
	(24.6)	(24.6)	(24.6)	(24.6)	(24.6)	(24.6)	(24.6)	(24.6)	(24.6)	(24.6)
	20.2	-8.4	11.7	49.8	33.6	-12.2	15.9	18.1	15.9	20.2
	4.558	-1.898	2.633	11.220	7.557	-2.754	3.588	4.071	3.588	4.558
c_O	113	124	120	105	121	117	122	103	118	103
	(25.5)	(27.9)	(27.0)	(23.6)	(27.3)	(26.4)	(27.5)	(23.2)	(26.6)	(23.2)
c_E	116.8	116.8	116.8	116.8	116.8	116.8	116.8	116.8	116.8	116.8
	(26.3)	(26.3)	(26.3)	(26.3)	(26.3)	(26.3)	(26.3)	(26.3)	(26.3)	(26.3)
	-7.5	14.9	6.5	-22.3	8.6	0.4	10.6	-25.9	2.4	-25.9
	-1.679	3.346	1.466	-5.033	1.930	0.095	2.398	-5.830	0.548	-5.830
N	444	444	444	444	444	444	444	444	444	444
X2	1.5	0.8	1.4	6.5	4.3	1.3	6.3	11.4	0.9	3.0
Gvalue	1.5	0.8	1.5	6.3	4.3	1.3	6.6	11.3	0.9	3.0
Gv/N	0.337	0.185	0.327	1.416	0.970	0.287	1.482	2.543	0.209	0.673
Prob	0.68348	0.84426	0.69382	0.09845	0.23030	0.73547	0.08662	0.01024	0.81837	0.39346

	80	79	78	77	76	75	74	73	72	71
a_O	103	105	114	108	113	109	104	95	121	110
	(23.2)	(23.6)	(25.7)	(24.3)	(25.5)	(24.5)	(23.4)	(21.4)	(27.3)	(24.8)
a_E	109.5	109.5	109.5	109.5	109.5	109.5	109.5	109.5	109.5	109.5
	(24.7)	(24.7)	(24.7)	(24.7)	(24.7)	(24.7)	(24.7)	(24.7)	(24.7)	(24.7)
	-12.7	-8.9	9.1	-3.0	7.0	-1.1	-10.8	-27.0	24.1	0.9
	-2.852	-1.998	2.054	-0.684	1.588	-0.238	-2.427	-6.090	5.428	0.212
t_O	97	99	112	105	96	103	94	112	102	126
	(21.8)	(22.3)	(25.2)	(23.6)	(21.6)	(23.2)	(21.2)	(25.2)	(23.0)	(28.4)
t_E	108.4	108.4	108.4	108.4	108.4	108.4	108.4	108.4	108.4	108.4
	(24.4)	(24.4)	(24.4)	(24.4)	(24.4)	(24.4)	(24.4)	(24.4)	(24.4)	(24.4)
	-21.5	-17.9	7.4	-6.7	-23.3	-10.5	-26.8	7.4	-12.4	38.0
	-4.847	-4.037	1.658	-1.499	-5.245	-2.362	-6.027	1.658	-2.788	8.550
g_O	126	120	103	119	108	118	119	122	107	99
	(28.4)	(27.0)	(23.2)	(26.8)	(24.3)	(26.6)	(26.8)	(27.5)	(24.1)	(22.3)
g_E	109.3	109.3	109.3	109.3	109.3	109.3	109.3	109.3	109.3	109.3
	(24.6)	(24.6)	(24.6)	(24.6)	(24.6)	(24.6)	(24.6)	(24.6)	(24.6)	(24.6)
	35.8	22.4	-12.2	20.2	-2.6	18.1	20.2	26.8	-4.6	-19.6
	8.070	5.048	-2.754	4.558	-0.582	4.071	4.558	6.041	-1.025	-4.414
c_O	118	120	115	112	127	114	127	115	114	109
	(26.6)	(27.0)	(25.9)	(25.2)	(28.6)	(25.7)	(28.6)	(25.9)	(25.7)	(24.5)
c_E	116.8	116.8	116.8	116.8	116.8	116.8	116.8	116.8	116.8	116.8
	(26.3)	(26.3)	(26.3)	(26.3)	(26.3)	(26.3)	(26.3)	(26.3)	(26.3)	(26.3)
	2.4	6.5	-3.6	-9.4	21.3	-5.5	21.3	-3.6	-5.5	-15.0
	0.548	1.466	-0.800	-2.113	4.795	-1.242	4.795	-0.800	-1.242	-3.389
N	444	444	444	444	444	444	444	444	444	444
X2	4.1	2.1	0.7	1.2	2.4	1.0	3.9	3.6	1.7	4.4
Gvalue	4.1	2.1	0.7	1.2	2.5	1.0	4.0	3.6	1.7	4.3
Gv/N	0.919	0.479	0.157	0.262	0.555	0.229	0.898	0.808	0.374	0.959
Prob	0.25300	0.54615	0.87380	0.76174	0.48197	0.79734	0.26292	0.30957	0.64578	0.23501

	70	69	68	67	66	65	64	63	62	61
a_O	106	110	92	101	113	122	117	113	113	106
	(23.9)	(24.8)	(20.7)	(22.7)	(25.5)	(27.5)	(26.4)	(25.5)	(25.5)	(23.9)
a_E	109.5	109.5	109.5	109.5	109.5	109.5	109.5	109.5	109.5	109.5
	(24.7)	(24.7)	(24.7)	(24.7)	(24.7)	(24.7)	(24.7)	(24.7)	(24.7)	(24.7)
	-6.9	0.9	-32.1	-16.4	7.0	26.3	15.4	7.0	7.0	-6.9
	-1.564	0.212	-7.228	-3.689	1.588	5.925	3.477	1.588	1.588	-1.564
t_O	124	115	103	100	108	94	113	101	86	99
	(27.9)	(25.9)	(23.2)	(22.5)	(24.3)	(21.2)	(25.5)	(22.7)	(19.4)	(22.3)

t_E	108.4	108.4	108.4	108.4	108.4	108.4	108.4	108.4	108.4	108.4
	(24.4)	(24.4)	(24.4)	(24.4)	(24.4)	(24.4)	(24.4)	(24.4)	(24.4)	(24.4)
	33.4	13.6	-10.5	-16.1	-0.8	-26.8	9.4	-14.2	-39.8	-17.9
	7.520	3.071	-2.362	-3.625	-0.171	-6.027	2.125	-3.208	-8.960	-4.037
g_O	99	99	127	111	112	101	115	123	127	112
	(22.3)	(22.3)	(28.6)	(25.0)	(25.2)	(22.7)	(25.9)	(27.7)	(28.6)	(25.2)
g_E	109.3	109.3	109.3	109.3	109.3	109.3	109.3	109.3	109.3	109.3
	(24.6)	(24.6)	(24.6)	(24.6)	(24.6)	(24.6)	(24.6)	(24.6)	(24.6)	(24.6)
	-19.6	-19.6	38.1	3.4	5.5	-16.0	11.7	29.0	38.1	5.5
	-4.414	-4.414	8.586	0.772	1.231	-3.593	2.633	6.543	8.586	1.231
c_O	115	120	122	132	111	127	99	107	118	127
	(25.9)	(27.0)	(27.5)	(29.7)	(25.0)	(28.6)	(22.3)	(24.1)	(26.6)	(28.6)
c_E	116.8	116.8	116.8	116.8	116.8	116.8	116.8	116.8	116.8	116.8
	(26.3)	(26.3)	(26.3)	(26.3)	(26.3)	(26.3)	(26.3)	(26.3)	(26.3)	(26.3)
	-3.6	6.5	10.6	32.3	-11.3	21.3	-32.7	-18.7	2.4	21.3
	-0.800	1.466	2.398	7.279	-2.542	4.795	-7.370	-4.220	0.548	4.795
N	444	444	444	444	444	444	444	444	444	444
X2	3.4	1.5	6.2	3.3	0.5	4.9	3.7	3.2	7.6	1.9
Gvalue	3.3	1.5	6.2	3.3	0.5	4.9	3.8	3.1	7.8	1.9
Gv/N	0.742	0.335	1.395	0.737	0.105	1.099	0.866	0.702	1.762	0.425
Prob	0.34828	0.68494	0.10262	0.35129	0.92582	0.18067	0.27885	0.37388	0.04985	0.59659

	60	59	58	57	56	55	54	53	52	51
a_O	105	92	102	103	94	128	111	100	126	101
	(23.6)	(20.7)	(23.0)	(23.2)	(21.2)	(28.8)	(25.0)	(22.5)	(28.4)	(22.7)
a_E	109.5	109.5	109.5	109.5	109.5	109.5	109.5	109.5	109.5	109.5
	(24.7)	(24.7)	(24.7)	(24.7)	(24.7)	(24.7)	(24.7)	(24.7)	(24.7)	(24.7)
	-8.9	-32.1	-14.5	-12.7	-28.7	39.9	3.0	-18.2	35.3	-16.4
	-1.998	-7.228	-3.273	-2.852	-6.474	8.985	0.667	-4.100	7.951	-3.689
t_O	106	127	118	106	95	85	123	107	108	112
	(23.9)	(28.6)	(26.6)	(23.9)	(21.4)	(19.1)	(27.7)	(24.1)	(24.3)	(25.2)
t_E	108.4	108.4	108.4	108.4	108.4	108.4	108.4	108.4	108.4	108.4
	(24.4)	(24.4)	(24.4)	(24.4)	(24.4)	(24.4)	(24.4)	(24.4)	(24.4)	(24.4)
	-4.7	40.3	20.1	-4.7	-25.0	-41.3	31.1	-2.7	-0.8	7.4
	-1.060	9.070	4.520	-1.060	-5.639	-9.304	7.011	-0.618	-0.171	1.658
g_O	109	104	117	124	116	115	104	99	108	115
	(24.5)	(23.4)	(26.4)	(27.9)	(26.1)	(25.9)	(23.4)	(22.3)	(24.3)	(25.9)
g_E	109.3	109.3	109.3	109.3	109.3	109.3	109.3	109.3	109.3	109.3
	(24.6)	(24.6)	(24.6)	(24.6)	(24.6)	(24.6)	(24.6)	(24.6)	(24.6)	(24.6)
	-0.6	-10.3	15.9	31.3	13.8	11.7	-10.3	-19.6	-2.6	11.7
	-0.135	-2.329	3.588	7.048	3.109	2.633	-2.329	-4.414	-0.582	2.633
c_O	124	121	107	111	139	116	106	138	102	116
	(27.9)	(27.3)	(24.1)	(25.0)	(31.3)	(26.1)	(23.9)	(31.1)	(23.0)	(26.1)
c_E	116.8	116.8	116.8	116.8	116.8	116.8	116.8	116.8	116.8	116.8
	(26.3)	(26.3)	(26.3)	(26.3)	(26.3)	(26.3)	(26.3)	(26.3)	(26.3)	(26.3)
	14.9	8.6	-18.7	-11.3	48.4	-1.6	-20.6	46.1	-27.6	-1.6
	3.346	1.930	-4.220	-2.542	10.901	-0.355	-4.629	10.373	-6.221	-0.355
N	444	444	444	444	444	444	444	444	444	444
X2	0.7	6.4	2.7	2.7	8.5	8.5	3.2	5.7	4.4	1.1
Gvalue	0.7	6.4	2.7	2.6	8.4	8.7	3.2	5.5	4.3	1.1
Gv/N	0.153	1.444	0.616	0.594	1.896	1.960	0.720	1.242	0.976	0.248
Prob	0.87797	0.09329	0.43441	0.45125	0.03809	0.03353	0.36195	0.13789	0.22746	0.77722

	50	49	48	47	46	45	44	43	42	41
a_O	118	114	111	107	109	118	106	105	91	87
	(26.6)	(25.7)	(25.0)	(24.1)	(24.5)	(26.6)	(23.9)	(23.6)	(20.5)	(19.6)
a_E	109.5	109.5	109.5	109.5	109.5	109.5	109.5	109.5	109.5	109.5
	(24.7)	(24.7)	(24.7)	(24.7)	(24.7)	(24.7)	(24.7)	(24.7)	(24.7)	(24.7)
	17.6	9.1	3.0	-5.0	-1.1	17.6	-6.9	-8.9	-33.7	-40.1
	3.959	2.054	0.667	-1.126	-0.238	3.959	-1.564	-1.998	-7.597	-9.025
t_O	102	94	110	112	102	113	114	108	103	123
	(23.0)	(21.2)	(24.8)	(25.2)	(23.0)	(25.5)	(25.7)	(24.3)	(23.2)	(27.7)
t_E	108.4	108.4	108.4	108.4	108.4	108.4	108.4	108.4	108.4	108.4
	(24.4)	(24.4)	(24.4)	(24.4)	(24.4)	(24.4)	(24.4)	(24.4)	(24.4)	(24.4)
	-12.4	-26.8	3.3	7.4	-12.4	9.4	11.5	-0.8	-10.5	31.1
	-2.788	-6.027	0.735	1.658	-2.788	2.125	2.596	-0.171	-2.362	7.011
g_O	117	114	110	108	108	126	121	100	128	118
	(26.4)	(25.7)	(24.8)	(24.3)	(24.3)	(28.4)	(27.3)	(22.5)	(28.8)	(26.6)
g_E	109.3	109.3	109.3	109.3	109.3	109.3	109.3	109.3	109.3	109.3
	(24.6)	(24.6)	(24.6)	(24.6)	(24.6)	(24.6)	(24.6)	(24.6)	(24.6)	(24.6)
	15.9	9.6	1.4	-2.6	-2.6	35.8	24.6	-17.8	40.4	18.1
	3.588	2.162	0.316	-0.582	-0.582	8.070	5.543	-4.006	9.106	4.071
c_O	107	122	113	117	125	87	103	131	122	116
	(24.1)	(27.5)	(25.5)	(26.4)	(28.2)	(19.6)	(23.2)	(29.5)	(27.5)	(26.1)
c_E	116.8	116.8	116.8	116.8	116.8	116.8	116.8	116.8	116.8	116.8
	(26.3)	(26.3)	(26.3)	(26.3)	(26.3)	(26.3)	(26.3)	(26.3)	(26.3)	(26.3)
	-18.7	10.6	-7.5	0.4	17.0	-51.2	-25.9	30.1	10.6	-1.6
	-4.220	2.398	-1.679	0.095	3.825	-11.540	-5.830	6.775	2.398	-0.355
N	444	444	444	444	444	444	444	444	444	444
X2	2.4	2.5	0.2	0.2	1.0	11.0	3.3	2.7	6.8	7.3
Gvalue	2.4	2.6	0.2	0.2	1.0	11.6	3.3	2.7	6.9	7.6
Gv/N	0.540	0.587	0.039	0.044	0.217	2.614	0.745	0.601	1.545	1.702
Prob	0.49426	0.45631	0.98189	0.97849	0.80964	0.00886	0.34654	0.44554	0.07650	0.05607

	40	39	38	37	36	35	34	33	32	31
a_O	116	114	118	109	94	96	109	95	106	131
	(26.1)	(25.7)	(26.6)	(24.5)	(21.2)	(21.6)	(24.5)	(21.4)	(23.9)	(29.5)
a_E	109.5	109.5	109.5	109.5	109.5	109.5	109.5	109.5	109.5	109.5
	(24.7)	(24.7)	(24.7)	(24.7)	(24.7)	(24.7)	(24.7)	(24.7)	(24.7)	(24.7)
	13.3	9.1	17.6	-1.1	-28.7	-25.3	-1.1	-27.0	-6.9	46.9
	2.999	2.054	3.959	-0.238	-6.474	-5.702	-0.238	-6.090	-1.564	10.563
t_O	111	113	111	130	112	133	125	123	107	99
	(25.0)	(25.5)	(25.0)	(29.3)	(25.2)	(30.0)	(28.2)	(27.7)	(24.1)	(22.3)
t_E	108.4	108.4	108.4	108.4	108.4	108.4	108.4	108.4	108.4	108.4
	(24.4)	(24.4)	(24.4)	(24.4)	(24.4)	(24.4)	(24.4)	(24.4)	(24.4)	(24.4)
	5.3	9.4	5.3	47.3	7.4	54.5	35.7	31.1	-2.7	-17.9
	1.194	2.125	1.194	10.651	1.658	12.264	8.033	7.011	-0.618	-4.037
g_O	106	101	109	107	125	98	114	125	128	103
	(23.9)	(22.7)	(24.5)	(24.1)	(28.2)	(22.1)	(25.7)	(28.2)	(28.8)	(23.2)
g_E	109.3	109.3	109.3	109.3	109.3	109.3	109.3	109.3	109.3	109.3
	(24.6)	(24.6)	(24.6)	(24.6)	(24.6)	(24.6)	(24.6)	(24.6)	(24.6)	(24.6)
	-6.5	-16.0	-0.6	-4.6	33.6	-21.4	9.6	33.6	40.4	-12.2
	-1.464	-3.593	-0.135	-1.025	7.557	-4.817	2.162	7.557	9.106	-2.754
c_O	111	116	106	98	113	117	96	101	103	111
	(25.0)	(26.1)	(23.9)	(22.1)	(25.5)	(26.4)	(21.6)	(22.7)	(23.2)	(25.0)
c_E	116.8	116.8	116.8	116.8	116.8	116.8	116.8	116.8	116.8	116.8

```
        ( 26.3) ( 26.3) ( 26.3) ( 26.3) ( 26.3) ( 26.3) ( 26.3) ( 26.3) ( 26.3) ( 26.3)
          -11.3    -1.6   -20.6   -34.4    -7.5     0.4   -37.6   -29.3   -25.9   -11.3
         -2.542  -0.355  -4.629  -7.743  -1.679   0.095  -8.477  -6.609  -5.830  -2.542

N           444     444     444     444     444     444     444     444     444     444
X2          0.8     1.0     1.7     7.4     4.7     8.4     6.5     8.3     5.0     5.7
Gvalue      0.8     1.0     1.7     7.3     4.7     8.2     6.6     8.3     4.9     5.5
Gv/N      0.187   0.231   0.390   1.645   1.061   1.840   1.480   1.869   1.095   1.229
Prob    0.84221 0.79486 0.62996 0.06285 0.19411 0.04267 0.08687 0.04020 0.18233 0.14128
        -------------------------------------------------------------------------------
             30      29      28      27      26      25      24      23      22      21
        -------------------------------------------------------------------------------
a_O         109     125     120     112     129     110     118     114     113      95
        ( 24.5) ( 28.2) ( 27.0) ( 25.2) ( 29.1) ( 24.8) ( 26.6) ( 25.7) ( 25.5) ( 21.4)
a_E       109.5   109.5   109.5   109.5   109.5   109.5   109.5   109.5   109.5   109.5
        ( 24.7) ( 24.7) ( 24.7) ( 24.7) ( 24.7) ( 24.7) ( 24.7) ( 24.7) ( 24.7) ( 24.7)
           -1.1    33.0    21.9     5.0    42.2     0.9    17.6     9.1     7.0   -27.0
         -0.238   7.439   4.935   1.125   9.507   0.212   3.959   2.054   1.588  -6.090
t_O         104      87     105     110      90      82     114      95     120     115
        ( 23.4) ( 19.6) ( 23.6) ( 24.8) ( 20.3) ( 18.5) ( 25.7) ( 21.4) ( 27.0) ( 25.9)
t_E       108.4   108.4   108.4   108.4   108.4   108.4   108.4   108.4   108.4   108.4
        ( 24.4) ( 24.4) ( 24.4) ( 24.4) ( 24.4) ( 24.4) ( 24.4) ( 24.4) ( 24.4) ( 24.4)
           -8.6   -38.2    -6.7     3.3   -33.5   -45.7    11.5   -25.0    24.4    13.6
         -1.933  -8.611  -1.499   0.735  -7.534 -10.303   2.596  -5.639   5.505   3.071
g_O         122     116     105     114     107     124     110     116     103     127
        ( 27.5) ( 26.1) ( 23.6) ( 25.7) ( 24.1) ( 27.9) ( 24.8) ( 26.1) ( 23.2) ( 28.6)
g_E       109.3   109.3   109.3   109.3   109.3   109.3   109.3   109.3   109.3   109.3
        ( 24.6) ( 24.6) ( 24.6) ( 24.6) ( 24.6) ( 24.6) ( 24.6) ( 24.6) ( 24.6) ( 24.6)
           26.8    13.8    -8.4     9.6    -4.6    31.3     1.4    13.8   -12.2    38.1
          6.041   3.109  -1.898   2.162  -1.025   7.048   0.316   3.109  -2.754   8.586
c_O         109     116     114     108     118     128     102     119     108     107
        ( 24.5) ( 26.1) ( 25.7) ( 24.3) ( 26.6) ( 28.8) ( 23.0) ( 26.8) ( 24.3) ( 24.1)
c_E       116.8   116.8   116.8   116.8   116.8   116.8   116.8   116.8   116.8   116.8
        ( 26.3) ( 26.3) ( 26.3) ( 26.3) ( 26.3) ( 26.3) ( 26.3) ( 26.3) ( 26.3) ( 26.3)
          -15.0    -1.6    -5.5   -16.9     2.4    23.5   -27.6     4.5   -16.9   -18.7
         -3.389  -0.355  -1.242  -3.807   0.548   5.284  -6.221   1.005  -3.807  -4.220

N           444     444     444     444     444     444     444     444     444     444
X2          2.2     6.8     1.3     0.9     6.6     9.5     2.8     2.3     2.4     6.0
Gvalue      2.1     7.0     1.3     1.0     6.6    10.0     2.9     2.3     2.4     6.0
Gv/N      0.481   1.582   0.296   0.216   1.496   2.242   0.650   0.529   0.532   1.347
Prob    0.54483 0.07118 0.72545 0.81154 0.08417 0.01895 0.40935 0.50330 0.50085 0.11245
        -------------------------------------------------------------------------------
             20      19      18      17      16      15      14      13      12      11
        -------------------------------------------------------------------------------
a_O         122     132      98     106     133     123     118      97     105     133
        ( 27.5) ( 29.7) ( 22.1) ( 23.9) ( 30.0) ( 27.7) ( 26.6) ( 21.8) ( 23.6) ( 30.0)
a_E       109.5   109.5   109.5   109.5   109.5   109.5   109.5   109.5   109.5   109.5
        ( 24.7) ( 24.7) ( 24.7) ( 24.7) ( 24.7) ( 24.7) ( 24.7) ( 24.7) ( 24.7) ( 24.7)
           26.3    49.3   -21.8    -6.9    51.6    28.5    17.6   -23.6    -8.9    51.6
          5.925  11.095  -4.910  -1.564  11.632   6.426   3.959  -5.308  -1.998  11.632
t_O         109     118     120     110     106     109     109     114     138     114
        ( 24.5) ( 26.6) ( 27.0) ( 24.8) ( 23.9) ( 24.5) ( 24.5) ( 25.7) ( 31.1) ( 25.7)
t_E       108.4   108.4   108.4   108.4   108.4   108.4   108.4   108.4   108.4   108.4
        ( 24.4) ( 24.4) ( 24.4) ( 24.4) ( 24.4) ( 24.4) ( 24.4) ( 24.4) ( 24.4) ( 24.4)
            1.2    20.1    24.4     3.3    -4.7     1.2     1.2    11.5    66.7    11.5
          0.280   4.520   5.505   0.735  -1.060   0.280   0.280   2.596  15.019   2.596
g_O         100      60     107      82      83      88      96      66      89      79
        ( 22.5) ( 13.5) ( 24.1) ( 18.5) ( 18.7) ( 19.8) ( 21.6) ( 14.9) ( 20.0) ( 17.8)
g_E       109.3   109.3   109.3   109.3   109.3   109.3   109.3   109.3   109.3   109.3
        ( 24.6) ( 24.6) ( 24.6) ( 24.6) ( 24.6) ( 24.6) ( 24.6) ( 24.6) ( 24.6) ( 24.6)
          -17.8   -72.0    -4.6   -47.1   -45.7   -38.1   -24.9   -66.6   -36.6   -51.3
         -4.006 -16.210  -1.025 -10.615 -10.291  -8.592  -5.611 -14.997  -8.237 -11.553
c_O         113     134     119     146     122     124     121     167     112     118
        ( 25.5) ( 30.2) ( 26.8) ( 32.9) ( 27.5) ( 27.9) ( 27.3) ( 37.6) ( 25.2) ( 26.6)
c_E       116.8   116.8   116.8   116.8   116.8   116.8   116.8   116.8   116.8   116.8
        ( 26.3) ( 26.3) ( 26.3) ( 26.3) ( 26.3) ( 26.3) ( 26.3) ( 26.3) ( 26.3) ( 26.3)
           -7.5    36.8     4.5    65.2    10.6    14.9     8.6   119.4    -9.4     2.4
         -1.679   8.297   1.005  14.681   2.398   3.346   1.930  26.902  -2.113   0.548

N           444     444     444     444     444     444     444     444     444     444
X2          2.3    30.2     2.5    14.3    11.6     6.3     2.4    40.5    12.2    13.7
Gvalue      2.3    34.2     2.6    14.4    11.9     6.5     2.5    40.8    11.9    14.3
Gv/N      0.521   7.703   0.575   3.237   2.679   1.460   0.559   9.193   2.672   3.223
Prob    0.51035 0.00000 0.46586 0.00244 0.00776 0.09035 0.47881 0.00000 0.00787 0.00251
        -------------------------------------------------------------------------------
             10       9       8       7       6       5       4       3       2       1
        -------------------------------------------------------------------------------
a_O         135     143     119     119     115     112      88     100     108     110
        ( 30.4) ( 32.2) ( 26.8) ( 26.8) ( 25.9) ( 25.2) ( 19.8) ( 22.5) ( 24.3) ( 24.8)
a_E       109.5   109.5   109.5   109.5   109.5   109.5   109.5   109.5   109.5   109.5
        ( 24.7) ( 24.7) ( 24.7) ( 24.7) ( 24.7) ( 24.7) ( 24.7) ( 24.7) ( 24.7) ( 24.7)
           56.5    76.3    19.7    19.7    11.2     5.0   -38.5   -18.2    -3.0     0.9
         12.714  17.176   4.445   4.445   2.524   1.125  -8.676  -4.100  -0.684   0.212
t_O         103     110     109     131     117     117     112      94     108     105
        ( 23.2) ( 24.8) ( 24.5) ( 29.5) ( 26.4) ( 26.4) ( 25.2) ( 21.2) ( 24.3) ( 23.6)
t_E       108.4   108.4   108.4   108.4   108.4   108.4   108.4   108.4   108.4   108.4
        ( 24.4) ( 24.4) ( 24.4) ( 24.4) ( 24.4) ( 24.4) ( 24.4) ( 24.4) ( 24.4) ( 24.4)
          -10.5     3.3     1.2    49.7    17.9    17.9     7.4   -26.8    -0.8    -6.7
         -2.362   0.735   0.280  11.185   4.033   4.033   1.658  -6.027  -0.171  -1.499
g_O          83      84      99      64      81      92     118     130      99      87
        ( 18.7) ( 18.9) ( 22.3) ( 14.4) ( 18.2) ( 20.7) ( 26.6) ( 29.3) ( 22.3) ( 19.6)
g_E       109.3   109.3   109.3   109.3   109.3   109.3   109.3   109.3   109.3   109.3
        ( 24.6) ( 24.6) ( 24.6) ( 24.6) ( 24.6) ( 24.6) ( 24.6) ( 24.6) ( 24.6) ( 24.6)
          -45.7   -44.2   -19.6   -68.5   -48.5   -31.7    18.1    45.1   -19.6   -39.7
        -10.291  -9.962  -4.414 -15.430 -10.933  -7.141   4.071  10.156  -4.414  -8.943
c_O         123     107     117     130     131     123     126     120     129     142
        ( 27.7) ( 24.1) ( 26.4) ( 29.3) ( 29.5) ( 27.7) ( 28.4) ( 27.0) ( 29.1) ( 32.0)
c_E       116.8   116.8   116.8   116.8   116.8   116.8   116.8   116.8   116.8   116.8
        ( 26.3) ( 26.3) ( 26.3) ( 26.3) ( 26.3) ( 26.3) ( 26.3) ( 26.3) ( 26.3) ( 26.3)
           12.7   -18.7     0.4    27.9    30.1    12.7    19.1     6.5    25.7    55.5
          2.870  -4.220   0.095   6.275   6.775   2.870   4.308   1.466   5.778  12.502

N           444     444     444     444     444     444     444     444     444     444
X2         12.8    16.9     1.8    25.8    10.0     3.8     5.8     6.7     2.3    10.1
Gvalue     13.0    16.6     1.8    28.8    10.7     3.9     6.0     6.6     2.3    10.1
Gv/N      2.931   3.729   0.406   6.476   2.400   0.888   1.361   1.494   0.509   2.273
Prob    0.00461 0.00087 0.61434 0.00000 0.01373 0.26766 0.10955 0.08452 0.52029 0.01780
        -------------------------------------------------------------------------------

CDS_INITIAL:
        -------------------------------------------------------------------------------
```

	1	2	3	4	5	6	7	8	9	10
a_O	274	0	0	169	115	104	125	134	107	114
	(61.7)	(0.0)	(0.0)	(38.1)	(25.9)	(23.4)	(28.2)	(30.2)	(24.1)	(25.7)
a_E	102.0	115.5	58.6	102.0	115.5	58.6	102.0	115.5	58.6	102.0
	(23.0)	(26.0)	(13.2)	(23.0)	(26.0)	(13.2)	(23.0)	(26.0)	(13.2)	(23.0)
	541.4	0.0	0.0	170.6	-1.1	119.4	50.8	39.7	128.9	25.3
	121.931	0.000	0.000	38.419	-0.240	26.887	11.435	8.949	29.033	5.699
t_O	106	444	0	54	107	103	77	108	103	79
	(23.9)	(100.0)	(0.0)	(12.2)	(24.1)	(23.2)	(17.3)	(24.3)	(23.2)	(17.8)
t_E	63.4	128.5	95.0	63.4	128.5	95.0	63.4	128.5	95.0	63.4
	(14.3)	(28.9)	(21.4)	(14.3)	(28.9)	(21.4)	(14.3)	(28.9)	(21.4)	(14.3)
	108.9	1101.0	0.0	-17.3	-39.2	16.6	29.9	-37.5	16.6	34.7
	24.533	247.979	0.000	-3.908	-8.825	3.738	6.735	-8.455	3.738	7.822
g_O	64	0	444	111	85	112	120	87	115	129
	(14.4)	(0.0)	(100.0)	(25.0)	(19.1)	(25.2)	(27.0)	(19.6)	(25.9)	(29.1)
g_E	163.4	86.5	136.8	163.4	86.5	136.8	163.4	86.5	136.8	163.4
	(36.8)	(19.5)	(30.8)	(36.8)	(19.5)	(30.8)	(36.8)	(19.5)	(30.8)	(36.8)
	-120.0	0.0	1045.6	-85.8	-3.0	-44.8	-74.0	1.0	-39.9	-60.9
	-27.016	0.000	235.498	-19.323	-0.675	-10.082	-16.676	0.220	-8.983	-13.724
c_O	0	0	0	110	137	125	122	115	119	122
	(0.0)	(0.0)	(0.0)	(24.8)	(30.9)	(28.2)	(27.5)	(25.9)	(26.8)	(27.5)
c_E	115.2	113.5	153.6	115.2	113.5	153.6	115.2	113.5	153.6	115.2
	(25.9)	(25.6)	(34.6)	(25.9)	(25.6)	(34.6)	(25.9)	(25.6)	(34.6)	(25.9)
	0.0	0.0	0.0	-10.2	51.7	-51.5	14.0	3.1	-60.8	14.0
	0.000	0.000	0.000	-2.287	11.639	-11.607	3.153	0.702	-13.686	3.153
N	444	444	444	444	444	444	444	444	444	444
X2	494.1	1090.1	997.3	62.4	8.5	45.7	20.0	6.2	51.9	12.9
Gvalue	530.3	1101.0	1045.6	57.3	8.4	39.7	20.6	6.3	44.9	13.1
Gv/N	119.448	247.979	235.498	12.901	1.898	8.937	4.647	1.416	10.103	2.950
Prob	0.00000	0.00000	0.00000	0.00000	0.03797	0.00000	0.00013	0.09837	0.00000	0.00443

	11	12	13	14	15	16	17	18	19	20
a_O	113	92	145	108	96	126	104	99	129	135
	(25.5)	(20.7)	(32.7)	(24.3)	(21.6)	(28.4)	(23.4)	(22.3)	(29.1)	(30.4)
a_E	115.5	58.6	102.0	115.5	58.6	102.0	115.5	58.6	102.0	115.5
	(26.0)	(13.2)	(23.0)	(26.0)	(13.2)	(23.0)	(26.0)	(13.2)	(23.0)	(26.0)
	-5.0	83.0	101.9	-14.6	94.8	53.2	-21.9	103.9	60.5	42.0
	-1.129	18.704	22.959	-3.281	21.358	11.979	-4.927	23.397	13.632	9.468
t_O	114	107	88	133	121	84	138	103	77	119
	(25.7)	(24.1)	(19.8)	(30.0)	(27.3)	(18.9)	(31.1)	(23.2)	(17.3)	(26.8)
t_E	128.5	95.0	63.4	128.5	95.0	63.4	128.5	95.0	63.4	128.5
	(28.9)	(21.4)	(14.3)	(28.9)	(21.4)	(14.3)	(28.9)	(21.4)	(14.3)	(28.9)
	-27.3	25.4	57.7	9.2	58.5	47.2	19.7	16.6	29.9	-18.3
	-6.148	5.720	12.990	2.062	13.170	10.640	4.434	3.738	6.735	-4.117
g_O	102	123	109	88	104	125	76	101	118	77
	(23.0)	(27.7)	(24.5)	(19.8)	(23.4)	(28.2)	(17.1)	(22.7)	(26.6)	(17.3)
g_E	86.5	136.8	163.4	86.5	136.8	163.4	86.5	136.8	163.4	86.5
	(19.5)	(30.8)	(36.8)	(19.5)	(30.8)	(36.8)	(19.5)	(30.8)	(36.8)	(19.5)
	33.6	-26.1	-88.2	3.0	-57.0	-66.9	-19.7	-61.2	-76.8	-17.9
	7.566	-5.881	-19.868	0.676	-12.833	-15.072	-4.435	-13.795	-17.292	-4.040
c_O	115	122	102	115	123	109	126	141	120	113
	(25.9)	(27.5)	(23.0)	(25.9)	(27.7)	(24.5)	(28.4)	(31.8)	(27.0)	(25.5)
c_E	113.5	153.6	115.2	113.5	153.6	115.2	113.5	153.6	115.2	113.5
	(25.6)	(34.6)	(25.9)	(25.6)	(34.6)	(25.9)	(25.6)	(34.6)	(25.9)	(25.6)
	3.1	-56.2	-24.8	3.1	-54.7	-12.1	26.4	-24.2	9.8	-0.9
	0.702	-12.663	-5.590	0.702	-12.315	-2.715	5.954	-5.442	2.208	-0.203
N	444	444	444	444	444	444	444	444	444	444
X2	4.5	28.5	47.2	0.7	45.0	21.7	4.5	38.9	22.8	5.0
Gvalue	4.4	26.1	46.6	0.7	41.6	21.5	4.5	35.1	23.5	4.9
Gv/N	0.991	5.879	10.491	0.159	9.380	4.831	1.025	7.898	5.283	1.108
Prob	0.22148	0.00001	0.00000	0.87211	0.00000	0.00008	0.20792	0.00000	0.00003	0.17789

	21	22	23	24	25	26	27	28	29	30
a_O	84	126	143	91	98	116	85	121	108	86
	(18.9)	(28.4)	(32.2)	(20.5)	(22.1)	(26.1)	(19.1)	(27.3)	(24.3)	(19.4)
a_E	58.6	102.0	115.5	58.6	102.0	115.5	58.6	102.0	115.5	58.6
	(13.2)	(23.0)	(26.0)	(13.2)	(23.0)	(26.0)	(13.2)	(23.0)	(26.0)	(13.2)
	60.5	53.2	61.0	80.2	-7.9	0.9	63.3	41.3	-14.6	66.0
	13.635	11.979	13.738	18.053	-1.777	0.210	14.251	9.297	-3.281	14.871
t_O	127	78	117	109	76	115	124	86	133	112
	(28.6)	(17.6)	(26.4)	(24.5)	(17.1)	(25.9)	(27.9)	(19.4)	(30.0)	(25.2)
t_E	95.0	63.4	128.5	95.0	63.4	128.5	95.0	63.4	128.5	95.0
	(21.4)	(14.3)	(28.9)	(21.4)	(14.3)	(28.9)	(21.4)	(14.3)	(28.9)	(21.4)
	73.7	32.3	-21.9	29.9	27.5	-25.5	66.0	52.4	9.2	36.8
	16.592	7.276	-4.941	6.736	6.200	-5.750	14.865	11.804	2.062	8.291
g_O	112	125	74	103	133	94	99	135	86	120
	(25.2)	(28.2)	(16.7)	(23.2)	(30.0)	(21.2)	(22.3)	(30.4)	(19.4)	(27.0)
g_E	136.8	163.4	86.5	136.8	163.4	86.5	136.8	163.4	86.5	136.8
	(30.8)	(36.8)	(19.5)	(30.8)	(36.8)	(19.5)	(30.8)	(36.8)	(19.5)	(30.8)
	-44.8	-66.9	-23.1	-58.4	-54.7	15.6	-64.0	-51.5	-1.0	-31.4
	-10.082	-15.072	-5.208	-13.158	-12.321	3.515	-14.414	-11.598	-0.230	-7.073
c_O	121	115	110	141	137	119	136	102	117	126
	(27.3)	(25.9)	(24.8)	(31.8)	(30.9)	(26.8)	(30.6)	(23.0)	(26.4)	(28.4)
c_E	153.6	115.2	113.5	153.6	115.2	113.5	153.6	115.2	113.5	153.6
	(34.6)	(25.9)	(25.6)	(34.6)	(25.9)	(25.6)	(34.6)	(25.9)	(25.6)	(34.6)
	-57.8	-0.4	-6.8	-24.2	47.5	11.4	-33.1	-24.8	7.2	-49.9
	-13.008	-0.089	-1.531	-5.442	10.697	2.559	-7.461	-5.590	1.623	-11.247
N	444	444	444	444	444	444	444	444	444	444
X2	33.2	18.0	9.5	29.4	12.4	2.3	33.2	18.0	0.8	22.9
Gvalue	31.7	18.2	9.1	27.5	12.4	2.4	32.1	17.4	0.8	21.5
Gv/N	7.137	4.094	2.058	6.188	2.799	0.534	7.240	3.913	0.174	4.843
Prob	0.00000	0.00040	0.02752	0.00000	0.00605	0.49930	0.00000	0.00059	0.85638	0.00008

	31	32	33	34	35	36	37	38	39	40
a_O	126	101	84	106	107	71	99	95	66	101
	(28.4)	(22.7)	(18.9)	(23.9)	(24.1)	(16.0)	(22.3)	(21.4)	(14.9)	(22.7)
a_E	102.0	115.5	58.6	102.0	115.5	58.6	102.0	115.5	58.6	102.0
	(23.0)	(26.0)	(13.2)	(23.0)	(26.0)	(13.2)	(23.0)	(26.0)	(13.2)	(23.0)
	53.2	-27.2	60.5	8.1	-16.4	27.3	-6.0	-37.2	15.7	-2.0
	11.979	-6.117	13.635	1.825	-3.699	6.148	-1.342	-8.374	3.544	-0.460
t_O	79	150	118	81	131	114	80	140	115	71
	(17.8)	(33.8)	(26.6)	(18.2)	(29.5)	(25.7)	(18.0)	(31.5)	(25.9)	(16.0)
t_E	63.4	128.5	95.0	63.4	128.5	95.0	63.4	128.5	95.0	63.4
	(14.3)	(28.9)	(21.4)	(14.3)	(28.9)	(21.4)	(14.3)	(28.9)	(21.4)	(14.3)
	34.7	46.4	51.1	39.7	5.0	41.5	37.2	24.0	43.9	16.1

	7.822	10.453	11.509	8.933	1.137	9.348	8.375	5.405	9.883	3.616
g_O	132	89	120	152	94	123	162	106	124	154
	(29.7)	(20.0)	(27.0)	(34.2)	(21.2)	(27.7)	(36.5)	(23.9)	(27.9)	(34.7)
g_E	163.4	86.5	136.8	163.4	86.5	136.8	163.4	86.5	136.8	163.4
	(36.8)	(19.5)	(30.8)	(36.8)	(19.5)	(30.8)	(36.8)	(19.5)	(30.8)	(36.8)
	−56.3	5.0	−31.4	−21.9	15.6	−26.1	−2.7	43.1	−24.3	−18.2
	−12.677	1.136	−7.073	−4.938	3.515	−5.881	−0.613	9.700	−5.477	−4.096
c_O	107	104	122	105	112	136	103	103	139	118
	(24.1)	(23.4)	(27.5)	(23.6)	(25.2)	(30.6)	(23.2)	(23.2)	(31.3)	(26.6)
c_E	115.2	113.5	153.6	115.2	113.5	153.6	115.2	113.5	153.6	115.2
	(25.9)	(25.6)	(34.6)	(25.9)	(25.6)	(34.6)	(25.9)	(25.6)	(34.6)	(25.9)
	−15.8	−18.1	−56.2	−19.5	−2.9	−33.1	−23.1	−19.9	−27.8	5.7
	−3.558	−4.075	−12.663	−4.384	−0.650	−7.461	−5.192	−4.484	−6.260	1.278
N	444	444	444	444	444	444	444	444	444	444
X2	16.1	6.3	25.1	6.7	1.3	9.8	5.7	10.0	7.7	1.5
Gvalue	15.8	6.2	24.0	6.4	1.3	9.6	5.4	10.0	7.5	1.5
Gv/N	3.567	1.397	5.409	1.436	0.303	2.153	1.227	2.246	1.690	0.338
Prob	0.00122	0.10212	0.00002	0.09472	0.71879	0.02270	0.14189	0.01879	0.05751	0.68231

	41	42	43	44	45	46	47	48	49	50
a_O	106	69	94	101	75	108	103	81	106	104
	(23.9)	(15.5)	(21.2)	(22.7)	(16.9)	(24.3)	(23.2)	(18.2)	(23.9)	(23.4)
a_E	115.5	58.6	102.0	115.5	58.6	102.0	115.5	58.6	102.0	115.5
	(26.0)	(13.2)	(23.0)	(26.0)	(13.2)	(23.0)	(26.0)	(13.2)	(23.0)	(26.0)
	−18.3	22.6	−15.4	−27.2	37.1	12.3	−23.7	52.5	8.1	−21.9
	−4.113	5.086	−3.469	−6.117	8.346	2.769	−5.328	11.821	1.825	−4.927
t_O	140	119	91	128	107	63	139	114	75	116
	(31.5)	(26.8)	(20.5)	(28.8)	(24.1)	(14.2)	(31.3)	(25.7)	(16.9)	(26.1)
t_E	128.5	95.0	63.4	128.5	95.0	63.4	128.5	95.0	63.4	128.5
	(28.9)	(21.4)	(14.3)	(28.9)	(21.4)	(14.3)	(28.9)	(21.4)	(14.3)	(28.9)
	24.0	53.5	65.7	−1.0	25.4	−0.8	21.8	41.5	25.2	−23.7
	5.405	12.059	14.807	−0.225	5.720	−0.184	4.918	9.348	5.671	−5.348
g_O	78	126	153	97	133	136	96	103	138	99
	(17.6)	(28.4)	(34.5)	(21.8)	(30.0)	(30.6)	(21.6)	(23.2)	(31.1)	(22.3)
g_E	86.5	136.8	163.4	86.5	136.8	163.4	86.5	136.8	163.4	86.5
	(19.5)	(30.8)	(36.8)	(19.5)	(30.8)	(36.8)	(19.5)	(30.8)	(36.8)	(19.5)
	−16.2	−20.7	−20.1	22.2	−7.4	−49.9	20.0	−58.4	−46.6	26.7
	−3.639	−4.657	−4.518	4.999	−1.677	−11.232	4.500	−13.158	−10.490	6.013
c_O	120	130	106	118	129	137	106	146	125	125
	(27.0)	(29.3)	(23.9)	(26.6)	(29.1)	(30.9)	(23.9)	(32.9)	(28.2)	(28.2)
c_E	113.5	153.6	115.2	113.5	153.6	115.2	113.5	153.6	115.2	113.5
	(25.6)	(34.6)	(25.9)	(25.6)	(34.6)	(25.9)	(25.6)	(34.6)	(25.9)	(25.6)
	13.5	−43.4	−17.6	9.3	−45.1	47.5	−14.4	−14.8	20.4	24.2
	3.033	−9.774	−3.973	2.089	−10.148	10.697	−3.244	−3.344	4.598	5.458
N	444	444	444	444	444	444	444	444	444	444
X2	3.0	12.4	14.0	3.3	10.2	9.1	3.7	21.1	7.0	5.3
Gvalue	3.0	12.1	12.6	3.3	9.9	9.1	3.8	20.7	7.1	5.3
Gv/N	0.686	2.714	2.847	0.747	2.241	2.049	0.845	4.667	1.605	1.196
Prob	0.38449	0.00721	0.00548	0.34554	0.01900	0.02801	0.28947	0.00012	0.06804	0.15056

	51	52	53	54	55	56	57	58	59	60
a_O	97	116	114	84	95	111	75	87	91	82
	(21.8)	(26.1)	(25.7)	(18.9)	(21.4)	(25.0)	(16.9)	(19.6)	(20.5)	(18.5)
a_E	58.6	102.0	115.5	58.6	102.0	115.5	58.6	102.0	115.5	58.6
	(13.2)	(23.0)	(26.0)	(13.2)	(23.0)	(26.0)	(13.2)	(23.0)	(26.0)	(13.2)
	97.8	29.8	−3.0	60.5	−13.6	−8.9	37.1	−27.7	−43.4	55.1
	22.033	6.708	−0.687	13.635	−3.053	−2.002	8.346	−6.243	−9.785	12.421
t_O	99	67	121	104	85	130	110	92	115	99
	(22.3)	(15.1)	(27.3)	(23.4)	(19.1)	(29.3)	(24.8)	(20.7)	(25.9)	(22.3)
t_E	95.0	63.4	128.5	95.0	63.4	128.5	95.0	63.4	128.5	95.0
	(21.4)	(14.3)	(28.9)	(21.4)	(14.3)	(28.9)	(21.4)	(14.3)	(28.9)	(21.4)
	8.1	7.4	−14.6	18.8	49.8	3.0	32.2	68.5	−25.5	8.1
	1.827	1.662	−3.278	4.227	11.219	0.679	7.250	15.423	−5.750	1.827
g_O	118	140	88	130	153	89	123	150	104	134
	(26.6)	(31.5)	(19.8)	(29.3)	(34.5)	(20.0)	(27.7)	(33.8)	(23.4)	(30.2)
g_E	136.8	163.4	86.5	136.8	163.4	86.5	136.8	163.4	86.5	136.8
	(30.8)	(36.8)	(19.5)	(30.8)	(36.8)	(19.5)	(30.8)	(36.8)	(19.5)	(30.8)
	−34.8	−43.2	3.0	−13.2	−20.1	5.0	−26.1	−25.6	38.3	−5.5
	−7.848	−9.734	0.676	−2.975	−4.518	1.136	−5.881	−5.768	8.624	−1.237
c_O	130	121	121	126	111	114	136	115	134	129
	(29.3)	(27.3)	(27.3)	(28.4)	(25.0)	(25.7)	(30.6)	(25.9)	(30.2)	(29.1)
c_E	153.6	115.2	113.5	153.6	115.2	113.5	153.6	115.2	113.5	153.6
	(34.6)	(25.9)	(25.6)	(34.6)	(25.9)	(25.6)	(34.6)	(25.9)	(25.6)	(34.6)
	−43.4	11.9	15.6	−49.9	−8.2	1.1	−33.1	−0.4	44.6	−45.1
	−9.774	2.679	3.511	−11.247	−1.856	0.247	−7.461	−0.089	10.048	−10.148
N	444	444	444	444	444	444	444	444	444	444
X2	31.6	5.8	1.0	17.2	8.6	0.3	10.4	16.2	13.9	13.5
Gvalue	27.7	5.8	1.0	16.2	8.0	0.3	10.0	14.8	13.9	12.7
Gv/N	6.237	1.314	0.222	3.640	1.792	0.061	2.253	3.323	3.137	2.862
Prob	0.00000	0.12000	0.80517	0.00105	0.04691	0.96534	0.01853	0.00204	0.00300	0.00531

	61	62	63	64	65	66	67	68	69	70
a_O	92	107	81	106	102	88	112	115	57	111
	(20.7)	(24.1)	(18.2)	(23.9)	(23.0)	(19.8)	(25.2)	(25.9)	(12.8)	(25.0)
a_E	102.0	115.5	58.6	102.0	115.5	58.6	102.0	115.5	58.6	102.0
	(23.0)	(26.0)	(13.2)	(23.0)	(26.0)	(13.2)	(23.0)	(26.0)	(13.2)	(23.0)
	−19.0	−16.4	52.5	8.1	−25.4	71.6	20.9	−1.1	−3.1	18.7
	−4.286	−3.699	11.821	1.825	−5.725	16.129	4.706	−0.240	−0.704	4.215
t_O	77	135	98	77	115	96	85	125	109	78
	(17.3)	(30.4)	(22.1)	(17.3)	(25.9)	(21.6)	(19.1)	(28.2)	(24.5)	(17.6)
t_E	63.4	128.5	95.0	63.4	128.5	95.0	63.4	128.5	95.0	63.4
	(14.3)	(28.9)	(21.4)	(14.3)	(28.9)	(21.4)	(14.3)	(28.9)	(21.4)	(14.3)
	29.9	13.3	6.0	29.9	−25.5	2.0	49.8	−6.9	29.9	32.3
	6.735	3.001	1.360	6.735	−5.750	0.441	11.219	−1.555	6.736	7.276
g_O	147	86	139	147	87	132	125	87	131	151
	(33.1)	(19.4)	(31.3)	(33.1)	(19.6)	(29.7)	(28.2)	(19.6)	(29.5)	(34.0)
g_E	163.4	86.5	136.8	163.4	86.5	136.8	163.4	86.5	136.8	163.4
	(36.8)	(19.5)	(30.8)	(36.8)	(19.5)	(30.8)	(36.8)	(19.5)	(30.8)	(36.8)
	−31.0	−1.0	4.5	−31.0	1.0	−9.4	−66.9	1.0	−11.3	−23.8
	−6.990	−0.230	1.010	−6.990	0.220	−2.113	−15.072	0.220	−2.546	−5.354
c_O	128	116	126	114	140	128	122	117	147	104
	(28.8)	(26.1)	(28.4)	(25.7)	(31.5)	(28.8)	(27.5)	(26.4)	(33.1)	(23.4)
c_E	115.2	113.5	153.6	115.2	113.5	153.6	115.2	113.5	153.6	115.2
	(25.9)	(25.6)	(34.6)	(25.9)	(25.6)	(34.6)	(25.9)	(25.6)	(34.6)	(25.9)
	27.0	5.2	−49.9	−2.4	58.9	−46.7	14.0	7.2	−12.9	−21.3
	6.076	1.160	−11.247	−0.536	13.260	−10.518	3.153	1.623	−2.915	−4.790

N	444	444	444	444	444	444	444	444	444	444
X2	7.0	1.0	13.7	4.7	9.2	19.2	17.7	0.2	2.6	6.2
Gvalue	6.8	1.0	13.1	4.6	8.9	17.5	17.8	0.2	2.5	6.0
Gv/N	1.535	0.232	2.945	1.033	2.005	3.939	4.006	0.048	0.572	1.347
Prob	0.07808	0.79404	0.00448	0.20467	0.03062	0.00056	0.00049	0.97575	0.46803	0.11264

	71	72	73	74	75	76	77	78	79	80
a_O	114	79	99	92	62	100	116	85	106	107
	(25.7)	(17.8)	(22.3)	(20.7)	(14.0)	(22.5)	(26.1)	(19.1)	(23.9)	(24.1)
a_E	115.5	58.6	102.0	115.5	58.6	102.0	115.5	58.6	102.0	115.5
	(26.0)	(13.2)	(23.0)	(26.0)	(13.2)	(23.0)	(26.0)	(13.2)	(23.0)	(26.0)
	−3.0	47.2	−6.0	−41.9	7.0	−4.0	0.9	63.3	8.1	−16.4
	−0.687	10.640	−1.342	−9.440	1.583	−0.903	0.210	14.251	1.825	−3.699
t_O	140	99	73	110	104	69	128	100	96	127
	(31.5)	(22.3)	(16.4)	(24.8)	(23.4)	(15.5)	(28.8)	(22.5)	(21.6)	(28.6)
t_E	128.5	95.0	63.4	128.5	95.0	63.4	128.5	95.0	63.4	128.5
	(28.9)	(21.4)	(14.3)	(28.9)	(21.4)	(14.3)	(28.9)	(21.4)	(14.3)	(28.9)
	24.0	8.1	20.6	−34.2	18.8	11.7	−1.0	10.2	79.6	−3.0
	5.405	1.827	4.631	−7.703	4.227	2.626	−0.225	2.298	17.934	−0.672
g_O	84	128	148	111	132	160	97	137	130	81
	(18.9)	(28.8)	(33.3)	(25.0)	(29.7)	(36.0)	(21.8)	(30.9)	(29.3)	(18.2)
g_E	86.5	136.8	163.4	86.5	136.8	163.4	86.5	136.8	163.4	86.5
	(19.5)	(30.8)	(36.8)	(19.5)	(30.8)	(36.8)	(19.5)	(30.8)	(36.8)	(19.5)
	−5.0	−17.0	−29.2	55.3	−9.4	−6.7	22.2	0.5	−59.4	−10.7
	−1.115	−3.823	−6.586	12.462	−2.113	−1.501	4.999	0.101	−13.379	−2.402
c_O	106	138	124	131	146	115	103	122	112	129
	(23.9)	(31.1)	(27.9)	(29.5)	(32.9)	(25.9)	(23.2)	(27.5)	(25.2)	(29.1)
c_E	113.5	153.6	115.2	113.5	153.6	115.2	113.5	153.6	115.2	113.5
	(25.6)	(34.6)	(25.9)	(25.6)	(34.6)	(25.9)	(25.6)	(34.6)	(25.9)	(25.6)
	−14.4	−29.6	18.3	37.7	−14.8	−0.4	−19.9	−56.2	−6.3	33.1
	−3.244	−6.663	4.113	8.486	−3.344	−0.089	−4.484	−12.663	−1.420	7.463
N	444	444	444	444	444	444	444	444	444	444
X2	1.6	9.4	3.7	17.1	1.6	0.6	2.2	18.7	23.8	3.1
Gvalue	1.6	8.8	3.6	16.9	1.6	0.6	2.2	17.7	22.0	3.1
Gv/N	0.359	1.980	0.816	3.806	0.354	0.133	0.500	3.987	4.960	0.690
Prob	0.66064	0.03220	0.30527	0.00074	0.66623	0.89883	0.52790	0.00051	0.00006	0.38205

	81	82	83	84	85	86	87	88	89	90
a_O	67	95	108	62	105	119	69	112	92	69
	(15.1)	(21.4)	(24.3)	(14.0)	(23.6)	(26.8)	(15.5)	(25.2)	(20.7)	(15.5)
a_E	58.6	102.0	115.5	58.6	102.0	115.5	58.6	102.0	115.5	58.6
	(13.2)	(23.0)	(26.0)	(13.2)	(23.0)	(26.0)	(13.2)	(23.0)	(26.0)	(13.2)
	18.0	−13.6	−14.6	7.0	6.0	7.0	22.6	20.9	−41.9	22.6
	4.051	−3.053	−3.281	1.583	1.359	1.584	5.086	4.706	−9.440	5.086
t_O	112	85	142	96	77	109	98	71	137	89
	(25.2)	(19.1)	(32.0)	(21.6)	(17.3)	(24.5)	(22.1)	(16.0)	(30.9)	(20.0)
t_E	95.0	63.4	128.5	95.0	63.4	128.5	95.0	63.4	128.5	95.0
	(21.4)	(14.3)	(28.9)	(21.4)	(14.3)	(28.9)	(21.4)	(14.3)	(28.9)	(21.4)
	36.8	49.8	28.4	2.0	29.9	−35.9	6.0	16.1	17.5	−11.7
	8.291	11.219	6.390	0.441	6.735	−8.081	1.360	3.616	3.953	−2.627
g_O	127	139	85	148	154	102	139	137	87	140
	(28.6)	(31.3)	(19.1)	(33.3)	(34.7)	(23.0)	(31.3)	(30.9)	(19.6)	(31.5)
g_E	136.8	163.4	86.5	136.8	163.4	86.5	136.8	163.4	86.5	136.8
	(30.8)	(36.8)	(19.5)	(30.8)	(36.8)	(19.5)	(30.8)	(36.8)	(19.5)	(30.8)
	−18.8	−44.9	−3.0	23.3	−18.2	33.6	4.5	−48.2	1.0	6.5
	−4.242	−10.114	−0.675	5.258	−4.096	7.566	1.010	−10.862	0.220	1.470
c_O	138	125	109	138	108	114	138	124	128	146
	(31.1)	(28.2)	(24.5)	(31.1)	(24.3)	(25.7)	(31.1)	(27.9)	(28.8)	(32.9)
c_E	153.6	115.2	113.5	153.6	115.2	113.5	153.6	115.2	113.5	153.6
	(34.6)	(25.9)	(25.6)	(34.6)	(25.9)	(25.6)	(34.6)	(25.9)	(25.6)	(34.6)
	−29.6	20.4	−8.7	−29.6	−13.9	1.1	−29.6	18.3	30.9	−14.8
	−6.663	4.598	−1.966	−6.663	−3.138	0.247	−6.663	4.113	6.956	−3.344
N	444	444	444	444	444	444	444	444	444	444
X2	6.5	12.3	2.1	2.7	4.0	5.8	3.6	6.8	7.2	2.7
Gvalue	6.4	11.8	2.1	2.7	3.8	5.8	3.5	7.0	7.5	2.6
Gv/N	1.437	2.651	0.468	0.619	0.860	1.317	0.794	1.572	1.689	0.586
Prob	0.09449	0.00821	0.55656	0.43240	0.28192	0.11928	0.31777	0.07253	0.05753	0.45731

	91	92	93	94	95	96	97	98	99	100
a_O	110	114	74	99	106	79	93	118	71	115
	(24.8)	(25.7)	(16.7)	(22.6)	(24.1)	(18.0)	(21.4)	(27.2)	(16.4)	(26.7)
a_E	102.0	115.5	58.6	100.9	114.2	57.9	99.7	112.9	57.3	99.0
	(23.0)	(26.0)	(13.2)	(23.0)	(26.0)	(13.2)	(23.0)	(26.0)	(13.2)	(23.0)
	16.6	−3.0	34.6	−3.7	−15.9	49.0	−13.0	10.4	30.5	34.4
	3.729	−0.687	7.787	−0.847	−3.613	11.169	−2.993	2.387	7.035	7.974
t_O	79	125	112	88	124	107	87	121	102	64
	(17.8)	(28.2)	(25.2)	(20.0)	(28.2)	(24.4)	(20.0)	(27.9)	(23.5)	(14.8)
t_E	63.4	128.5	95.0	62.7	127.1	94.0	62.0	125.6	92.9	61.6
	(14.3)	(28.9)	(21.4)	(14.3)	(28.9)	(21.4)	(14.3)	(28.9)	(21.4)	(14.3)
	34.7	−6.9	36.8	59.7	−6.0	27.8	59.0	−9.0	19.1	5.0
	7.822	−1.555	8.291	13.592	−1.374	6.337	13.594	−2.083	4.399	1.157
g_O	138	92	120	137	96	122	127	83	123	163
	(31.1)	(20.7)	(27.0)	(31.2)	(21.9)	(27.8)	(29.3)	(19.1)	(28.3)	(37.8)
g_E	163.4	86.5	136.8	161.5	85.5	135.2	159.7	84.6	133.7	158.6
	(36.8)	(19.5)	(30.8)	(36.8)	(19.5)	(30.8)	(36.8)	(19.5)	(30.8)	(36.8)
	−46.6	11.3	−31.4	−45.1	22.2	−25.1	−58.2	−3.1	−20.5	9.0
	−10.490	2.549	−7.073	−10.279	5.046	−5.724	−13.404	−0.714	−4.726	2.078
c_O	117	113	138	115	113	131	127	112	138	89
	(26.4)	(25.5)	(31.1)	(26.2)	(25.7)	(29.8)	(29.3)	(25.8)	(31.8)	(20.6)
c_E	115.2	113.5	153.6	113.9	112.2	151.9	112.6	110.9	150.2	111.8
	(25.9)	(25.6)	(34.6)	(25.9)	(25.6)	(34.6)	(25.9)	(25.6)	(34.6)	(25.9)
	3.6	−0.9	−29.6	2.2	1.7	−38.8	30.6	2.2	−23.3	−40.6
	0.818	−0.203	−6.663	0.504	0.377	−8.828	7.042	0.511	−5.368	−9.428
N	444	444	444	439	439	439	434	434	434	431
X2	8.4	0.5	10.7	14.0	2.0	13.6	19.1	0.4	6.0	7.5
Gvalue	8.3	0.5	10.4	13.0	1.9	13.0	18.4	0.4	5.8	7.7
Gv/N	1.880	0.103	2.342	2.970	0.437	2.953	4.238	0.100	1.340	1.781
Prob	0.03933	0.92771	0.01547	0.00456	0.58948	0.00472	0.00036	0.93290	0.12090	0.05323

5UTR:

	100	99	98	97	96	95	94	93	92	91
a_O	230	230	227	223	231	229	234	232	206	260
	(17.1)	(17.1)	(16.9)	(16.6)	(17.2)	(17.0)	(17.4)	(17.2)	(15.3)	(19.3)
a_E	238.3	238.3	238.3	238.3	238.3	238.3	238.3	238.3	238.3	238.3
	(17.7)	(17.7)	(17.7)	(17.7)	(17.7)	(17.7)	(17.7)	(17.7)	(17.7)	(17.7)
	-16.2	-16.2	-22.0	-29.5	-14.3	-18.2	-8.4	-12.4	-59.9	45.4
	-1.207	-1.207	-1.634	-2.195	-1.063	-1.350	-0.628	-0.919	-4.457	3.376
t_O	247	246	226	245	219	201	247	210	211	218
	(18.4)	(18.3)	(16.8)	(18.2)	(16.3)	(14.9)	(18.4)	(15.6)	(15.7)	(16.2)
t_E	238.3	238.3	238.3	238.3	238.3	238.3	238.3	238.3	238.3	238.3
	(17.7)	(17.7)	(17.7)	(17.7)	(17.7)	(17.7)	(17.7)	(17.7)	(17.7)	(17.7)
	17.7	15.6	-24.0	13.6	-37.0	-68.4	17.7	-53.1	-51.4	-38.8
	1.315	1.162	-1.782	1.009	-2.752	-5.089	1.315	-3.949	-3.819	-2.888
g_O	436	418	452	422	489	453	420	449	433	435
	(32.4)	(31.1)	(33.6)	(31.4)	(36.4)	(33.7)	(31.2)	(33.4)	(32.2)	(32.3)
g_E	408.8	408.8	408.8	408.8	408.8	408.8	408.8	408.8	408.8	408.8
	(30.4)	(30.4)	(30.4)	(30.4)	(30.4)	(30.4)	(30.4)	(30.4)	(30.4)	(30.4)
	56.3	18.7	90.9	26.9	175.3	93.1	22.8	84.3	49.9	54.1
	4.183	1.389	6.758	2.000	13.033	6.922	1.694	6.269	3.709	4.024
c_O	432	451	440	455	406	462	444	454	495	432
	(32.1)	(33.5)	(32.7)	(33.8)	(30.2)	(34.3)	(33.0)	(33.8)	(36.8)	(32.1)
c_E	459.7	459.7	459.7	459.7	459.7	459.7	459.7	459.7	459.7	459.7
	(34.2)	(34.2)	(34.2)	(34.2)	(34.2)	(34.2)	(34.2)	(34.2)	(34.2)	(34.2)
	-53.6	-17.2	-38.5	-9.3	-100.8	4.7	-30.8	-11.3	73.3	-53.6
	-3.988	-1.277	-2.861	-0.691	-7.495	0.347	-2.290	-0.838	5.450	-3.988
N	1345	1345	1345	1345	1345	1345	1345	1345	1345	1345
X2	4.1	0.9	6.6	1.6	23.8	11.0	1.2	7.6	11.7	7.1
Gvalue	4.1	0.9	6.5	1.7	23.2	11.2	1.2	7.6	11.9	7.1
Gv/N	0.303	0.067	0.480	0.123	1.723	0.831	0.092	0.563	0.884	0.525
Prob	0.25304	0.82371	0.09115	0.64656	0.00004	0.01083	0.74430	0.05558	0.00775	0.07008

	90	89	88	87	86	85	84	83	82	81
a_O	220	234	235	220	229	249	223	223	250	210
	(16.4)	(17.4)	(17.5)	(16.4)	(17.0)	(18.5)	(16.6)	(16.6)	(18.6)	(15.6)
a_E	238.3	238.3	238.3	238.3	238.3	238.3	238.3	238.3	238.3	238.3
	(17.7)	(17.7)	(17.7)	(17.7)	(17.7)	(17.7)	(17.7)	(17.7)	(17.7)	(17.7)
	-35.1	-8.4	-6.5	-35.1	-18.2	22.0	-29.5	-29.5	24.0	-53.0
	-2.608	-0.628	-0.481	-2.608	-1.350	1.632	-2.195	-2.195	1.788	-3.943
t_O	237	225	242	245	219	261	224	220	242	231
	(17.6)	(16.7)	(18.0)	(18.2)	(16.3)	(19.4)	(16.7)	(16.4)	(18.0)	(17.2)
t_E	238.3	238.3	238.3	238.3	238.3	238.3	238.3	238.3	238.3	238.3
	(17.7)	(17.7)	(17.7)	(17.7)	(17.7)	(17.7)	(17.7)	(17.7)	(17.7)	(17.7)
	-2.6	-25.9	7.4	13.6	-37.0	47.5	-27.7	-35.2	7.4	-14.4
	-0.194	-1.923	0.553	1.009	-2.752	3.530	-2.063	-2.615	0.553	-1.070
g_O	457	406	438	412	455	431	459	454	430	452
	(34.0)	(30.2)	(32.6)	(30.6)	(33.8)	(32.0)	(34.1)	(33.8)	(32.0)	(33.6)
g_E	408.8	408.8	408.8	408.8	408.8	408.8	408.8	408.8	408.8	408.8
	(30.4)	(30.4)	(30.4)	(30.4)	(30.4)	(30.4)	(30.4)	(30.4)	(30.4)	(30.4)
	102.0	-5.5	60.5	6.5	97.5	45.7	106.4	95.3	43.6	90.9
	7.581	-0.409	4.500	0.484	7.251	3.395	7.912	7.086	3.239	6.758
c_O	431	480	430	468	442	404	439	448	423	452
	(32.0)	(35.7)	(32.0)	(34.8)	(32.9)	(30.0)	(32.6)	(33.3)	(31.4)	(33.6)
c_E	459.7	459.7	459.7	459.7	459.7	459.7	459.7	459.7	459.7	459.7
	(34.2)	(34.2)	(34.2)	(34.2)	(34.2)	(34.2)	(34.2)	(34.2)	(34.2)	(34.2)
	-55.5	41.5	-57.4	16.8	-34.7	-104.3	-40.4	-23.0	-70.3	-15.2
	-4.127	3.089	-4.266	1.250	-2.576	-7.755	-3.003	-1.713	-5.229	-1.131
N	1345	1345	1345	1345	1345	1345	1345	1345	1345	1345
X2	8.9	1.7	4.1	1.8	7.8	10.6	8.9	7.7	4.7	8.3
Gvalue	8.8	1.7	4.1	1.8	7.7	10.8	8.8	7.6	4.7	8.3
Gv/N	0.651	0.129	0.305	0.134	0.573	0.802	0.651	0.563	0.351	0.615
Prob	0.03278	0.62828	0.25058	0.61529	0.05251	0.01290	0.03272	0.05569	0.19369	0.04077

	80	79	78	77	76	75	74	73	72	71
a_O	234	247	222	211	246	240	224	256	235	253
	(17.4)	(18.4)	(16.5)	(15.7)	(18.3)	(17.8)	(16.7)	(19.0)	(17.5)	(18.8)
a_E	238.3	238.3	238.3	238.3	238.3	238.3	238.3	238.3	238.3	238.3
	(17.7)	(17.7)	(17.7)	(17.7)	(17.7)	(17.7)	(17.7)	(17.7)	(17.7)	(17.7)
	-8.4	17.8	-31.4	-51.3	15.7	3.5	-27.6	36.8	-6.5	30.4
	-0.628	1.323	-2.333	-3.812	1.169	0.260	-2.056	2.734	-0.481	2.258
t_O	200	233	254	200	243	241	226	209	220	201
	(14.9)	(17.3)	(18.9)	(14.9)	(18.1)	(17.9)	(16.8)	(15.5)	(16.4)	(14.9)
t_E	238.3	238.3	238.3	238.3	238.3	238.3	238.3	238.3	238.3	238.3
	(17.7)	(17.7)	(17.7)	(17.7)	(17.7)	(17.7)	(17.7)	(17.7)	(17.7)	(17.7)
	-70.1	-10.5	32.4	-70.1	9.5	5.4	-24.0	-54.9	-35.2	-68.4
	-5.212	-0.781	2.408	-5.212	0.704	0.402	-1.782	-4.079	-2.615	-5.089
g_O	450	420	437	438	436	410	434	423	438	441
	(33.5)	(31.2)	(32.5)	(32.6)	(32.4)	(30.5)	(32.3)	(31.4)	(32.6)	(32.8)
g_E	408.8	408.8	408.8	408.8	408.8	408.8	408.8	408.8	408.8	408.8
	(30.4)	(30.4)	(30.4)	(30.4)	(30.4)	(30.4)	(30.4)	(30.4)	(30.4)	(30.4)
	86.5	22.8	58.4	60.5	56.3	2.5	52.0	29.0	60.5	67.0
	6.432	1.694	4.341	4.500	4.183	0.185	3.867	2.154	4.500	4.978
c_O	461	445	432	496	420	454	461	457	452	450
	(34.3)	(33.1)	(32.1)	(36.9)	(31.2)	(33.8)	(34.3)	(34.0)	(33.6)	(33.5)
c_E	459.7	459.7	459.7	459.7	459.7	459.7	459.7	459.7	459.7	459.7
	(34.2)	(34.2)	(34.2)	(34.2)	(34.2)	(34.2)	(34.2)	(34.2)	(34.2)	(34.2)
	2.7	-28.9	-53.6	75.5	-75.8	-11.3	2.7	-5.3	-15.2	-19.1
	0.198	-2.146	-3.988	5.610	-5.637	-0.838	0.198	-0.396	-1.131	-1.423
N	1345	1345	1345	1345	1345	1345	1345	1345	1345	1345
X2	10.4	1.2	5.8	14.2	5.6	0.1	3.1	5.4	3.7	9.5
Gvalue	10.6	1.2	5.8	14.6	5.6	0.1	3.0	5.6	3.7	9.7
Gv/N	0.790	0.090	0.428	1.086	0.420	0.009	0.227	0.413	0.272	0.725
Prob	0.01393	0.74929	0.12418	0.00219	0.13032	0.98973	0.38407	0.13523	0.30044	0.02082

	70	69	68	67	66	65	64	63	62	61
a_O	230	223	212	229	228	250	253	229	236	214
	(17.1)	(16.6)	(15.8)	(17.0)	(17.0)	(18.6)	(18.8)	(17.0)	(17.5)	(15.9)
a_E	238.3	238.3	238.3	238.3	238.3	238.3	238.3	238.3	238.3	238.3
	(17.7)	(17.7)	(17.7)	(17.7)	(17.7)	(17.7)	(17.7)	(17.7)	(17.7)	(17.7)
	-16.2	-29.5	-49.5	-18.2	-20.1	24.0	30.4	-18.2	-4.5	-46.0
	-1.207	-2.195	-3.681	-1.350	-1.492	1.788	2.258	-1.350	-0.334	-3.417
t_O	232	232	208	236	218	233	239	237	223	252
	(17.2)	(17.2)	(15.5)	(17.5)	(16.2)	(17.3)	(17.8)	(17.6)	(16.6)	(18.7)
t_E	238.3	238.3	238.3	238.3	238.3	238.3	238.3	238.3	238.3	238.3

	(17.7)	(17.7)	(17.7)	(17.7)	(17.7)	(17.7)	(17.7)	(17.7)	(17.7)	(17.7)
	-12.5	-12.5	-56.6	-4.6	-38.8	-10.5	1.4	-2.6	-29.6	28.2
	-0.926	-0.926	-4.207	-0.342	-2.888	-0.781	0.103	-0.194	-2.202	2.093
g_O	464	437	451	429	431	417	419	418	429	460
	(34.5)	(32.5)	(33.5)	(31.9)	(32.0)	(31.0)	(31.2)	(31.1)	(31.9)	(34.2)
g_E	408.8	408.8	408.8	408.8	408.8	408.8	408.8	408.8	408.8	408.8
	(30.4)	(30.4)	(30.4)	(30.4)	(30.4)	(30.4)	(30.4)	(30.4)	(30.4)	(30.4)
	117.6	58.4	88.7	41.5	45.7	16.6	20.7	18.7	41.5	108.7
	8.746	4.341	6.595	3.083	3.395	1.238	1.542	1.389	3.083	8.078
c_O	419	453	474	451	468	445	434	461	457	419
	(31.2)	(33.7)	(35.2)	(33.5)	(34.8)	(33.1)	(32.3)	(34.3)	(34.0)	(31.2)
c_E	459.7	459.7	459.7	459.7	459.7	459.7	459.7	459.7	459.7	459.7
	(34.2)	(34.2)	(34.2)	(34.2)	(34.2)	(34.2)	(34.2)	(34.2)	(34.2)	(34.2)
	-77.6	-13.2	29.1	-17.2	16.8	-28.9	-49.9	2.7	-5.3	-77.6
	-5.772	-0.985	2.164	-1.277	1.250	-2.146	-3.708	0.198	-0.396	-5.772
N	1345	1345	1345	1345	1345	1345	1345	1345	1345	1345
X2	11.5	3.2	11.6	1.5	3.5	1.3	2.6	0.6	2.0	13.3
Gvalue	11.3	3.2	11.7	1.5	3.6	1.3	2.6	0.6	2.0	13.2
Gv/N	0.841	0.236	0.870	0.114	0.265	0.099	0.194	0.043	0.151	0.982
Prob	0.01013	0.36581	0.00848	0.67359	0.31188	0.72281	0.45556	0.90031	0.56653	0.00420

	60	59	58	57	56	55	54	53	52	51
a_O	208	250	243	223	248	268	213	258	249	225
	(15.5)	(18.6)	(18.1)	(16.6)	(18.4)	(19.9)	(15.8)	(19.2)	(18.5)	(16.7)
a_E	238.3	238.3	238.3	238.3	238.3	238.3	238.3	238.3	238.3	238.3
	(17.7)	(17.7)	(17.7)	(17.7)	(17.7)	(17.7)	(17.7)	(17.7)	(17.7)	(17.7)
	-56.5	24.0	9.6	-29.5	19.9	63.0	-47.7	41.1	22.0	-25.8
	-4.201	1.788	0.712	-2.195	1.478	4.687	-3.550	3.054	1.632	-1.916
t_O	228	227	246	222	213	228	246	195	233	249
	(17.0)	(16.9)	(18.3)	(16.5)	(15.8)	(17.0)	(18.3)	(14.5)	(17.3)	(18.5)
t_E	238.3	238.3	238.3	238.3	238.3	238.3	238.3	238.3	238.3	238.3
	(17.7)	(17.7)	(17.7)	(17.7)	(17.7)	(17.7)	(17.7)	(17.7)	(17.7)	(17.7)
	-20.2	-22.1	15.6	-31.5	-47.8	-20.2	15.6	-78.2	-10.5	21.9
	-1.499	-1.641	1.162	-2.340	-3.556	-1.499	1.162	-5.816	-0.781	1.625
g_O	445	421	405	442	435	420	431	454	422	423
	(33.1)	(31.3)	(30.1)	(32.9)	(32.3)	(31.2)	(32.0)	(33.8)	(31.4)	(31.4)
g_E	408.8	408.8	408.8	408.8	408.8	408.8	408.8	408.8	408.8	408.8
	(30.4)	(30.4)	(30.4)	(30.4)	(30.4)	(30.4)	(30.4)	(30.4)	(30.4)	(30.4)
	75.6	24.8	-7.5	69.1	54.1	22.8	45.7	95.3	26.9	29.0
	5.621	1.847	-0.557	5.138	4.024	1.694	3.395	7.086	2.000	2.154
c_O	464	447	451	458	449	429	455	438	441	448
	(34.5)	(33.2)	(33.5)	(34.1)	(33.4)	(31.9)	(33.8)	(32.6)	(32.8)	(33.3)
c_E	459.7	459.7	459.7	459.7	459.7	459.7	459.7	459.7	459.7	459.7
	(34.2)	(34.2)	(34.2)	(34.2)	(34.2)	(34.2)	(34.2)	(34.2)	(34.2)	(34.2)
	8.7	-25.0	-17.2	-3.3	-21.1	-59.2	-9.3	-42.3	-36.6	-23.0
	0.647	-1.858	-1.277	-0.248	-1.568	-4.405	-0.691	-3.145	-2.719	-1.713
N	1345	1345	1345	1345	1345	1345	1345	1345	1345	1345
X2	7.5	1.8	0.5	4.8	5.0	6.5	4.2	15.5	1.8	2.0
Gvalue	7.6	1.8	0.5	4.8	5.1	6.4	4.3	15.9	1.8	2.0
Gv/N	0.567	0.136	0.040	0.355	0.378	0.477	0.317	1.179	0.133	0.150
Prob	0.05425	0.60842	0.91036	0.18861	0.16599	0.09291	0.23474	0.00121	0.61727	0.56955

	50	49	48	47	46	45	44	43	42	41
a_O	257	220	215	246	252	214	242	244	207	240
	(19.1)	(16.4)	(16.0)	(18.3)	(18.7)	(15.9)	(18.0)	(18.1)	(15.4)	(17.8)
a_E	238.3	238.3	238.3	238.3	238.3	238.3	238.3	238.3	238.3	238.3
	(17.7)	(17.7)	(17.7)	(17.7)	(17.7)	(17.7)	(17.7)	(17.7)	(17.7)	(17.7)
	38.9	-35.1	-44.2	15.7	28.3	-46.0	7.5	11.6	-58.2	3.5
	2.893	-2.608	-3.284	1.169	2.101	-3.417	0.560	0.864	-4.329	0.260
t_O	219	235	209	210	216	237	210	220	218	219
	(16.3)	(17.5)	(15.5)	(15.6)	(16.1)	(17.6)	(15.6)	(16.4)	(16.2)	(16.3)
t_E	238.3	238.3	238.3	238.3	238.3	238.3	238.3	238.3	238.3	238.3
	(17.7)	(17.7)	(17.7)	(17.7)	(17.7)	(17.7)	(17.7)	(17.7)	(17.7)	(17.7)
	-37.0	-6.6	-54.9	-53.1	-42.5	-2.6	-53.1	-35.2	-38.8	-37.0
	-2.752	-0.489	-4.079	-3.949	-3.157	-0.194	-3.949	-2.615	-2.888	-2.752
g_O	428	432	470	435	432	440	456	387	445	440
	(31.8)	(32.1)	(34.9)	(32.3)	(32.1)	(32.7)	(33.9)	(28.8)	(33.1)	(32.7)
g_E	408.8	408.8	408.8	408.8	408.8	408.8	408.8	408.8	408.8	408.8
	(30.4)	(30.4)	(30.4)	(30.4)	(30.4)	(30.4)	(30.4)	(30.4)	(30.4)	(30.4)
	39.4	47.8	131.2	54.1	47.8	64.8	99.7	-42.3	75.6	64.8
	2.927	3.552	9.757	4.024	3.552	4.819	7.416	-3.148	5.621	4.819
c_O	441	458	451	454	445	454	437	494	475	446
	(32.8)	(34.1)	(33.5)	(33.8)	(33.1)	(33.8)	(32.5)	(36.7)	(35.3)	(33.2)
c_E	459.7	459.7	459.7	459.7	459.7	459.7	459.7	459.7	459.7	459.7
	(34.2)	(34.2)	(34.2)	(34.2)	(34.2)	(34.2)	(34.2)	(34.2)	(34.2)	(34.2)
	-36.6	-3.3	-17.2	-11.3	-28.9	-11.3	-44.2	71.2	31.2	-26.9
	-2.719	-0.248	-1.277	-0.838	-2.146	-0.838	-3.286	5.291	2.317	-2.002
N	1345	1345	1345	1345	1345	1345	1345	1345	1345	1345
X2	4.7	2.8	15.2	5.4	4.7	4.9	10.0	5.3	9.6	4.4
Gvalue	4.7	2.8	15.0	5.5	4.7	5.0	10.0	5.3	9.7	4.4
Gv/N	0.350	0.207	1.117	0.407	0.350	0.369	0.741	0.391	0.721	0.324
Prob	0.19463	0.42584	0.00180	0.14025	0.19474	0.17438	0.01890	0.15350	0.02126	0.22503

	40	39	38	37	36	35	34	33	32	31
a_O	237	231	242	249	228	213	208	200	232	263
	(17.6)	(17.2)	(18.0)	(18.5)	(17.0)	(15.8)	(15.5)	(14.9)	(17.2)	(19.6)
a_E	238.3	238.3	238.3	238.3	238.3	238.3	238.3	238.3	238.3	238.3
	(17.7)	(17.7)	(17.7)	(17.7)	(17.7)	(17.7)	(17.7)	(17.7)	(17.7)	(17.7)
	-2.5	-14.3	7.5	22.0	-20.1	-47.7	-56.5	-70.0	-12.4	52.0
	-0.187	-1.063	0.560	1.632	-1.492	-3.550	-4.201	-5.206	-0.919	3.864
t_O	262	236	217	259	283	262	290	288	241	251
	(19.5)	(17.5)	(16.1)	(19.3)	(21.0)	(19.5)	(21.6)	(21.4)	(17.9)	(18.7)
t_E	238.3	238.3	238.3	238.3	238.3	238.3	238.3	238.3	238.3	238.3
	(17.7)	(17.7)	(17.7)	(17.7)	(17.7)	(17.7)	(17.7)	(17.7)	(17.7)	(17.7)
	49.7	-4.6	-40.7	43.1	97.3	49.7	113.9	109.1	5.4	26.0
	3.692	-0.342	-3.023	3.206	7.233	3.692	8.465	8.111	0.402	1.936
g_O	420	403	426	398	416	409	384	402	420	407
	(31.2)	(30.0)	(31.7)	(29.6)	(30.9)	(30.4)	(28.6)	(29.9)	(31.2)	(30.3)
g_E	408.8	408.8	408.8	408.8	408.8	408.8	408.8	408.8	408.8	408.8
	(30.4)	(30.4)	(30.4)	(30.4)	(30.4)	(30.4)	(30.4)	(30.4)	(30.4)	(30.4)
	22.8	-11.4	35.2	-21.2	14.6	0.5	-48.0	-13.4	22.8	-3.5
	1.694	-0.850	2.617	-1.579	1.086	0.036	-3.568	-0.997	1.694	-0.261
c_O	426	475	460	439	418	461	463	455	452	424
	(31.7)	(35.3)	(34.2)	(32.6)	(31.1)	(34.3)	(34.4)	(33.8)	(33.6)	(31.5)
c_E	459.7	459.7	459.7	459.7	459.7	459.7	459.7	459.7	459.7	459.7
	(34.2)	(34.2)	(34.2)	(34.2)	(34.2)	(34.2)	(34.2)	(34.2)	(34.2)	(34.2)

	-64.8	31.2	0.7	-40.4	-79.4	2.7	6.7	-9.3	-15.2	-68.5
	-4.819	2.317	0.049	-3.003	-5.907	0.198	0.497	-0.691	-1.131	-5.093
N	1345	1345	1345	1345	1345	1345	1345	1345	1345	1345
X2	5.1	0.8	2.7	3.5	12.7	5.0	16.6	16.7	0.6	6.0
Gvalue	5.1	0.8	2.7	3.5	12.4	5.1	16.1	16.4	0.6	6.0
Gv/N	0.381	0.062	0.204	0.257	0.920	0.376	1.193	1.217	0.047	0.446
Prob	0.16305	0.84152	0.43334	0.32692	0.00621	0.16727	0.00111	0.00095	0.88926	0.11169

	30	29	28	27	26	25	24	23	22	21
a_O	233	259	254	257	254	225	224	238	254	219
	(17.3)	(19.3)	(18.9)	(19.1)	(18.9)	(16.7)	(16.7)	(17.7)	(18.9)	(16.3)
a_E	238.3	238.3	238.3	238.3	238.3	238.3	238.3	238.3	238.3	238.3
	(17.7)	(17.7)	(17.7)	(17.7)	(17.7)	(17.7)	(17.7)	(17.7)	(17.7)	(17.7)
	-10.4	43.2	32.5	38.9	32.5	-25.8	-27.6	-0.5	32.5	-36.9
	-0.773	3.215	2.416	2.893	2.416	-1.916	-2.056	-0.039	2.416	-2.745
t_O	232	223	266	253	266	259	264	238	258	245
	(17.2)	(16.6)	(19.8)	(18.8)	(19.8)	(19.3)	(19.6)	(17.7)	(19.2)	(18.2)
t_E	238.3	238.3	238.3	238.3	238.3	238.3	238.3	238.3	238.3	238.3
	(17.7)	(17.7)	(17.7)	(17.7)	(17.7)	(17.7)	(17.7)	(17.7)	(17.7)	(17.7)
	-12.5	-29.6	58.5	30.3	58.5	43.1	54.1	-0.6	41.0	13.6
	-0.926	-2.202	4.348	2.250	4.348	3.206	4.019	-0.046	3.046	1.009
g_O	388	397	375	385	403	402	369	406	366	408
	(28.8)	(29.5)	(27.9)	(28.6)	(30.0)	(29.9)	(27.4)	(30.2)	(27.2)	(30.3)
g_E	408.8	408.8	408.8	408.8	408.8	408.8	408.8	408.8	408.8	408.8
	(30.4)	(30.4)	(30.4)	(30.4)	(30.4)	(30.4)	(30.4)	(30.4)	(30.4)	(30.4)
	-40.4	-23.2	-64.7	-46.1	-11.4	-13.4	-75.5	-5.5	-80.9	-1.5
	-3.007	-1.723	-4.807	-3.428	-0.850	-0.997	-5.615	-0.409	-6.014	-0.113
c_O	492	466	450	450	422	459	488	463	467	473
	(36.6)	(34.6)	(33.5)	(33.5)	(31.4)	(34.1)	(36.3)	(34.4)	(34.7)	(35.2)
c_E	459.7	459.7	459.7	459.7	459.7	459.7	459.7	459.7	459.7	459.7
	(34.2)	(34.2)	(34.2)	(34.2)	(34.2)	(34.2)	(34.2)	(34.2)	(34.2)	(34.2)
	66.9	12.7	-19.1	-19.1	-72.2	-1.3	58.4	6.7	14.8	27.0
	4.973	0.948	-1.423	-1.423	-5.365	-0.100	4.340	0.497	1.099	2.011
N	1345	1345	1345	1345	1345	1345	1345	1345	1345	1345
X2	3.6	3.2	7.2	4.0	7.4	2.6	9.2	0.0	7.3	2.1
Gvalue	3.6	3.2	7.2	3.9	7.4	2.6	9.3	0.0	7.4	2.2
Gv/N	0.266	0.237	0.535	0.293	0.548	0.194	0.688	0.003	0.547	0.161
Prob	0.31046	0.36341	0.06607	0.26826	0.06089	0.45541	0.02607	0.99762	0.06139	0.53768

	20	19	18	17	16	15	14	13	12	11
a_O	259	251	256	246	241	223	249	272	259	289
	(19.3)	(18.7)	(19.0)	(18.3)	(17.9)	(16.6)	(18.5)	(20.2)	(19.3)	(21.5)
a_E	238.3	238.3	238.3	238.3	238.3	238.3	238.3	238.3	238.3	238.3
	(17.7)	(17.7)	(17.7)	(17.7)	(17.7)	(17.7)	(17.7)	(17.7)	(17.7)	(17.7)
	43.2	26.1	36.8	15.7	5.5	-29.5	22.0	72.0	43.2	111.6
	3.215	1.944	2.734	1.169	0.410	-2.195	1.632	5.357	3.215	8.297
t_O	259	296	271	273	287	283	270	249	276	248
	(19.3)	(22.0)	(20.1)	(20.3)	(21.3)	(21.0)	(20.1)	(18.5)	(20.5)	(18.4)
t_E	238.3	238.3	238.3	238.3	238.3	238.3	238.3	238.3	238.3	238.3
	(17.7)	(17.7)	(17.7)	(17.7)	(17.7)	(17.7)	(17.7)	(17.7)	(17.7)	(17.7)
	43.1	128.3	69.7	74.2	106.7	97.3	67.4	21.9	81.0	19.8
	3.206	9.542	5.180	5.517	7.934	7.233	5.013	1.625	6.026	1.470
g_O	330	318	313	325	326	321	337	315	345	331
	(24.5)	(23.6)	(23.3)	(24.2)	(24.2)	(23.9)	(25.1)	(23.4)	(25.7)	(24.6)
g_E	408.8	408.8	408.8	408.8	408.8	408.8	408.8	408.8	408.8	408.8
	(30.4)	(30.4)	(30.4)	(30.4)	(30.4)	(30.4)	(30.4)	(30.4)	(30.4)	(30.4)
	-141.3	-159.7	-167.1	-149.0	-147.5	-155.2	-130.1	-164.1	-117.0	-139.7
	-10.503	-11.872	-12.423	-11.082	-10.967	-11.536	-9.674	-12.204	-8.700	-10.386
c_O	497	480	505	501	491	518	489	509	465	477
	(37.0)	(35.7)	(37.5)	(37.2)	(36.5)	(38.5)	(36.4)	(37.8)	(34.6)	(35.5)
c_E	459.7	459.7	459.7	459.7	459.7	459.7	459.7	459.7	459.7	459.7
	(34.2)	(34.2)	(34.2)	(34.2)	(34.2)	(34.2)	(34.2)	(34.2)	(34.2)	(34.2)
	77.6	41.5	95.0	86.3	64.7	123.8	60.5	103.8	10.7	35.3
	5.770	3.089	7.062	6.414	4.814	9.202	4.498	7.716	0.797	2.625
N	1345	1345	1345	1345	1345	1345	1345	1345	1345	1345
X2	21.8	35.7	32.7	26.2	28.9	35.6	19.2	32.1	17.8	26.6
Gvalue	22.7	36.3	34.3	27.2	29.5	36.4	19.8	33.5	18.0	27.0
Gv/N	1.689	2.703	2.553	2.019	2.191	2.704	1.469	2.492	1.338	2.006
Prob	0.00005	0.00000	0.00000	0.00001	0.00000	0.00000	0.00019	0.00000	0.00044	0.00001

	10	9	8	7	6	5	4	3	2	1
a_O	245	268	274	285	295	261	238	298	222	186
	(18.2)	(19.9)	(20.4)	(21.2)	(21.9)	(19.4)	(17.7)	(22.2)	(16.5)	(13.8)
a_E	238.3	238.3	238.3	238.3	238.3	238.3	238.3	238.3	238.3	238.3
	(17.7)	(17.7)	(17.7)	(17.7)	(17.7)	(17.7)	(17.7)	(17.7)	(17.7)	(17.7)
	13.7	63.0	76.6	102.1	126.0	47.6	-0.5	133.3	-31.4	-92.1
	1.016	4.687	5.695	7.591	9.370	3.538	-0.039	9.914	-2.333	-6.849
t_O	220	238	252	259	248	274	242	238	248	174
	(16.4)	(17.7)	(18.7)	(19.3)	(18.4)	(20.4)	(18.0)	(17.7)	(18.4)	(12.9)
t_E	238.3	238.3	238.3	238.3	238.3	238.3	238.3	238.3	238.3	238.3
	(17.7)	(17.7)	(17.7)	(17.7)	(17.7)	(17.7)	(17.7)	(17.7)	(17.7)	(17.7)
	-35.2	-0.6	28.2	43.1	19.8	76.5	7.4	-0.6	19.8	-109.5
	-2.615	-0.046	2.093	3.206	1.470	5.686	0.553	-0.046	1.470	-8.138
g_O	366	371	374	297	327	301	362	398	329	360
	(27.2)	(27.6)	(27.8)	(22.1)	(24.3)	(22.4)	(26.9)	(29.6)	(24.5)	(26.8)
g_E	408.8	408.8	408.8	408.8	408.8	408.8	408.8	408.8	408.8	408.8
	(30.4)	(30.4)	(30.4)	(30.4)	(30.4)	(30.4)	(30.4)	(30.4)	(30.4)	(30.4)
	-80.9	-71.9	-66.5	-189.7	-146.0	-184.2	-88.0	-21.2	-142.8	-91.5
	-6.014	-5.347	-4.942	-14.106	-10.851	-13.697	-6.539	-1.579	-10.620	-6.800
c_O	514	468	445	504	475	509	503	411	546	625
	(38.2)	(34.8)	(33.1)	(37.5)	(35.3)	(37.8)	(37.4)	(30.6)	(40.6)	(46.5)
c_E	459.7	459.7	459.7	459.7	459.7	459.7	459.7	459.7	459.7	459.7
	(34.2)	(34.2)	(34.2)	(34.2)	(34.2)	(34.2)	(34.2)	(34.2)	(34.2)	(34.2)
	114.8	16.8	-28.9	92.8	31.2	103.8	90.6	-92.0	187.9	384.1
	8.538	1.250	-2.146	6.900	2.317	7.716	6.738	-6.840	13.974	28.554
N	1345	1345	1345	1345	1345	1345	1345	1345	1345	1345
X2	12.5	7.4	9.6	45.8	30.8	41.2	9.5	20.4	33.3	94.1
Gvalue	12.5	7.3	9.4	48.3	31.0	43.6	9.6	19.5	33.5	91.0
Gv/N	0.926	0.544	0.699	3.592	2.306	3.243	0.713	1.449	2.490	6.768
Prob	0.00598	0.06242	0.02442	0.00000	0.00000	0.00000	0.02245	0.00022	0.00000	0.00000

CDS_INITIAL:

	1	2	3	4	5	6	7	8	9	10

a_O	943	0	0	468	345	213	353	340	246	328
	(70.1)	(0.0)	(0.0)	(34.8)	(25.7)	(15.8)	(26.2)	(25.3)	(18.3)	(24.4)
a_E	247.8	314.6	74.0	247.8	314.6	74.0	247.8	314.6	74.0	247.8
	(18.4)	(23.4)	(5.5)	(18.4)	(23.4)	(5.5)	(18.4)	(23.4)	(5.5)	(18.4)
	2520.4	0.0	0.0	595.1	63.6	450.3	249.8	52.7	591.0	183.9
	187.393	0.000	0.000	44.246	4.725	33.483	18.571	3.919	43.939	13.673
t_O	162	1345	0	168	190	232	166	286	244	227
	(12.0)	(100.0)	(0.0)	(12.5)	(14.1)	(17.2)	(12.3)	(21.3)	(18.1)	(16.9)
t_E	170.0	366.7	101.0	170.0	366.7	101.0	170.0	366.7	101.0	170.0
	(12.6)	(27.3)	(7.5)	(12.6)	(27.3)	(7.5)	(12.6)	(27.3)	(7.5)	(12.6)
	-15.7	3495.7	0.0	-4.1	-249.9	386.0	-8.0	-142.2	430.6	131.2
	-1.167	259.900	0.000	-0.302	-18.580	28.699	-0.594	-10.575	32.013	9.752
g_O	240	0	1345	246	313	374	414	293	338	365
	(17.8)	(0.0)	(100.0)	(18.3)	(23.3)	(27.8)	(30.8)	(21.8)	(25.1)	(27.1)
g_E	540.7	275.9	562.1	540.7	275.9	562.1	540.7	275.9	562.1	540.7
	(40.2)	(20.5)	(41.8)	(40.2)	(20.5)	(41.8)	(40.2)	(20.5)	(41.8)	(40.2)
	-389.8	0.0	2347.0	-387.4	78.9	-304.7	-221.0	35.2	-343.8	-286.8
	-28.984	0.000	174.501	-28.805	5.868	-22.656	-16.432	2.616	-25.562	-21.324
c_O	0	0	0	463	497	526	412	426	517	425
	(0.0)	(0.0)	(0.0)	(34.4)	(37.0)	(39.1)	(30.6)	(31.7)	(38.4)	(31.6)
c_E	386.5	387.7	607.9	386.5	387.7	607.9	386.5	387.7	607.9	386.5
	(28.7)	(28.8)	(45.2)	(28.7)	(28.8)	(45.2)	(28.7)	(28.8)	(45.2)	(28.7)
	0.0	0.0	0.0	167.3	246.9	-152.3	52.7	80.3	-167.5	80.7
	0.000	0.000	0.000	12.436	18.355	-11.324	3.916	5.968	-12.457	6.003
N	1345	1345	1345	1345	1345	1345	1345	1345	1345	1345
X2	2504.3	3587.7	1873.4	371.4	123.9	505.1	76.1	24.7	705.3	105.9
Gvalue	2114.9	3495.7	2347.0	370.9	139.5	379.3	73.5	25.9	510.2	109.0
Gv/N	157.242	259.900	174.501	27.574	10.369	28.202	5.461	1.928	37.933	8.104
Prob	1.00000	1.00000	1.00000	0.00000	0.00000	0.00000	0.00000	0.00001	0.00000	0.00000

	11	12	13	14	15	16	17	18	19	20
a_O	298	203	318	265	186	304	294	167	253	288
	(22.2)	(15.1)	(23.6)	(19.7)	(13.8)	(22.6)	(21.9)	(12.4)	(18.8)	(21.4)
a_E	314.6	74.0	247.8	314.6	74.0	247.8	314.6	74.0	247.8	314.6
	(23.4)	(5.5)	(18.4)	(23.4)	(5.5)	(18.4)	(23.4)	(5.5)	(18.4)	(23.4)
	-32.4	409.7	158.6	-91.0	342.8	124.2	-39.9	271.8	10.5	-51.0
	-2.408	30.460	11.792	-6.766	25.490	9.238	-2.966	20.210	0.779	-3.789
t_O	288	217	205	354	222	213	324	250	230	319
	(21.4)	(16.1)	(15.2)	(26.3)	(16.5)	(15.8)	(24.1)	(18.6)	(17.1)	(23.7)
t_E	366.7	101.0	170.0	366.7	101.0	170.0	366.7	101.0	170.0	366.7
	(27.3)	(7.5)	(12.6)	(27.3)	(7.5)	(12.6)	(27.3)	(7.5)	(12.6)	(27.3)
	-139.2	332.0	76.7	-25.0	349.8	96.0	-80.3	453.3	138.9	-89.0
	-10.350	24.687	5.699	-1.861	26.008	7.134	-5.970	33.704	10.330	-6.615
g_O	283	360	393	270	363	429	261	359	424	294
	(21.0)	(26.8)	(29.2)	(20.1)	(27.0)	(31.9)	(19.4)	(26.7)	(31.5)	(21.9)
g_E	275.9	562.1	540.7	275.9	562.1	540.7	275.9	562.1	540.7	275.9
	(20.5)	(41.8)	(40.2)	(20.5)	(41.8)	(40.2)	(20.5)	(41.8)	(40.2)	(20.5)
	14.3	-320.8	-250.7	-11.7	-317.4	-198.5	-29.0	-321.9	-206.1	37.3
	1.066	-23.851	-18.640	-0.871	-23.601	-14.757	-2.158	-23.933	-15.324	2.774
c_O	476	565	429	456	574	399	466	569	438	444
	(35.4)	(42.0)	(31.9)	(33.9)	(42.7)	(29.7)	(34.6)	(42.3)	(32.6)	(33.0)
c_E	387.7	607.9	386.5	387.7	607.9	386.5	387.7	607.9	386.5	387.7
	(28.8)	(45.2)	(28.7)	(28.8)	(45.2)	(28.7)	(28.8)	(45.2)	(28.7)	(28.8)
	195.3	-82.8	89.5	148.0	-66.0	25.4	171.5	-75.3	109.6	120.4
	14.524	-6.154	6.657	11.003	-4.904	1.890	12.747	-5.601	8.149	8.953
N	1345	1345	1345	1345	1345	1345	1345	1345	1345	1345
X2	38.1	433.9	72.1	20.4	387.0	47.1	23.0	412.7	53.3	17.8
Gvalue	38.1	338.2	74.1	20.2	309.2	47.1	22.2	327.9	52.9	17.8
Gv/N	2.831	25.141	5.508	1.505	22.992	3.505	1.653	24.380	3.934	1.323
Prob	0.00000	0.00000	0.00000	0.00015	0.00000	0.00000	0.00006	0.00000	0.00000	0.00049

	21	22	23	24	25	26	27	28	29	30
a_O	178	292	251	162	265	270	169	235	258	152
	(13.2)	(21.7)	(18.7)	(12.0)	(19.7)	(20.1)	(12.6)	(17.5)	(19.2)	(11.3)
a_E	74.0	247.8	314.6	74.0	247.8	314.6	74.0	247.8	314.6	74.0
	(5.5)	(18.4)	(23.4)	(5.5)	(18.4)	(23.4)	(5.5)	(18.4)	(23.4)	(5.5)
	312.4	95.8	-113.4	253.8	35.5	-82.6	279.1	-25.0	-102.4	218.8
	23.230	7.124	-8.434	18.873	2.642	-6.143	20.751	-1.855	-7.614	16.268
t_O	225	203	330	209	192	320	220	198	339	187
	(16.7)	(15.1)	(24.5)	(15.5)	(14.3)	(23.8)	(16.4)	(14.7)	(25.2)	(13.9)
t_E	101.0	170.0	366.7	101.0	170.0	366.7	101.0	170.0	366.7	101.0
	(7.5)	(12.6)	(27.3)	(7.5)	(12.6)	(27.3)	(7.5)	(12.6)	(27.3)	(7.5)
	360.6	71.9	-69.7	304.1	46.6	-87.3	342.7	60.3	-53.3	230.5
	26.808	5.348	-5.180	22.609	3.467	-6.487	25.477	4.482	-3.965	17.137
g_O	386	393	276	431	452	292	408	463	316	439
	(28.7)	(29.2)	(20.5)	(32.0)	(33.6)	(21.7)	(30.3)	(34.4)	(23.5)	(32.6)
g_E	562.1	540.7	275.9	562.1	540.7	275.9	562.1	540.7	275.9	562.1
	(41.8)	(40.2)	(20.5)	(41.8)	(40.2)	(20.5)	(41.8)	(40.2)	(20.5)	(41.8)
	-290.1	-250.7	0.2	-228.9	-161.9	33.1	-261.4	-143.6	85.7	-217.0
	-21.571	-18.640	0.011	-17.018	-12.038	2.459	-19.437	-10.675	6.373	-16.133
c_O	556	457	488	543	436	463	548	449	432	567
	(41.3)	(34.0)	(36.3)	(40.4)	(32.4)	(34.4)	(40.7)	(33.4)	(32.1)	(42.2)
c_E	607.9	386.5	387.7	607.9	386.5	387.7	607.9	386.5	387.7	607.9
	(45.2)	(28.7)	(28.8)	(45.2)	(28.7)	(28.8)	(45.2)	(28.7)	(28.8)	(45.2)
	-99.3	153.2	224.6	-122.7	105.1	164.4	-113.8	134.6	93.5	-79.1
	-7.384	11.388	16.697	-9.122	7.815	12.221	-8.459	10.010	6.951	-5.878
N	1345	1345	1345	1345	1345	1345	1345	1345	1345	1345
X2	358.1	67.5	42.5	257.7	24.9	27.9	310.4	26.5	23.2	185.2
Gvalue	283.6	70.2	41.6	206.4	25.4	27.6	246.6	26.4	23.5	153.2
Gv/N	21.083	5.220	3.094	15.342	1.887	2.049	18.333	1.961	1.744	11.393
Prob	0.00000	0.00000	0.00000	0.00000	0.00001	0.00000	0.00000	0.00001	0.00003	0.00000

	31	32	33	34	35	36	37	38	39	40
a_O	242	258	149	232	247	144	259	257	131	207
	(18.0)	(19.2)	(11.1)	(17.2)	(18.4)	(10.7)	(19.3)	(19.1)	(9.7)	(15.4)
a_E	247.8	314.6	74.0	247.8	314.6	74.0	247.8	314.6	74.0	247.8
	(18.4)	(23.4)	(5.5)	(18.4)	(23.4)	(5.5)	(18.4)	(23.4)	(5.5)	(18.4)
	-11.5	-102.4	208.5	-30.6	-119.6	191.7	22.9	-104.0	149.6	-74.5
	-0.854	-7.614	15.505	-2.275	-8.890	14.254	1.701	-7.733	11.124	-5.539
t_O	190	357	196	210	340	175	201	337	180	188
	(14.1)	(26.5)	(14.6)	(15.6)	(25.3)	(13.0)	(14.9)	(25.1)	(13.4)	(14.0)
t_E	170.0	366.7	101.0	170.0	366.7	101.0	170.0	366.7	101.0	170.0
	(12.6)	(27.3)	(7.5)	(12.6)	(27.3)	(7.5)	(12.6)	(27.3)	(7.5)	(12.6)
	42.2	-19.2	260.0	88.6	-51.5	192.5	67.2	-57.0	208.1	37.7
	3.135	-1.429	19.331	6.591	-3.827	14.311	4.999	-4.238	15.474	2.806

g_O	470	273	443	457	312	443	480	294	438	520
	(34.9)	(20.3)	(32.9)	(34.0)	(23.2)	(32.9)	(35.7)	(21.9)	(32.6)	(38.7)
g_E	540.7	275.9	562.1	540.7	275.9	562.1	540.7	275.9	562.1	540.7
	(40.2)	(20.5)	(41.8)	(40.2)	(20.5)	(41.8)	(40.2)	(20.5)	(41.8)	(40.2)
	-131.6	-5.8	-210.9	-153.6	76.7	-210.9	-114.2	37.3	-218.5	-40.5
	-9.788	-0.432	-15.683	-11.423	5.701	-15.683	-8.494	2.774	-16.245	-3.012
c_O	443	457	557	446	446	583	405	457	596	430
	(32.9)	(34.0)	(41.4)	(33.2)	(33.2)	(43.3)	(30.1)	(34.0)	(44.3)	(32.0)
c_E	386.5	387.7	607.9	386.5	387.7	607.9	386.5	387.7	607.9	386.5
	(28.7)	(28.8)	(45.2)	(28.7)	(28.8)	(45.2)	(28.7)	(28.8)	(45.2)	(28.7)
	120.9	150.3	-97.5	127.8	125.0	-48.8	37.9	150.3	-23.6	91.7
	8.990	11.176	-7.248	9.498	9.291	-3.632	2.818	11.176	-1.758	6.821
N	1345	1345	1345	1345	1345	1345	1345	1345	1345	1345
X2	20.0	22.9	194.9	32.5	30.0	146.7	13.8	26.5	133.4	14.3
Gvalue	19.9	22.9	160.1	32.2	30.6	124.4	13.8	26.6	115.6	14.5
Gv/N	1.483	1.701	11.905	2.391	2.275	9.250	1.024	1.979	8.594	1.077
Prob	0.00017	0.00004	0.00000	0.00000	0.00000	0.00000	0.00324	0.00001	0.00000	0.00232

	41	42	43	44	45	46	47	48	49	50
a_O	244	137	249	286	154	228	253	134	236	276
	(18.1)	(10.2)	(18.5)	(21.3)	(11.4)	(17.0)	(18.8)	(10.0)	(17.5)	(20.5)
a_E	314.6	74.0	247.8	314.6	74.0	247.8	314.6	74.0	247.8	314.6
	(23.4)	(5.5)	(18.4)	(23.4)	(5.5)	(18.4)	(23.4)	(5.5)	(18.4)	(23.4)
	-124.1	168.7	2.4	-54.6	225.7	-38.0	-110.3	159.1	-23.1	-72.3
	-9.225	12.546	0.177	-4.059	16.781	-2.825	-8.203	11.830	-1.714	-5.378
t_O	371	175	192	318	185	161	369	156	180	336
	(27.6)	(13.0)	(14.3)	(23.6)	(13.8)	(12.0)	(27.4)	(11.6)	(13.4)	(25.0)
t_E	366.7	101.0	170.0	366.7	101.0	170.0	366.7	101.0	170.0	366.7
	(27.3)	(7.5)	(12.6)	(27.3)	(7.5)	(12.6)	(27.3)	(7.5)	(12.6)	(27.3)
	8.6	192.5	46.6	-90.7	224.0	-17.6	4.5	135.7	20.5	-58.8
	0.637	14.311	3.467	-6.743	16.657	-1.308	0.337	10.091	1.523	-4.374
g_O	292	457	493	313	413	509	293	462	504	288
	(21.7)	(34.0)	(36.7)	(23.3)	(30.7)	(37.8)	(21.8)	(34.3)	(37.5)	(21.4)
g_E	275.9	562.1	540.7	275.9	562.1	540.7	275.9	562.1	540.7	275.9
	(20.5)	(41.8)	(40.2)	(20.5)	(41.8)	(40.2)	(20.5)	(41.8)	(40.2)	(20.5)
	33.1	-189.2	-91.0	78.9	-254.6	-61.4	35.2	-181.2	-70.8	24.7
	2.459	-14.064	-6.765	5.868	-18.927	-4.567	2.616	-13.471	-5.262	1.834
c_O	438	576	411	428	593	447	430	593	425	445
	(32.6)	(42.8)	(30.6)	(31.8)	(44.1)	(33.2)	(32.0)	(44.1)	(31.6)	(33.1)
c_E	387.7	607.9	386.5	387.7	607.9	386.5	387.7	607.9	386.5	387.7
	(28.8)	(45.2)	(28.7)	(28.8)	(45.2)	(28.7)	(28.8)	(45.2)	(28.7)	(28.8)
	106.9	-62.2	50.5	84.7	-29.5	130.0	89.1	-29.5	80.7	122.7
	7.946	-4.623	3.758	6.294	-2.194	9.668	6.622	-2.194	6.003	9.122
N	1345	1345	1345	1345	1345	1345	1345	1345	1345	1345
X2	23.4	129.2	8.6	18.3	196.3	13.4	17.8	96.8	7.5	16.3
Gvalue	24.4	109.9	8.6	18.3	165.7	13.0	18.5	84.1	7.4	16.2
Gv/N	1.816	8.170	0.638	1.361	12.317	0.968	1.372	6.256	0.550	1.205
Prob	0.00002	0.00000	0.03545	0.00038	0.00000	0.00459	0.00035	0.00000	0.06009	0.00103

	51	52	53	54	55	56	57	58	59	60
a_O	147	231	258	116	232	252	126	255	264	128
	(10.9)	(17.2)	(19.2)	(8.6)	(17.2)	(18.7)	(9.4)	(19.0)	(19.6)	(9.5)
a_E	74.0	247.8	314.6	74.0	247.8	314.6	74.0	247.8	314.6	74.0
	(5.5)	(18.4)	(23.4)	(5.5)	(18.4)	(23.4)	(5.5)	(18.4)	(23.4)	(5.5)
	201.8	-32.5	-102.4	104.3	-30.6	-111.9	134.1	14.6	-92.7	140.3
	15.001	-2.413	-7.614	7.753	-2.275	-8.319	9.970	1.084	-6.889	10.428
t_O	188	197	379	163	177	343	160	182	346	151
	(14.0)	(14.6)	(28.2)	(12.1)	(13.2)	(25.5)	(11.9)	(13.5)	(25.7)	(11.2)
t_E	101.0	170.0	366.7	101.0	170.0	366.7	101.0	170.0	366.7	101.0
	(7.5)	(12.6)	(27.3)	(7.5)	(12.6)	(27.3)	(7.5)	(12.6)	(27.3)	(7.5)
	233.7	58.0	24.9	156.1	14.2	-45.9	147.3	24.7	-40.3	121.5
	17.377	4.311	1.853	11.608	1.055	-3.413	10.952	1.839	-2.995	9.036
g_O	450	493	288	475	535	305	458	485	315	473
	(33.5)	(36.7)	(21.4)	(35.3)	(39.8)	(22.7)	(34.1)	(36.1)	(23.4)	(35.2)
g_E	562.1	540.7	275.9	562.1	540.7	275.9	562.1	540.7	275.9	562.1
	(41.8)	(40.2)	(20.5)	(41.8)	(40.2)	(20.5)	(41.8)	(40.2)	(20.5)	(41.8)
	-200.2	-91.0	24.7	-159.9	-11.3	61.1	-187.6	-105.4	83.4	-163.2
	-14.882	-6.765	1.834	-11.890	-0.837	4.544	-13.946	-7.835	6.204	-12.136
c_O	560	424	420	591	401	445	601	423	420	593
	(41.6)	(31.5)	(31.2)	(43.9)	(29.8)	(33.1)	(44.7)	(31.4)	(31.2)	(44.1)
c_E	607.9	386.5	387.7	607.9	386.5	387.7	607.9	386.5	387.7	607.9
	(45.2)	(28.7)	(28.8)	(45.2)	(28.7)	(28.8)	(45.2)	(28.7)	(28.8)	(45.2)
	-92.0	78.6	67.2	-33.4	29.6	122.7	-13.8	76.4	67.2	-29.5
	-6.840	5.840	4.998	-2.484	2.198	9.122	-1.026	5.678	4.998	-2.194
N	1345	1345	1345	1345	1345	1345	1345	1345	1345	1345
X2	173.1	13.3	13.8	75.9	1.9	25.5	90.4	10.2	17.5	78.7
Gvalue	143.3	13.1	14.4	67.1	1.9	26.0	80.0	10.3	17.7	69.1
Gv/N	10.657	0.973	1.072	4.987	0.142	1.934	5.950	0.767	1.318	5.134
Prob	0.00000	0.00445	0.00239	0.00000	0.59140	0.00001	0.00000	0.01610	0.00050	0.00000

	61	62	63	64	65	66	67	68	69	70
a_O	231	249	131	235	272	126	232	269	136	212
	(17.2)	(18.5)	(9.7)	(17.5)	(20.2)	(9.4)	(17.2)	(20.0)	(10.1)	(15.8)
a_E	247.8	314.6	74.0	247.8	314.6	74.0	247.8	314.6	74.0	247.8
	(18.4)	(23.4)	(5.5)	(18.4)	(23.4)	(5.5)	(18.4)	(23.4)	(5.5)	(18.4)
	-32.5	-116.5	149.6	-25.0	-79.2	134.1	-30.6	-84.3	165.5	-66.2
	-2.413	-8.663	11.124	-1.855	-5.890	9.970	-2.275	-6.269	12.306	-4.920
t_O	190	352	154	170	344	150	175	323	172	198
	(14.1)	(26.2)	(11.4)	(12.6)	(25.6)	(11.2)	(13.0)	(24.0)	(12.8)	(14.7)
t_E	170.0	366.7	101.0	170.0	366.7	101.0	170.0	366.7	101.0	170.0
	(12.6)	(27.3)	(7.5)	(12.6)	(27.3)	(7.5)	(12.6)	(27.3)	(7.5)	(12.6)
	42.2	-28.9	130.0	-0.1	-44.0	118.7	10.1	-82.0	183.2	60.3
	3.135	-2.147	9.666	-0.006	-3.274	8.828	0.748	-6.100	13.623	4.482
g_O	481	300	487	528	304	475	517	276	506	502
	(35.8)	(22.3)	(36.2)	(39.3)	(22.6)	(35.3)	(38.4)	(20.5)	(37.6)	(37.3)
g_E	540.7	275.9	562.1	540.7	275.9	562.1	540.7	275.9	562.1	540.7
	(40.2)	(20.5)	(41.8)	(40.2)	(20.5)	(41.8)	(40.2)	(20.5)	(41.8)	(40.2)
	-112.5	50.2	-139.7	-25.0	58.9	-159.9	-46.3	0.2	-106.4	-74.5
	-8.362	3.732	-10.383	-1.860	4.380	-11.890	-3.440	0.011	-7.909	-5.538
c_O	443	444	573	412	425	594	421	477	531	433
	(32.9)	(33.0)	(42.6)	(30.6)	(31.6)	(44.2)	(31.3)	(35.5)	(39.5)	(32.2)
c_E	386.5	387.7	607.9	386.5	387.7	607.9	386.5	387.7	607.9	386.5
	(28.7)	(28.8)	(45.2)	(28.7)	(28.8)	(45.2)	(28.7)	(28.8)	(45.2)	(28.7)
	120.9	120.4	-67.8	52.7	78.1	-27.6	72.0	197.8	-143.7	98.4
	8.990	8.953	-5.044	3.916	5.806	-2.049	5.354	14.703	-10.685	7.317

N	1345	1345	1345	1345	1345	1345	1345	1345	1345	1345
X2	18.3	24.6	83.8	2.6	13.6	74.1	5.3	32.4	117.2	18.1
Gvalue	18.1	25.2	72.1	2.6	13.7	65.4	5.2	31.6	98.7	18.0
Gv/N	1.349	1.874	5.363	0.195	1.022	4.860	0.388	2.346	7.336	1.340
Prob	0.00041	0.00001	0.00000	0.45383	0.00328	0.00000	0.15647	0.00000	0.00000	0.00043

	71	72	73	74	75	76	77	78	79	80
a_O	269	121	245	275	128	220	280	121	258	281
	(20.0)	(9.0)	(18.2)	(20.4)	(9.5)	(16.4)	(20.8)	(9.0)	(19.2)	(20.9)
a_E	314.6	74.0	247.8	314.6	74.0	247.8	314.6	74.0	247.8	314.6
	(23.4)	(5.5)	(18.4)	(23.4)	(5.5)	(18.4)	(23.4)	(5.5)	(18.4)	(23.4)
	-84.3	119.0	-5.6	-74.1	140.3	-52.4	-65.3	119.0	20.8	-63.5
	-6.269	8.846	-0.416	-5.507	10.428	-3.894	-4.857	8.846	1.546	-4.725
t_O	340	176	174	353	148	198	330	157	188	340
	(25.3)	(13.1)	(12.9)	(26.2)	(11.0)	(14.7)	(24.5)	(11.7)	(14.0)	(25.3)
t_E	366.7	101.0	170.0	366.7	101.0	170.0	366.7	101.0	170.0	366.7
	(27.3)	(7.5)	(12.6)	(27.3)	(7.5)	(12.6)	(27.3)	(7.5)	(12.6)	(27.3)
	-51.5	195.6	8.0	-27.0	113.2	60.3	-69.7	138.6	37.7	-51.5
	-3.827	14.542	0.595	-2.004	8.415	4.482	-5.180	10.305	2.806	-3.827
g_O	295	467	481	299	482	497	320	484	505	306
	(21.9)	(34.7)	(35.8)	(22.2)	(35.8)	(37.0)	(23.8)	(36.0)	(37.5)	(22.8)
g_E	275.9	562.1	540.7	275.9	562.1	540.7	275.9	562.1	540.7	275.9
	(20.5)	(41.8)	(40.2)	(20.5)	(41.8)	(40.2)	(20.5)	(41.8)	(40.2)	(20.5)
	39.4	-173.1	-112.5	48.0	-148.2	-83.7	94.8	-144.8	-68.9	63.3
	2.932	-12.869	-8.362	3.571	-11.016	-6.222	7.052	-10.764	-5.123	4.708
c_O	441	581	445	418	587	430	415	583	394	418
	(32.8)	(43.2)	(33.1)	(31.1)	(43.6)	(32.0)	(30.9)	(43.3)	(29.3)	(31.1)
c_E	387.7	607.9	386.5	387.7	607.9	386.5	387.7	607.9	386.5	387.7
	(28.8)	(45.2)	(28.7)	(28.8)	(45.2)	(28.7)	(28.8)	(45.2)	(28.7)	(28.8)
	113.6	-52.7	125.5	62.9	-41.2	91.7	56.5	-48.8	15.2	62.9
	8.448	-3.916	9.328	4.678	-3.060	6.821	4.200	-3.632	1.128	4.678
N	1345	1345	1345	1345	1345	1345	1345	1345	1345	1345
X2	17.2	102.9	15.6	9.8	73.4	16.1	16.5	72.8	4.8	11.2
Gvalue	17.3	88.8	15.4	9.9	64.1	16.0	16.3	64.0	4.8	11.2
Gv/N	1.284	6.603	1.145	0.738	4.767	1.186	1.215	4.755	0.357	0.833
Prob	0.00062	0.00000	0.00150	0.01920	0.00000	0.00116	0.00096	0.00000	0.18735	0.01066

	81	82	83	84	85	86	87	88	89	90
a_O	103	235	256	93	242	275	110	230	312	128
	(7.7)	(17.5)	(19.0)	(6.9)	(18.0)	(20.4)	(8.2)	(17.1)	(23.2)	(9.5)
a_E	74.0	247.8	314.6	74.0	247.8	314.6	74.0	247.8	314.6	74.0
	(5.5)	(18.4)	(23.4)	(5.5)	(18.4)	(23.4)	(5.5)	(18.4)	(23.4)	(5.5)
	68.1	-25.0	-105.6	42.5	-11.5	-74.1	87.2	-34.3	-5.3	140.3
	5.063	-1.855	-7.852	3.159	-0.854	-5.507	6.483	-2.551	-0.391	10.428
t_O	169	200	350	150	175	334	140	192	327	144
	(12.6)	(14.9)	(26.0)	(11.2)	(13.0)	(24.8)	(10.4)	(14.3)	(24.3)	(10.7)
t_E	101.0	170.0	366.7	101.0	170.0	366.7	101.0	170.0	366.7	101.0
	(7.5)	(12.6)	(27.3)	(7.5)	(12.6)	(27.3)	(7.5)	(12.6)	(27.3)	(7.5)
	174.1	64.9	-32.7	118.7	10.1	-62.5	91.5	46.6	-75.0	102.2
	12.944	4.826	-2.431	8.828	0.748	-4.644	6.803	3.467	-5.577	7.601
g_O	457	499	276	489	528	314	512	505	284	459
	(34.0)	(37.1)	(20.5)	(36.4)	(39.3)	(23.3)	(38.1)	(37.5)	(21.1)	(34.1)
g_E	562.1	540.7	275.9	562.1	540.7	275.9	562.1	540.7	275.9	562.1
	(41.8)	(40.2)	(20.5)	(41.8)	(40.2)	(20.5)	(41.8)	(40.2)	(20.5)	(41.8)
	-189.2	-80.0	0.2	-136.2	-25.0	81.2	-95.6	-68.9	16.4	-186.0
	-14.064	-5.949	0.011	-10.128	-1.860	6.036	-7.105	-5.123	1.218	-13.828
c_O	616	411	463	613	400	422	583	418	422	614
	(45.8)	(30.6)	(34.4)	(45.6)	(29.7)	(31.4)	(43.3)	(31.1)	(31.4)	(45.7)
c_E	607.9	386.5	387.7	607.9	386.5	387.7	607.9	386.5	387.7	607.9
	(45.2)	(28.7)	(28.8)	(45.2)	(28.7)	(28.8)	(45.2)	(28.7)	(28.8)	(45.2)
	16.2	50.5	164.4	10.2	27.5	71.6	-48.8	65.5	71.6	12.2
	1.206	3.758	12.221	0.755	2.044	5.320	-3.632	4.872	5.320	0.905
N	1345	1345	1345	1345	1345	1345	1345	1345	1345	1345
X2	76.9	10.7	26.3	38.2	1.0	16.2	38.1	9.0	7.6	76.7
[truncated: 816,809 more chars]
